# Sucrose-induced Receptor Kinase 1 is Modulated by an Interacting Kinase with Short Extracellular Domain*[Fn FN2]

**DOI:** 10.1074/mcp.RA119.001336

**Published:** 2019-05-30

**Authors:** Xu Na Wu, Liangcui Chu, Lin Xi, Heidi Pertl-Obermeyer, Zhi Li, Kamil Sklodowski, Clara Sanchez-Rodriguez, Gerhard Obermeyer, Waltraud X. Schulze

**Affiliations:** ‡Department of Plant Systems Biology, University of Hohenheim, 70593 Stuttgart, Germany; §Molecular Plant Biophysics and Biochemistry, Department of Biosciences, University of Salzburg, 5020 Salzburg, Austria; ¶Department of Biology, ETH Zürich, Universitätsstrasse 2, 8092 Zürich, Switzerland

**Keywords:** Phosphoproteome, Plant Biology*, Protein kinases*, Signal Transduction*, Protein-Protein Interactions*, Plasma membrane

## Abstract

Sucrose as a product of photosynthesis is the major carbohydrate translocated from photosynthetic leaves to growing nonphotosynthetic organs such as roots and seeds. These growing tissues, besides carbohydrate supply, require uptake of water through aquaporins to enhance cell expansion during growth. Previous work revealed Sucrose Induced Receptor Kinase, SIRK1, to control aquaporin activity via phosphorylation in response to external sucrose stimulation. Here, we present the regulatory role of AT3G02880 (QSK1), a receptor kinase with a short external domain, in modulation of SIRK1 activity. Our results suggest that SIRK1 autophosphorylates at Ser-744 after sucrose treatment. Autophosphorylated SIRK1 then interacts with and transphosphorylates QSK1 and QSK2. Upon interaction with QSK1, SIRK1 phosphorylates aquaporins at their regulatory C-terminal phosphorylation sites. Consequently, in root protoplast swelling assays, the *qsk1qsk2* mutant showed reduced water influx rates under iso-osmotic sucrose stimulation, confirming an involvement in the same signaling pathway as the receptor kinase SIRK1. Large-scale phosphoproteomics comparing single mutant *sirk1*, *qsk1*, and double mutant *sirk1 qsk1* revealed that aquaporins were regulated by phosphorylation depending on an activated receptor kinase complex of SIRK1, as well as QSK1. QSK1 thereby acts as a coreceptor stabilizing and enhancing SIRK1 activity and recruiting substrate proteins, such as aquaporins.

## Abstract (precis)

The activation of aquaporins by a receptor kinase complex of SIRK1 and QSK1 was studied in detail. Based on phosphoproteomics, pulldown studies and physiological experiments we conclude that SIRK1 may function as a main receptor which forms a complex with coreceptor QSK1. SIRK1 can autophosphorylate and then trans-phosphorylate QSK1. Phosphorylated QSK1 enhanced and stabilized the interaction with aquaporins as substrates of the receptor kinase complex.

## Graphical Abstract

**Figure:**
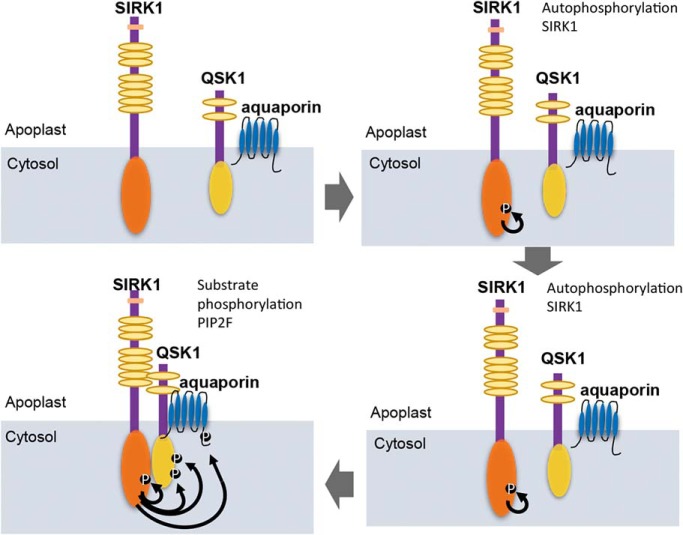

**Highlights**

- Functional role of a yet uncharacterized receptor kinase QSK1.
- Activation model for SIRK1 receptor kinase in a heteromer with QSK1.
- Role of QSK1 in substrate recruitment and stabilization of the complex.

Growth and development of a plant require precise control of carbon assimilation, transport and storage (1). In this context, sucrose as a main product of photosynthesis in most plant species is the major carbohydrate translocated within the phloem to serve as carbon supply for nonphotosynthetic tissues such as roots or seeds. Sucrose is used for the maintenance of cellular metabolism, as precursor for cell wall biosynthesis, and a major storage sugar in vacuoles. Mechanisms of how sucrose is loaded into the phloem (2, 3) and distributed within the plant (4) are well understood and were completed with discovery and characterization of sucrose-exporting SWEET family (5). Besides sucrose, expansion of cells during growth and storage requires the influx of water. Since the discovery of aquaporins as water channels within membranes (6), their regulation through C-terminal phosphorylation was unraveled (7–9). Aquaporins play important roles during lateral root growth (10, 11) and seed development (12).

Arabidopsis contains ∼600 receptor like kinases which play critical roles in regulation of general signal perception and transduction as well as plant growth and defense (13). There are about 223 LRR receptor-like kinases in Arabidopsis (14), and only about 60 of these have been functionally characterized (15). Receptor kinases with a large extracellular domain are considered to play key roles in ligand binding and perception, being specific to a single signaling pathways (16). In contrast, receptor kinases with short extracellular domains are often found to be involved in more than one signaling pathway and have coreceptor functions. For example, BRASSINOSTEROID INSENSITIVE 1 (BRI1) and BRI1-ASSOCIATION RECEPTOR KINASE 1 (BAK1, also known as SOMATIC EMBRYOGENESIS RECEPTOR KINASE 3, SERK3) function as brassinosteroid (BR)^1^ receptor and coreceptor, respectively. In the BR signaling pathway, BR binding induces a basal activation of the receptor BRI1 for binding BAK1, and transactivation occurs between BRI1 and BAK1 to fully activate BRI1 to enhance the phosphorylation of downstream substrate (17–24). BAK1 is also coreceptor recruited to receptor kinase FLAGELLIN-SENSING 2 (FLS2) after perception of the flagellin peptide (flg22). The formation of a complex of receptor FLS2, coreceptor BAK1 and ligand flg22 leads to a full activation of downstream immune defense signaling (25–28). In addition, SERKs including BAK1 function as coreceptors of IDA-receptors HAE/HLS2 and EPF-receptor ERECTA in regulation of floral organ abscission and stomatal patterning (29, 30), as well as in phytosulfokine signaling (31) and other pathways.

Recently, several receptor kinases were shown to directly interact with and regulate plasma membrane transmembrane transporters, channels and proton pumps. For example, different LRR-receptor kinases, besides linking to cytoplasmic signaling cascades, directly regulate the plasma membrane H^+^-ATPases (32–34), Ca^2+^-ATPases (35) or aquaporins (36). The recent discovery of such short, direct regulatory circuits within the plasma membrane between receptor kinases and transporters or channels suggests that this is a generic modular principle allowing plants to very rapidly adjust to changing environments. A prerequisite for the function of such short signaling circuits are highly dynamic protein-protein interactions between kinases and the respective transporters or channels. Based on existing evidence from ABA-induced membrane protein complex dynamics in membrane nanodomains (37), also lateral segregation within the membrane may play a key role in the dynamics of such regulatory interactions.

In previous research (36), we found that Sucrose-induced receptor kinase SIRK1 (AT5G10020) can regulate aquaporins through phosphorylation under conditions of external sucrose supply. Upon sucrose stimulation, SIRK1 was found to form a complex with another yet uncharacterized receptor kinase with a short extracellular domain (AT3G02880). Based on our findings in this work we here name this protein QSK1, qiān shŏu, (Chinese: 千 手) “thousand hands” kinase.

Interestingly, the localization of QSK1 within the plasma membrane between detergent resistant membrane fractions (DRM) and detergent soluble fractions (DSM) was previously found to be highly dependent on cytoskeleton integrity (38). Disruption of actin filaments resulted in a depletion of QSK1 from DRM and increase in DSM location which was visible by enhanced appearance of the punctate location of QSK1 (39). In contrast, disruption of microtubules resulted in a more uniform and less structured localization and was accompanied by general internalization processes or reduced secretion (38). However, the role of QSK1 in regulation of receptor kinase SIRK1 and aquaporins remained to be elucidated.

In the present study, we now explore the activation mechanism of SIRK1 and QSK1 using aquaporins as a known phosphorylation and activation target of the SIRK1/QSK1 complex. We conclude that QSK1 functions in stabilizing SIRK1 activity like a coreceptor in other receptor kinase signaling pathways.

## EXPERIMENTAL PROCEDURES

### 

#### 

##### Experimental Design and Statistical Rationale

To investigate the role of *QSK1* (*At3g02880*) and *QSK2* (*At5g16590*) in the regulation of aquaporins, we performed different experiments involving mass-spectrometry based proteomic analyses. First, a comparative phosphoproteomic analysis of wild type, *qsk1* mutant, *sirk1* mutant, and *sirk1 qsk1* was performed under sucrose starvation and sucrose resupplied conditions. Secondly, pull-down experiments were carried out using SIRK1-GFP or QSK1-GFP as bait proteins. Furthermore, *in vitro* phosphorylation assays were done using purified recombinant QSK1 and SIRK1 kinase domains and synthetic peptides as phosphorylation targets. All these experiments were carried out with at least three biological replicates per genotype or treatment and results are presented as averages from these biological replicates. We used the MaxQuant/Perseus data analysis platform for quantitative analysis in all proteomics experiments.

##### Plant Materials and Growth Conditions

Arabidopsis seeds of wild type (col-0), *sirk1* single mutant (SALK_125543), *qsk1* single mutant (SALK_019840), *qsk2* single mutant (WiscDsLoxHs082_03E), *sirk1 qsk1* double mutant (crossing of the *sirk1* and *qsk1* T-DNA insertion lines), *sirk1 qsk1 qsk2* triple mutant (crossing of *sirk1*, *qsk1*, and *qsk2* T-DNA insertional lines), as well as overexpression lines *^35^S::SIRK1-GFP* and *^35^S::QSK1-GFP* were used. Homozygous T-DNA insertional mutants *sirk1*, *qsk1* and double mutant *sirk1 qsk1* were confirmed via PCR amplification using T-DNA border primer LBb1.3 (5′-ATTTTGCCGATTTCGGAAC-3′) and gene-specific primers (SIRK1-RP: 5′-TTTCCAGCATTTCCAACACTC-3′, SIRK1-LP: 5′-CACTAAGCTTGTTGAGGTCGC-3′; QSK1-RP: 5′-CAAACCAGGTCCATCAAGATC-3′, QSK1-LP: 5′-GAGATTCCGTCGCTTCTCTTC-3′). Lack of *SIRK1* gene and protein expression was already characterized previously for the *sirk1* mutant (36), and Lack of *QSK1* expression was demonstrated for the *qsk1* mutant (supplemental Fig. S1). Mutants of *qsk2* and *qsk1 qsk2* were confirmed via PCR amplification using T-DNA border primer LB (5′-TGATCCATGTAGATTTCCCGGACATGAAG-3) and gene specific primer (QSK2-RP: 5′-TTCCATTCACTGCAGTCTGC-3′, QSK2-LP: 5′-GCAGAAGCTTTCAGCAATCC-3′).

Plants were germinated and grown under 16/8 day/night (22 °C, 120 μE/s*m^2^) in ½ MS medium plus 0.5% sucrose in a hydroponic cultivation system (39). After 19 days, seedlings were starved by changing the growth medium to a sucrose-free medium and leaving the culture vessels in the dark for 48 h as described (40). Sucrose was then resupplied to a final concentration of 30 mm for 3 min before roots were harvested for microsomal protein preparation.

##### Quantitative Real-time PCR

Total RNA was extracted from the *qsk1* plants using the Plant RNA Mini Kit (peQlab, Germany) according to the instructions provided by the manufacturer. RNA was digested with DNaseI (Roche Diagnostics, Germany) to remove the genomic DNA. First-strand cDNA was synthesized using PrimeScript RT reagent kit (TaKaRa). Quantitative real-time PCR analysis was performed using the Bio-Rad CFX Connect real-time PCR system (BioRad Laboratories; Munich, Germany) with the SYBR green detection protocol (TaKaRa, Saint-Germain-en-Laye, France). The Actin and Tubulin genes were used as reference genes, and the relative expression of the gene of interest was calculated by the 2∧-ΔΔCq method. RT Primers for QSK1 was F 5-TGAGTCATGCCAATCTCGTGAC-3, R 5-GCAATATCGCAGACAAGCTTCC-3. Primers used for Actin and Tubulin were: Actin-F 5-ACTTTCATCAGCCGTTTTGA-3, Actin-R-5-ACGATTGGTTGAATATCATCAG-3 and Tubulin-F 5-ACCTACTGGTCTGAAGATGGCAT-3 and Tubulin-R5-TTTCTCCTGAACATAGCTGTGAAC-3.

##### Constructs

For ratiometric bimolecular Fluorescence Complementation (rBiFC) of Arabidopsis proteins, cDNAs of the following genes were cloned into rBiFC plasmids (41): SIRK1, or SIRK1 with phosphorylation site mutations SIRK1S744A, SIRK1S744D, QSK1, QSK1 with phosphorylation site mutations QSK1S621AS626A, QSK1S621DS626D, QSK2 and PIP2;4. SIRK1, SIRK1S744A and SIRK1S744D were cloned as fusions with the C-terminal half of YFP, whereas QSK1, QSK1S621AS626A, QSK1S621DS626D, QSK2 and PIP2;4 were cloned as fusions with the N-terminal half of YFP. All constructs were transformed into *Agrobacterium tumefaciens* strain GV3101 by electroporation. Positive colonies were confirmed by spectinomycin and rifampicin resistance and colony PCR.

To produce overexpression lines of SIRK1 and QSK1 for pull-down analysis (*^35^S::SIRK1-GFP* and *^35^S::QSK1-GFP*), cDNA of the SIRK1 gene and QSK1 gene without a stop code were cloned into the plant transformation vector pEZR(H)-LN (36) and fused with GFP coding sequence. Homozygous T2 transgenic lines were further selected by means of segregation analysis, and T3 seeds were used for experiments. The fluorescence of GFP in the transgenic lines was checked using confocal laser-scanning microscopy (TCS SP5, Leica Microsystems CMS GmbH, Wetzlar, Germany).

For purification of the cytoplasmic domain of SIRK1 protein (SIRK1-CD, amino acid 625–1048) and QSK1 protein (QSK1-CD, amino acid 276–627) to be used in *in vitro* kinase assays, SIRK1-CD and QSK1-CD were cloned into *Escherichia coli* BL21(DE3) expressing plasmid pETGST 1a and fused with His and GST tags, resulting in the plasmid His-GST-SIRK1-CD and His-GST-QSK1-CD.

##### Protein Expression and Purification

Plasmids His-GST-SIRK1-CD and His-GST-QSK1-CD were transformed into *Escherichia coli* BL21 (DE3). After 5 h induction by IPTG (isopropyl β-d-thiogalactopyranoside), cells were harvested and lysed using BugBuster Protein Extraction Reagent (Novagen, Nottingham, UK), soluble fractions were used over gravity flow Ni^2+^-NTA Sepharose columns (1 ml, IBA GmbH, Goettingen, Germany) for His-GST-SIRK1-CD and His-GST-QSK1-CD protein purification.

##### Microsomal Membrane Preparation, Tryptic Digestion, and Phosphopeptide Enrichment

A total of 1 to 1.5 g of roots (fresh weight) was homogenized in 10 ml ice-cold extraction buffer (330 mm mannitol, 100 mm KCl, 1 mm EDTA, 50 mm Tris-MES, fresh 5 mm DTT, and 1 mm phenylmethylsulfonylfluoride, pH 7.5) (42) in the presence of 0.5% v/v proteinase inhibitor mixture (Sigma-Aldrich, Taufkirchen, Germany) and phosphatase inhibitors (25 mm NaF, 1 mm Na3VO4, 1 mm benzamidin, 3 μm proteinase inhibitor leupeptin). The homogenate was centrifuged for 15 min at 7500 × *g* at 4 °C. The pellet was discarded, and the supernatant was centrifuged for 75 min at 48,000 × *g* at 4 °C. The microsomal pellet was resuspended in 100 μl of membrane buffer (330 mm mannitol, 25 mm Tris-MES, 0.5 mm DTT) or UTU (6 m urea, 2 m thiourea, pH 8). Further tryptic digestion, desalting over C18 and enrichment of phosphopeptides over titanium dioxide beads was performed as described (43).

##### Pull-downs of GFP-tagged SIRK1 and QSK1

Root microsomal proteins (100 μg) isolated as described above was incubated with 25 μl of anti-GFP agarose beads (Chromotek, Planegg, Germany) for two hours on a rotating wheel at 4 °C (36). After incubation, the beads were collected by centrifugation and washed two times with 500 μl wash buffer (10 mm Tris/HCl pH 7.5, 150 mm NaCl, 0.5 mm EDTA, 0.01% IGEPAL). For protein-protein interaction assays, the proteins were eluted from the beads with 100 μl UTU (6 m urea, 2 m thiourea), pH 8, before in-solution tryptic digestion. For kinase activity assays, three more washing steps were carried out with one-time wash buffer (10 mm Tris/HCl, pH 7.5, 300 mm NaCl, 0.5 mm EDTA) and two times kinase reaction buffer (40 mm Tris/HCl pH 7.5, 10 mm MgCl_2_, 0.1% BSA, 2 mm DTT).

##### Kinase Activity Assay

SIRK1-GFP and QSK1-GFP fusion proteins were affinity purified over anti-GFP beads (see above). A luciferase-based kinase activity assay was performed as described (36). The agarose beads with GFP-tagged proteins were re-suspended into 30 μl kinase reaction buffer with ATP and the generic kinase substrate myelin basic protein (40 mm Tris/HCl pH 7.5, 10 mm MgCl_2_, 0.1% BSA, 2 mm DTT, 100 μm ATP, 0.4 μg/μl myelin basic protein). After incubation for one hour, 30 μl ADP-GLO Reagents (Promega, Mannheim, Germany) was added and incubated for 40 min. Then Kinase Detection Reagents were added and incubated for another hour. Luminescence as a measure of ATP conversion from ADP was recorded with a luminometer (Tecan M200 Pro, Crailsheim, Germany). In general, protein isolations from three independent batches of roots were tested, and the average activity value is presented.

##### In Vitro Peptide Phosphorylation Assay

Kinase activity assays were performed as described above, except that 10 pmol of the peptides FSDQPVMLDVYSPDR (SIRK1), LIEEVSHSSGSPNPVSD (QSK1), or ALGSFGSFGSFR (PIP2;4) were used as a substrate and His-GST-SIRK1-CD and His-GST-QSK1-CD protein were used as kinases. All peptides were synthesized based on experimentally identified phosphopeptides in this work or previous studies (36, 40). After exposure to the target peptide, the reaction mixture was acidified with 2 μl TFA. Acidified peptide mixtures were then desalted over C18 prior to mass spectrometric analysis. The phosphorylation of target peptides was confirmed via mass spectrometry (see above) (44). Phosphorylation was quantified based on signature fragment ion sums using MaxQant. The nonphosphorylated input peptide was used for normalization. The same three recombinant kinase preparations were used in the activity assays with all substrate peptides.

##### Transient Expression of Ratiometric Bimolecular Fluorescence Complementation (rBiFC) Constructs

Positive colonies of *Agrobacterium tumefaciens* harboring the relevant constructs described above were propagated in LB medium at 28 °C for 2 days, and then diluted as 1:500 into new medium for overnight culture. *Agrobacterium* pellets were collected by centrifugation at 1500 × *g* for 15 min and resuspended in resuspension buffer (10 mm MES, 10 mm MgCl_2_, 0.15 mm acetosyringone, pH 5.8), the suspension was diluted to OD600 of 0.5. The suspensions were injected into 5 to 6 weeks old *Nicotiana benthamiana* leaves with 1 ml syringe for 2 days before observation. Fluorescence was observed using a Zeiss LSM700 confocal microscope (20× 0.75-NA objectives). In all cases, excitation intensities, filter settings, photomultiplier gains and other parameters were standardized. The YFP and RFP fluorochromes were excited with 488 nm and 561 nm, respectively. Emitted light was collected at a range of 500–560 nm for YFP and 575–625 nm for RFP. All images throughout all experiments were collected using the same settings. The collected images were processed and both YFP and RFP intensity was measured using the FIJI software (45) and the YFP/RFP ratio was calculated. 10–30 different cells from leaves of the 2 plants were analyzed. Statistical significance was determined using Welch Two Sample *t* test. To calibrate YFP/RFP ratios, known interaction of CBL9 with CIPK23 was used as positive control, whereas the interaction of CBL9 with CIPK14 was used as negative control (46).

##### Protoplast Swelling Assay

Seedlings were grown on vertical plates with ½ MS without sucrose for 7 days and roots were cut into small pieces. The root fractions were incubated in 2 ml enzymatic digestion medium (ES-300M: 300 mm mannitol, 10 mm MES/KOH, pH 5.8, 10 mm CaCl_2_, 10 mm KCl; plus 1% (w/v) cellulase Onozuka R10 (Duchefa, Haarlem, The Netherlands) and 1% (w/v) macerozyme R10 (Duchefa) at room temperature. Protoplasts were harvested after 4 h of gentle shaking via a 50 μm nylon filter. The washing steps were done by three times ice cold ES-300 M (without enzymes) and centrifugation at 80 × *g*. The protoplasts were finally resuspended in 150 μl ES-300 M buffer.

The protoplast swelling assay was performed as described previously (36, 47, 48). Approximately 20 μl protoplasts were pipetted into a perfusion chamber with 200 μl ES-300M. After 10 min the protoplasts settled down and the chamber was perfused with 3 ml of ES-300 M to flush away protoplasts not sticking to the chamber glass bottom. The hypotonic challenge was performed by changing ES-300 M to ES-150 M solution with reduced mannitol to 150 mm. Images were taken every 3 s with a video-camera-equipped microscope for 5 min. For the sucrose treatment, protoplasts not stuck to the glass slide were firstly washed away by ES-300M, and then perfusion solution was changed to ES-270M-30S (270 mm mannitol, 30 mm sucrose, 10 mm MES/KOH, pH 5.8, 10 mm CaCl_2_, 10 mm KCl) for 5 min. The hypotonic perfusion was done with ES-120M-30S. The osmolarities of each solution was measured with a cryoscopic osmometer (Osmomat 030, Gonotec): ES-300 M 0.350 osmol/kg; ES-150 M 0.197 osmol/kg; ES-270M-30S 0.353 osmol/kg; ES-120M-30S, 0.197 osmol/kg. The diameter of the protoplasts was measured using imageJ software. Diameters were converted to micrometers, and the volume, surface area, and relative volume changes were calculated for each time point. A regression line was fitted to the steepest part of the swelling curve to determine the maximal water volume flux density, which corresponds to aquaporin opening status. Statistical analysis was performed with Sigma Plot (Jandel Scientific).

##### LC-MS/MS Analysis of Peptides and Phosphopeptides

Peptides mixtures were analyzed by nanoflow Easy-nLC (Thermo Scientific) and Orbitrap hybrid mass spectrometer (Q-exactive, Thermo Scientific). Peptides were eluted from a 75 μm × 50 cm analytical C_18_ column (PepMan, Thermo Scientific) on a linear gradient running from 4% to 64% acetonitrile over 135 min. Proteins were identified based on the information-dependent acquisition of fragmentation spectra of multiple charged peptides. Up to twelve data-dependent MS/MS spectra were acquired in the linear ion trap for each full-scan spectrum acquired at 70,000 full-width half-maximum (FWHM) resolution.

##### Protein Identification and Label Free Quantification of Protein Intensities

MaxQuant version 1.5.3.8 (49) was used for raw file peak extraction and protein identification against the UniProt Arabidopsis database UP000006548 (release 2017_10, 39,389 entries). Protein quantification was performed in MaxQuant using the label free quantification (LFQ) algorithm (50). The following parameters were applied: trypsin as cleaving enzyme; minimum peptide length of seven amino acids; maximal two missed cleavages; carbamidomethylation of cysteine as a fixed modification; N-terminal protein acetylation, oxidation of methionine as variable modifications. For phosphopeptide identification also the phosphorylation of serine, threonine, and tyrosine was included as variable modifications. Peptide mass tolerance was set to 20 ppm and 0.5 Da was used as MS/MS tolerance. Further settings were: “label-free quantification” marked, multiplicity set to 1; “match between runs” marked with time window 2 min; peptide and protein false discovery rates (FDR) set to 0.01; common contaminants (trypsin, keratin, etc.) excluded. Phosphorylation sites were determined by the site-scanning algorithm search engine Andromeda (51). Phosphopeptide identifications were submitted to the PhosPhAt database (52), and are supplied in supplemental Fig. S6. The raw MS data from this study was deposited at the ProteomeXchange Consortium (http://proteomecentral.proteomexchange.org) via the PRIDE partner repository with the identifier PXD011284 (phosphorylation profiling) and PXD011265 (pulldowns).

##### Statistical Analyses and Data Visualization

The proteomic data from pulldowns (evidence.txt) derived from MaxQuant was normalized and quantified by the R based script cRacker (53) (version 1.496). Within each sample, all peptide intensities were normalized to fraction of total ion-intensity sums. Subsequently, peptide intensities were scaled across samples or treatments. For each peptide, values from three biological replicates were averaged after normalization and mean scaling. Protein group averages were calculated from proteotypic peptides.

Phosphosites data (Phospho(STY)Sites.txt) were analyzed by Perseus software (54). Briefly, phosphosites quantified in at least 50% in all of samples were analyzed by ANOVA. For comparison analysis between sucrose-resupplied sample and sucrose-starved samples, the log_2_ ratios (sucrose/starvation) were calculated across the different genotypes and the student *t*-tests were used to determine the significant difference in two conditions.

Functional classification of proteins was done based on MAPMAN (55). Information about subcellular location was derived from SUBA3 (56). Detailed protein function was manually updated with the support of TAIR (57). Other statistical analyses were carried out with Sigma Plot (version 11.0) and Excel (Microsoft, 2013). Overrepresentation analysis was done via Fisher's exact test, *p* values were adjusted using Bonferroni correction.

## RESULTS

This work aimed at a close investigation of the functional role of QSK1 (AT3G02880) and its close homolog QSK2 (AT5G16590) in the complex with SUCROSE INDUCED RECEPTOR KINASE 1 (SIRK1, AT5G10020) during regulation of aquaporins in response to external changes in sucrose concentrations.

### 

#### 

##### Interaction of SIRK1 and QSK1 is Phosphorylation-dependent

**Fig. 1.**
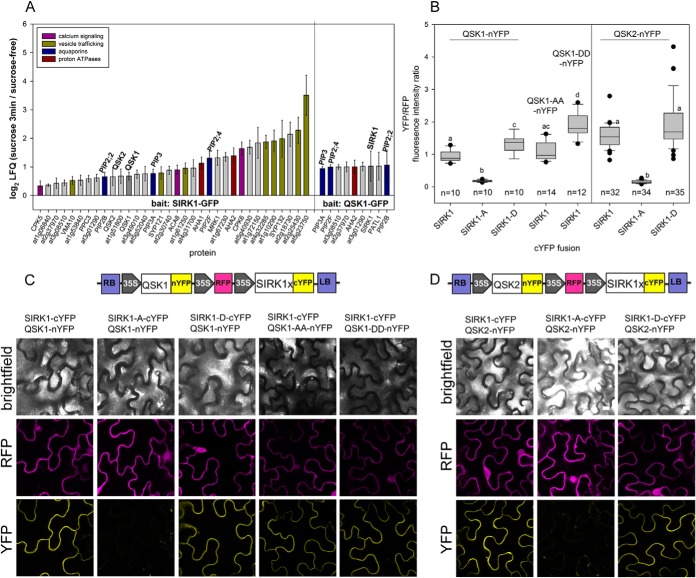
**Interaction of SIRK1 with QSK1 and QSK2.** *A*, Inverse pull-downs using either SIRK1-GFP or with QSK1-GFP as bait. Bars are proportional to the sucrose-induced interaction as measured by the summed ratio of ion intensities under sucrose supply compared with sucrose free condition. Error bars indicate standard deviations of ratios calculated from three biological replicates. *B*, Quantitative YFP/RFP fluorescence intensity ratio describing the interaction of SIRK1 with QSK1 and QSK2. Phosphodead (S to A) and phosphomimic (S to D) versions of SIRK1 and QSK1 were included. 10–30 randomly selected cells were quantified. Center lines of boxes represent medians, black dots indicate outliers. Small letters indicate significant differences (*p* < 0.05; pairwise rank-sum test). *C*, Representative images of epidermal cells showing expression of the illustrated constructs (RFP-channel) and interaction of SIRK1 with QSK1 by rBiFC (YFP-channel). *D*, Representative images of epidermal cells showing expression of the illustrated constructs (RFP-channel) and interaction of SIRK1 with QSK2 by rBiFC (YFP-channel). Cartoon shows the respective T-DNA of the rBiFC-2in1-NC vector QSK1-nYFP or QSK2-nYFP and different versions of SIRK1-cYFP. In (*C*) and (*D*): Scale bar, 10 μm.

To confirm the sucrose-induced complex formation of SIRK1 with QSK1, we performed inverse single-step pull-downs of endogenously expressed SIRK1-GFP or QSK1-GFP as a bait under sucrose-starved and sucrose-supplied conditions using an affinity enrichment-mass spectrometry approach (58). After LC-MS/MS and intensity-based label-free quantitation analysis, each pulldowns contained large numbers of background binders, which were additionally defined in a GFP-only pulldown experiment under sucrose stimulated and sucrose free condition (supplemental Table S1). Roughly, 10% less proteins were found in the GFP-only pulldown compared with the kinase-GFP experiments (supplemental Table S3). To further filter for relevant interactors of SIRK1 and QSK1 under the sucrose-induced condition, we (1) focused on plasma membrane proteins and (2) performed pairwise *t* test between sucrose-supplied condition and control treatment and between GFP-only binders and baits as described (58). The ratio of normalized ion intensity sums (LFQ-values) of each protein identified under sucrose supplied conditions *versus* sucrose starvation condition was used as a measure of sucrose-dependent interaction based on a statistical comparison (*p* < 0.05, pairwise *t* test, Fig. 1*A*). In total, 11 out of the 47 sucrose-induced interactors to SIRK1 and QSK1 were also found in GFP-only pulldowns, but with lower abundance (supplemental Table S1). QSK1 (AT3G02880) as well as its close homolog QSK2 (AT5G16590), were found in a complex with SIRK1-GFP with stronger interaction under sucrose supply, and not in the GFP-only pulldown. In the inverse pulldowns using QSK1 as bait, consistently SIRK1 was found as part of the complex with QSK1 (Fig. 1A, supplemental Table S1). These results confirm that SIRK1 forms a complex with QSK1, and this interaction is enhanced under sucrose stimulation. In contrast to previous work (36) 33 out of the 45 sucrose-induced interaction partners listed for the SIRK1-GFP bait were putative novel interactors to SIRK1, not identified previously. In the present study, root tissue was used, whereas previously published experiments were carried out on whole seedlings. These newly identified interaction partners mainly were proteins with functions in vesicle trafficking (*e.g.* SYPs), and calcium signaling proteins (*e.g.* CPK5, CPK6, ACA8).In addition, and confirming previous findings, SIRK1-GFP was found to be in a complex with aquaporins and plasma membrane proton ATPases (Fig. 1*A*; supplemental Table S1). QSK1-GFP was also found to be in a complex with aquaporins.

The direct interaction of SIRK1 and QSK1 was confirmed by ratiometric bimolecular fluorescence complementation assays (Fig. 1*B*, 1*C*). To calibrate YFP/RFP ratios, the known interaction of Calcineurin B-like Calcium Sensor Protein CBL9 (AT5G47100) with CBL-interacting protein kinase CIPK23 (AT4G17615) was used as positive control, whereas the known absence of interaction of CBL9 with CIPK14 (AT5G01820) was used as negative control (supplemental Fig. S2). Thus, YFP/RFP florescence ratios greater than 1 suggest interaction of the two proteins tested, whereas fluorescence ratios lower than 1 indicate weak or no interaction. Based on this calibration, we found that the interaction of SIRK1-^c^YFP with QSK1-^N^YFP was enhanced by phosphorylation mimicking mutation at SIRK1 Ser-744 (SIRK1-D), which is the phosphorylation site previously identified under sucrose supply (36, 40). In contrast, SIRK1 with Ser-744 mutated to phosphorylation-dead alanine (SIRK1-A), showed no interaction with QSK1. Furthermore, the interaction of SIRK1 and QSK1 was affected by phosphorylation of the two C-terminal serines (Ser-621 and Ser-626) of QSK1 (Fig. 1 *B, C*). When the C-terminal serines were mutated to phosphomimicking aspartic acids, the interaction of QSK1 with SIRK1 was significantly enhanced. When these C-terminal serines of QSK1 were mutated to alanines, the interaction SIRK1 and QSK1 of both proteins was still well detectable, similarly to the interaction of nonmutated QSK1 (Fig. 1 *C*). QSK2 also interacted with SIRK1 in the bimolecular fluorescence complementation assay (Fig. 1*B, D*), and this interaction was abolished by a phosphodead mutation of SIRK1 Ser-744 to alanine. Interaction behavior of SIRK1 with sucrose-induced substrates identified by the pulldown analysis was additionally tested in seedling tissue of *sirk1 qsk1* mutant background (Supplemental Fig. S3). Both, SIRK1 and QSK1 are expressed in seedlings (59), therefore, by using the double mutant background we confirm interaction of the two proteins in a background without endogenous protein expression levels (supplemental Fig. S3*A*). Furthermore, we also used the transient expression in *sirk1 qsk1* mutant background to test the newly identified interaction of SIRK1 with putative substrate protein Calcium ATPase ACA8 (supplemental Fig. S3*B*).

##### SIRK1 and QSK1 Form an Active Complex Under Sucrose Stimulation

**Fig. 2.**
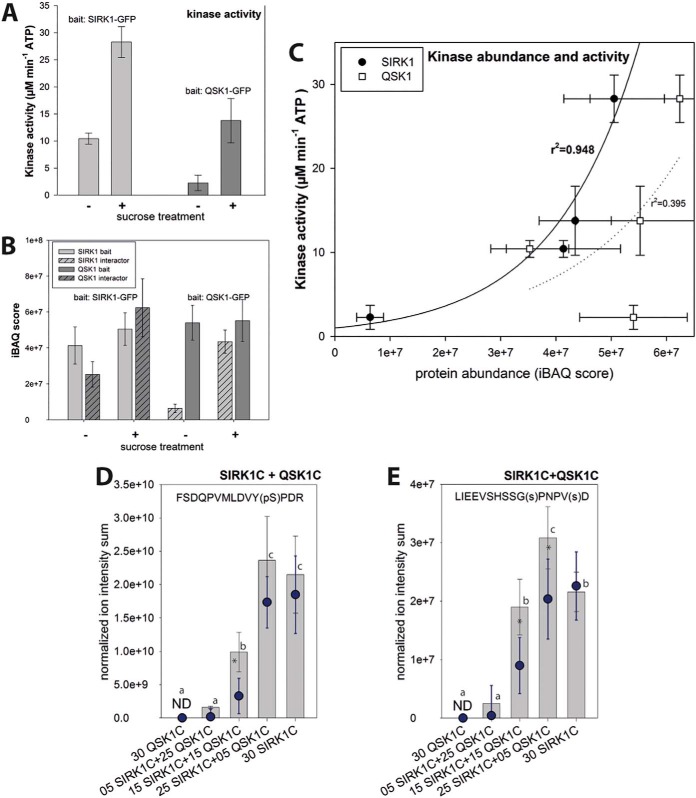
**Kinase activity and phosphorylation of SIRK1 and QSK1.** *A*, Kinase activity of SIRK1 or QSK1 pulldowns under sucrose starvation (-) or 30 mm sucrose supply (+). *B*, Abundance of bait kinases and interacting partners were assessed by quantitative Intensity based absolute quantification (iBAQ) scores (83). Hatched bars represent the interaction partner abundance in pull-downs with respective bait proteins. *C*, Correlation of protein abundance (iBAQ) for either SIRK1 or QSK1, as shown in (*B*), with the activity of isolated kinase complexes, as shown in (*A*). *D*, Peptide phosphorylation assay for SIRK1 Ser-744 in presence of different mixing ratios of SIRK1 and QSK1 recombinant kinase domains. *E*, Peptide phosphorylation assay for QSK1 C terminus in presence of different mixing ratios of SIRK1 and QSK1 recombinant kinase domains. In (*D*) and (*E*) dots indicate PIP2;4-phosphorylation respective amounts of SIRK1 kinase domain only, asterisks mark significant differences between SIRK1 only and SIRK1/QSK1 combinations (*p* < 0.05, pairwise *t* test). In all panels, means with standard deviations of three independent isolations are shown, small letters indicate significant differences based on ONEWAY-ANOVA pairwise comparisons (*p* < 0.05). ND: not detected.

We then used kinase activity assays and *in vitro* phosphorylation assays (44) to study the functional relationship between SIRK1 and QSK1 based on the SIRK1/QSK1 complexes enriched through pulldowns of tagged SIRK1 or QSK1 (Fig. 1*A*). We showed that SIRK1-GFP or QSK1-GFP complexes showed higher kinase activity toward the generic substrate myelin basic protein when enriched from roots supplied with external sucrose compared with isolations from roots grown in sucrose-free medium (Fig. 2*A*). Thereby, the average amount of enriched SIRK1 was similar in the SIRK1-GFP pulldowns under sucrose supplied and sucrose free conditions (Fig. 2*B*). Likewise, comparable amounts of QSK1 were enriched in QSK1-GFP pulldowns under both conditions (Fig. 2*B*). However, the abundance of QSK1 in SIRK1-GFP pulldowns and the abundance of SIRK1 in QSK1-GFP pulldowns was higher under sucrose supplied conditions (Fig. 2*B*). This confirmed that the interaction of SIRK1 and QSK1 was enhanced by external sucrose, not their total amounts within the membrane. Kinase activity correlated with the abundance of SIRK1 in the complex enrichments (r^2^ = 0.948), but not with the abundance of QSK1 (r^2^ = 0.395). This suggests that SIRK1 was the active kinase within the complex (Fig. 2*C*).

##### SIRK1 Autophosphorylates at Ser-744 and Transphosphorylates the C Terminus of QSK1

Previous work has revealed sucrose-induced phosphorylation at Ser-744 of SIRK1, and at Ser-621 and Ser-626 of QSK1 (40). Using *in vitro* phosphorylation assays, we exposed synthetic peptides covering these experimentally identified sucrose-induced phosphorylation sites of SIRK1 and QSK1 to different dilutions of recombinant kinase domains of SIRK1 and QSK1. Recombinant intracellular domains of SIRK1 (SIRK1C) and QSK1 (QSK1C) were used in order to avoid effects by co-purified proteins present from *in planta* pulldowns of SIRK1-GFP or QSK1-GFP. We found that SIRK1 Ser-744 phosphorylation (supplemental Fig. S4) by recombinant SIRK1 kinase domain, but not by recombinant QSK1 alone (Fig. 2*D*). However, presence of QSK1 together with SIRK1 resulted in increased substrate phosphorylation compared with reactions with recombinant SIRK1 alone (Fig. 2*D*). Also, the C-terminal QSK1 peptide was phosphorylated (supplemental Fig. S4) in the presence of recombinant SIRK1 intracellular domain, but not with QSK1 intracellular domain alone (Fig. 2*E*). Moreover, an enhanced kinase activity toward the QSK1 C-terminal peptides was observed when recombinant SIRK1 was combined with recombinant QSK1 (Fig. 2*E*). The normalized ion intensities of phosphorylated QSK1 substrate peptide did not decrease with decreasing amounts of recombinant SIRK1 when up to equal amounts of recombinant QSK1 were present in the assay only in those phosphorylation assays, in which recombinant SIRK1 intracellular domain (SIRK1C) was less abundant than recombinant QSK1, low substrate phosphorylation was observed (Fig. 2*E*). This effect was less pronounced for SIRK1 autophosphorylation (Fig. 2*D*). *In vitro* kinase assays were further confirmed by using SIRK1-GFP expressed and purified in sirk1 background as an active kinase together with different amounts of recombinant QSK1 kinase domain (supplemental Fig. S5). These assay with full-length SIRK1 as a kinase confirmed autophosphorylation of SIRK1, as well as trans-phosphorylation of QSK1.

In support of the above *in vitro* kinase activity assays, the C-terminal phosphopeptide of QSK1 was identified only in wild type, and neither in *sirk1* nor *qsk1* mutants (supplemental Table S2). Furthermore, our results agree with other findings that QSK1 does not undergo autophosphorylation (60) and suggest a sucrose-induced trans-phosphorylation of the QSK1 C terminus at two serines by receptor kinase SIRK1.

##### Aquaporins as Phosphorylation Substrates to SIRK1/QSK1 Complex

In a phosphoproteome profiling experiment comparing *sirk1*, *qsk1* and *sirk1 qsk1* double mutant we aimed at finding preferential substrates for each of the two kinases. In total, 1714 phosphosites were identified and 1029 phosphosites were scored with a localization probability > 0.75 (class I sites). We obtained quantitative data (MaxQuant LFQ-values (50)) for 669 of the class I phosphosites in at least two biological replicates from at least one of the two treatments, sucrose starvation and sucrose resupply (supplemental Table S2, supplemental Fig. S6). Sucrose stimulation at a final concentration of 30 mm for 3 min was used, as this was previously identified as the time point for maximal transient phosphorylation of SIRK1 (36, 40). Thereby, particularly those phosphosites with reduced phosphorylation or no phosphorylation in the *sirk1 qsk1* double mutant compared with wild type or either single mutant (*sirk1* or *qsk1*) were considered as dependent on a functional SIRK1/QSK1 complex. Proteins with transport functions (aquaporins, plasma membrane H+-ATPases, sucrose exporters; *p* = 9.68 × 10–11, Fisher's exact test) constituted the major functional group within those putative targets of the SIRK1/QSK1 complex. These putative substrates included calcium ATPase ACA8 (AT5G57110), members of the SYP-family (syntaxin of plants, SYP122 AT3G52400, SYP121 AT3G11820) or the plasma membrane proton ATPase AHA2 (AT4G30190) (supplemental Table S2). The phosphopeptide LIEEVSRSPA(pS)PGPLSD, matching a close homolog of QSK1 C-terminal peptide (QSK2, AT5G16590) was also found as putative phosphorylation target of the SIRK1/QSK1 complex. Further, among the putative substrates of the SIRK1/QSK1 complex, we found the two well-known conserved and regulatory serines (61) in the C terminus of aquaporins at well characterized sites Ser-273 and Ser-276 in PIP3 (AT4G35100); Ser-283 and Ser-286 in PIP2;4 (AT5G60660); Ser-278 and Ser-281 (AT2G37170) in PIP2;2). Because aquaporins were also identified as part of this putative SIRK1/QSK1 complex in pulldown experiments (Fig. 1*A*), we explored in more detail the functional role of SIRK1 and QSK1 phosphorylation during interactions between SIRK1, QSK1 and their aquaporin substrates.

##### Differential Roles of SIRK1 and QSK1 in Regulation of PIP2;4

We specifically quantified C-terminal phosphorylation of PIP2;4 by sucrose treatment in the different genotypes. Thereby, Ser-283 of PIP2;4 is conserved and correspond to the pore gating Ser-274 of aquaporin in spinach SoPIP2;1 (61). Serine Ser-286 of PIP2;4 is conserved with Ser-286 of PIP2;1 (AT3G53420) controlling plasma membrane localization (8). Thus, phosphorylation of these sites is expected to result in higher water channel activity and plasma membrane localization, respectively. Dephosphorylation is expected to relate to pore closing and removal from plasma membrane.

**Fig. 3.**
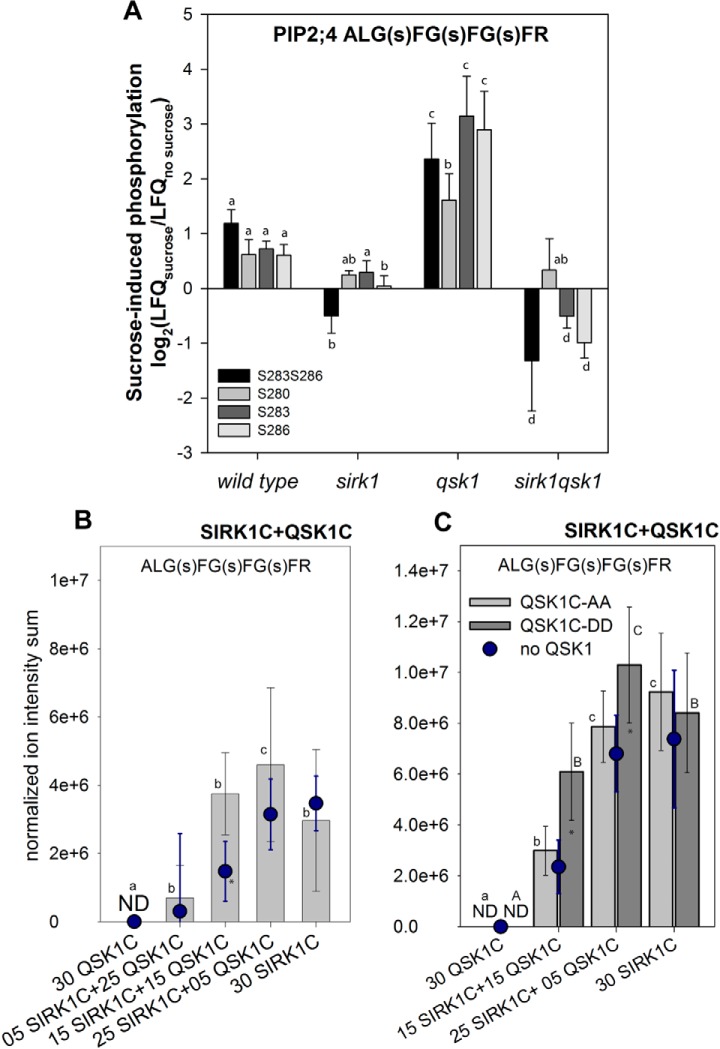
**SIRK1/QSK1 complex modulates aquaporin phosphorylation.** *A*, Sucrose-induced phosphorylation of PIP2;4 in wild type, *sirk1*, *qsk1* and *sirk1 qsk1* double mutant shown as mean with standard deviation of three replicates. Small letters indicate significant differences (*p* < 0.05; pairwise *t* test) for each phosphopeptide between genotypes. *B*, Peptide phosphorylation assay for PIP2;4 C-terminal peptide in presence of different mixing ratios of recombinant SIRK1 and QSK1. *C*, Peptide phosphorylation assay for PIP2;4 C-terminal peptide in presence of different mixing ratios of recombinant SIRK1 and recombinant QSK1 with phosphodead (QSK1-AA) or phosphomimicked (QSK1-DD) mutations. In (*B*) and (*C*) ion intensity sums of phosphorylated forms of the peptide are shown as means with standard deviations of three independent assays. Dots indicate PIP2;4-phosphorylation respective amounts of SIRK1 kinase domain only. Small letters indicate significant differences based on ONEWAY-ANOVA pairwise comparisons (*p* < 0.05). Asterisks mark significant differences between SIRK1 only and SIRK1/QSK1 or SIRK1/QSK1-DD combinations (*p* < 0.05, pairwise *t* test). ND: not detected. LFQ: label-free quantitation within the peptide sequence ALGSFGSFGSFR, (s) indicates sites of phosphorylation in combinations as explained in the legends.

Our results here confirm reduced C-terminal aquaporin phosphorylation in *sirk1* (36). In contrast, in the *qsk1* mutant we found a higher than wild type phosphorylation status of aquaporin PIP2;4 at singly and doubly phosphorylated sites. In the *sirk1 qsk1* double mutant, sucrose-induced aquaporin phosphorylation was even lower than in the *sirk1* single mutant (Fig. 3*A*). Thus, the loss of QSK1 alone did not result in drastic phosphorylation changes in sucrose-induced aquaporin phosphorylation, but loss of QSK1 in the background of *sirk1* enhanced the *sirk1* phenotype by significantly lower sucrose-induced aquaporin phosphorylation. To further investigate the contributions of SIRK1 and QSK1 to phosphorylation of aquaporins, a synthetic C-terminal peptide of PIP2;4 was exposed to different mixtures of recombinant SIRK1 and QSK1 intracellular domains (Fig. 3*B*). Although recombinant QSK1 alone did not result in phosphorylation of PIP2;4, recombinant SIRK1 alone was clearly able to phosphorylate the substrate peptide at Ser-283 and Ser-286 as shown previously (36). Moreover, when SIRK1 intracellular domain was exposed with the substrate peptides in presence of recombinant QSK1 intracellular domain, QSK1 contributed to a significantly enhanced activity of recombinant SIRK1, possibly through stabilization of an active complex. This became visible through significantly higher substrate phosphorylation ion intensity sums in the respective assay. Introducing phosphodead alanine mutations to recombinant QSK1 C-terminal phosphorylation sites (QSK1C-AA) resulted in target peptide phosphorylation intensities proportional to reactions in the presence of only recombinant SIRK1 intracellular domain (Fig. 3*C*). In contrast, phosphomimicking aspartate mutations within recombinant QSK1 (QSK1C-DD) resulted in significantly enhanced phosphorylation of the PIP2;4 target peptide compared with the substrate phosphorylation by recombinant SIRK1 alone (Fig. 3*C*). We conclude that QSK1, particularly in its phosphorylated state, enhanced PIP2;4 phosphorylation by SIRK1, possibly through complex formation of SIRK1 and QSK1 kinase domains. The enhanced phosphorylation of substrate peptide of PIP2;4 in presence of QSK1 kinase domain was also confirmed when SIRK1-GFP enriched from expression in the sirk1 mutant background was used as a kinase substrate (supplemental Fig. S7).

**Fig. 4.**
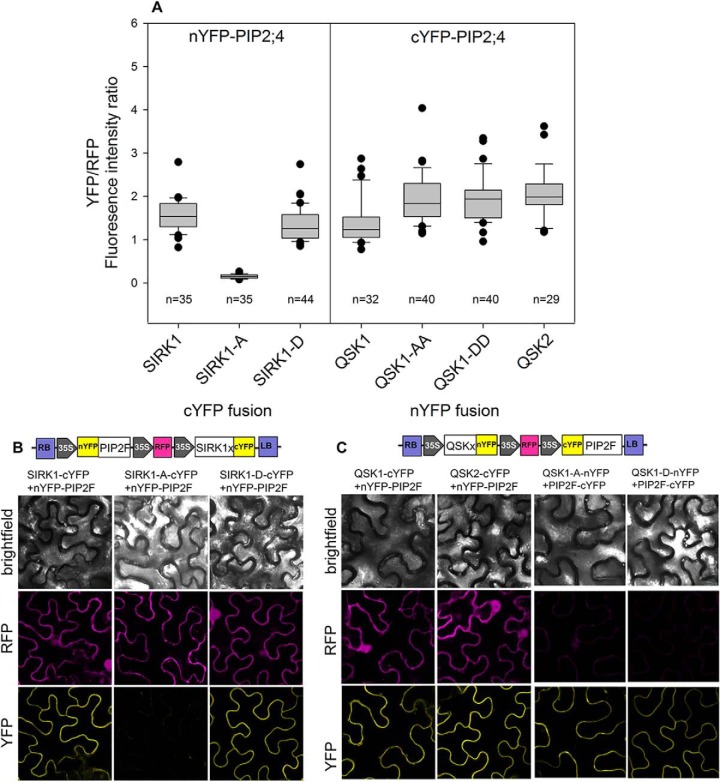
**Interaction of SIRK1, QSK1 and QSK2 with PIP2;4/PIP2F.** *A*, Quantification of YFP/RFP mean fluorescence intensities ratio from around 30 randomly selected cells. The interactions of SIRK1 phosphodead (SIRK1-A) and phosphomimic (SIRK1-D) with PIP2;4 as well as the interaction of QSK1 phosphodead (QSK1-AA) and phosphomimic (QSK1-DD) with PIP2;4 were tested. Center lines of boxes represent medians, black dots indicate outliers. Small letters indicate significant differences (*p* < 0.05; pairwise *t* test). *B*, Representative images of epidermal cell showing expression of the constructs (RFP-channel) and BIFC (YFP-channel) from transiently transformed *Nicotiana benthamiana* leaves. Cartoon shows the T-DNA of the rBiFC-2in1-NC vector containing nYFP-PIP2;4 and different versions of SIRK1-cYFP. Scale bar: 10 μm. *C*, Representative images of the rBiFc analysis of PIP2;4 with QSK1 and QSK2. Cartoon shows the T-DNA of rBiFC-2in1-CN vector containing versions of QSK1-nYFP and cYFP-PIP2;4.

The interaction of SIRK1 with PIP2;4 was abolished when the SIRK1 autophosphorylation site Ser-744 was mutated to nonphosphorylated alanine (SIRK1-A), but not when mutated to phosphomimicking aspartate (SIRK1-D), as shown by ratiometric bimolecular fluorescence complementation (Fig. 4*A*). Interestingly, also QSK1 and QSK2 were able to interact with PIP2;4 (Fig. 4*A*, 4*C*). Mutation of QSK1 C-terminal phosphorylation sites to phosphodead alanine (QSK1-AA) or phosphomimicking aspartic acid (QSK1-DD) remained with strong interaction with PIP2;4 in both cases. Thus, the interaction of QSK1 with PIP2;4 was not dependent on the phosphorylation of the two C-terminal serines of QSK1 (Fig. 4*A*). However, the interaction of SIRK1 with QSK1 was enhanced by C-terminal QSK1 phosphorylation (Fig. 1*B*), and phosphorylated QSK1 enhanced SIRK1 kinase activity (Fig. 3*C*).

**Fig. 5.**
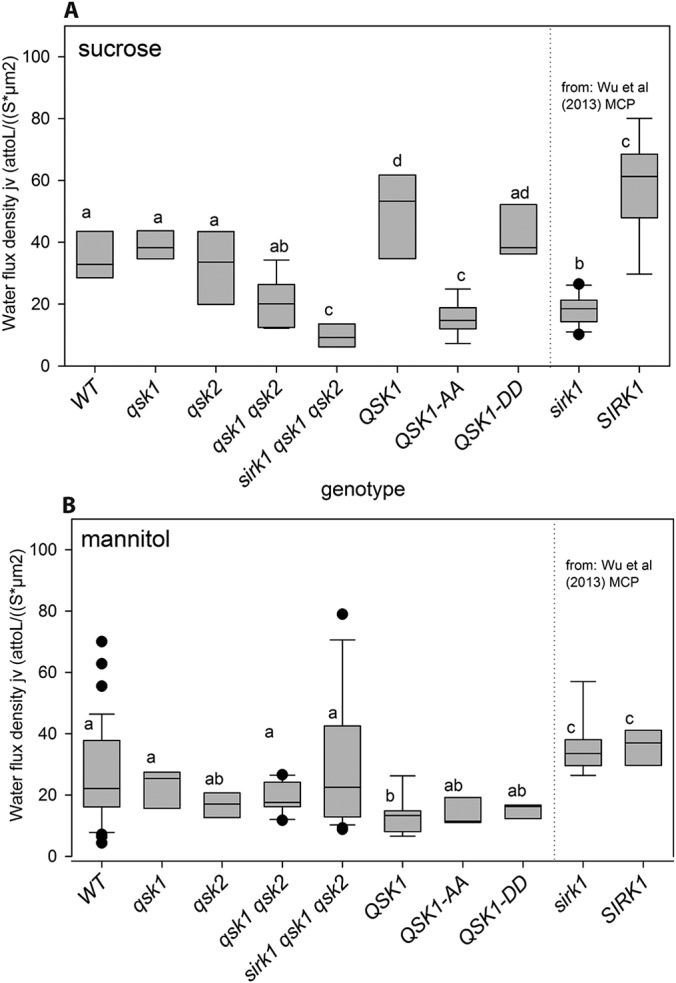
**SIRK1/QSK1 complex modulates aquaporin gating as indicated by root protoplast swelling assays.** Boxplots of water flux densities (*j_v_*) as calculated from 6 to 10 individual protoplasts and genotype. *A*, Sucrose was included into the hypoosmotic medium. *B*, Only mannitol was used as osmoticum with the same osmolarity as in (*A*). Center lines of boxes represent medians, with showing each outer. Small letters indicate significant differences (*p* < 0.05; rank sum test) within one figure panel. Data for water flux density of *sirk1* mutant were obtained from seedlings in previous work (36).

To further dissect the role of QSK1 in the regulation of aquaporins, we performed swelling assays on root protoplasts of different *qsk1* and *qsk2* mutants and we used the *sirk1* mutant from previous work as reference (Fig. 5*A*). When sucrose was included in the hyperosmotic medium, small alterations of water influx densities were observed in the *qsk1* mutant compared with wild type (WT), but the *qsk1 qsk2* double mutant showed a slightly reduced water influx density. This suggests that QSK1 and QSK2 have some degree of functional redundancy. Knock-out of *sirk1* was shown to result in significantly lower water influx into seedling protoplasts (36), and in combination with *qsk1 qsk2* knock-out mutants (*sirk1 qsk1 qsk2*), water flux into root protoplasts was highly impaired. Overexpression of QSK1 in *qsk1* background restored water influx to flux densities higher than wild type. Expression of a QSK1 phosphodead mutant (QSK1-AA) resulted in significantly lower water influxes, whereas expression of a QSK1 phosphomimic mutant (QSK1-DD) significantly increased the water influx density. Thus, although phosphorylation of QSK1 did not influence the interaction properties with aquaporin PIP2;4/PIP2F (Fig. 4*A*, 4*C*), the phosphorylation status of QSK1 seemed to be important for the regulation of aquaporins through inducing the formation and activation of the SIRK1/QSK1 complex. No large differences in water uptake were observed with mannitol only in the hypoosmotic medium (Fig. 5*B*), except for the triple mutant *sirk1 qsk1 qsk2*, which showed highest variability of water influx densities under mannitol (Fig. 5*B*). We conclude that the observed responses were induced by sucrose and not by general osmolarity changes because sucrose-containing and mannitol-containing solutions had the same osmolality.

## DISCUSSION

Phosphorylation of SIRK1 at Ser-744 was identified in several experiments under sucrose resupply (36, 40), and a slightly higher *in vit*ro kinase activity was observed for a phosphomimic version SIRK1-S744D (36). Thus, in this work we further studied the role Ser-744 in context of formation of an active complex with interaction partner QSK1.

### 

#### 

##### Role of SIRK1 Ser-744 Phosphorylation

Autophosphorylation of receptor kinases on specific serine and/or threonine residues in the cytoplasmic domain plays a key role in their activation mechanism and signaling transduction. The activation of brassinosteroid receptor BRI1 and its coreceptor BAK1 in regulation of plant growth (20, 21, 62, 63) as well as for FLS2 and BAK1 in plant defense responses (64) are well studied examples. A meta-analysis of known autophosphorylation sites revealed an overrepresentation in the juxtamembrane domain (JM) and the C-terminal (CT) region (65). JM and CT autophosphorylation sites are known to generate docking sites for downstream kinase substrates in receptor kinase signaling pathway or enhance the possibility for (hetero)dimerization (66). For example, BRI1 autophosphorylation sites in the JM domain did not result in loss of general kinase activity. However, they strongly affected downstream substrates (20, 21).

**Fig. 6.**
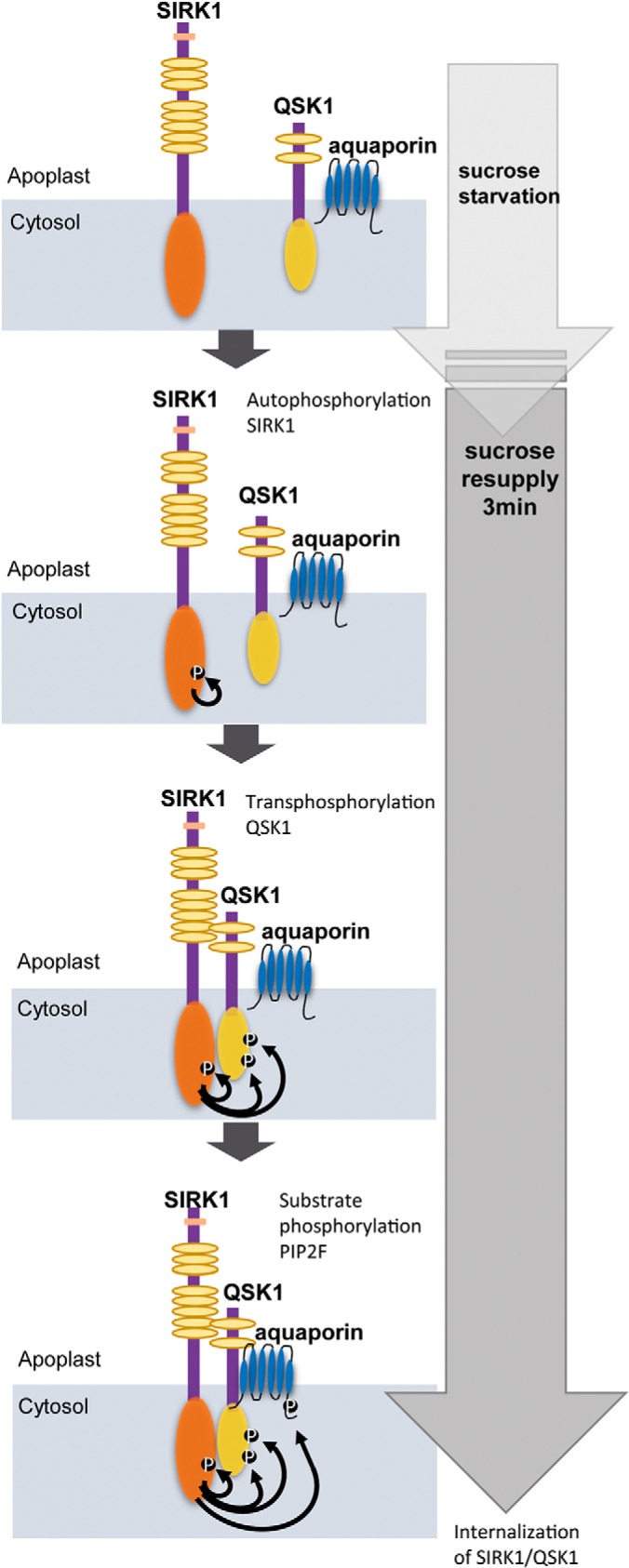
**Functional model of the SIRK1/QSK1 complex at the plasma membrane.** SIRK1 is autophosphorylated after sucrose resupply, leading to an active SIRK1 that interacts with and transpho sphorylates QSK1. The complex of SIRK1and QSK1 then regulates the activity of aquaporins via phosphorylation. Thereby, QSK1 may be involved in recruitment of substrate proteins and enhances SIRK1 kinase activity to downstream substrates such as PIP2;4.

In this study we could identify Ser-744 as an autophosphorylation site in the juxtamembrane region of SIRK1 (Fig. 2). Our results here clearly show that SIRK1 phosphorylation at Ser-744 enhanced the interaction with QSK1 as well as with substrate PIP2;4 (Fig. 1, 4). However, in previous experiments, no significant difference of water-influx under sucrose treatment was observed in SIRK1-D and SIRK1-A, but expression of SIRK1-D increased water influx compared with wild type (36). Thus, the autophosphorylation of SIRK1 can be considered as first step (Fig. 6) in formation of an active signaling complex which in a second step leads to trans-phosphorylation of interacting kinase QSK1 (Fig. 6). This complex of SIRK1 and QSK1 (possibly also with QSK2) then interacts with and phosphorylates downstream substrates such as PIP2;4 (Fig. 6). Thus, aquaporin phosphorylation most likely was strongly dependent on the complex formation of SIRK1 and QSK1, as well as on QSK1 C-terminal phosphorylation.

Previously, a role for Ser-744 was already established in internalization of SIRK1 (36). This role is further supported here by the high number of vesicle trafficking proteins (SYPs, clathrins) identified as interactors and substrates to the SIRK1/QSK1 complex. Also observed high *in vivo* aquaporin phosphorylation (Fig. 3) in the *qsk1* mutant may point to an involvement of QSK1 in SIRK1 internalization and thus removal from the plasma membrane. This process could be interrupted by missing *qsk1* thus retaining phosphorylated PIP2;4 at the plasma membrane. However, these processes require further exploration in future.

##### QSKs Show Functional Redundancy and May Function as a Coreceptor for SIRK1

Many receptor kinases are involved in sensing and transducing extracellular signals, such as hormones, secreted peptides, and pathogens, ultimately leading to respective physiological responses (67). Recently, direct regulations of transmembrane transporters by receptor kinases became evident, such as regulation of H+-ATPases by receptor kinase FERONIA (68), phytosulfokine receptor PSKR1 (34) or brassinosteroid receptor BRI1 (33), Ca^2+^-ATPases by FLS2 (35) or aquaporins by SIRK1 (36). Most of these receptor kinases do not work alone but are part of larger complexes (69). The well characterized coreceptor BAK1 (SERK3; AT4G33430) plays an important role in assembly of active signaling complexes (70). Moreover, there is increasing evidence that BAK1 is part of several receptor kinase complexes and plays an enhancing and additive role in signaling pathways ranging from brassinosteroid or phytosulfokine hormone signaling to defense or light responses, or developmental processes (29, 31, 71, 72). In addition, recent structural analyses revealed that the coreceptor BAK1 (SERK3) is crucial for cooperative binding of ligands to the respective primary receptor kinases of different receptor-coreceptor heteromers (73, 74). Thus, coreceptors mediate cooperative enhancement of the receptor signal and stabilize the receptor complex during ligand binding. Here, for SIR1 we propose that perception of an unknown ligand by receptor kinase SIRK1 could induce SIRK1 autophosphorylation at Ser-744 (Fig. 2), induce interaction with QSK1 (Fig. 1), and result in transphosphorylation of QSK1 at Ser-621 and Ser-626 (Fig. 2). Finally, sucrose-induced complex formation of SIRK1 and QSK1 enhanced kinase activity of SIRK1, but not of QSK1. This is in high analogy of activation processes described for the BR-induced association between receptor kinase BRI1 and its coreceptor BAK1 (21).

To genetically determine whether QSK1/QSK2 function in regulation of sucrose-induced aquaporin activity as SIRK1, we test water influx (swelling assay) with the single mutants *qsk1* and *qsk2* as well as double mutant *qsk1 qsk2* and triple mutant *sirk1 qsk1 qsk2* (Fig. 5). In fact, both *qsk* single mutants displayed no observable phenotype, whereas the double mutant *qsk1 qsk2*, showed reduced water influx rates, and were further significantly reduced in the *sirk1 qsk1 qsk2* triple mutant. This indicated functional redundancy of QSK1 and QSK2. Overexpression of either QSK1 or QSK1-DD in the *qsk1 qsk2* mutant background restored water influx, but overexpression of QSK1-AA resulted in similarly low water influx as in the *qsk1qsk2* mutant. These results suggest synergistic interactions of SIRK1 and QSK1 (also QSK2) affecting interactions with substrate proteins (*e.g.* PIP2;4) by subsequent phosphorylation. Based on our findings of enhanced SIRK1 activity during interaction with (phosphorylated) QKS1 (Fig. 3) and enhanced complex formation of SIRK1 with (phosphorylated) QSK1 (Fig. 4) we propose that SIRK1 and QSK1/QSK2 act in the same signaling pathway and function together to activate water influx in response to sucrose stimulation.

The kinase domain of LRR-receptor kinase SIRK1 is a member of the same evolutional monophyletic clade as kinase domain of BRI1 (13). QSK1 (AT3G02880) and QSK2 (AT5G16590) are also located within that same clade, but cluster into a different subclade containing four close homologs together with AT3G17840 and AT1G48480 (supplemental Fig. S7*A*). Ligand binding requires larger extracellular structures. Thus, receptor kinases with extracellular domains larger than ∼400 amino acids were considered as ligand-binding kinases whereas those with shorter extracellular domains could be rather considered as having coreceptor functions (16). SIRK1 has a large extracellular domain of 599 amino acids, whereas QSK1 and QSK2 have short extracellular domains of 252 and 245 amino acids, respectively (75). The structure model of SIRK1 suggests that it is a typical LRR receptor kinase with several N-terminal extracellular LRR domains like other well characterized LRR receptor kinases, such as BRI1 or FLS2. In contrast, QSK1 and QSK2 have short extracellular domains, like the SERK coreceptors (supplemental Fig. S7*B*). Interestingly, CIKs, a group of LRR-receptor kinases with short extracellular domains were characterized as additional coreceptors in the CLAVATA3/EMBRYO SURROUNDING REGION (CLE) -signaling pathway (76). Thus, given over 100 receptor kinases with short extracellular domains, the identification of further coreceptors in context of different signaling pathways is highly likely.

##### Aquaporins as Highly Regulated Water Channels

Aquaporin pore gating is known to be regulated by phosphorylation. Particularly, the phosphorylation status of two serines, one in a cytosolic loop (S115 in SoPIP2;1) and one in the C terminus (S274 of SoPIP2;1 is equivalent to S283 in Arabidopsis PIP2A) is critical for the channel pore opening (61). In the dephosphorylated status the pore is closed, whereas phosphorylated aquaporins enable water transport. Further, there is evidence that phosphorylation of S286, but not S283, is involved in the targeting of PIP2;1 to the plasma membrane (8). Treatment with 100 mm NaCl reduced phosphorylation at S283 and induced relocation of PIP2A to intracellular membranes (77). A decrease in phosphorylation of PIP2;1 at S283 and S286 was observed upon treatment with abscisic acid, leading to a reduction in water flux through pore closing (dephosphorylation of S283) and removal of PIP2A from the membrane (dephosphorylation of S286) (78). In contrast, ethylene increases water transport rate by enhancing phosphorylation of PIP2;1 at S283 and S286 (79). In our experiments we confirmed already published increase of phosphorylation at these regulatory serines upon sucrose treatment (36, 40). Analysis of *sirk1* and *qsk1* mutants revealed new regulatory roles of both proteins in context of aquaporin pore gating and aquaporin membrane localization. QSK1 was recently also identified as interaction partner to aquaporins in pull-downs with GFP-tagged PIP2;1 (80).

Removal of PIPs from the plasma membrane under strong osmotic exposure (8) could also involve internalization of the proteins and their interaction partners. Sucrose-induced internalization of the (phosphorylated) SIRK1 complex was already shown (36), and this process could also include interacting QSK1 (and QSK2) and possibly aquaporins as their substrates.

##### Functional Model of SIRK1 and QSK1 Signaling

Thus, considering our biochemical and physiological results we conclude that SIRK1 acts as the main receptor perceiving the sucrose signal through a yet unknown ligand and this signal perception induces SIRK1 autophosphorylation at Ser-744 (Fig. 6). Based on the short extracellular domain structure of QSK1 and QSK2, and particularly because of our findings that QSK1 did not show kinase activity by itself but that (phosphorylated) QSK1 enhanced SIRK1 activity (Fig. 2, 4), we propose that QSK1 and QSK2 most likely function as coreceptors to SIRK1. The coreceptors QSK1 and QSK2 are first trans-phosphorylated by activated and autophosphorylated SIRK1 (Fig. 6) and phosphorylated QSK1 then stabilizes the complex with SIRK1 through enhanced interaction with SIRK1 (Fig. 1). This complex formation of SIRK1 and phosphorylated QSK1 also results in interactions with downstream substrate proteins, such as PIP2;4 (Fig. 3). The weak water influx rates of *qsk1* mutants compared with mutants in the receptor kinase *sirk1* (Fig. 5) point to an additive, signal enhancing function of QSK1 as well as to functional redundancy with QSK2. This is in striking analogy to weak phenotypes and additive signal enhancing effects shown for BAK1 in context of brassinosteroid signaling through BRI1 (21) and the family of SERKs as coreceptors for the IDA-receptors HAE/HLS2 (30).

The C-terminal phosphopeptide of QSK1 was identified in many proteomic studies under various experimental conditions (PhosPhAt (52)). QSK1 was recently also identified as a protein segregating between sterol-rich and sterol-depleted membrane phases depending on a functional cytoskeleton (38). We therefore propose that QSK1, possibly through the interaction with cytoskeletal components, could have a major role in directing receptor complexes and required substrates to their correct membrane environment action. Although this role of QSK1 requires further testing, dynamic assembly of signaling components could already be shown for the ion channel SLAH3 and the kinase CPK21 (37). The interactions of SIRK1/QSK1 with vesicle trafficking proteins point to an involvement of SNARE proteins and clathrins in delivery and/or internalization of the complexes. Phosphorylation-dependent internalization was already shown for SIRK1 (36). QSK1, as a putative coreceptor, most probably is involved in more than one signaling pathway and, like BAK1, might associate with more than one receptor kinase. A role of QSK1 in regulation of potassium transport was recently published (81), that strongly supports the proposal of its involvement in different signaling pathways.

## CONCLUSION

The above results revealed that SIRK1 Ser-744 is a sucrose-induced autophosphorylation site and important for the interaction of SIRK1 with QSK1 and the substrate protein PIP2;4. Cooperatively enhanced kinase activity of SIRK1 was observed when recombinant QSK1 was jointly submitted to activity assays. Phosphorylation of QSK1 enhanced the activity of the SIRK1/QSK1 complex but was not required for interaction with the substrate proteins. Water influx assays suggested that QSK1 and QSK2 contributed to the overall water influx rates besides known receptor kinase SIRK1.

This work proposes a key role for a yet uncharacterized receptor kinase, AT3G02880 in signal-dependent regulation of cellular water influx. We named the protein QSK1 qiān shŏu (chinese: 千 手 “thousand hands”) kinase, describing the dual interaction with receptor kinase SIRK1 as well as with substrate proteins (aquaporins). We propose that QSK1 not only cooperatively enhances activity of receptor kinase SIRK1, but also may have recruiting function of kinase and substrate proteins within the membrane. Because QSK1 is a very abundant protein identified and phosphorylated under a variety of biotic and abiotic stresses, we propose the generic model of QSK1 as a coreceptor may recruit different substrates to the respective signaling complexes also with other (receptor)kinases.

## DATA AVAILABILITY

The mass spectrometry proteomics data have been deposited to the ProteomeXchange Consortium via the PRIDE (82) partner repository (http://www.ebi.ac.uk/pride) with the dataset identifier PXD011284 (phosphorylation profiling) and PXD011265 (pulldowns).

## Supplementary Material

Supplementary Material Information

Supplementary Table S1

Supplementary Table S2

Supplementary Table S3

Supplementary Figure S1

Supplementary Figure S2

Supplementary Figure S3

Supplementary Figure S4

Supplementary Figure S5

Supplementary Figure S6a

Supplementary Figure S6b

Supplementary Figure S6c

Supplementary Figure S6d

Supplementary Figure S6e

Supplementry Figure 6f

Supplementary Figure S6g

Supplementay Figure S6h

Supplementary Figure S6i

Supplementary Figure S7

## Footnotes

^1^ The abbreviations used are:

BR: brassinosteroid
SIRK 1: sucrose-induced receptor kinase
FWHM: full width half maximum
FDR: false discovery rates.
