# Supplementary material for: Sucrose-induced Receptor Kinase 1 is Modulated by an Interacting Kinase with Short Extracellular Domain
Source: Mol Cell Proteomics. 2019 May 30;18(8):1556–71. doi: 10.1074/mcp.RA119.001336 (PMC6683012; doi:10.1074/mcp.RA119.001336)
Supplement: Supplementary Material Information [file 143141_1_supp_311863_ppmw8f.pdf]

SUCROSE-INDUCED RECEPTOR KINASE 1 is modulated by an interacting kinase with short extracellular domain

Xu Na Wu [1], Liangcui Chu [1], Lin Xi [1], Heidi Pertl-Obermeyer [2], Zhi Li [1], Kamil Sklodowski [3] Clara Sanchez-Rodriguez [3], Gerhard Obermeyer [2], Waltraud X. Schulze [1]

[1] Department of Plant Systems Biology, University of Hohenheim, 70593 Stuttgart, Germany

[2] Molecular Plant Biophysics and Biochemistry, Department of Biosciences, University of Salzburg, 5020 Salzburg, Austria

[3] Department of Biology, ETH Zürich, Universitätstrasse 2, 8092 Zürich, Switzerland

Supplementary Material

Supplementary Table S1: Interaction candidates for SIRK1 and QSK1 in sucrose starvation and resupplied conditions. Results are from ANOVA t-tests on each protein identified in bait pull-downs for the different bait constructs (SIRK1 and QSK1) under sucrose resupply condition (3 min) relative to sucrose starvation condition (0 min).

Supplementary Table S2: Quantitative phospho-profiling in wild type (Col-0), sirk1 mutant, qsk1 mutant, qsk1 qsk2 double mutant and sirk1 qsk1 double mutant mutants in sucrose-starvation and resupplied experiments. Averaged sucrose induced phosphorylation from three biological replicates are expressed as Log2 (ion intensity ratios) at sucrose-resupplied condition (3 minutes) relative to sucrose-starved condition (0 minutes).

Supplementary Table S3: List of identified proteins and normalized LFQ-values of GFP only ("beadome") as well as bait-GFP-fusion pulldowns as derived from the list of identified proteins groups (protein groups.txt).

Figure S1: qPCR analysis of knock-out mutants used. (**A**) *SIRK1* gene expression and protein abundance in the *sirk1* mutant. (**B**) *QSK1* gene expression and protein expression in the *qsk1* mutant. Gene expression was analyzed in the respective mutant background using qPCR, protein abundance was estimated based on ion intensity sums of identified peptides. Averages of three biological replicates of each genotype are shown. Letters indicate significant ($p<0.05$, pairwise t-test) differences within a panel. ND: not detected.

Figure S2: Calibration of the rBIFC system. Known interaction of CBL9 with CIPK23 was used as positive control, while the published absence of interaction of CBL9 with CIPK14 was used as negative control (1). Center lines of boxes represent medians, black dots indicate outliers.

Figure S3: Confirmation of the interaction of SIRK1 and QSK1, as well as verification of the interaction of SIRK1 with calcium-related proteins ACA8 in transiently transformed *sirk1 sak1* Arabidopsis seedlings. (**A**) SIRK1-QSK1 interaction. (**B**) SIRK1-ACA8 interaction. Representative images of the *in vivo* interactions BiFC analysis (z-stacks were merged in order to show signal from a whole cell). Water infiltrated leaves were used as negative control (autofluorescence). Data represent at least 30

randomly selected cells from 2 plants (Welch Two Sample t-test compared to wild type SIRK1 interaction (*** p-value < 0.001).

Figure S4: Representative spectra of phosphorylated substrate peptides identified in the *in vitro* kinase reactions with combinations of recombinant SIRK1 and QSK1.

Figure S5: Peptide phosphorylation assays in presence of different mixing ratios of SIRK1-GFP purified from root tissue and recombinant QSK1 kinase domain. (**A**) SIRK1 autophosphorylation. (**B**) QSK1 transphosphorylation. (**C**) Phosphorylation of substrate protein PIP2F.

Figure S6: Spectra of all identified phosphopeptides.

Figure S7: Proposed function of QSK1 as a coreceptor. (A) Phylogeny of the LRR receptor kinase clade (2) including the SERK subfamily, kinase FLS2 as well as BRI1, SIRK1 and the QSK1/QSK2. SIRK1 and QSKs are in the same subclade as is BRI1 and BIR1. (B) Domain structure models obtained from SMART (3) for SIRK1 and its suggested coreceptors QSK1 and QSK2 in comparison with established receptors FLS2, BRI1 and their coreceptors of the SERK family.

1. Cheong, Y. H., Pandey, G. K., Grant, J. J., Batistic, O., Li, L., Kim, B. G., Lee, S. C., Kudla, J., and Luan, S. (2007) Two calcineurin B-like calcium sensors, interacting with protein kinase CIPK23, regulate leaf transpiration and root potassium uptake in Arabidopsis. *The Plant journal : for cell and molecular biology* 52, 223-239
2. Zulawski, M., Schulze, G., Braginets, R., Hartmann, S., and Schulze, W. X. (2014) The Arabidopsis Kinome: Phylogeny and evolutionary insights into functional diversification. *BMC Genomics* 15, 548
3. Schultz, J., Milpertz, F., Bork, P., and Ponting, C. P. (1998) SMART, a simple modular architecture research tool: Identification of signaling domains. *Proc. Natl. Acad. Sci. USA* 95, 5857-5864
