# Supplementary figures and images for: Sucrose-induced Receptor Kinase 1 is Modulated by an Interacting Kinase with Short Extracellular Domain
Source: Mol Cell Proteomics. 2019 May 30;18(8):1556–71. doi: 10.1074/mcp.RA119.001336 (PMC6683012; doi:10.1074/mcp.RA119.001336)

**A*****sirk1* mutant**

data from Wu XN et al (2013) Molecular and Cellular Proteomics

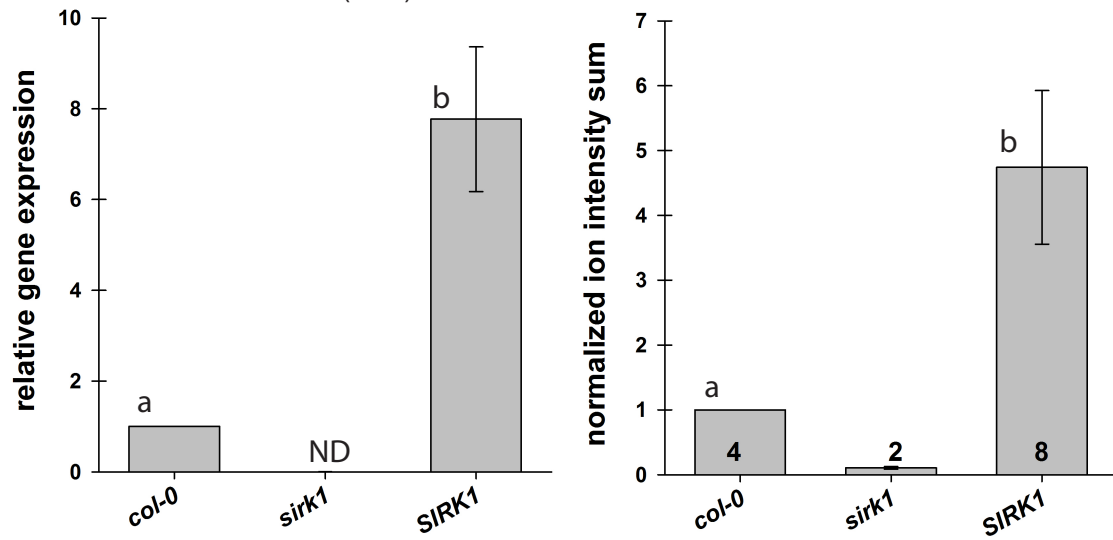**B*****qsk1* mutant**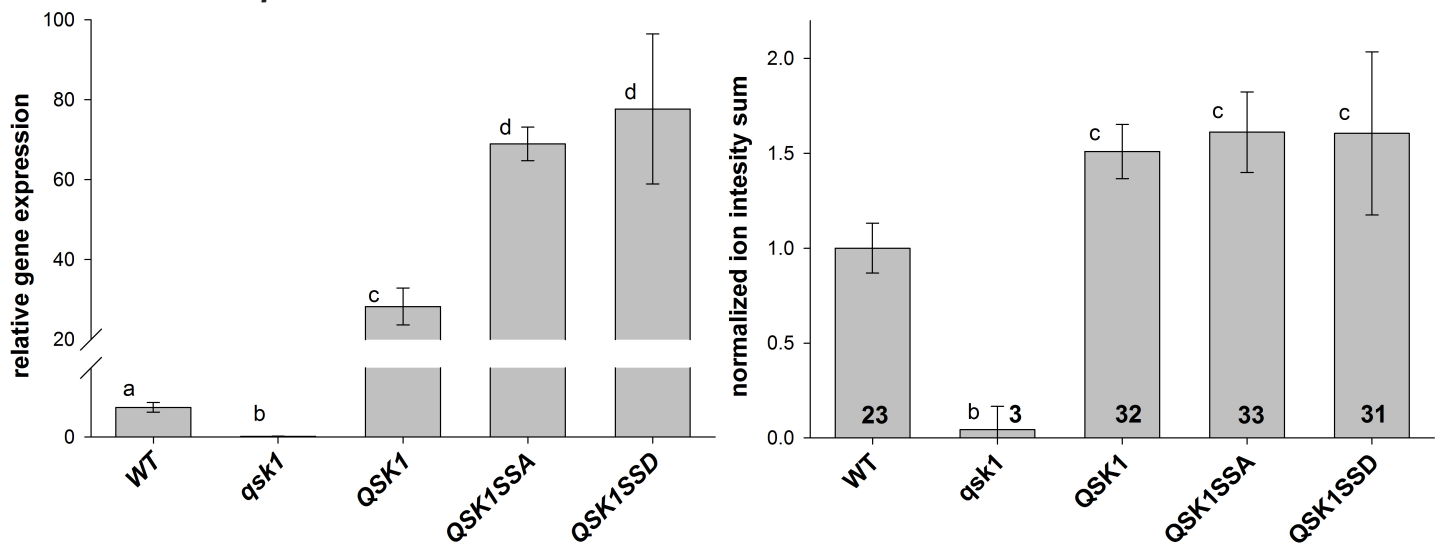

Figure S1

Supplement: Supplementary Figure S1 [file 143141_1_supp_311877_ps5kkw.pdf]

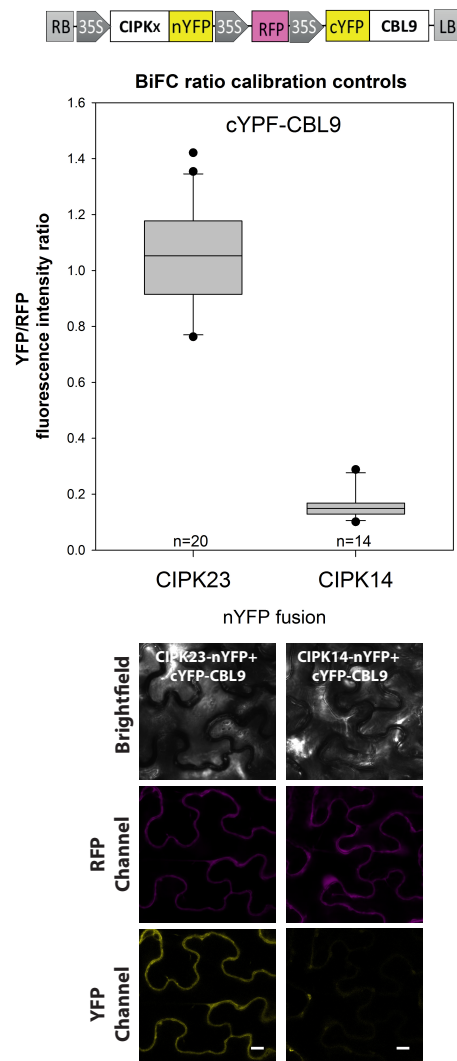

Figure S2

Supplement: Supplementary Figure S2 [file 143141_1_supp_311879_ps5hkw.pdf]

A interaction of SIRT1 and QSK1

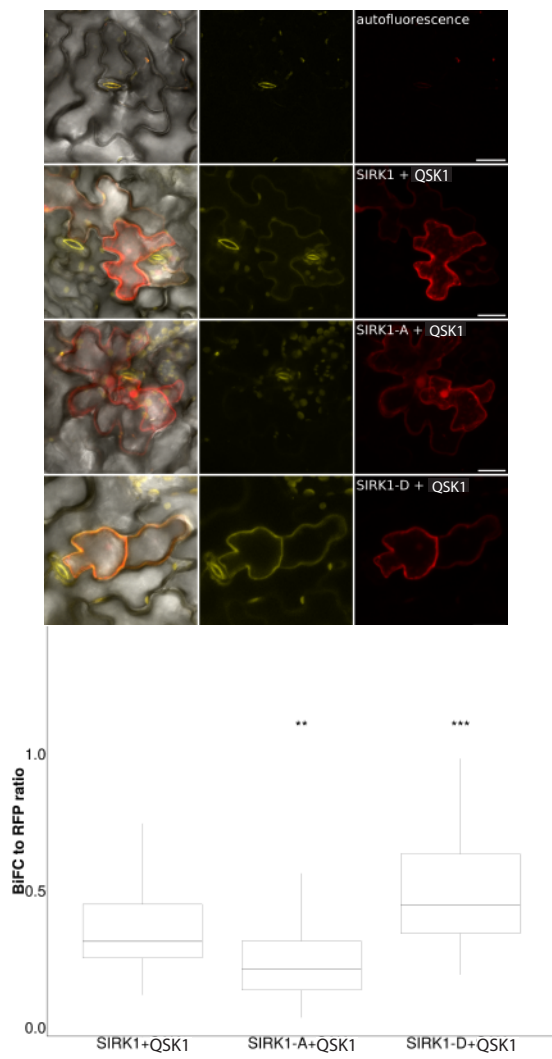

B interaction of SIRT1 and ACA8

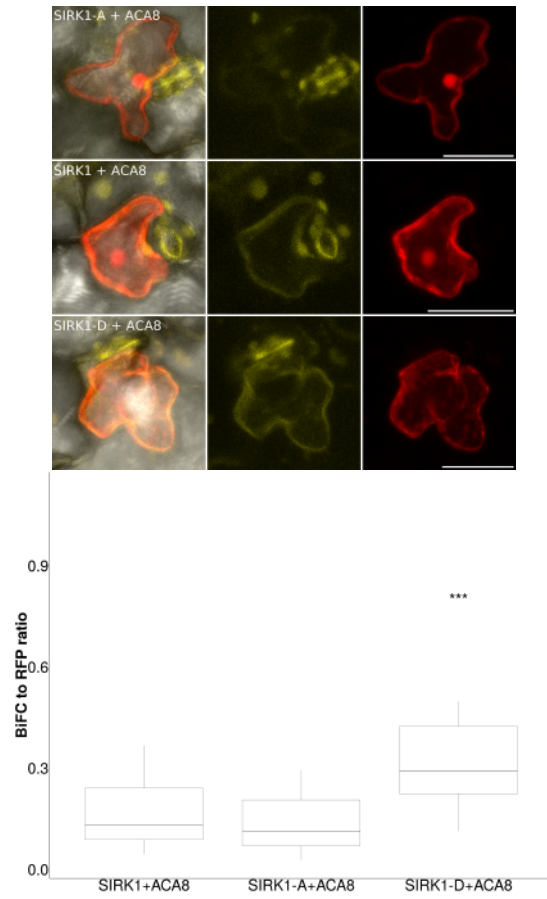

Figure S3

Supplement: Supplementary Figure S3 [file 143141_1_supp_337913_ps5fkz.pdf]

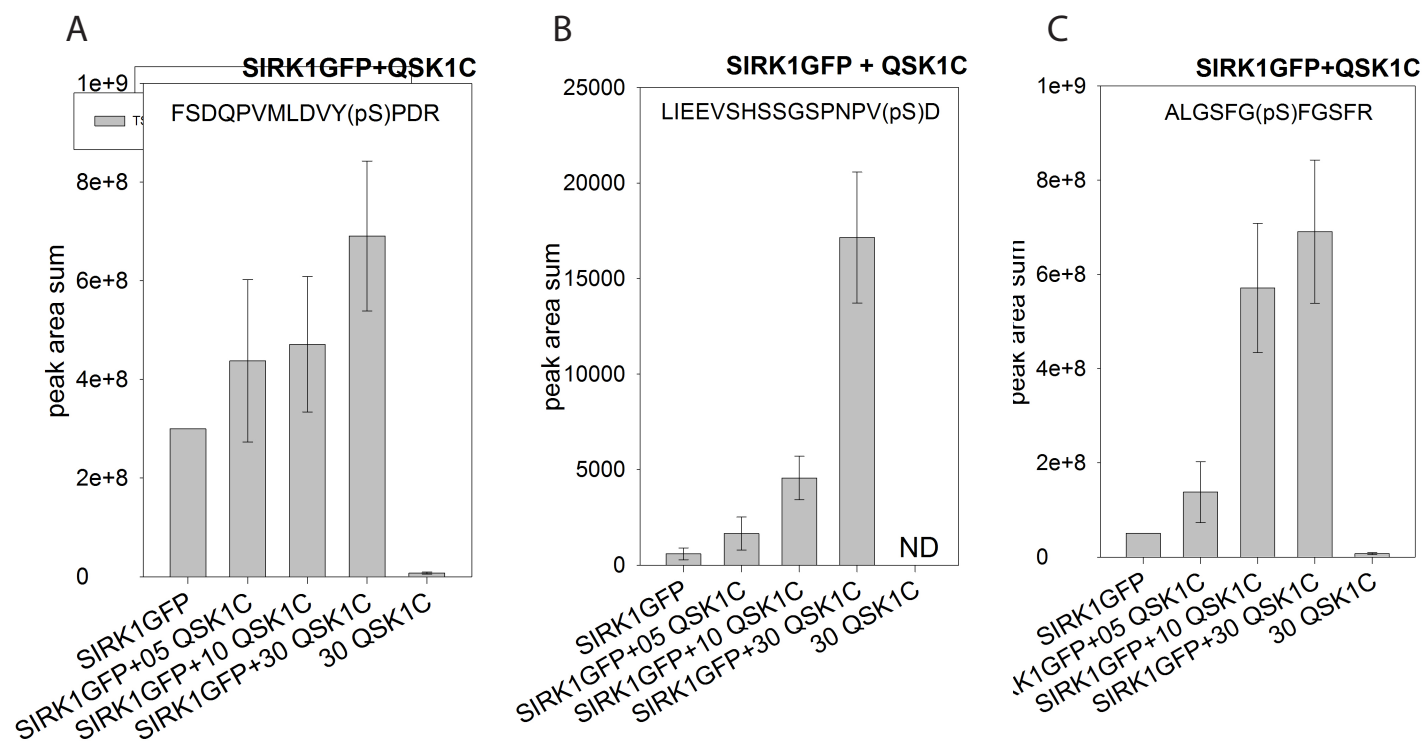

Figure S5

Supplement: Supplementary Figure S5 [file 143141_1_supp_311881_ps5hkx.pdf]

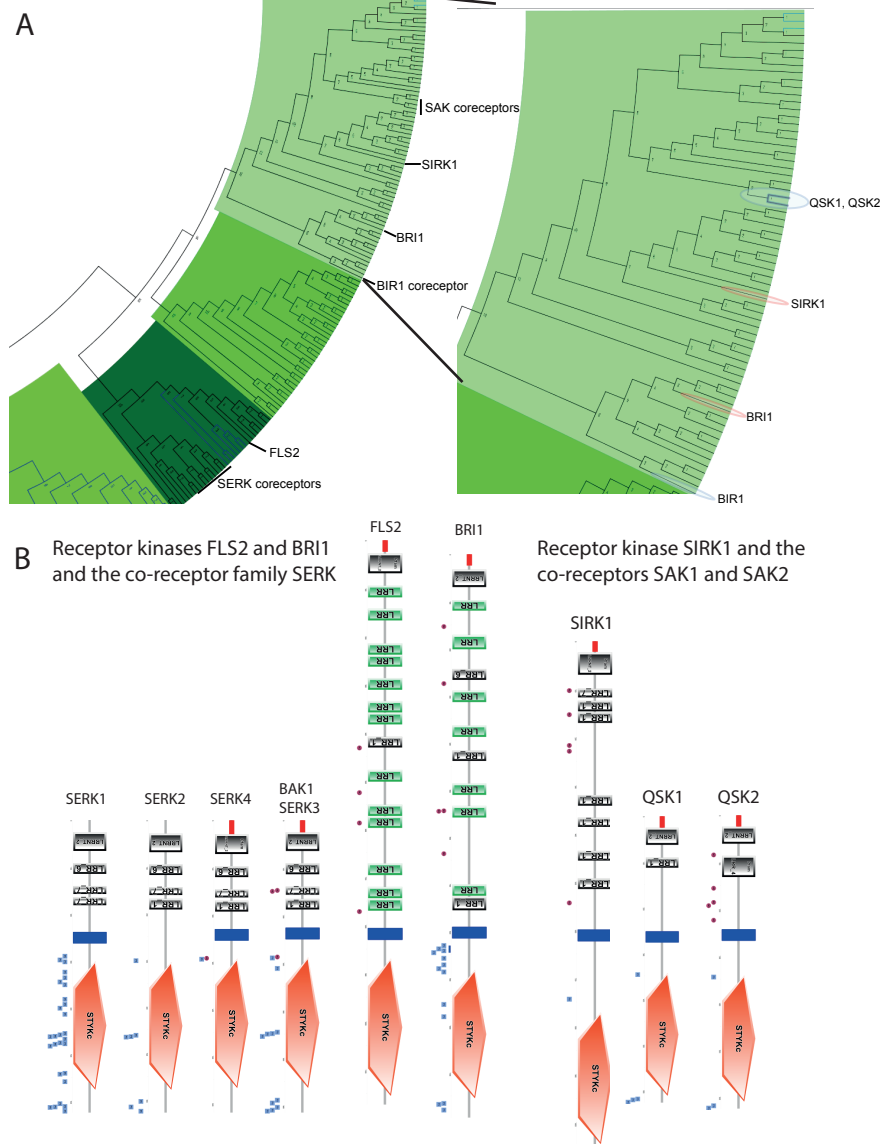

Figure S7

Supplement: Supplementary Figure S7 [file 143141_1_supp_311882_ps5bkz.pdf]
