# Supplementary material for: Sucrose-induced Receptor Kinase 1 is Modulated by an Interacting Kinase with Short Extracellular Domain
Source: Mol Cell Proteomics. 2019 May 30;18(8):1556–71. doi: 10.1074/mcp.RA119.001336 (PMC6683012; doi:10.1074/mcp.RA119.001336)

Figure S4:

Representative spectra of phosphorylated substrate peptides identified in the *in vitro* kinase reactions with combinations of recombinant SIRK1 and QSK1.

Raw file	Scan	Method	Score	m/z
SIRK1pp_30ugSIRK1C_3	13263	FTMS; HCD	40.97	924.9

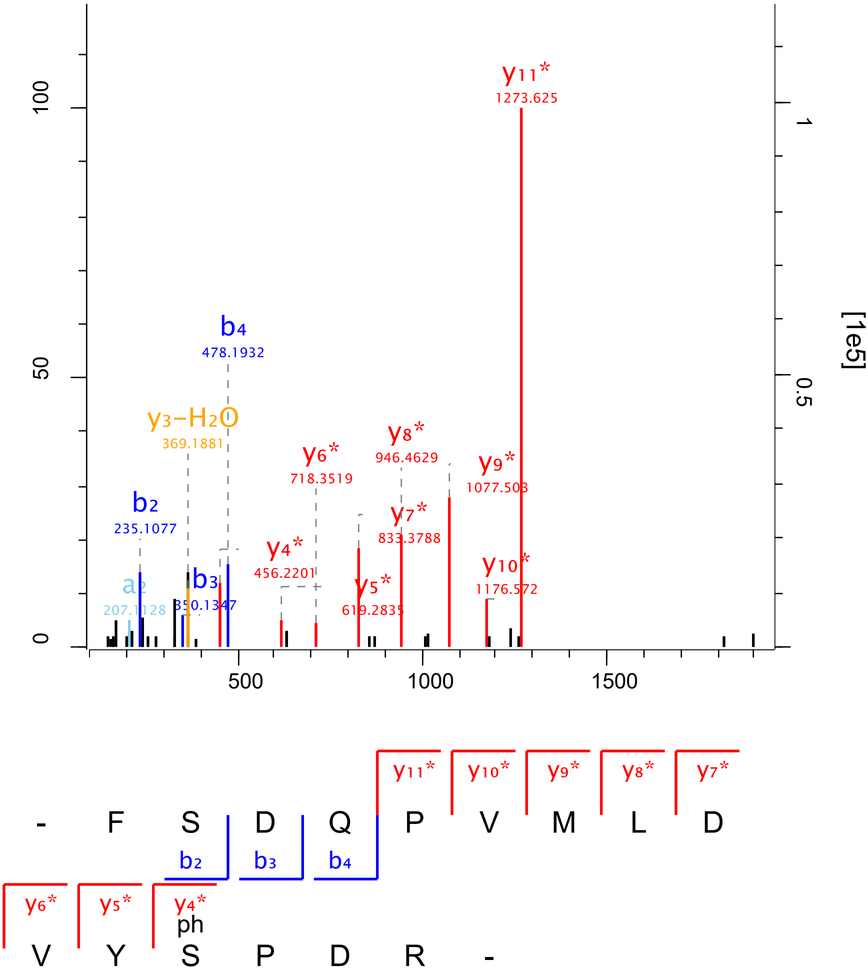

Raw file	Scan	Method	Score	m/z
SAK1pp-30SAK1C-3	7464	FTMS; HCD	128.92	917.4

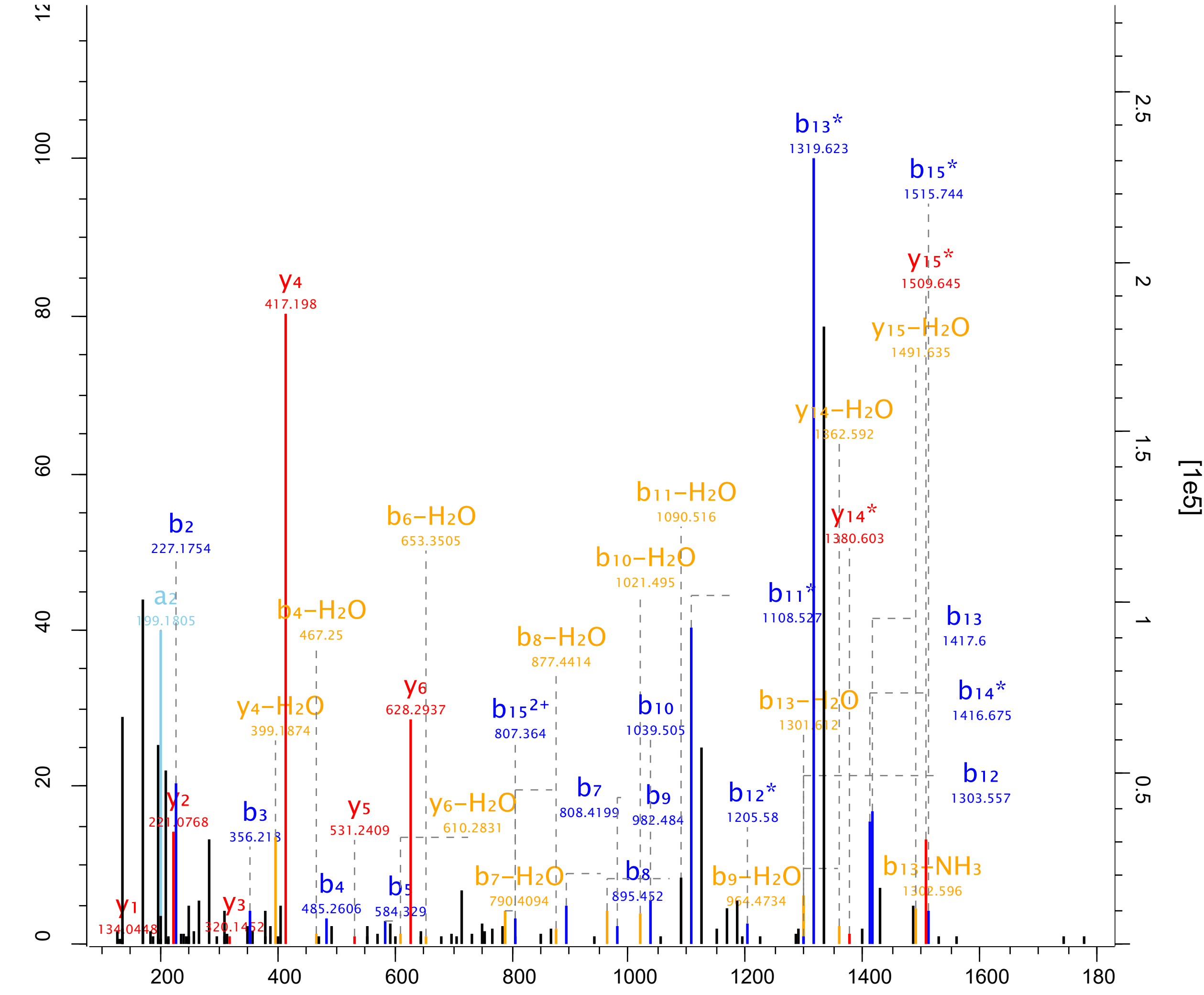

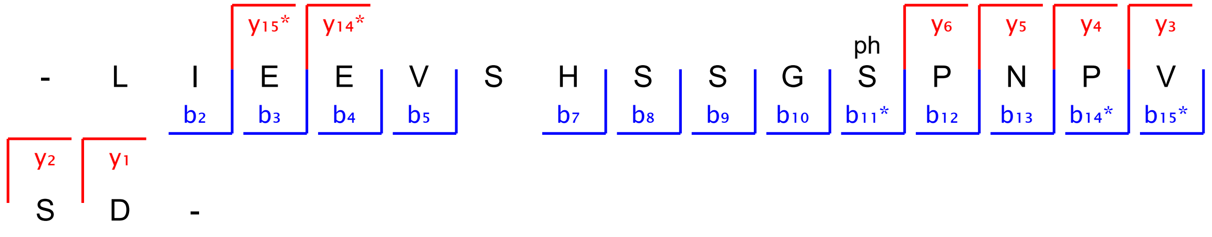

Raw file	Scan	Method	Score	m/z
SAK1C-QQ4-5-3	11777	FTMS; HCD	94.49	656.79

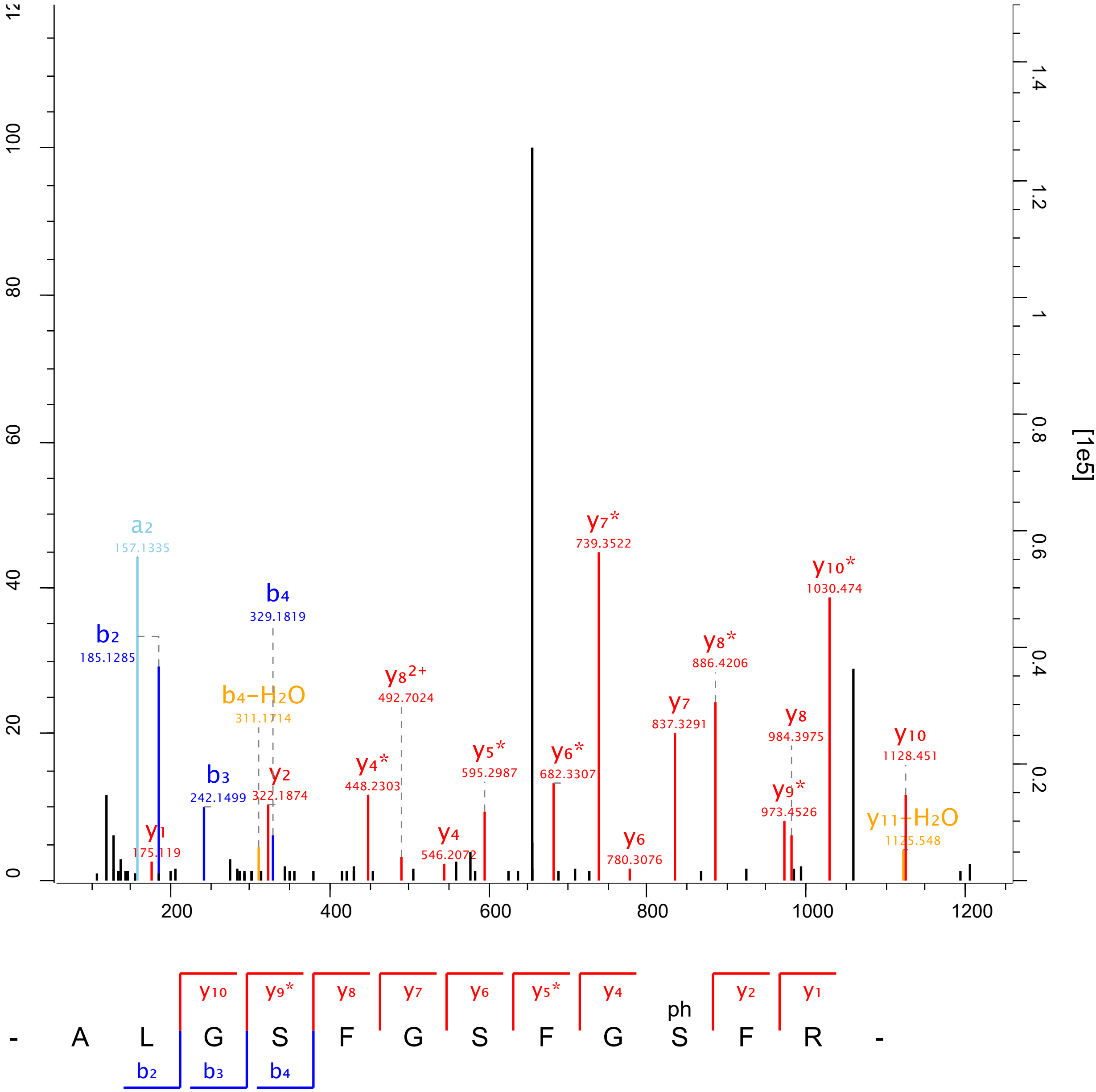

Supplement: Supplementary Figure S4 [file 143141_1_supp_311880_ps5xkw.pdf]
