# Supplementary material for: Sucrose-induced Receptor Kinase 1 is Modulated by an Interacting Kinase with Short Extracellular Domain
Source: Mol Cell Proteomics. 2019 May 30;18(8):1556–71. doi: 10.1074/mcp.RA119.001336 (PMC6683012; doi:10.1074/mcp.RA119.001336)
Supplement: Supplementary Figure S6a [file 143141_1_supp_311928_ps5dkx.pdf]

Figure S6:

Spectra of all identified phosphopeptides.

Raw file	Scan	Method	Score	m/z	Gene names
0523_1	1436	FTMS; HCD	64.1	437.86	U2AF35B

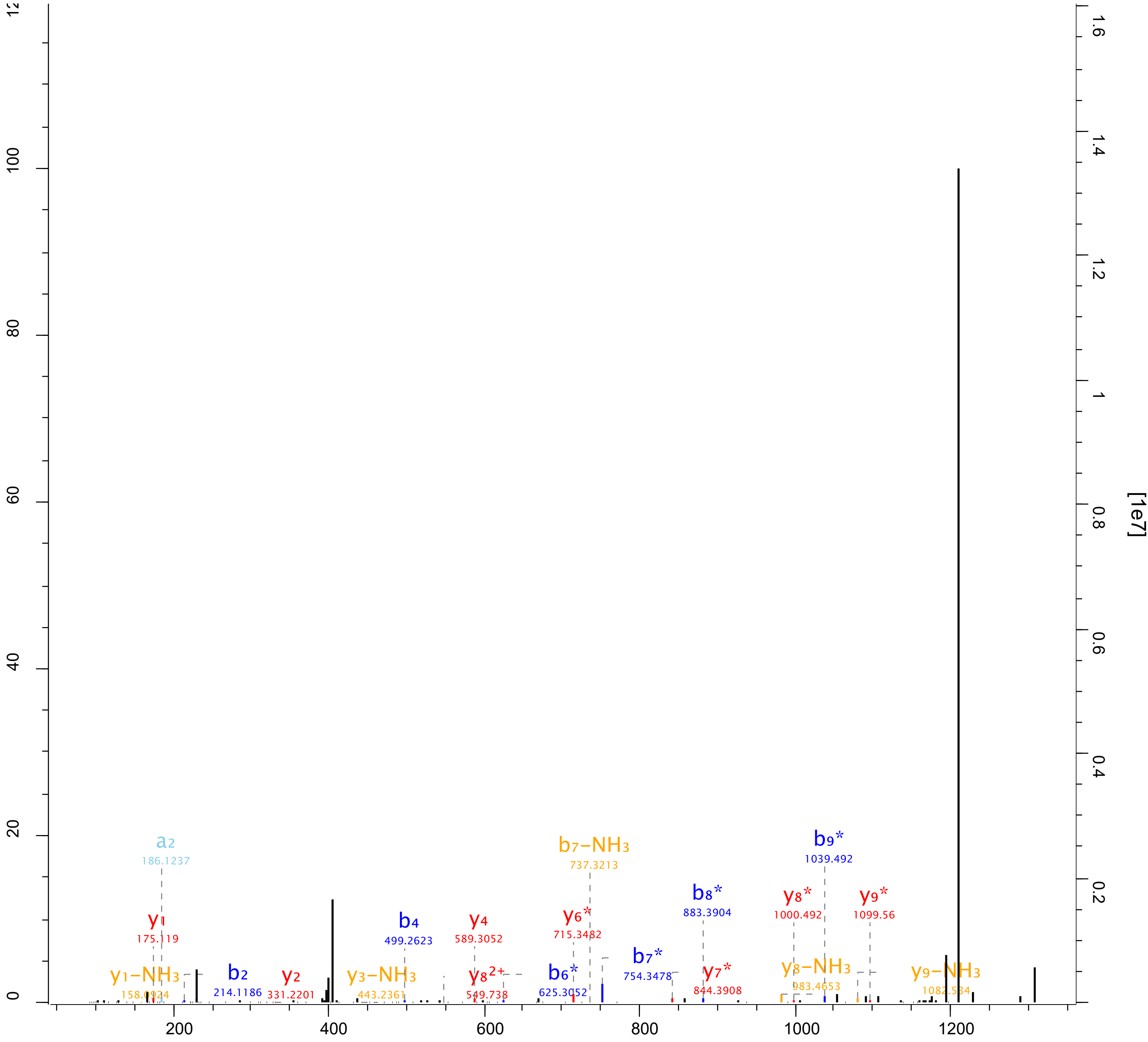

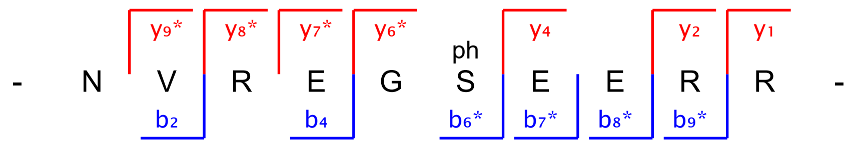

Sample output to test PDF Combine only

Raw file	Scan	Method	Score	m/z	Gene names
0523_1	1450	FTMS; HCD	55.81	345.5	SR45

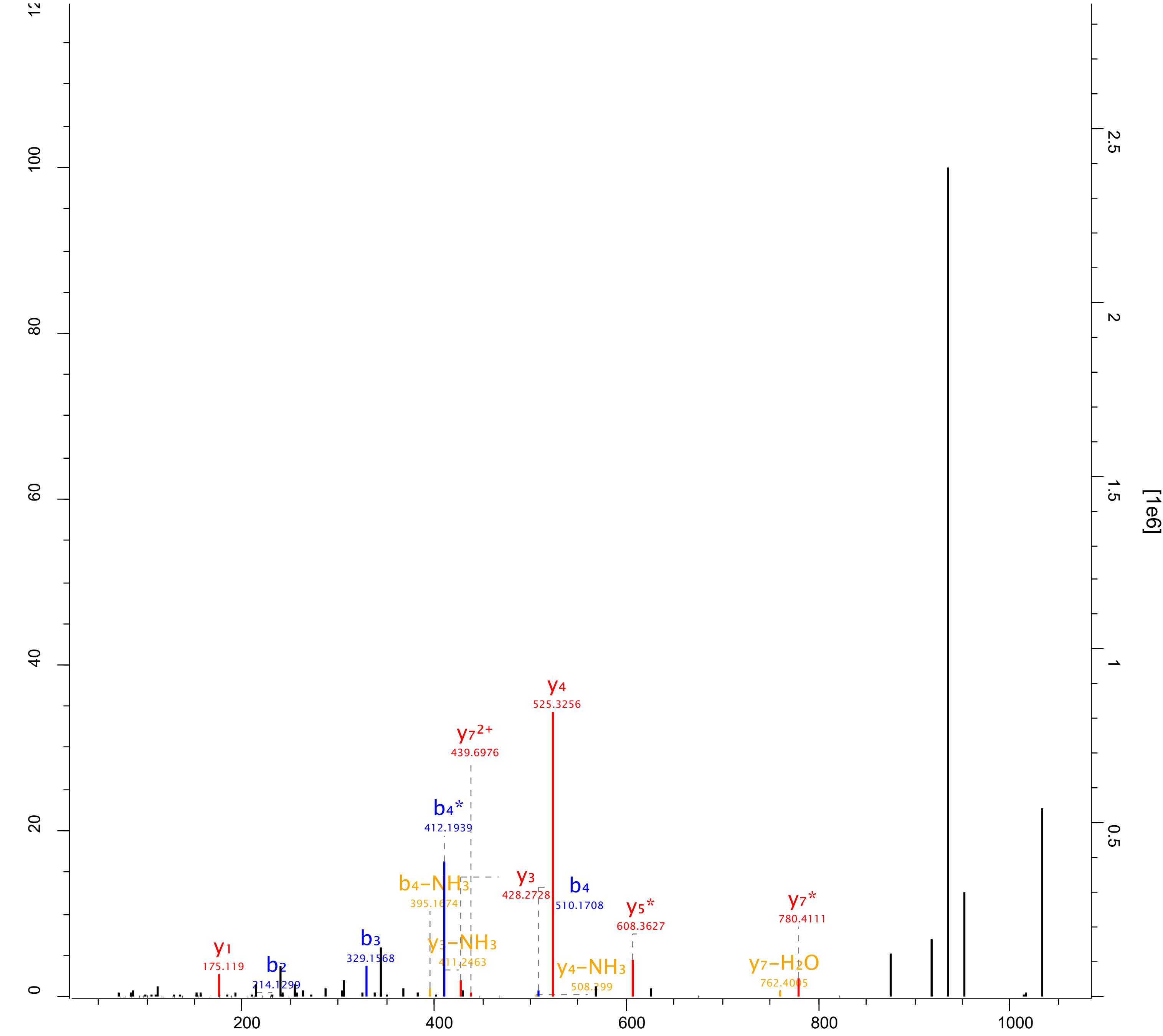

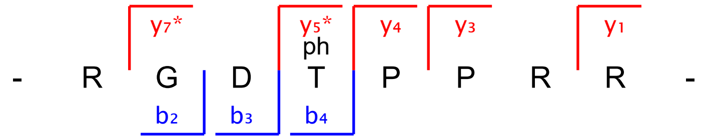

Sample output to test PDF Combine only

Raw file	Scan	Method	Score	m/z	Gene names
0523_1	1451	FTMS; HCD	138.76	337.49	PHOS34

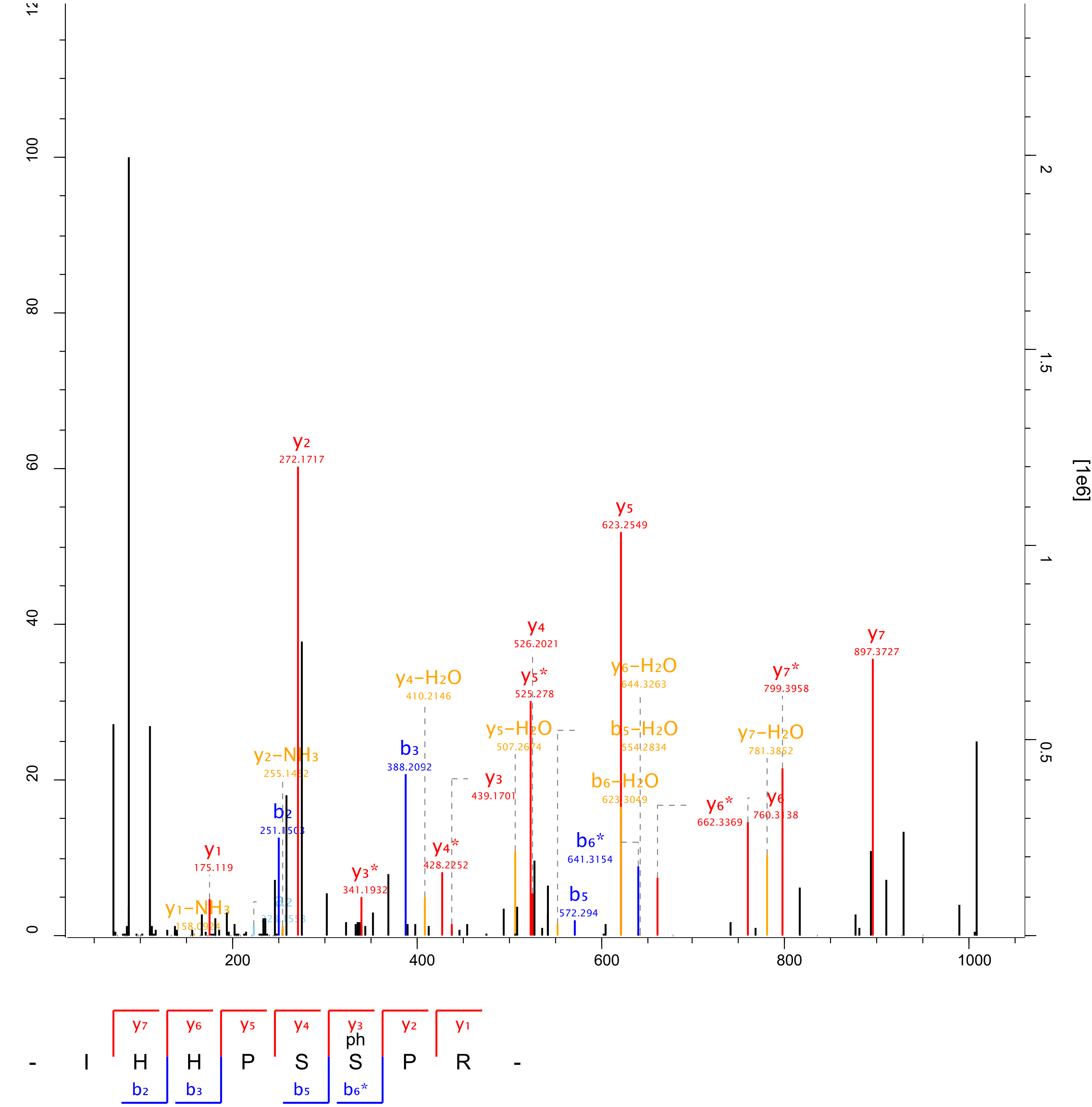

Sample output to test PDF Combine only

Raw file	Scan	Method	Score	m/z	Gene names
0523_1	1454	FTMS; HCD	46.35	377.2	At1g53633

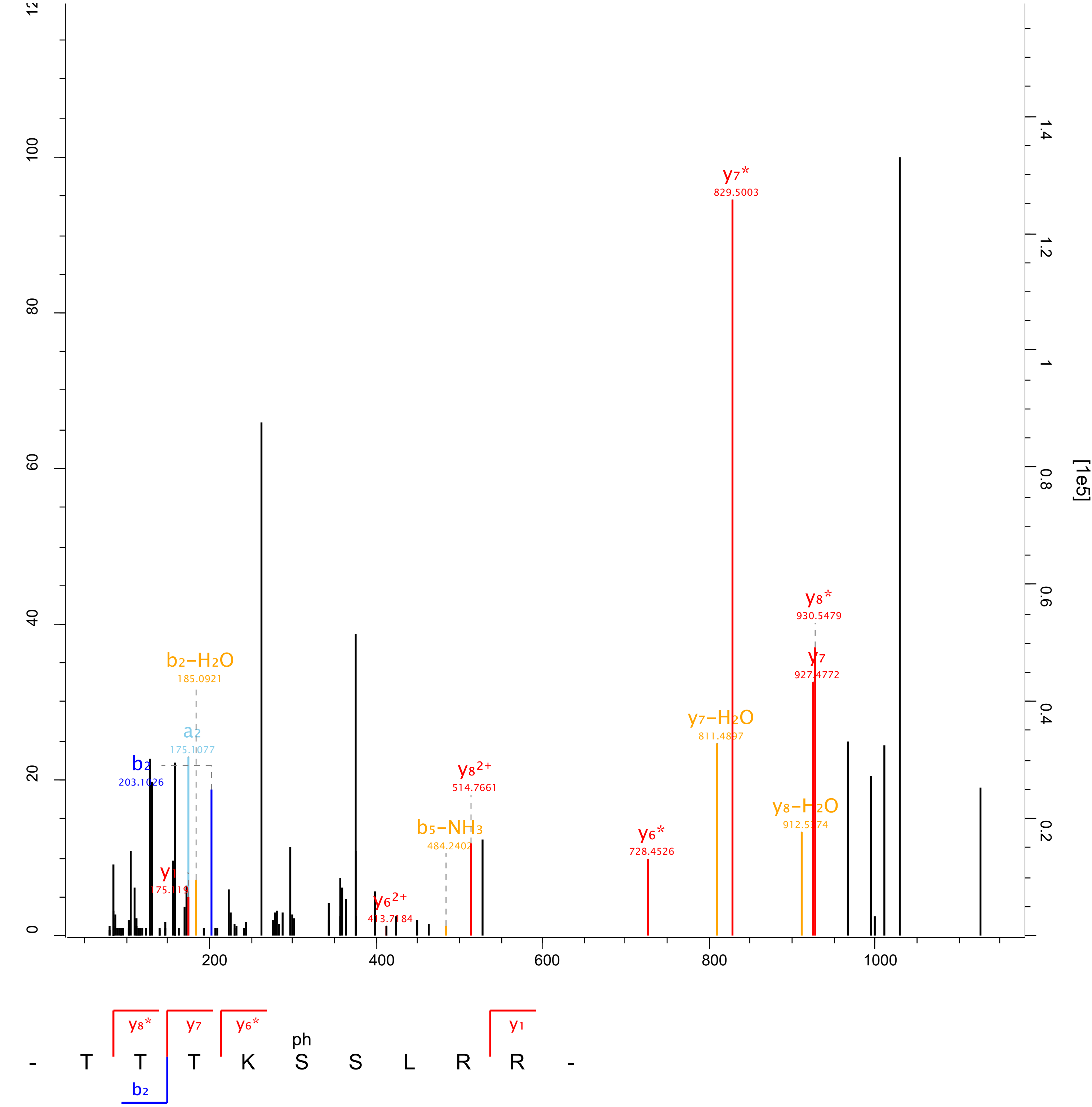

Sample output to test PDF Combine only

Raw file	Scan	Method	Score	m/z	Gene names
0523_1	1493	FTMS; HCD	83.95	417.19	CLASP

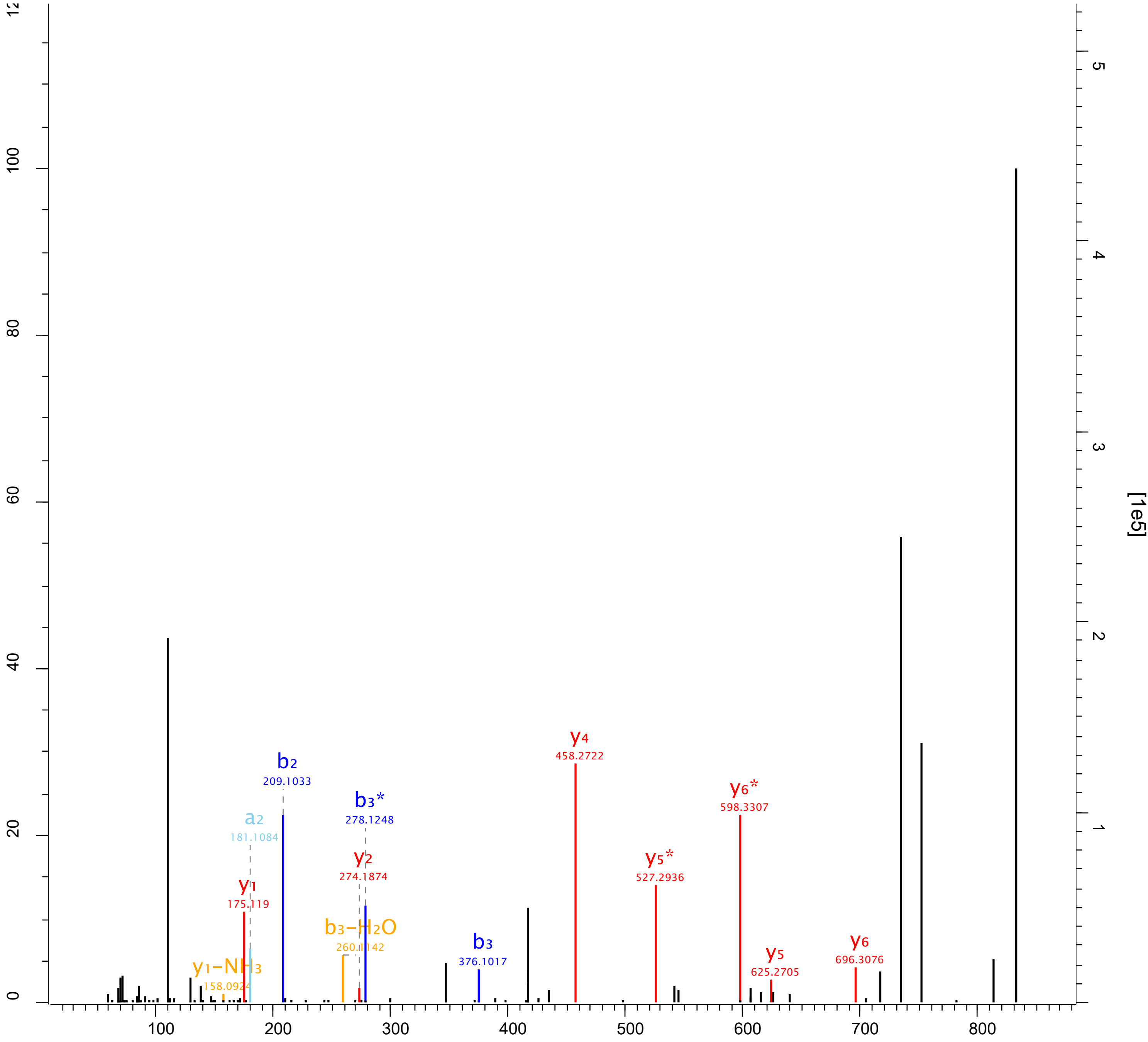

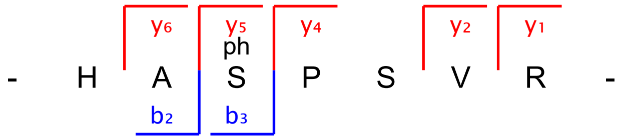

Sample output to test PDF Combine only

Raw file	Scan	Method	Score	m/z	Gene names
0523_1	1524	FTMS; HCD	122.66	543.74	ACA8

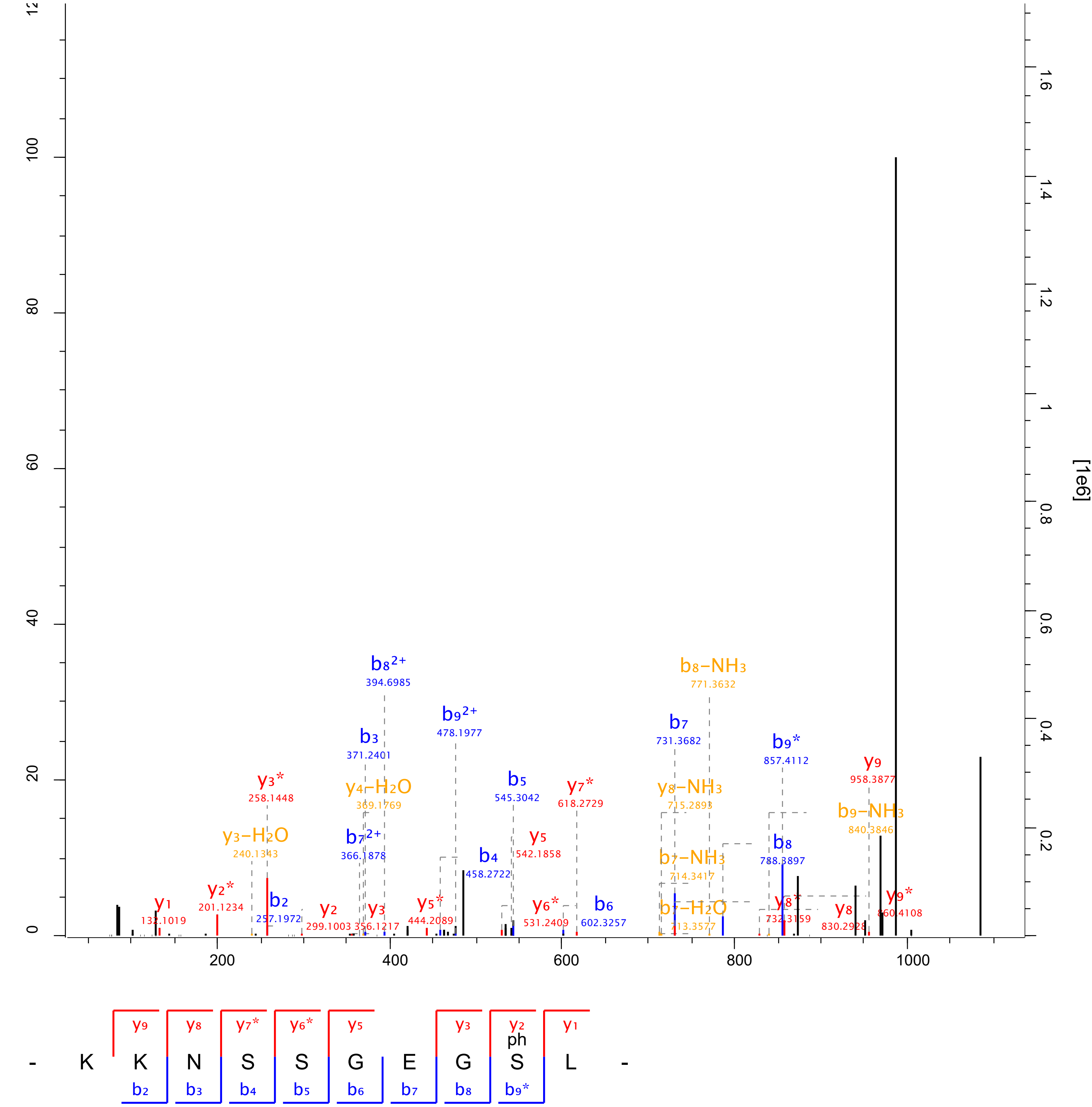

Sample output to test PDF Combine only

Raw file	Scan	Method	Score	m/z	Gene names
0523_1	1529	FTMS; HCD	55.55	564.59	SUN2

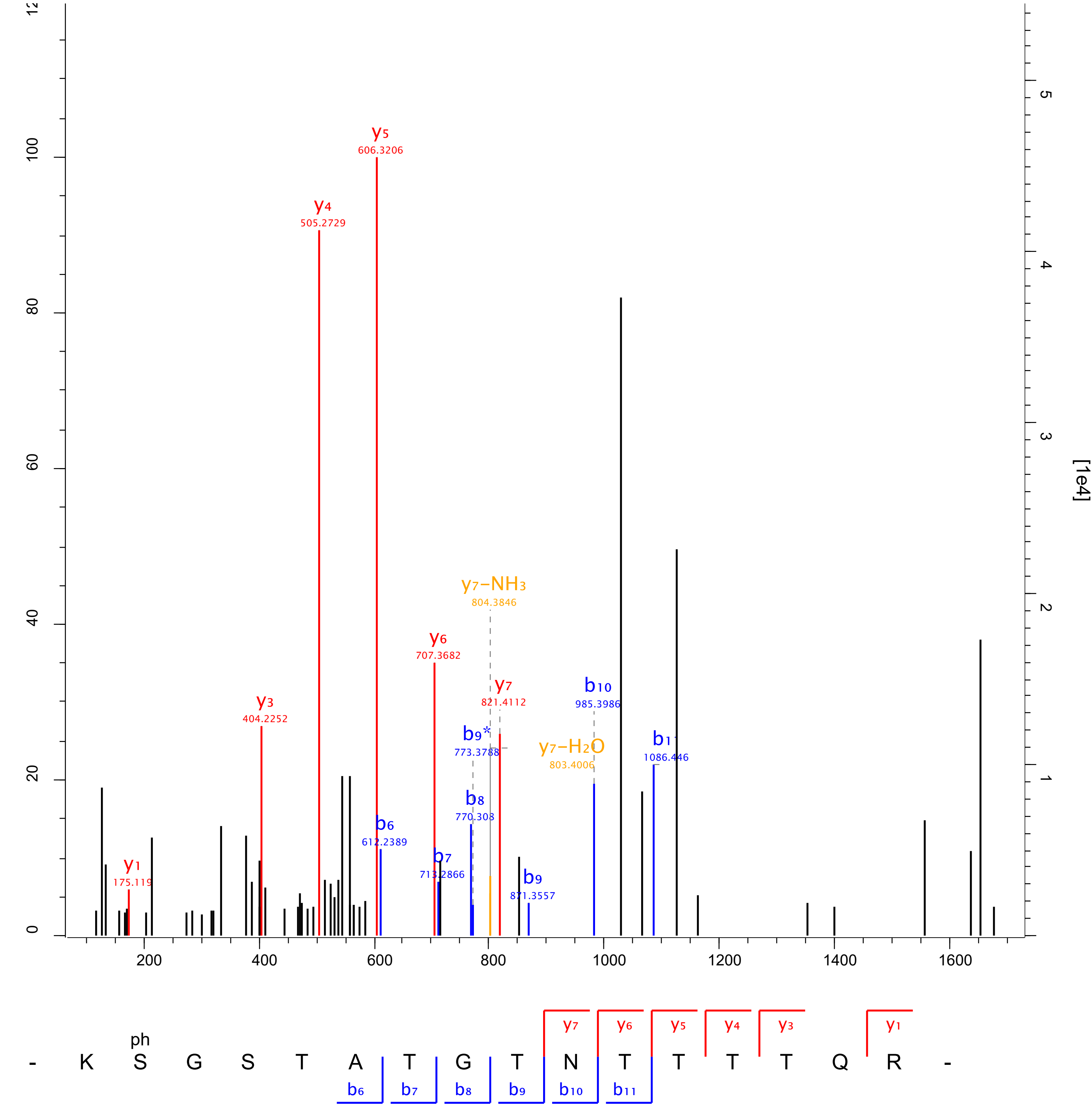

Sample output to test PDF Combine only

Raw file	Scan	Method	Score	m/z	Gene names
0523_1	3308	FTMS; HCD	72.2	442.2	F9K20.7

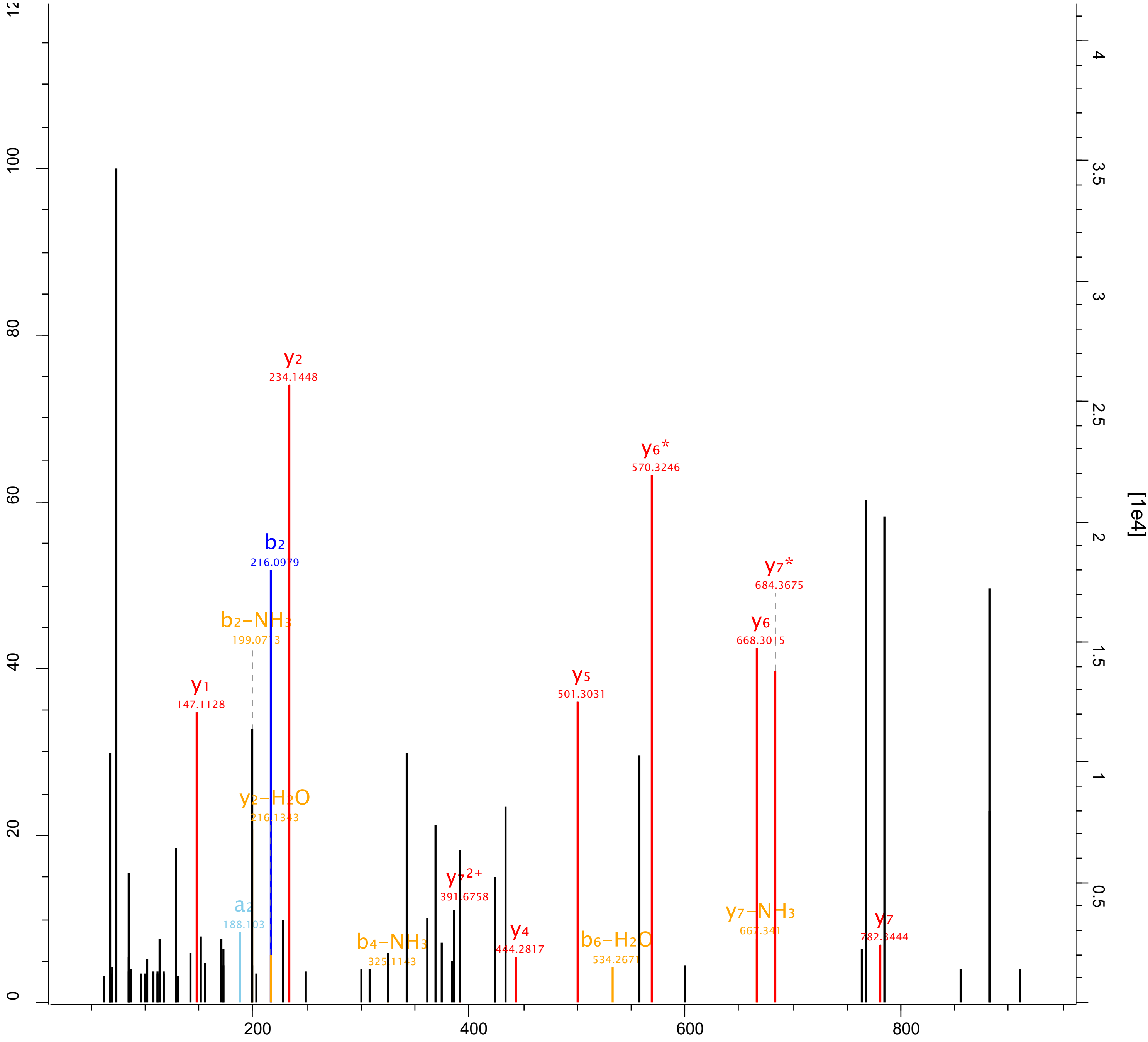

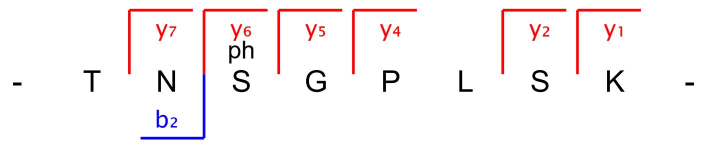

Sample output to test PDF Combine only

Raw file	Scan	Method	Score	m/z	Gene names
0523_1	8775	FTMS; HCD	82.45	597.23	AMT1-3

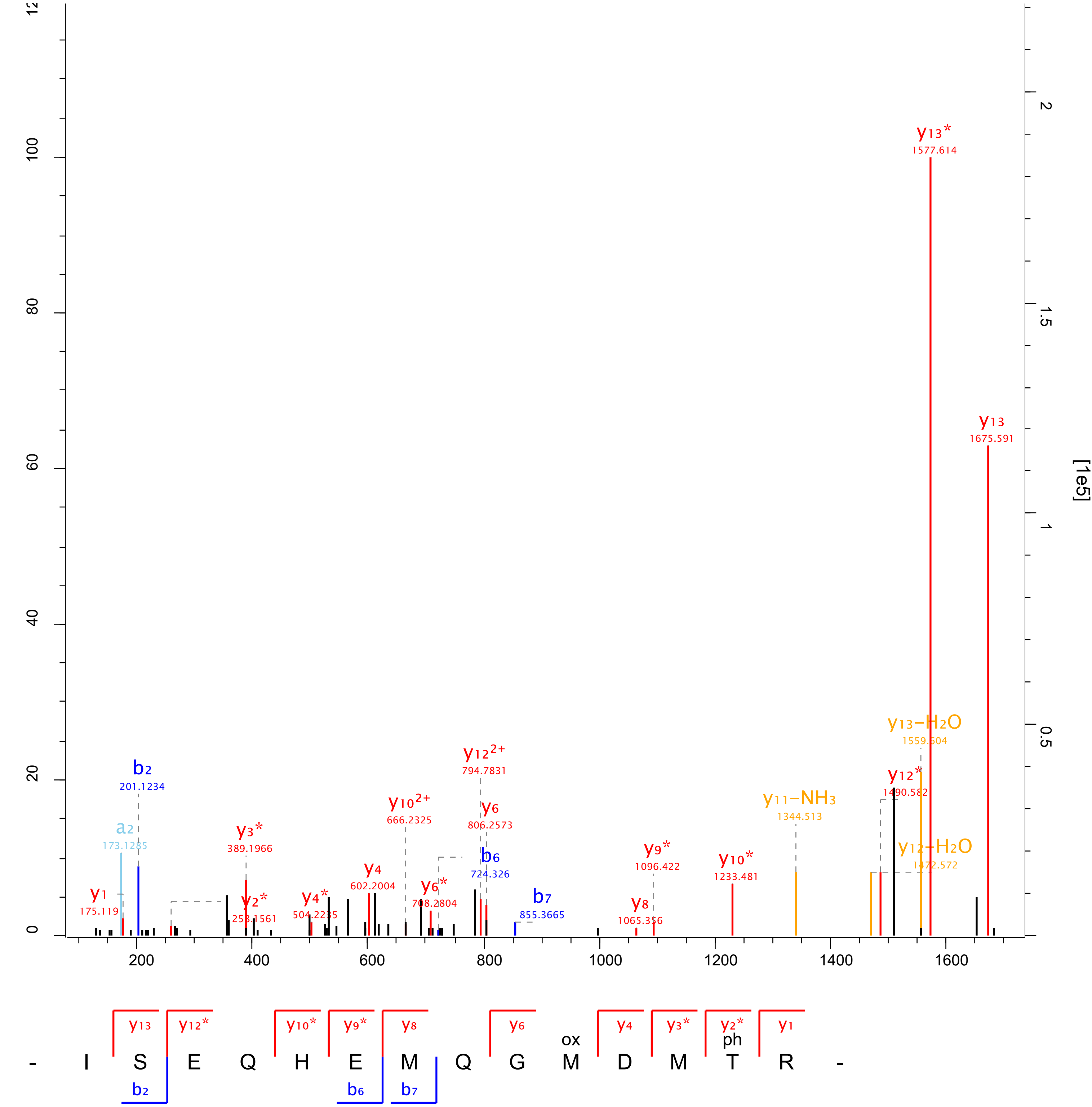

Sample output to test PDF Combine only

Raw file	Scan	Method	Score	m/z	Gene names
0523_1	9828	FTMS; HCD	43.59	528.58	F5M6.12

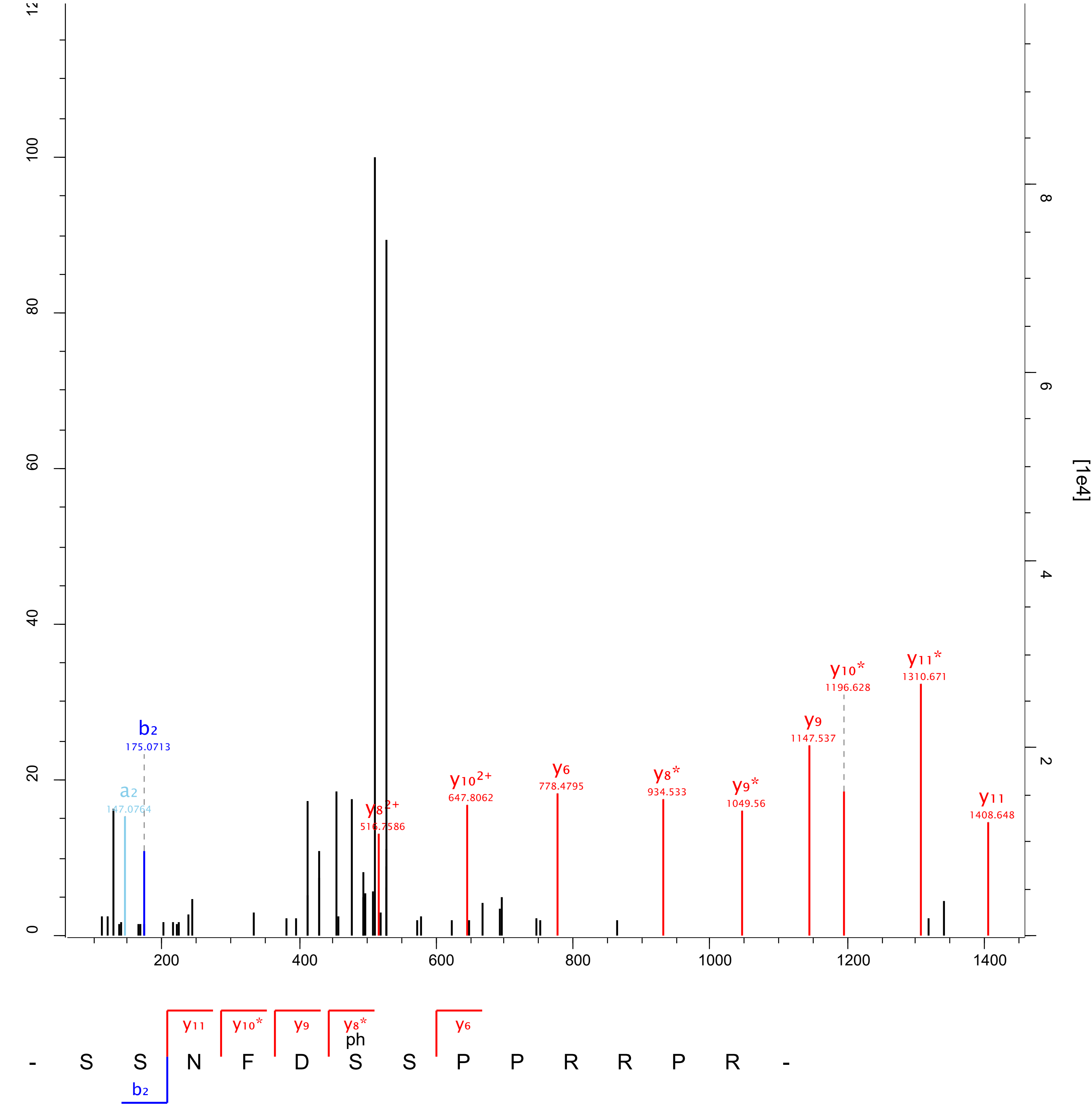

Sample output to test PDF Combine only

Raw file	Scan	Method	Score	m/z	Gene names
0523_1	9846	FTMS; HCD	107.78	677.66	AMT1-1

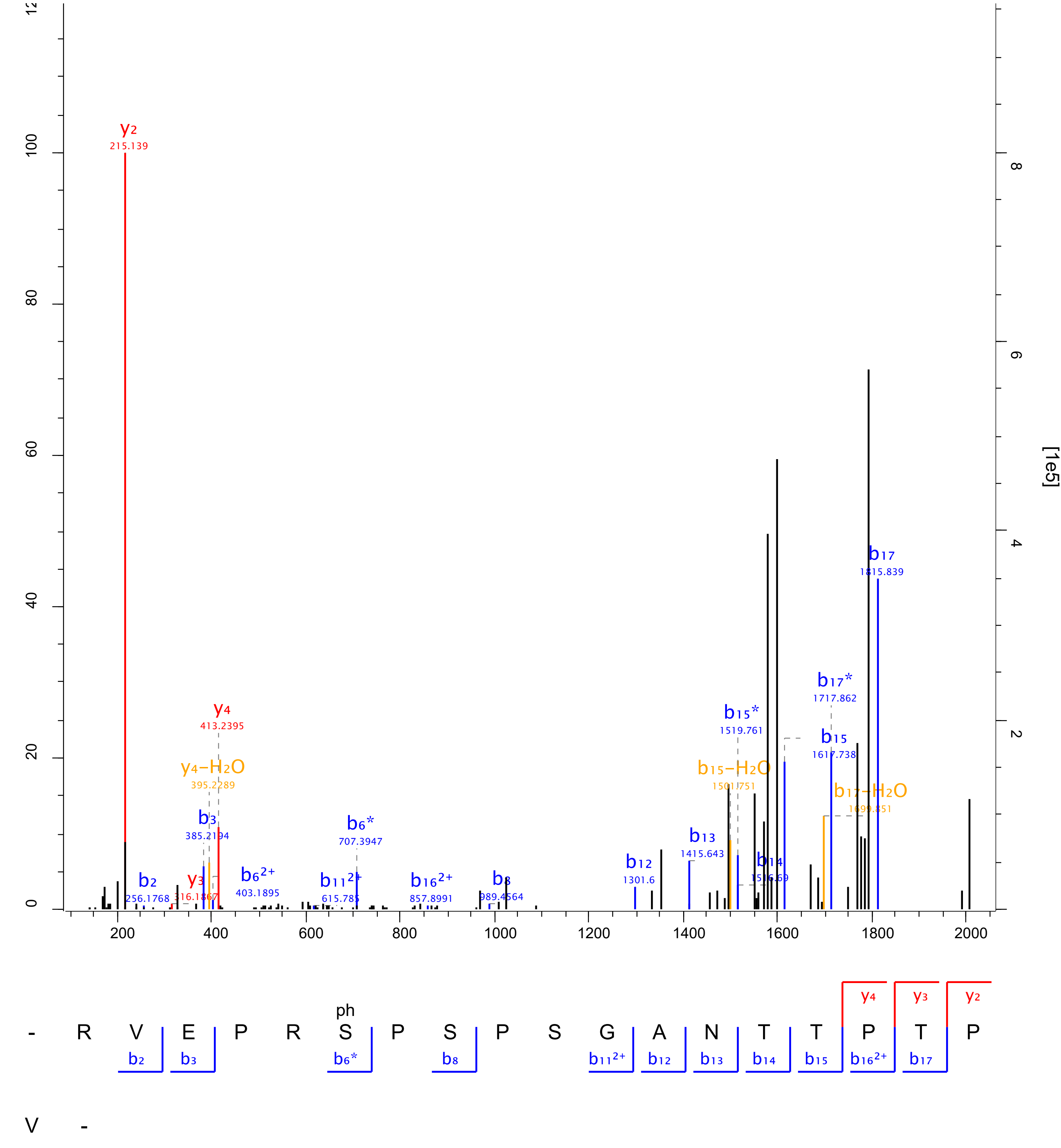

Sample output to test PDF Combine only

Raw file	Scan	Method	Score	m/z	Gene names
0523_1	10656	FTMS; HCD	57.48	671.3	At1g51850

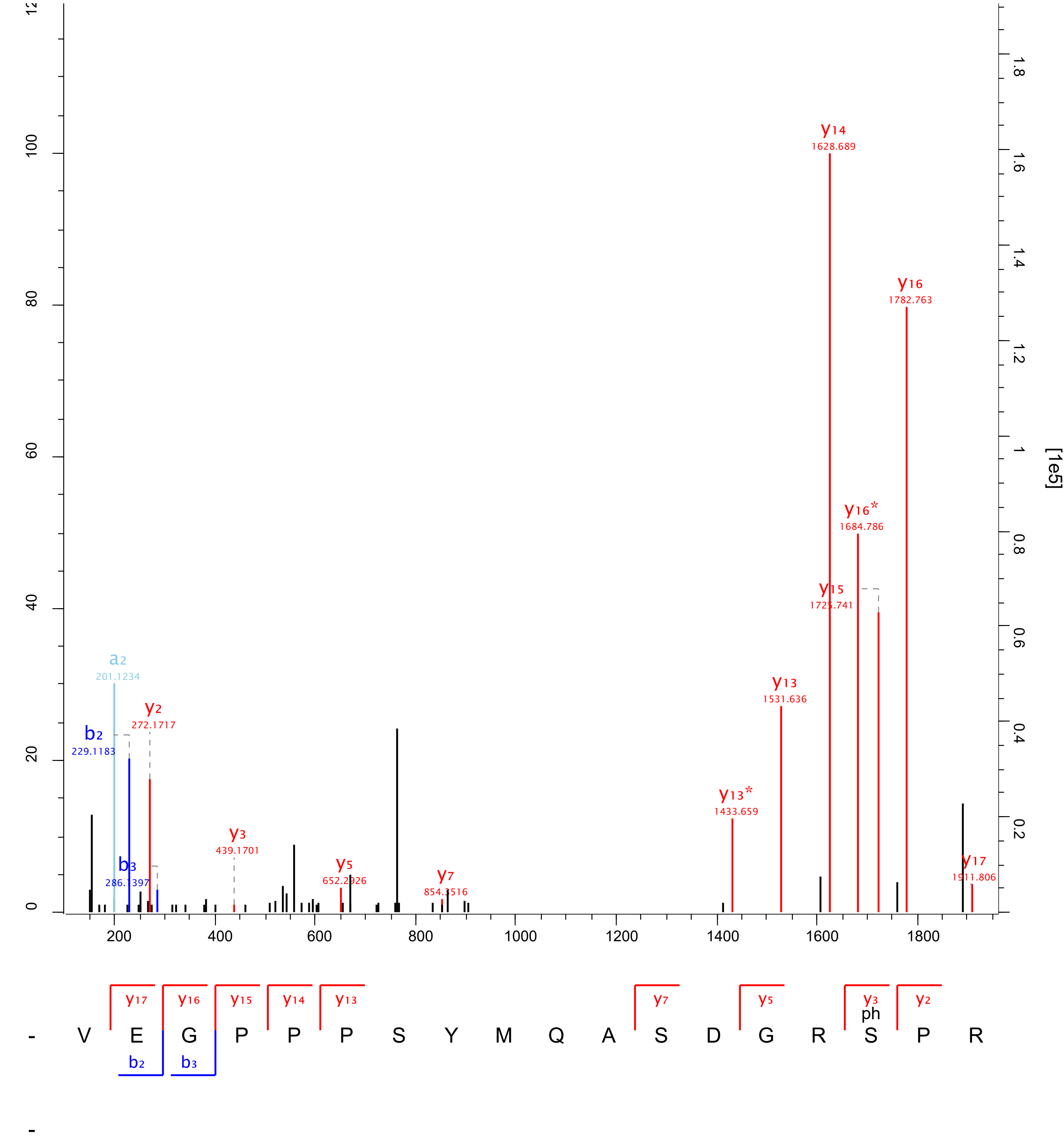

Sample output to test PDF Combine only

Raw file	Scan	Method	Score	m/z	Gene names
0523_1	11022	FTMS; HCD	87.65	523.94	F19K6.7;At1g52320

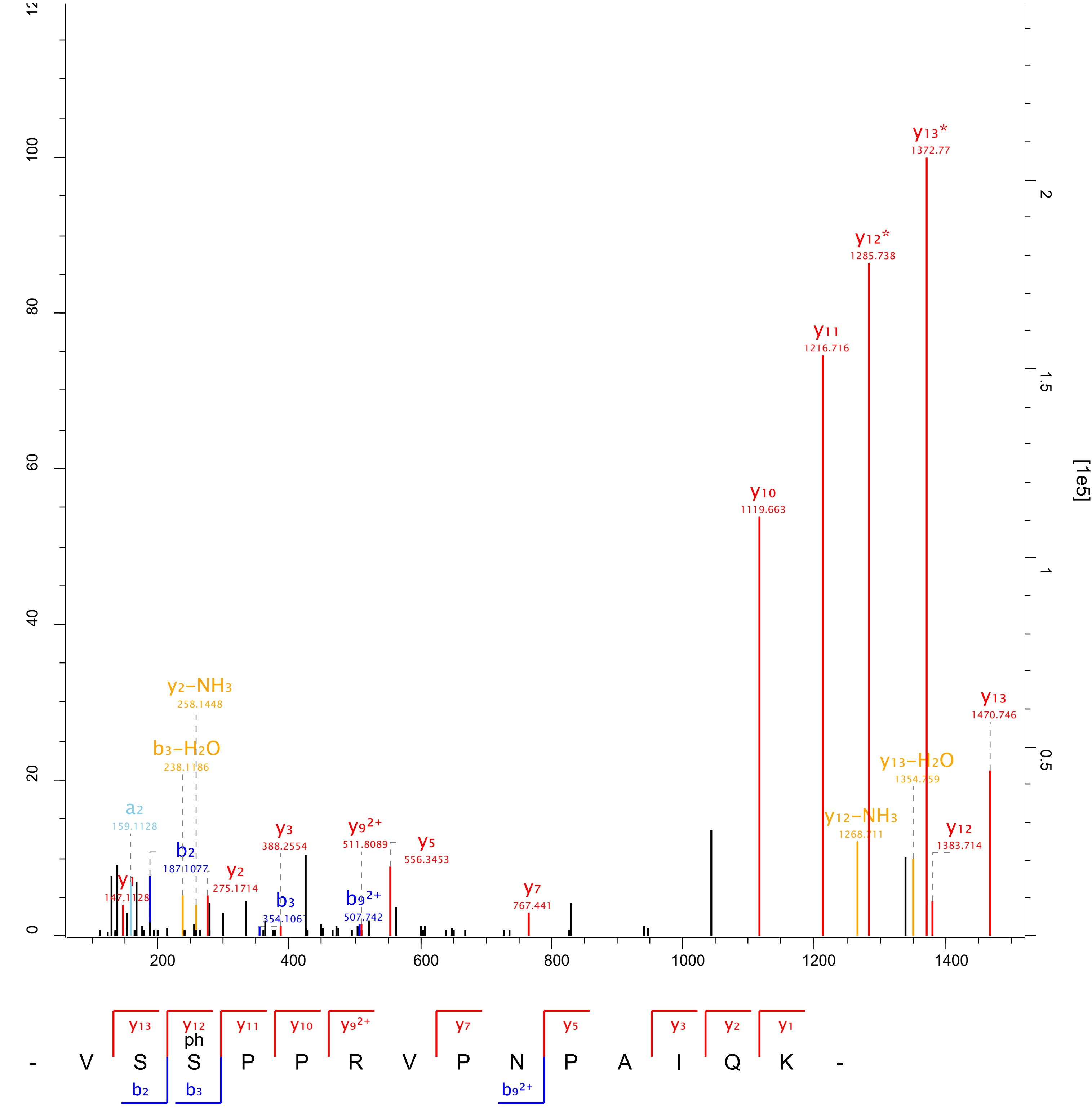

Sample output to test PDF Combine only

Raw file	Scan	Method	Score	m/z
0523_1	11138	FTMS; HCD	41.23	472.23

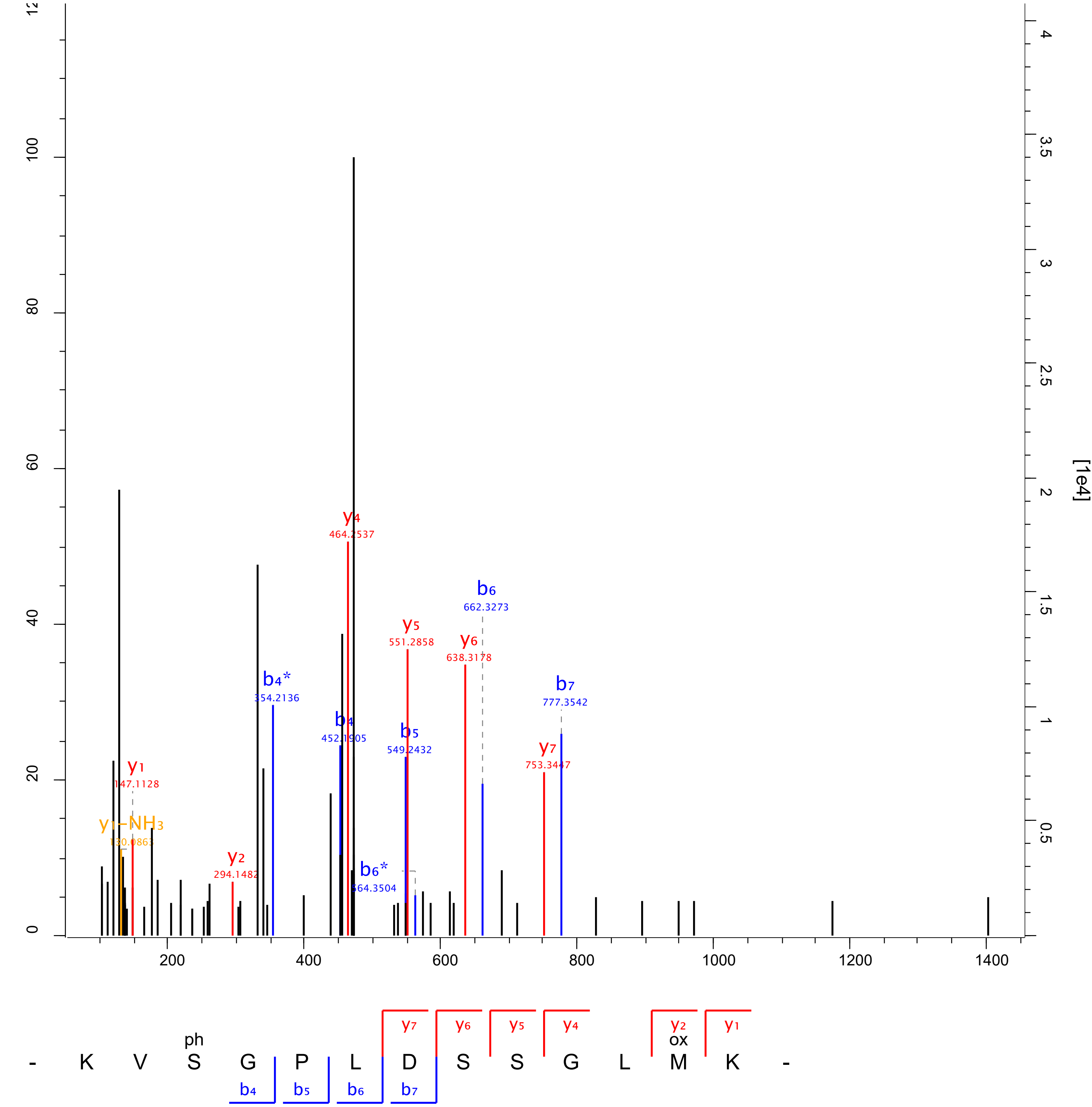

Sample output to test PDF Combine only

Raw file	Scan	Method	Score	m/z
0523_1	11467	FTMS; HCD	298.45	917.4

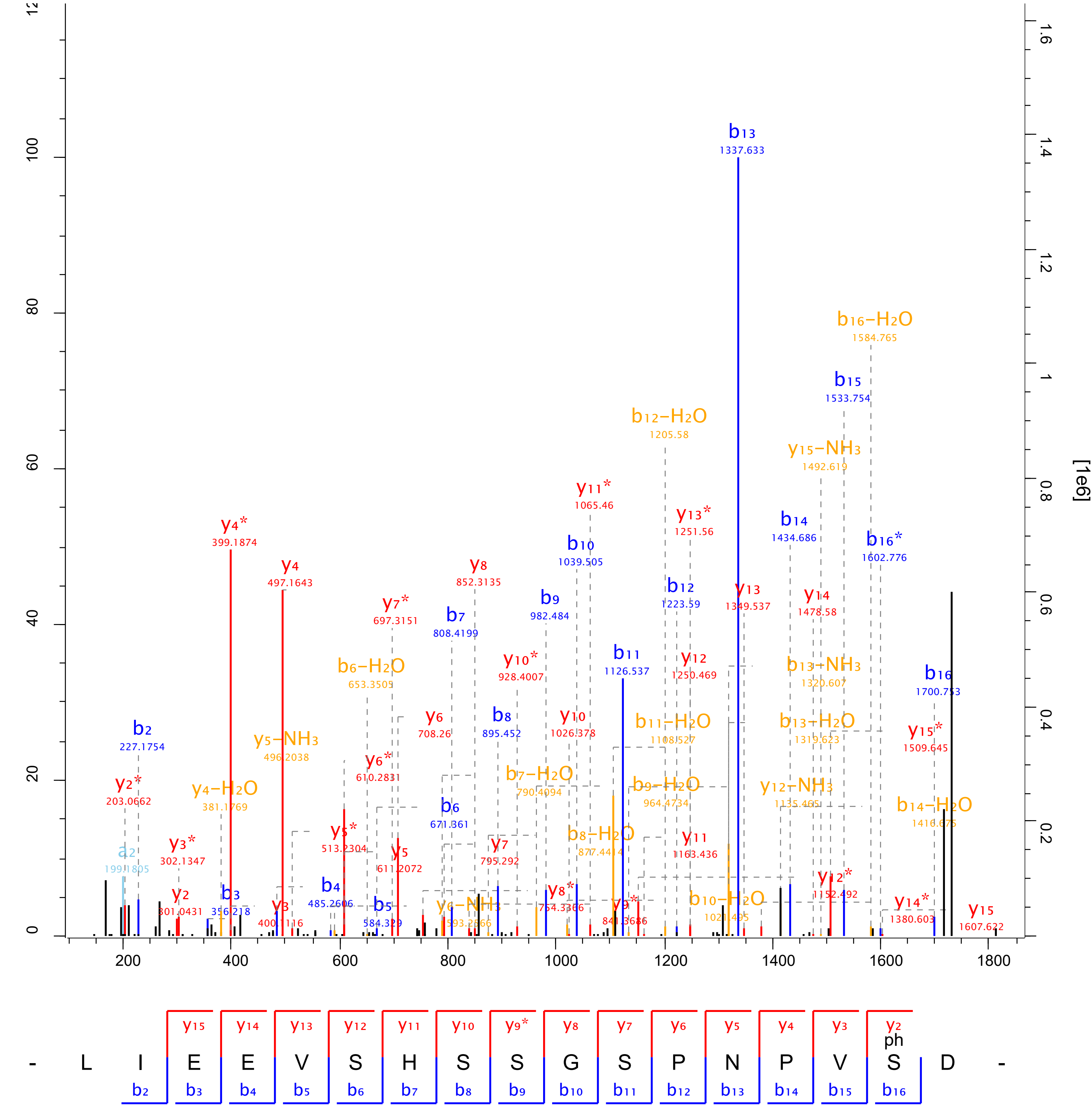

Sample output to test PDF Combine only

Raw file	Scan	Method	Score	m/z	Gene names
0523_1	11483	FTMS; HCD	41.51	570.96	IQD14

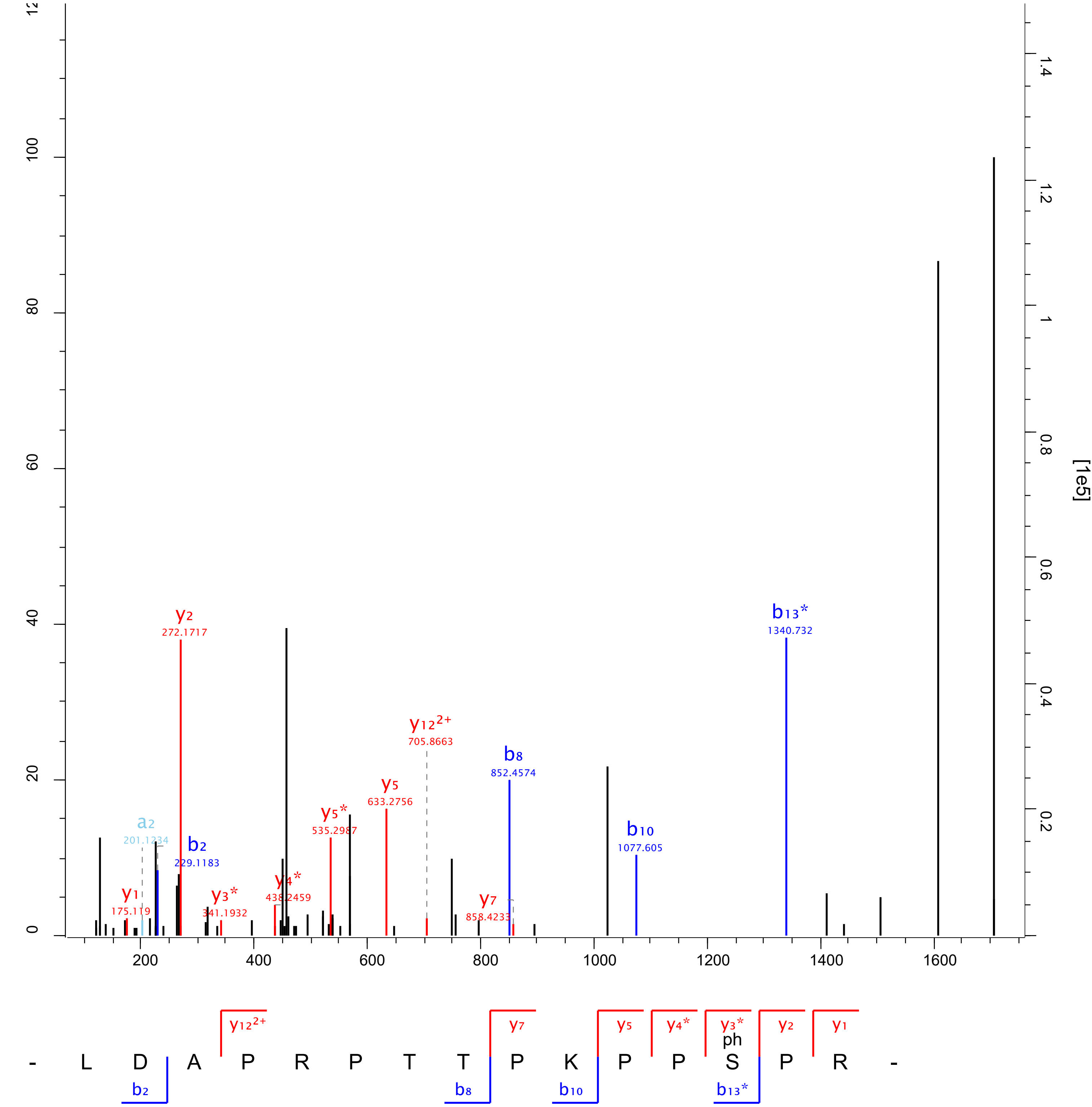

Sample output to test PDF Combine only

Raw file	Scan	Method	Score	m/z
0523_1	11666	FTMS; HCD	70.56	632.62

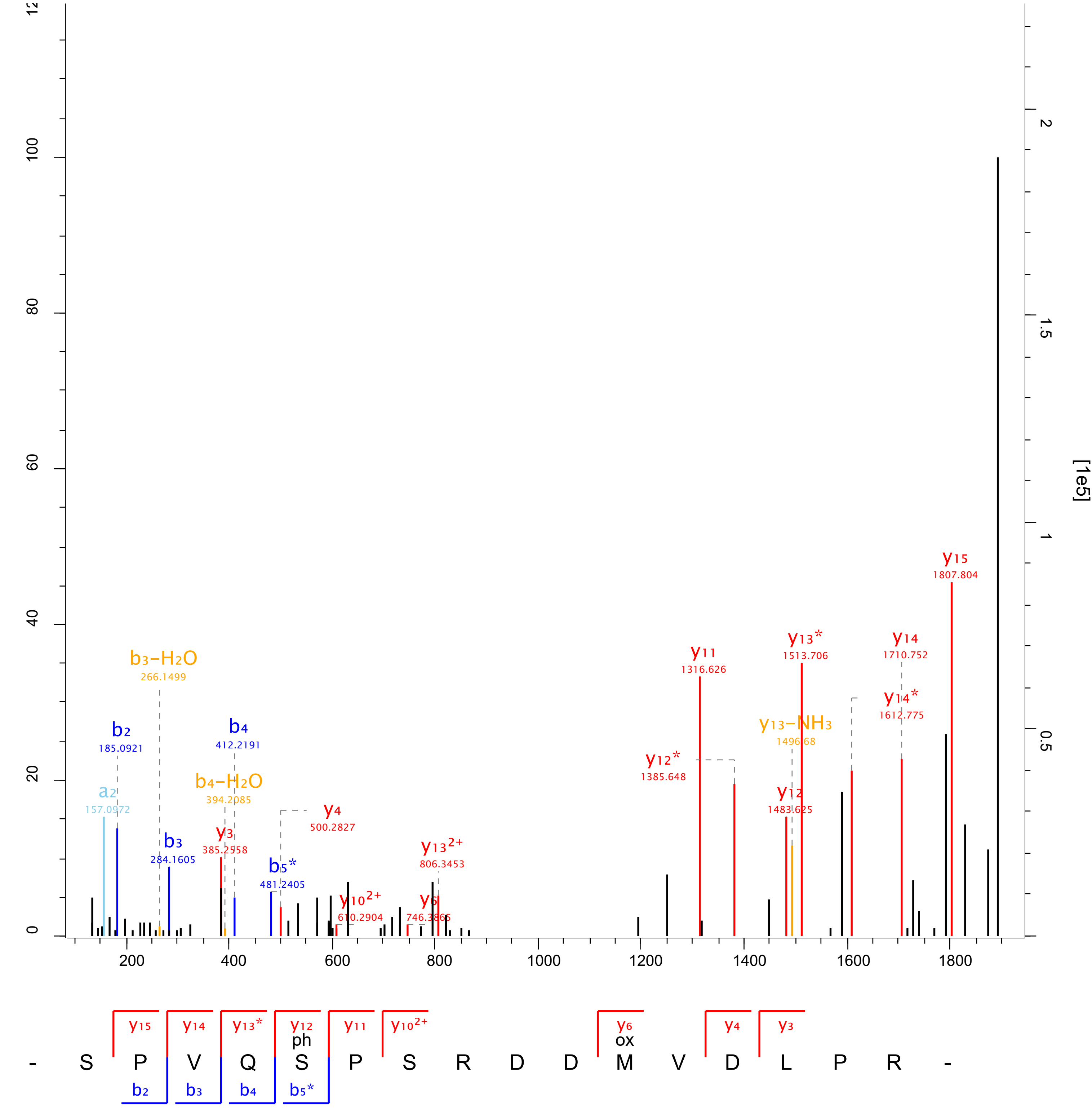

Sample output to test PDF Combine only

Raw file	Scan	Method	Score	m/z	Gene names
0523_1	12075	FTMS; HCD	51.57	603.97	F17M5.160

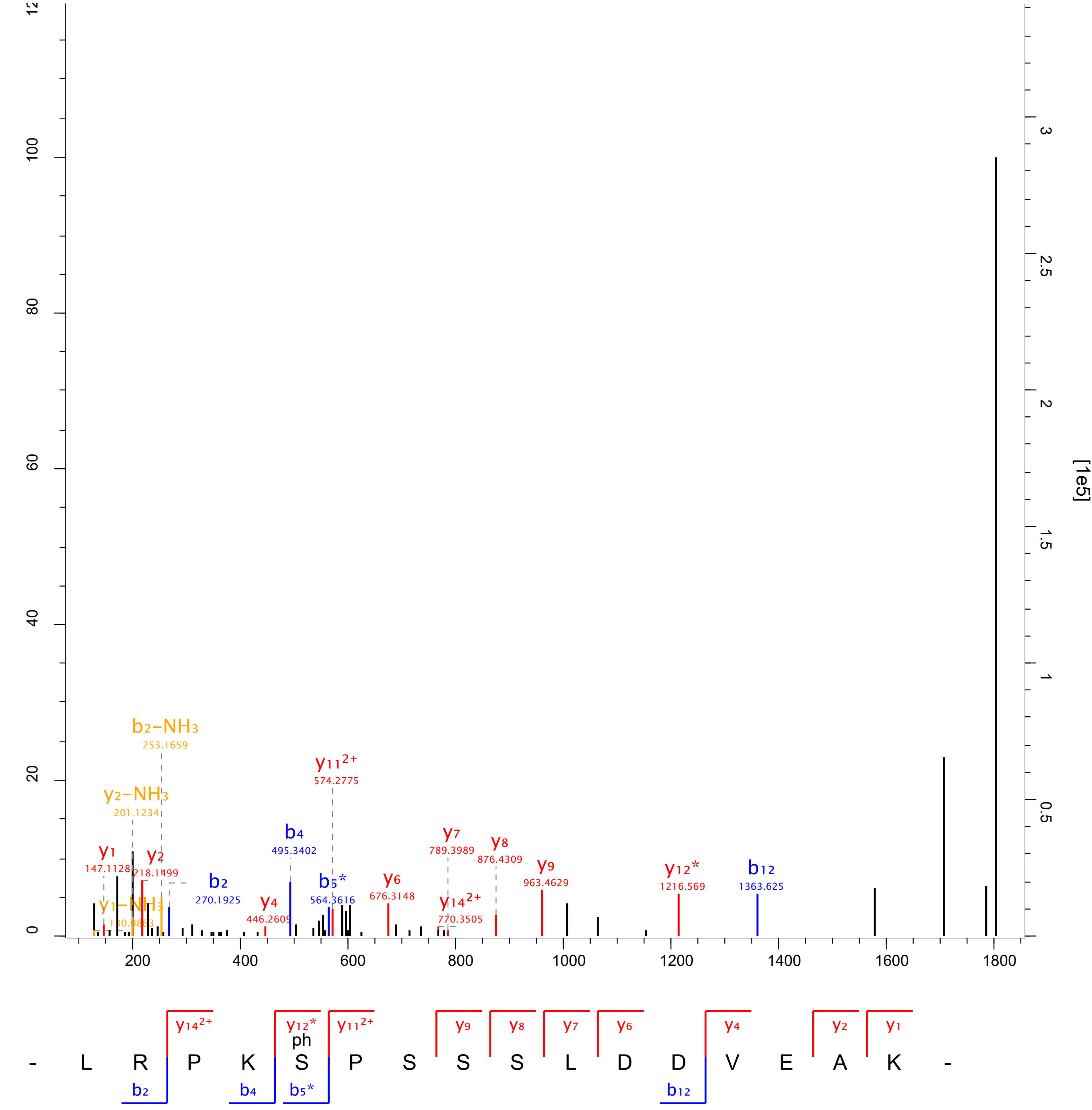

Sample output to test PDF Combine only

Raw file	Scan	Method	Score	m/z	Gene names
0523_1	12171	FTMS; HCD	161.04	601.29	F19C24.3

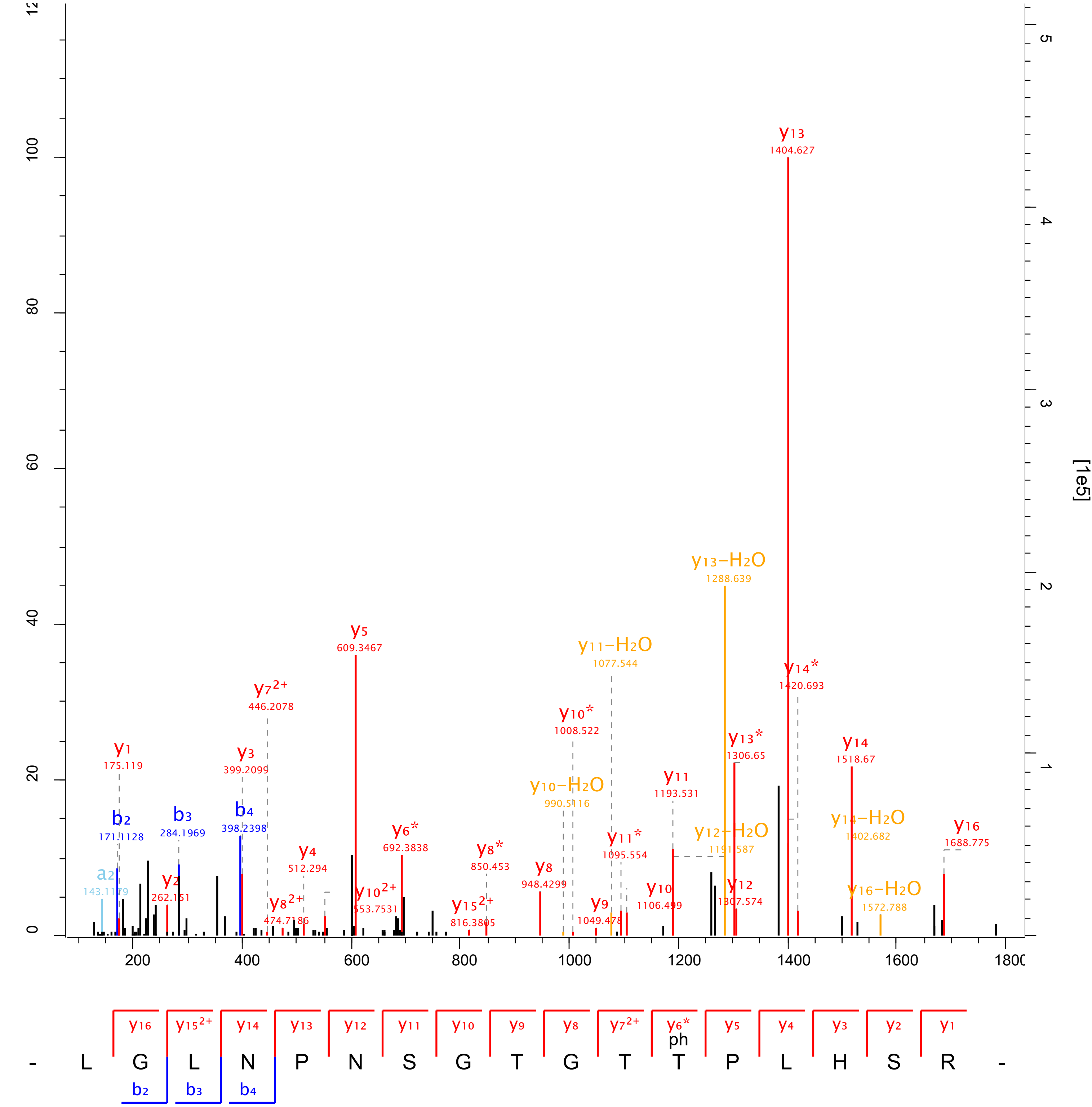

Sample output to test PDF Combine only

Raw file	Scan	Method	Score	m/z	Gene names
0523_1	12220	FTMS; HCD	55.05	716.99	ABCG34

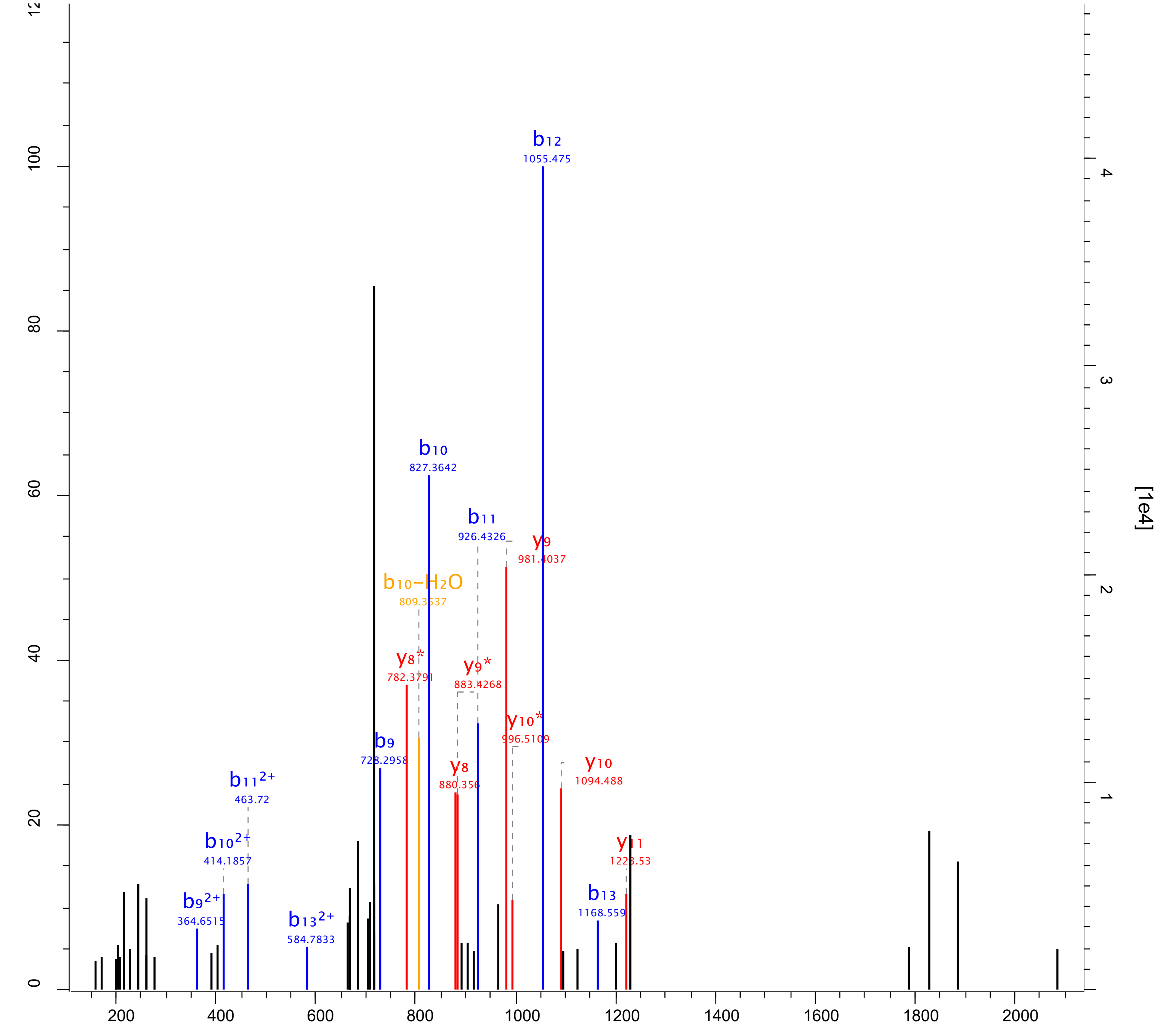

- G S H S G T G G S V V E L T S T S S

H G P K -

y11 y10 y9 y8

ph

b9 b10 b11 b12 b13

Sample output to test PDF Combine only

Raw file	Scan	Method	Score	m/z	Gene names
0523_1	12326	FTMS; HCD	45.62	528.27	CPK2;CPK1

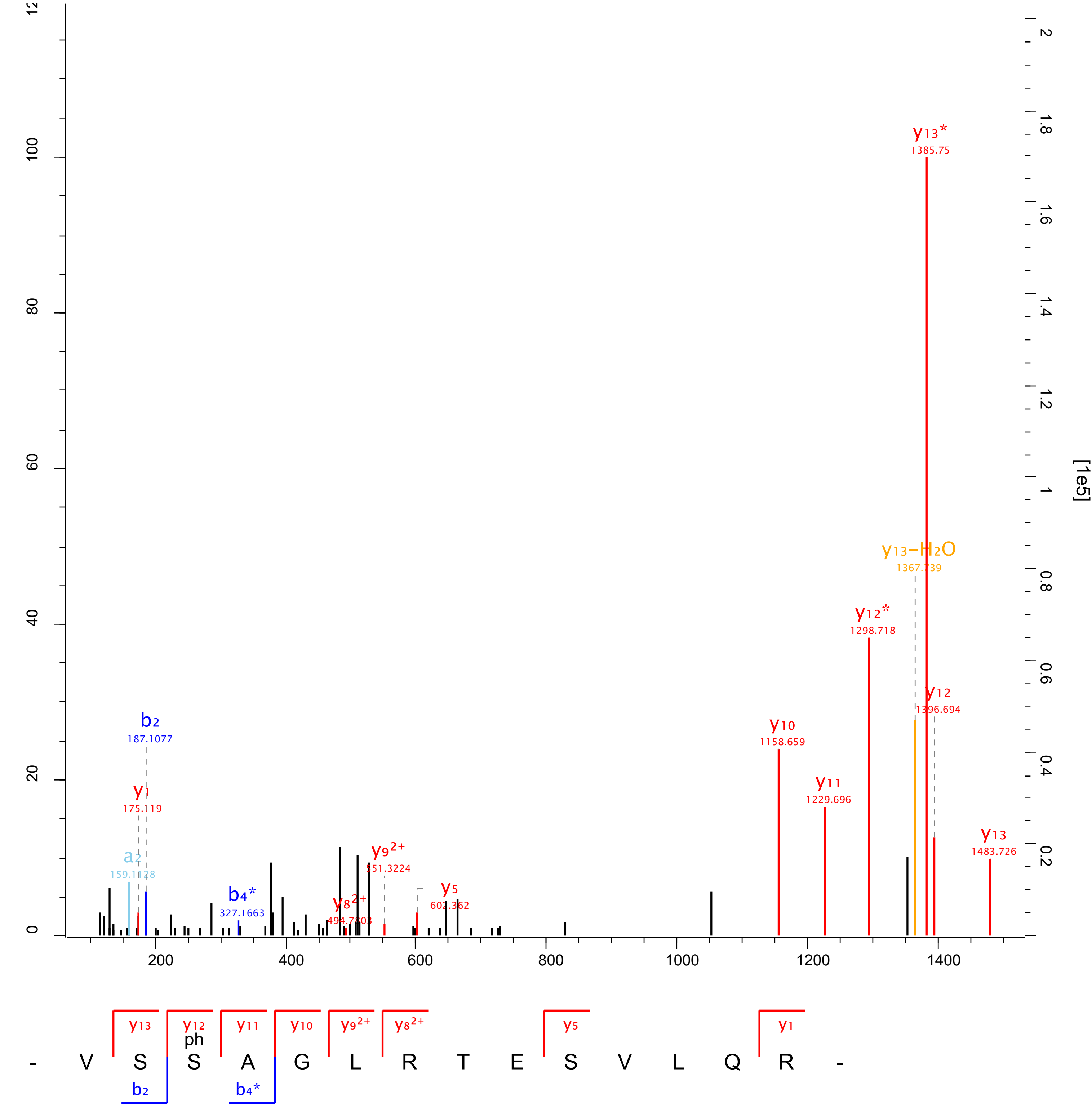

Sample output to test PDF Combine only

Raw file	Scan	Method	Score	m/z	Gene names
0523_1	12348	FTMS; HCD	65.39	767.69	PRP40A

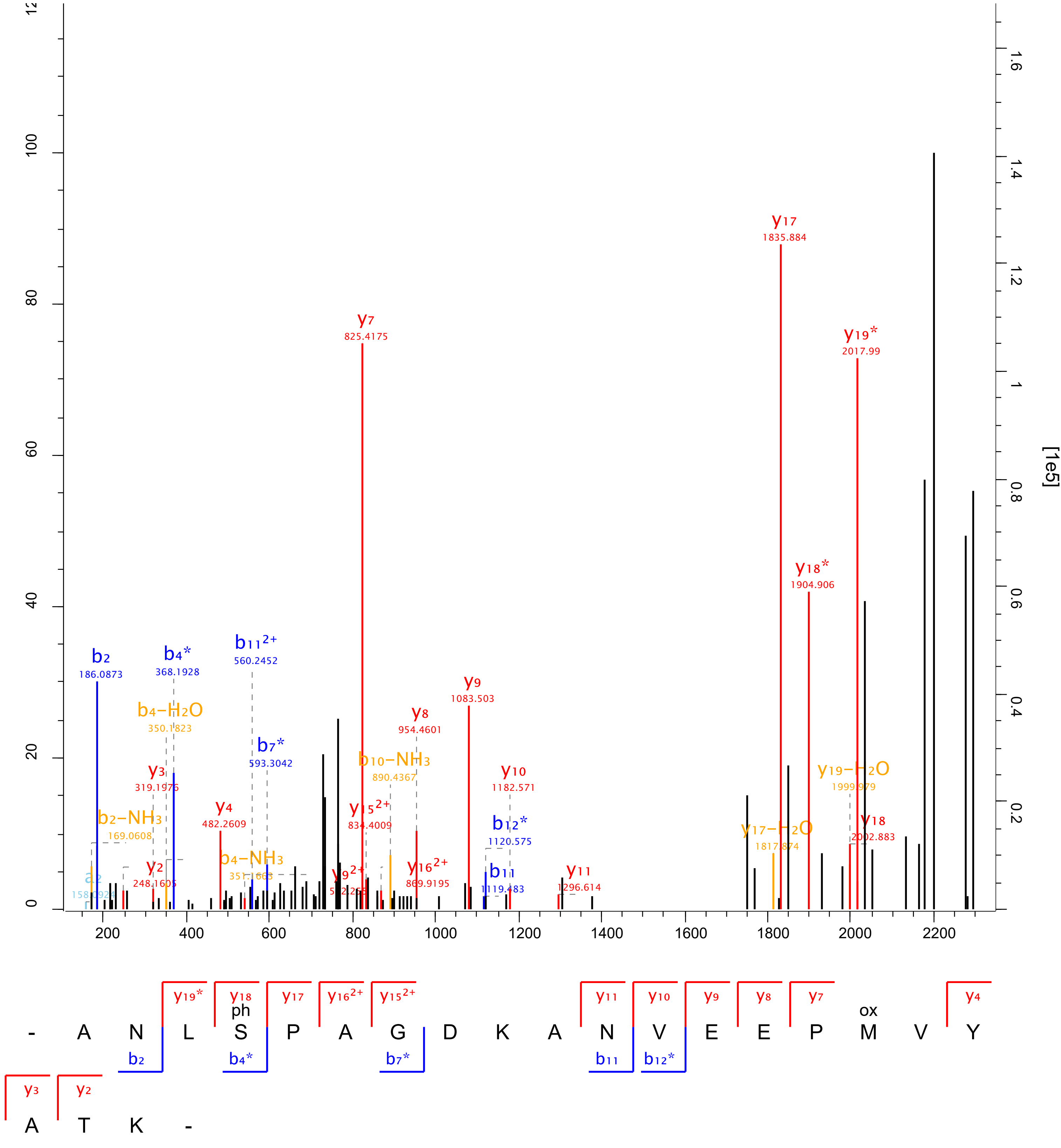

Sample output to test PDF Combine only

Raw file	Scan	Method	Score	m/z	Gene names
0523_1	12639	FTMS; HCD	143.4	634.93	ALA10

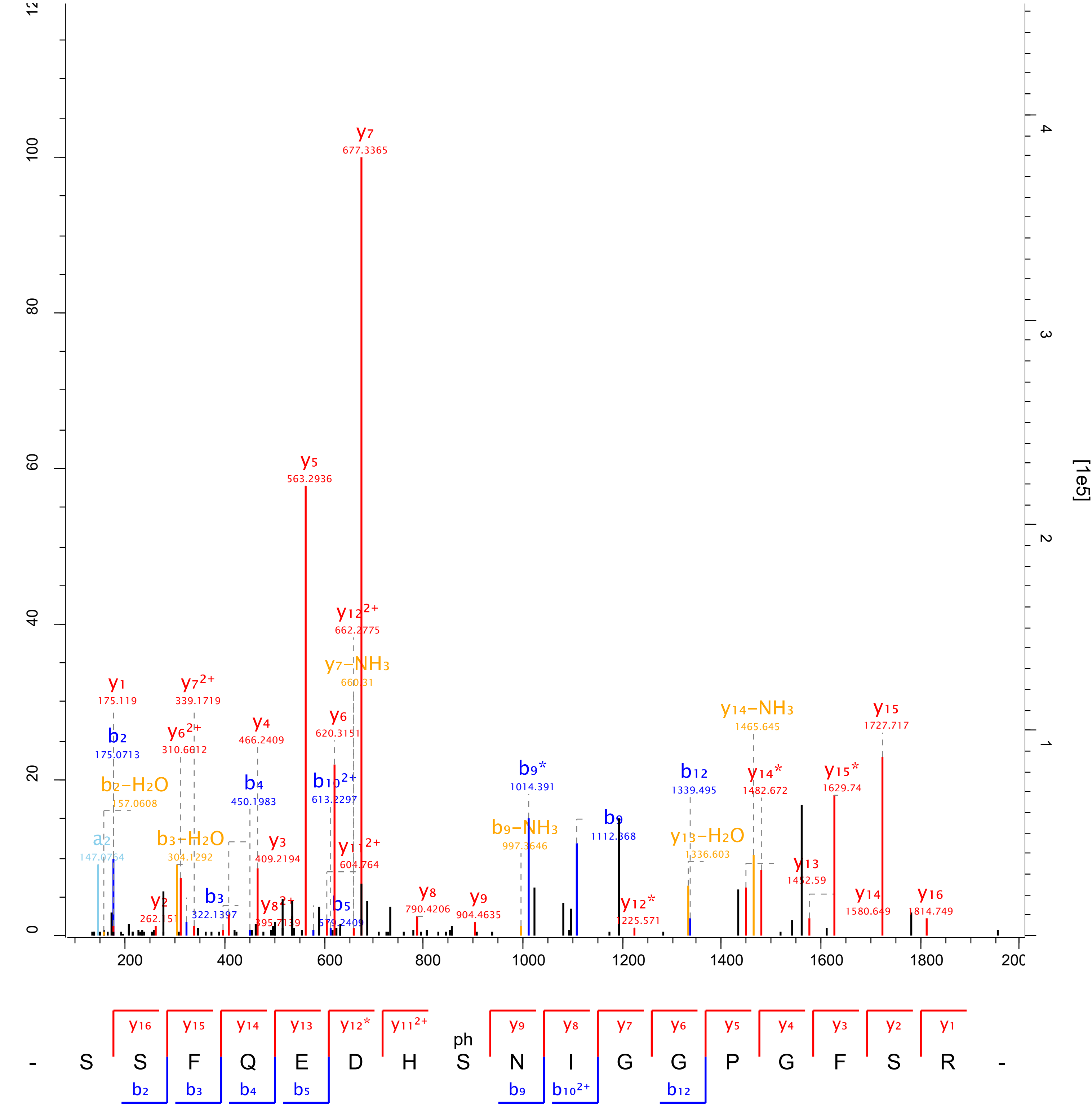

Sample output to test PDF Combine only

Raw file	Scan	Method	Score	m/z
0523_1	12700	FTMS; HCD	45.87	530.28

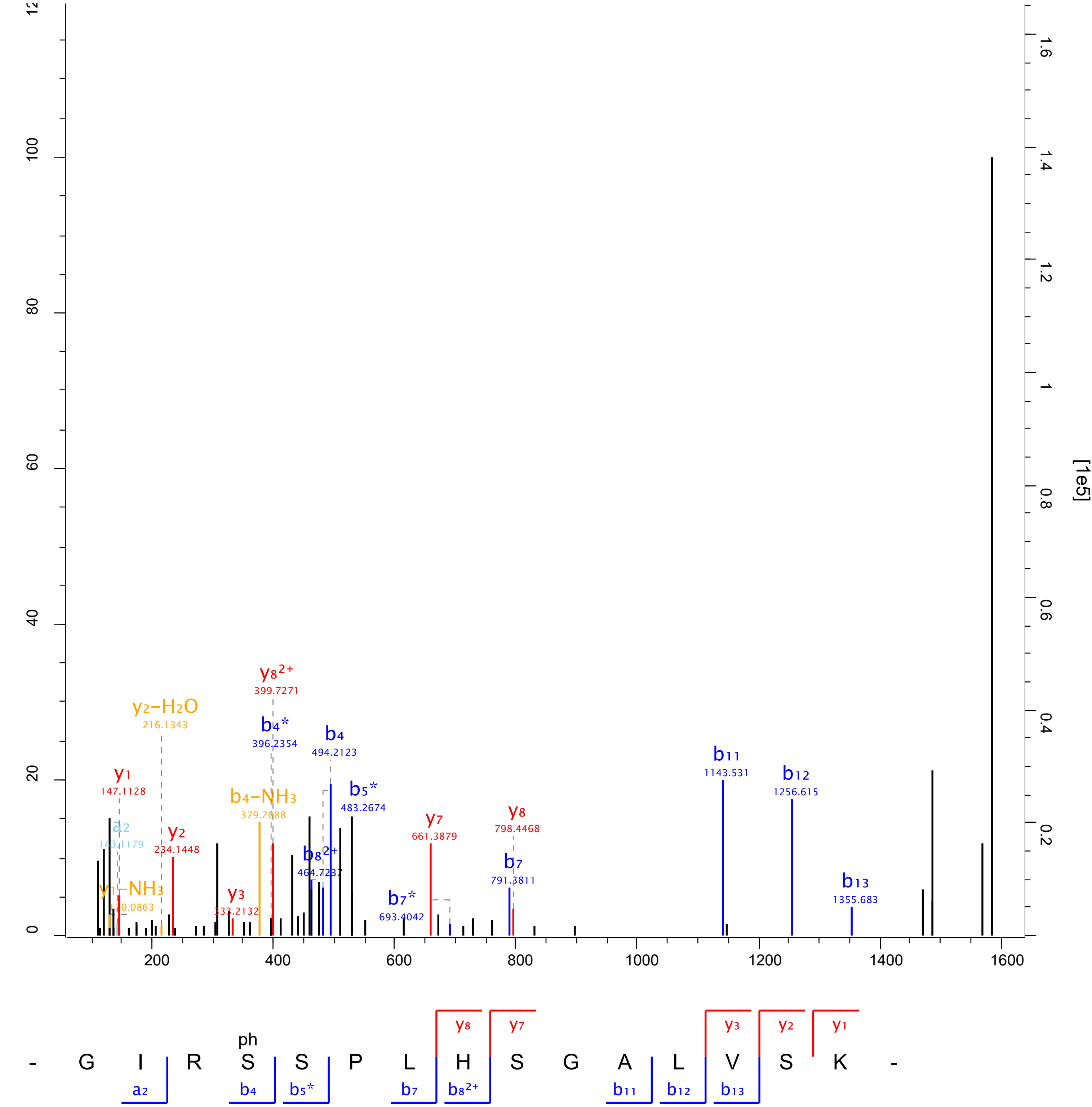

Sample output to test PDF Combine only

Raw file	Scan	Method	Score	m/z	Gene names
0523_1	12899	FTMS; HCD	107.75	838.06	LRS1

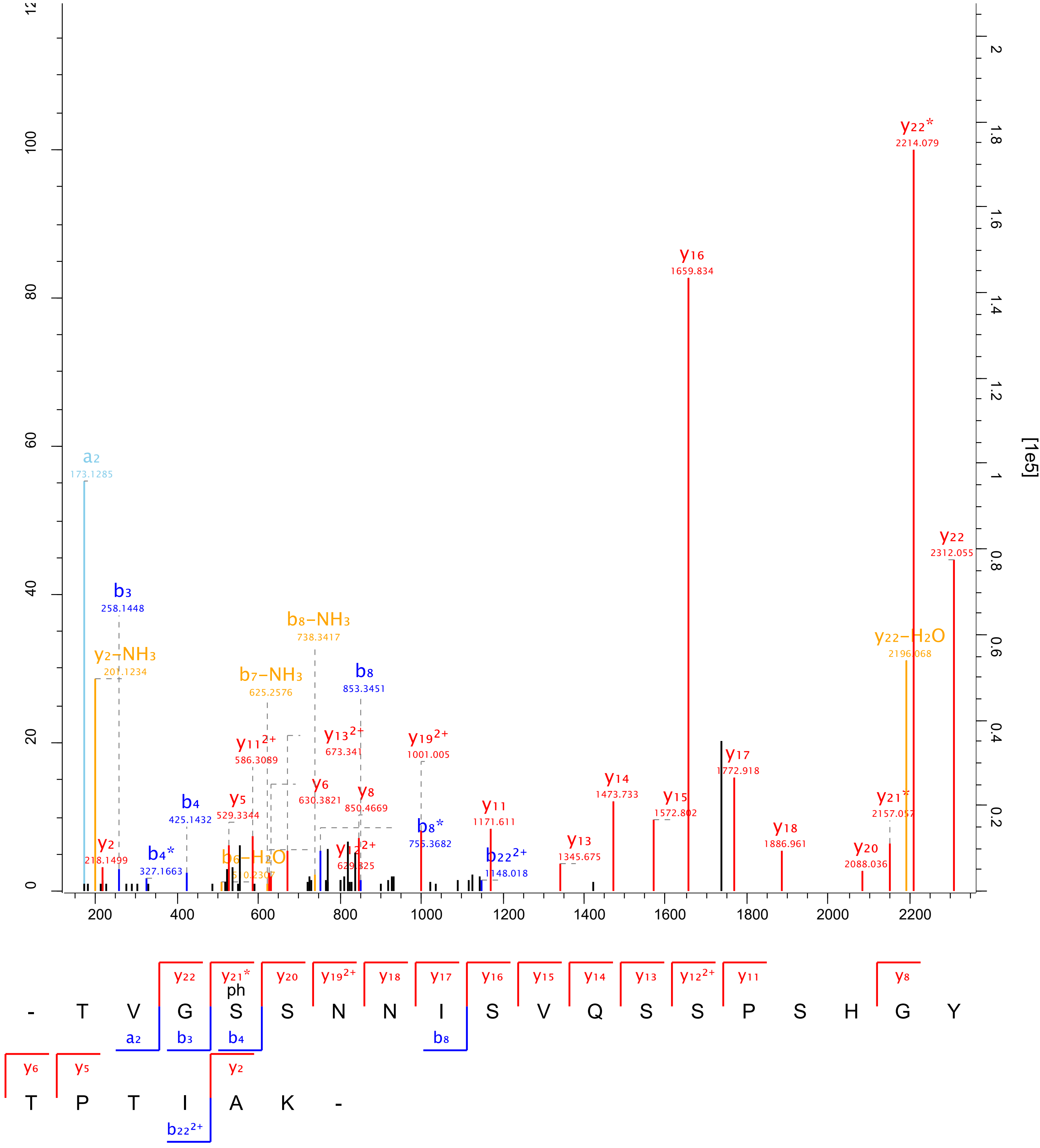

Sample output to test PDF Combine only

Raw file	Scan	Method	Score	m/z
0523_1	12921	FTMS; HCD	86	544.24

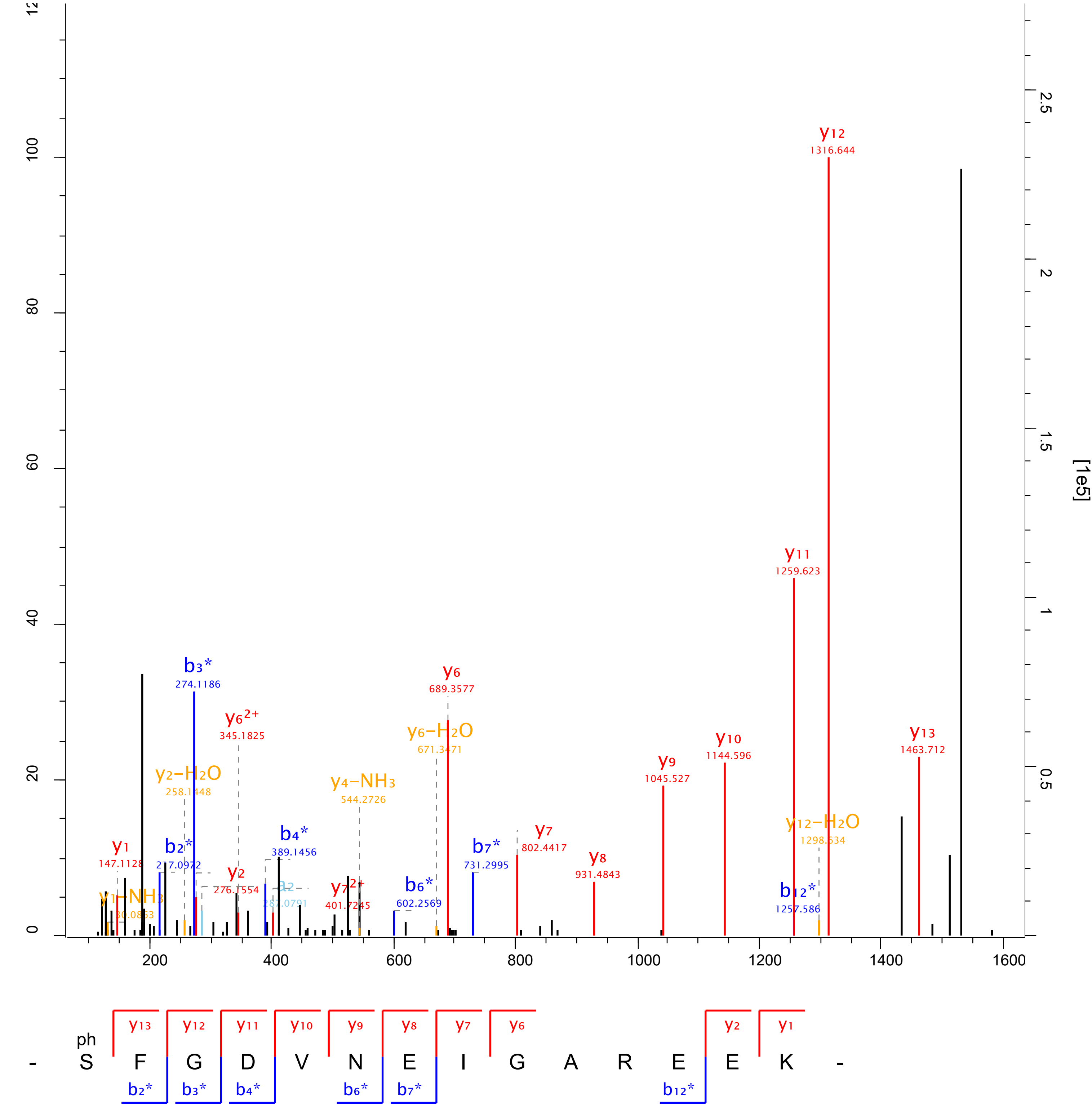

Sample output to test PDF Combine only

Raw file	Scan	Method	Score	m/z
0523_1	12976	FTMS; HCD	118.44	625.29

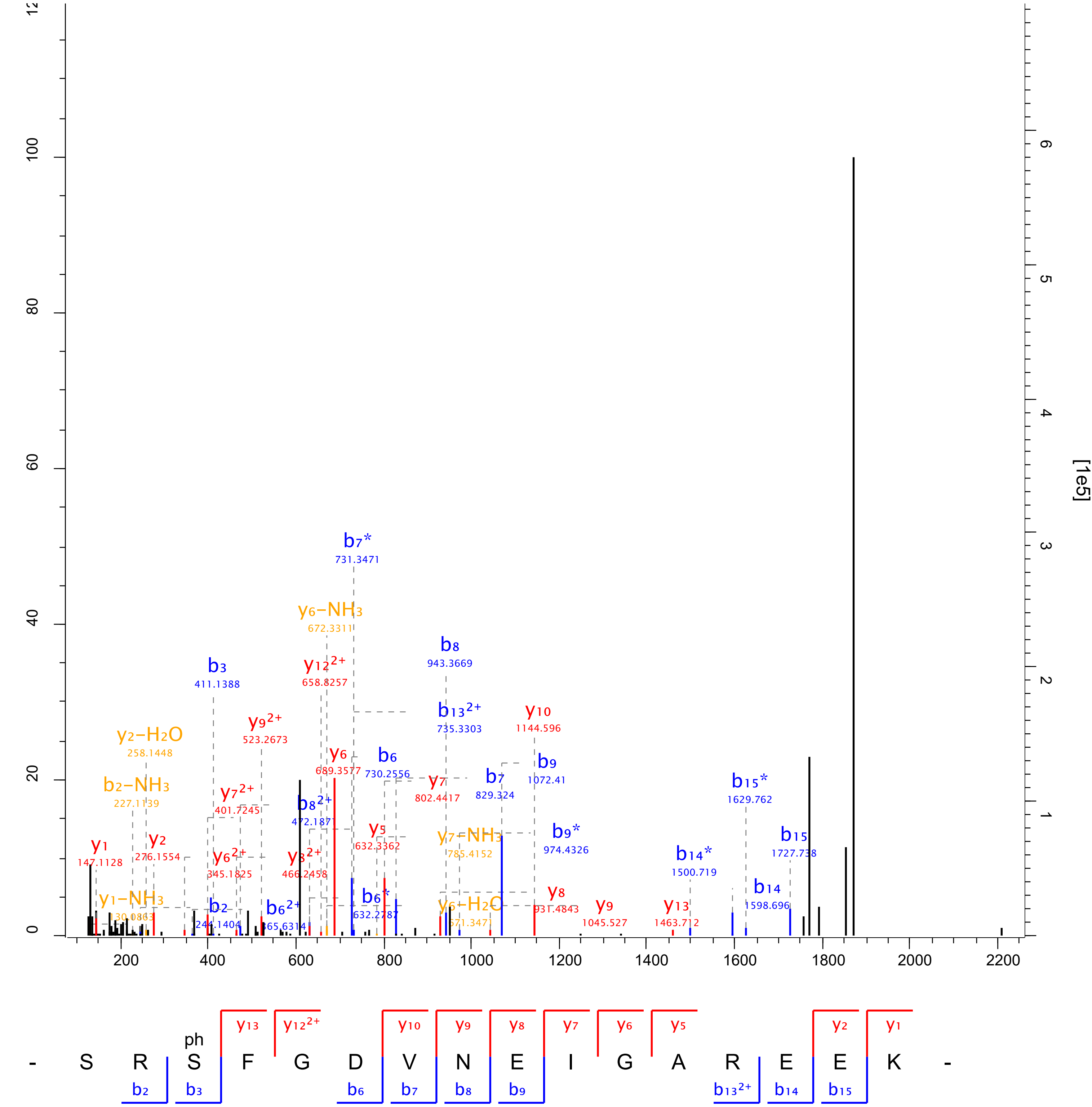

Sample output to test PDF Combine only

Raw file	Scan	Method	Score	m/z
0523_1	13040	FTMS; HCD	164.27	808.66

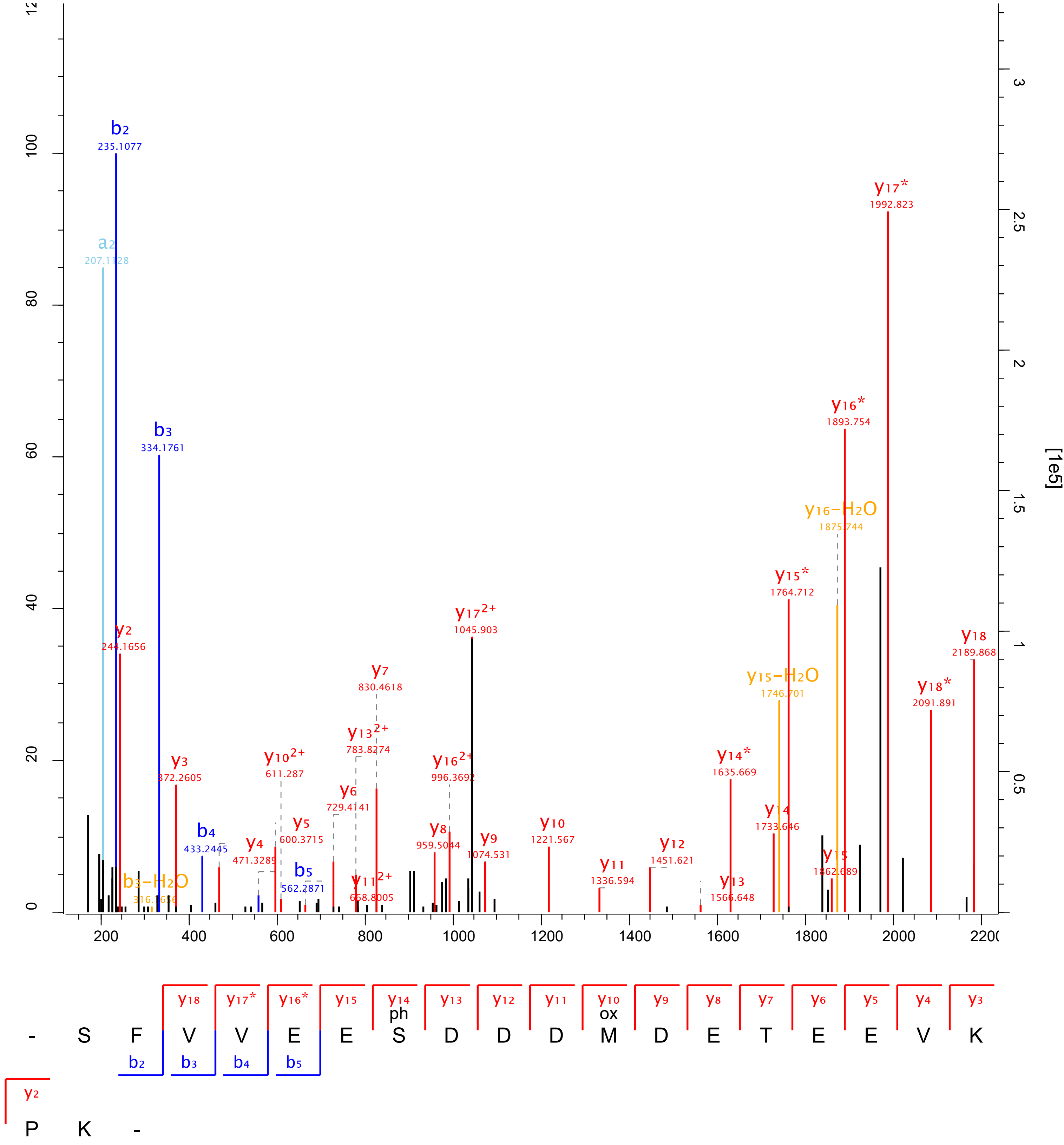

Sample output to test PDF Combine only

Raw file	Scan	Method	Score	m/z	Gene names
0523_1	13081	FTMS; HCD	79.49	641.97	IQD32

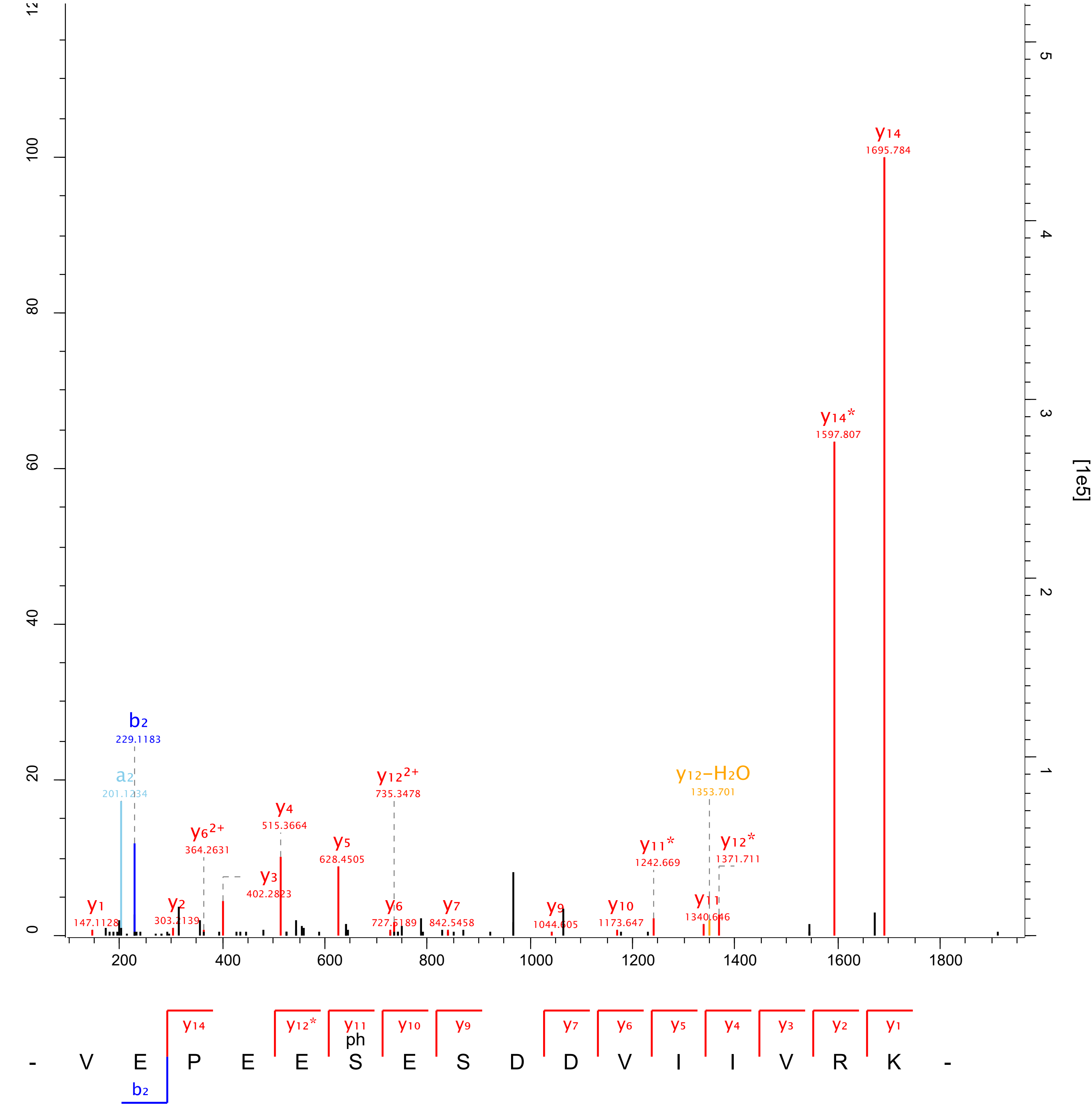

Sample output to test PDF Combine only

Raw file	Scan	Method	Score	m/z	Gene names
0523_1	13266	FTMS; HCD	82.63	667.96	TRS130

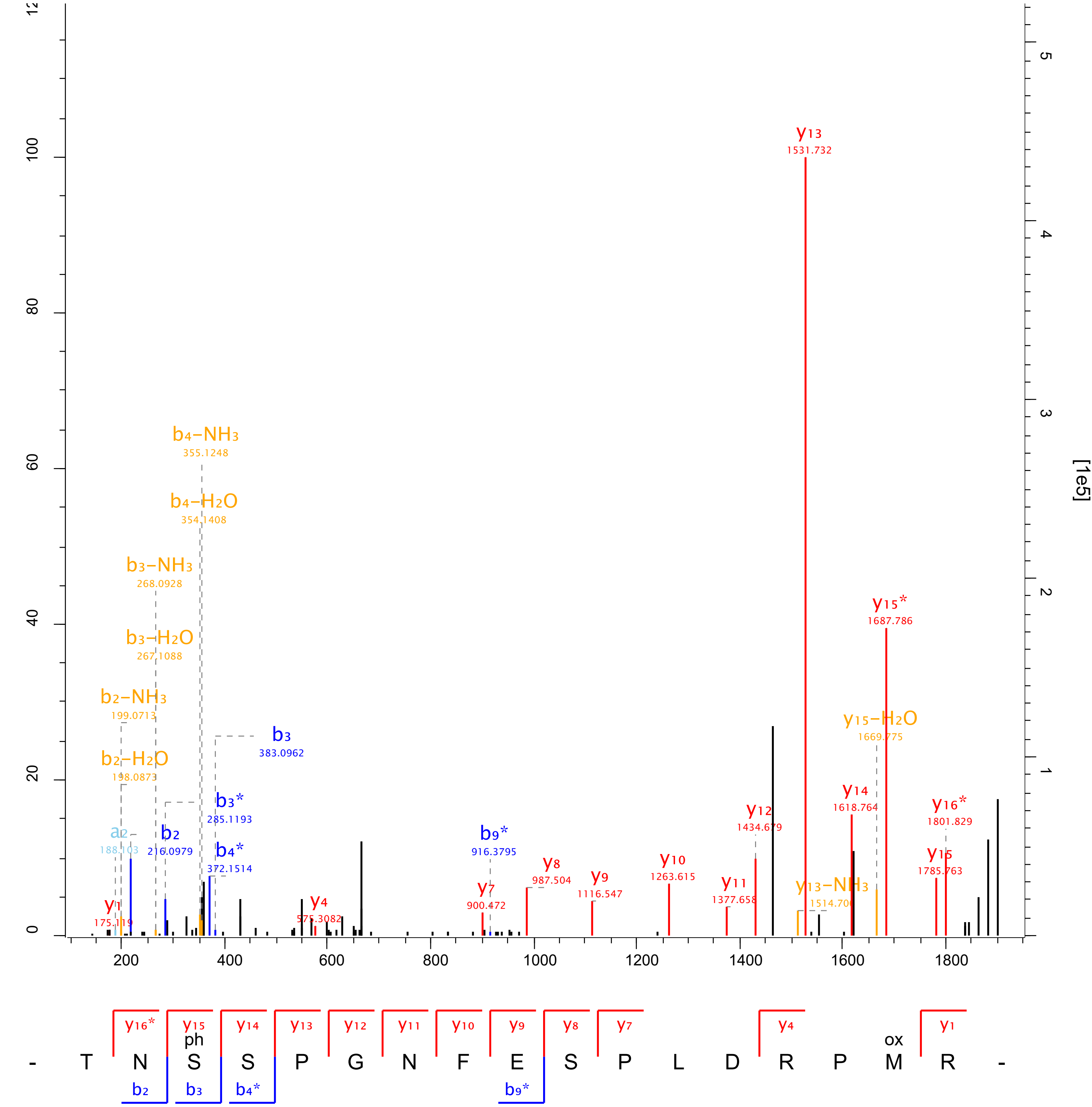

Sample output to test PDF Combine only

Raw file	Scan	Method	Score	m/z	Gene names
0523_1	13388	FTMS; HCD	56.72	623.63	At4g11860

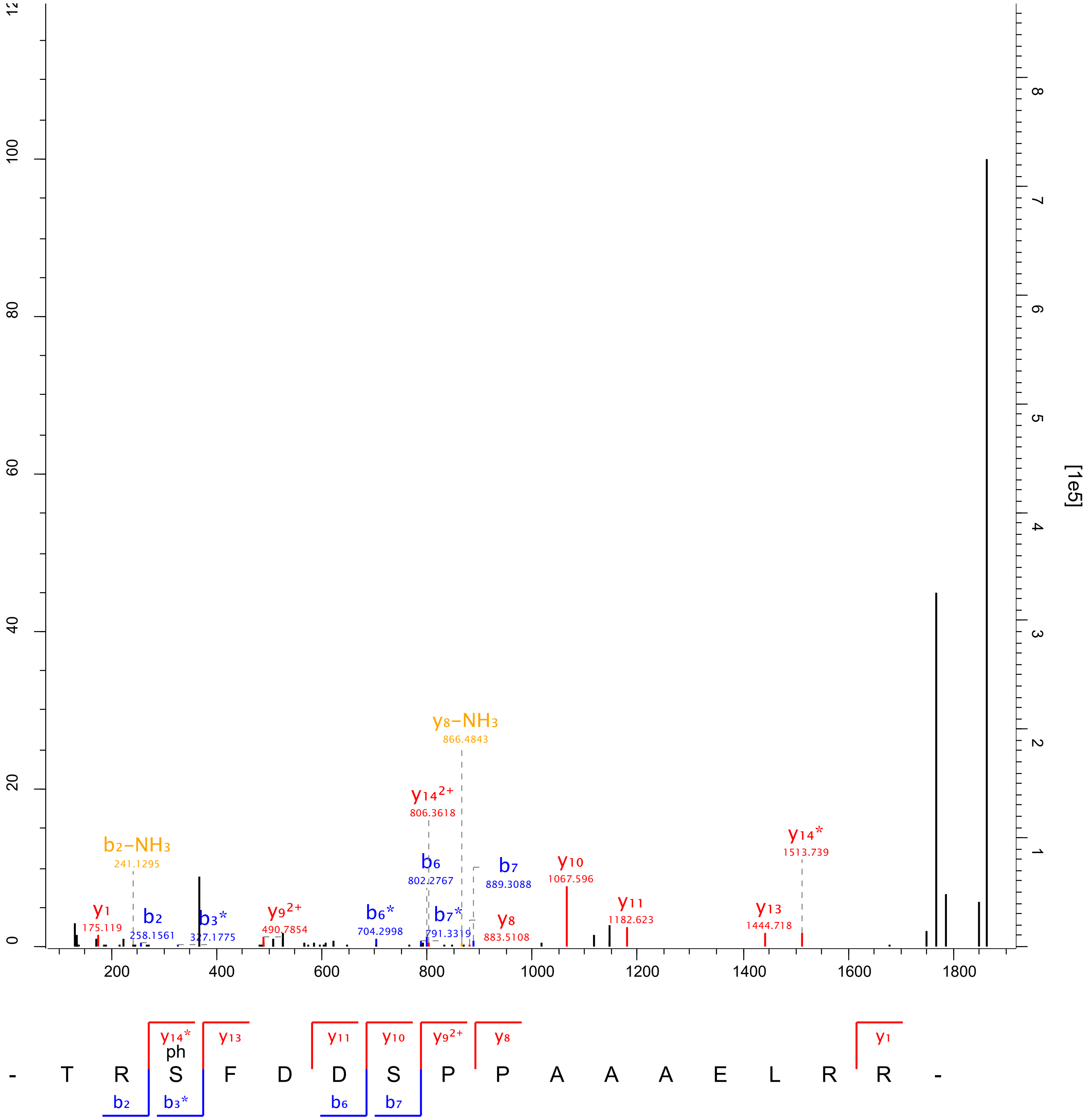

Sample output to test PDF Combine only

Raw file	Scan	Method	Score	m/z	Gene names
0523_1	13402	FTMS; HCD	44.63	802.37	At5g06440

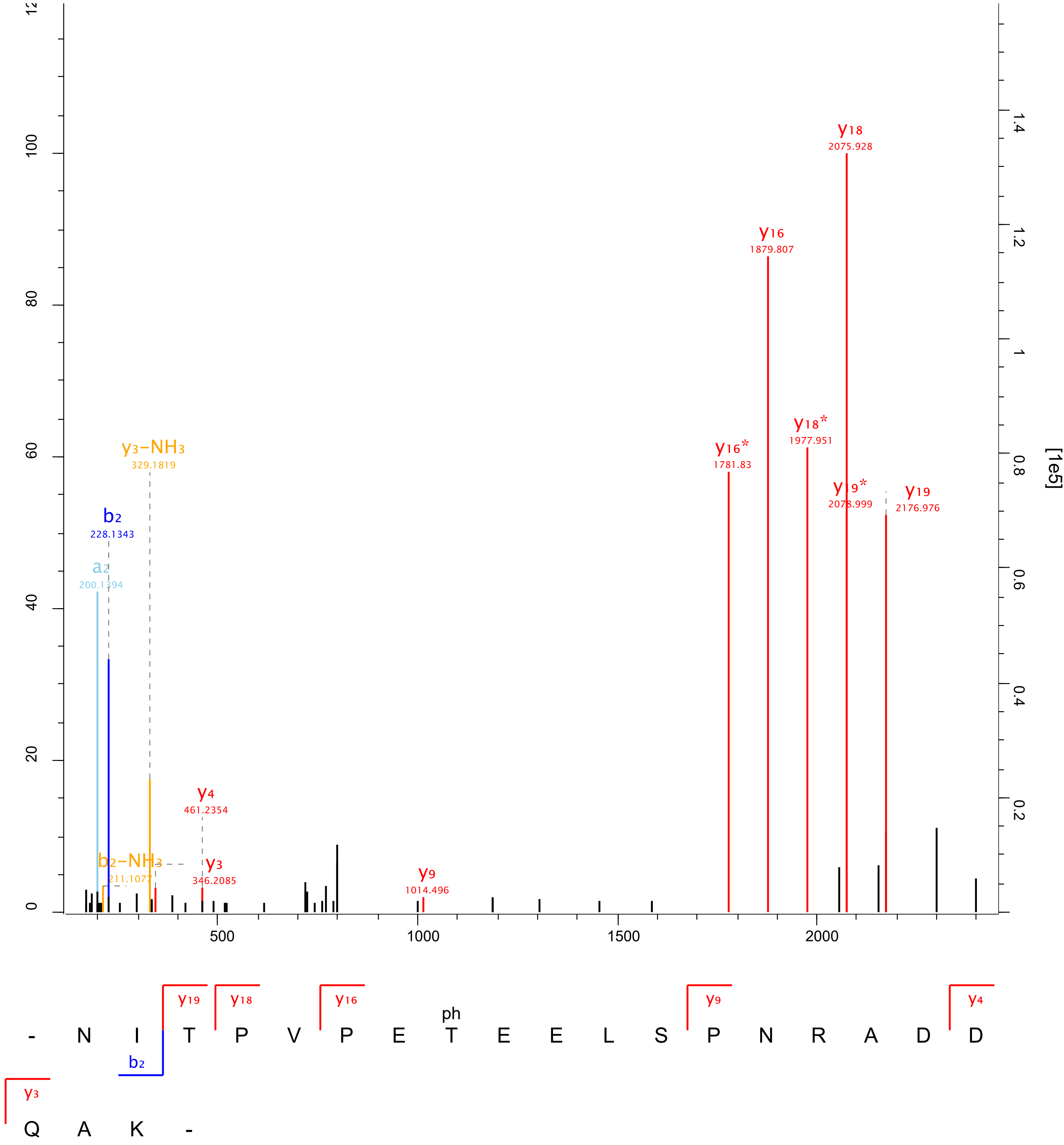

Sample output to test PDF Combine only

Raw file	Scan	Method	Score	m/z	Gene names
0523_1	13542	FTMS; HCD	177.02	626.62	PATL3

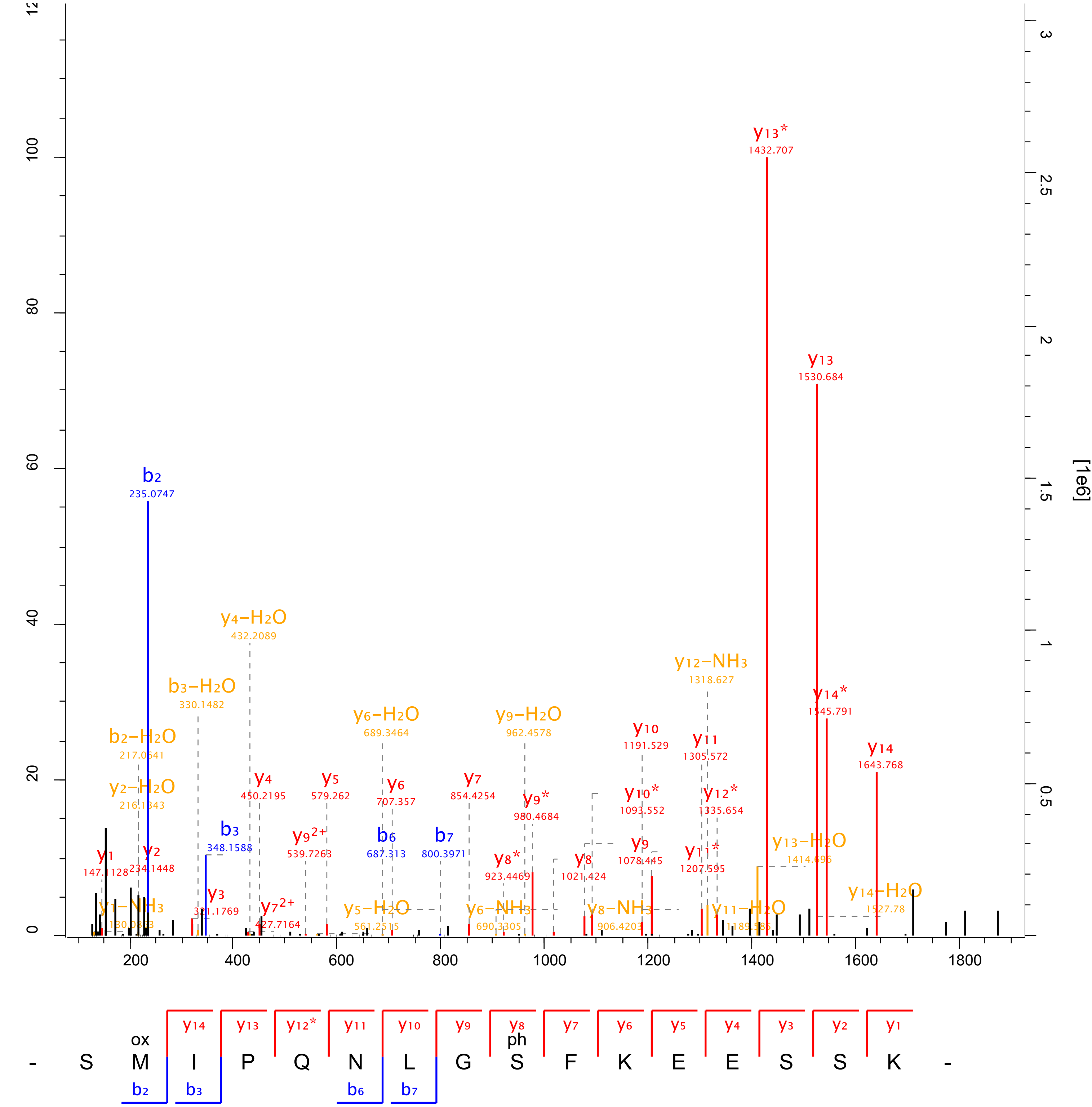

Sample output to test PDF Combine only

Raw file	Scan	Method	Score	m/z	Gene names
0523_1	13749	FTMS; HCD	102.51	904.09	PVA12

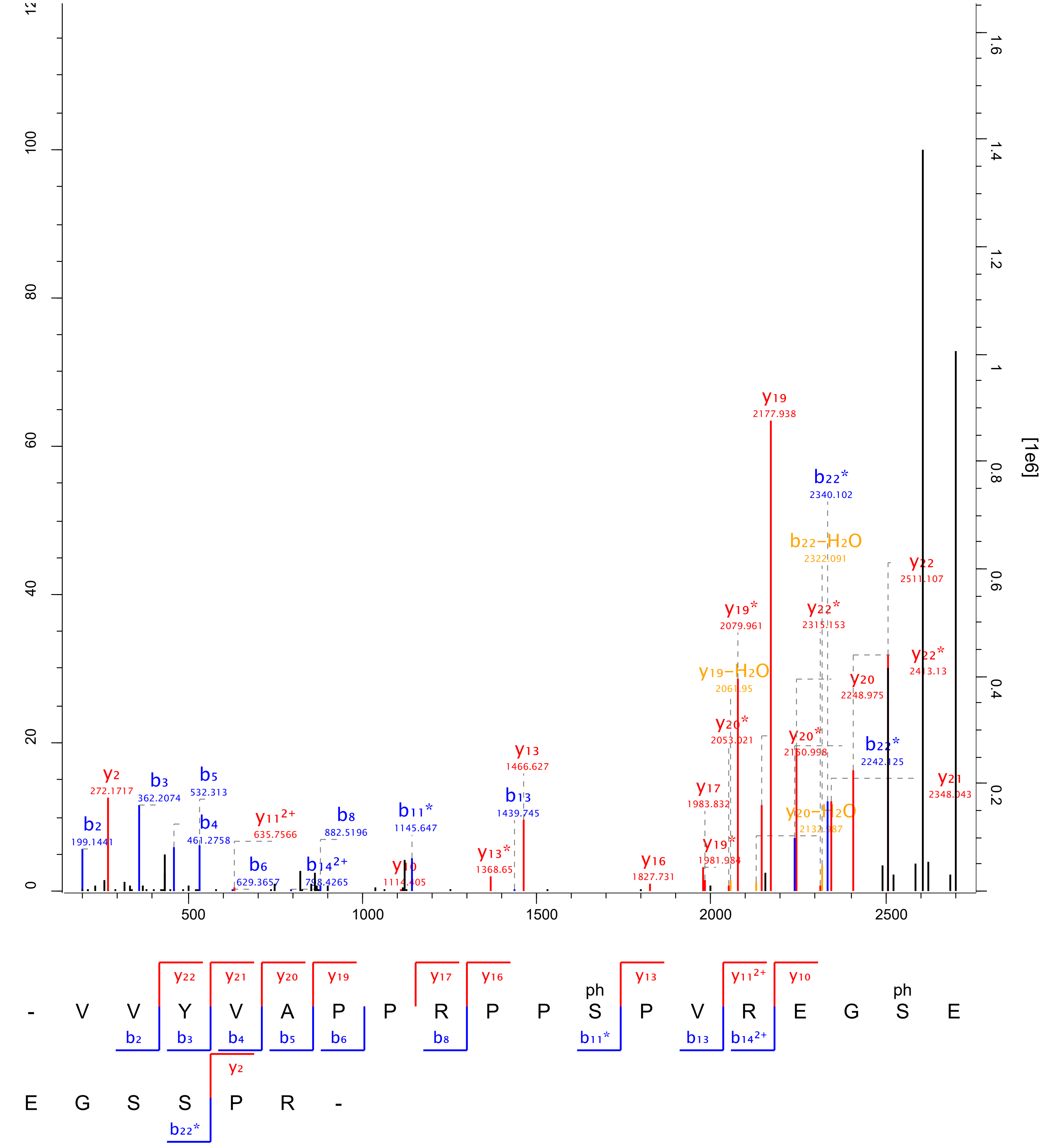

Sample output to test PDF Combine only

Raw file	Scan	Method	Score	m/z	Gene names
0523_1	13955	FTMS; HCD	240.83	1165.85	PPI1

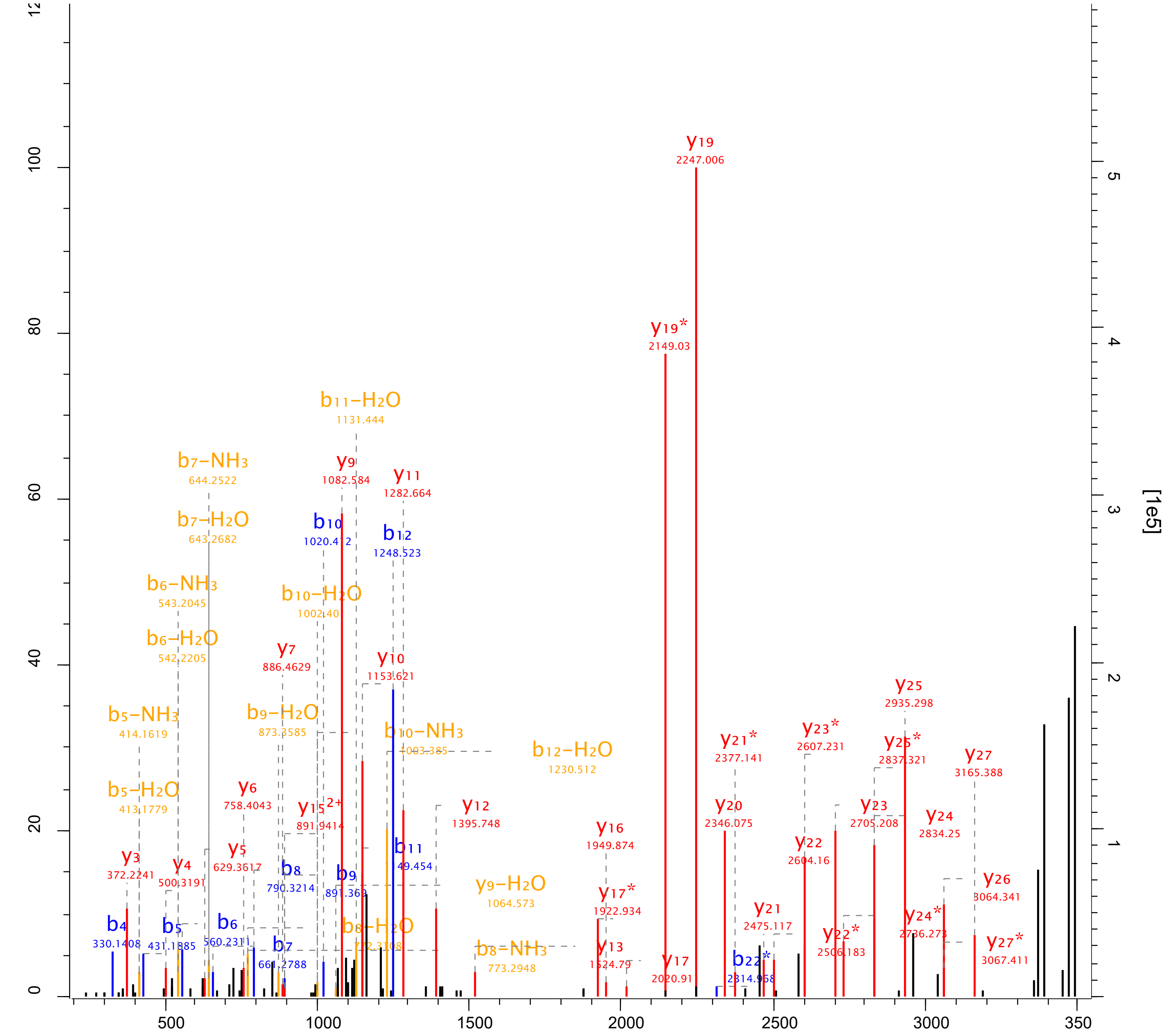

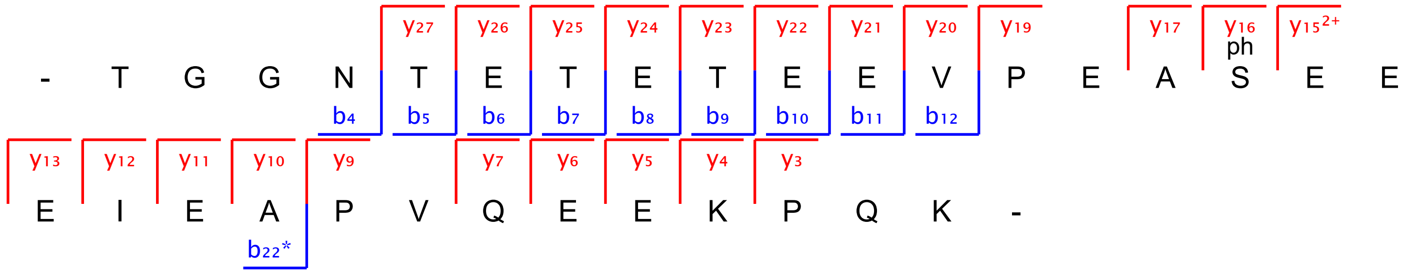

Sample output to test PDF Combine only

Raw file	Scan	Method	Score	m/z
0523_1	13959	FTMS; HCD	81.81	814.7

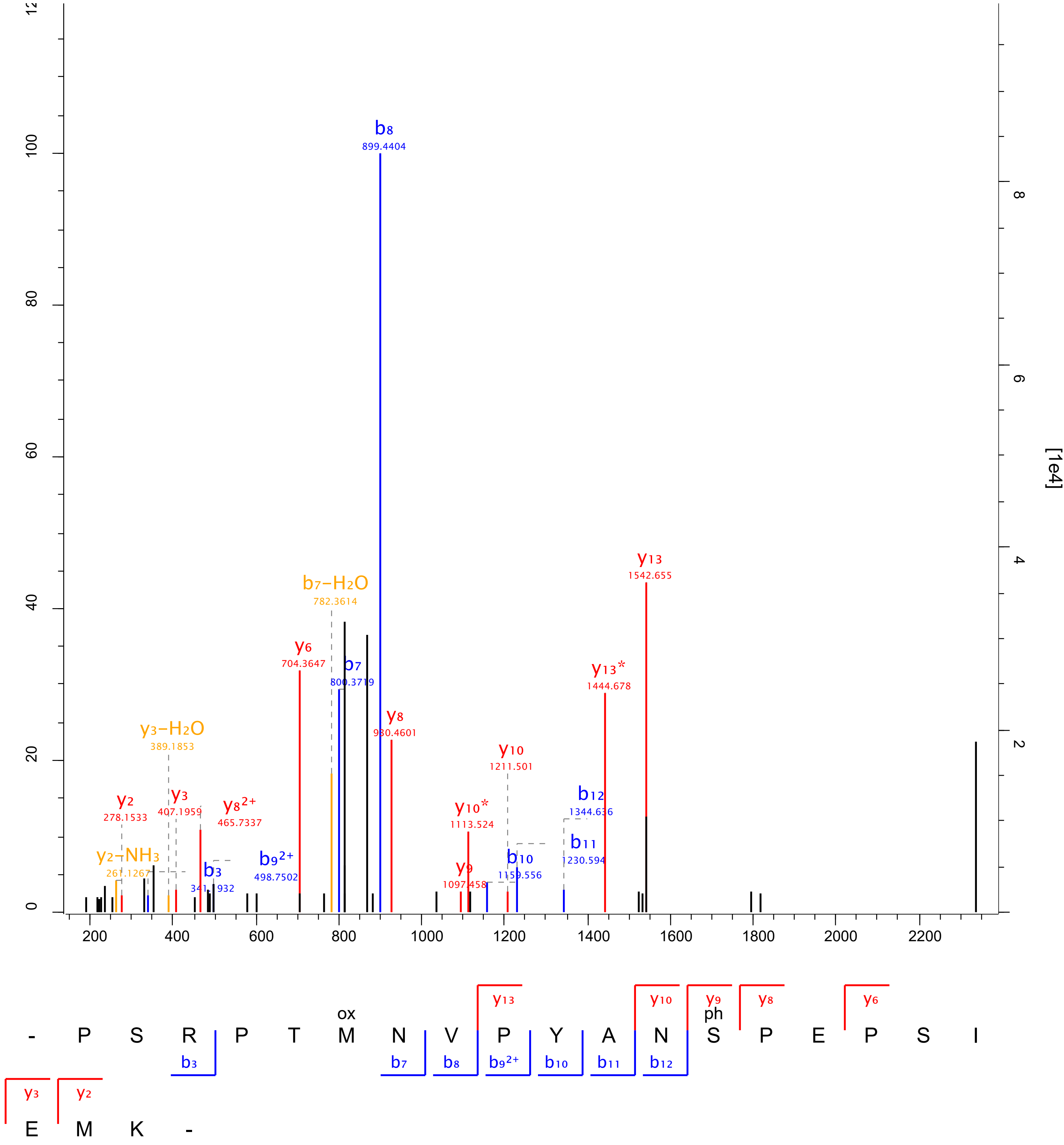

Sample output to test PDF Combine only

Raw file	Scan	Method	Score	m/z	Gene names
0523_1	14107	FTMS; HCD	83.99	779.01	F20B18.240

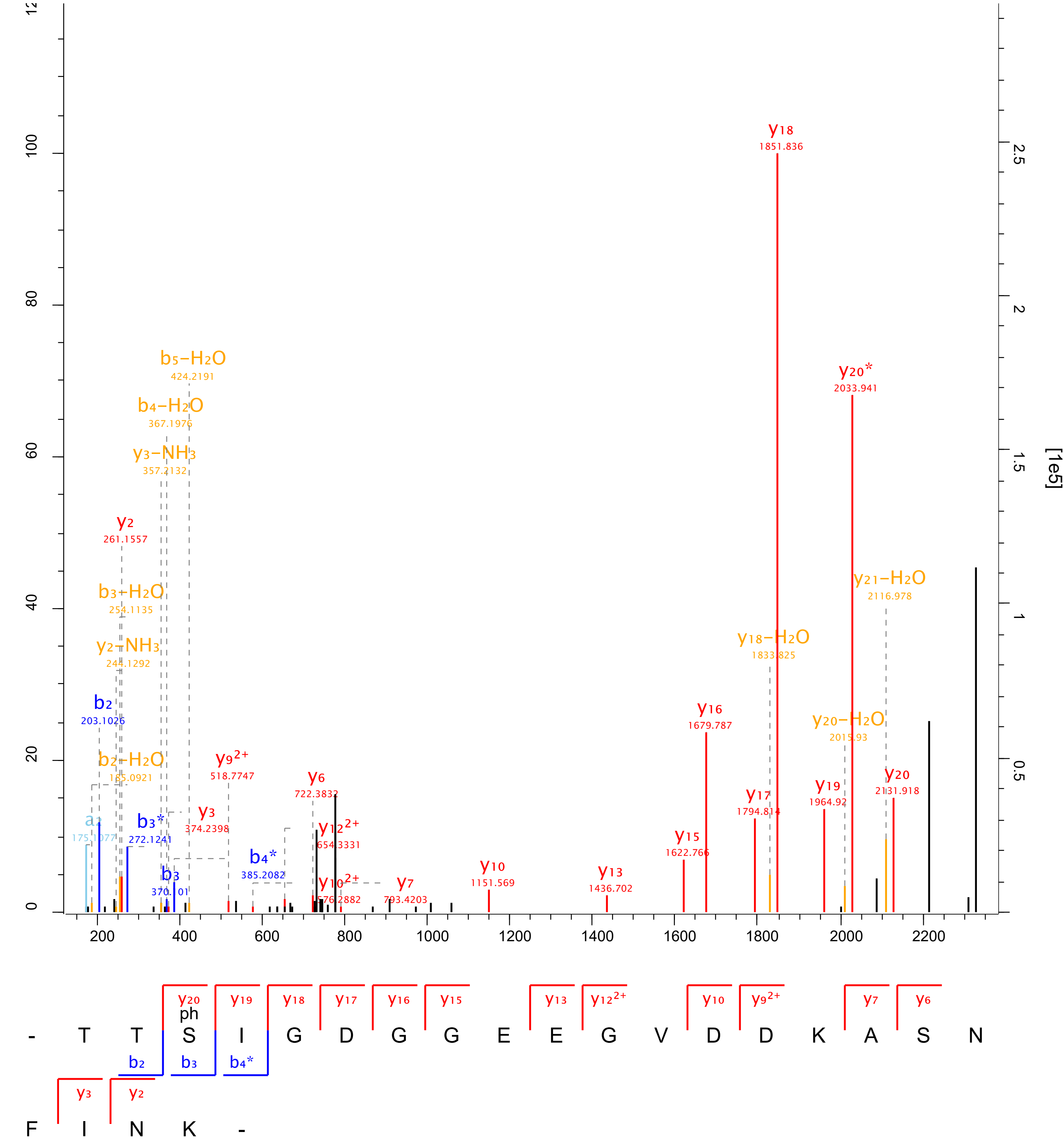

Sample output to test PDF Combine only

Raw file	Scan	Method	Score	m/z	Gene names
0523_1	14133	FTMS; HCD	43.16	705.06	PGM1

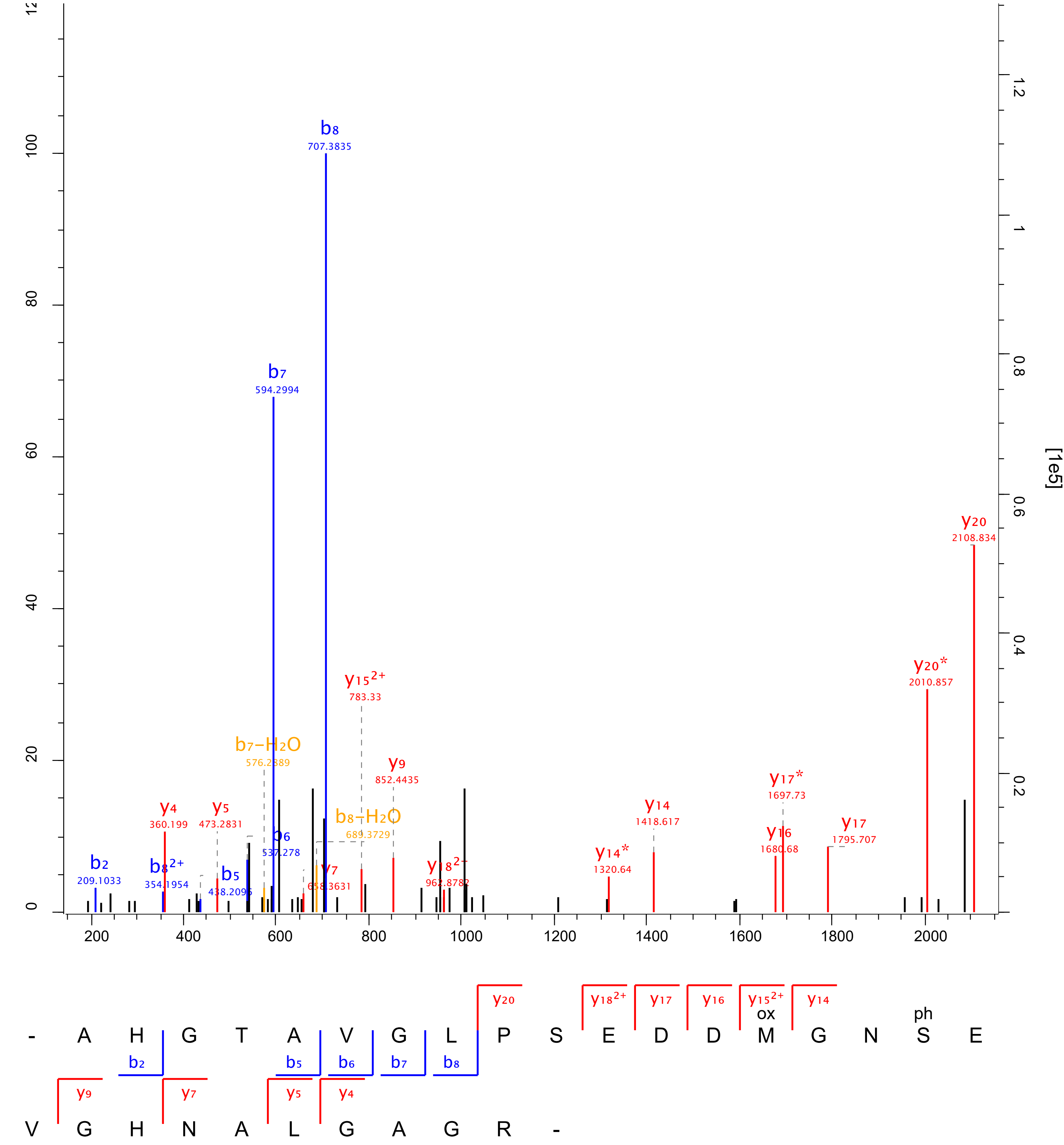

Sample output to test PDF Combine only

Raw file	Scan	Method	Score	m/z	Gene names
0523_1	14287	FTMS; HCD	99.22	802.71	MOR1

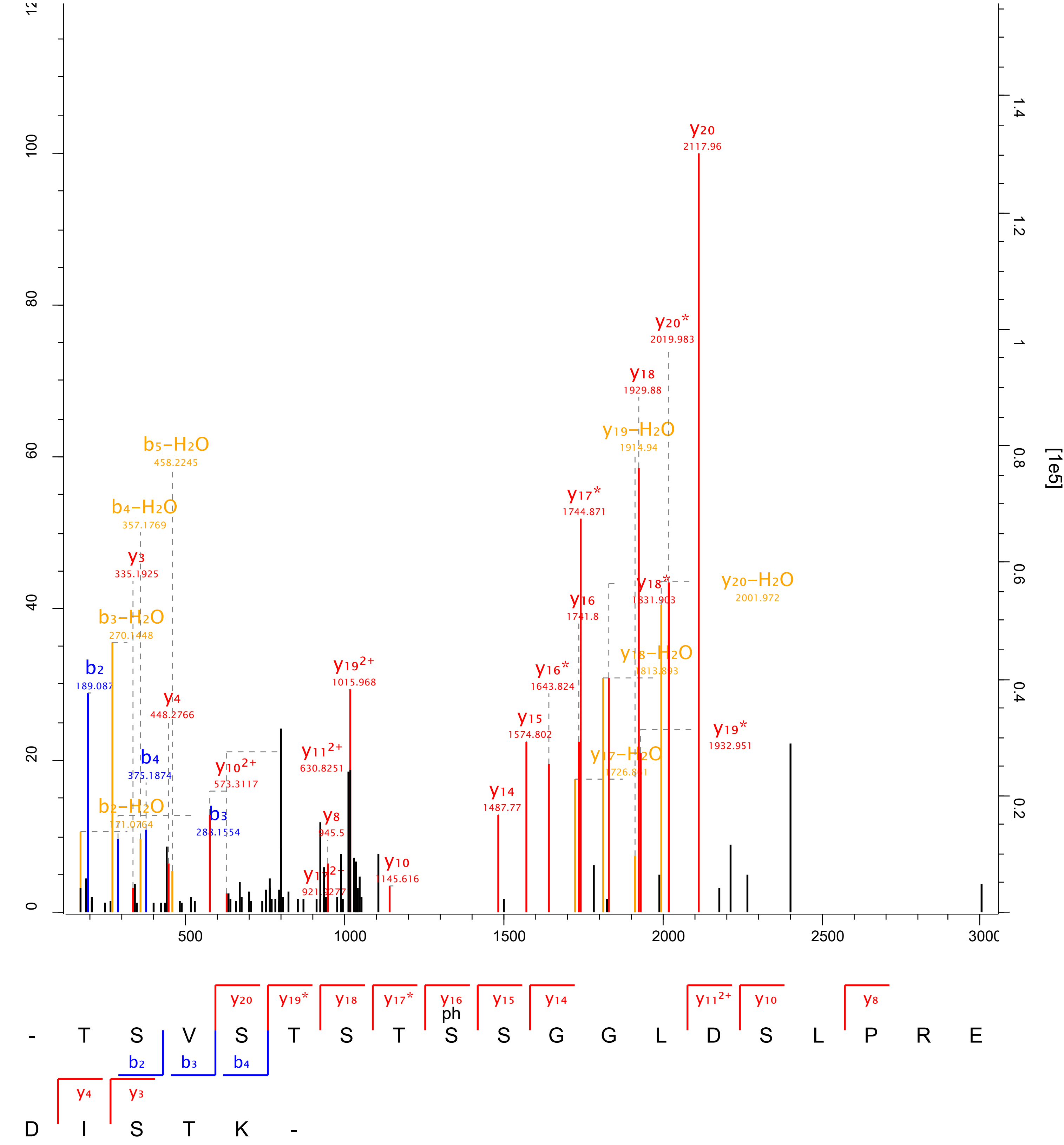

Sample output to test PDF Combine only

Raw file	Scan	Method	Score	m/z	Gene names
0523_1	14373	FTMS; HCD	45.89	633.99	At3g62900

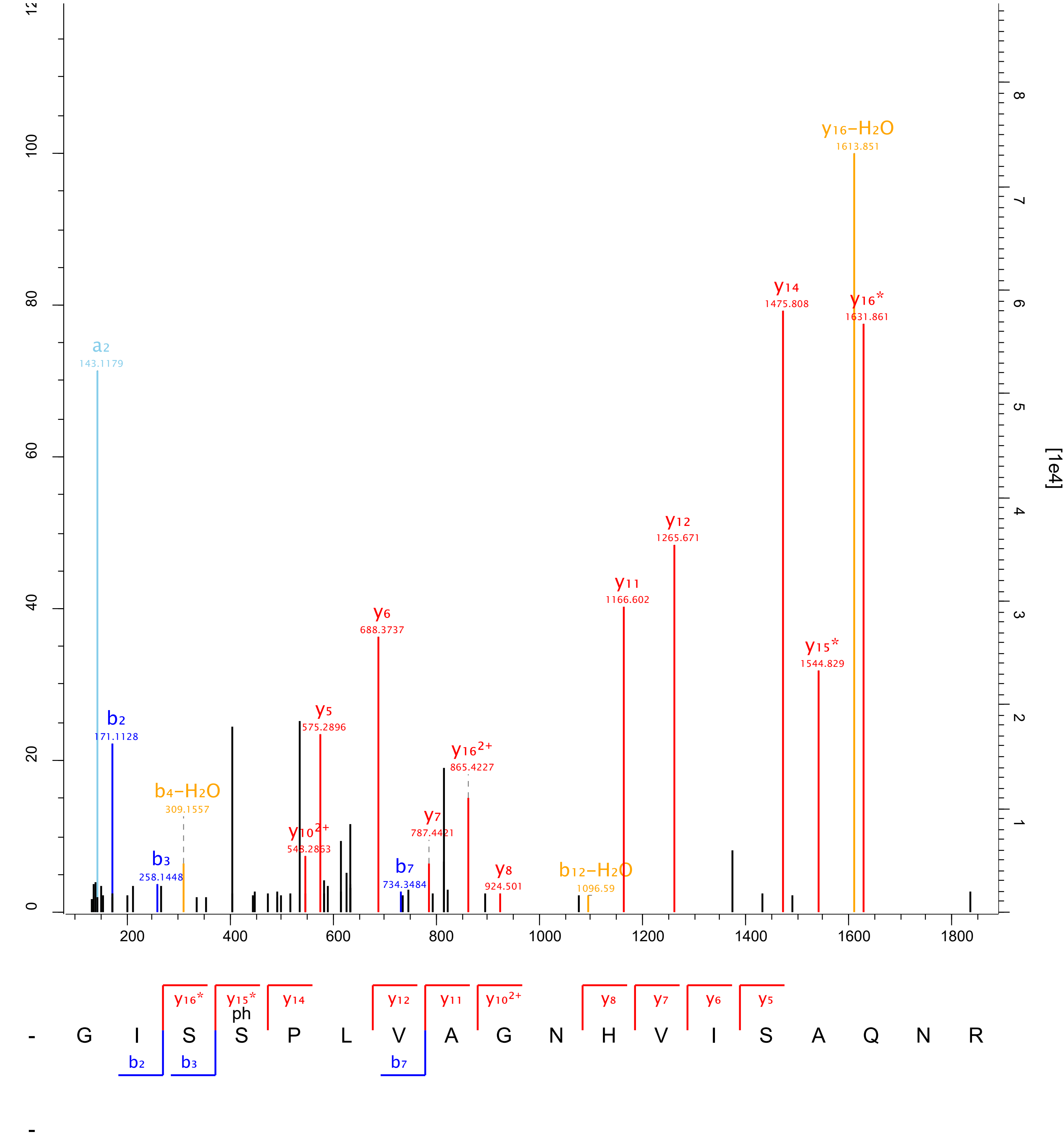

Sample output to test PDF Combine only

Raw file	Scan	Method	Score	m/z	Gene names
0523_1	14611	FTMS; HCD	96.14	604.62	SAC5

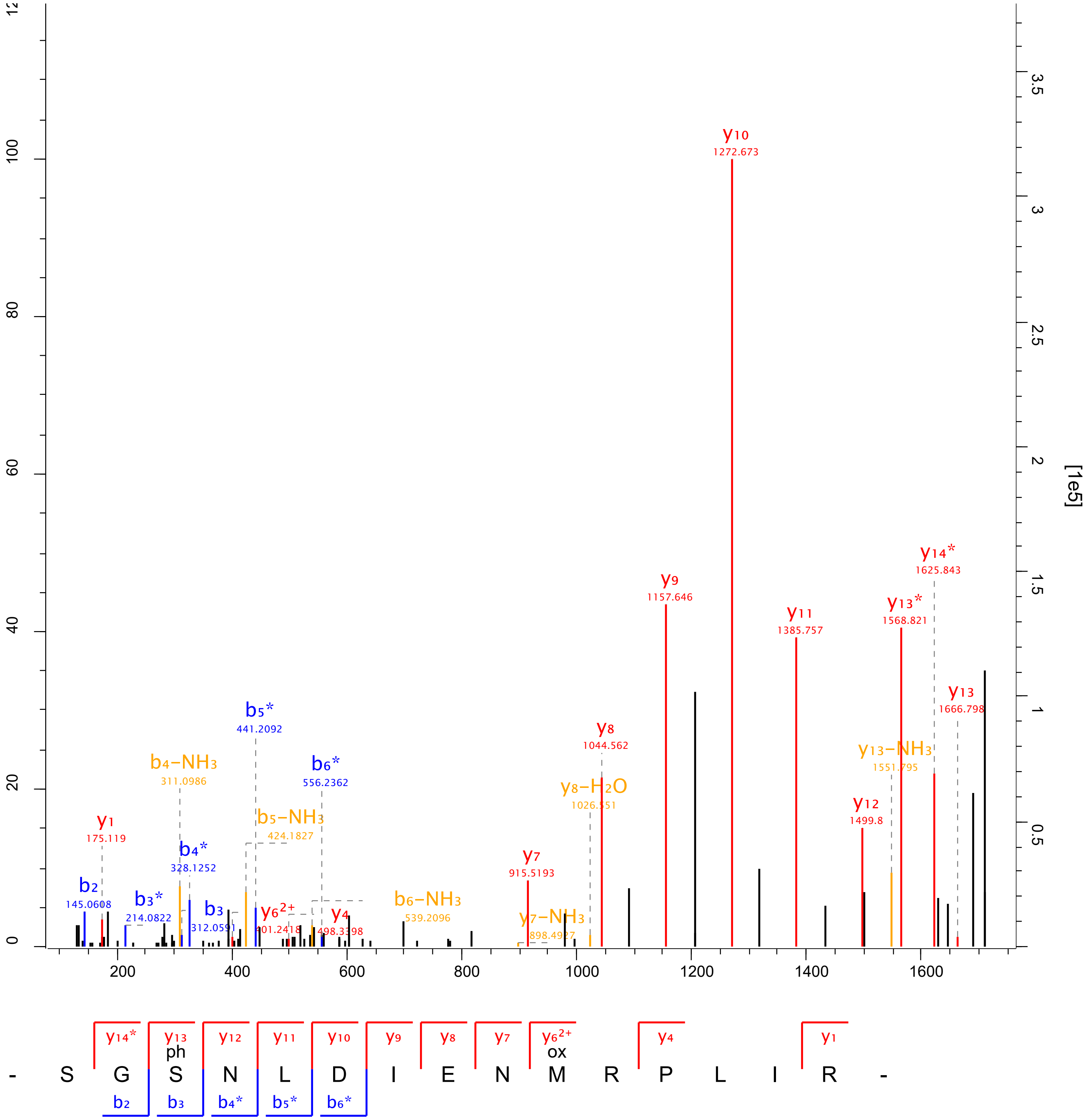

Sample output to test PDF Combine only

Raw file	Scan	Method	Score	m/z	Gene names
0523_1	14658	FTMS; HCD	147	916.96	TRX9

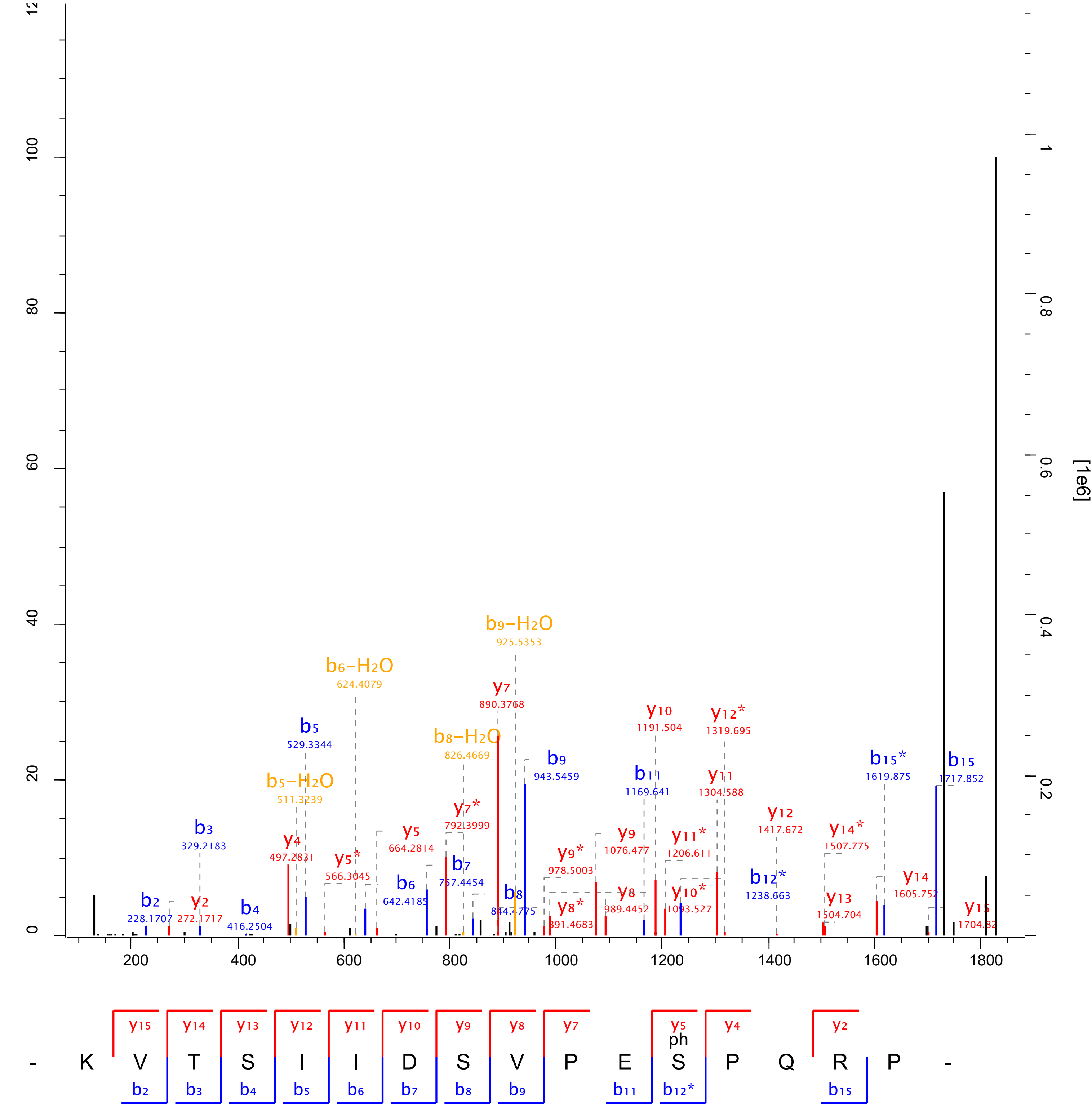

Sample output to test PDF Combine only

Raw file	Scan	Method	Score	m/z	Gene names
0523_1	14664	FTMS; HCD	42.79	762.35	PRP40A

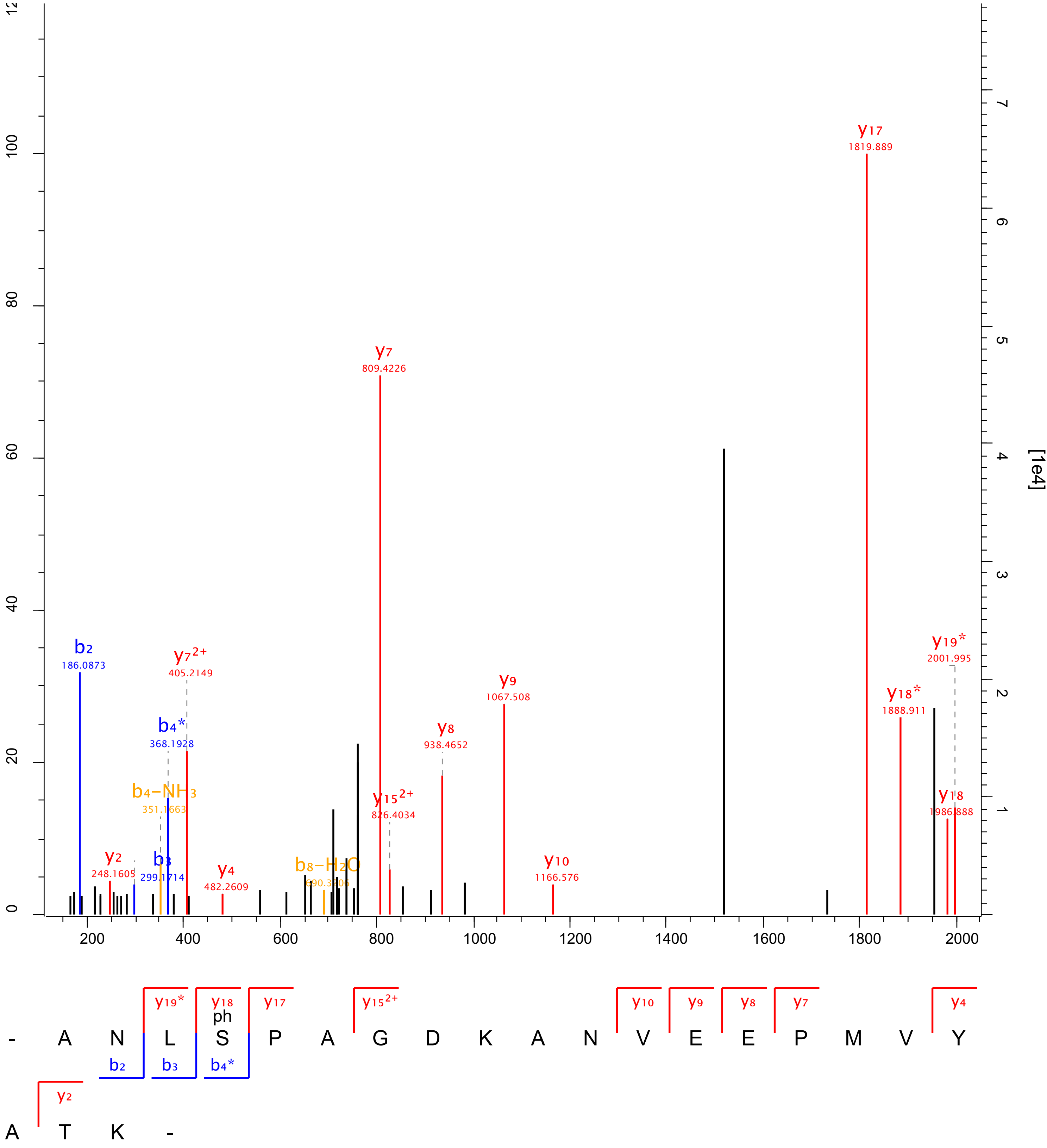

Sample output to test PDF Combine only

Raw file	Scan	Method	Score	m/z	Gene names
0523_1	14849	FTMS; HCD	155.09	466.89	F19C24.3

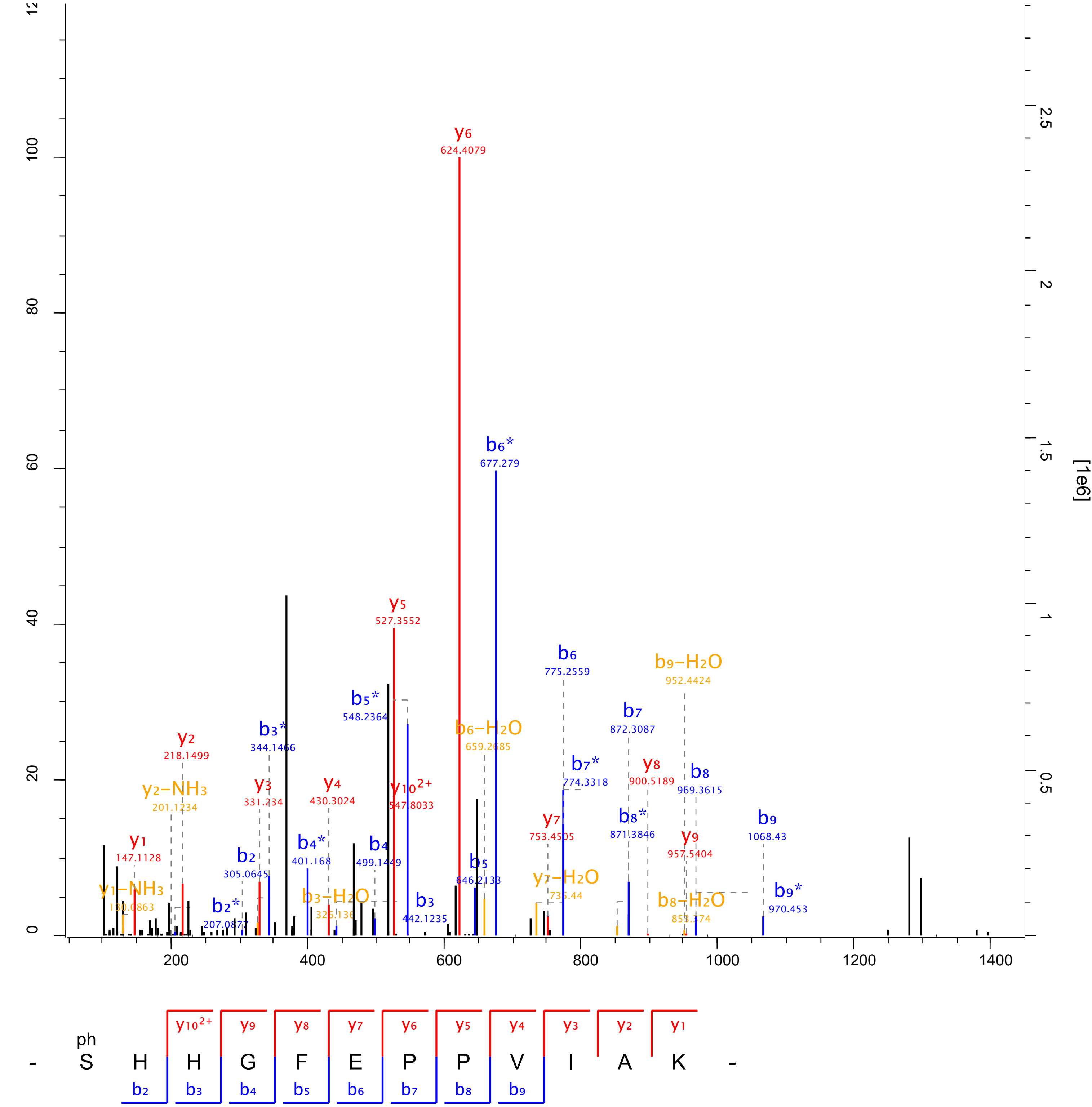

Sample output to test PDF Combine only

Raw file	Scan	Method	Score	m/z
0523_1	15010	FTMS; HCD	152.34	734.36

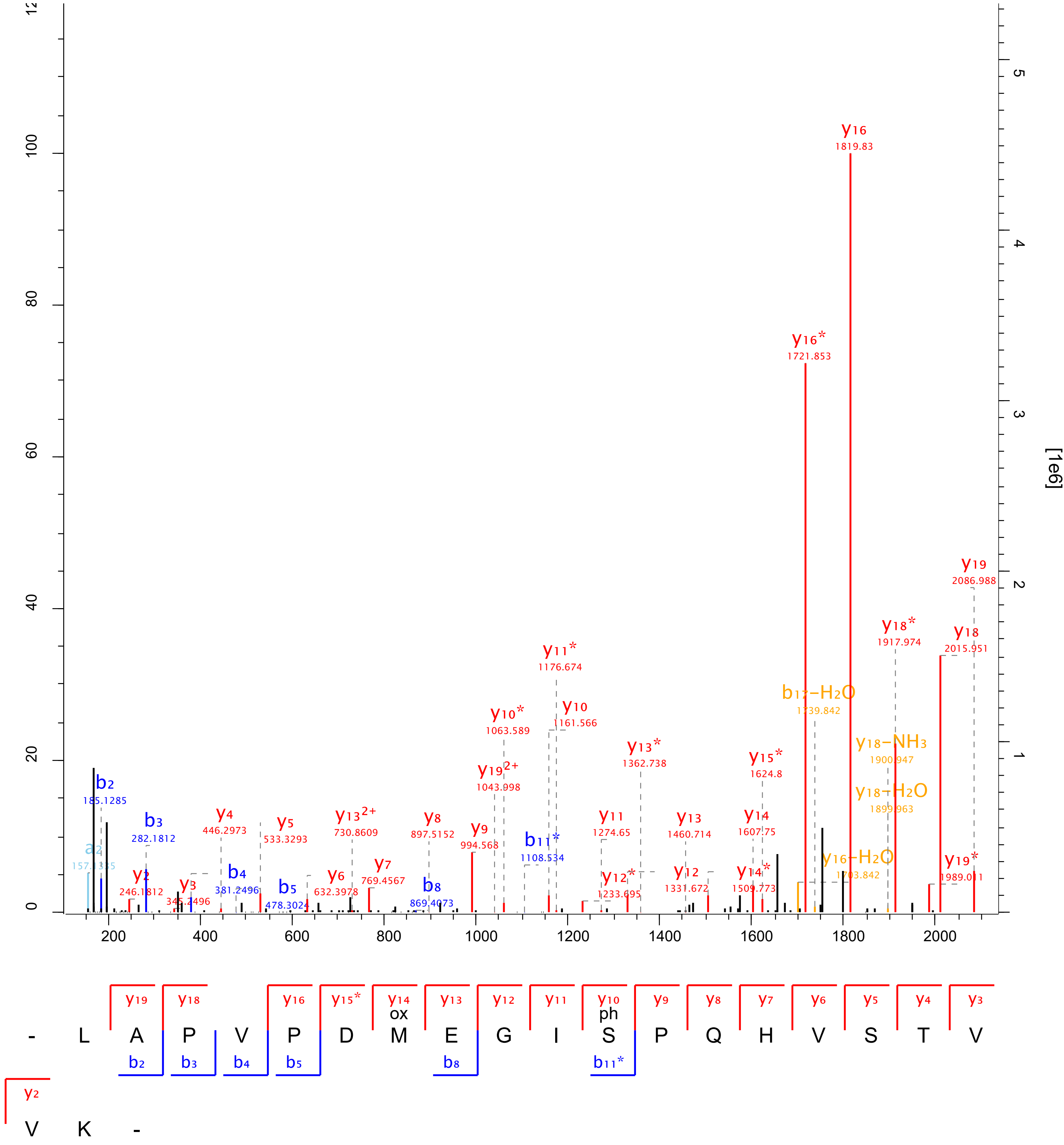

Sample output to test PDF Combine only

Raw file	Scan	Method	Score	m/z	Gene names
0523_1	15309	FTMS; HCD	43.31	919.11	AATP2

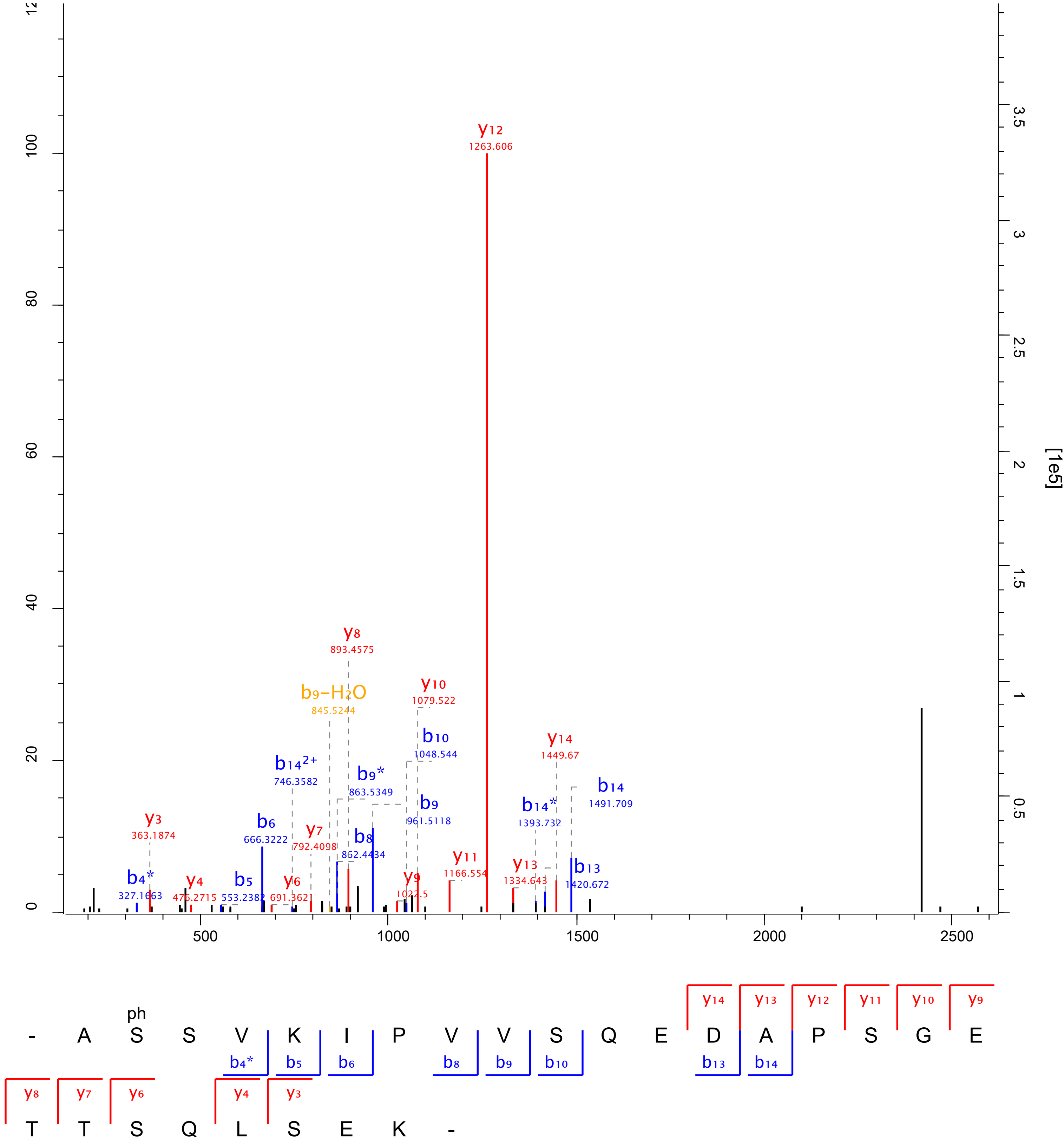

Sample output to test PDF Combine only

Raw file	Scan	Method	Score	m/z	Gene names
0523_1	15408	FTMS; HCD	44.25	878.38	PVA12

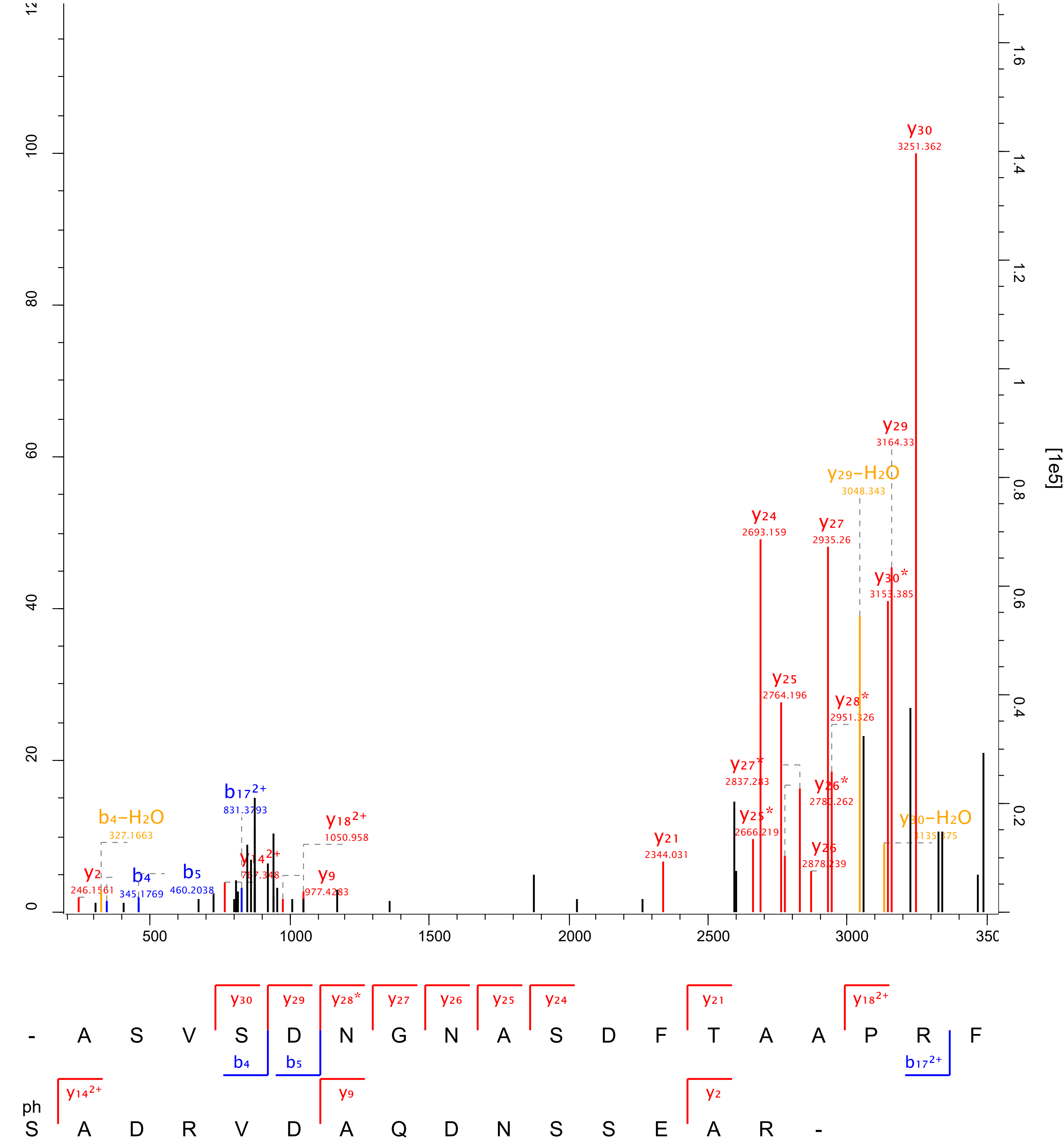

Sample output to test PDF Combine only

Raw file	Scan	Method	Score	m/z	Gene names
0523_1	15487	FTMS; HCD	60.55	589.26	MLO6

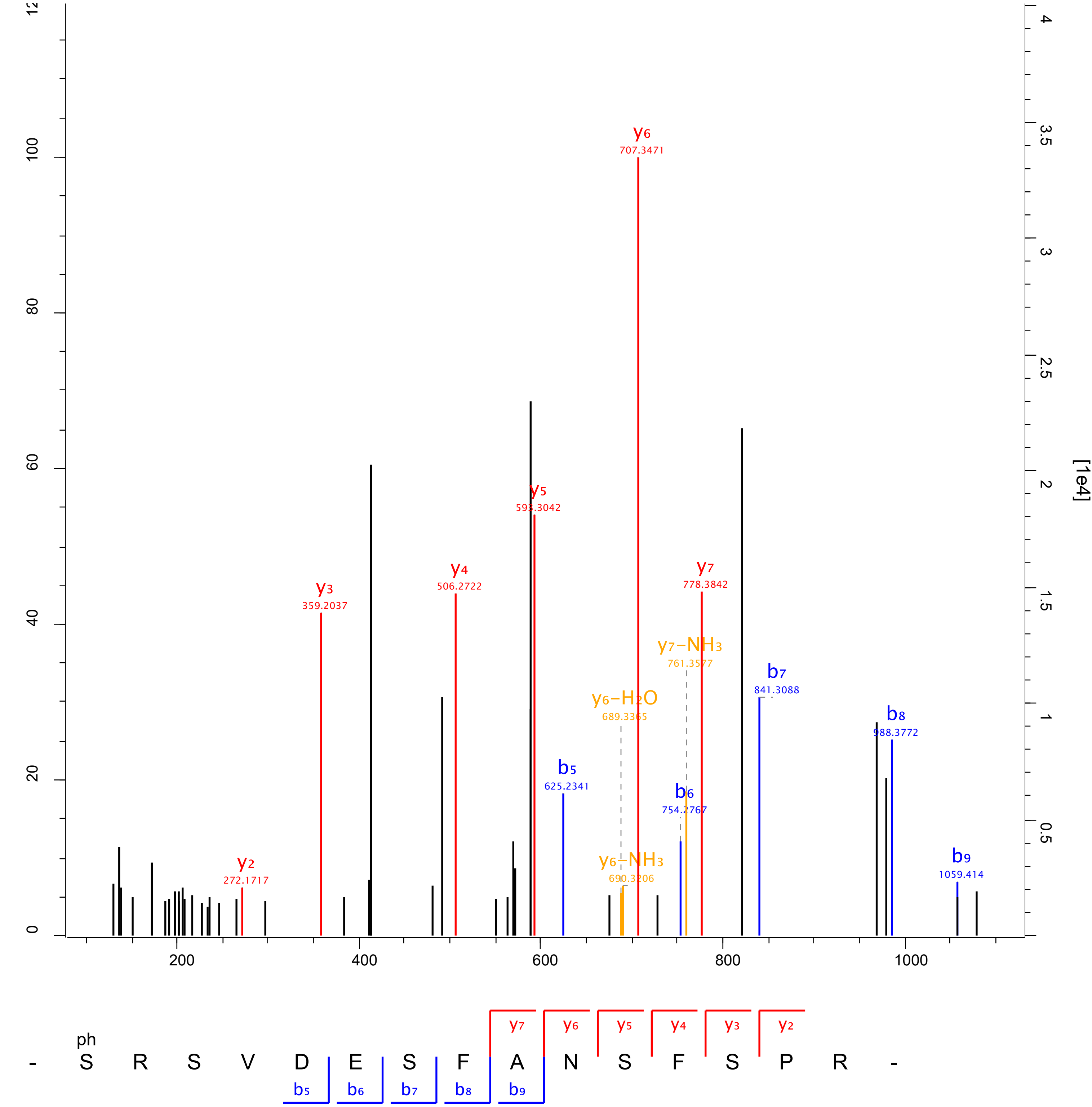

Sample output to test PDF Combine only

Raw file	Scan	Method	Score	m/z
0523_1	15503	FTMS; HCD	181.56	727.35

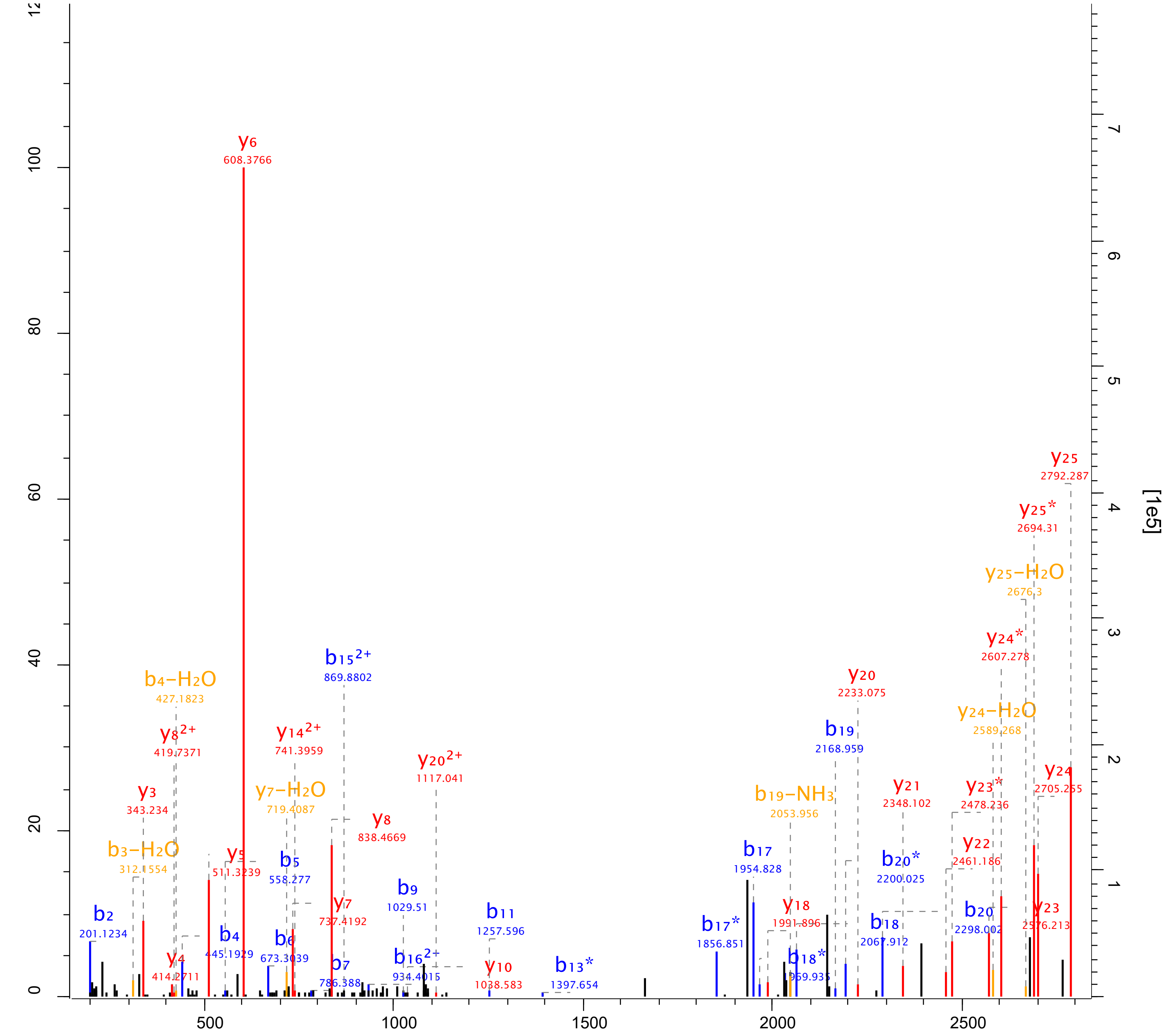

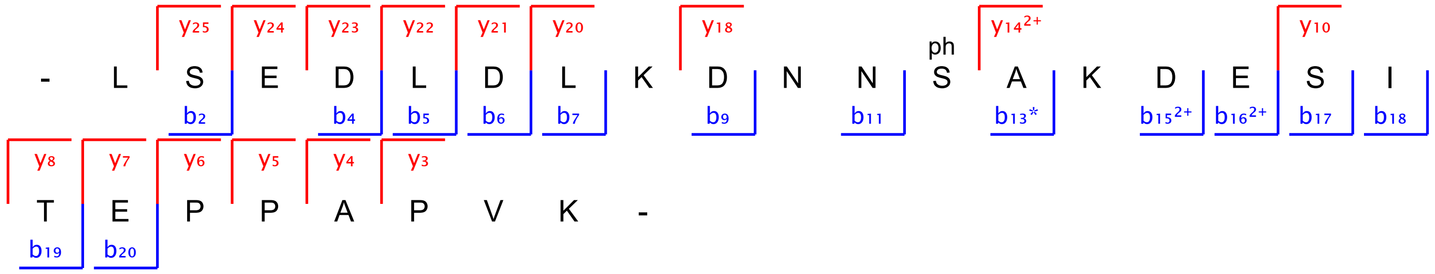

Sample output to test PDF Combine only

Raw file	Scan	Method	Score	m/z	Gene names
0523_1	15773	FTMS; HCD	92.47	770.01	At5g53440

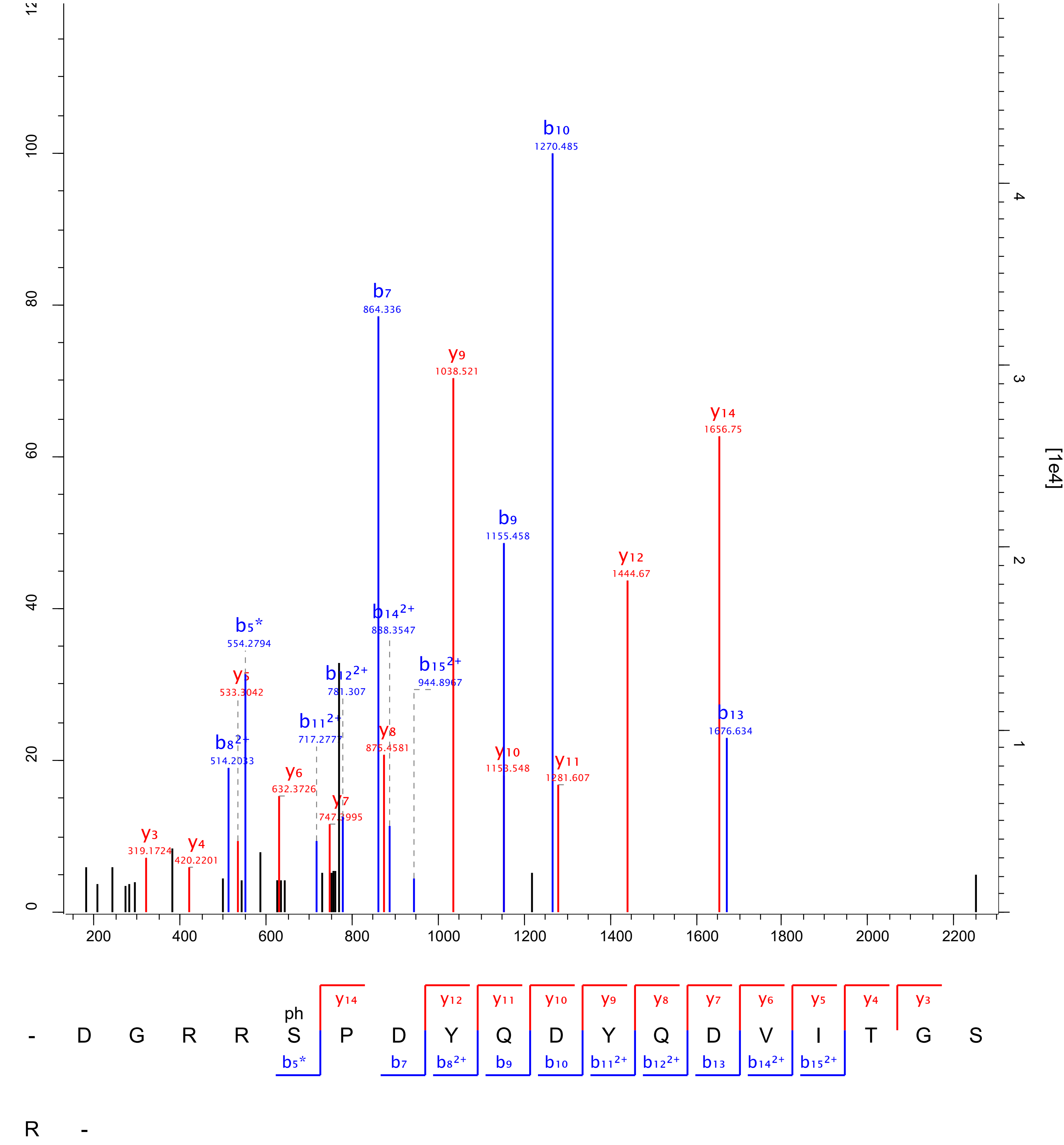

Sample output to test PDF Combine only

Raw file	Scan	Method	Score	m/z	Gene names
0523_1	16079	FTMS; HCD	72.3	821.71	F13I12.260

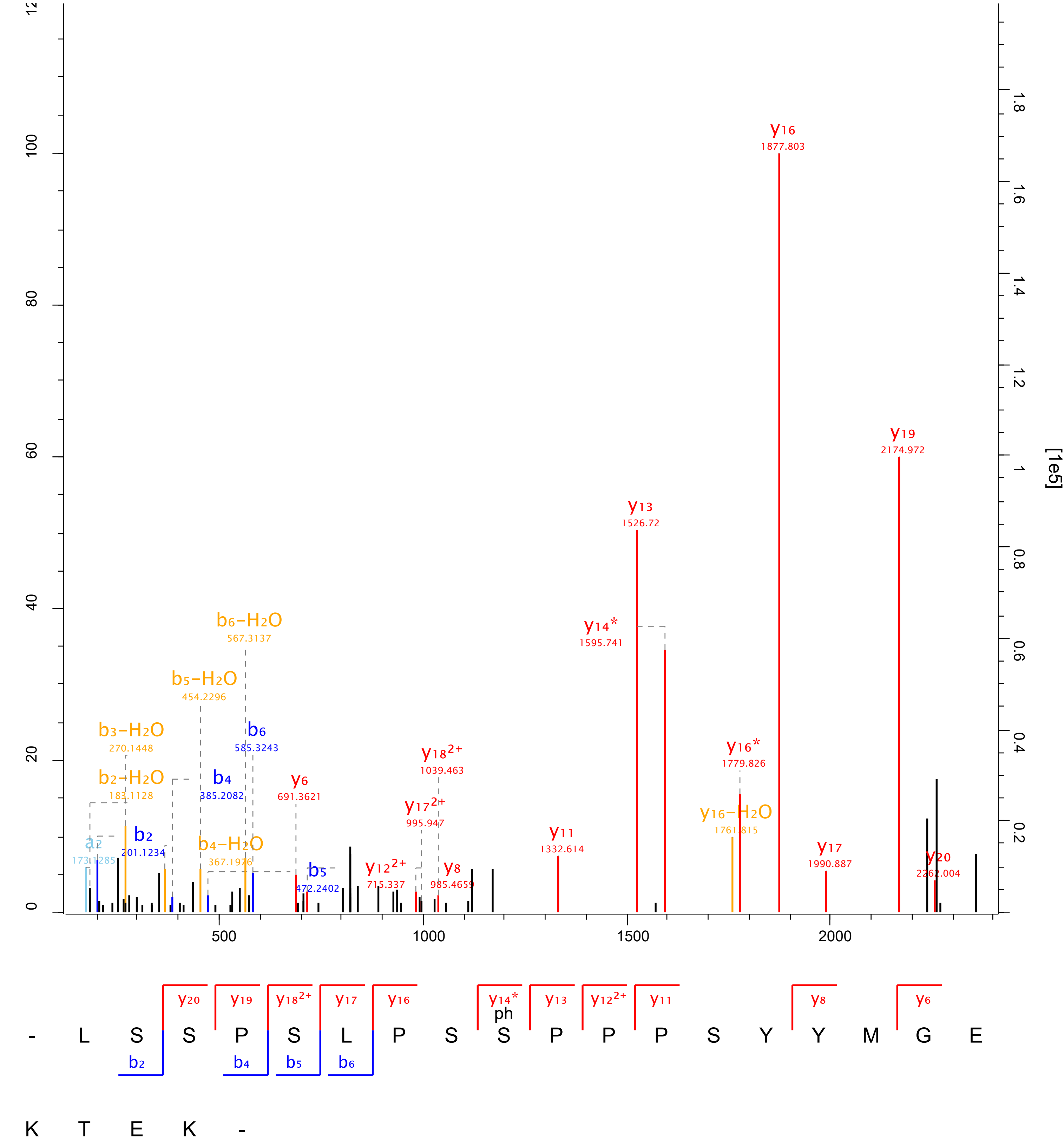

Sample output to test PDF Combine only

Raw file	Scan	Method	Score	m/z	Gene names
0523_1	16297	FTMS; HCD	80.84	666.96	CASP

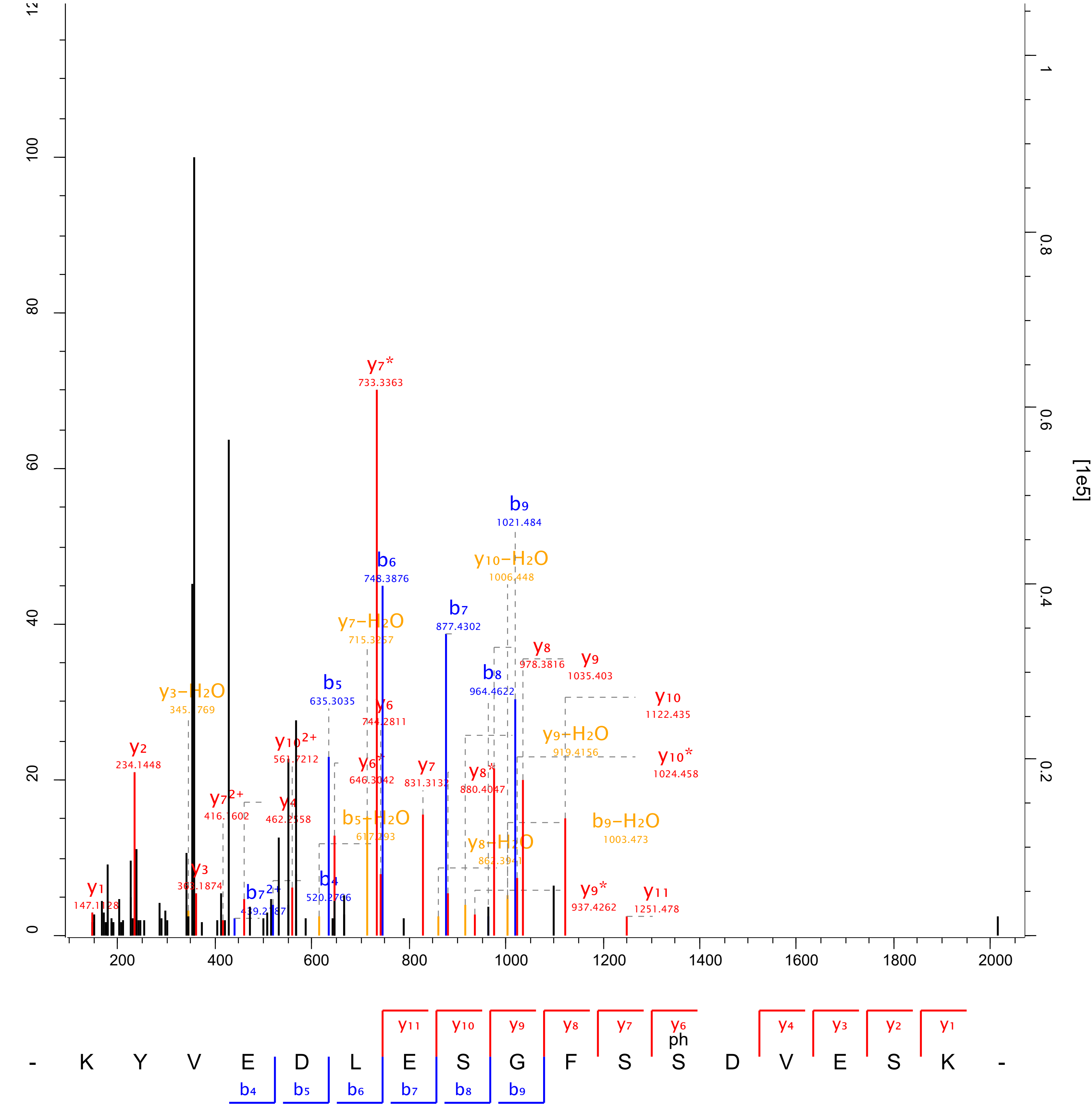

Sample output to test PDF Combine only

Raw file	Scan	Method	Score	m/z	Gene names
0523_1	16321	FTMS; HCD	183.64	877.39	DUR3

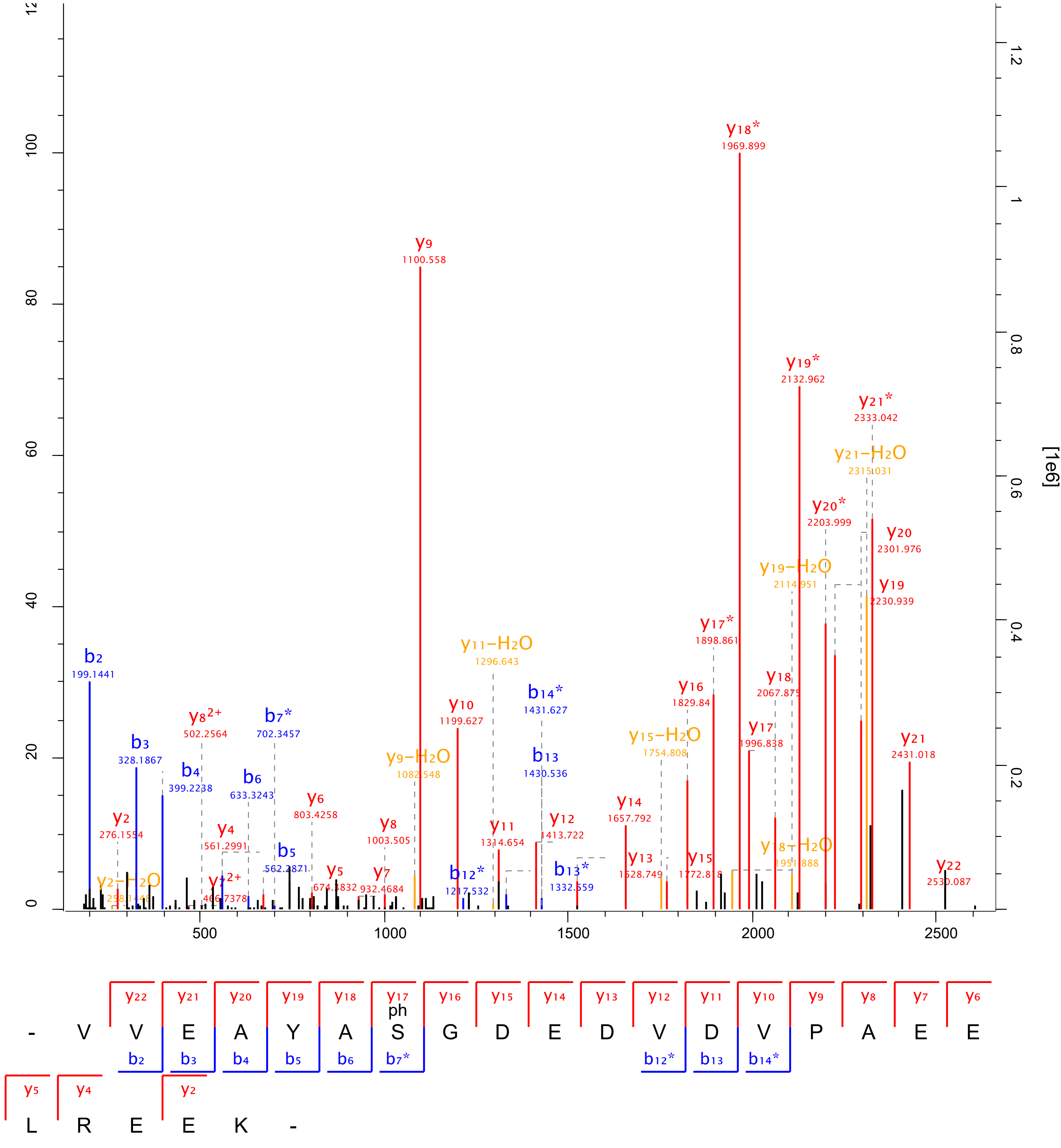

Sample output to test PDF Combine only

Raw file	Scan	Method	Score	m/z	Gene names
0523_1	16438	FTMS; HCD	214.19	843.72	RBL15

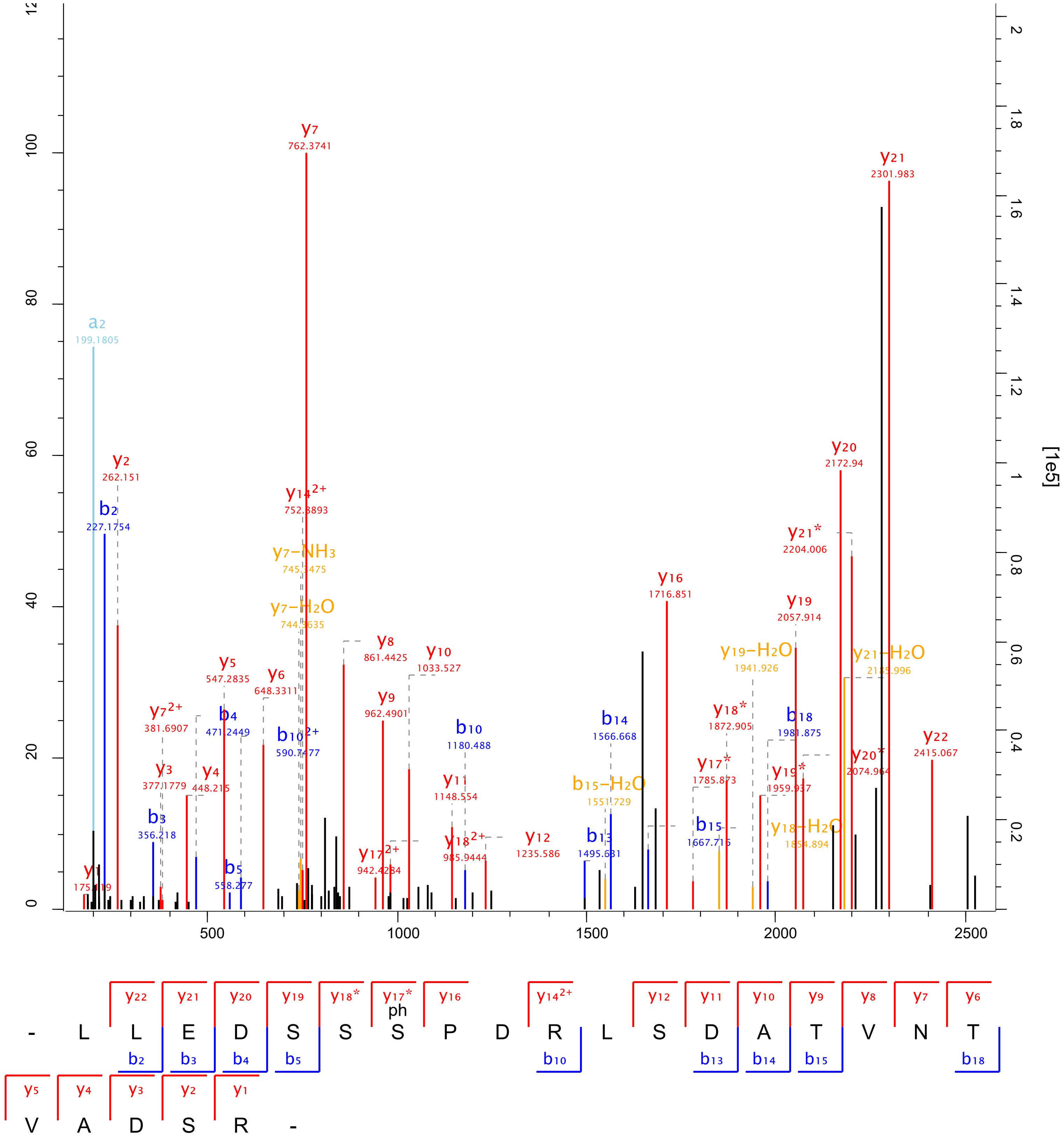

Sample output to test PDF Combine only

Raw file	Scan	Method	Score	m/z	Gene names
0523_1	16491	FTMS; HCD	75.53	674.67	BSL2

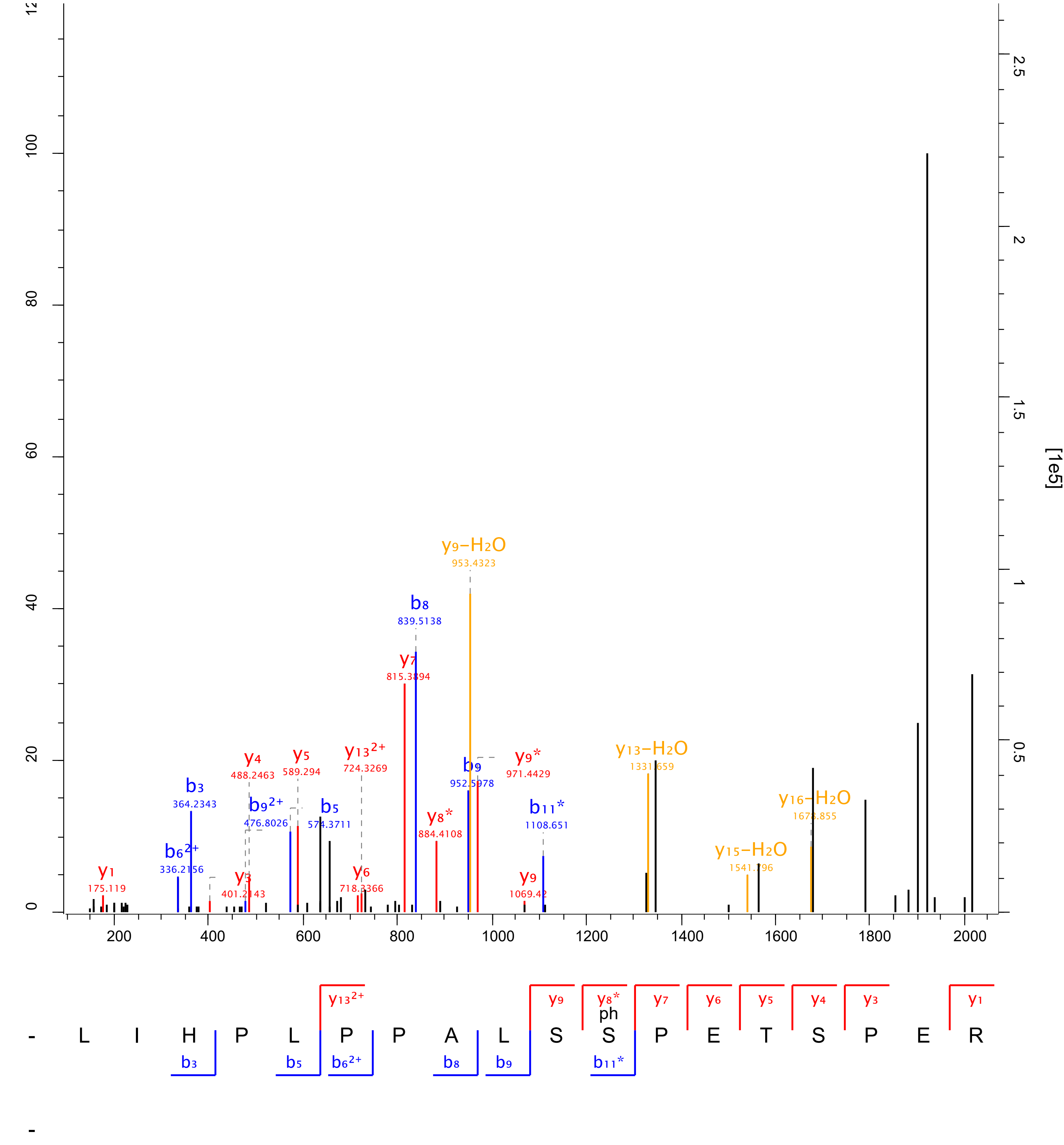

Sample output to test PDF Combine only

Raw file	Scan	Method	Score	m/z	Gene names
0523_1	16712	FTMS; HCD	75.53	679.35	BSL3

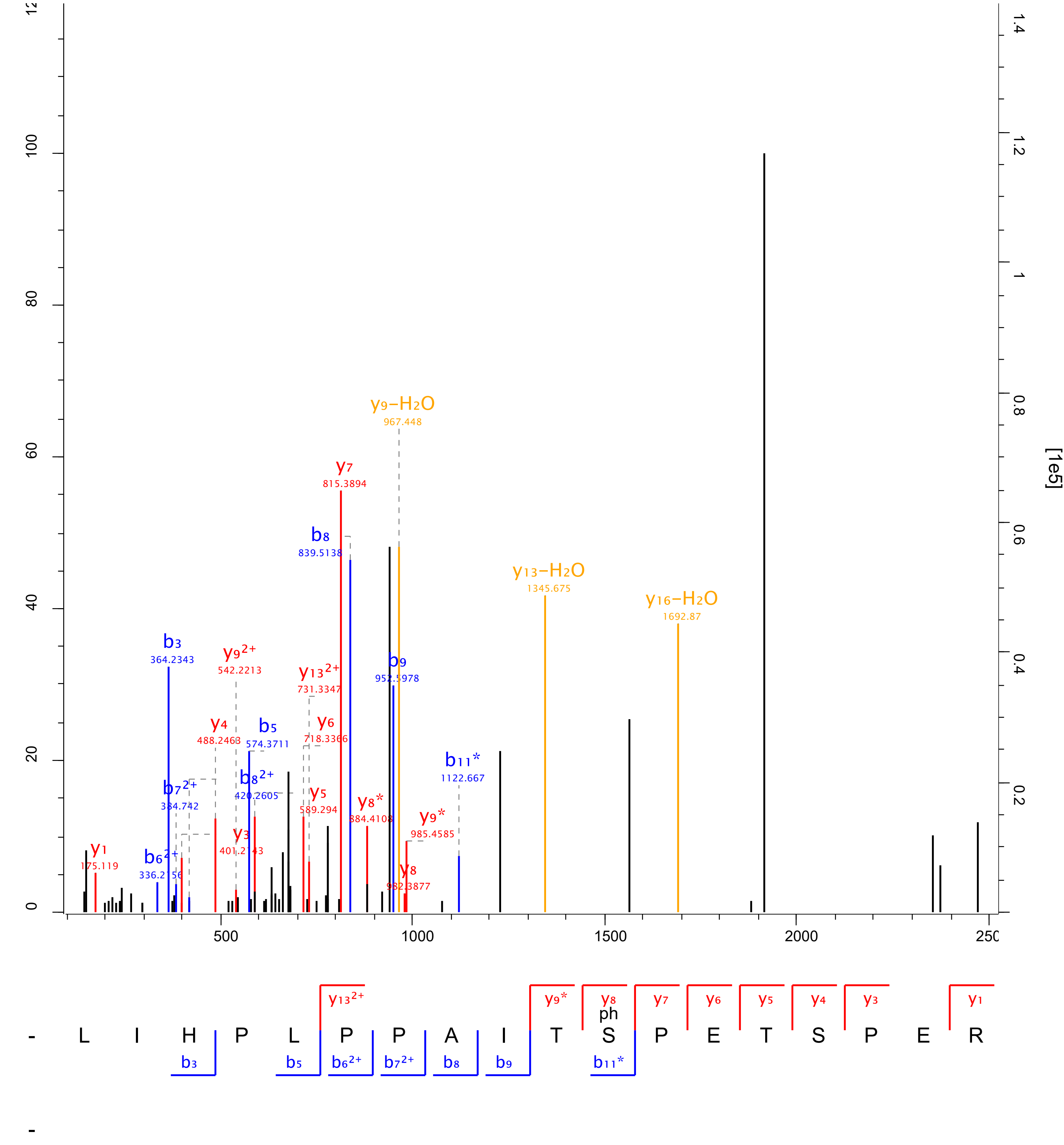

Sample output to test PDF Combine only

Raw file	Scan	Method	Score	m/z	Gene names
0523_1	16961	FTMS; HCD	89.86	619.98	PAT07

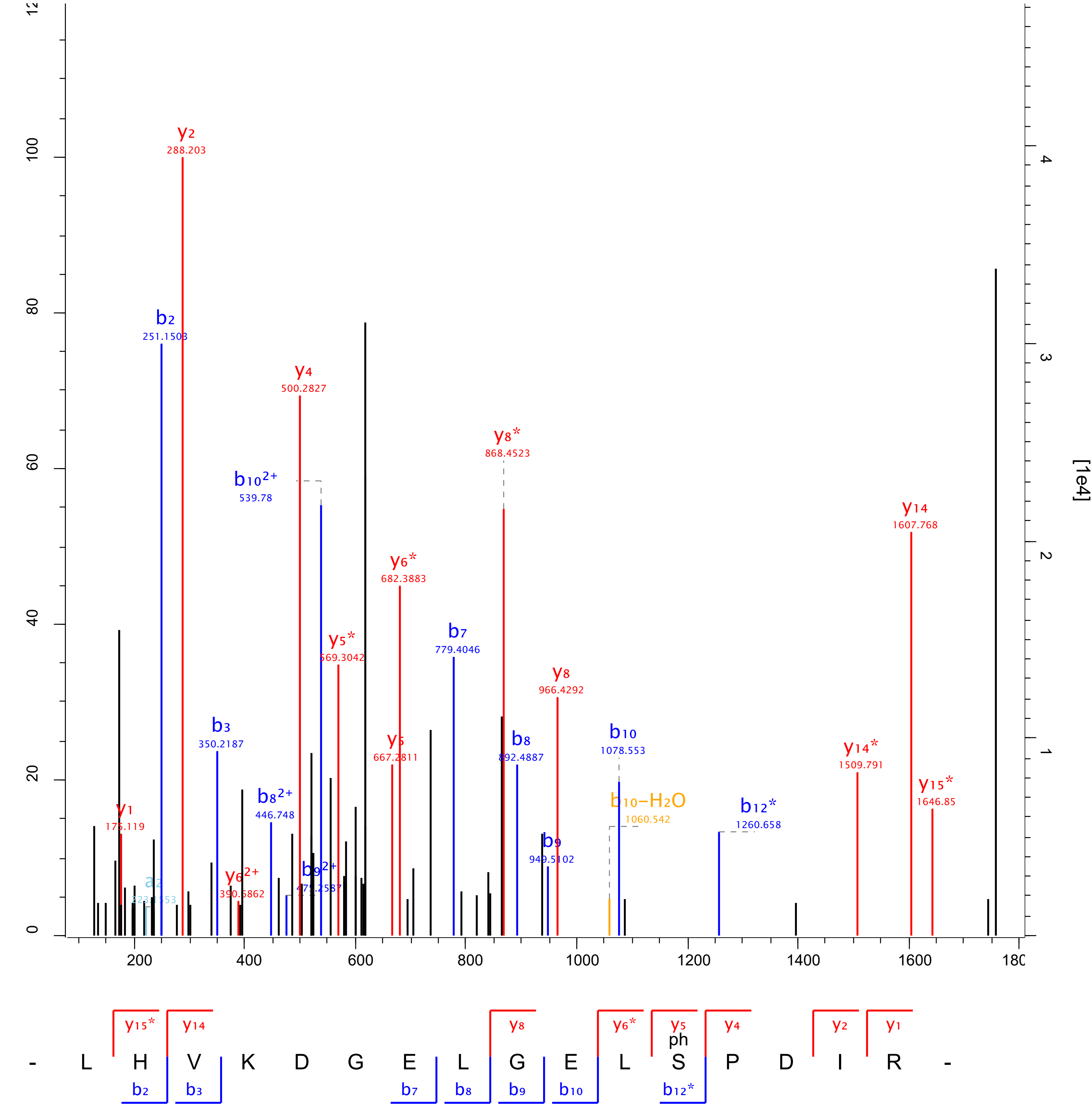

Raw file	Scan	Method	Score	m/z
0523_1	17267	FTMS; HCD	71.15	585.75

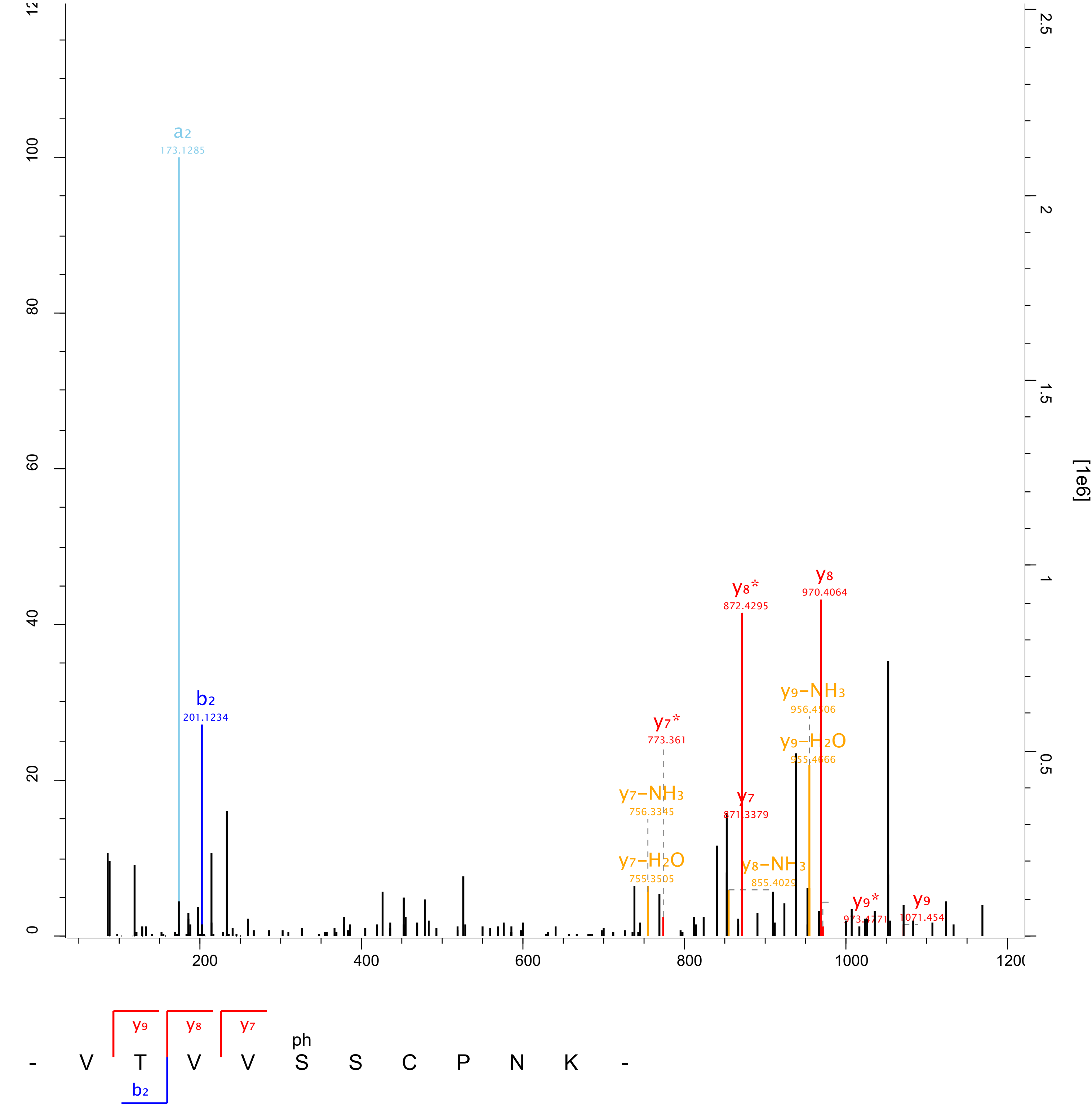

Sample output to test PDF Combine only

Raw file	Scan	Method	Score	m/z	Gene names
0523_1	17436	FTMS; HCD	81.02	648.99	MKP1

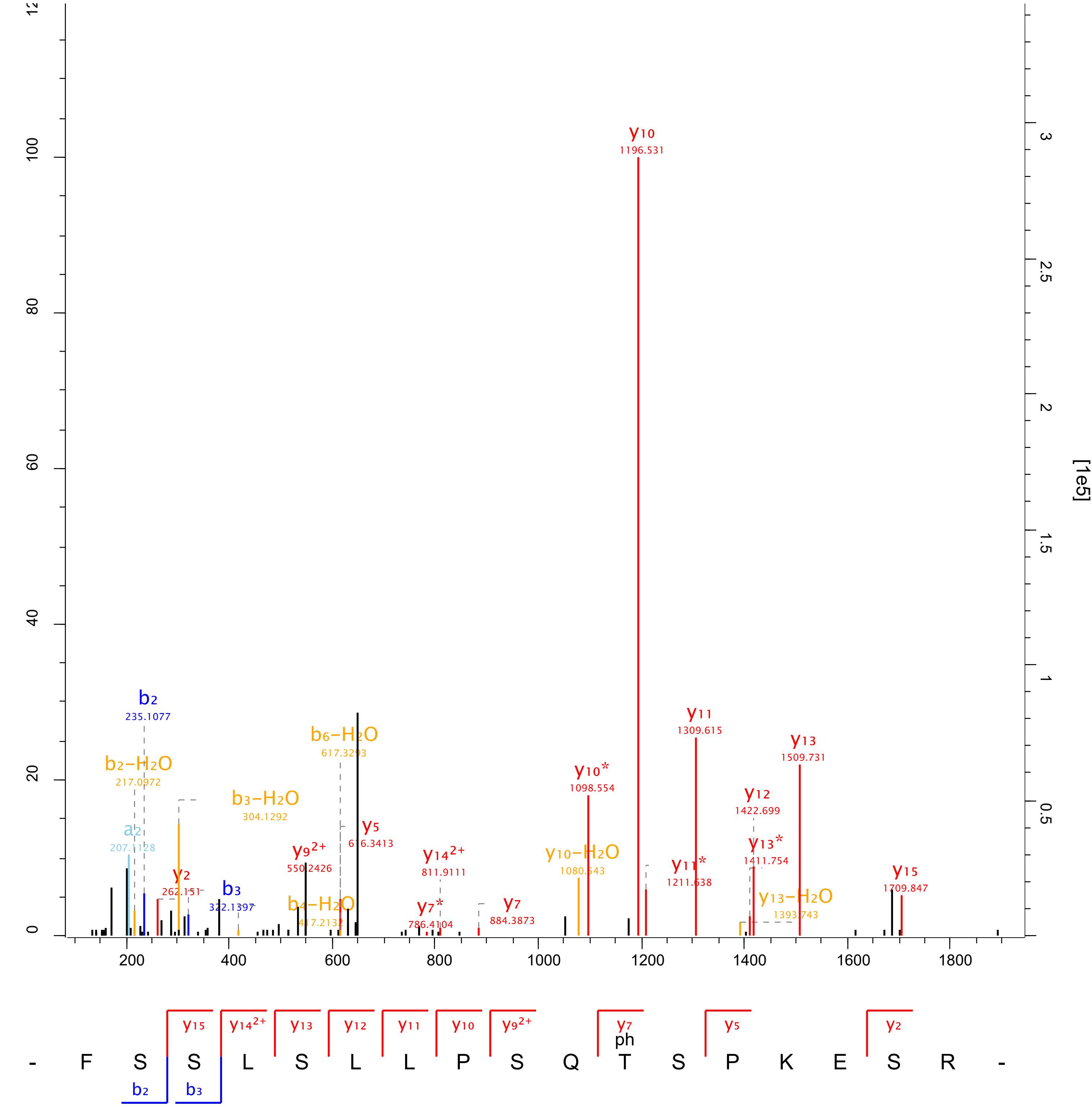

Sample output to test PDF Combine only

Raw file	Scan	Method	Score	m/z	Gene names
0523_1	17472	FTMS; HCD	57.09	762.08	PWP2

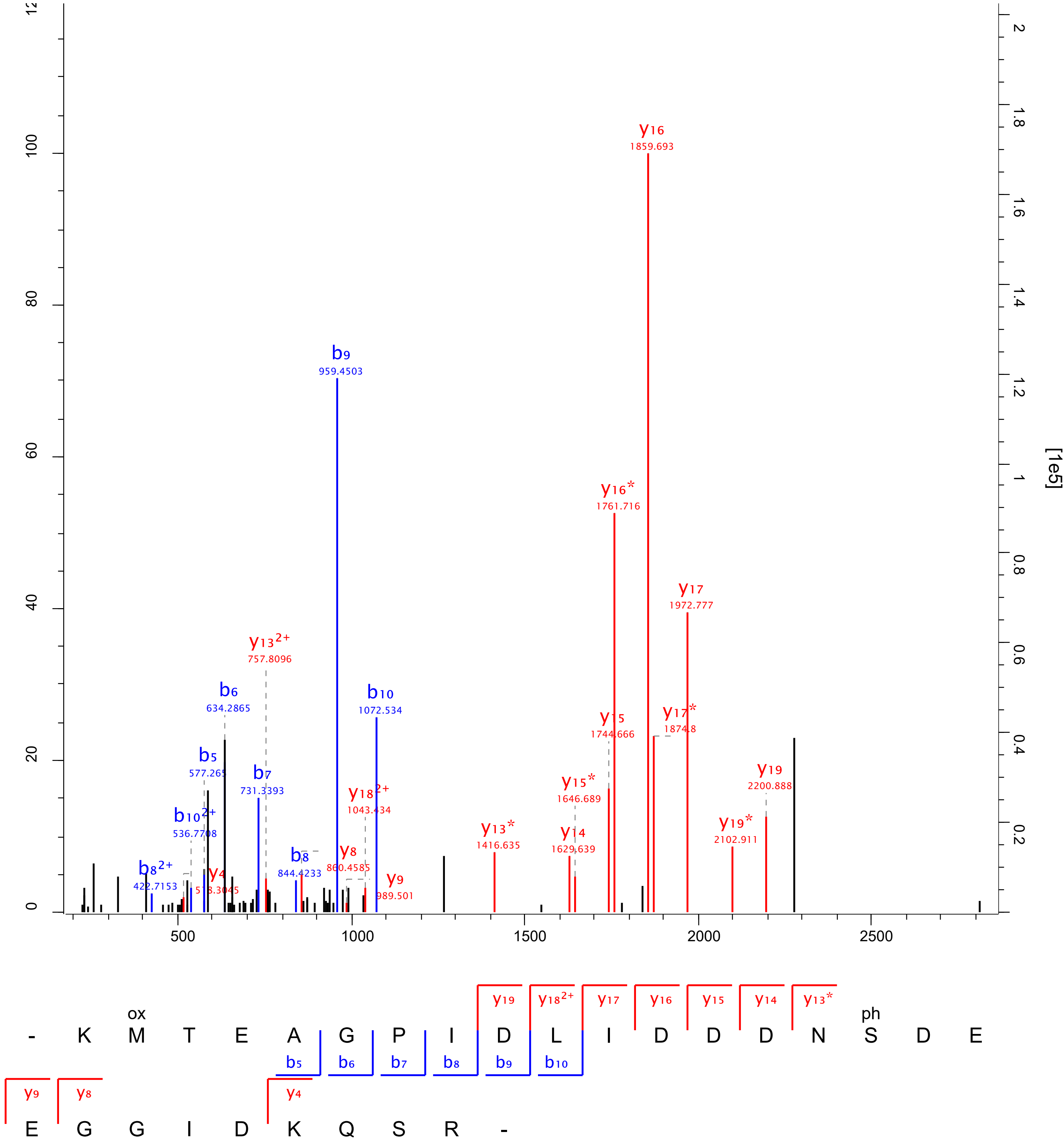

Sample output to test PDF Combine only

Raw file	Scan	Method	Score	m/z	Gene names
0523_1	17843	FTMS; HCD	80.41	713.34	RPT3

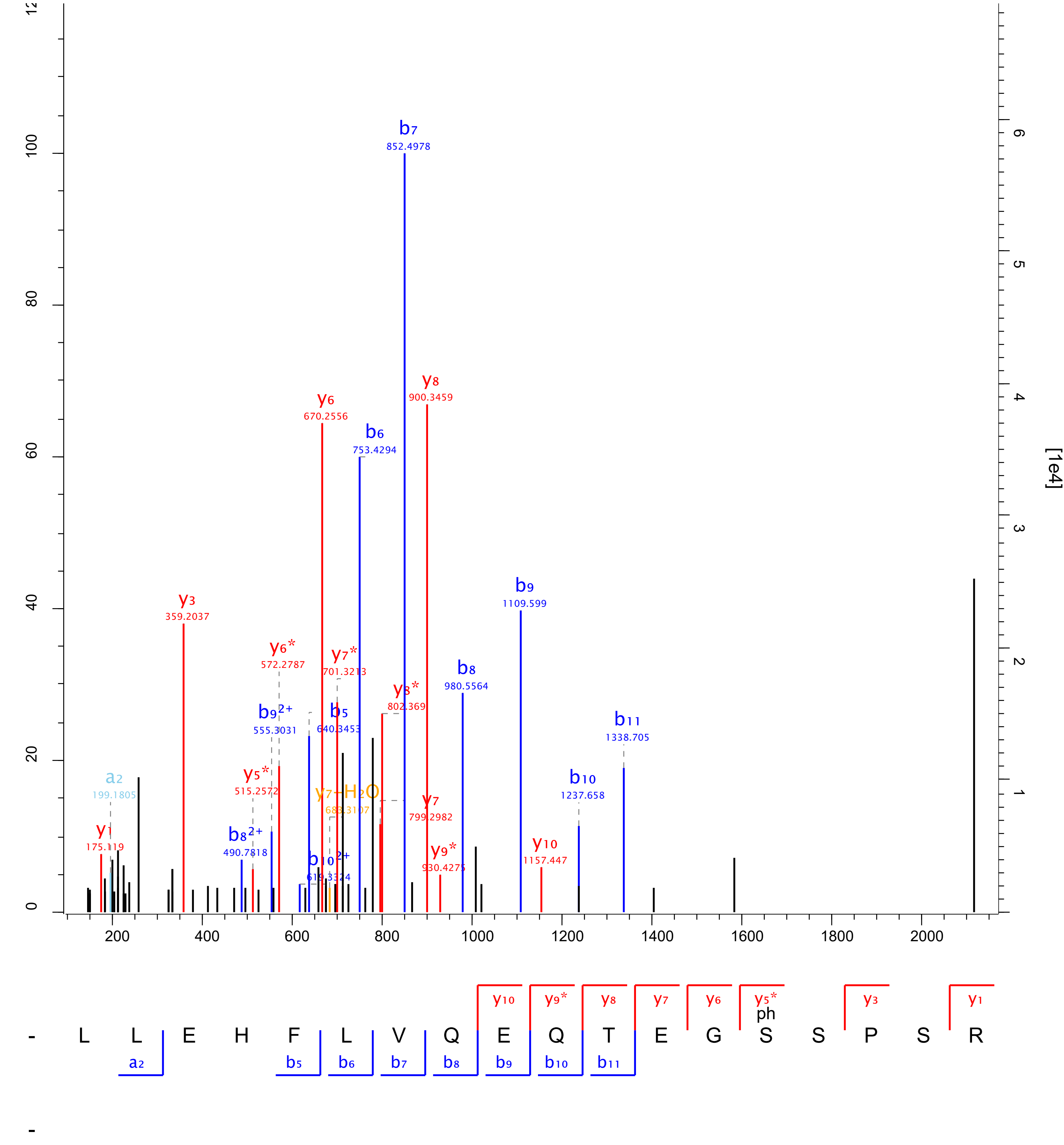

Sample output to test PDF Combine only

Raw file	Scan	Method	Score	m/z	Gene names
0523_1	17844	FTMS; HCD	88.39	1044.48	F12E4_50

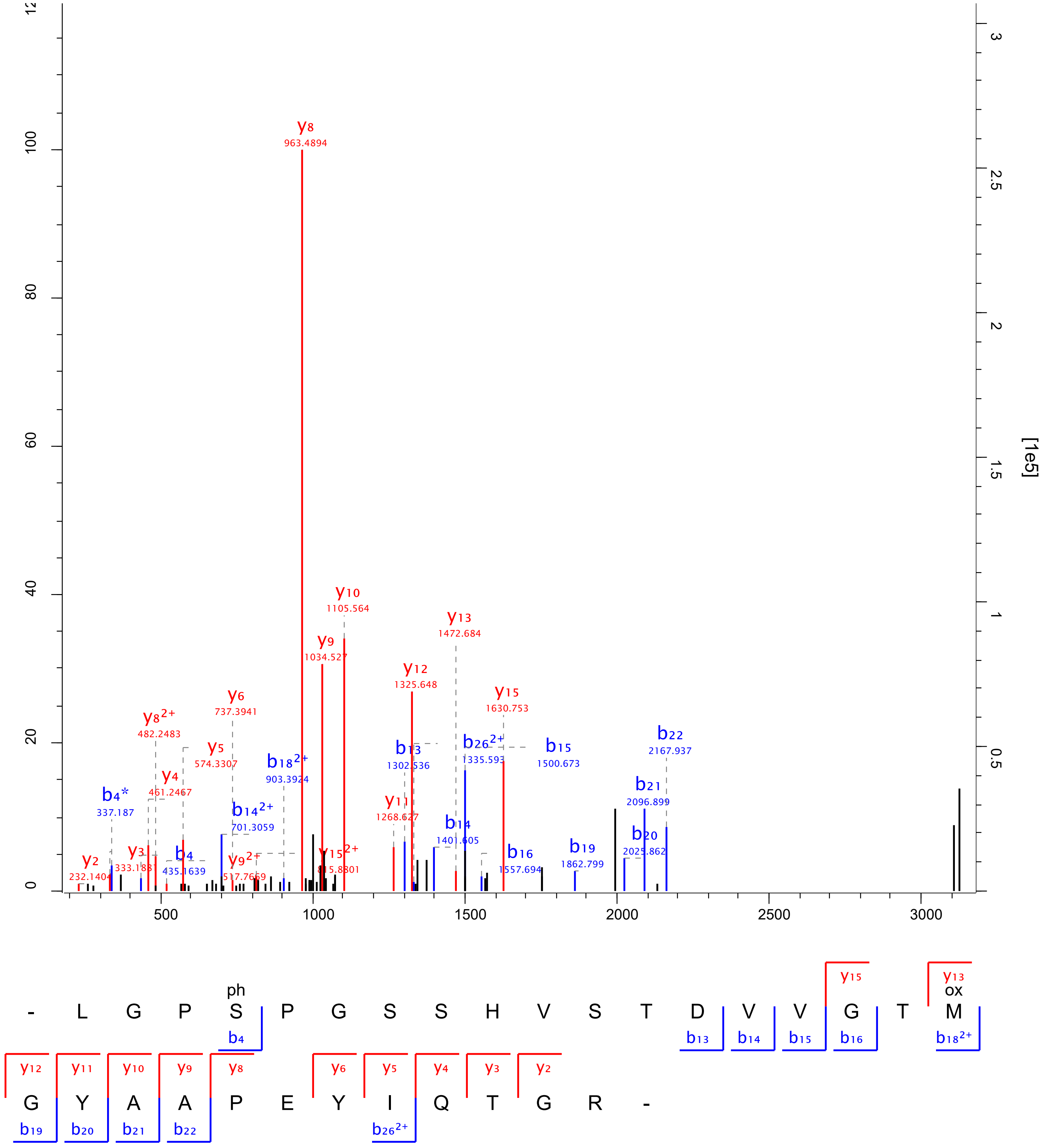

Sample output to test PDF Combine only

Raw file	Scan	Method	Score	m/z	Gene names
0523_1	17919	FTMS; HCD	230.82	827.4	AHA2;HA2

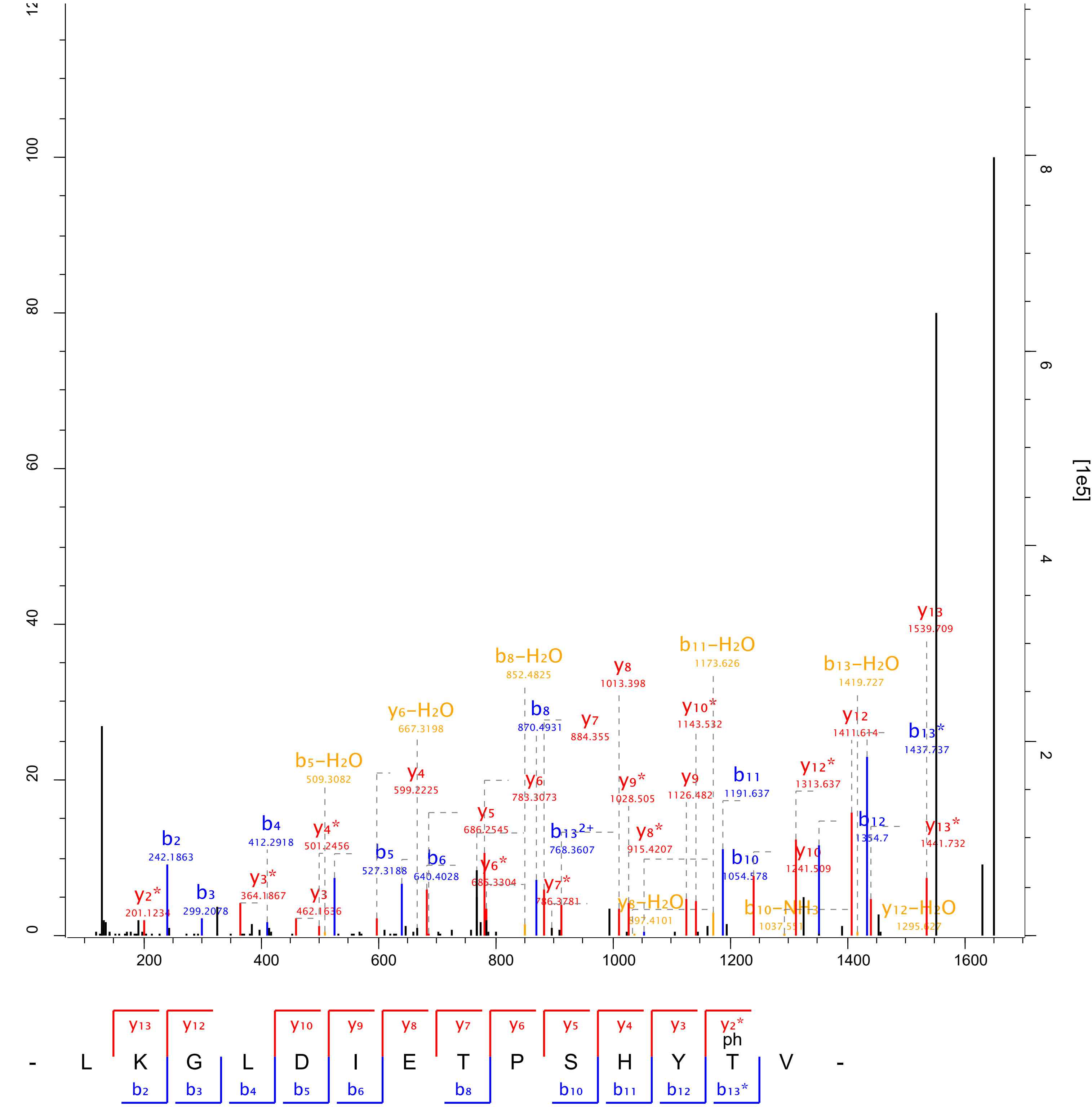

Sample output to test PDF Combine only

Raw file	Scan	Method	Score	m/z	Gene names
0523_1	17979	FTMS; HCD	117.53	729.34	At5g65950

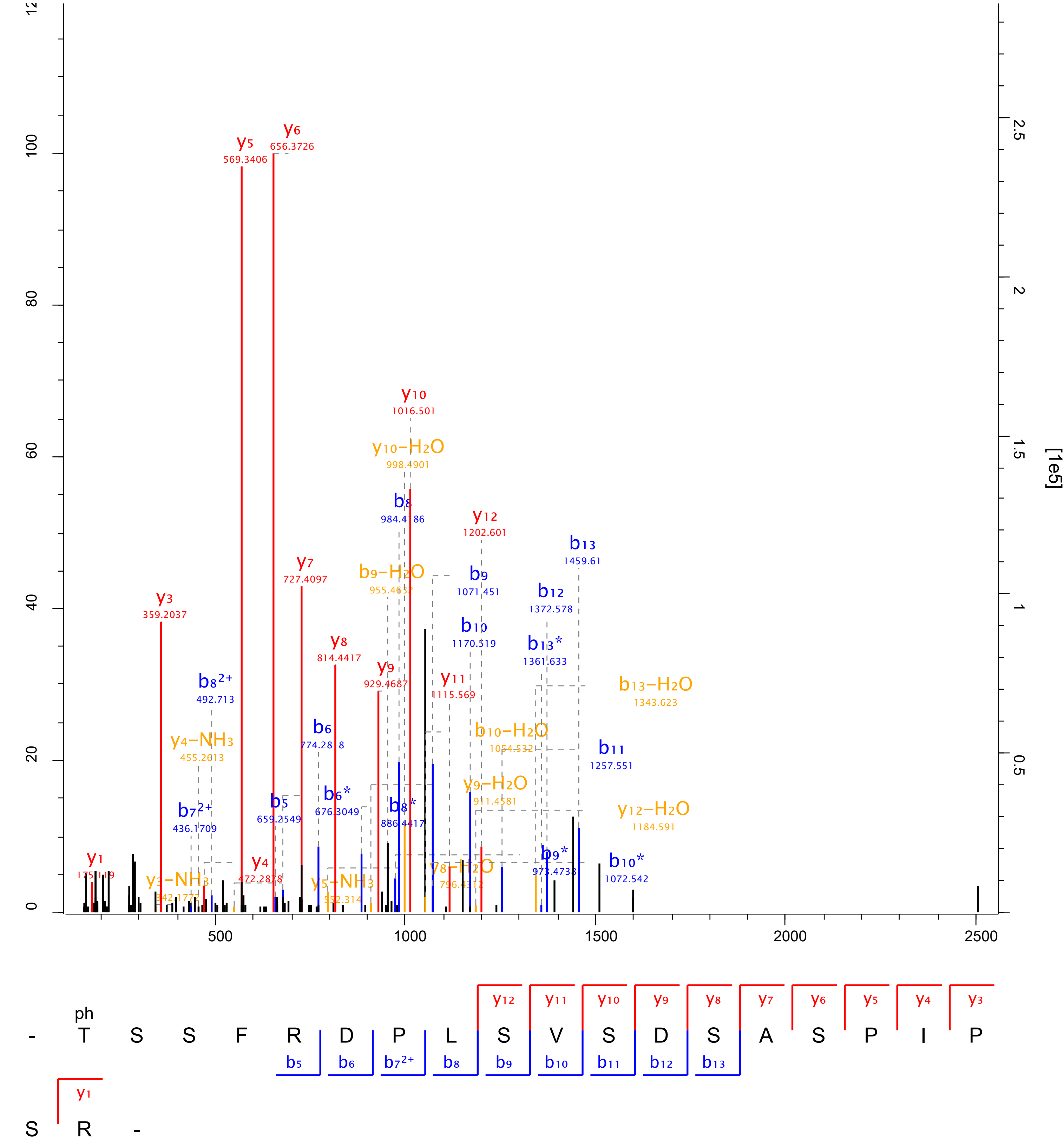

Sample output to test PDF Combine only

Raw file	Scan	Method	Score	m/z	Gene names
0523_1	18013	FTMS; HCD	49.43	702.32	F13I12.260

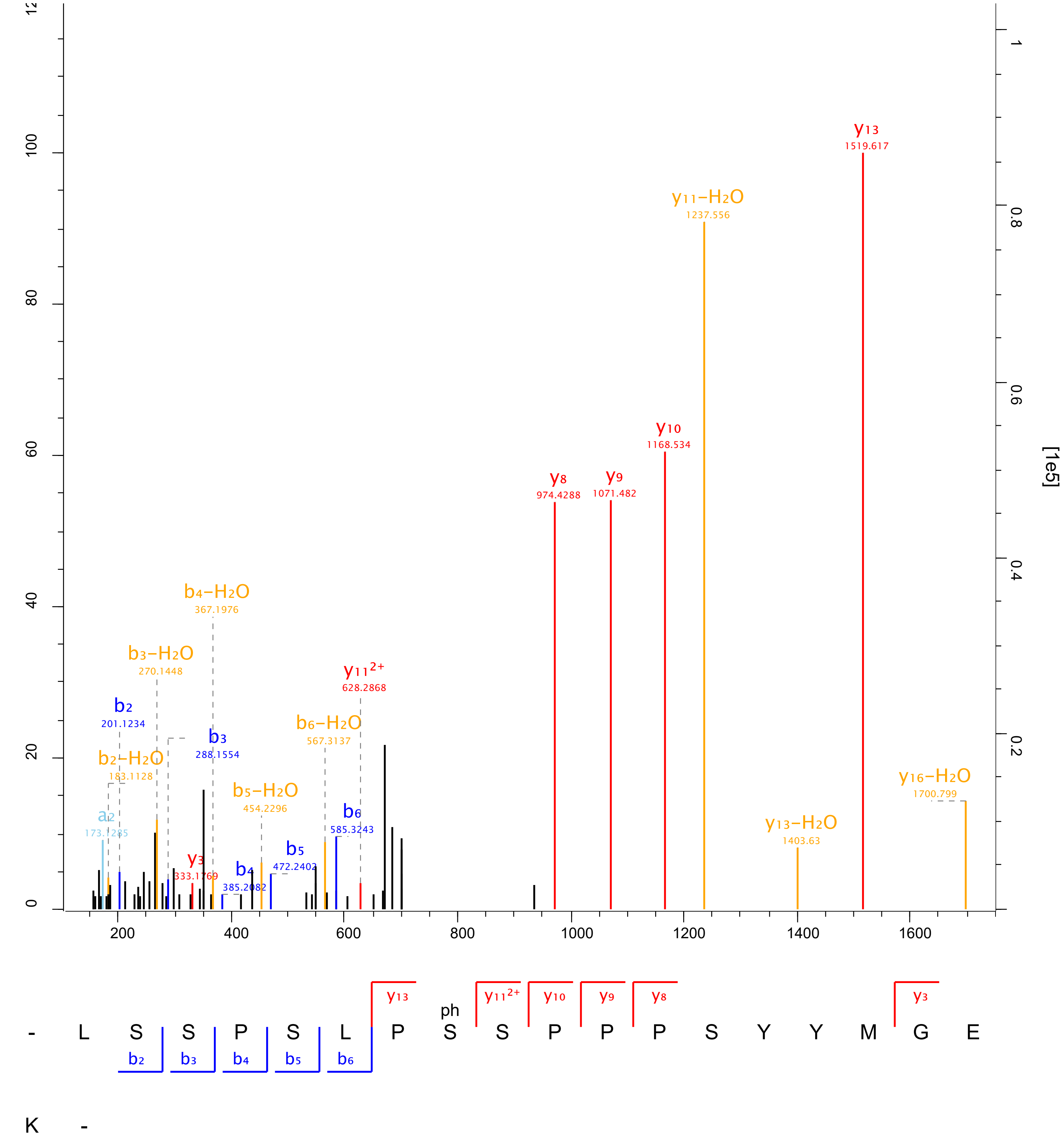

Sample output to test PDF Combine only

Raw file	Scan	Method	Score	m/z
0523_1	18322	FTMS; HCD	44.85	716.36

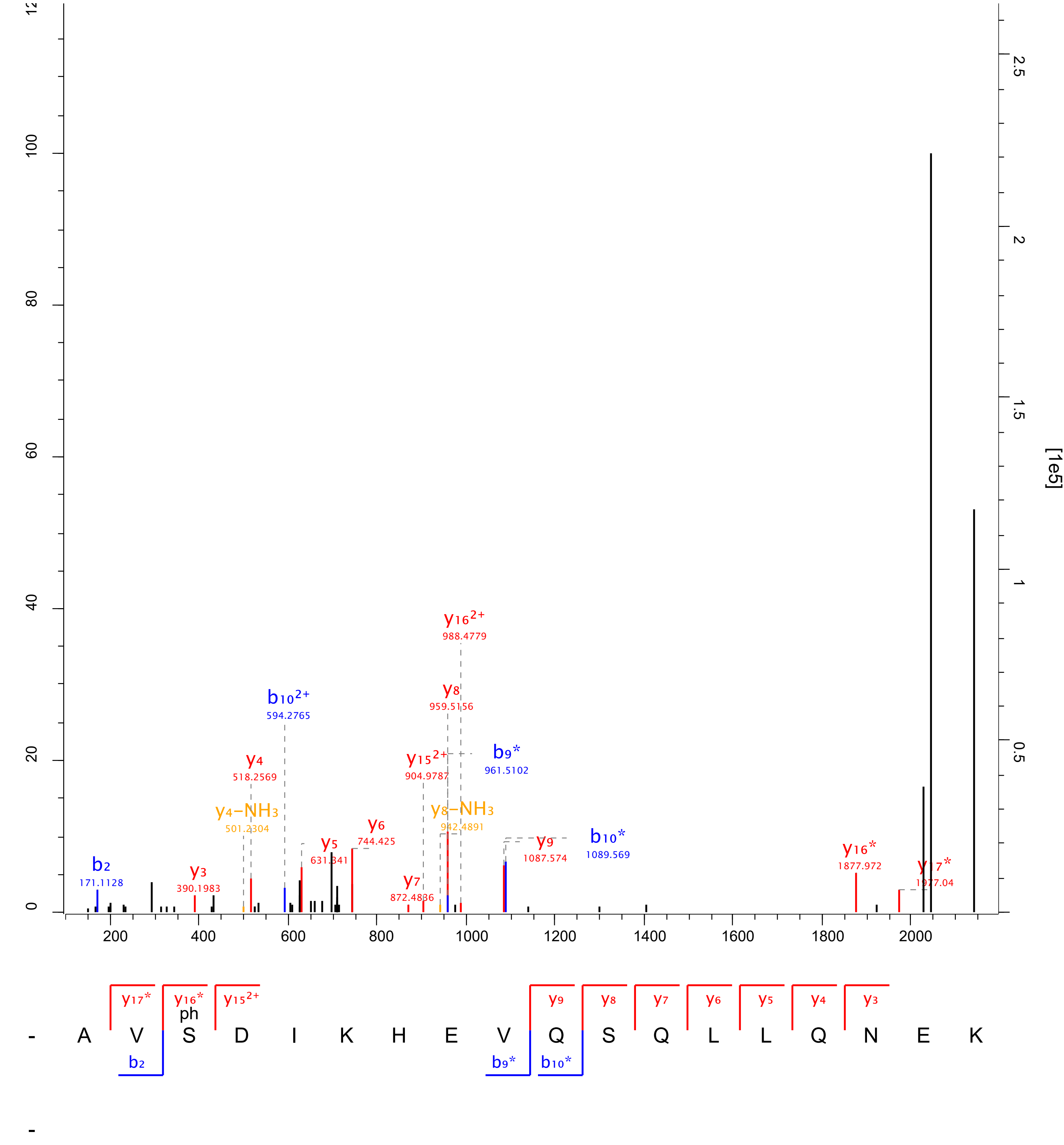

Sample output to test PDF Combine only

Raw file	Scan	Method	Score	m/z	Gene names
0523_1	19138	FTMS; HCD	167.34	957.11	F13I12.260

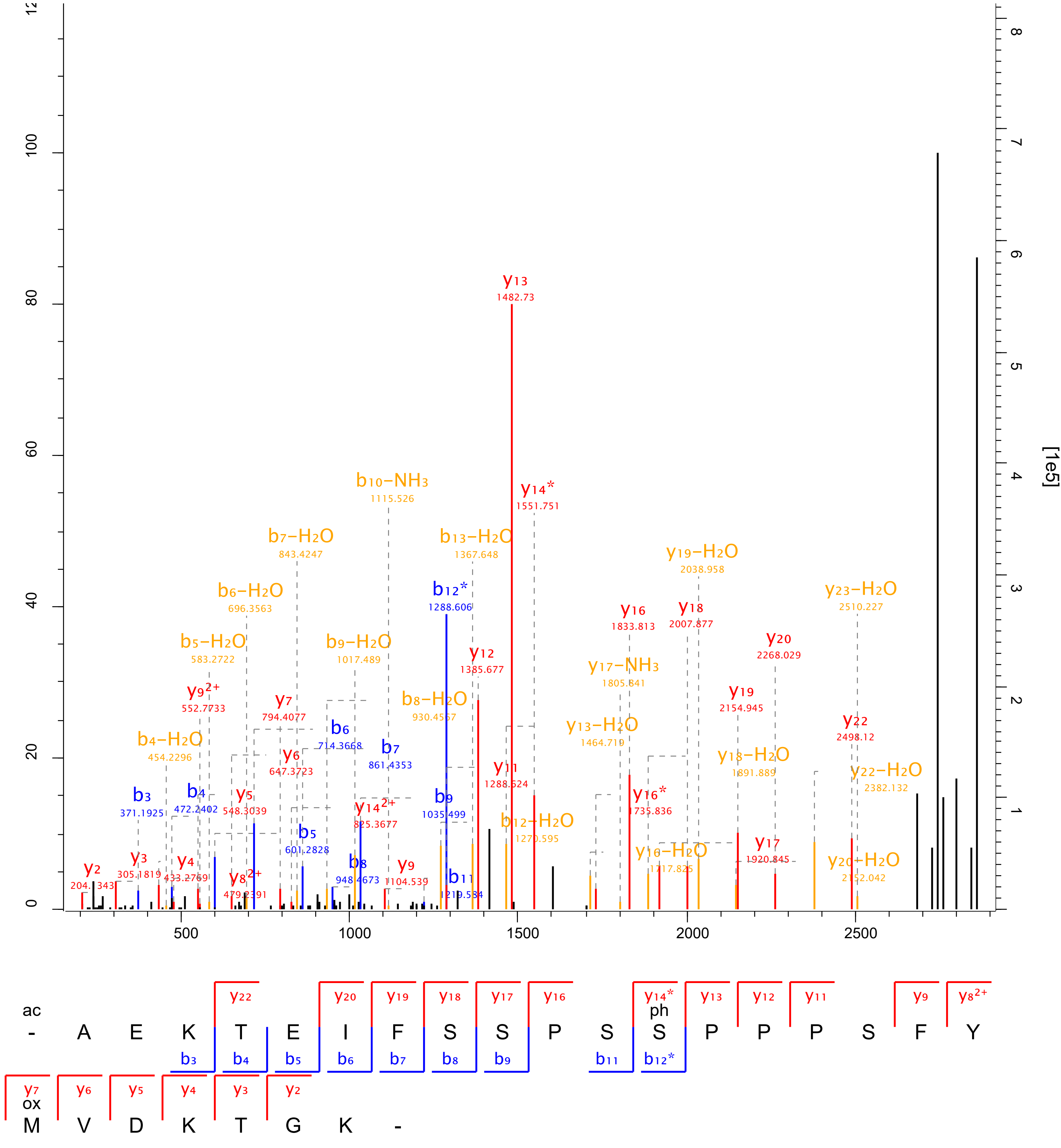

Sample output to test PDF Combine only

Raw file	Scan	Method	Score	m/z	Gene names
0523_1	19243	FTMS; HCD	133.4	828.06	PRA1F3

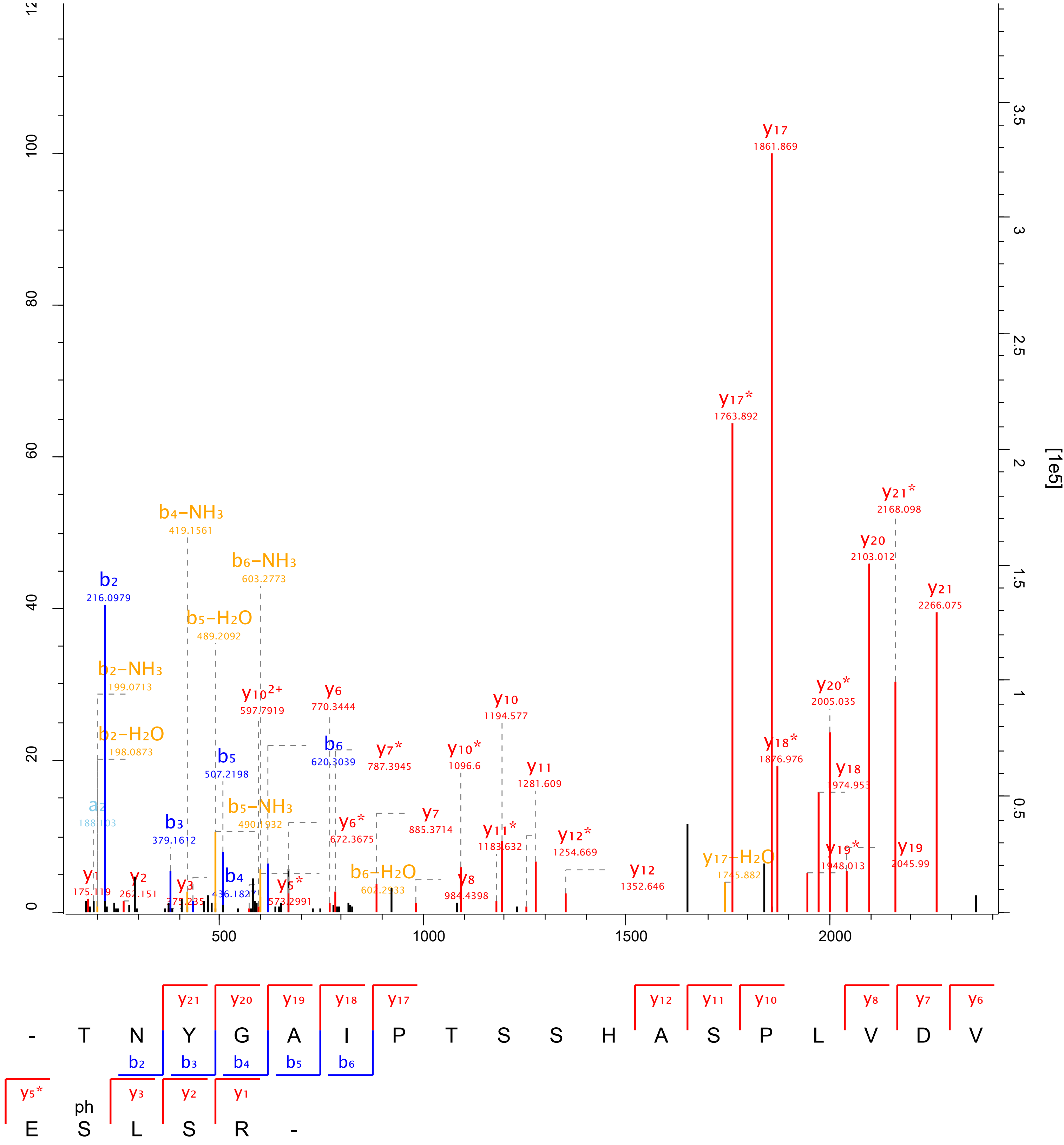

Sample output to test PDF Combine only

Raw file	Scan	Method	Score	m/z	Gene names
0523_1	19449	FTMS; HCD	64.45	691.35	PVA22

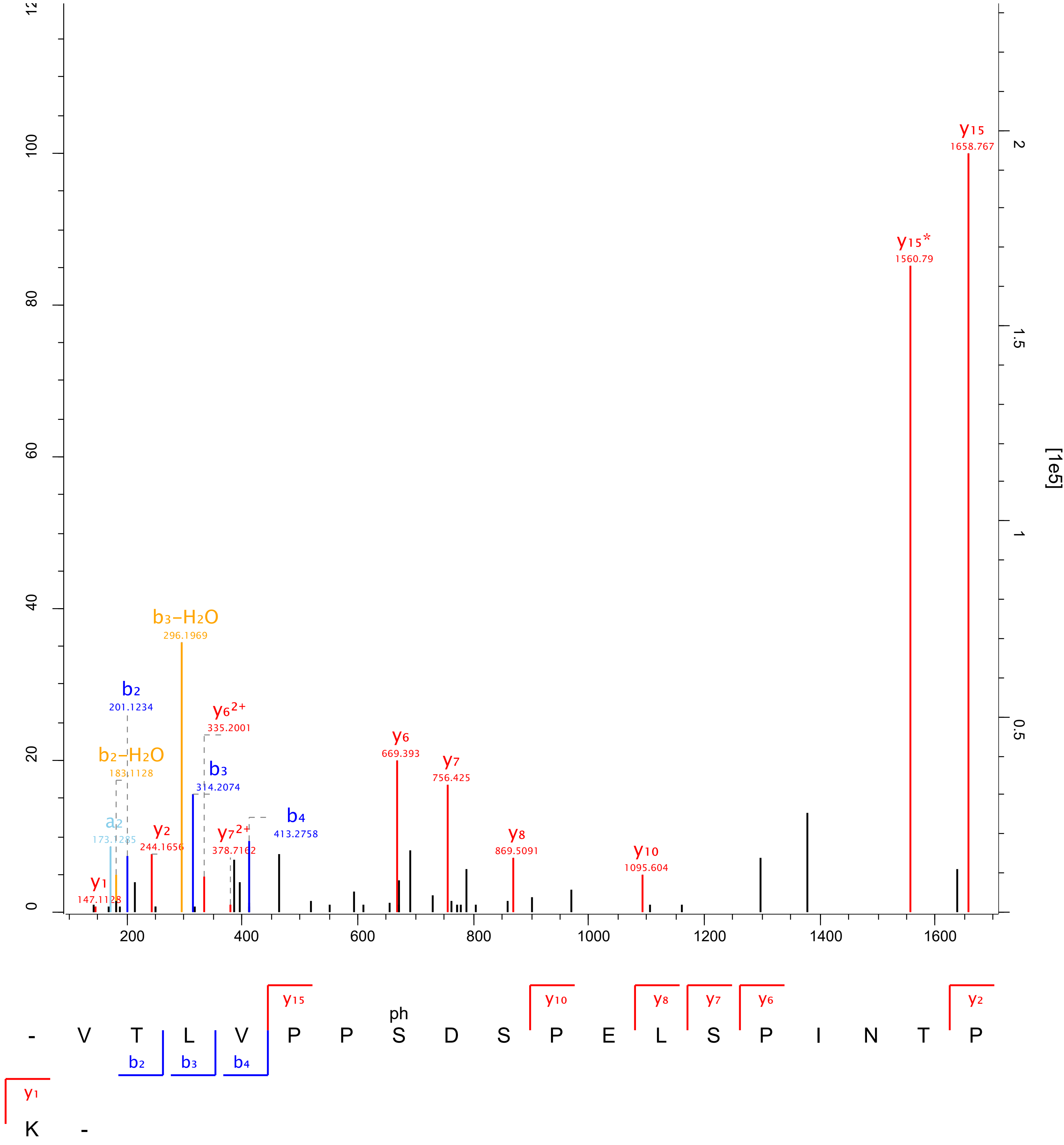

Sample output to test PDF Combine only

Raw file	Scan	Method	Score	m/z	Gene names
0523_1	19743	FTMS; HCD	116.38	751.06	TPR1

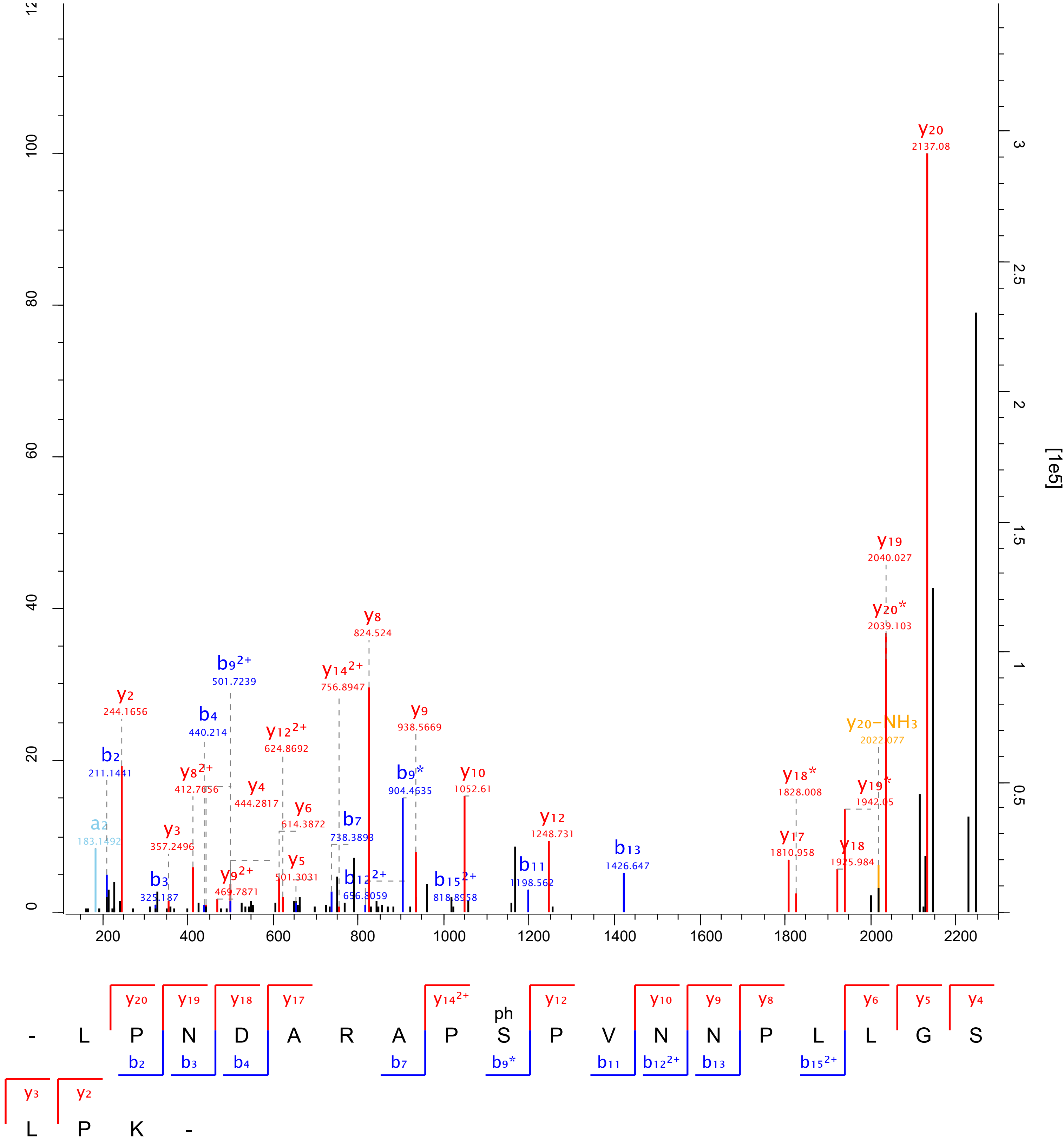

Raw file	Scan	Method	Score	m/z	Gene names
0523_1	19827	FTMS; HCD	88.57	1047.41	At4g18905

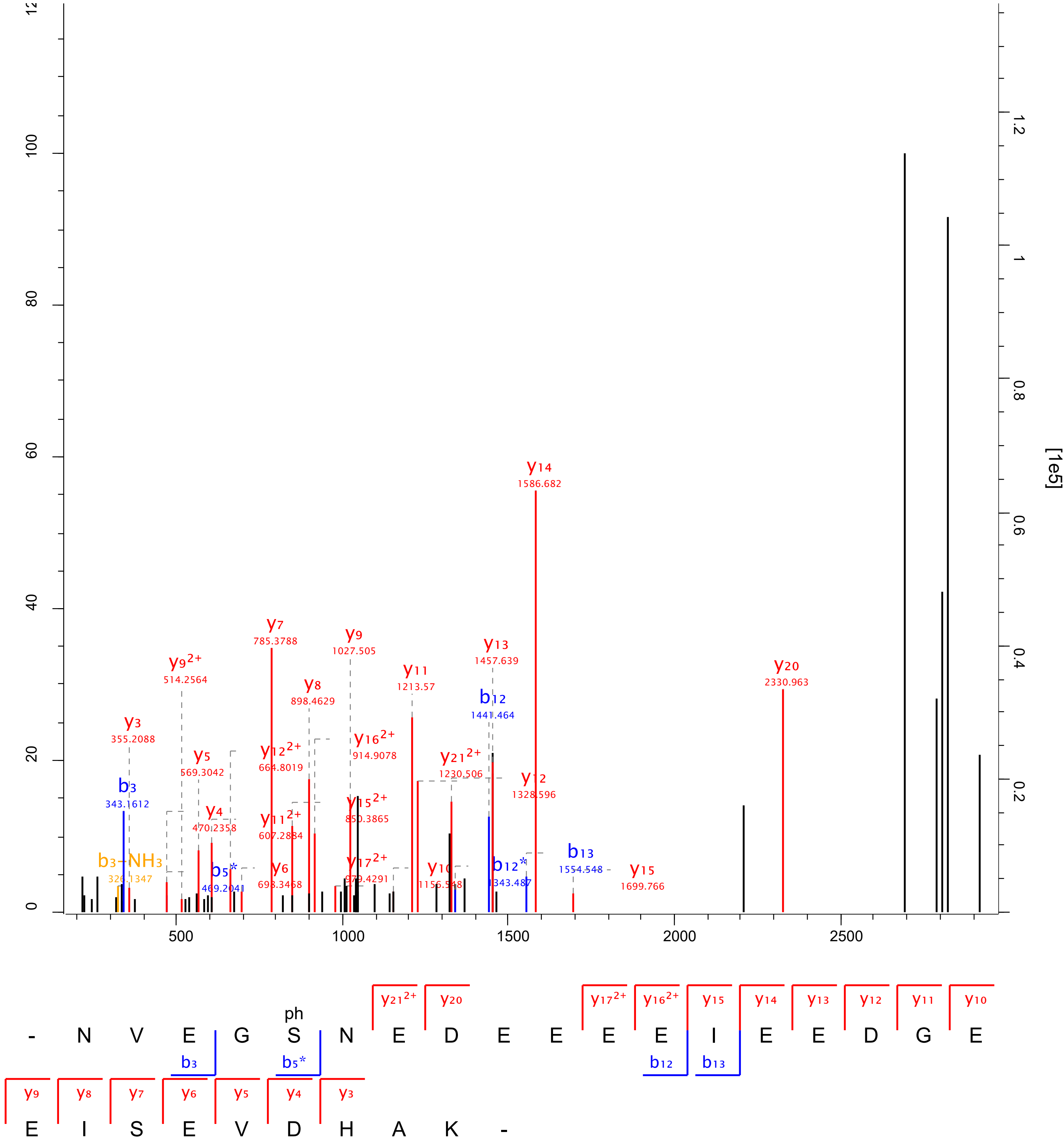

Sample output to test PDF Combine only

Raw file	Scan	Method	Score	m/z	Gene names
0523_1	19967	FTMS; HCD	245.48	777.41	TPL

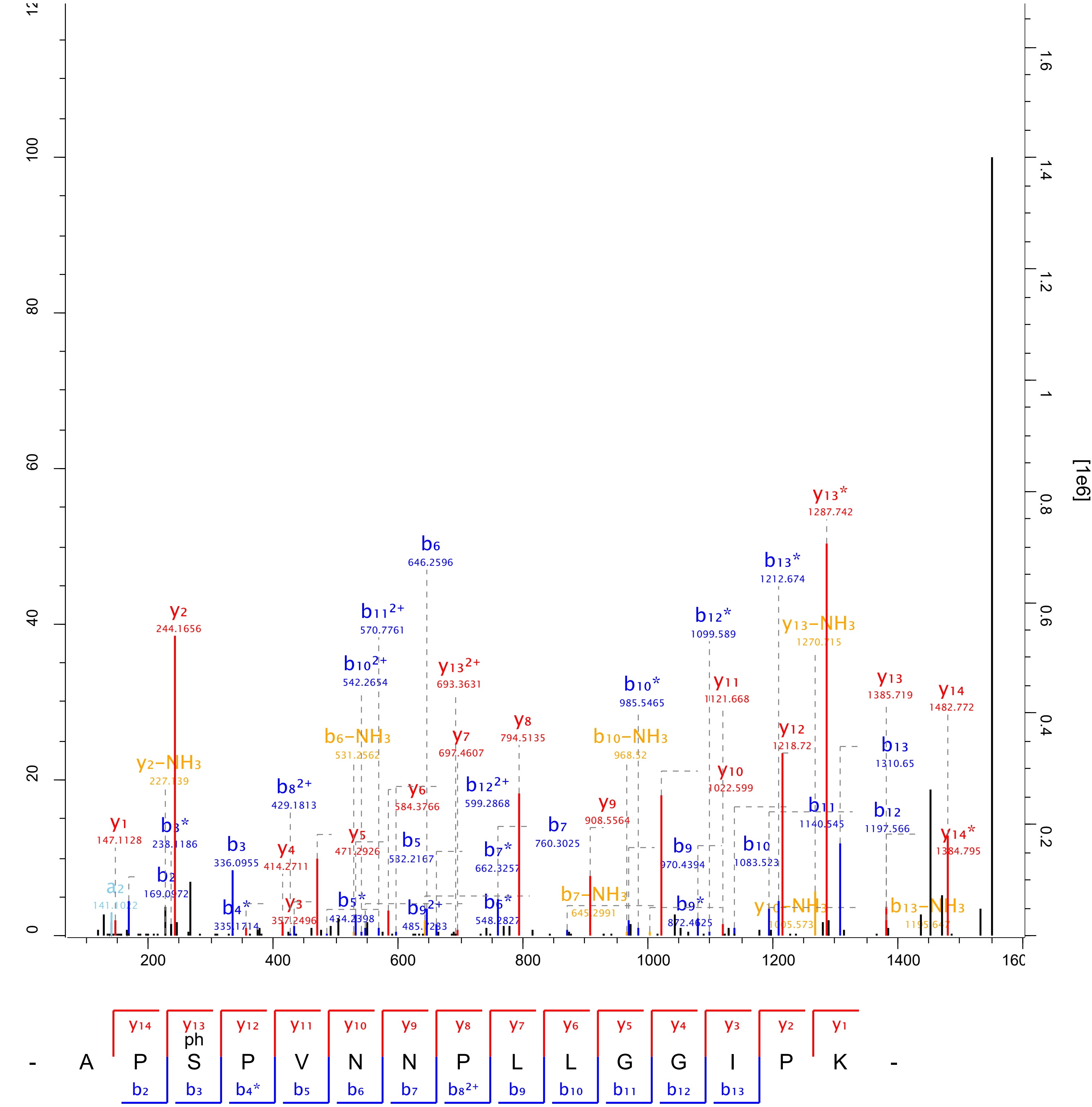

Sample output to test PDF Combine only

Raw file	Scan	Method	Score	m/z	Gene names
0523_1	20250	FTMS; HCD	58.27	779.33	At4g12780;At4g12770

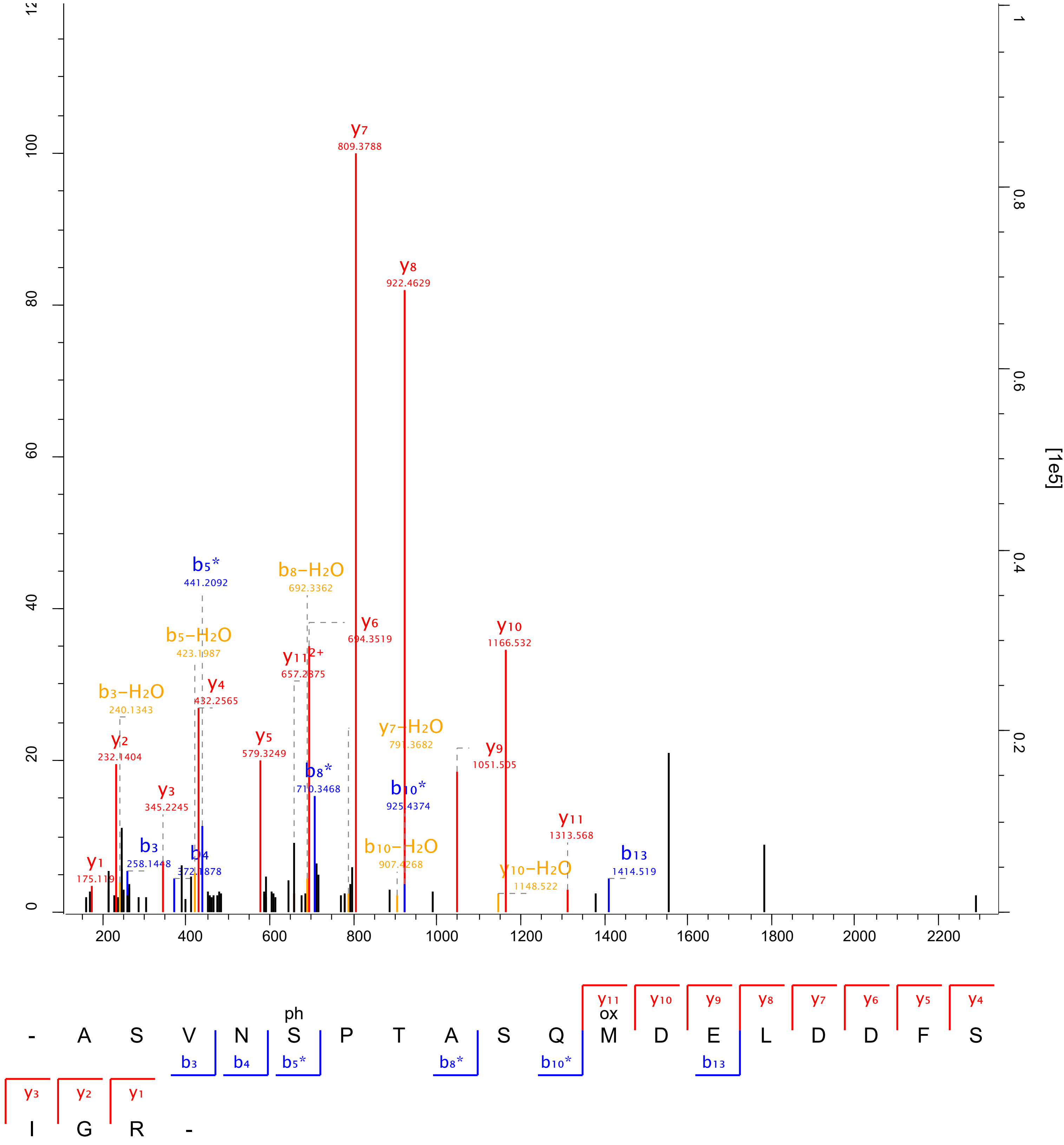

Sample output to test PDF Combine only

Raw file	Scan	Method	Score	m/z	Gene names
0523_1	20506	FTMS; HCD	40.99	741.36	NHX1

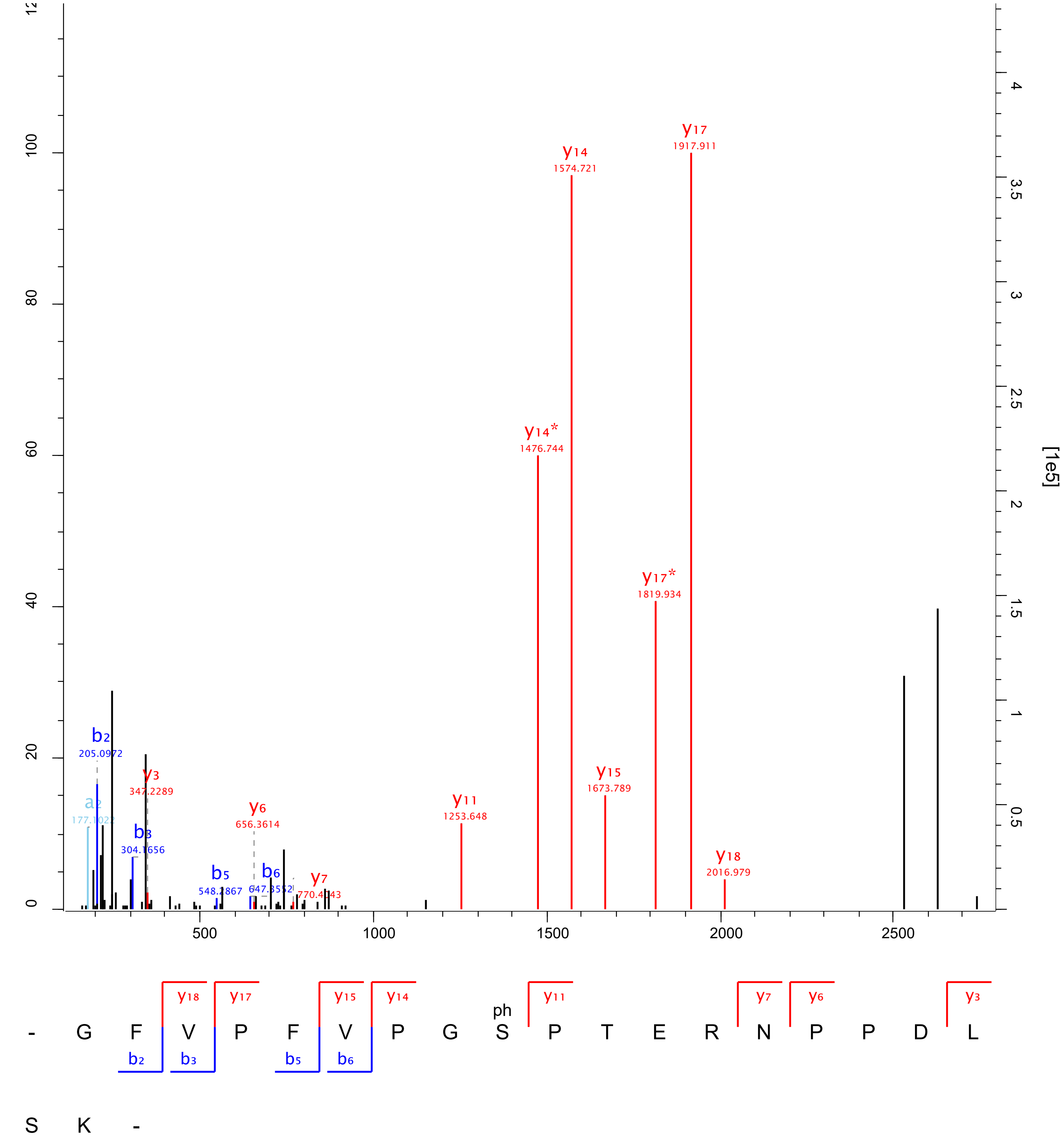

Sample output to test PDF Combine only

Raw file	Scan	Method	Score	m/z	Gene names
0523_1	20726	FTMS; HCD	107.65	812.39	F9F8.20

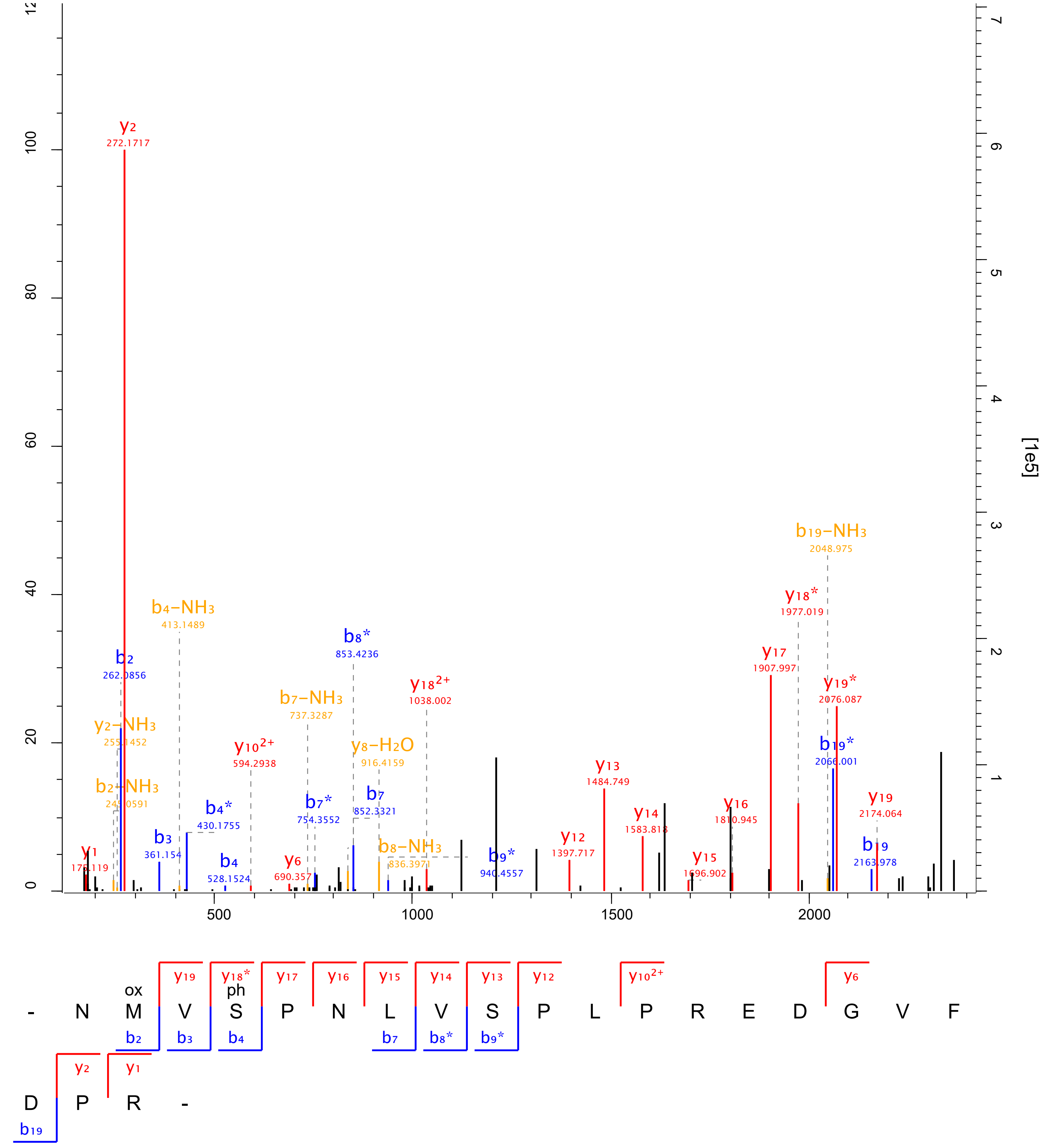

Sample output to test PDF Combine only

Raw file	Scan	Method	Score	m/z	Gene names
0523_1	21304	FTMS; HCD	148.57	674.82	FKFBP

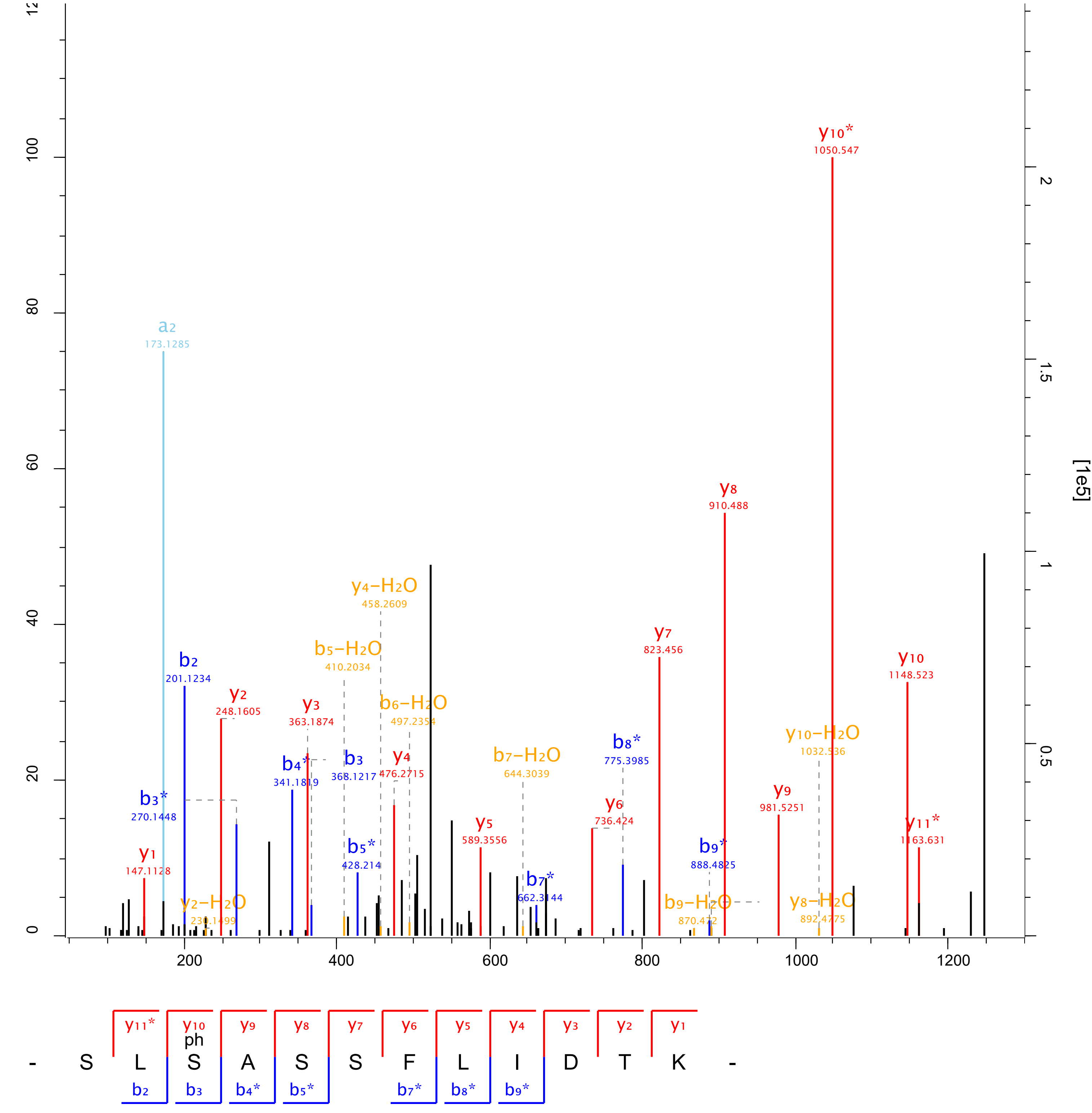

Sample output to test PDF Combine only

Raw file	Scan	Method	Score	m/z	Gene names
0523_1	21909	FTMS; HCD	116.24	861.72	F13I12.260

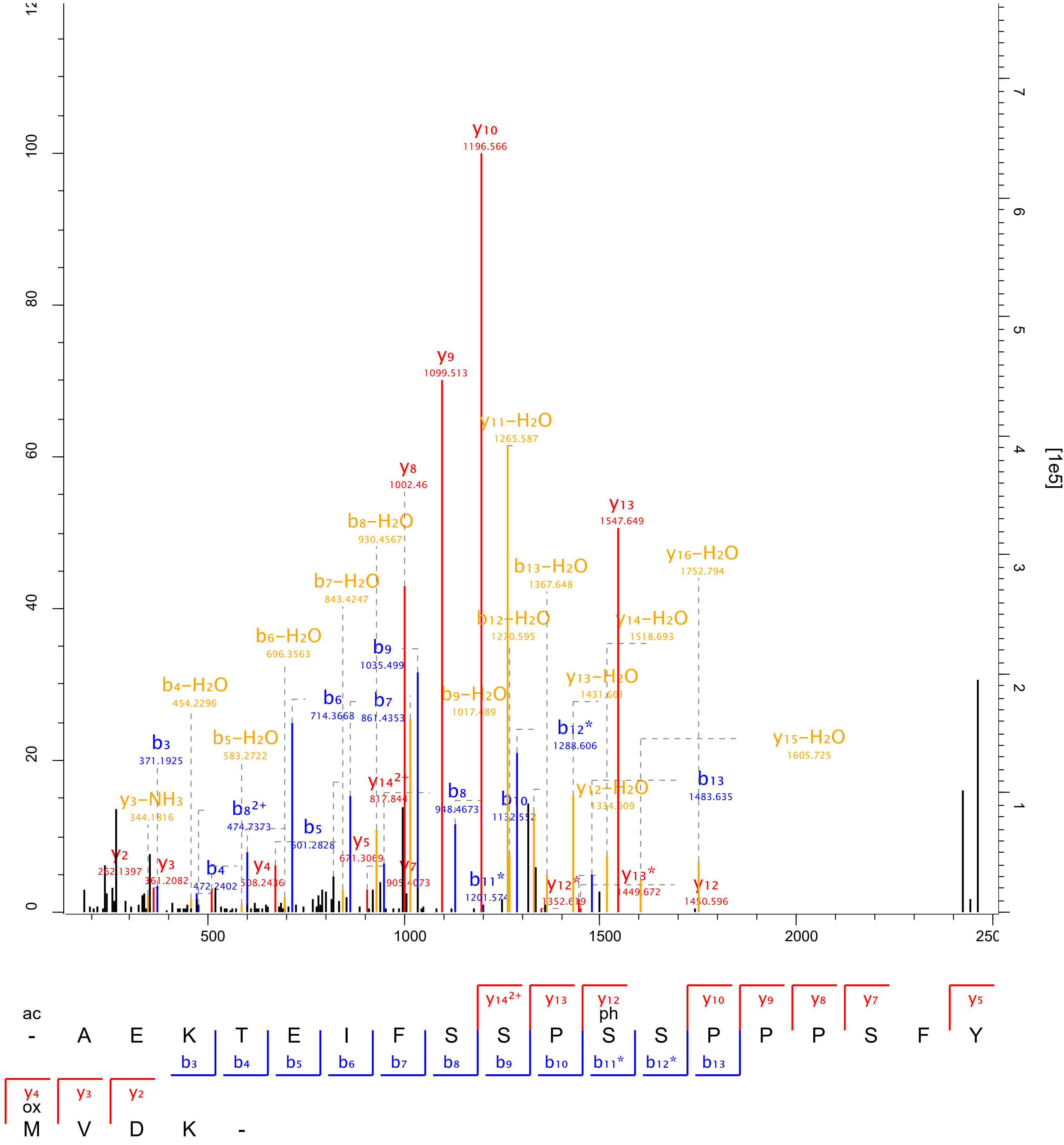

Sample output to test PDF Combine only

Raw file	Scan	Method	Score	m/z	Gene names
0523_1	22112	FTMS; HCD	45.56	839.91	POT13

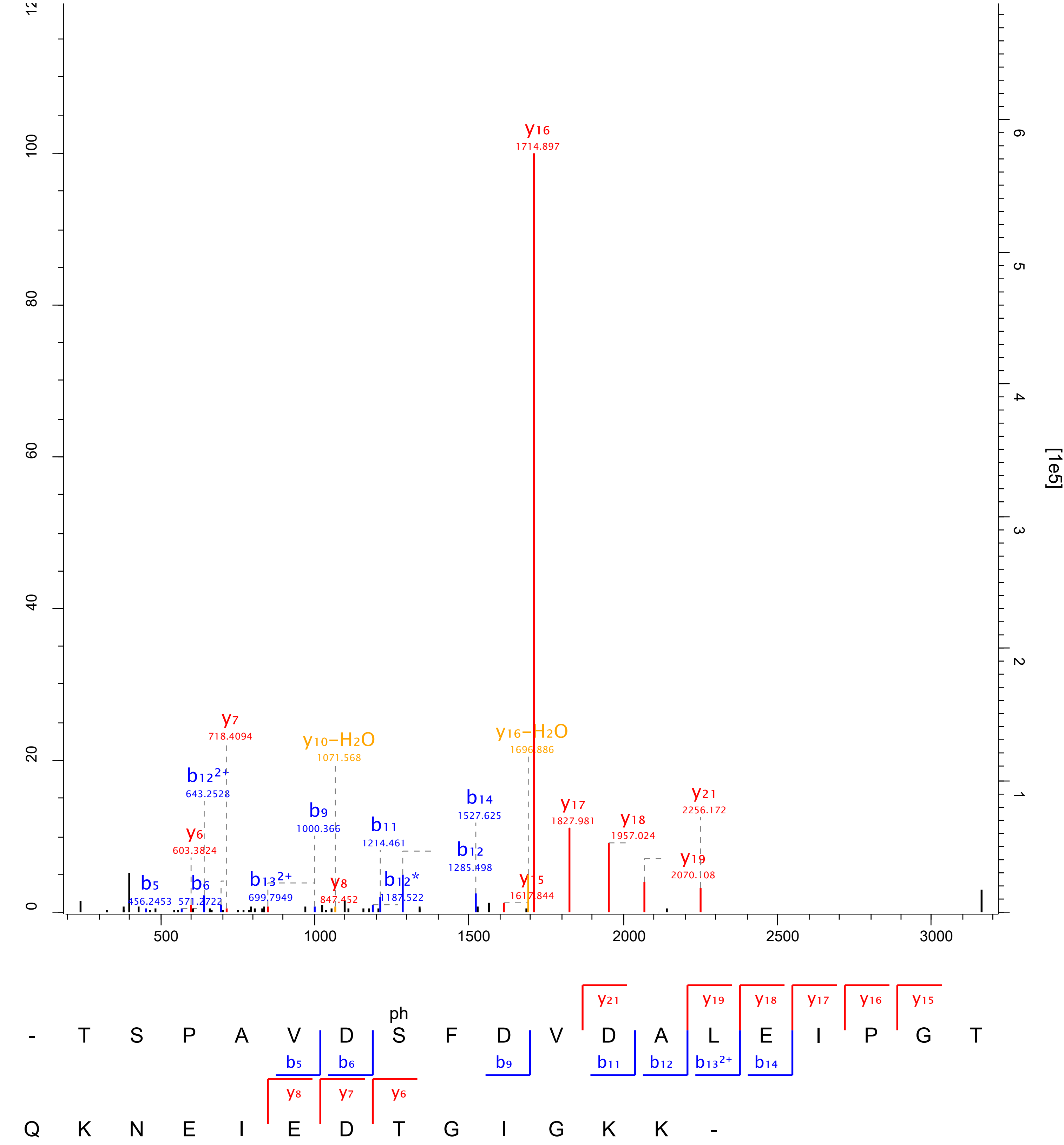

Sample output to test PDF Combine only

Raw file	Scan	Method	Score	m/z	Gene names
0523_1	22119	FTMS; HCD	97.77	790.38	TRS120

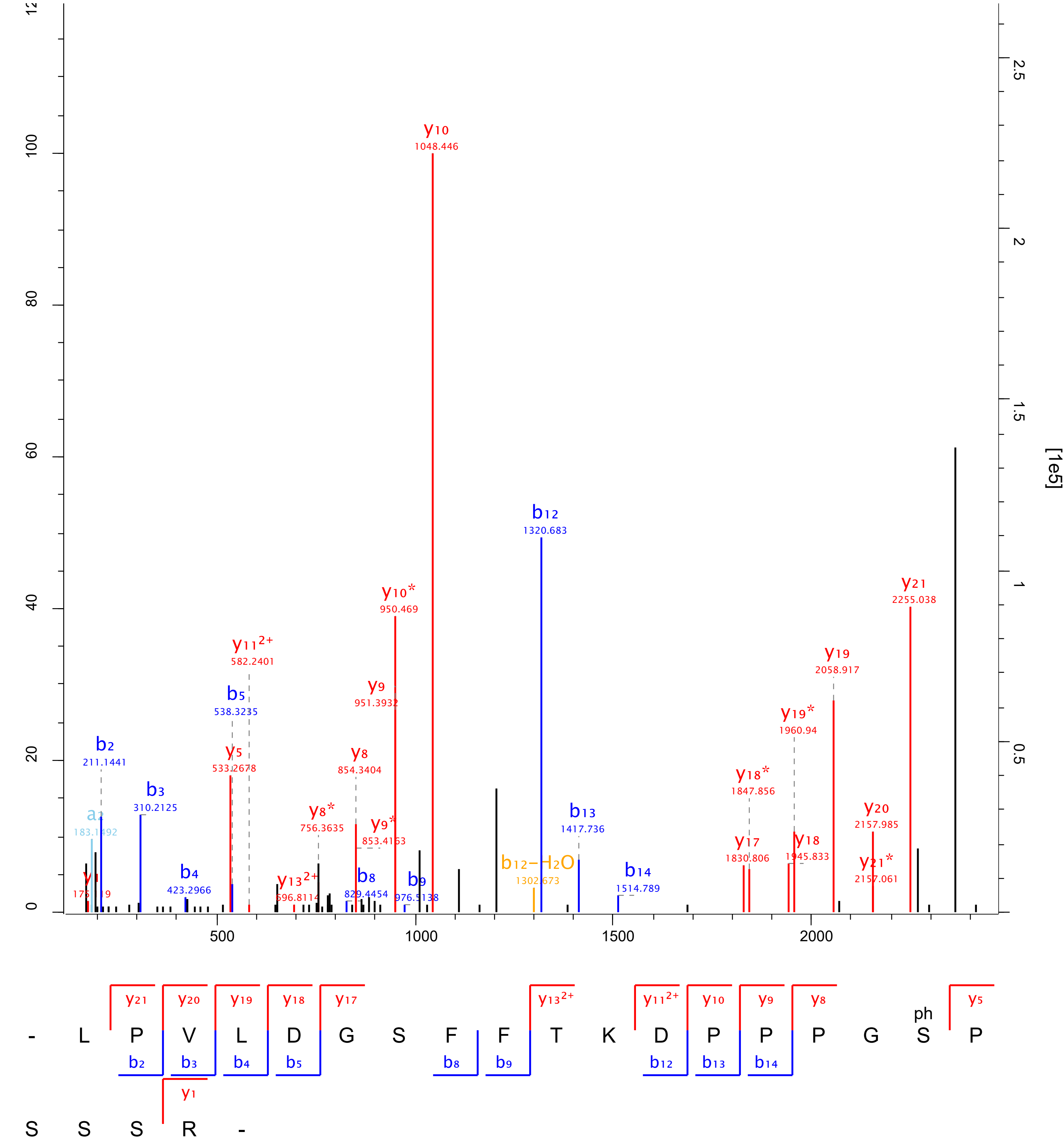

Sample output to test PDF Combine only

Raw file	Scan	Method	Score	m/z	Gene names
0523_1	22336	FTMS; HCD	186.97	1009.95	NPF6.3

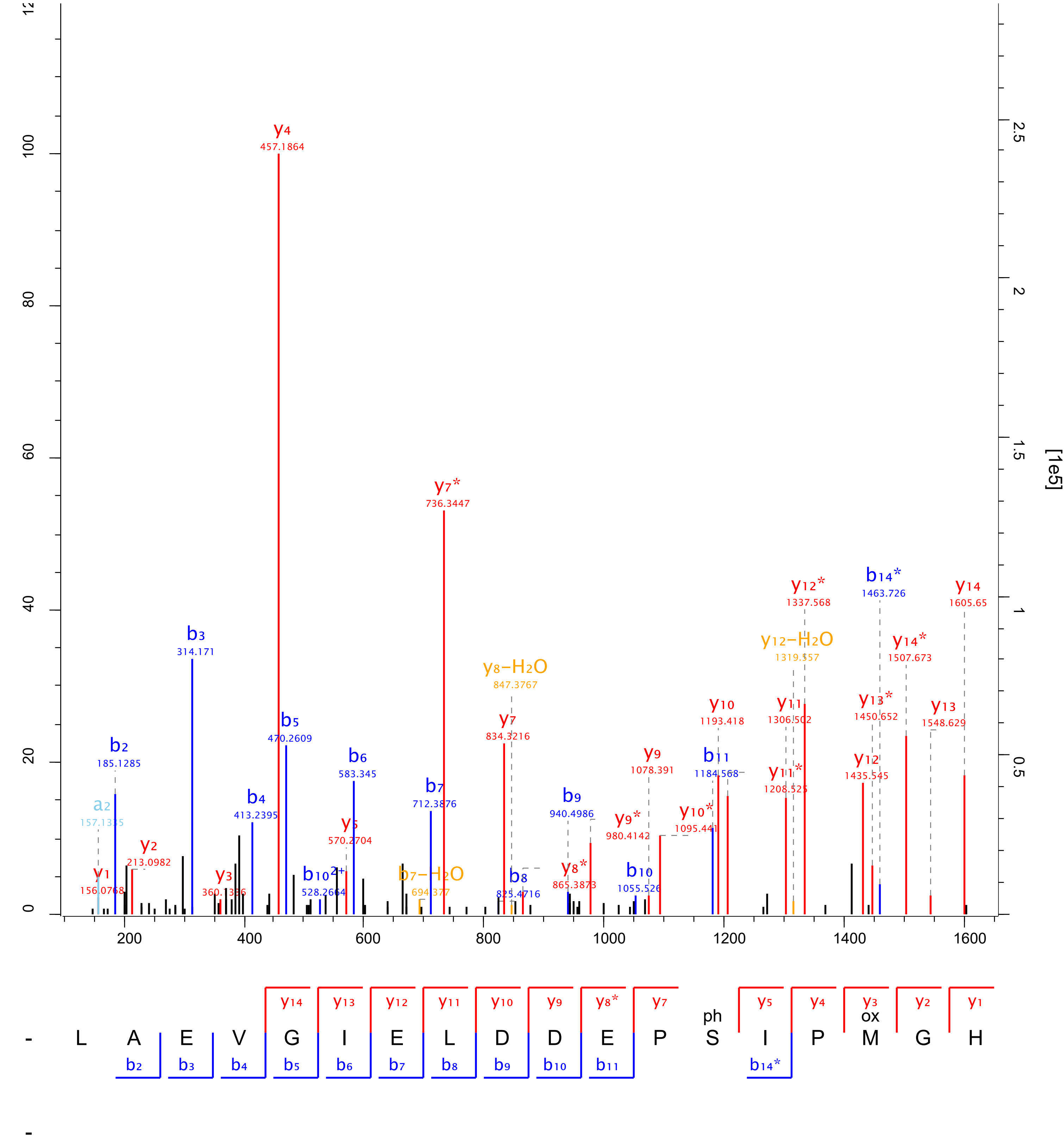

Sample output to test PDF Combine only

Raw file	Scan	Method	Score	m/z	Gene names
0523_1	22532	FTMS; HCD	103.42	1102.86	GPAT9

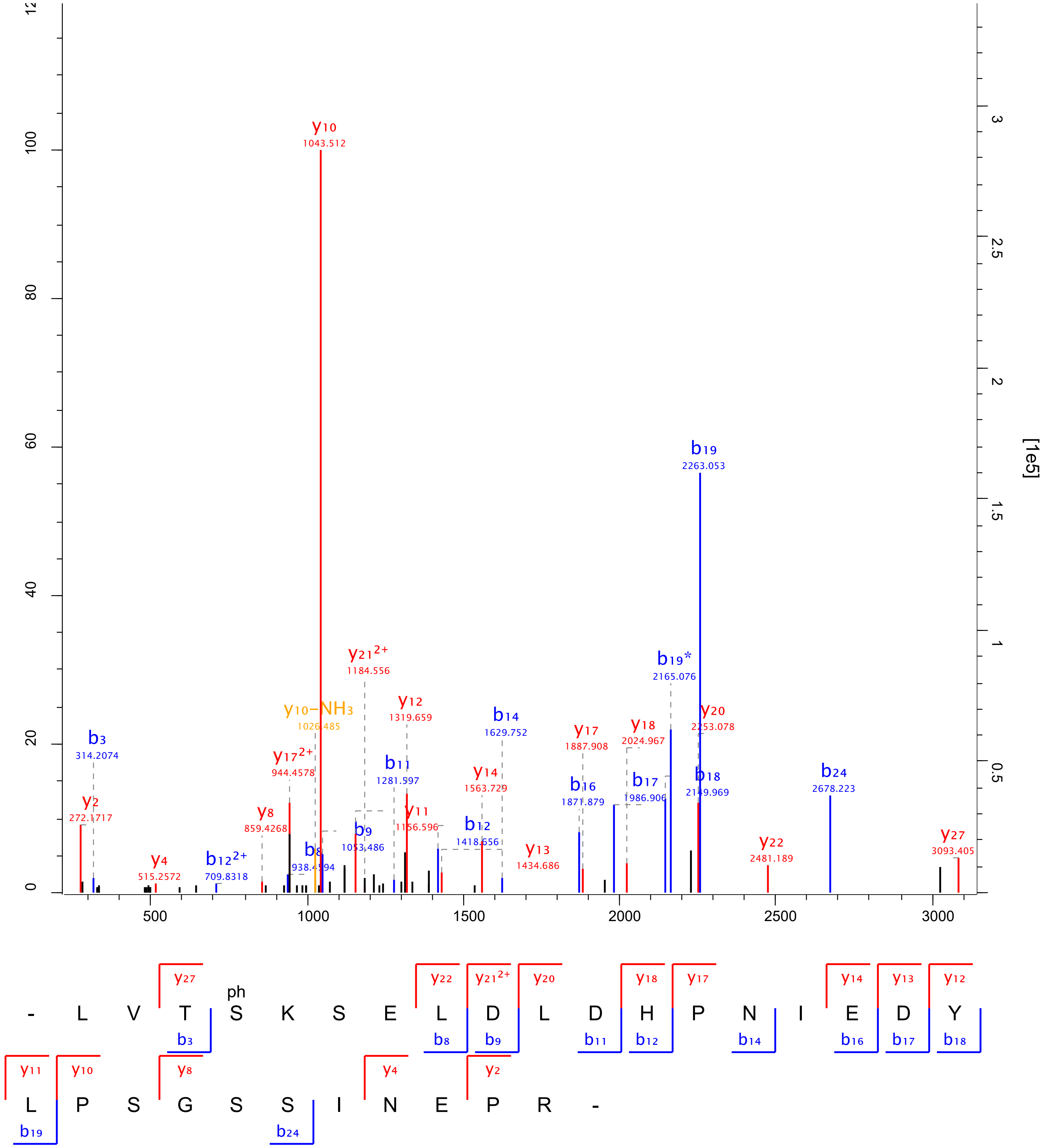

Sample output to test PDF Combine only

Raw file	Scan	Method	Score	m/z	Gene names
0523_1	22648	FTMS; HCD	221.45	965.08	SYP132

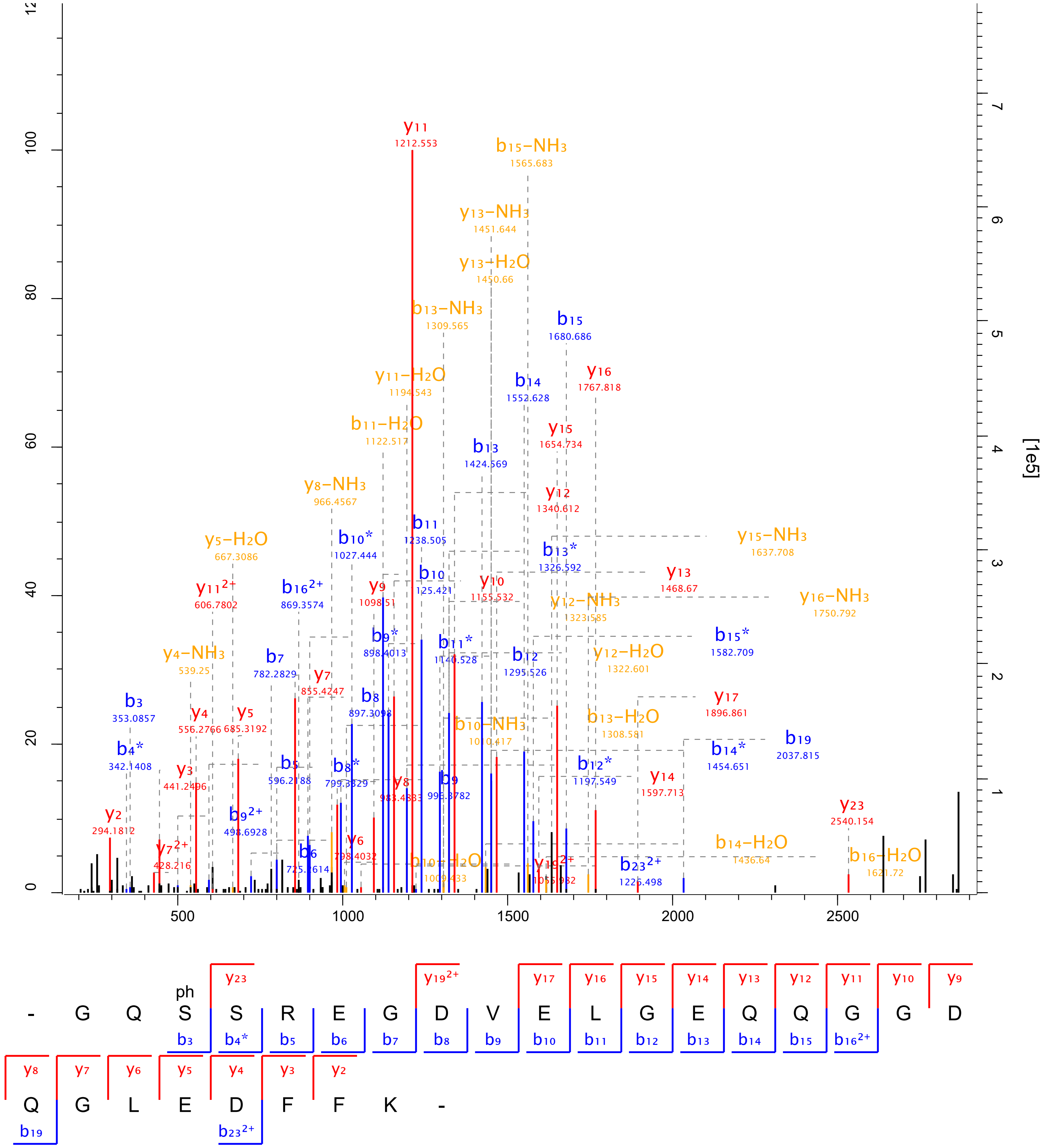

Sample output to test PDF Combine only

Raw file	Scan	Method	Score	m/z	Gene names
0523_1	22827	FTMS; HCD	51.83	802.74	At4g14350;At3g23310;At1g03920

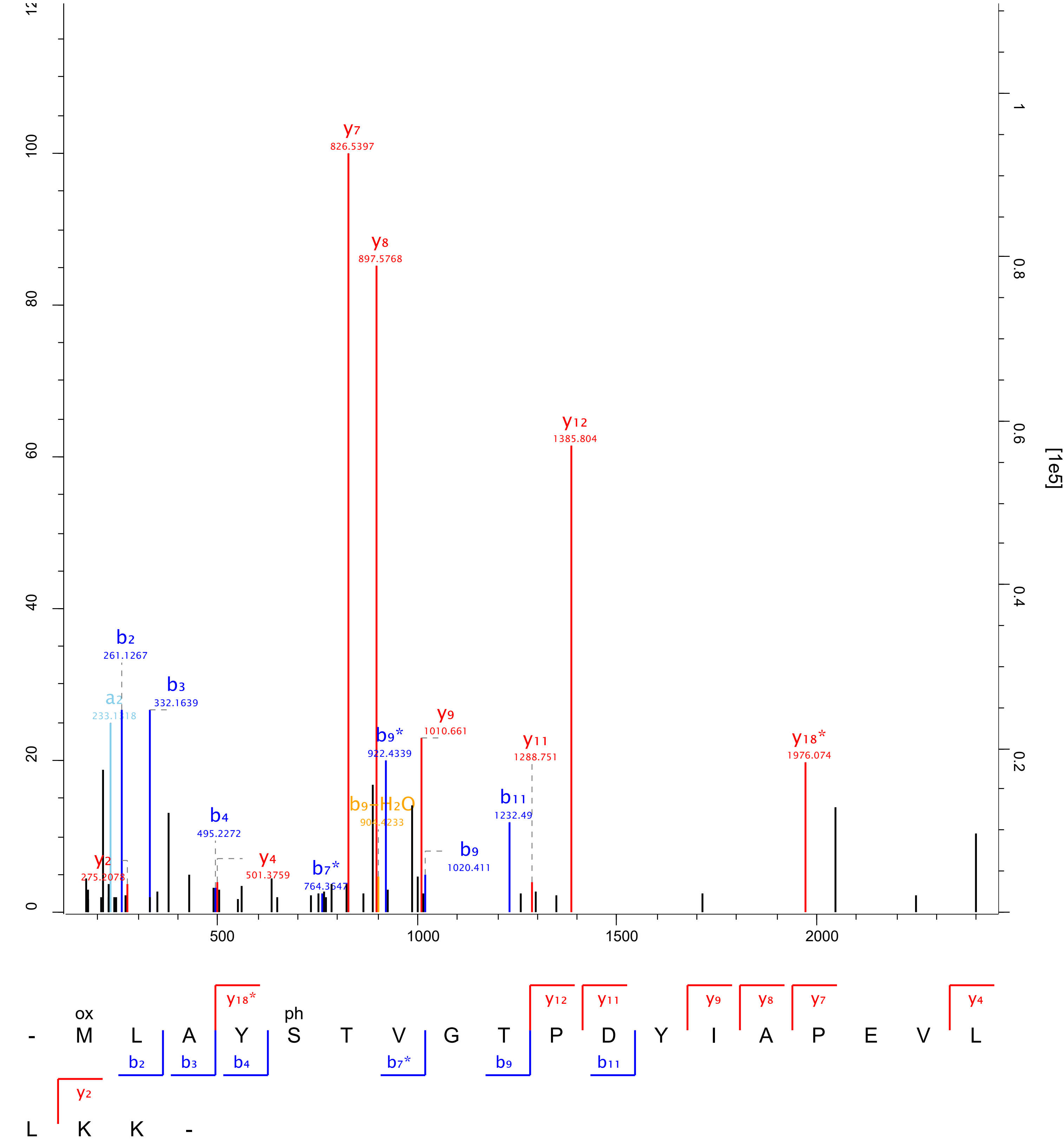

Sample output to test PDF Combine only

Raw file	Scan	Method	Score	m/z	Gene names
0523_1	22836	FTMS; HCD	143.07	841.73	PRA1F3

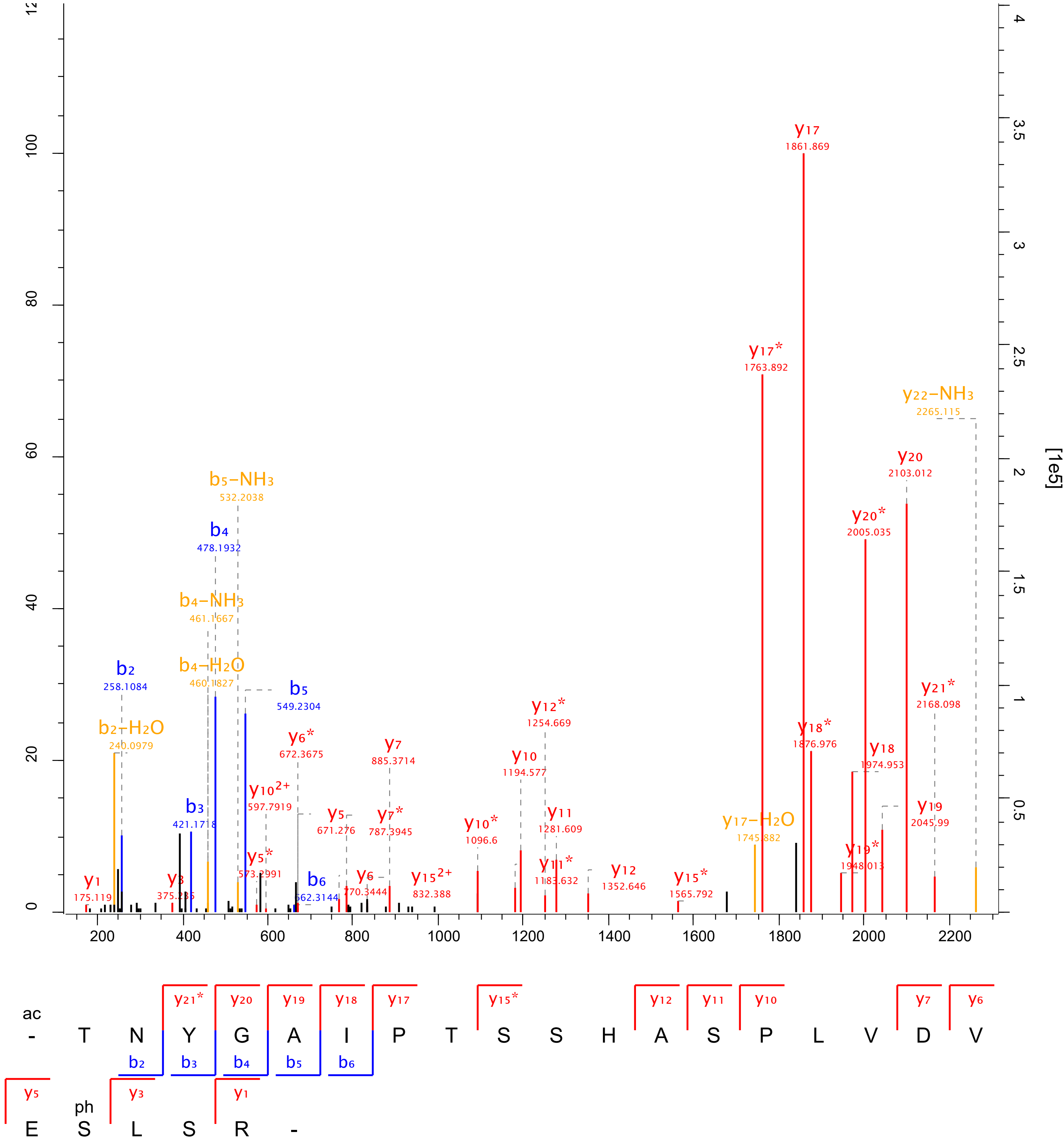

Sample output to test PDF Combine only

Raw file	Scan	Method	Score	m/z	Gene names
0523_1	23248	FTMS; HCD	61.42	761.02	At1g58025

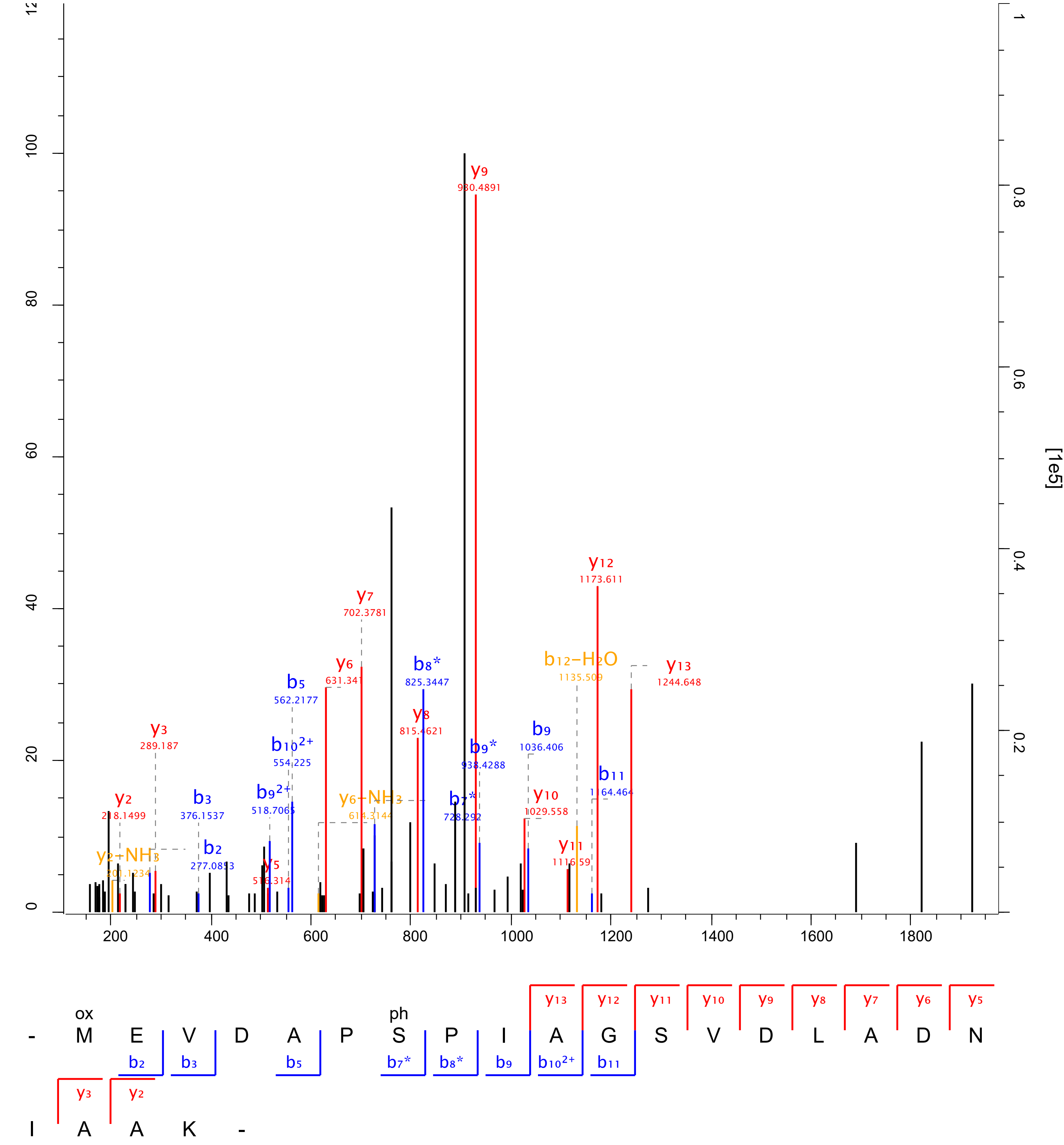

Sample output to test PDF Combine only

Raw file	Scan	Method	Score	m/z	Gene names
0523_1	23478	FTMS; HCD	63.78	1054.44	At1g76810

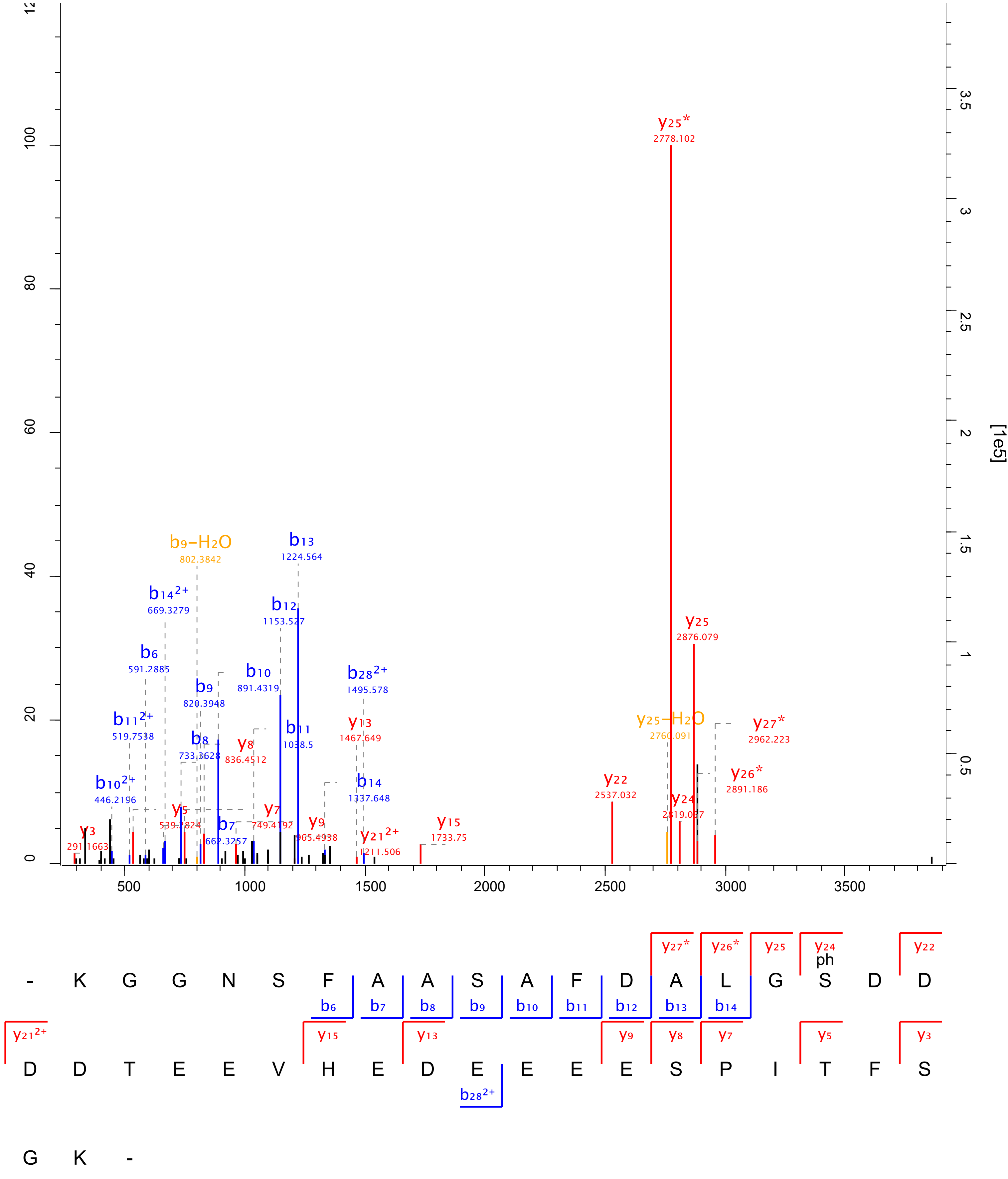

Sample output to test PDF Combine only

Raw file	Scan	Method	Score	m/z
0523_1	24961	FTMS; HCD	96.38	819.01

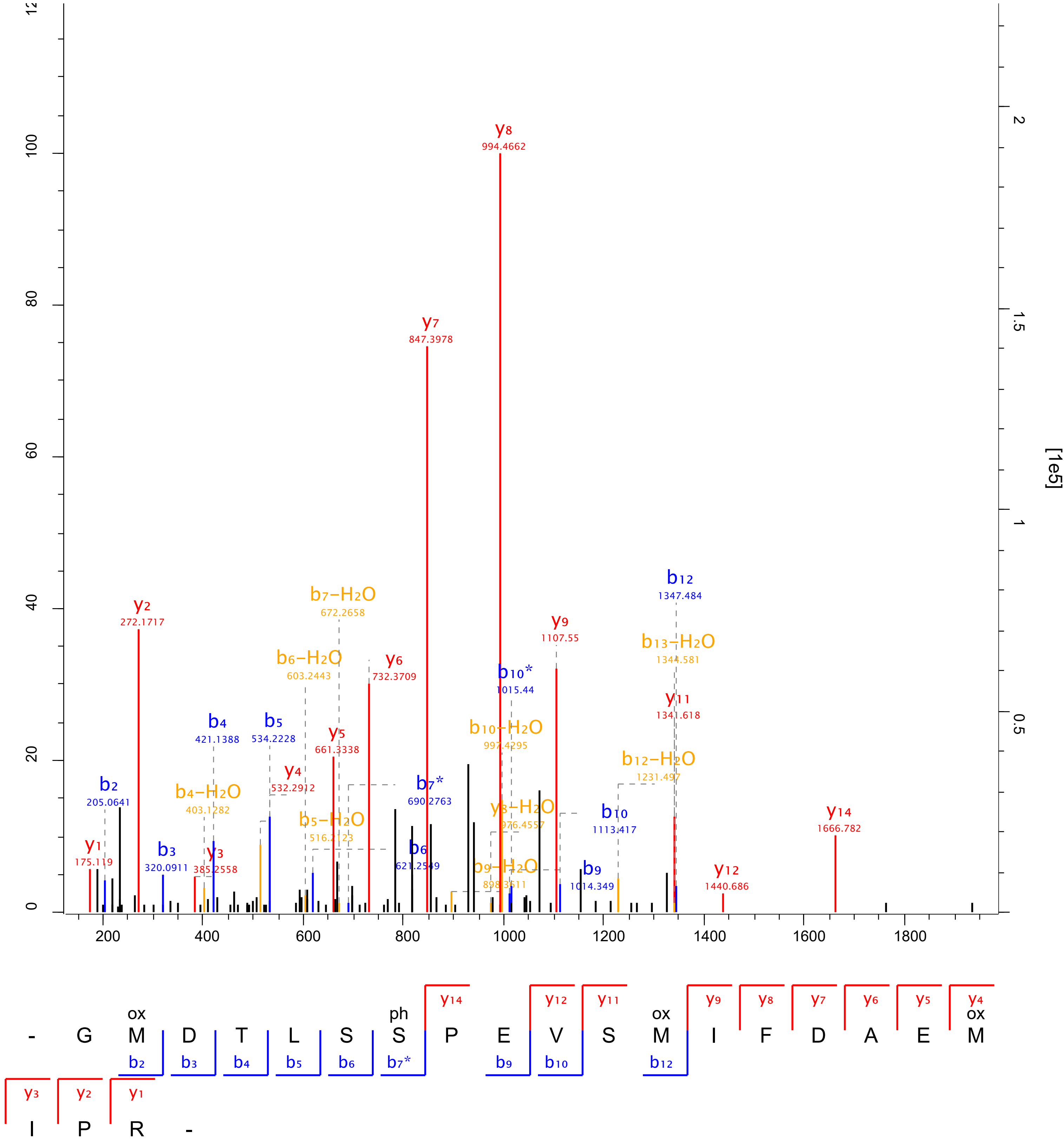

Sample output to test PDF Combine only

Raw file	Scan	Method	Score	m/z	Gene names
0523_1	25401	FTMS; HCD	118.51	1196.52	At4g31880

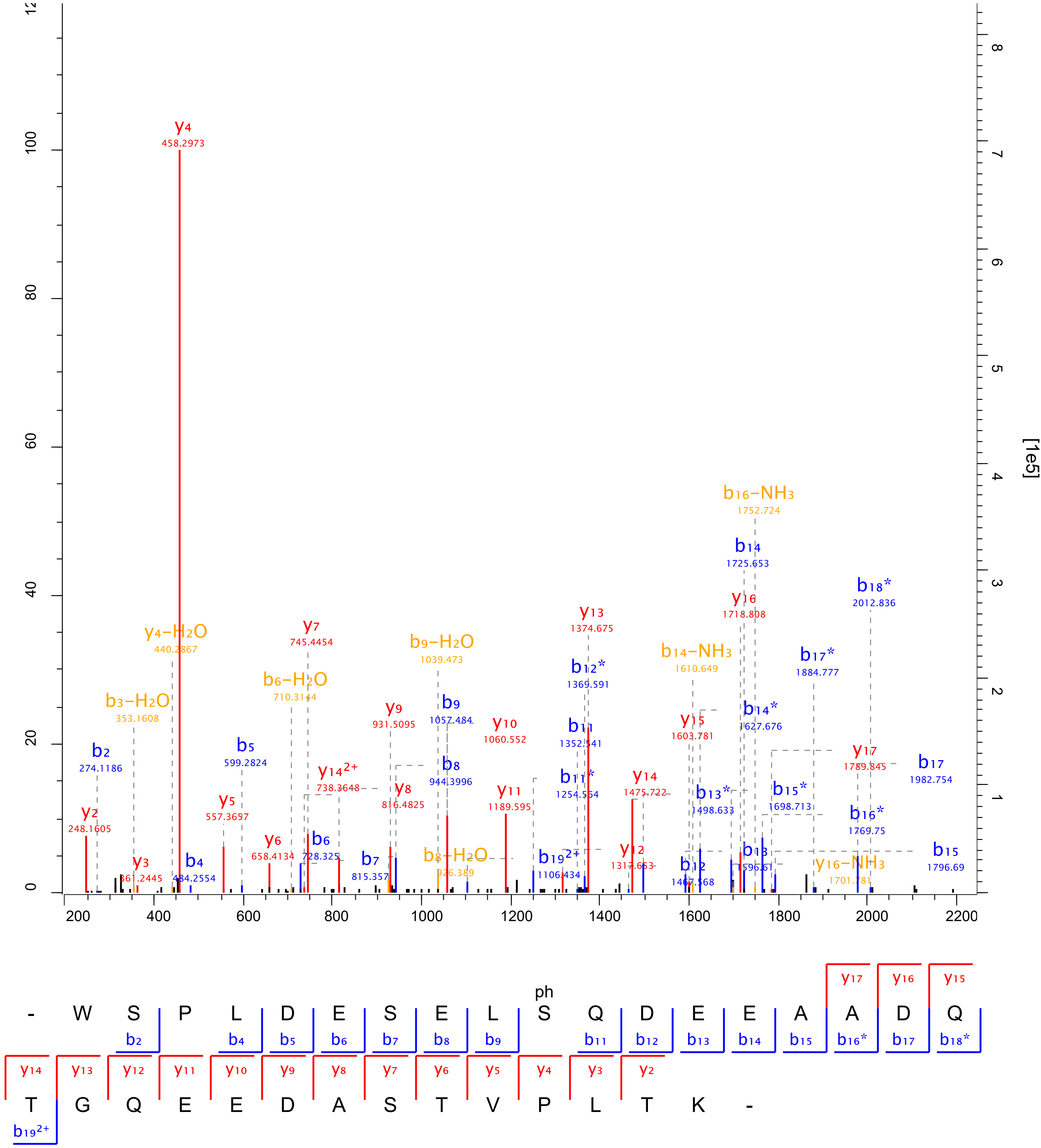

Sample output to test PDF Combine only

Raw file	Scan	Method	Score	m/z	Gene names
0523_1	25530	FTMS; HCD	67.23	820.03	ANN2;ANNAT2

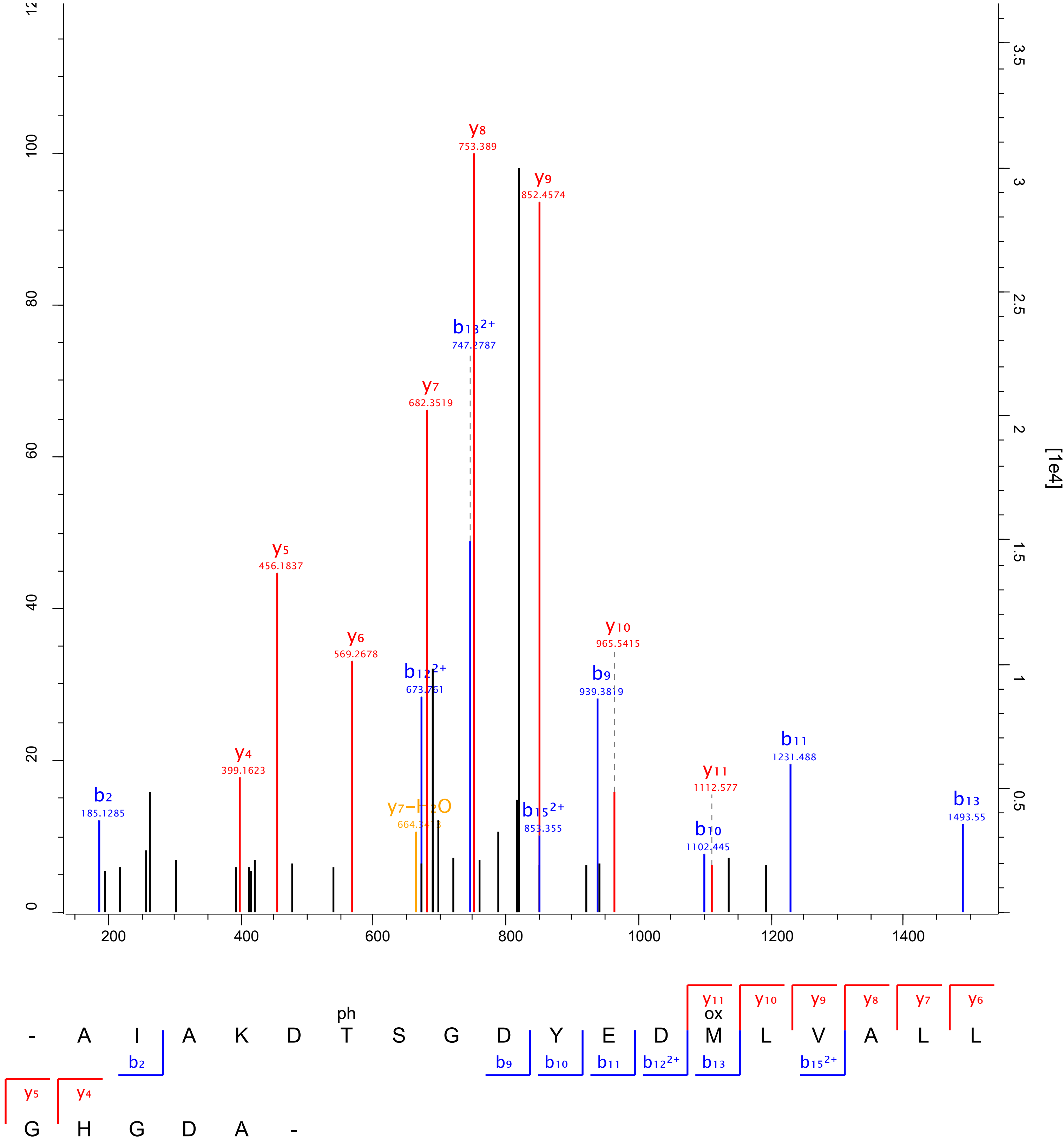

Sample output to test PDF Combine only

Raw file	Scan	Method	Score	m/z	Gene names
0523_1	25844	FTMS; HCD	88.87	831.74	T28J14_190

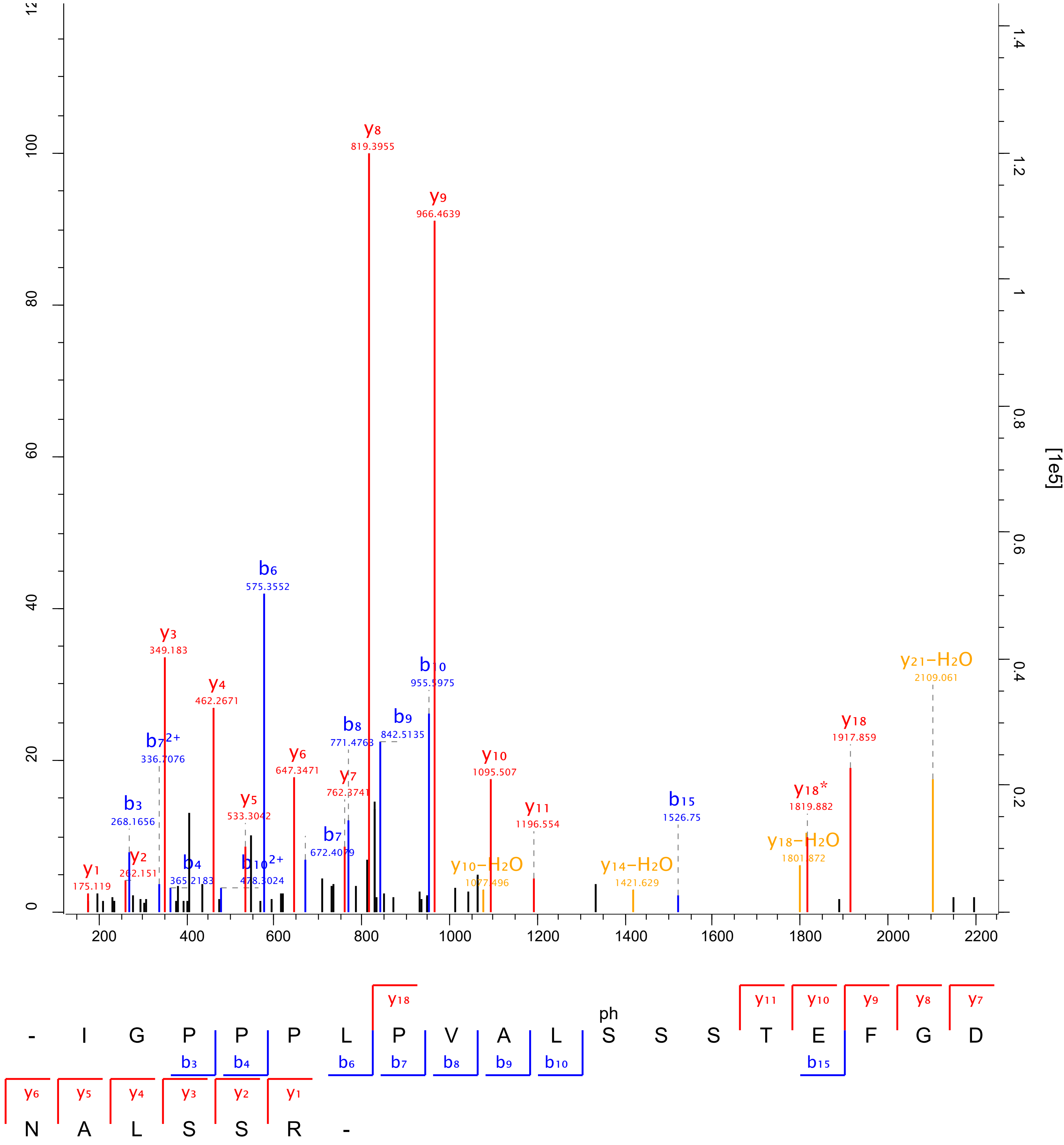

Sample output to test PDF Combine only

Raw file	Scan	Method	Score	m/z	Gene names
0523_1	26160	FTMS; HCD	58.37	803.33	RPP1B

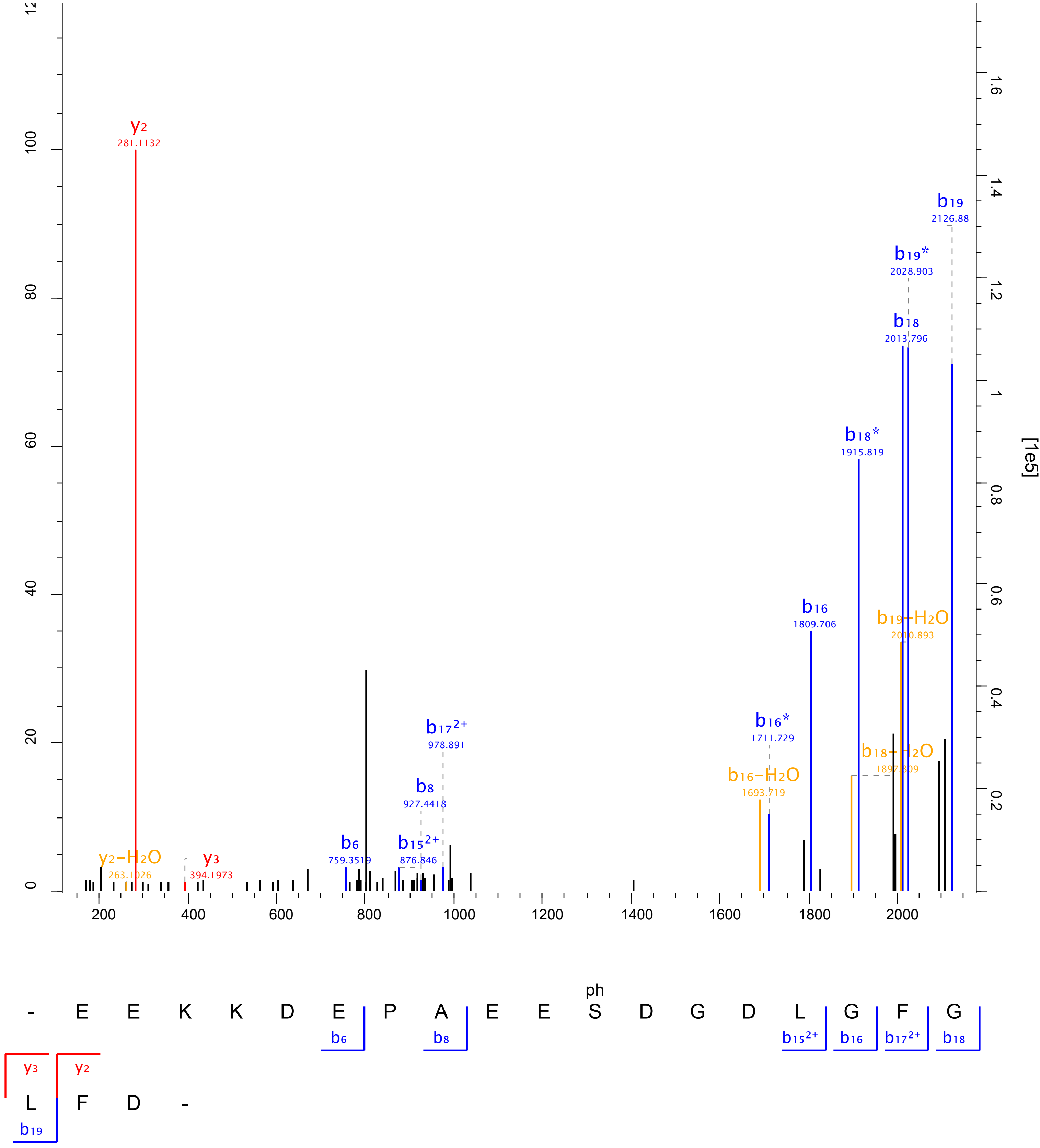

Sample output to test PDF Combine only

Raw file	Scan	Method	Score	m/z	Gene names
0523_1	27243	FTMS; HCD	183.09	1088.96	PIP2-2

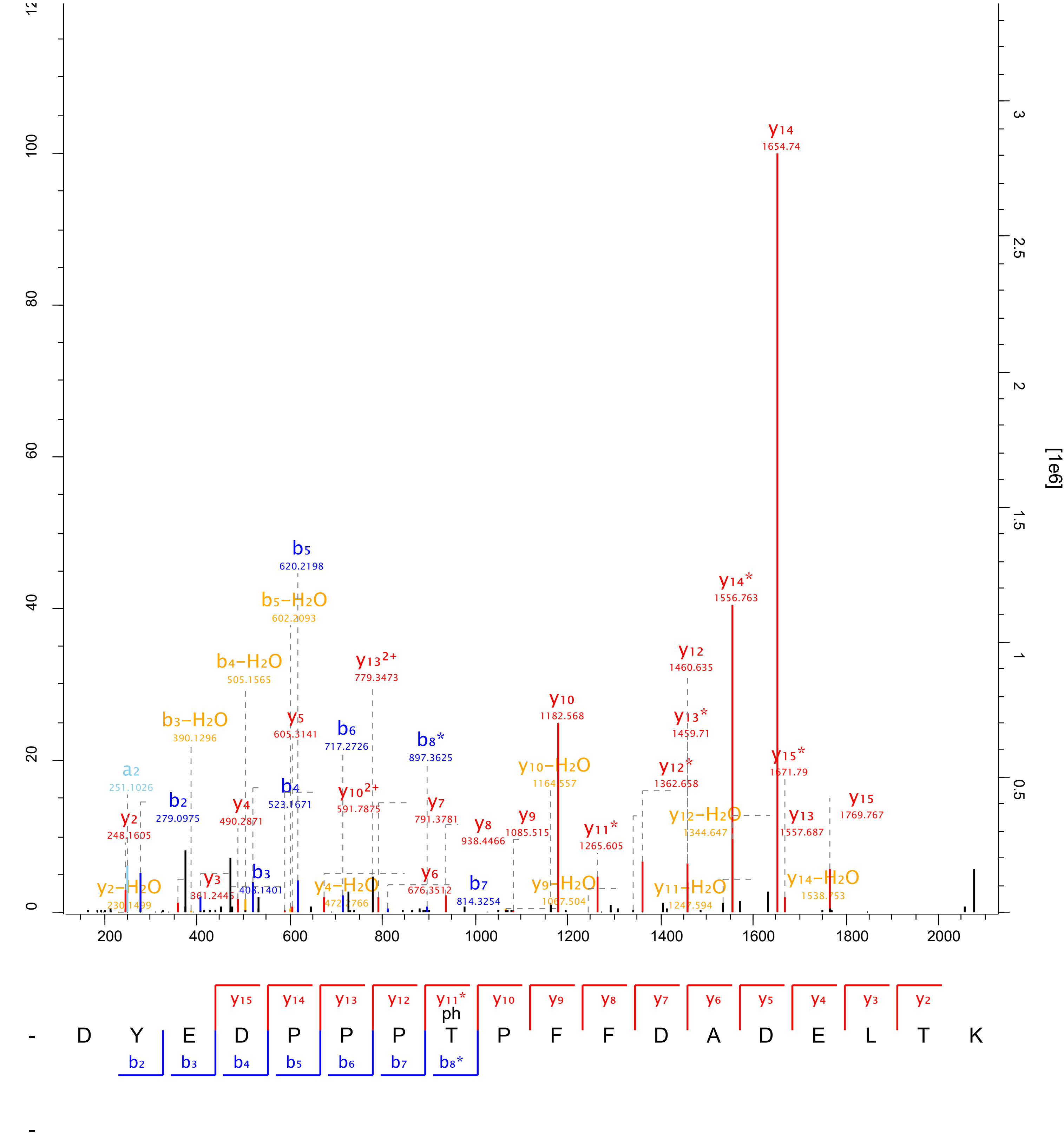

Sample output to test PDF Combine only

Raw file	Scan	Method	Score	m/z	Gene names
0523_1	27473	FTMS; HCD	100.95	824.38	F23M19.10;At1g34220

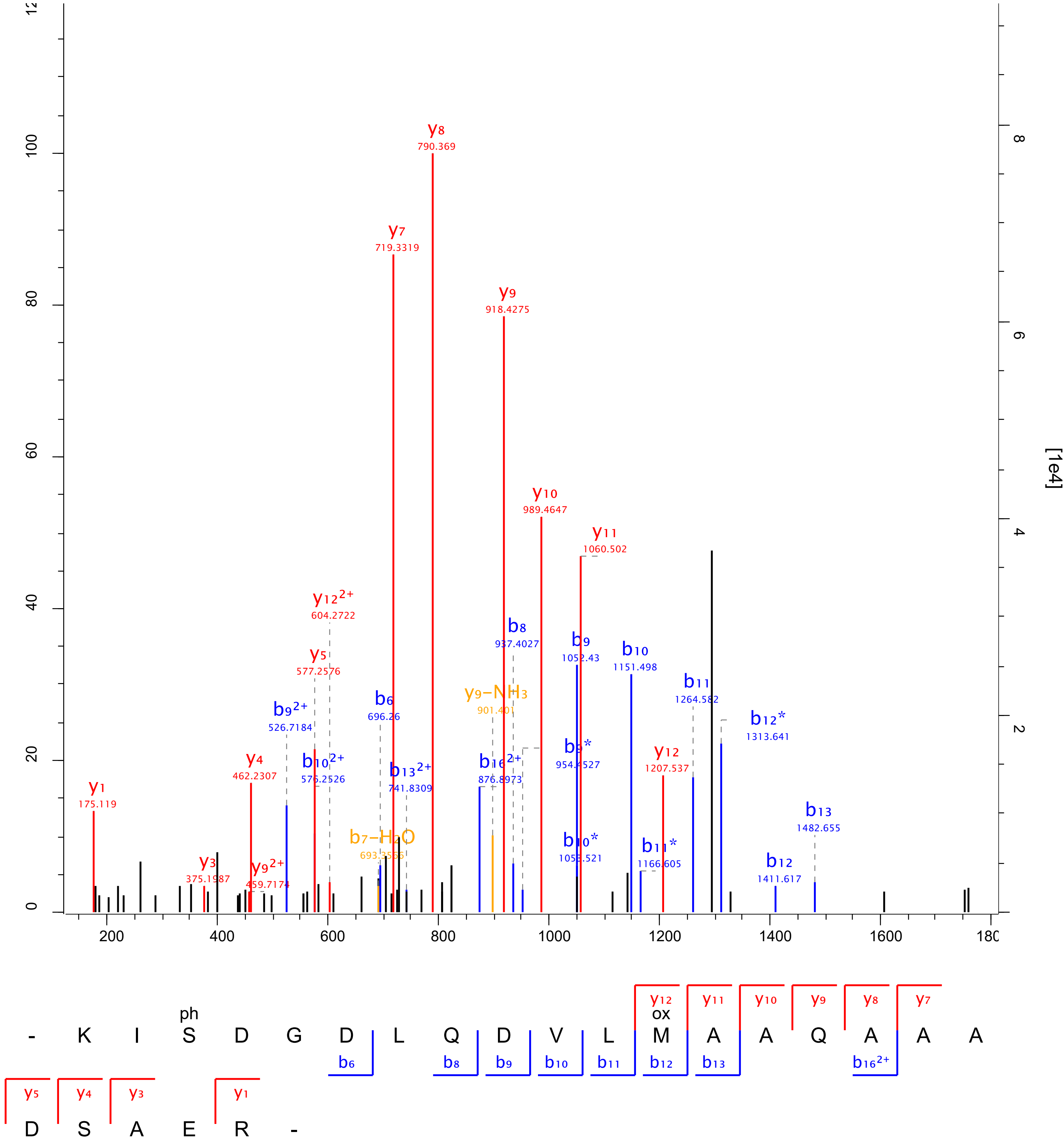

Sample output to test PDF Combine only

Raw file	Scan	Method	Score	m/z	Gene names
0523_1	27524	FTMS; HCD	93.5	821.39	SYP132

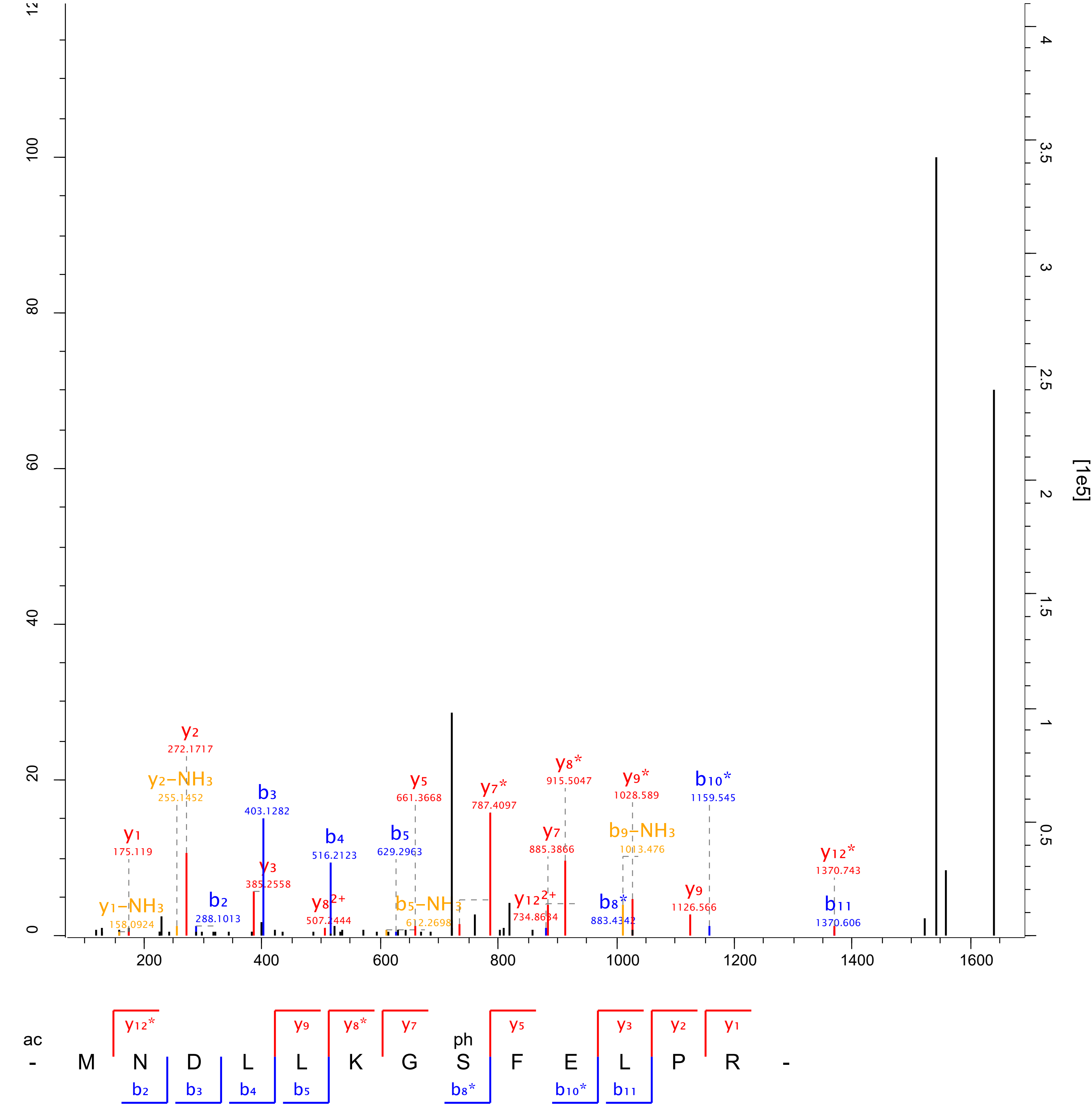

Sample output to test PDF Combine only

Raw file	Scan	Method	Score	m/z	Gene names
0523_1	27590	FTMS; HCD	100.85	1096.46	PIP2-3

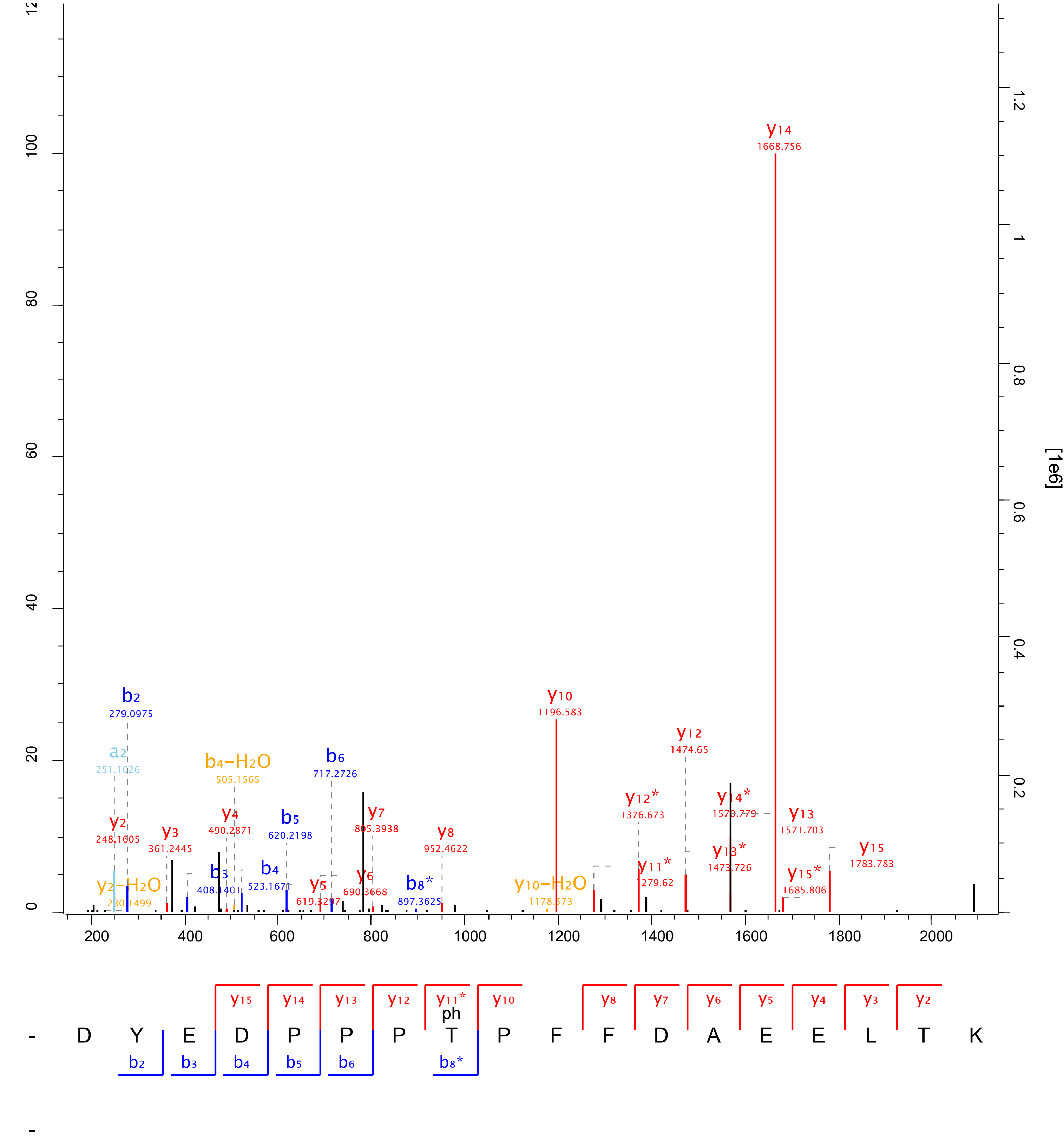

Sample output to test PDF Combine only

Raw file	Scan	Method	Score	m/z
0523_1	28436	FTMS; HCD	181.88	1047.87

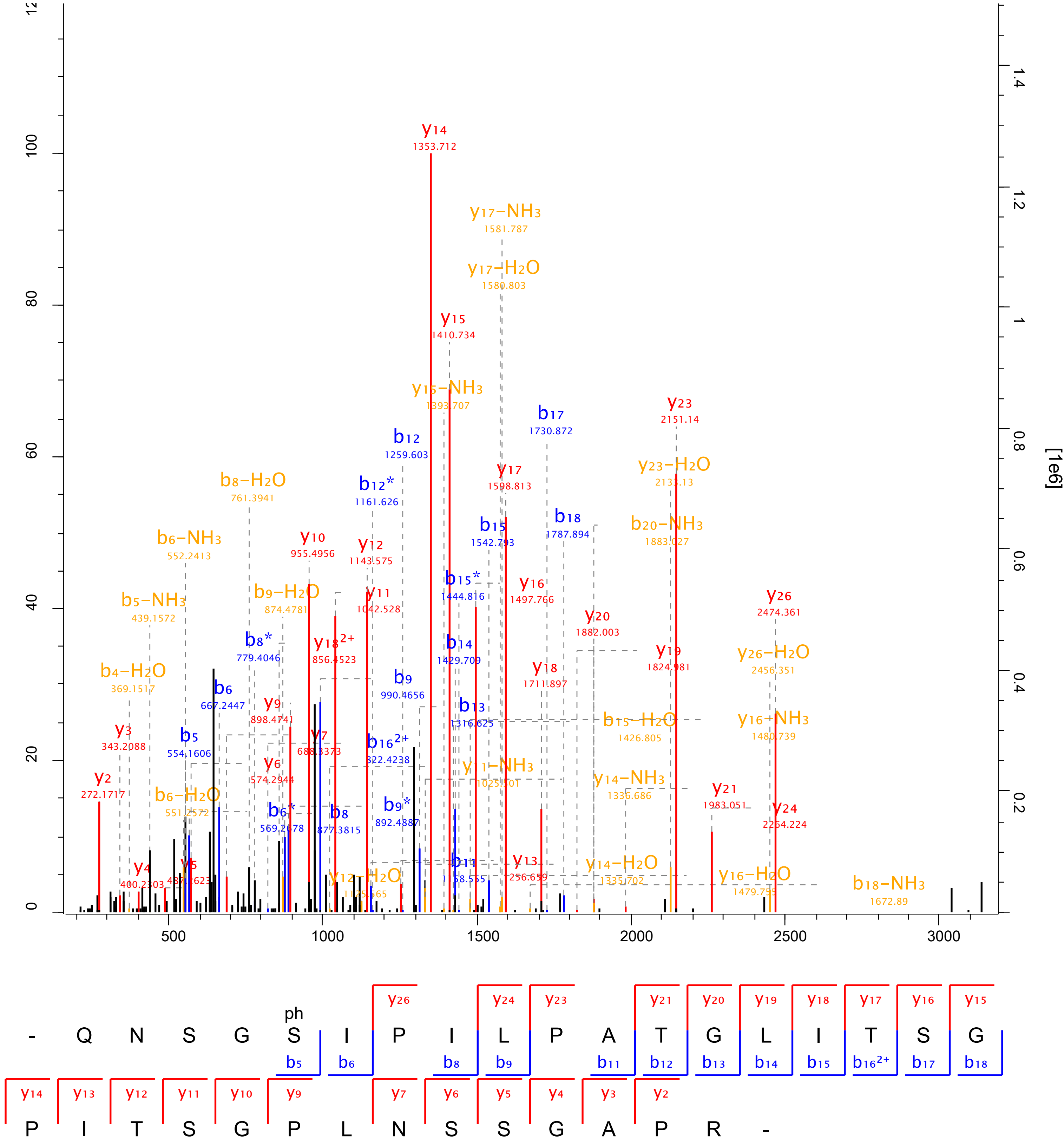

Sample output to test PDF Combine only

Raw file	Scan	Method	Score	m/z	Gene names
0523_1	30270	FTMS; HCD	275.2	1011.41	RPP1C;At1g01100;RPP1A;RPP1B

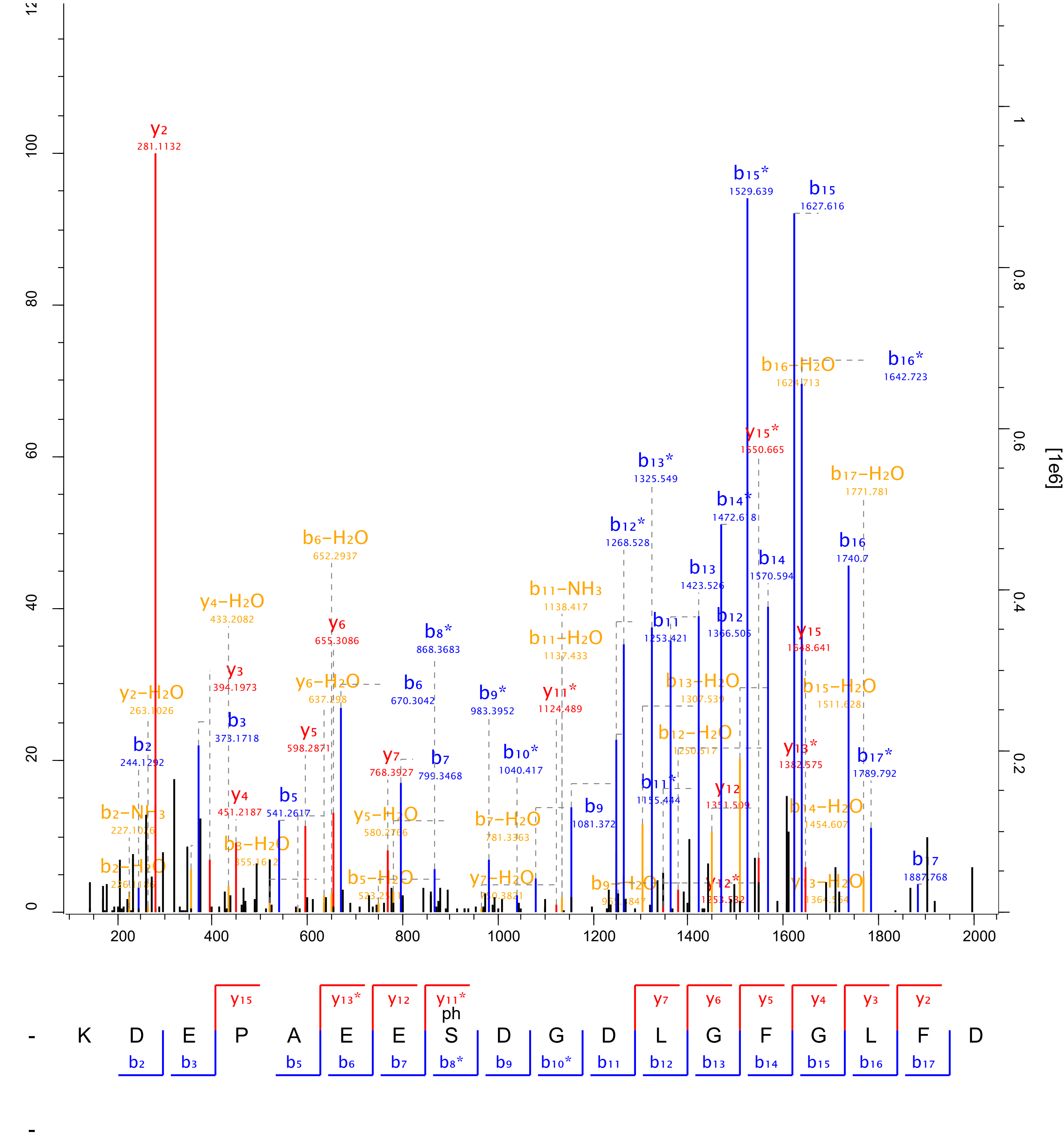

Sample output to test PDF Combine only

Raw file	Scan	Method	Score	m/z	Gene names
0523_1	31260	FTMS; HCD	115.54	1076.47	CBL2;CBL3

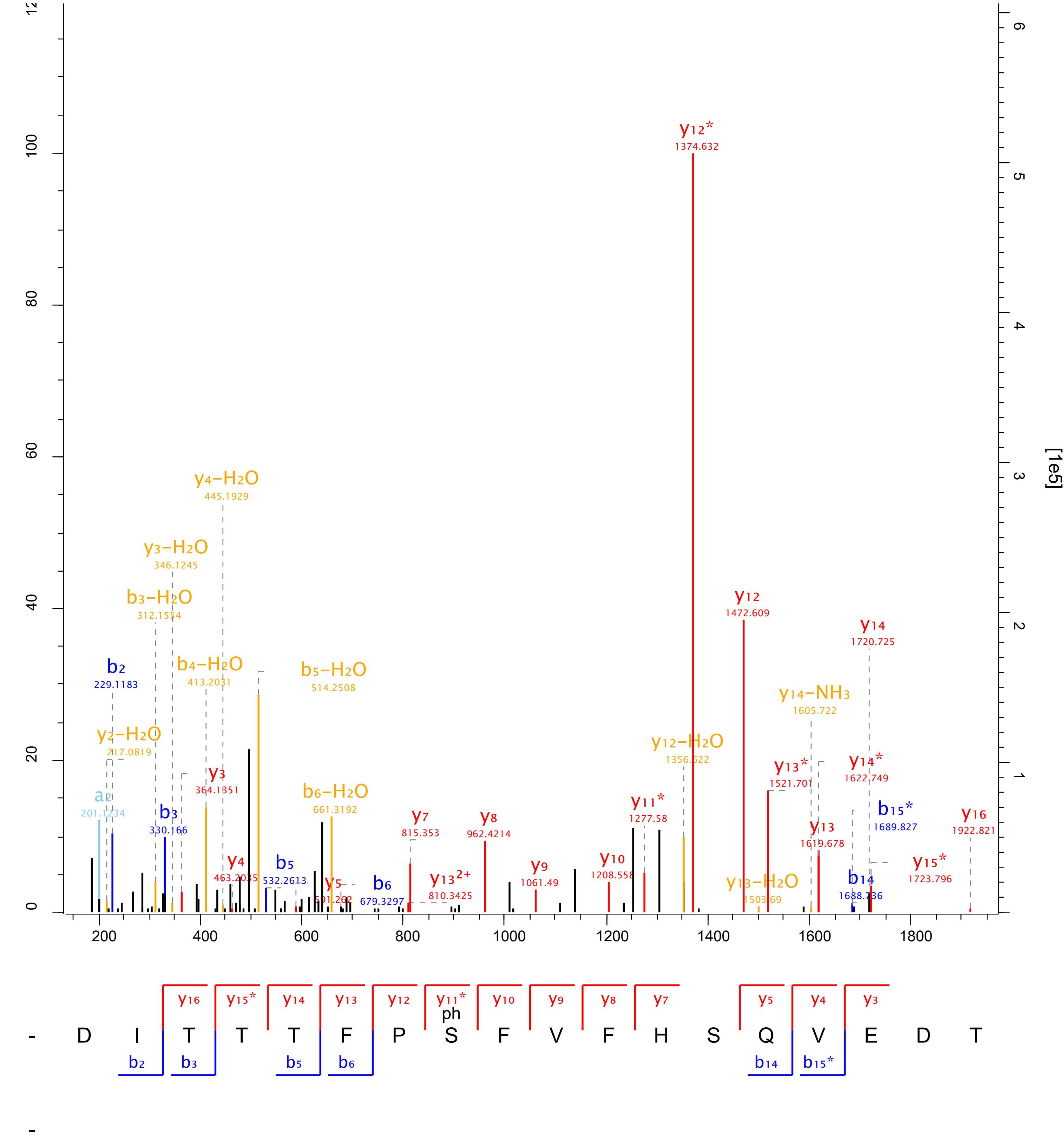

Sample output to test PDF Combine only

Raw file	Scan	Method	Score	m/z	Gene names
0523_1	31374	FTMS; HCD	118.27	1036.18	CALS2

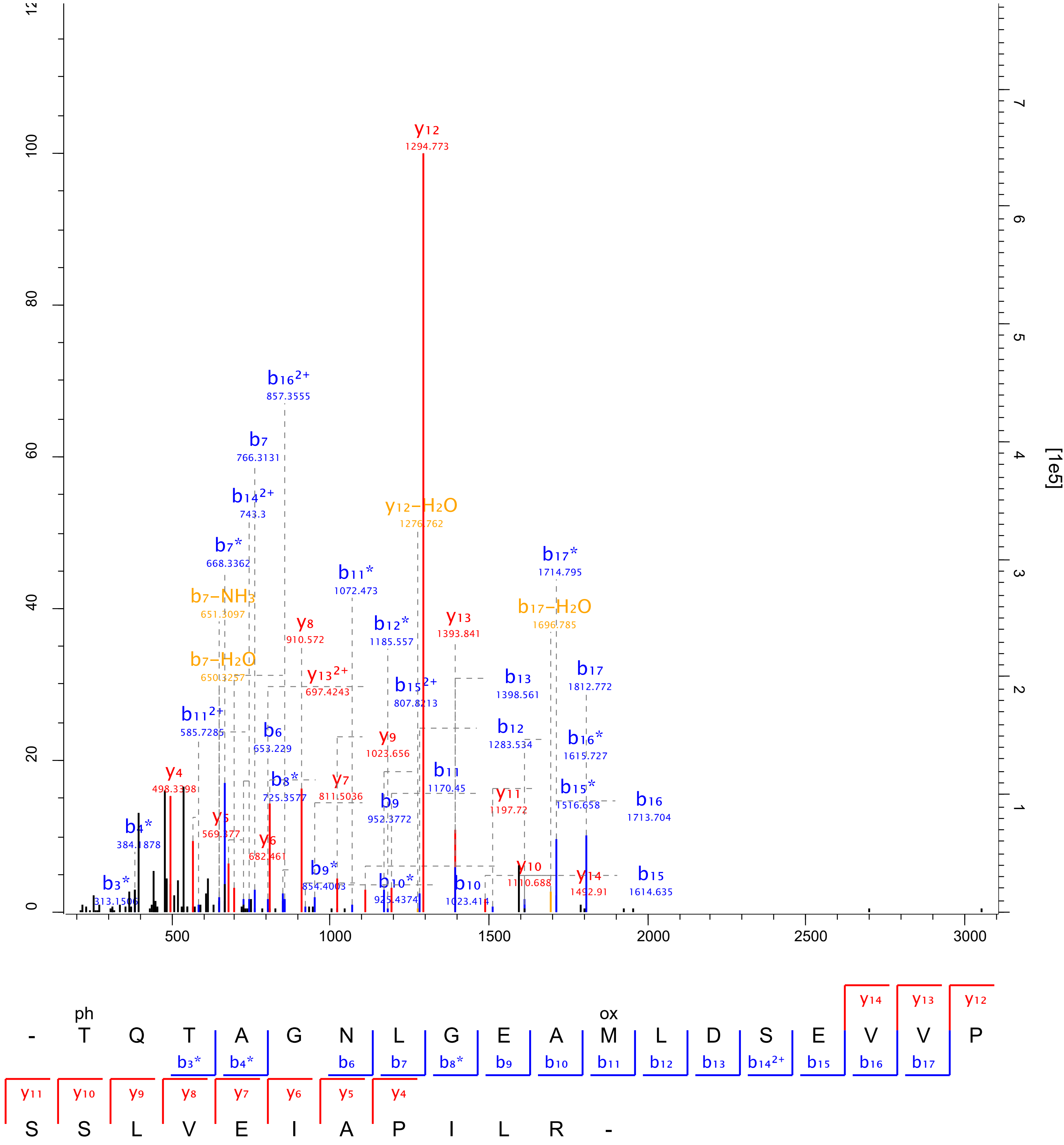

Sample output to test PDF Combine only

Raw file	Scan	Method	Score	m/z	Gene names
0523_1	31836	FTMS; HCD	90.56	1037.19	CALS1

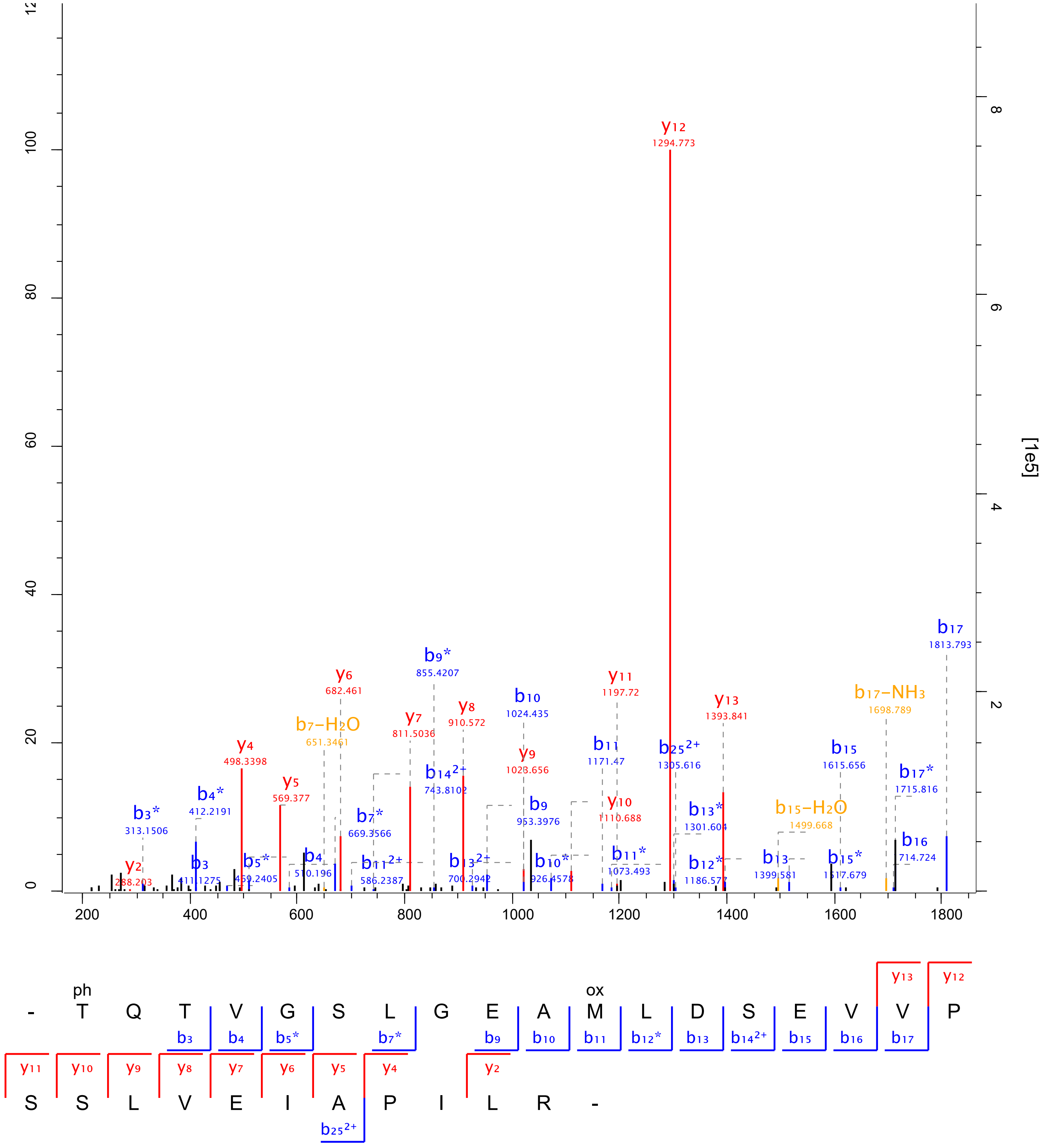

Sample output to test PDF Combine only

Raw file	Scan	Method	Score	m/z	Gene names
0523_2	1463	FTMS; HCD	244.68	605.26	At5g40450

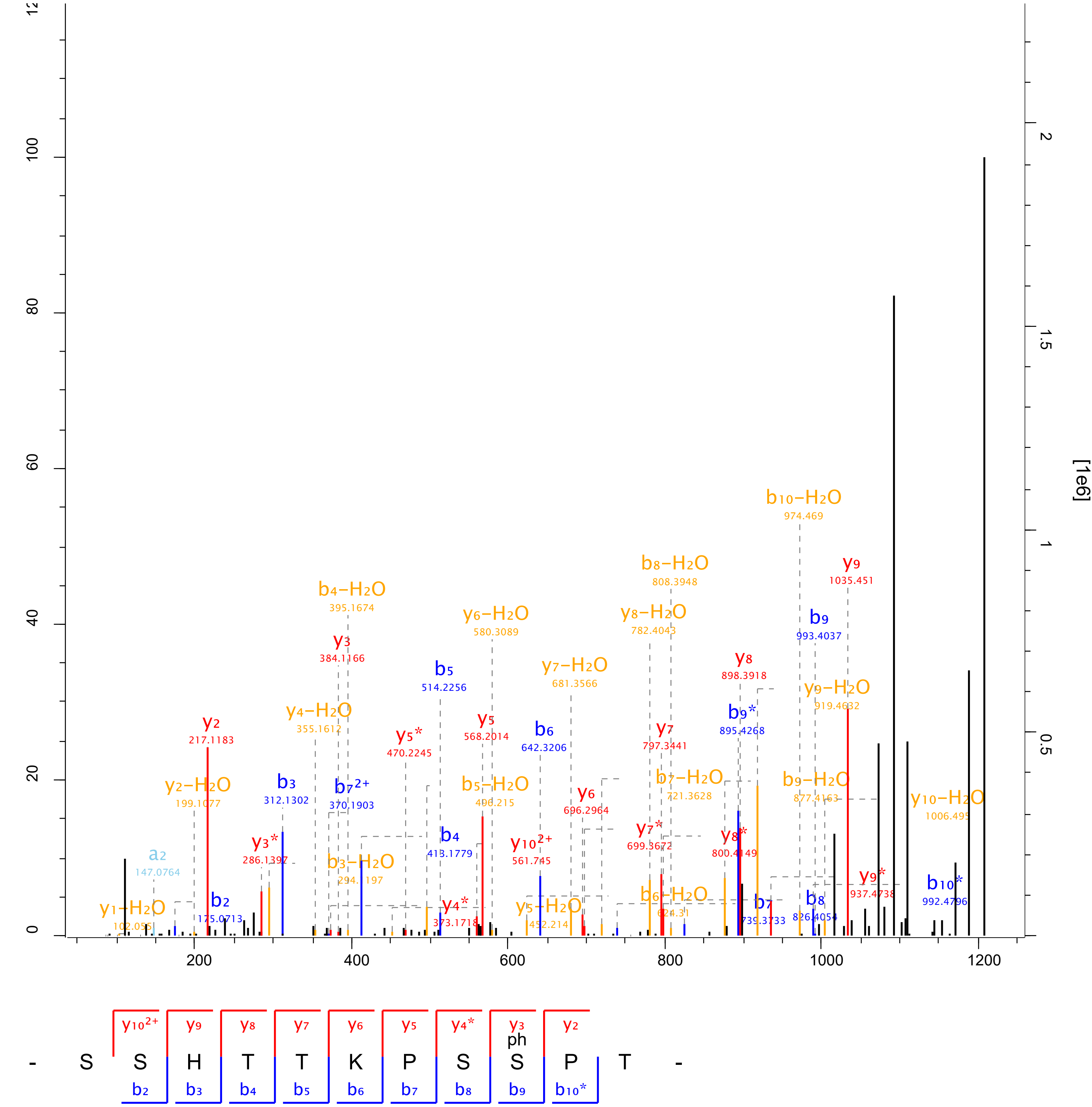

Sample output to test PDF Combine only

Raw file	Scan	Method	Score	m/z	Gene names
0523_2	3363	FTMS; HCD	73.5	494.69	ABCC14

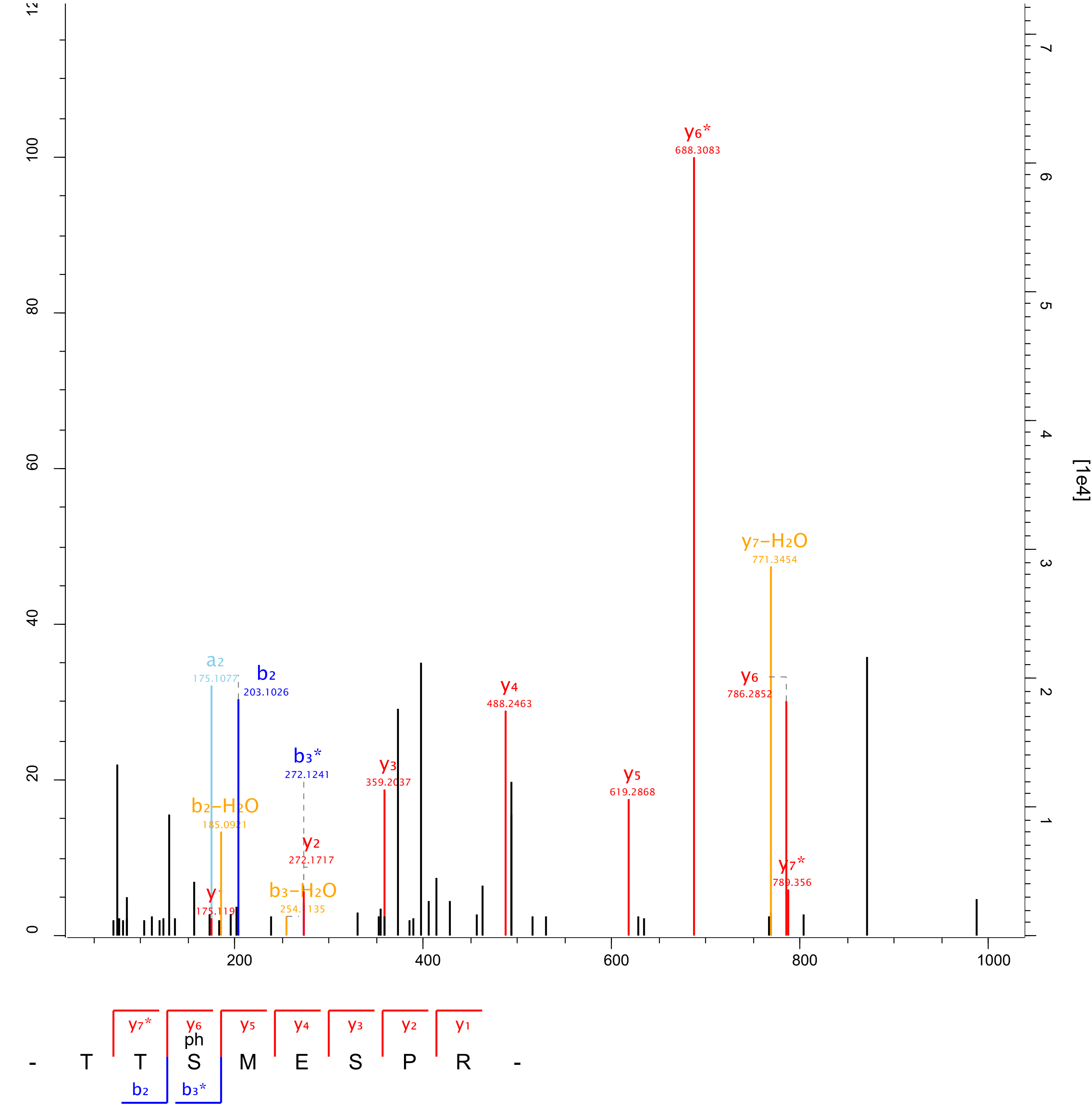

Sample output to test PDF Combine only

Raw file	Scan	Method	Score	m/z	Gene names
0523_2	4468	FTMS; HCD	51.01	367.18	SR45

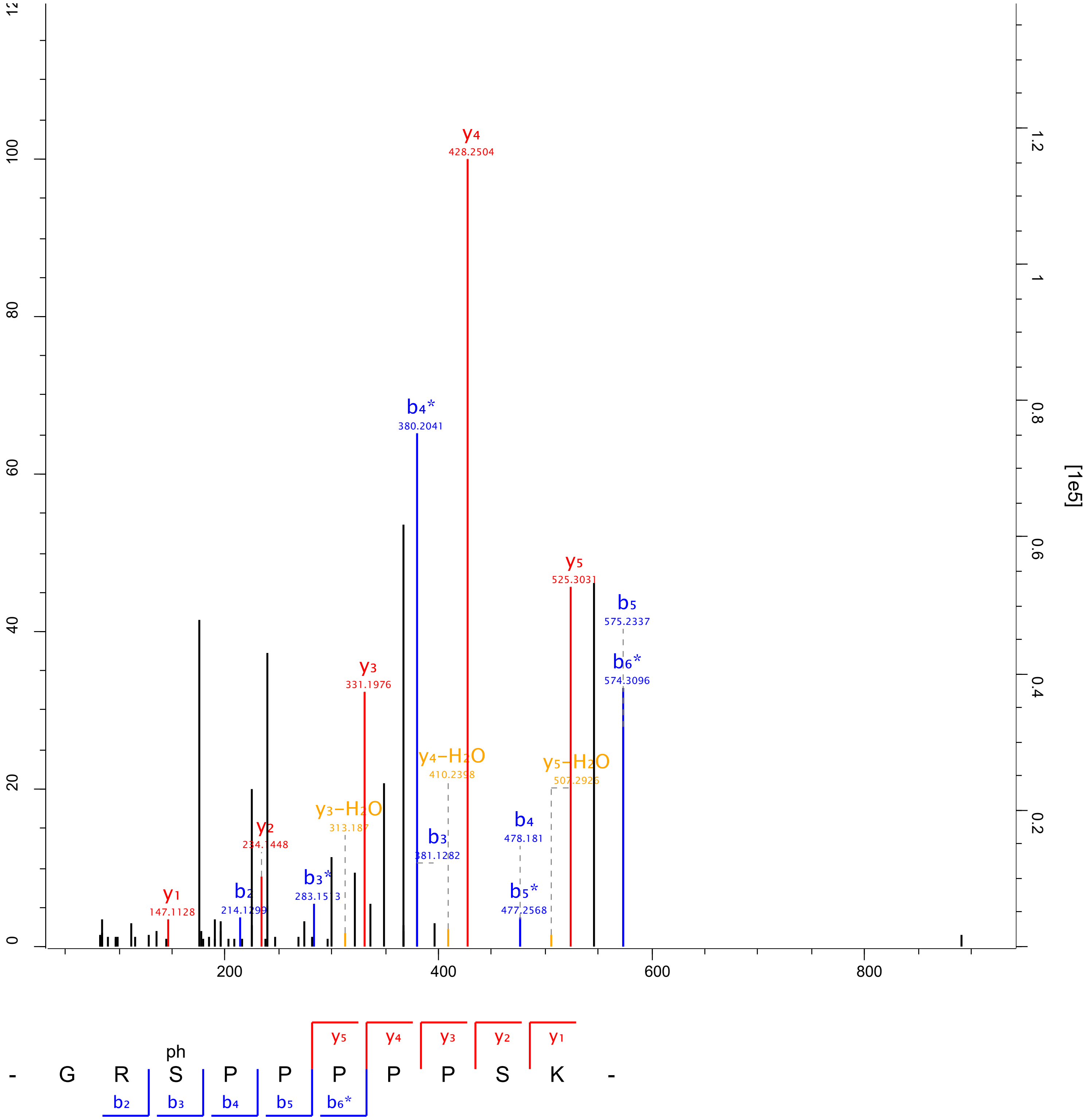

Sample output to test PDF Combine only

Raw file	Scan	Method	Score	m/z	Gene names
0523_2	6788	FTMS; HCD	45.61	576.25	SE

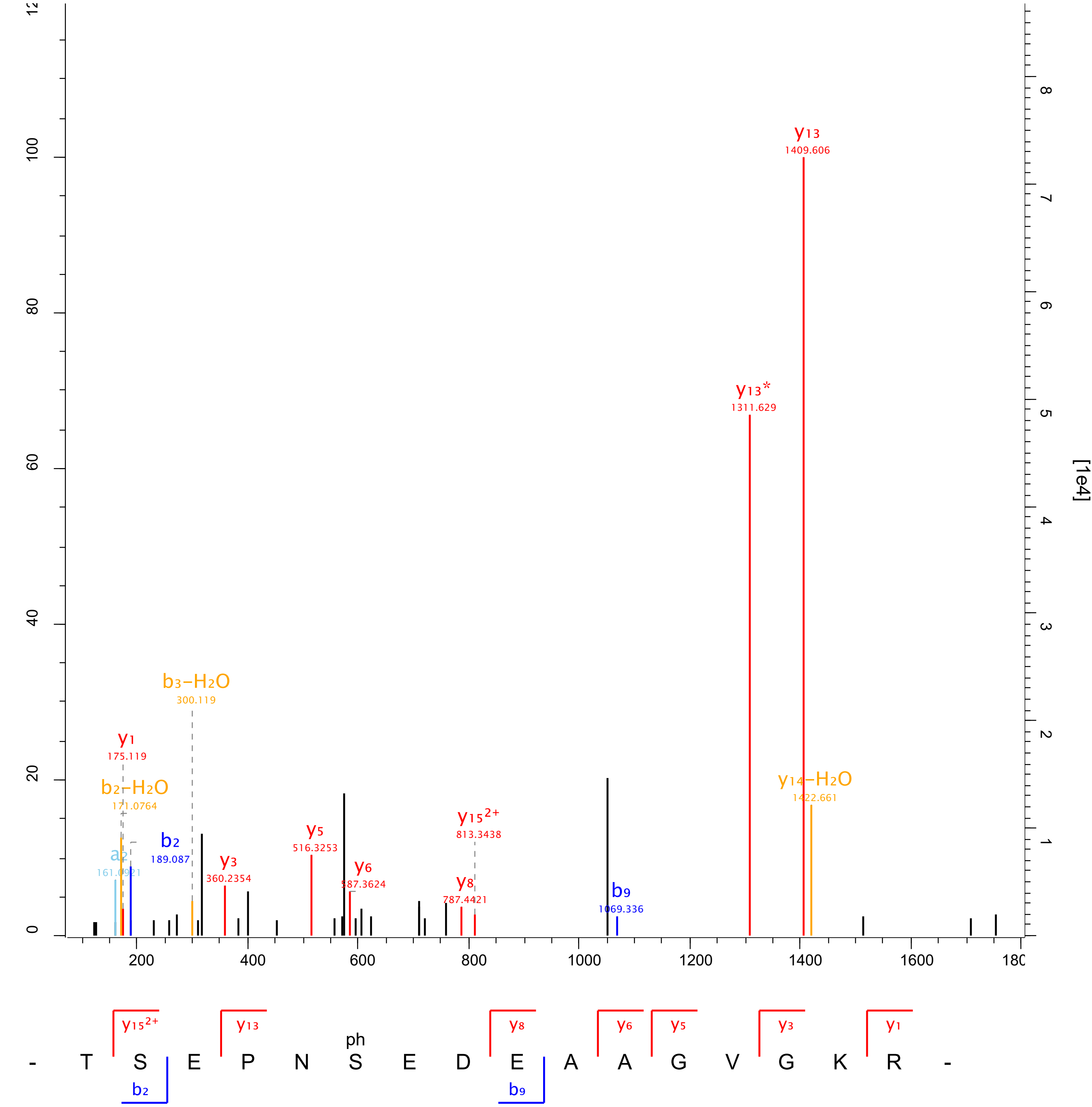

Sample output to test PDF Combine only

Raw file	Scan	Method	Score	m/z	Gene names
0523_2	6858	FTMS; HCD	52.9	702.95	SE

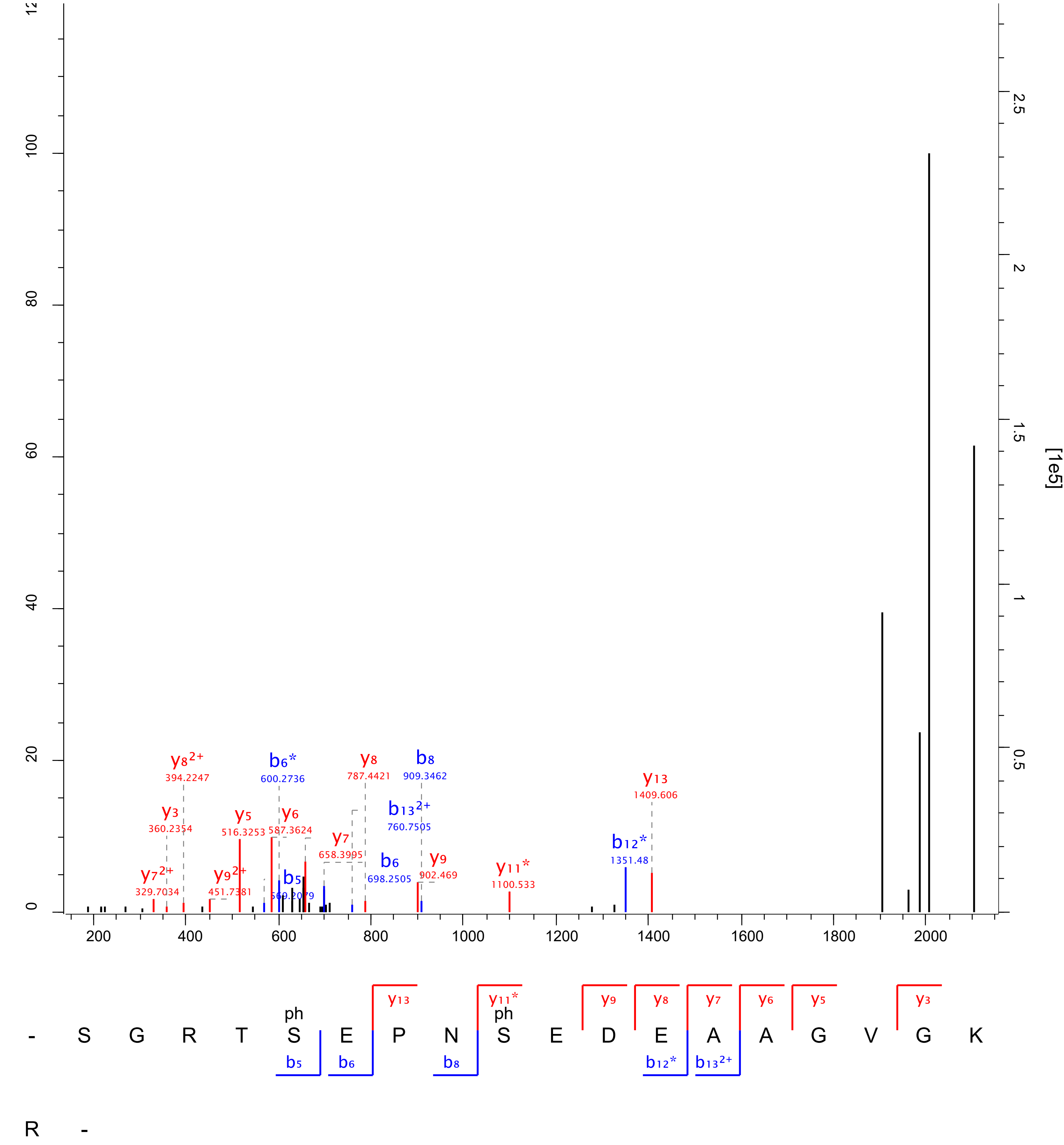

Sample output to test PDF Combine only

Raw file	Scan	Method	Score	m/z	Gene names
0523_2	7046	FTMS; HCD	91.59	602.23	AMT1-3

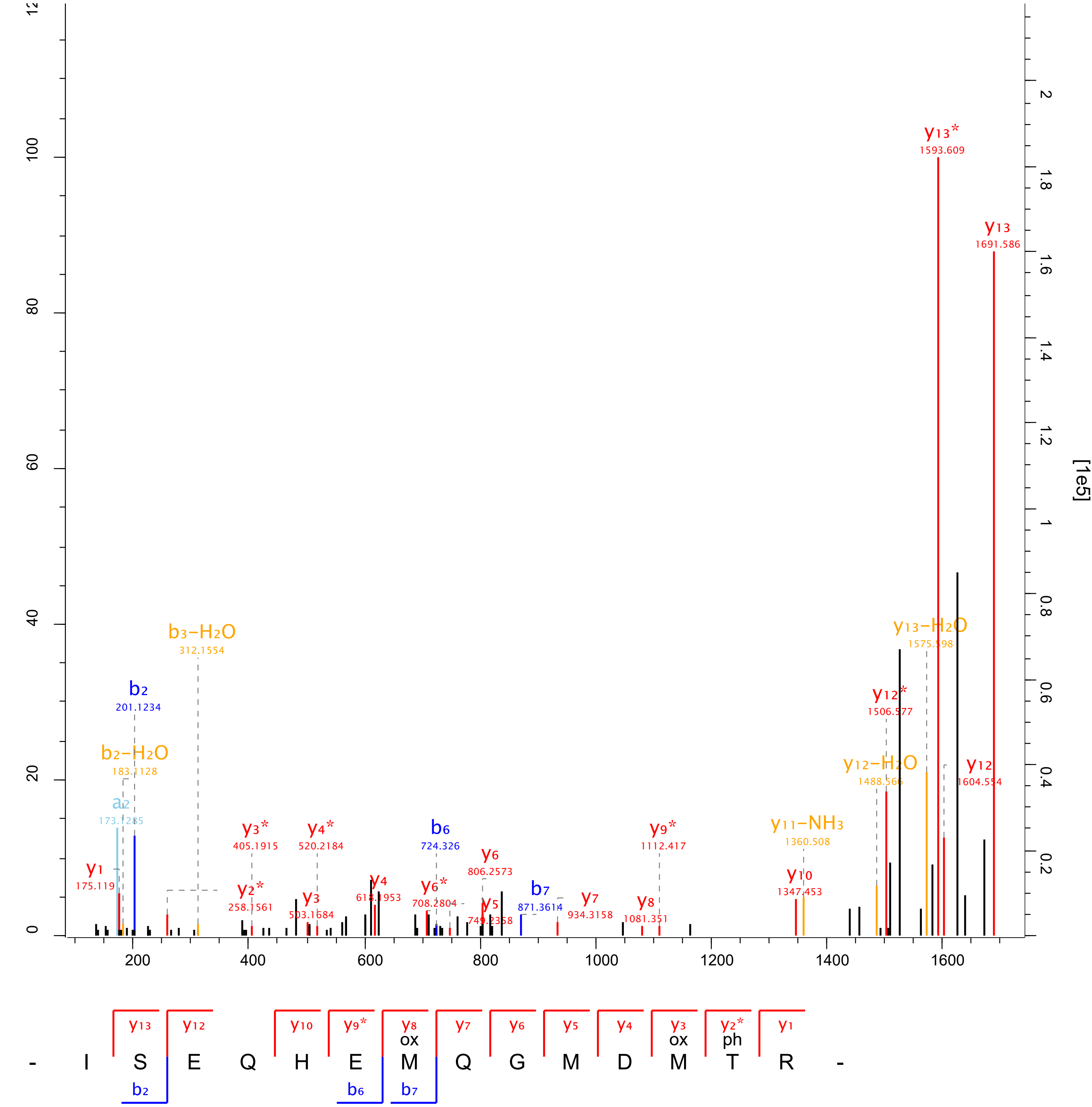

Sample output to test PDF Combine only

Raw file	Scan	Method	Score	m/z	Gene names
0523_2	8592	FTMS; HCD	190.39	609.27	PIP2-7

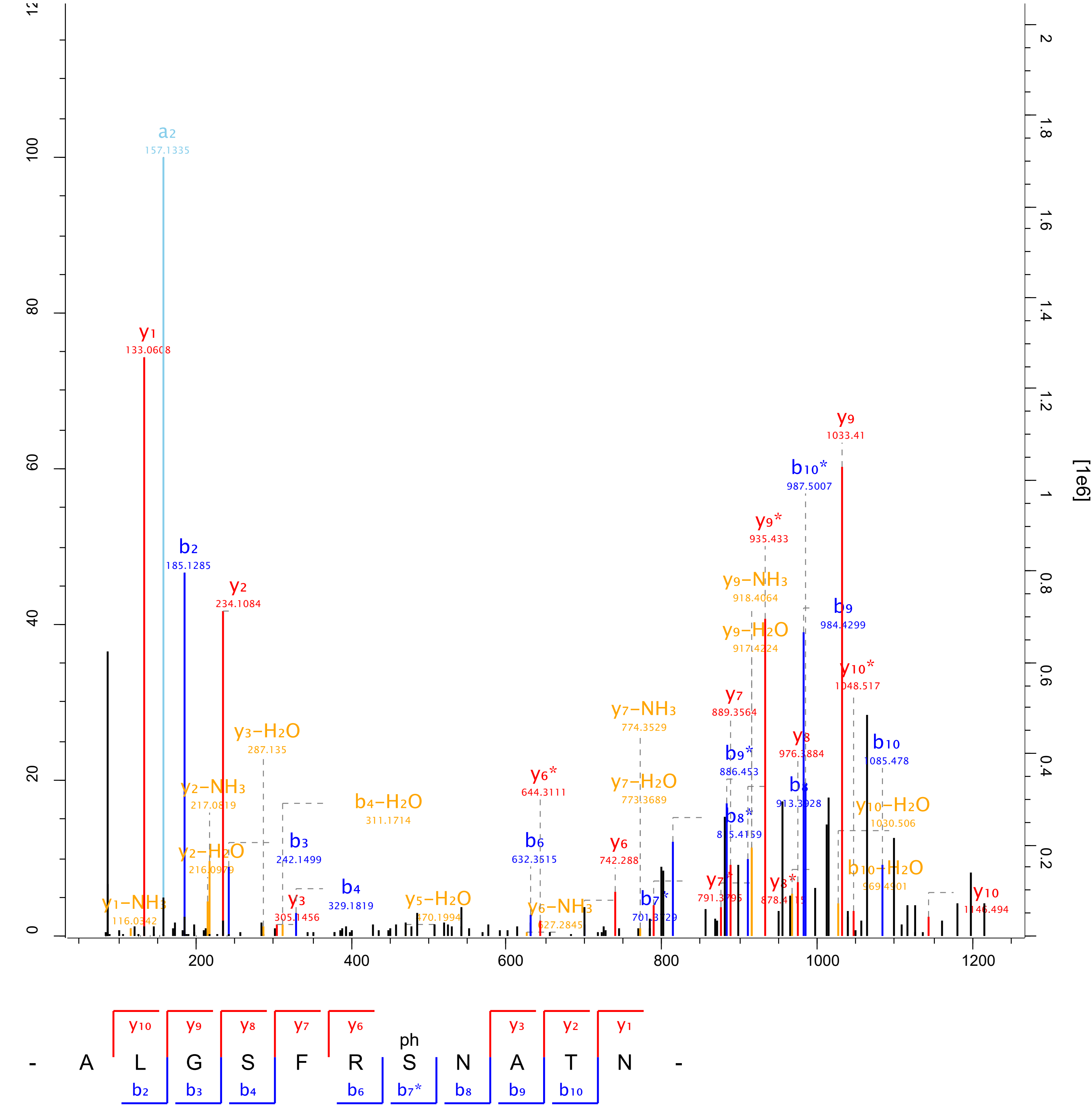

Sample output to test PDF Combine only

Raw file	Scan	Method	Score	m/z	Gene names
0523_2	9065	FTMS; HCD	79.2	484.73	ABCC14

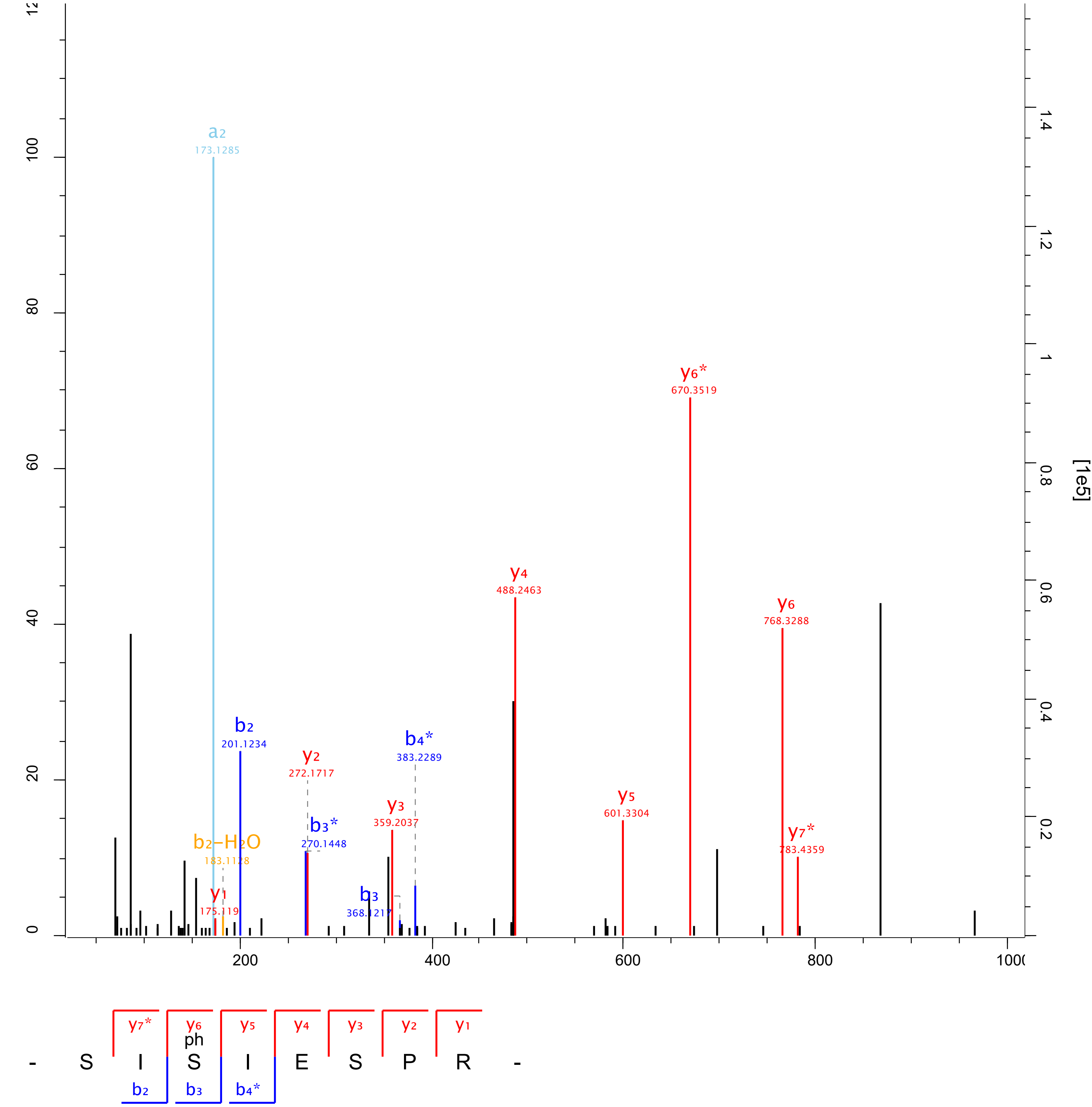

Sample output to test PDF Combine only

Raw file	Scan	Method	Score	m/z	Gene names
0523_2	9258	FTMS; HCD	74.85	436.7	F9F8.20

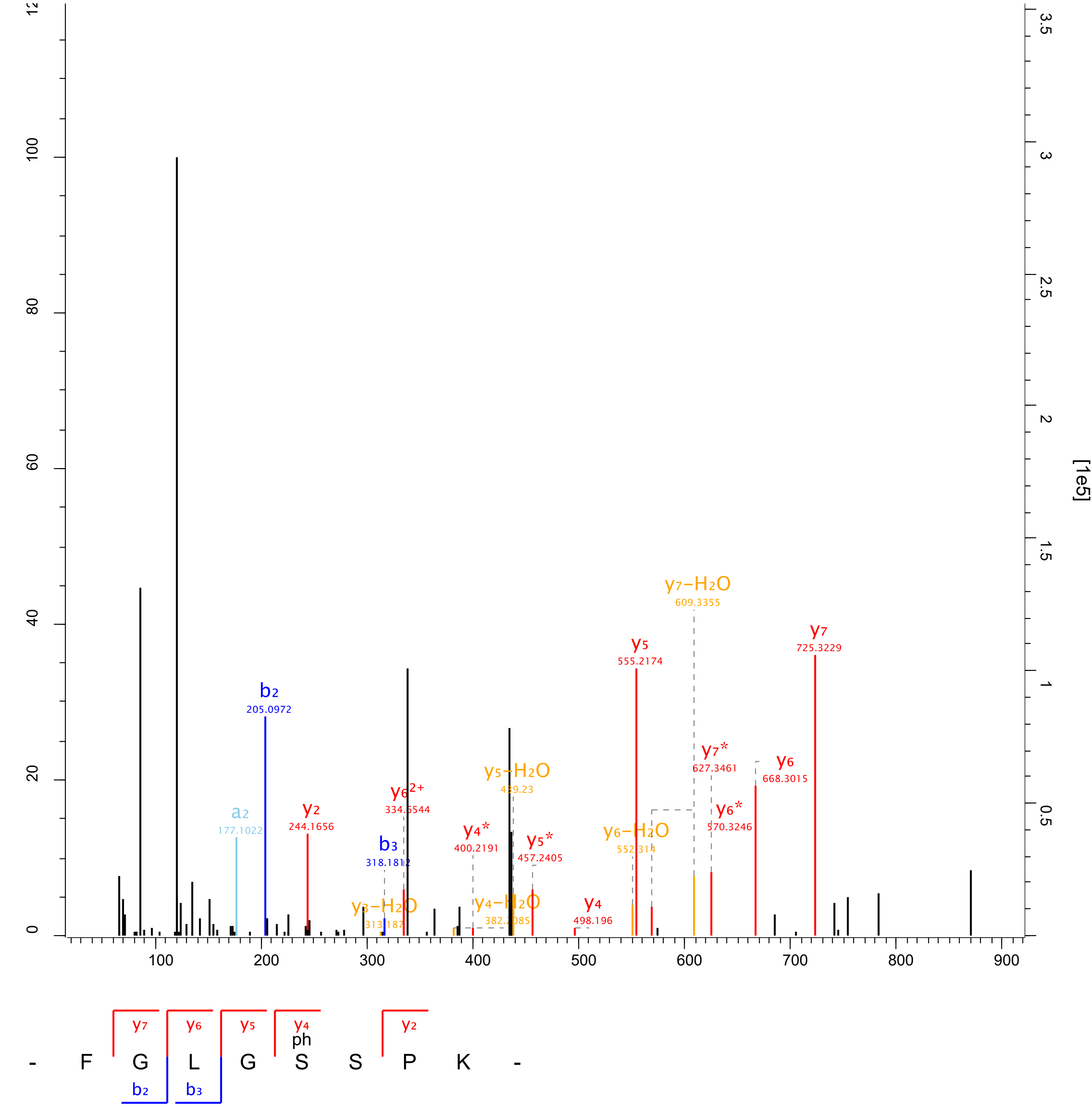

Sample output to test PDF Combine only

Raw file	Scan	Method	Score	m/z	Gene names
0523_2	9331	FTMS; HCD	58.11	676.29	At1g51850

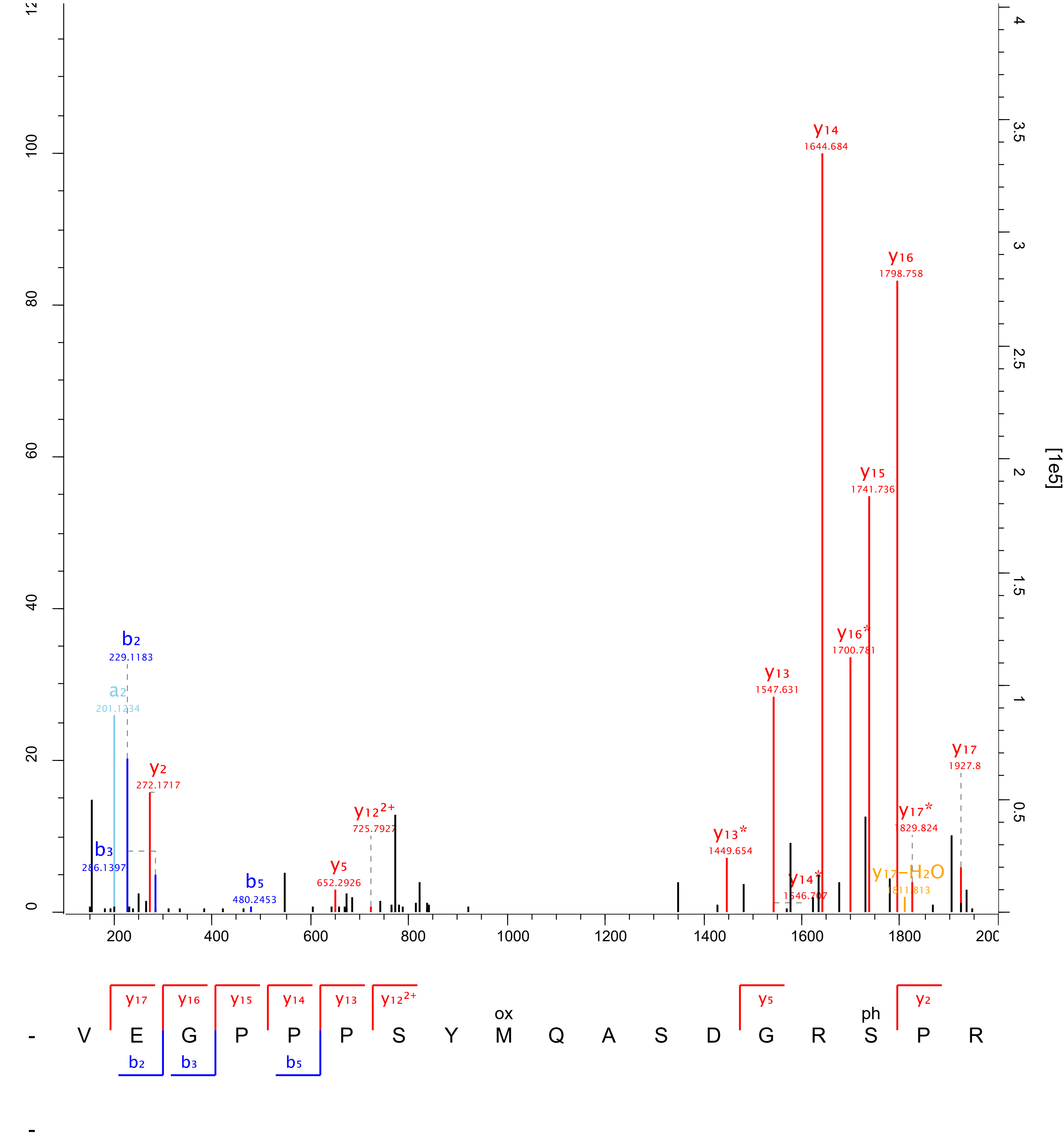

Sample output to test PDF Combine only

Raw file	Scan	Method	Score	m/z	Gene names
0523_2	9926	FTMS; HCD	56.72	516.92	At5g63270

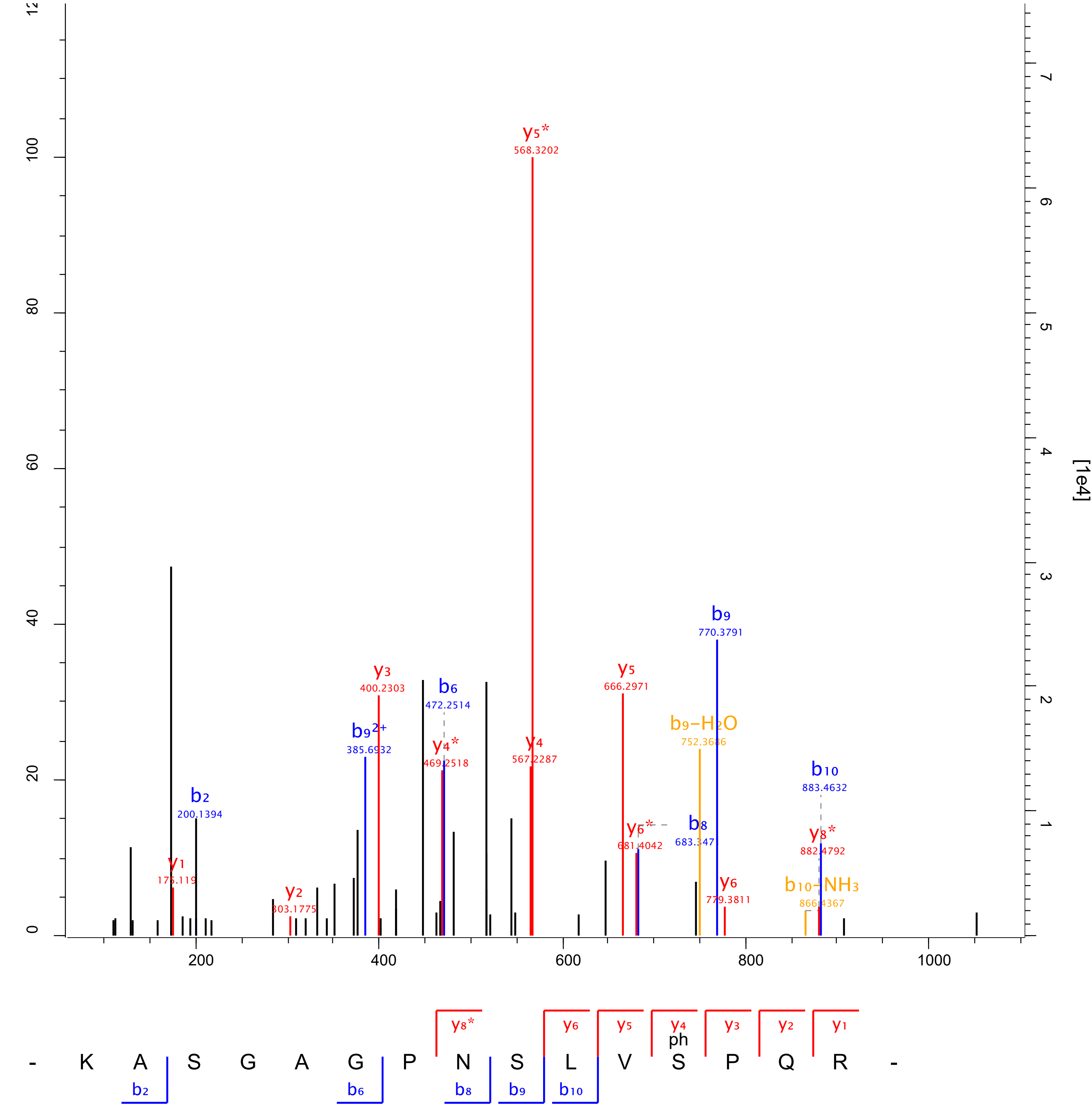

Sample output to test PDF Combine only

Raw file	Scan	Method	Score	m/z	Gene names
0523_2	10155	FTMS; HCD	71.85	681.64	WEB1

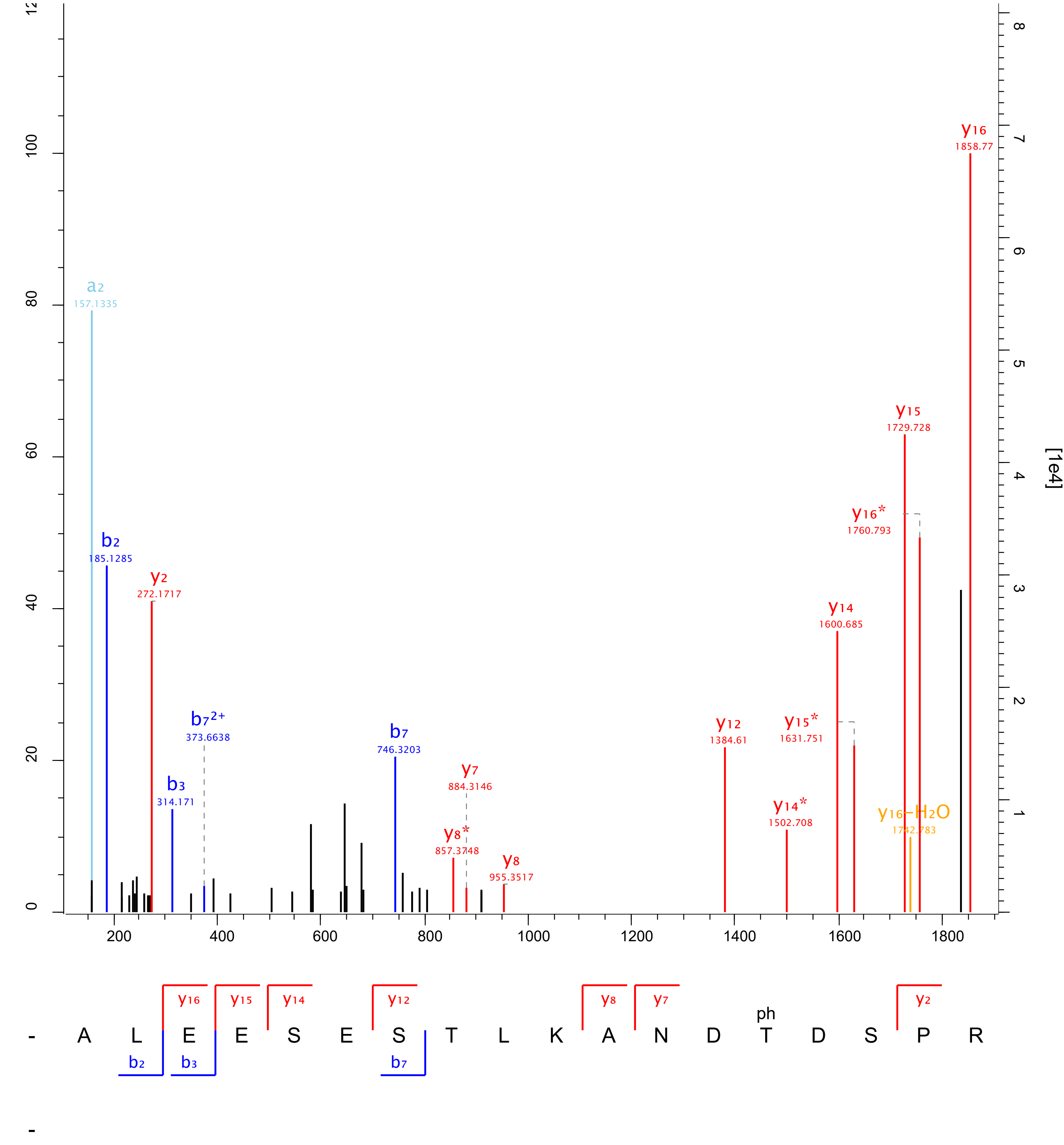

Sample output to test PDF Combine only

Raw file	Scan	Method	Score	m/z	Gene names
0523_2	10642	FTMS; HCD	73.67	597.62	IQD14

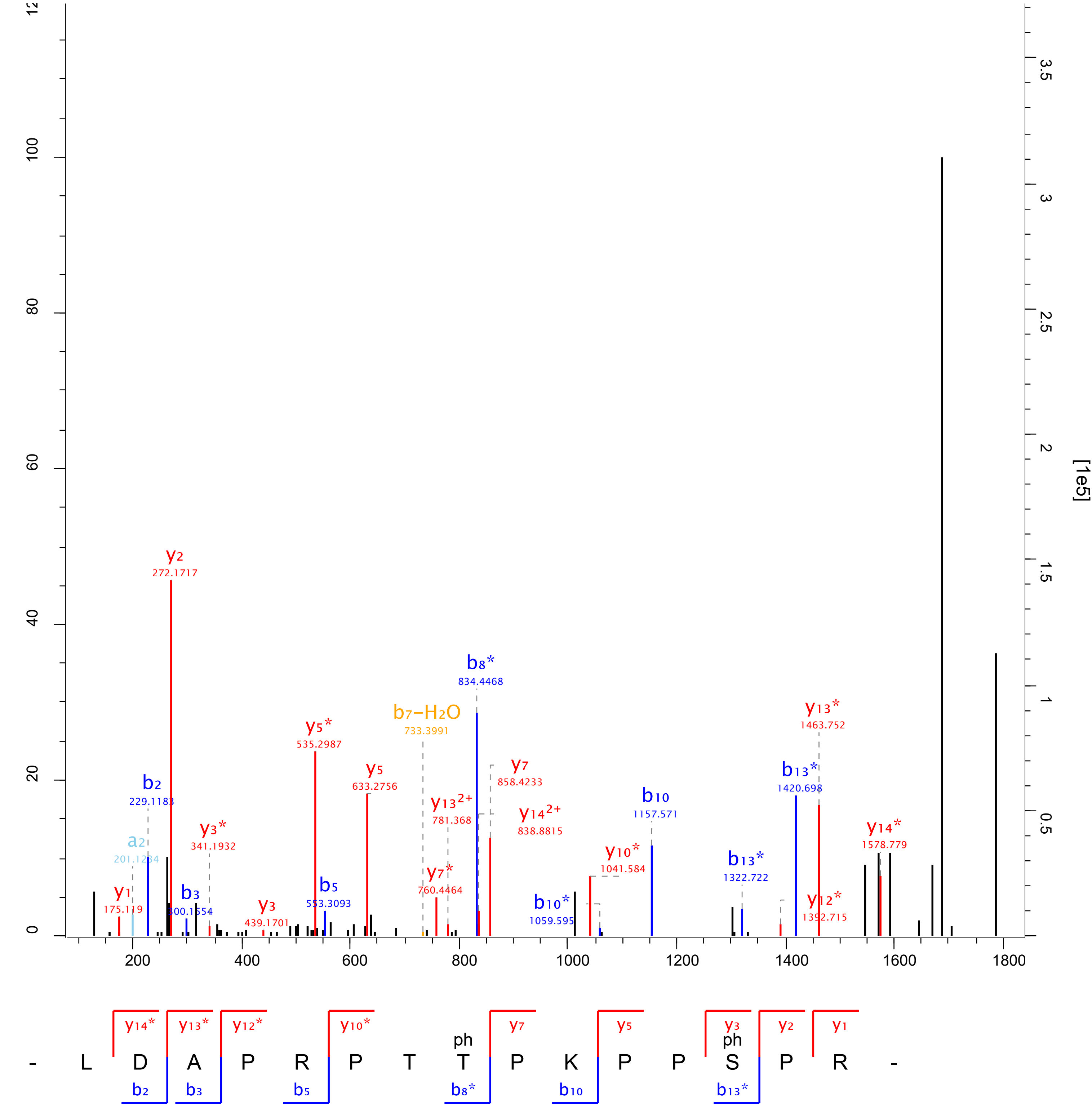

Sample output to test PDF Combine only

Raw file	Scan	Method	Score	m/z
0523_2	11506	FTMS; HCD	58.89	486.9

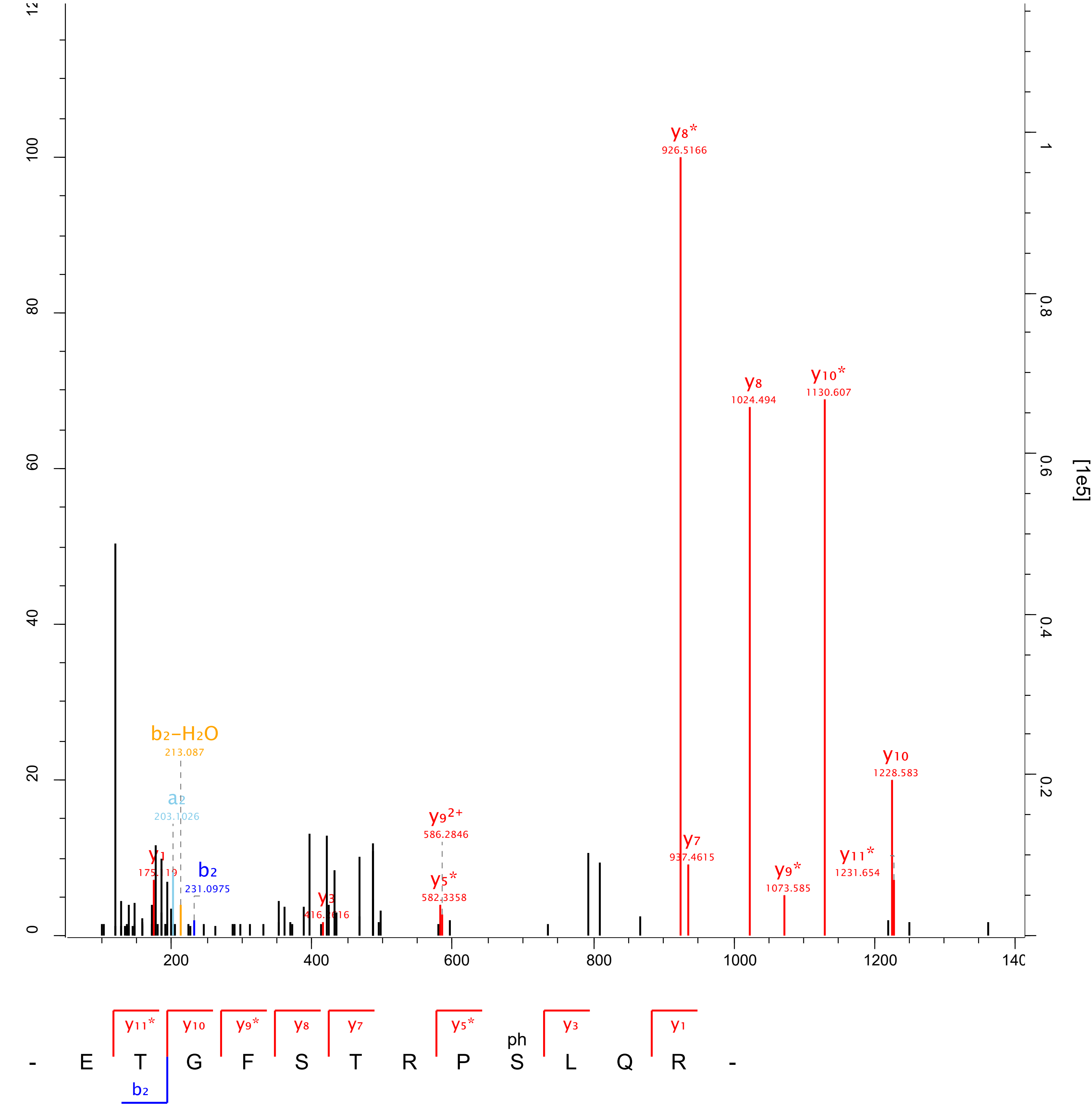

Sample output to test PDF Combine only

Raw file	Scan	Method	Score	m/z	Gene names
0523_2	11586	FTMS; HCD	76.01	454.55	At5g40450

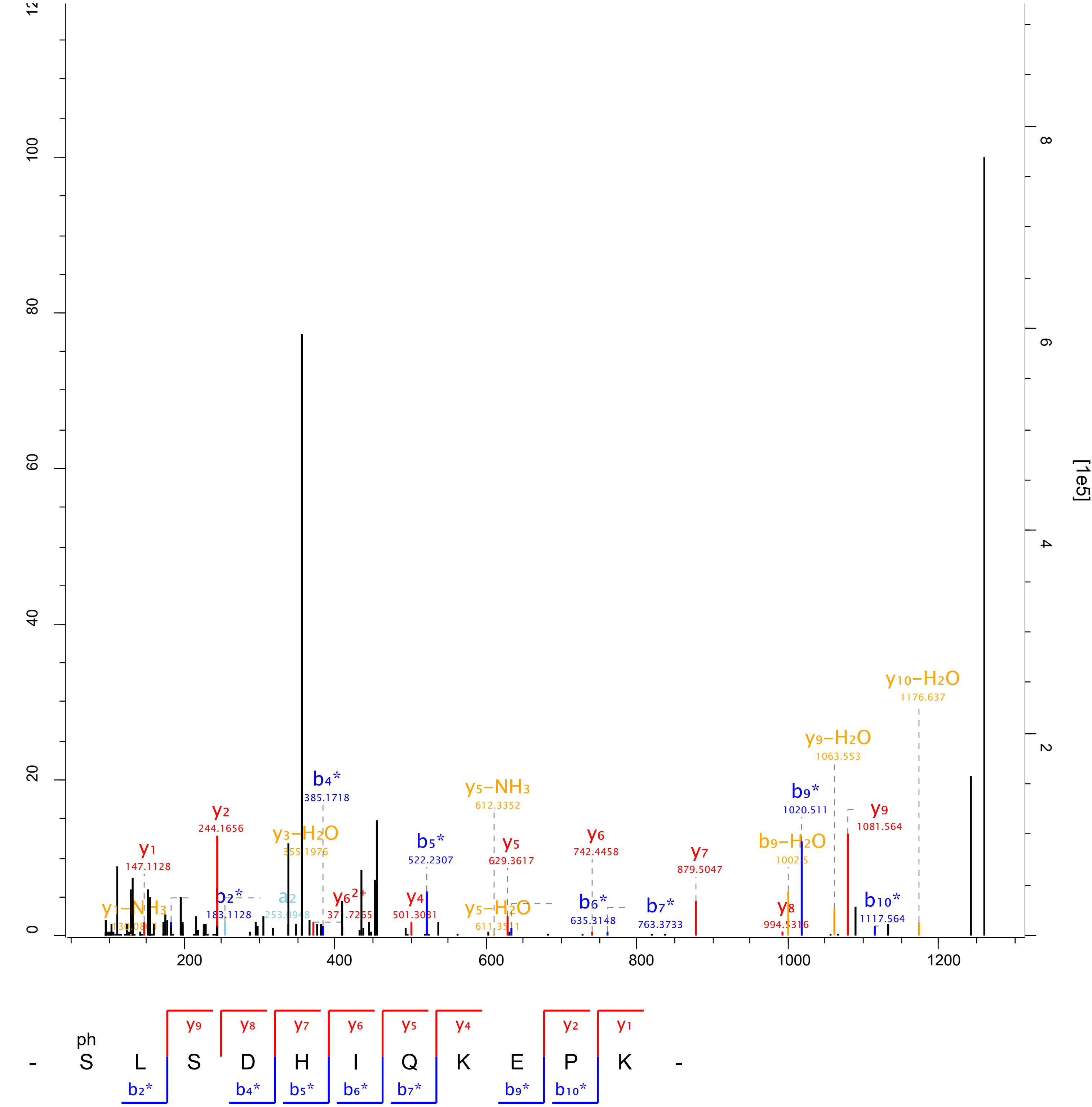

Sample output to test PDF Combine only

Raw file	Scan	Method	Score	m/z	Gene names
0523_2	11966	FTMS; HCD	44.31	819.64	RS41

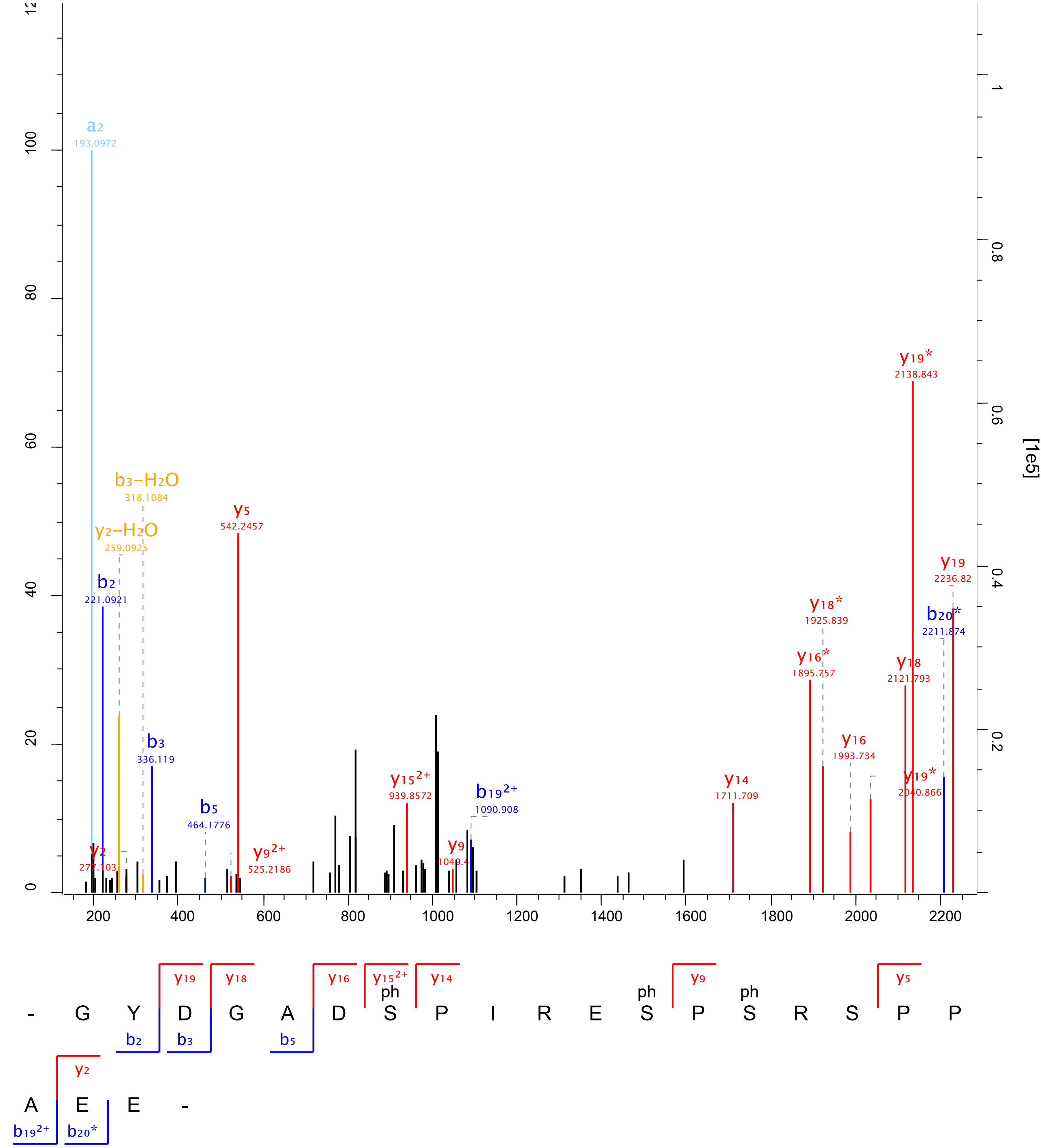

Sample output to test PDF Combine only

Raw file	Scan	Method	Score	m/z
0523_2	12156	FTMS; HCD	109.04	620.6

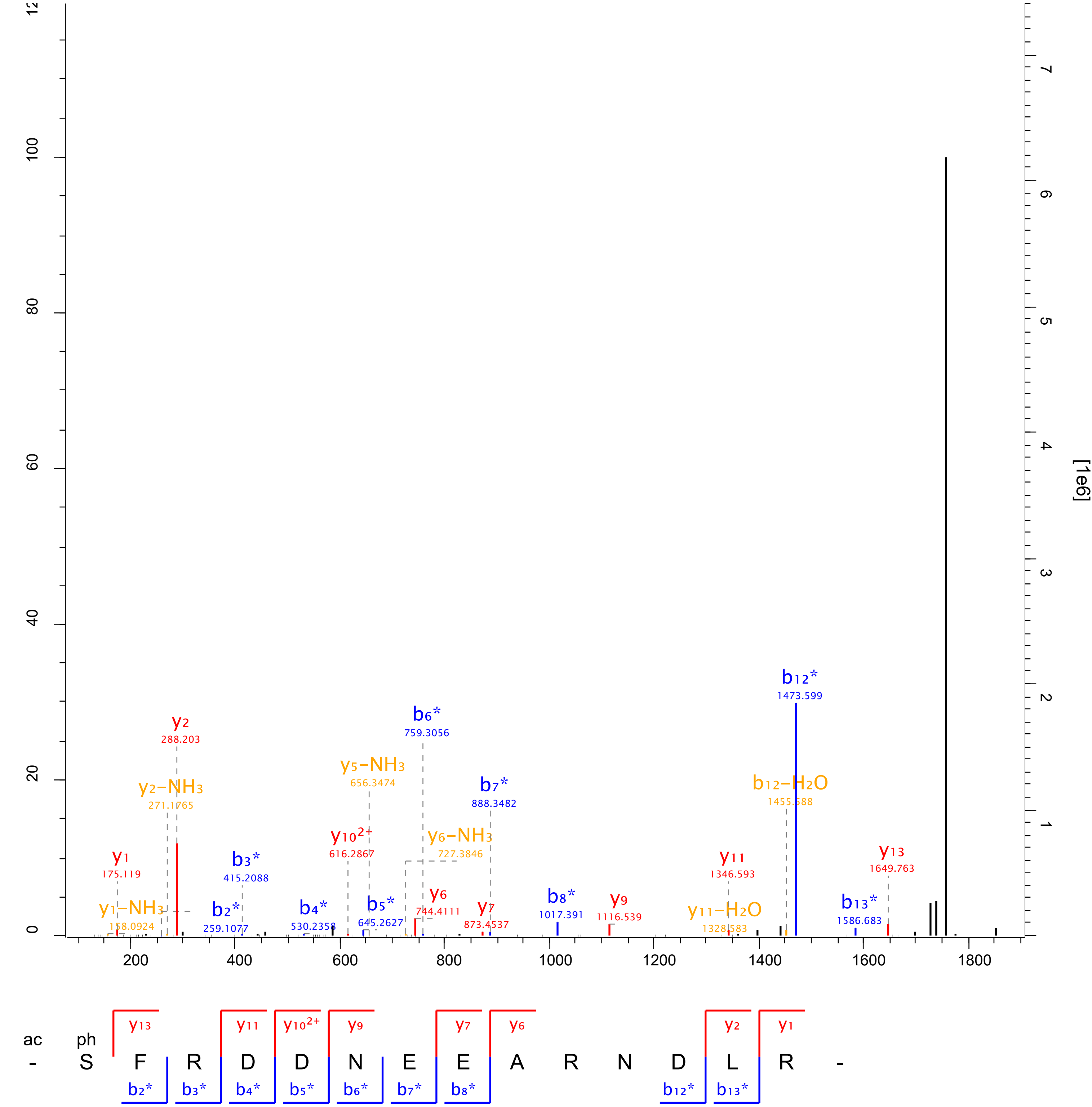

Sample output to test PDF Combine only

Raw file	Scan	Method	Score	m/z
0523_2	12388	FTMS; HCD	71.59	575.92

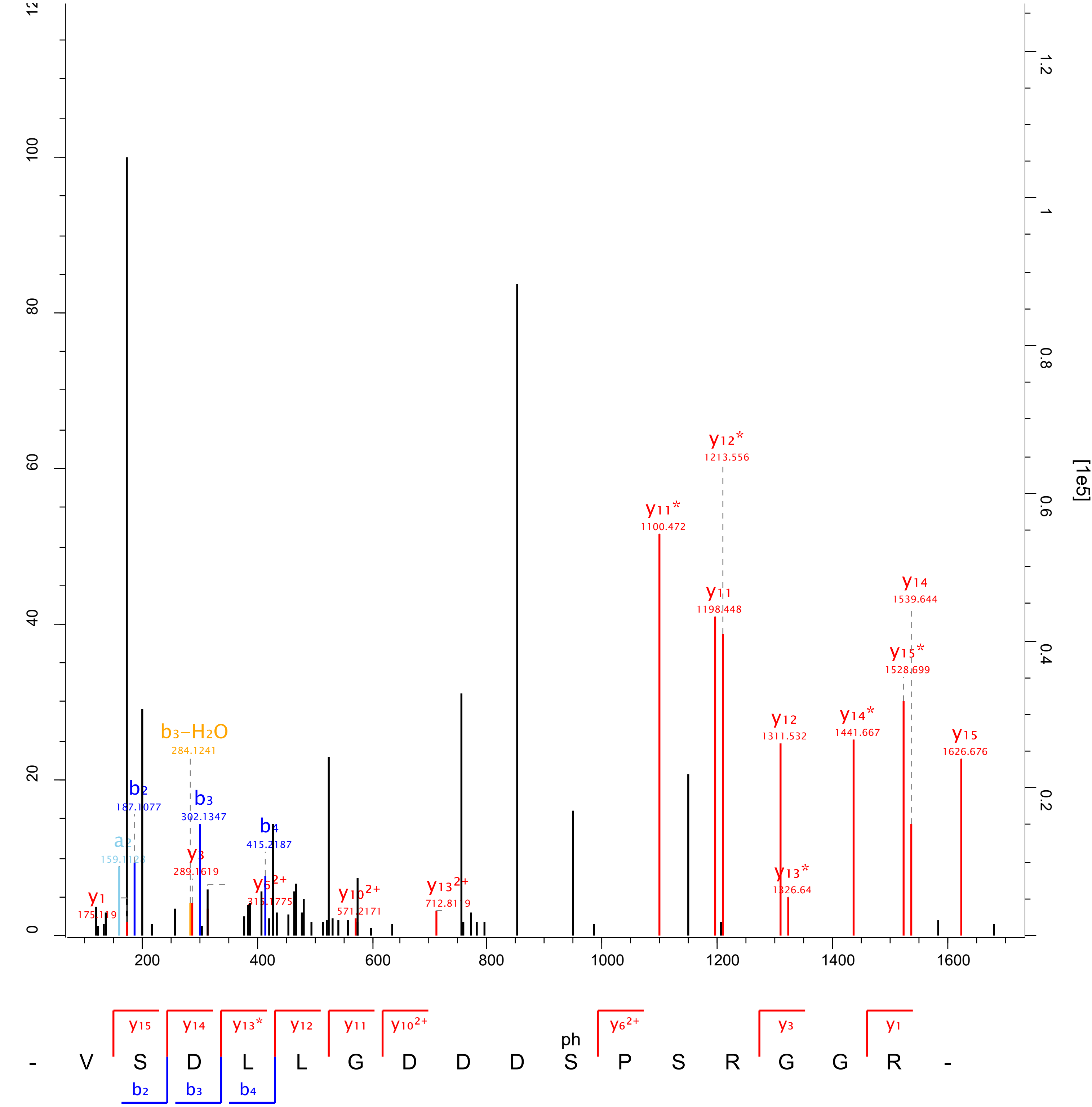

Sample output to test PDF Combine only

Raw file	Scan	Method	Score	m/z	Gene names
0523_2	12465	FTMS; HCD	59.29	667.35	DIM

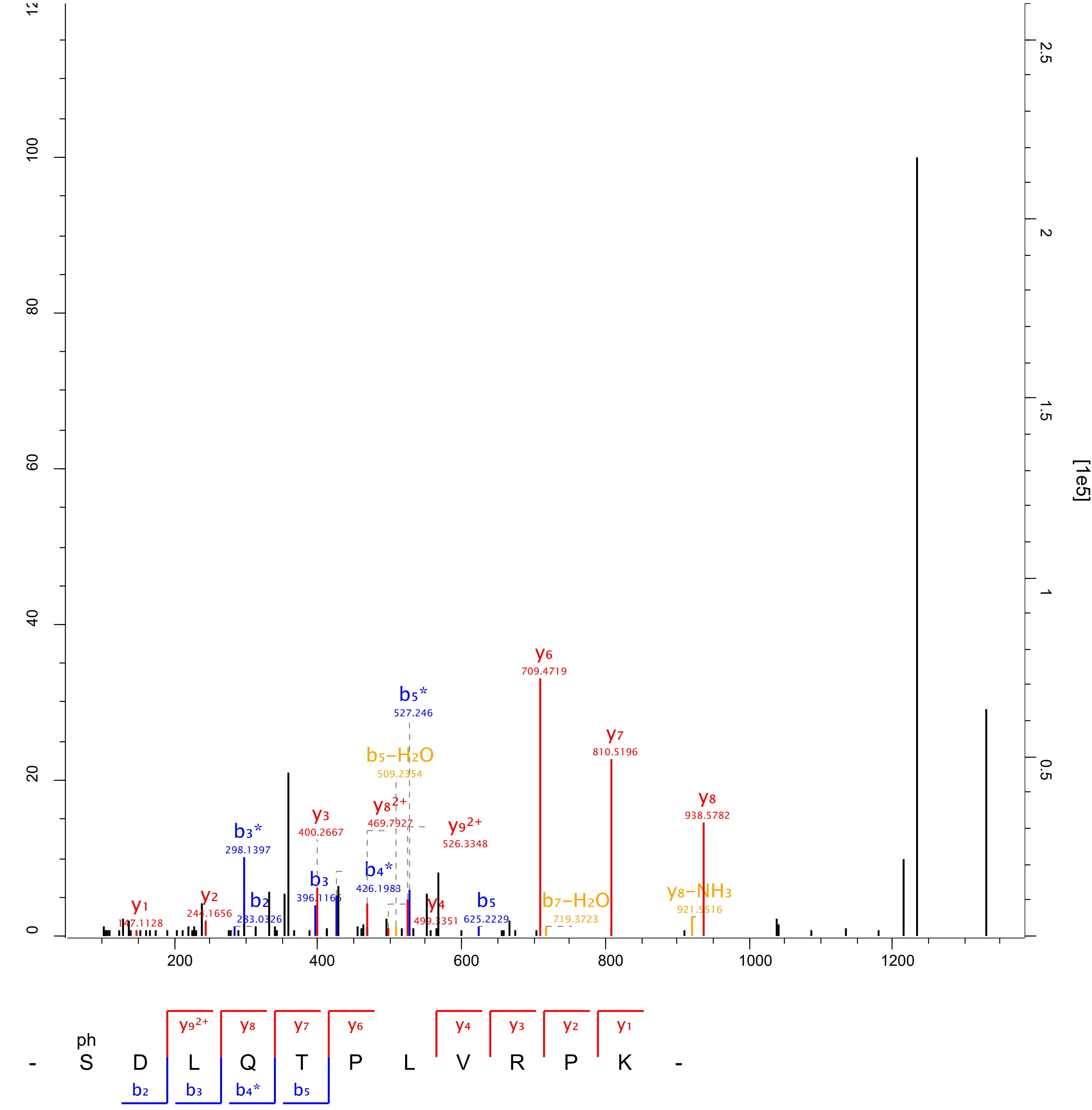

Sample output to test PDF Combine only

Raw file	Scan	Method	Score	m/z	Gene names
0523_2	12983	FTMS; HCD	50.36	641.64	ABCB11

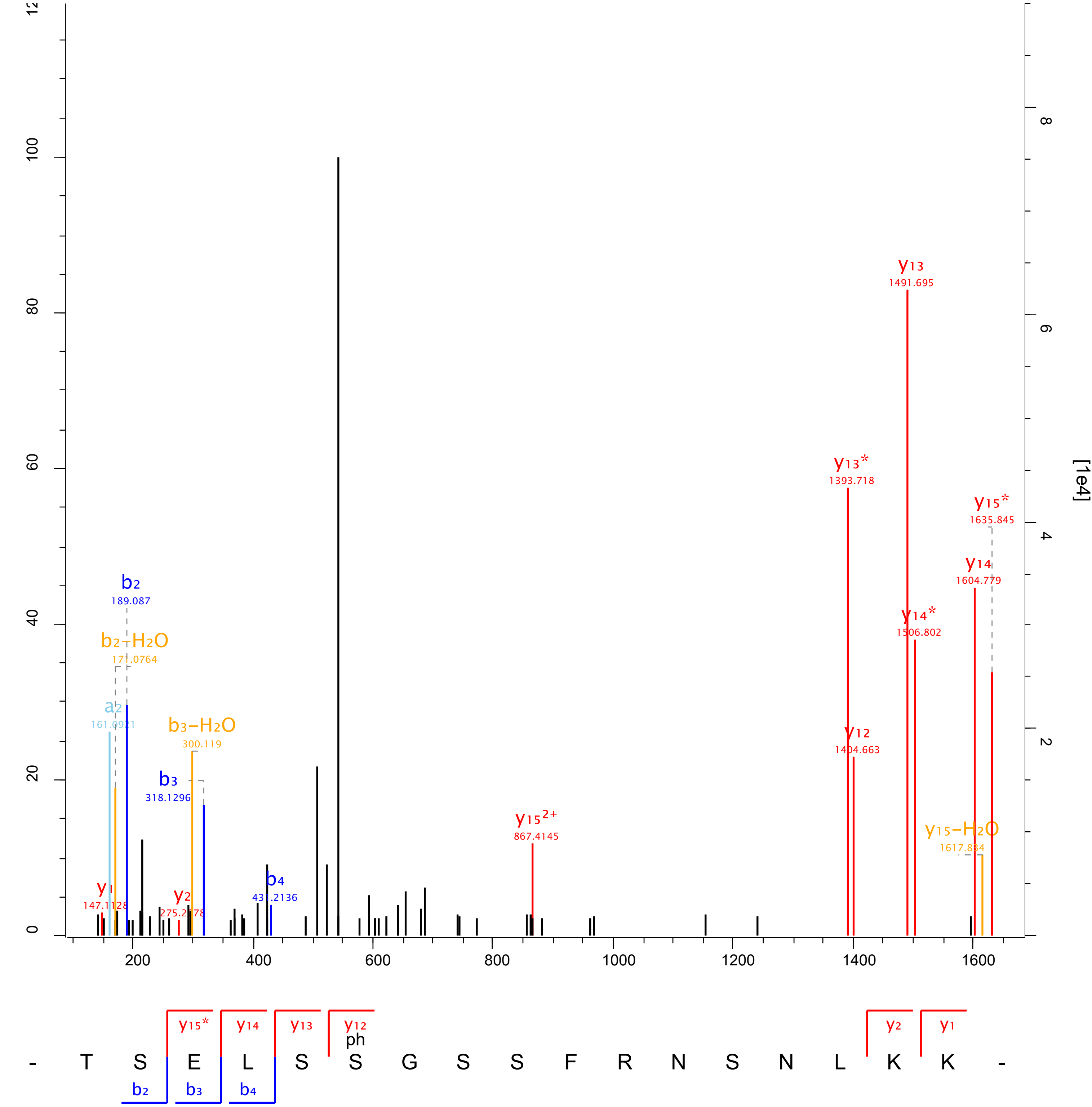

Sample output to test PDF Combine only

Raw file	Scan	Method	Score	m/z
0523_2	13338	FTMS; HCD	95.98	496.56

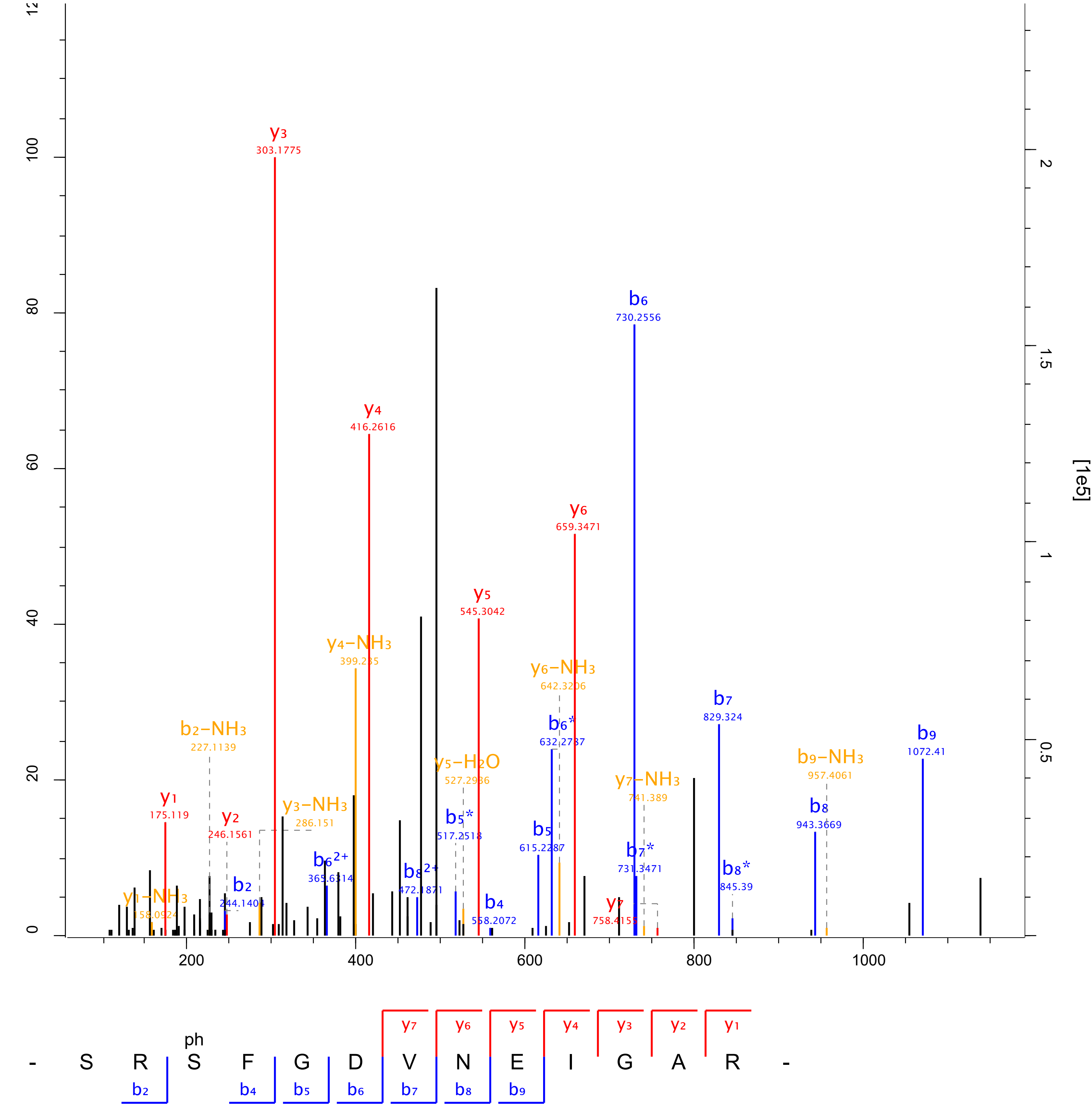

Sample output to test PDF Combine only

Raw file	Scan	Method	Score	m/z	Gene names
0523_2	13415	FTMS; HCD	374	919.89	PHT1-2;PHT1-1

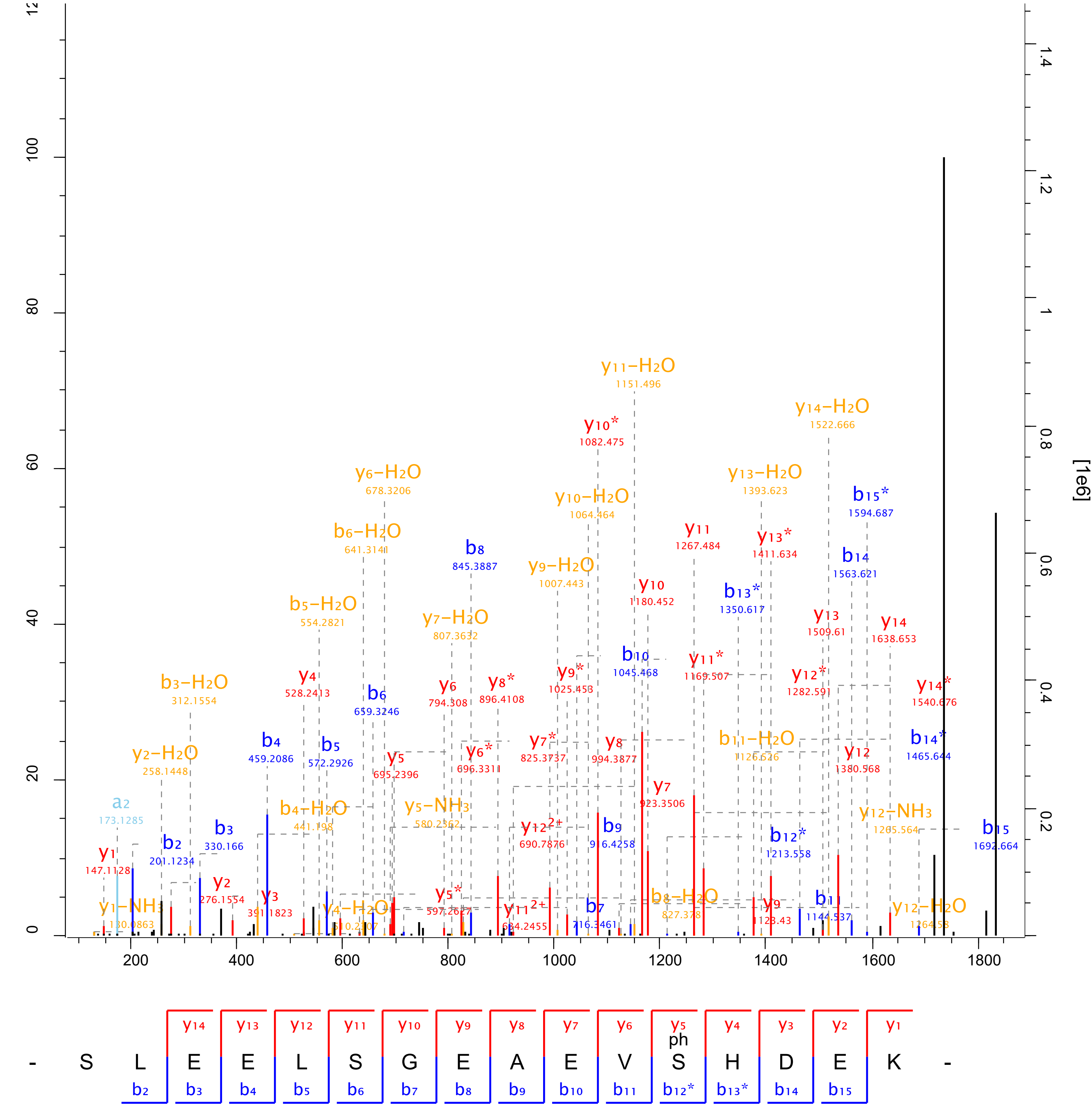

Sample output to test PDF Combine only

Raw file	Scan	Method	Score	m/z	Gene names
0523_2	13453	FTMS; HCD	212.09	781.34	POT8

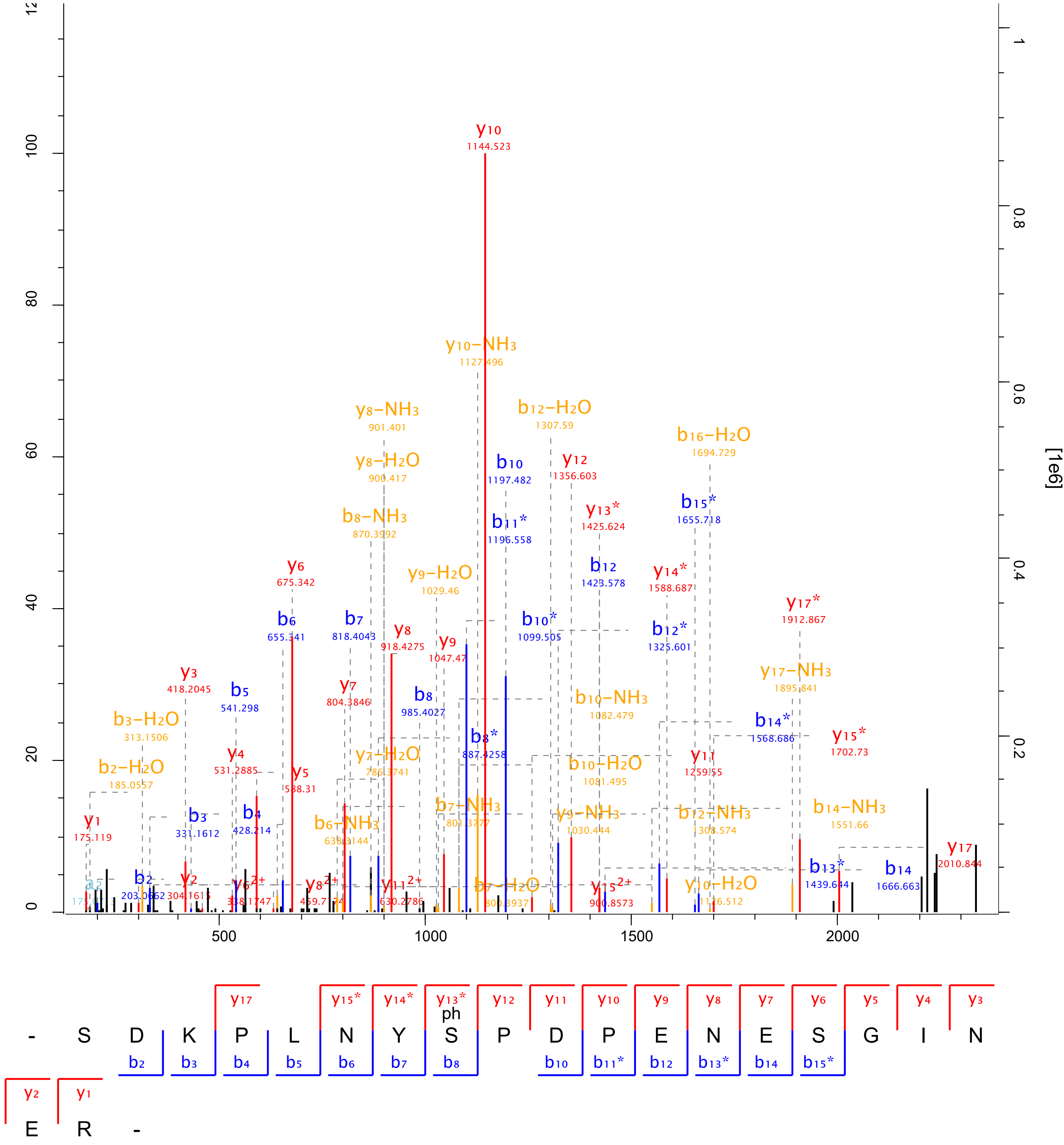

Sample output to test PDF Combine only

Raw file	Scan	Method	Score	m/z	Gene names
0523_2	13672	FTMS; HCD	86.36	683.65	PAPP2C

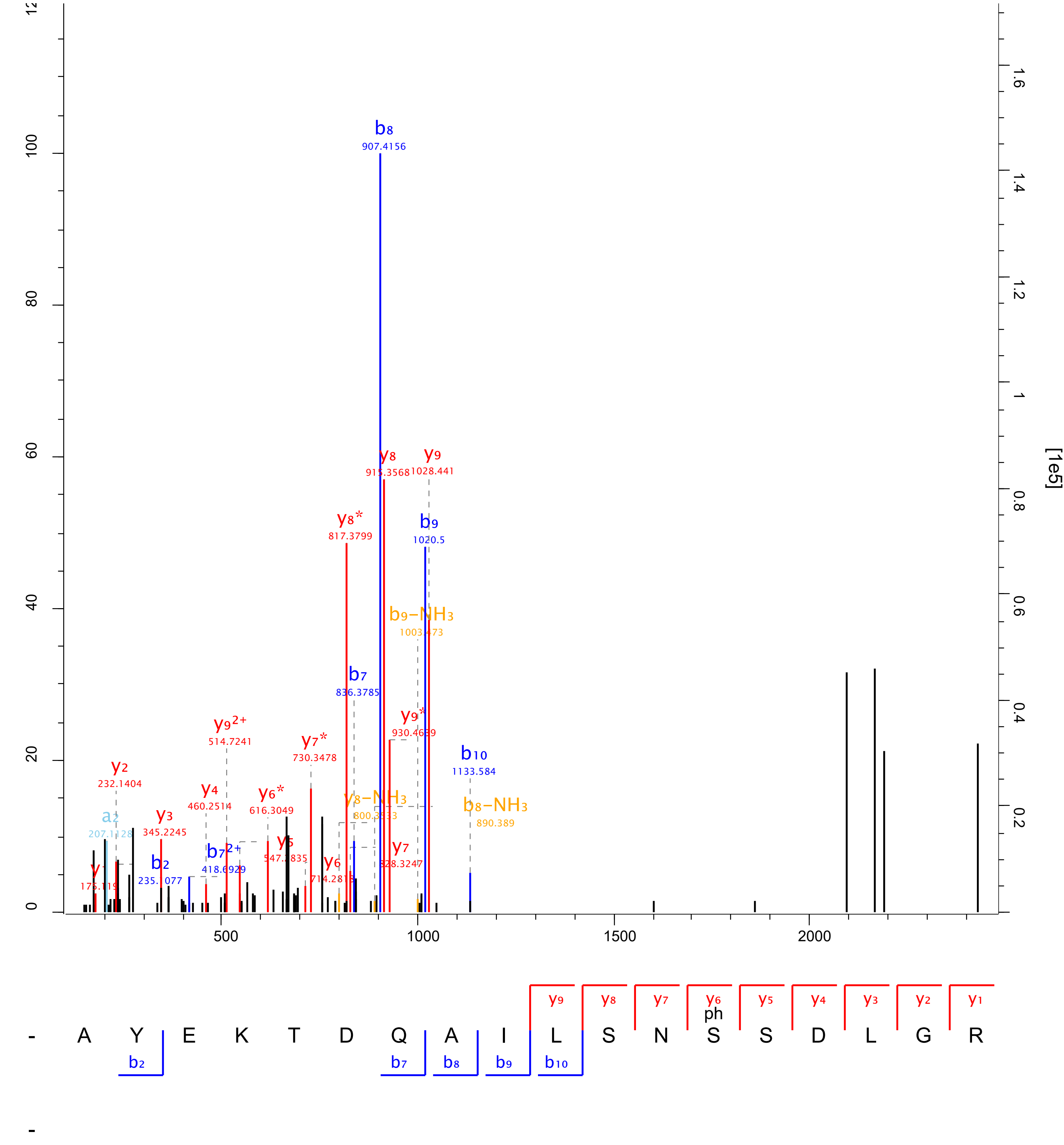

Sample output to test PDF Combine only

Raw file	Scan	Method	Score	m/z	Gene names
0523_2	14395	FTMS; HCD	51.32	984.81	HON5

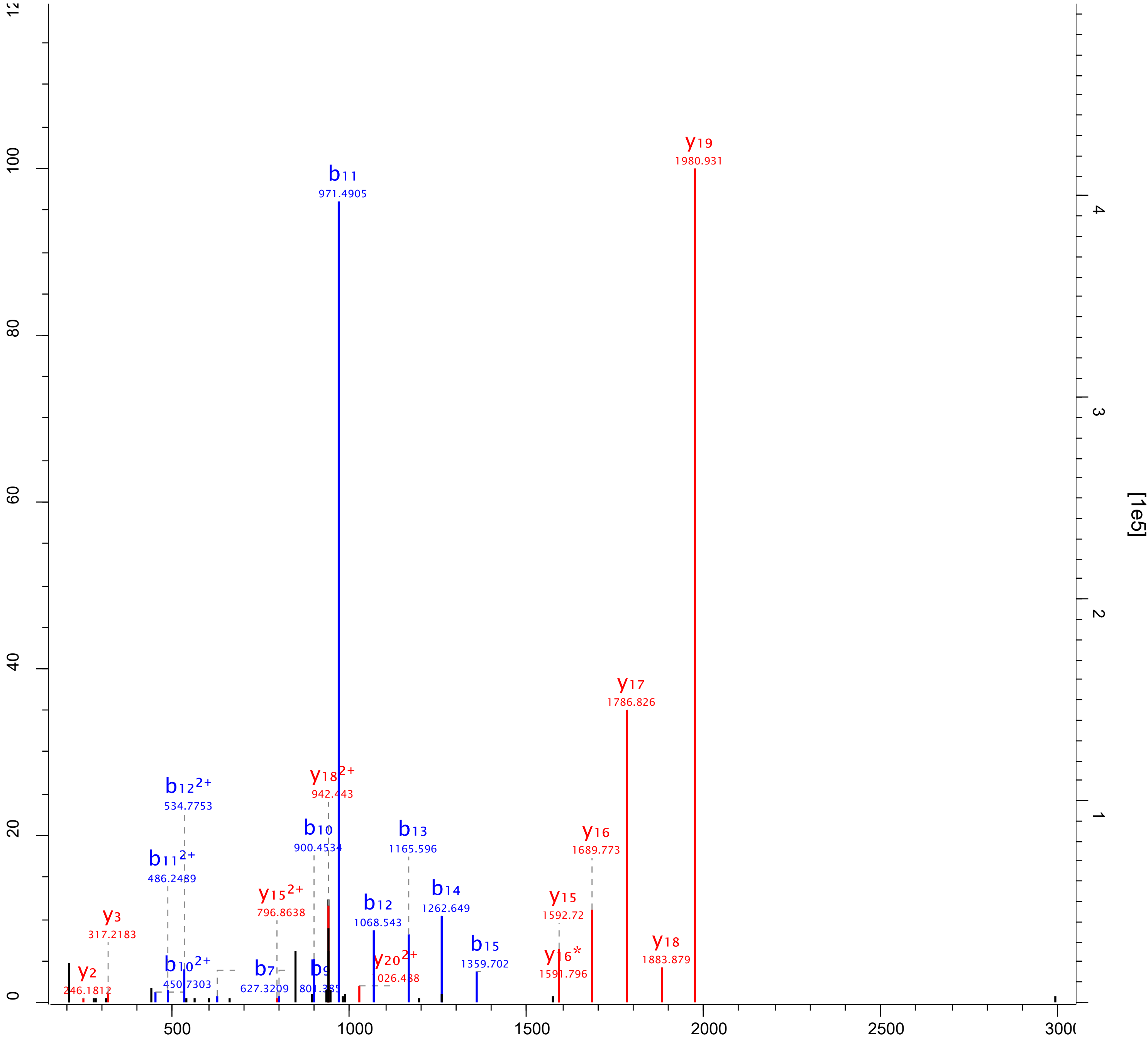

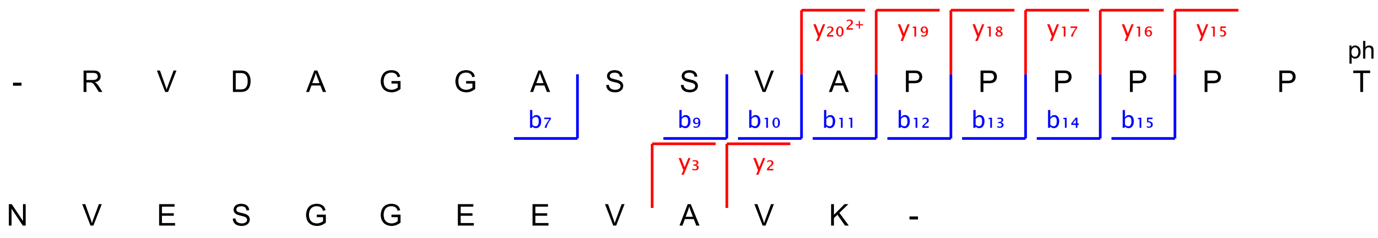

Sample output to test PDF Combine only

Raw file	Scan	Method	Score	m/z	Gene names
0523_2	14957	FTMS; HCD	124.12	632.31	At4g35785

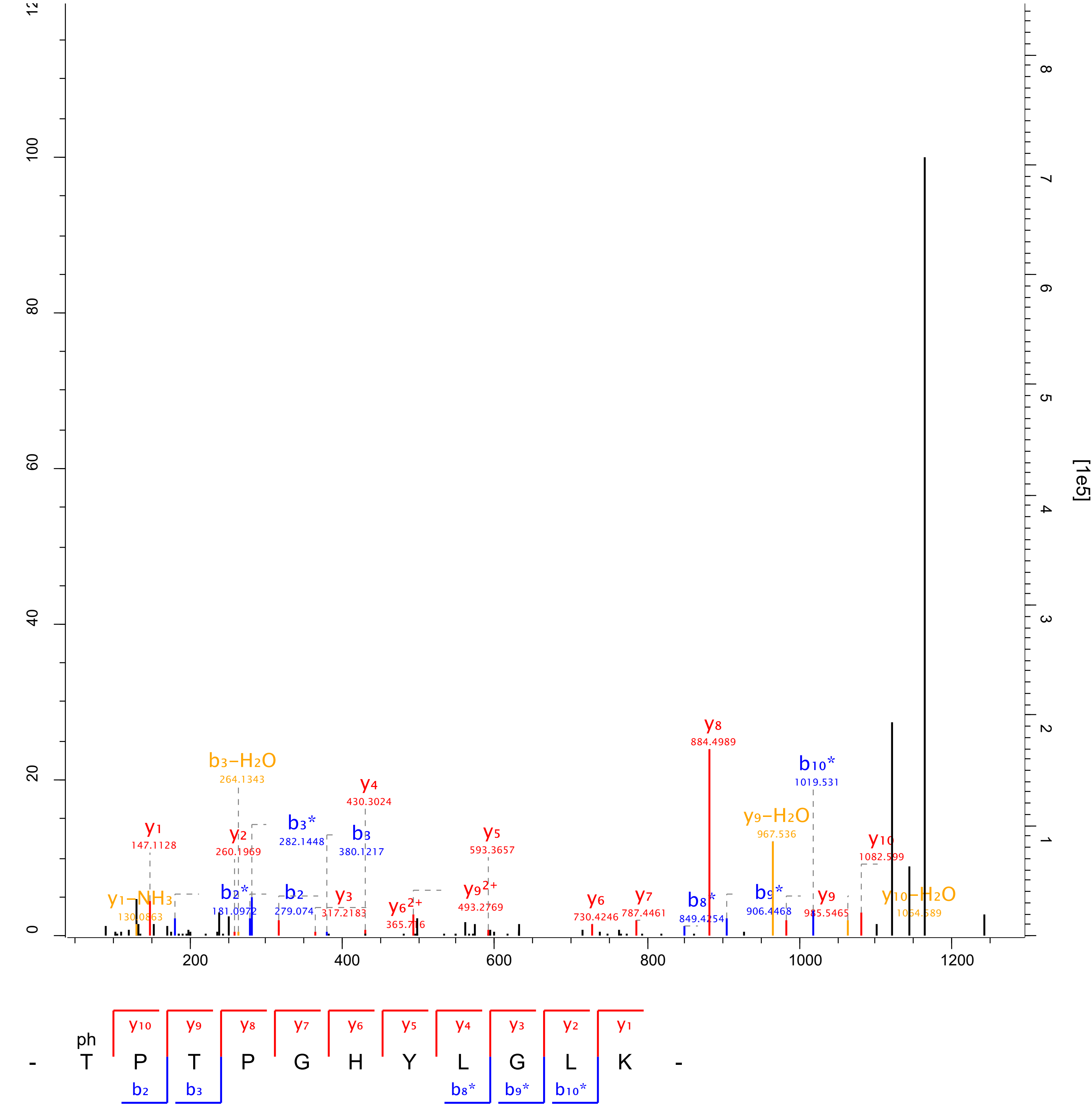

Sample output to test PDF Combine only

Raw file	Scan	Method	Score	m/z	Gene names
0523_2	15088	FTMS; HCD	63.3	627.25	ACA8

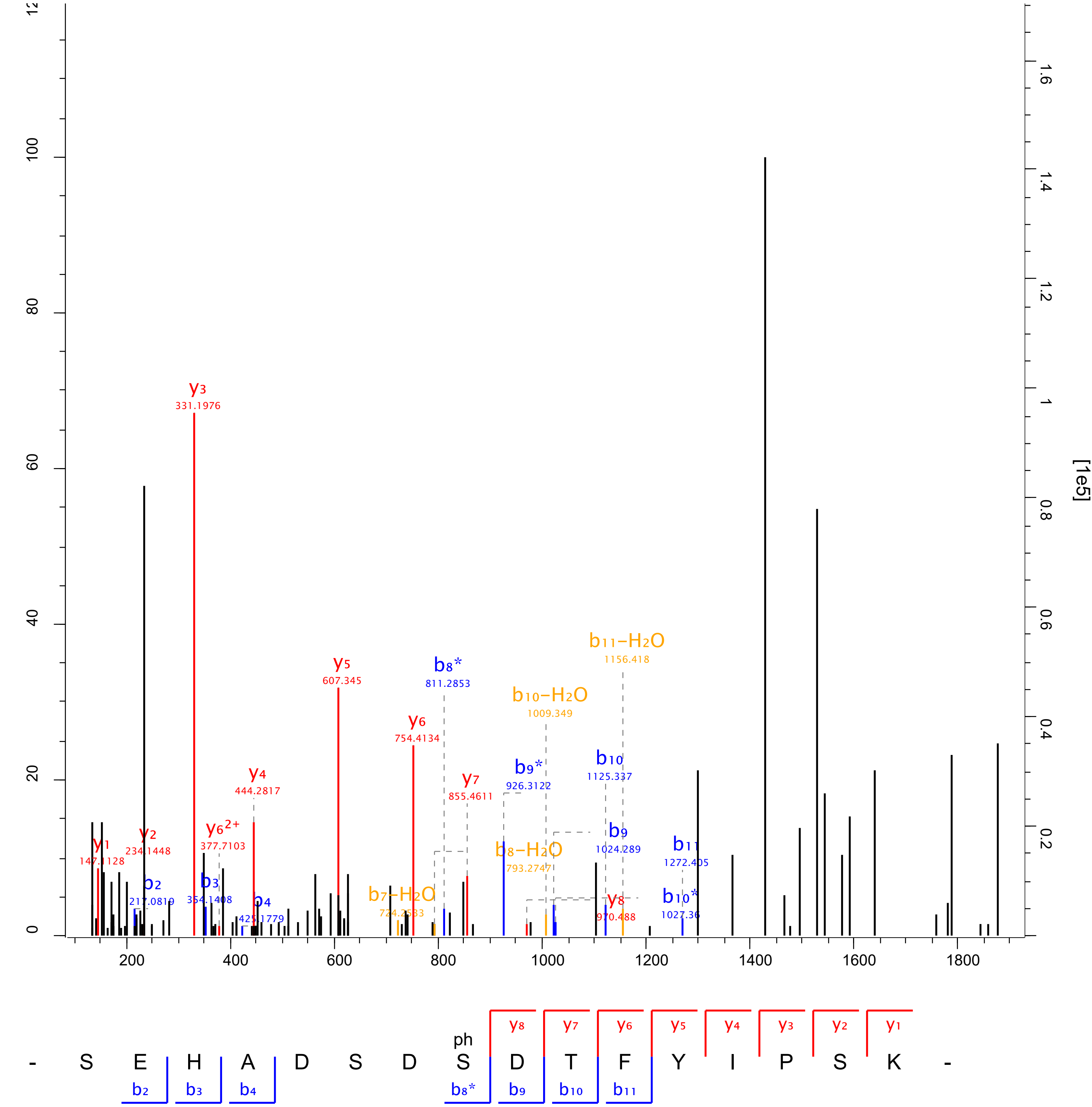

Sample output to test PDF Combine only

Raw file	Scan	Method	Score	m/z	Gene names
0523_2	15510	FTMS; HCD	87.88	778.05	At1g20670

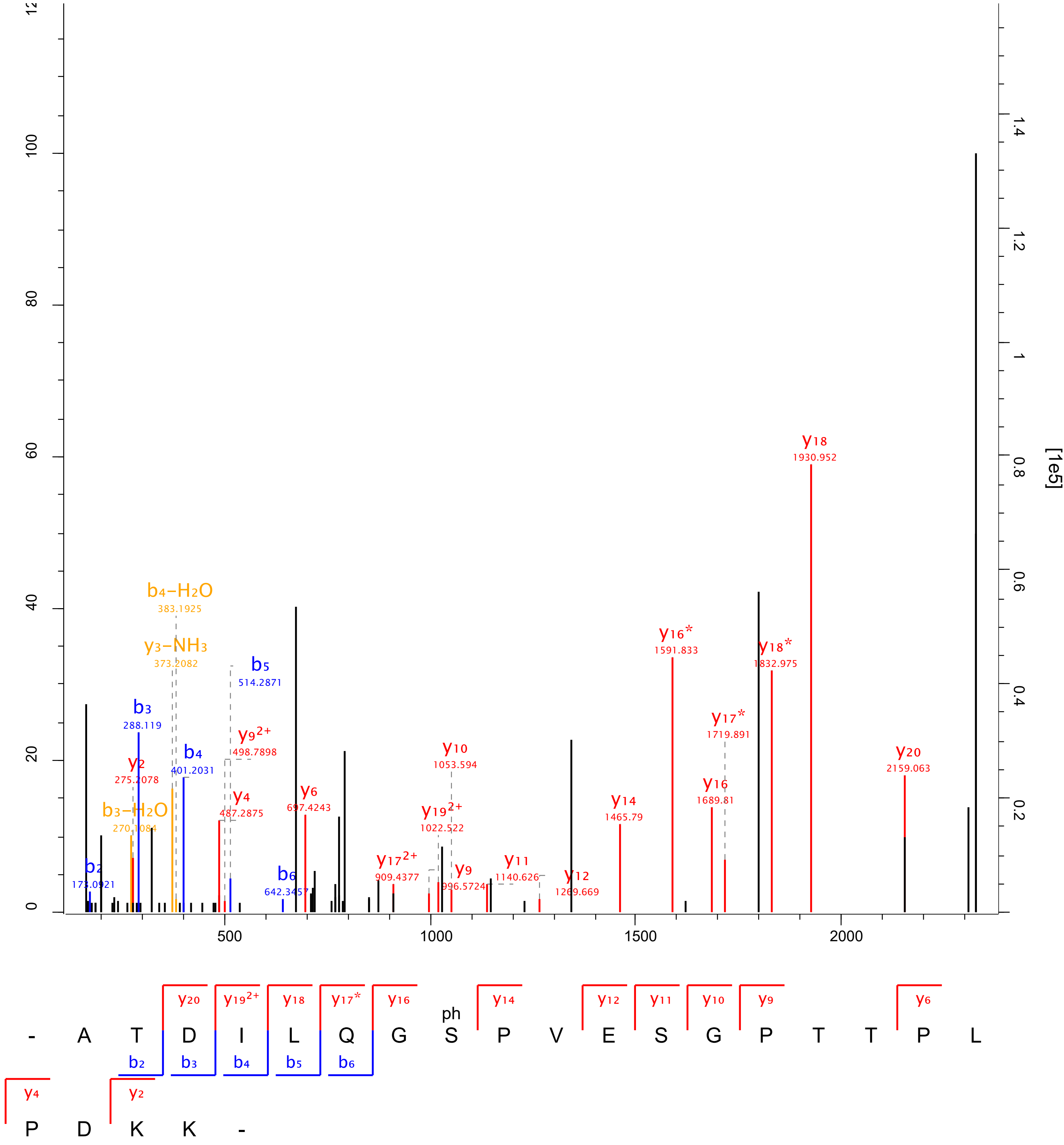

Sample output to test PDF Combine only

Raw file	Scan	Method	Score	m/z	Gene names
0523_2	15572	FTMS; HCD	96.79	870.36	ACA8

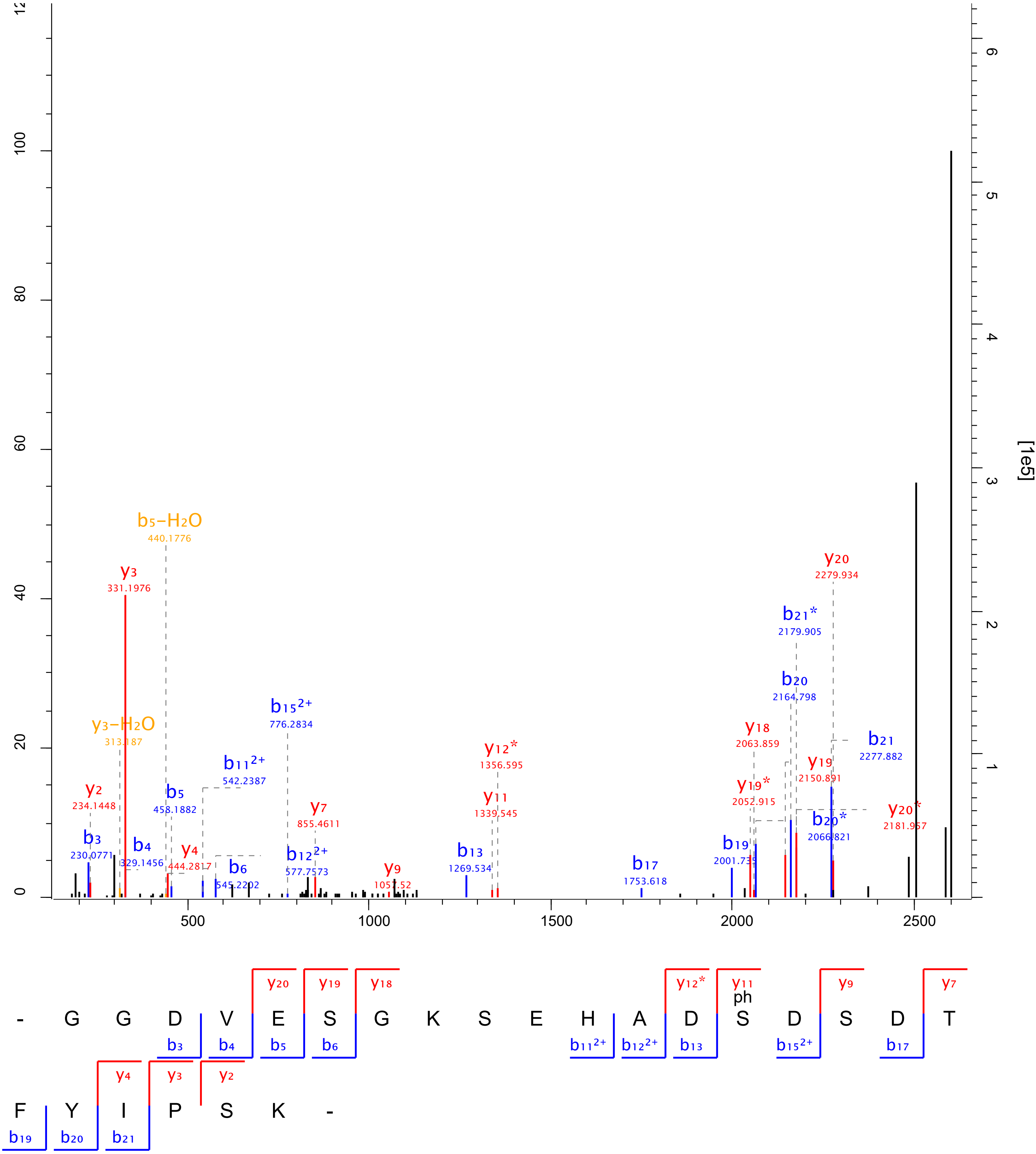

Sample output to test PDF Combine only

Raw file	Scan	Method	Score	m/z
0523_2	15609	FTMS; HCD	156.23	803.33

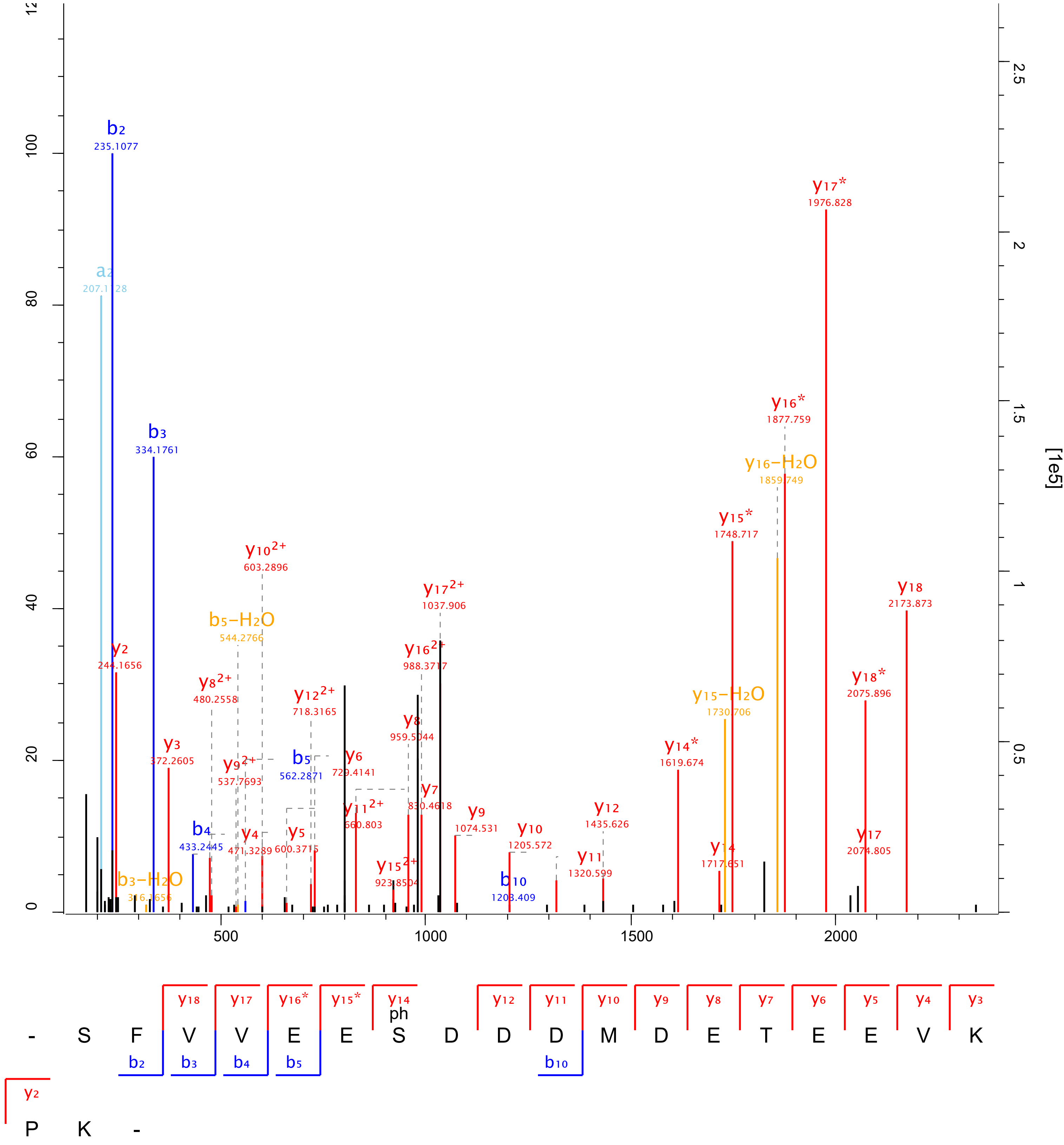

Sample output to test PDF Combine only

Raw file	Scan	Method	Score	m/z	Gene names
0523_2	15787	FTMS; HCD	44.16	846.38	RS41

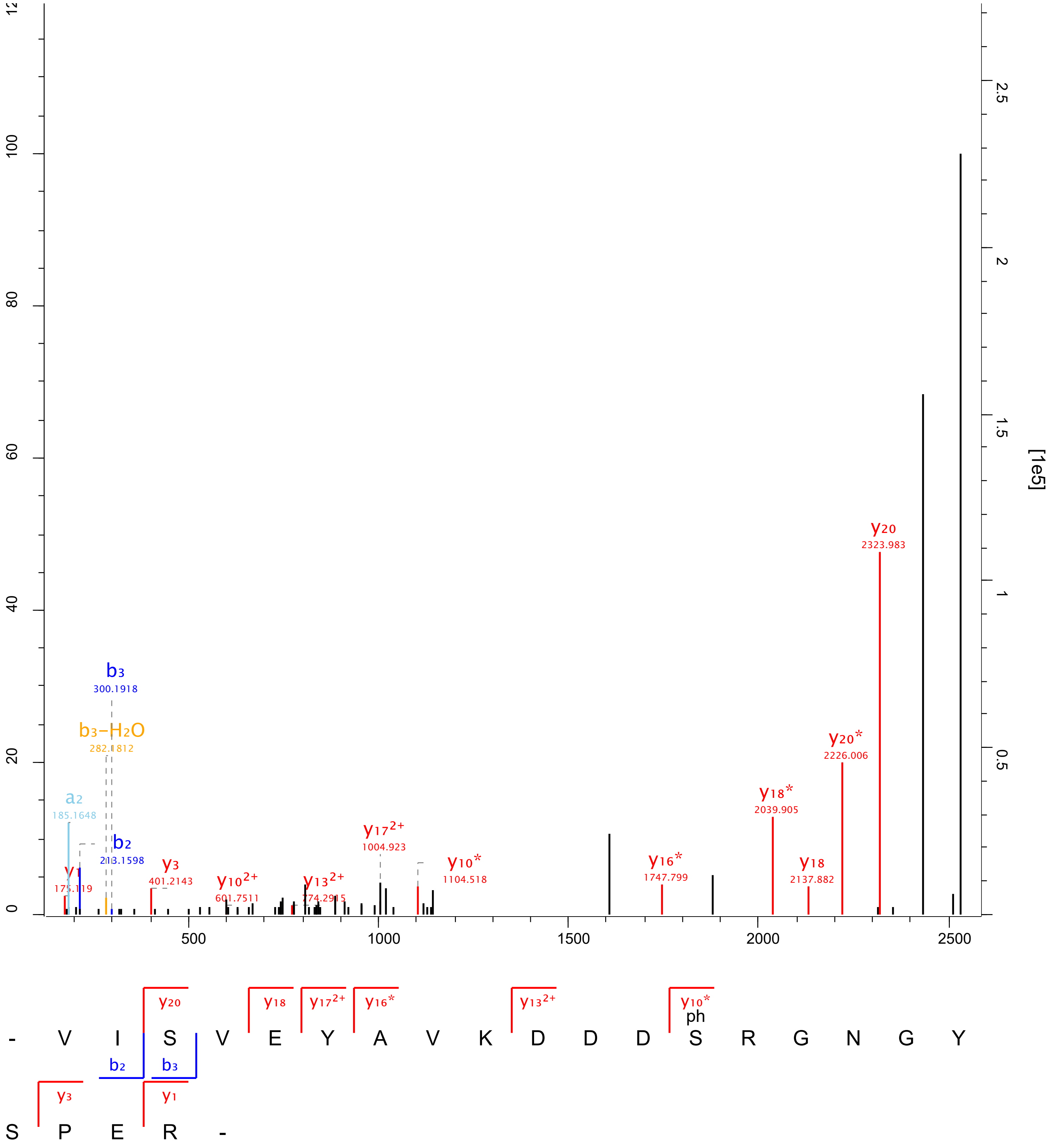

Sample output to test PDF Combine only

Raw file	Scan	Method	Score	m/z	Gene names
0523_2	15814	FTMS; HCD	243.97	852.91	TRX9

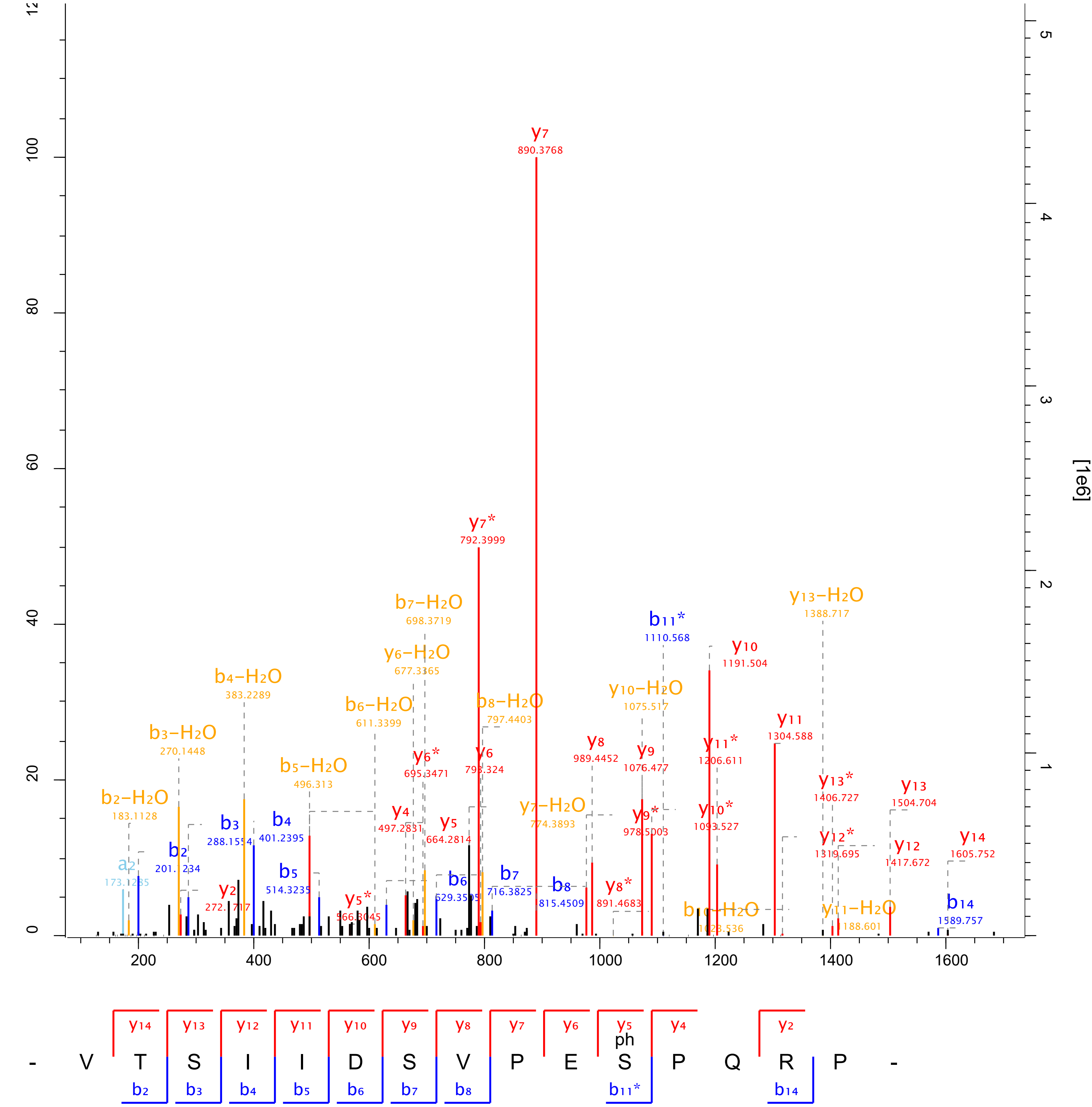

Sample output to test PDF Combine only

Raw file	Scan	Method	Score	m/z	Gene names
0523_2	16585	FTMS; HCD	85.18	823.07	At1g64110

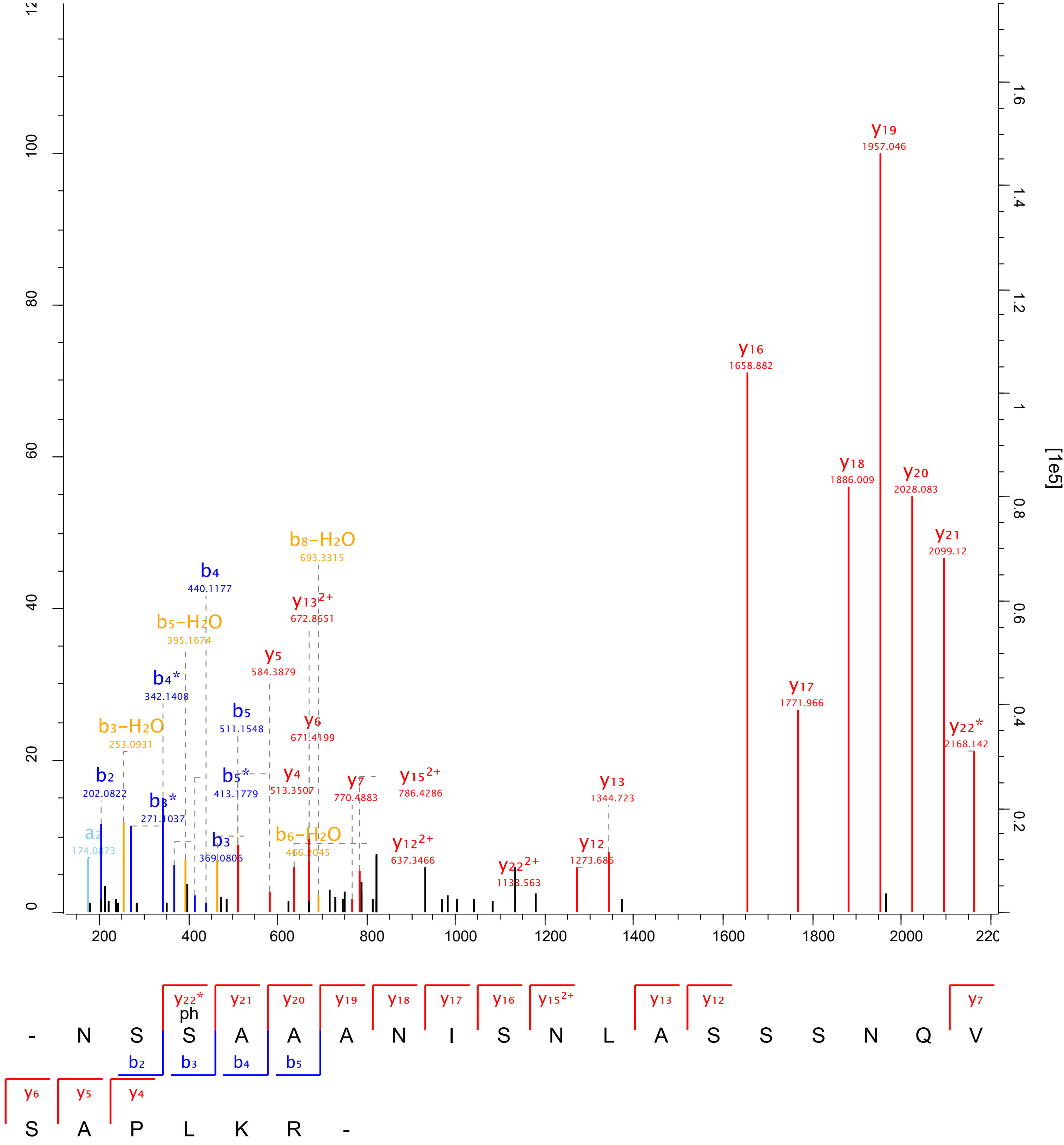

Sample output to test PDF Combine only

Raw file	Scan	Method	Score	m/z	Gene names
0523_2	16946	FTMS; HCD	157.42	792.64	At2g38410

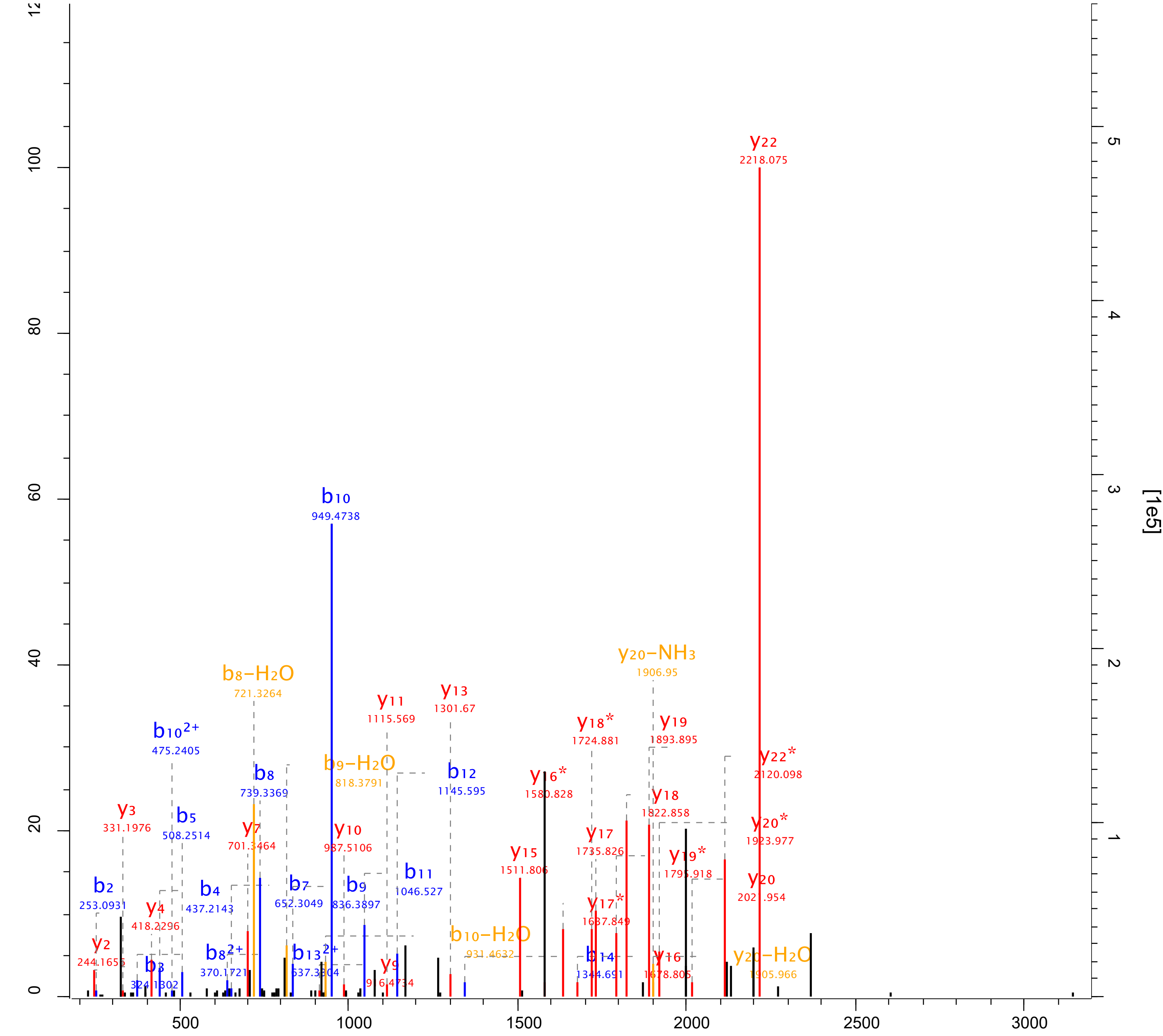

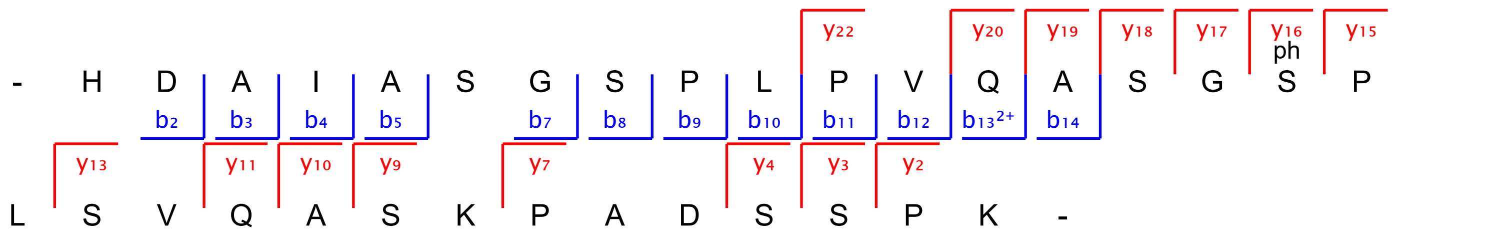

Sample output to test PDF Combine only

Raw file	Scan	Method	Score	m/z	Gene names
0523_2	17280	FTMS; HCD	178.7	942.08	FRO2

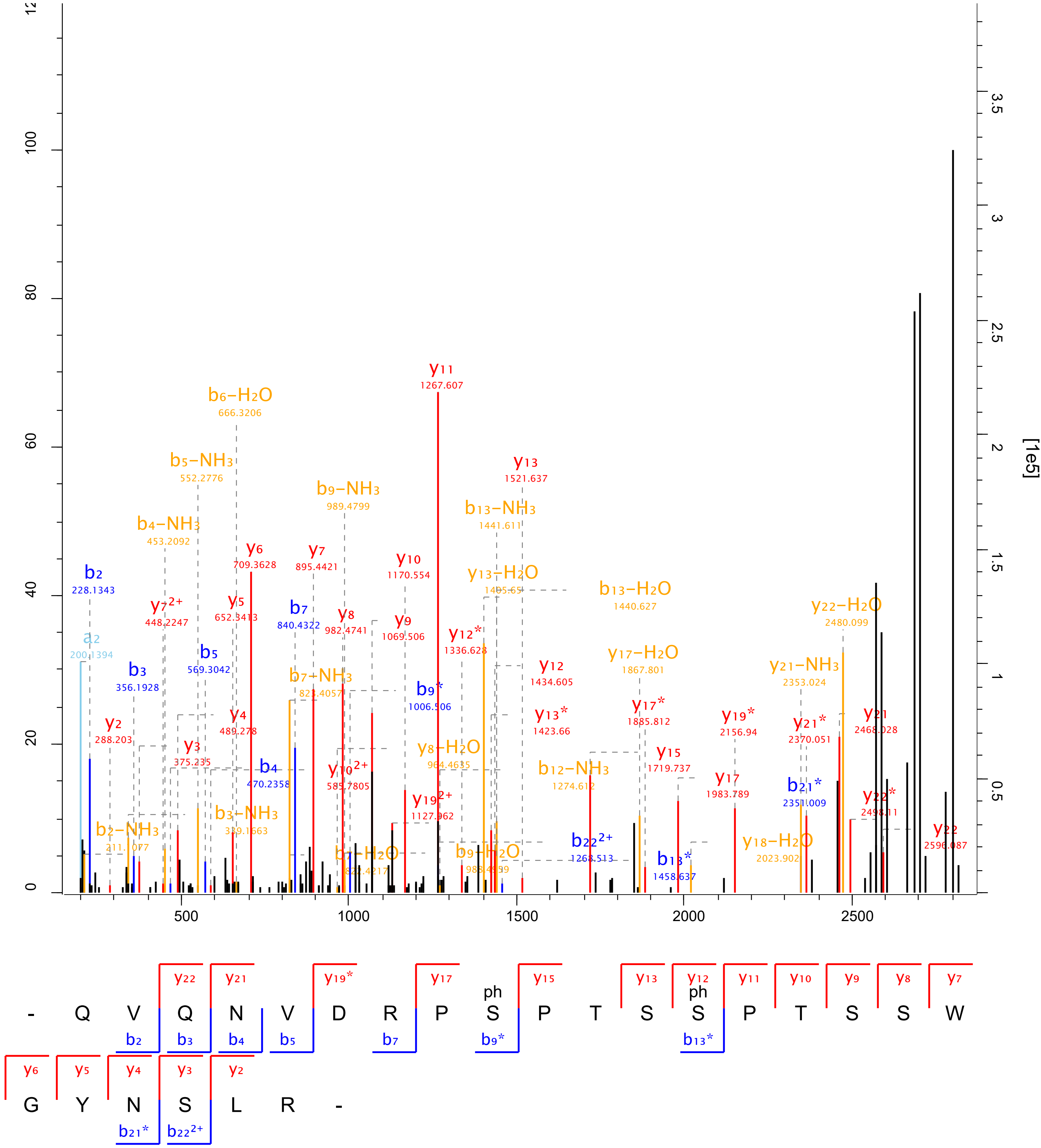

Sample output to test PDF Combine only

Raw file	Scan	Method	Score	m/z
0523_2	17332	FTMS; HCD	61.54	743.33

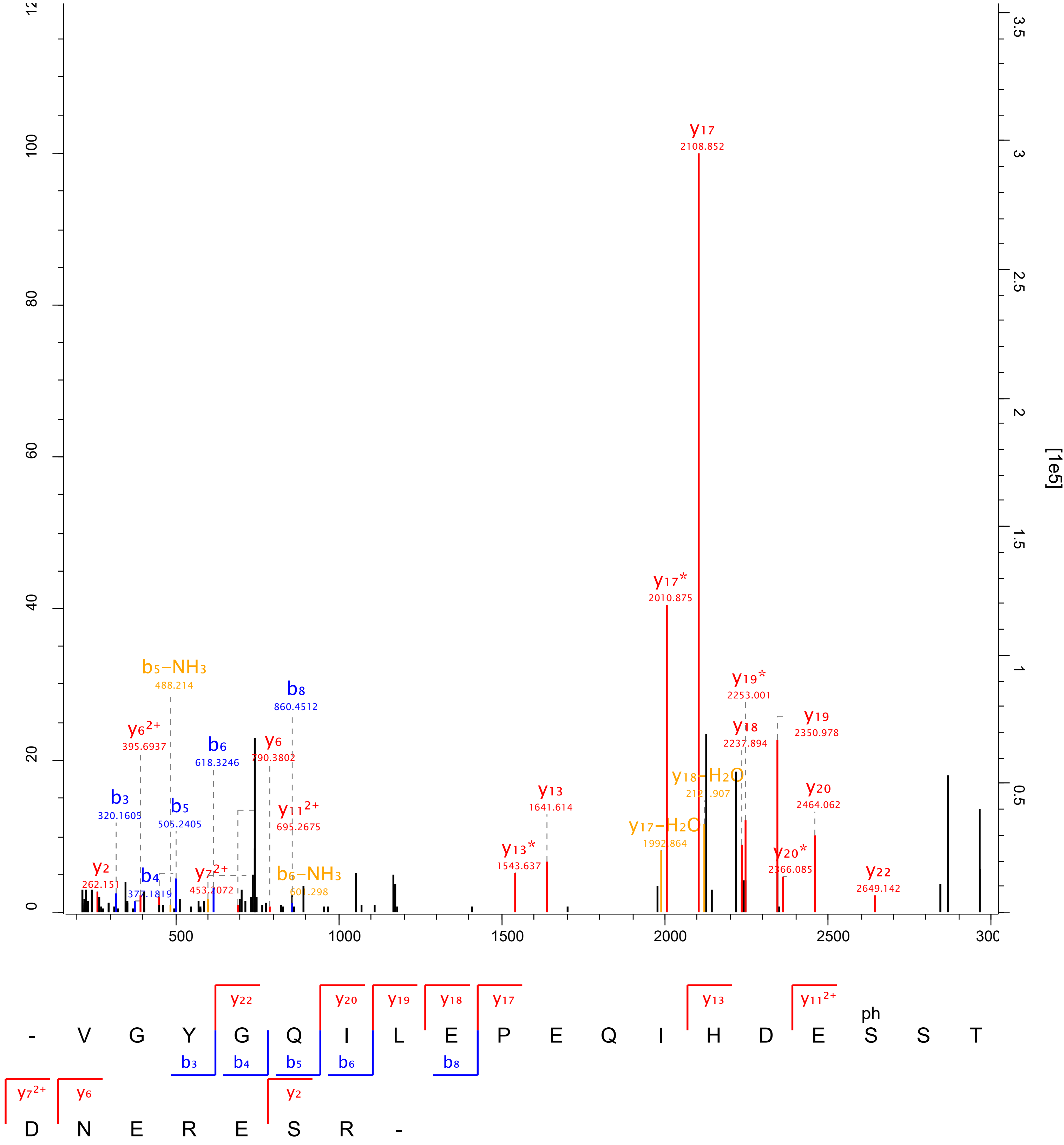

Sample output to test PDF Combine only

Raw file	Scan	Method	Score	m/z	Gene names
0523_2	17443	FTMS; HCD	49.12	742.34	At1g12760

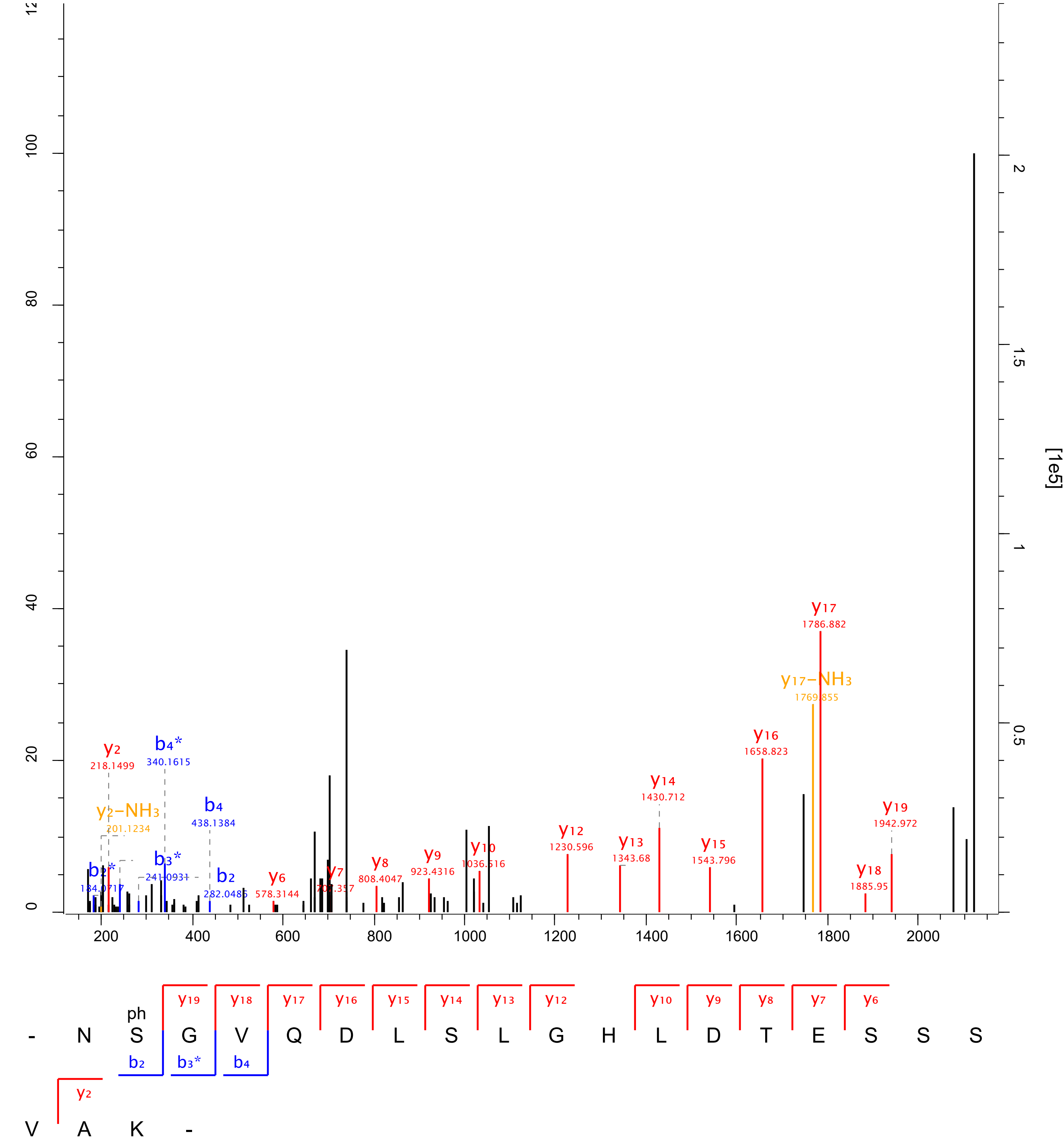

Sample output to test PDF Combine only

Raw file	Scan	Method	Score	m/z	Gene names
0523_2	17648	FTMS; HCD	194.06	794.38	At1g12760

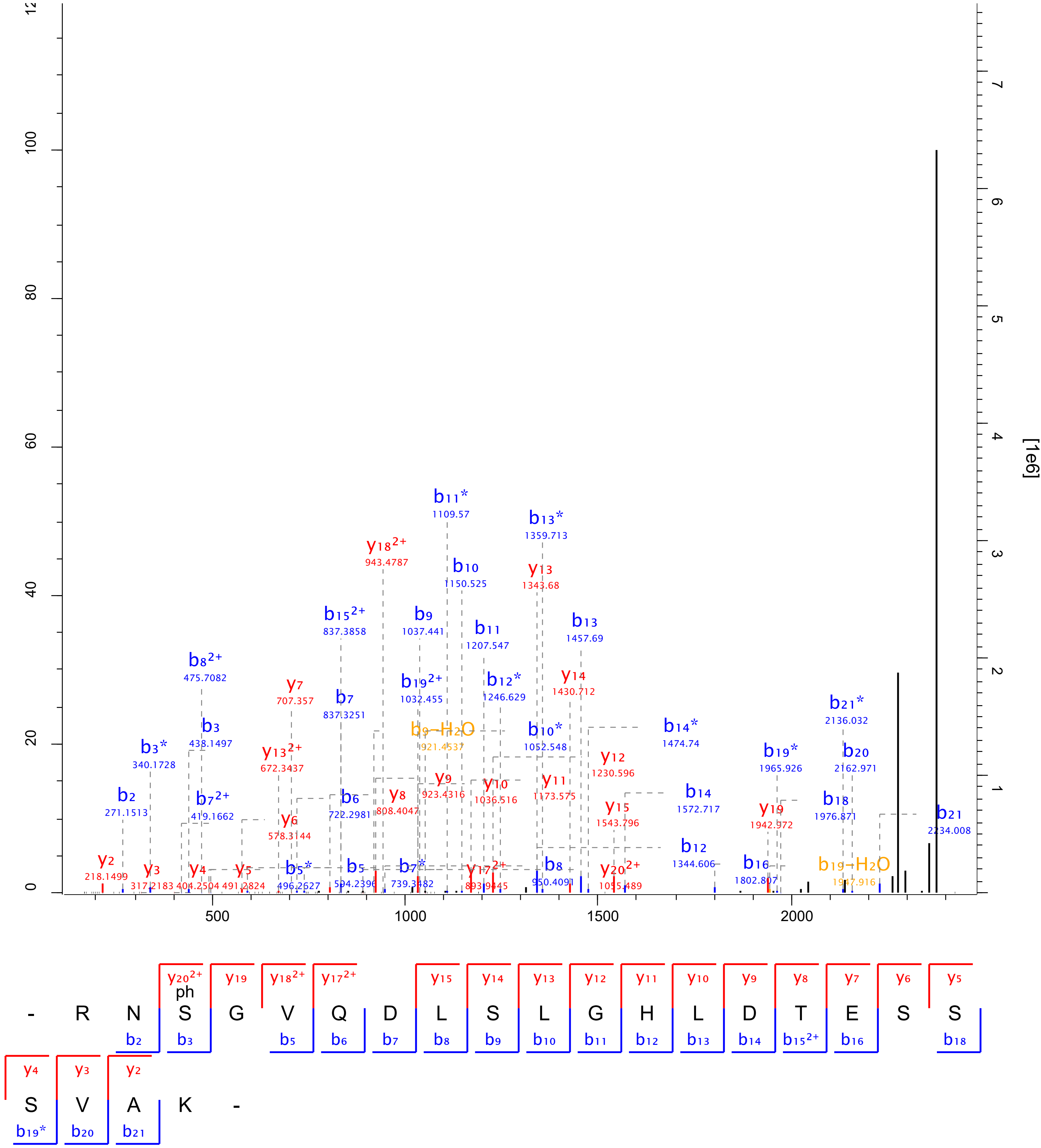

Sample output to test PDF Combine only

Raw file	Scan	Method	Score	m/z	Gene names
0523_2	17650	FTMS; HCD	252.23	1090.99	CTN

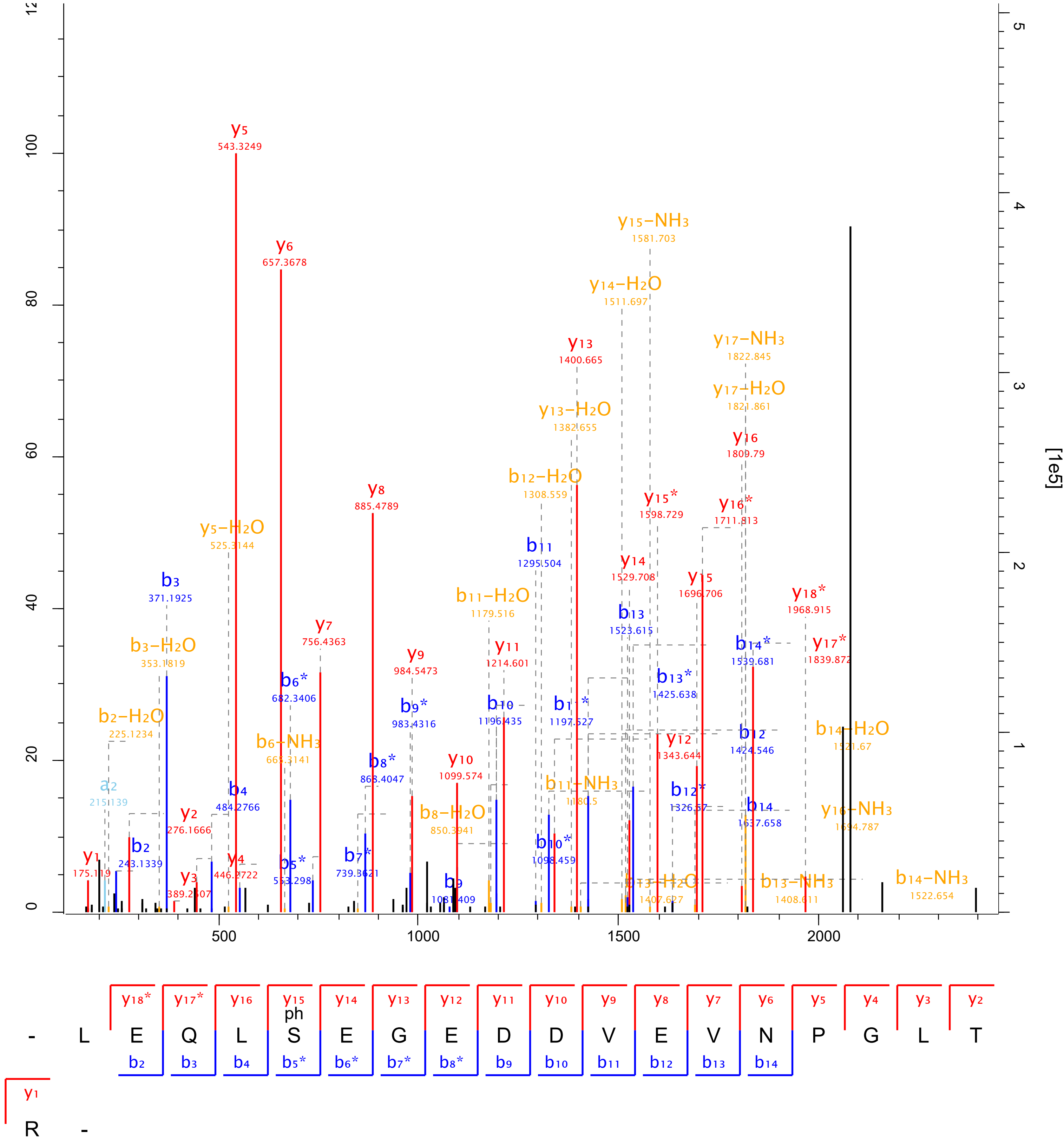

Sample output to test PDF Combine only

Raw file	Scan	Method	Score	m/z	Gene names
0523_2	17707	FTMS; HCD	121.1	868.4	At5g55210

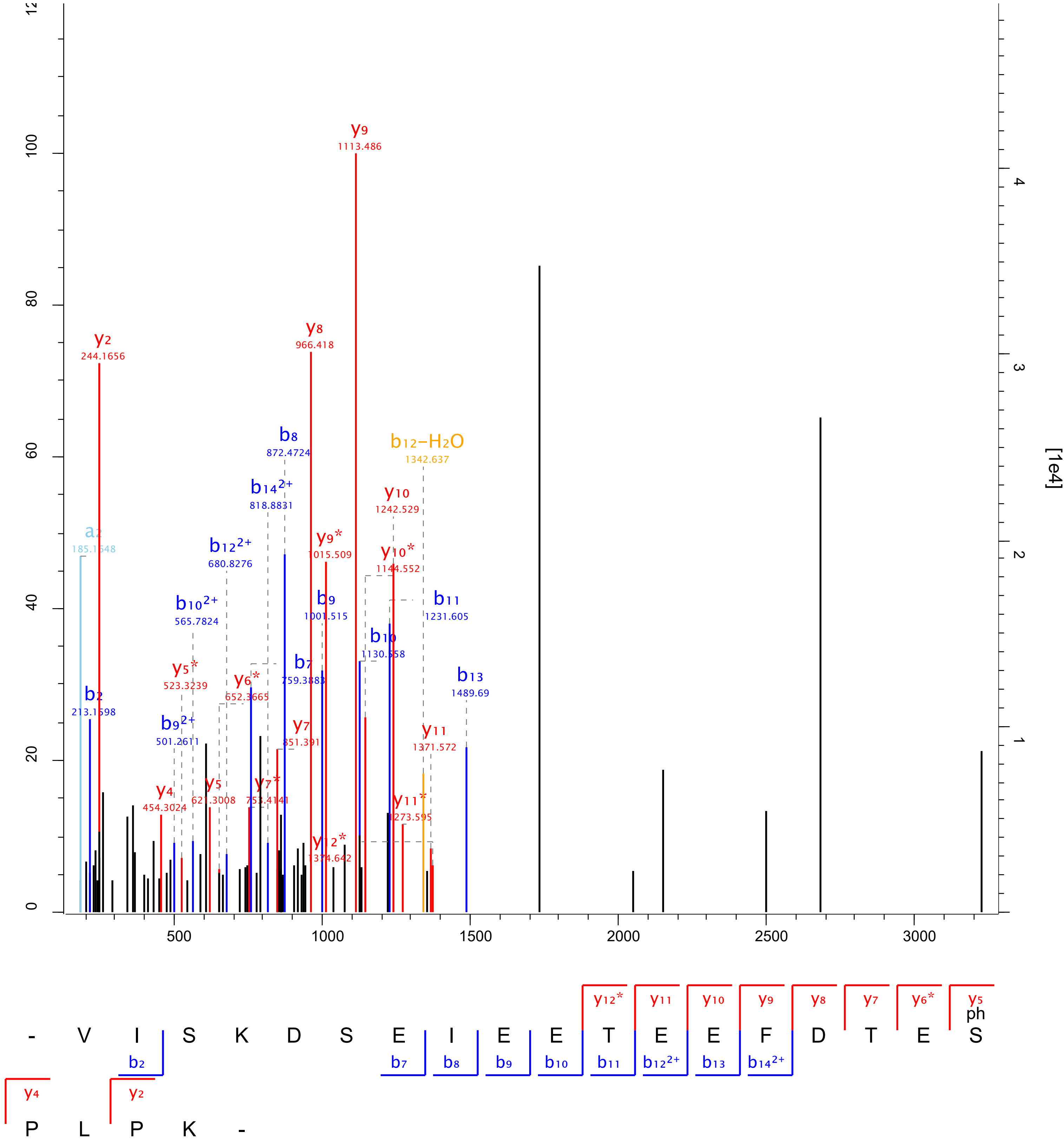

Sample output to test PDF Combine only

Raw file	Scan	Method	Score	m/z	Gene names
0523_2	18194	FTMS; HCD	85.94	766.71	P5CS1;P5CSA

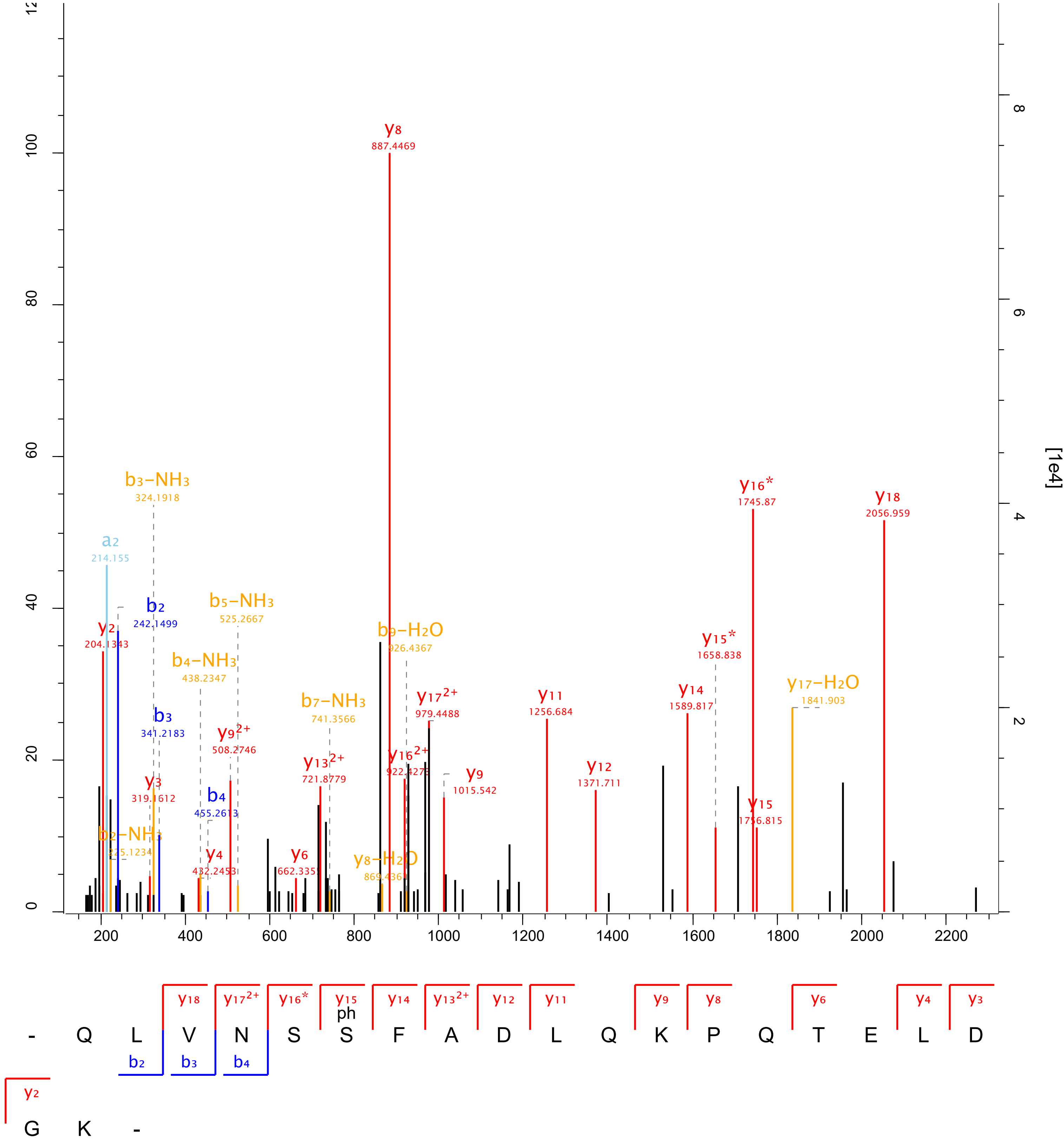

Sample output to test PDF Combine only

Raw file	Scan	Method	Score	m/z	Gene names
0523_2	18285	FTMS; HCD	177.99	652.55	TRX9

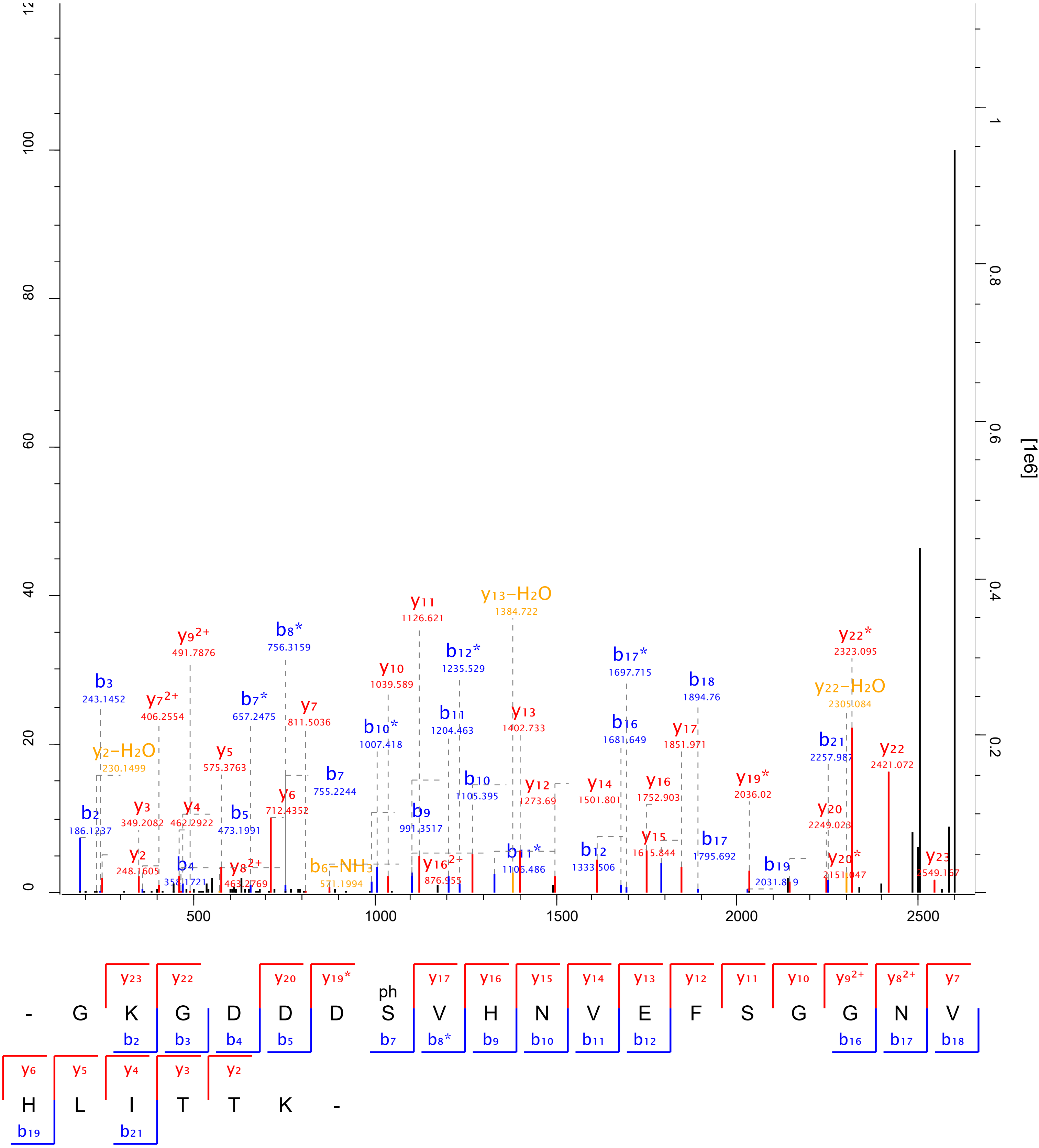

Sample output to test PDF Combine only

Raw file	Scan	Method	Score	m/z
0523_2	19055	FTMS; HCD	141.36	729.03

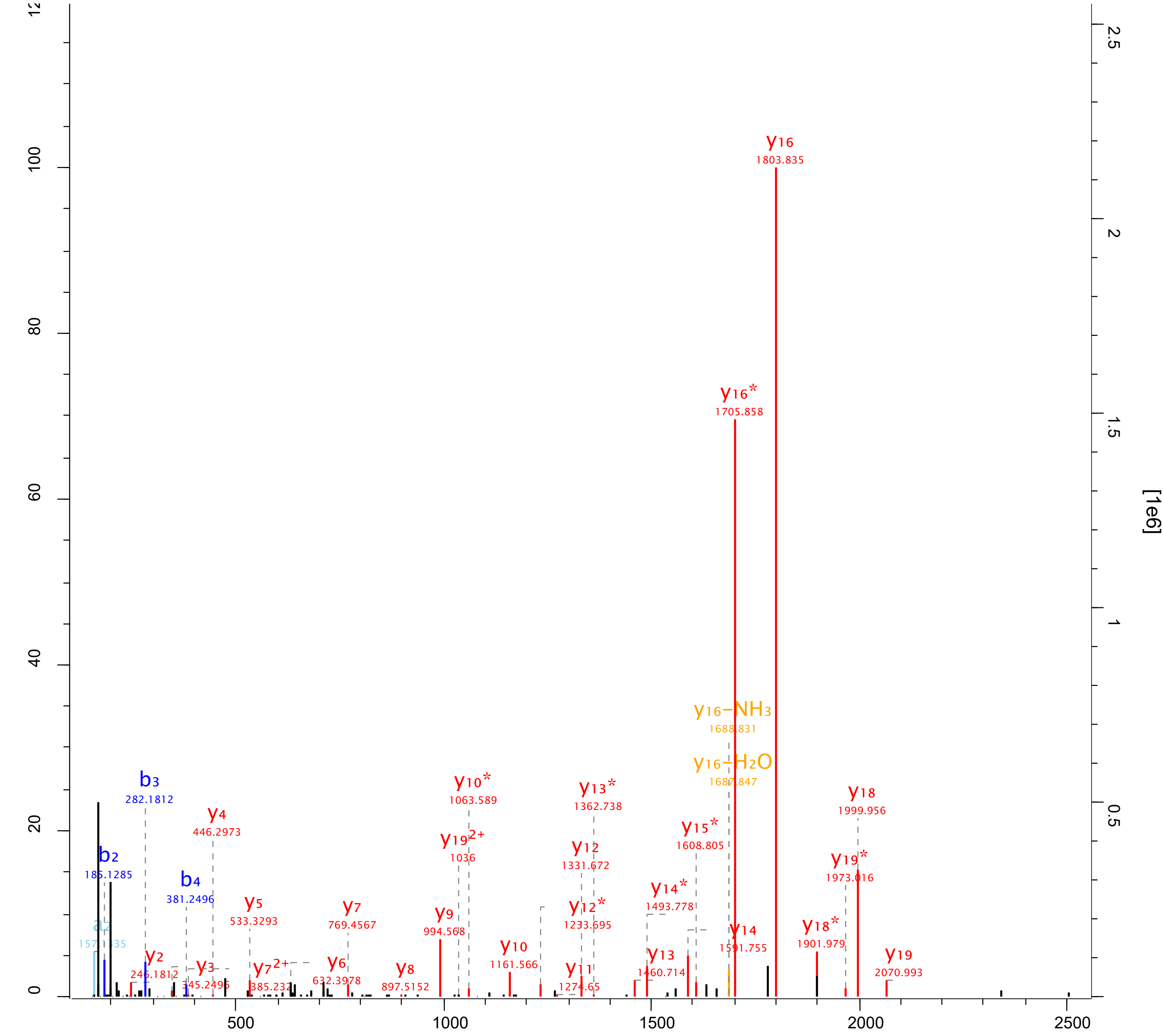

		y19	y18		y16	y15*	y14	y13	y12	y11	y10 ph	y9	y8	y7	y6	y5	y4	y3
-	L	A	P	V	P	D	M	E	G	I	S	P	Q	H	V	S	T	V
		b2	b3	b4														

y2		
V	K	-

Sample output to test PDF Combine only

Raw file	Scan	Method	Score	m/z	Gene names
0523_2	19082	FTMS; HCD	95.21	665	ACBP4

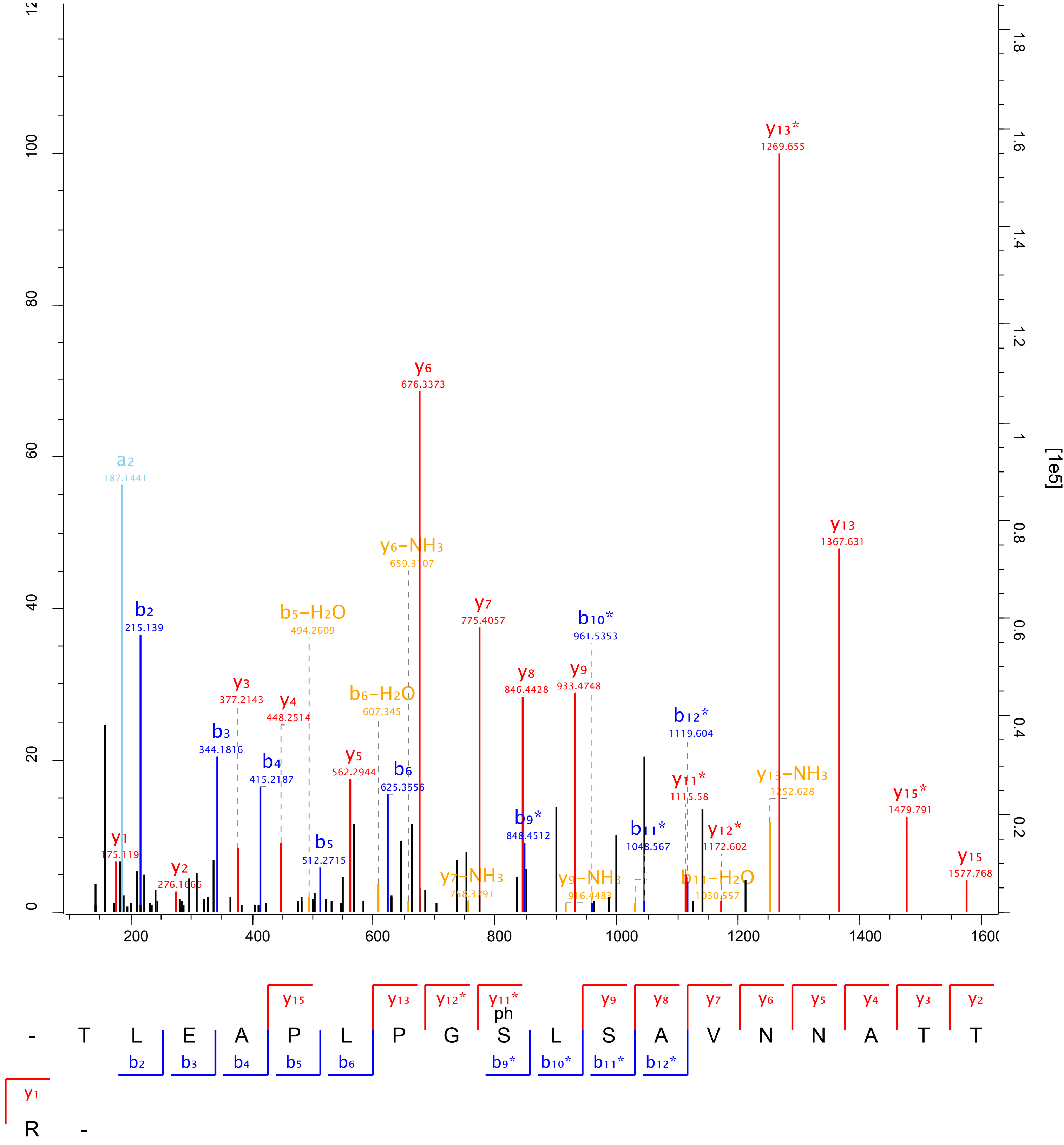

Sample output to test PDF Combine only

Raw file	Scan	Method	Score	m/z	Gene names
0523_2	19585	FTMS; HCD	98.66	1011.11	ABCB21

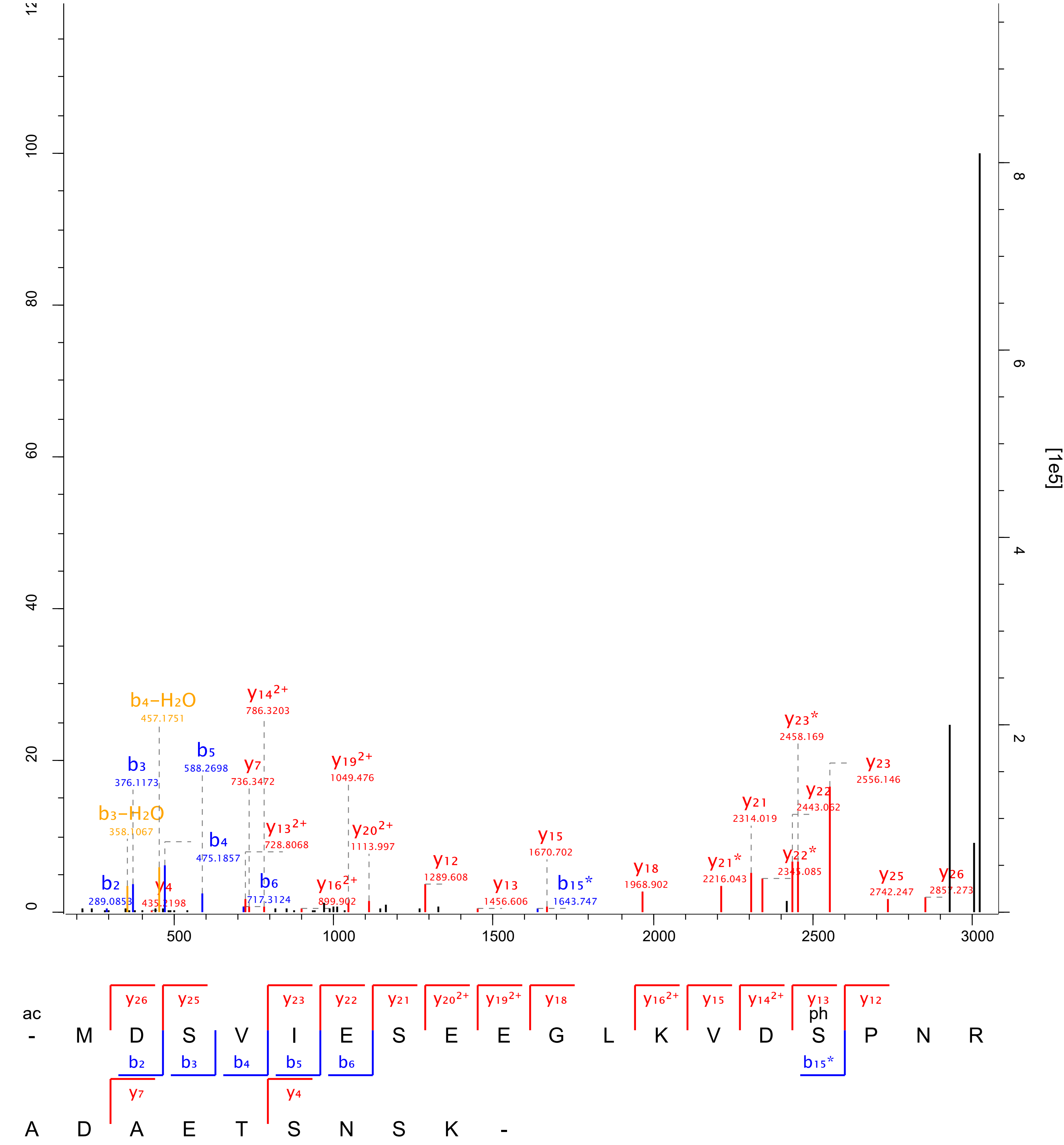

Sample output to test PDF Combine only

Raw file	Scan	Method	Score	m/z	Gene names
0523_2	20812	FTMS; HCD	139.67	788.72	AMPD

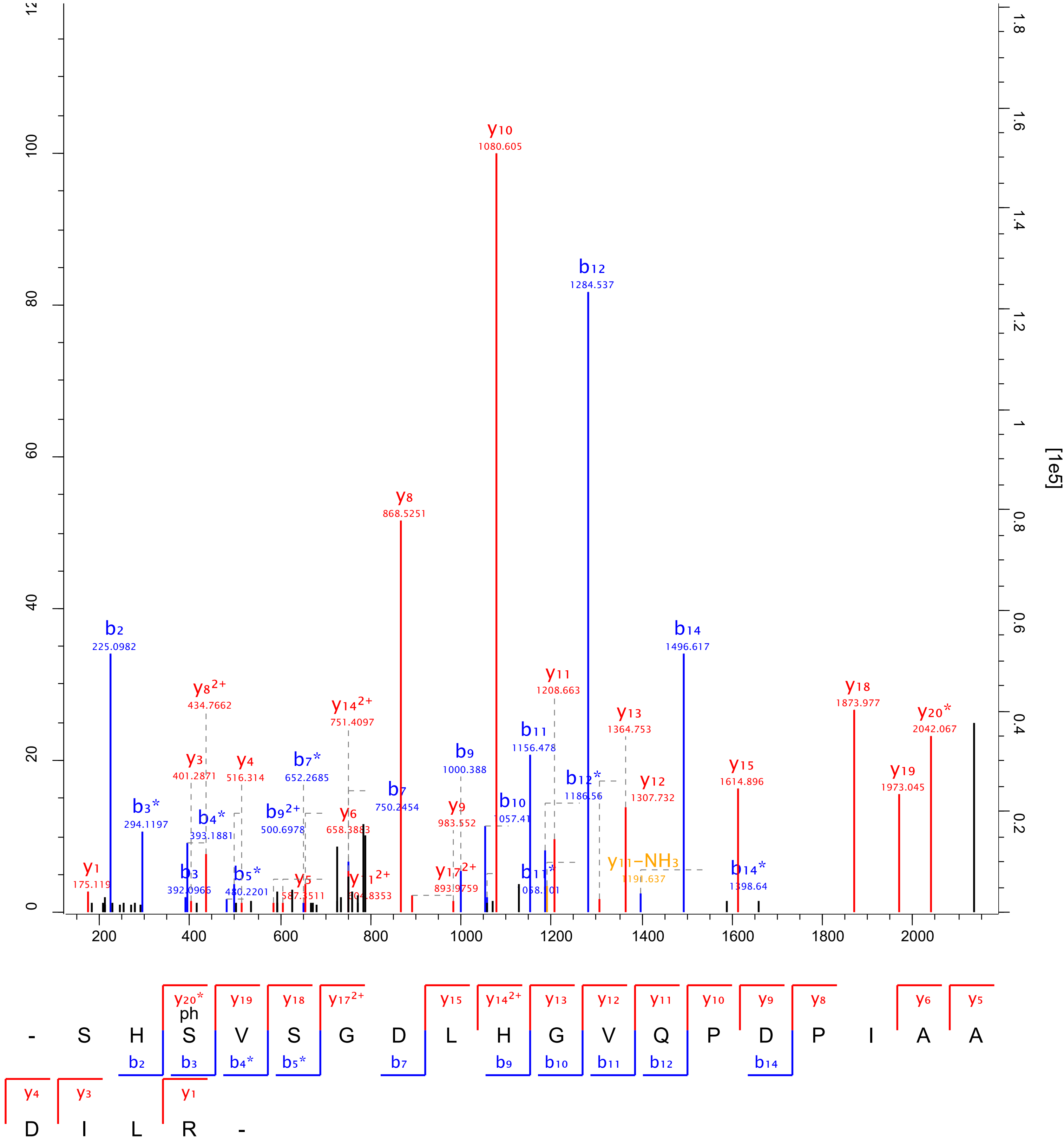

Sample output to test PDF Combine only

Raw file	Scan	Method	Score	m/z	Gene names
0523_2	20855	FTMS; HCD	52.97	814.37	RH20

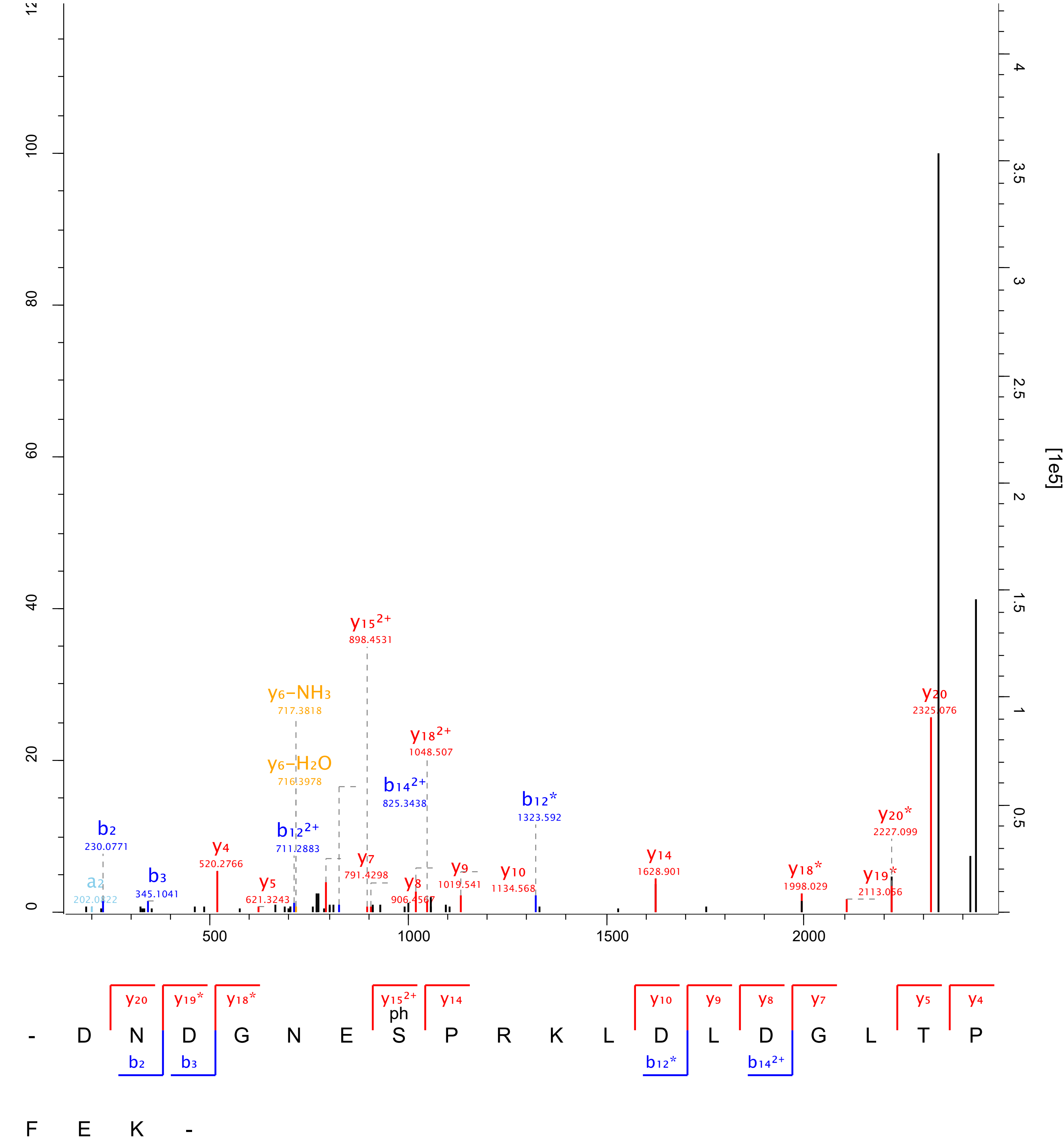

Sample output to test PDF Combine only

Raw file	Scan	Method	Score	m/z	Gene names
0523_2	23326	FTMS; HCD	351.69	1066.93	OPT4

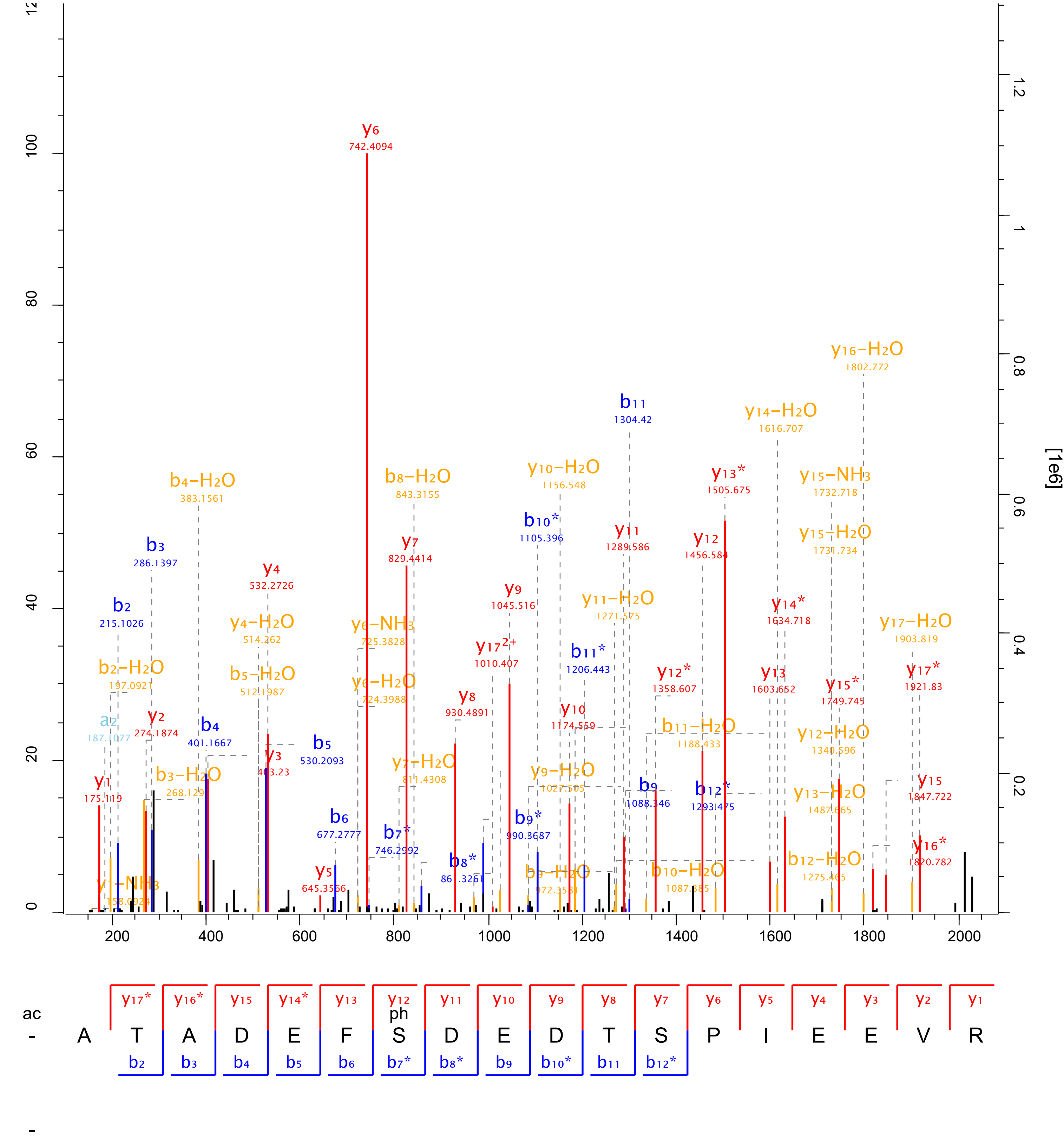

Sample output to test PDF Combine only

Raw file	Scan	Method	Score	m/z
0523_2	23394	FTMS; HCD	126.9	865.37

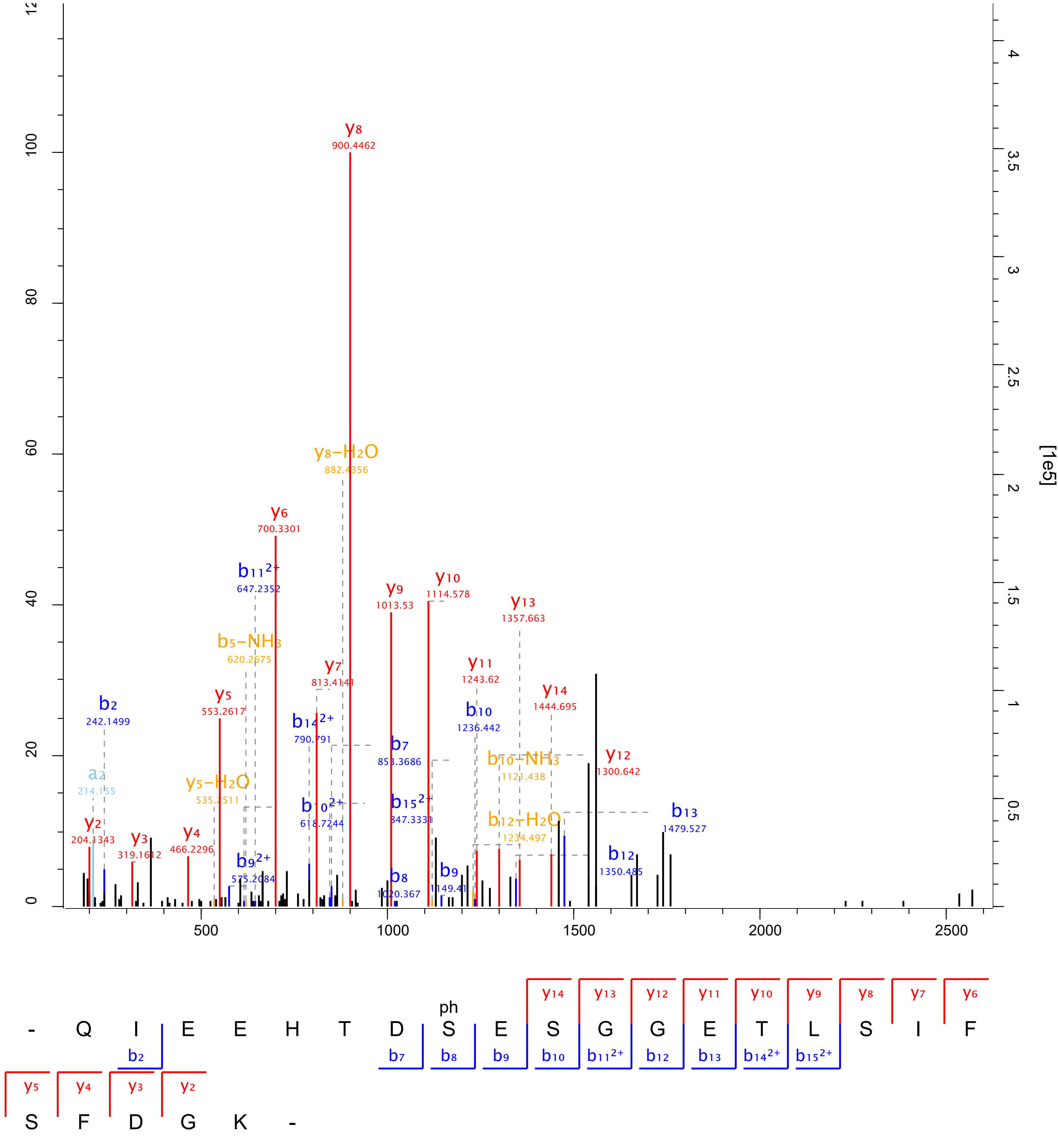

Sample output to test PDF Combine only

Raw file	Scan	Method	Score	m/z	Gene names
0523_2	23465	FTMS; HCD	78.76	790.03	ABCB4

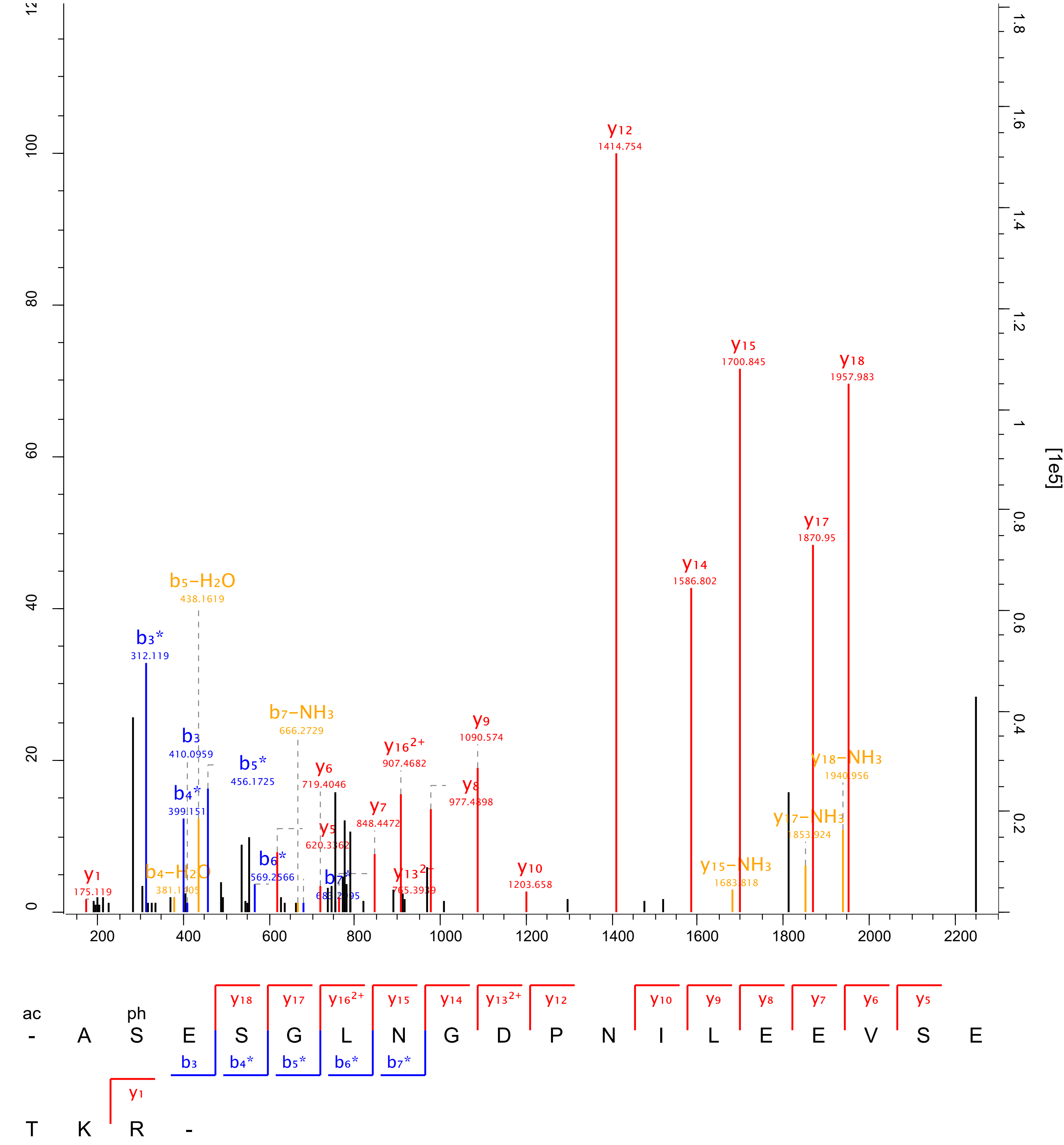

Sample output to test PDF Combine only

Raw file	Scan	Method	Score	m/z	Gene names
0523_2	26845	FTMS; HCD	40.2	814.08	LCBK1

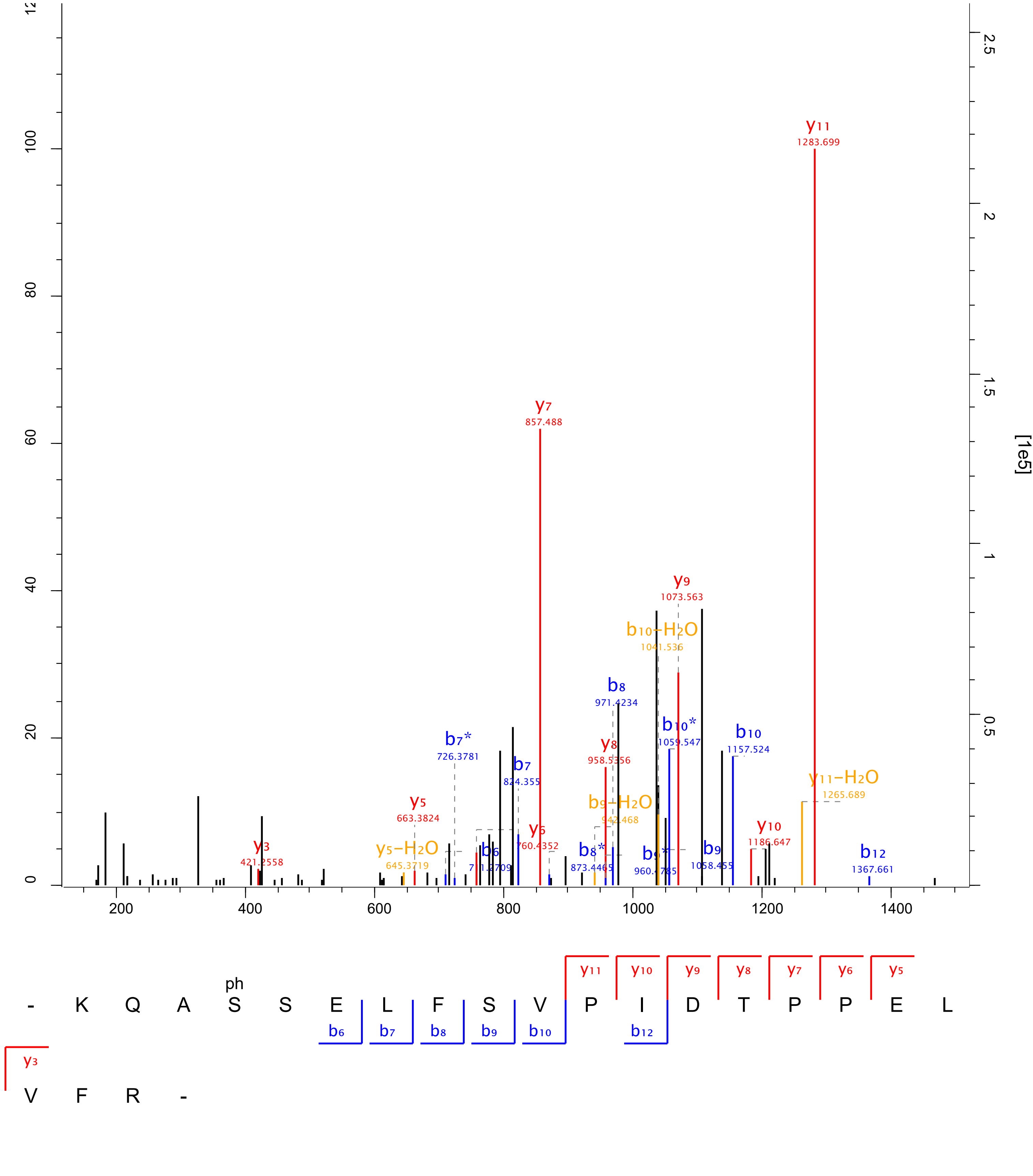

Sample output to test PDF Combine only

Raw file	Scan	Method	Score	m/z	Gene names
0523_2	27055	FTMS; HCD	232.87	1152.42	RPP0C

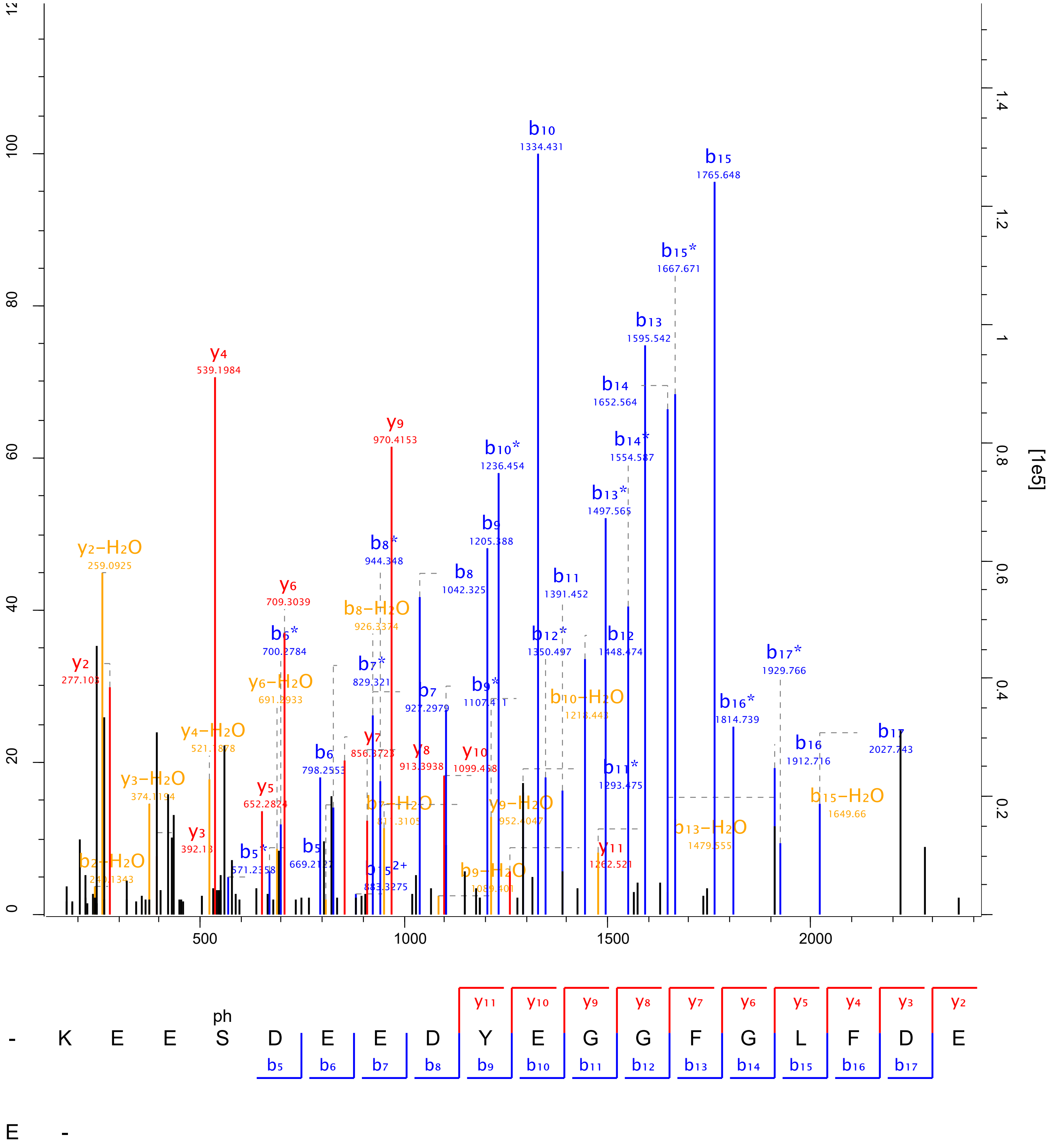

Sample output to test PDF Combine only

Raw file	Scan	Method	Score	m/z	Gene names
0523_2	27861	FTMS; HCD	133.36	1166.91	GRV2

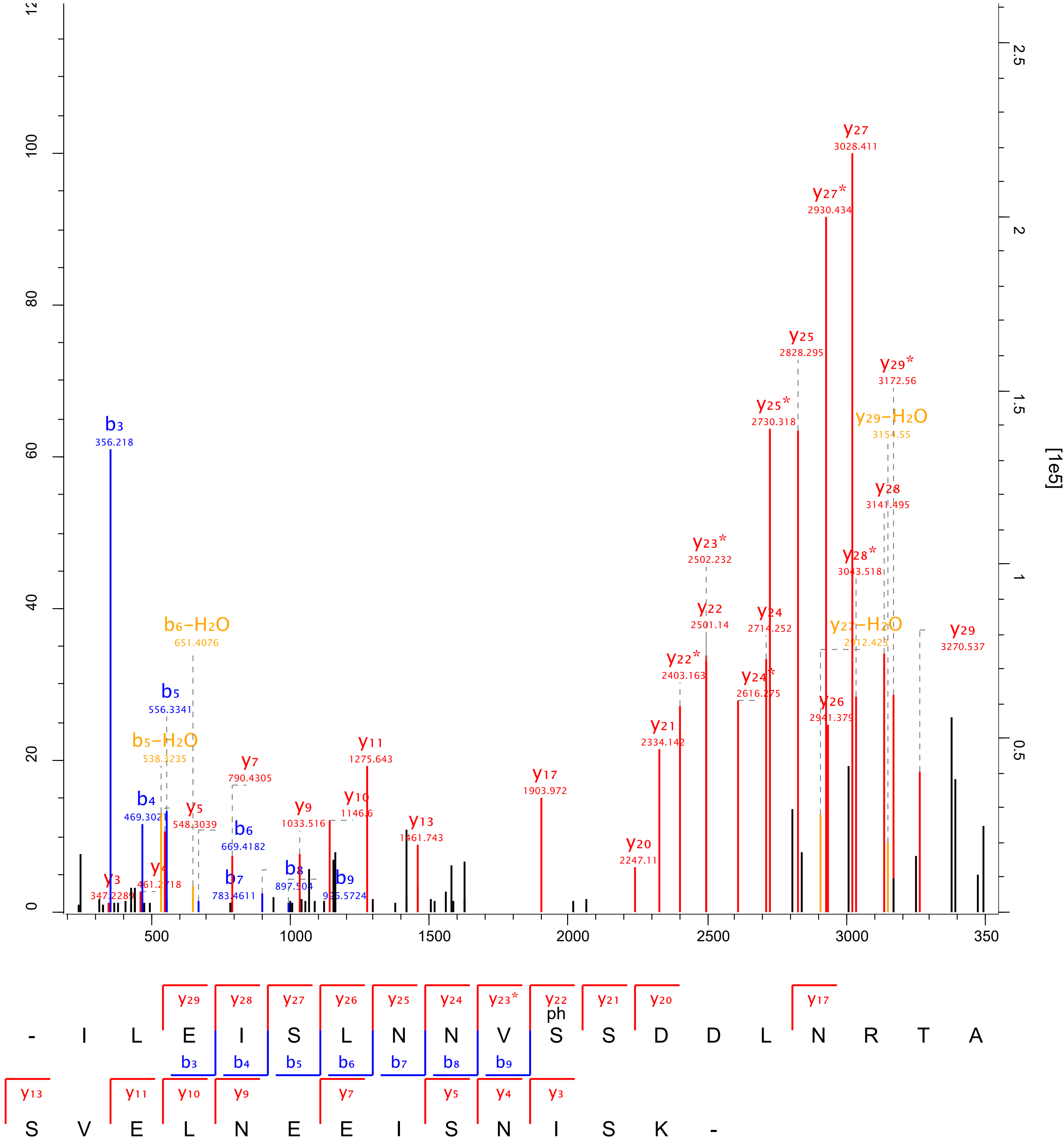

Sample output to test PDF Combine only

Raw file	Scan	Method	Score	m/z	Gene names
0523_2	31292	FTMS; HCD	51.97	843.05	F8L10.9

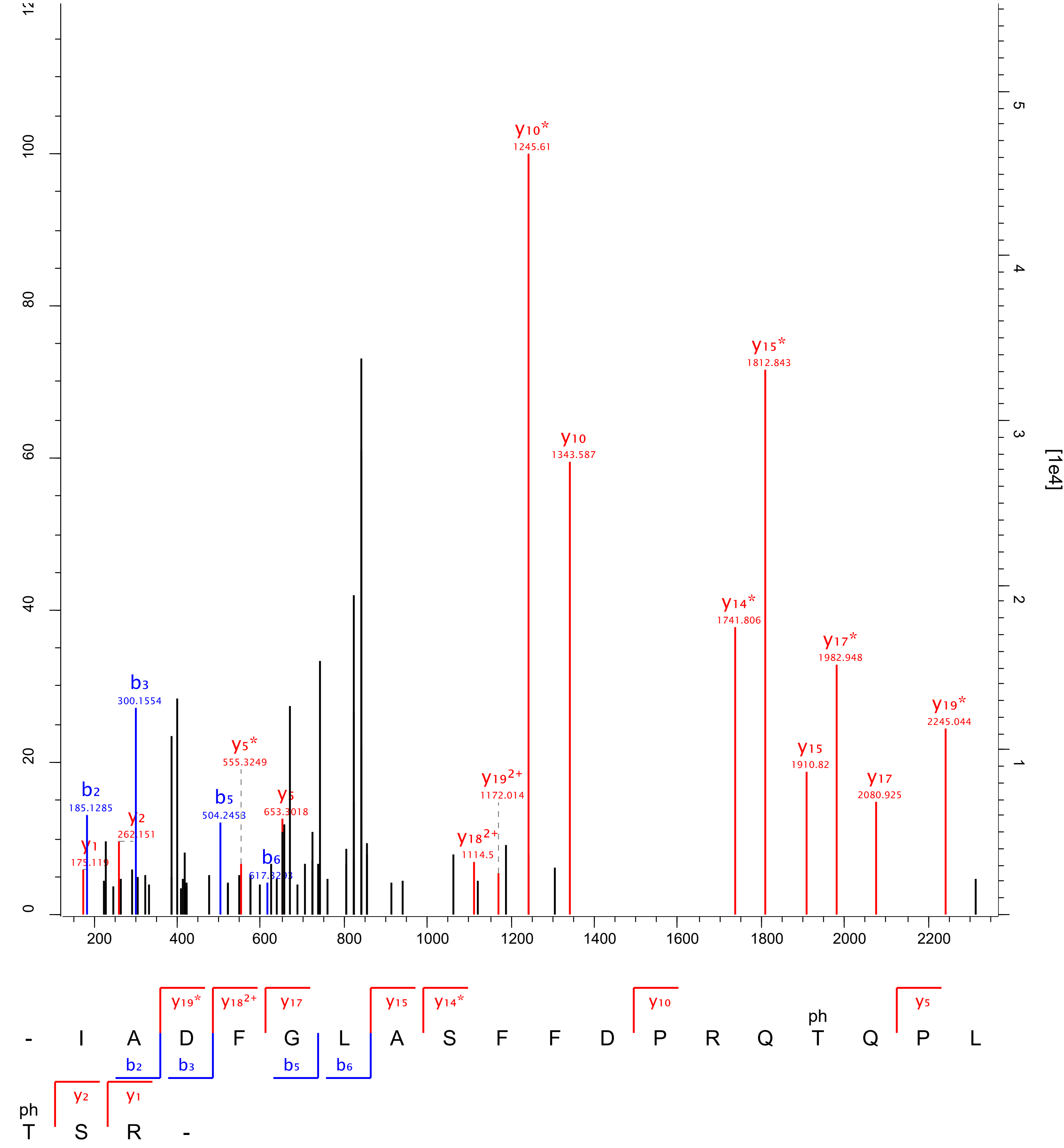

Sample output to test PDF Combine only

Raw file	Scan	Method	Score	m/z	Gene names
0523_2	31557	FTMS; HCD	69.98	994.43	ZDP

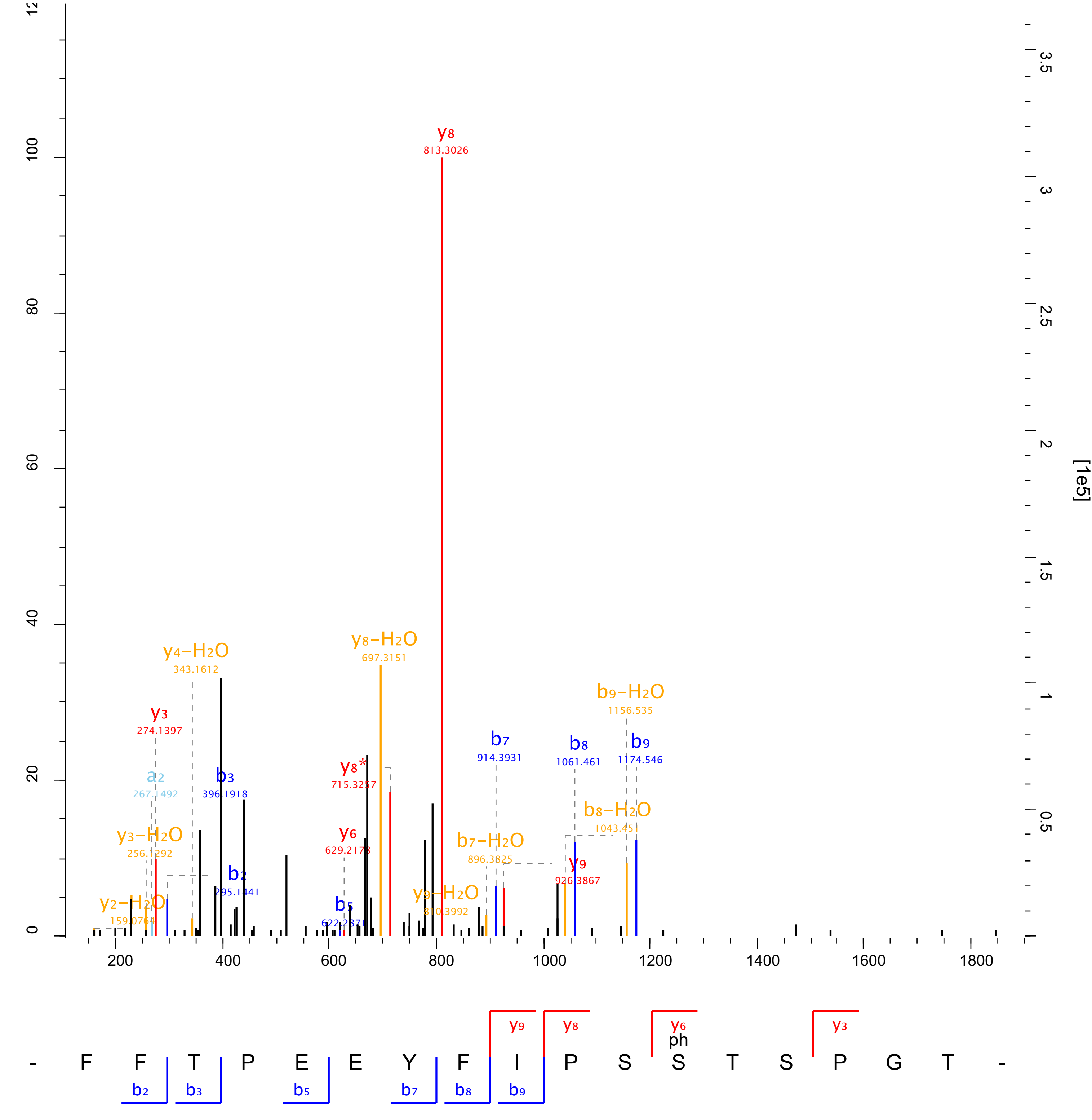

Sample output to test PDF Combine only

Raw file	Scan	Method	Score	m/z	Gene names
0523_2	31661	FTMS; HCD	110.9	1087.87	F9K20.7

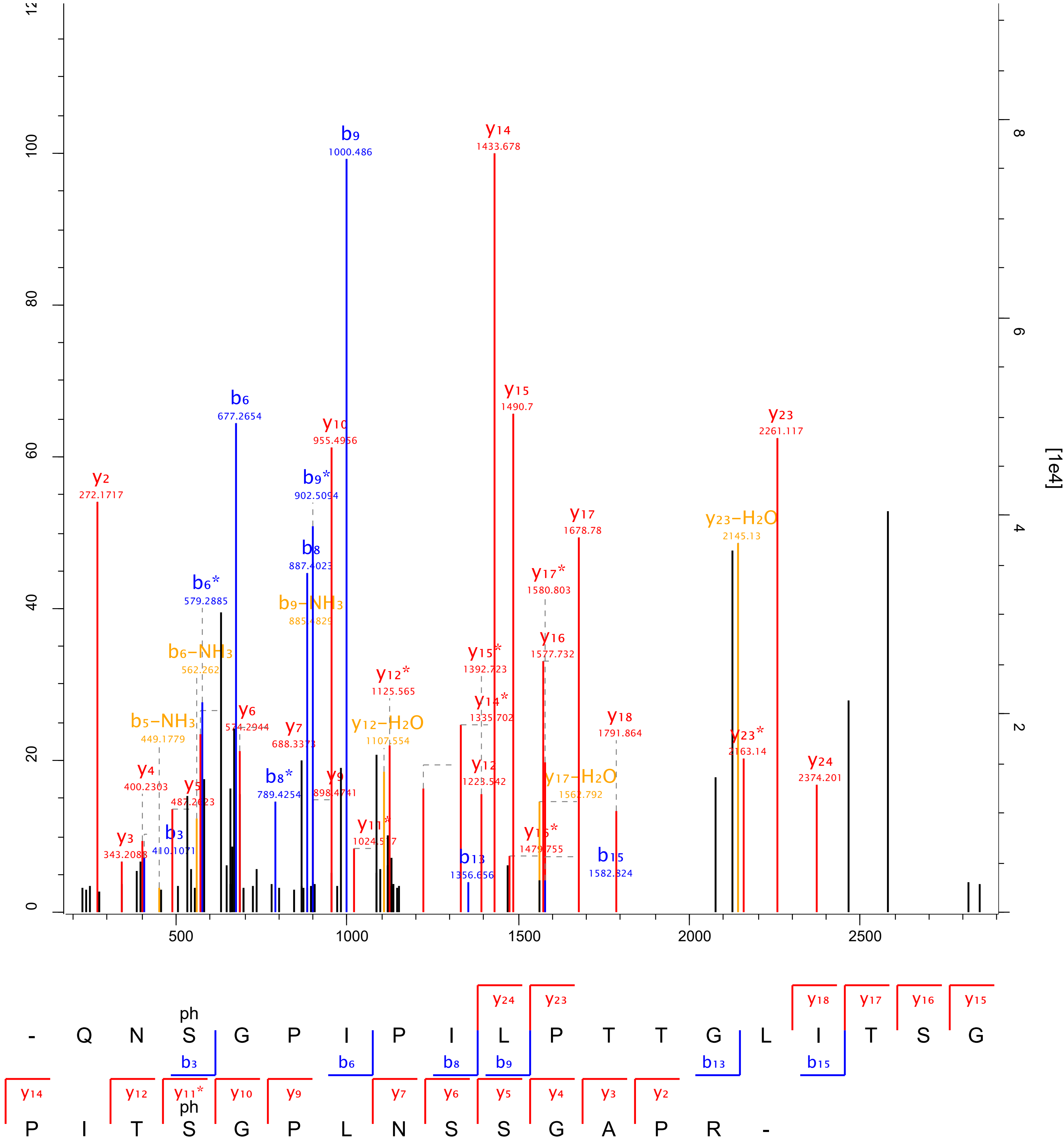

Sample output to test PDF Combine only

Raw file	Scan	Method	Score	m/z	Gene names
0523_2	32647	FTMS; HCD	78	1054.81	At3g08020

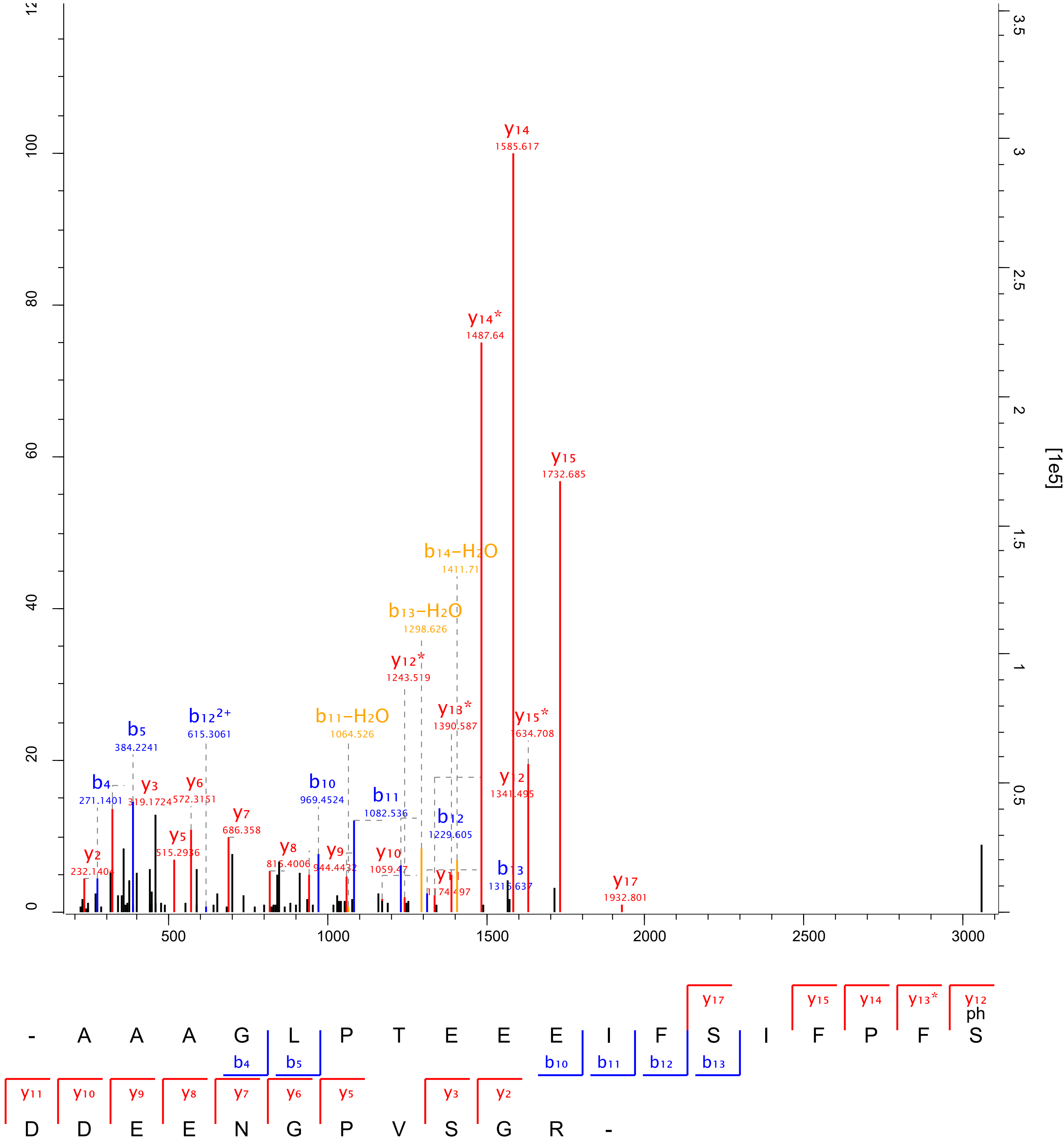

Sample output to test PDF Combine only

Raw file	Scan	Method	Score	m/z	Gene names
0523_3	3249	FTMS; HCD	145.23	535.24	ARL

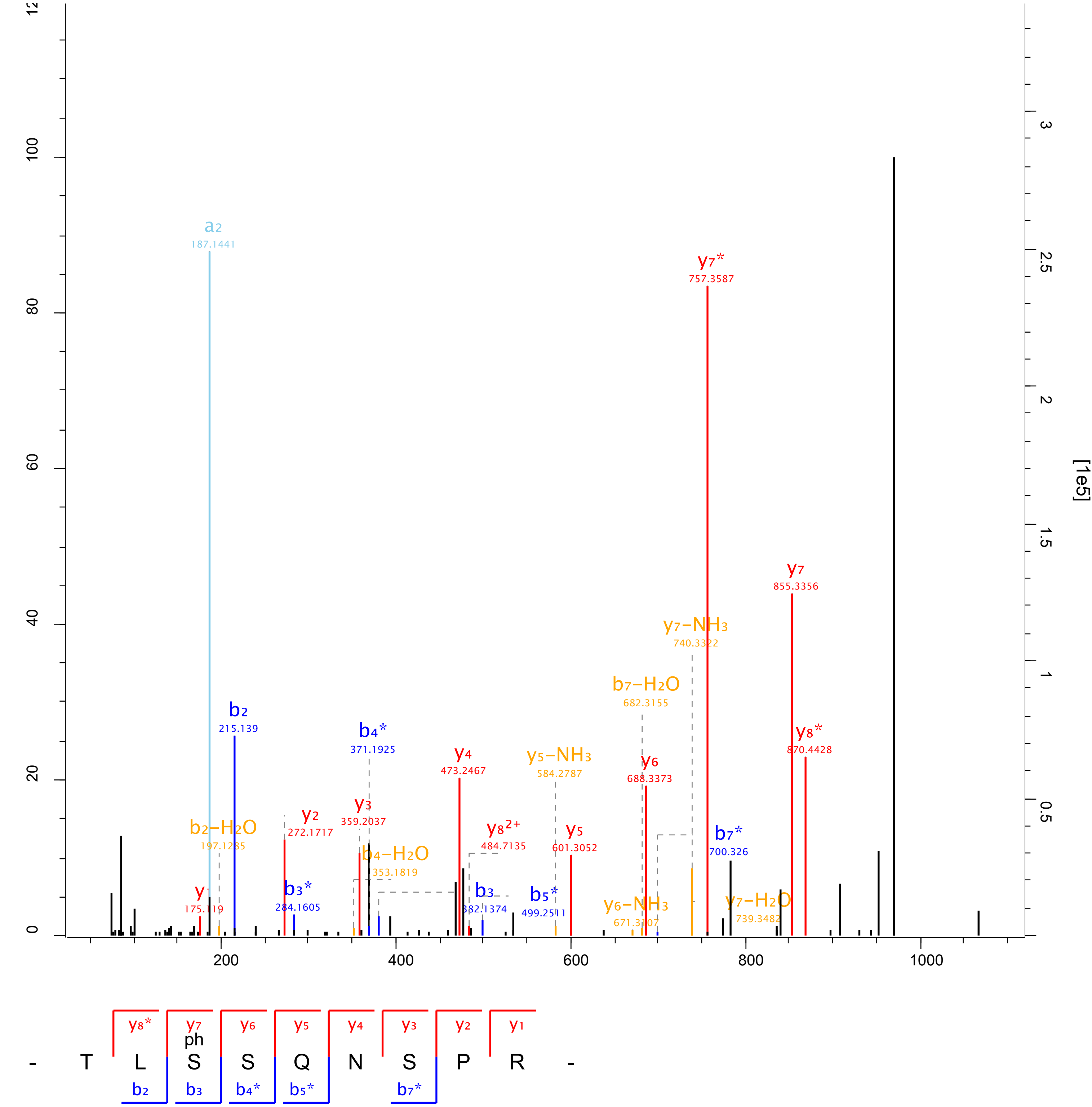

Sample output to test PDF Combine only

Raw file	Scan	Method	Score	m/z	Gene names
0523_3	3406	FTMS; HCD	142.65	713.28	NFYB10

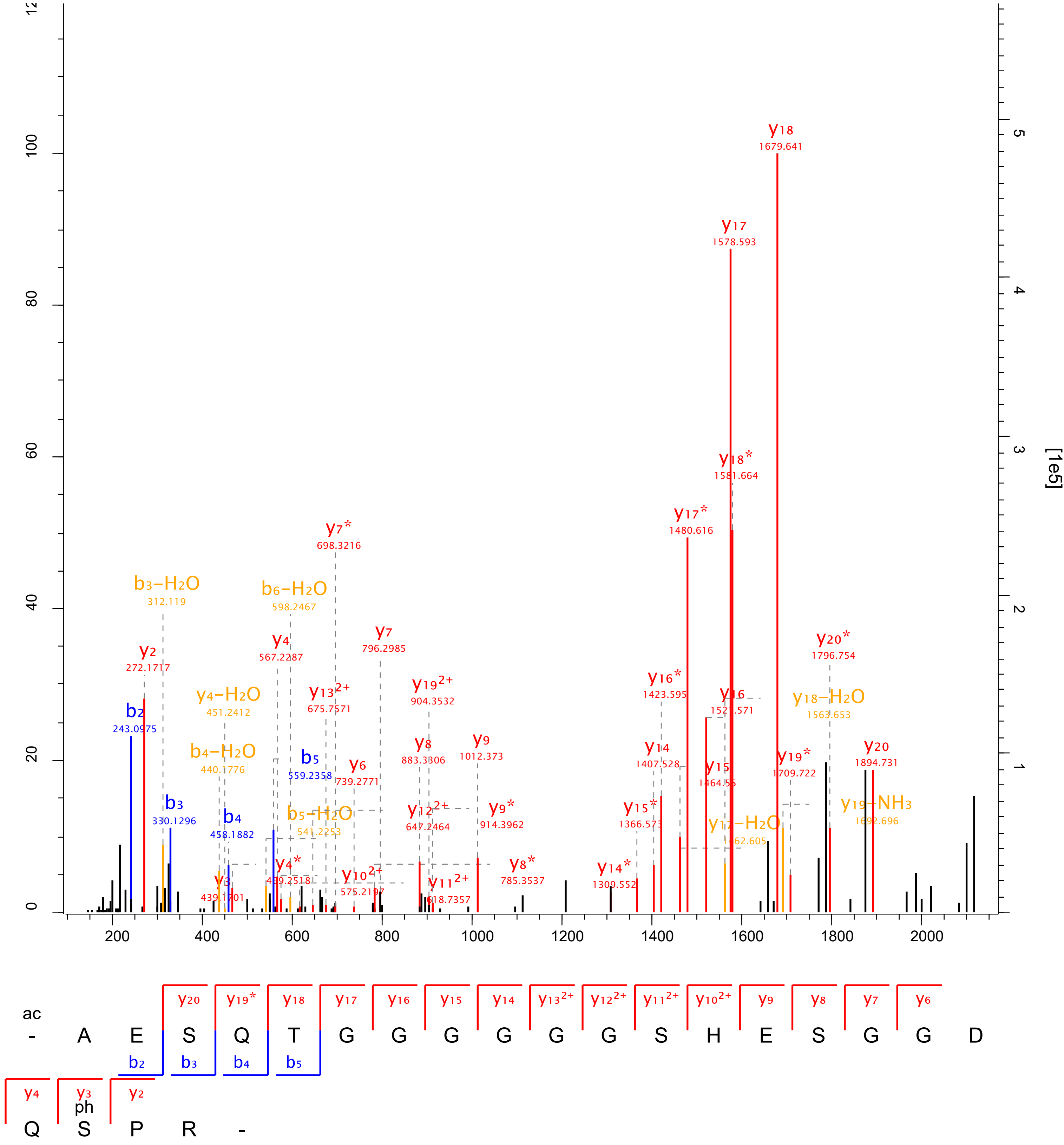

Raw file	Scan	Method	Score	m/z	Gene names
0523_3	3564	FTMS; HCD	130.01	720.8	At4g00238

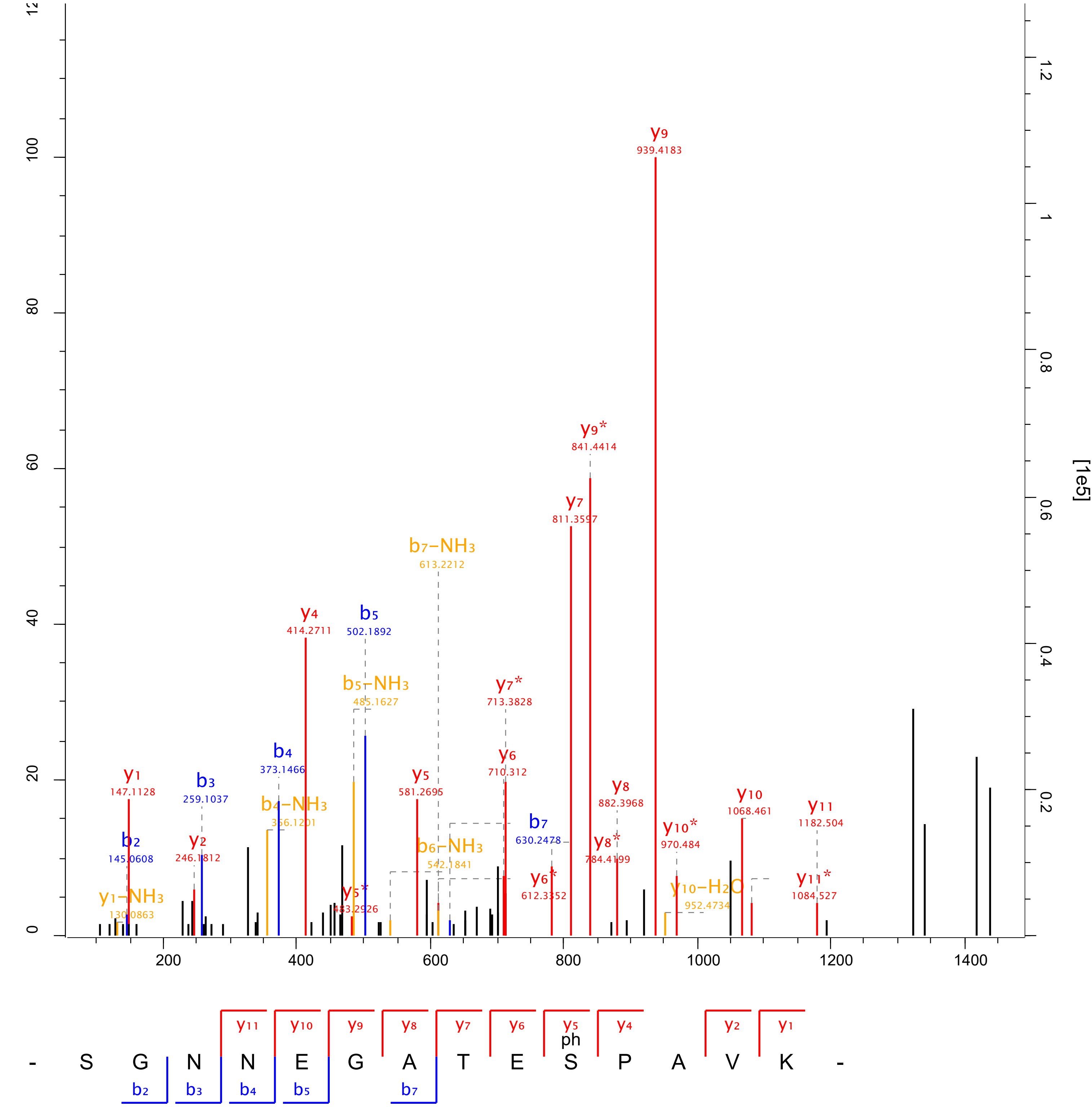

Sample output to test PDF Combine only

Raw file	Scan	Method	Score	m/z	Gene names
0523_3	5272	FTMS; HCD	210.12	842.8	AMT1-2

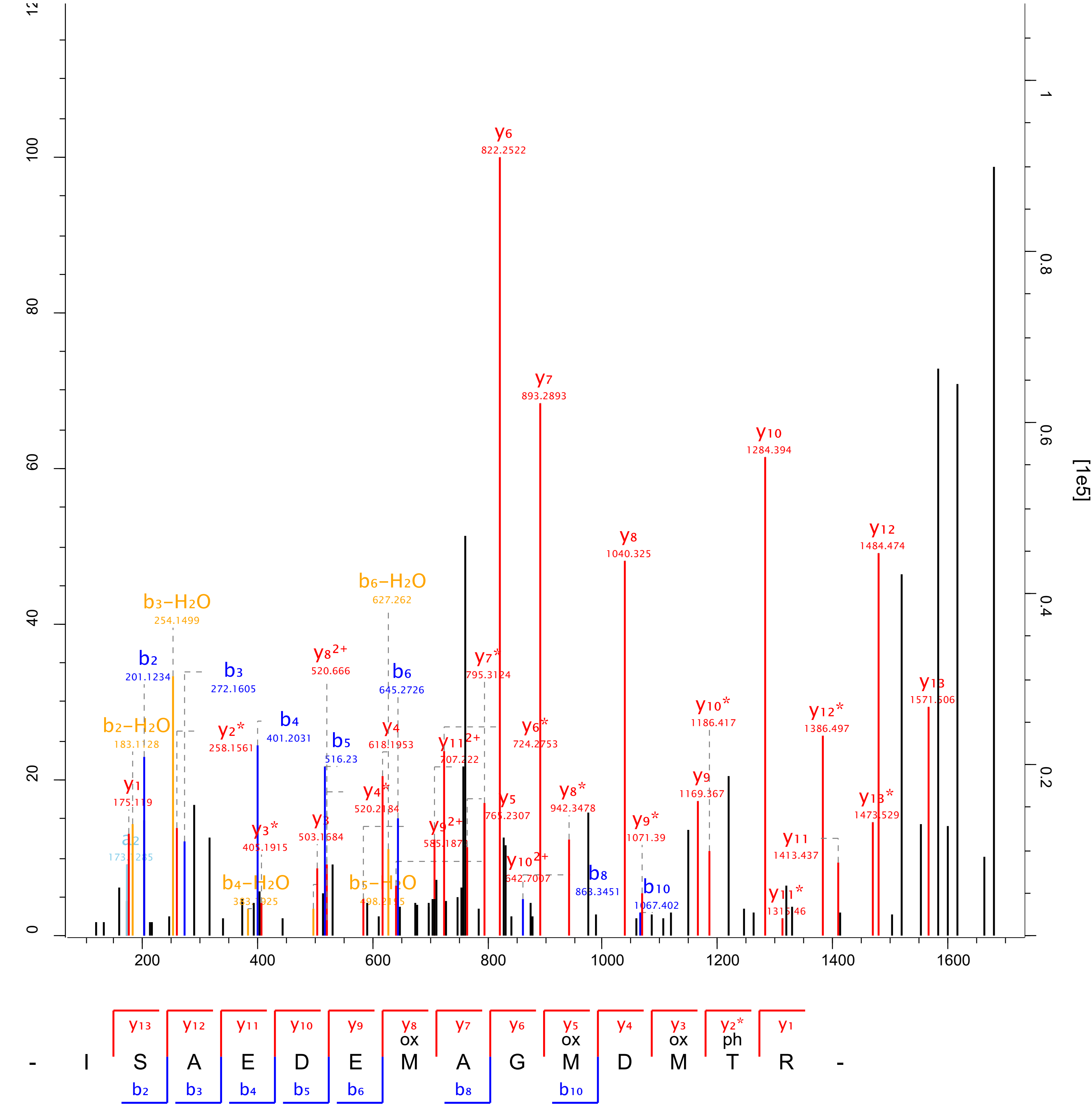

Sample output to test PDF Combine only

Raw file	Scan	Method	Score	m/z	Gene names
0523_3	5371	FTMS; HCD	68.66	427.7	RLK902

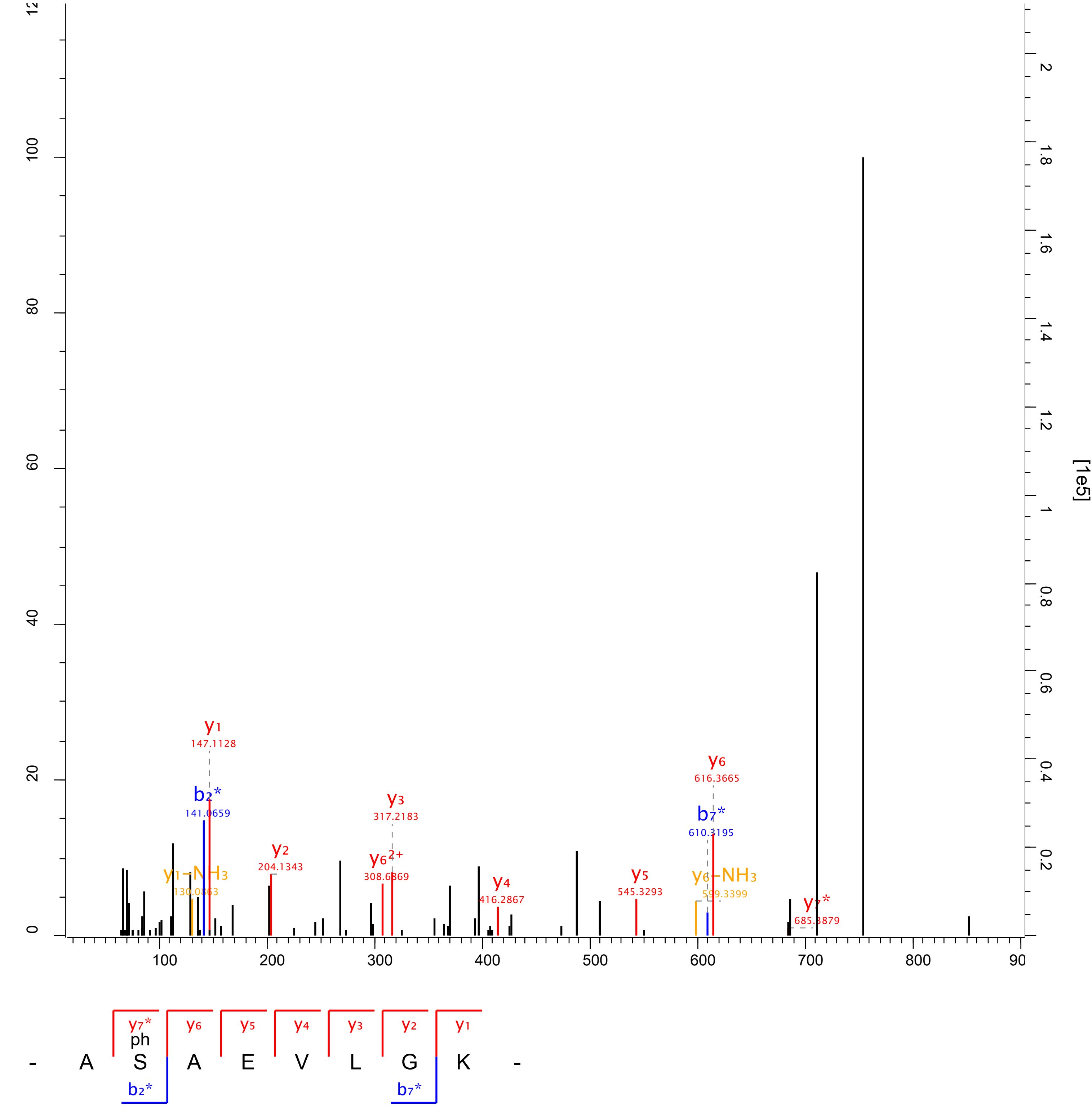

Sample output to test PDF Combine only

Raw file	Scan	Method	Score	m/z	Gene names
0523_3	5511	FTMS; HCD	71.98	667.81	At2g17700

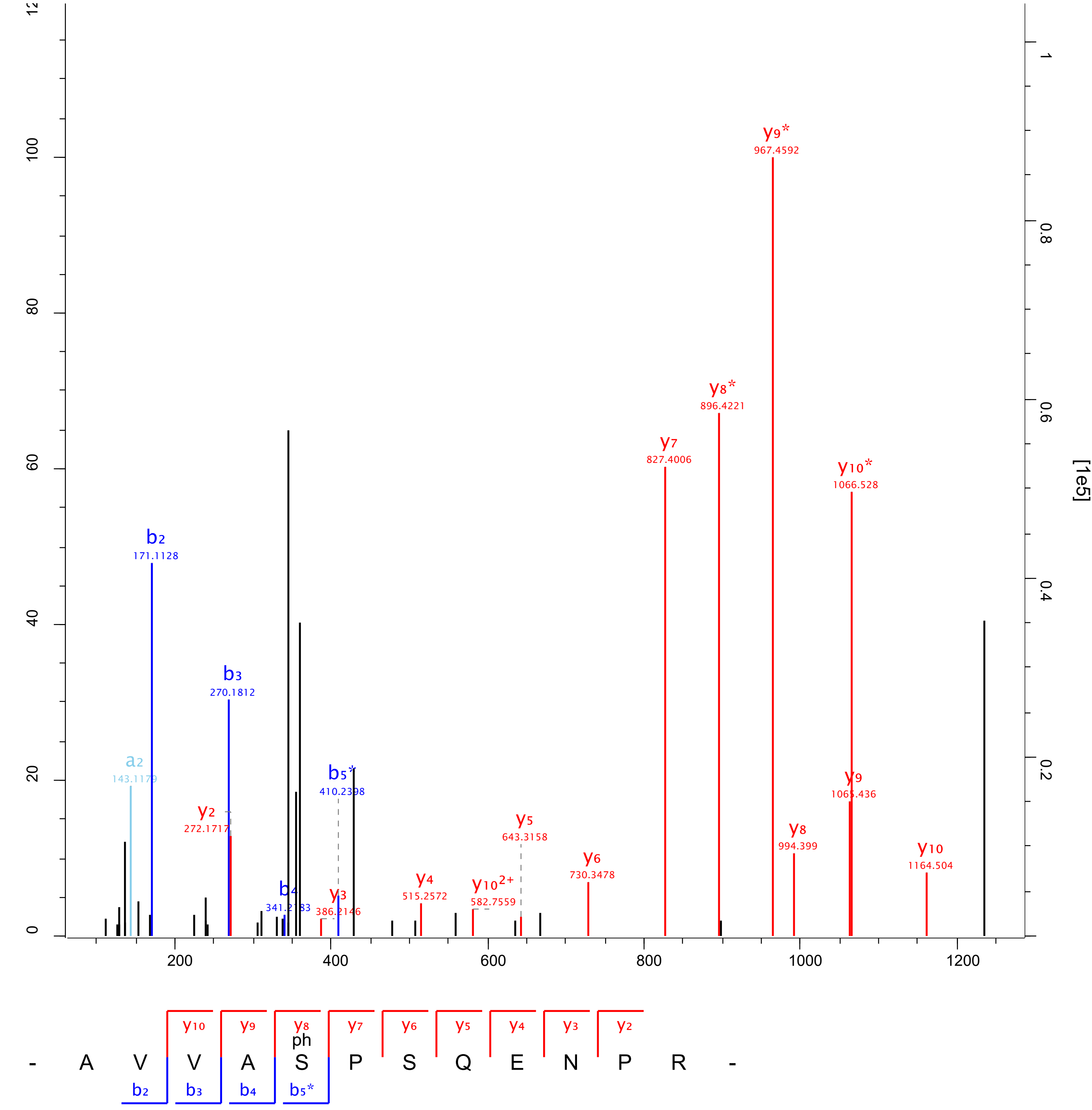

Sample output to test PDF Combine only

Raw file	Scan	Method	Score	m/z	Gene names
0523_3	5818	FTMS; HCD	149.36	786.84	NPC3

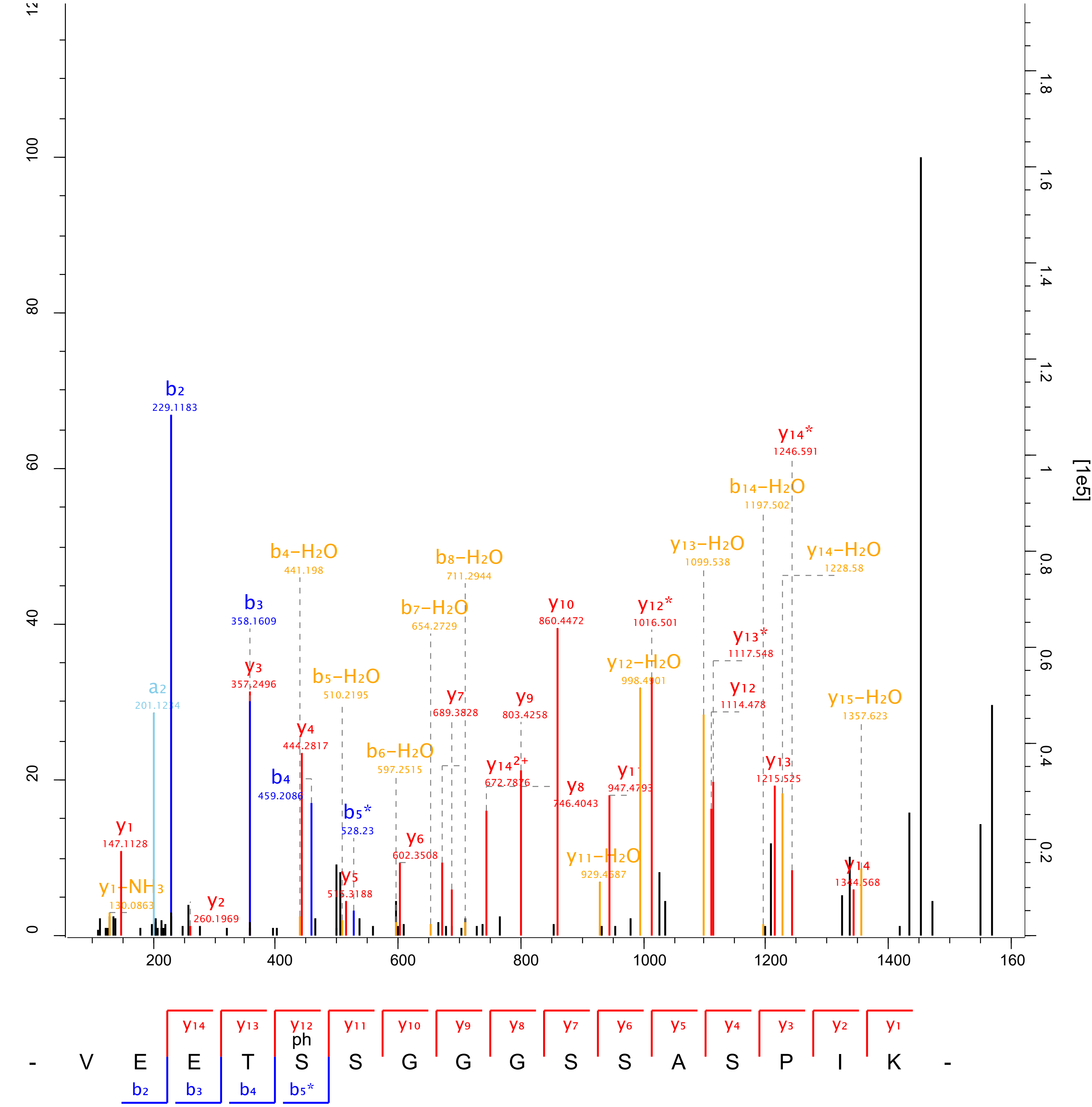

Sample output to test PDF Combine only

Raw file	Scan	Method	Score	m/z	Gene names
0523_3	8009	FTMS; HCD	84.33	913.36	NF-YB1;NFYB1

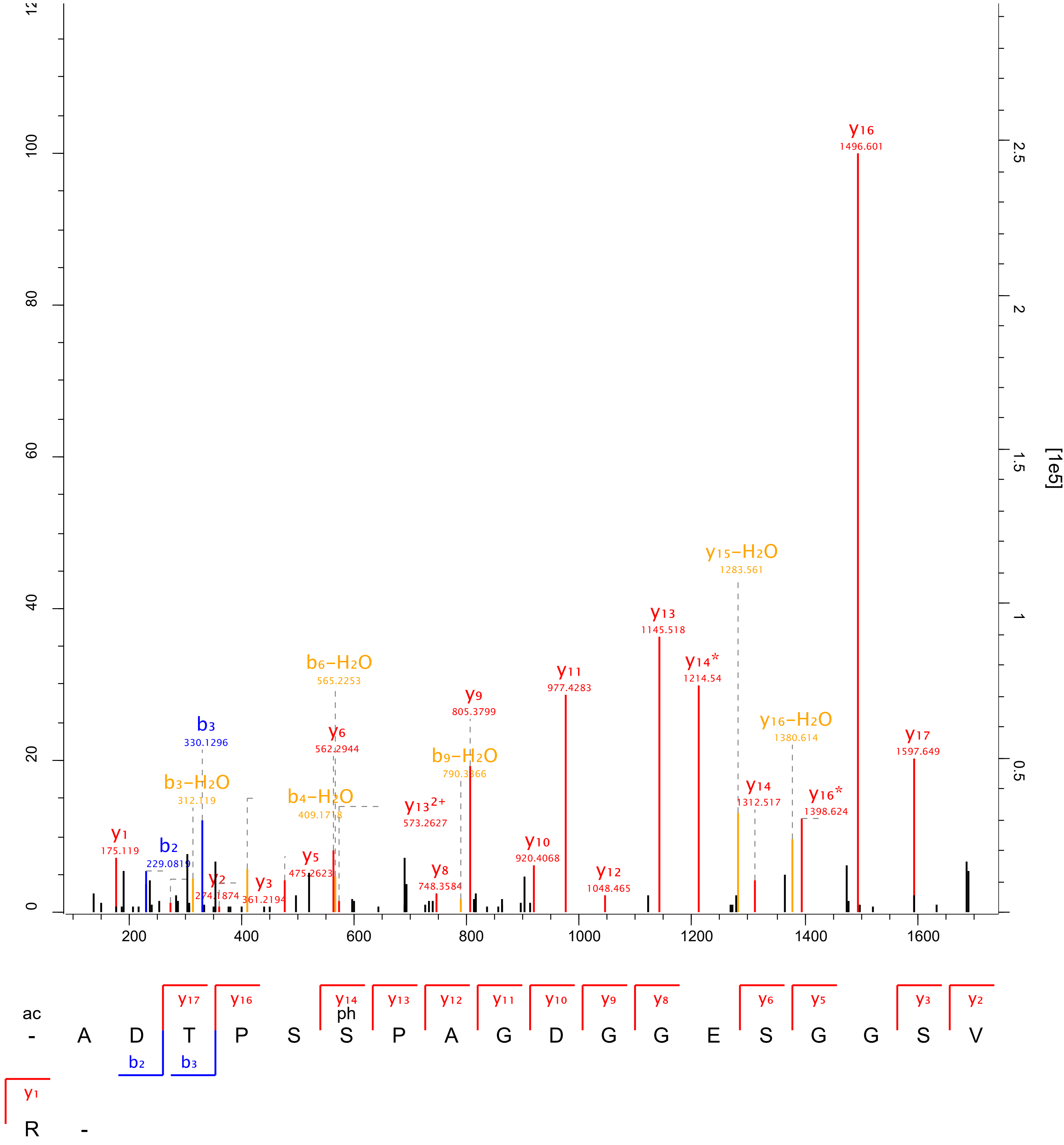

Sample output to test PDF Combine only

Raw file	Scan	Method	Score	m/z	Gene names
0523_3	8330	FTMS; HCD	77.12	570.92	At2g23520

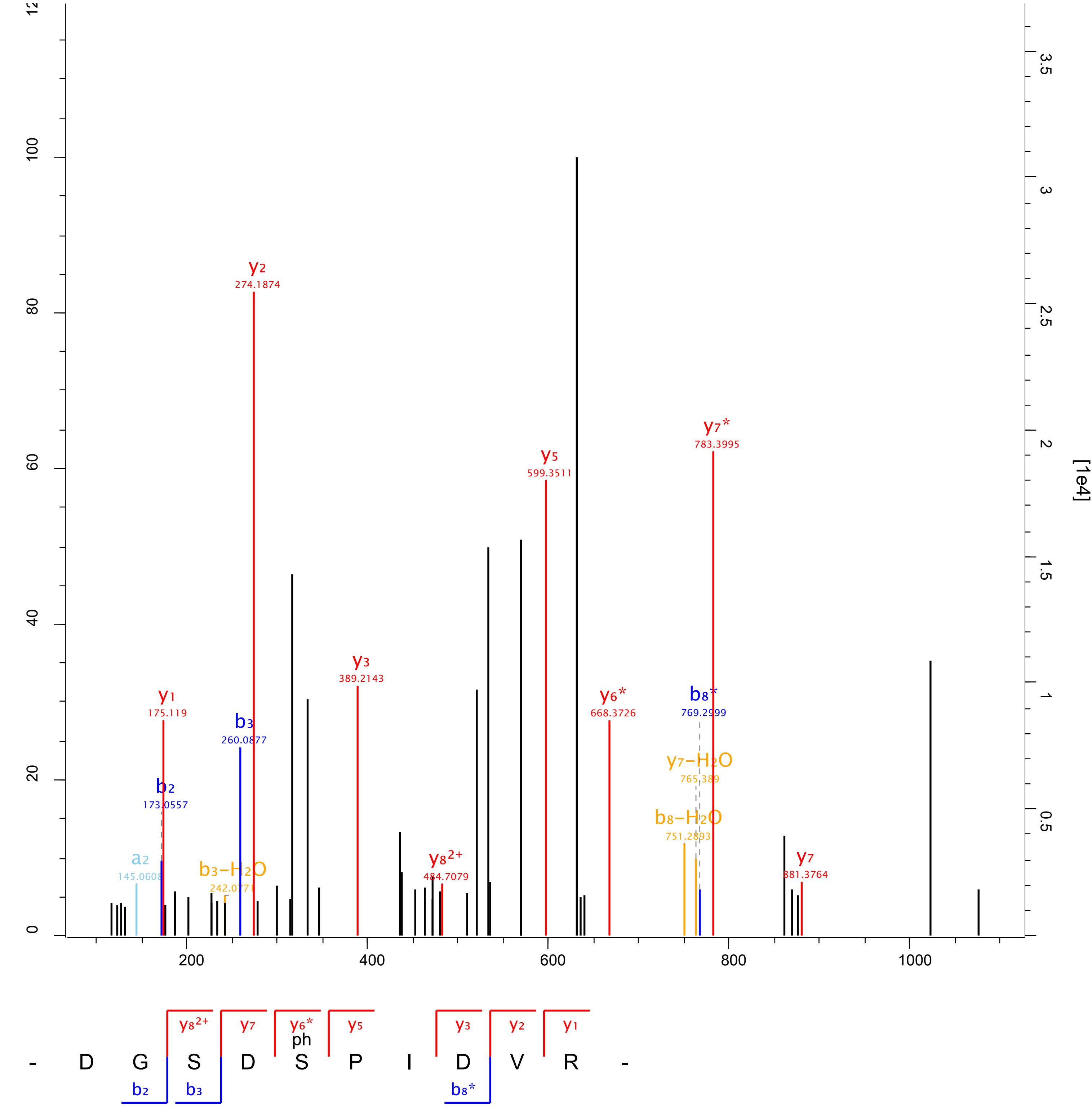

Sample output to test PDF Combine only

Raw file	Scan	Method	Score	m/z	Gene names
0523_3	8797	FTMS; HCD	89.86	877.34	NRT2.1

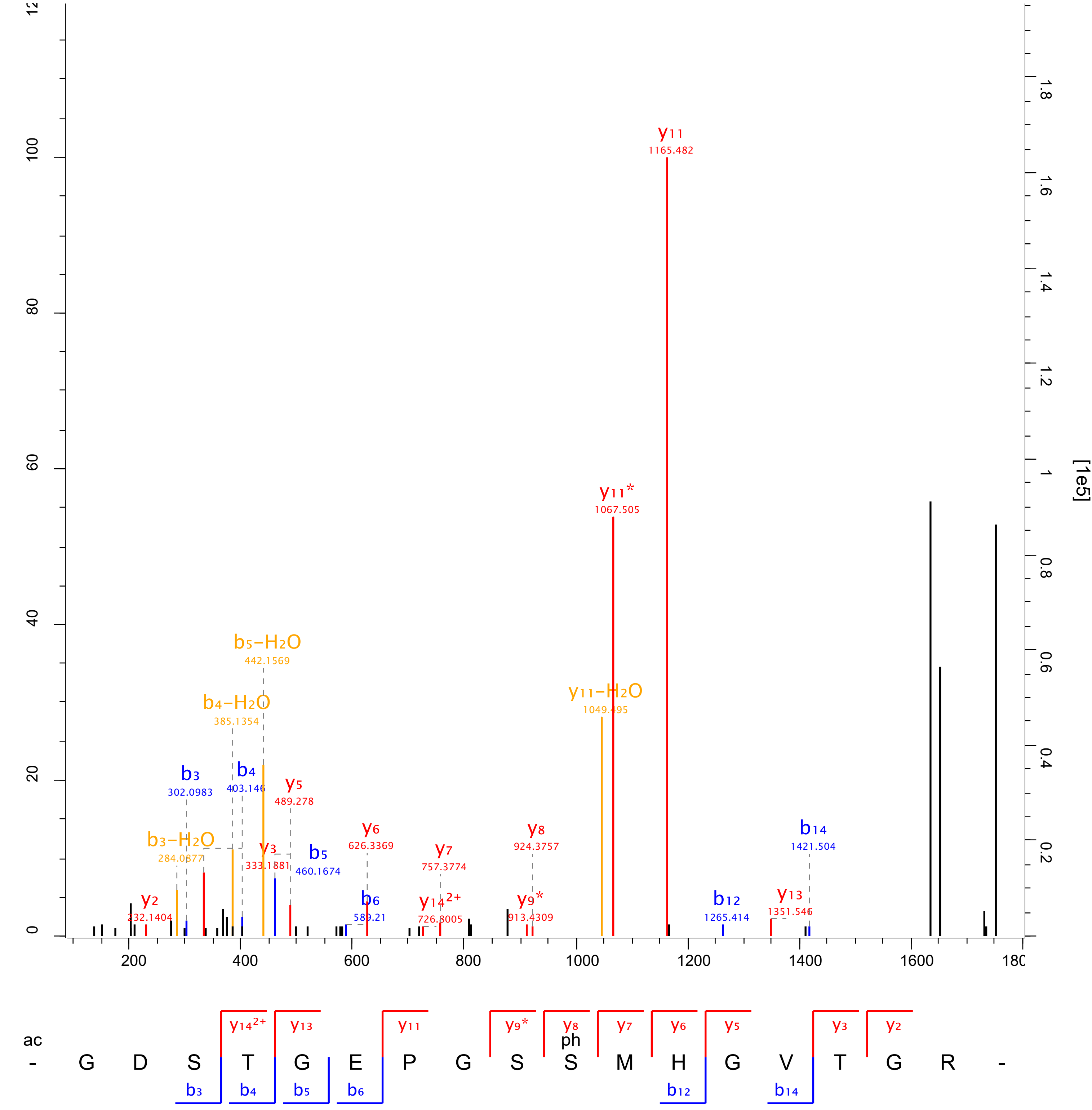

Sample output to test PDF Combine only

Raw file	Scan	Method	Score	m/z	Gene names
0523_3	8868	FTMS; HCD	51.29	516.7	NIP2-1

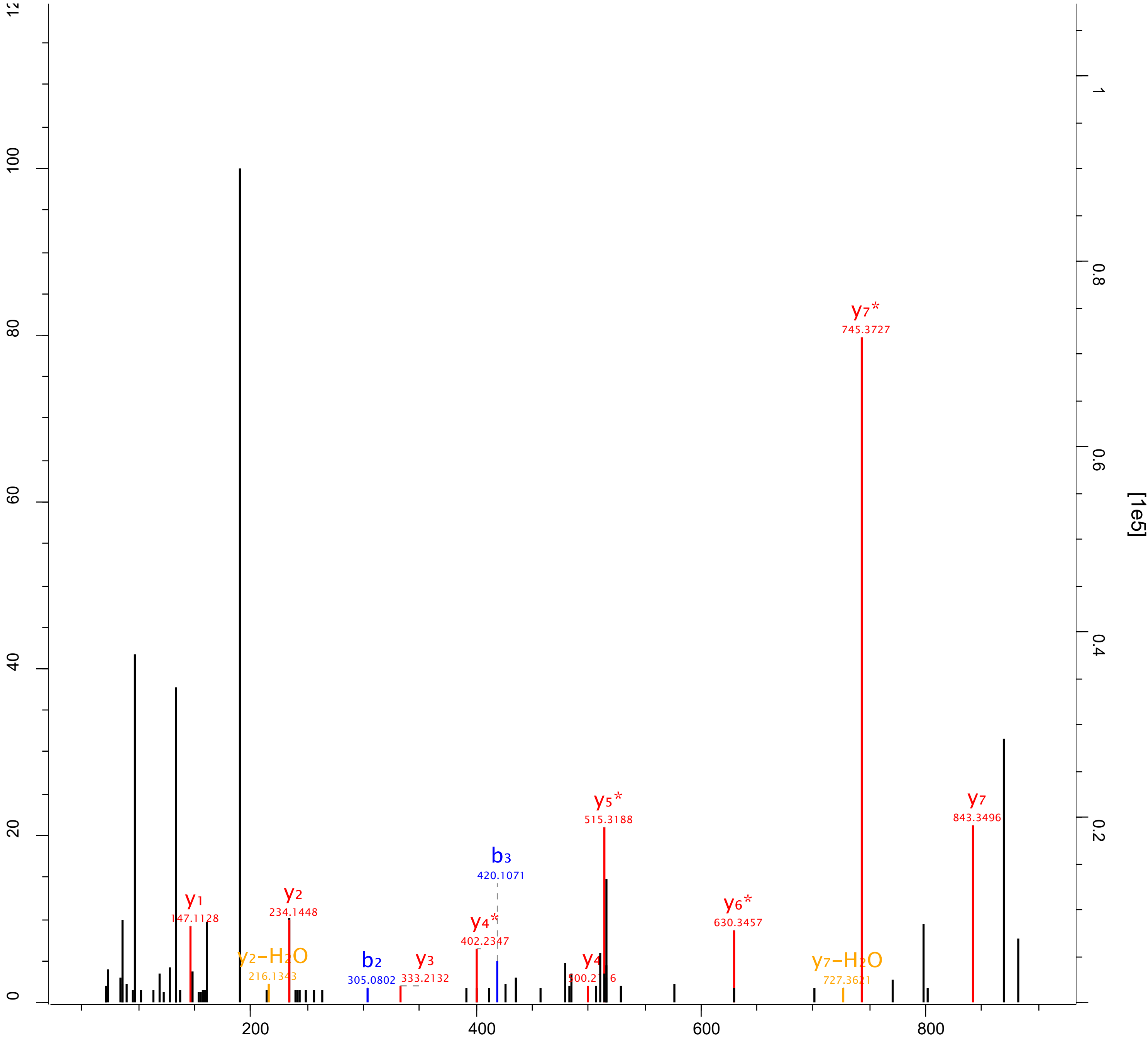

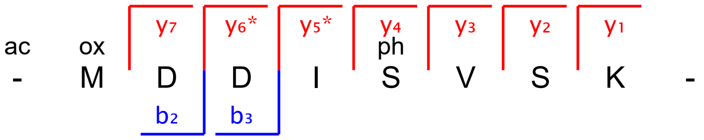

Sample output to test PDF Combine only

Raw file	Scan	Method	Score	m/z	Gene names
0523_3	9140	FTMS; HCD	66.39	641.78	SCC3

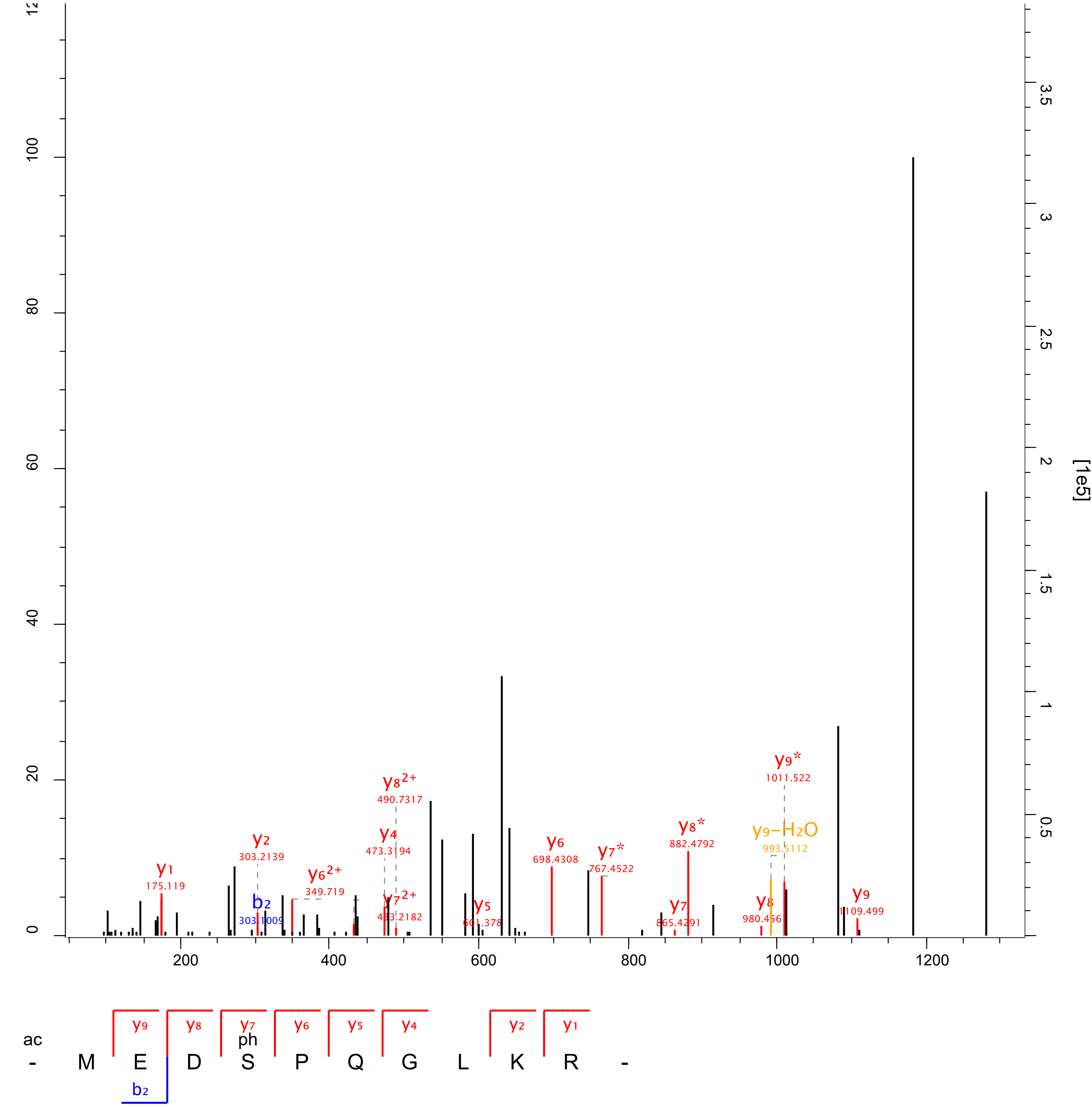

Sample output to test PDF Combine only

Raw file	Scan	Method	Score	m/z	Gene names
0523_3	9687	FTMS; HCD	195.98	696.81	AMT1-1

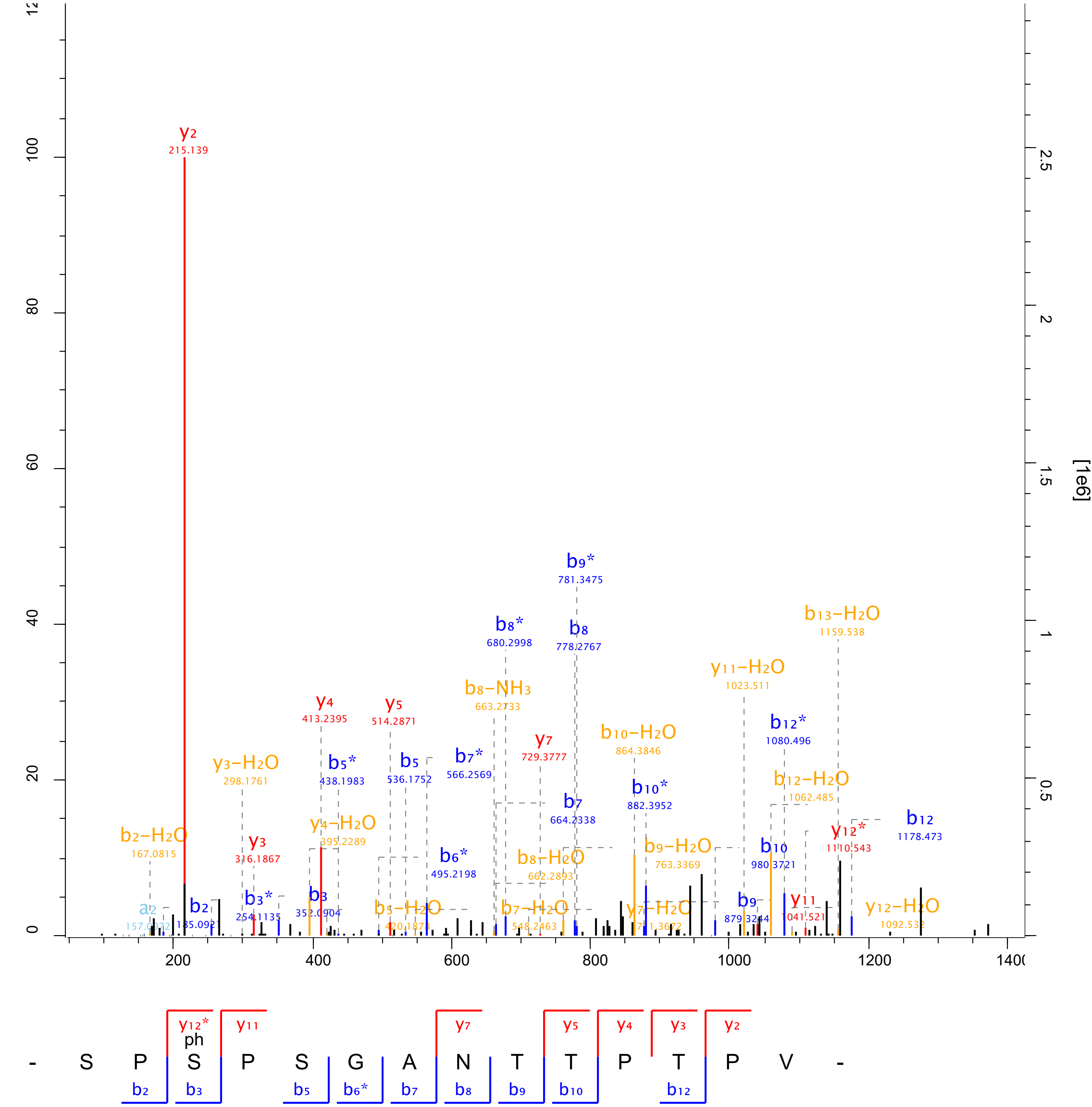

Sample output to test PDF Combine only

Raw file	Scan	Method	Score	m/z	Gene names
0523_3	9943	FTMS; HCD	114.96	725.33	PFP-BETA1

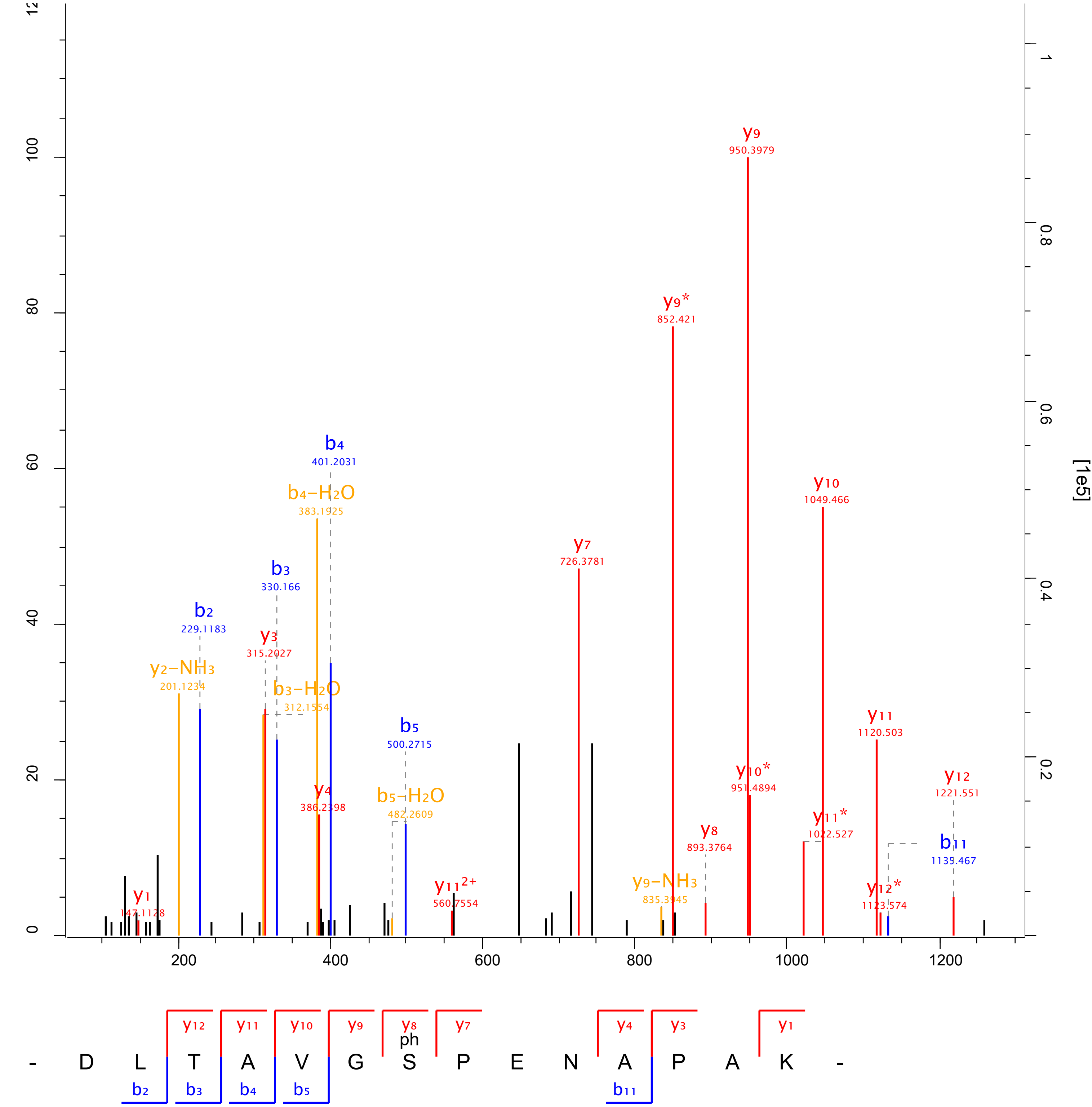

Sample output to test PDF Combine only

Raw file	Scan	Method	Score	m/z	Gene names
0523_3	10331	FTMS; HCD	67.91	818.29	POT13

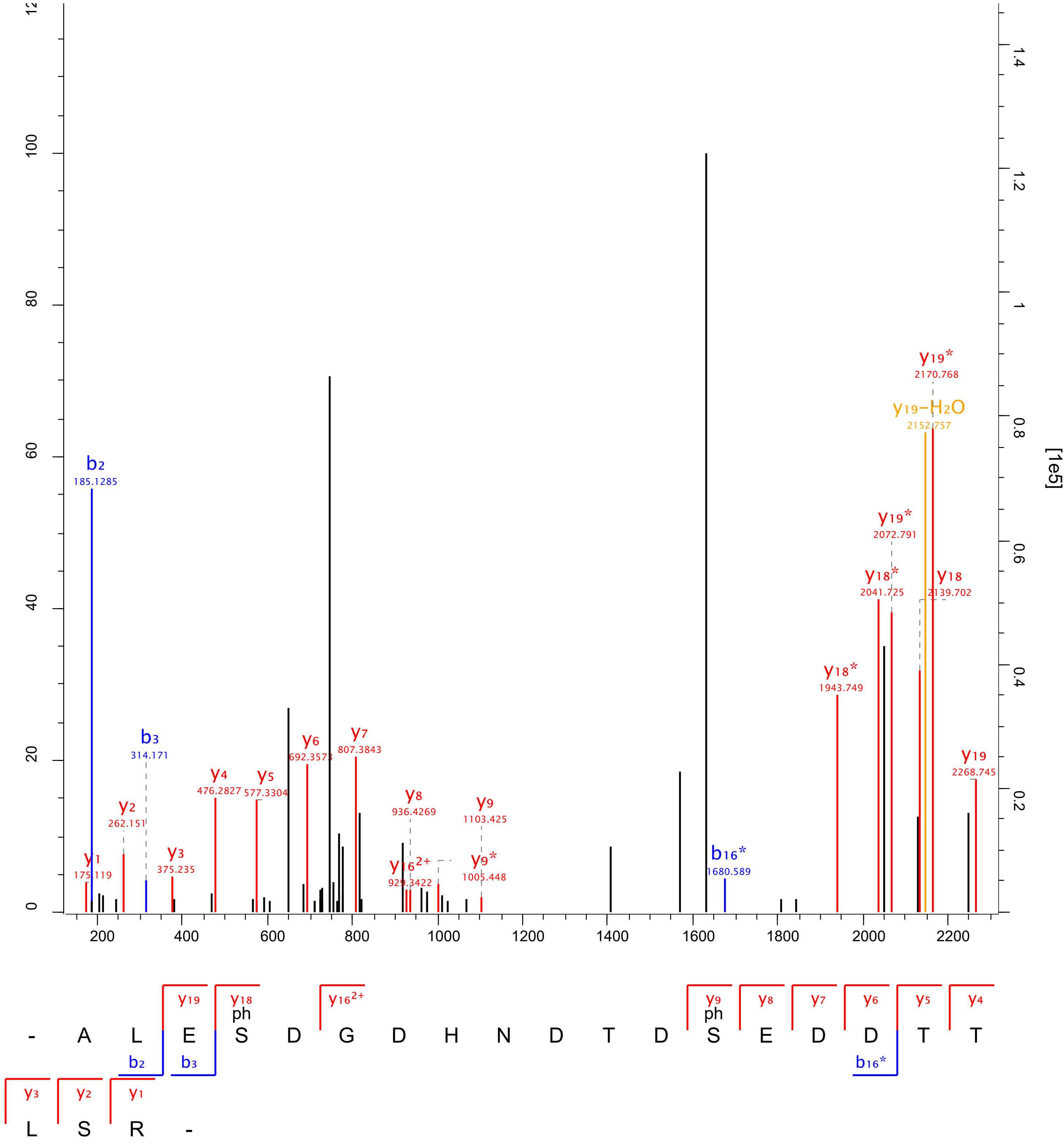

Sample output to test PDF Combine only

Raw file	Scan	Method	Score	m/z	Gene names
0523_3	10360	FTMS; HCD	155.64	645.78	CSLC6

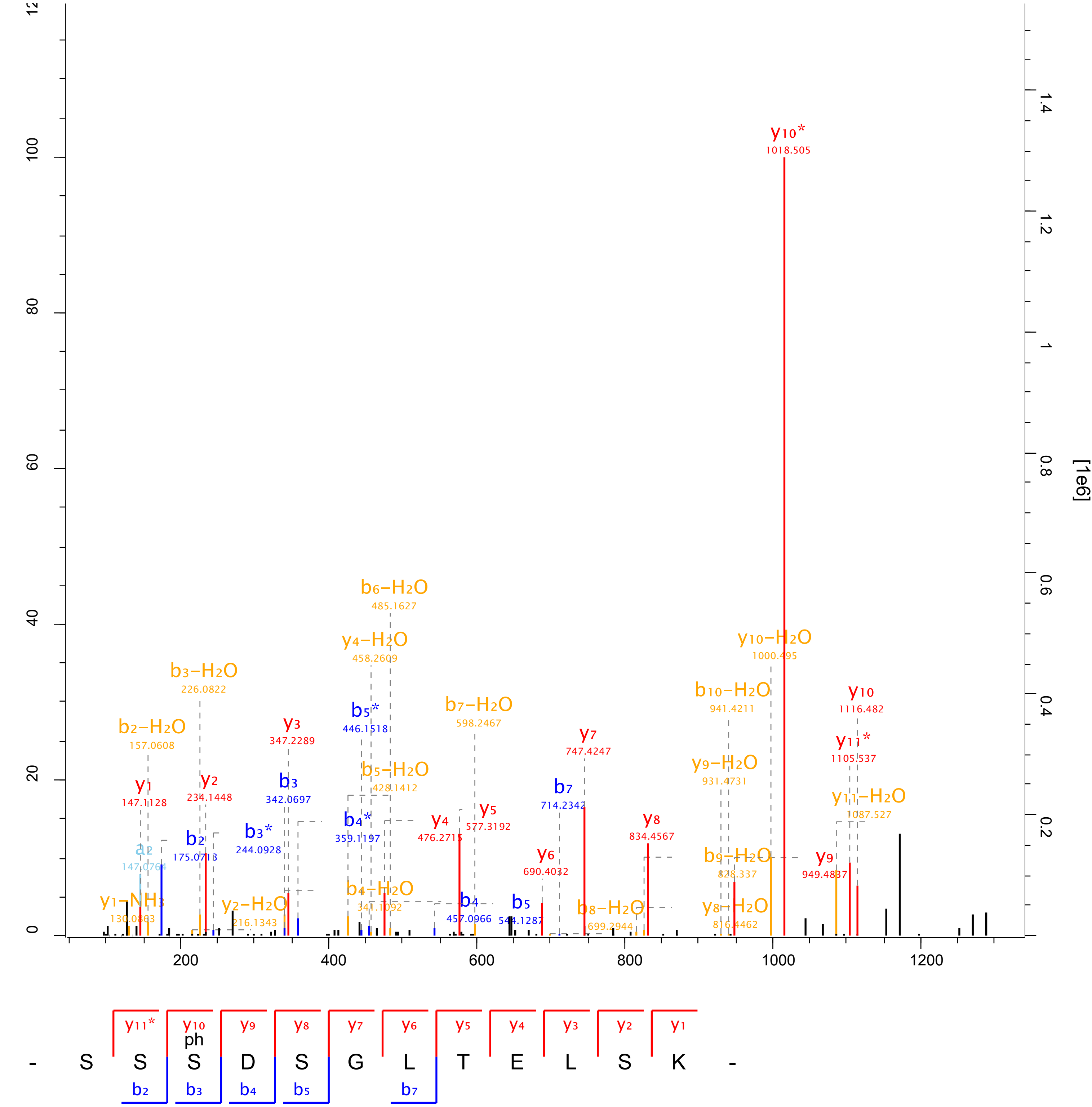

Sample output to test PDF Combine only

Raw file	Scan	Method	Score	m/z	Gene names
0523_3	10618	FTMS; HCD	68.17	833.4	LRR-RLK

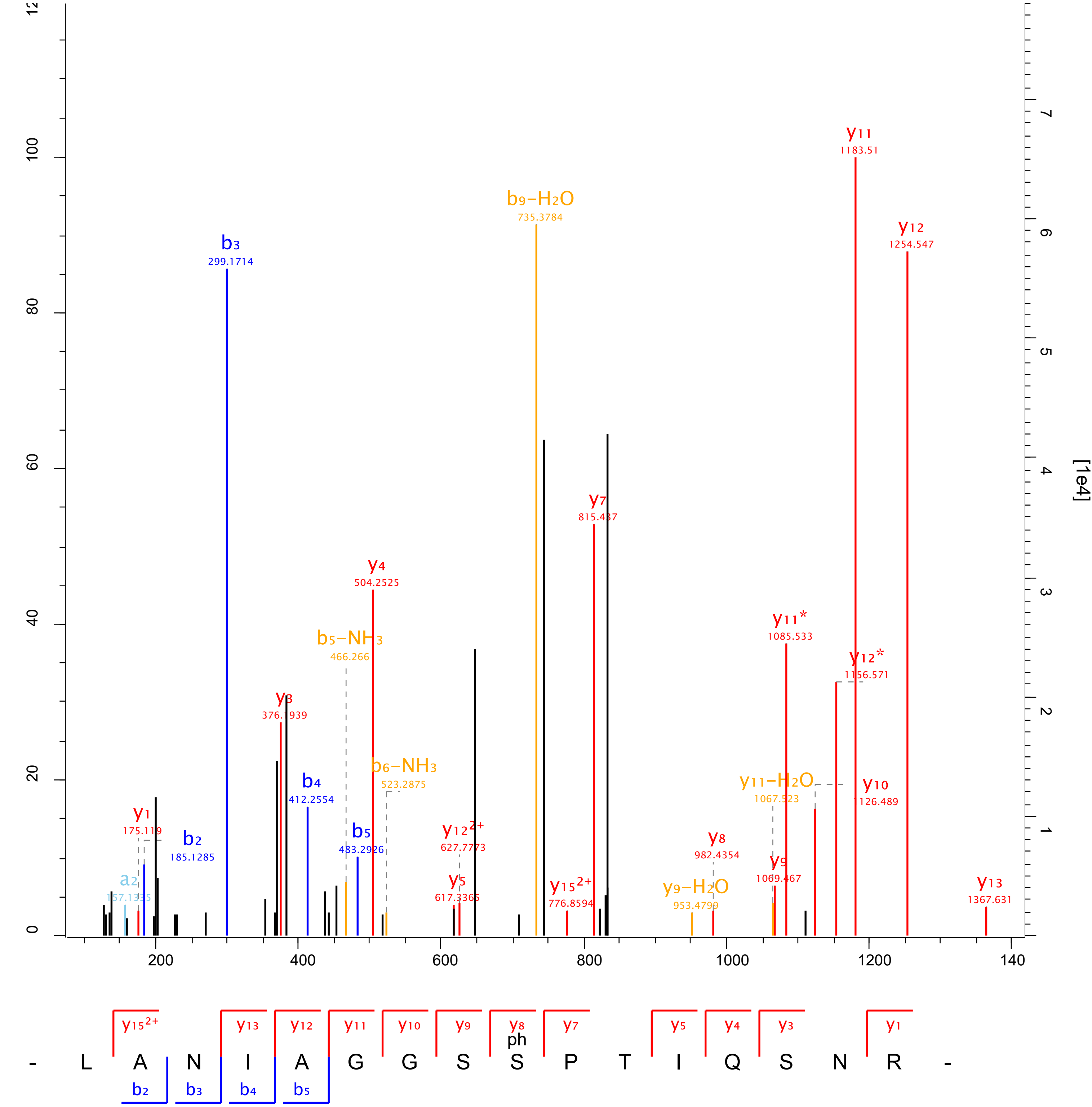

Sample output to test PDF Combine only

Raw file	Scan	Method	Score	m/z	Gene names
0523_3	10848	FTMS; HCD	78.81	631.8	F19B15.20;At4g28990

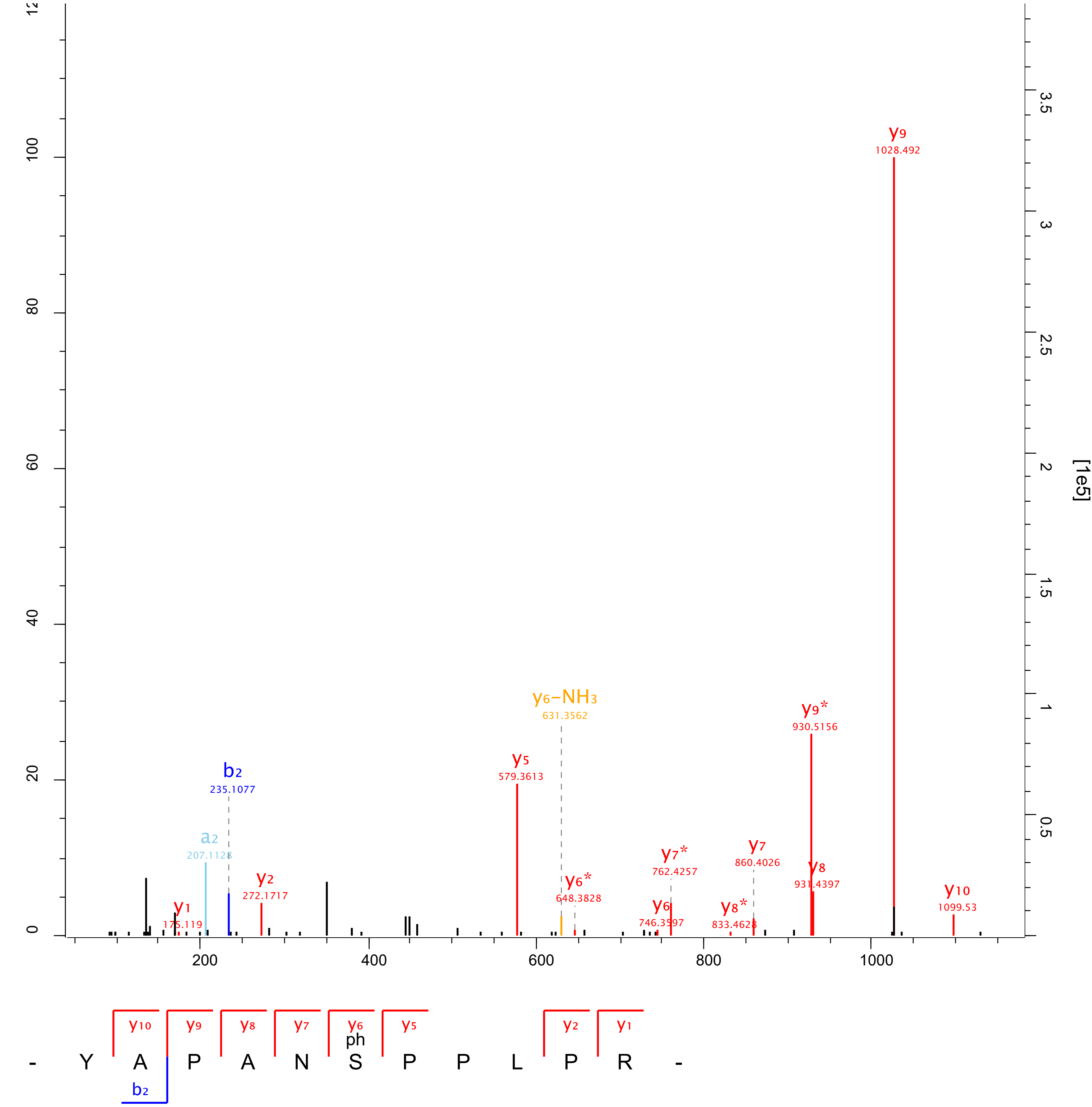

Sample output to test PDF Combine only

Raw file	Scan	Method	Score	m/z	Gene names
0523_3	10921	FTMS; HCD	203.26	848.36	MSI4

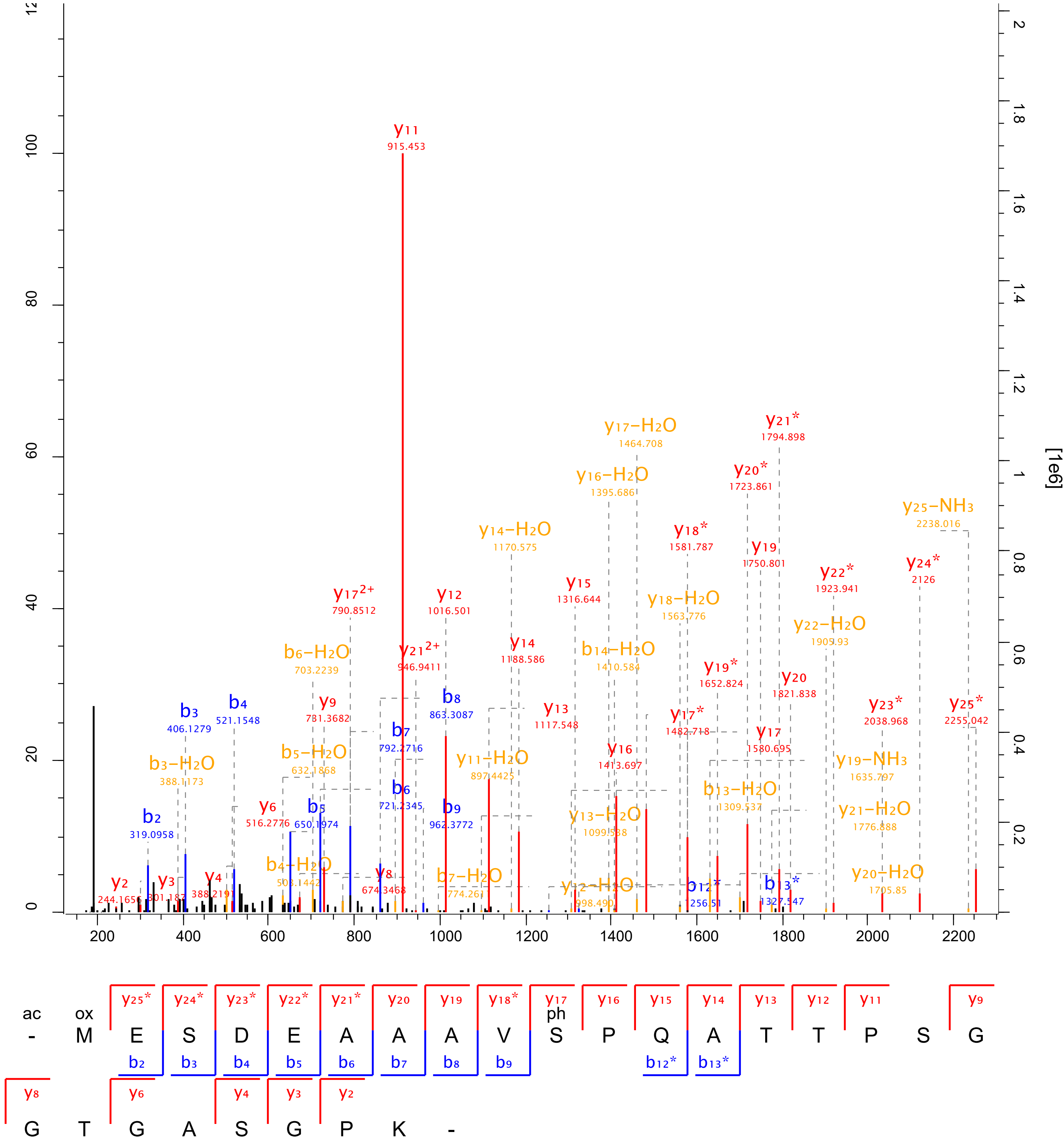

Sample output to test PDF Combine only

Raw file	Scan	Method	Score	m/z	Gene names
0523_3	11052	FTMS; HCD	186.85	719.28	KEU

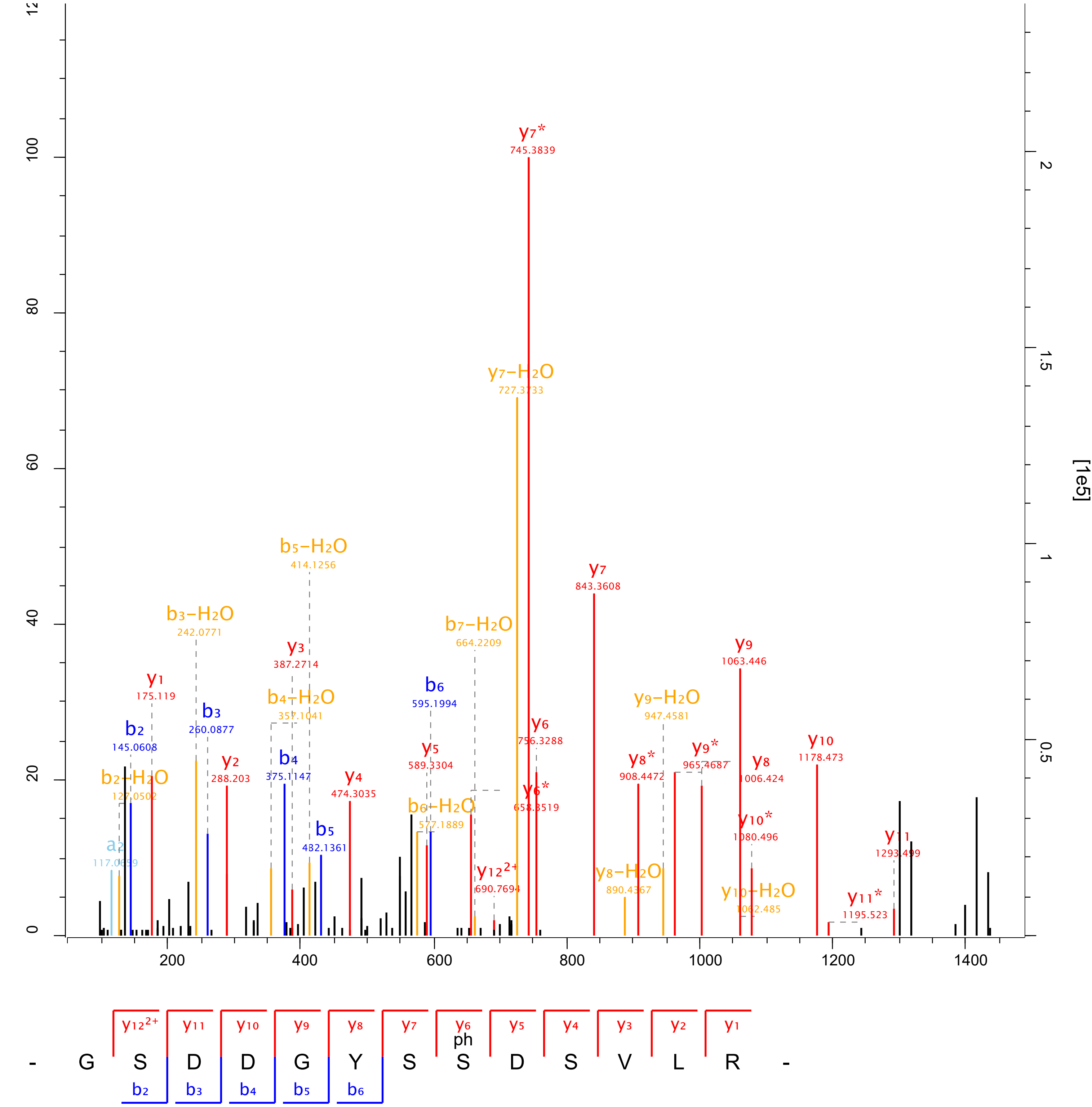

Sample output to test PDF Combine only

Raw file	Scan	Method	Score	m/z
0523_3	11117	FTMS; HCD	213.36	755.35

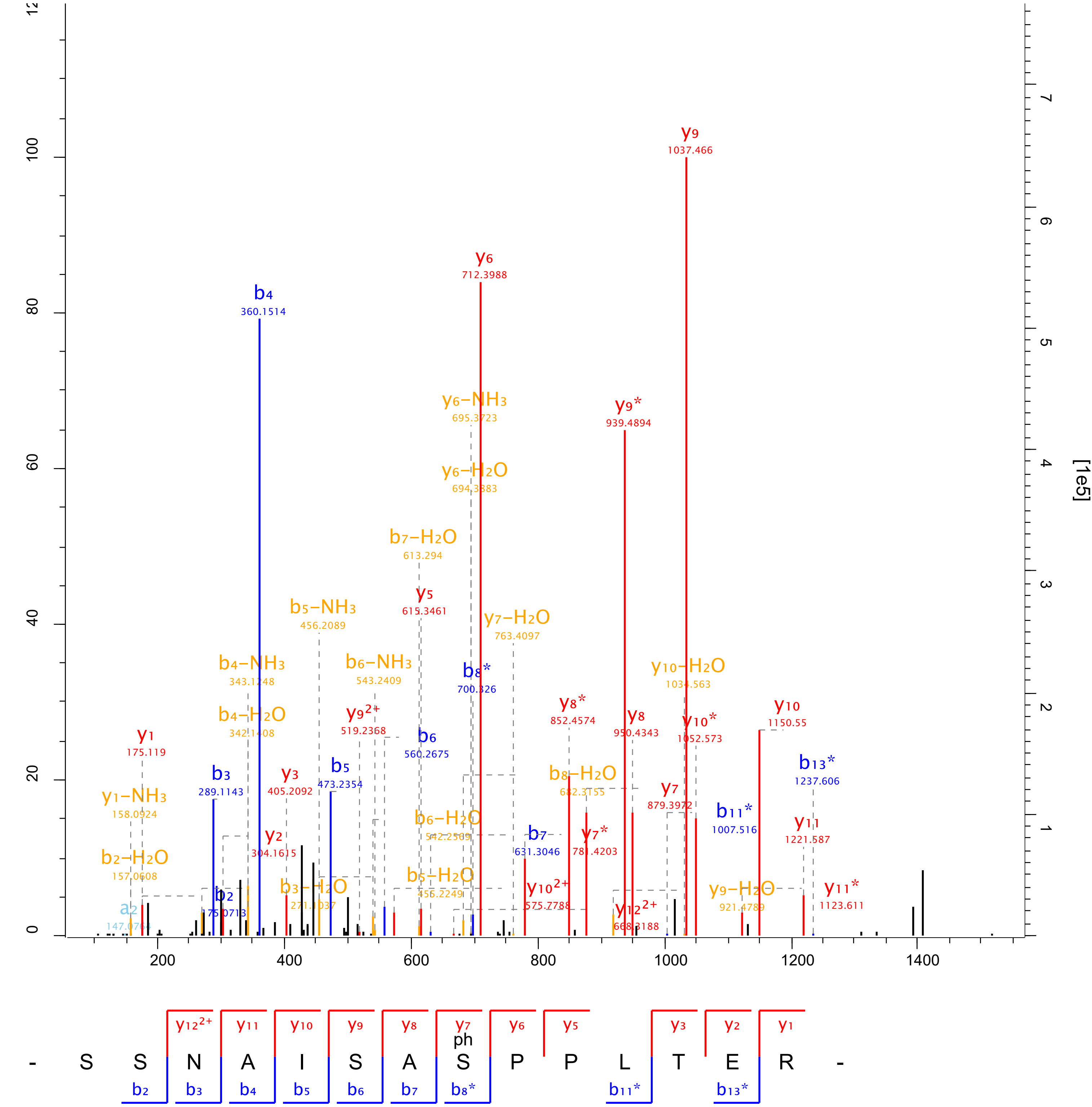

Sample output to test PDF Combine only

Raw file	Scan	Method	Score	m/z	Gene names
0523_3	11176	FTMS; HCD	50.03	963.42	F7A19.9

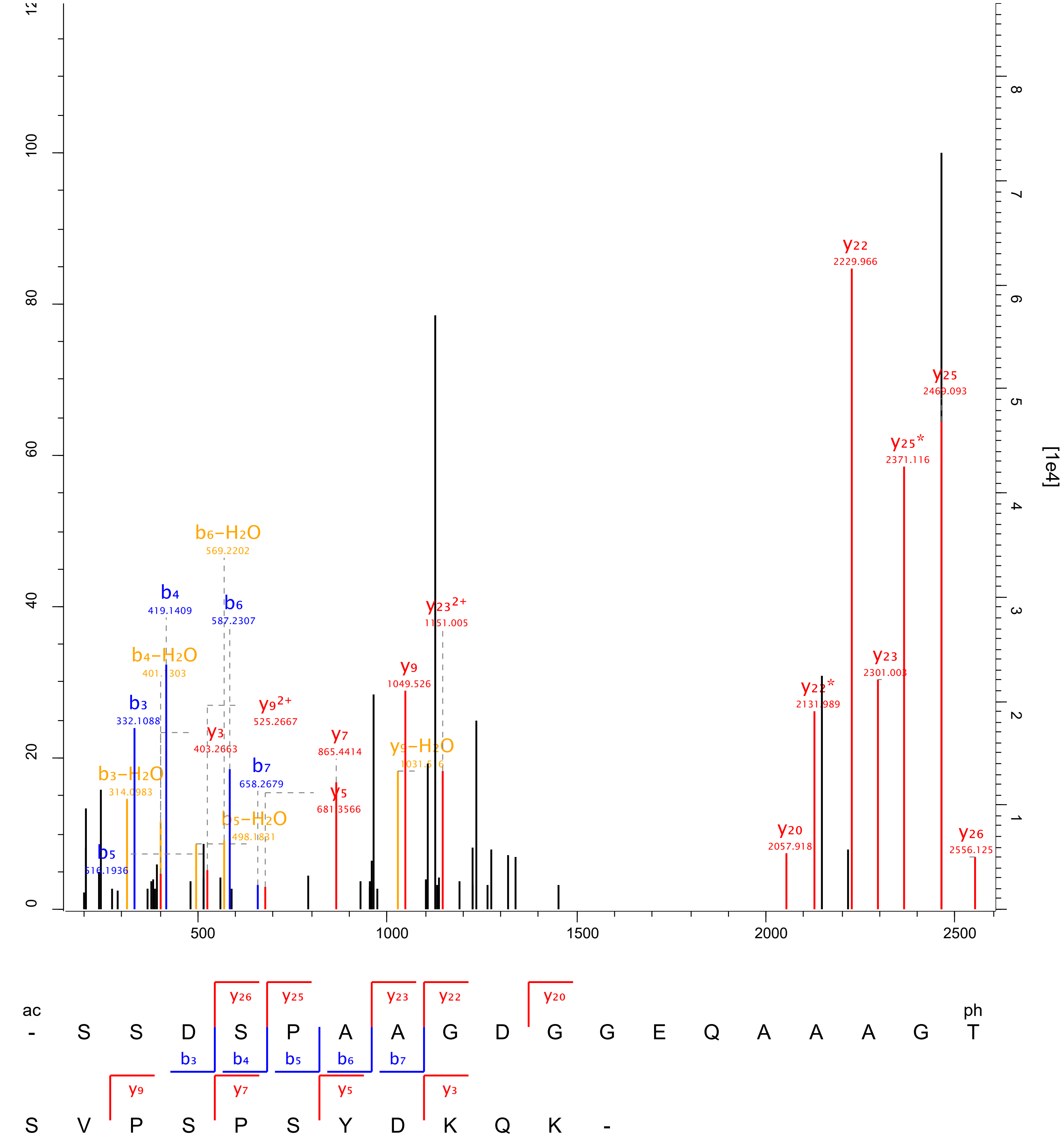

Sample output to test PDF Combine only

Raw file	Scan	Method	Score	m/z	Gene names
0523_3	11345	FTMS; HCD	73.5	853.87	GRF5

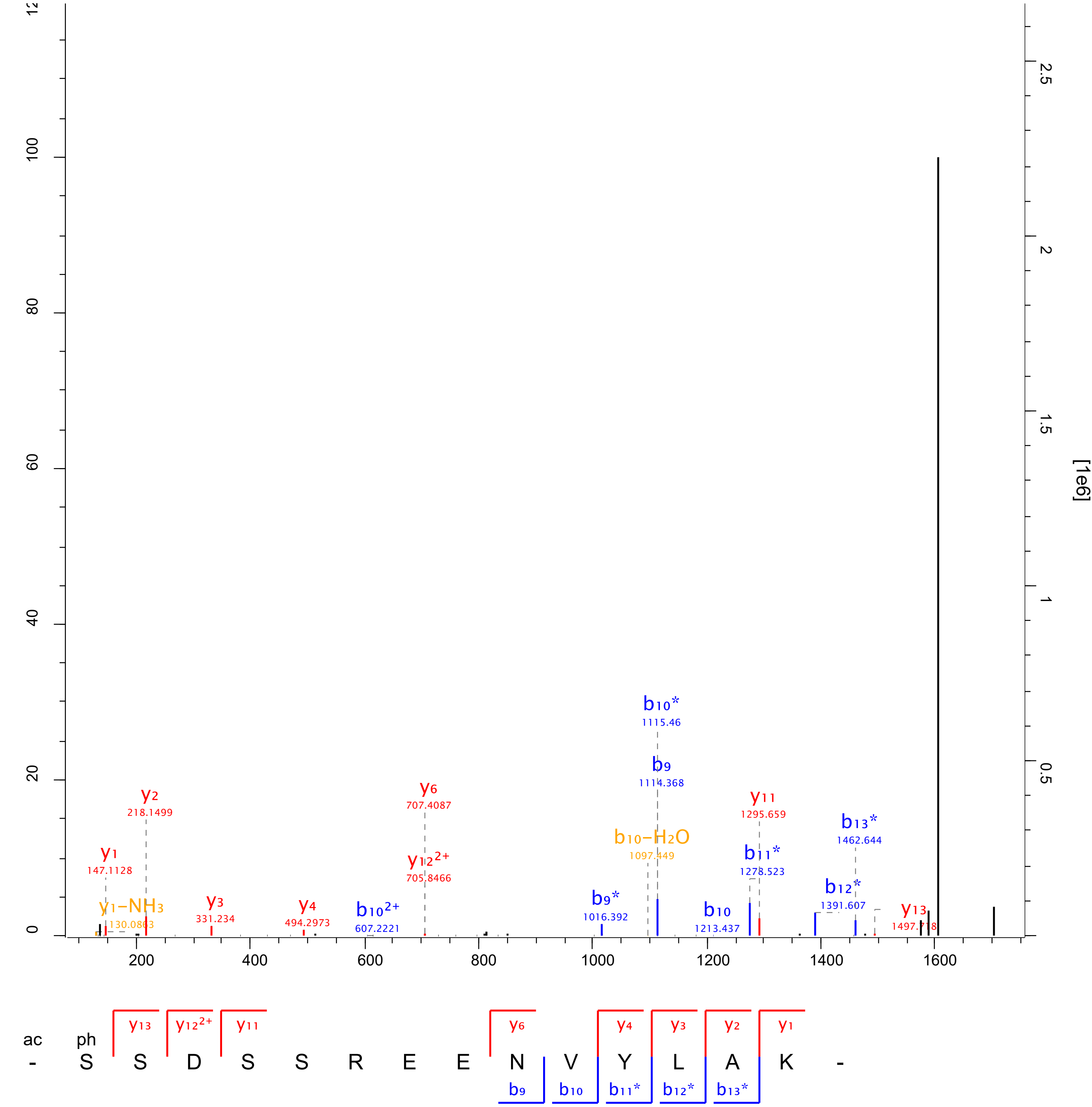

Sample output to test PDF Combine only

Raw file	Scan	Method	Score	m/z	Gene names
0523_3	11512	FTMS; HCD	84.25	723.32	At5g52882

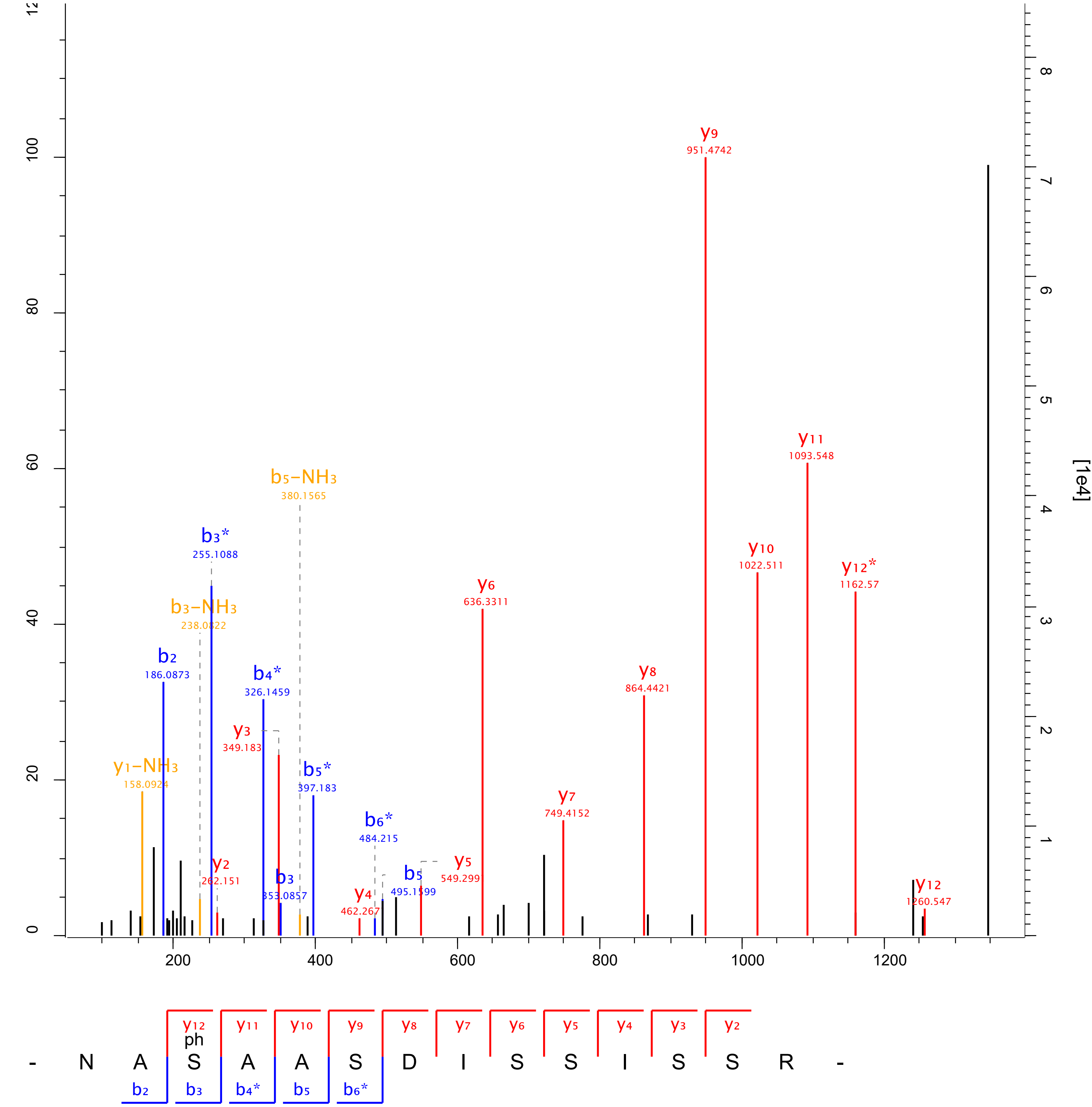

Sample output to test PDF Combine only

Raw file	Scan	Method	Score	m/z	Gene names
0523_3	11523	FTMS; HCD	111.64	673.27	SYP71

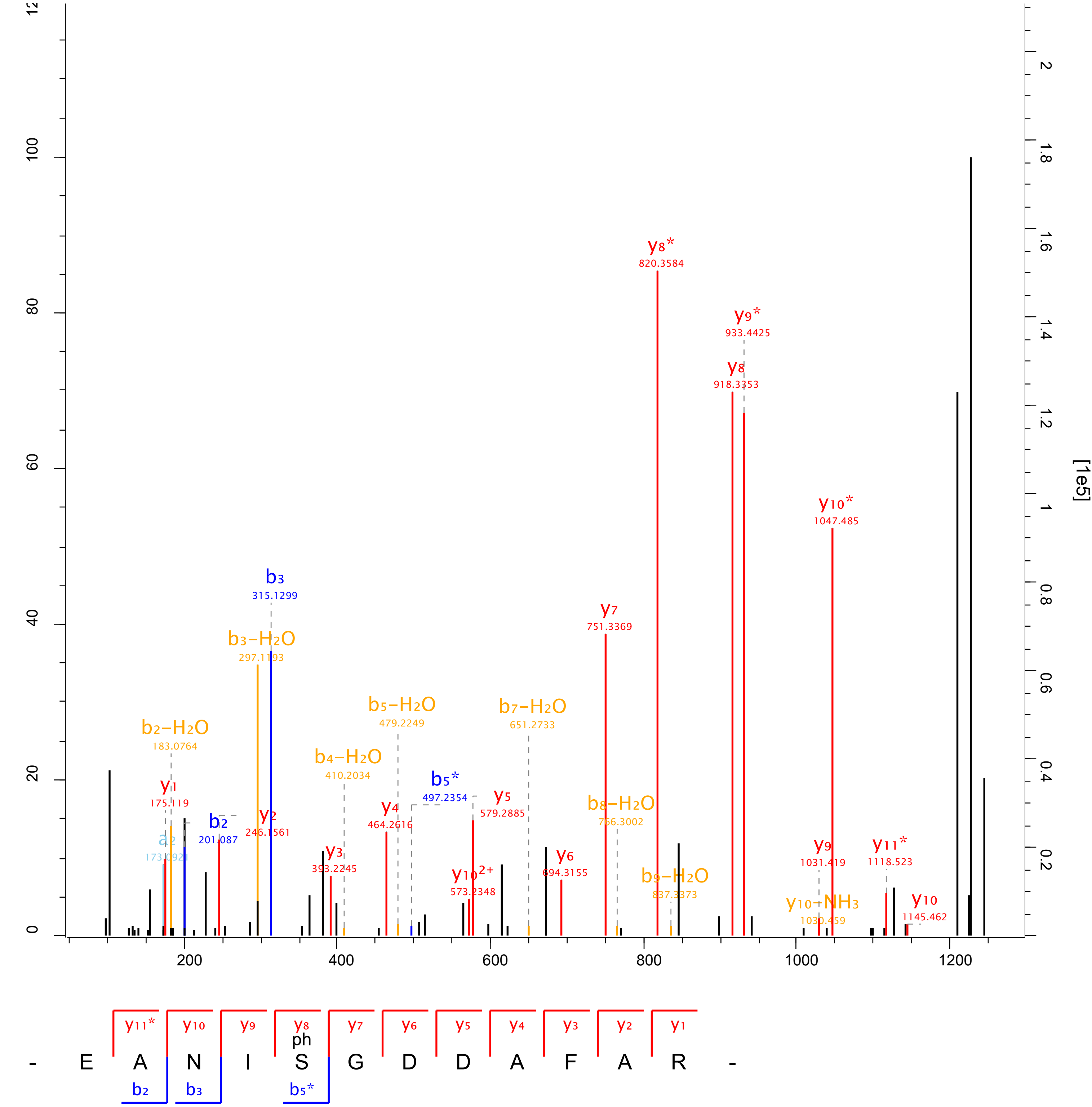

Sample output to test PDF Combine only
