# Supplementary material for: Sucrose-induced Receptor Kinase 1 is Modulated by an Interacting Kinase with Short Extracellular Domain
Source: Mol Cell Proteomics. 2019 May 30;18(8):1556–71. doi: 10.1074/mcp.RA119.001336 (PMC6683012; doi:10.1074/mcp.RA119.001336)
Supplement: Supplementary Figure S6b [file 143141_1_supp_311929_ps5mkx.pdf]

Figure S6:

Spectra of all identified phosphopeptides.

Raw file	Scan	Method	Score	m/z	Gene names
0523_3	12104	FTMS; HCD	52.81	638.3	LRR-RLK

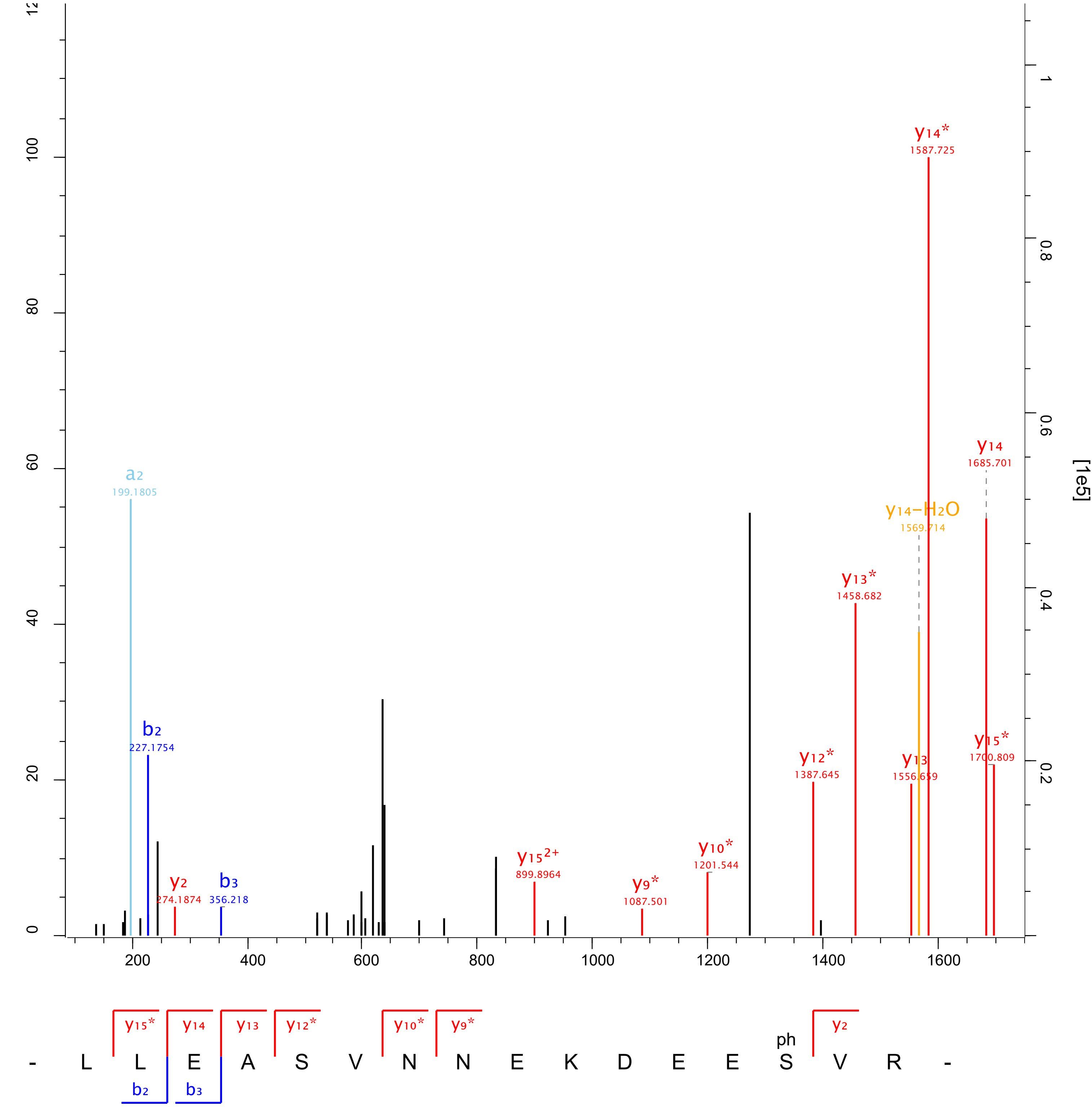

Sample output to test PDF Combine only

Raw file	Scan	Method	Score	m/z	Gene names
0523_3	12151	FTMS; HCD	68.04	632.79	At5g16280

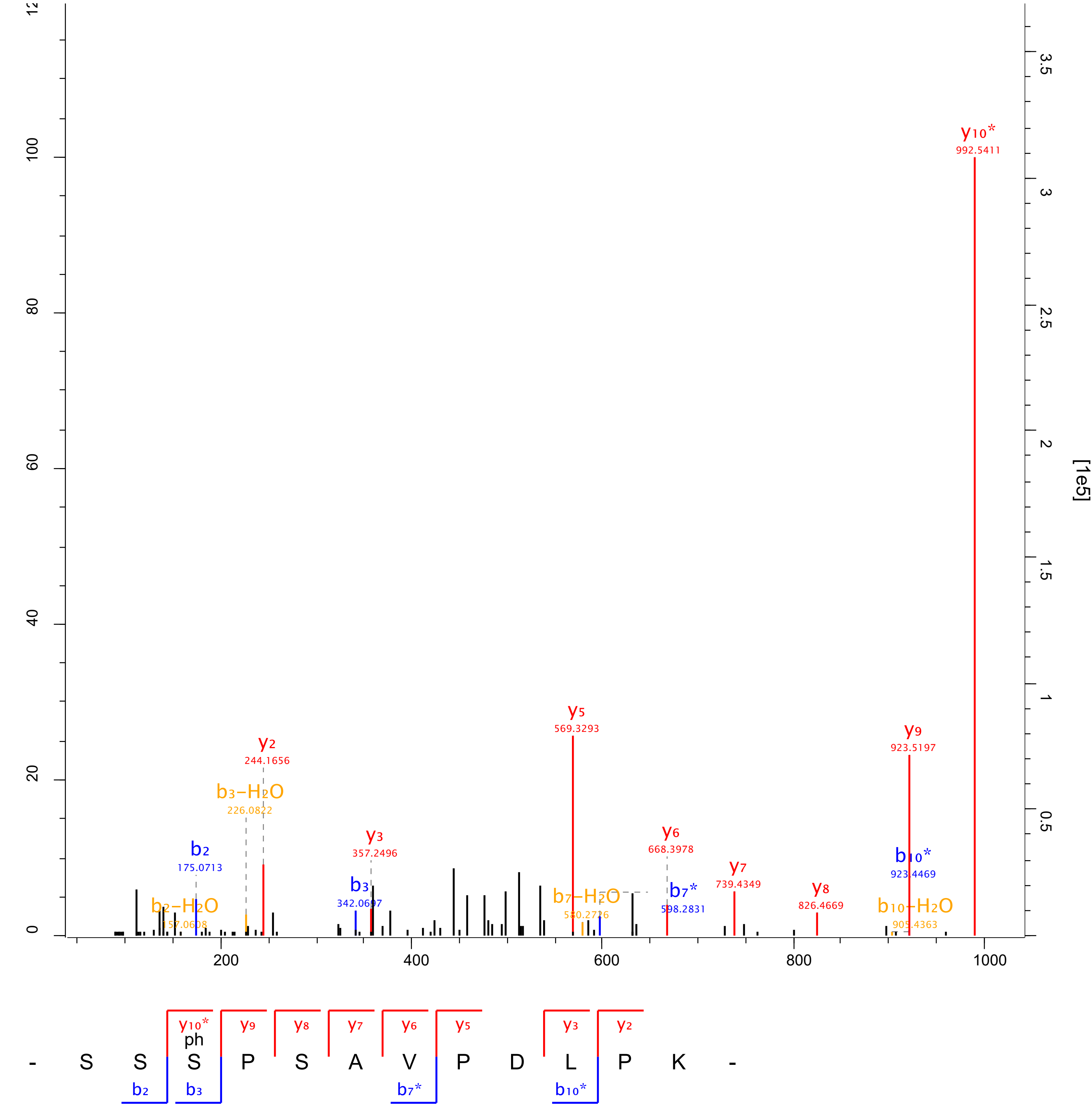

Sample output to test PDF Combine only

Raw file	Scan	Method	Score	m/z	Gene names
0523_3	12198	FTMS; HCD	76.3	807.85	NPC3

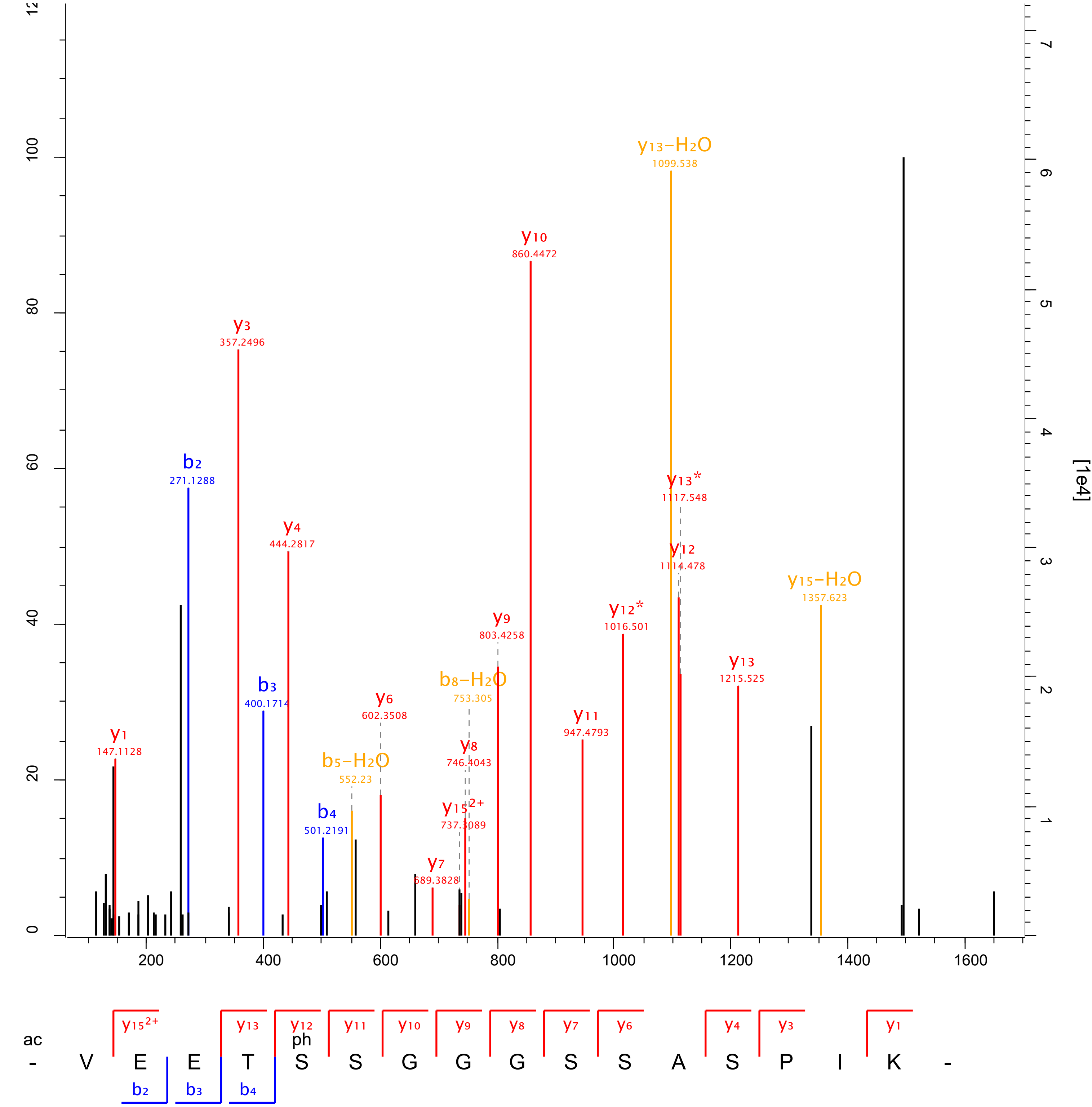

Sample output to test PDF Combine only

Raw file	Scan	Method	Score	m/z	Gene names
0523_3	12201	FTMS; HCD	59.23	559.75	At3g17410;PTI12;PTI13

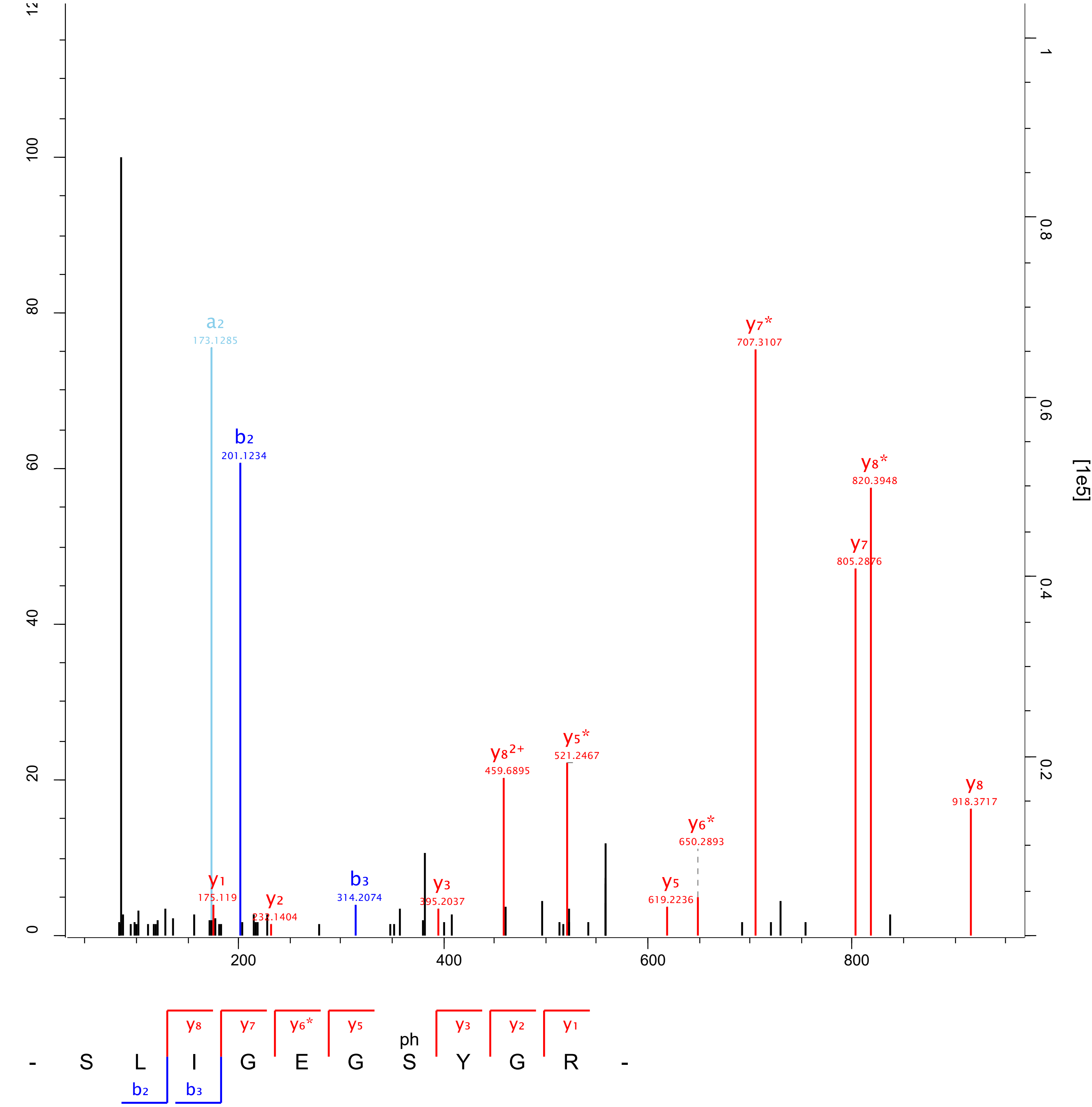

Sample output to test PDF Combine only

Raw file	Scan	Method	Score	m/z	Gene names
0523_3	12345	FTMS; HCD	48.08	792.67	CRWN3

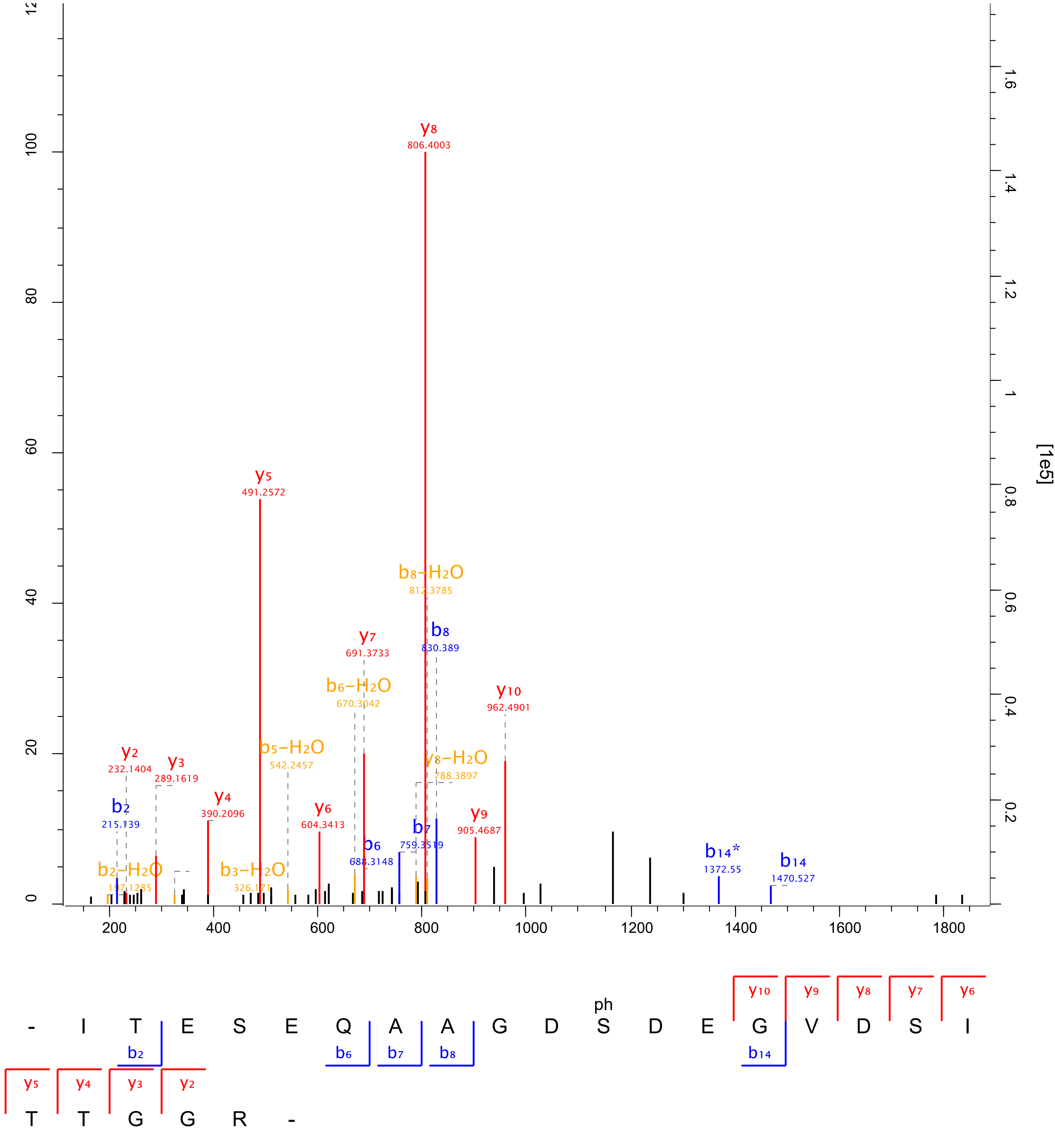

Raw file	Scan	Method	Score	m/z	Gene names
0523_3	12537	FTMS; HCD	132.79	778.85	PAPP2C

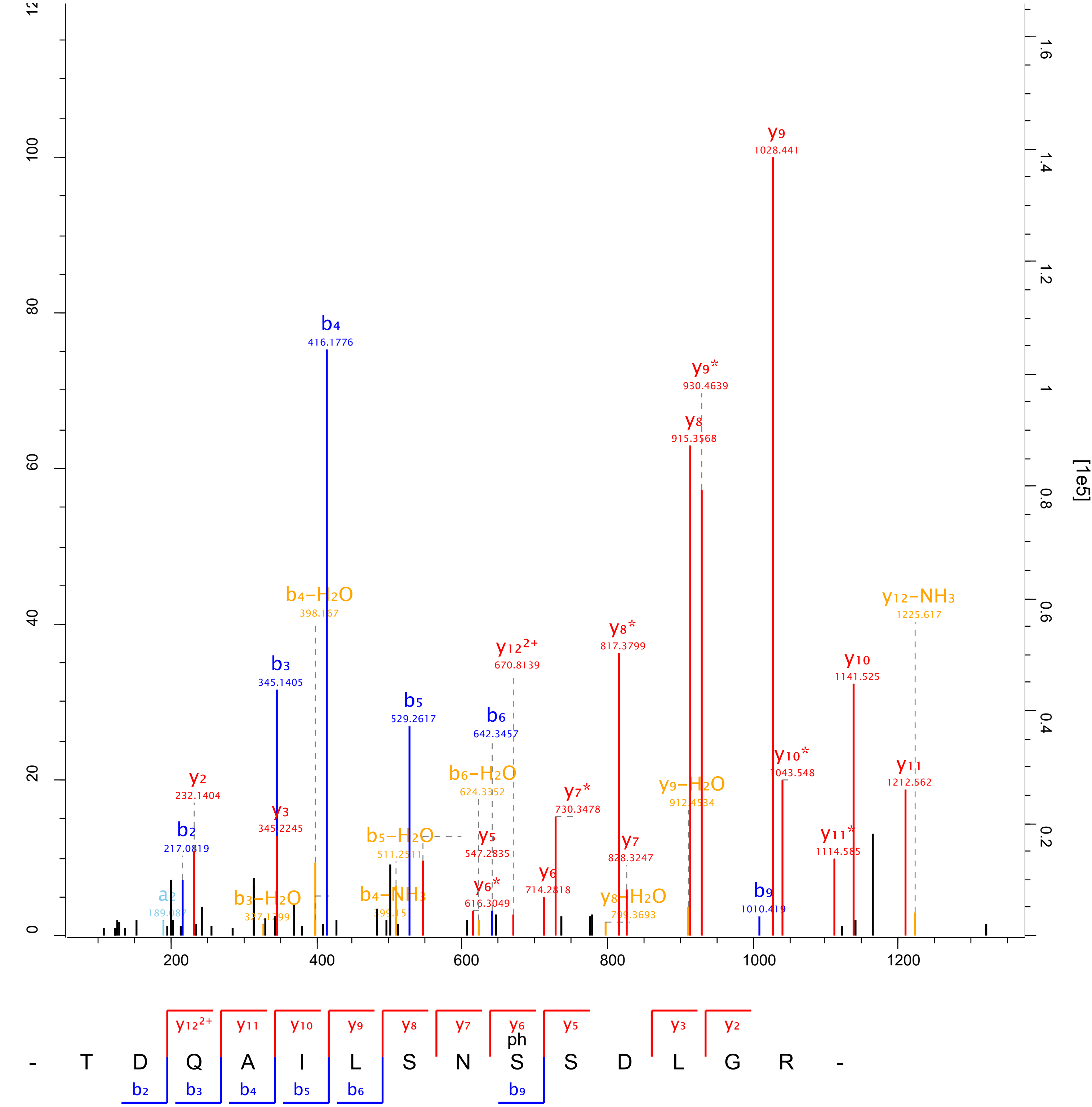

Sample output to test PDF Combine only

Raw file	Scan	Method	Score	m/z	Gene names
0523_3	12722	FTMS; HCD	86.77	649.8	SUN2

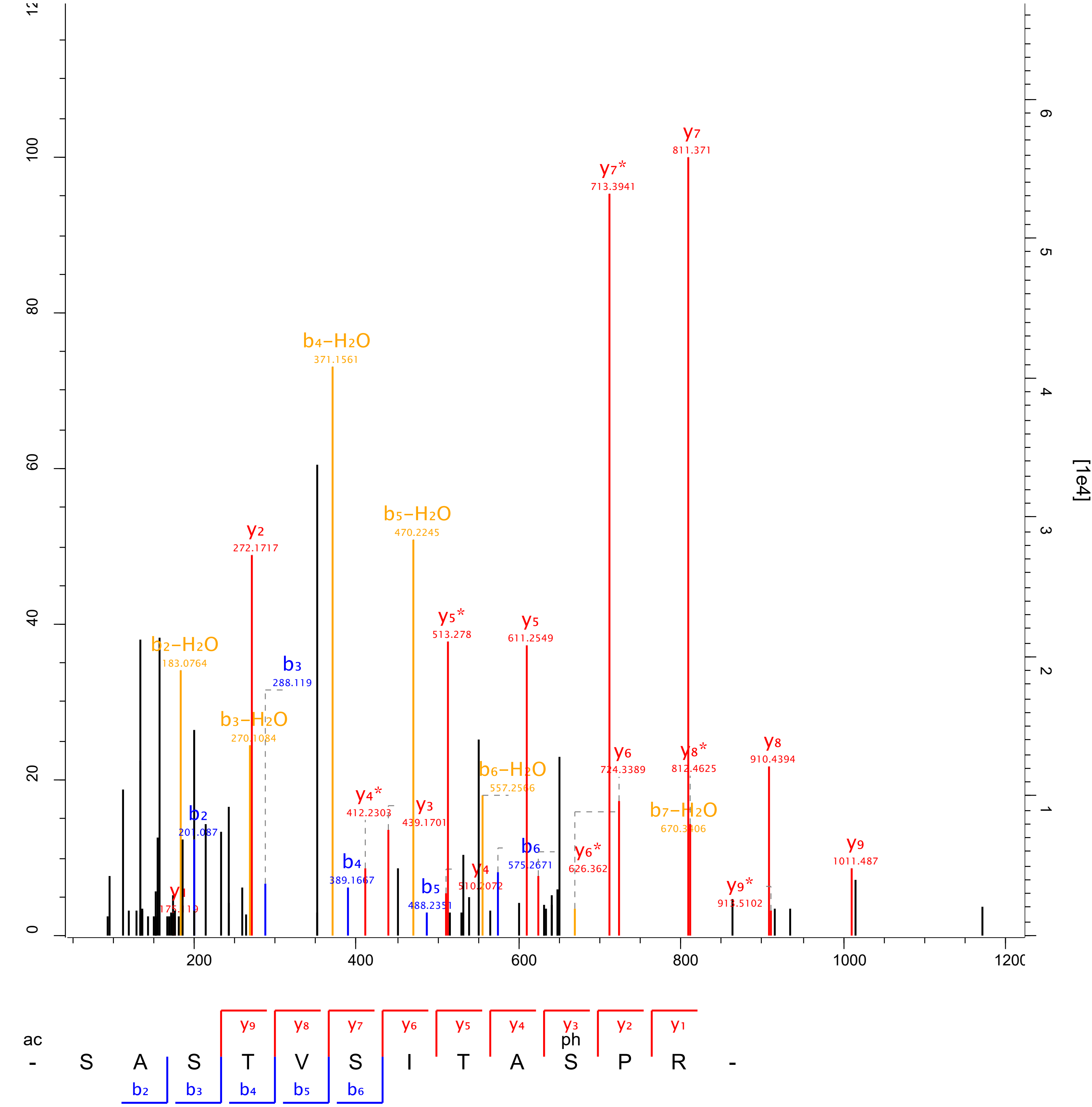

Sample output to test PDF Combine only

Raw file	Scan	Method	Score	m/z	Gene names
0523_3	13004	FTMS; HCD	154.34	887.74	EXO70A1

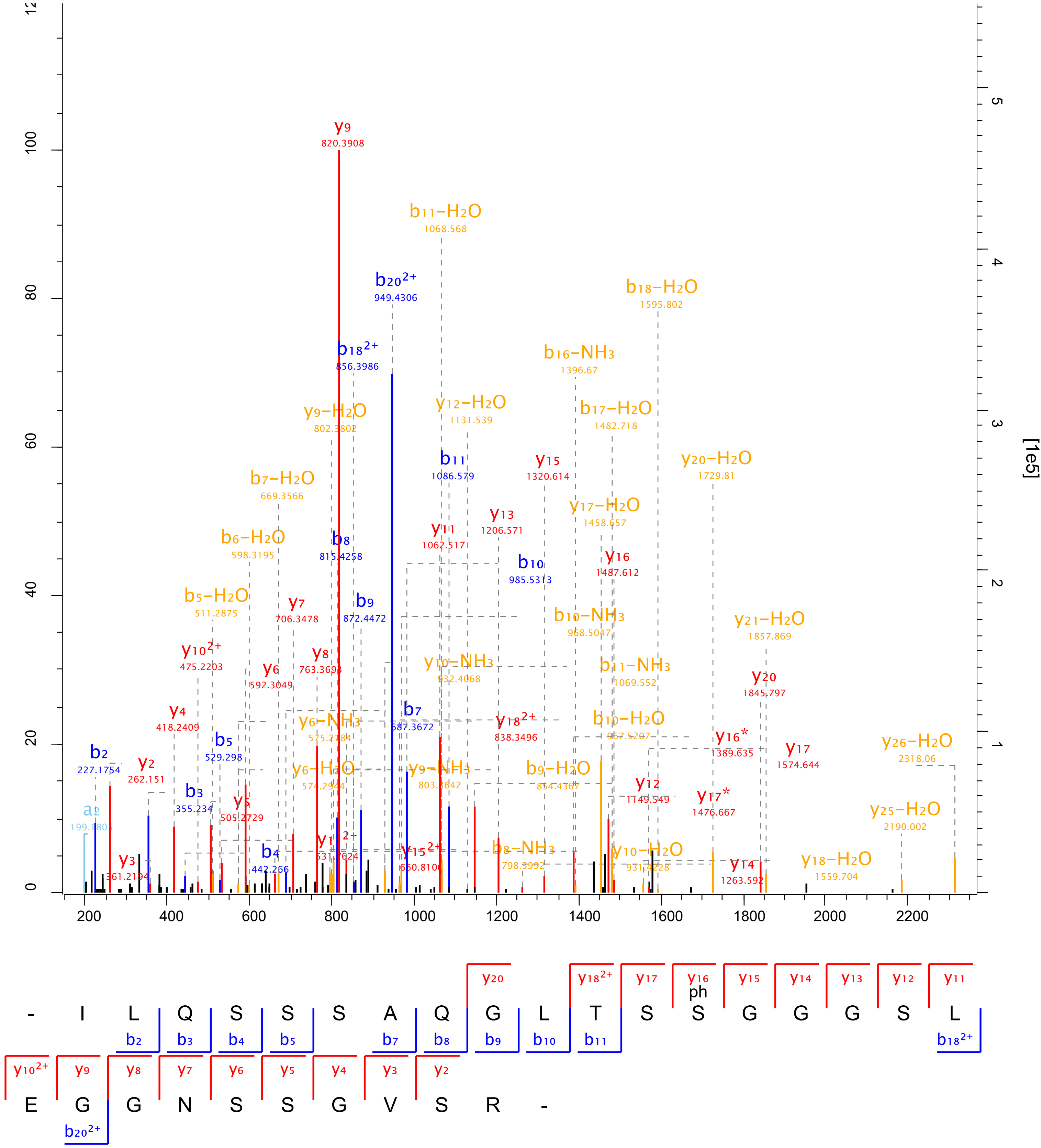

Raw file	Scan	Method	Score	m/z	Gene names
0523_3	13073	FTMS; HCD	120.23	912.43	NUP50A

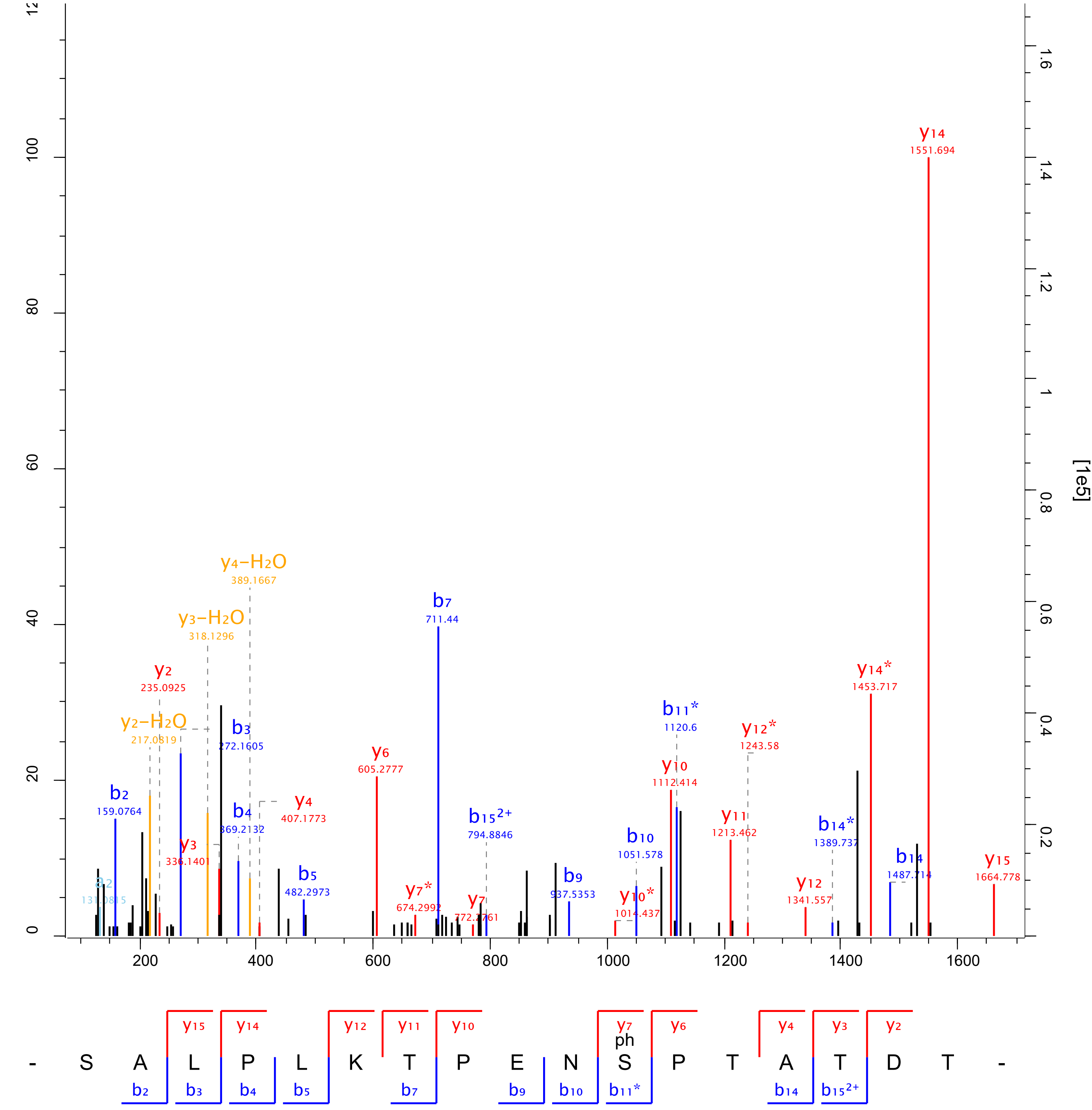

Sample output to test PDF Combine only

Raw file	Scan	Method	Score	m/z	Gene names
0523_3	13328	FTMS; HCD	130.01	729.32	F12P19.15

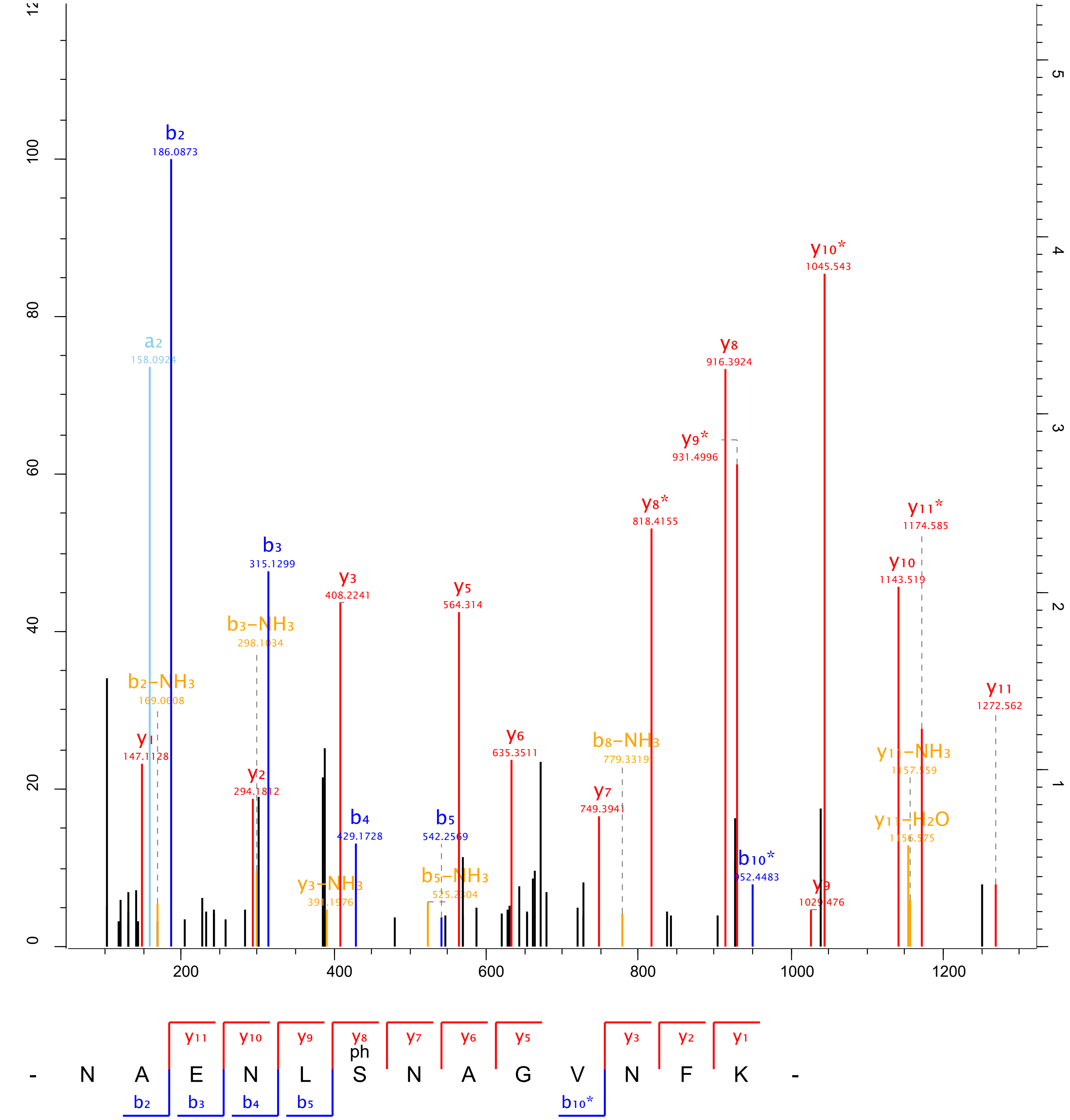

Sample output to test PDF Combine only

Raw file	Scan	Method	Score	m/z	Gene names
0523_3	13396	FTMS; HCD	169.04	663.3	ALA4

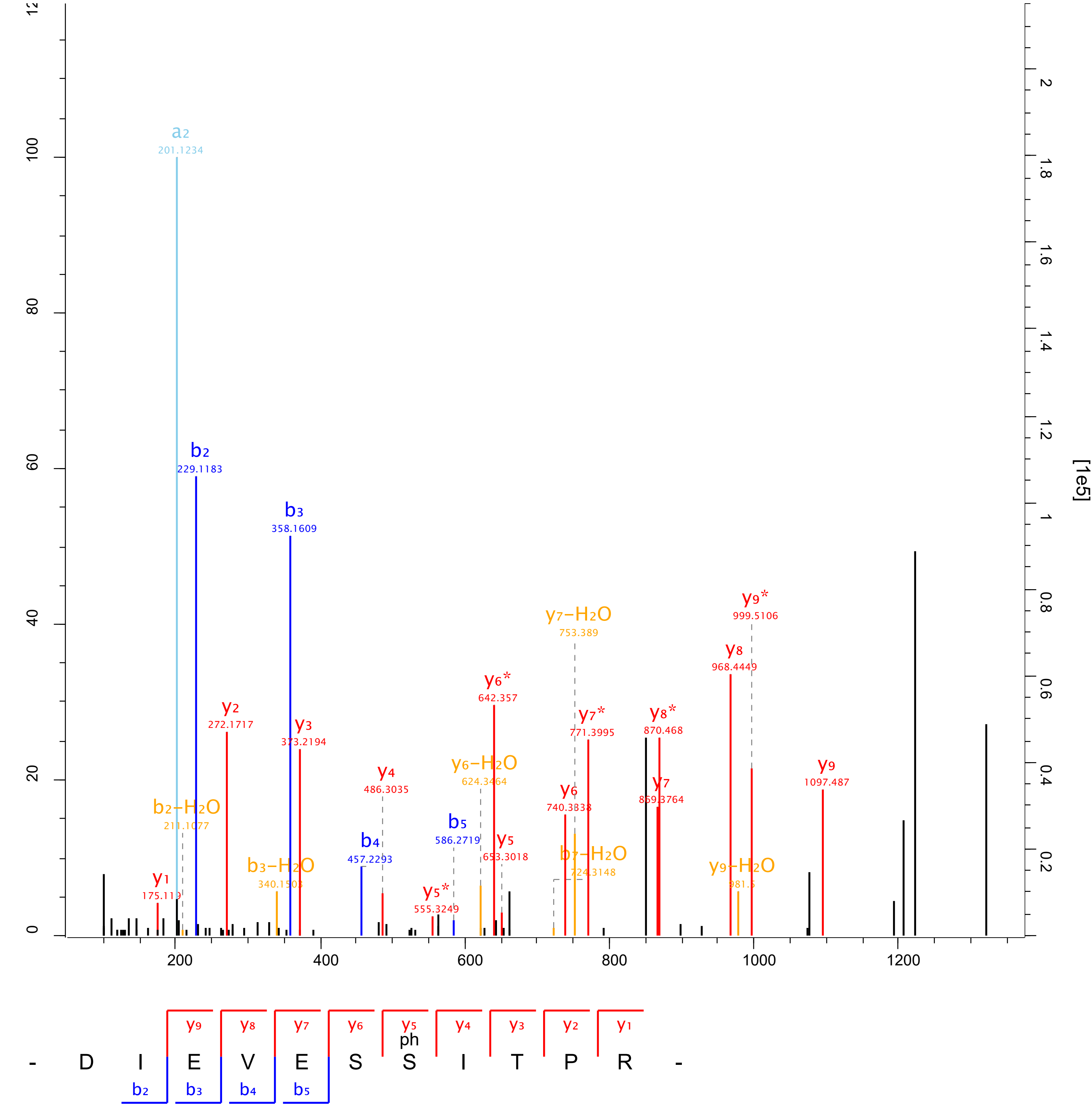

Sample output to test PDF Combine only

Raw file	Scan	Method	Score	m/z	Gene names
0523_3	13473	FTMS; HCD	45.43	563.74	SUMO2

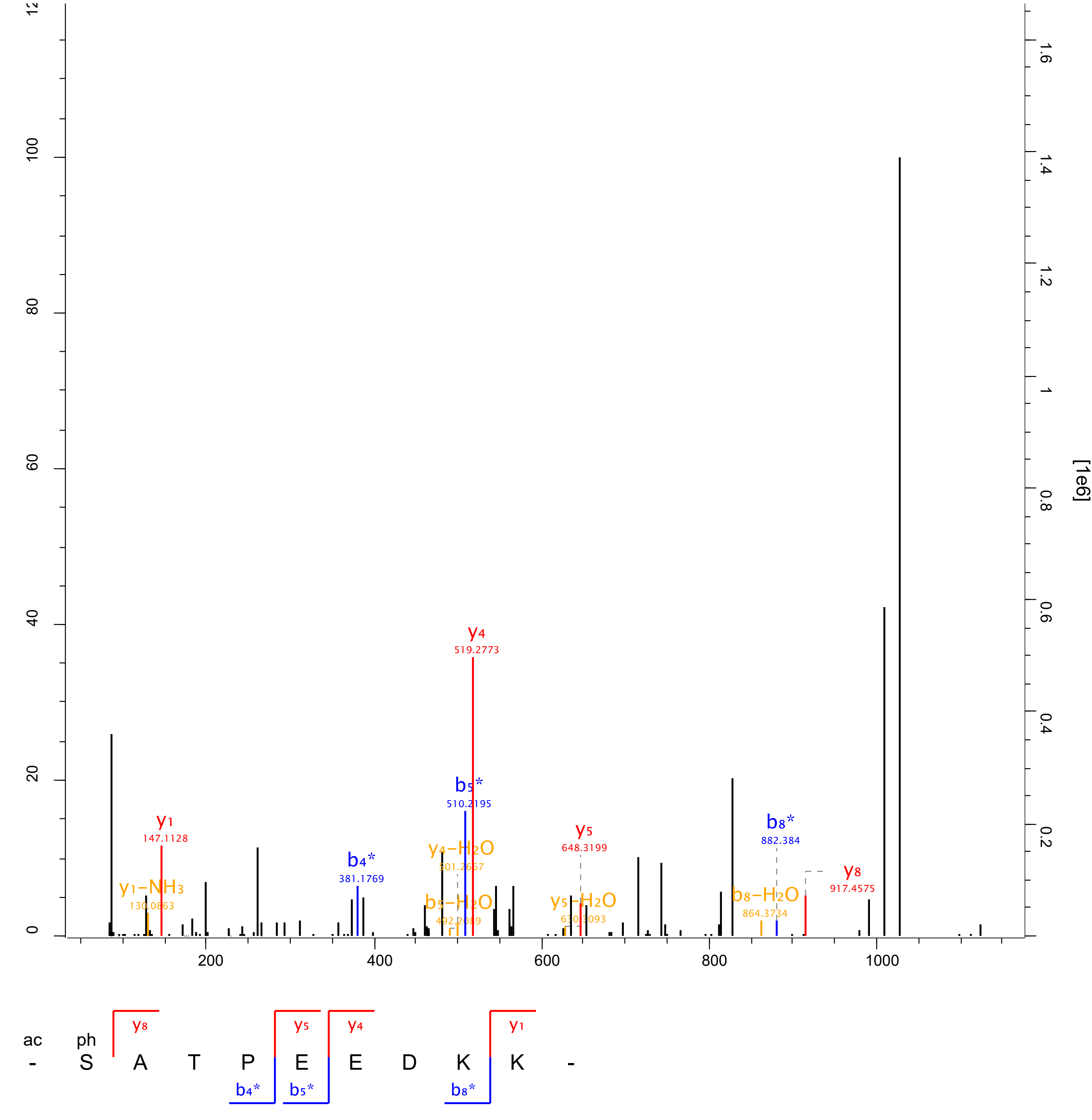

Sample output to test PDF Combine only

Raw file	Scan	Method	Score	m/z	Gene names
0523_3	13635	FTMS; HCD	177.44	792.38	PLDGAMMA1

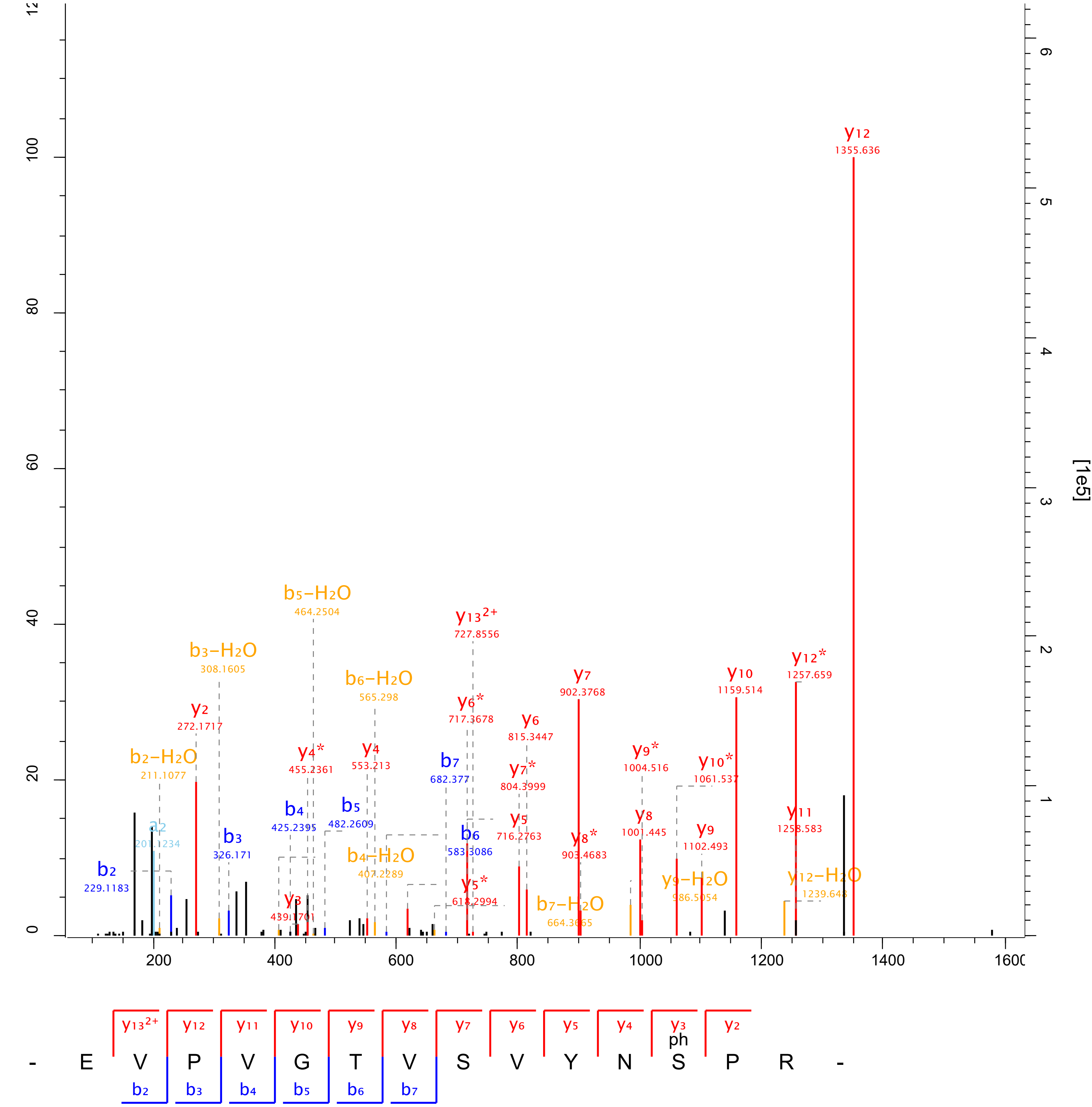

Sample output to test PDF Combine only

Raw file	Scan	Method	Score	m/z	Gene names
0523_3	13689	FTMS; HCD	137.64	701.32	At4g25770

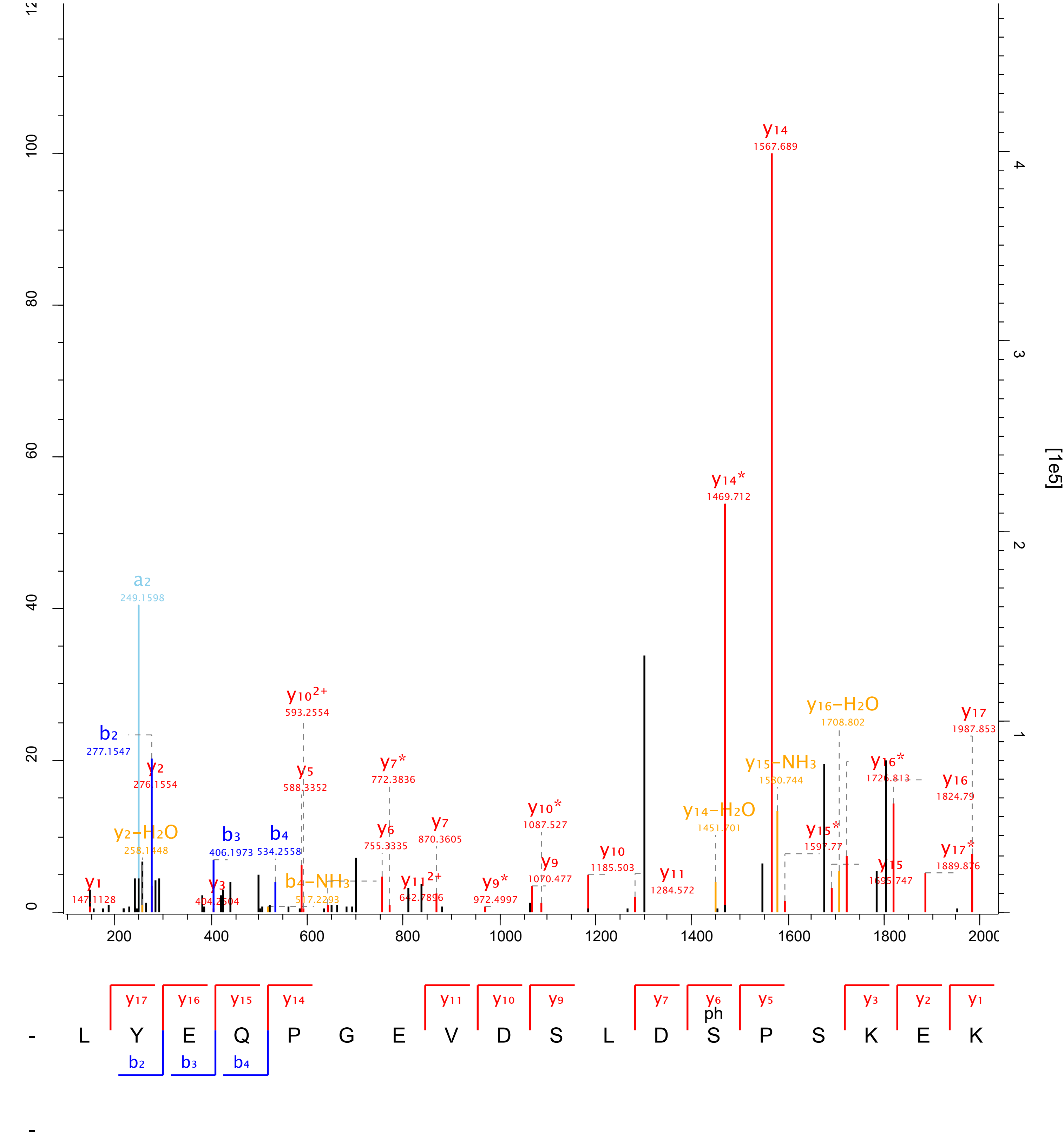

Sample output to test PDF Combine only

Raw file	Scan	Method	Score	m/z	Gene names
0523_3	13736	FTMS; HCD	52.48	632.78	At2g01690;VAC14

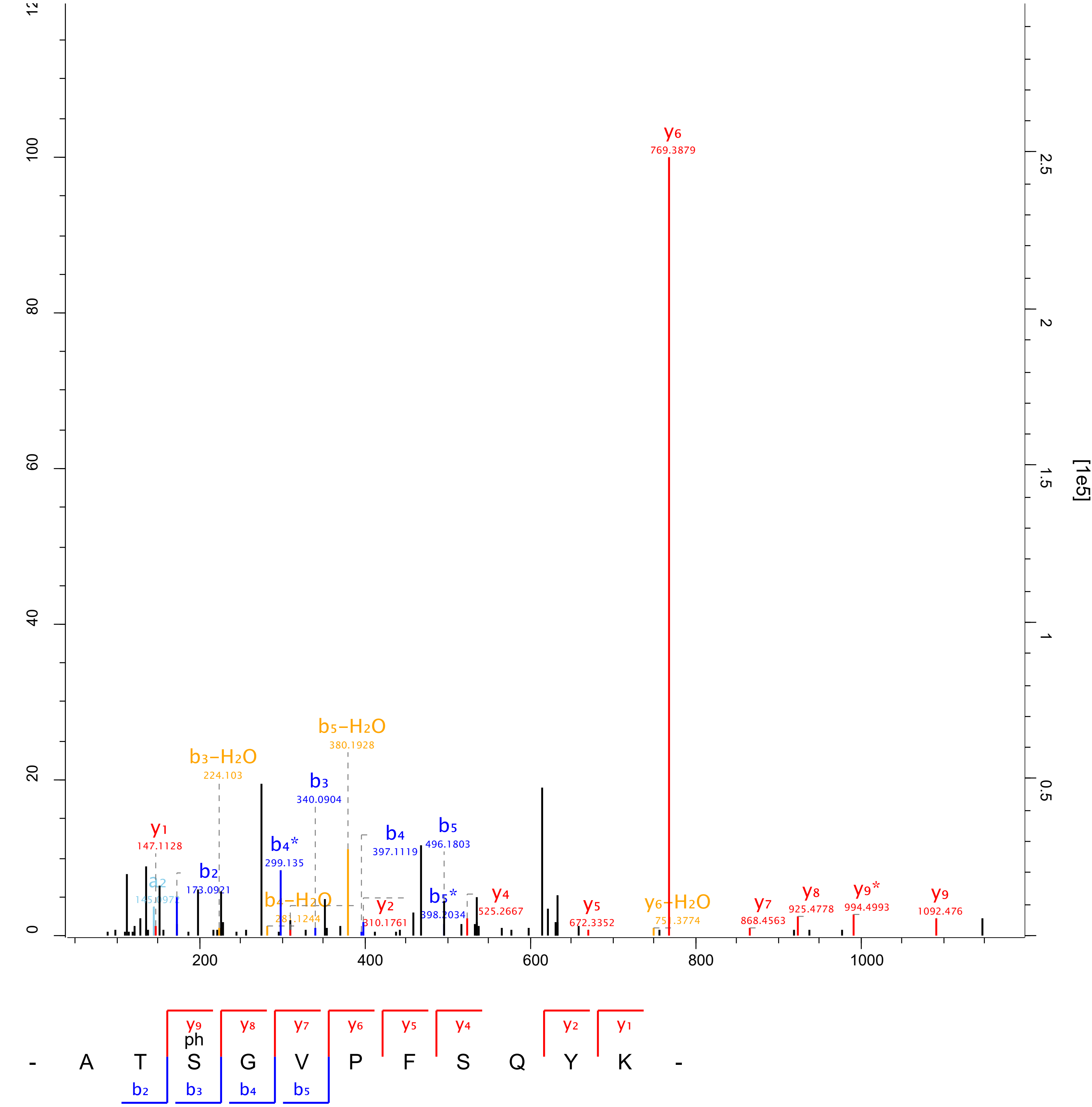

Sample output to test PDF Combine only

Raw file	Scan	Method	Score	m/z
0523_3	13828	FTMS; HCD	129.02	871.39

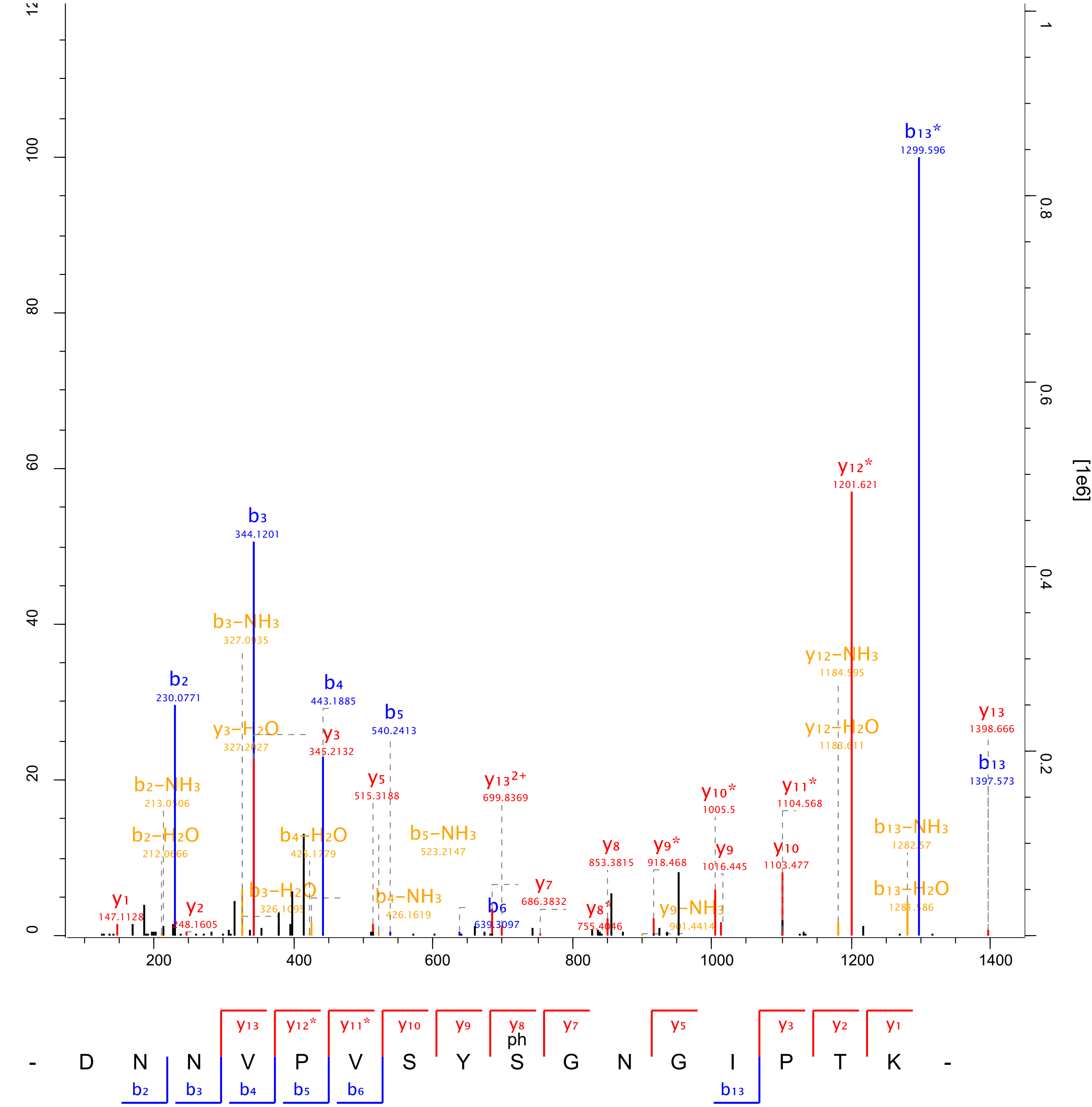

Sample output to test PDF Combine only

Raw file	Scan	Method	Score	m/z	Gene names
0523_3	14121	FTMS; HCD	107.71	843.03	MSI4

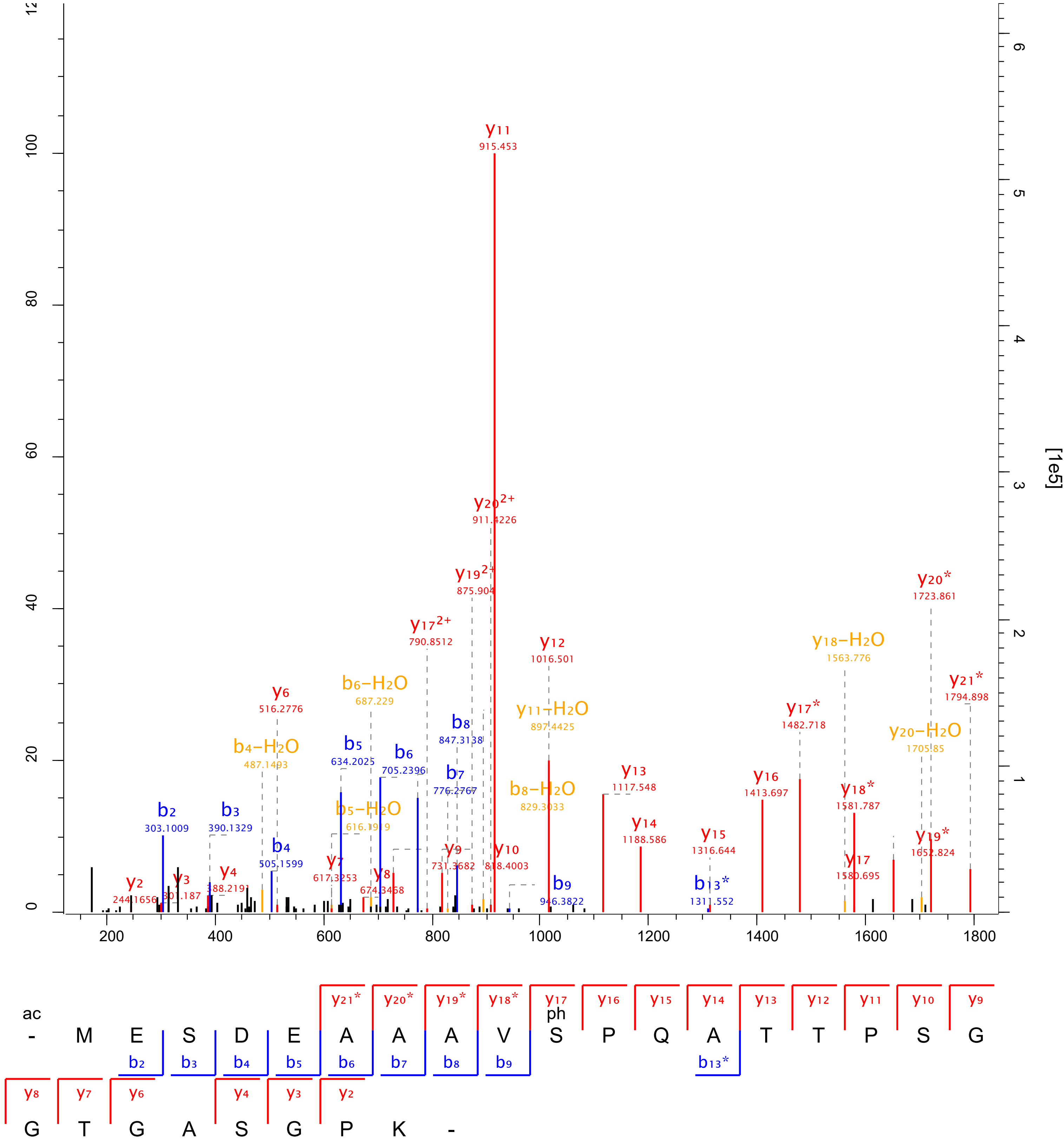

Raw file	Scan	Method	Score	m/z	Gene names
0523_3	14152	FTMS; HCD	82.59	735.35	MBK5.12

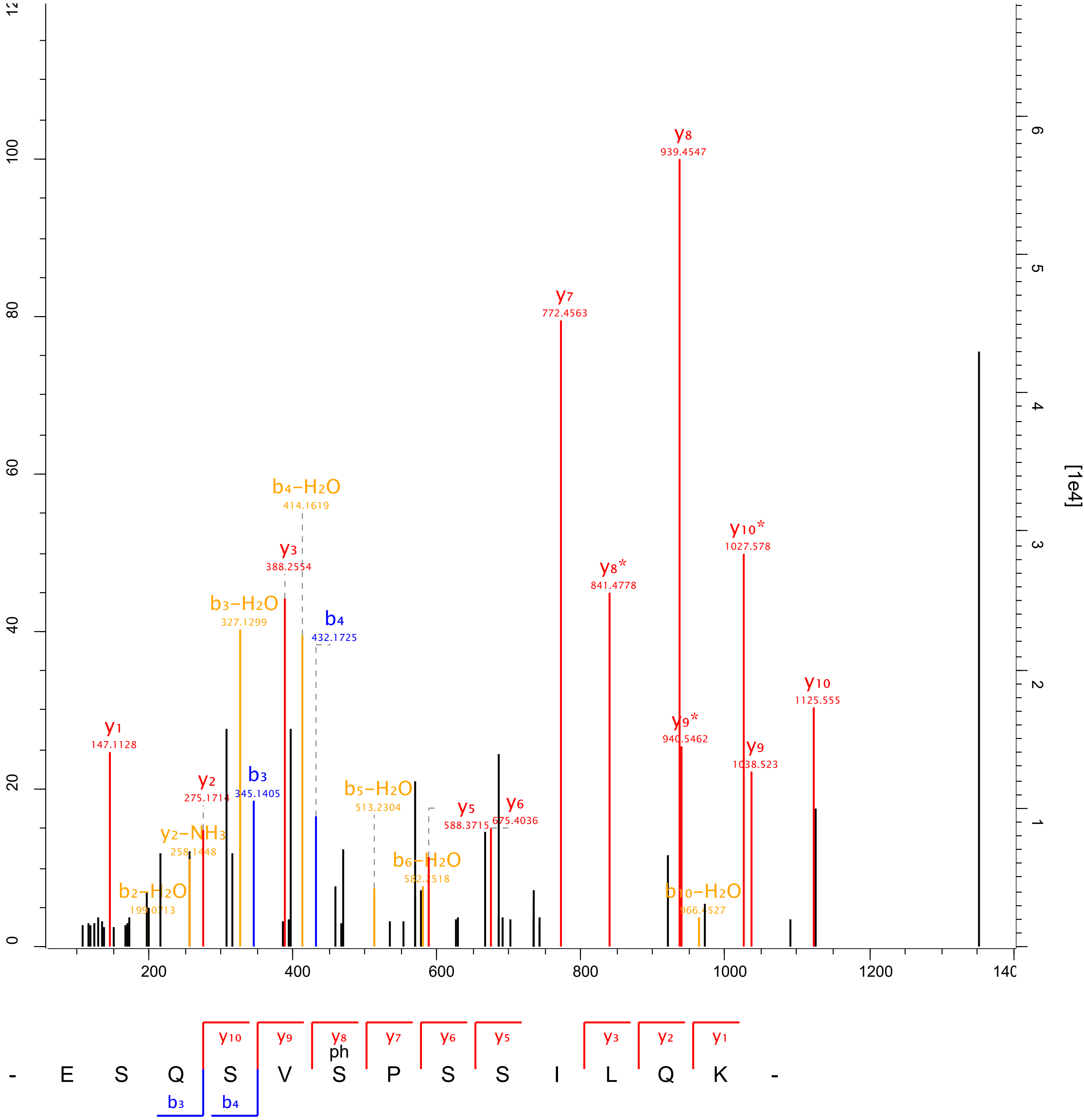

Sample output to test PDF Combine only

Raw file	Scan	Method	Score	m/z	Gene names
0523_3	14222	FTMS; HCD	203.71	799.87	RPS2B;RPS2A

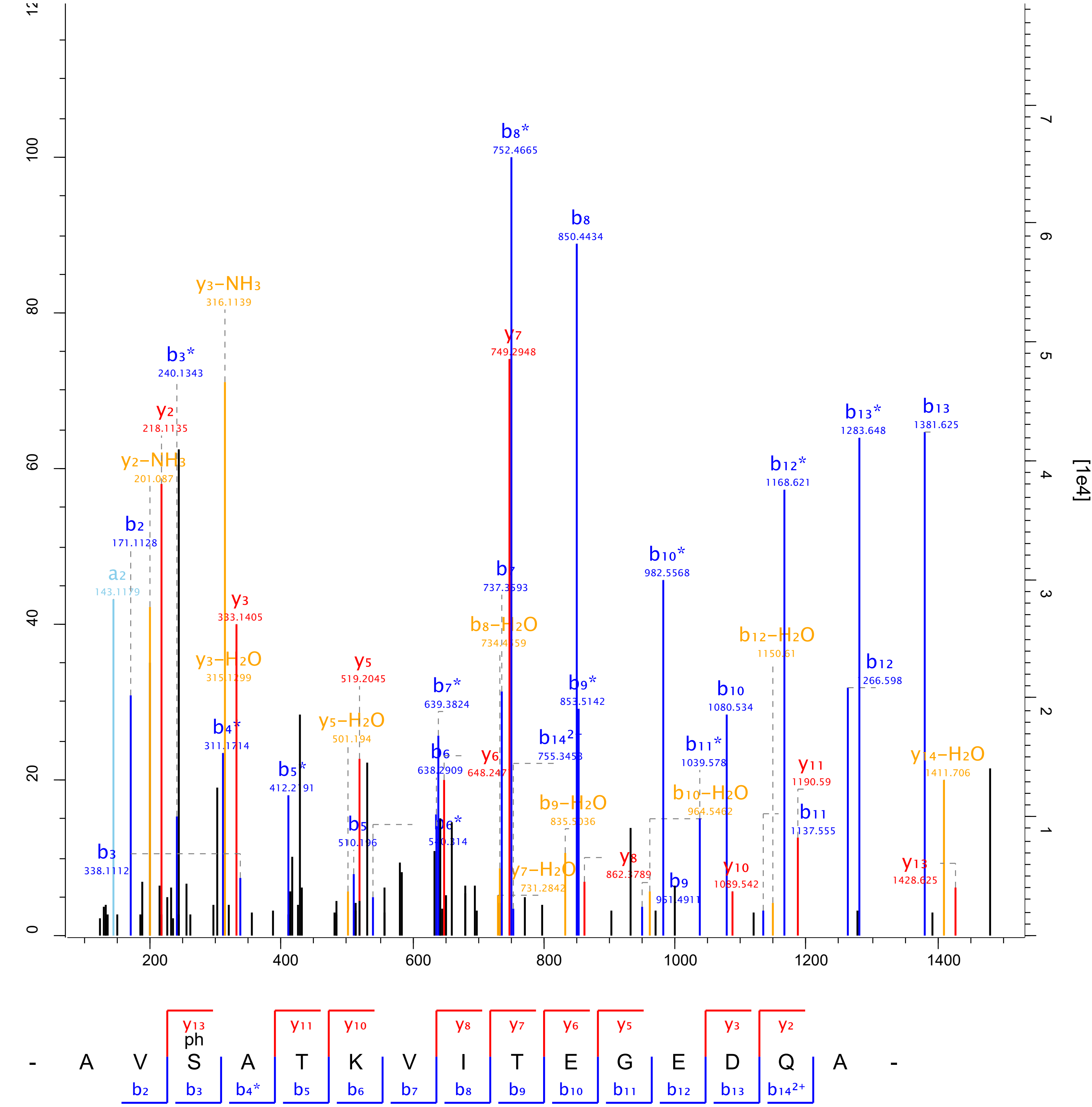

Sample output to test PDF Combine only

Raw file	Scan	Method	Score	m/z	Gene names
0523_3	14609	FTMS; HCD	63.49	866.39	At2g40980

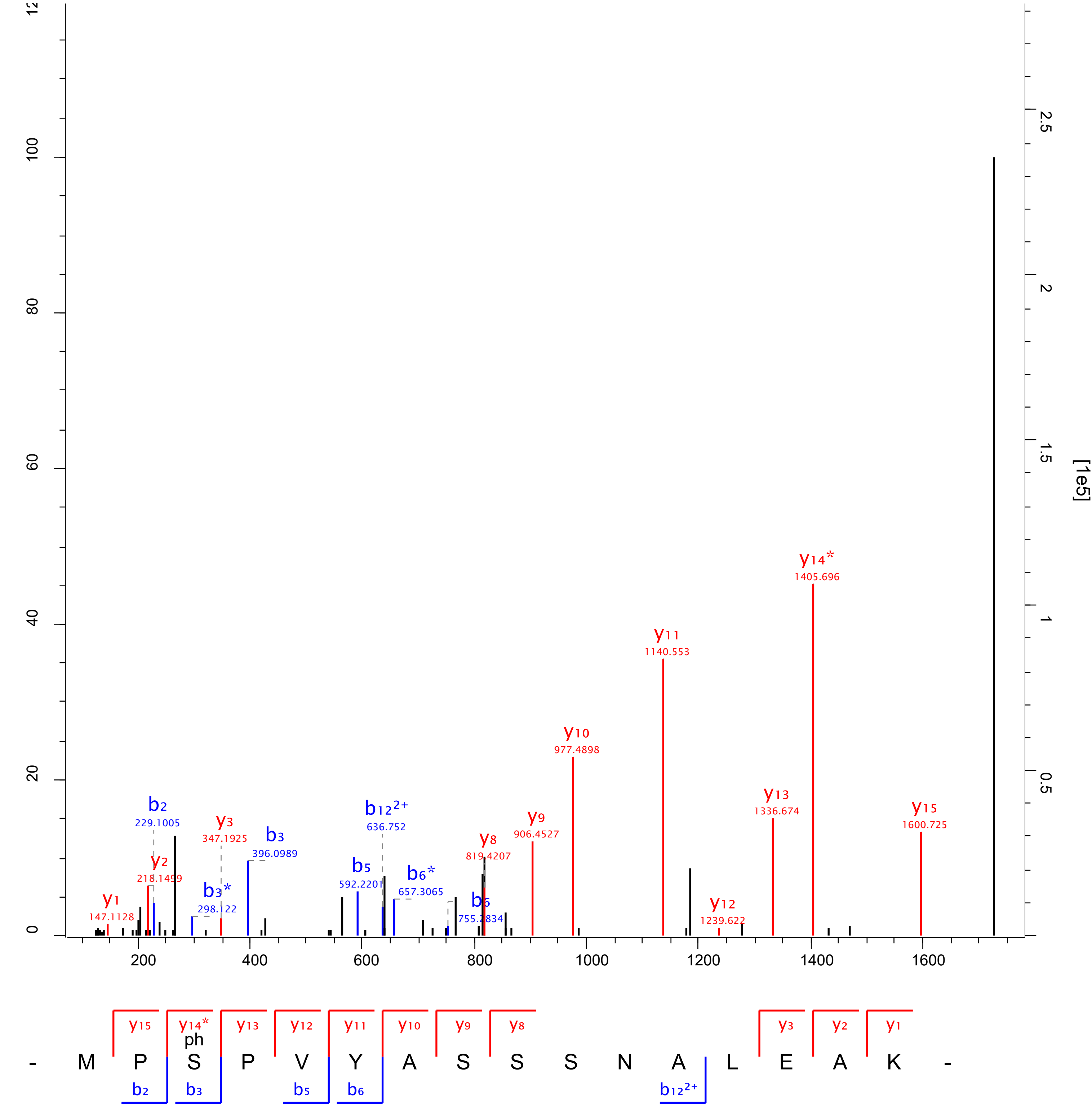

Sample output to test PDF Combine only

Raw file	Scan	Method	Score	m/z	Gene names
0523_3	14617	FTMS; HCD	102.64	707.8	F9P14.7

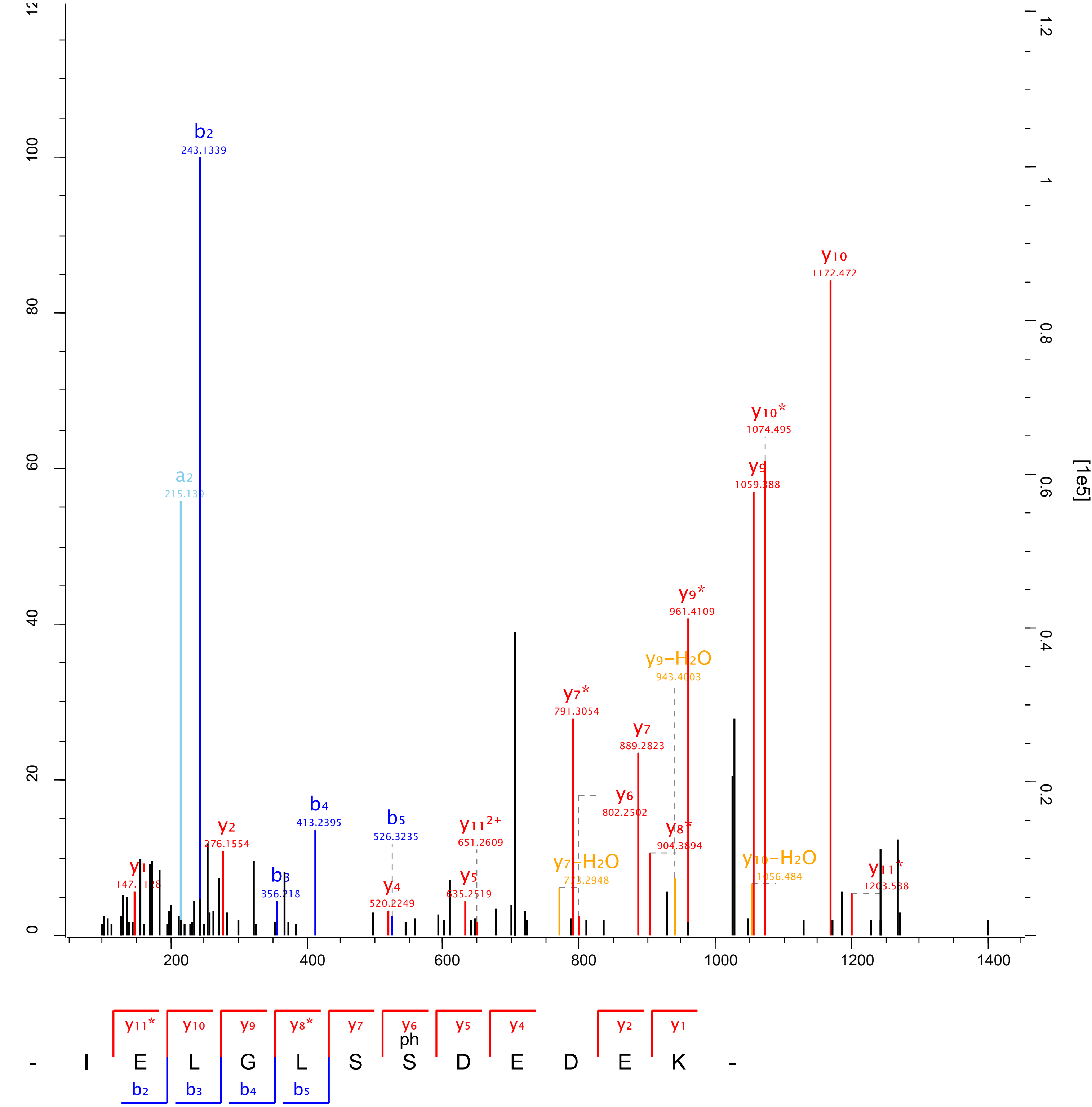

Sample output to test PDF Combine only

Raw file	Scan	Method	Score	m/z	Gene names
0523_3	15033	FTMS; HCD	62.16	643.28	At5g15680

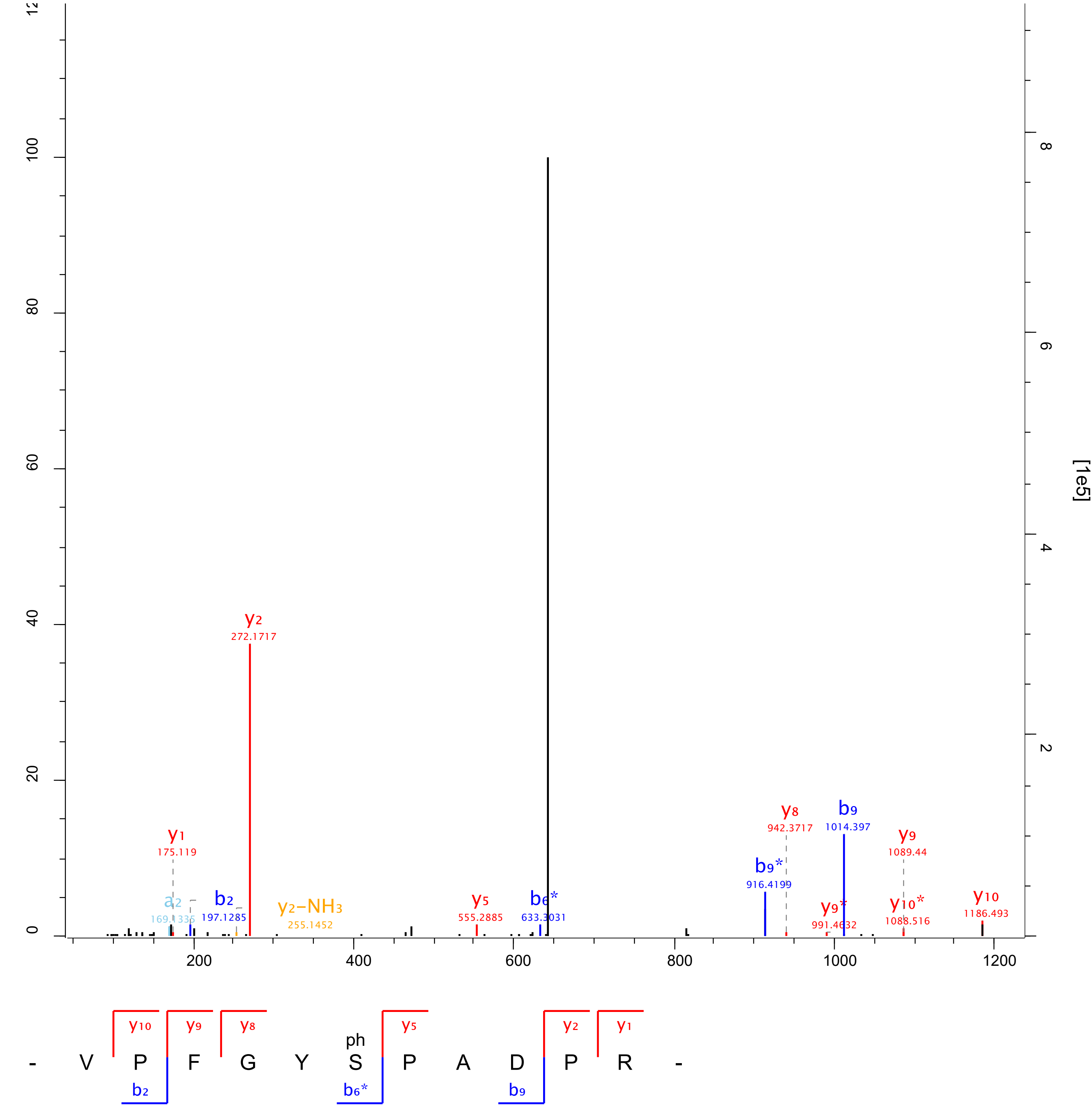

Sample output to test PDF Combine only

Raw file	Scan	Method	Score	m/z	Gene names
0523_3	15821	FTMS; HCD	300.24	1019.43	HDT1

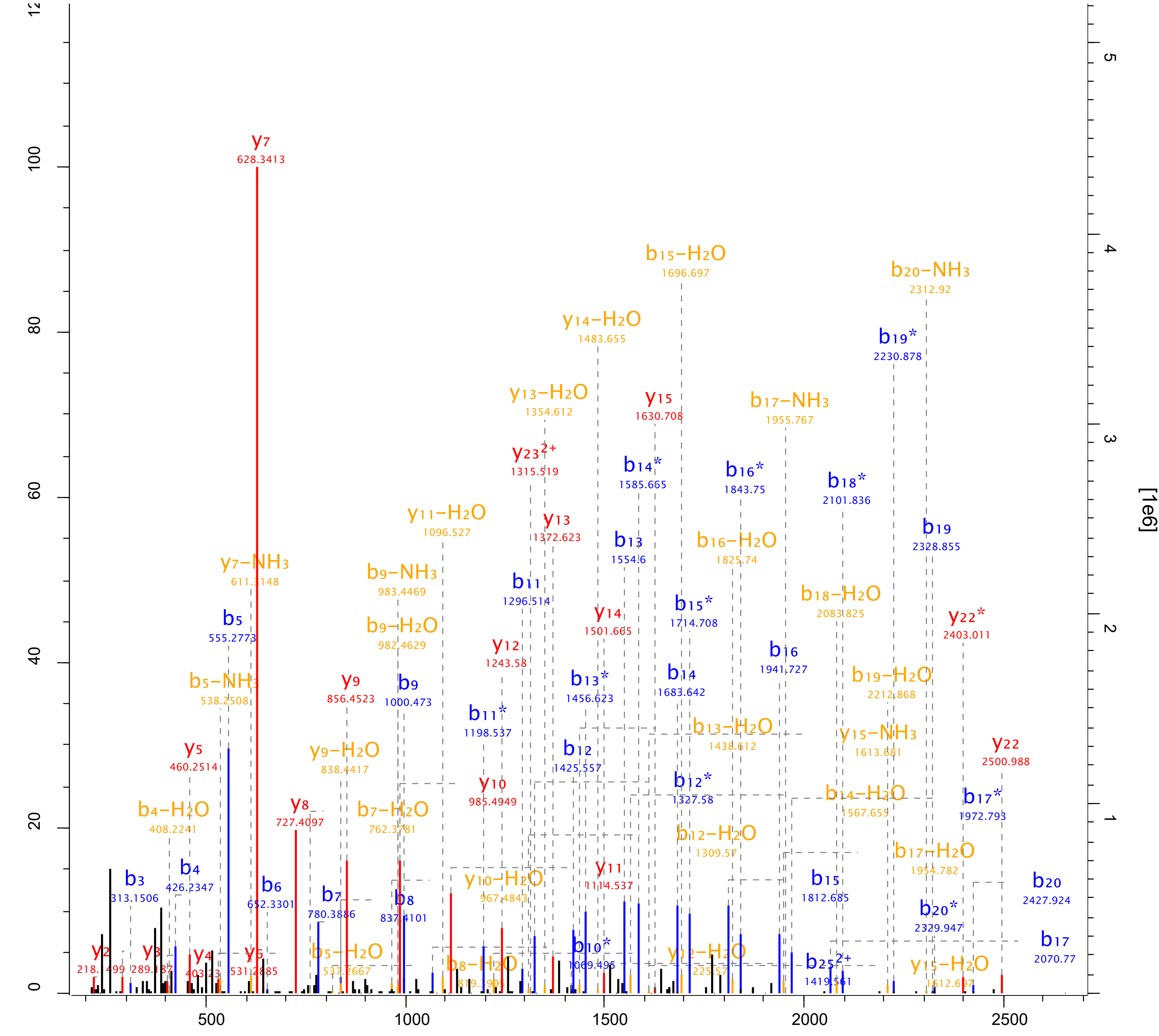

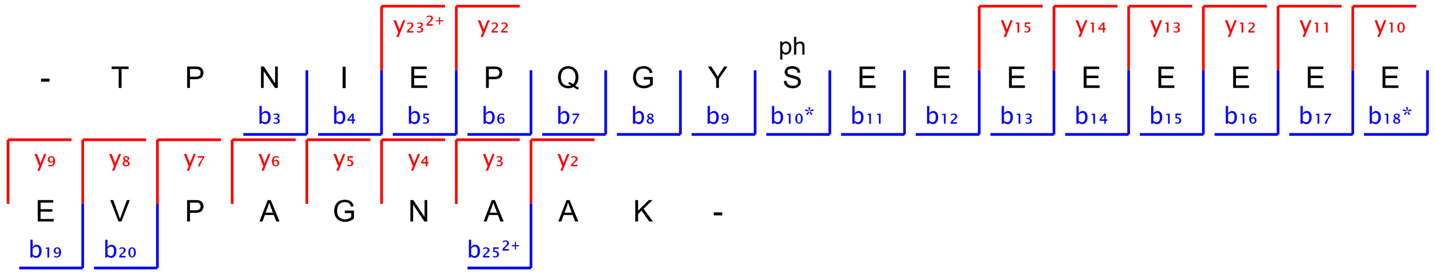

Sample output to test PDF Combine only

Raw file	Scan	Method	Score	m/z	Gene names
0523_3	15863	FTMS; HCD	41.45	737.68	BPM5

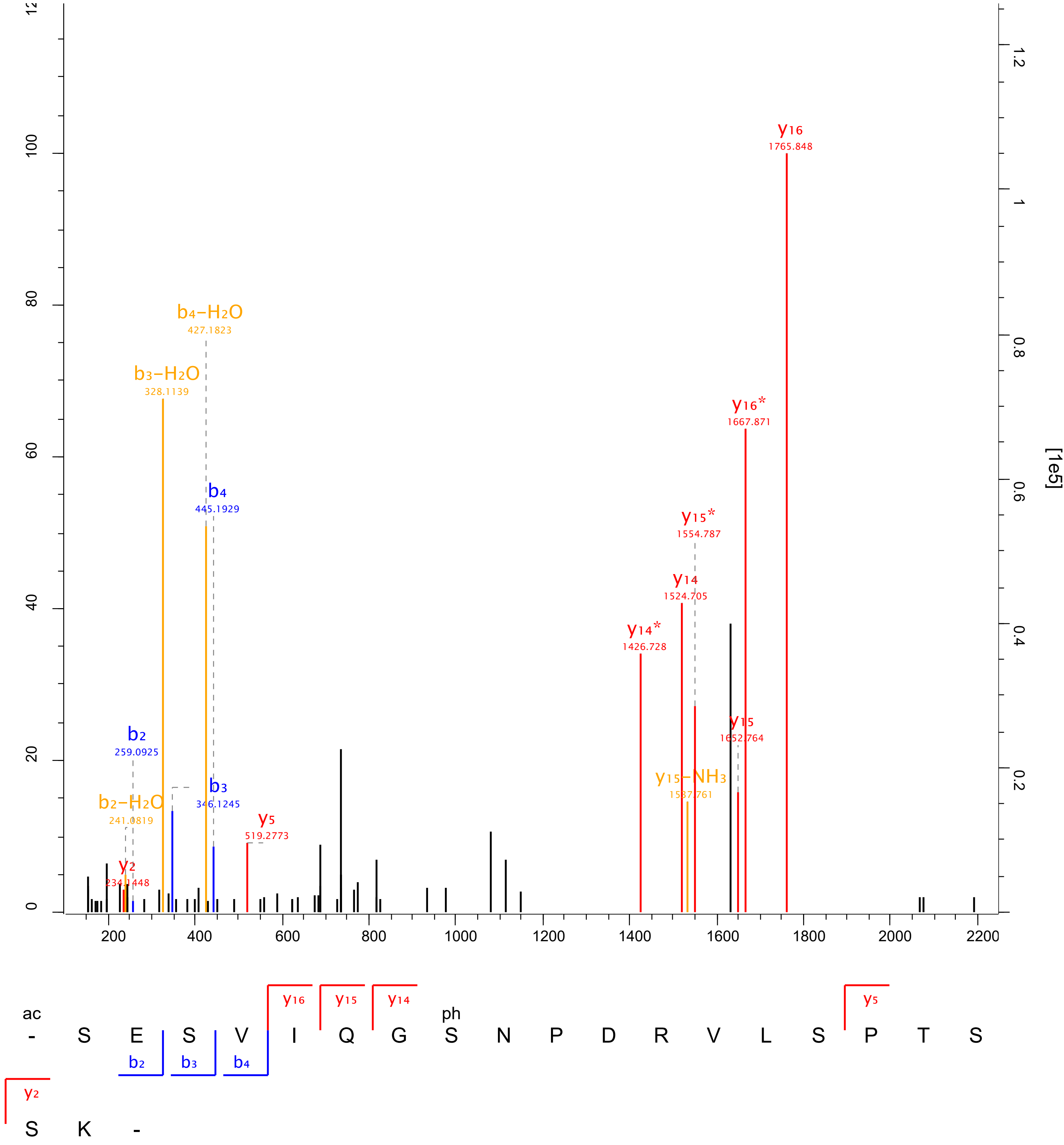

Raw file	Scan	Method	Score	m/z	Gene names
0523_3	16139	FTMS; HCD	50.03	704.32	At5g67220

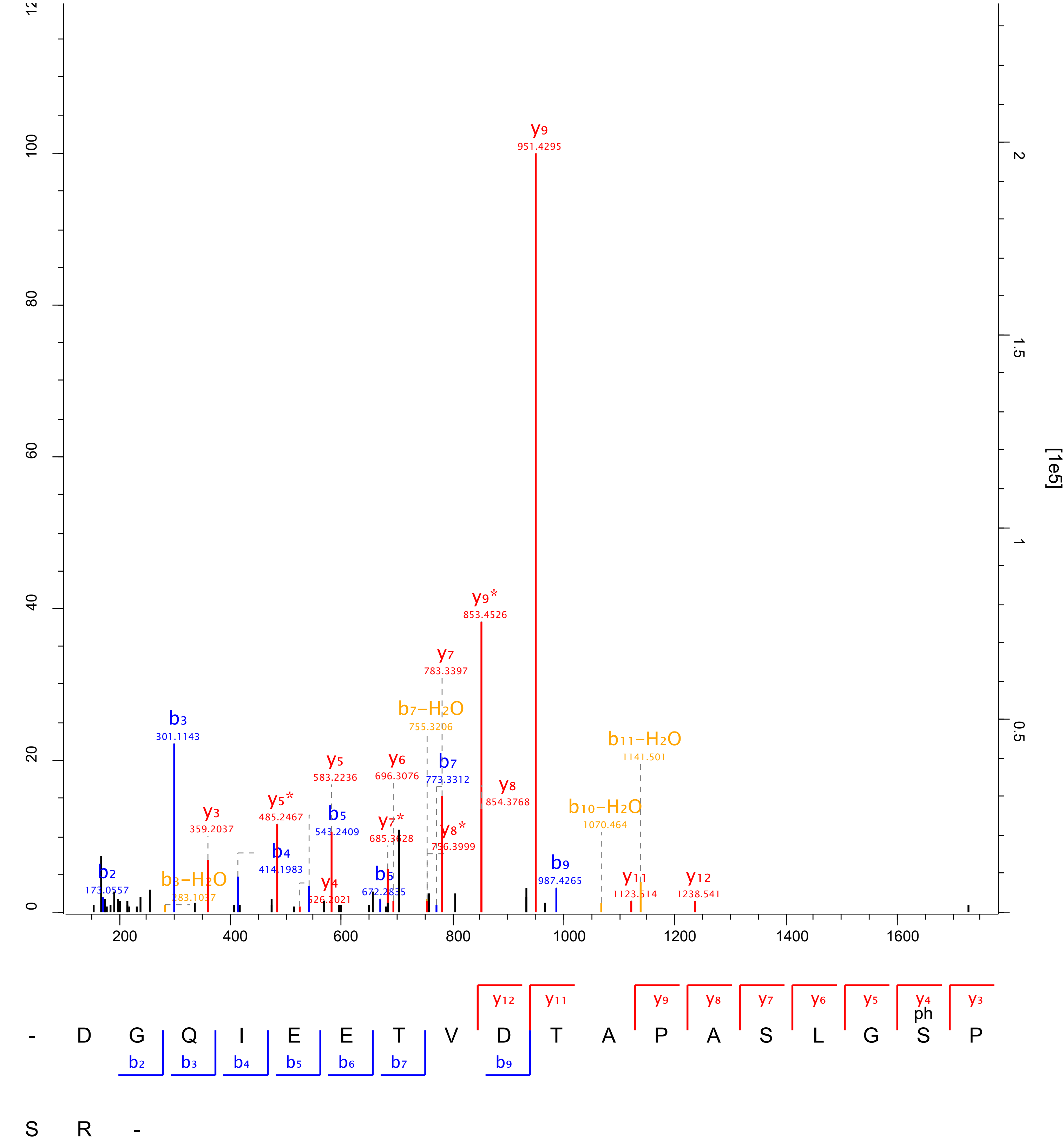

Sample output to test PDF Combine only

Raw file	Scan	Method	Score	m/z	Gene names
0523_3	16330	FTMS; HCD	58.32	650.3	At1g27540;T17H3.4

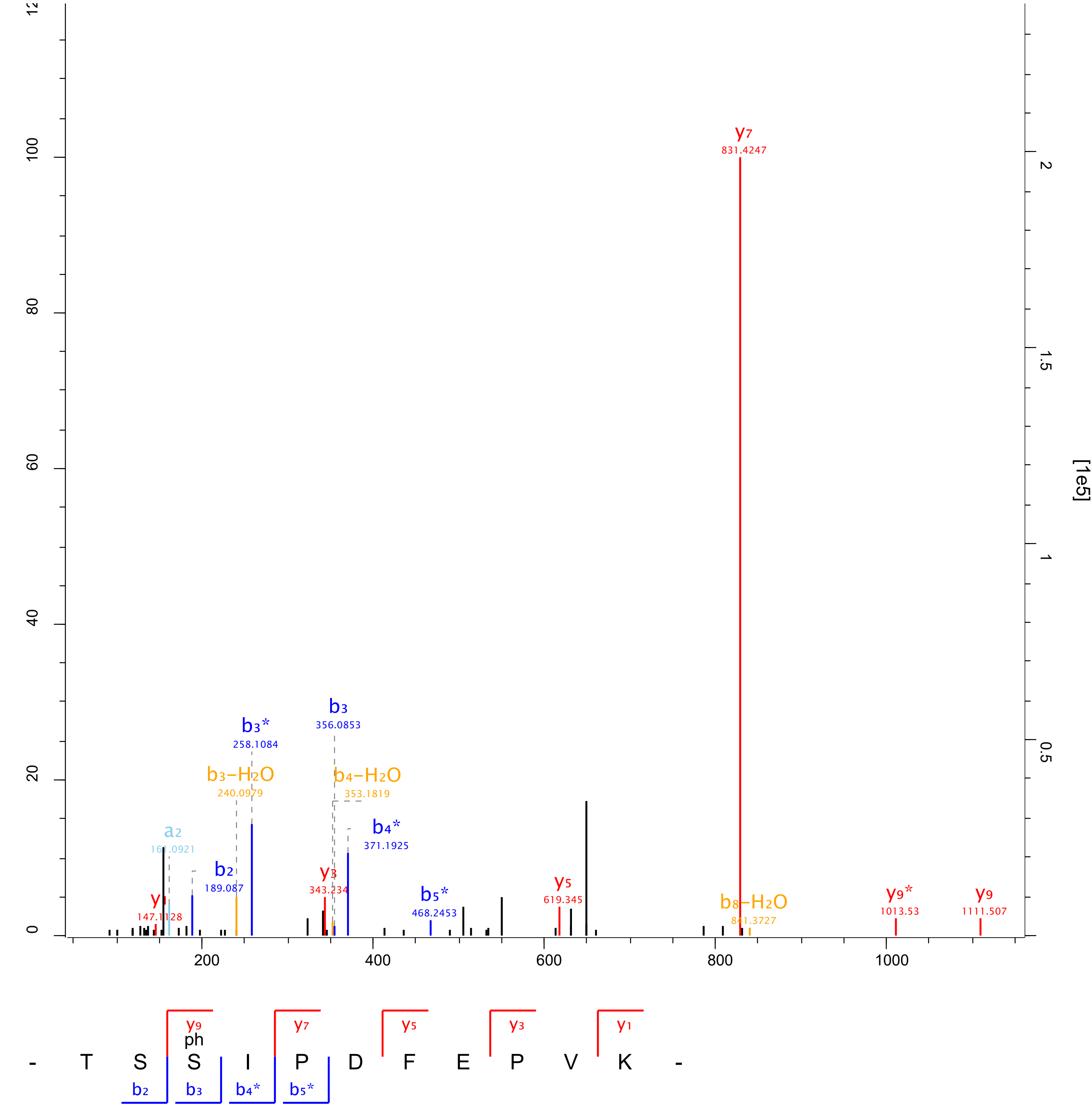

Sample output to test PDF Combine only

Raw file	Scan	Method	Score	m/z	Gene names
0523_3	16423	FTMS; HCD	68.97	612.8	ZIP2

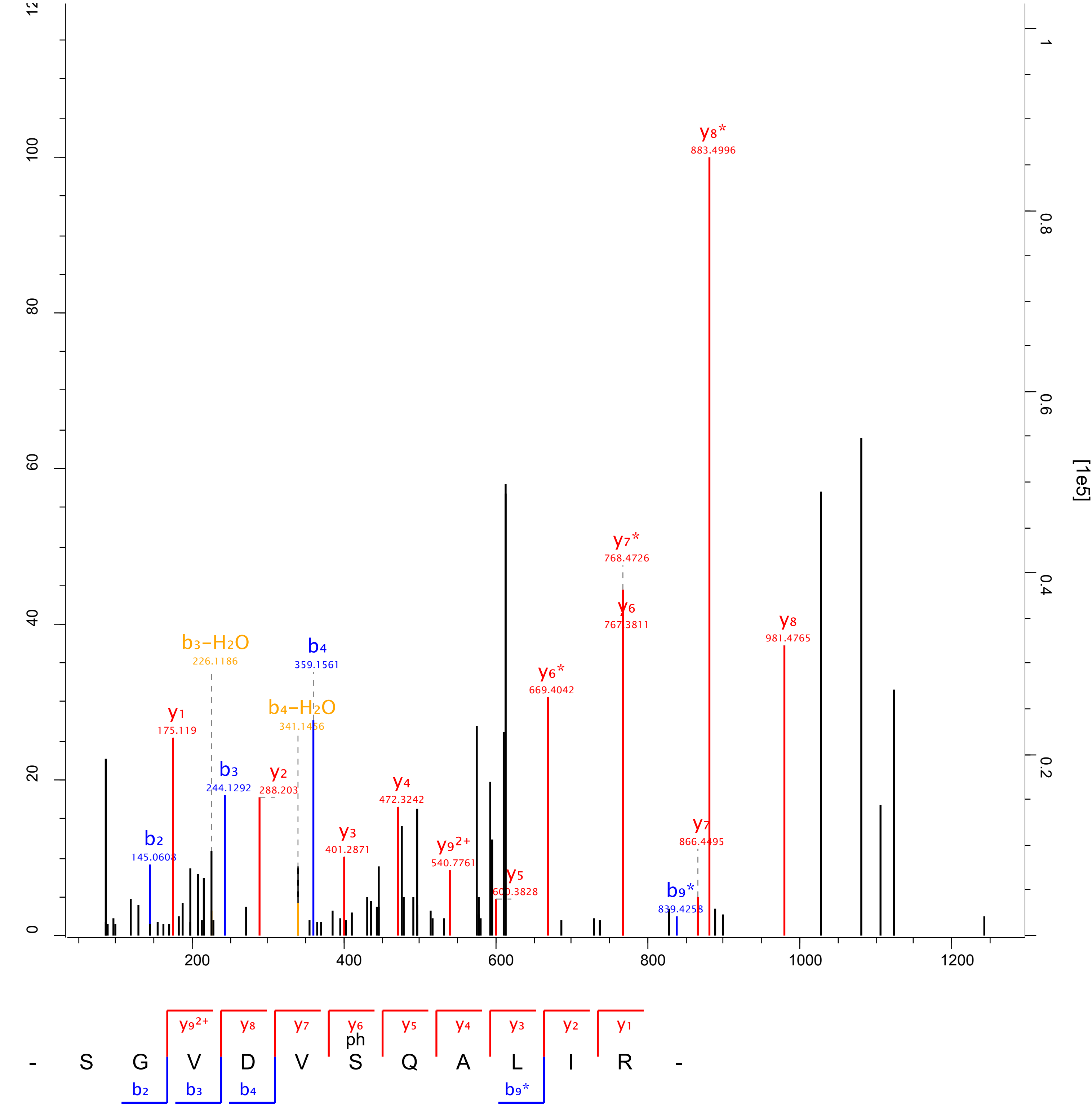

Sample output to test PDF Combine only

Raw file	Scan	Method	Score	m/z	Gene names
0523_3	17314	FTMS; HCD	84.48	888.74	AHL13

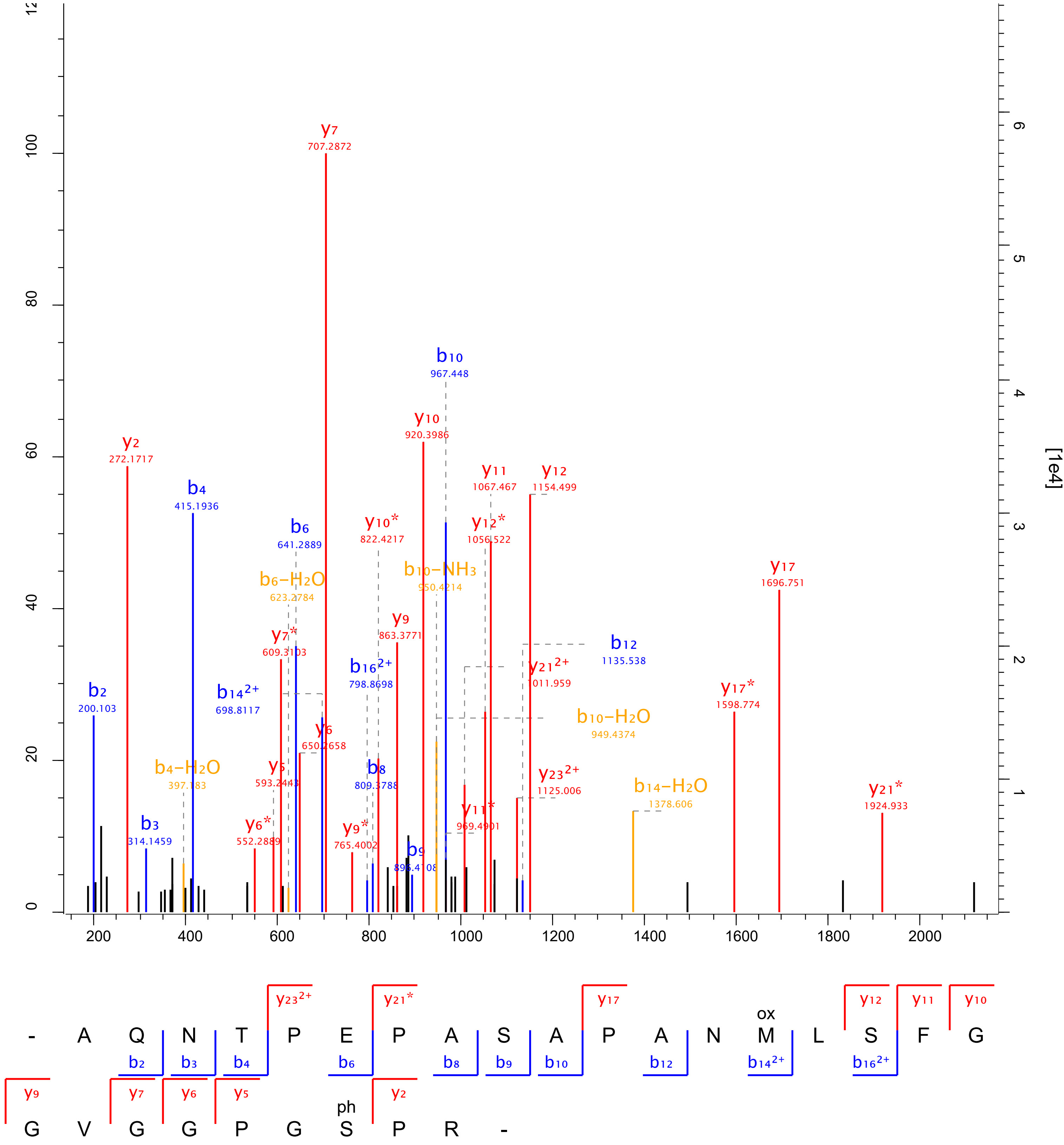

Sample output to test PDF Combine only

Raw file	Scan	Method	Score	m/z	Gene names
0523_3	17648	FTMS; HCD	52.81	757.87	GAPC2;GAPC1

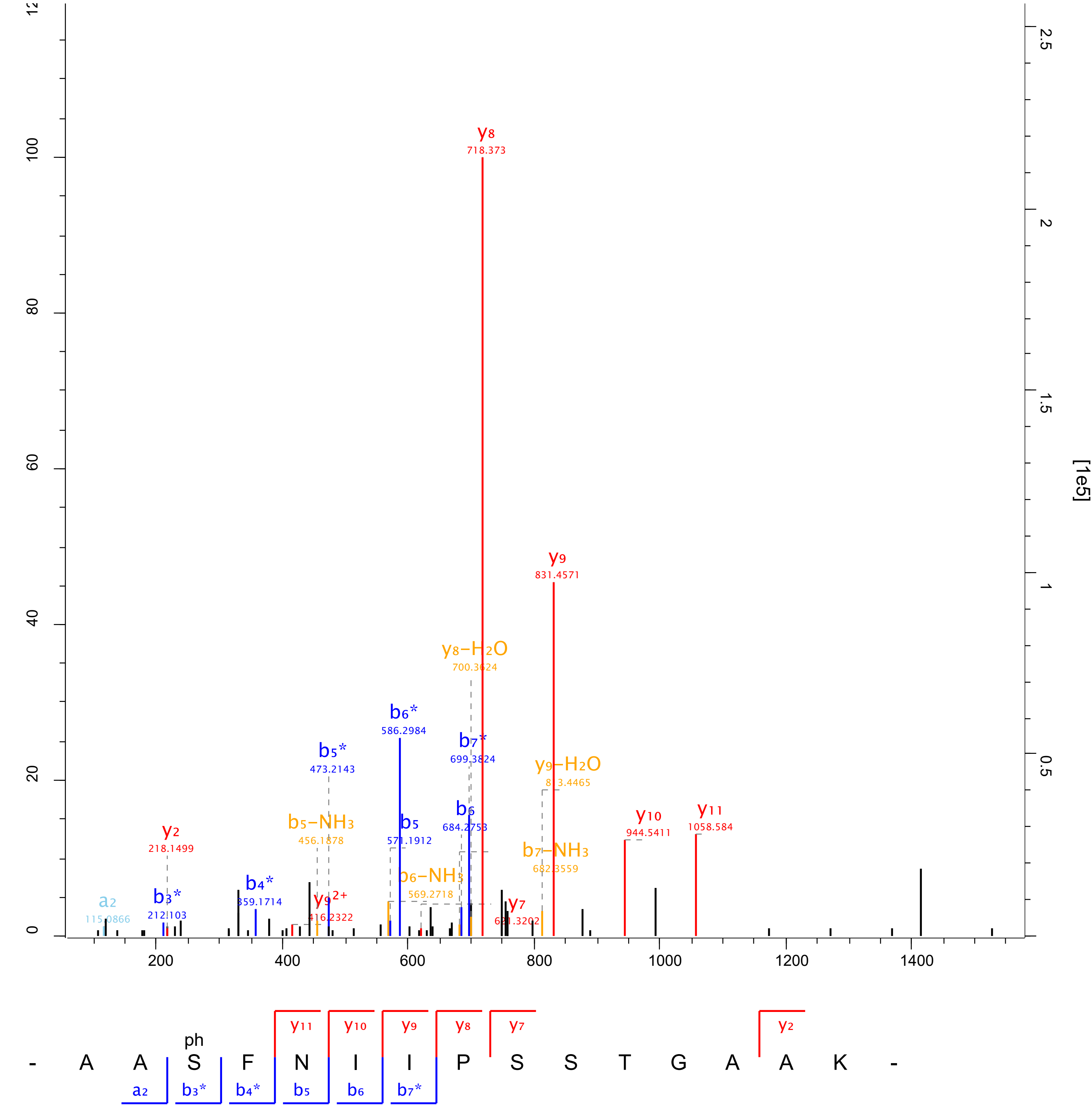

Sample output to test PDF Combine only

Raw file	Scan	Method	Score	m/z	Gene names
0523_3	19015	FTMS; HCD	170.33	930.42	RPL13D

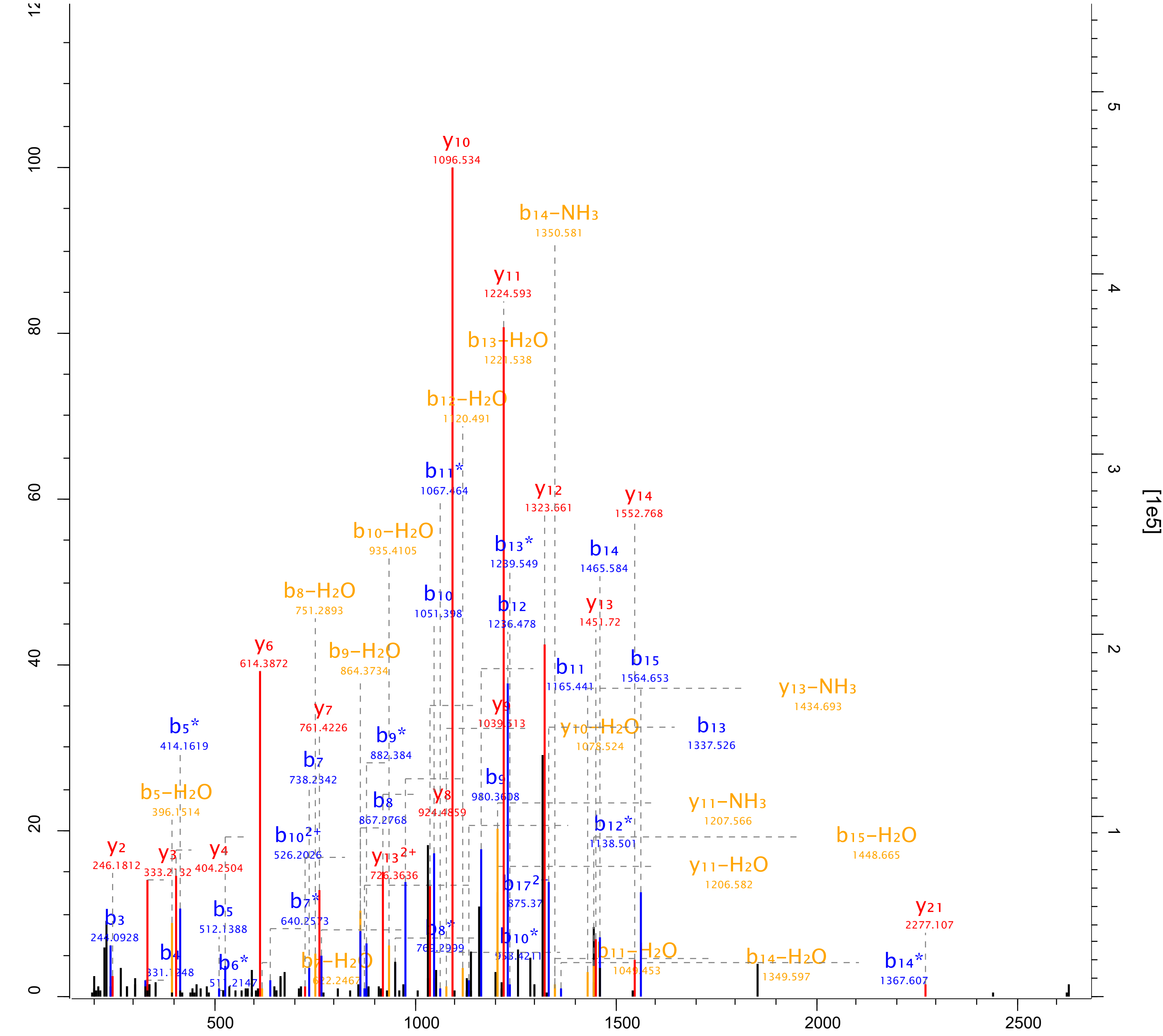

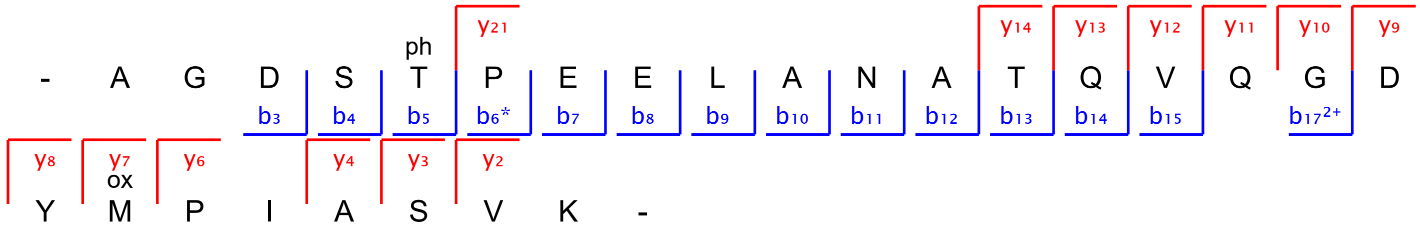

Sample output to test PDF Combine only

Raw file	Scan	Method	Score	m/z	Gene names
0523_3	23037	FTMS; HCD	80.17	900.44	BSL2

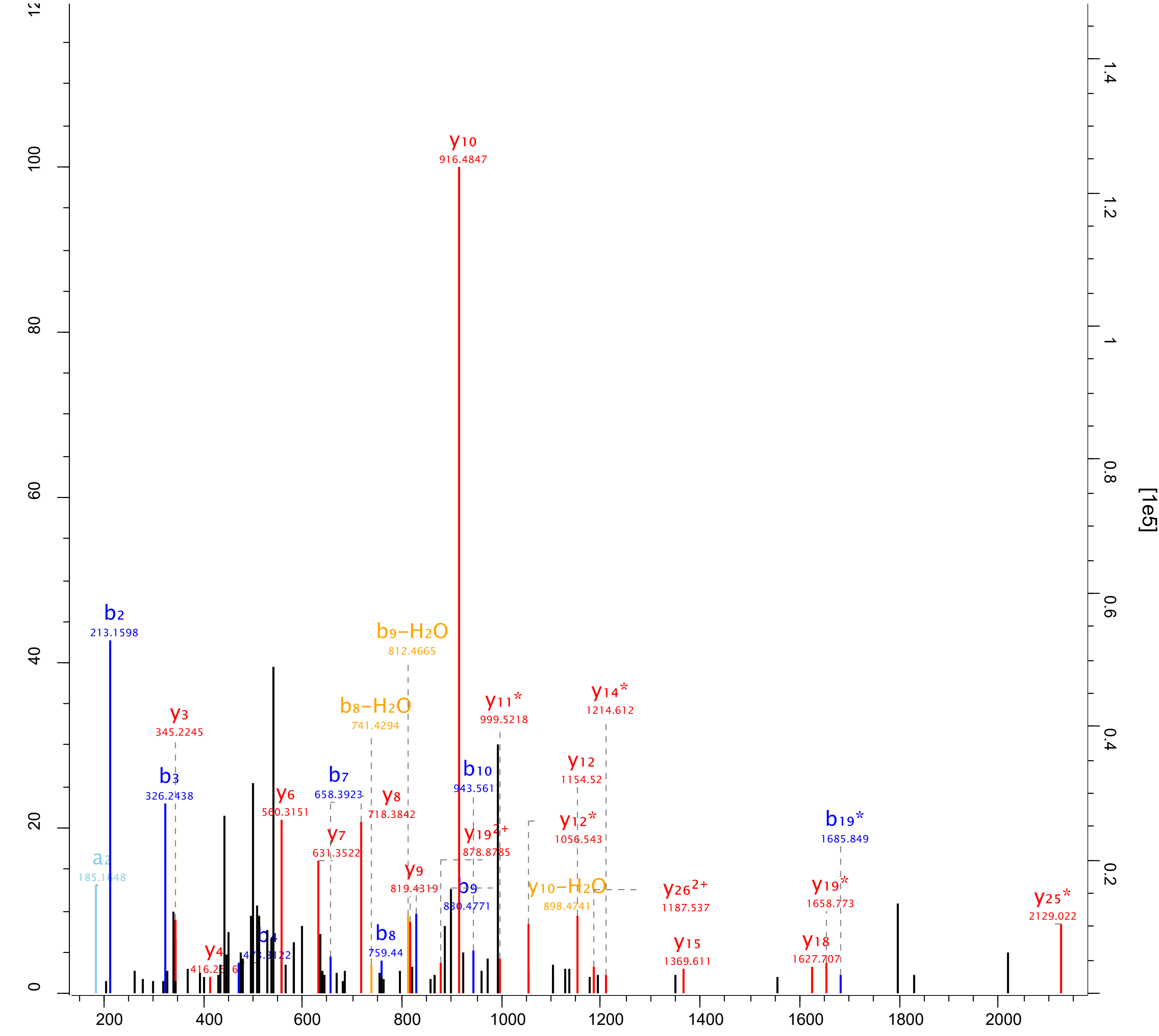

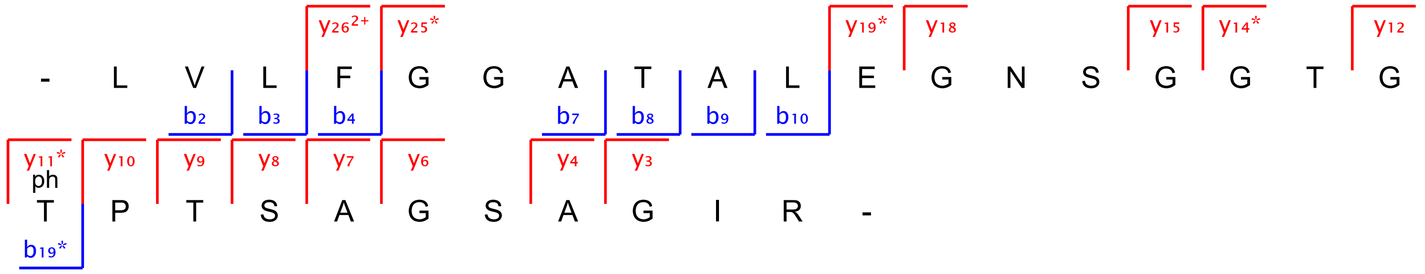

Sample output to test PDF Combine only

Raw file	Scan	Method	Score	m/z	Gene names
0523_3	23909	FTMS; HCD	45.14	892.42	JKD

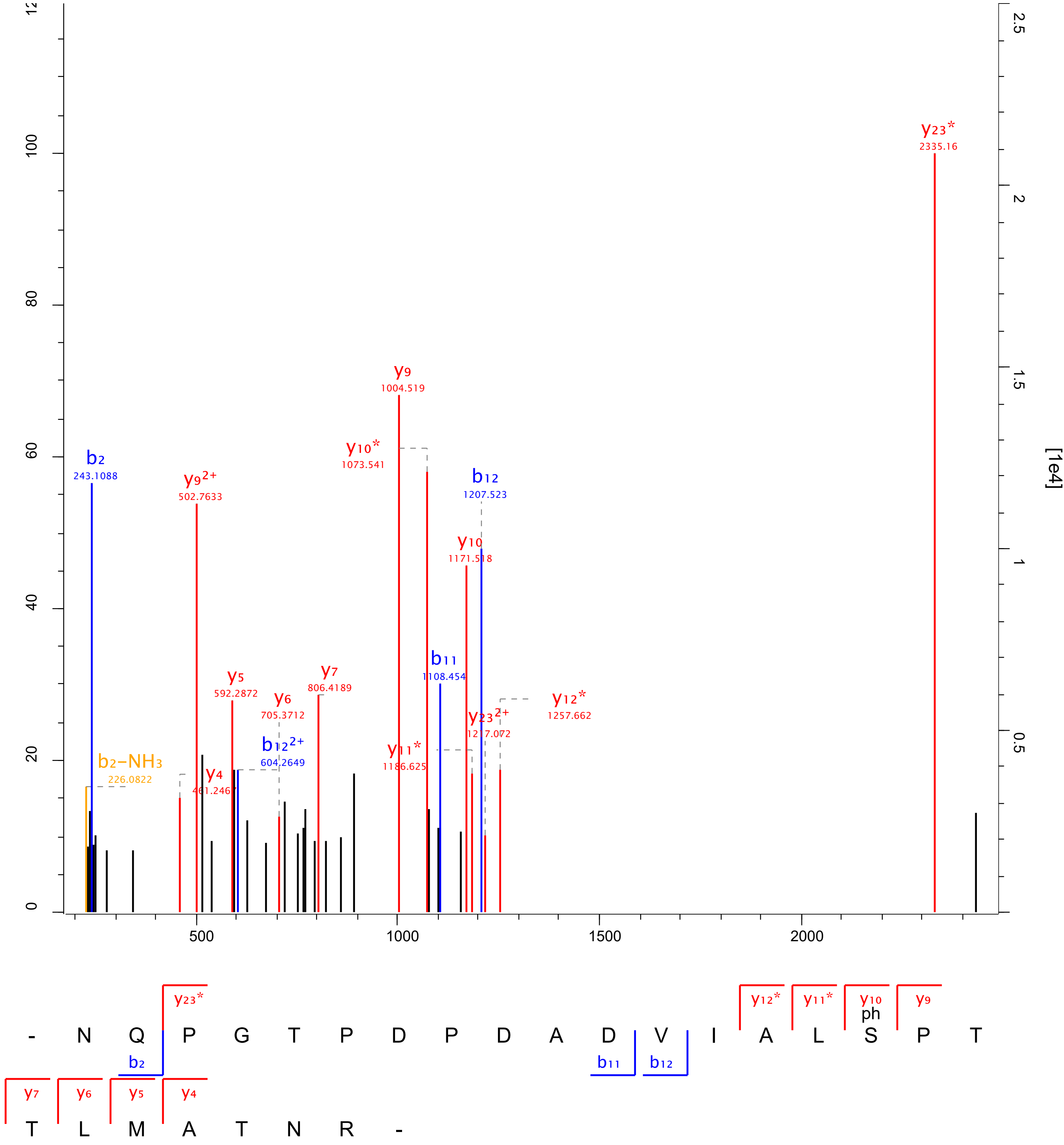

Sample output to test PDF Combine only

Raw file	Scan	Method	Score	m/z	Gene names
0523_3	27616	FTMS; HCD	57.35	1112.48	GN

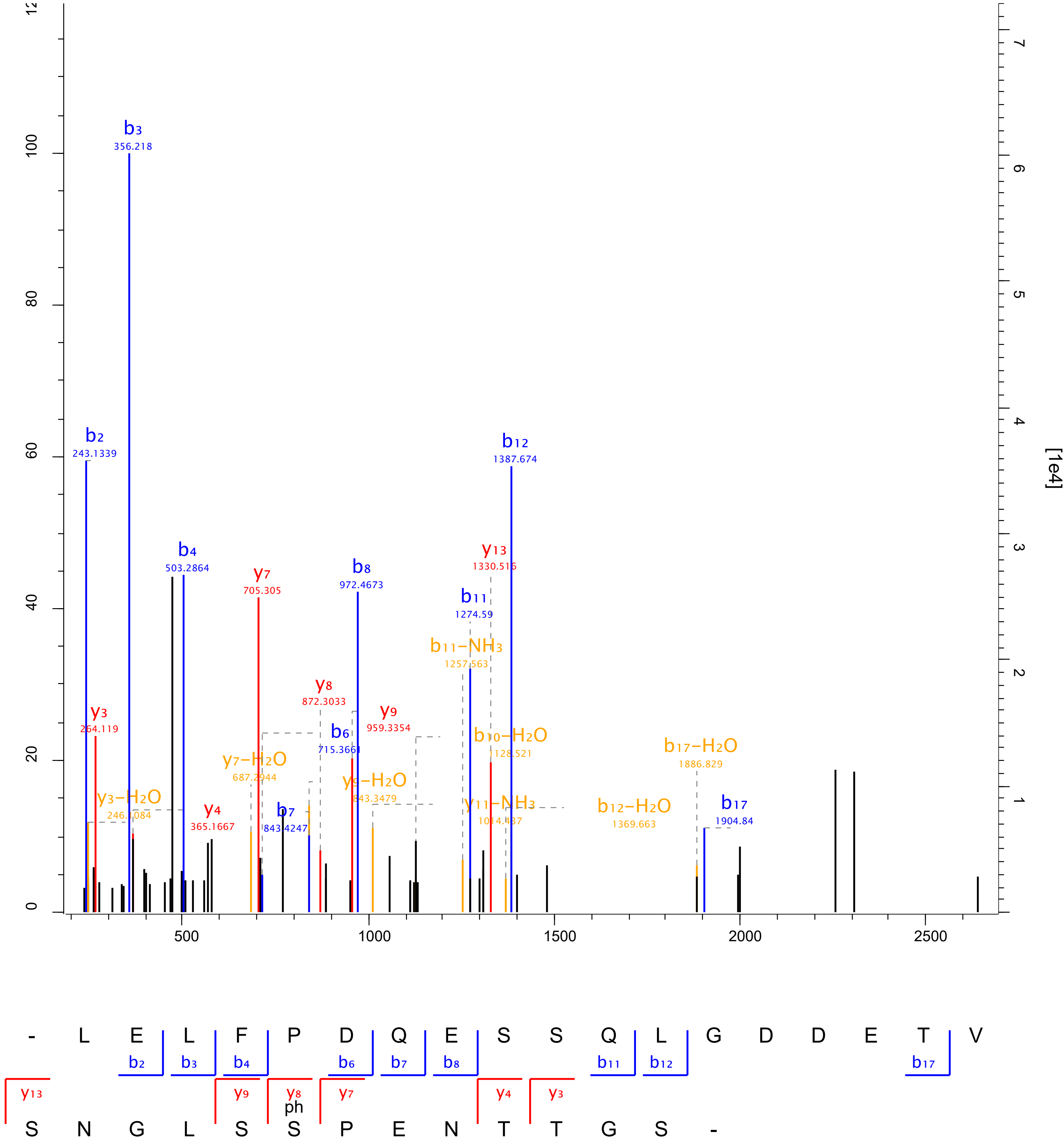

Sample output to test PDF Combine only

Raw file	Scan	Method	Score	m/z	Gene names
0523_3	32451	FTMS; HCD	46.61	971.73	RPS12A

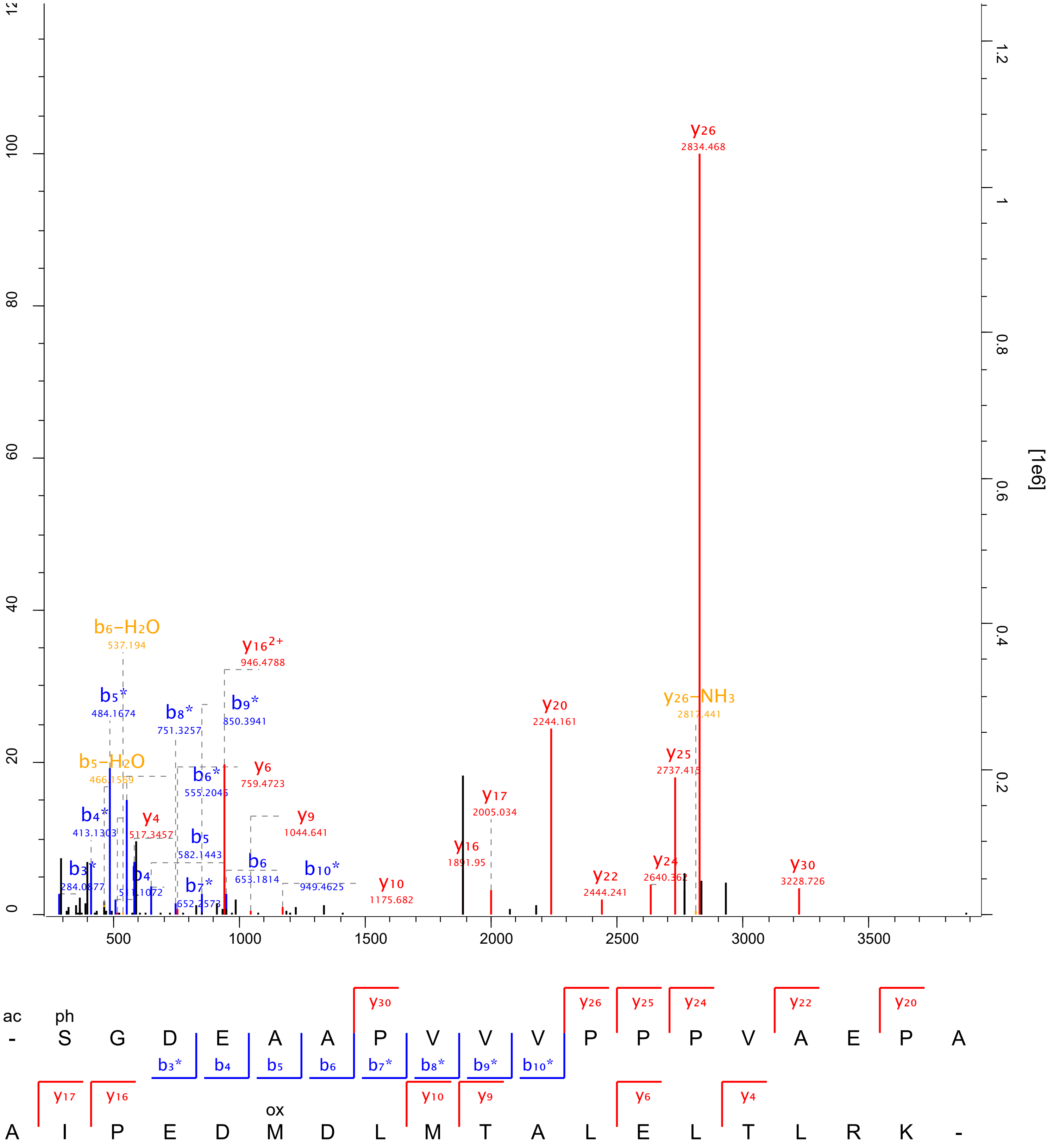

Sample output to test PDF Combine only

Raw file	Scan	Method	Score	m/z	Gene names
0523_4	1420	FTMS; HCD	73.59	454.21	RTNLB1

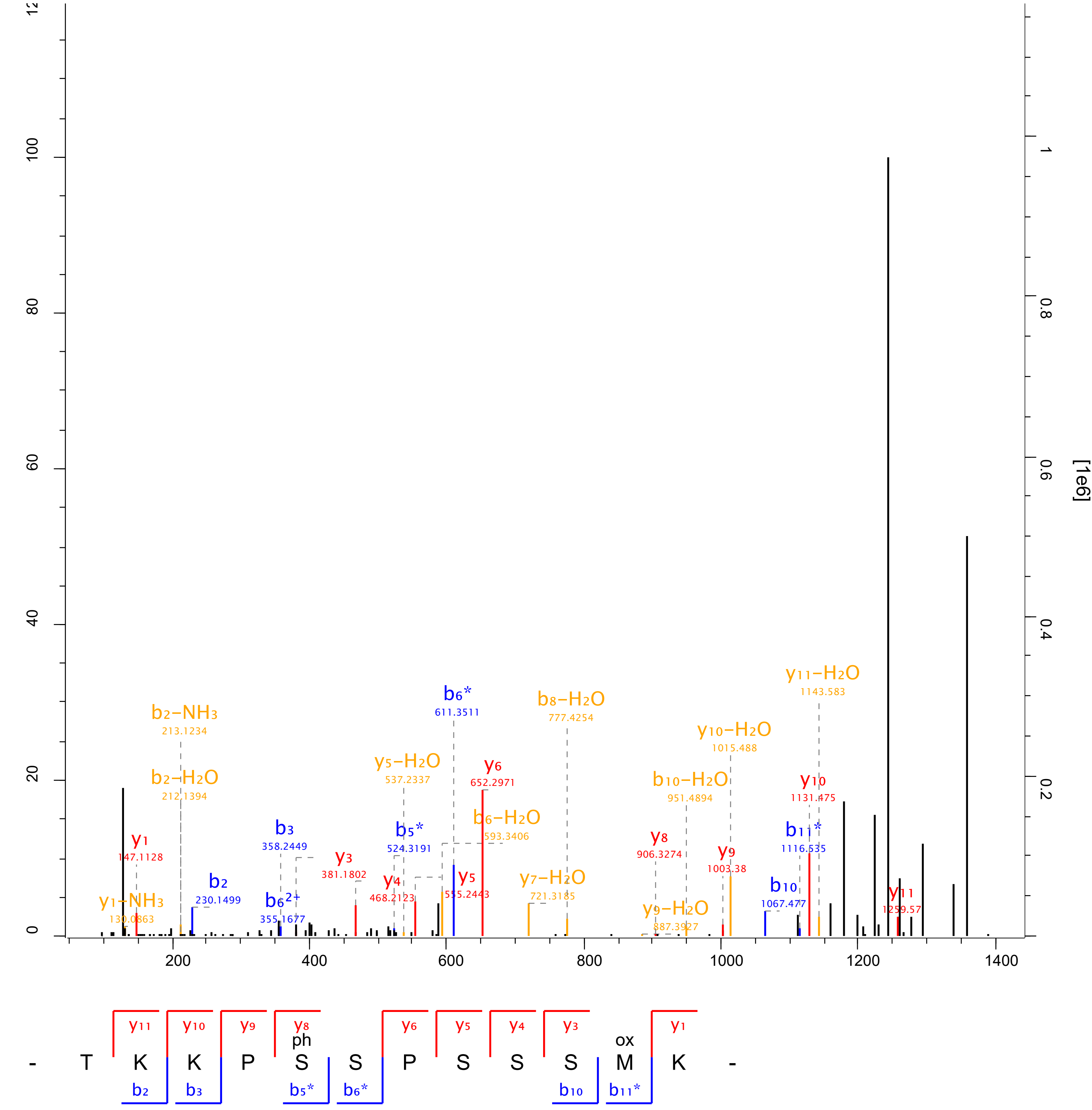

Sample output to test PDF Combine only

Raw file	Scan	Method	Score	m/z	Gene names
0523_4	1423	FTMS; HCD	69.26	593.76	PLP1

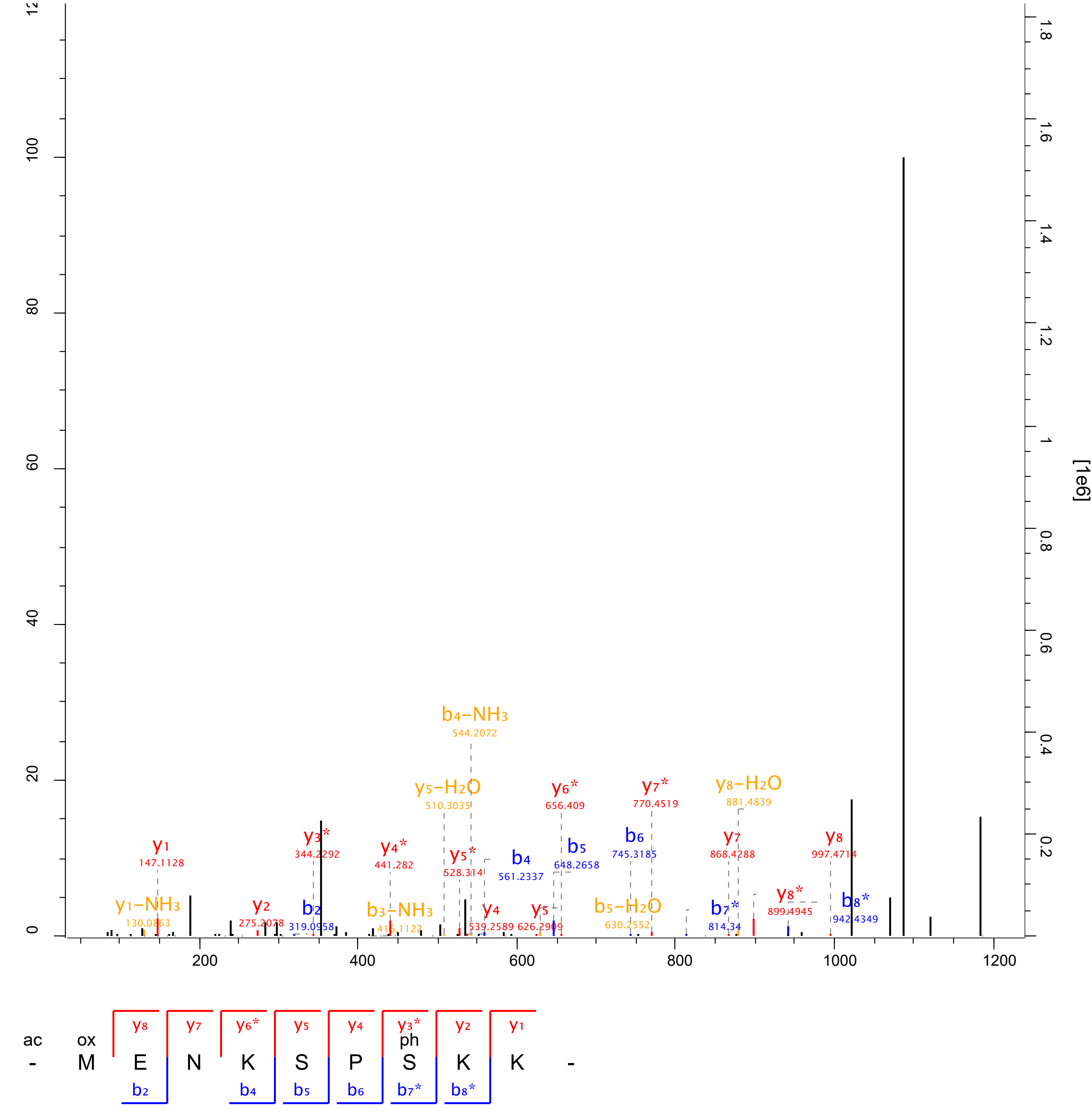

Sample output to test PDF Combine only

Raw file	Scan	Method	Score	m/z	Gene names
0523_4	1452	FTMS; HCD	88.16	460.72	SR45

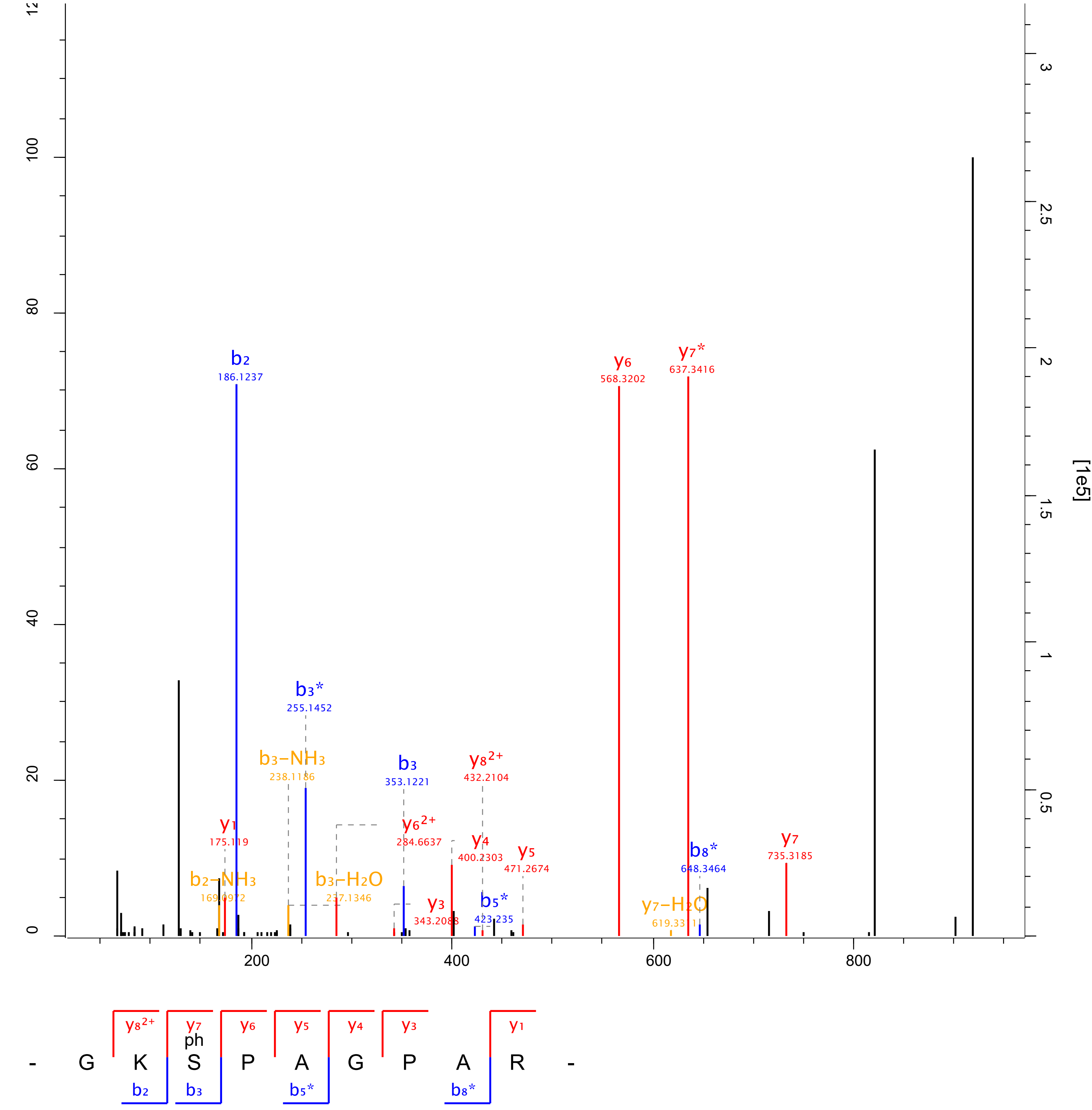

Sample output to test PDF Combine only

Raw file	Scan	Method	Score	m/z	Gene names
0523_4	1464	FTMS; HCD	58.17	385.83	U2AF35B

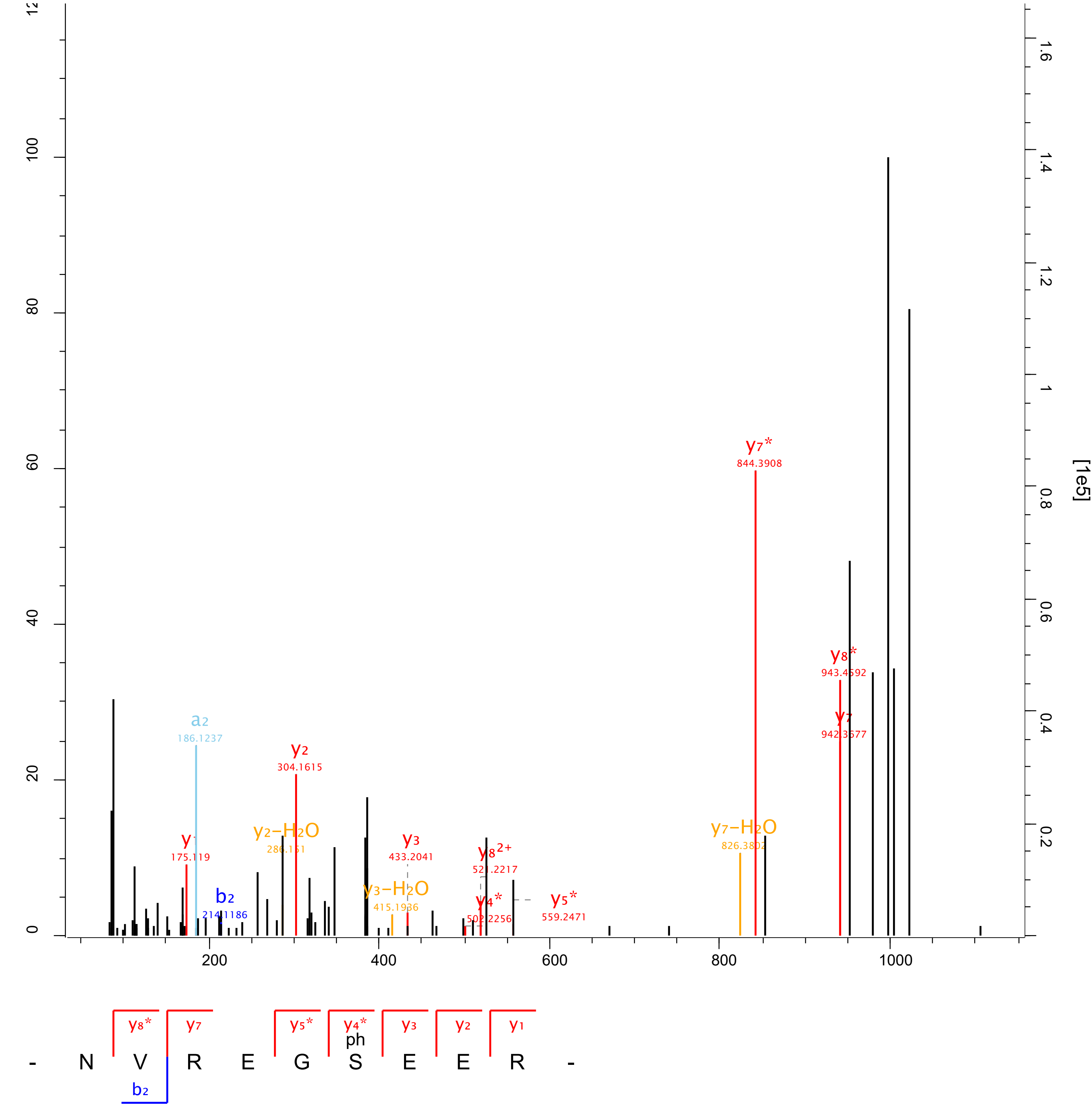

Sample output to test PDF Combine only

Raw file	Scan	Method	Score	m/z	Gene names
0523_4	1701	FTMS; HCD	68.13	502.69	ABCC14

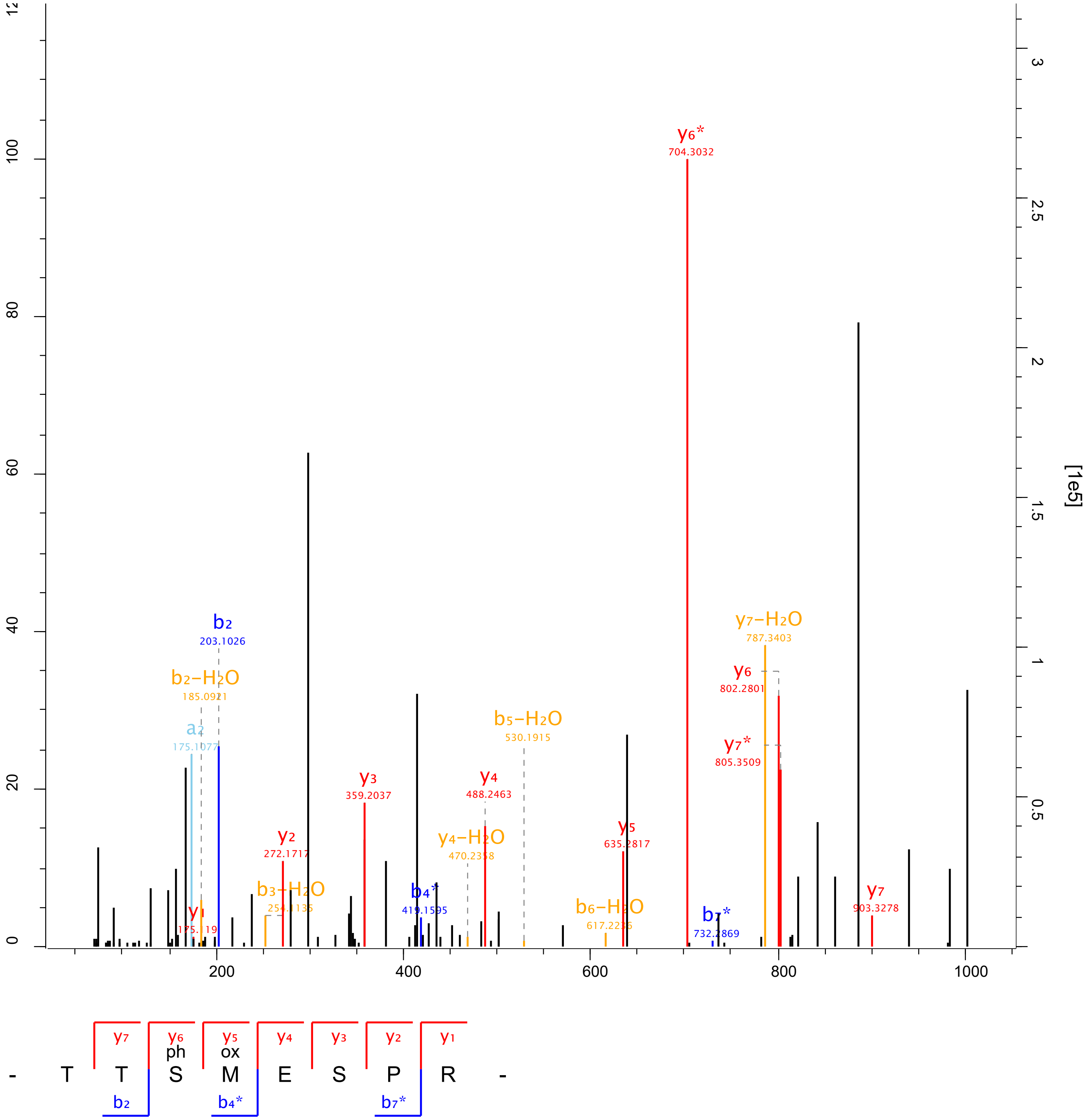

Sample output to test PDF Combine only

Raw file	Scan	Method	Score	m/z	Gene names
0523_4	2911	FTMS; HCD	127.57	707.3	At4g31880

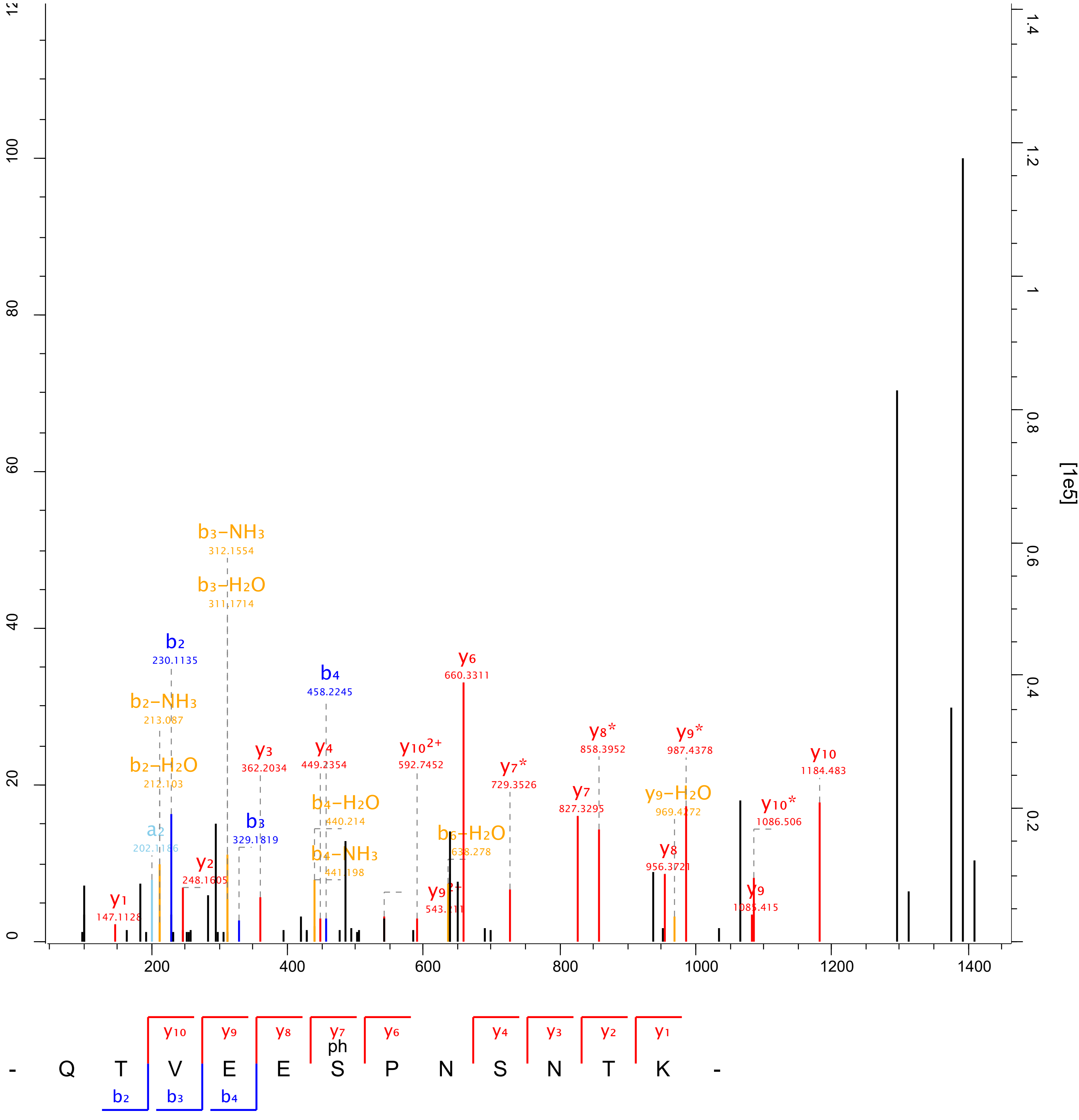

Sample output to test PDF Combine only

Raw file	Scan	Method	Score	m/z	Gene names
0523_4	2994	FTMS; HCD	66.27	495.72	At2g42310

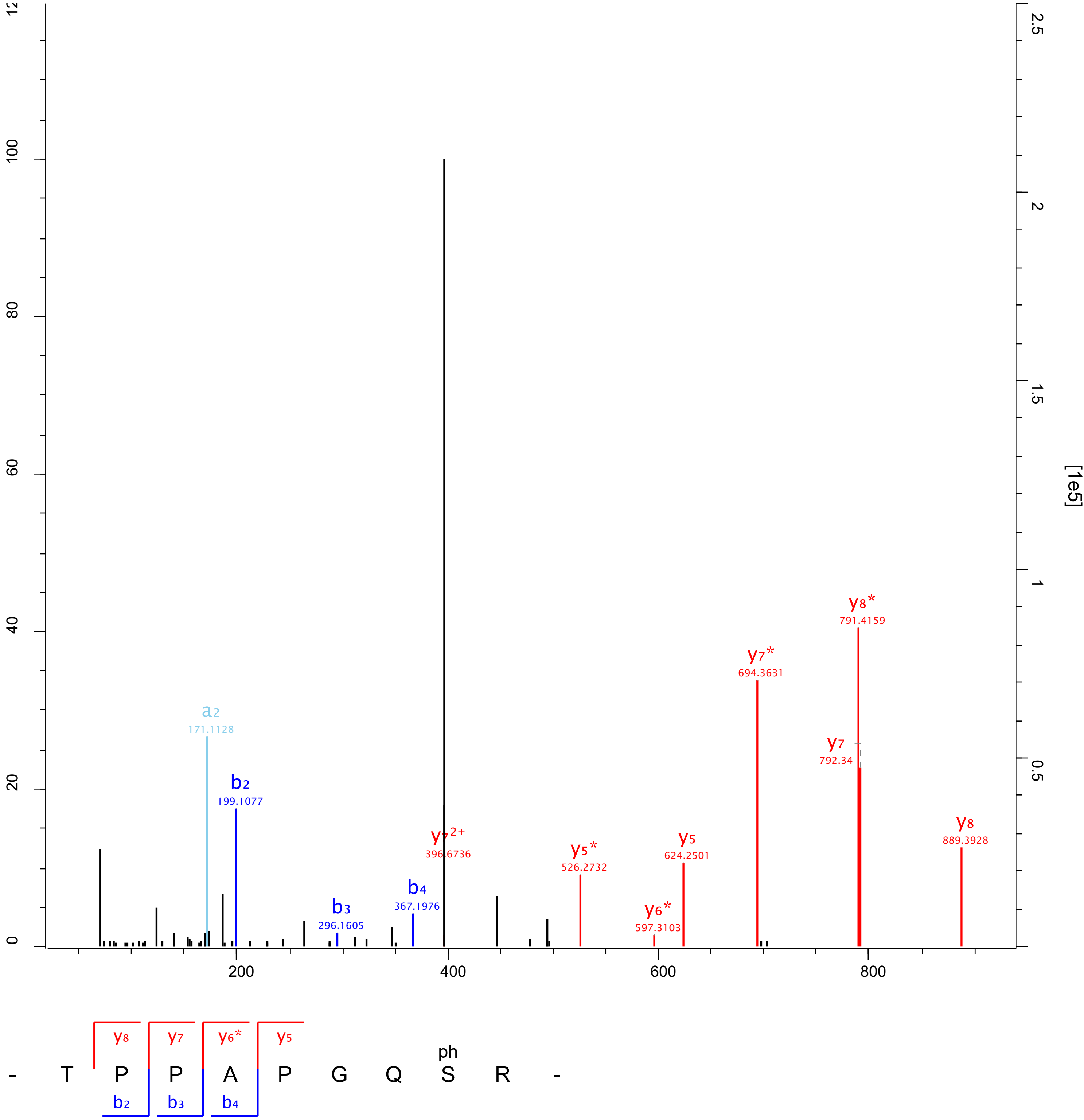

Sample output to test PDF Combine only

Raw file	Scan	Method	Score	m/z	Gene names
0523_4	3088	FTMS; HCD	171.11	617.26	F26G5_140

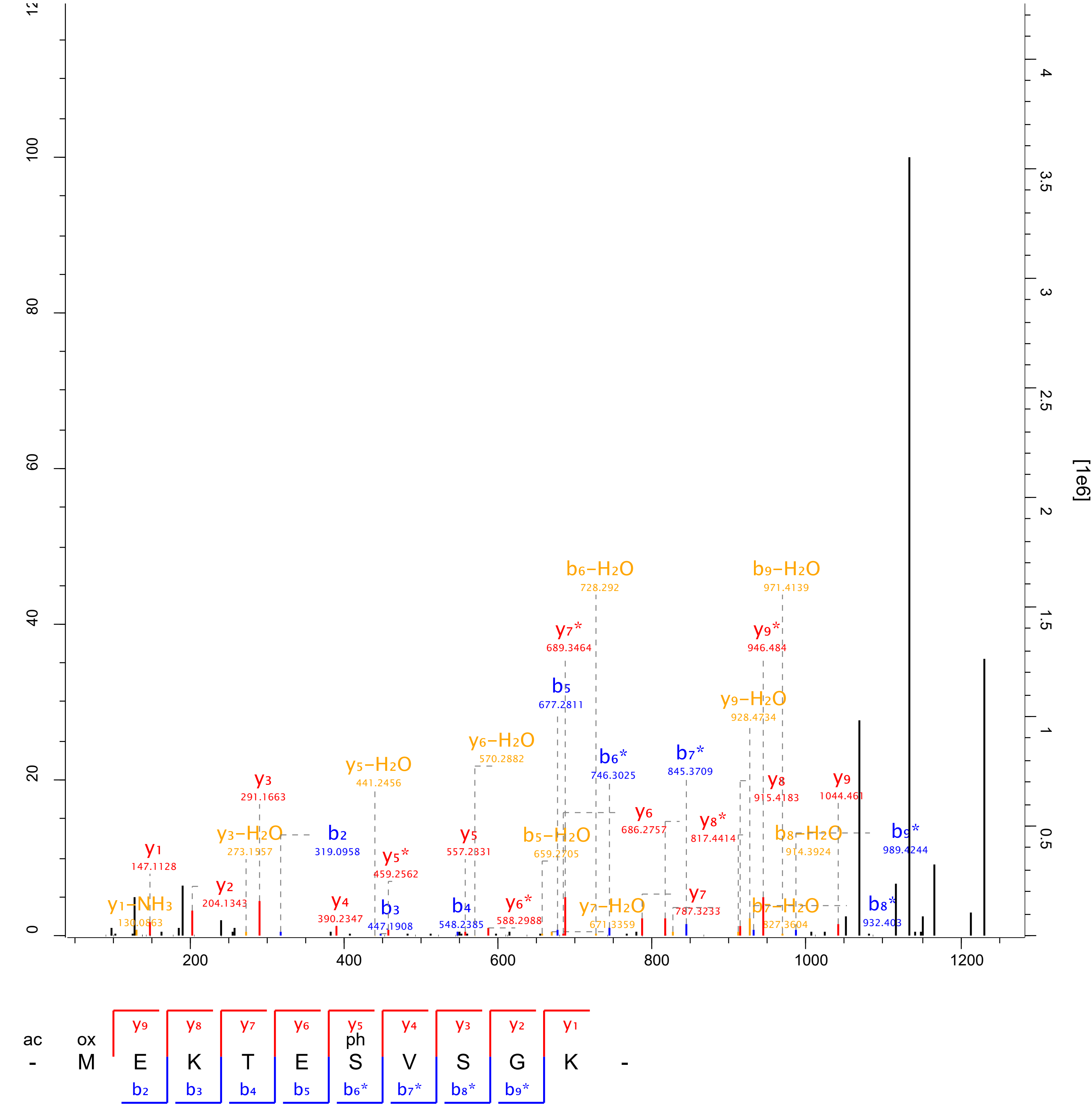

Sample output to test PDF Combine only

Raw file	Scan	Method	Score	m/z	Gene names
0523_4	3098	FTMS; HCD	192.62	784.3	At1g67325

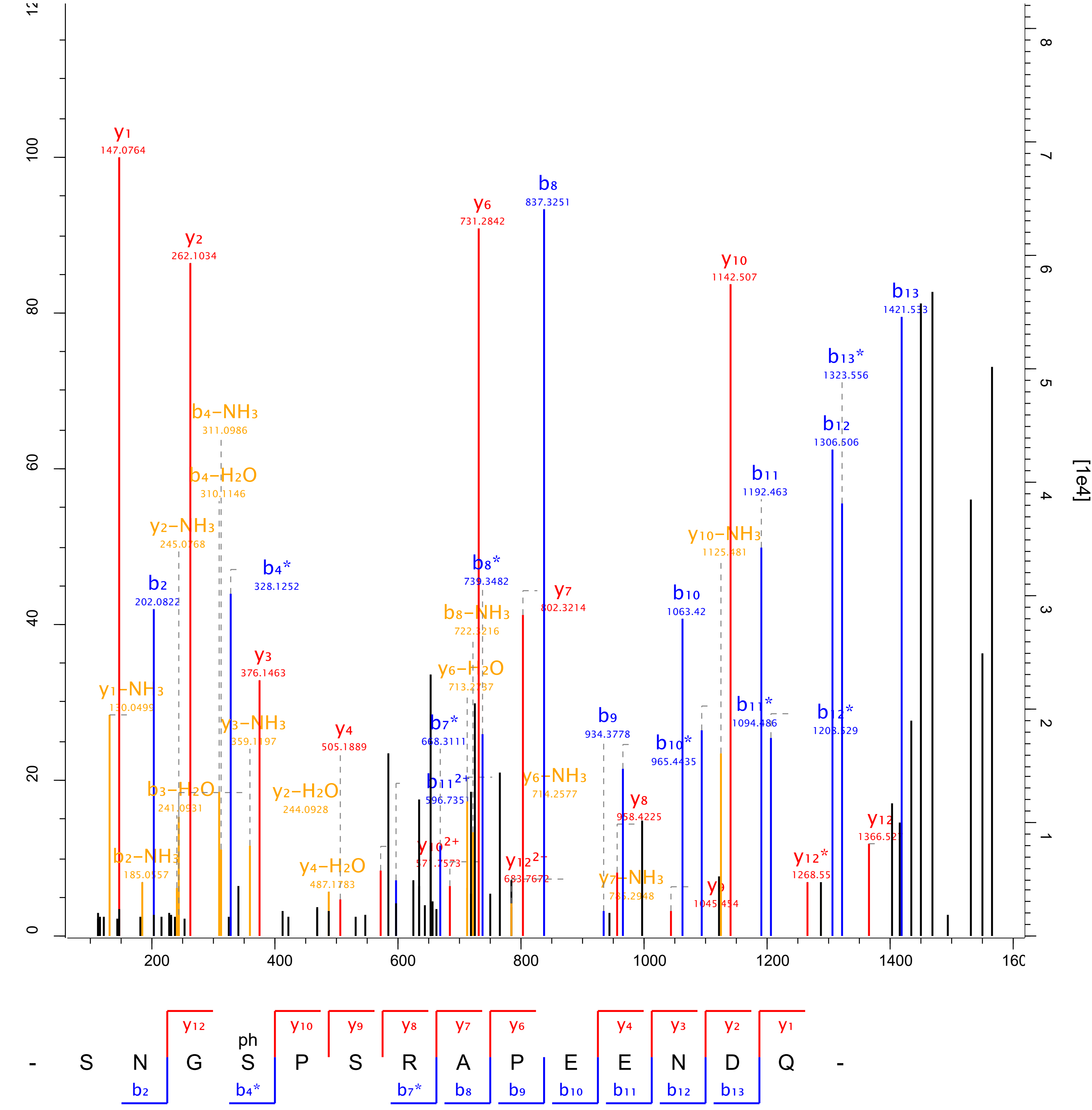

Sample output to test PDF Combine only

Raw file	Scan	Method	Score	m/z	Gene names
0523_4	3154	FTMS; HCD	60.27	602.55	RS2Z33

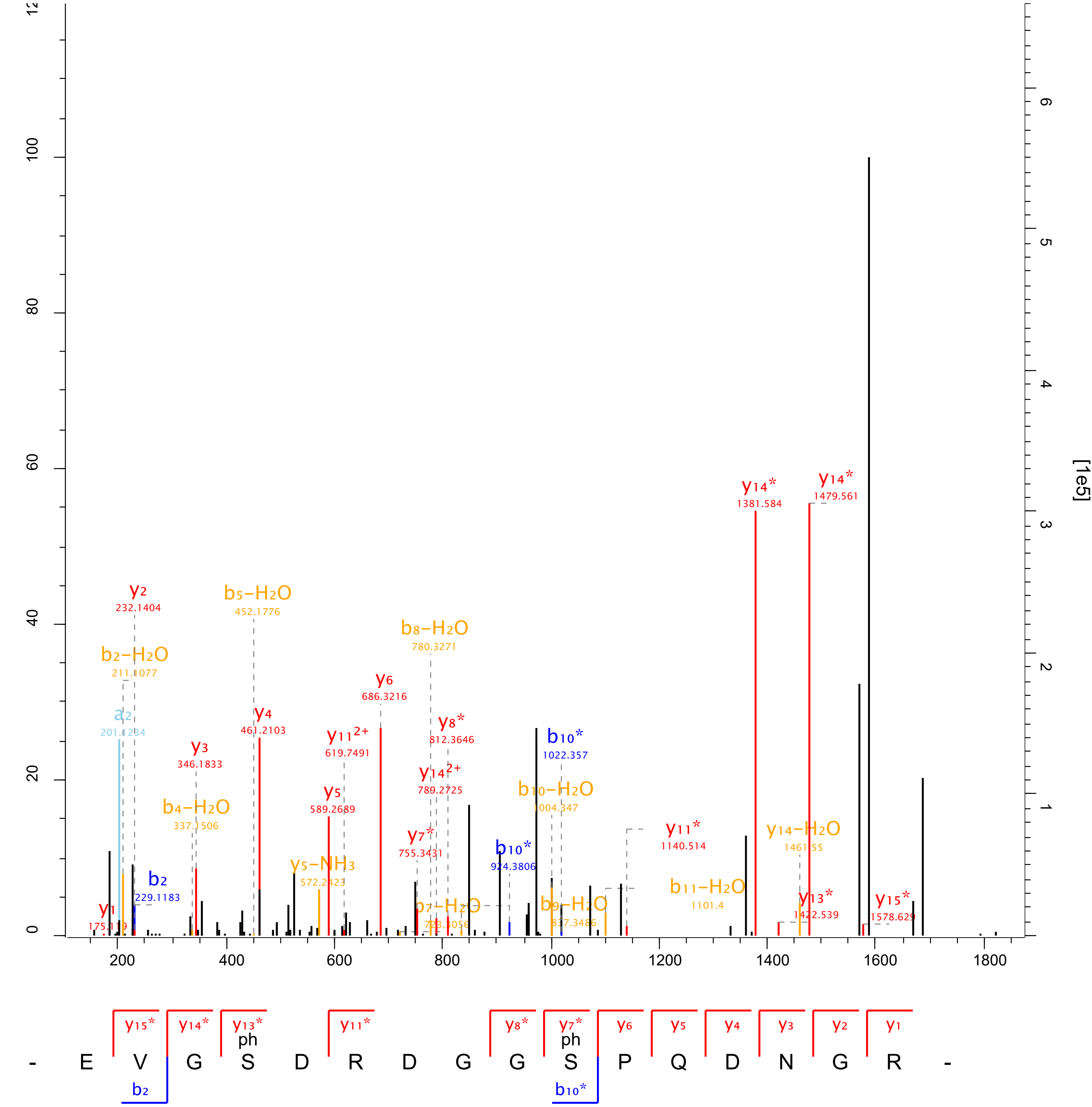

Sample output to test PDF Combine only

Raw file	Scan	Method	Score	m/z	Gene names
0523_4	3193	FTMS; HCD	143.46	533.23	SPK1

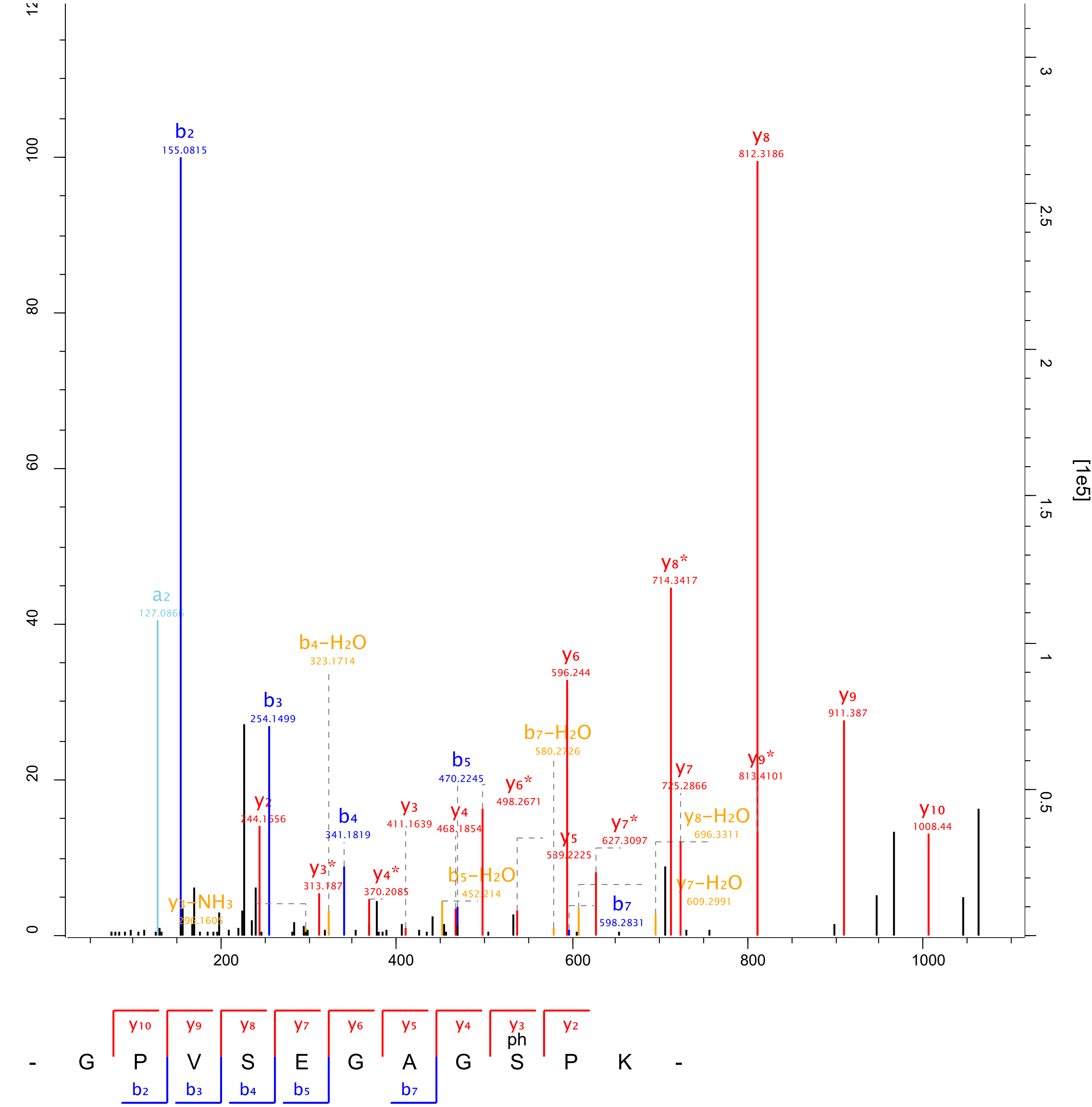

Sample output to test PDF Combine only

Raw file	Scan	Method	Score	m/z	Gene names
0523_4	3252	FTMS; HCD	59.73	679.76	RS40

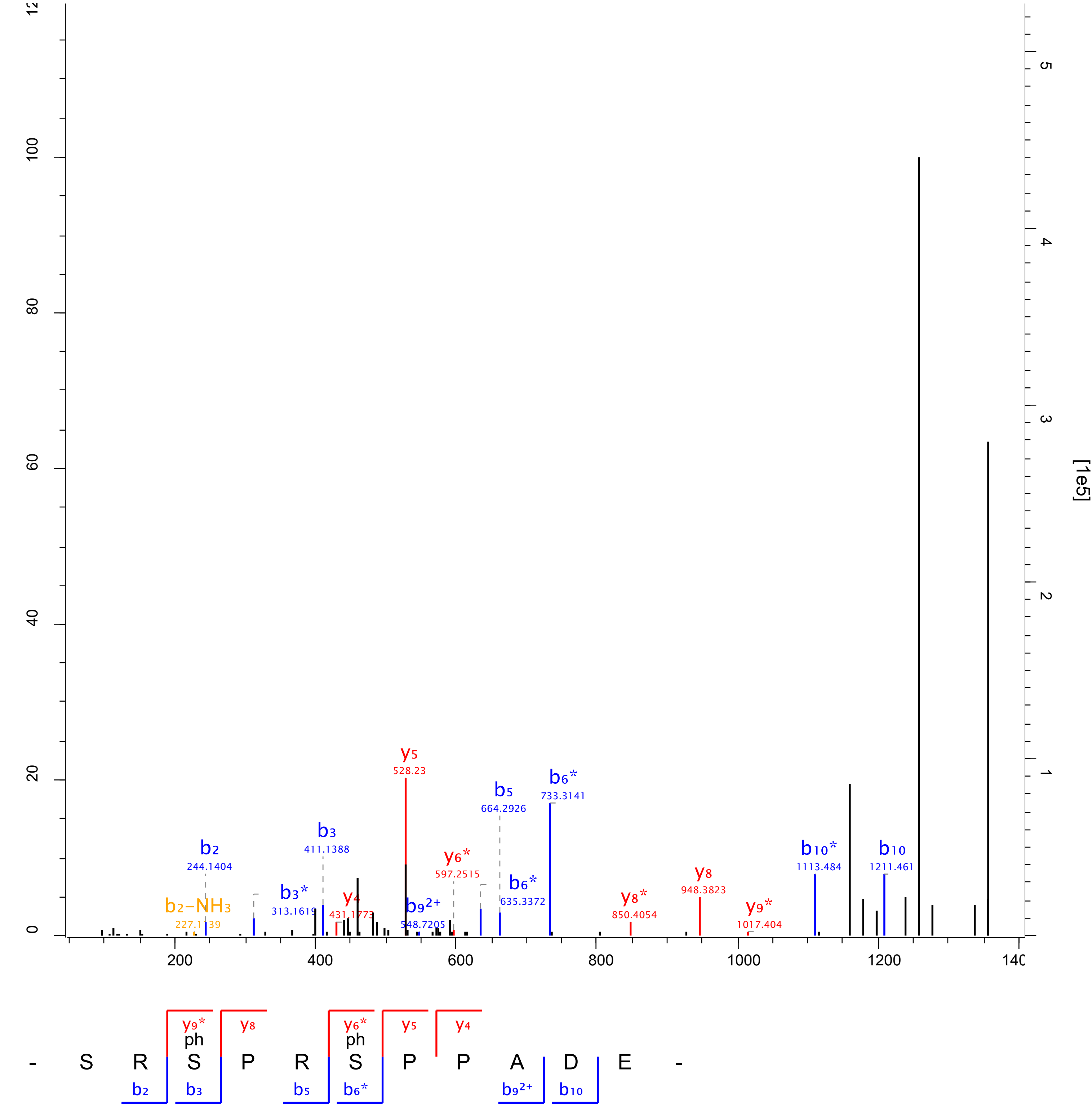

Sample output to test PDF Combine only

Raw file	Scan	Method	Score	m/z	Gene names
0523_4	3316	FTMS; HCD	126.71	614.27	IQD14

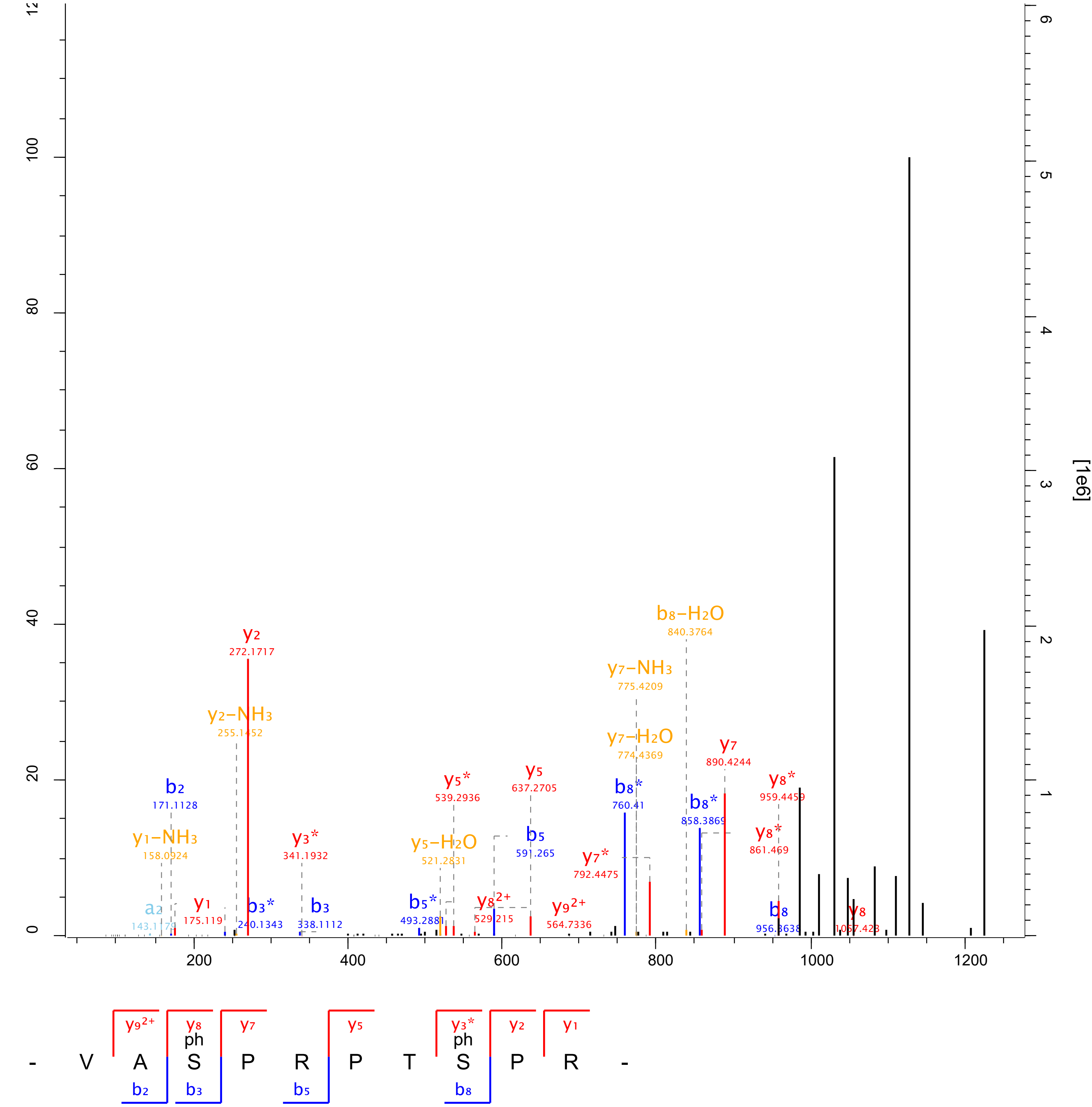

Sample output to test PDF Combine only

Raw file	Scan	Method	Score	m/z
0523_4	3435	FTMS; HCD	83.37	707.31

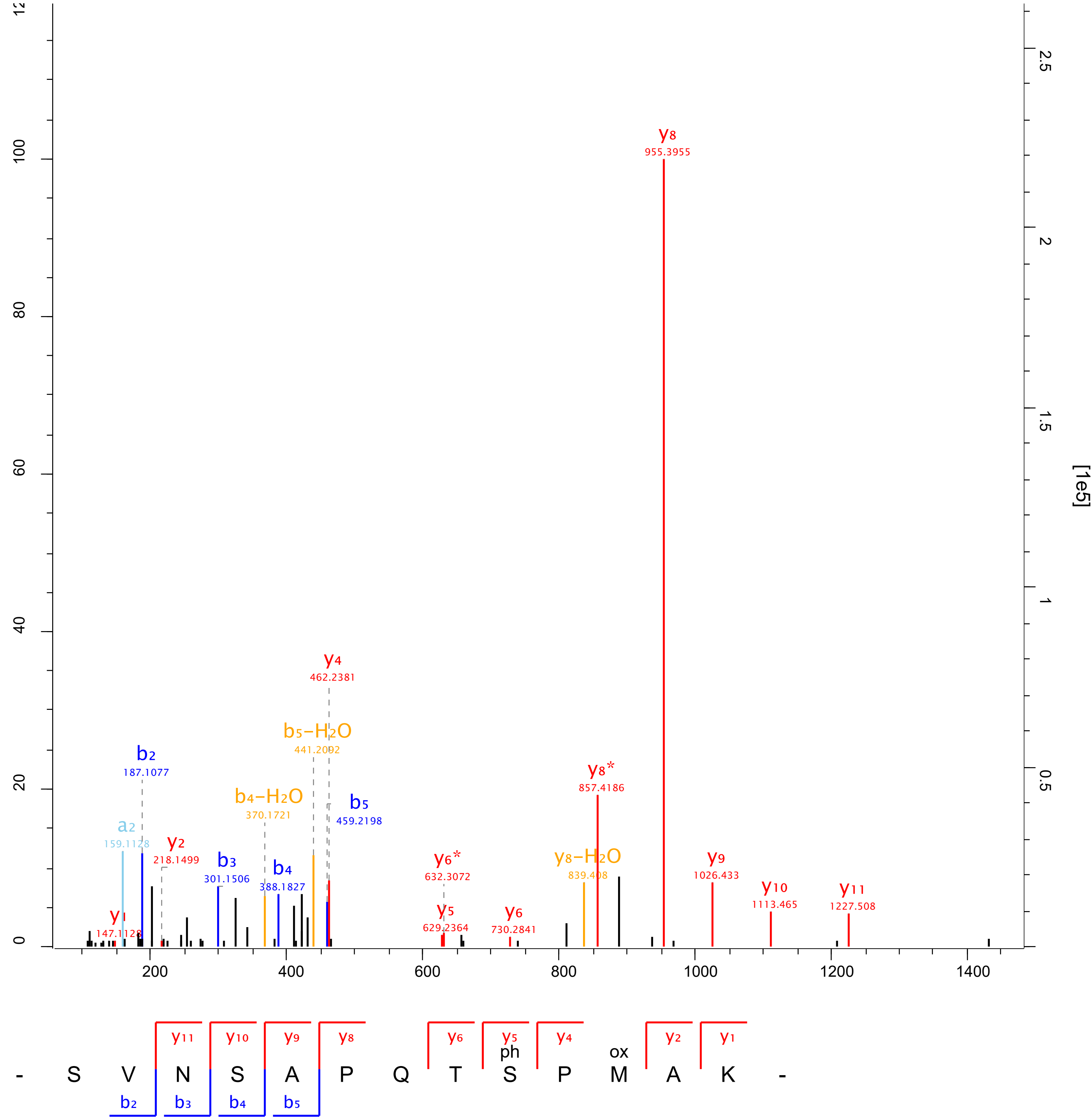

Sample output to test PDF Combine only

Raw file	Scan	Method	Score	m/z	Gene names
0523_4	3537	FTMS; HCD	142.08	500.71	ABCG36

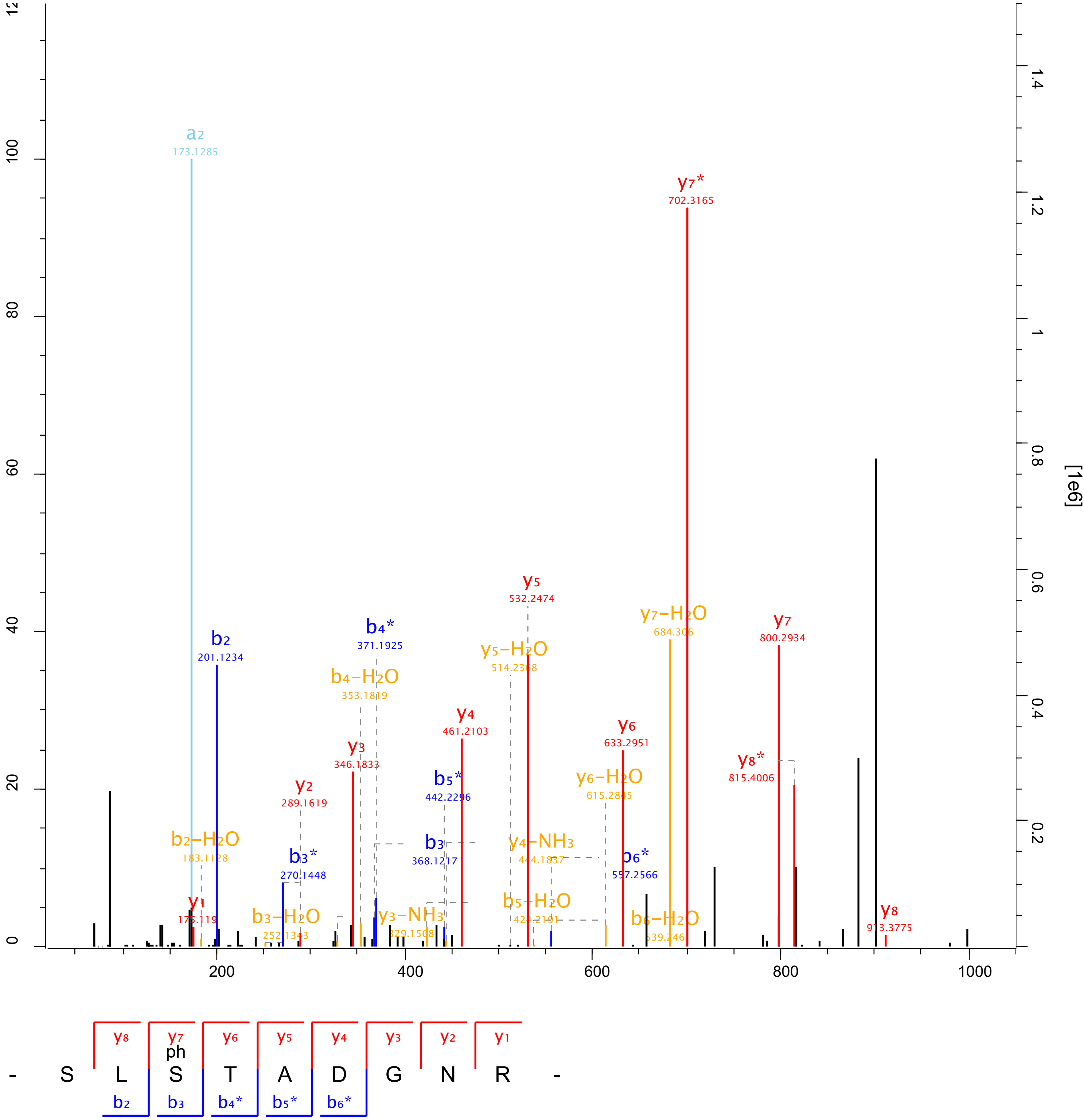

Sample output to test PDF Combine only

Raw file	Scan	Method	Score	m/z	Gene names
0523_4	3665	FTMS; HCD	43.24	684.95	At2g05940

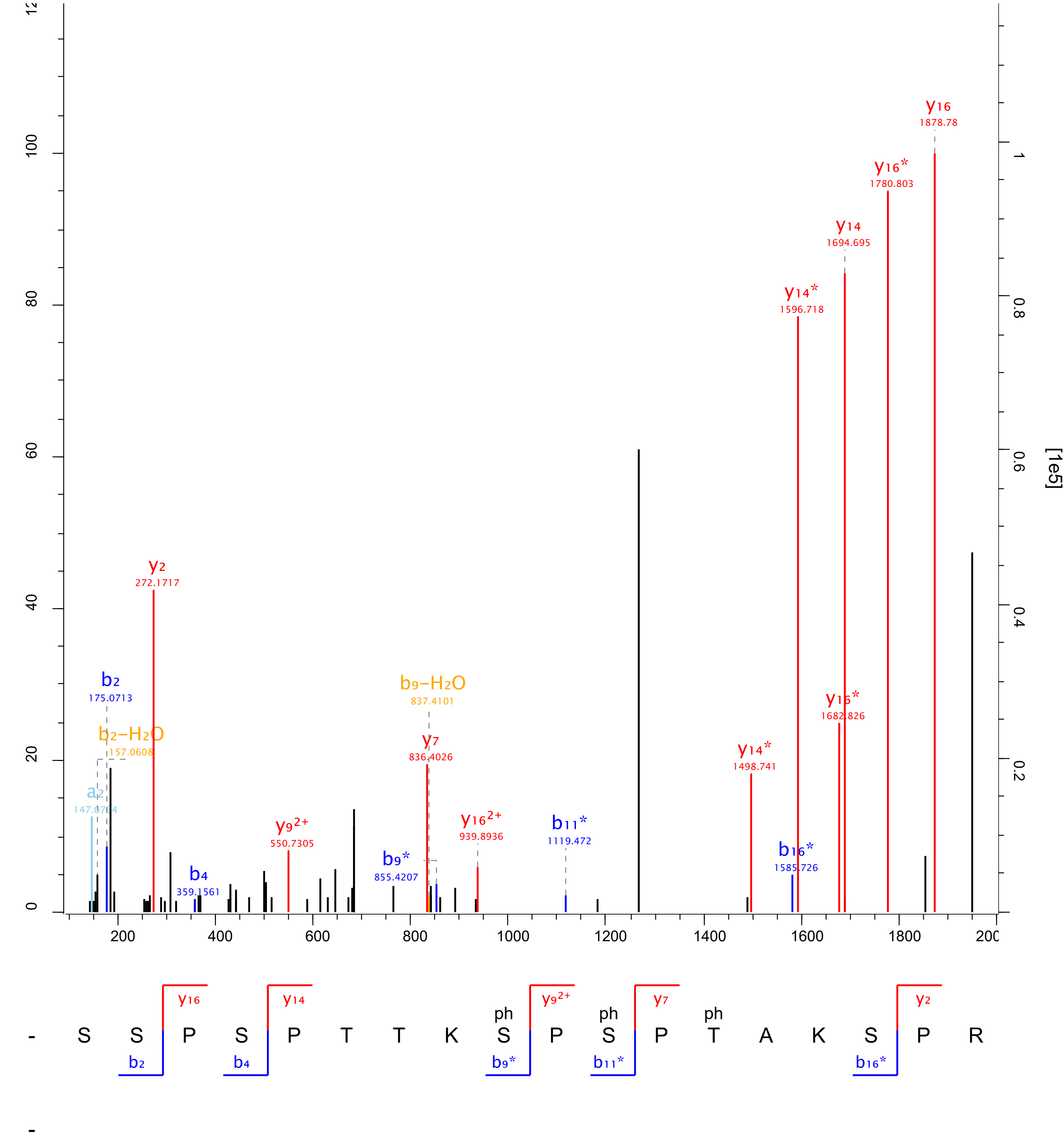

Sample output to test PDF Combine only

Raw file	Scan	Method	Score	m/z
0523_4	3855	FTMS; HCD	100.45	663.27

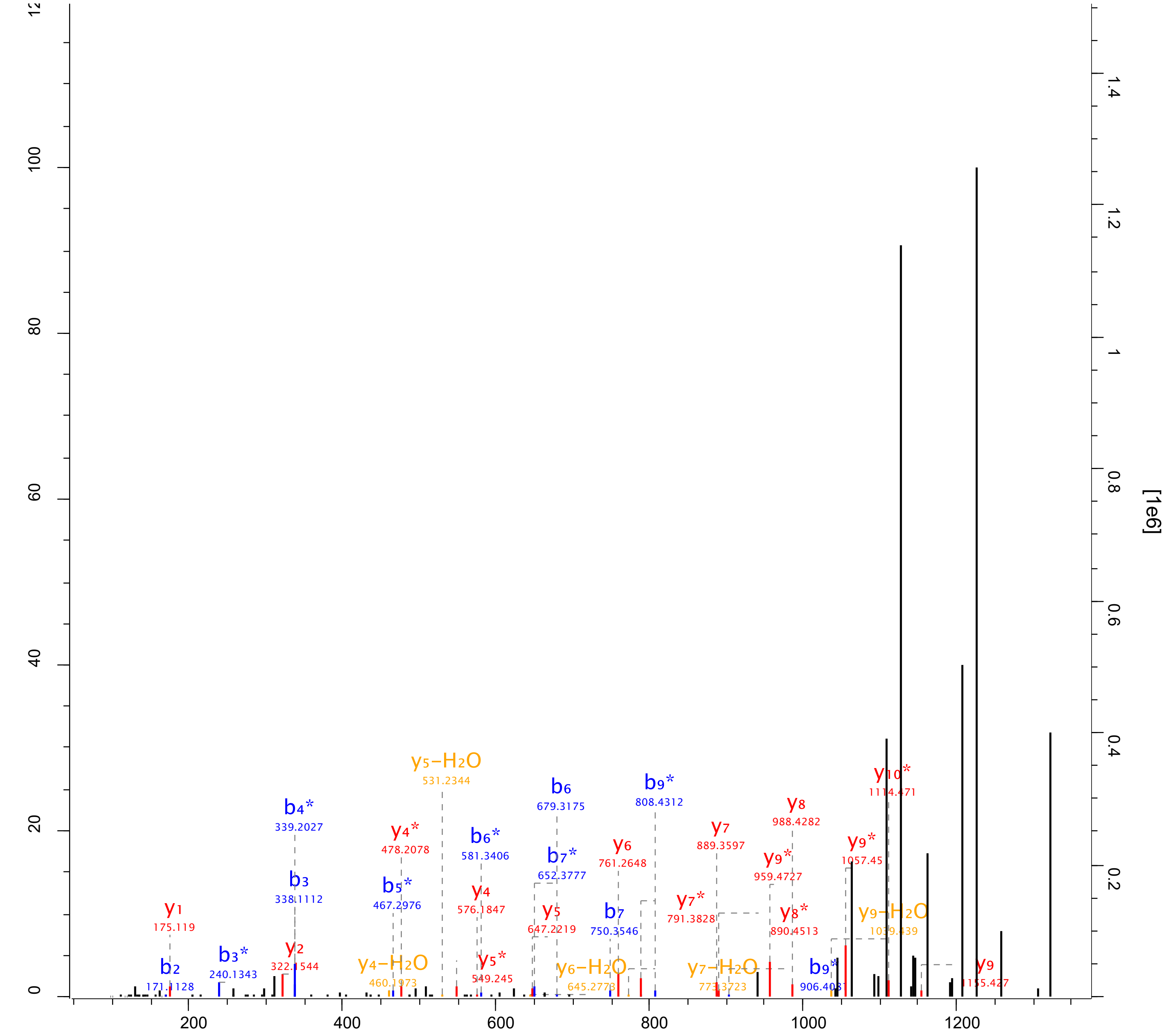

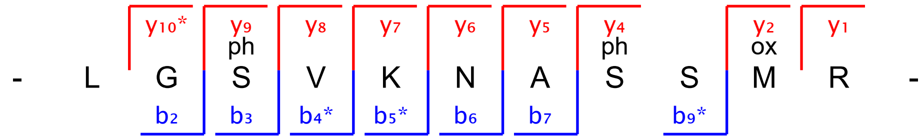

Sample output to test PDF Combine only

Raw file	Scan	Method	Score	m/z	Gene names
0523_4	3877	FTMS; HCD	96.6	506.21	At1g21630

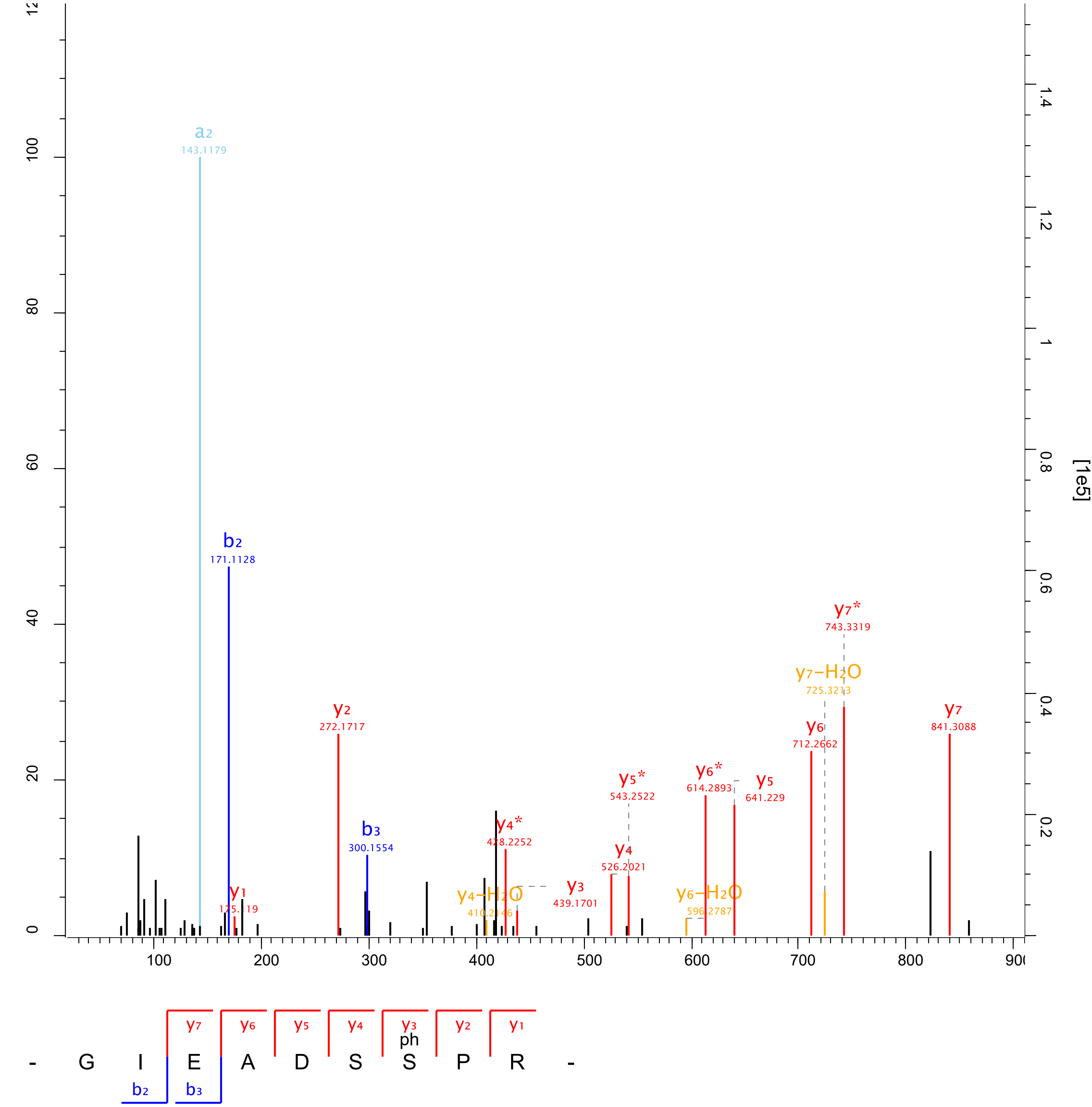

Sample output to test PDF Combine only

Raw file	Scan	Method	Score	m/z
0523_4	3986	FTMS; HCD	96.34	493.7

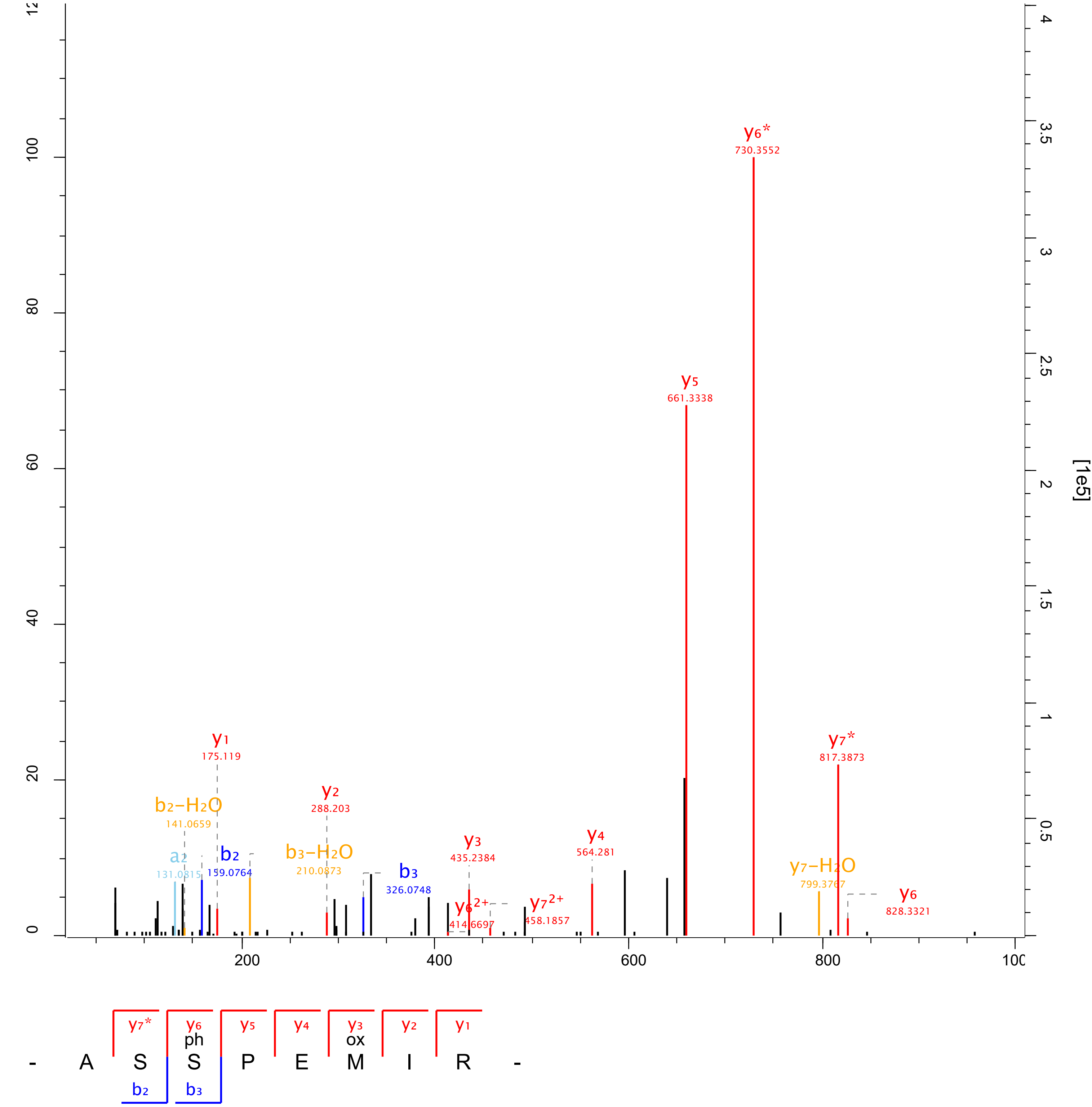

Sample output to test PDF Combine only

Raw file	Scan	Method	Score	m/z	Gene names
0523_4	3990	FTMS; HCD	100.11	654.78	At2g27060

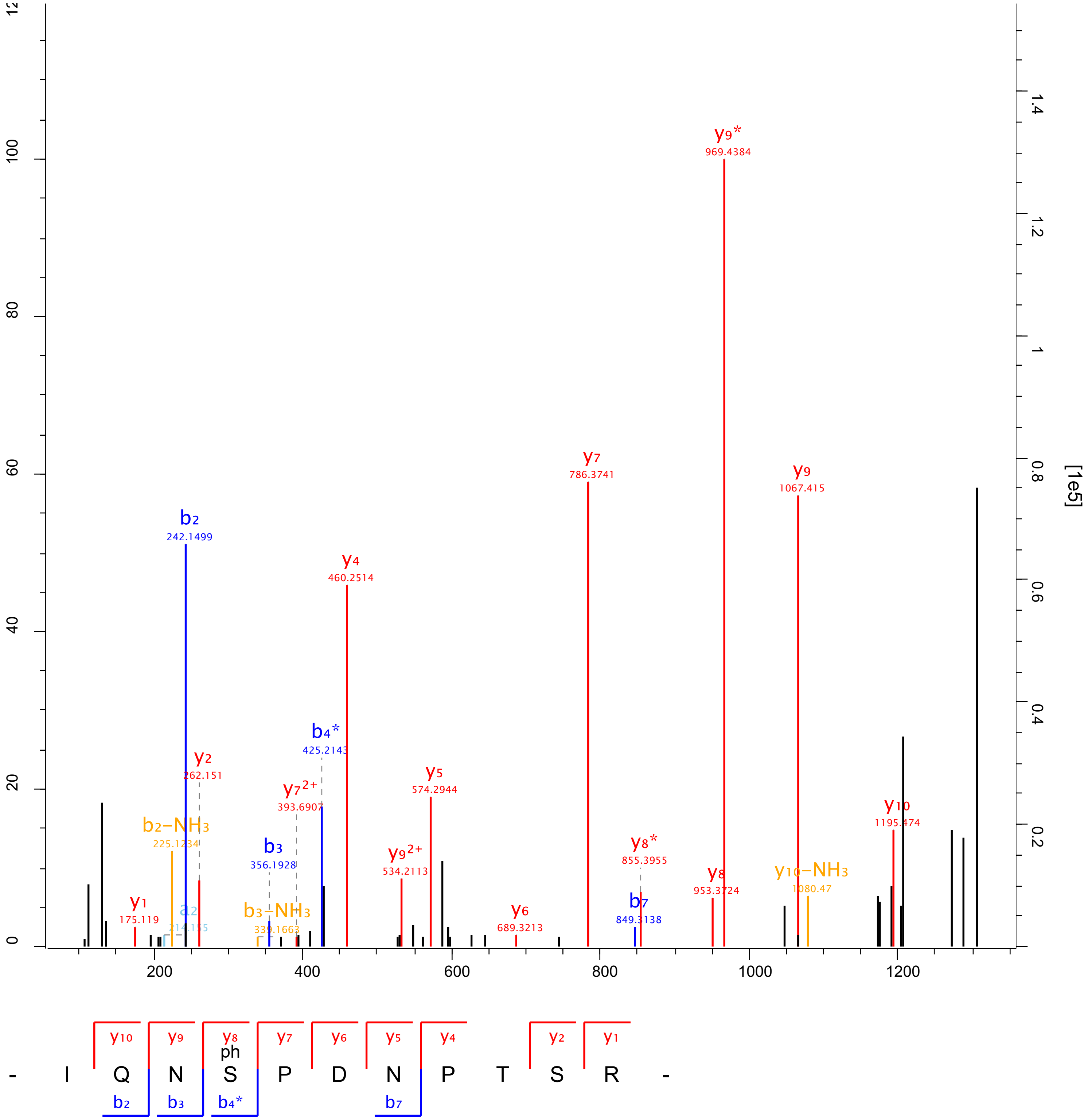

Sample output to test PDF Combine only

Raw file	Scan	Method	Score	m/z	Gene names
0523_4	3996	FTMS; HCD	187.78	702.29	DGK3

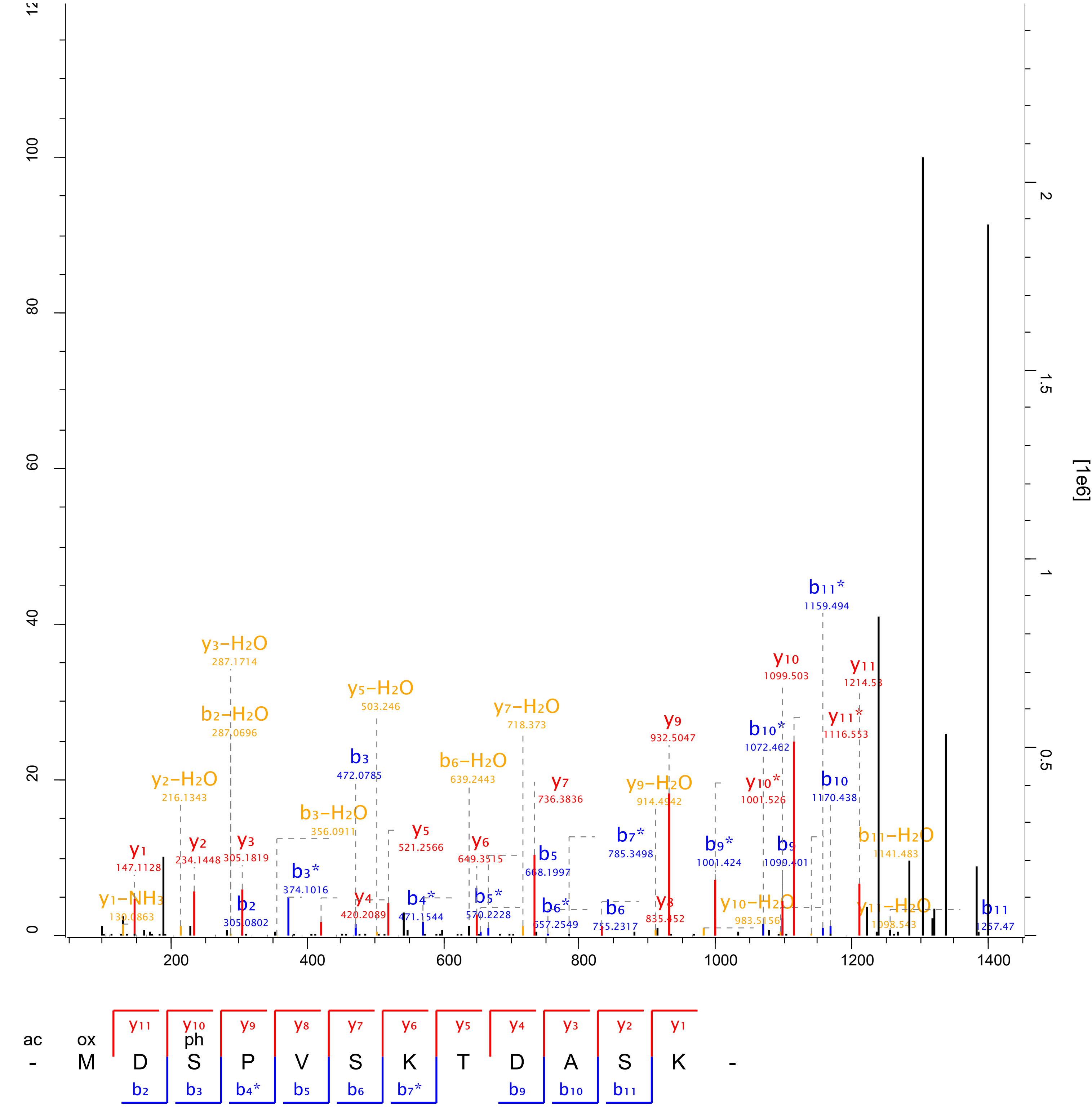

Sample output to test PDF Combine only

Raw file	Scan	Method	Score	m/z	Gene names
0523_4	3999	FTMS; HCD	63.18	557.75	VLN3

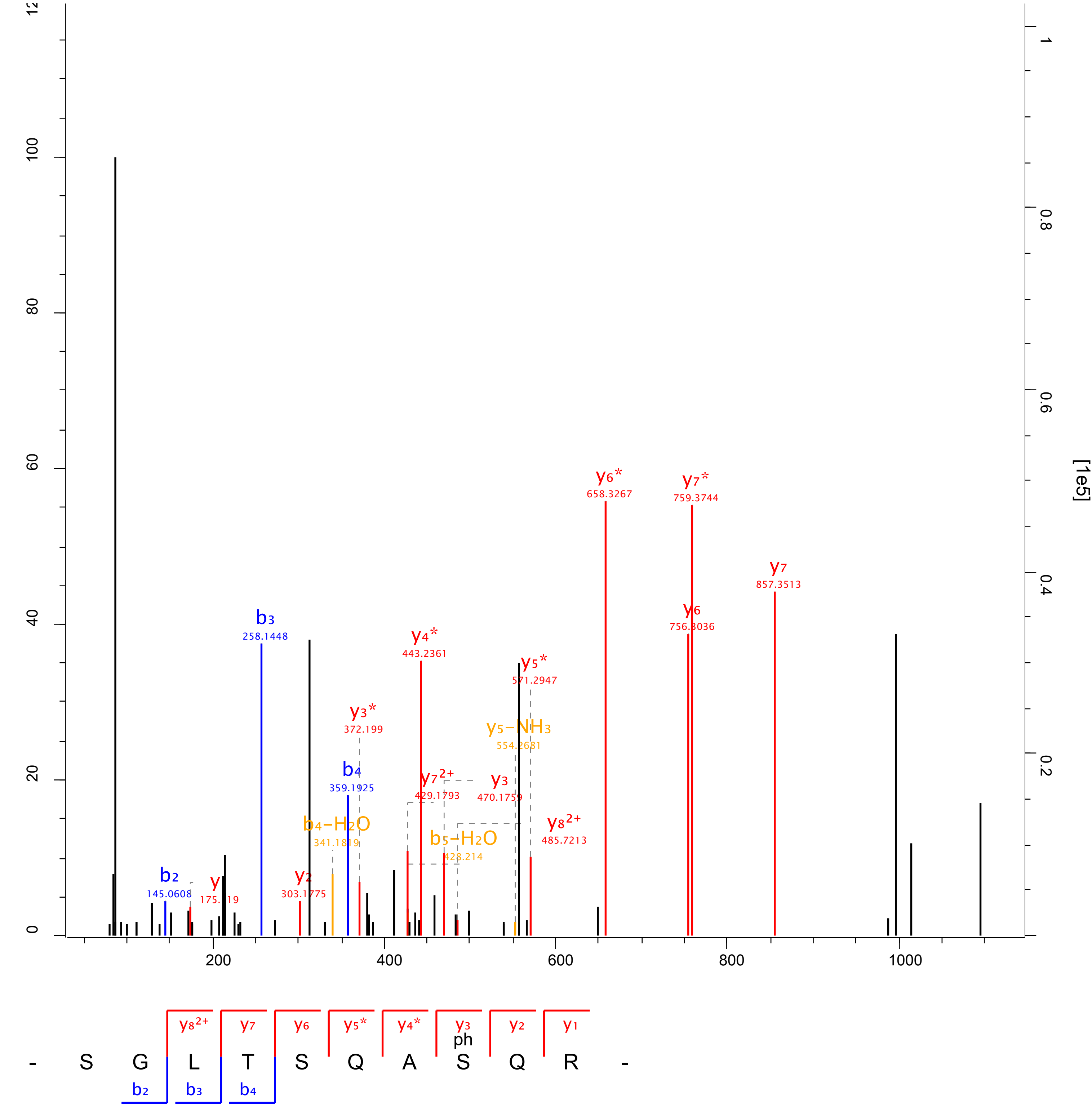

Sample output to test PDF Combine only

Raw file	Scan	Method	Score	m/z	Gene names
0523_4	4009	FTMS; HCD	265.37	716.32	KCA2

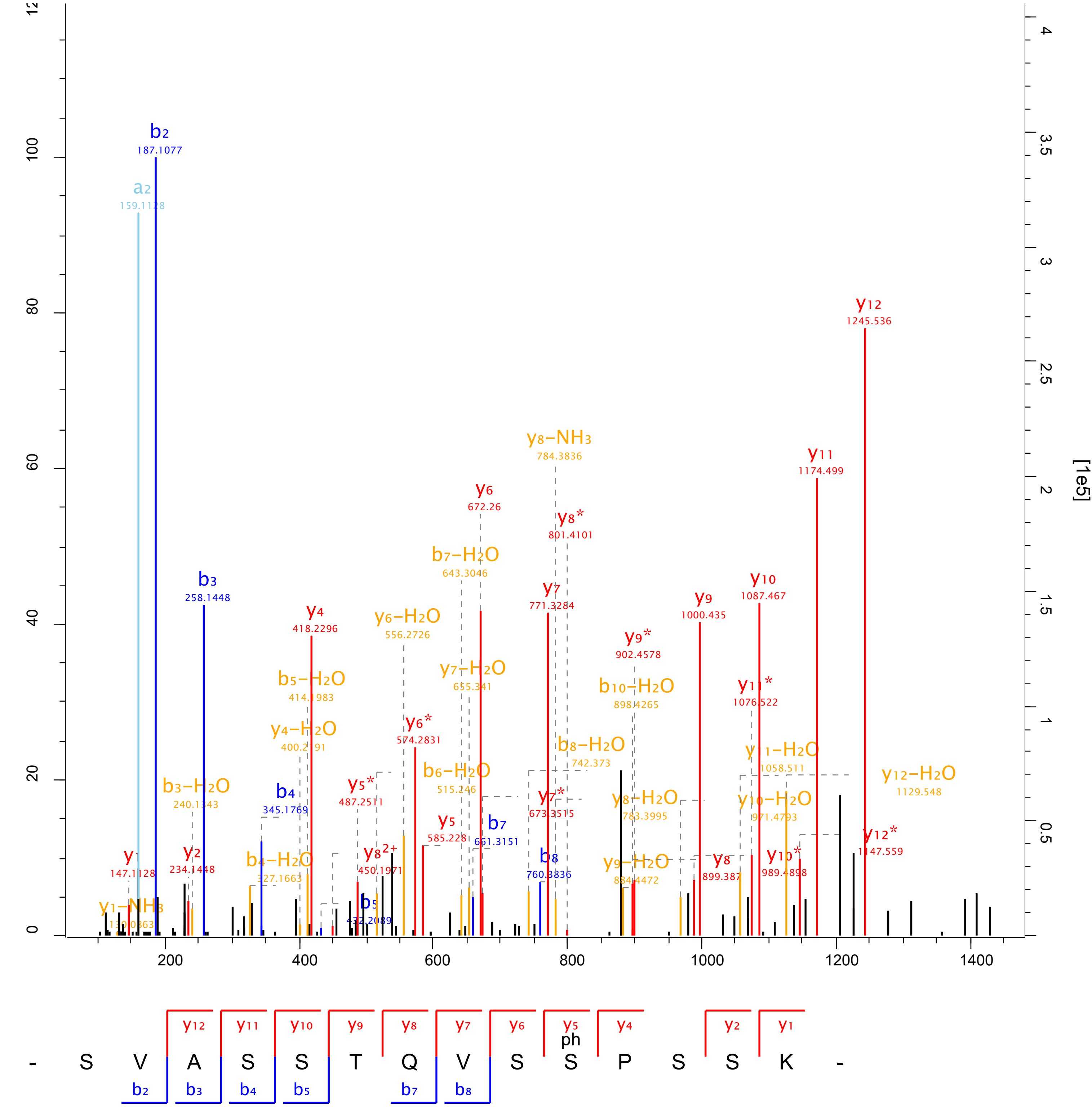

Sample output to test PDF Combine only

Raw file	Scan	Method	Score	m/z	Gene names
0523_4	4063	FTMS; HCD	101.89	506.73	At2g07360

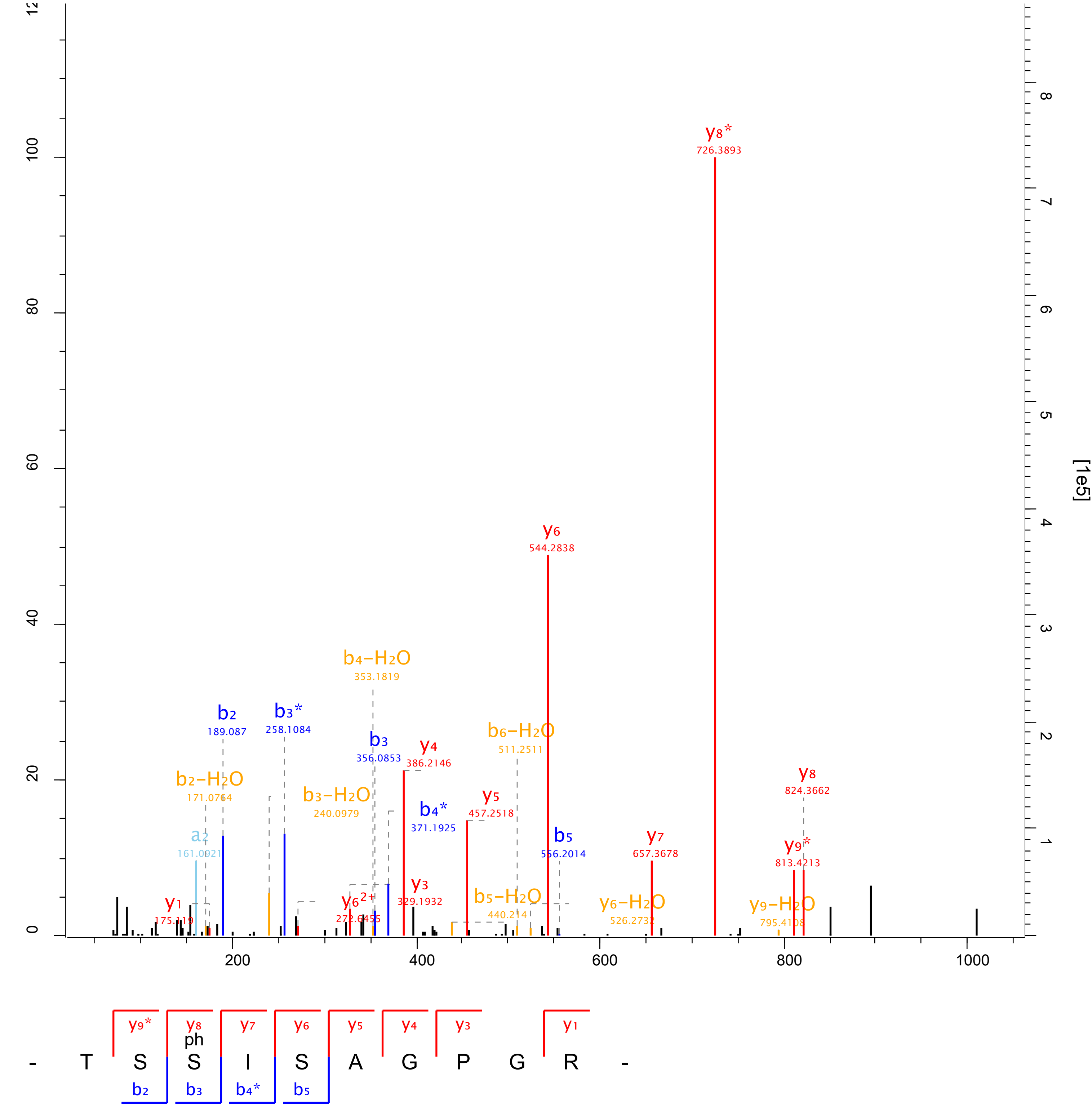

Sample output to test PDF Combine only

Raw file	Scan	Method	Score	m/z	Gene names
0523_4	4136	FTMS; HCD	120.27	449.21	NET1D

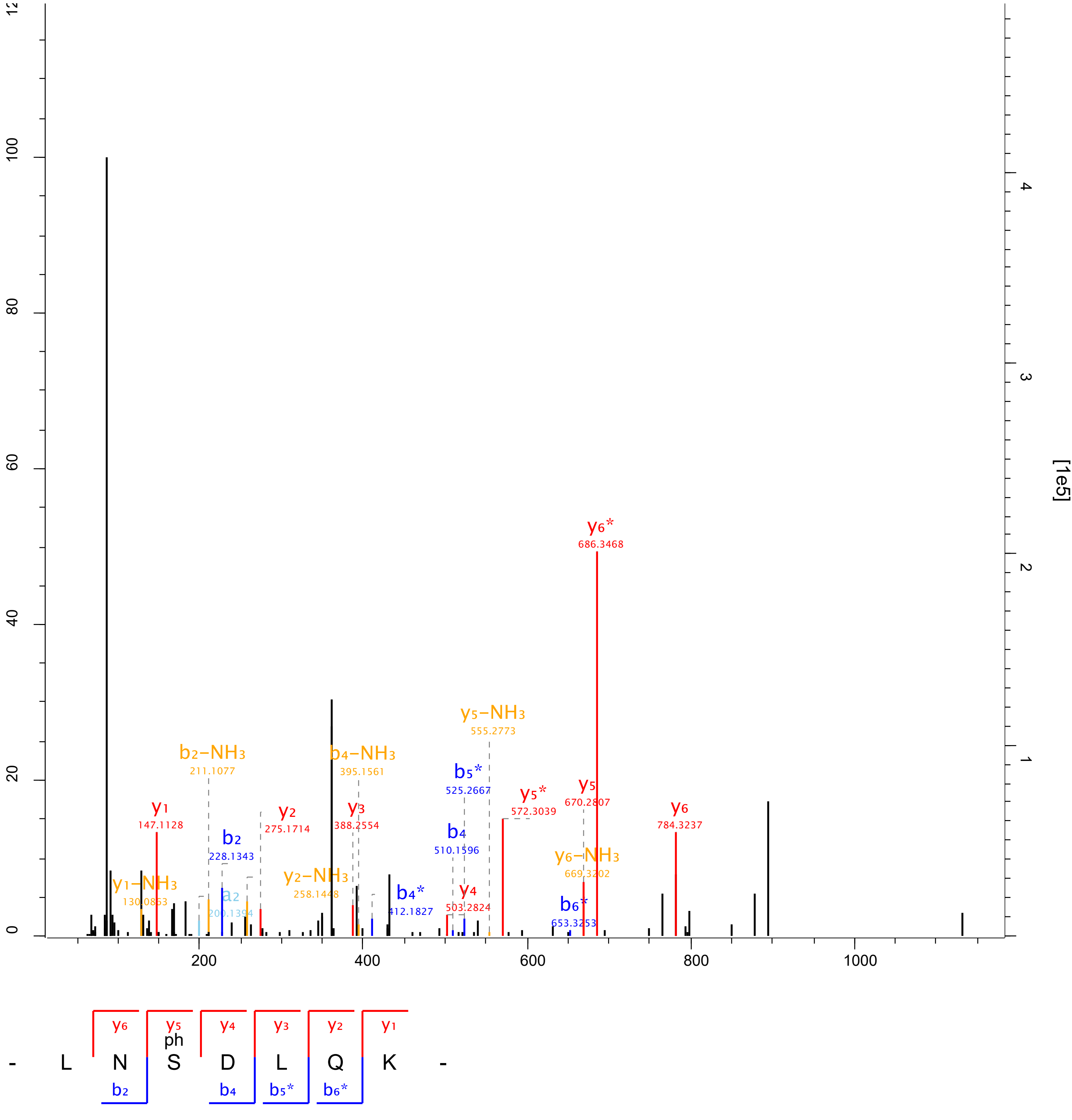

Sample output to test PDF Combine only

Raw file	Scan	Method	Score	m/z	Gene names
0523_4	4155	FTMS; HCD	182.37	540.25	RS41

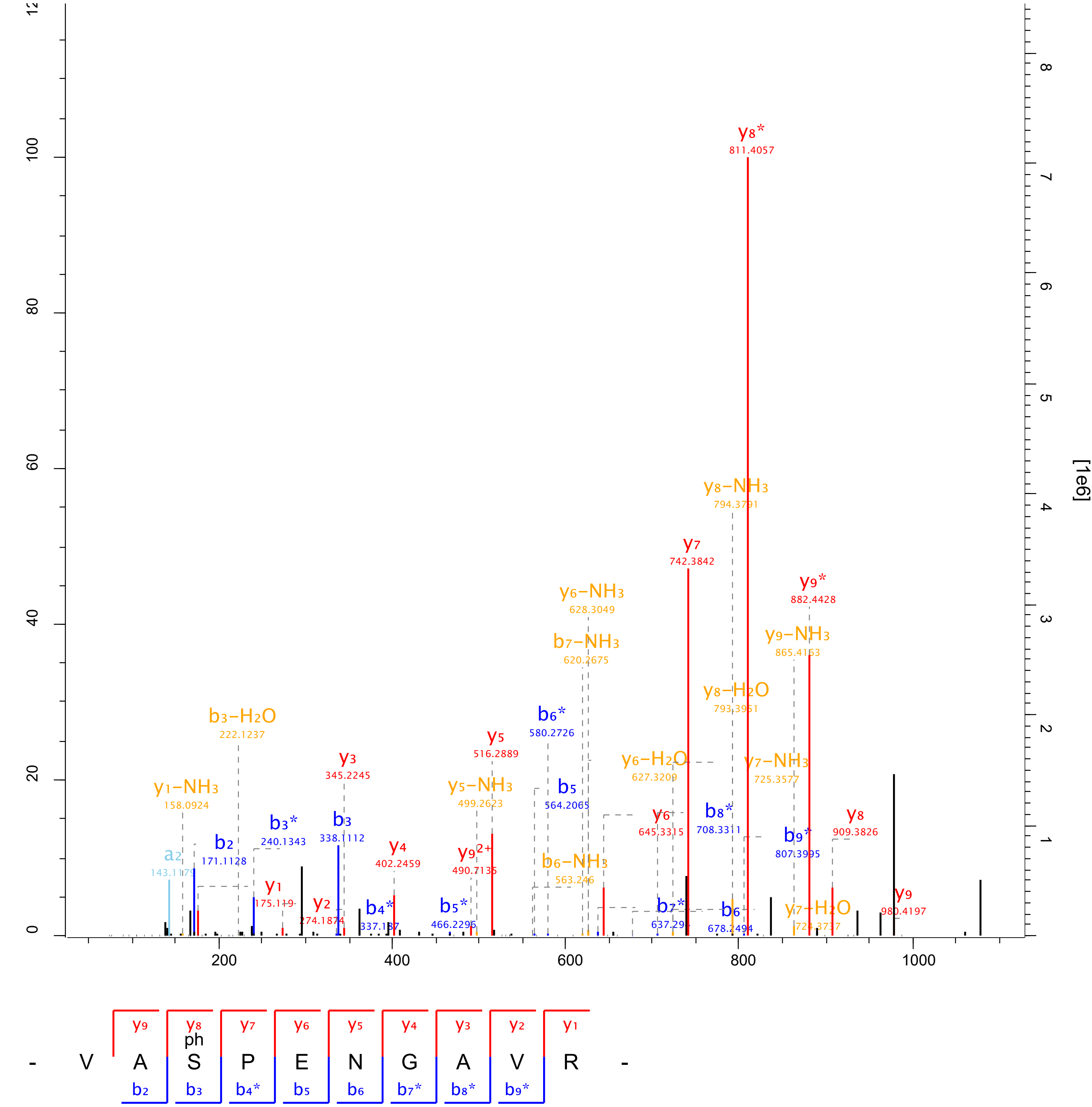

Sample output to test PDF Combine only

Raw file	Scan	Method	Score	m/z	Gene names
0523_4	4194	FTMS; HCD	62.86	620.27	F9F8.20

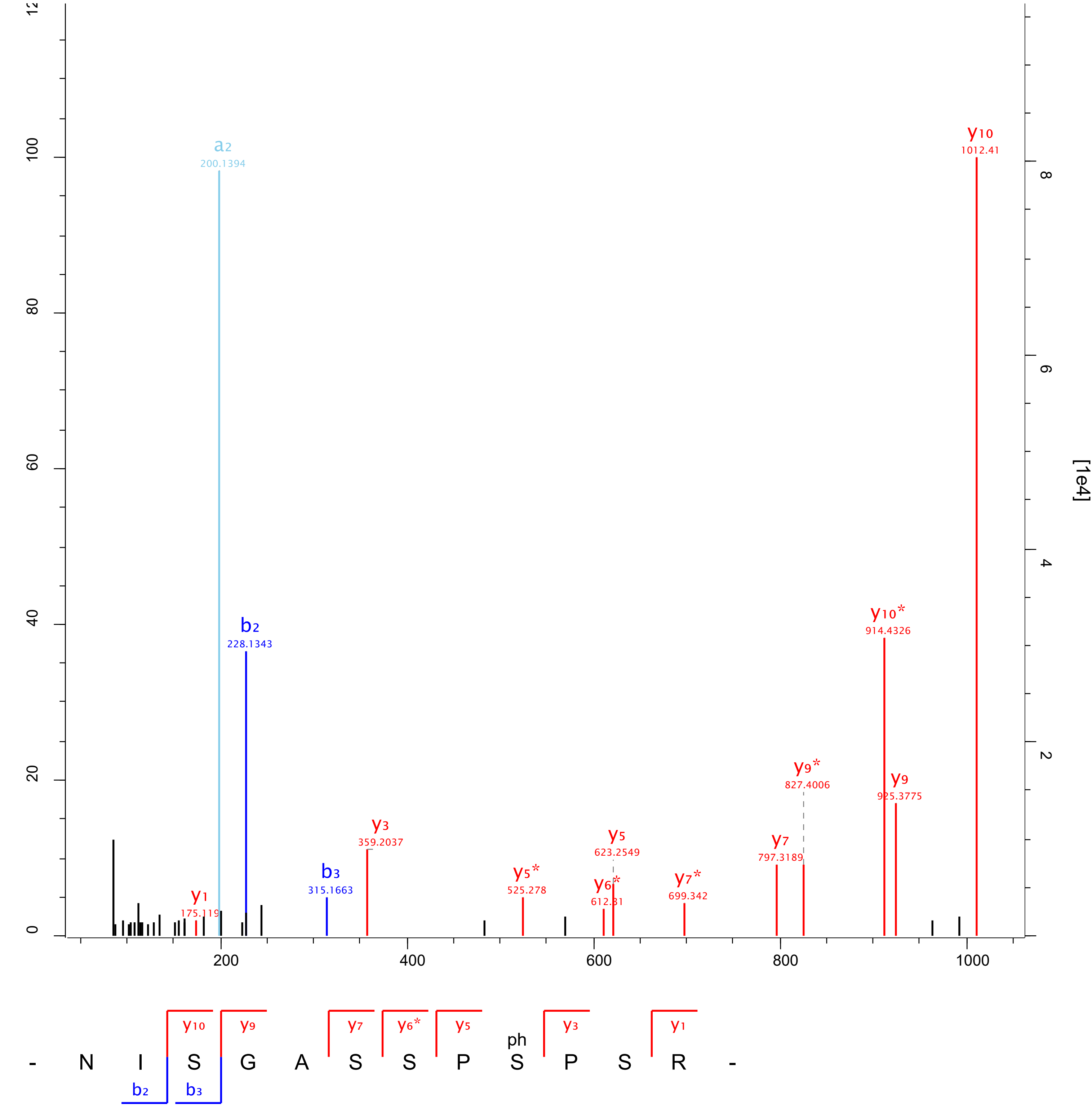

Sample output to test PDF Combine only

Raw file	Scan	Method	Score	m/z	Gene names
0523_4	4228	FTMS; HCD	65.37	651.78	At4g14990

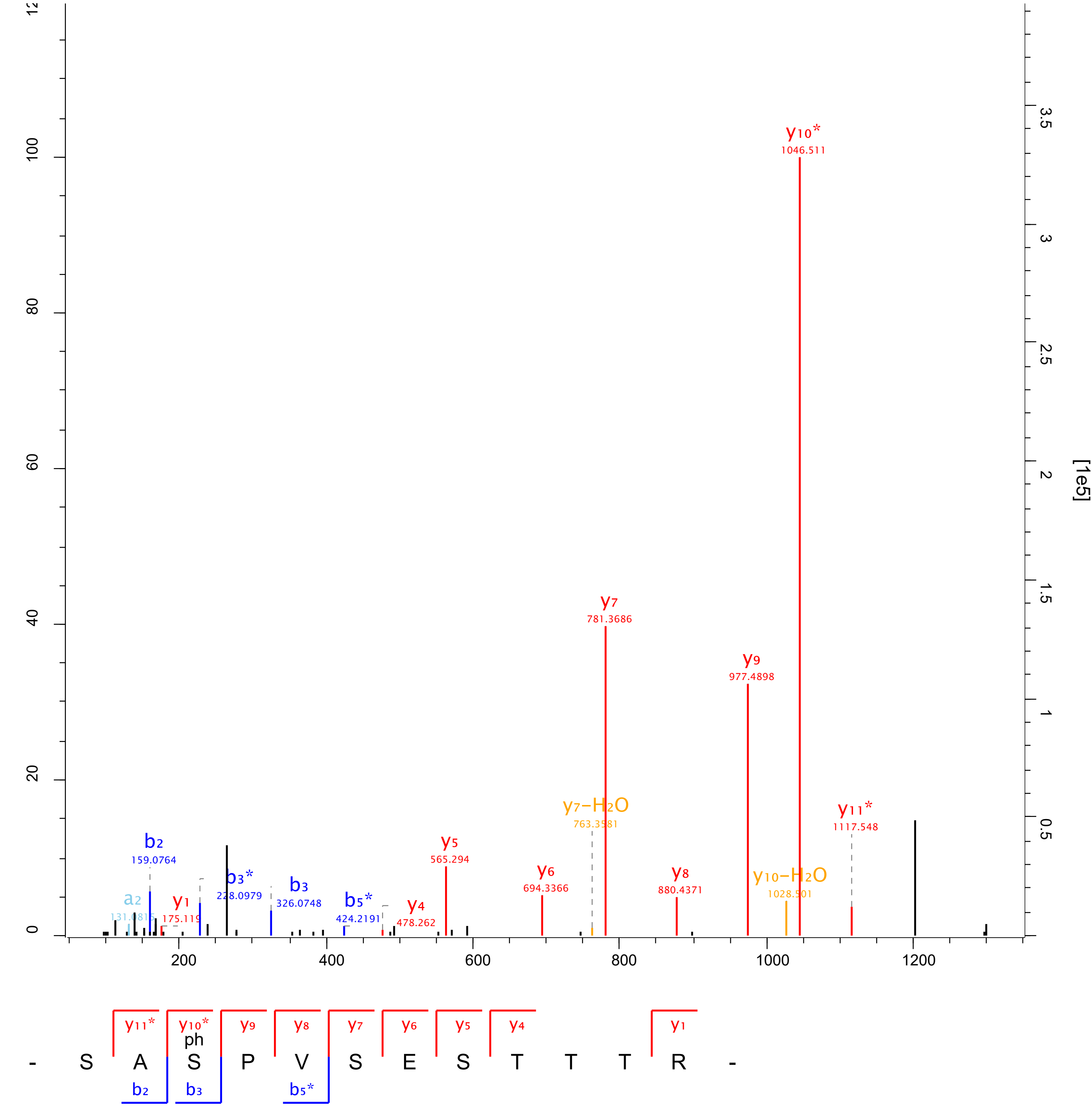

Sample output to test PDF Combine only

Raw file	Scan	Method	Score	m/z	Gene names
0523_4	4250	FTMS; HCD	89.48	627.3	HAG1

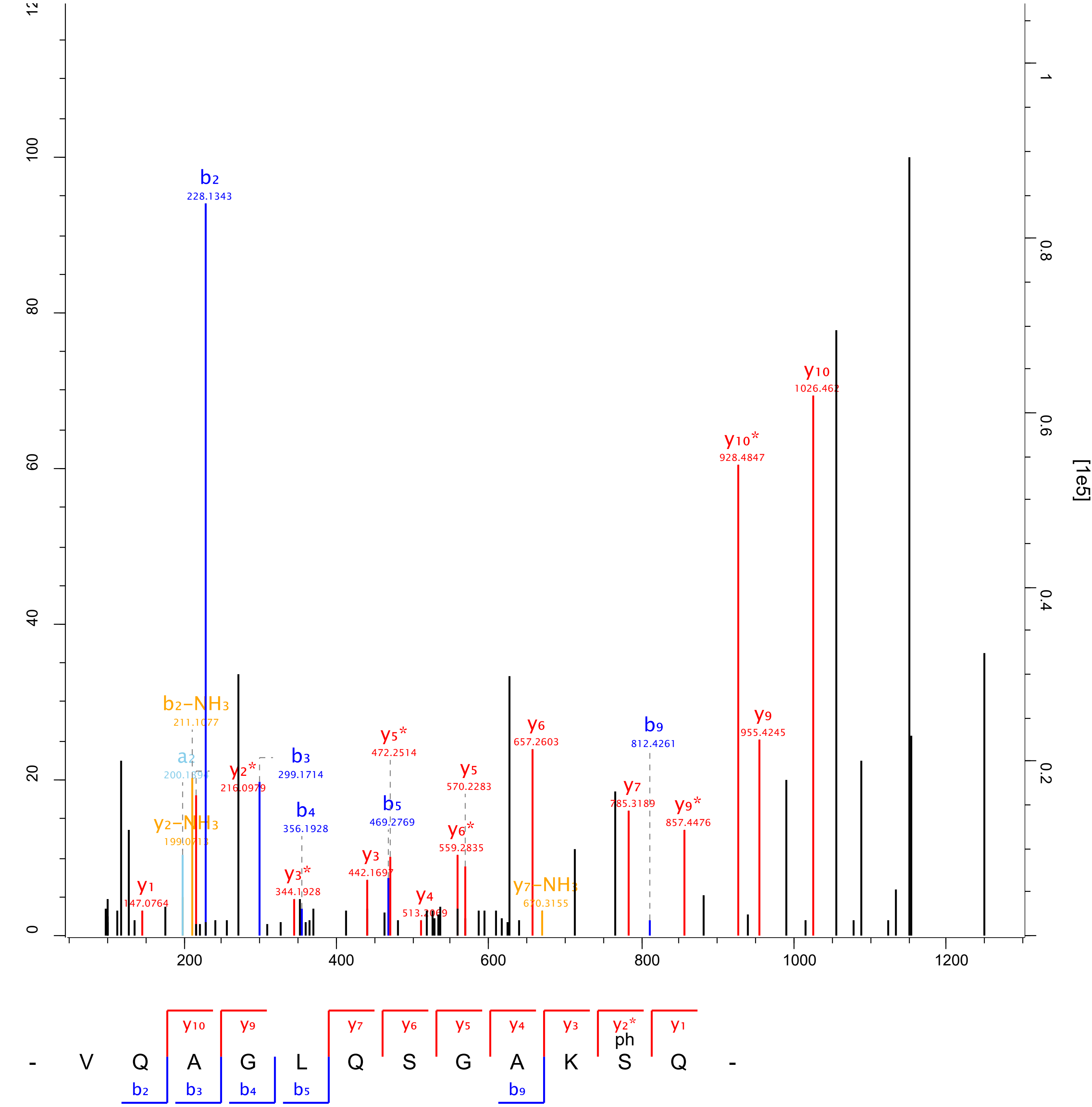

Sample output to test PDF Combine only

Raw file	Scan	Method	Score	m/z	Gene names
0523_4	4254	FTMS; HCD	241.13	850.8	AMT1-1

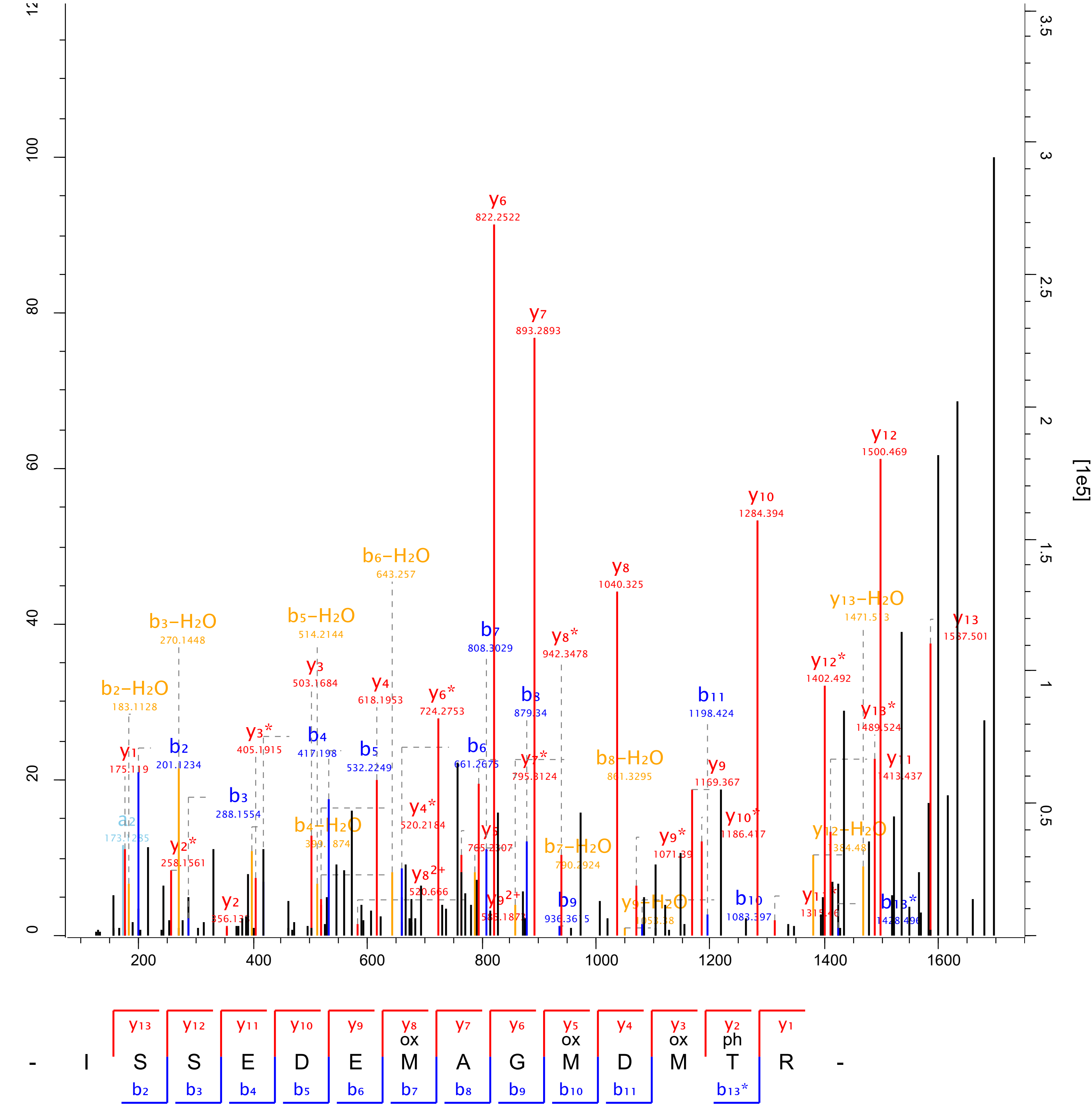

Sample output to test PDF Combine only

Raw file	Scan	Method	Score	m/z	Gene names
0523_4	4317	FTMS; HCD	234.21	751.79	T32M21_20

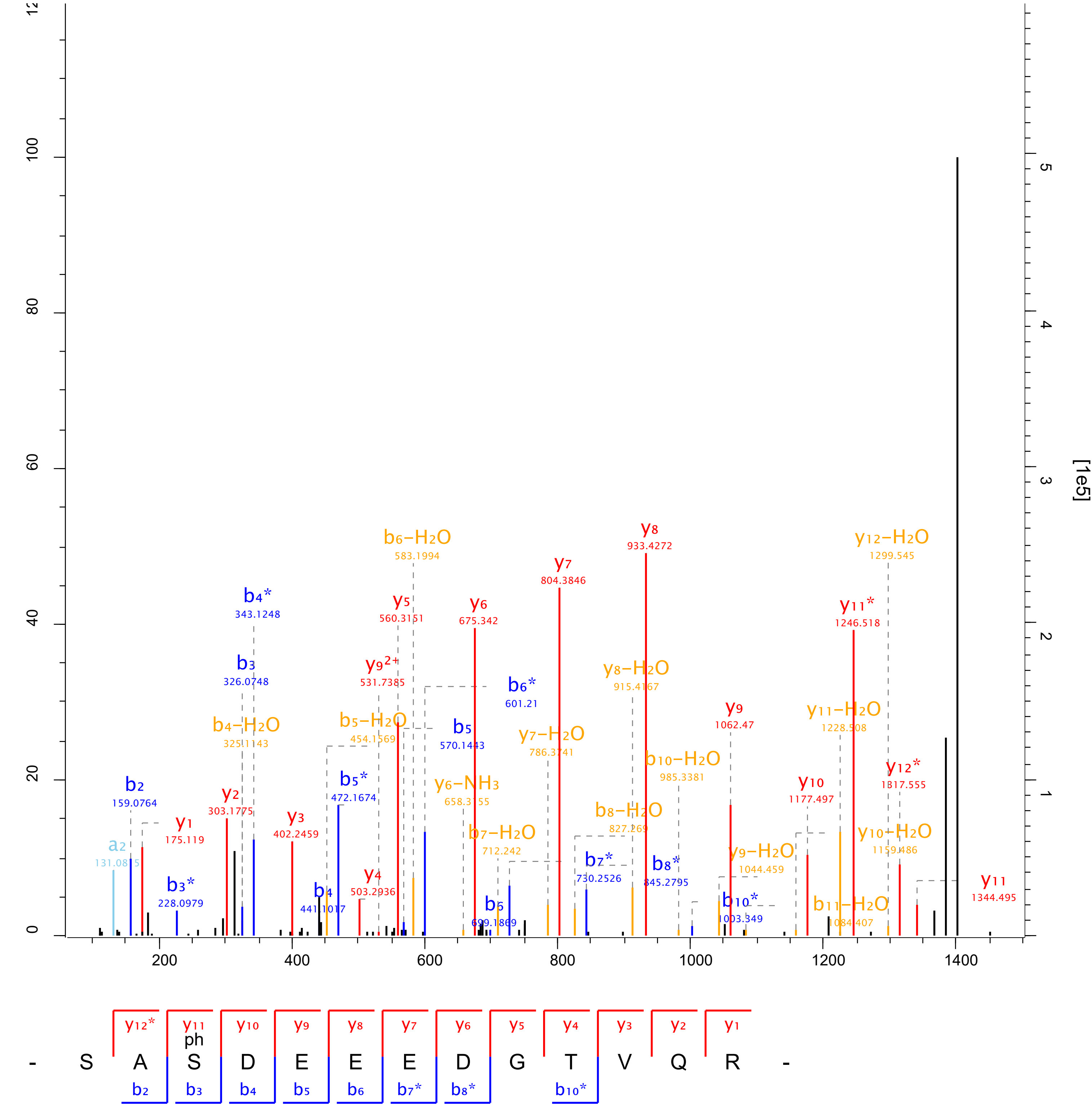

Sample output to test PDF Combine only

Raw file	Scan	Method	Score	m/z	Gene names
0523_4	4458	FTMS; HCD	58.89	626.72	RS2Z33

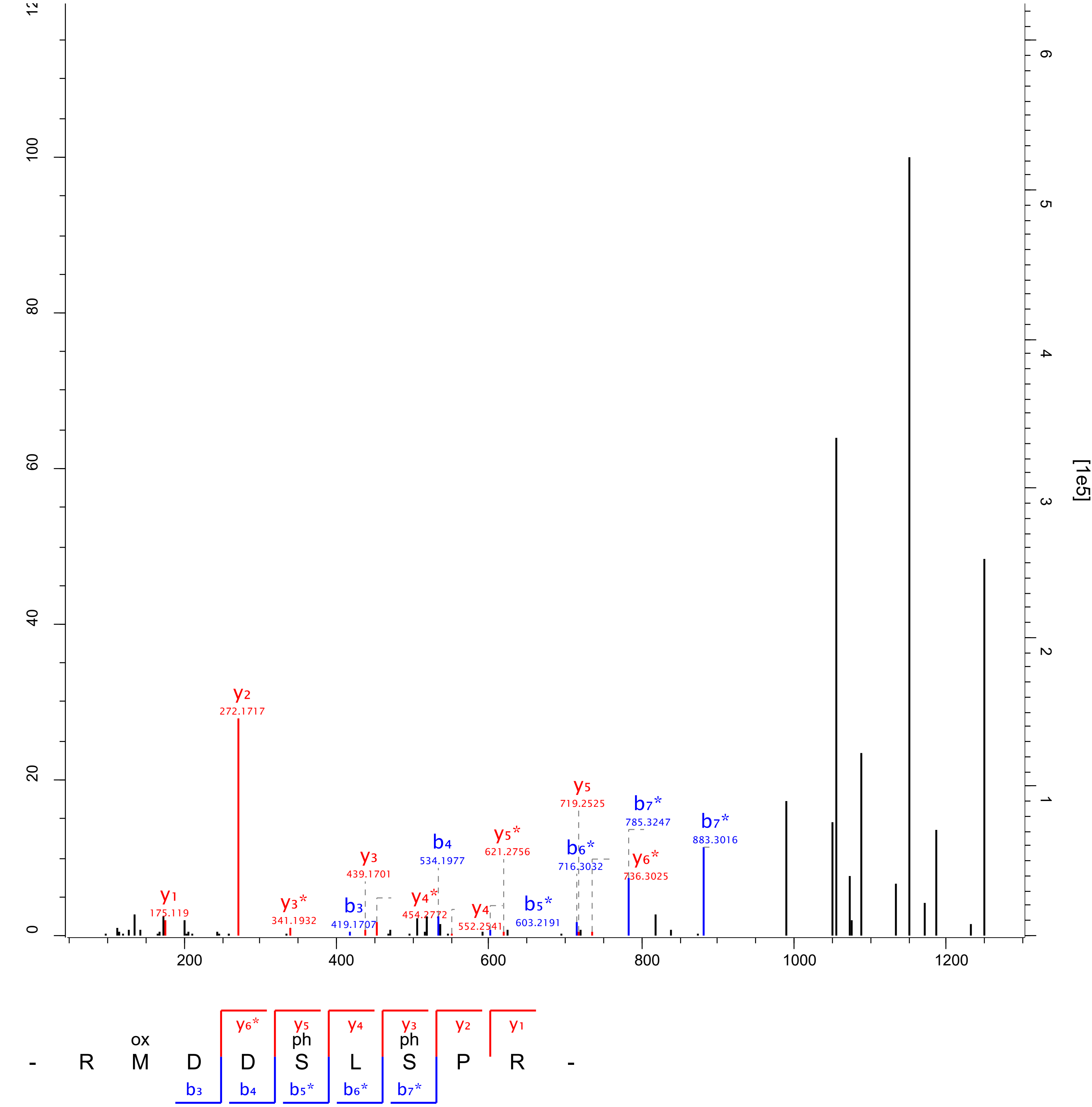

Sample output to test PDF Combine only

Raw file	Scan	Method	Score	m/z	Gene names
0523_4	4461	FTMS; HCD	166.79	697.77	At1g52780

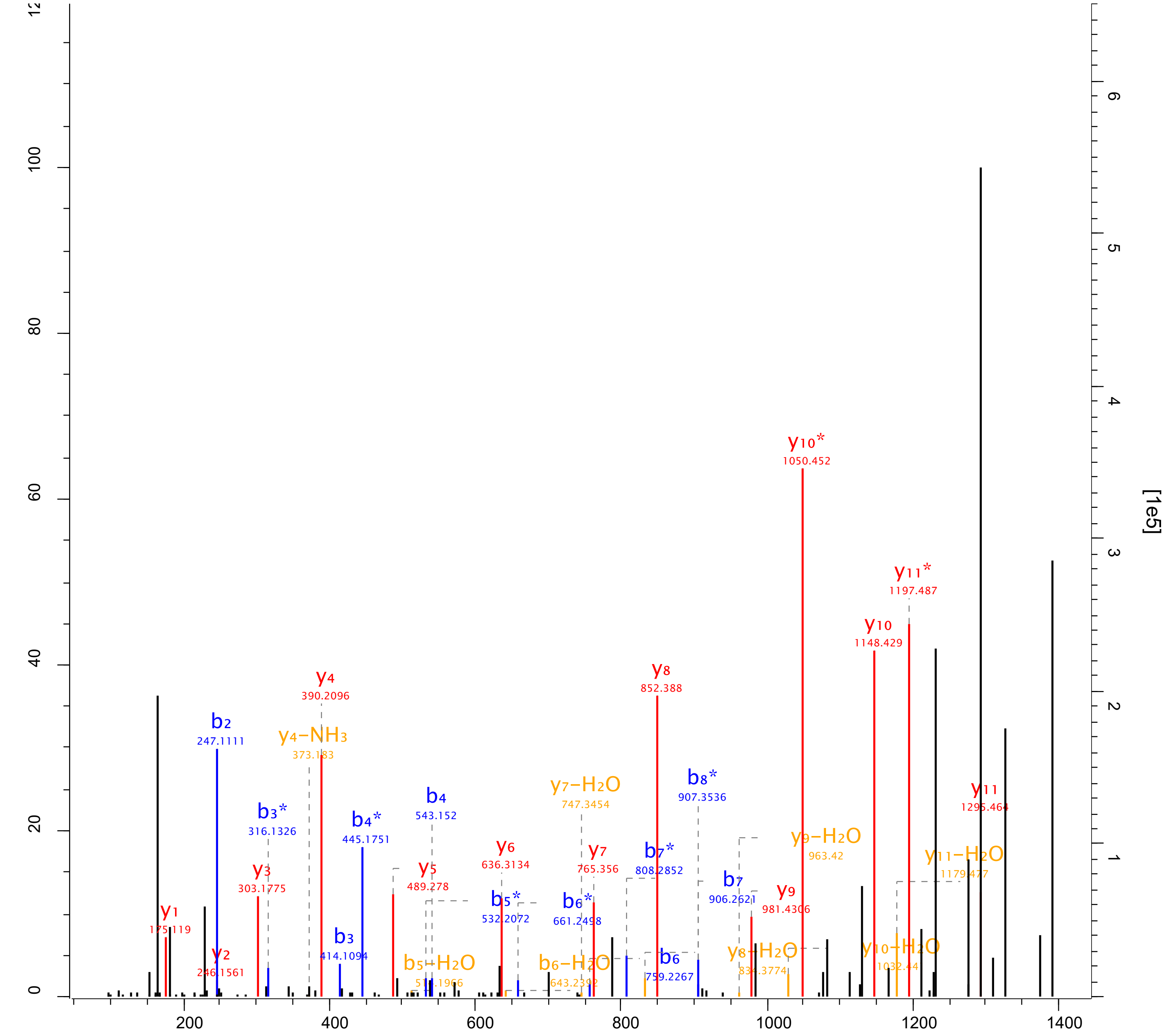

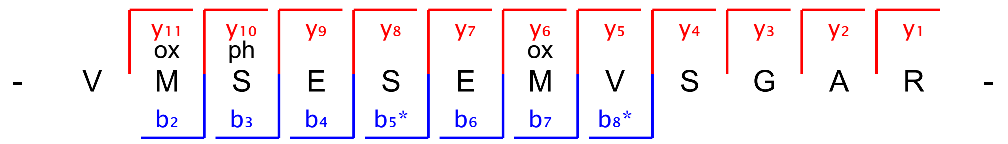

Sample output to test PDF Combine only

Raw file	Scan	Method	Score	m/z	Gene names
0523_4	4480	FTMS; HCD	45.35	651.22	CPSF30

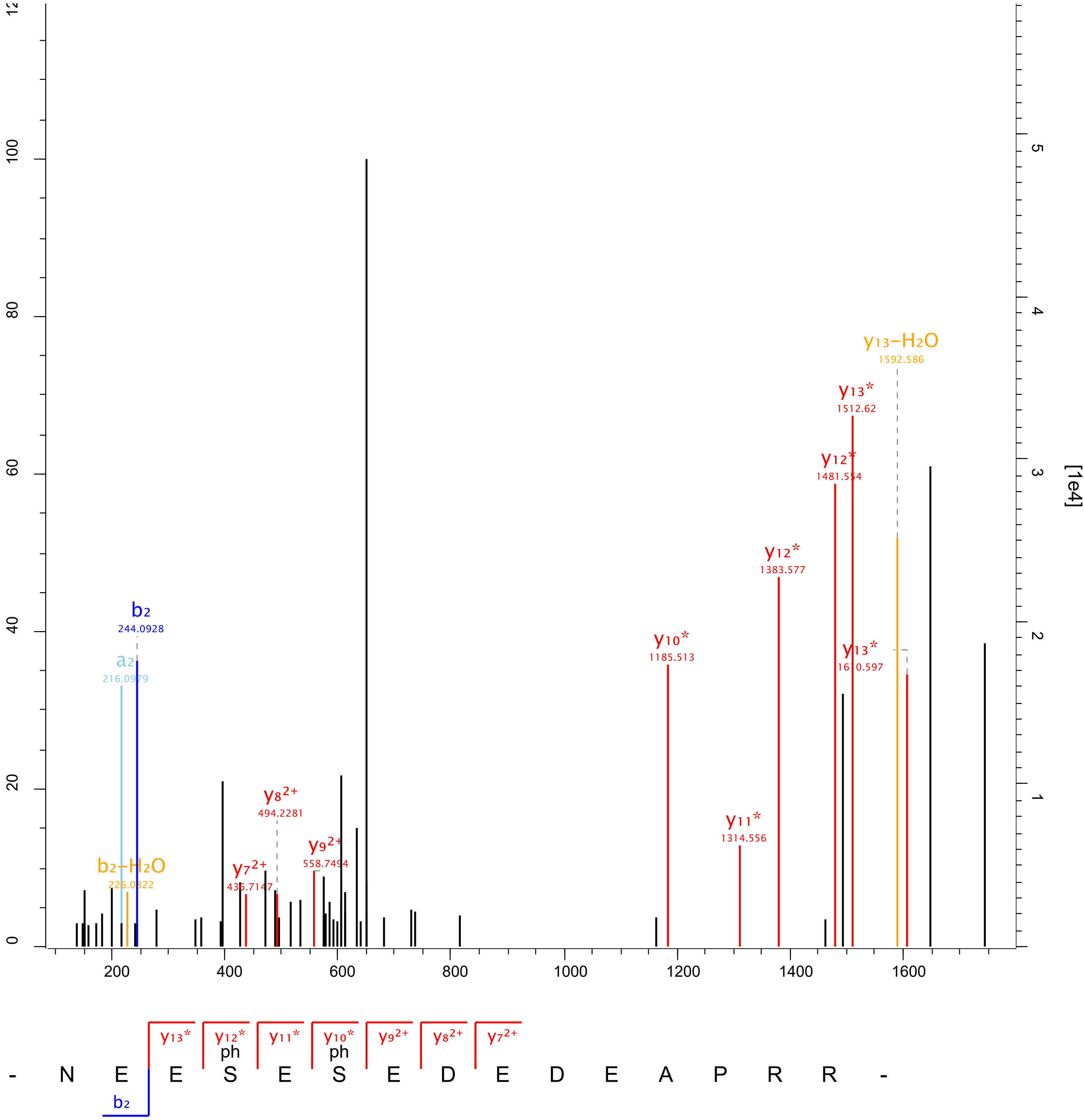

Sample output to test PDF Combine only

Raw file	Scan	Method	Score	m/z	Gene names
0523_4	4499	FTMS; HCD	359.9	910.35	MLO1

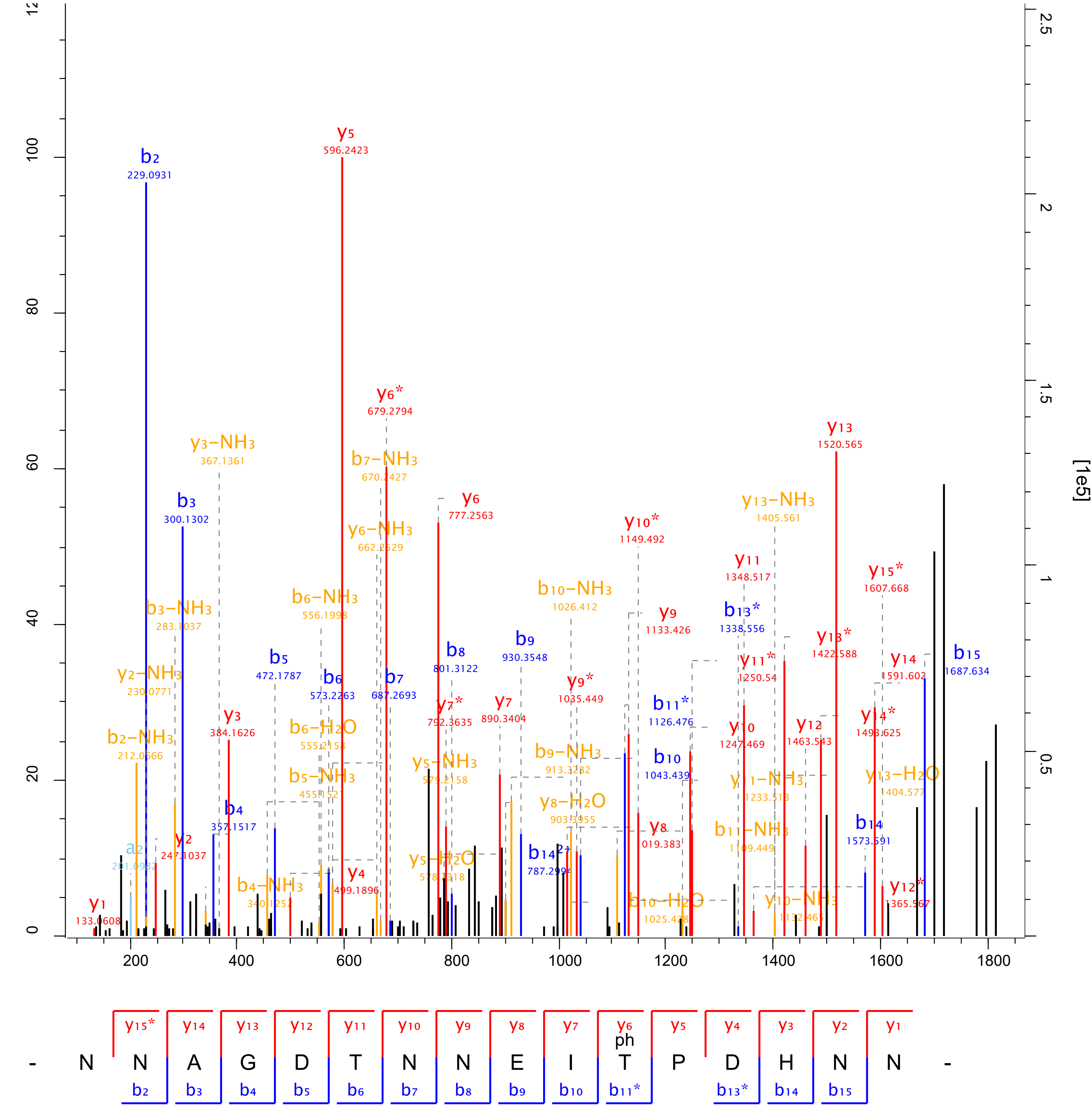

Sample output to test PDF Combine only

Raw file	Scan	Method	Score	m/z	Gene names
0523_4	4546	FTMS; HCD	124.29	804.3	EMB2754

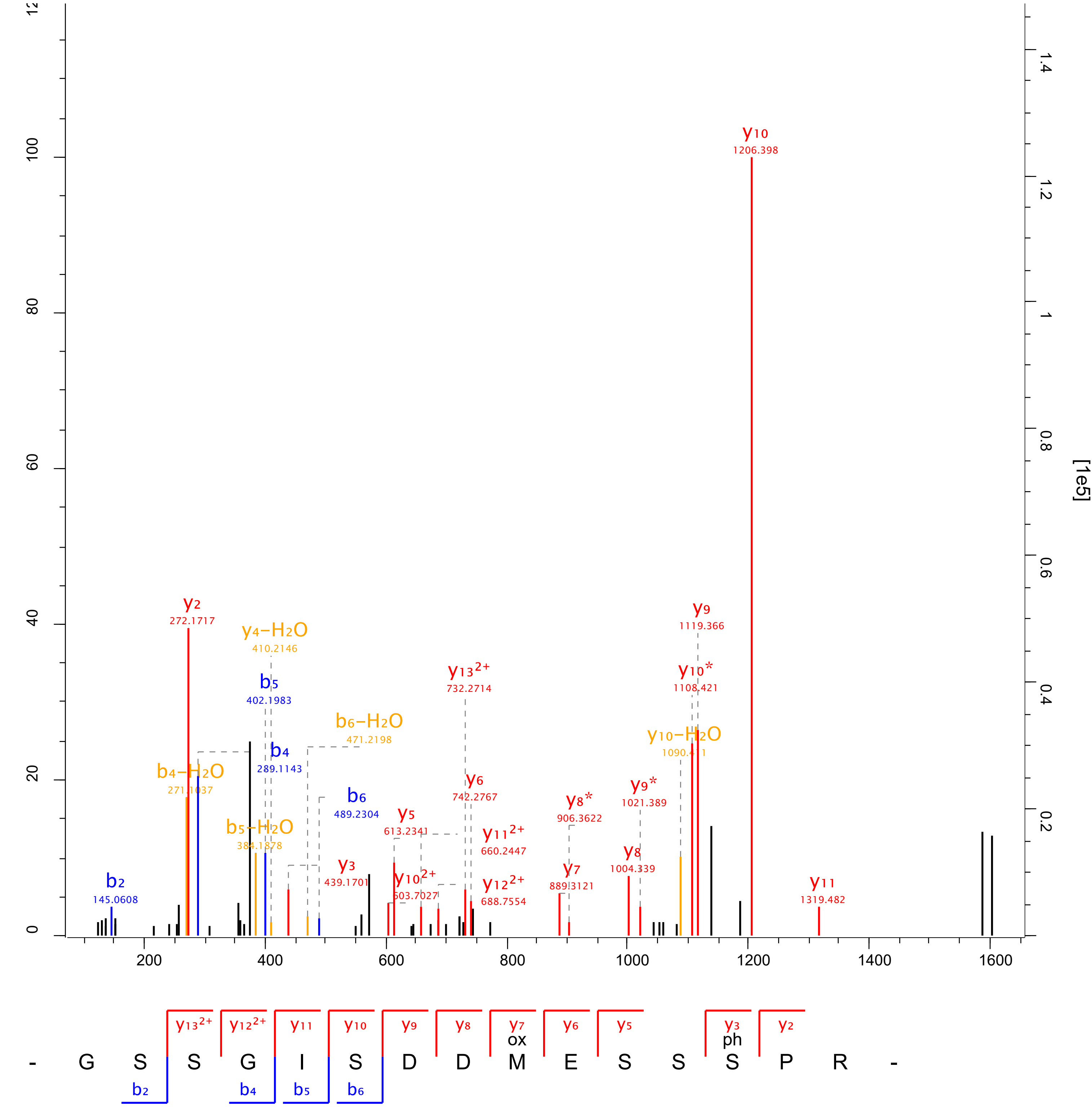

Sample output to test PDF Combine only

Raw file	Scan	Method	Score	m/z
0523_4	4574	FTMS; HCD	137.01	479.22

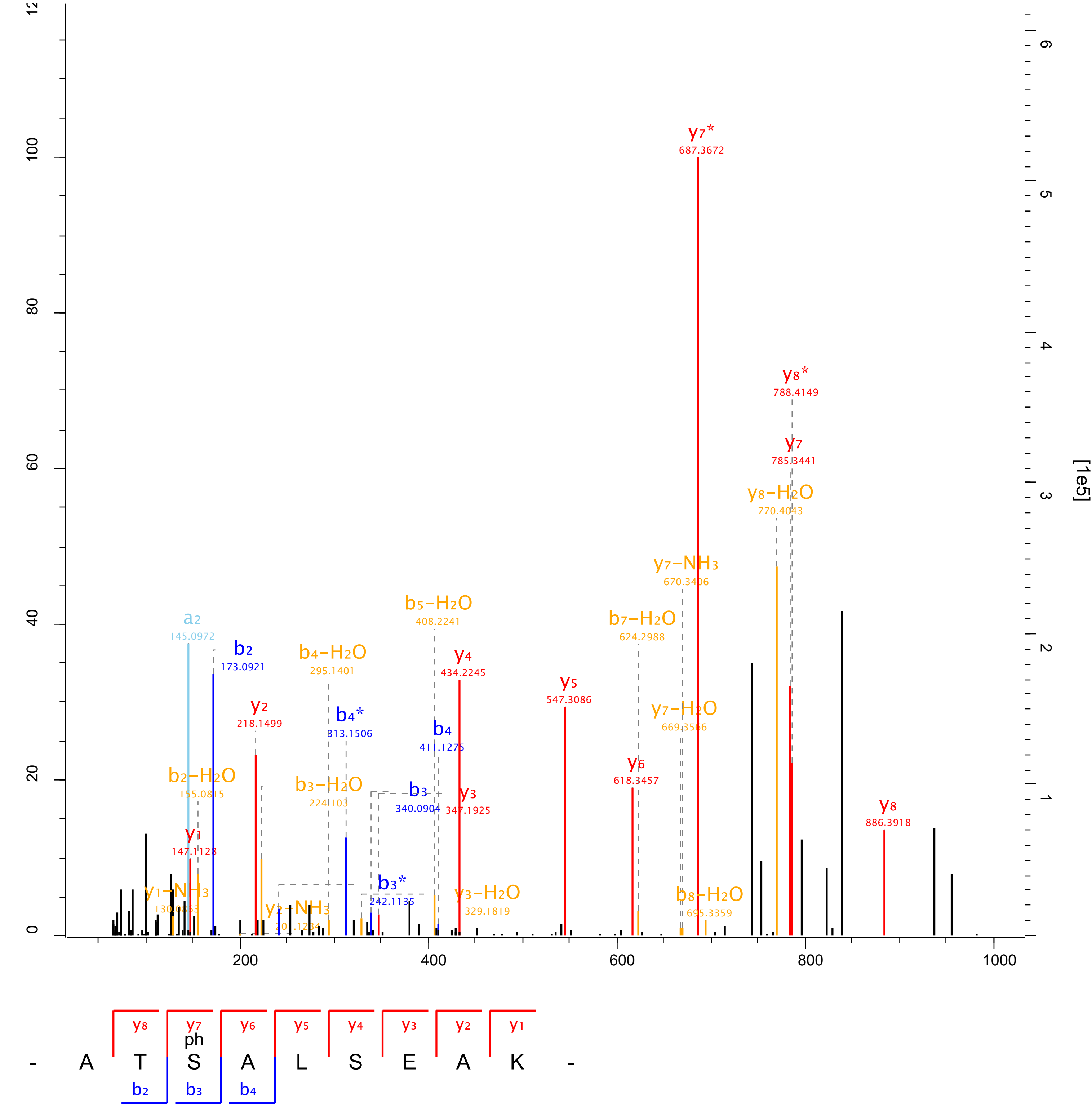

Sample output to test PDF Combine only

Raw file	Scan	Method	Score	m/z	Gene names
0523_4	4638	FTMS; HCD	83.99	724.28	At5g51300

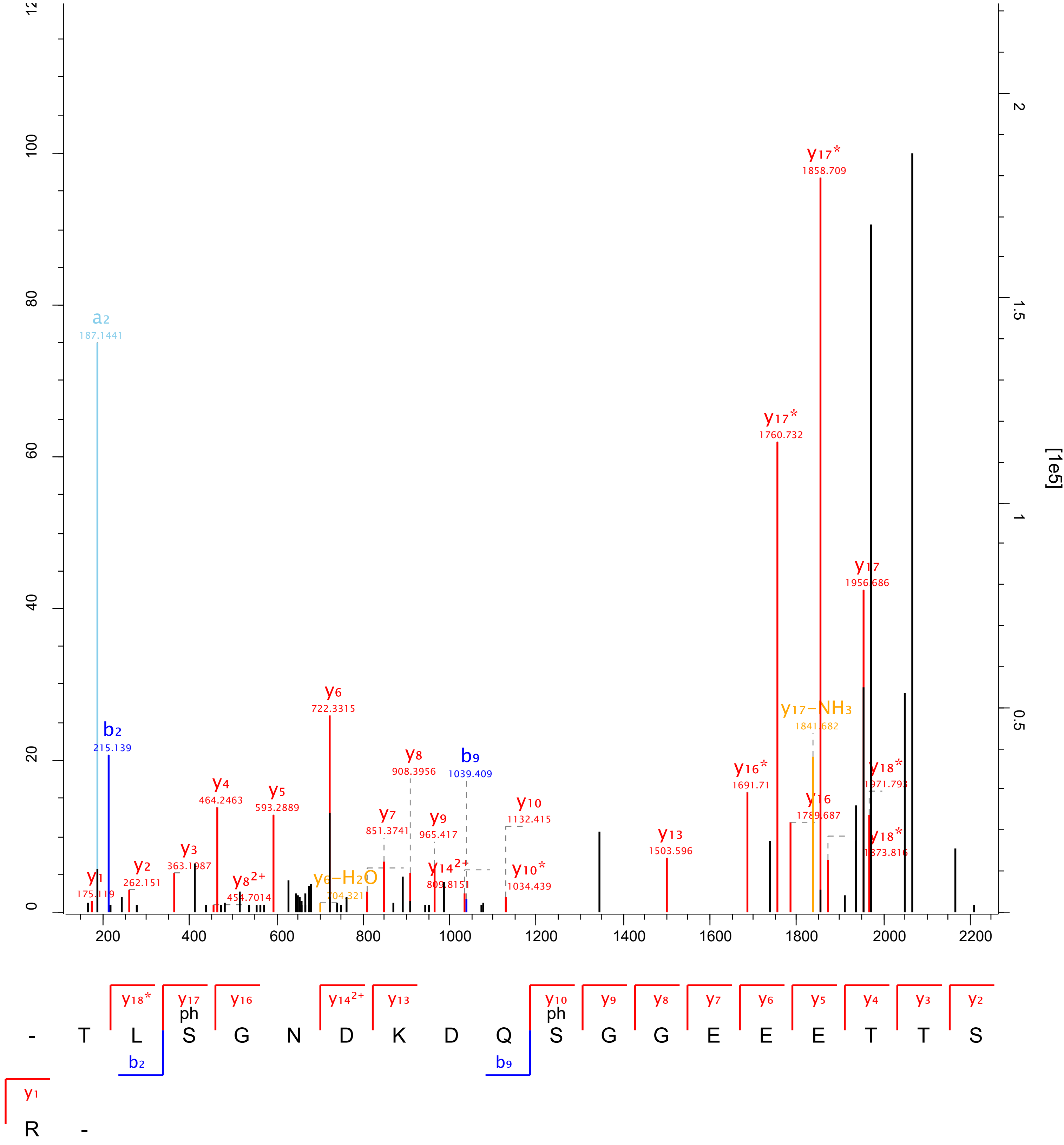

Sample output to test PDF Combine only

Raw file	Scan	Method	Score	m/z	Gene names
0523_4	4672	FTMS; HCD	131.82	701.31	PAT05

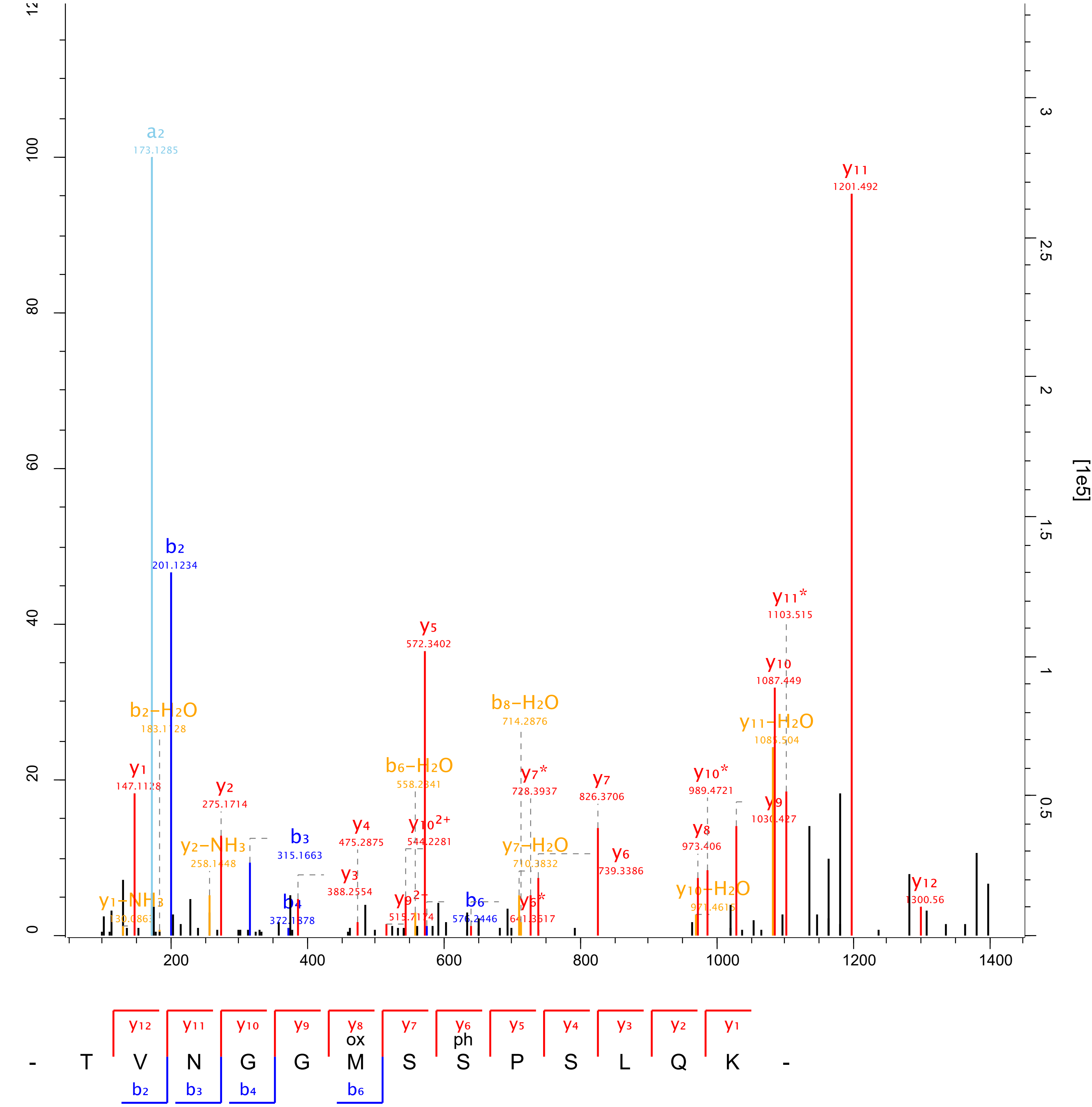

Sample output to test PDF Combine only

Raw file	Scan	Method	Score	m/z	Gene names
0523_4	4701	FTMS; HCD	287.16	744.28	T15K4.8

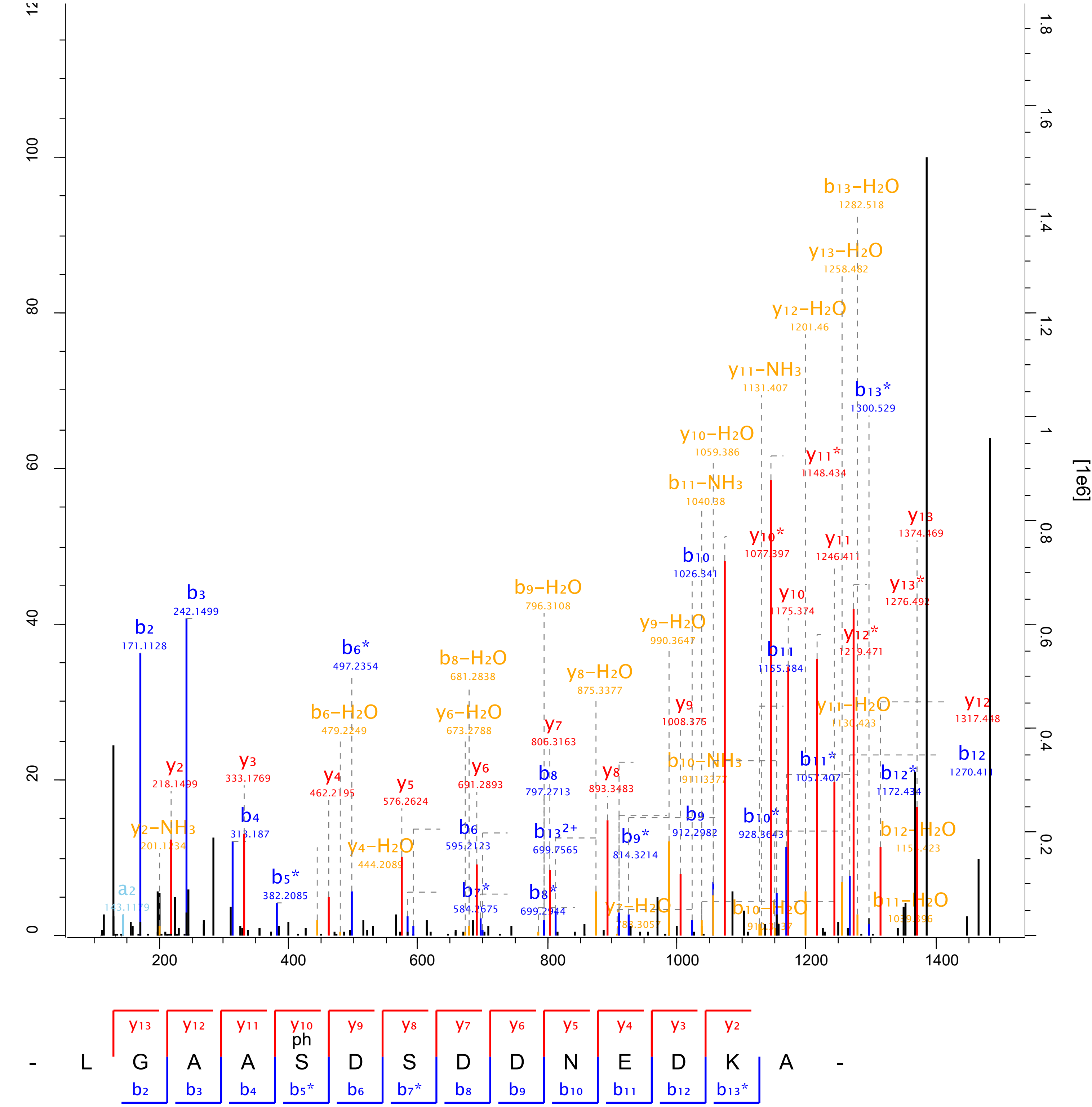

Sample output to test PDF Combine only

Raw file	Scan	Method	Score	m/z	Gene names
0523_4	4711	FTMS; HCD	331.52	919.37	DRP2A

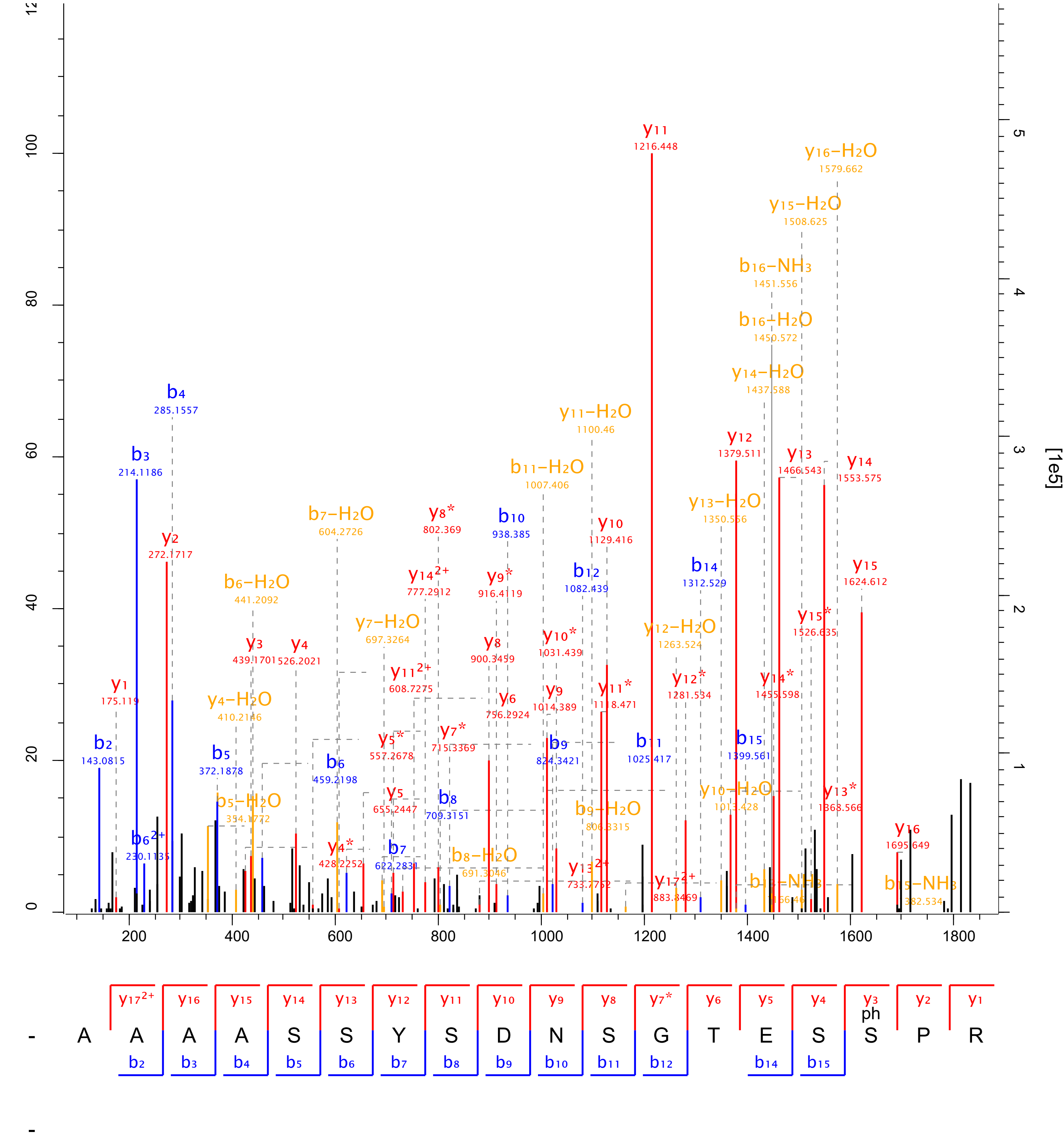

Sample output to test PDF Combine only

Raw file	Scan	Method	Score	m/z	Gene names
0523_4	4730	FTMS; HCD	61.52	607.89	AMT1-3

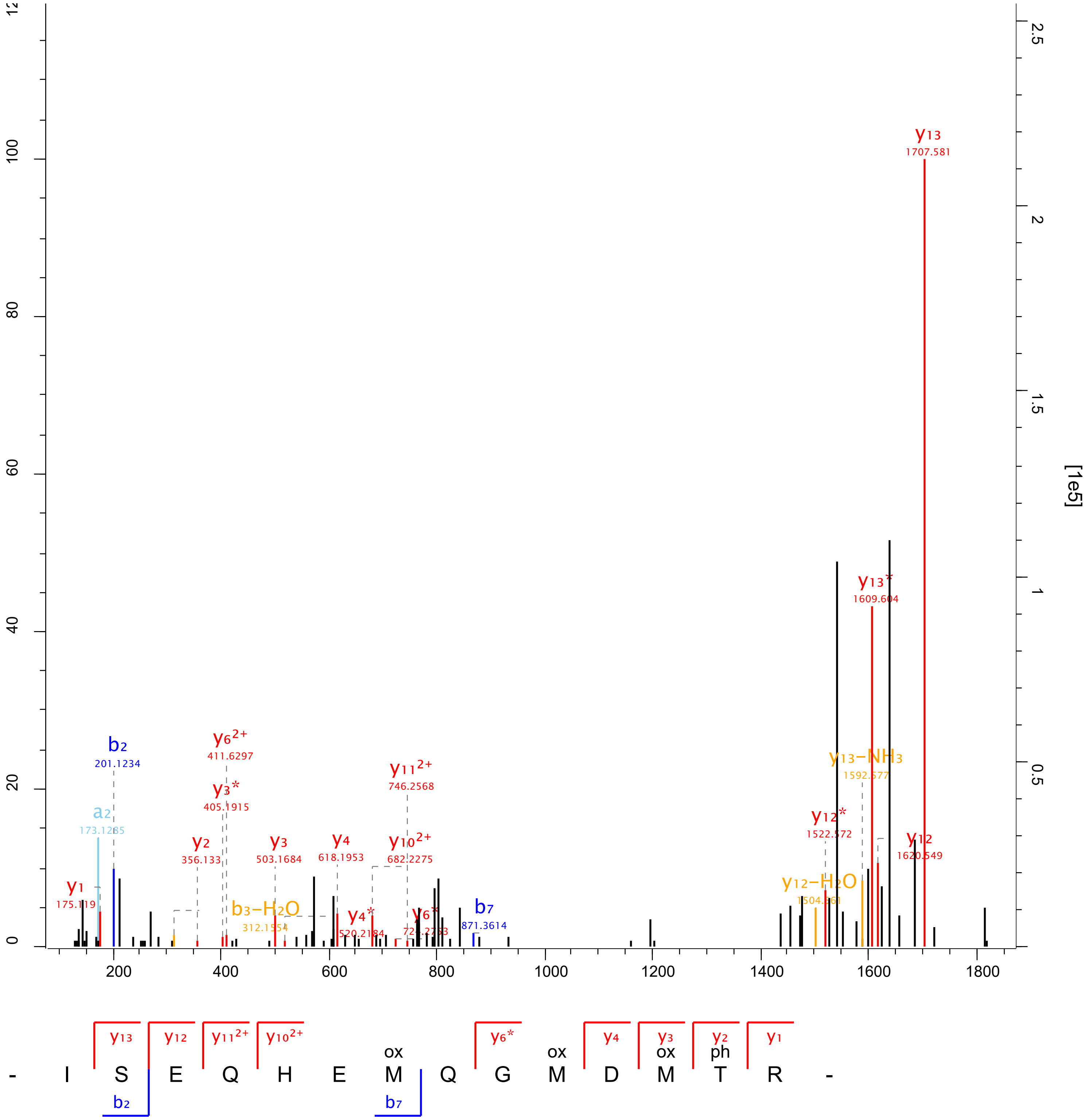

Sample output to test PDF Combine only

Raw file	Scan	Method	Score	m/z
0523_4	4791	FTMS; HCD	148.96	610.73

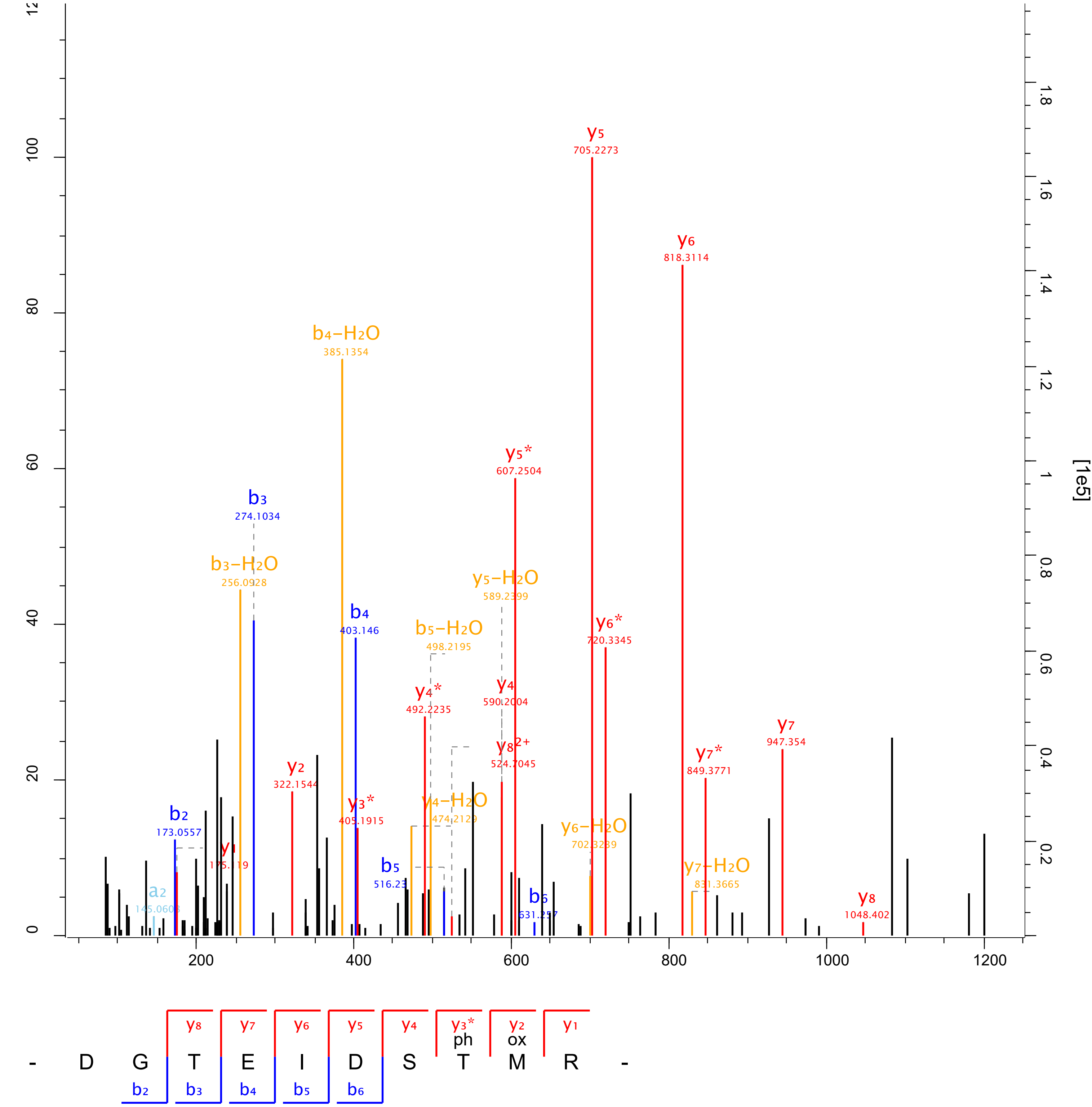

Sample output to test PDF Combine only

Raw file	Scan	Method	Score	m/z	Gene names
0523_4	4853	FTMS; HCD	84.82	427.68	At1g20760

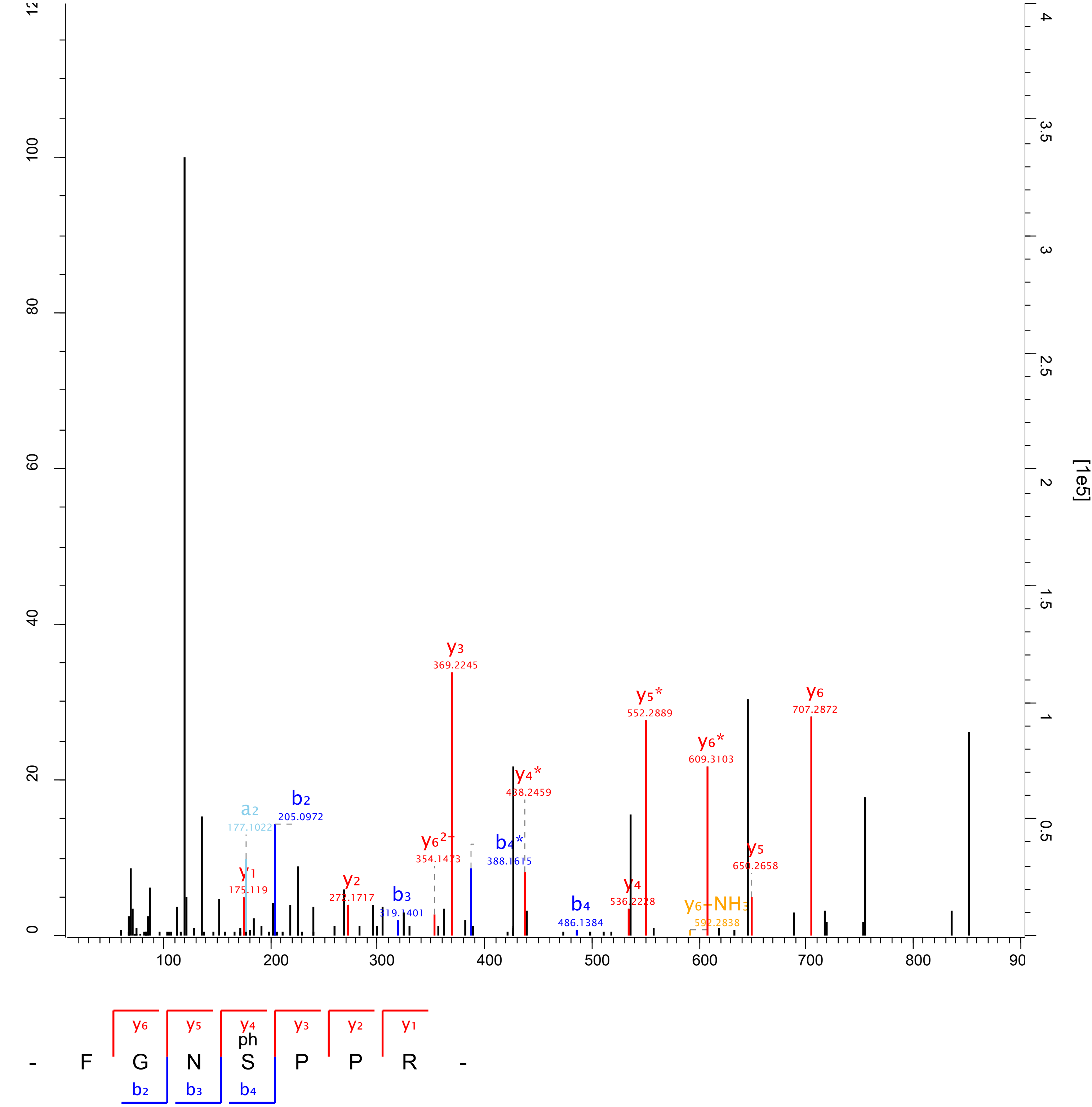

Sample output to test PDF Combine only

Raw file	Scan	Method	Score	m/z	Gene names
0523_4	4863	FTMS; HCD	223.65	773.35	SUN1

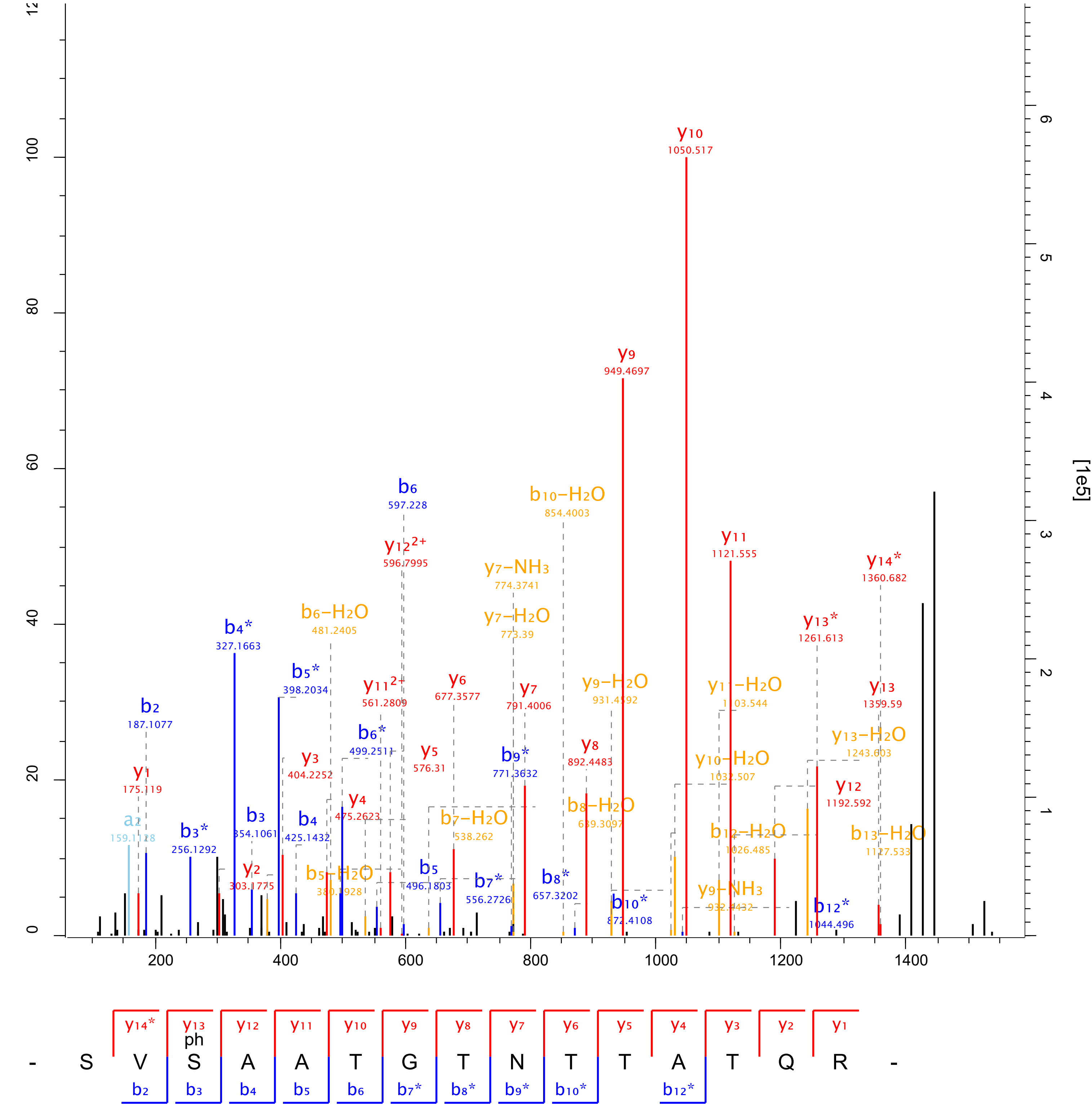

Sample output to test PDF Combine only

Raw file	Scan	Method	Score	m/z	Gene names
0523_4	5148	FTMS; HCD	134.75	550.73	FRO2

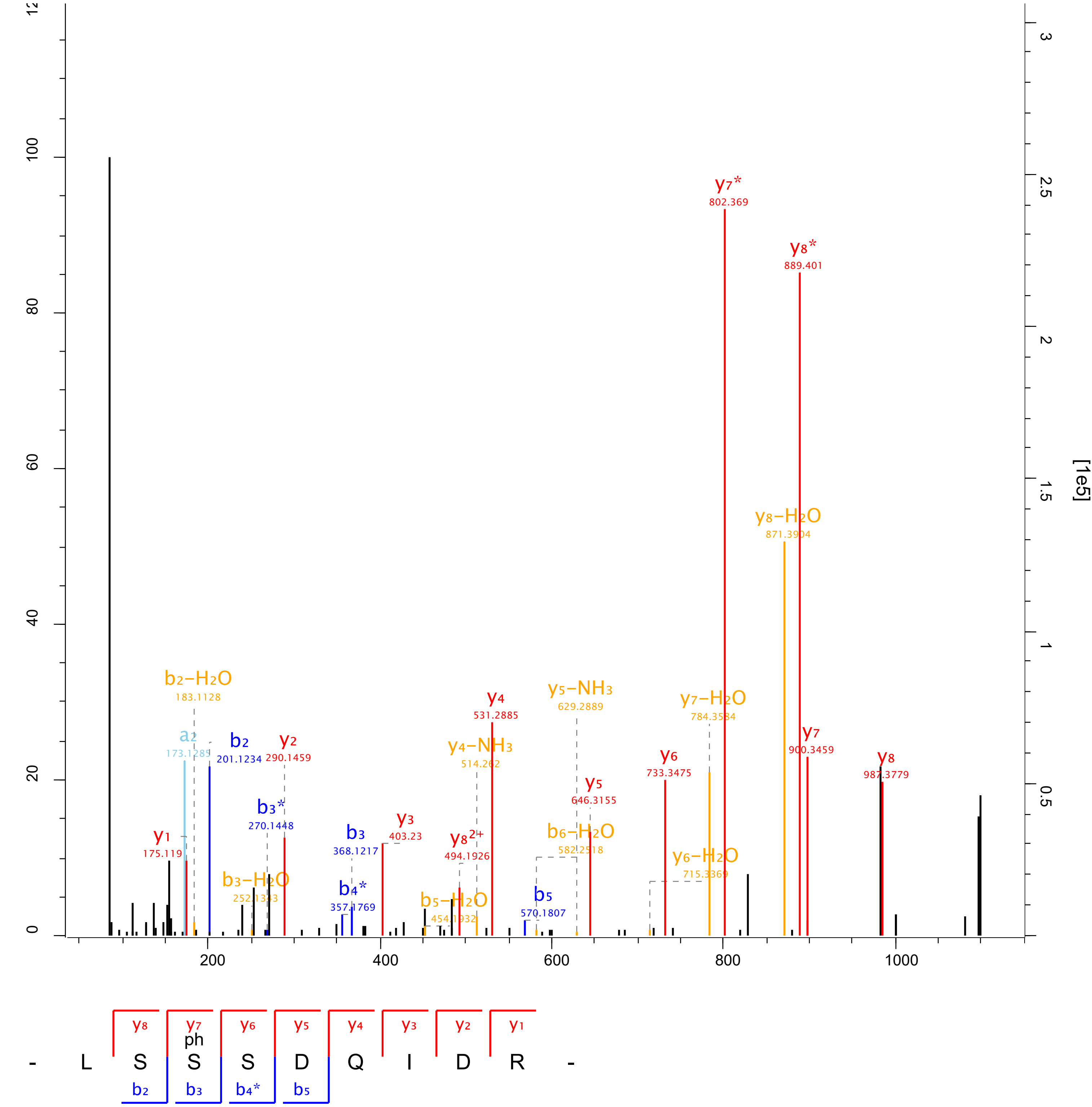

Sample output to test PDF Combine only

Raw file	Scan	Method	Score	m/z	Gene names
0523_4	5155	FTMS; HCD	91.4	703.77	RS41

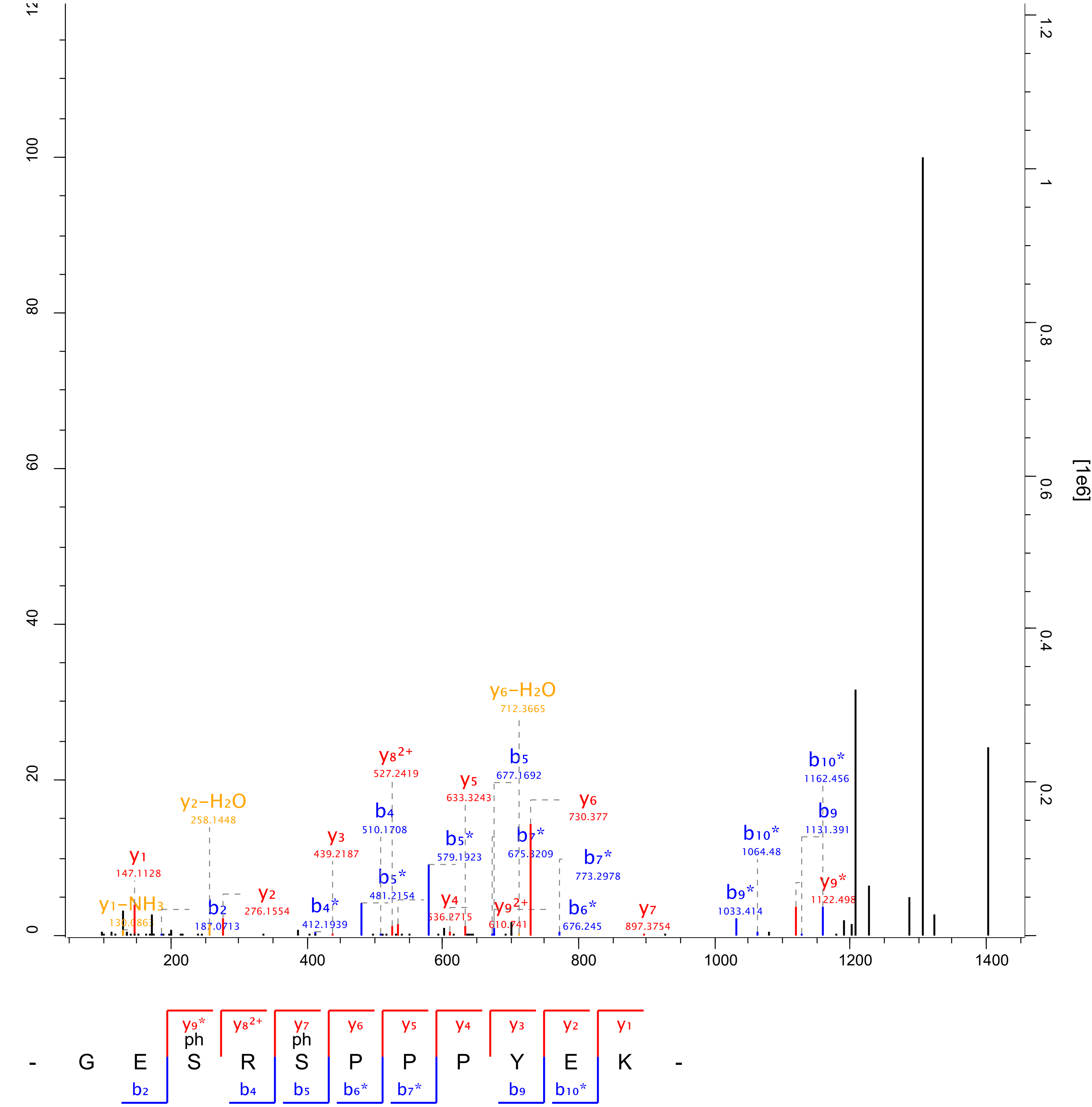

Sample output to test PDF Combine only

Raw file	Scan	Method	Score	m/z	Gene names
0523_4	5184	FTMS; HCD	83.6	650.92	SE

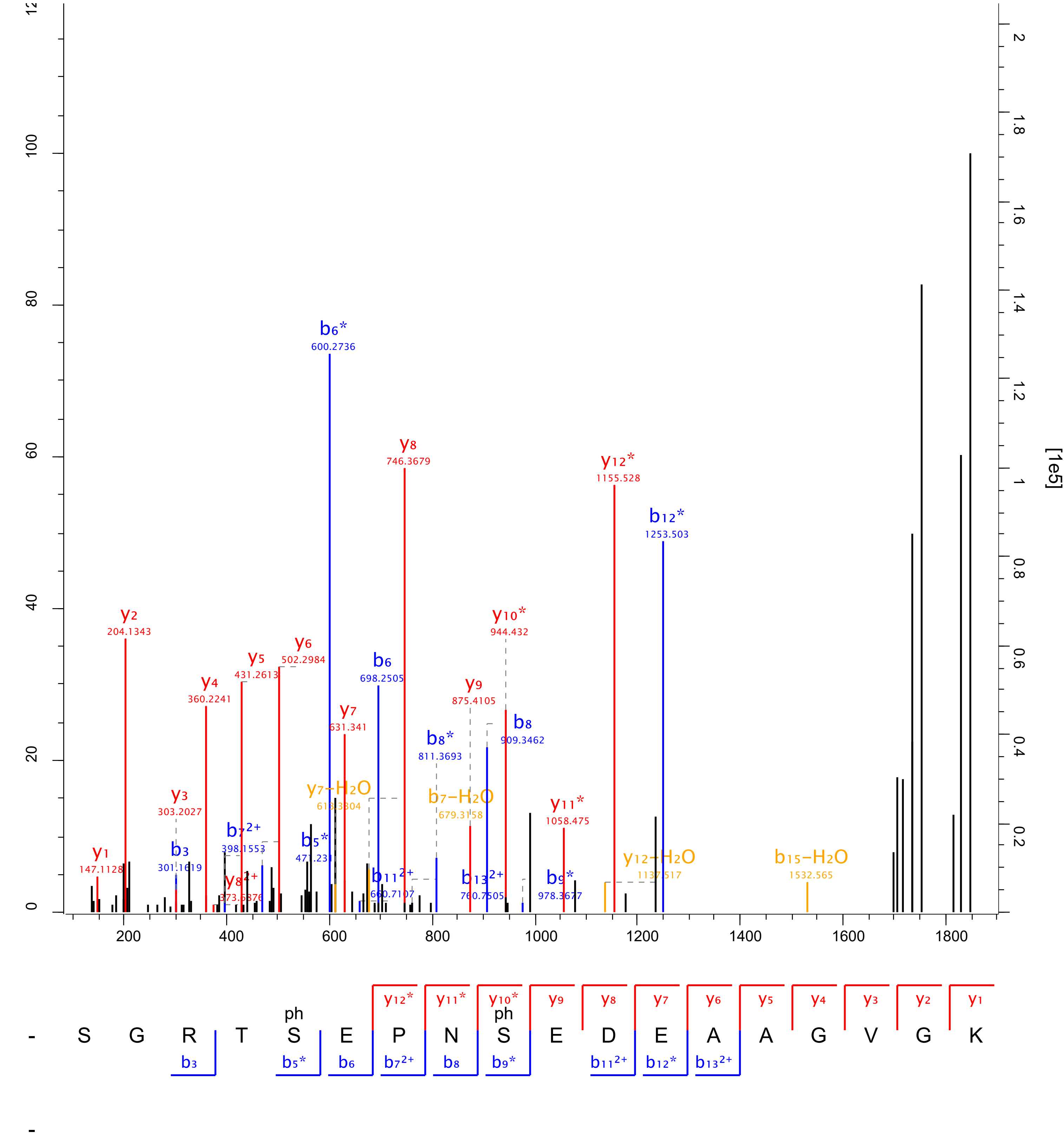

Sample output to test PDF Combine only

Raw file	Scan	Method	Score	m/z	Gene names
0523_4	5207	FTMS; HCD	152.01	826.79	RS40

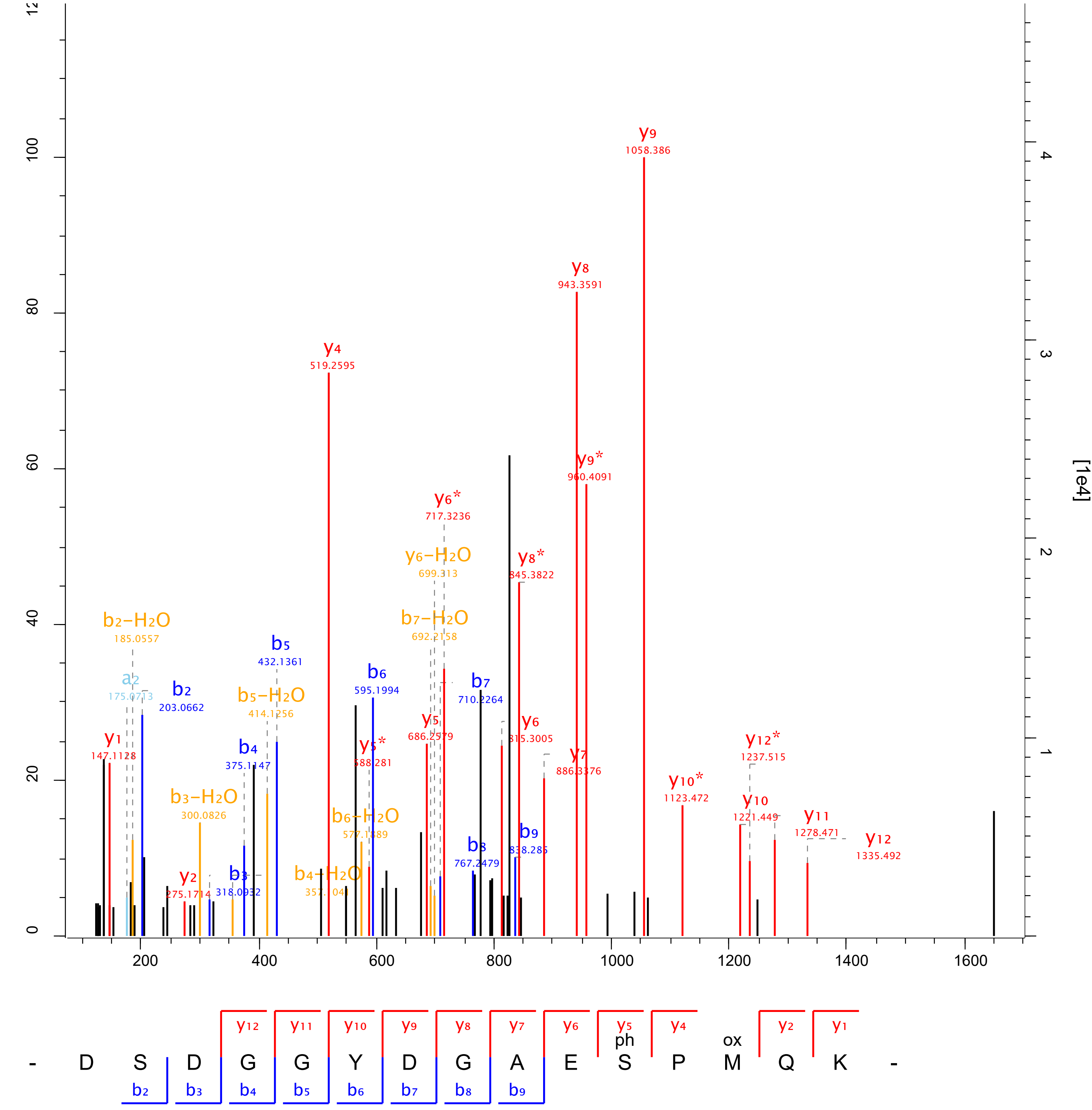

Sample output to test PDF Combine only

Raw file	Scan	Method	Score	m/z	Gene names
0523_4	5255	FTMS; HCD	68	537.75	BIG3

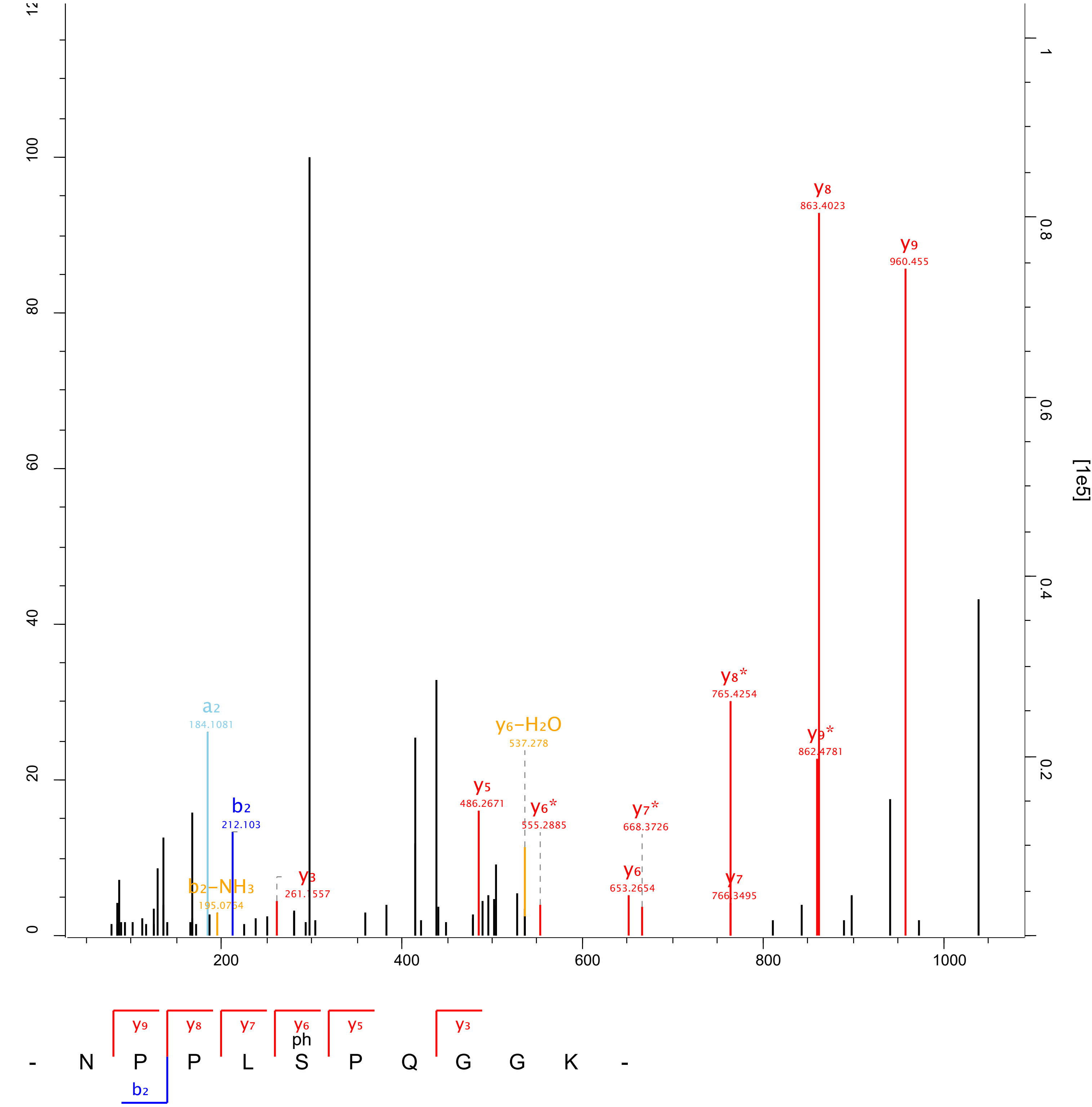

Sample output to test PDF Combine only

Raw file	Scan	Method	Score	m/z	Gene names
0523_4	5296	FTMS; HCD	79.2	498.72	ATL15

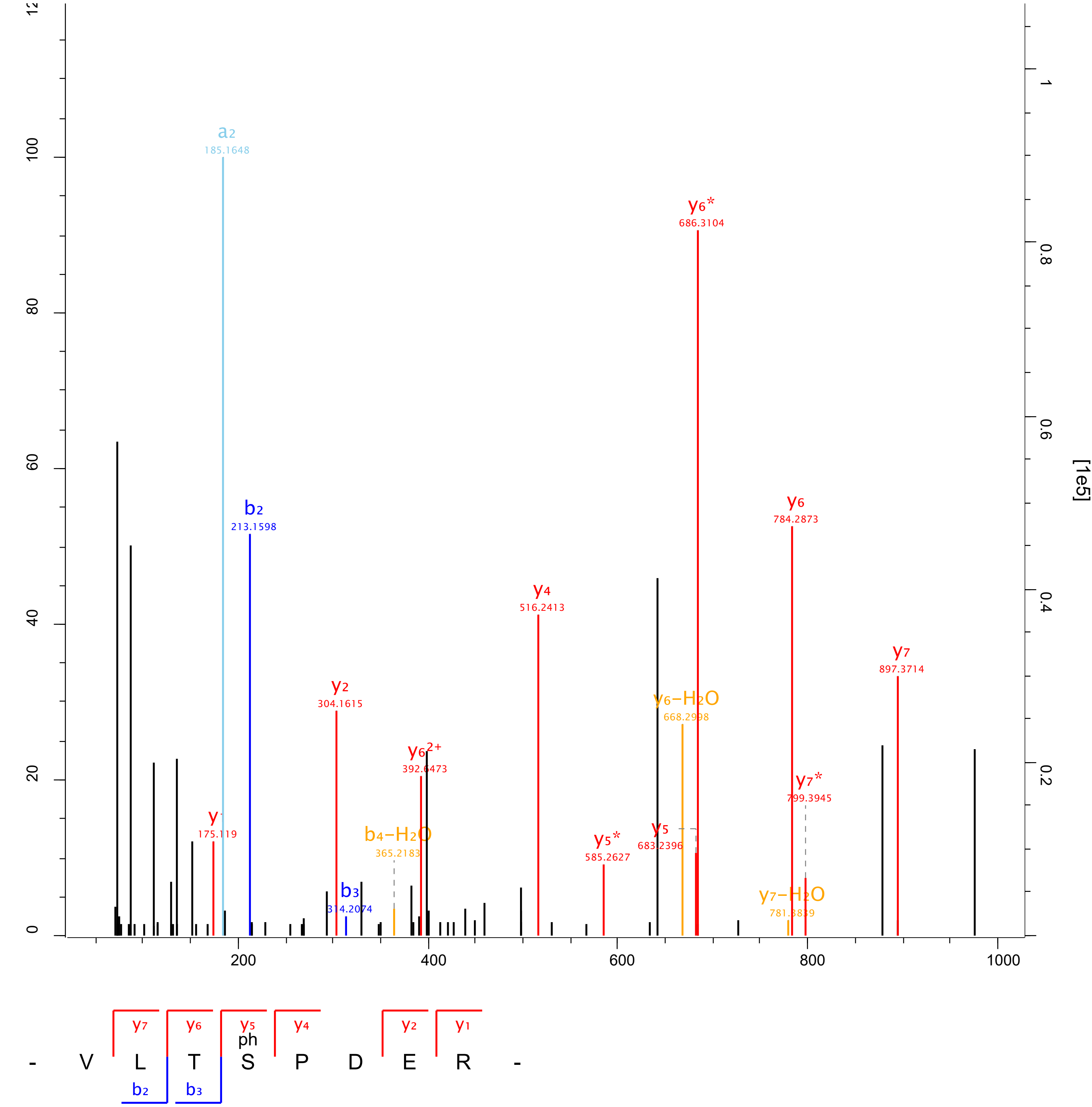

Sample output to test PDF Combine only

Raw file	Scan	Method	Score	m/z	Gene names
0523_4	5307	FTMS; HCD	126.77	672.75	MSBP2

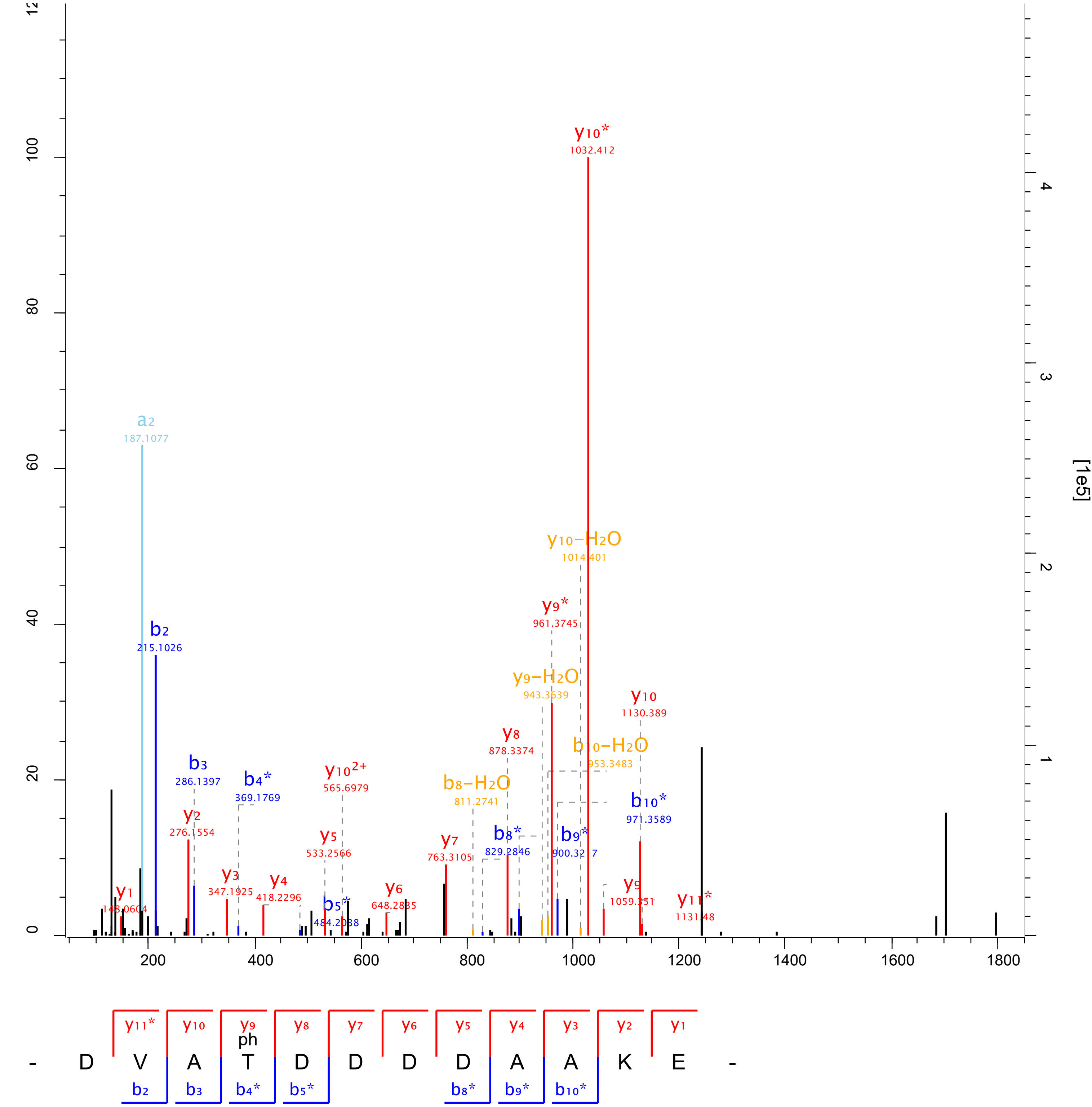

Sample output to test PDF Combine only

Raw file	Scan	Method	Score	m/z	Gene names
0523_4	5315	FTMS; HCD	109.79	657.8	DPL1

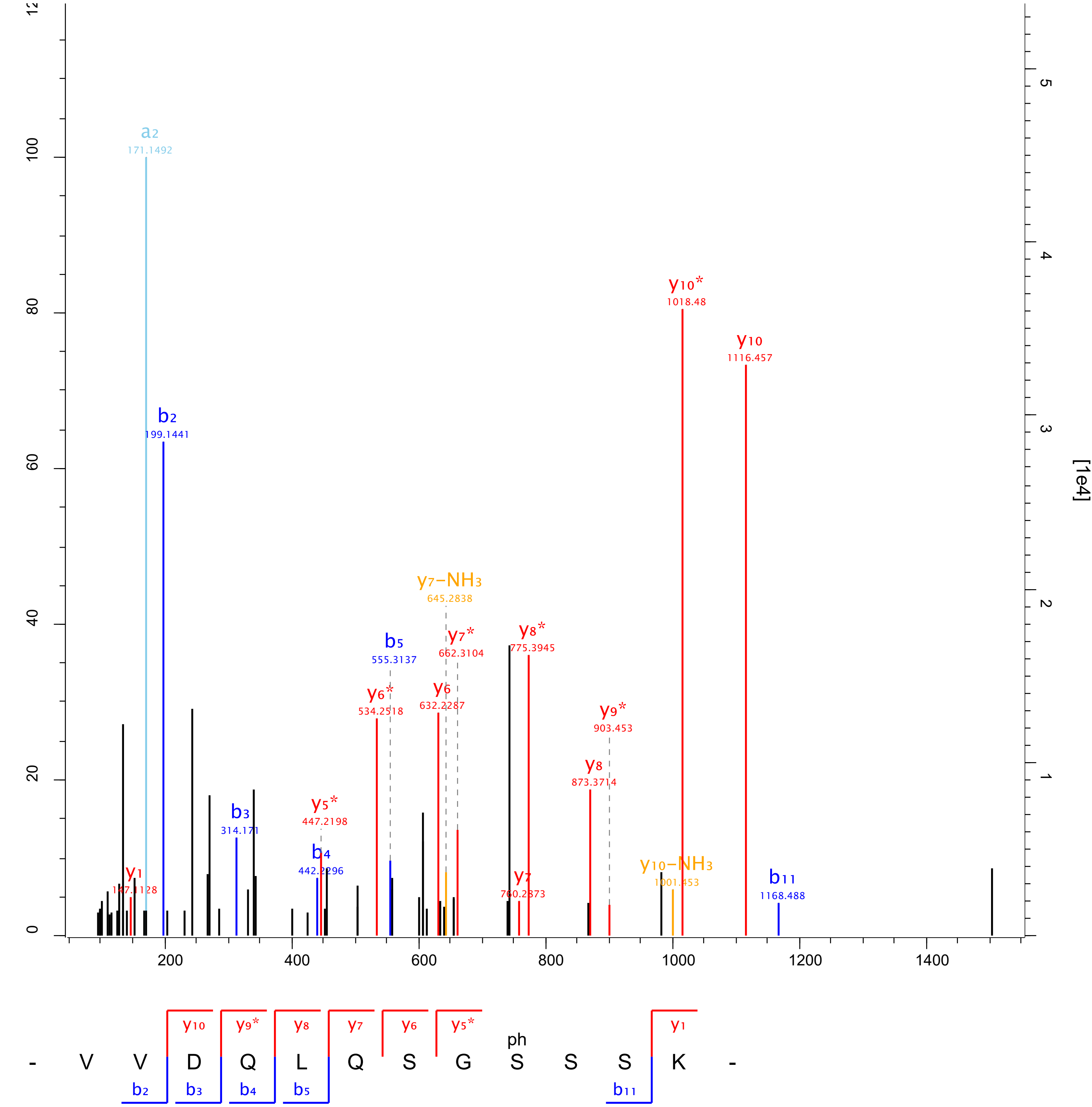

Sample output to test PDF Combine only

Raw file	Scan	Method	Score	m/z	Gene names
0523_4	5316	FTMS; HCD	55.66	785.82	SE

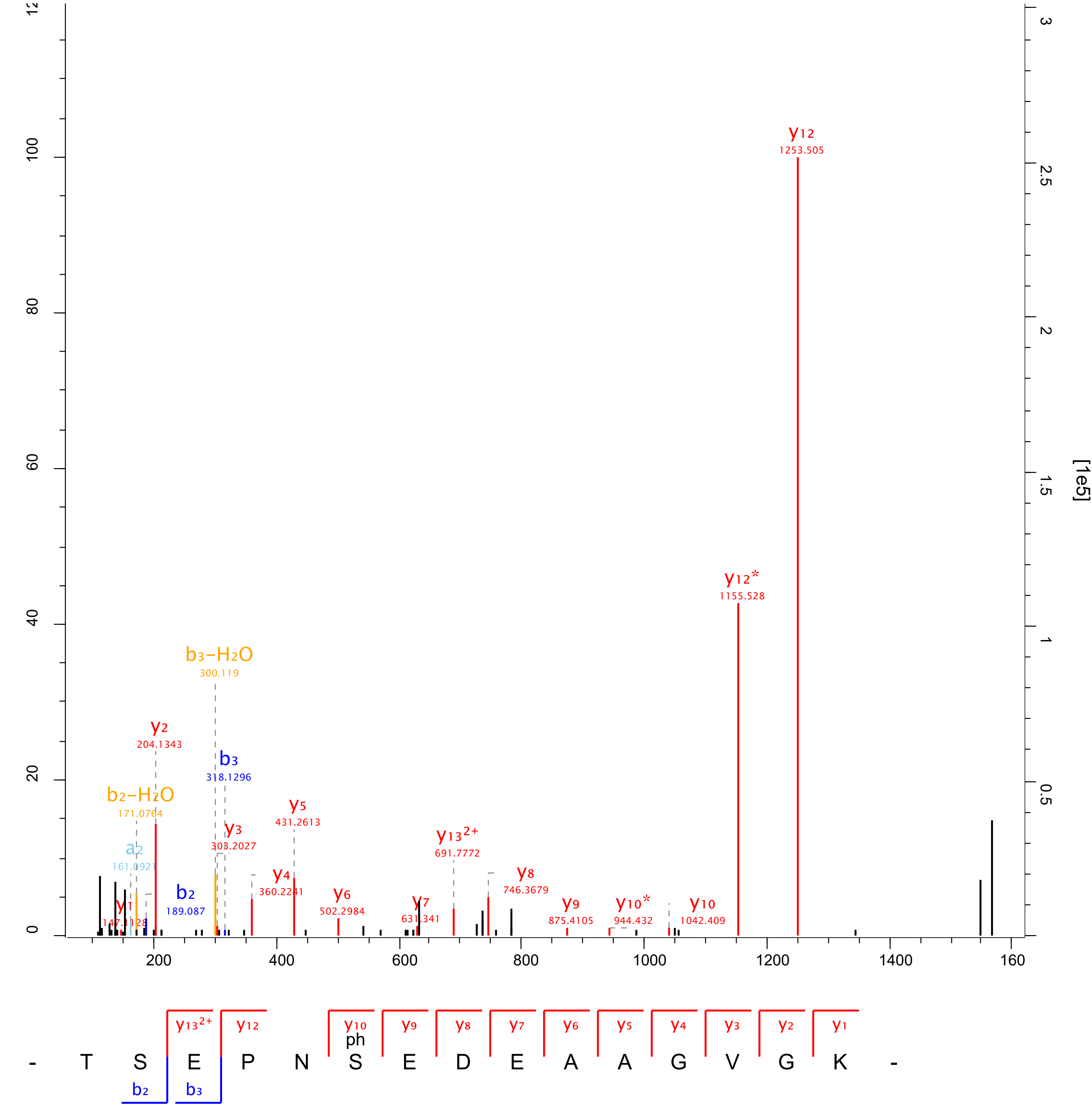

Sample output to test PDF Combine only

Raw file	Scan	Method	Score	m/z	Gene names
0523_4	5325	FTMS; HCD	104.21	554.24	DGK3

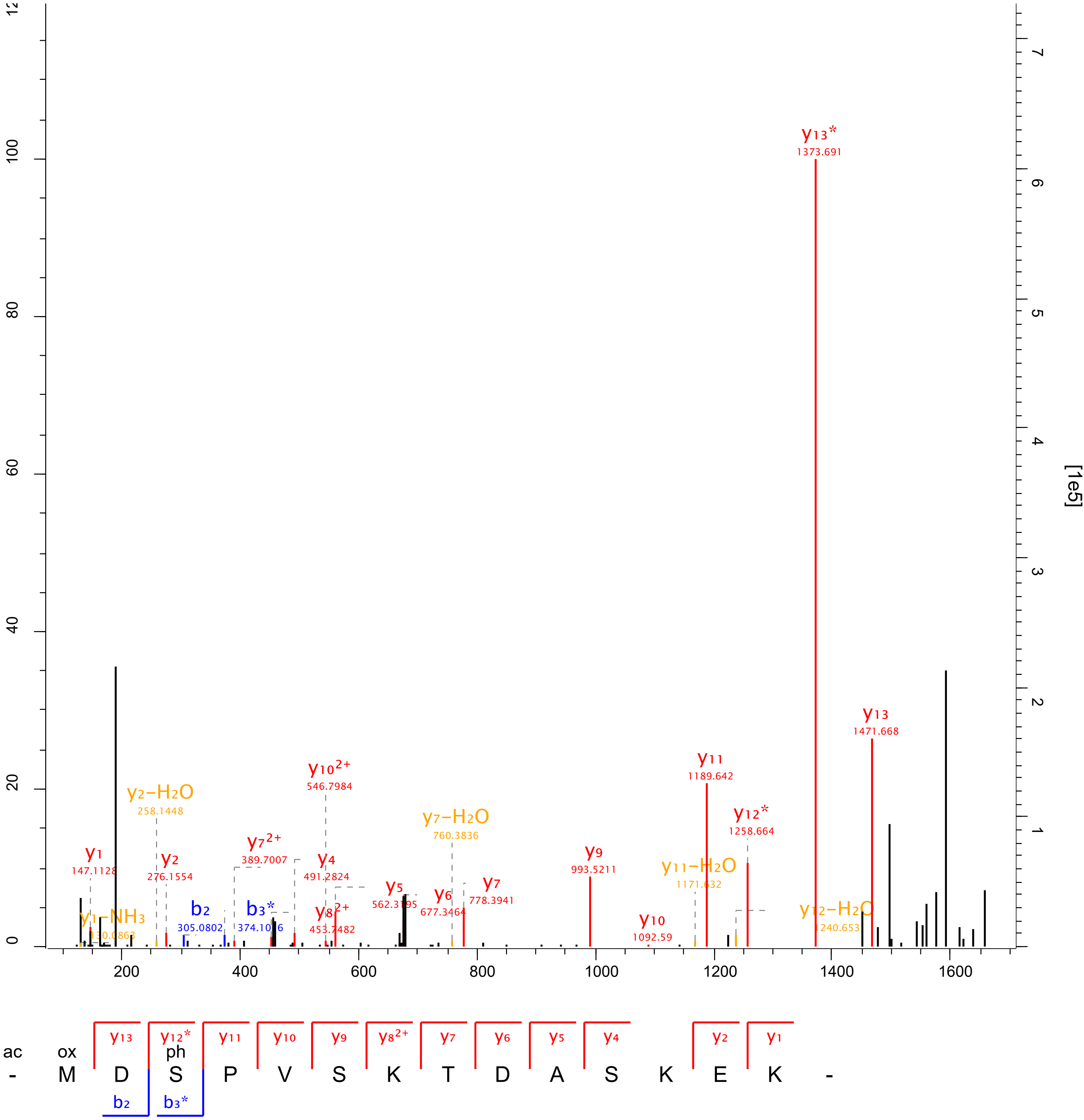

Sample output to test PDF Combine only

Raw file	Scan	Method	Score	m/z
0523_4	5371	FTMS; HCD	54	677.25

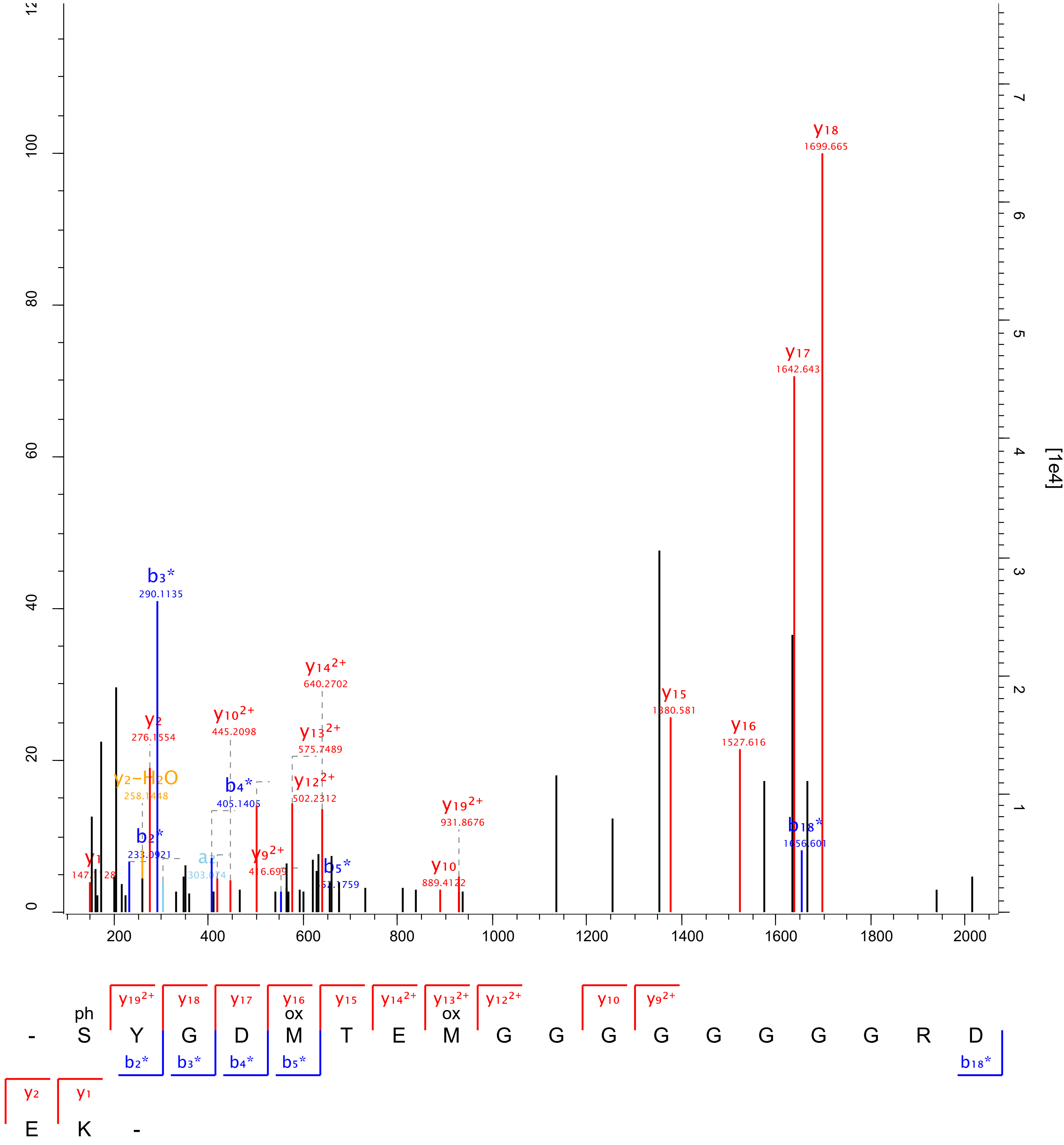

Sample output to test PDF Combine only

Raw file	Scan	Method	Score	m/z	Gene names
0523_4	5446	FTMS; HCD	69.82	512.25	At3g59090

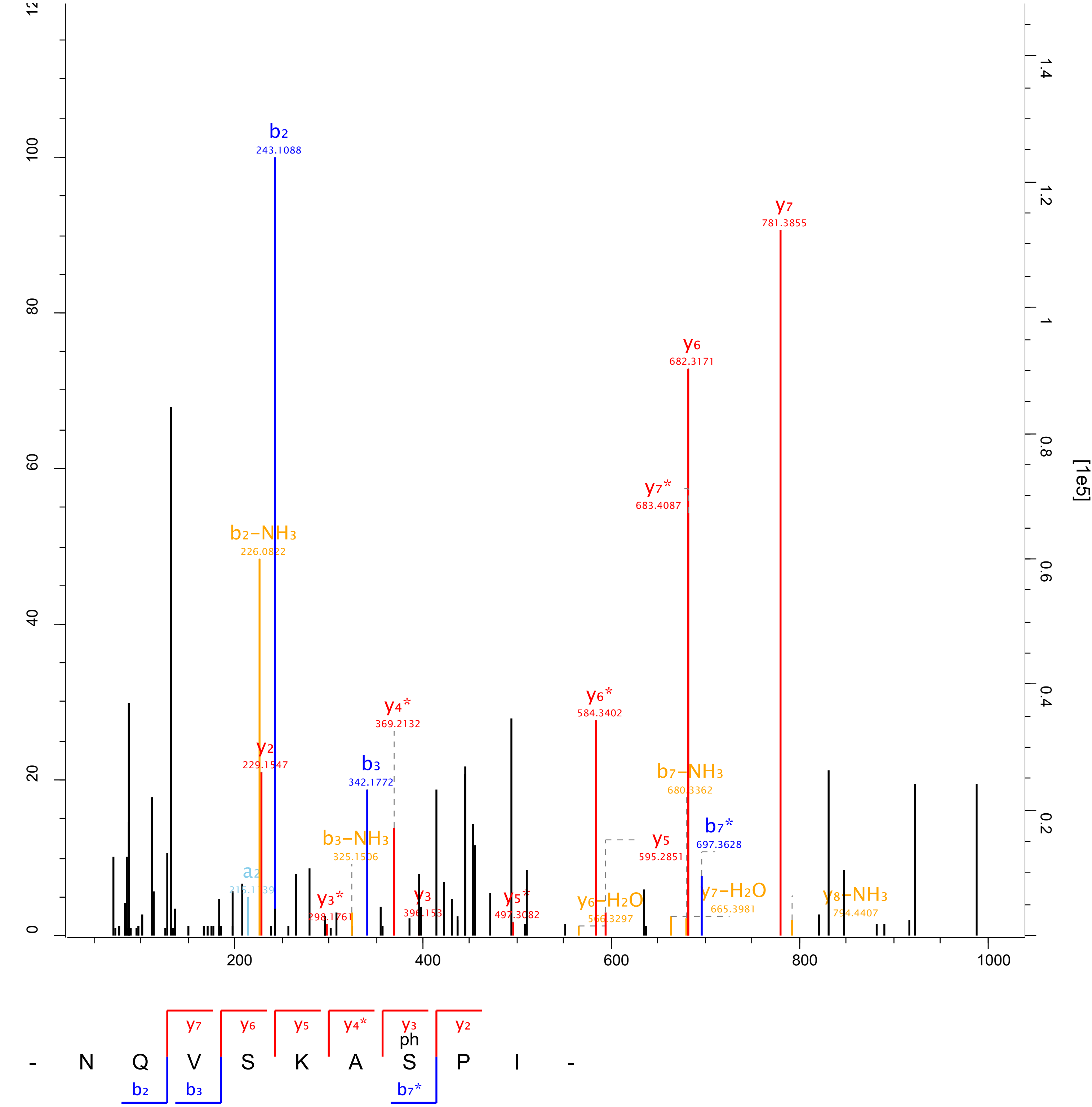

Sample output to test PDF Combine only

Raw file	Scan	Method	Score	m/z	Gene names
0523_4	5580	FTMS; HCD	180.15	488.69	At5g06970

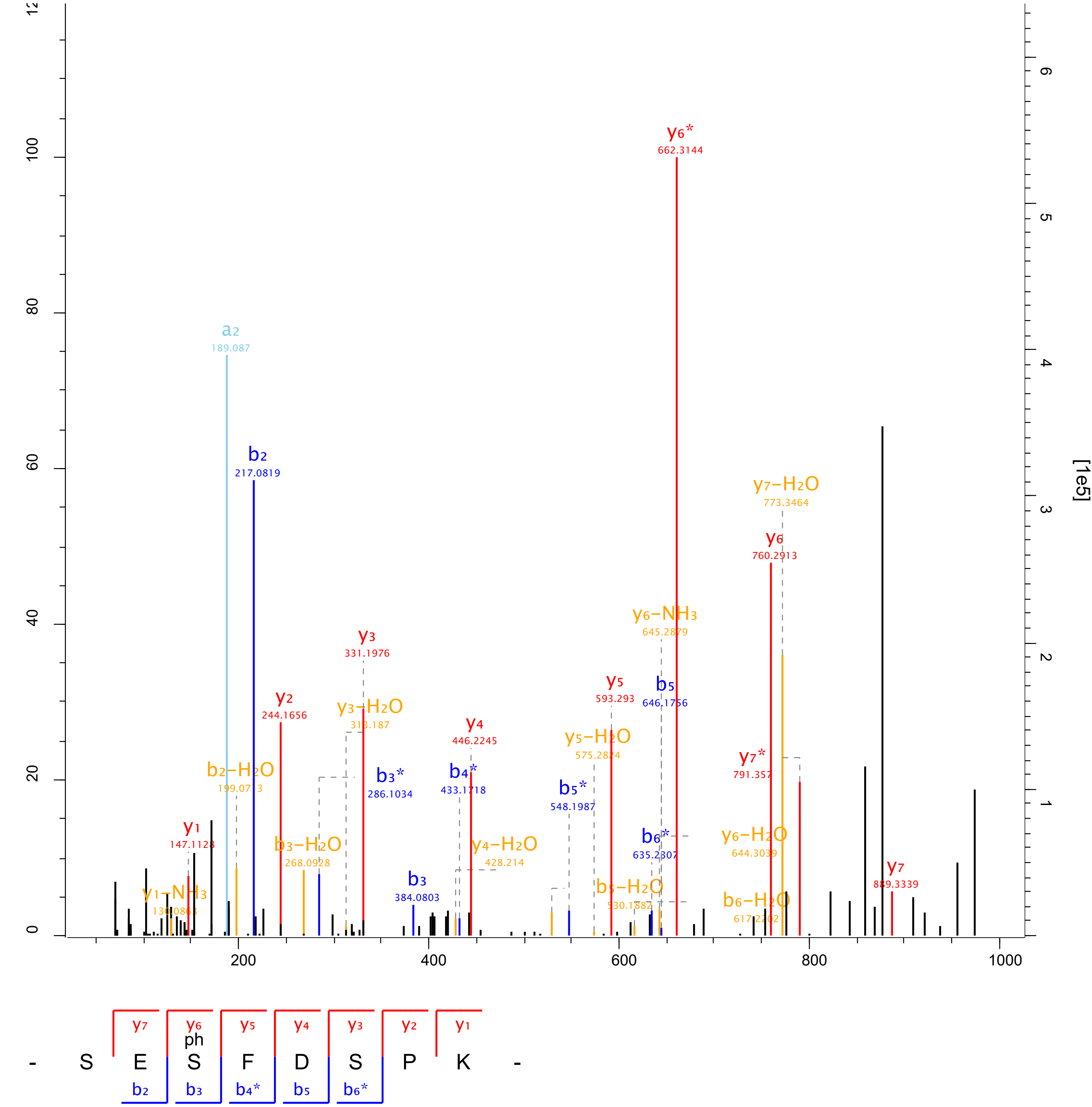

Sample output to test PDF Combine only

Raw file	Scan	Method	Score	m/z	Gene names
0523_4	5658	FTMS; HCD	99.93	601.26	F25P22.8

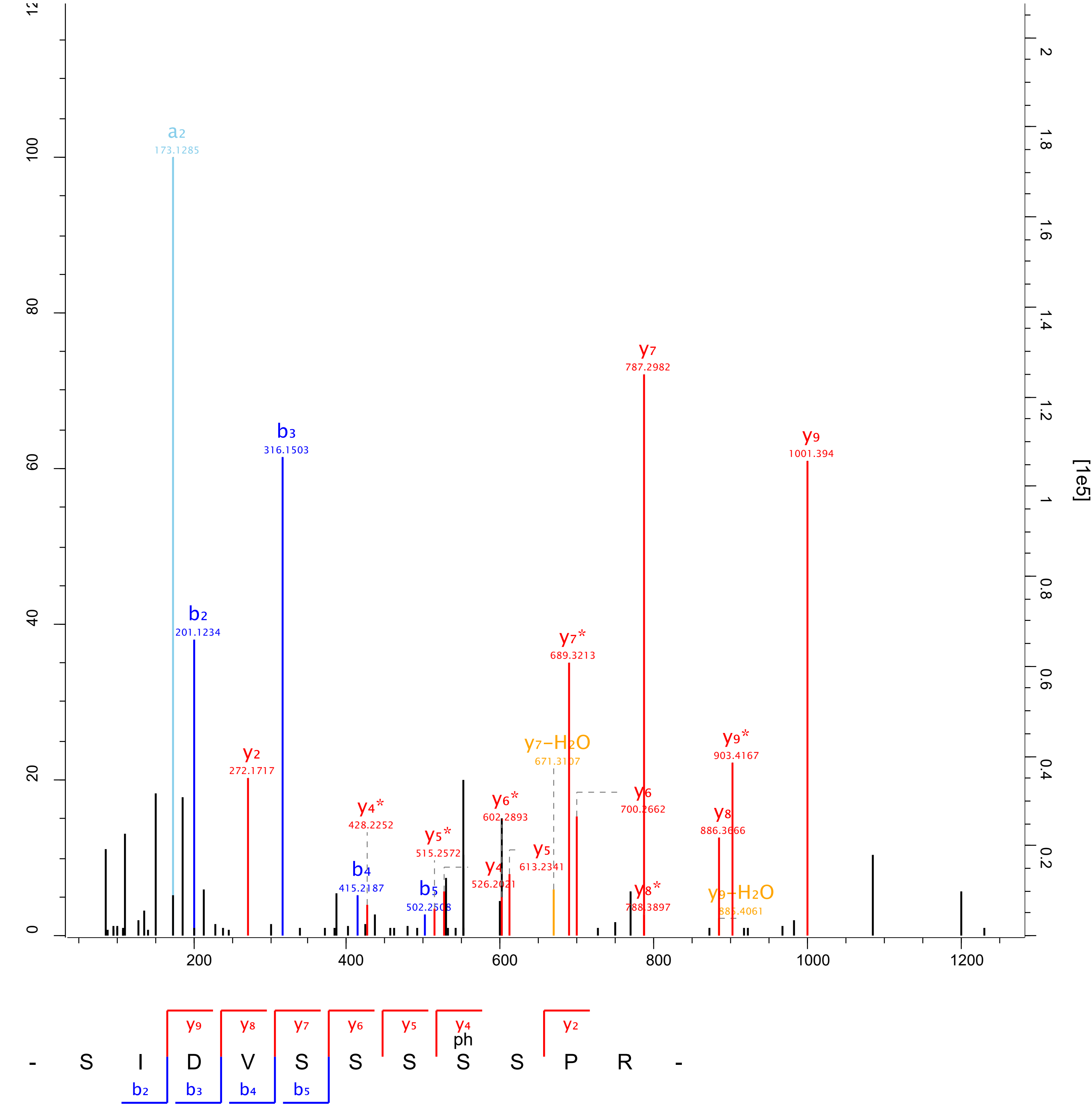

Sample output to test PDF Combine only

Raw file	Scan	Method	Score	m/z	Gene names
0523_4	5664	FTMS; HCD	55.55	671.79	ATL31

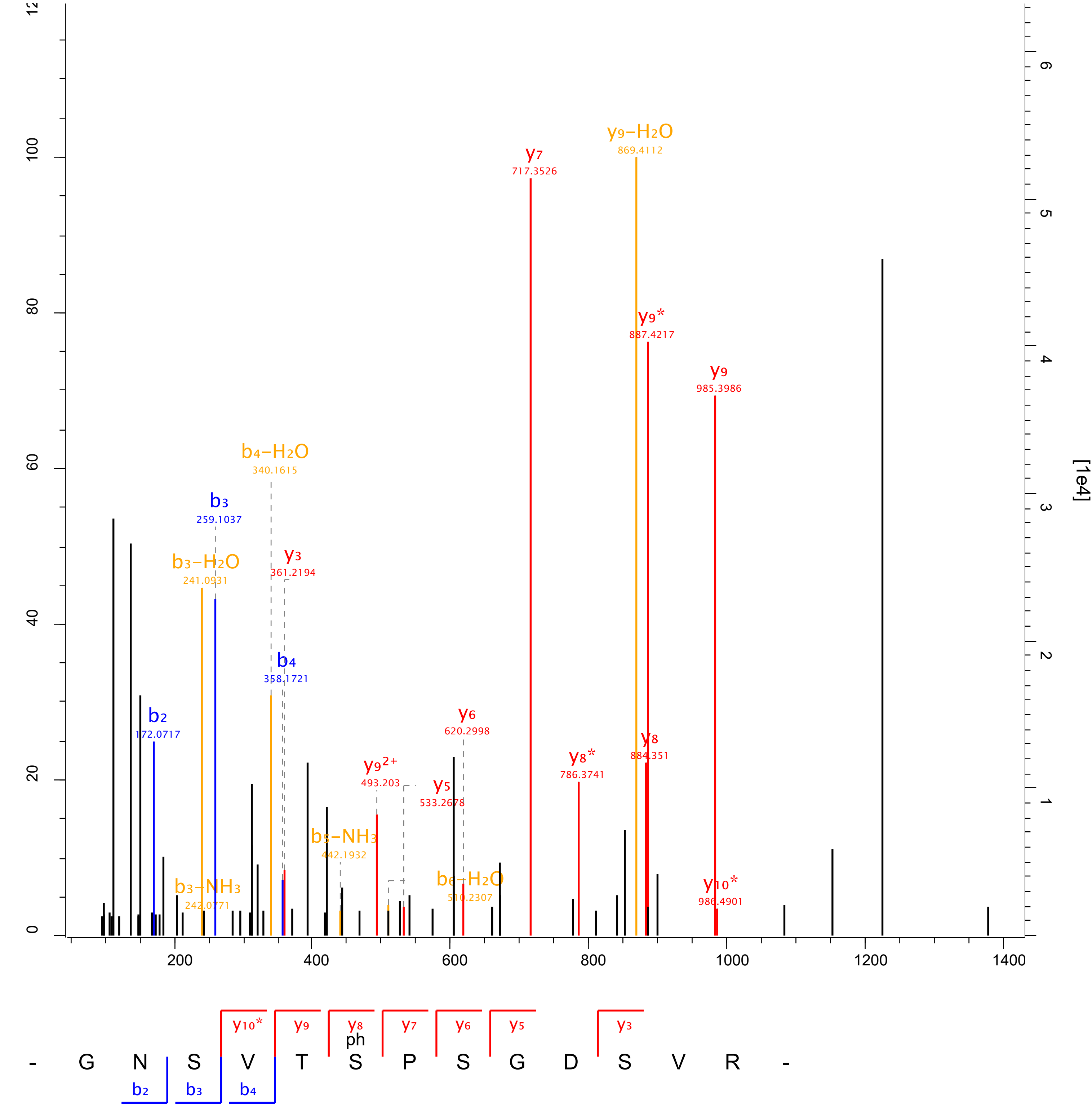

Sample output to test PDF Combine only

Raw file	Scan	Method	Score	m/z	Gene names
0523_4	5725	FTMS; HCD	58.59	452.72	RABF1

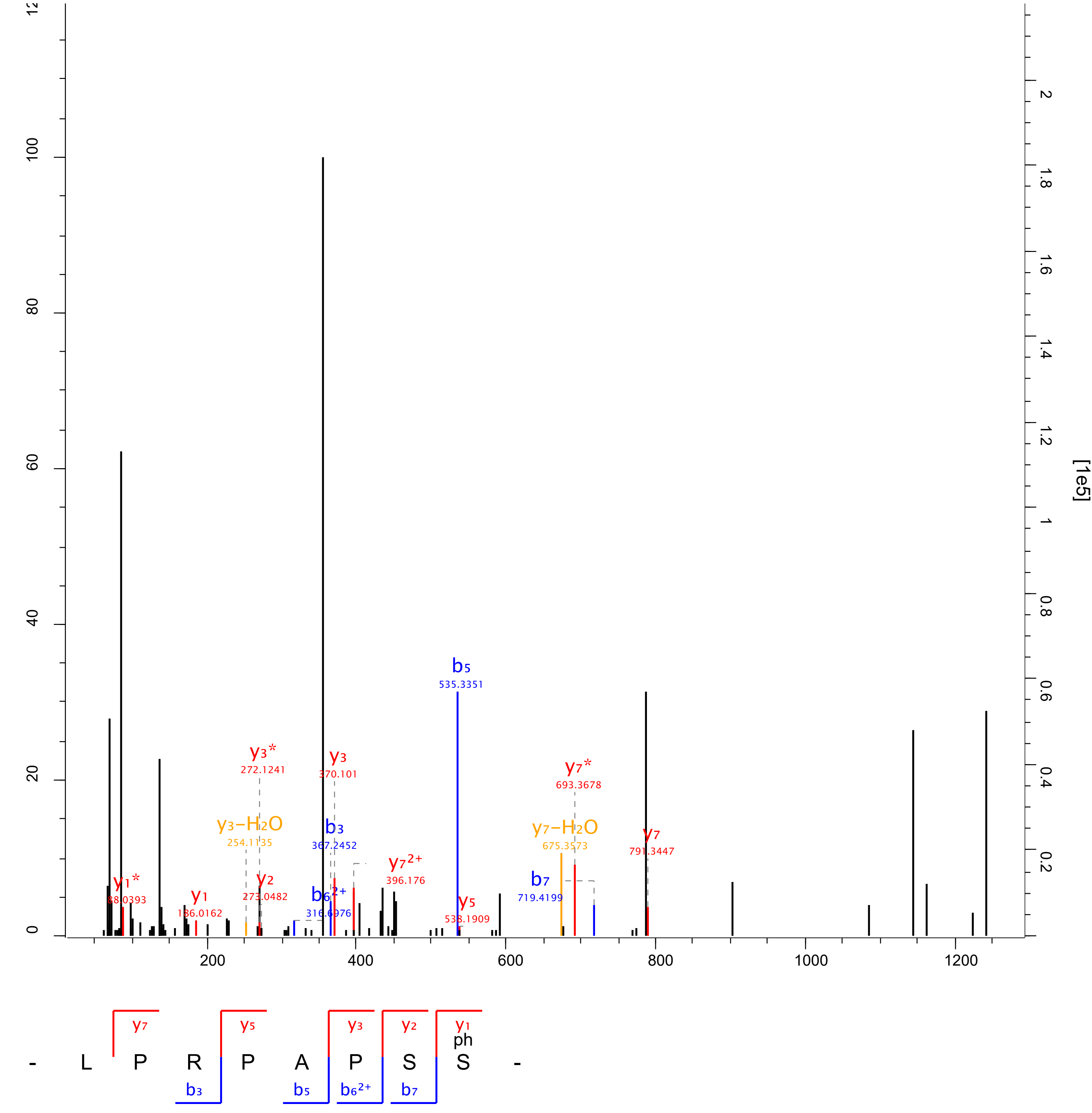

Sample output to test PDF Combine only

Raw file	Scan	Method	Score	m/z	Gene names
0523_4	5786	FTMS; HCD	103.4	885.34	NRT2.1

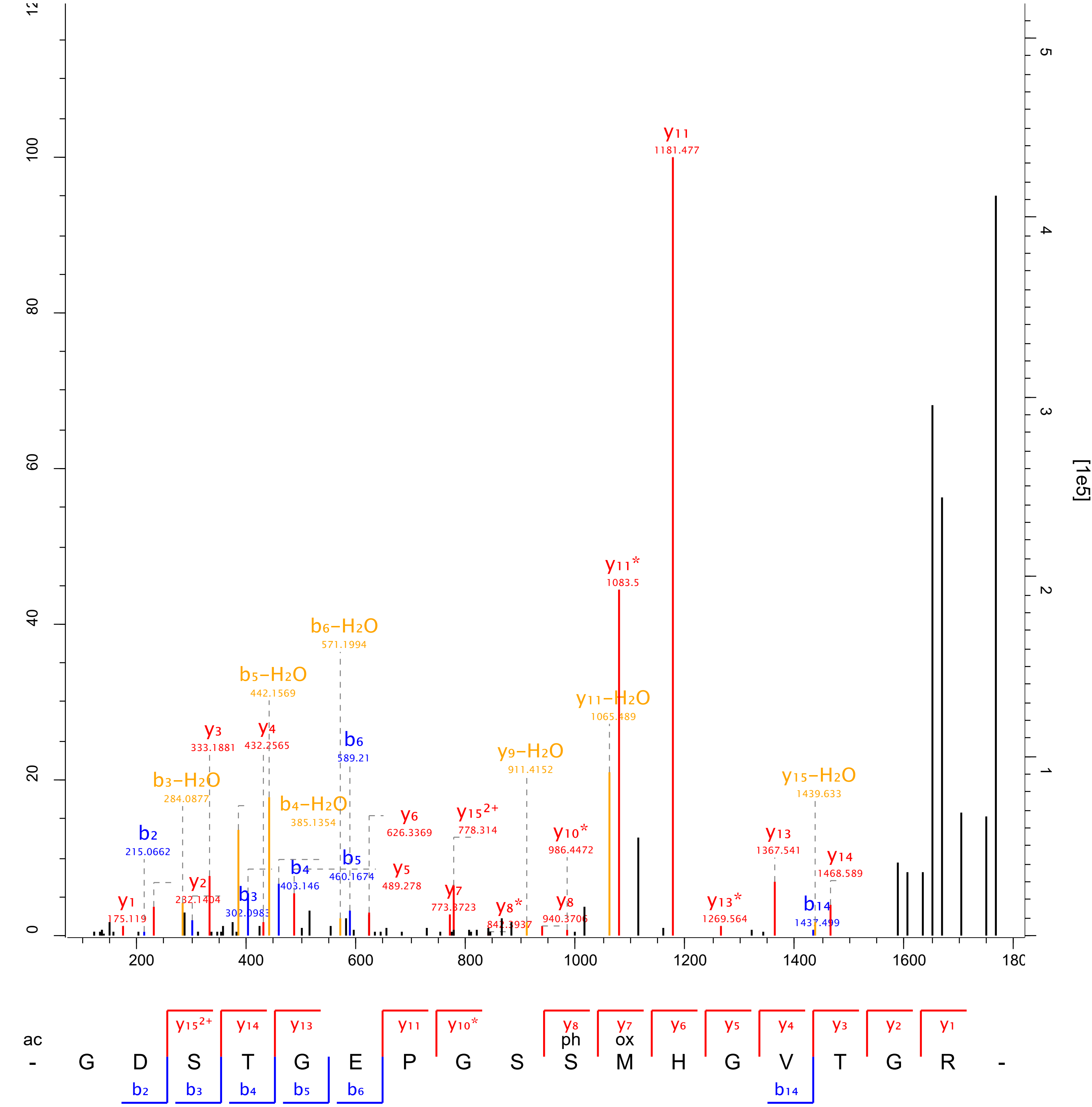

Sample output to test PDF Combine only

Raw file	Scan	Method	Score	m/z
0523_4	5913	FTMS; HCD	111.79	558.27

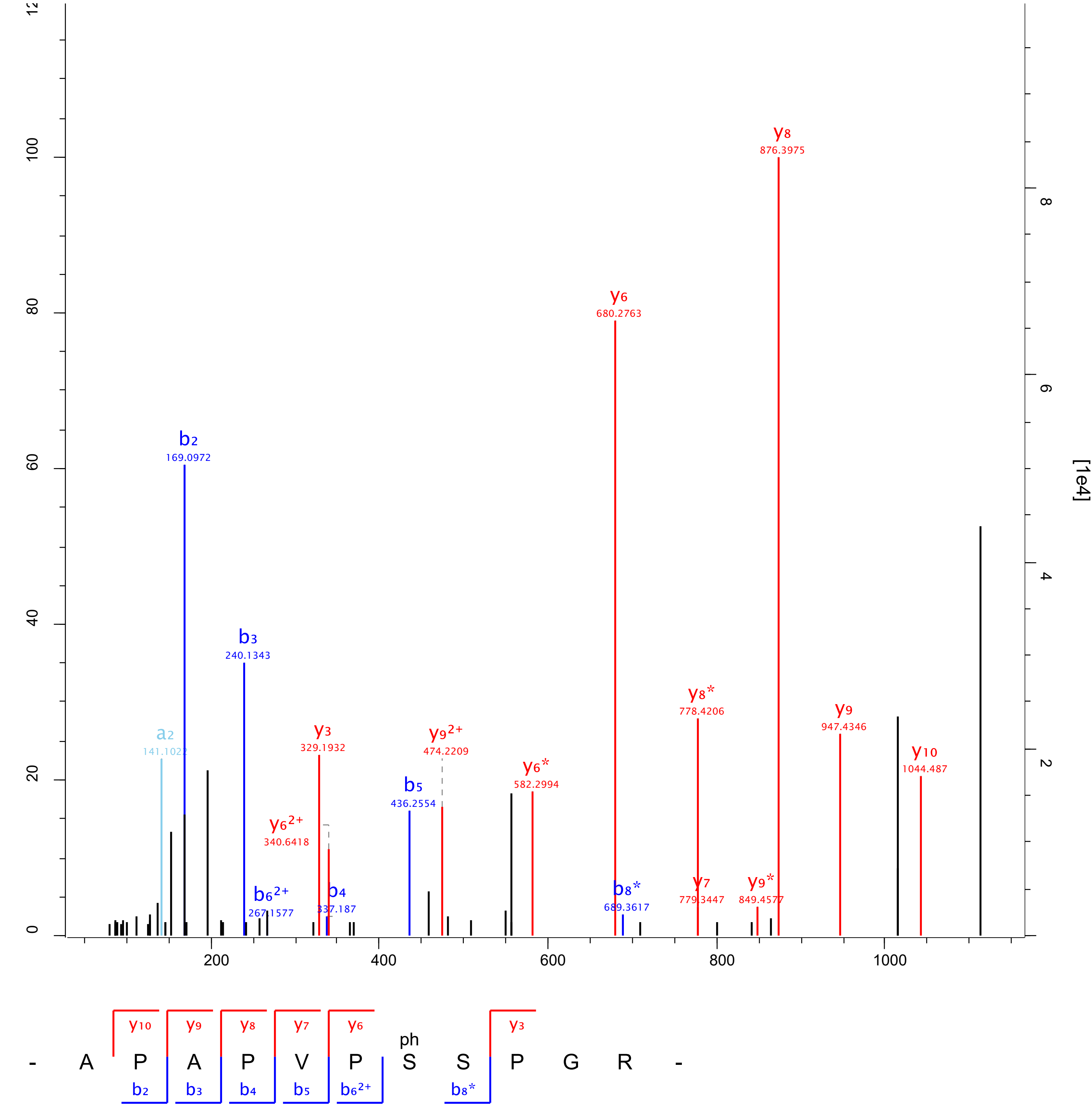

Sample output to test PDF Combine only

Raw file	Scan	Method	Score	m/z	Gene names
0523_4	5987	FTMS; HCD	128.22	636.24	F23A5.29

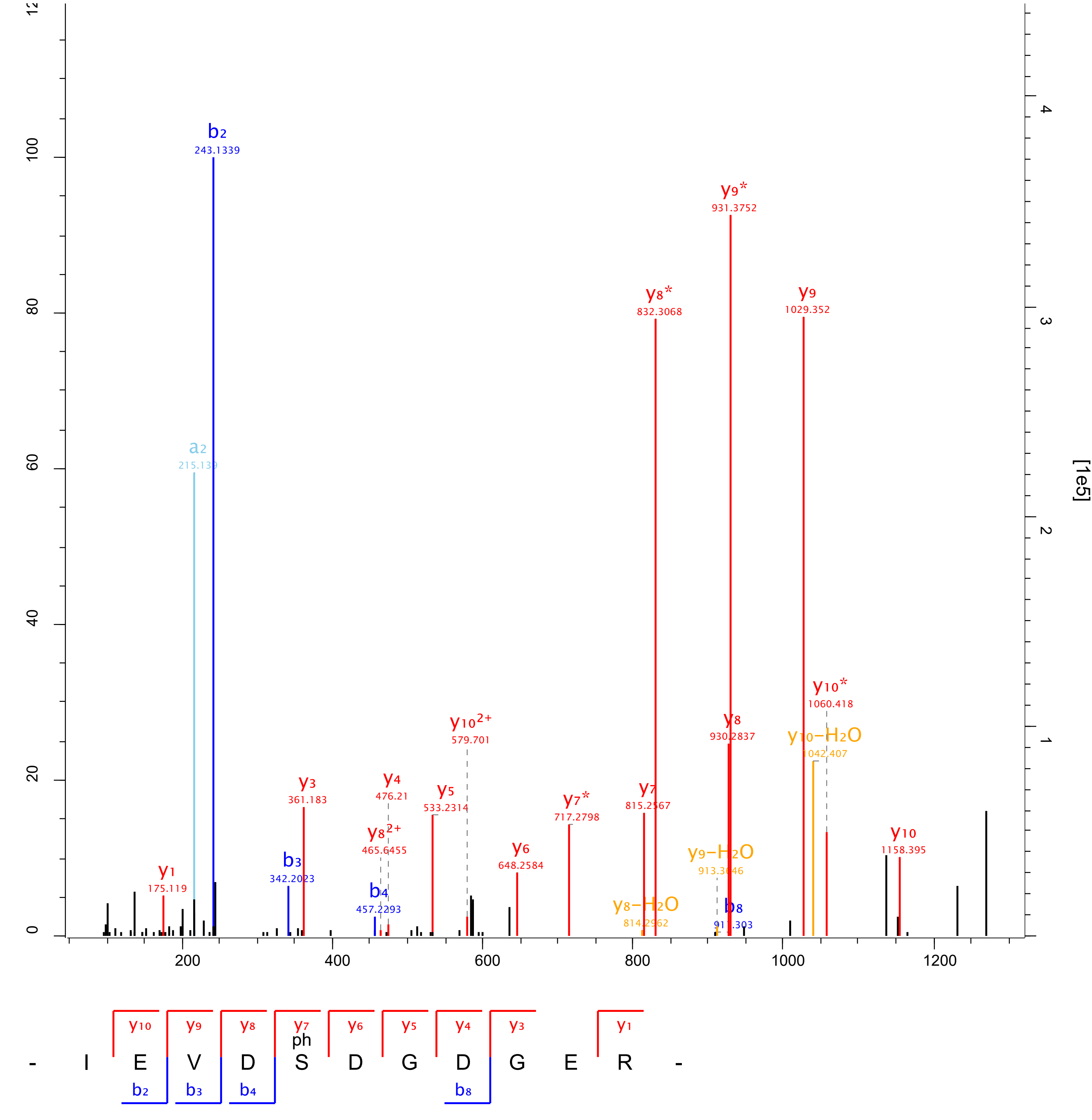

Sample output to test PDF Combine only

Raw file	Scan	Method	Score	m/z	Gene names
0523_4	6055	FTMS; HCD	157.56	649.78	SCC3

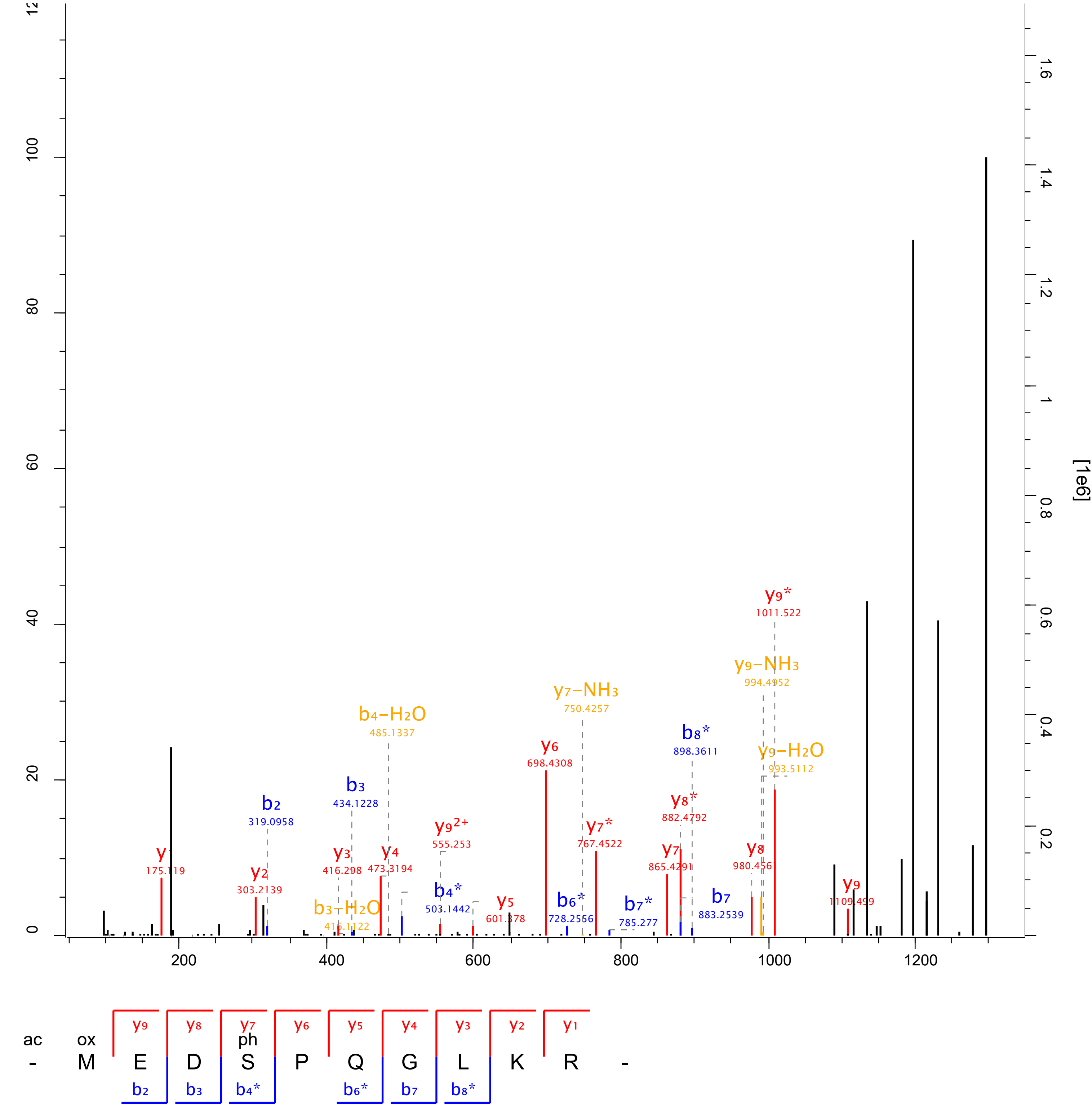

Sample output to test PDF Combine only

Raw file	Scan	Method	Score	m/z	Gene names
0523_4	6128	FTMS; HCD	142.97	528.72	ABCB6

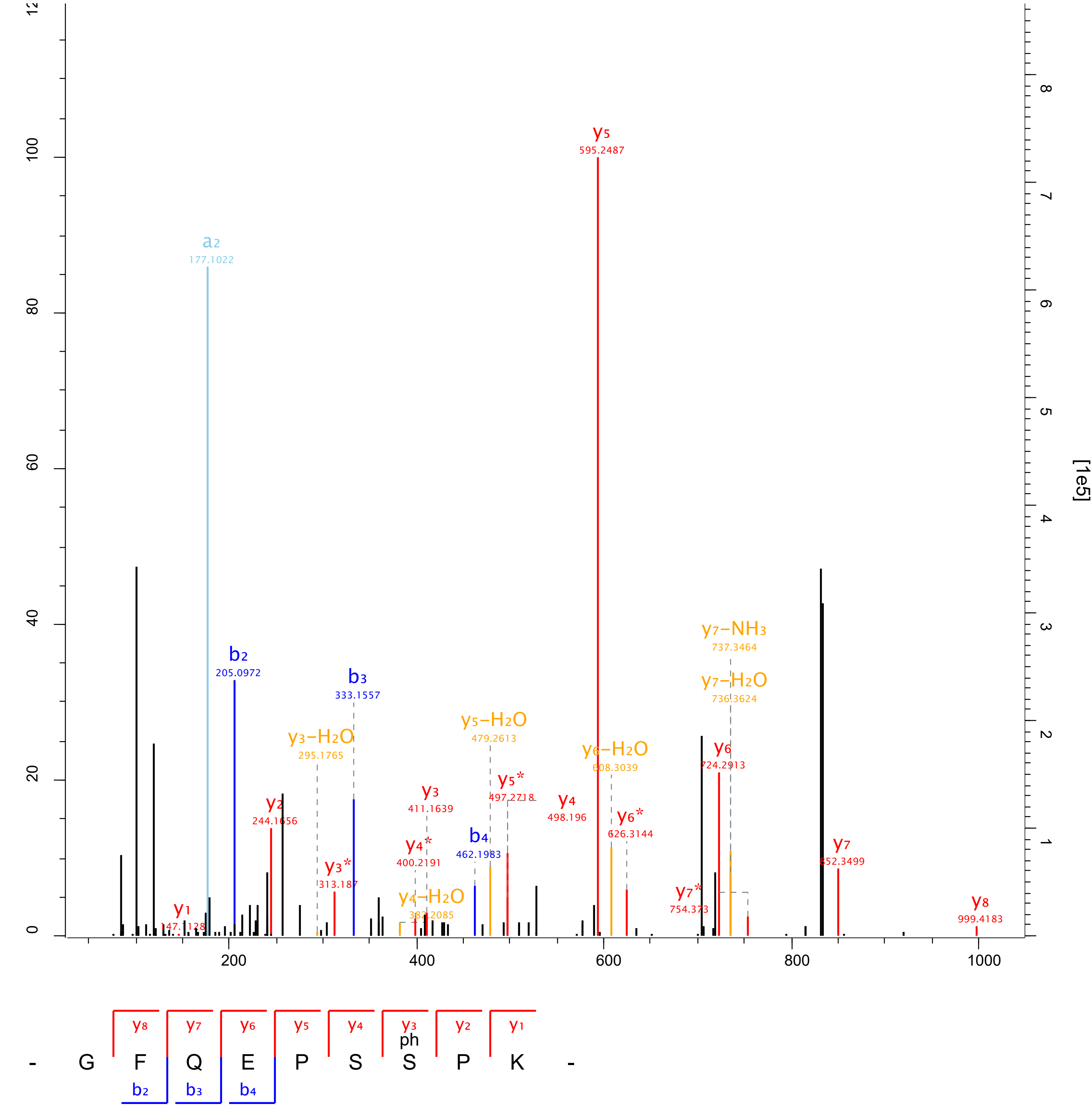

Sample output to test PDF Combine only

Raw file	Scan	Method	Score	m/z
0523_4	6136	FTMS; HCD	104.88	751.77

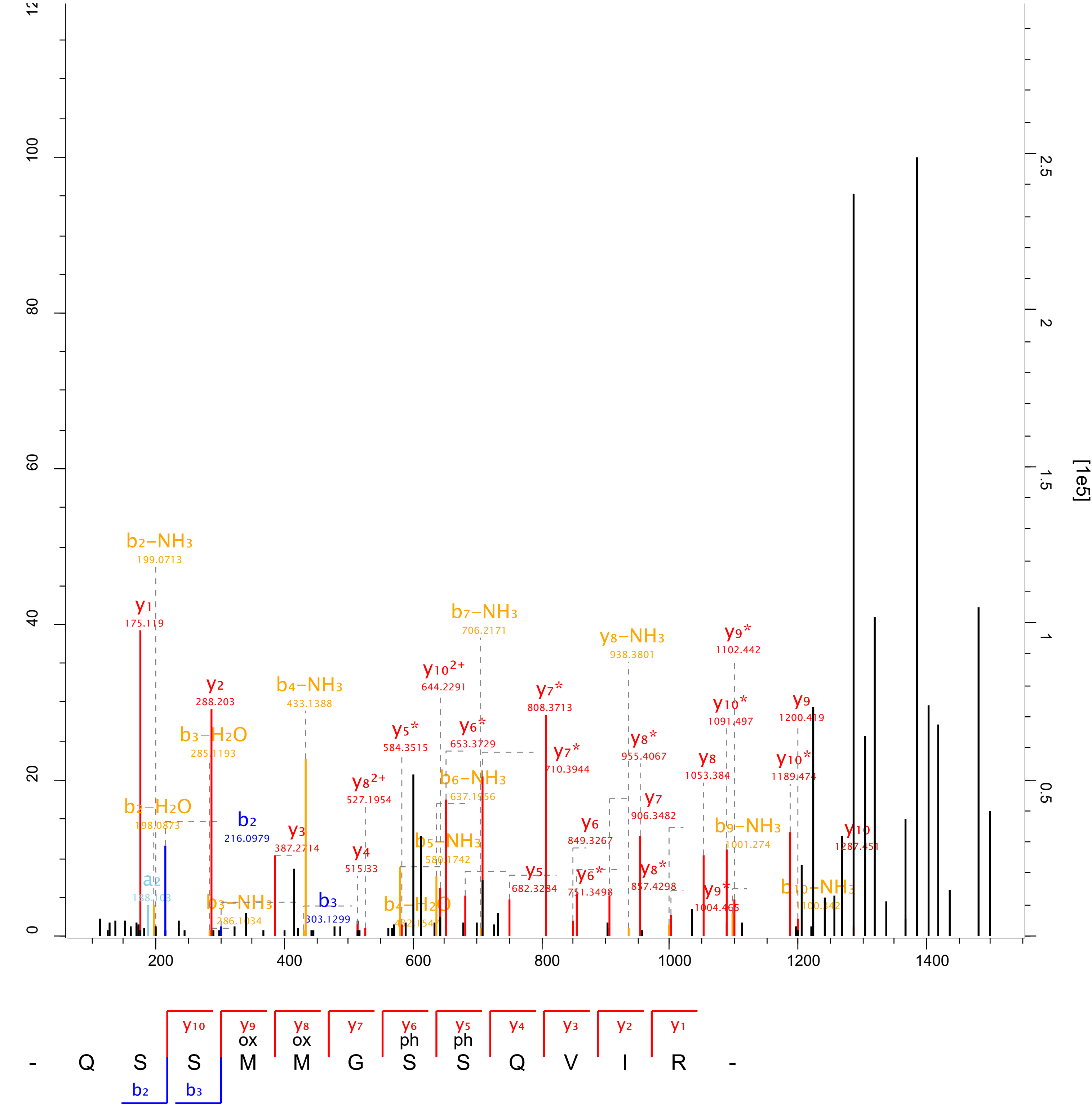

Sample output to test PDF Combine only

Raw file	Scan	Method	Score	m/z
0523_4	6163	FTMS; HCD	127.18	458.2

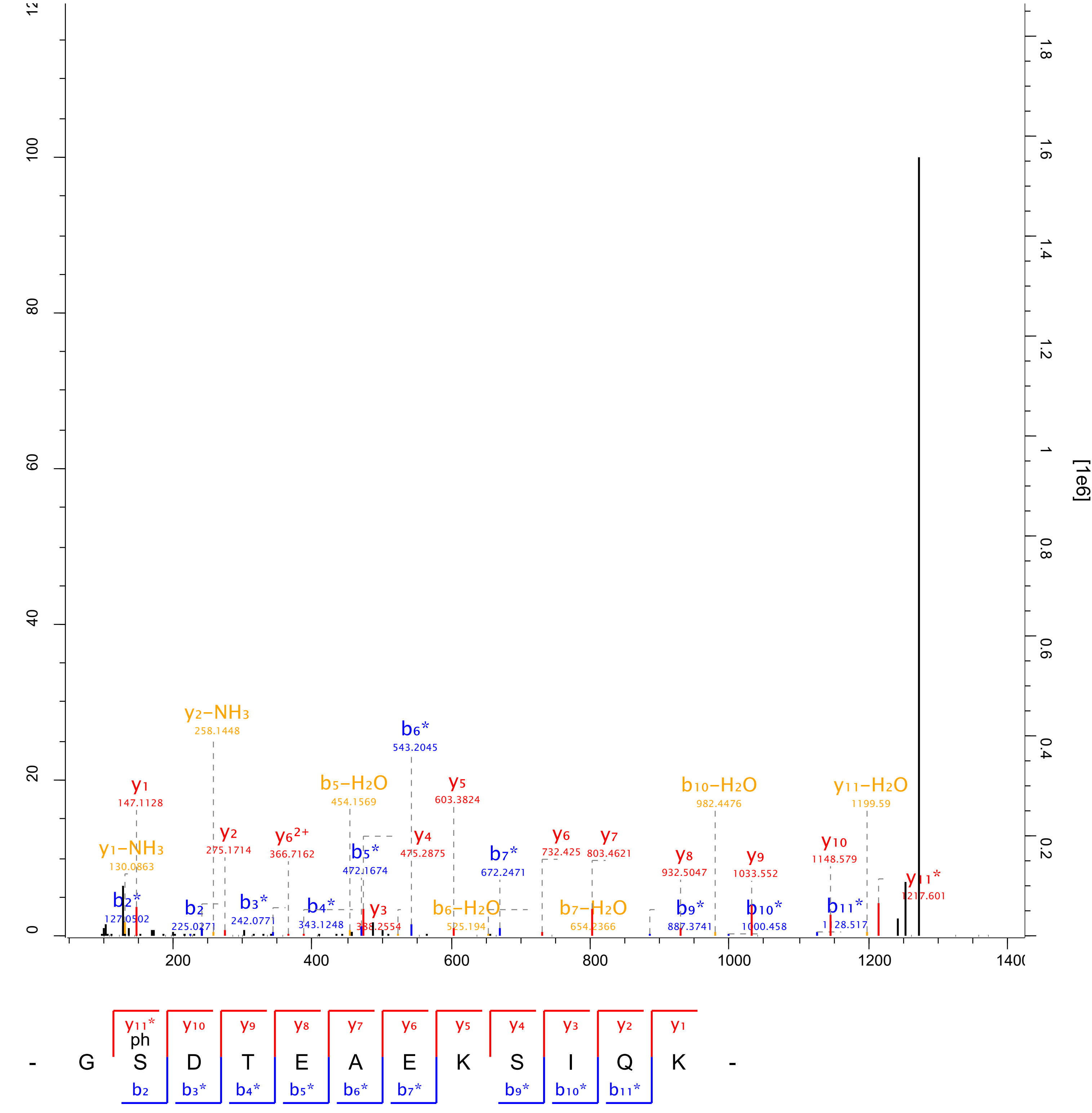

Sample output to test PDF Combine only

Raw file	Scan	Method	Score	m/z	Gene names
0523_4	6187	FTMS; HCD	235.62	833.87	DRP2B

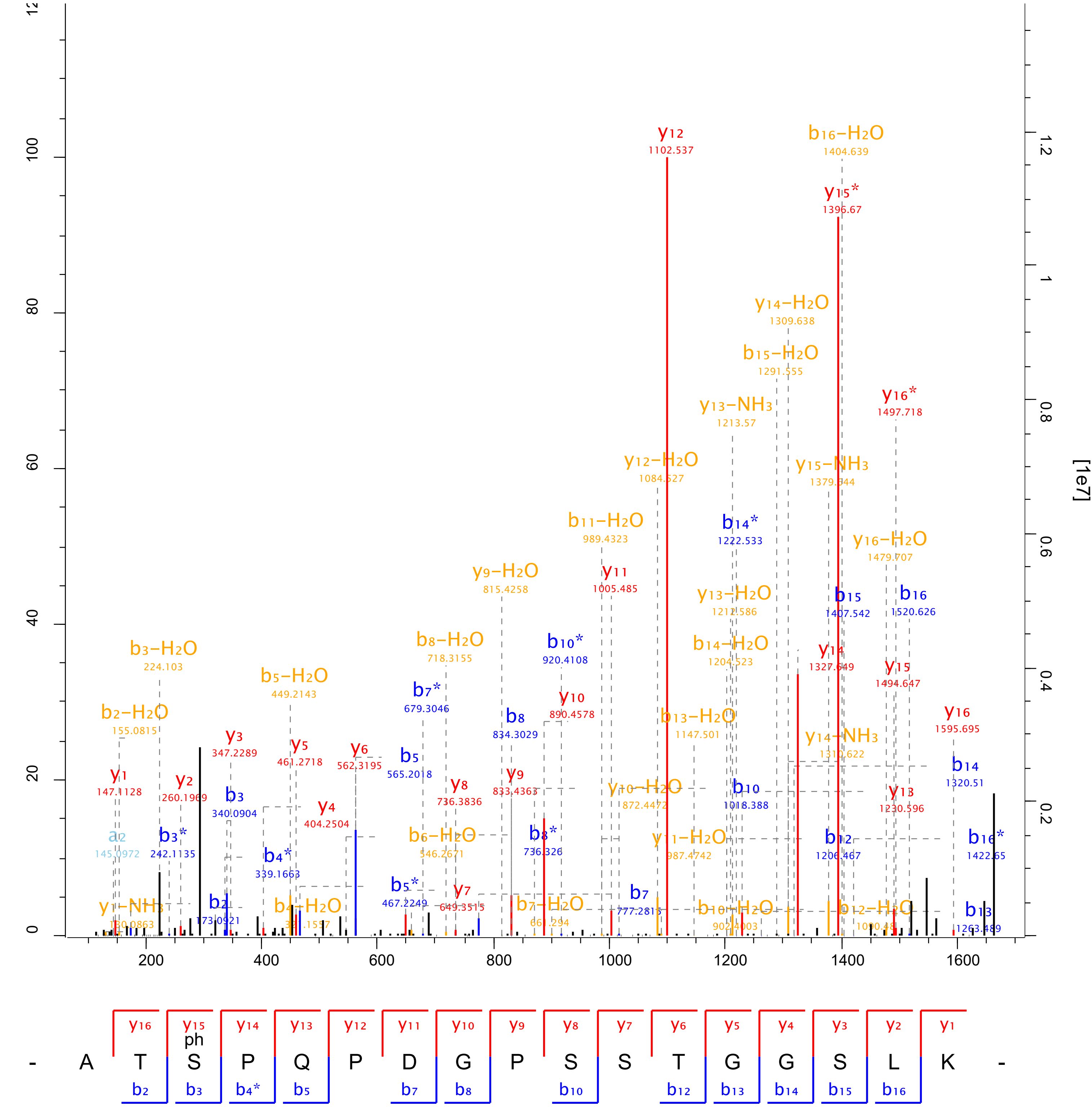

Sample output to test PDF Combine only

Raw file	Scan	Method	Score	m/z	Gene names
0523_4	6191	FTMS; HCD	133.16	784.26	T15K4.8

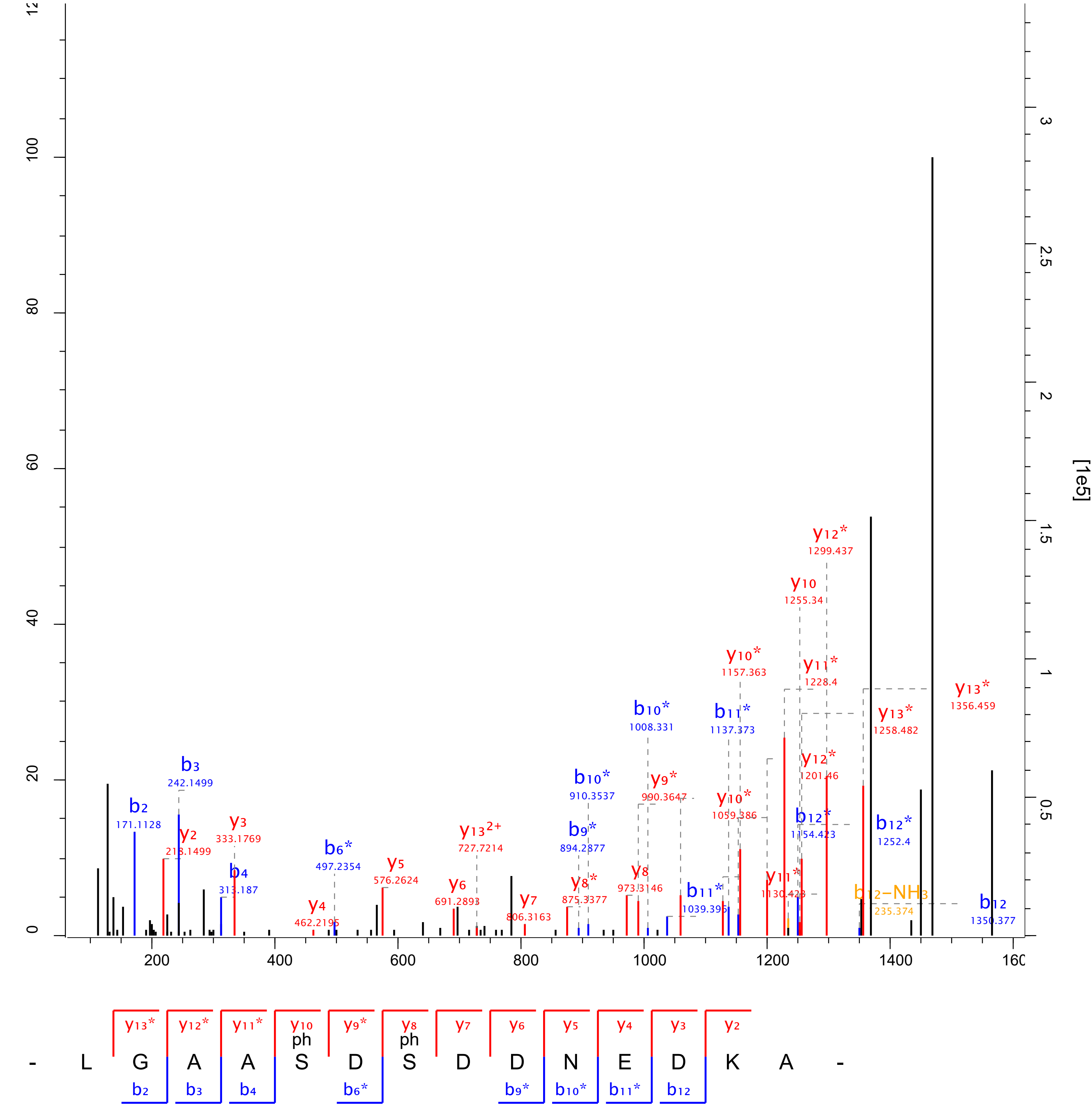

Sample output to test PDF Combine only

Raw file	Scan	Method	Score	m/z	Gene names
0523_4	6200	FTMS; HCD	171.26	491.87	At2g04410

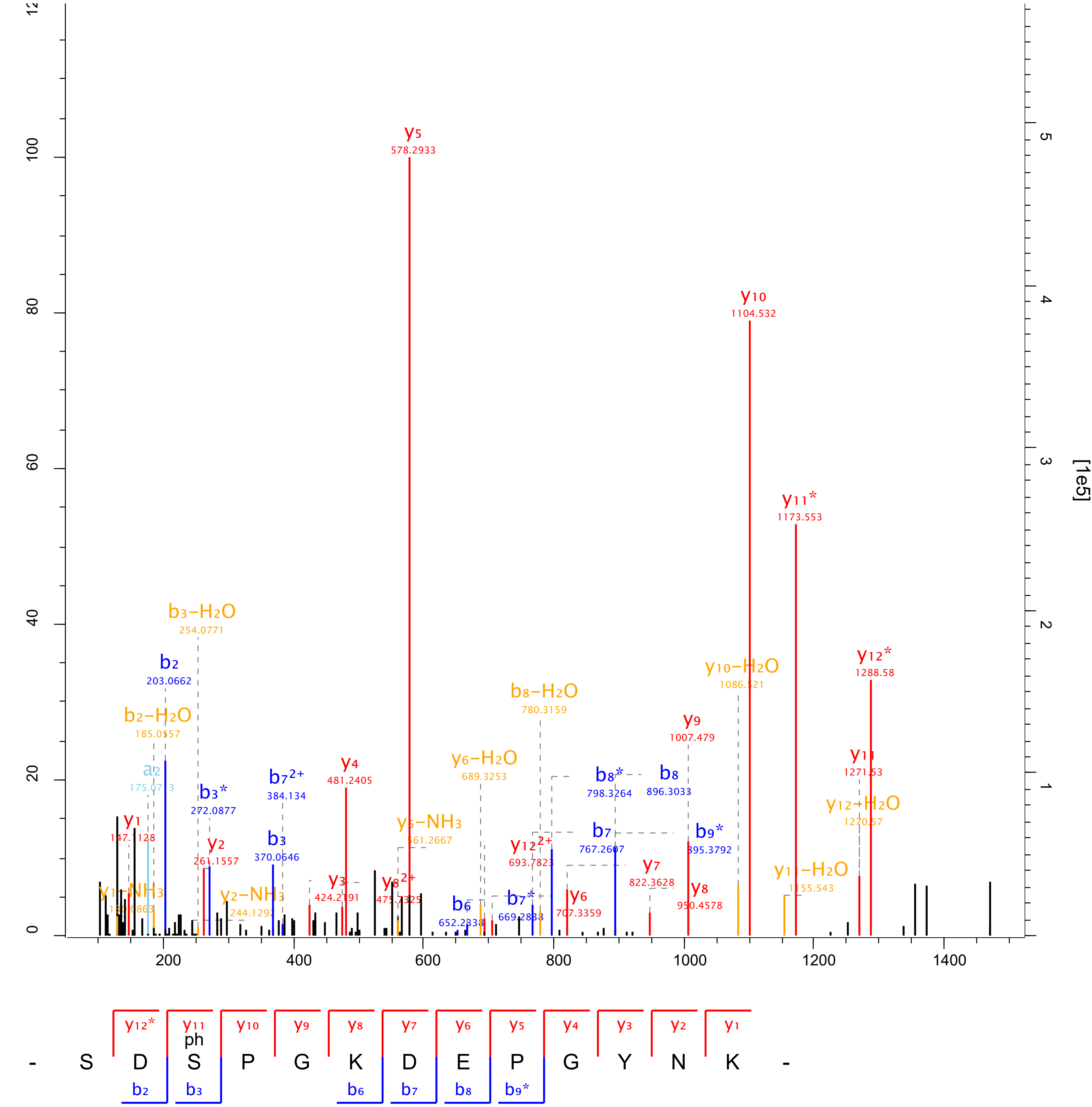

Sample output to test PDF Combine only

Raw file	Scan	Method	Score	m/z	Gene names
0523_4	6228	FTMS; HCD	89.47	689.81	F9K20.7

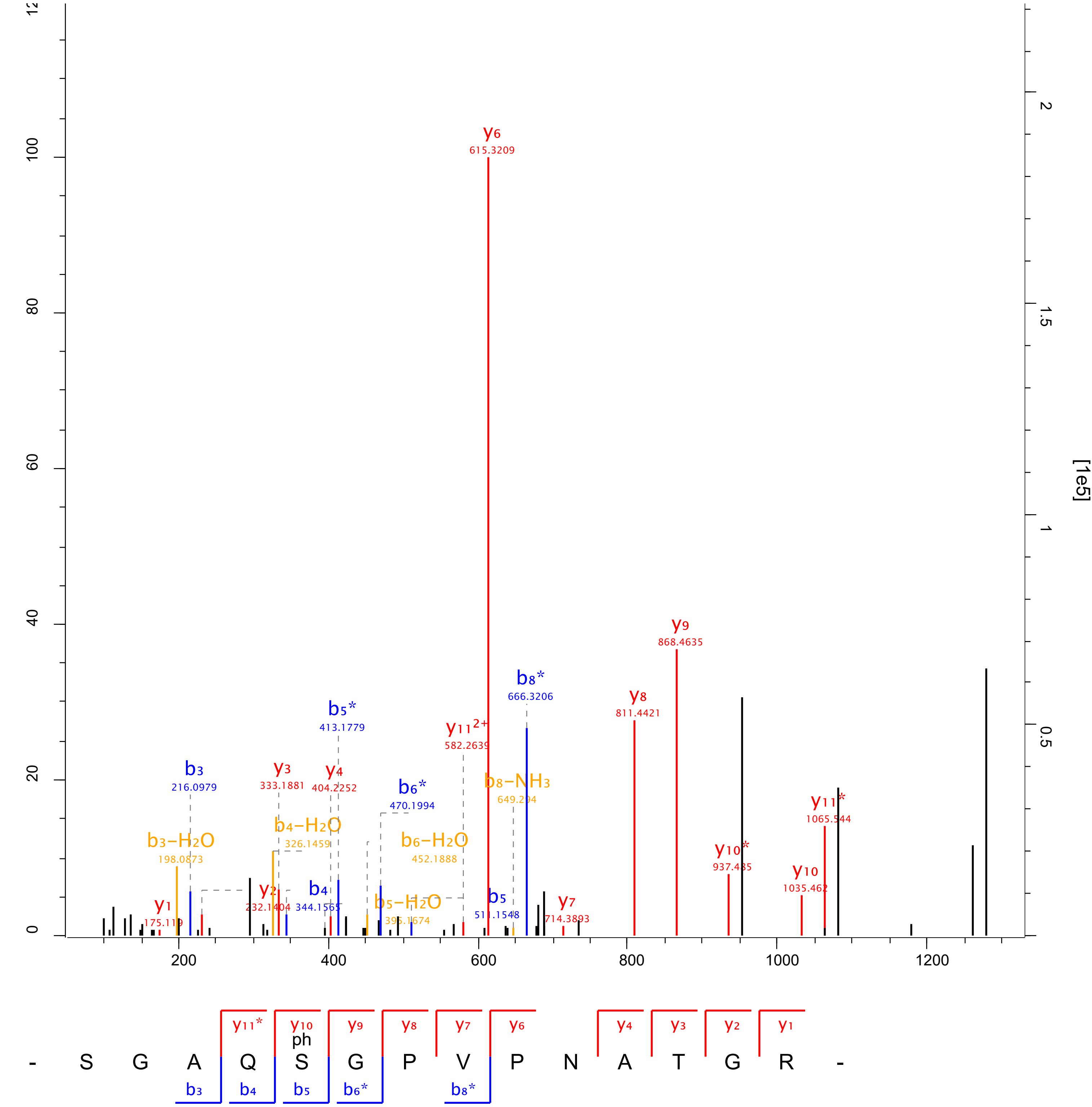

Sample output to test PDF Combine only

Raw file	Scan	Method	Score	m/z	Gene names
0523_4	6451	FTMS; HCD	270.54	1002.86	TIF3C1

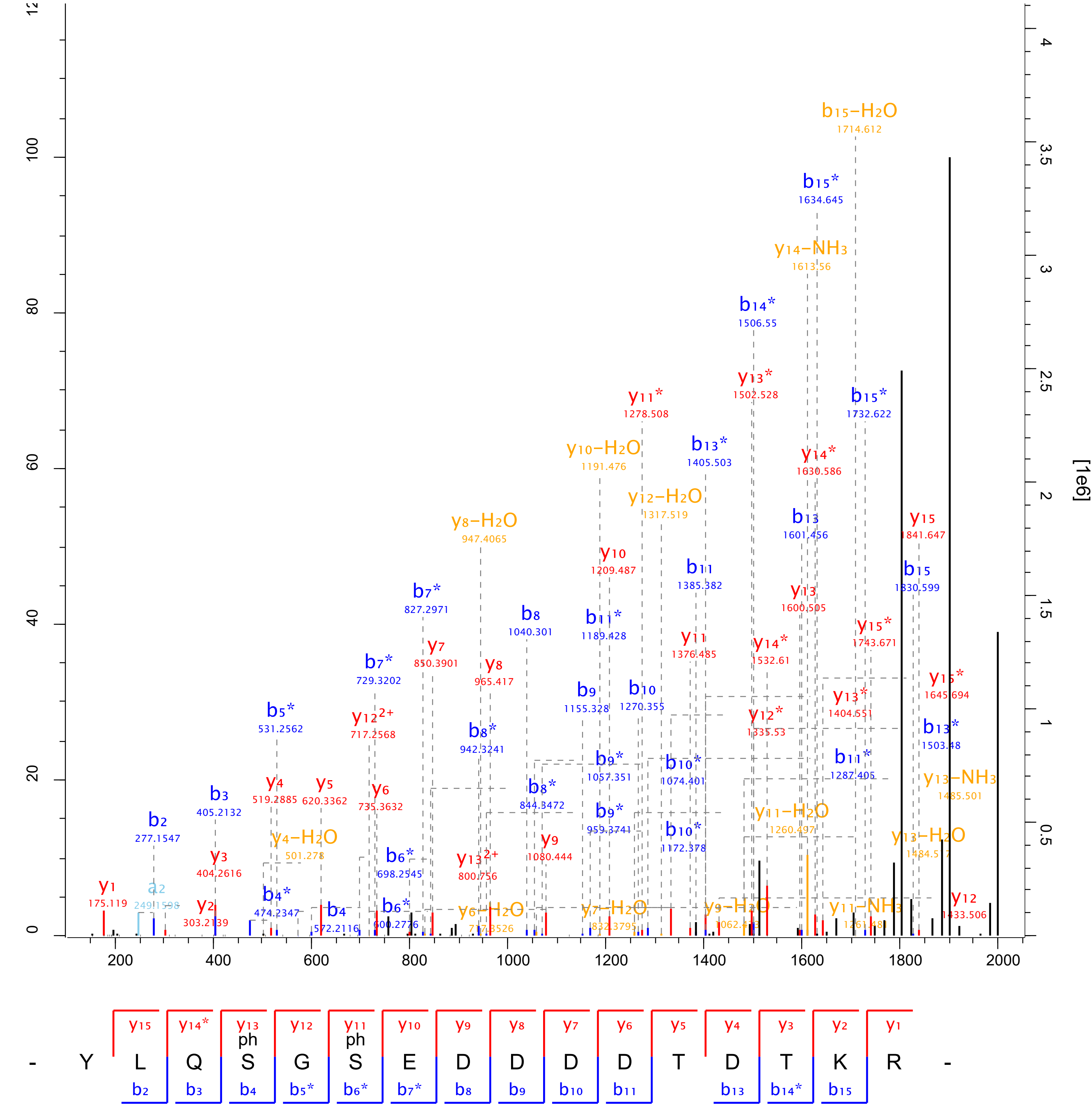

Sample output to test PDF Combine only

Raw file	Scan	Method	Score	m/z	Gene names
0523_4	6458	FTMS; HCD	46.41	868	At4g31880

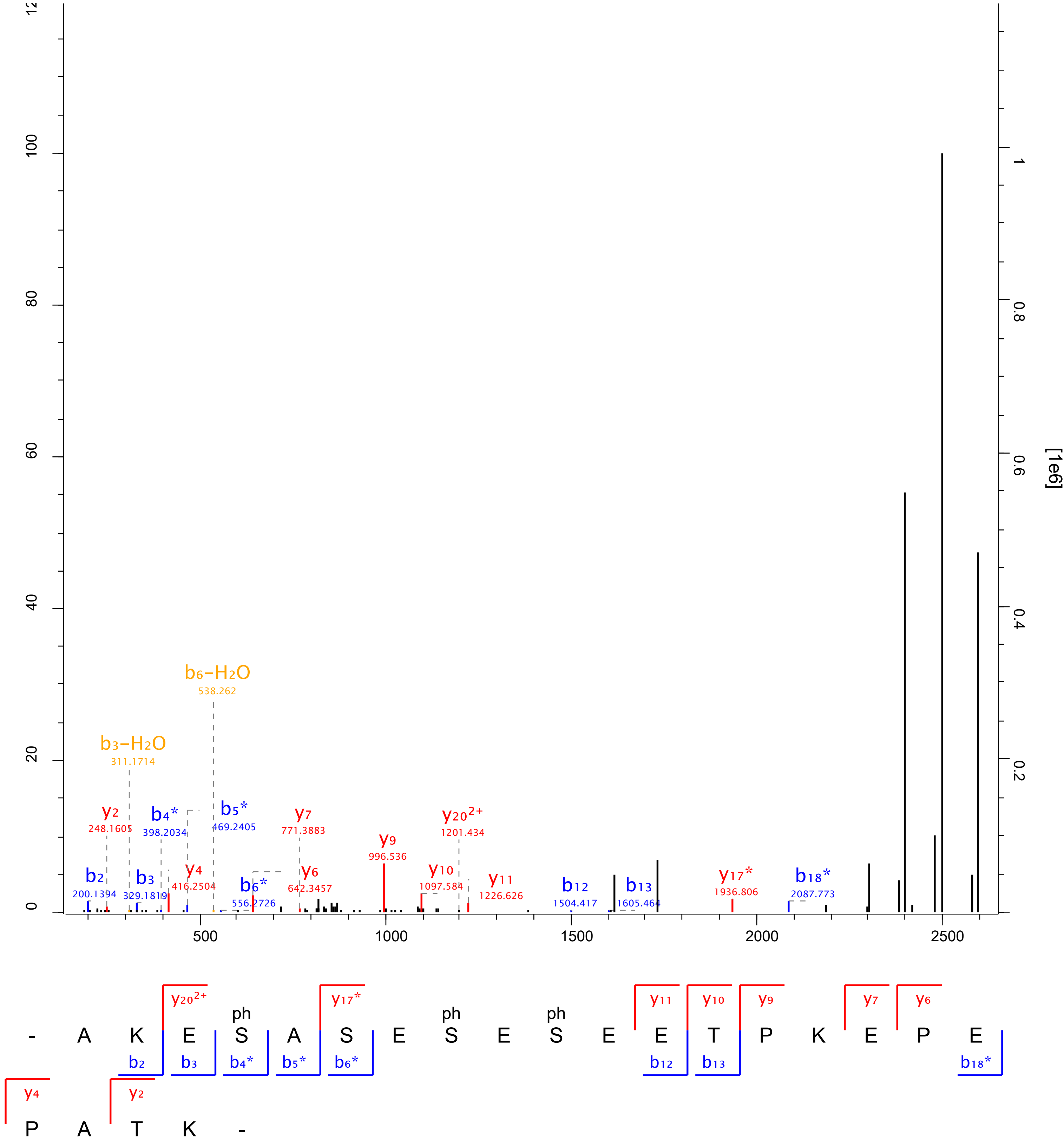

Sample output to test PDF Combine only

Raw file	Scan	Method	Score	m/z	Gene names
0523_4	6491	FTMS; HCD	67.14	643.78	CMTA4

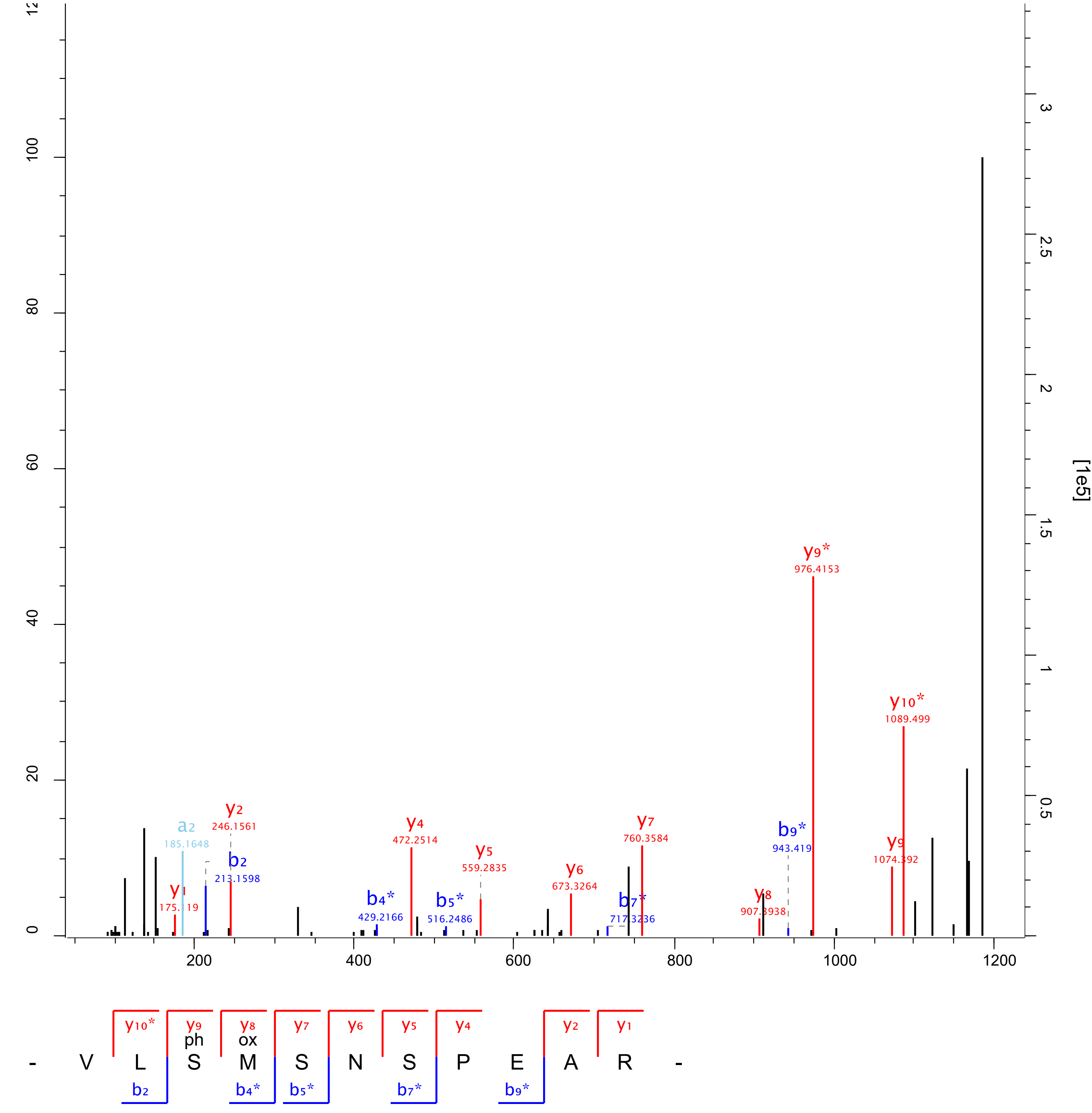

Sample output to test PDF Combine only

Raw file	Scan	Method	Score	m/z	Gene names
0523_4	6506	FTMS; HCD	65.04	594.26	ABCB1

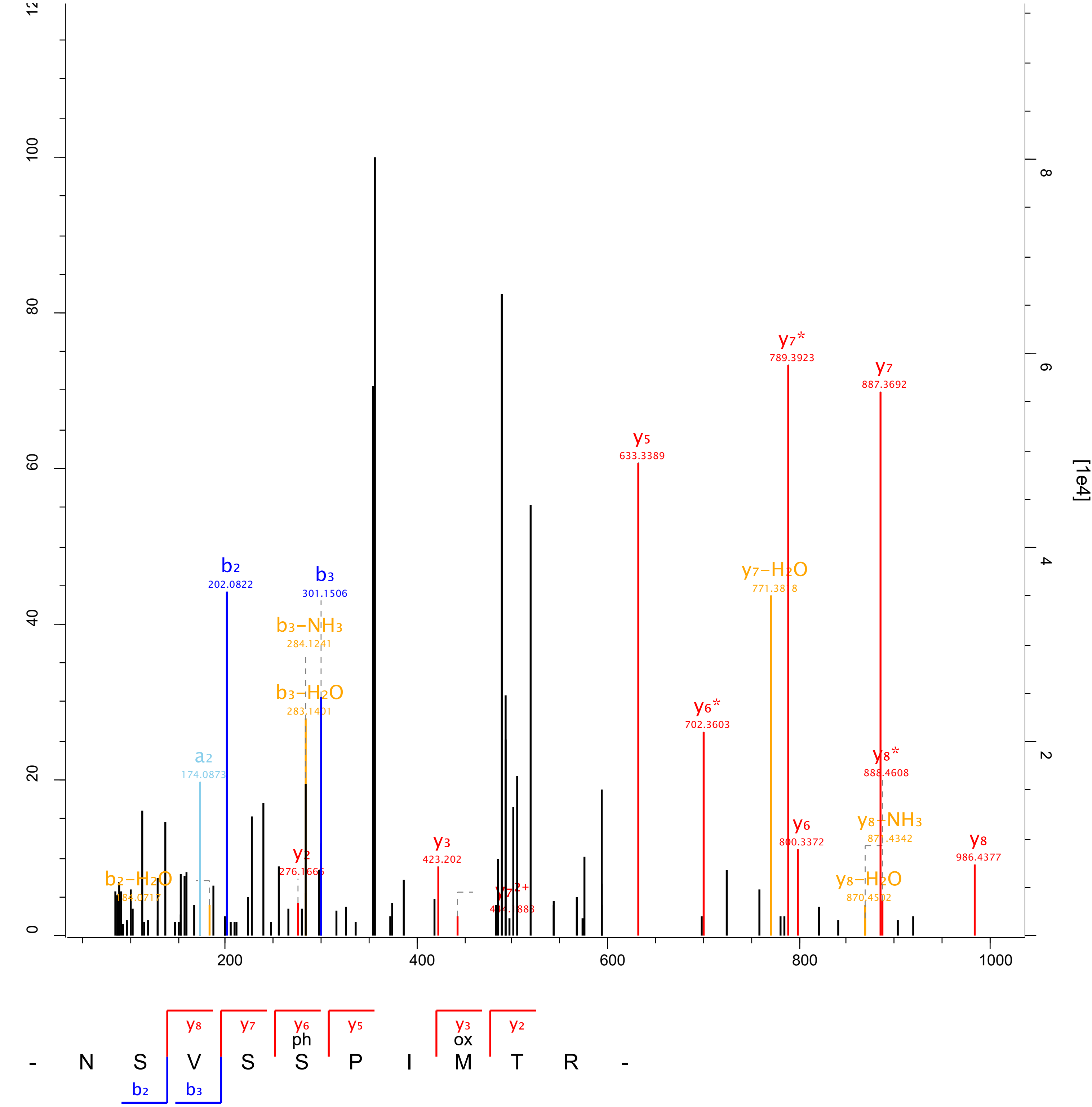

Sample output to test PDF Combine only

Raw file	Scan	Method	Score	m/z	Gene names
0523_4	6510	FTMS; HCD	192.6	803.32	GG2

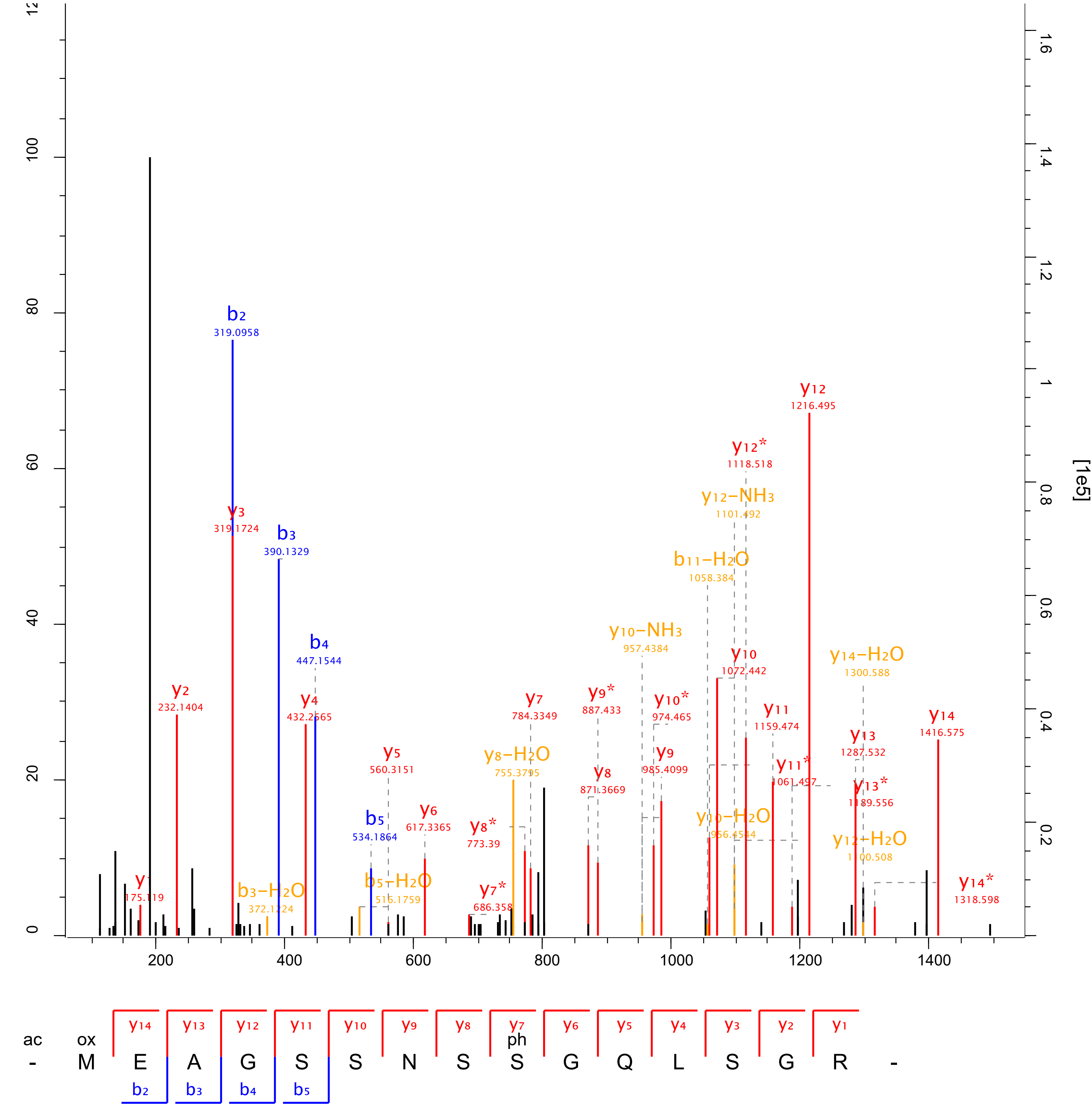

Sample output to test PDF Combine only

Raw file	Scan	Method	Score	m/z	Gene names
0523_4	6548	FTMS; HCD	55.04	506.24	At1g51850;At1g51805

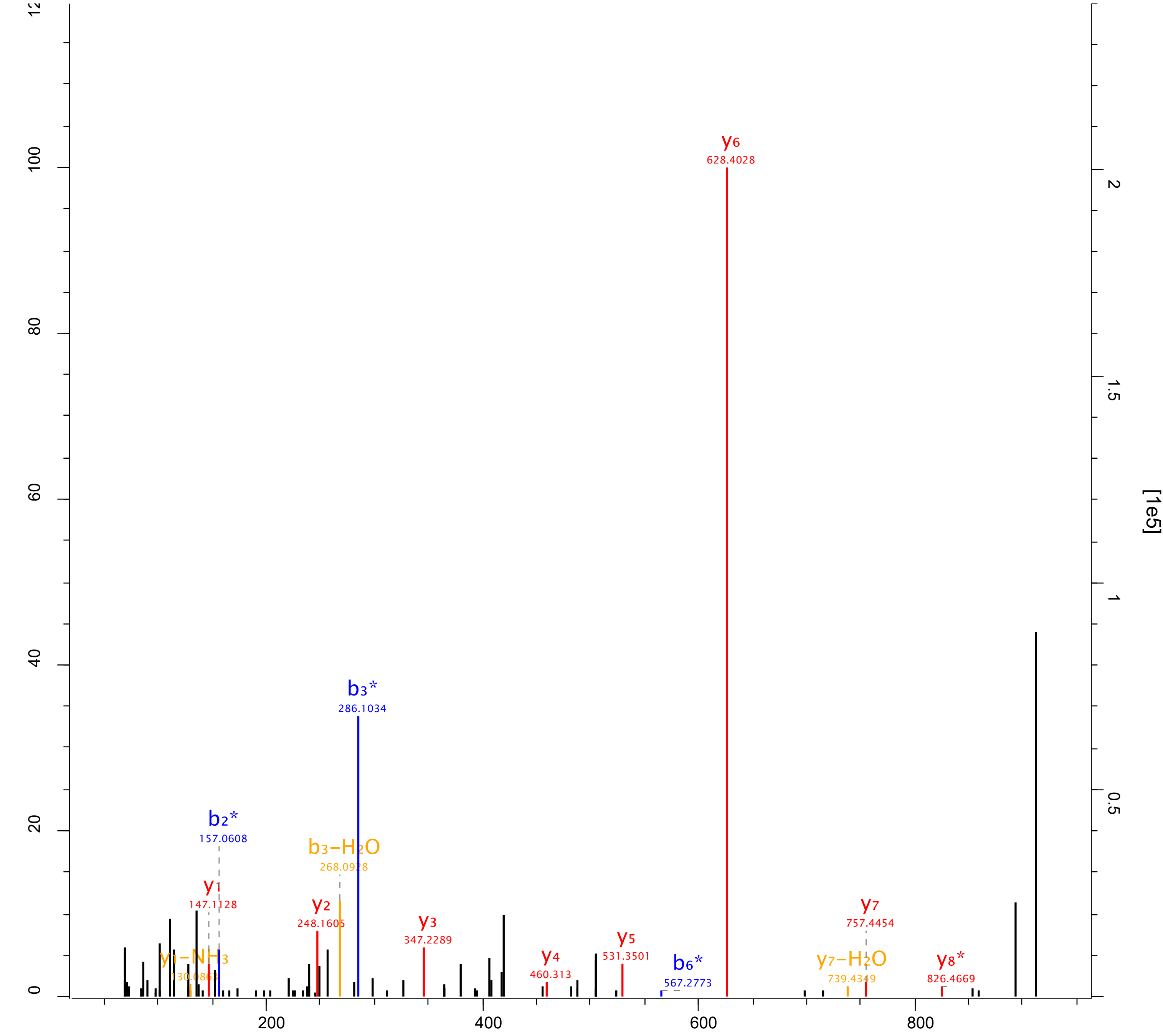

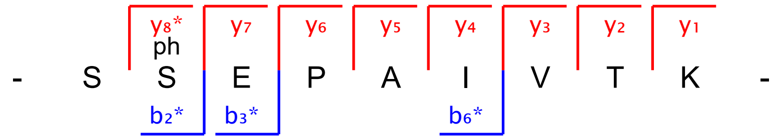

Sample output to test PDF Combine only

Raw file	Scan	Method	Score	m/z	Gene names
0523_4	6566	FTMS; HCD	227.06	716.31	EIN2

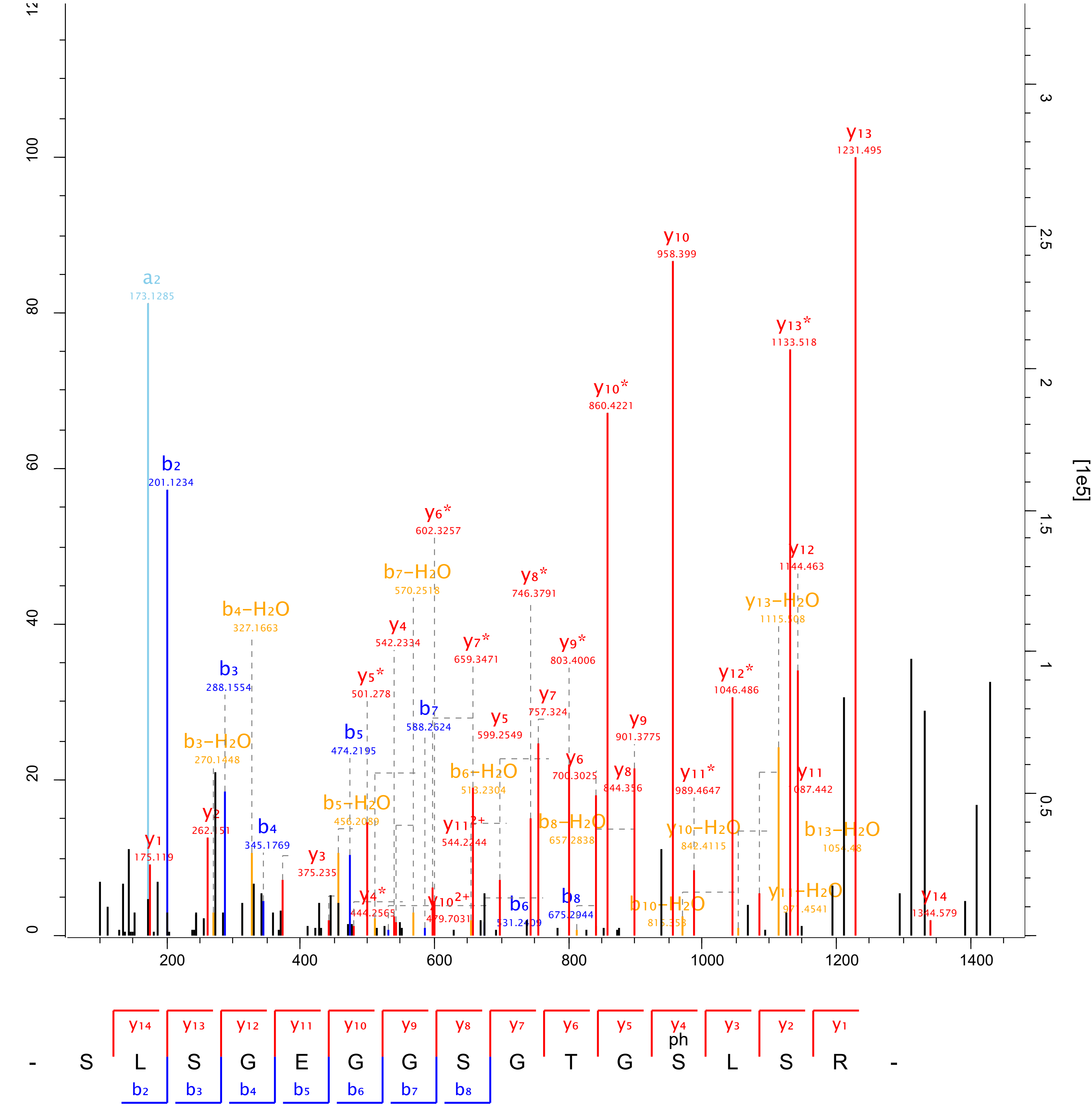

Sample output to test PDF Combine only

Raw file	Scan	Method	Score	m/z	Gene names
0523_4	6623	FTMS; HCD	70.78	763.75	CXIP4

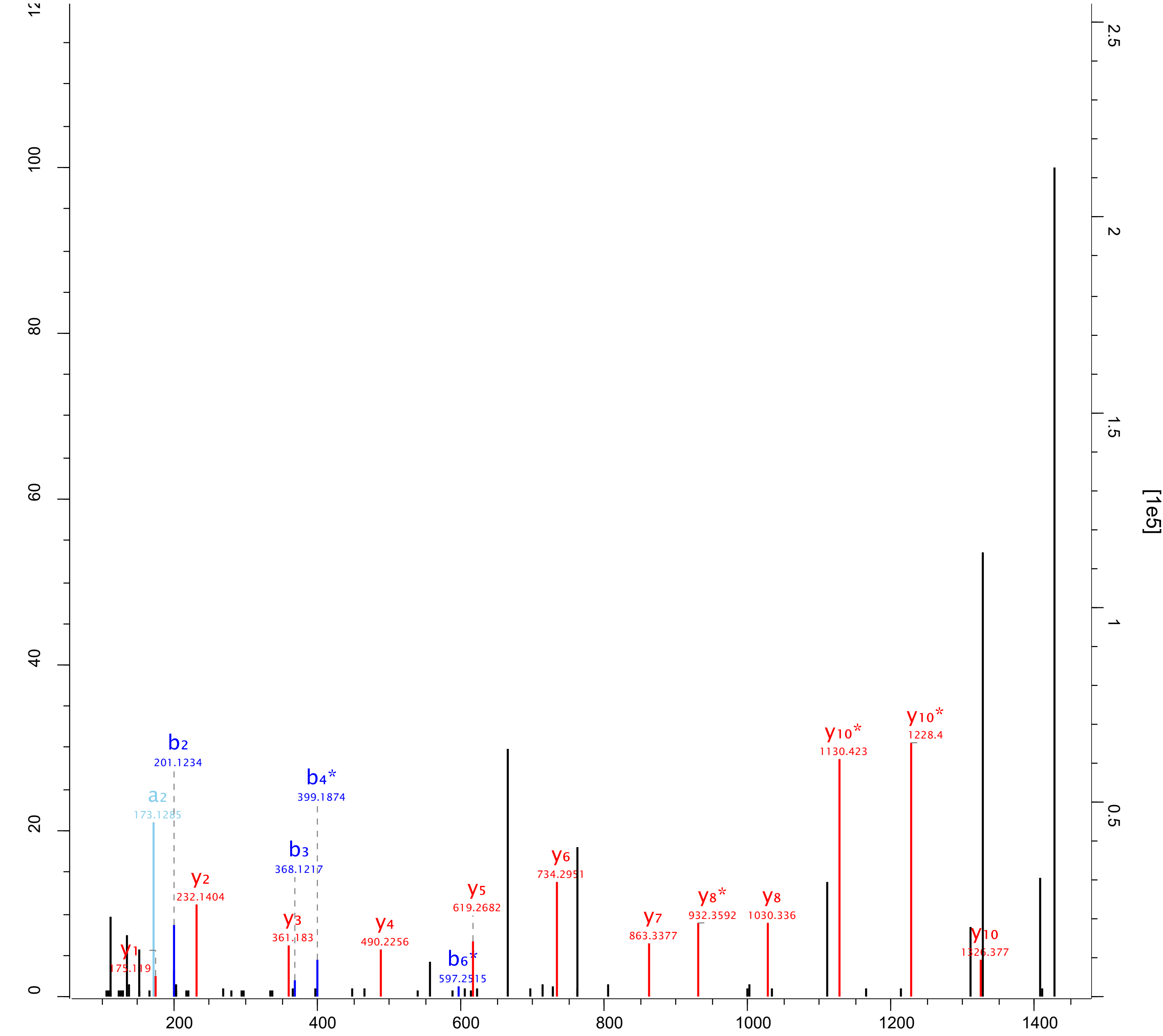

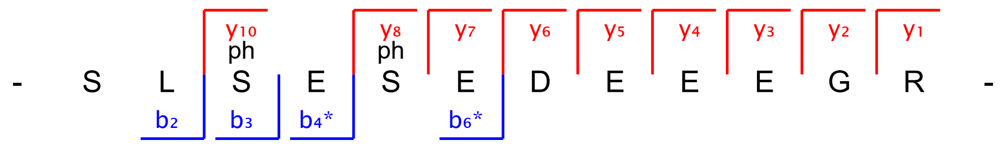

Sample output to test PDF Combine only

Raw file	Scan	Method	Score	m/z
0523_4	6644	FTMS; HCD	54.1	549.91

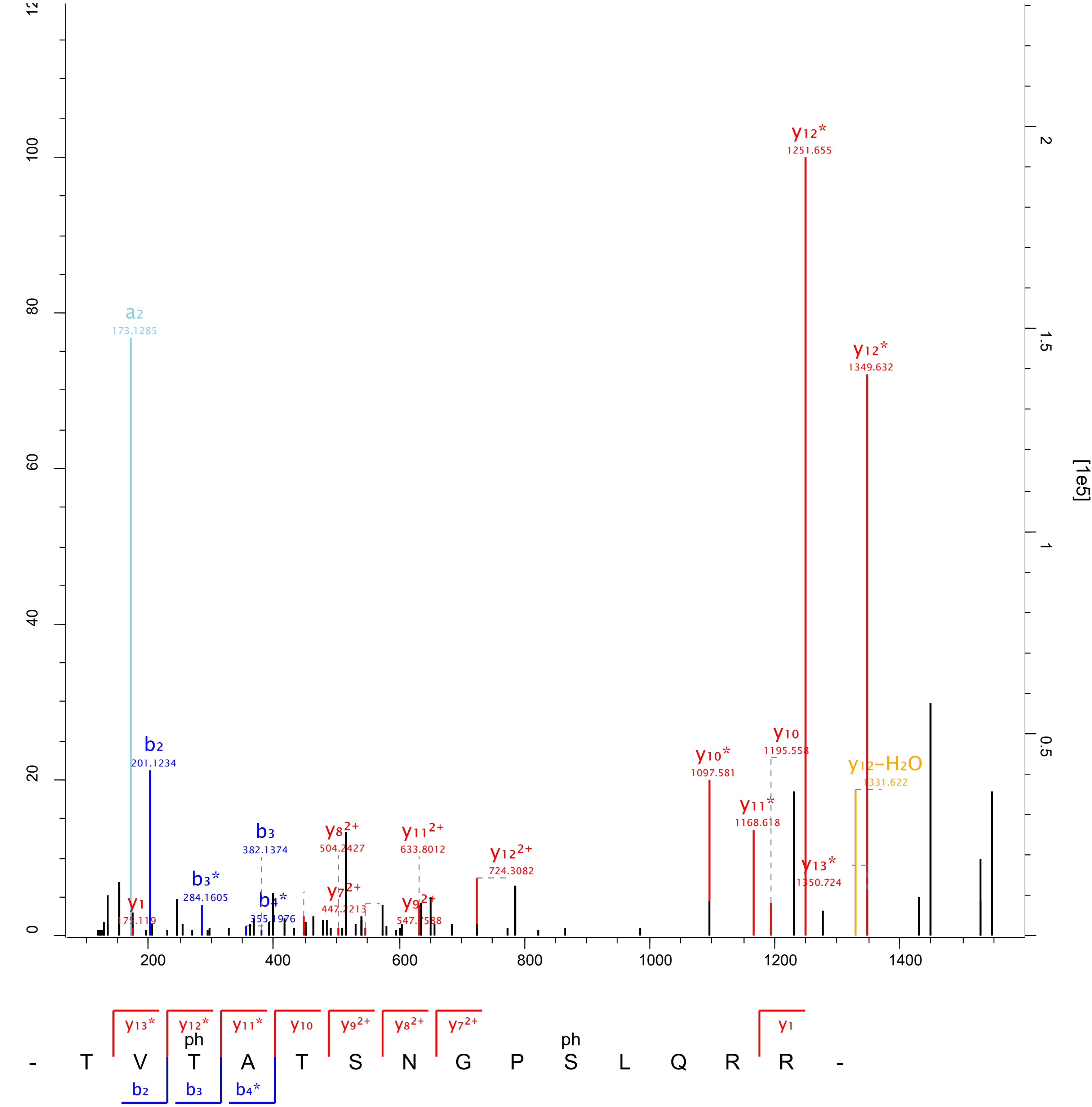

Sample output to test PDF Combine only

Raw file	Scan	Method	Score	m/z	Gene names
0523_4	6669	FTMS; HCD	121.94	743.96	LIG4

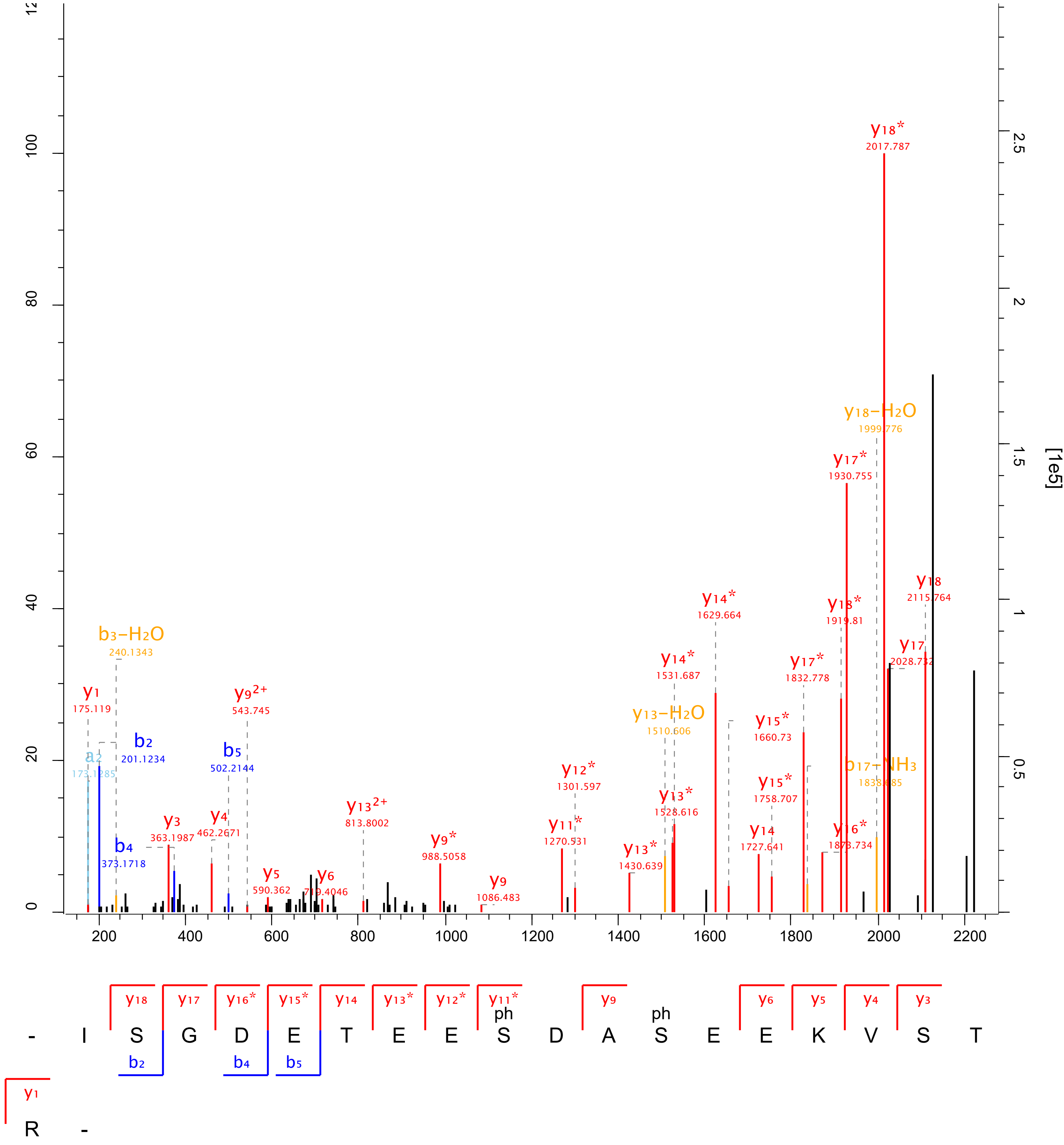

Sample output to test PDF Combine only

Raw file	Scan	Method	Score	m/z	Gene names
0523_4	6748	FTMS; HCD	205.24	900.36	At2g23520

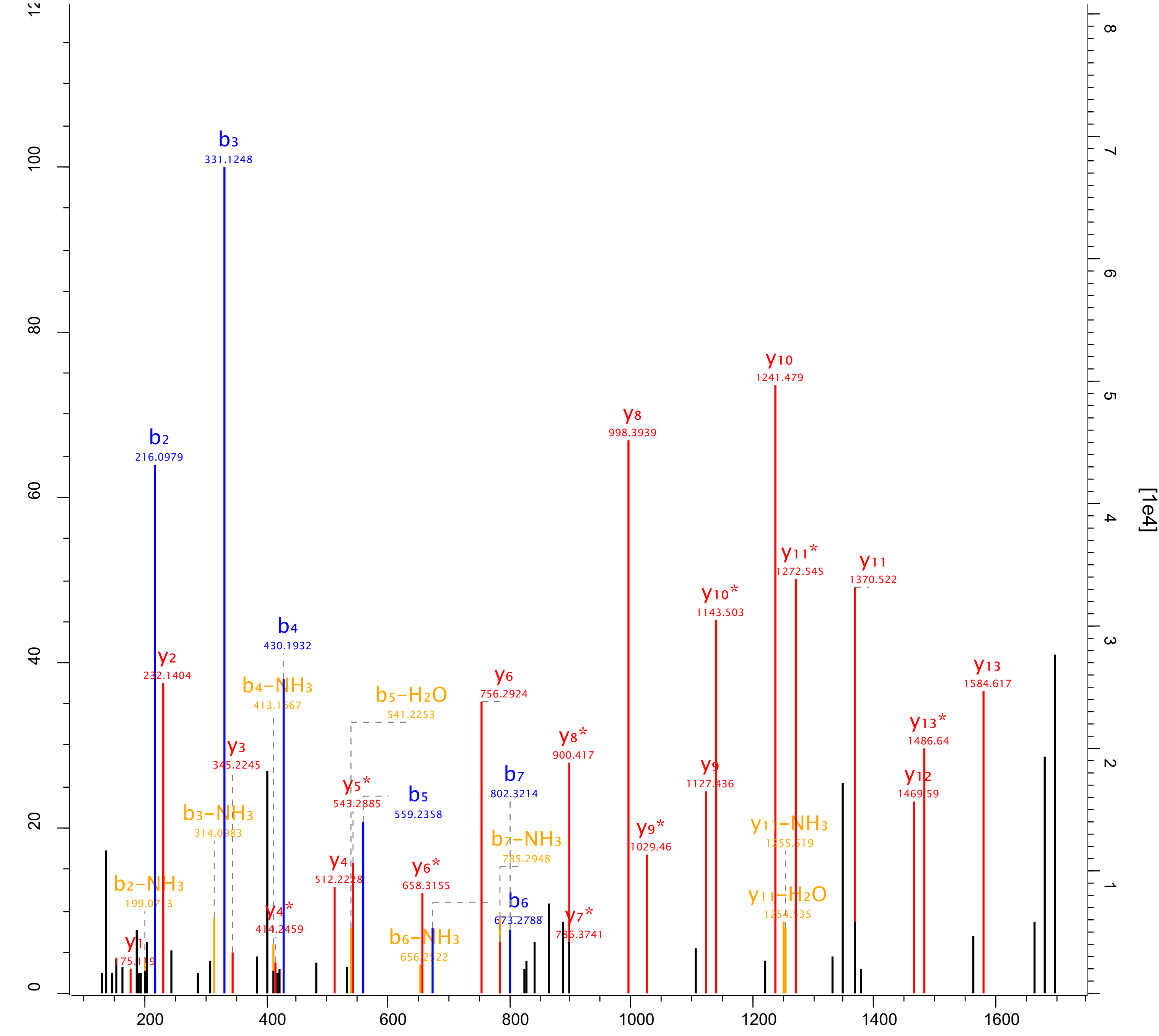

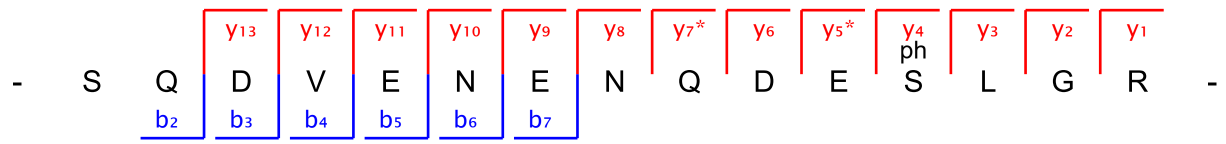

Sample output to test PDF Combine only

Raw file	Scan	Method	Score	m/z	Gene names
0523_4	6820	FTMS; HCD	115.63	728.32	MLO8

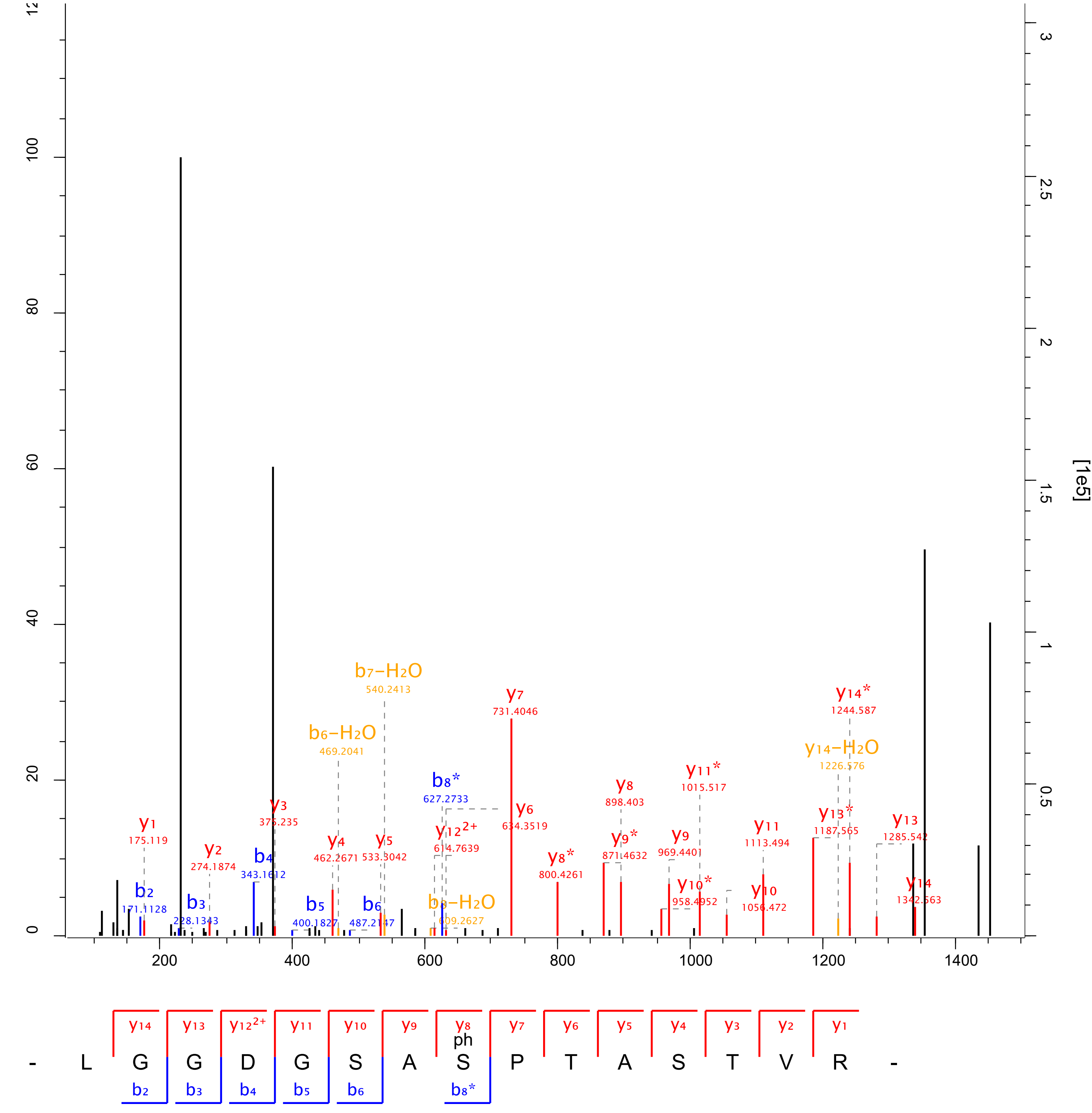

Sample output to test PDF Combine only

Raw file	Scan	Method	Score	m/z	Gene names
0523_4	6828	FTMS; HCD	118.2	611.71	At1g71710

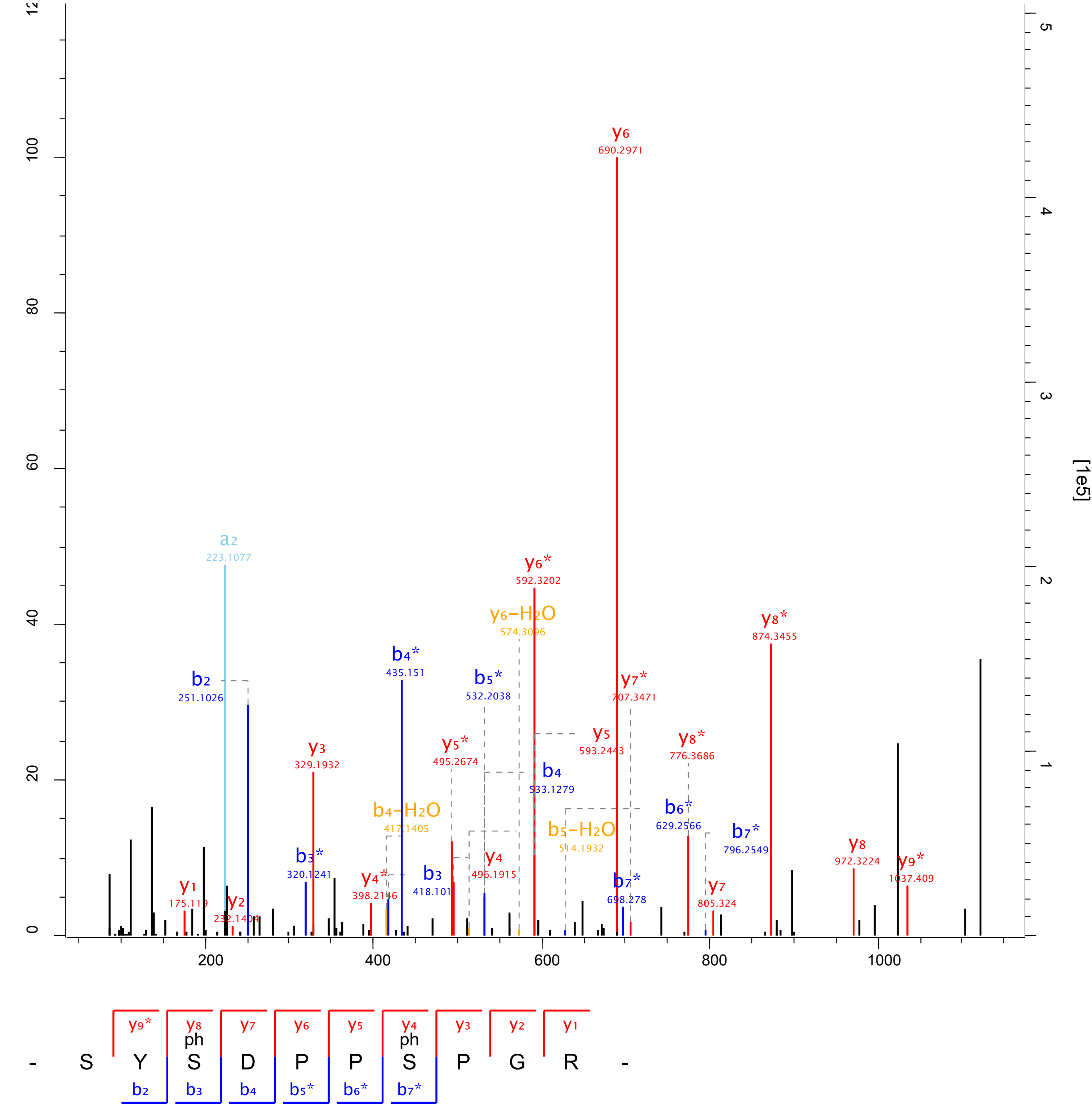

Sample output to test PDF Combine only

Raw file	Scan	Method	Score	m/z	Gene names
0523_4	6857	FTMS; HCD	92.03	708.57	LAG1

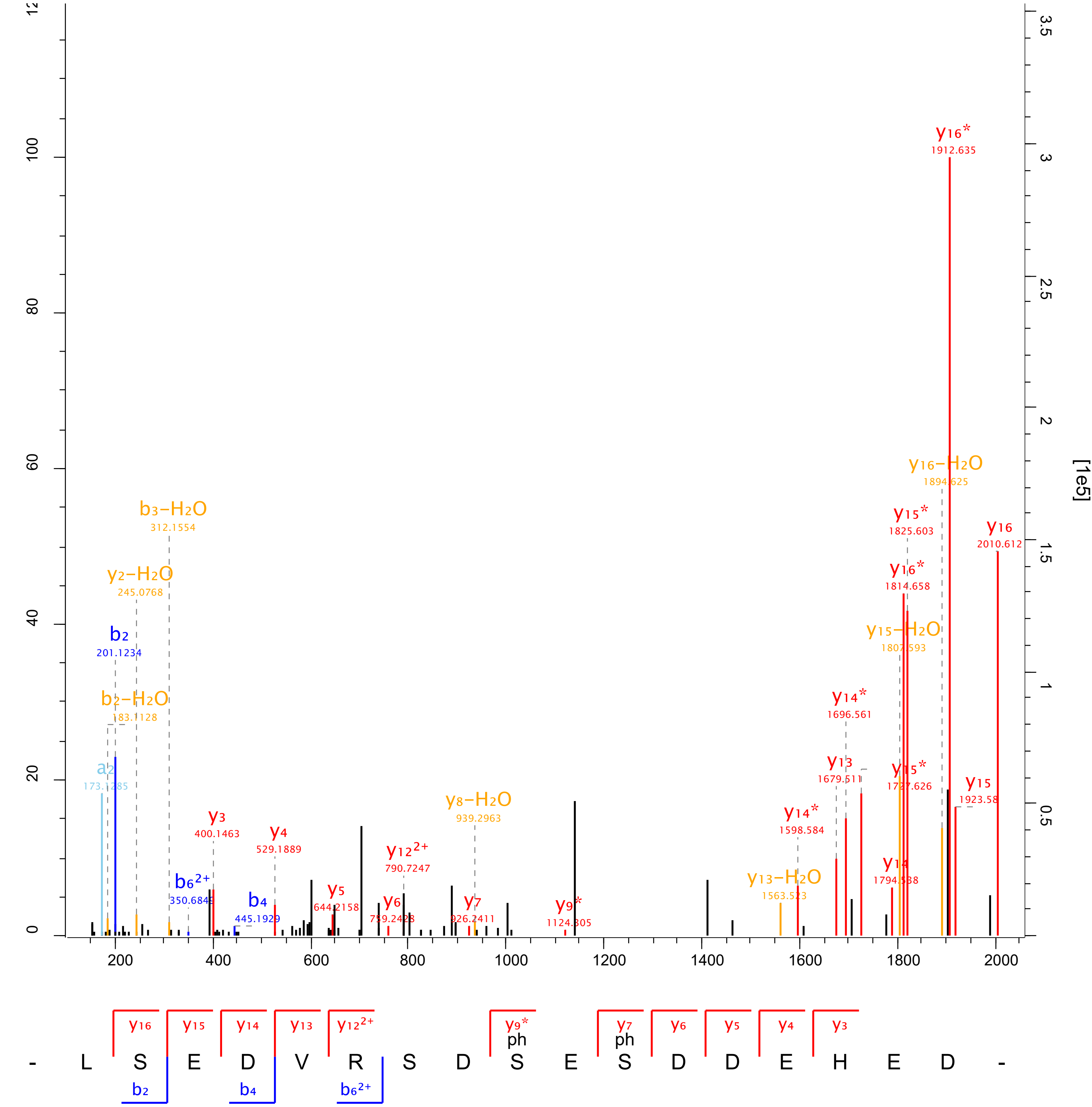

Sample output to test PDF Combine only

Raw file	Scan	Method	Score	m/z	Gene names
0523_4	6884	FTMS; HCD	80.54	732.8	NMT1

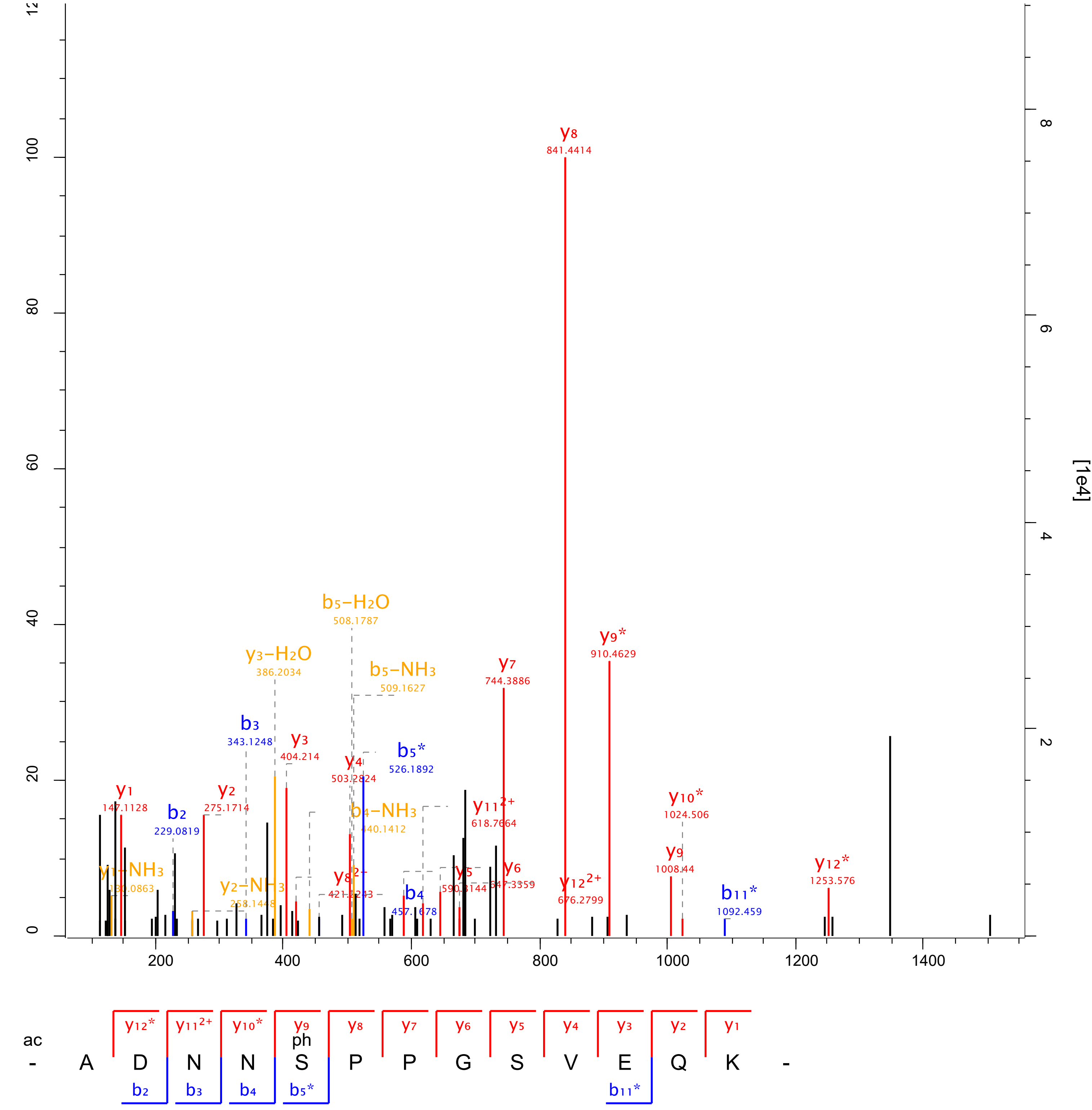

Sample output to test PDF Combine only

Raw file	Scan	Method	Score	m/z	Gene names
0523_4	6957	FTMS; HCD	60.27	910.34	SC35

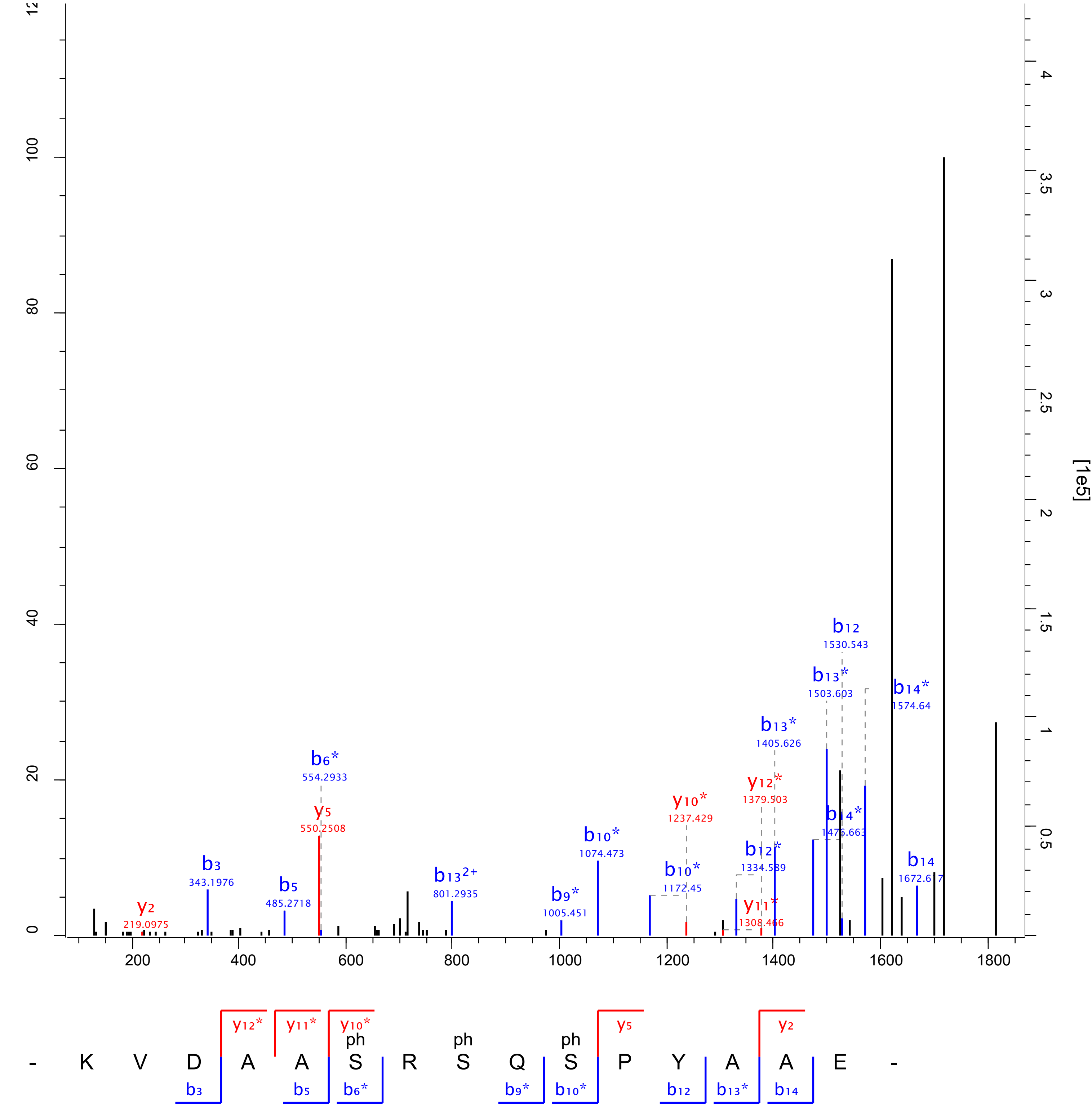

Sample output to test PDF Combine only

Raw file	Scan	Method	Score	m/z	Gene names
0523_4	6994	FTMS; HCD	274.68	823.79	LAG2

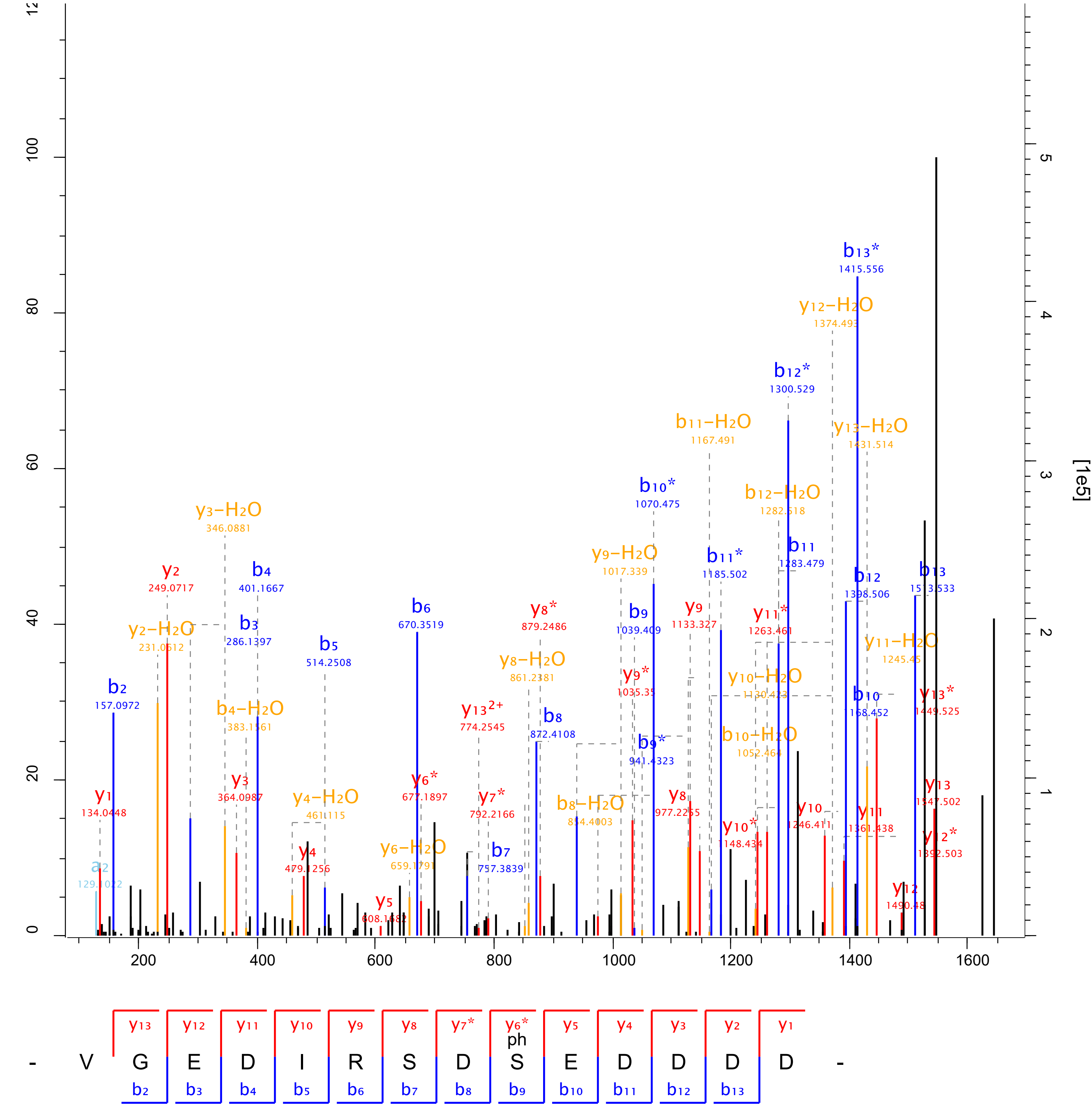

Sample output to test PDF Combine only

Raw file	Scan	Method	Score	m/z	Gene names
0523_4	7017	FTMS; HCD	65.22	422.21	At2g29210

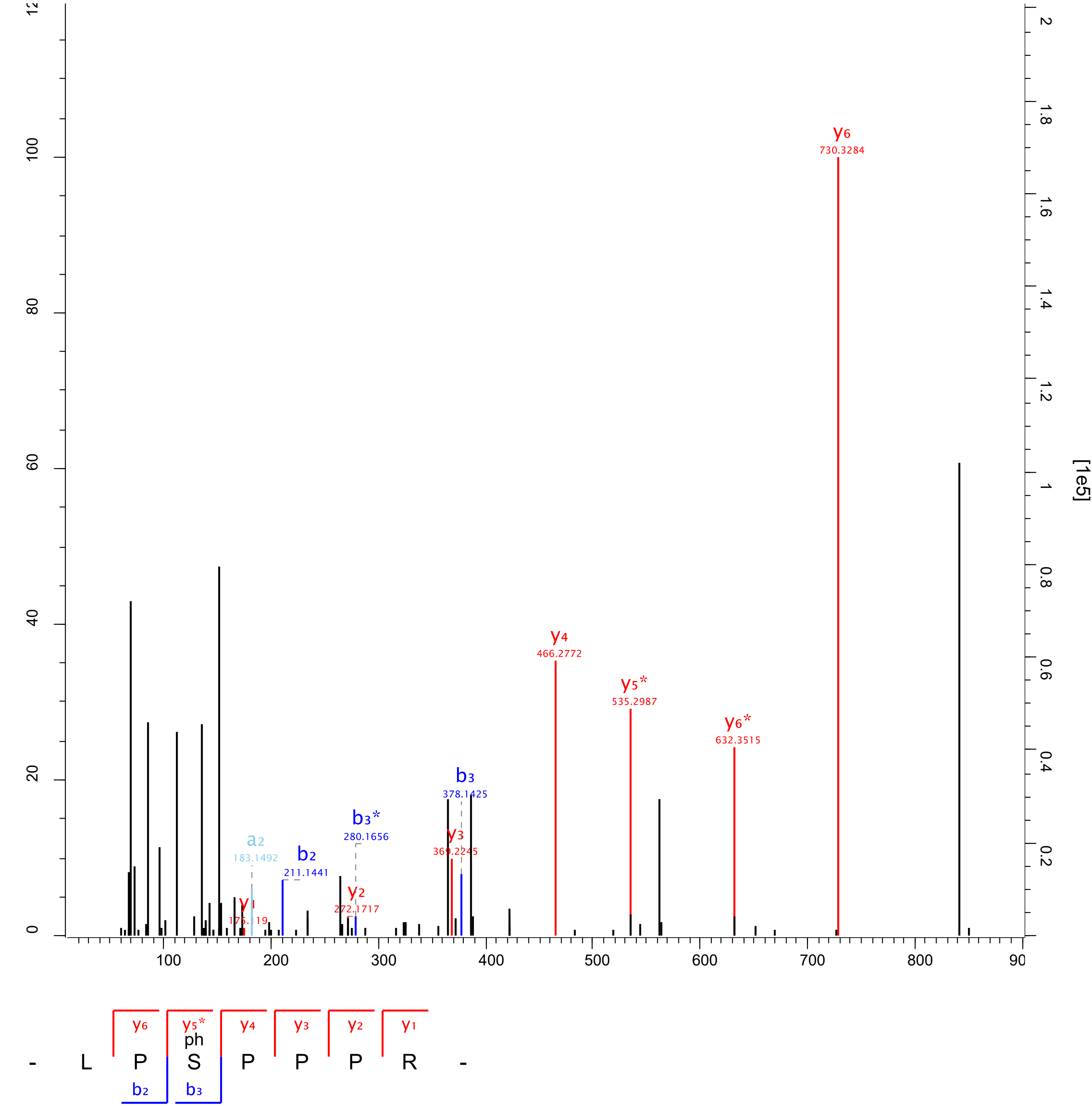

Sample output to test PDF Combine only

Raw file	Scan	Method	Score	m/z	Gene names
0523_4	7033	FTMS; HCD	399.05	899.87	At1g10140

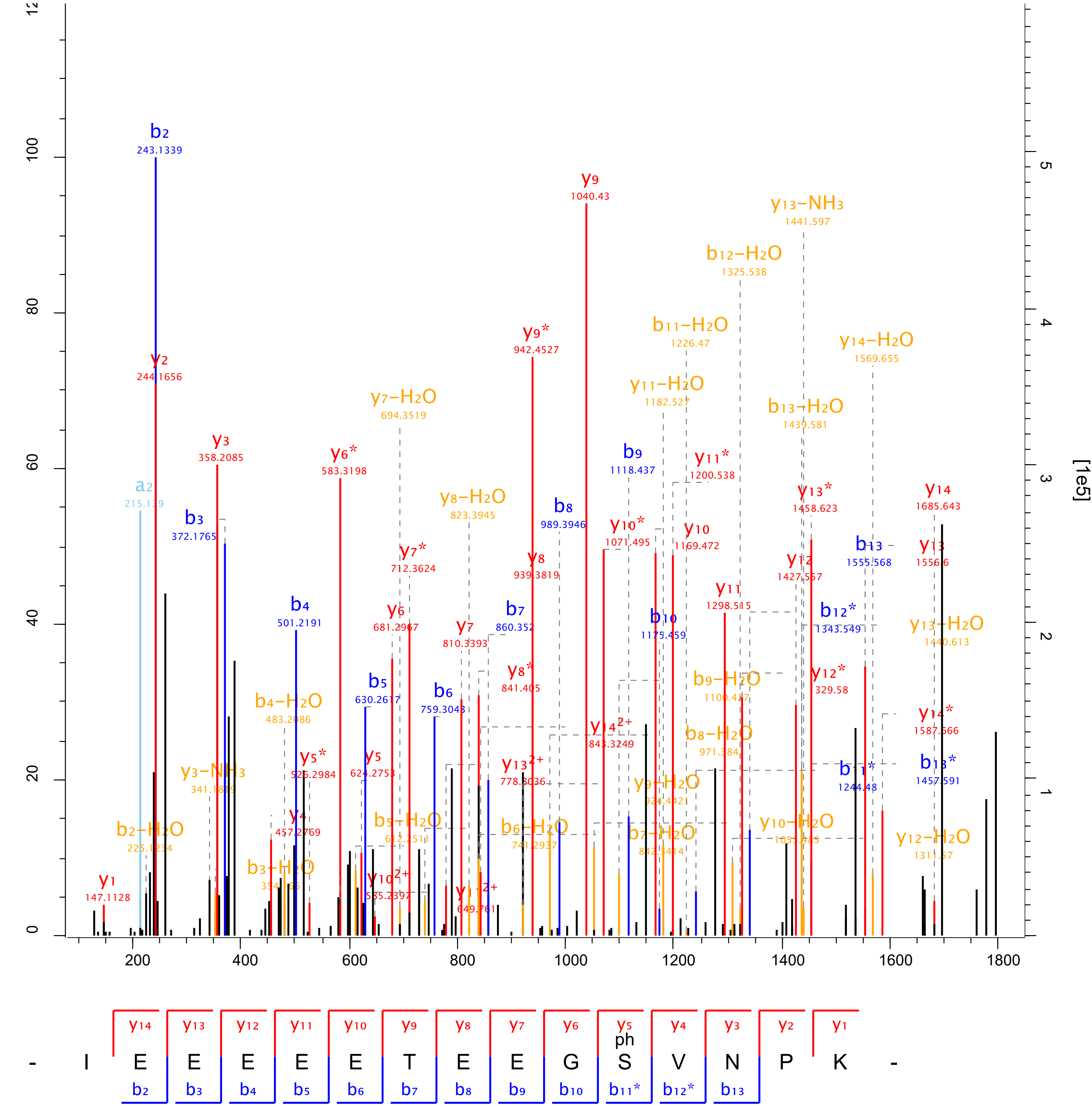

Sample output to test PDF Combine only

Raw file	Scan	Method	Score	m/z	Gene names
0523_4	7078	FTMS; HCD	203.37	782.36	DRP2A

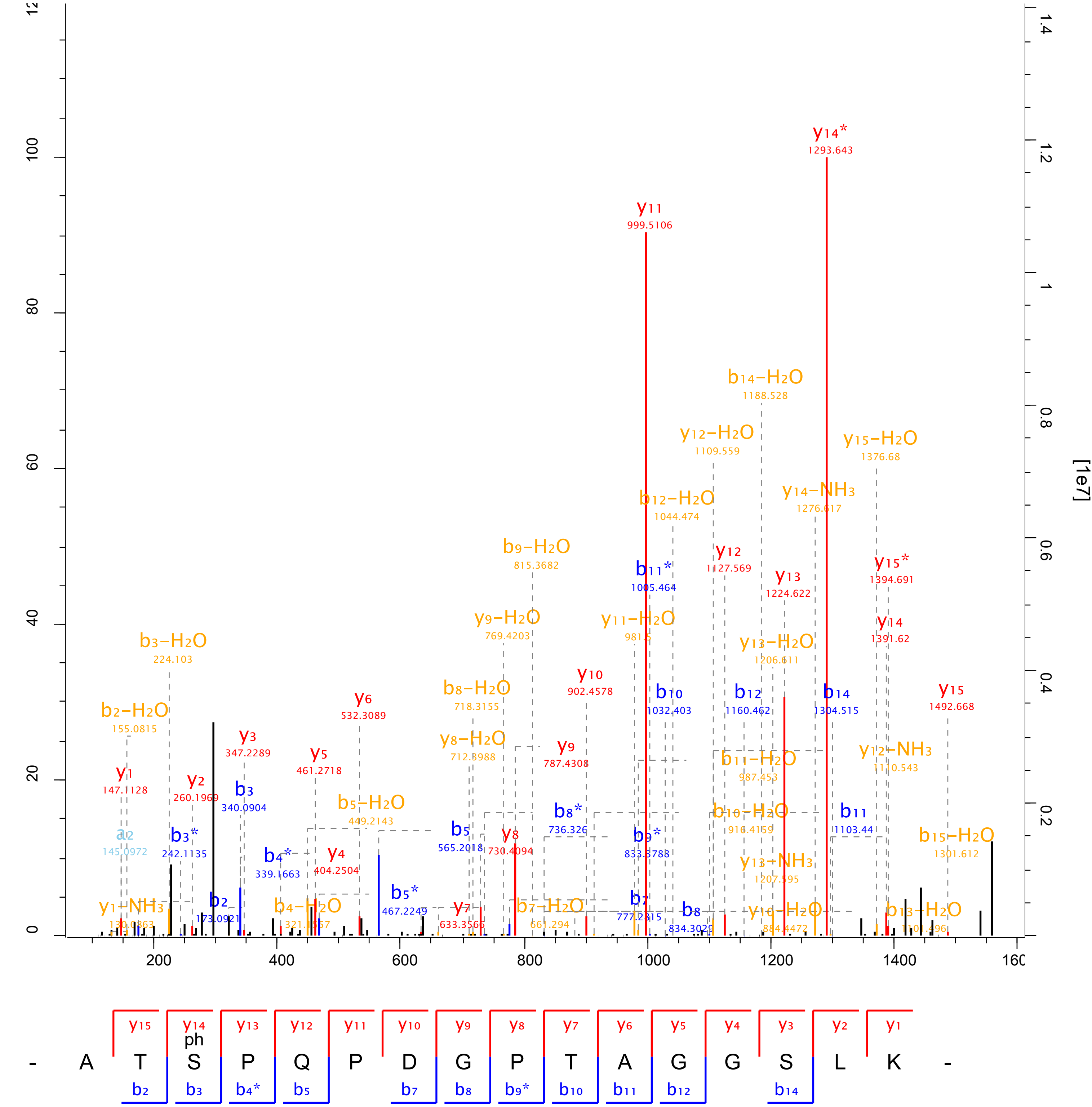

Sample output to test PDF Combine only

Raw file	Scan	Method	Score	m/z	Gene names
0523_4	7085	FTMS; HCD	57.62	708.32	ABCB15

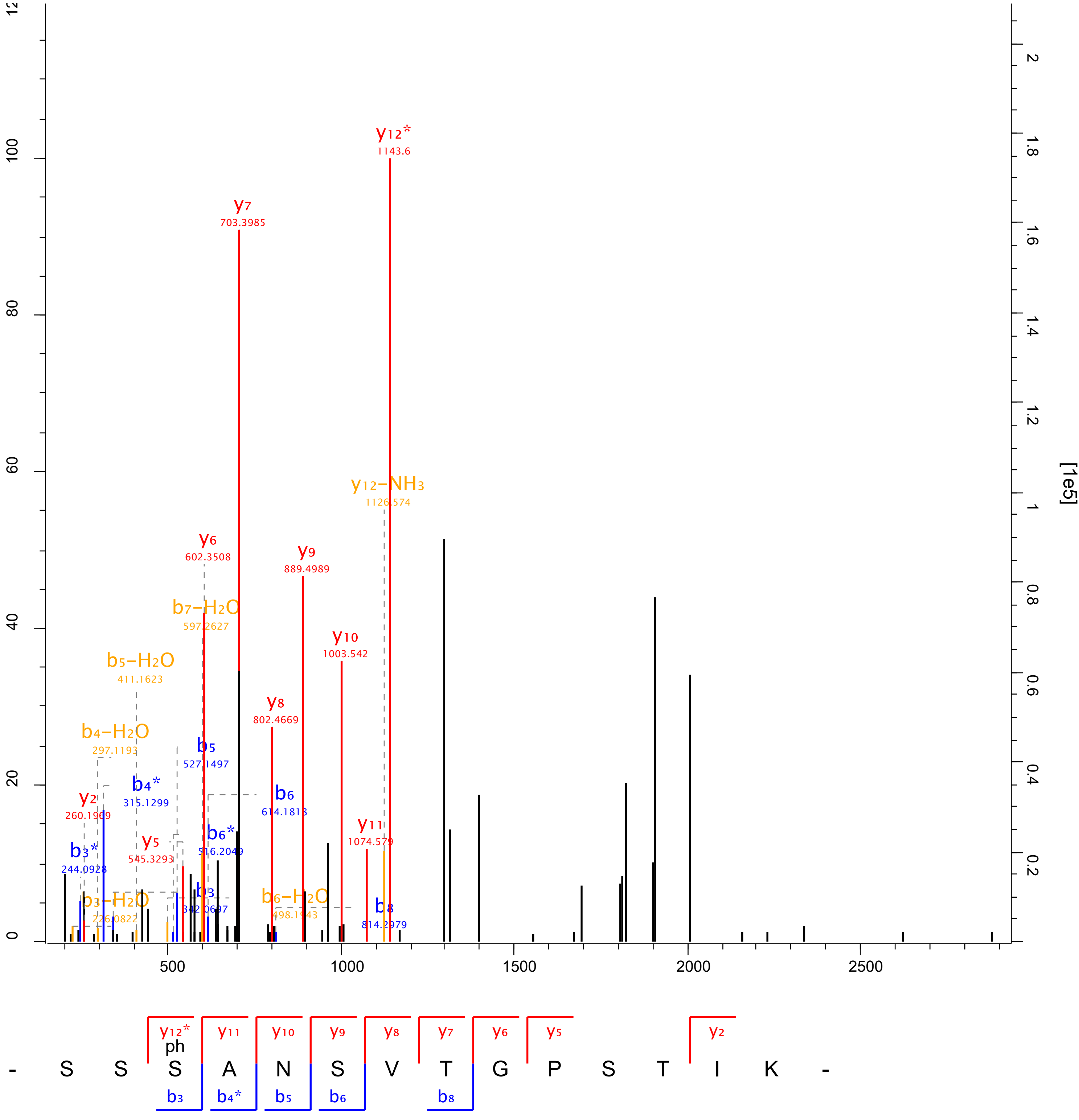

Sample output to test PDF Combine only

Raw file	Scan	Method	Score	m/z	Gene names
0523_4	7093	FTMS; HCD	187.57	828.83	C7A10.260

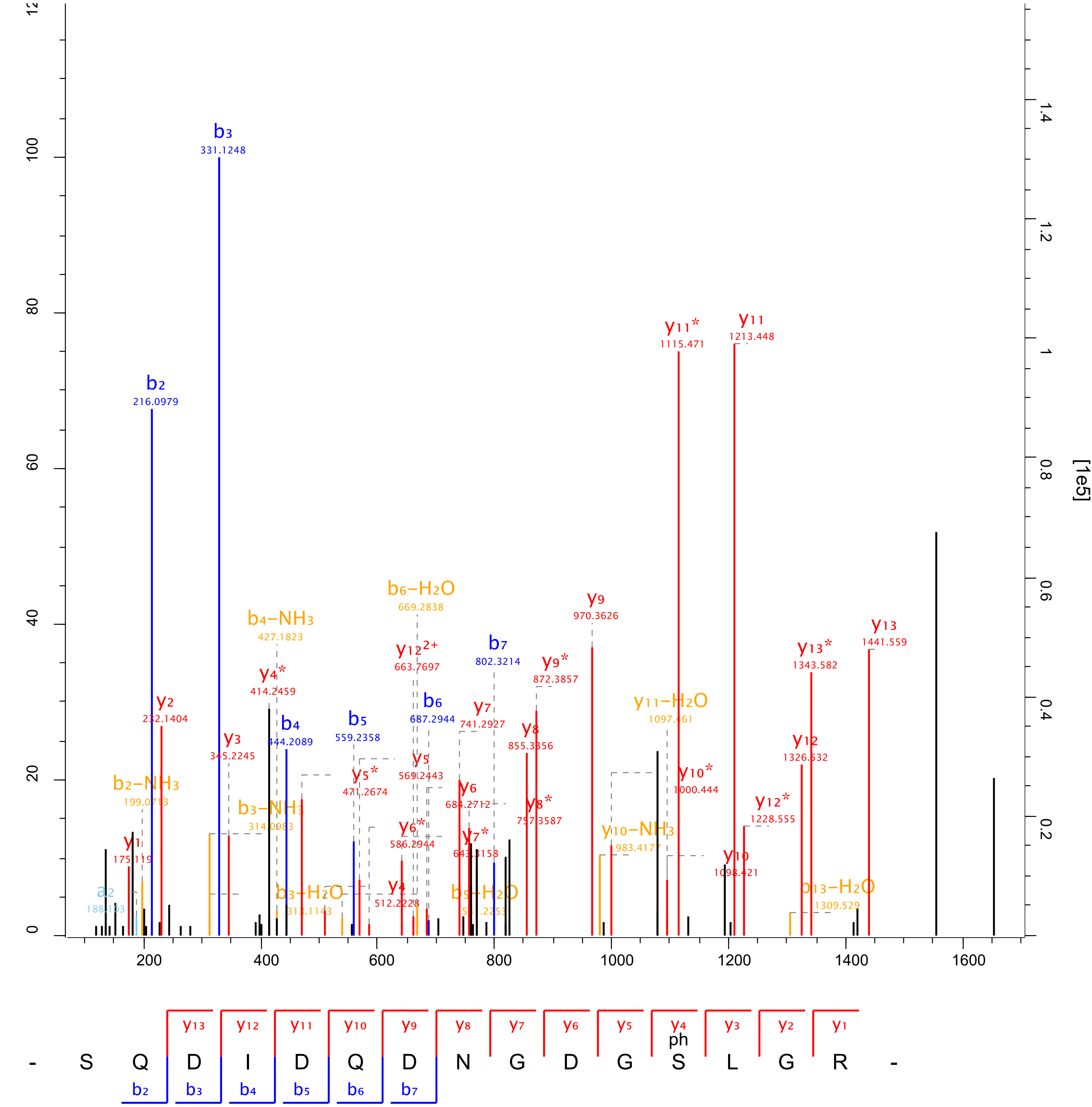

Raw file	Scan	Method	Score	m/z	Gene names
0523_4	7173	FTMS; HCD	119.47	694.29	DGK3

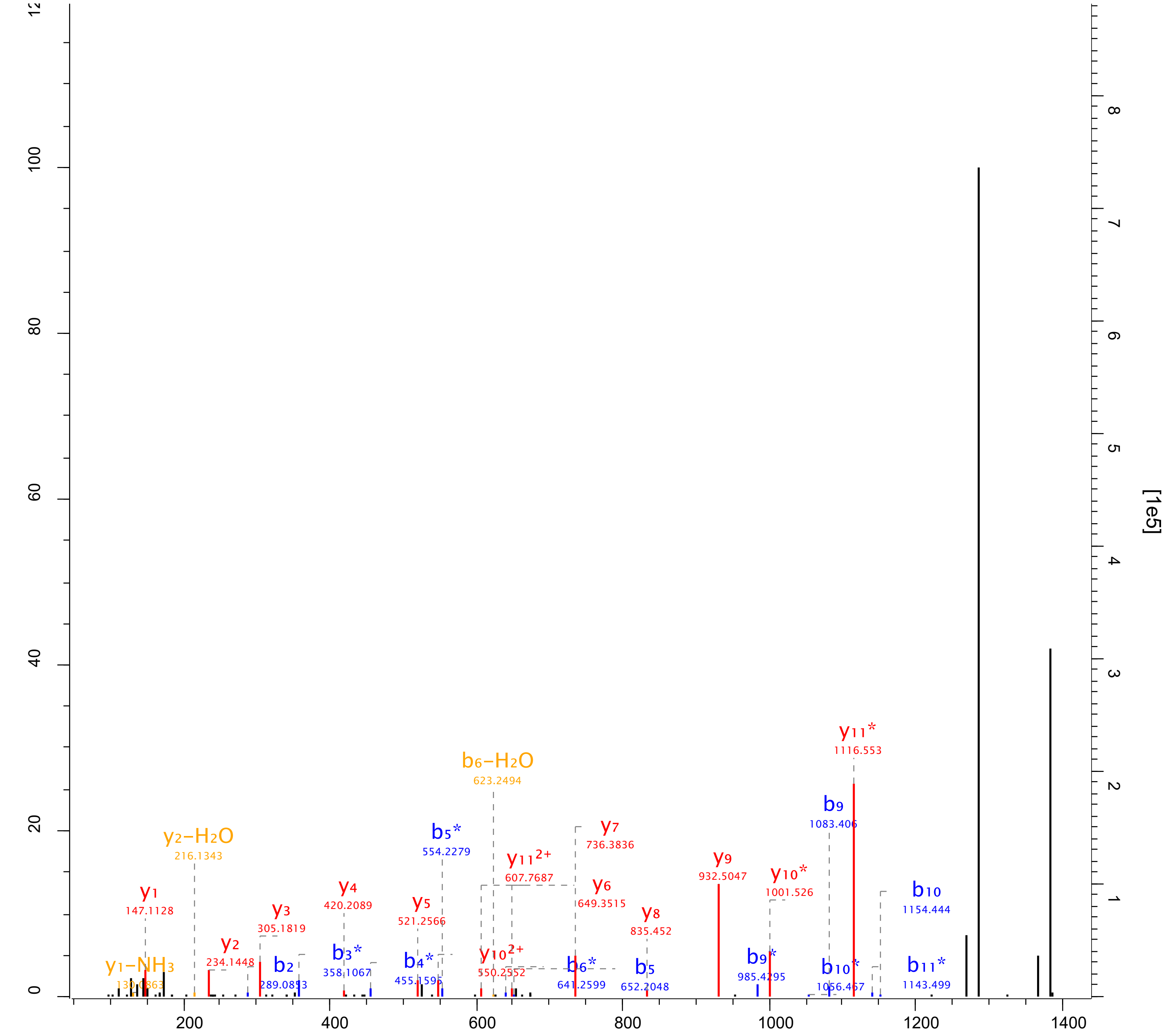

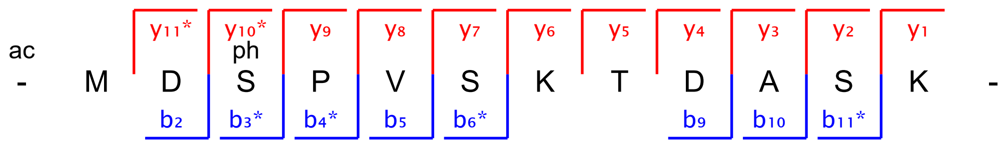

Sample output to test PDF Combine only

Raw file	Scan	Method	Score	m/z	Gene names
0523_4	7192	FTMS; HCD	87.72	564.73	ACA10

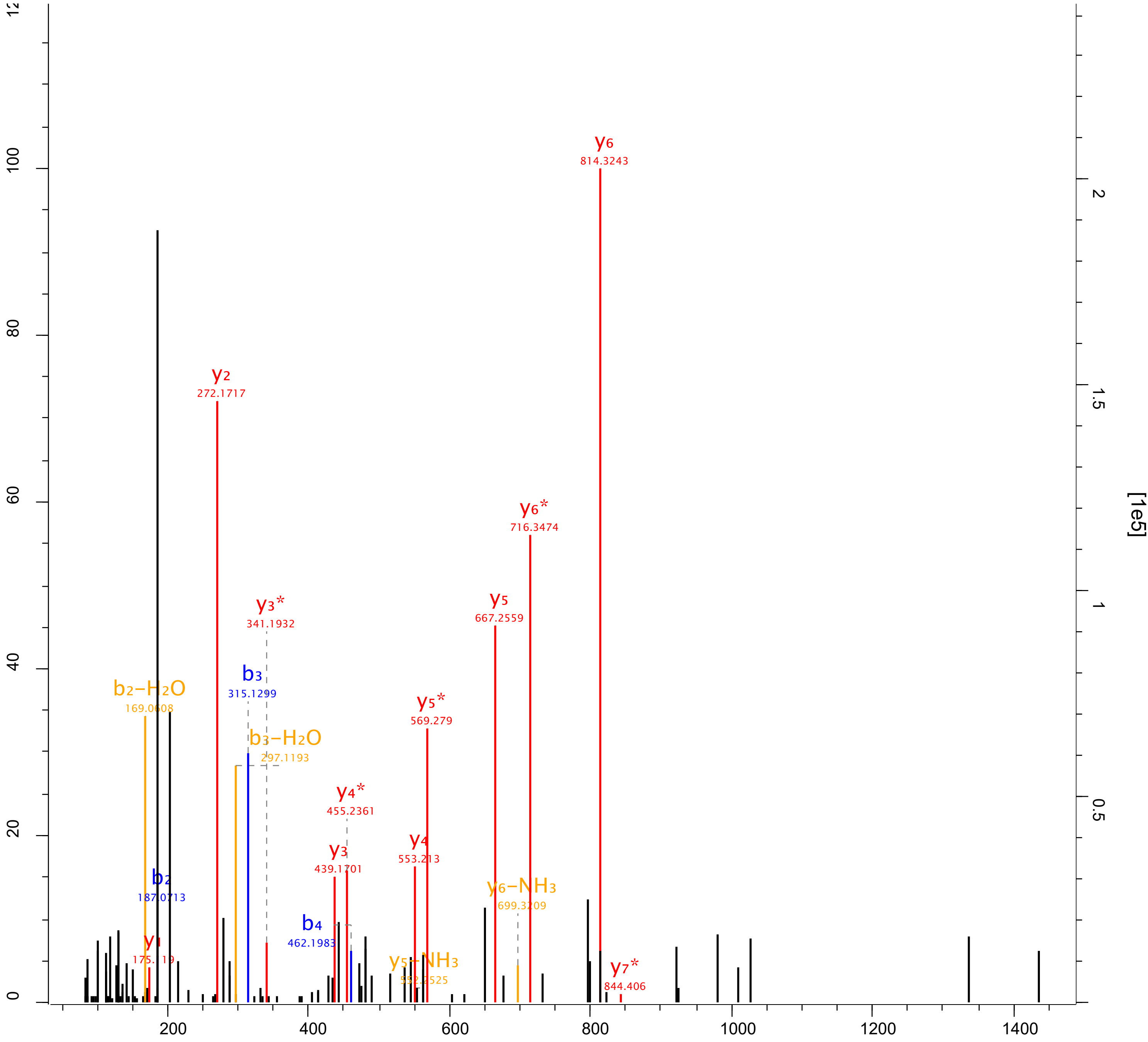

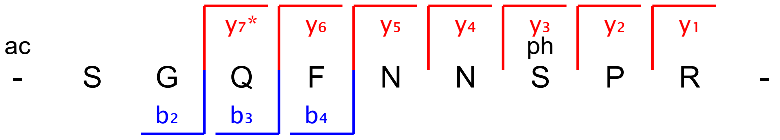

Sample output to test PDF Combine only

Raw file	Scan	Method	Score	m/z	Gene names
0523_4	7256	FTMS; HCD	86.49	831.87	BRM

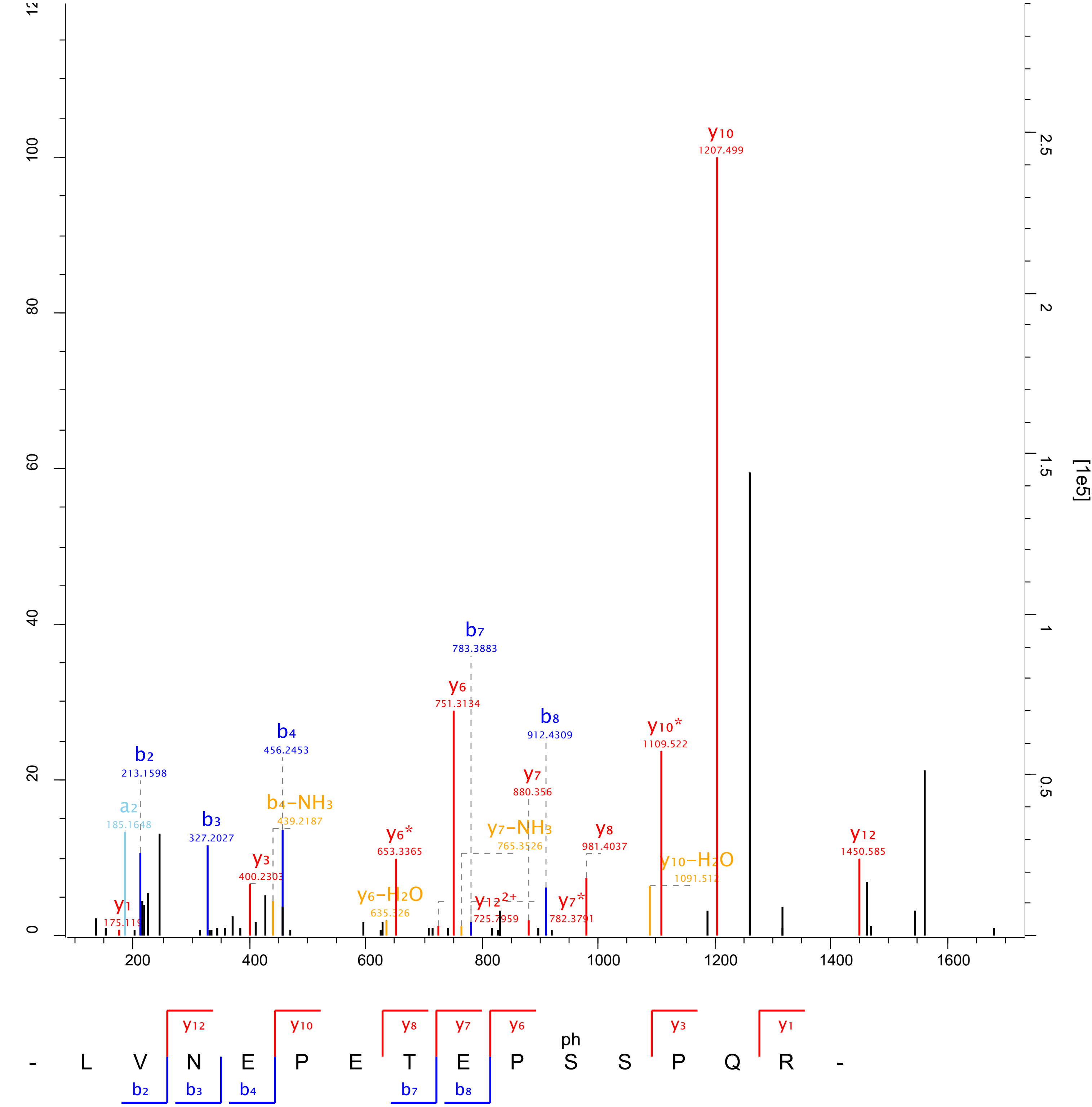

Sample output to test PDF Combine only

Raw file	Scan	Method	Score	m/z	Gene names
0523_4	7258	FTMS; HCD	221.46	792.84	CCC1

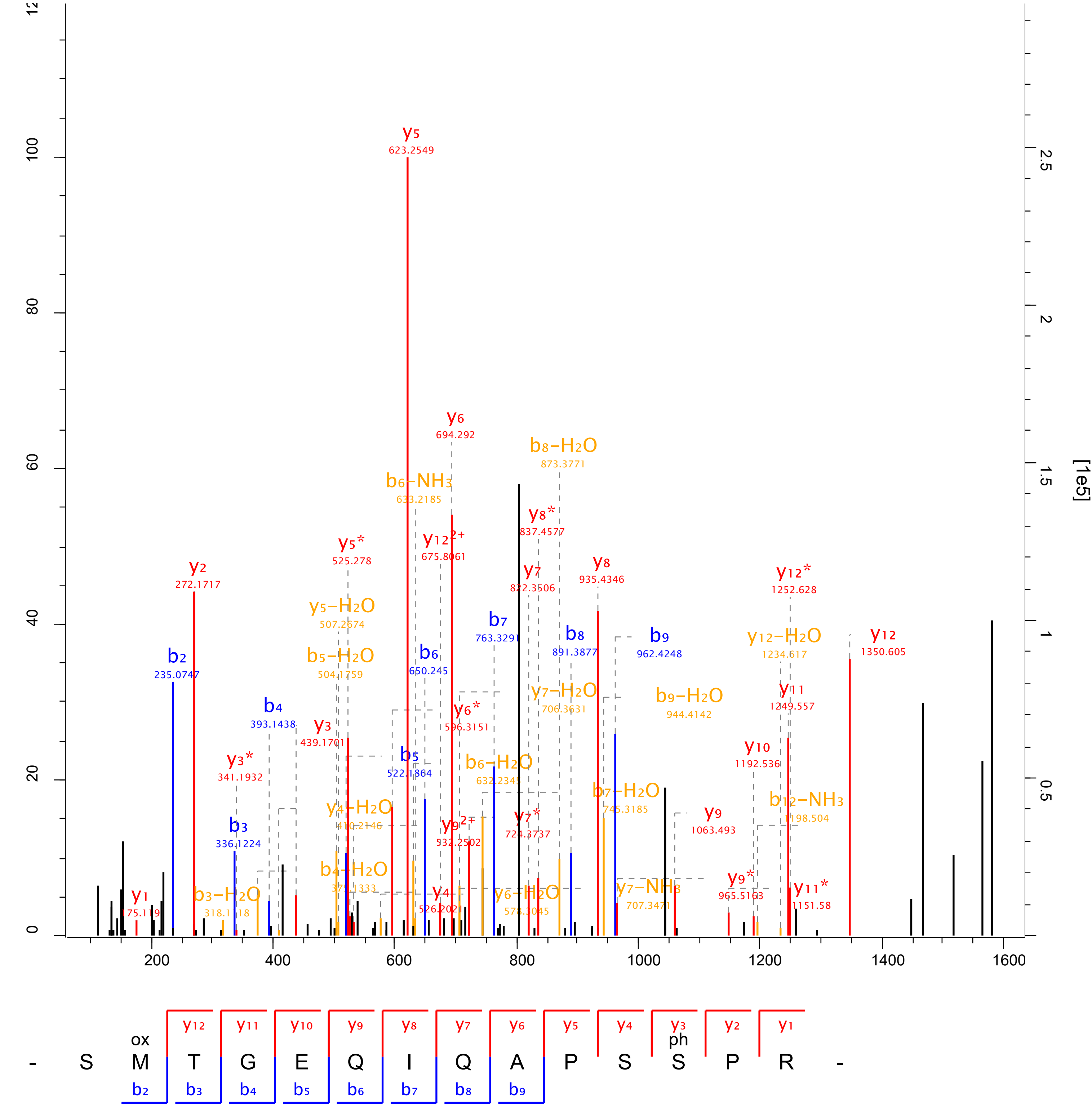

Sample output to test PDF Combine only

Raw file	Scan	Method	Score	m/z
0523_4	7285	FTMS; HCD	164.22	680.76

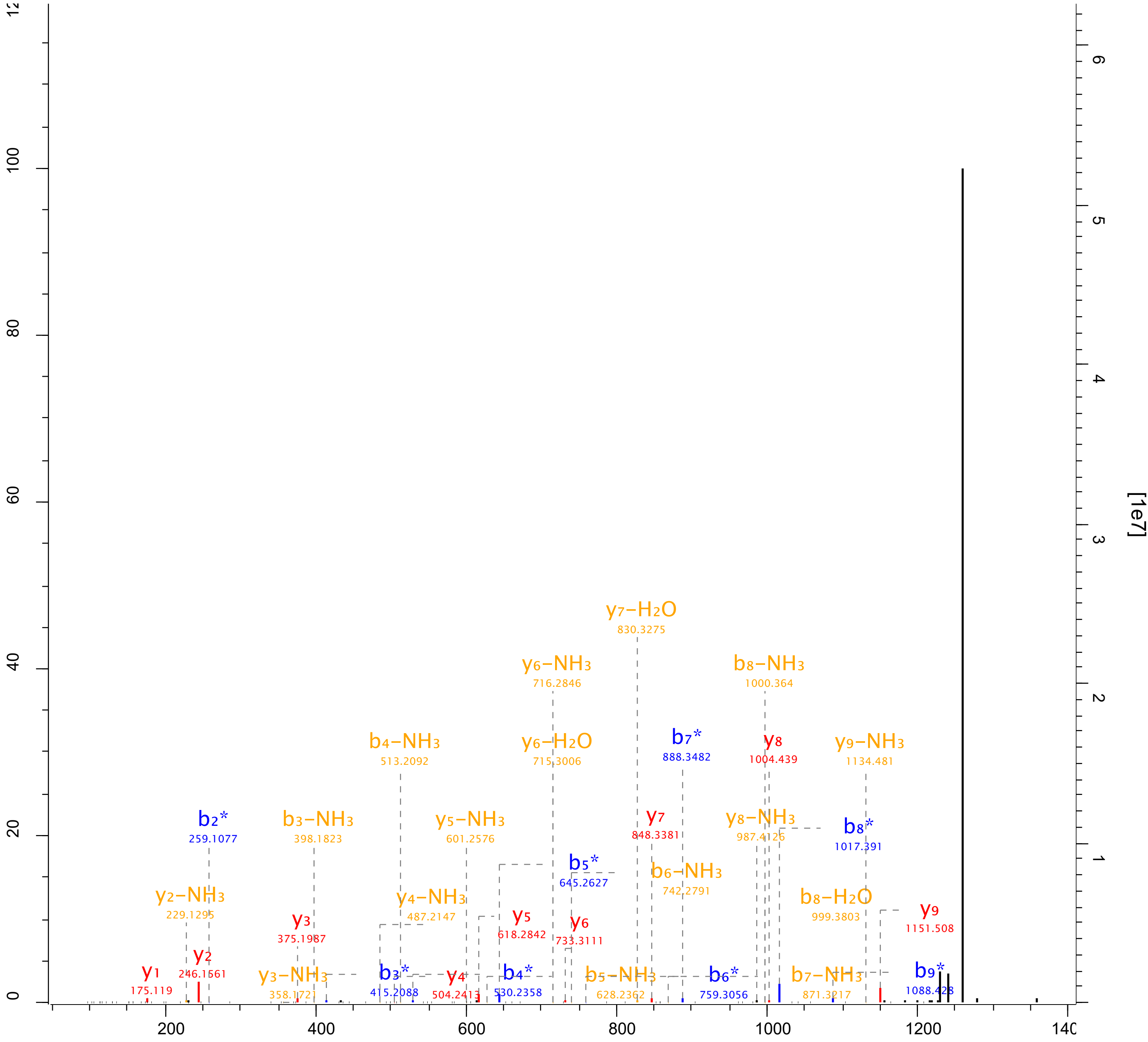

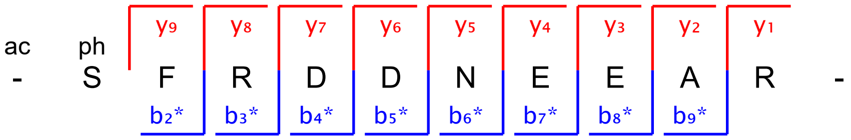

Sample output to test PDF Combine only

Raw file	Scan	Method	Score	m/z	Gene names
0523_4	7305	FTMS; HCD	95.43	619.25	SNX2A

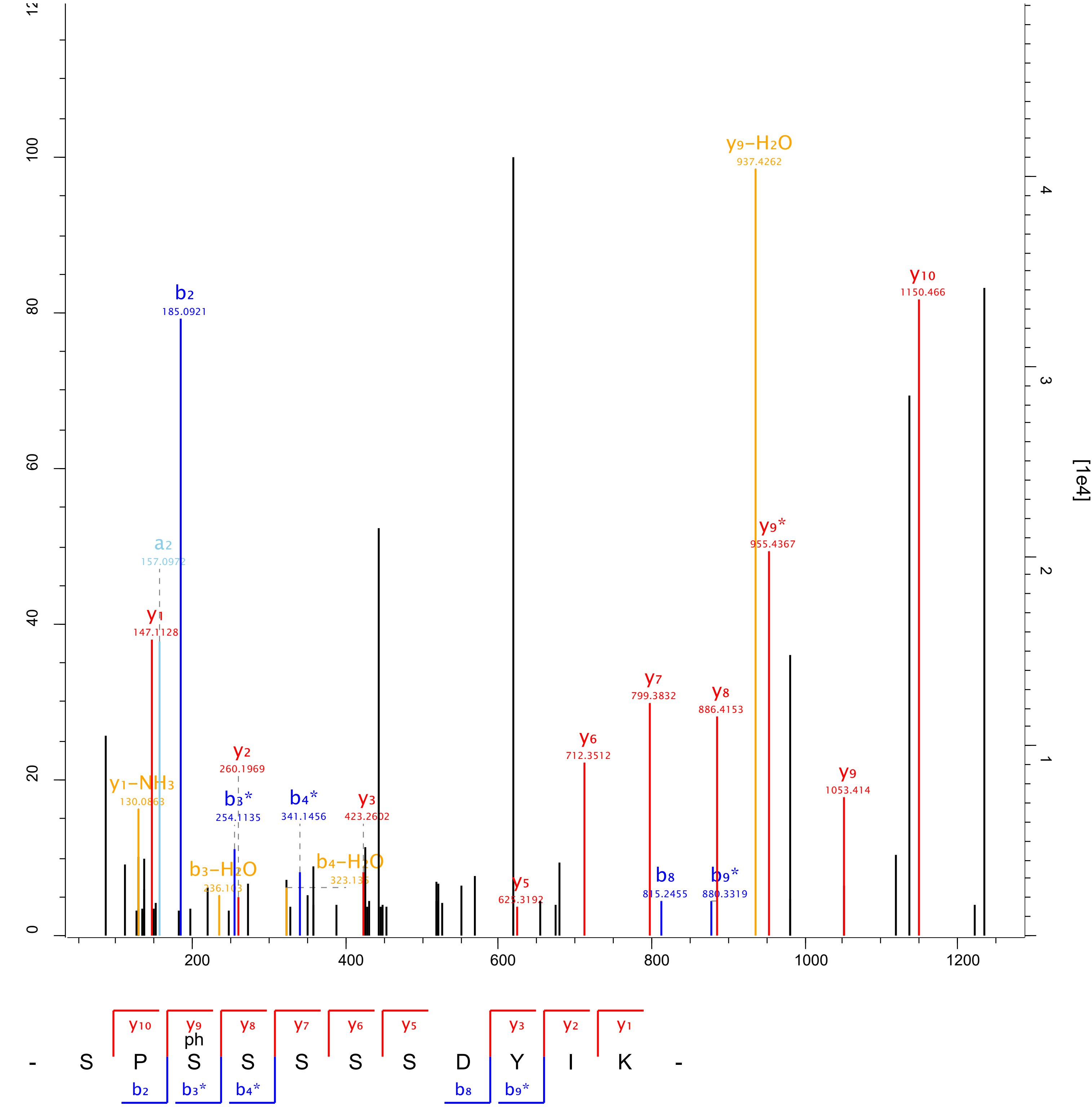

Sample output to test PDF Combine only

Raw file	Scan	Method	Score	m/z	Gene names
0523_4	7369	FTMS; HCD	125.79	806.31	SC35

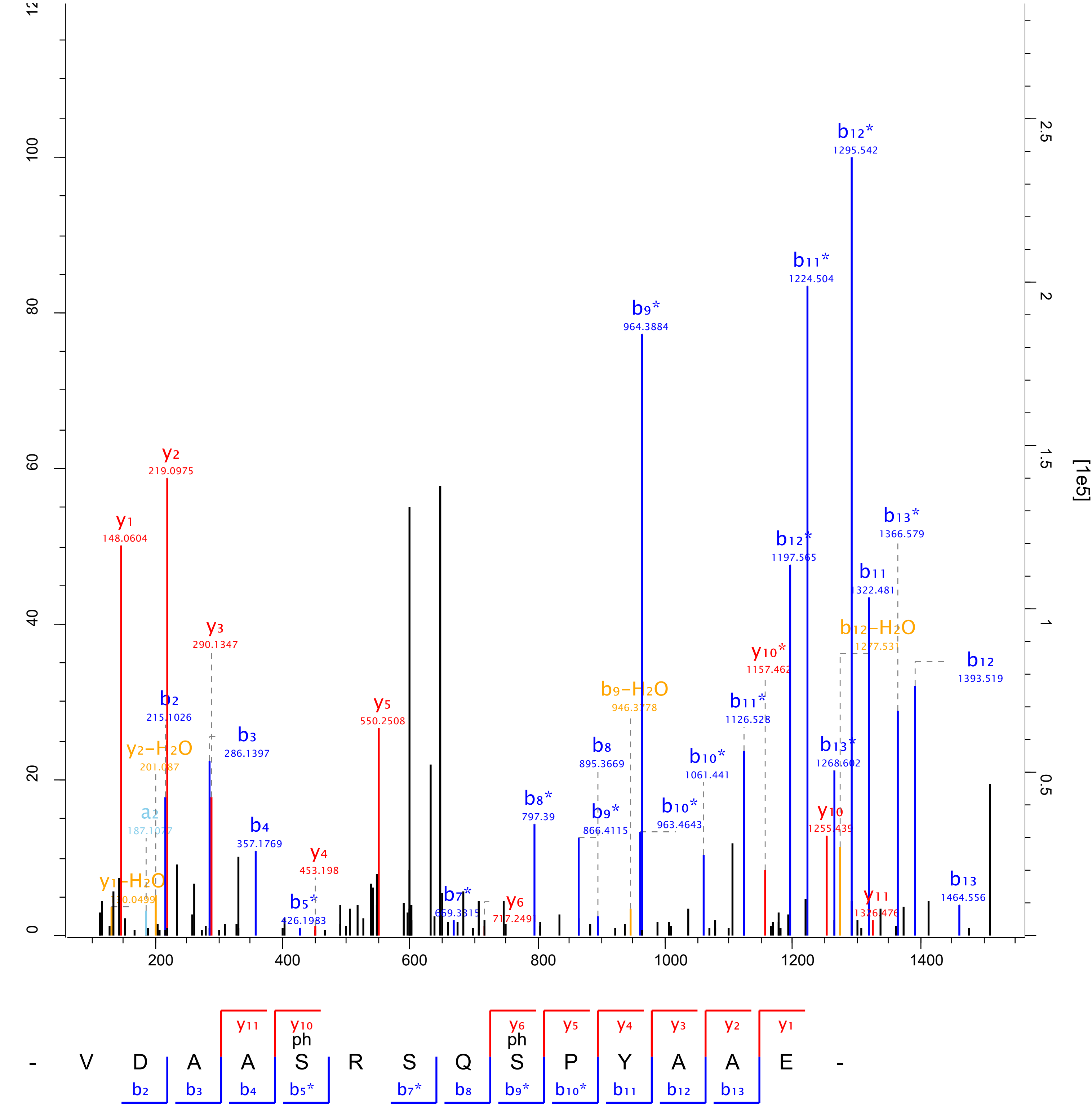

Sample output to test PDF Combine only

Raw file	Scan	Method	Score	m/z	Gene names
0523_4	7395	FTMS; HCD	85.55	563.25	CALS12

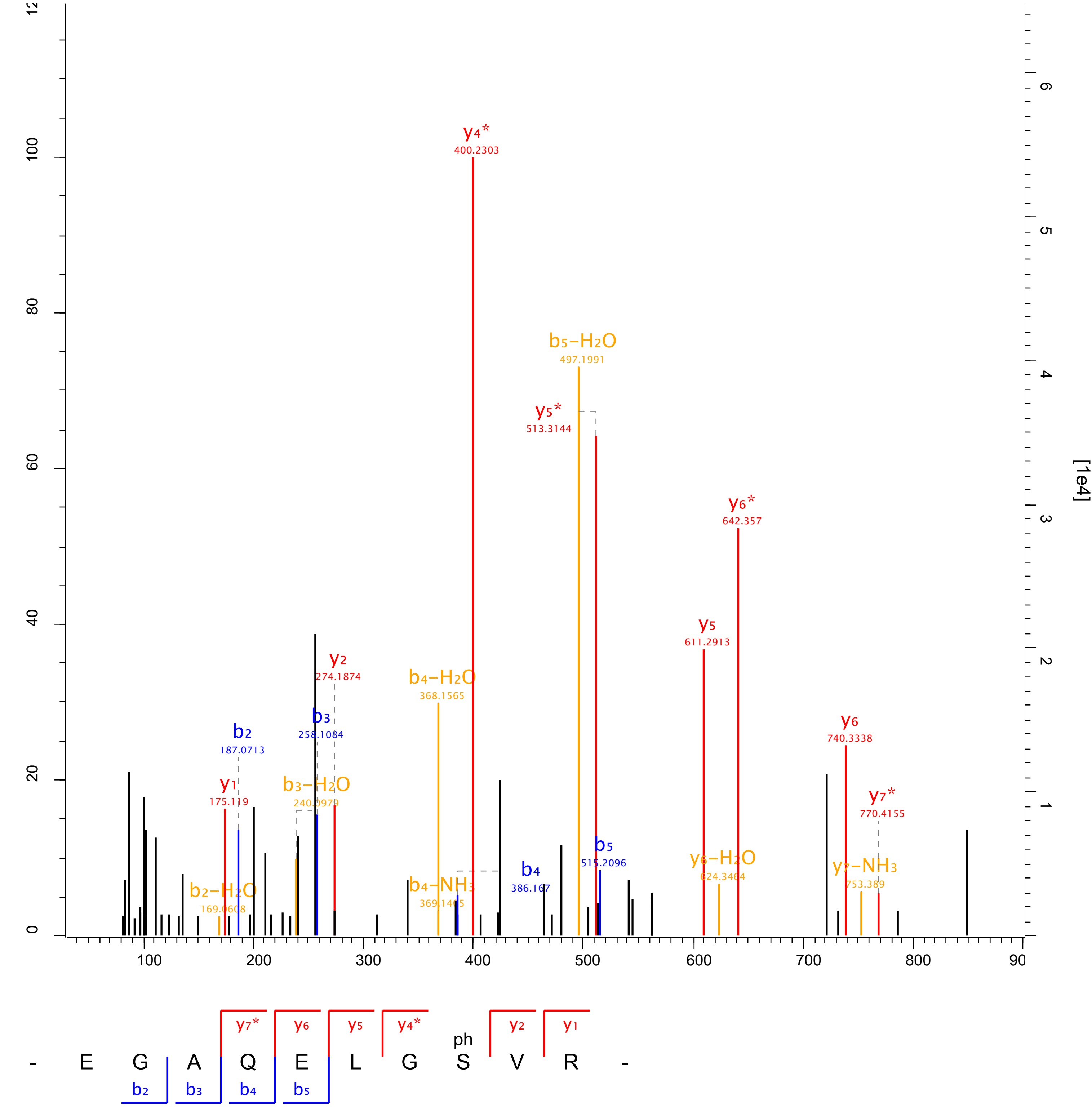

Sample output to test PDF Combine only

Raw file	Scan	Method	Score	m/z
0523_4	7400	FTMS; HCD	60.55	724.28

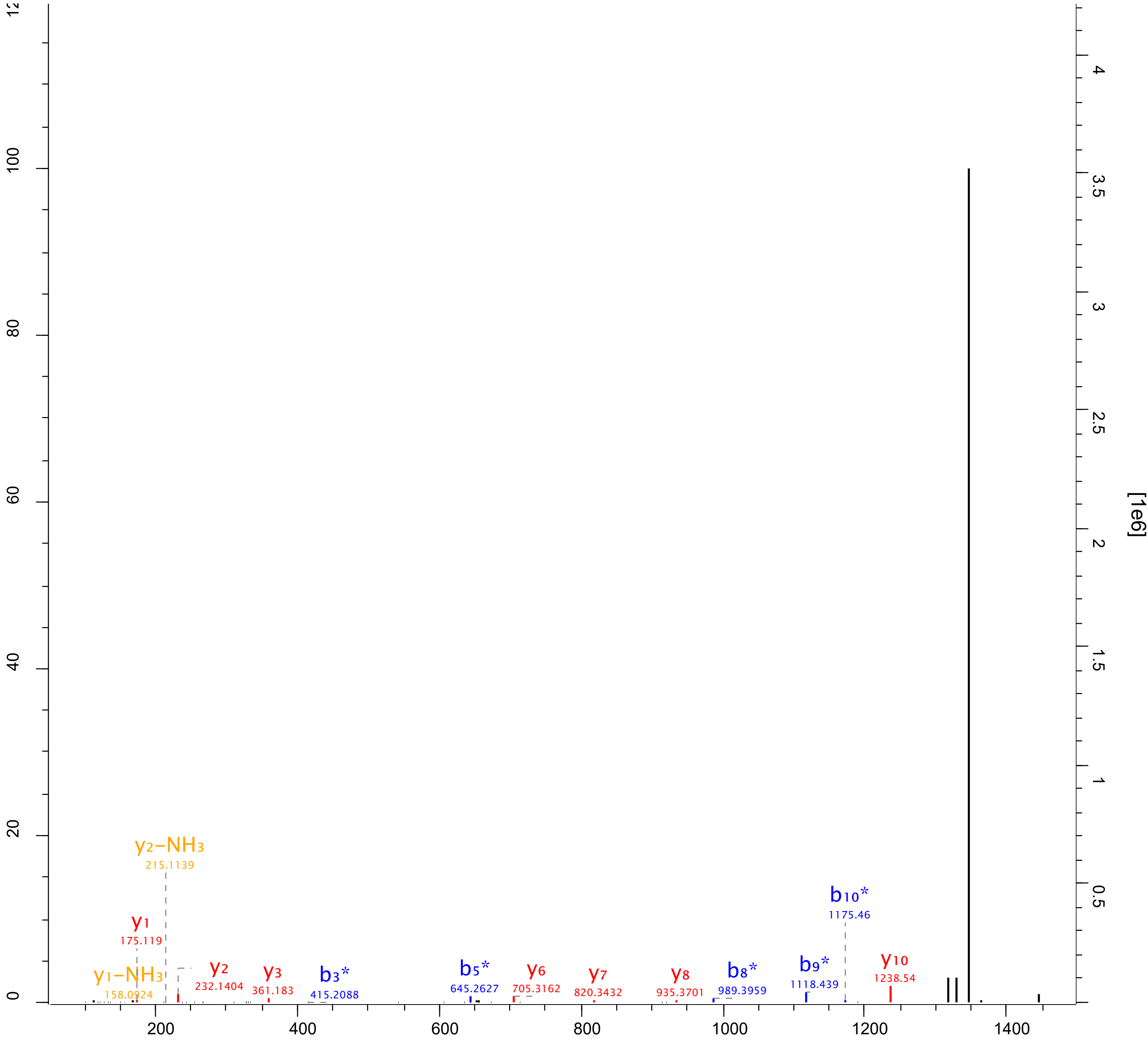

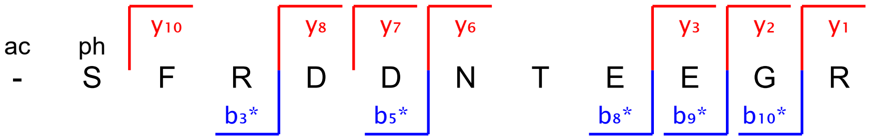

Sample output to test PDF Combine only

Raw file	Scan	Method	Score	m/z	Gene names
0523_4	7402	FTMS; HCD	70.2	695.81	T16L24.240

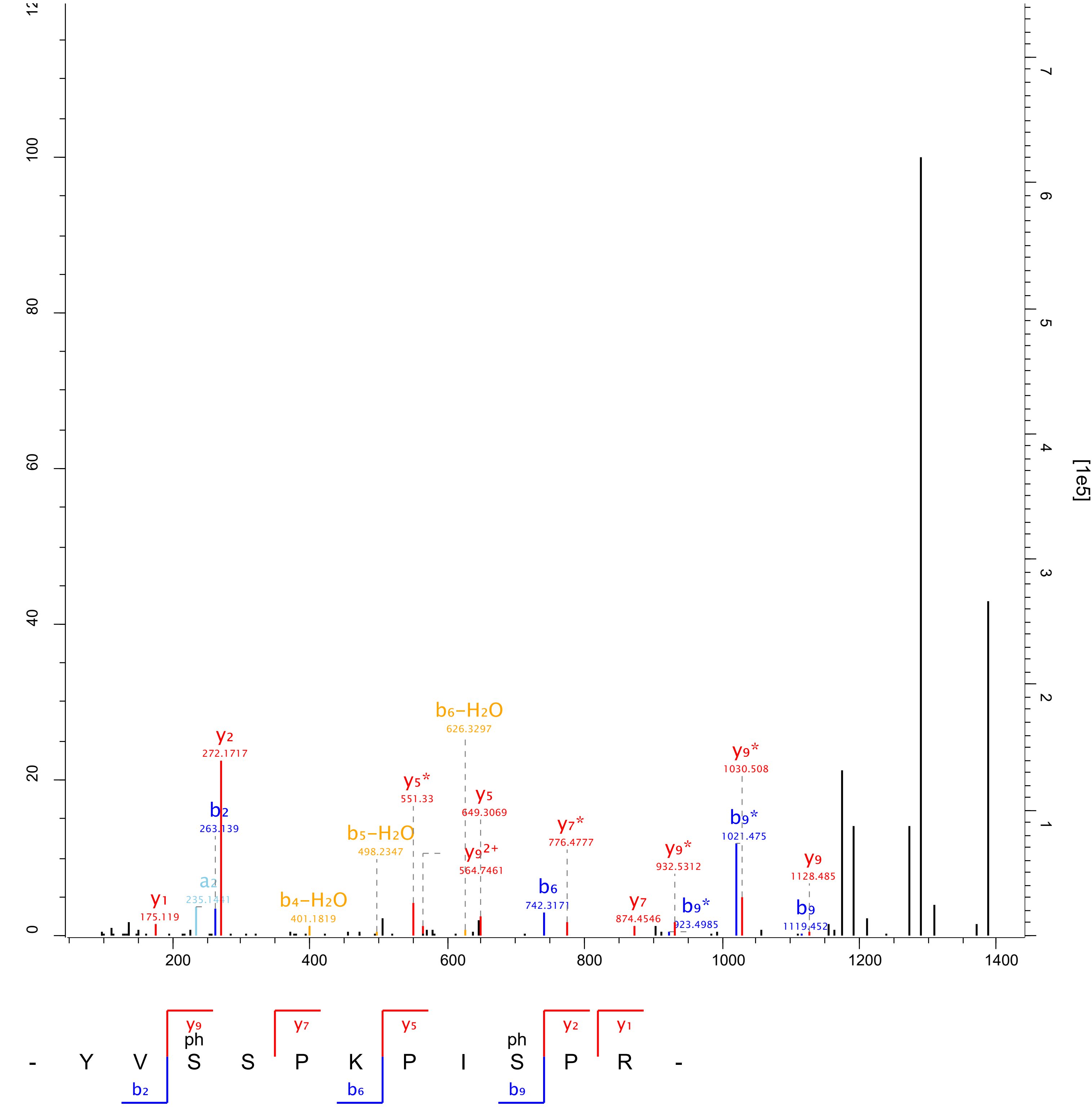

Sample output to test PDF Combine only

Raw file	Scan	Method	Score	m/z	Gene names
0523_4	7429	FTMS; HCD	181.87	1037.41	PYL1

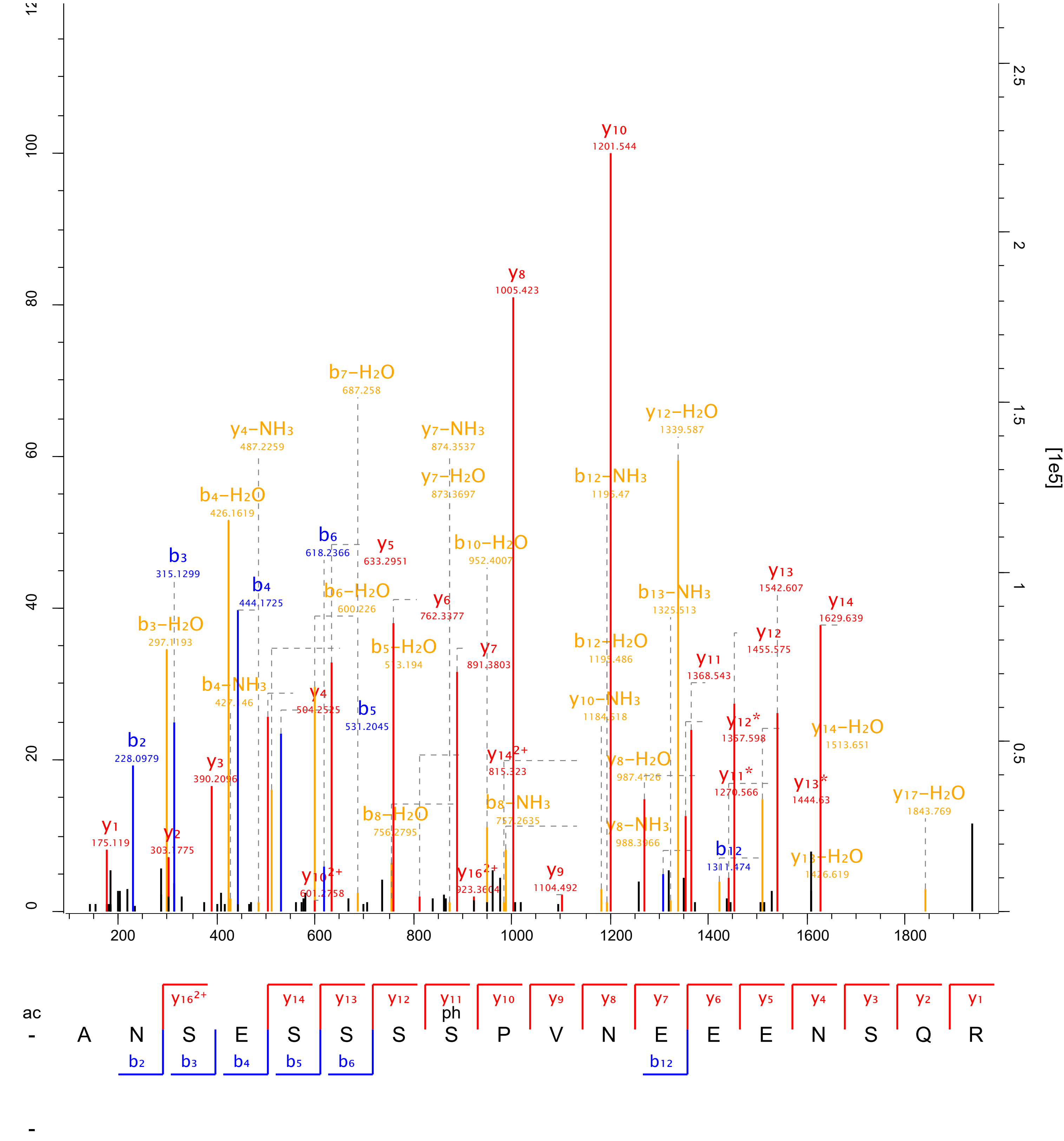

Sample output to test PDF Combine only

Raw file	Scan	Method	Score	m/z	Gene names
0523_4	7503	FTMS; HCD	82.61	798.85	At3g45190

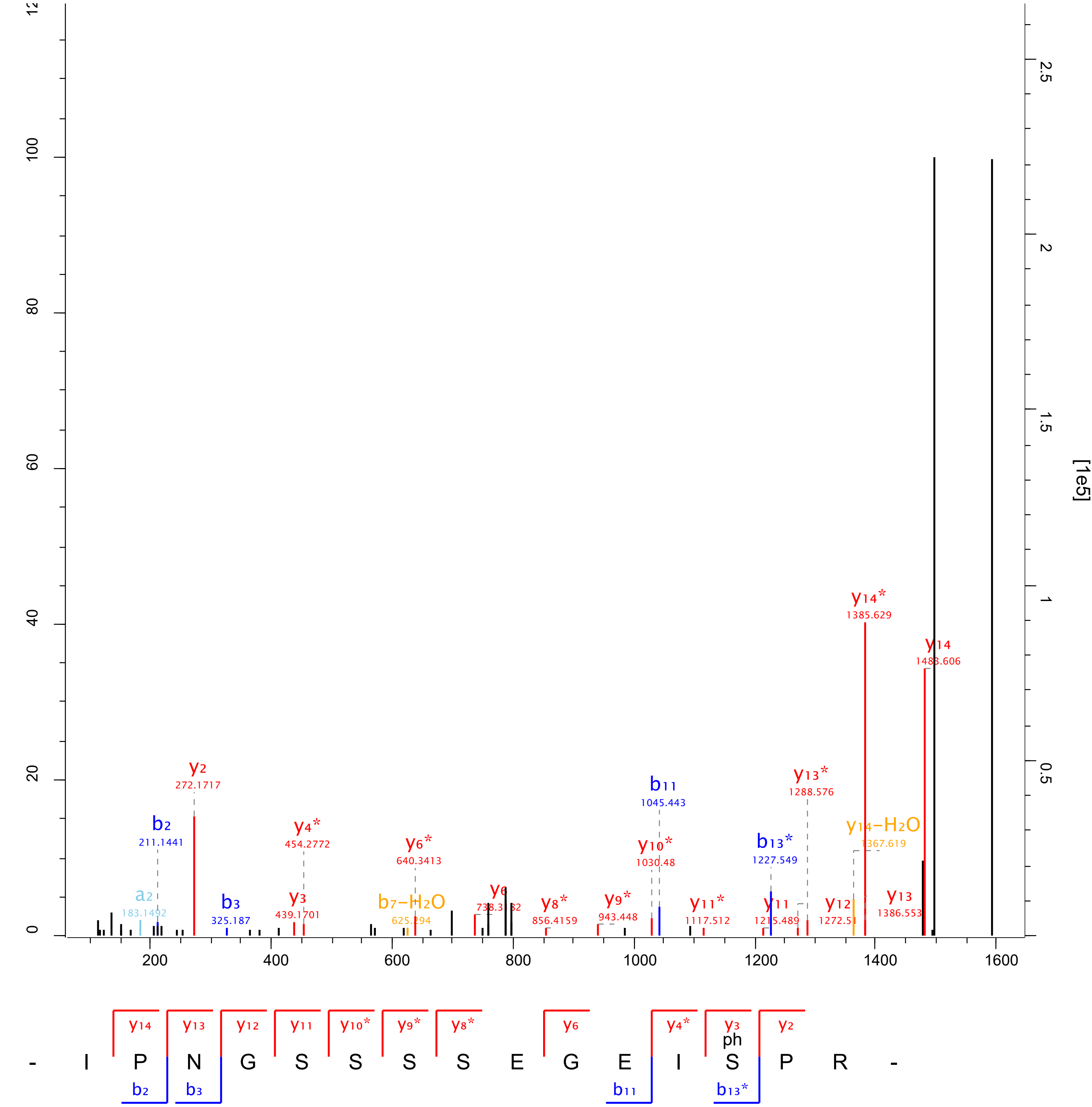

Sample output to test PDF Combine only

Raw file	Scan	Method	Score	m/z	Gene names
0523_4	7520	FTMS; HCD	44.51	625.26	At2g29210

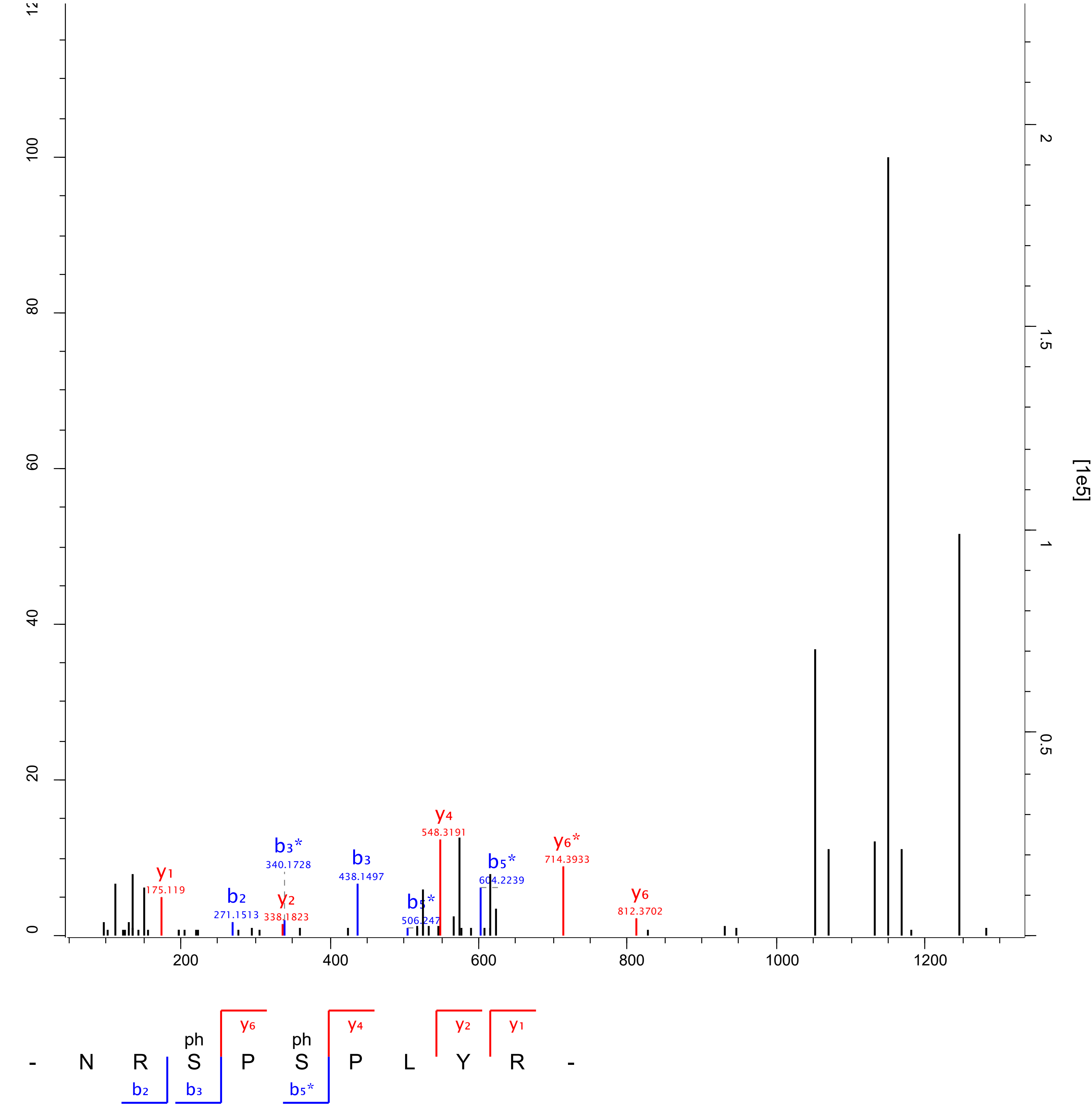

Sample output to test PDF Combine only

Raw file	Scan	Method	Score	m/z
0523_4	7536	FTMS; HCD	142.38	588.24

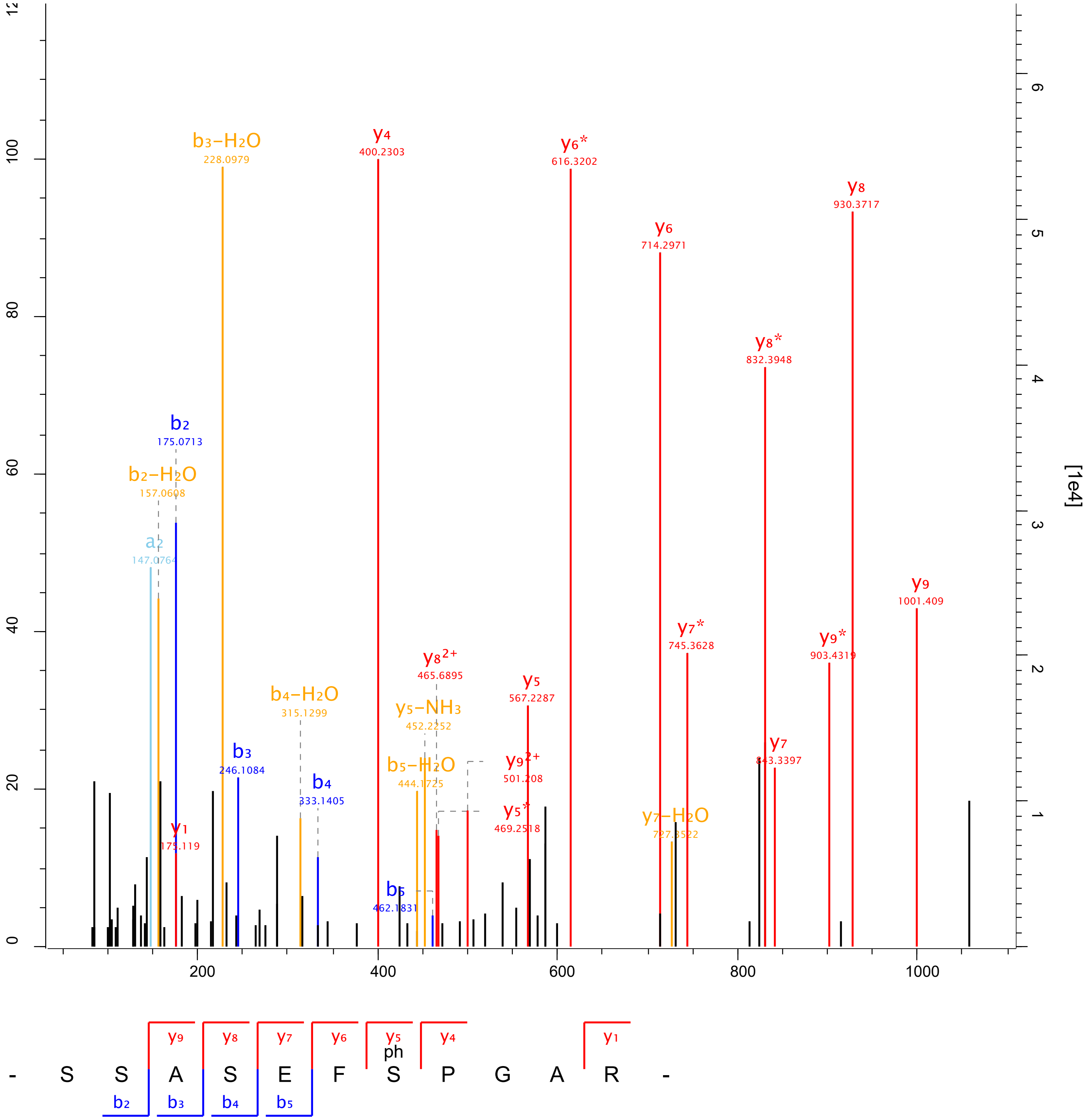

Sample output to test PDF Combine only

Raw file	Scan	Method	Score	m/z	Gene names
0523_4	7560	FTMS; HCD	128.71	622.78	WEB1

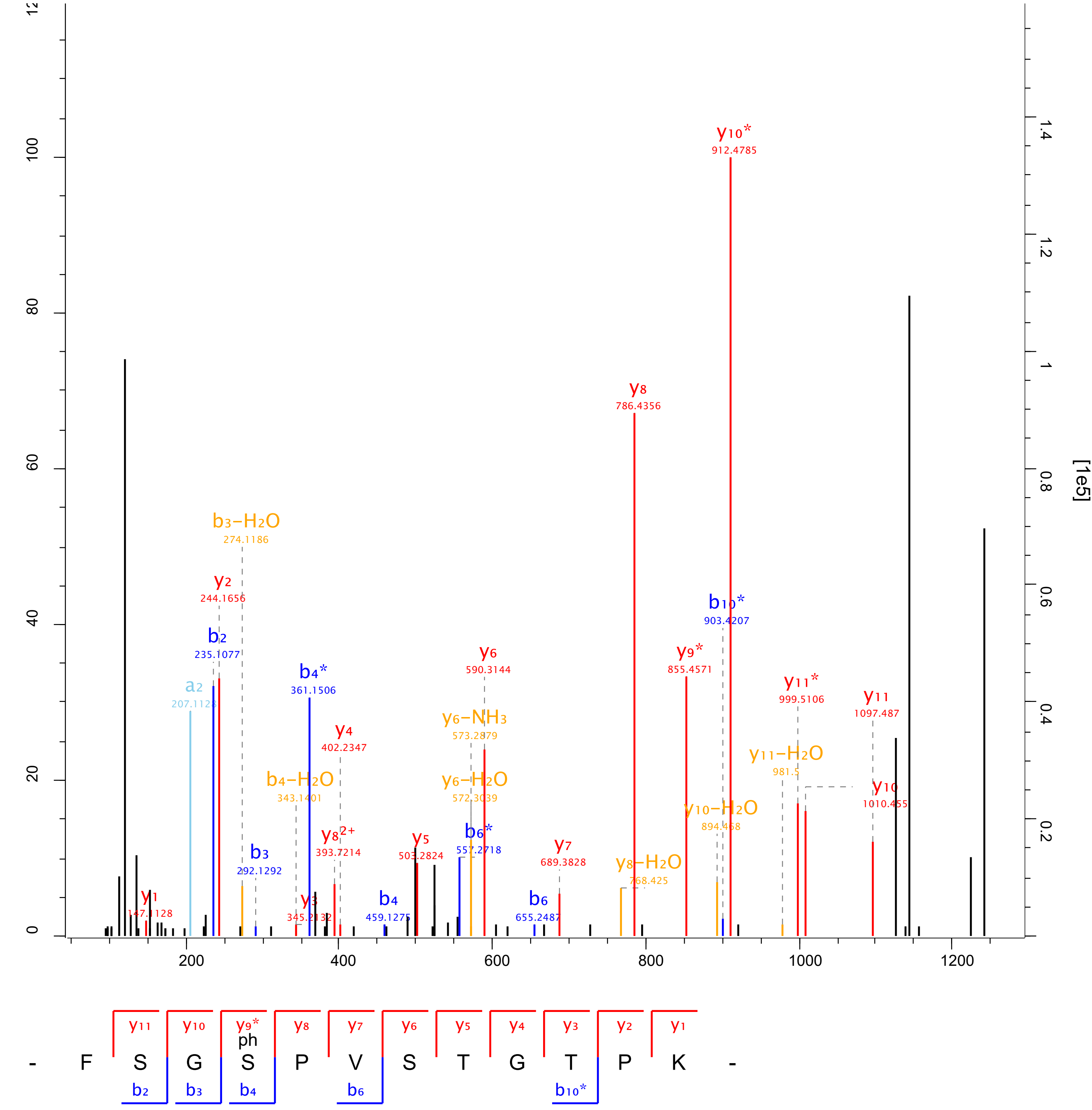

Sample output to test PDF Combine only

Raw file	Scan	Method	Score	m/z	Gene names
0523_4	7577	FTMS; HCD	118.31	684.31	T14L22.4

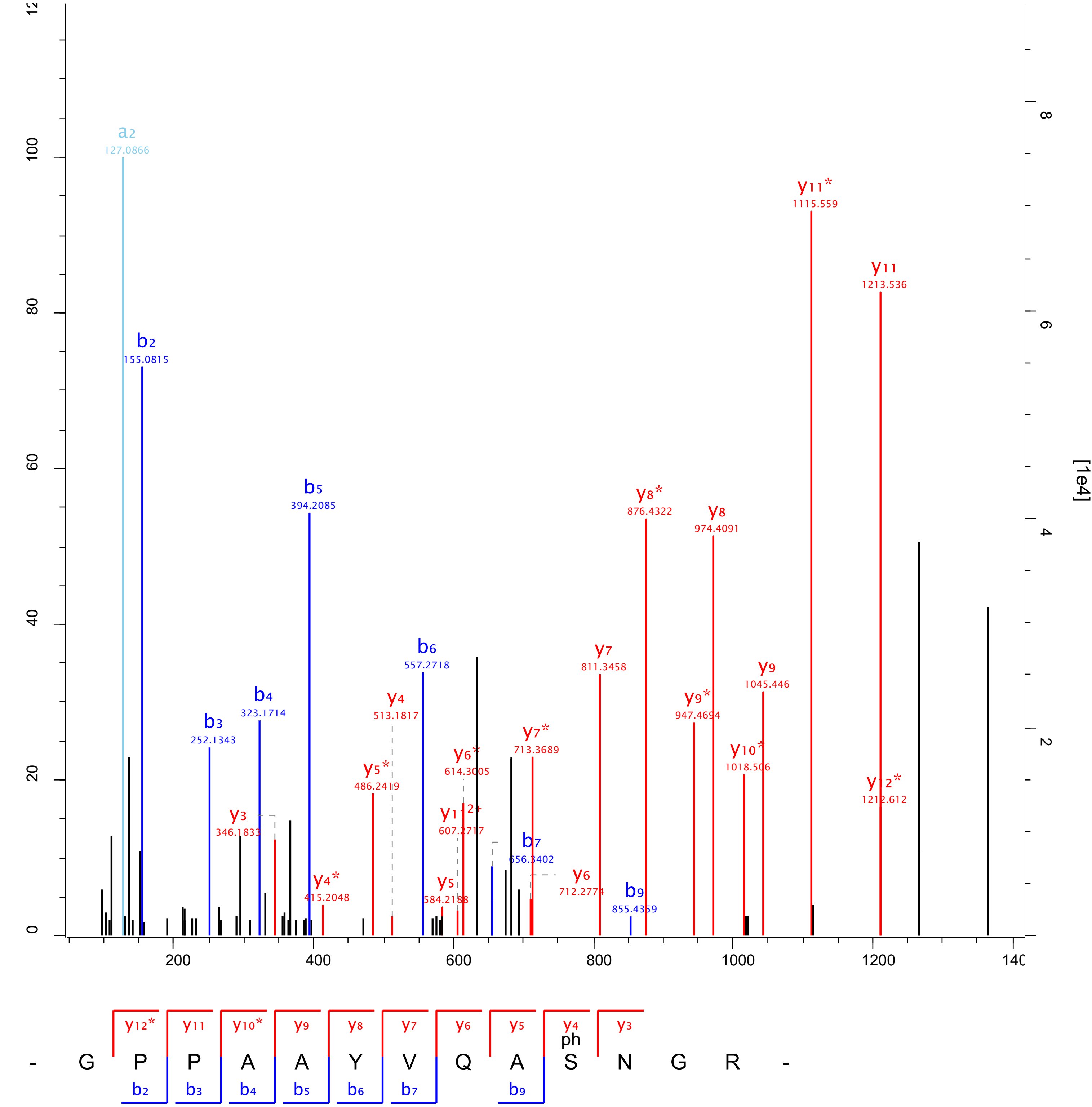

Sample output to test PDF Combine only

Raw file	Scan	Method	Score	m/z	Gene names
0523_4	7634	FTMS; HCD	148.06	791.33	At4g38470

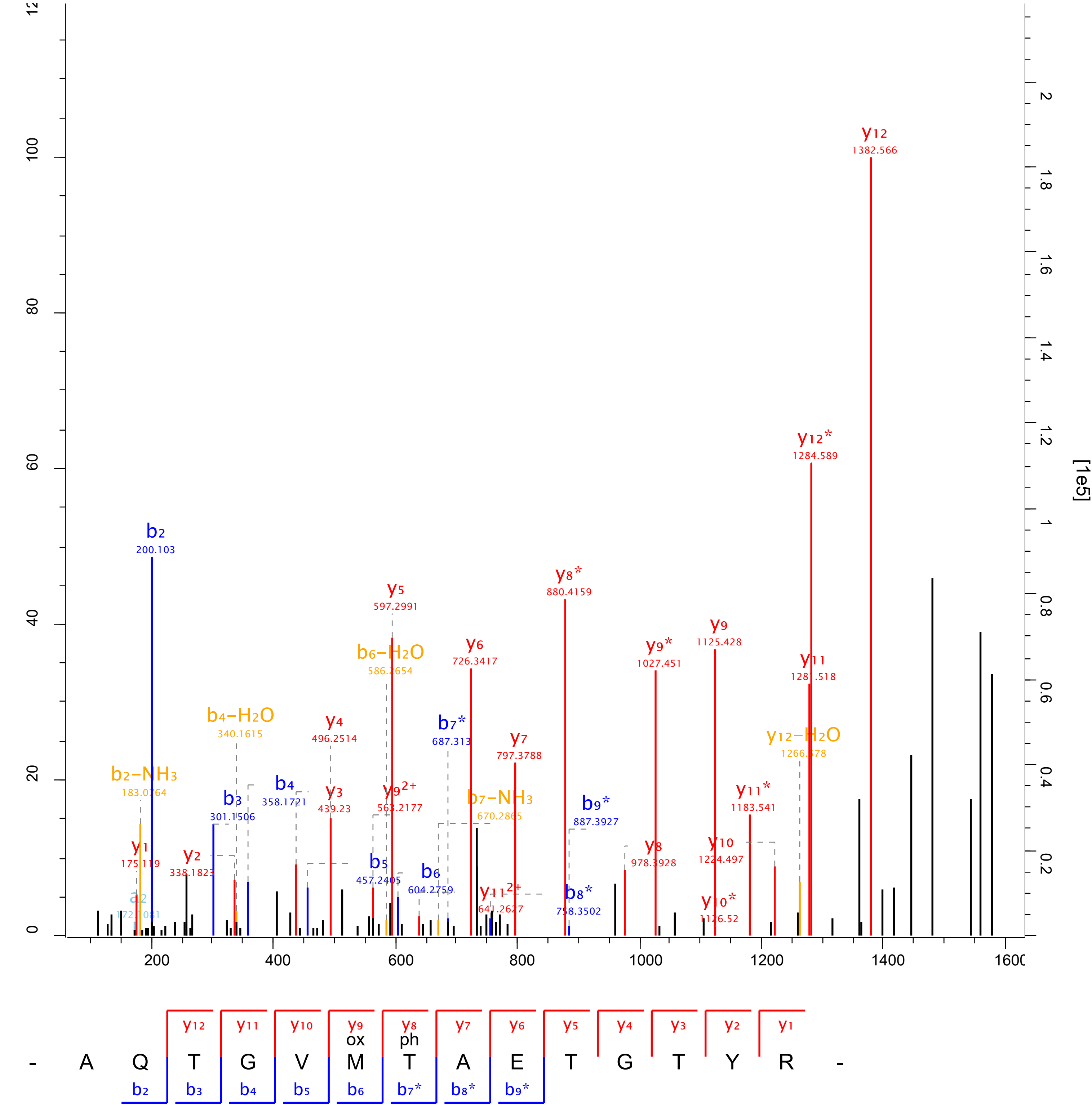

Sample output to test PDF Combine only

Raw file	Scan	Method	Score	m/z	Gene names
0523_4	7646	FTMS; HCD	106.6	666.3	KAT3

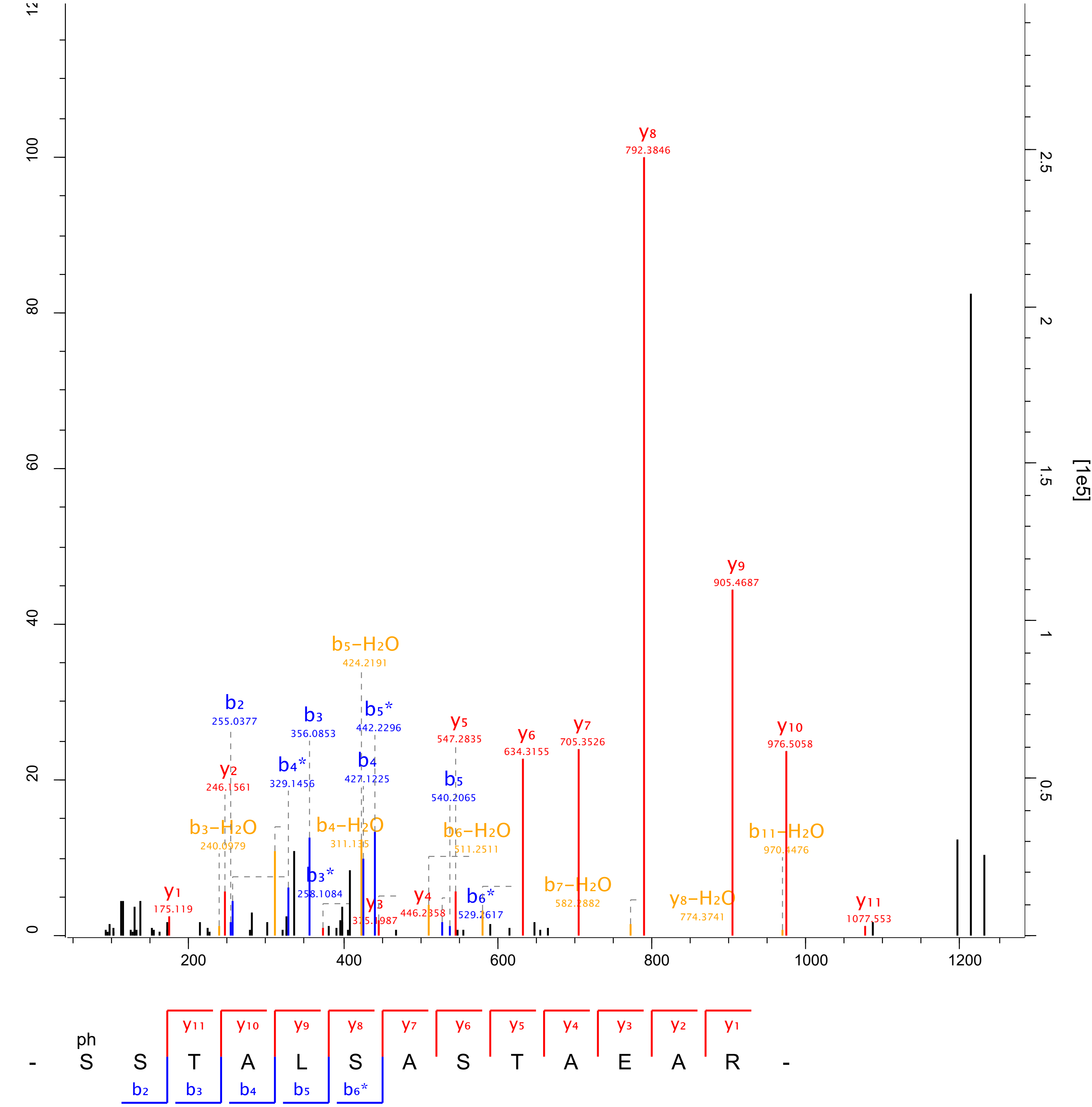

Sample output to test PDF Combine only

Raw file	Scan	Method	Score	m/z	Gene names
0523_4	7687	FTMS; HCD	66.47	694.93	ESP3

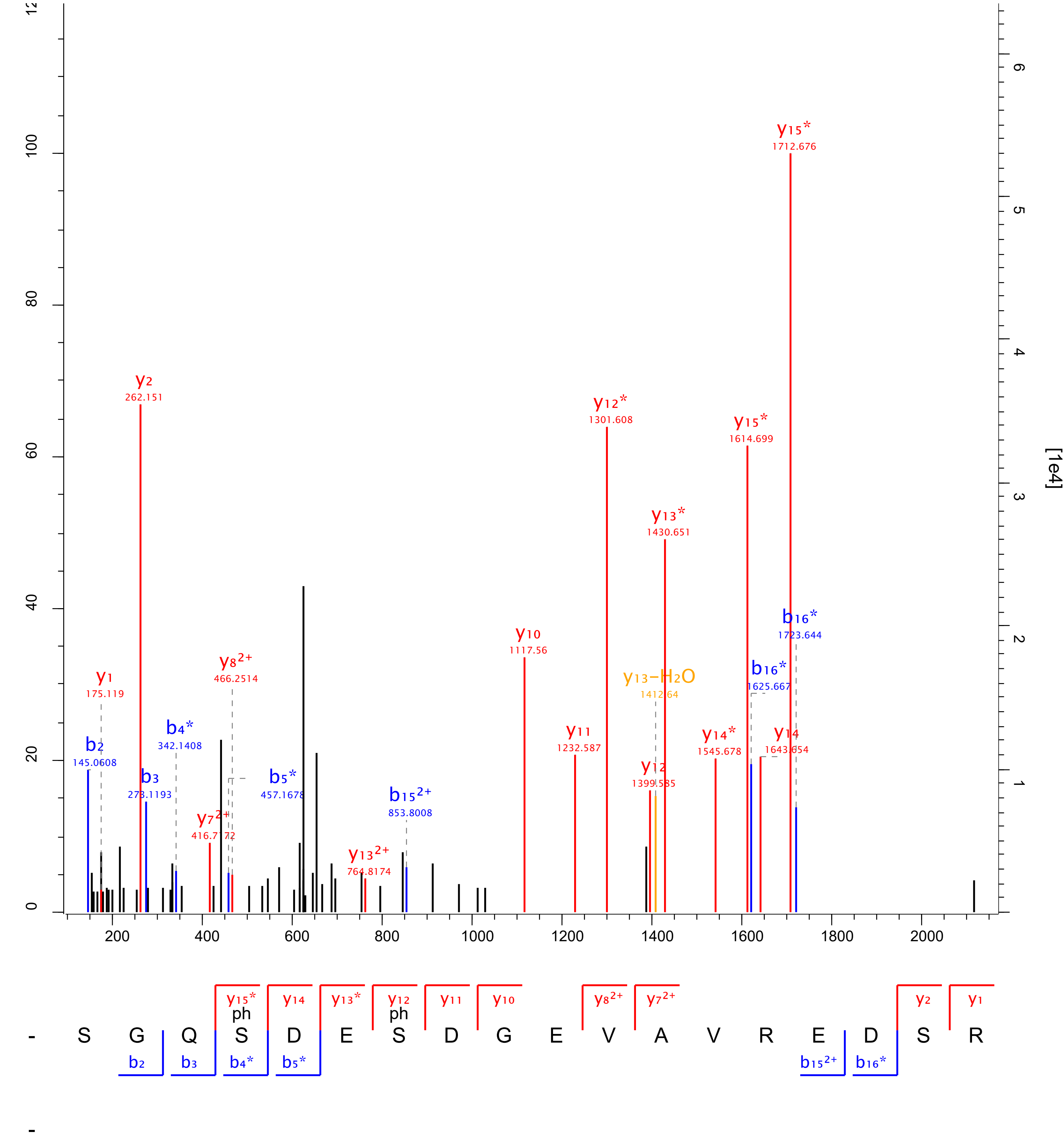

Sample output to test PDF Combine only

Raw file	Scan	Method	Score	m/z	Gene names
0523_4	7709	FTMS; HCD	68.48	697.78	PAT07

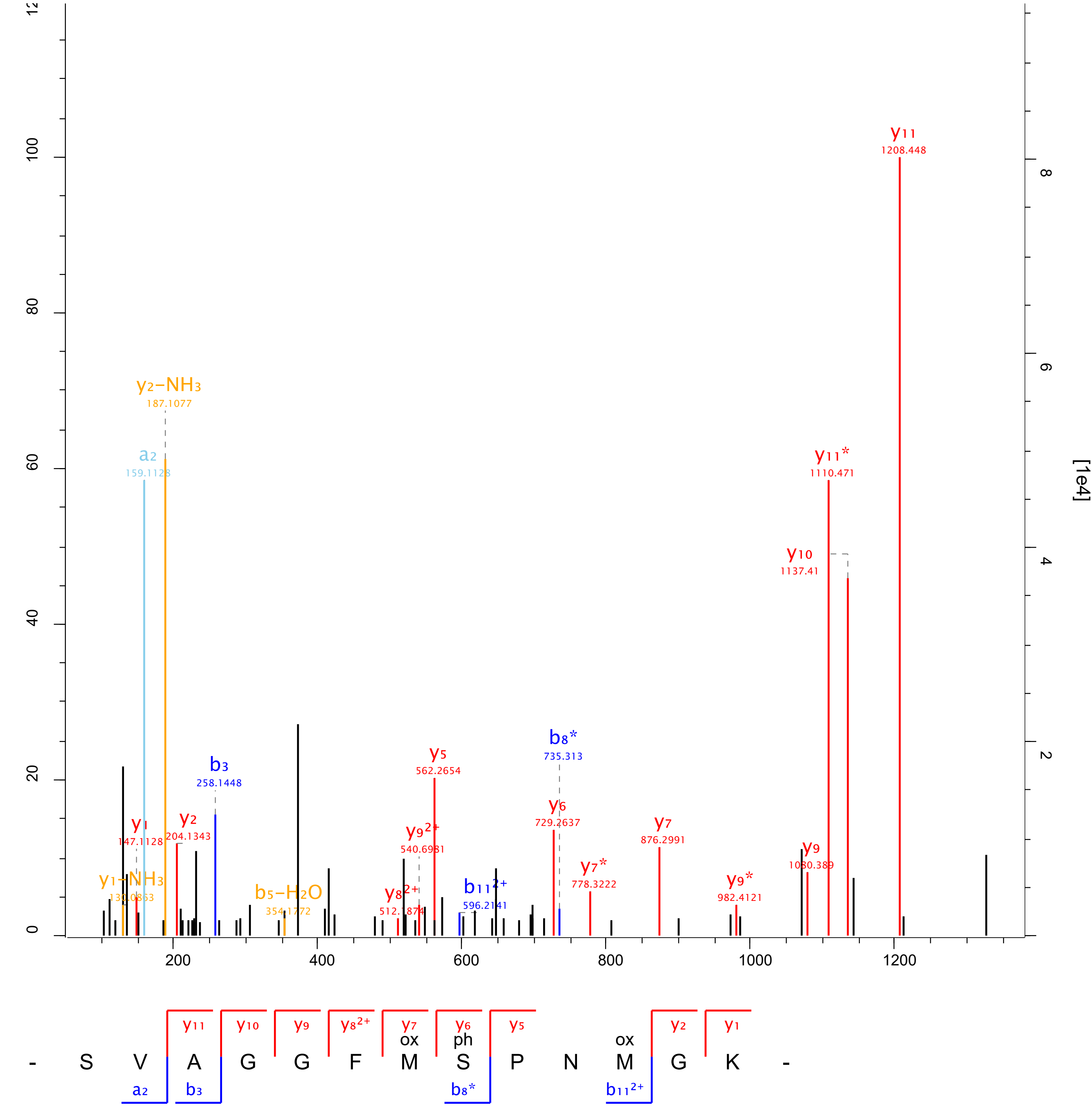

Sample output to test PDF Combine only

Raw file	Scan	Method	Score	m/z	Gene names
0523_4	7733	FTMS; HCD	157.98	642.25	TIF3C1

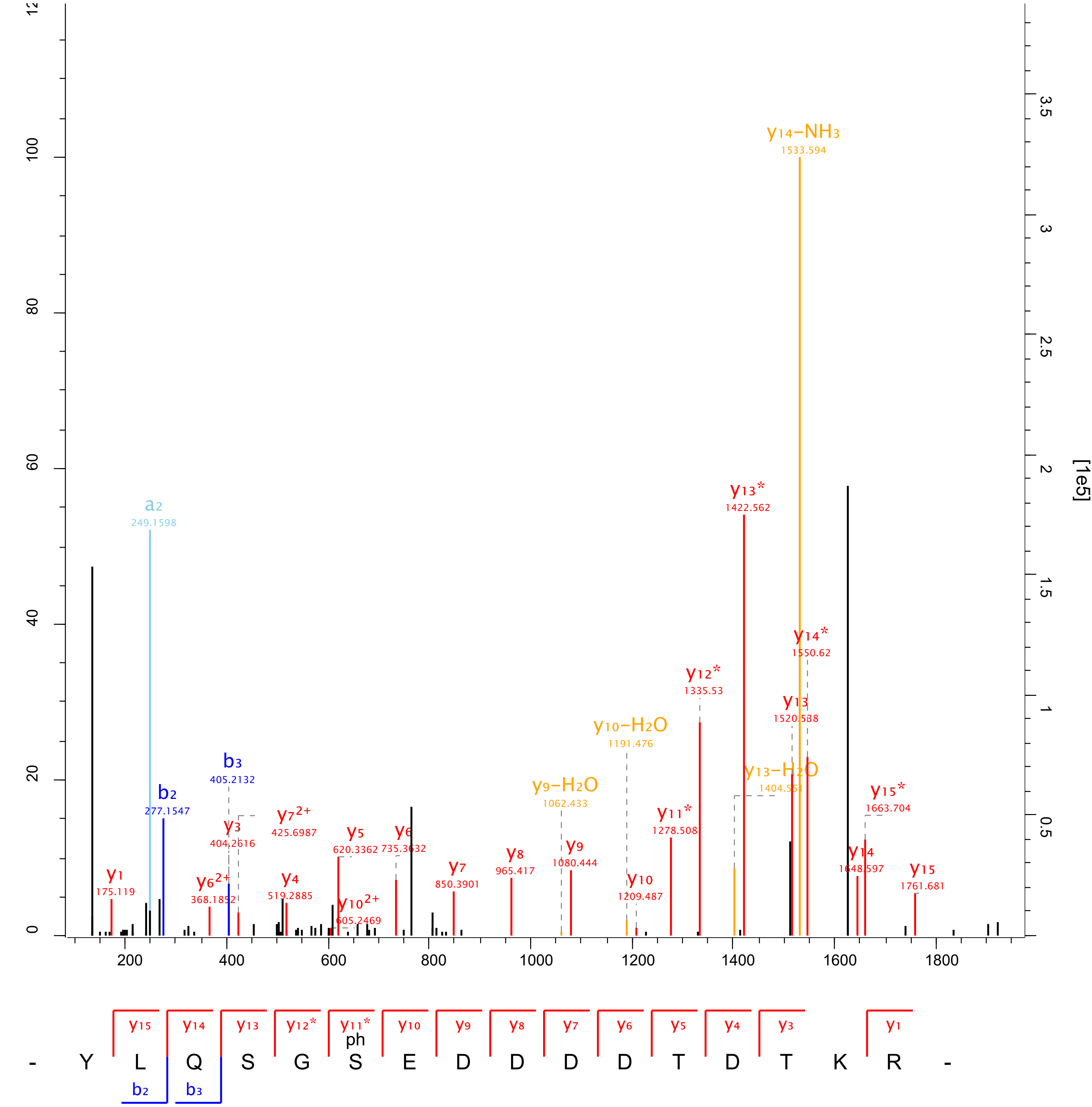

Sample output to test PDF Combine only

Raw file	Scan	Method	Score	m/z	Gene names
0523_4	7734	FTMS; HCD	74.53	732.96	SCL30A

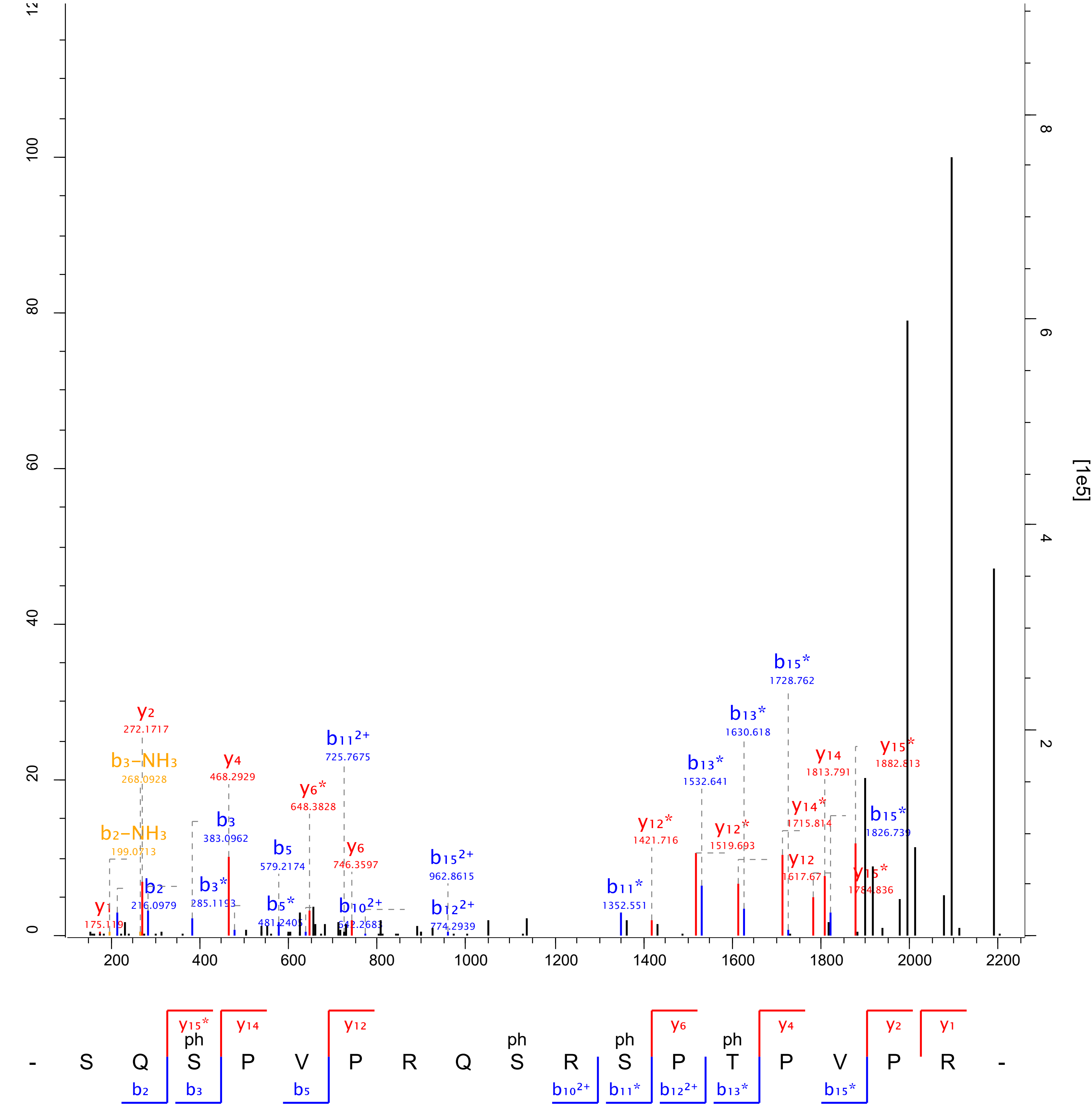

Sample output to test PDF Combine only

Raw file	Scan	Method	Score	m/z	Gene names
0523_4	7801	FTMS; HCD	61.34	515.72	CPK6;CPK5

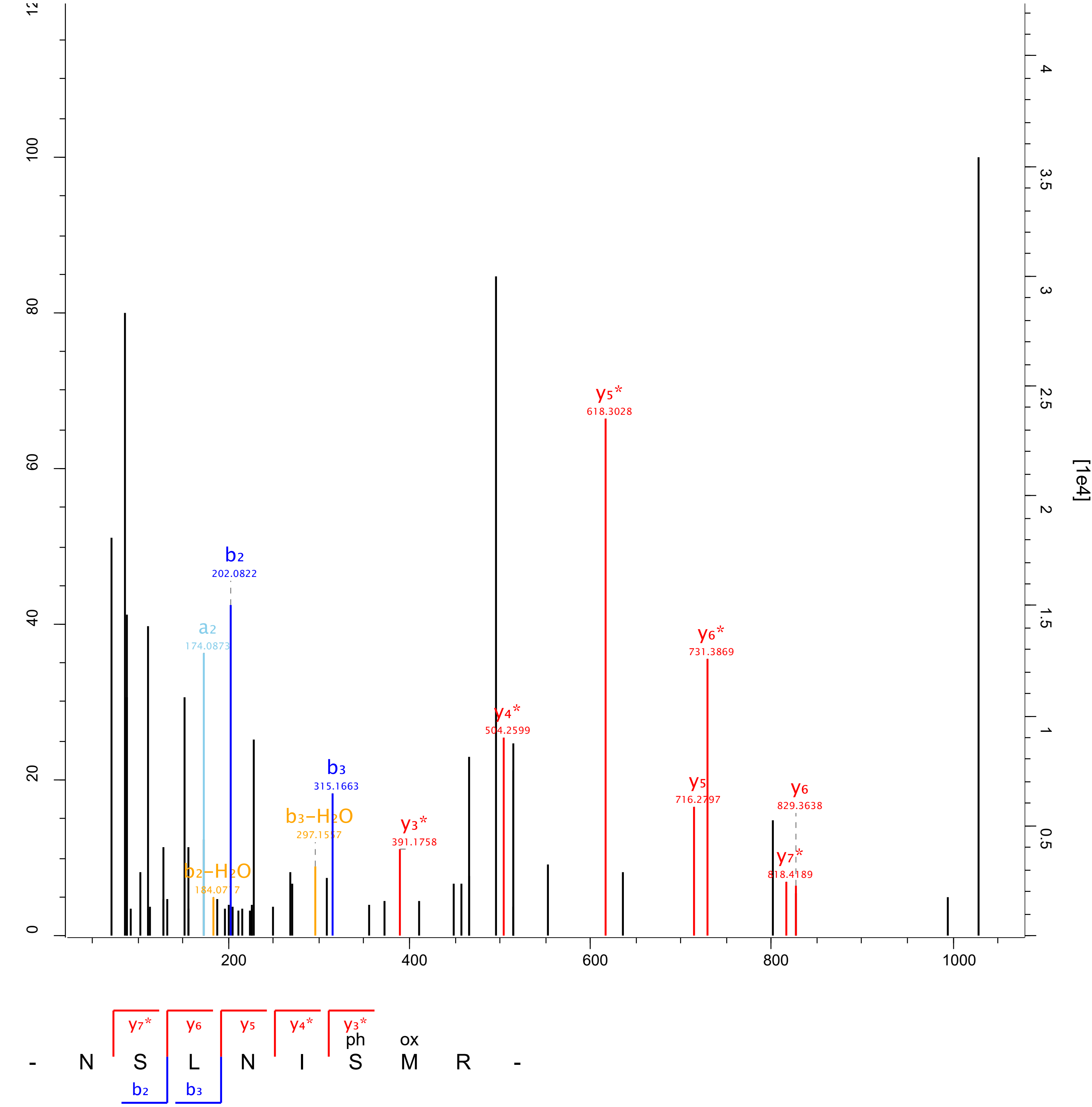

Sample output to test PDF Combine only

Raw file	Scan	Method	Score	m/z	Gene names
0523_4	7858	FTMS; HCD	74.39	523.76	PAT24

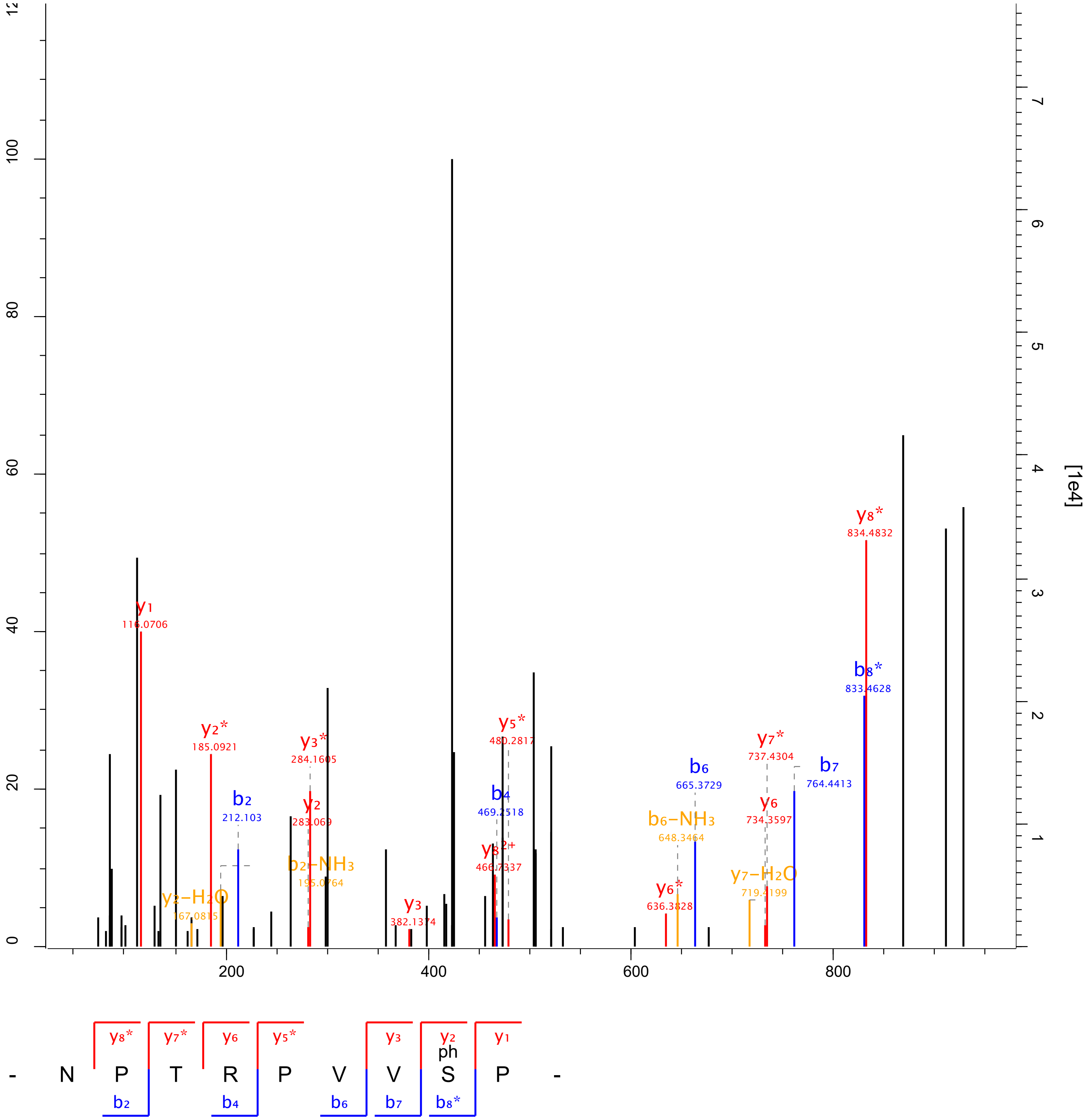

Sample output to test PDF Combine only

Raw file	Scan	Method	Score	m/z	Gene names
0523_4	7861	FTMS; HCD	44.34	657.95	MUR1

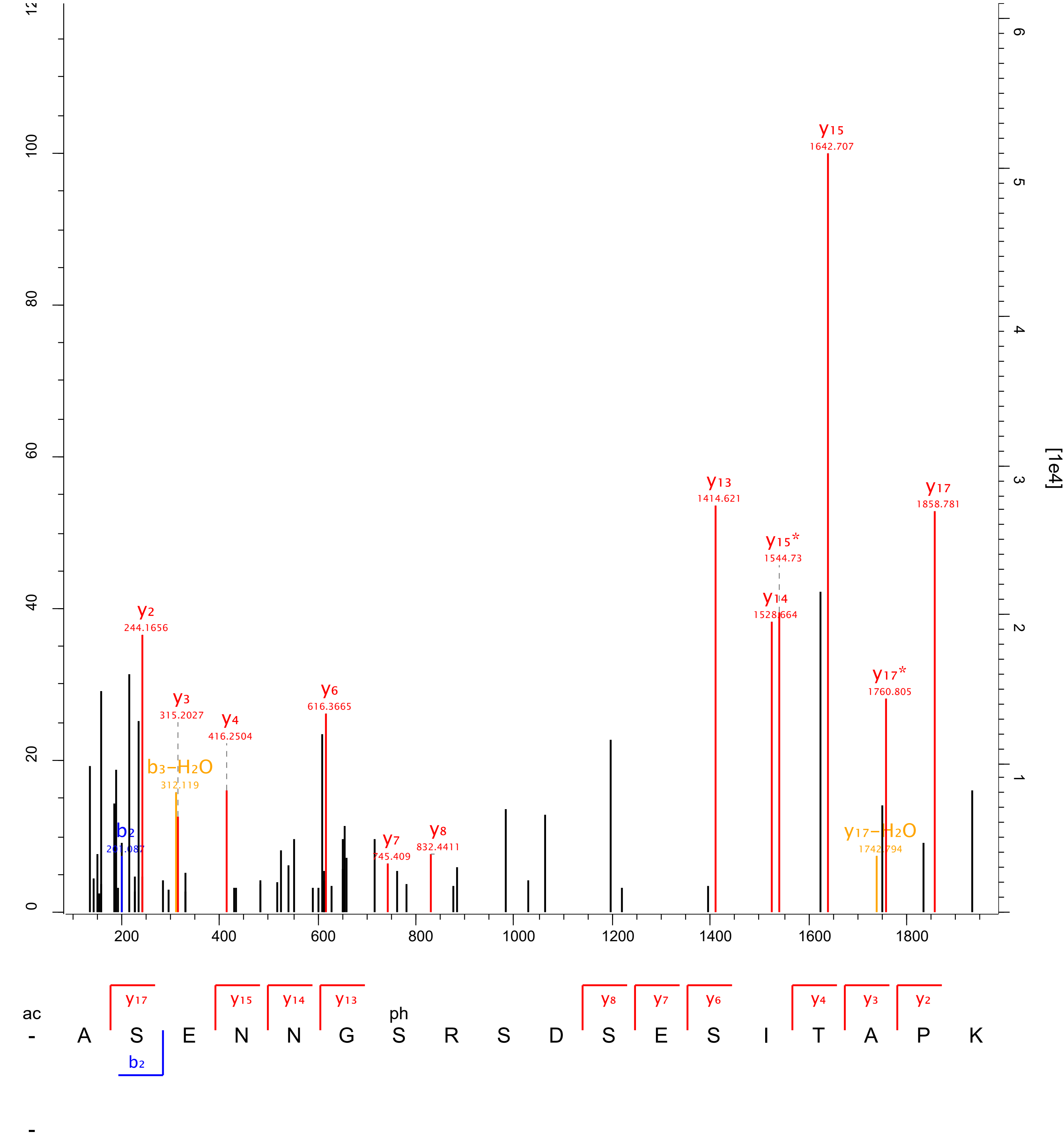

Sample output to test PDF Combine only

Raw file	Scan	Method	Score	m/z	Gene names
0523_4	7884	FTMS; HCD	73.36	765.29	At3g50590

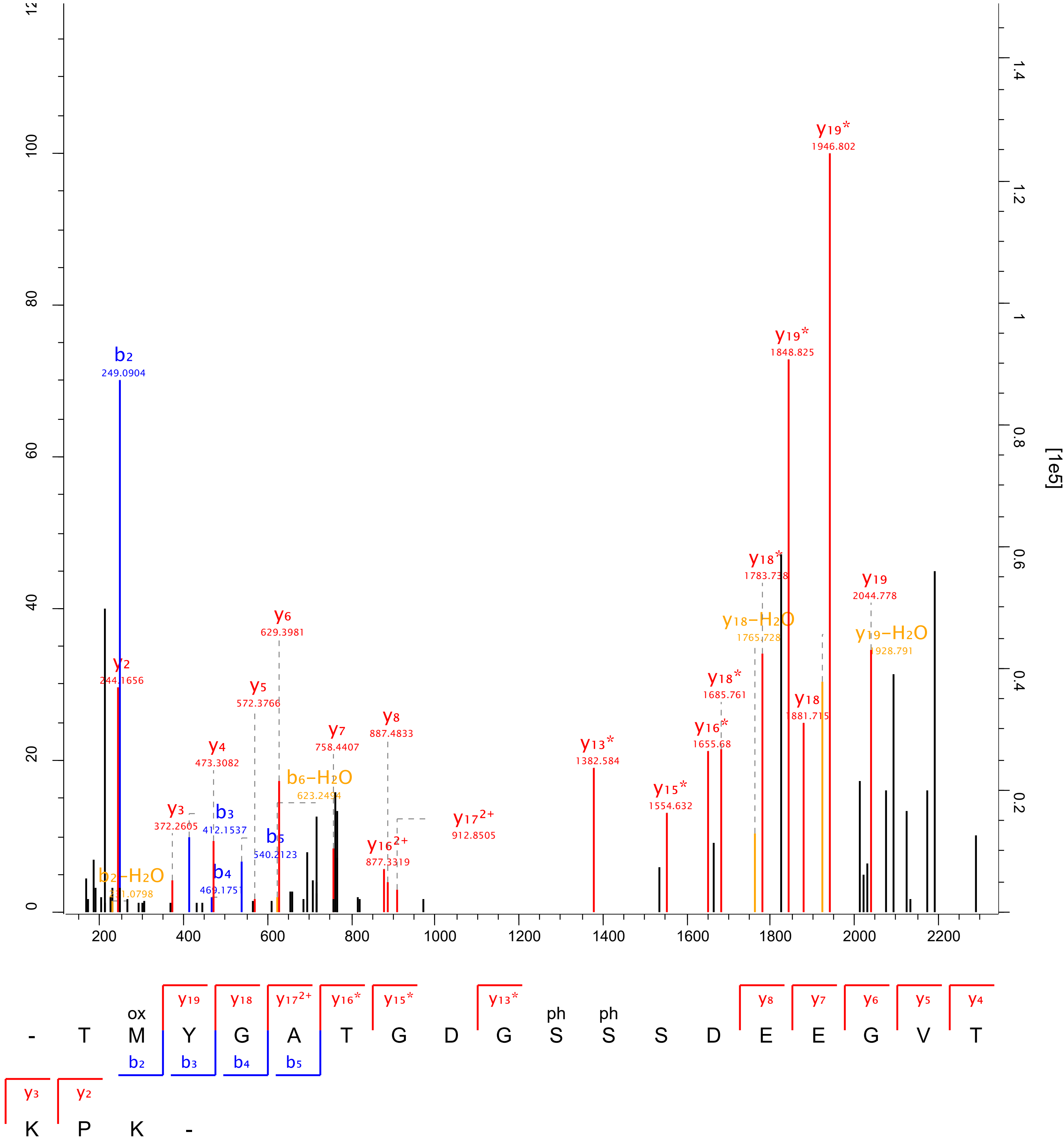

Sample output to test PDF Combine only

Raw file	Scan	Method	Score	m/z	Gene names
0523_4	7930	FTMS; HCD	57.21	796.93	LAG1

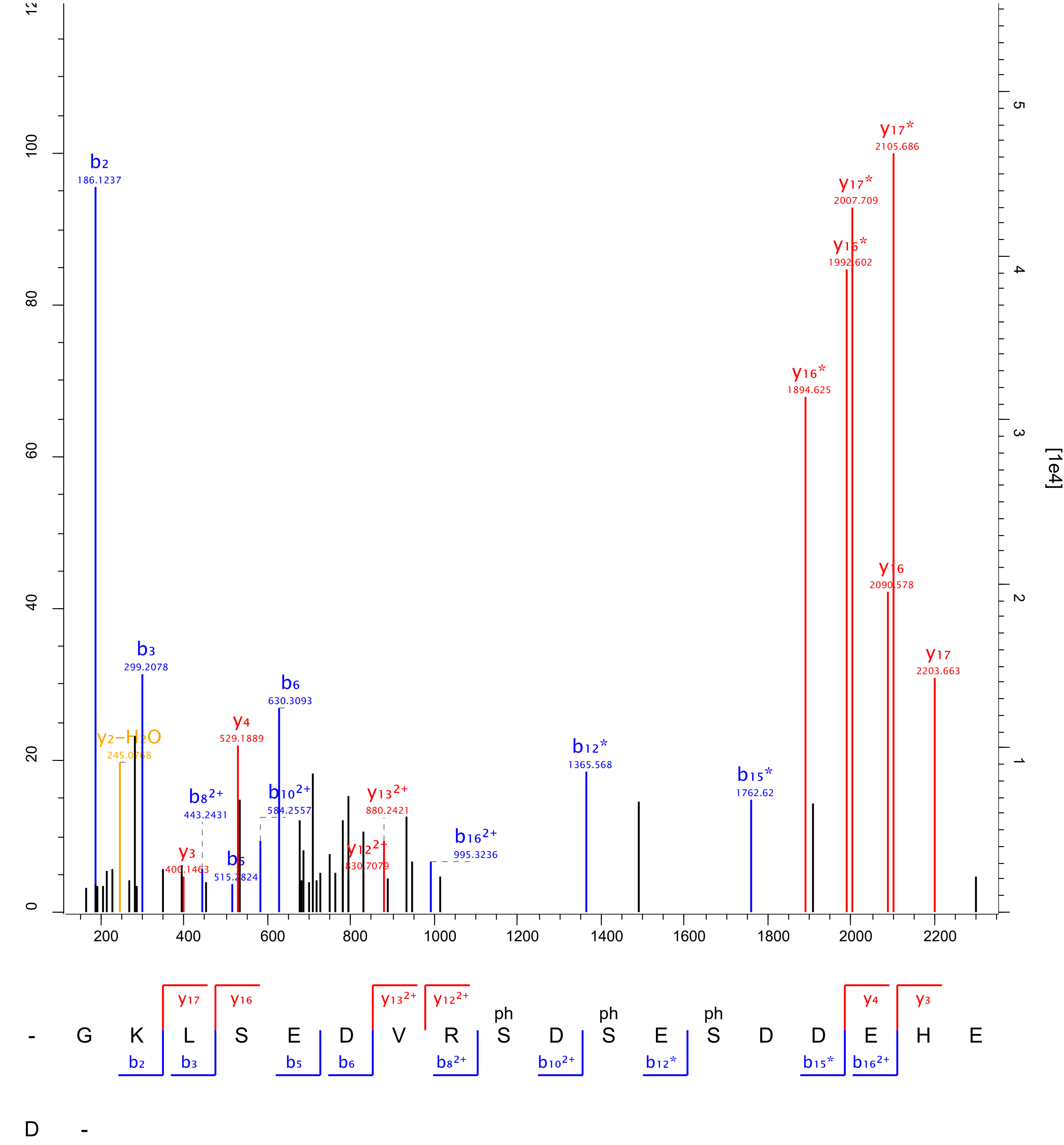

Sample output to test PDF Combine only

Raw file	Scan	Method	Score	m/z	Gene names
0523_4	7952	FTMS; HCD	102.65	689.77	At1g52780

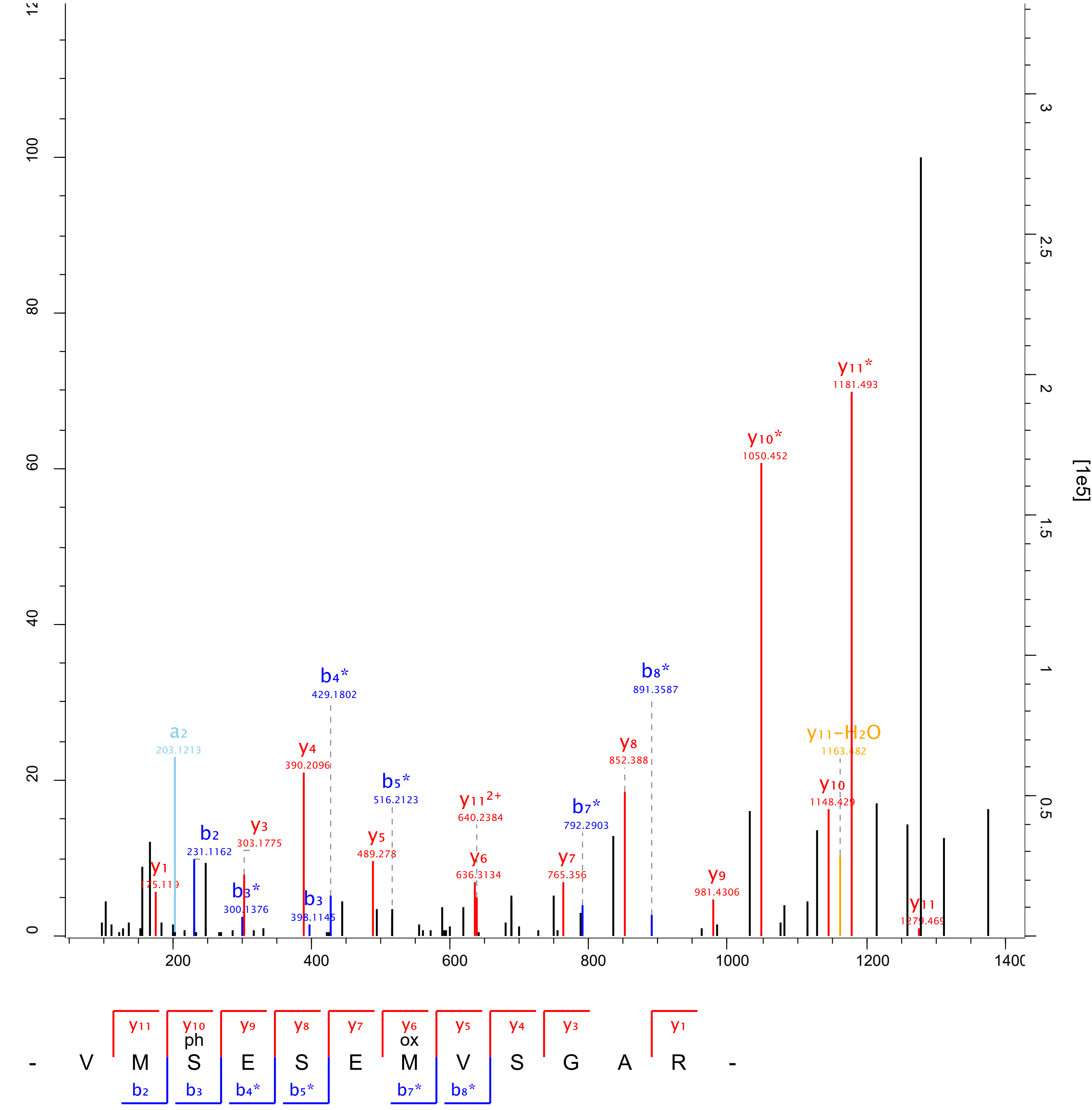

Sample output to test PDF Combine only

Raw file	Scan	Method	Score	m/z	Gene names
0523_4	7970	FTMS; HCD	138.75	834.8	AMT1-2

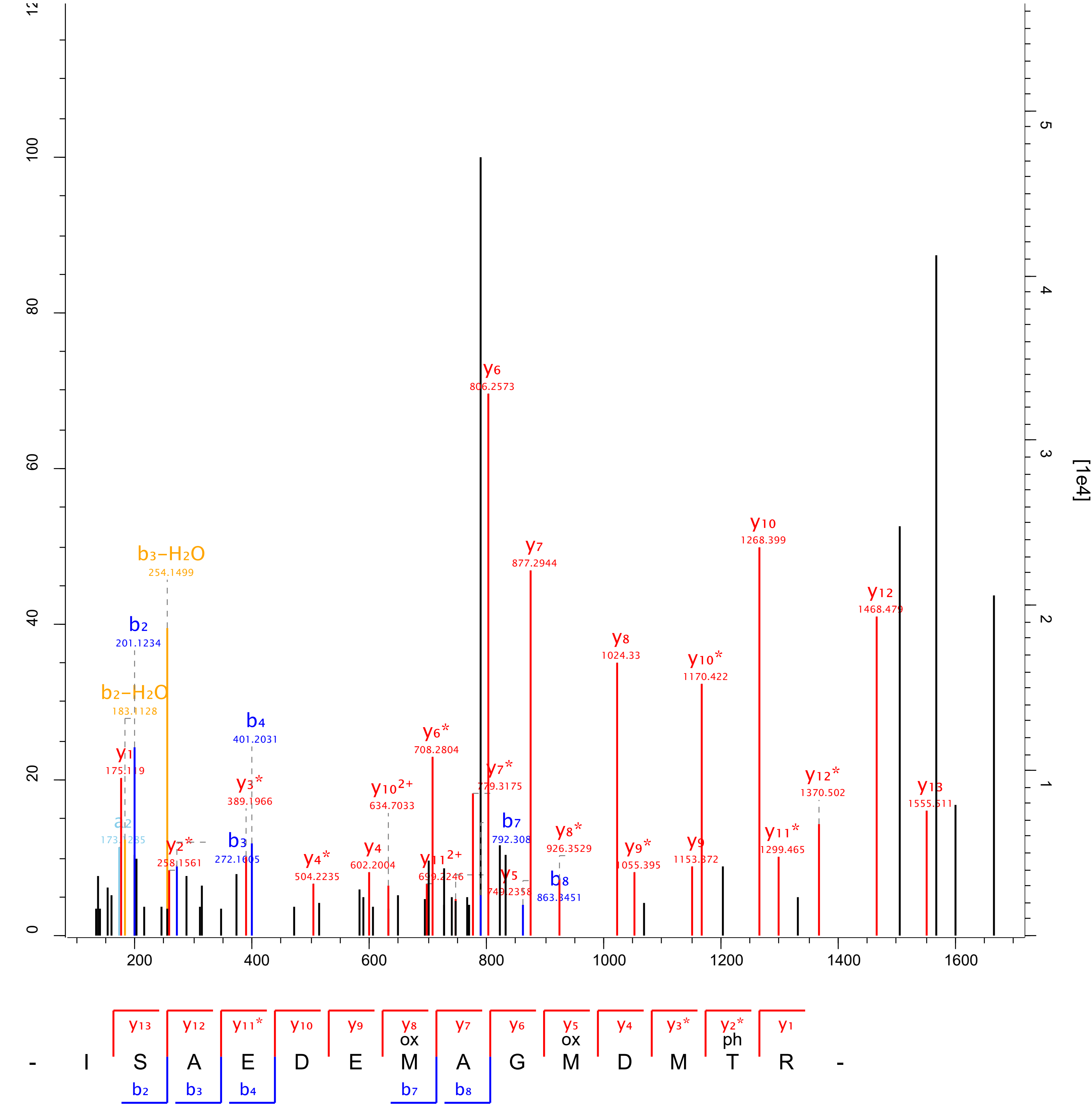

Sample output to test PDF Combine only

Raw file	Scan	Method	Score	m/z	Gene names
0523_4	7990	FTMS; HCD	57.17	572.28	RBOHD

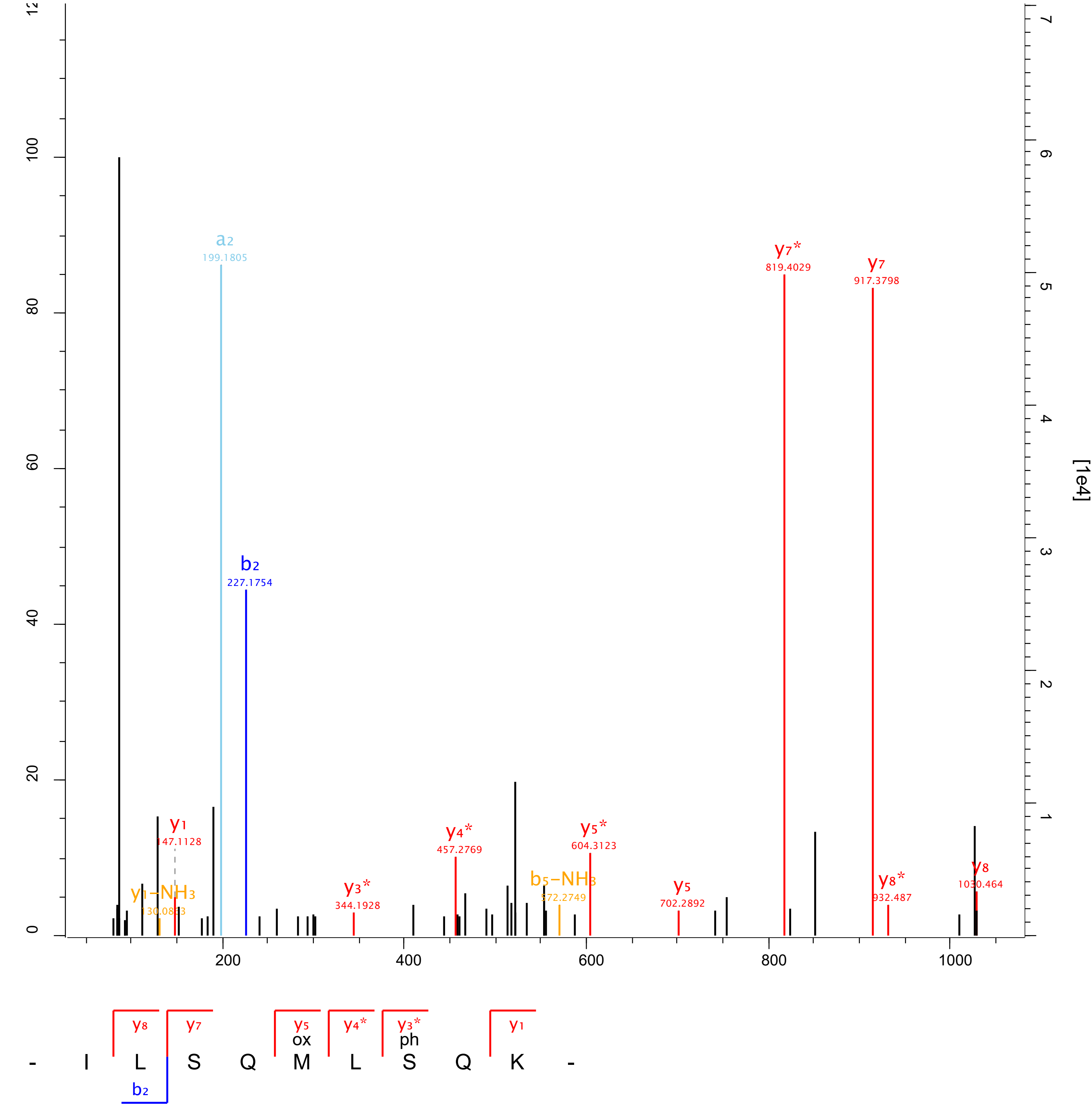

Sample output to test PDF Combine only

Raw file	Scan	Method	Score	m/z	Gene names
0523_4	7995	FTMS; HCD	233.56	756.29	EIN2

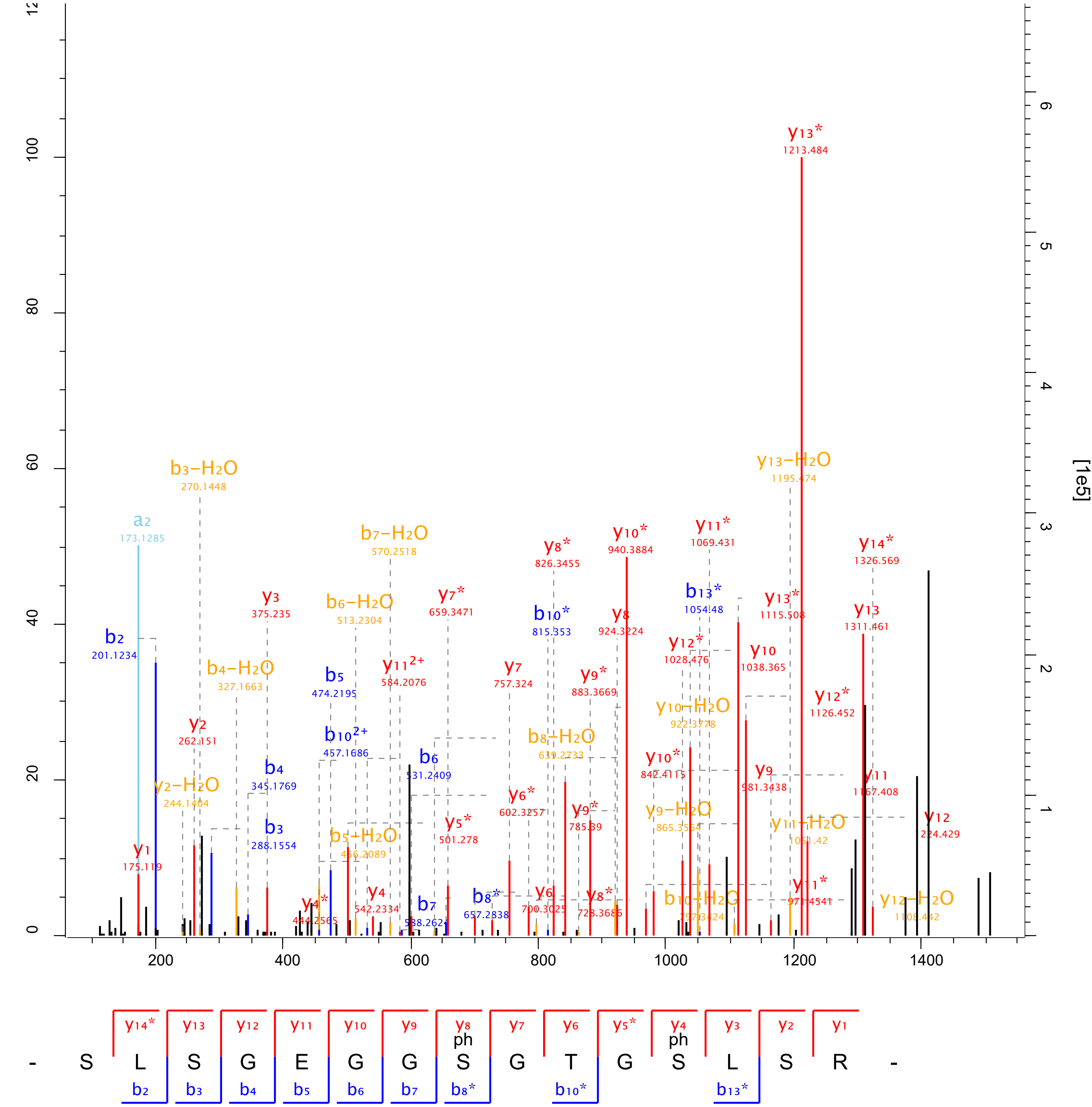

Sample output to test PDF Combine only

Raw file	Scan	Method	Score	m/z	Gene names
0523_4	8025	FTMS; HCD	55.01	662.76	WEB1

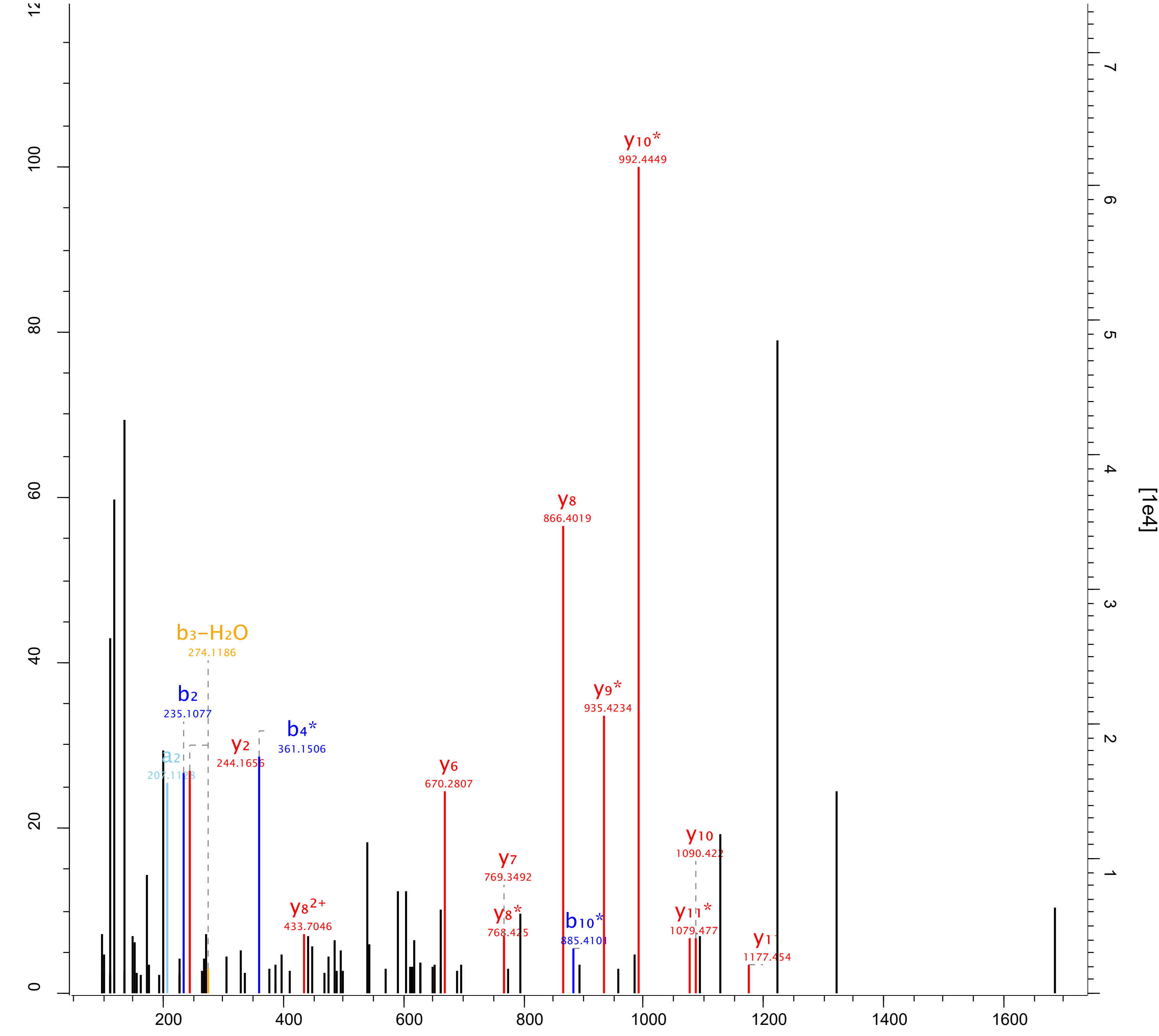

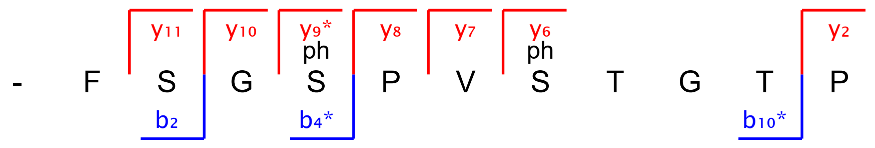

Sample output to test PDF Combine only

Raw file	Scan	Method	Score	m/z
0523_4	8081	FTMS; HCD	126.4	746.32

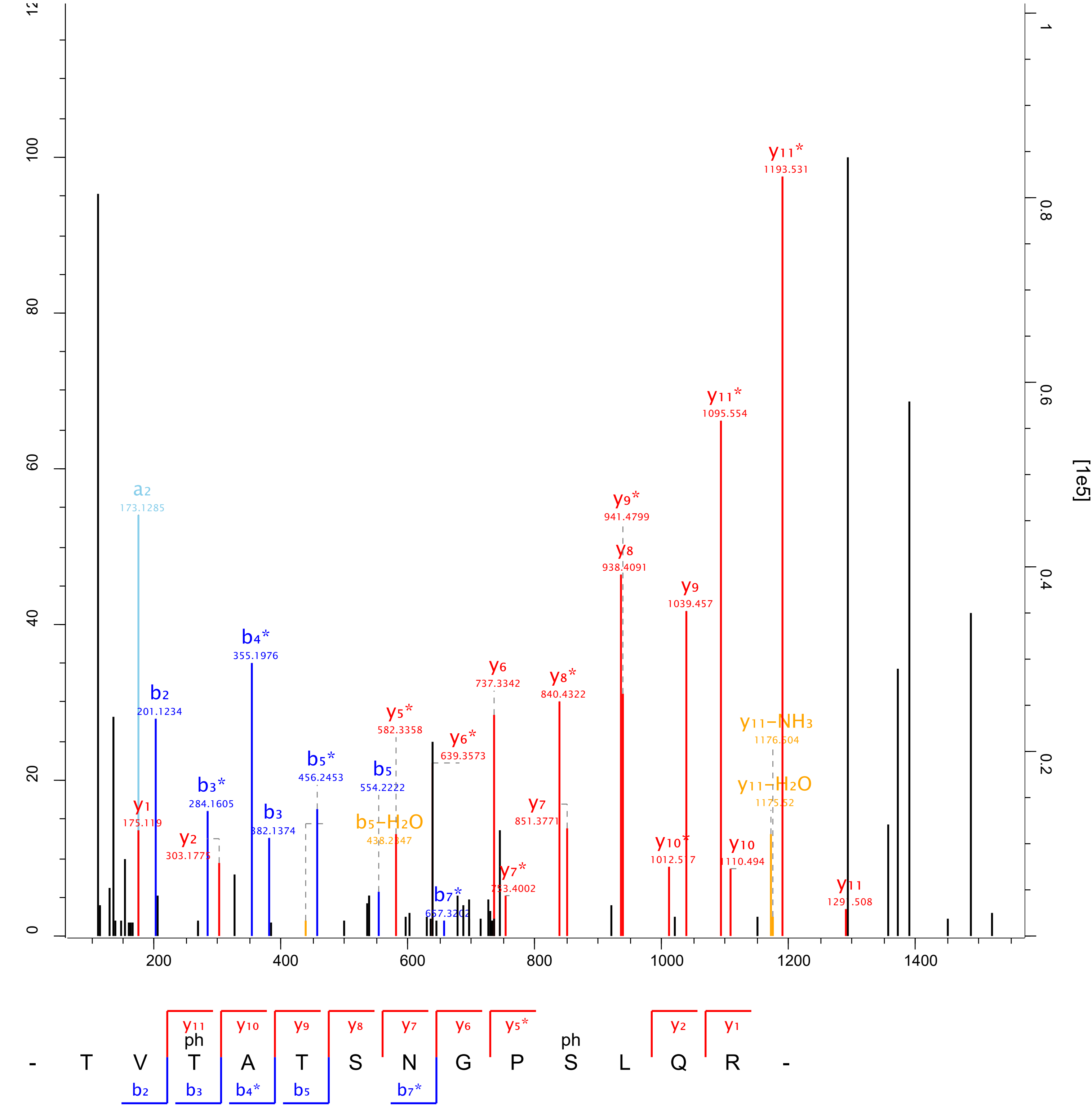

Sample output to test PDF Combine only

Raw file	Scan	Method	Score	m/z	Gene names
0523_4	8111	FTMS; HCD	183.37	506.25	At2g40270;At5g45840;MRH1

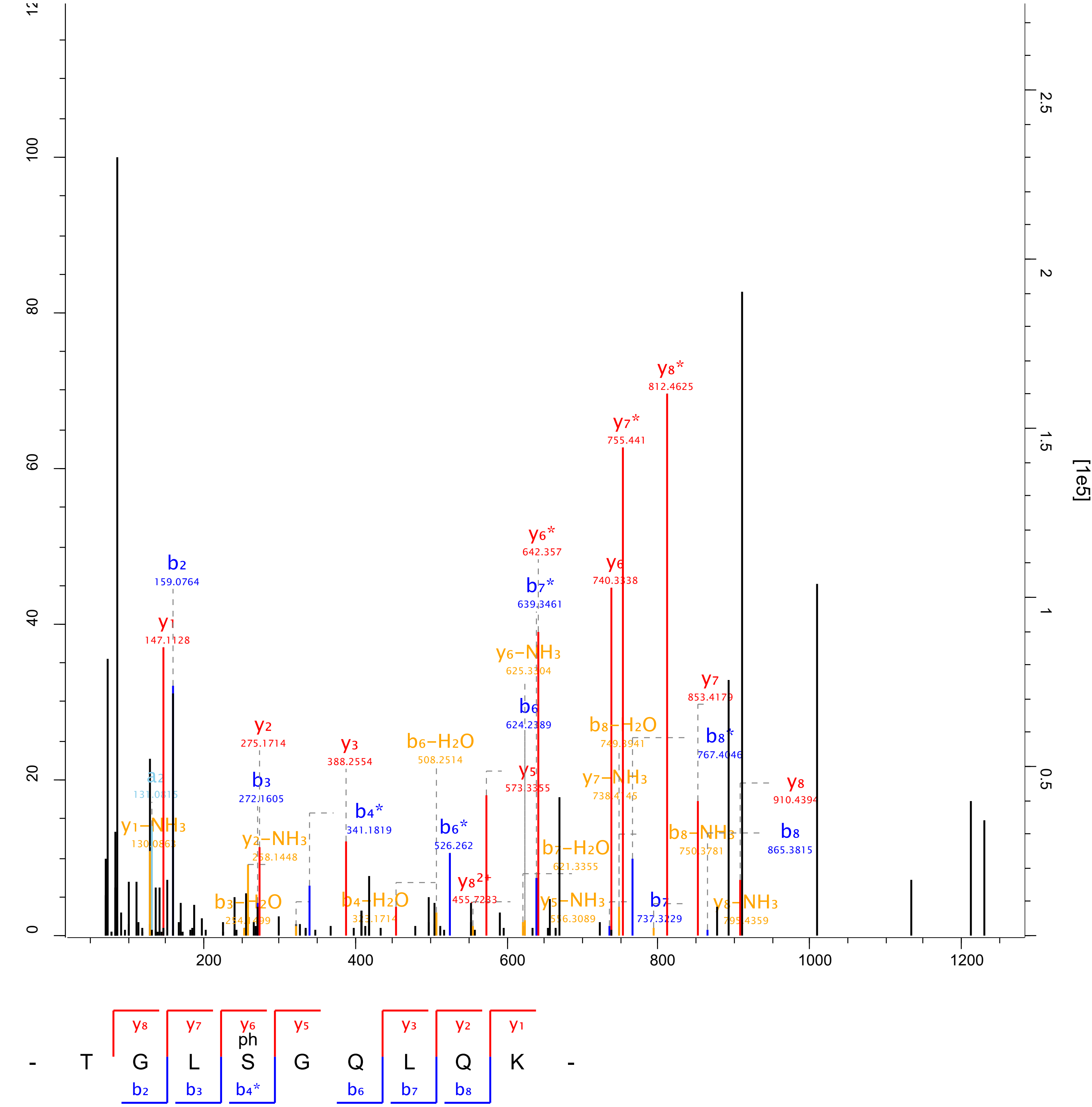

Sample output to test PDF Combine only

Raw file	Scan	Method	Score	m/z	Gene names
0523_4	8258	FTMS; HCD	50.18	562.27	At1g67590;F12B7.14

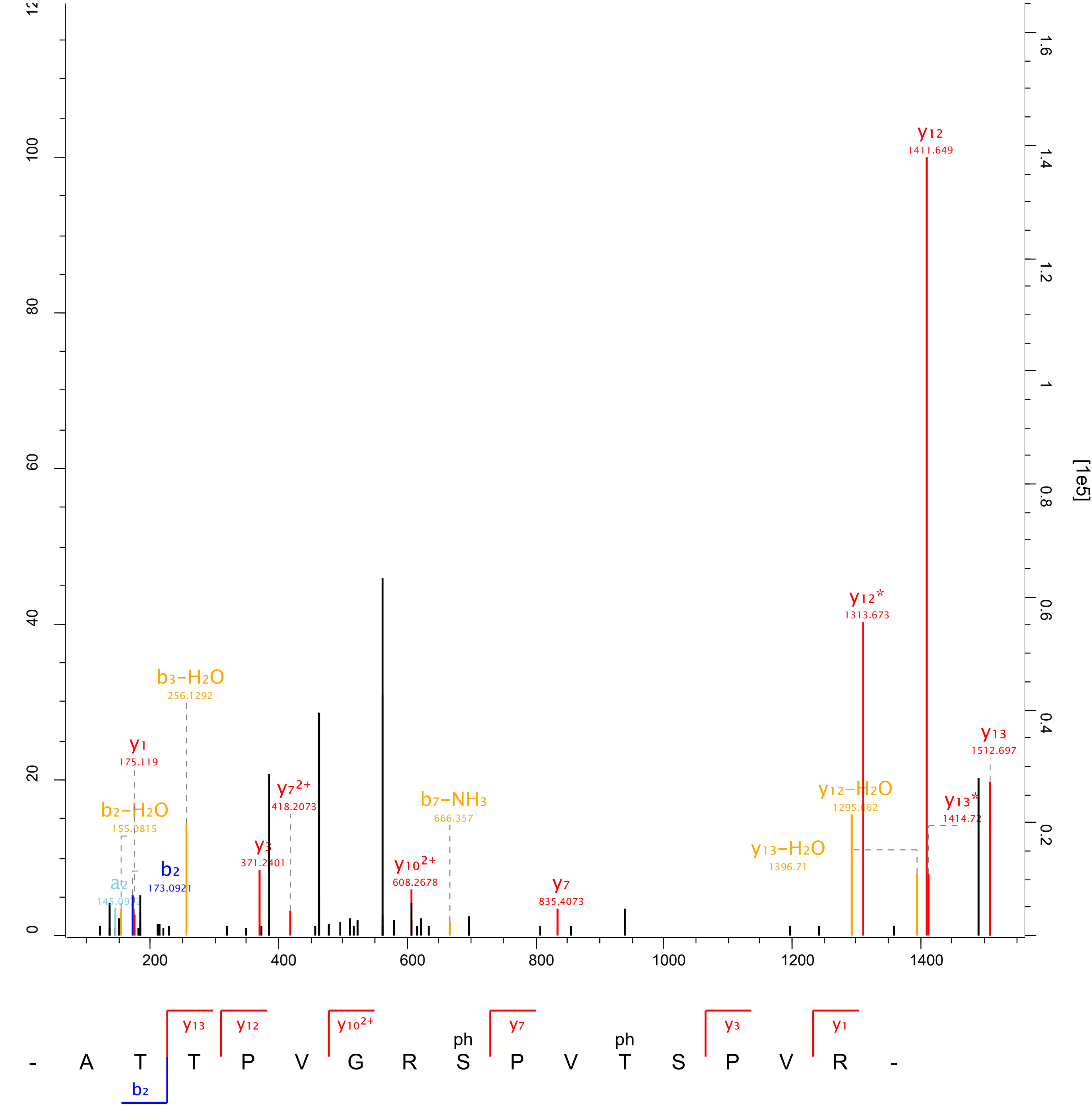

Sample output to test PDF Combine only

Raw file	Scan	Method	Score	m/z	Gene names
0523_4	8315	FTMS; HCD	110.35	781.32	At5g12420

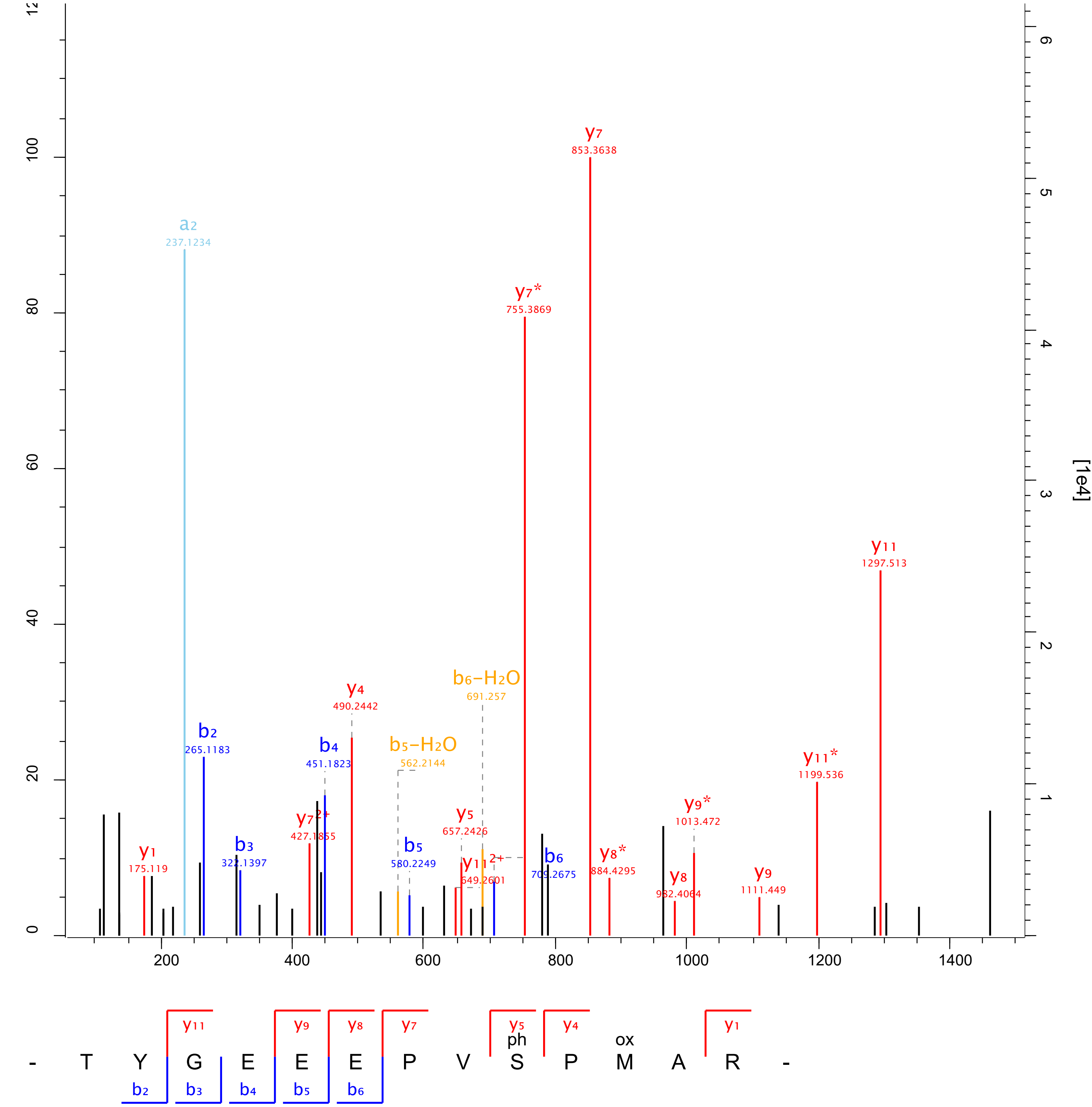

Sample output to test PDF Combine only

Raw file	Scan	Method	Score	m/z	Gene names
0523_4	8319	FTMS; HCD	45.35	748.3	ABCB15

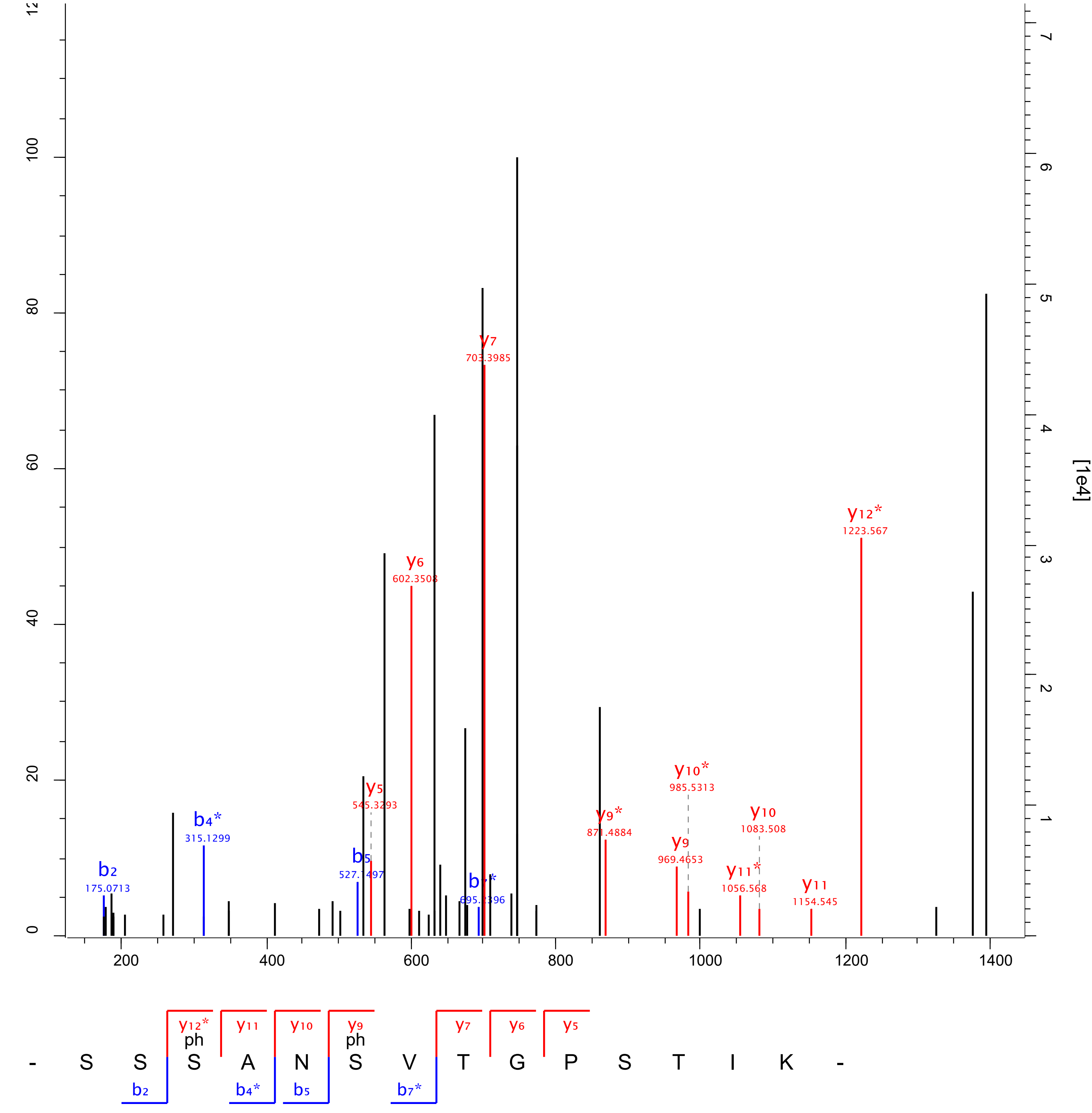

Sample output to test PDF Combine only

Raw file	Scan	Method	Score	m/z	Gene names
0523_4	8439	FTMS; HCD	151.11	687.82	iqd2

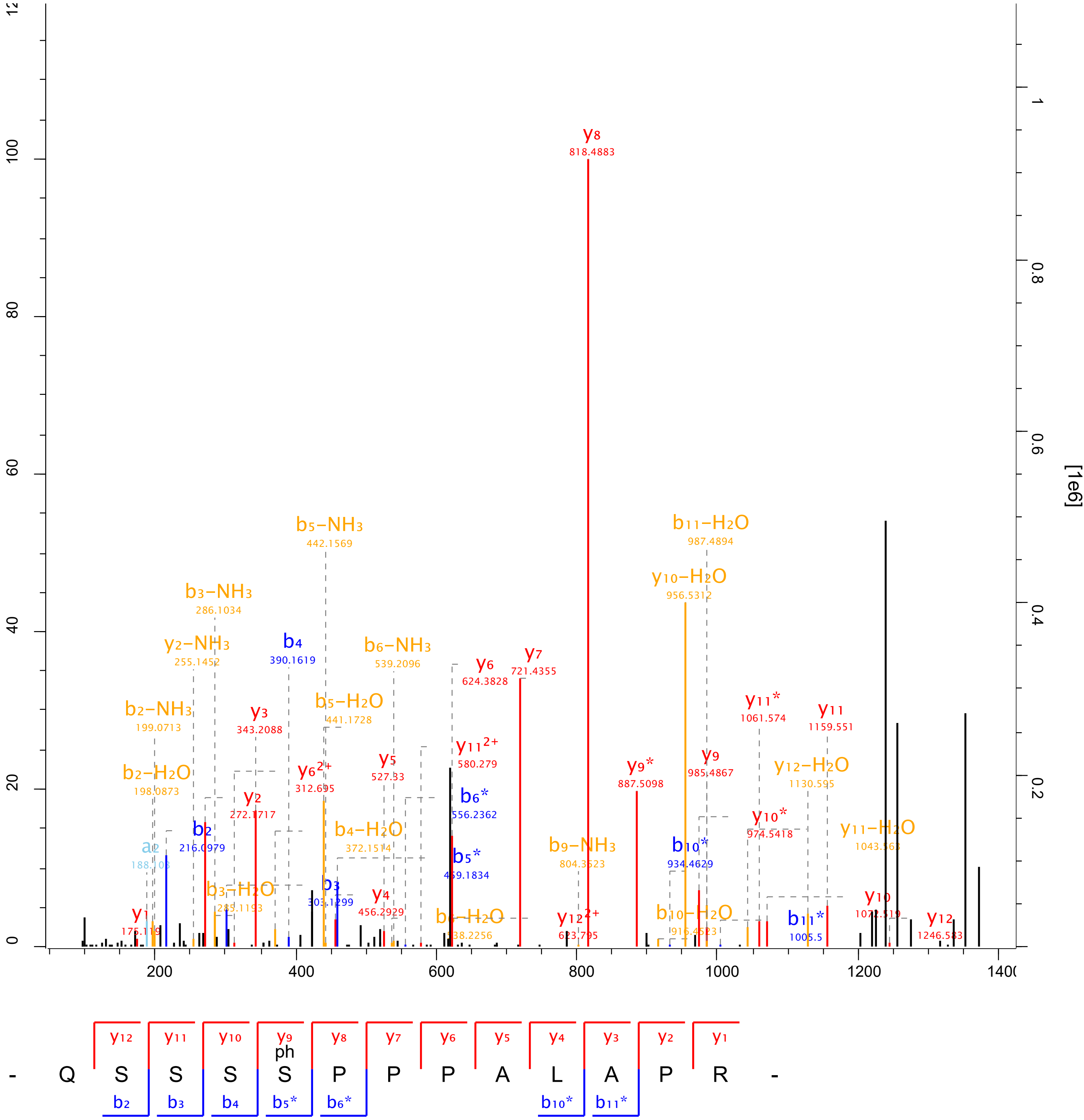

Sample output to test PDF Combine only

Raw file	Scan	Method	Score	m/z	Gene names
0523_4	8448	FTMS; HCD	70.44	621.76	IP5PI;IP5P1

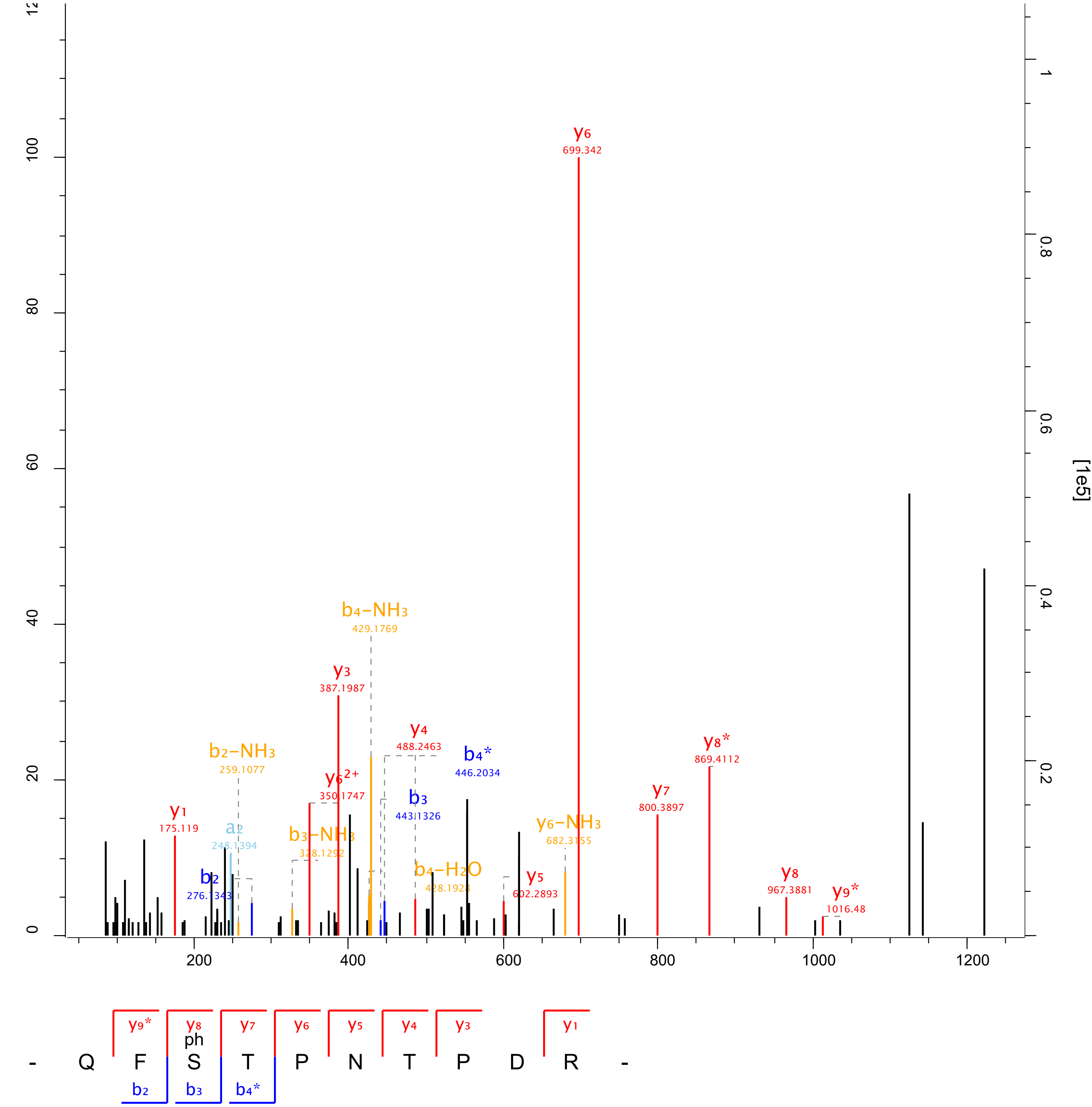

Sample output to test PDF Combine only

Raw file	Scan	Method	Score	m/z	Gene names
0523_4	8464	FTMS; HCD	141.7	855.37	F18B13.26

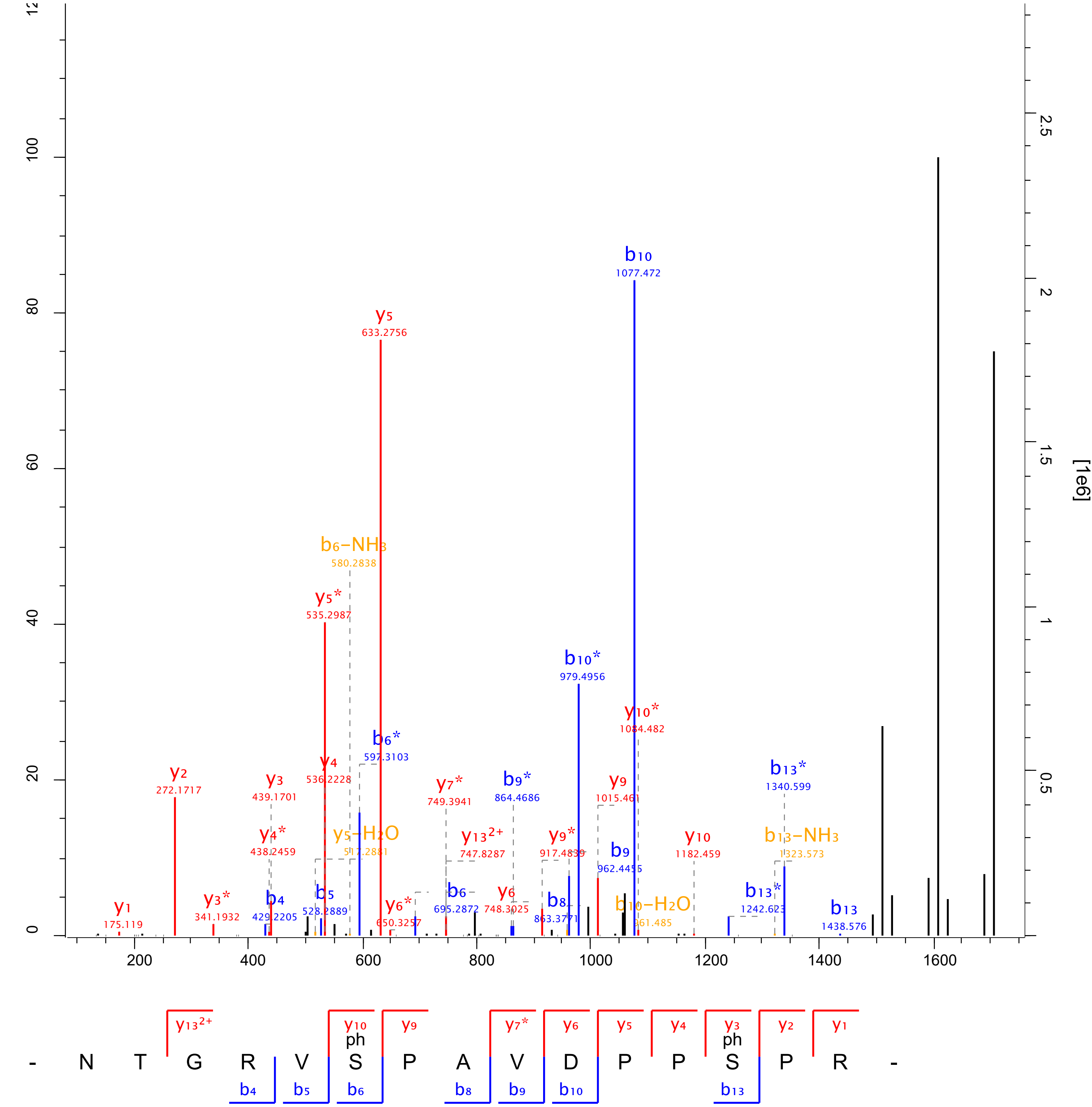

Raw file	Scan	Method	Score	m/z
0523_4	8474	FTMS; HCD	53.45	642.3

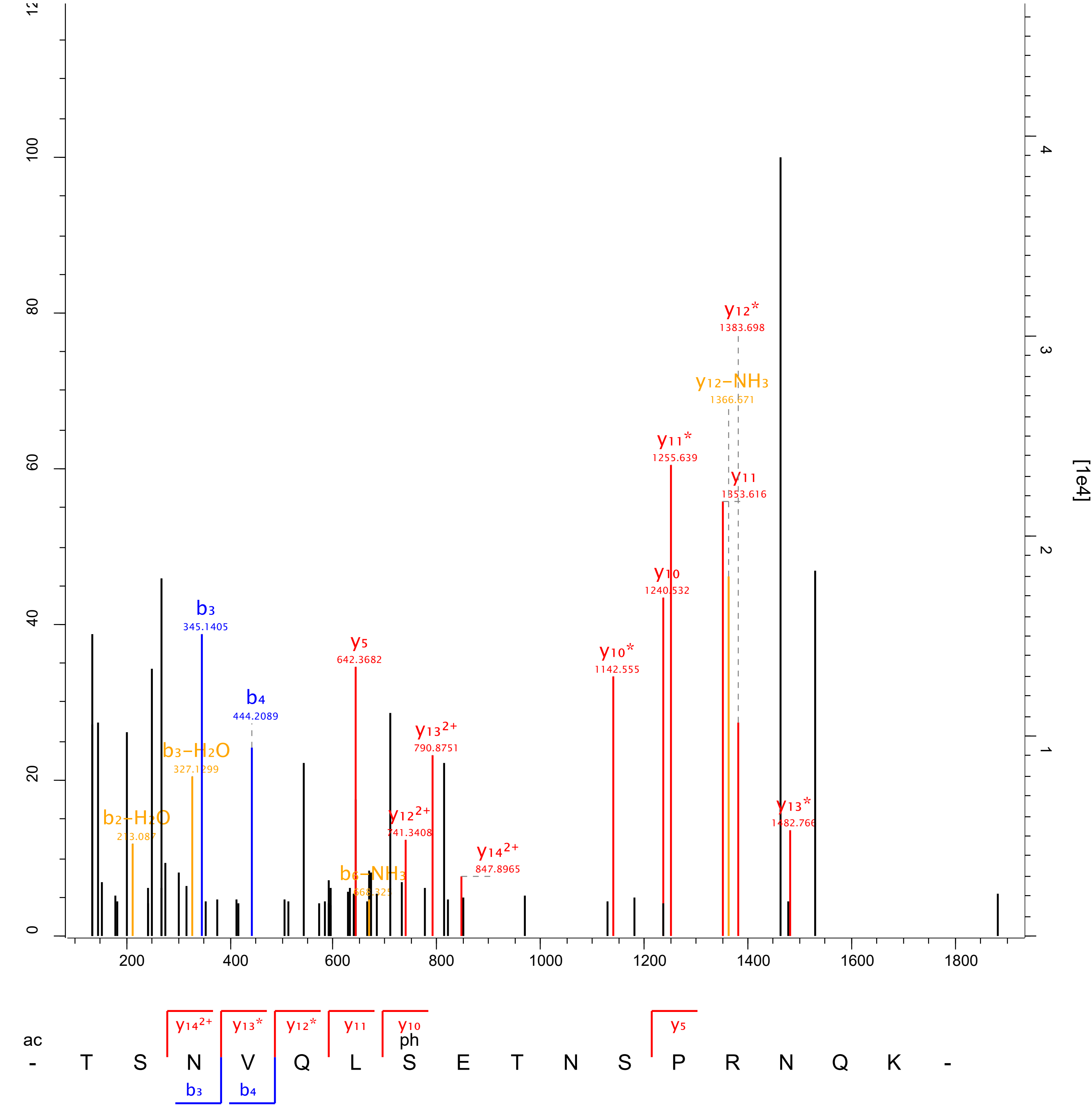

Sample output to test PDF Combine only

Raw file	Scan	Method	Score	m/z	Gene names
0523_4	8508	FTMS; HCD	124.29	786.35	MEBL

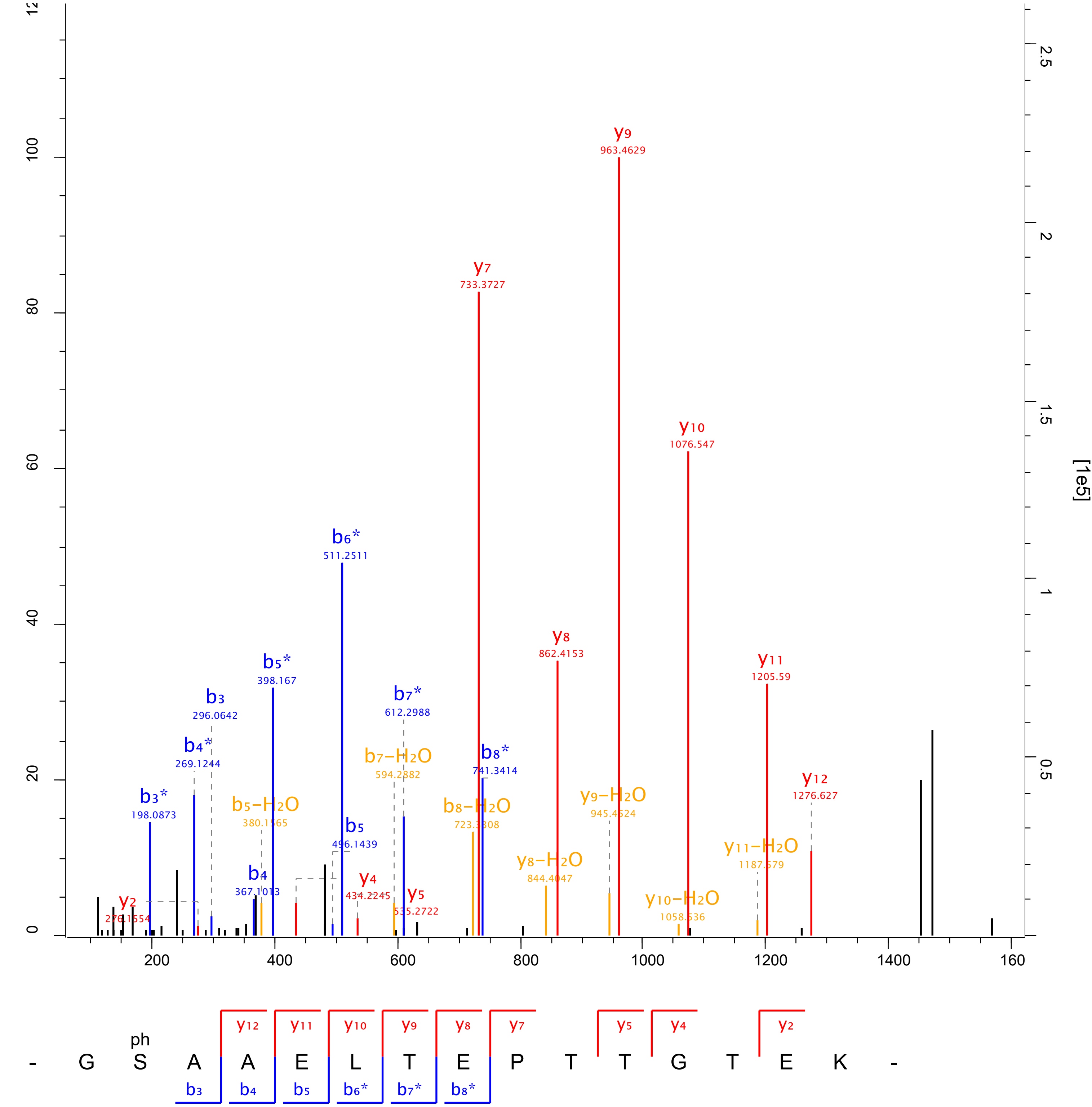

Sample output to test PDF Combine only

Raw file	Scan	Method	Score	m/z
0523_4	8550	FTMS; HCD	66.02	980.47

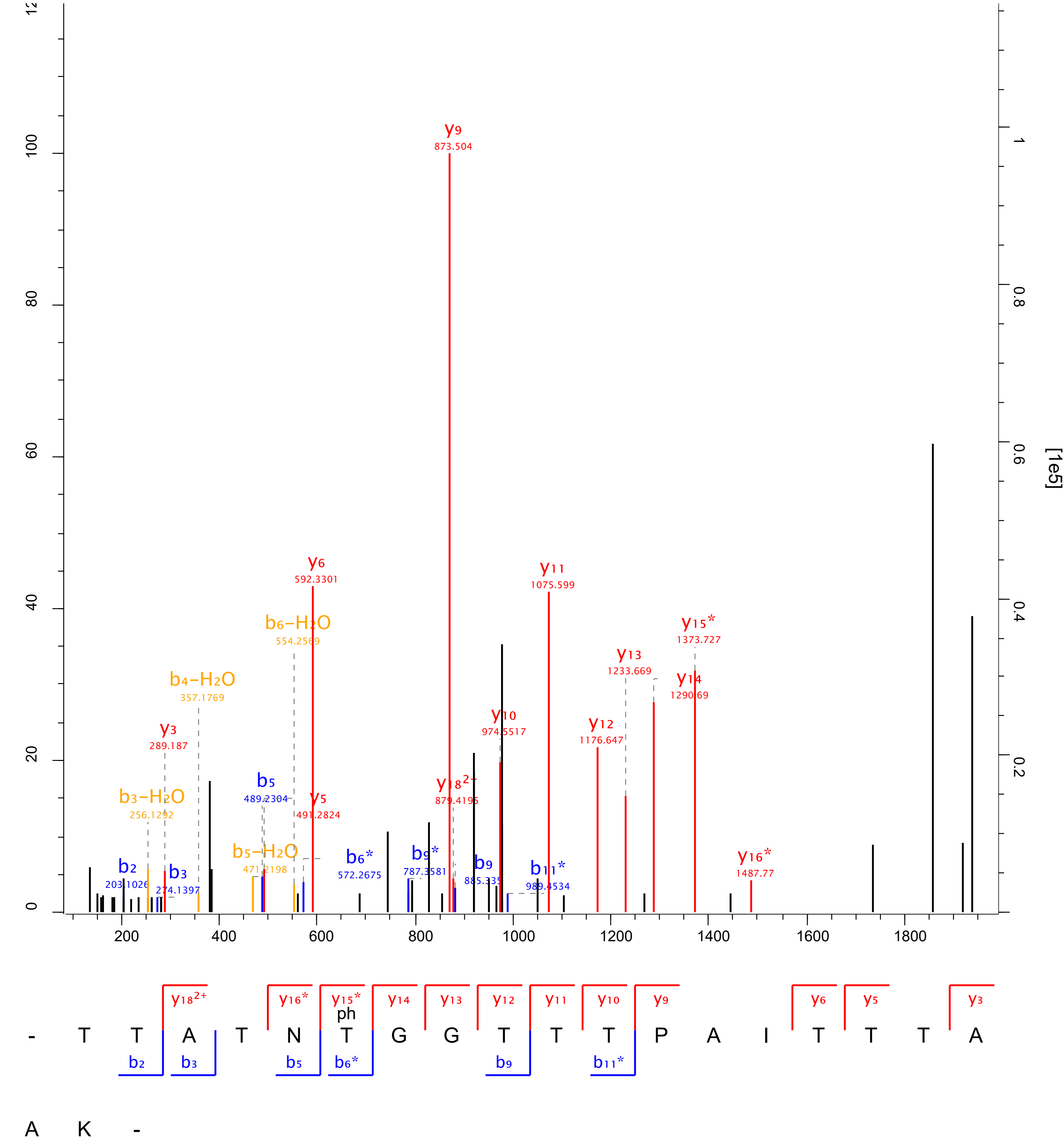

Sample output to test PDF Combine only

Raw file	Scan	Method	Score	m/z	Gene names
0523_4	8577	FTMS; HCD	93.88	828.99	STL2P

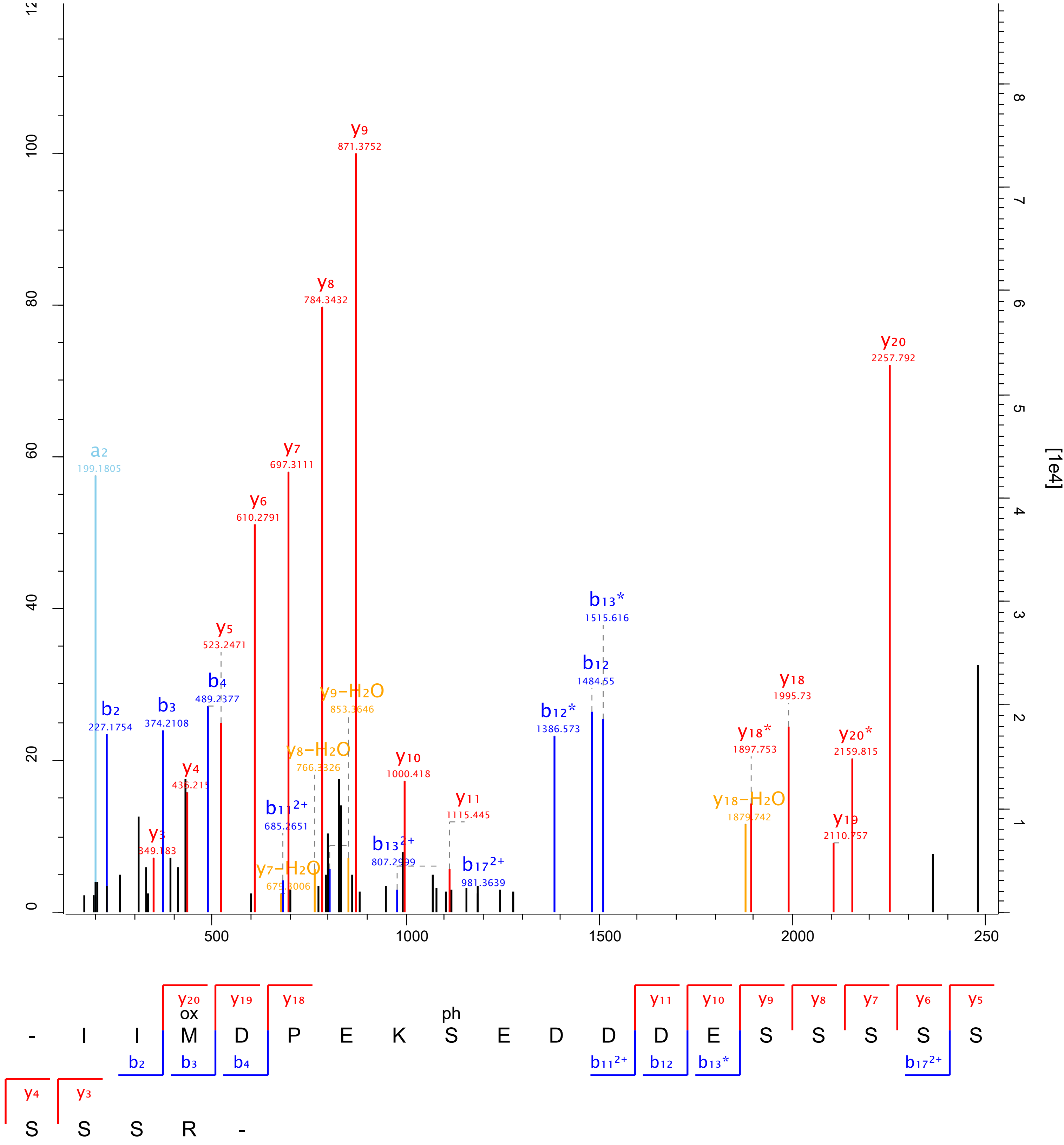

Raw file	Scan	Method	Score	m/z
0523_4	8604	FTMS; HCD	124.21	566.25

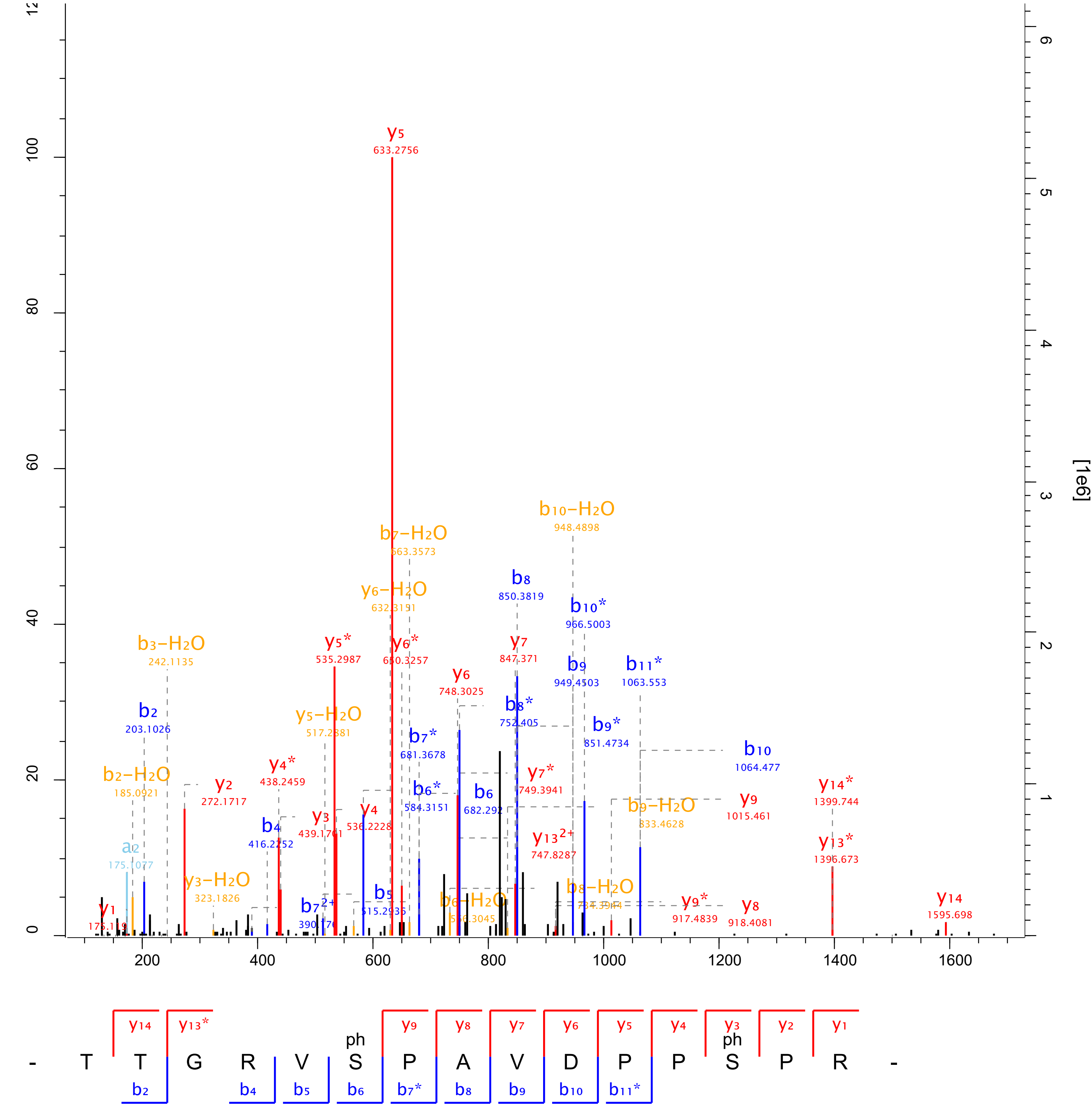

Sample output to test PDF Combine only

Raw file	Scan	Method	Score	m/z	Gene names
0523_4	8616	FTMS; HCD	53.77	522.56	At4g06534

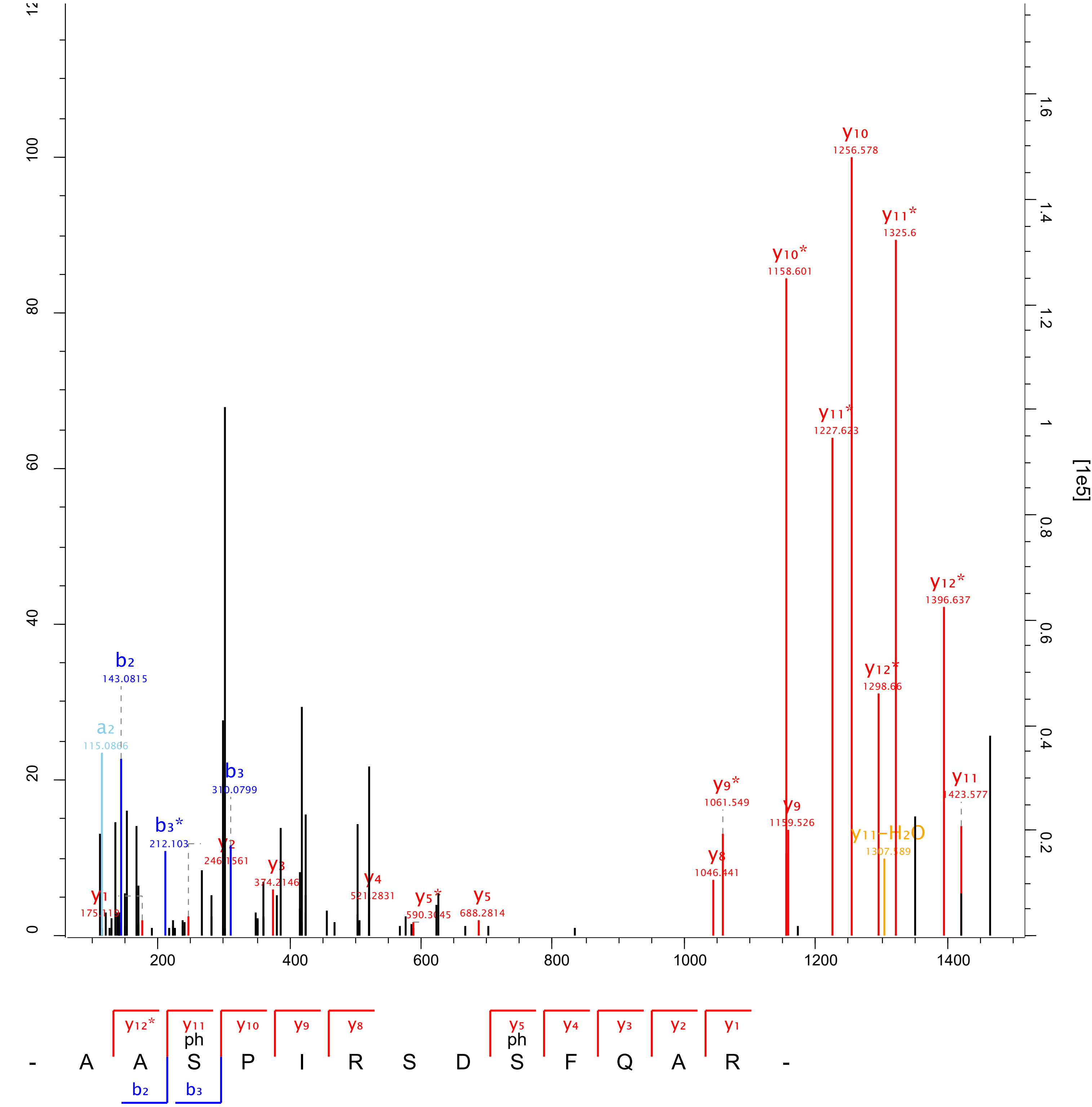

Sample output to test PDF Combine only

Raw file	Scan	Method	Score	m/z	Gene names
0523_4	8846	FTMS; HCD	289.4	710.83	At5g63270

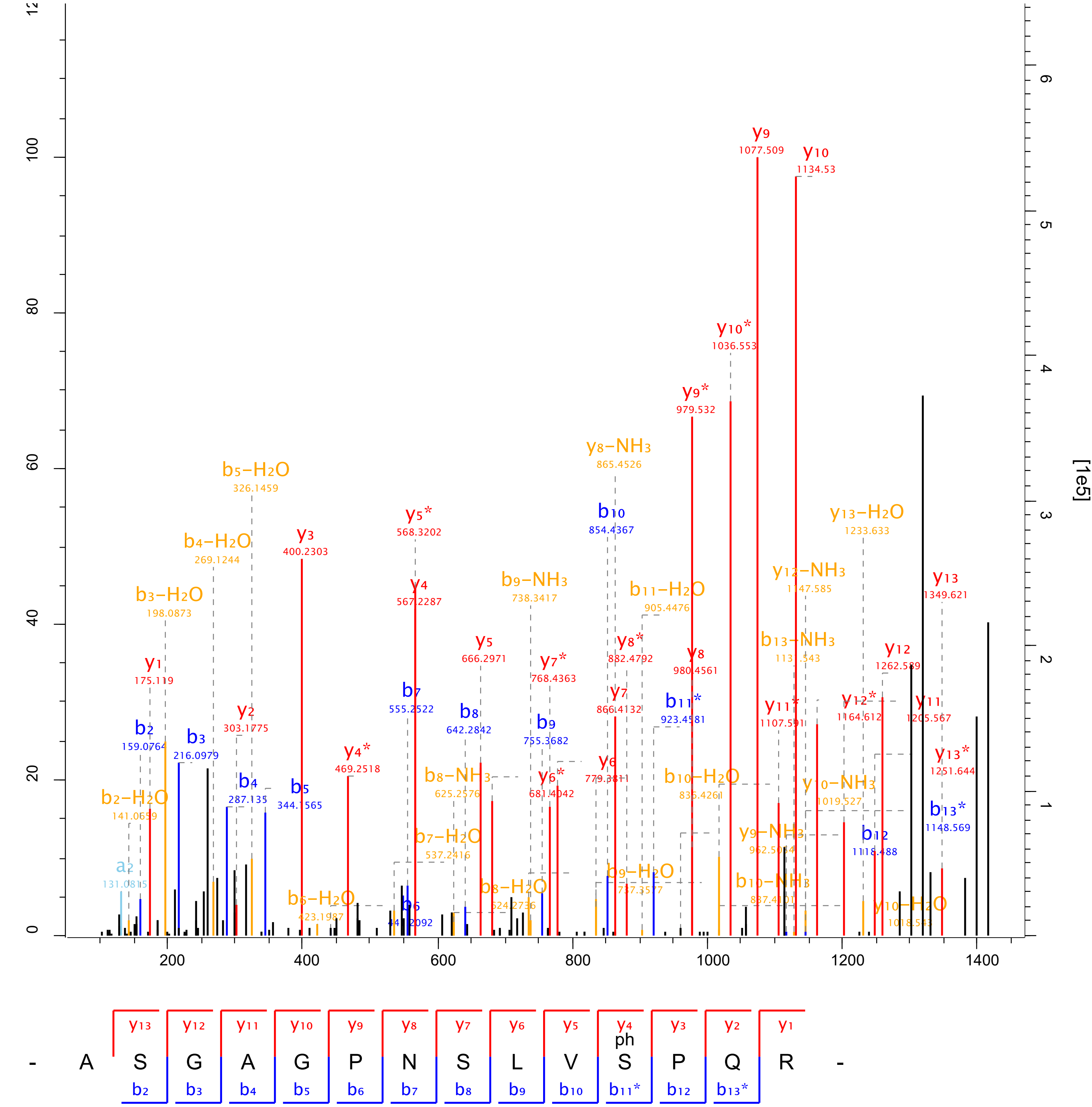

Sample output to test PDF Combine only

Raw file	Scan	Method	Score	m/z	Gene names
0523_4	8864	FTMS; HCD	116.63	608.76	CPK5

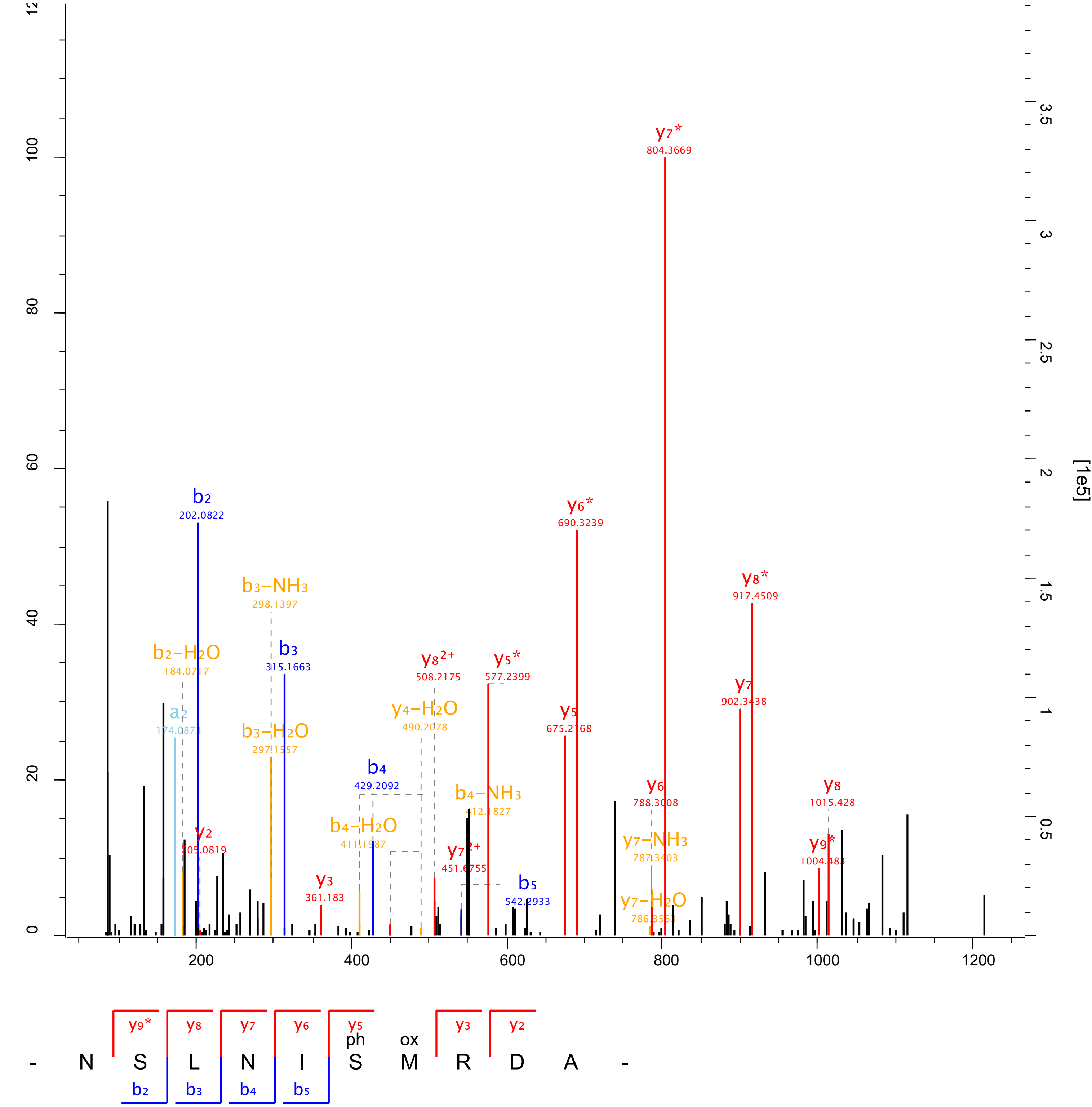

Sample output to test PDF Combine only

Raw file	Scan	Method	Score	m/z	Gene names
0523_4	8879	FTMS; HCD	59.29	650.79	BON2

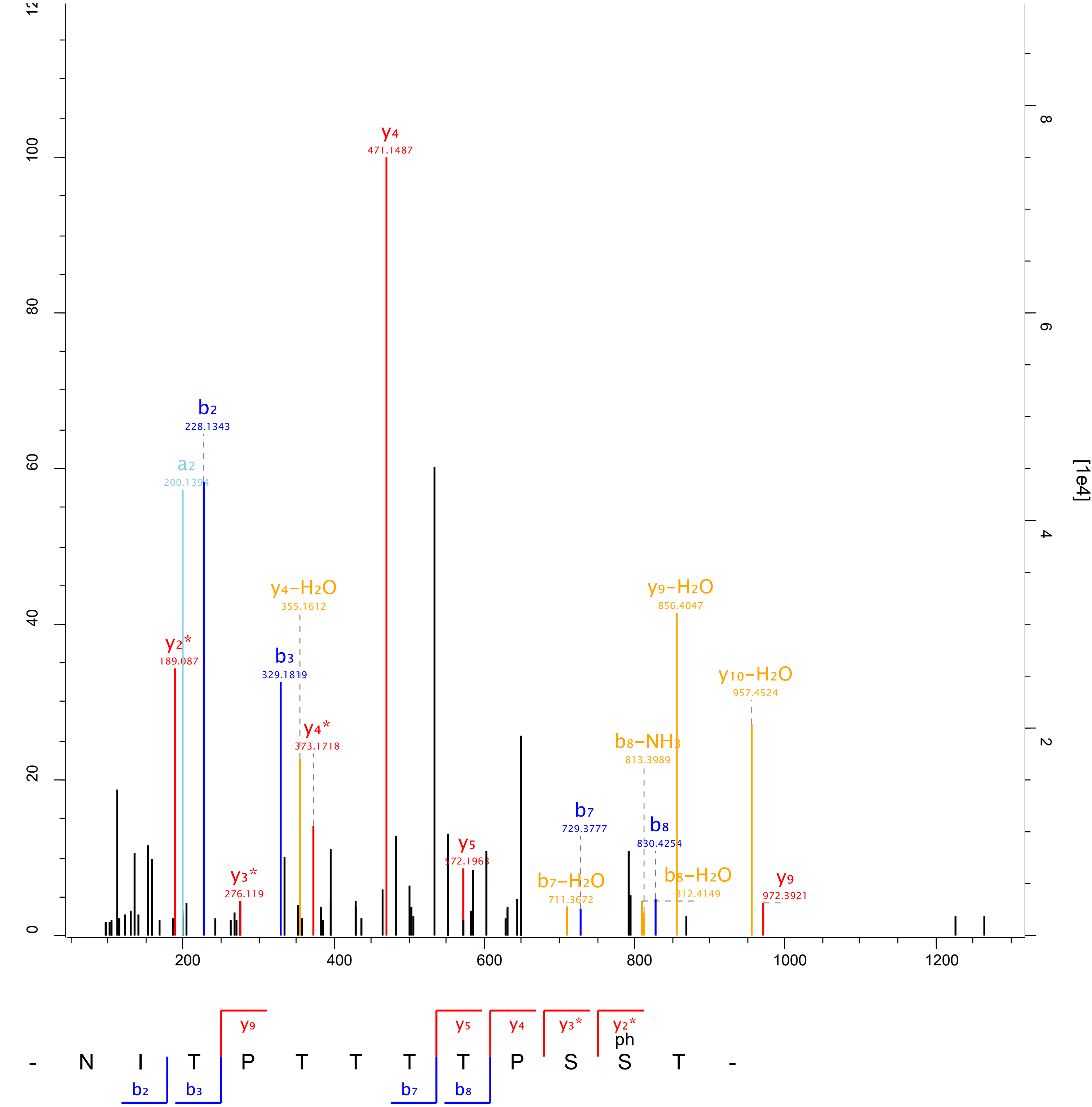

Sample output to test PDF Combine only

Raw file	Scan	Method	Score	m/z	Gene names
0523_4	8880	FTMS; HCD	119.65	891.06	MSI4

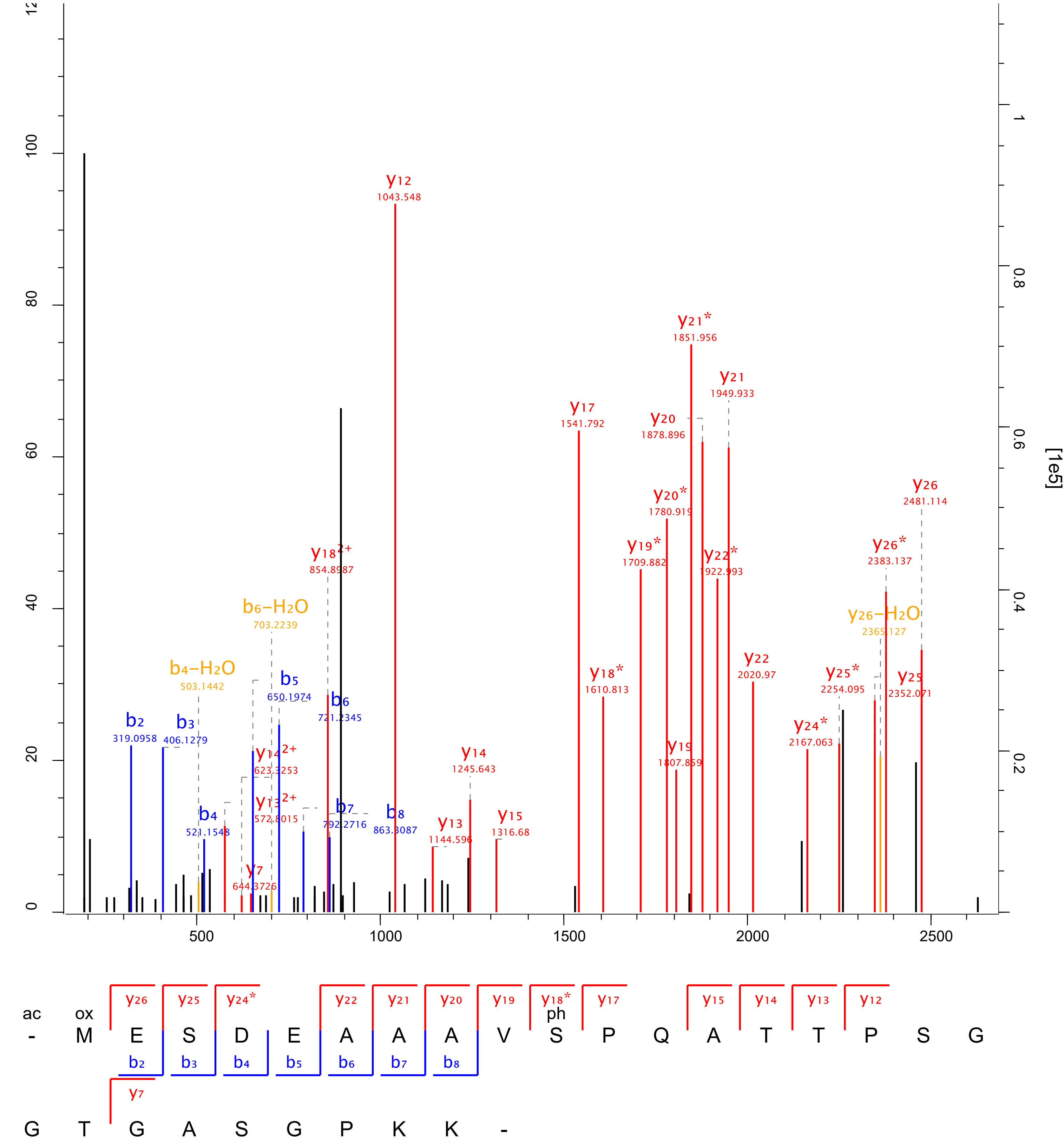

Sample output to test PDF Combine only

Raw file	Scan	Method	Score	m/z	Gene names
0523_4	8926	FTMS; HCD	81.14	589.27	PEX14

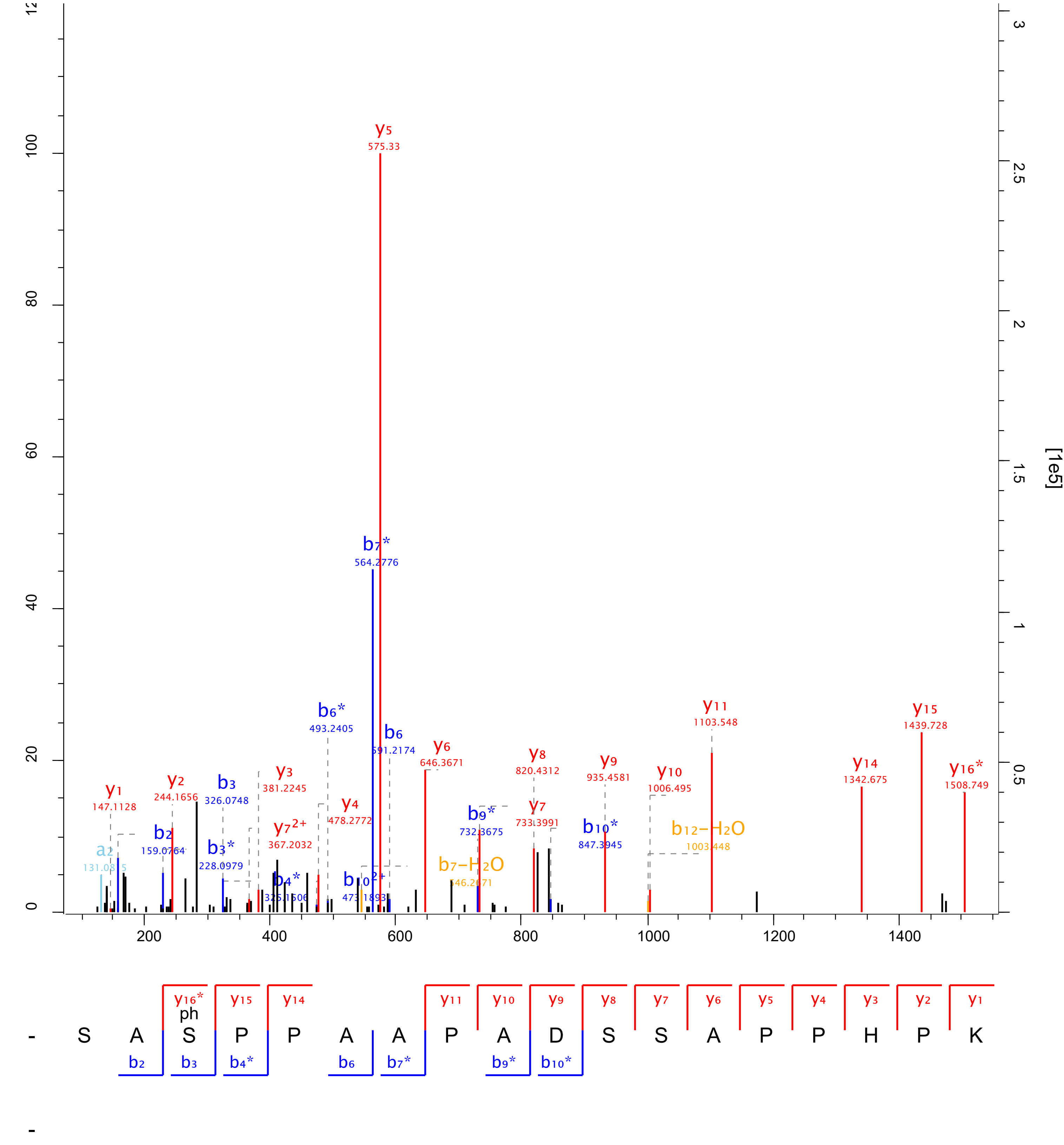

Sample output to test PDF Combine only

Raw file	Scan	Method	Score	m/z	Gene names
0523_4	8935	FTMS; HCD	57.29	672.85	SR45

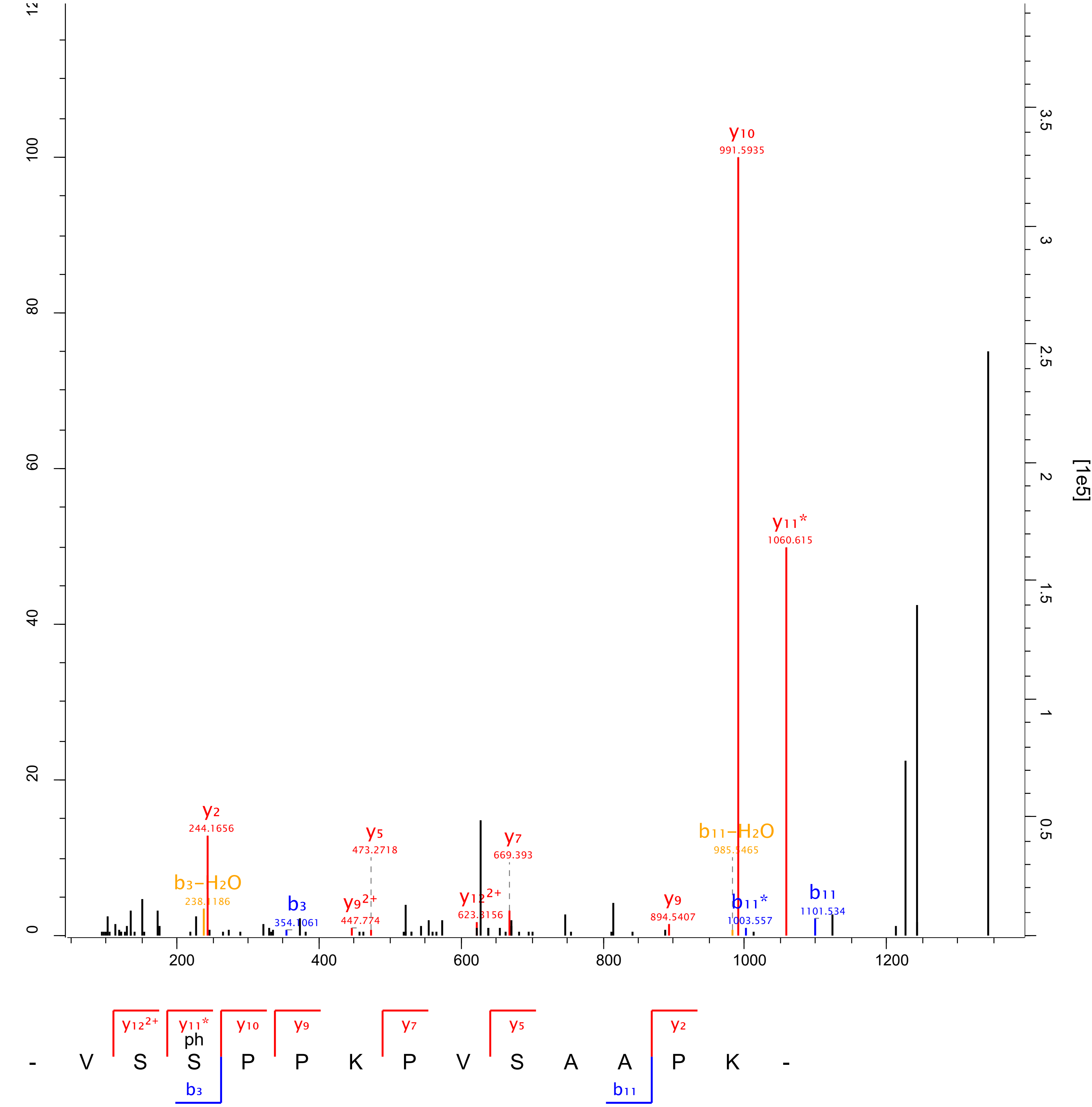

Sample output to test PDF Combine only

Raw file	Scan	Method	Score	m/z	Gene names
0523_4	8966	FTMS; HCD	86.5	701.31	ABCC14

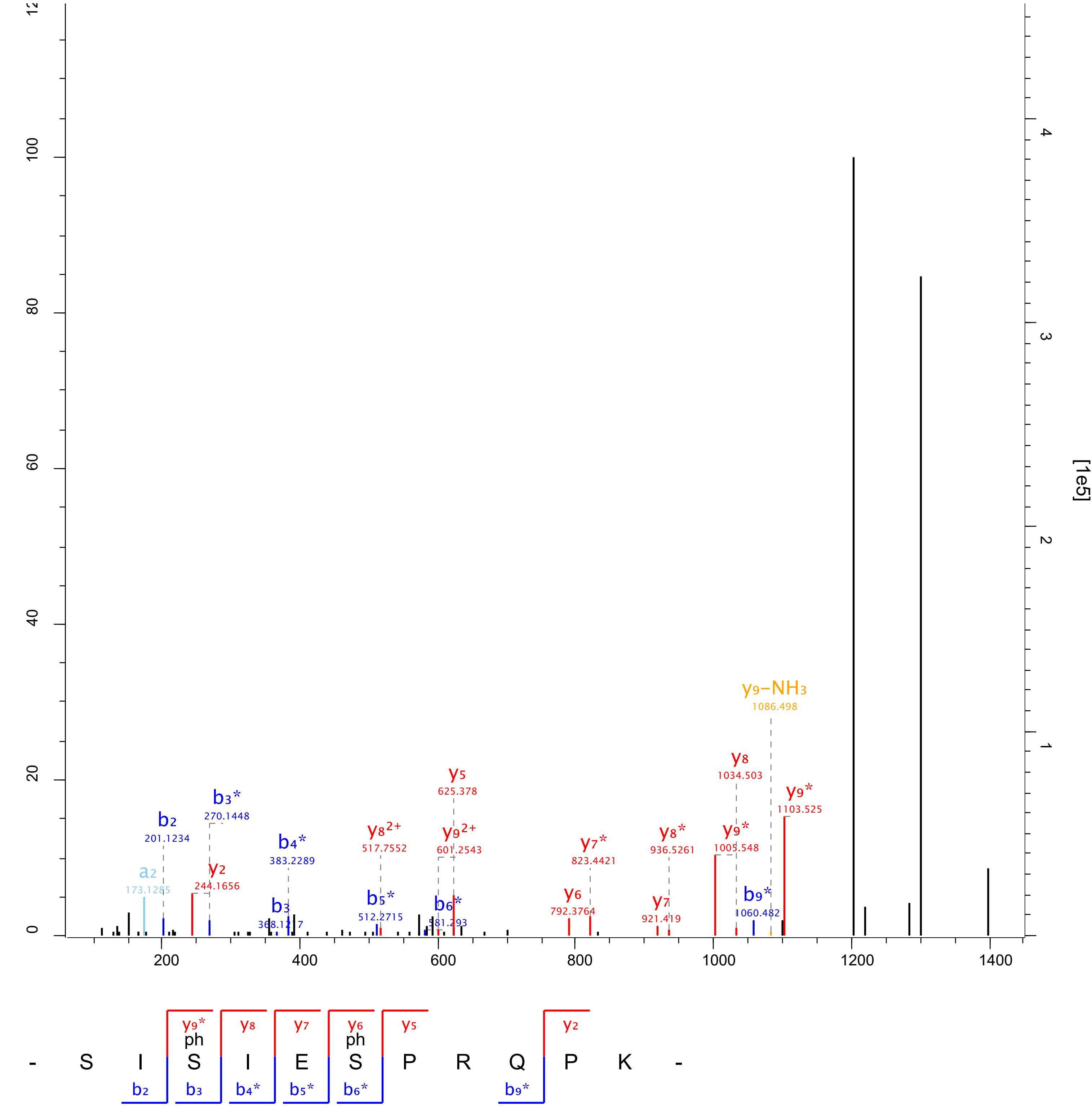

Sample output to test PDF Combine only

Raw file	Scan	Method	Score	m/z
0523_4	9012	FTMS; HCD	54.77	743.78

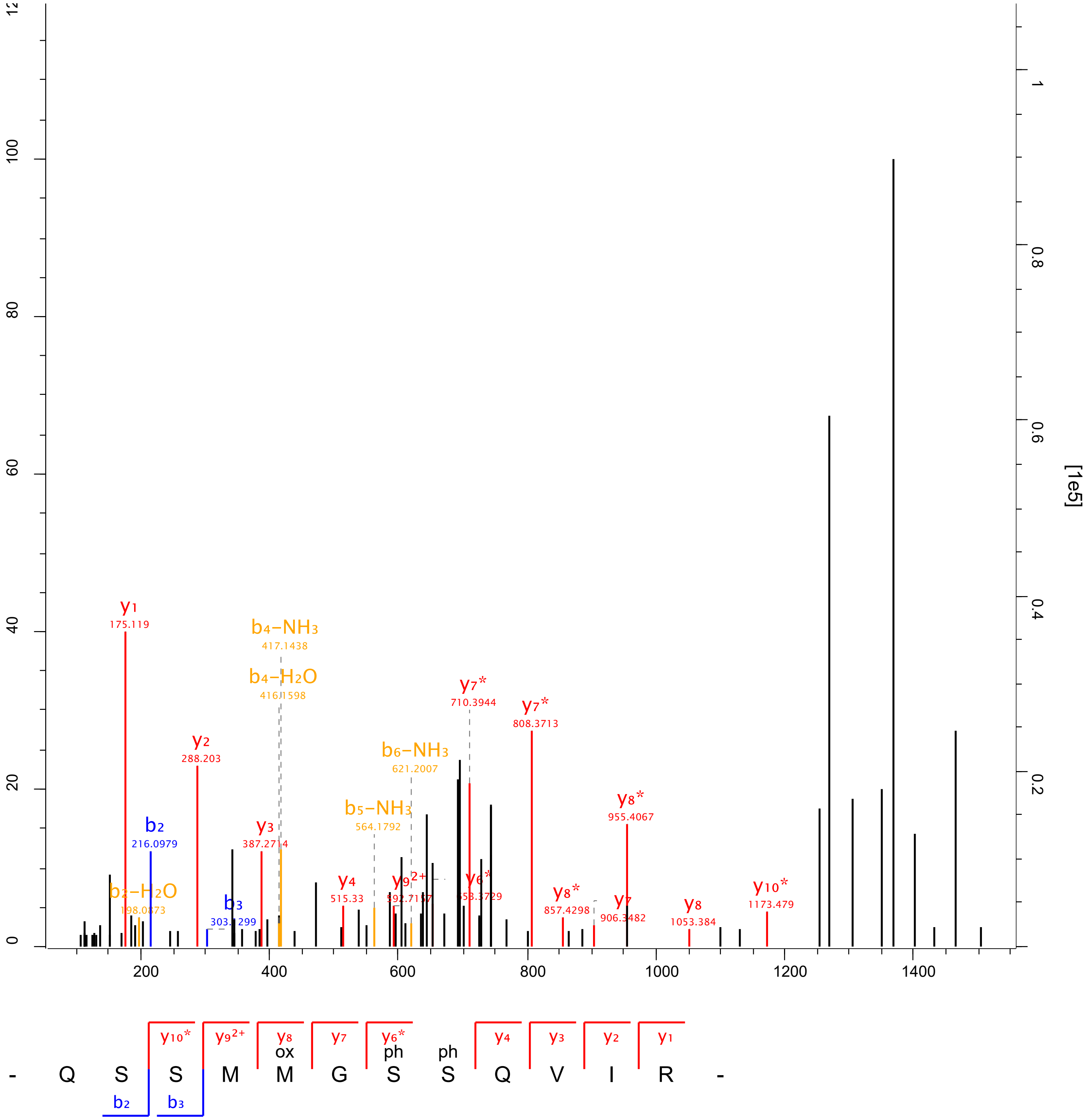

Sample output to test PDF Combine only
