# Supplementary material for: Sucrose-induced Receptor Kinase 1 is Modulated by an Interacting Kinase with Short Extracellular Domain
Source: Mol Cell Proteomics. 2019 May 30;18(8):1556–71. doi: 10.1074/mcp.RA119.001336 (PMC6683012; doi:10.1074/mcp.RA119.001336)
Supplement: Supplementary Figure S6c [file 143141_1_supp_311930_ps52kx.pdf]

Figure S6:

Spectra of all identified phosphopeptides.

Raw file	Scan	Method	Score	m/z	Gene names
0523_4	9136	FTMS; HCD	82.03	587.93	TOM20-4

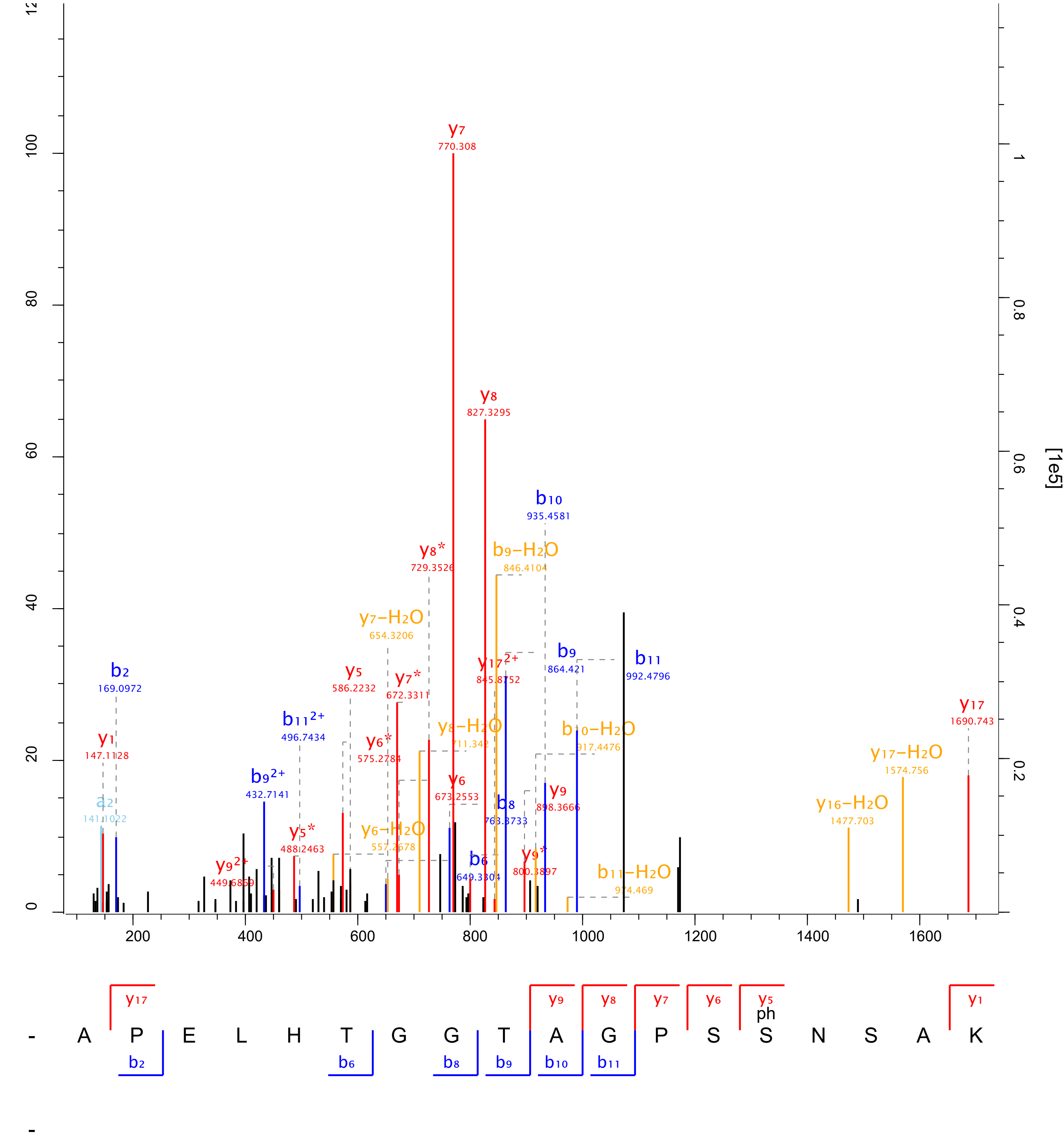

Sample output to test PDF Combine only

Raw file	Scan	Method	Score	m/z	Gene names
0523_4	9220	FTMS; HCD	202.16	827.83	DDL

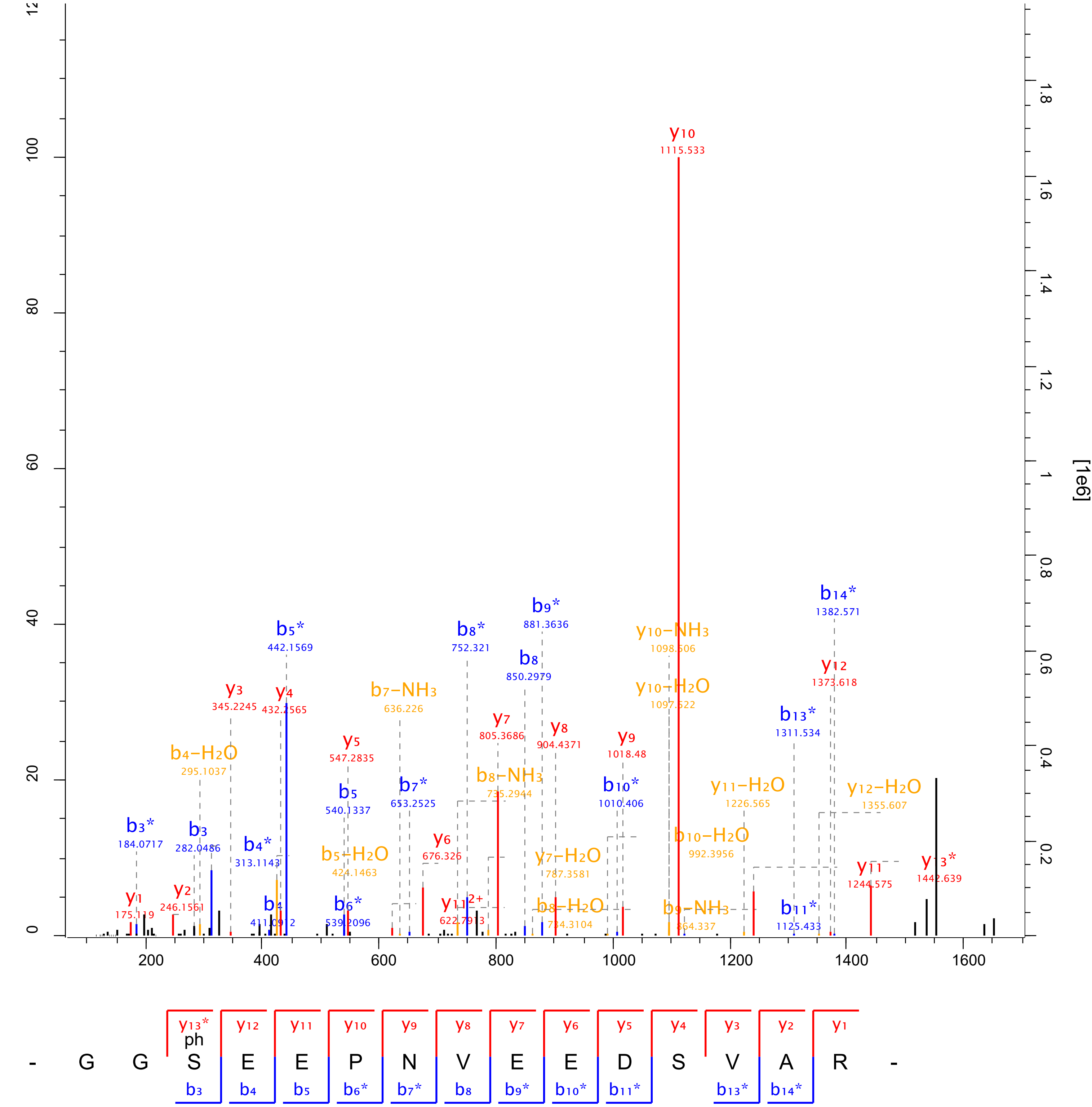

Sample output to test PDF Combine only

Raw file	Scan	Method	Score	m/z
0523_4	9223	FTMS; HCD	115.89	1045.45

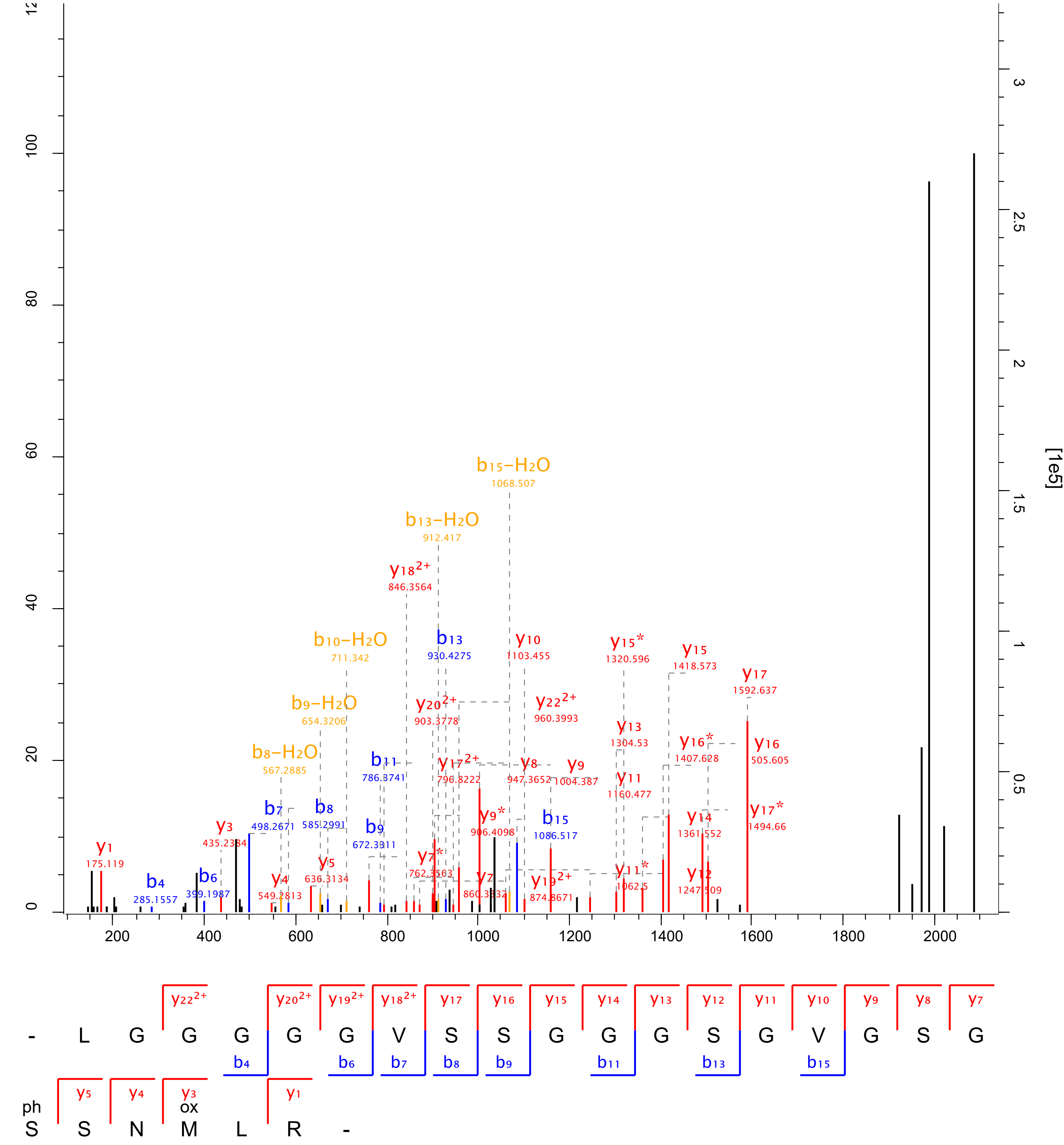

Sample output to test PDF Combine only

Raw file	Scan	Method	Score	m/z	Gene names
0523_4	9251	FTMS; HCD	96.21	717.34	At3g18230

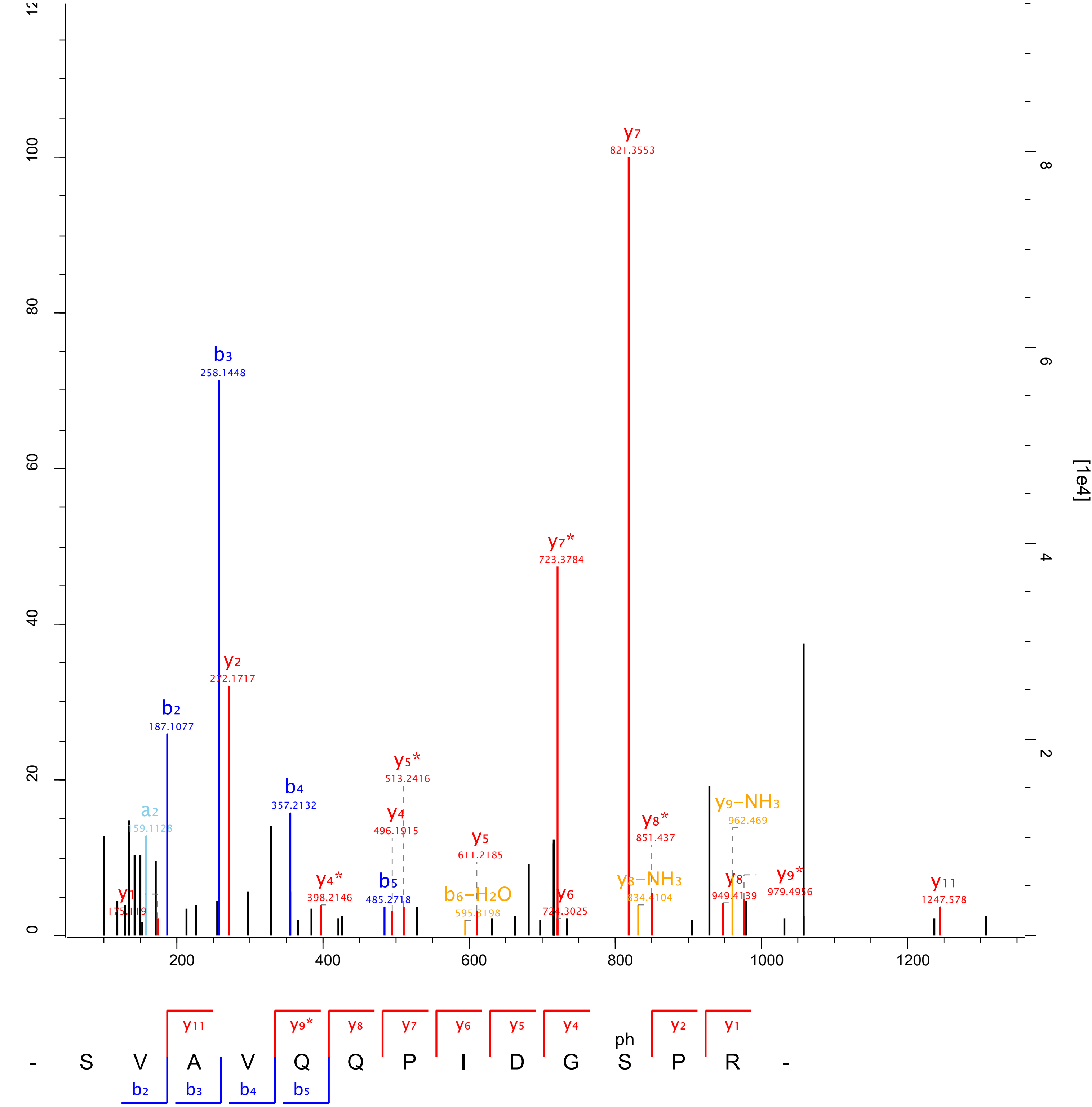

Sample output to test PDF Combine only

Raw file	Scan	Method	Score	m/z	Gene names
0523_4	9257	FTMS; HCD	63.69	703.8	CYP63

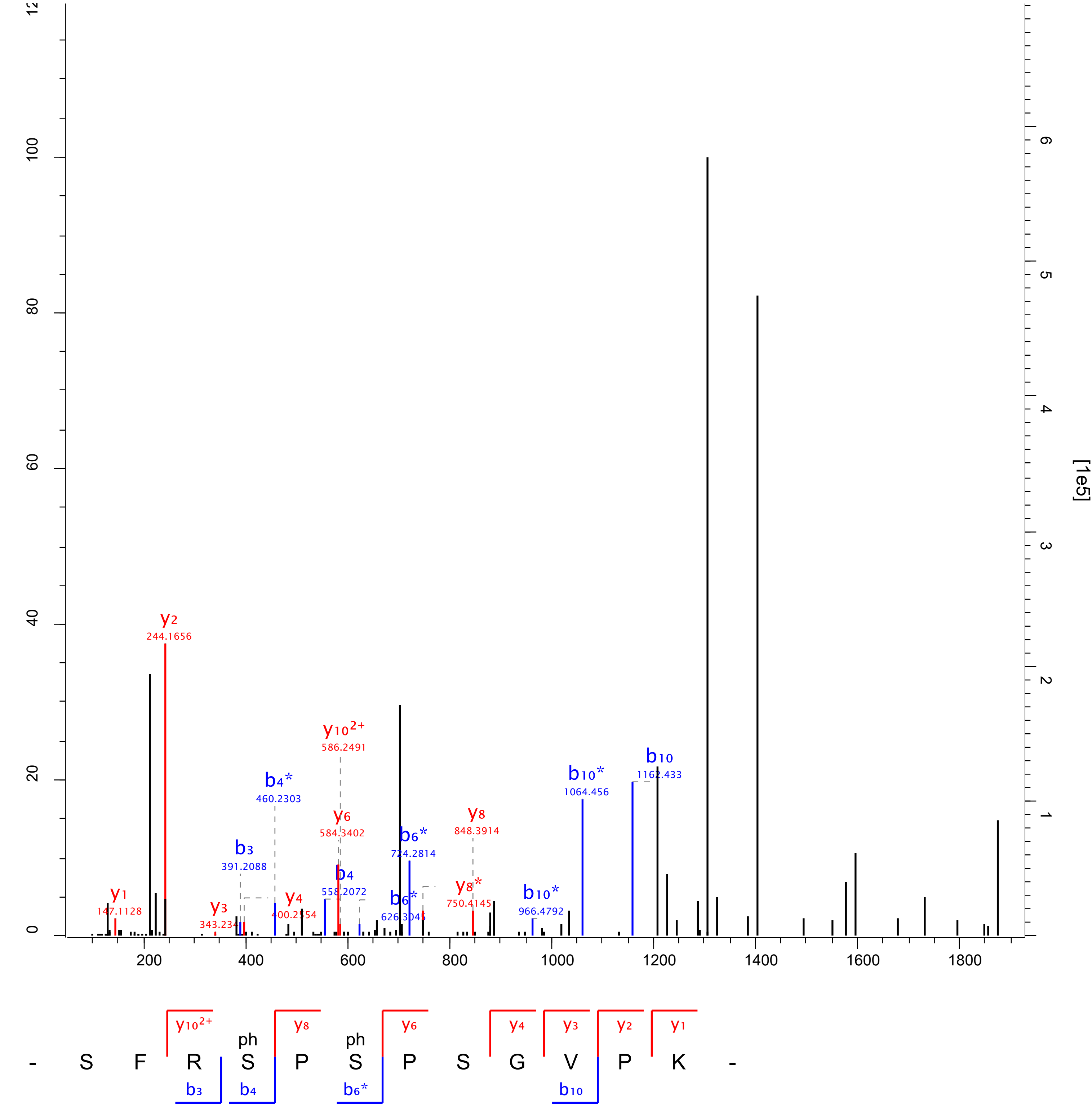

Sample output to test PDF Combine only

Raw file	Scan	Method	Score	m/z	Gene names
0523_4	9295	FTMS; HCD	125.45	732.78	At5g56980

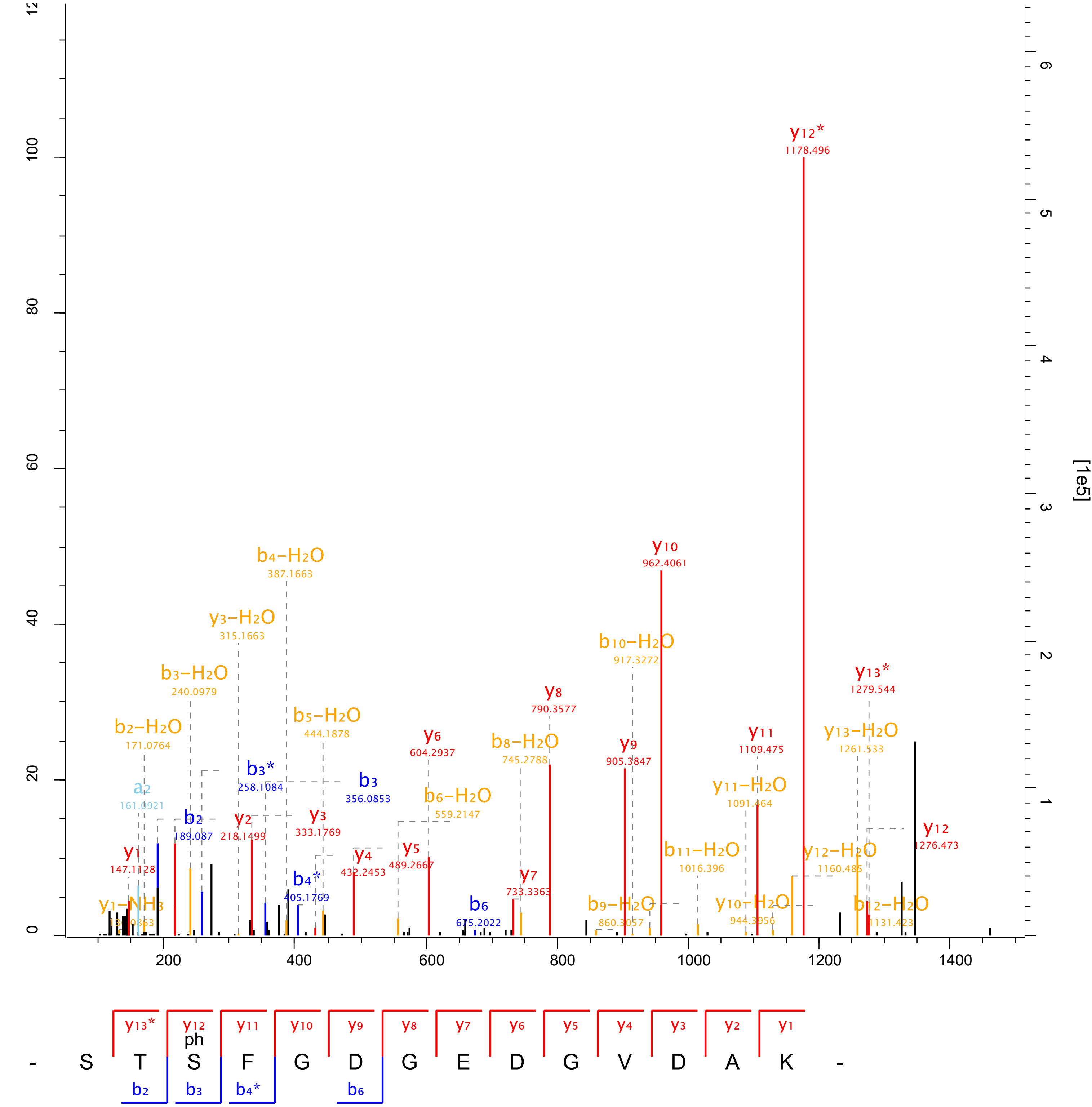

Raw file	Scan	Method	Score	m/z
0523_4	9403	FTMS; HCD	145.44	819.81

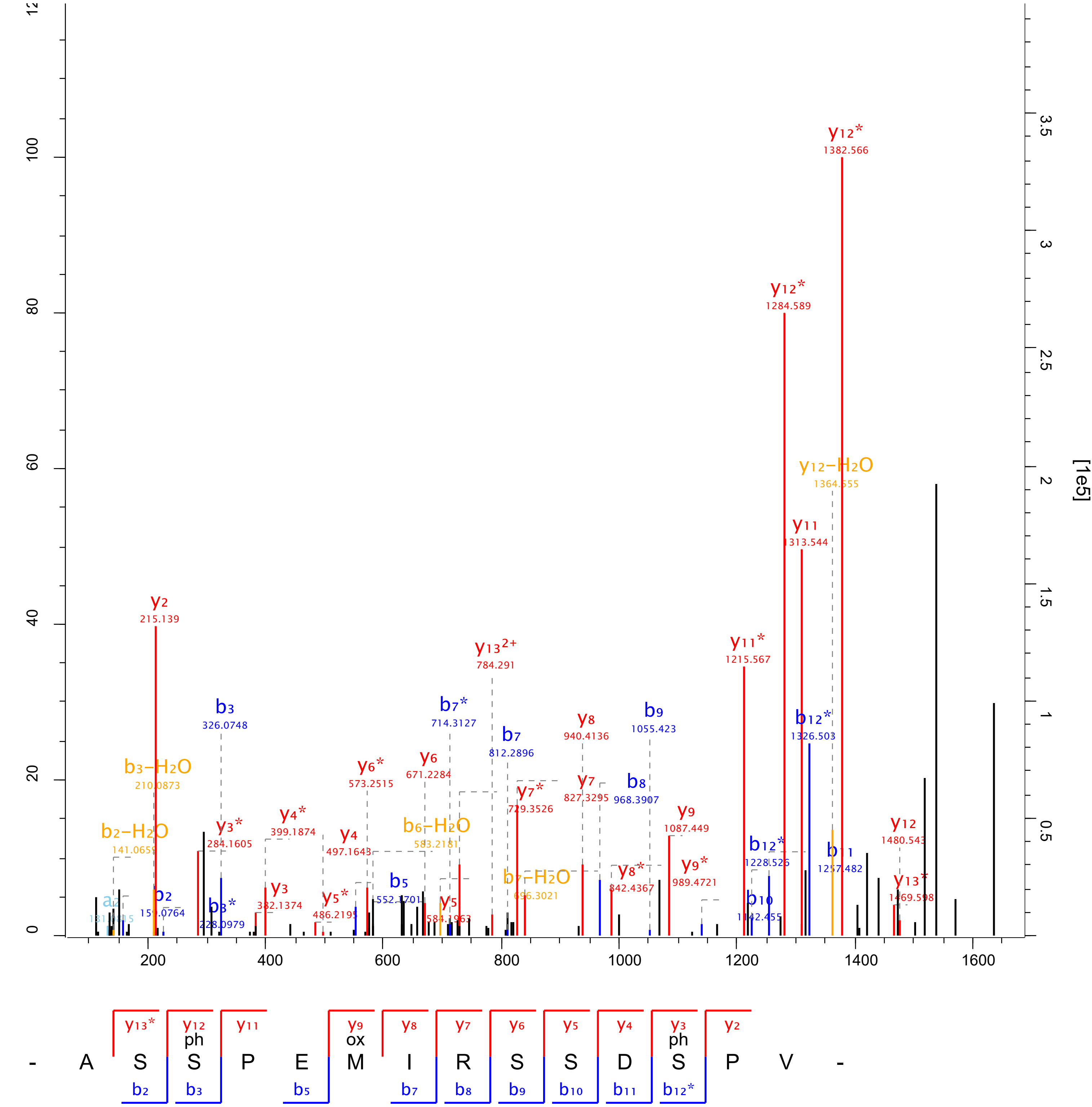

Sample output to test PDF Combine only

Raw file	Scan	Method	Score	m/z	Gene names
0523_4	9420	FTMS; HCD	51.88	692.28	CBF5

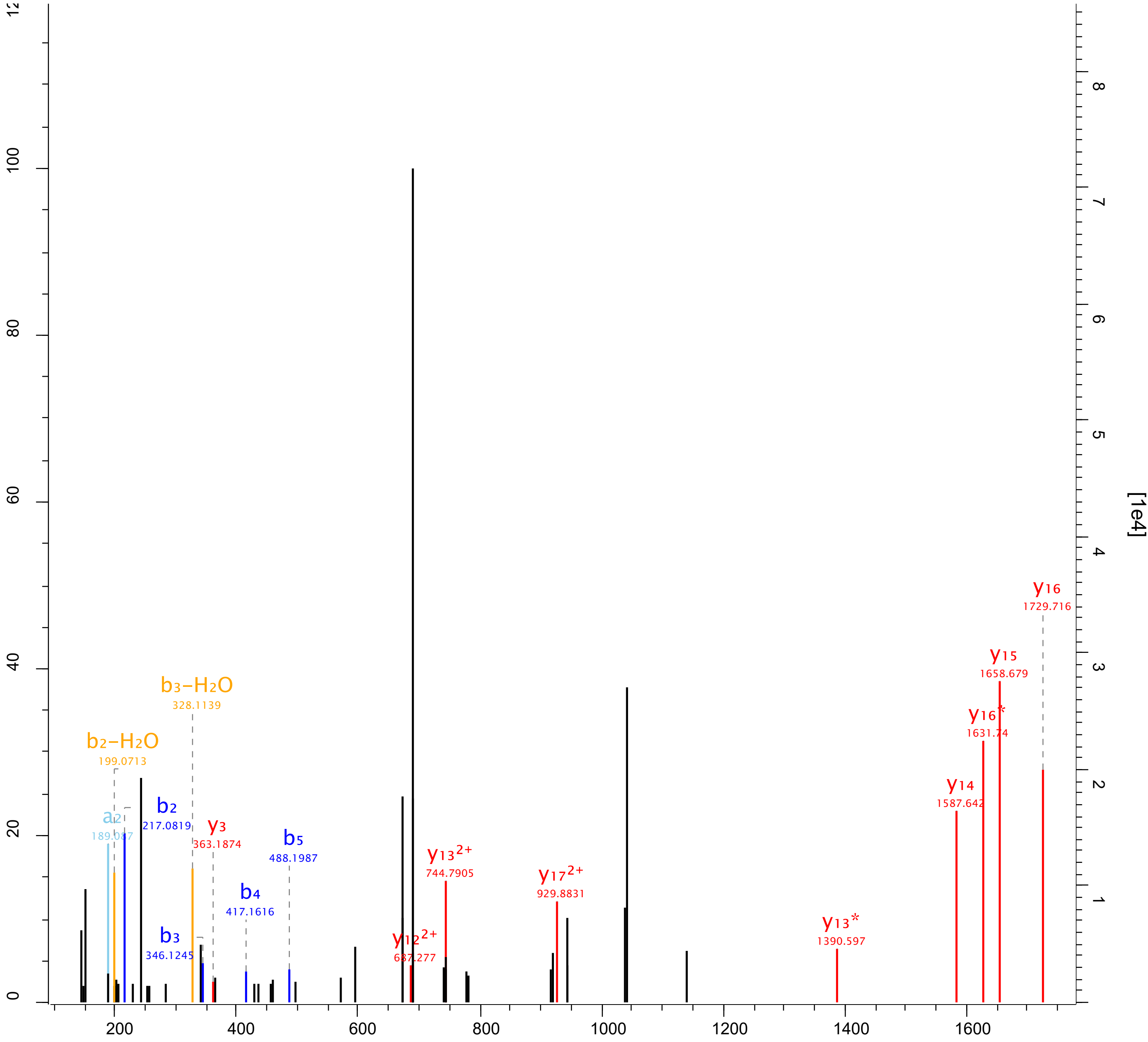

- D T E A A V D A E D E S A A E K S E
K -

ph: S; y ions: y17 2+, y16, y15, y14, y13*, y12 2+, y3; b ions: b2, b3, b4, b5

Sample output to test PDF Combine only

Raw file	Scan	Method	Score	m/z	Gene names
0523_4	9465	FTMS; HCD	66.89	756.94	AGO1

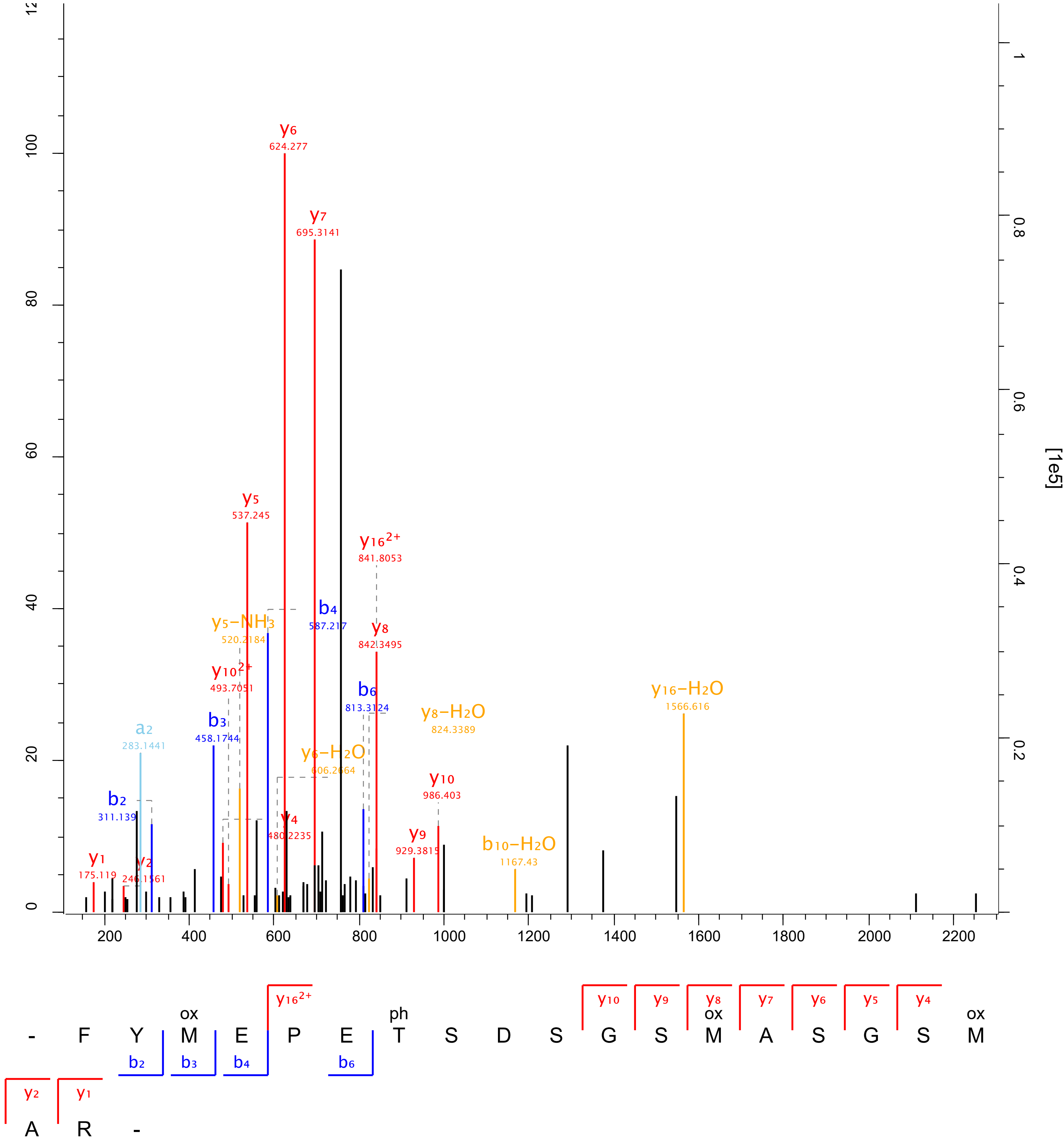

Sample output to test PDF Combine only

Raw file	Scan	Method	Score	m/z	Gene names
0523_4	9531	FTMS; HCD	120.9	740.84	LPP1

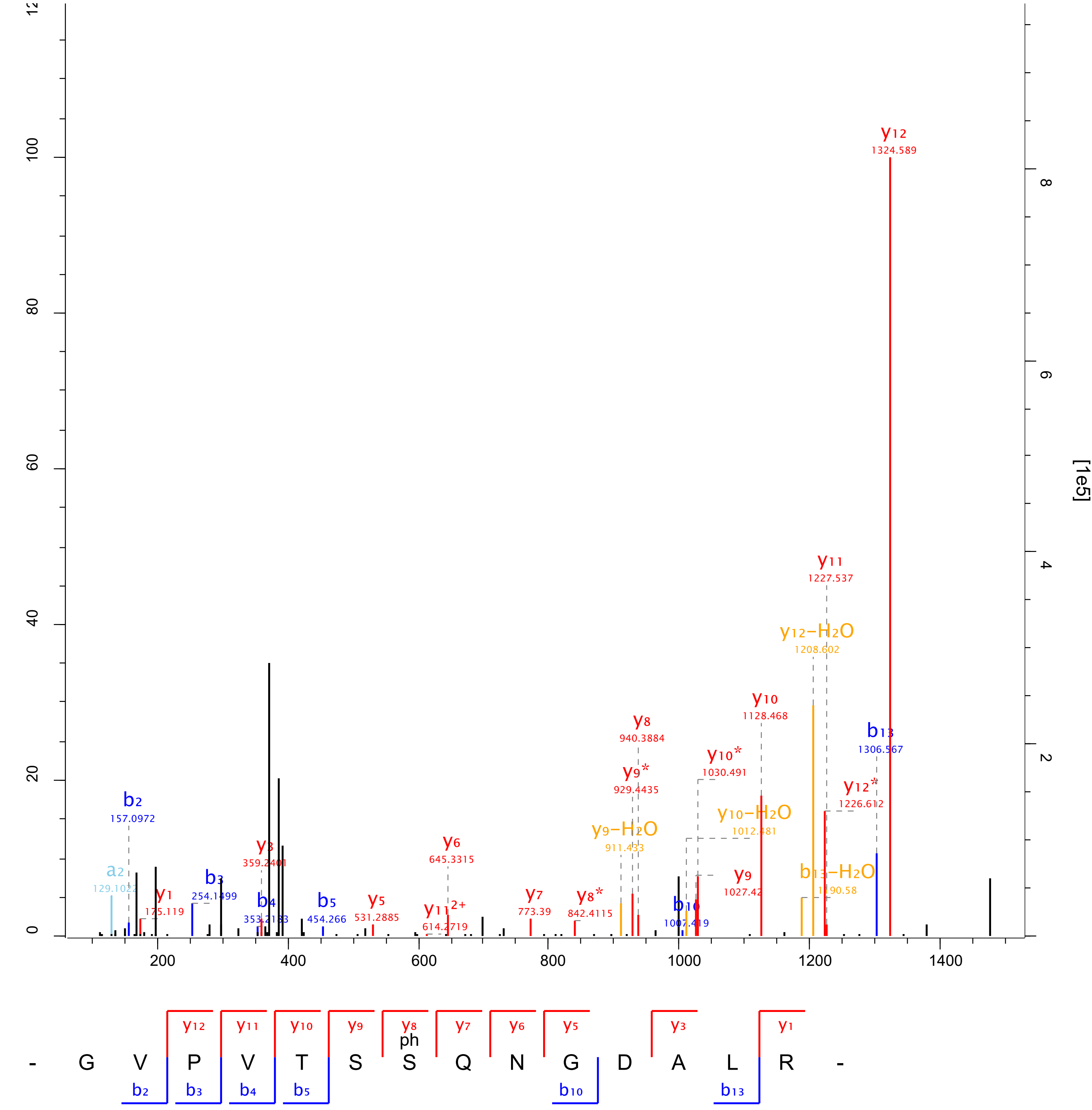

Sample output to test PDF Combine only

Raw file	Scan	Method	Score	m/z	Gene names
0523_4	9571	FTMS; HCD	88.49	737.32	MTM1

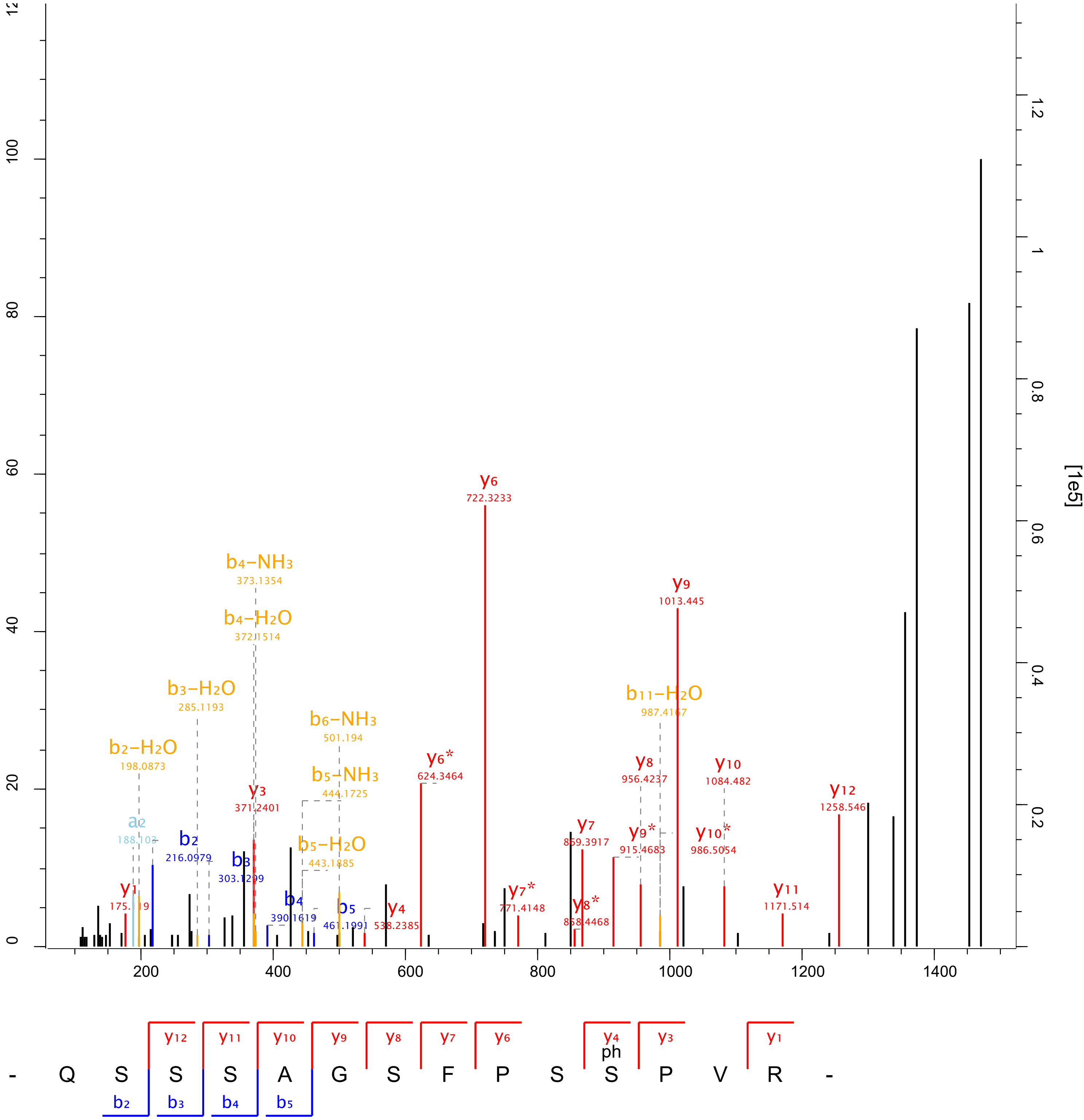

Sample output to test PDF Combine only

Raw file	Scan	Method	Score	m/z
0523_4	9574	FTMS; HCD	208.03	504.21

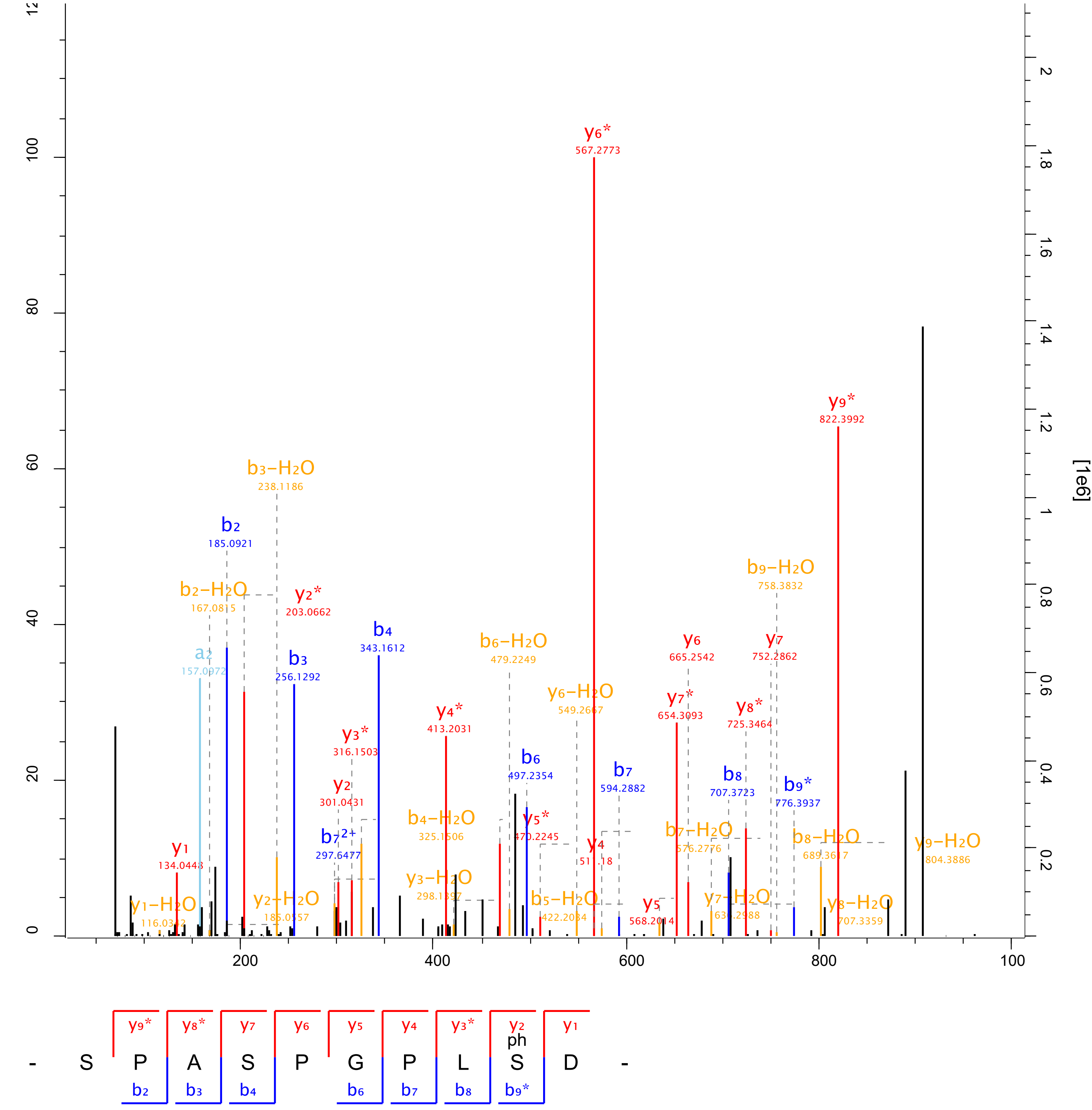

Sample output to test PDF Combine only

Raw file	Scan	Method	Score	m/z
0523_4	9638	FTMS; HCD	205.12	726.35

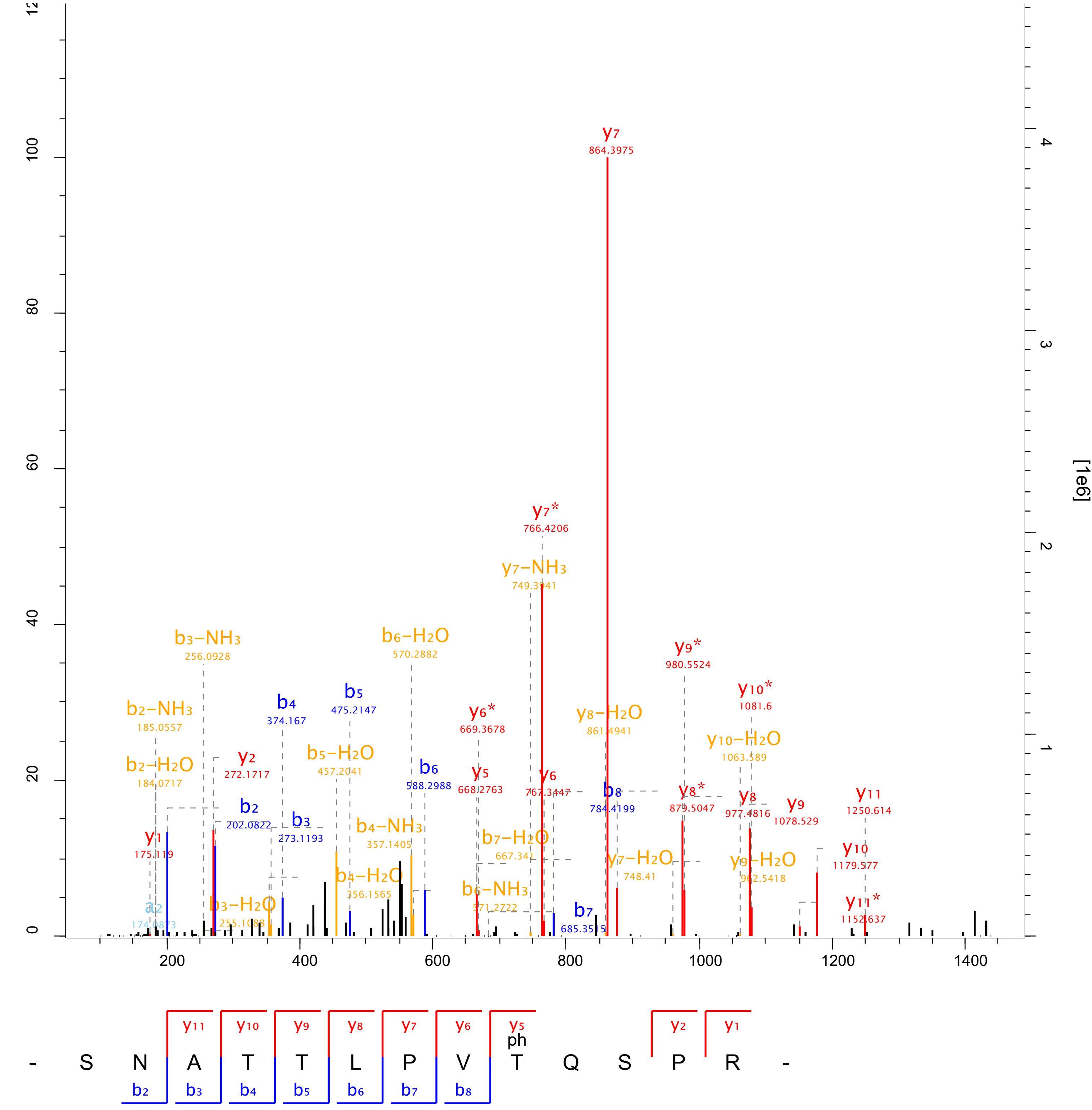

Sample output to test PDF Combine only

Raw file	Scan	Method	Score	m/z	Gene names
0523_4	9701	FTMS; HCD	106.39	636.6	PLP1

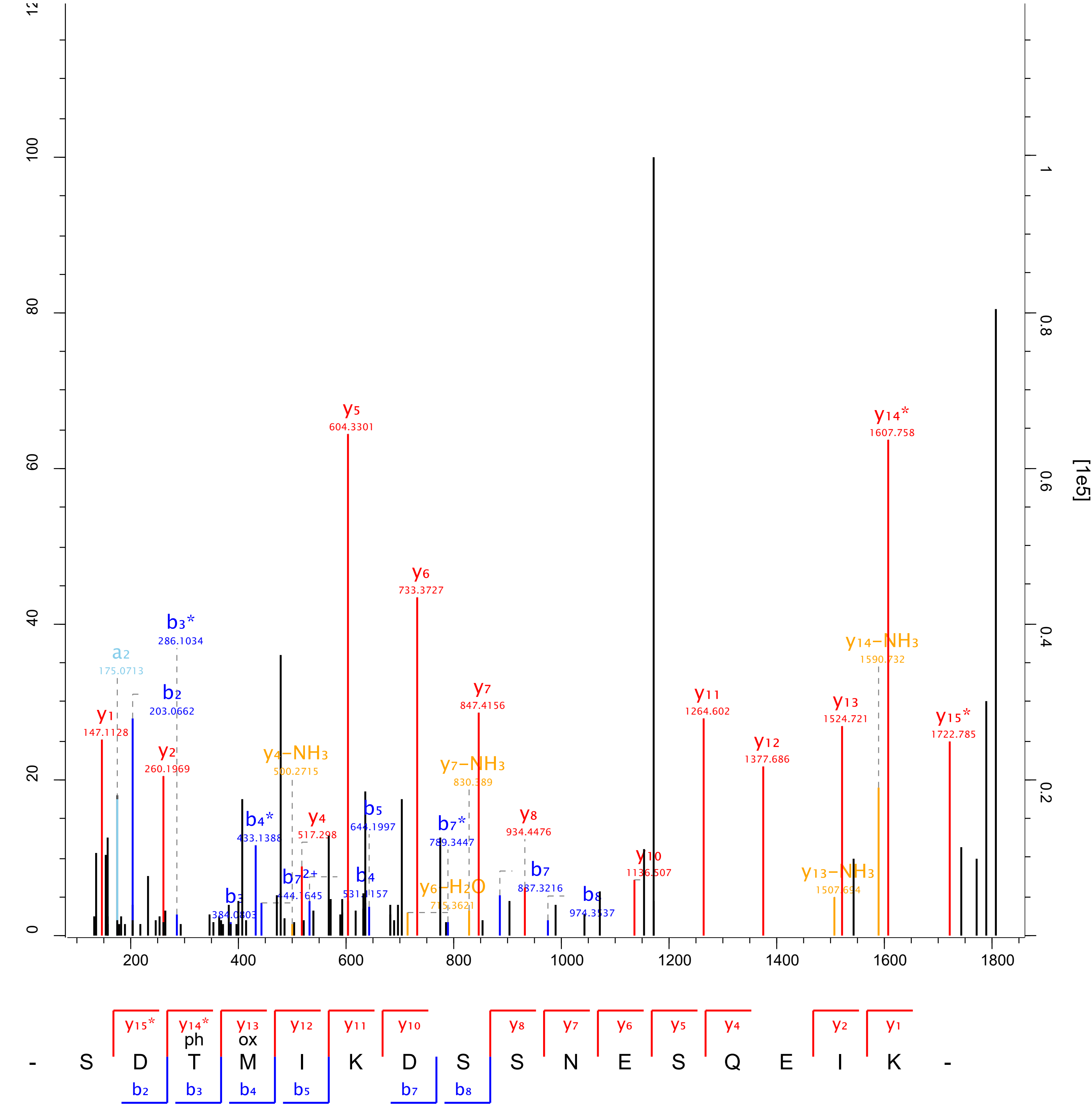

Sample output to test PDF Combine only

Raw file	Scan	Method	Score	m/z	Gene names
0523_4	9776	FTMS; HCD	88.1	599.78	At5g35180

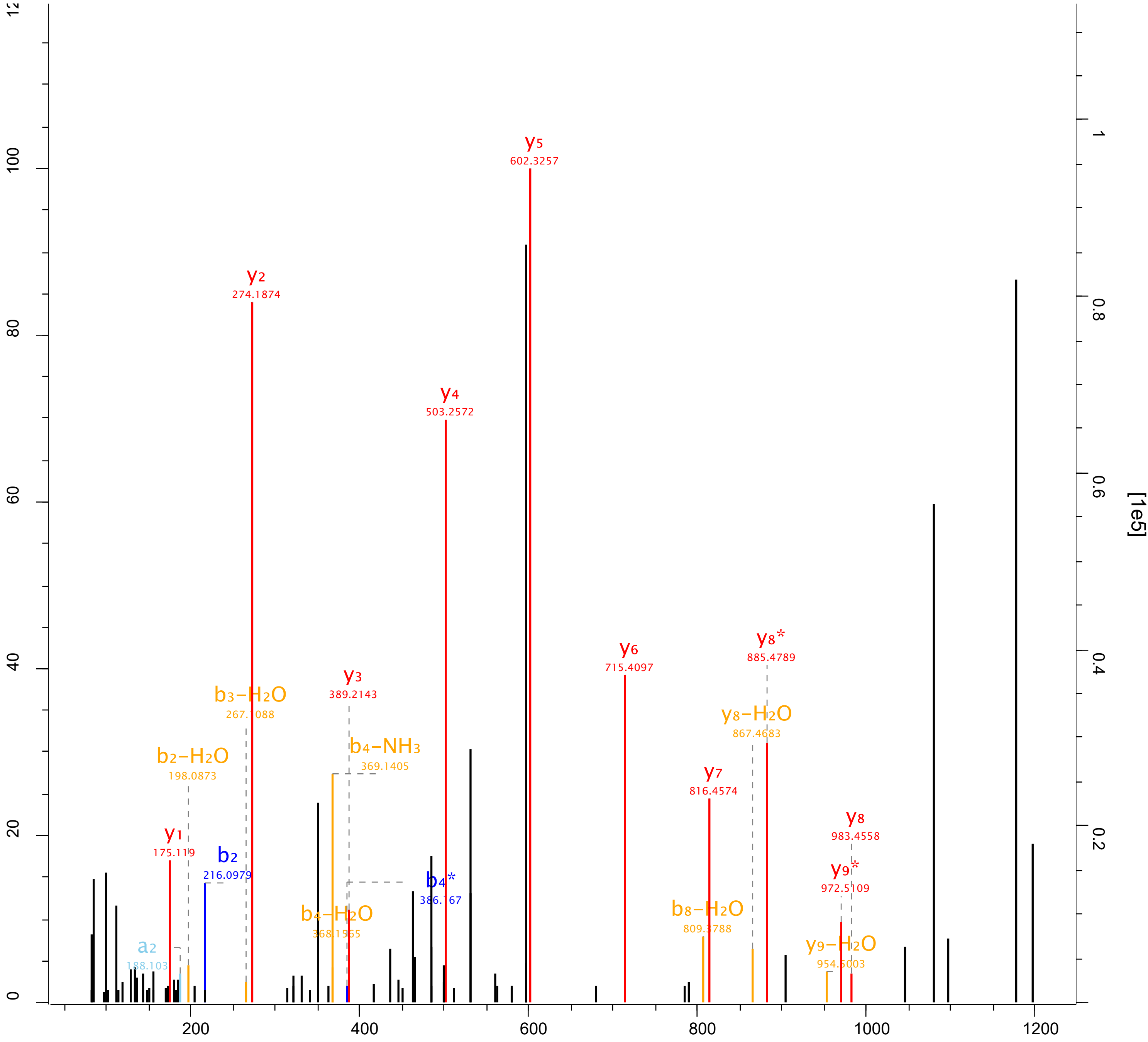

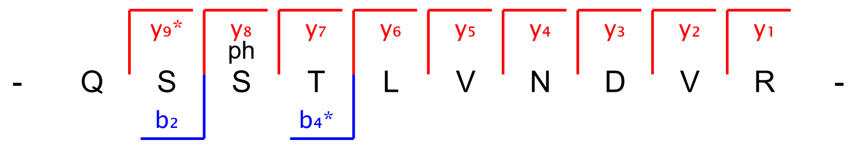

Sample output to test PDF Combine only

Raw file	Scan	Method	Score	m/z	Gene names
0523_4	9799	FTMS; HCD	54	1095.95	AMT1-1

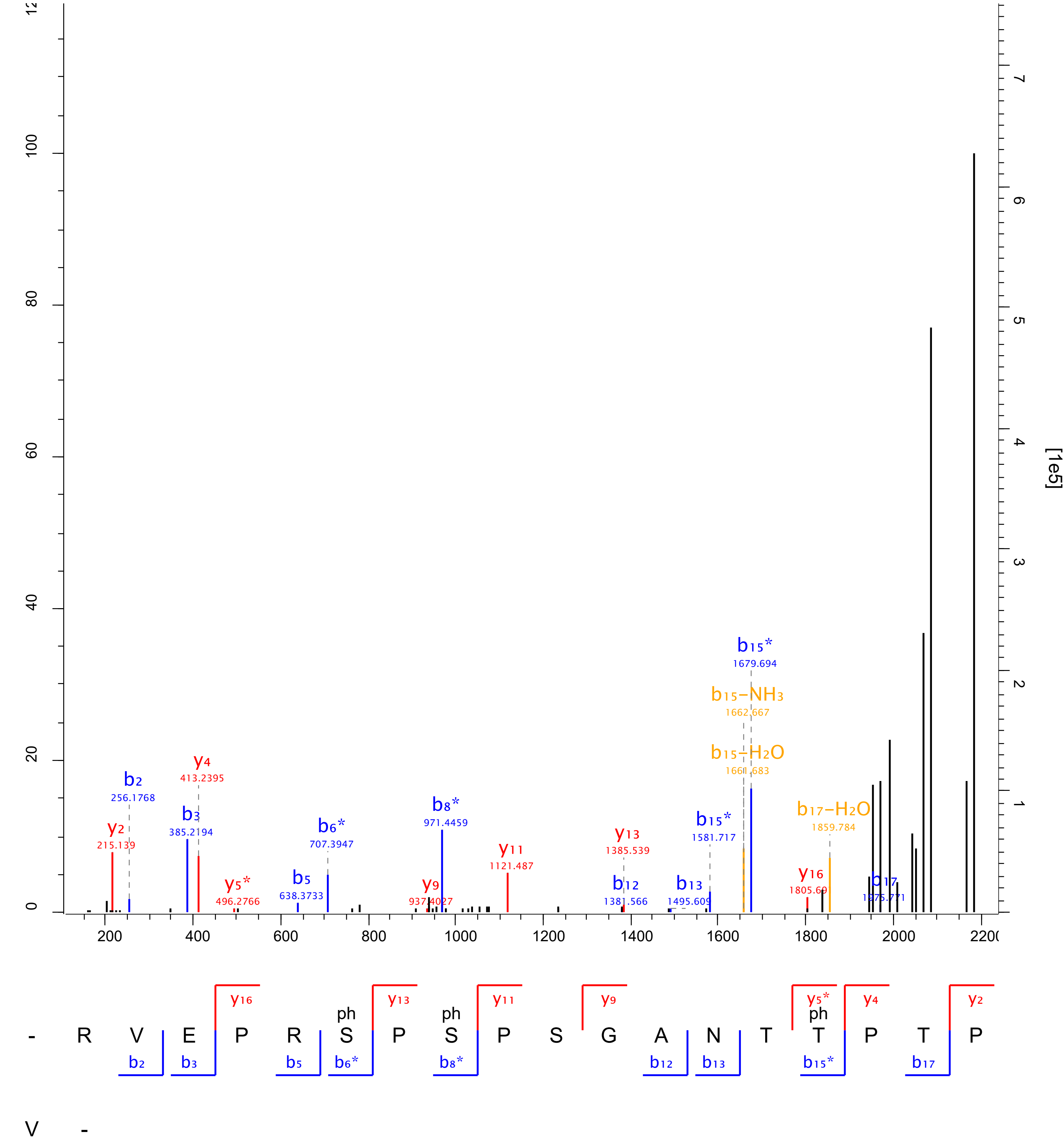

Sample output to test PDF Combine only

Raw file	Scan	Method	Score	m/z	Gene names
0523_4	9840	FTMS; HCD	290.82	745.3	F5A18.5

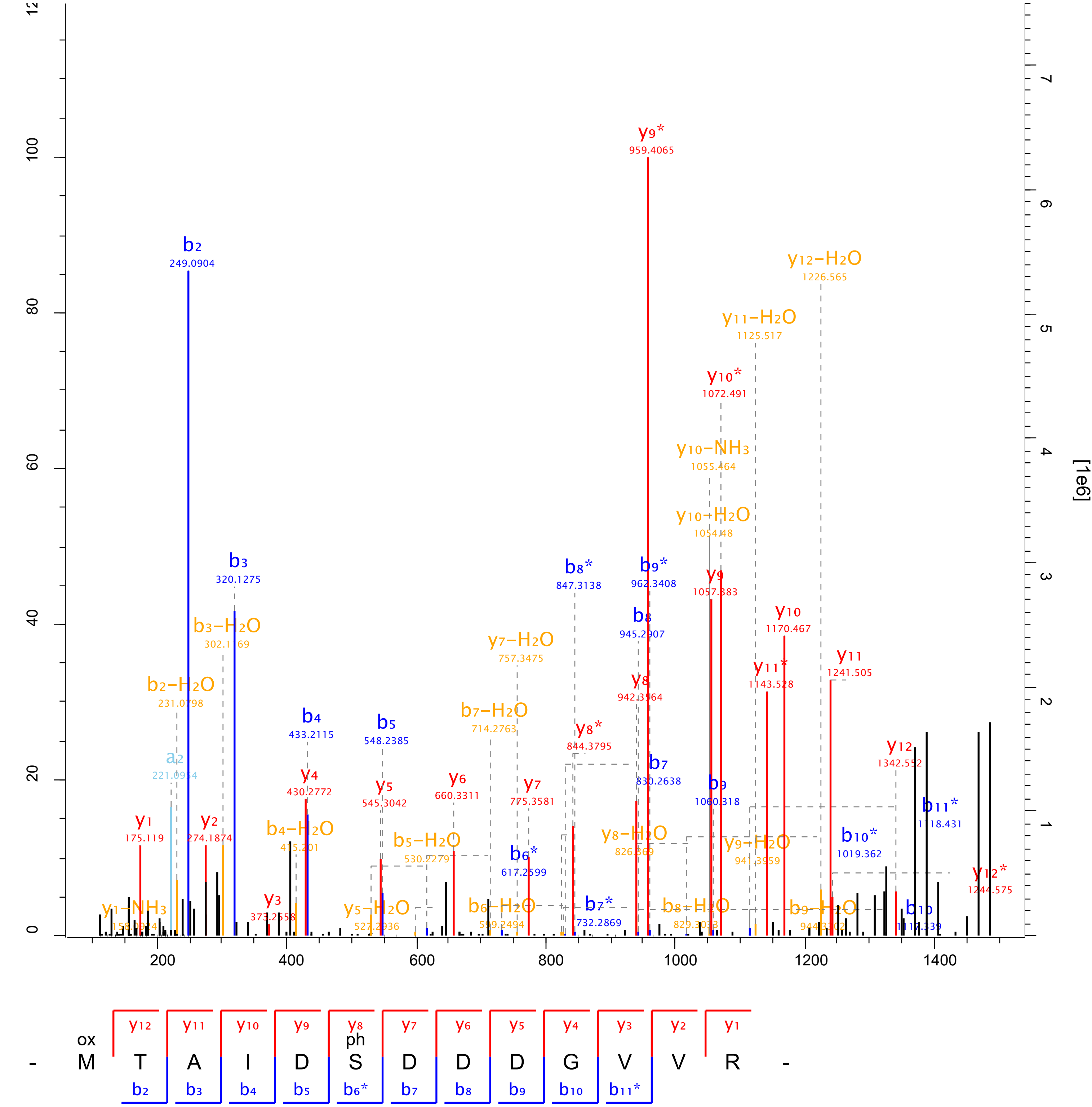

Sample output to test PDF Combine only

Raw file	Scan	Method	Score	m/z	Gene names
0523_4	9929	FTMS; HCD	103.01	629.26	PIP2-1;PIP2-3;PIP2-2

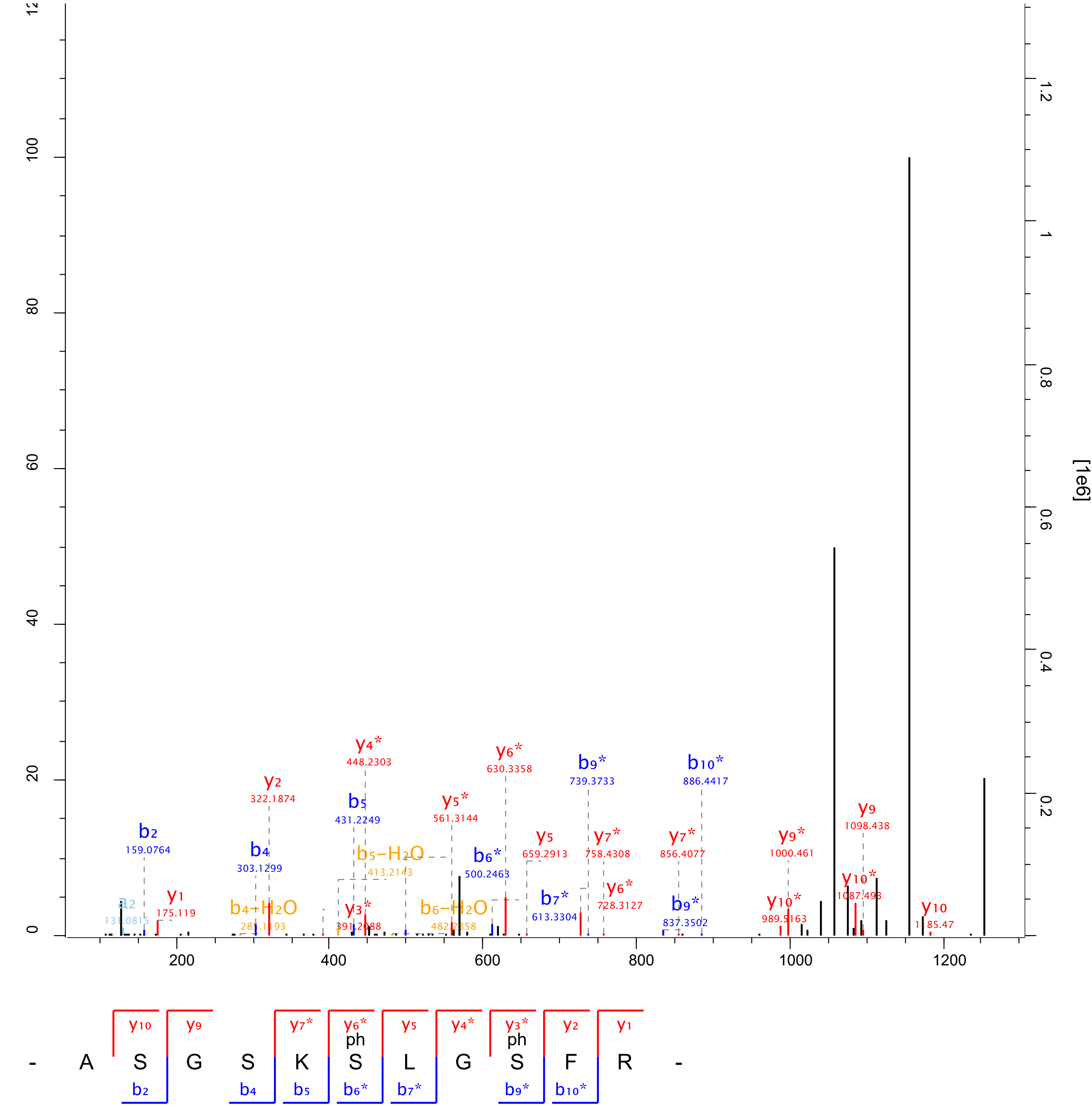

Sample output to test PDF Combine only

Raw file	Scan	Method	Score	m/z	Gene names
0523_4	9974	FTMS; HCD	74.22	762.64	CPK6

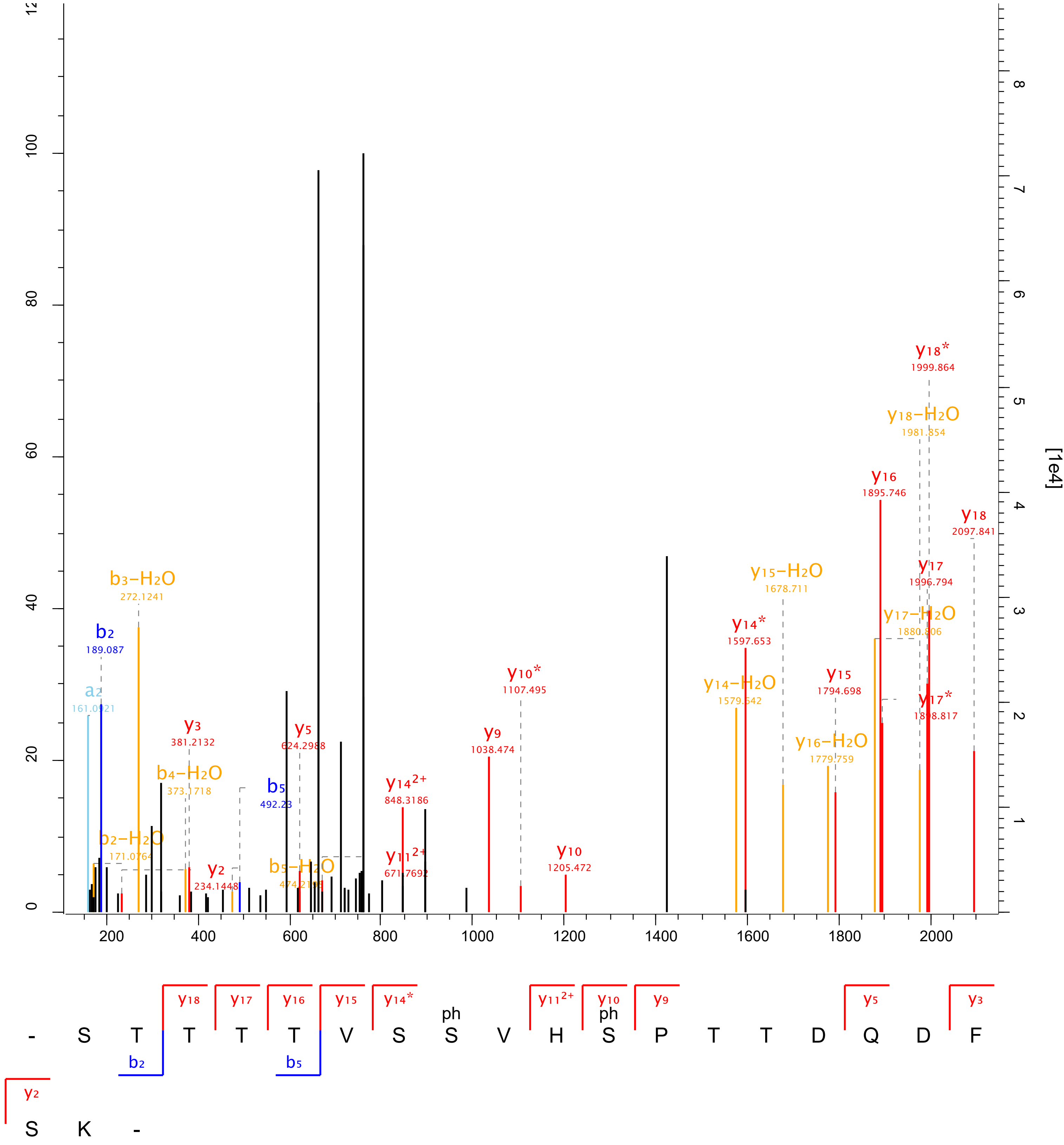

Sample output to test PDF Combine only

Raw file	Scan	Method	Score	m/z	Gene names
0523_4	10356	FTMS; HCD	88.28	636.26	At1g35220

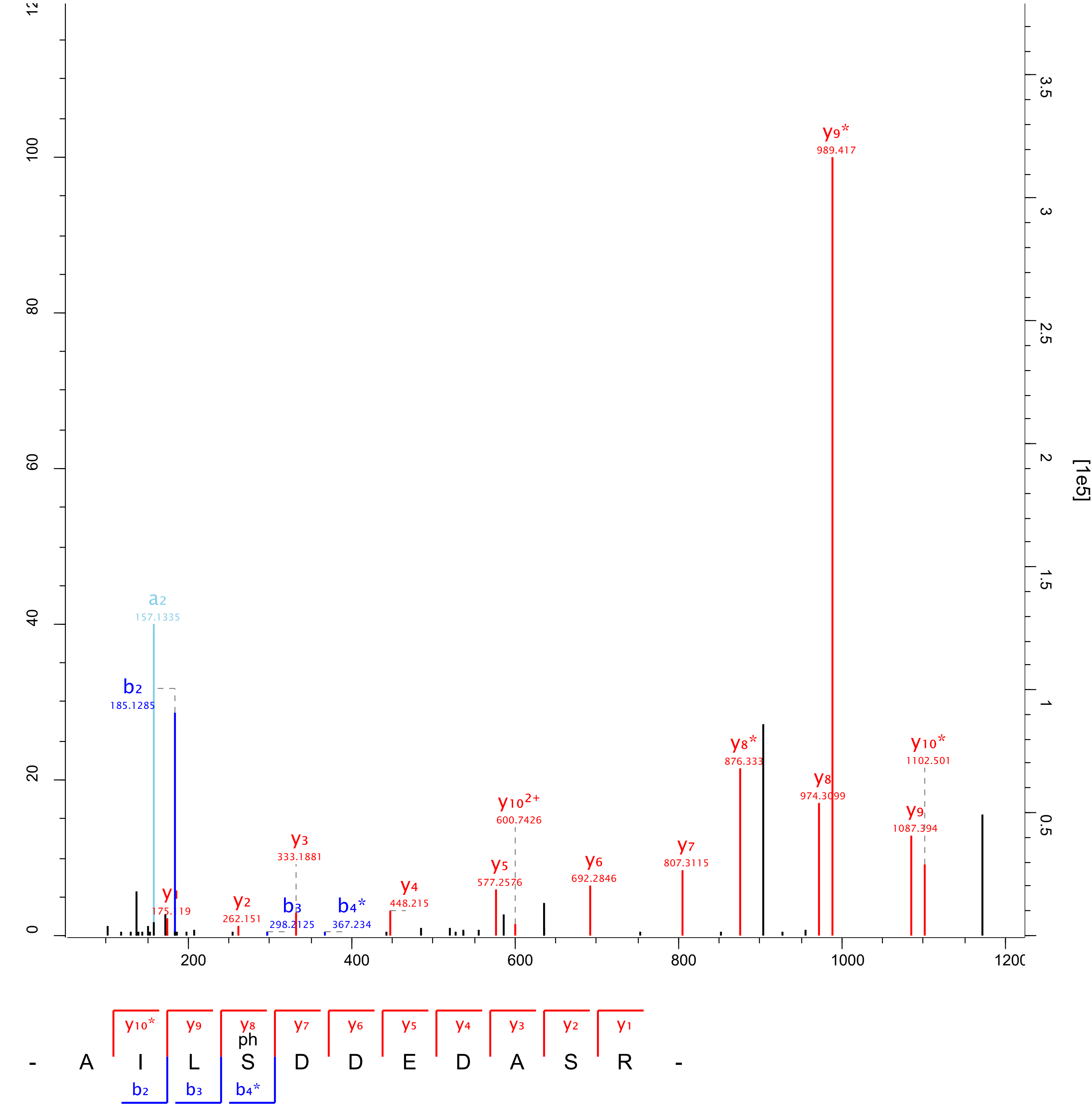

Sample output to test PDF Combine only

Raw file	Scan	Method	Score	m/z	Gene names
0523_4	10493	FTMS; HCD	113.4	683.31	RS40

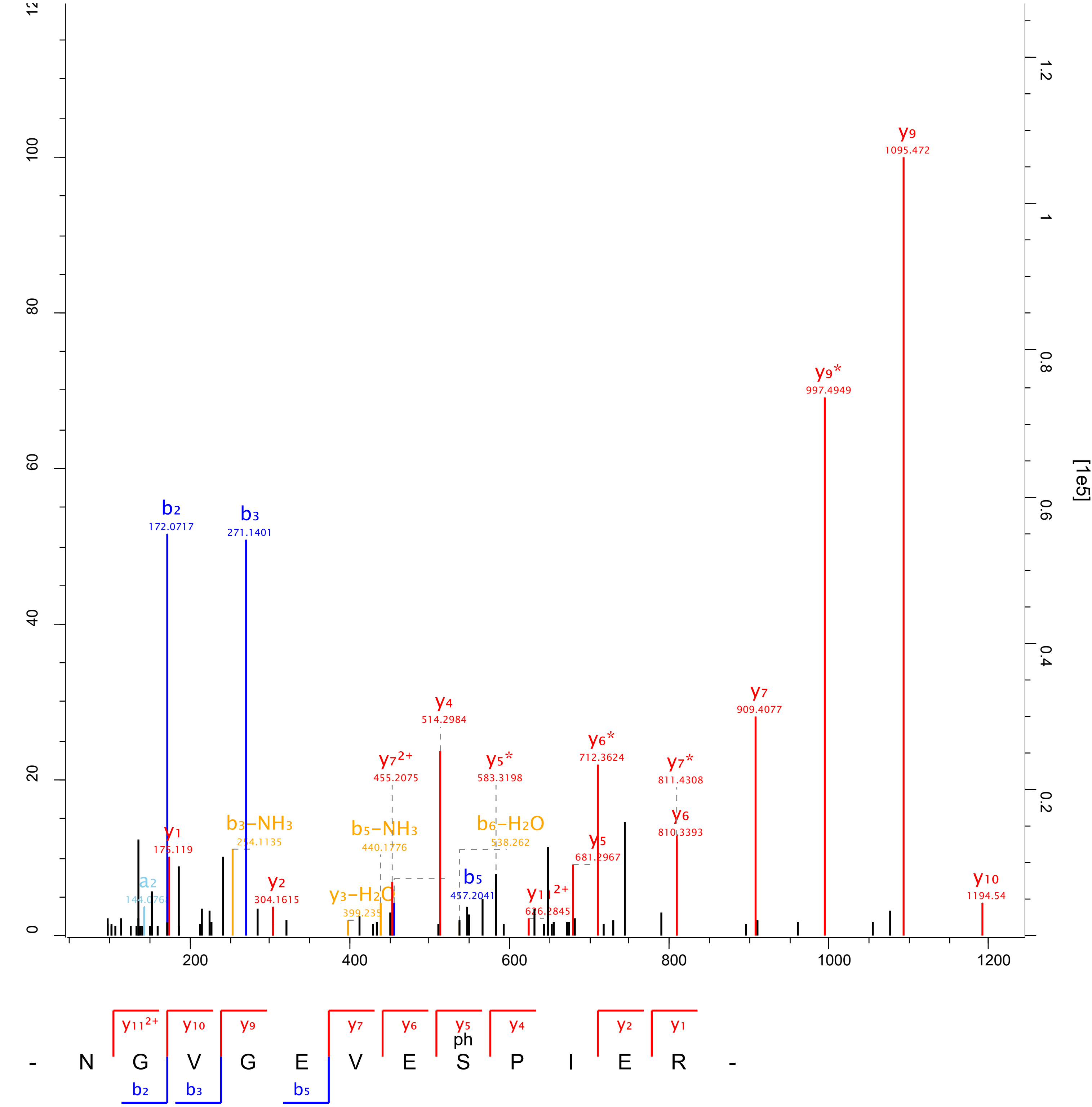

Sample output to test PDF Combine only

Raw file	Scan	Method	Score	m/z	Gene names
0523_4	10668	FTMS; HCD	43.84	664.62	At1g45688;F2G19.25

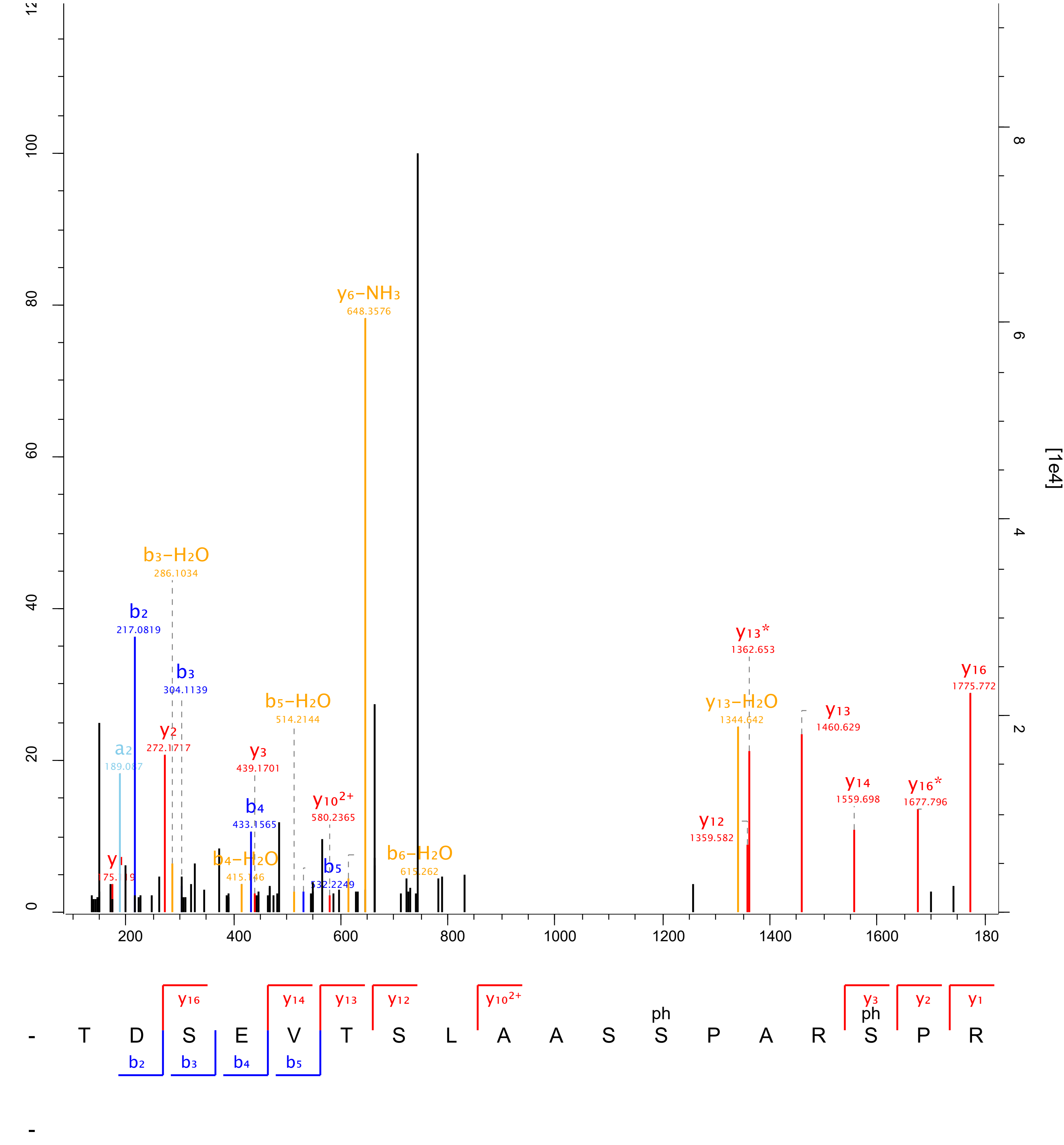

Sample output to test PDF Combine only

Raw file	Scan	Method	Score	m/z	Gene names
0523_4	10705	FTMS; HCD	348.79	723.3	PVA42;At4g21450

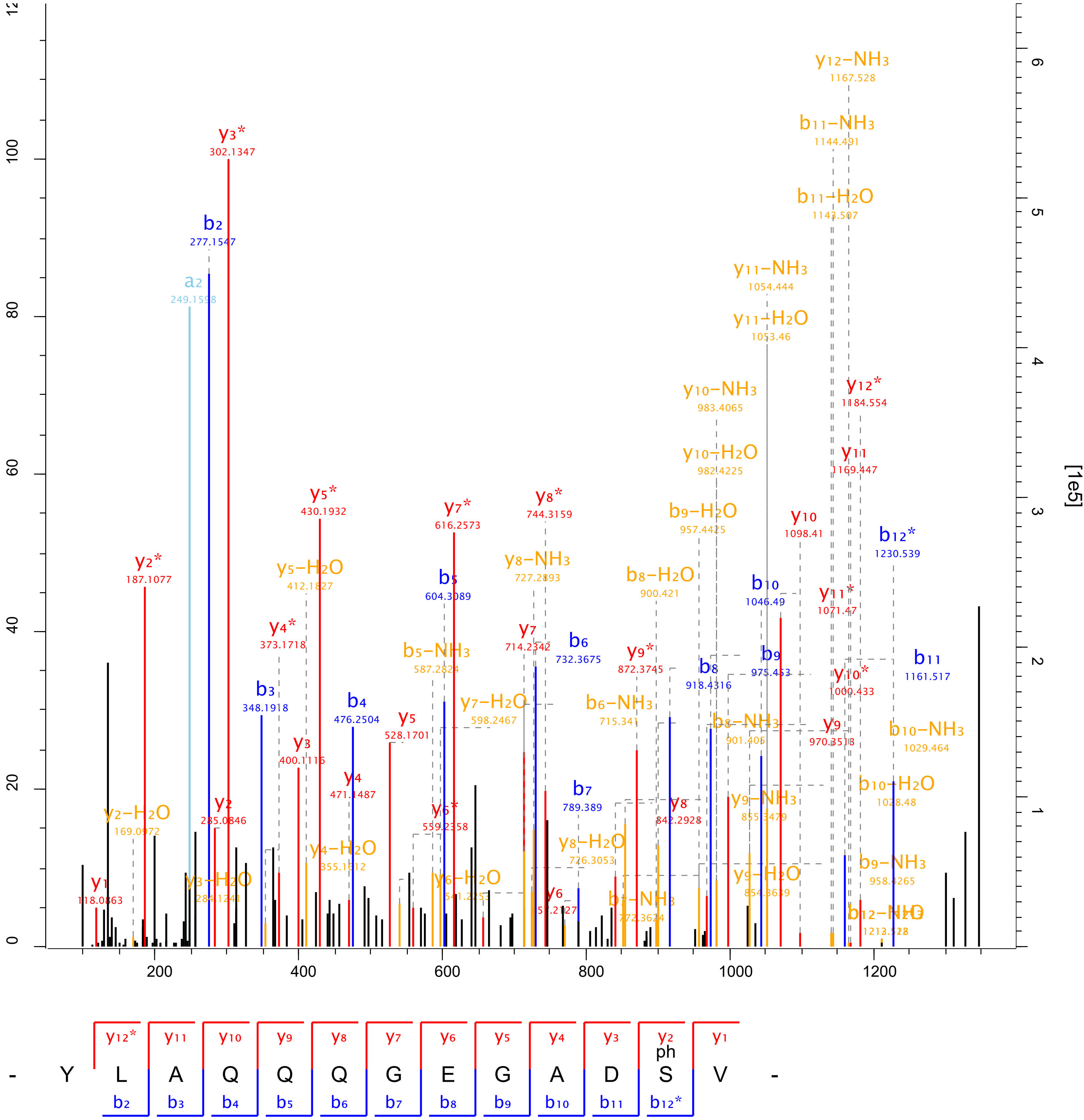

Sample output to test PDF Combine only

Raw file	Scan	Method	Score	m/z
0523_4	10985	FTMS; HCD	150.09	830.85

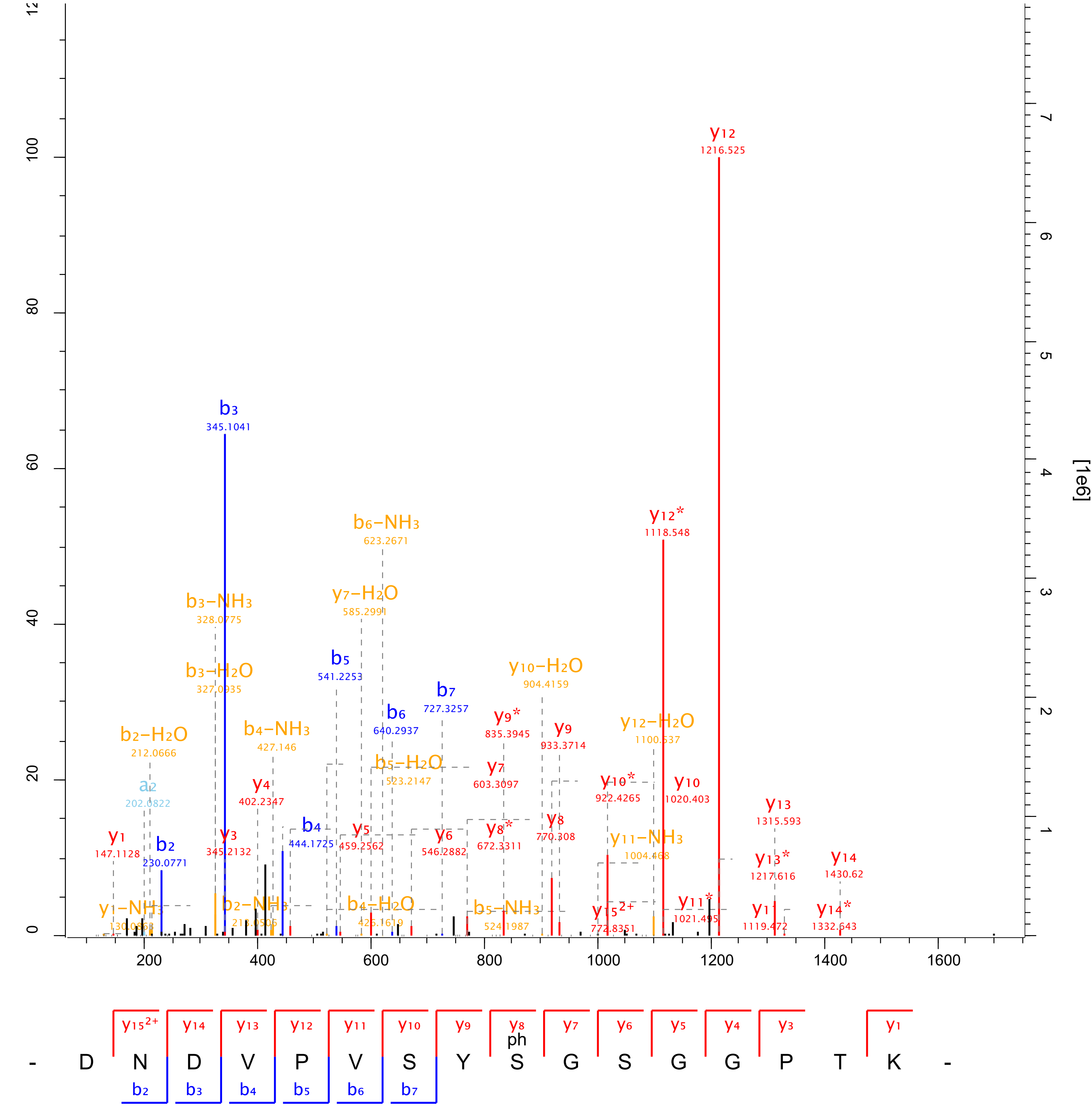

Sample output to test PDF Combine only

Raw file	Scan	Method	Score	m/z
0523_4	11006	FTMS; HCD	79.51	770.81

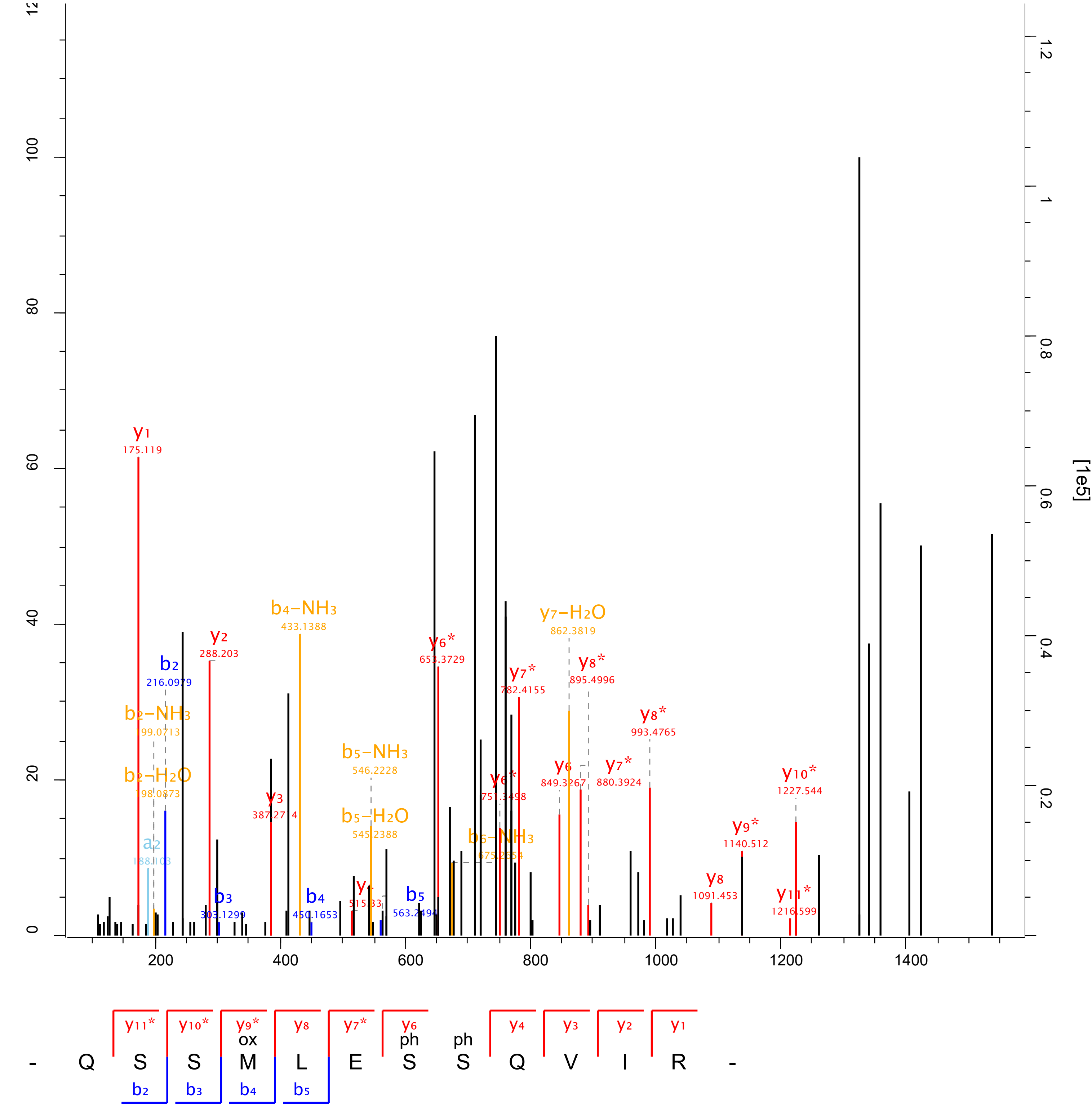

Sample output to test PDF Combine only

Raw file	Scan	Method	Score	m/z	Gene names
0523_4	11121	FTMS; HCD	143.04	863.77	LAG2

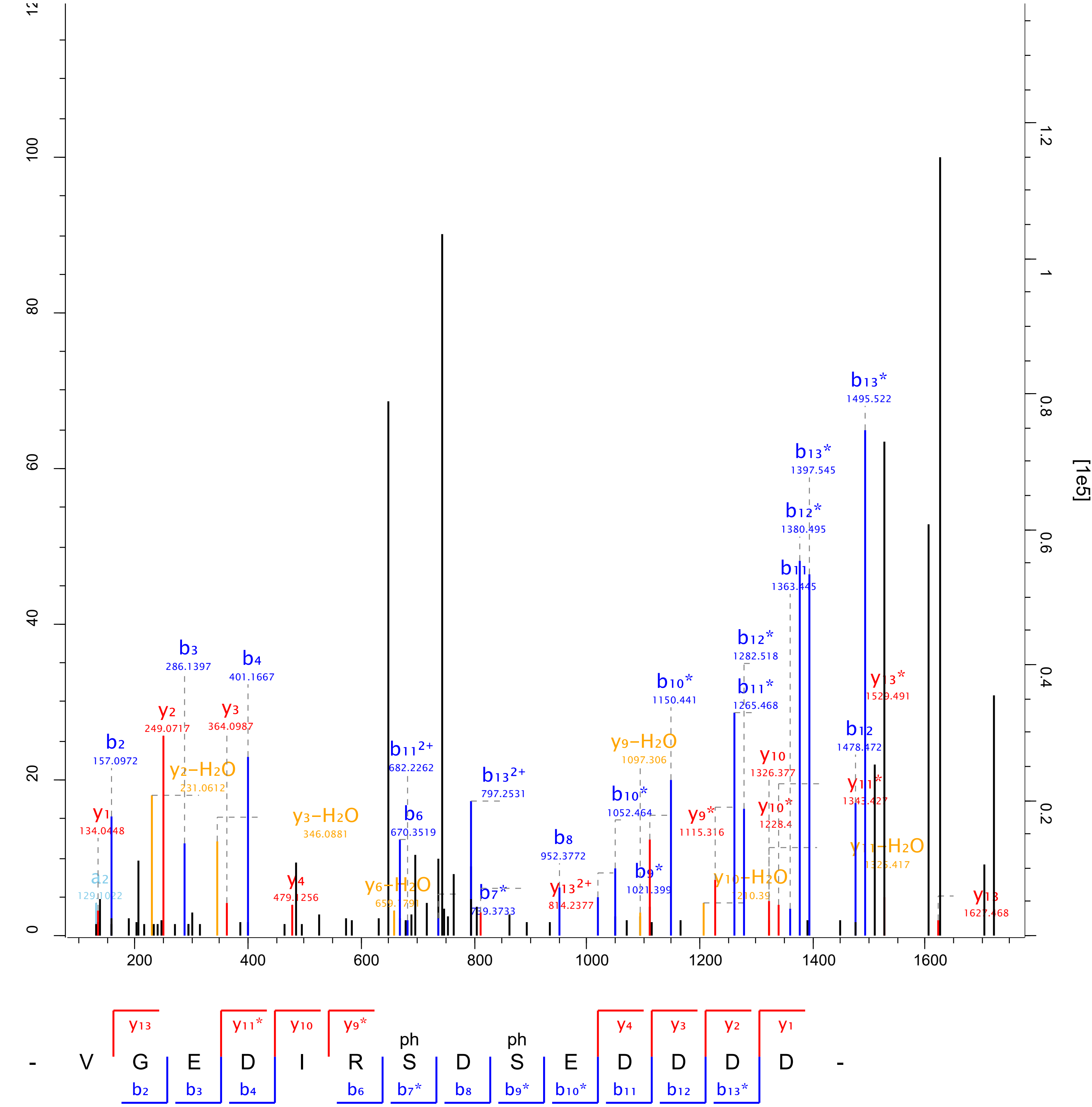

Sample output to test PDF Combine only

Raw file	Scan	Method	Score	m/z	Gene names
0523_4	11511	FTMS; HCD	109.44	977.42	AMT1-1

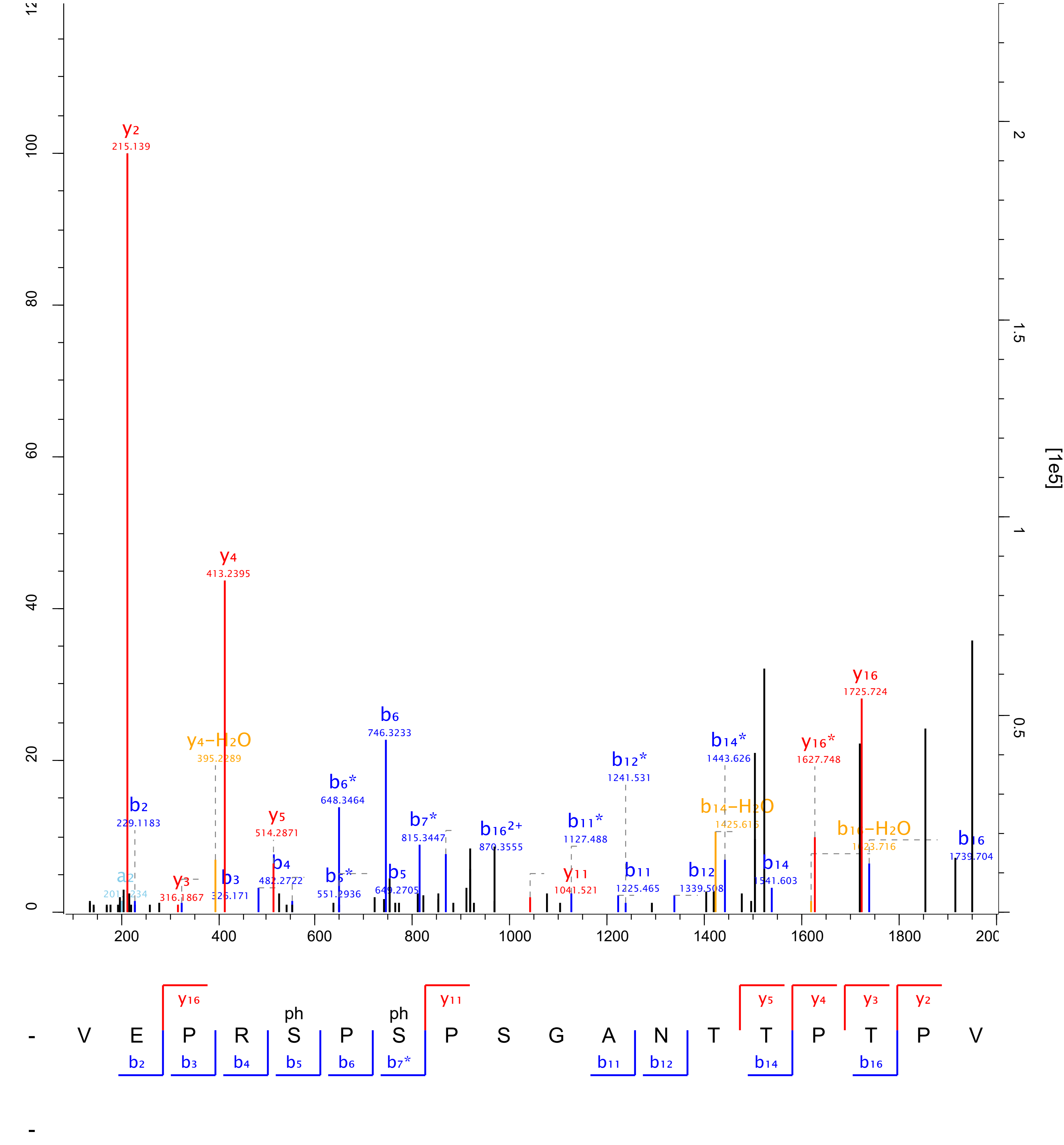

Sample output to test PDF Combine only

Raw file	Scan	Method	Score	m/z	Gene names
0523_4	11519	FTMS; HCD	111.83	702.28	PECT1

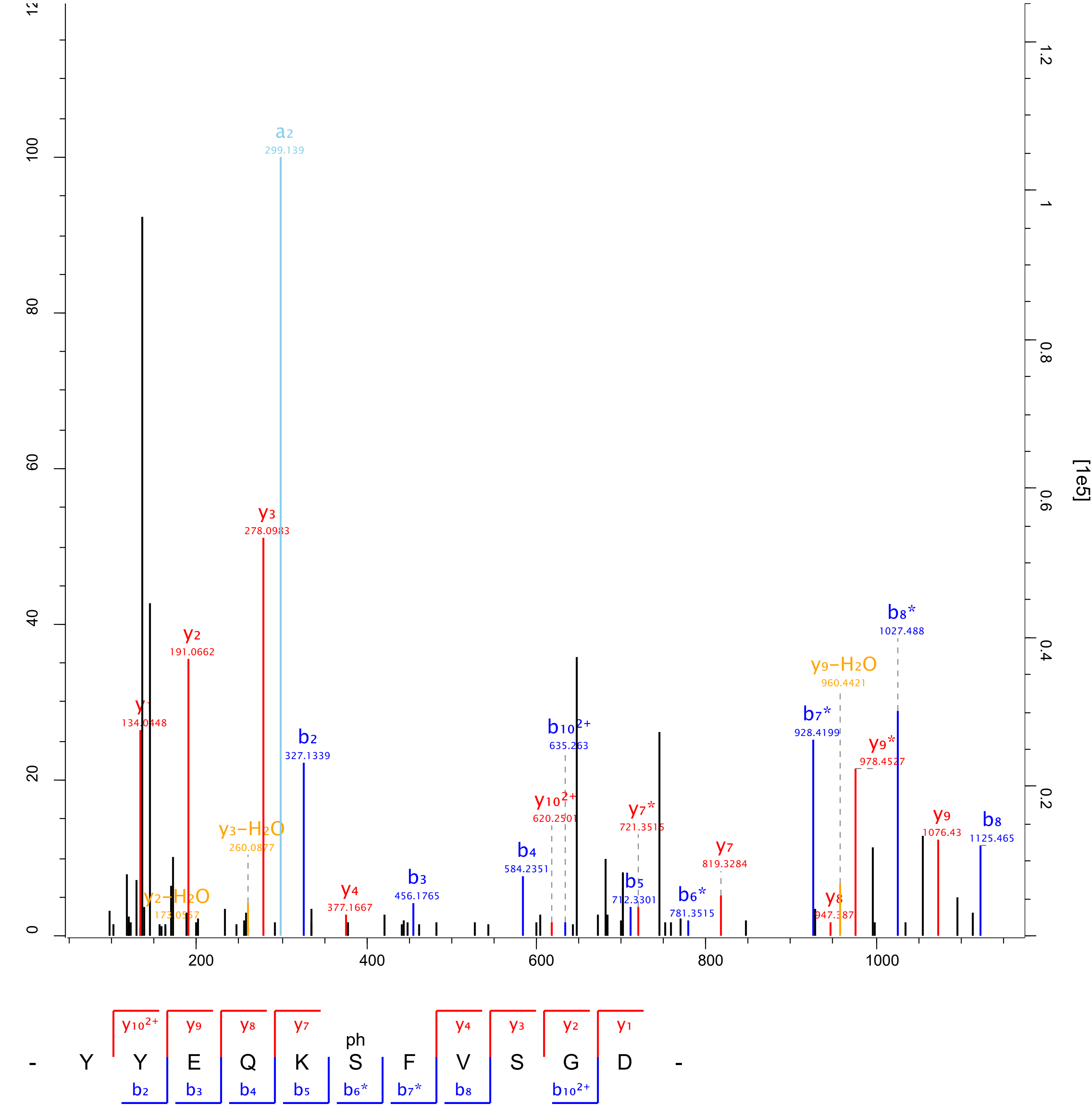

Sample output to test PDF Combine only

Raw file	Scan	Method	Score	m/z	Gene names
0523_4	11521	FTMS; HCD	57.48	880.37	PVA12

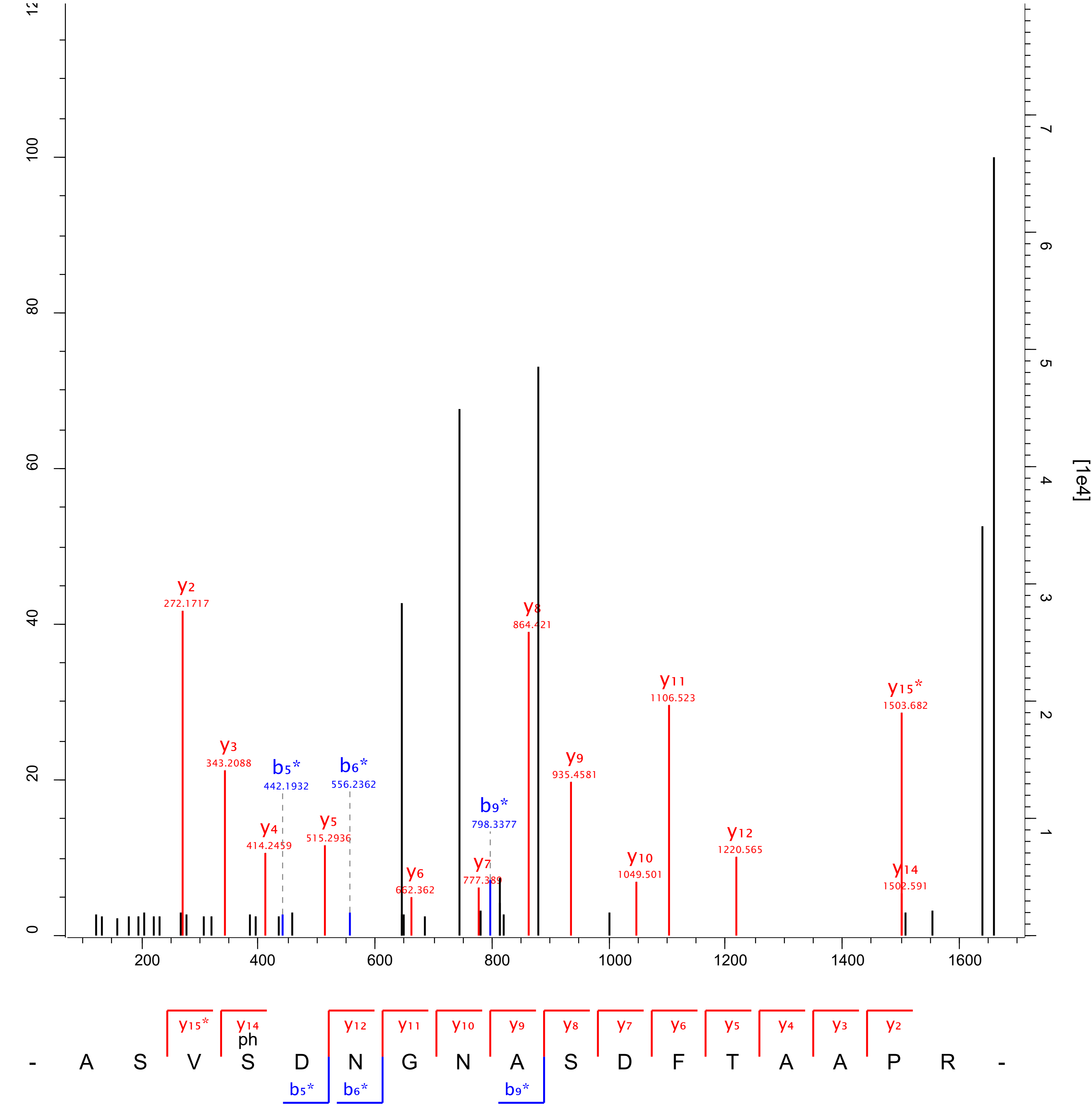

Sample output to test PDF Combine only

Raw file	Scan	Method	Score	m/z
0523_4	11640	FTMS; HCD	155.63	777.84

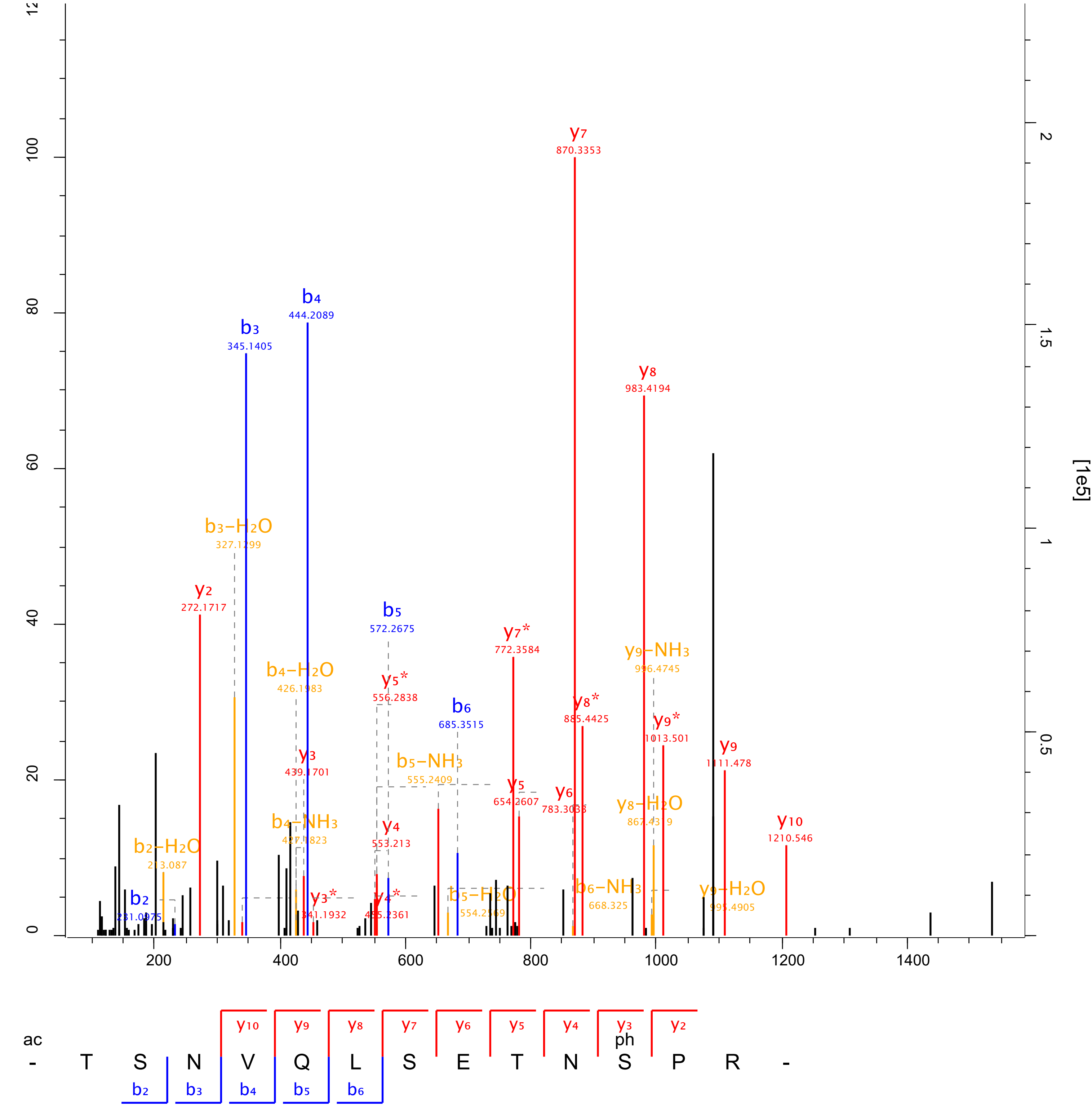

Sample output to test PDF Combine only

Raw file	Scan	Method	Score	m/z	Gene names
0523_4	11671	FTMS; HCD	424.54	971.9	ILA

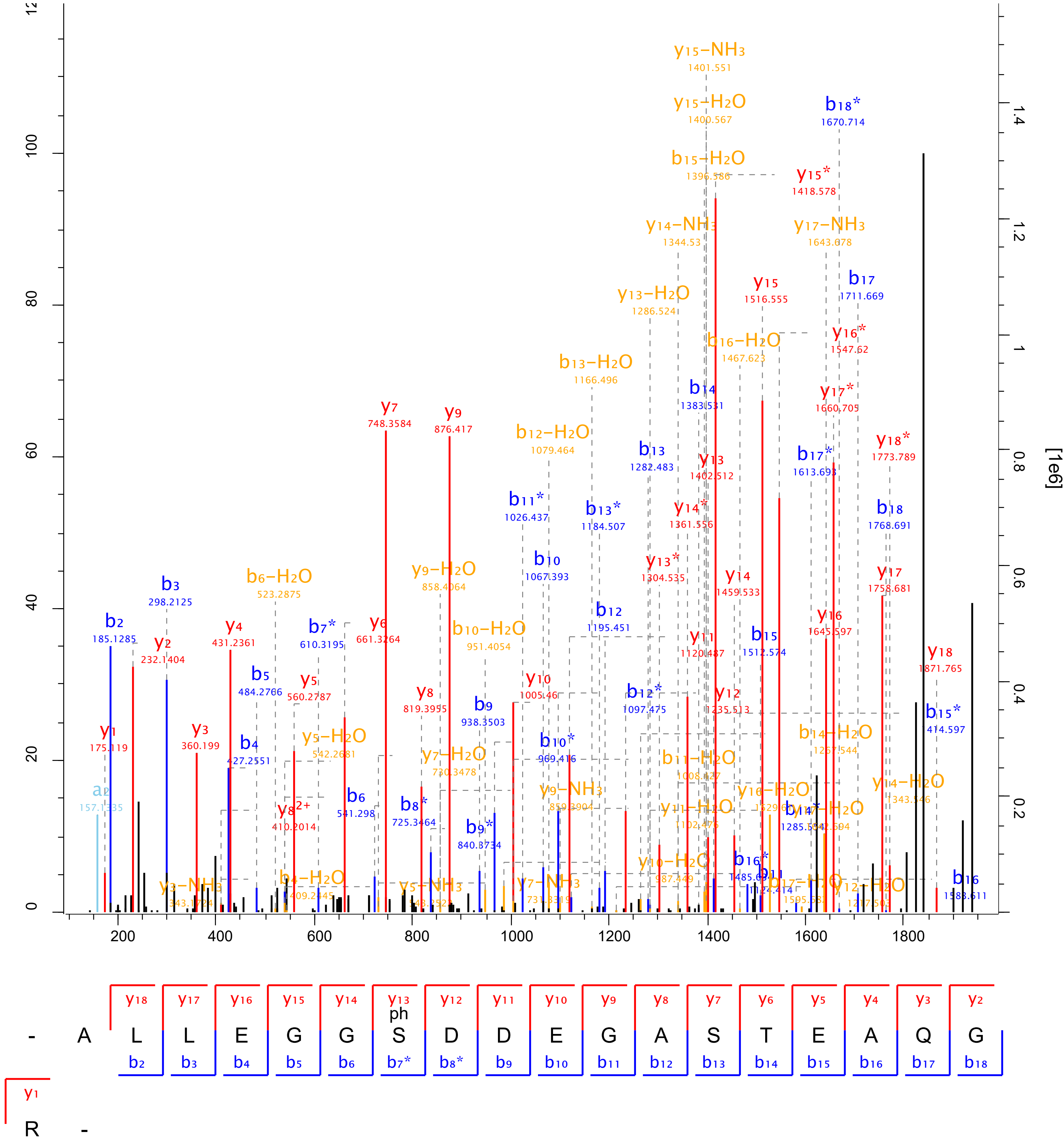

Sample output to test PDF Combine only

Raw file	Scan	Method	Score	m/z
0523_4	11694	FTMS; HCD	80.24	531.7

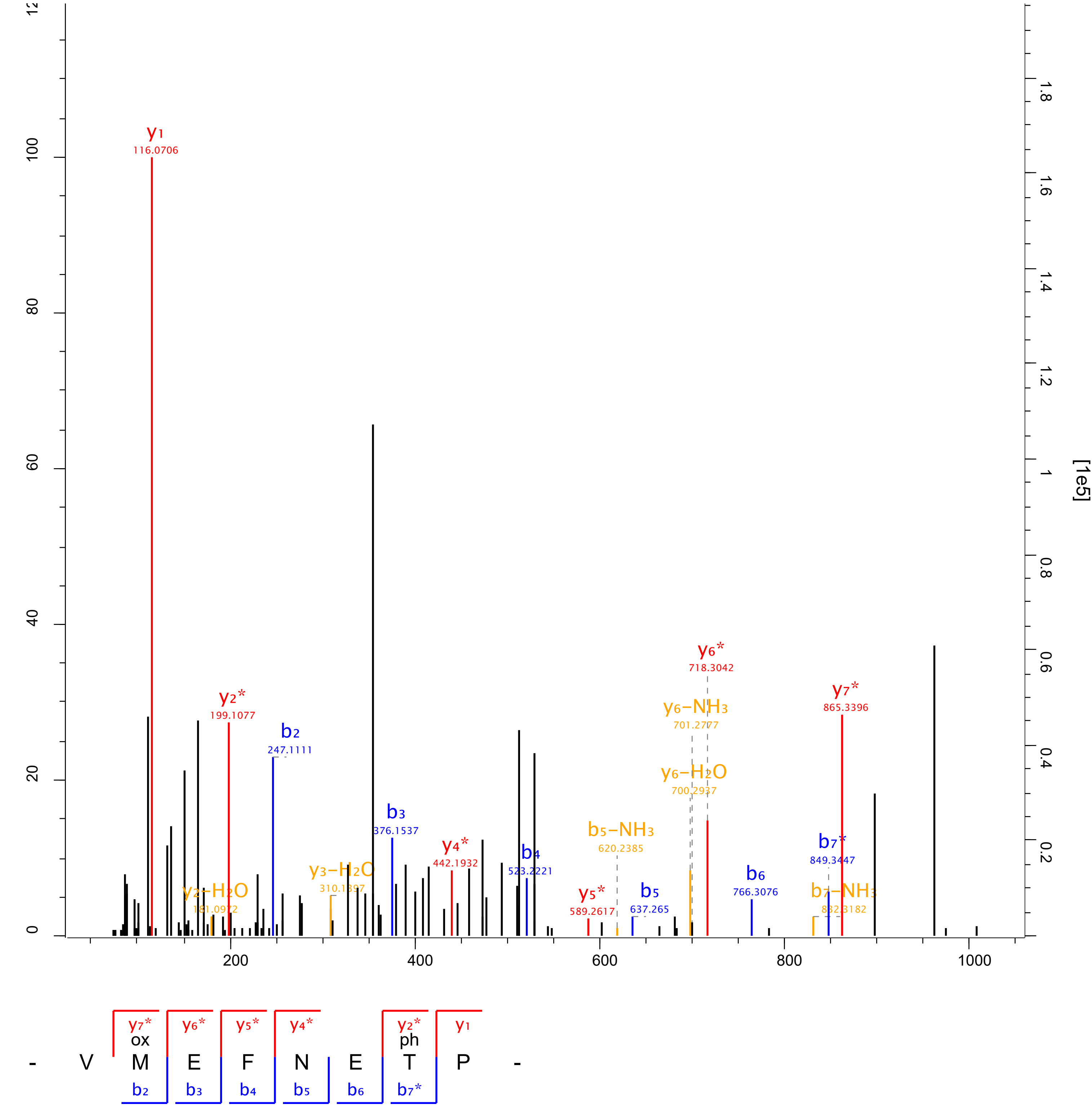

Sample output to test PDF Combine only

Raw file	Scan	Method	Score	m/z	Gene names
0523_4	11754	FTMS; HCD	202.62	818.88	RPS2C

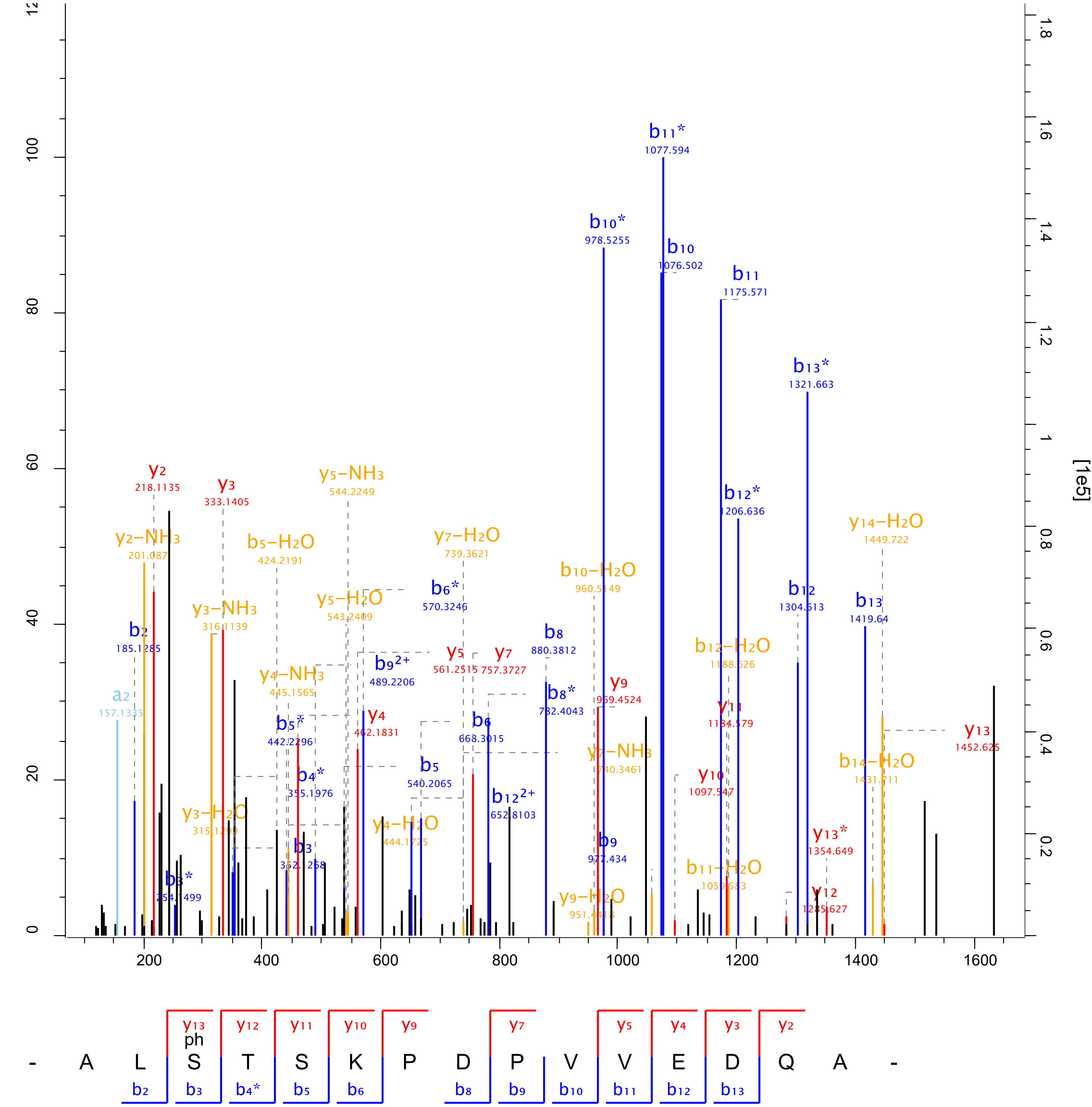

Sample output to test PDF Combine only

Raw file	Scan	Method	Score	m/z	Gene names
0523_4	11795	FTMS; HCD	60.73	642.29	F23H11.2

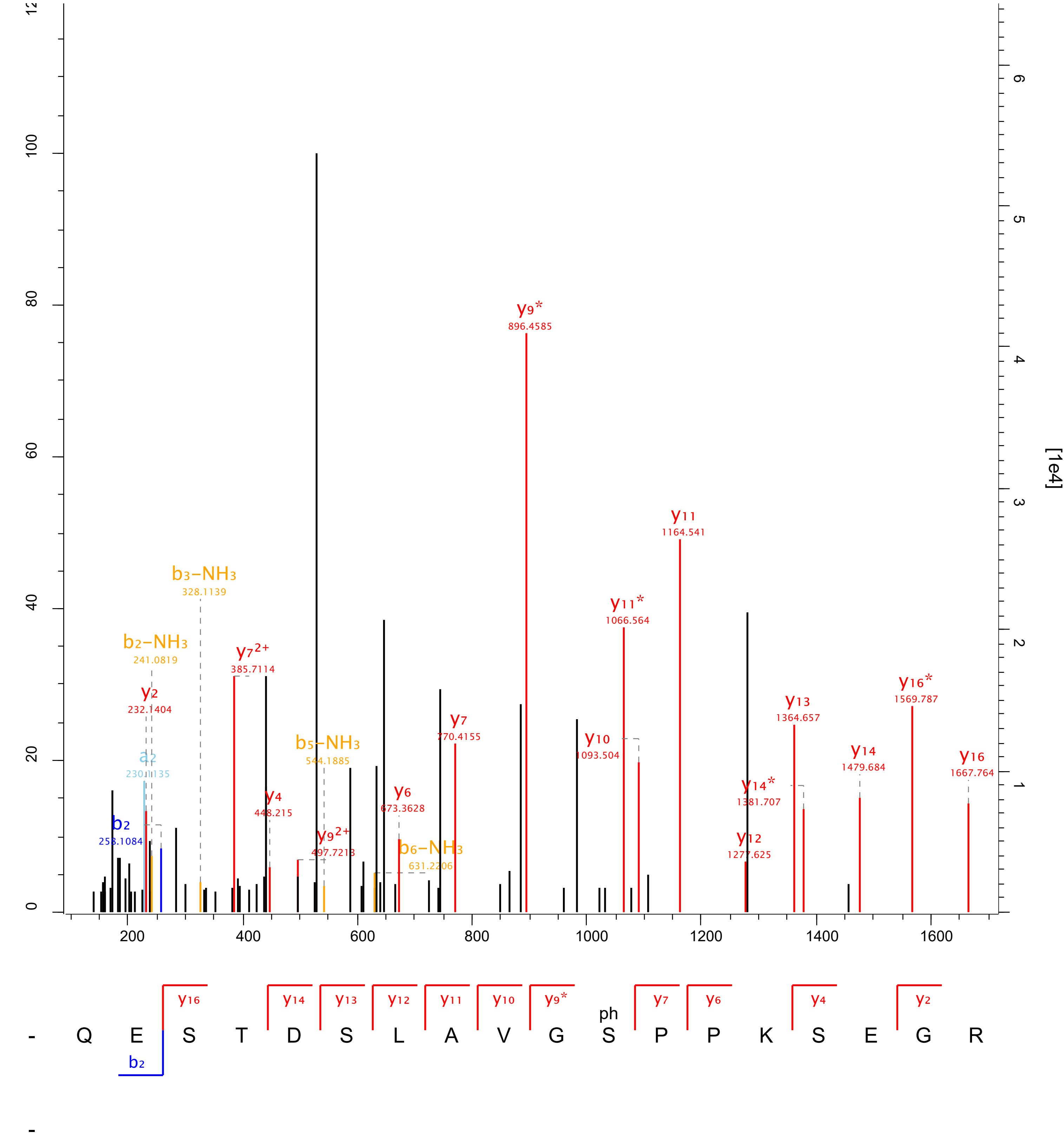

Sample output to test PDF Combine only

Raw file	Scan	Method	Score	m/z	Gene names
0523_4	11830	FTMS; HCD	174.39	725.84	TOM2AH2

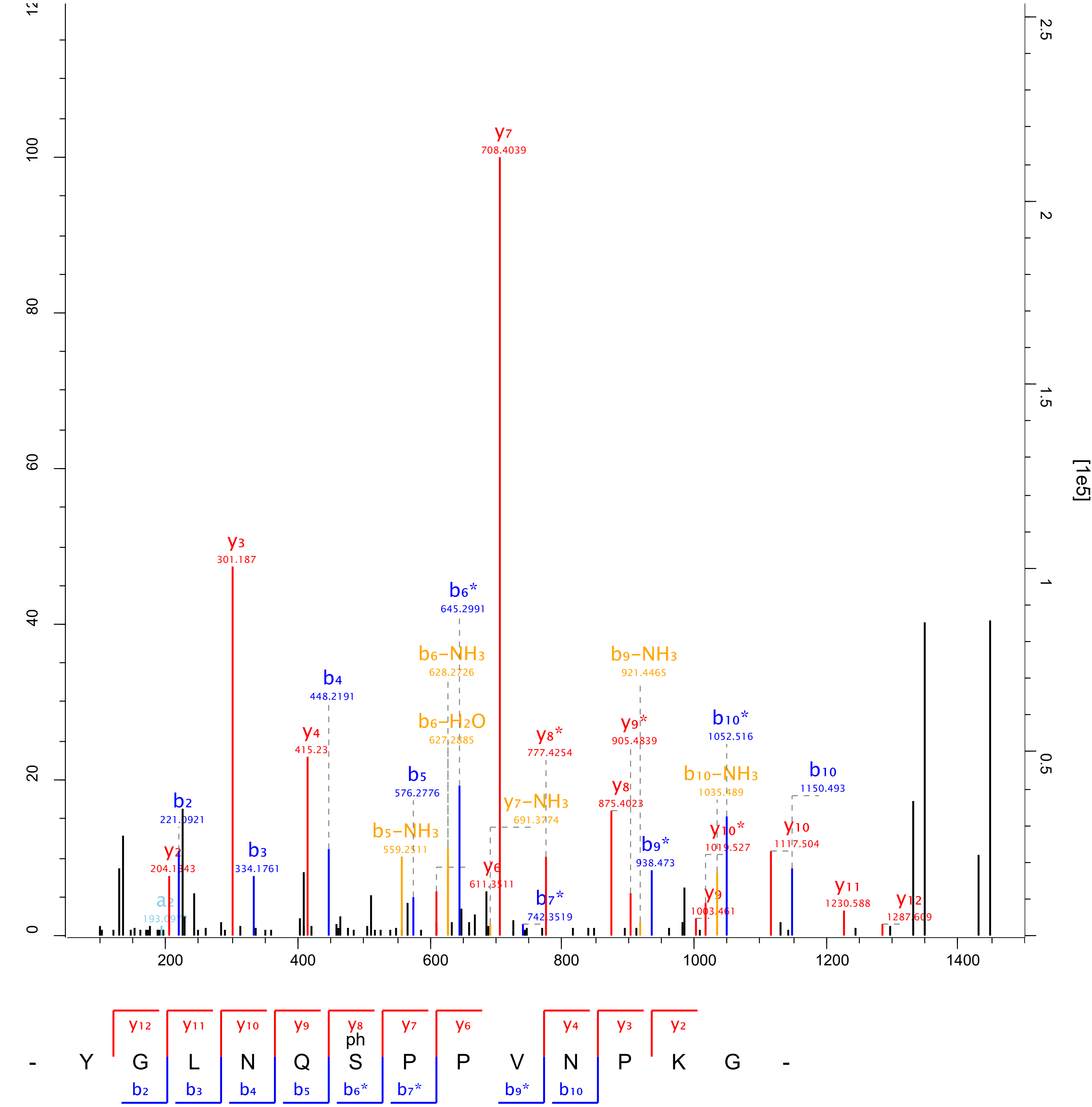

Sample output to test PDF Combine only

Raw file	Scan	Method	Score	m/z	Gene names
0523_4	11916	FTMS; HCD	110.12	786.34	CPK8

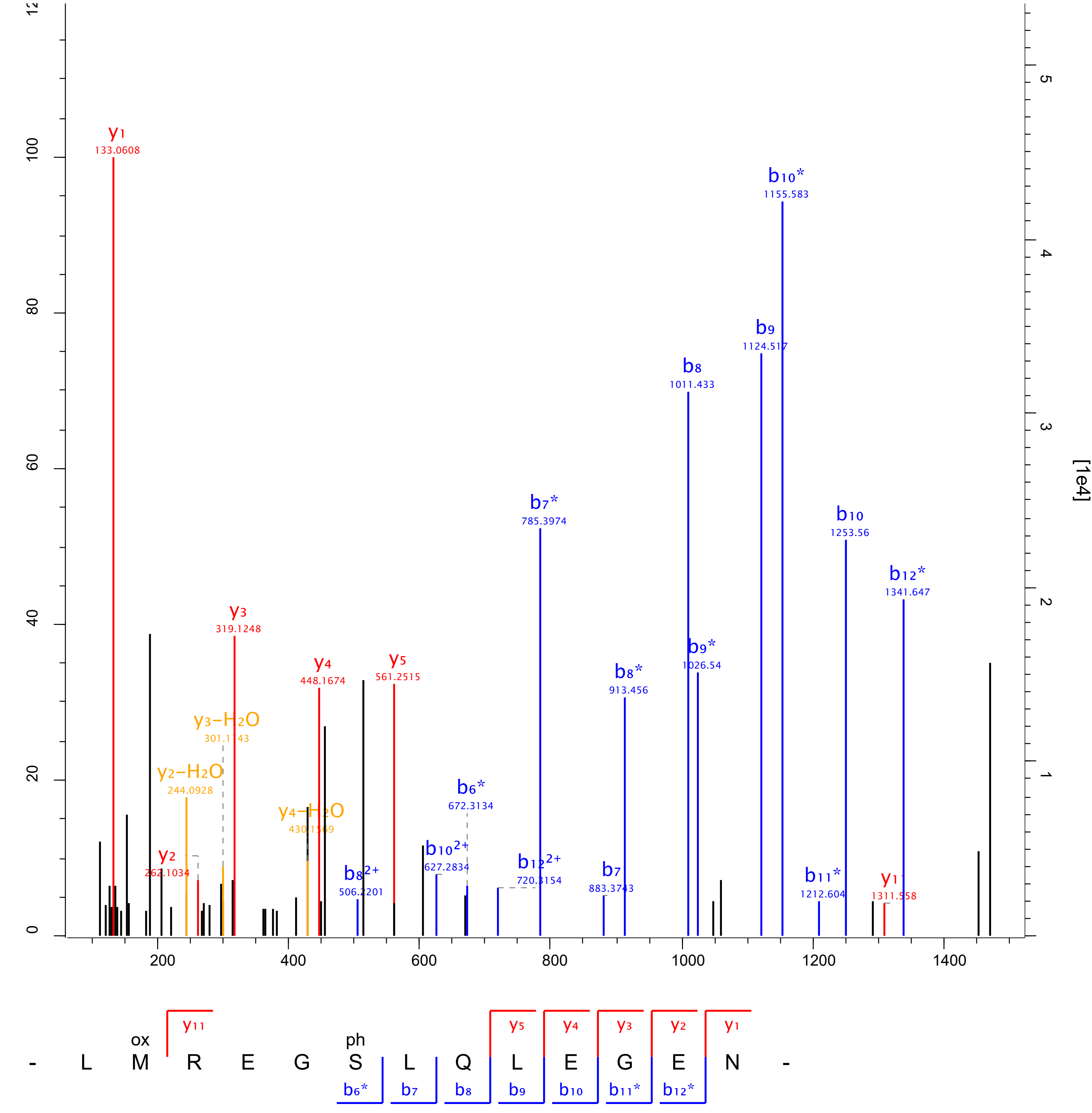

Sample output to test PDF Combine only

Raw file	Scan	Method	Score	m/z	Gene names
0523_4	11933	FTMS; HCD	55.35	464.17	RBOHB

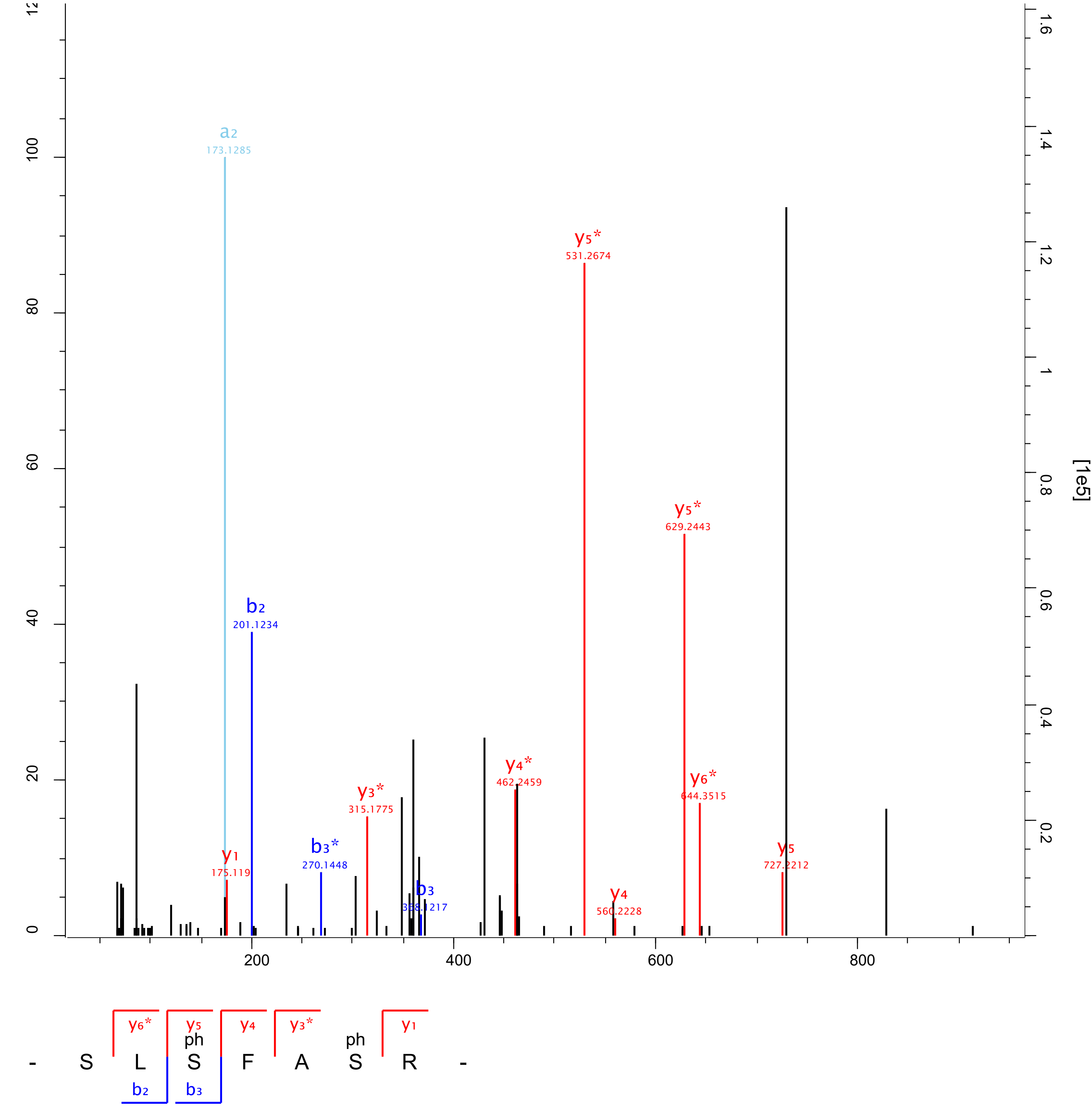

Sample output to test PDF Combine only

Raw file	Scan	Method	Score	m/z	Gene names
0523_4	12004	FTMS; HCD	187.79	980.43	PCR8

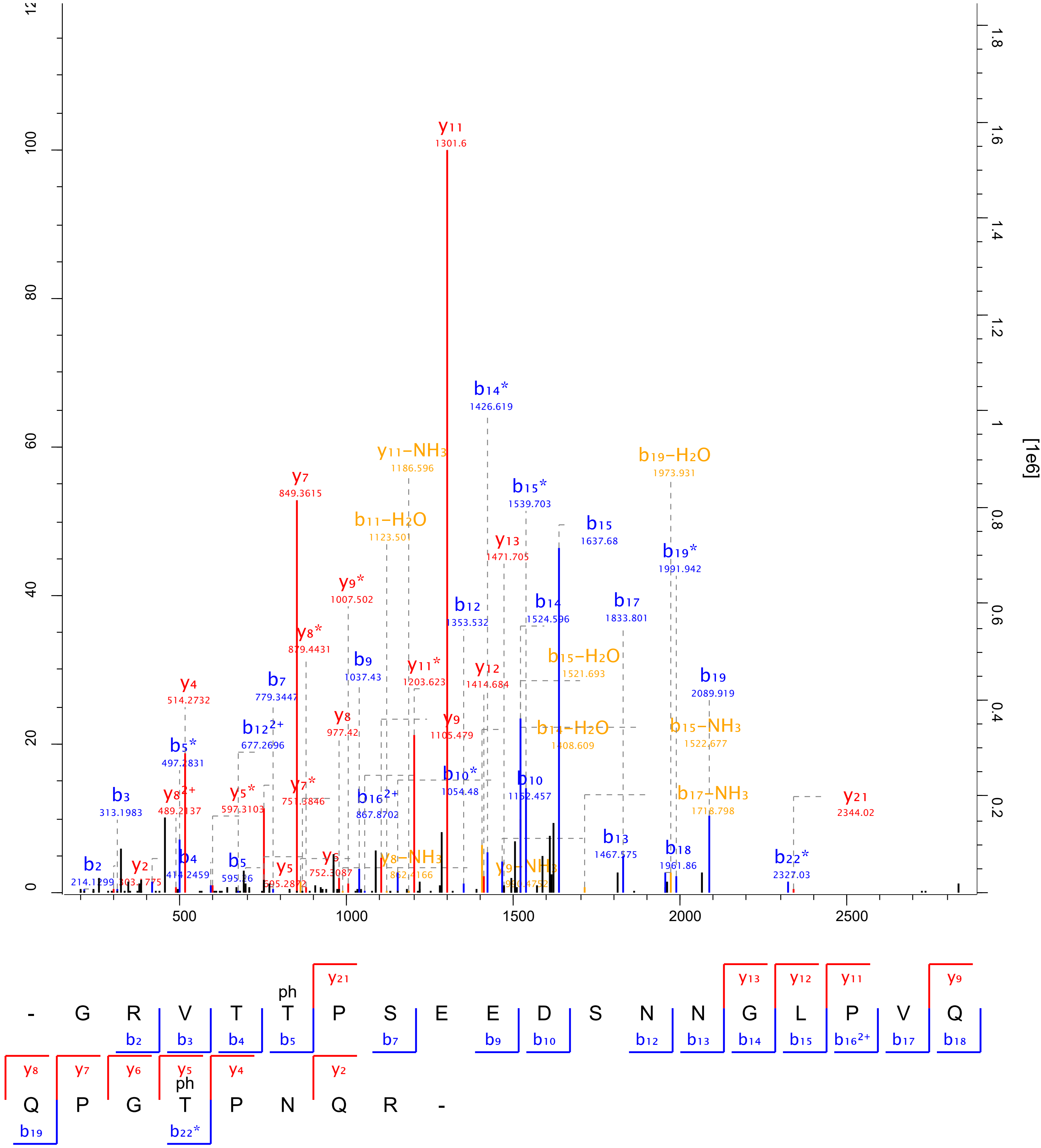

Sample output to test PDF Combine only

Raw file	Scan	Method	Score	m/z	Gene names
0523_4	12136	FTMS; HCD	125.06	679.64	At1g62330

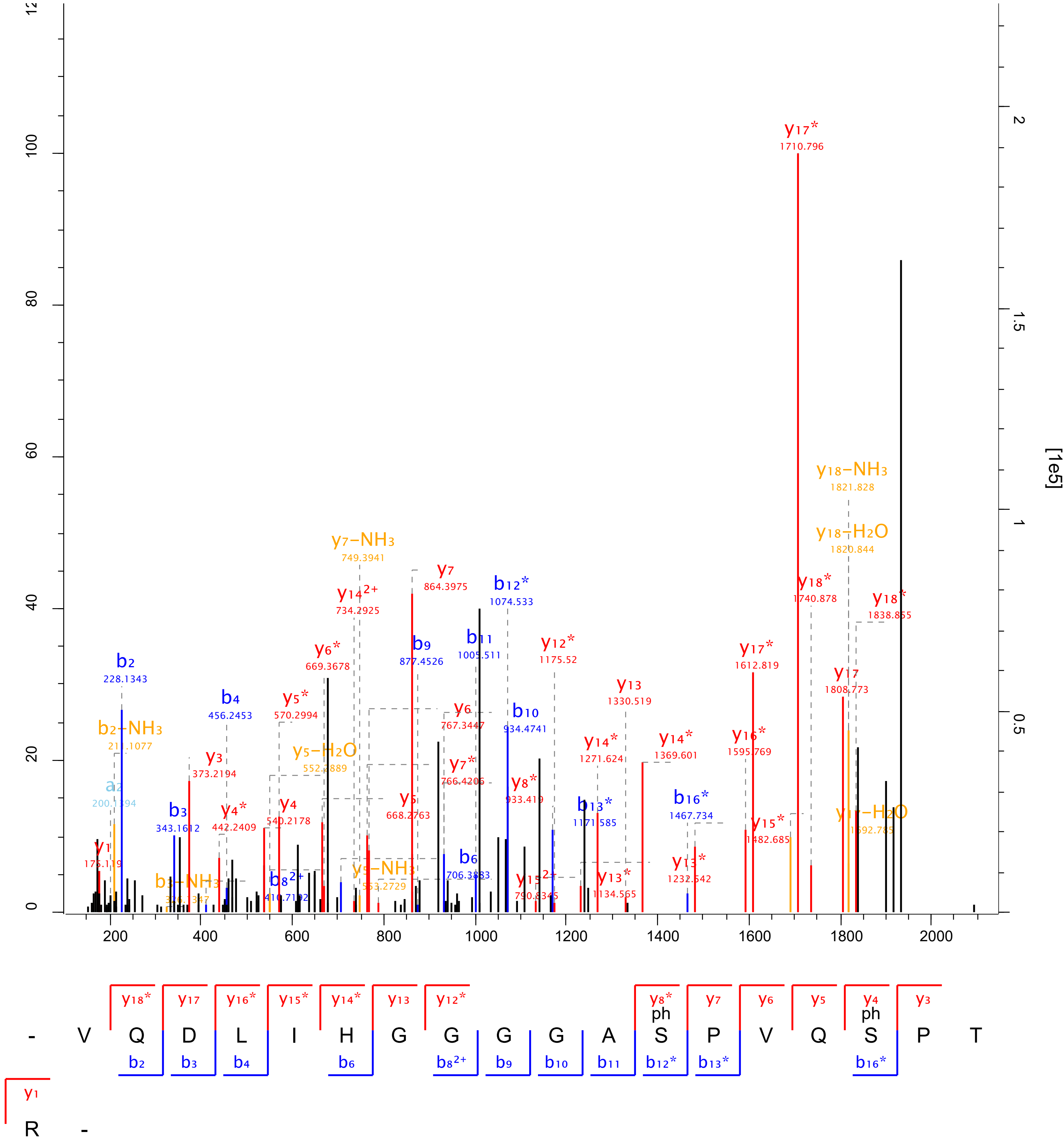

Sample output to test PDF Combine only

Raw file	Scan	Method	Score	m/z	Gene names
0523_4	12154	FTMS; HCD	59.54	751.67	CCT1

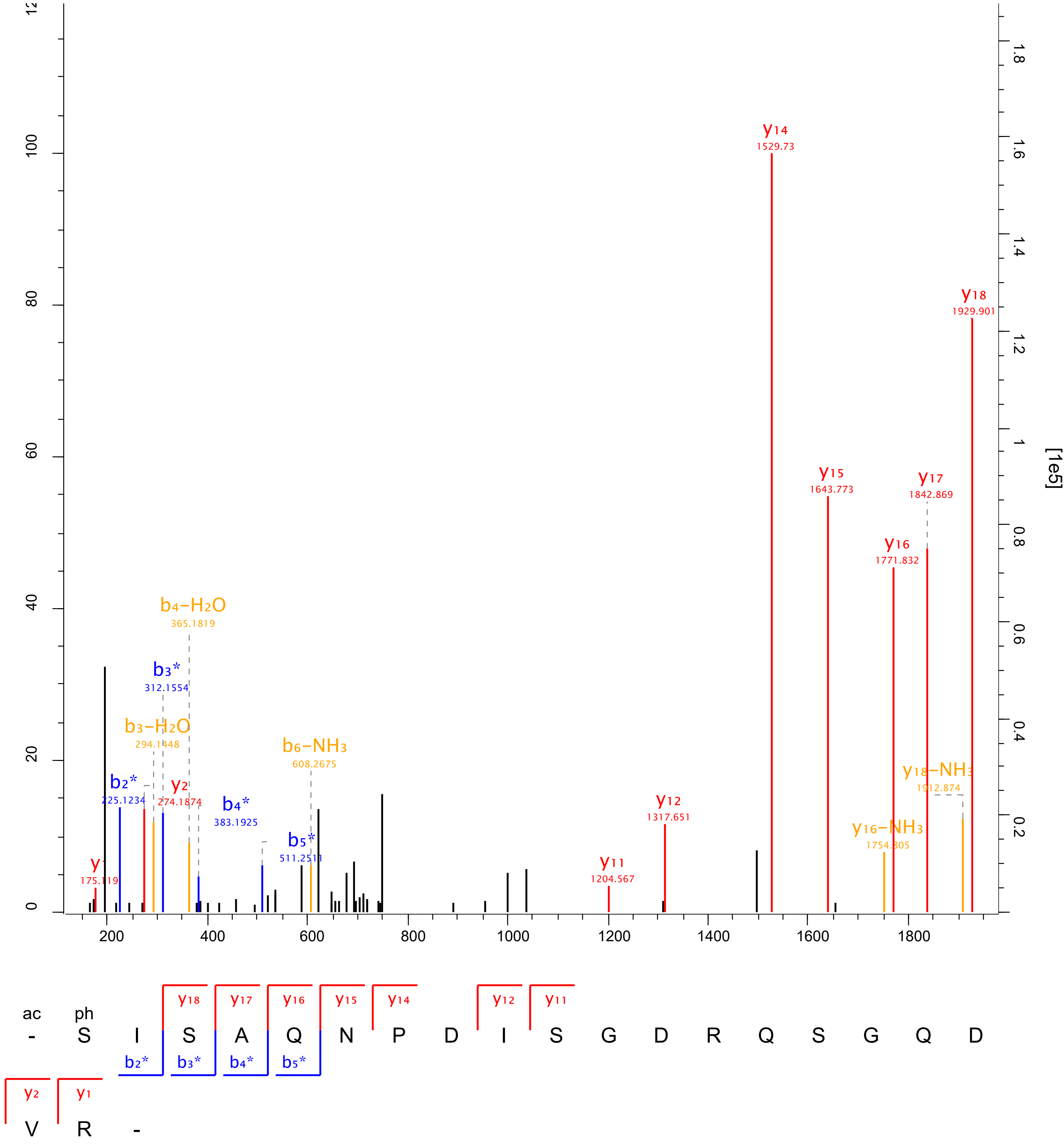

Sample output to test PDF Combine only

Raw file	Scan	Method	Score	m/z	Gene names
0523_4	12300	FTMS; HCD	74.99	550.77	LECRK64

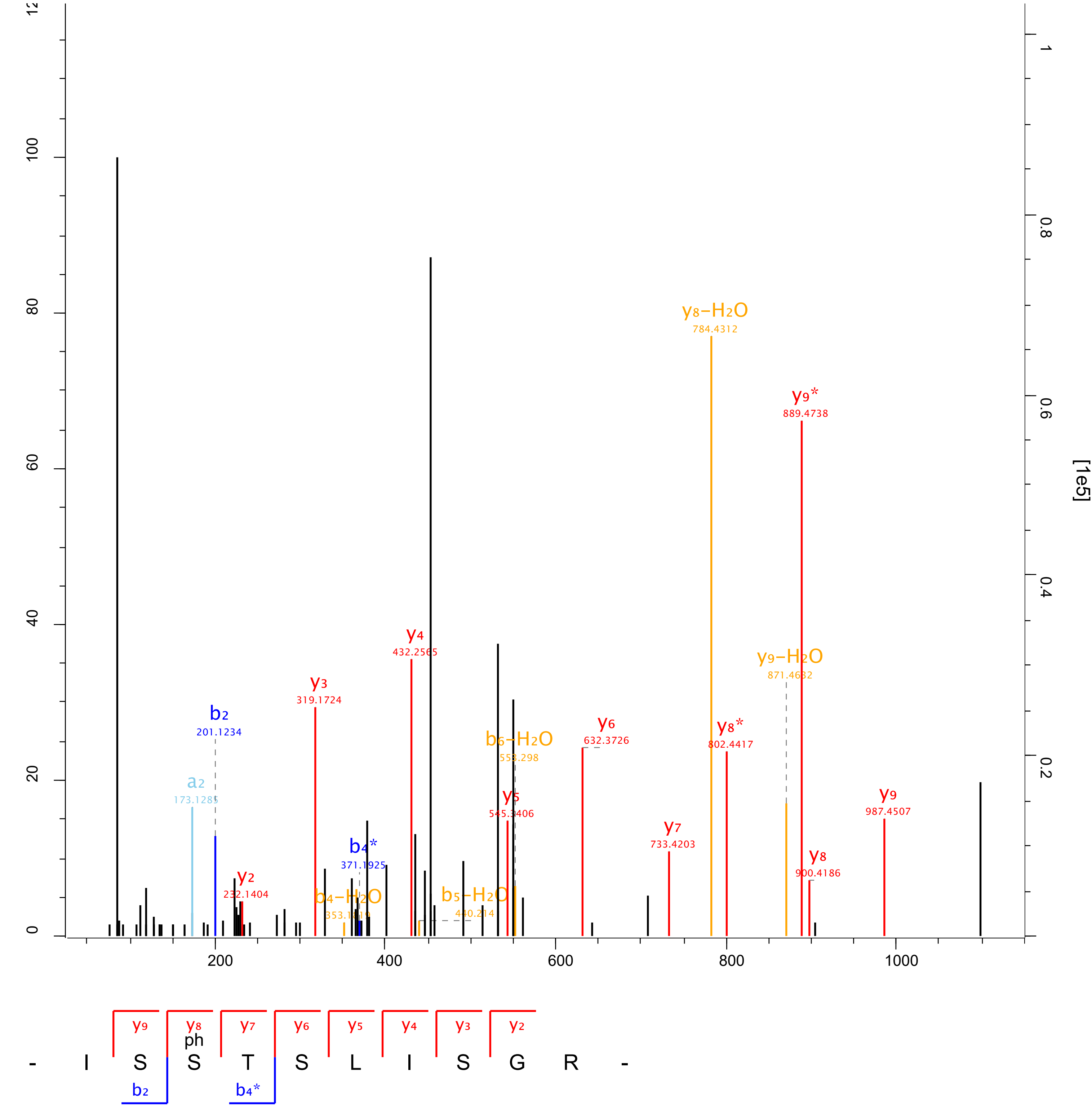

Sample output to test PDF Combine only

Raw file	Scan	Method	Score	m/z	Gene names
0523_4	12303	FTMS; HCD	112.67	718.79	PIRL4

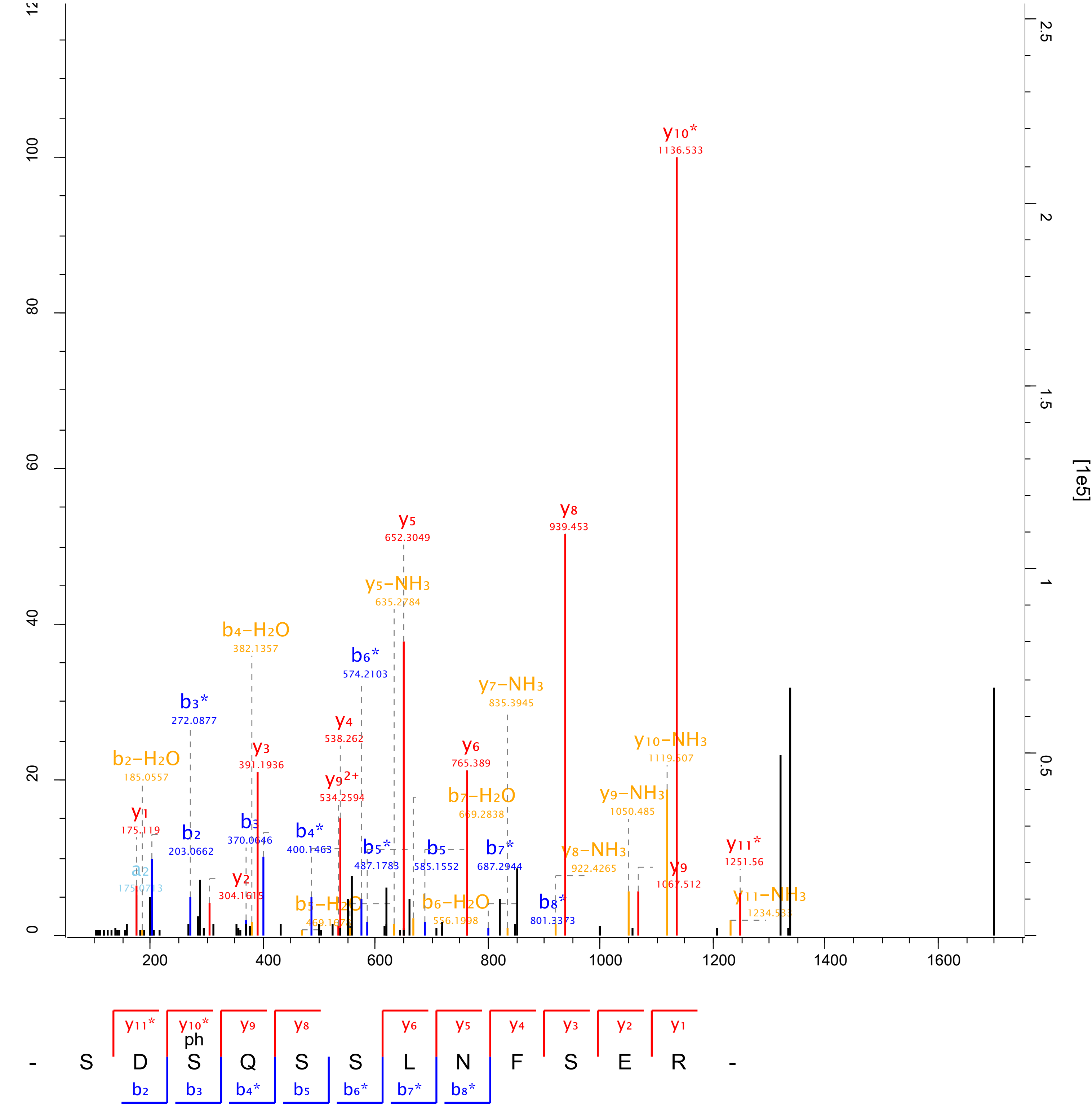

Sample output to test PDF Combine only

Raw file	Scan	Method	Score	m/z	Gene names
0523_4	12827	FTMS; HCD	137.81	622.77	CPK6

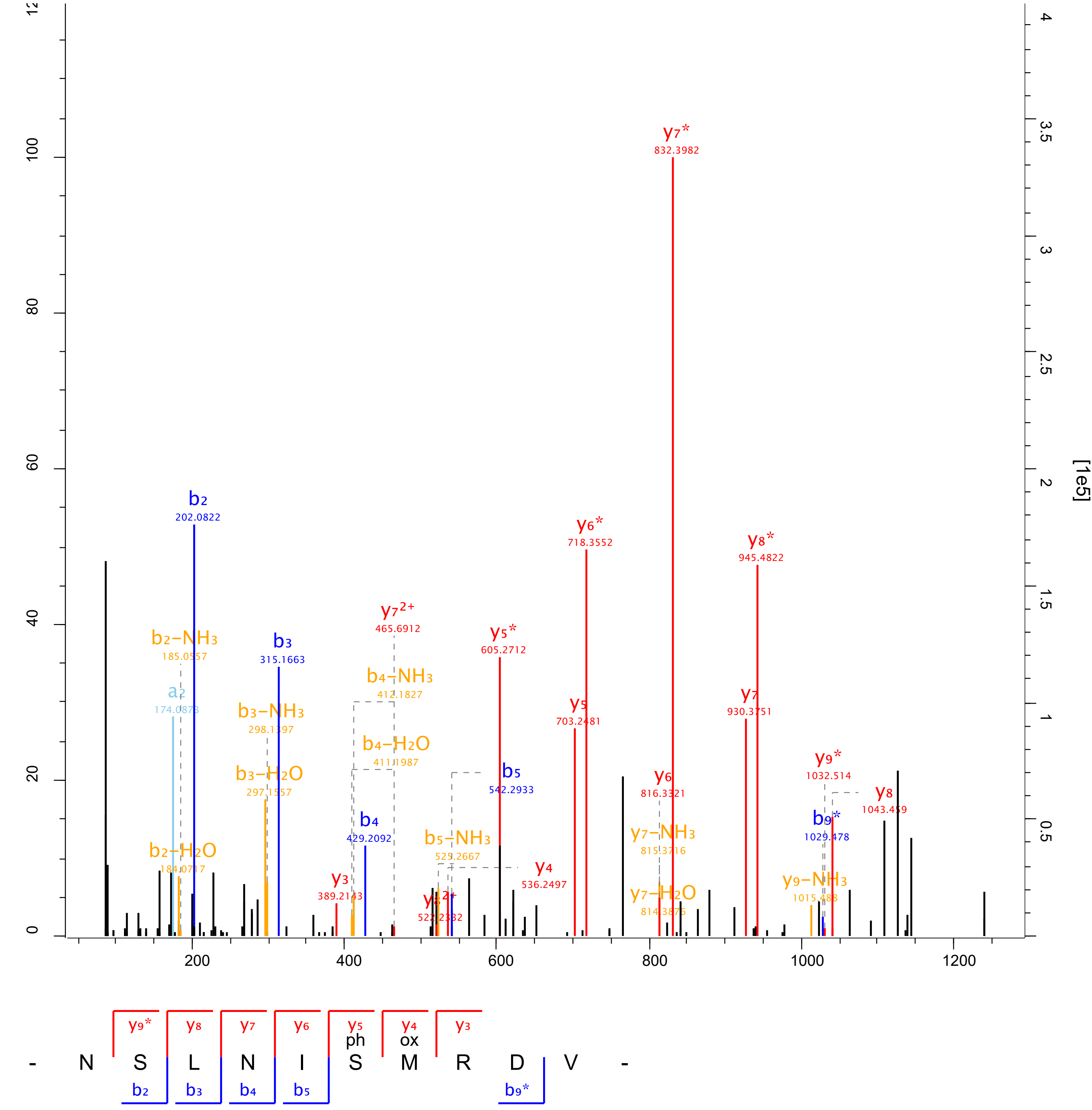

Sample output to test PDF Combine only

Raw file	Scan	Method	Score	m/z	Gene names
0523_4	12848	FTMS; HCD	265.56	737.3	F5A18.5

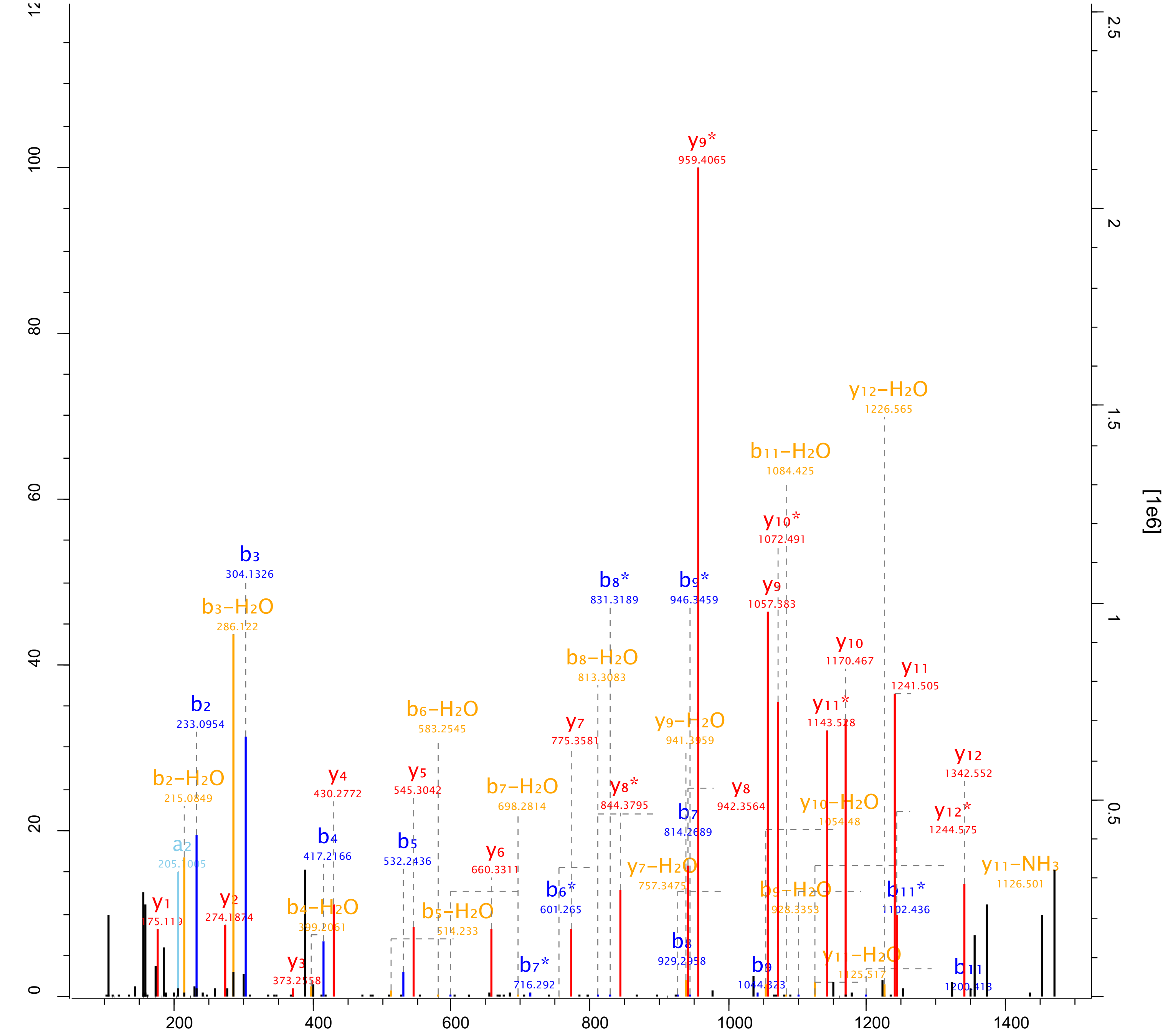

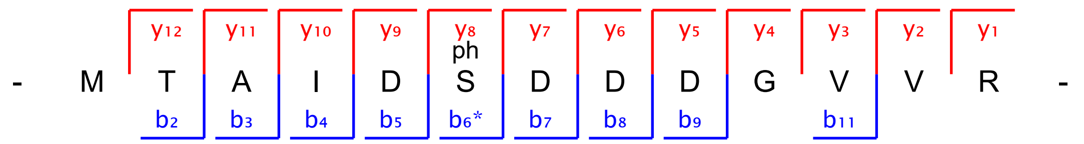

Sample output to test PDF Combine only

Raw file	Scan	Method	Score	m/z	Gene names
0523_4	12930	FTMS; HCD	47.55	707.82	At1g69980

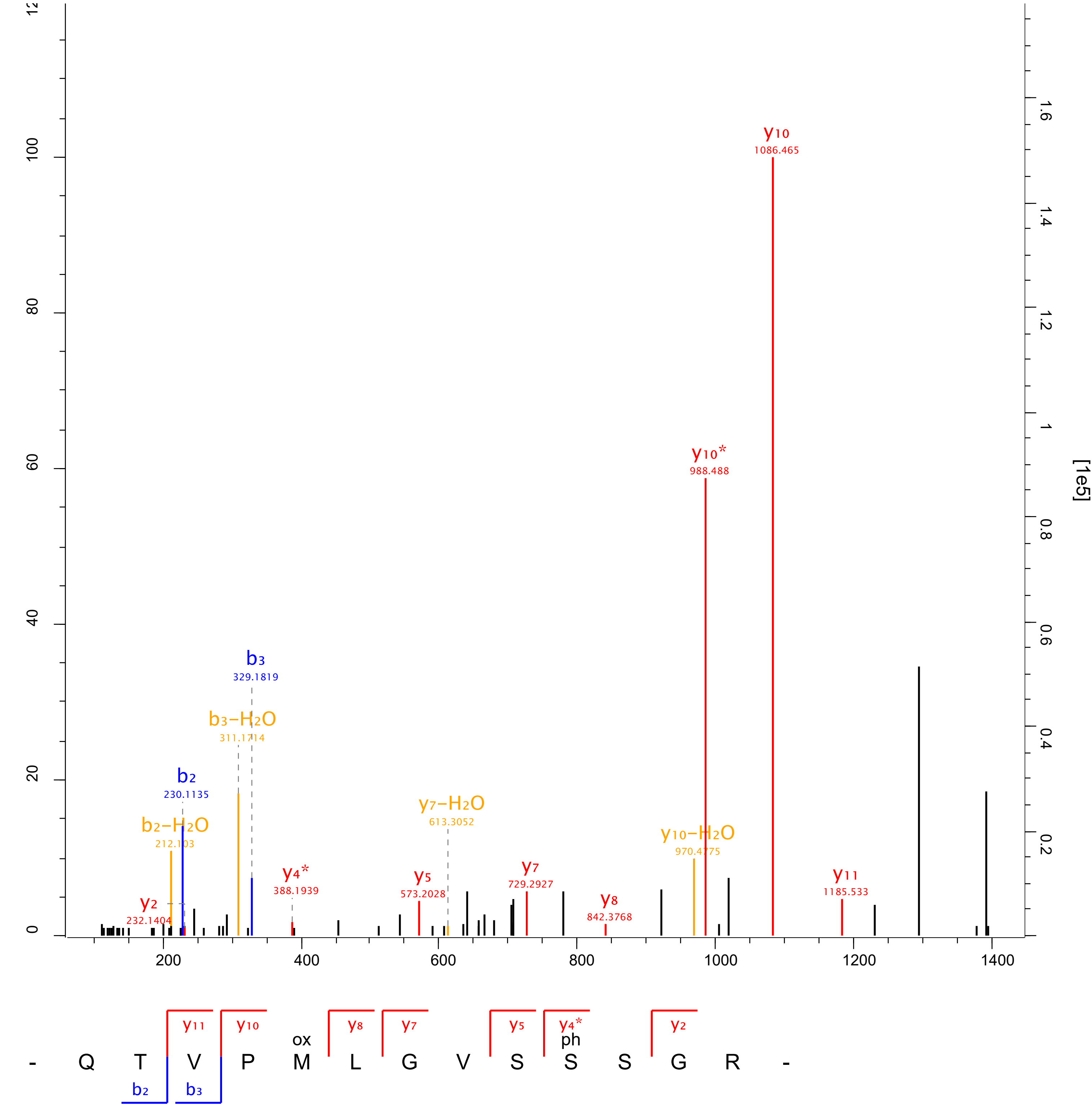

Sample output to test PDF Combine only

Raw file	Scan	Method	Score	m/z
0523_4	13066	FTMS; HCD	84.17	811.81

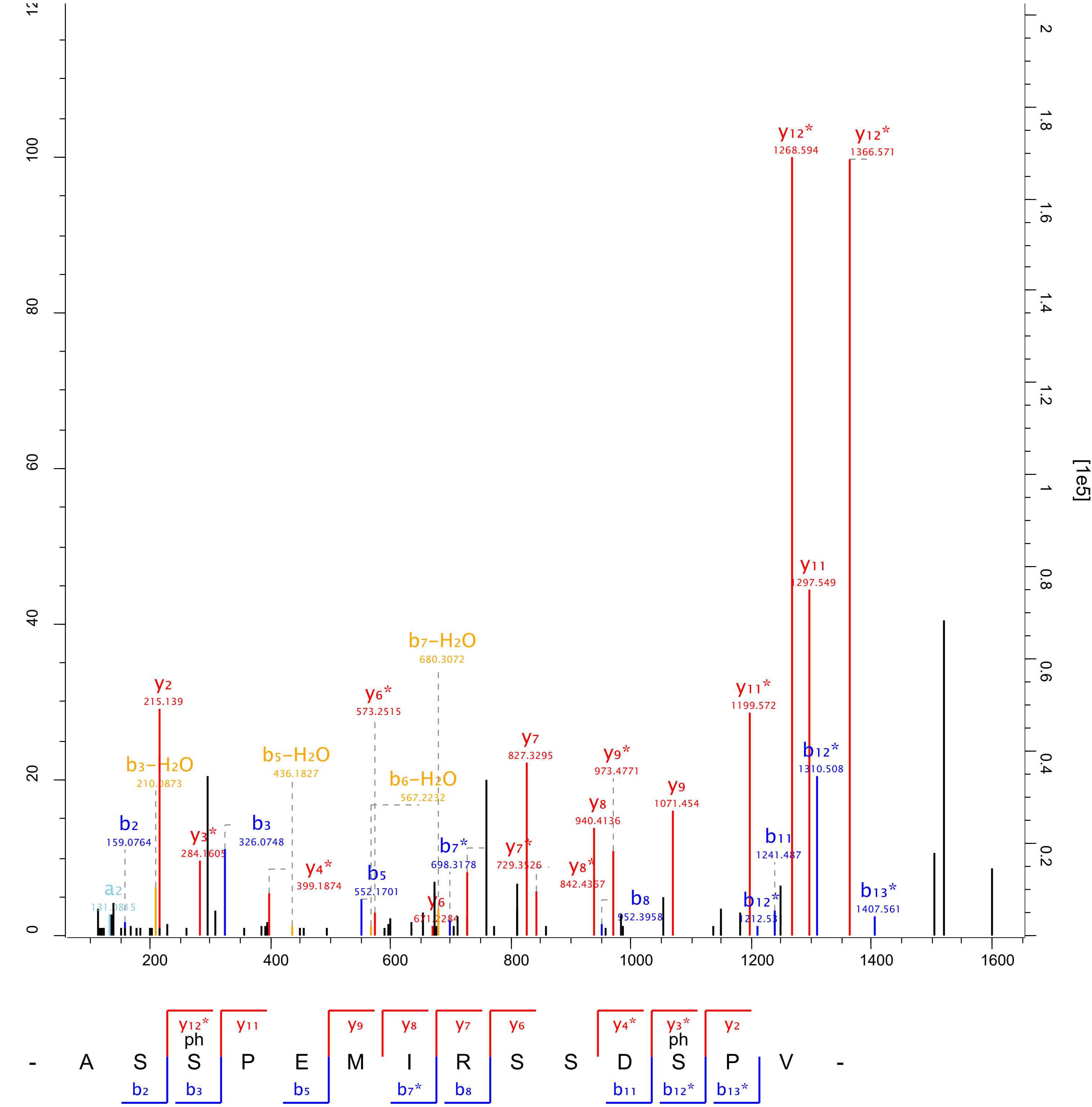

Sample output to test PDF Combine only

Raw file	Scan	Method	Score	m/z	Gene names
0523_4	13183	FTMS; HCD	60.17	855.36	At4g17140

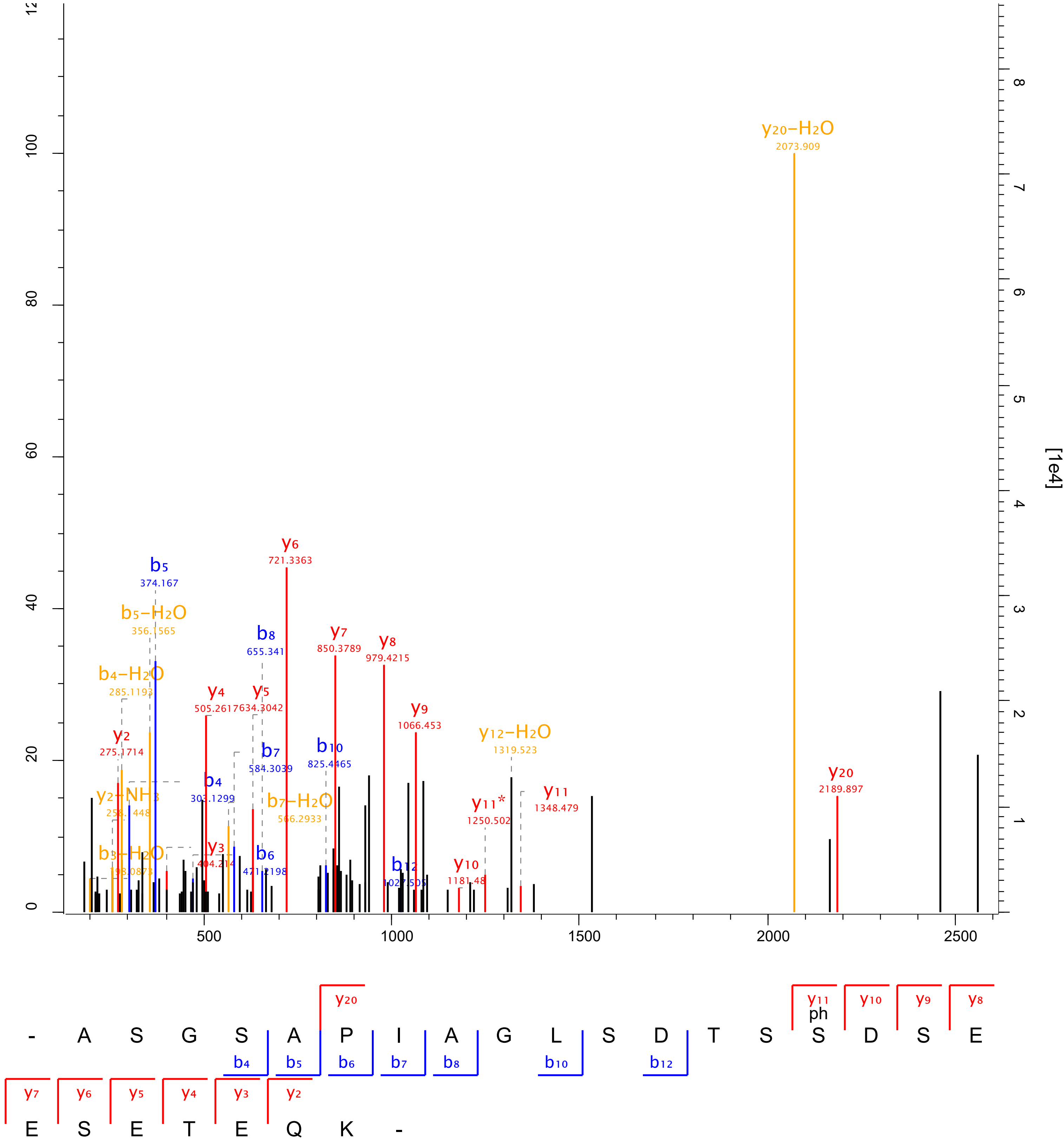

Sample output to test PDF Combine only

Raw file	Scan	Method	Score	m/z
0523_4	13185	FTMS; HCD	131.06	544.19

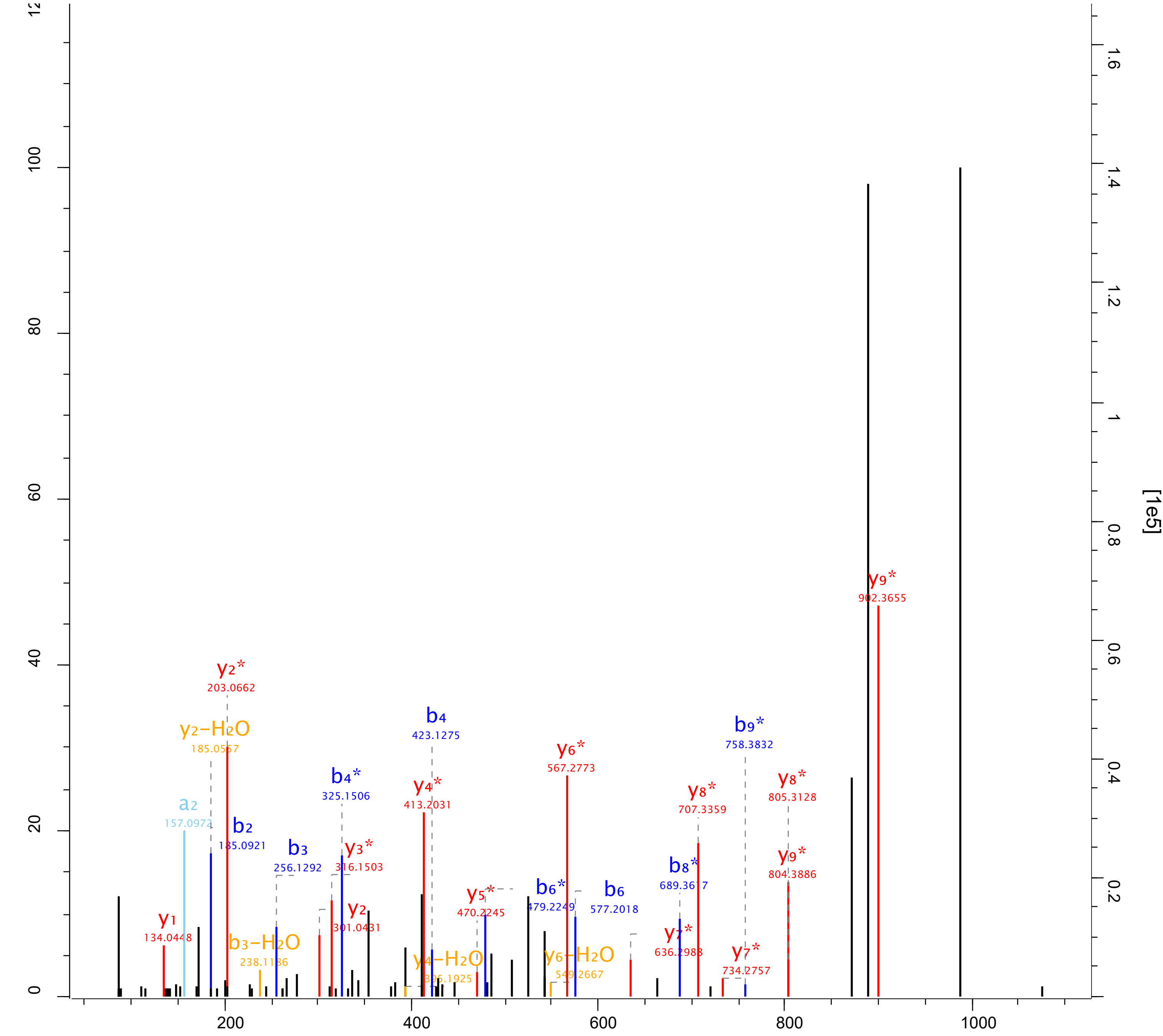

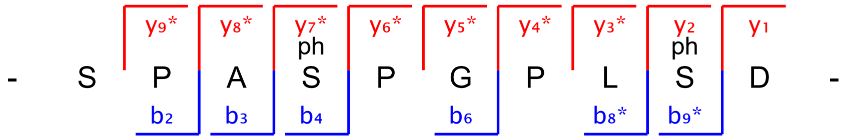

Sample output to test PDF Combine only

Raw file	Scan	Method	Score	m/z	Gene names
0523_4	13188	FTMS; HCD	159.77	578.23	CPK8

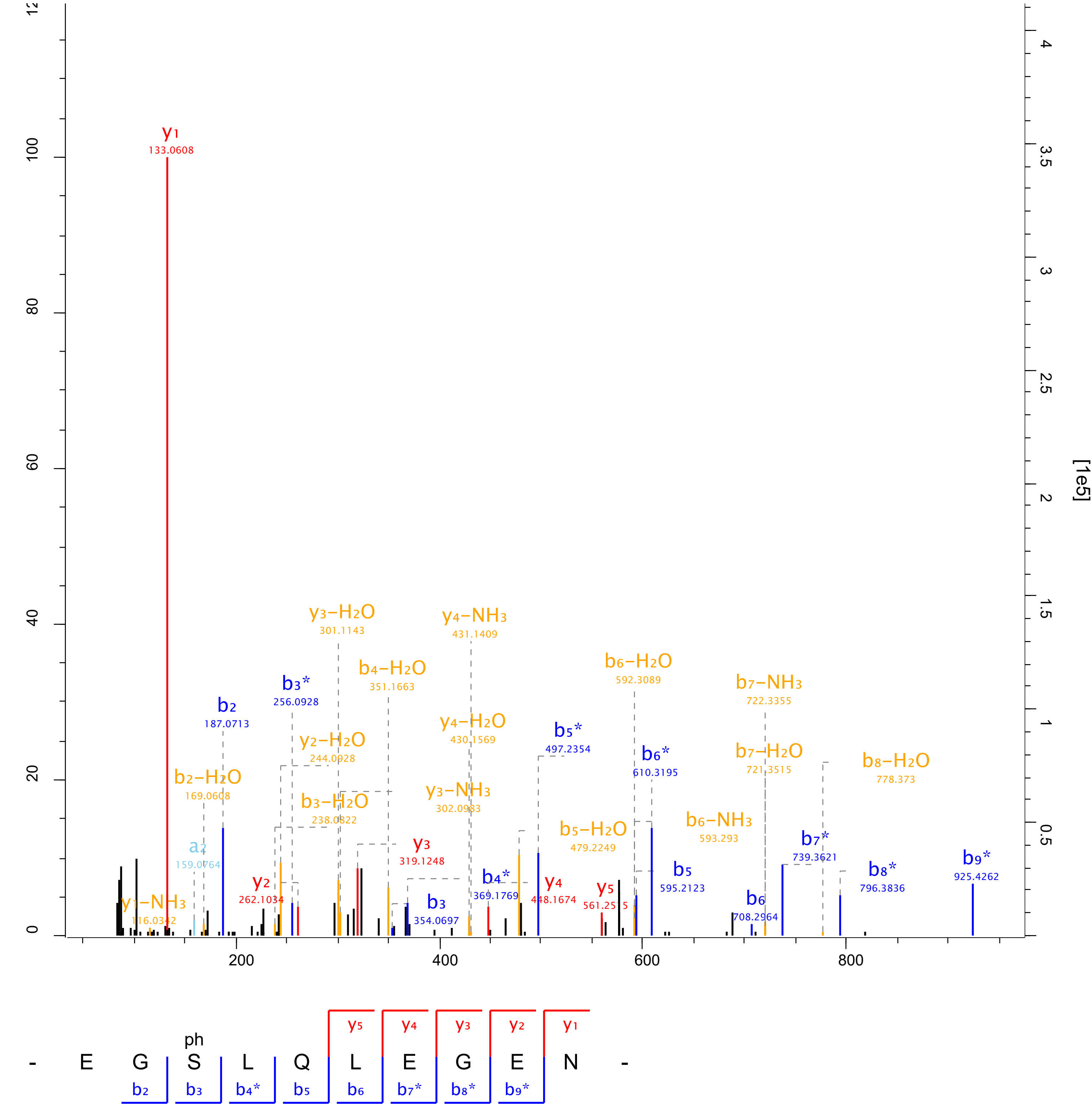

Sample output to test PDF Combine only

Raw file	Scan	Method	Score	m/z	Gene names
0523_4	13335	FTMS; HCD	78.98	763.33	At5g13890

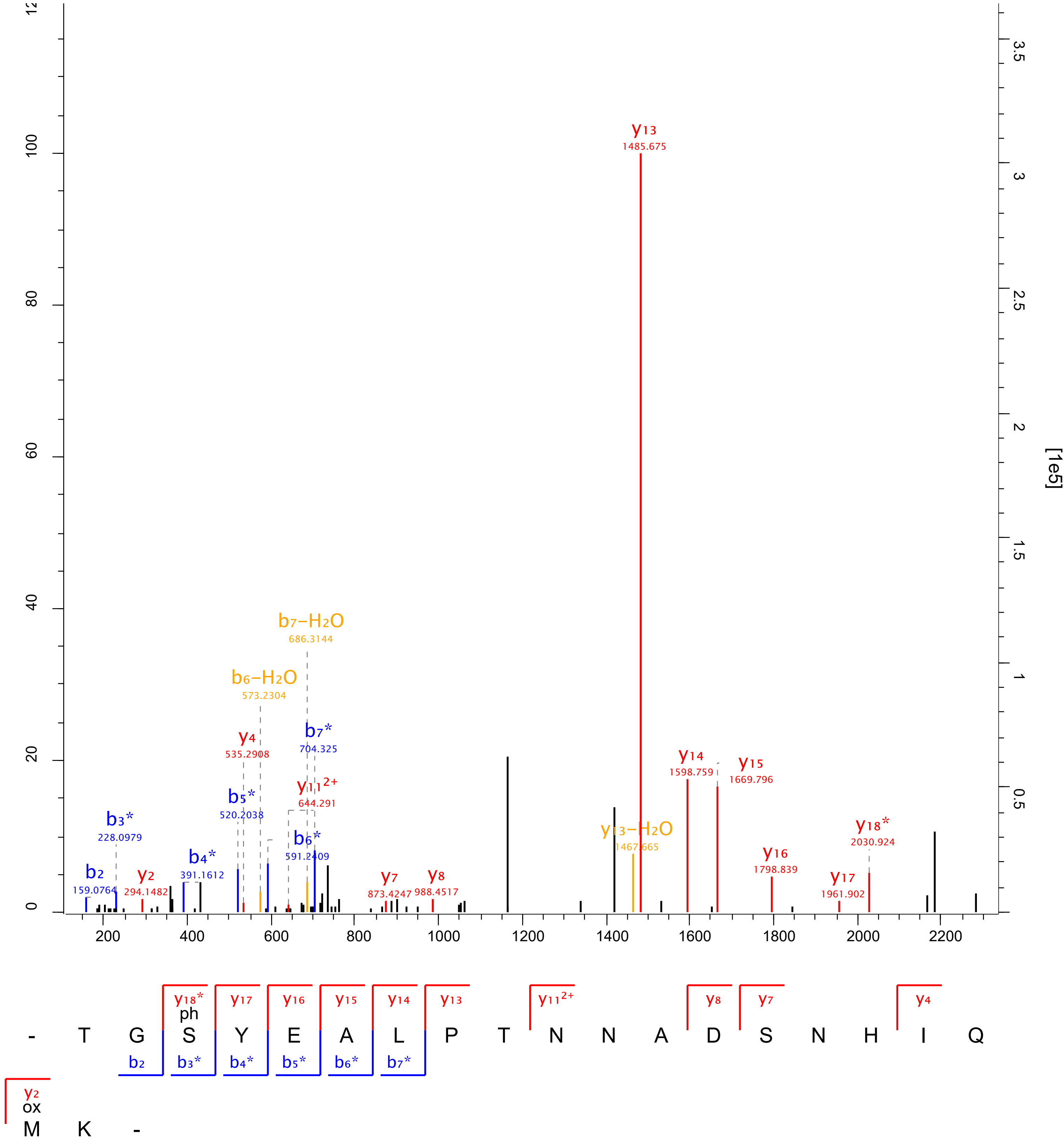

Sample output to test PDF Combine only

Raw file	Scan	Method	Score	m/z	Gene names
0523_4	13365	FTMS; HCD	77.78	694.33	TOM7-1

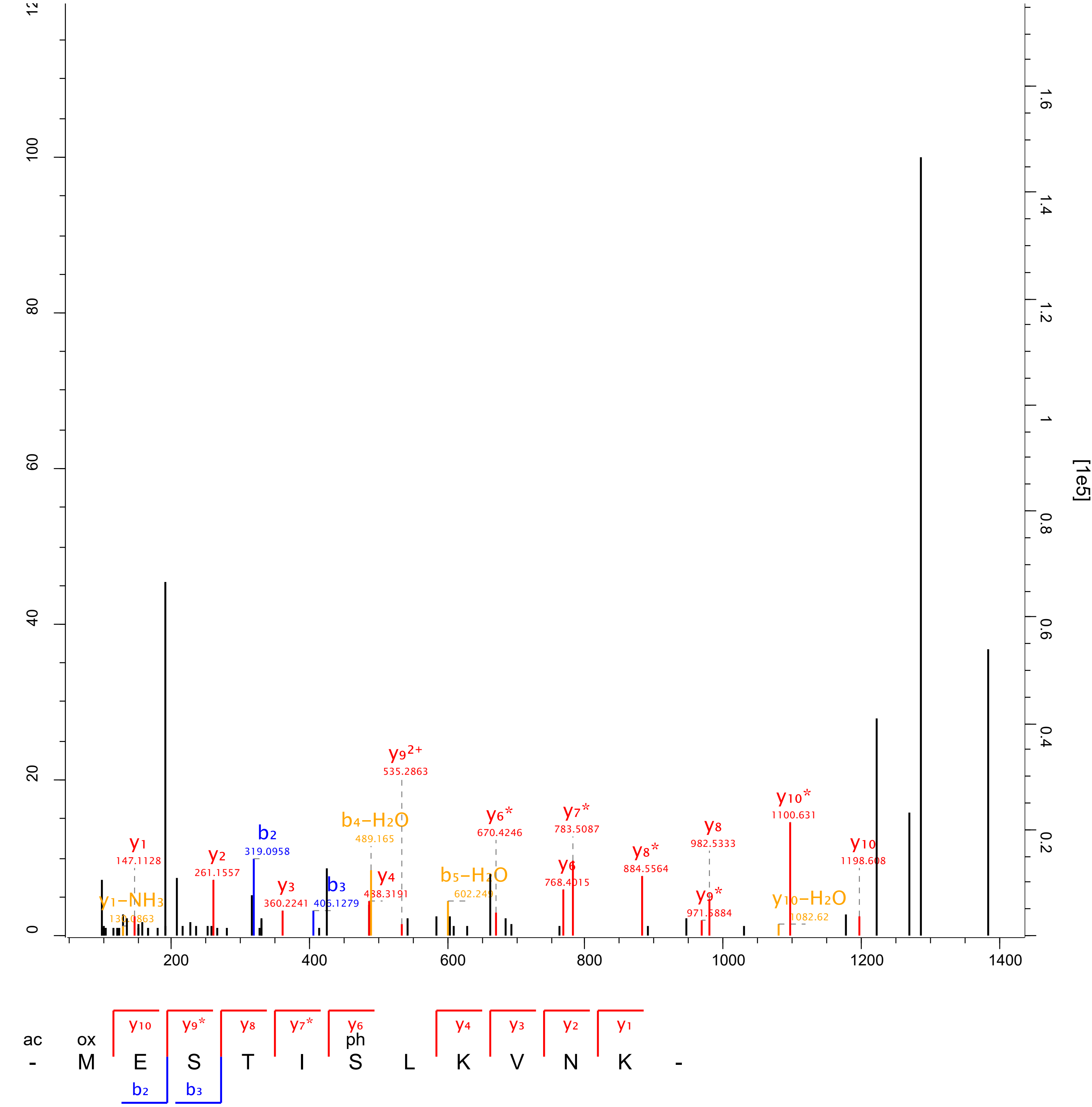

Sample output to test PDF Combine only

Raw file	Scan	Method	Score	m/z
0523_4	13370	FTMS; HCD	77.58	894.89

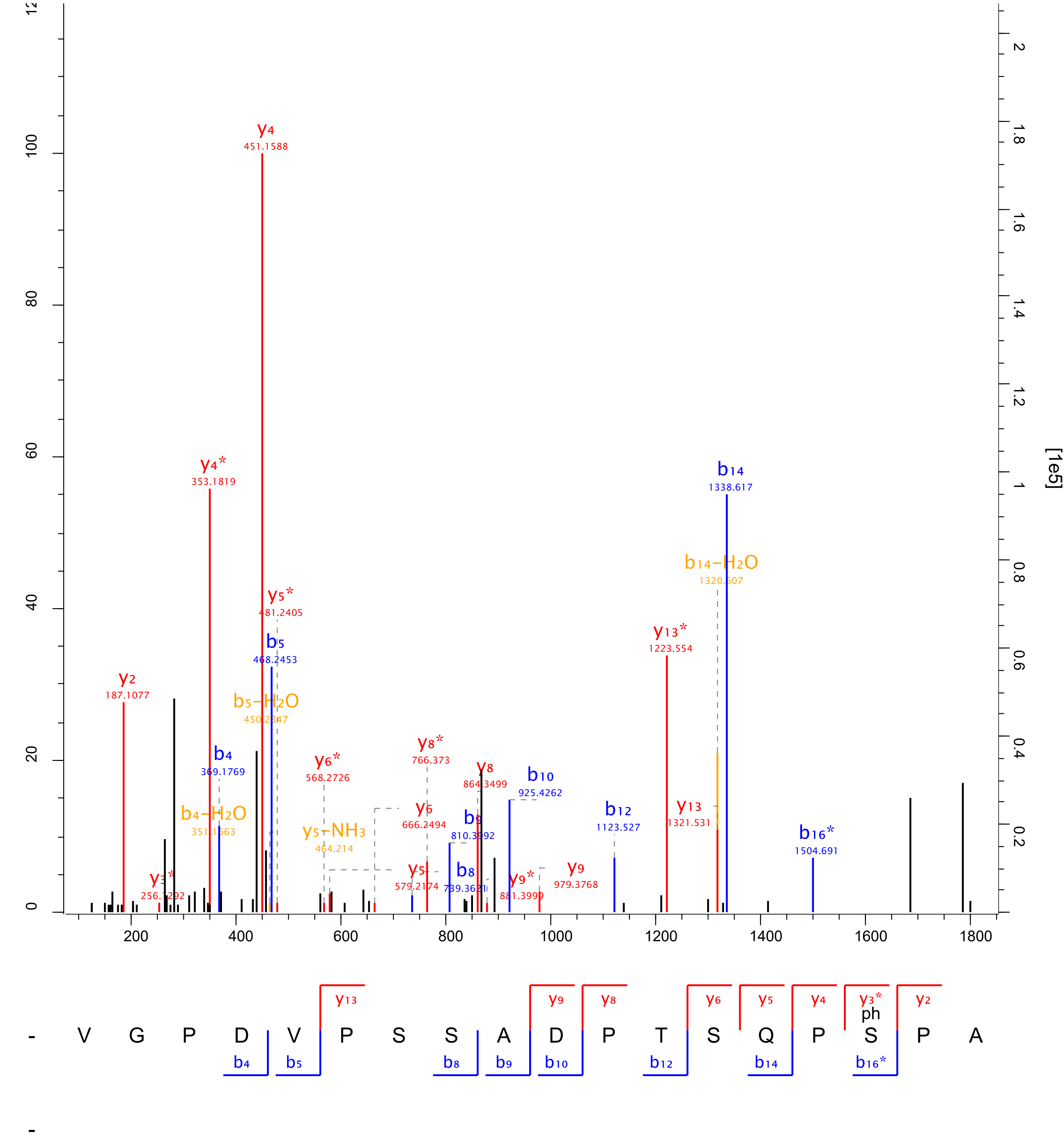

Sample output to test PDF Combine only

Raw file	Scan	Method	Score	m/z
0523_4	13377	FTMS; HCD	102.89	819.83

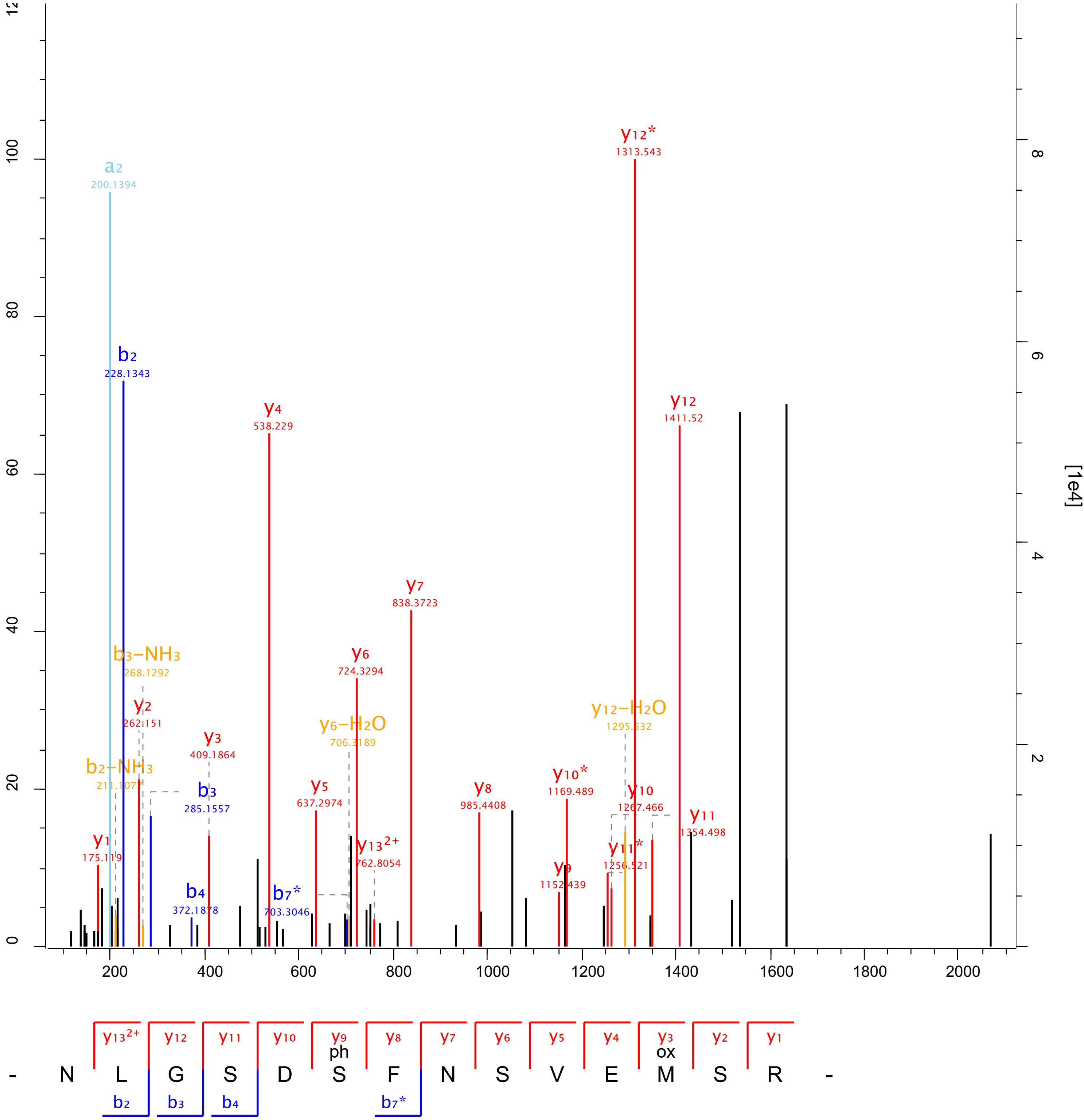

Sample output to test PDF Combine only

Raw file	Scan	Method	Score	m/z	Gene names
0523_4	13417	FTMS; HCD	66.64	900.41	MLO12

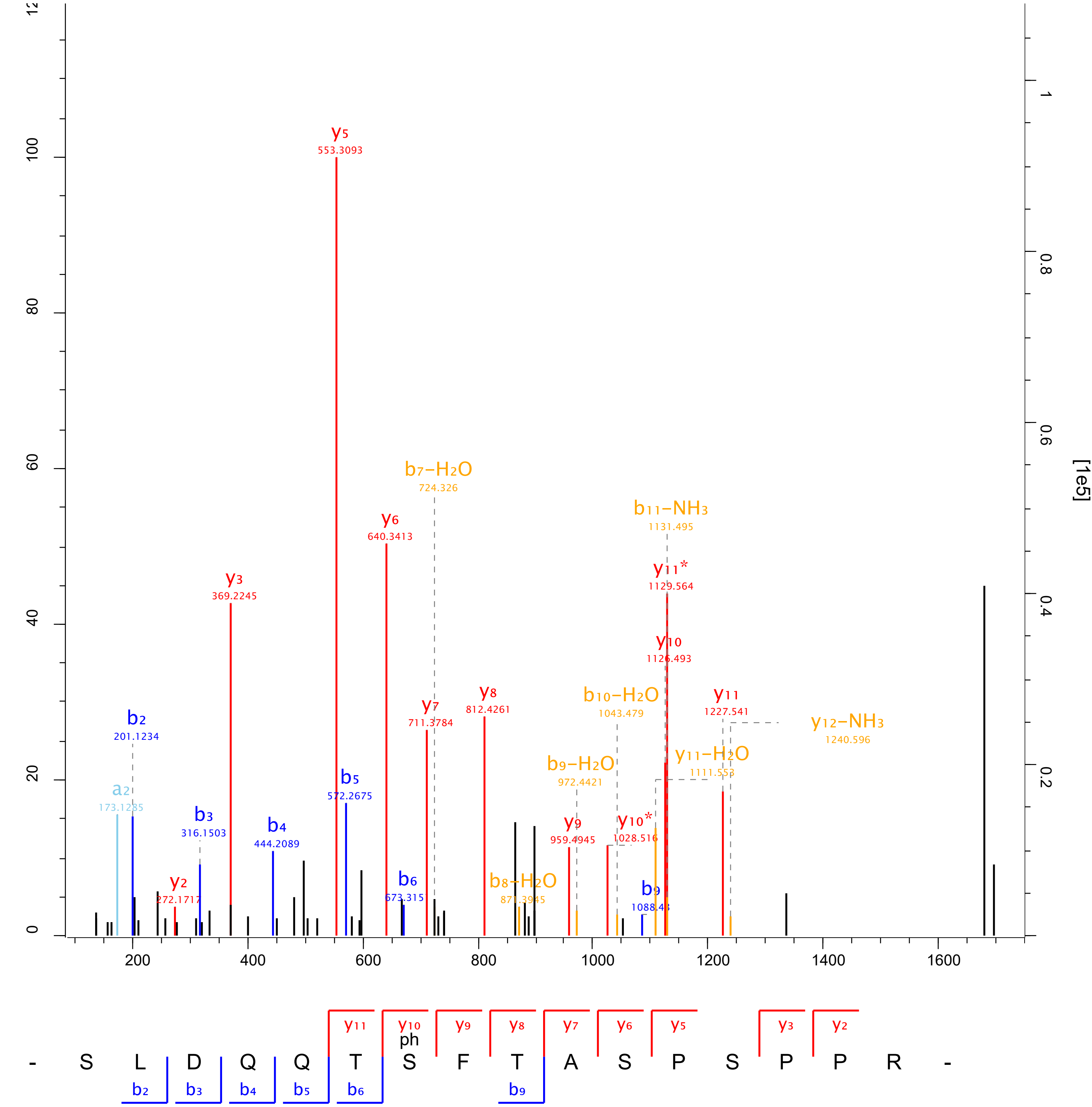

Sample output to test PDF Combine only

Raw file	Scan	Method	Score	m/z
0523_4	13443	FTMS; HCD	340.36	957.38

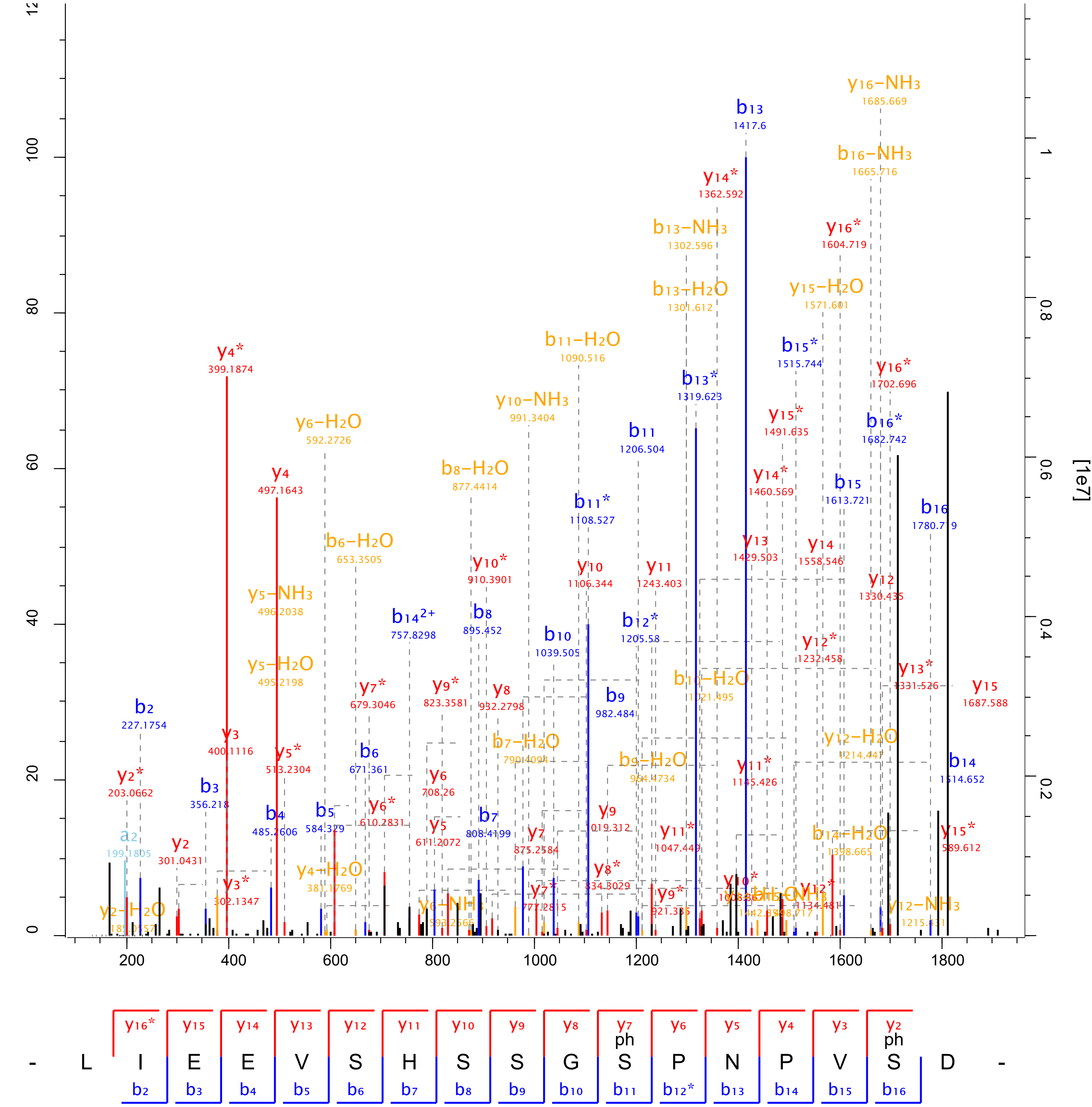

Sample output to test PDF Combine only

Raw file	Scan	Method	Score	m/z	Gene names
0523_4	13538	FTMS; HCD	48.57	524.21	At2g37050;LRR-RLK

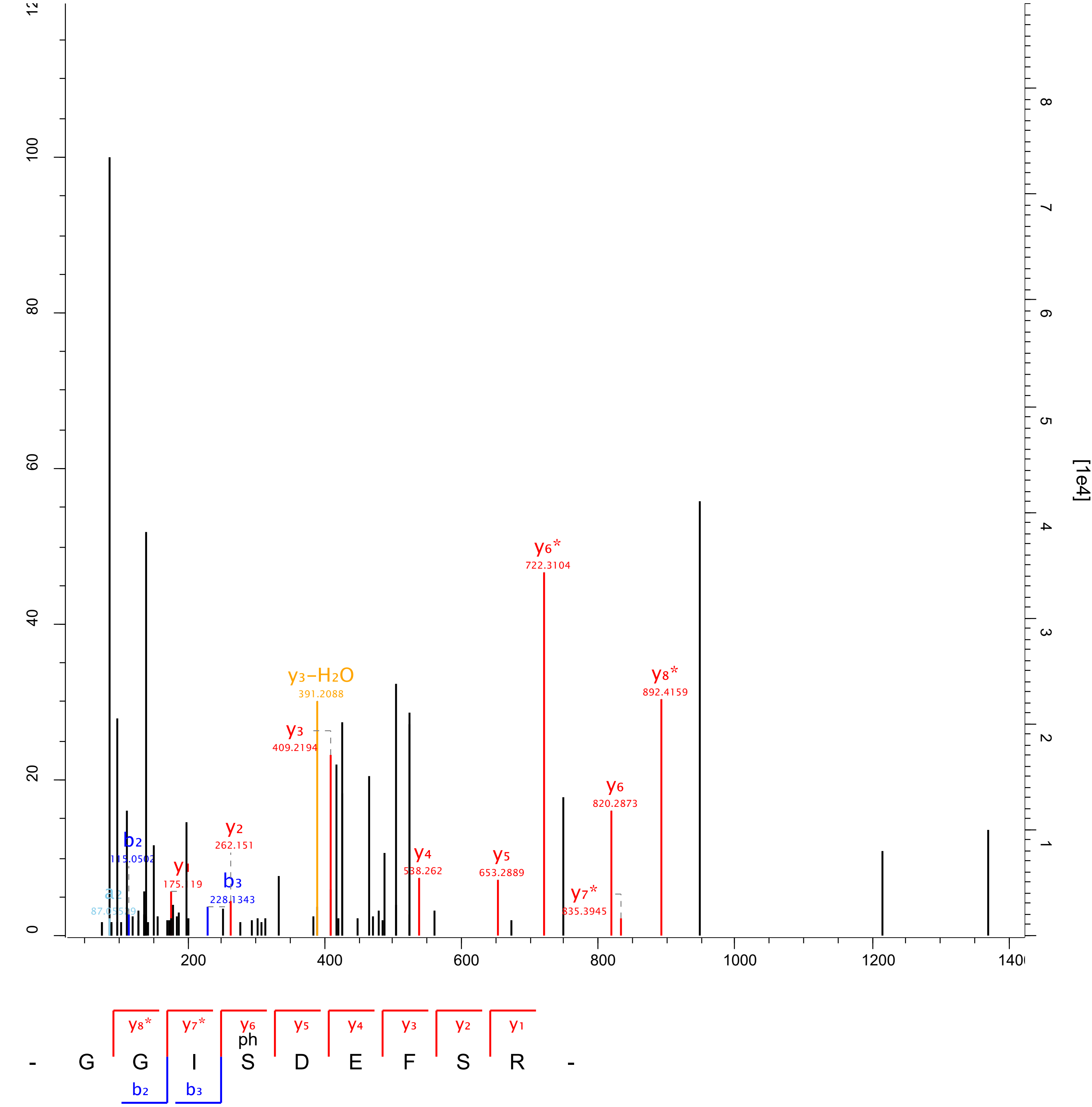

Sample output to test PDF Combine only

Raw file	Scan	Method	Score	m/z	Gene names
0523_4	13543	FTMS; HCD	43.24	968.9	CRK6

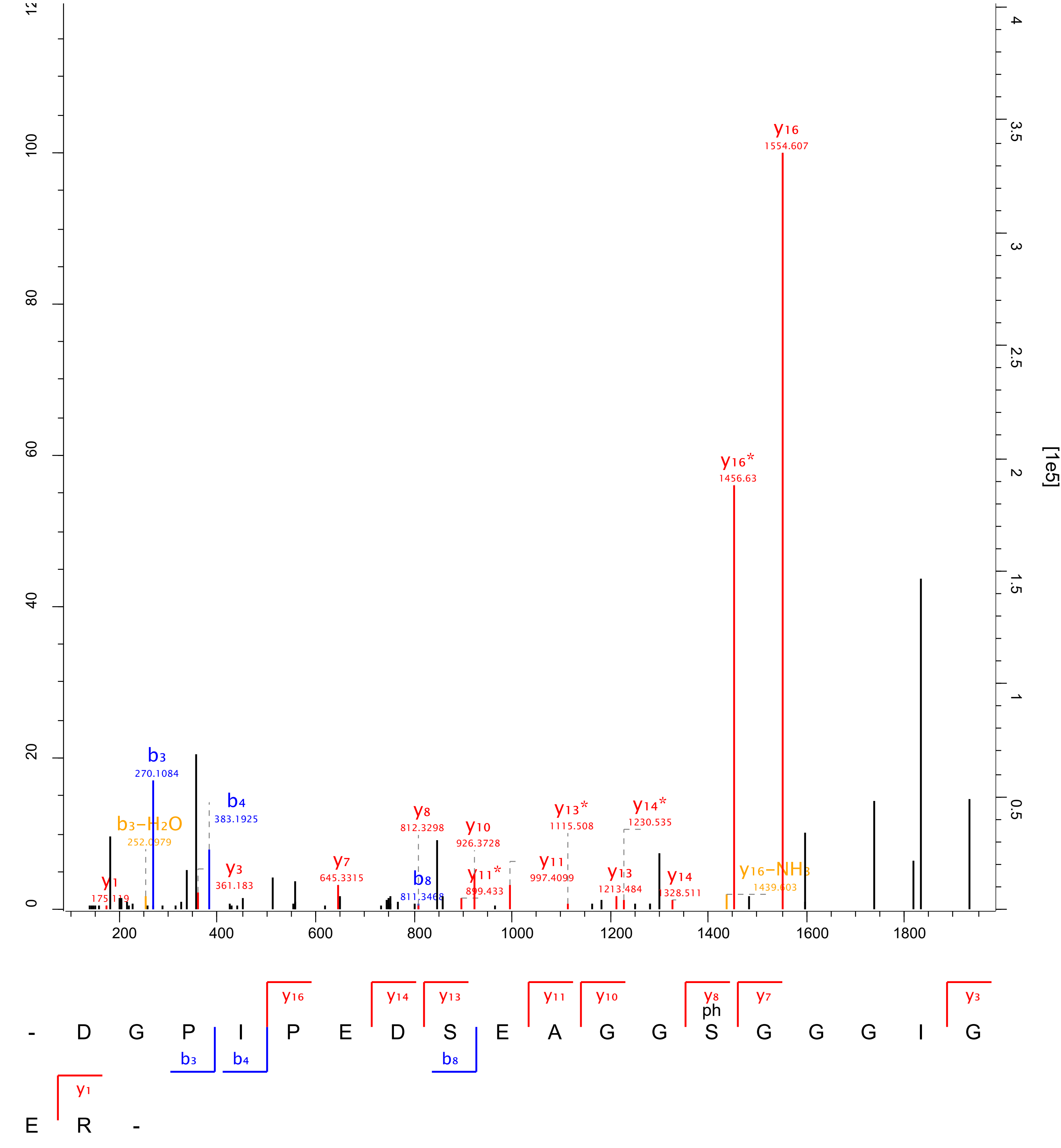

Sample output to test PDF Combine only

Raw file	Scan	Method	Score	m/z	Gene names
0523_4	13556	FTMS; HCD	189.99	965.45	At2g38410

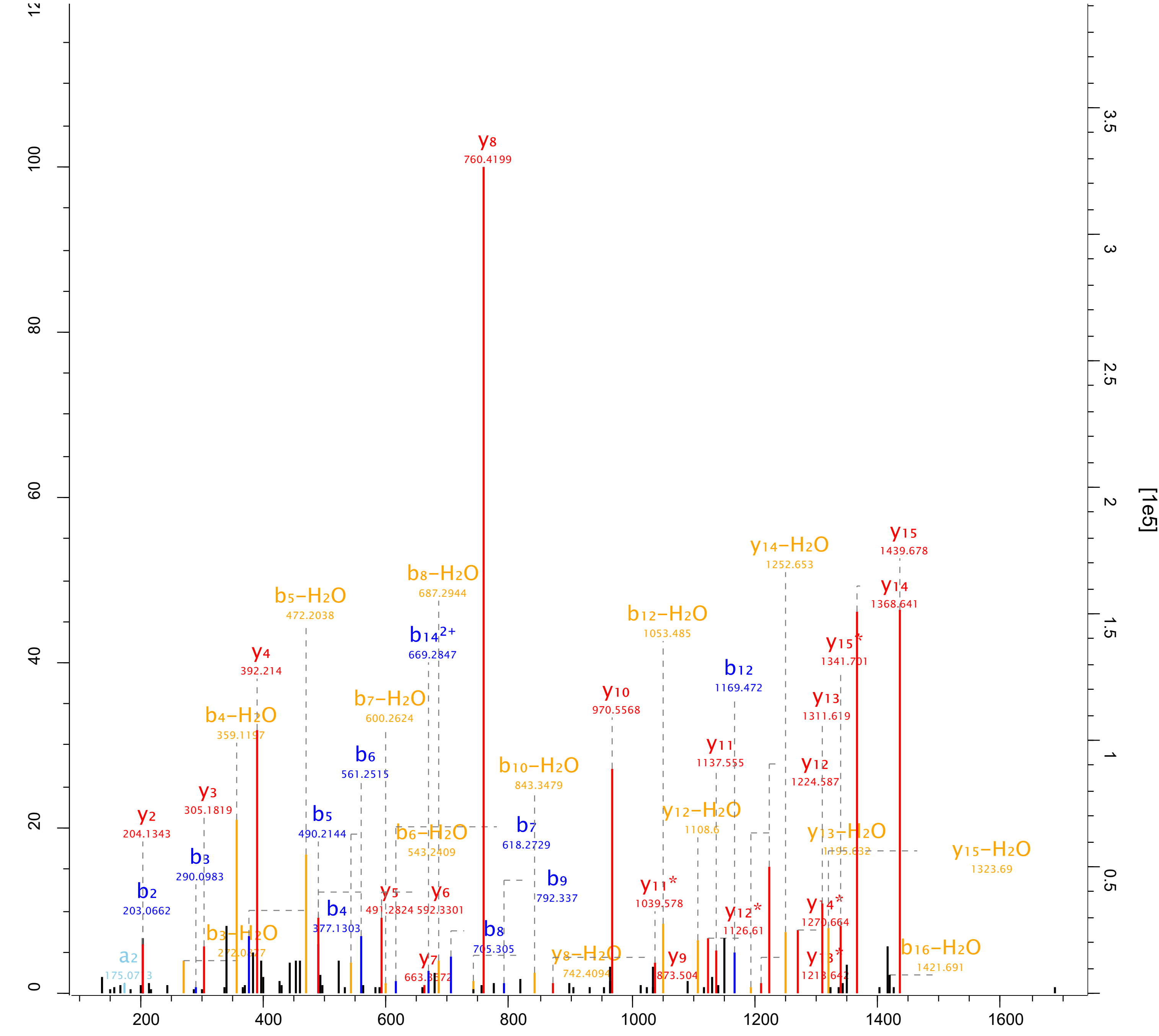

y15 y14 y13 y12 y11 y10 y9 y8 y7 y6 y5 y4 y3

ph

\- D S S S I A G S S S P I P A T V S T

b2 b3 b4 b5 b6 b7 b8 b9 b12 b14 2+

y2

G K -

Sample output to test PDF Combine only

Raw file	Scan	Method	Score	m/z	Gene names
0523_4	13654	FTMS; HCD	147.09	692.8	F5M6.12

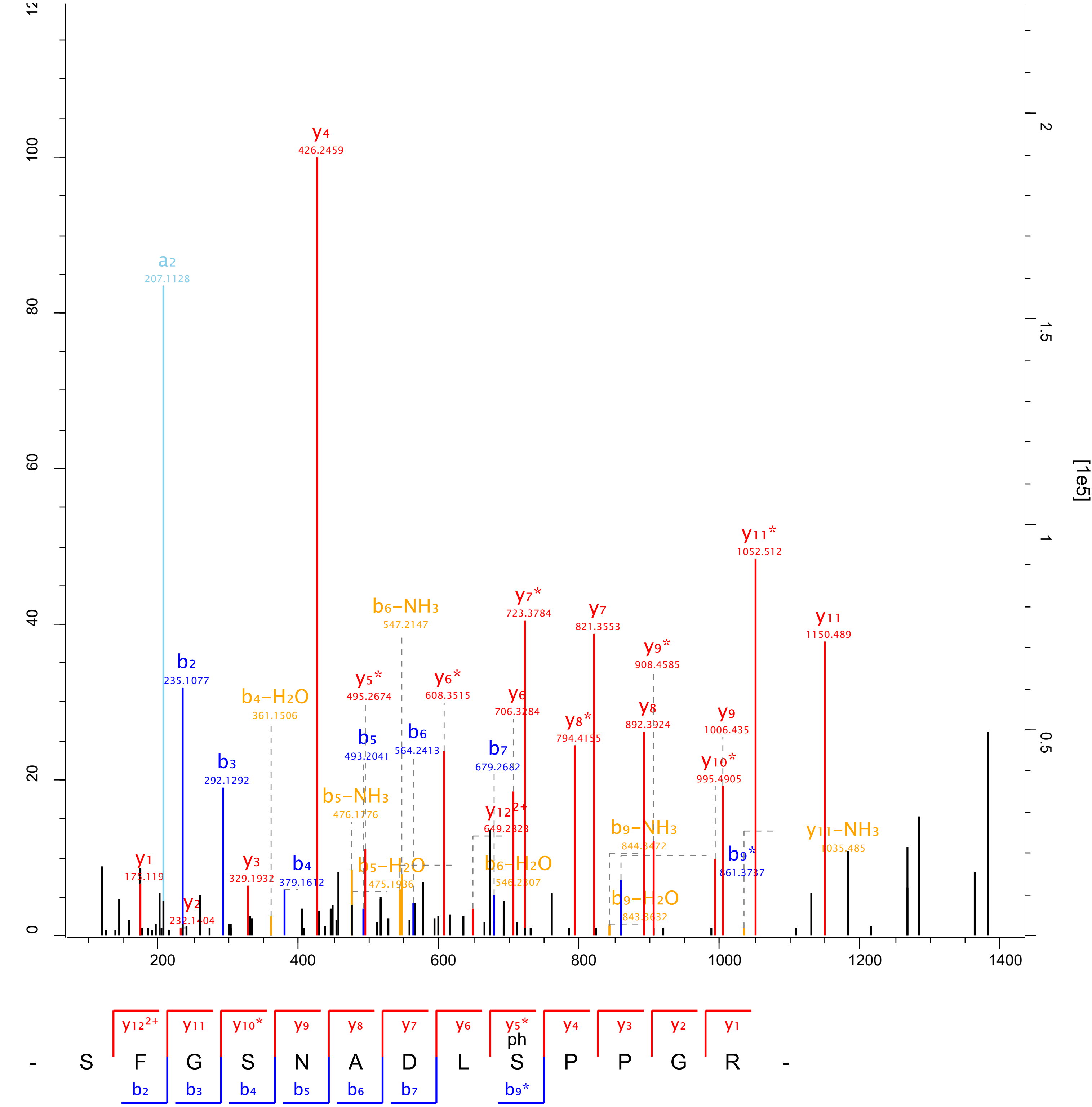

Sample output to test PDF Combine only

Raw file	Scan	Method	Score	m/z	Gene names
0523_4	13726	FTMS; HCD	73.8	909.39	PCR8

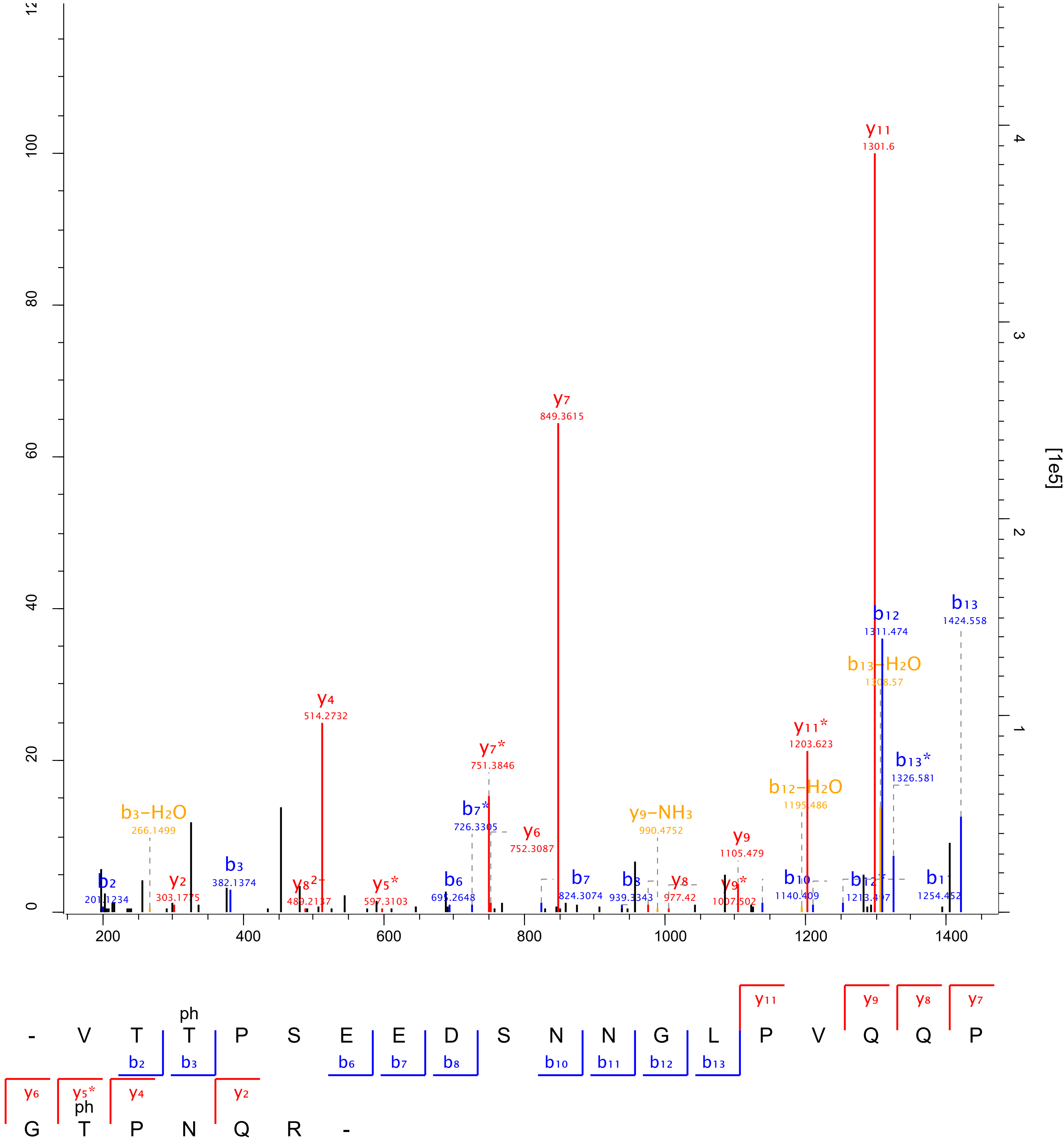

Sample output to test PDF Combine only

Raw file	Scan	Method	Score	m/z
0523_4	13751	FTMS; HCD	68.95	902.35

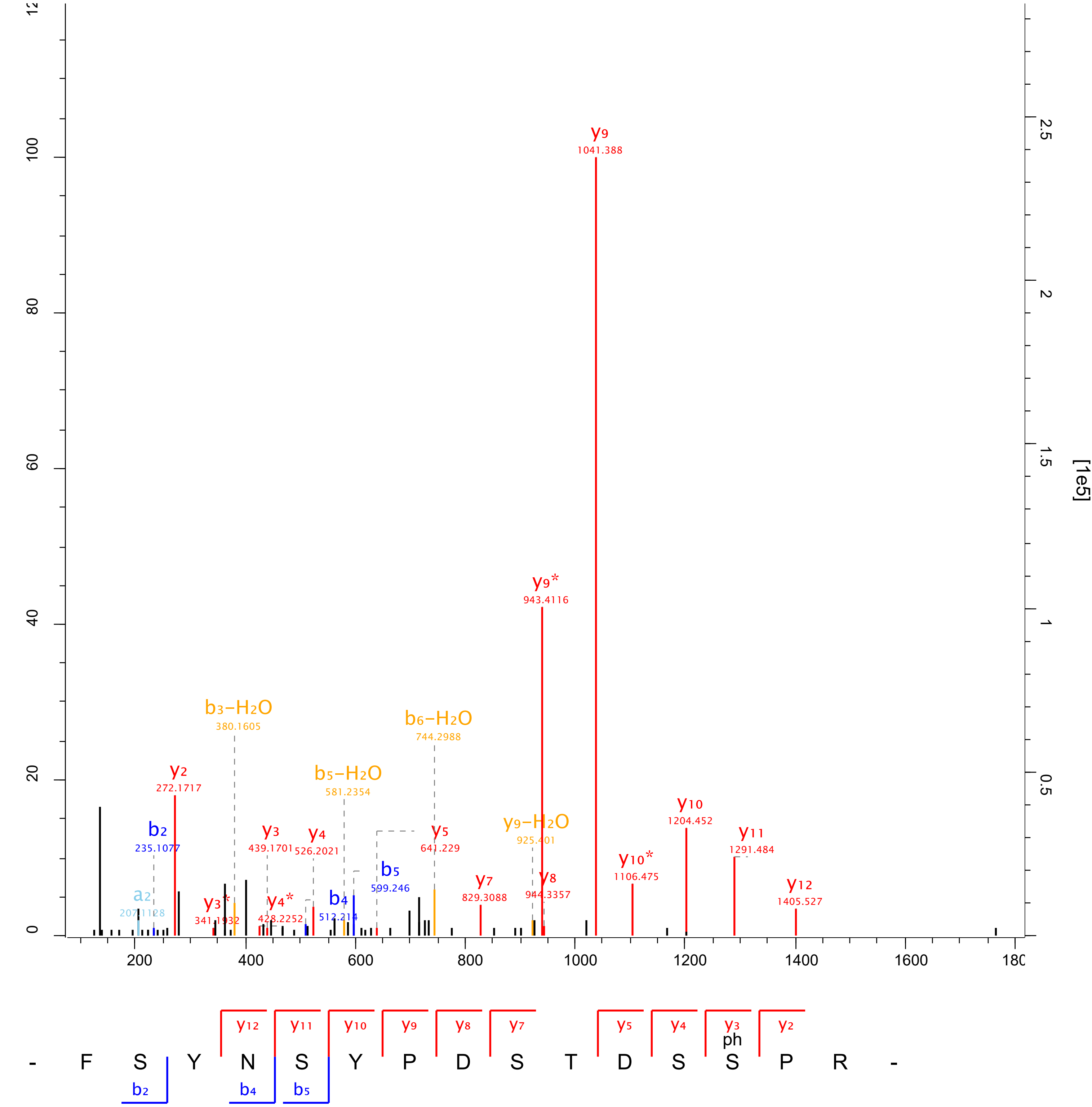

Sample output to test PDF Combine only

Raw file	Scan	Method	Score	m/z	Gene names
0523_4	13823	FTMS; HCD	74.82	690.31	F16M2_110;ATMRK1

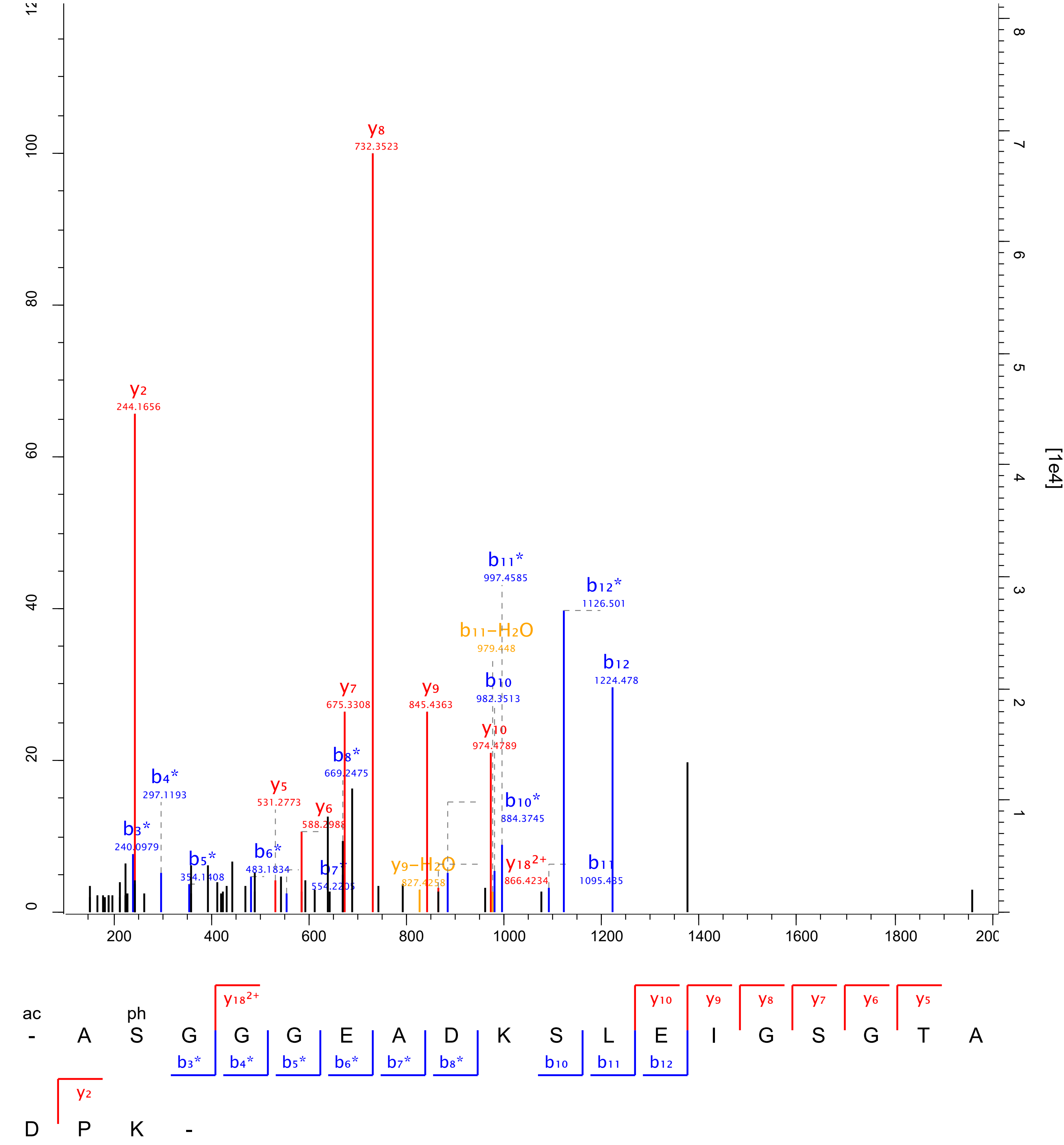

Sample output to test PDF Combine only

Raw file	Scan	Method	Score	m/z
0523_4	13934	FTMS; HCD	93.17	869.38

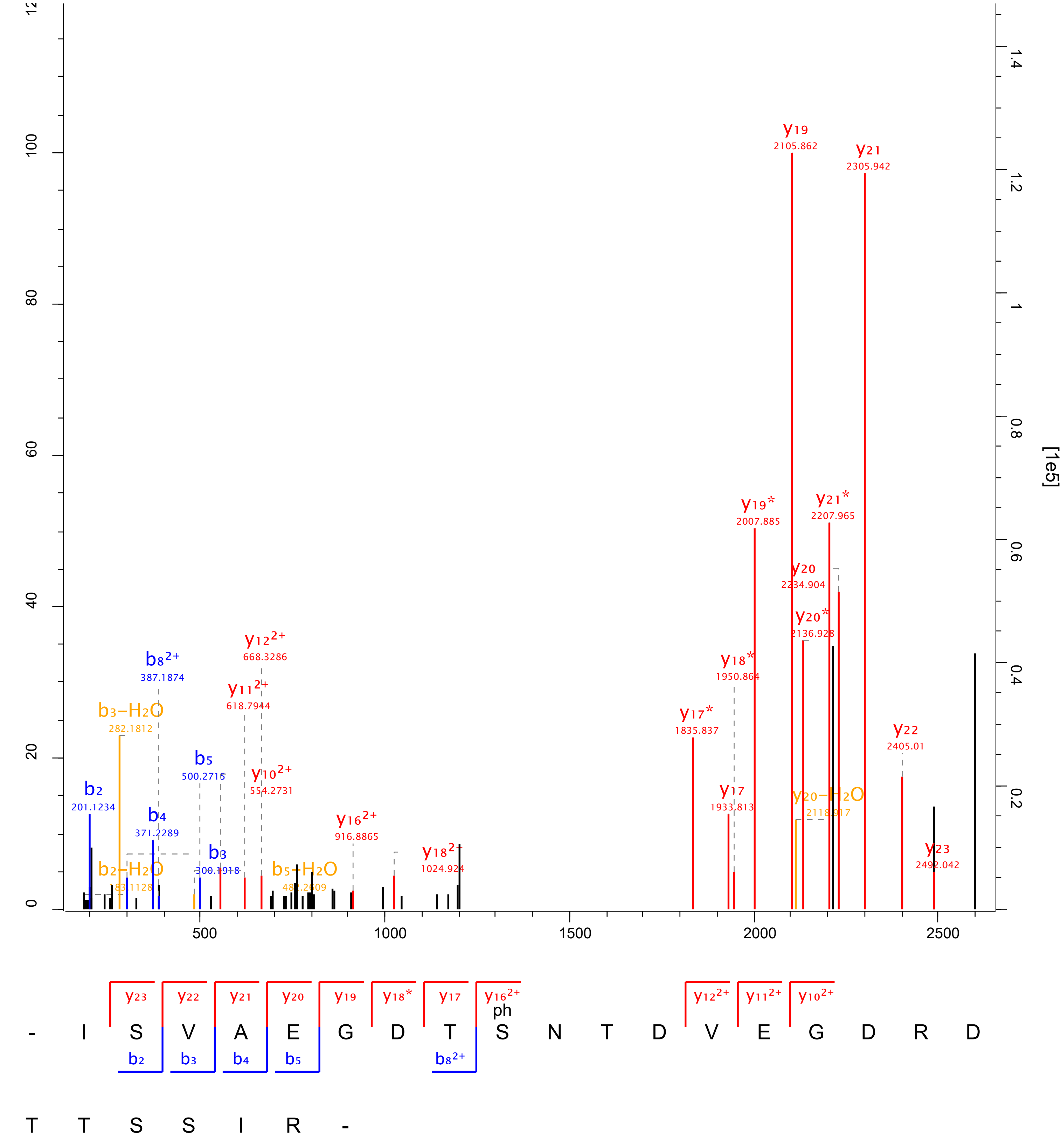

Sample output to test PDF Combine only

Raw file	Scan	Method	Score	m/z	Gene names
0523_4	14019	FTMS; HCD	55.05	725.32	CMTA3

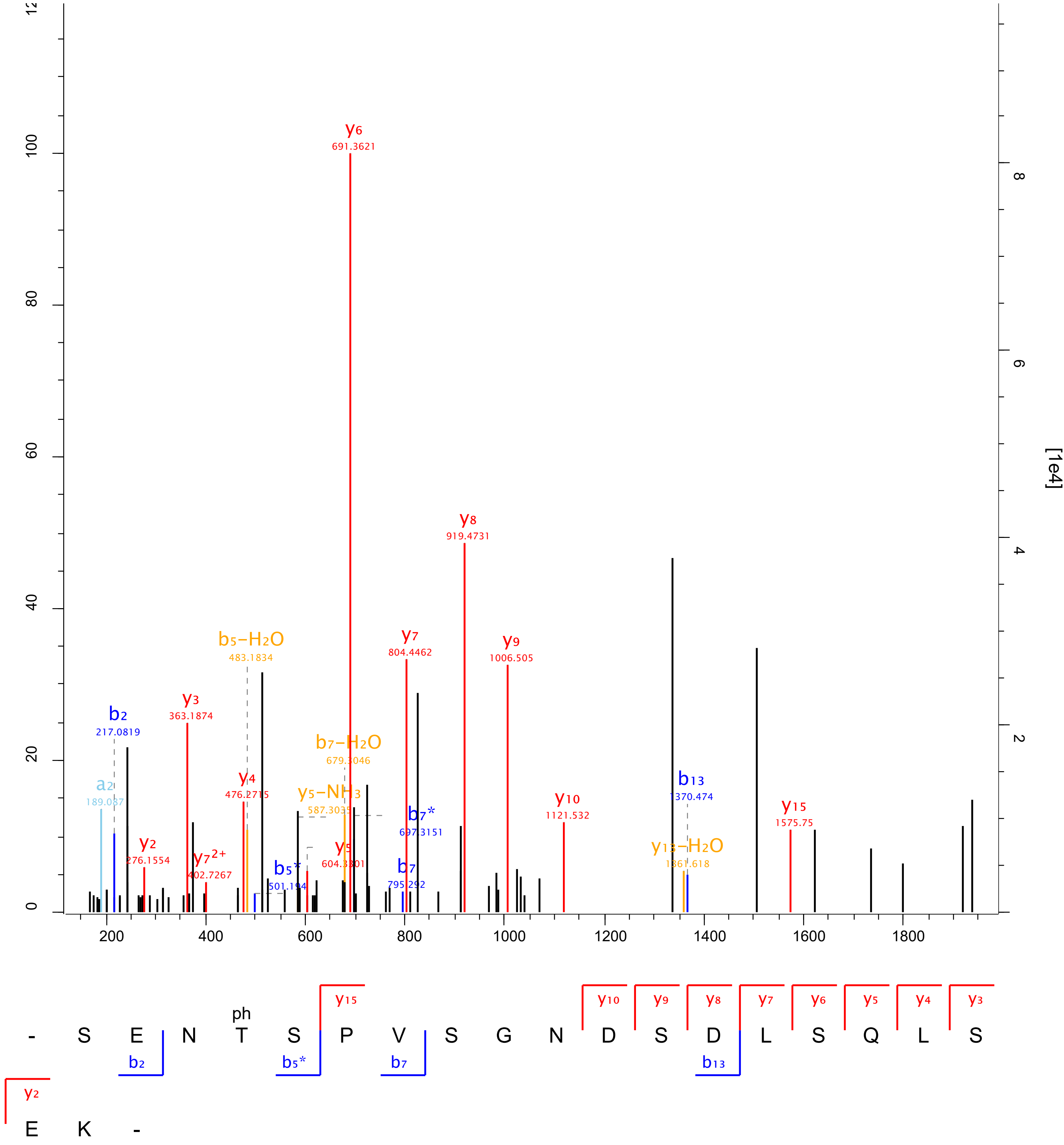

Sample output to test PDF Combine only

Raw file	Scan	Method	Score	m/z
0523_4	14078	FTMS; HCD	157.54	799.84

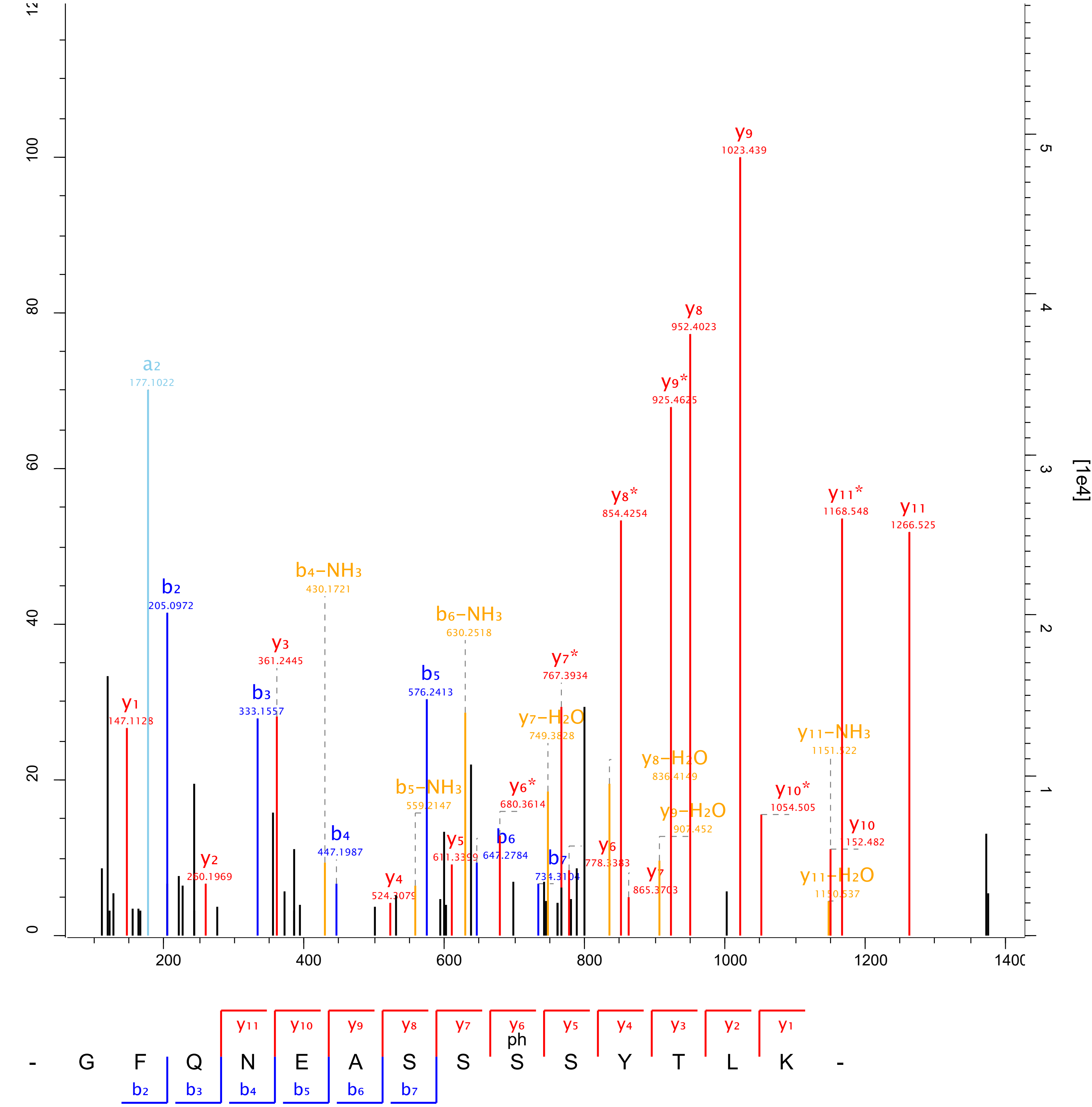

Sample output to test PDF Combine only

Raw file	Scan	Method	Score	m/z	Gene names
0523_4	14190	FTMS; HCD	77.97	783.36	T24D18.26

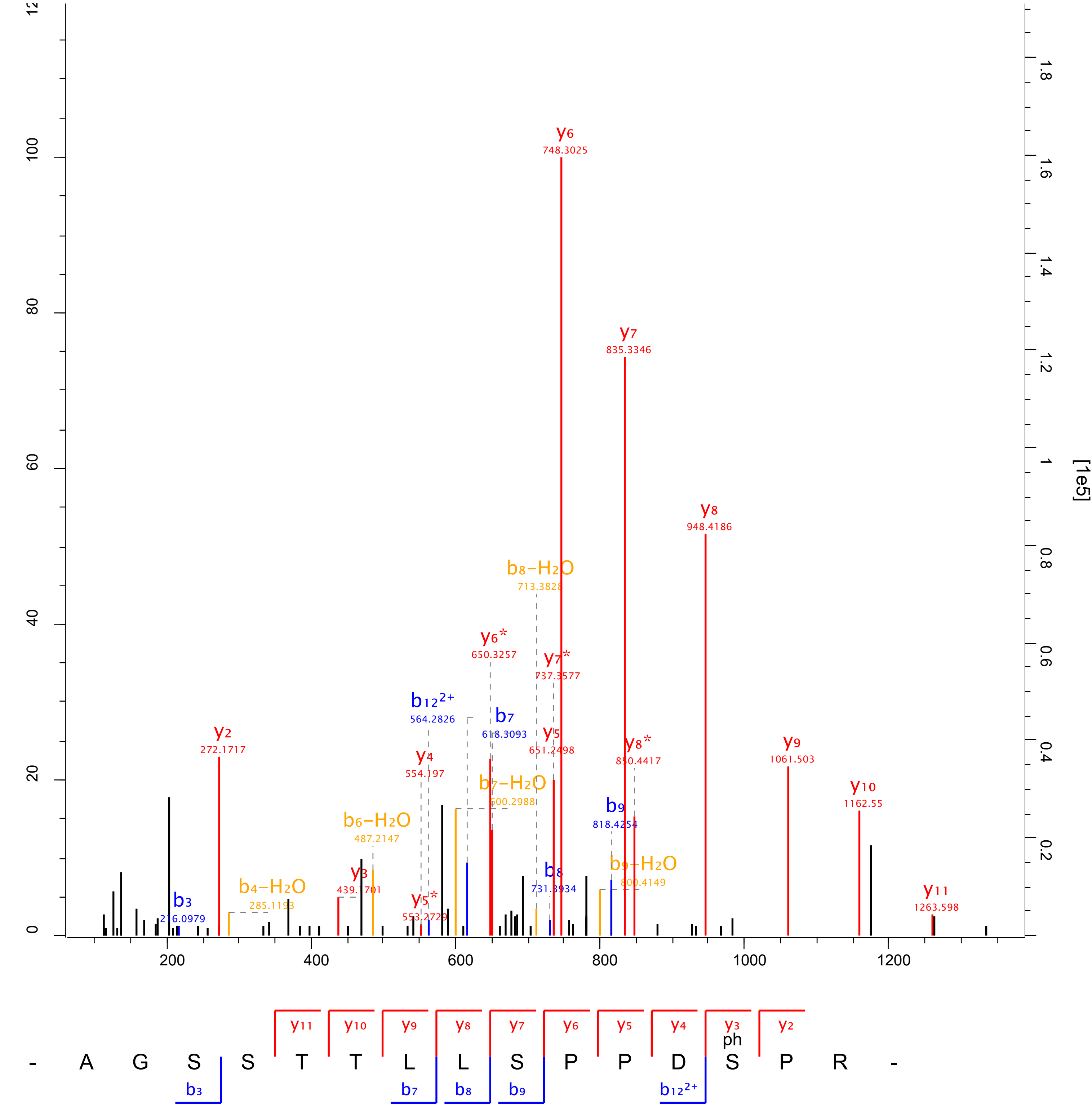

Sample output to test PDF Combine only

Raw file	Scan	Method	Score	m/z	Gene names
0523_4	14253	FTMS; HCD	68.28	632.28	SNX2B

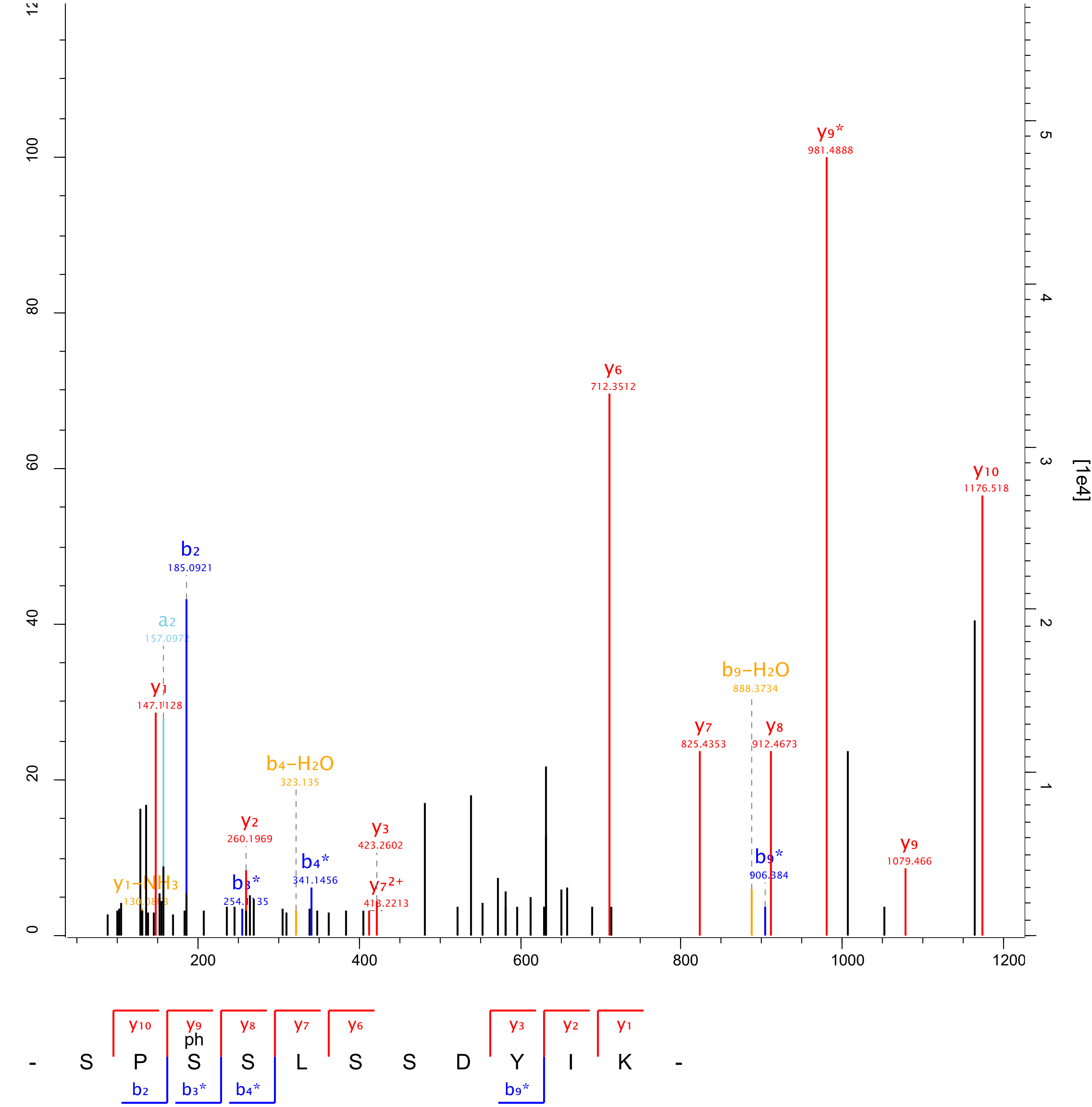

Sample output to test PDF Combine only

Raw file	Scan	Method	Score	m/z	Gene names
0523_4	14394	FTMS; HCD	42.64	553.89	At4g38550

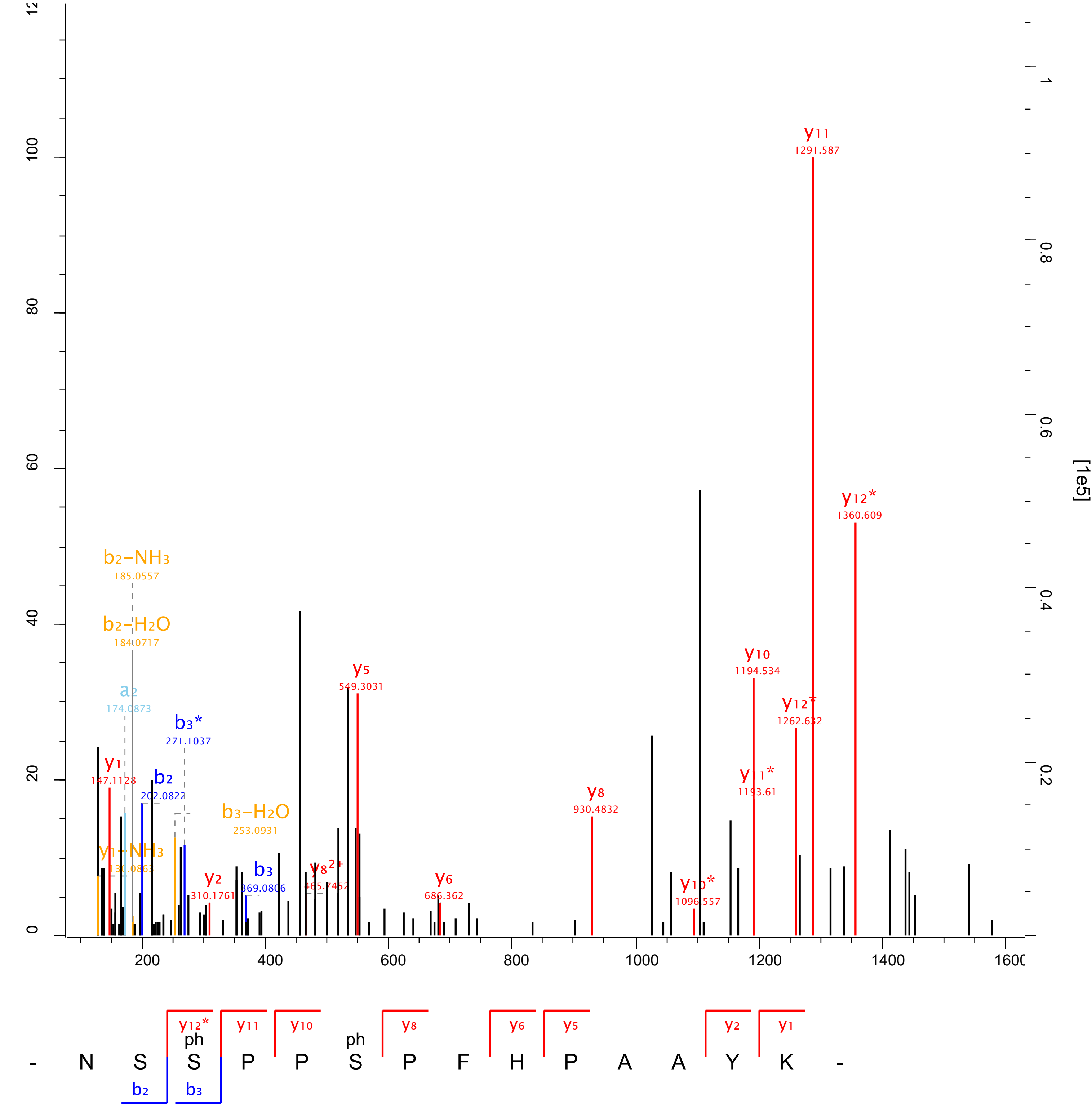

Sample output to test PDF Combine only

Raw file	Scan	Method	Score	m/z
0523_4	14440	FTMS; HCD	213.69	799.36

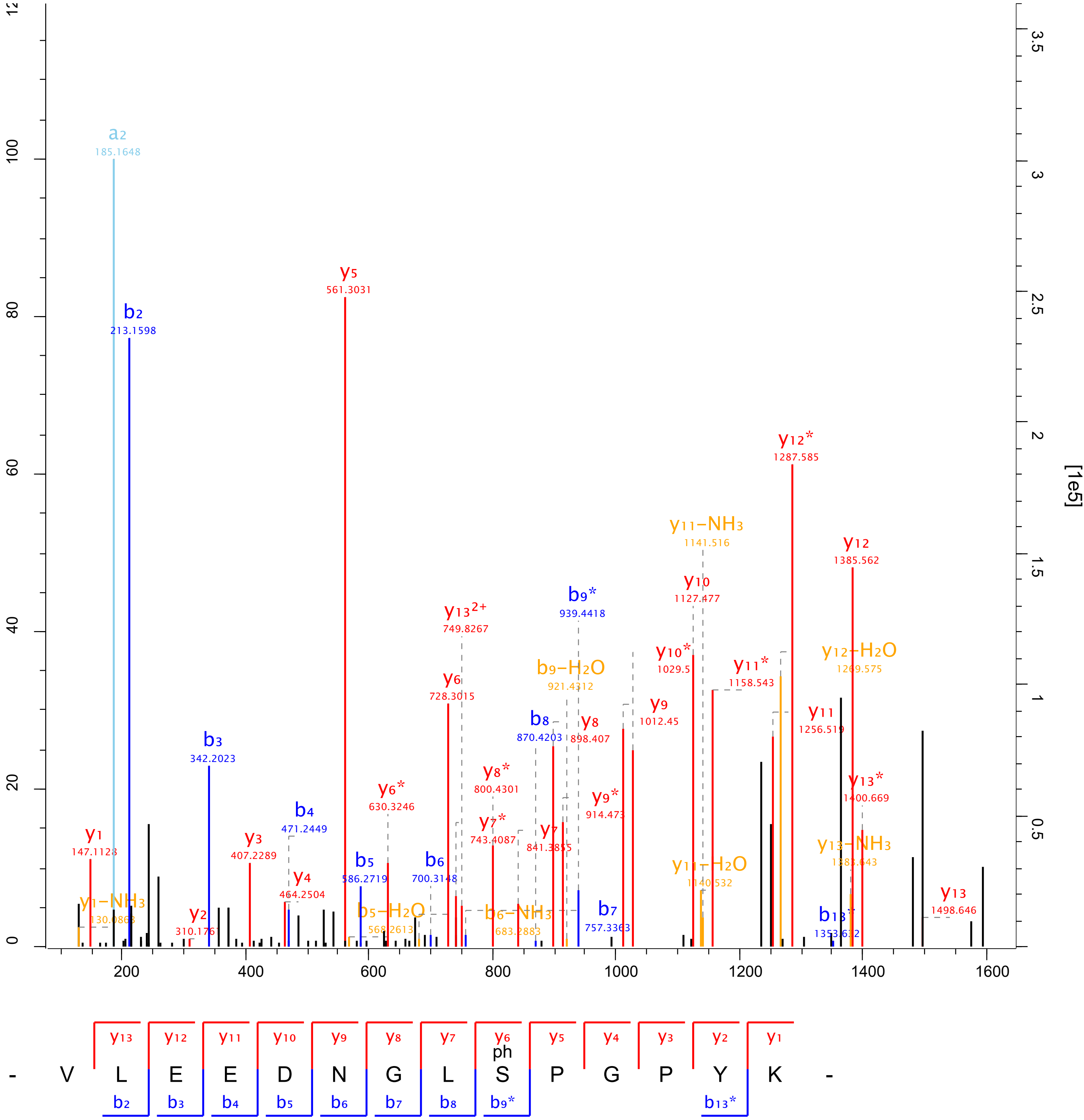

Sample output to test PDF Combine only

Raw file	Scan	Method	Score	m/z	Gene names
0523_4	14474	FTMS; HCD	45.35	844.95	POT13

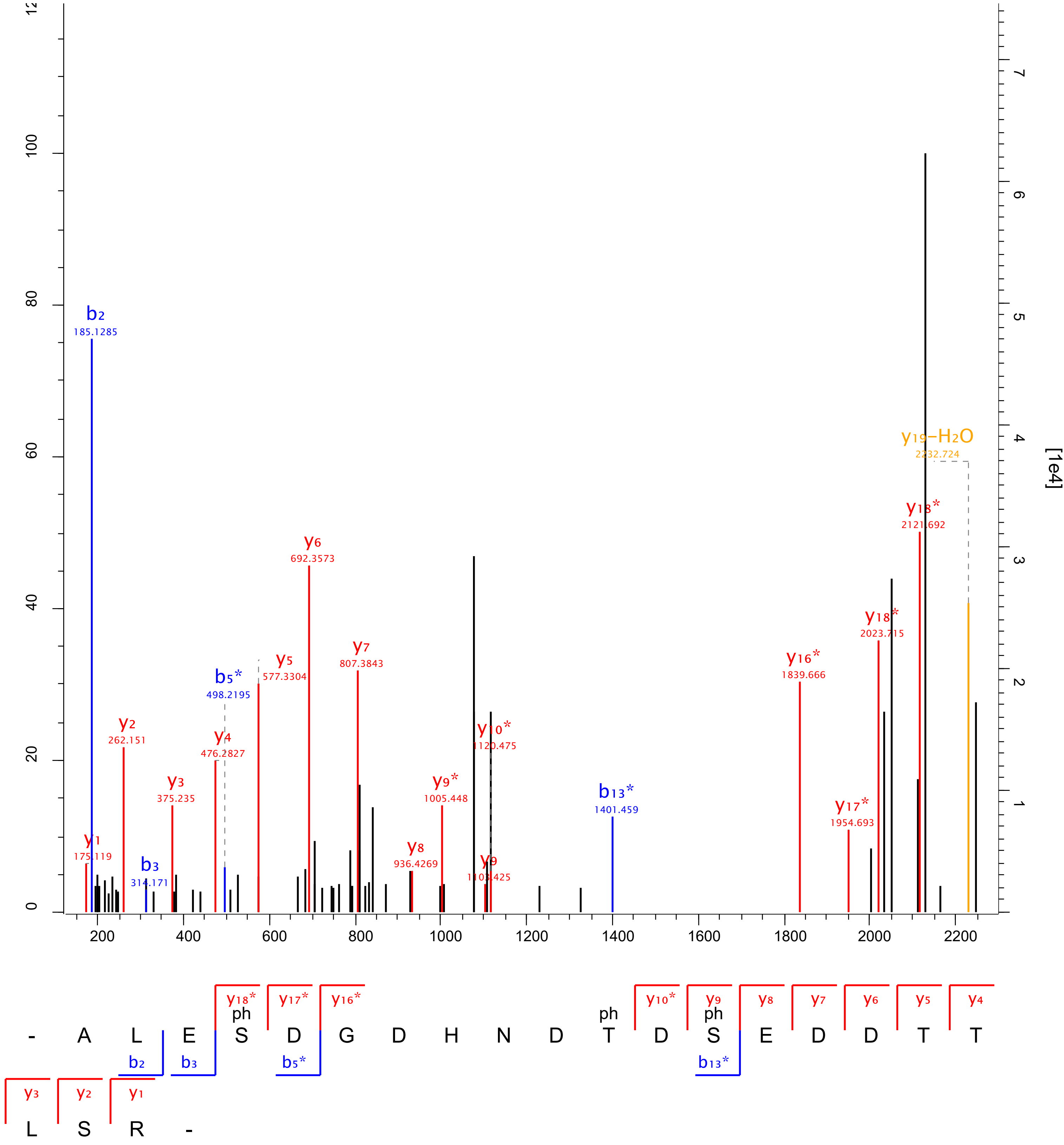

Sample output to test PDF Combine only

Raw file	Scan	Method	Score	m/z	Gene names
0523_4	14496	FTMS; HCD	68	602.25	ATL15

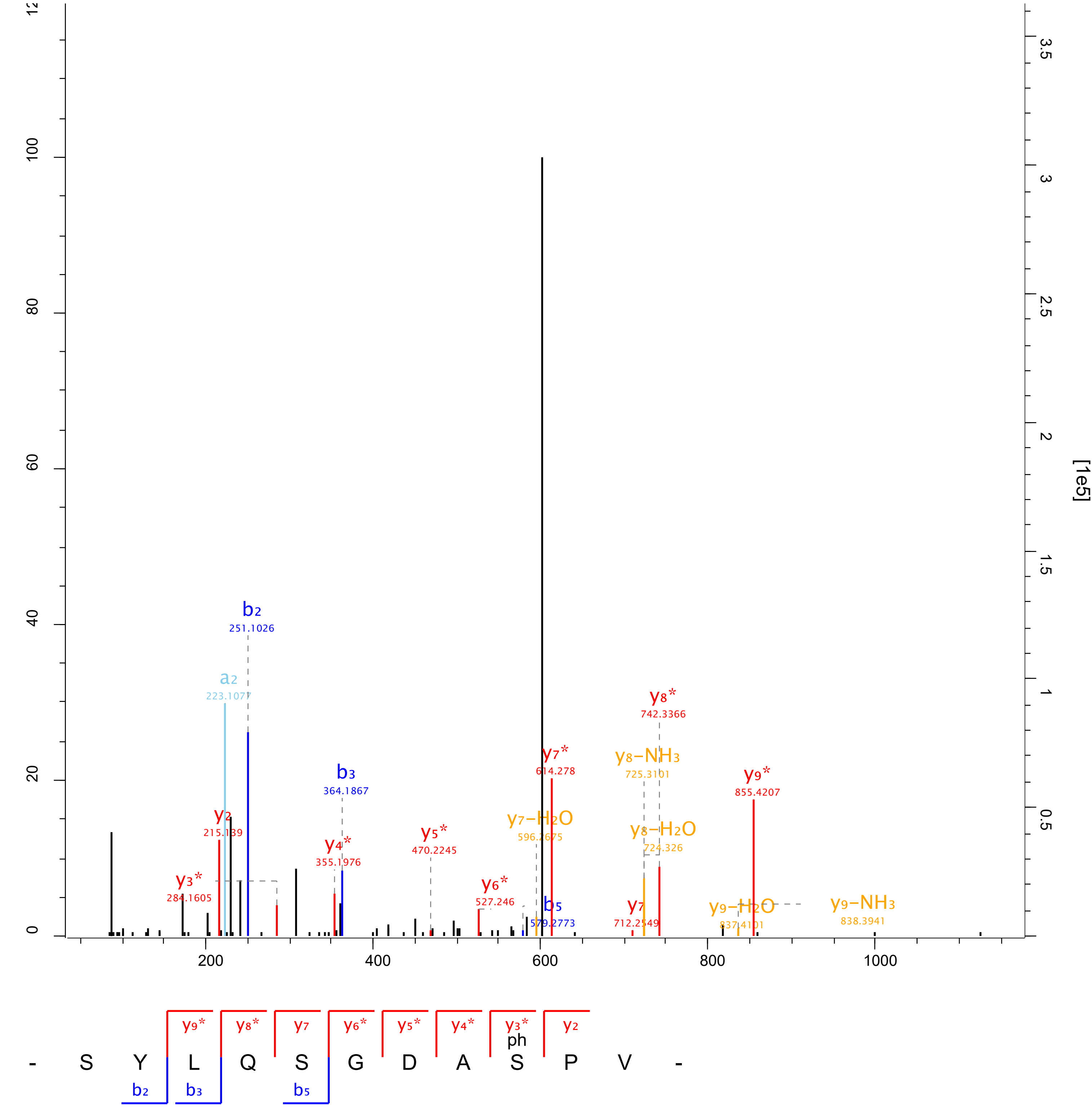

Sample output to test PDF Combine only

Raw file	Scan	Method	Score	m/z	Gene names
0523_4	14602	FTMS; HCD	107.65	711.34	M3KE1

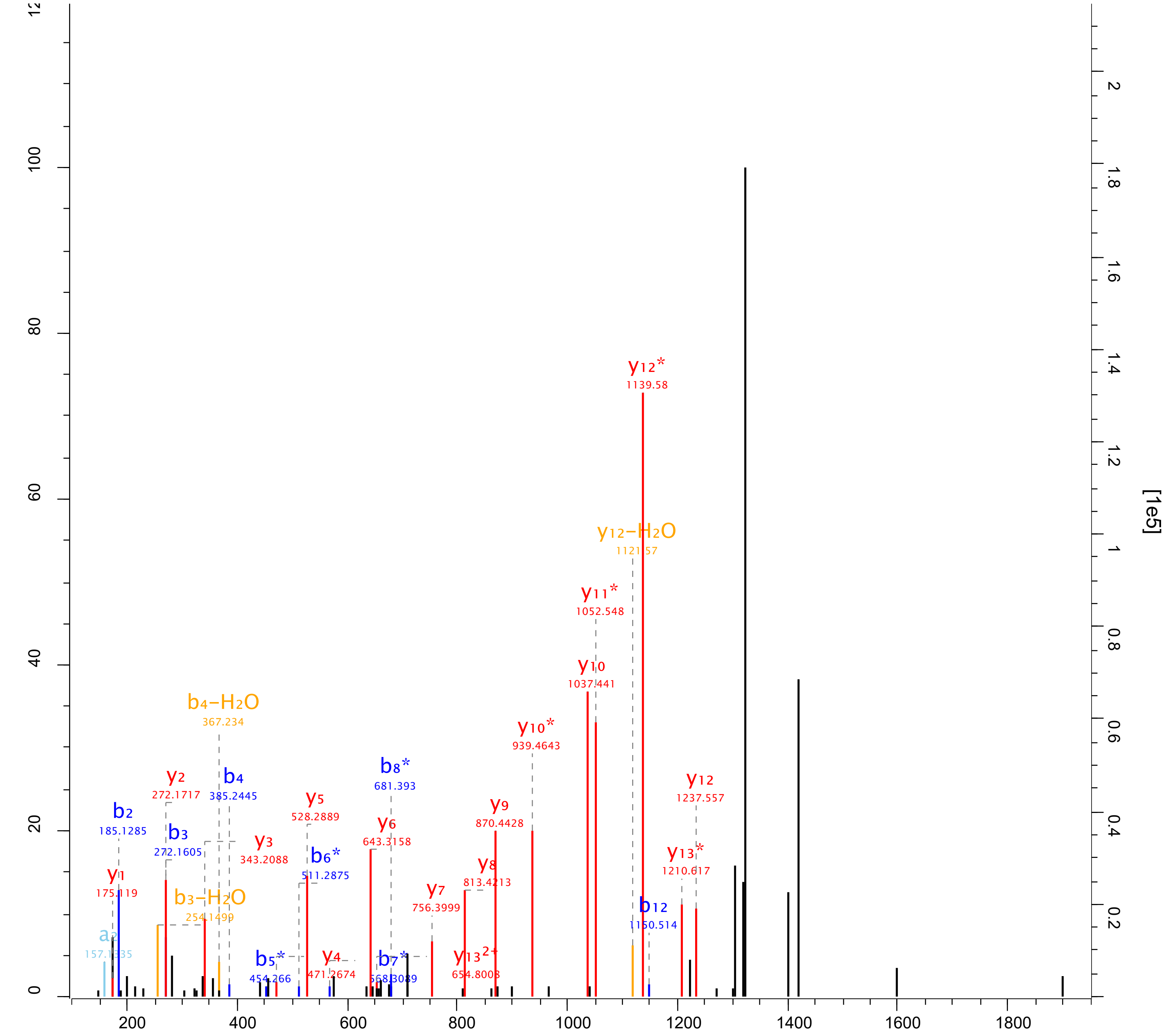

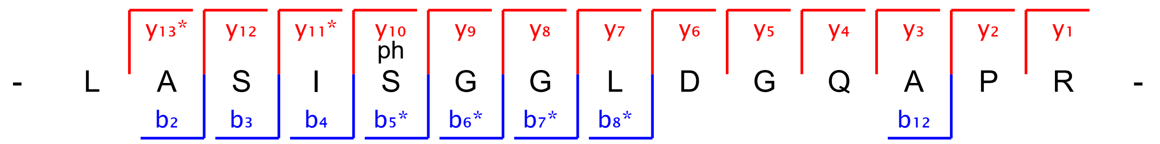

Sample output to test PDF Combine only

Raw file	Scan	Method	Score	m/z
0523_4	14702	FTMS; HCD	252.41	887.86

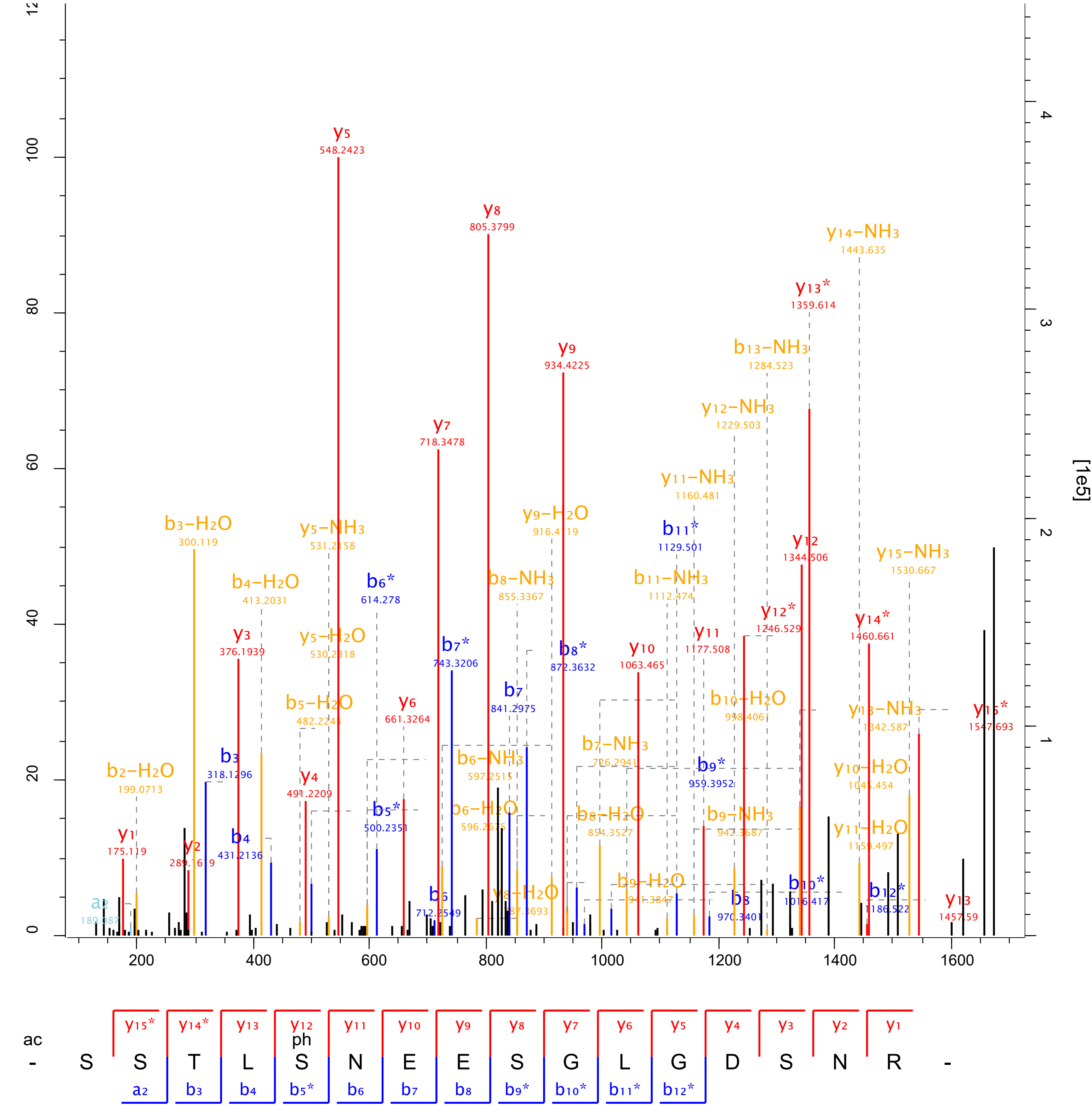

Sample output to test PDF Combine only

Raw file	Scan	Method	Score	m/z	Gene names
0523_4	14704	FTMS; HCD	163.29	925.05	PVA11

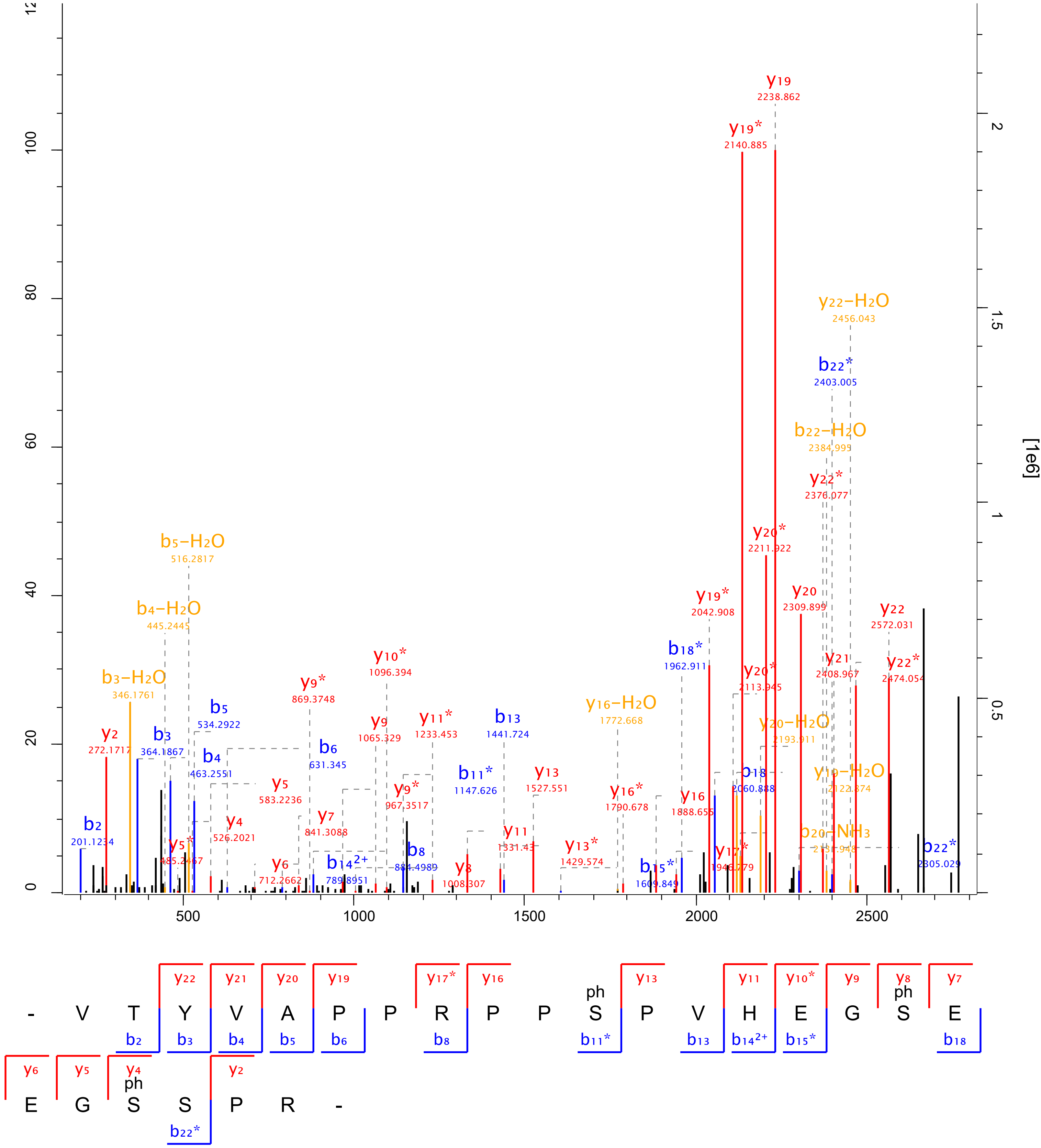

Sample output to test PDF Combine only

Raw file	Scan	Method	Score	m/z	Gene names
0523_4	14717	FTMS; HCD	48.61	914.04	At5g17523;MEX1

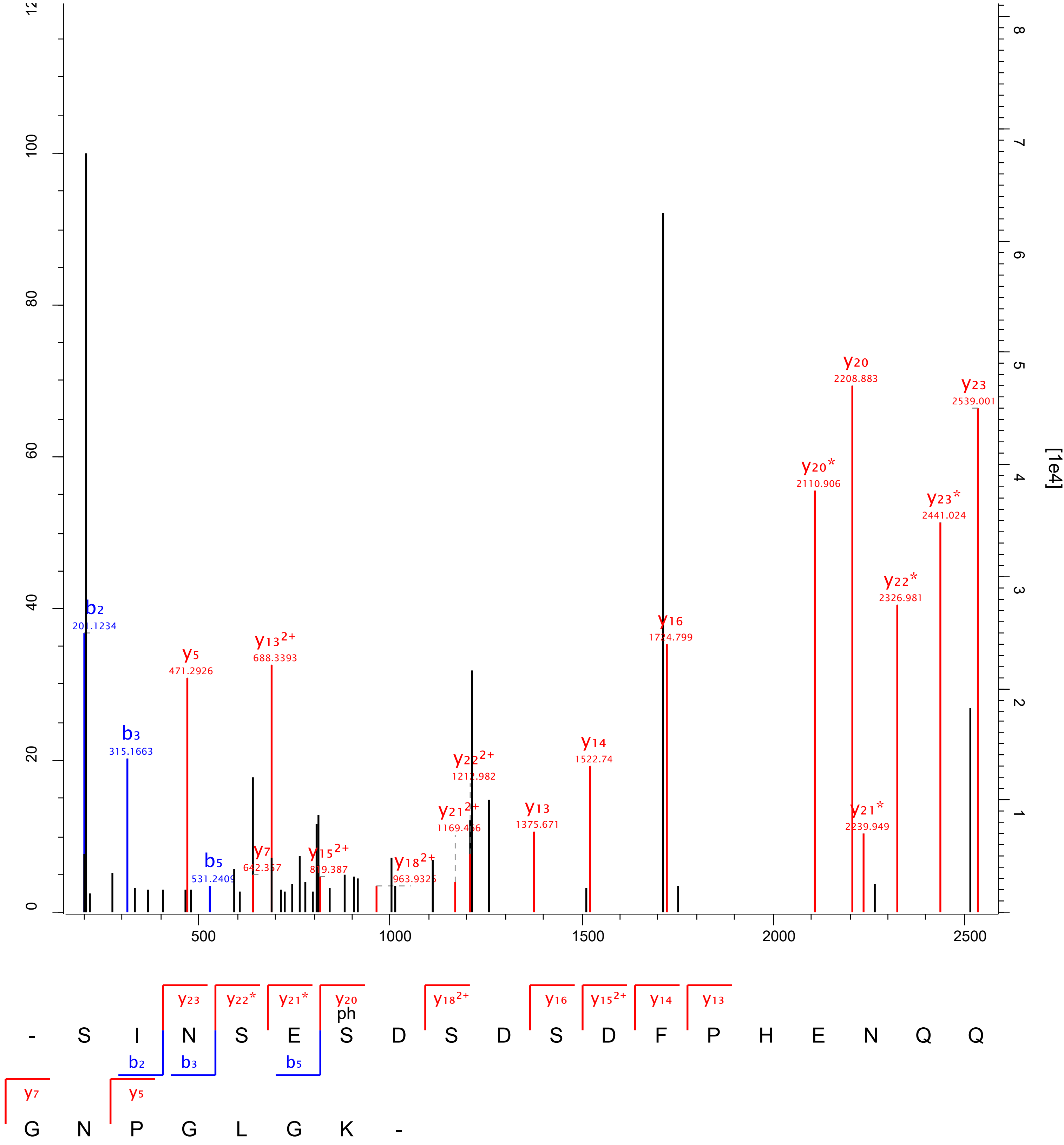

Sample output to test PDF Combine only

Raw file	Scan	Method	Score	m/z	Gene names
0523_4	14924	FTMS; HCD	57.41	679.79	PIP2-5

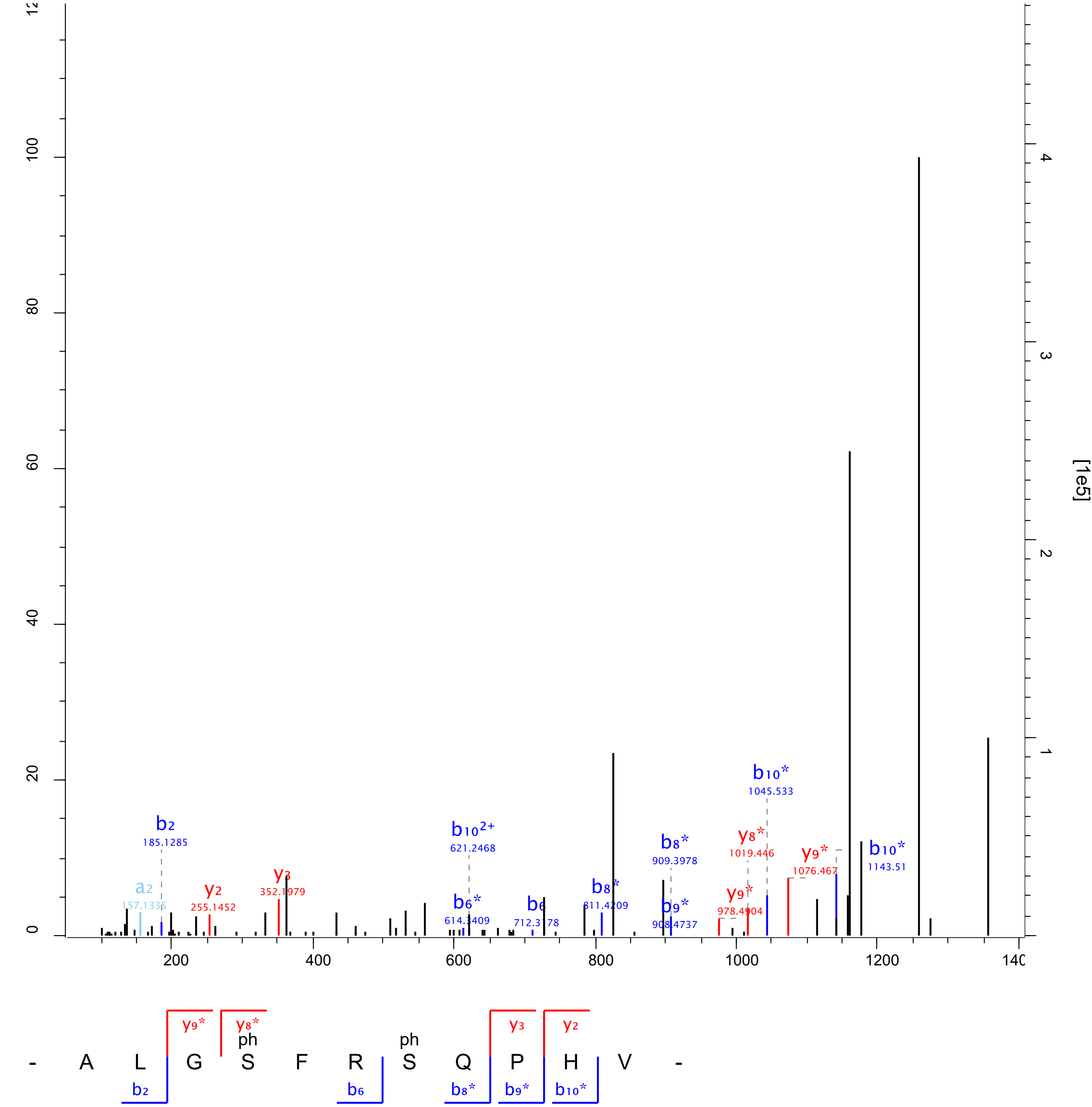

Sample output to test PDF Combine only

Raw file	Scan	Method	Score	m/z	Gene names
0523_4	14948	FTMS; HCD	85.78	896.39	At4g31880

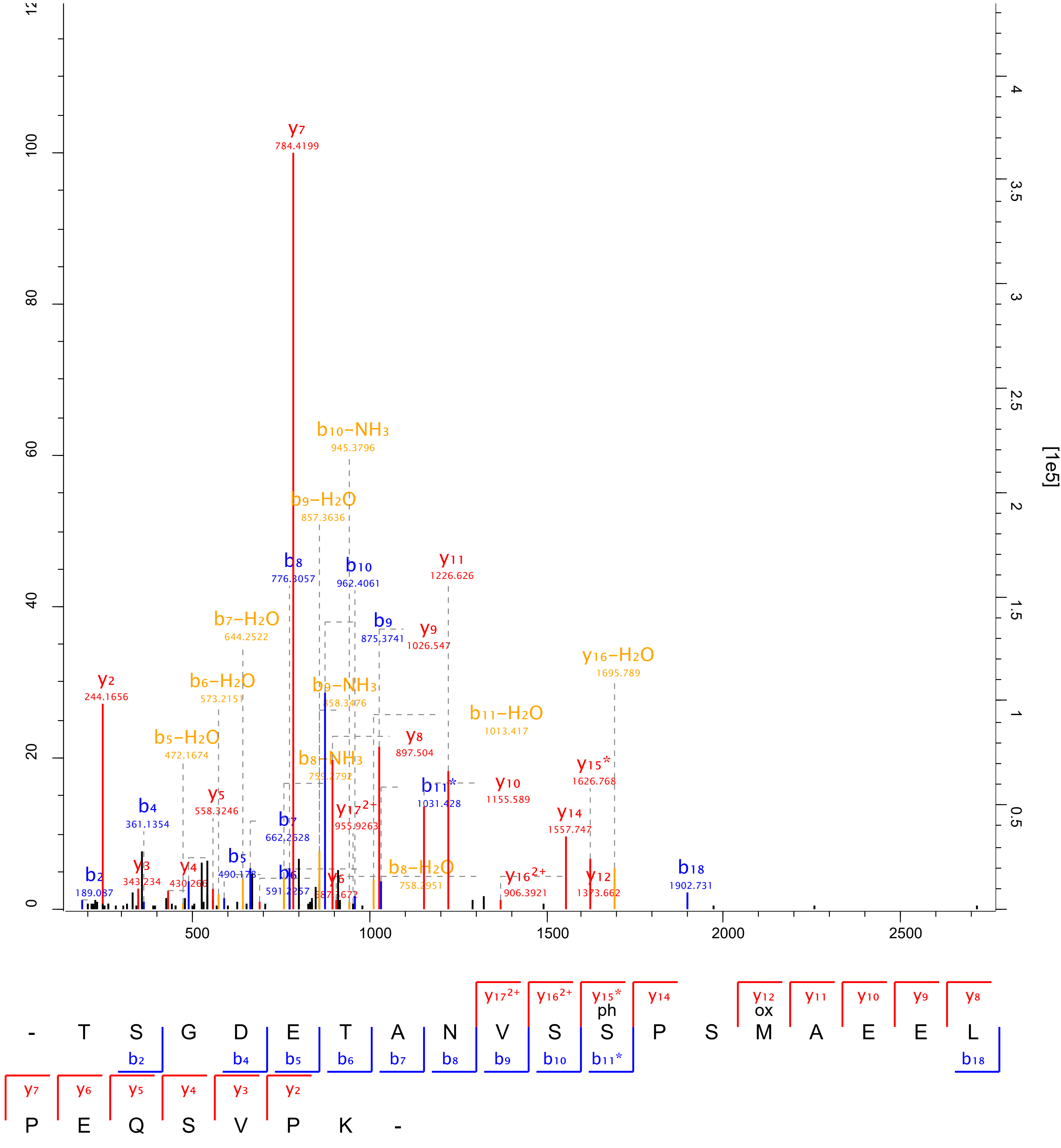

Sample output to test PDF Combine only

Raw file	Scan	Method	Score	m/z	Gene names
0523_4	15054	FTMS; HCD	102.51	865.05	LRS1

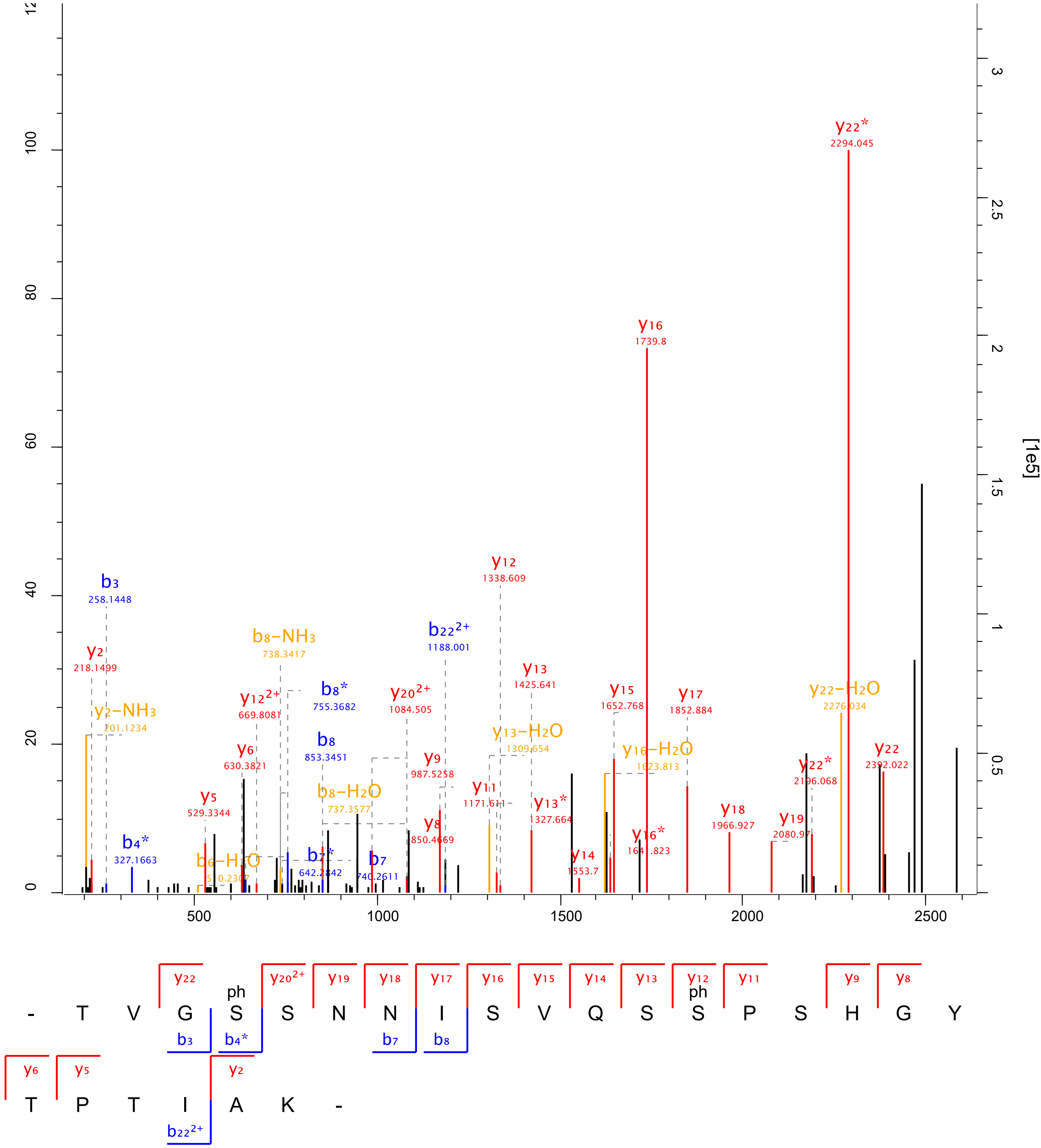

Sample output to test PDF Combine only

Raw file	Scan	Method	Score	m/z	Gene names
0523_4	15211	FTMS; HCD	124.66	951.91	NUP50A

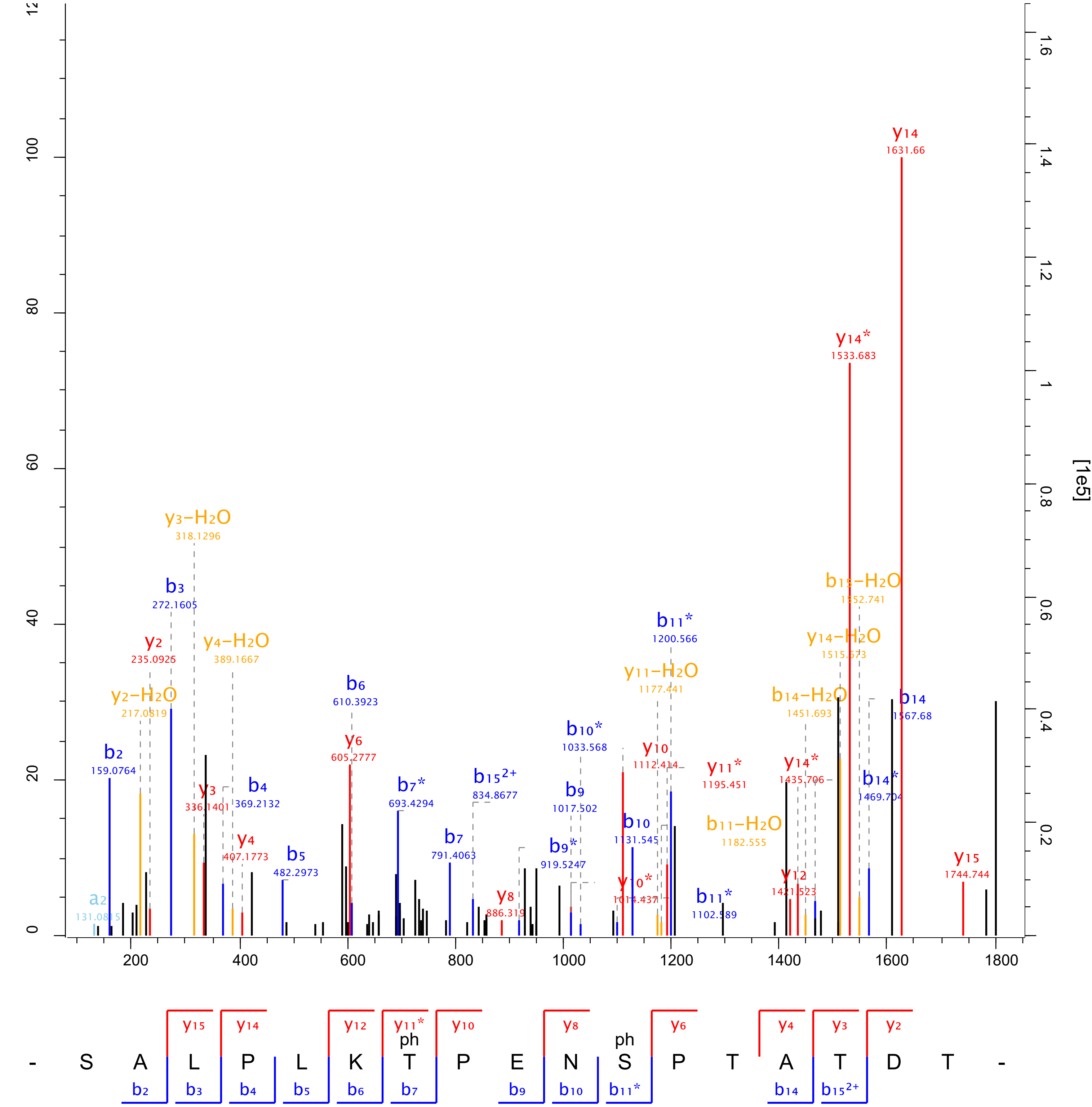

Sample output to test PDF Combine only

Raw file	Scan	Method	Score	m/z	Gene names
0523_4	15214	FTMS; HCD	110.56	866.39	T9C5.180;At3g49590

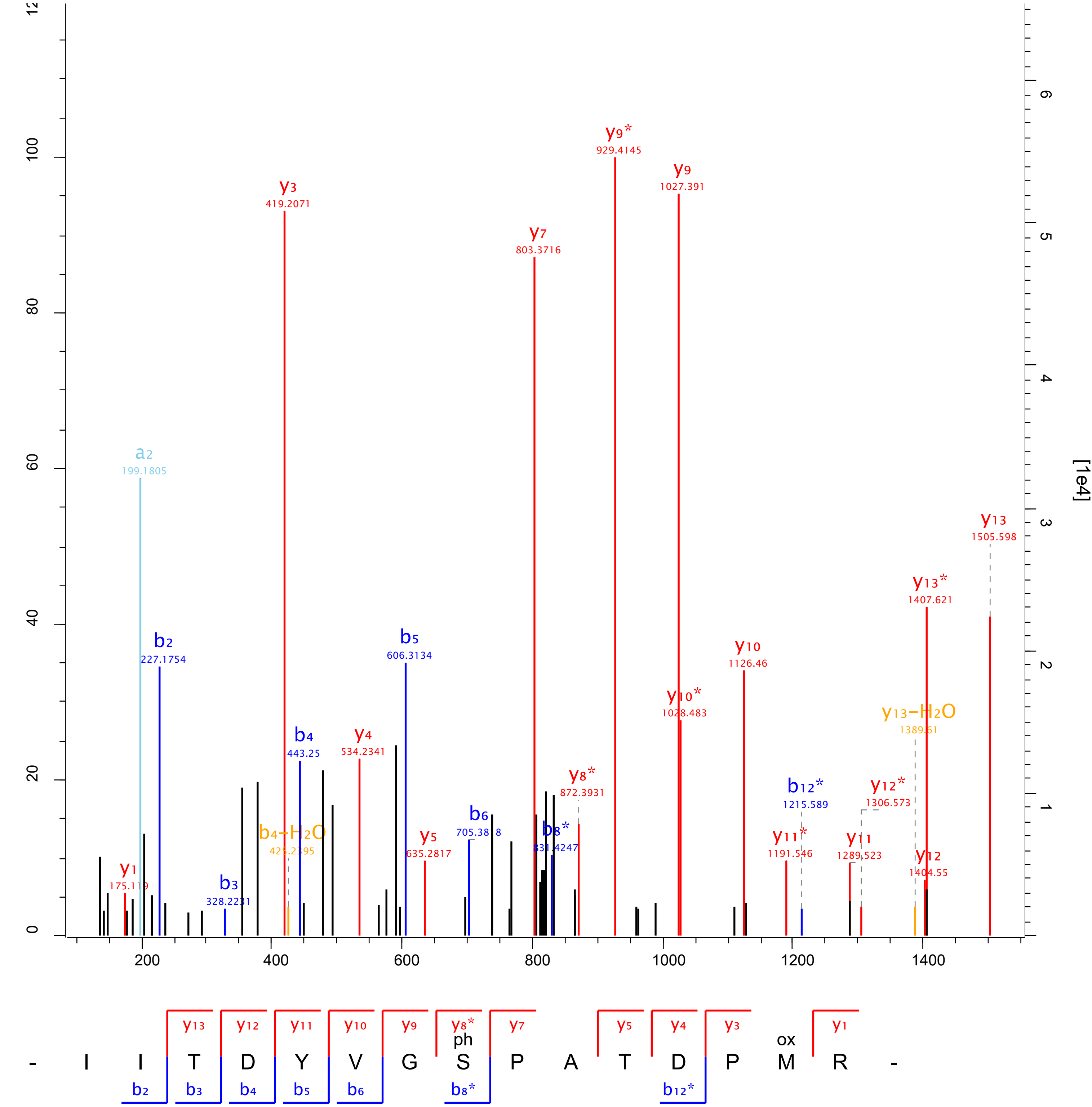

Sample output to test PDF Combine only

Raw file	Scan	Method	Score	m/z	Gene names
0523_4	15241	FTMS; HCD	197.38	981.38	At1g15280

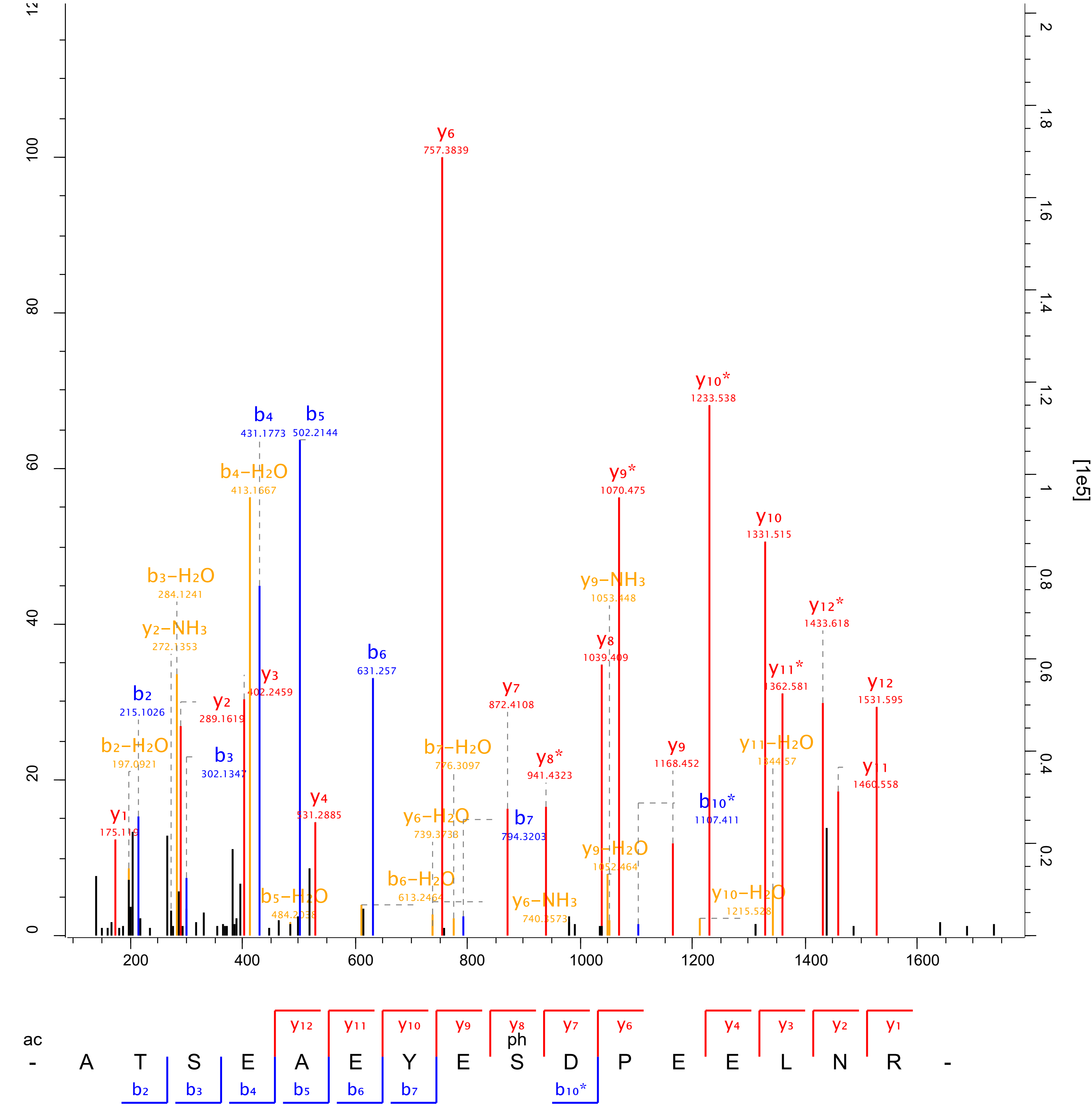

Sample output to test PDF Combine only

Raw file	Scan	Method	Score	m/z	Gene names
0523_4	15252	FTMS; HCD	65.8	853.7	F13I12.260

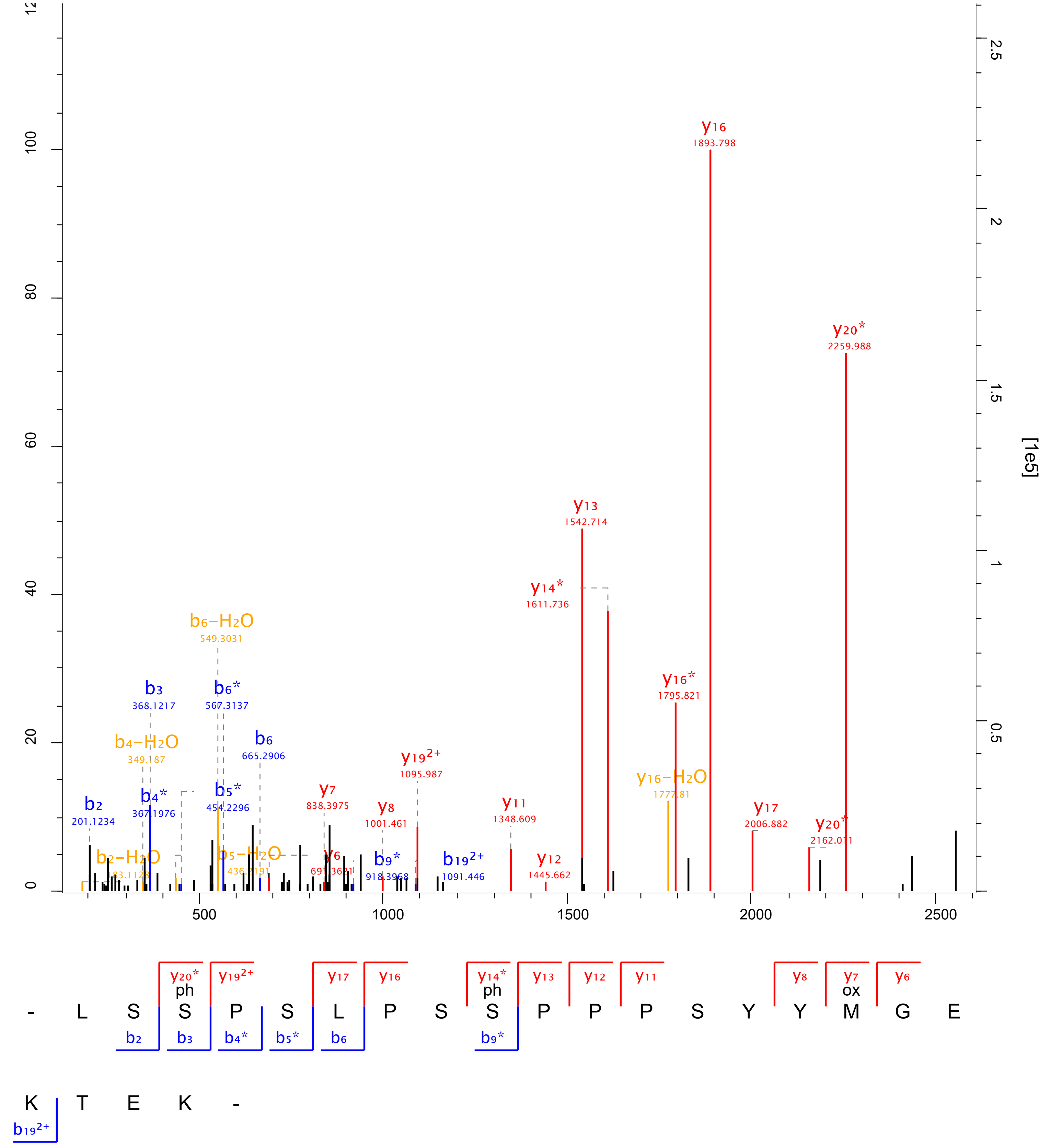

Sample output to test PDF Combine only

Raw file	Scan	Method	Score	m/z	Gene names
0523_4	15268	FTMS; HCD	56.98	834.36	ABCB20

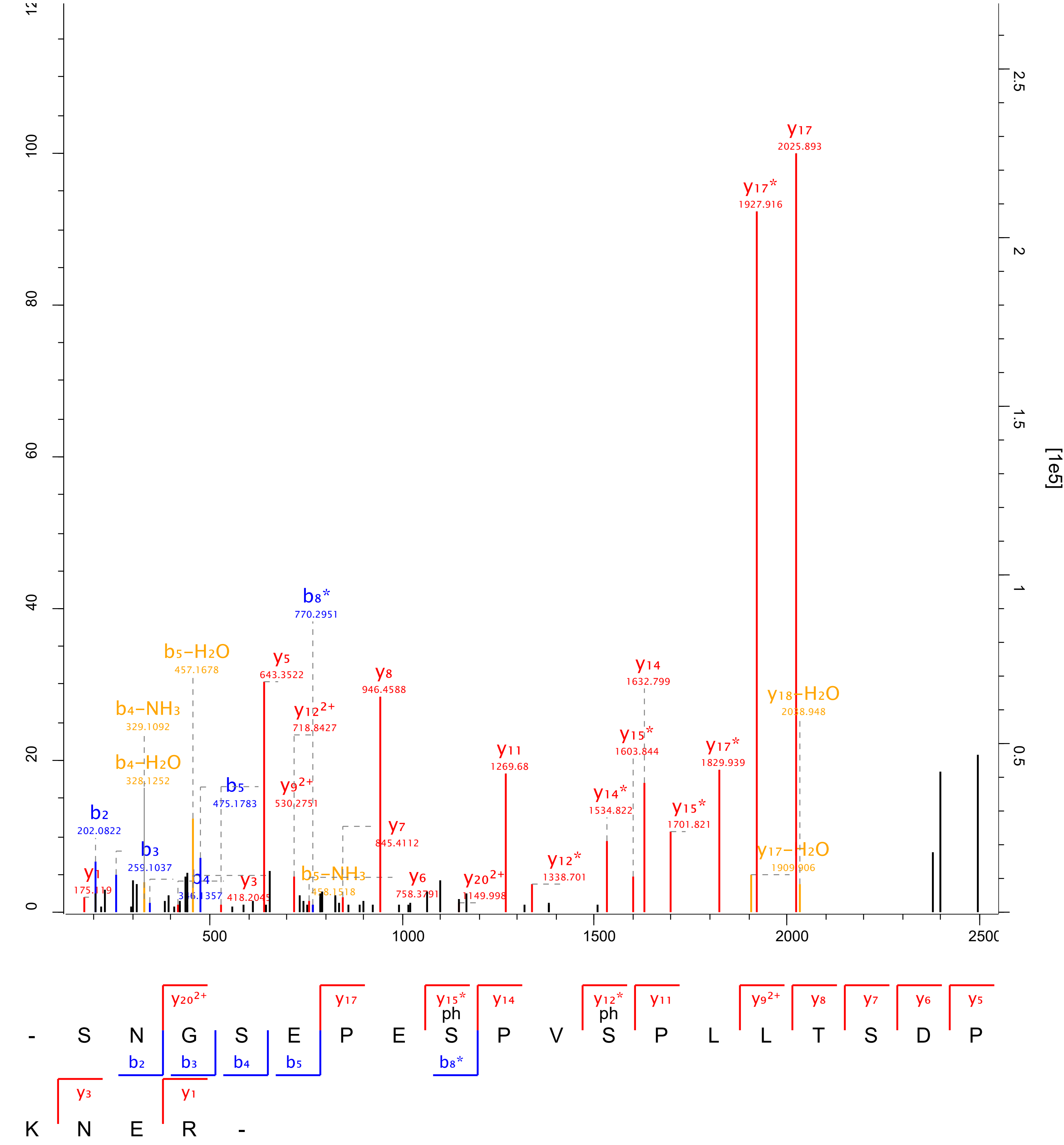

Sample output to test PDF Combine only

Raw file	Scan	Method	Score	m/z
0523_4	15315	FTMS; HCD	131.33	697.67

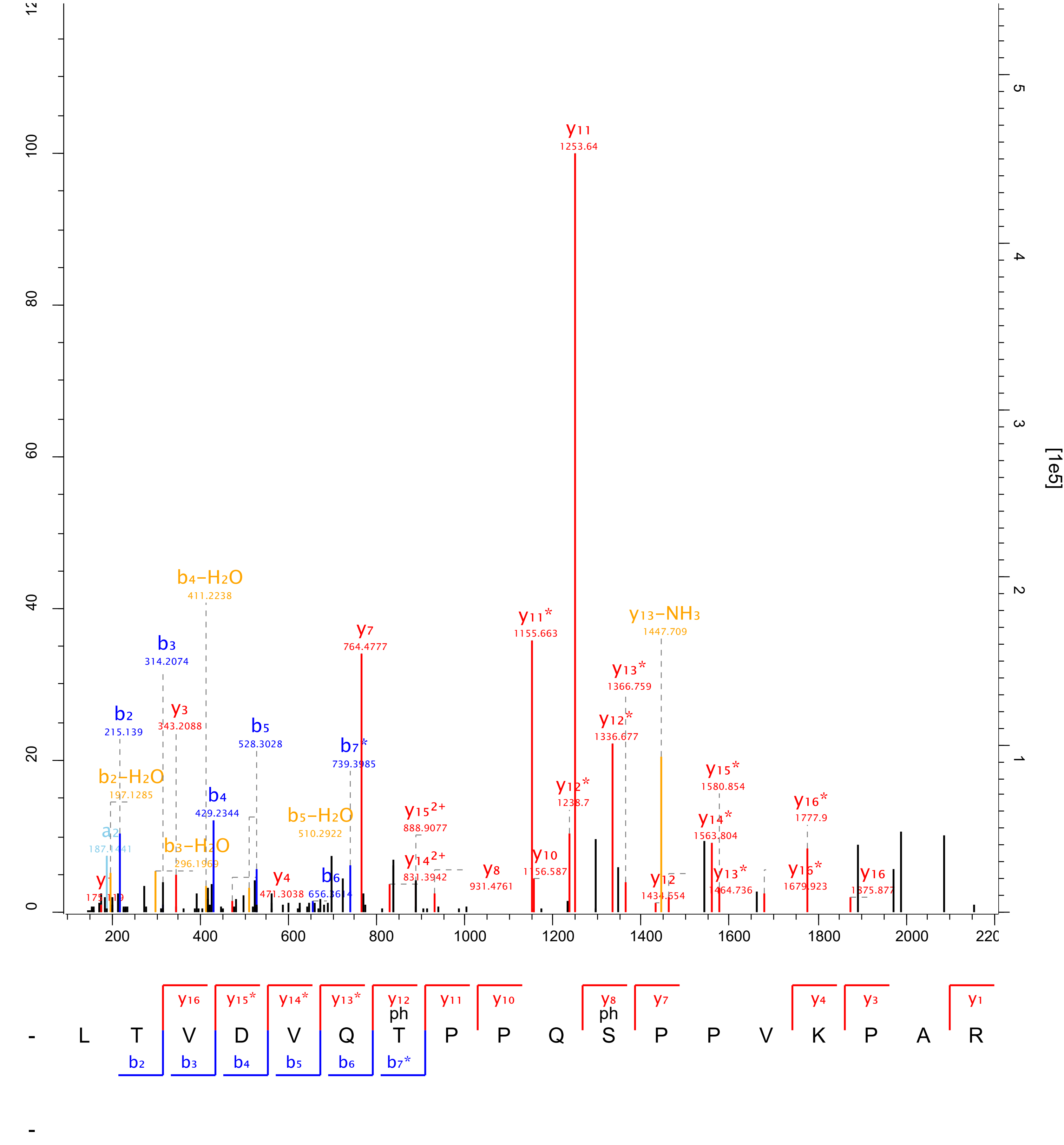

Sample output to test PDF Combine only

Raw file	Scan	Method	Score	m/z	Gene names
0523_4	15381	FTMS; HCD	91.42	827.73	T20N10_10

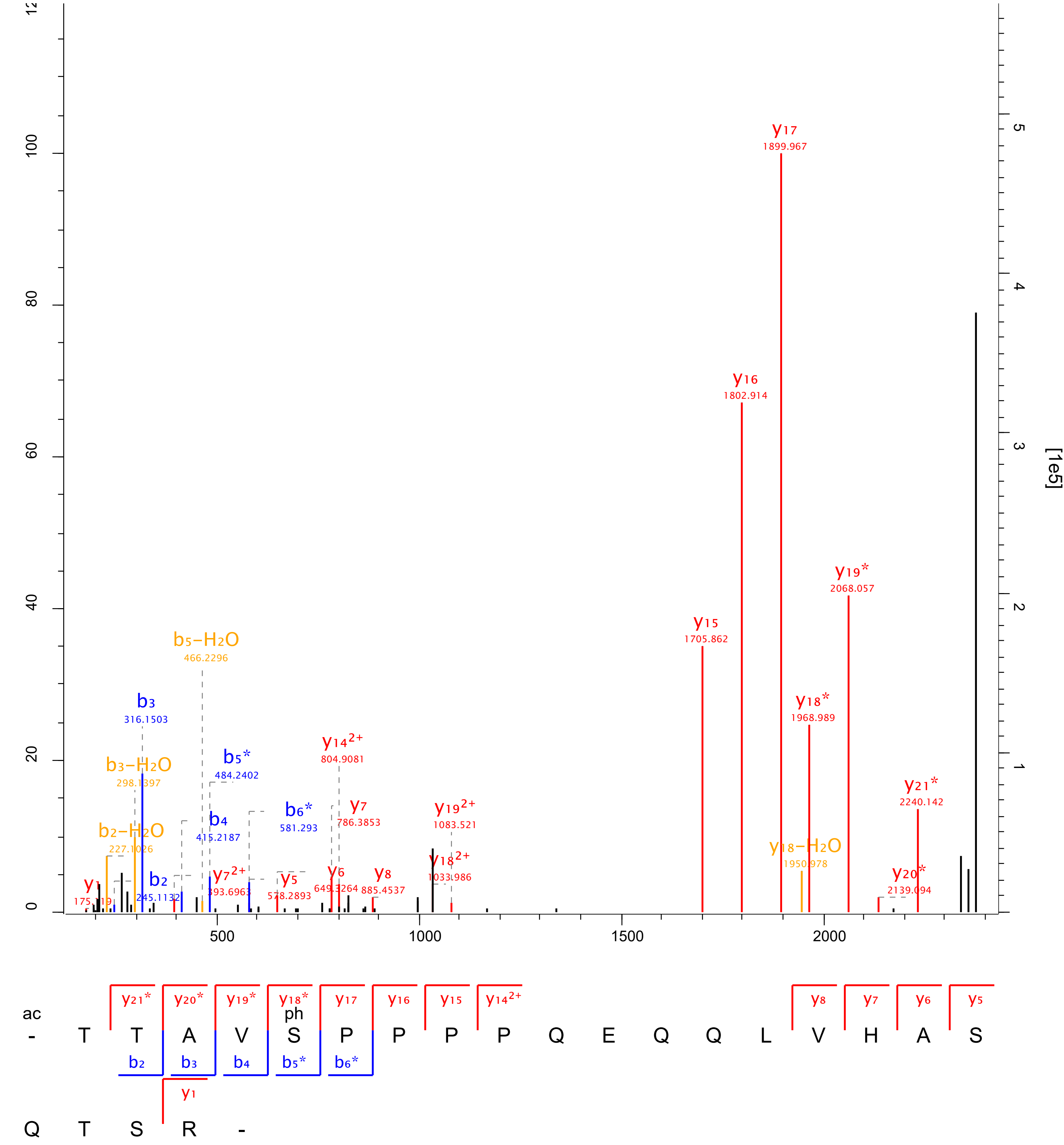

Sample output to test PDF Combine only

Raw file	Scan	Method	Score	m/z
0523_4	15439	FTMS; HCD	183.7	622.77

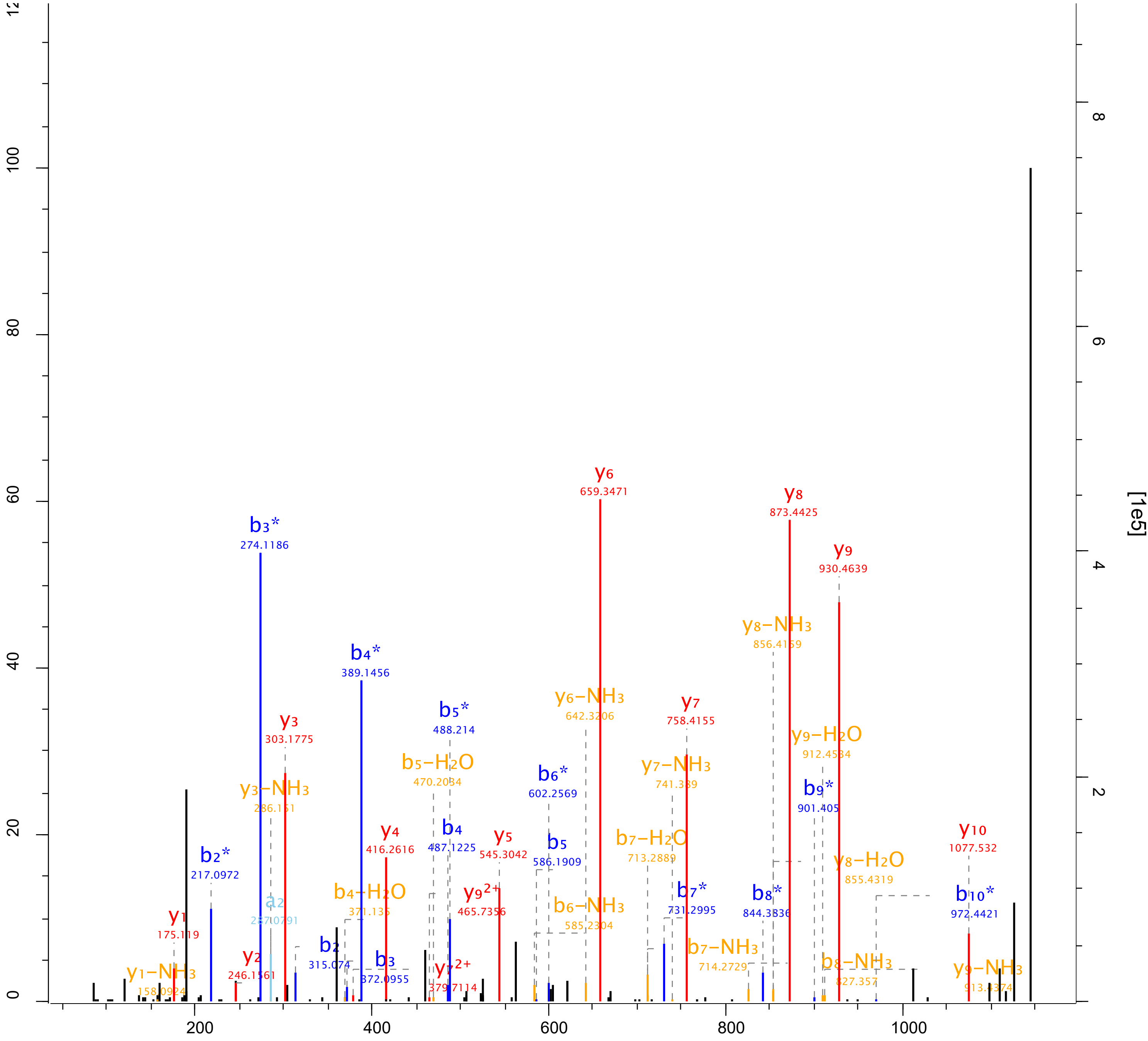

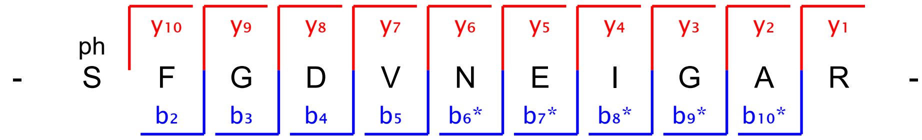

Sample output to test PDF Combine only

Raw file	Scan	Method	Score	m/z	Gene names
0523_4	15448	FTMS; HCD	142.93	963.94	At4g08330

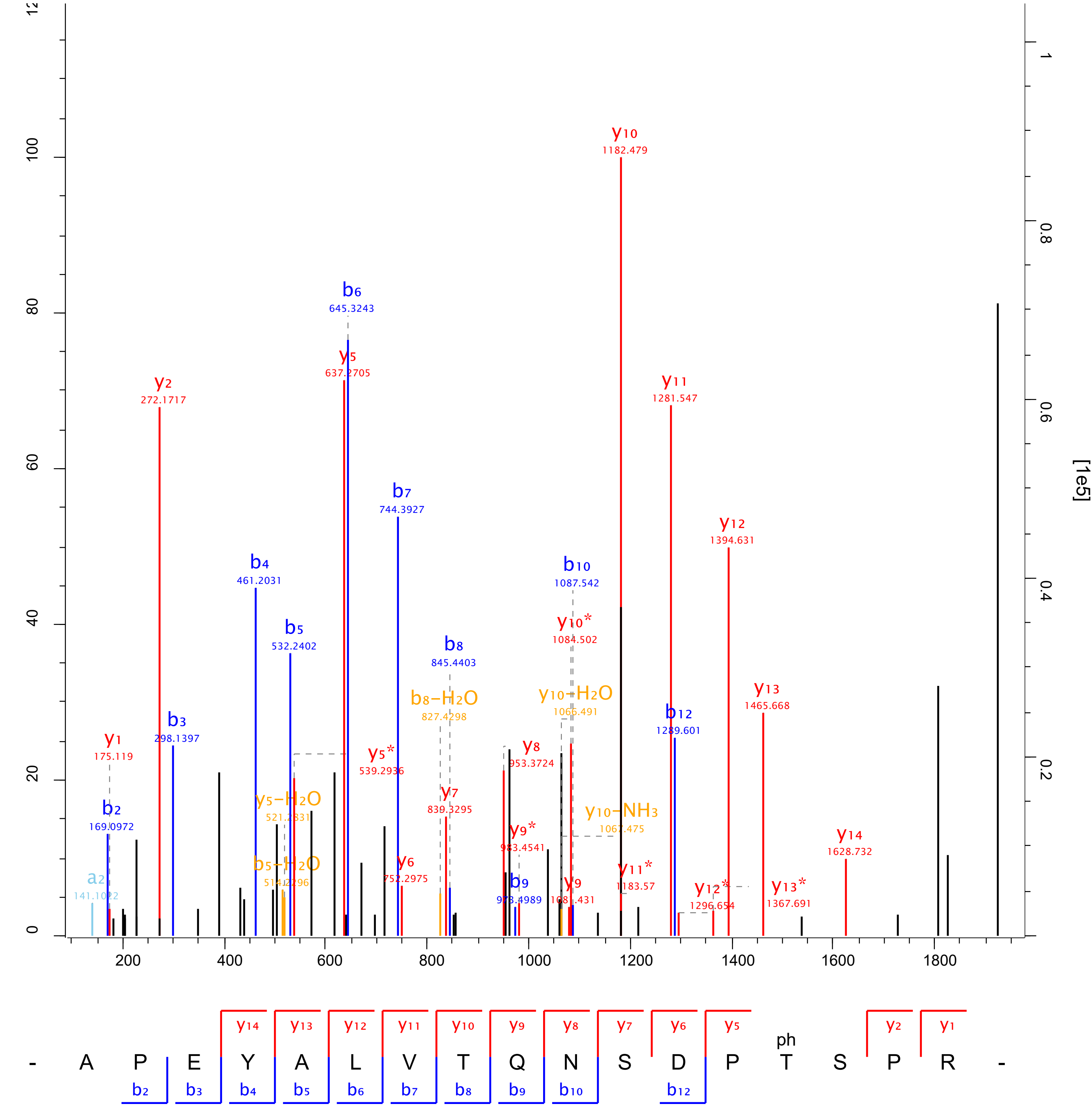

Sample output to test PDF Combine only

Raw file	Scan	Method	Score	m/z	Gene names
0523_4	15506	FTMS; HCD	165.61	898.4	PVA11

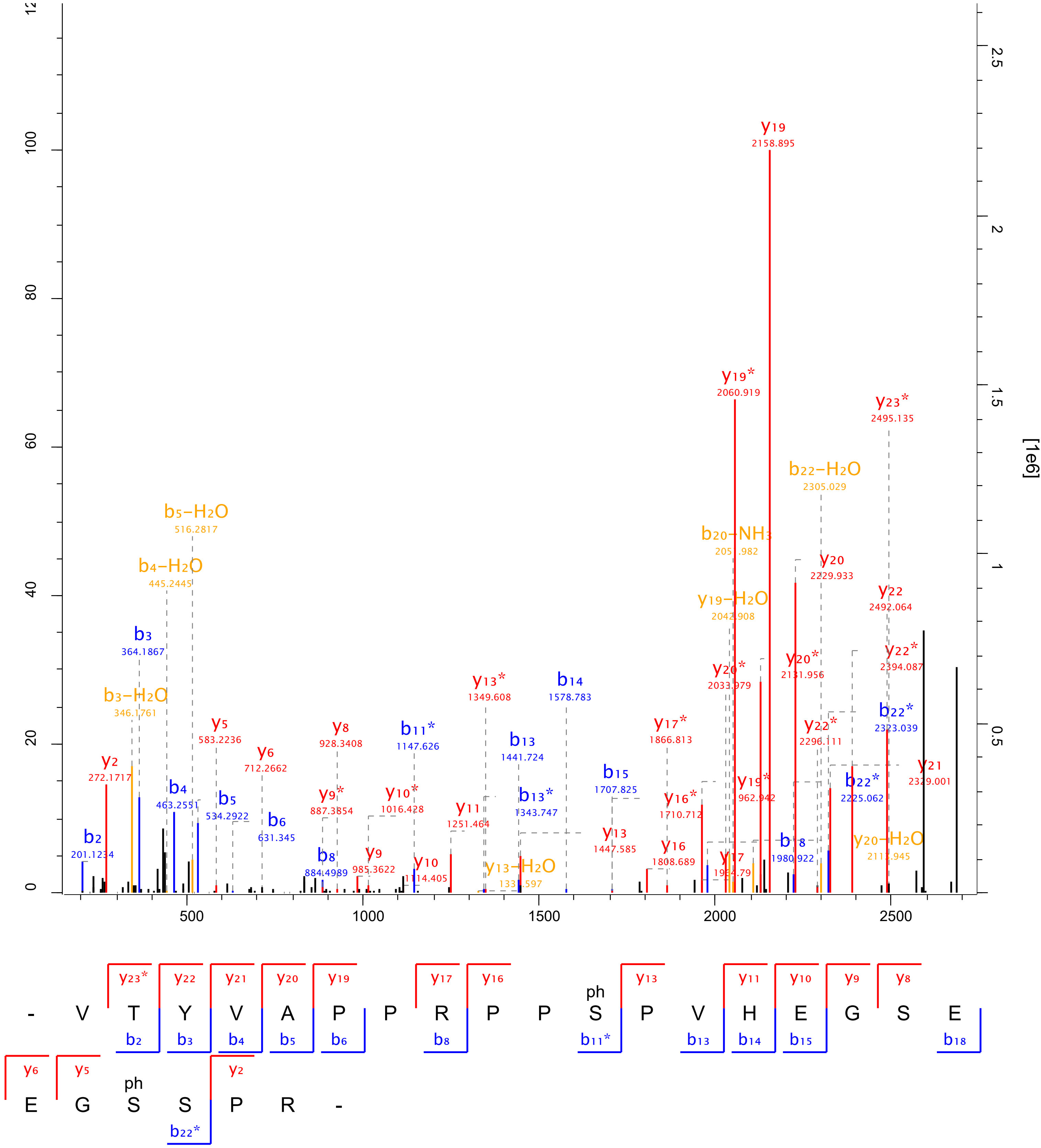

Sample output to test PDF Combine only

Raw file	Scan	Method	Score	m/z	Gene names
0523_4	15520	FTMS; HCD	40.62	866.86	MYOB7

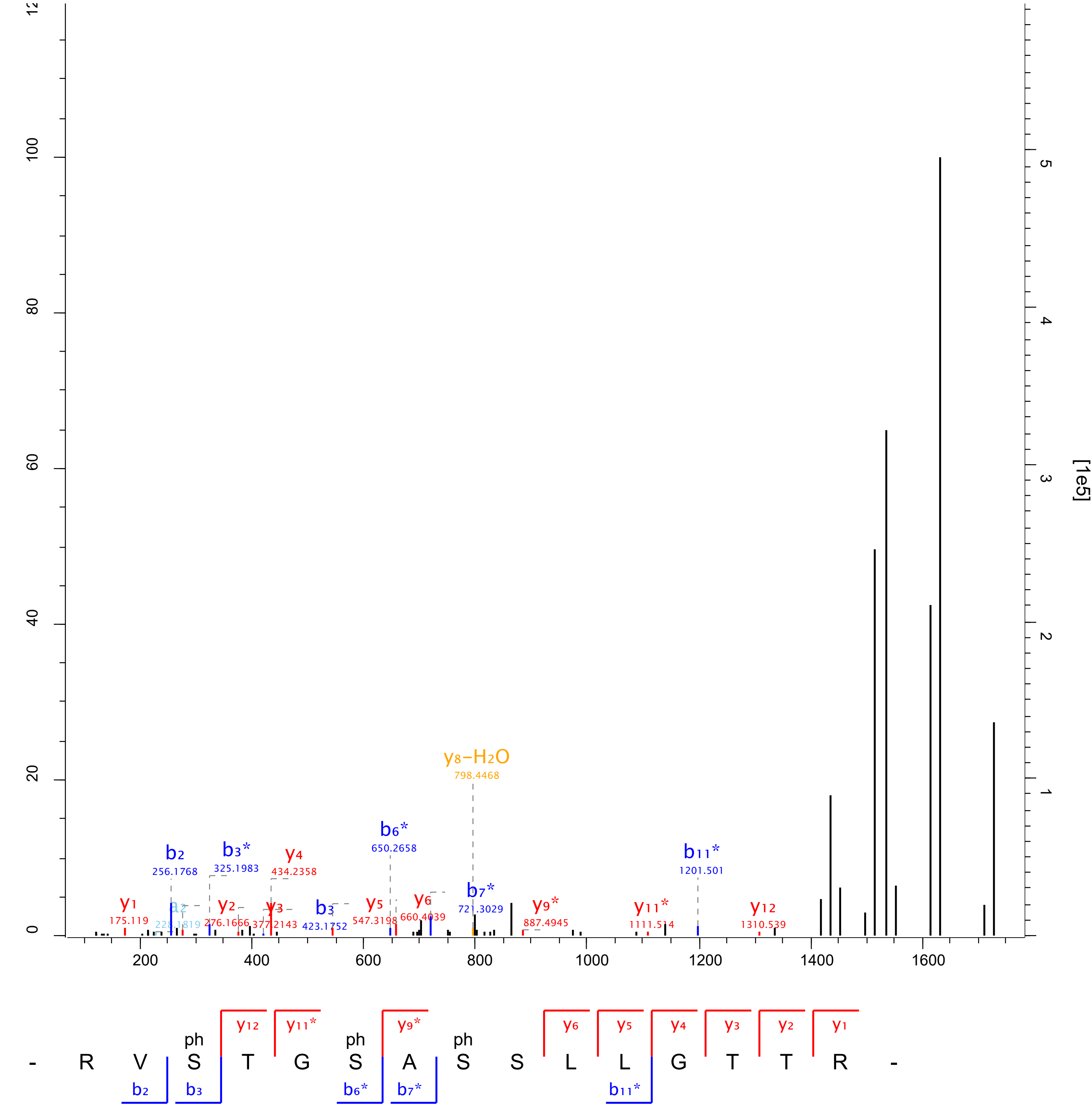

Sample output to test PDF Combine only

Raw file	Scan	Method	Score	m/z	Gene names
0523_4	15539	FTMS; HCD	110.74	797.36	At4g24840

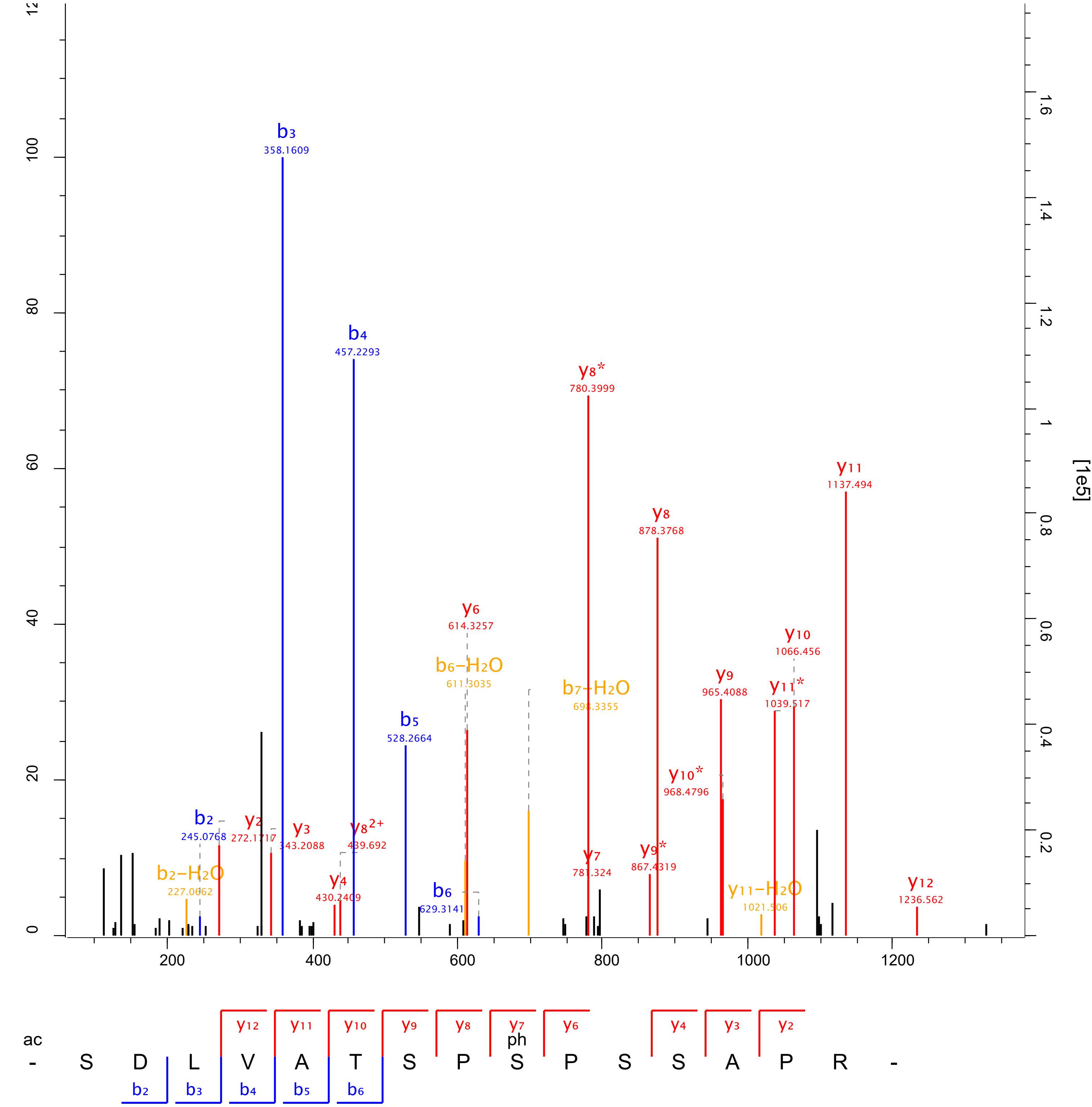

Sample output to test PDF Combine only

Raw file	Scan	Method	Score	m/z	Gene names
0523_4	15610	FTMS; HCD	130	709.25	PIP2-8

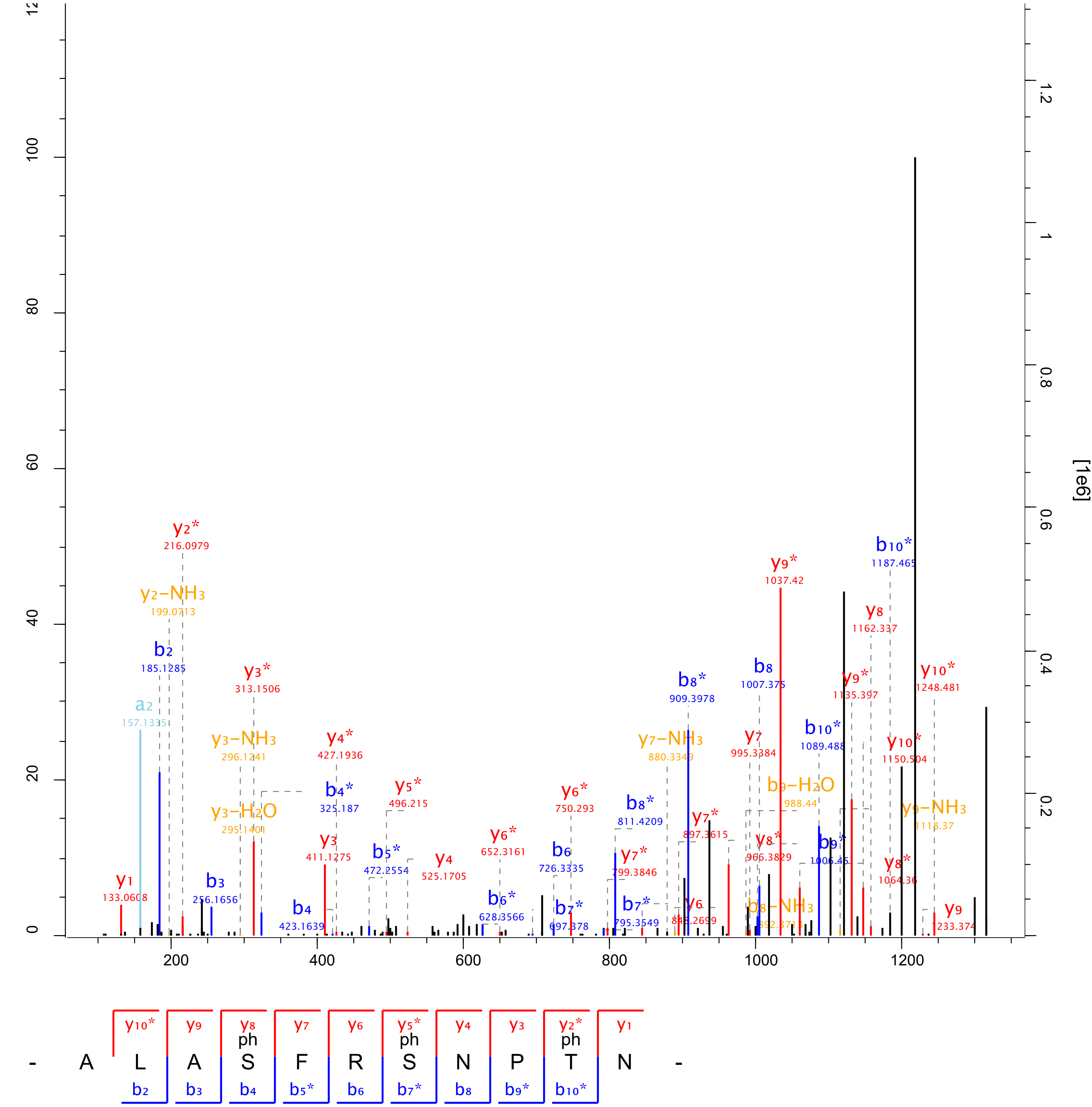

Sample output to test PDF Combine only

Raw file	Scan	Method	Score	m/z	Gene names
0523_4	15659	FTMS; HCD	56.02	823.35	T24D18.26

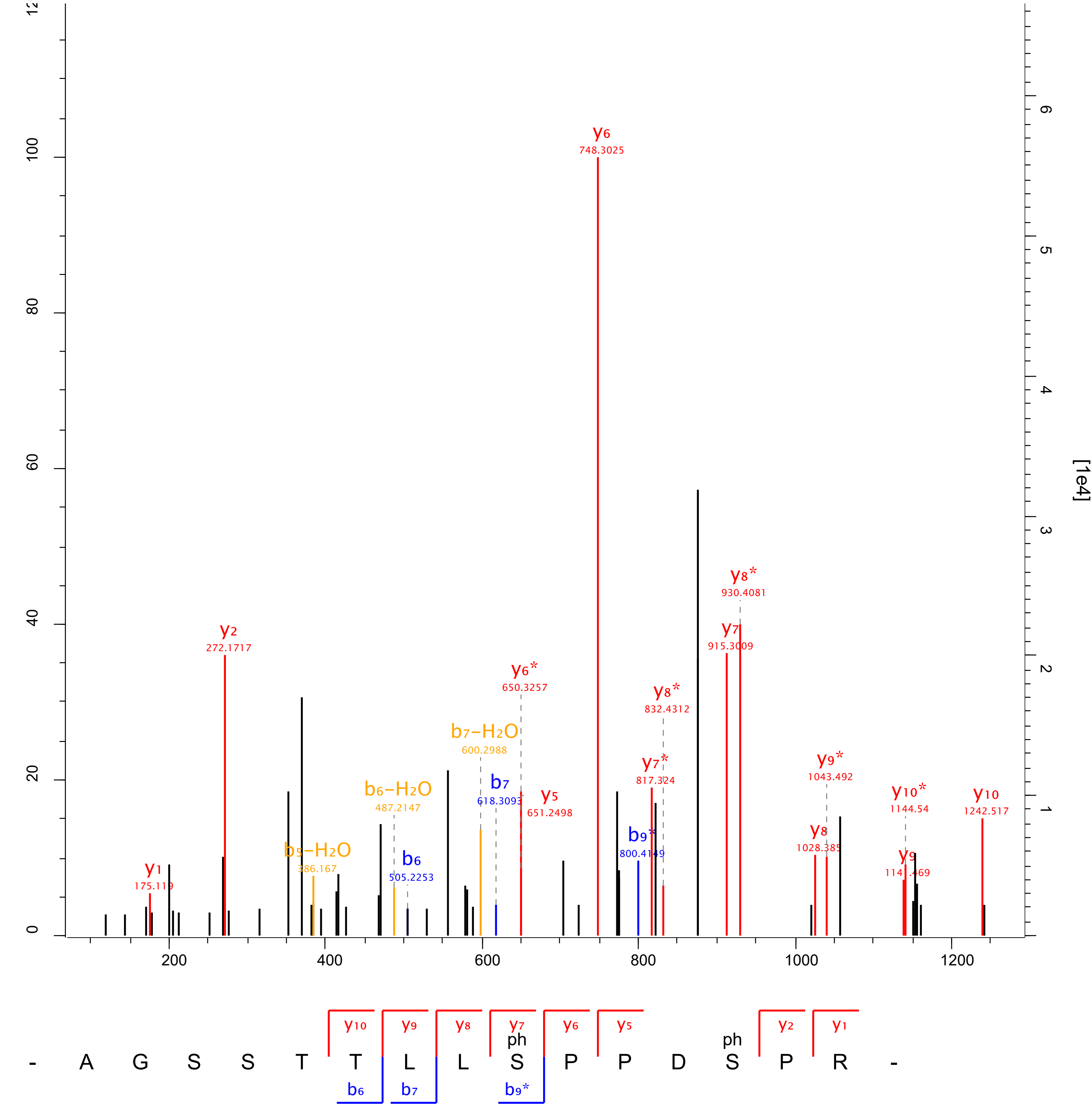

Sample output to test PDF Combine only

Raw file	Scan	Method	Score	m/z	Gene names
0523_4	15677	FTMS; HCD	69.92	706.83	At3g58640

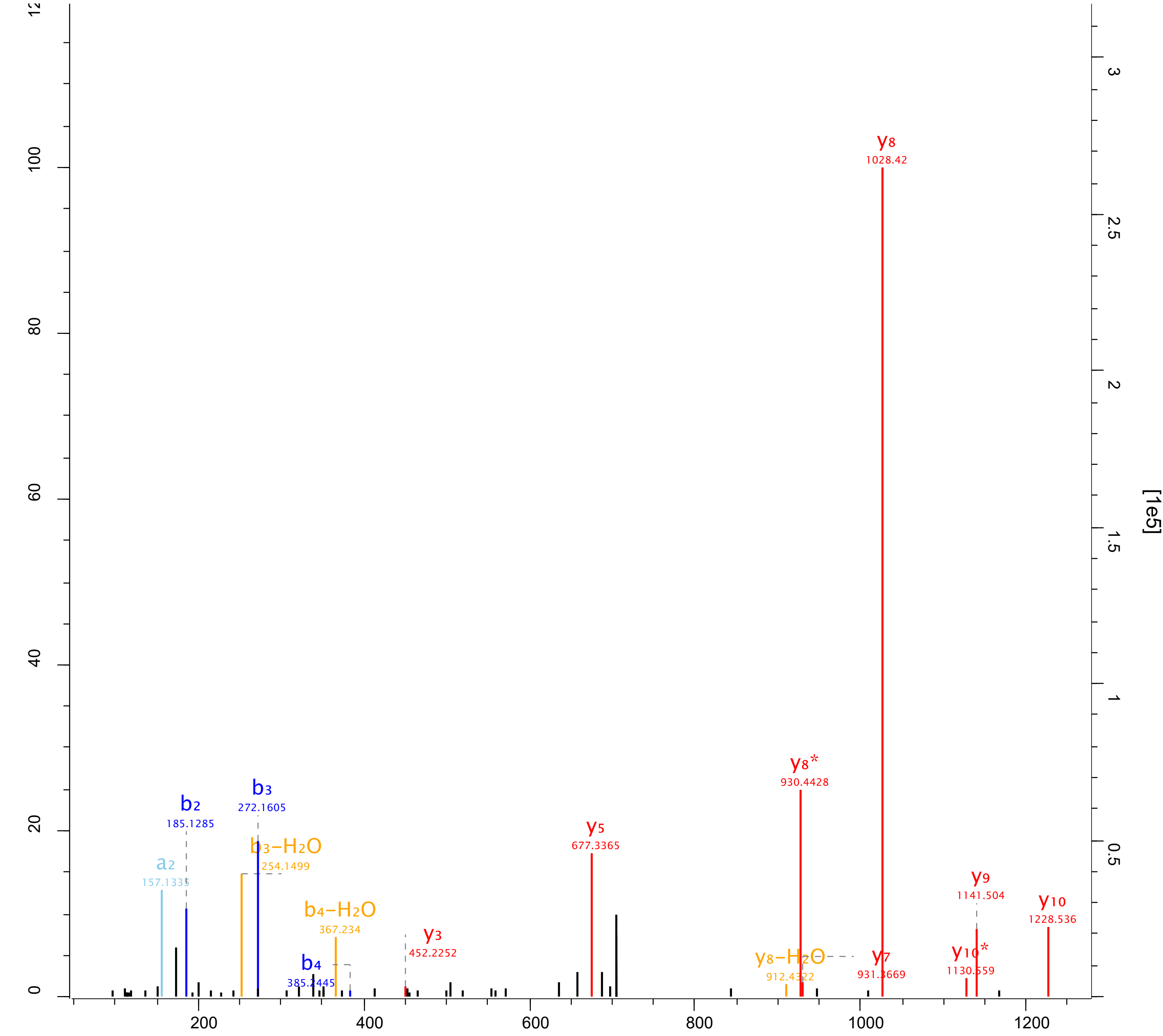

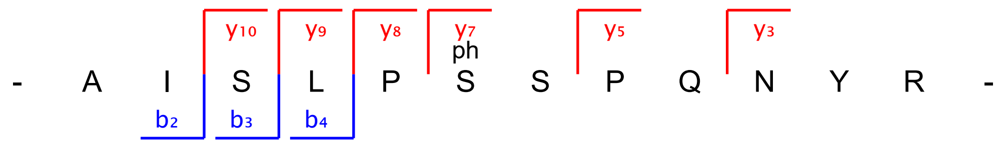

Sample output to test PDF Combine only

Raw file	Scan	Method	Score	m/z	Gene names
0523_4	15771	FTMS; HCD	96.16	930.74	PVA12

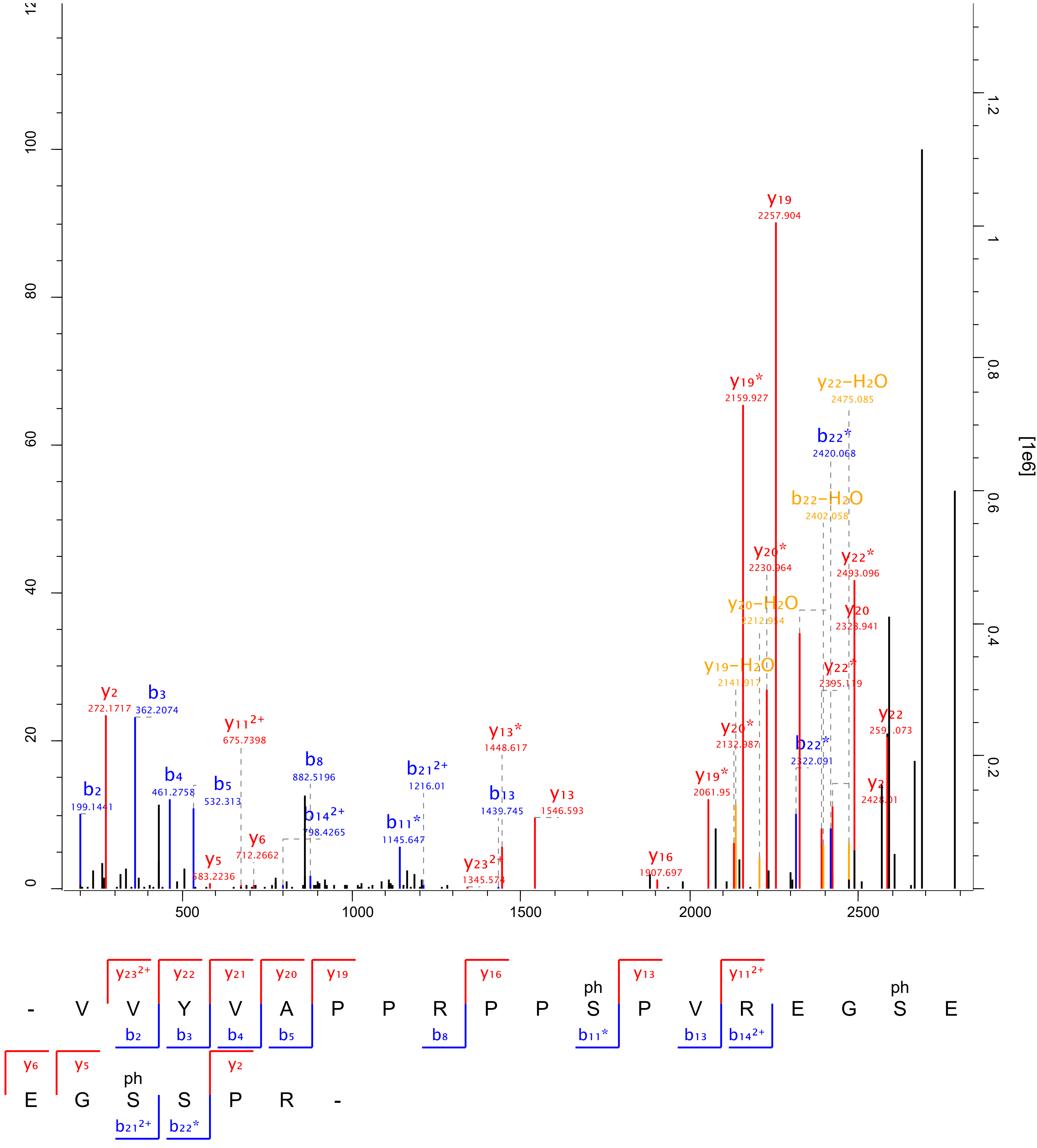

Sample output to test PDF Combine only

Raw file	Scan	Method	Score	m/z	Gene names
0523_4	15783	FTMS; HCD	148.41	688.35	DIM

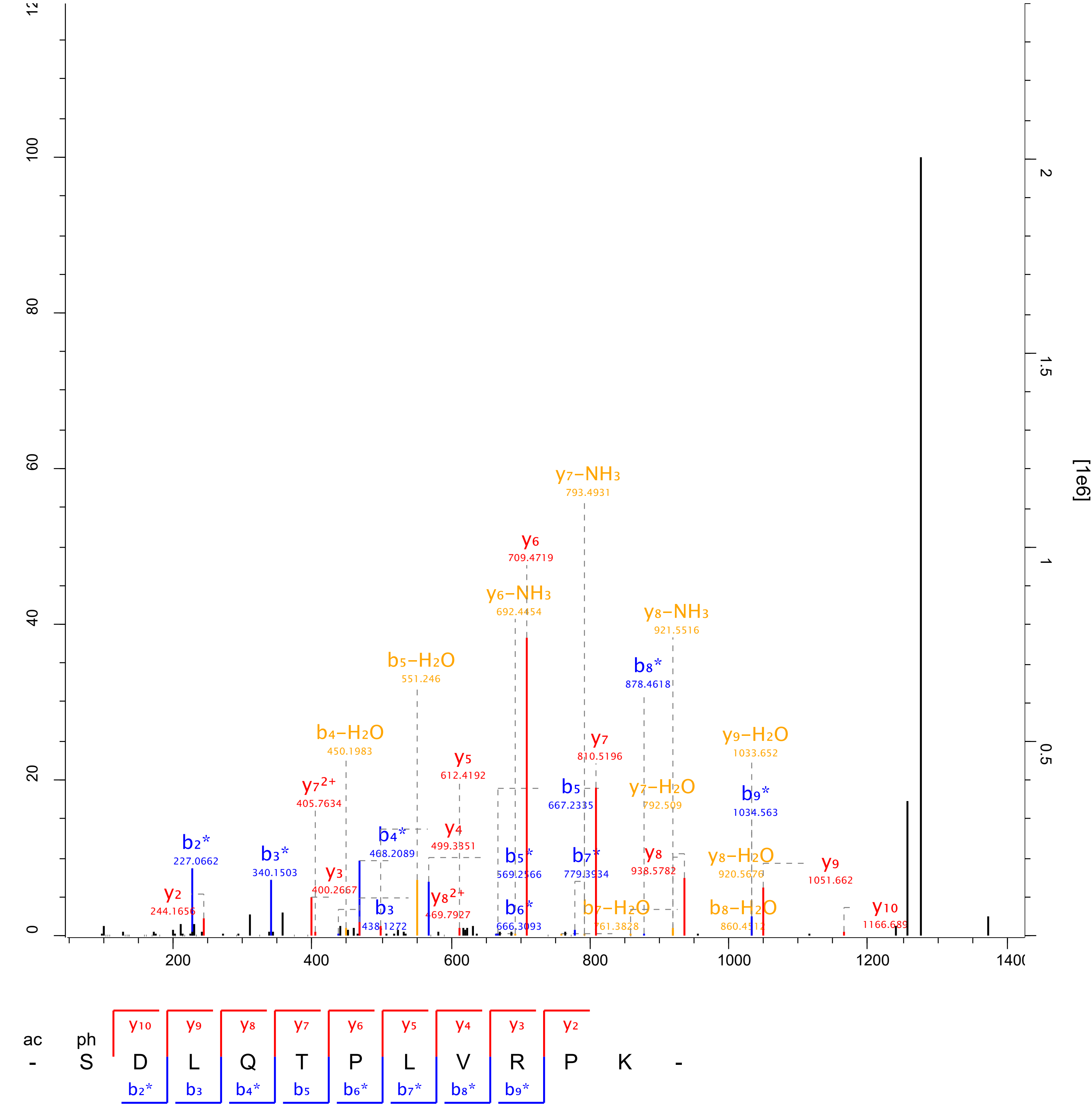

Sample output to test PDF Combine only

Raw file	Scan	Method	Score	m/z	Gene names
0523_4	15932	FTMS; HCD	96.79	805.65	At3g56720

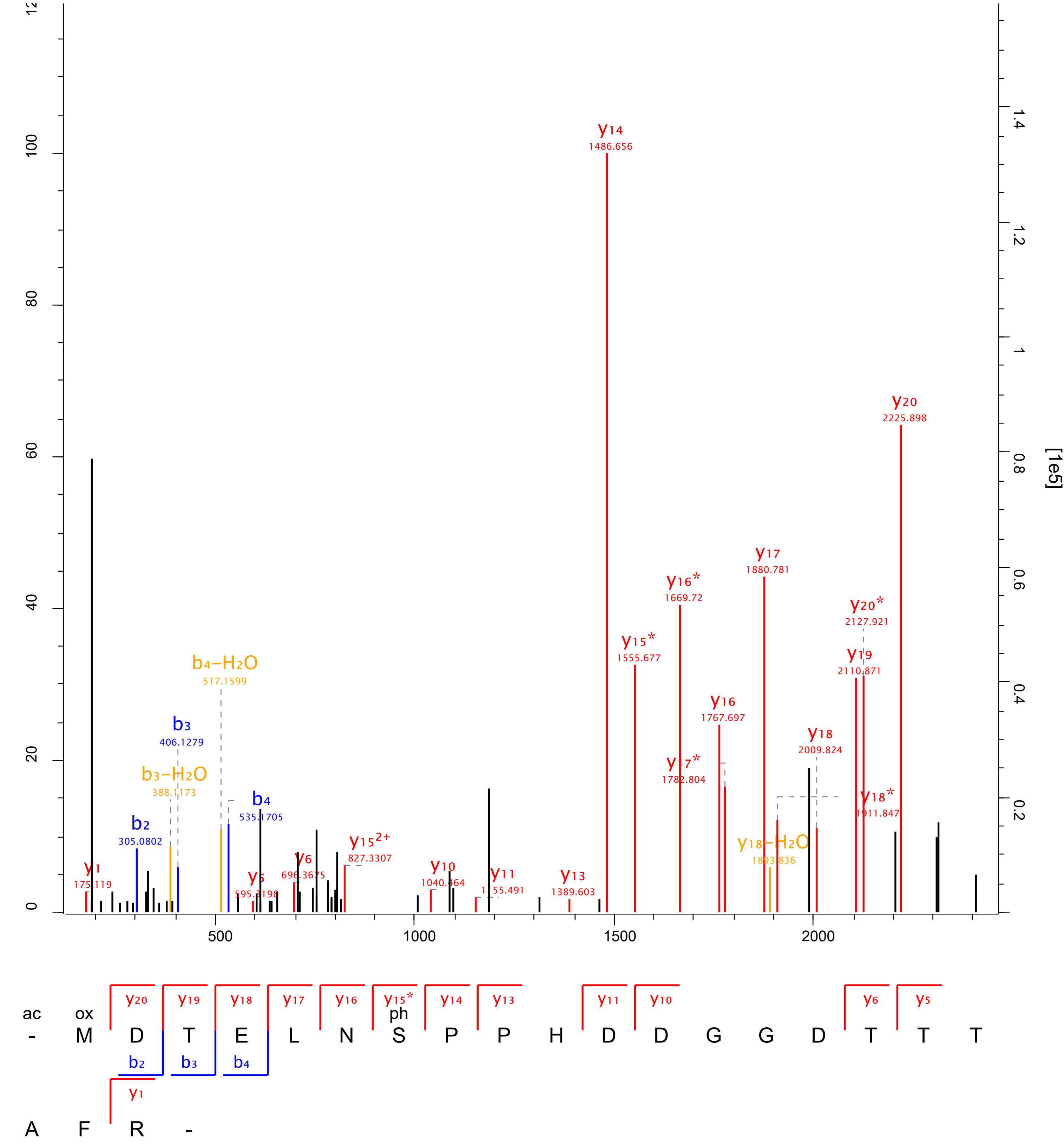

Sample output to test PDF Combine only

Raw file	Scan	Method	Score	m/z	Gene names
0523_4	15968	FTMS; HCD	40.14	650.95	At4g31130

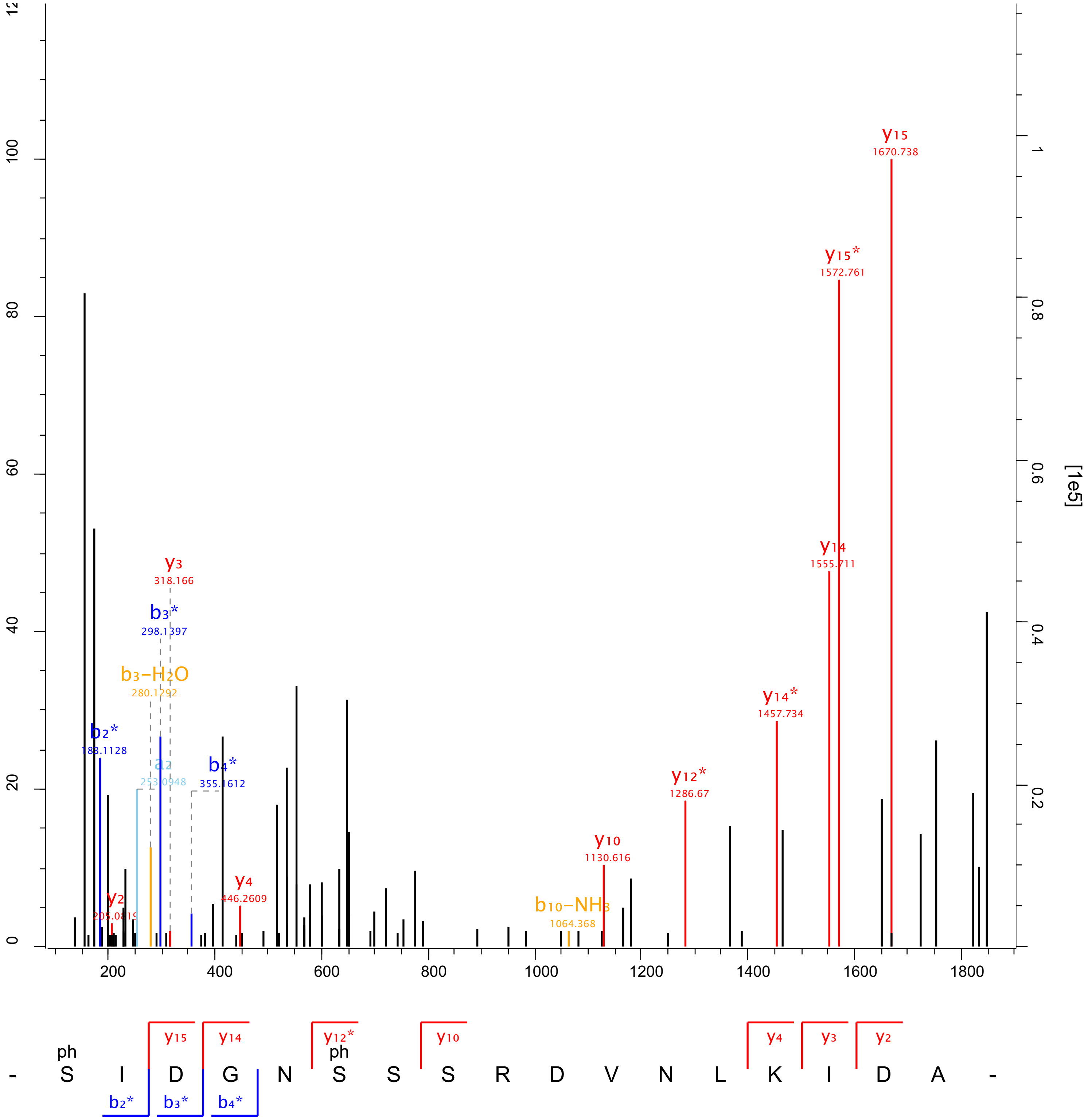

Sample output to test PDF Combine only

Raw file	Scan	Method	Score	m/z	Gene names
0523_4	16151	FTMS; HCD	132.65	969.39	BTR1

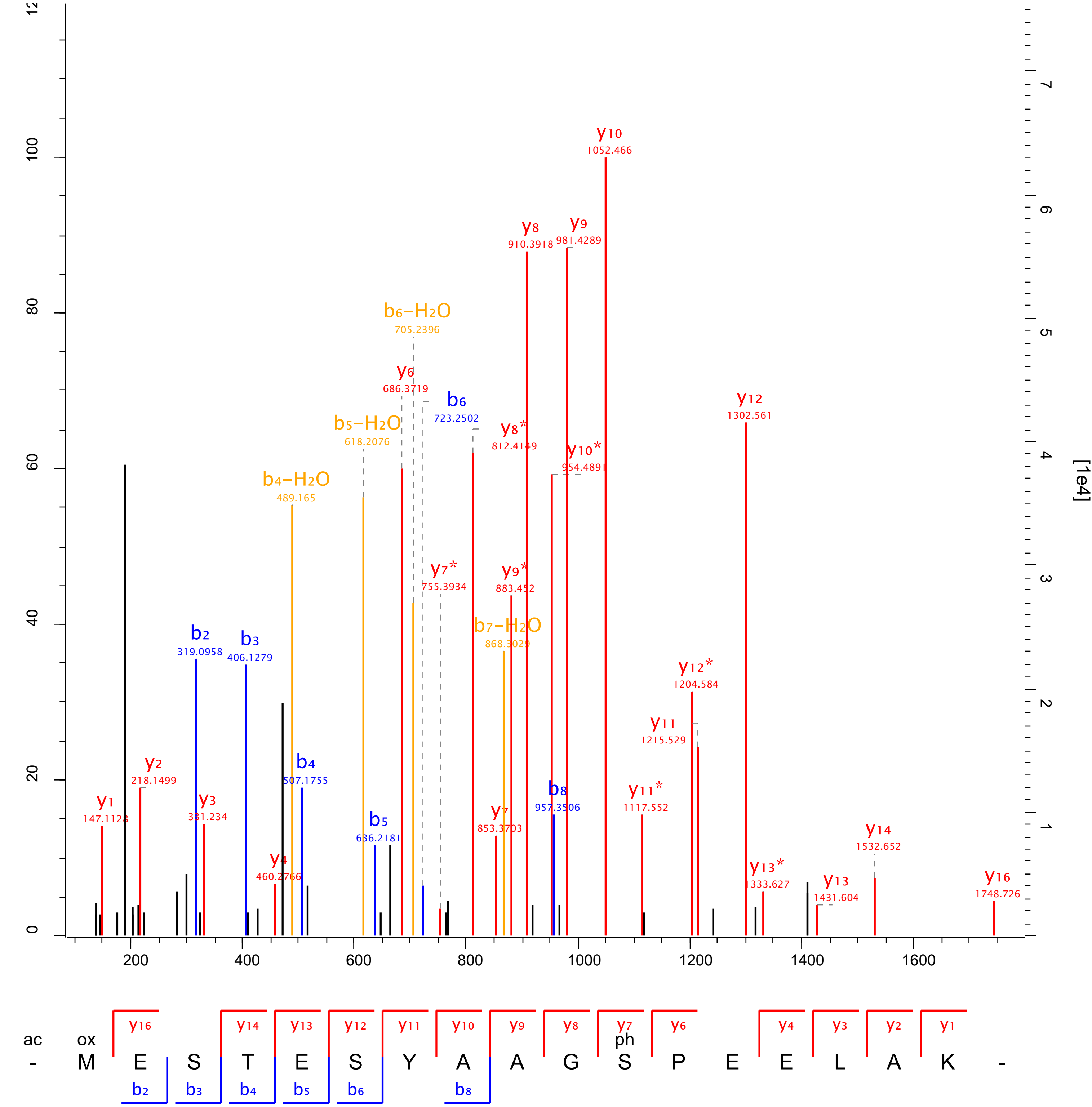

Sample output to test PDF Combine only

Raw file	Scan	Method	Score	m/z	Gene names
0523_4	16309	FTMS; HCD	199.41	822.39	SUN1

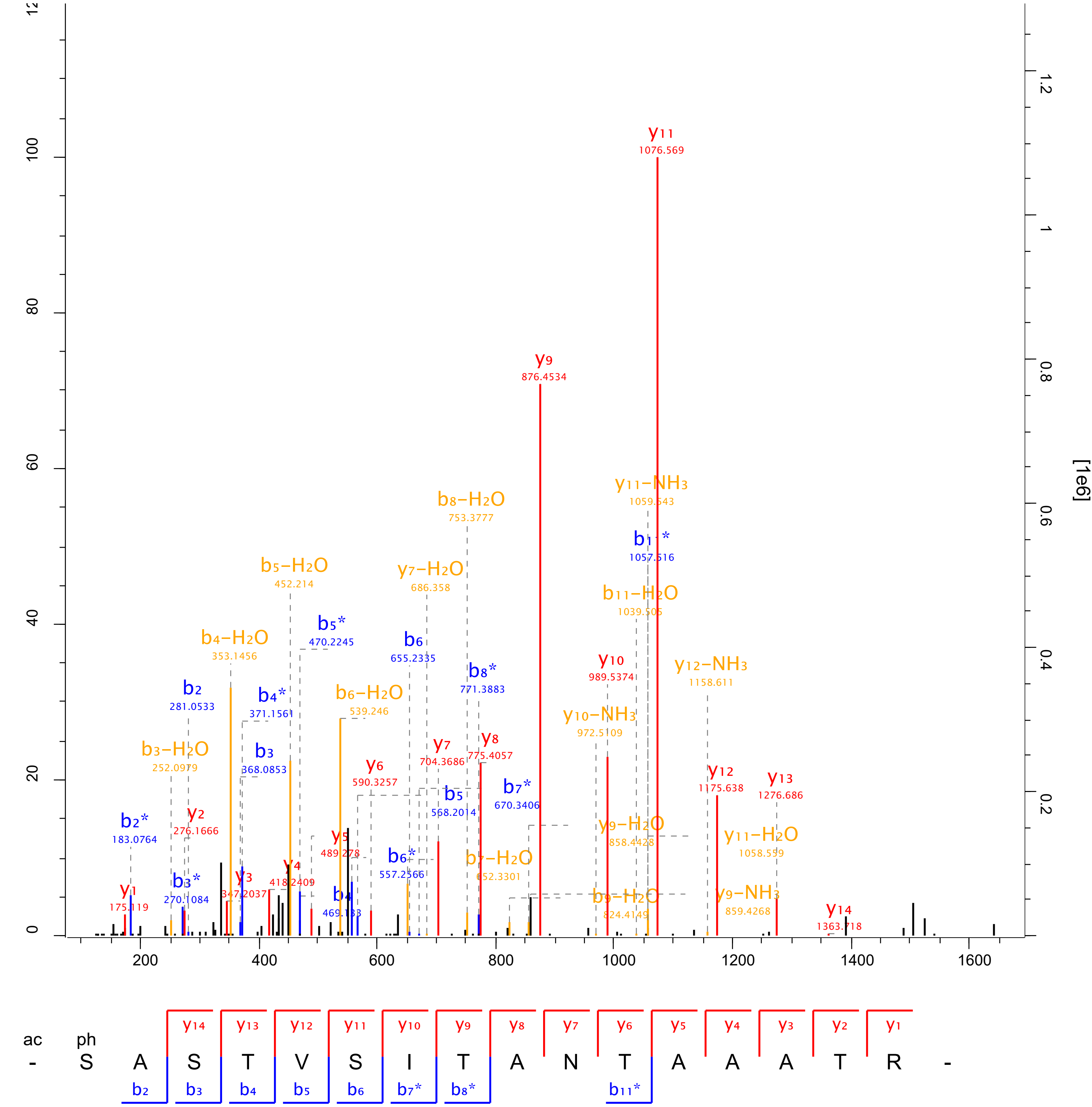

Sample output to test PDF Combine only

Raw file	Scan	Method	Score	m/z	Gene names
0523_4	16529	FTMS; HCD	169.92	778.83	ACBP4

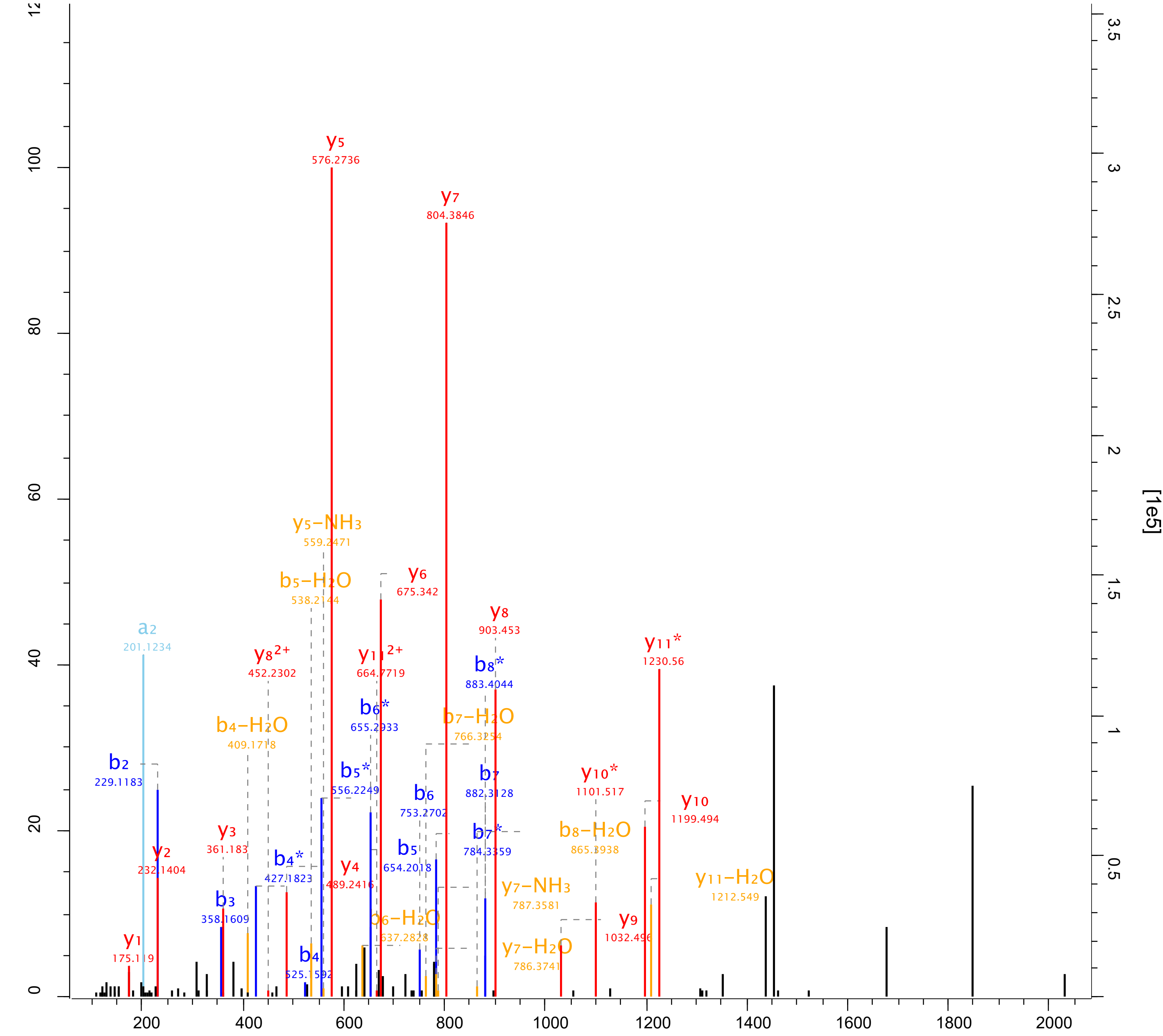

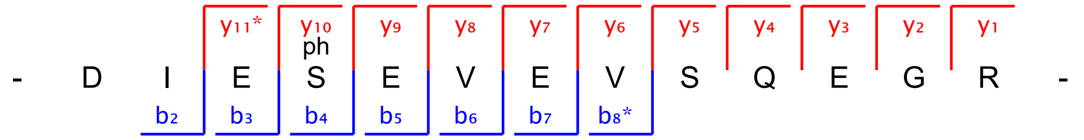

Sample output to test PDF Combine only

Raw file	Scan	Method	Score	m/z	Gene names
0523_4	16619	FTMS; HCD	126.68	1028.46	CRWN1

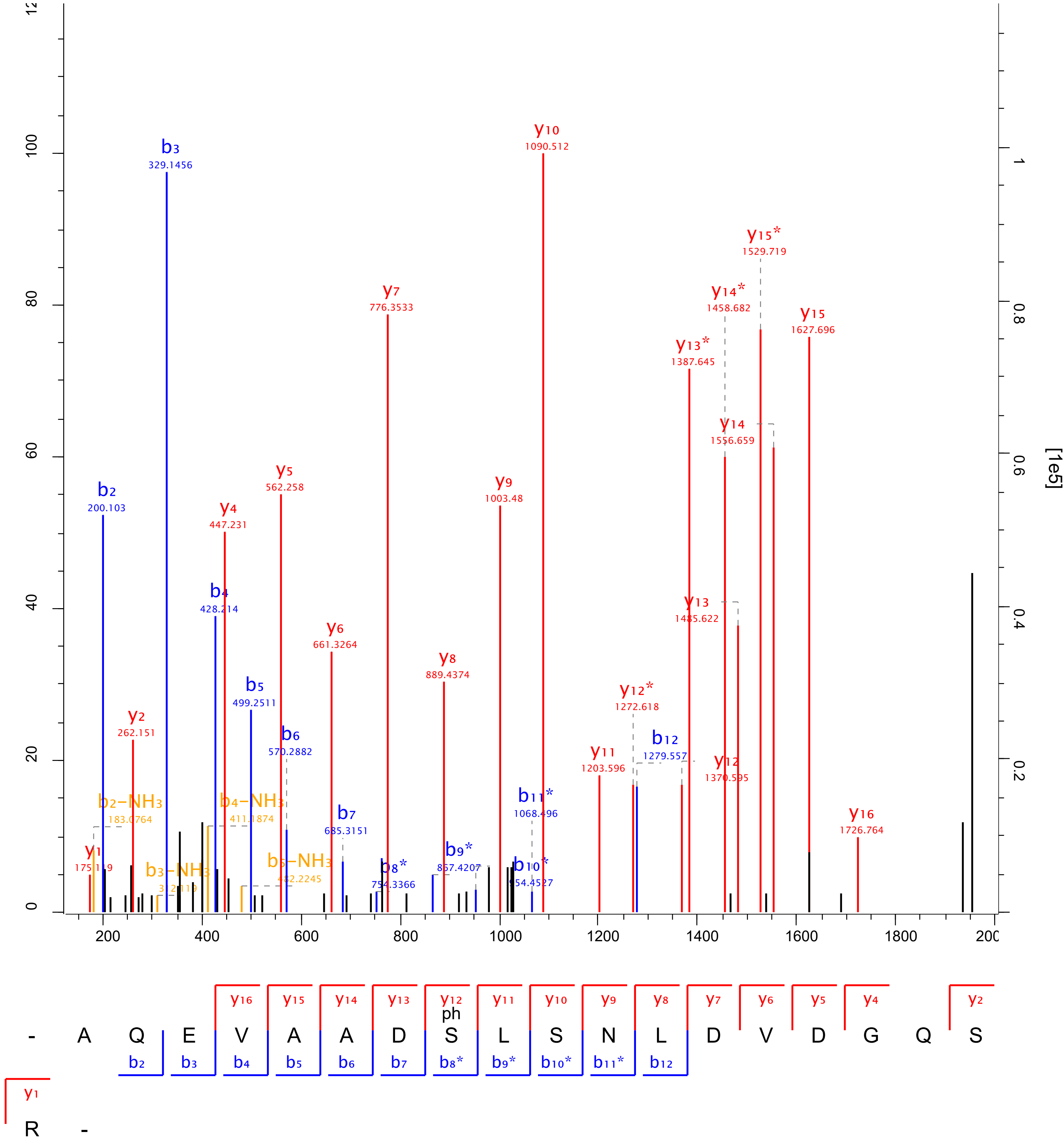

Sample output to test PDF Combine only

Raw file	Scan	Method	Score	m/z
0523_4	16637	FTMS; HCD	251.56	917.94

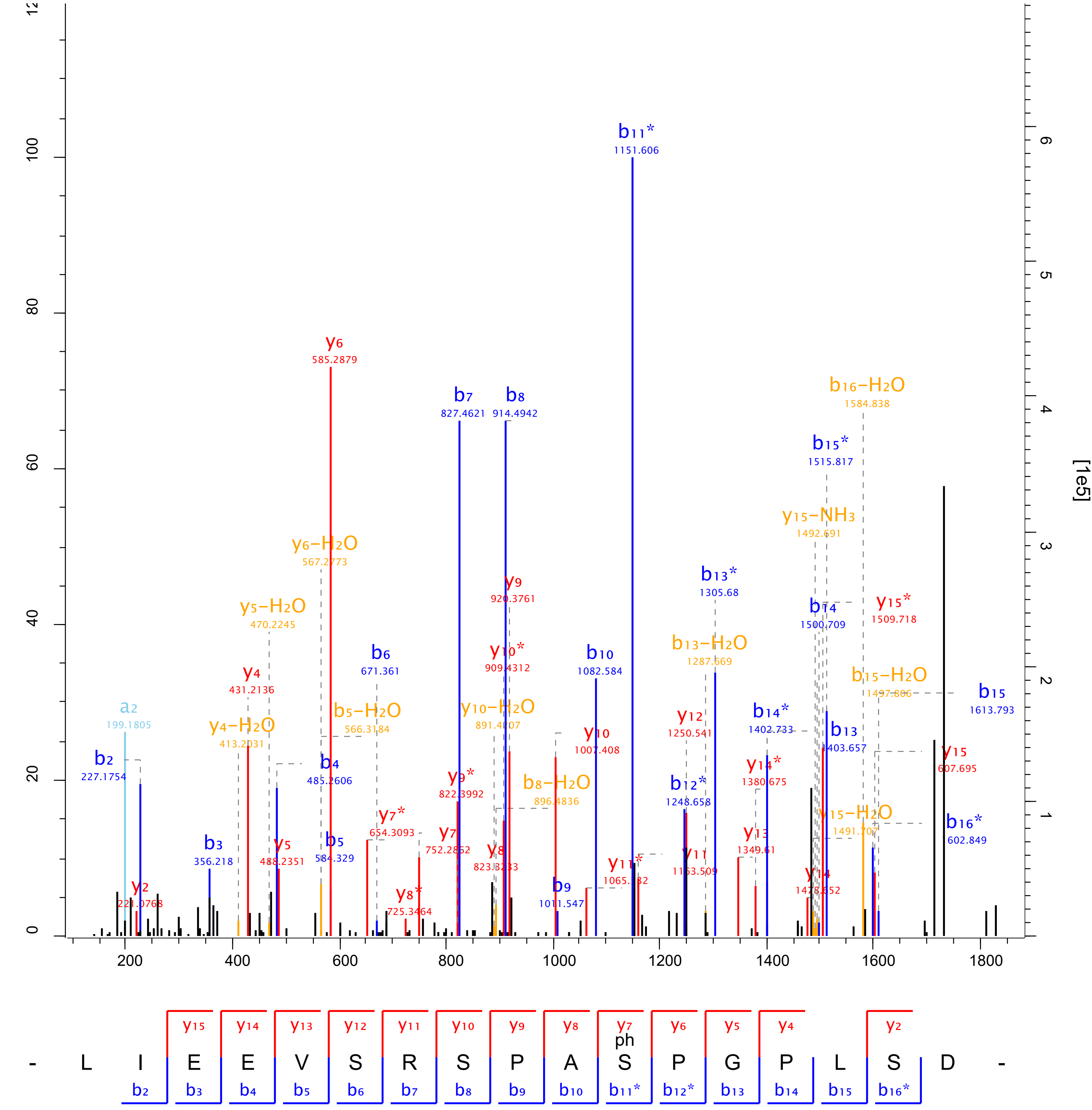

Sample output to test PDF Combine only

Raw file	Scan	Method	Score	m/z	Gene names
0523_4	16663	FTMS; HCD	88.24	623.98	At2g29210

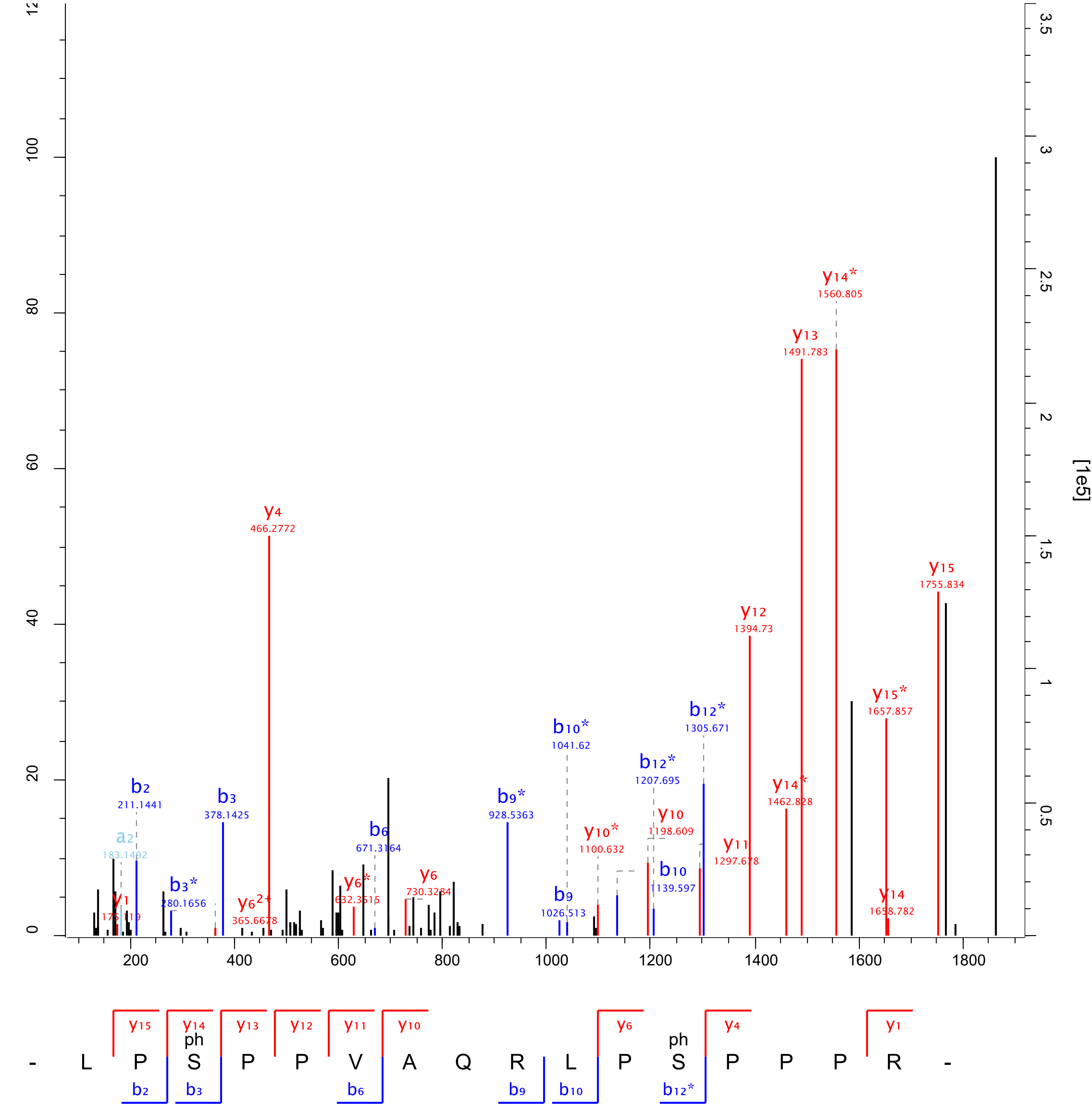

Sample output to test PDF Combine only

Raw file	Scan	Method	Score	m/z	Gene names
0523_4	16677	FTMS; HCD	181.51	727.8	PME1

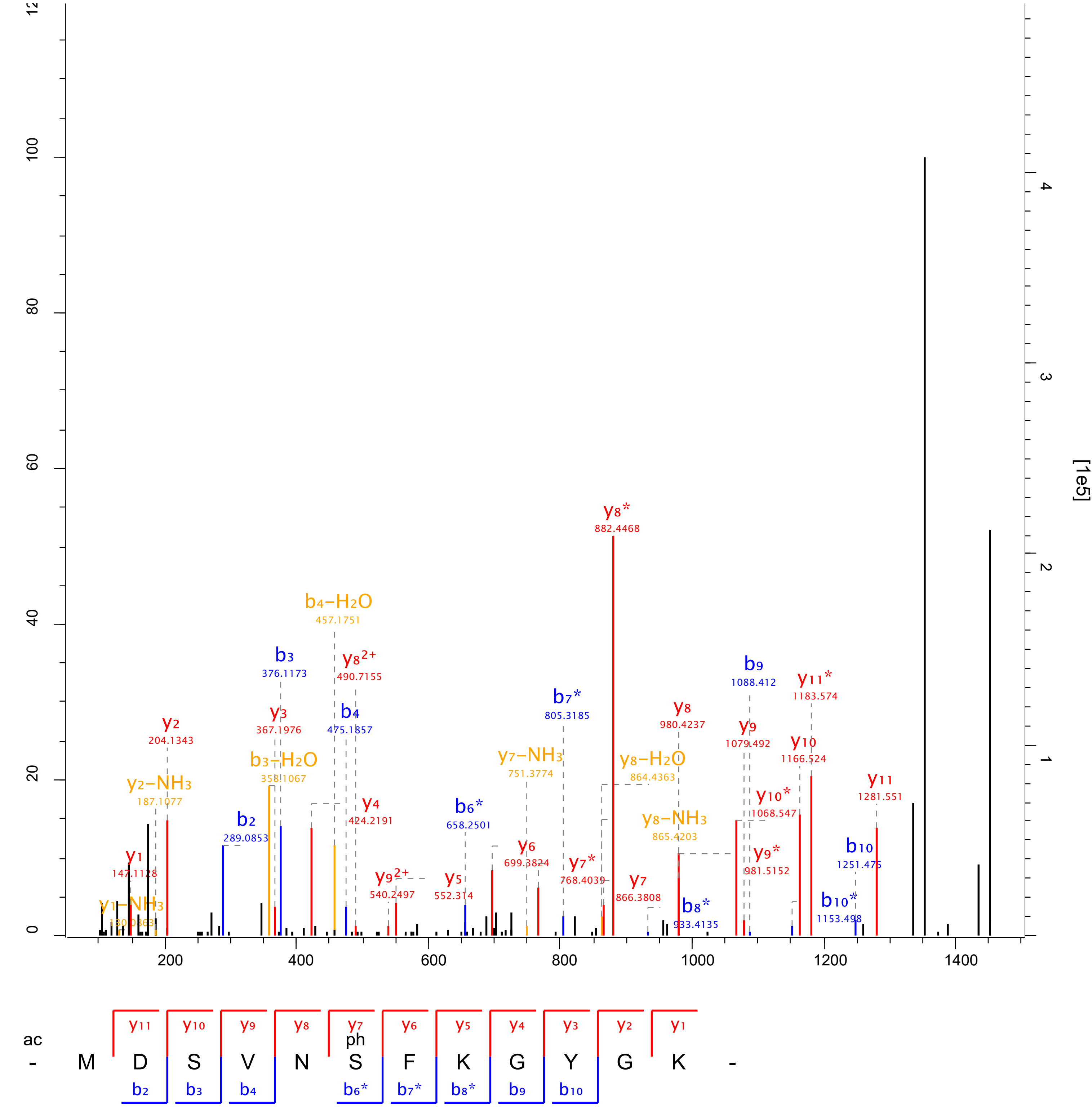

Sample output to test PDF Combine only

Raw file	Scan	Method	Score	m/z	Gene names
0523_4	16973	FTMS; HCD	95.24	946.45	F18K10.11

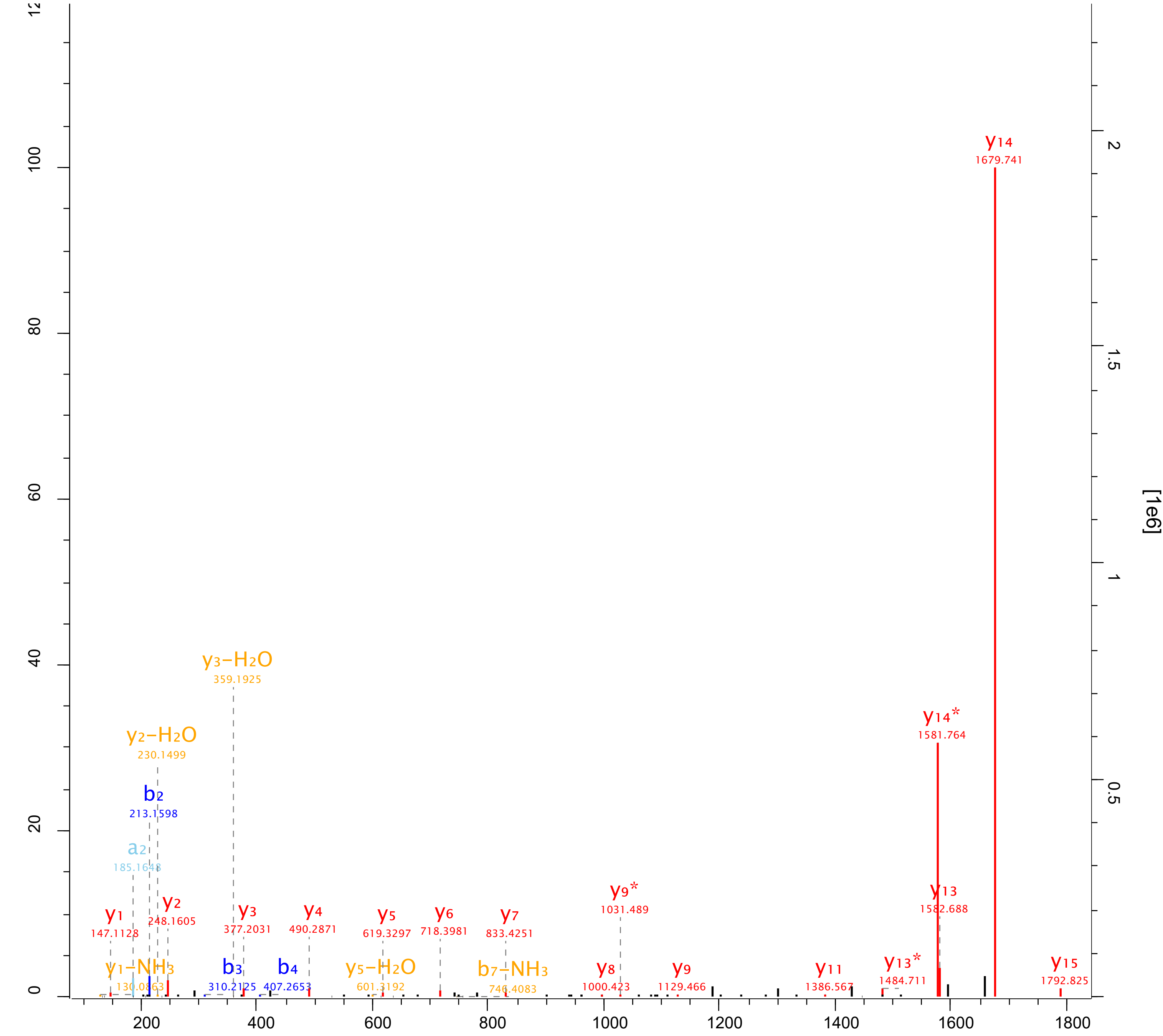

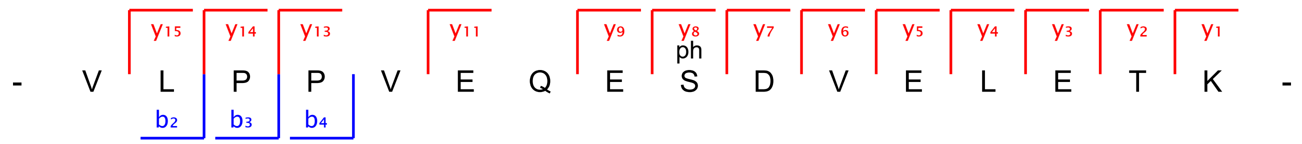

Sample output to test PDF Combine only

Raw file	Scan	Method	Score	m/z	Gene names
0523_4	17013	FTMS; HCD	84.65	689.79	SUN2

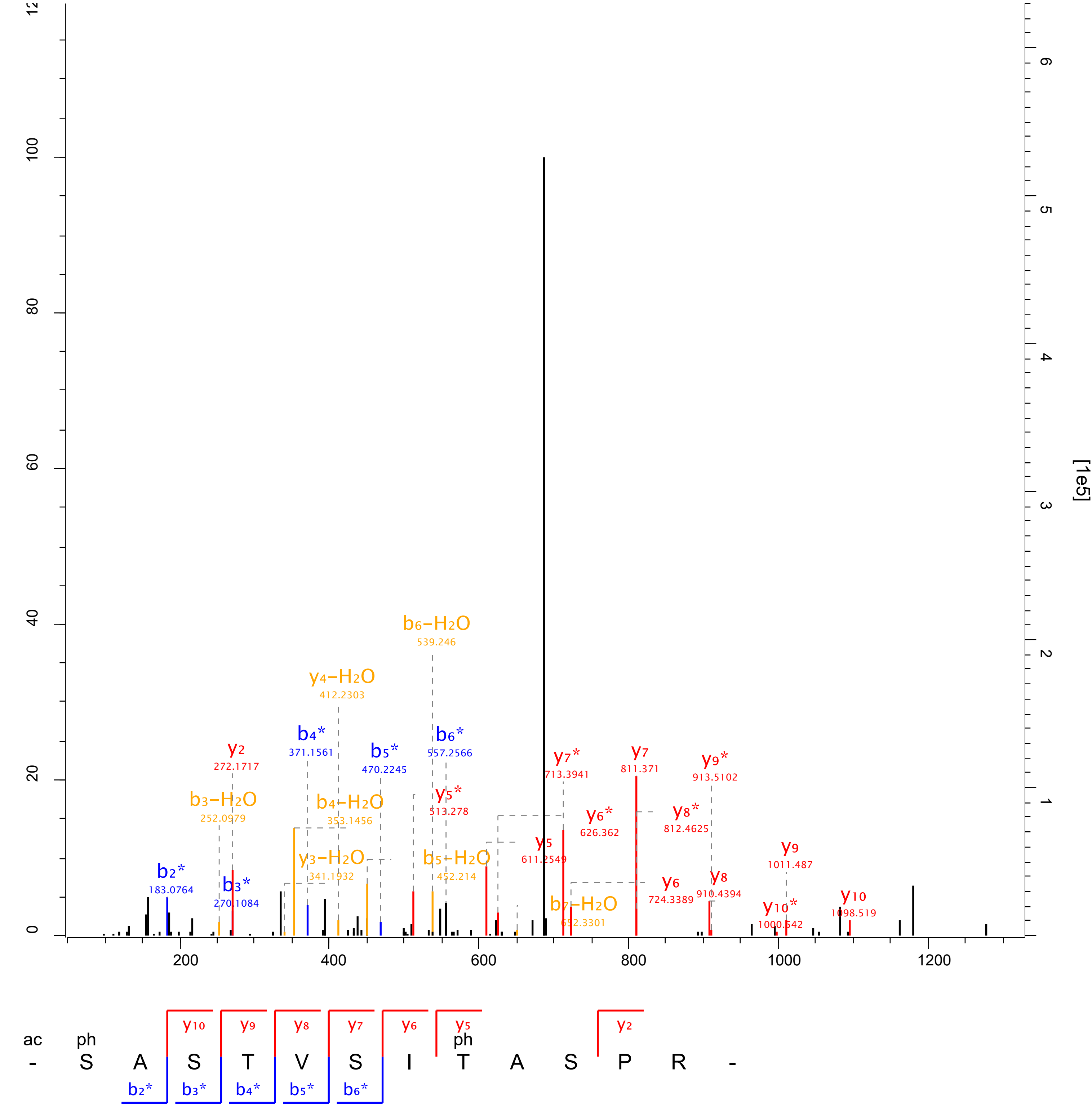

Sample output to test PDF Combine only

Raw file	Scan	Method	Score	m/z	Gene names
0523_4	17089	FTMS; HCD	116.35	1060.97	F13I12.260

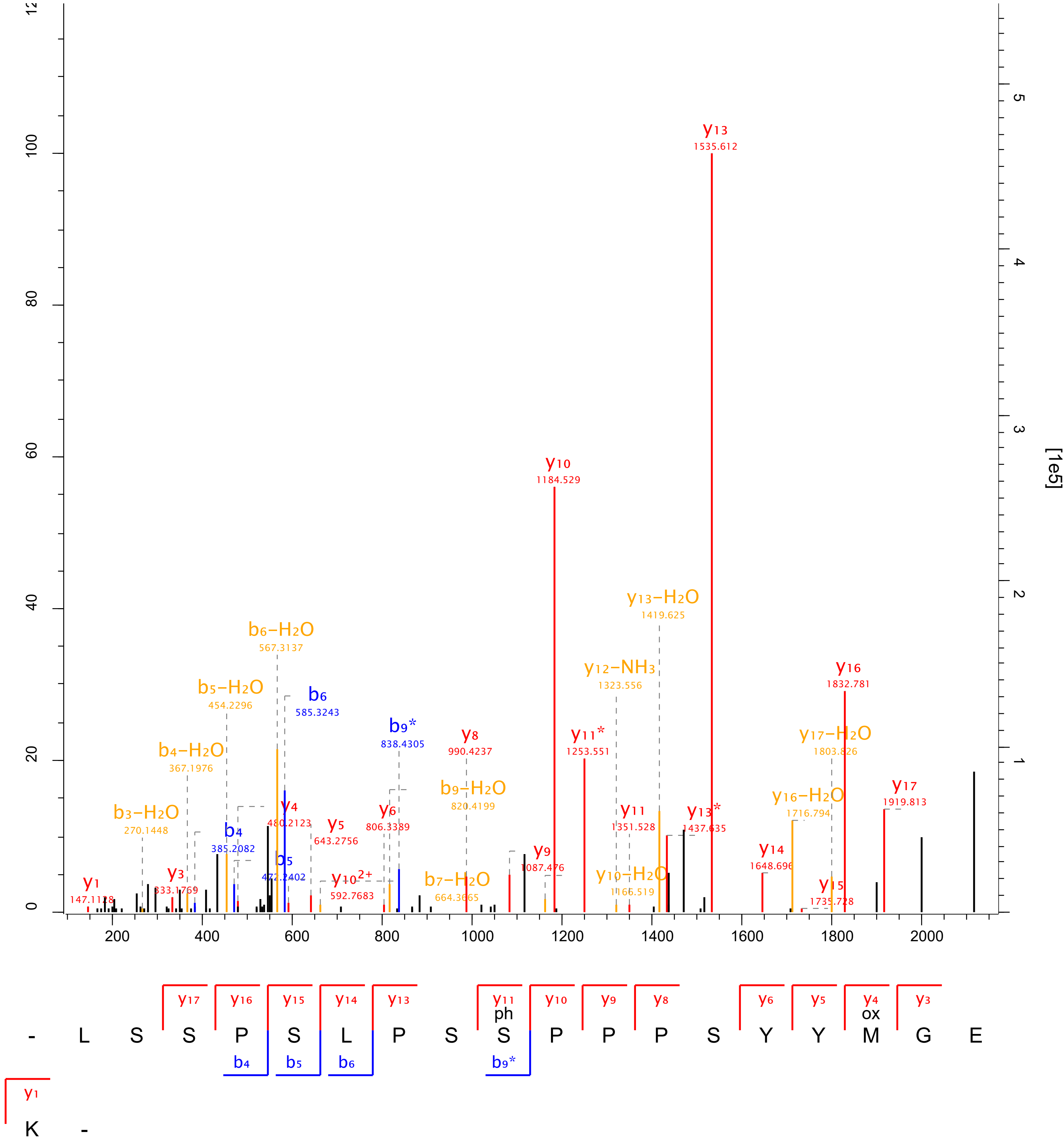

Sample output to test PDF Combine only

Raw file	Scan	Method	Score	m/z	Gene names
0523_4	17138	FTMS; HCD	77.58	786.33	At3g61480

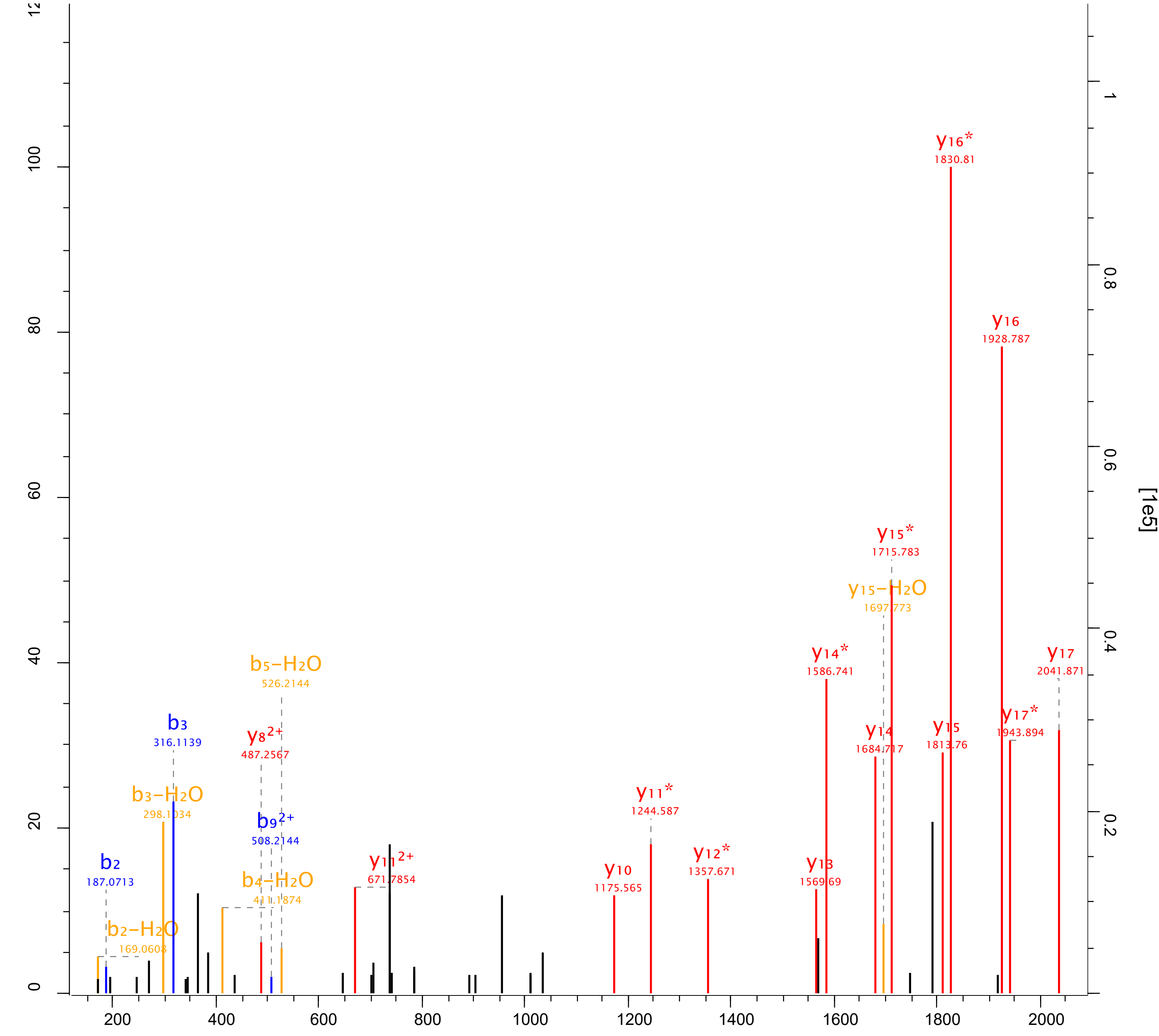

- E G E L D E D N L S S D L S E R E P
S R -

y17 y16 y15 y14 y13 y12* y11* ph y10 y8 2+

b2 b3 b9 2+

Sample output to test PDF Combine only

Raw file	Scan	Method	Score	m/z	Gene names
0523_4	17178	FTMS; HCD	75.7	793.65	CPK1

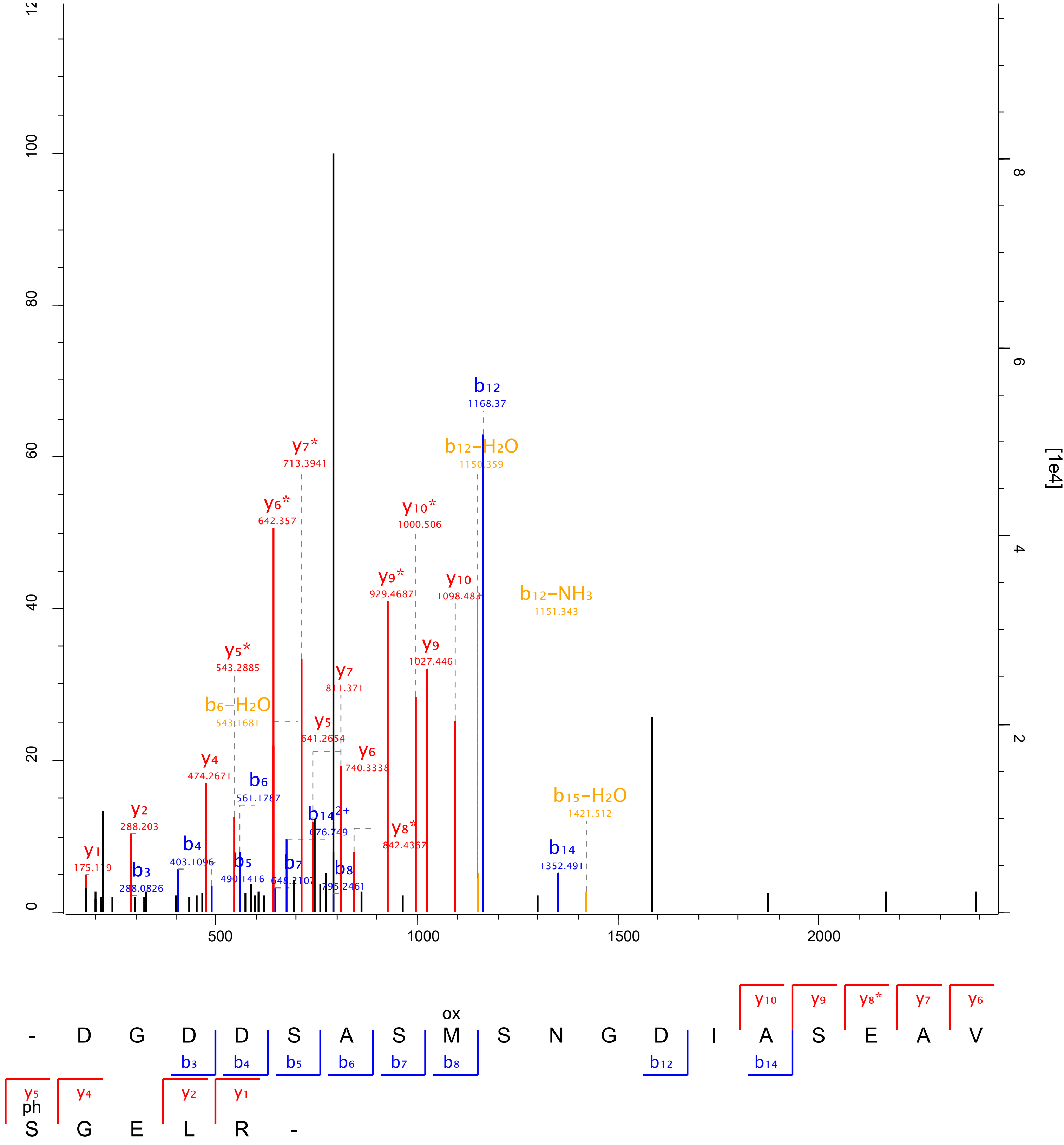

Sample output to test PDF Combine only

Raw file	Scan	Method	Score	m/z	Gene names
0523_4	17179	FTMS; HCD	70.26	746.81	At3g58640

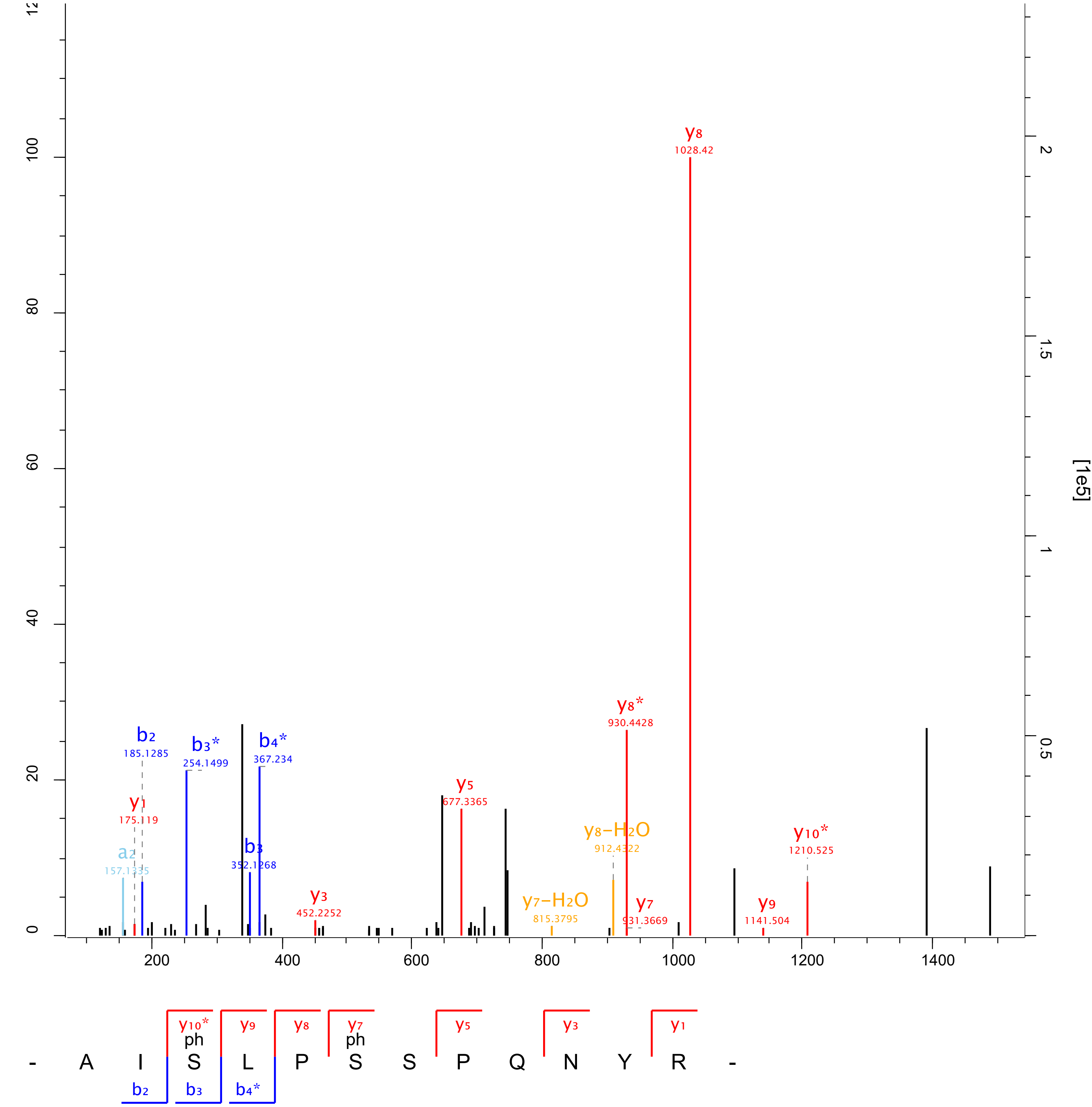

Sample output to test PDF Combine only

Raw file	Scan	Method	Score	m/z	Gene names
0523_4	17198	FTMS; HCD	282.85	858.35	CHX17

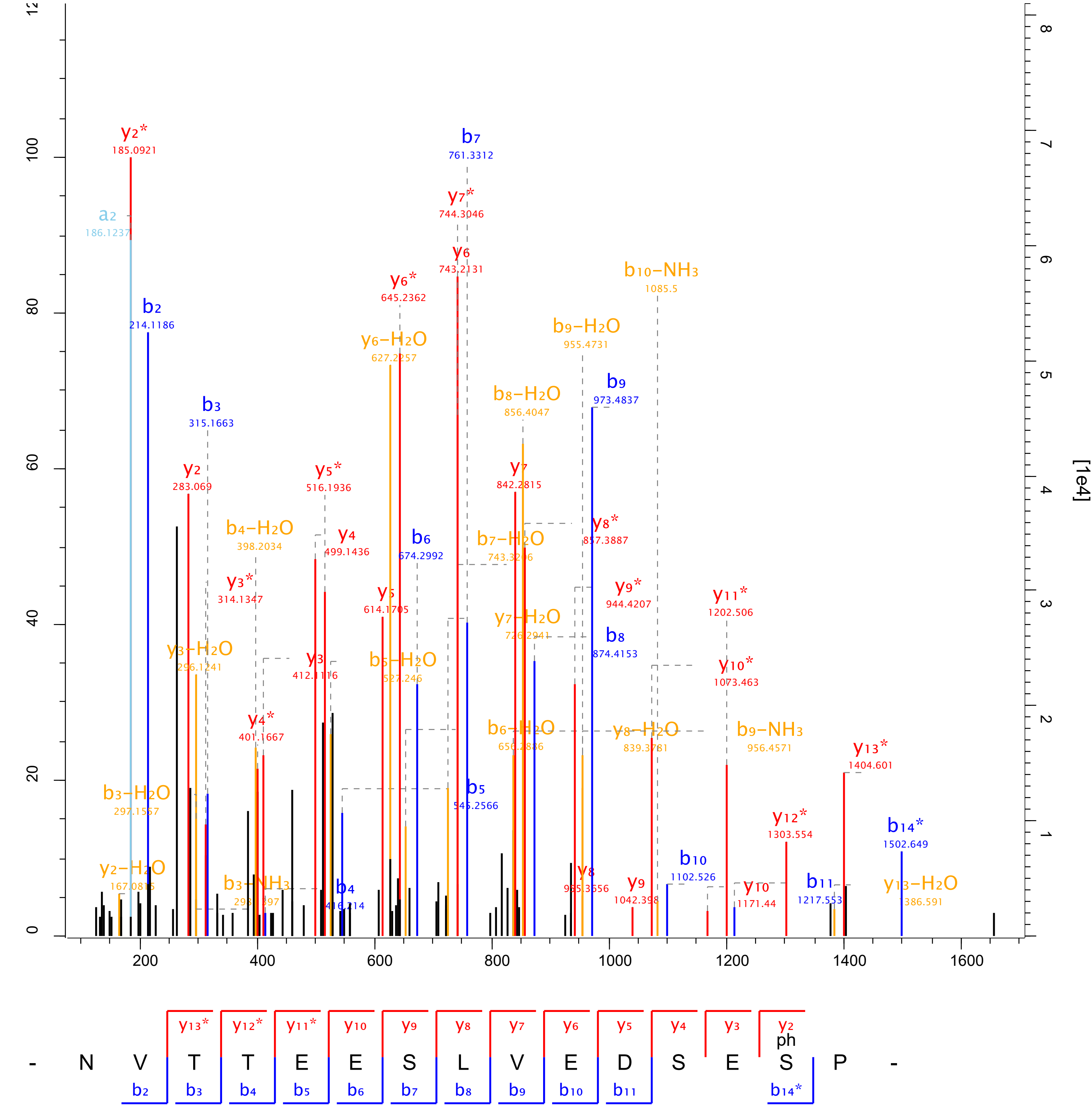

Sample output to test PDF Combine only

Raw file	Scan	Method	Score	m/z	Gene names
0523_4	17210	FTMS; HCD	185.71	1018.43	PLP1

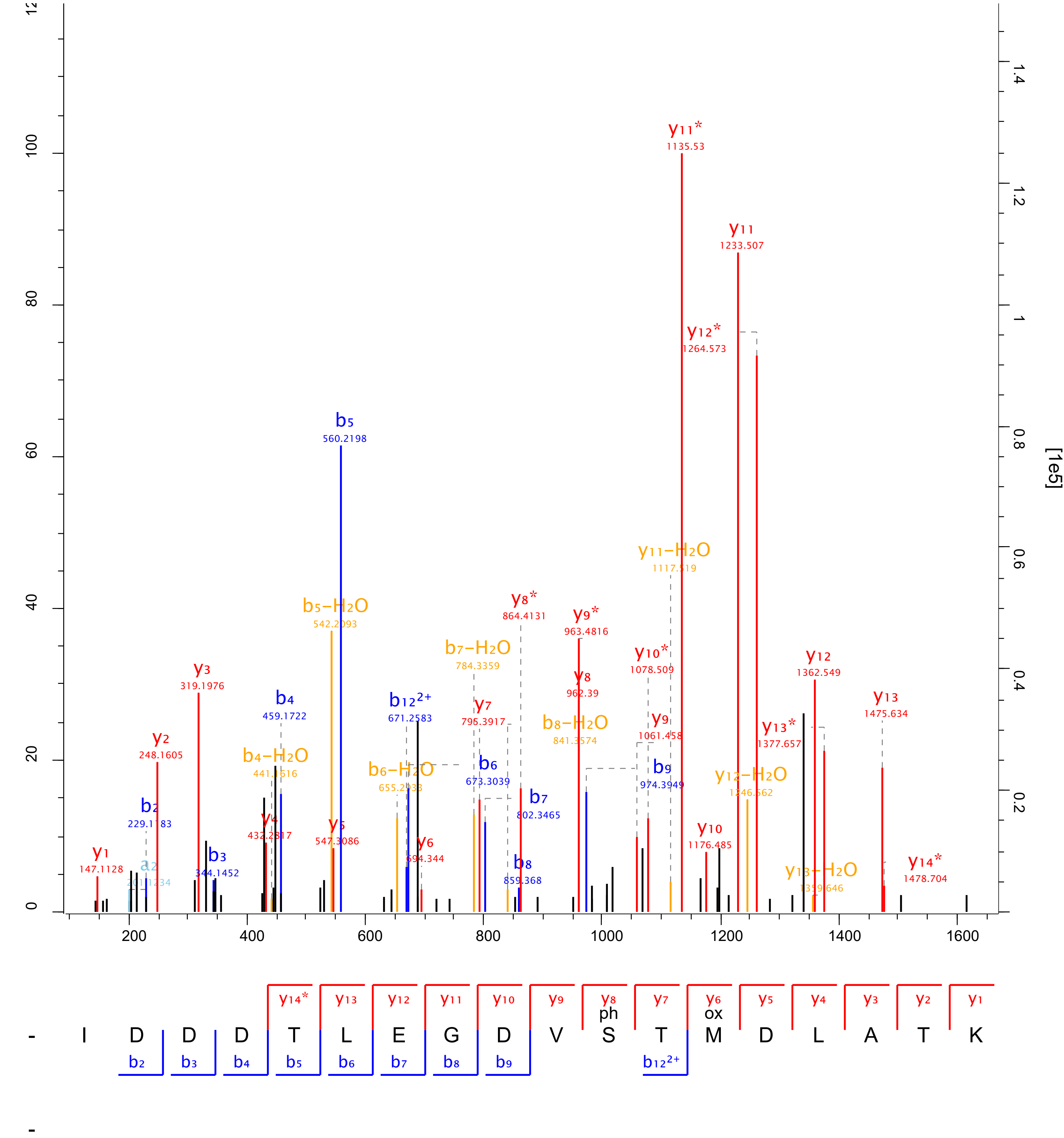

Sample output to test PDF Combine only

Raw file	Scan	Method	Score	m/z	Gene names
0523_4	17274	FTMS; HCD	153.74	665.78	CDC27B

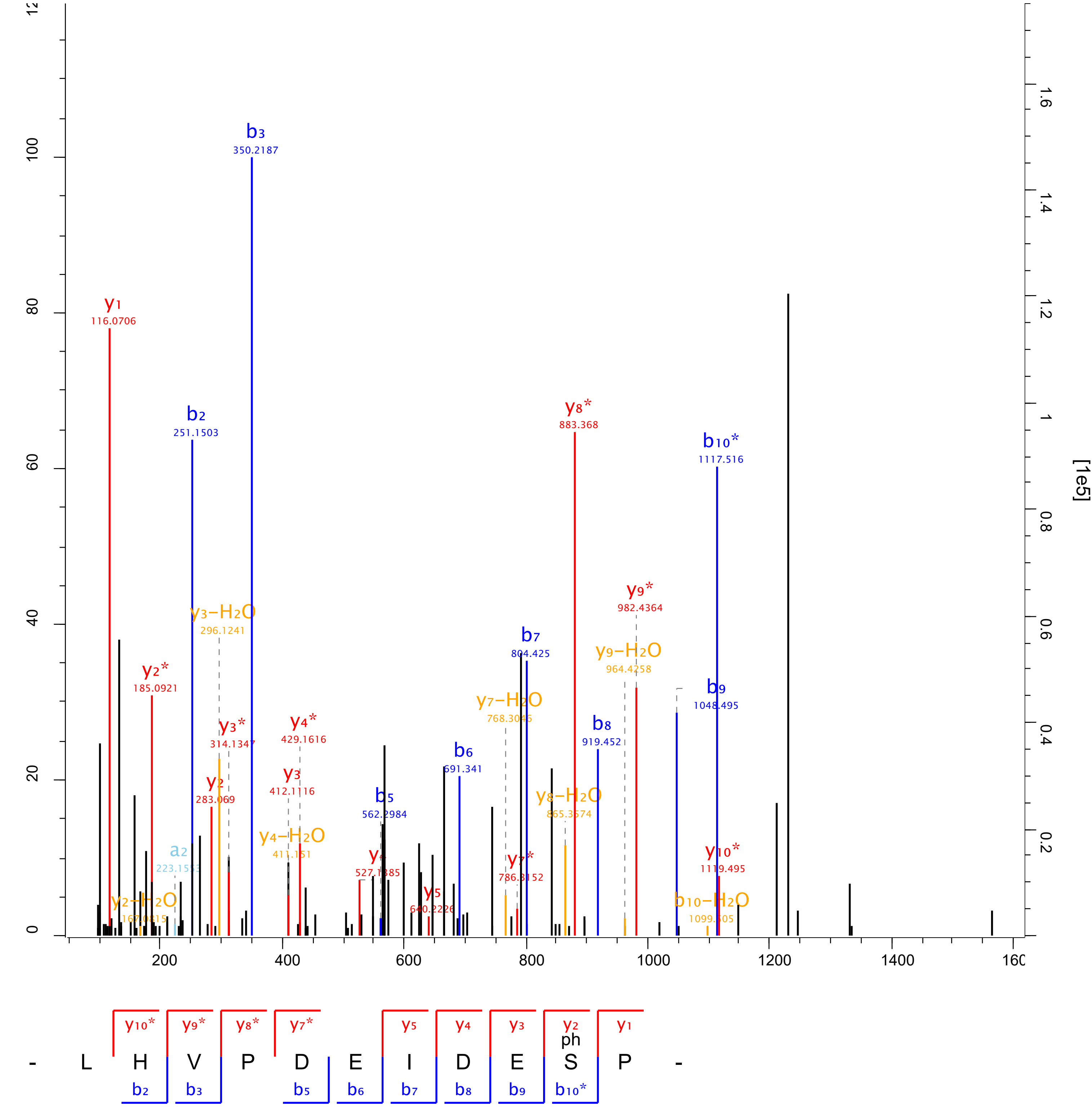

Sample output to test PDF Combine only

Raw file	Scan	Method	Score	m/z	Gene names
0523_4	17364	FTMS; HCD	82.01	916.91	AMT1-2

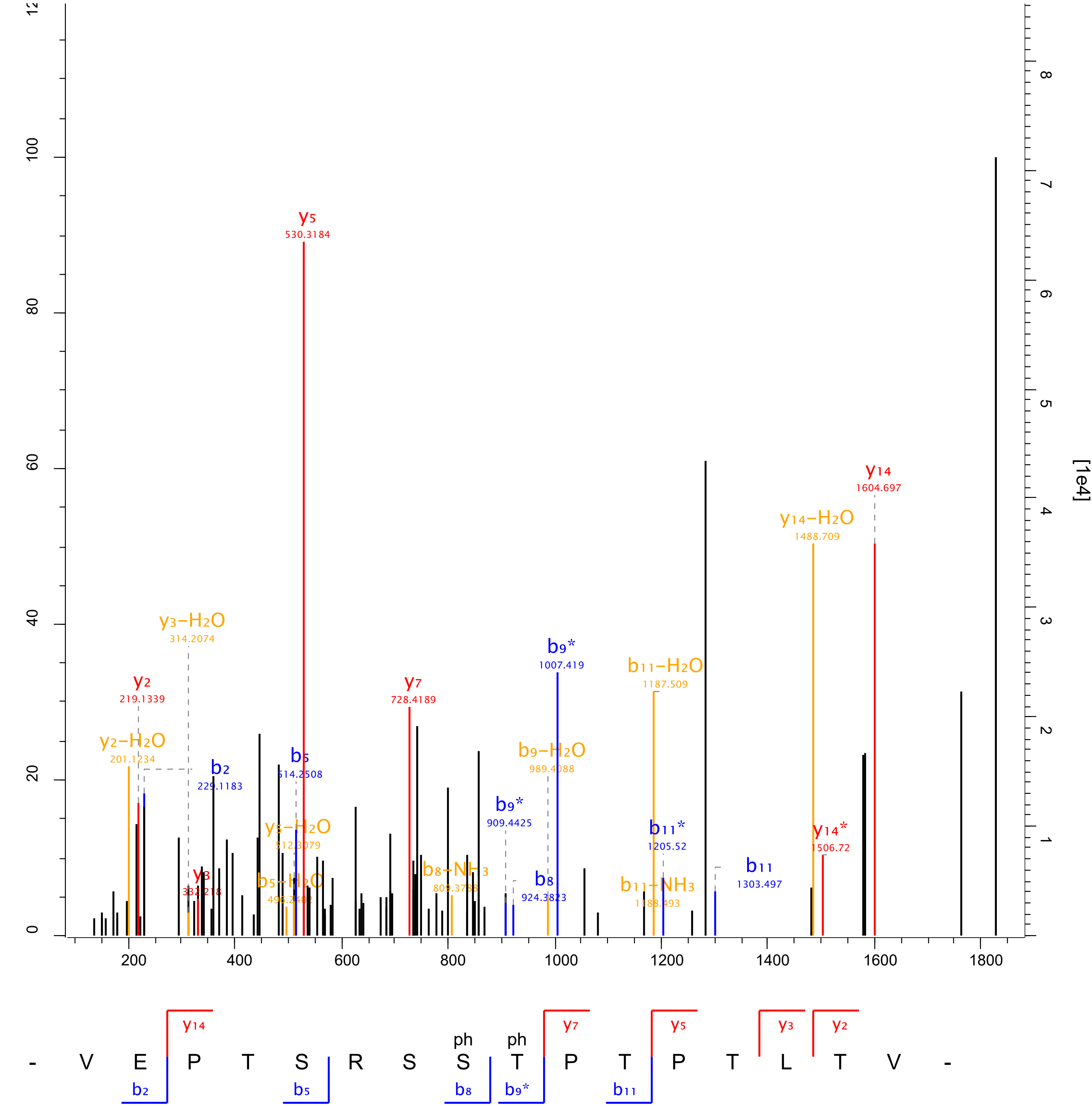

Sample output to test PDF Combine only

Raw file	Scan	Method	Score	m/z	Gene names
0523_4	17496	FTMS; HCD	63.2	804.71	At1g20670

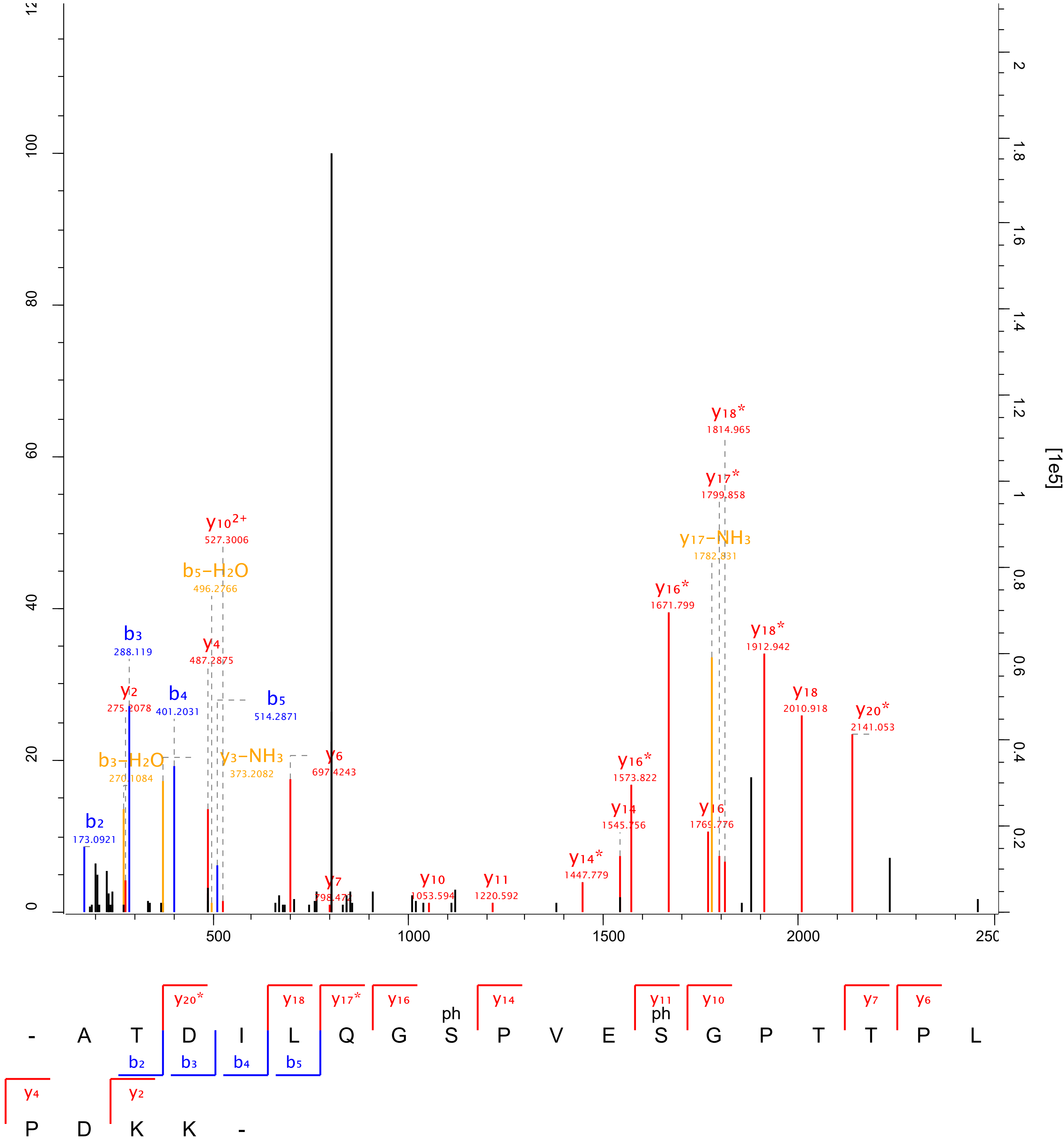

Sample output to test PDF Combine only

Raw file	Scan	Method	Score	m/z	Gene names
0523_4	17501	FTMS; HCD	50.9	637.3	F19P19.28;At1g04280

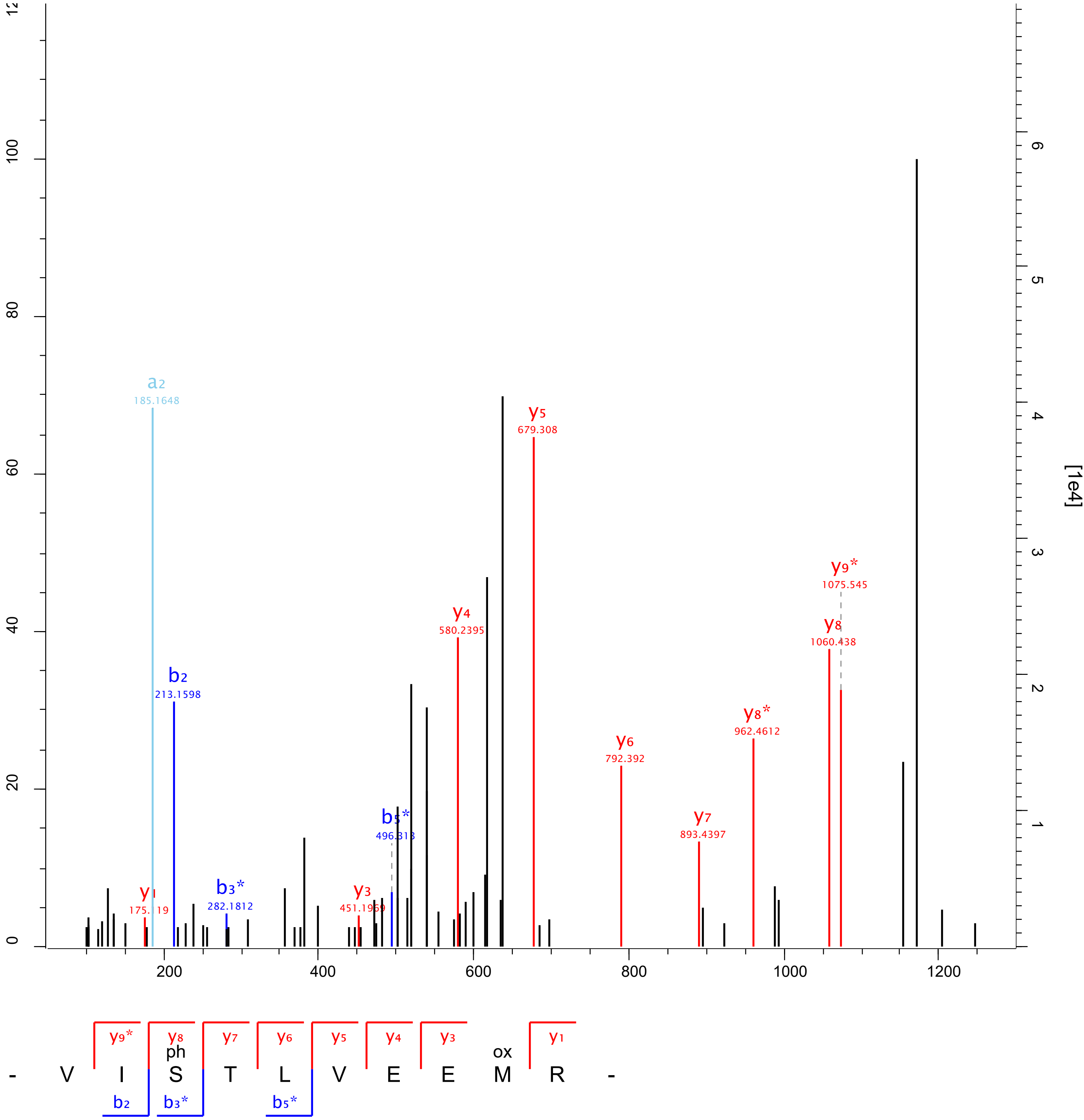

Sample output to test PDF Combine only

Raw file	Scan	Method	Score	m/z
0523_4	17514	FTMS; HCD	137.69	788.9

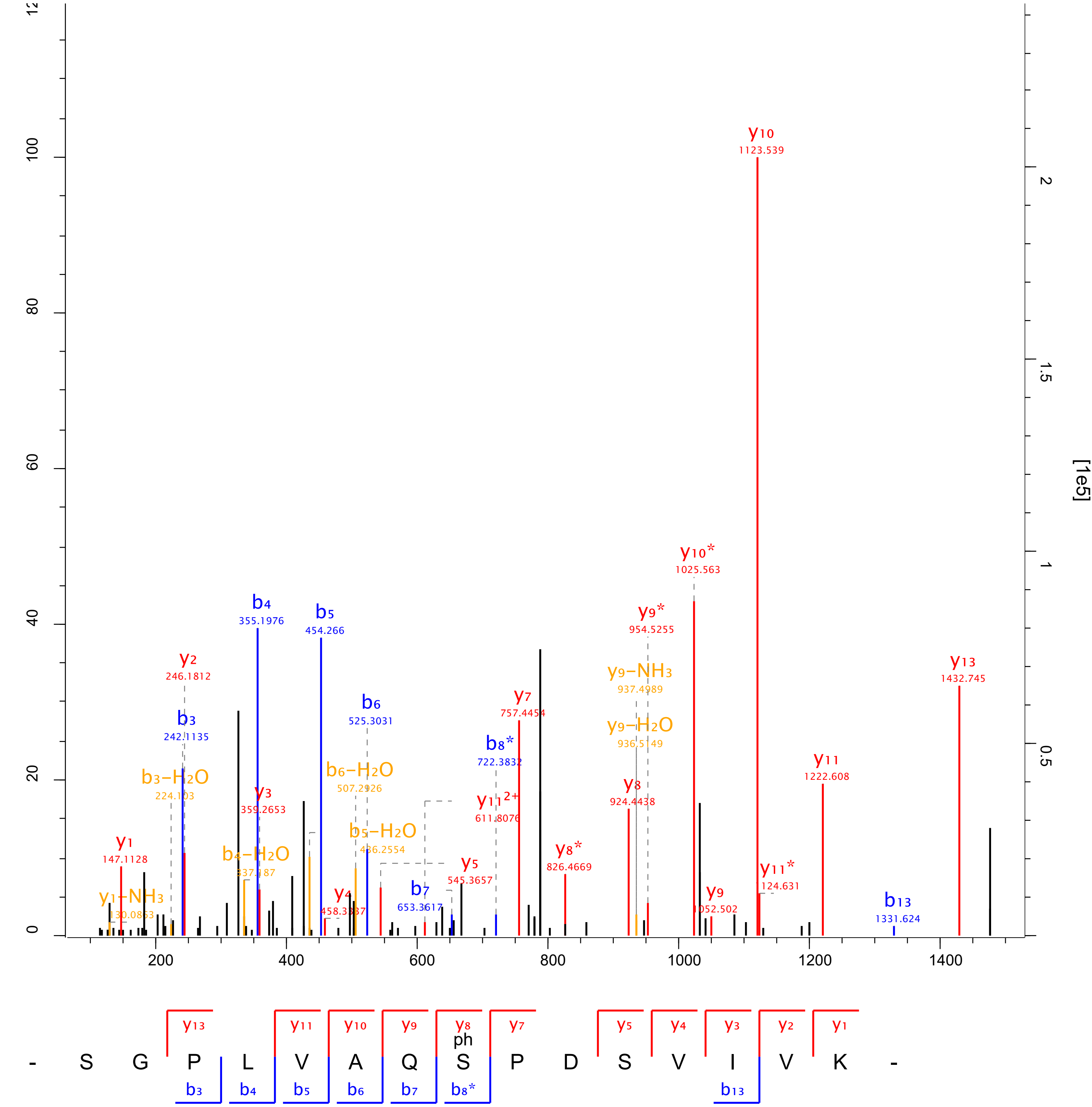

Sample output to test PDF Combine only

Raw file	Scan	Method	Score	m/z	Gene names
0523_4	17541	FTMS; HCD	89.47	898.91	IQD32

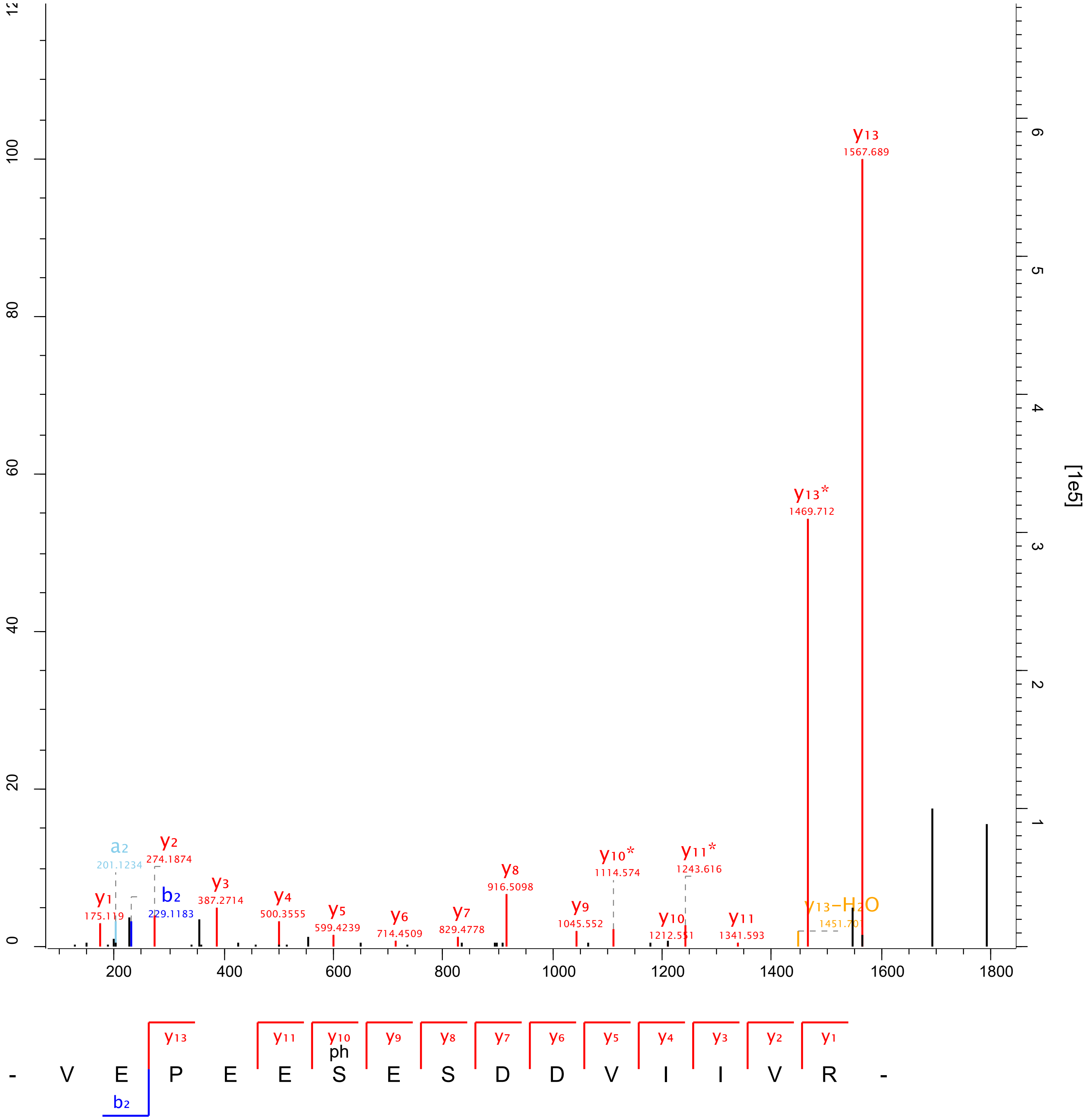

Sample output to test PDF Combine only

Raw file	Scan	Method	Score	m/z	Gene names
0523_4	17646	FTMS; HCD	194.24	893.4	TRX9

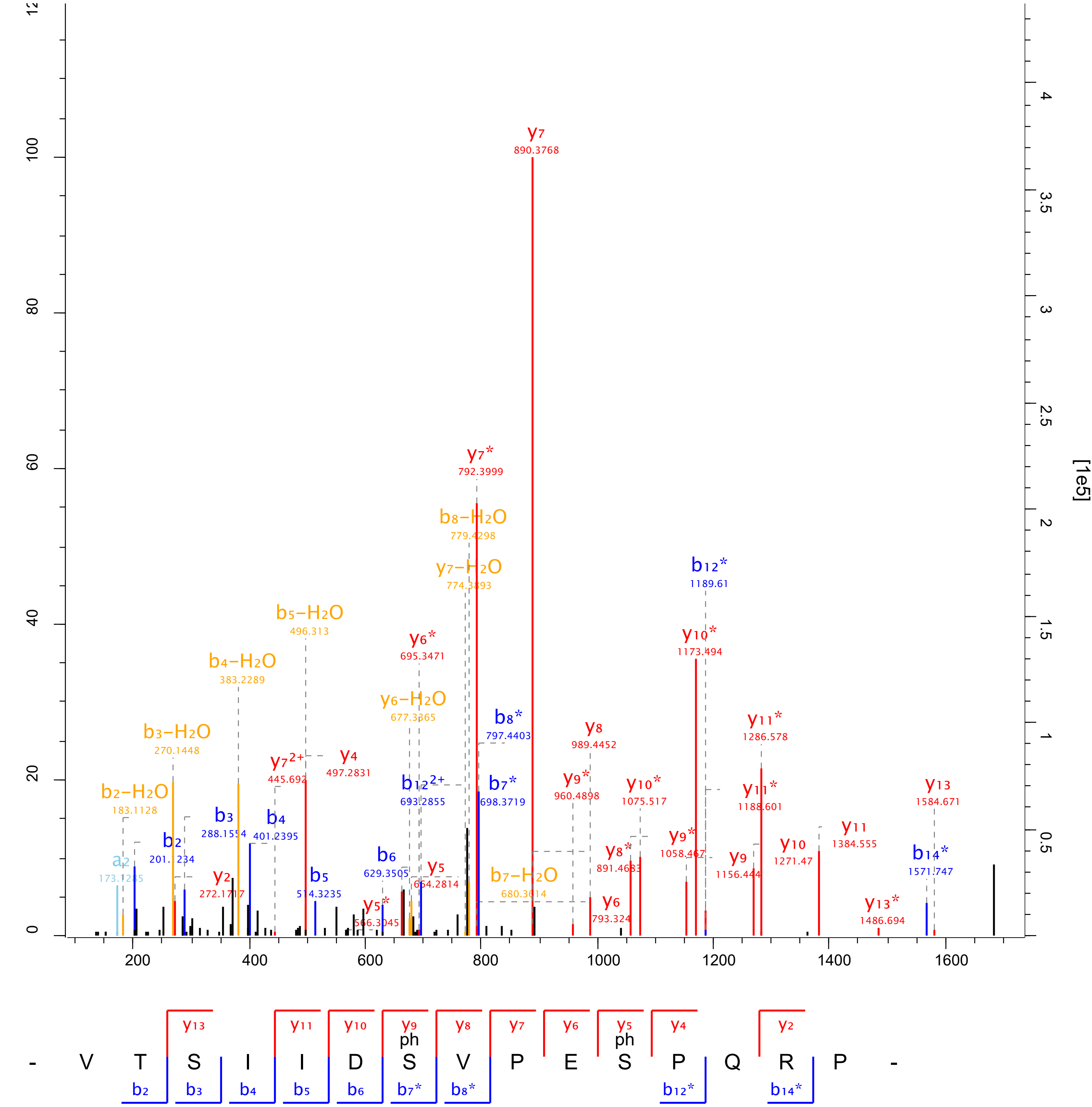

Sample output to test PDF Combine only

Raw file	Scan	Method	Score	m/z
0523_4	17700	FTMS; HCD	330.91	957.42

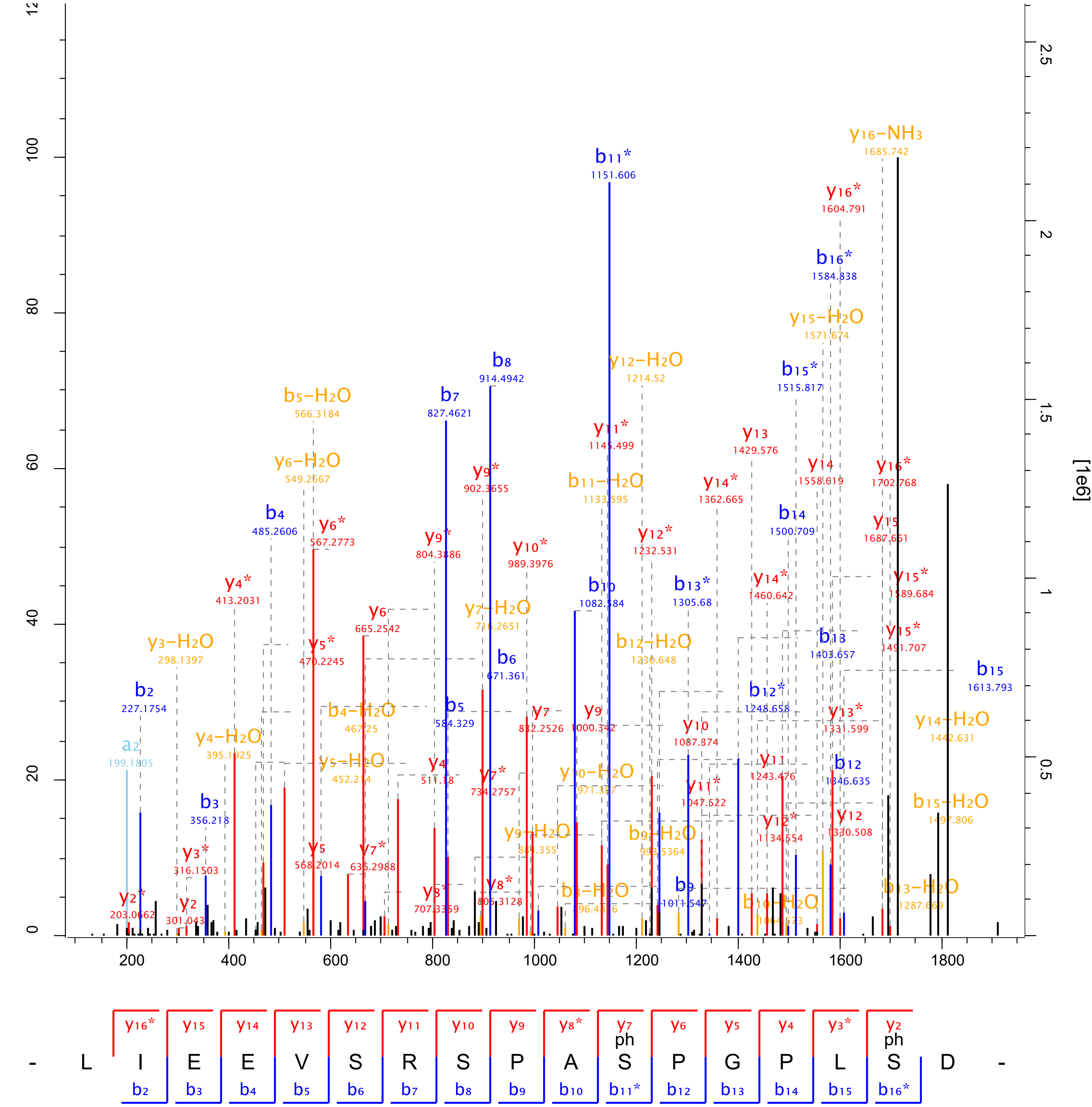

Sample output to test PDF Combine only

Raw file	Scan	Method	Score	m/z	Gene names
0523_4	17745	FTMS; HCD	179.58	913.4	T23K23.8

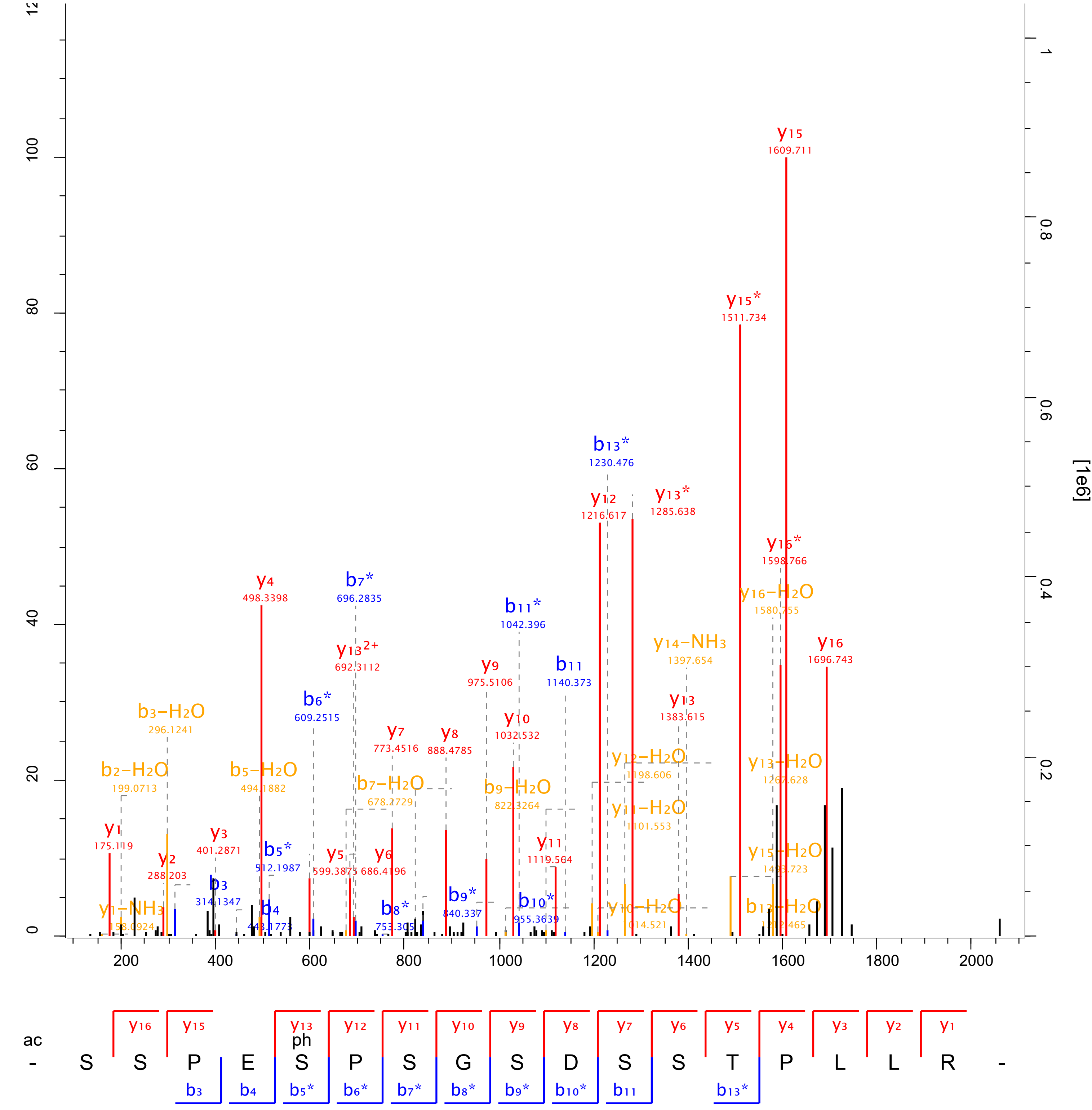

Sample output to test PDF Combine only

Raw file	Scan	Method	Score	m/z	Gene names
0523_4	18063	FTMS; HCD	223.49	1159.39	POT7

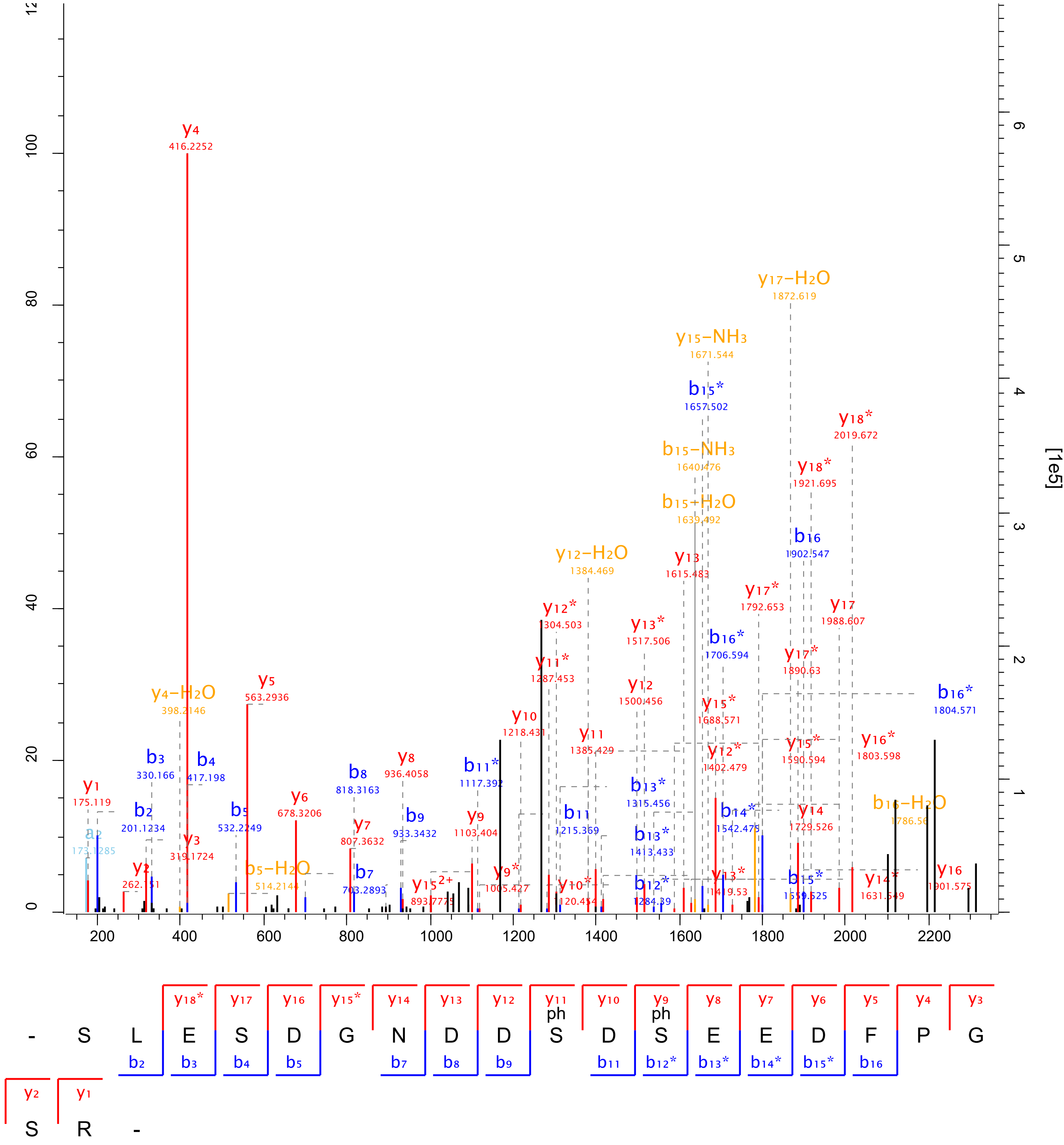

Sample output to test PDF Combine only

Raw file	Scan	Method	Score	m/z	Gene names
0523_4	18096	FTMS; HCD	40.19	760.37	F9F8.20

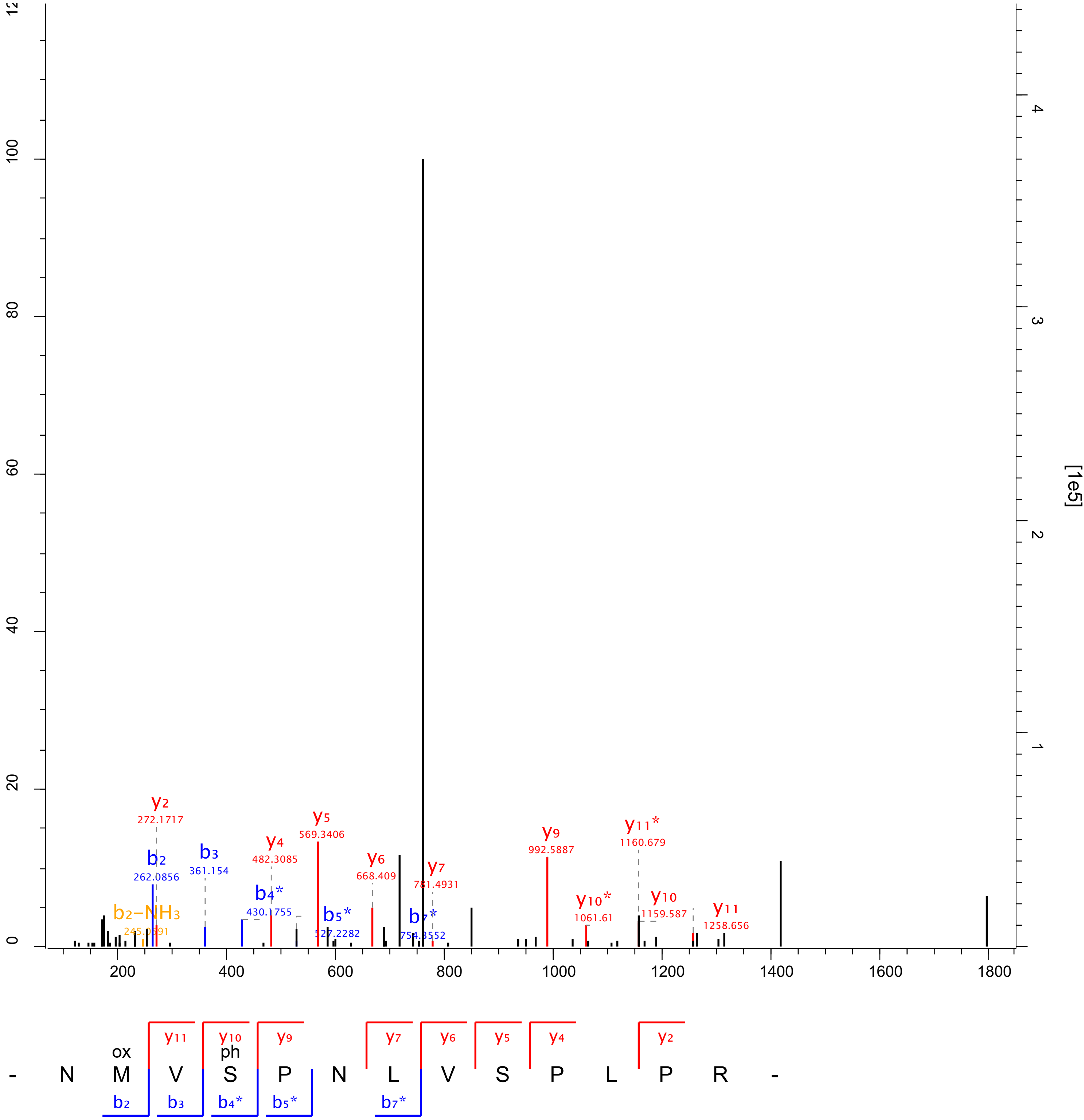

Sample output to test PDF Combine only

Raw file	Scan	Method	Score	m/z	Gene names
0523_4	18106	FTMS; HCD	77.53	933.38	LYK4

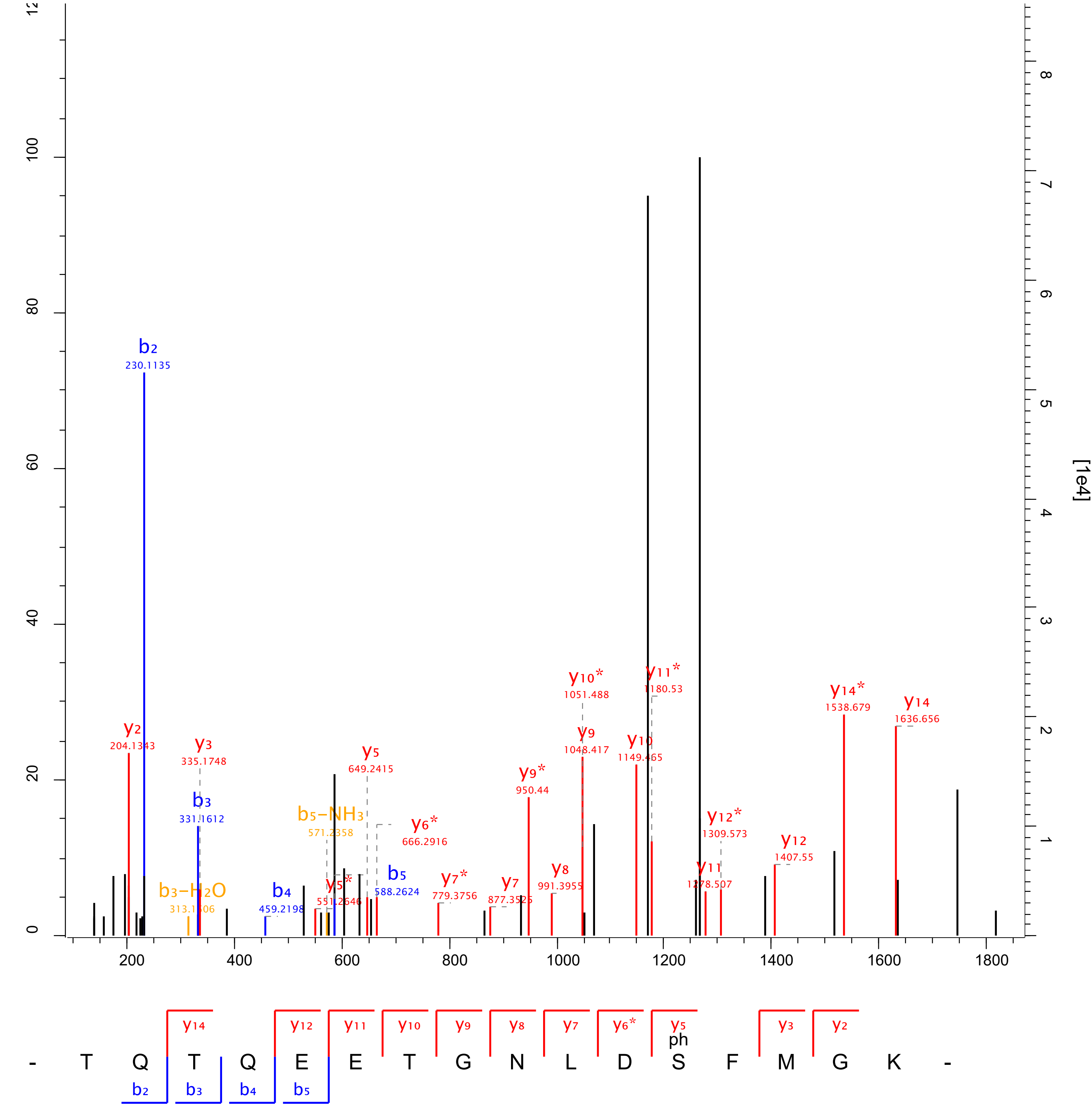

Sample output to test PDF Combine only

Raw file	Scan	Method	Score	m/z	Gene names
0523_4	18111	FTMS; HCD	190.64	1032.4	CSLD3

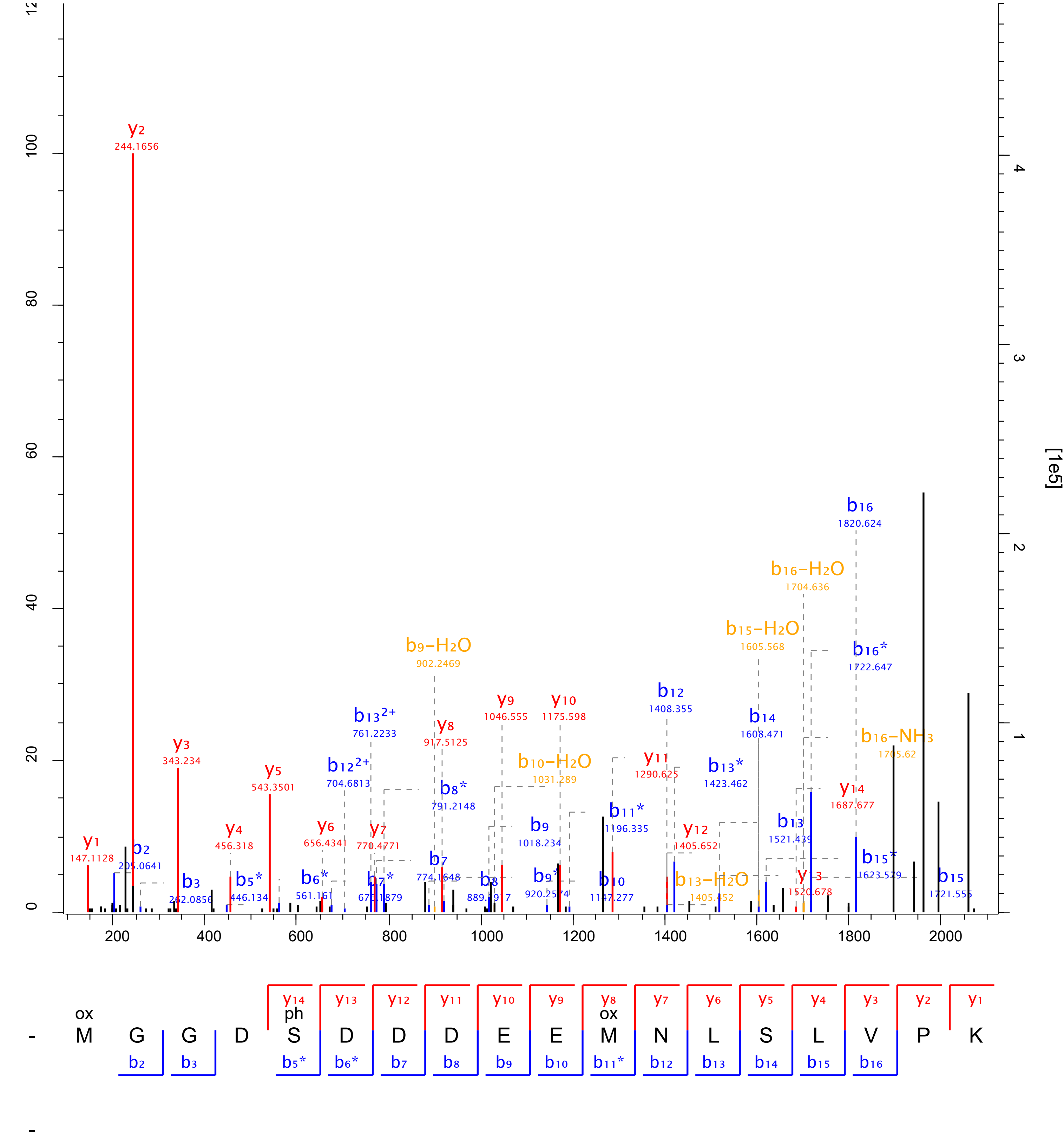

Raw file	Scan	Method	Score	m/z
0523_4	18114	FTMS; HCD	290.36	985.49

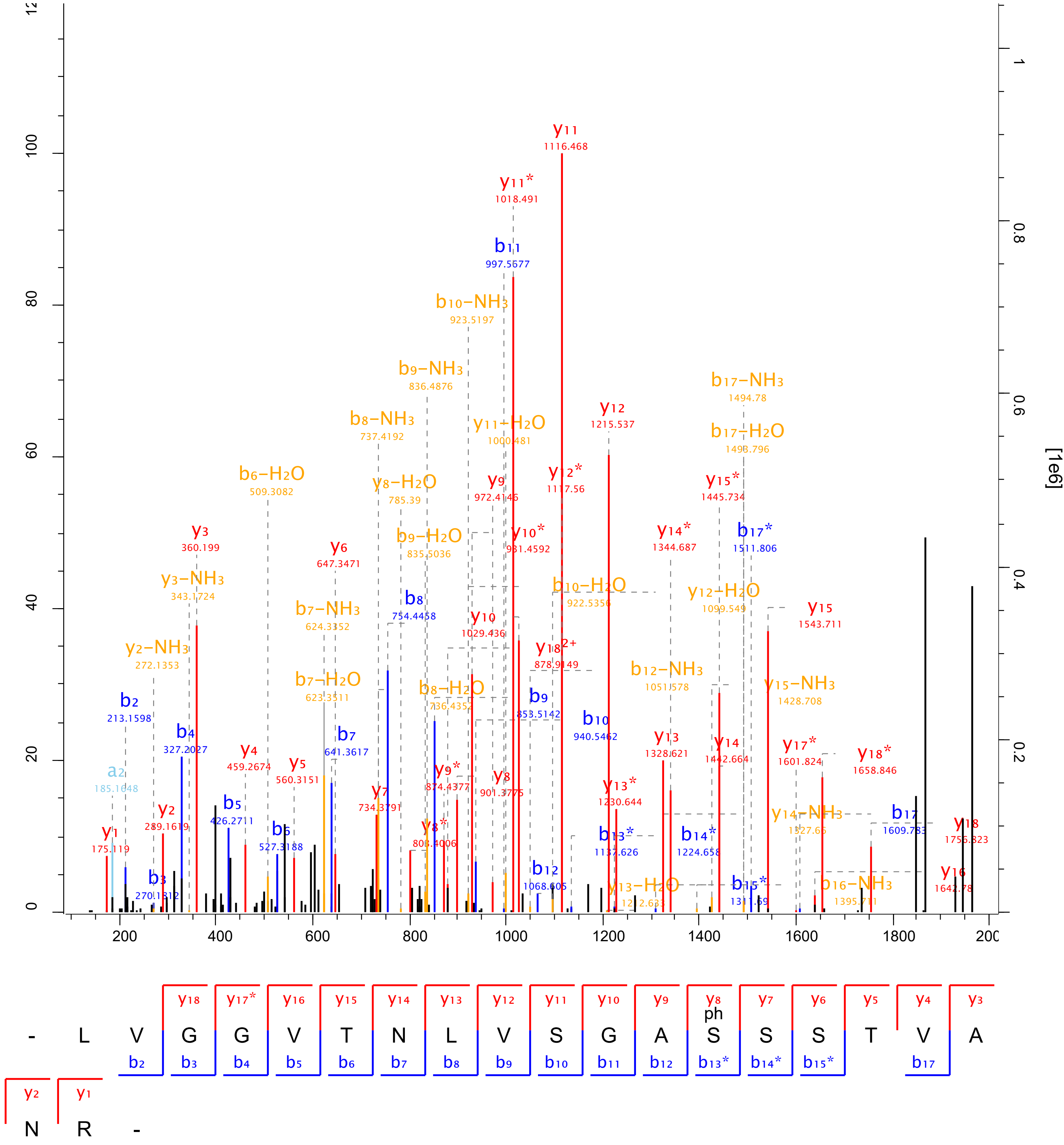

Sample output to test PDF Combine only

Raw file	Scan	Method	Score	m/z	Gene names
0523_4	18141	FTMS; HCD	185.99	747.79	F9P14.7

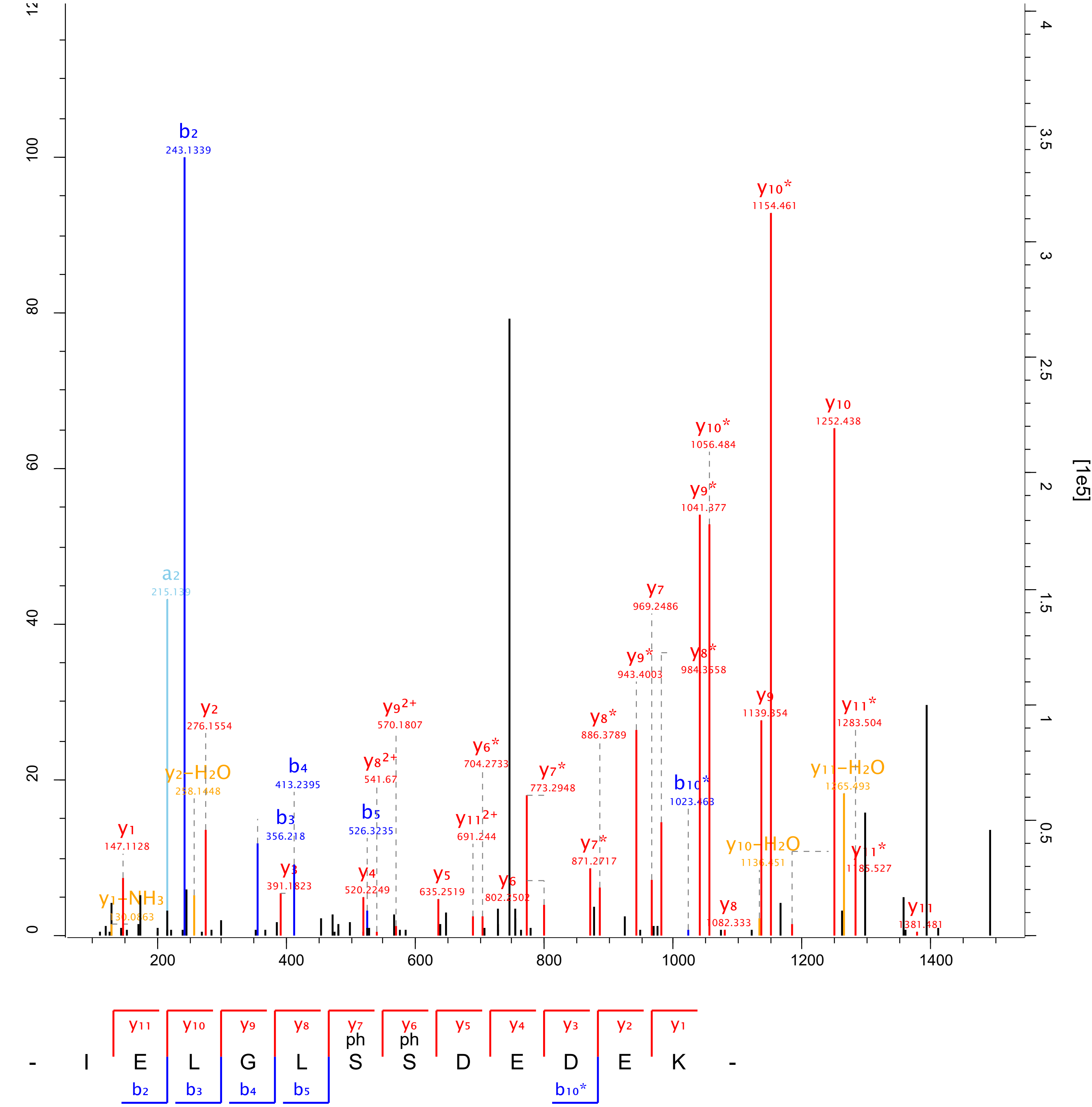

Sample output to test PDF Combine only

Raw file	Scan	Method	Score	m/z	Gene names
0523_4	18161	FTMS; HCD	89.72	709.66	At1g27090

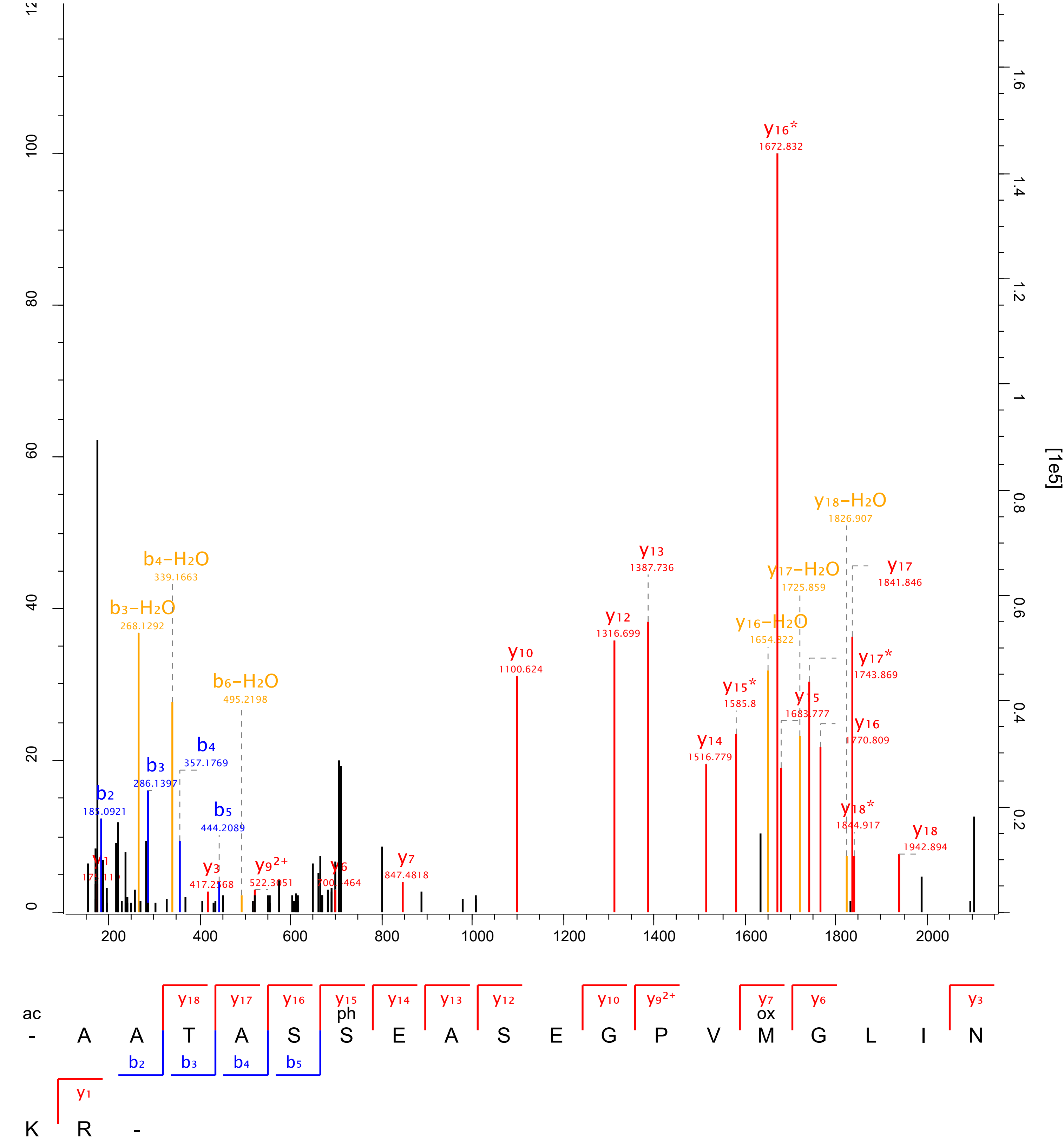

Sample output to test PDF Combine only

Raw file	Scan	Method	Score	m/z	Gene names
0523_4	18183	FTMS; HCD	67.65	806.9	RPL11A;RPL11B

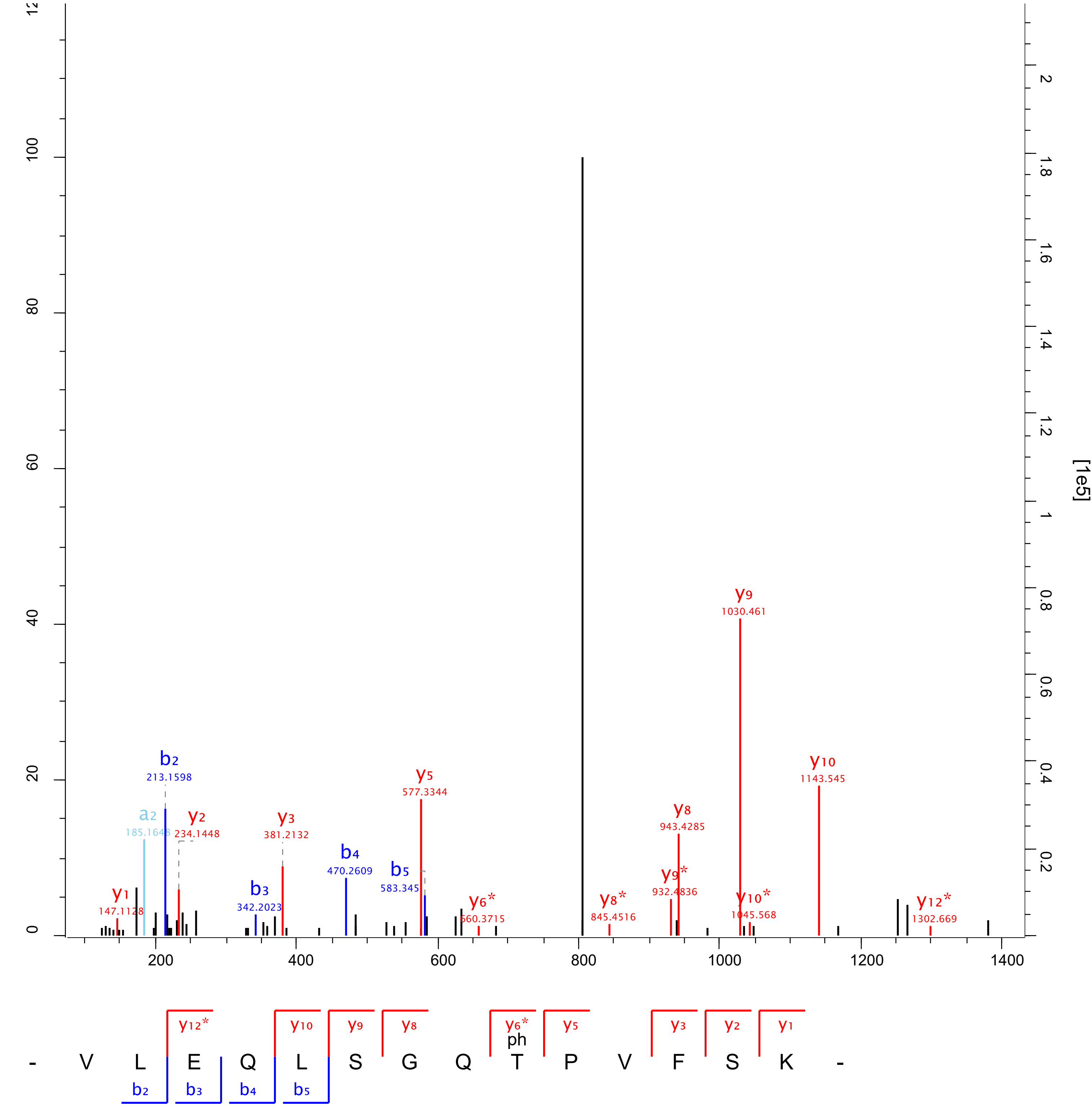

Sample output to test PDF Combine only

Raw file	Scan	Method	Score	m/z	Gene names
0523_4	18282	FTMS; HCD	52.25	870.05	RBL15

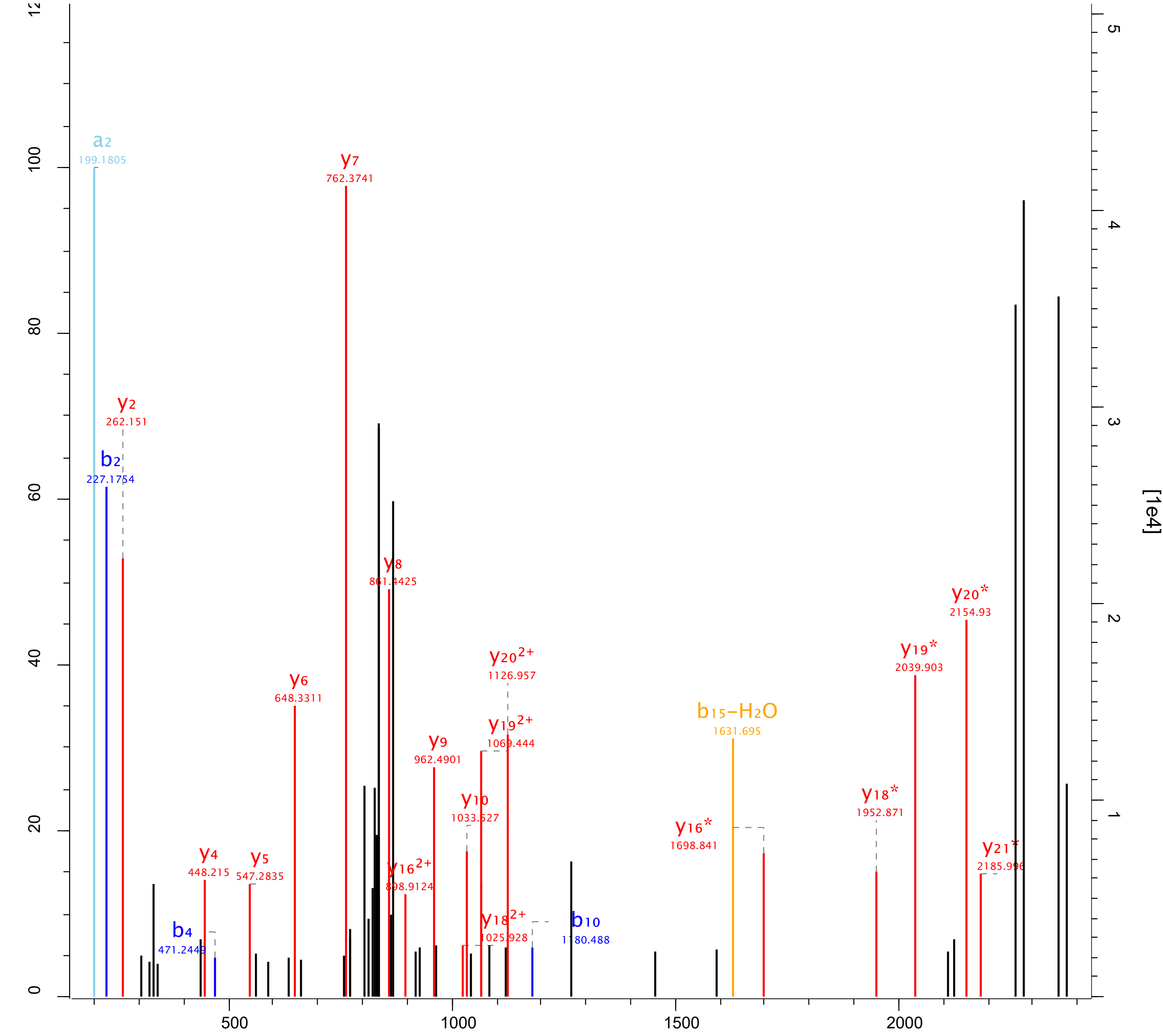

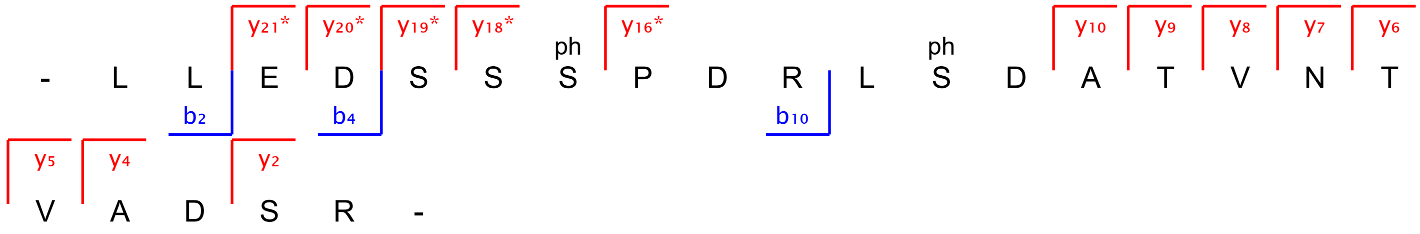

Sample output to test PDF Combine only

Raw file	Scan	Method	Score	m/z	Gene names
0523_4	18290	FTMS; HCD	77.75	699.85	NHX3

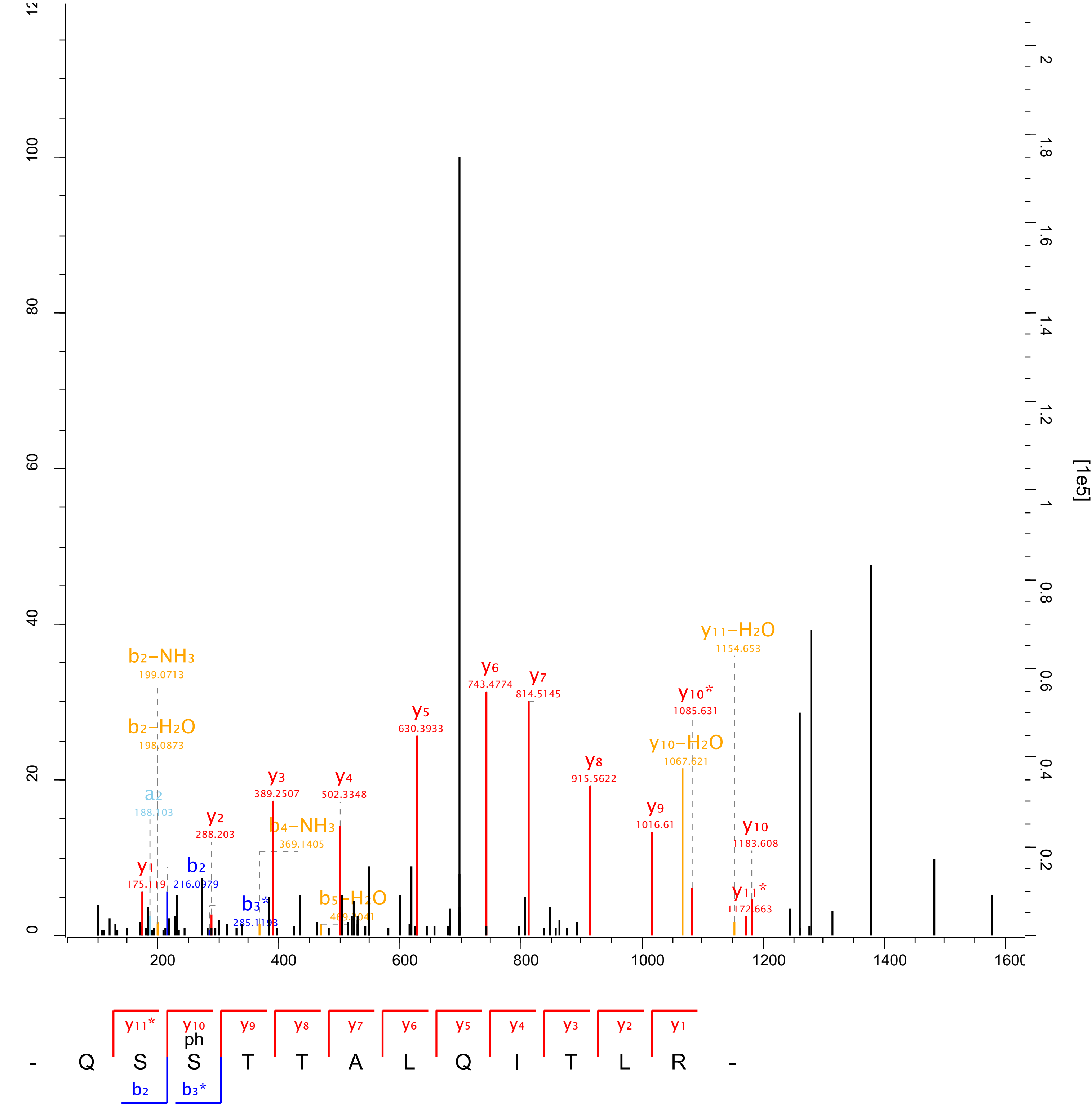

Sample output to test PDF Combine only

Raw file	Scan	Method	Score	m/z	Gene names
0523_4	18409	FTMS; HCD	126.42	1068.44	CRWN1

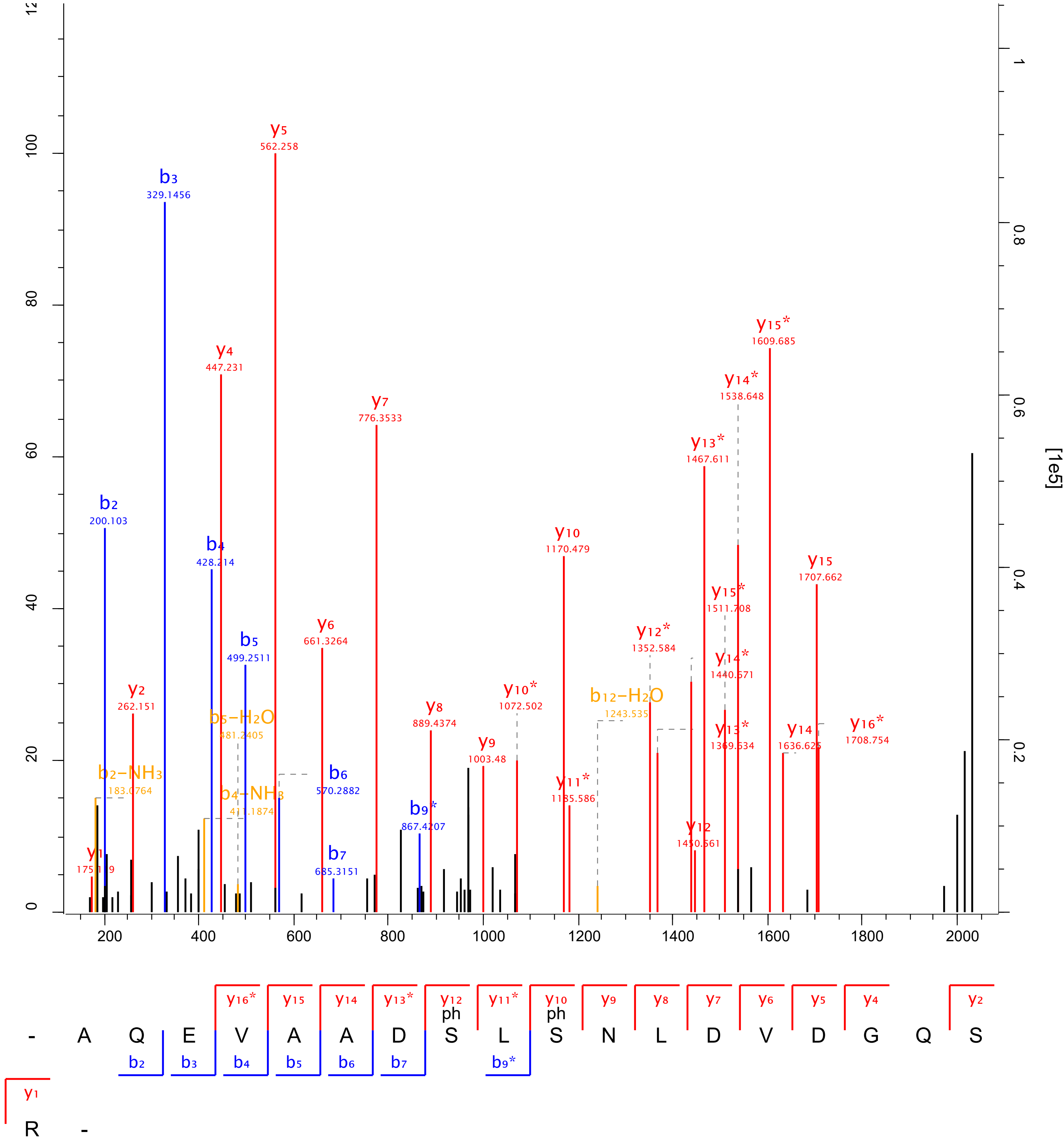

Sample output to test PDF Combine only

Raw file	Scan	Method	Score	m/z	Gene names
0523_4	18418	FTMS; HCD	135.12	1039.02	F8A24.12

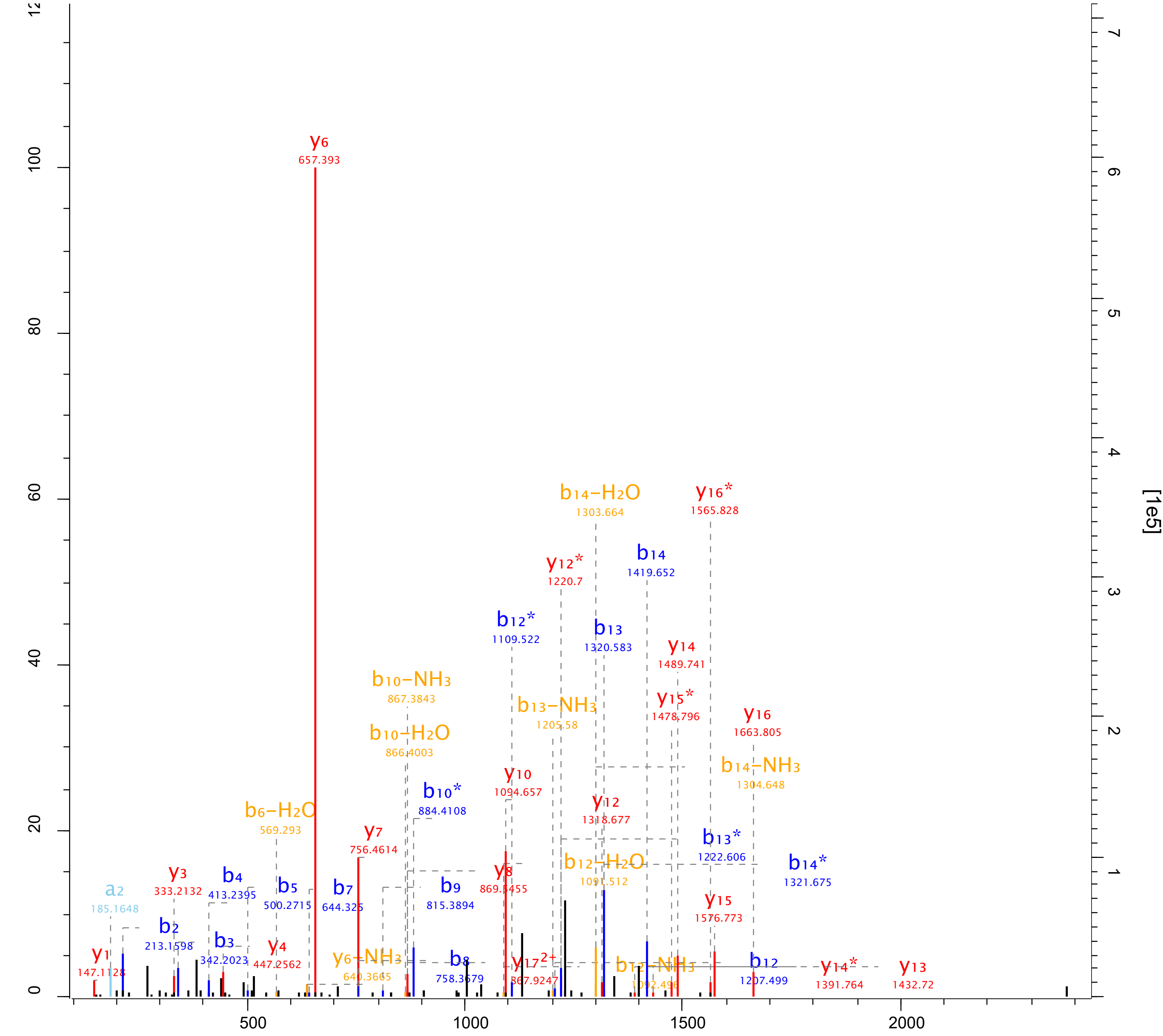

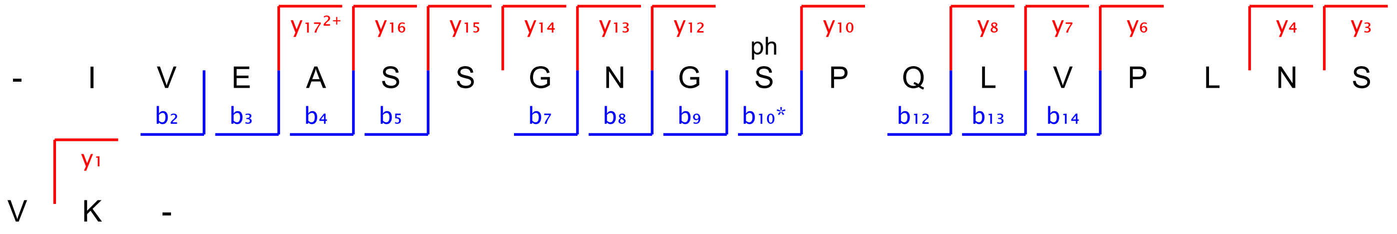

Sample output to test PDF Combine only

Raw file	Scan	Method	Score	m/z	Gene names
0523_4	18419	FTMS; HCD	160.57	1046.43	OPT4

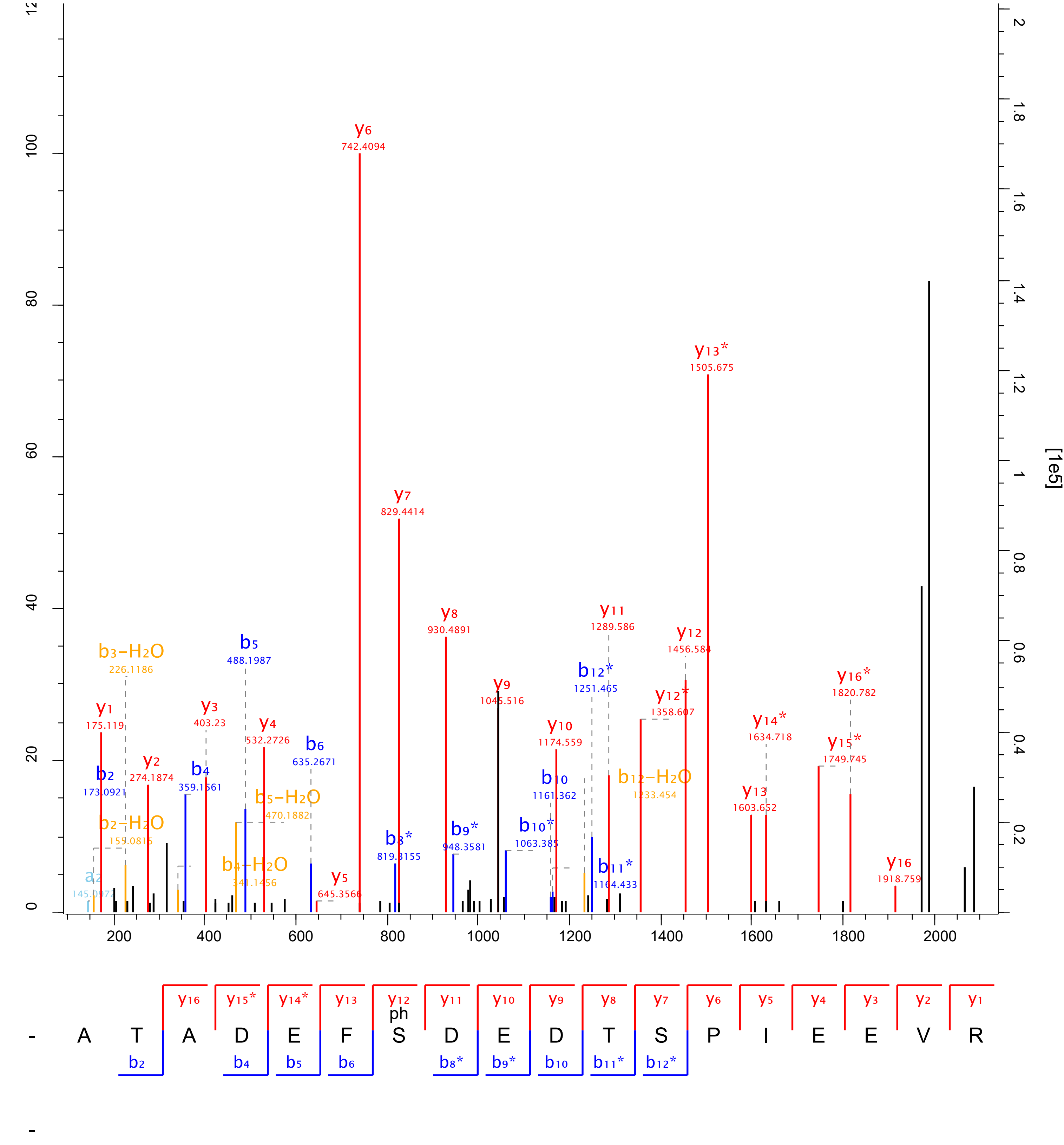

Sample output to test PDF Combine only

Raw file	Scan	Method	Score	m/z	Gene names
0523_4	18653	FTMS; HCD	89.72	984.39	At4g11560

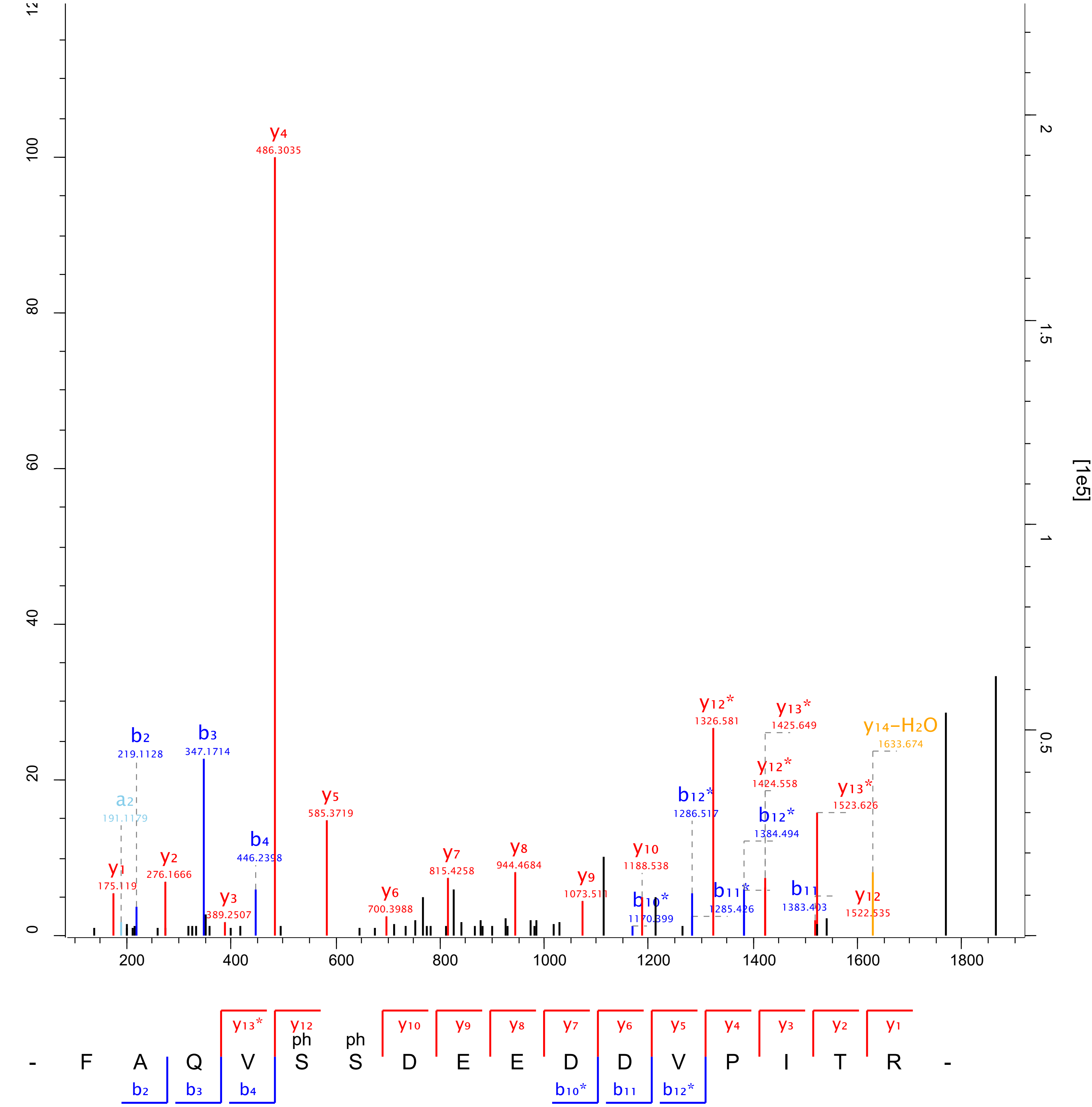

Sample output to test PDF Combine only

Raw file	Scan	Method	Score	m/z	Gene names
0523_4	18680	FTMS; HCD	79.84	776.35	DCAF1

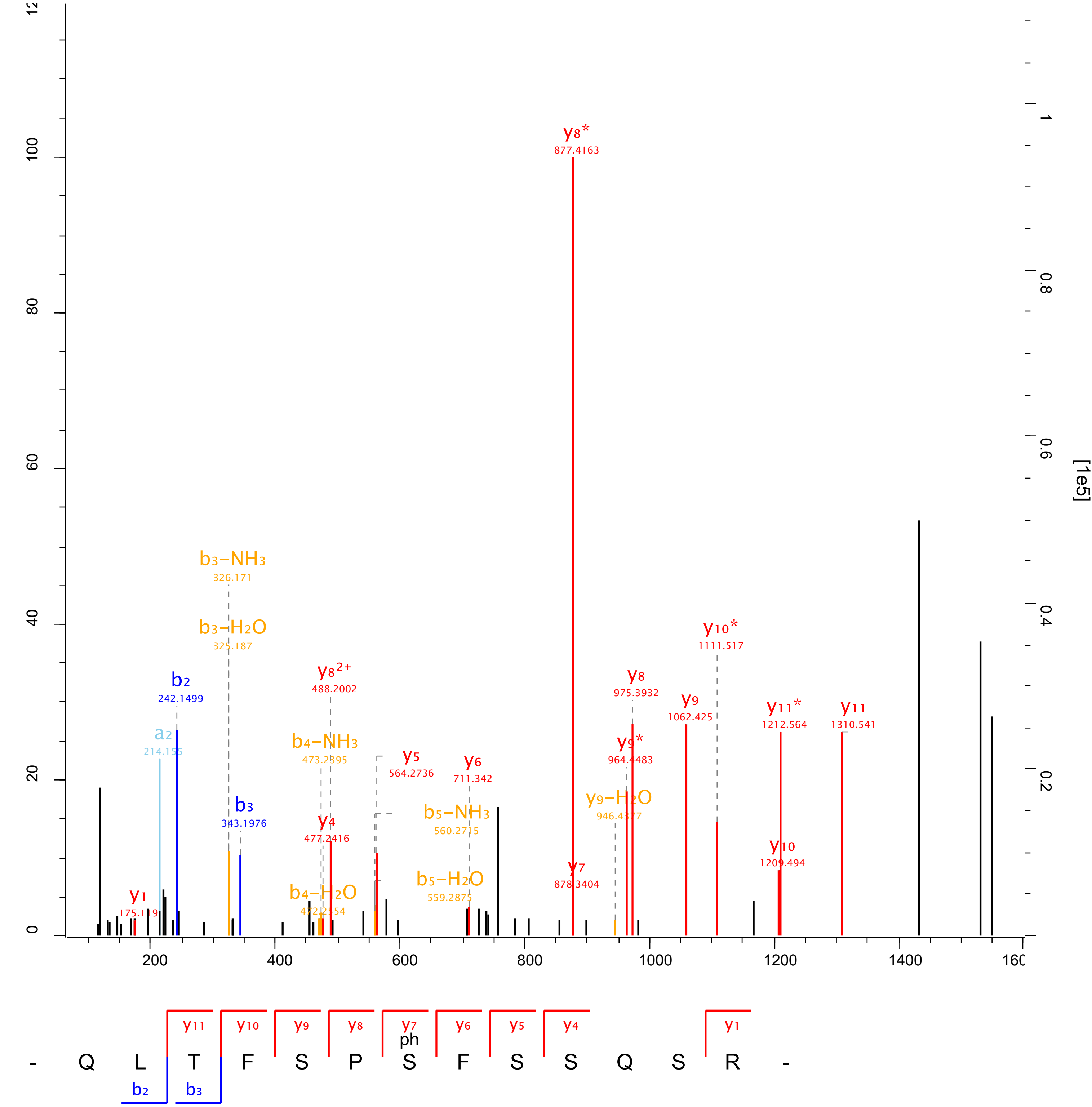

Sample output to test PDF Combine only

Raw file	Scan	Method	Score	m/z	Gene names
0523_4	18800	FTMS; HCD	132.84	848.84	RABA5E

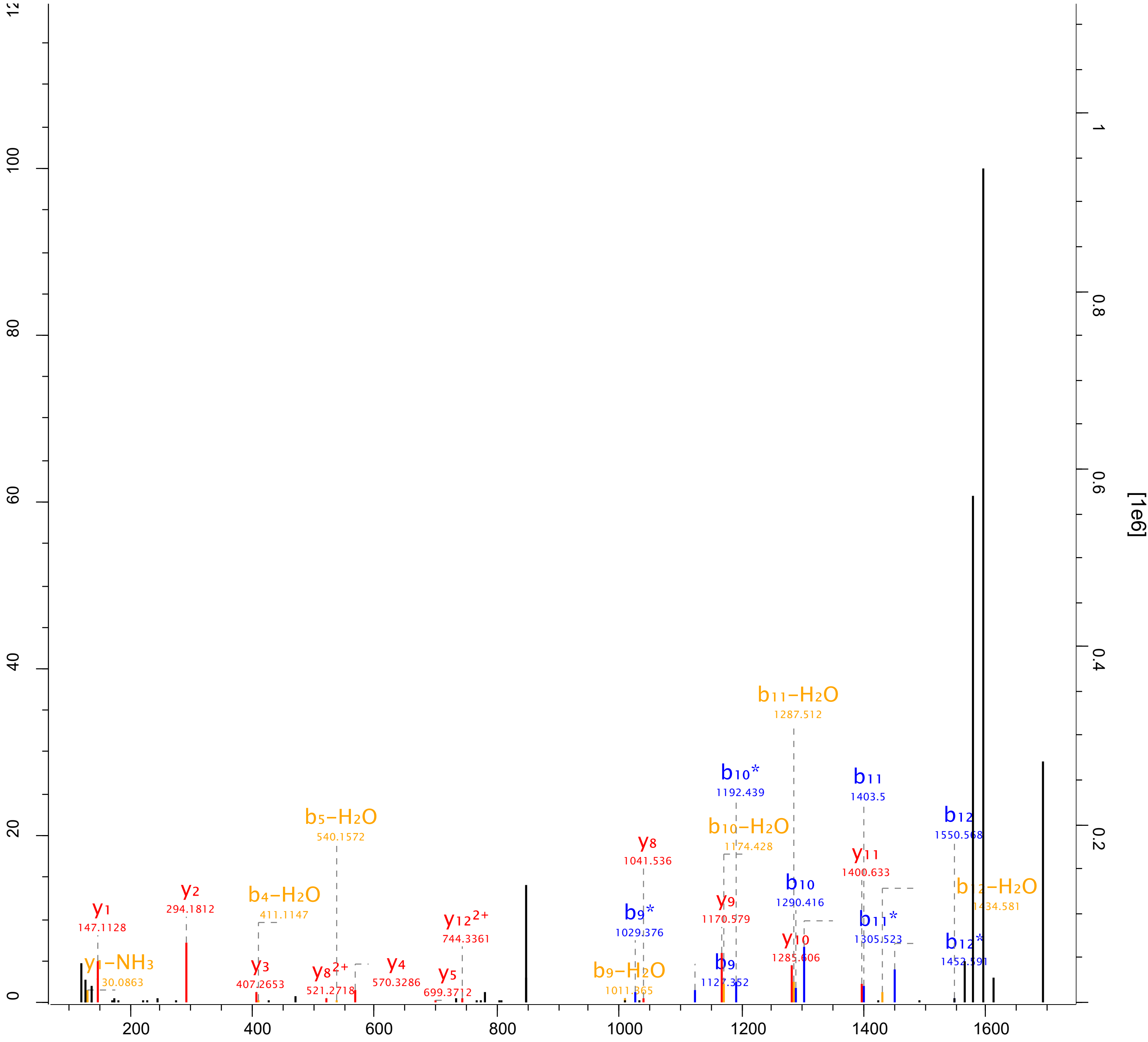

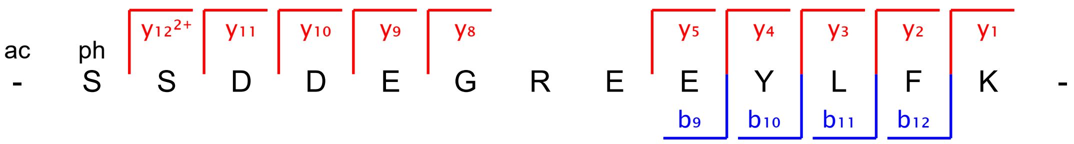

Sample output to test PDF Combine only

Raw file	Scan	Method	Score	m/z	Gene names
0523_4	18804	FTMS; HCD	52.37	728.99	ABCG36

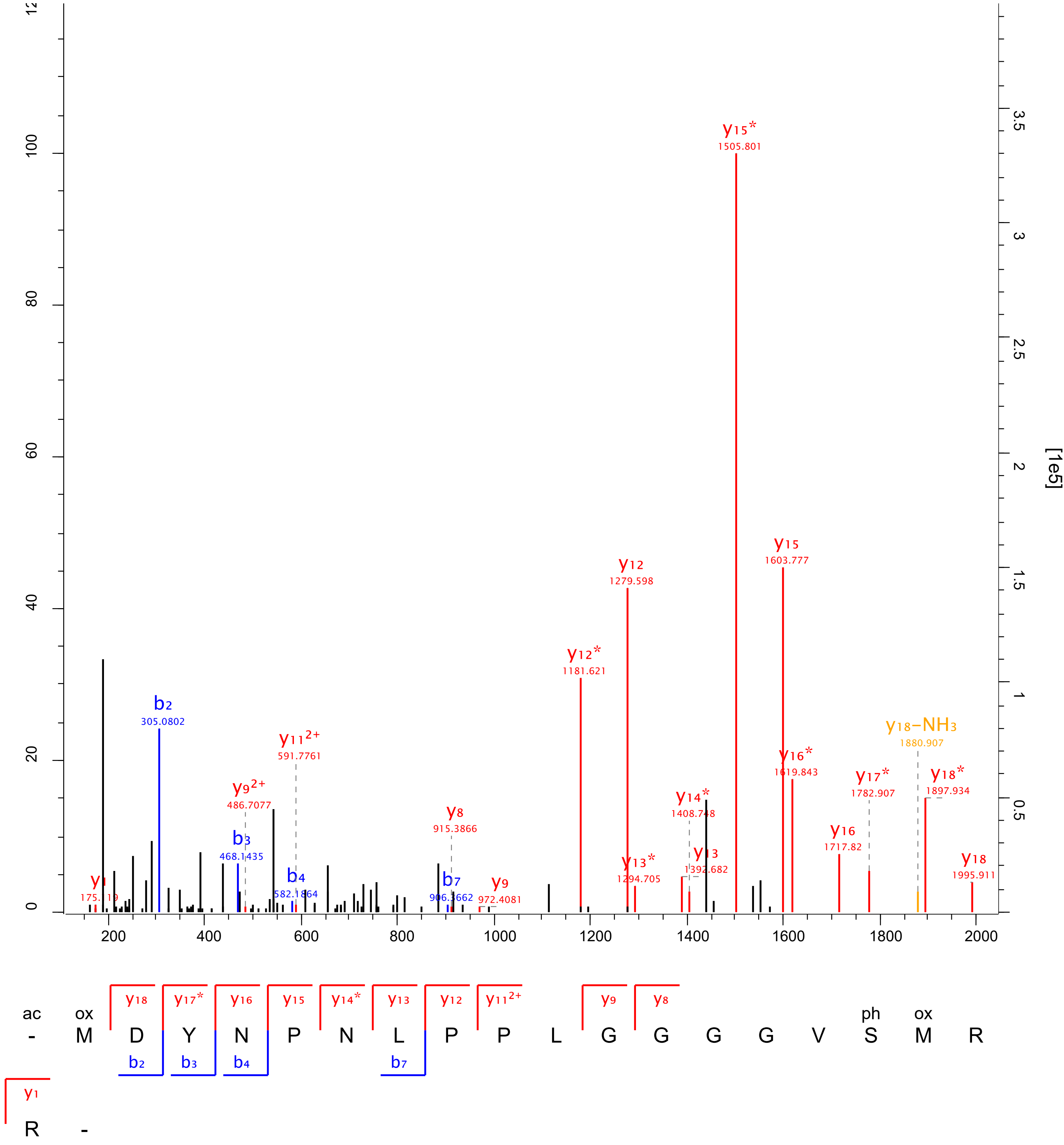

Sample output to test PDF Combine only

Raw file	Scan	Method	Score	m/z	Gene names
0523_4	18837	FTMS; HCD	136.57	1044.46	ATL15

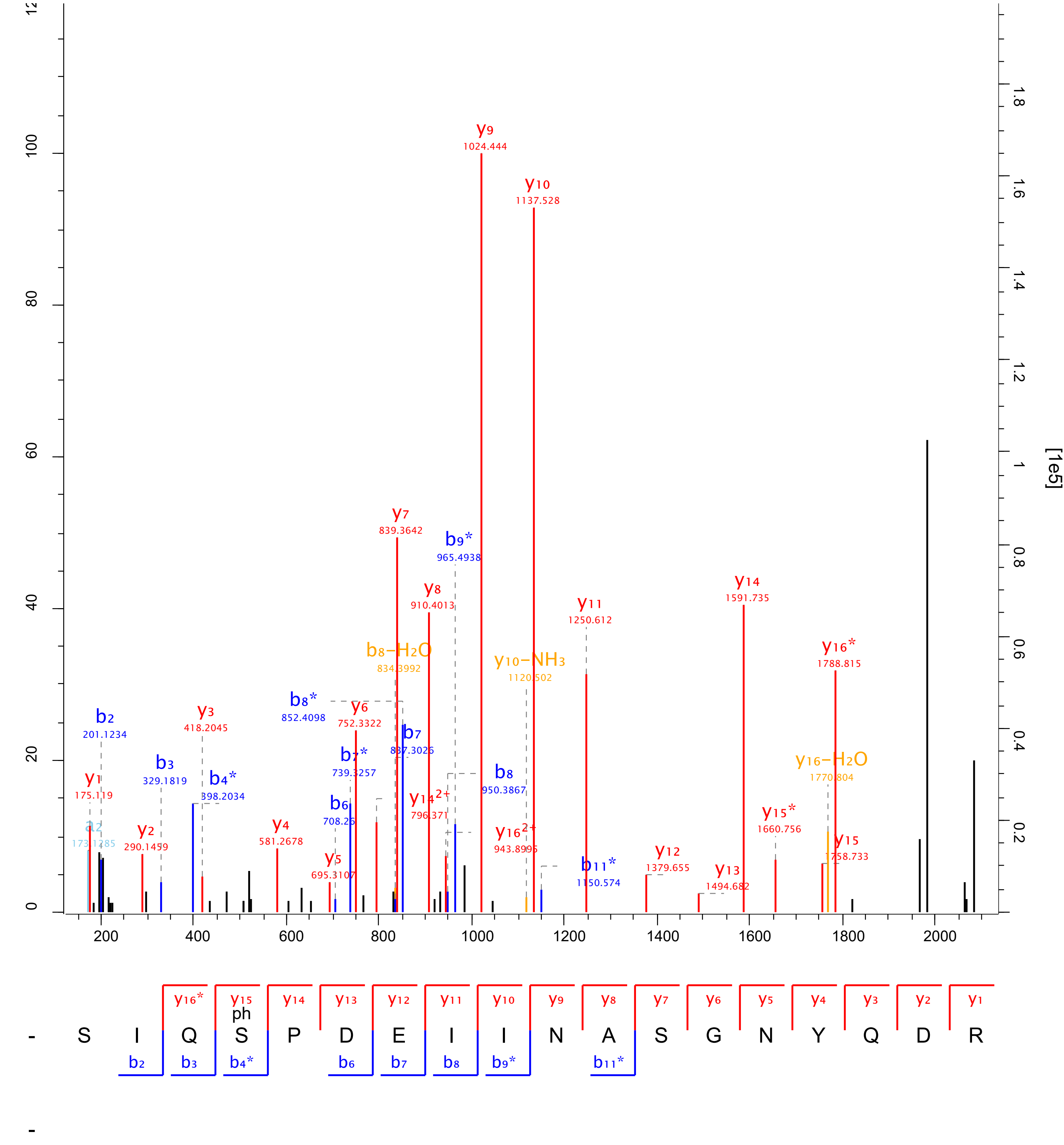

Sample output to test PDF Combine only

Raw file	Scan	Method	Score	m/z	Gene names
0523_4	18847	FTMS; HCD	118.9	711.68	At2g29210

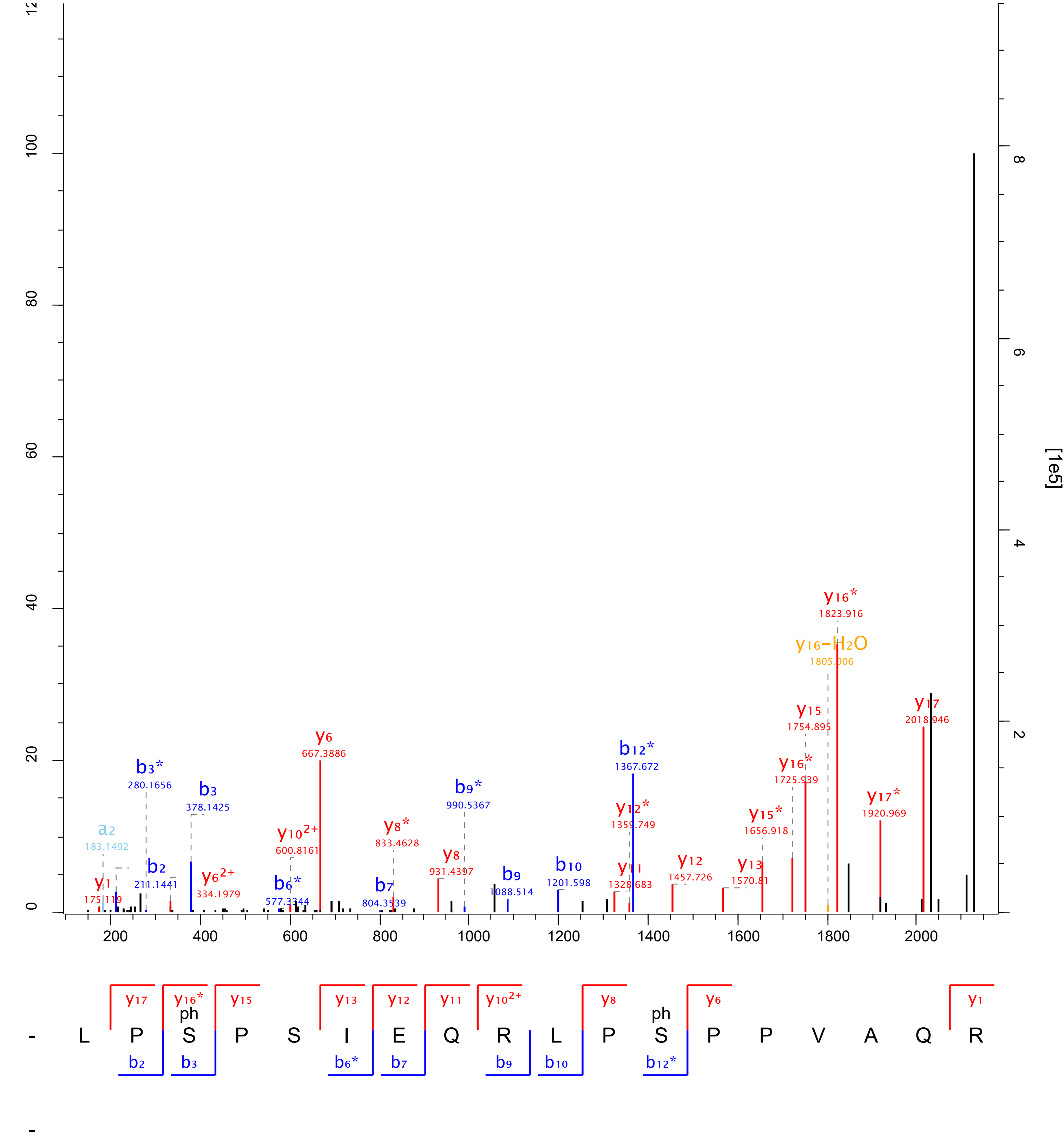

Sample output to test PDF Combine only

Raw file	Scan	Method	Score	m/z	Gene names
0523_4	19170	FTMS; HCD	99.8	758.33	BSL2;BSL3

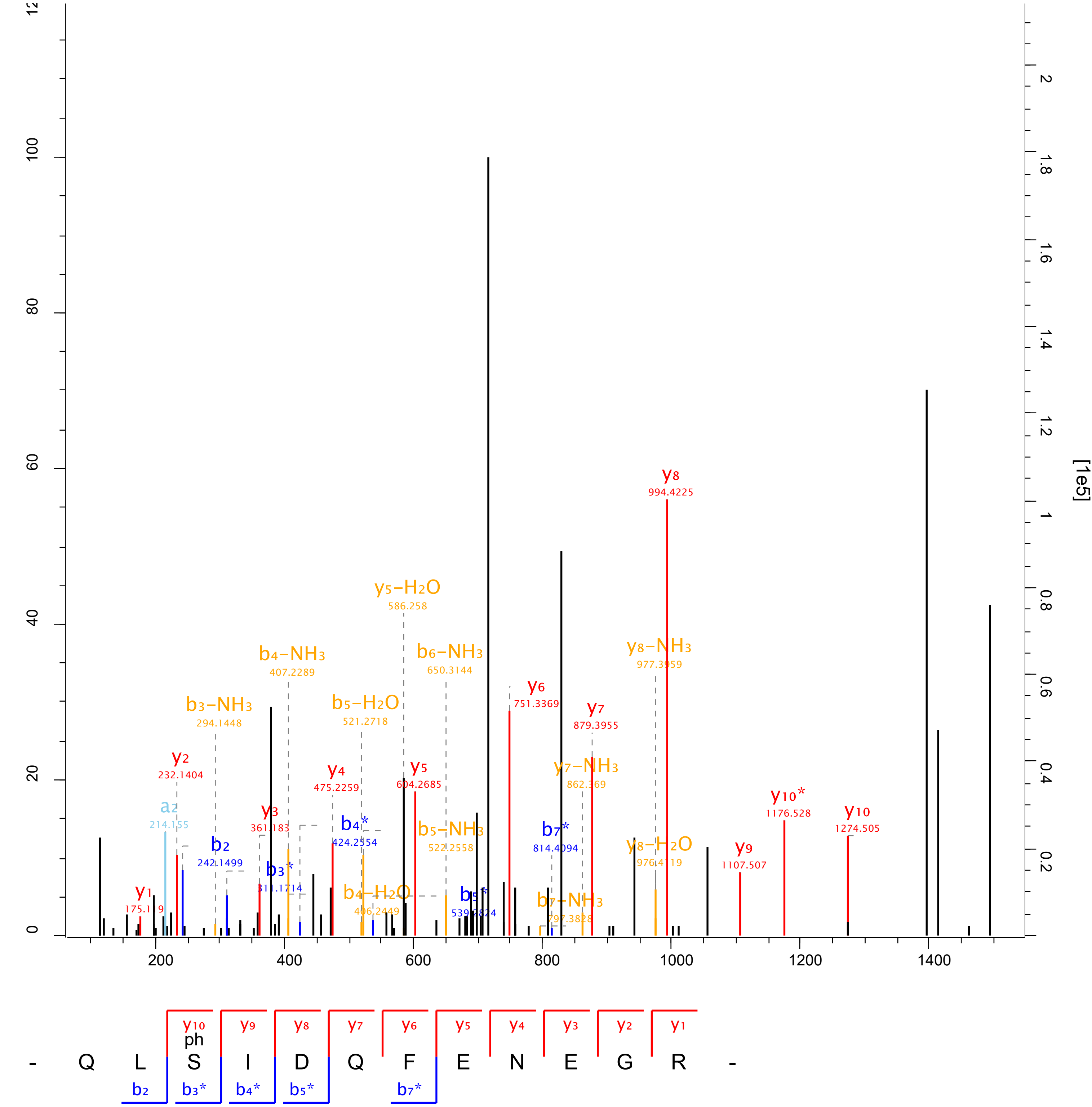

Sample output to test PDF Combine only

Raw file	Scan	Method	Score	m/z	Gene names
0523_4	19210	FTMS; HCD	259.49	935.89	CASP

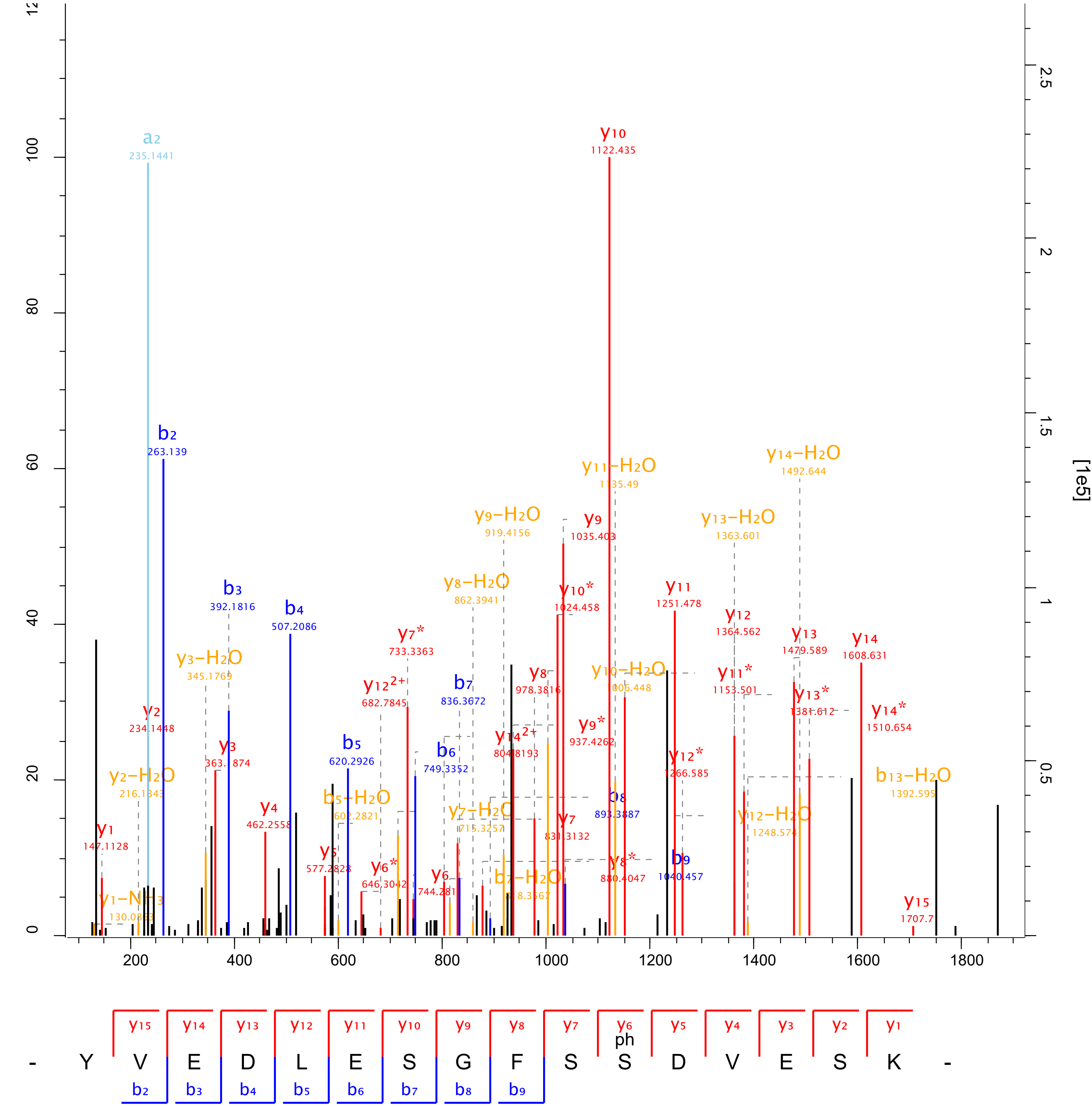

Sample output to test PDF Combine only

Raw file	Scan	Method	Score	m/z	Gene names
0523_4	19253	FTMS; HCD	150.14	955.95	STP7

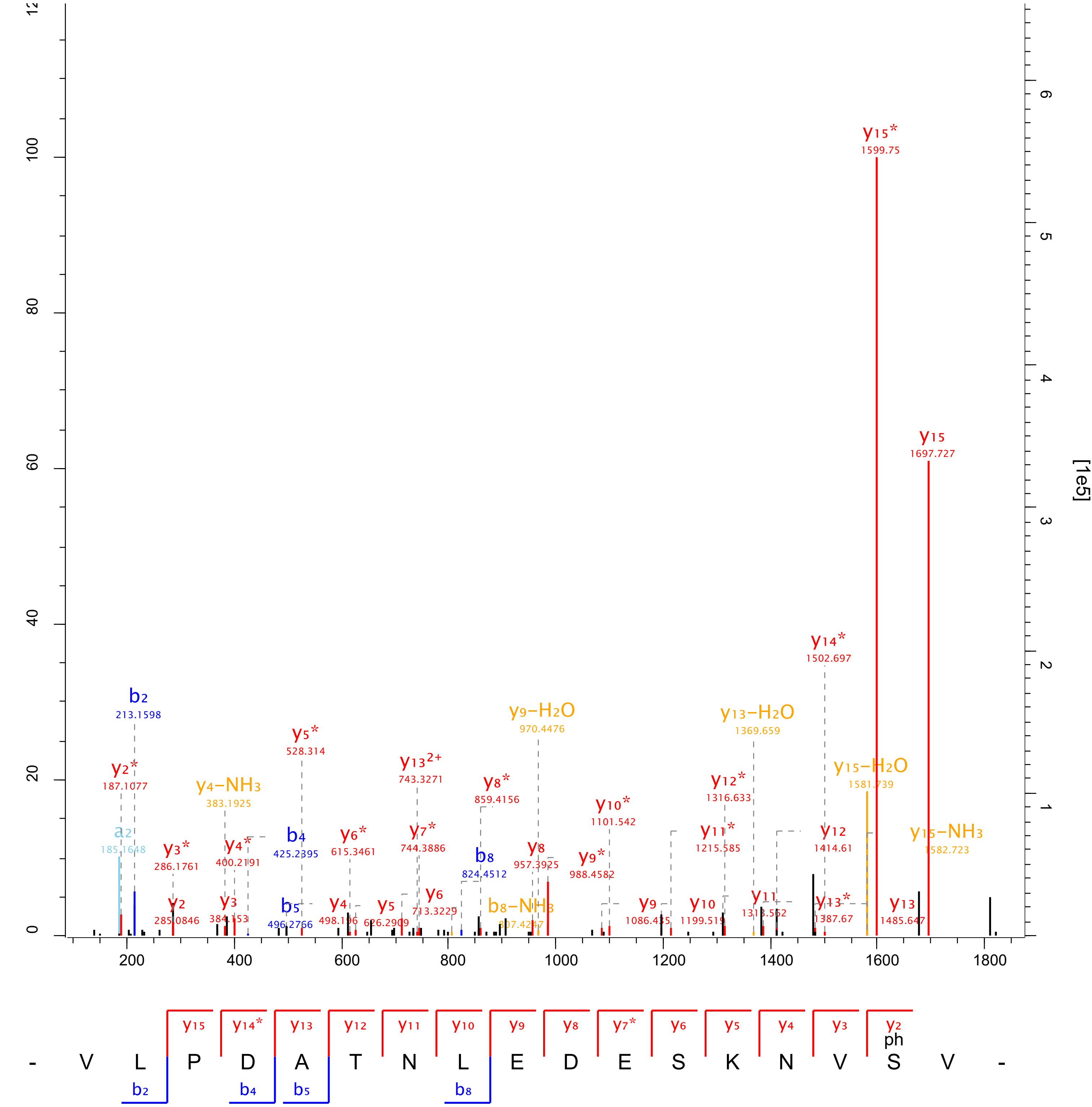

Sample output to test PDF Combine only

Raw file	Scan	Method	Score	m/z	Gene names
0523_4	19358	FTMS; HCD	66.92	789.01	At3g62860

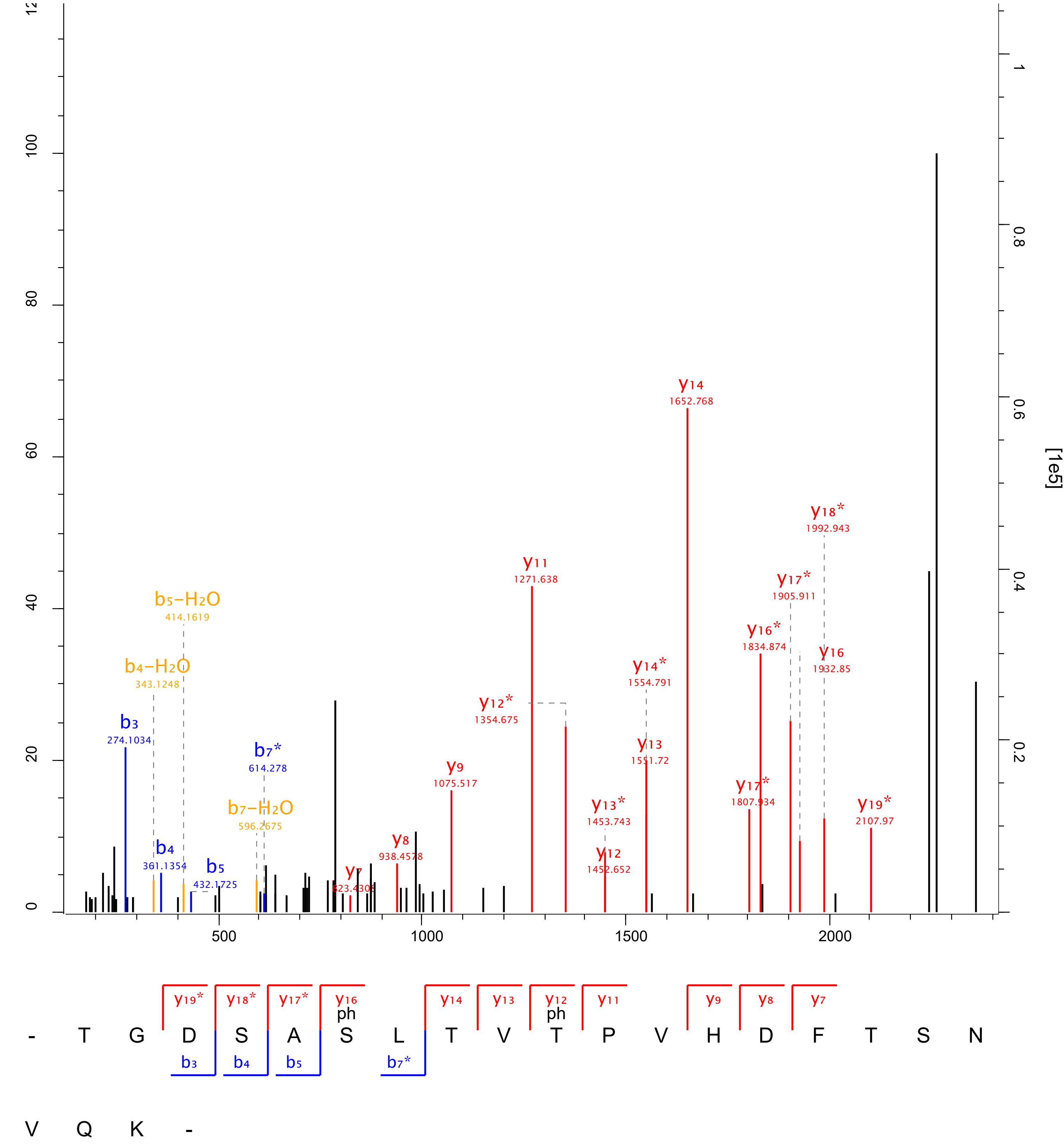

Sample output to test PDF Combine only

Raw file	Scan	Method	Score	m/z	Gene names
0523_4	19471	FTMS; HCD	185.71	1024.4	CSLD3

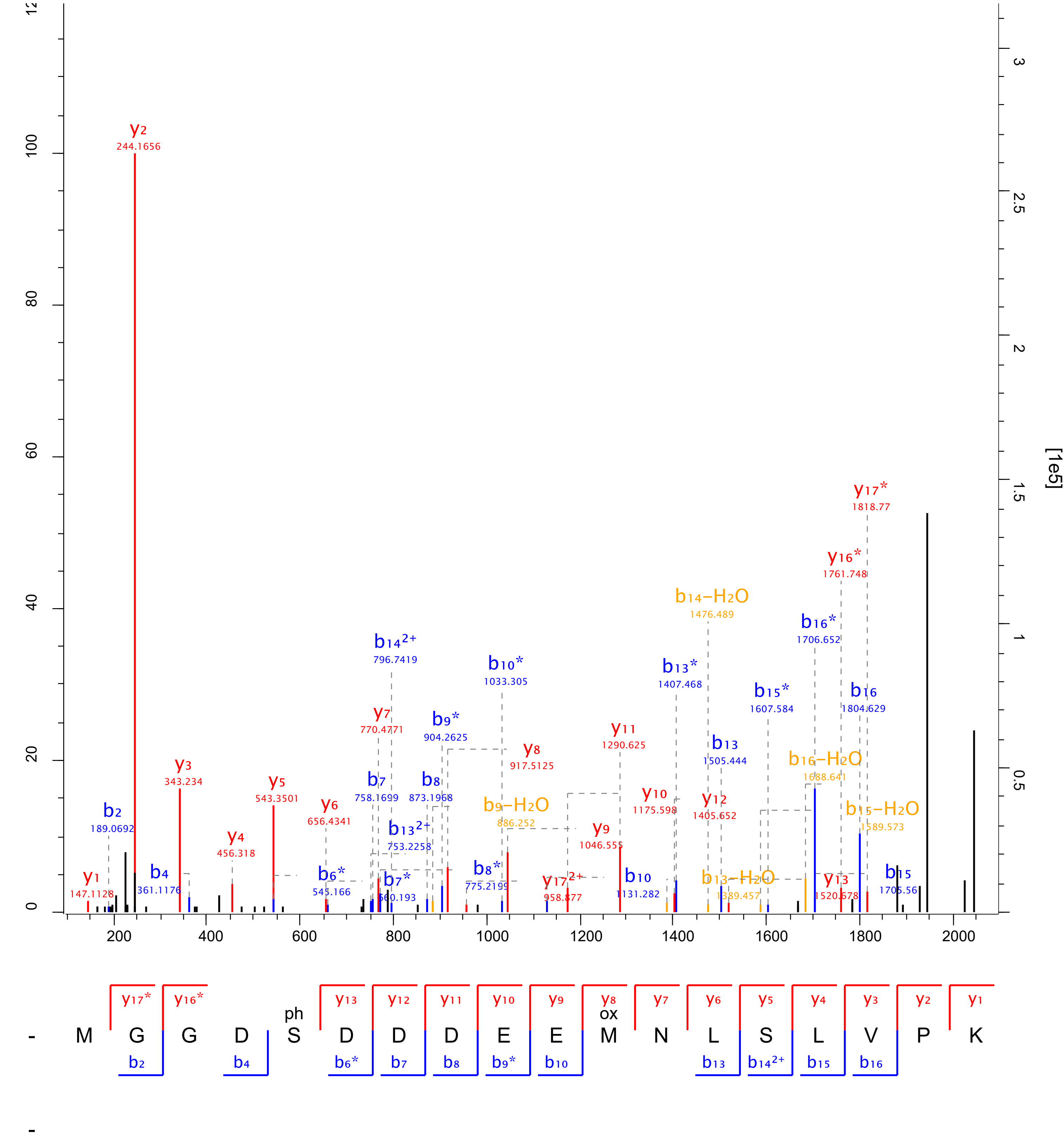

Sample output to test PDF Combine only

Raw file	Scan	Method	Score	m/z	Gene names
0523_4	19511	FTMS; HCD	99.06	761.81	MLO6

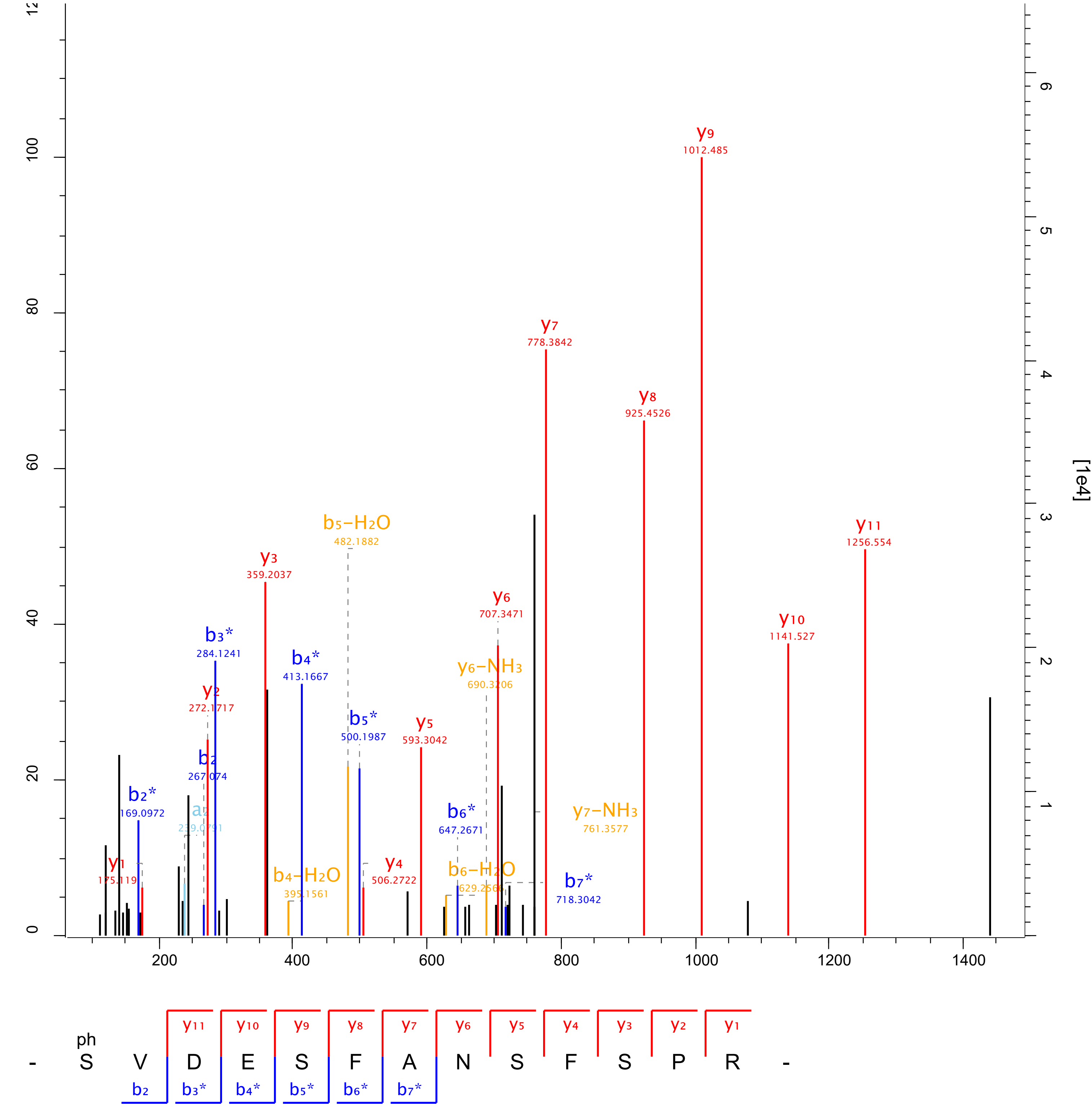

Sample output to test PDF Combine only

Raw file	Scan	Method	Score	m/z	Gene names
0523_4	19683	FTMS; HCD	119.92	723.35	At1g60890;PIP5K8

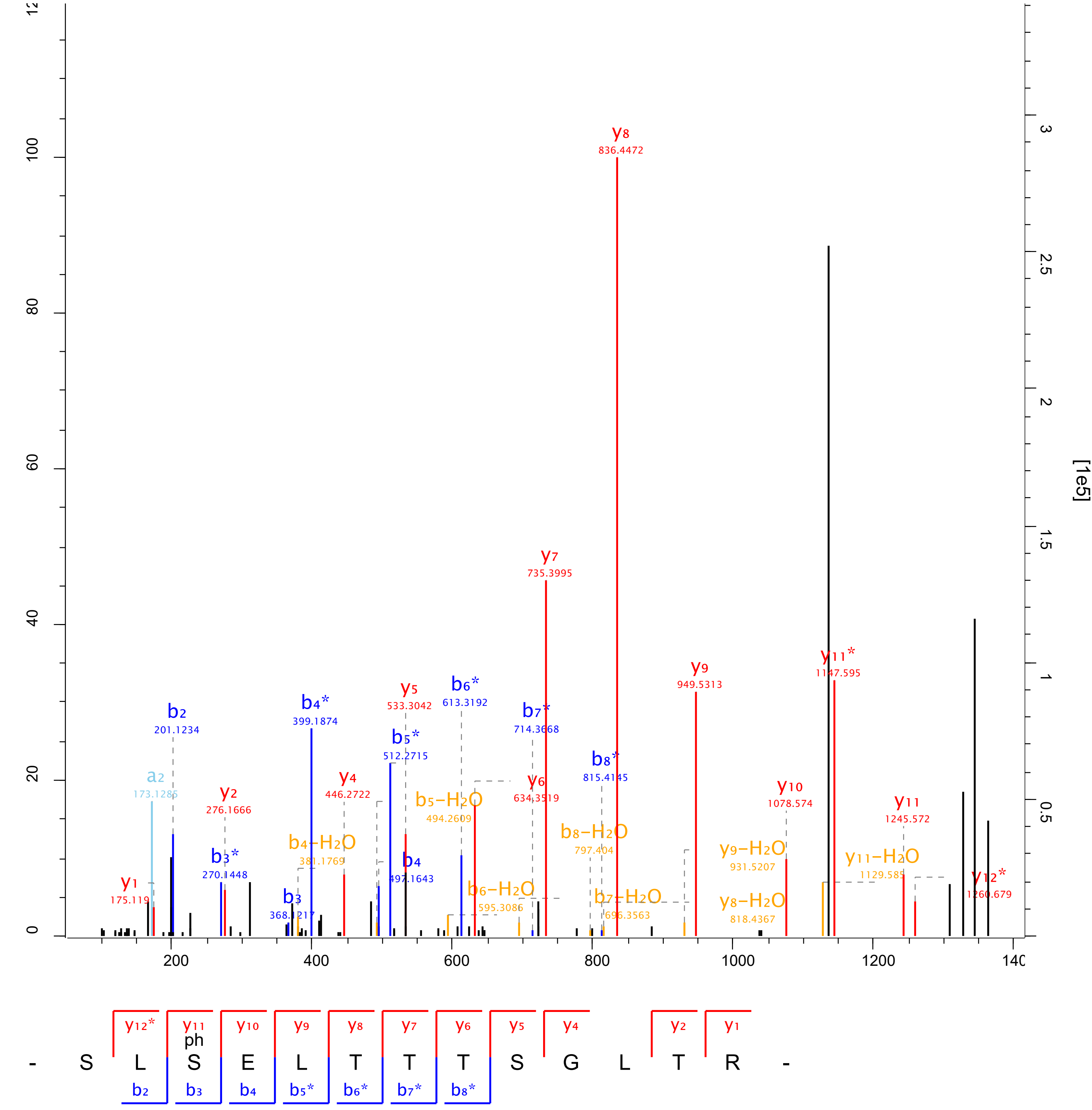

Raw file	Scan	Method	Score	m/z	Gene names
0523_4	19800	FTMS; HCD	166.9	862.84	RABA5C

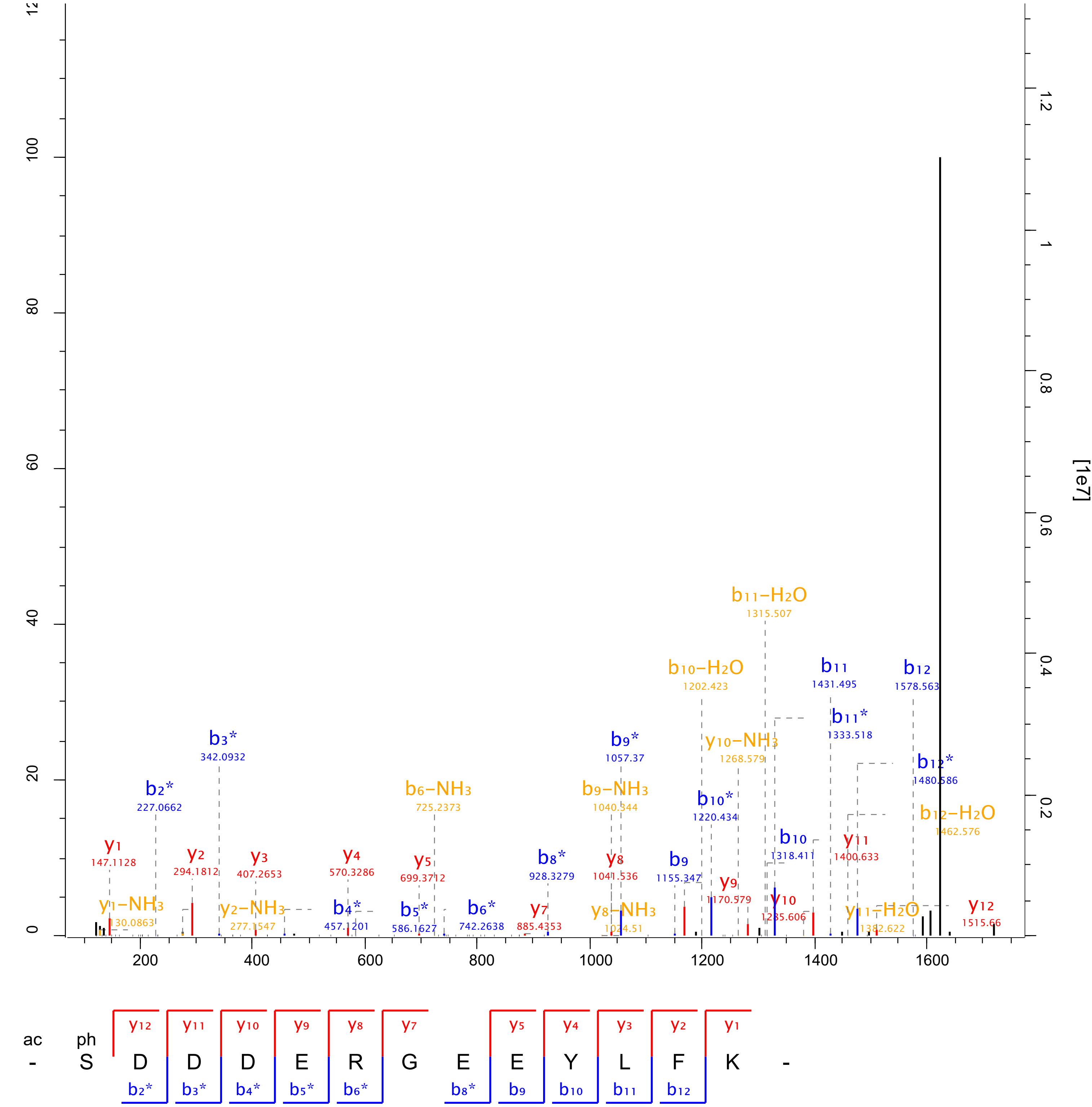

Sample output to test PDF Combine only

Raw file	Scan	Method	Score	m/z	Gene names
0523_4	19924	FTMS; HCD	89.47	795.84	At5g40450

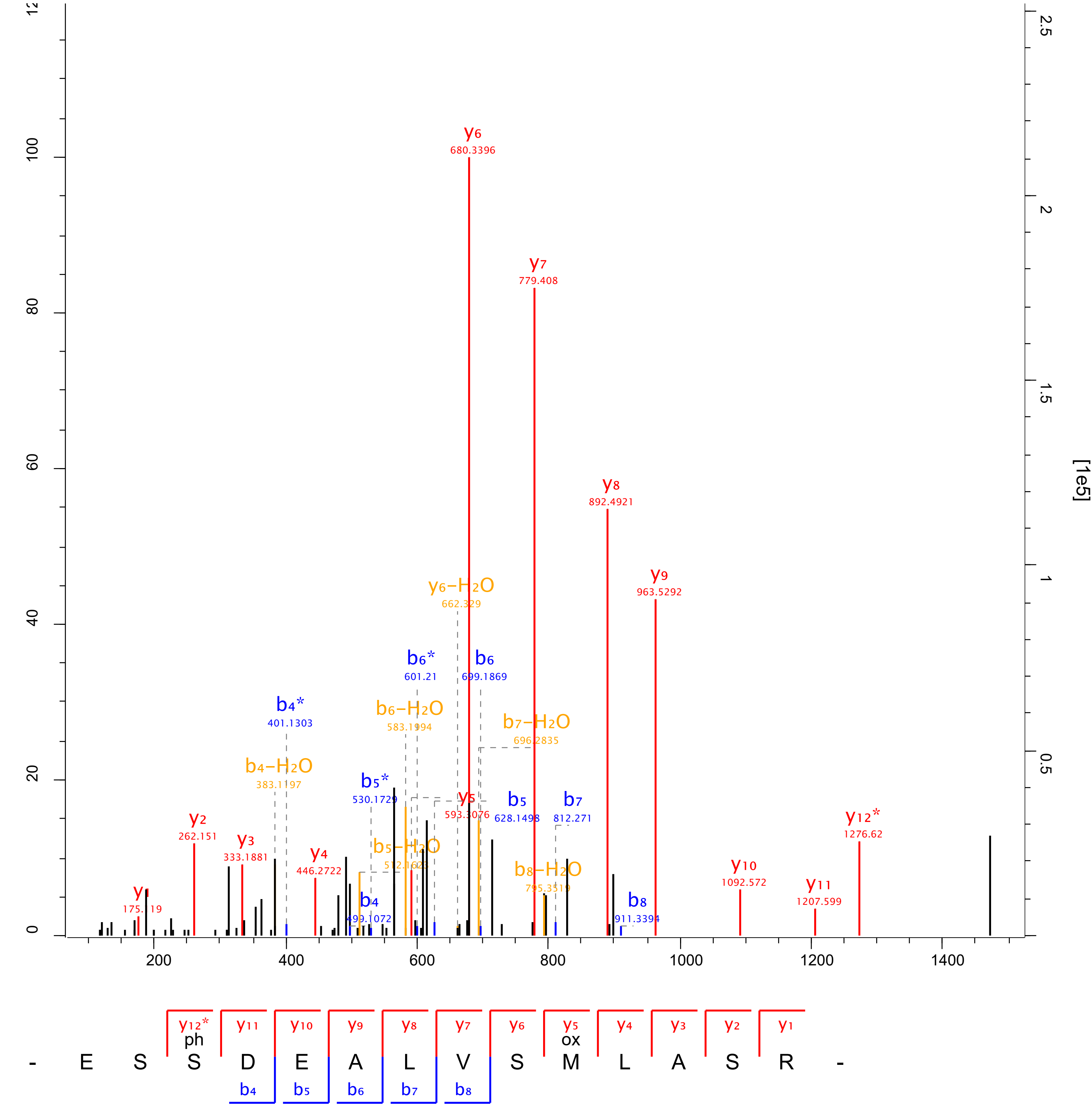

Sample output to test PDF Combine only

Raw file	Scan	Method	Score	m/z	Gene names
0523_4	20035	FTMS; HCD	162.26	1054.44	CPK8

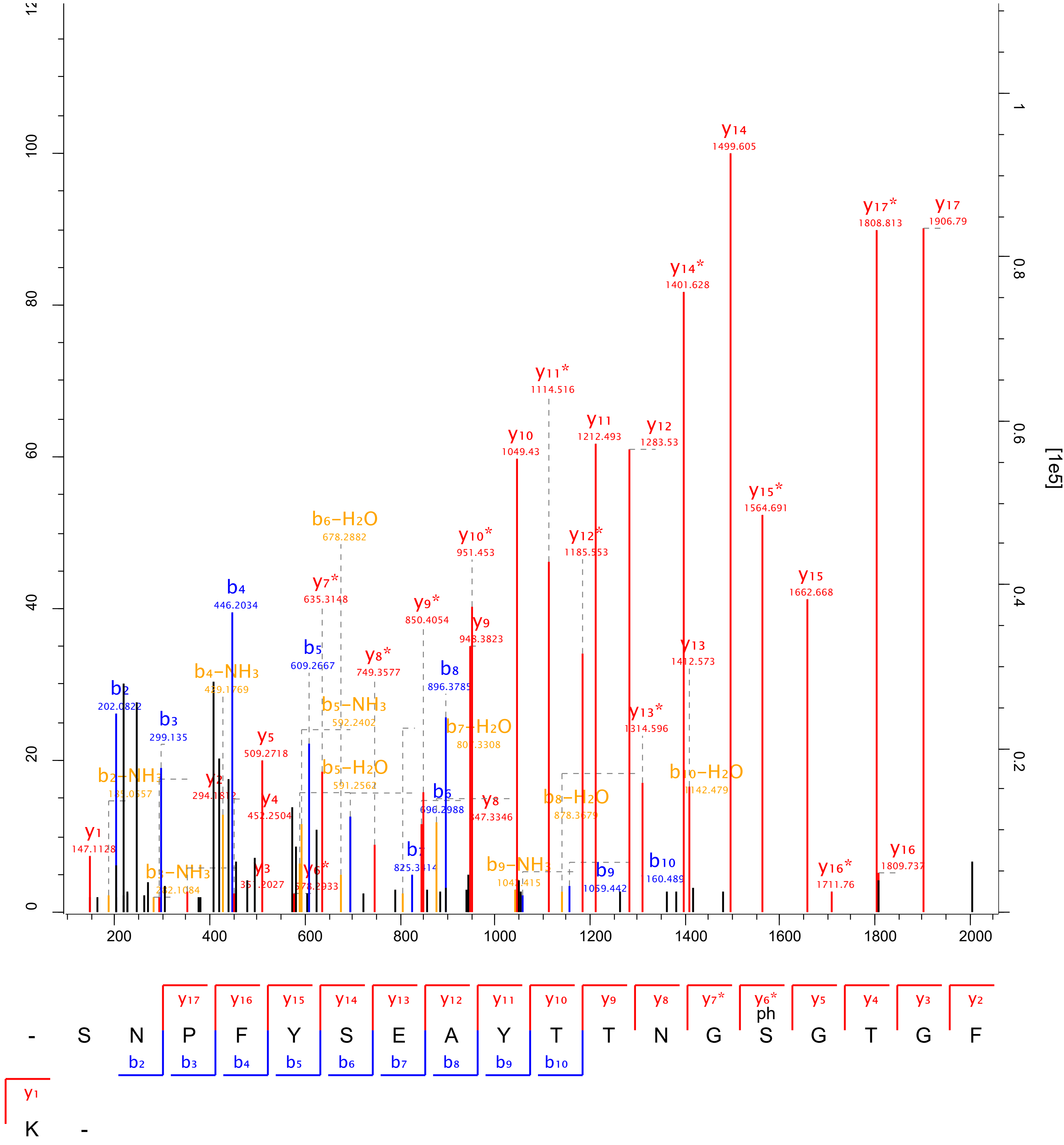

Raw file	Scan	Method	Score	m/z	Gene names
0523_4	20122	FTMS; HCD	48.91	771.81	RABC2A;RABC2B

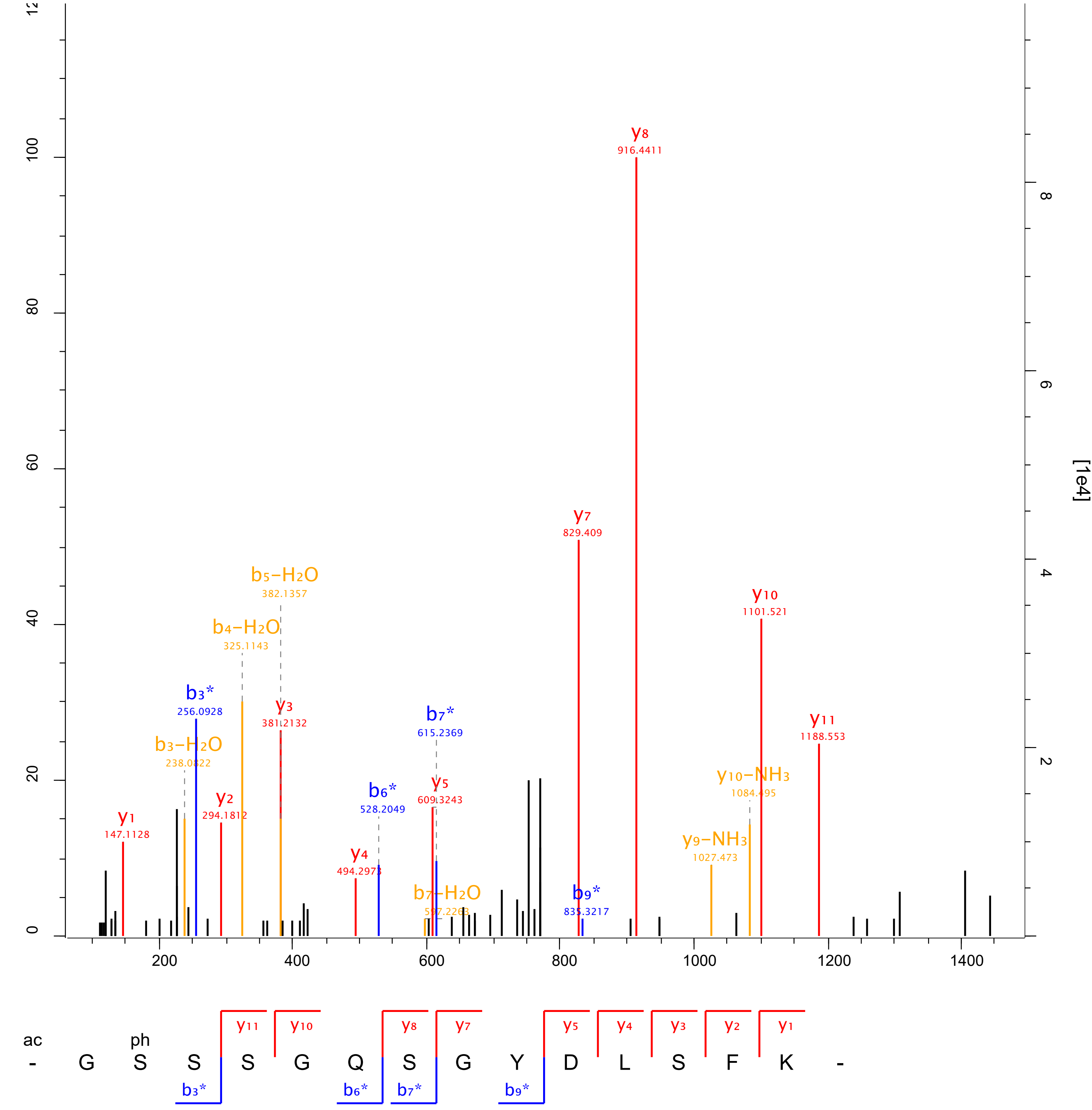

Sample output to test PDF Combine only

Raw file	Scan	Method	Score	m/z	Gene names
0523_4	20173	FTMS; HCD	356.12	928.42	At5g37710

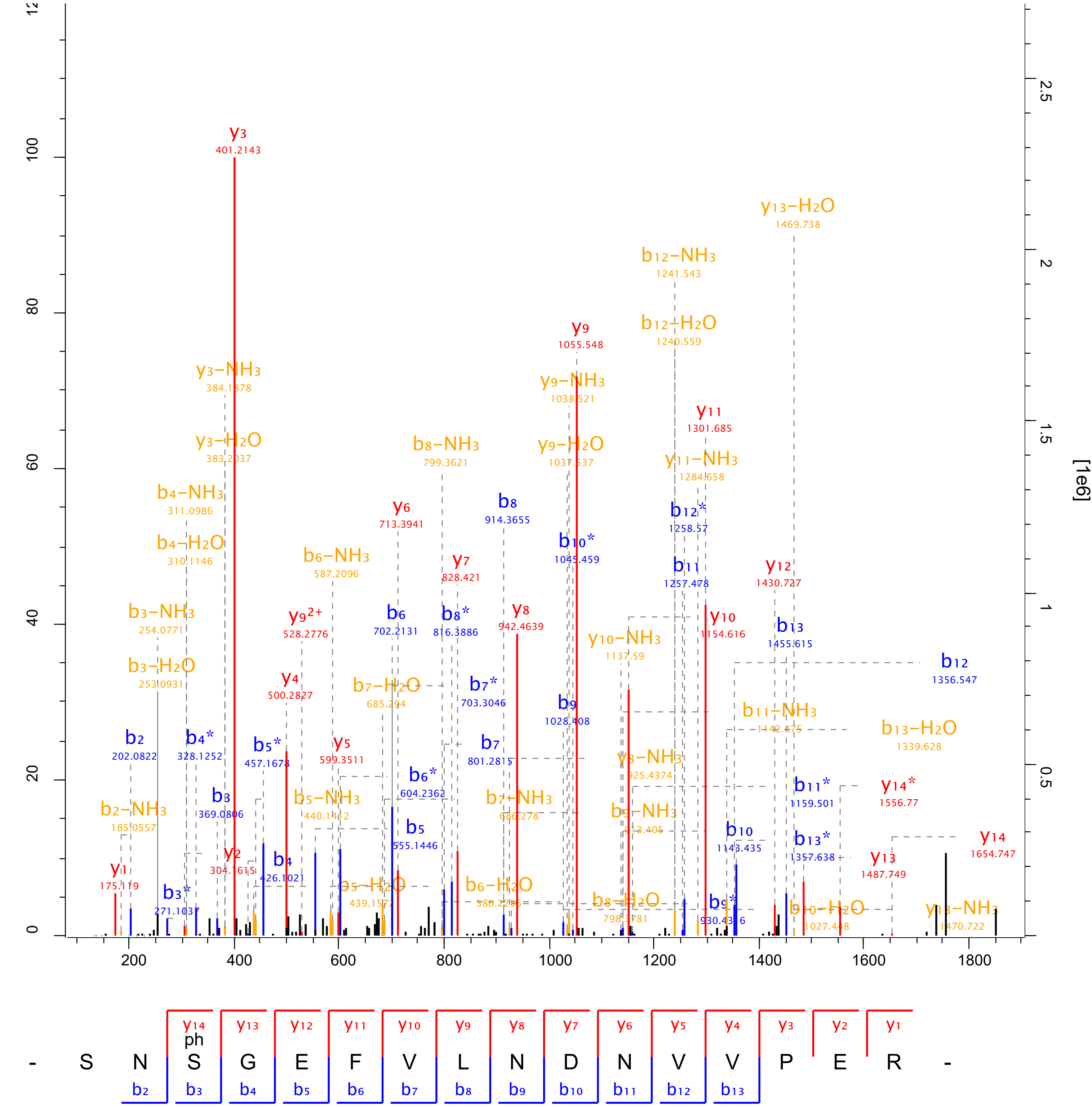

Raw file	Scan	Method	Score	m/z	Gene names
0523_4	20192	FTMS; HCD	84.65	918.43	At5g01450

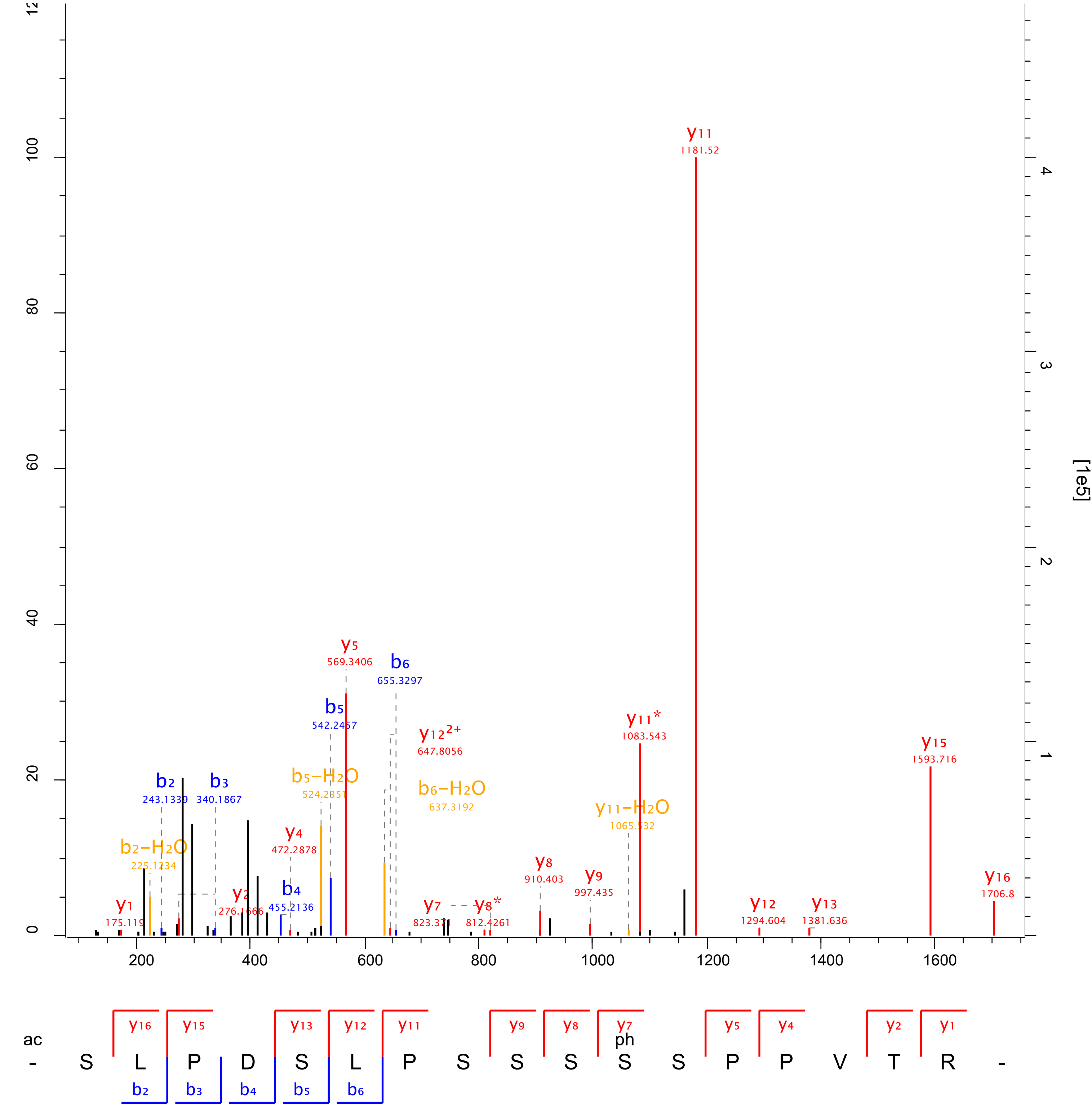

Sample output to test PDF Combine only

Raw file	Scan	Method	Score	m/z	Gene names
0523_4	20252	FTMS; HCD	98.94	645.79	At2g32240

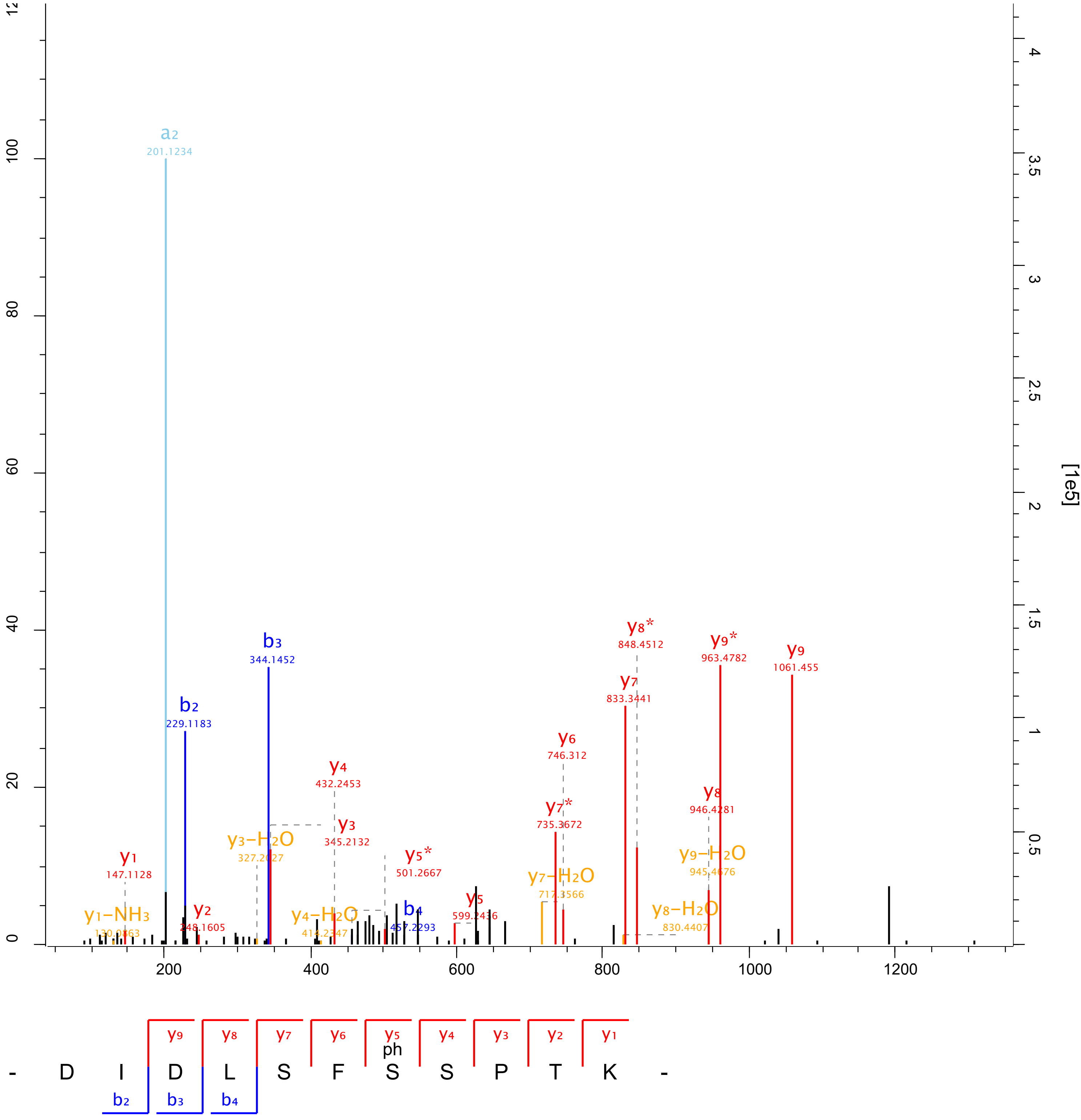

Sample output to test PDF Combine only

Raw file	Scan	Method	Score	m/z	Gene names
0523_4	20336	FTMS; HCD	210.32	992.44	MSL9

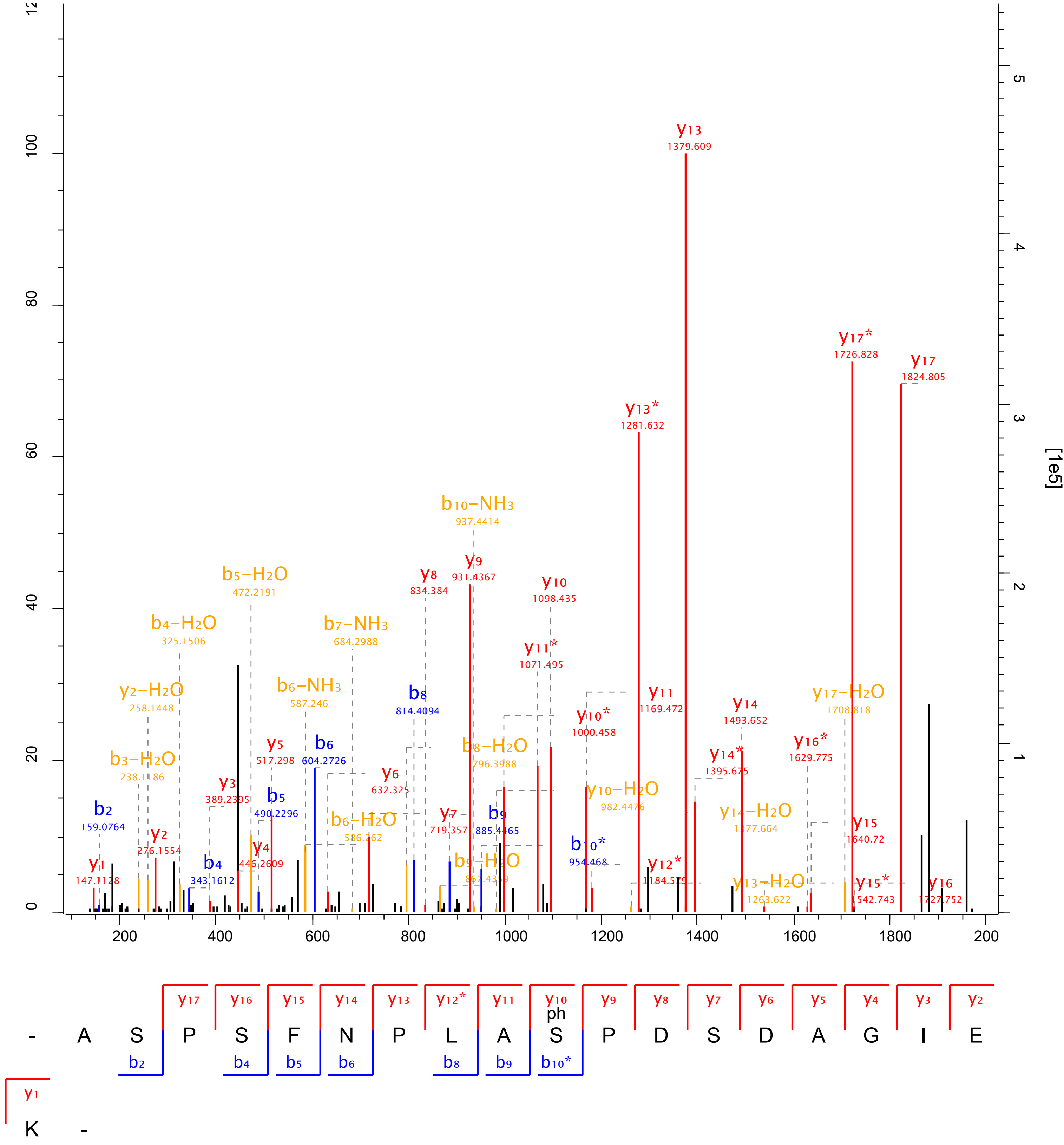

Raw file	Scan	Method	Score	m/z
0523_4	20349	FTMS; HCD	202.96	900.87

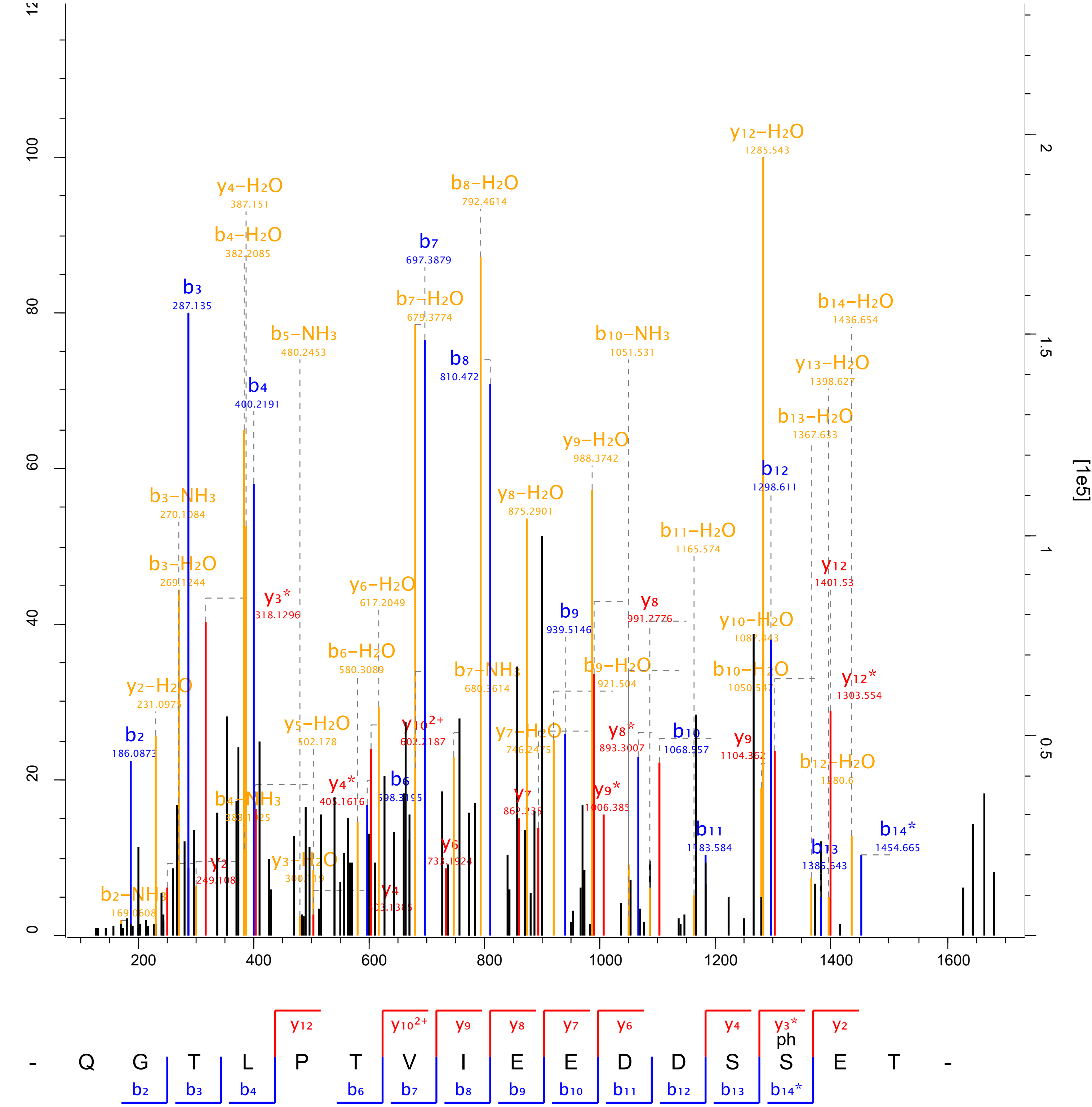

Sample output to test PDF Combine only

Raw file	Scan	Method	Score	m/z	Gene names
0523_4	20466	FTMS; HCD	69.81	706.86	WIP3

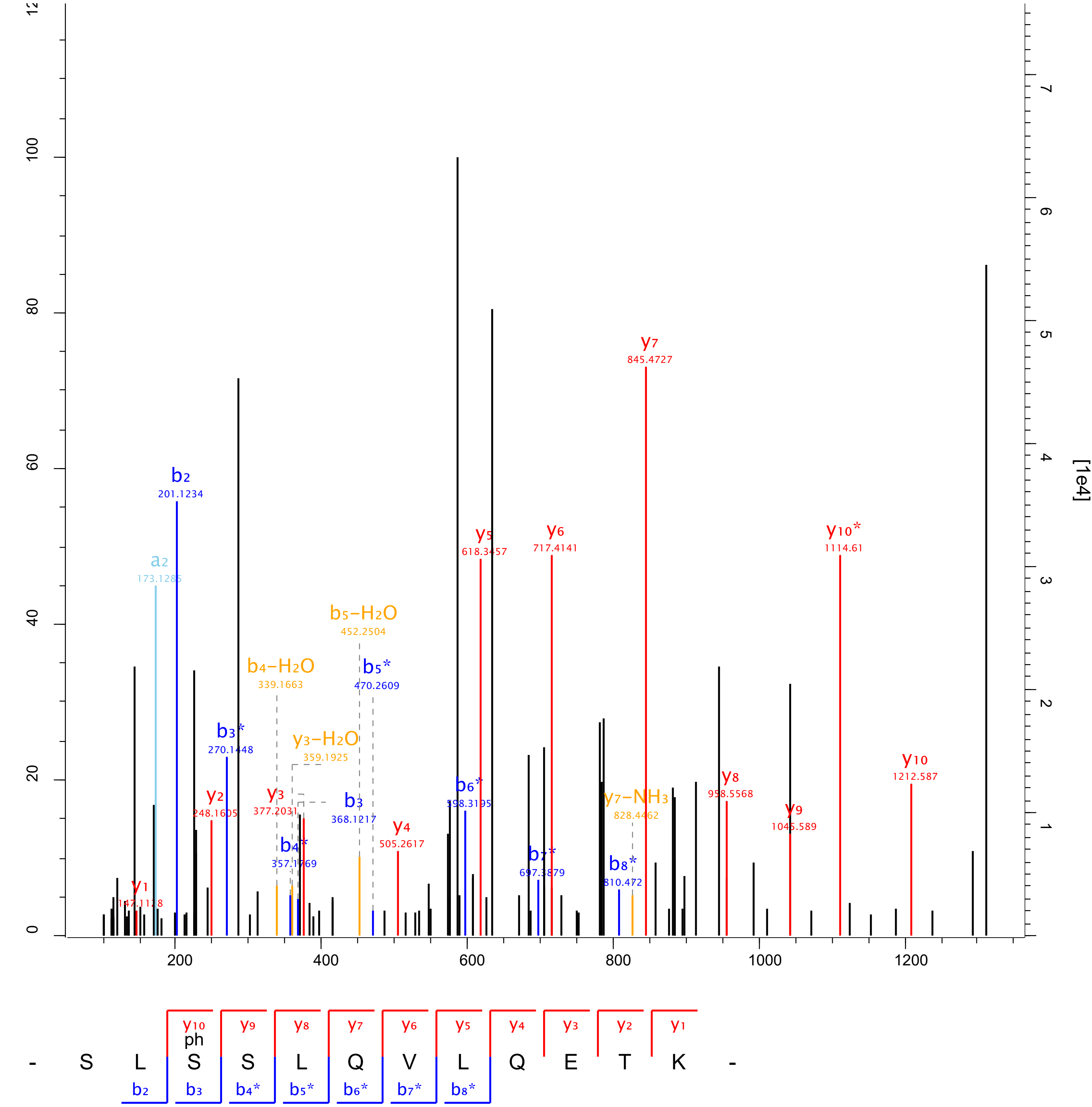

Sample output to test PDF Combine only

Raw file	Scan	Method	Score	m/z	Gene names
0523_4	20604	FTMS; HCD	87.65	648.27	At1g27100

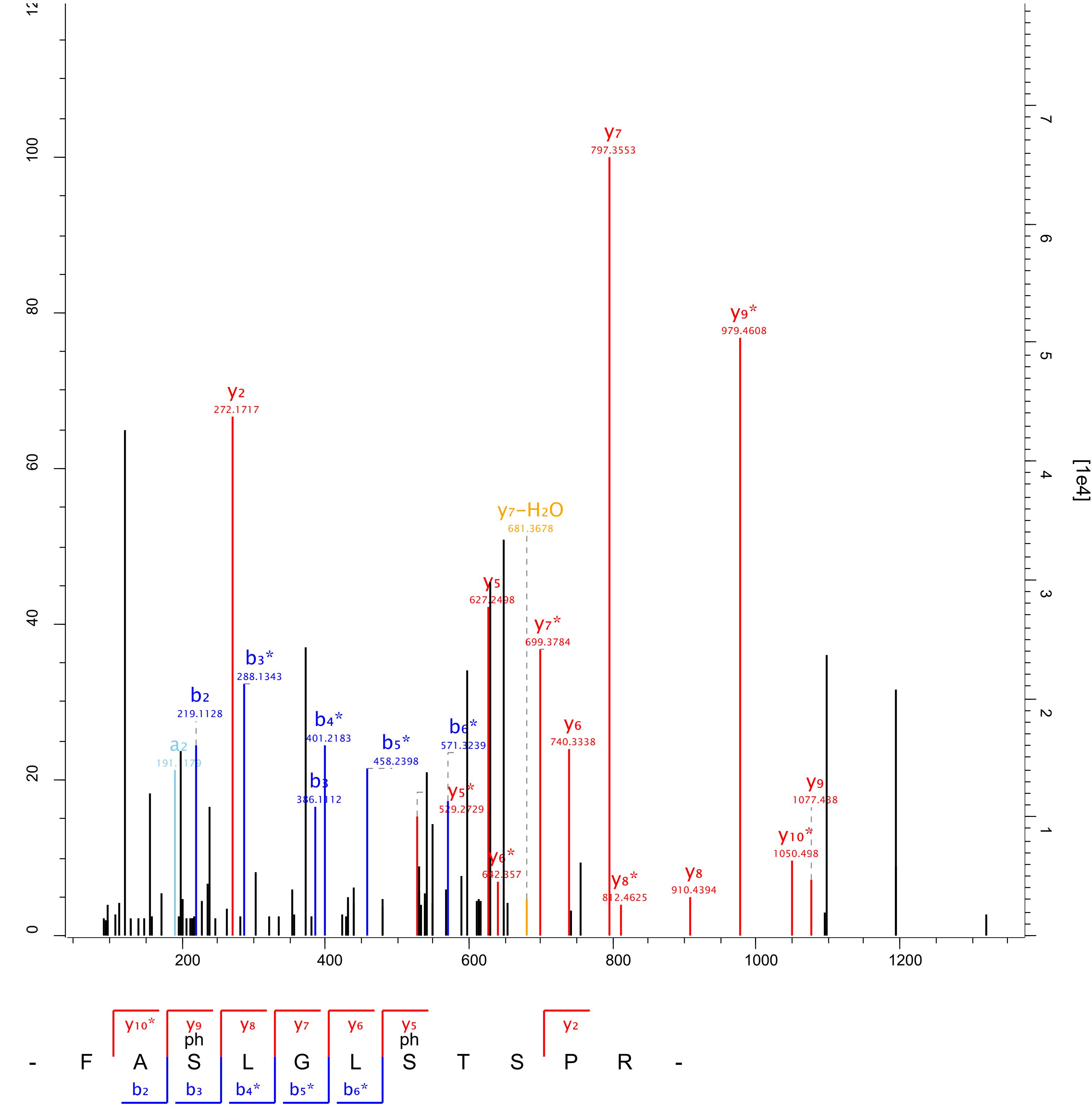

Sample output to test PDF Combine only

Raw file	Scan	Method	Score	m/z	Gene names
0523_4	20801	FTMS; HCD	226.79	732.8	At1g56230;F14G9.16

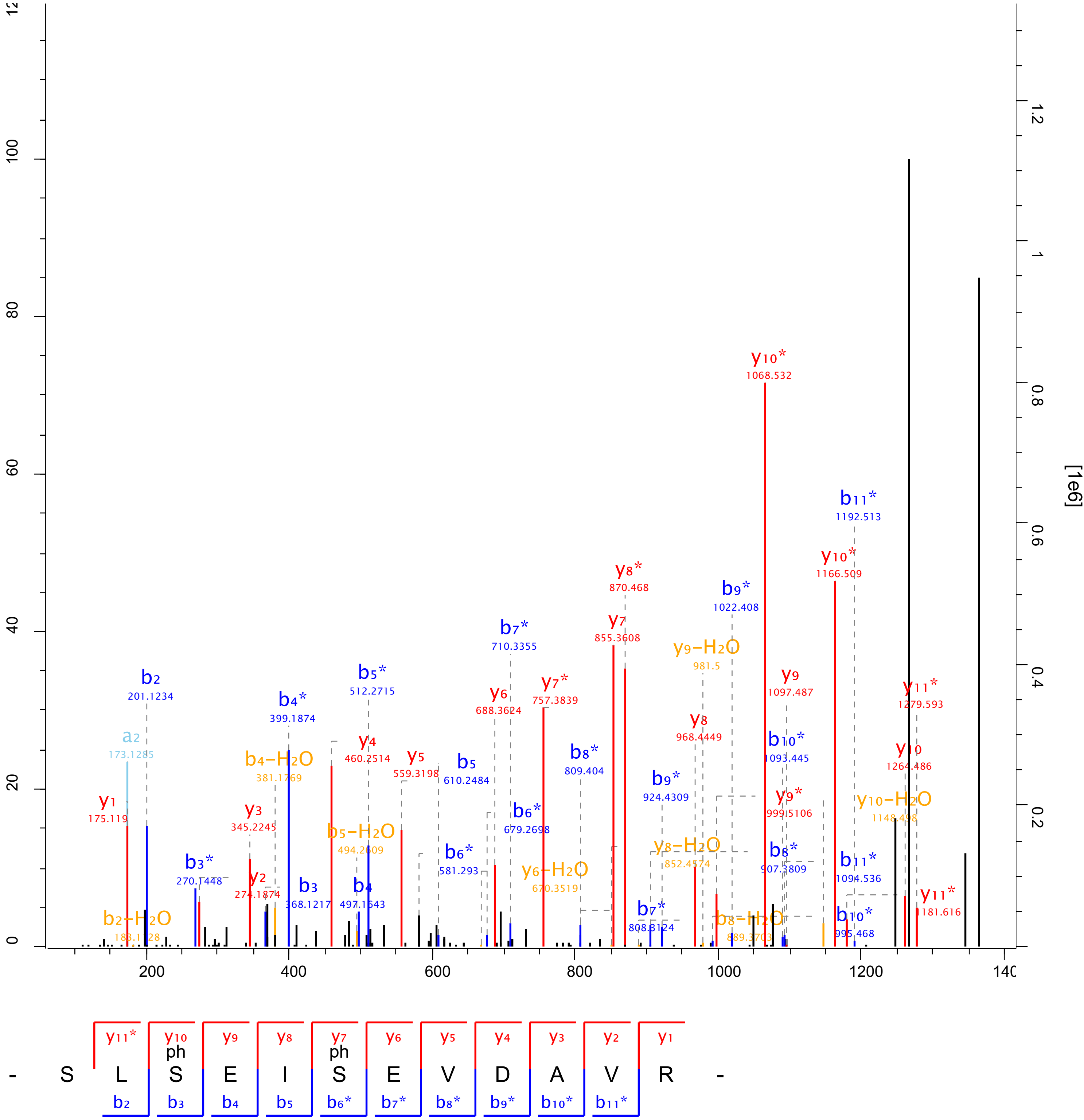

Sample output to test PDF Combine only

Raw file	Scan	Method	Score	m/z	Gene names
0523_4	21101	FTMS; HCD	130.27	806.89	At3g28690

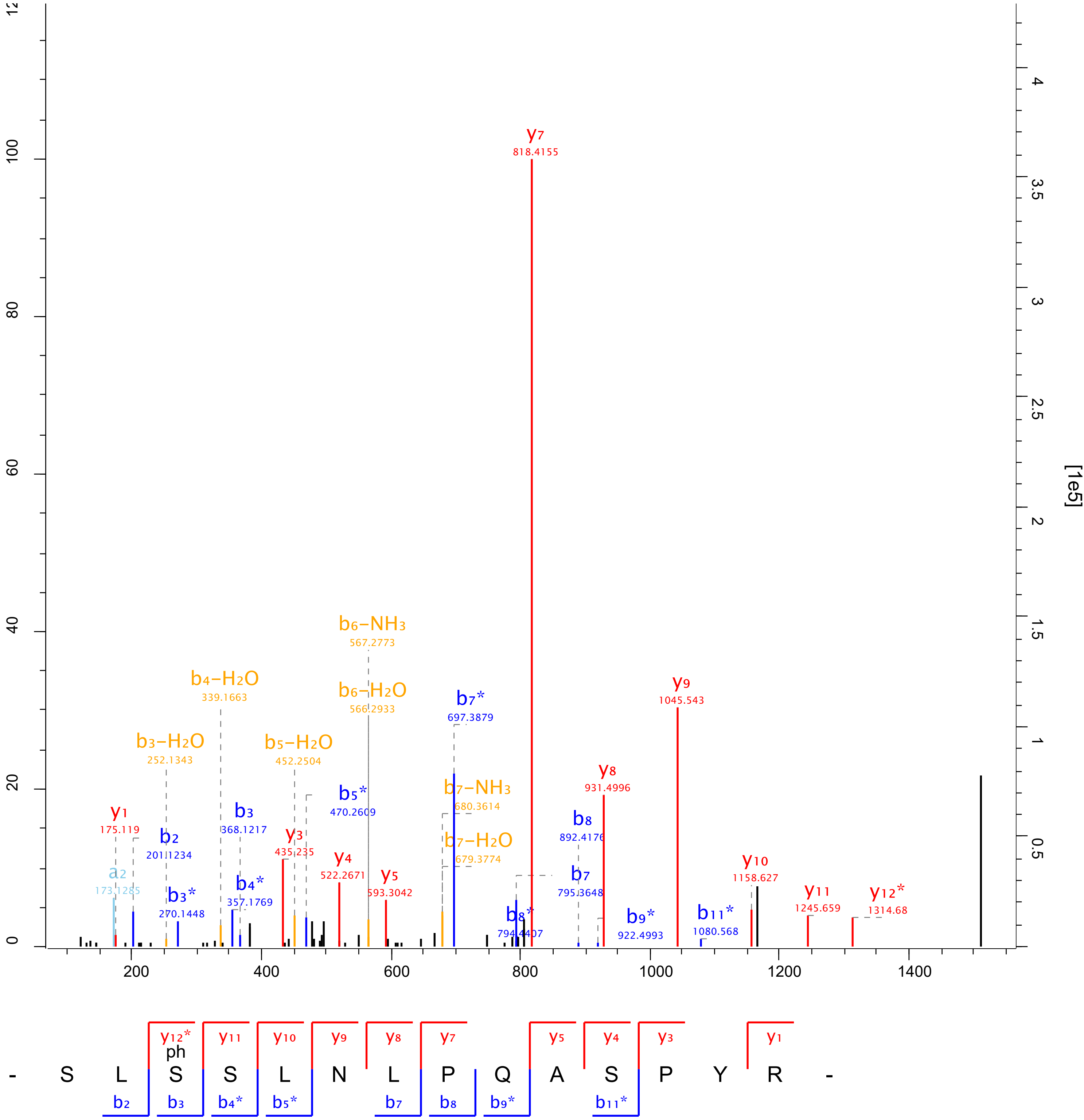

Sample output to test PDF Combine only

Raw file	Scan	Method	Score	m/z	Gene names
0523_4	21122	FTMS; HCD	237.33	796.7	NIP2-1

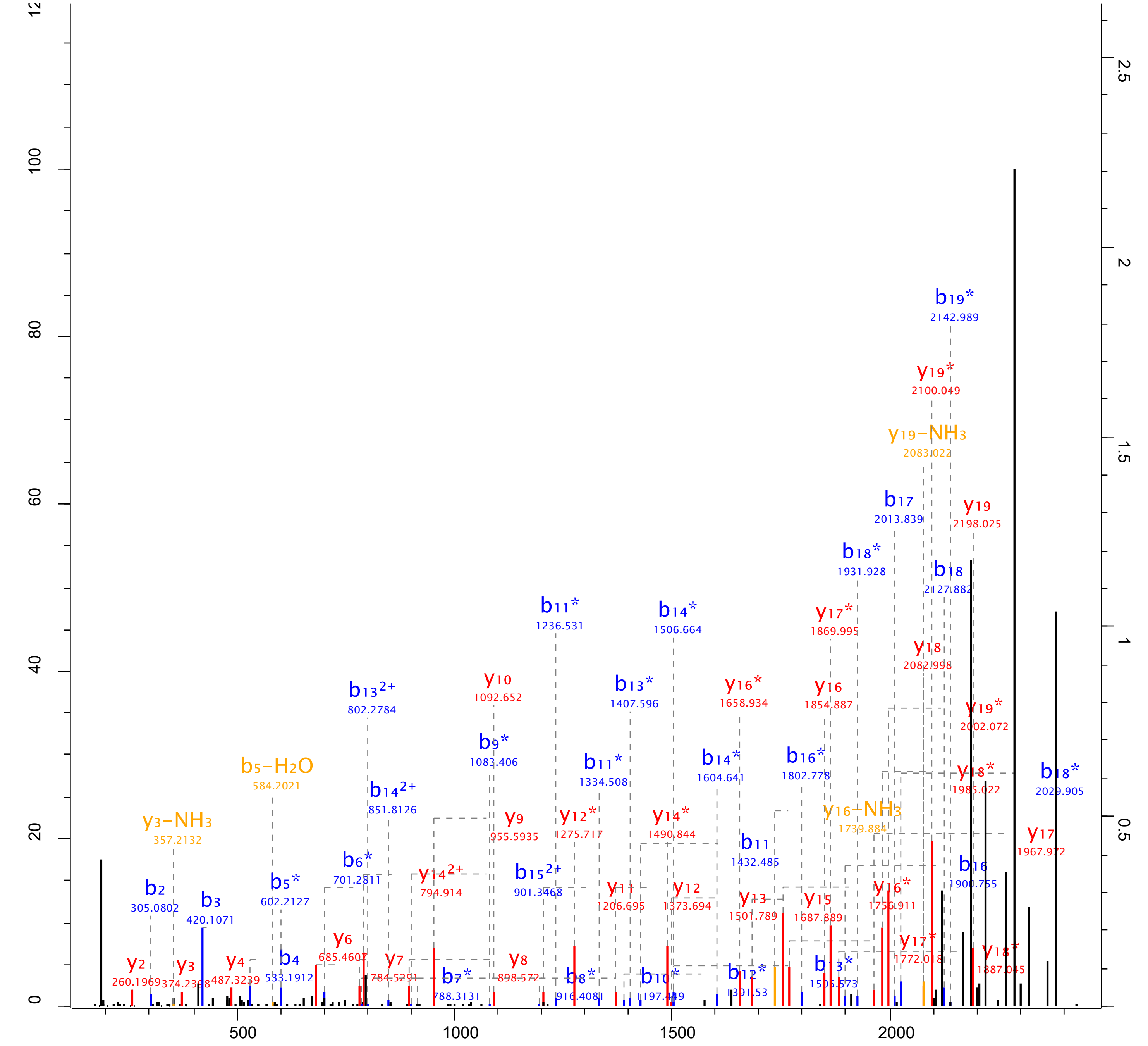

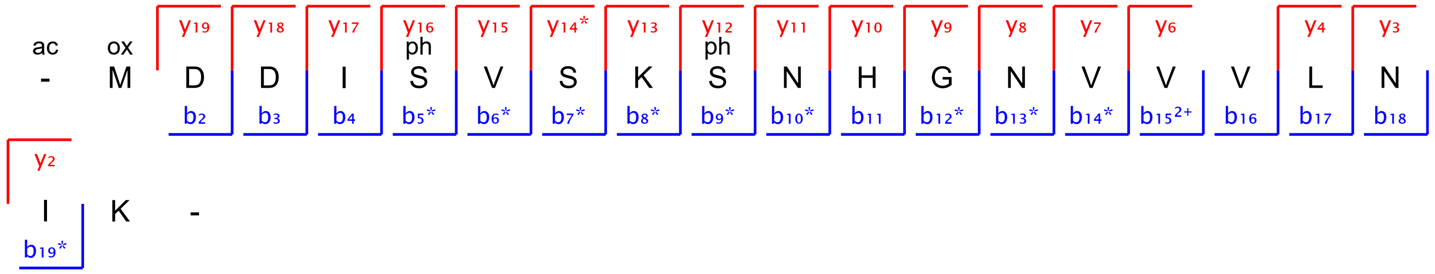

Sample output to test PDF Combine only

Raw file	Scan	Method	Score	m/z	Gene names
0523_4	21140	FTMS; HCD	59.34	929.87	At1g79090

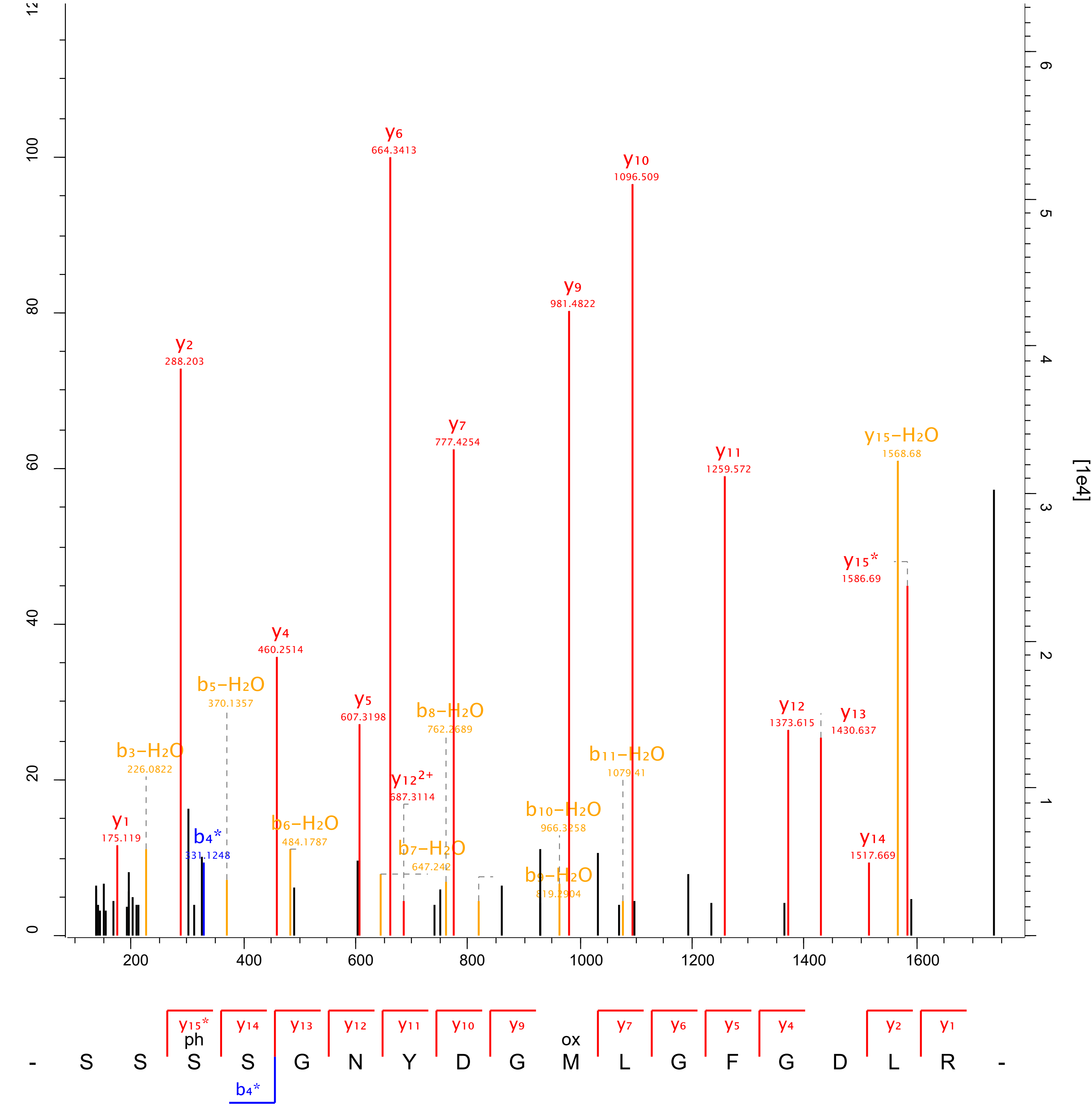

Sample output to test PDF Combine only

Raw file	Scan	Method	Score	m/z	Gene names
0523_4	21219	FTMS; HCD	40.21	794.02	T9C5.180;At3g49590

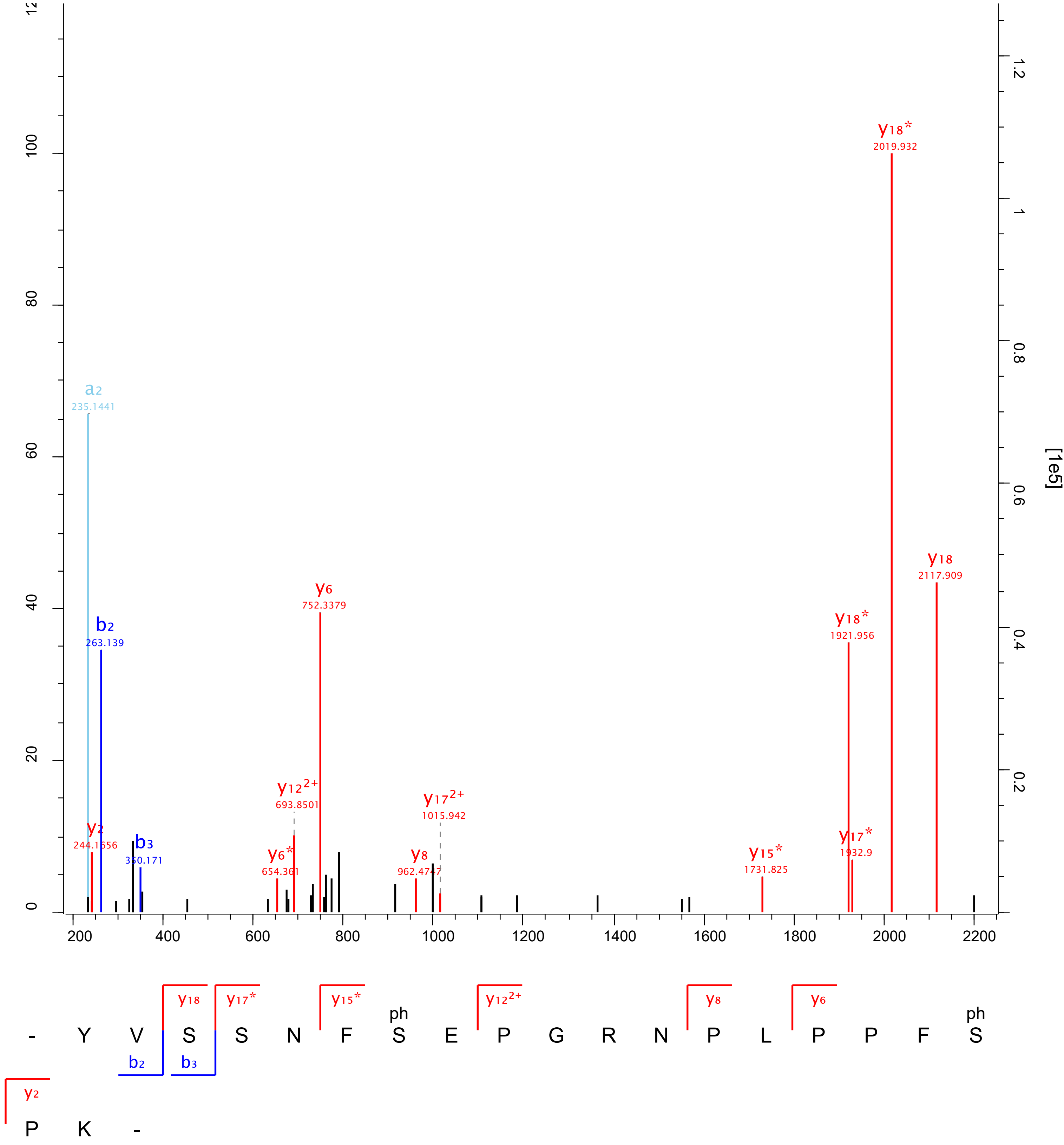

Sample output to test PDF Combine only

Raw file	Scan	Method	Score	m/z	Gene names
0523_4	21298	FTMS; HCD	194.26	957.42	IQD31

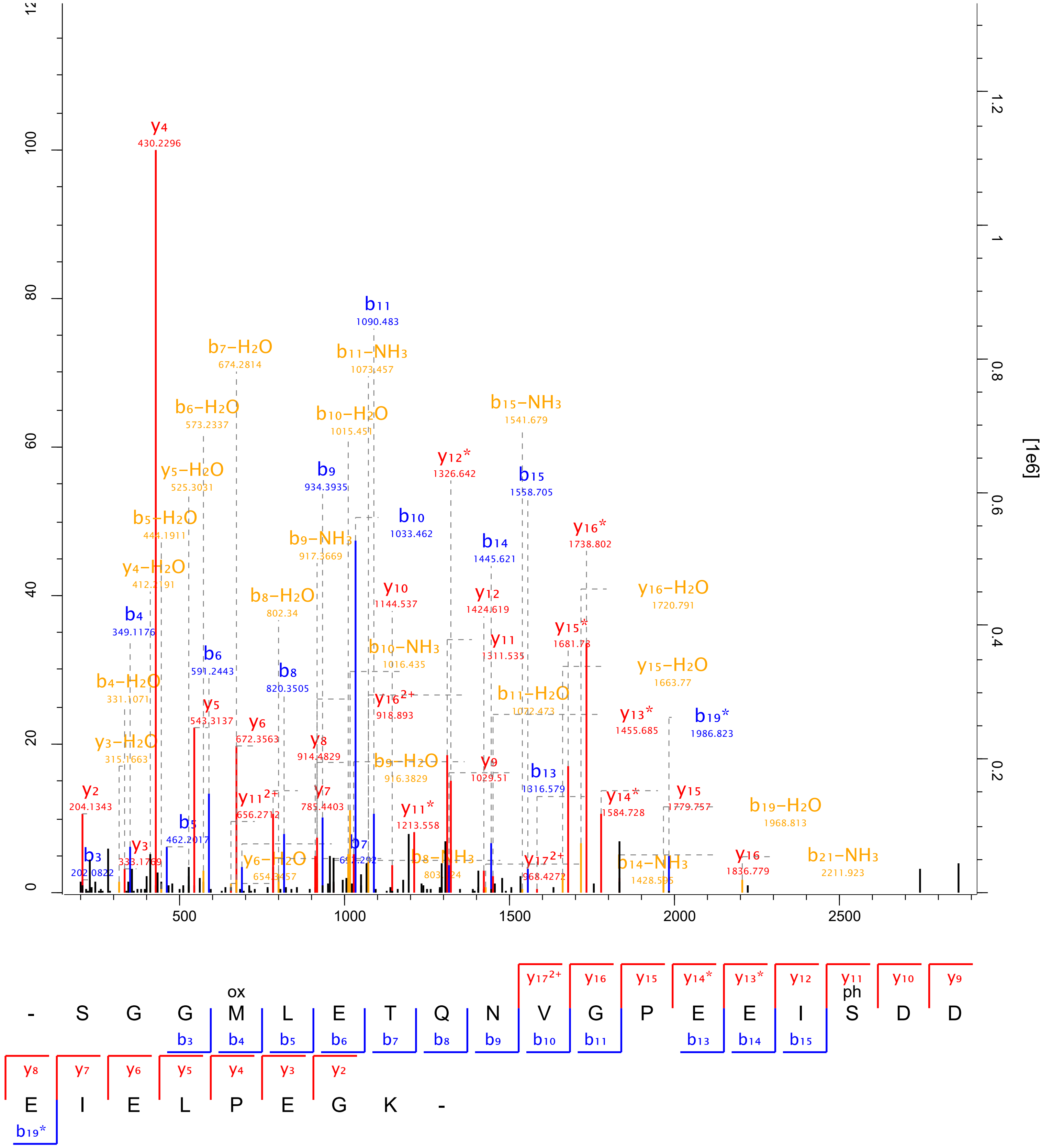

Sample output to test PDF Combine only

Raw file	Scan	Method	Score	m/z
0523_4	21323	FTMS; HCD	72.47	1042.37

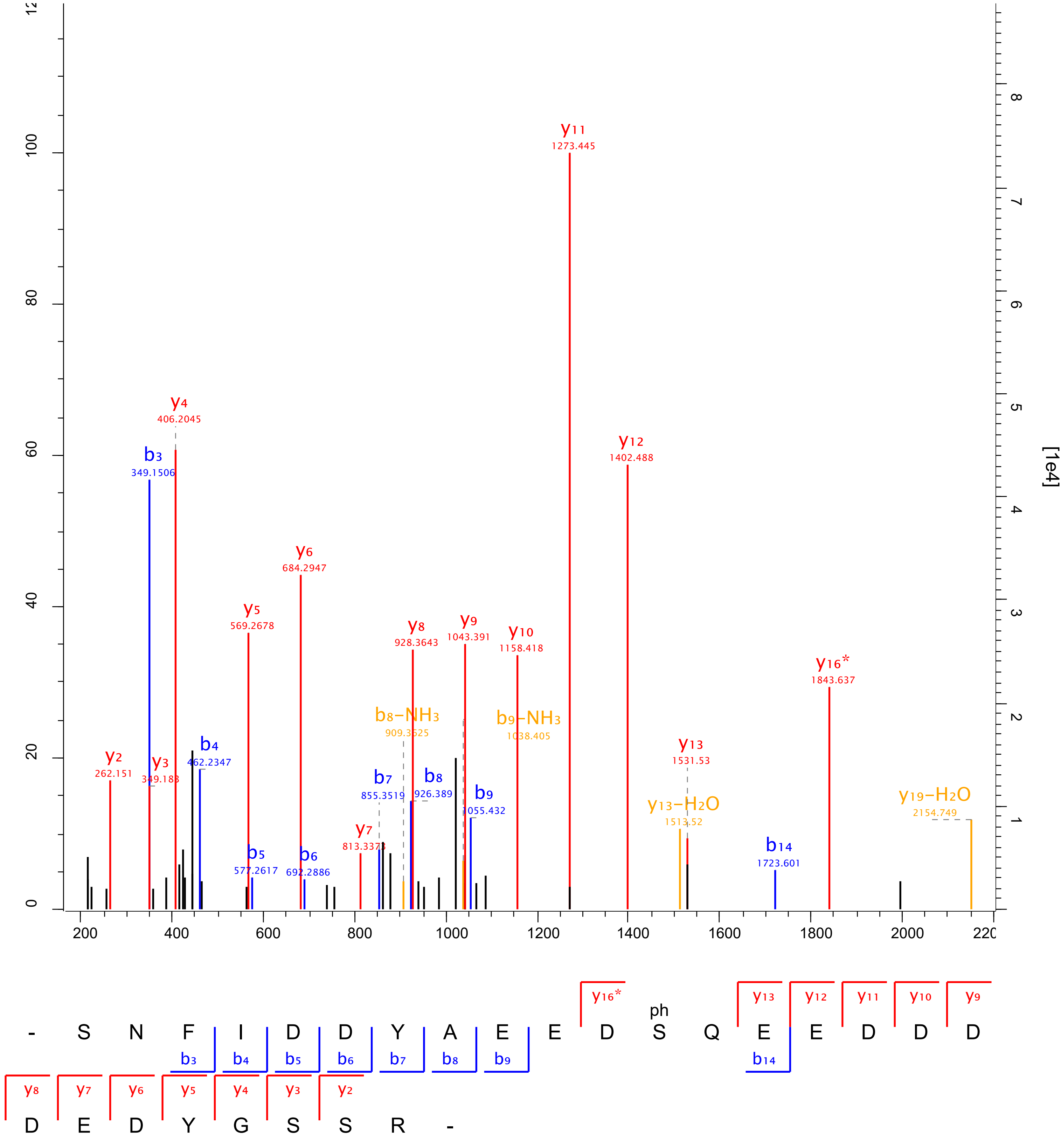

Sample output to test PDF Combine only

Raw file	Scan	Method	Score	m/z	Gene names
0523_4	21359	FTMS; HCD	50.18	882.94	VCS

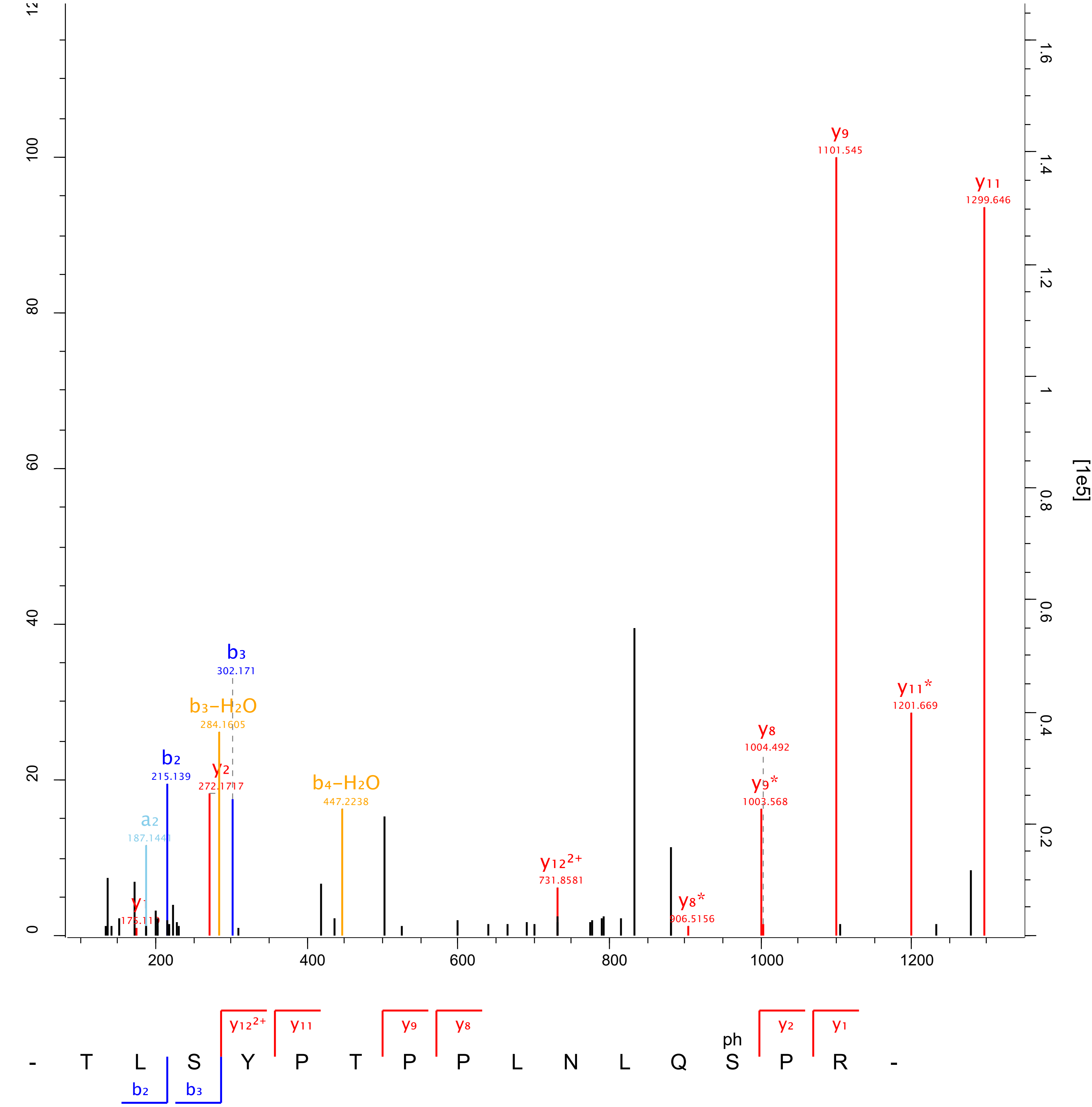

Sample output to test PDF Combine only

Raw file	Scan	Method	Score	m/z
0523_4	21383	FTMS; HCD	103.13	820.84

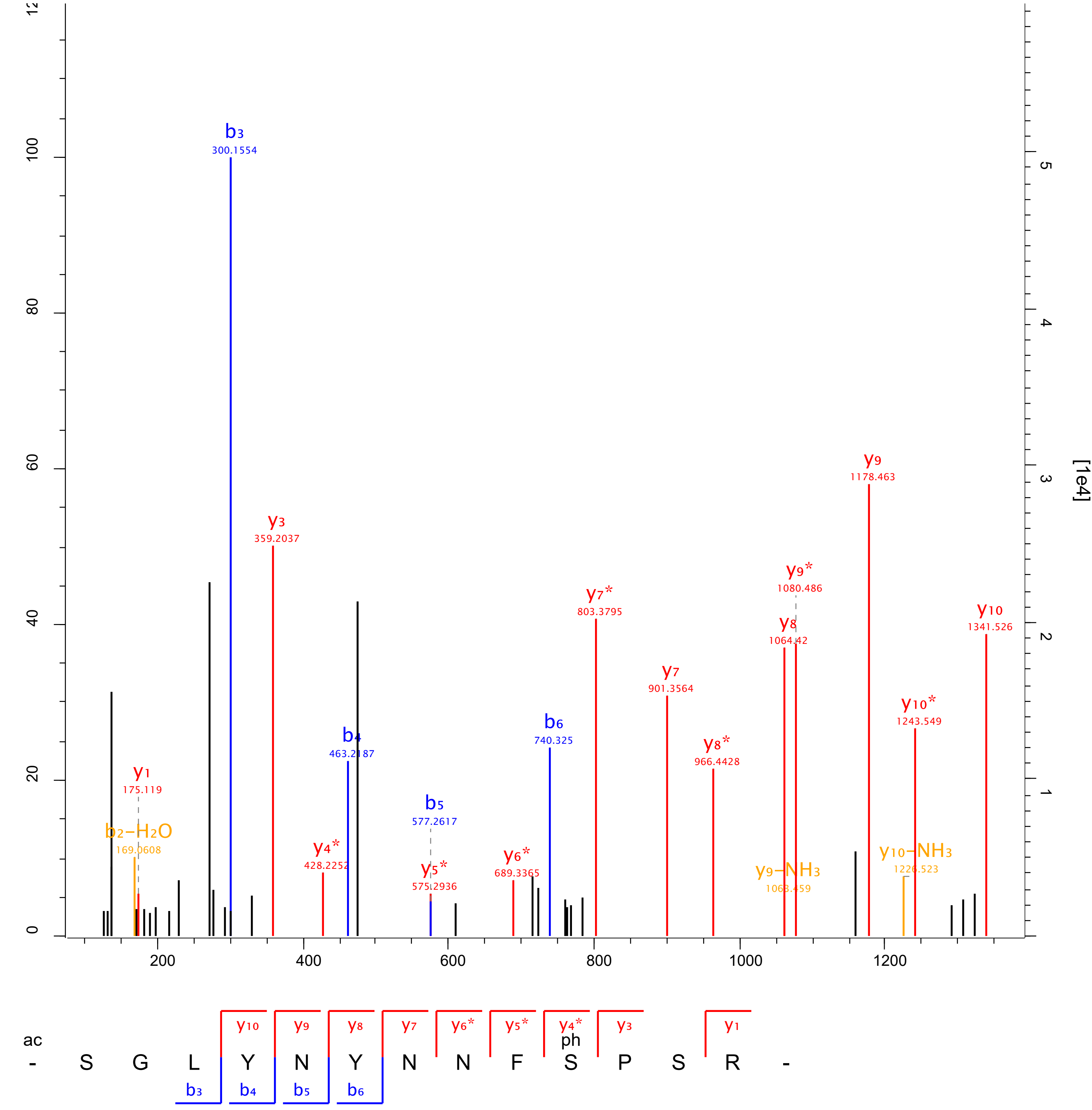

Sample output to test PDF Combine only

Raw file	Scan	Method	Score	m/z	Gene names
0523_4	21516	FTMS; HCD	206.72	805.36	At4g12780;At4g12770

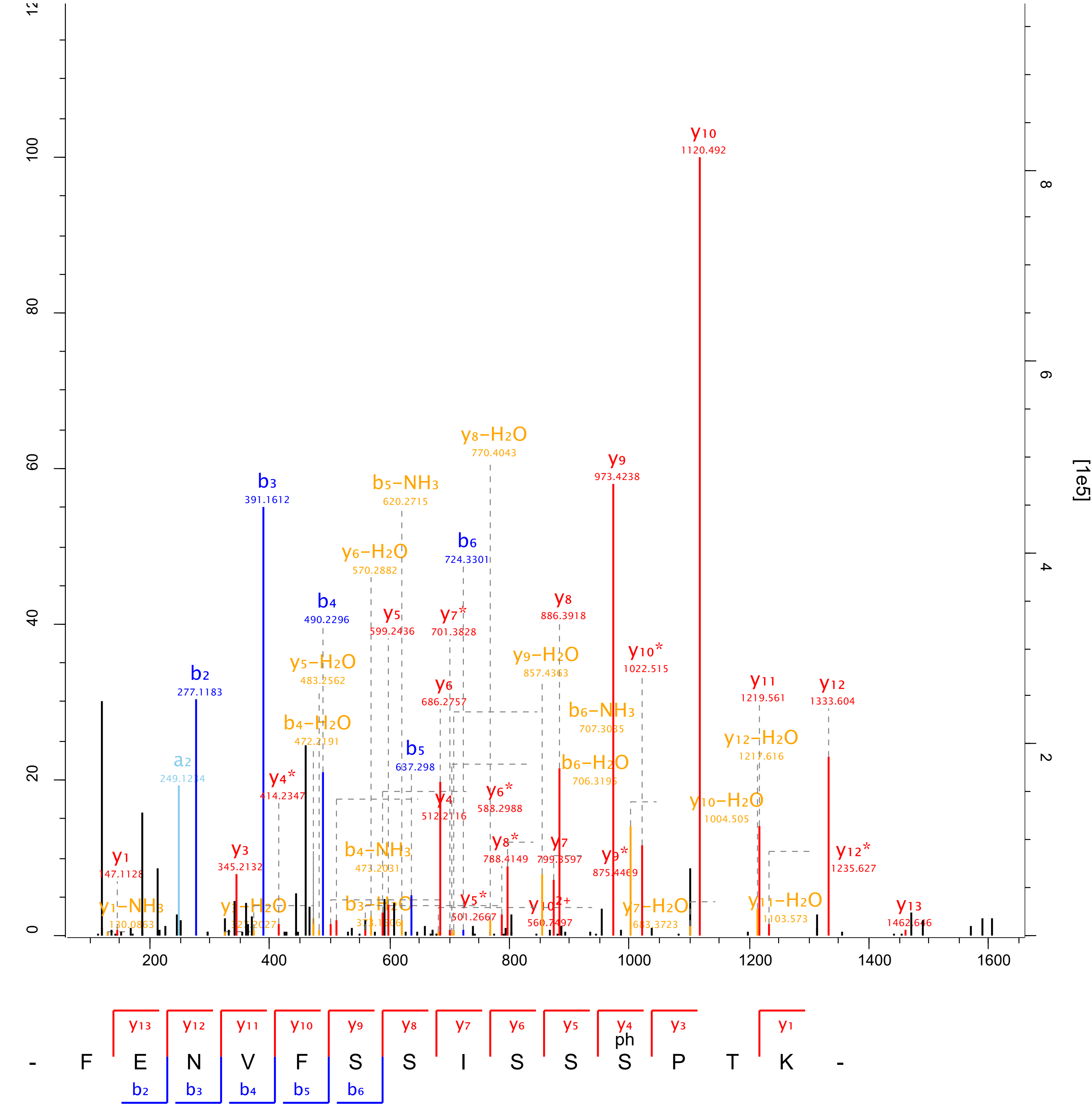

Sample output to test PDF Combine only

Raw file	Scan	Method	Score	m/z
0523_4	21589	FTMS; HCD	102.51	1049.43

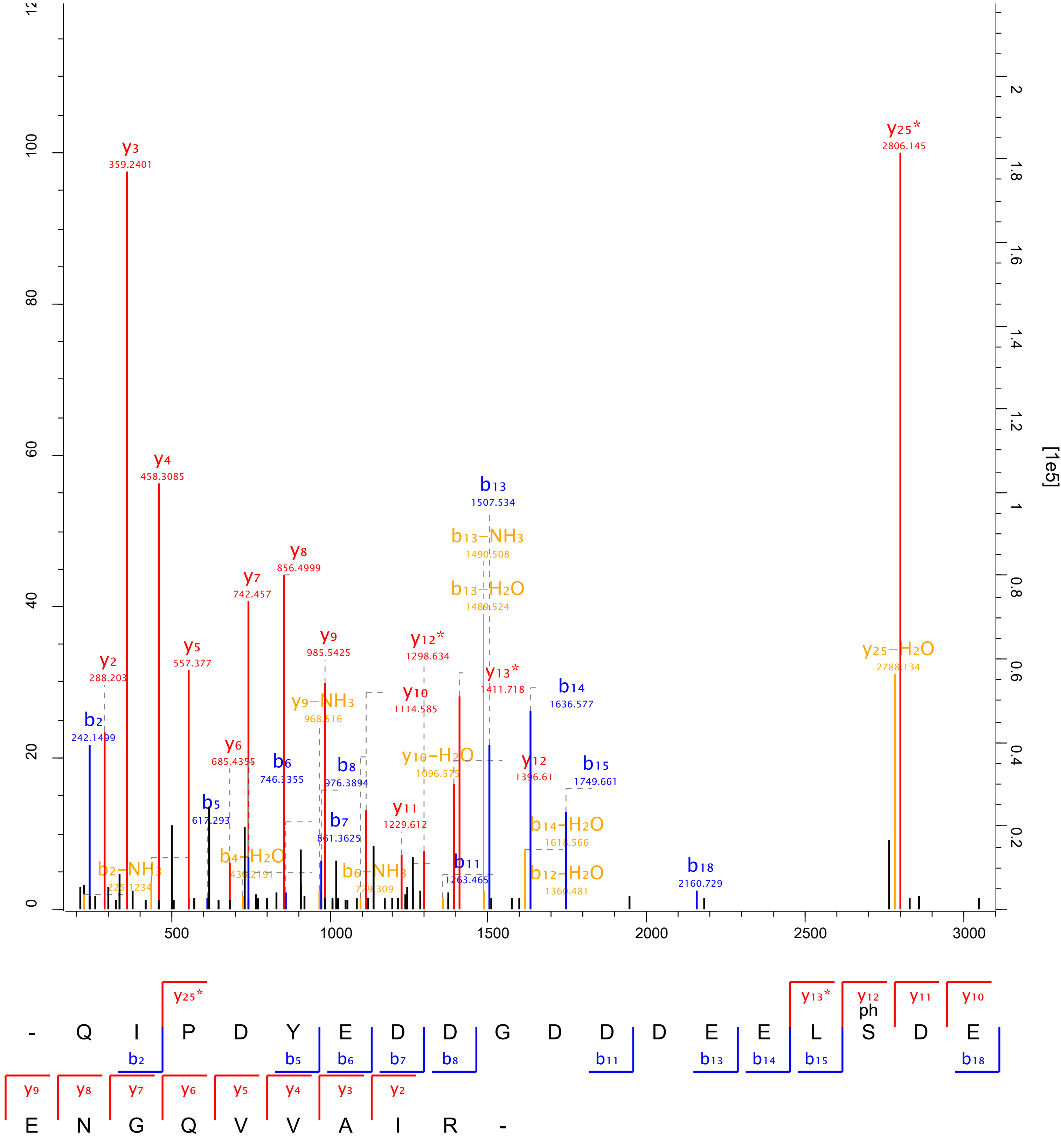

Sample output to test PDF Combine only

Raw file	Scan	Method	Score	m/z	Gene names
0523_4	21623	FTMS; HCD	76.8	783.67	UBP24

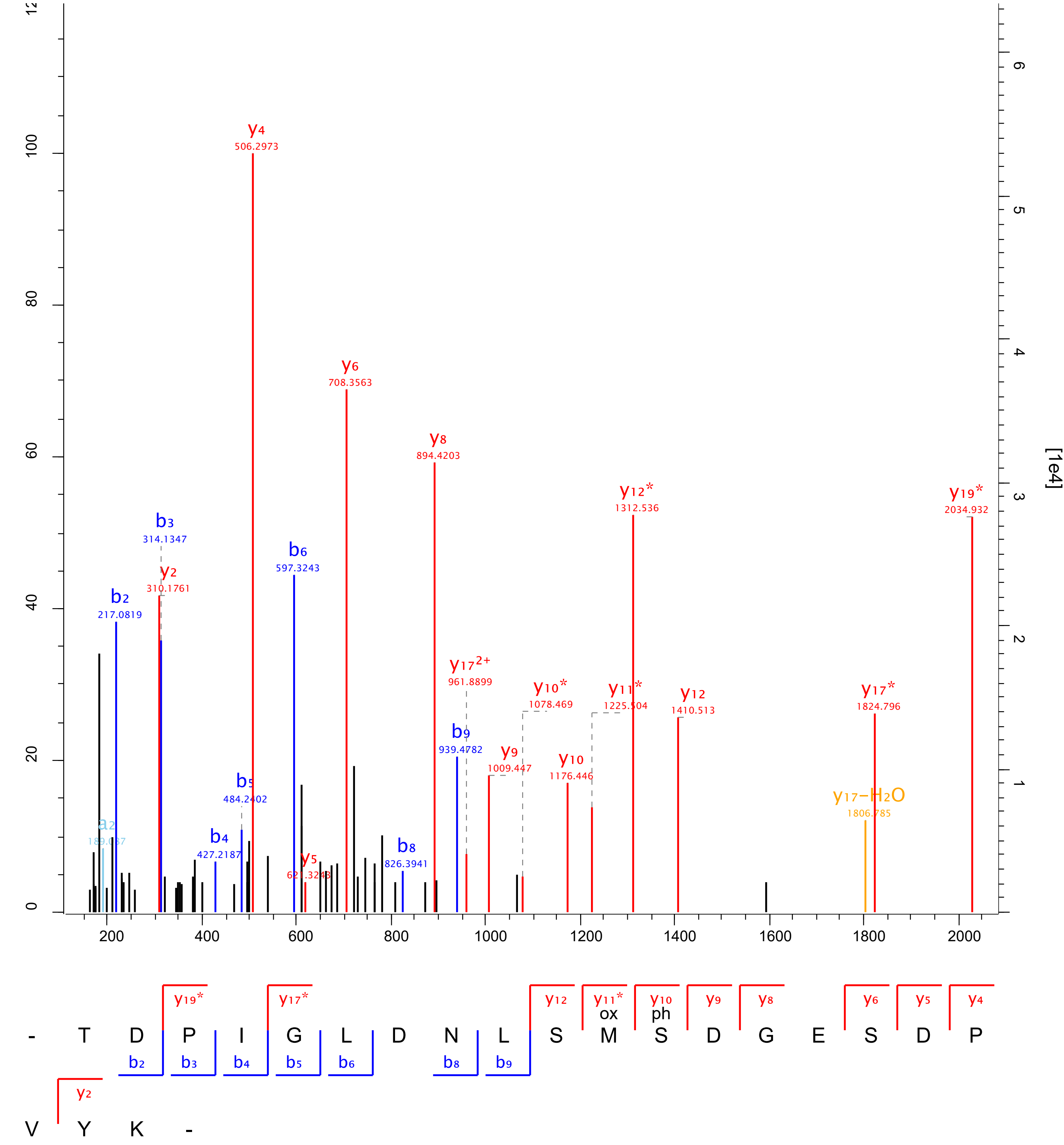

Sample output to test PDF Combine only

Raw file	Scan	Method	Score	m/z	Gene names
0523_4	21810	FTMS; HCD	124.2	931.93	AGD4

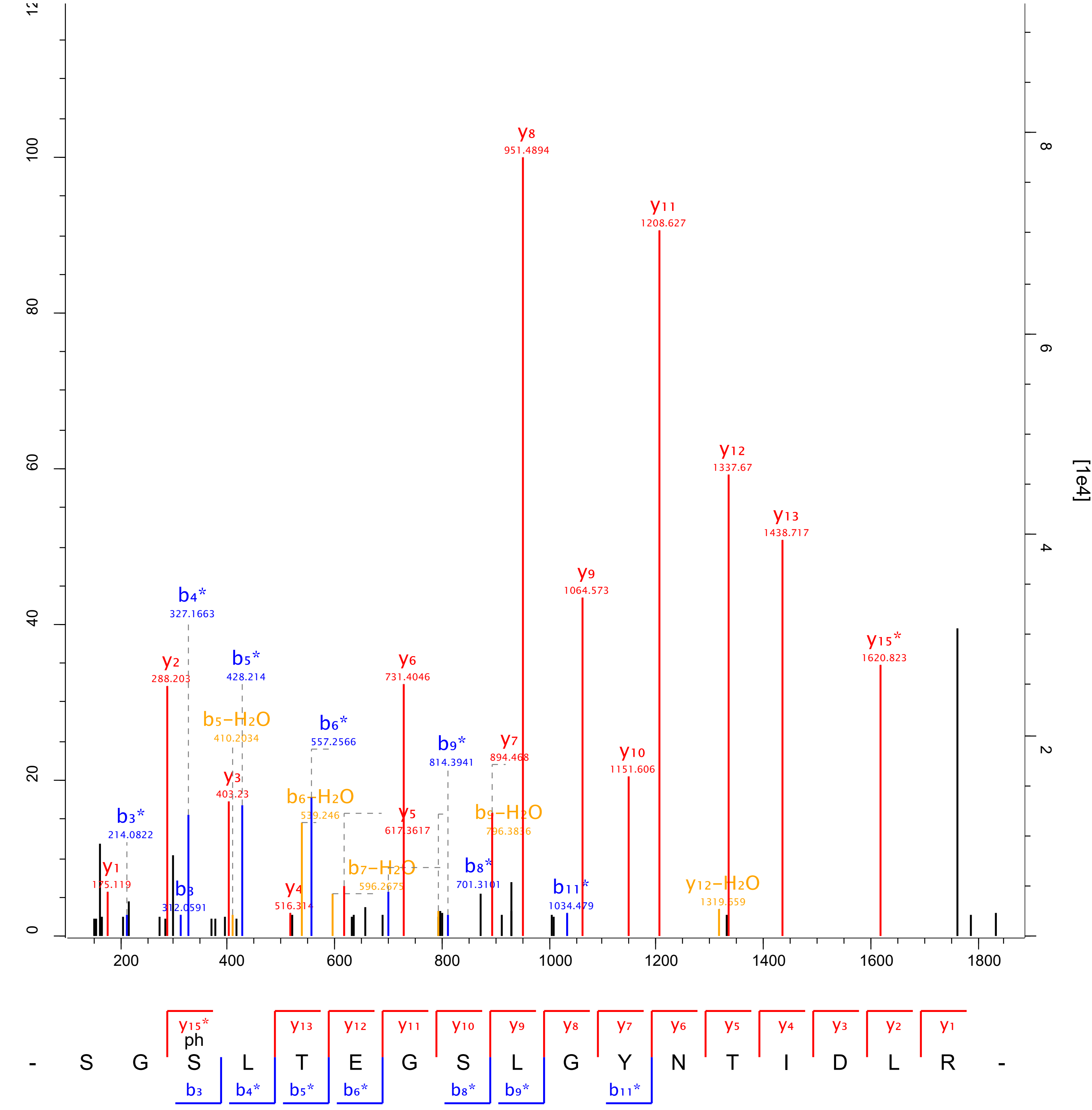

Sample output to test PDF Combine only

Raw file	Scan	Method	Score	m/z	Gene names
0523_4	21815	FTMS; HCD	145.69	900.83	ERF1-3

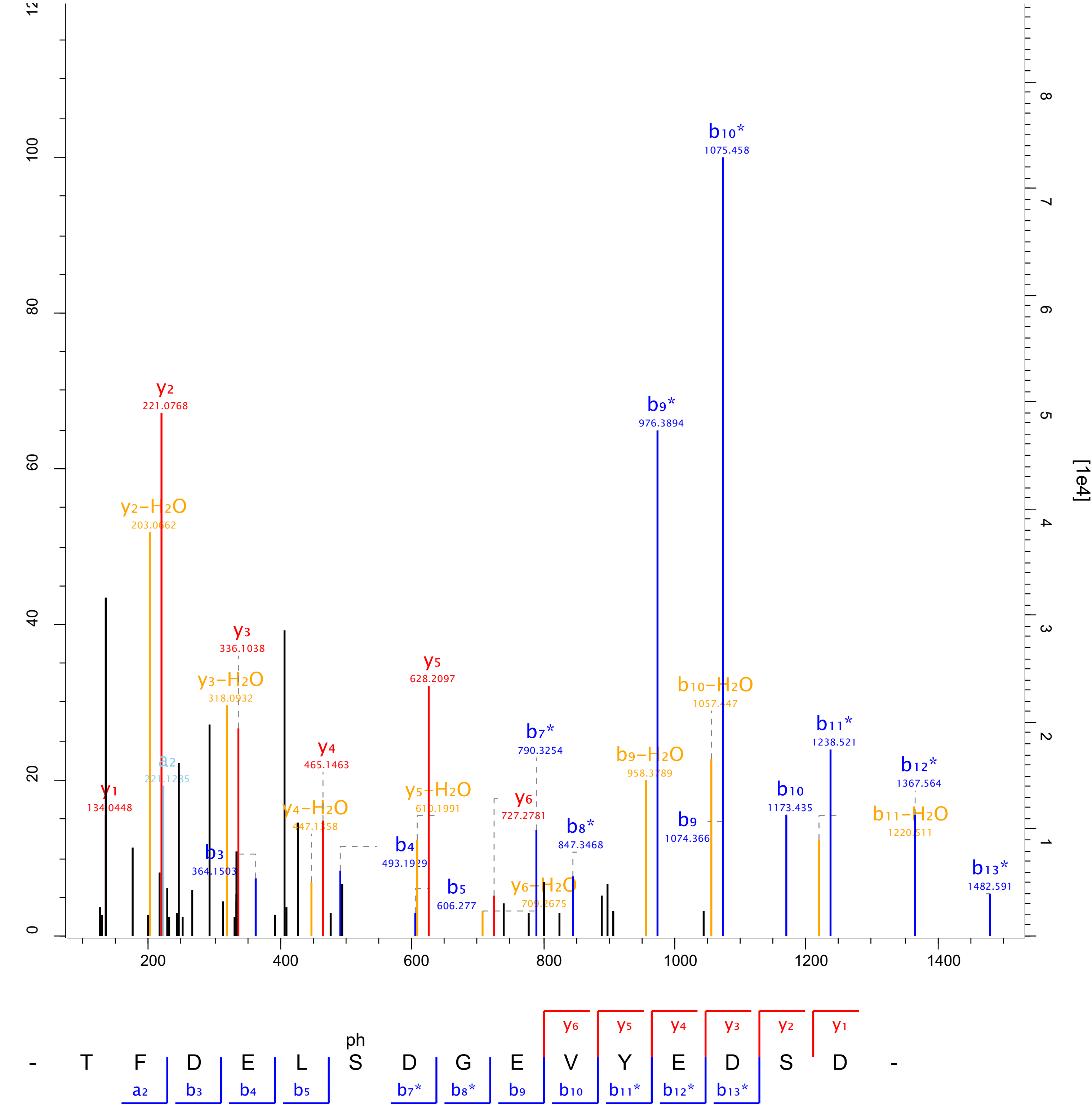

Sample output to test PDF Combine only

Raw file	Scan	Method	Score	m/z
0523_4	21842	FTMS; HCD	106.63	916.93

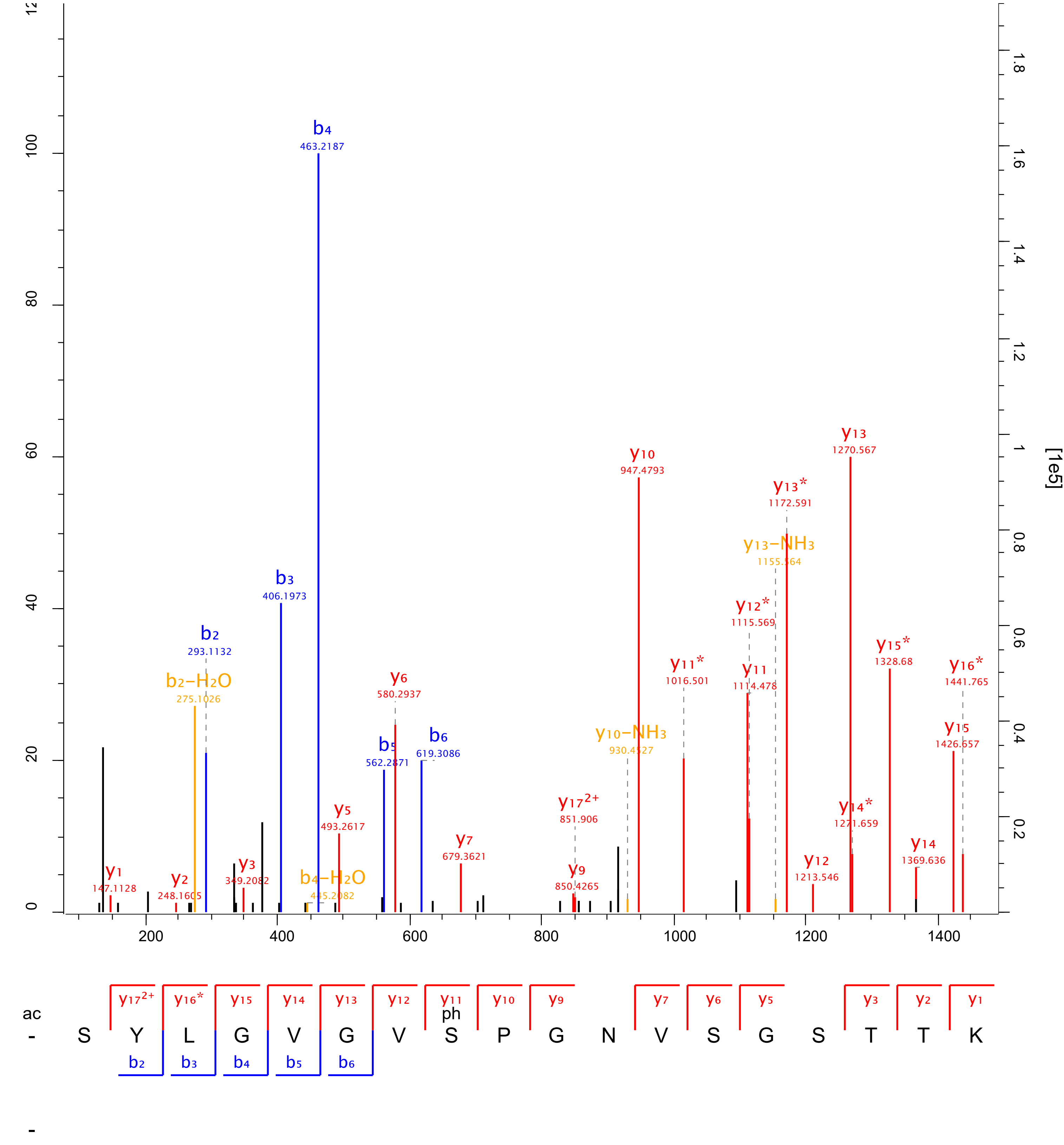

Sample output to test PDF Combine only

Raw file	Scan	Method	Score	m/z	Gene names
0523_4	21896	FTMS; HCD	88.57	888.82	RABA5E

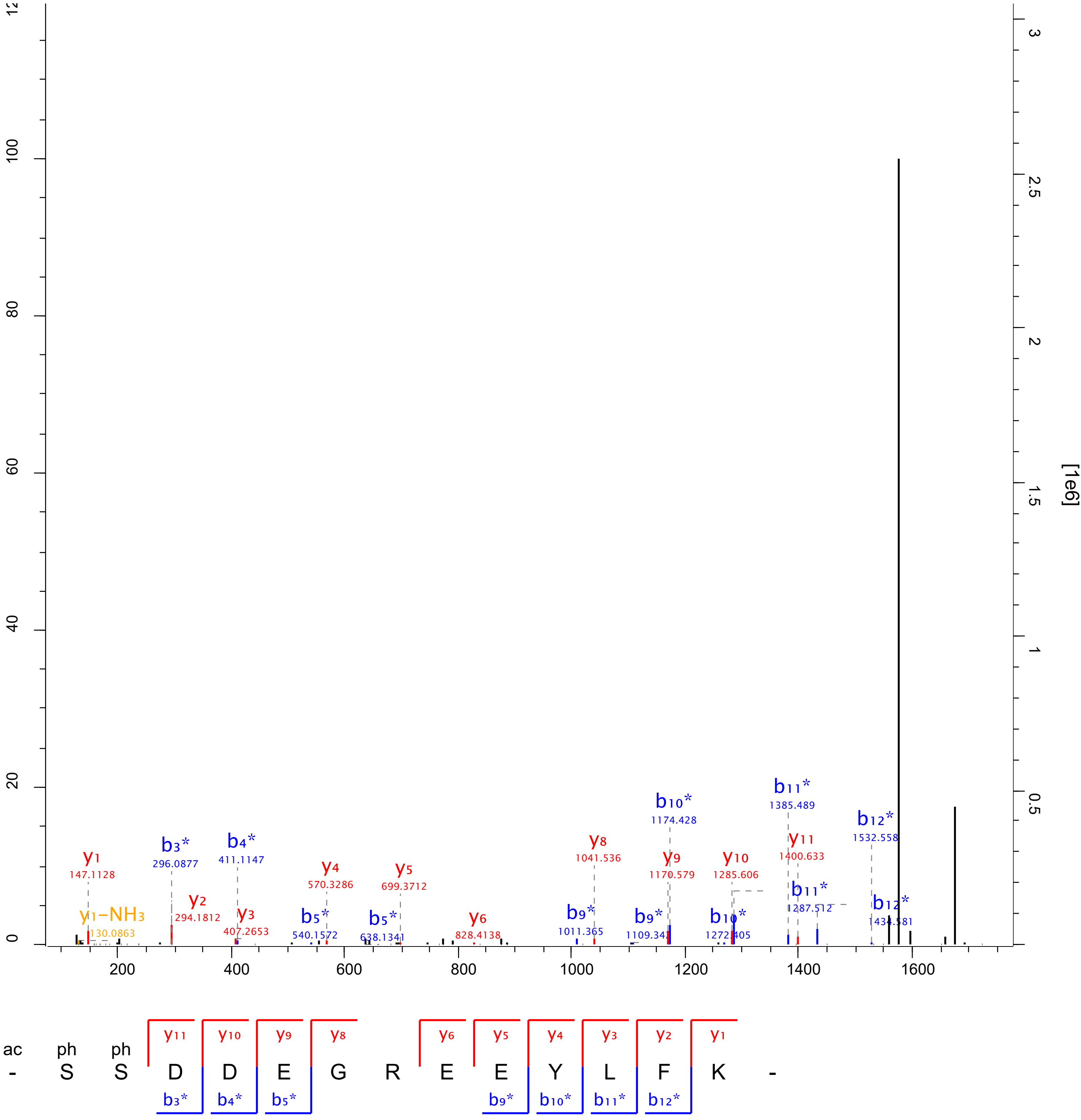

Sample output to test PDF Combine only

Raw file	Scan	Method	Score	m/z	Gene names
0523_4	21934	FTMS; HCD	117.94	914.4	At5g47430

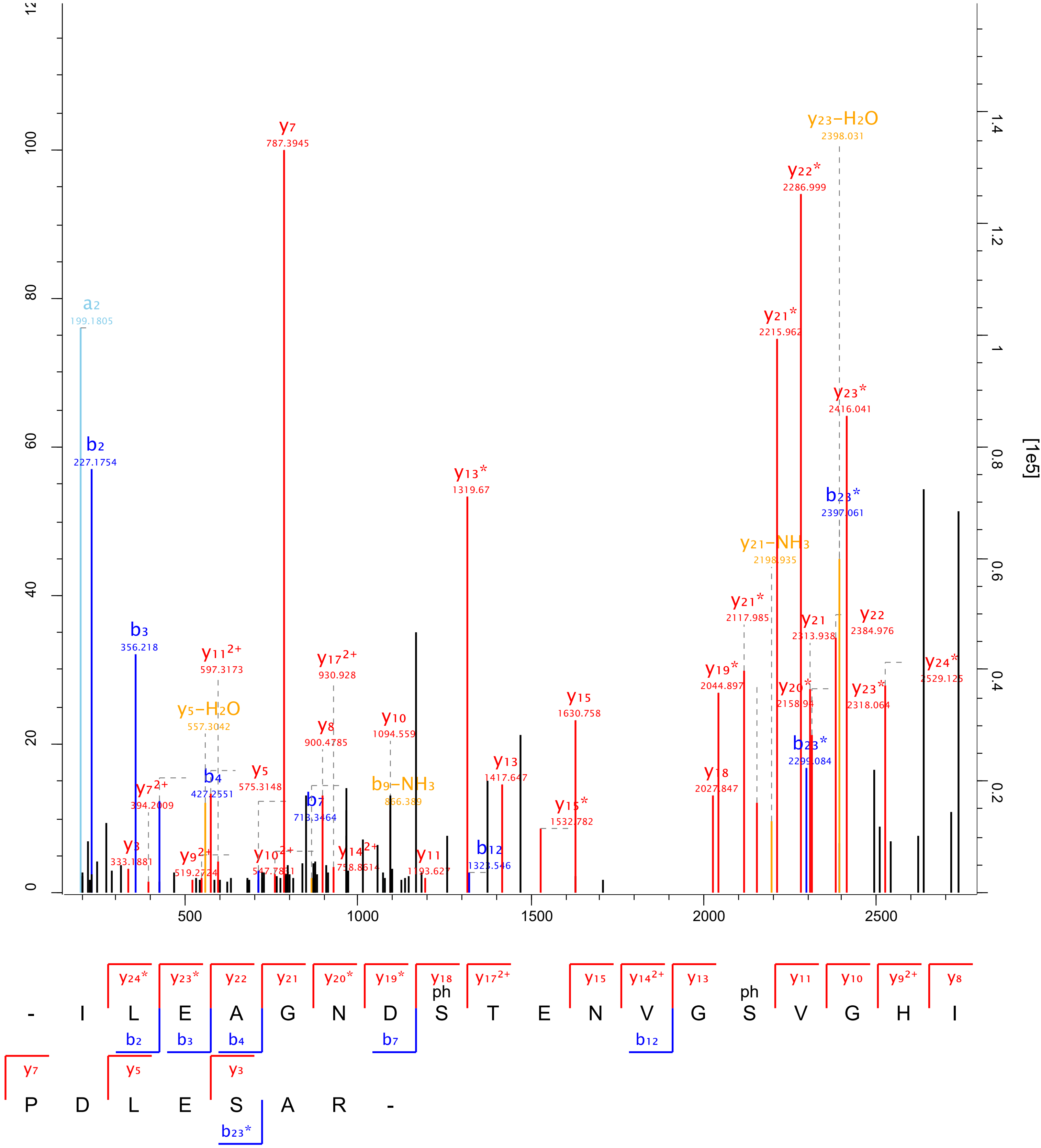

Sample output to test PDF Combine only

Raw file	Scan	Method	Score	m/z	Gene names
0523_4	22007	FTMS; HCD	86.57	818.37	VHA-a3

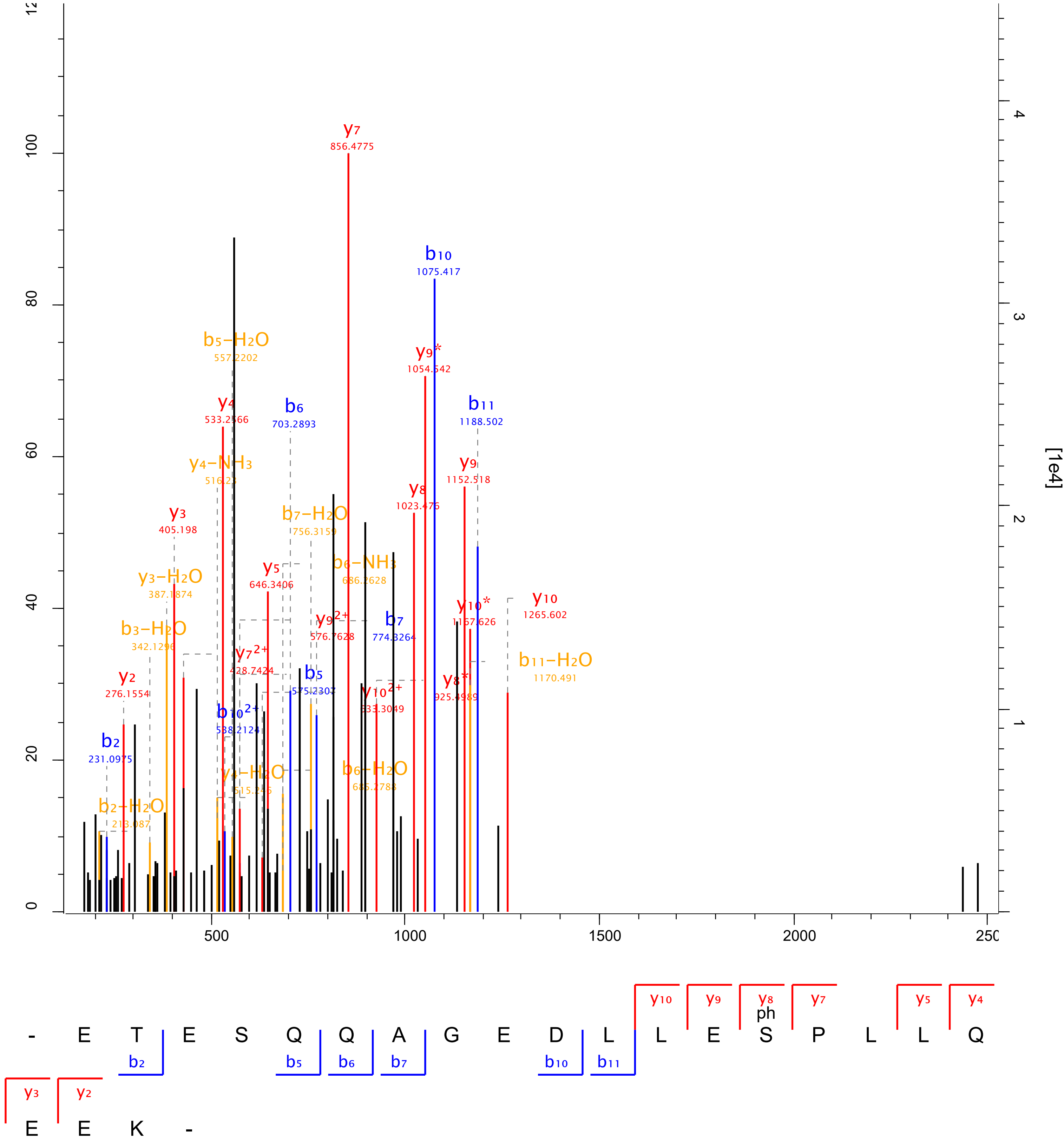

Sample output to test PDF Combine only

Raw file	Scan	Method	Score	m/z	Gene names
0523_4	22156	FTMS; HCD	86.58	919.06	CRWN1

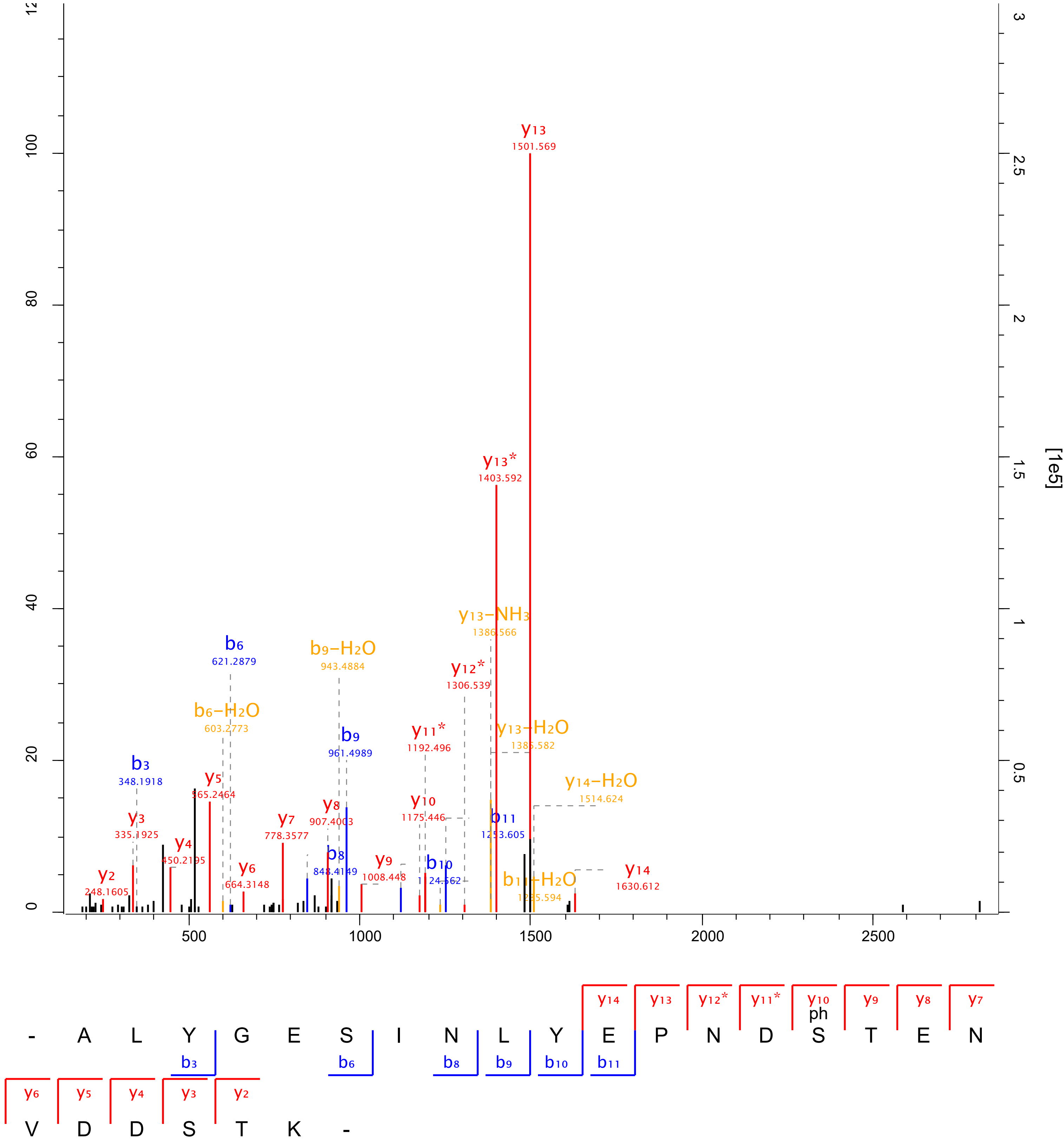

Sample output to test PDF Combine only

Raw file	Scan	Method	Score	m/z	Gene names
0523_4	22238	FTMS; HCD	110.12	815.03	LRR-RLK

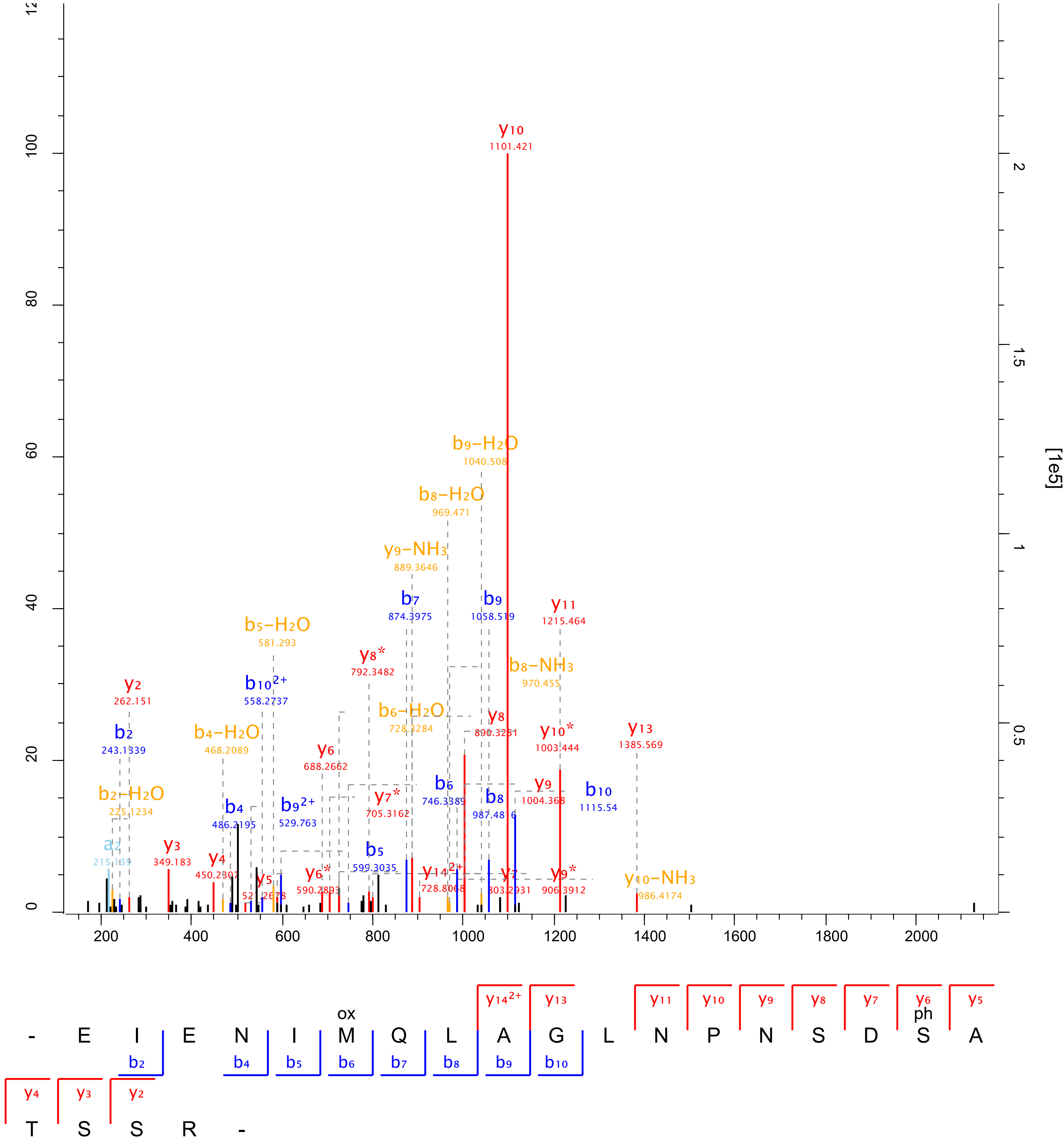

Sample output to test PDF Combine only

Raw file	Scan	Method	Score	m/z	Gene names
0523_4	22283	FTMS; HCD	164.46	1031.92	MSL9

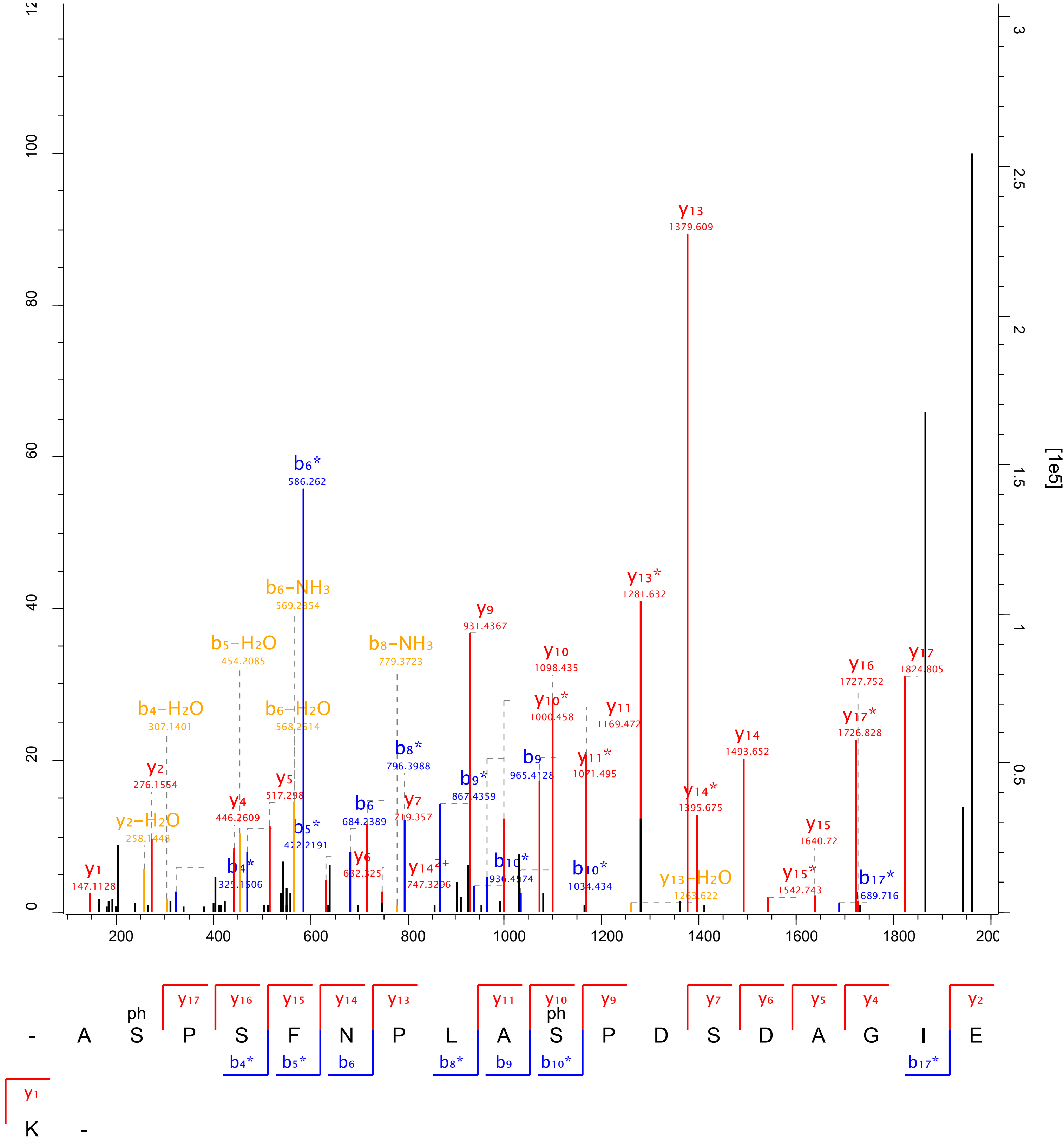

Sample output to test PDF Combine only

Raw file	Scan	Method	Score	m/z	Gene names
0523_4	22452	FTMS; HCD	246.44	1097.01	ALA10

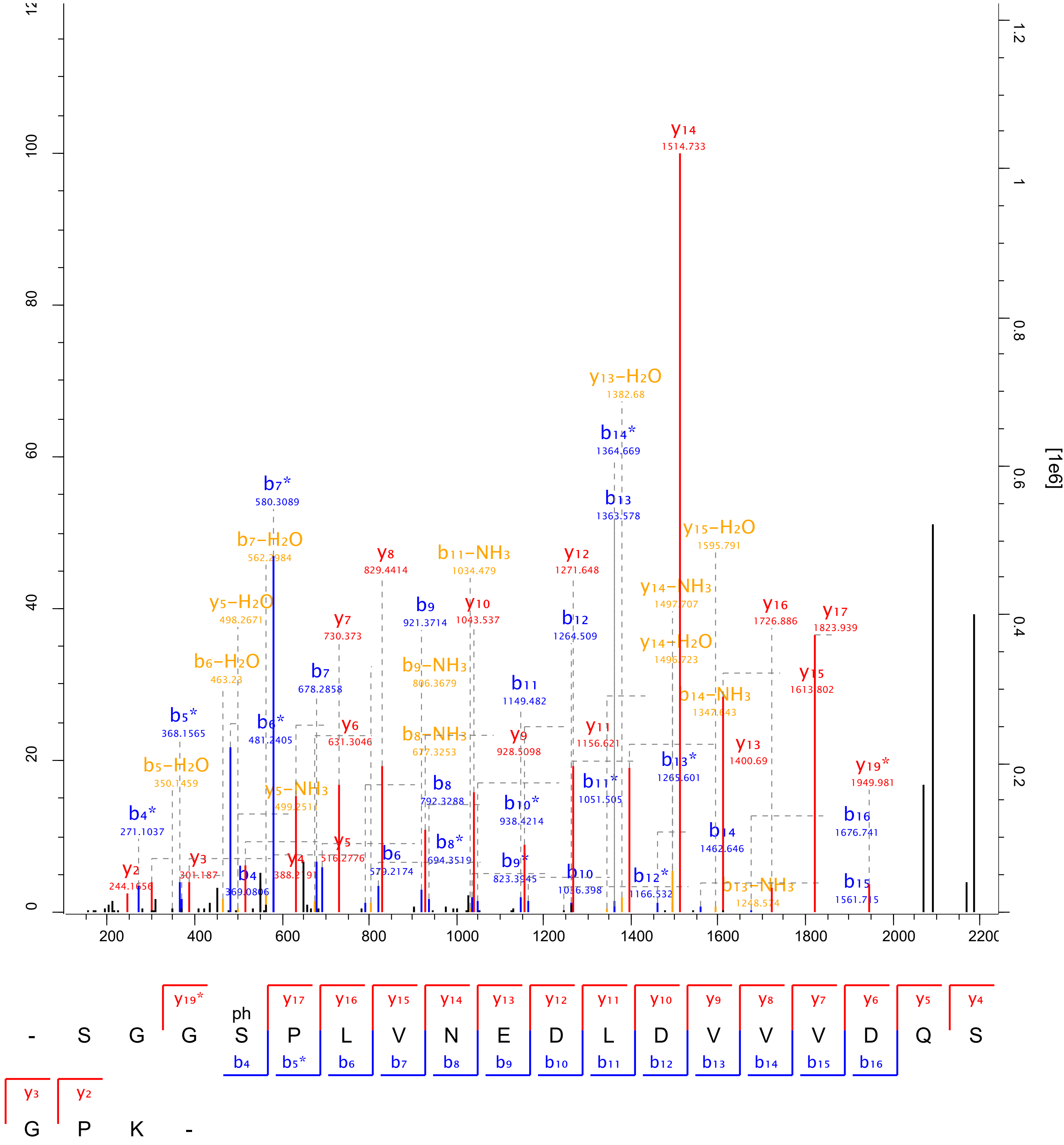

Sample output to test PDF Combine only

Raw file	Scan	Method	Score	m/z
0523_4	22470	FTMS; HCD	71.06	781

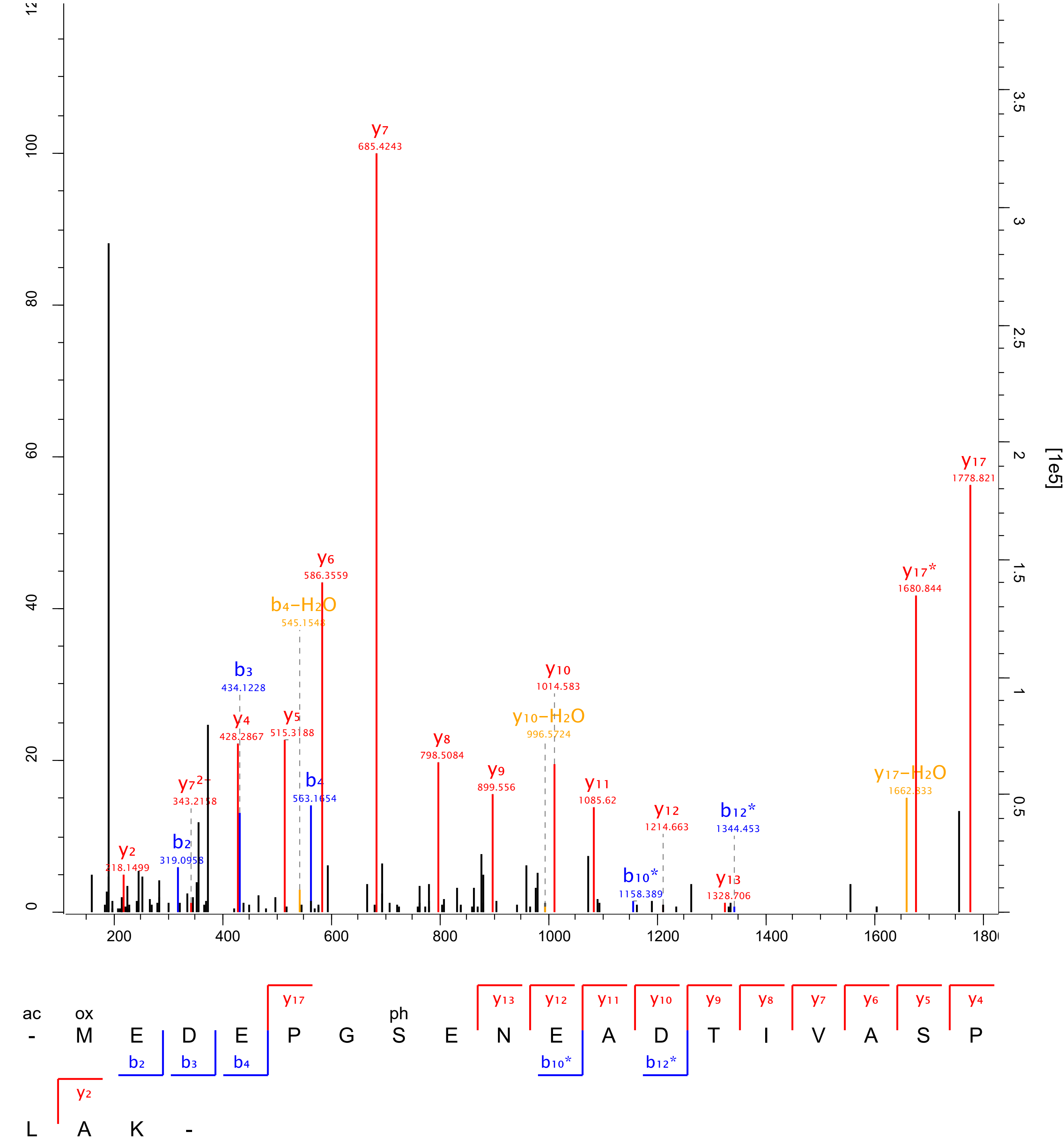

Sample output to test PDF Combine only

Raw file	Scan	Method	Score	m/z	Gene names
0523_4	22511	FTMS; HCD	90.71	674.74	PIP2-1;PIP2-3;PIP2-2

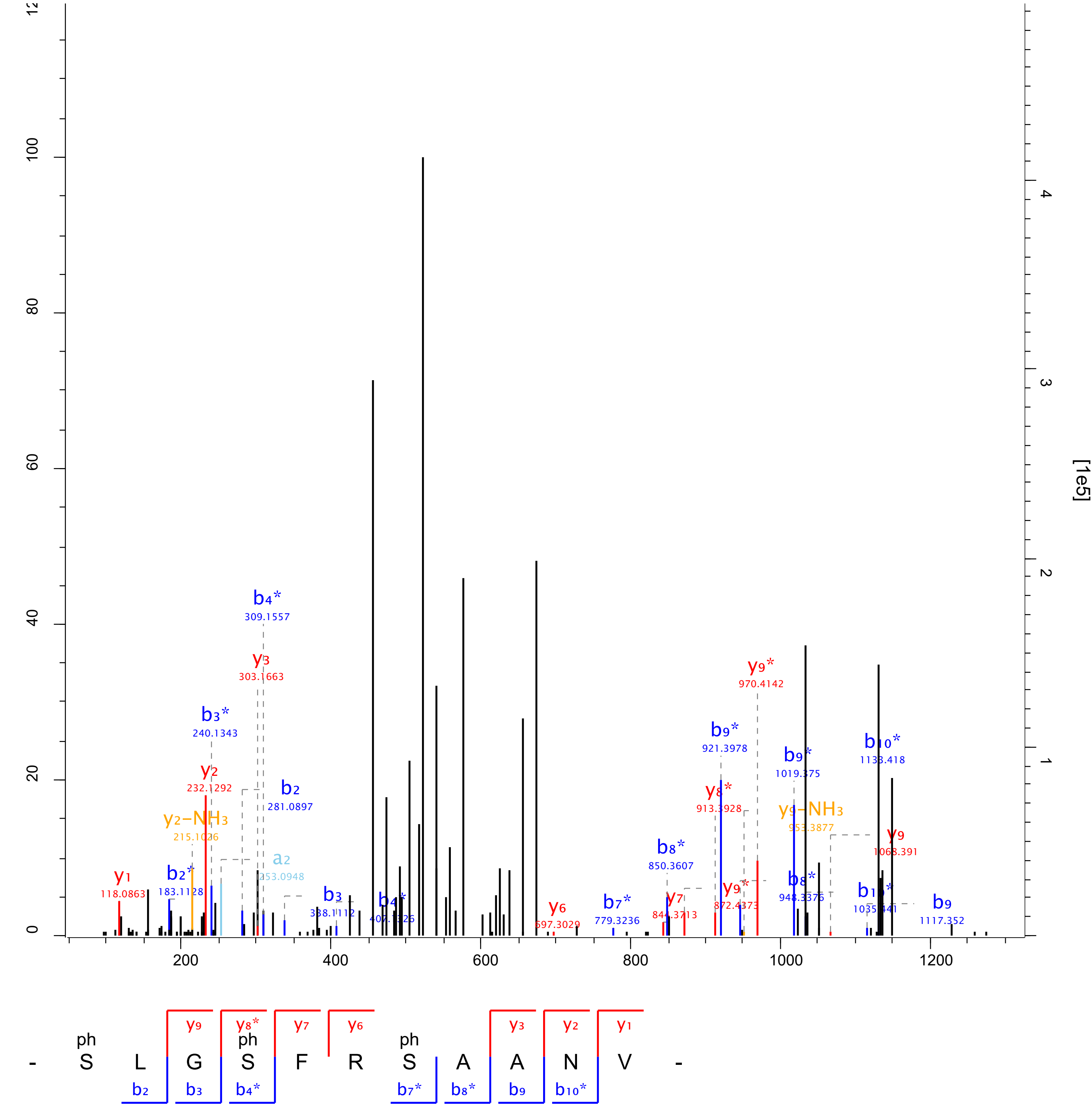

Sample output to test PDF Combine only
