# Supplementary material for: Sucrose-induced Receptor Kinase 1 is Modulated by an Interacting Kinase with Short Extracellular Domain
Source: Mol Cell Proteomics. 2019 May 30;18(8):1556–71. doi: 10.1074/mcp.RA119.001336 (PMC6683012; doi:10.1074/mcp.RA119.001336)
Supplement: Supplementary Figure S6d [file 143141_1_supp_311901_ps58ky.pdf]

Figure S6:

Spectra of all identified phosphopeptides.

Raw file	Scan	Method	Score	m/z	Gene names
0523_4	22706	FTMS; HCD	419	1217.97	F5A18.5

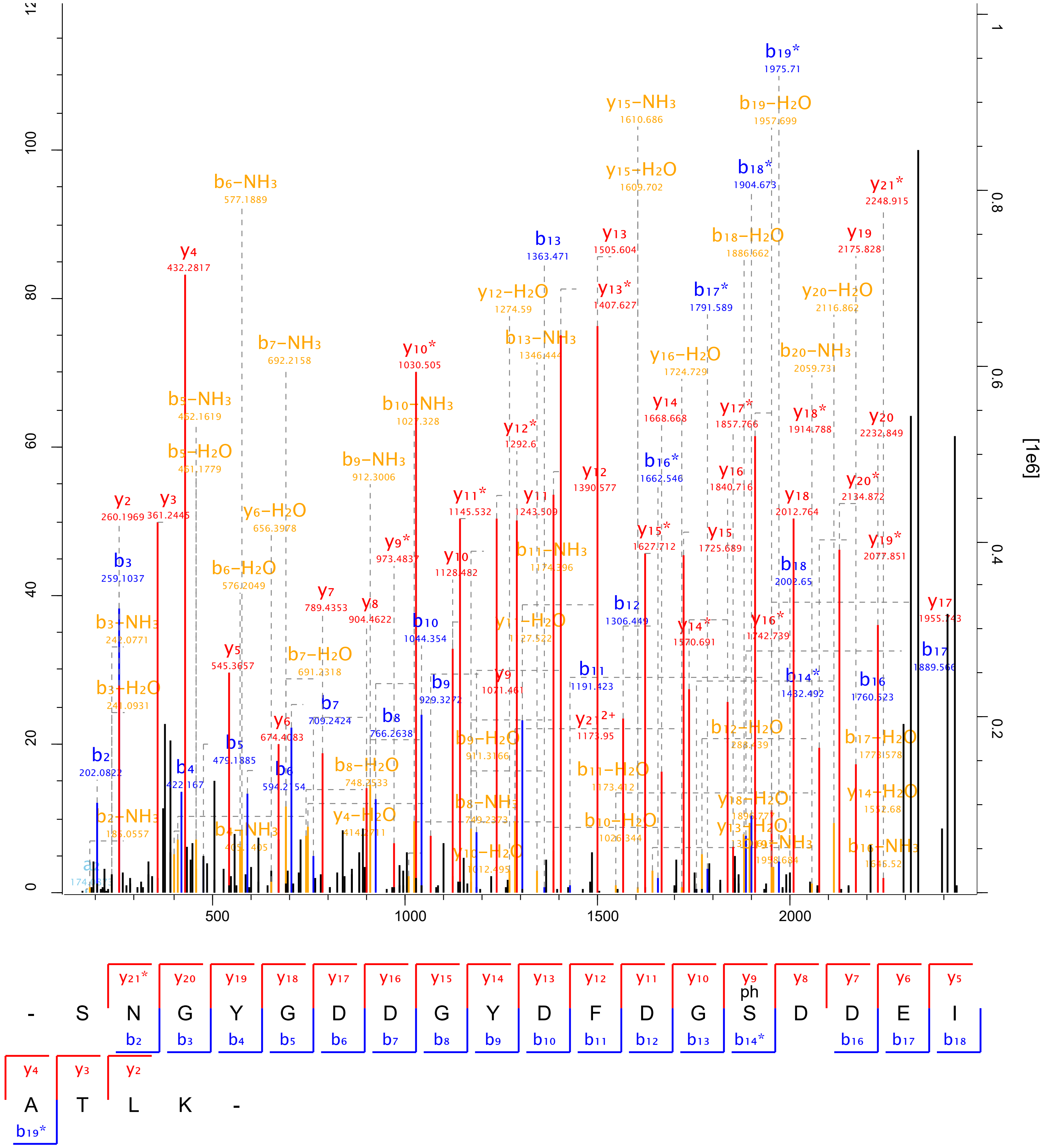

Sample output to test PDF Combine only

Raw file	Scan	Method	Score	m/z	Gene names
0523_4	22791	FTMS; HCD	144.55	826.38	MED26C

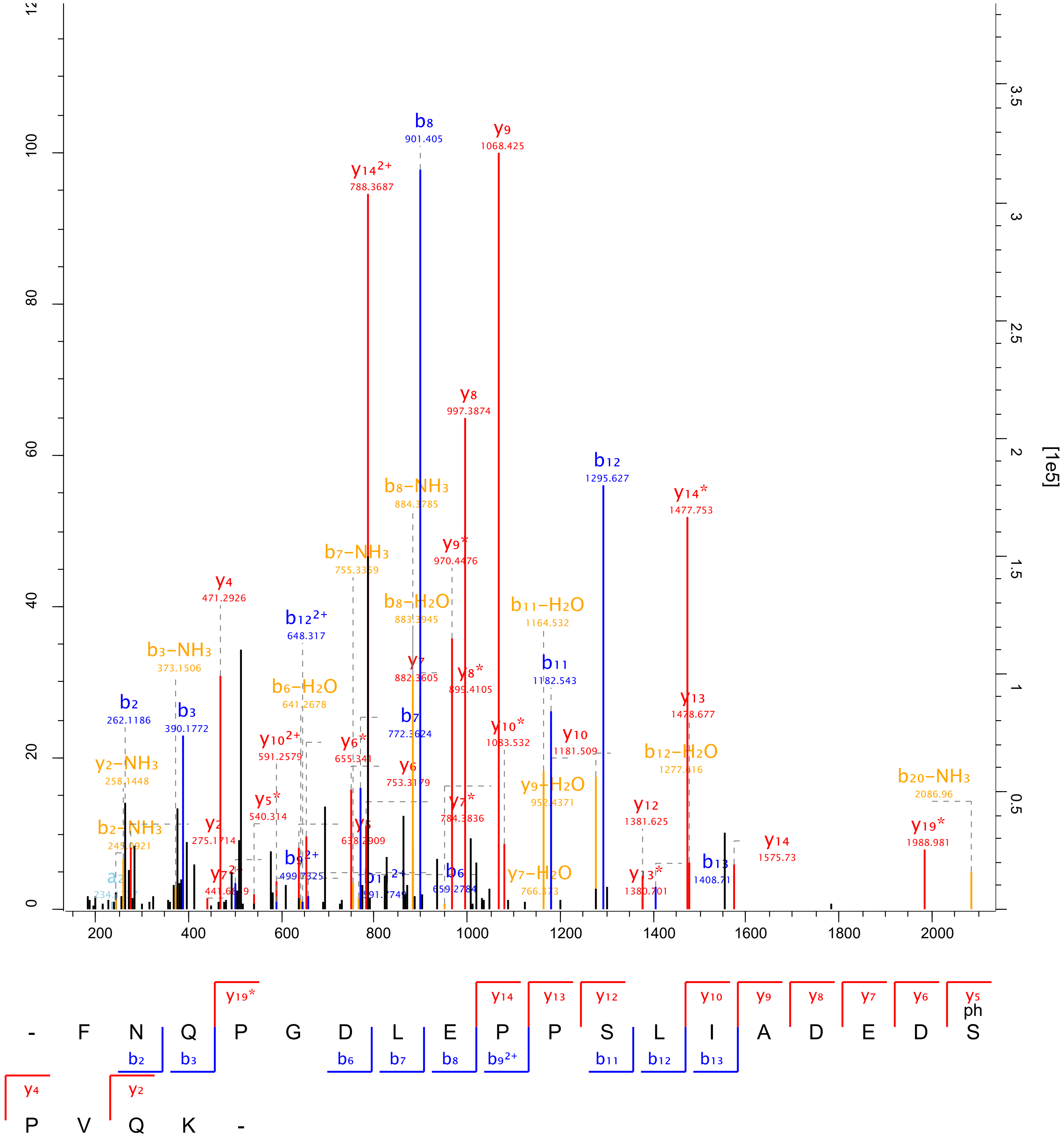

Sample output to test PDF Combine only

Raw file	Scan	Method	Score	m/z	Gene names
0523_4	22808	FTMS; HCD	109.41	920.74	KCA2

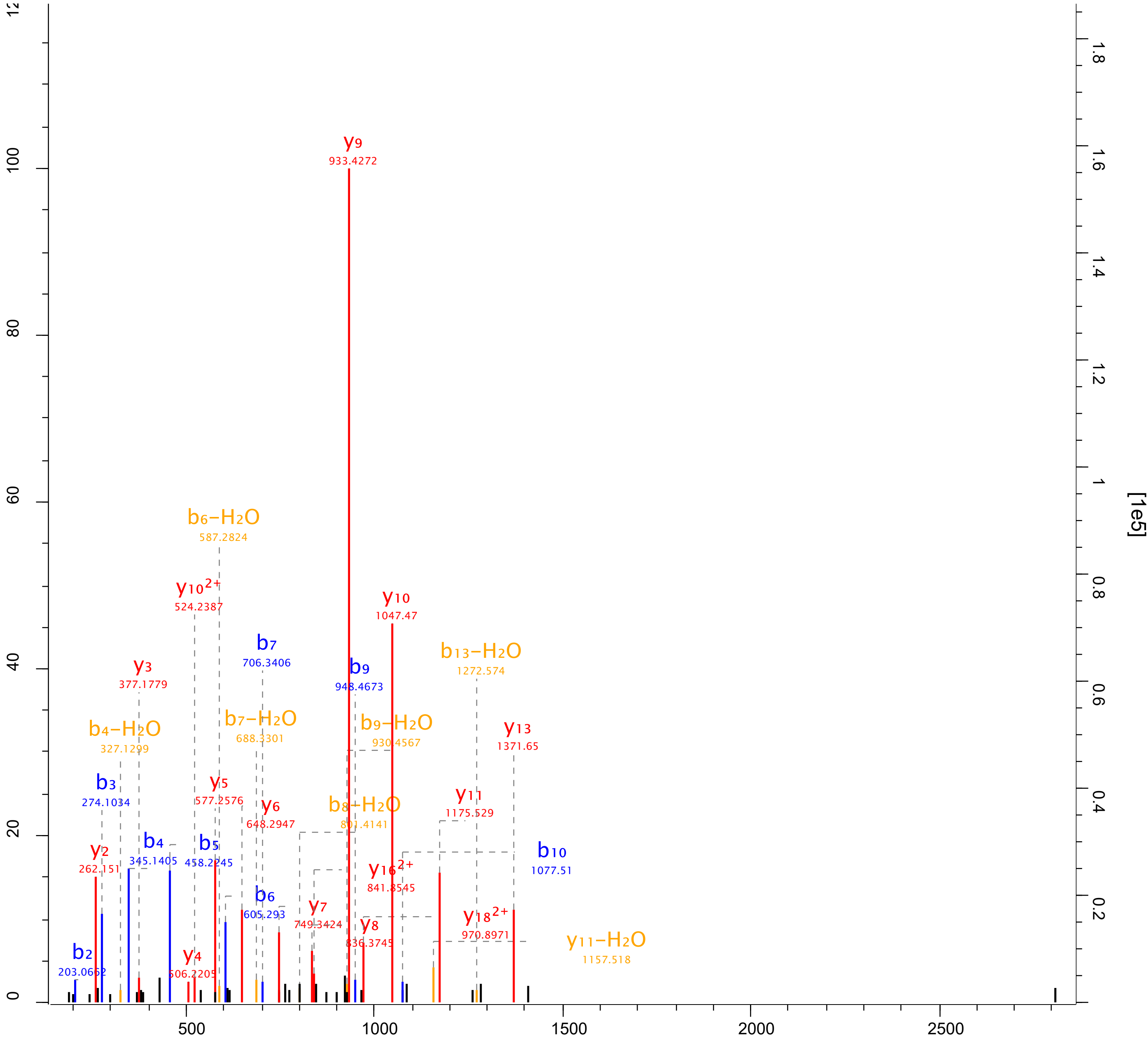

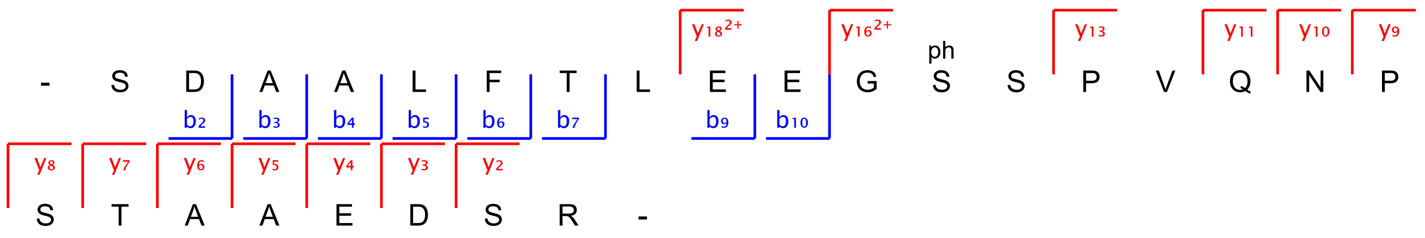

Sample output to test PDF Combine only

Raw file	Scan	Method	Score	m/z	Gene names
0523_4	22867	FTMS; HCD	43.35	897.02	At2g17410;ARID3

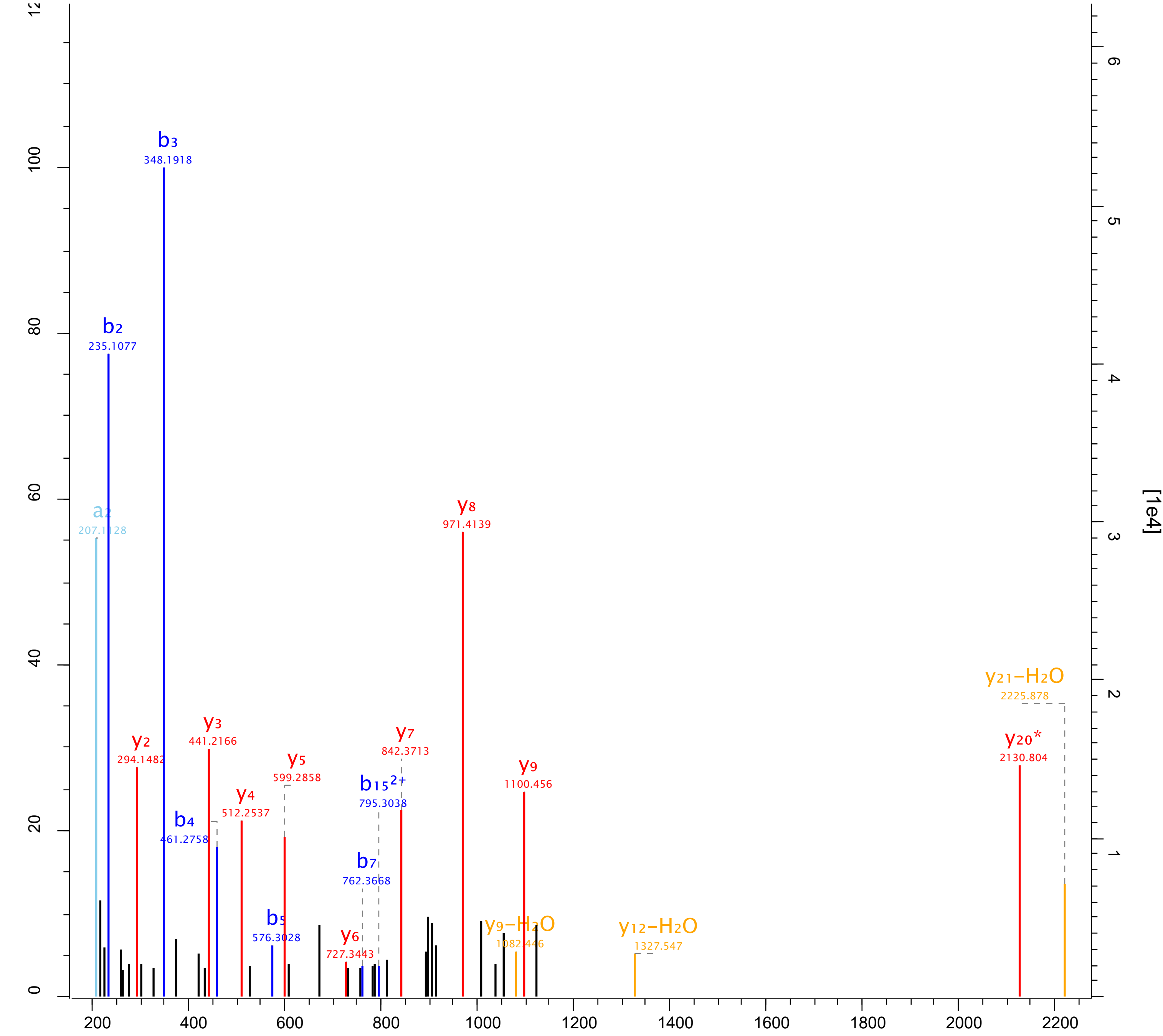

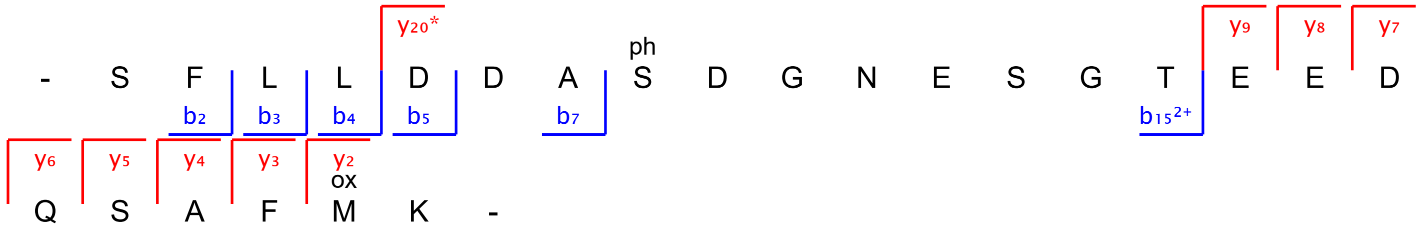

Sample output to test PDF Combine only

Raw file	Scan	Method	Score	m/z	Gene names
0523_4	22988	FTMS; HCD	161.04	1062.52	SAC2

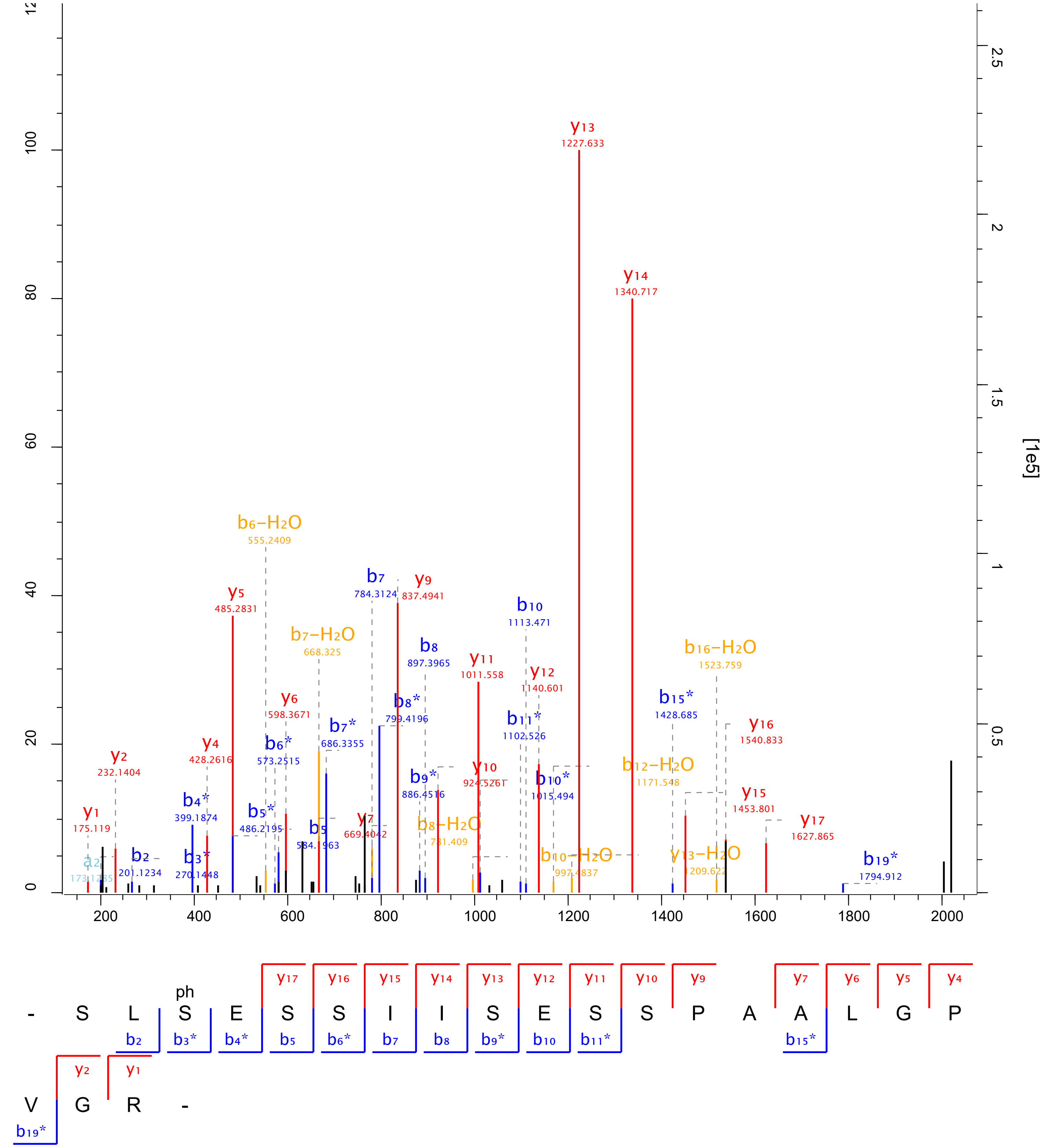

Sample output to test PDF Combine only

Raw file	Scan	Method	Score	m/z	Gene names
0523_4	23017	FTMS; HCD	249.41	923.34	ERF1-2

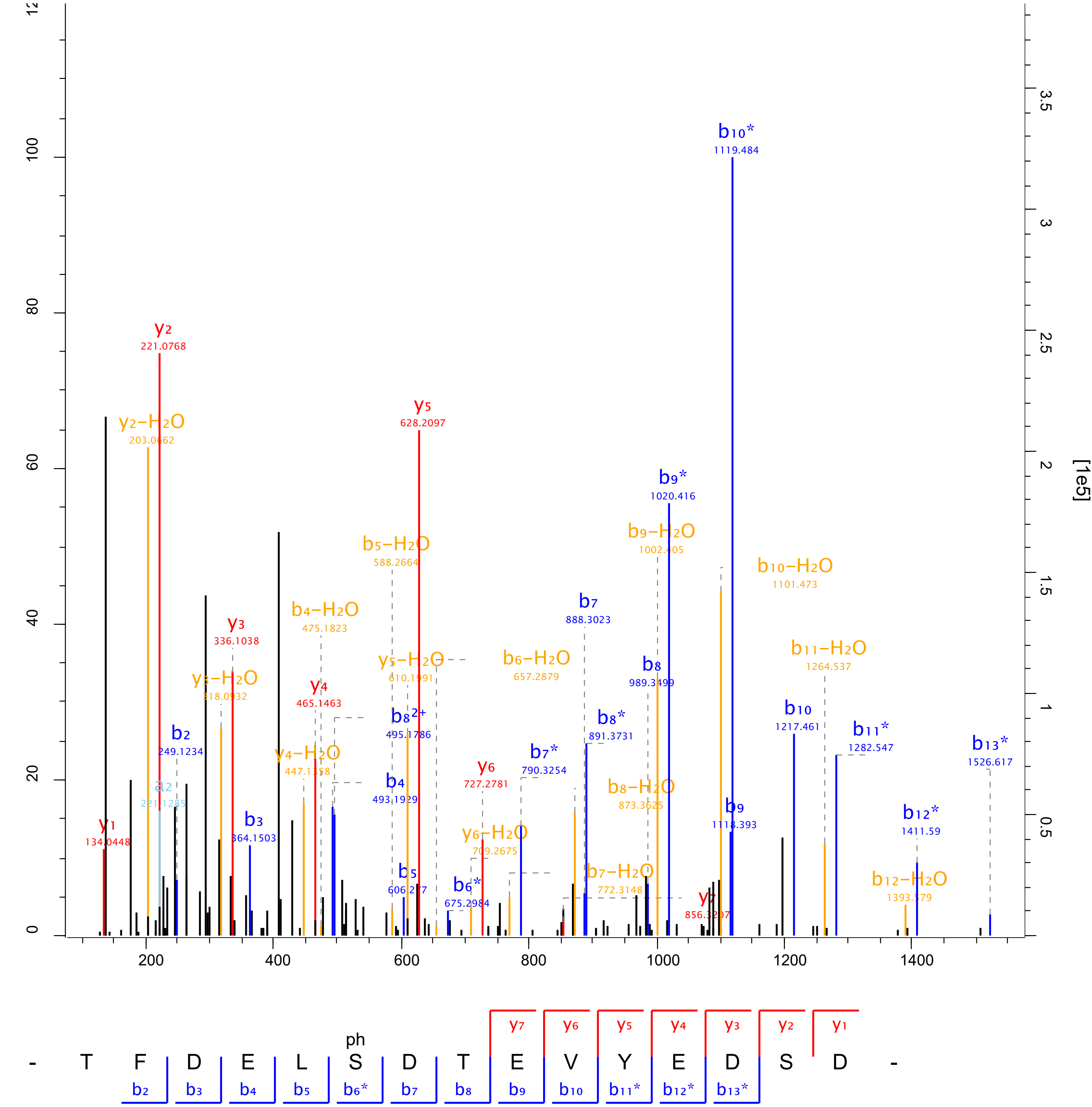

Sample output to test PDF Combine only

Raw file	Scan	Method	Score	m/z	Gene names
0523_4	23030	FTMS; HCD	43.31	833.7	IQD32

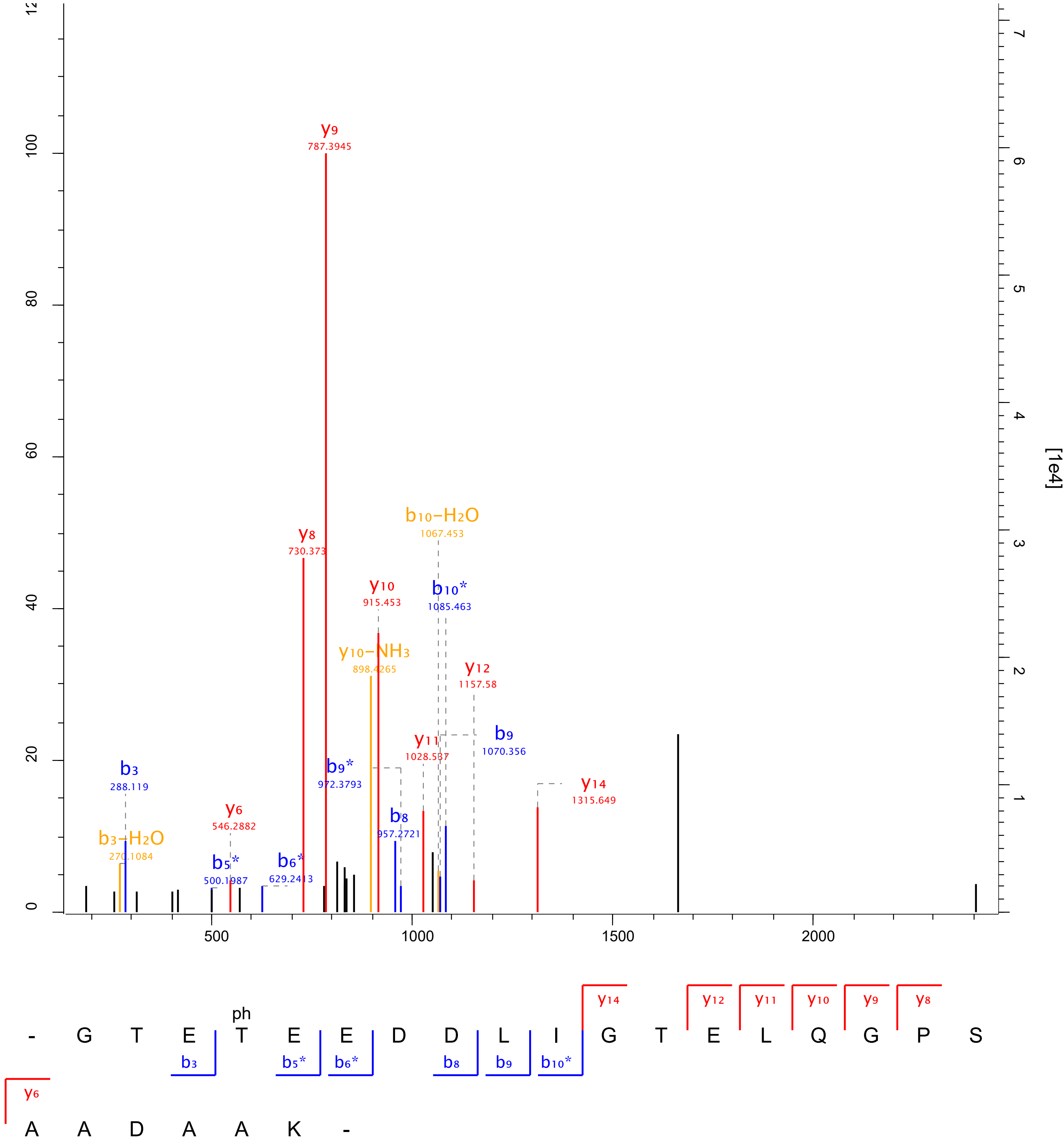

Sample output to test PDF Combine only

Raw file	Scan	Method	Score	m/z	Gene names
0523_4	23226	FTMS; HCD	96.85	953.38	T23K23.8

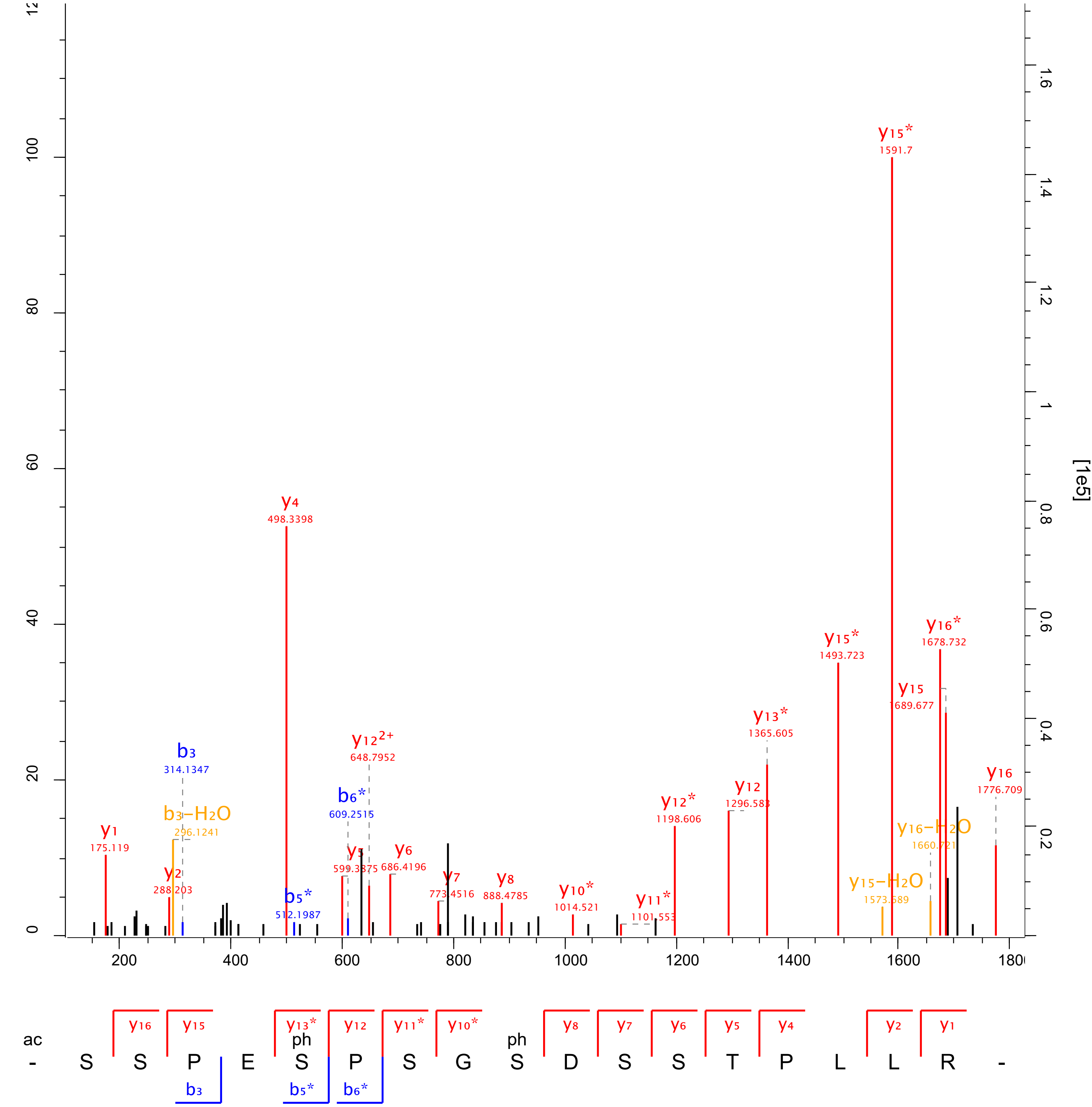

Sample output to test PDF Combine only

Raw file	Scan	Method	Score	m/z	Gene names
0523_4	23229	FTMS; HCD	167.35	957.89	At2g23520

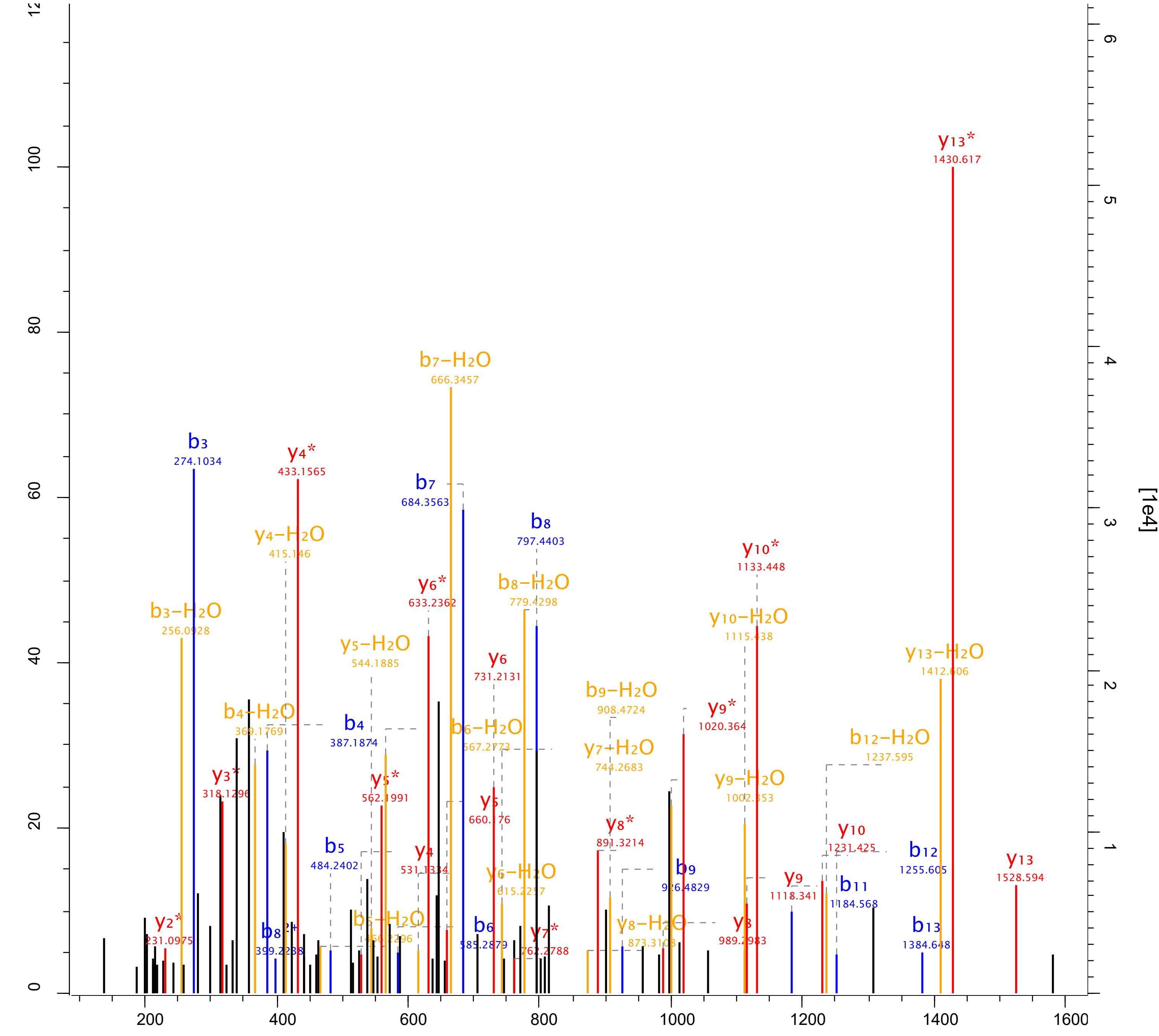

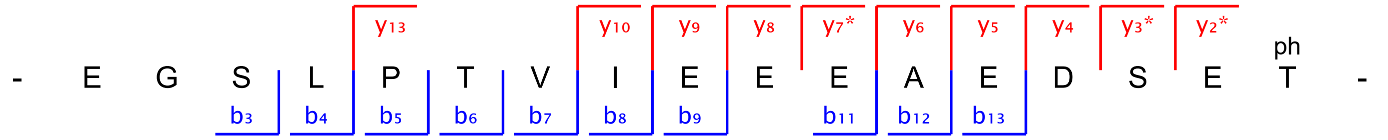

Sample output to test PDF Combine only

Raw file	Scan	Method	Score	m/z	Gene names
0523_4	23262	FTMS; HCD	242.07	799.3	RABA5D

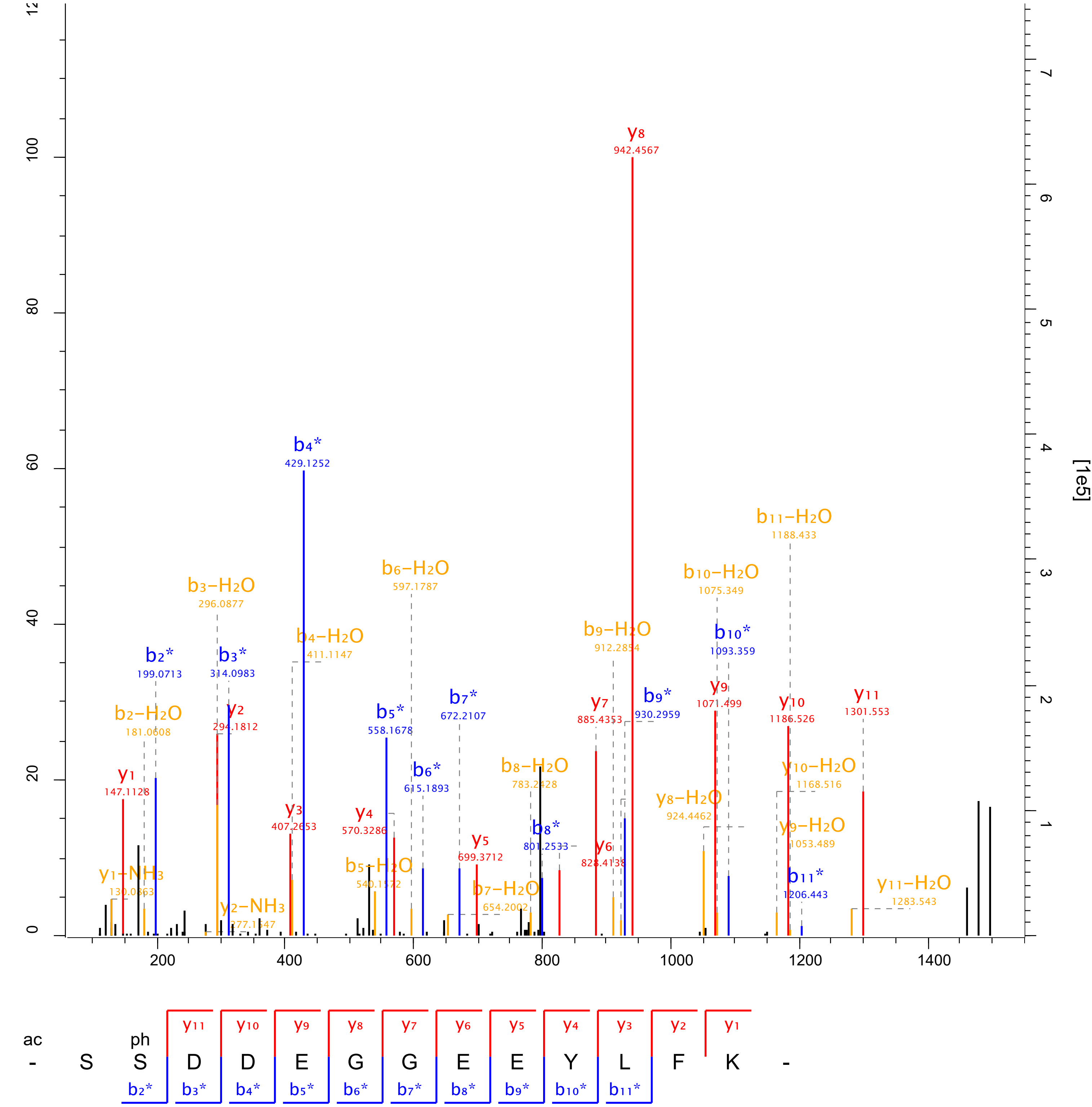

Sample output to test PDF Combine only

Raw file	Scan	Method	Score	m/z	Gene names
0523_4	23761	FTMS; HCD	135.98	759.98	At4g37090;C7A10.270

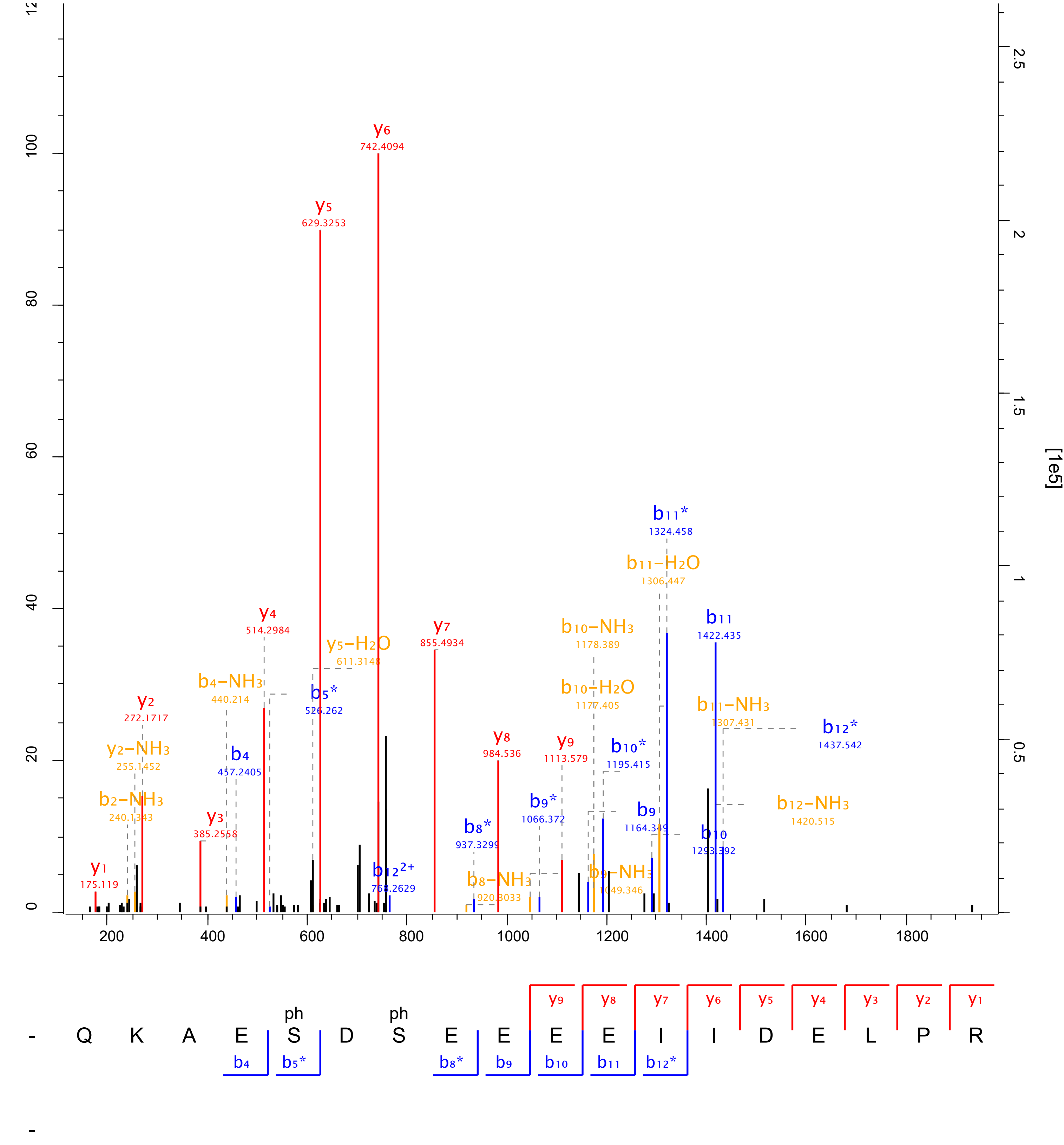

Sample output to test PDF Combine only

Raw file	Scan	Method	Score	m/z	Gene names
0523_4	23859	FTMS; HCD	123.32	797.35	At3g50370

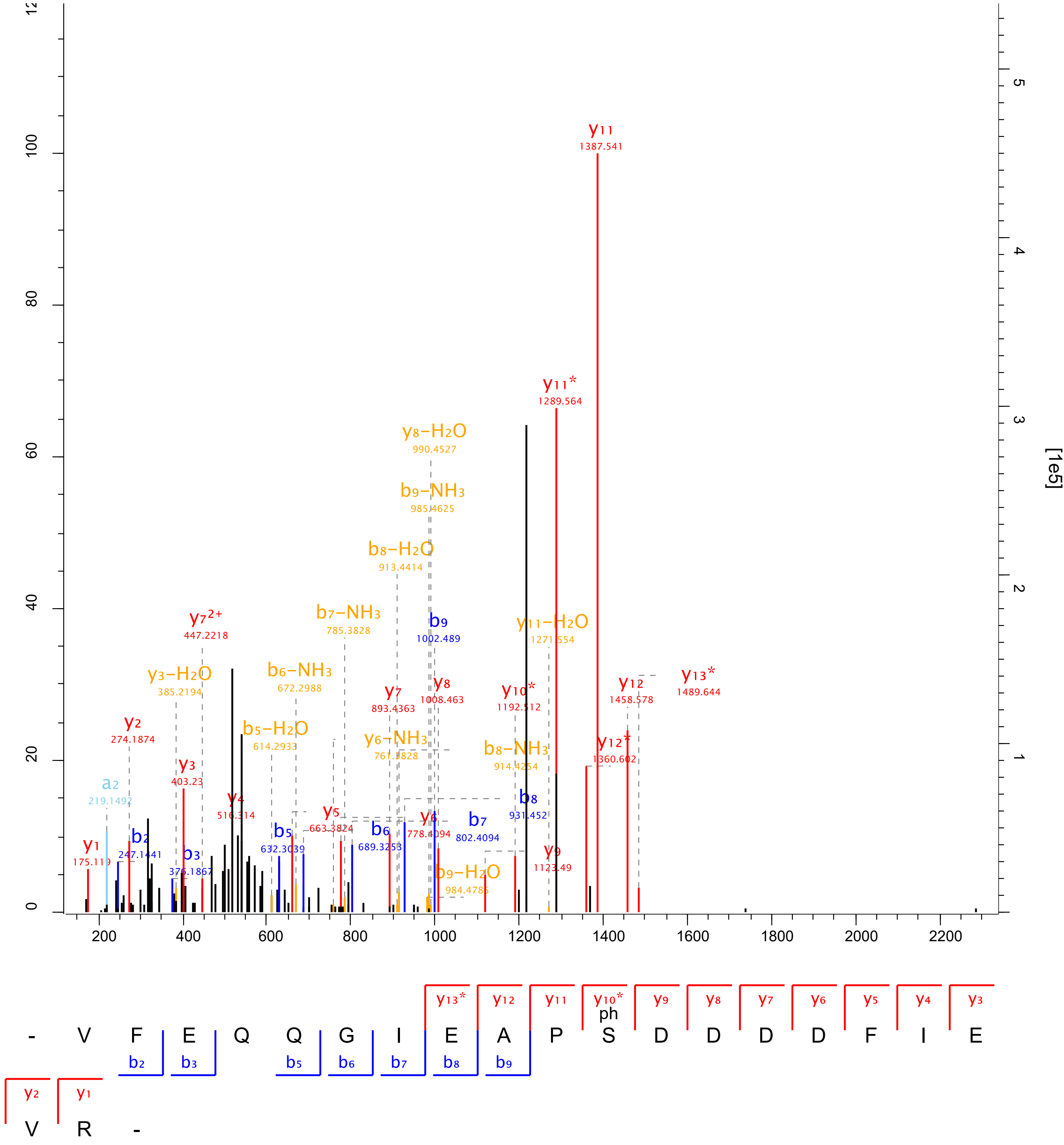

Sample output to test PDF Combine only

Raw file	Scan	Method	Score	m/z	Gene names
0523_4	23866	FTMS; HCD	47.29	838.03	ABCB6

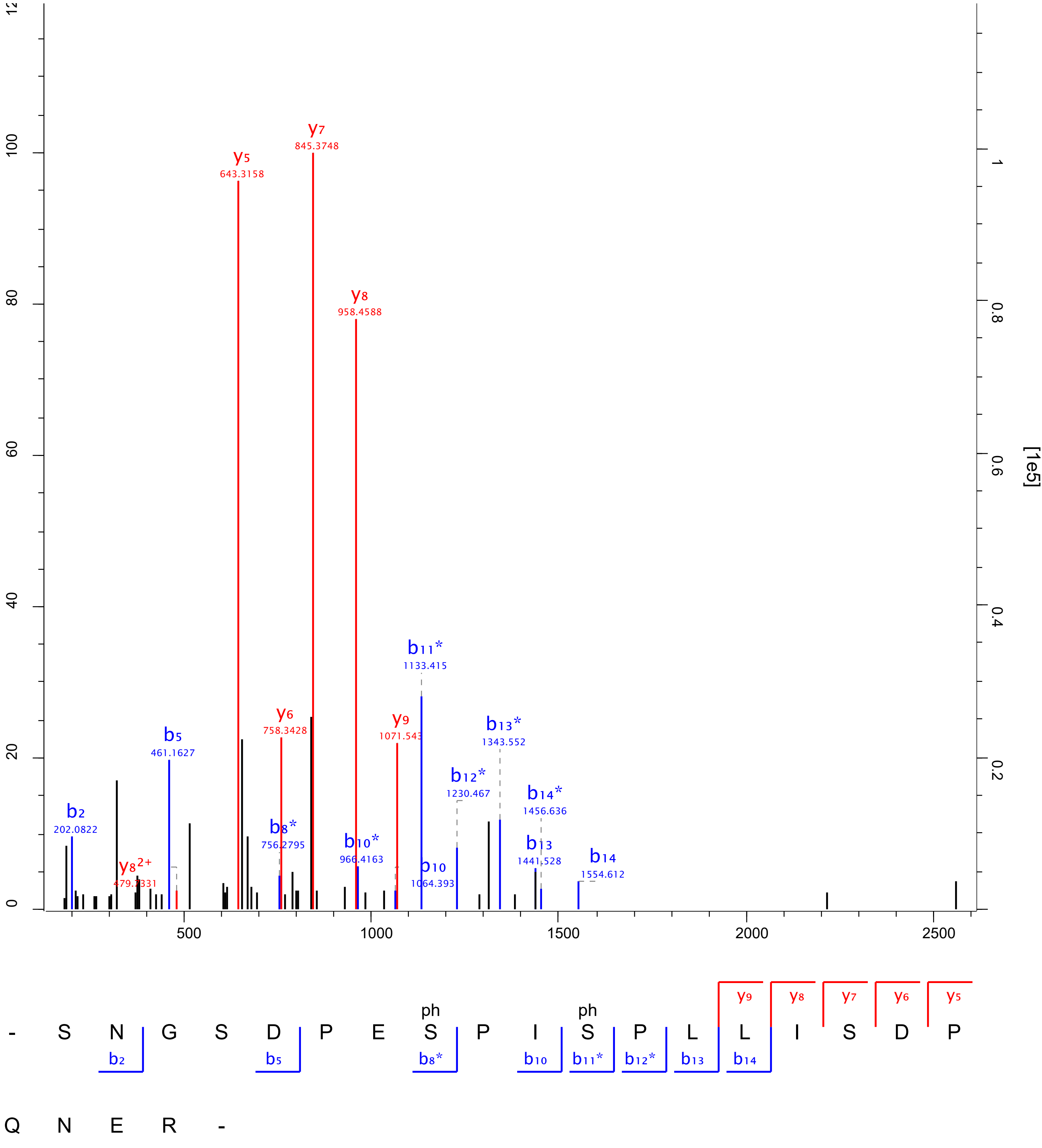

Sample output to test PDF Combine only

Raw file	Scan	Method	Score	m/z	Gene names
0523_4	23908	FTMS; HCD	183.8	1148.78	POT13

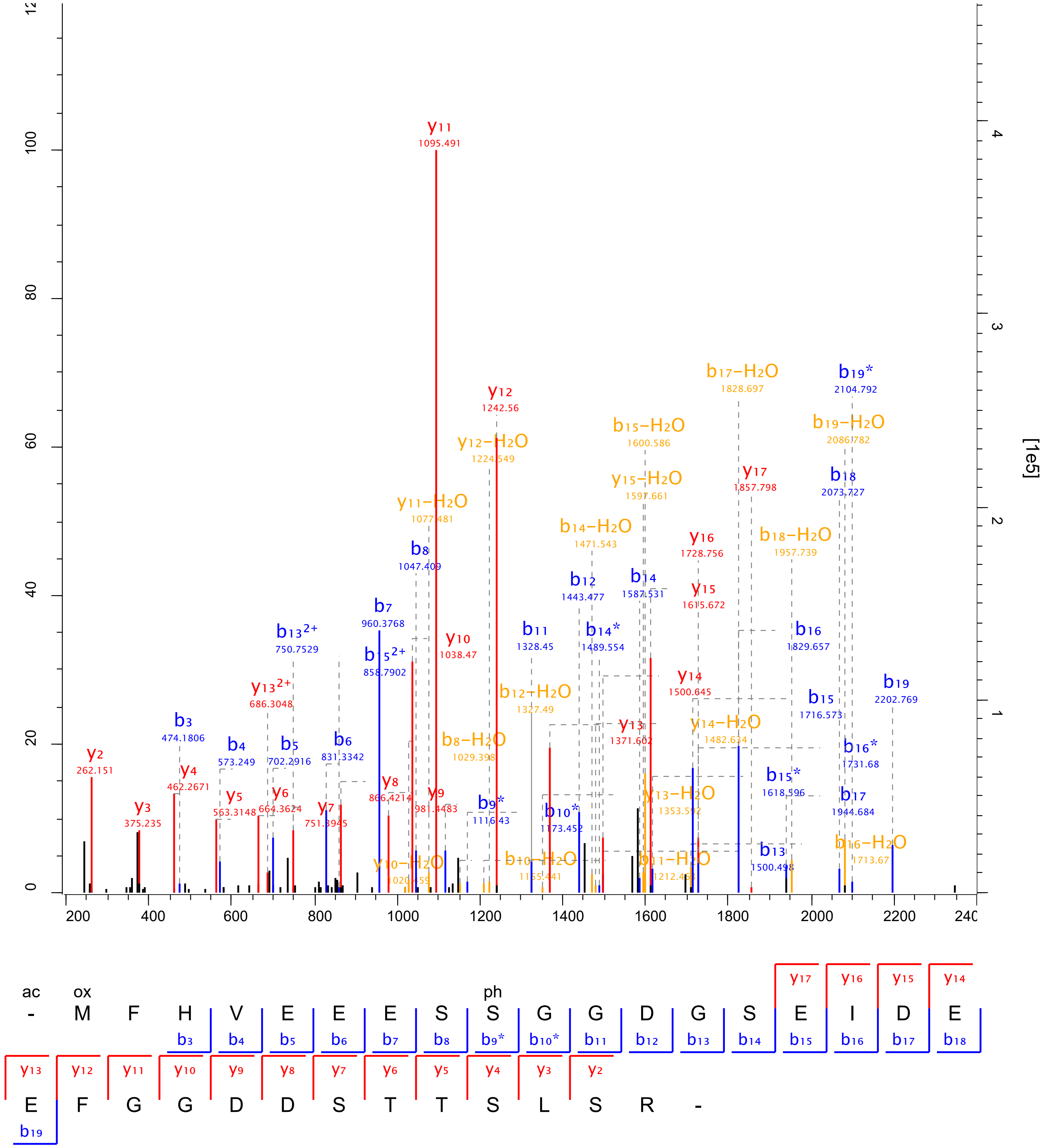

Sample output to test PDF Combine only

Raw file	Scan	Method	Score	m/z	Gene names
0523_4	24262	FTMS; HCD	139.69	1007.95	dl4560c

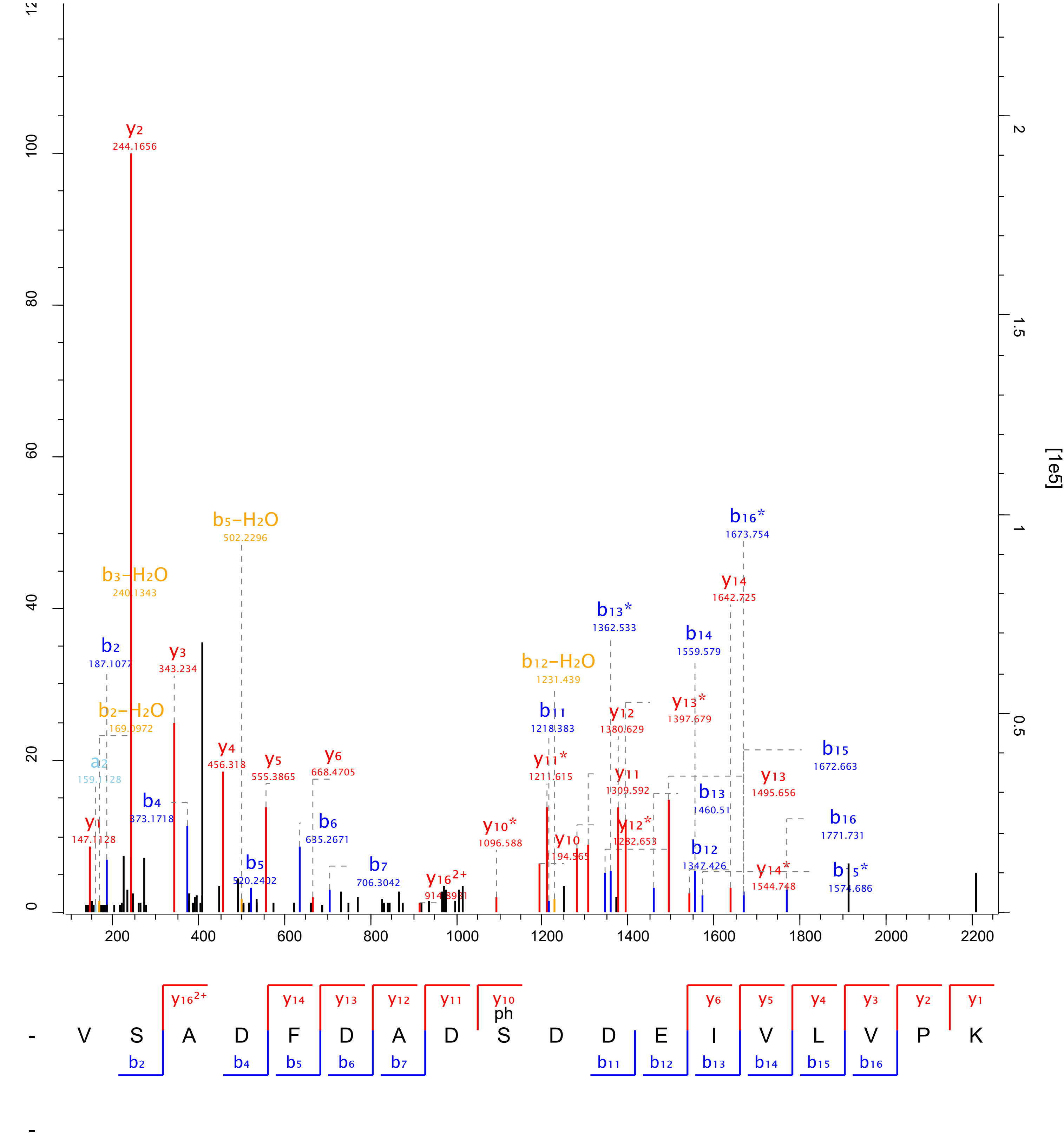

Sample output to test PDF Combine only

Raw file	Scan	Method	Score	m/z	Gene names
0523_4	24380	FTMS; HCD	154.26	829.39	SYP132

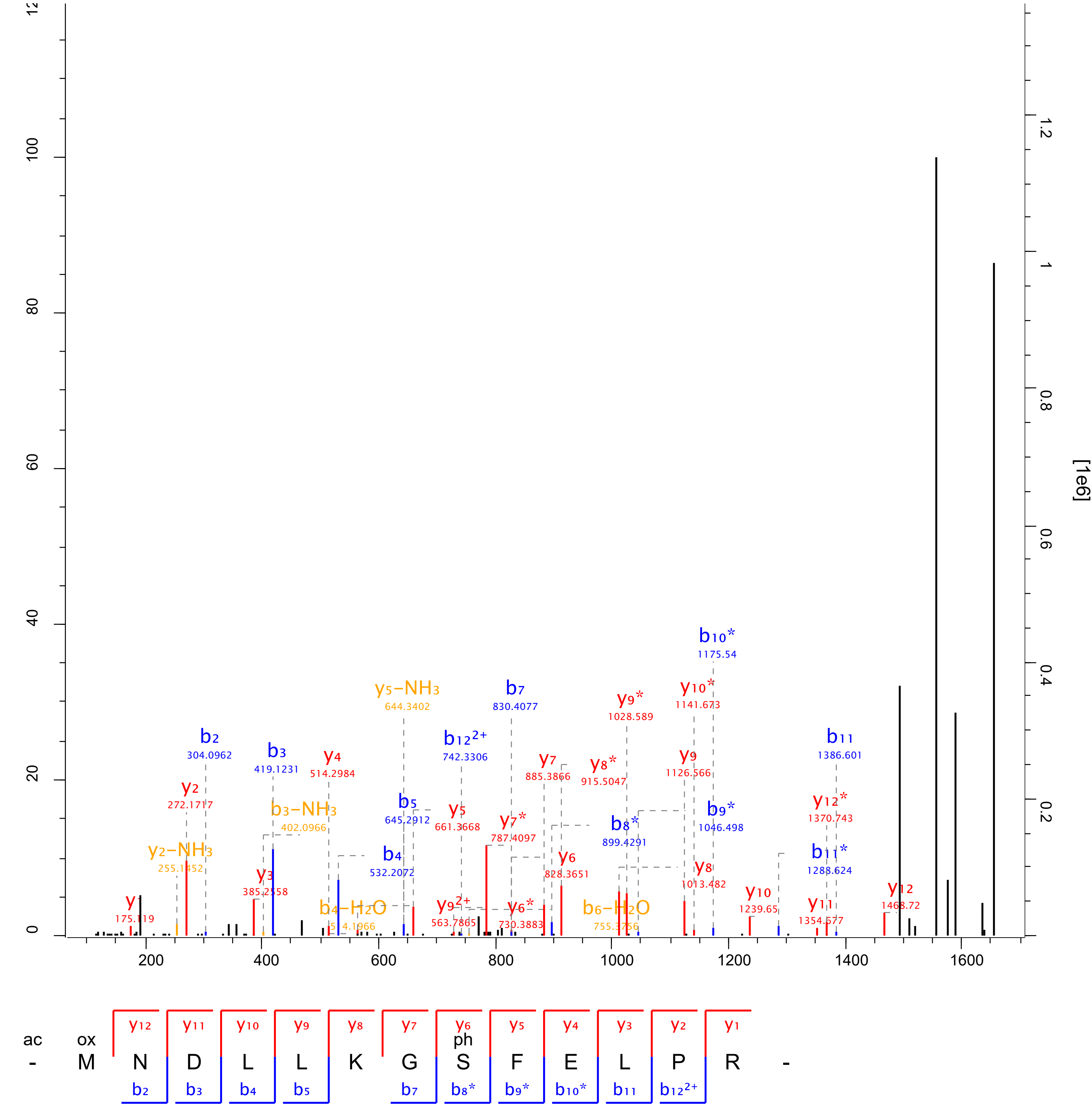

Sample output to test PDF Combine only

Raw file	Scan	Method	Score	m/z	Gene names
0523_4	24442	FTMS; HCD	197.28	1231.44	ERF1-1

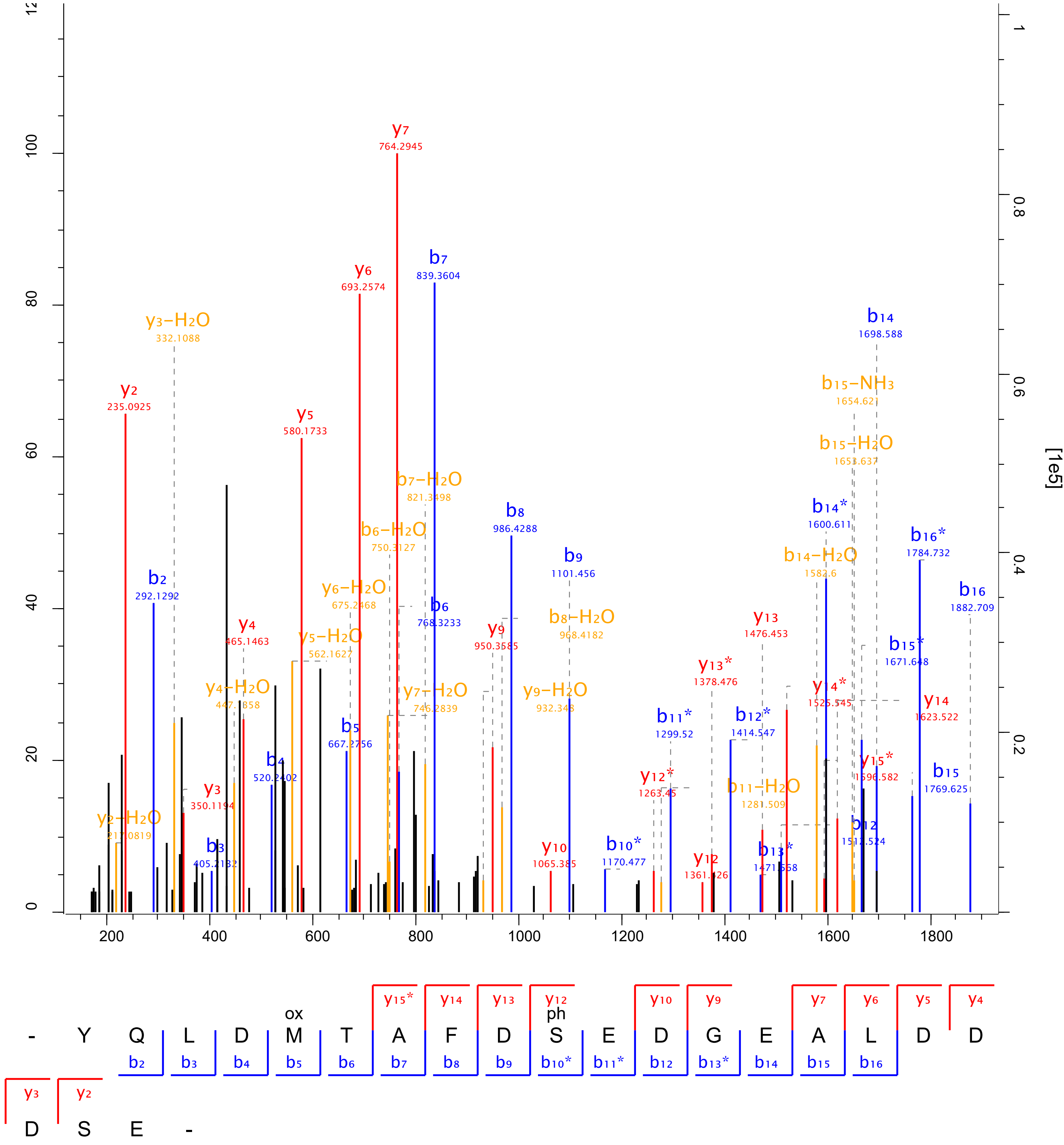

Sample output to test PDF Combine only

Raw file	Scan	Method	Score	m/z	Gene names
0523_4	24803	FTMS; HCD	47.06	855.06	F17M5.160

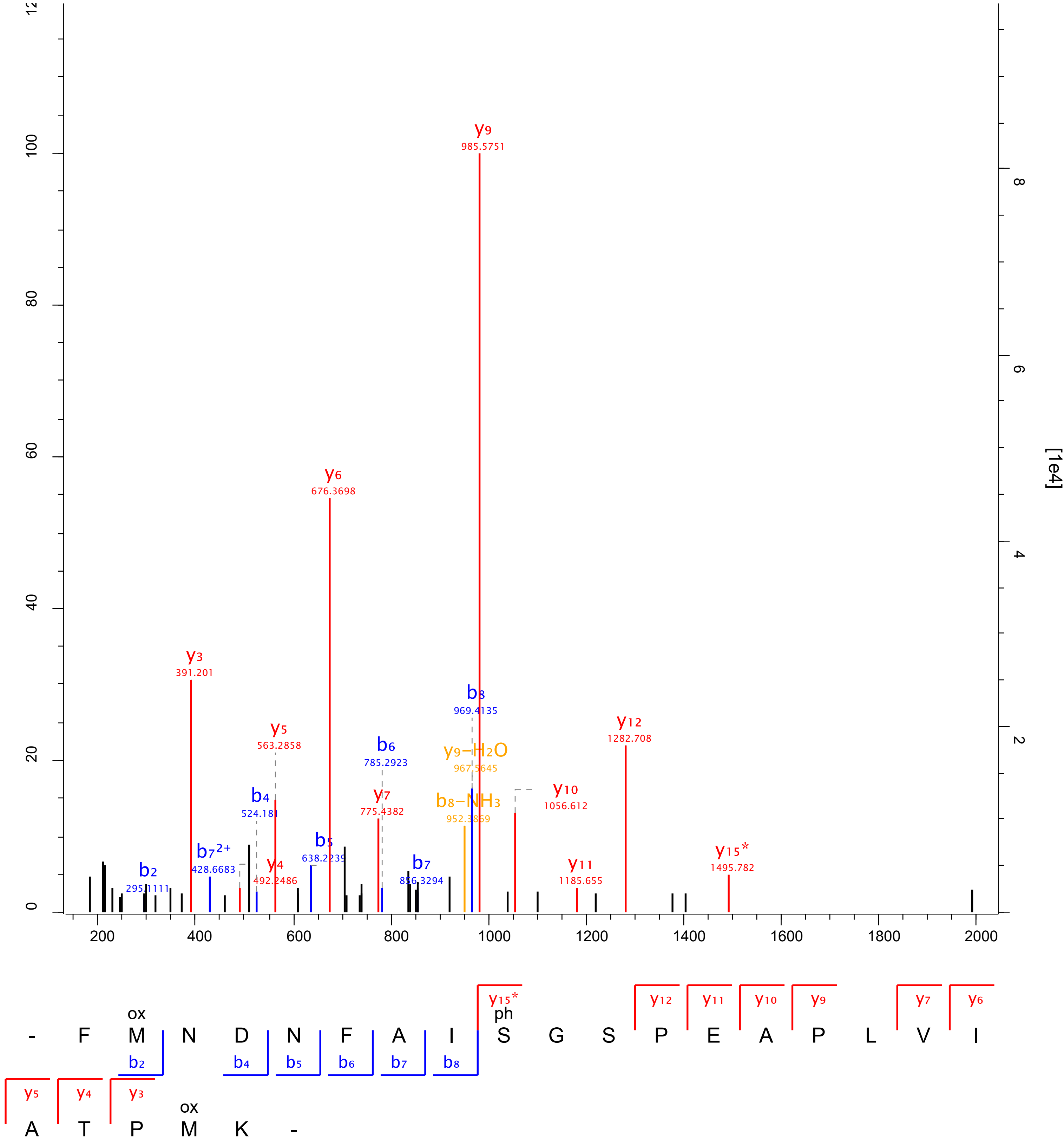

Sample output to test PDF Combine only

Raw file	Scan	Method	Score	m/z	Gene names
0523_4	24940	FTMS; HCD	76.59	788.34	At5g40450

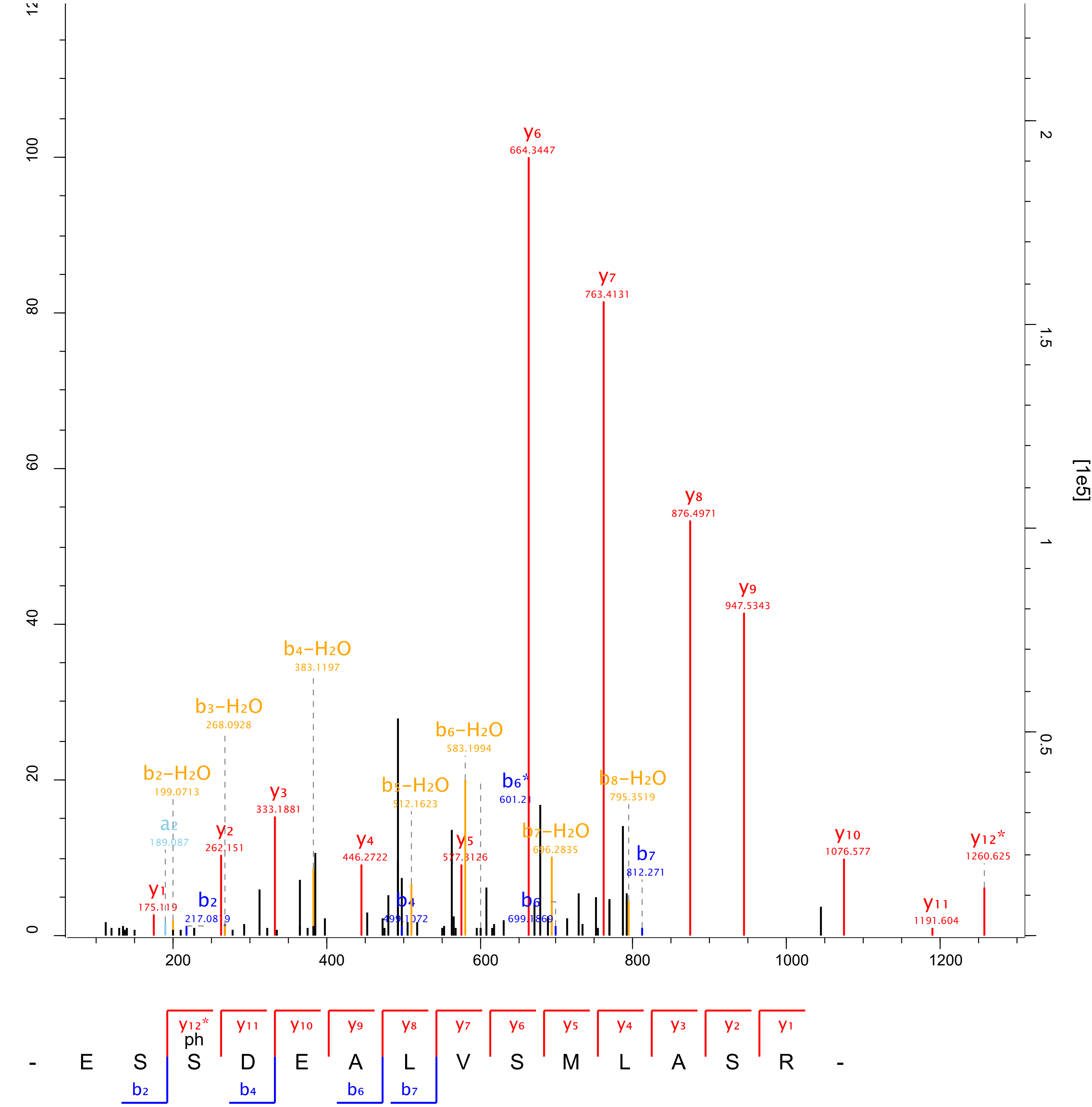

Sample output to test PDF Combine only

Raw file	Scan	Method	Score	m/z	Gene names
0523_4	25128	FTMS; HCD	306.39	1159.49	Tudor1

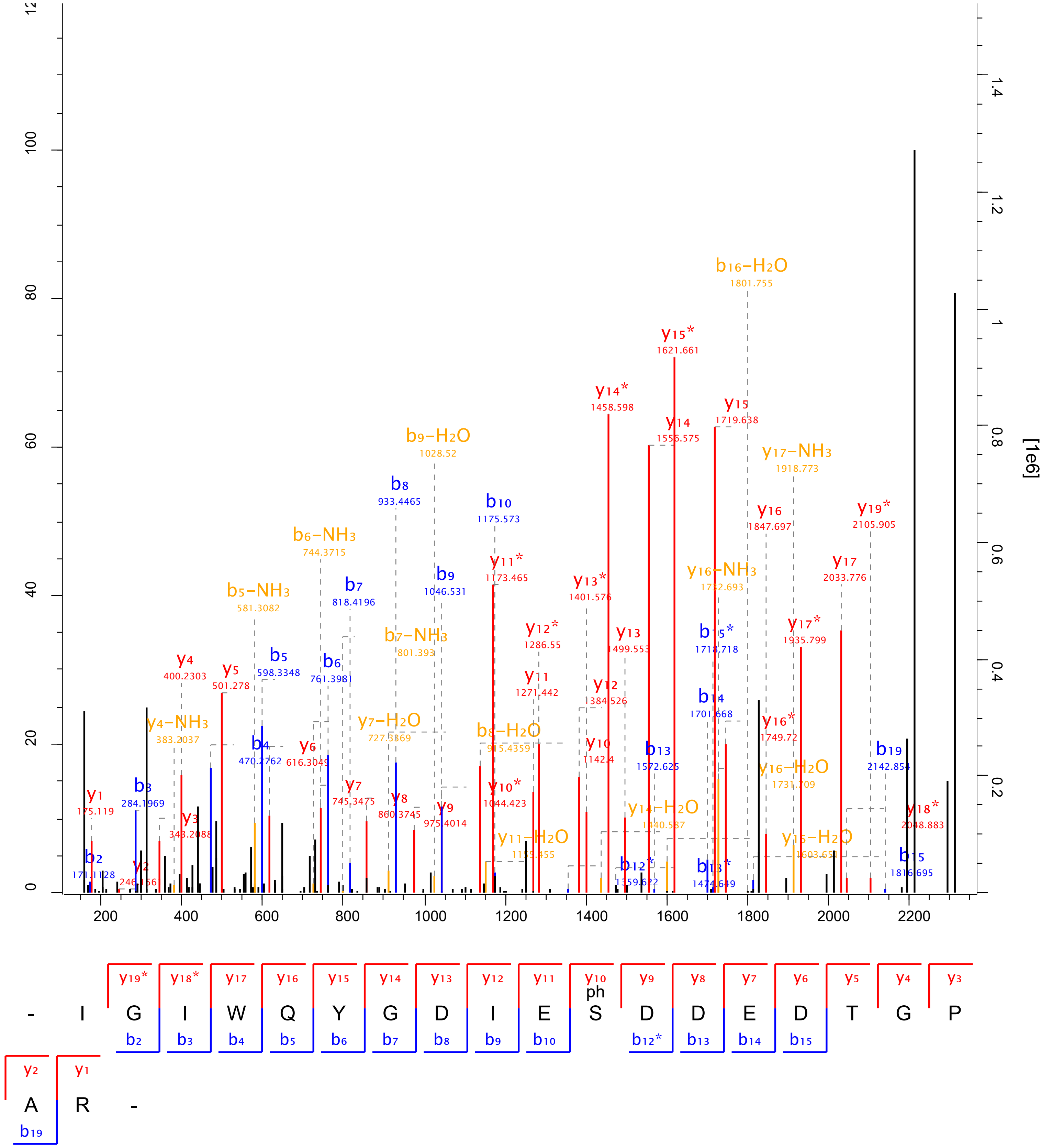

Sample output to test PDF Combine only

Raw file	Scan	Method	Score	m/z	Gene names
0523_4	25478	FTMS; HCD	83.4	1002.36	RPP2D;RPP2A;At2g27720;RPP2B;At2g27710

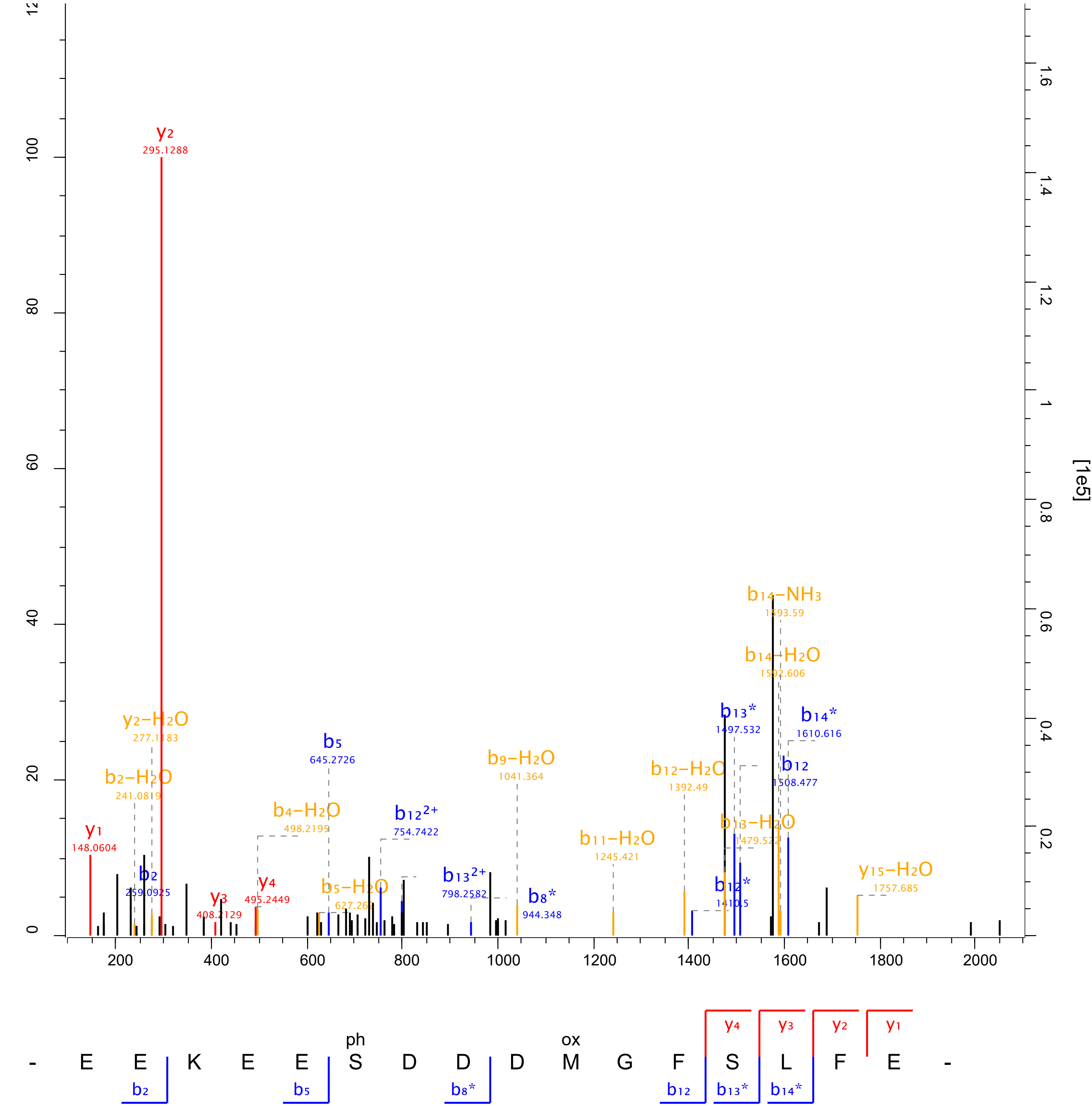

Sample output to test PDF Combine only

Raw file	Scan	Method	Score	m/z	Gene names
0523_4	25698	FTMS; HCD	255.69	1040.94	At1g25260

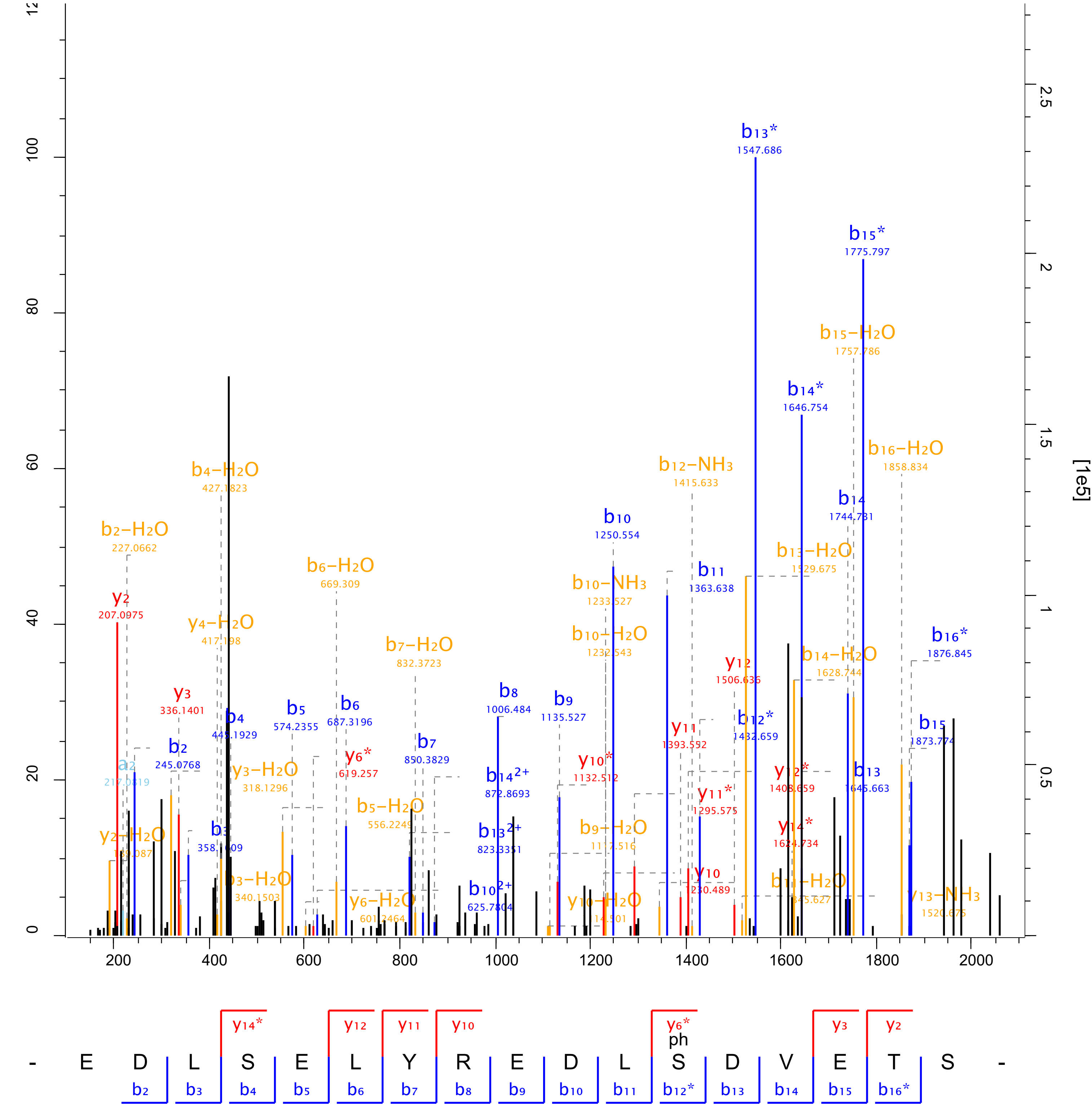

Sample output to test PDF Combine only

Raw file	Scan	Method	Score	m/z	Gene names
0523_4	25779	FTMS; HCD	177.91	1178.52	At2g18690

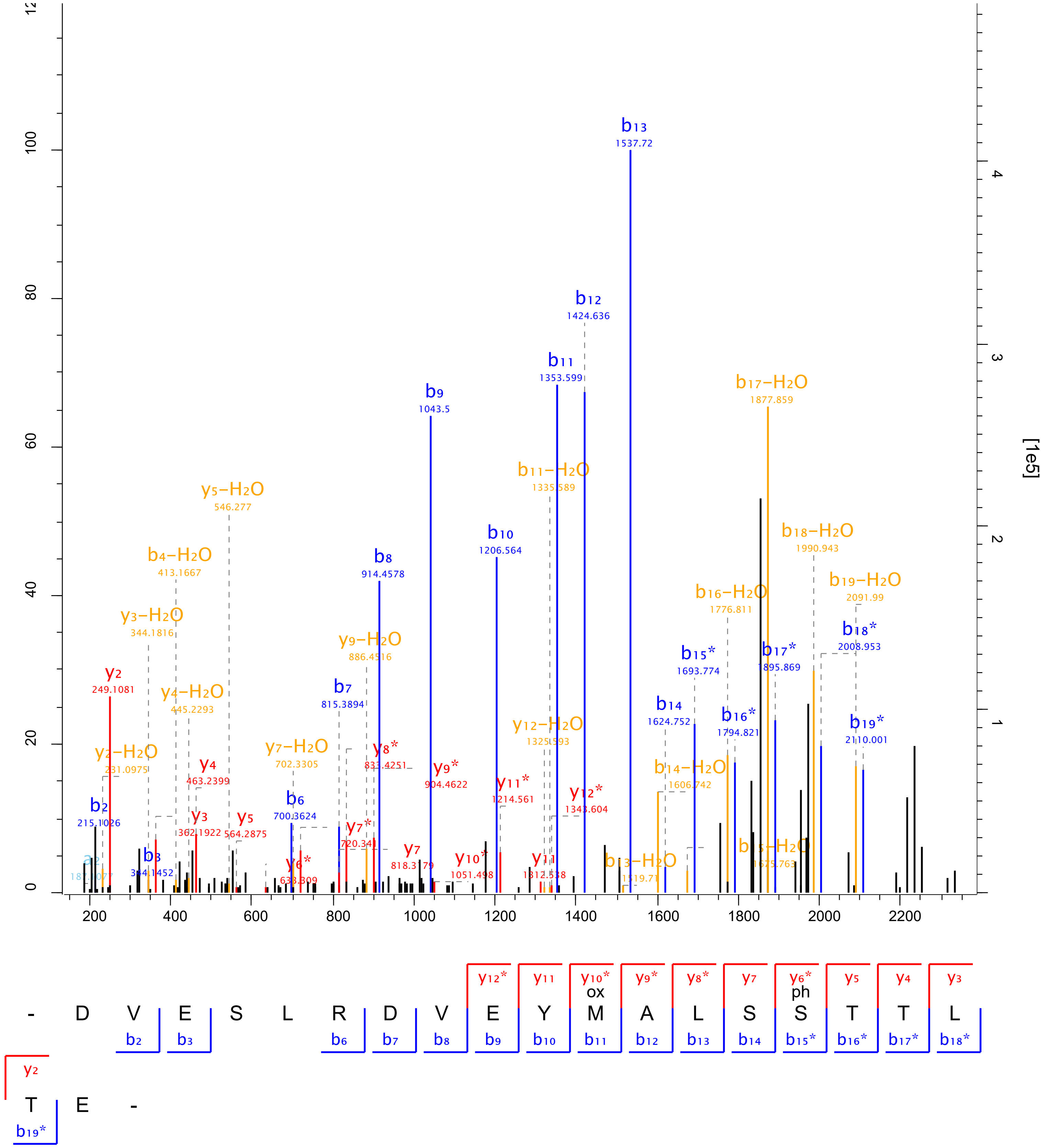

Raw file	Scan	Method	Score	m/z	Gene names
0523_4	26028	FTMS; HCD	131.04	1085.5	POT13

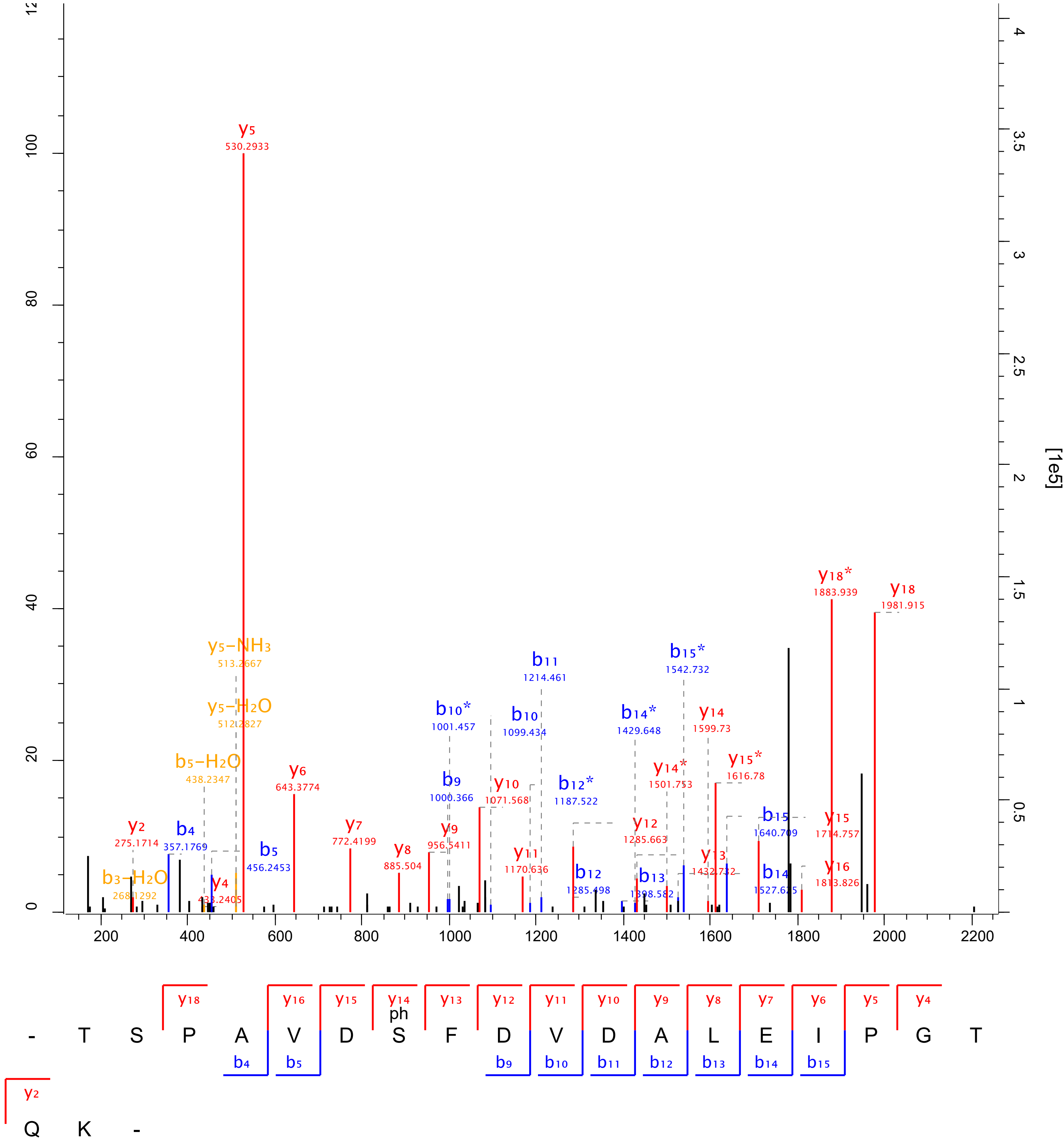

Sample output to test PDF Combine only

Raw file	Scan	Method	Score	m/z	Gene names
0523_4	26087	FTMS; HCD	80.79	1129	At1g75640

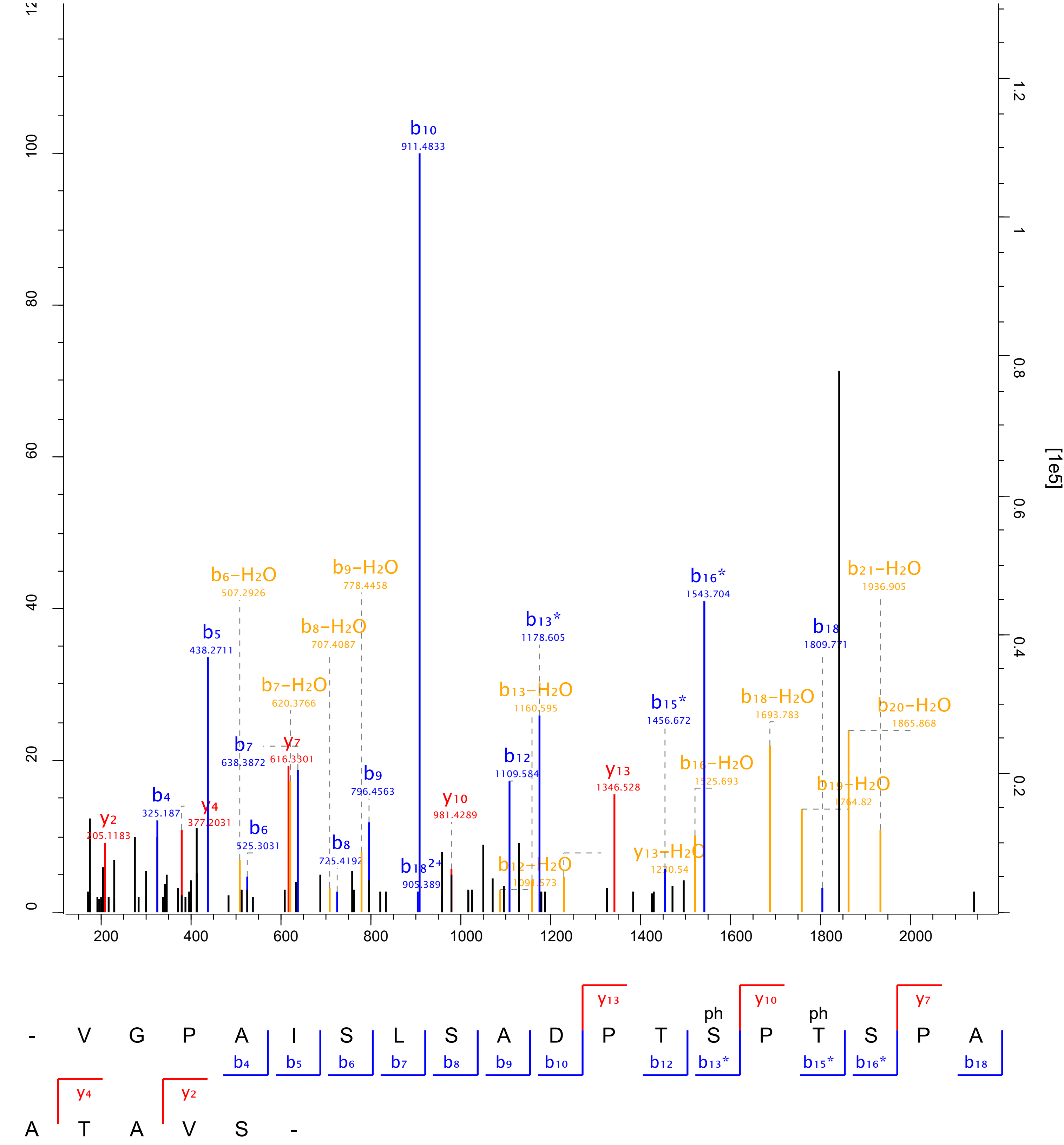

Sample output to test PDF Combine only

Raw file	Scan	Method	Score	m/z	Gene names
0523_4	26091	FTMS; HCD	145.52	765.86	AHA11;AHA4

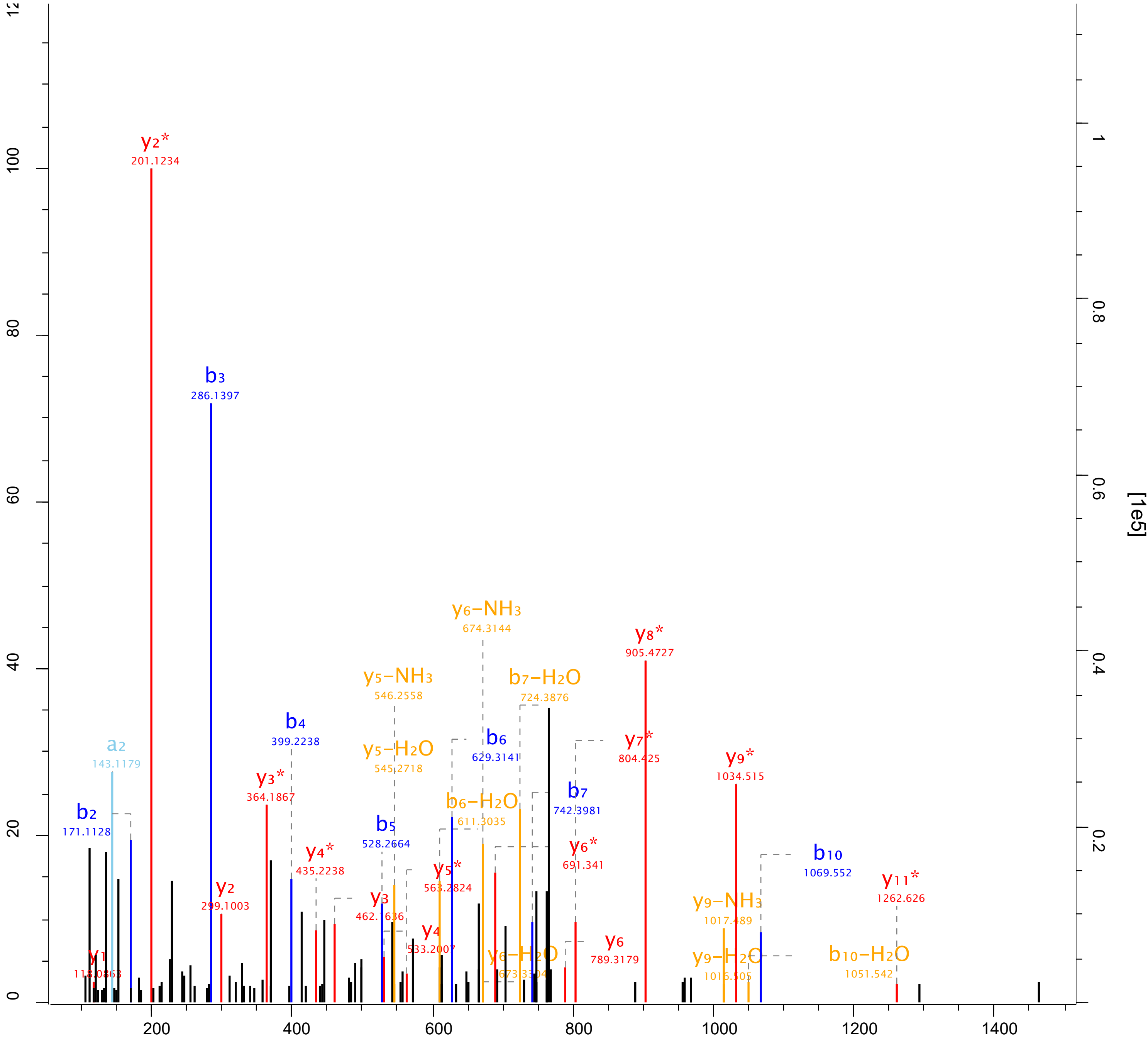

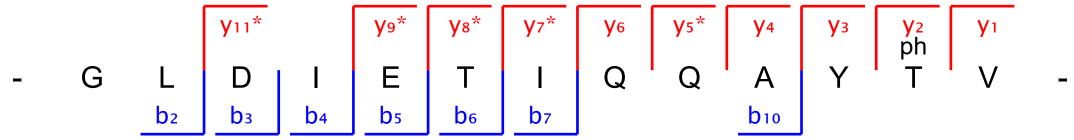

Sample output to test PDF Combine only

Raw file	Scan	Method	Score	m/z	Gene names
0523_4	26791	FTMS; HCD	117.5	831.04	FER

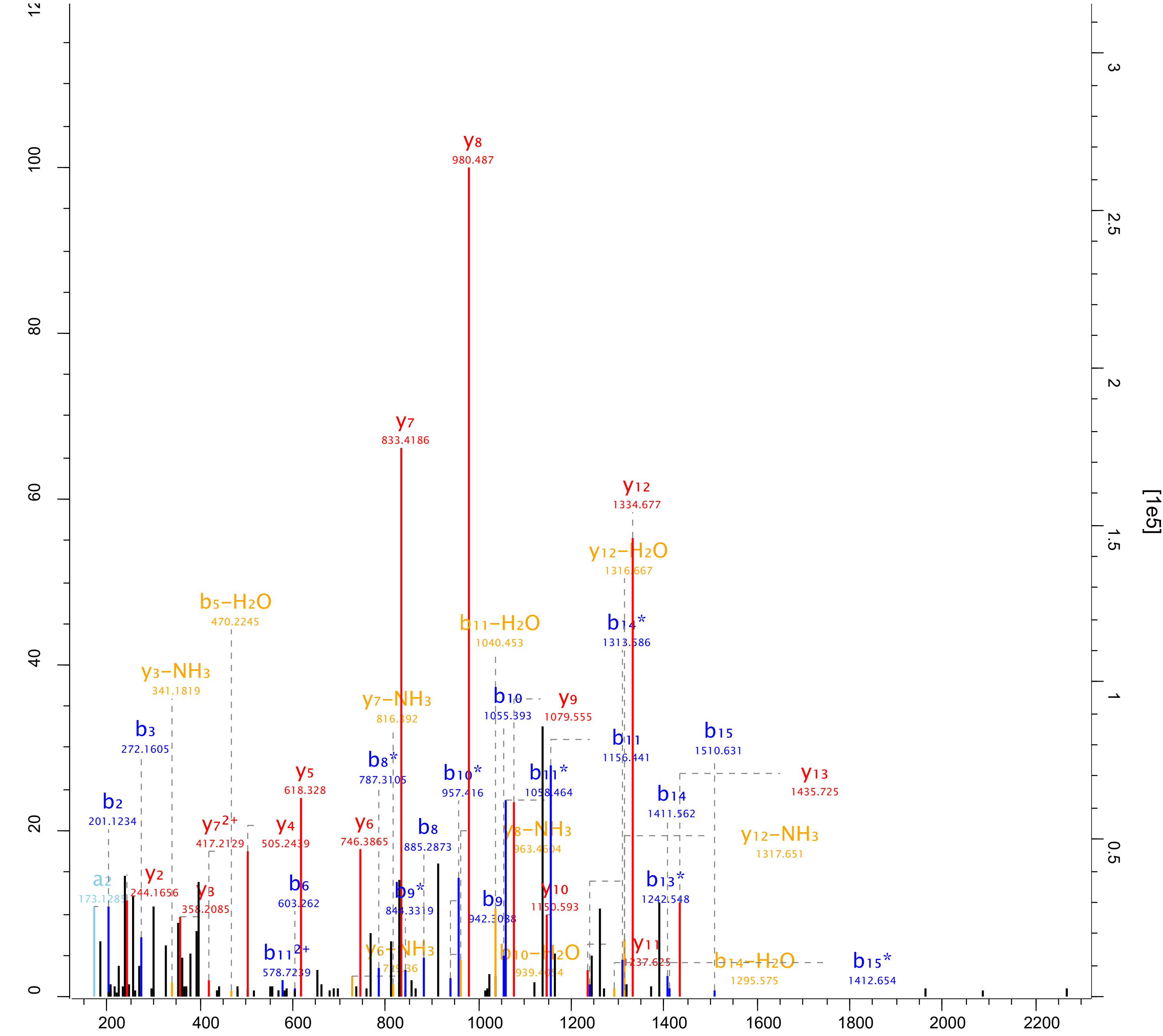

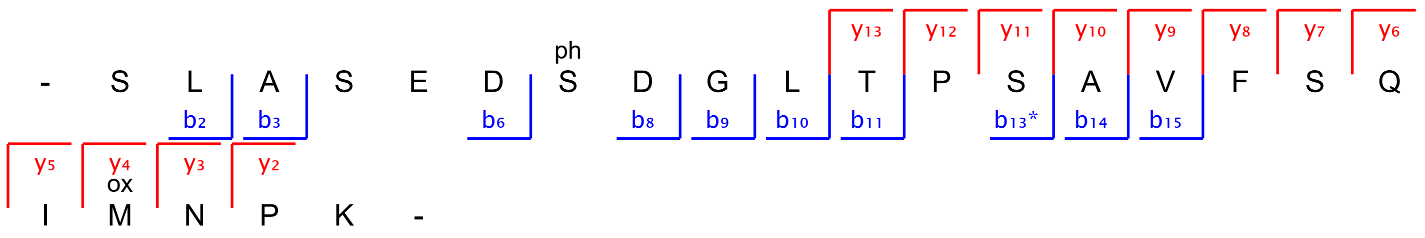

Sample output to test PDF Combine only

Raw file	Scan	Method	Score	m/z
0523_4	27383	FTMS; HCD	62.86	801.39

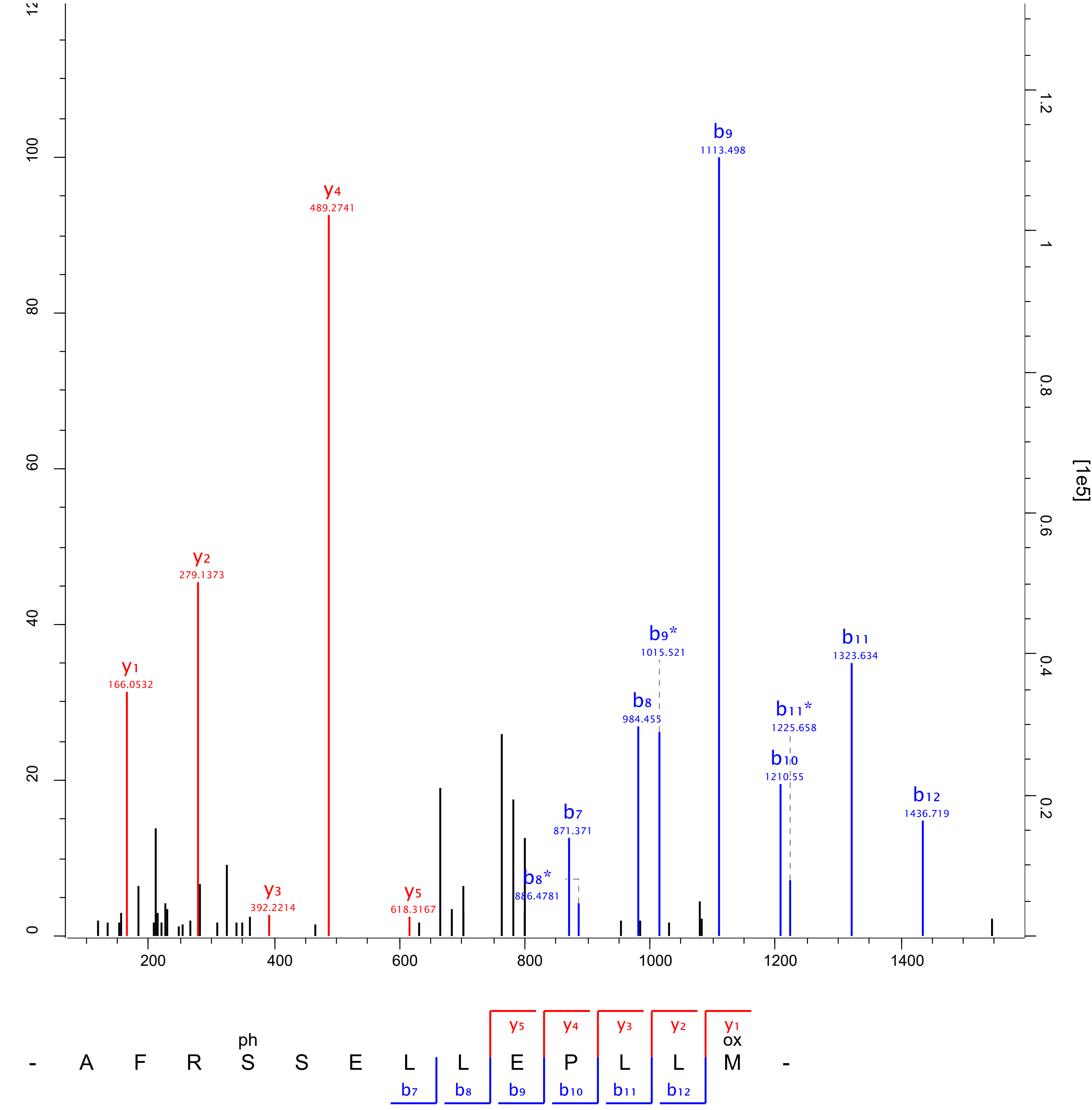

Sample output to test PDF Combine only

Raw file	Scan	Method	Score	m/z	Gene names
0523_4	28836	FTMS; HCD	187.94	1083.81	F23N20.8

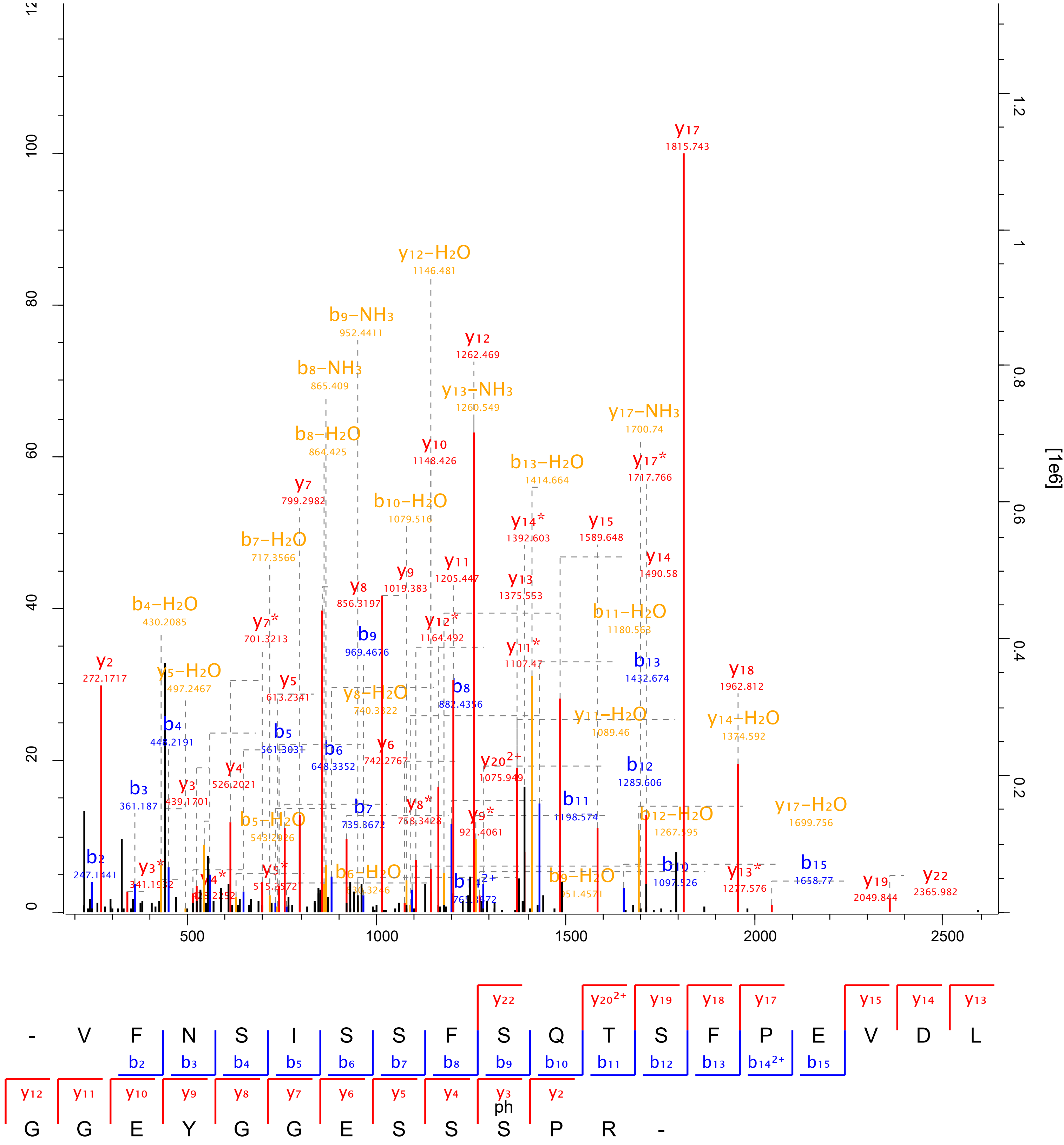

Sample output to test PDF Combine only

Raw file	Scan	Method	Score	m/z	Gene names
0523_4	29286	FTMS; HCD	111.22	839.28	RABA5D

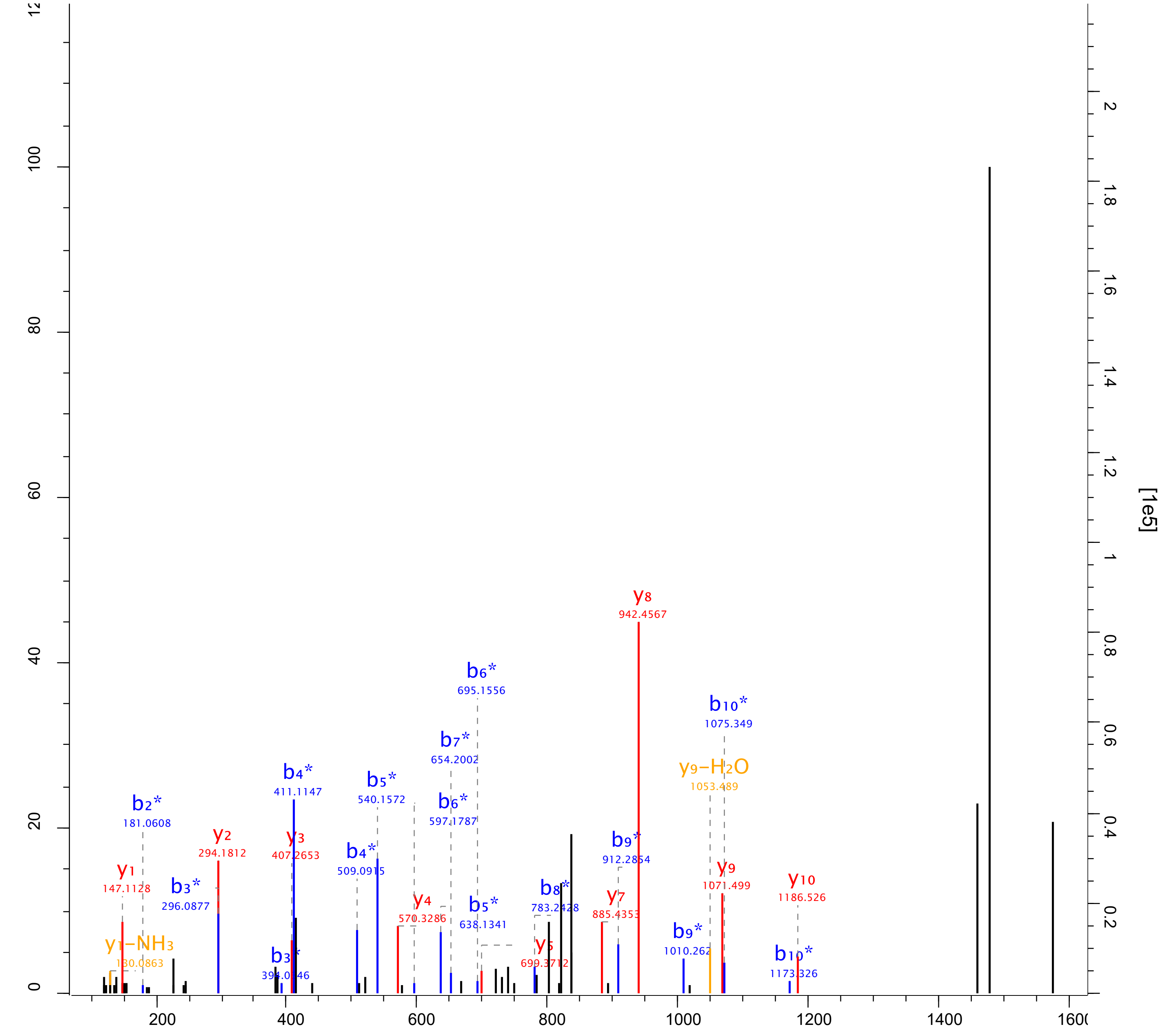

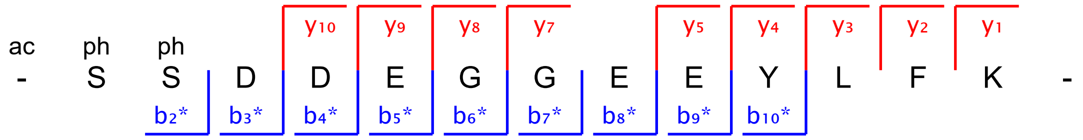

Sample output to test PDF Combine only

Raw file	Scan	Method	Score	m/z	Gene names
0523_4	29397	FTMS; HCD	89.91	660.32	BAM1

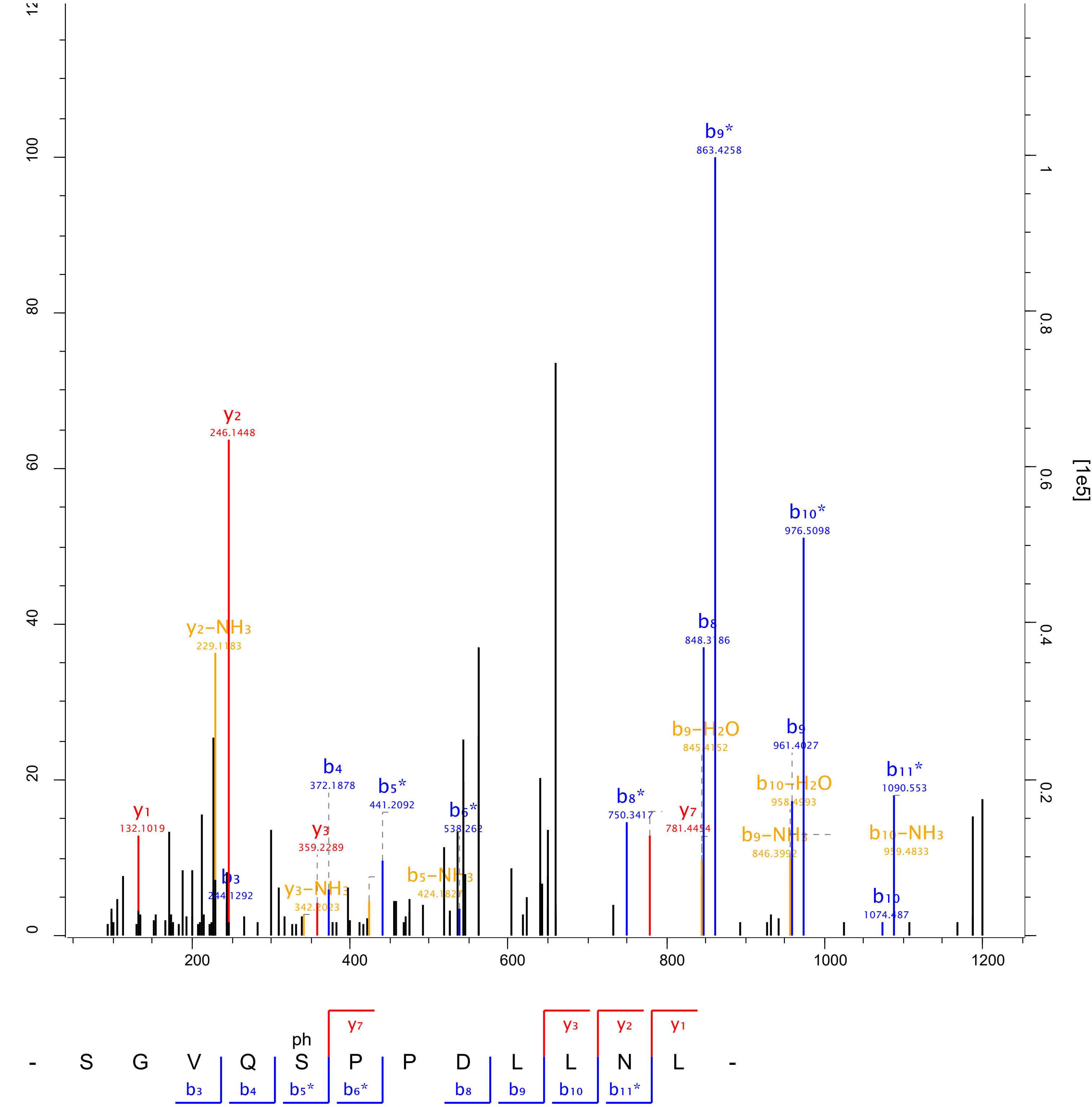

Sample output to test PDF Combine only

Raw file	Scan	Method	Score	m/z	Gene names
0523_4	29788	FTMS; HCD	68.05	971.8	ACA10

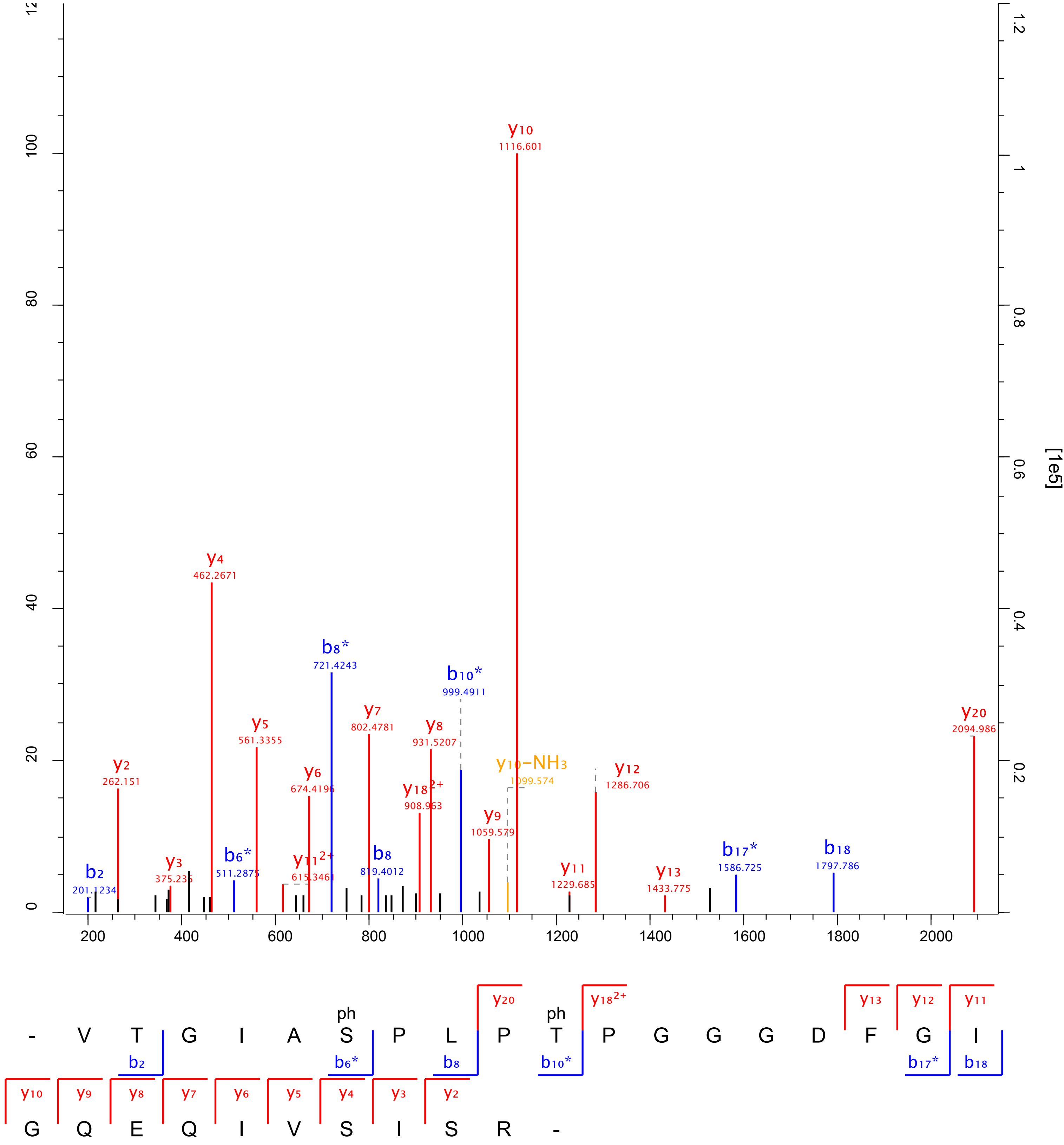

Sample output to test PDF Combine only

Raw file	Scan	Method	Score	m/z	Gene names
0523_4	29892	FTMS; HCD	180.79	884.4	At1g51790

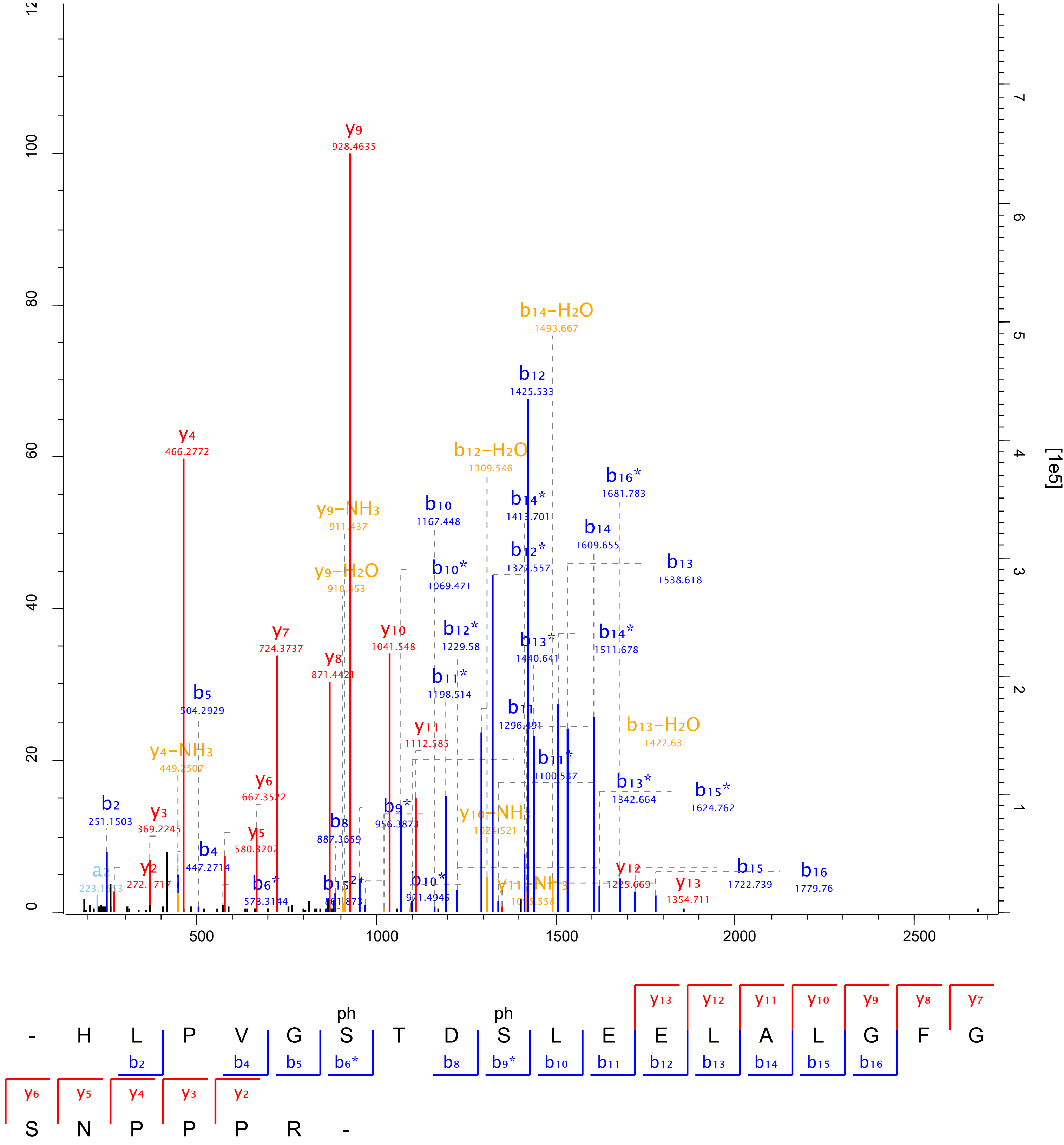

Raw file	Scan	Method	Score	m/z	Gene names
0523_4	31260	FTMS; HCD	108.23	1082.49	ASY2

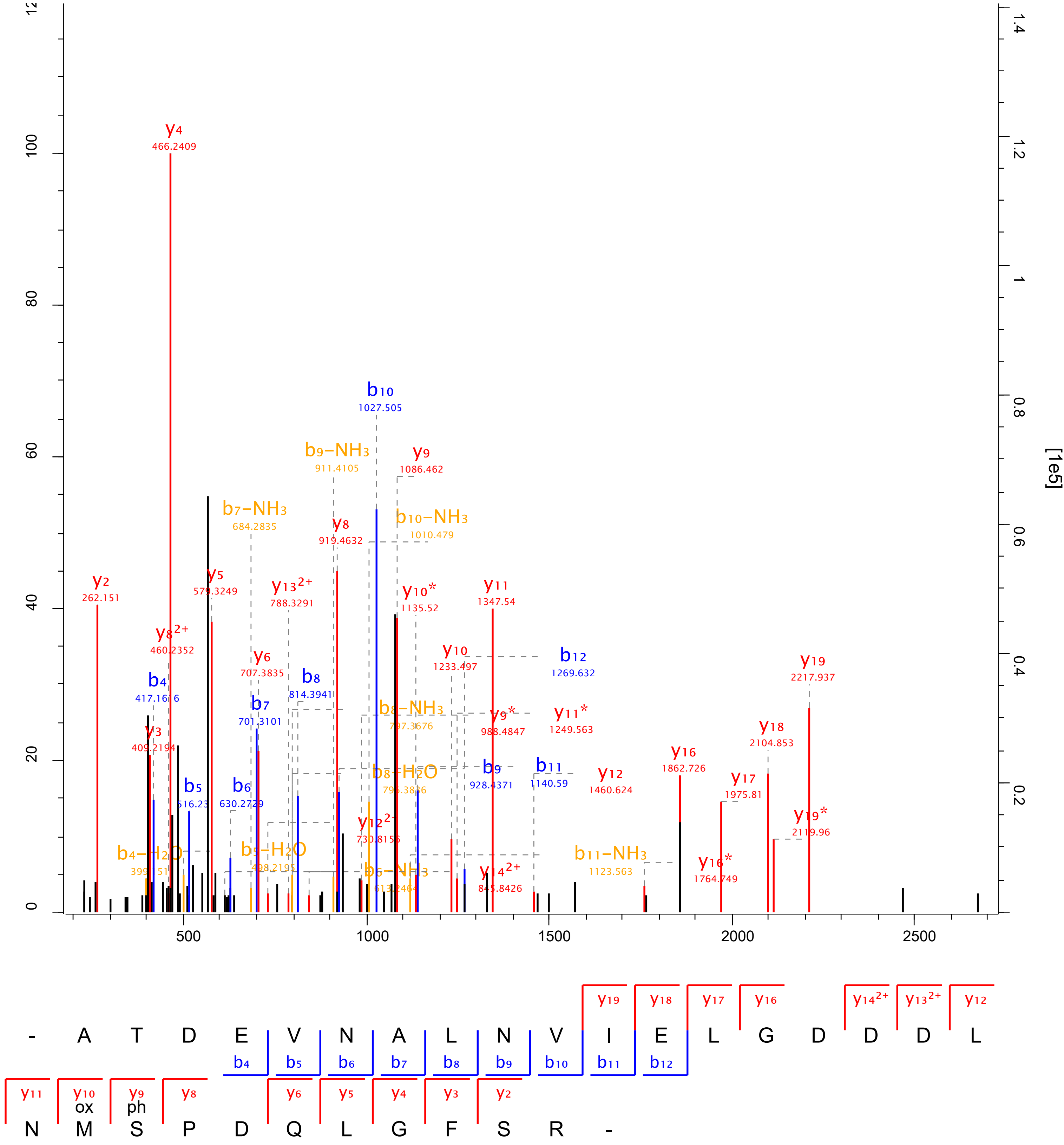

Sample output to test PDF Combine only

Raw file	Scan	Method	Score	m/z	Gene names
0523_4	31537	FTMS; HCD	187.45	912.42	RH24

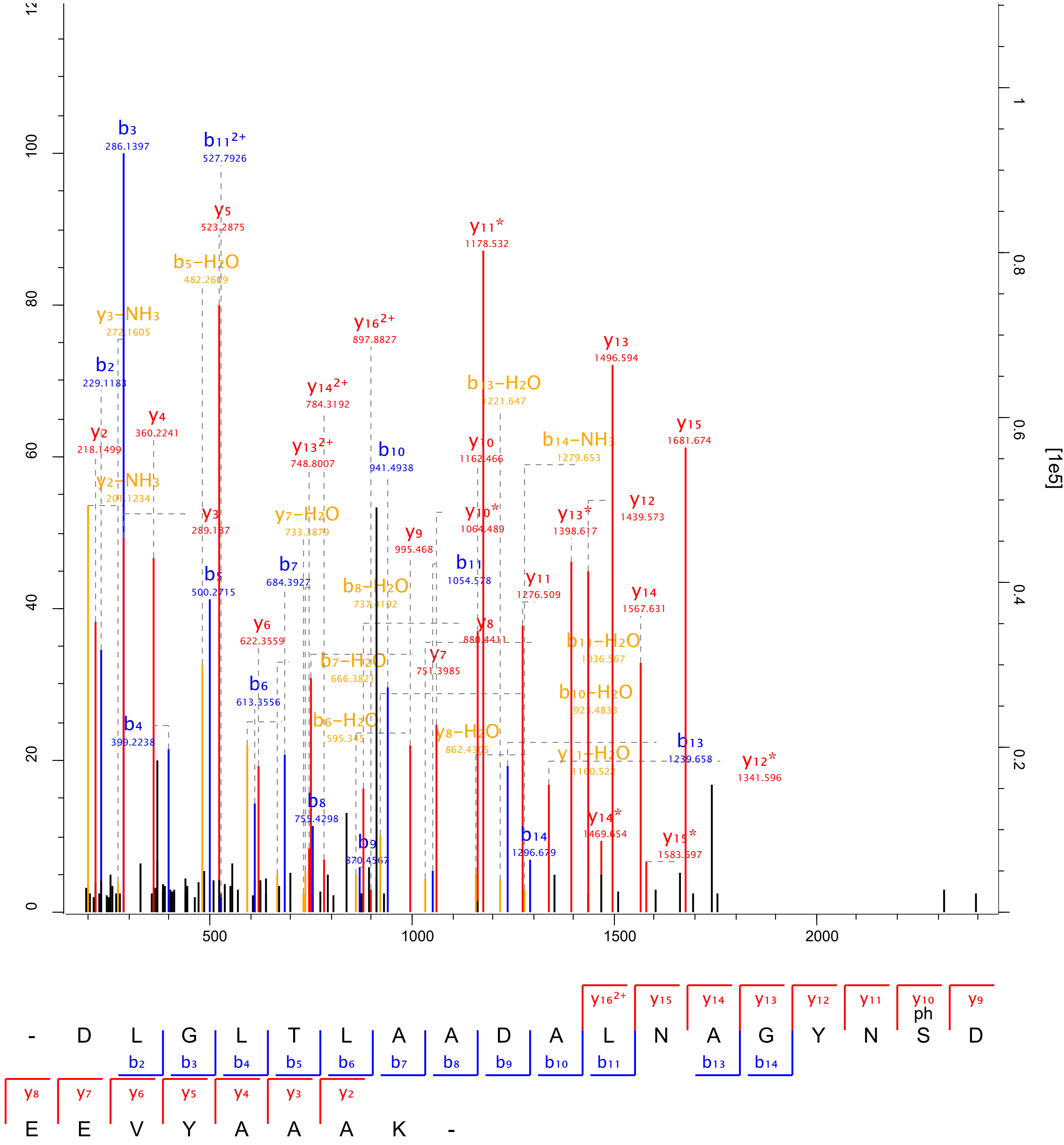

Sample output to test PDF Combine only

Raw file	Scan	Method	Score	m/z	Gene names
0523_4	31809	FTMS; HCD	134.34	1110.13	F23N20.8

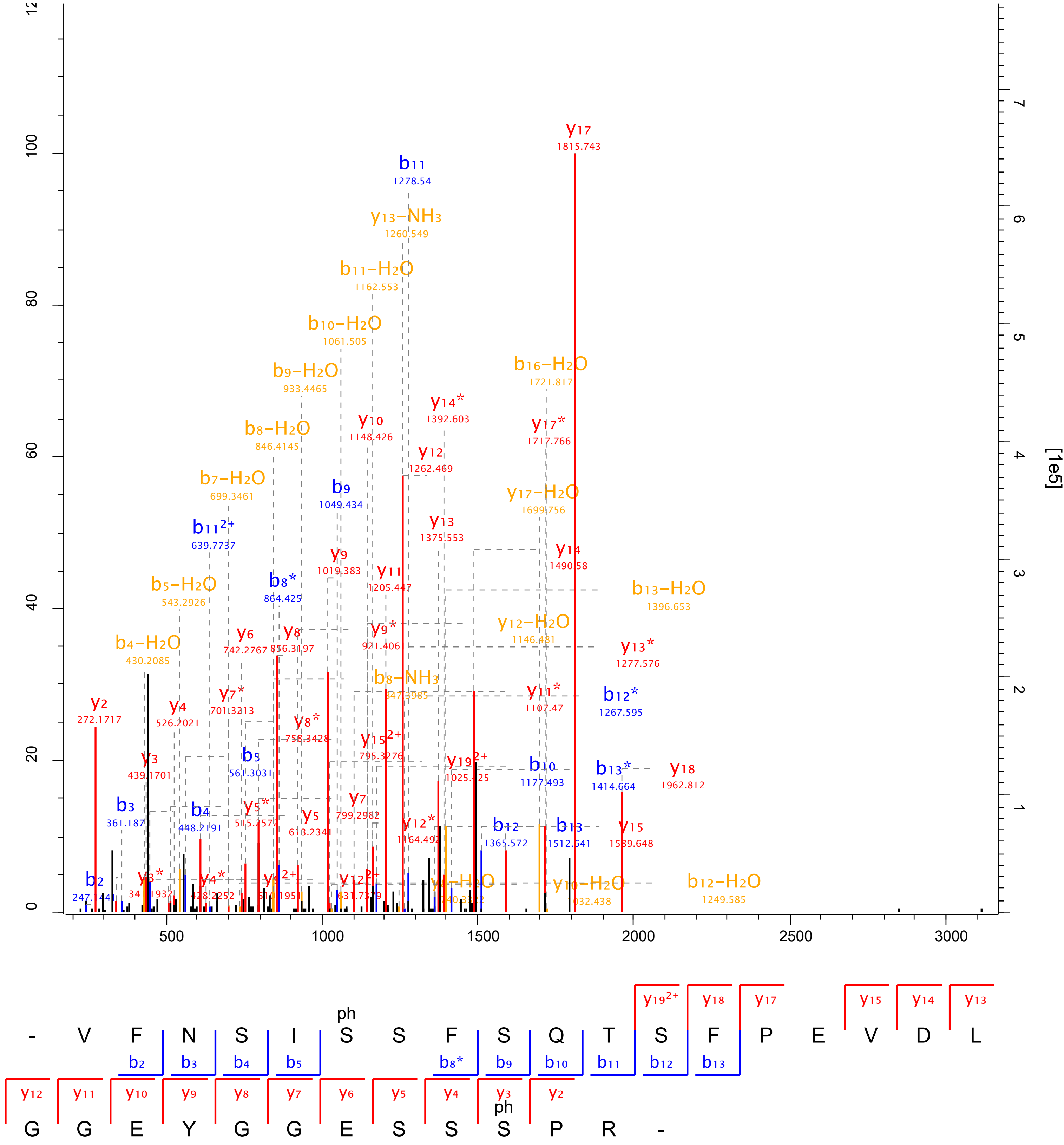

Sample output to test PDF Combine only

Raw file	Scan	Method	Score	m/z	Gene names
0523_4	32176	FTMS; HCD	313.58	849.34	PIP2-4

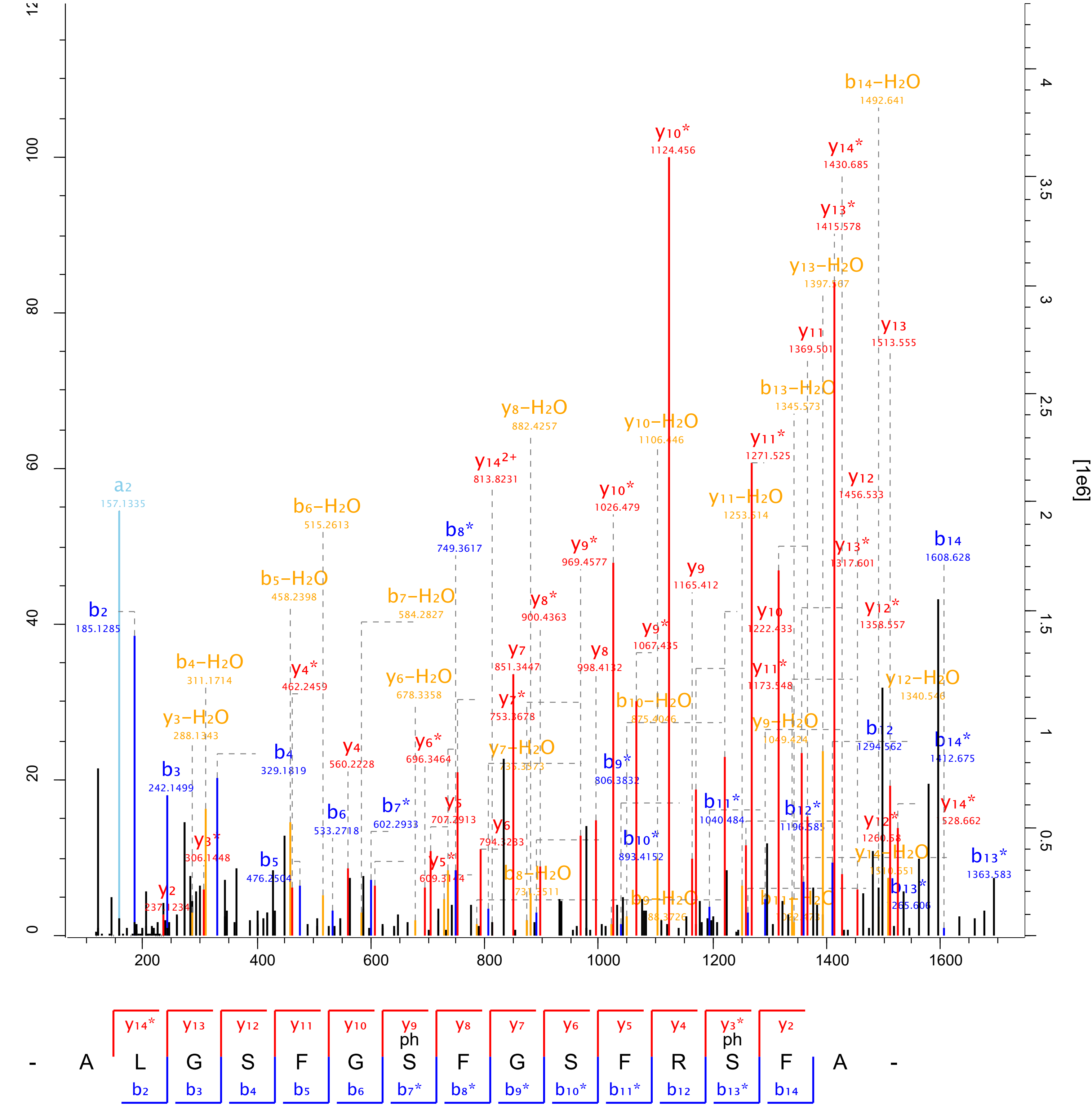

Sample output to test PDF Combine only

Raw file	Scan	Method	Score	m/z	Gene names
0523_4	32292	FTMS; HCD	73.67	971.97	SEC10;AT5G12370;SEC10b

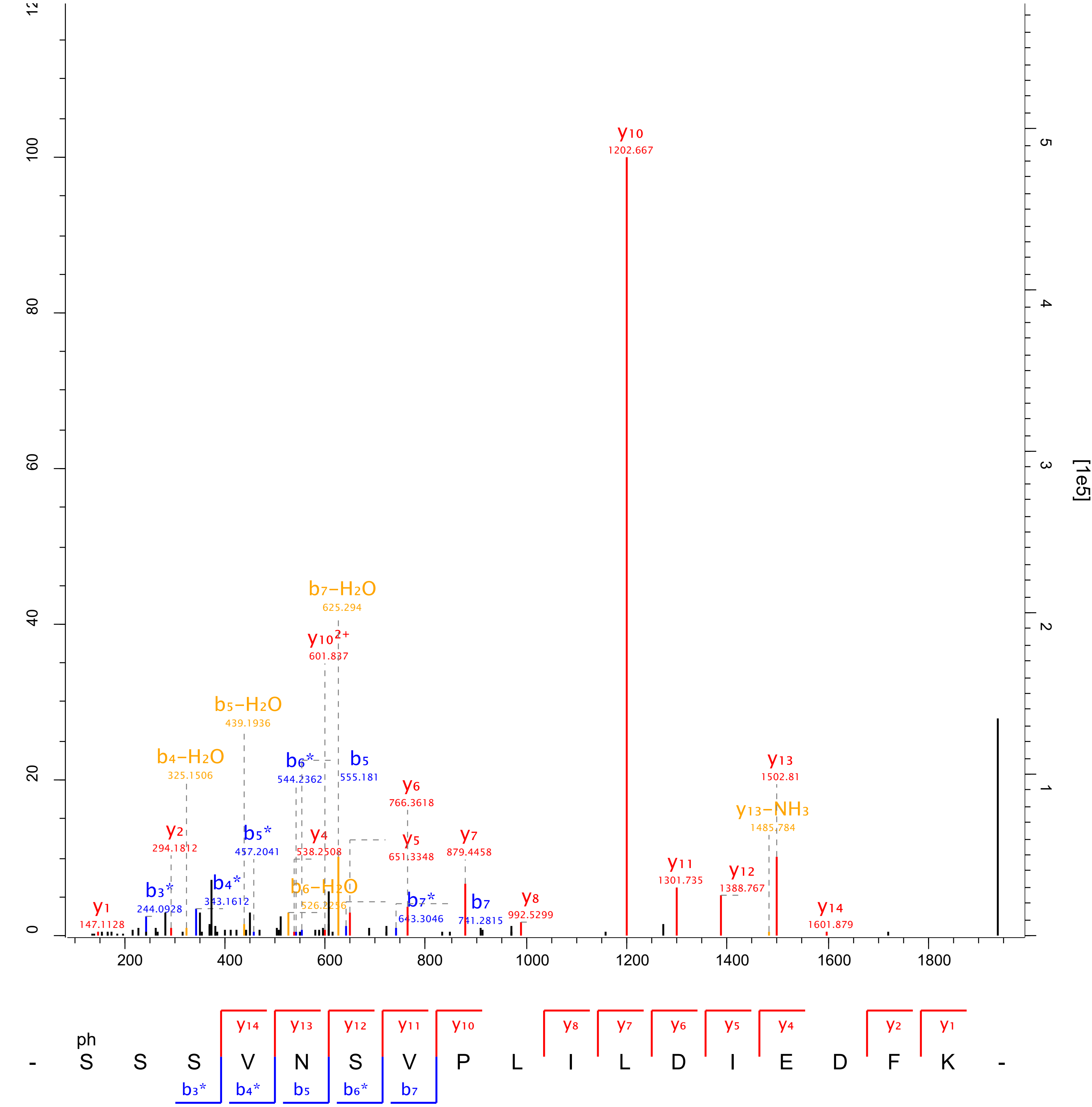

Sample output to test PDF Combine only

Raw file	Scan	Method	Score	m/z	Gene names
0523_4	32465	FTMS; HCD	359.58	1072.47	BAG7

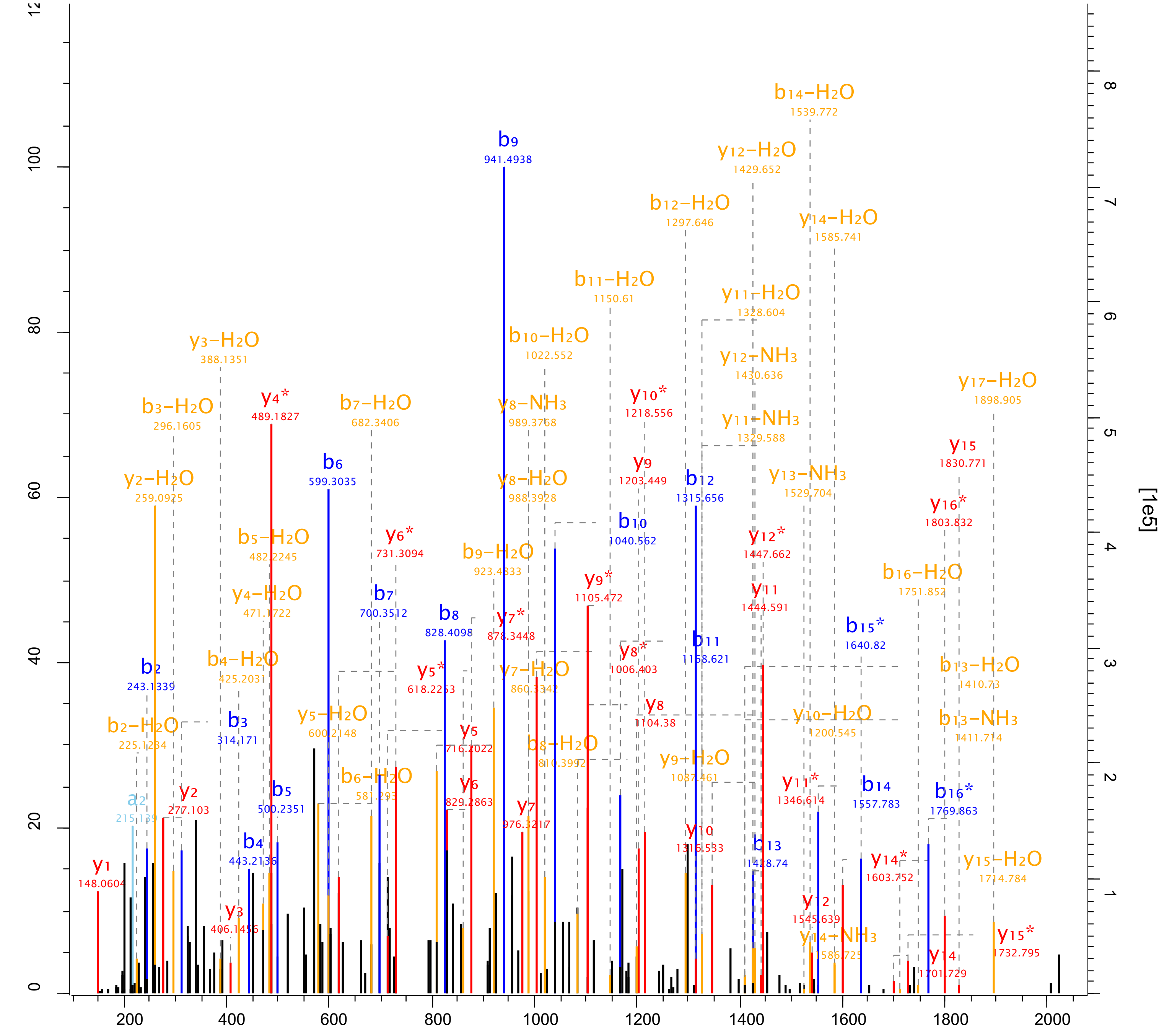

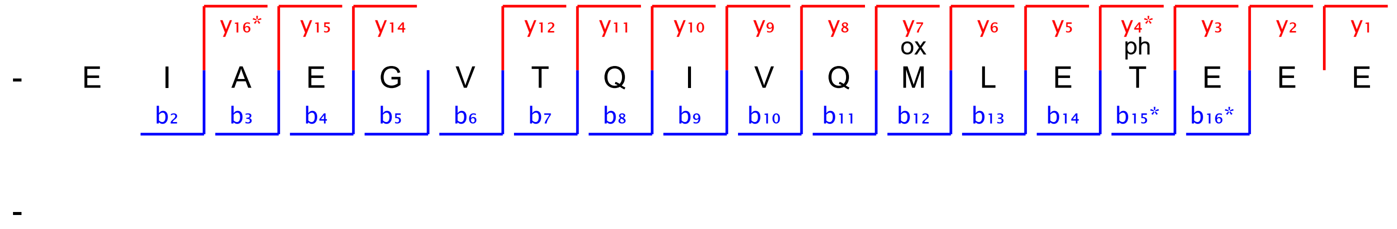

Sample output to test PDF Combine only

Raw file	Scan	Method	Score	m/z	Gene names
0523_4	32729	FTMS; HCD	79.21	1262.62	RPS12C

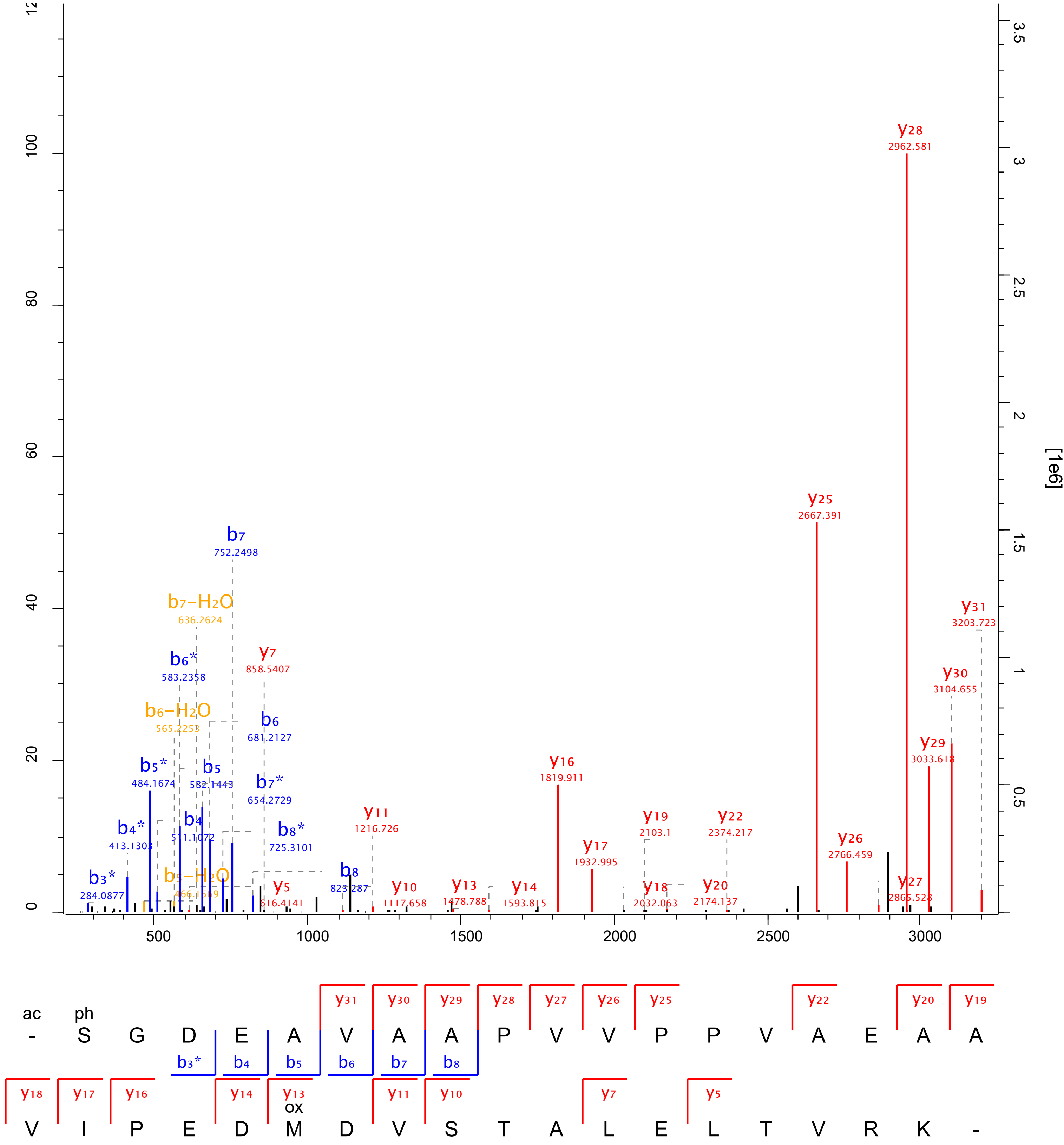

Sample output to test PDF Combine only

Raw file	Scan	Method	Score	m/z	Gene names
0523_4	33149	FTMS; HCD	71.21	1004.17	GSL12;CALS3

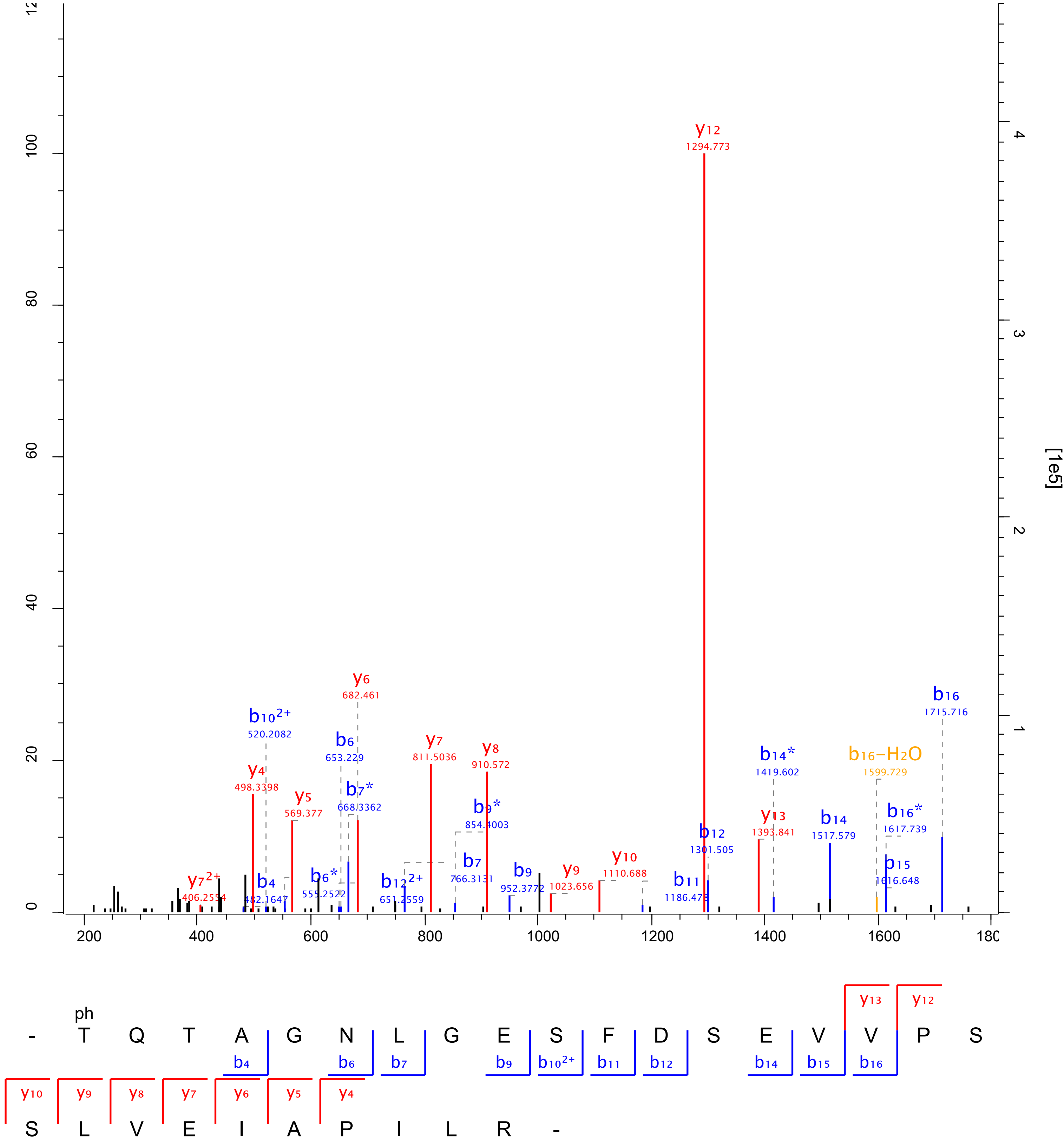

Sample output to test PDF Combine only

Raw file	Scan	Method	Score	m/z	Gene names
0523_4	33361	FTMS; HCD	124.88	1229.84	Y14

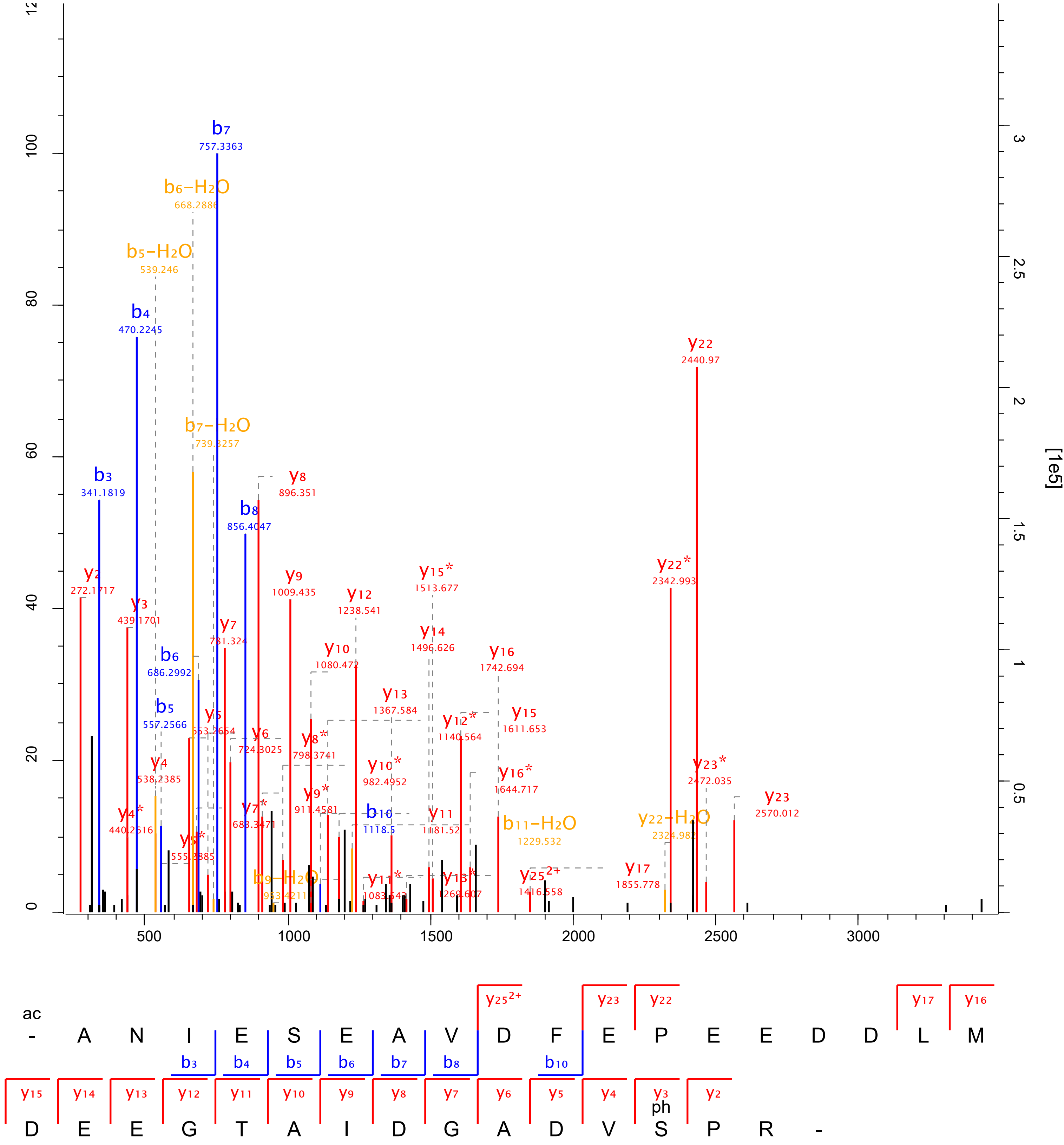

Sample output to test PDF Combine only

Raw file	Scan	Method	Score	m/z	Gene names
0523_4	33419	FTMS; HCD	119.88	975.23	RPS12A

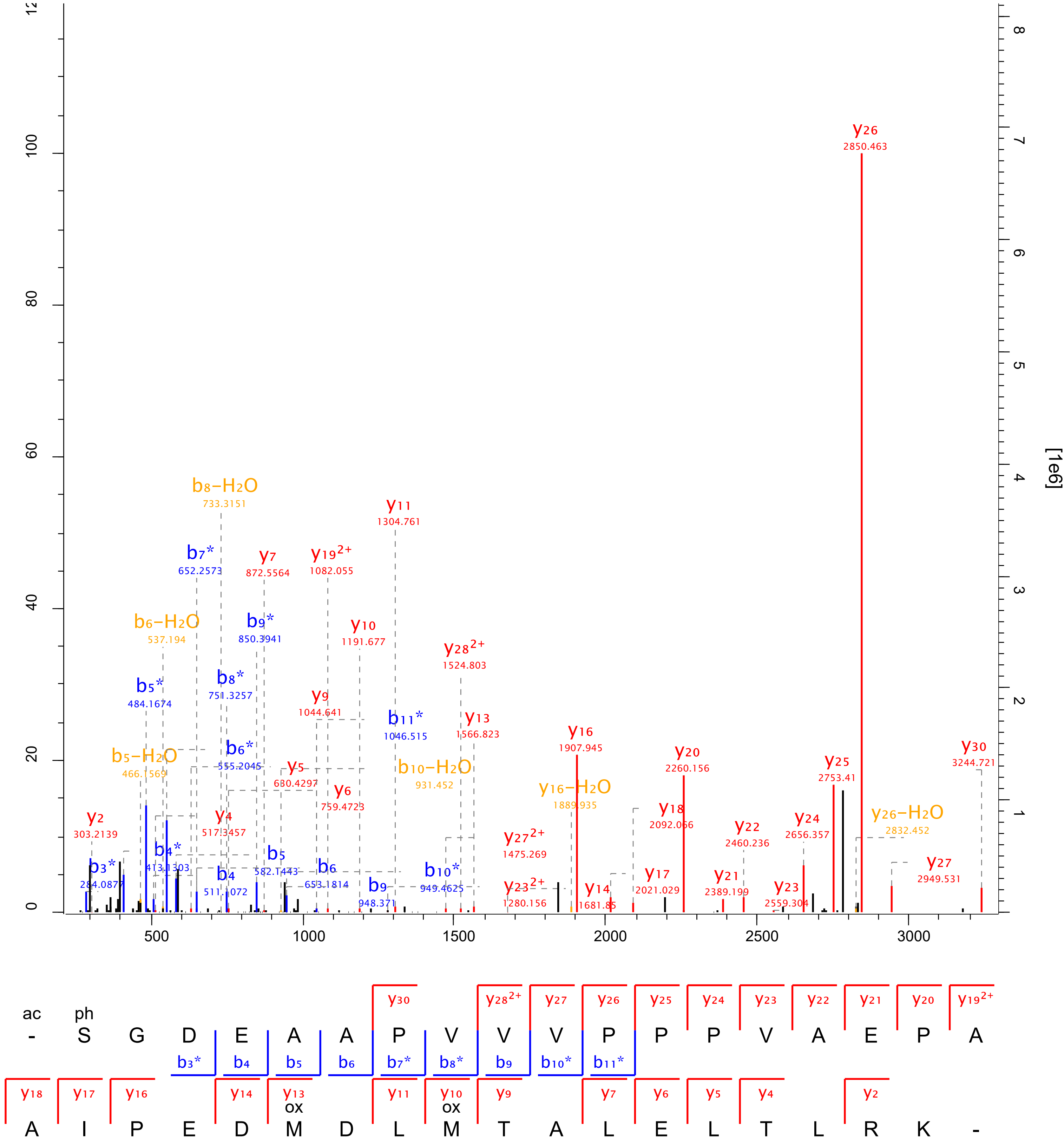

Sample output to test PDF Combine only

Raw file	Scan	Method	Score	m/z	Gene names
0523_4	34356	FTMS; HCD	60.34	1131	PVA13

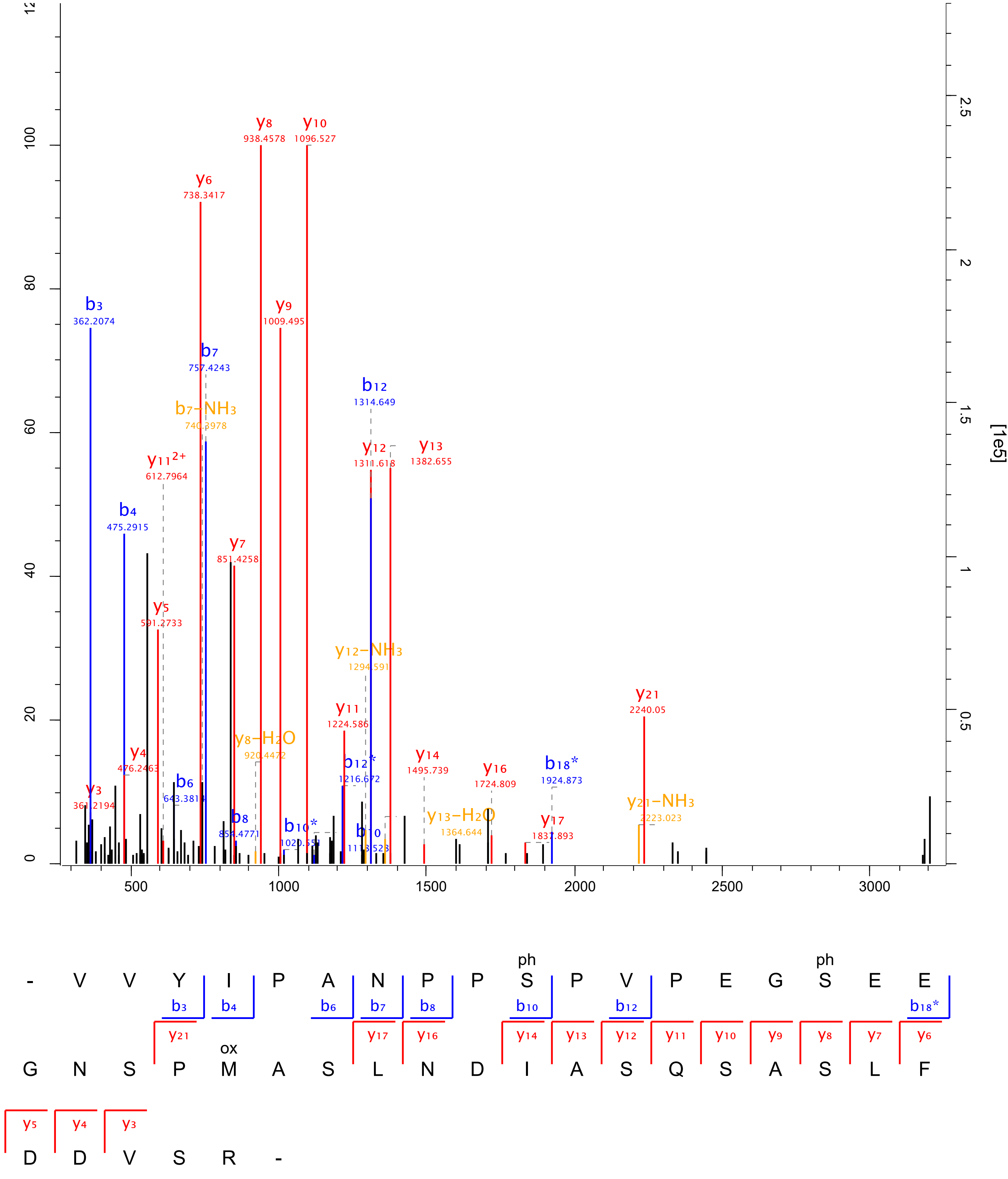

Sample output to test PDF Combine only

Raw file	Scan	Method	Score	m/z	Gene names
0523_4	35334	FTMS; HCD	69.52	1219.59	RPS12C

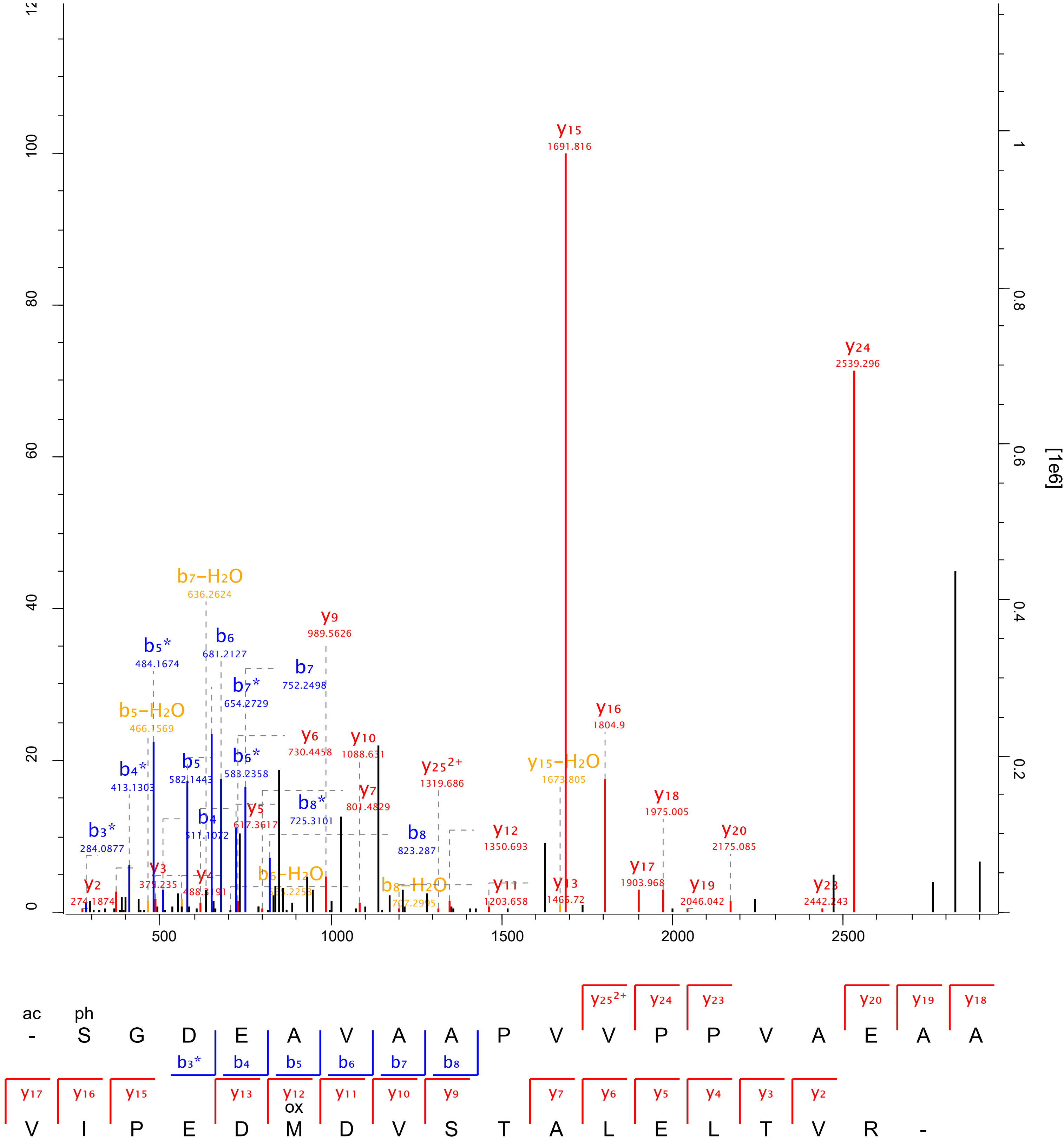

Raw file	Scan	Method	Score	m/z	Gene names
0523_5	1430	FTMS; HCD	96.38	515.22	At4g39680

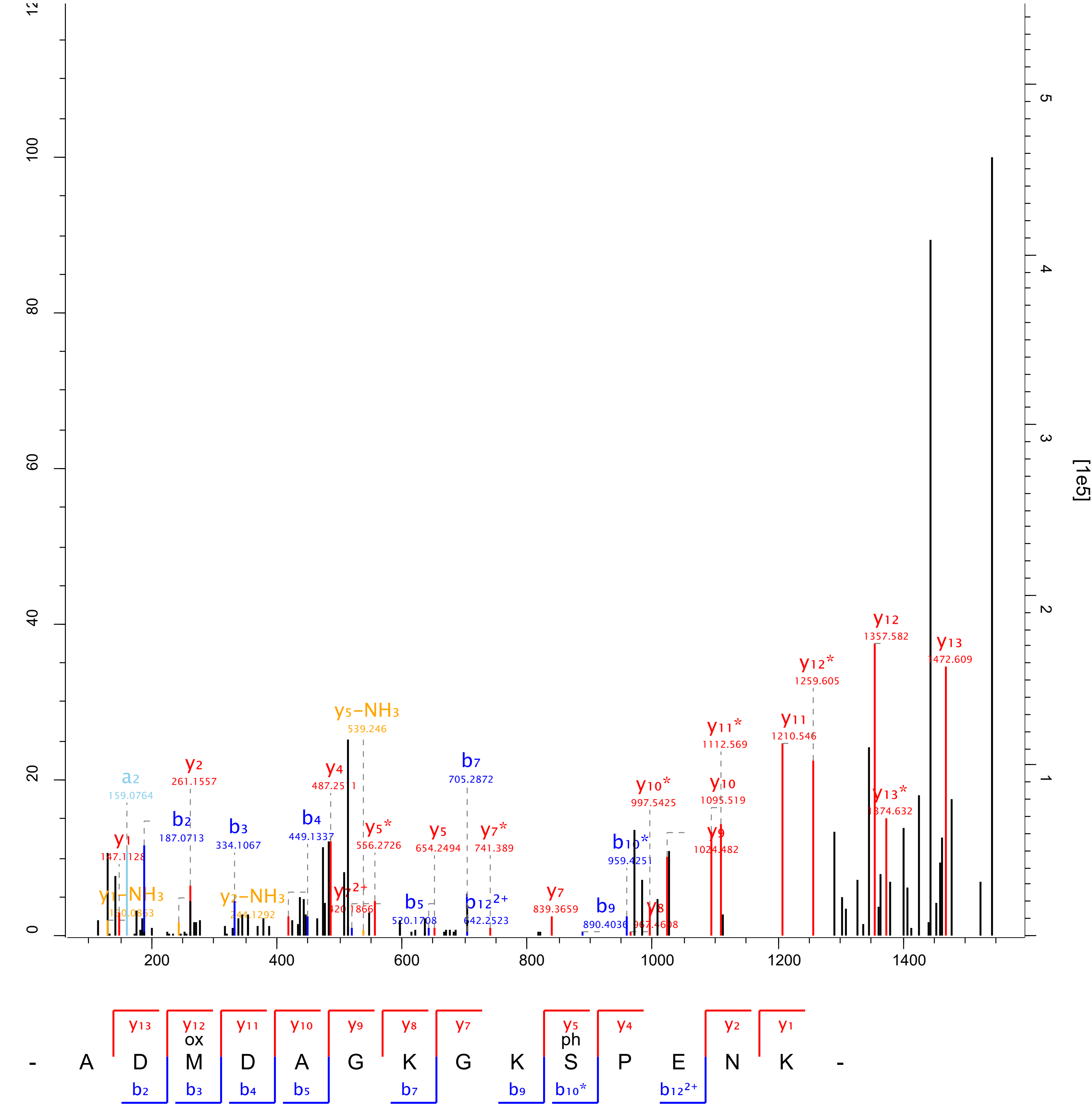

Sample output to test PDF Combine only

Raw file	Scan	Method	Score	m/z	Gene names
0523_5	1439	FTMS; HCD	97.73	334.83	RS41

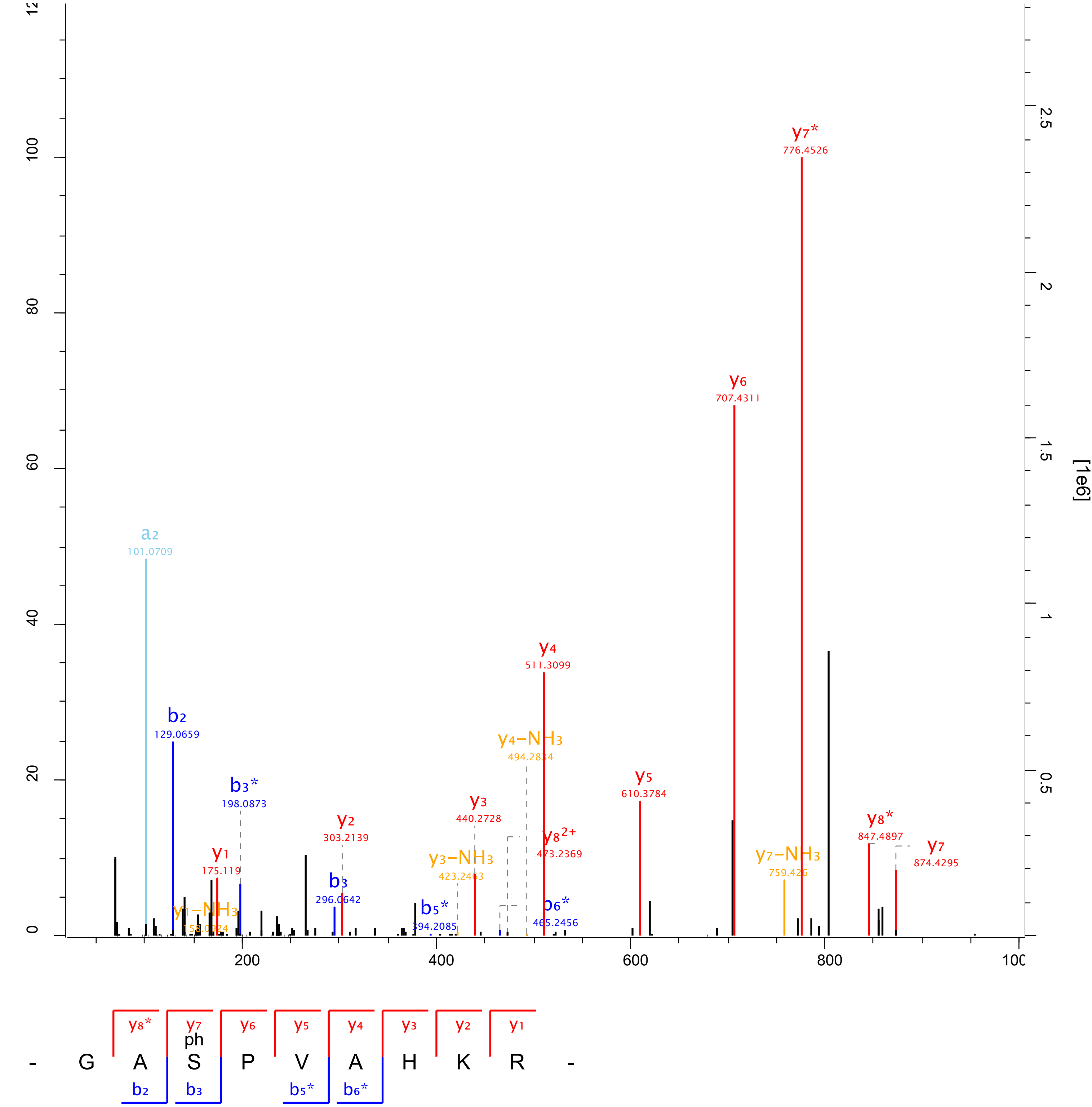

Sample output to test PDF Combine only

Raw file	Scan	Method	Score	m/z	Gene names
0523_5	1440	FTMS; HCD	55.89	359.52	SR45

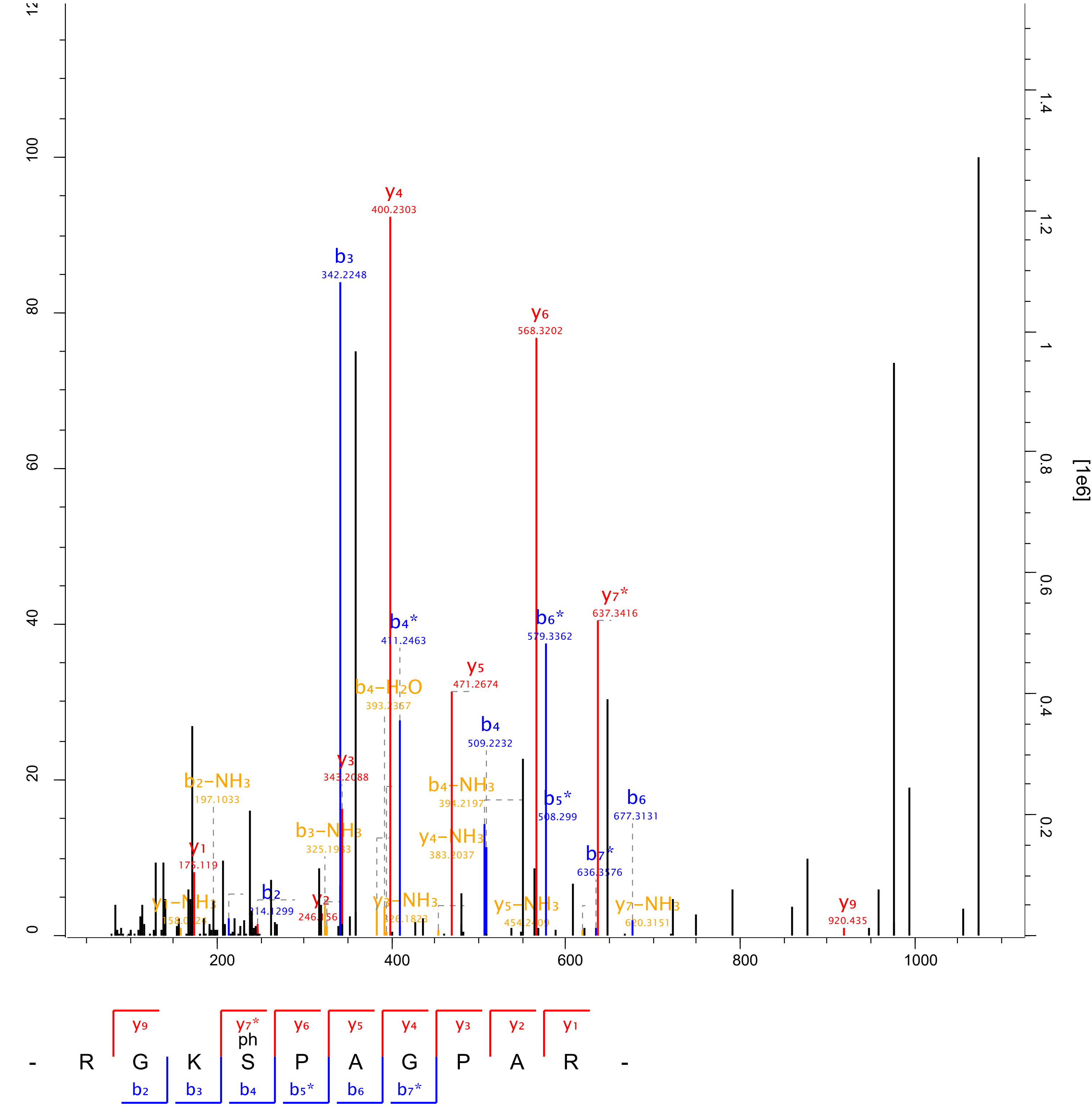

Sample output to test PDF Combine only

Raw file	Scan	Method	Score	m/z	Gene names
0523_5	1453	FTMS; HCD	80.31	408.49	CPK13

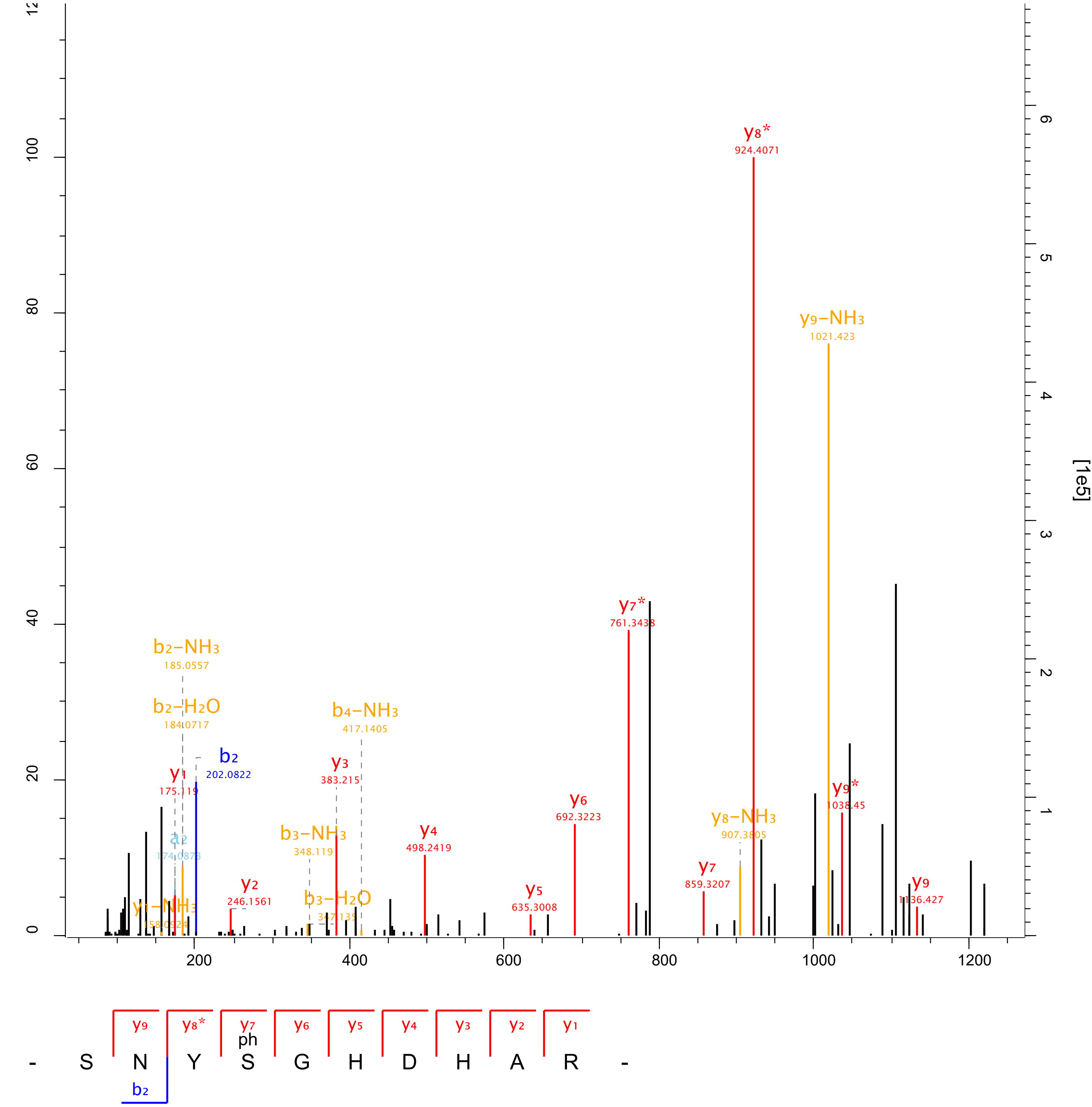

Sample output to test PDF Combine only

Raw file	Scan	Method	Score	m/z	Gene names
0523_5	1460	FTMS; HCD	43.9	364.2	At2g23520

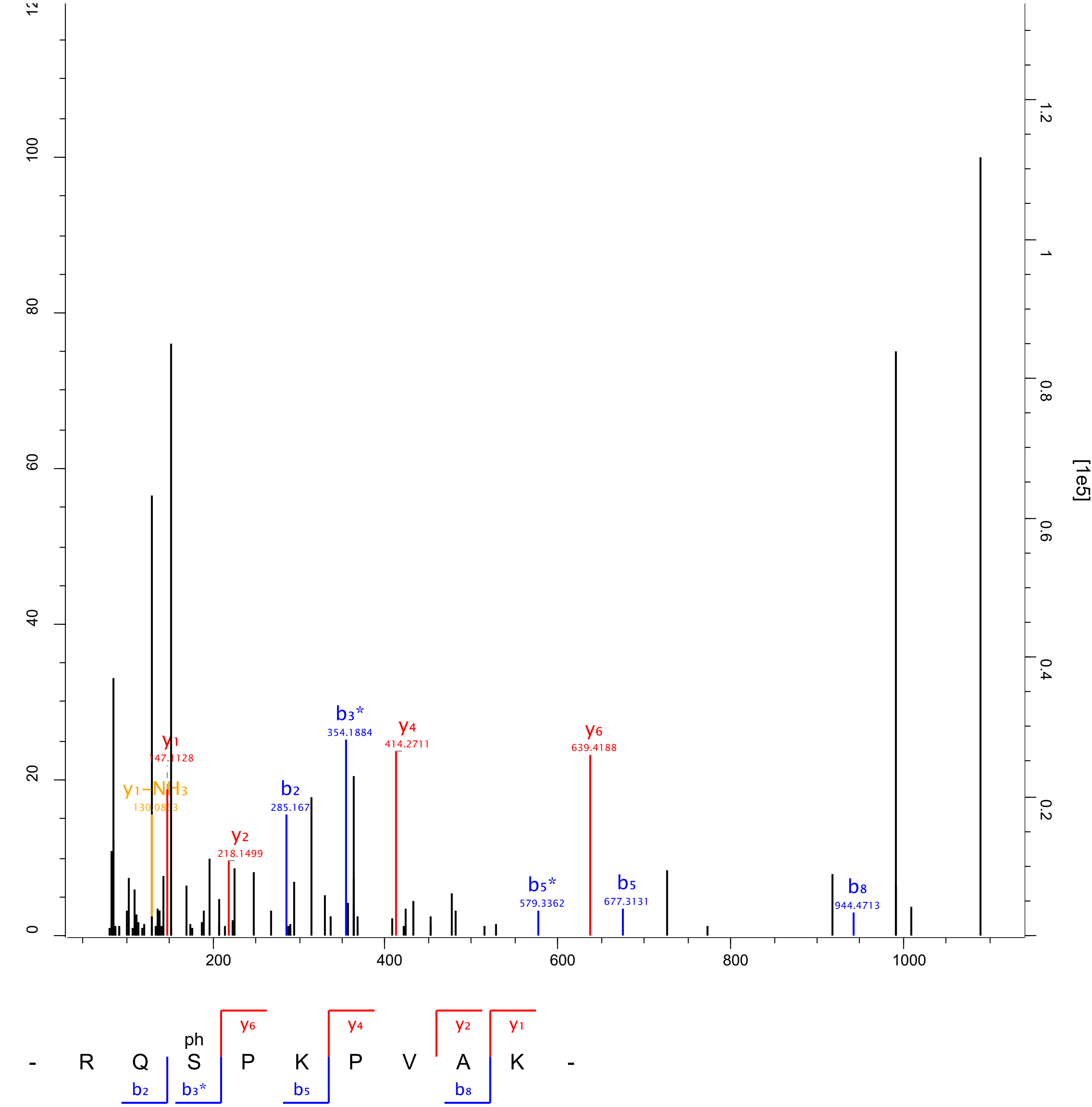

Sample output to test PDF Combine only

Raw file	Scan	Method	Score	m/z	Gene names
0523_5	1466	FTMS; HCD	73.39	479.72	ABCB4

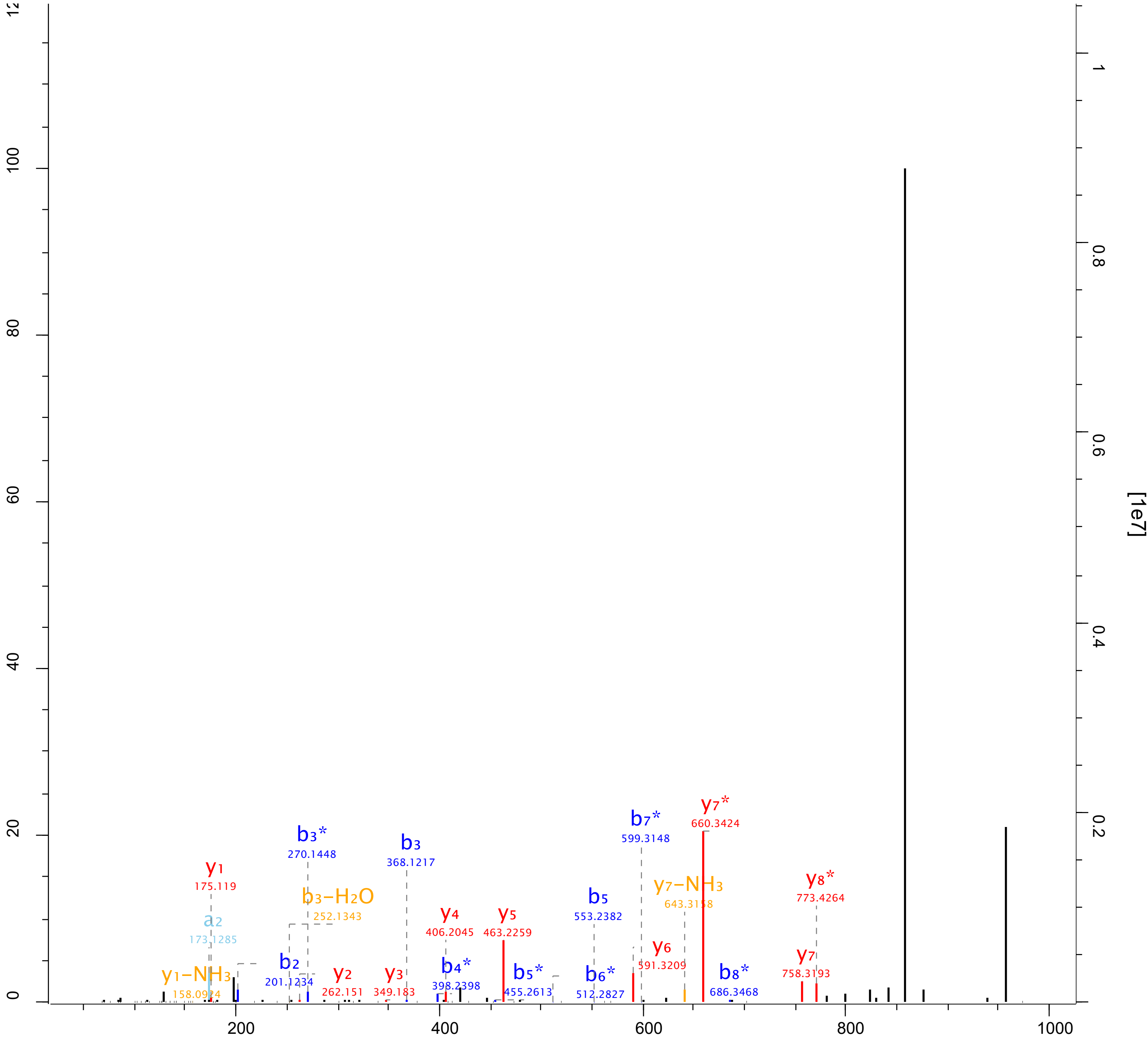

- S L S K G G S S R -

y8* y7 y6 y5 y4 y3 y2 y1

ph

b2 b3 b4* b5 b6* b7* b8*

Sample output to test PDF Combine only

Raw file	Scan	Method	Score	m/z
0523_5	1480	FTMS; HCD	97.73	411.71

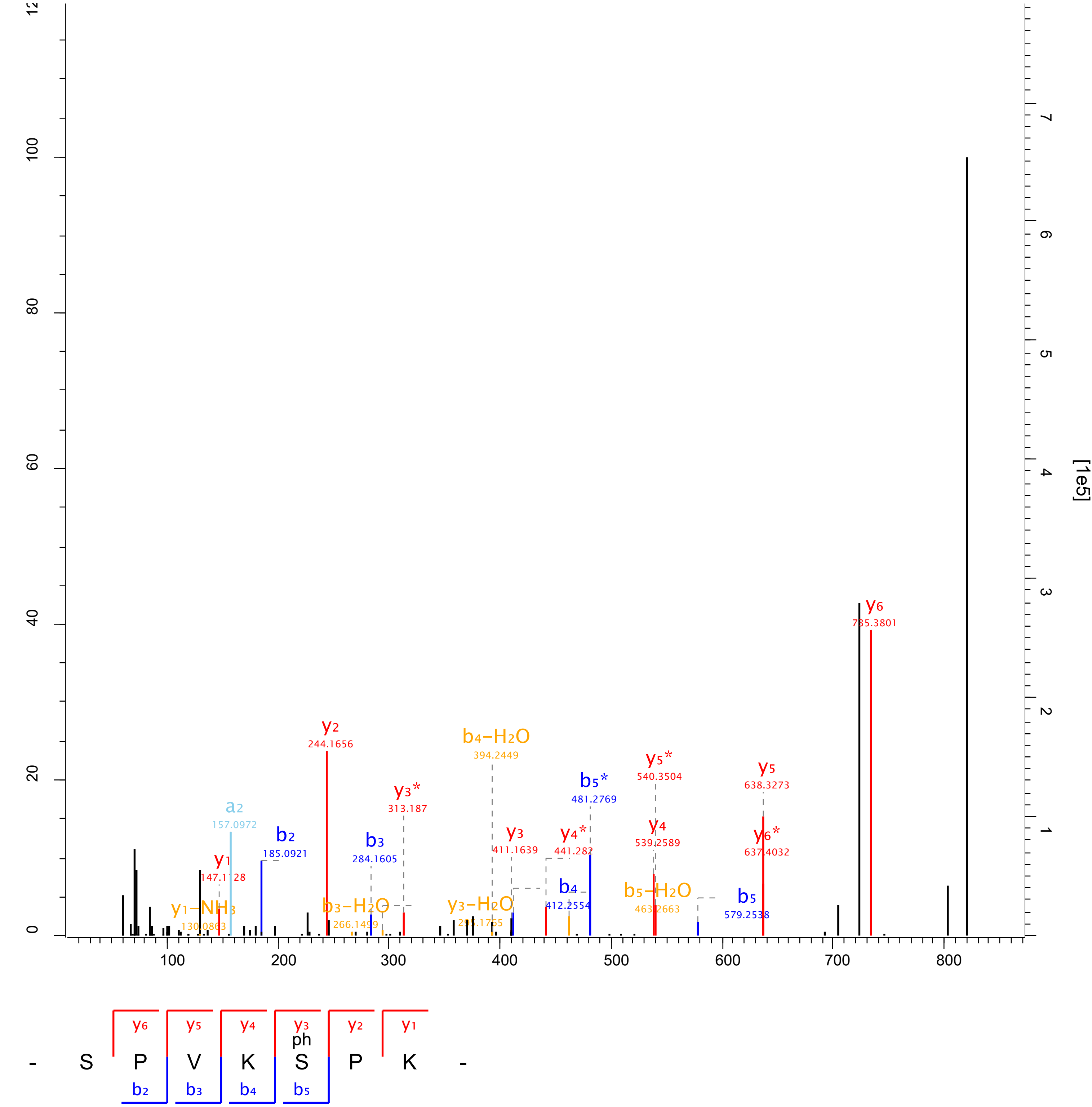

Sample output to test PDF Combine only

Raw file	Scan	Method	Score	m/z	Gene names
0523_5	1491	FTMS; HCD	75.22	549.88	U2AF35A;ATU2AF35A

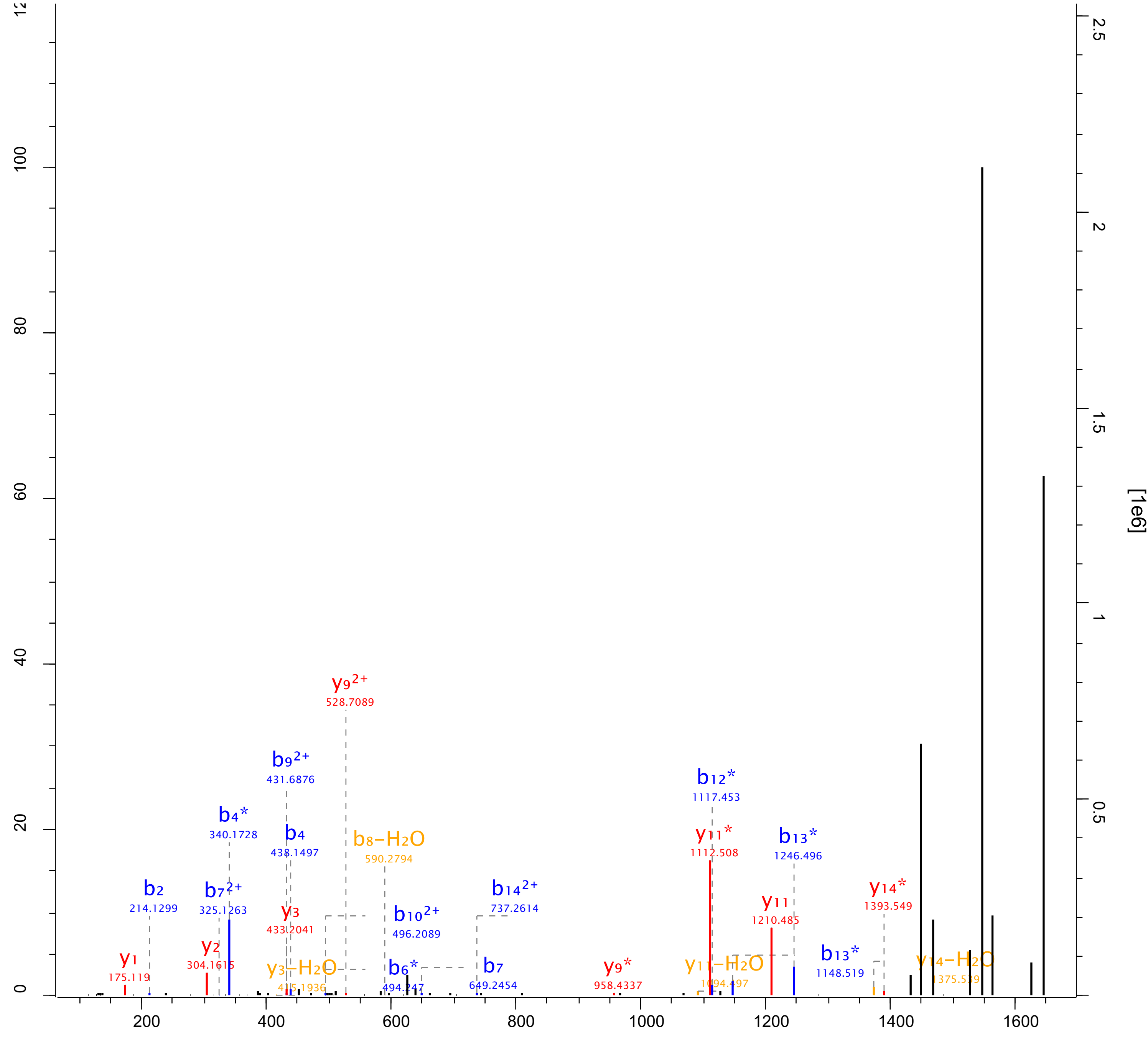

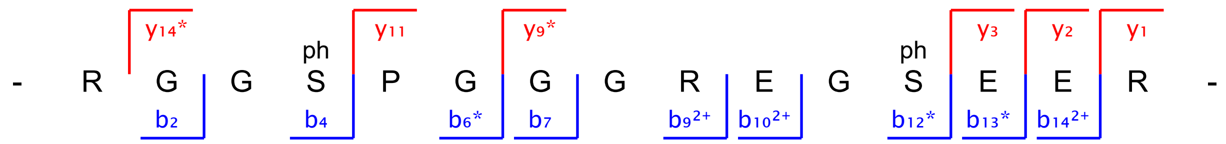

Sample output to test PDF Combine only

Raw file	Scan	Method	Score	m/z	Gene names
0523_5	1495	FTMS; HCD	50.81	408.52	At1g70180;F20P5.10

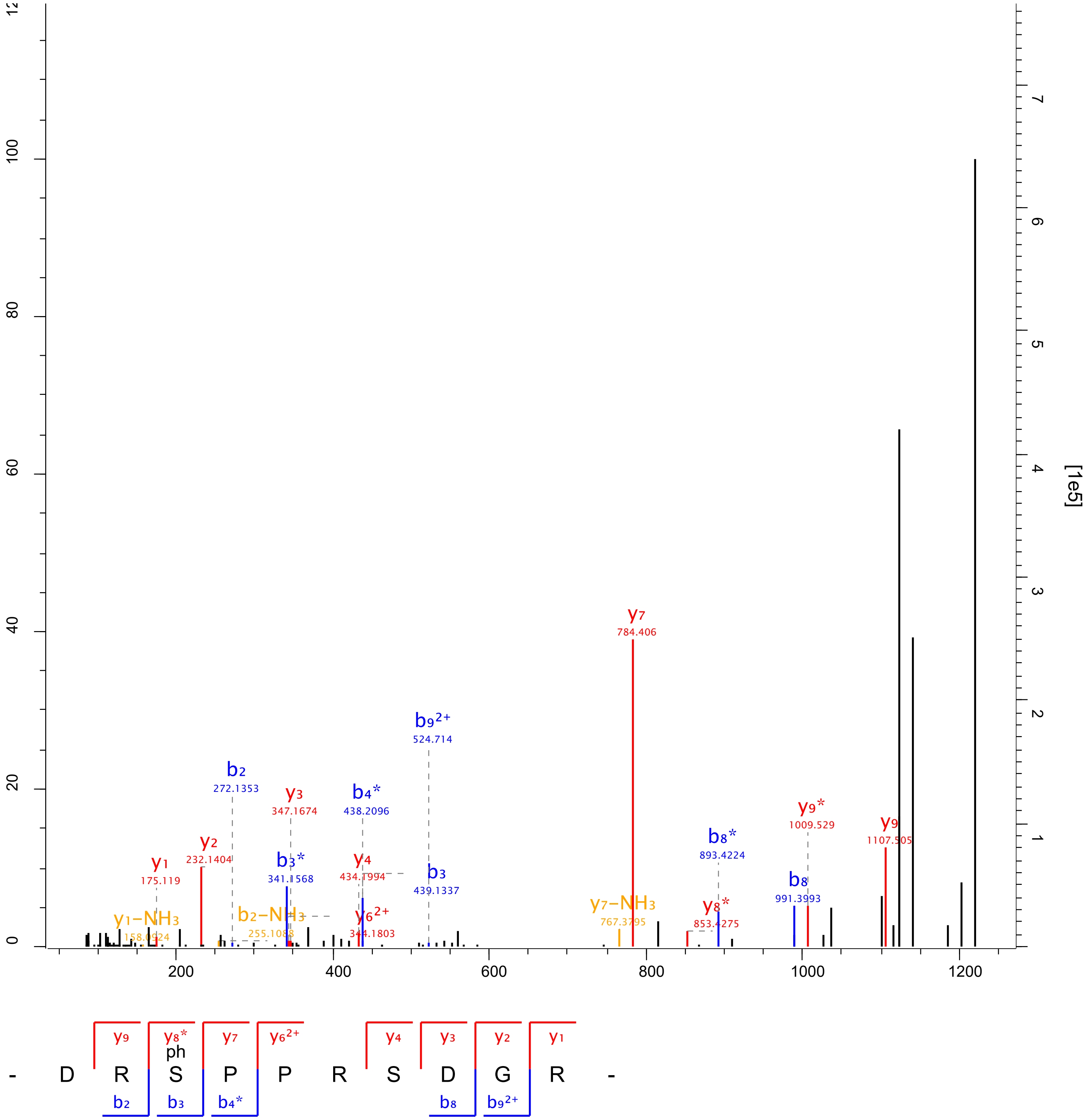

Sample output to test PDF Combine only

Raw file	Scan	Method	Score	m/z	Gene names
0523_5	1558	FTMS; HCD	123.5	415.18	M4E13.120;HIPP26

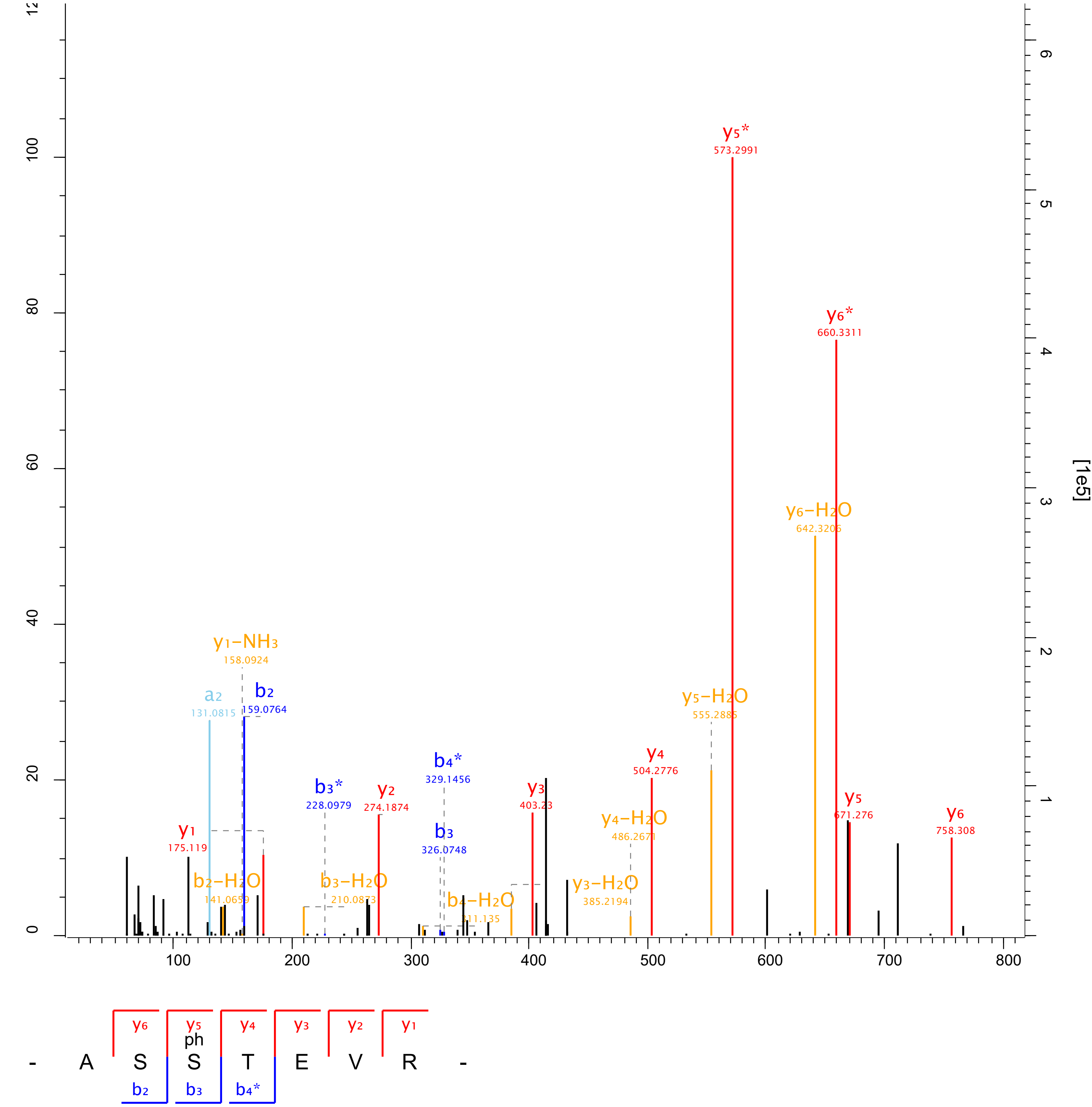

Sample output to test PDF Combine only

Raw file	Scan	Method	Score	m/z	Gene names
0523_5	1725	FTMS; HCD	48.44	353.49	RS31

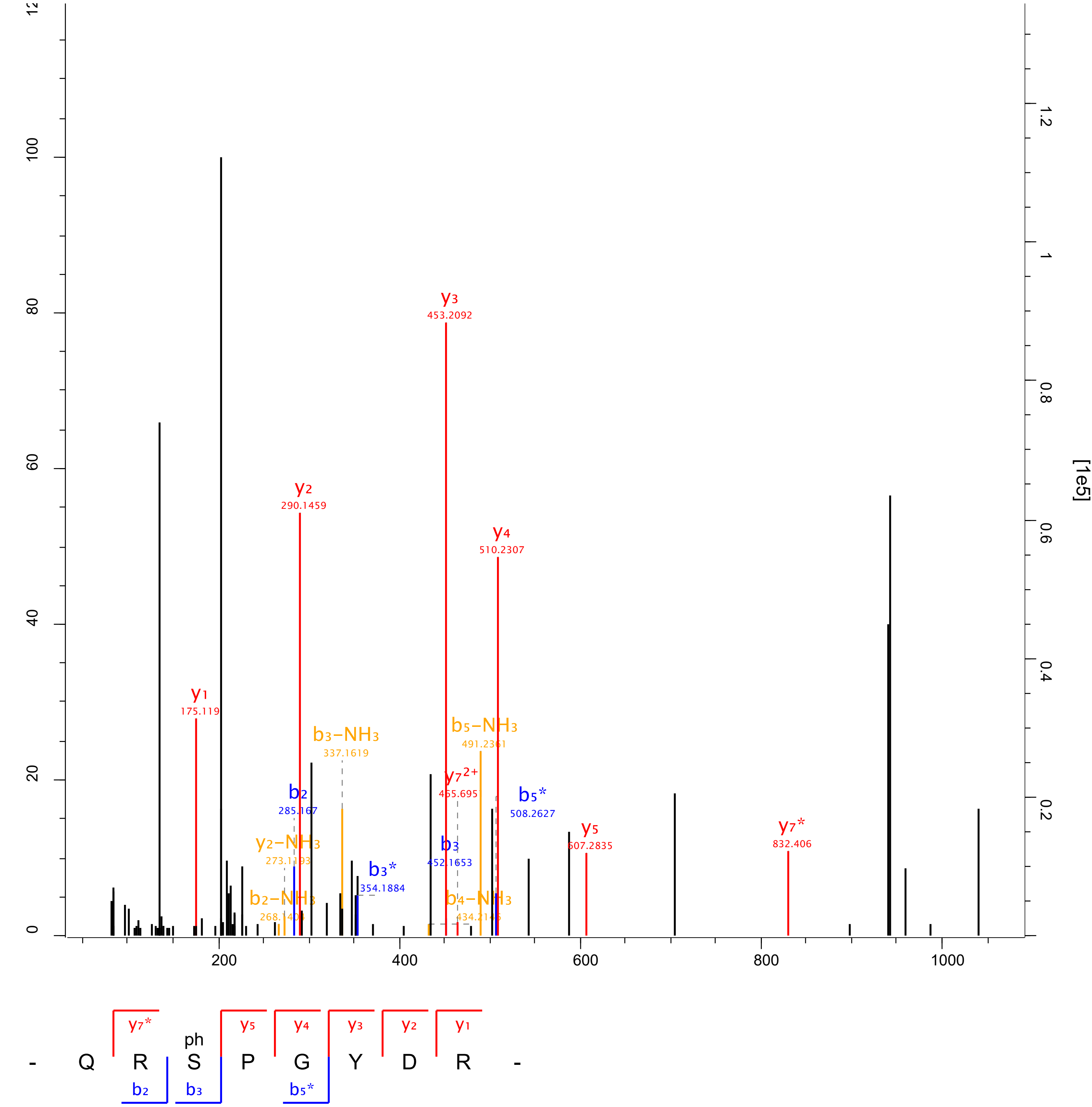

Sample output to test PDF Combine only

Raw file	Scan	Method	Score	m/z	Gene names
0523_5	2477	FTMS; HCD	72.29	500.21	KING1

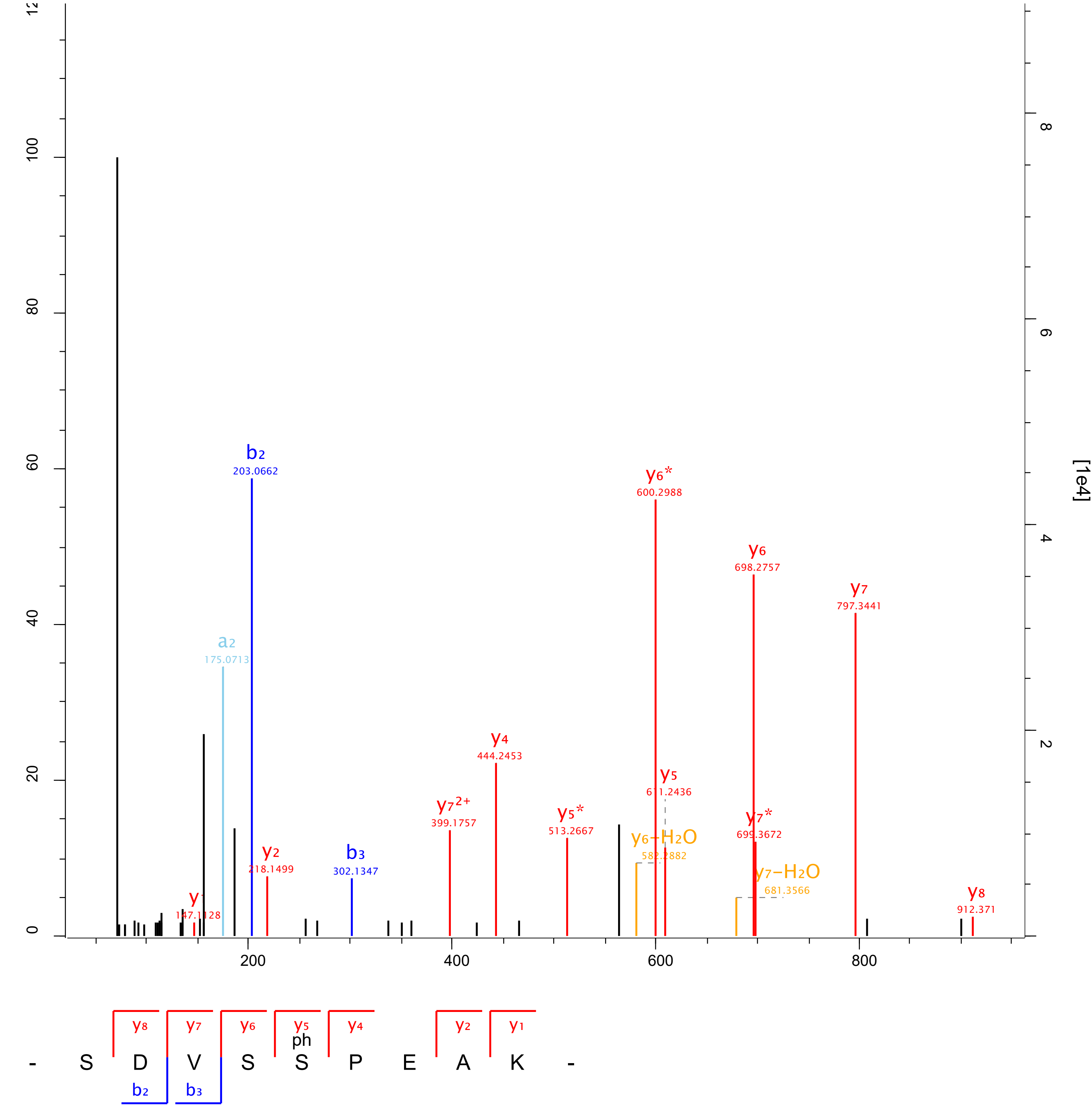

Sample output to test PDF Combine only

Raw file	Scan	Method	Score	m/z	Gene names
0523_5	14818	FTMS; HCD	79.78	692.05	ACA8

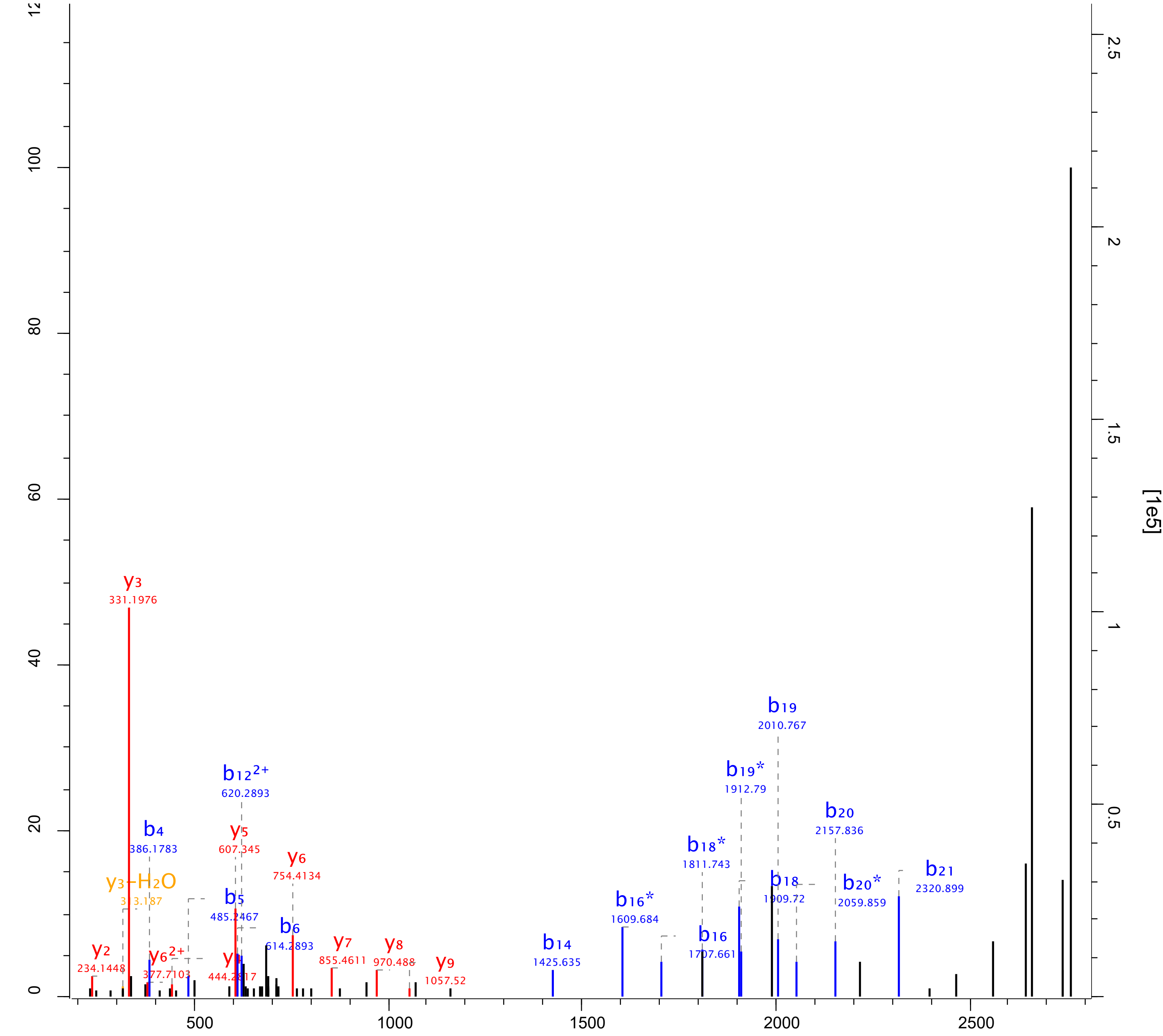

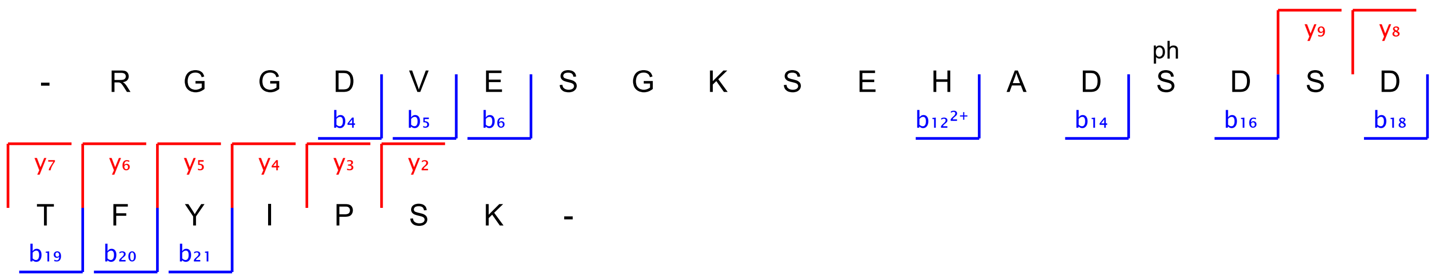

Sample output to test PDF Combine only

Raw file	Scan	Method	Score	m/z	Gene names
0523_5	15273	FTMS; HCD	360.58	829.71	EBP1

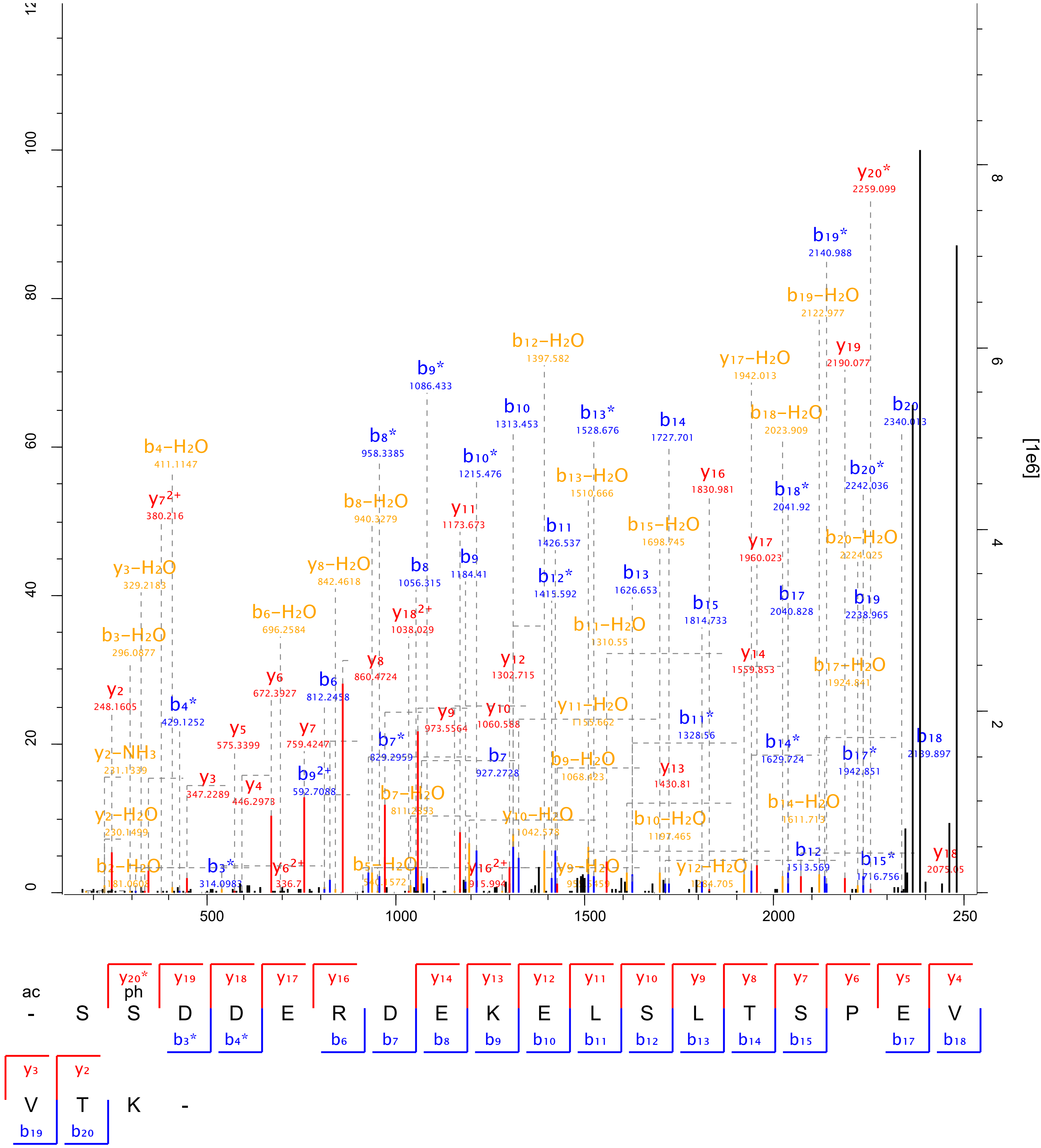

Sample output to test PDF Combine only

Raw file	Scan	Method	Score	m/z	Gene names
0523_5	15431	FTMS; HCD	61.54	868.42	CCC1

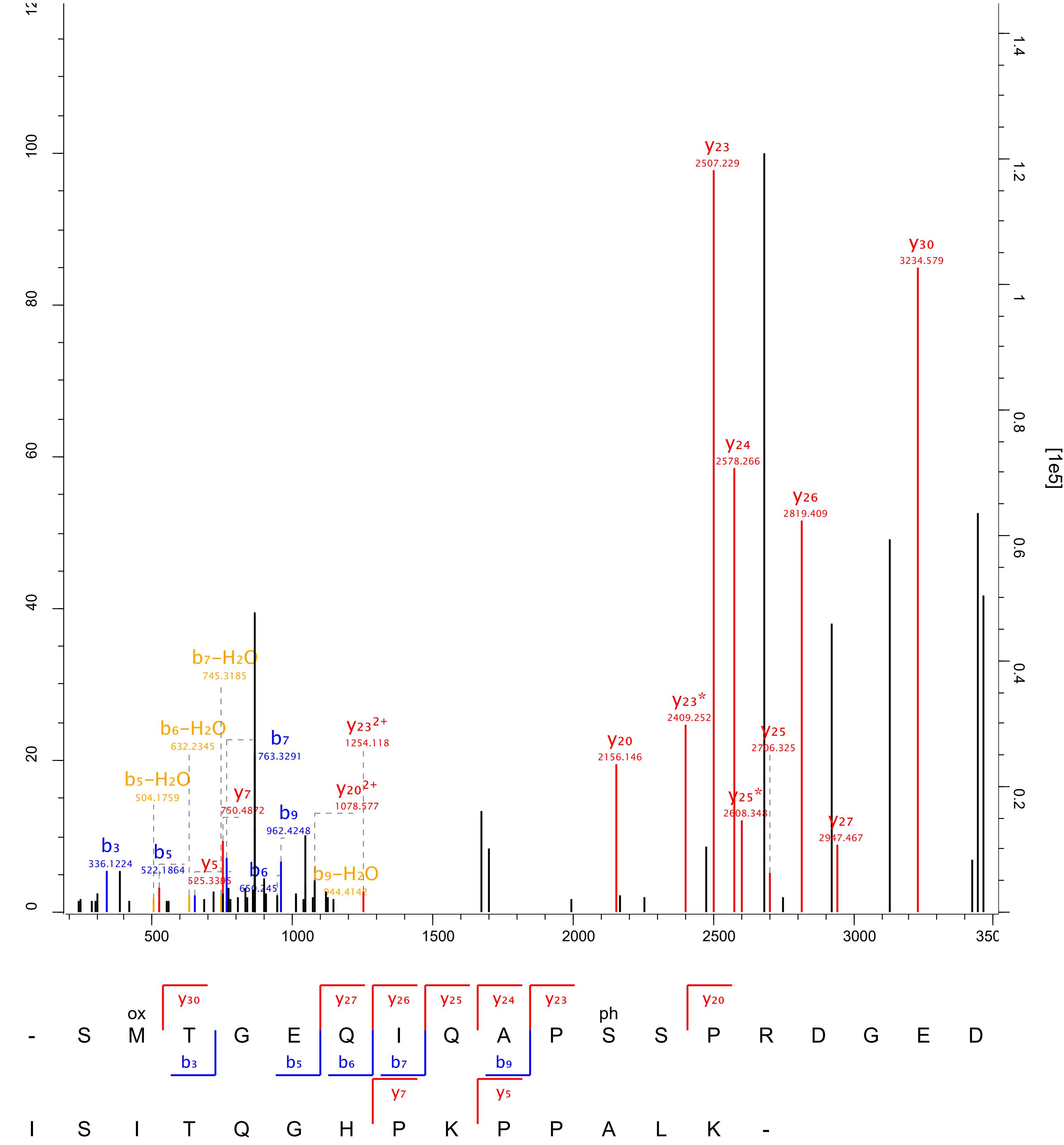

- S M(ox) T G E Q I Q A P S(ph) S P R D G E D

I S I T Q G H P K P P A L K -

Sample output to test PDF Combine only

Raw file	Scan	Method	Score	m/z
0523_5	15740	FTMS; HCD	75.54	737.35

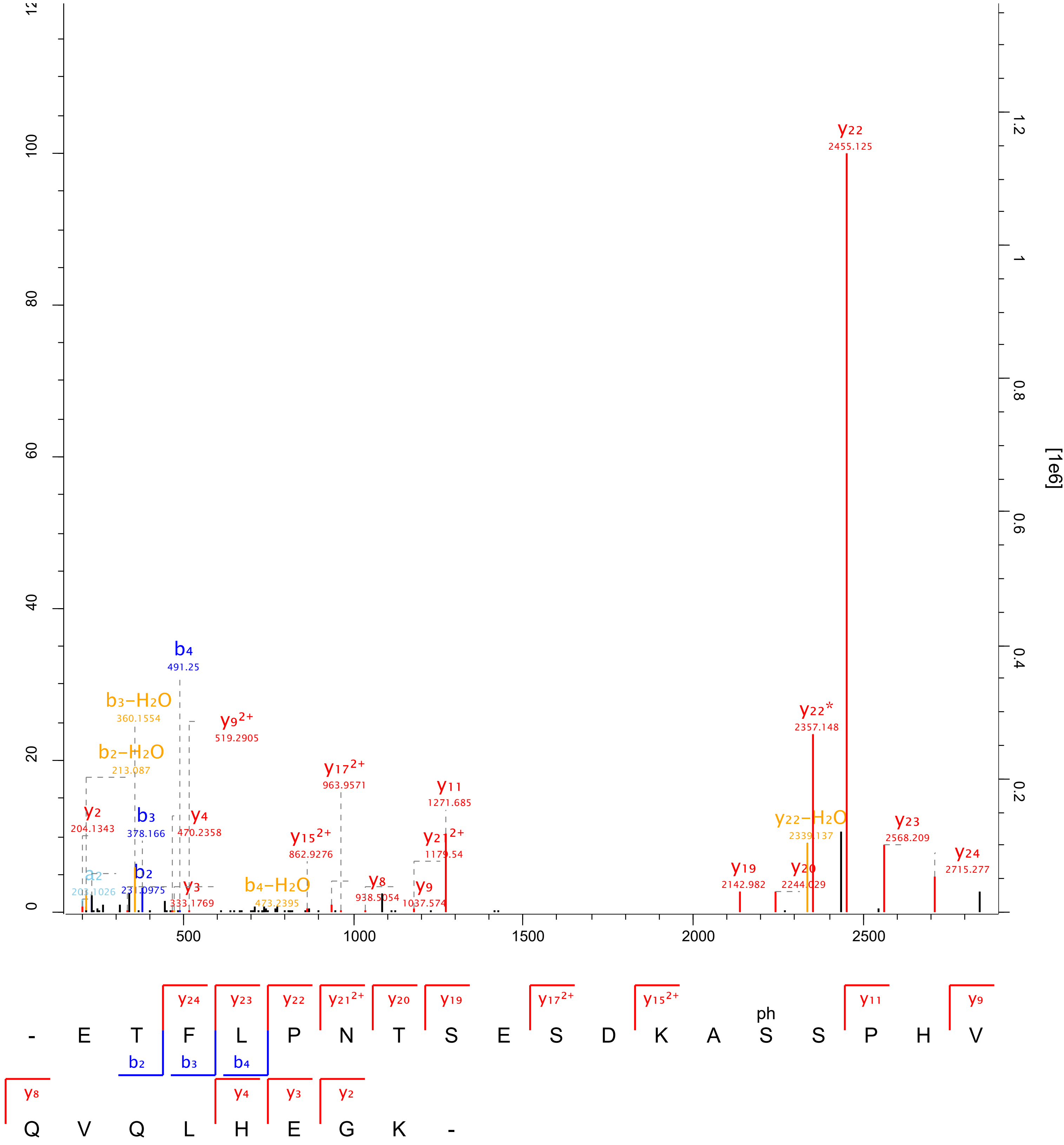

Sample output to test PDF Combine only

Raw file	Scan	Method	Score	m/z	Gene names
0523_5	16956	FTMS; HCD	163.49	728.35	MSSP1

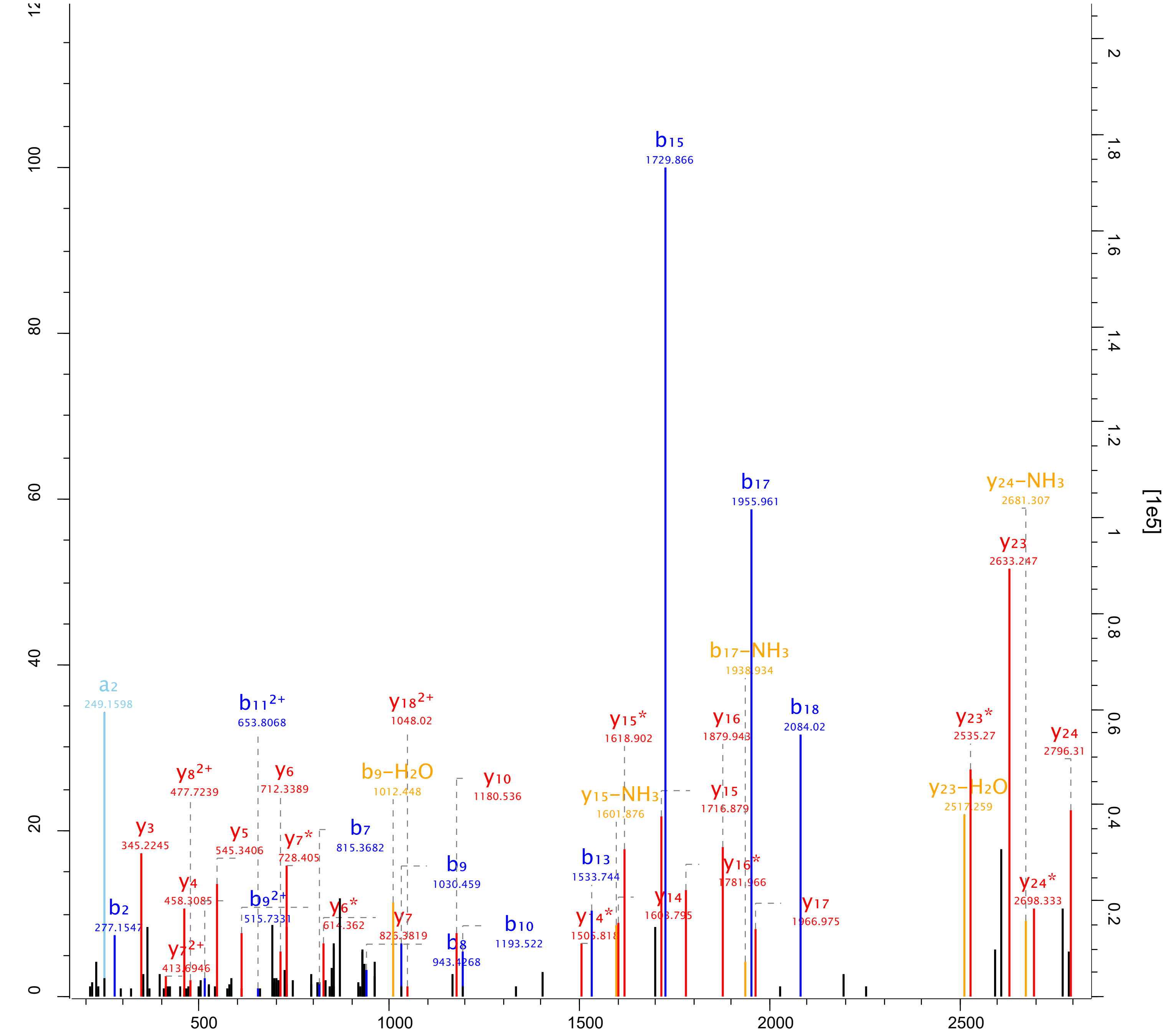

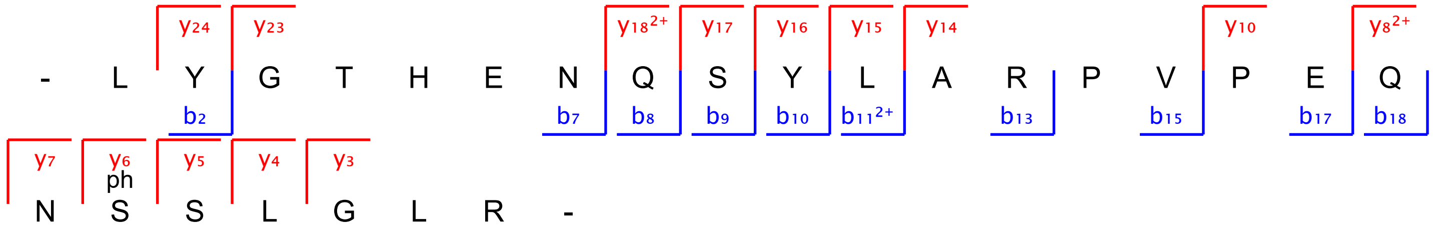

Sample output to test PDF Combine only

Raw file	Scan	Method	Score	m/z
0523_5	17555	FTMS; HCD	149.53	607.96

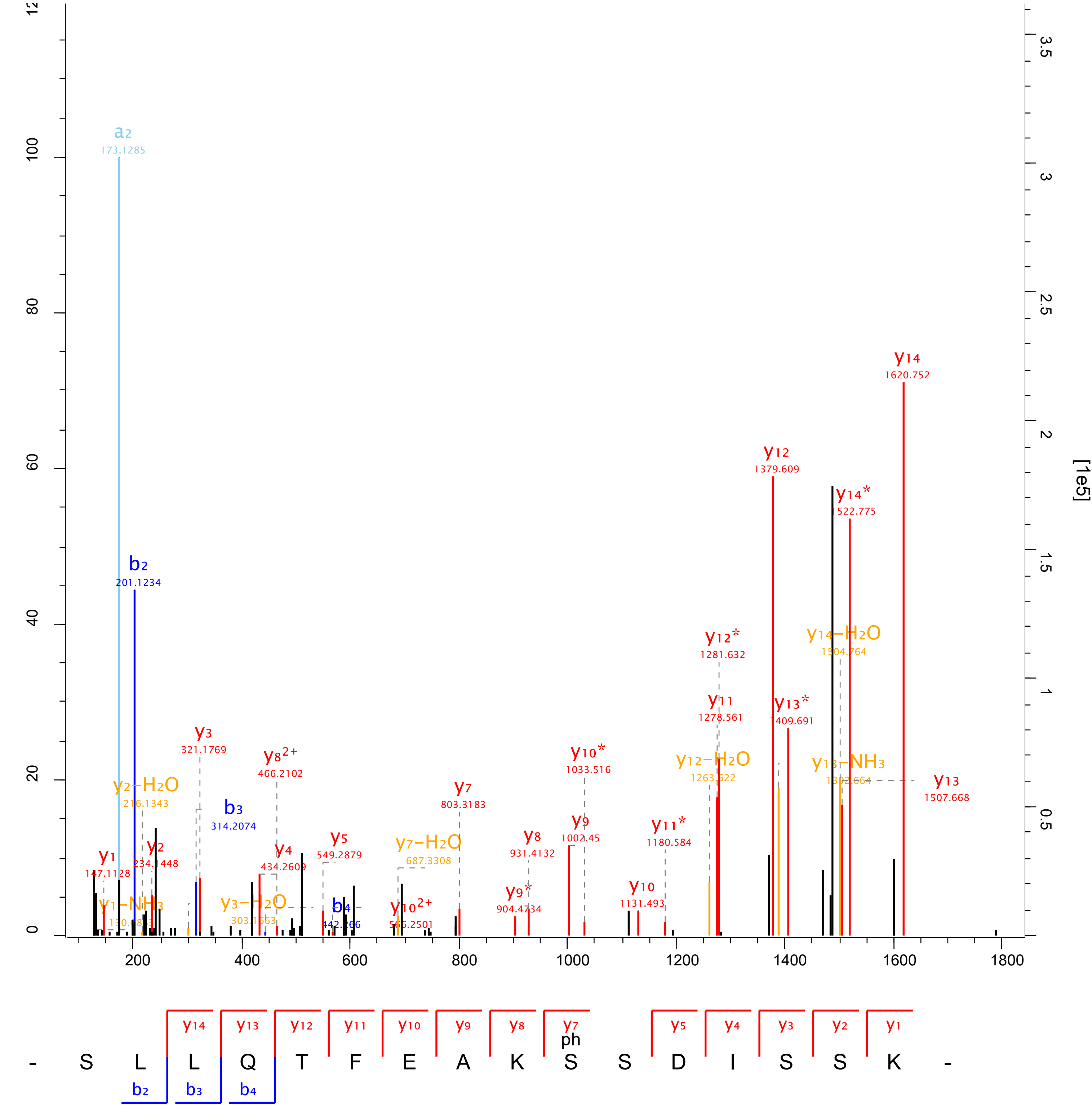

Sample output to test PDF Combine only

Raw file	Scan	Method	Score	m/z
0523_5	17621	FTMS; HCD	149.99	790.7

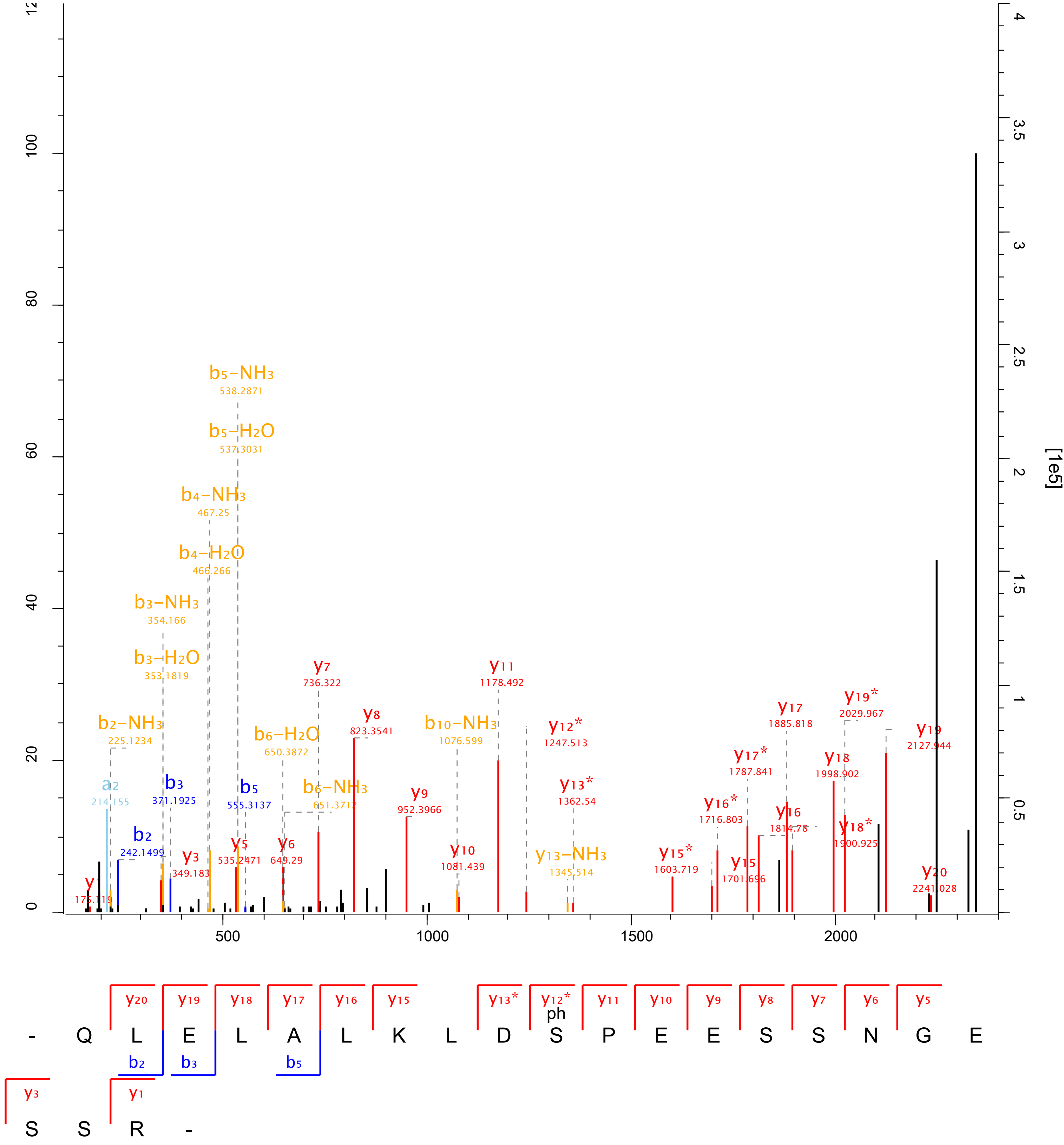

Sample output to test PDF Combine only

Raw file	Scan	Method	Score	m/z	Gene names
0523_5	17837	FTMS; HCD	71.65	753.97	PAH1

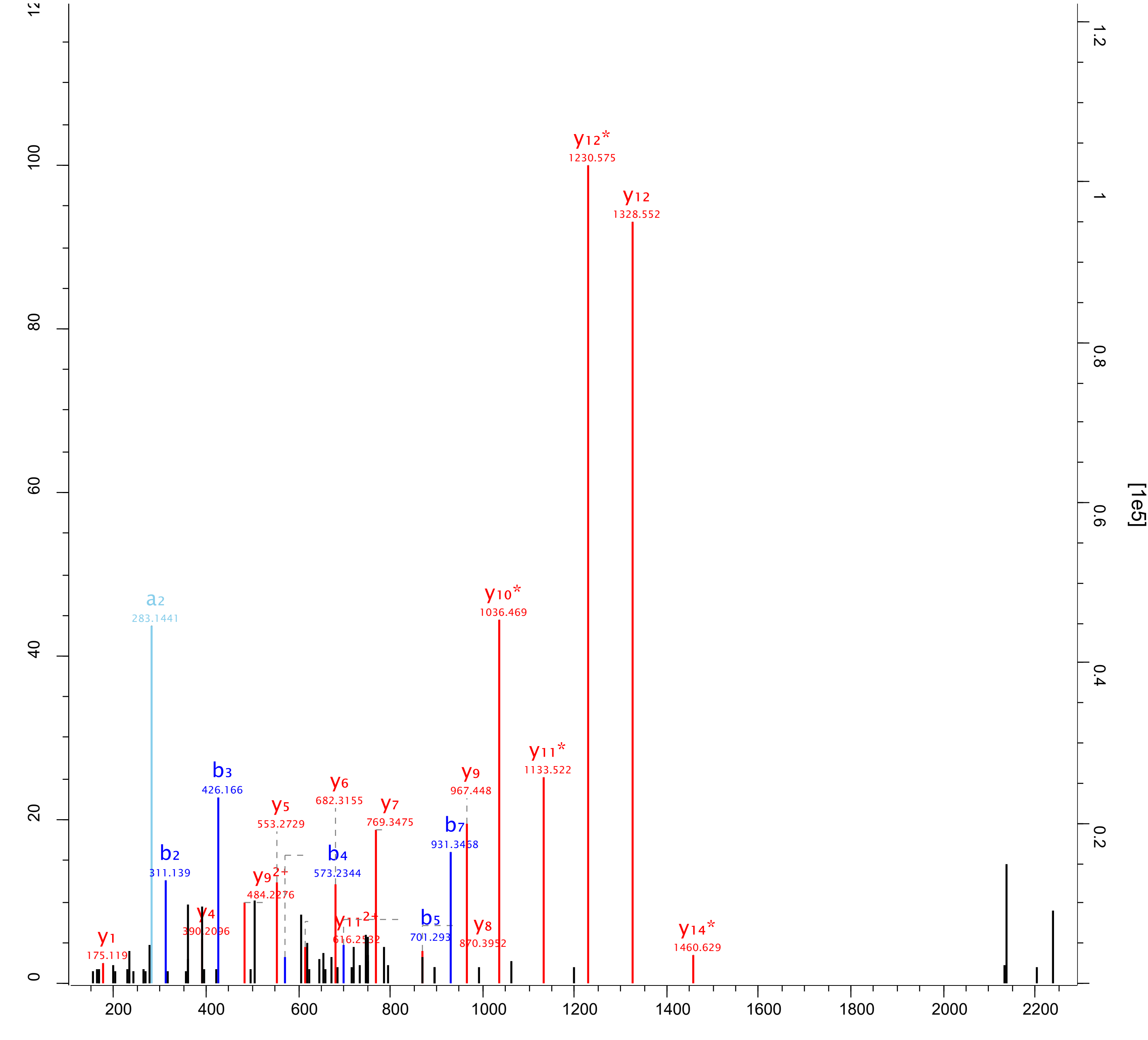

- F Y D F Q D D P P S P T S E Y G S A

R -

(y14*, y12, y11*, y10*, y9, y8, y7, y6, y5, y4, y1; b2, b3, b4, b5, b7; ph on S)

Sample output to test PDF Combine only

Raw file	Scan	Method	Score	m/z	Gene names
0523_5	17862	FTMS; HCD	62.49	749.1	F20M13.200

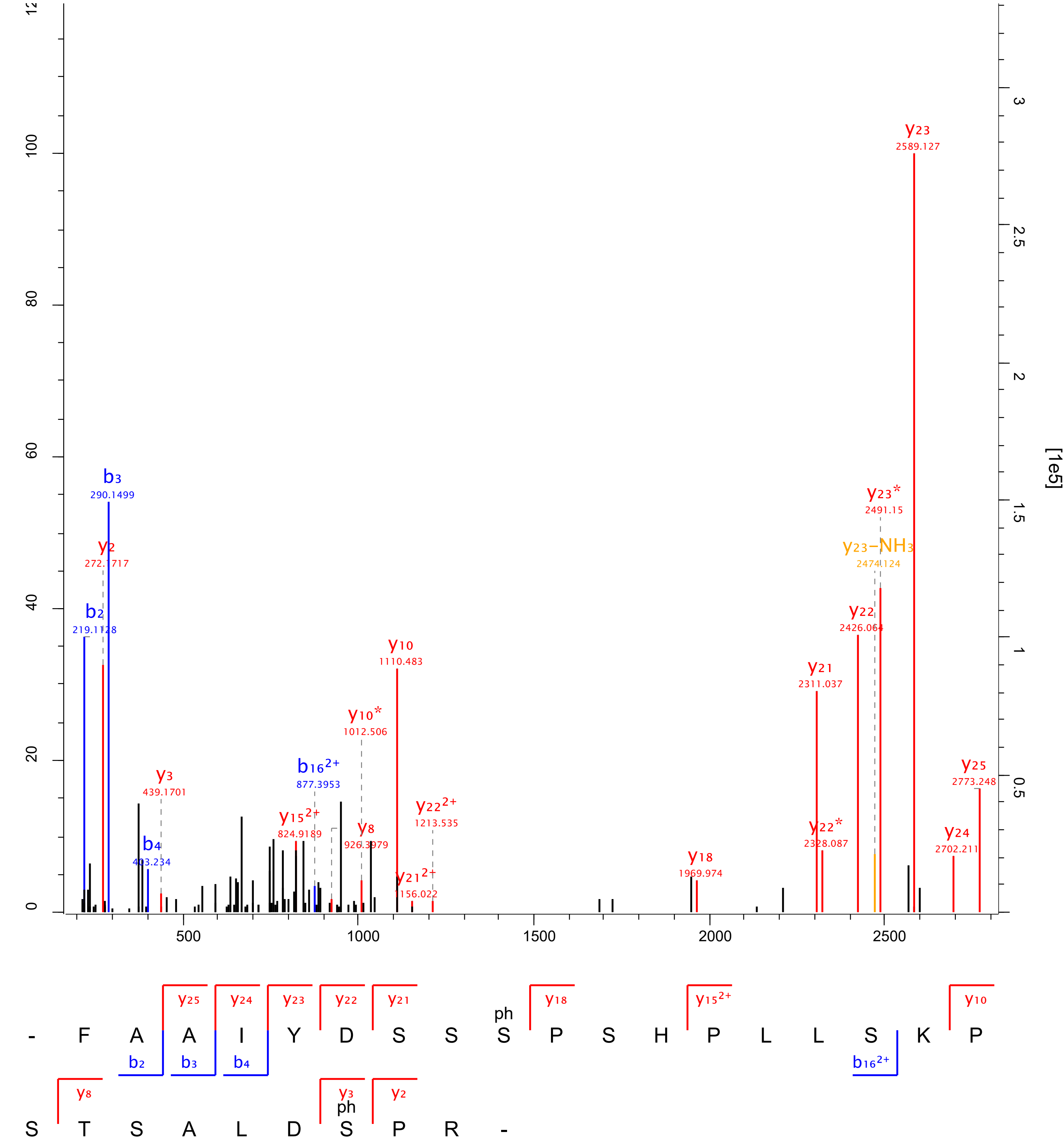

Sample output to test PDF Combine only

Raw file	Scan	Method	Score	m/z	Gene names
0523_5	18603	FTMS; HCD	46.41	726.8	FRO4

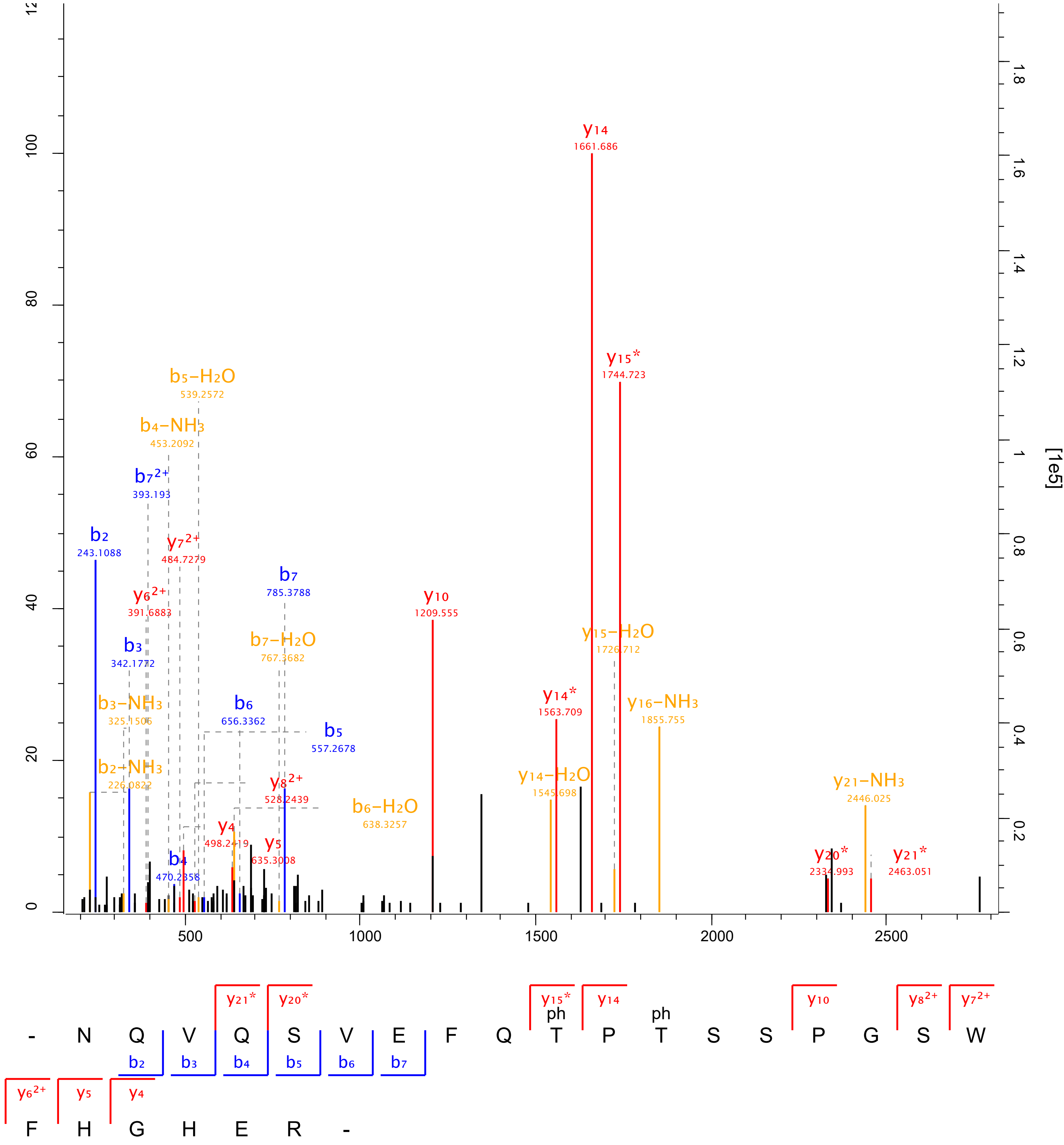

Sample output to test PDF Combine only

Raw file	Scan	Method	Score	m/z	Gene names
0523_5	18754	FTMS; HCD	105.36	740.11	At2g29210

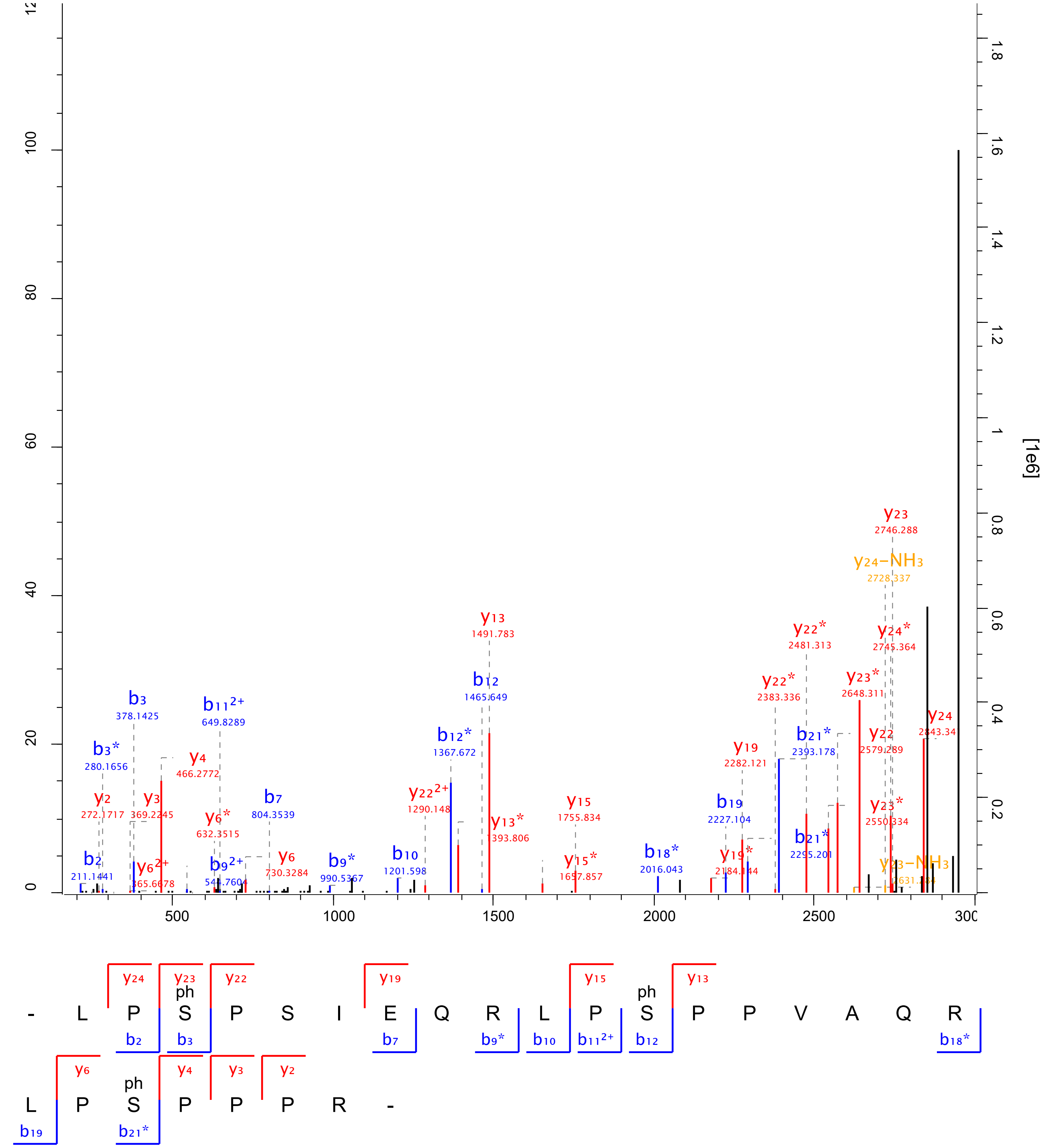

Sample output to test PDF Combine only

Raw file	Scan	Method	Score	m/z
0523_5	19199	FTMS; HCD	95.2	651.31

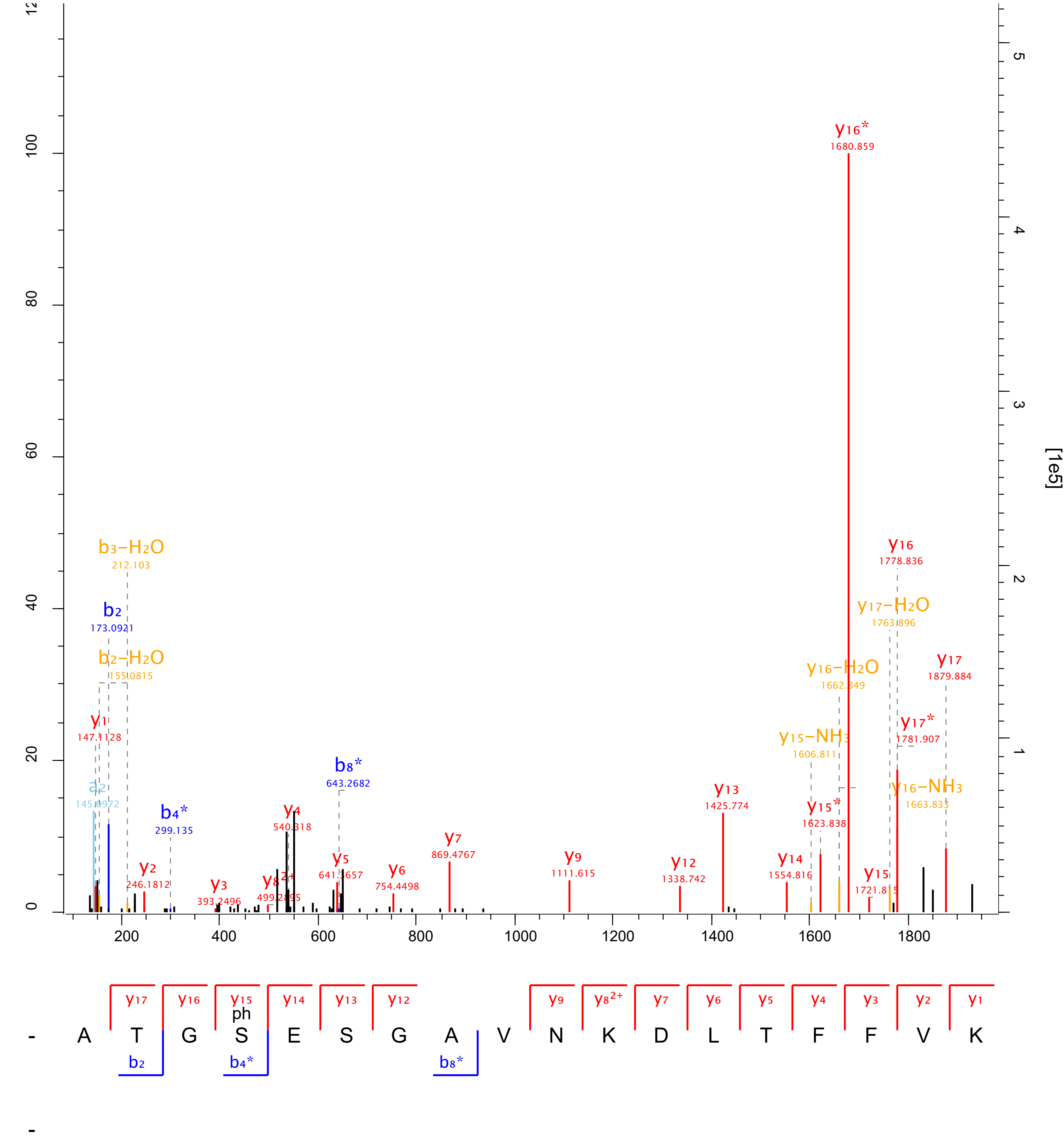

Sample output to test PDF Combine only

Raw file	Scan	Method	Score	m/z	Gene names
0523_5	19490	FTMS; HCD	105.21	1056.82	EMB1135

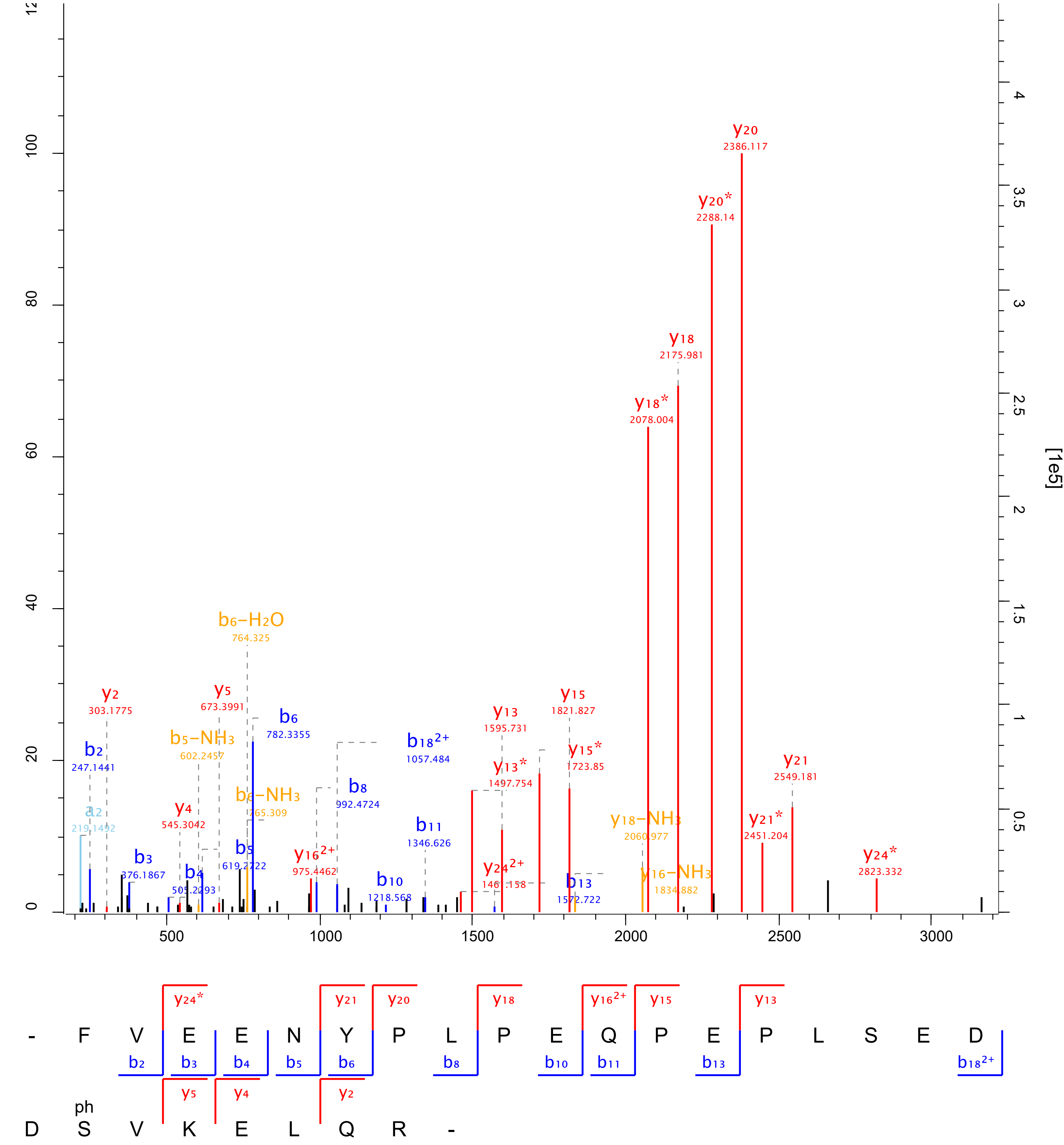

Sample output to test PDF Combine only

Raw file	Scan	Method	Score	m/z	Gene names
0523_5	19576	FTMS; HCD	84.53	708.61	HIR3

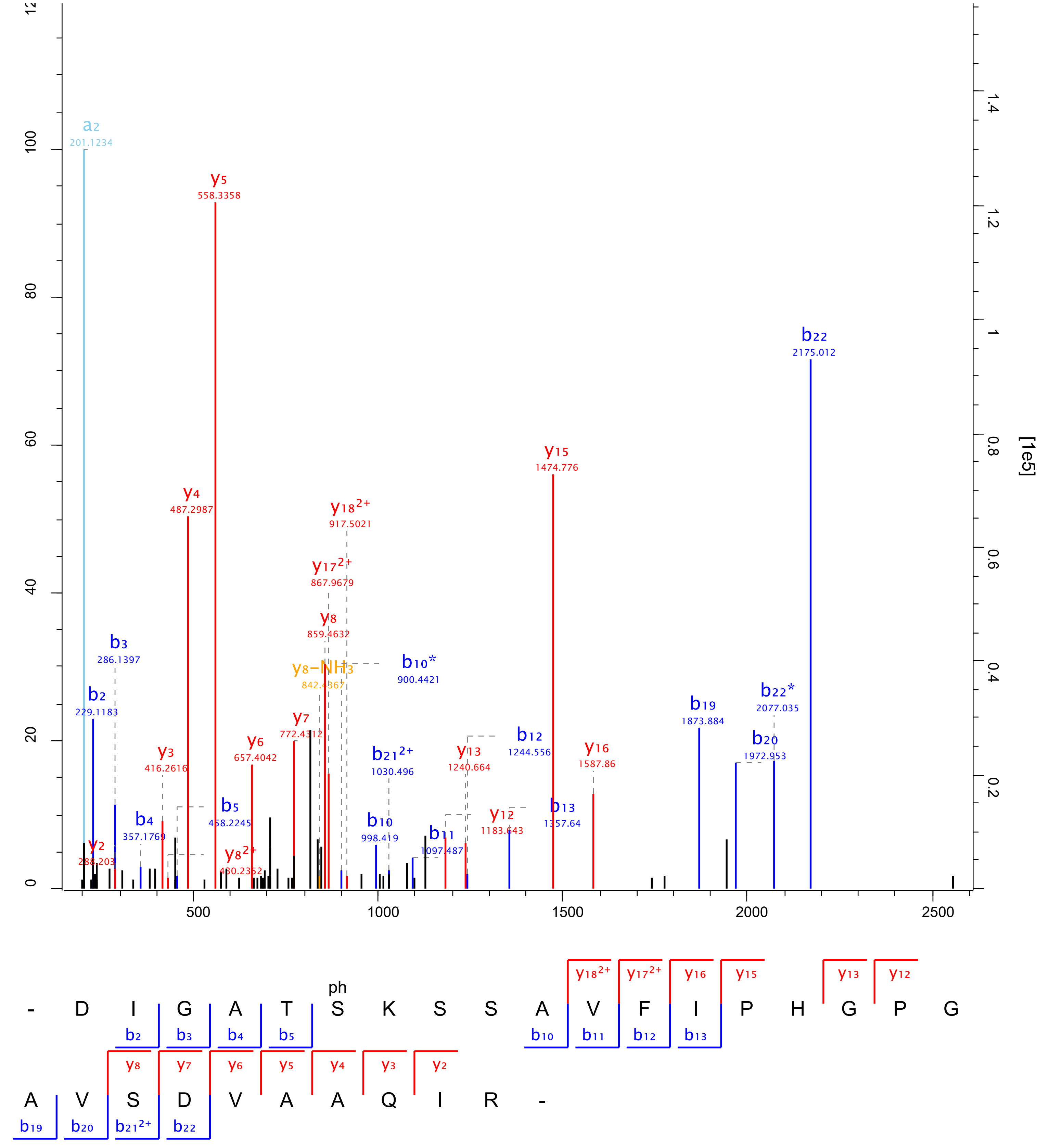

Sample output to test PDF Combine only

Raw file	Scan	Method	Score	m/z	Gene names
0523_5	19625	FTMS; HCD	71.71	787.32	AMT1-3

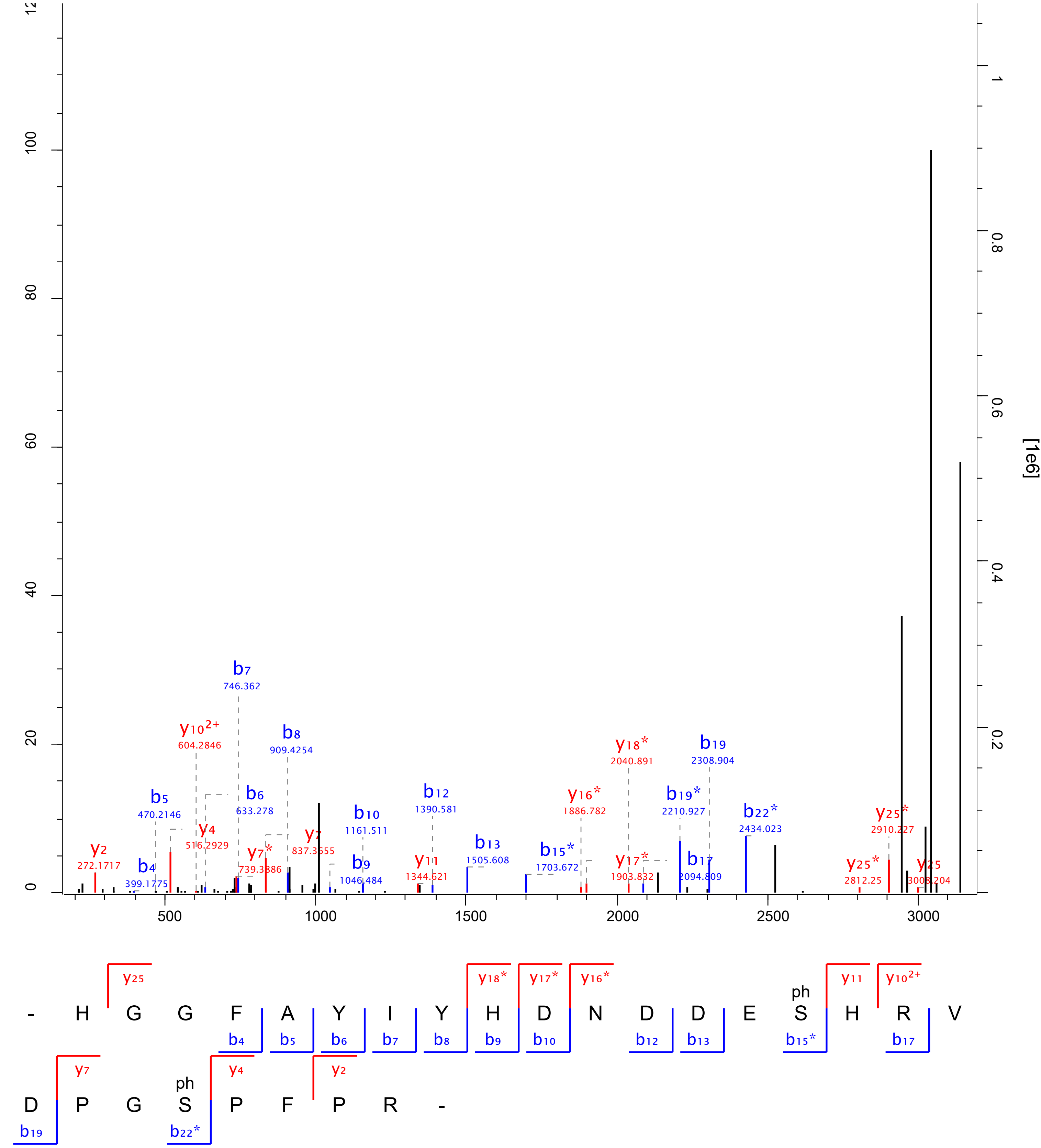

Sample output to test PDF Combine only

Raw file	Scan	Method	Score	m/z	Gene names
0523_5	19641	FTMS; HCD	258.32	1007.78	SYP132

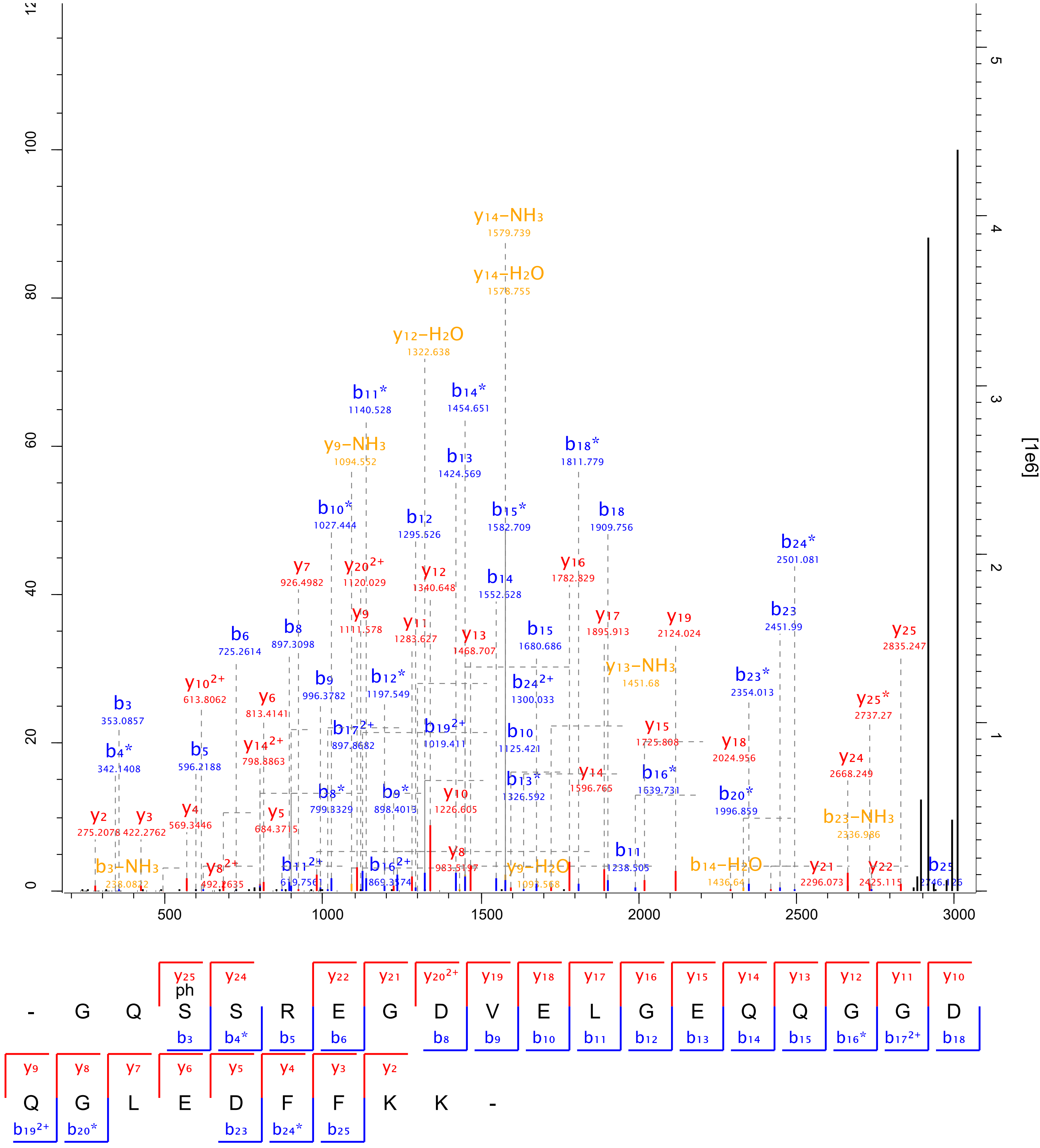

Sample output to test PDF Combine only

Raw file	Scan	Method	Score	m/z	Gene names
0523_5	19895	FTMS; HCD	53.09	834.05	ALA10

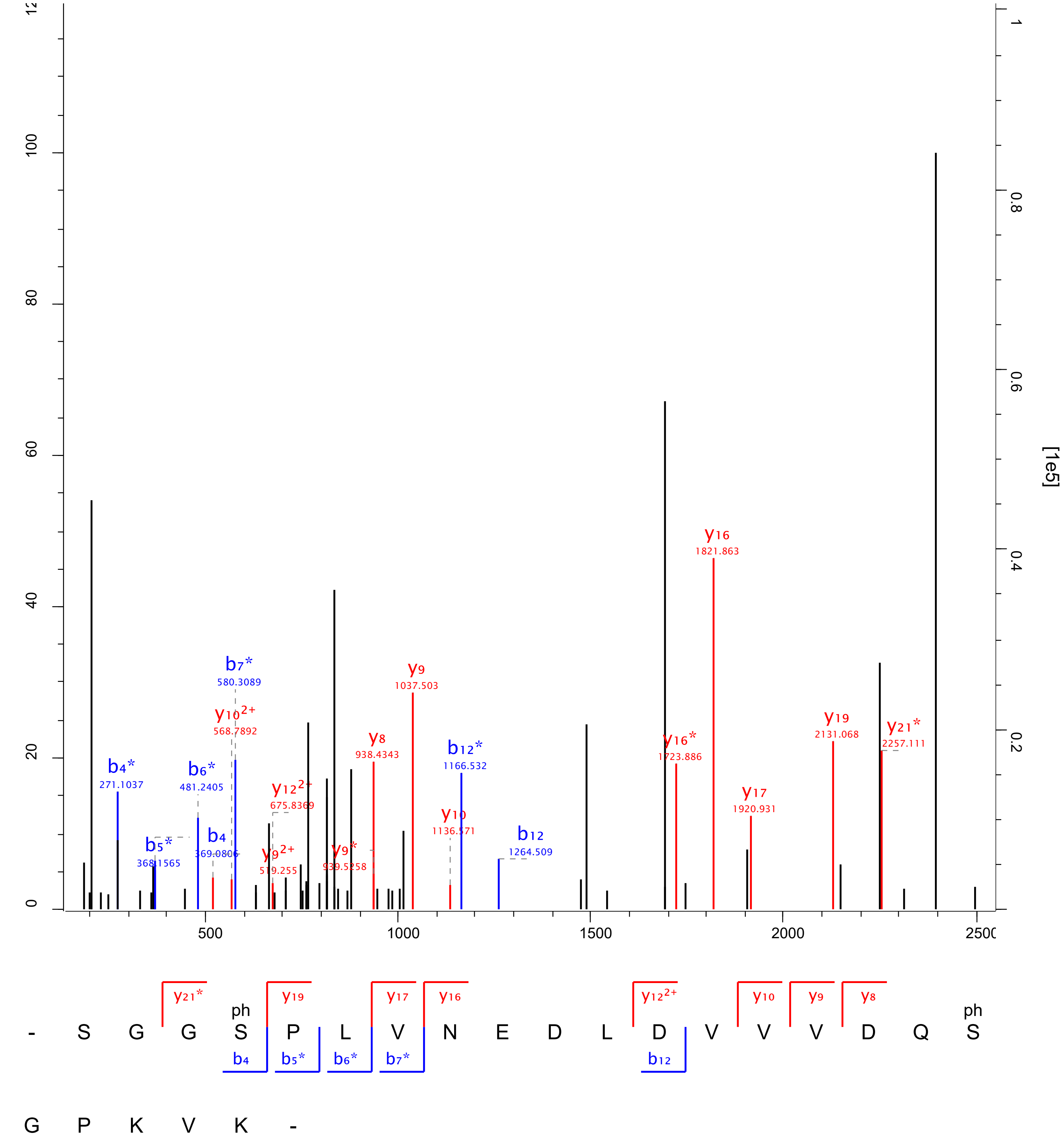

Sample output to test PDF Combine only

Raw file	Scan	Method	Score	m/z	Gene names
0523_5	20096	FTMS; HCD	105.24	951.77	F13I12.260

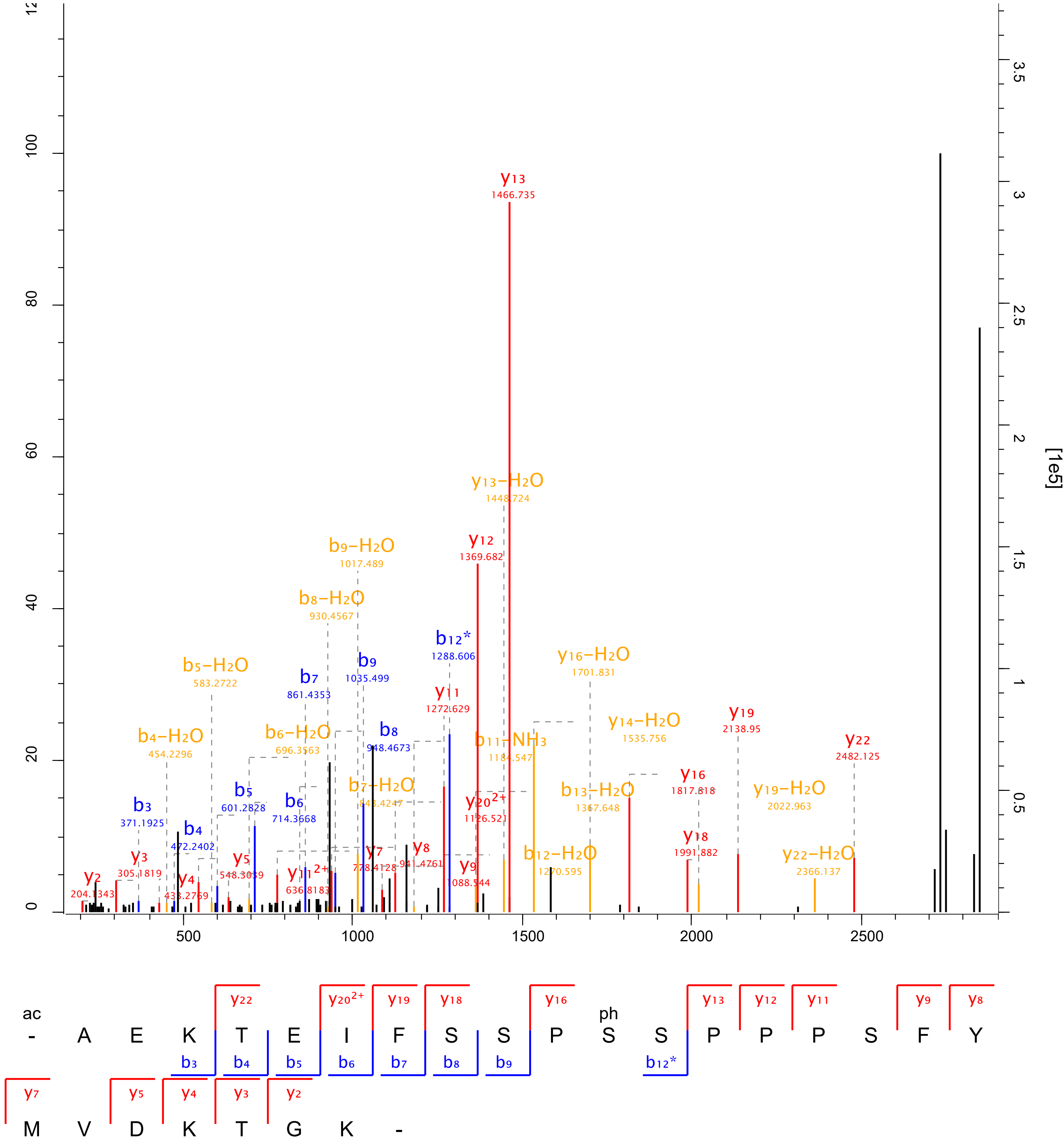

Sample output to test PDF Combine only

Raw file	Scan	Method	Score	m/z	Gene names
0523_5	20185	FTMS; HCD	173.05	854.4	FHY3

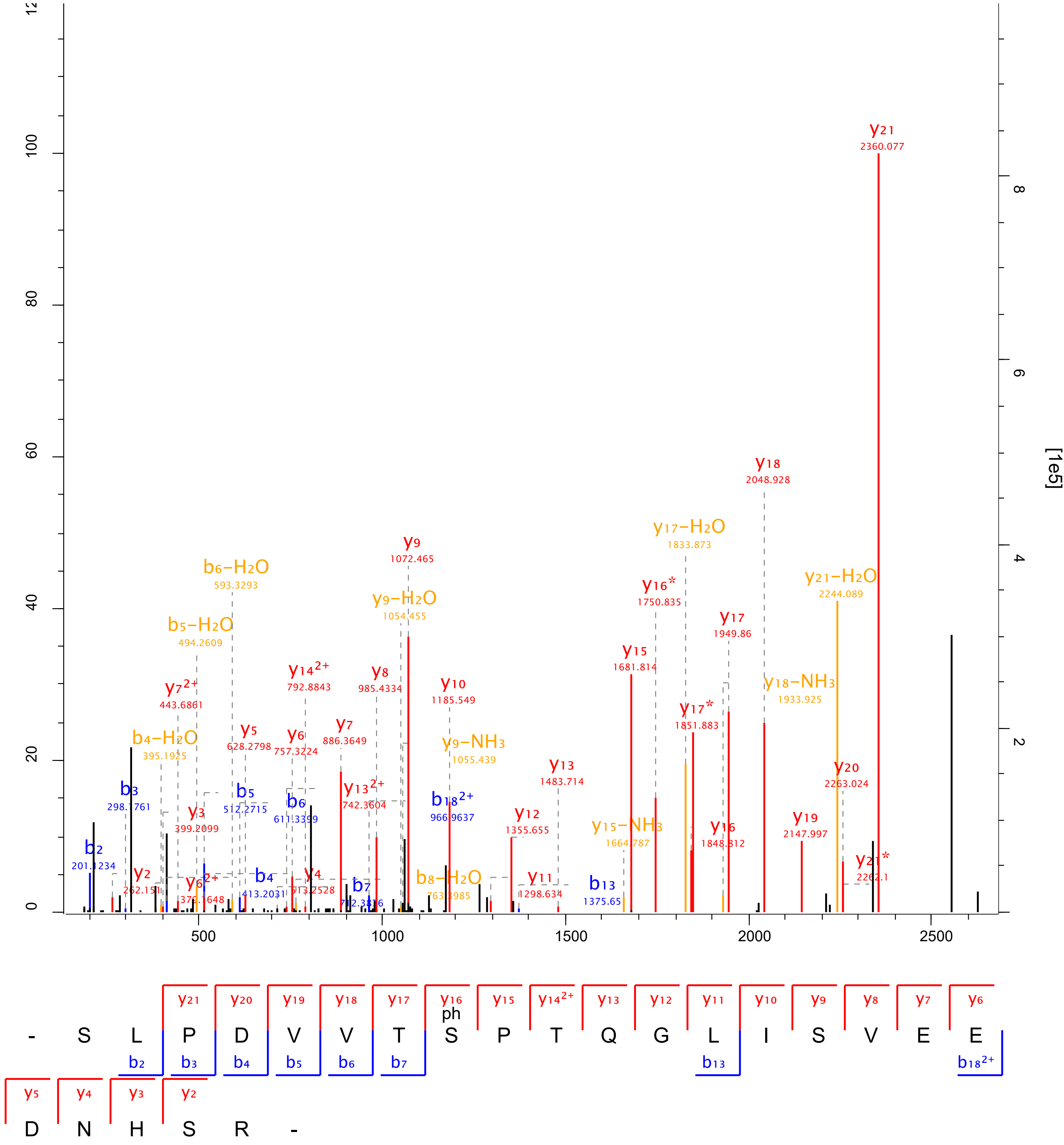

Sample output to test PDF Combine only

Raw file	Scan	Method	Score	m/z	Gene names
0523_5	20340	FTMS; HCD	350.49	941.95	GRV2

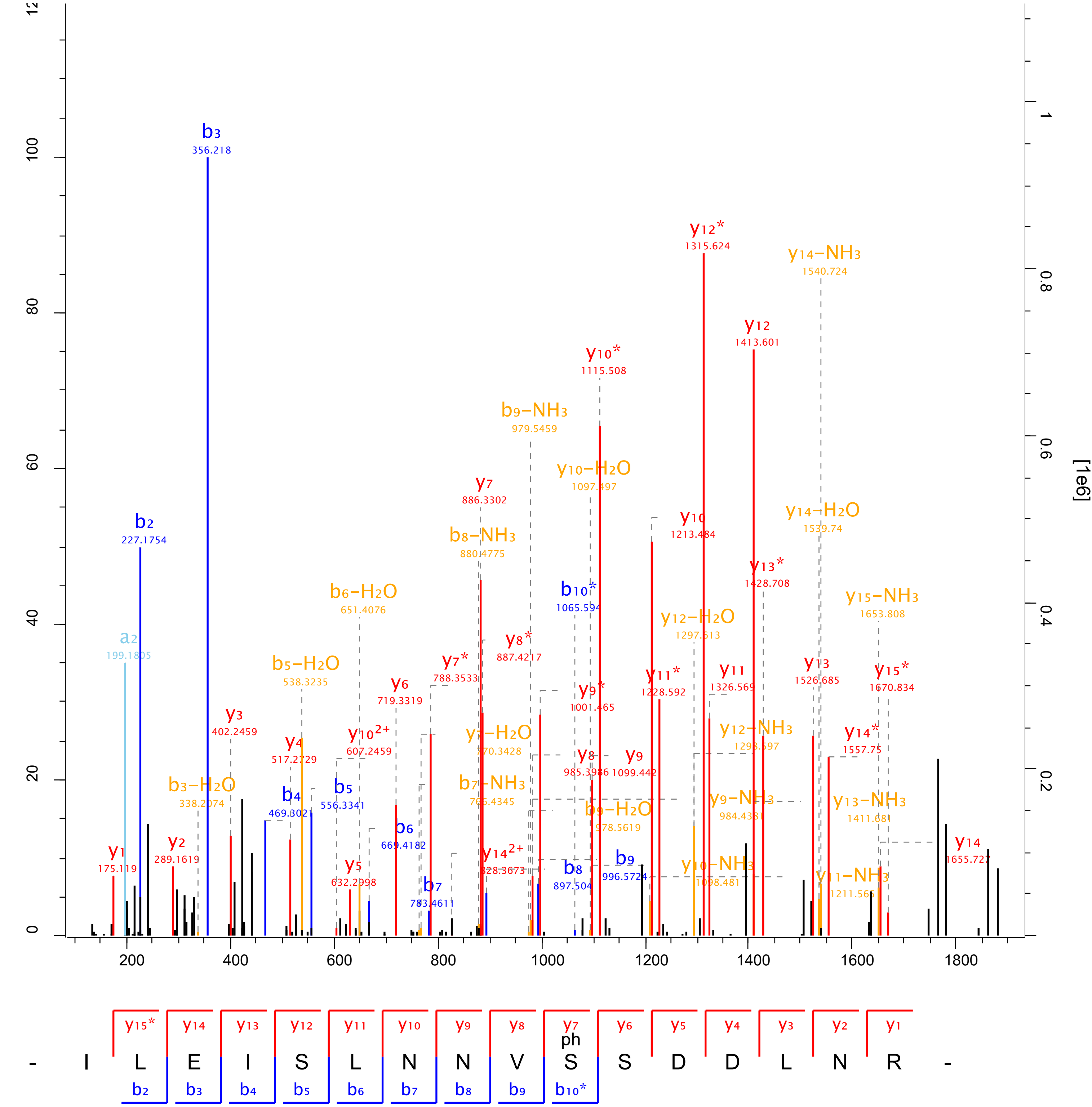

Sample output to test PDF Combine only

Raw file	Scan	Method	Score	m/z	Gene names
0523_5	21445	FTMS; HCD	98.98	839.04	F9F8.20

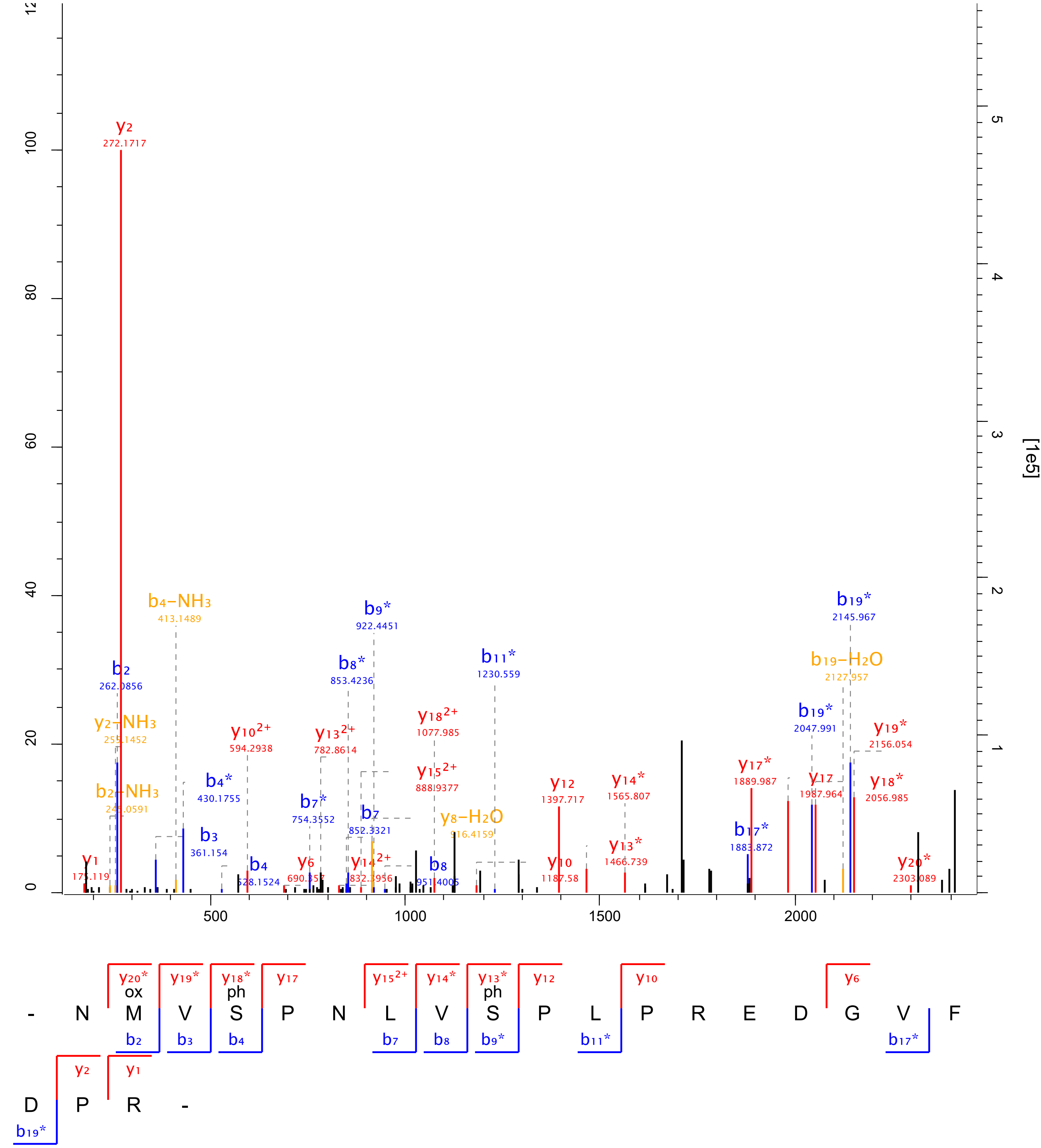

Sample output to test PDF Combine only

Raw file	Scan	Method	Score	m/z	Gene names
0523_5	21558	FTMS; HCD	76.42	956.76	ATL15

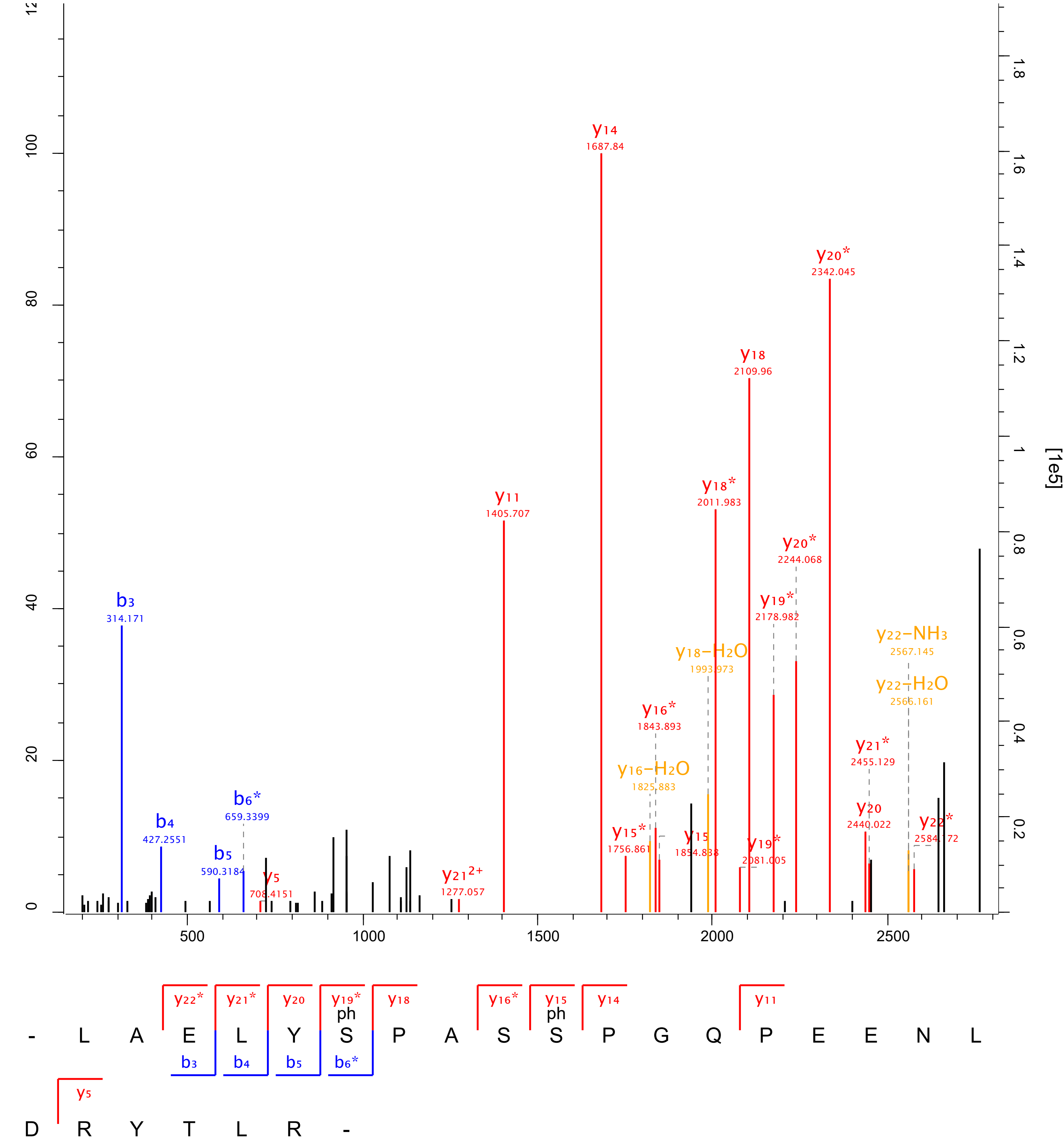

Sample output to test PDF Combine only

Raw file	Scan	Method	Score	m/z	Gene names
0523_5	21673	FTMS; HCD	187.98	791.03	NIP2-1

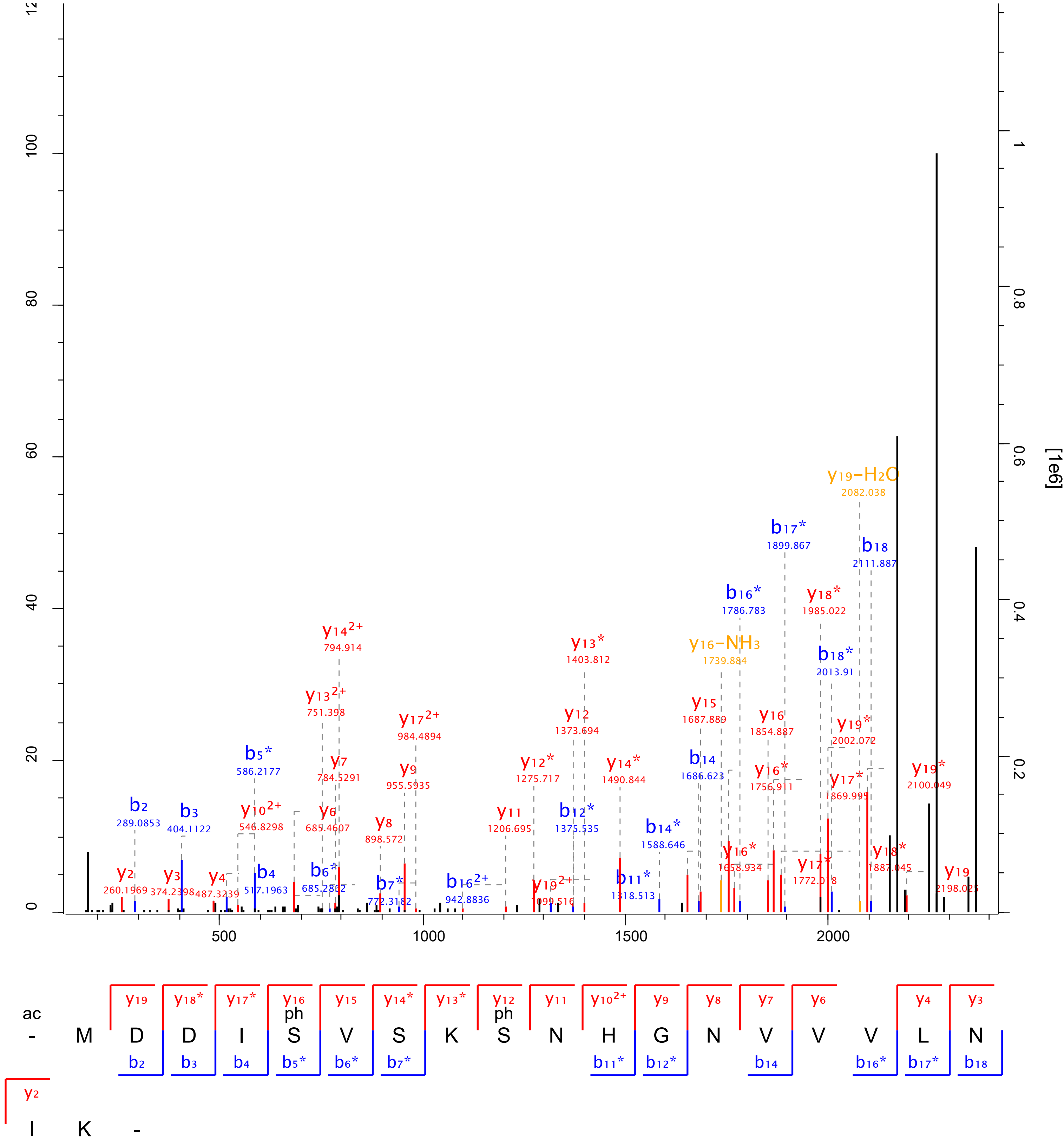

Sample output to test PDF Combine only

Raw file	Scan	Method	Score	m/z	Gene names
0523_5	21683	FTMS; HCD	45.07	976.94	F20M13.200

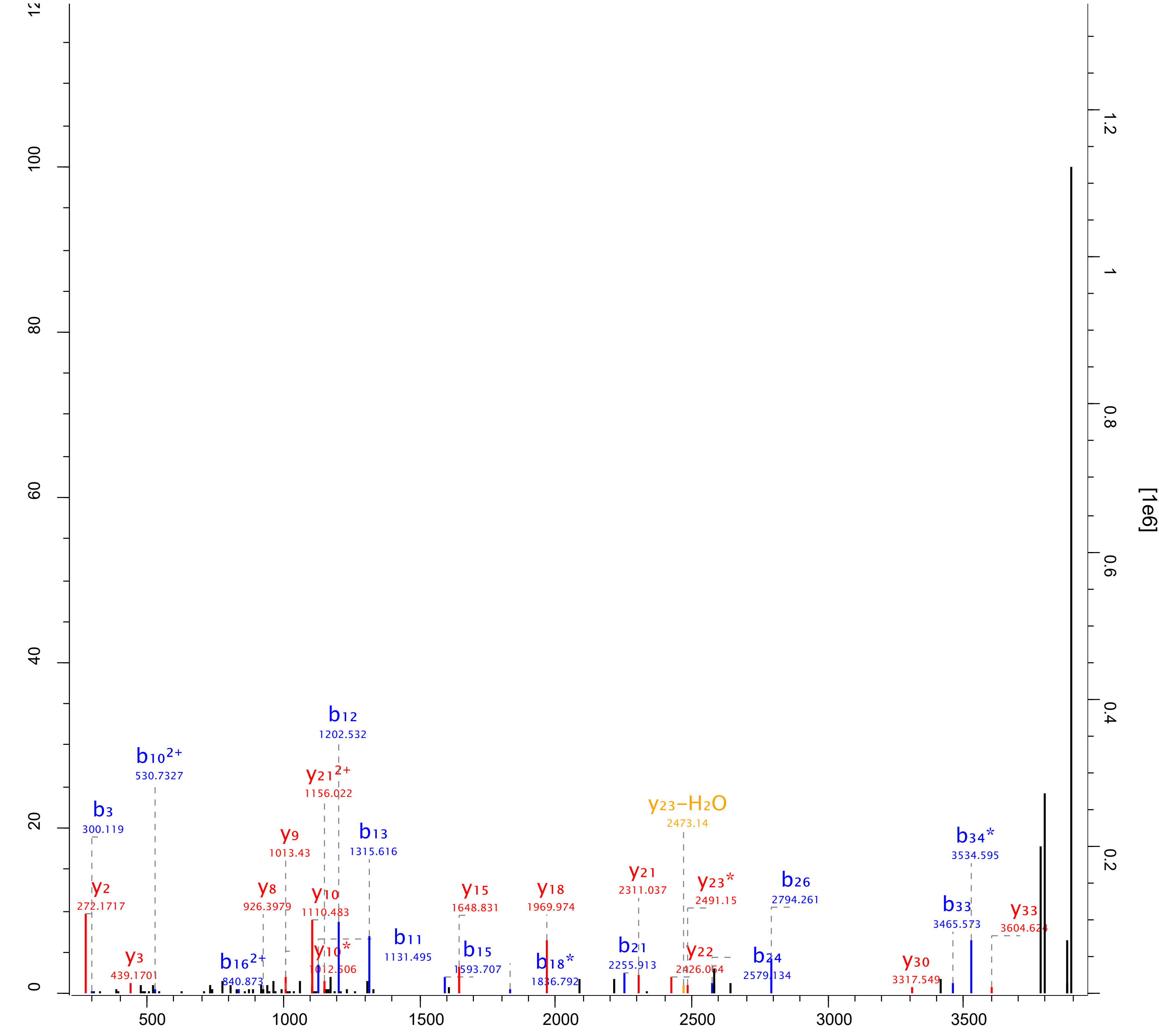

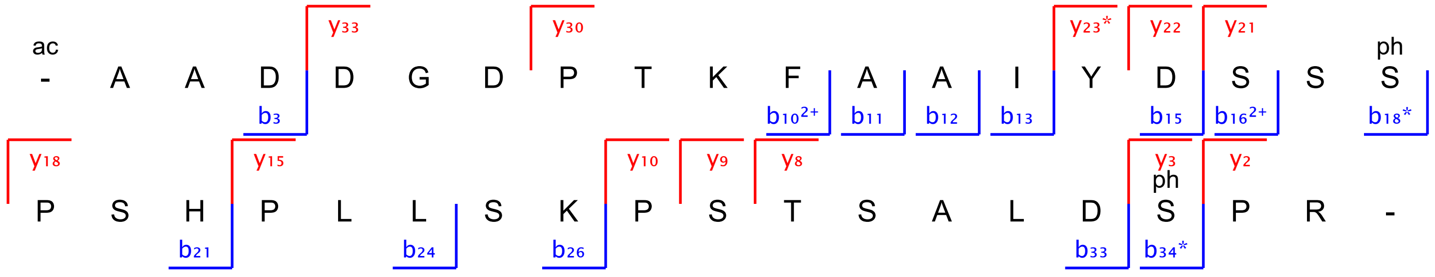

Sample output to test PDF Combine only

Raw file	Scan	Method	Score	m/z	Gene names
0523_5	21731	FTMS; HCD	135.52	880.1	VCS

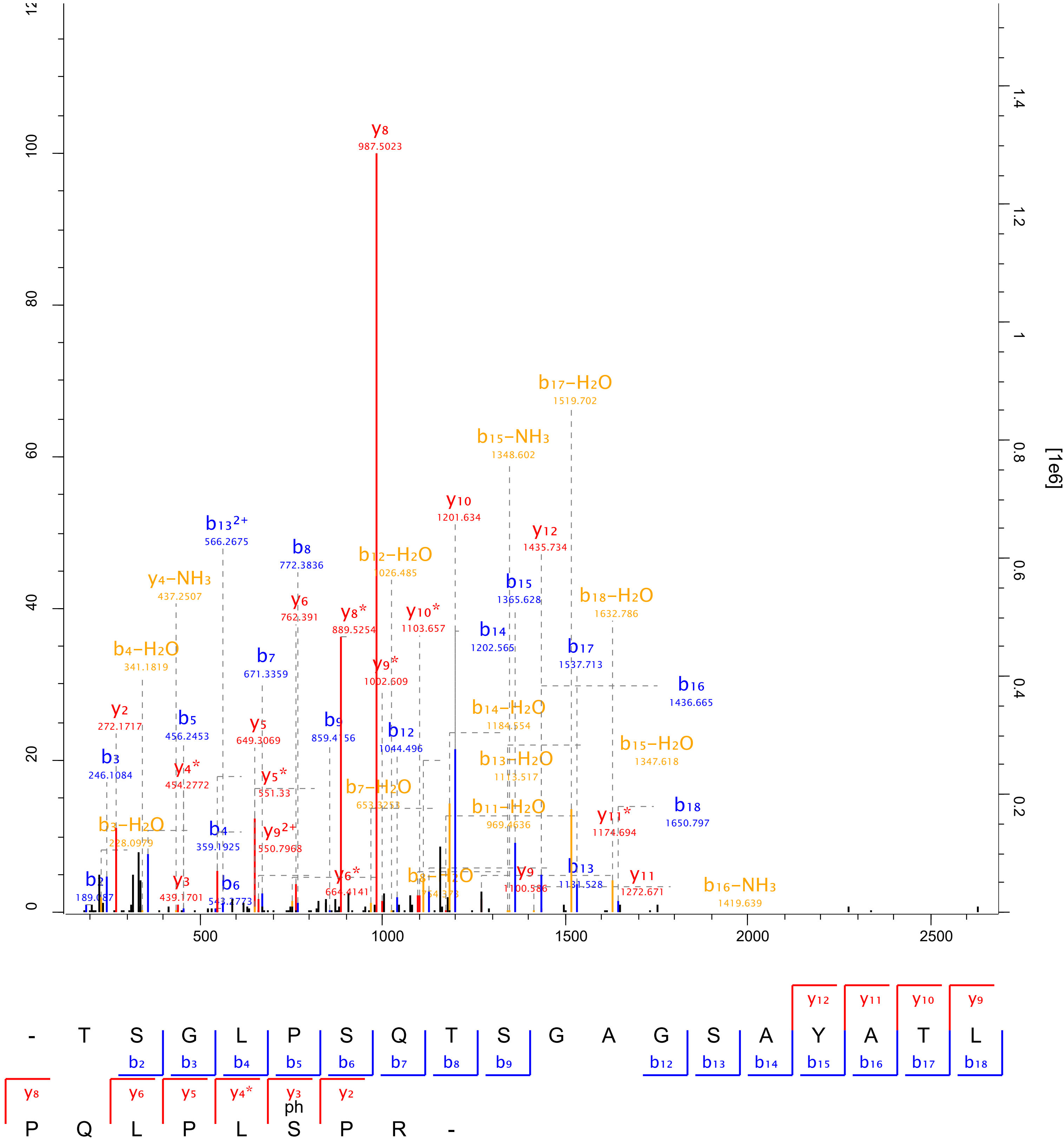

Sample output to test PDF Combine only

Raw file	Scan	Method	Score	m/z	Gene names
0523_5	22049	FTMS; HCD	129.05	977.13	F25C20.18

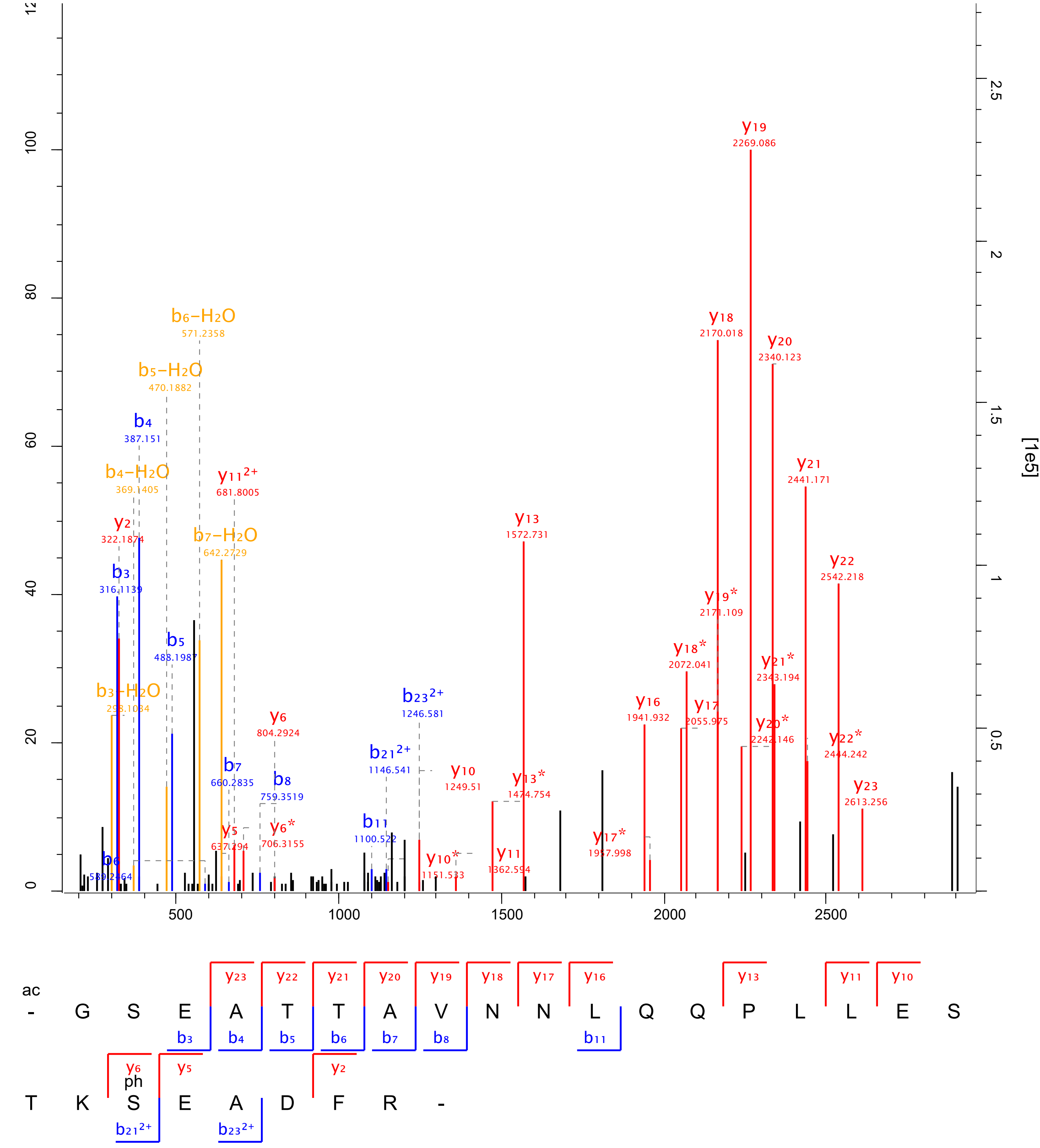

Sample output to test PDF Combine only

Raw file	Scan	Method	Score	m/z	Gene names
0523_5	22462	FTMS; HCD	408.33	1346.24	ACA10

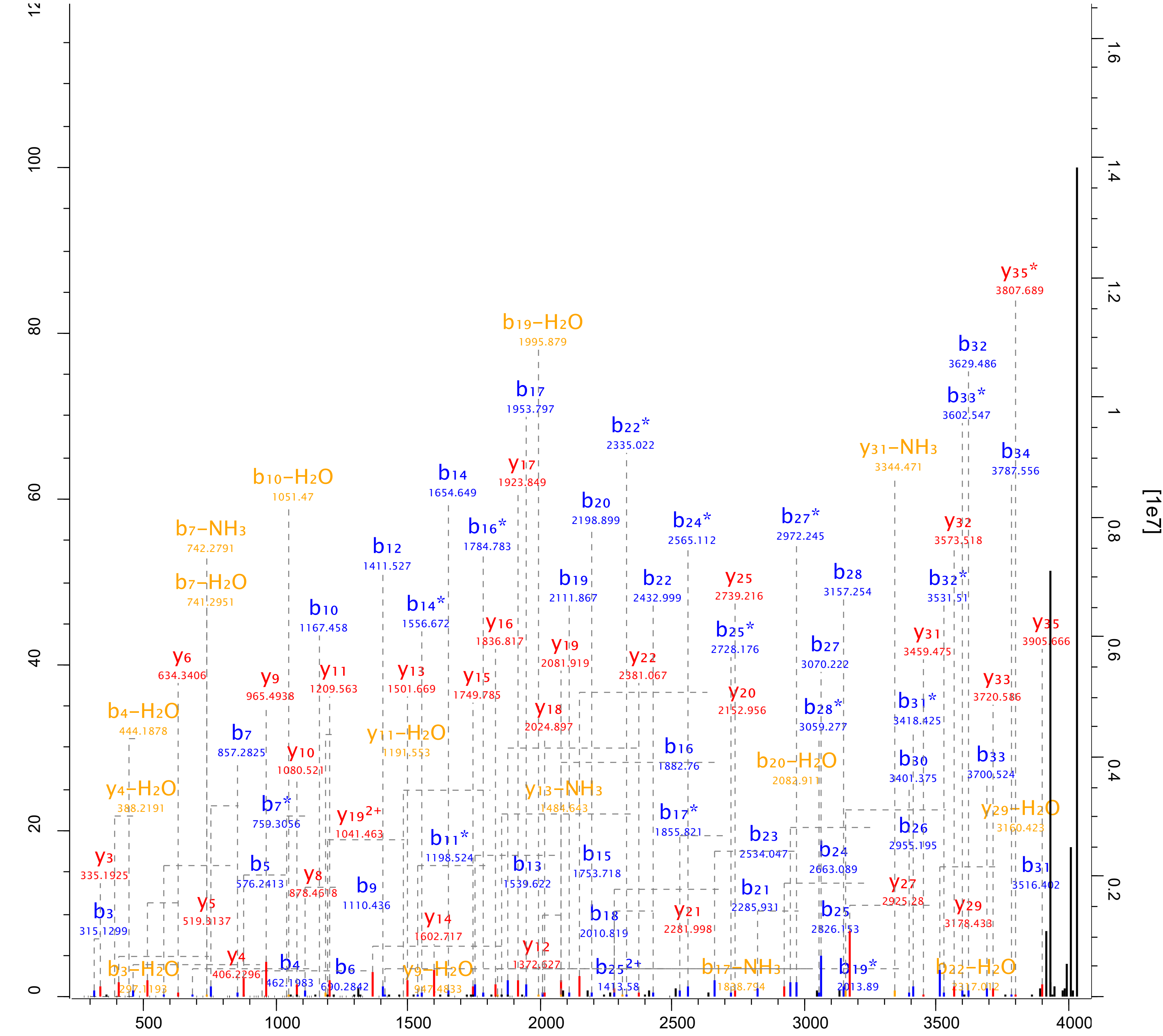

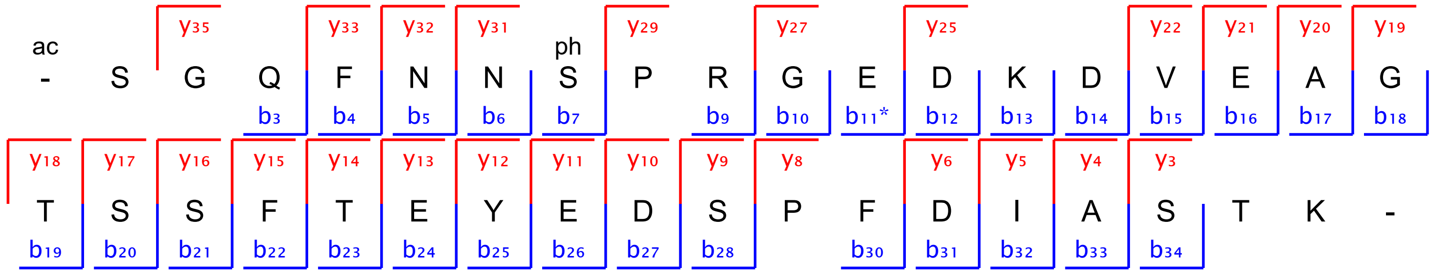

Sample output to test PDF Combine only

Raw file	Scan	Method	Score	m/z	Gene names
0523_5	22567	FTMS; HCD	198.1	754.68	CRRSP1

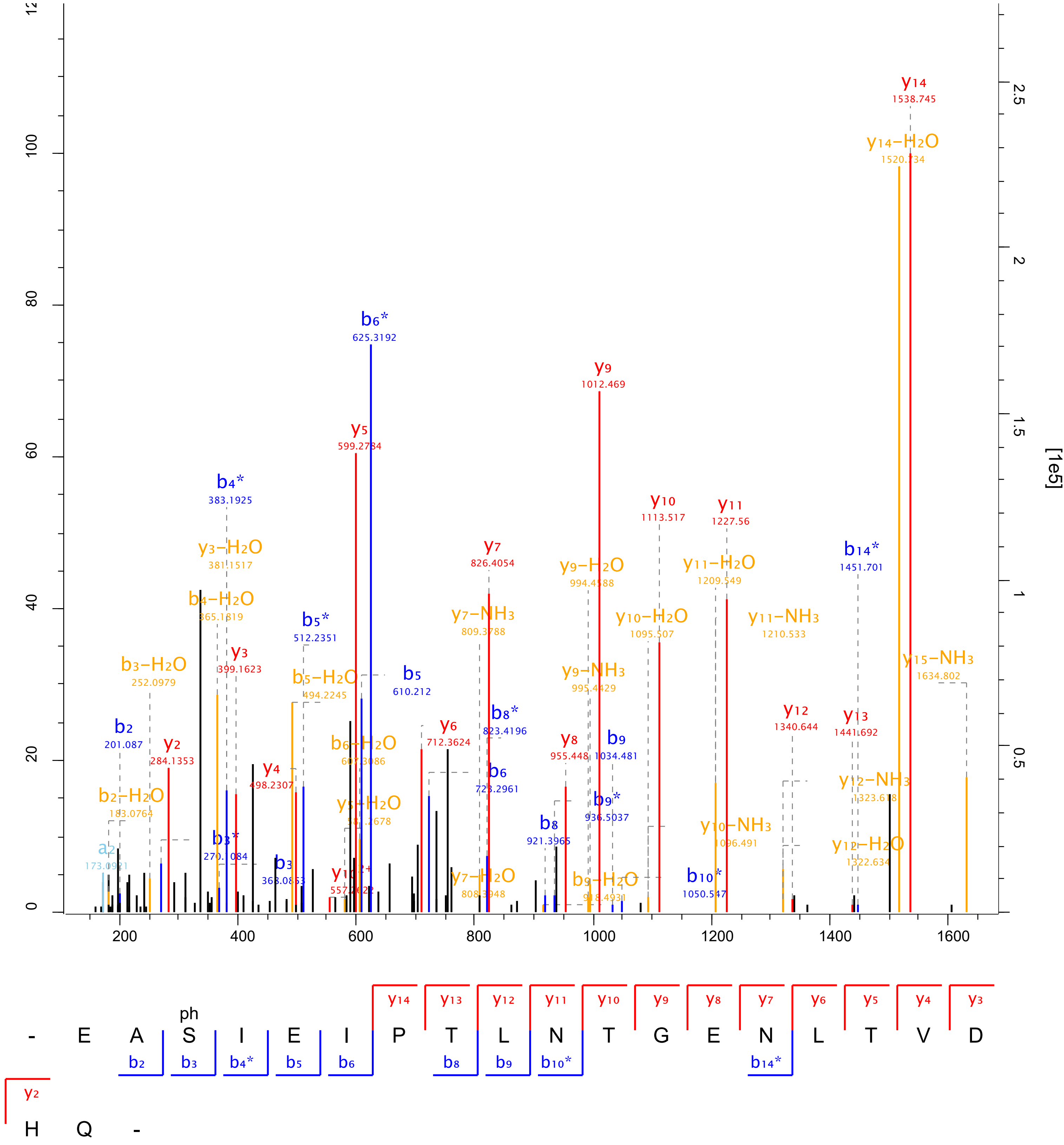

Sample output to test PDF Combine only

Raw file	Scan	Method	Score	m/z	Gene names
0523_5	22670	FTMS; HCD	143.47	1039.46	At2g34970

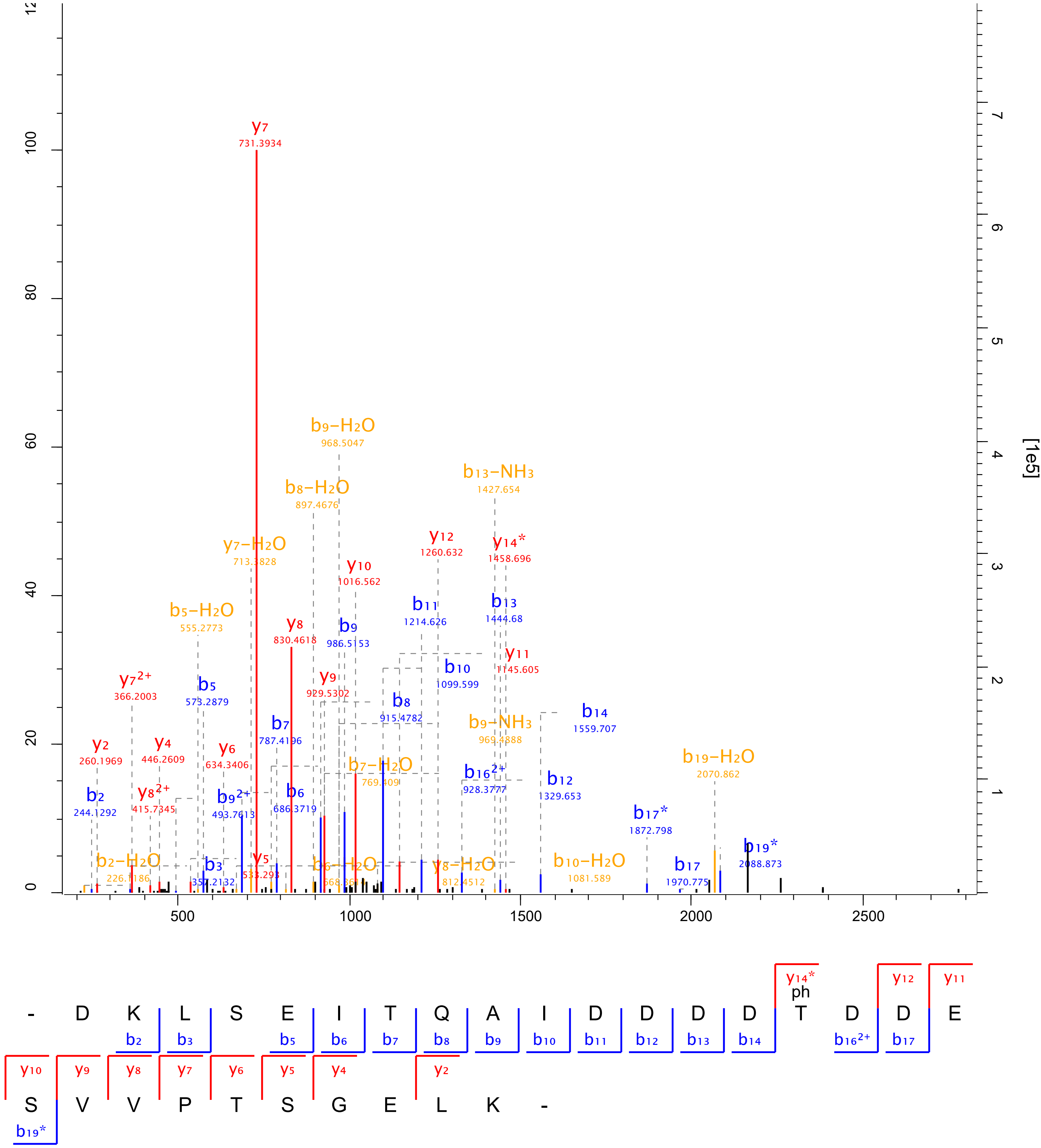

Sample output to test PDF Combine only

Raw file	Scan	Method	Score	m/z	Gene names
0523_5	22808	FTMS; HCD	249.28	705.94	RPP2D;RPP2A;At2g27720;RPP2B;At2g27710

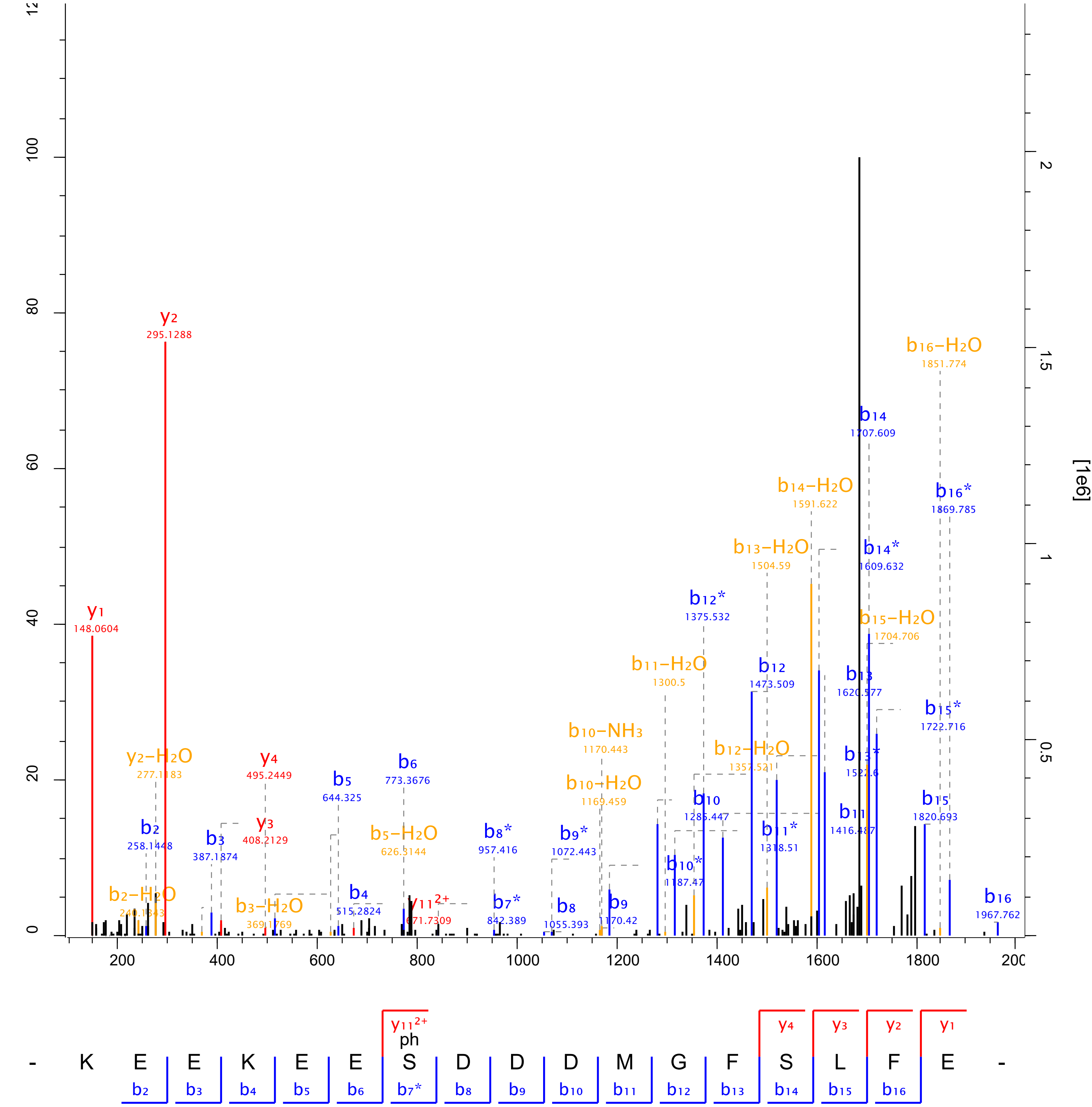

Sample output to test PDF Combine only

Raw file	Scan	Method	Score	m/z
0523_5	22824	FTMS; HCD	139.6	877.95

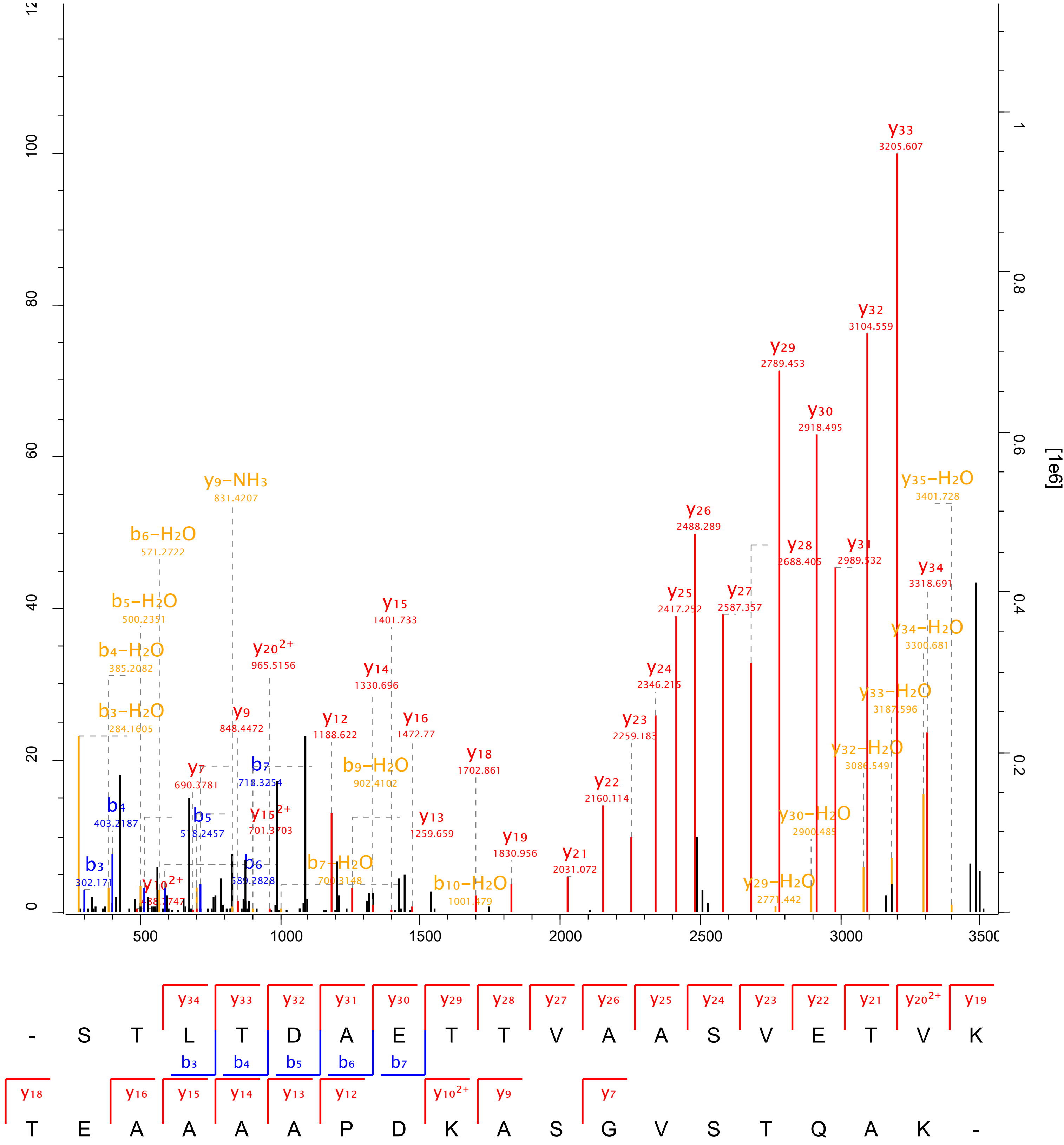

Sample output to test PDF Combine only

Raw file	Scan	Method	Score	m/z
0523_5	23603	FTMS; HCD	99.6	834.38

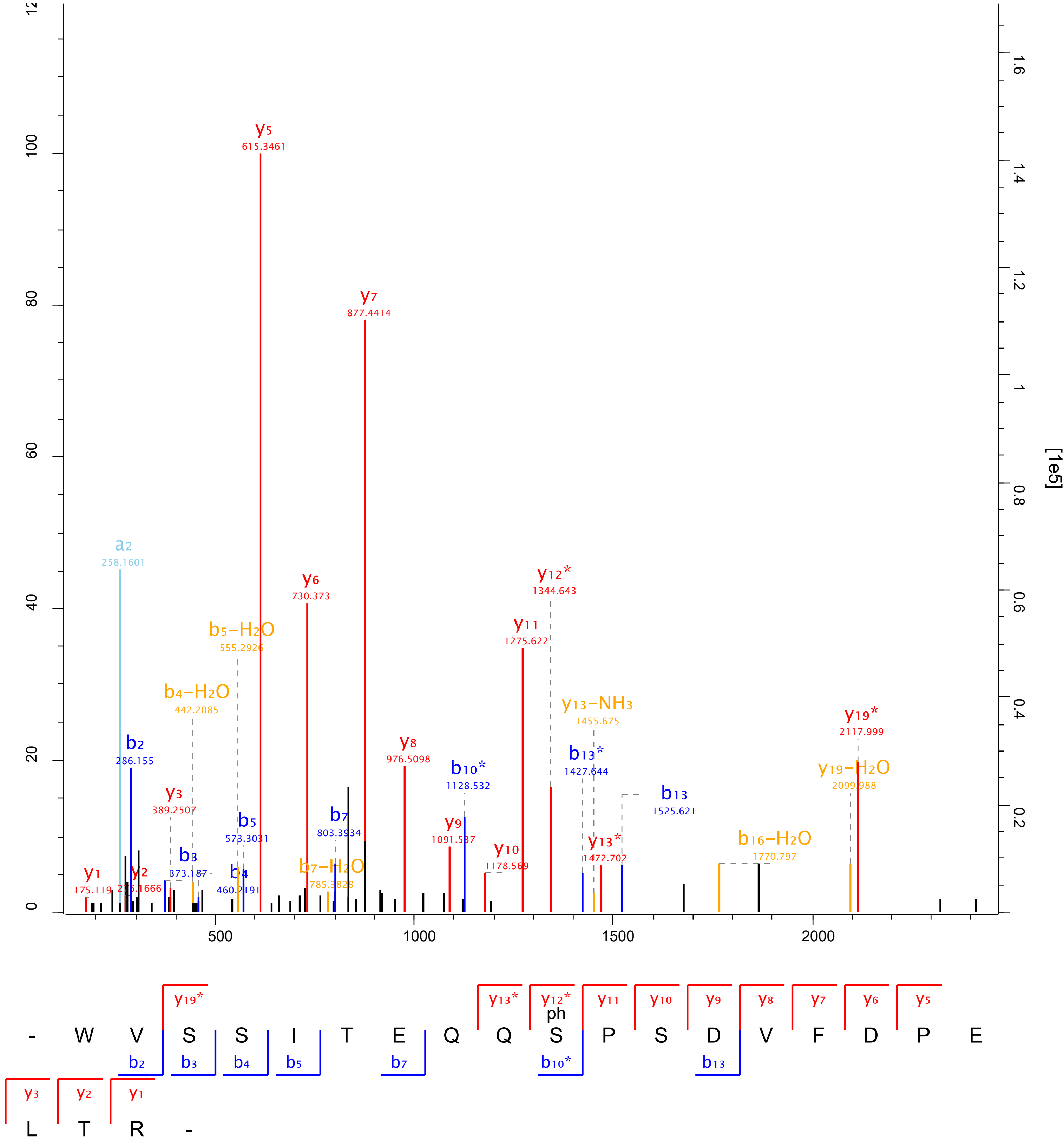

Sample output to test PDF Combine only

Raw file	Scan	Method	Score	m/z	Gene names
0523_5	23716	FTMS; HCD	309.43	1075.46	RPP1C;At1g01100;RPP1A

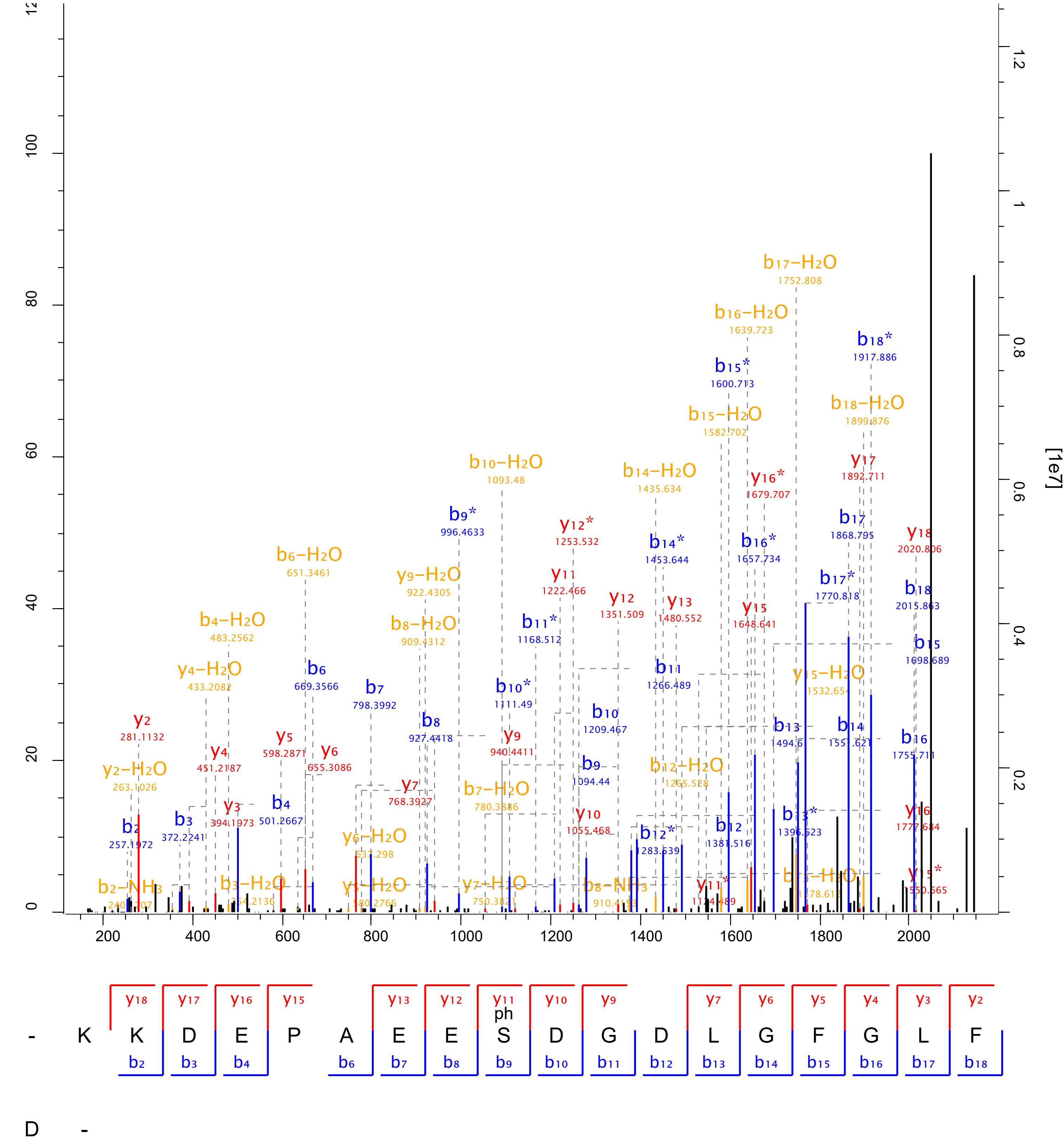

Sample output to test PDF Combine only

Raw file	Scan	Method	Score	m/z	Gene names
0523_5	24260	FTMS; HCD	72.57	971.75	CMTA5

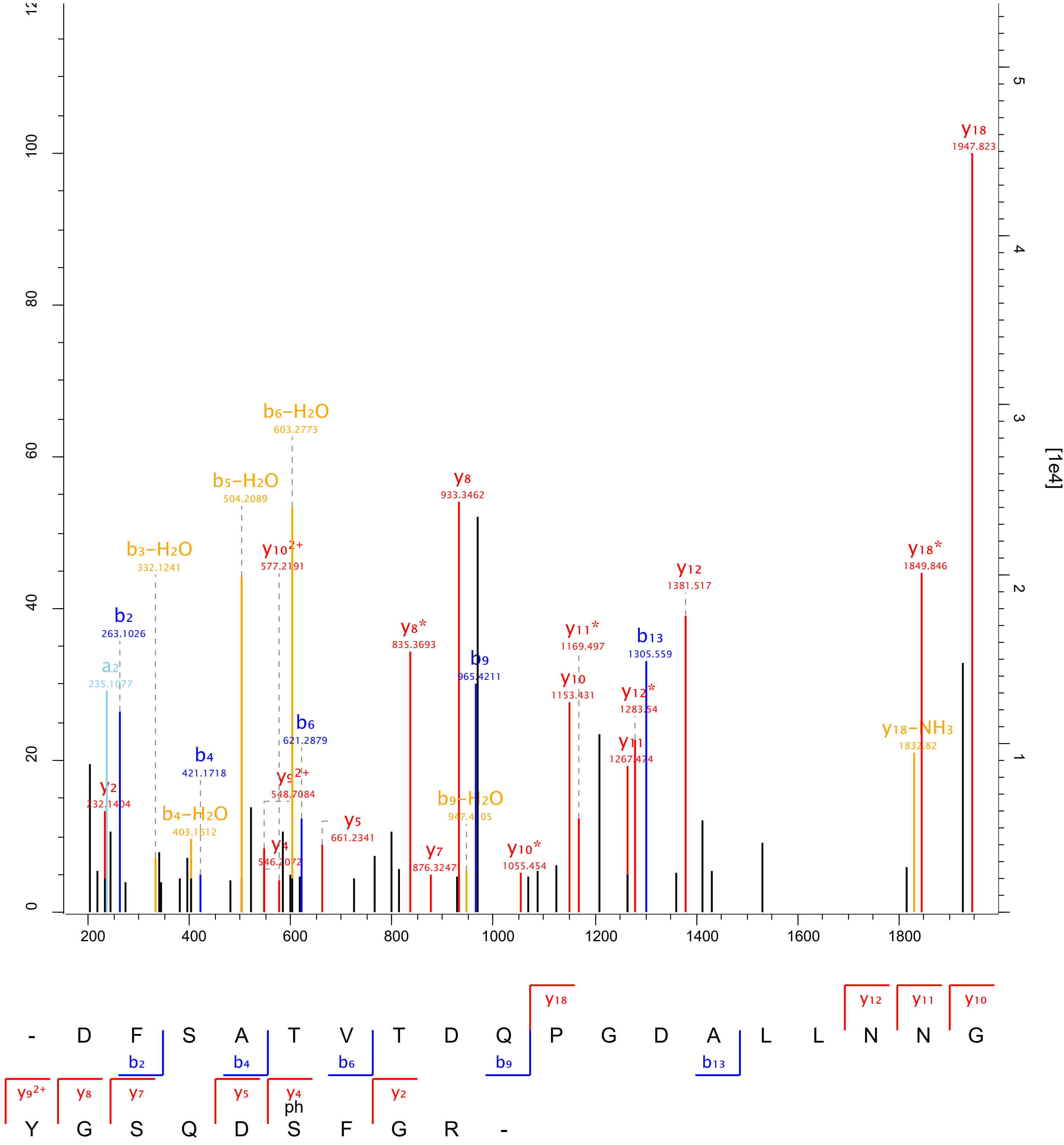

Sample output to test PDF Combine only

Raw file	Scan	Method	Score	m/z	Gene names
0523_5	24509	FTMS; HCD	152.85	1050.78	At5g21160;LARP1A

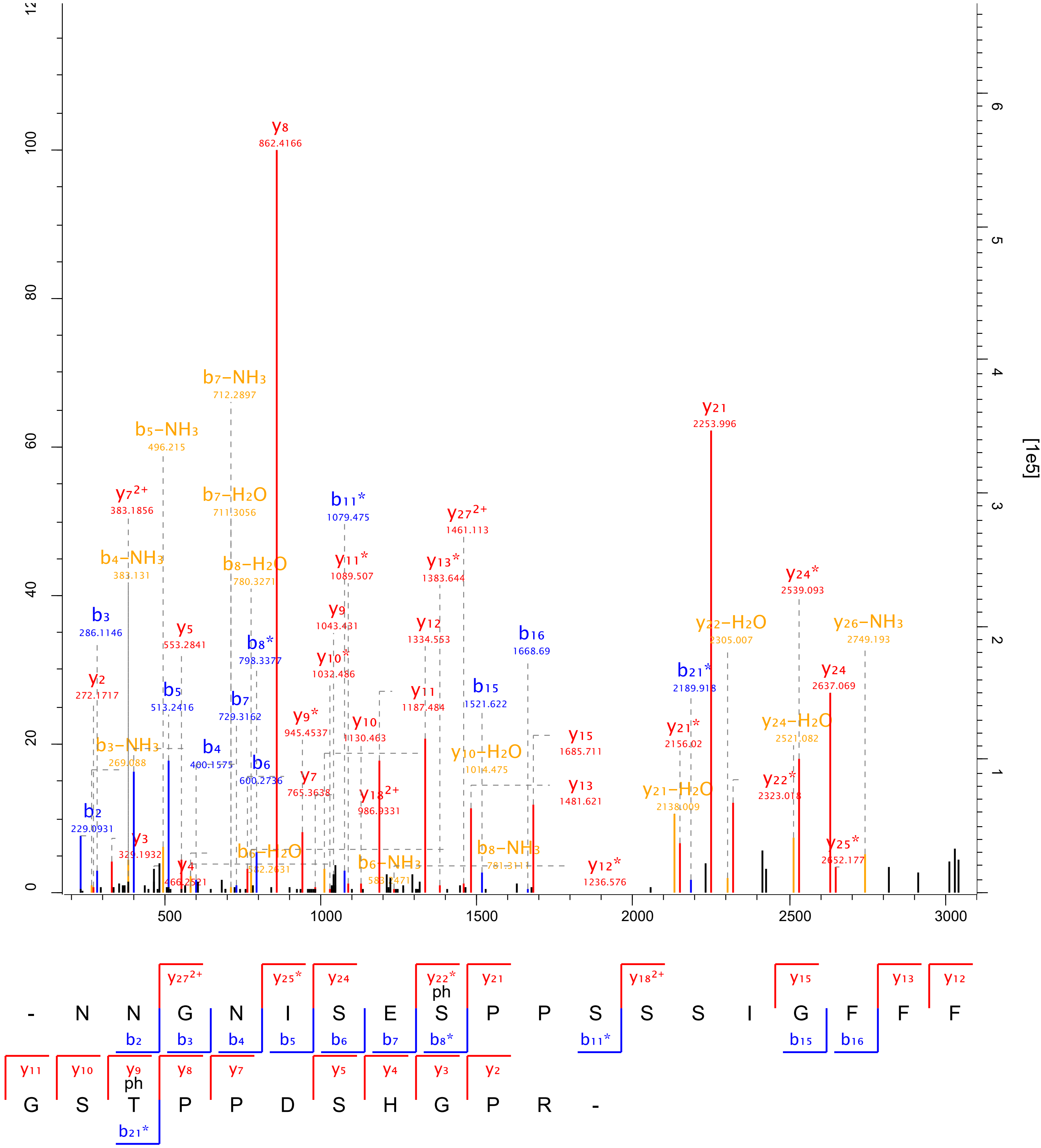

Raw file	Scan	Method	Score	m/z	Gene names
0523_5	24674	FTMS; HCD	253.1	992.95	F24J8.3

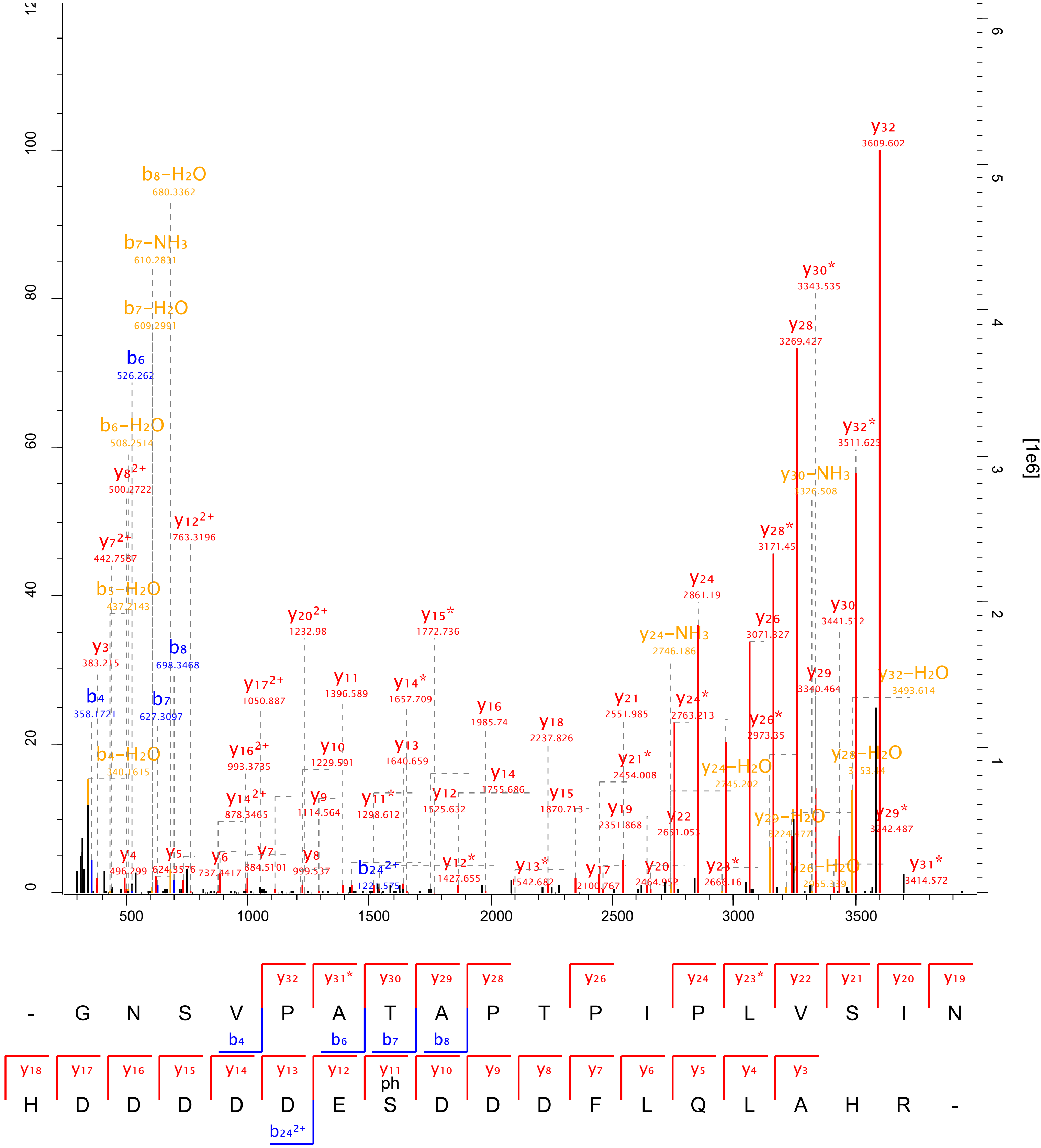

Sample output to test PDF Combine only

Raw file	Scan	Method	Score	m/z
0523_5	24772	FTMS; HCD	191.04	936.47

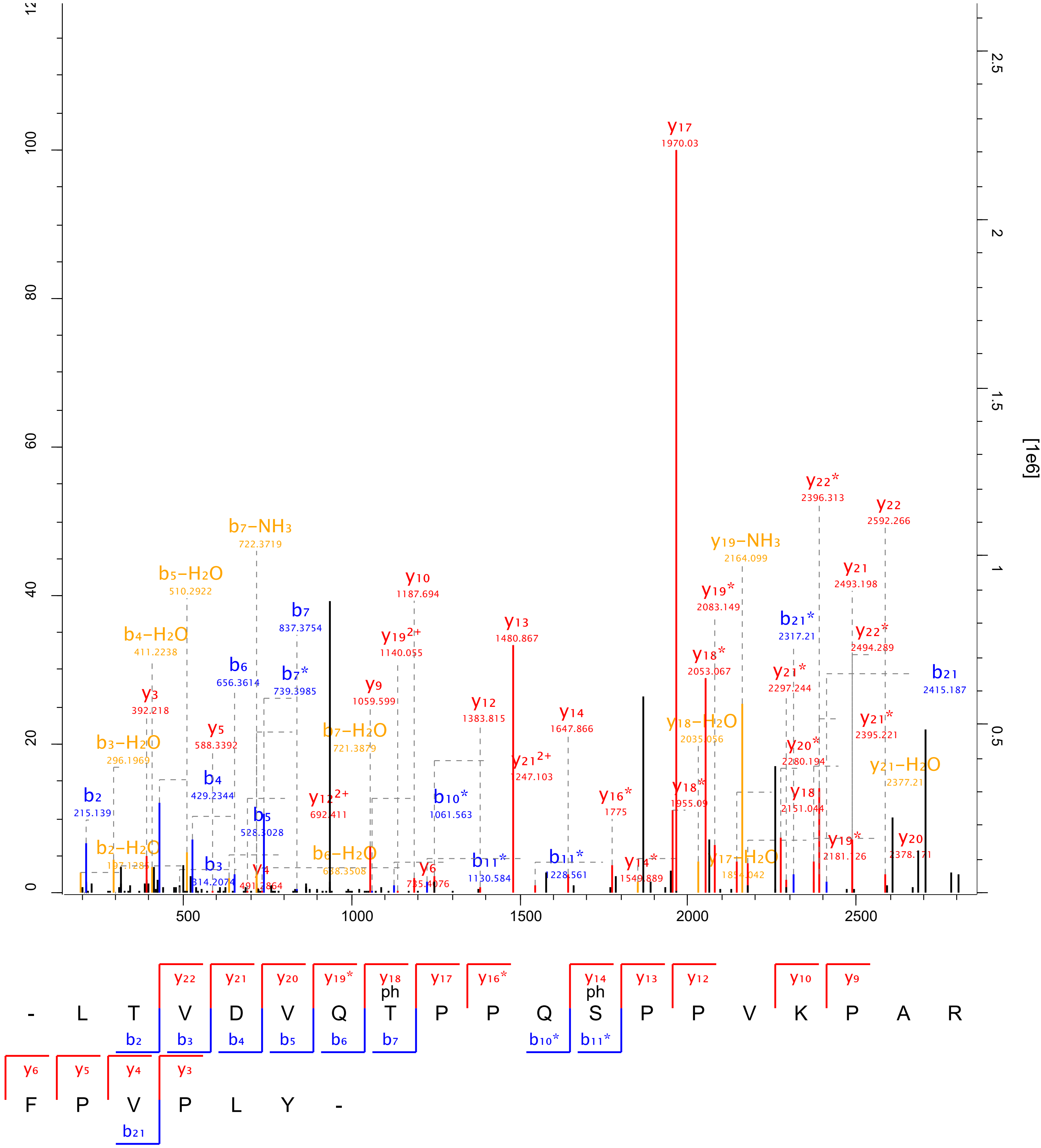

Sample output to test PDF Combine only

Raw file	Scan	Method	Score	m/z
0523_5	24997	FTMS; HCD	80.79	1056.51

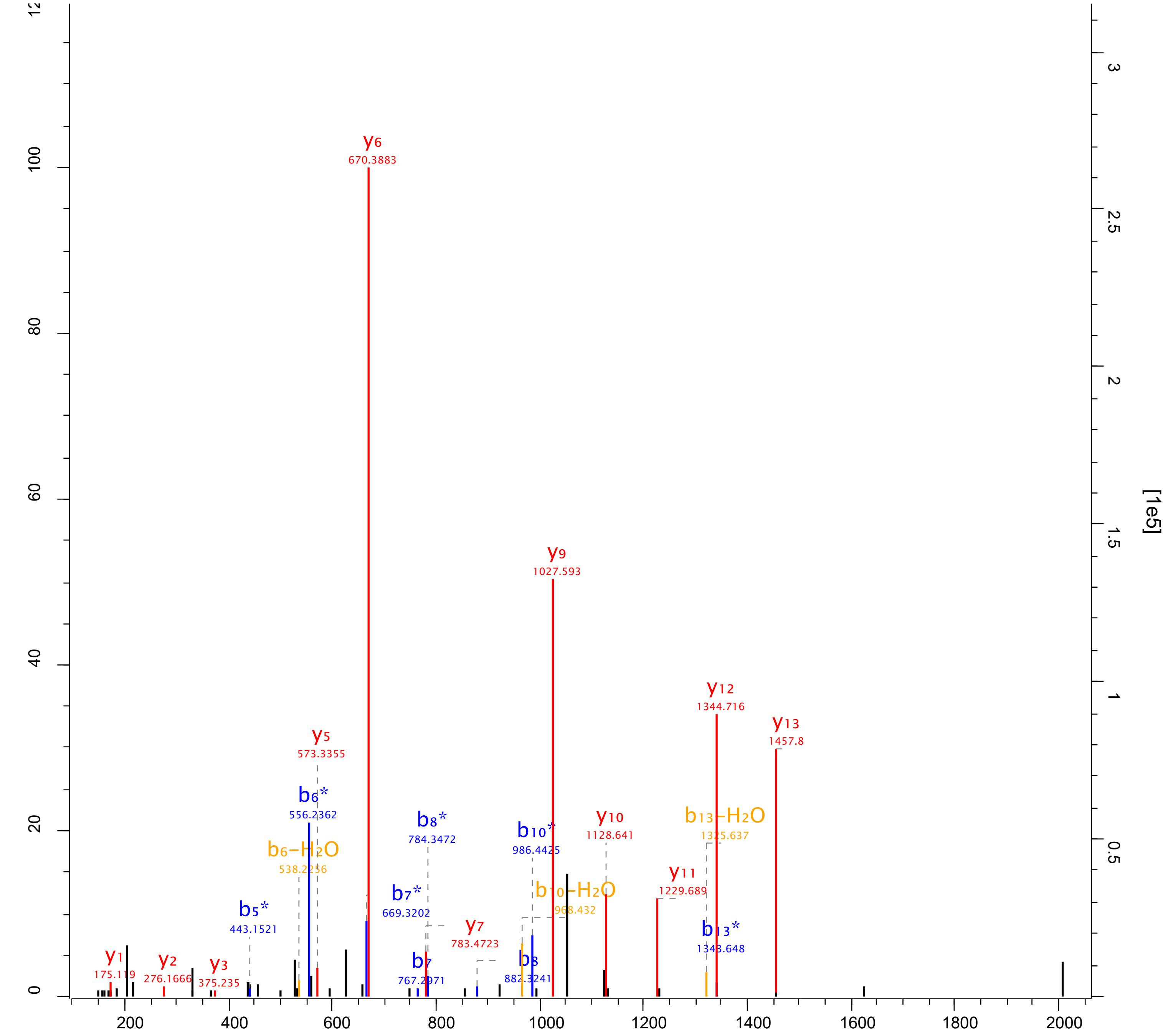

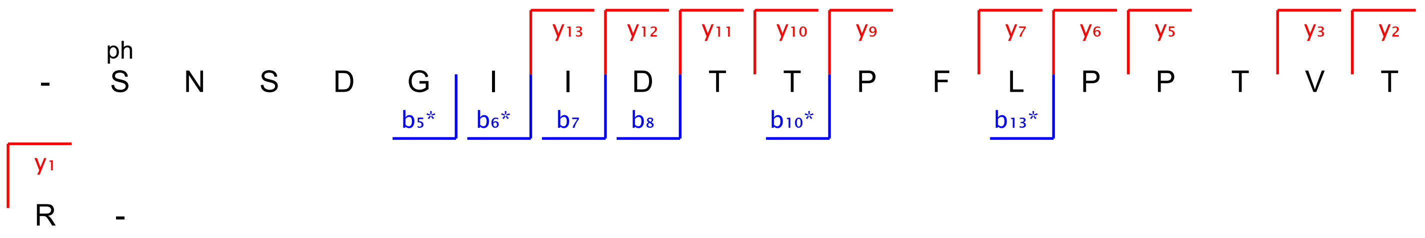

Sample output to test PDF Combine only

Raw file	Scan	Method	Score	m/z	Gene names
0523_5	25496	FTMS; HCD	47.17	1086.81	At4g26620

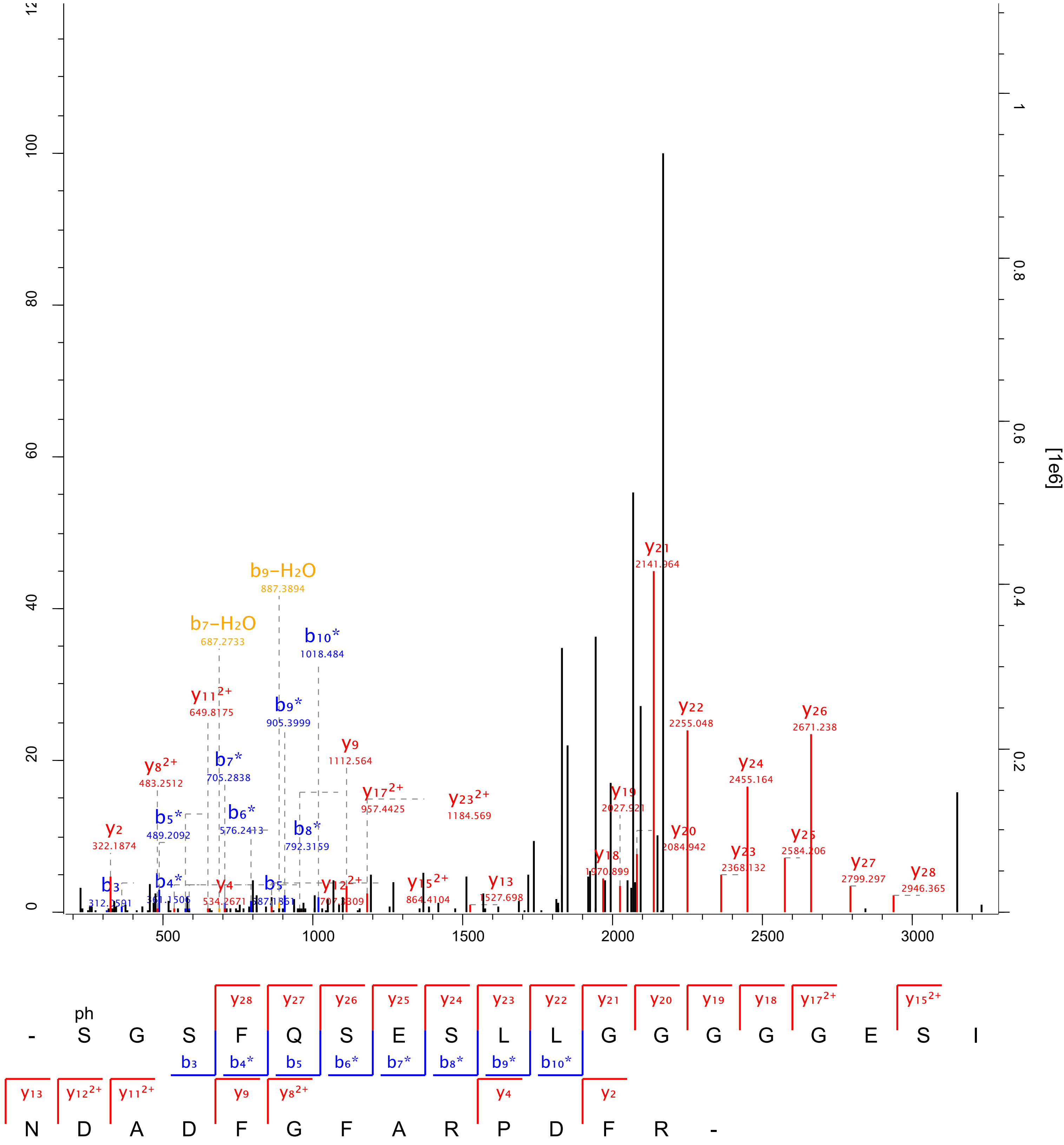

Sample output to test PDF Combine only

Raw file	Scan	Method	Score	m/z	Gene names
0523_5	25542	FTMS; HCD	75.1	818.72	KAT3

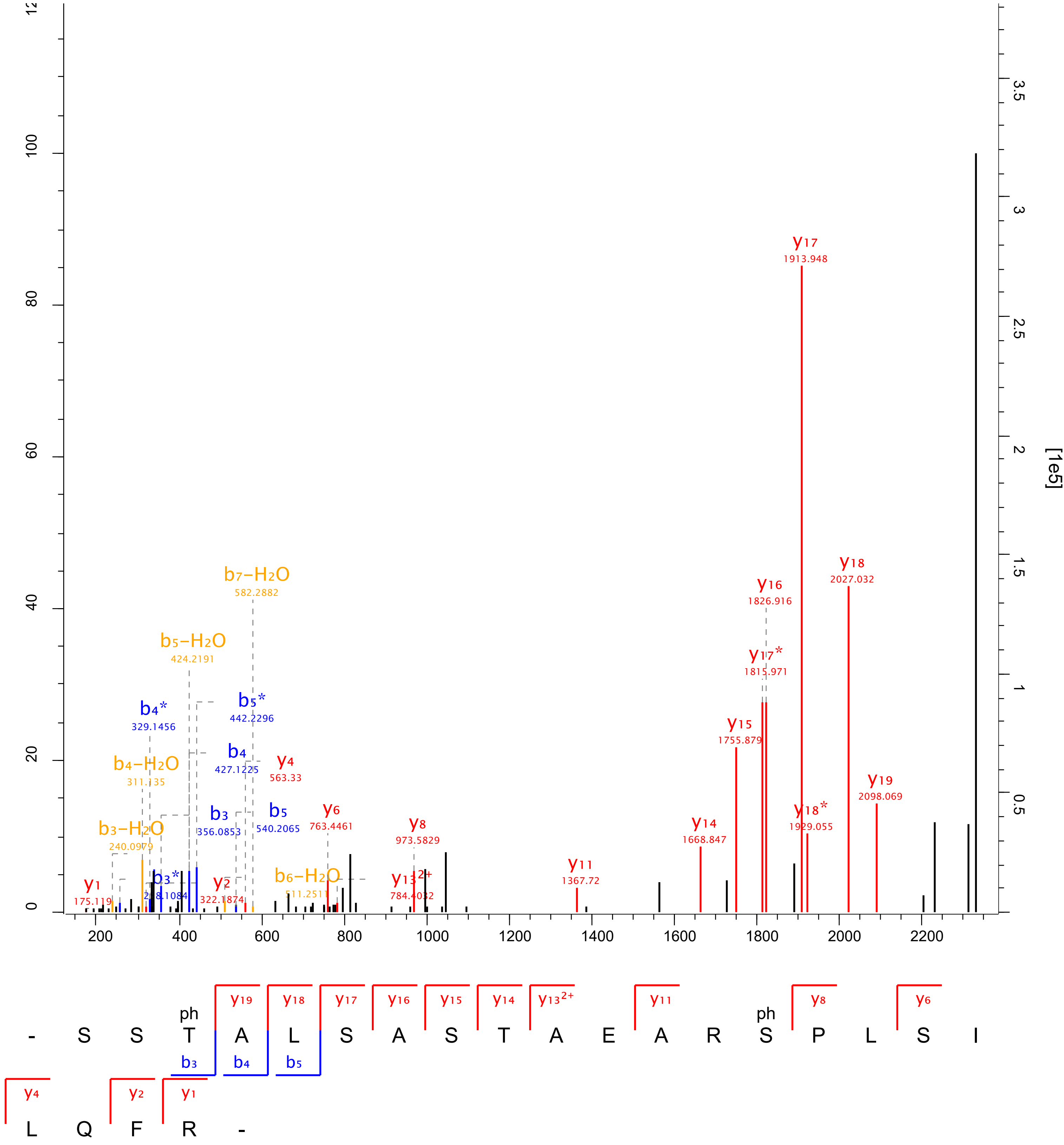

Sample output to test PDF Combine only

Raw file	Scan	Method	Score	m/z	Gene names
0523_5	25797	FTMS; HCD	90.36	976.1	At4g28300

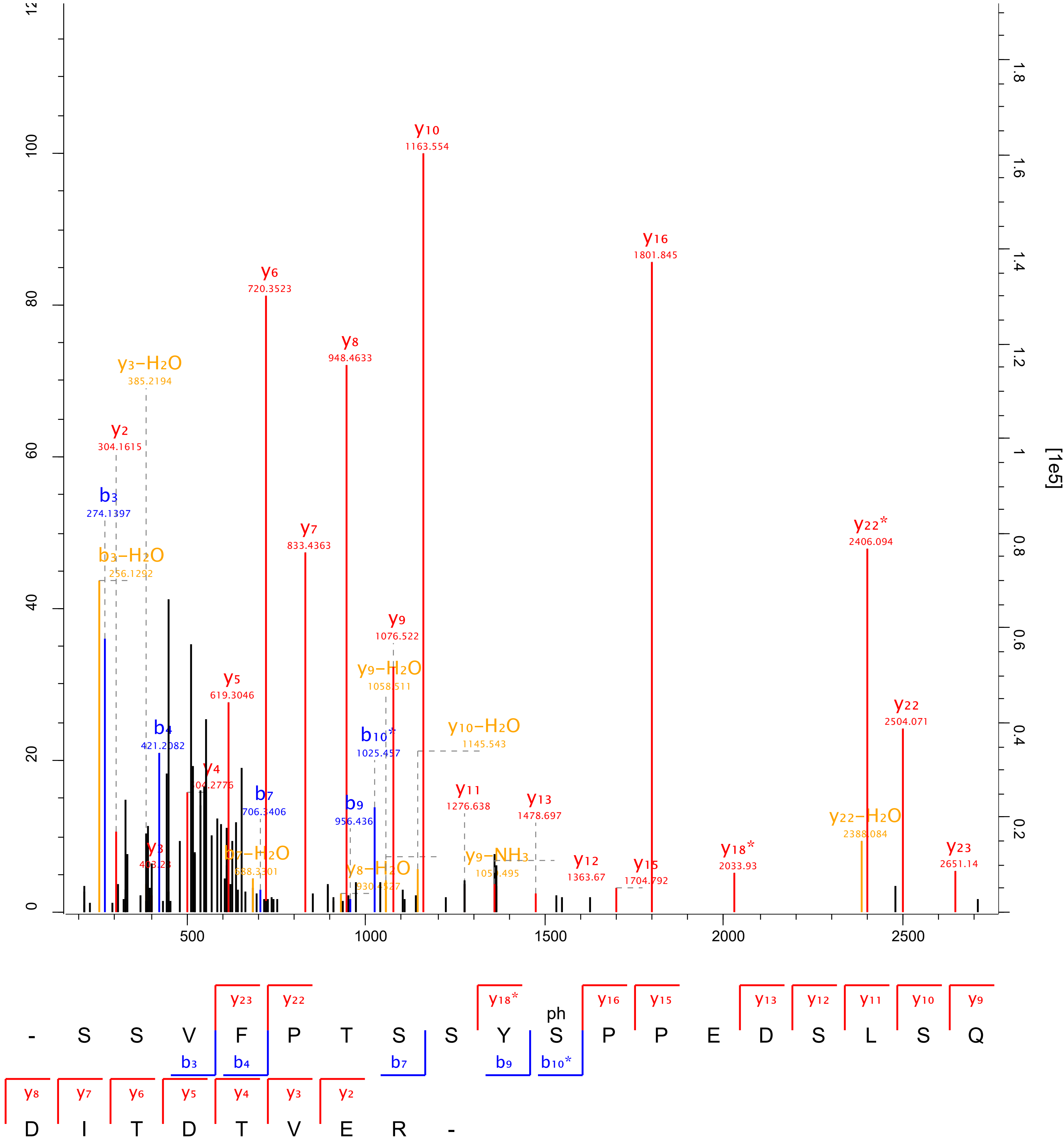

Sample output to test PDF Combine only

Raw file	Scan	Method	Score	m/z	Gene names
0523_5	26790	FTMS; HCD	150.63	810.04	LRR-RLK

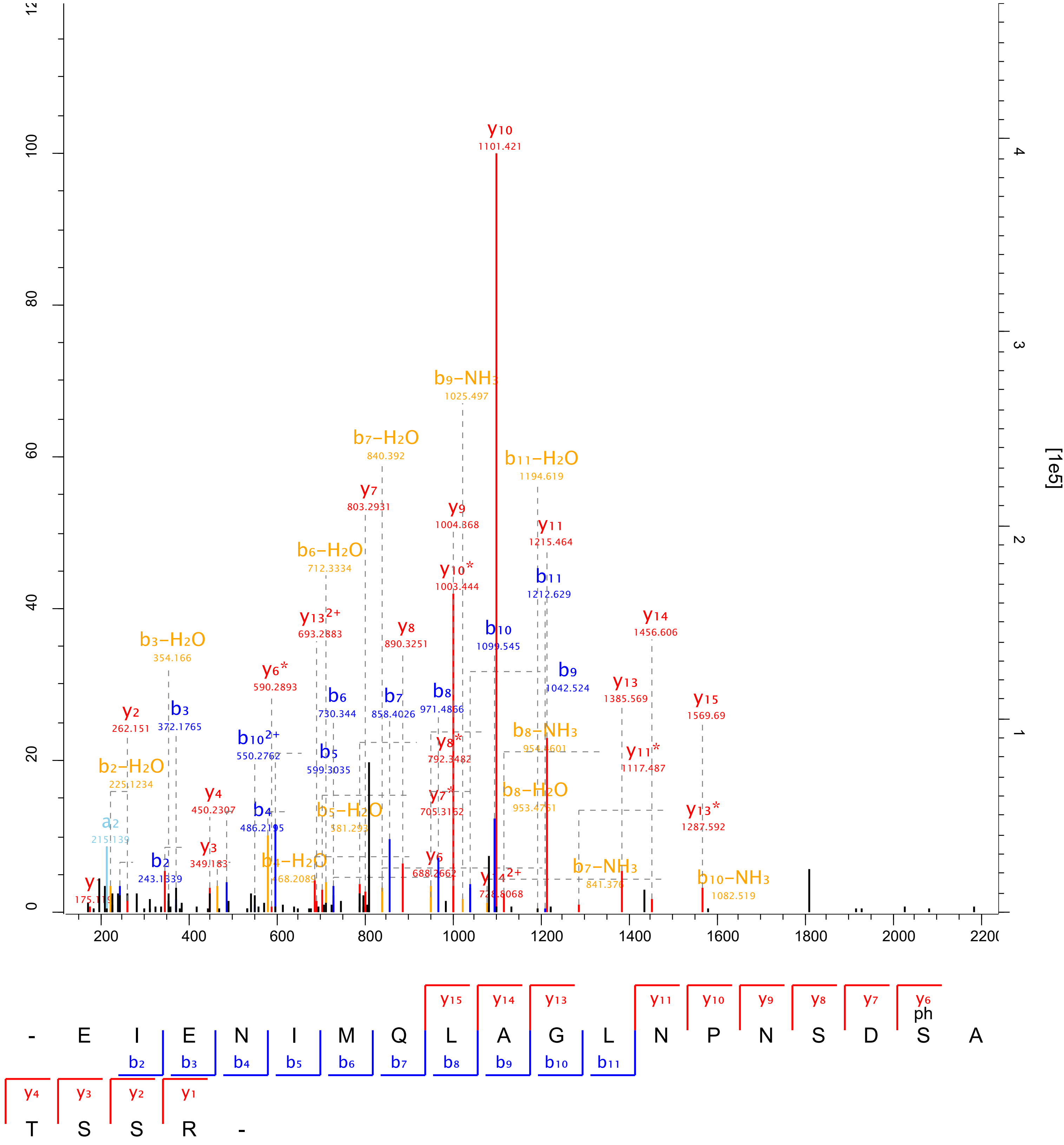

Raw file	Scan	Method	Score	m/z
0523_5	27346	FTMS; HCD	54.17	978.18

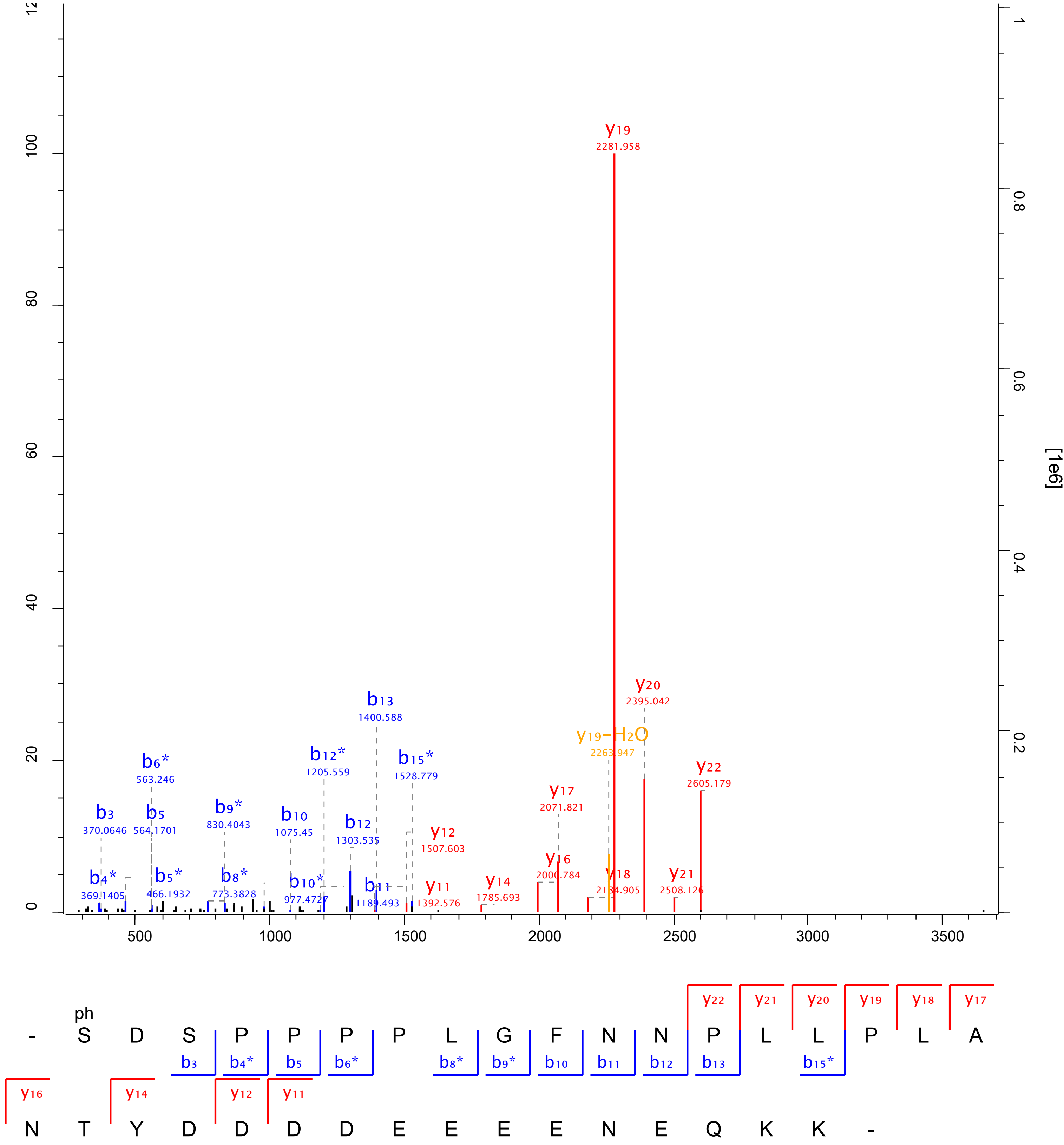

Sample output to test PDF Combine only

Raw file	Scan	Method	Score	m/z	Gene names
0523_5	28163	FTMS; HCD	161.17	1122.99	At3g09200;RPP0B

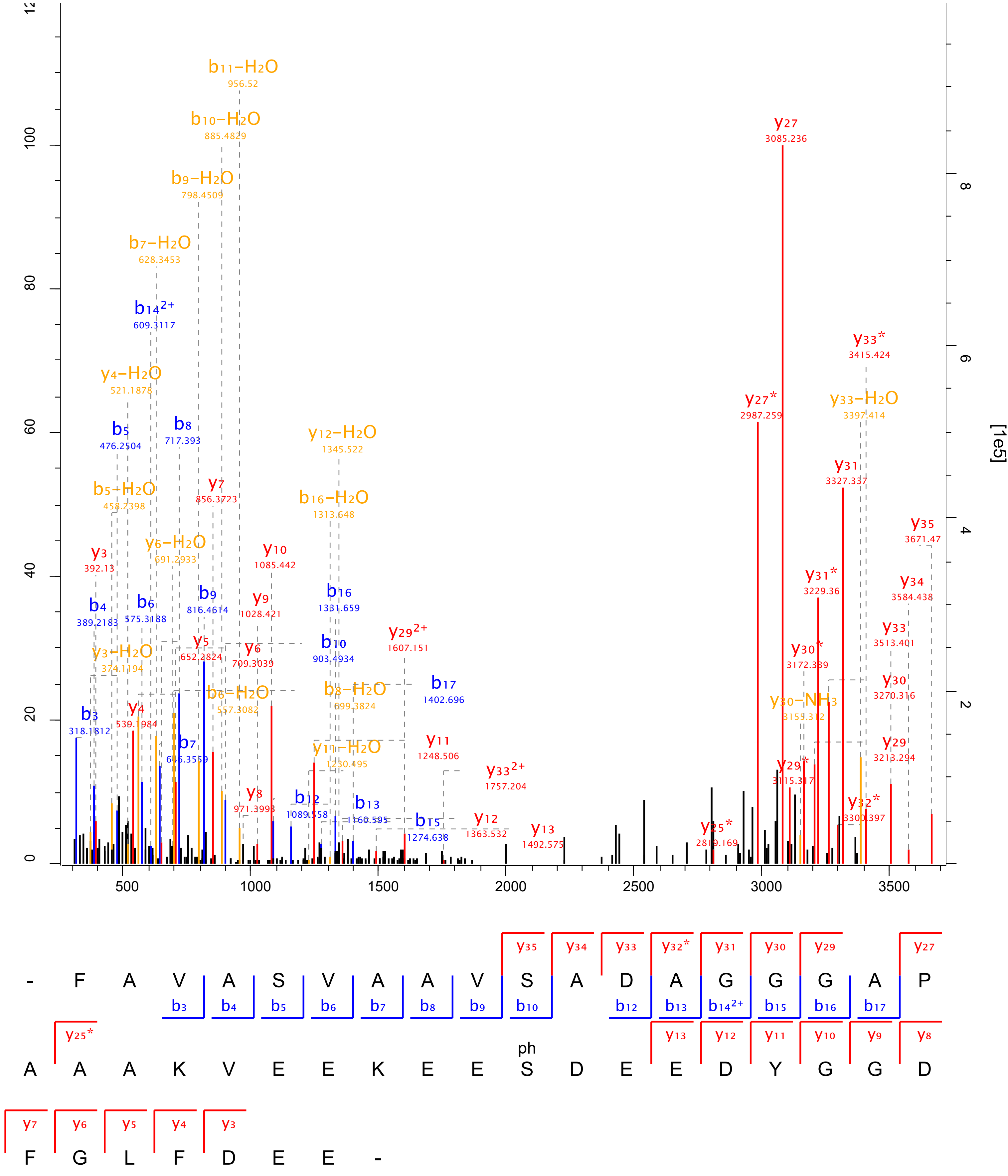

Sample output to test PDF Combine only

Raw file	Scan	Method	Score	m/z	Gene names
0523_5	28933	FTMS; HCD	69.73	1108.74	At5g43960

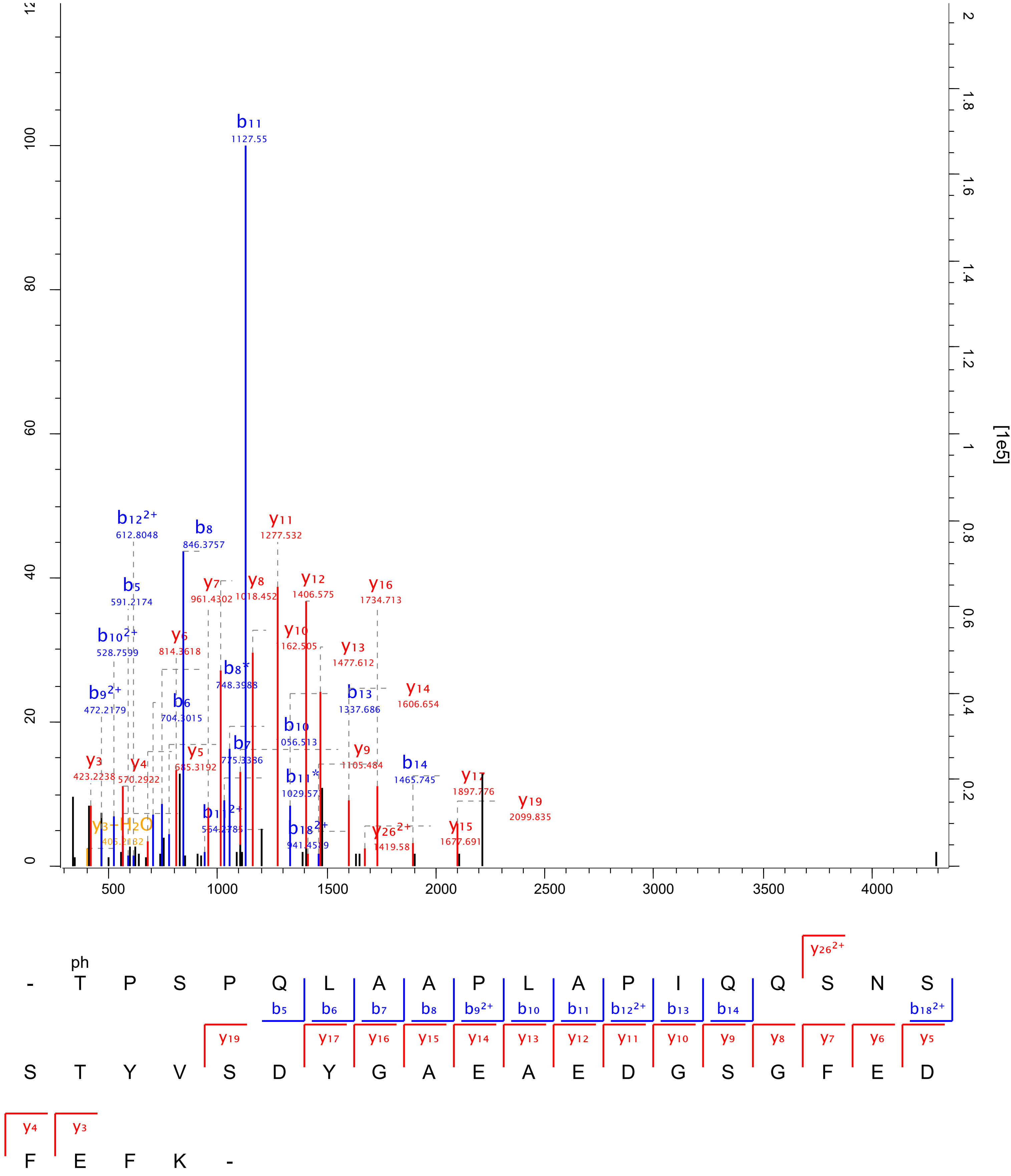

Sample output to test PDF Combine only

Raw file	Scan	Method	Score	m/z	Gene names
0523_5	29416	FTMS; HCD	290.25	1142.01	At4g24800

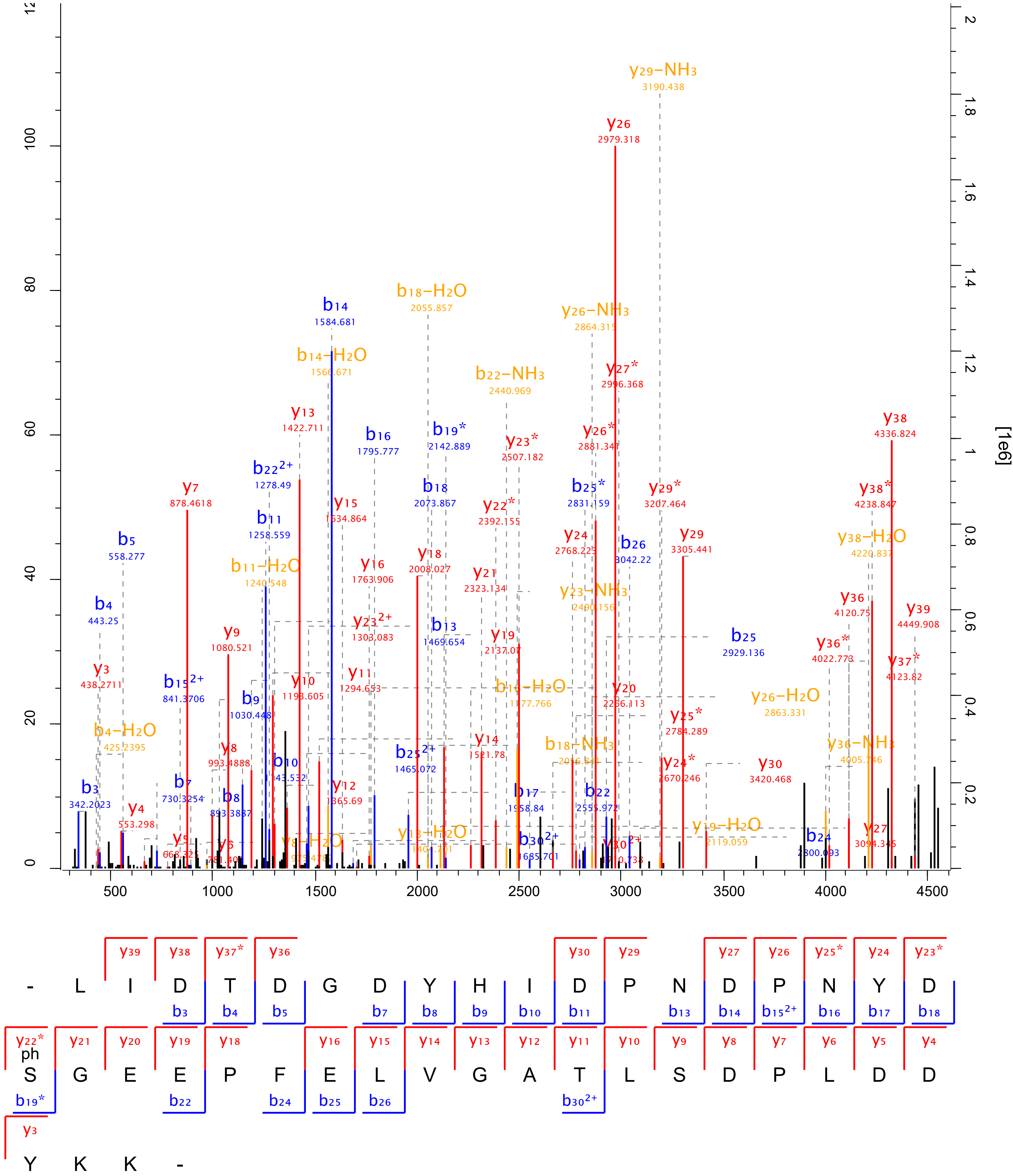

Raw file	Scan	Method	Score	m/z	Gene names
0523_5	29436	FTMS; HCD	247.08	978.08	LRR-RLK

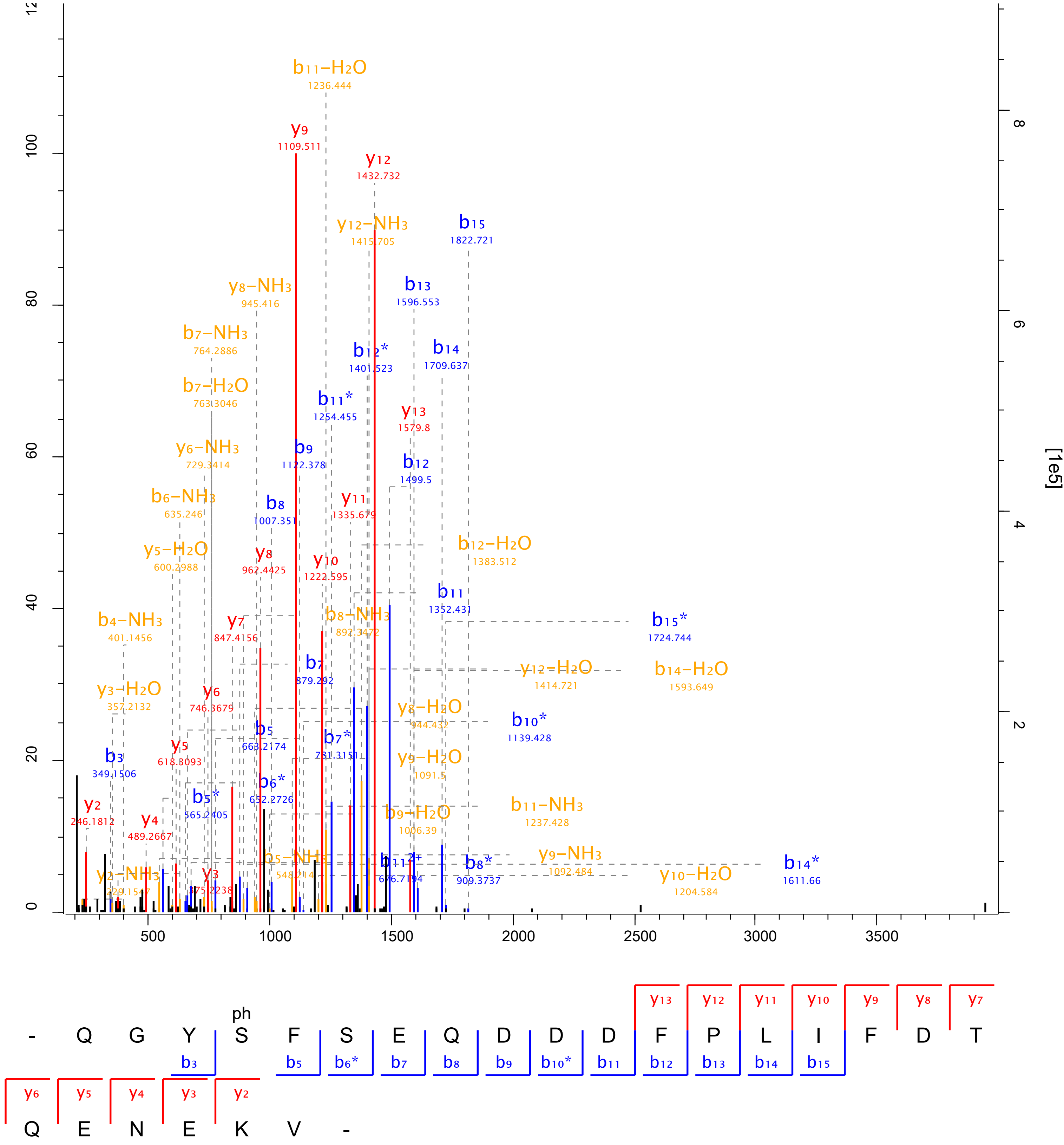

Sample output to test PDF Combine only

Raw file	Scan	Method	Score	m/z	Gene names
0523_5	30001	FTMS; HCD	51.47	825.71	FER

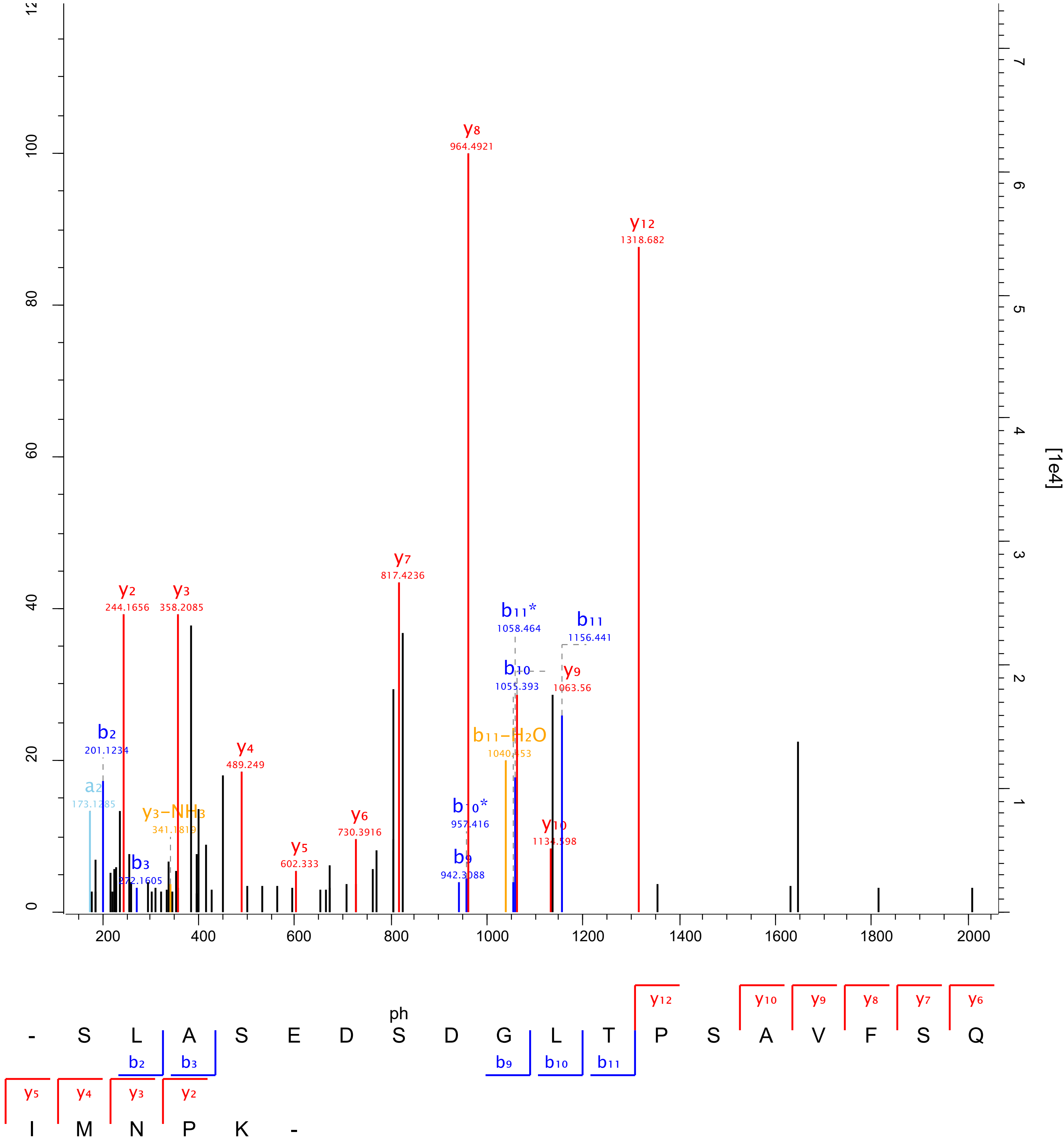

Sample output to test PDF Combine only

Raw file	Scan	Method	Score	m/z	Gene names
0523_5	30118	FTMS; HCD	253.89	1235.84	Y14

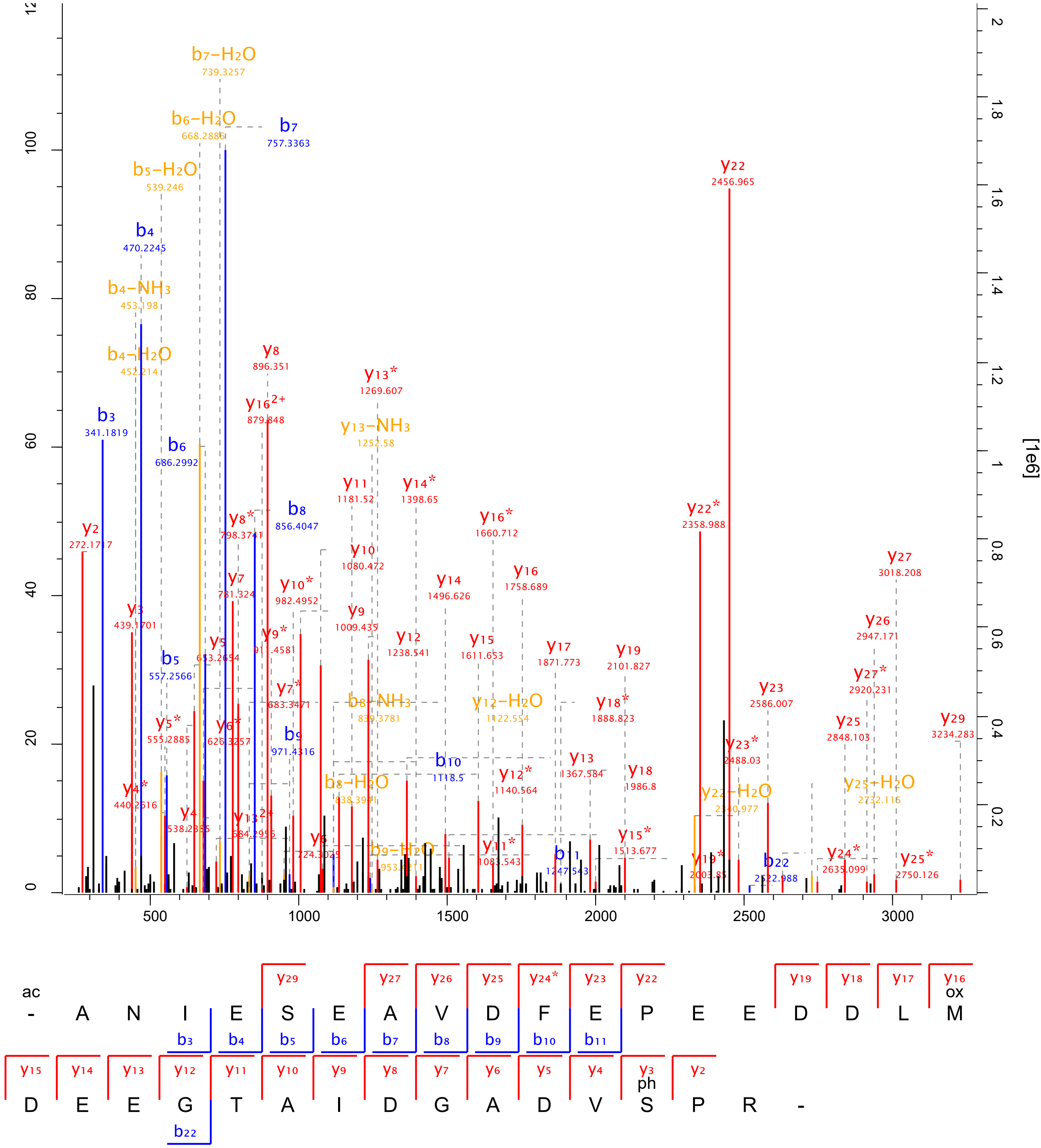

Sample output to test PDF Combine only

Raw file	Scan	Method	Score	m/z
0523_5	30648	FTMS; HCD	146.36	1074.2

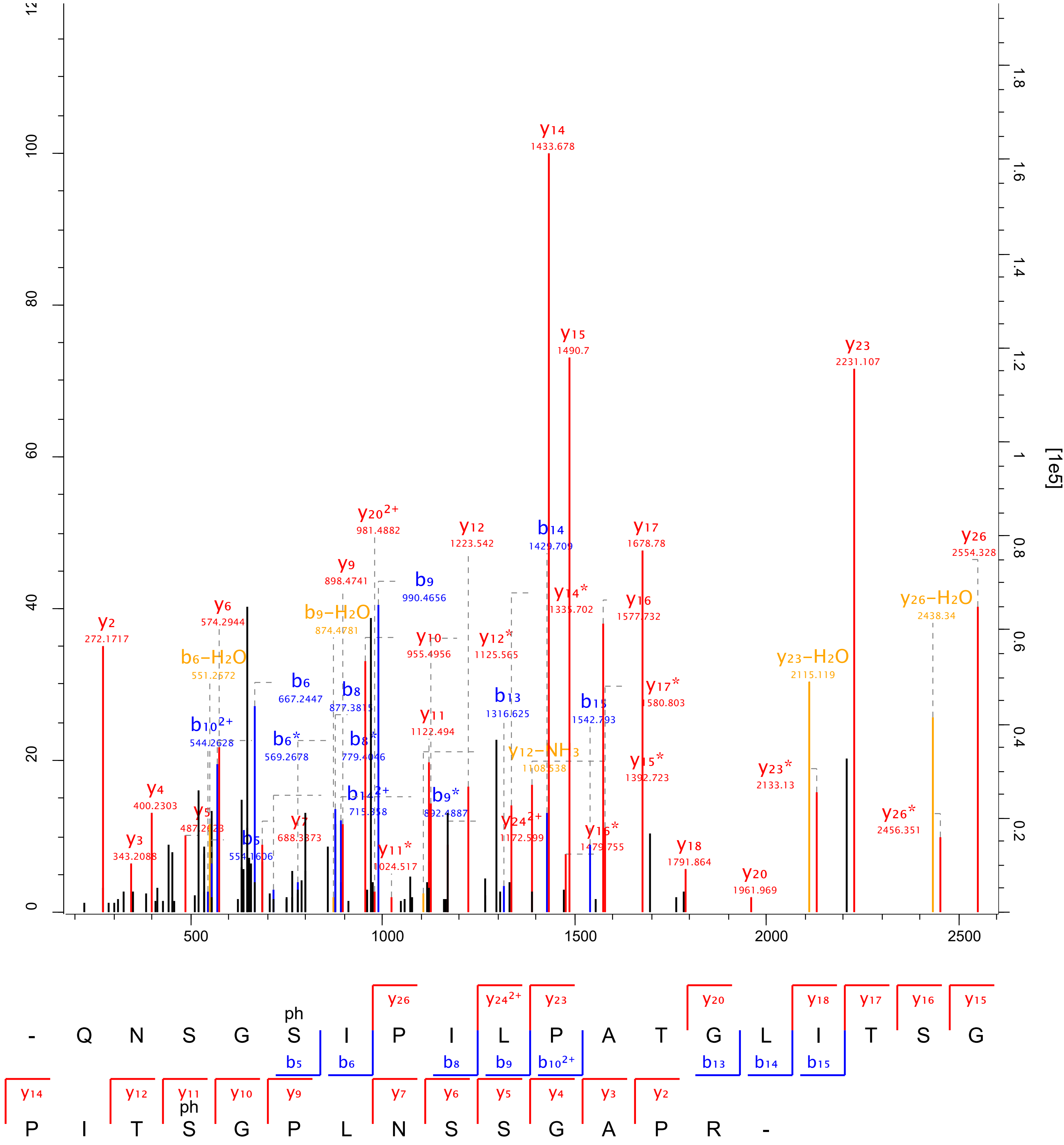

Sample output to test PDF Combine only

Raw file	Scan	Method	Score	m/z	Gene names
0523_5	30858	FTMS; HCD	74.97	885.81	ATL70

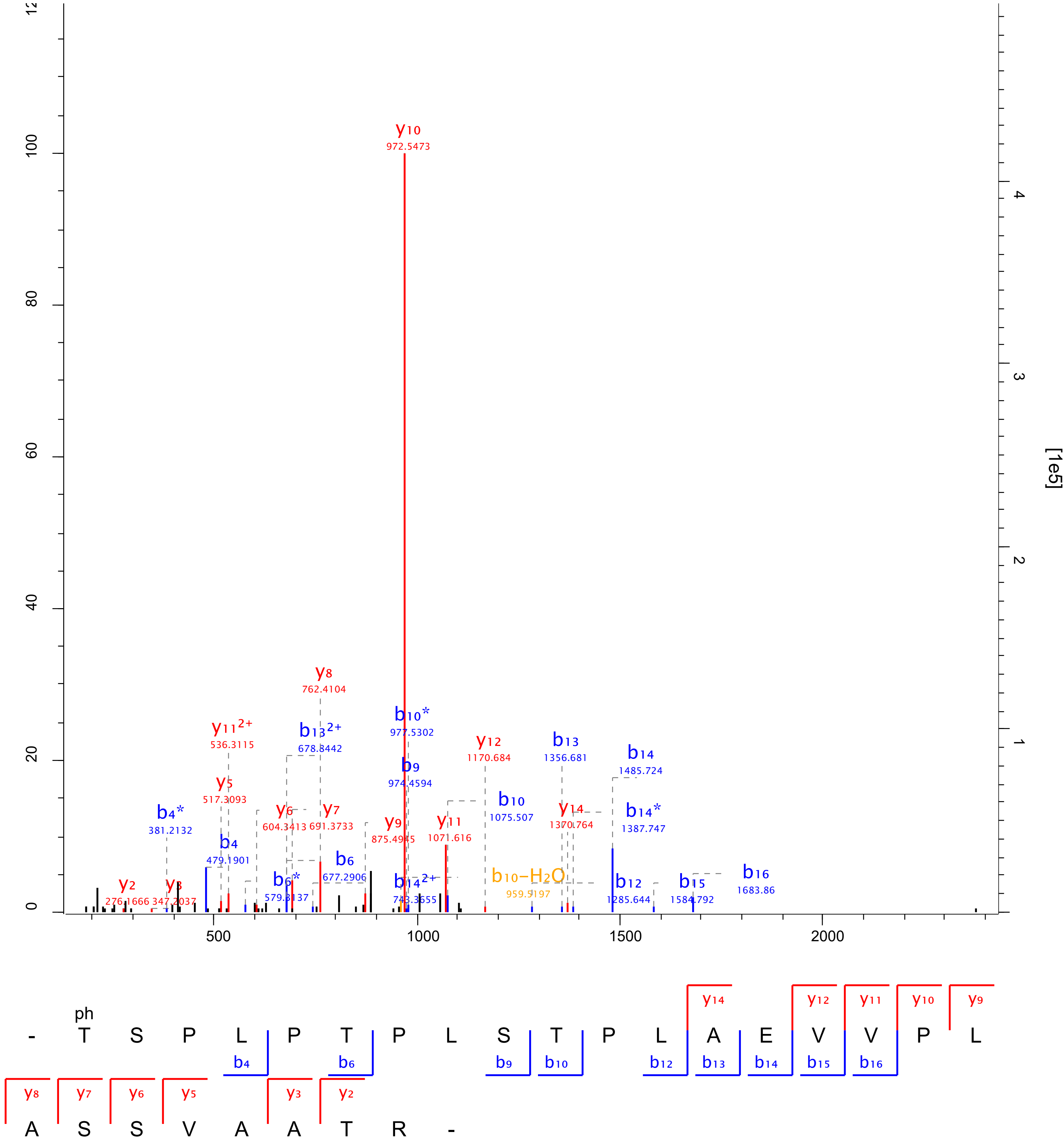

Sample output to test PDF Combine only

Raw file	Scan	Method	Score	m/z
0523_5	31250	FTMS; HCD	45.14	966.14

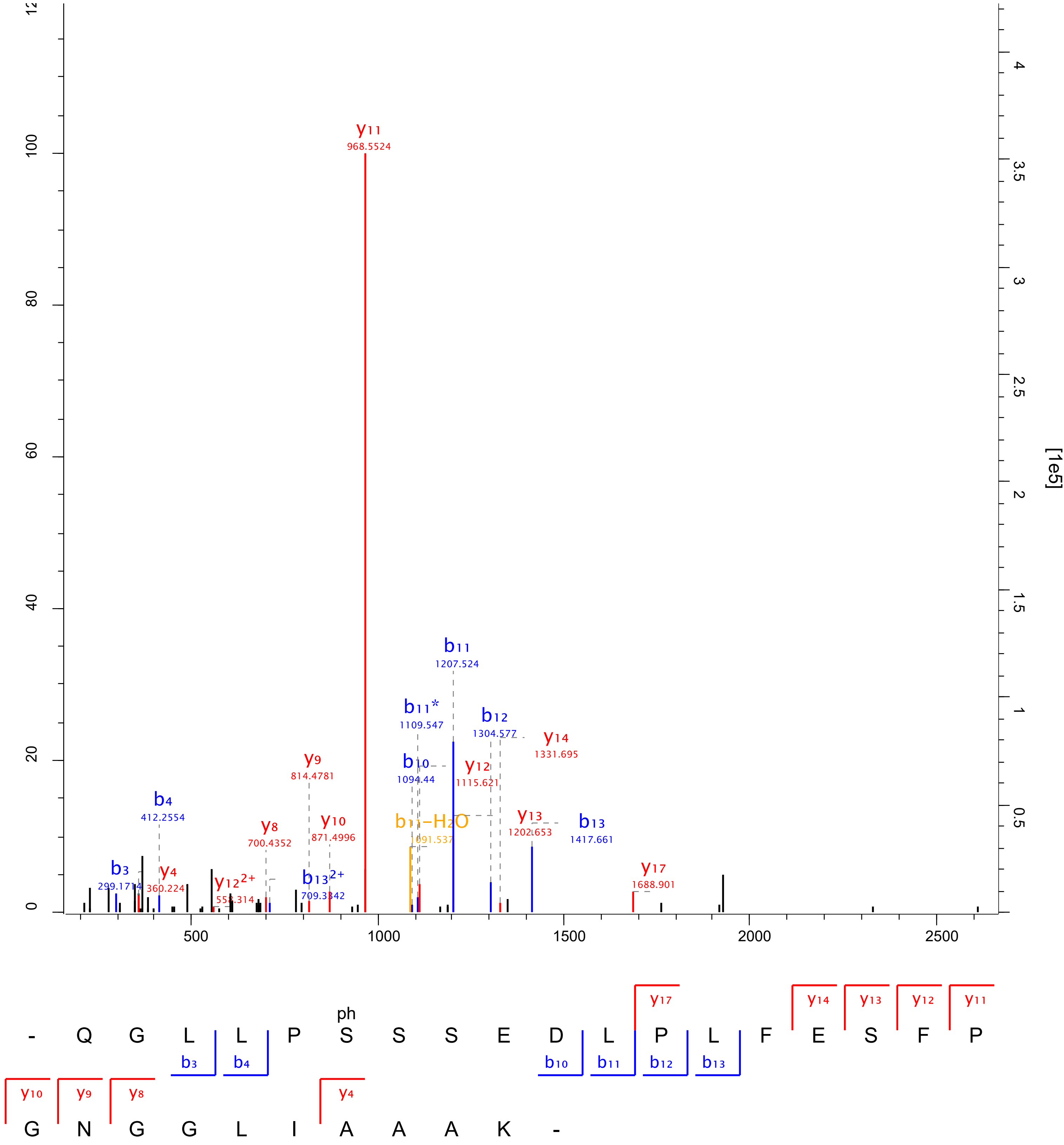

Sample output to test PDF Combine only

Raw file	Scan	Method	Score	m/z
0523_5	31313	FTMS; HCD	87.39	794.86

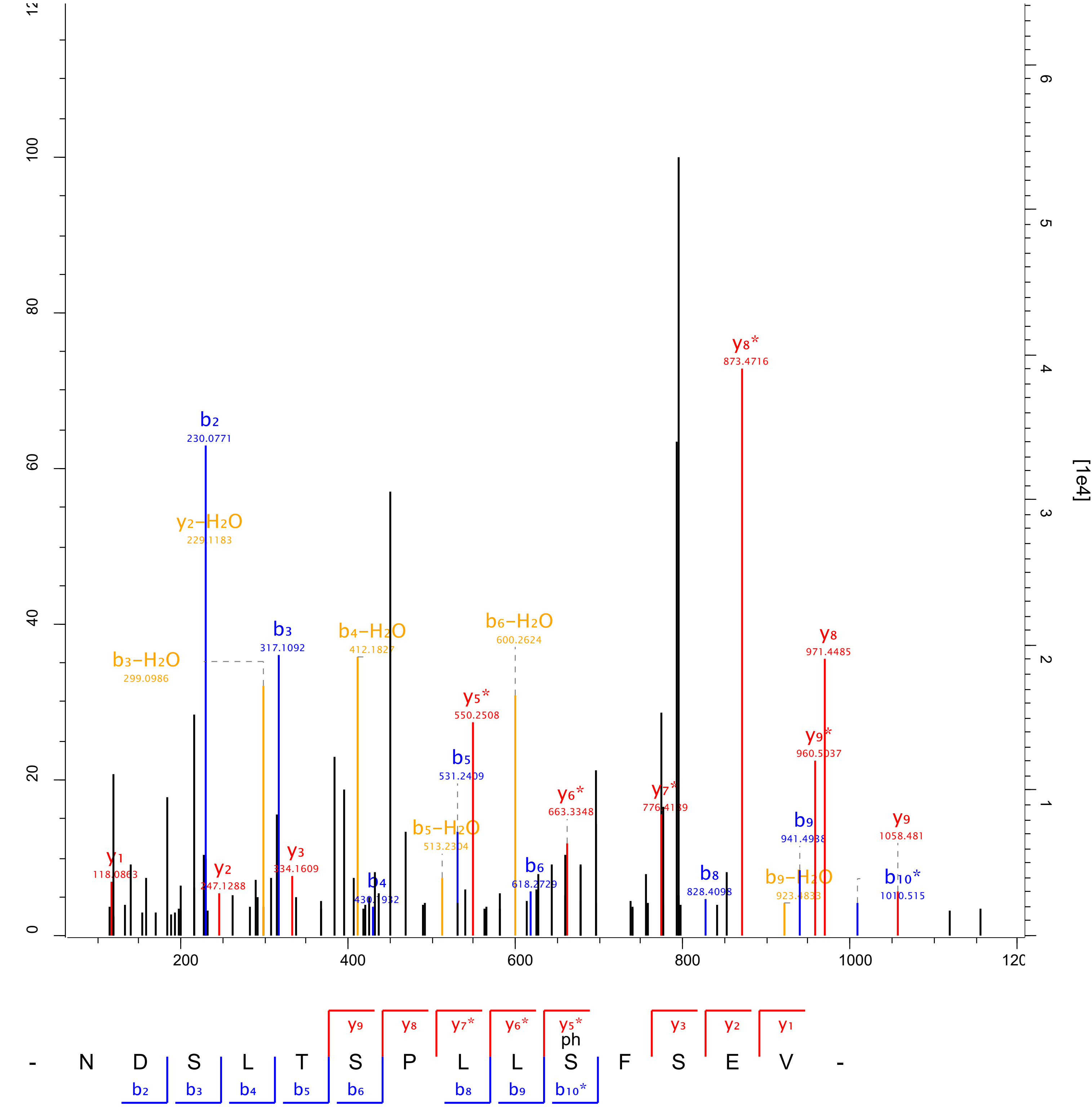

Sample output to test PDF Combine only

Raw file	Scan	Method	Score	m/z	Gene names
0523_5	31890	FTMS; HCD	94.63	1290.25	At4g26620

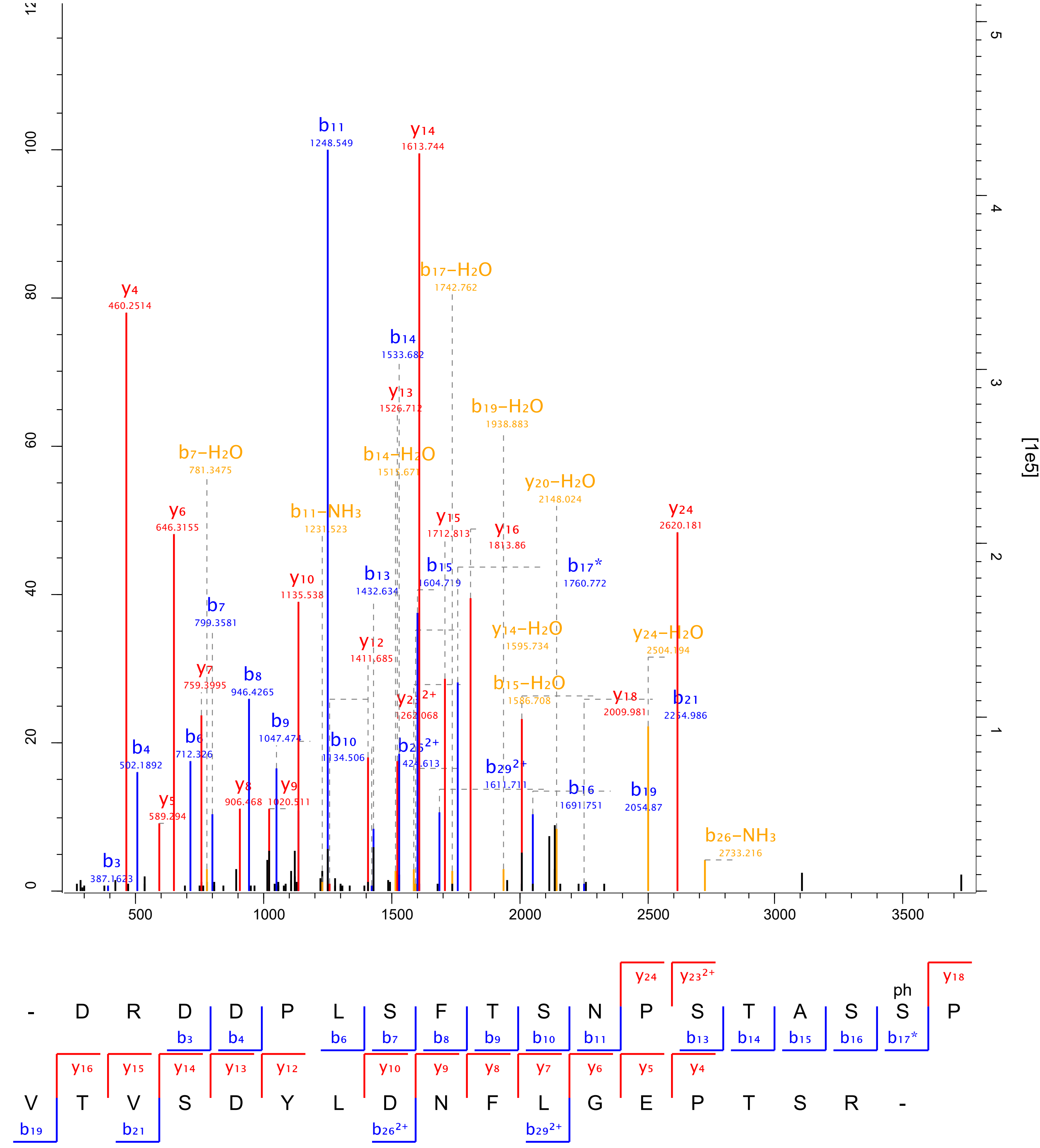

Sample output to test PDF Combine only

Raw file	Scan	Method	Score	m/z	Gene names
0523_5	32154	FTMS; HCD	54.94	1107.26	PVA13

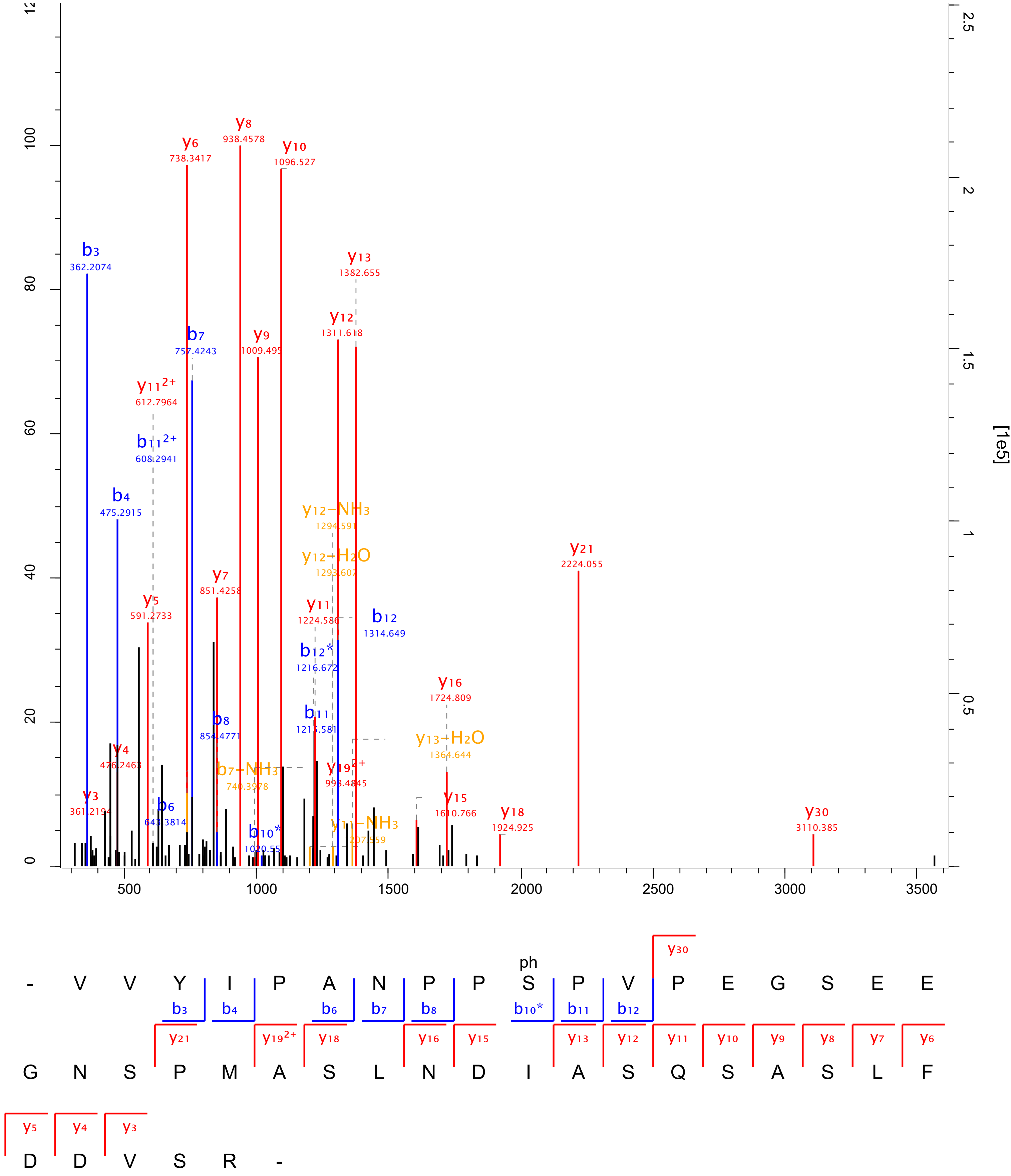

Sample output to test PDF Combine only

Raw file	Scan	Method	Score	m/z	Gene names
0523_5	32248	FTMS; HCD	55.76	840.72	BHLH128

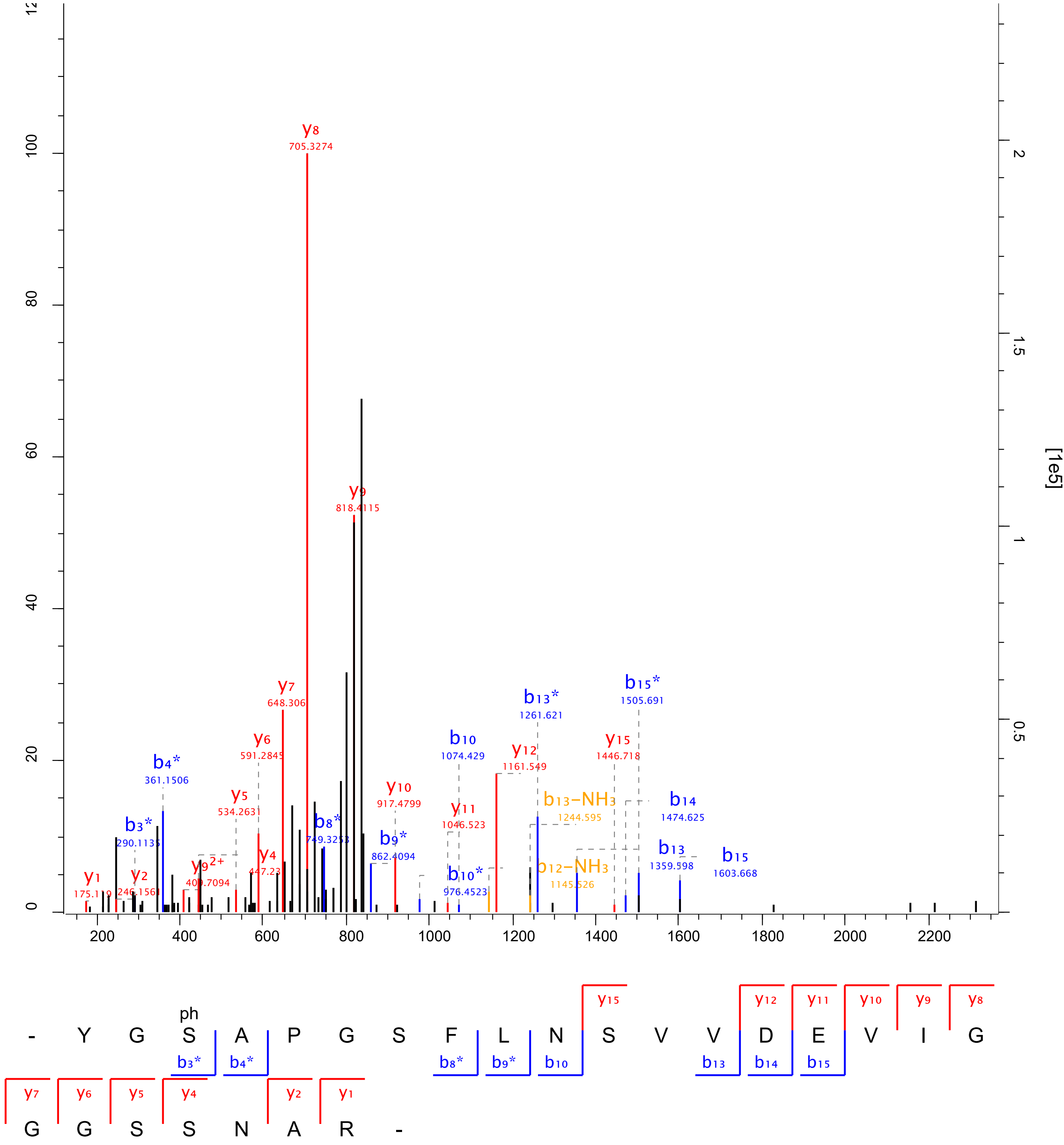

Sample output to test PDF Combine only

Raw file	Scan	Method	Score	m/z	Gene names
0523_5	33131	FTMS; HCD	261.17	1064.47	BAG7

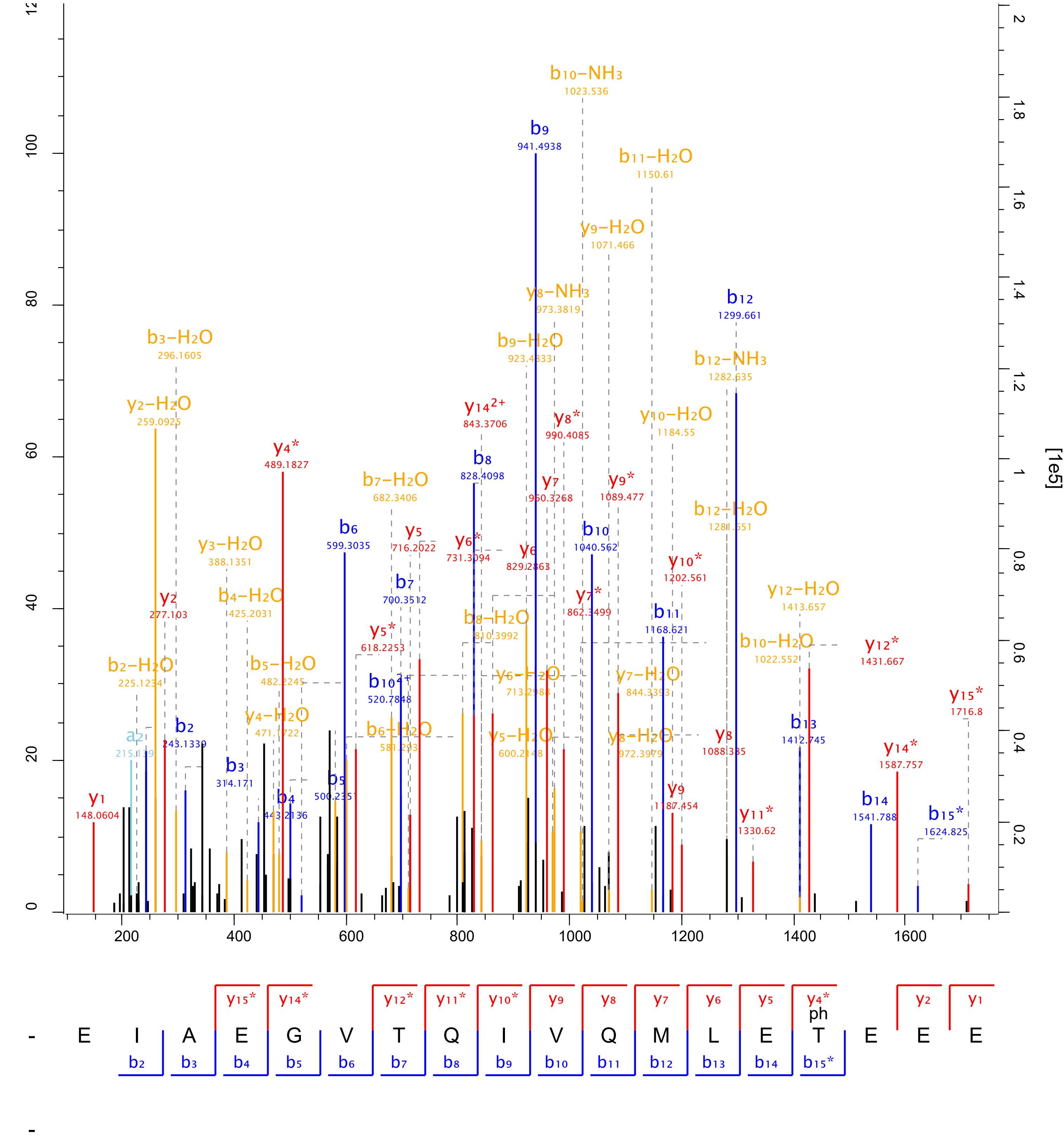

Sample output to test PDF Combine only

Raw file	Scan	Method	Score	m/z	Gene names
0523_6	1455	FTMS; HCD	156.02	379.85	CASP1

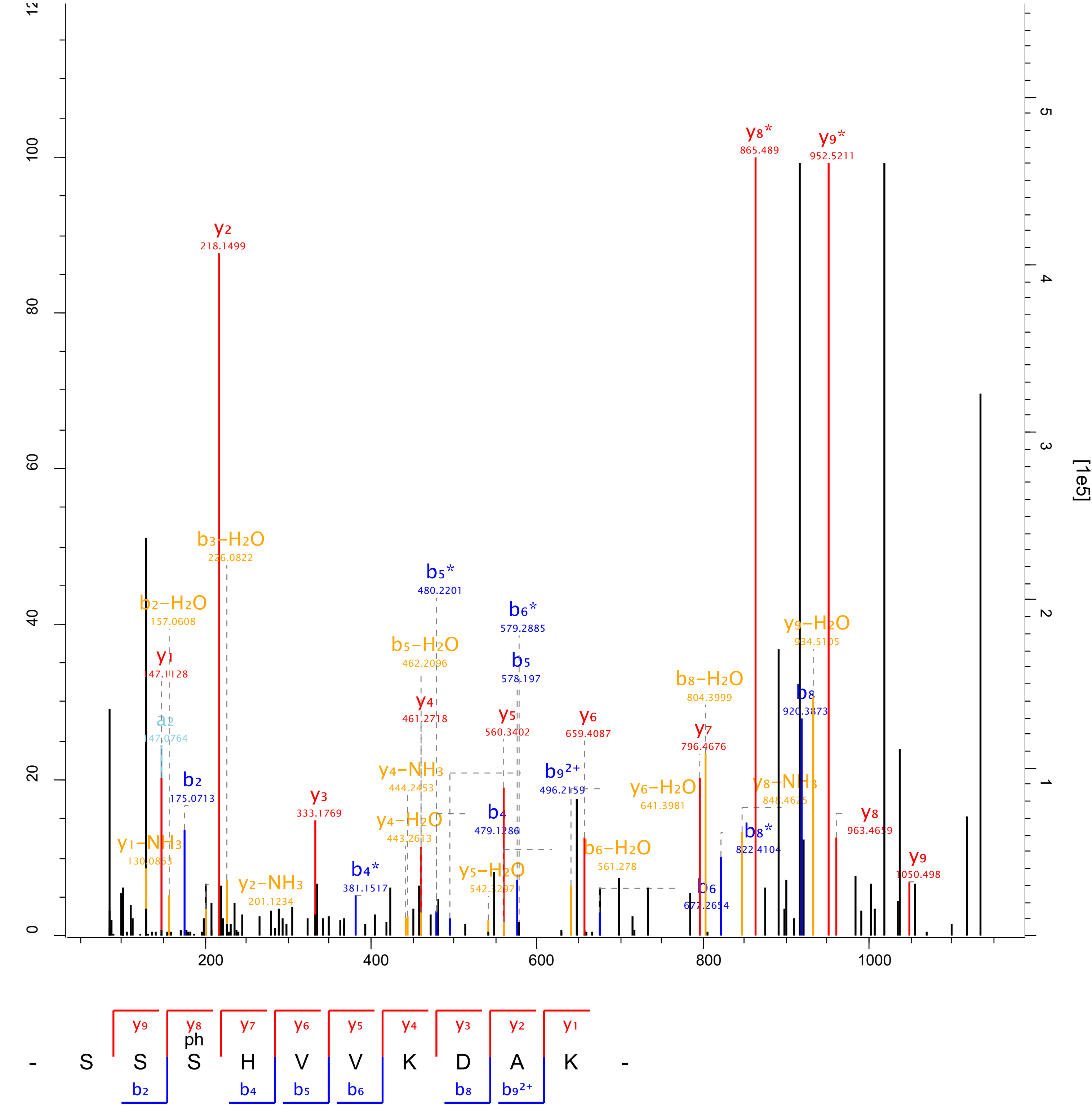

Sample output to test PDF Combine only

Raw file	Scan	Method	Score	m/z	Gene names
0523_6	1482	FTMS; HCD	74.39	492.23	CBP60C

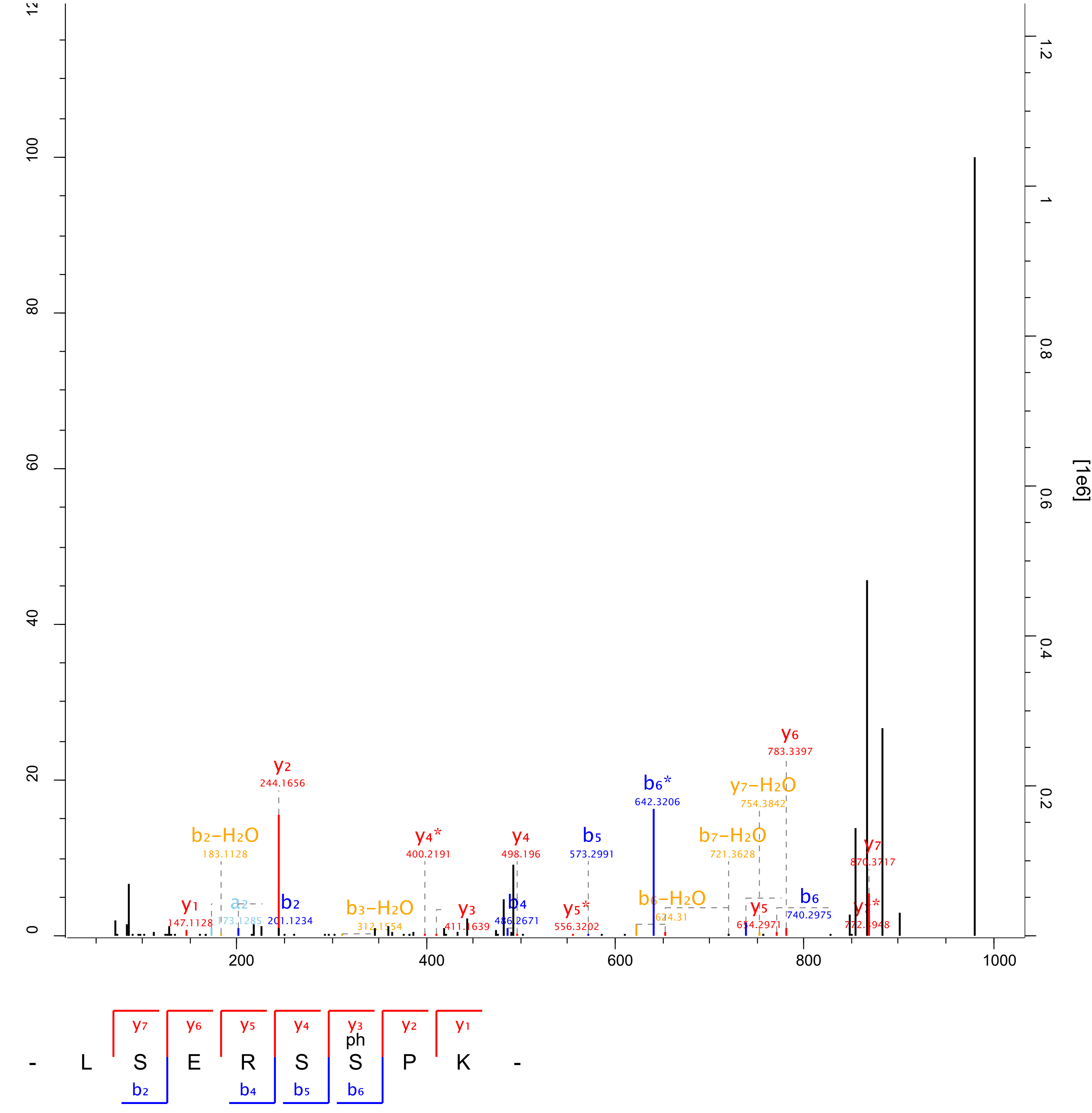

Sample output to test PDF Combine only

Raw file	Scan	Method	Score	m/z	Gene names
0523_6	5979	FTMS; HCD	96.5	642.57	KING1

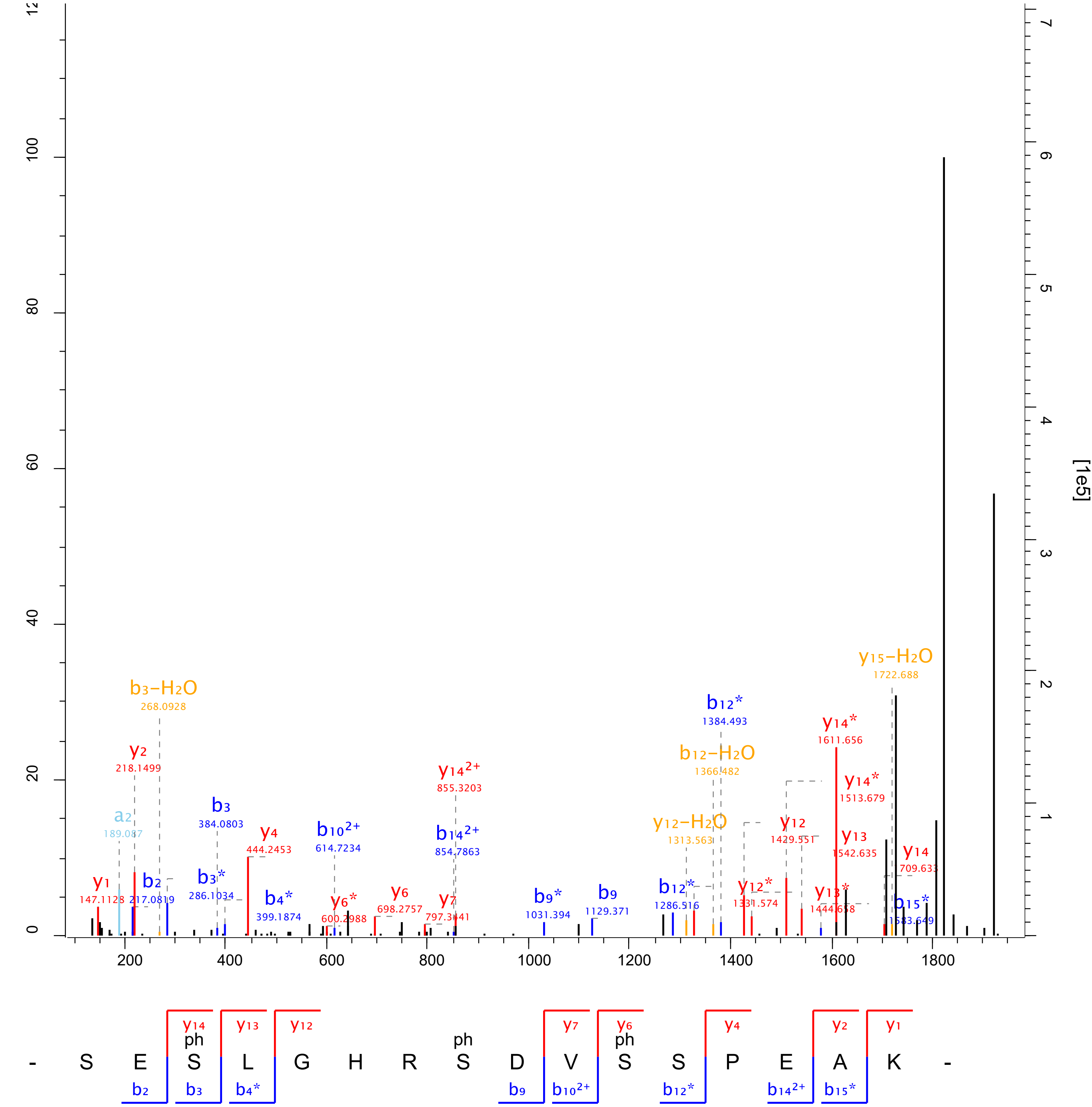

Sample output to test PDF Combine only

Raw file	Scan	Method	Score	m/z
0523_6	6958	FTMS; HCD	65.21	703.24

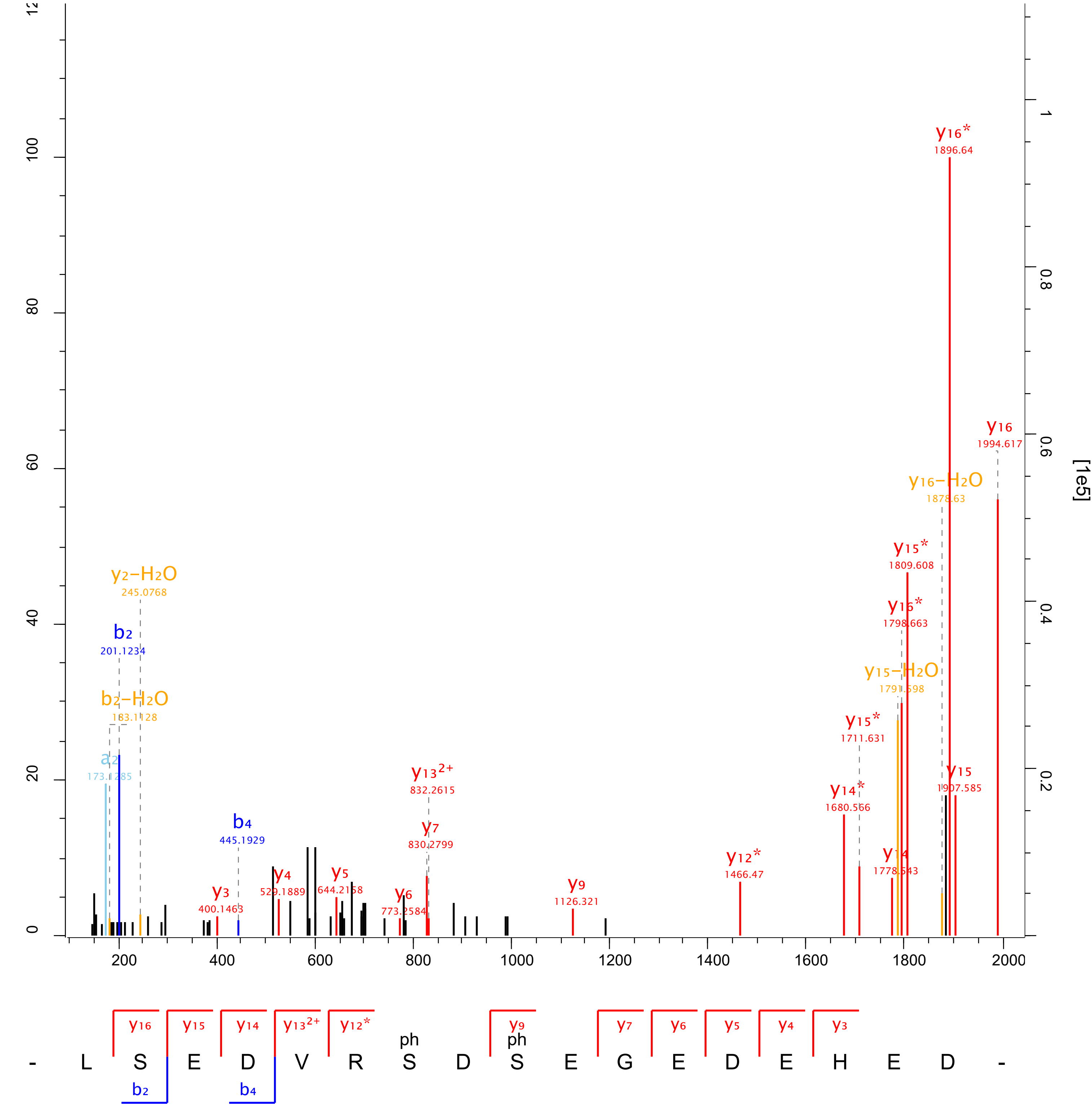

Sample output to test PDF Combine only

Raw file	Scan	Method	Score	m/z	Gene names
0523_6	7084	FTMS; HCD	65.04	466.19	RSZ22A

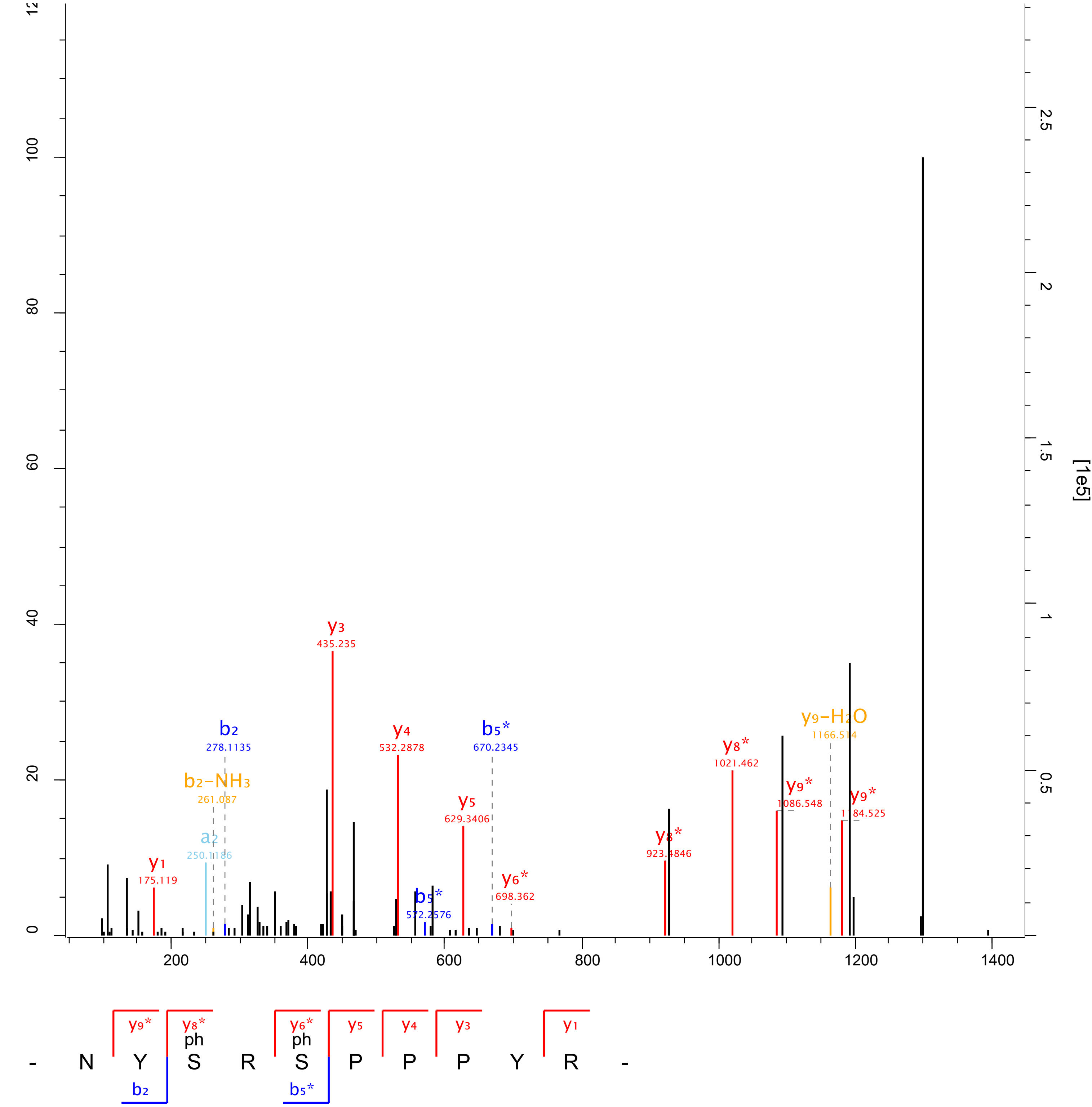

Sample output to test PDF Combine only

Raw file	Scan	Method	Score	m/z	Gene names
0523_6	8358	FTMS; HCD	74.44	616.25	KING1

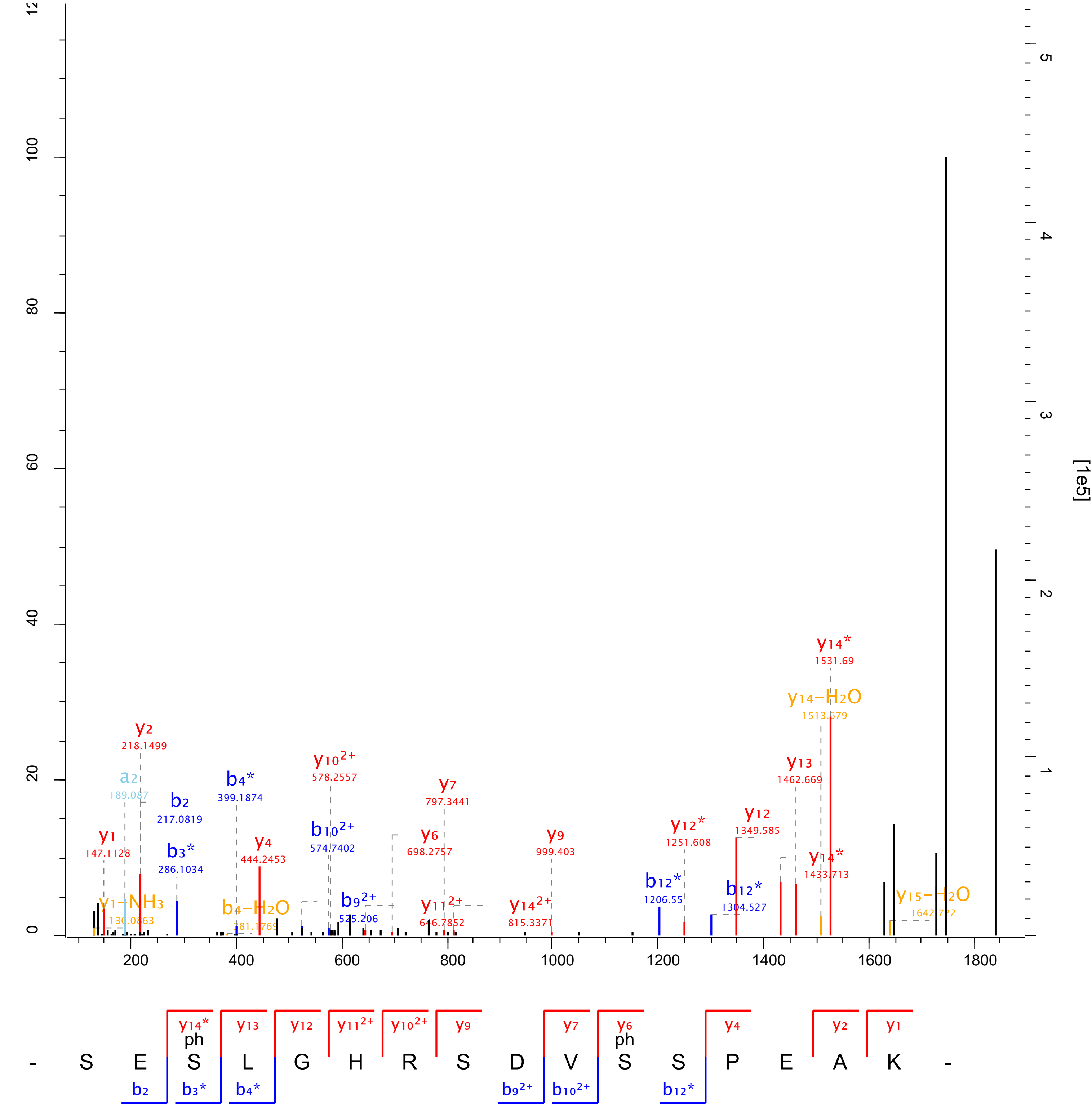

Sample output to test PDF Combine only

Raw file	Scan	Method	Score	m/z	Gene names
0523_6	8766	FTMS; HCD	40.97	578.26	At5g41950

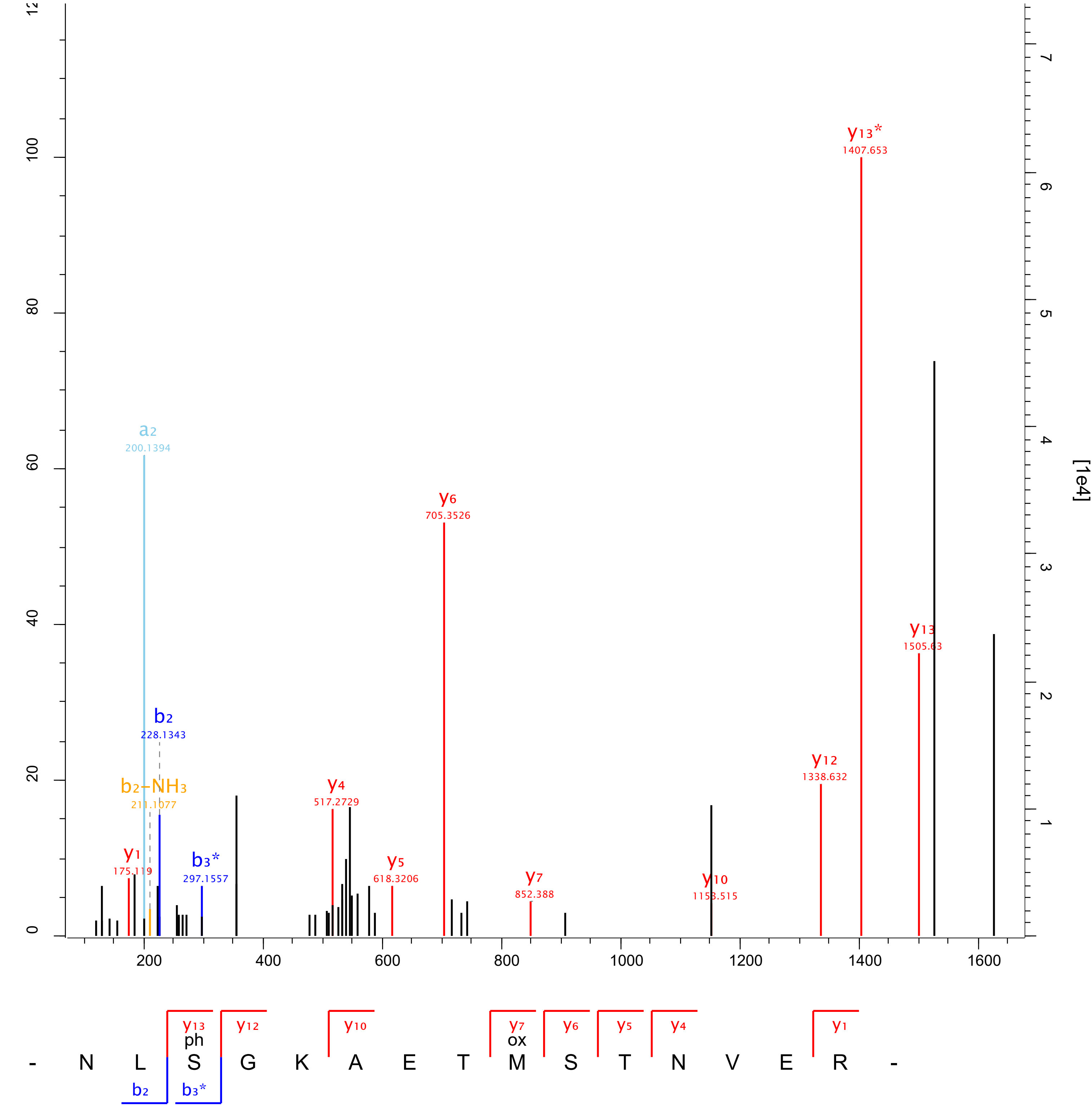

Sample output to test PDF Combine only

Raw file	Scan	Method	Score	m/z	Gene names
0523_6	12176	FTMS; HCD	156.86	669.27	PIP2-8

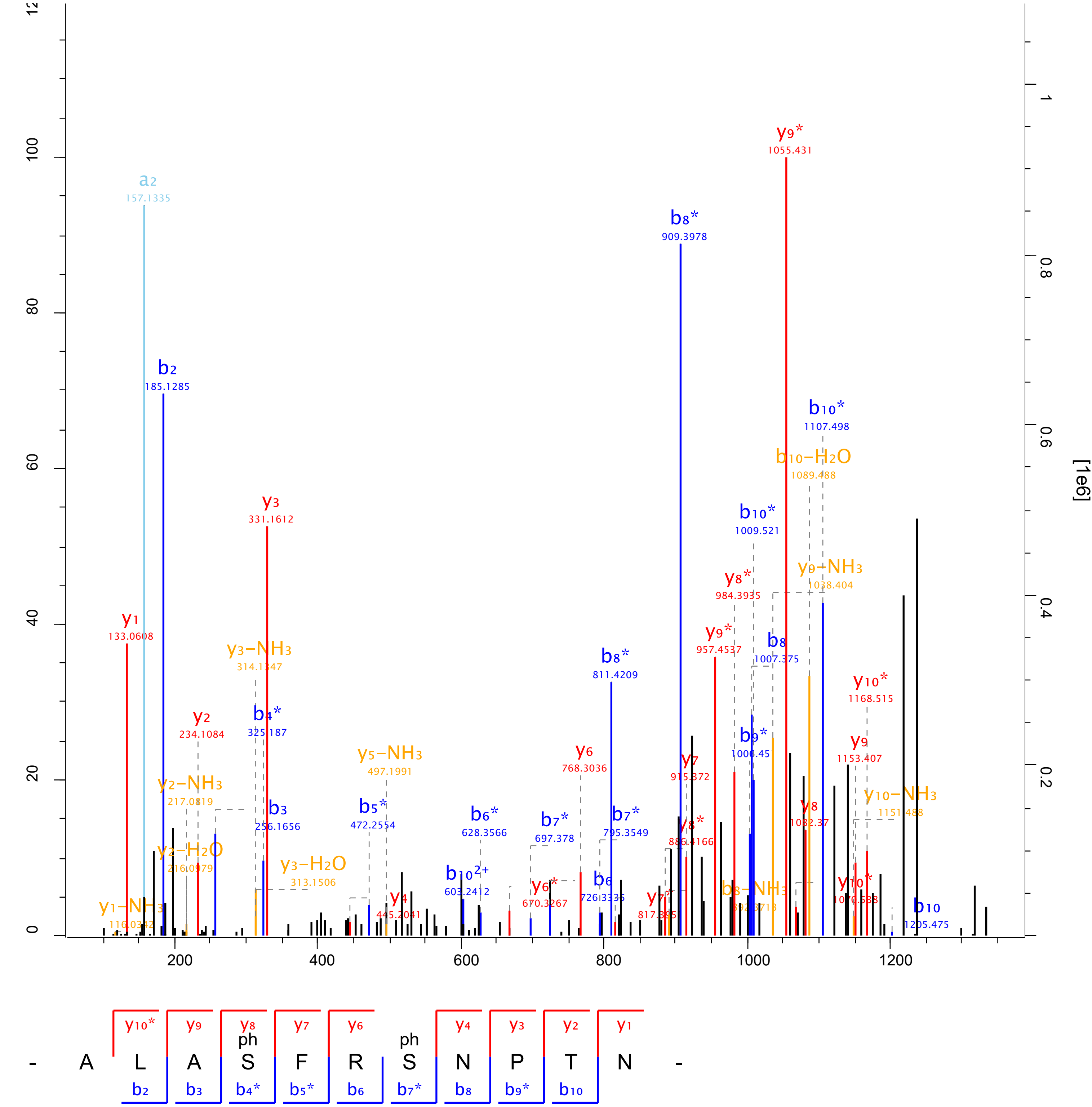

Sample output to test PDF Combine only

Raw file	Scan	Method	Score	m/z
0523_6	12922	FTMS; HCD	54	723.98

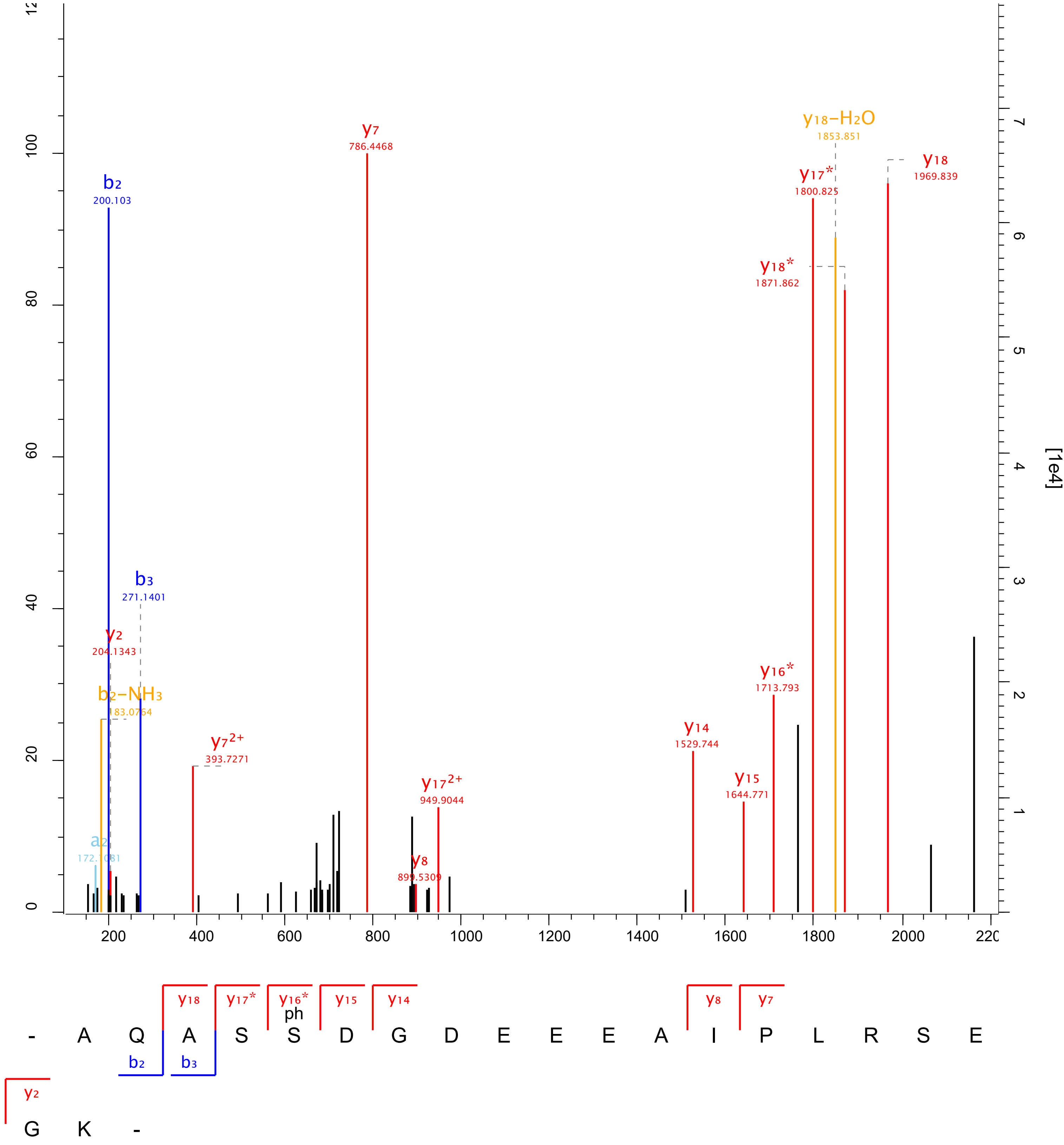

Sample output to test PDF Combine only

Raw file	Scan	Method	Score	m/z	Gene names
0523_6	14507	FTMS; HCD	93.57	935.7	At5g28040

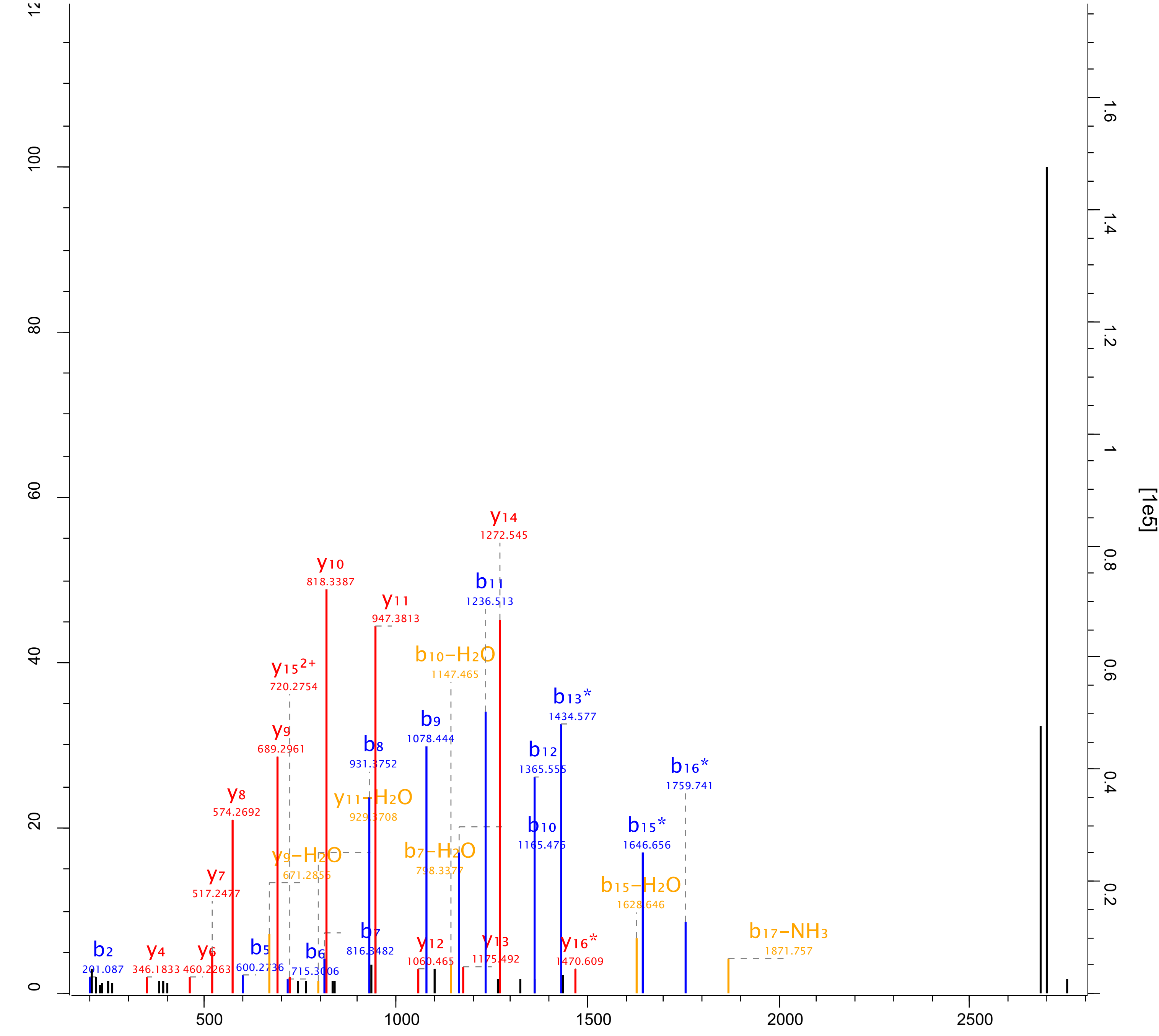

ac ph

- A S D Q R D T D F S A E S P D L E E

b2 b5 b6 b7 b8 b9 b10 b11 b12 b13* b15* b16*

y16* y15 2+ y14 y13 y12 y11 y10

D G G G G G G G R -

y9 y8 y7 y6 y4

Sample output to test PDF Combine only

Raw file	Scan	Method	Score	m/z	Gene names
0523_6	15015	FTMS; HCD	186.44	889.85	PIP2-1;PIP2-3;PIP2-2

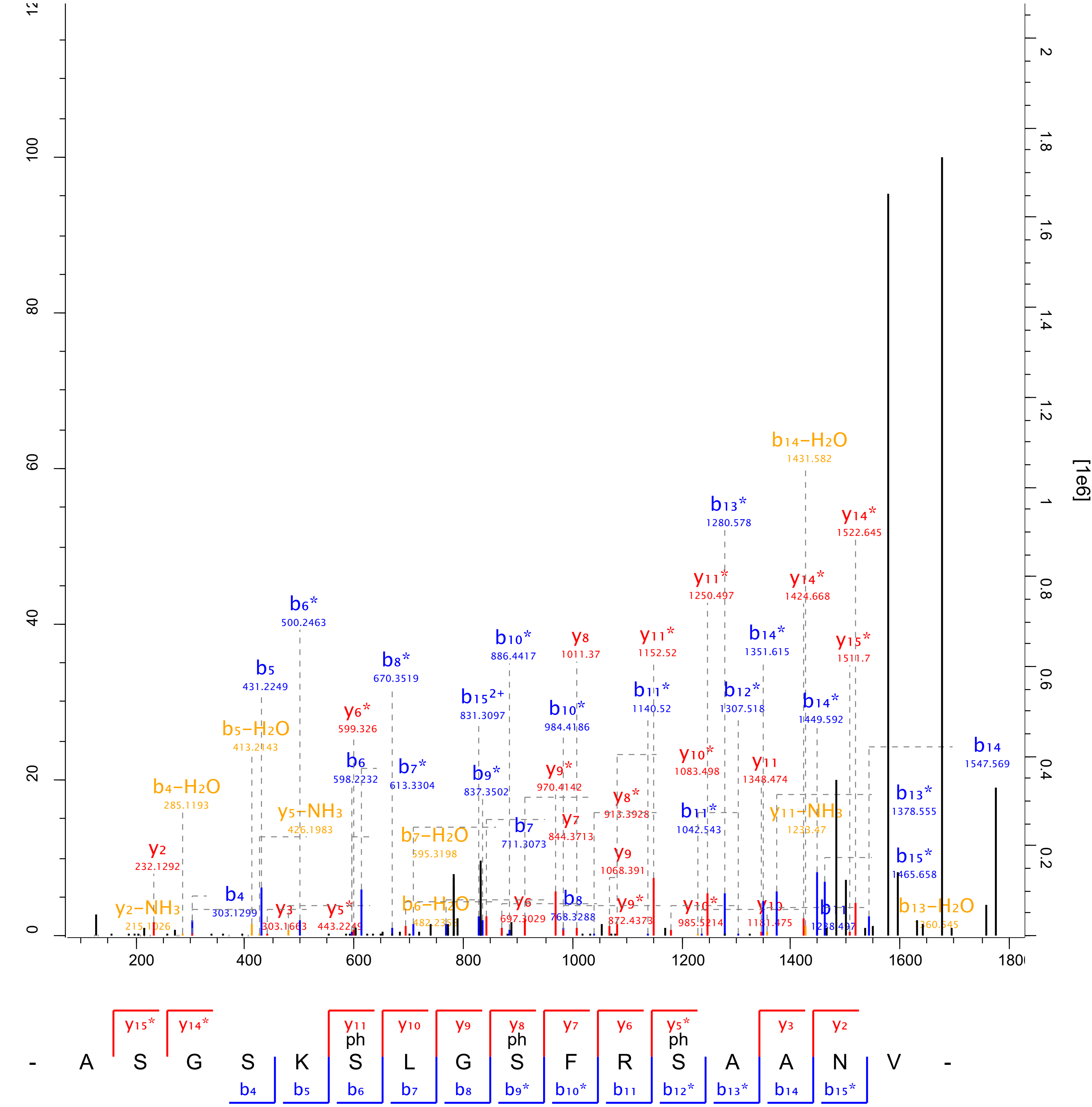

Sample output to test PDF Combine only

Raw file	Scan	Method	Score	m/z
0523_6	16665	FTMS; HCD	54.66	761.02

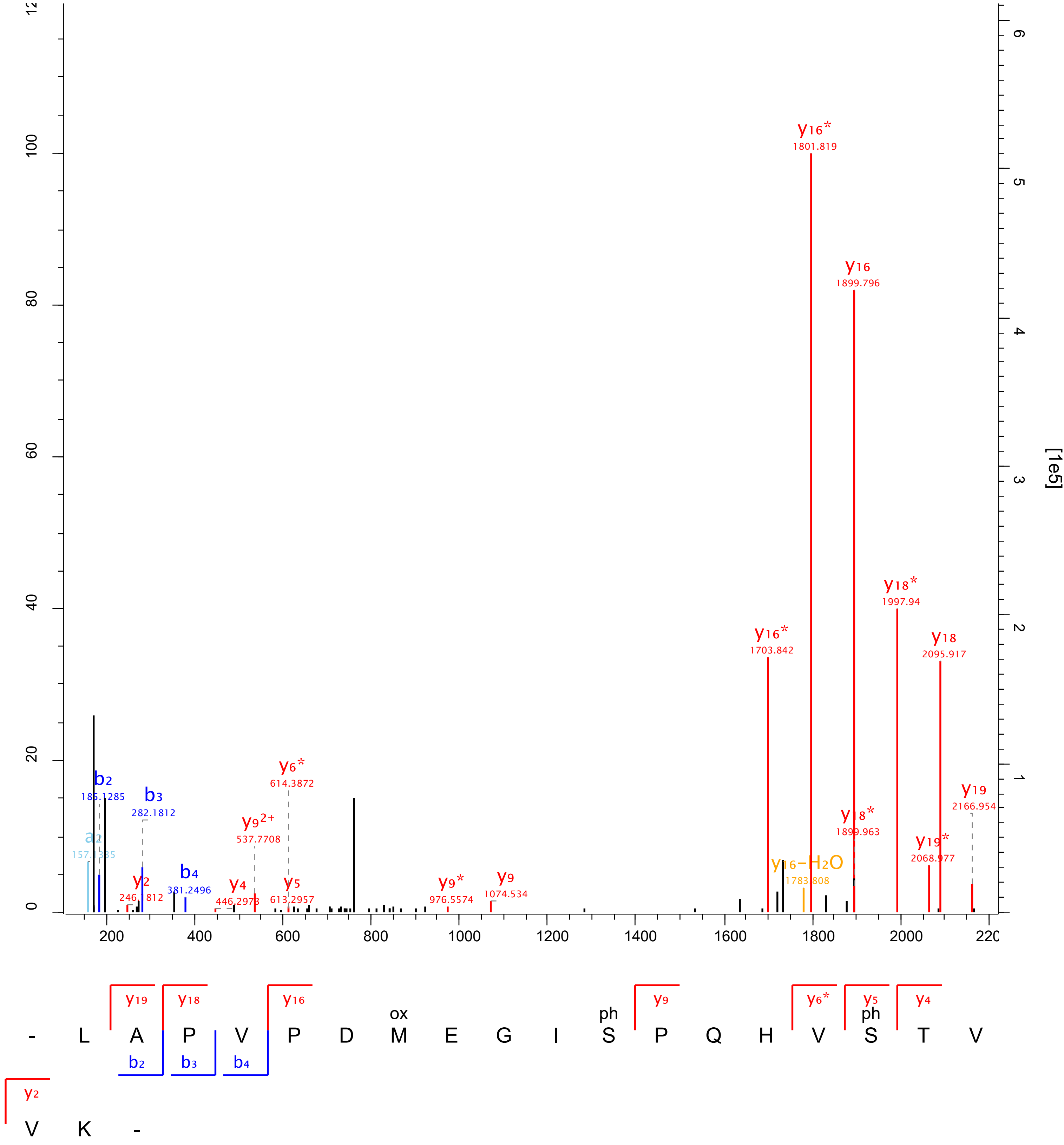

Sample output to test PDF Combine only

Raw file	Scan	Method	Score	m/z	Gene names
0523_6	18224	FTMS; HCD	139.5	942.05	RS2Z33

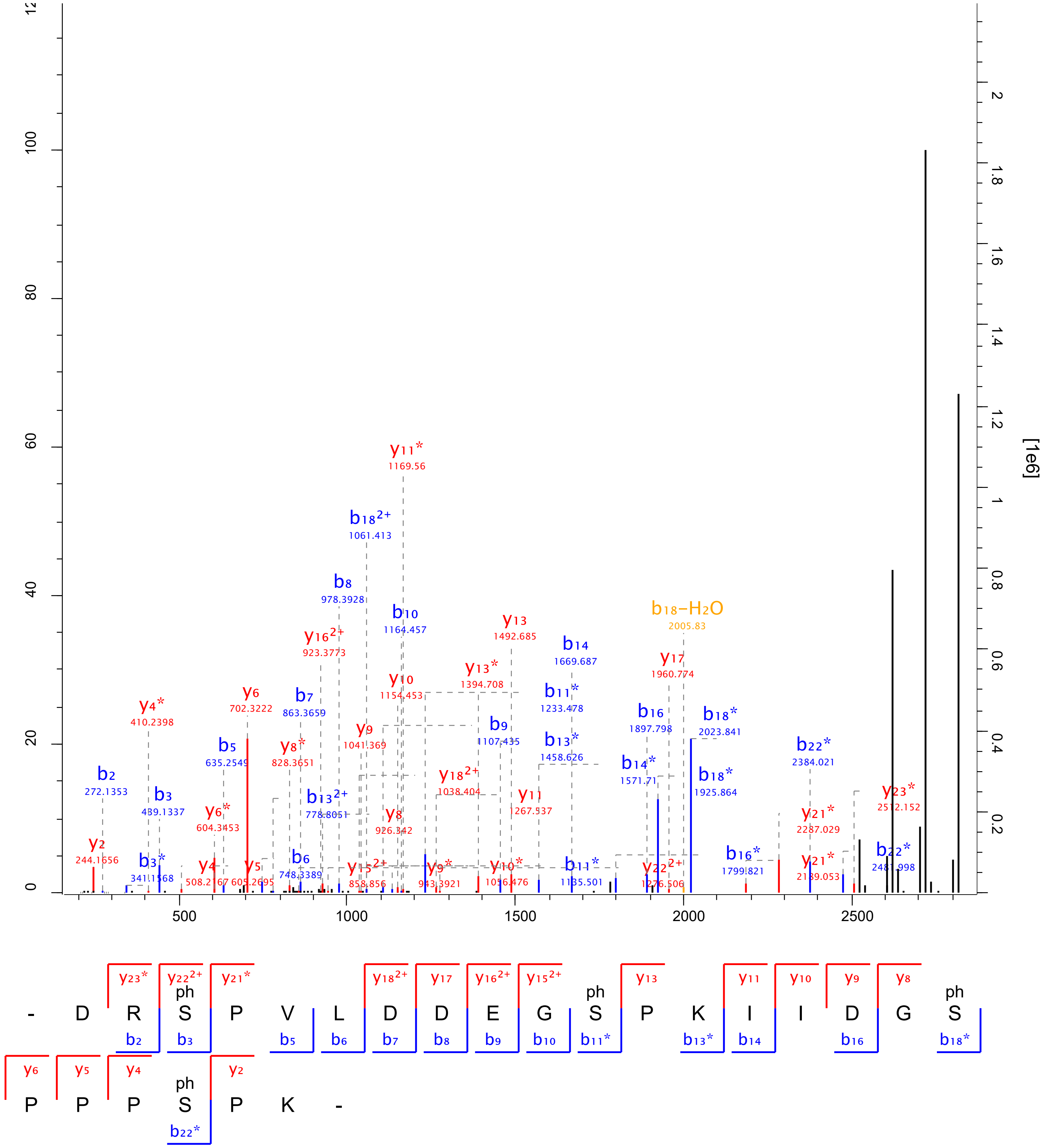

Sample output to test PDF Combine only

Raw file	Scan	Method	Score	m/z	Gene names
0523_6	19466	FTMS; HCD	208.47	793.84	NUP107

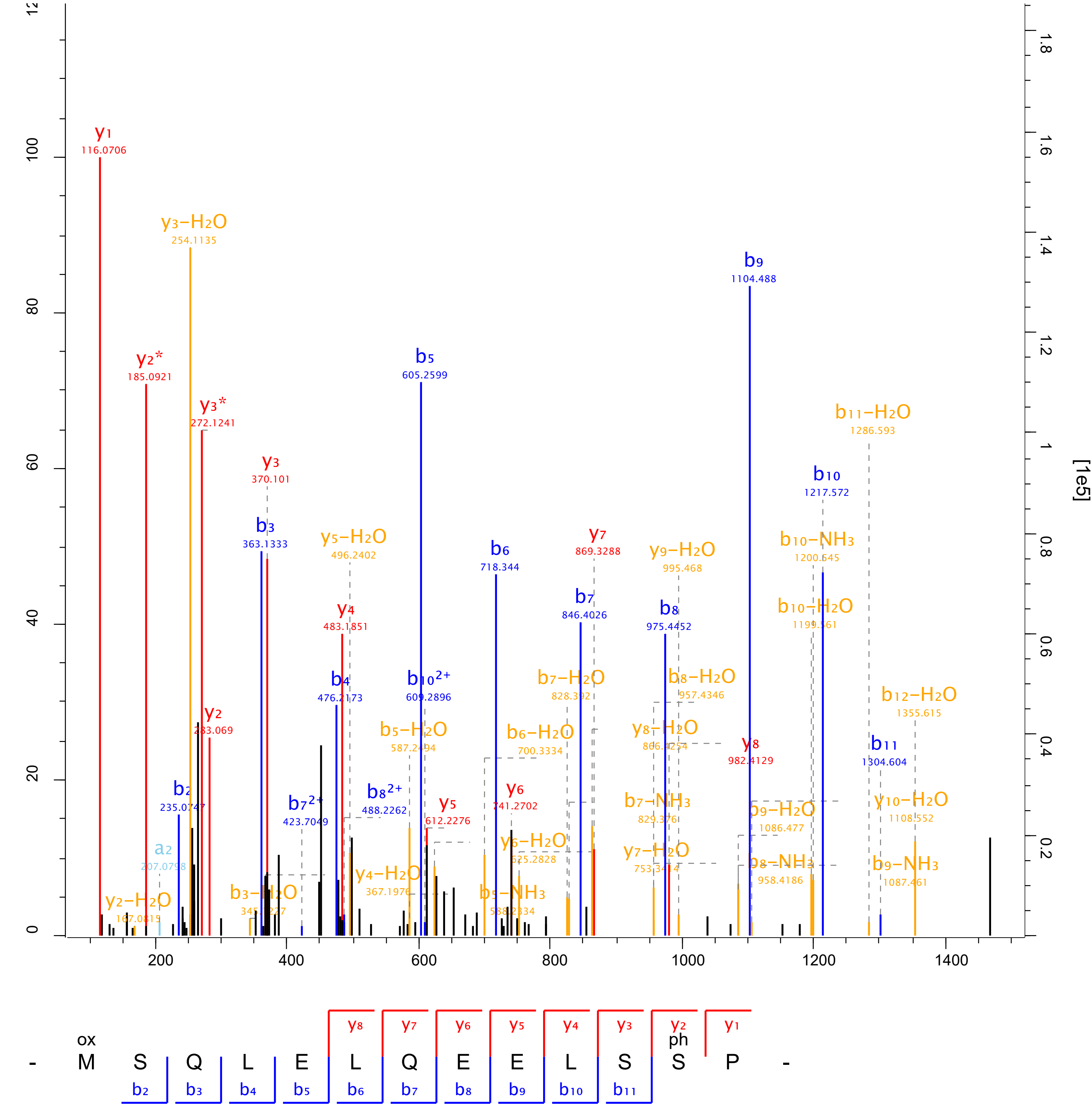

Sample output to test PDF Combine only

Raw file	Scan	Method	Score	m/z	Gene names
0523_6	19618	FTMS; HCD	49.32	778.68	VPS9A

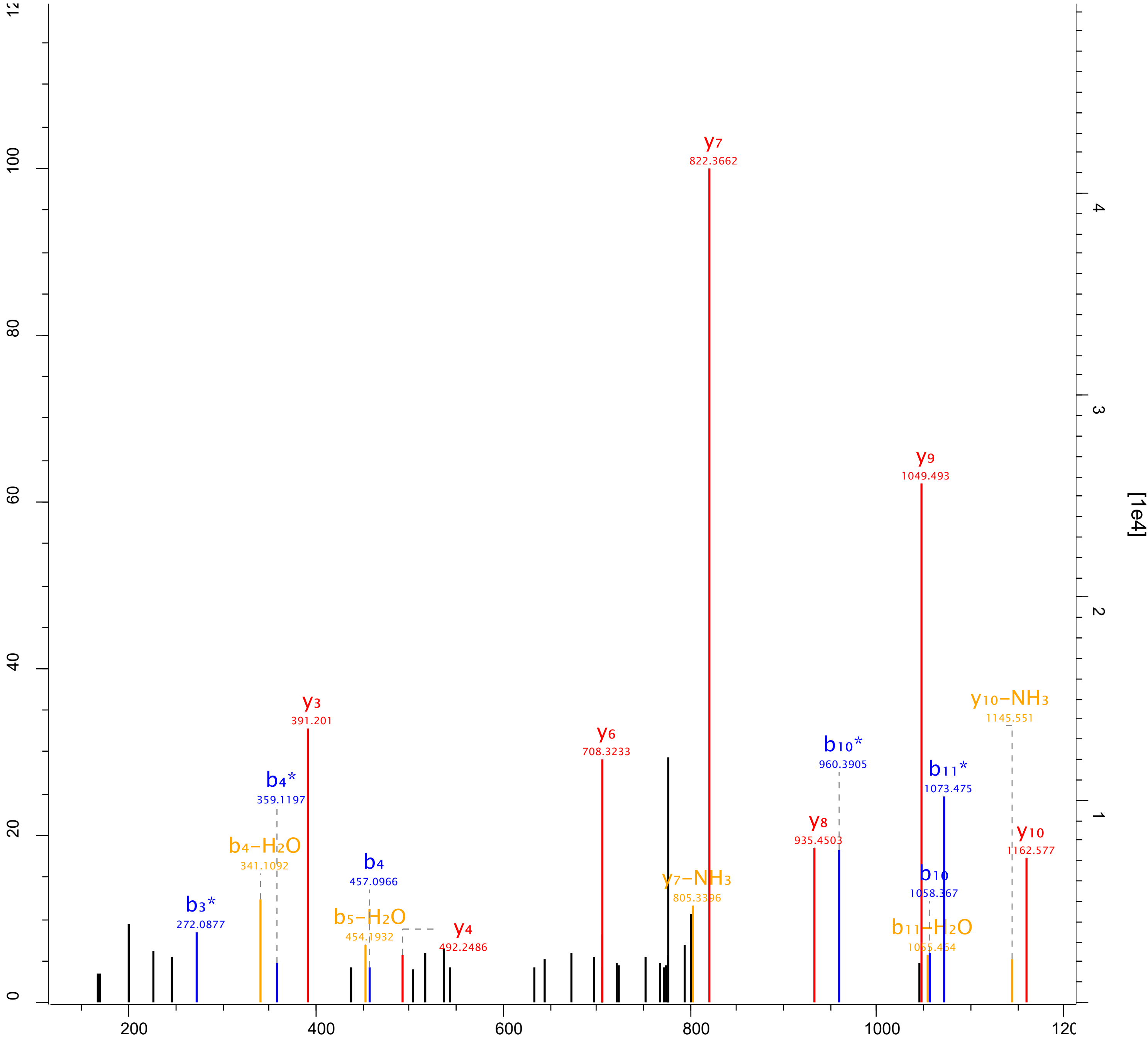

Sample output to test PDF Combine only

Raw file	Scan	Method	Score	m/z	Gene names
0523_6	23416	FTMS; HCD	133.21	1042.41	APUM5

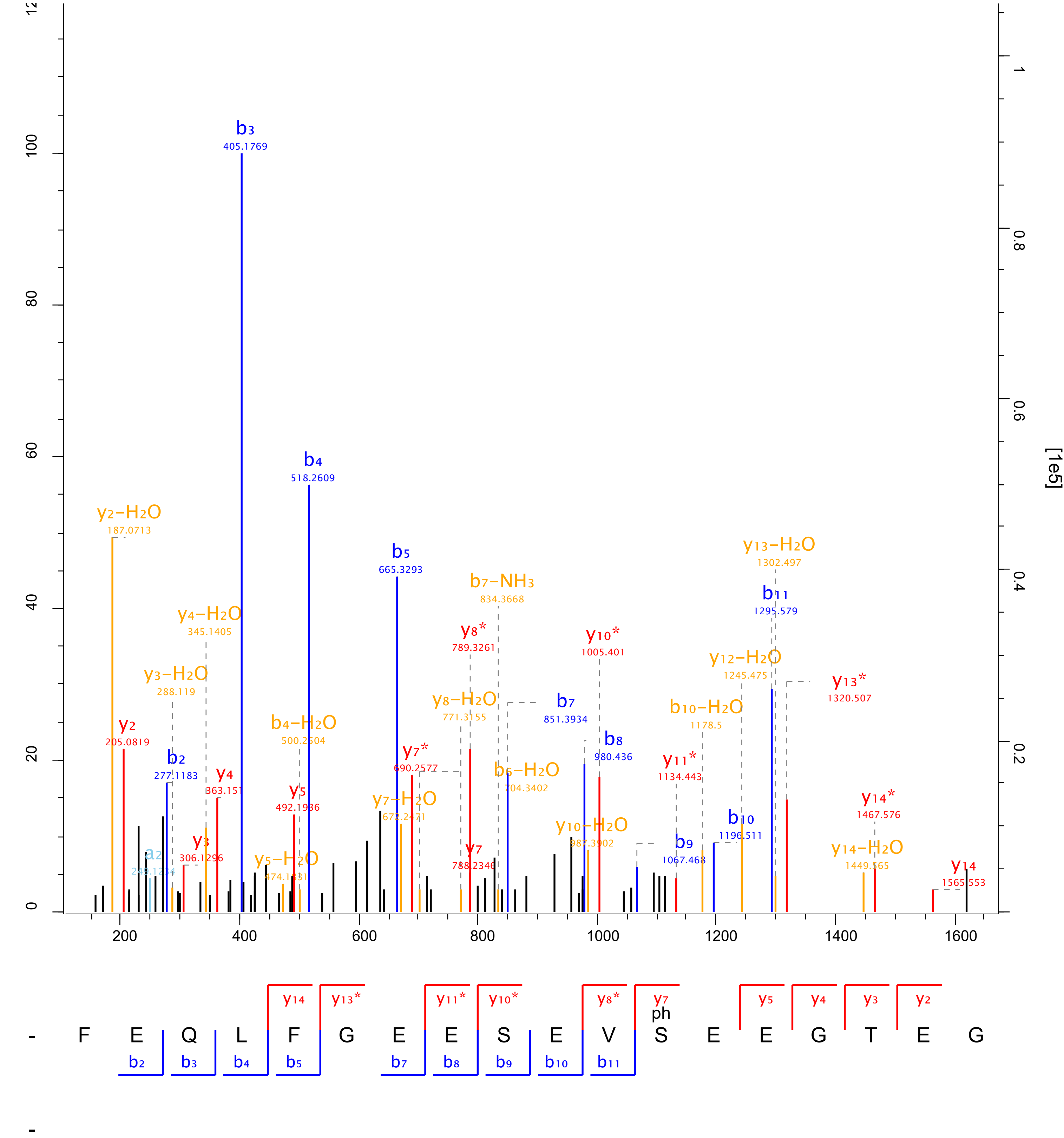

Sample output to test PDF Combine only

Raw file	Scan	Method	Score	m/z	Gene names
0523_6	25487	FTMS; HCD	382.29	1323.99	At3g09200;RPP0B

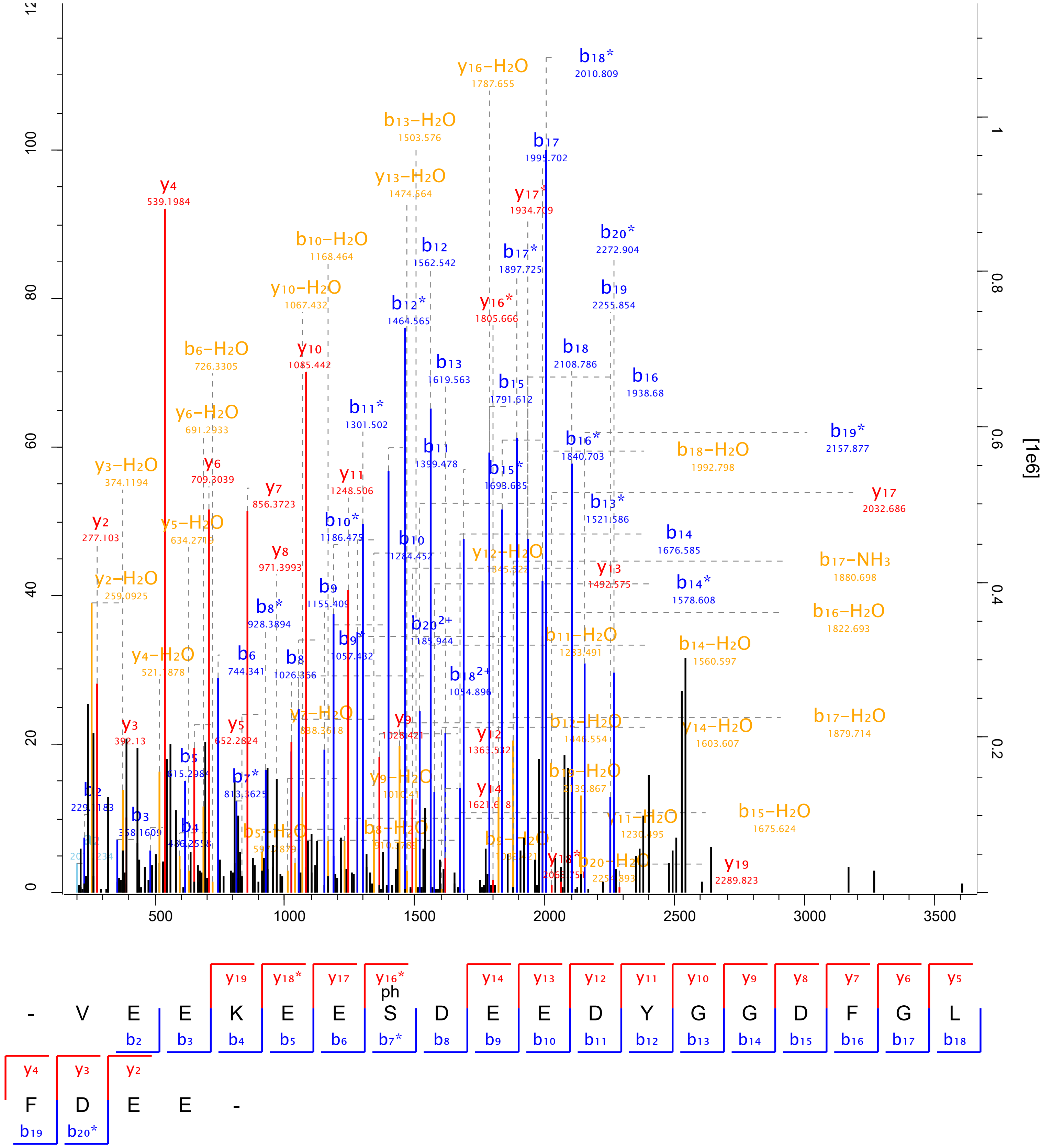

Sample output to test PDF Combine only

Raw file	Scan	Method	Score	m/z	Gene names
0523_10	5919	FTMS; HCD	101.46	404.19	AMT1-3

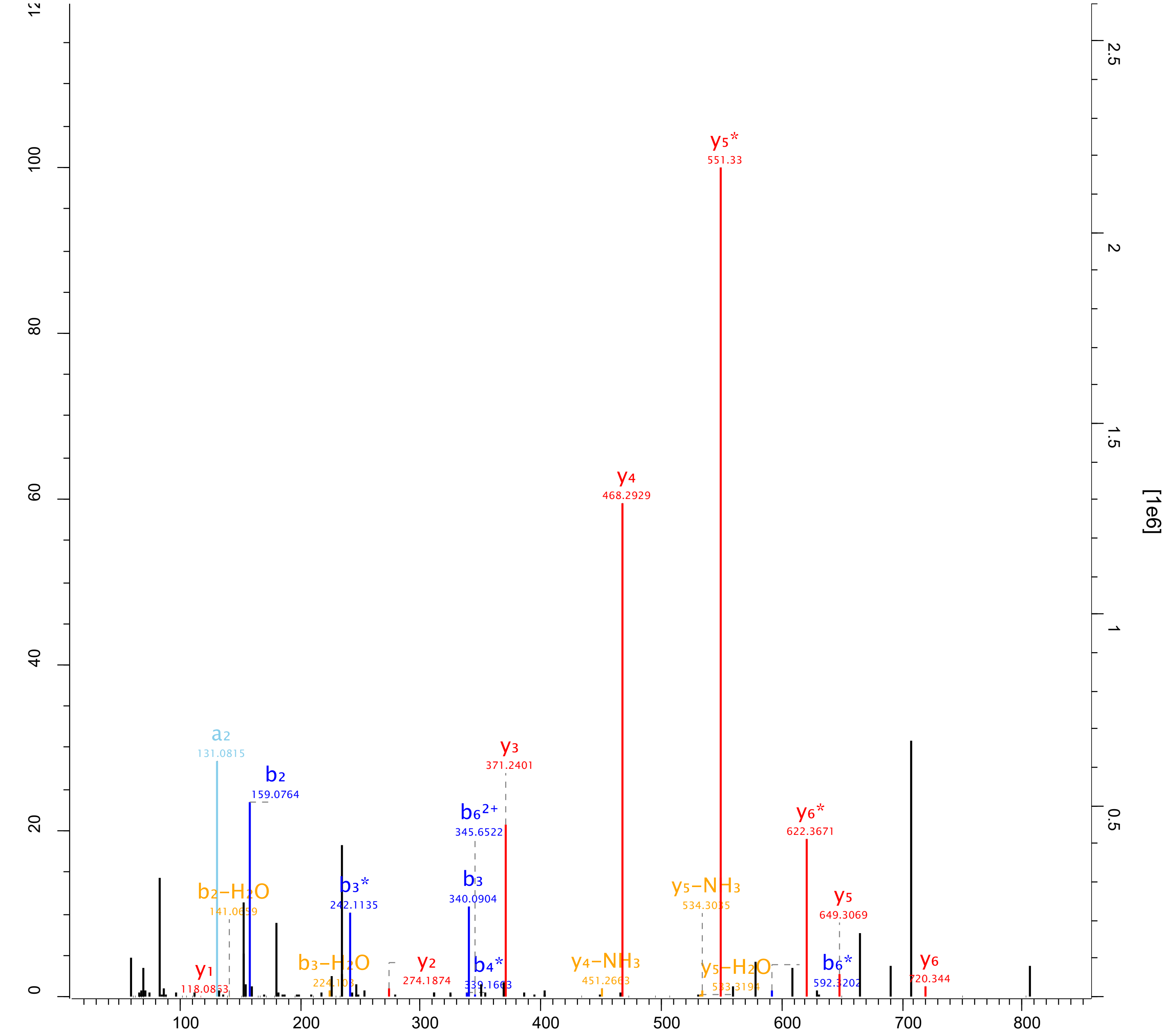

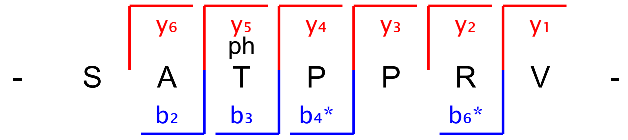

Sample output to test PDF Combine only

Raw file	Scan	Method	Score	m/z	Gene names
0523_10	6890	FTMS; HCD	103.58	787.81	RS2Z33

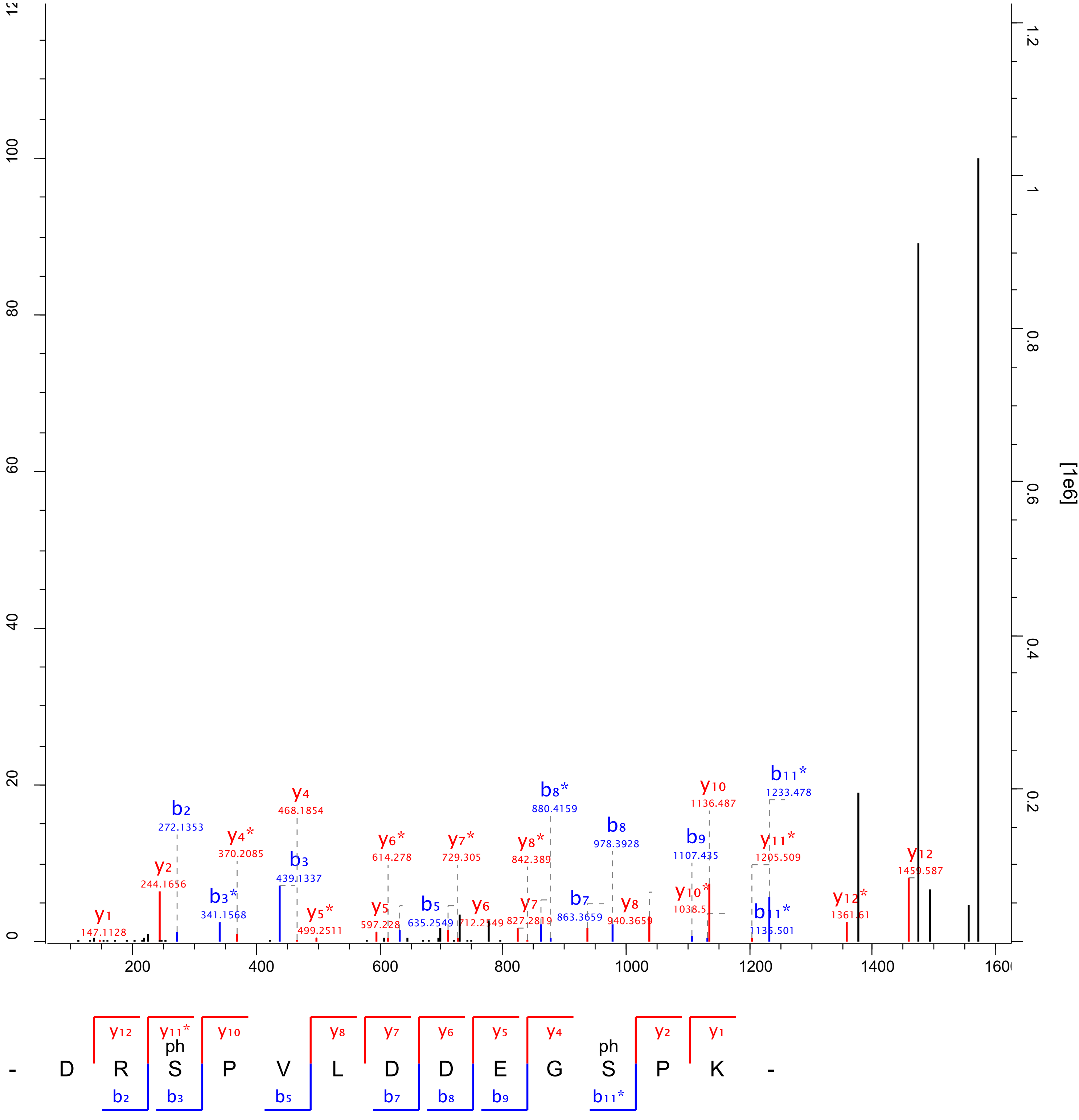

Sample output to test PDF Combine only

Raw file	Scan	Method	Score	m/z	Gene names
0523_10	7160	FTMS; HCD	84.76	620.28	IQD14

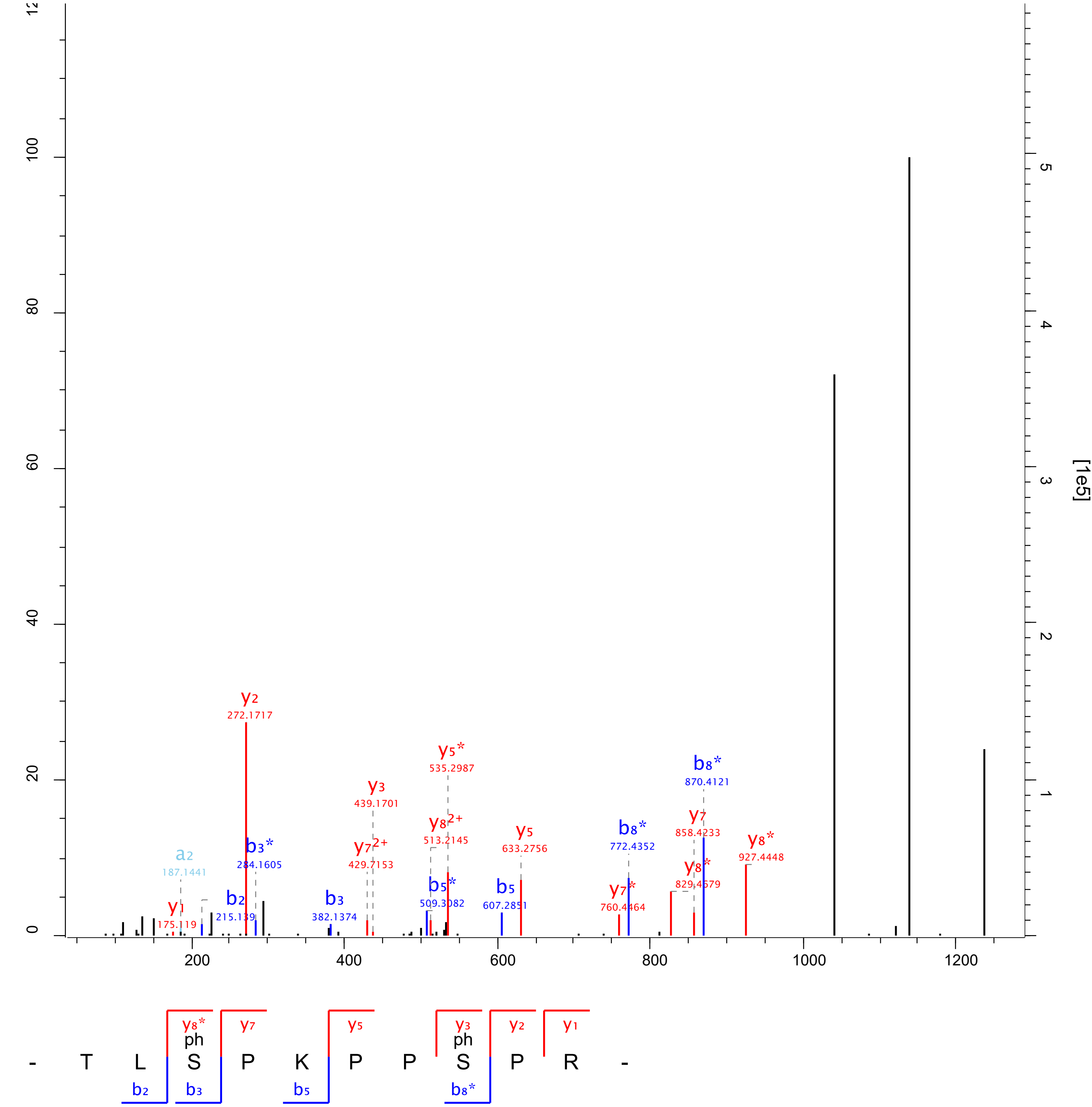

Sample output to test PDF Combine only

Raw file	Scan	Method	Score	m/z	Gene names
0523_10	7315	FTMS; HCD	49.19	409.19	IQD14

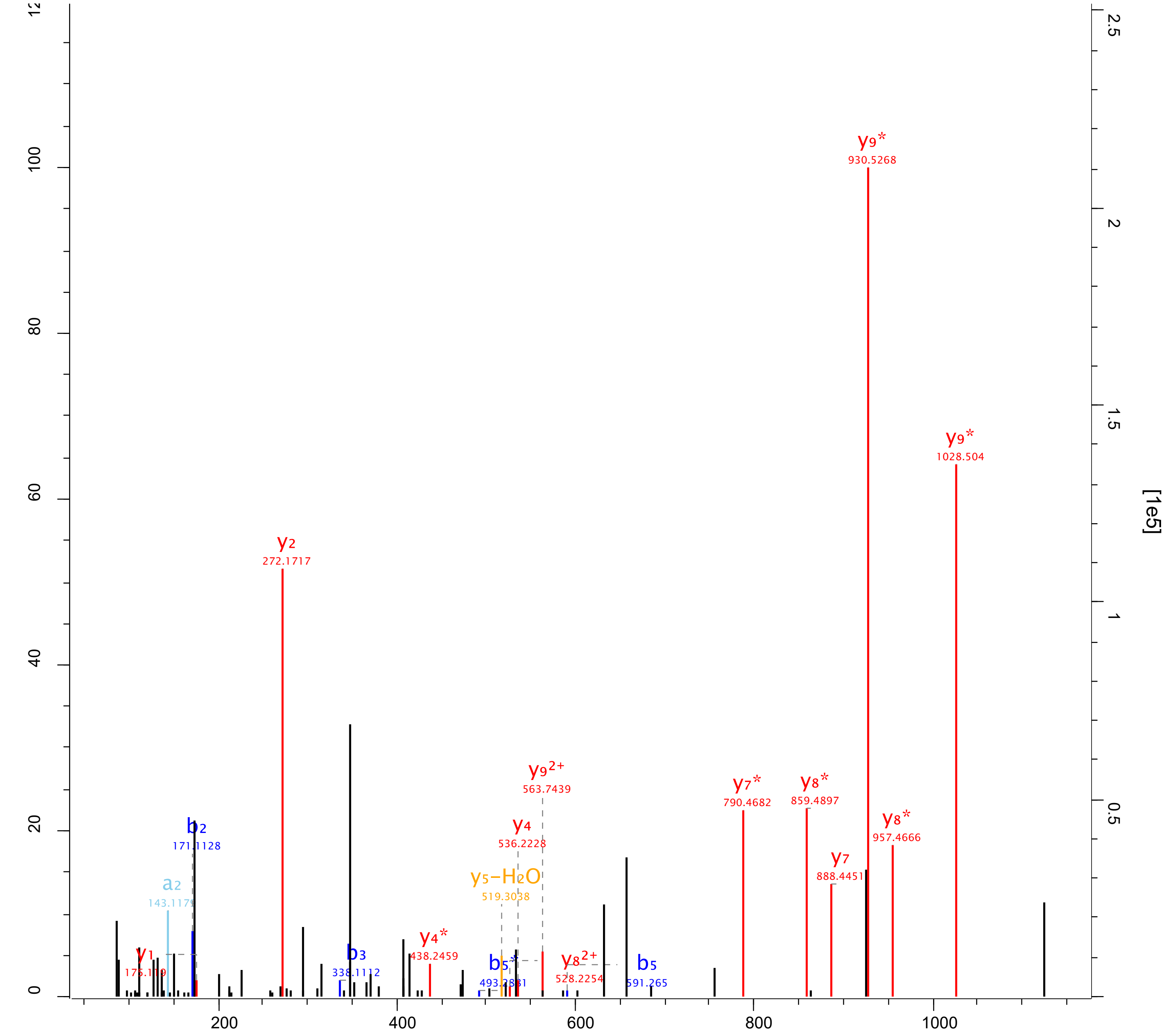

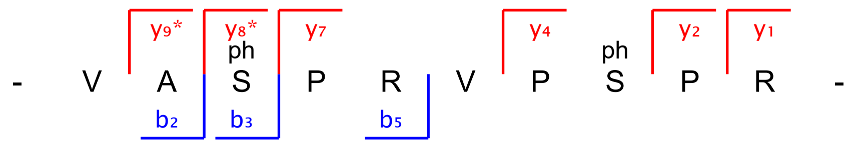

Sample output to test PDF Combine only

Raw file	Scan	Method	Score	m/z	Gene names
0523_10	7779	FTMS; HCD	46.88	540.68	RS2Z33

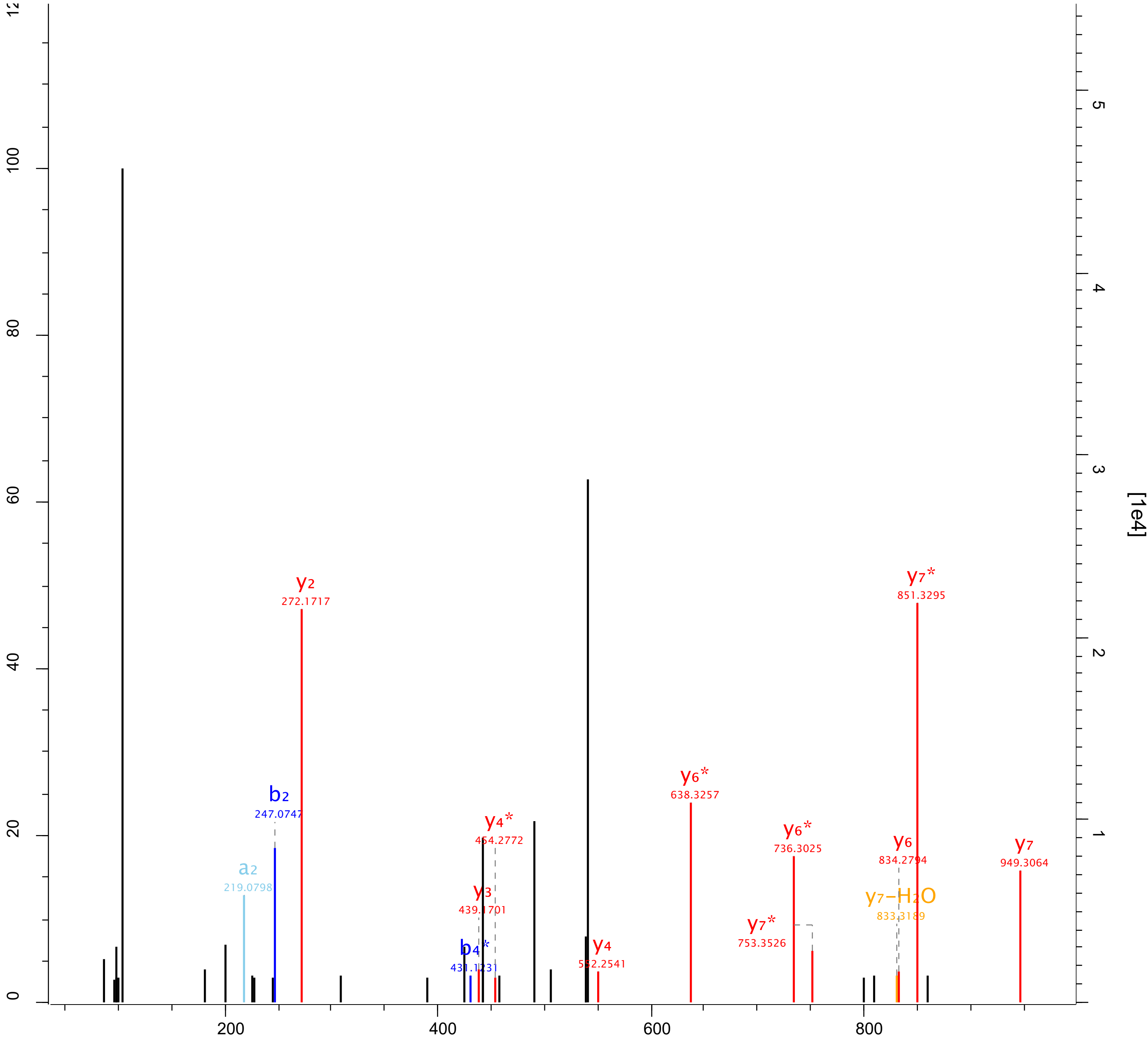

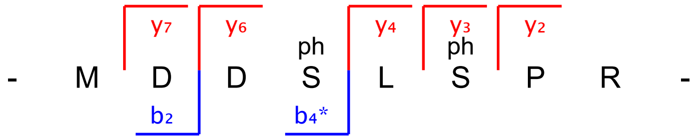

Sample output to test PDF Combine only

Raw file	Scan	Method	Score	m/z	Gene names
0523_10	7916	FTMS; HCD	51.29	532.57	SCL30A;SCL33

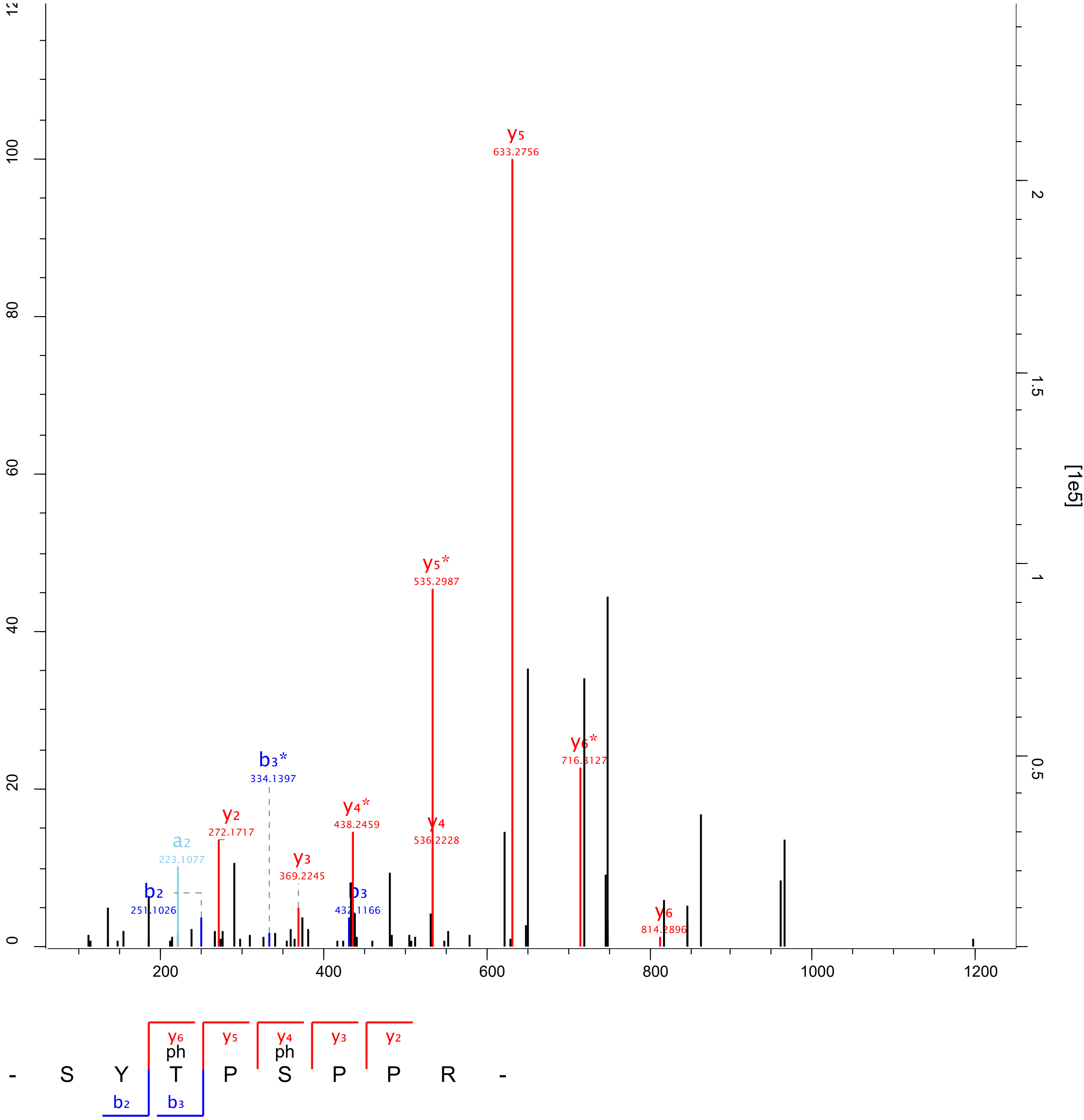

Sample output to test PDF Combine only

Raw file	Scan	Method	Score	m/z	Gene names
0523_10	7922	FTMS; HCD	156.11	634.27	RS2Z33

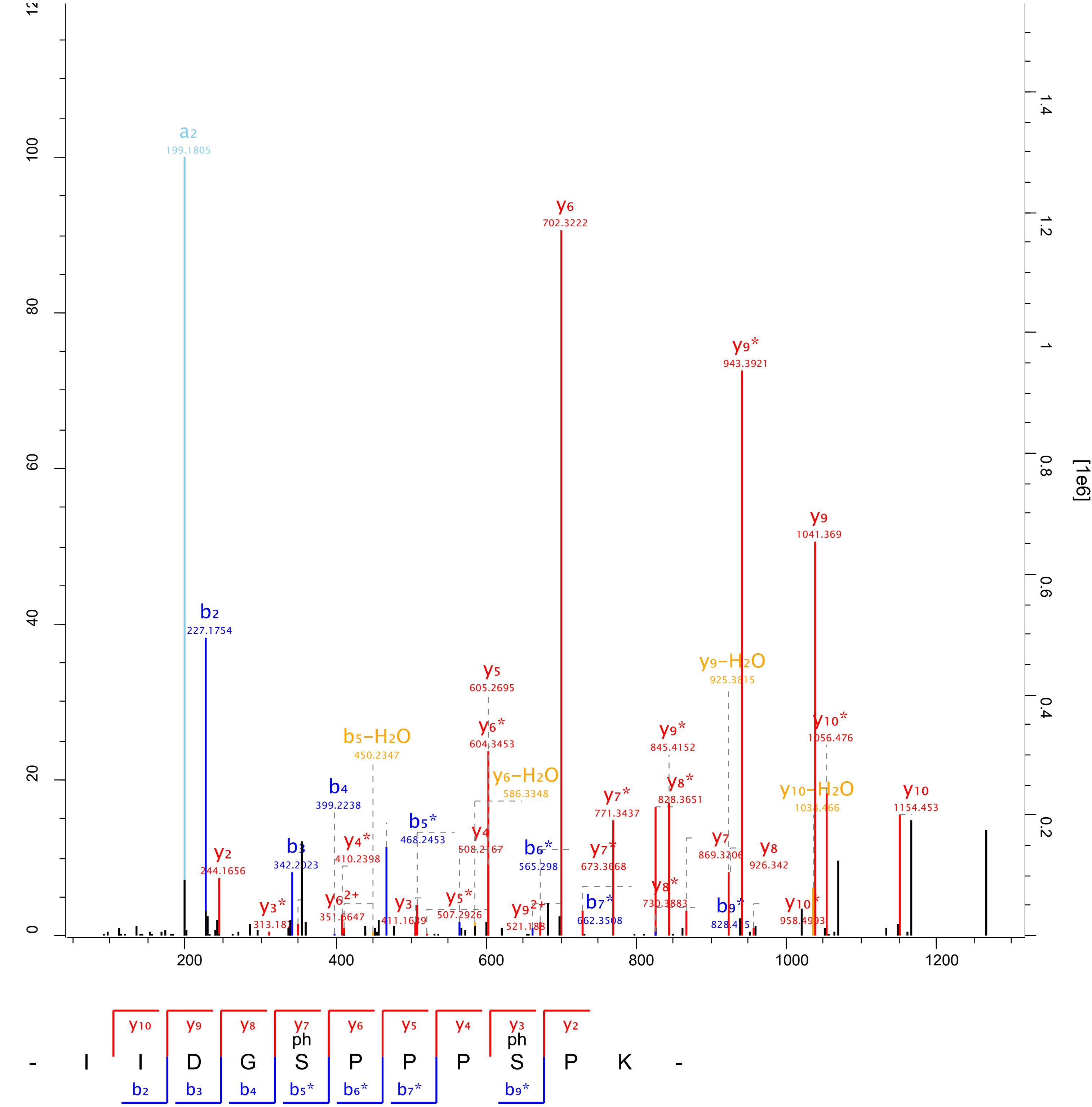

Sample output to test PDF Combine only

Raw file	Scan	Method	Score	m/z	Gene names
0523_10	8994	FTMS; HCD	117.78	641.27	F18B13.26

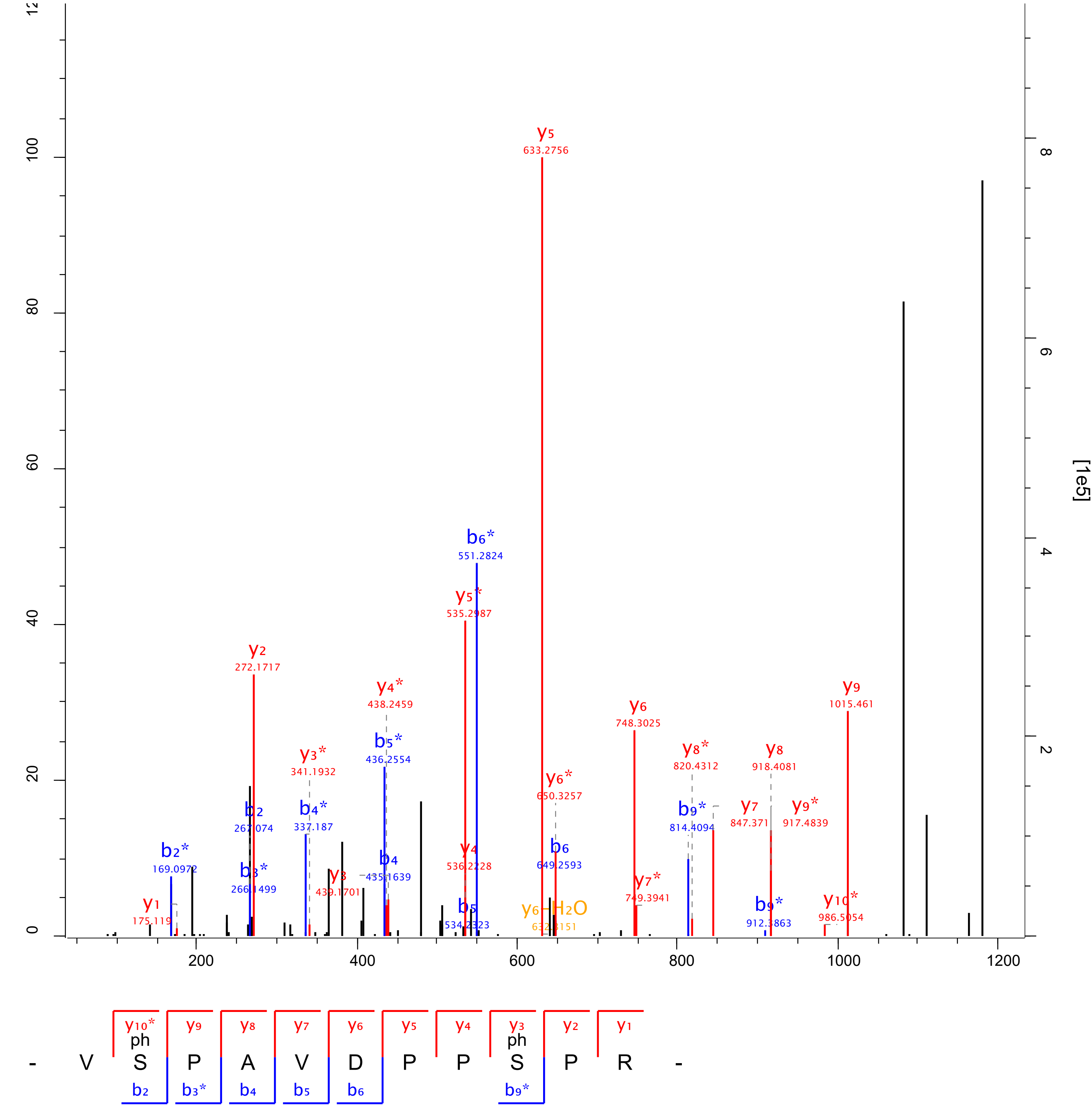

Sample output to test PDF Combine only

Raw file	Scan	Method	Score	m/z	Gene names
0523_10	12653	FTMS; HCD	232.84	897.34	EIF3B-2;TIF3B1

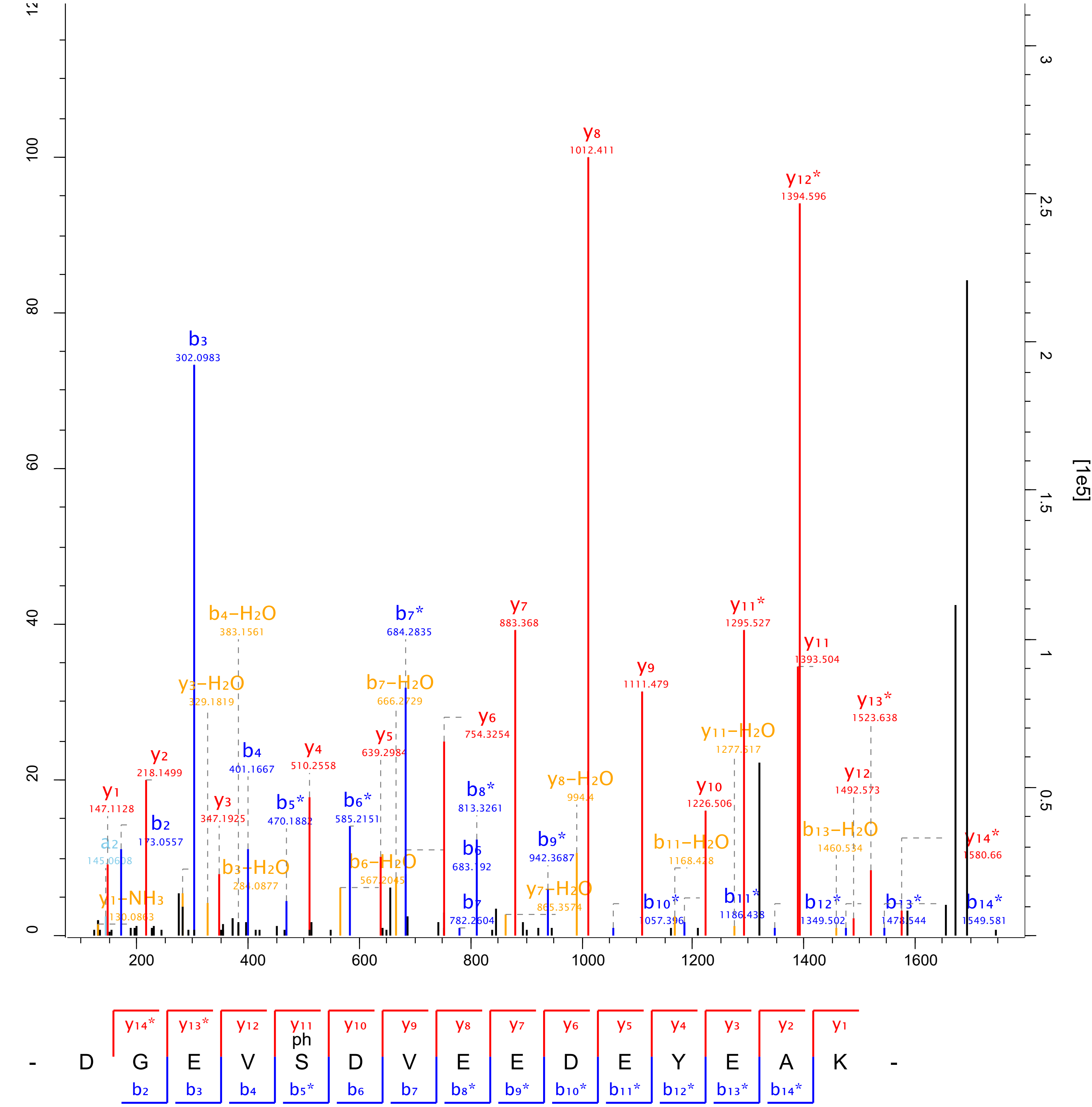

Sample output to test PDF Combine only

Raw file	Scan	Method	Score	m/z
0523_10	13114	FTMS; HCD	168.05	735.78

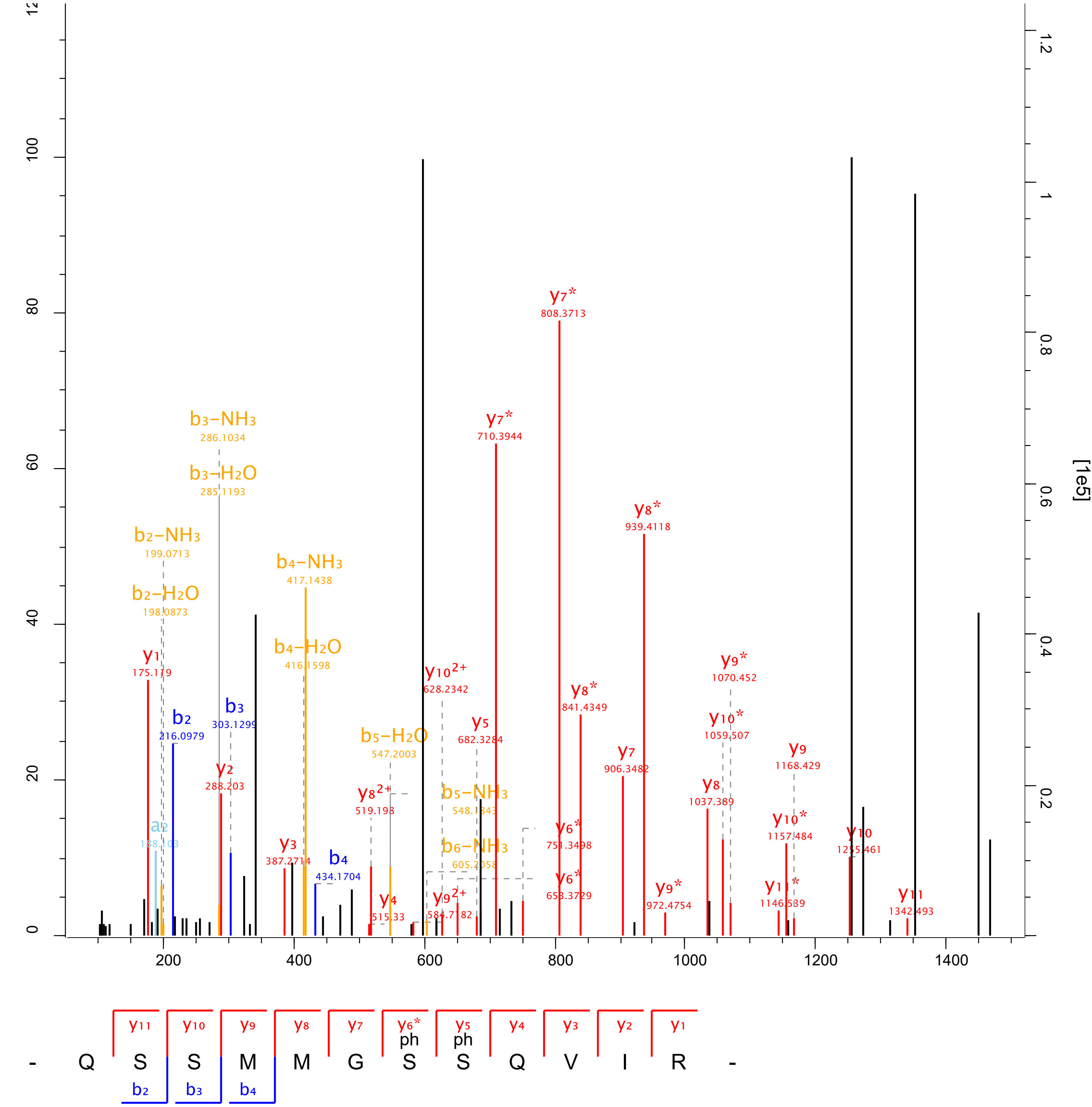

Sample output to test PDF Combine only

Raw file	Scan	Method	Score	m/z	Gene names
0523_10	13453	FTMS; HCD	89.36	736.79	AMT1-1

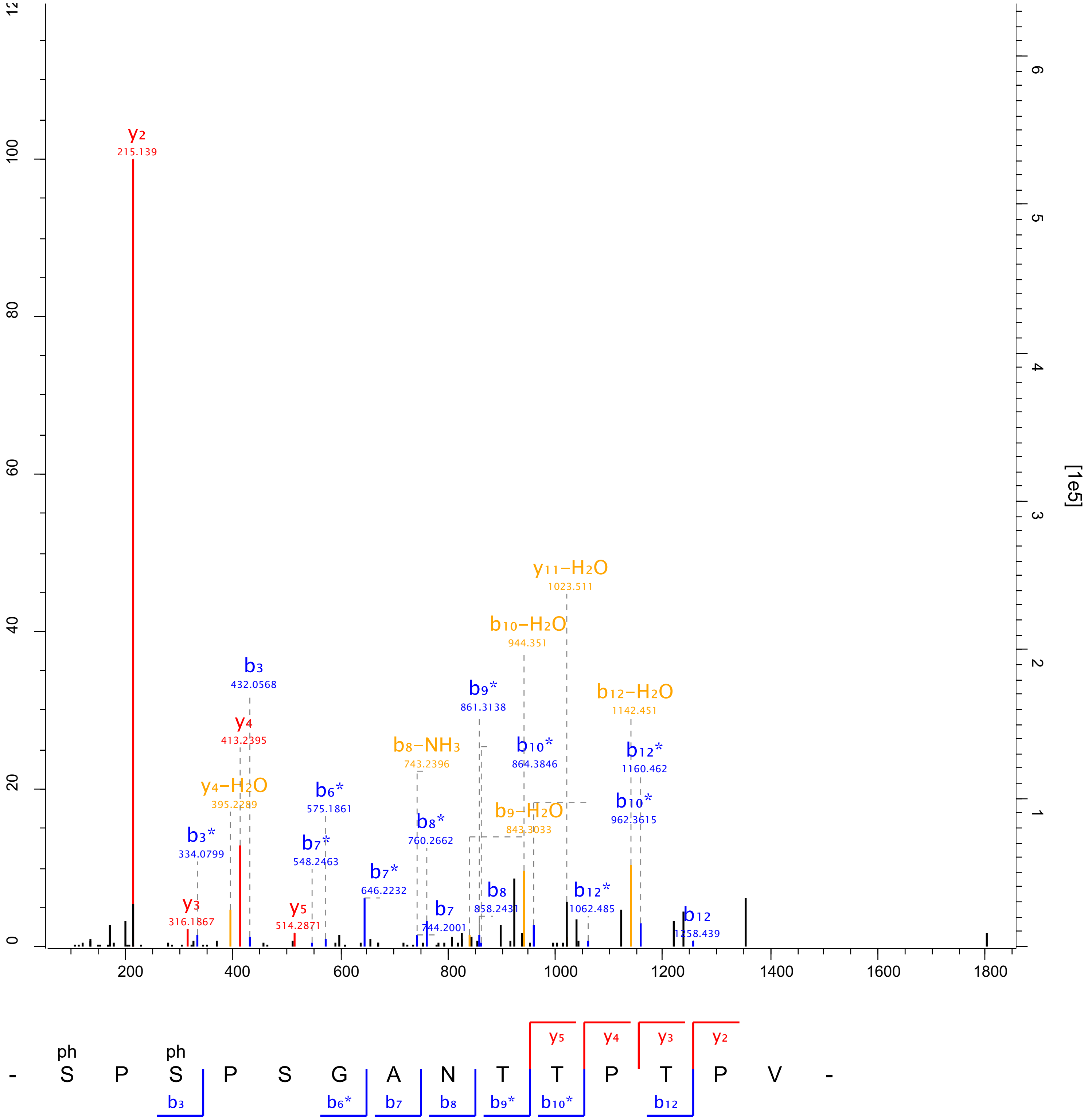

Sample output to test PDF Combine only

Raw file	Scan	Method	Score	m/z
0523_10	13794	FTMS; HCD	44.31	876.35

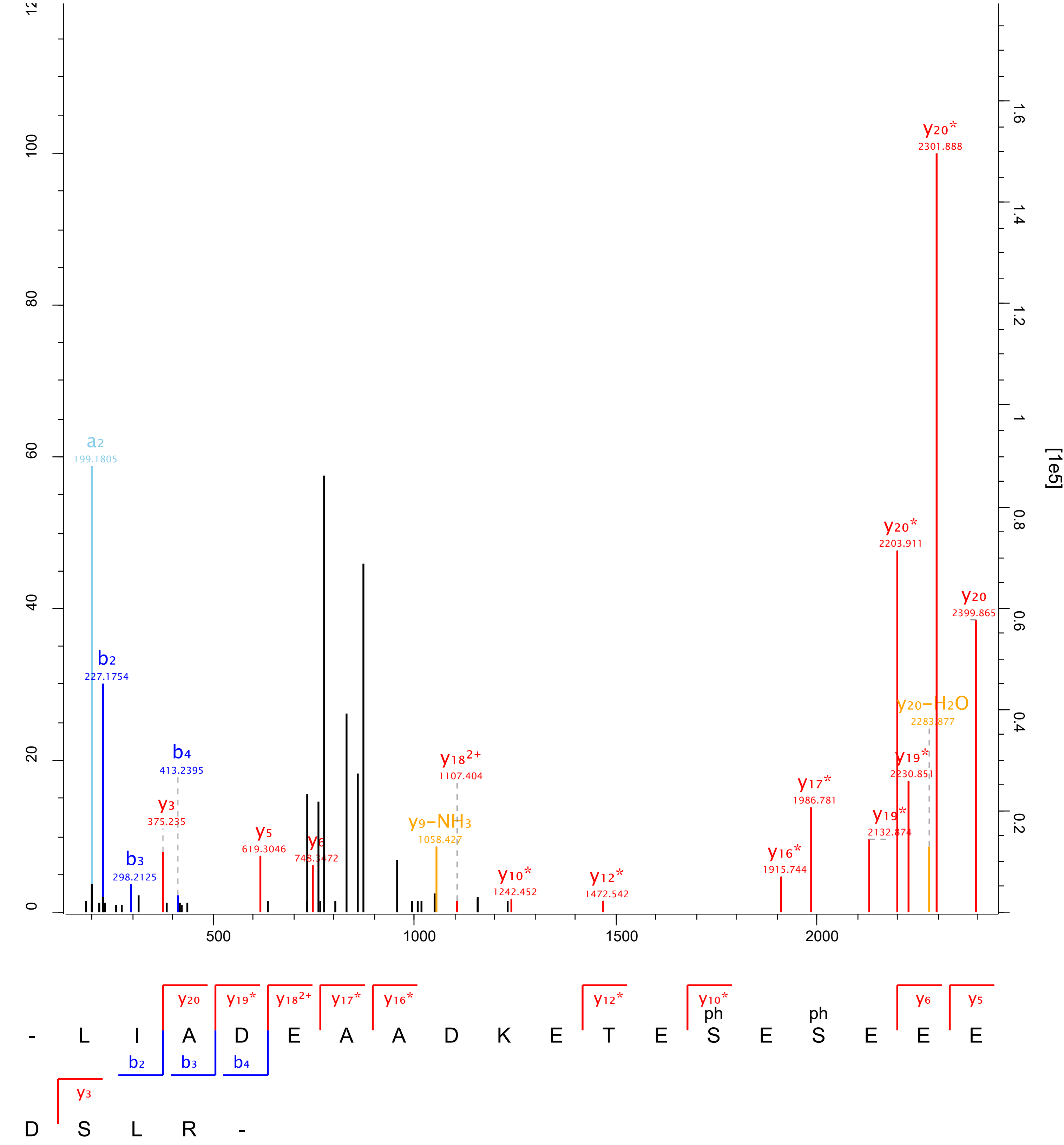

Sample output to test PDF Combine only

Raw file	Scan	Method	Score	m/z	Gene names
0523_10	13852	FTMS; HCD	85.09	591.9	AMT1-3

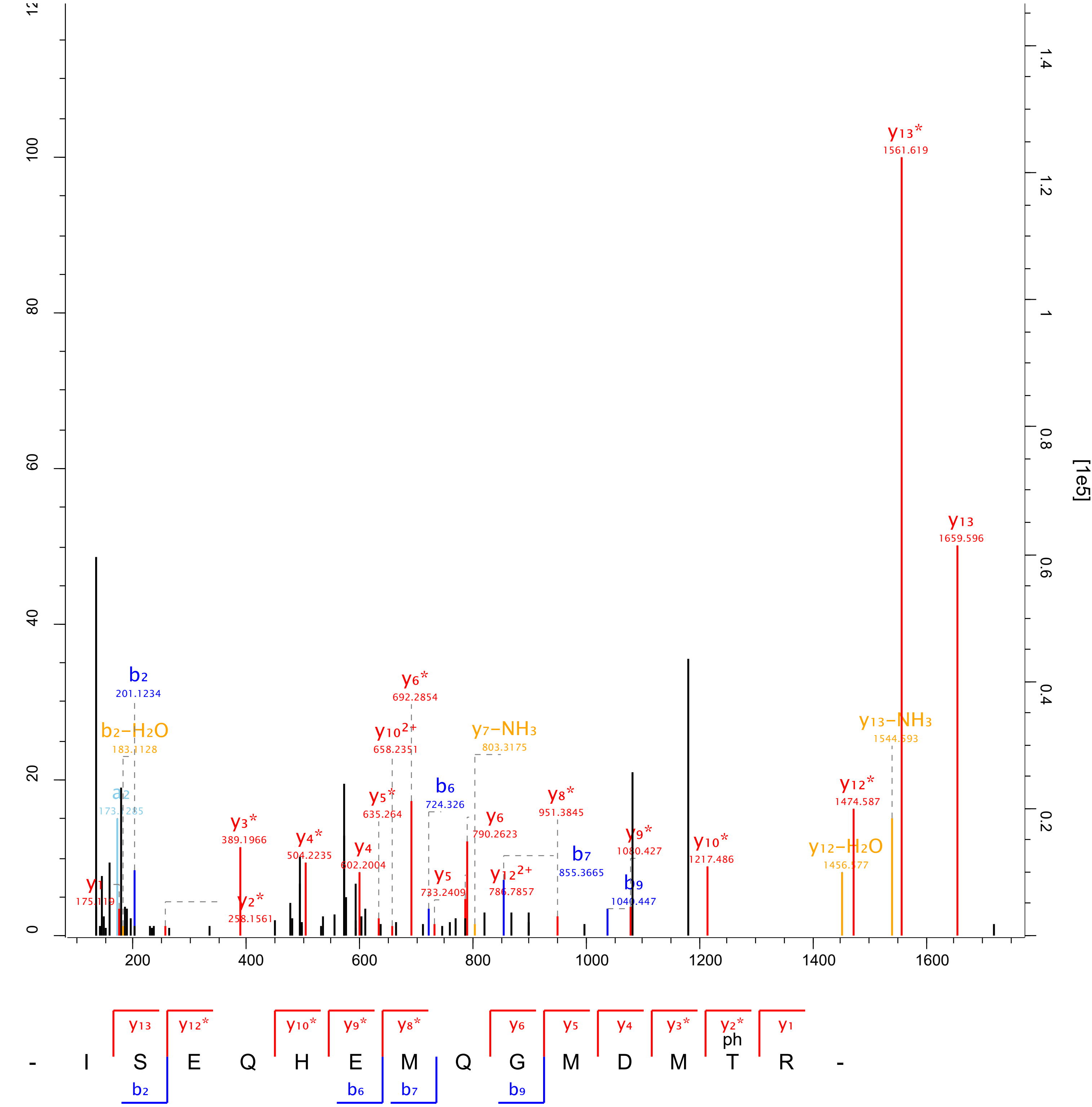

Sample output to test PDF Combine only

Raw file	Scan	Method	Score	m/z	Gene names
0523_10	13906	FTMS; HCD	204.37	689.24	PIP2-7

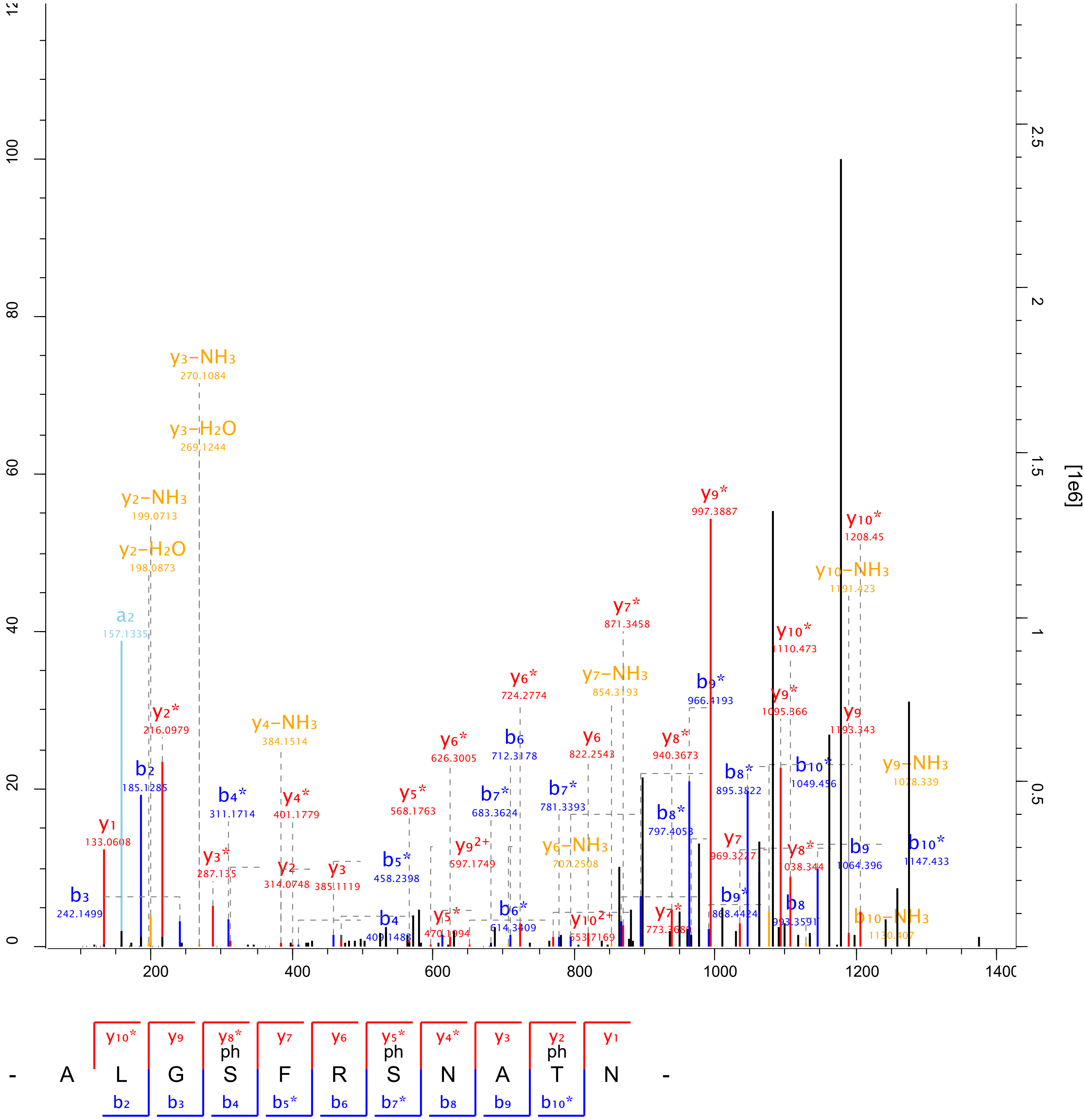

Sample output to test PDF Combine only

Raw file	Scan	Method	Score	m/z	Gene names
0523_10	14641	FTMS; HCD	76.8	725.31	BIG3

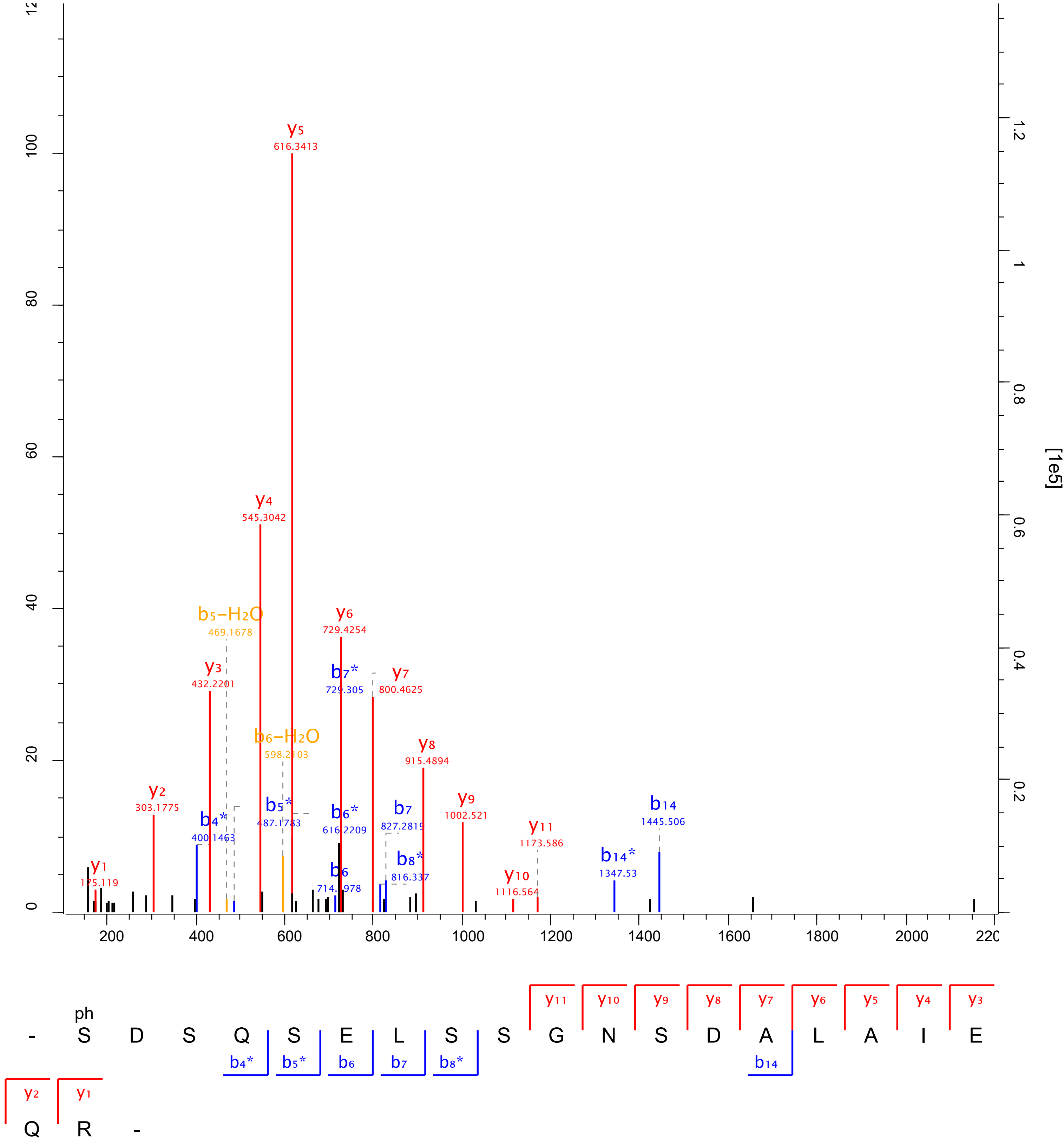

Sample output to test PDF Combine only

Raw file	Scan	Method	Score	m/z	Gene names
0523_10	14688	FTMS; HCD	178.72	826.8	AMT1-1

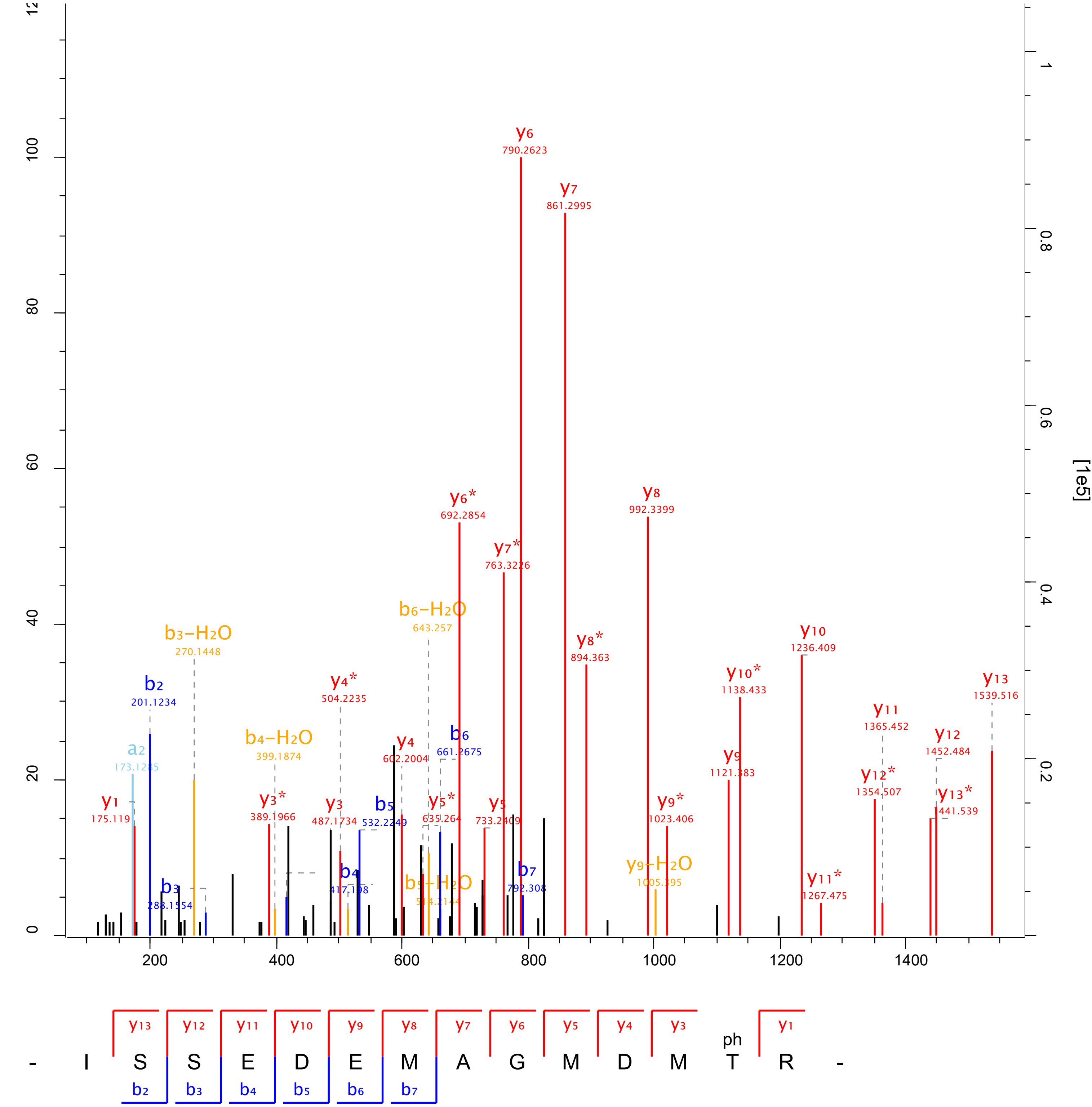

Sample output to test PDF Combine only

Raw file	Scan	Method	Score	m/z	Gene names
0523_10	14914	FTMS; HCD	98.94	671.77	EML3

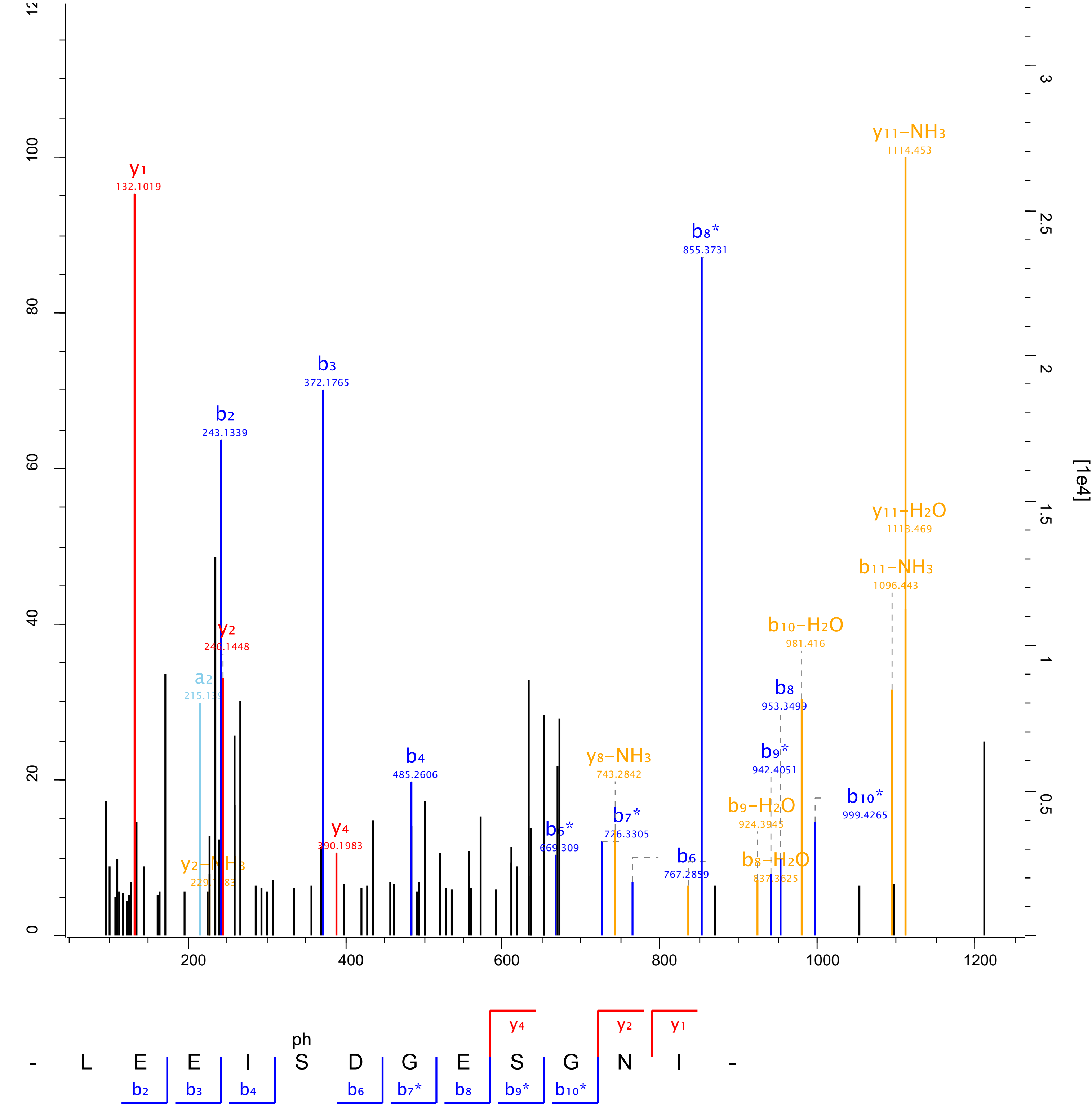

Sample output to test PDF Combine only

Raw file	Scan	Method	Score	m/z	Gene names
0523_10	15364	FTMS; HCD	82.18	911.87	At1g15280

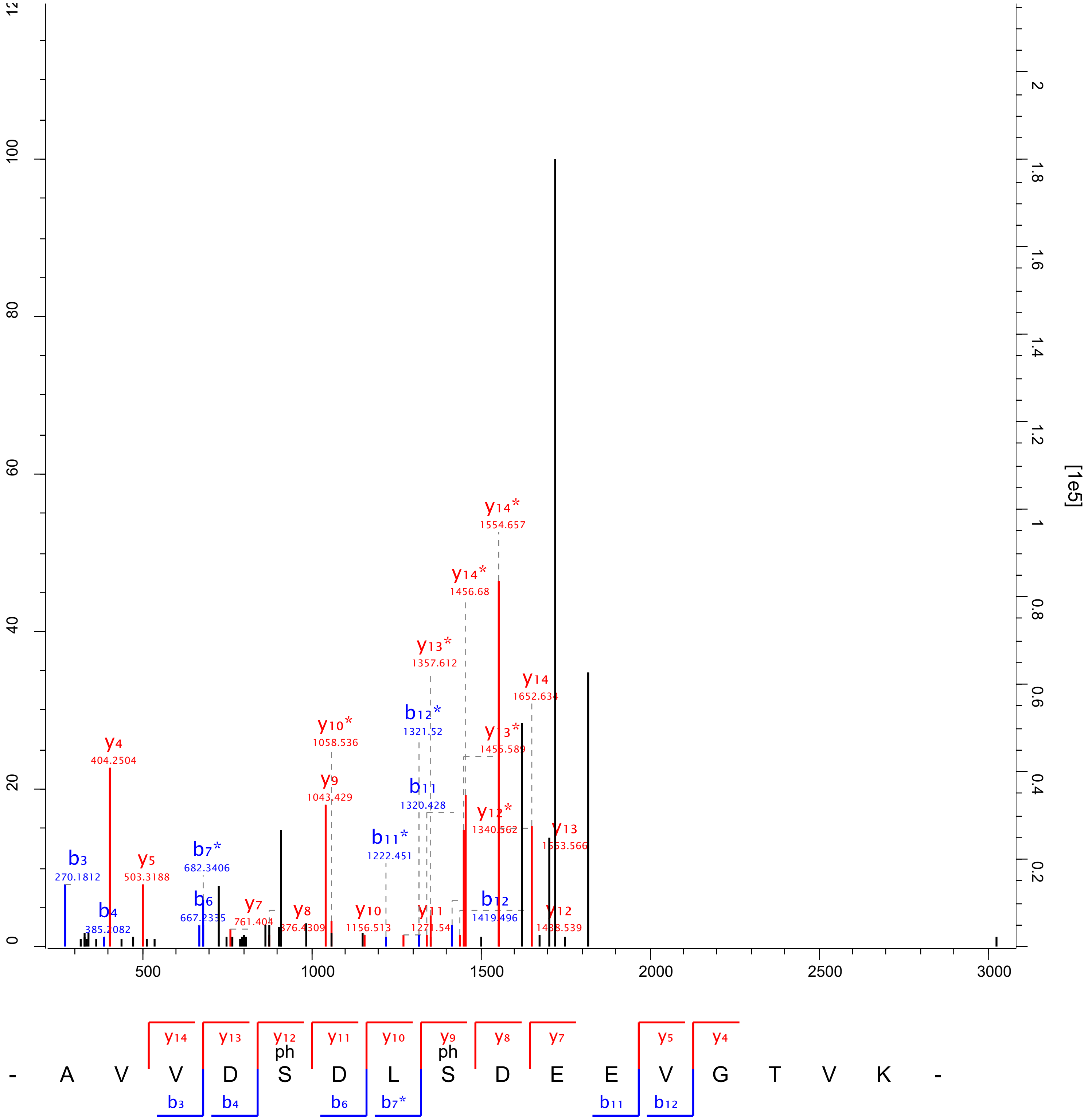

Sample output to test PDF Combine only

Raw file	Scan	Method	Score	m/z	Gene names
0523_10	15555	FTMS; HCD	177.29	826.34	At3g06170

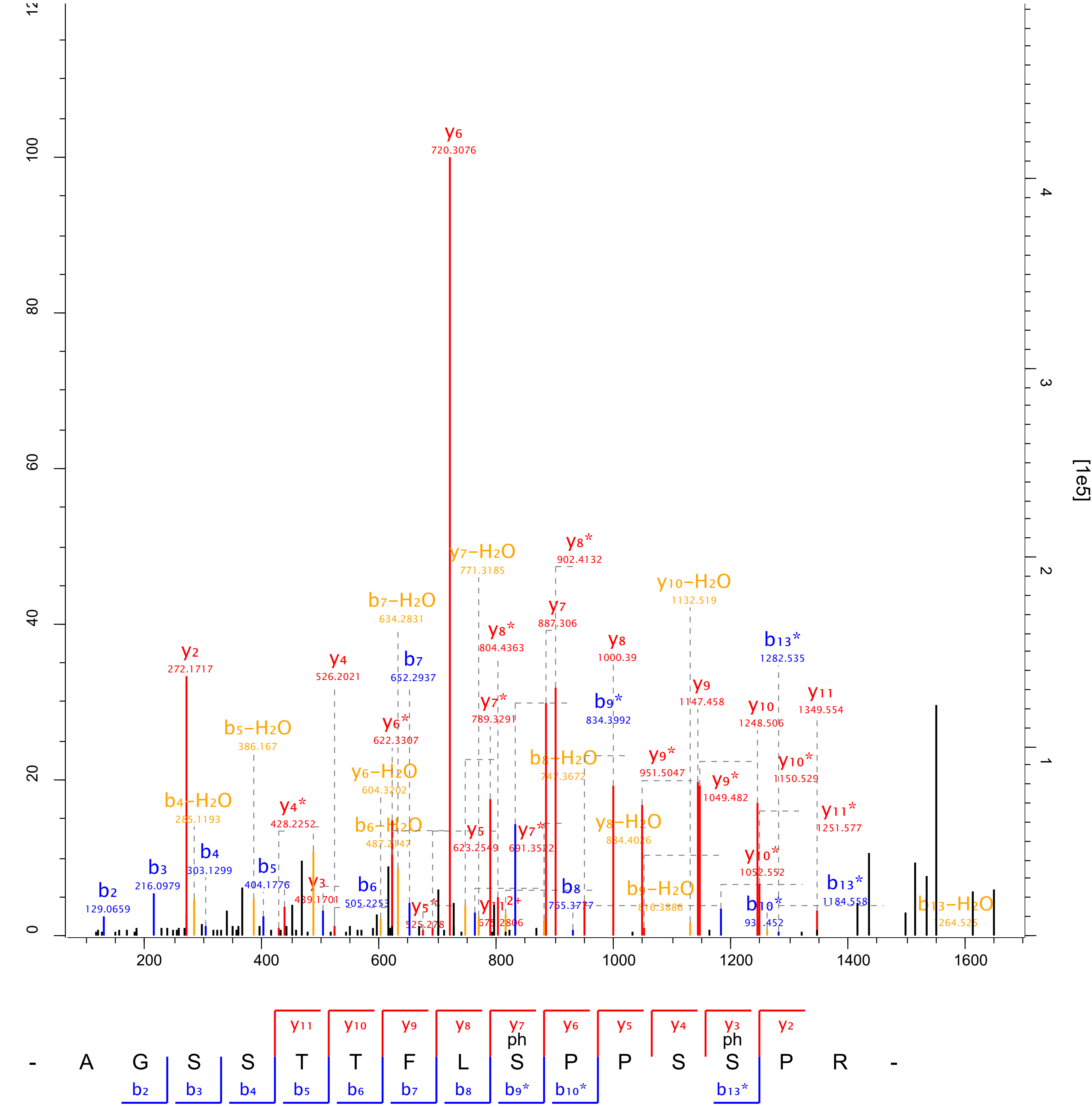

Sample output to test PDF Combine only

Raw file	Scan	Method	Score	m/z	Gene names
0523_10	15845	FTMS; HCD	309.43	1058.43	HMGB1

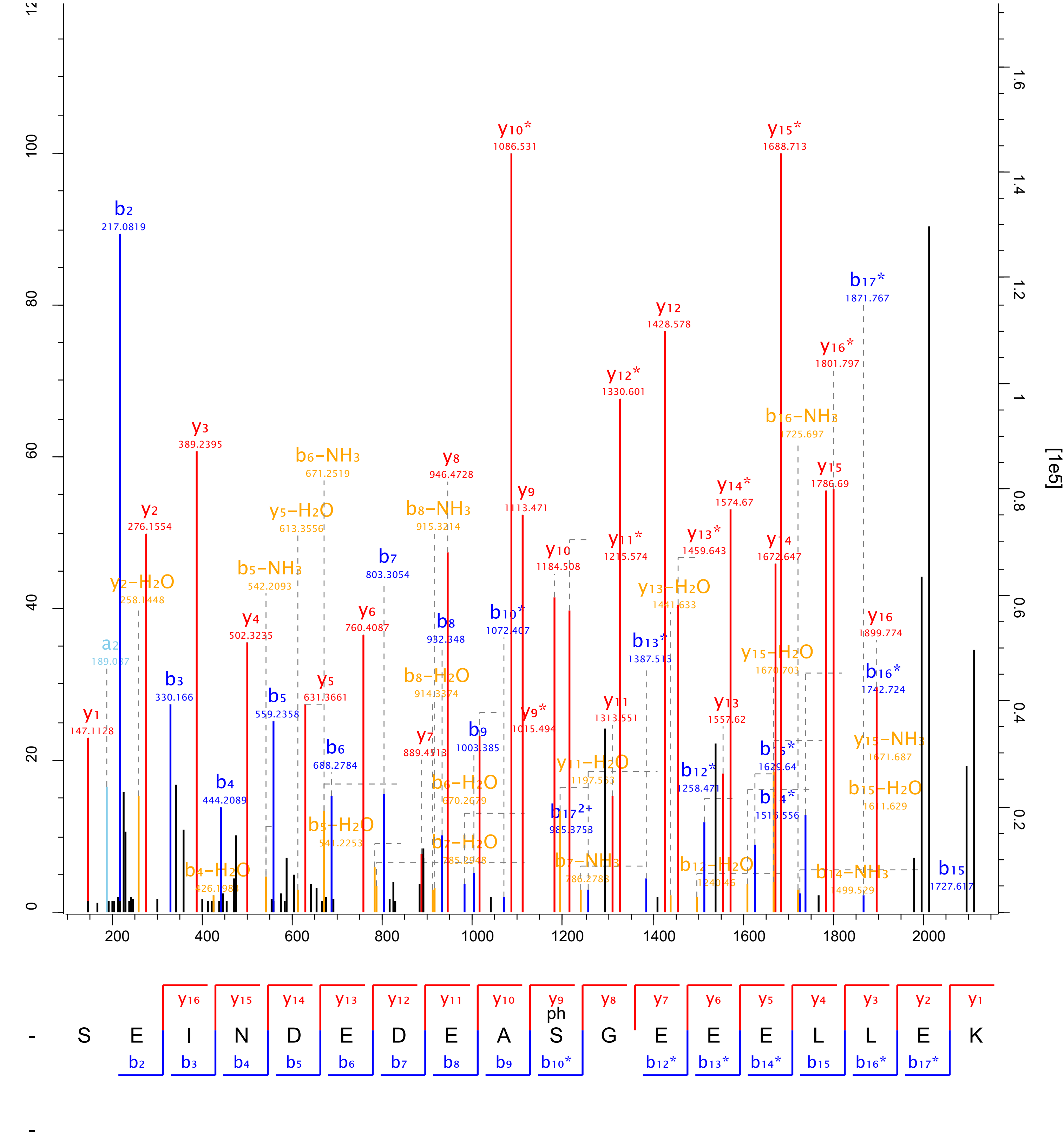

Sample output to test PDF Combine only

Raw file	Scan	Method	Score	m/z	Gene names
0523_10	15869	FTMS; HCD	86.5	738.31	At4g27450

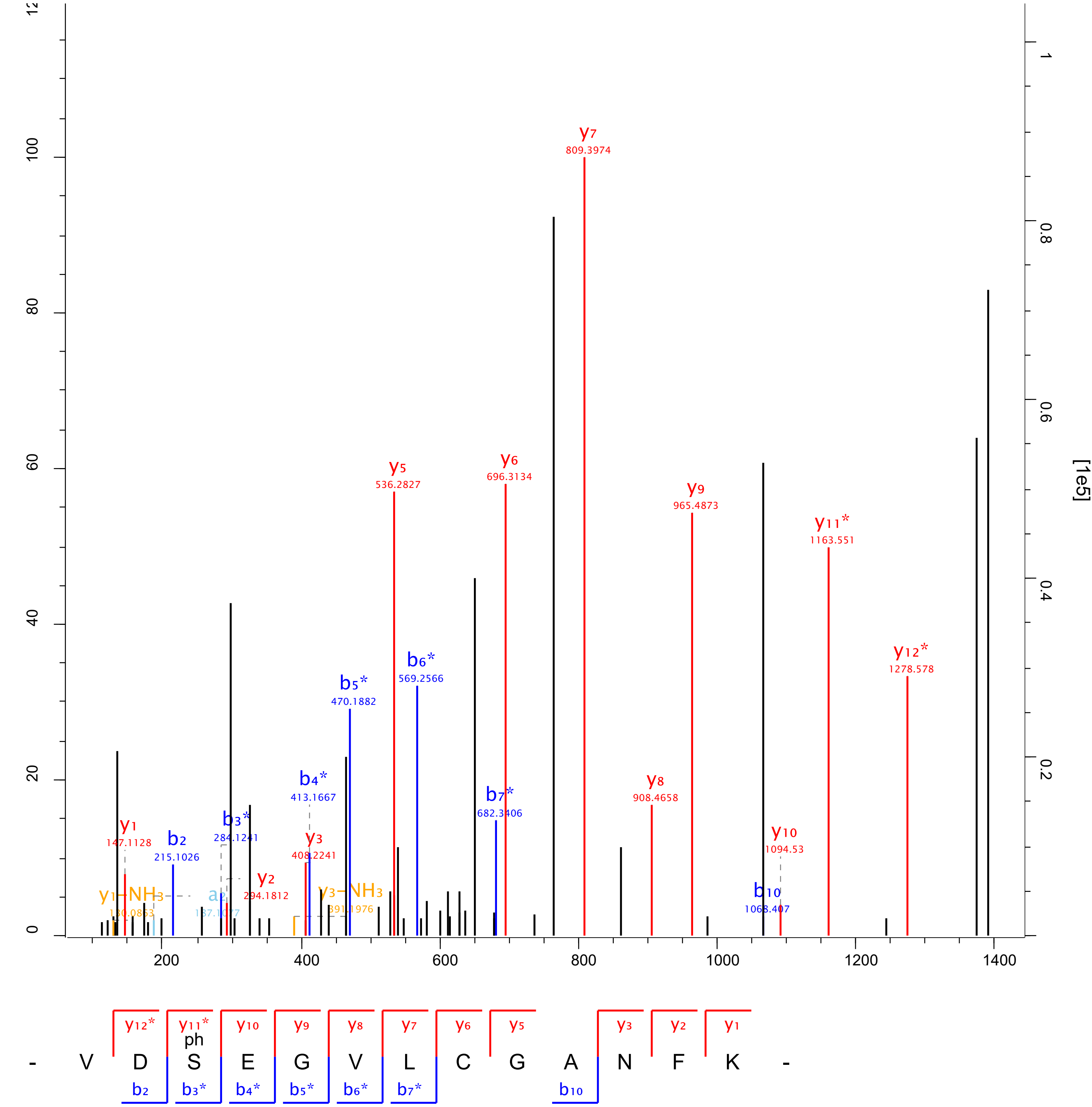

Sample output to test PDF Combine only

Raw file	Scan	Method	Score	m/z	Gene names
0523_10	16614	FTMS; HCD	159.75	818.81	AMT1-2

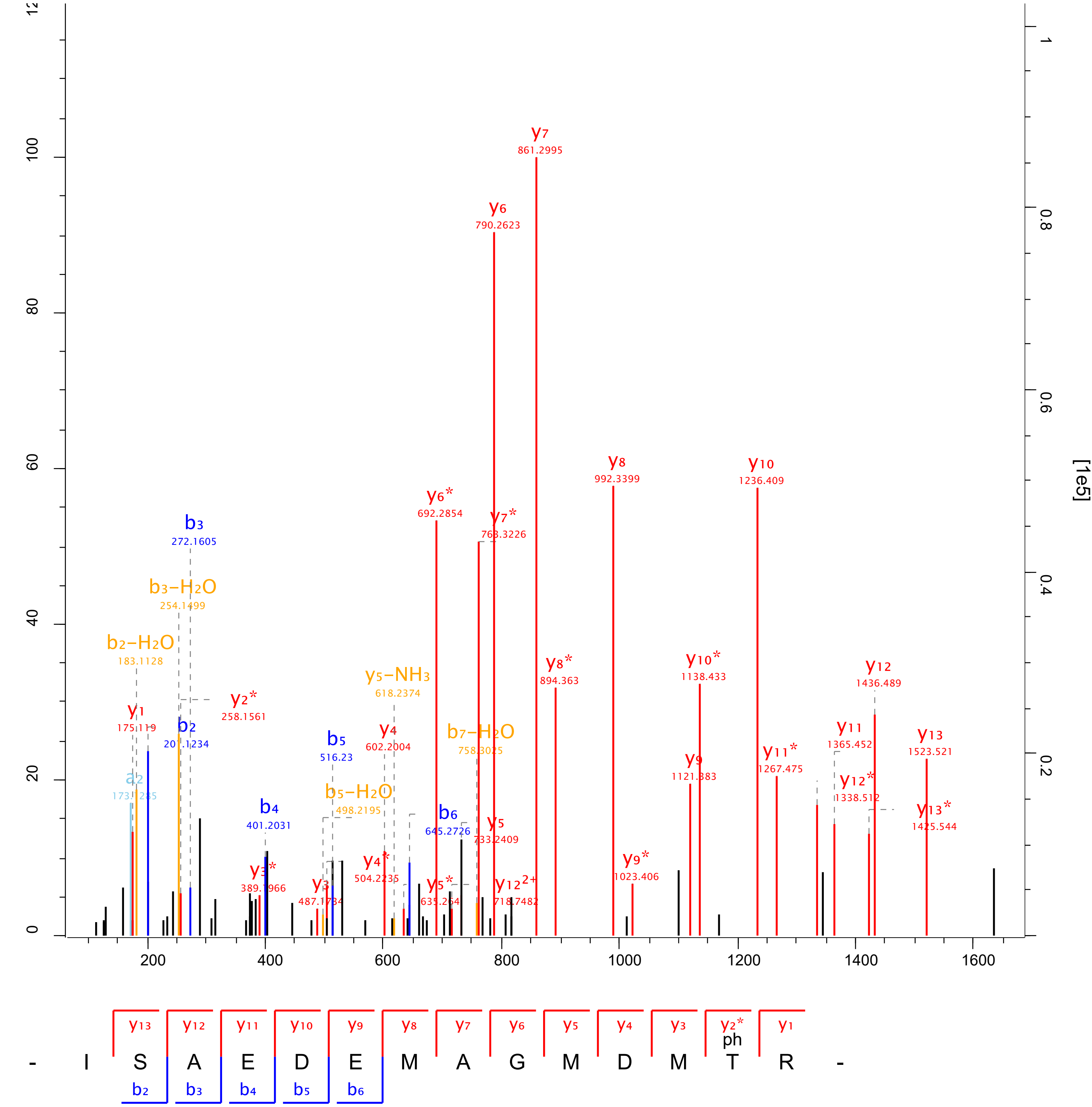

Sample output to test PDF Combine only

Raw file	Scan	Method	Score	m/z	Gene names
0523_10	17650	FTMS; HCD	227.24	856.37	EBP1

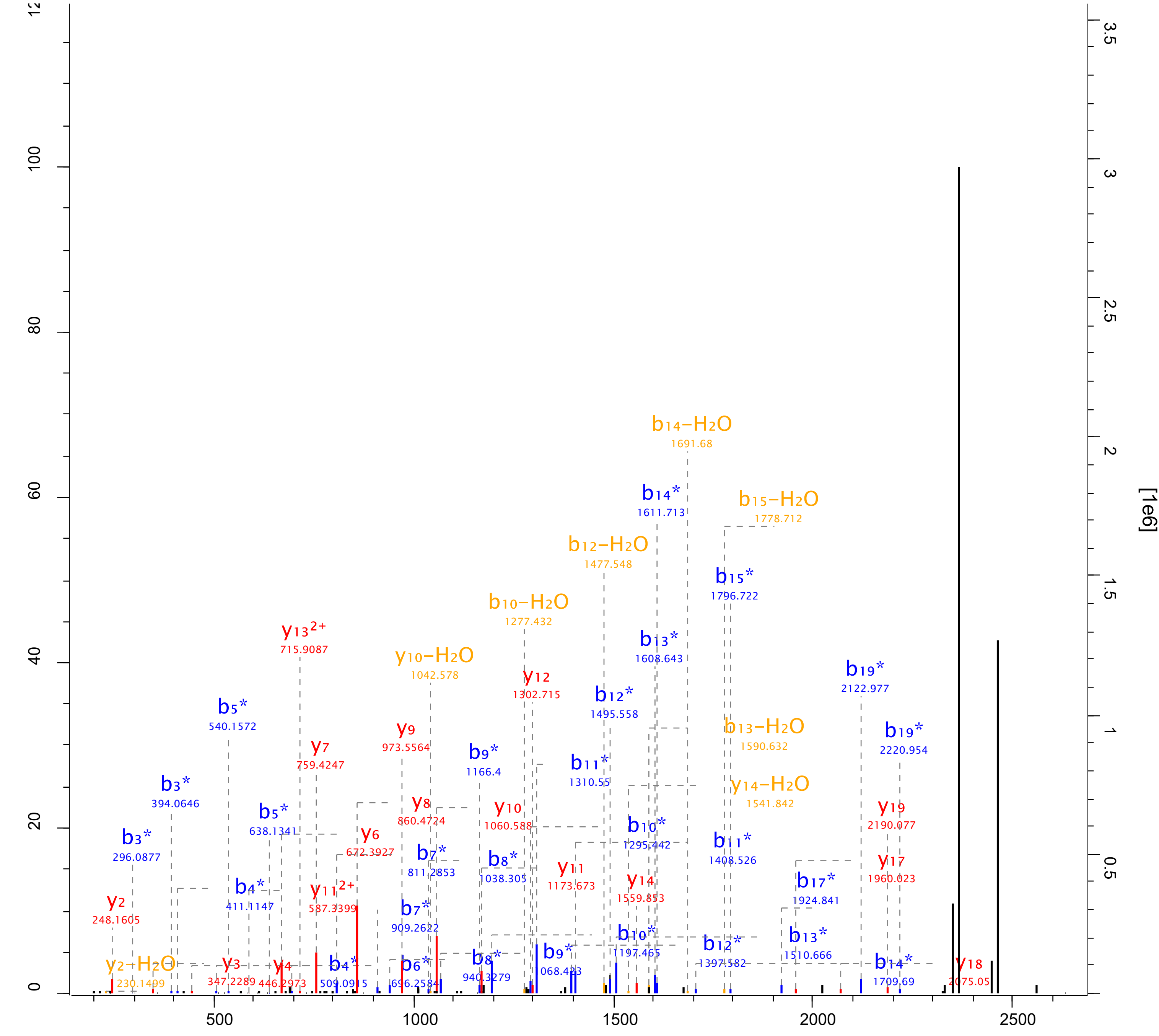

ac - | ph S | ph S | D | D | E | R | D | E | K | E | L | S | L | T | S | P | E | V | V | T | K | -

y19 y18 y17 y14 y13 2+ y12 y11 y10 y9 y8 y7 y6 y4 y3 y2

b3* b4* b5* b6* b7* b8* b9* b10* b11* b12* b13* b14* b15* b17* b19*

Sample output to test PDF Combine only

Raw file	Scan	Method	Score	m/z	Gene names
0523_10	17711	FTMS; HCD	44.16	719.67	F8A24.12

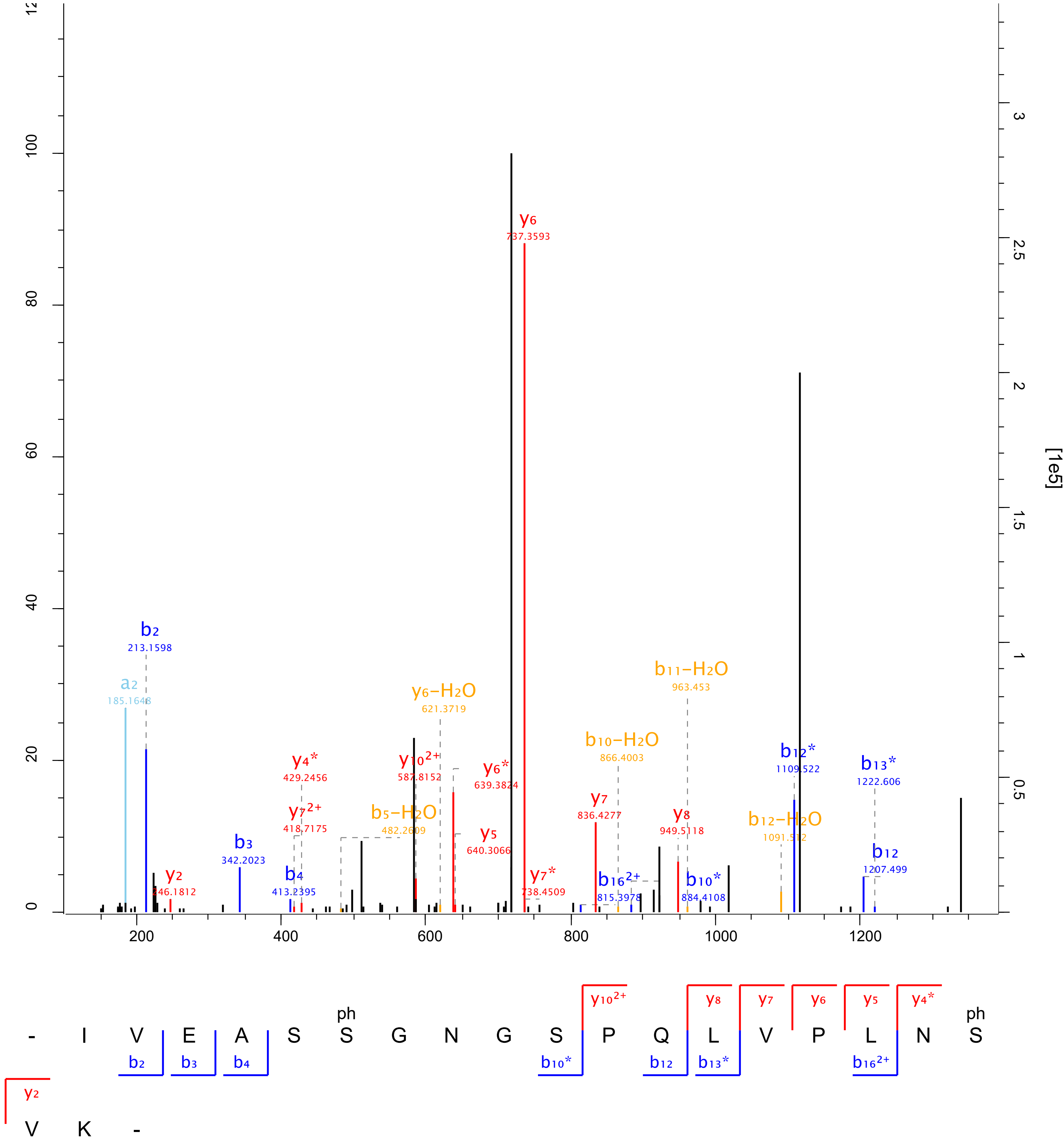

Sample output to test PDF Combine only

Raw file	Scan	Method	Score	m/z	Gene names
0523_10	17719	FTMS; HCD	111.64	690.81	PAT07

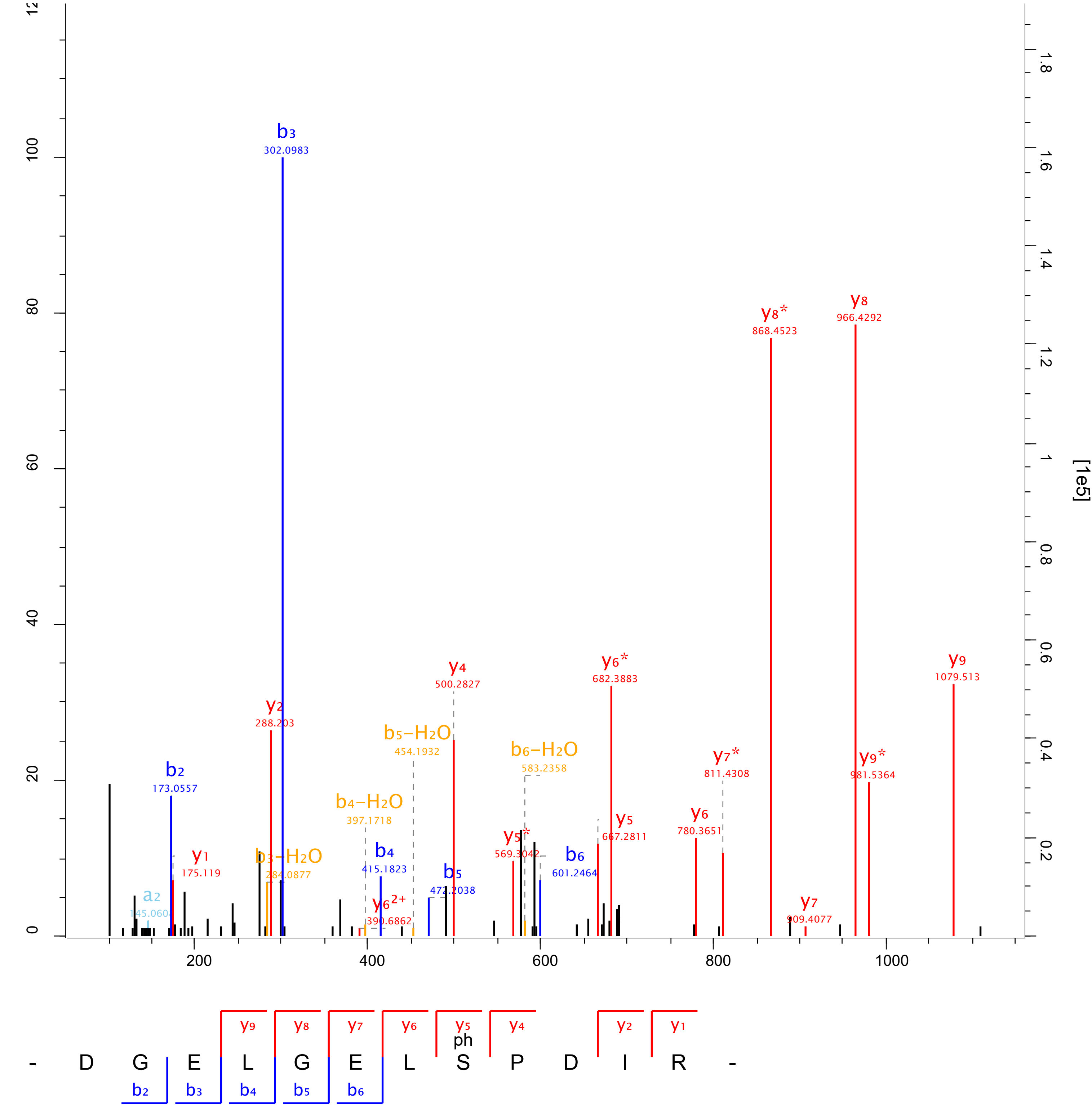

Raw file	Scan	Method	Score	m/z	Gene names
0523_10	17814	FTMS; HCD	54.28	610.76	ML5

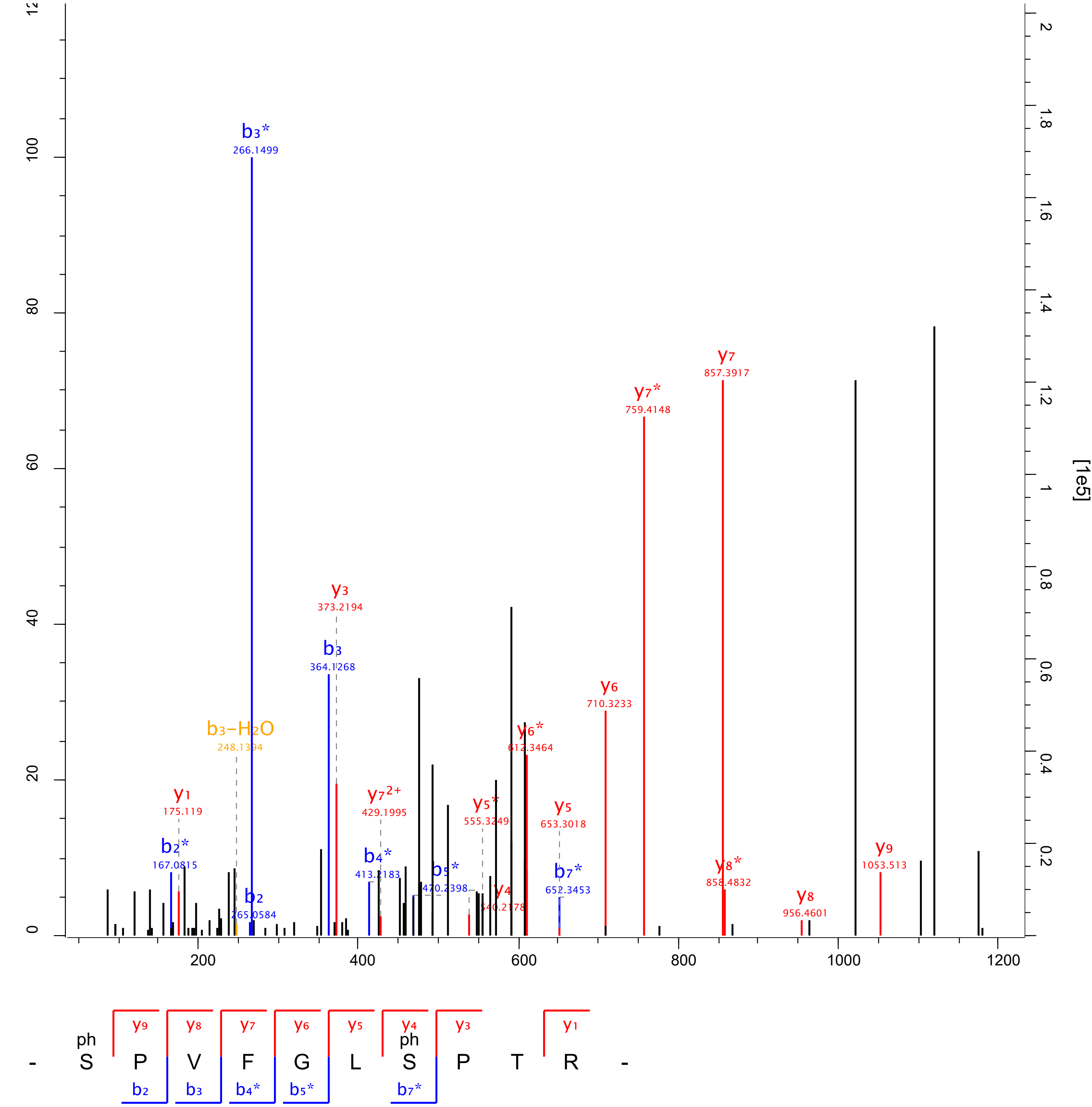

Sample output to test PDF Combine only

Raw file	Scan	Method	Score	m/z	Gene names
0523_10	17941	FTMS; HCD	59.71	679.29	PVA11

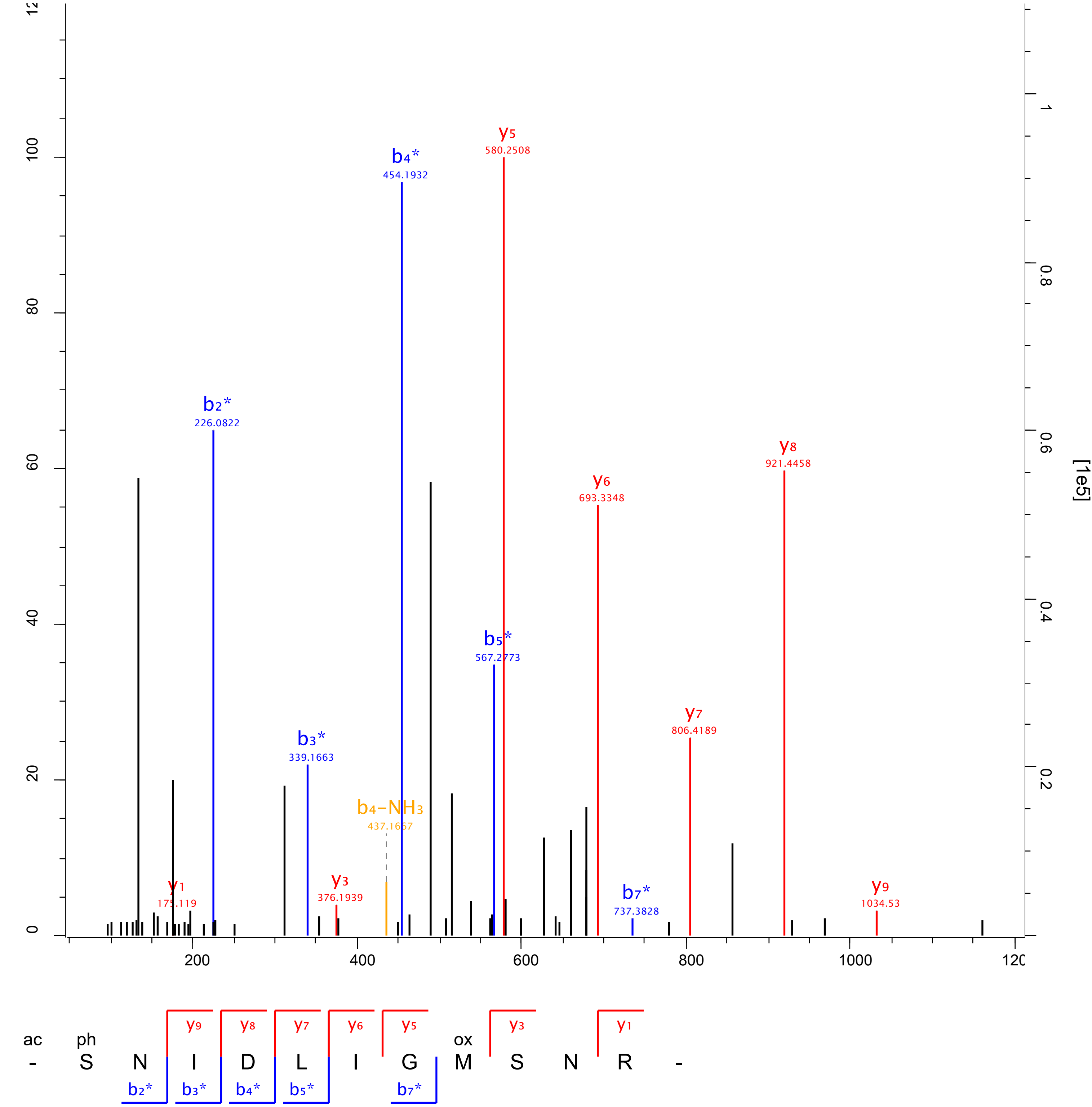

Sample output to test PDF Combine only

Raw file	Scan	Method	Score	m/z	Gene names
0523_10	18397	FTMS; HCD	47.13	967.1	At4g00238

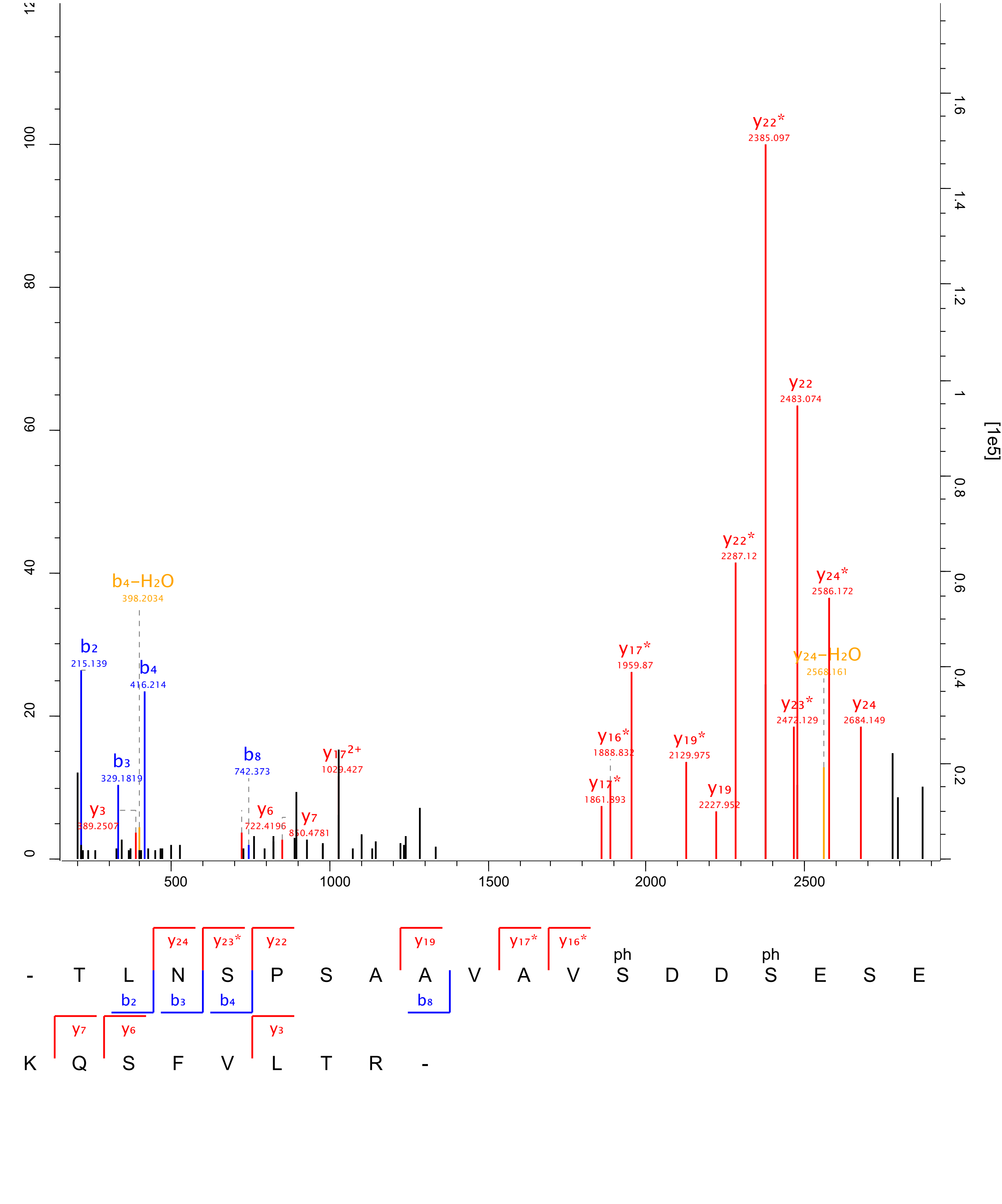

- T L N S P S A A V A V S D D S E S E
K Q S F V L T R -

Sample output to test PDF Combine only

Raw file	Scan	Method	Score	m/z
0523_10	19038	FTMS; HCD	104.39	898.37

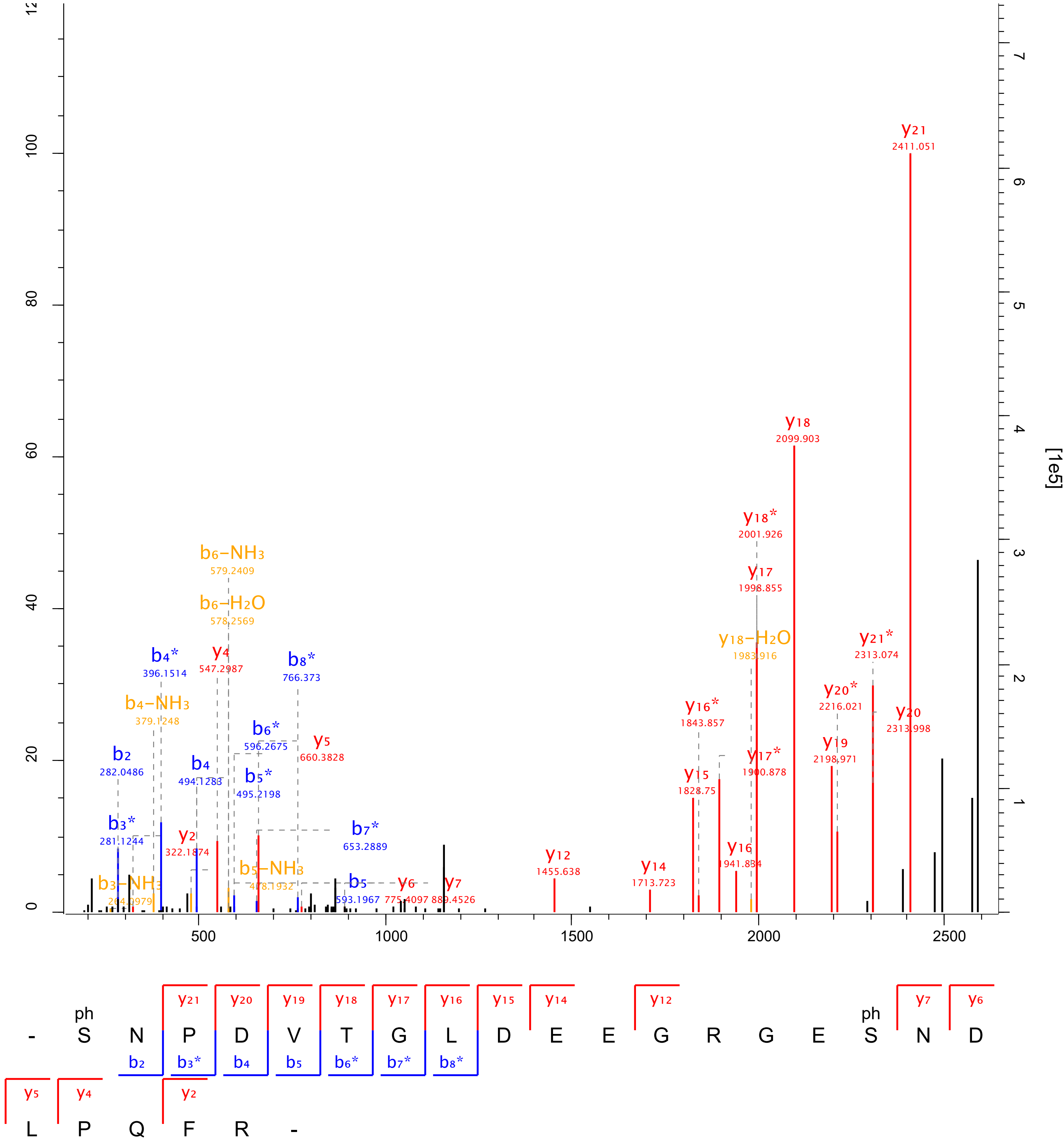

Sample output to test PDF Combine only

Raw file	Scan	Method	Score	m/z	Gene names
0523_10	19130	FTMS; HCD	45.49	728.97	F13I12.260

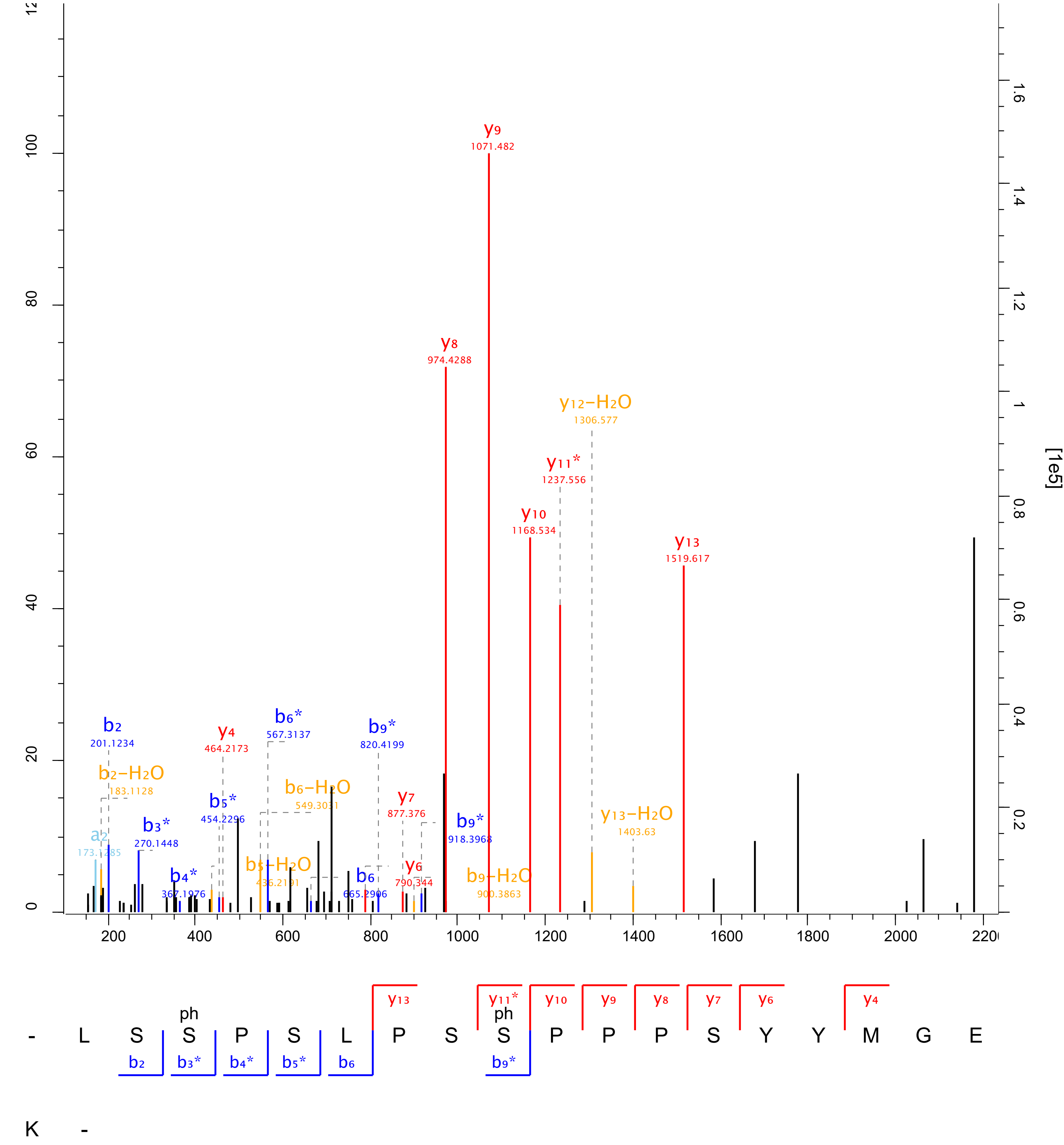

Sample output to test PDF Combine only

Raw file	Scan	Method	Score	m/z
0523_10	19859	FTMS; HCD	144.24	1208.17

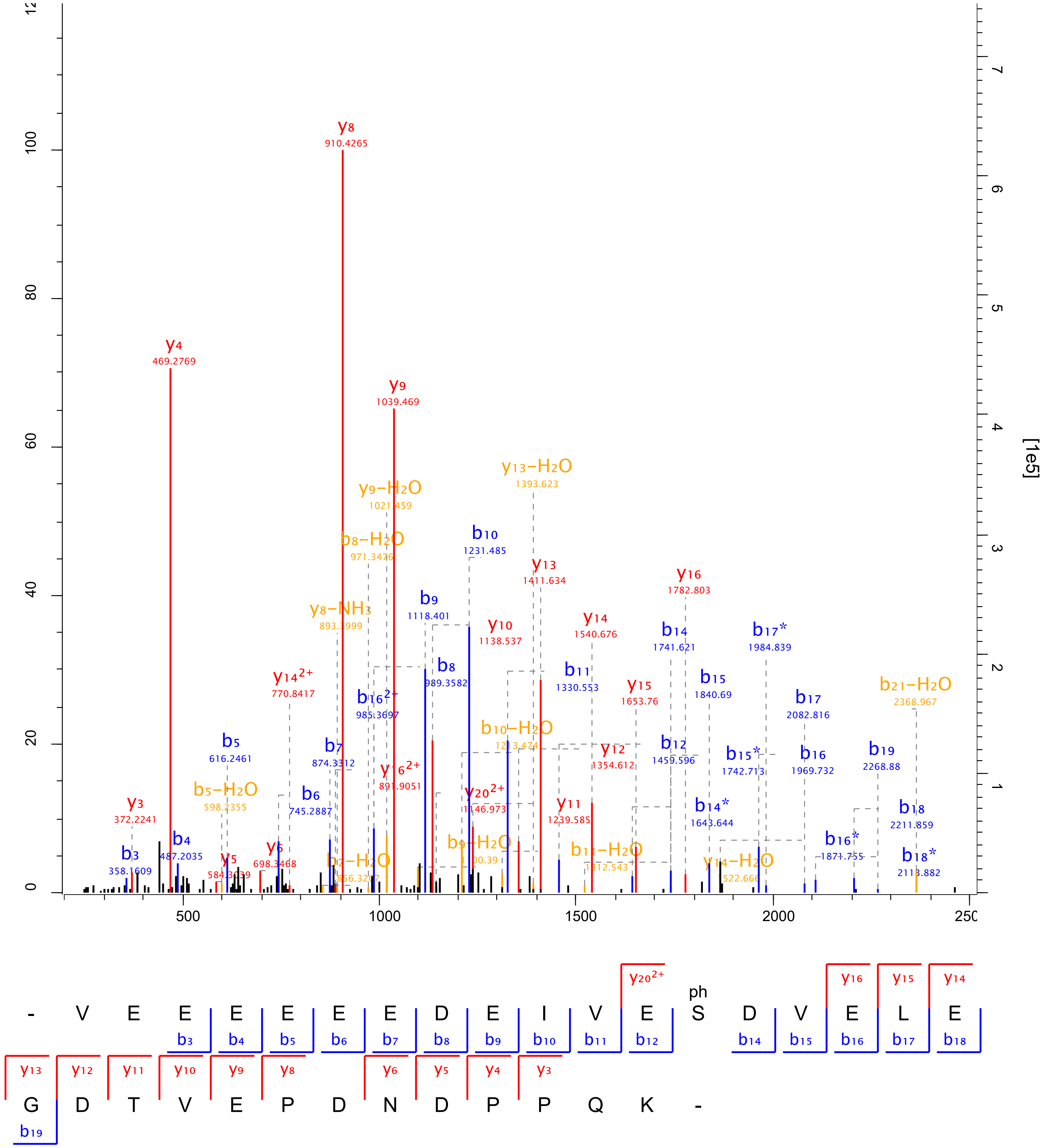

Sample output to test PDF Combine only

Raw file	Scan	Method	Score	m/z	Gene names
0523_10	20831	FTMS; HCD	163.8	785.84	NUP107

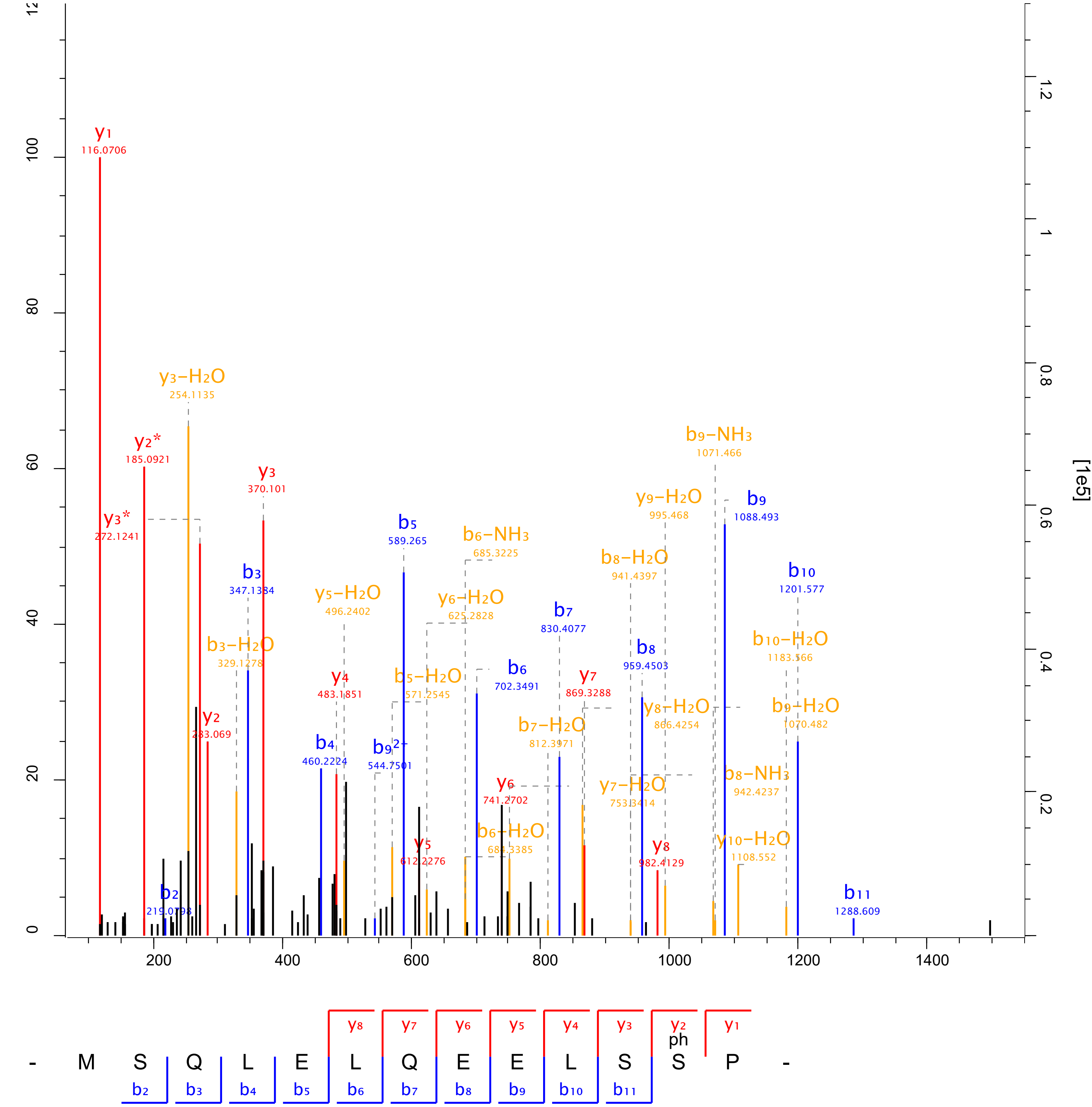

Sample output to test PDF Combine only

Raw file	Scan	Method	Score	m/z	Gene names
0523_10	21364	FTMS; HCD	117.83	1036.48	ACBP4

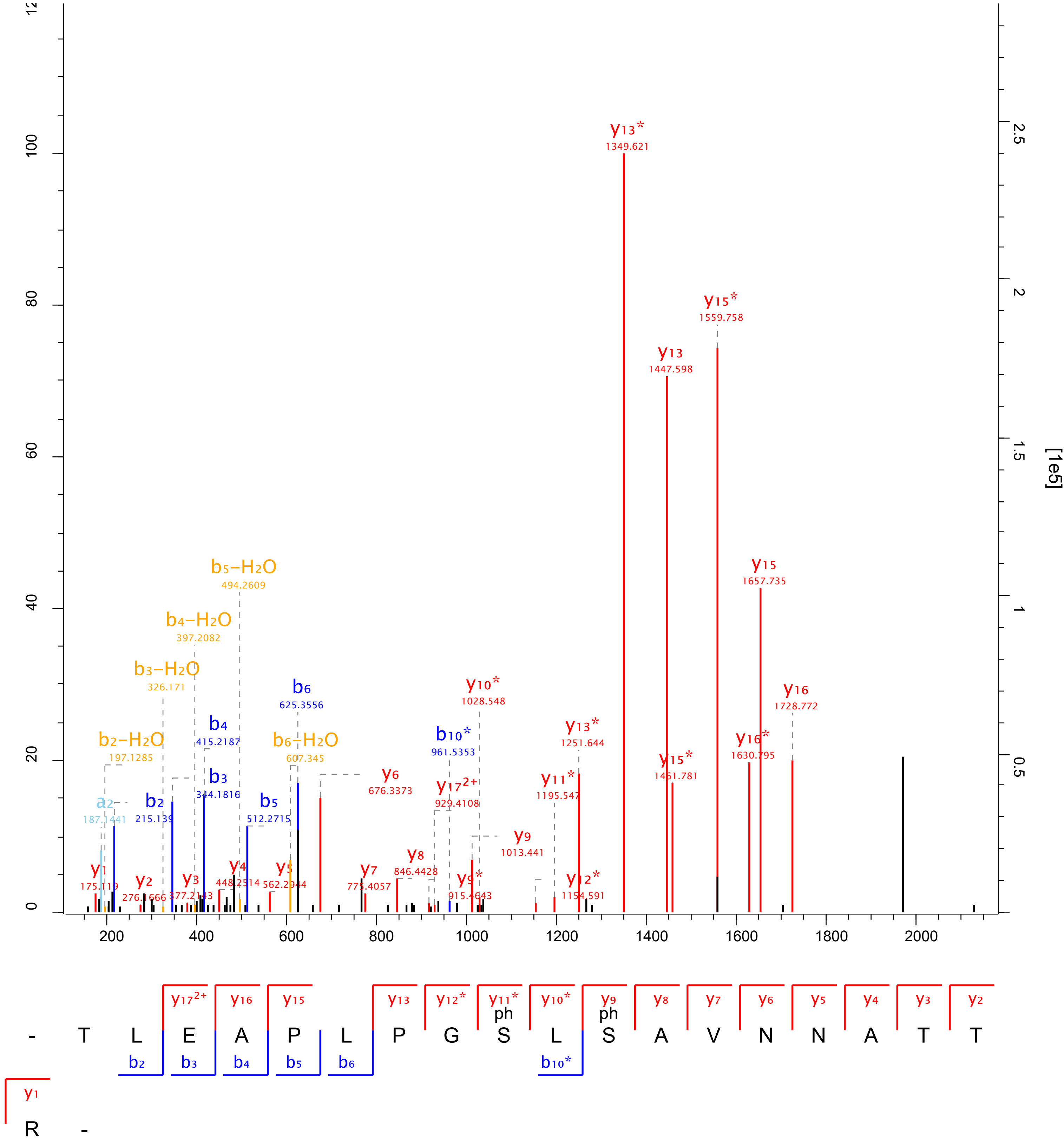

Sample output to test PDF Combine only

Raw file	Scan	Method	Score	m/z	Gene names
0523_10	22233	FTMS; HCD	40.14	951.4	T27C4.12

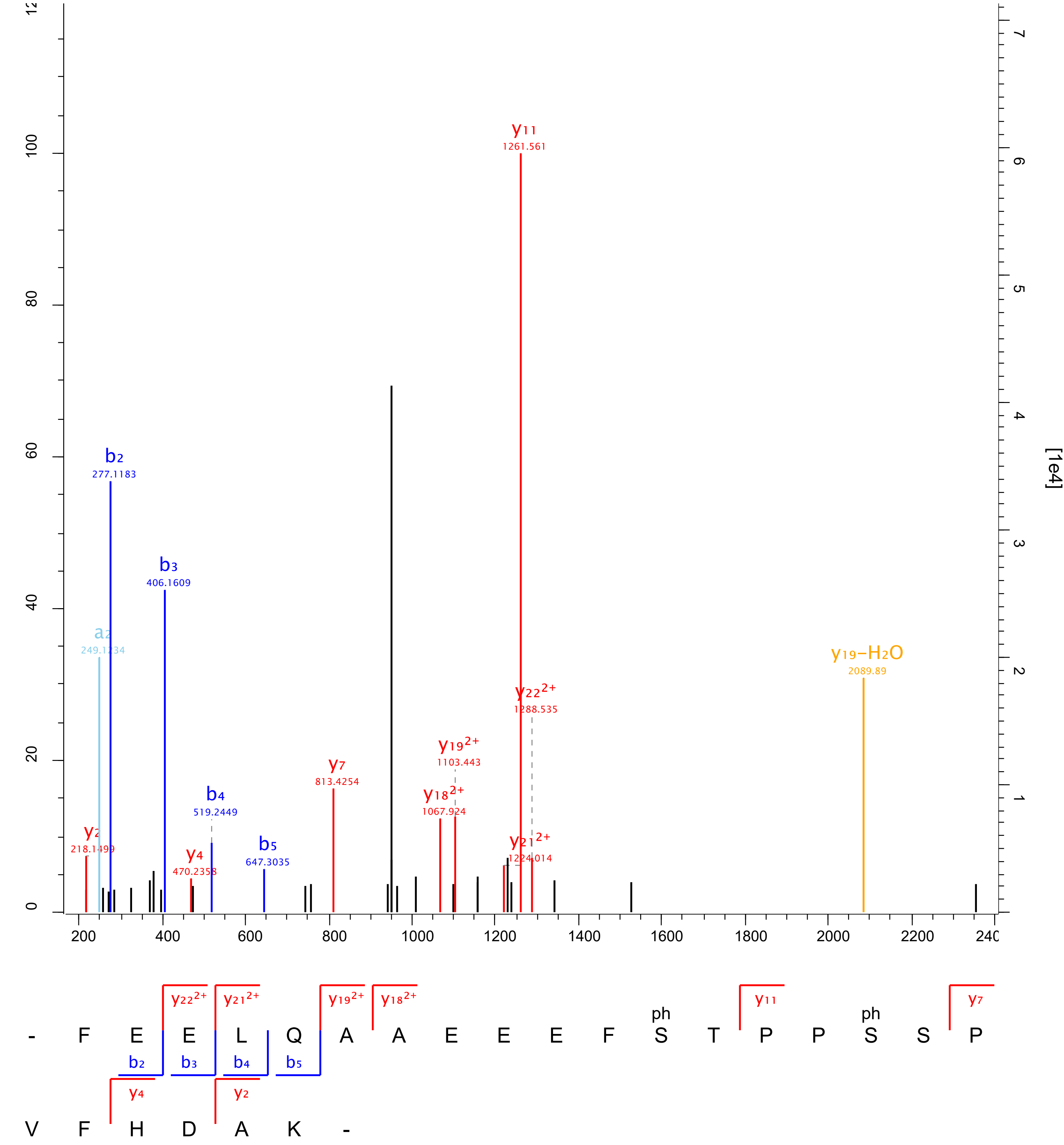

Sample output to test PDF Combine only

Raw file	Scan	Method	Score	m/z	Gene names
0523_10	22326	FTMS; HCD	202.43	797.01	Tudor2

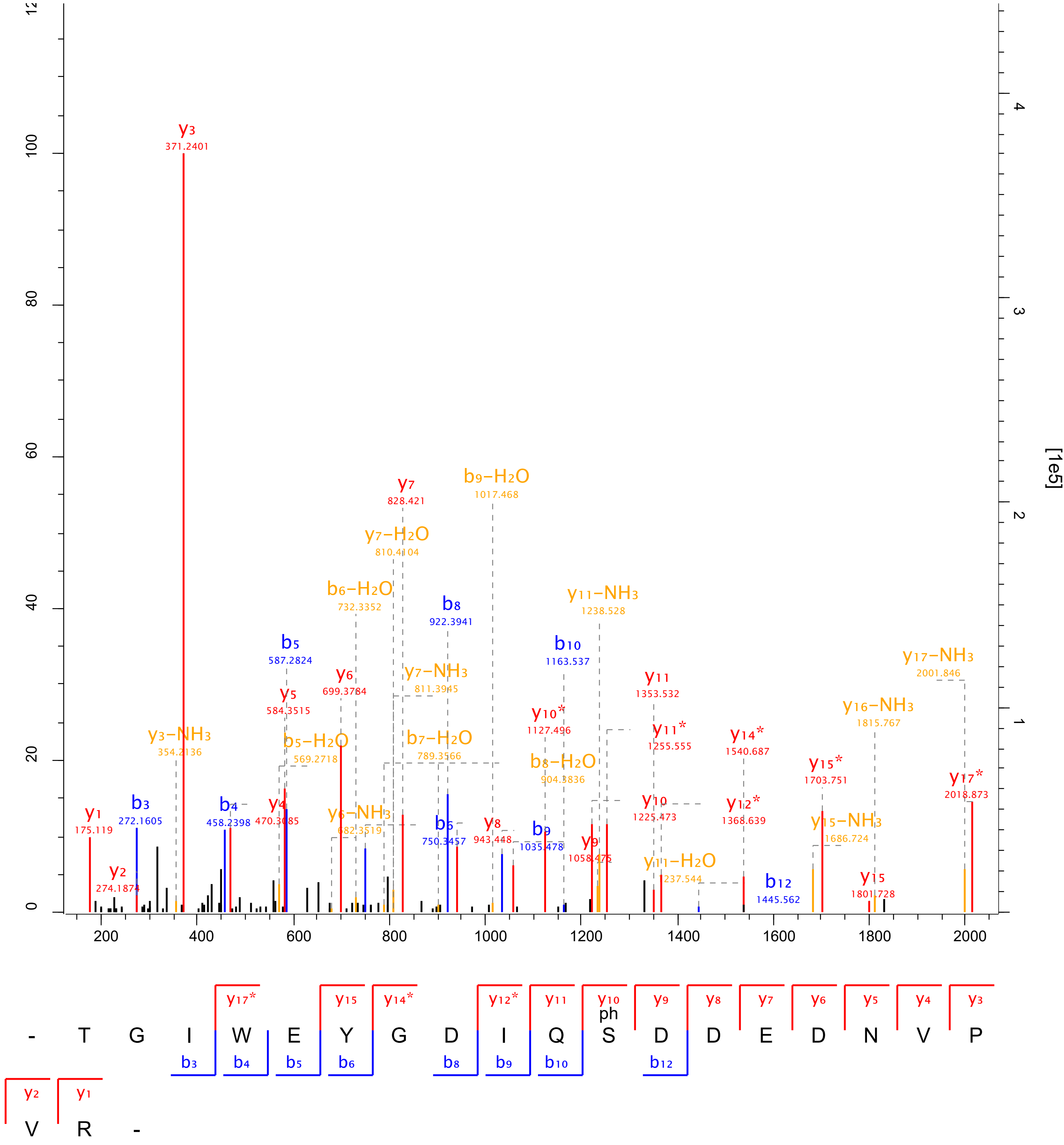

Sample output to test PDF Combine only

Raw file	Scan	Method	Score	m/z	Gene names
0523_10	22953	FTMS; HCD	160.67	750.31	LRS1

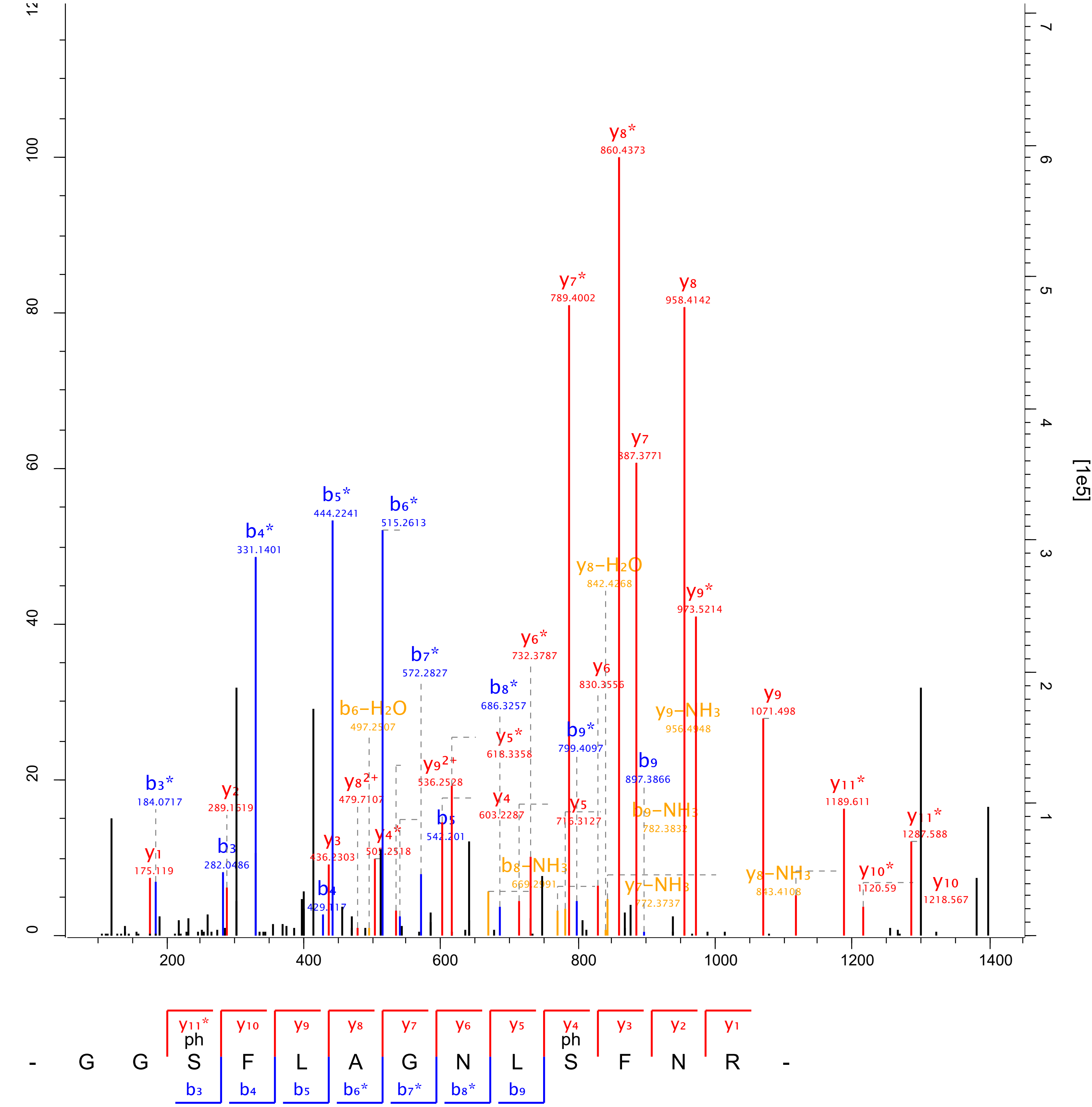

Sample output to test PDF Combine only

Raw file	Scan	Method	Score	m/z	Gene names
0523_10	26947	FTMS; HCD	47.64	617.26	SYP122

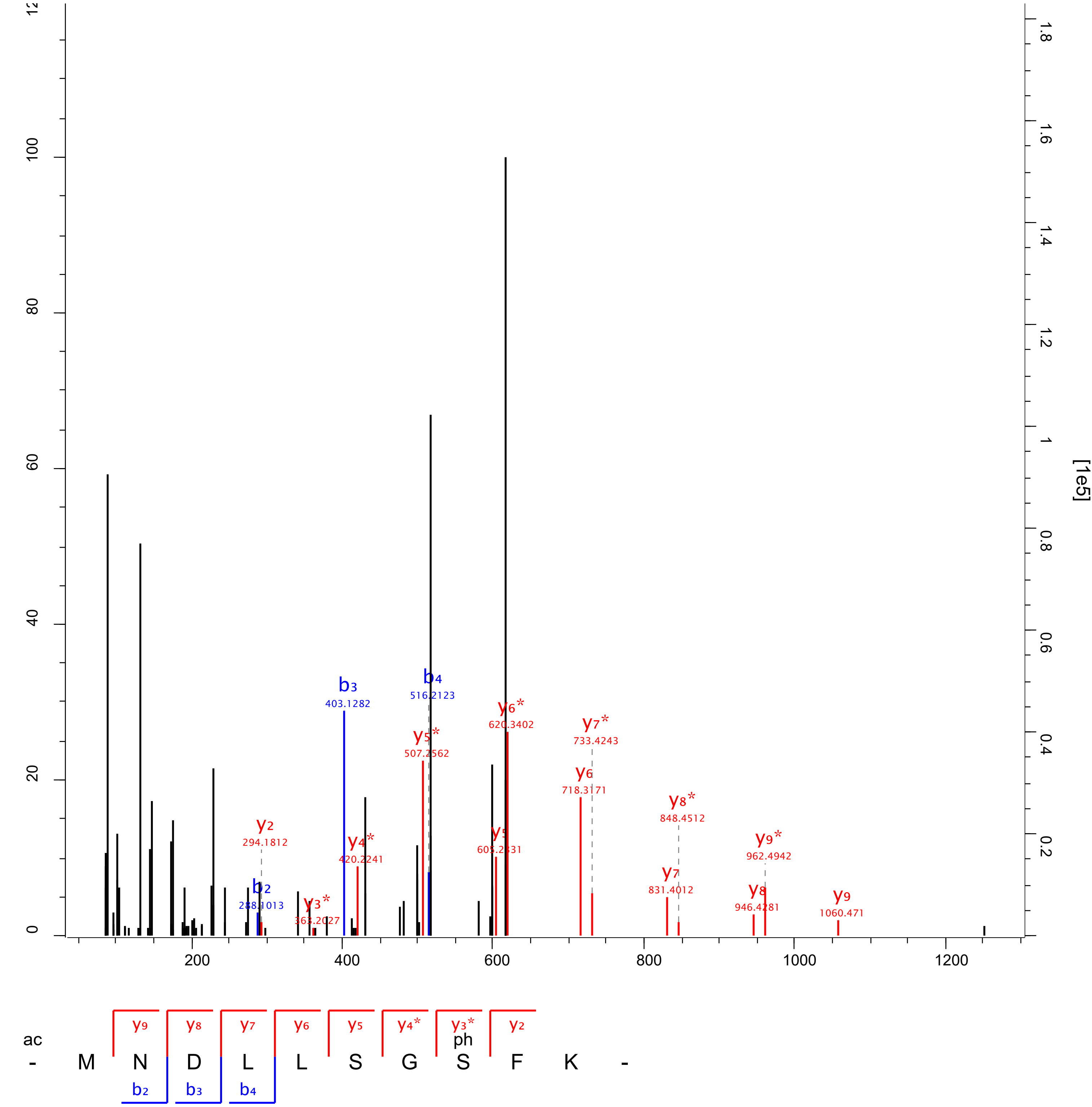

Sample output to test PDF Combine only

Raw file	Scan	Method	Score	m/z	Gene names
0523_10	28954	FTMS; HCD	93.63	1081.89	APUM5

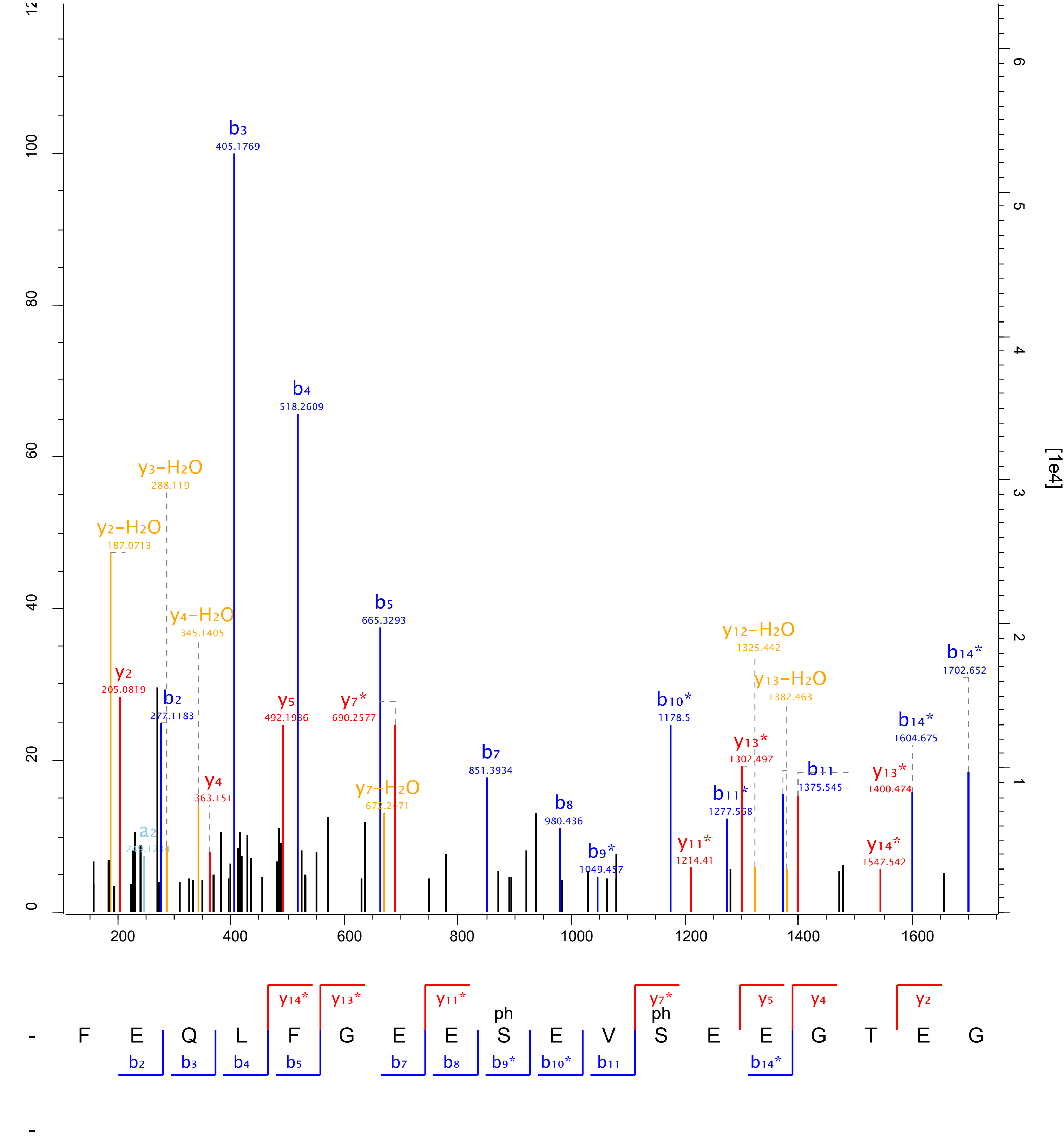

Raw file	Scan	Method	Score	m/z	Gene names
0523_10	29394	FTMS; HCD	112.08	808.77	RPP2D;RPP2A;At2g27720;RPP2B;At2g27710

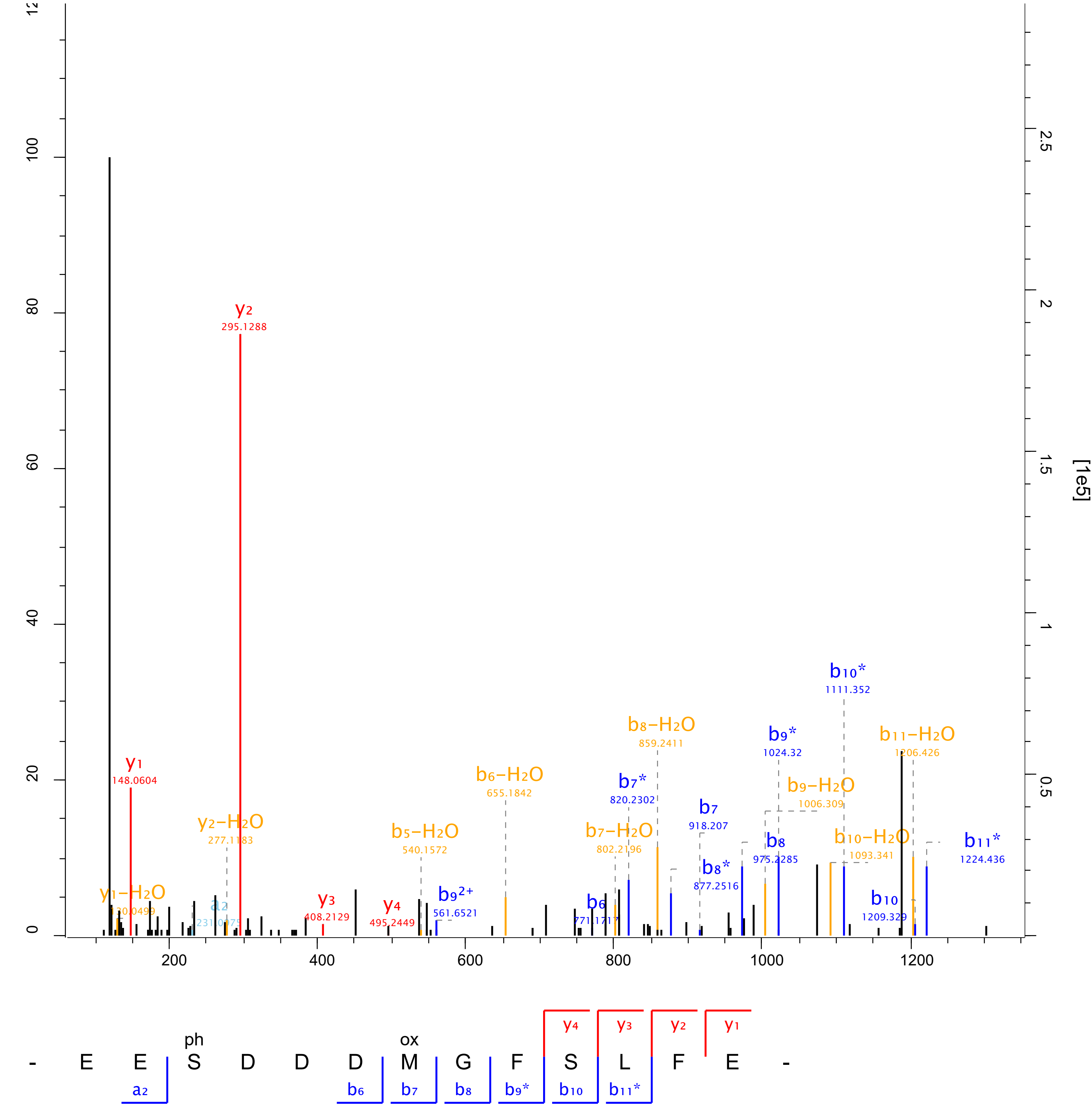

Sample output to test PDF Combine only

Raw file	Scan	Method	Score	m/z	Gene names
0523_10	30687	FTMS; HCD	116.73	736.76	PIP2-4

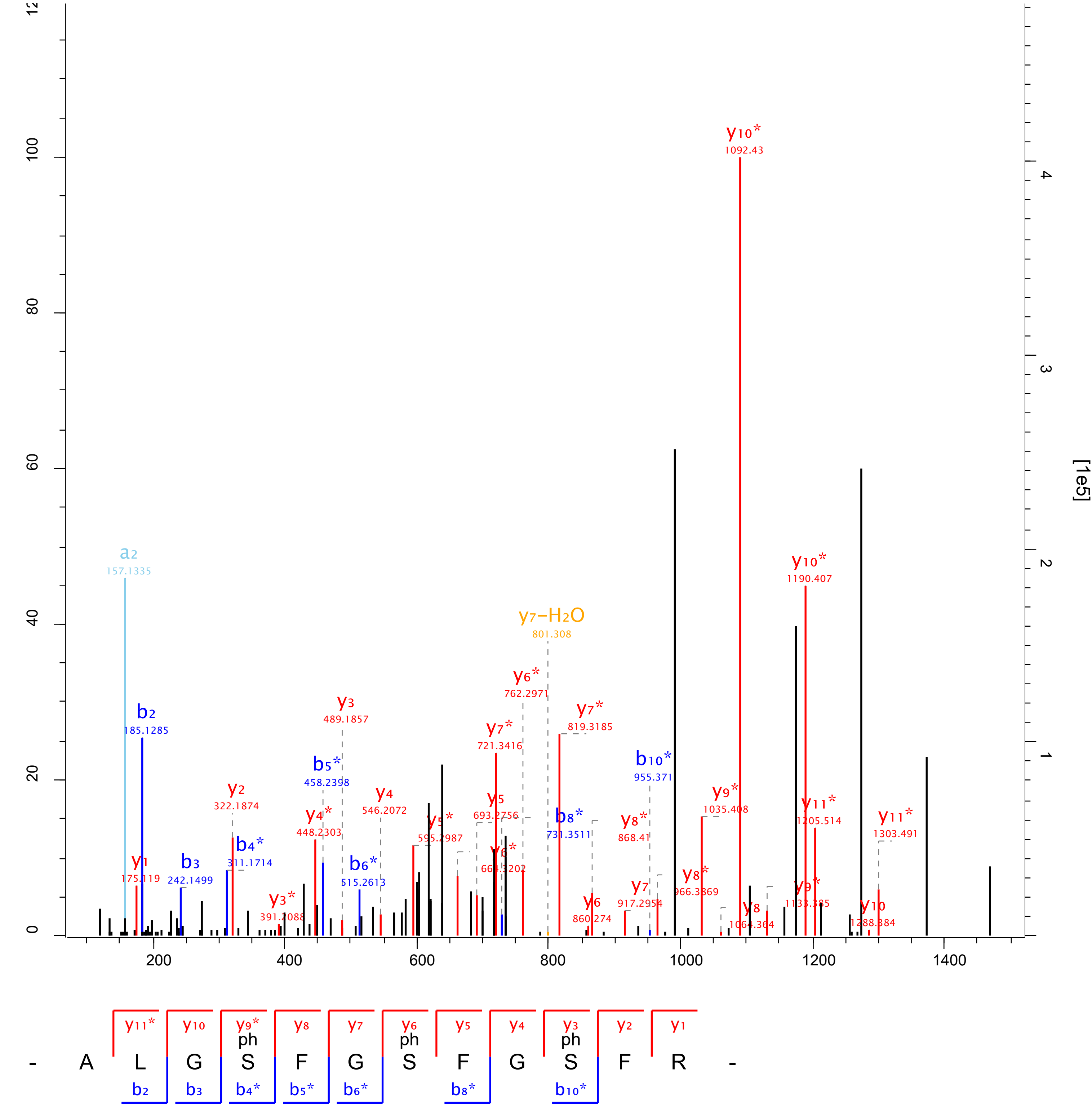

Sample output to test PDF Combine only

Raw file	Scan	Method	Score	m/z	Gene names
0523_10	30861	FTMS; HCD	162.37	1081.37	At3g09200;RPP0B

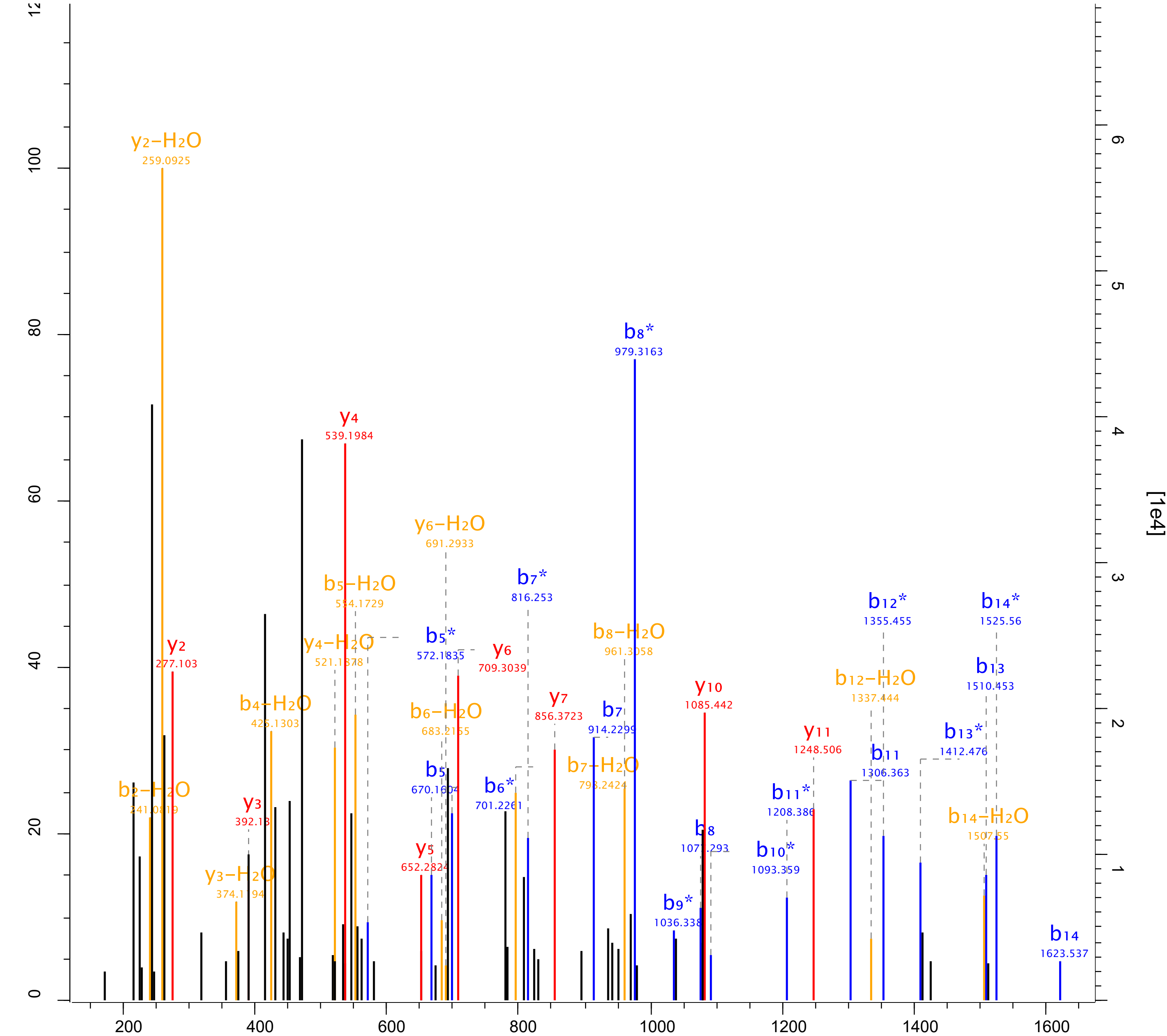

- E E S D E E D Y G G D F G L F D E E

ph (on S)

y11 y10 y7 y6 y5 y4 y3 y2

b5 b6* b7 b8 b9* b10* b11 b12* b13 b14

-

Sample output to test PDF Combine only

Raw file	Scan	Method	Score	m/z	Gene names
0523_11	1399	FTMS; HCD	178.46	718.95	ERD14

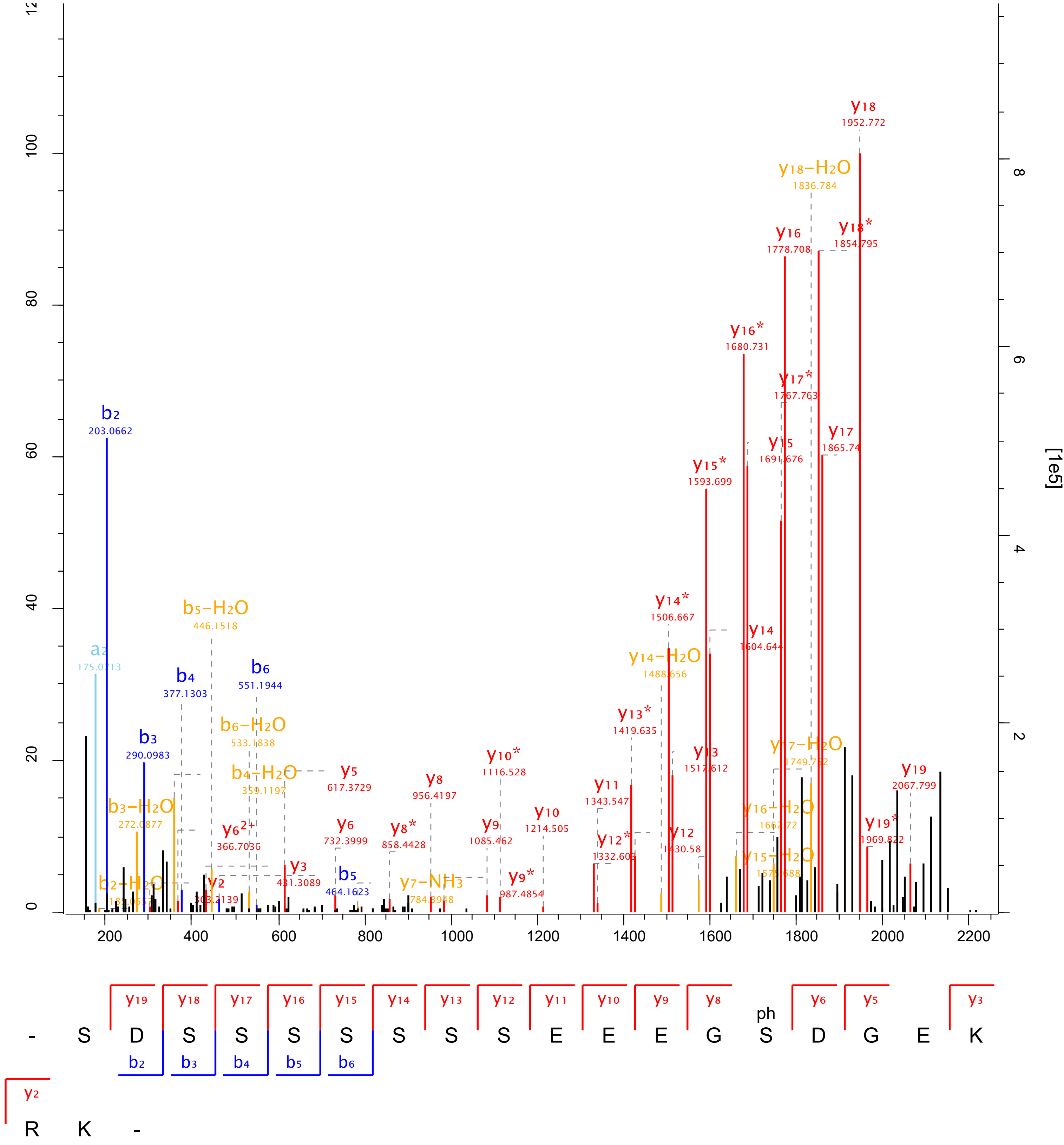

Sample output to test PDF Combine only

Raw file	Scan	Method	Score	m/z	Gene names
0523_11	1449	FTMS; HCD	119.34	423.2	At5g55850;NOI

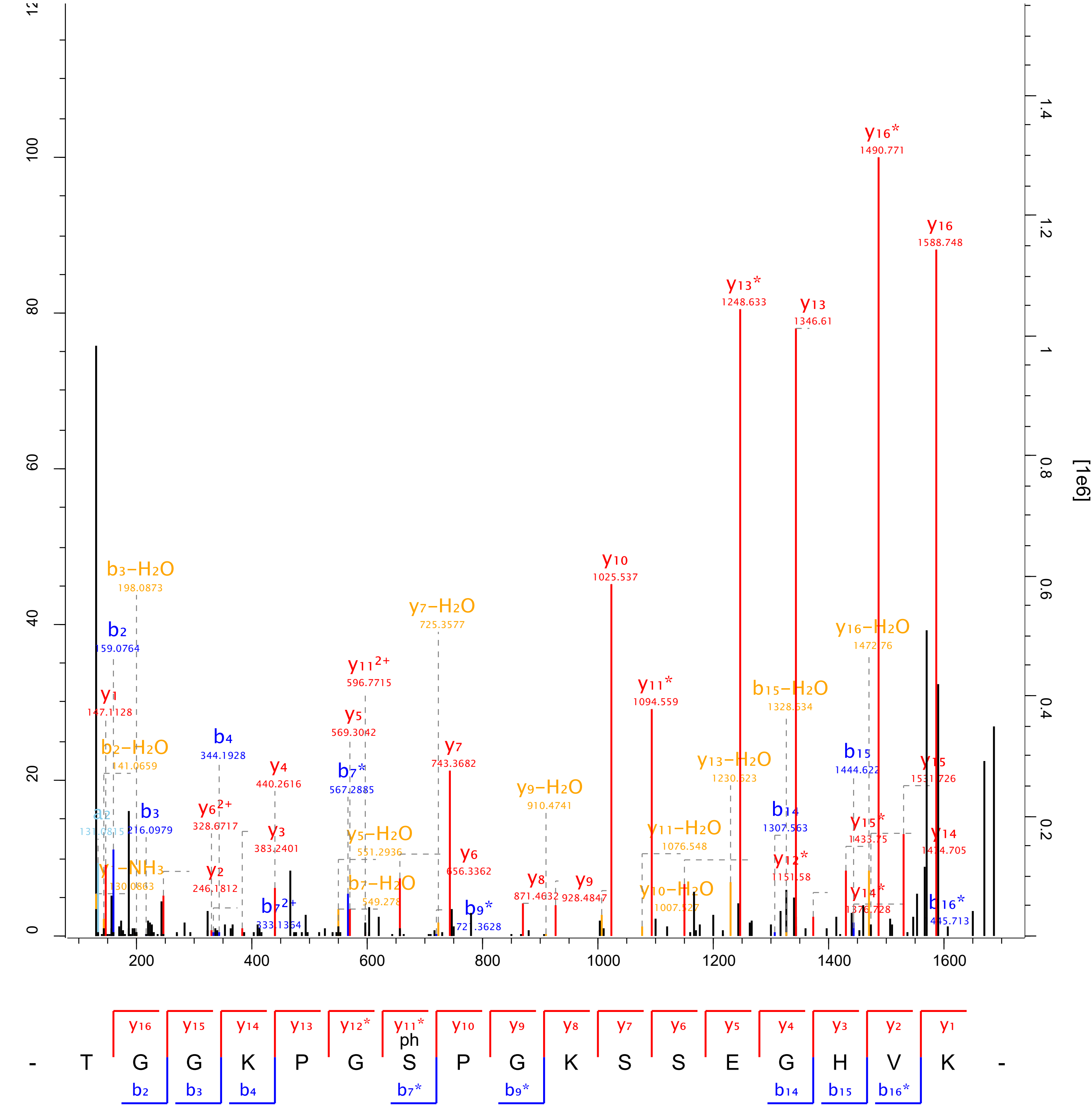

Sample output to test PDF Combine only

Raw file	Scan	Method	Score	m/z	Gene names
0523_11	1488	FTMS; HCD	58.51	382.51	IP5PI;IP5P1

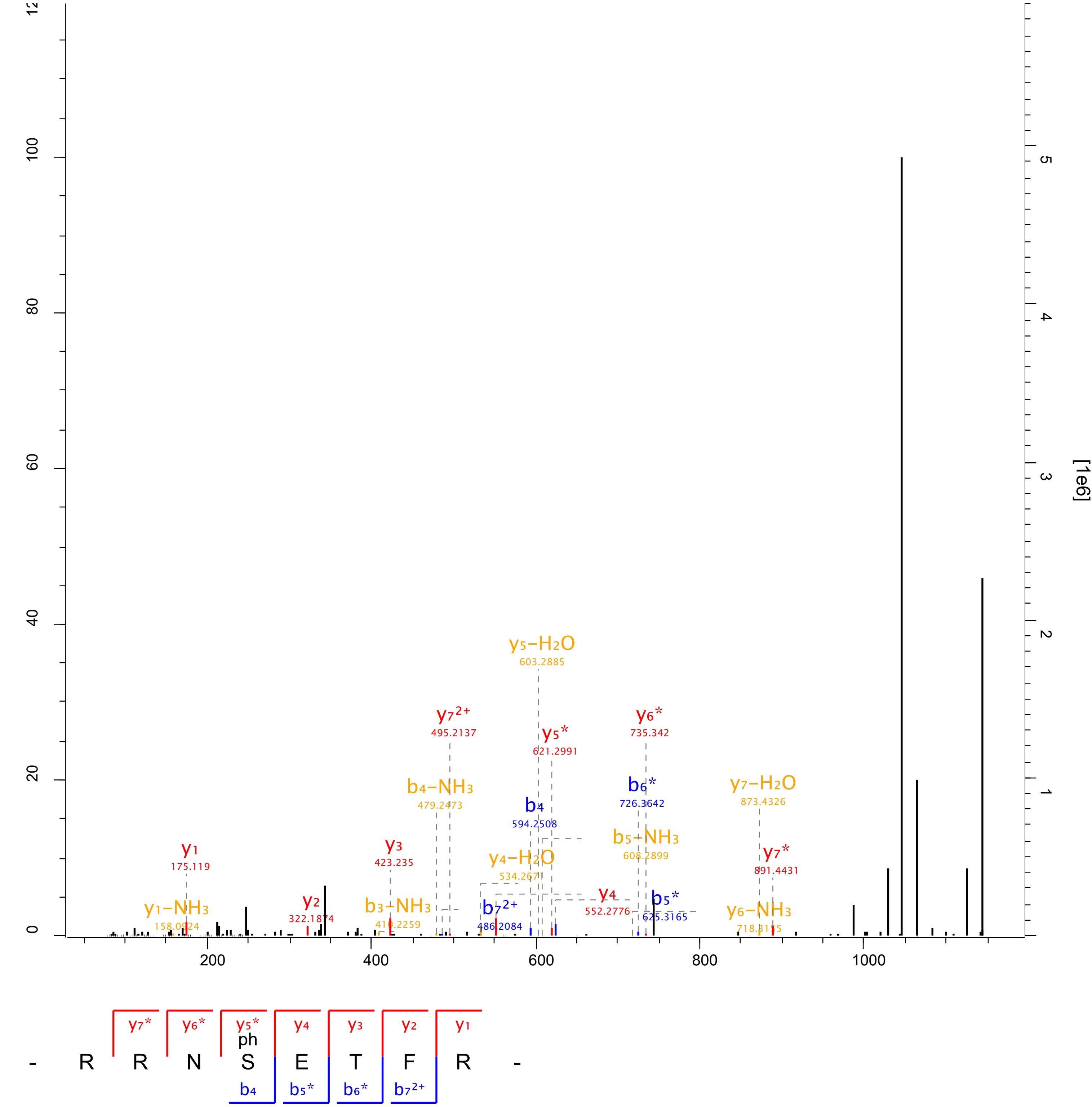

Sample output to test PDF Combine only

Raw file	Scan	Method	Score	m/z
0523_11	1492	FTMS; HCD	97.35	551.22

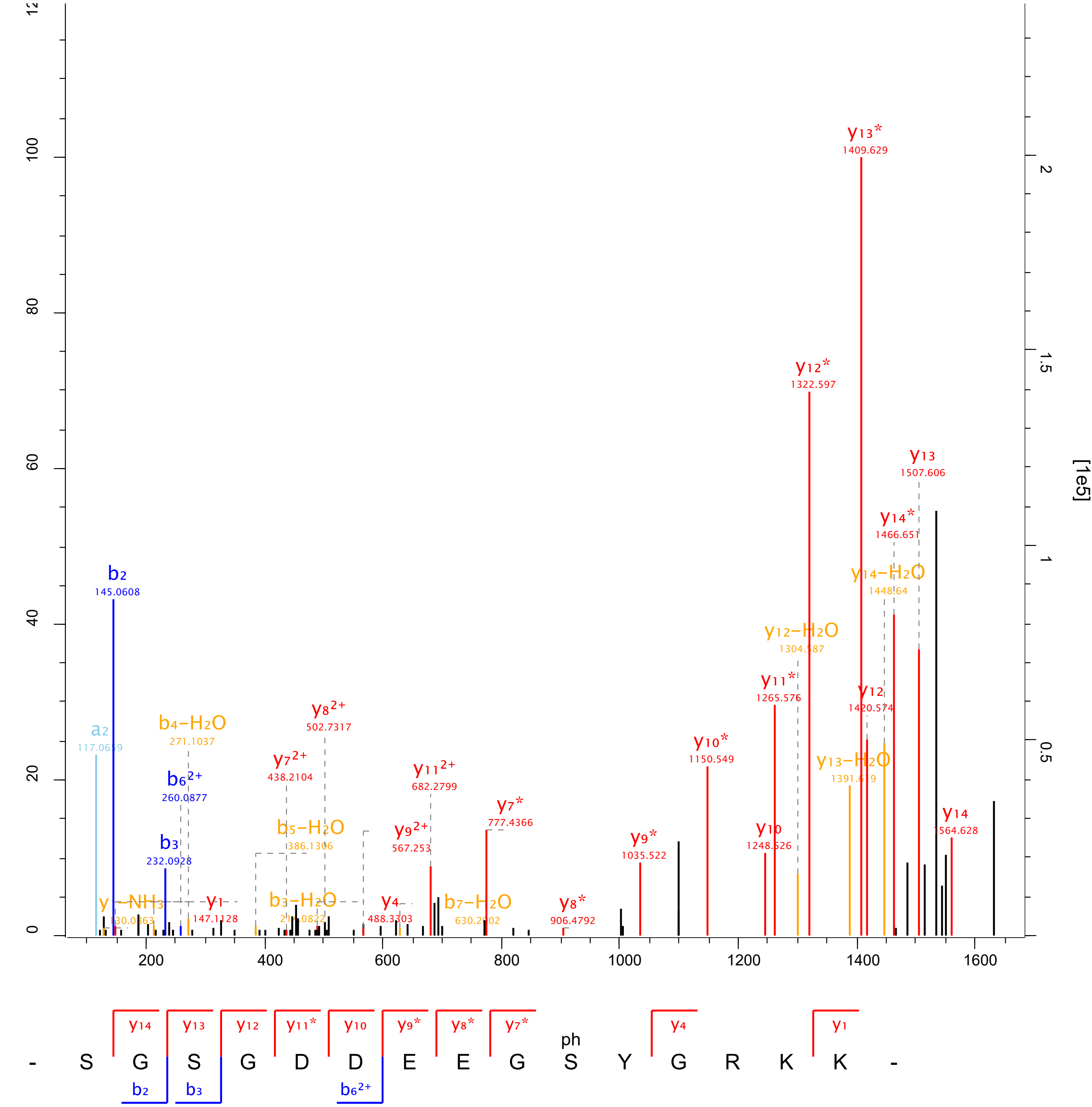

Sample output to test PDF Combine only

Raw file	Scan	Method	Score	m/z	Gene names
0523_11	1497	FTMS; HCD	61.41	424.85	RS40

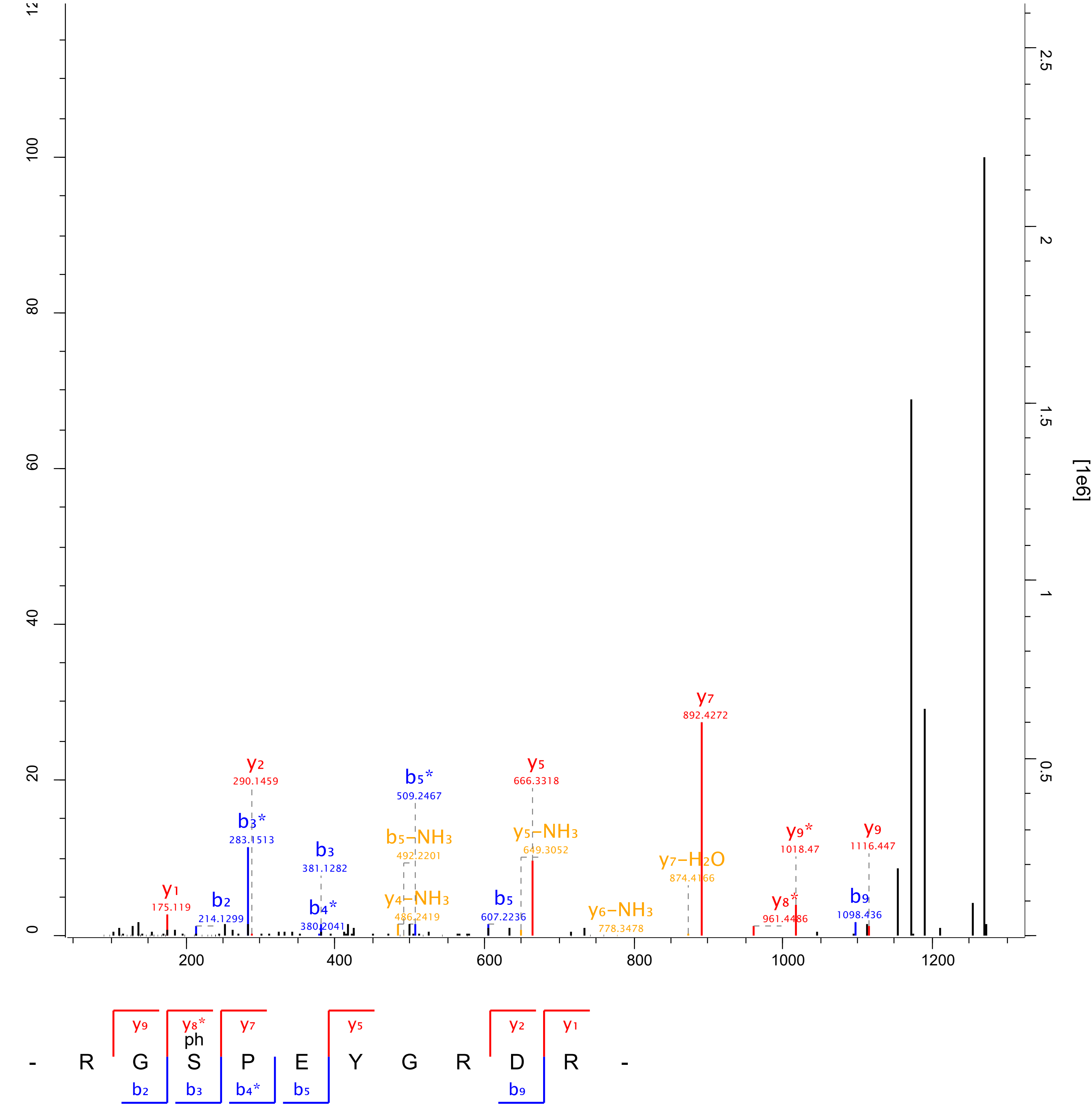

Sample output to test PDF Combine only

Raw file	Scan	Method	Score	m/z	Gene names
0523_11	1501	FTMS; HCD	97.45	340.83	PHOS32

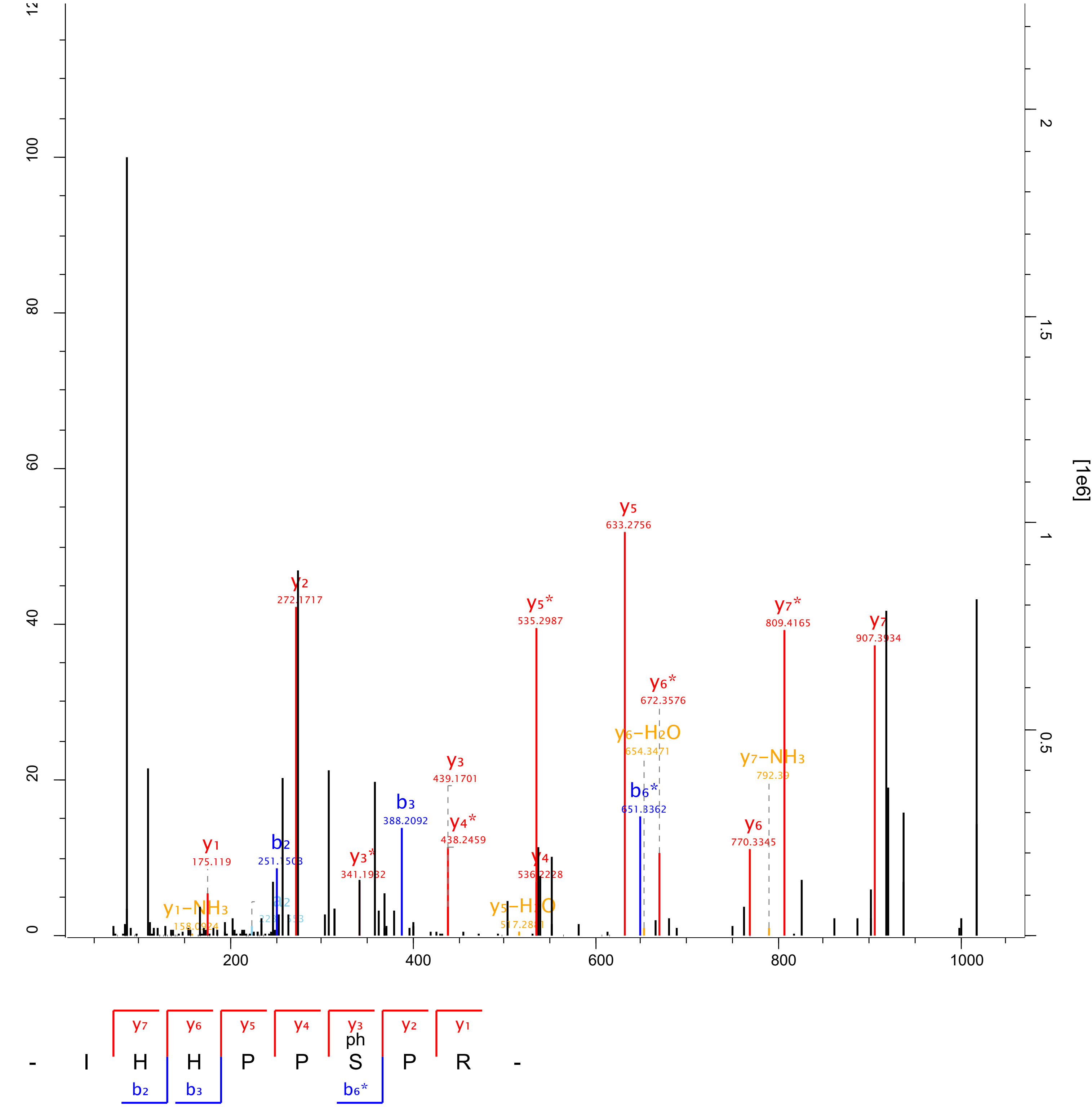

Sample output to test PDF Combine only

Raw file	Scan	Method	Score	m/z	Gene names
0523_11	1502	FTMS; HCD	44.39	321.17	SR45

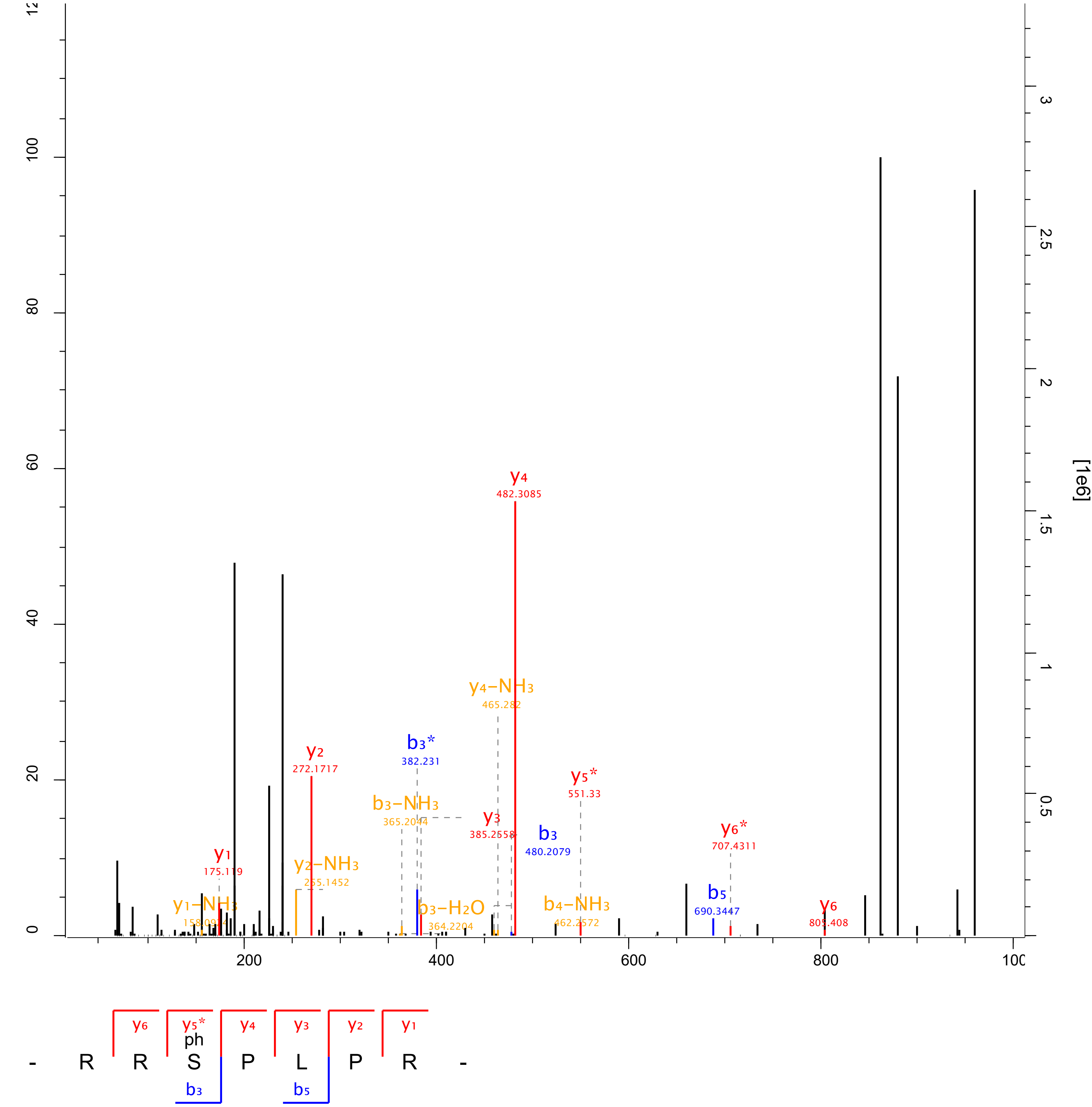

Raw file	Scan	Method	Score	m/z	Gene names
0523_11	1504	FTMS; HCD	50.09	403.17	RS41

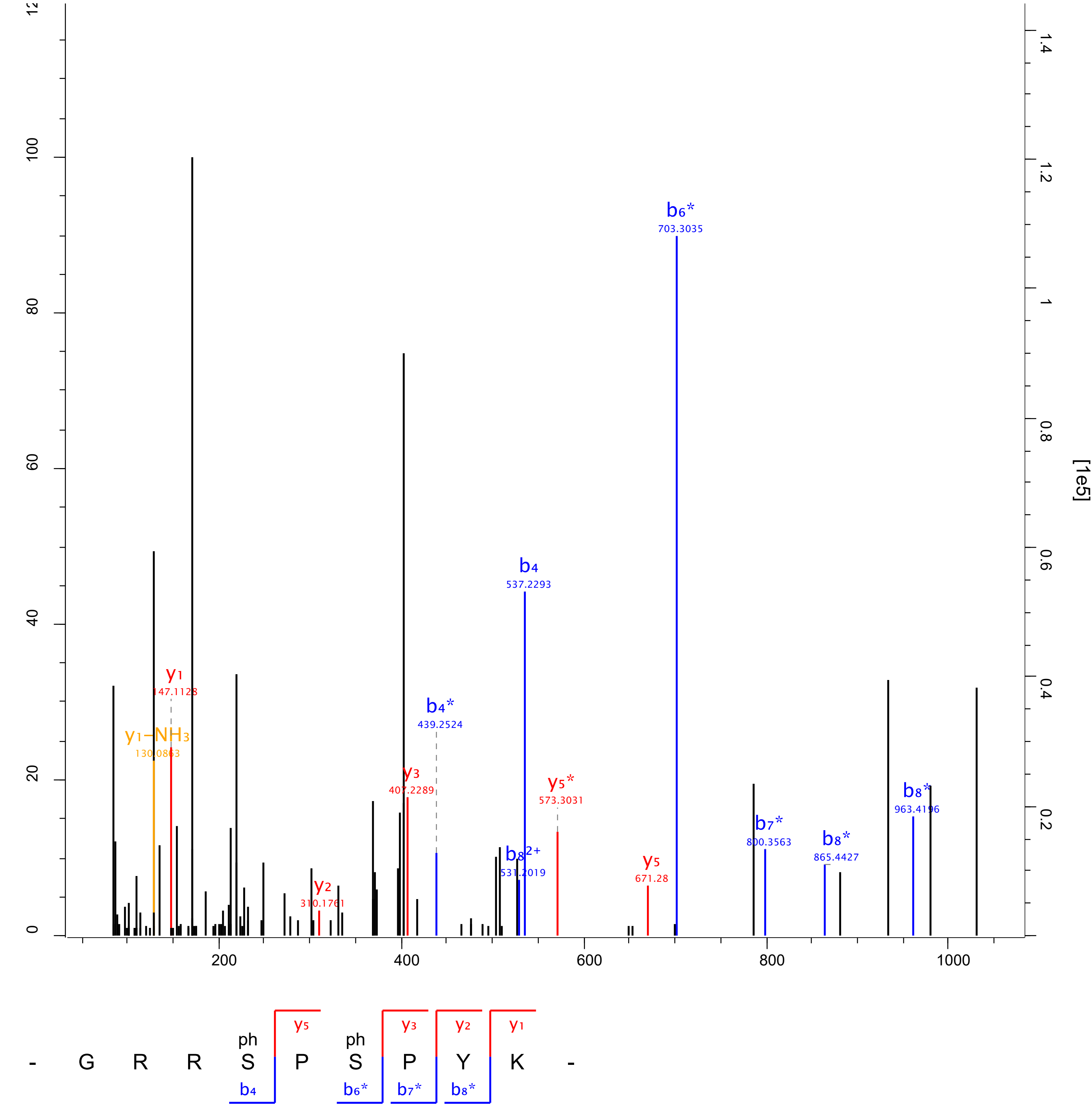

Sample output to test PDF Combine only

Raw file	Scan	Method	Score	m/z	Gene names
0523_11	1505	FTMS; HCD	210.32	661.96	RIN4

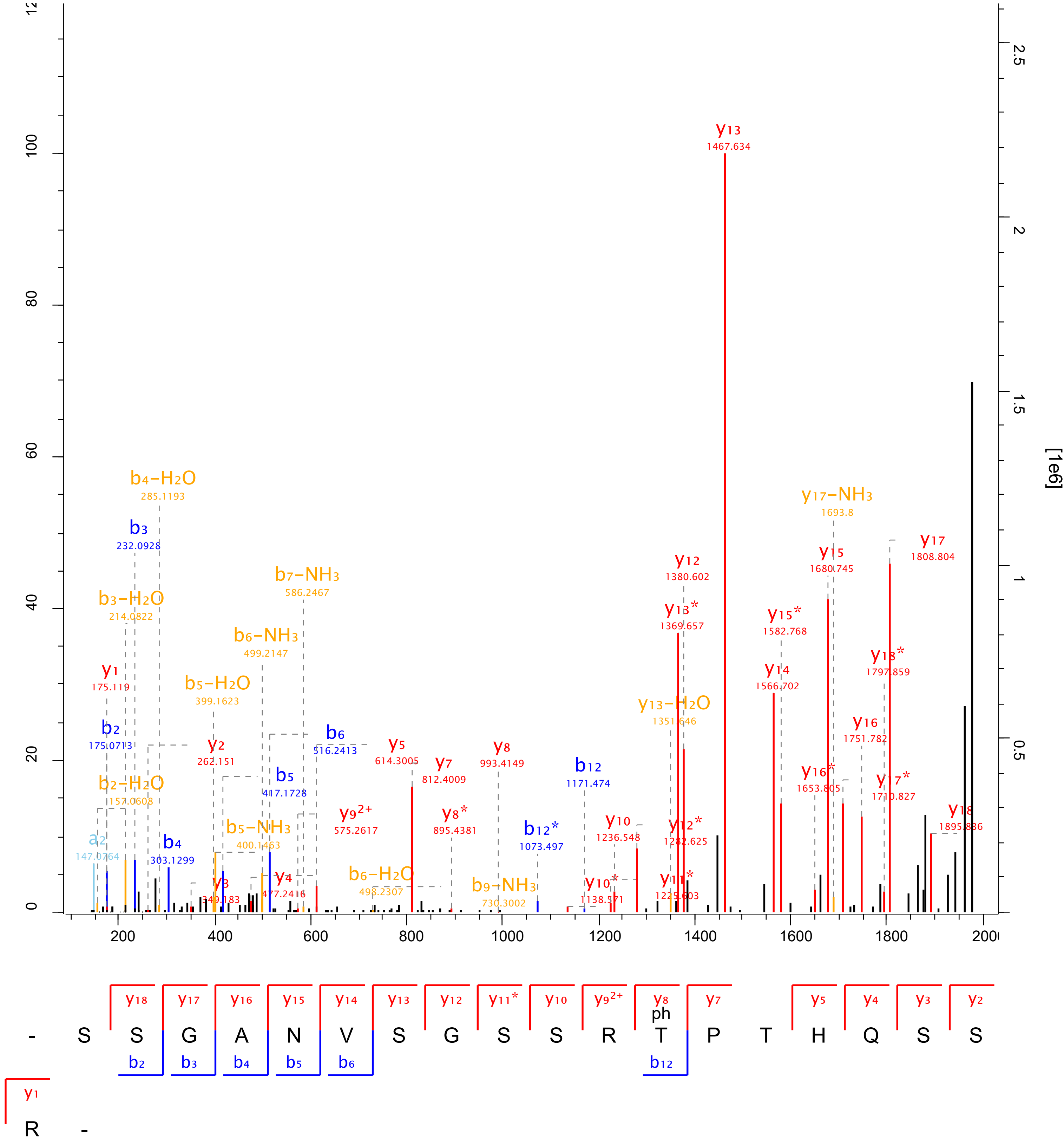

Sample output to test PDF Combine only

Raw file	Scan	Method	Score	m/z	Gene names
0523_11	1506	FTMS; HCD	108.14	419.21	SR45

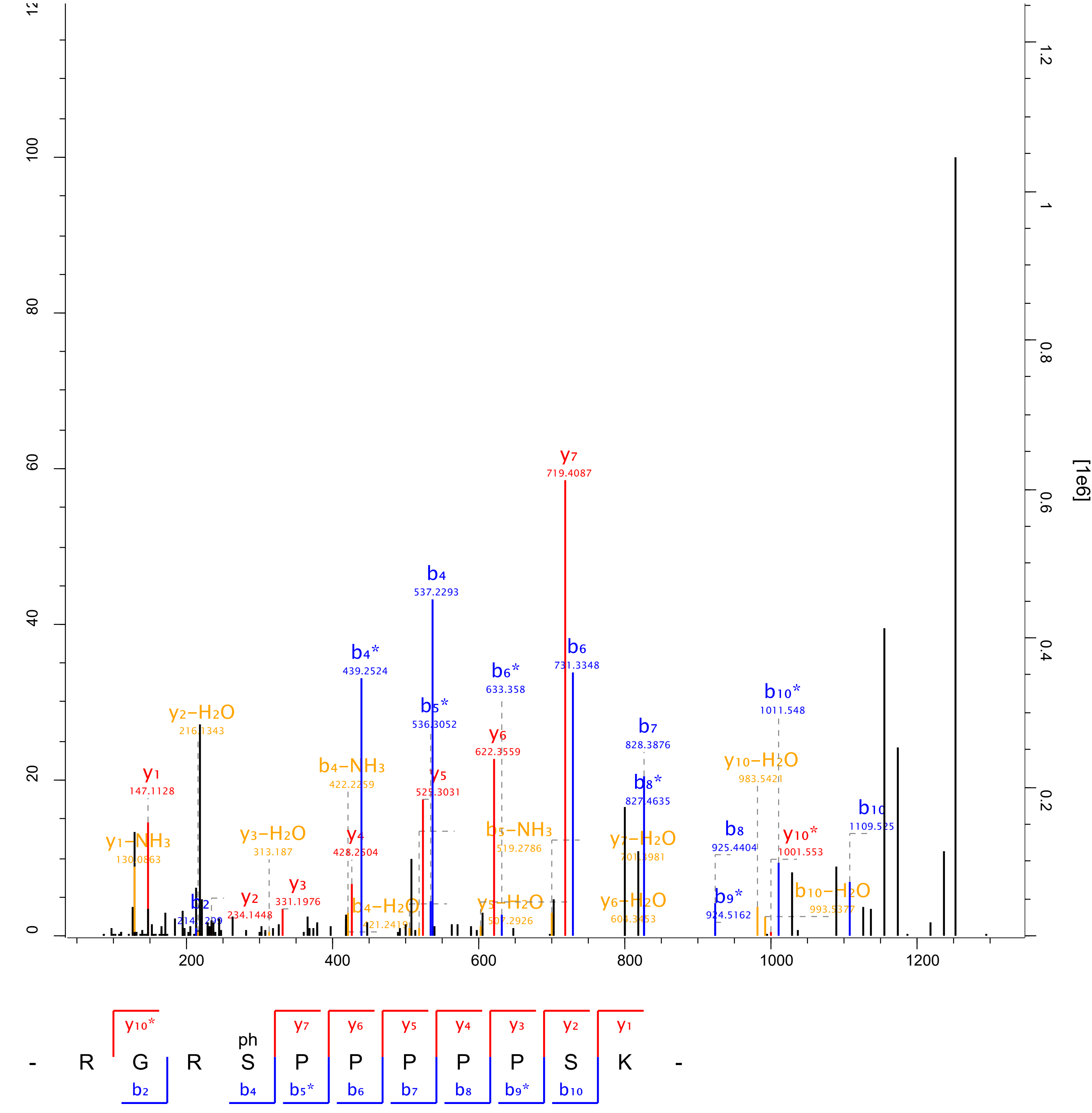

Sample output to test PDF Combine only
