# Supplementary material for: Sucrose-induced Receptor Kinase 1 is Modulated by an Interacting Kinase with Short Extracellular Domain
Source: Mol Cell Proteomics. 2019 May 30;18(8):1556–71. doi: 10.1074/mcp.RA119.001336 (PMC6683012; doi:10.1074/mcp.RA119.001336)
Supplement: Supplementary Figure S6e [file 143141_1_supp_311902_ps5wky.pdf]

Figure S6:

Spectra of all identified phosphopeptides.

Raw file	Scan	Method	Score	m/z	Gene names
0523_11	1515	FTMS; HCD	109.24	510.56	ABCG34

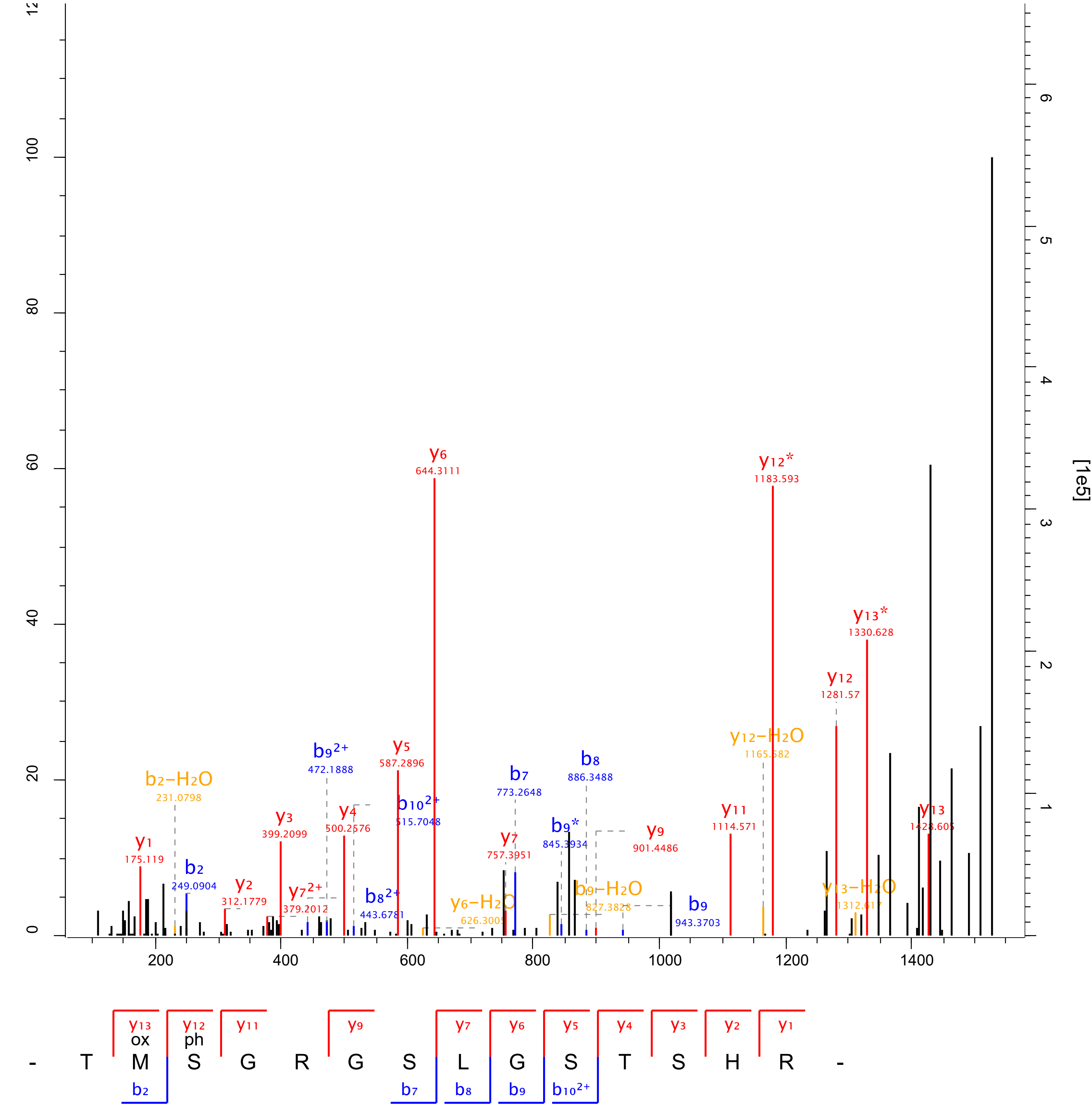

Sample output to test PDF Combine only

Raw file	Scan	Method	Score	m/z	Gene names
0523_11	1527	FTMS; HCD	135.38	590.25	At2g32240

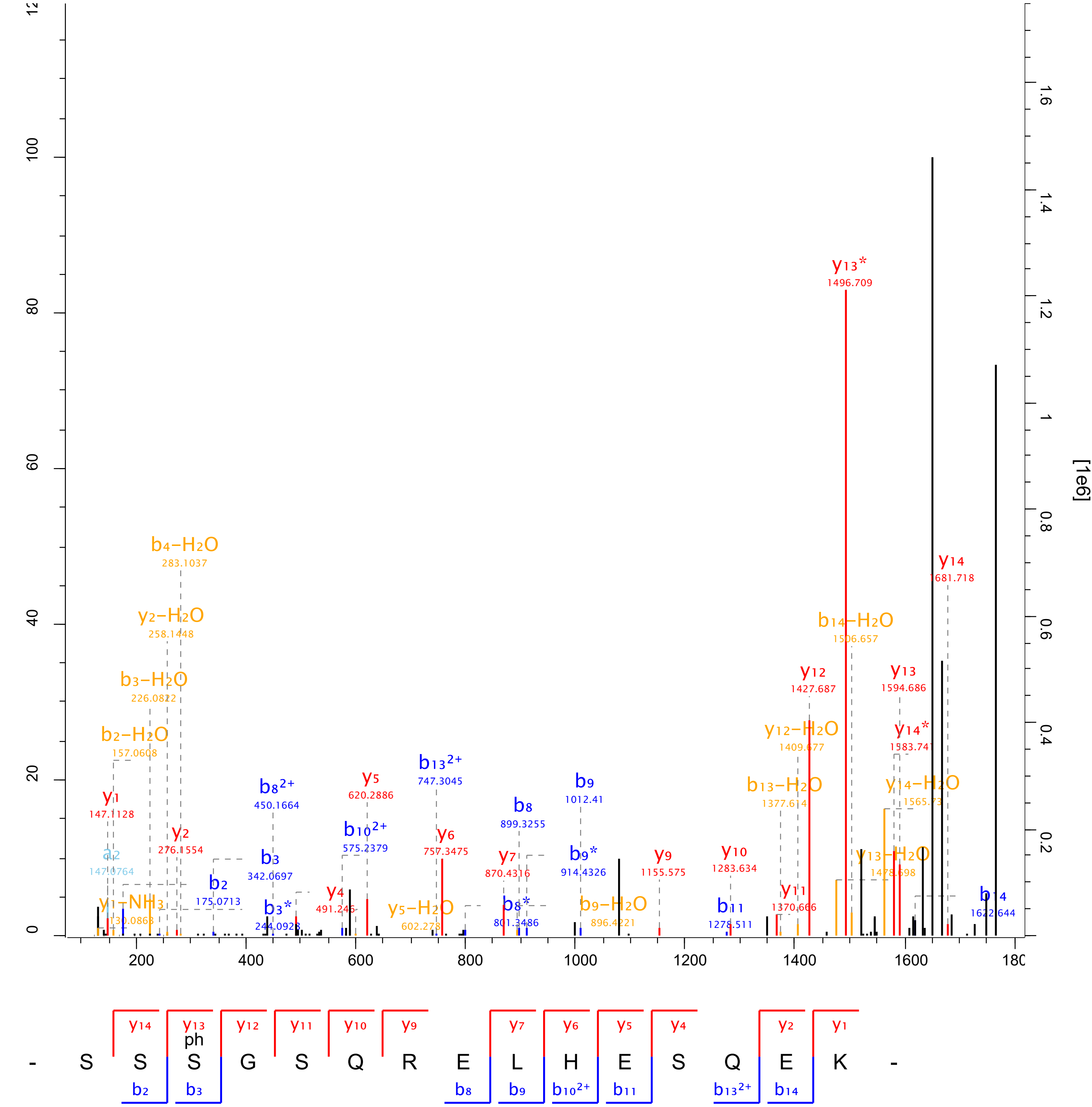

Sample output to test PDF Combine only

Raw file	Scan	Method	Score	m/z	Gene names
0523_11	1528	FTMS; HCD	62.81	427.53	RSZ22

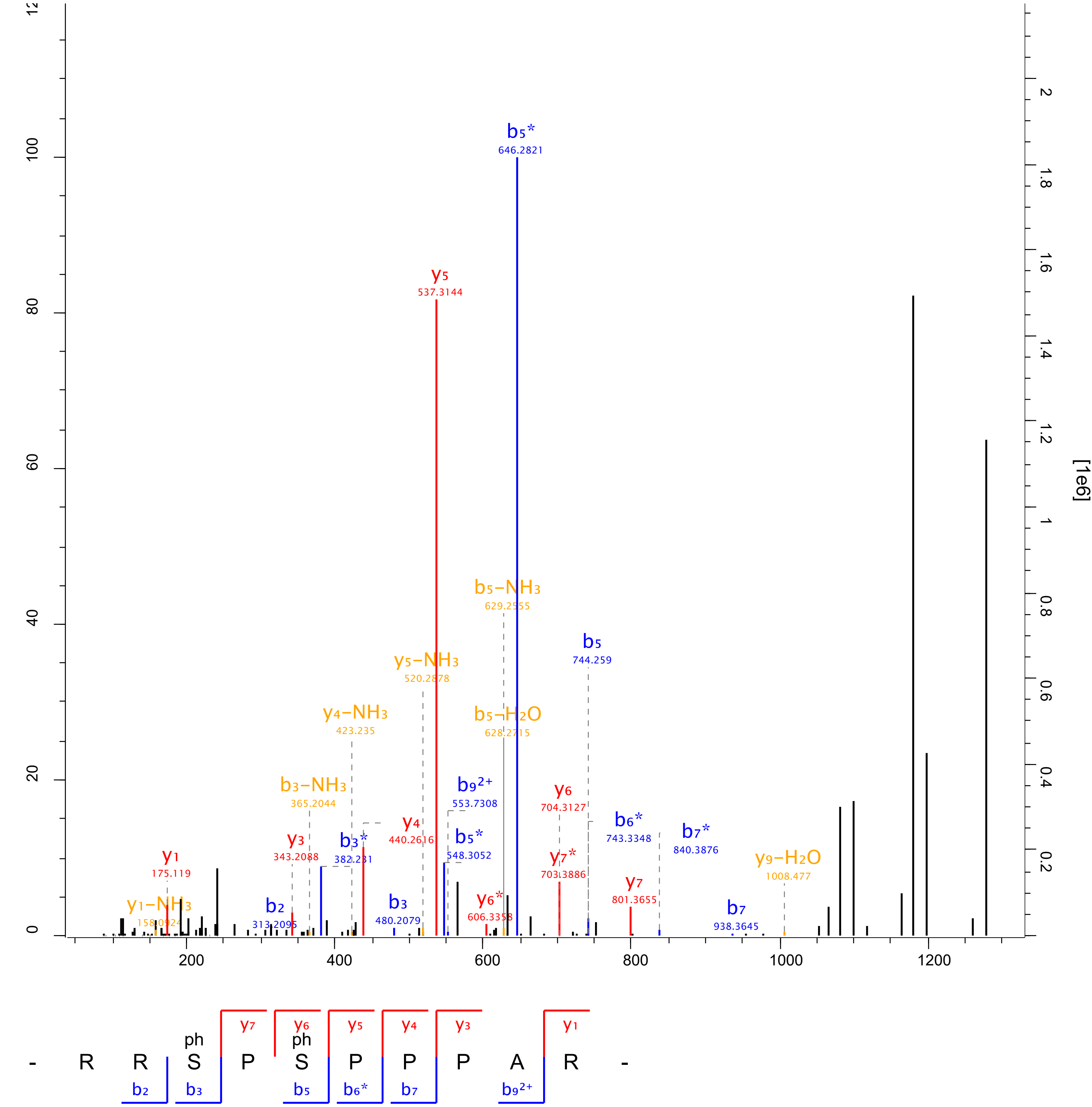

Sample output to test PDF Combine only

Raw file	Scan	Method	Score	m/z	Gene names
0523_11	1554	FTMS; HCD	59.71	440.9	At5g55660

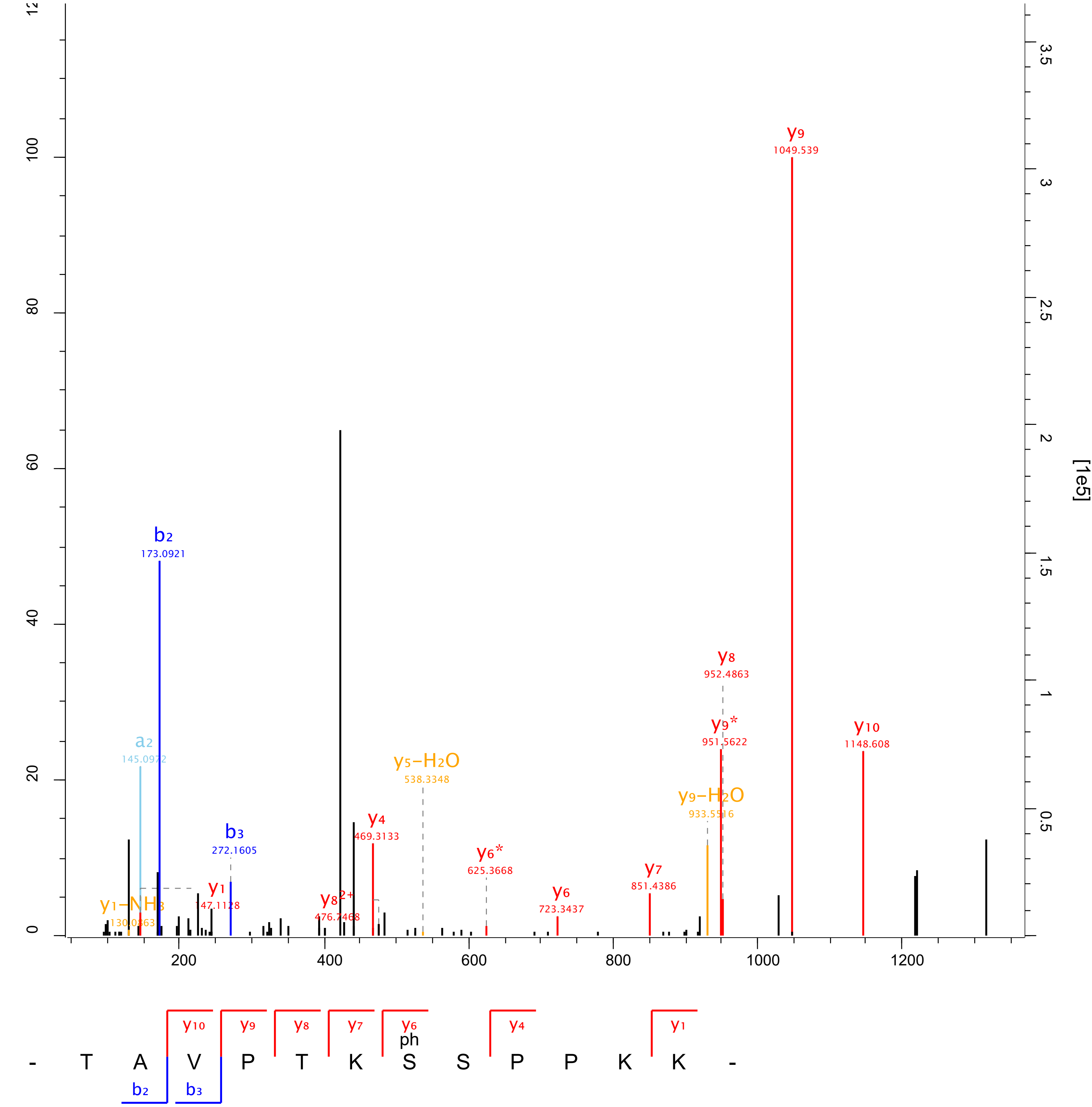

Sample output to test PDF Combine only

Raw file	Scan	Method	Score	m/z	Gene names
0523_11	1579	FTMS; HCD	111.97	676.25	ERD14

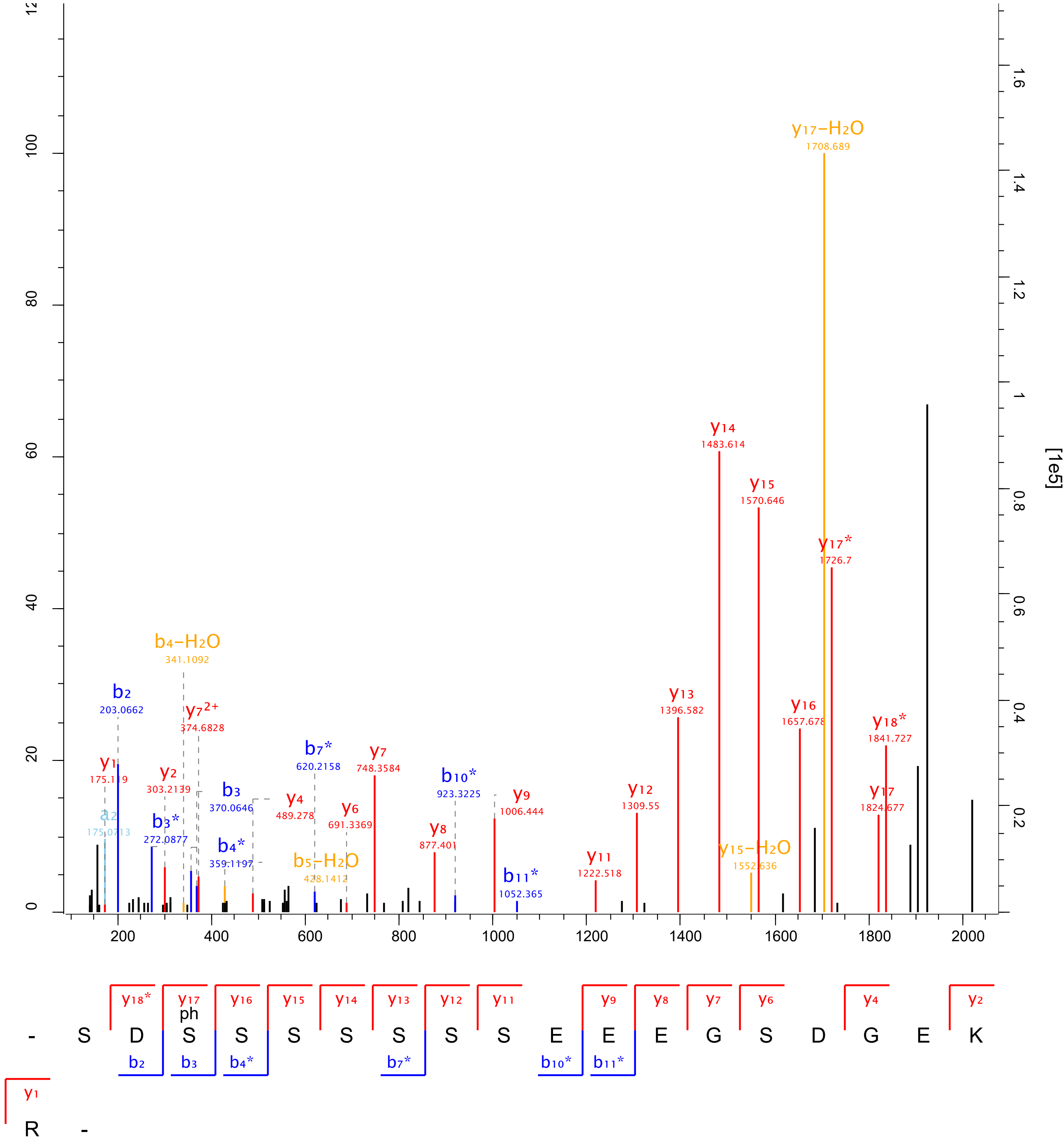

Sample output to test PDF Combine only

Raw file	Scan	Method	Score	m/z	Gene names
0523_11	2222	FTMS; HCD	57.43	407.51	EPSIN2

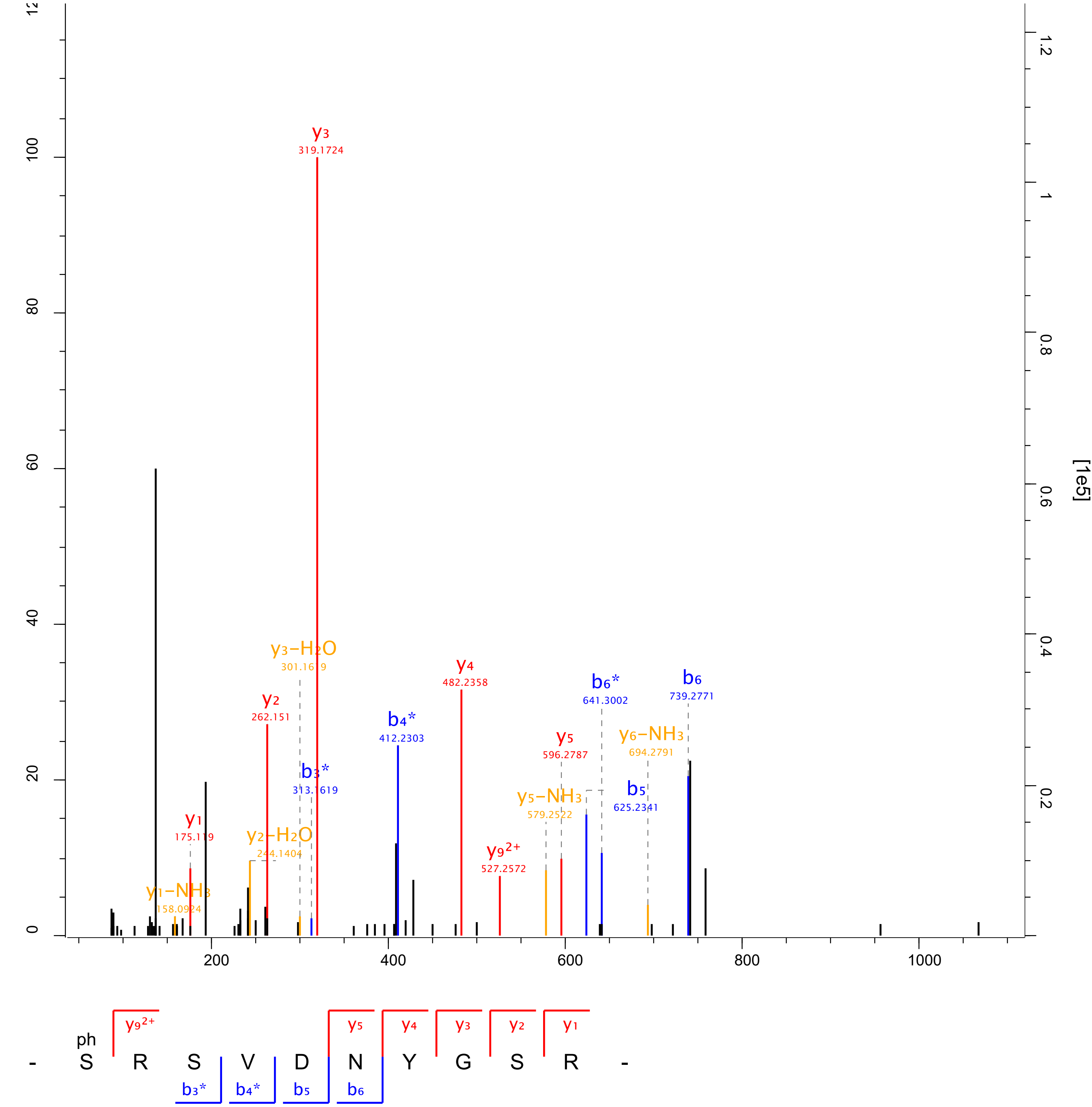

Sample output to test PDF Combine only

Raw file	Scan	Method	Score	m/z	Gene names
0523_11	2741	FTMS; HCD	48.22	523.55	EPSIN2

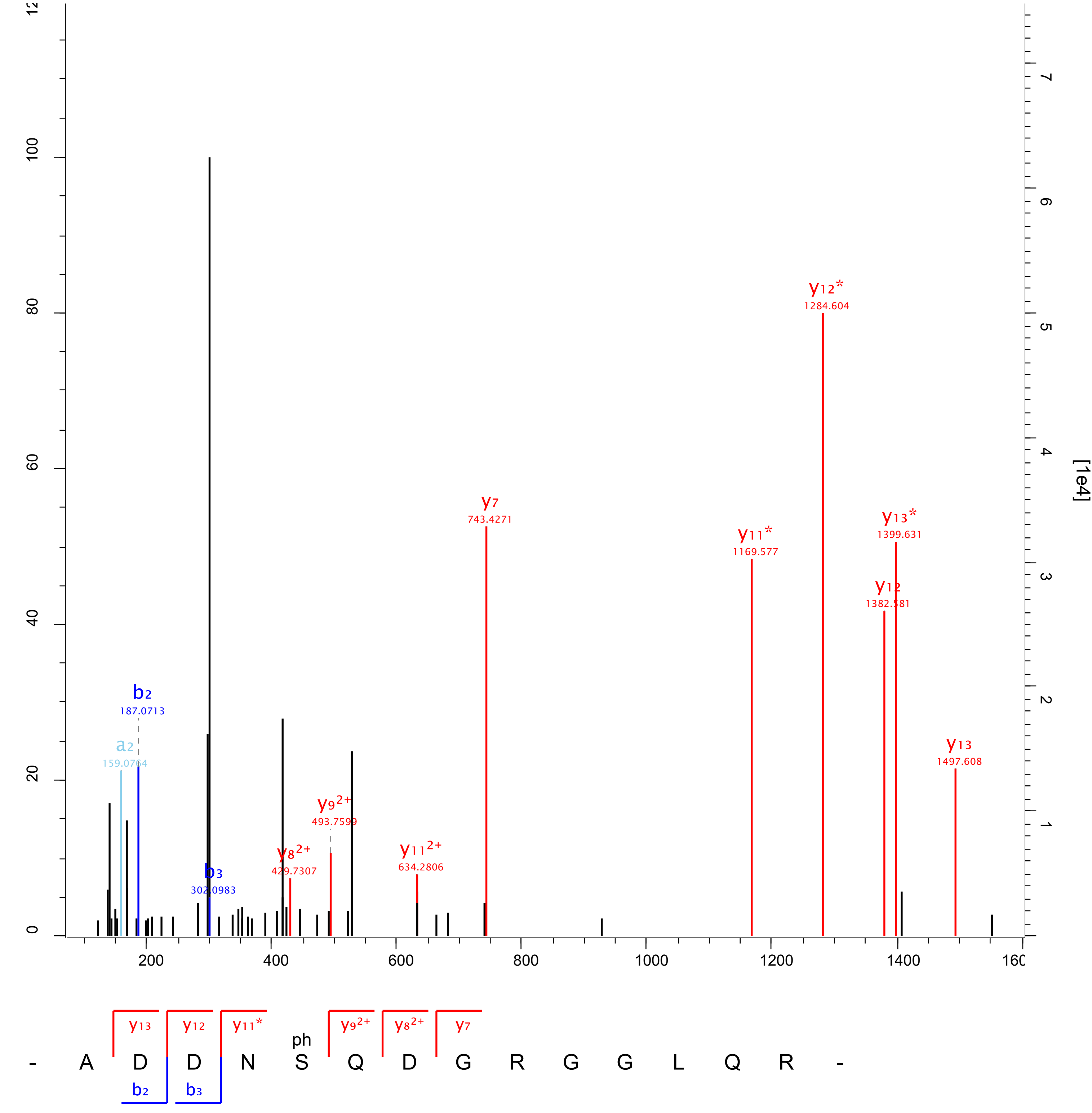

Sample output to test PDF Combine only

Raw file	Scan	Method	Score	m/z	Gene names
0523_11	2885	FTMS; HCD	70.4	467.72	AHA2;HA2;AHA5;AHA3

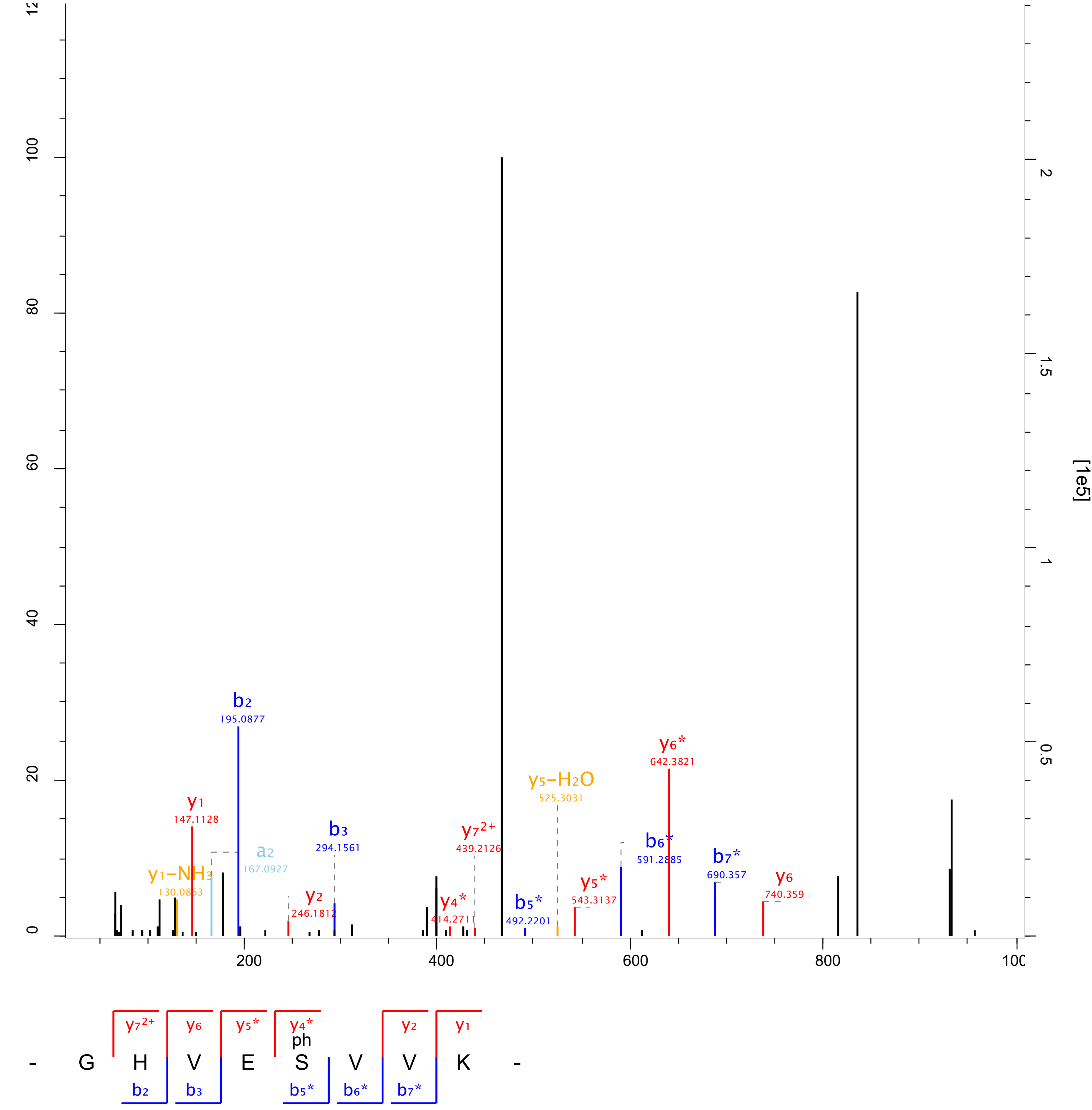

Sample output to test PDF Combine only

Raw file	Scan	Method	Score	m/z	Gene names
0523_11	3324	FTMS; HCD	62.29	626.27	AGD7

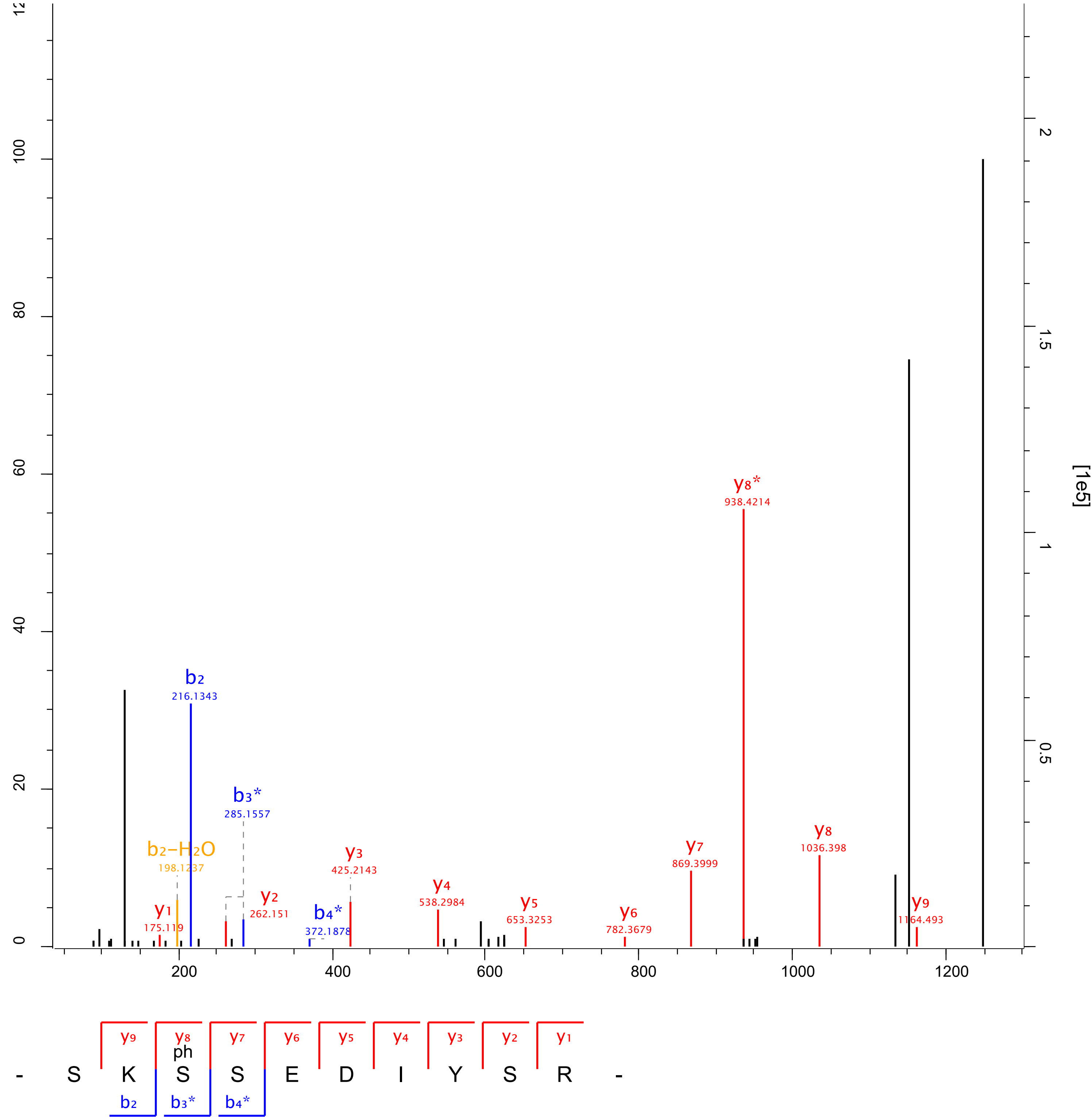

Sample output to test PDF Combine only

Raw file	Scan	Method	Score	m/z	Gene names
0523_11	9111	FTMS; HCD	172.94	953.77	PCR8

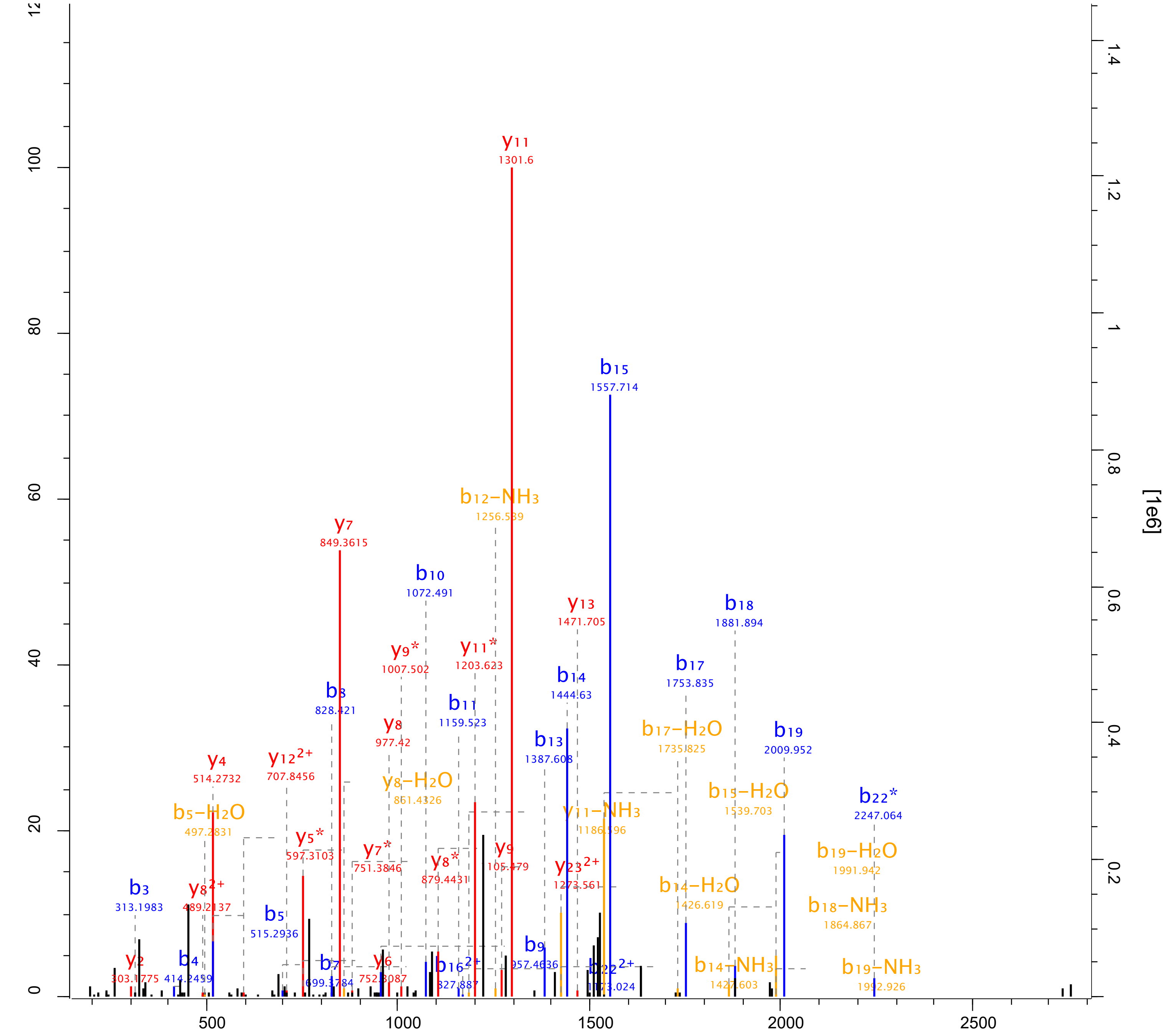

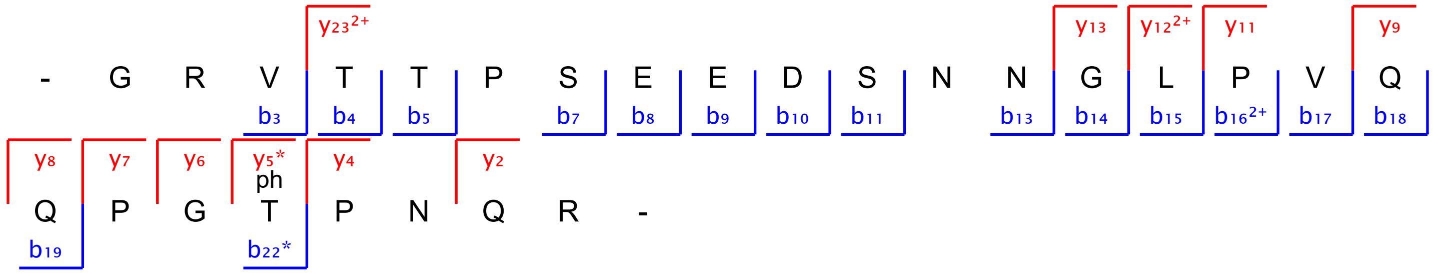

Sample output to test PDF Combine only

Raw file	Scan	Method	Score	m/z	Gene names
0523_11	10124	FTMS; HCD	69.7	762.32	FLS5

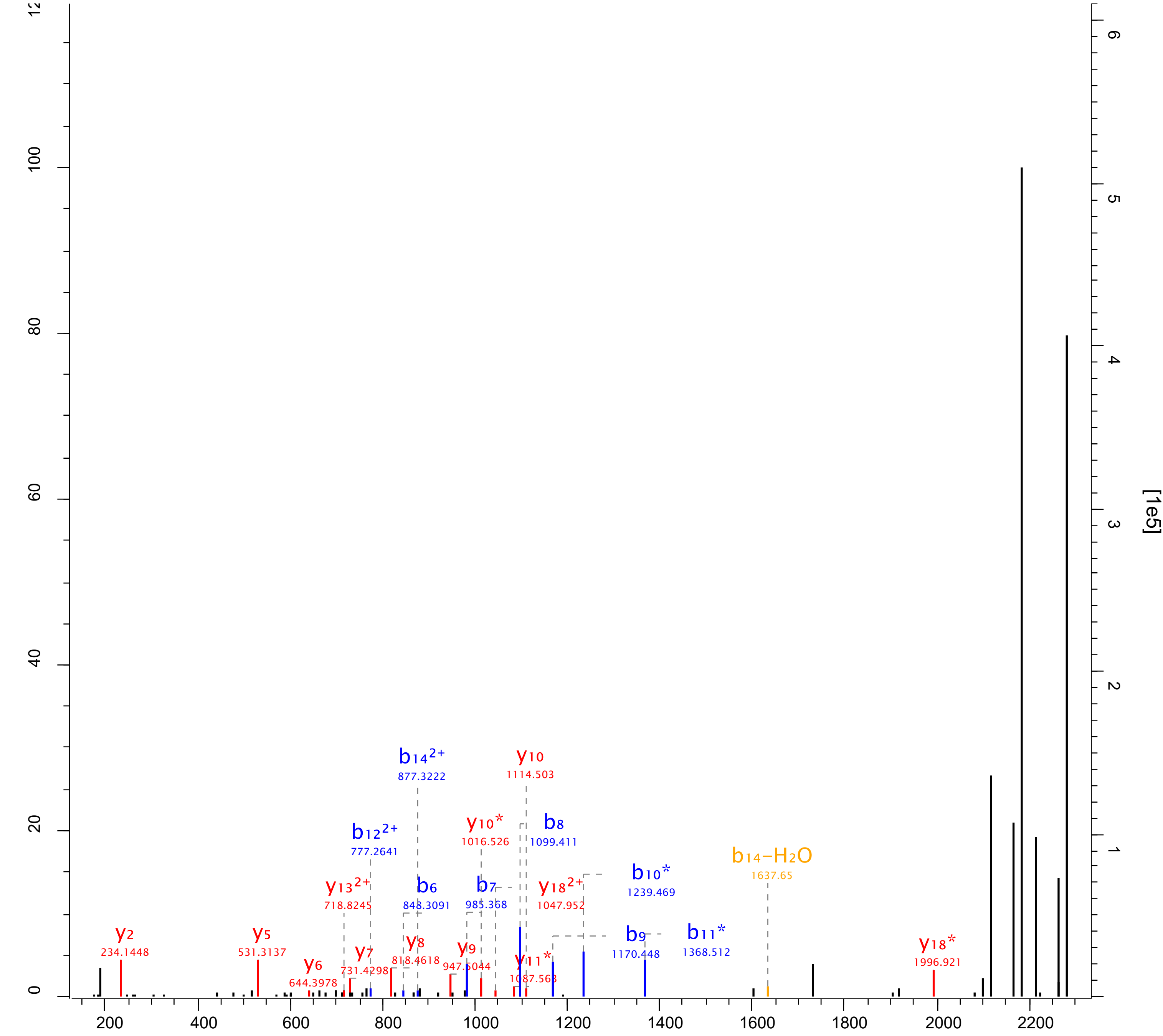

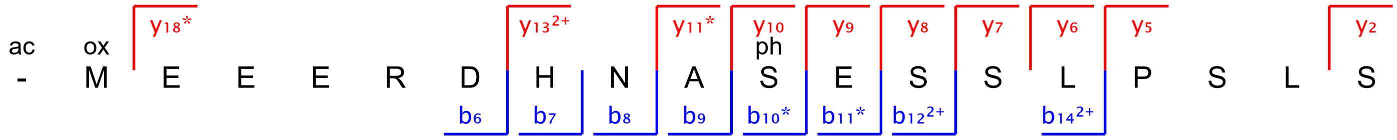

K -

Sample output to test PDF Combine only

Raw file	Scan	Method	Score	m/z	Gene names
0523_11	10513	FTMS; HCD	111.31	654.06	PVA11

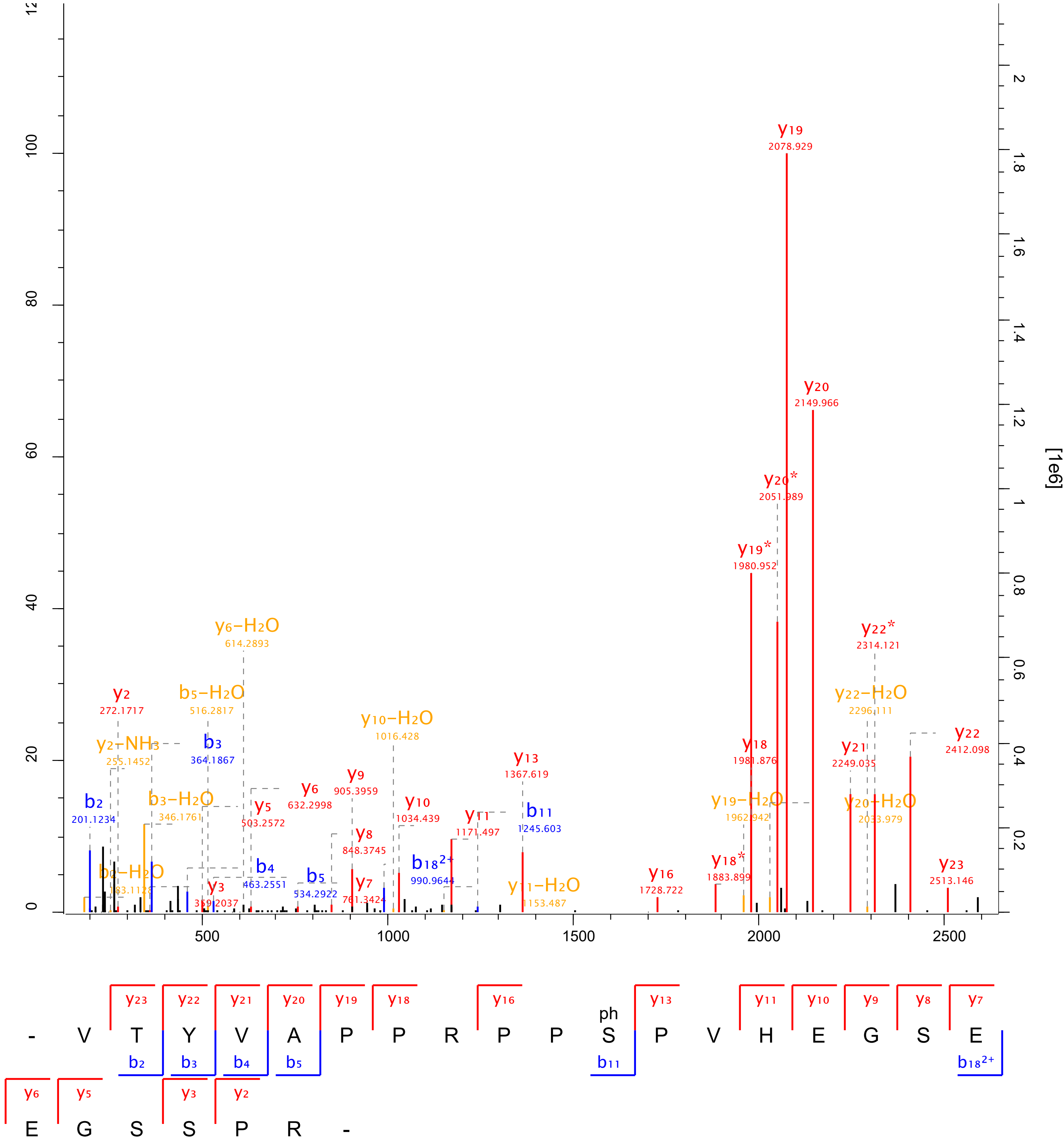

Sample output to test PDF Combine only

Raw file	Scan	Method	Score	m/z	Gene names
0523_11	11304	FTMS; HCD	43.31	877.43	PVA12

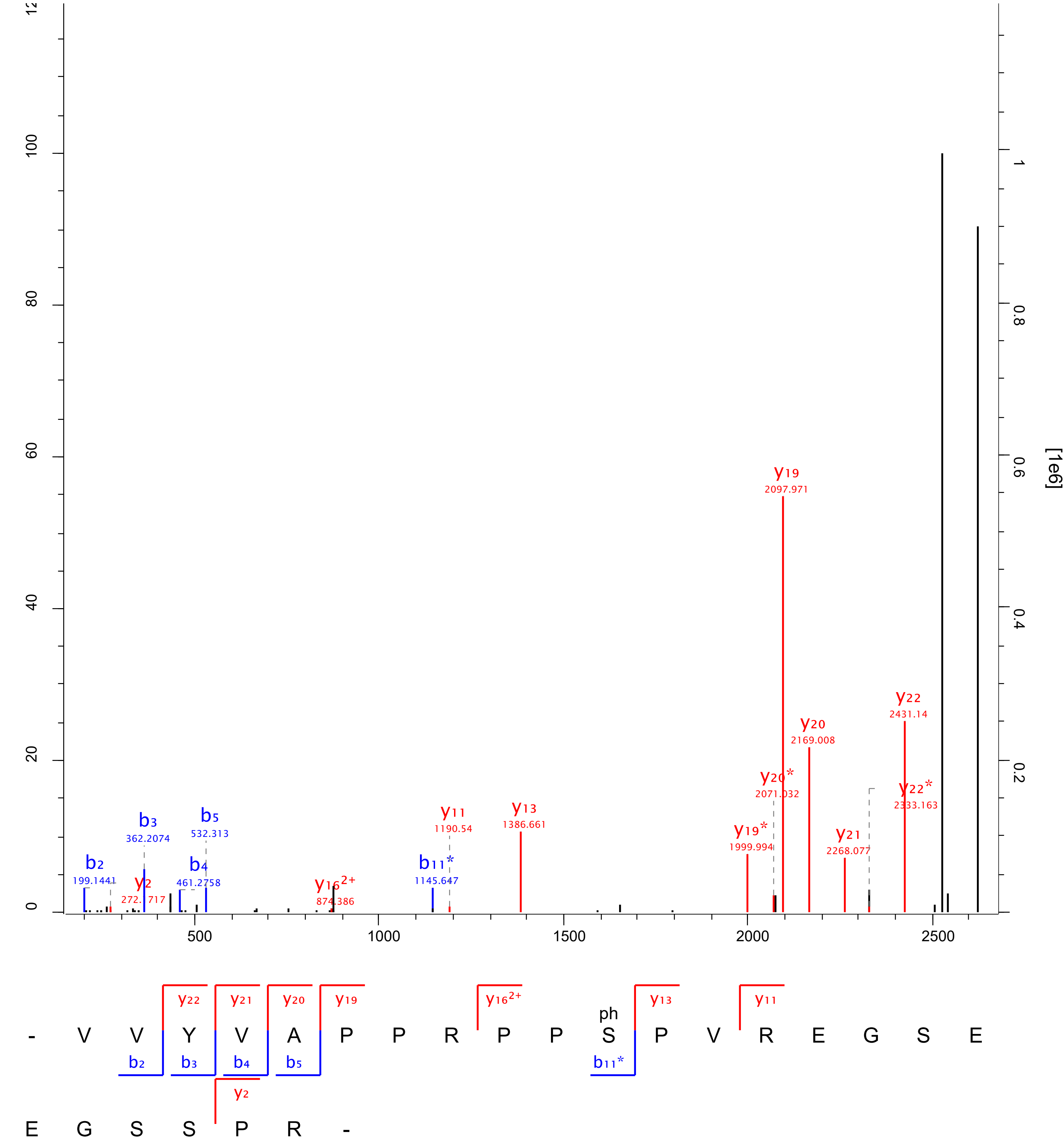

Sample output to test PDF Combine only

Raw file	Scan	Method	Score	m/z	Gene names
0523_11	11619	FTMS; HCD	235.57	759.33	GAD1

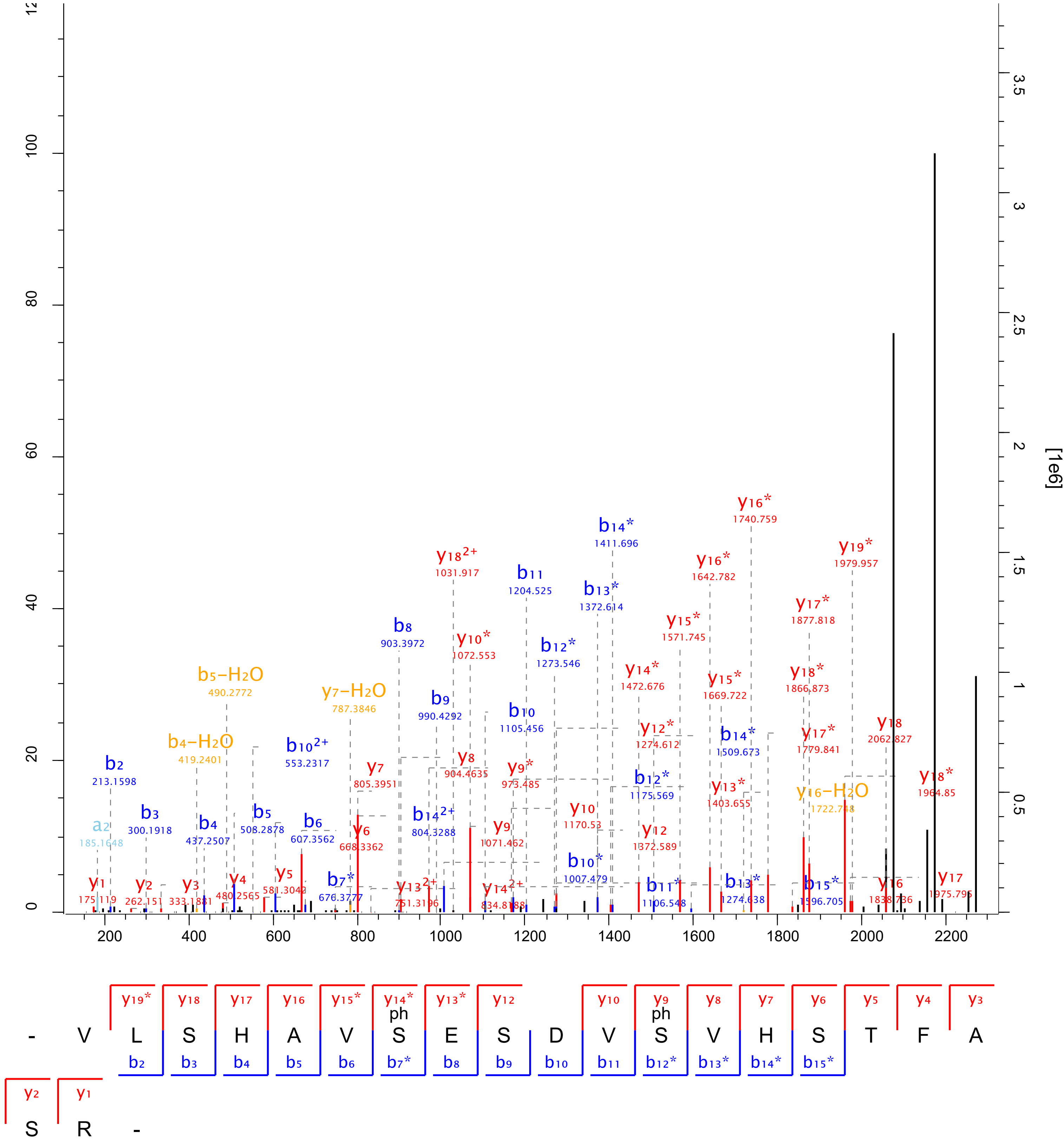

Sample output to test PDF Combine only

Raw file	Scan	Method	Score	m/z	Gene names
0523_11	11832	FTMS; HCD	96.77	775.86	UPL2

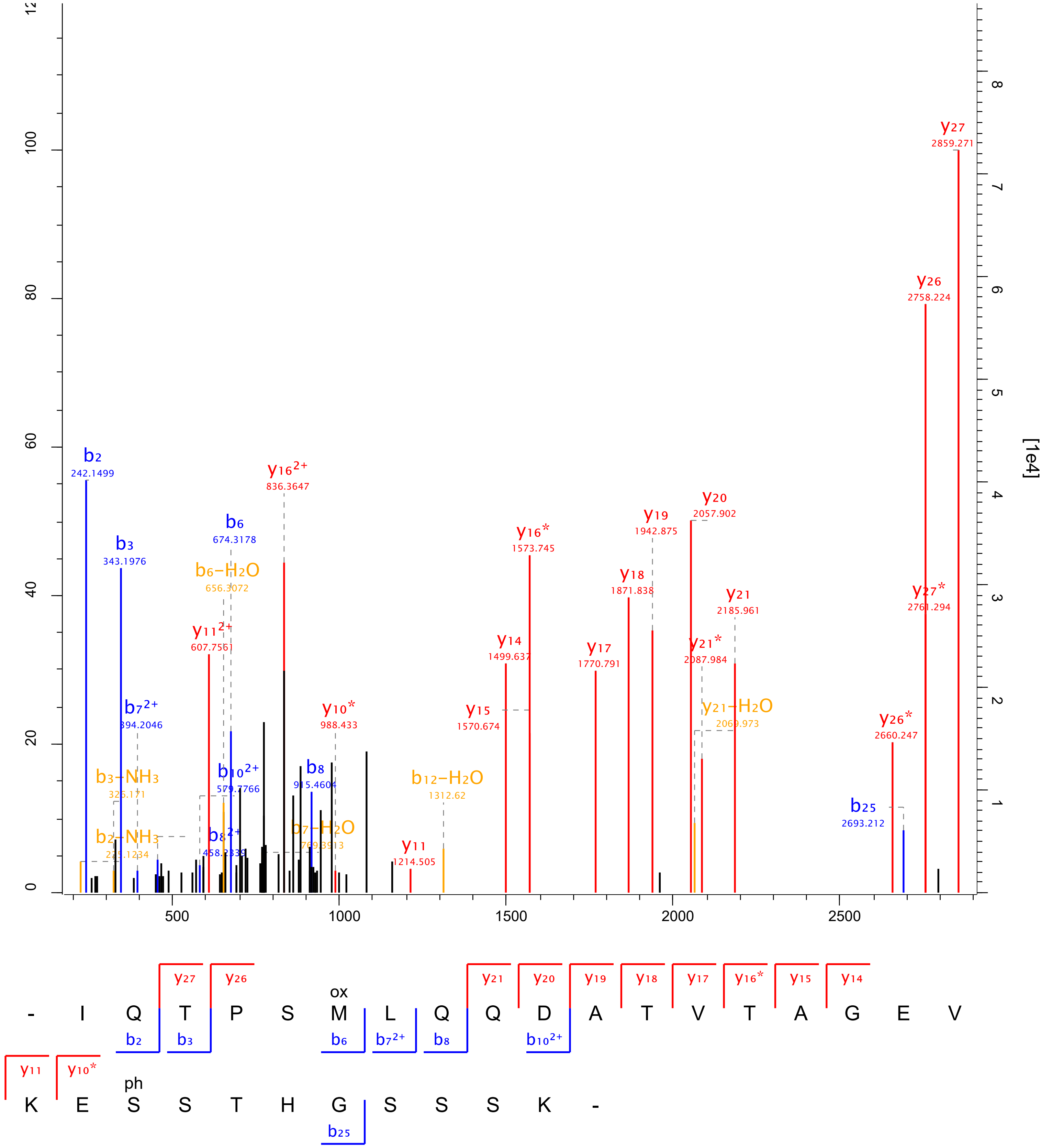

Sample output to test PDF Combine only

Raw file	Scan	Method	Score	m/z	Gene names
0523_11	11840	FTMS; HCD	235.76	732.34	GAD1

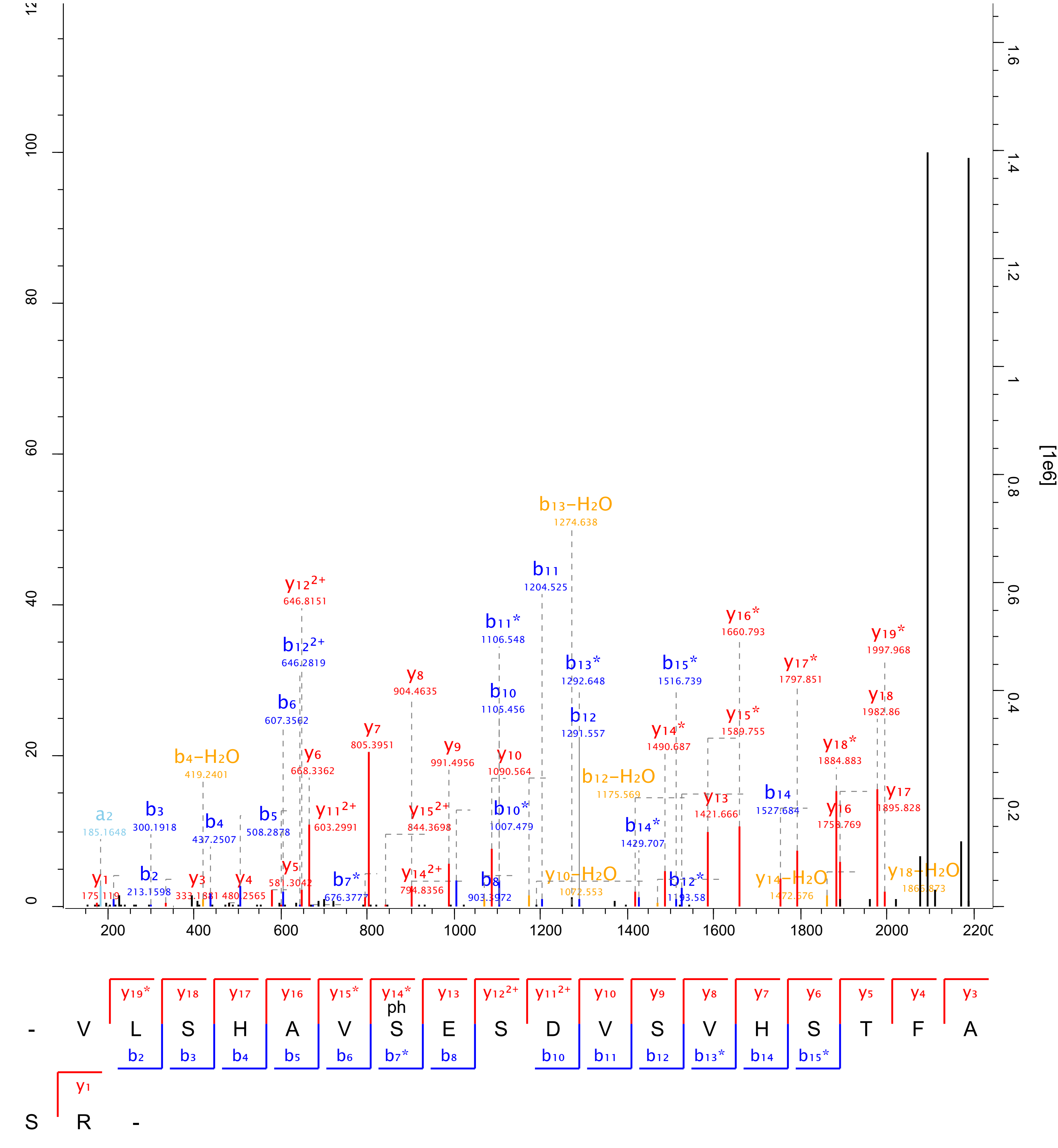

Sample output to test PDF Combine only

Raw file	Scan	Method	Score	m/z	Gene names
0523_11	12059	FTMS; HCD	70.72	631.28	NIP2-1

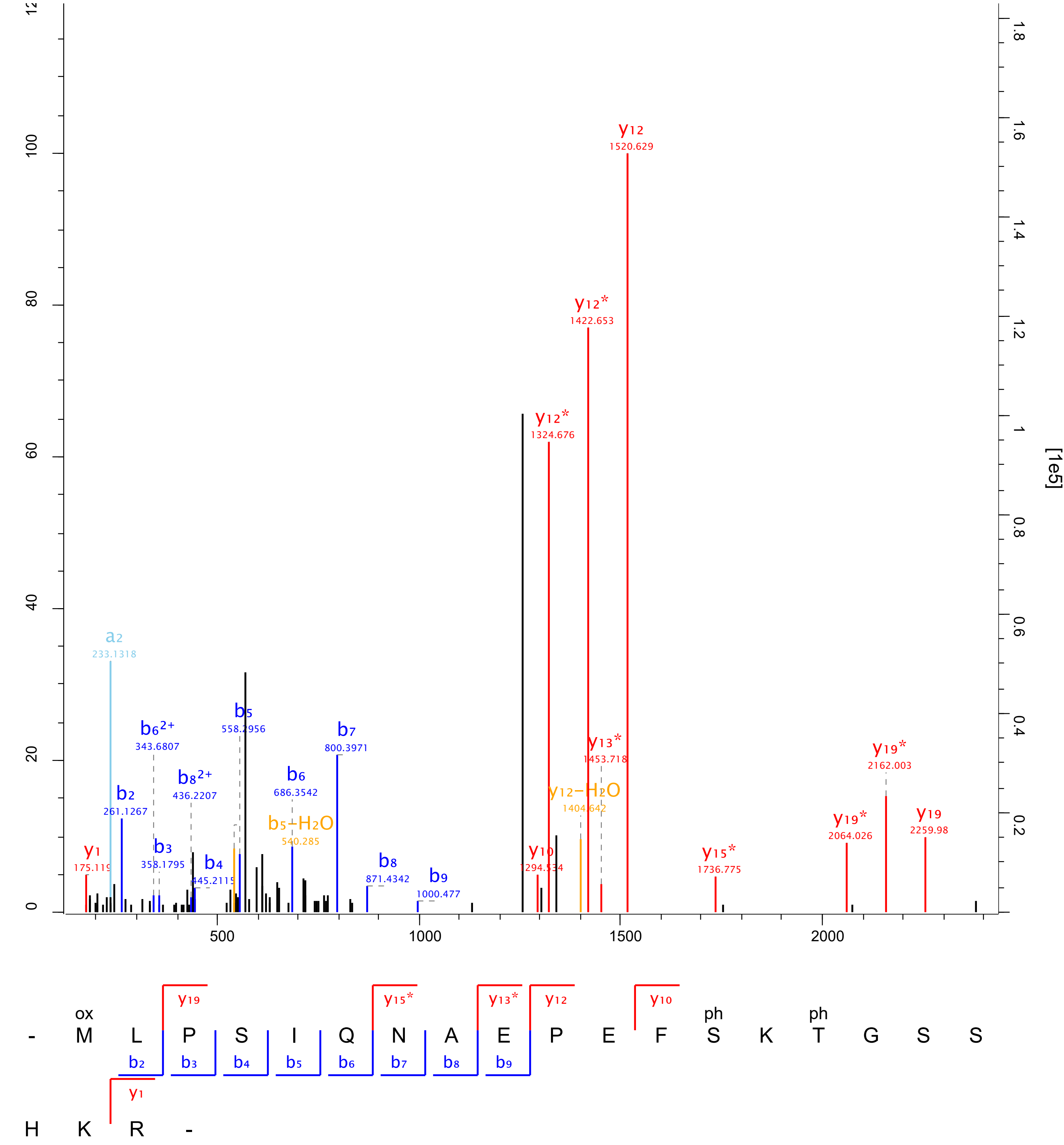

Sample output to test PDF Combine only

Raw file	Scan	Method	Score	m/z	Gene names
0523_11	12548	FTMS; HCD	43.7	649.62	At4g27450

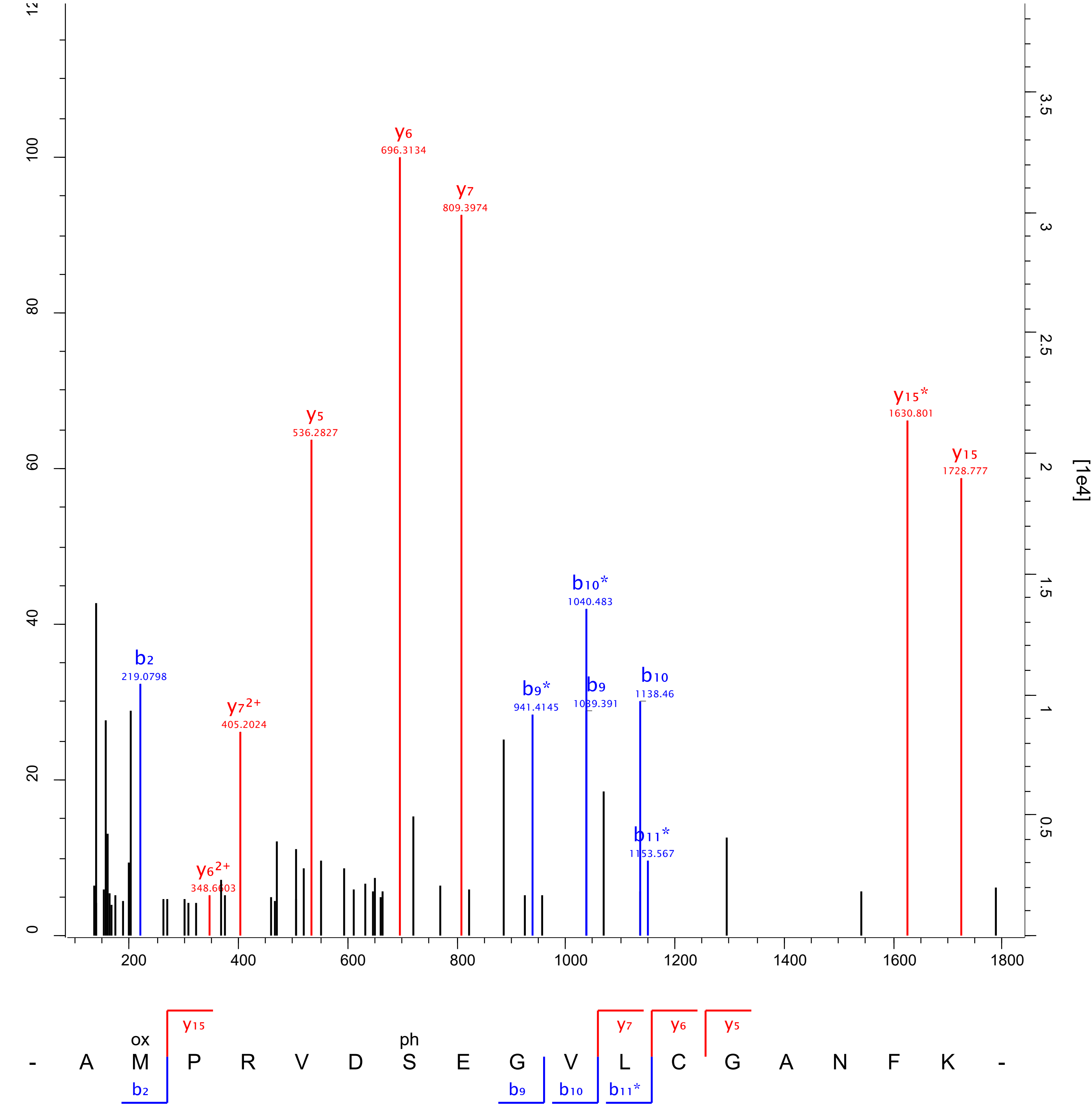

Sample output to test PDF Combine only

Raw file	Scan	Method	Score	m/z	Gene names
0523_11	12893	FTMS; HCD	56.37	653.32	IQD32

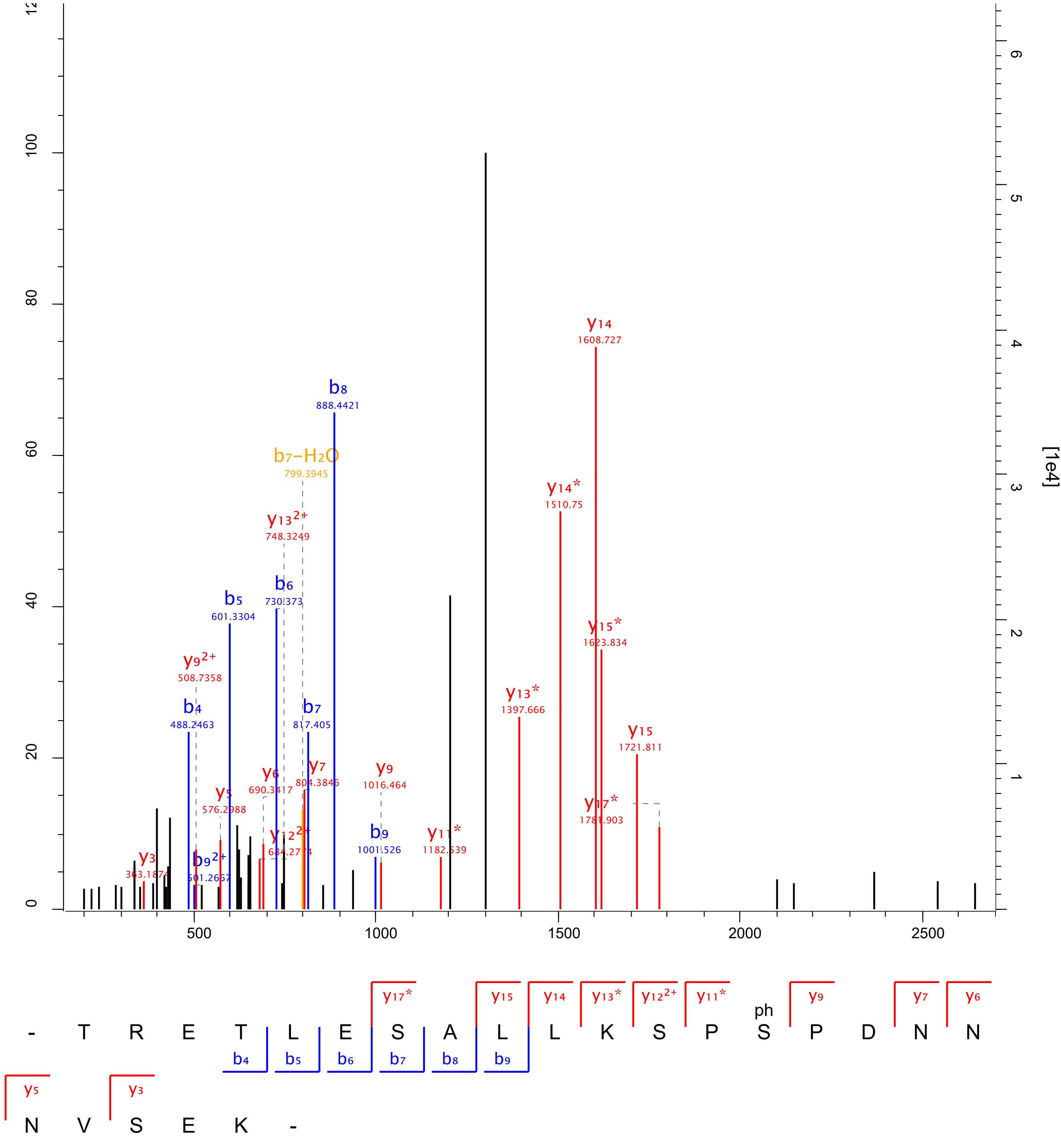

Sample output to test PDF Combine only

Raw file	Scan	Method	Score	m/z	Gene names
0523_11	12916	FTMS; HCD	42.32	437.54	RBOHB

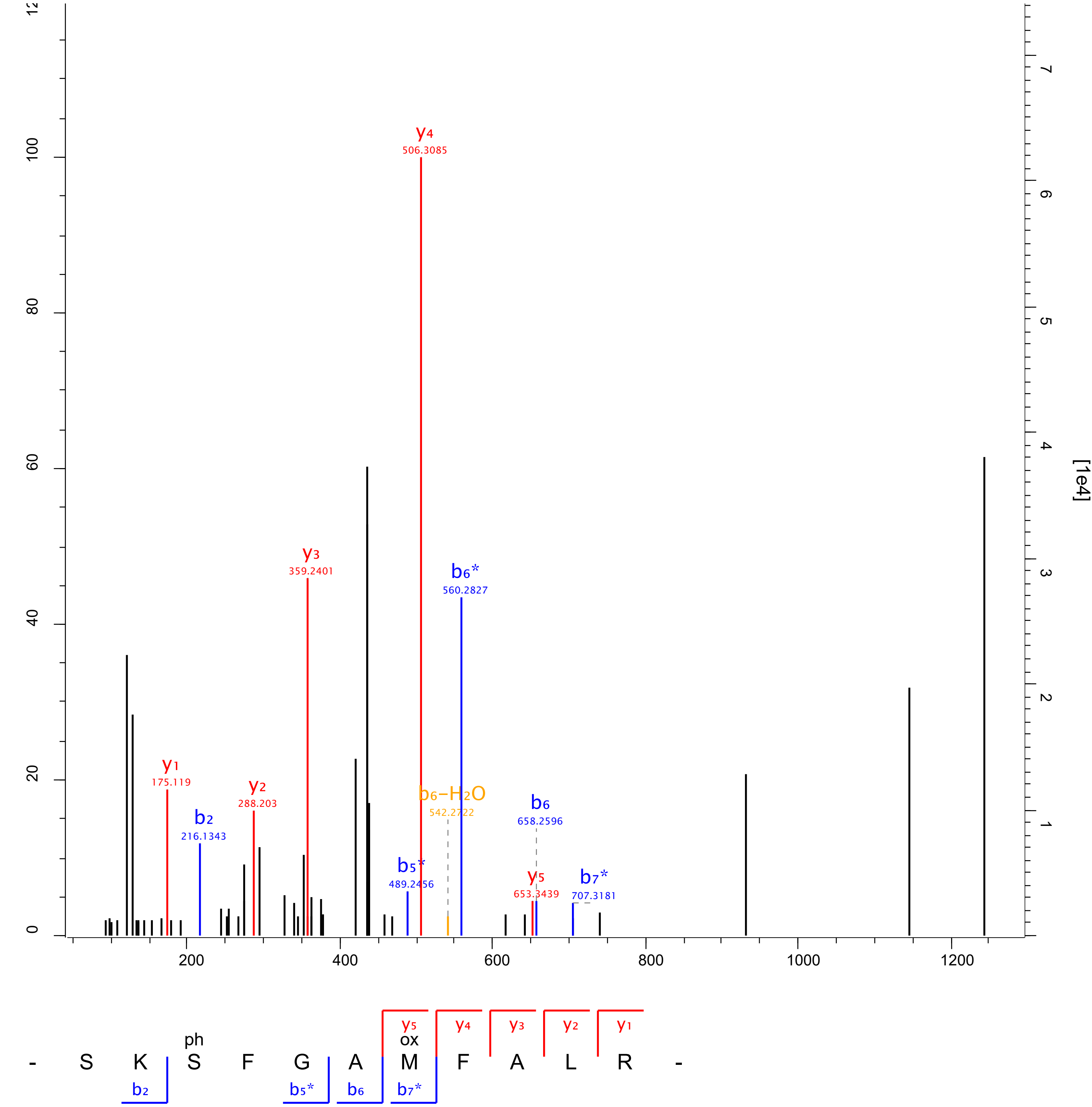

Sample output to test PDF Combine only

Raw file	Scan	Method	Score	m/z	Gene names
0523_11	13494	FTMS; HCD	112.08	843.61	At1g14170;F7A19.25

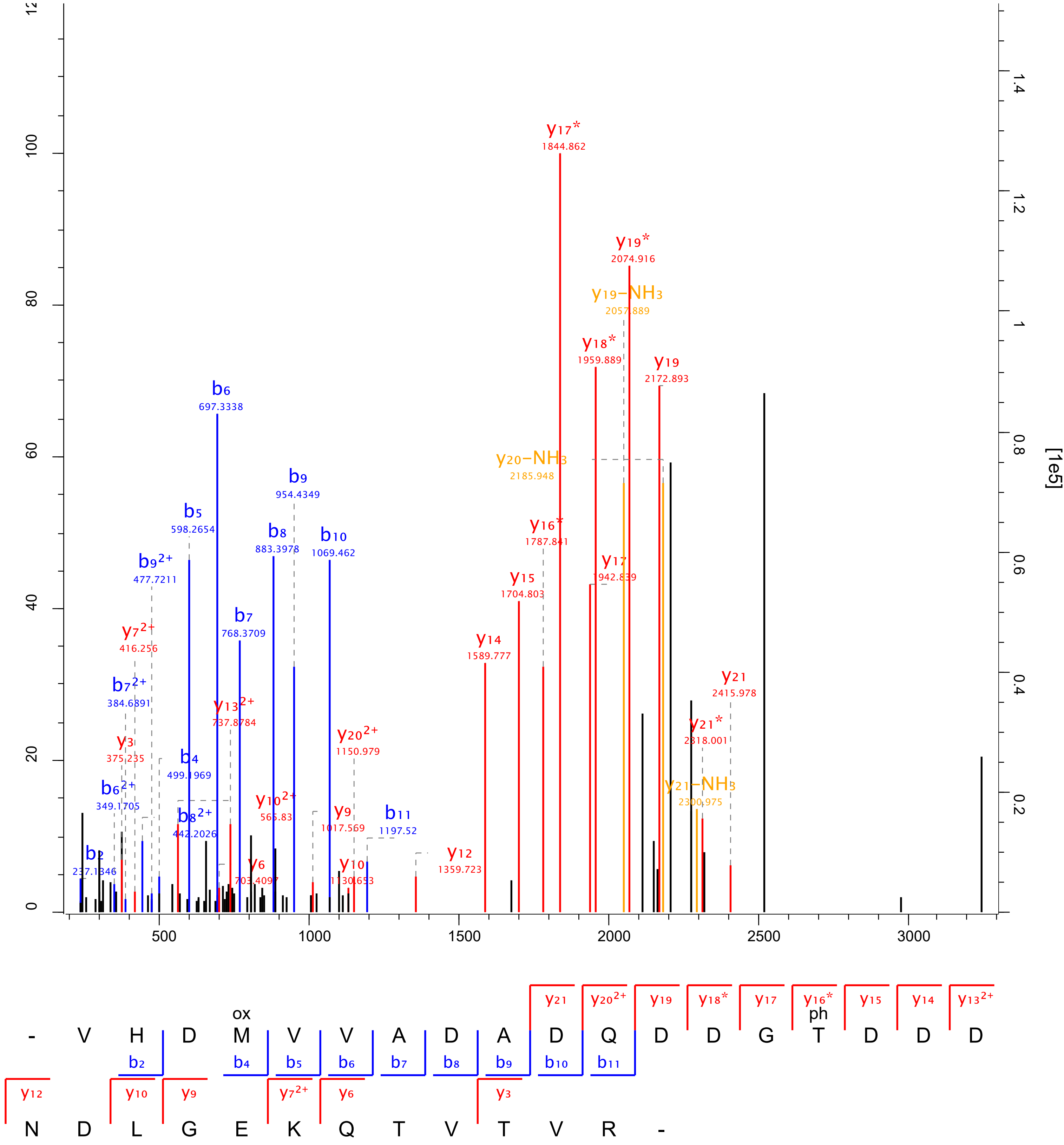

Sample output to test PDF Combine only

Raw file	Scan	Method	Score	m/z	Gene names
0523_11	14126	FTMS; HCD	54.68	1065.21	At1g30880

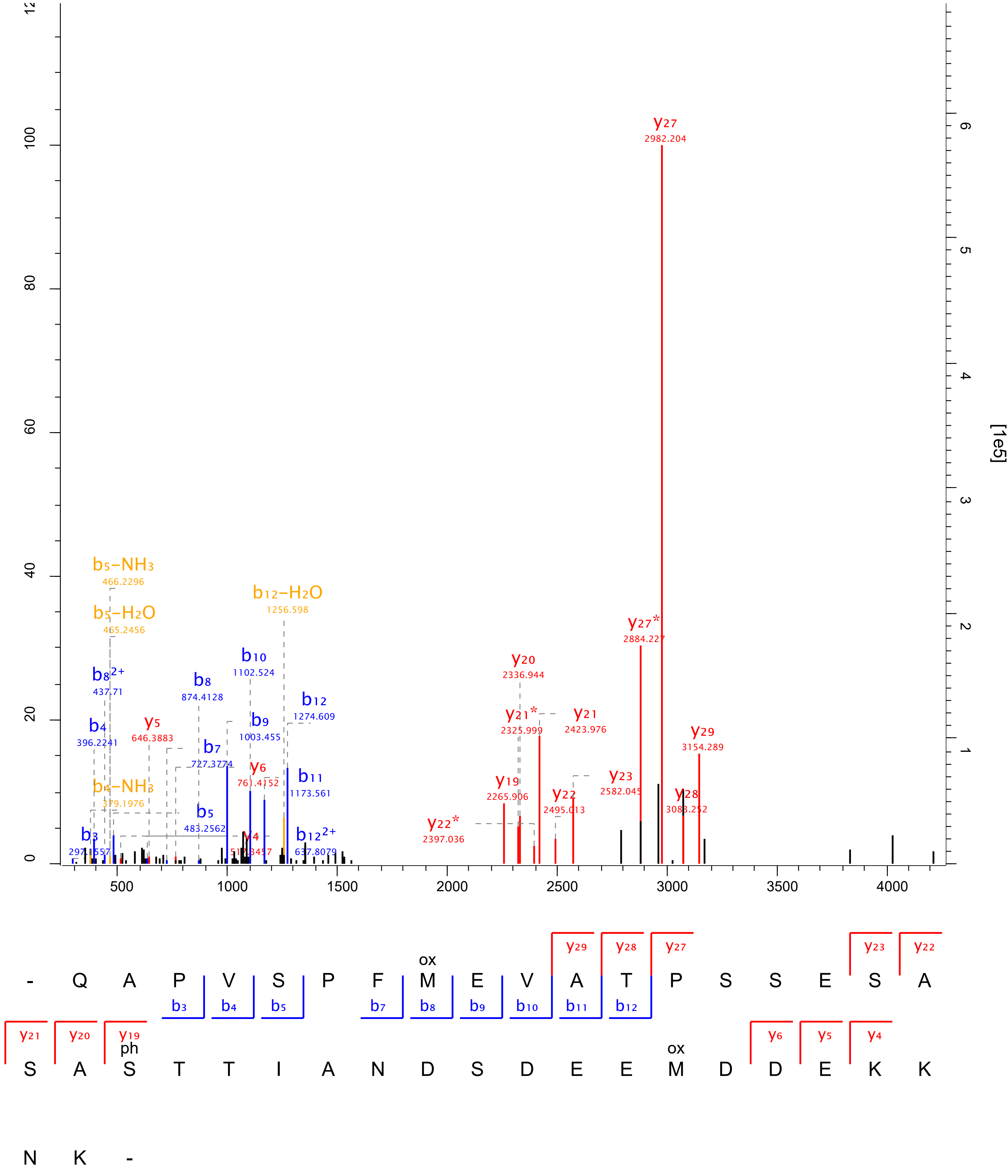

Sample output to test PDF Combine only

Raw file	Scan	Method	Score	m/z	Gene names
0523_11	14524	FTMS; HCD	40.46	682.07	TOC159

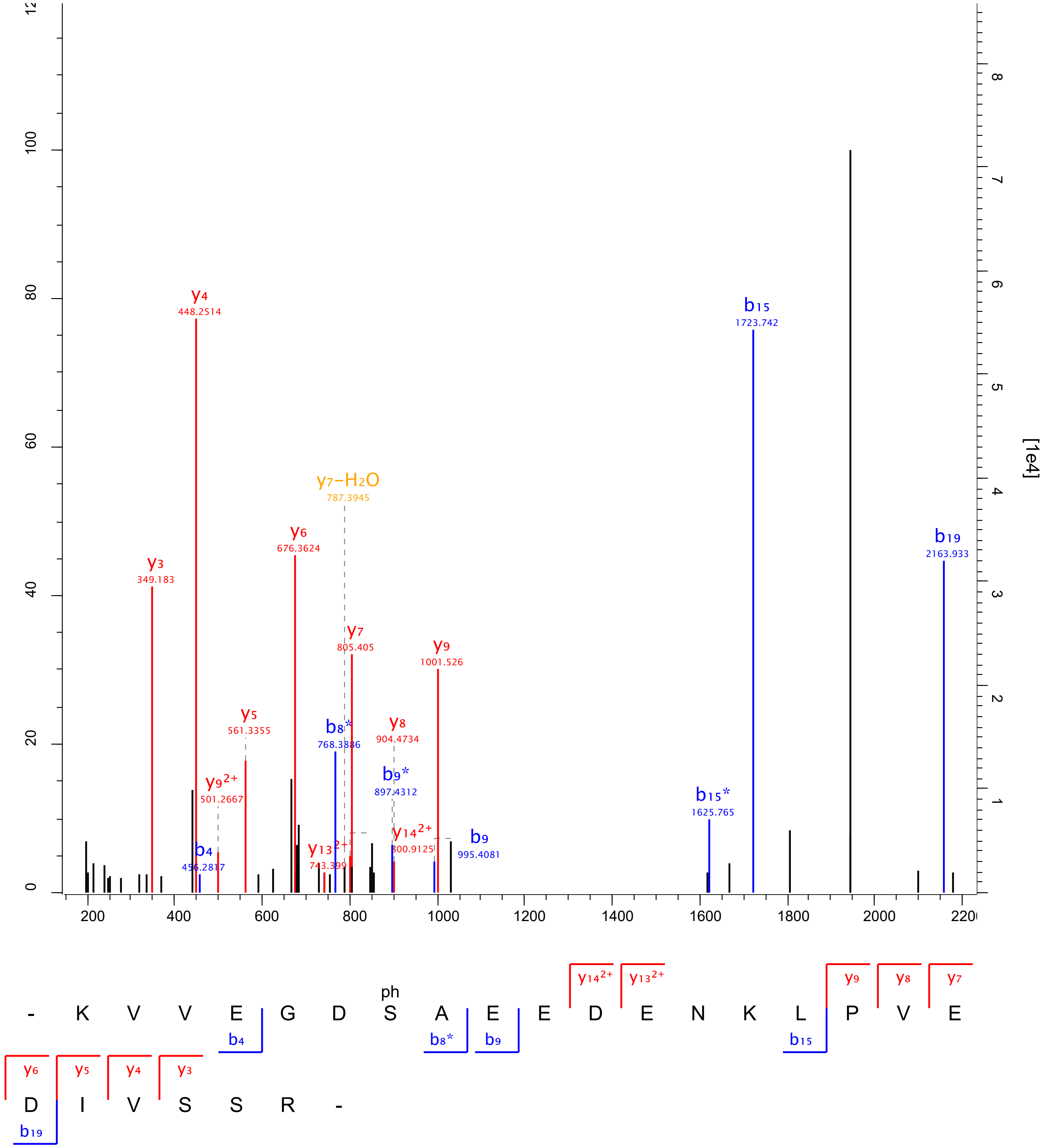

Sample output to test PDF Combine only

Raw file	Scan	Method	Score	m/z	Gene names
0523_11	14599	FTMS; HCD	41.93	691.31	At5g56980

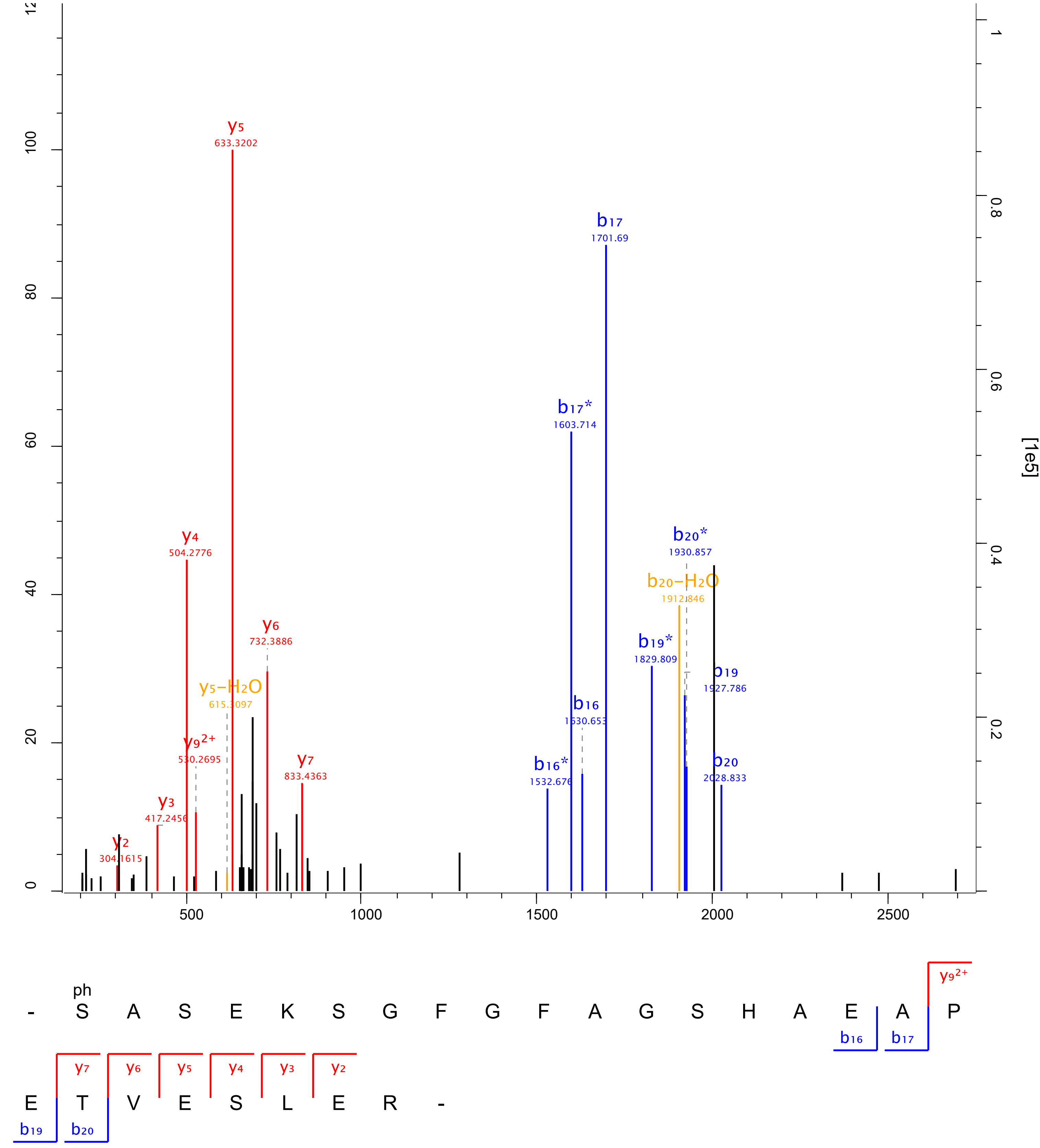

Sample output to test PDF Combine only

Raw file	Scan	Method	Score	m/z	Gene names
0523_11	14760	FTMS; HCD	60.64	954.21	MAP65-1

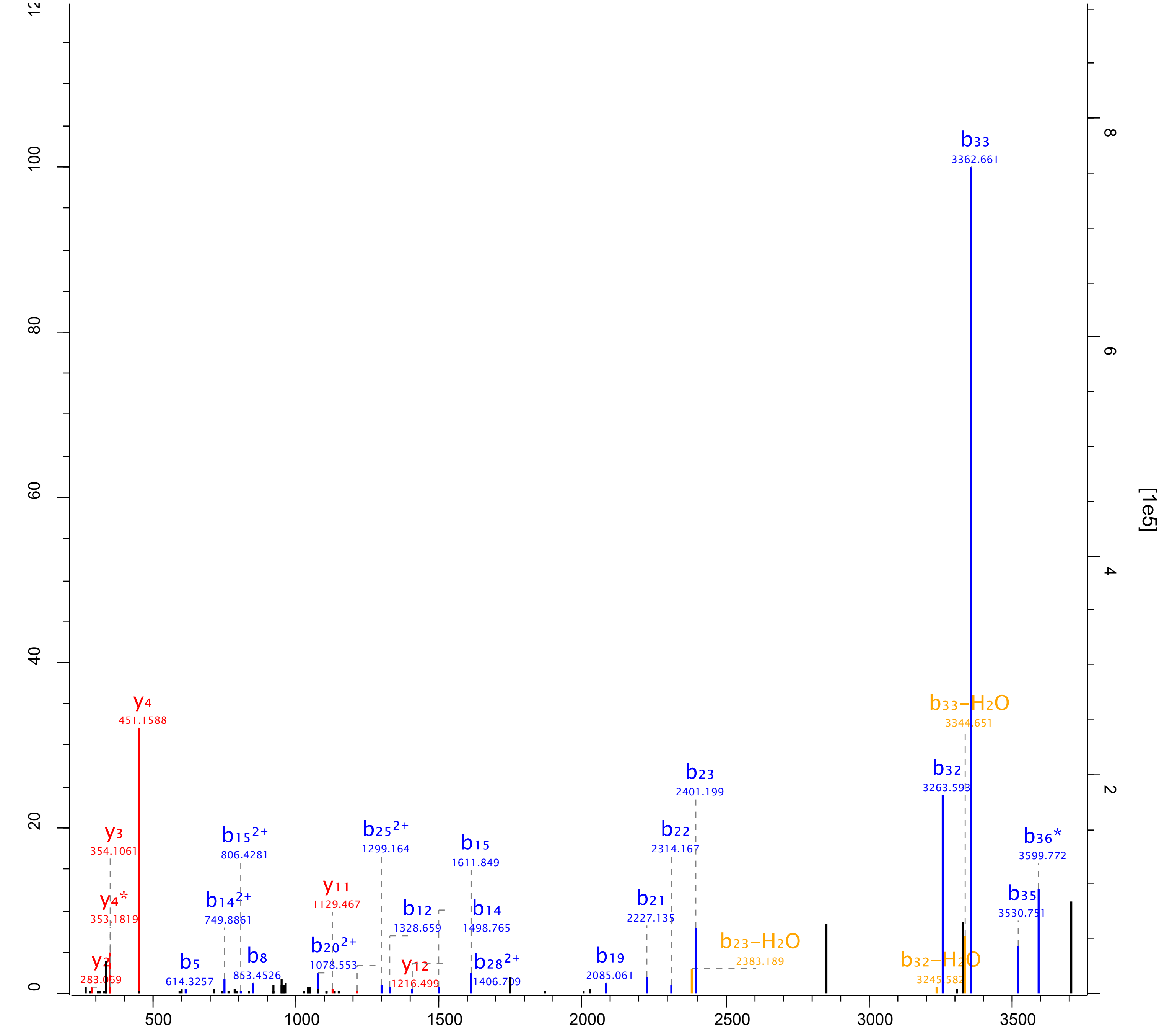

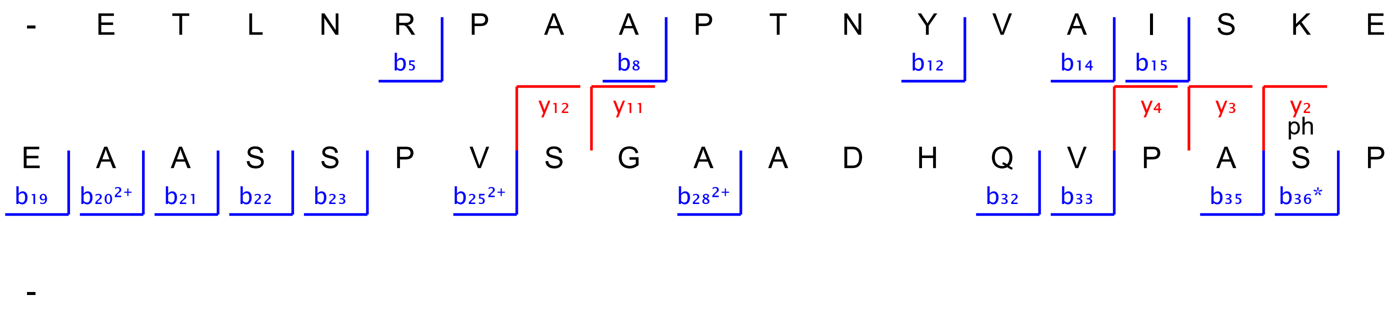

Sample output to test PDF Combine only

Raw file	Scan	Method	Score	m/z	Gene names
0523_11	14806	FTMS; HCD	143.32	784.7	IQD32

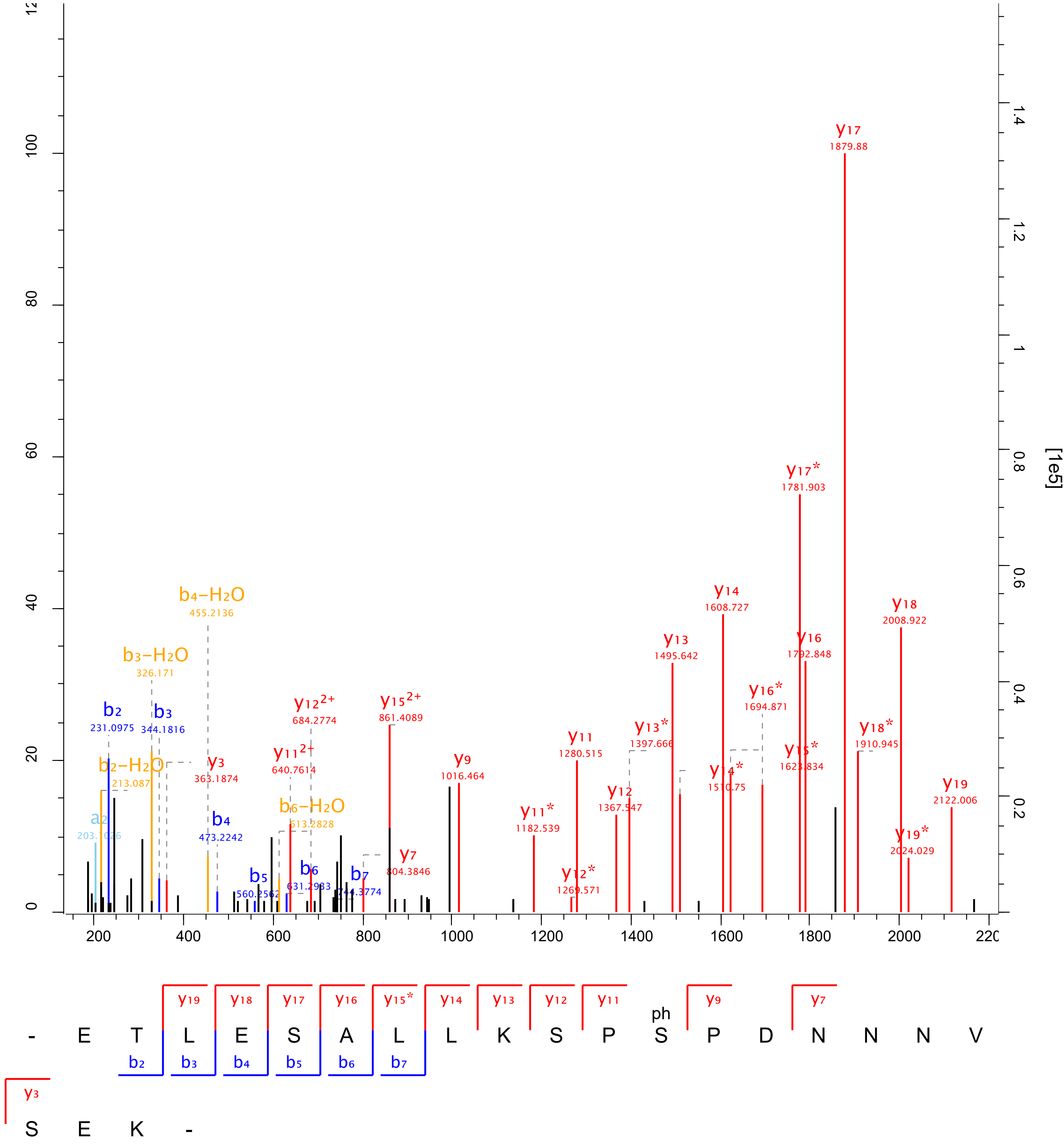

Sample output to test PDF Combine only

Raw file	Scan	Method	Score	m/z	Gene names
0523_11	14915	FTMS; HCD	65.43	738.04	EIN4

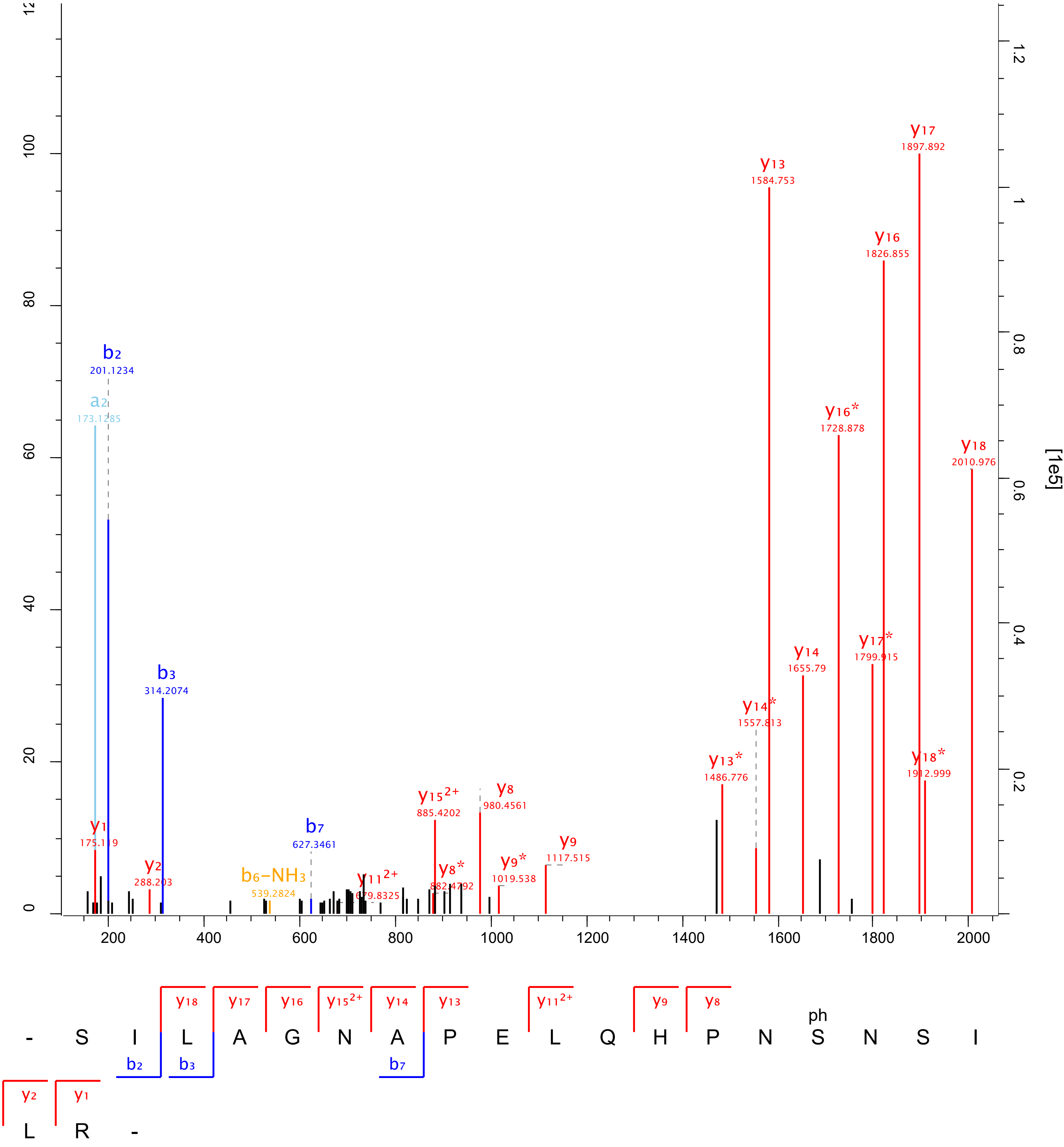

Sample output to test PDF Combine only

Raw file	Scan	Method	Score	m/z	Gene names
0523_11	15005	FTMS; HCD	90.31	681.32	At5g58950

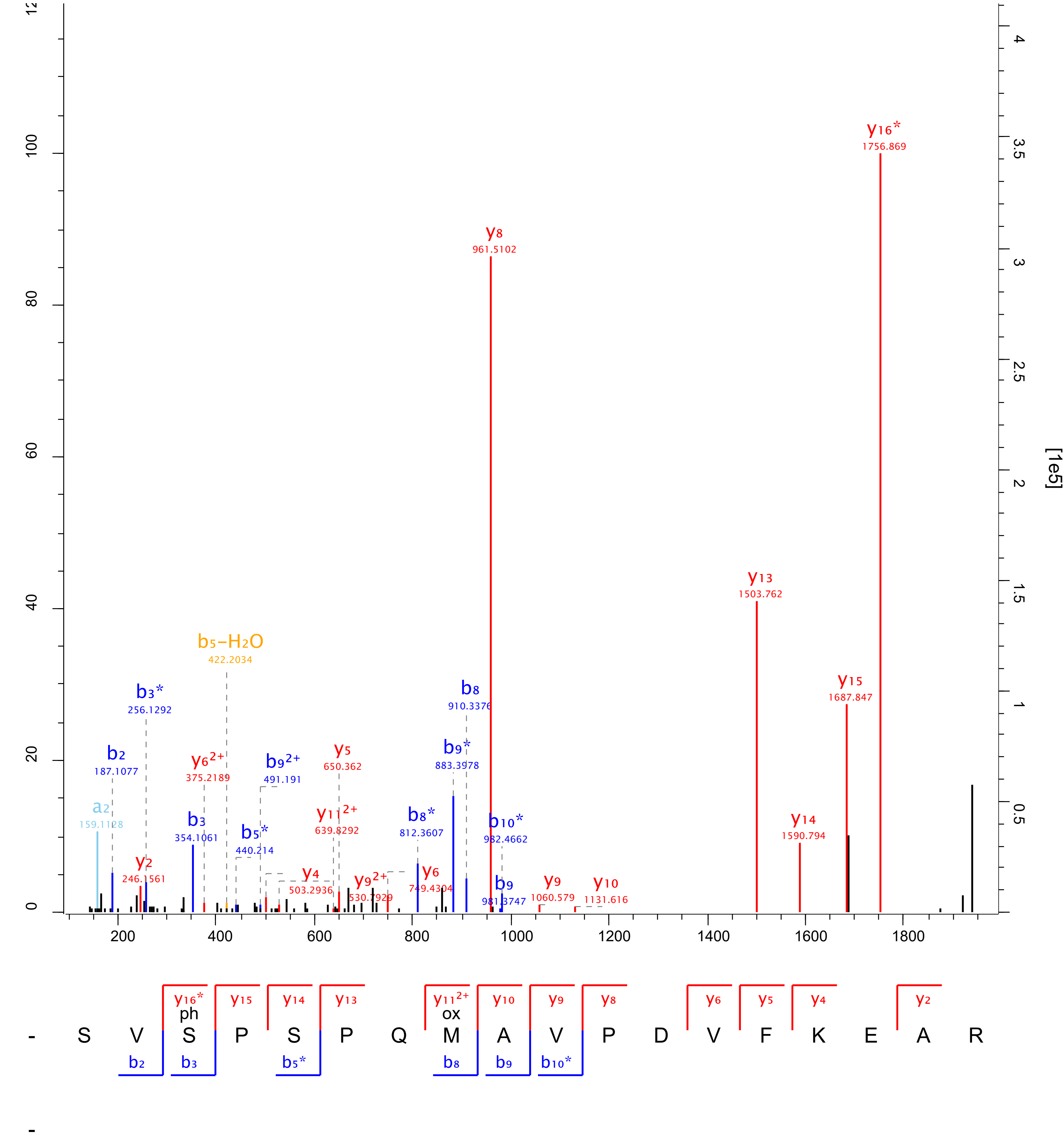

Sample output to test PDF Combine only

Raw file	Scan	Method	Score	m/z	Gene names
0523_11	15112	FTMS; HCD	187.49	960.13	T9E8.90

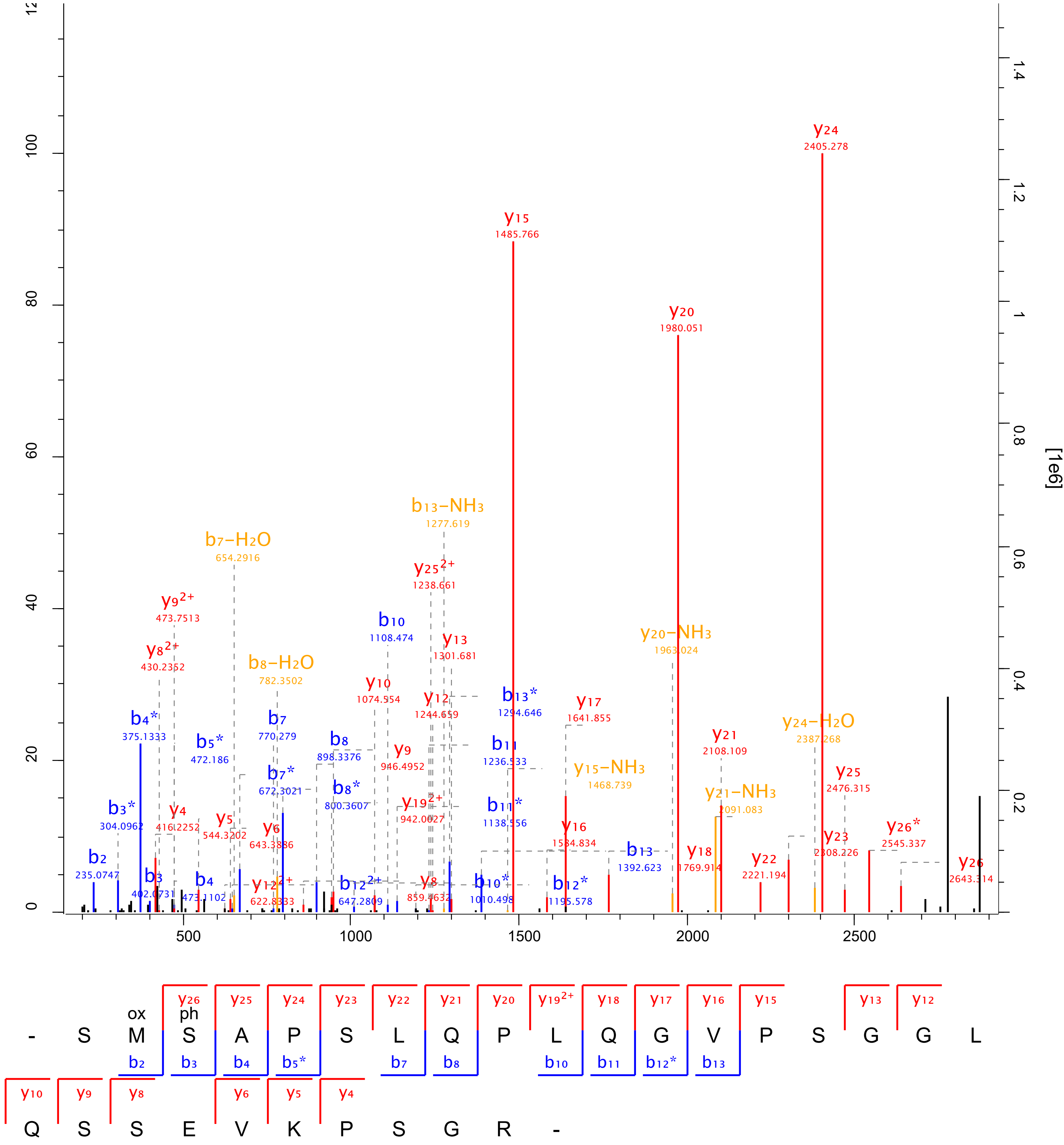

Sample output to test PDF Combine only

Raw file	Scan	Method	Score	m/z	Gene names
0523_11	15130	FTMS; HCD	48.25	1065.97	At4g11380;BETAB-AD

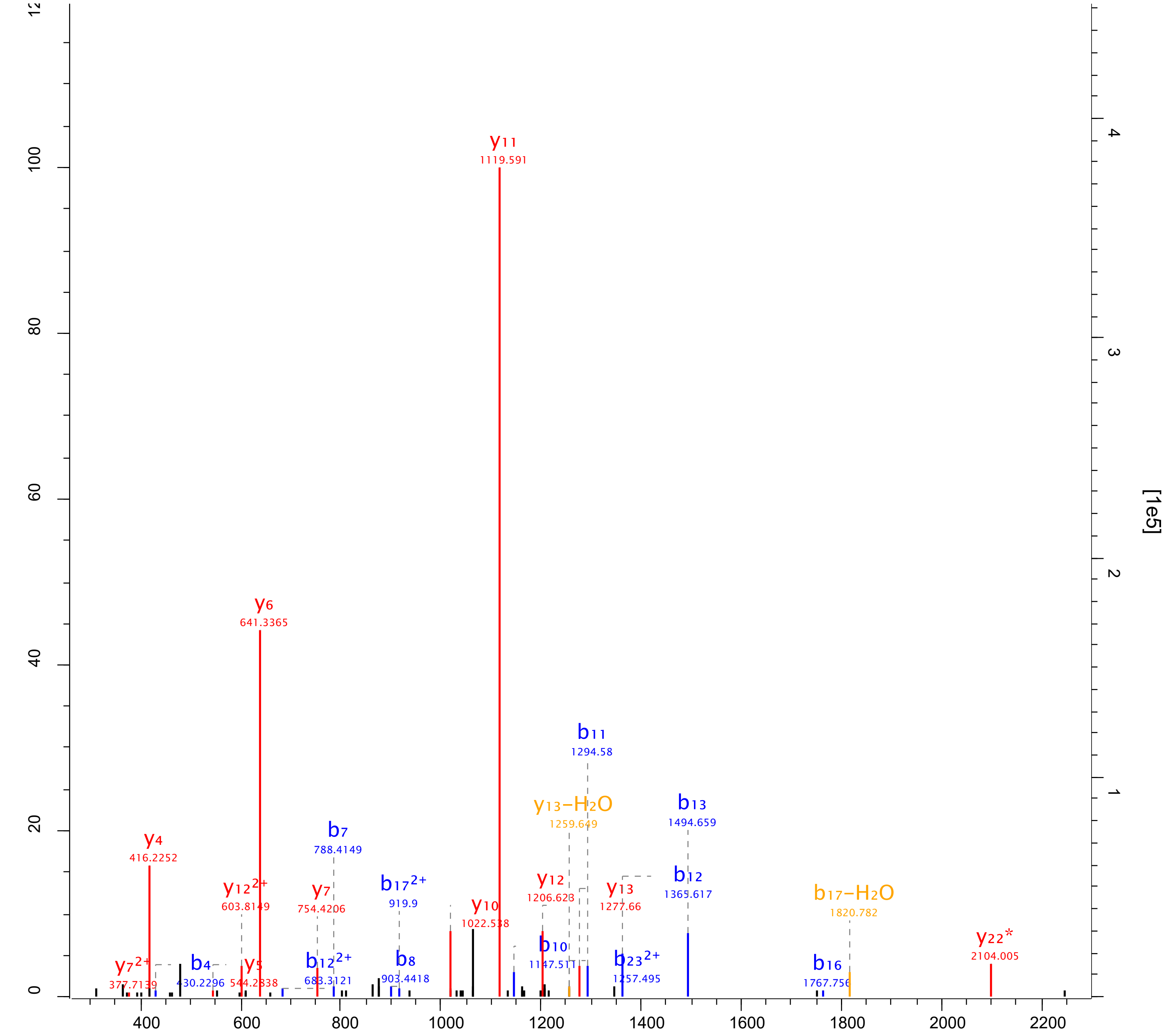

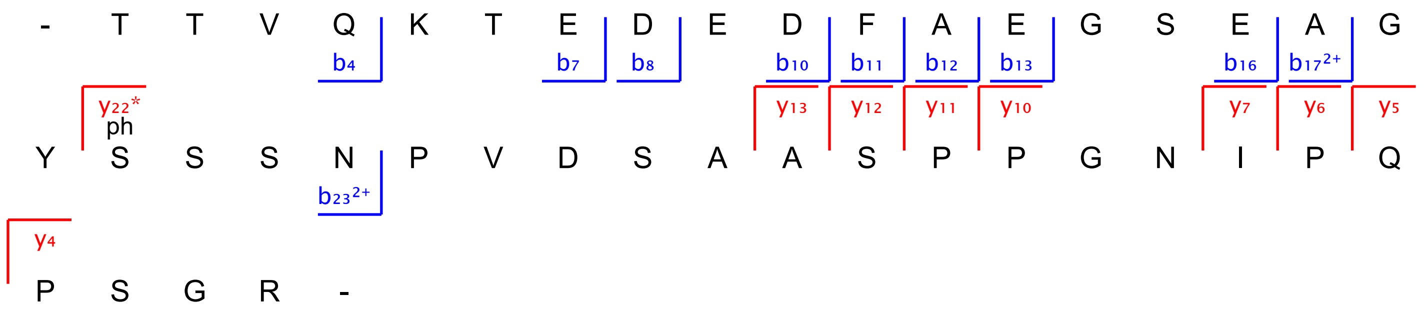

Sample output to test PDF Combine only

Raw file	Scan	Method	Score	m/z	Gene names
0523_11	15306	FTMS; HCD	50.03	607.05	VLN3

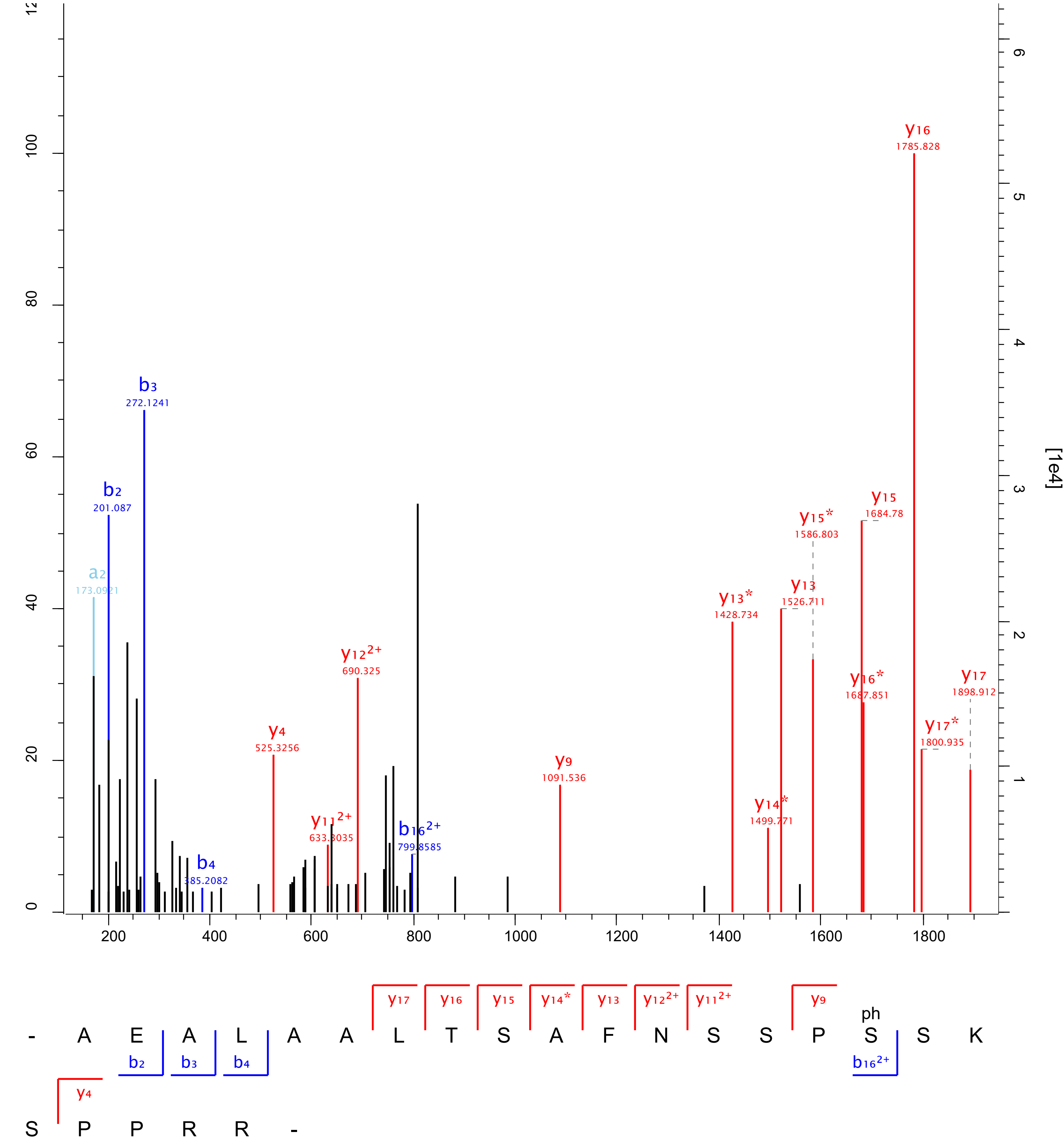

Raw file	Scan	Method	Score	m/z	Gene names
0523_11	15437	FTMS; HCD	258.27	770.04	NIP2-1

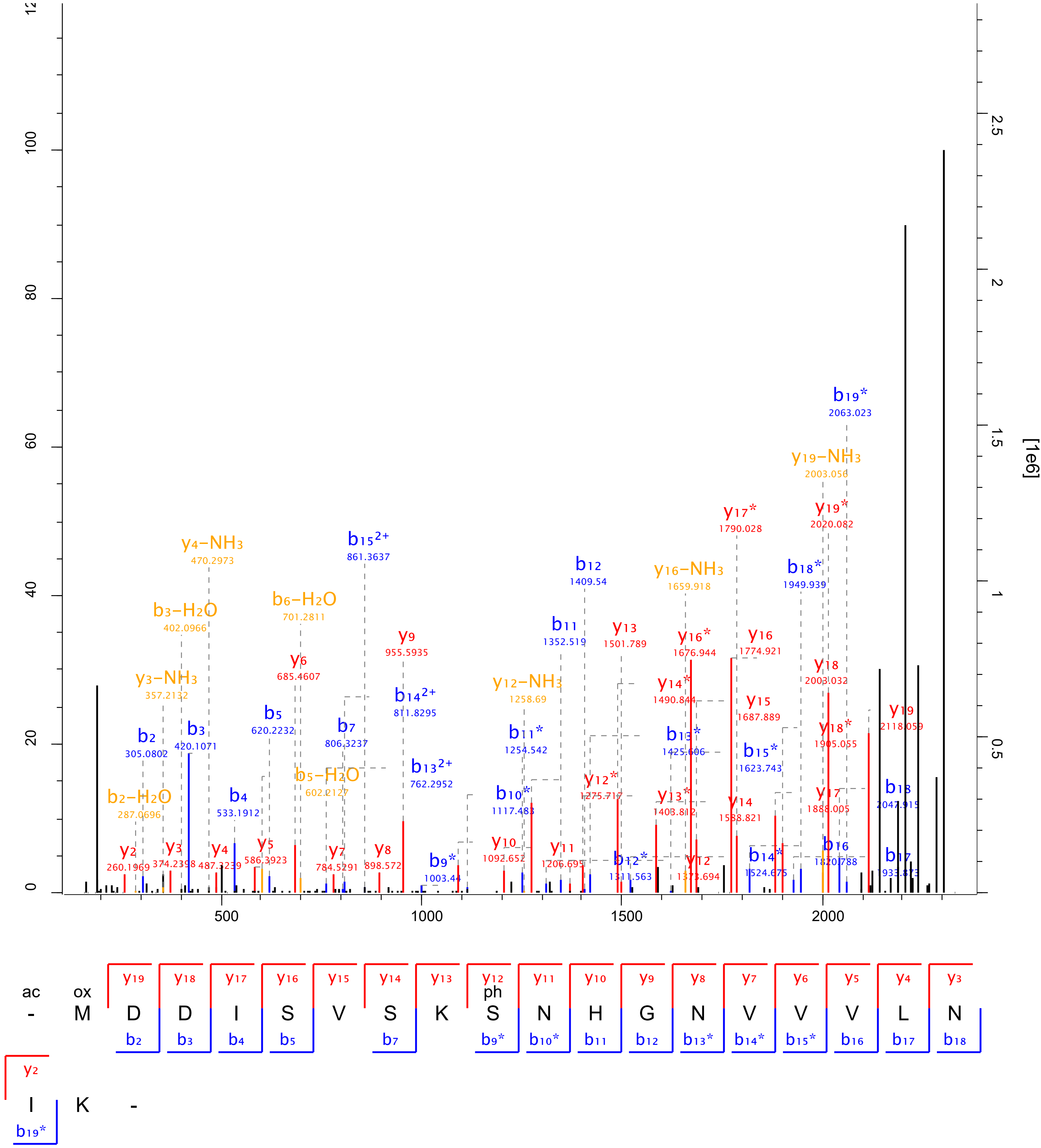

Sample output to test PDF Combine only

Raw file	Scan	Method	Score	m/z	Gene names
0523_11	15506	FTMS; HCD	56.17	761.38	RH38

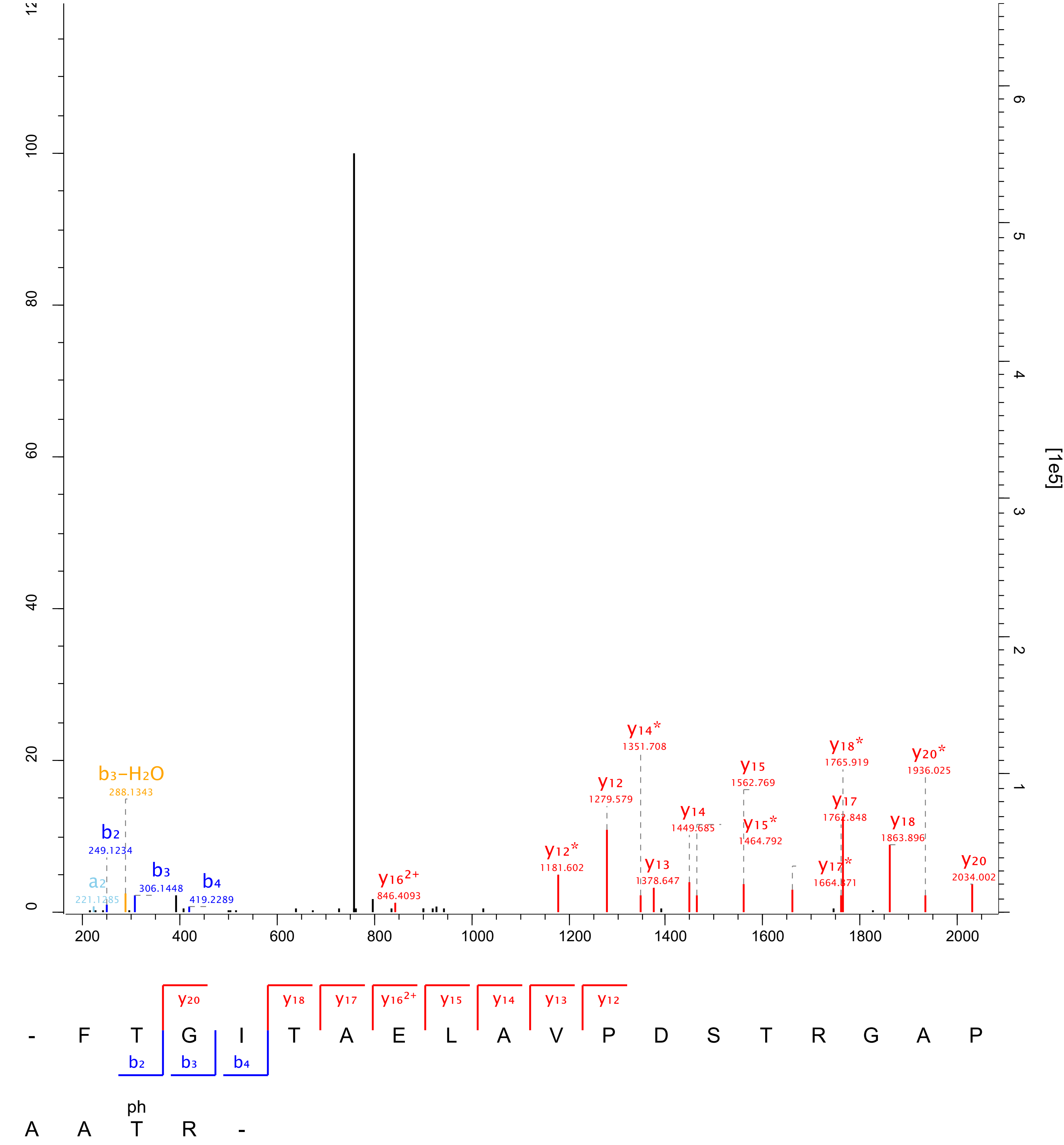

Sample output to test PDF Combine only

Raw file	Scan	Method	Score	m/z	Gene names
0523_11	15660	FTMS; HCD	141.19	782.37	P5CS2;P5CSB

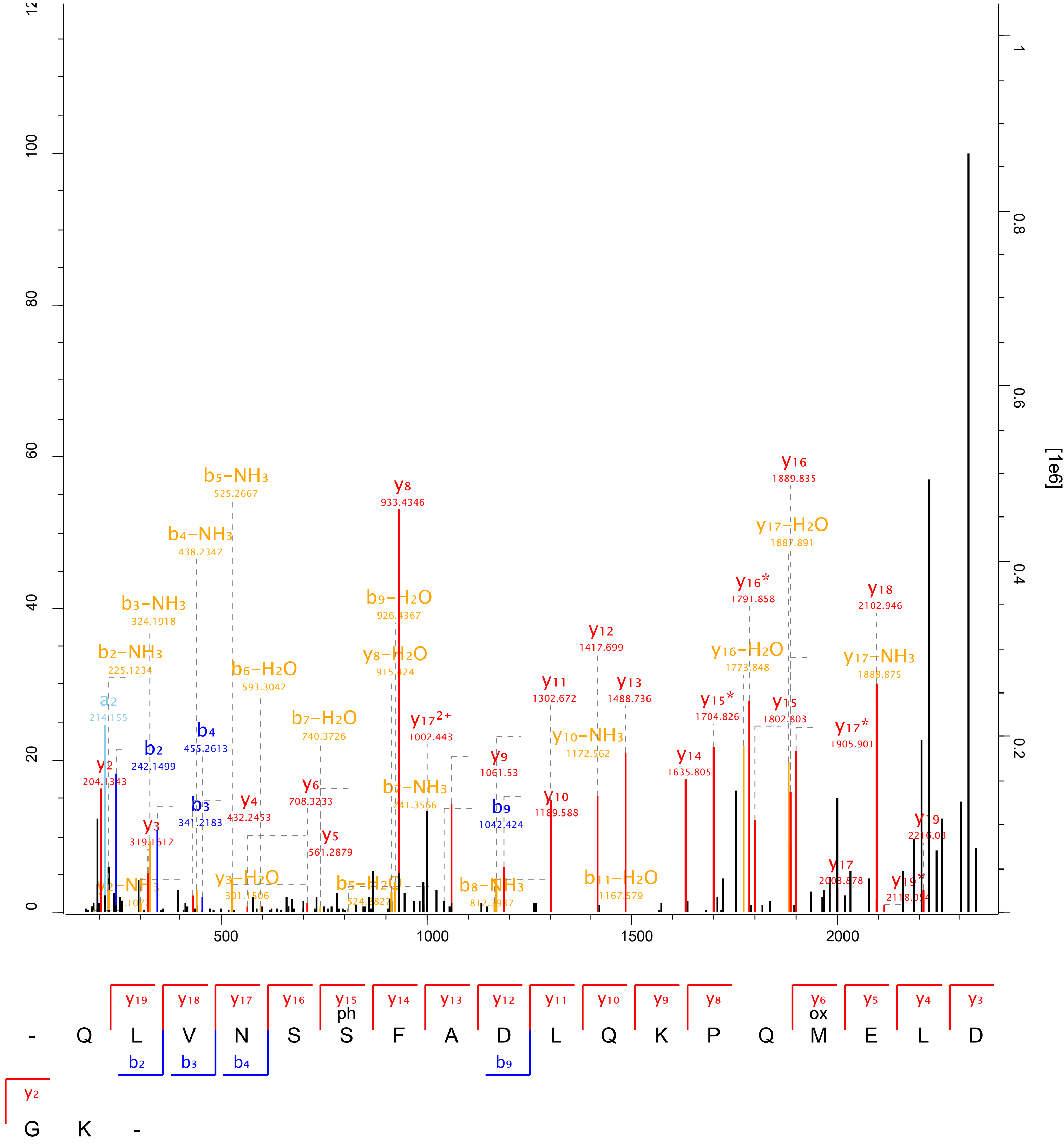

Sample output to test PDF Combine only

Raw file	Scan	Method	Score	m/z	Gene names
0523_11	15816	FTMS; HCD	77.69	727.01	VPS54

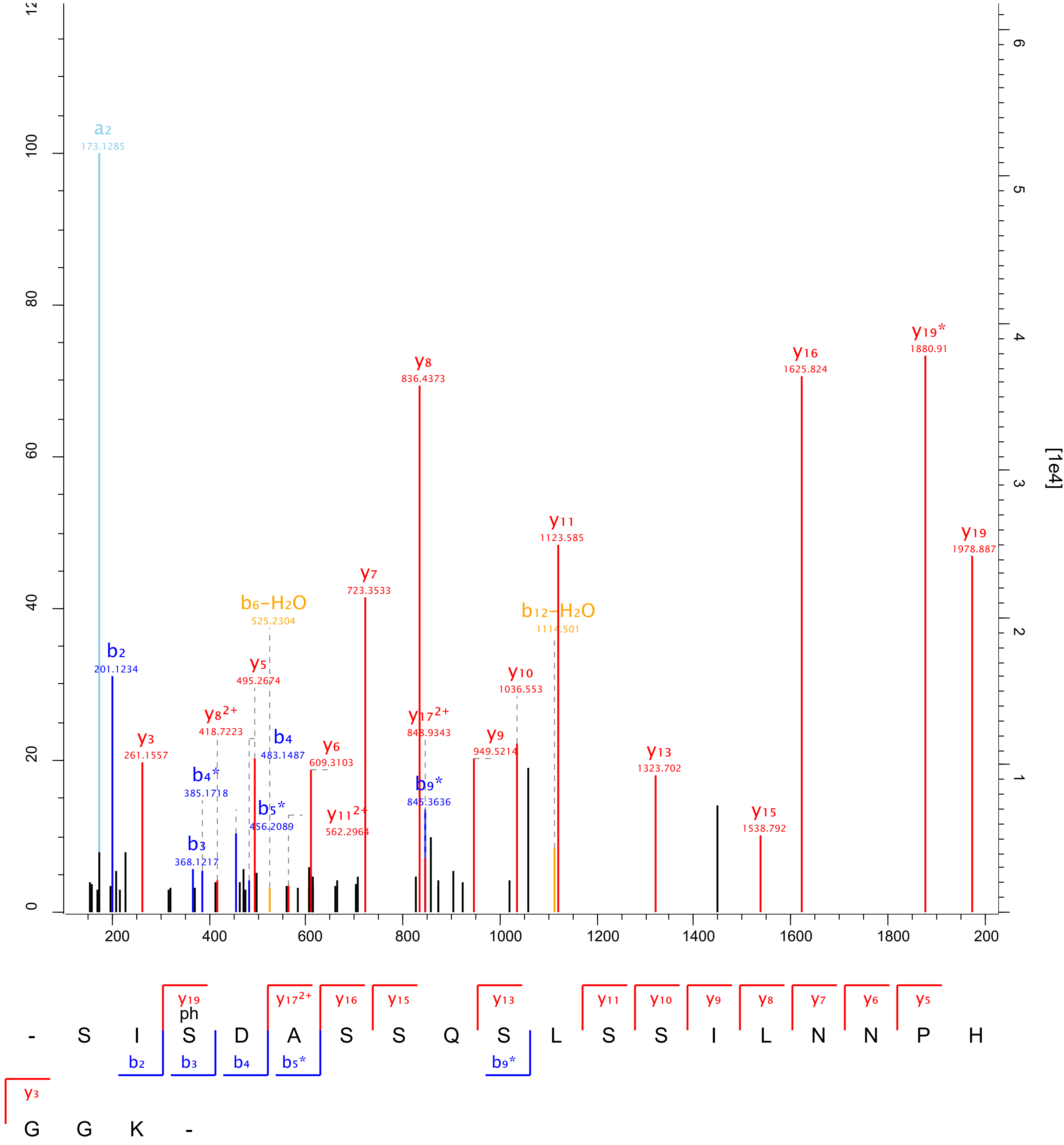

Sample output to test PDF Combine only

Raw file	Scan	Method	Score	m/z	Gene names
0523_11	15916	FTMS; HCD	59.82	933.65	PLDGAMMA1

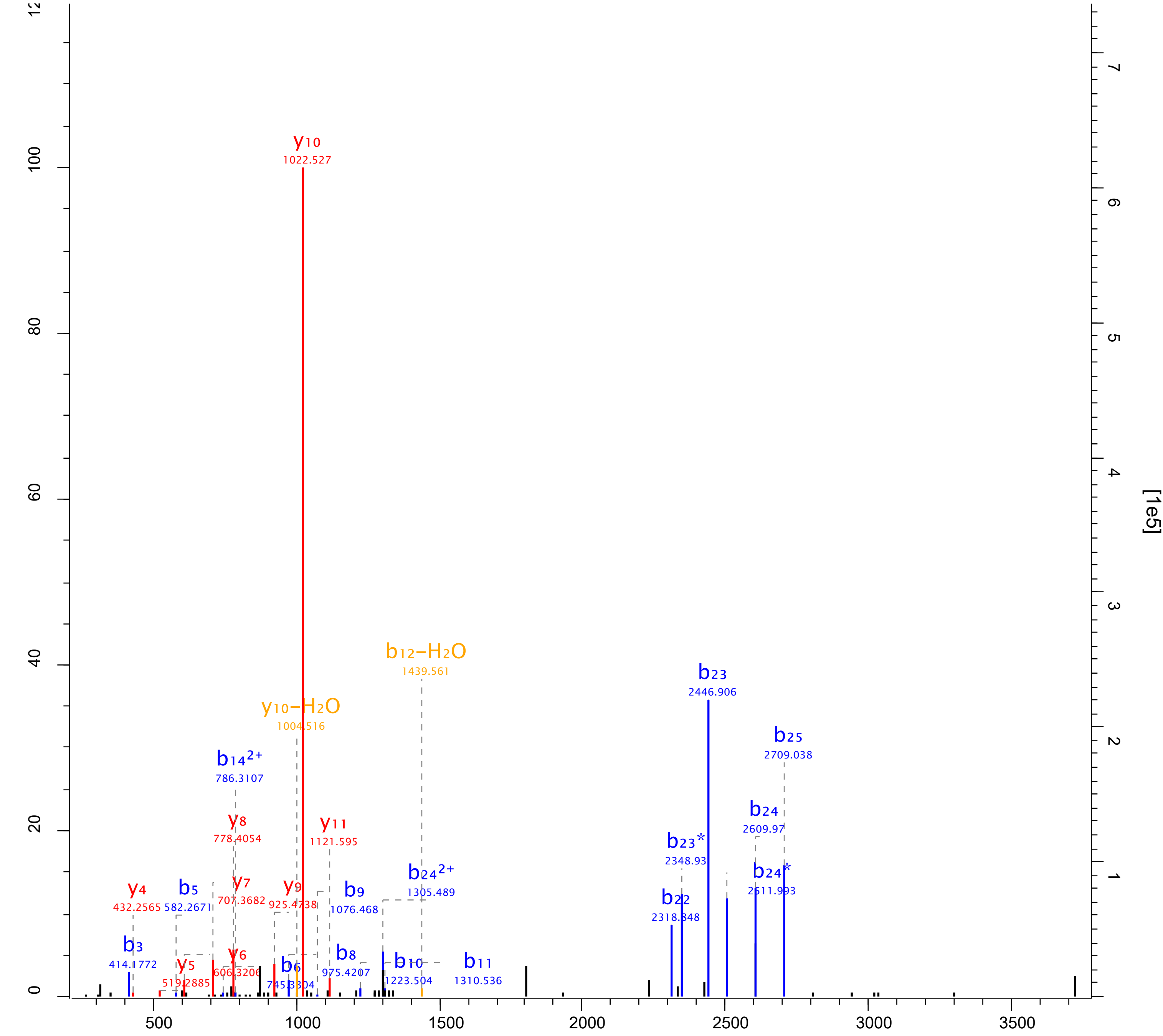

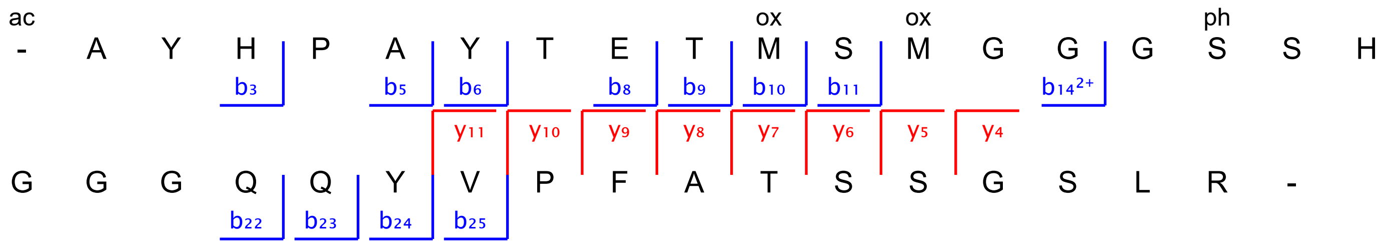

Sample output to test PDF Combine only

Raw file	Scan	Method	Score	m/z	Gene names
0523_11	15983	FTMS; HCD	68.58	925.69	SCAMP4

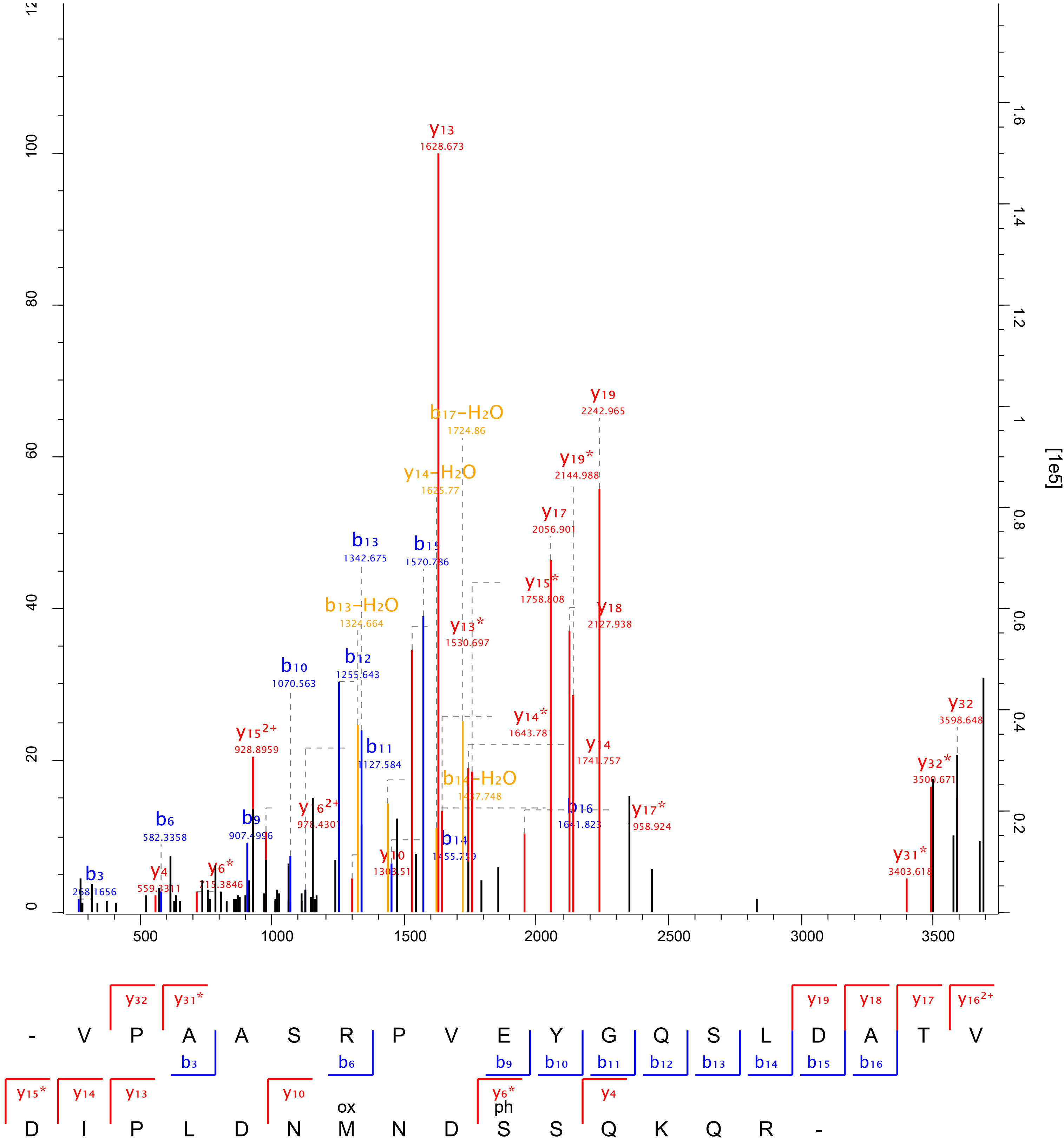

Sample output to test PDF Combine only

Raw file	Scan	Method	Score	m/z	Gene names
0523_11	15990	FTMS; HCD	92.89	728	UPL2;UPL1

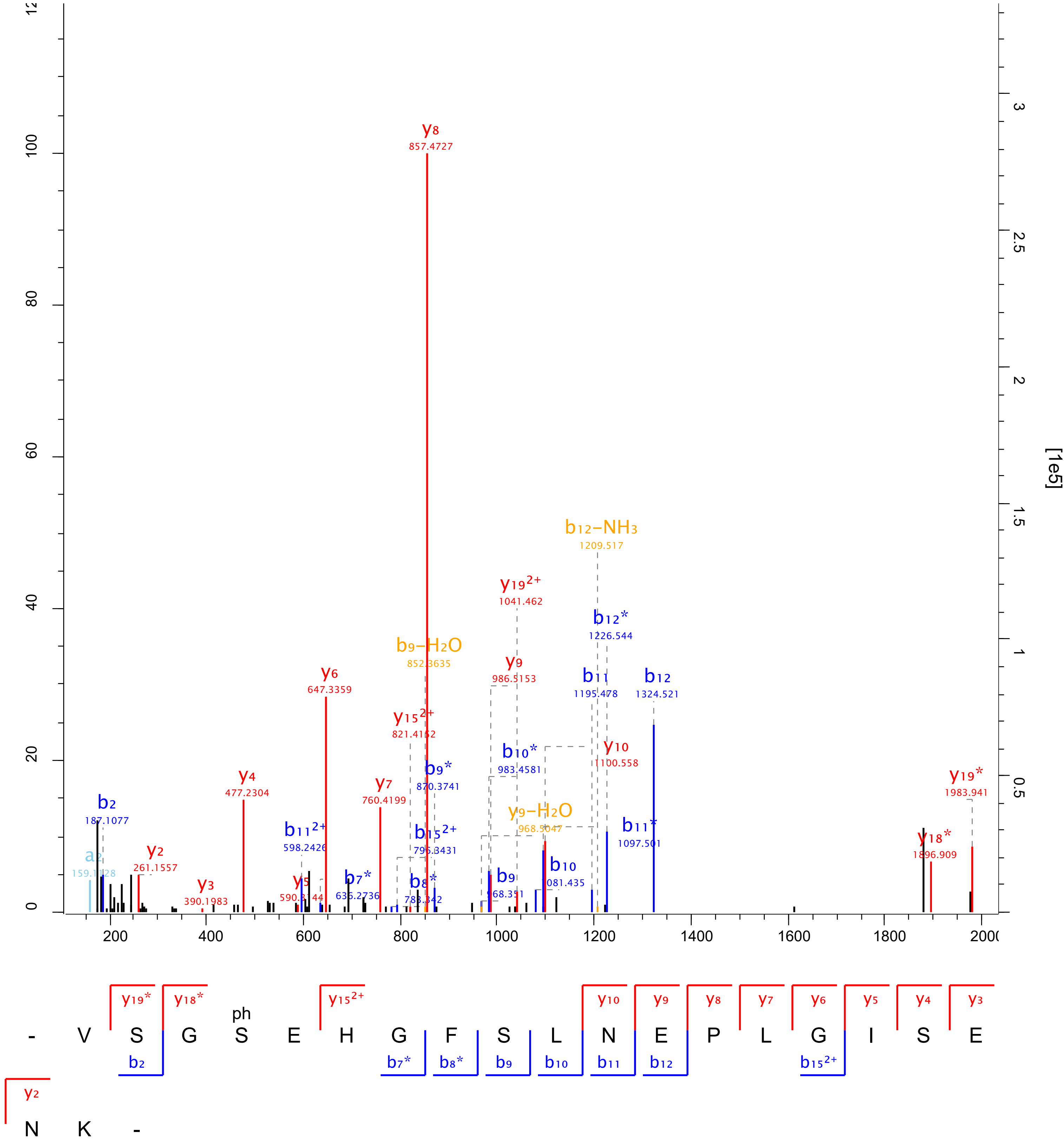

Sample output to test PDF Combine only

Raw file	Scan	Method	Score	m/z	Gene names
0523_11	16133	FTMS; HCD	99.41	686.99	LYK5

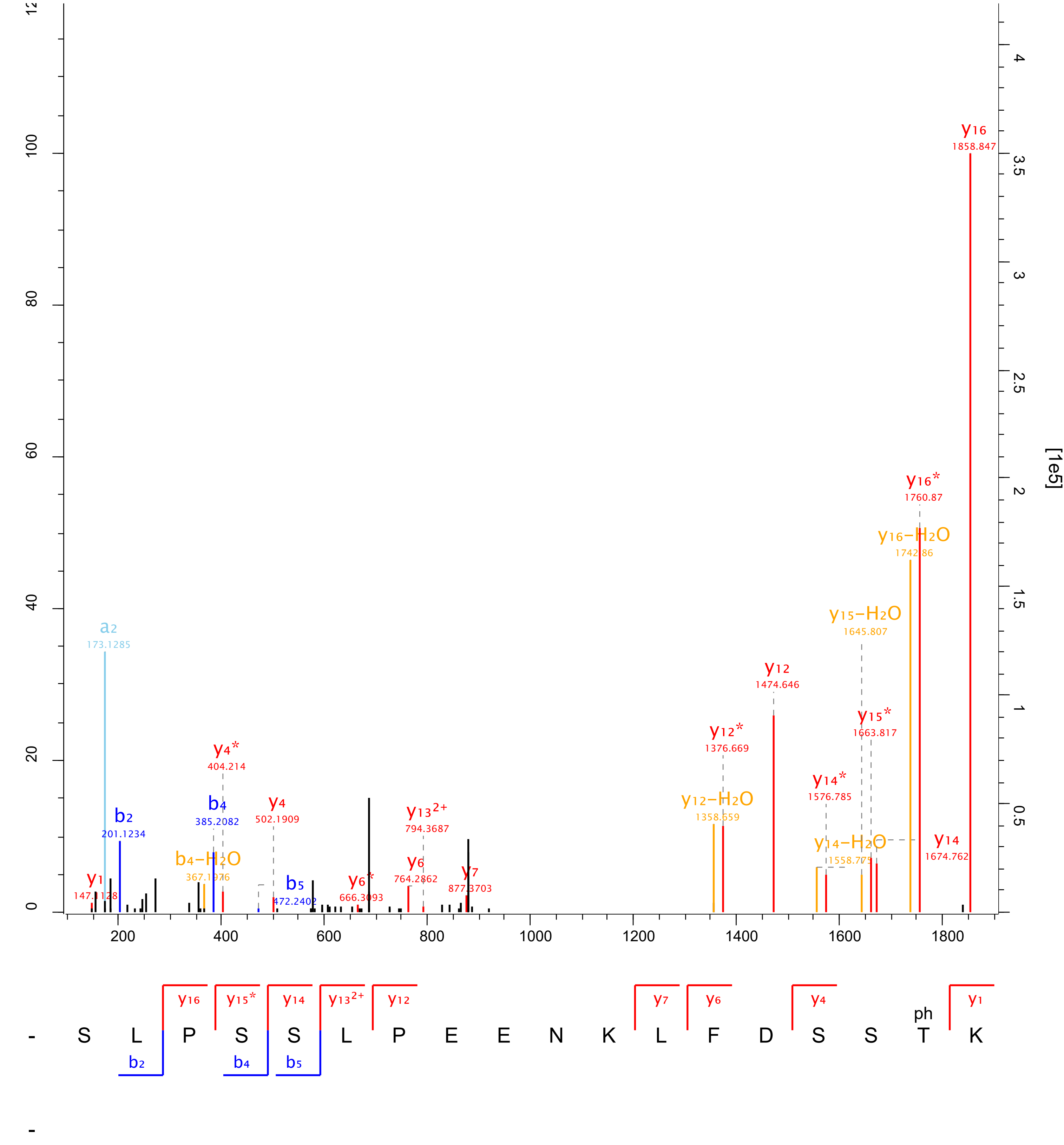

Sample output to test PDF Combine only

Raw file	Scan	Method	Score	m/z
0523_11	16300	FTMS; HCD	189.45	871.71

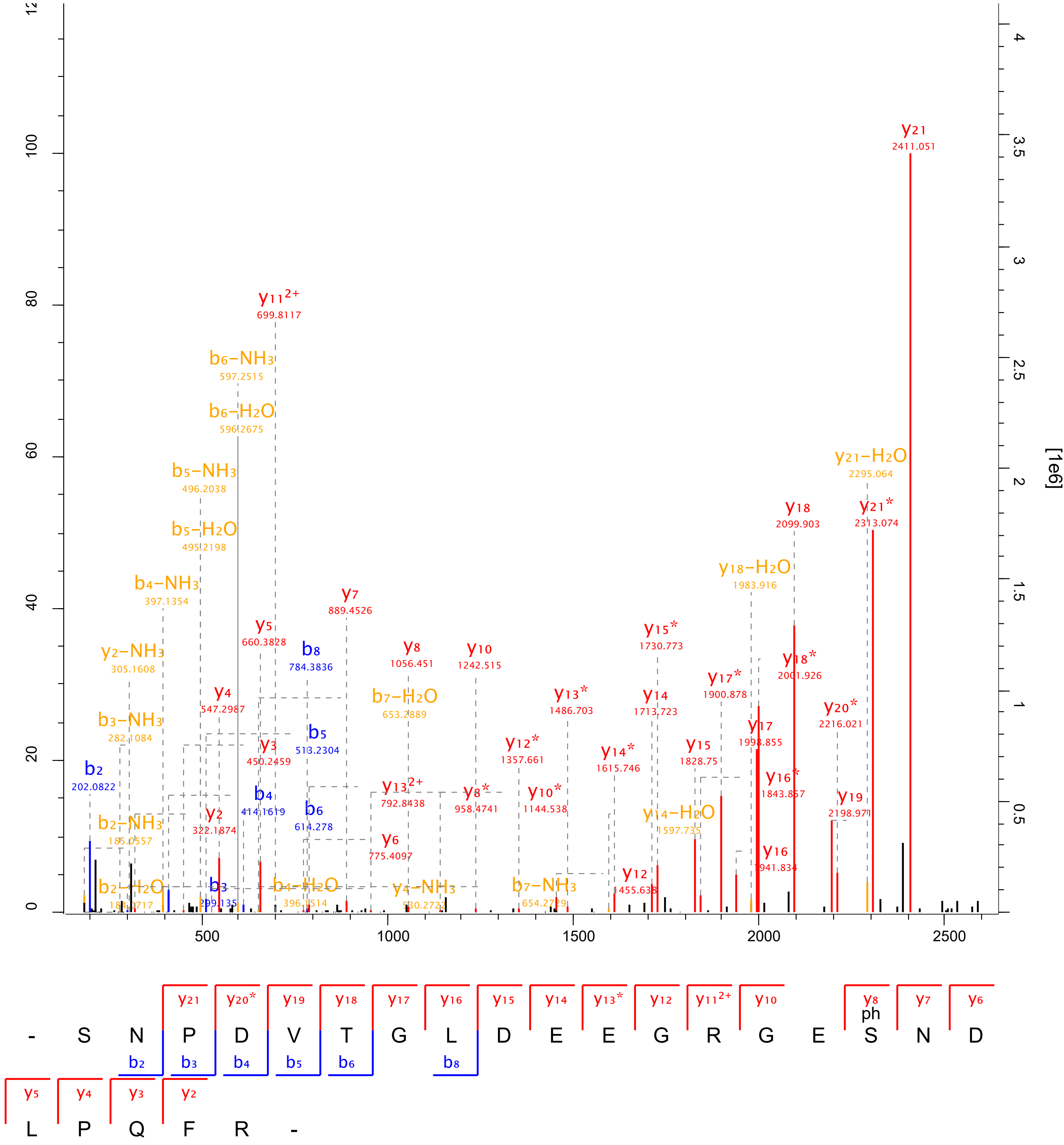

Sample output to test PDF Combine only

Raw file	Scan	Method	Score	m/z	Gene names
0523_11	16320	FTMS; HCD	124.82	713.58	PATL3

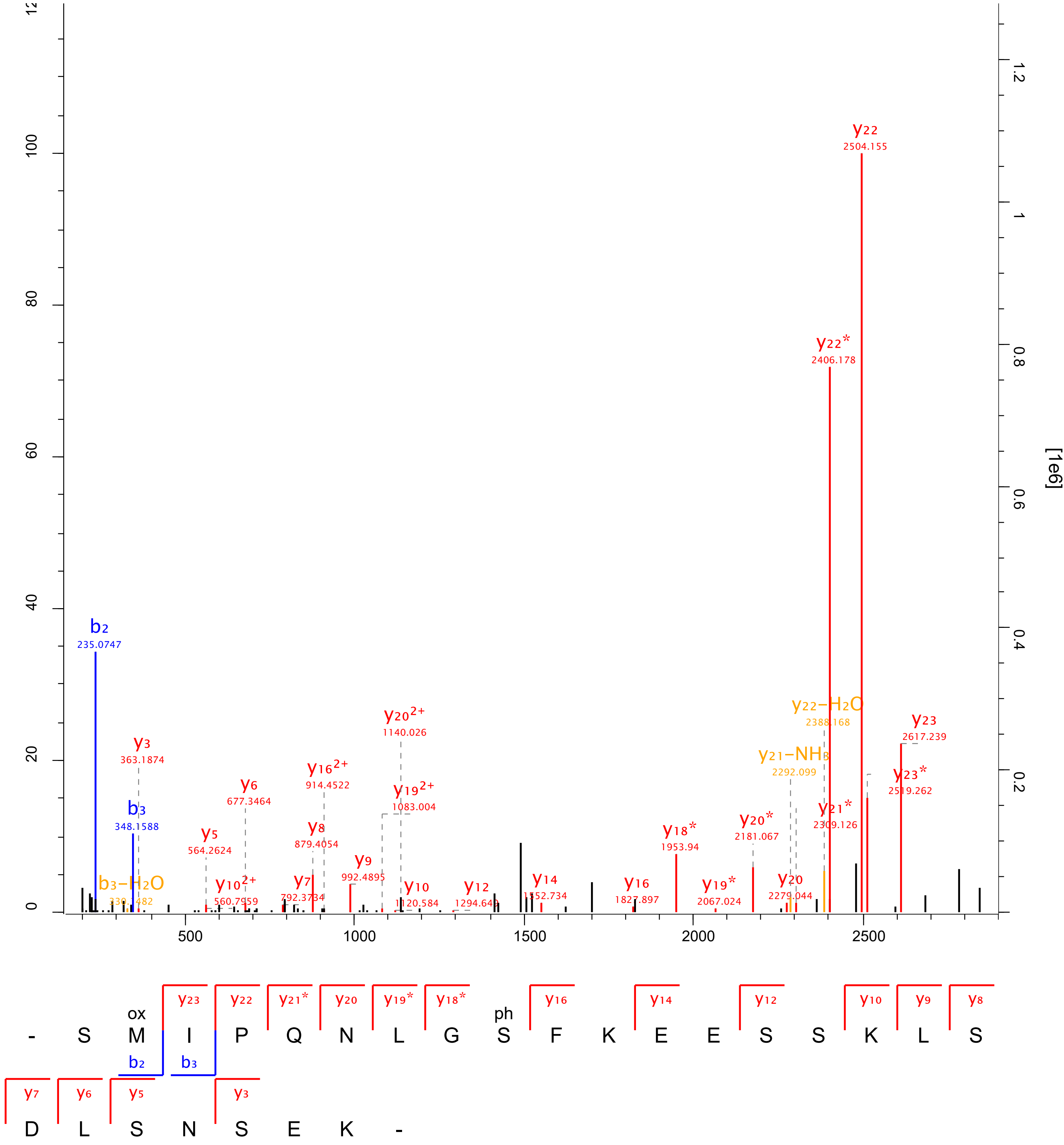

Sample output to test PDF Combine only

Raw file	Scan	Method	Score	m/z	Gene names
0523_11	16394	FTMS; HCD	71.67	856.11	IWS1

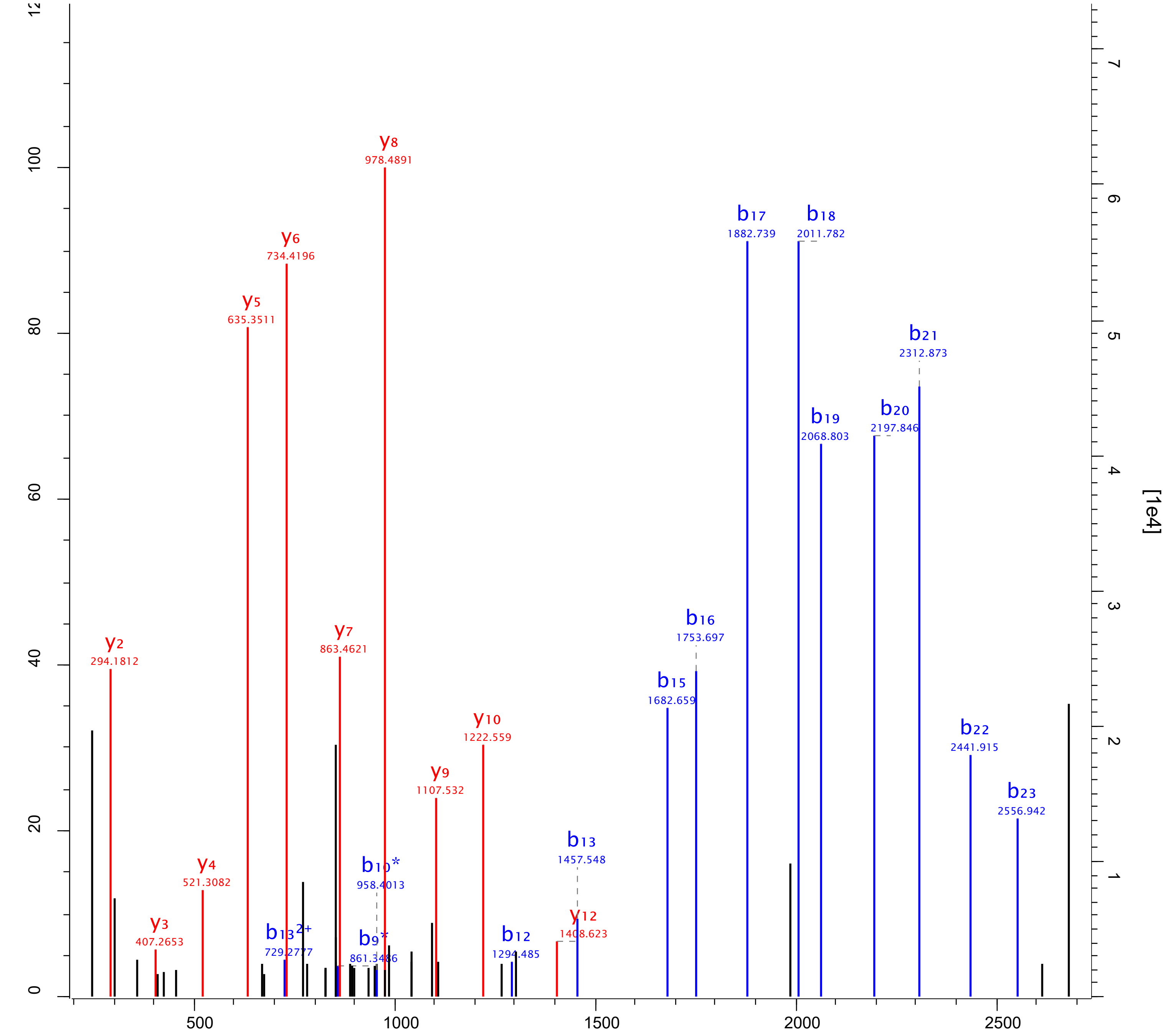

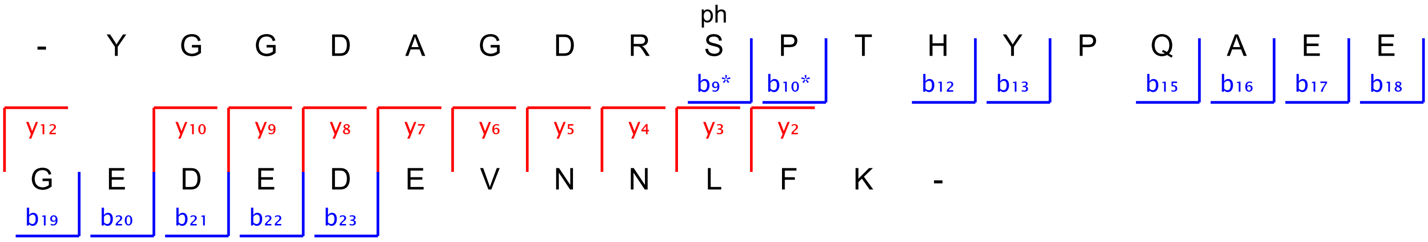

Sample output to test PDF Combine only

Raw file	Scan	Method	Score	m/z	Gene names
0523_11	16600	FTMS; HCD	107.01	877.05	At4g38550

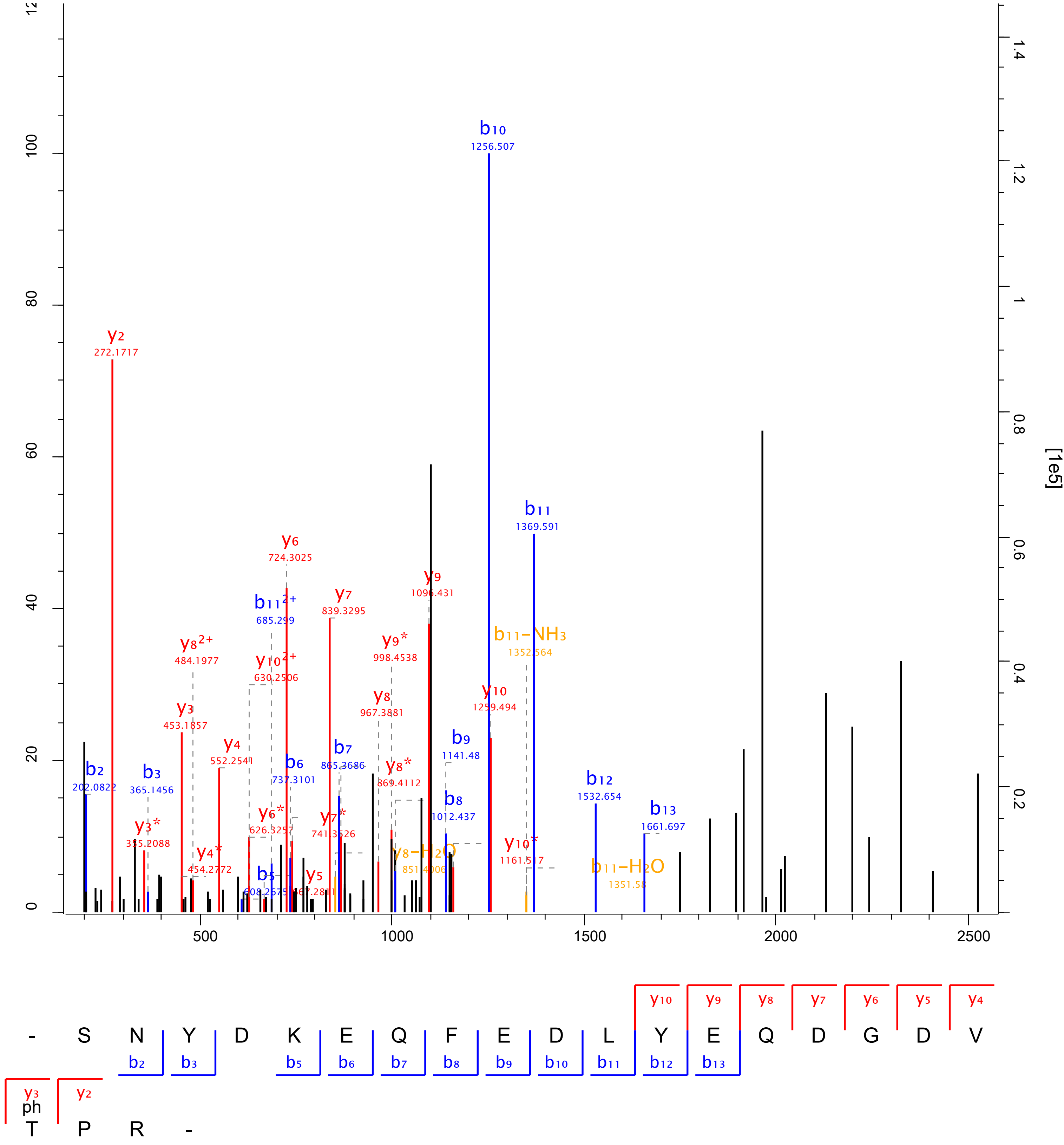

Sample output to test PDF Combine only

Raw file	Scan	Method	Score	m/z	Gene names
0523_11	16744	FTMS; HCD	177.49	1150.23	At2g40730

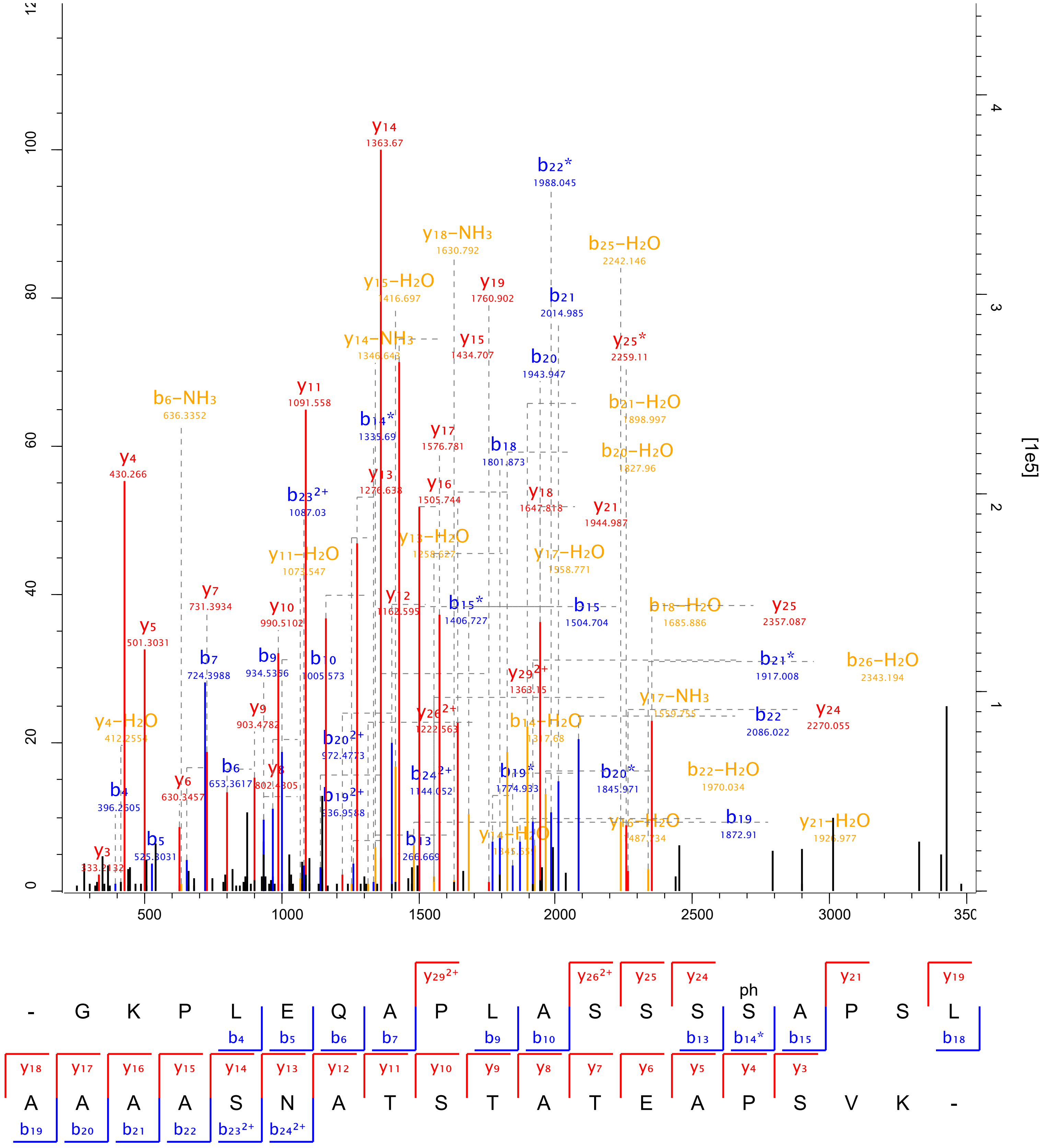

Sample output to test PDF Combine only

Raw file	Scan	Method	Score	m/z	Gene names
0523_11	16756	FTMS; HCD	80.41	667.31	NRT2.1

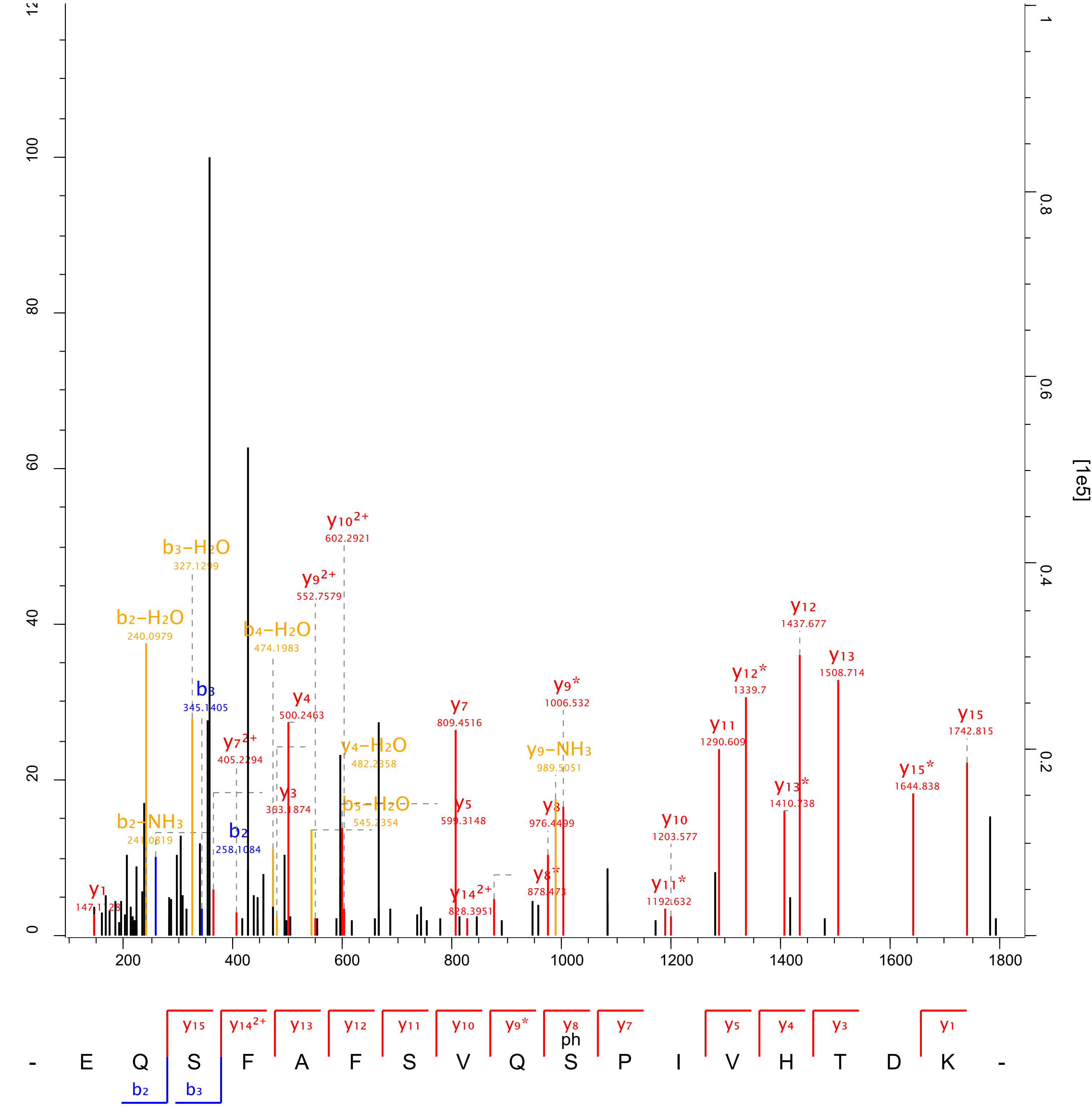

Sample output to test PDF Combine only

Raw file	Scan	Method	Score	m/z	Gene names
0523_11	17177	FTMS; HCD	111.65	696.66	VLN4

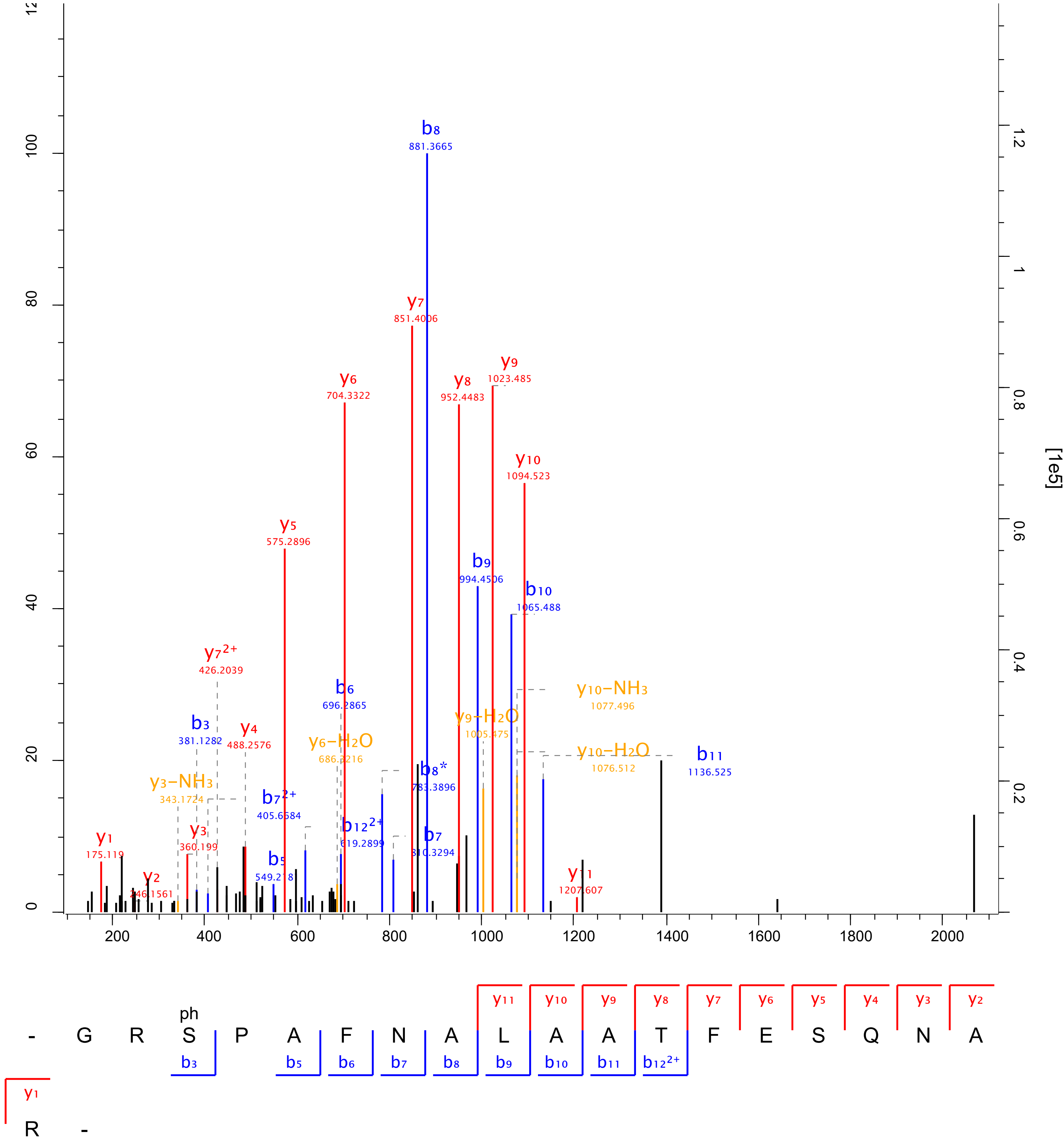

Sample output to test PDF Combine only

Raw file	Scan	Method	Score	m/z	Gene names
0523_11	17214	FTMS; HCD	69.36	768.03	EXO70D3

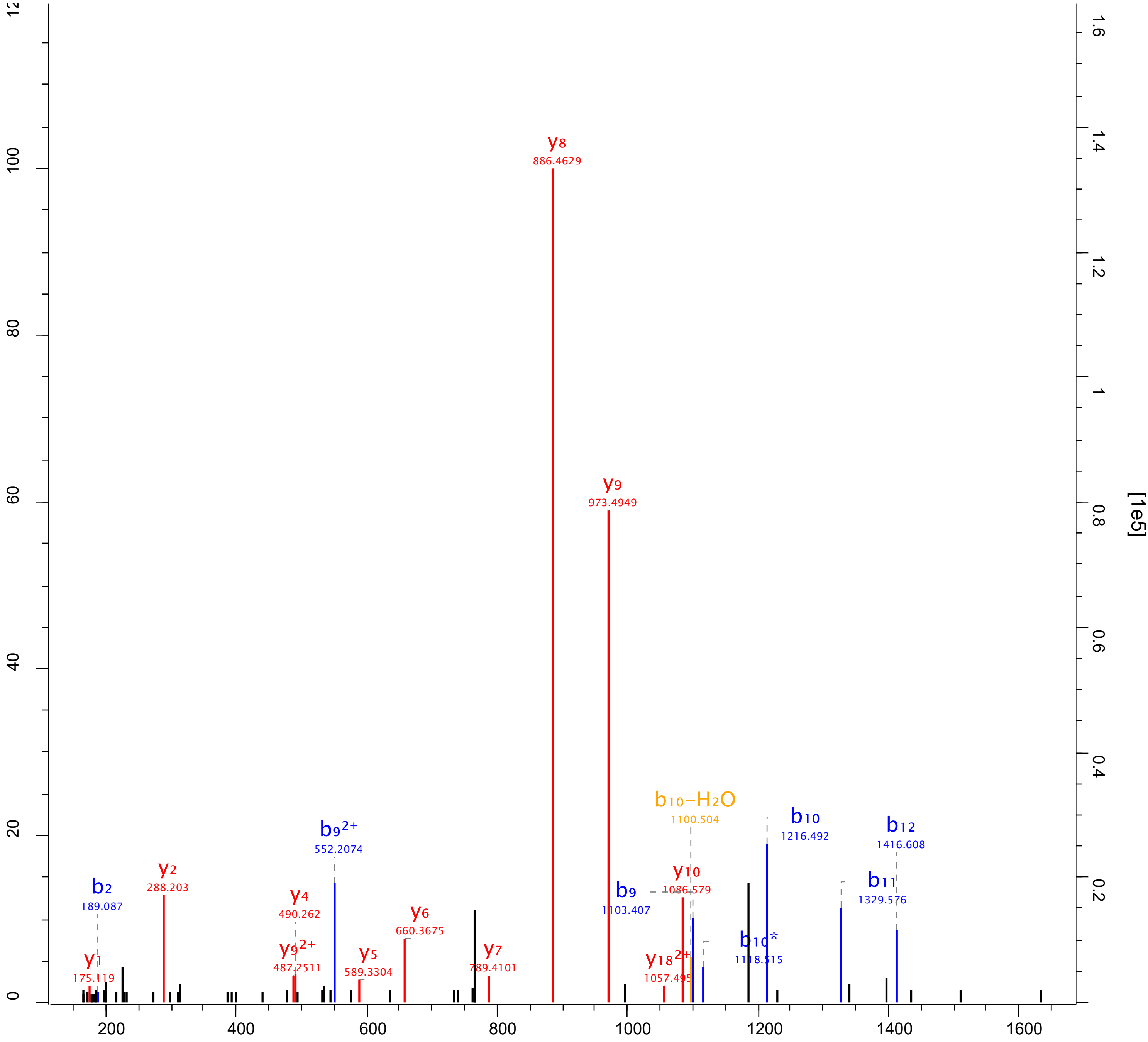

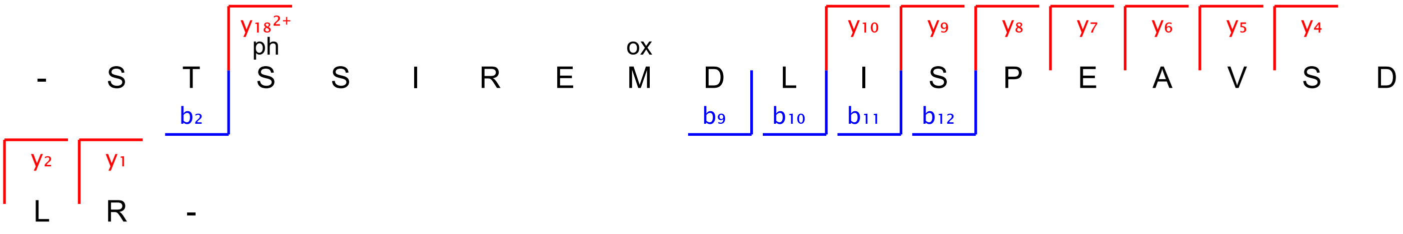

Sample output to test PDF Combine only

Raw file	Scan	Method	Score	m/z	Gene names
0523_11	17380	FTMS; HCD	67.91	950.45	AGO4

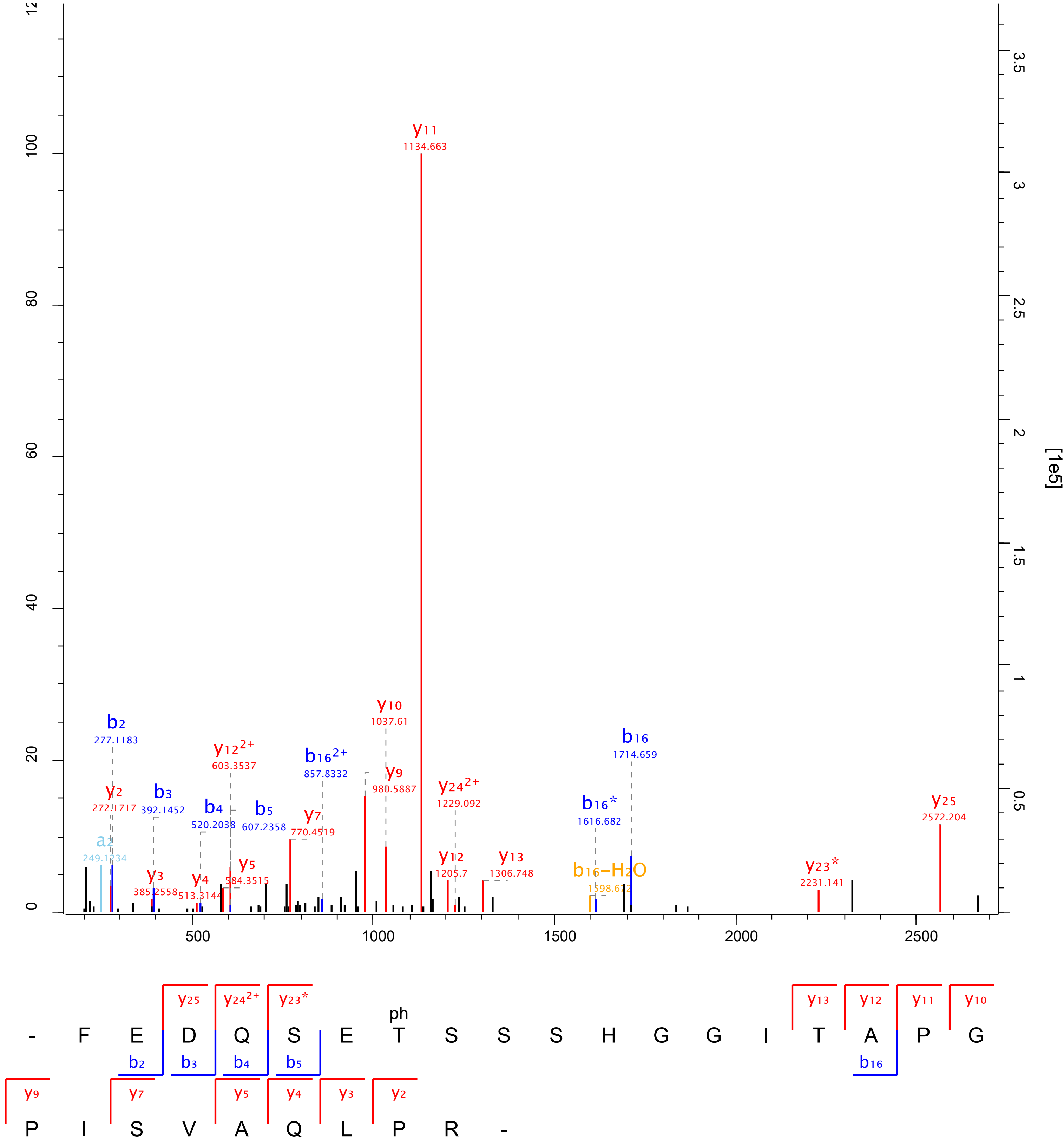

Sample output to test PDF Combine only

Raw file	Scan	Method	Score	m/z	Gene names
0523_11	17438	FTMS; HCD	152.07	777.04	P5CS2;P5CSB

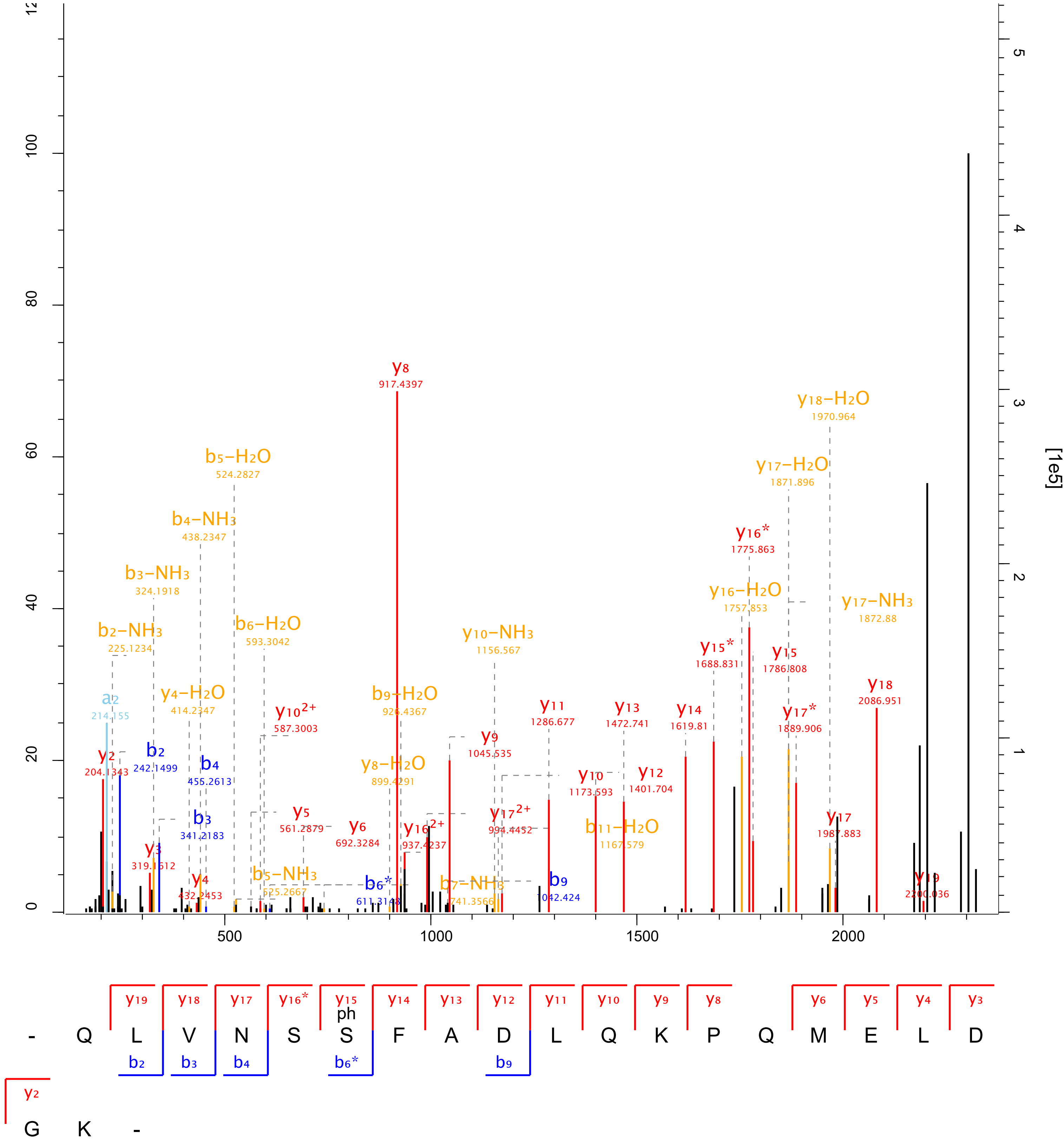

Sample output to test PDF Combine only

Raw file	Scan	Method	Score	m/z	Gene names
0523_11	17528	FTMS; HCD	45.75	954.42	SCAMP1

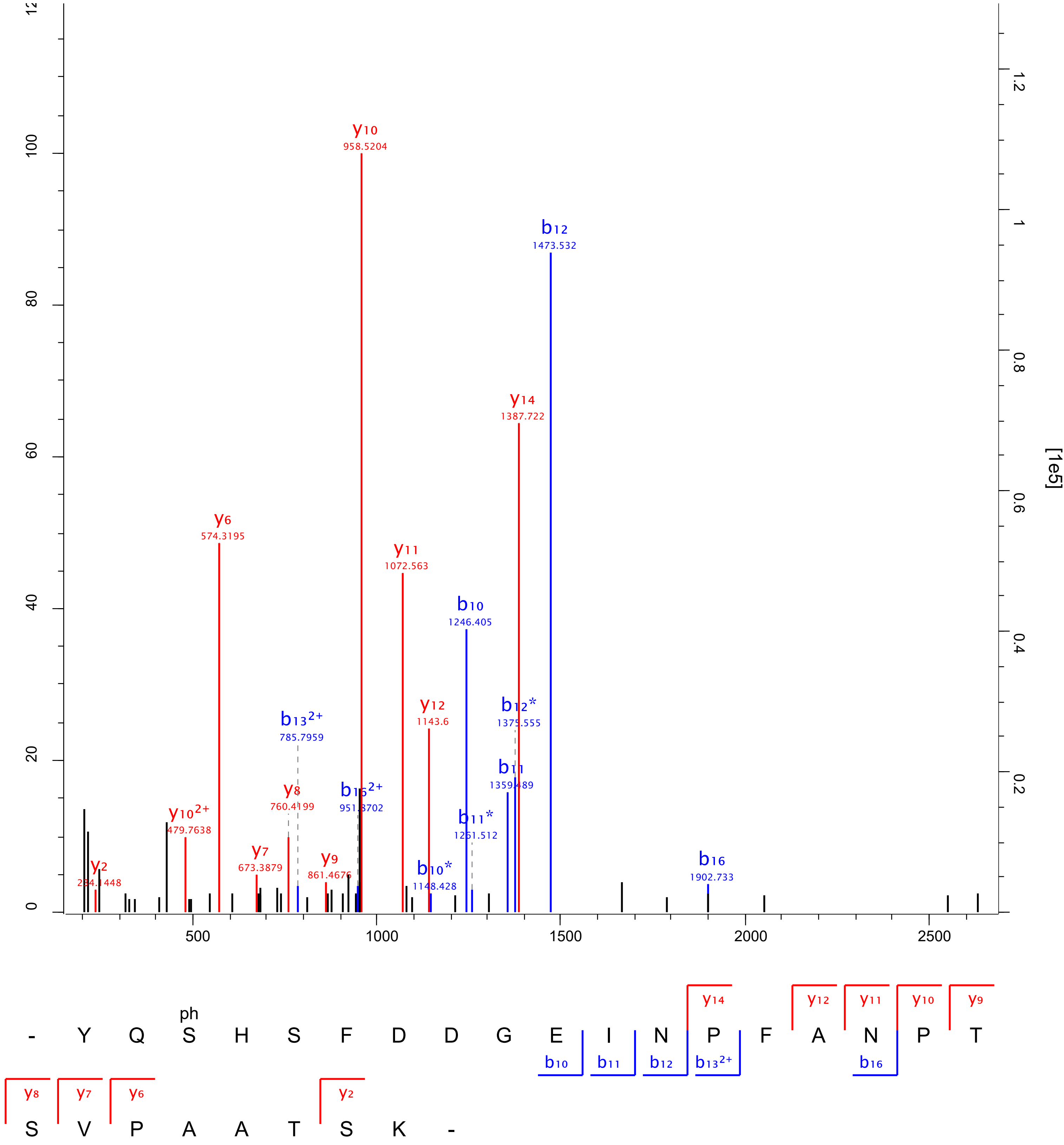

Sample output to test PDF Combine only

Raw file	Scan	Method	Score	m/z
0523_11	17715	FTMS; HCD	47.75	676.96

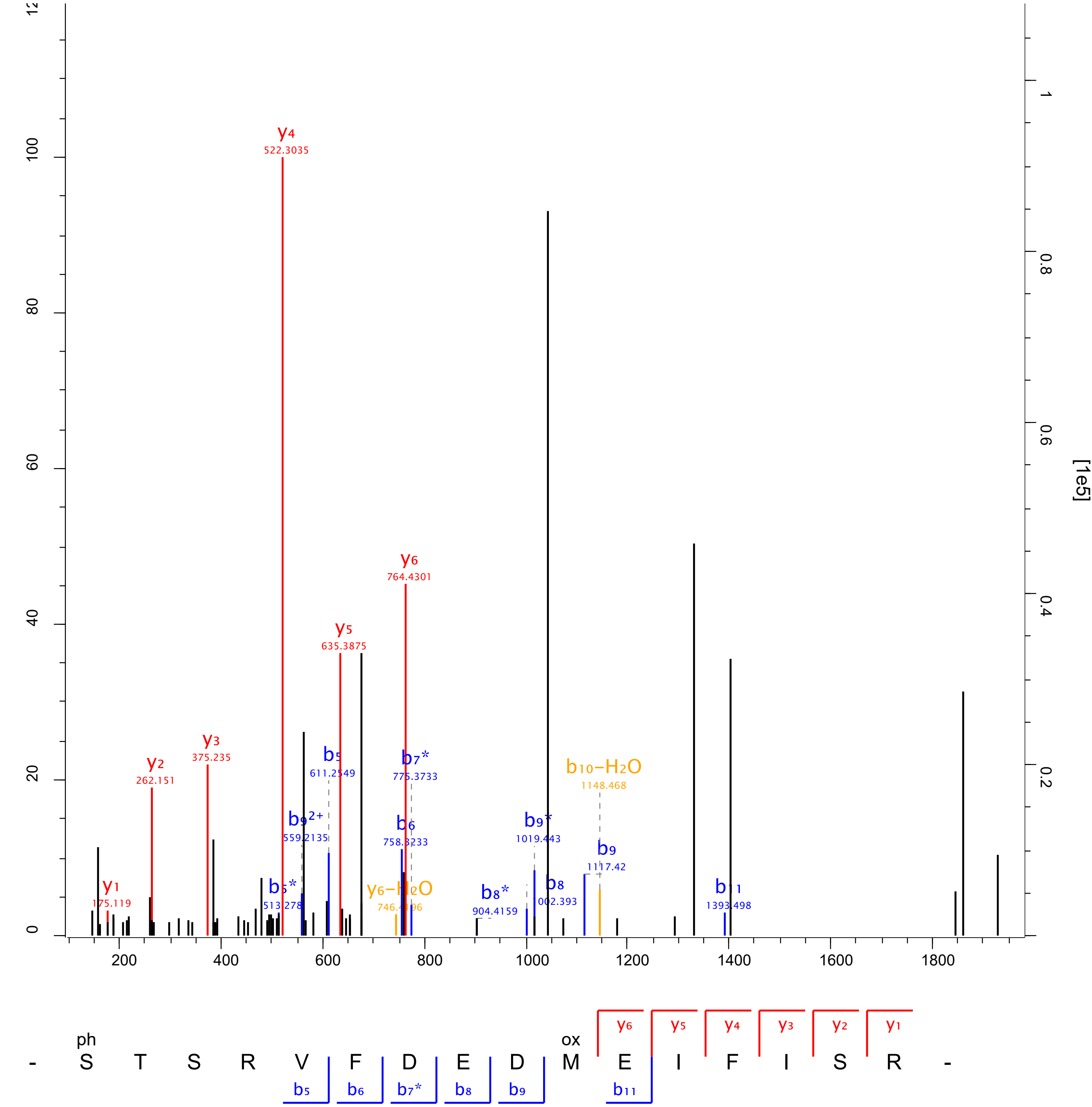

Sample output to test PDF Combine only

Raw file	Scan	Method	Score	m/z	Gene names
0523_11	17791	FTMS; HCD	93.5	936.79	T24D18.26

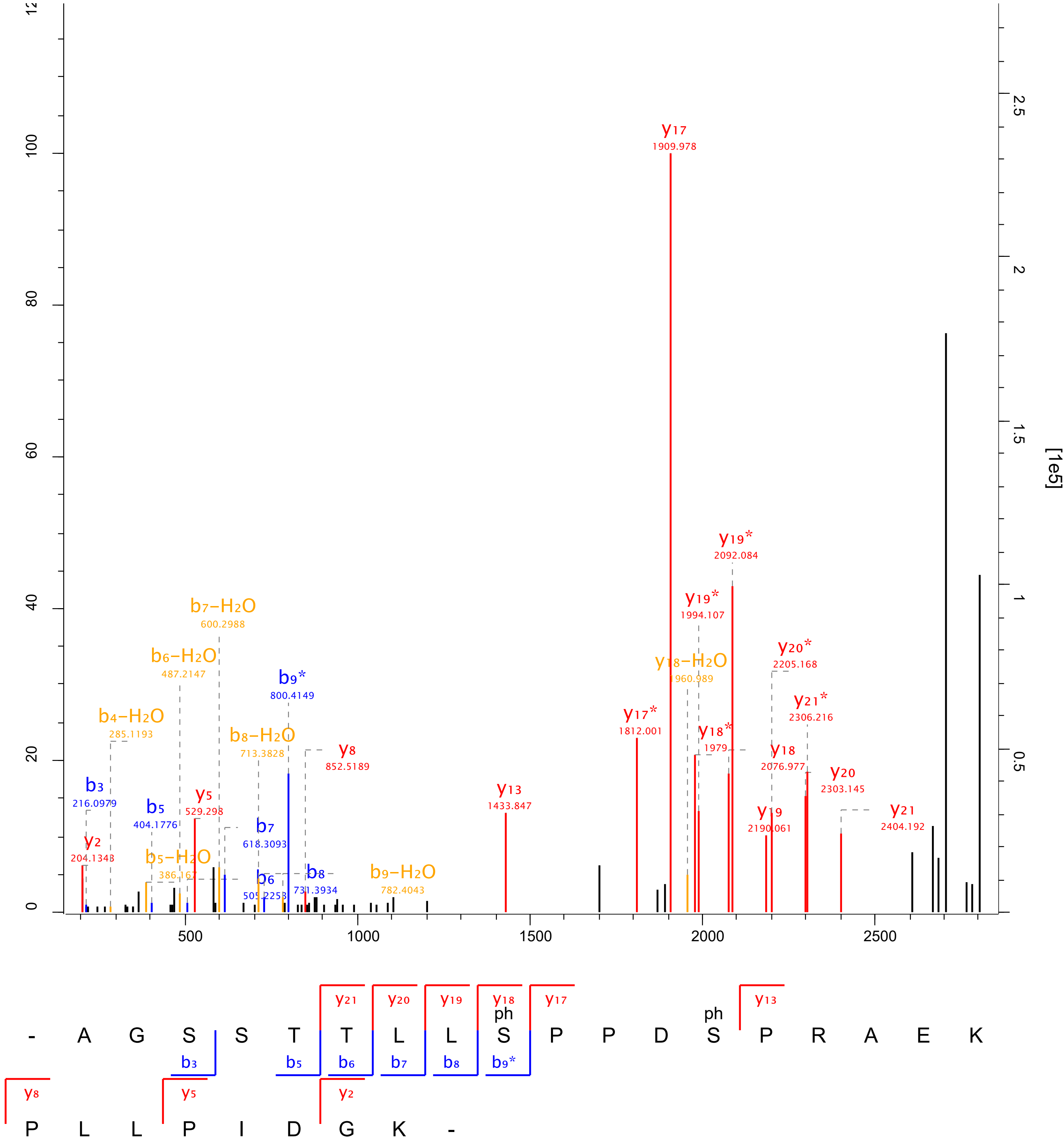

Sample output to test PDF Combine only

Raw file	Scan	Method	Score	m/z	Gene names
0523_11	18365	FTMS; HCD	179.5	764.71	NIP2-1

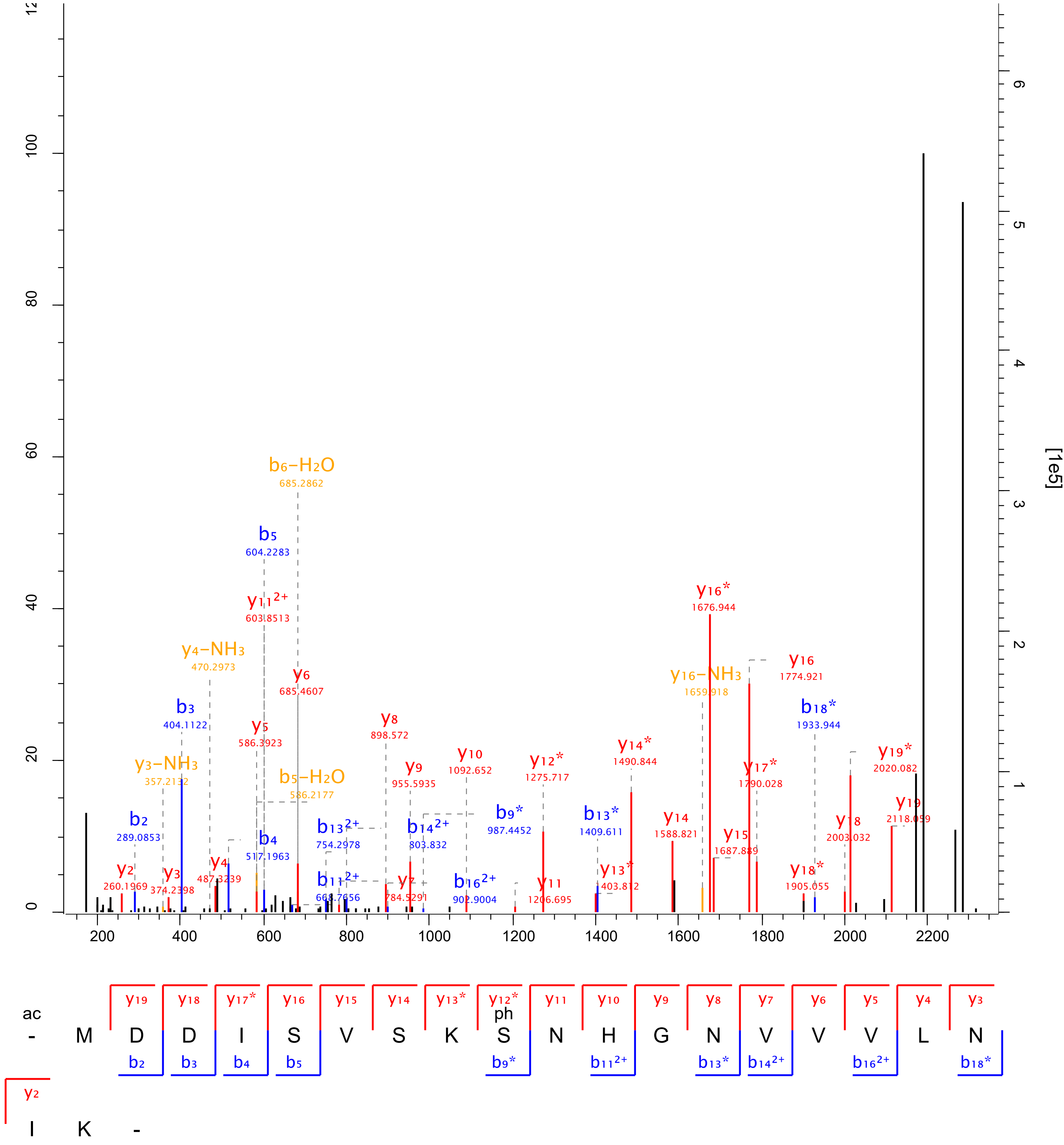

Sample output to test PDF Combine only

Raw file	Scan	Method	Score	m/z	Gene names
0523_11	18621	FTMS; HCD	141.97	1036.47	TRS130

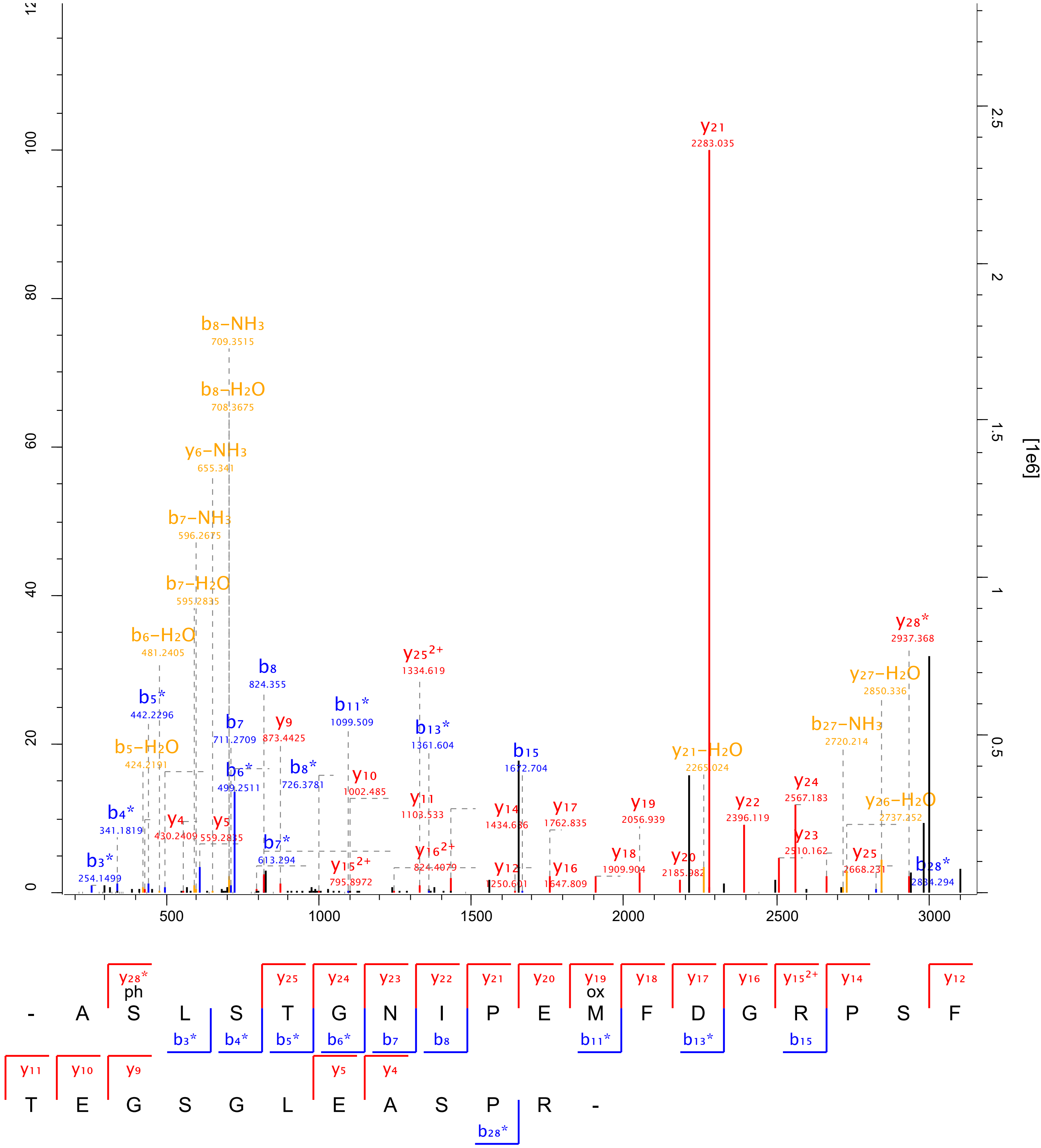

Sample output to test PDF Combine only

Raw file	Scan	Method	Score	m/z	Gene names
0523_11	18625	FTMS; HCD	98	991.07	XCT

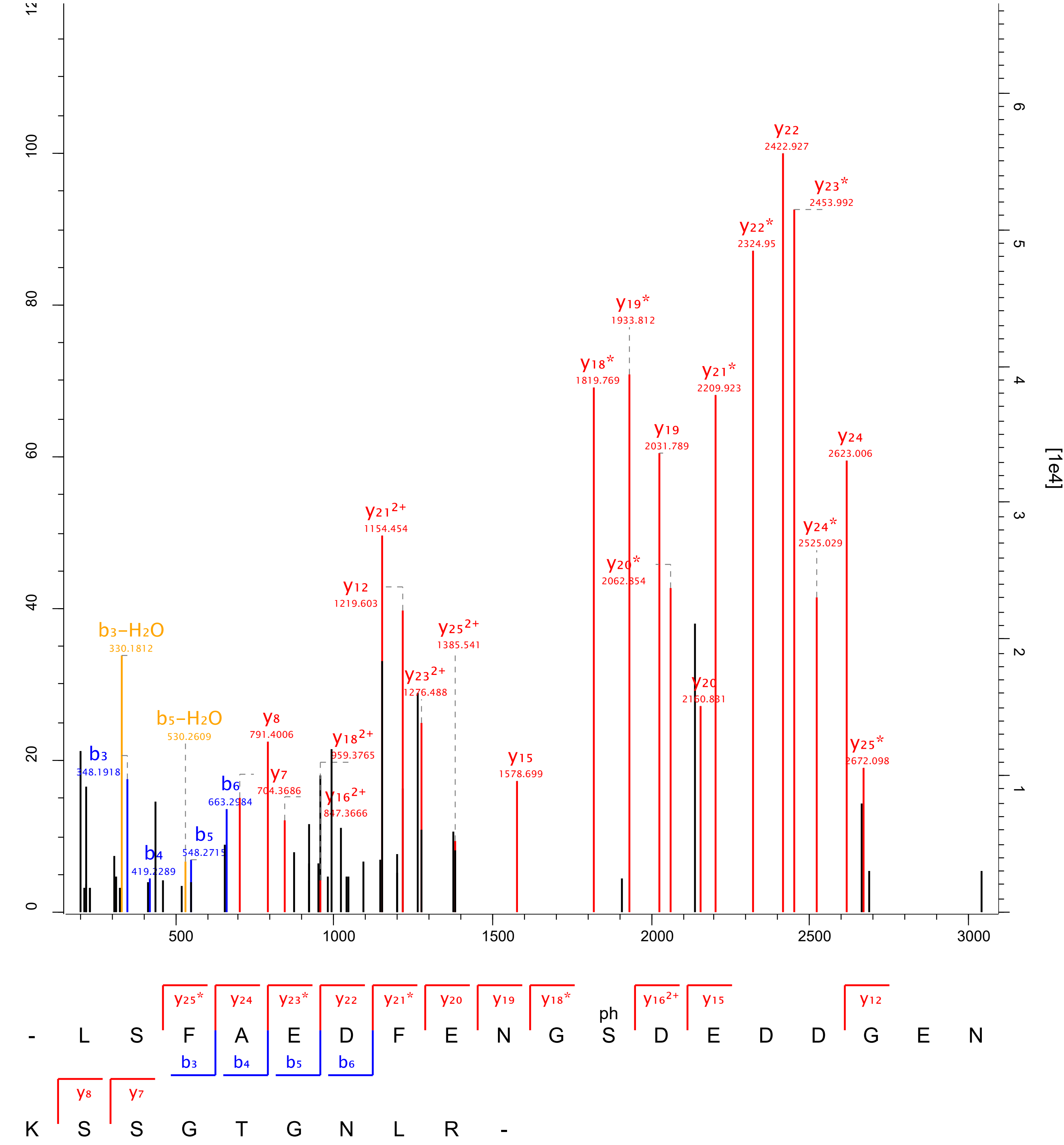

Sample output to test PDF Combine only

Raw file	Scan	Method	Score	m/z	Gene names
0523_11	18652	FTMS; HCD	173.95	894.76	PCKA

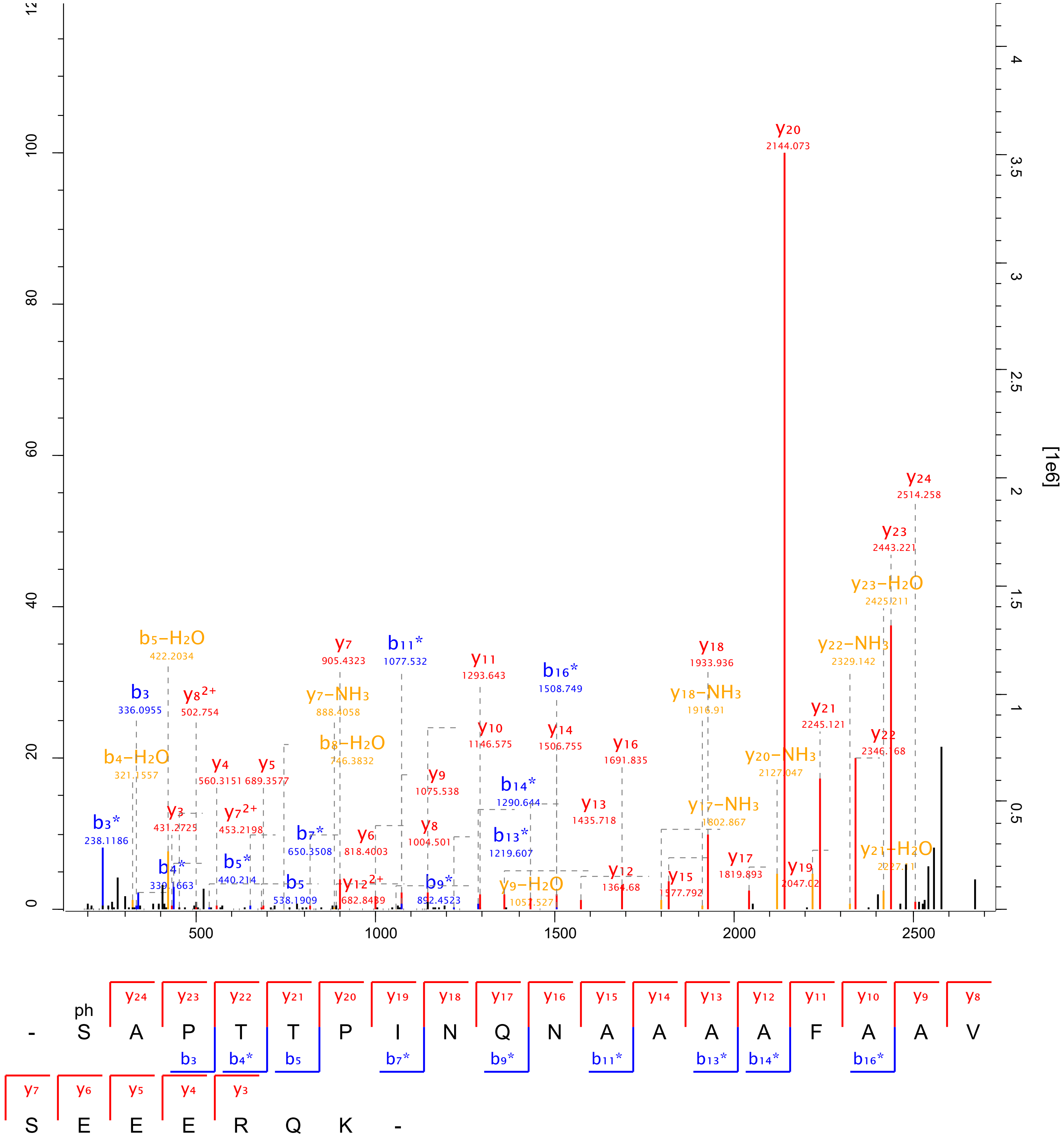

Sample output to test PDF Combine only

Raw file	Scan	Method	Score	m/z
0523_11	19015	FTMS; HCD	89.14	947.47

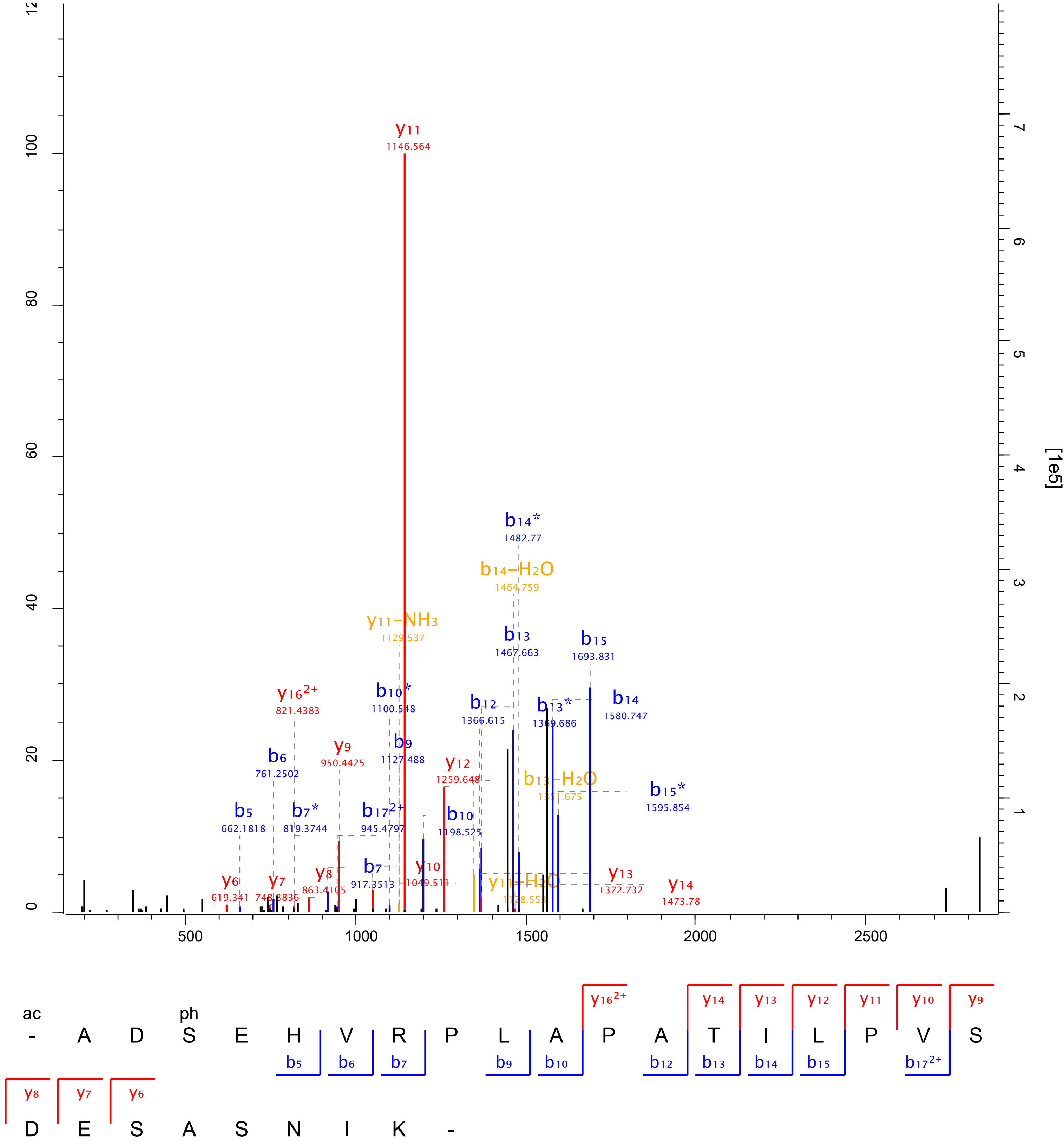

Sample output to test PDF Combine only

Raw file	Scan	Method	Score	m/z	Gene names
0523_11	19241	FTMS; HCD	117.8	852.86	ERD7

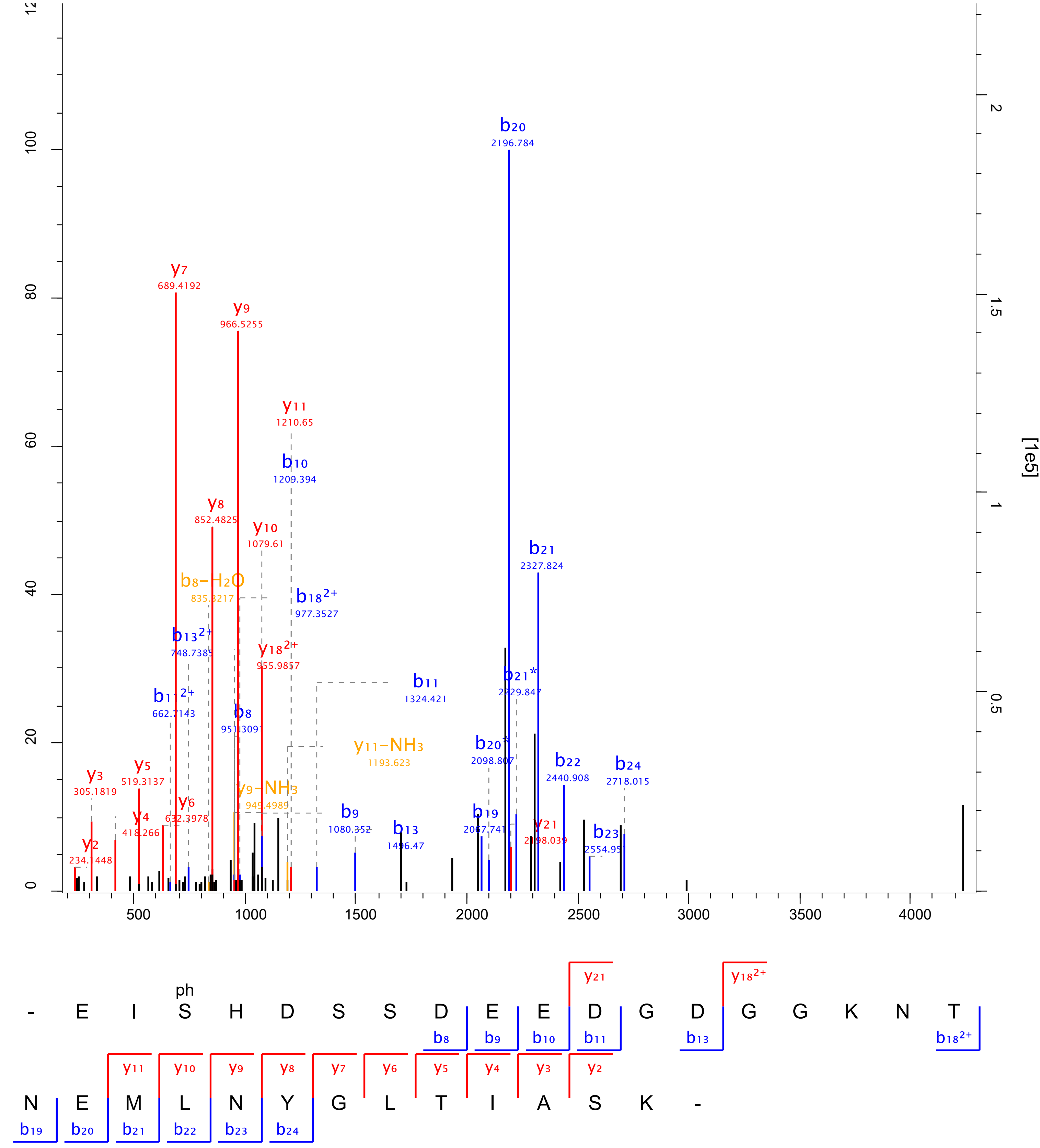

Sample output to test PDF Combine only

Raw file	Scan	Method	Score	m/z	Gene names
0523_11	19577	FTMS; HCD	97.5	647.27	VHA-D

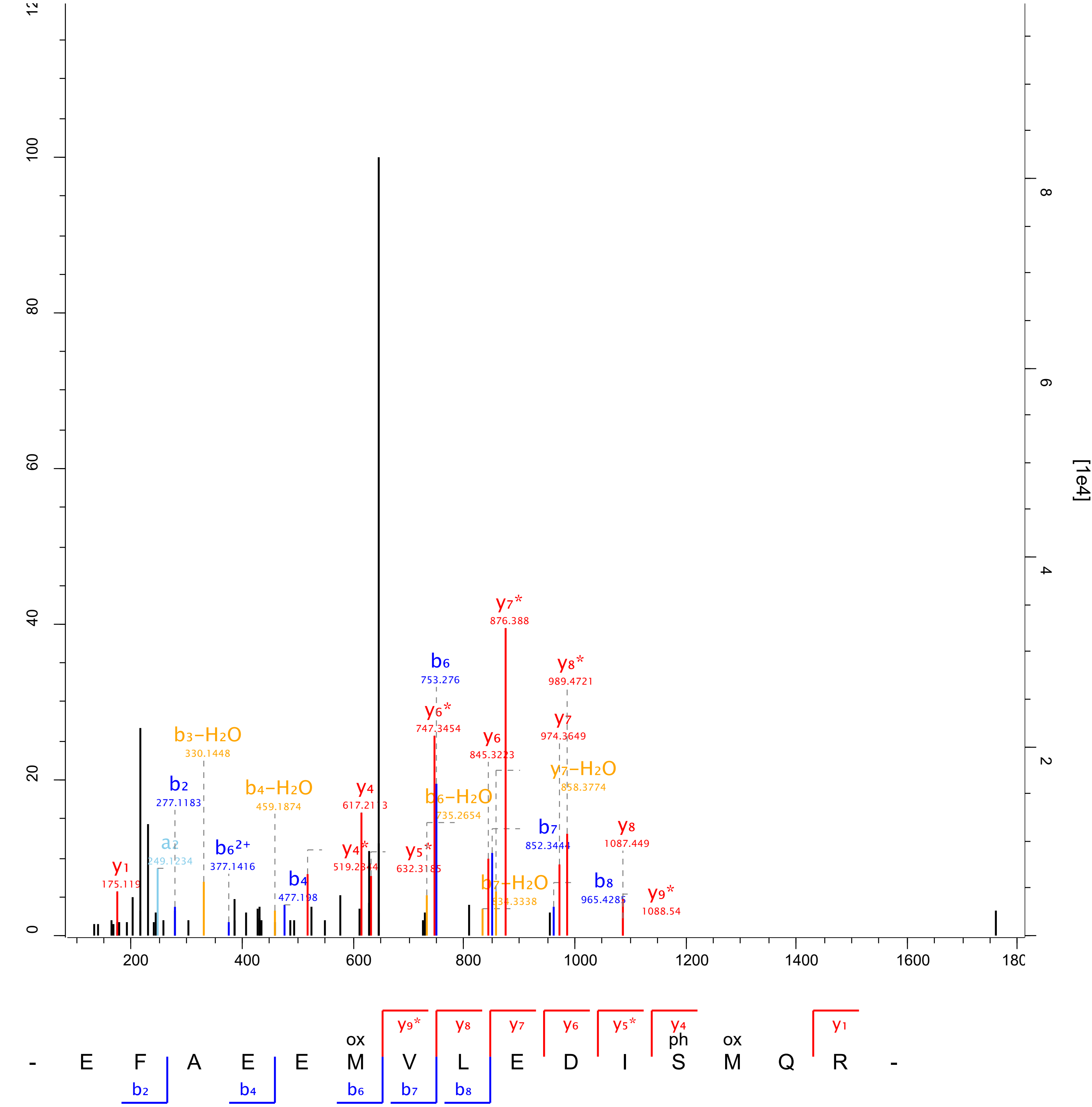

Sample output to test PDF Combine only

Raw file	Scan	Method	Score	m/z	Gene names
0523_11	19583	FTMS; HCD	43.1	923.38	At4g18950

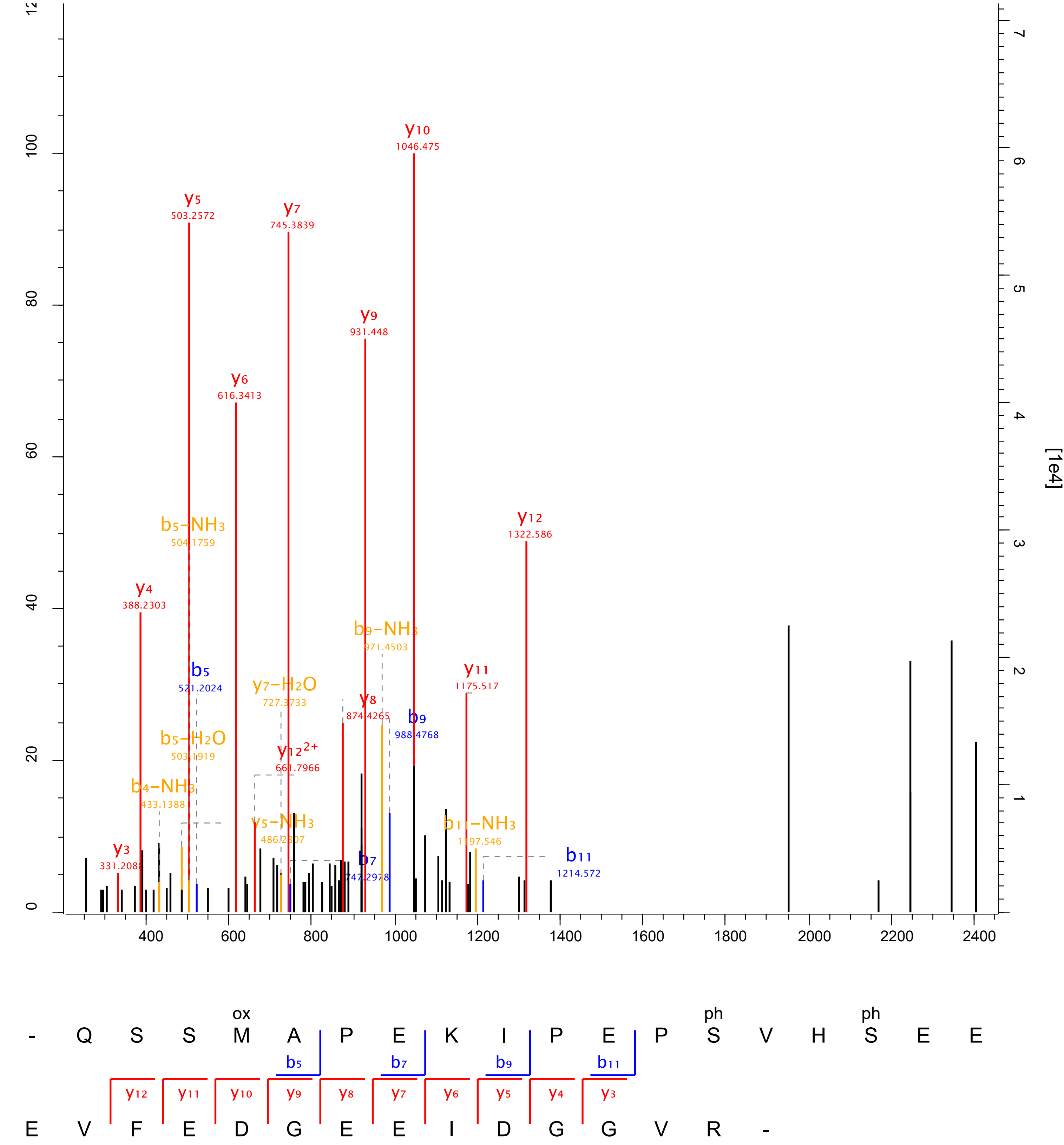

Sample output to test PDF Combine only

Raw file	Scan	Method	Score	m/z
0523_11	19618	FTMS; HCD	46.46	1137.03

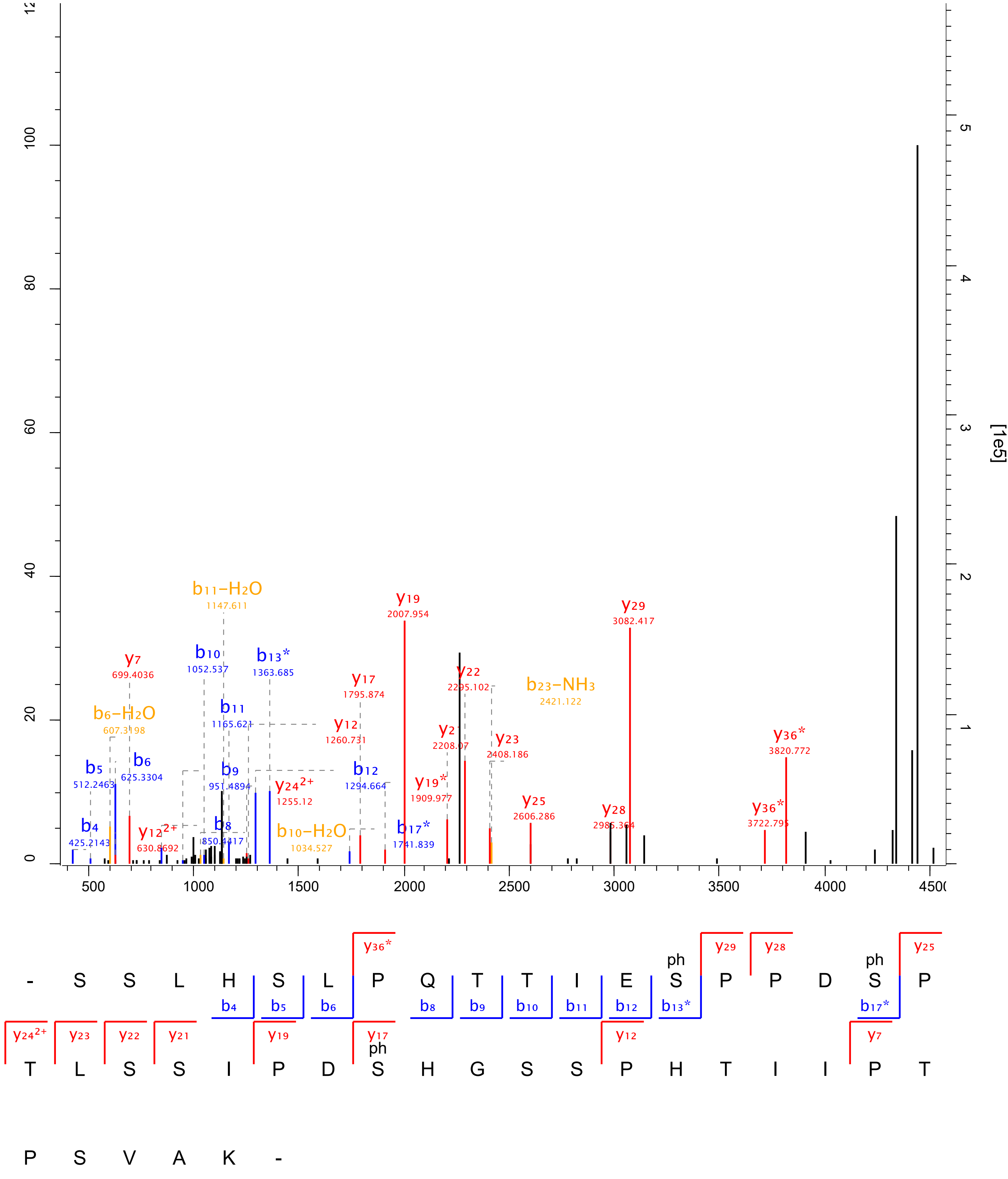

Sample output to test PDF Combine only

Raw file	Scan	Method	Score	m/z	Gene names
0523_11	19619	FTMS; HCD	49.27	965.12	HSBP

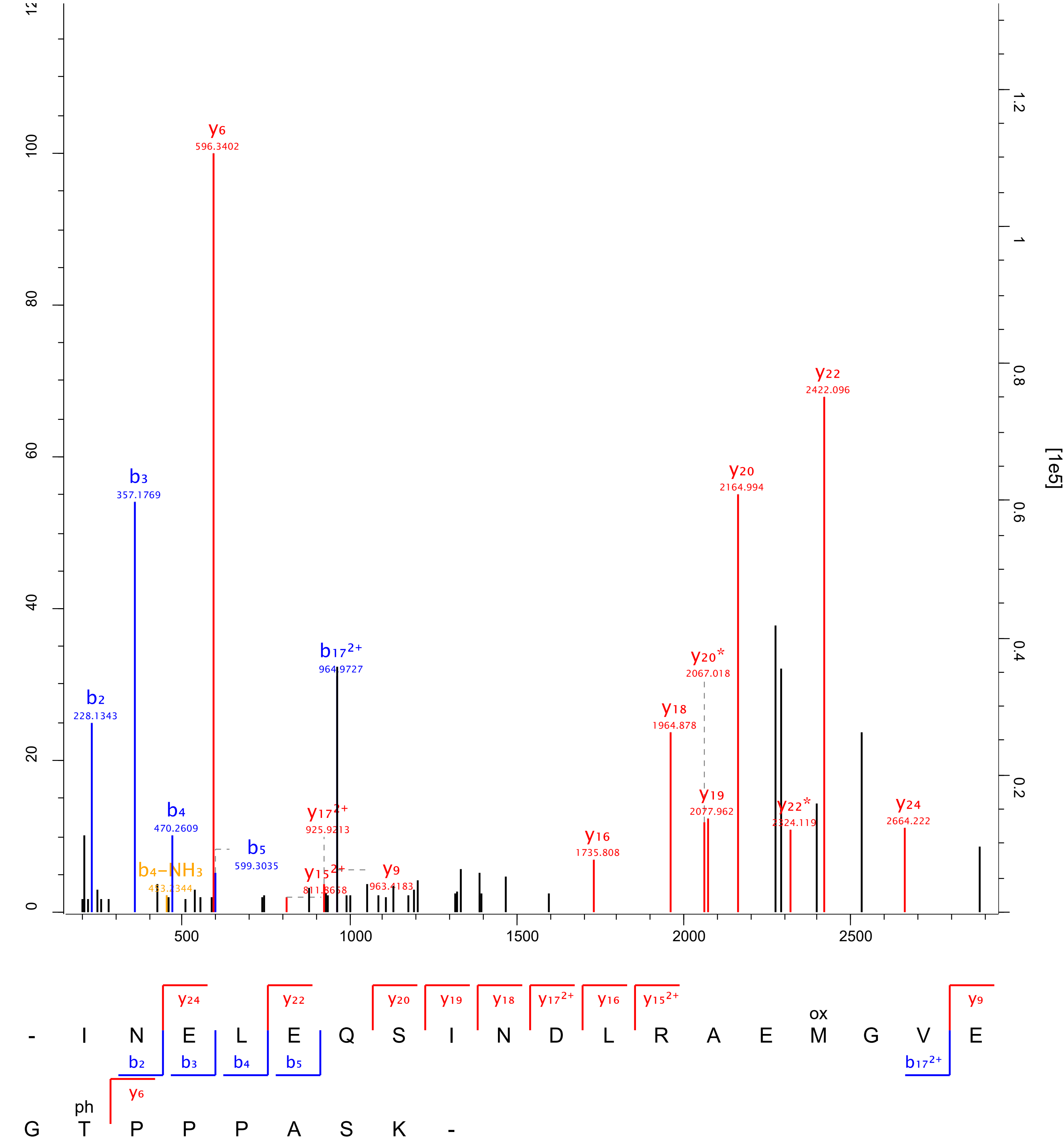

Sample output to test PDF Combine only

Raw file	Scan	Method	Score	m/z	Gene names
0523_11	19622	FTMS; HCD	128.89	957.2	F20M13.200

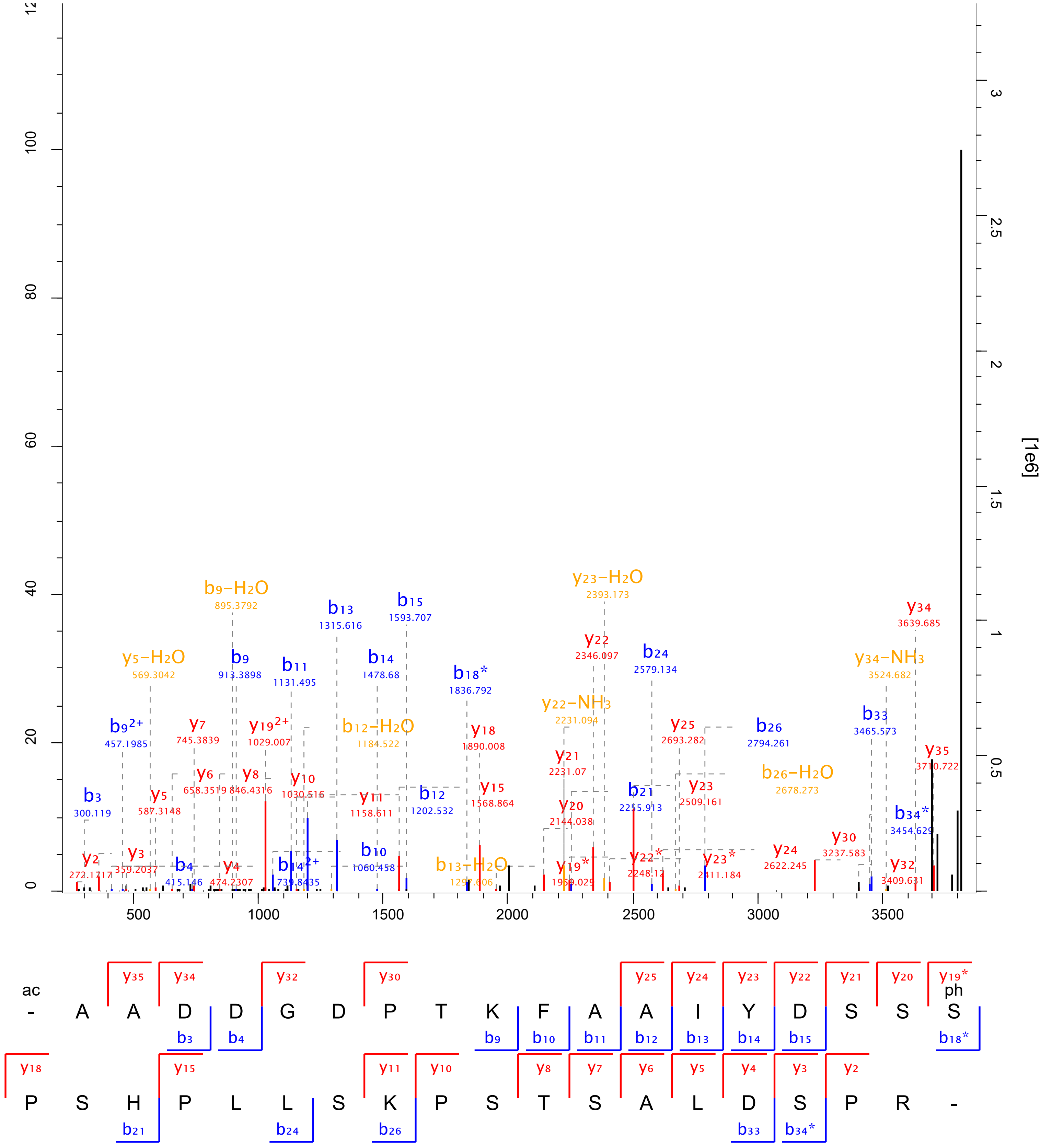

Sample output to test PDF Combine only

Raw file	Scan	Method	Score	m/z	Gene names
0523_11	19966	FTMS; HCD	59.04	722.35	UGT80A2

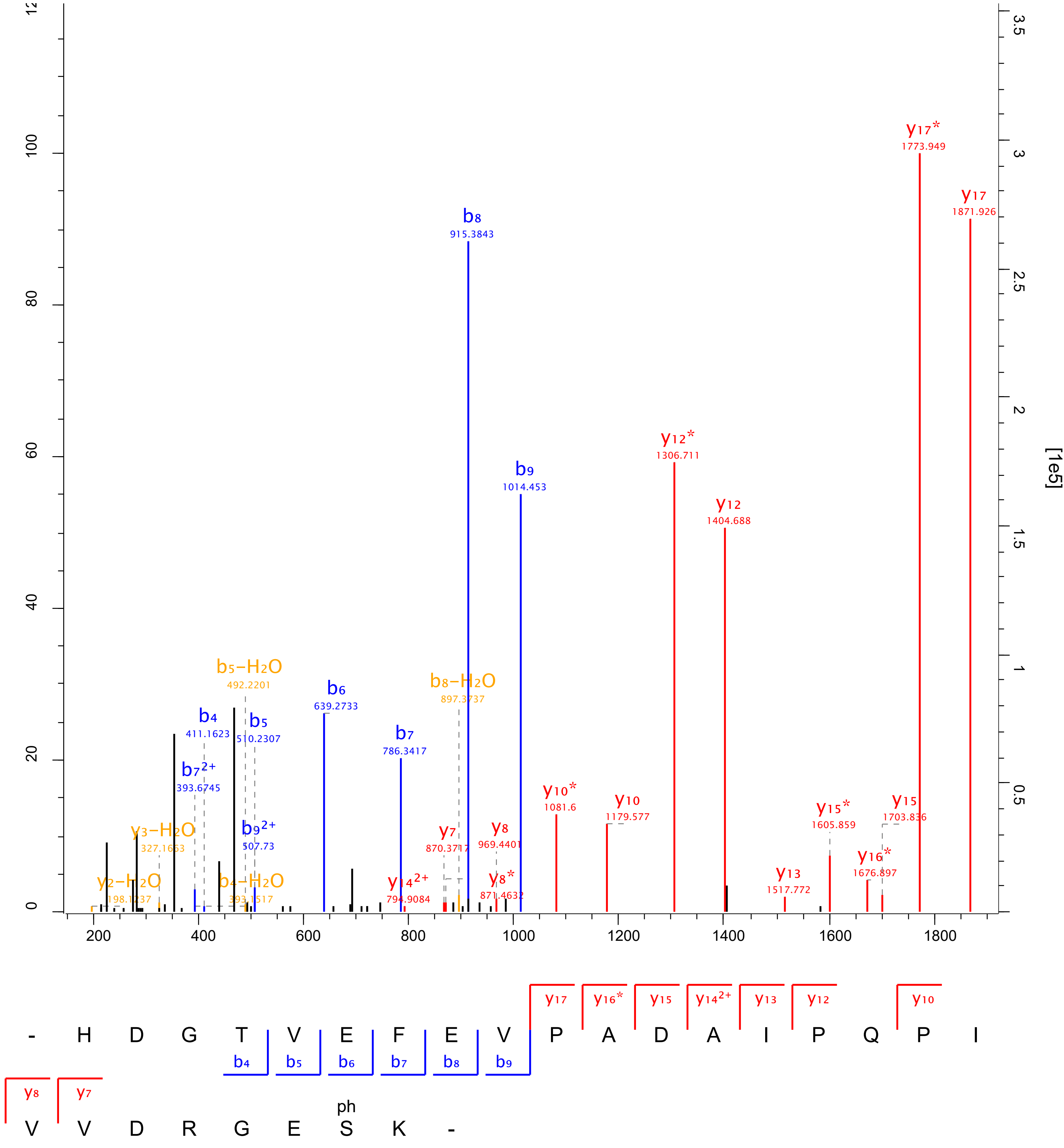

Sample output to test PDF Combine only

Raw file	Scan	Method	Score	m/z	Gene names
0523_11	19987	FTMS; HCD	86.56	717.32	CML38

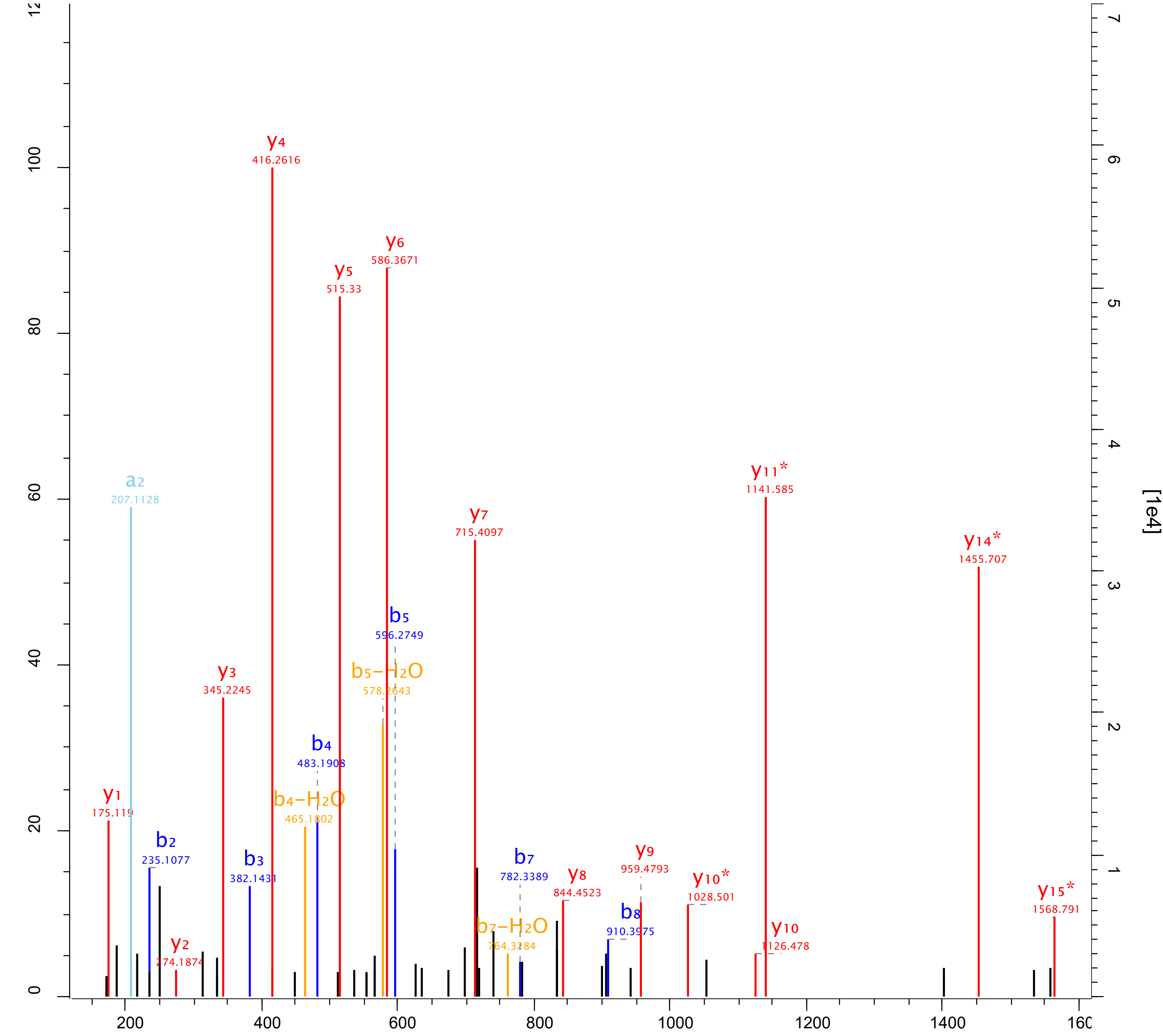

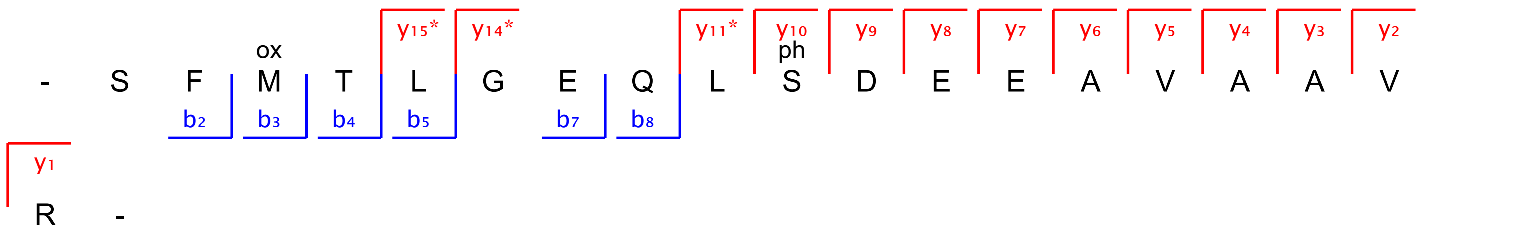

Sample output to test PDF Combine only

Raw file	Scan	Method	Score	m/z	Gene names
0523_11	20010	FTMS; HCD	45.04	806.7	SPK1

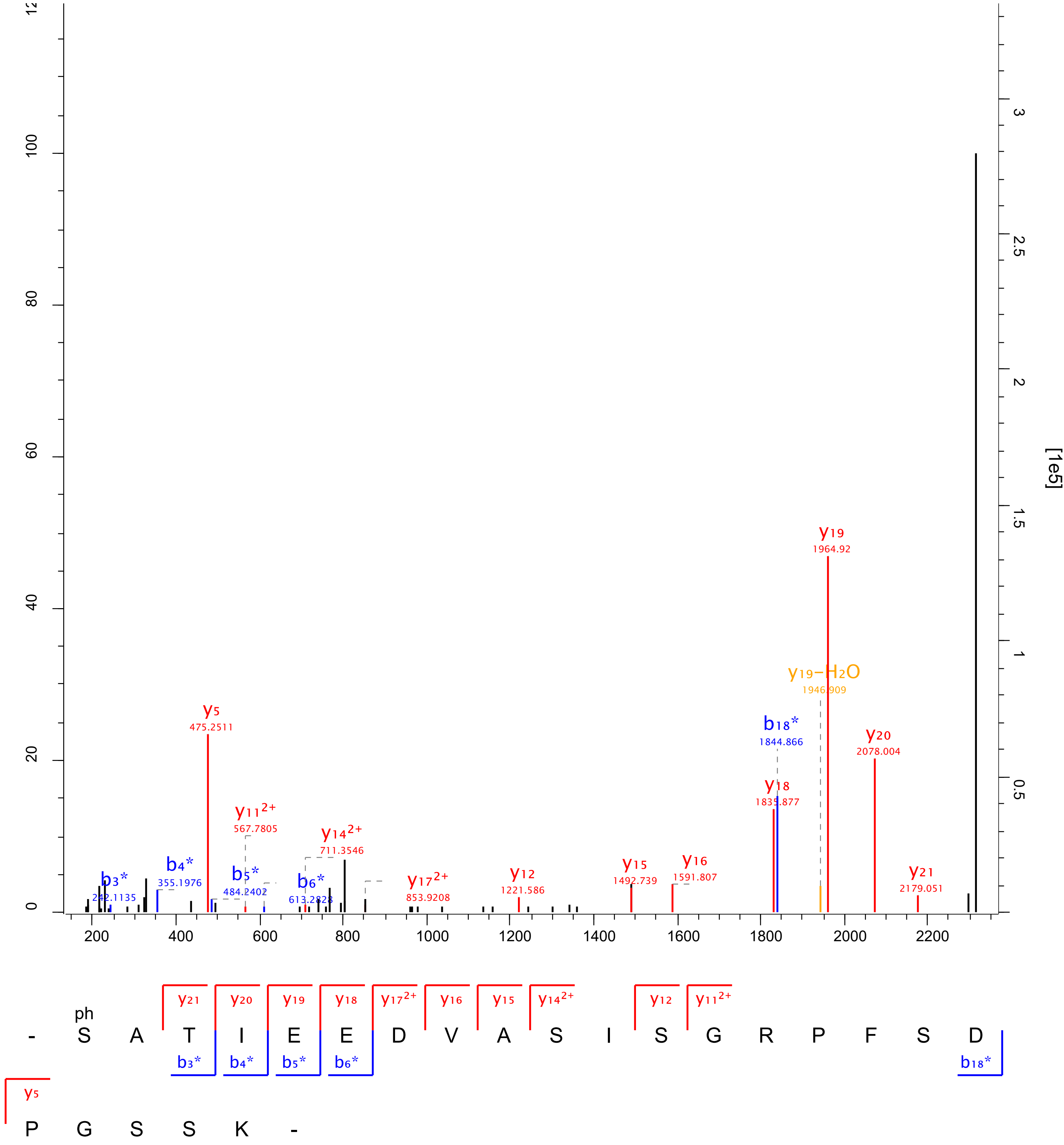

Sample output to test PDF Combine only

Raw file	Scan	Method	Score	m/z	Gene names
0523_11	20200	FTMS; HCD	131.95	768.38	CLC-C

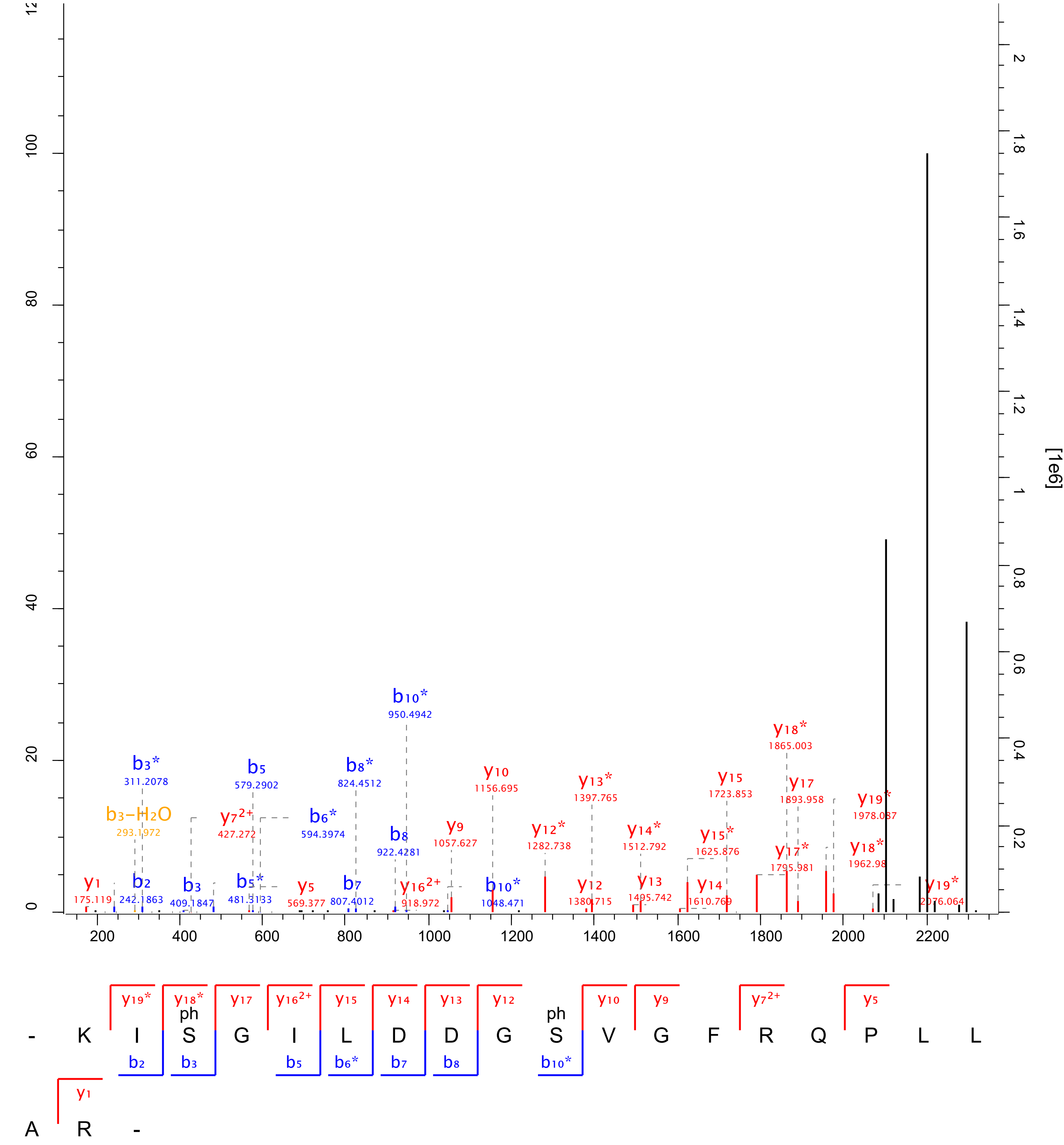

Sample output to test PDF Combine only

Raw file	Scan	Method	Score	m/z	Gene names
0523_11	20806	FTMS; HCD	66.29	967.1	ABCB21

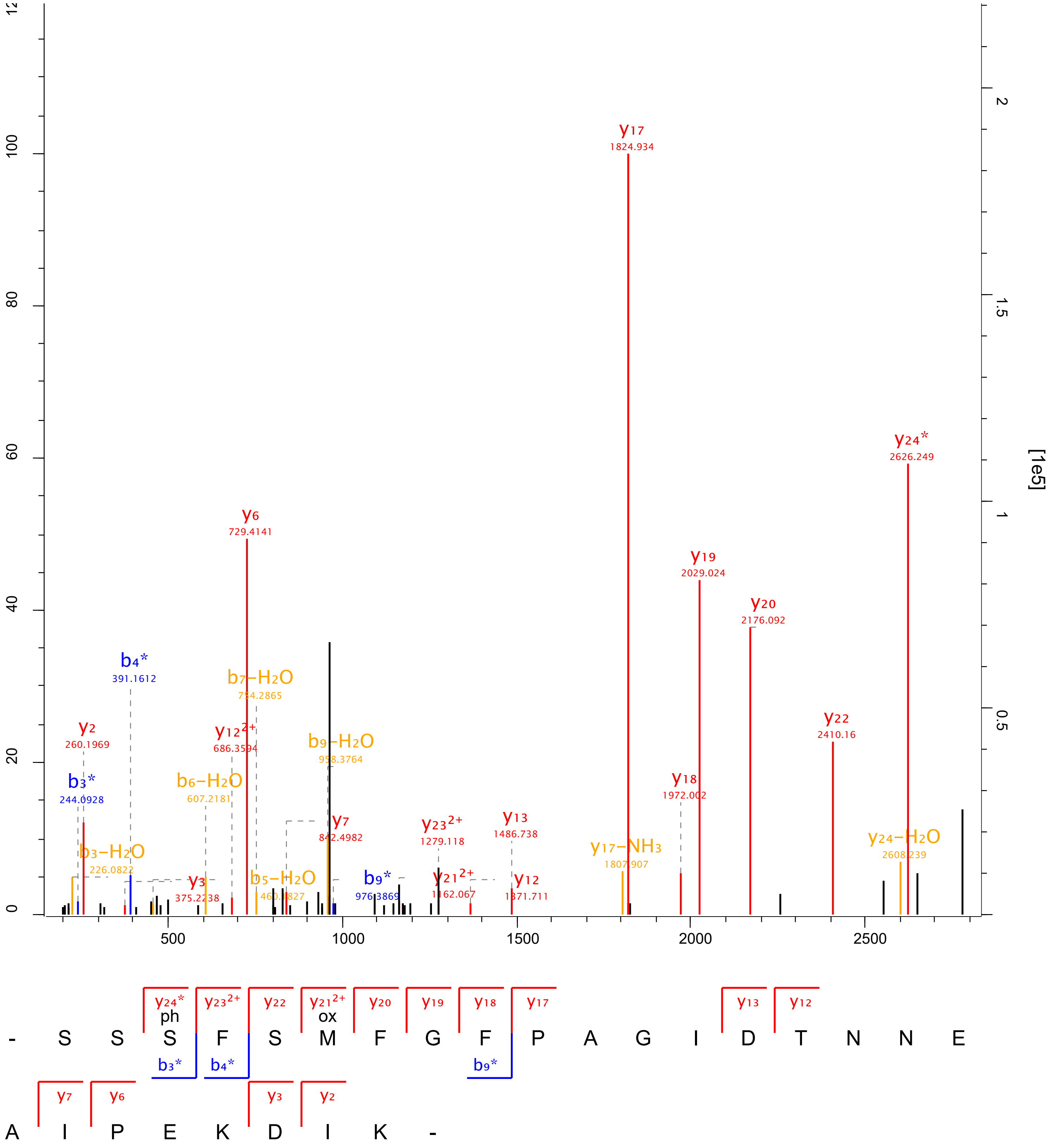

Sample output to test PDF Combine only

Raw file	Scan	Method	Score	m/z	Gene names
0523_11	21004	FTMS; HCD	42.32	921.41	PCKA

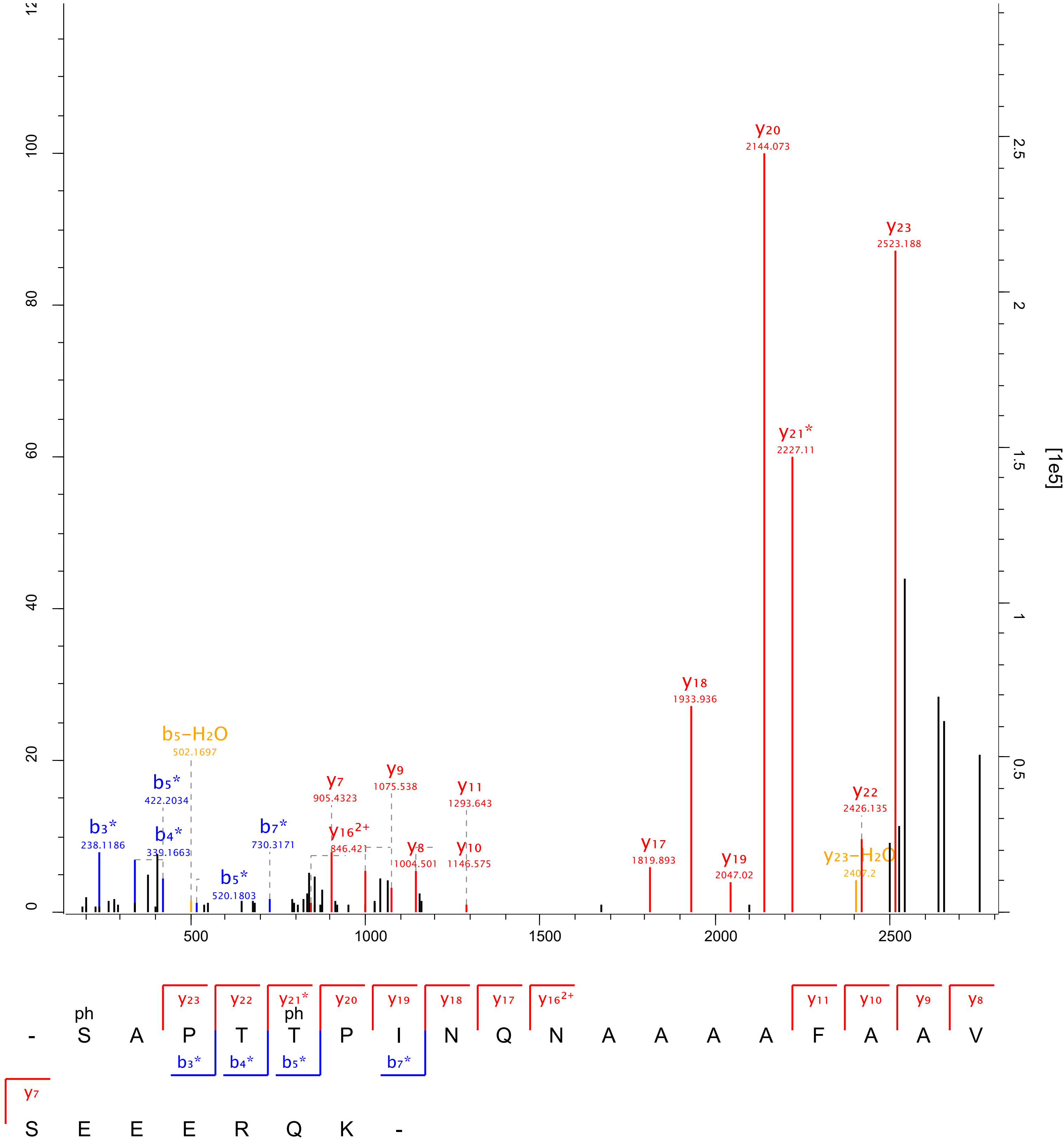

Sample output to test PDF Combine only

Raw file	Scan	Method	Score	m/z	Gene names
0523_11	21136	FTMS; HCD	68.66	925.41	SEC5A

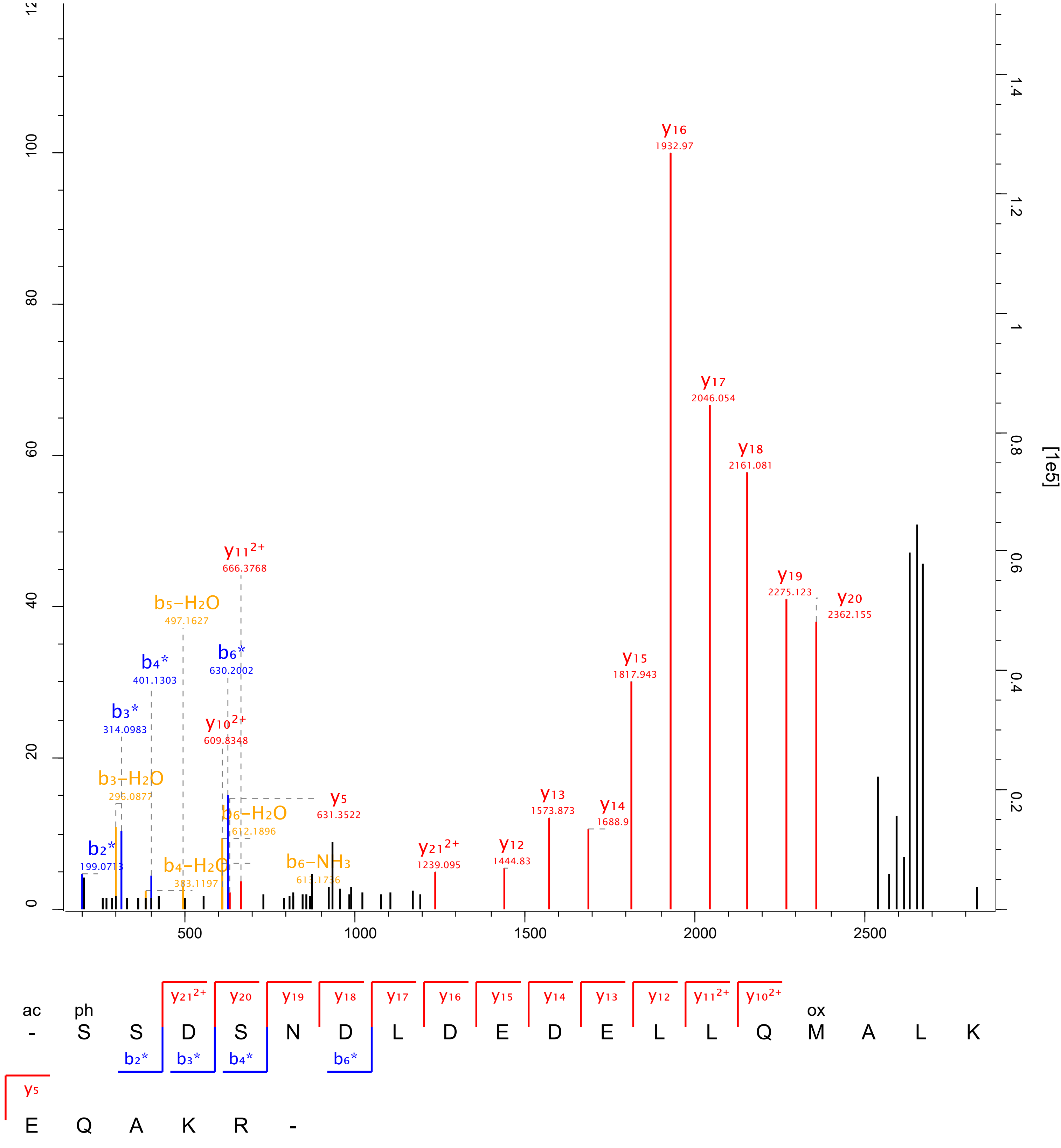

Sample output to test PDF Combine only

Raw file	Scan	Method	Score	m/z	Gene names
0523_11	21314	FTMS; HCD	99.45	1139.19	WIN2

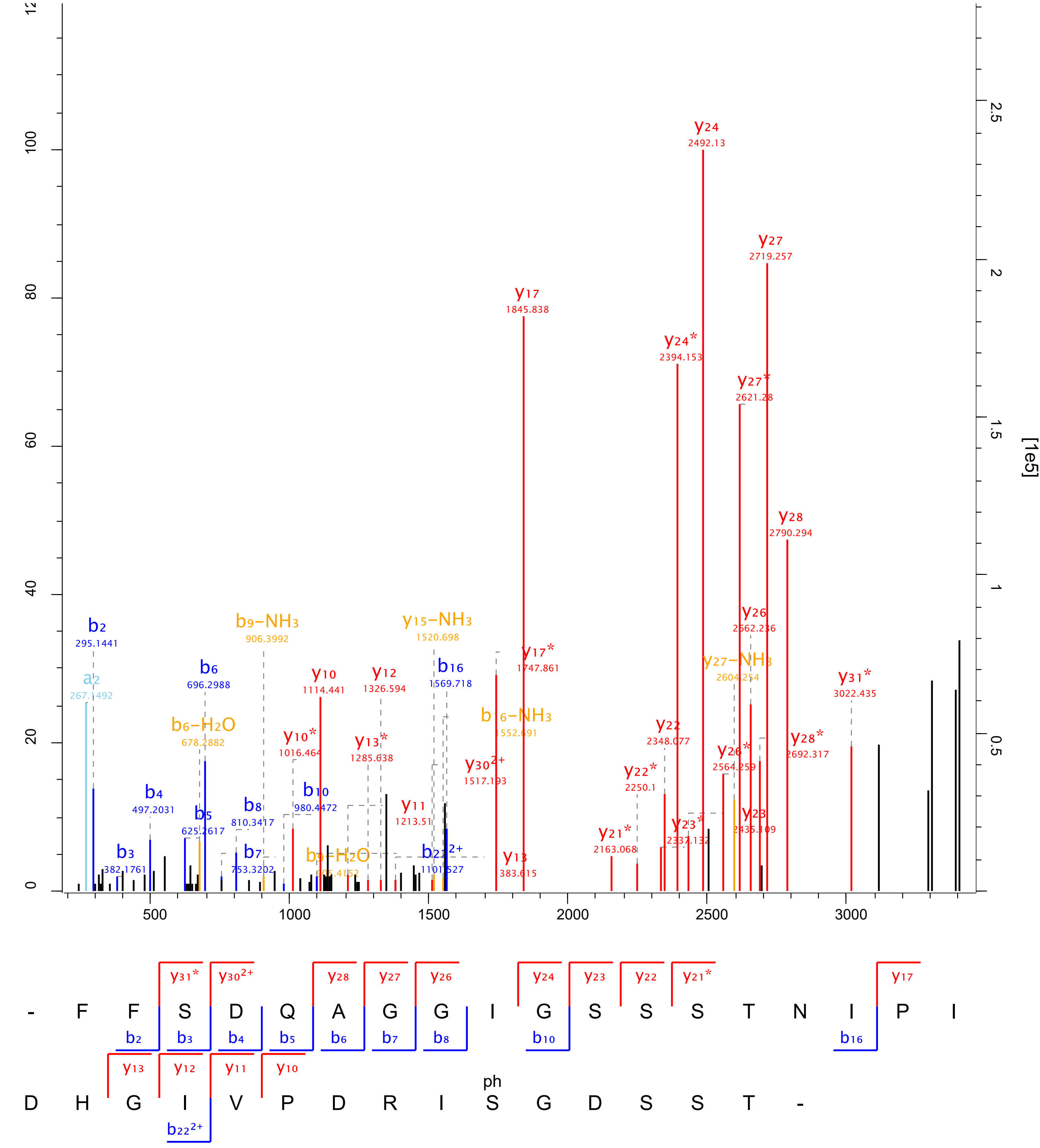

Sample output to test PDF Combine only

Raw file	Scan	Method	Score	m/z	Gene names
0523_11	21469	FTMS; HCD	118.36	995.45	NPC3

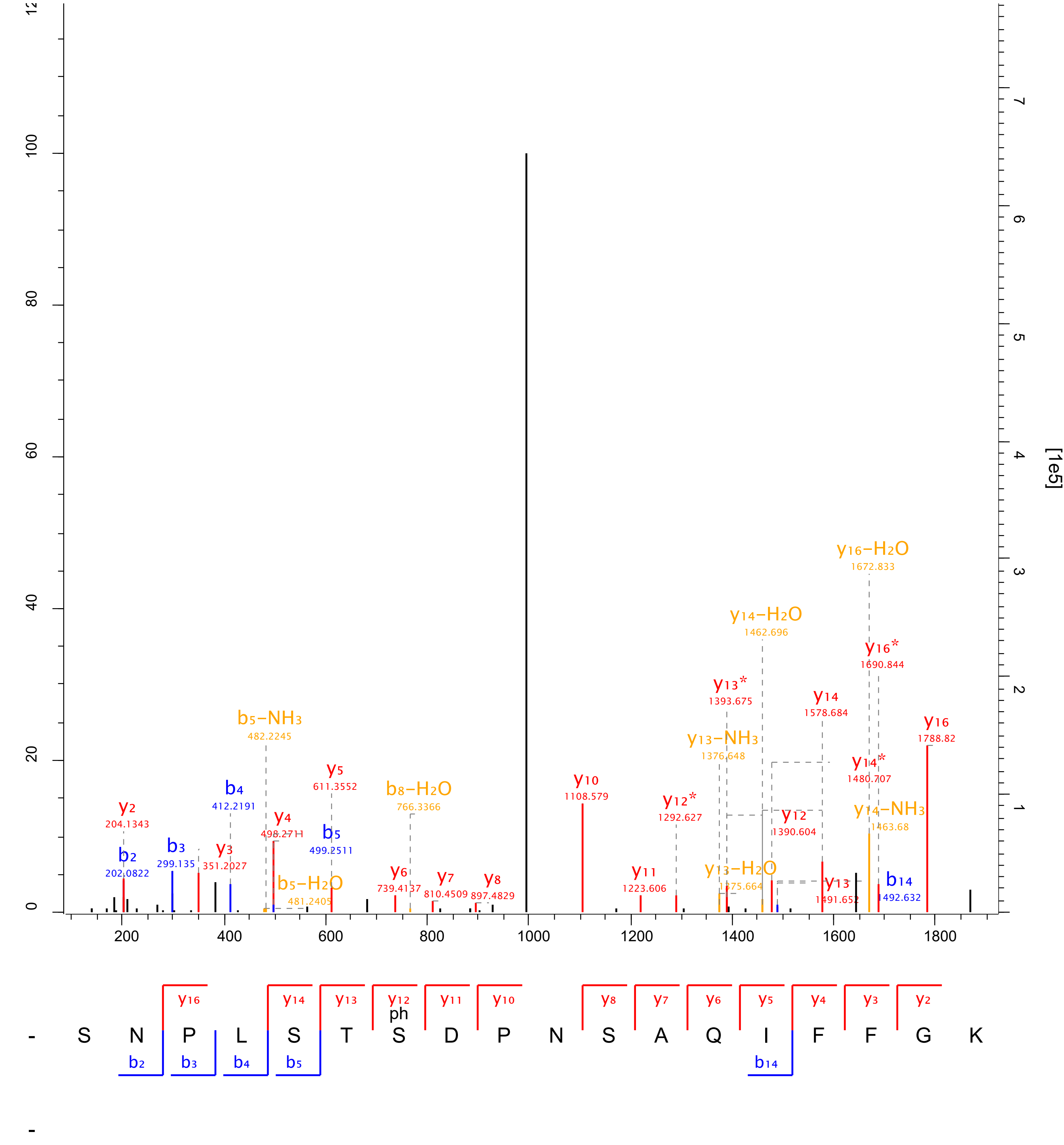

Sample output to test PDF Combine only

Raw file	Scan	Method	Score	m/z	Gene names
0523_11	21591	FTMS; HCD	103.05	925.09	RPL13D

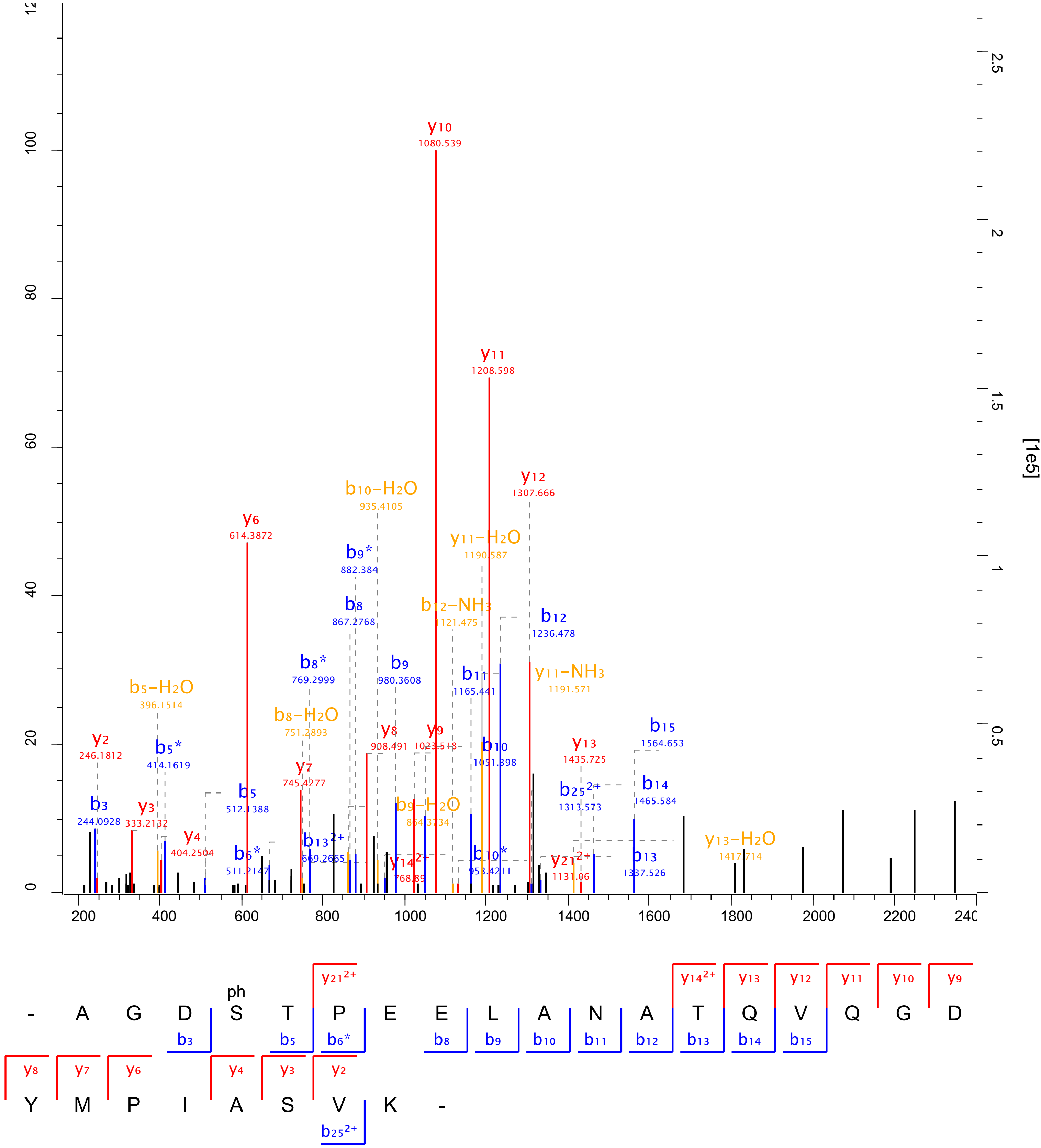

Sample output to test PDF Combine only

Raw file	Scan	Method	Score	m/z	Gene names
0523_11	21599	FTMS; HCD	113.43	809.71	PCKA

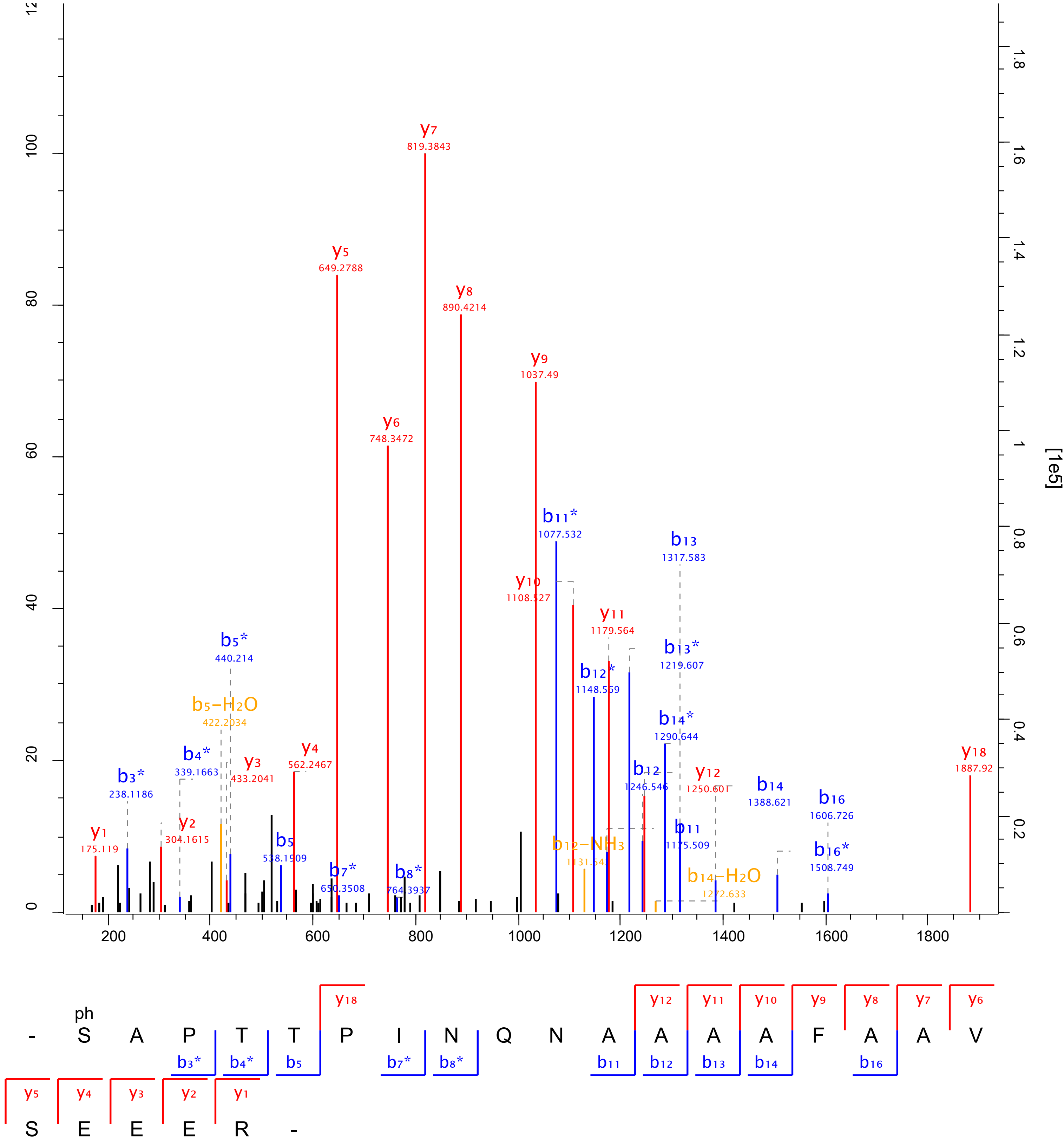

Sample output to test PDF Combine only

Raw file	Scan	Method	Score	m/z	Gene names
0523_11	21640	FTMS; HCD	63.43	1031.13	TRS130

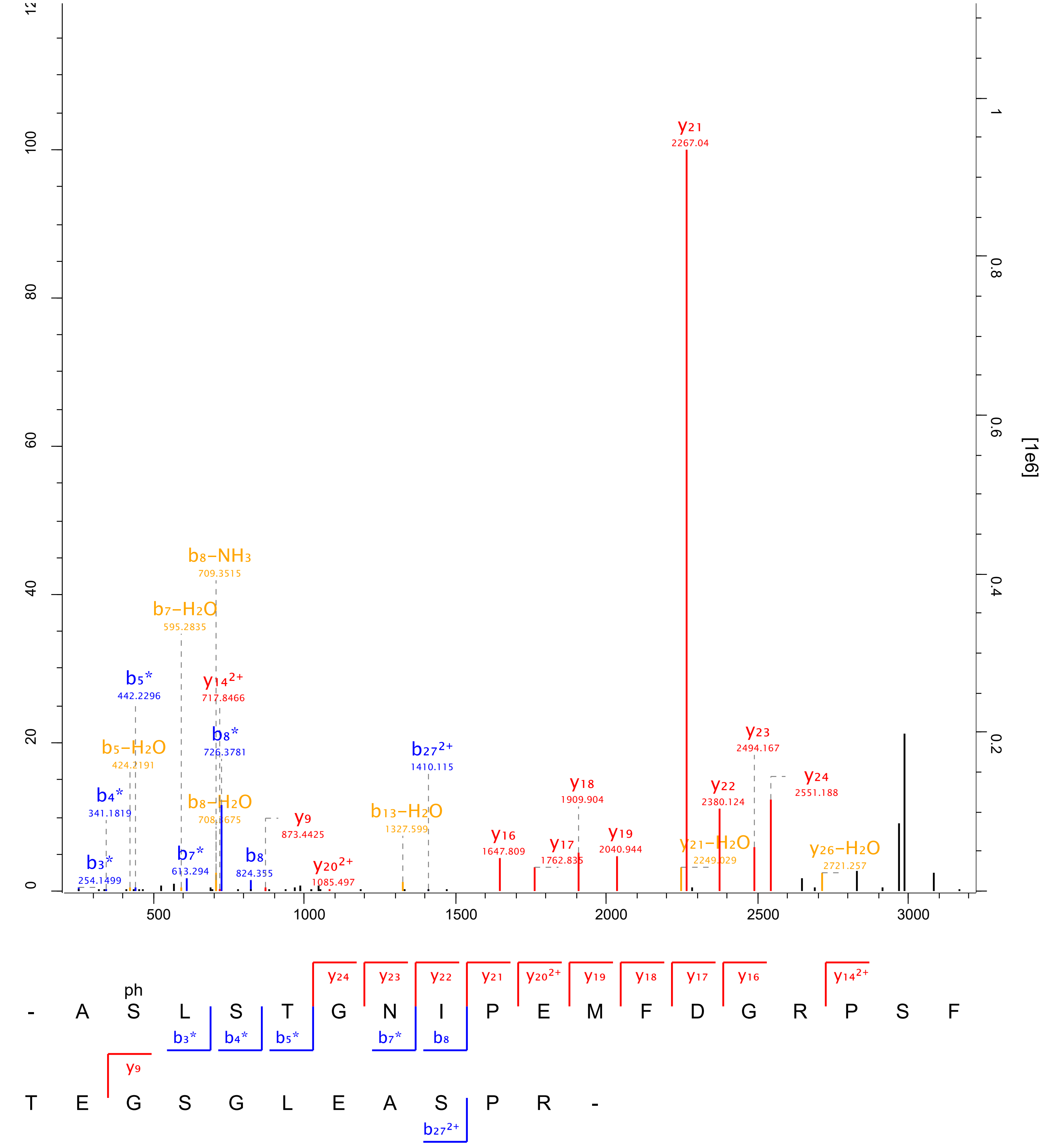

Sample output to test PDF Combine only

Raw file	Scan	Method	Score	m/z	Gene names
0523_11	21767	FTMS; HCD	65.23	698.34	At1g24300

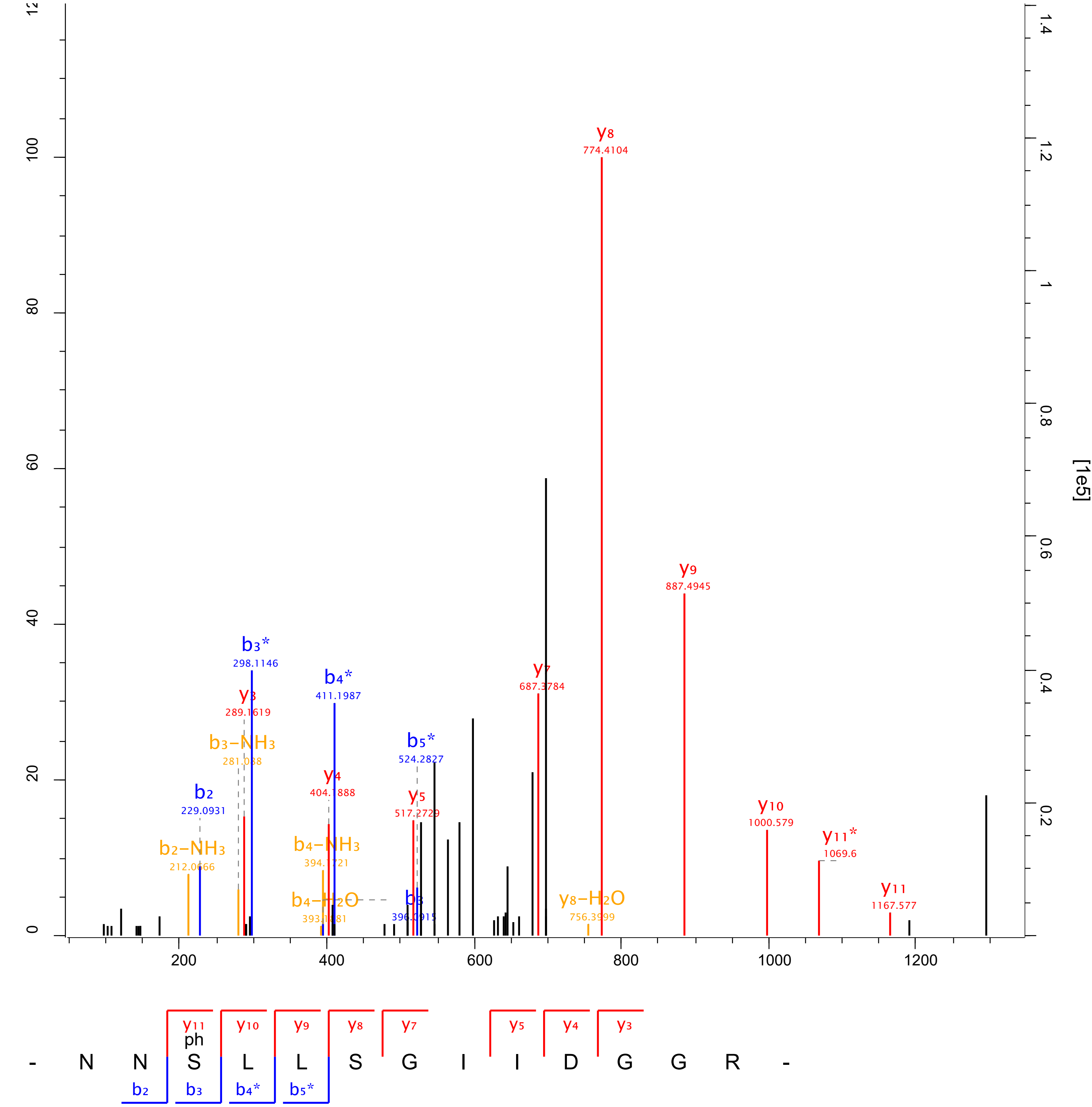

Sample output to test PDF Combine only

Raw file	Scan	Method	Score	m/z	Gene names
0523_11	21985	FTMS; HCD	107.59	916.45	At4g05150

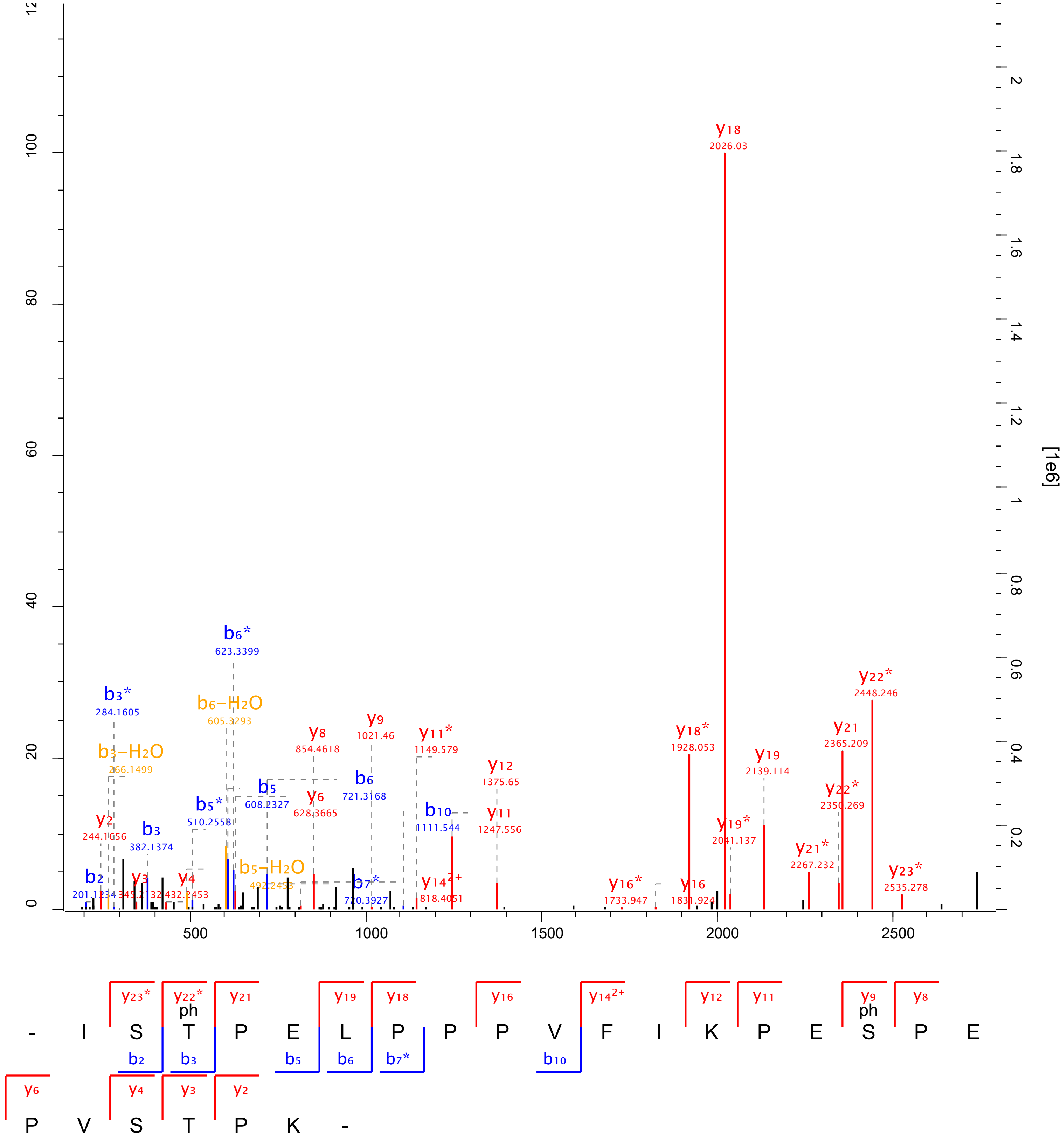

Sample output to test PDF Combine only

Raw file	Scan	Method	Score	m/z	Gene names
0523_11	22328	FTMS; HCD	308.57	1013.48	At5g63220

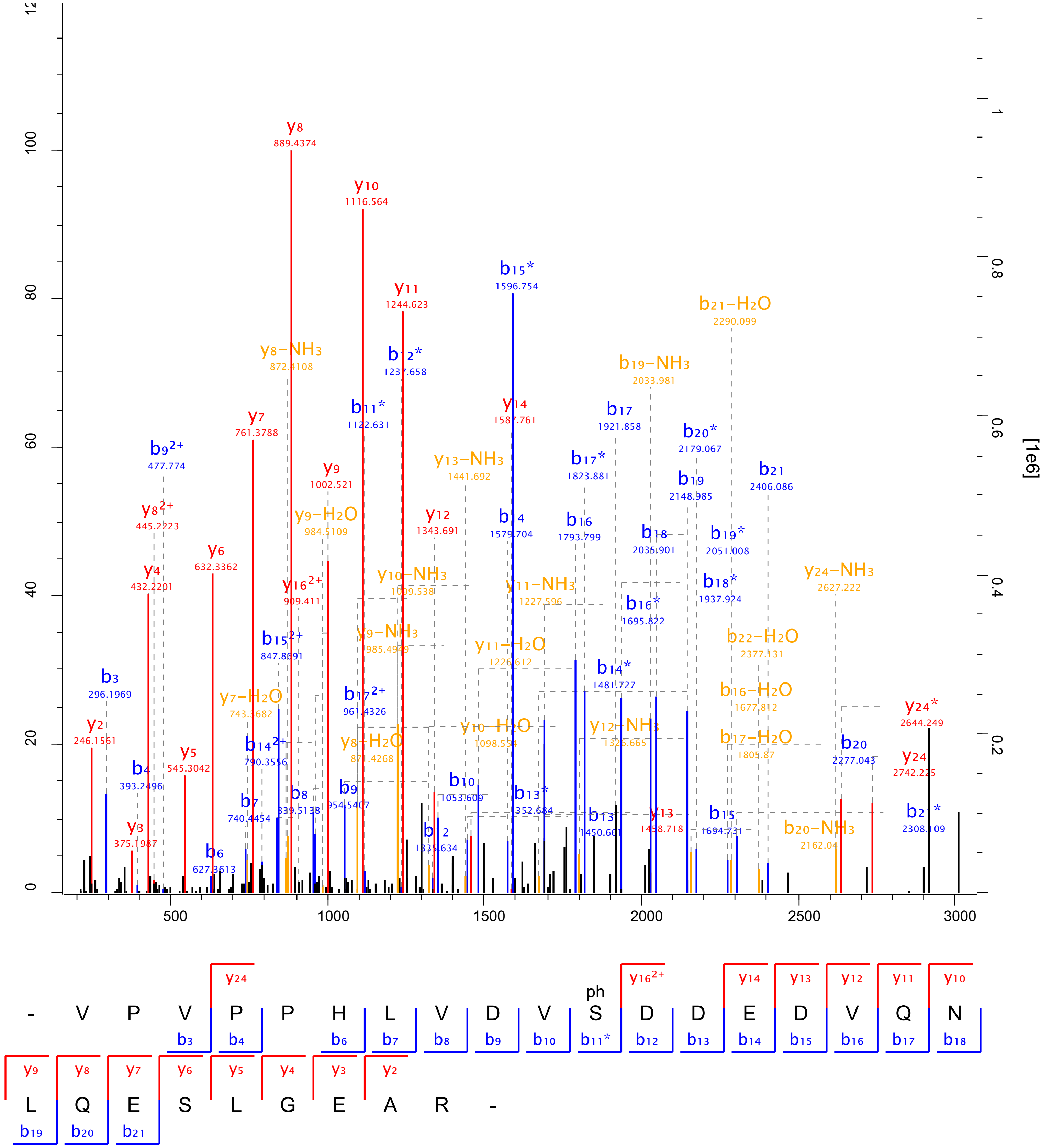

Sample output to test PDF Combine only

Raw file	Scan	Method	Score	m/z	Gene names
0523_11	22373	FTMS; HCD	87.74	873.41	T13J8.10

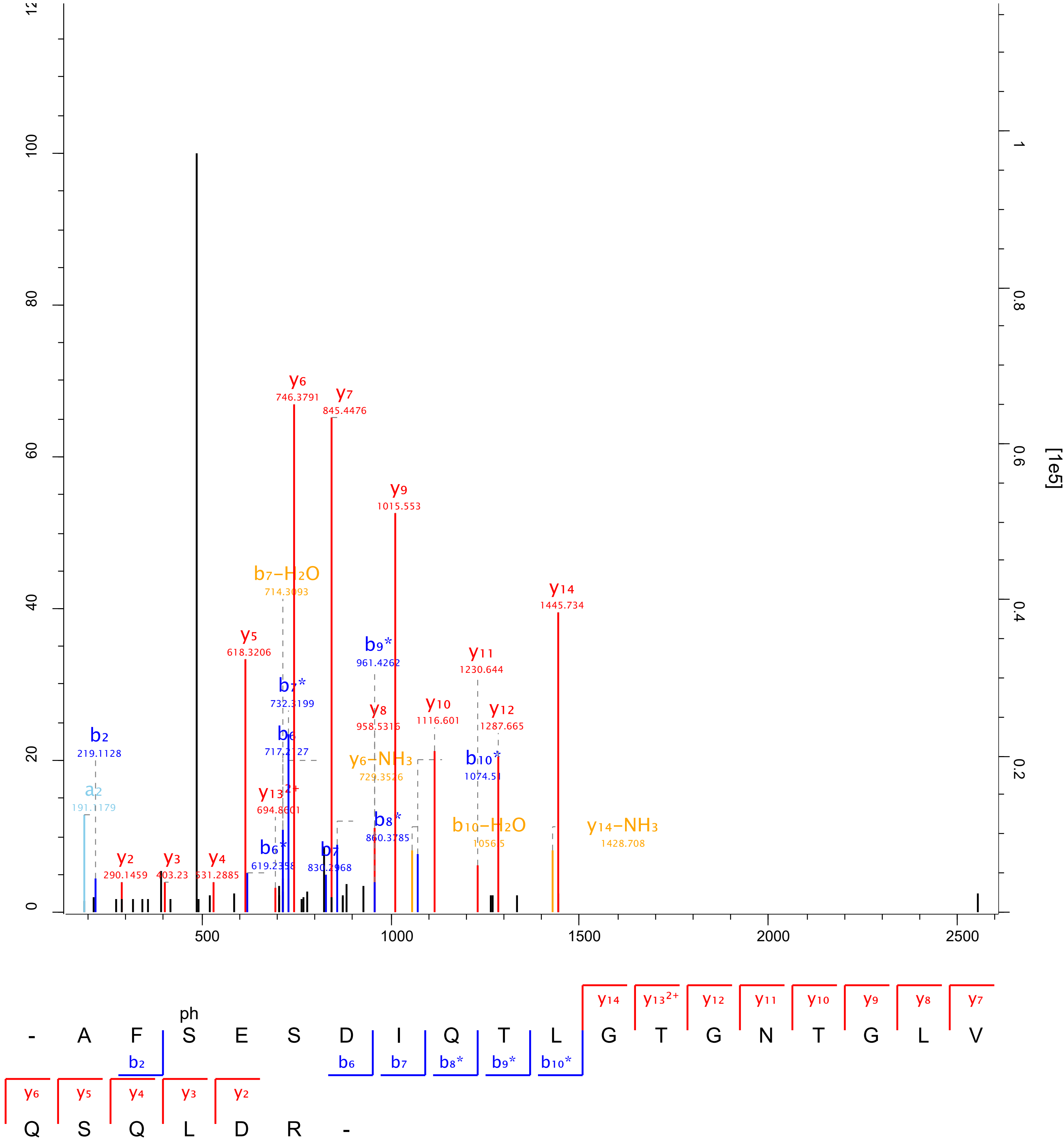

Sample output to test PDF Combine only

Raw file	Scan	Method	Score	m/z	Gene names
0523_11	22466	FTMS; HCD	99.6	908.08	KCA1

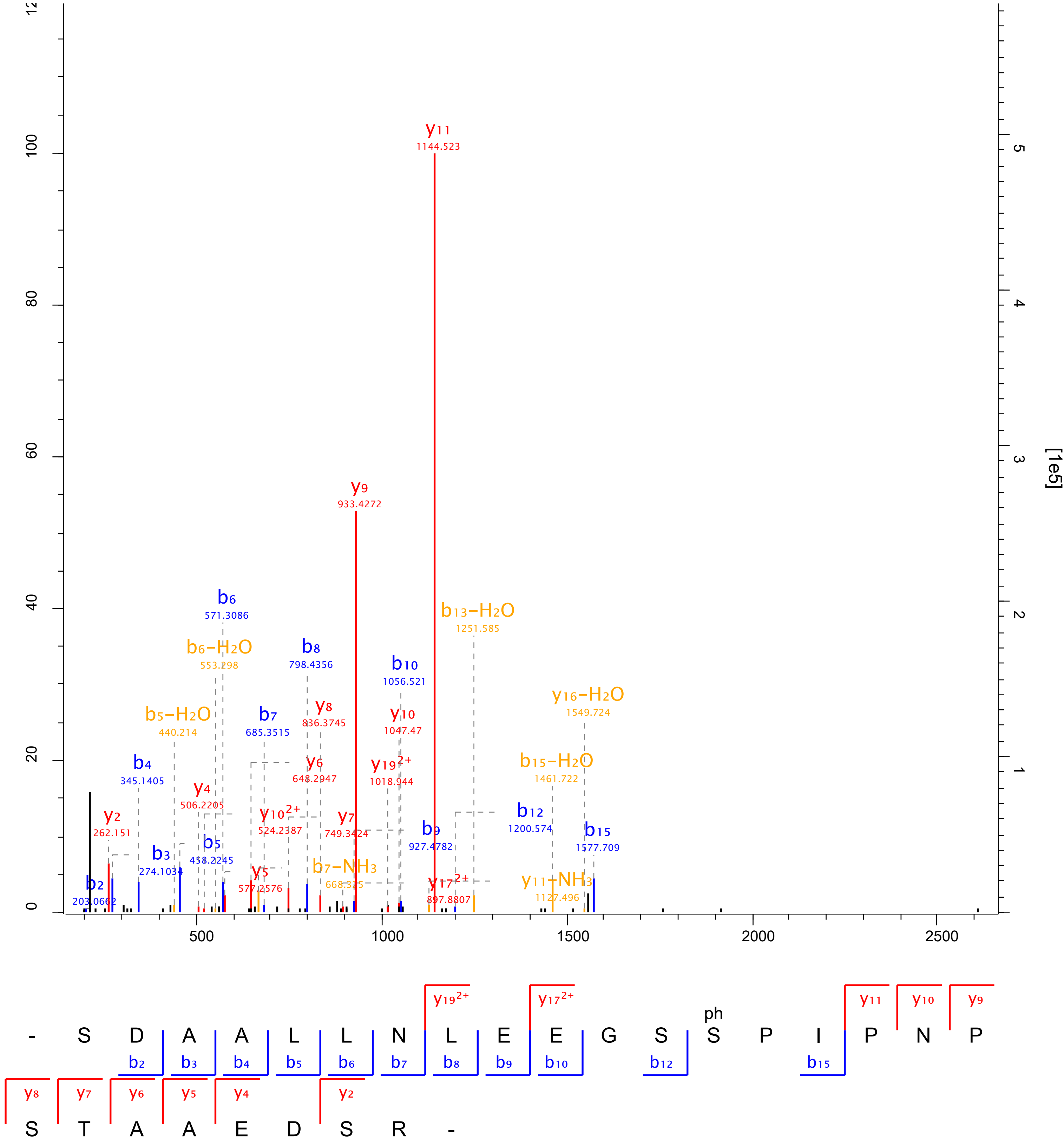

Sample output to test PDF Combine only

Raw file	Scan	Method	Score	m/z	Gene names
0523_11	22477	FTMS; HCD	61.49	1002.76	ACA10

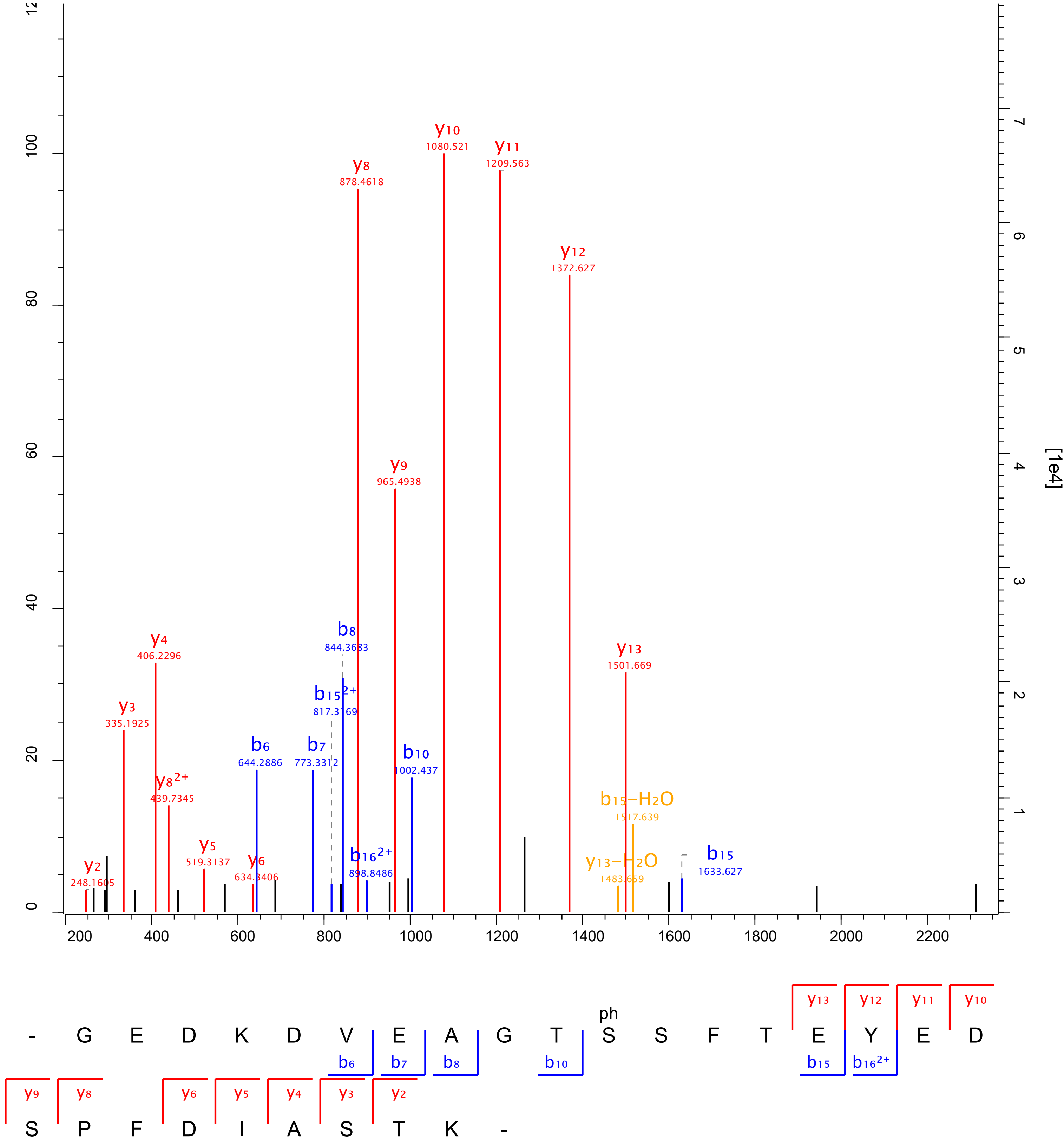

Sample output to test PDF Combine only

Raw file	Scan	Method	Score	m/z	Gene names
0523_11	22825	FTMS; HCD	186.11	975.81	F9K20.7

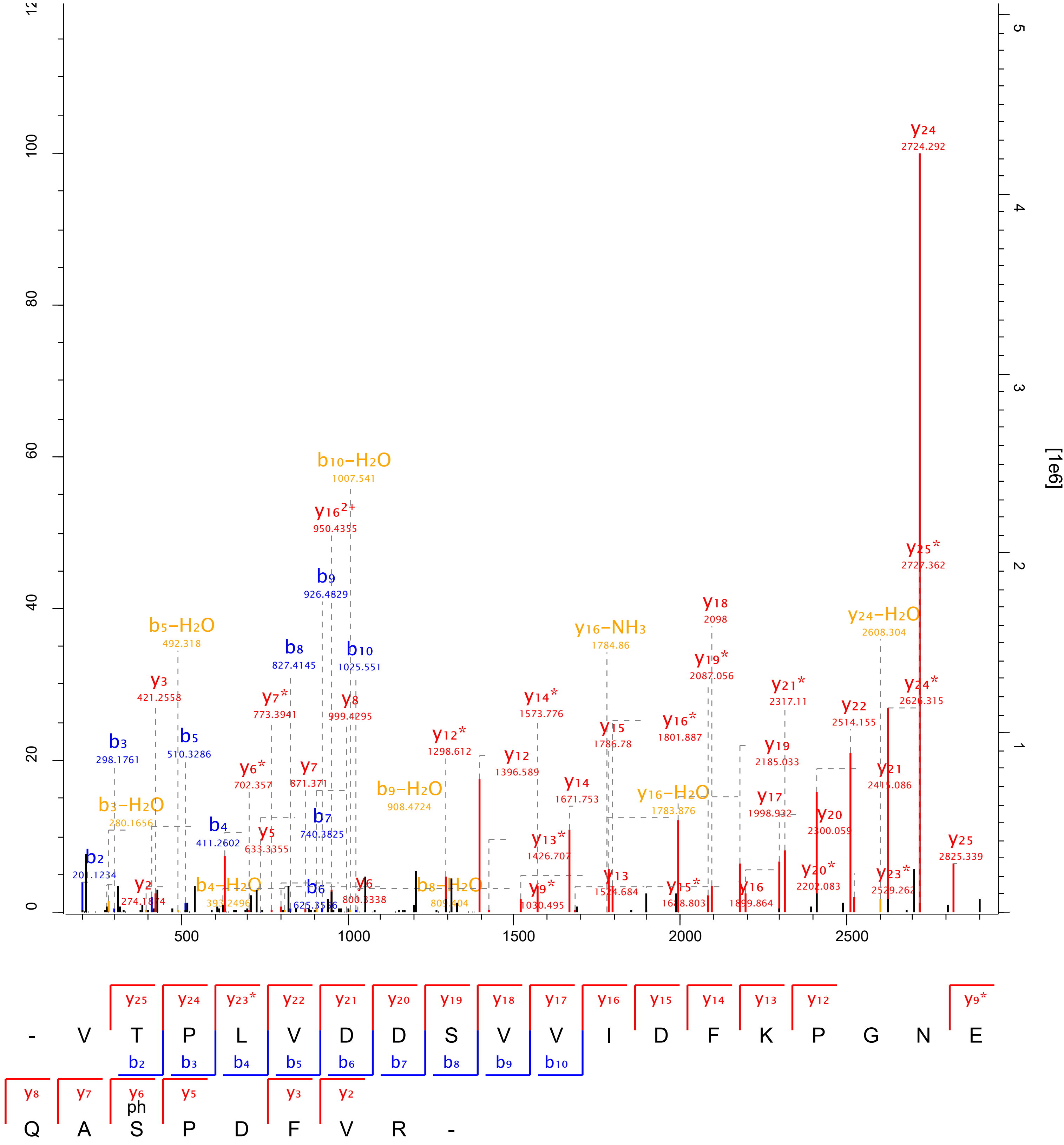

Sample output to test PDF Combine only

Raw file	Scan	Method	Score	m/z	Gene names
0523_11	23001	FTMS; HCD	180.97	1081.23	At2g34970

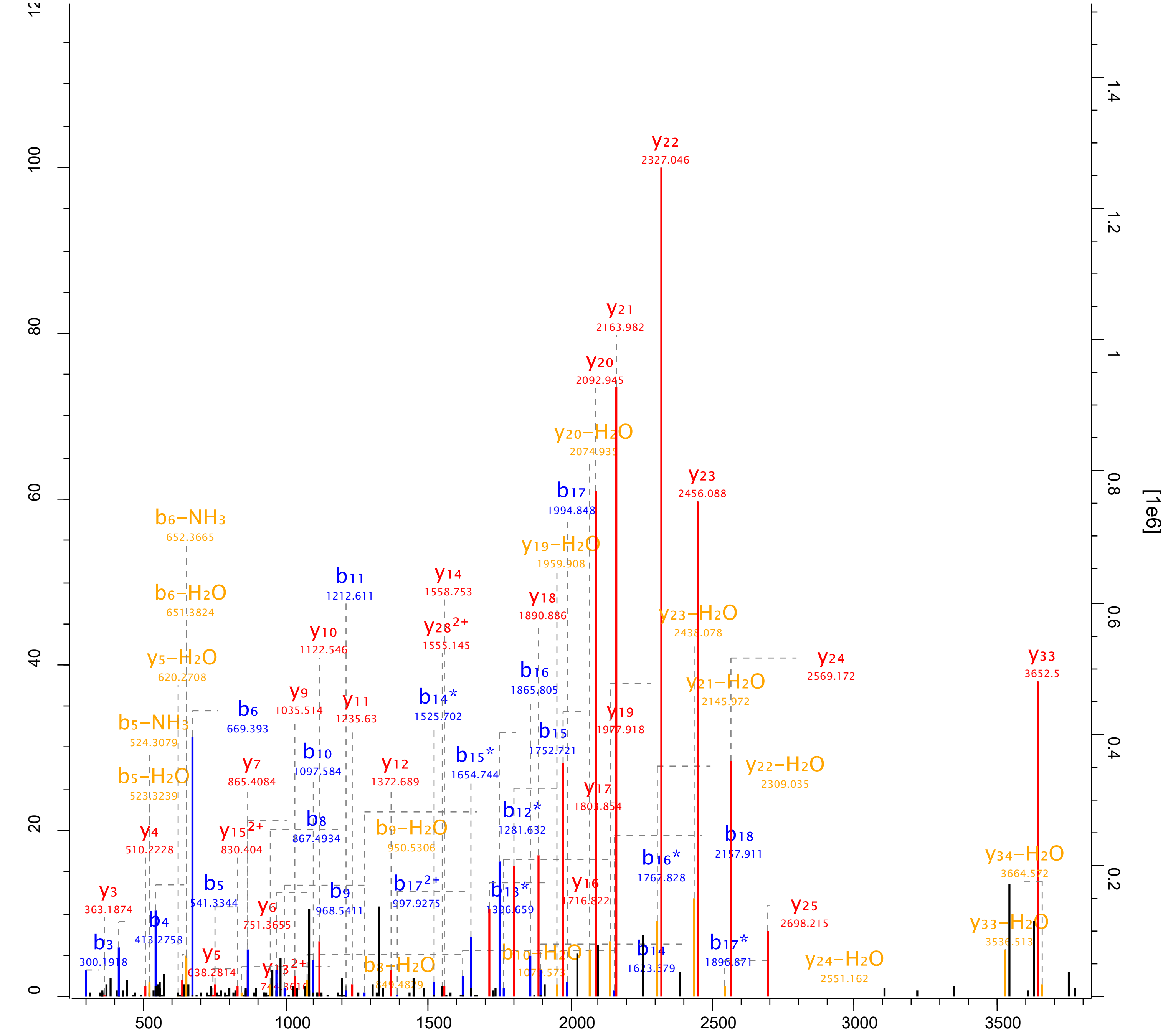

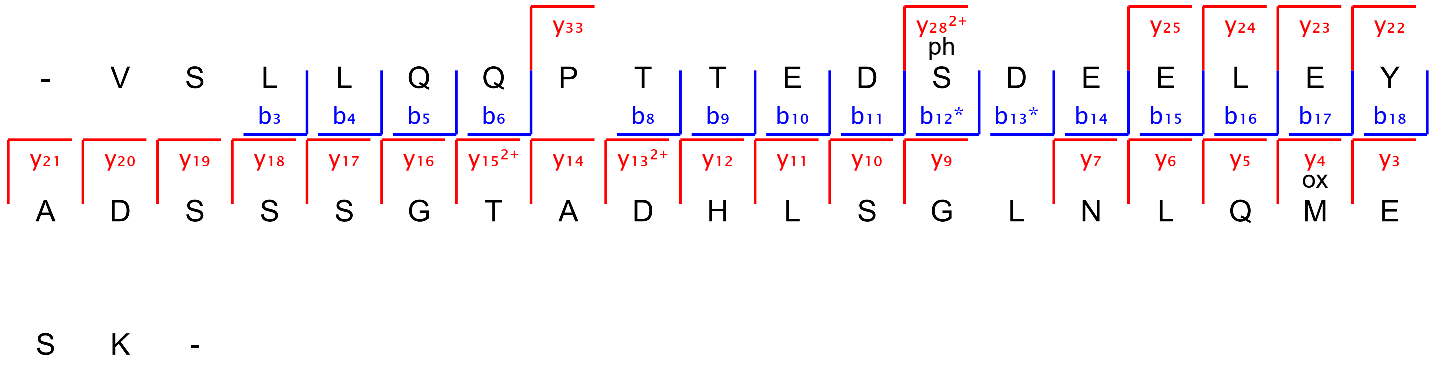

Sample output to test PDF Combine only

Raw file	Scan	Method	Score	m/z	Gene names
0523_11	23140	FTMS; HCD	110.83	815.09	SAC5

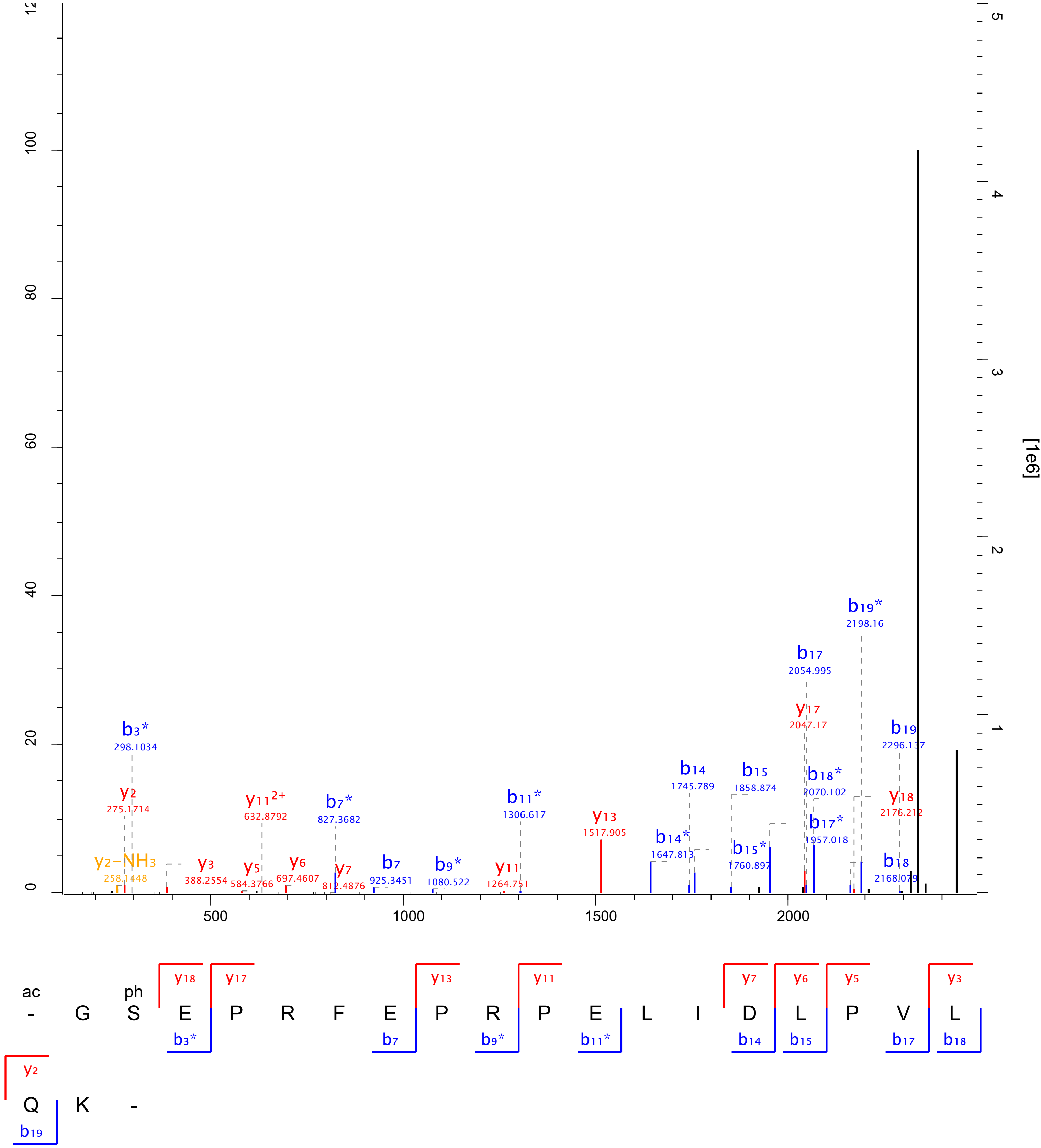

Sample output to test PDF Combine only

Raw file	Scan	Method	Score	m/z	Gene names
0523_11	23241	FTMS; HCD	280.41	1313.11	RPL13B;BBC1

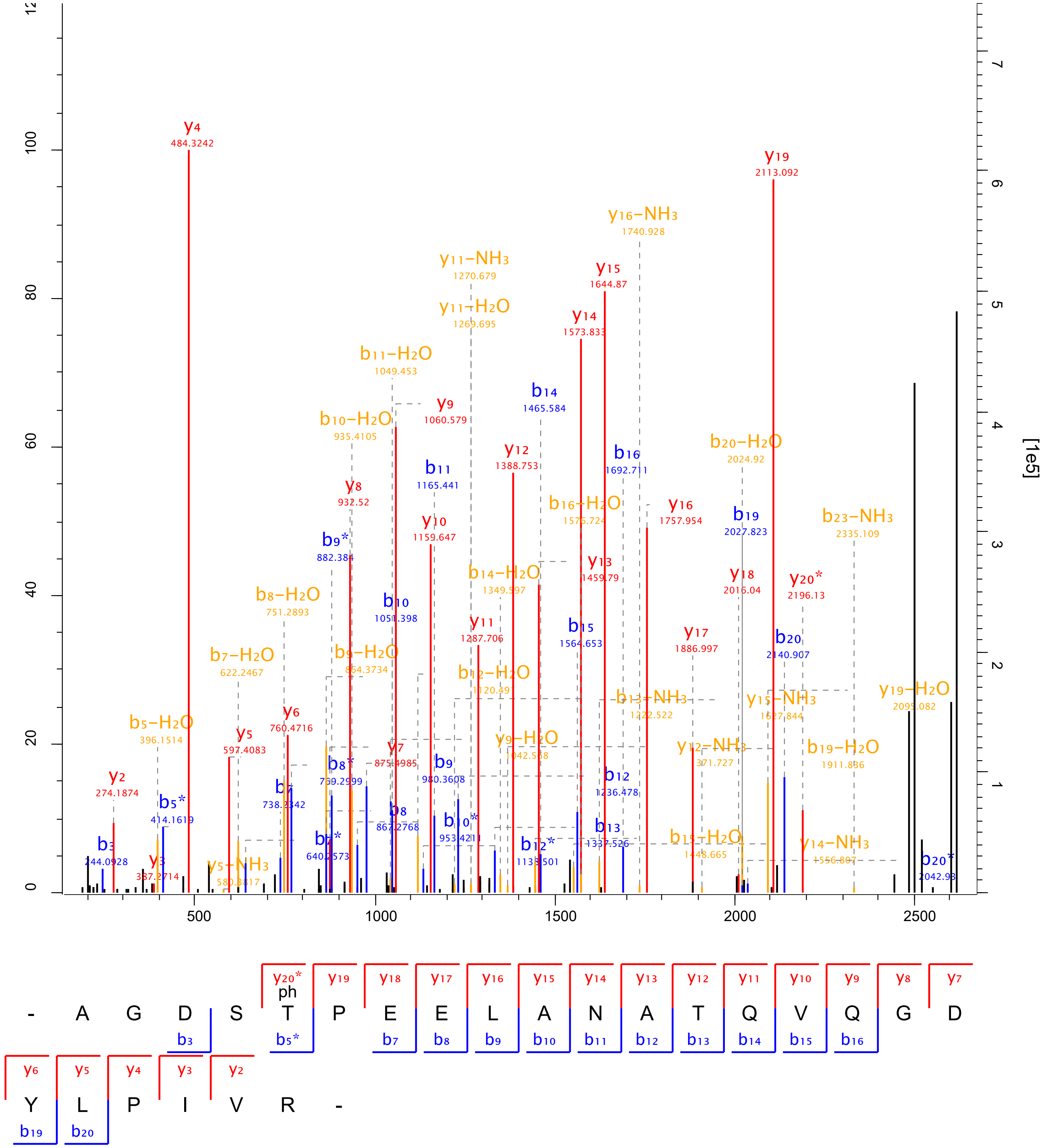

Sample output to test PDF Combine only

Raw file	Scan	Method	Score	m/z	Gene names
0523_11	23897	FTMS; HCD	135.91	886.07	F6N23.9

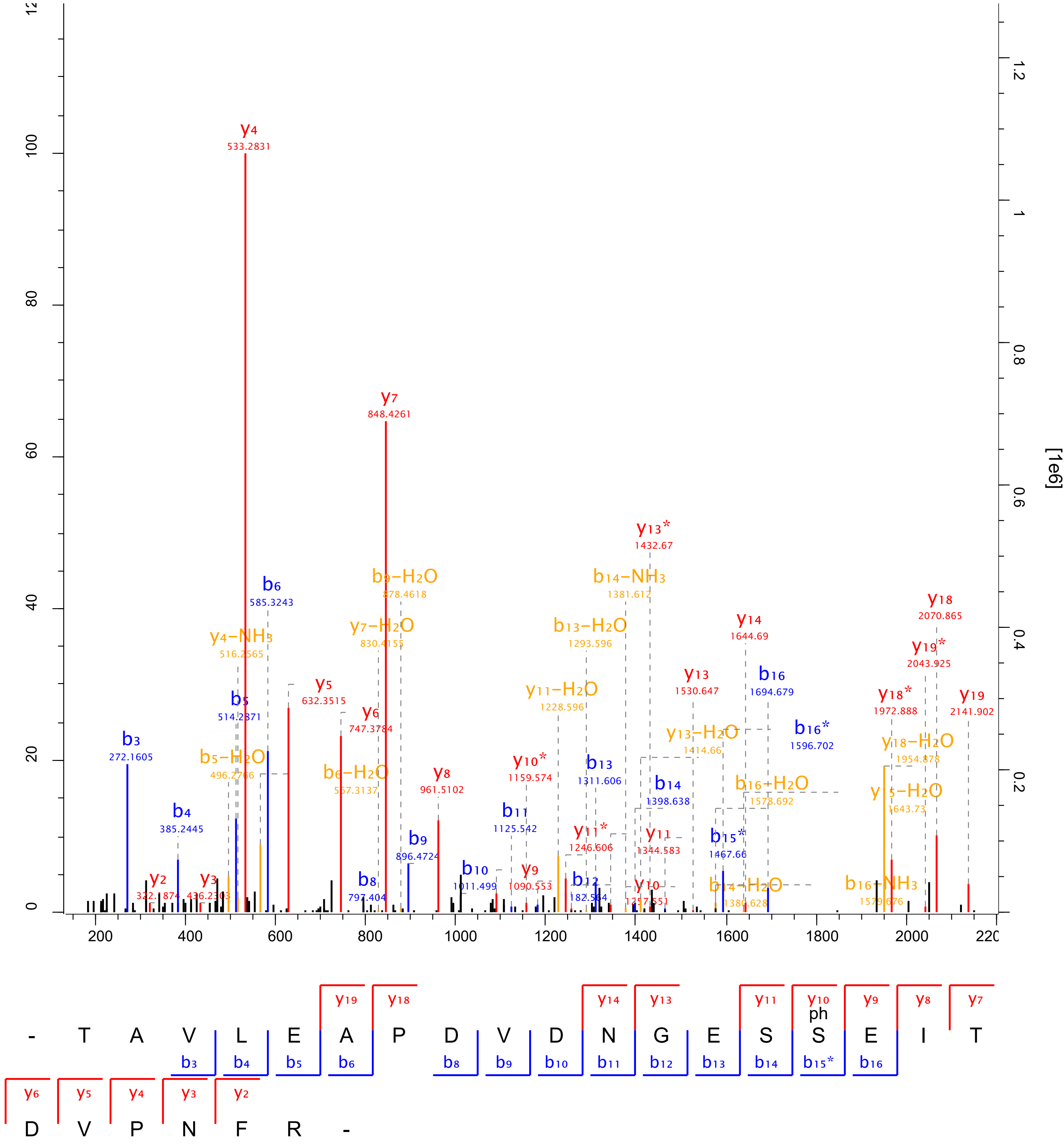

Sample output to test PDF Combine only

Raw file	Scan	Method	Score	m/z	Gene names
0523_11	24040	FTMS; HCD	61.28	799.37	PCR8

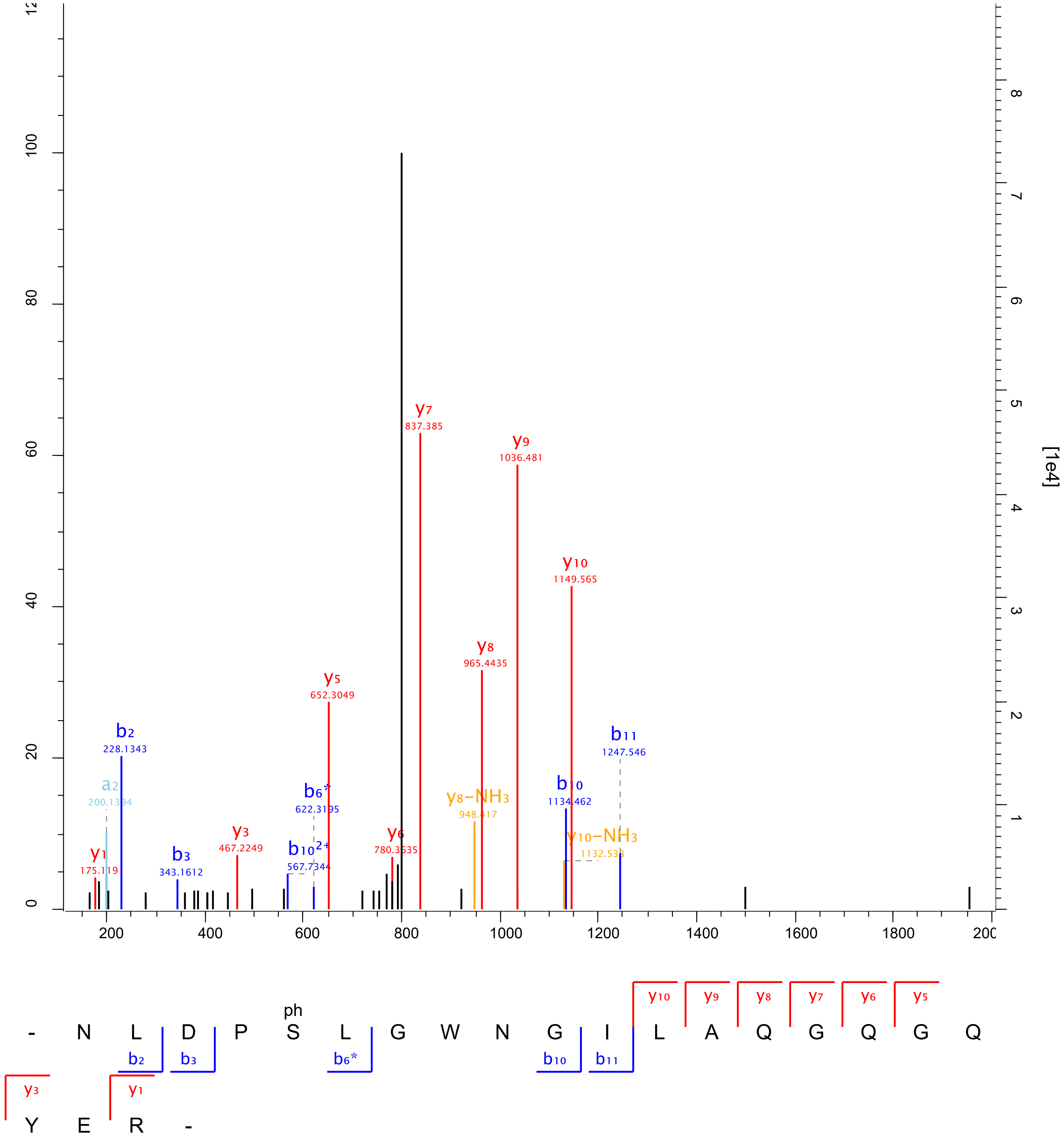

Sample output to test PDF Combine only

Raw file	Scan	Method	Score	m/z	Gene names
0523_11	24174	FTMS; HCD	73.29	757.36	T8K14.1

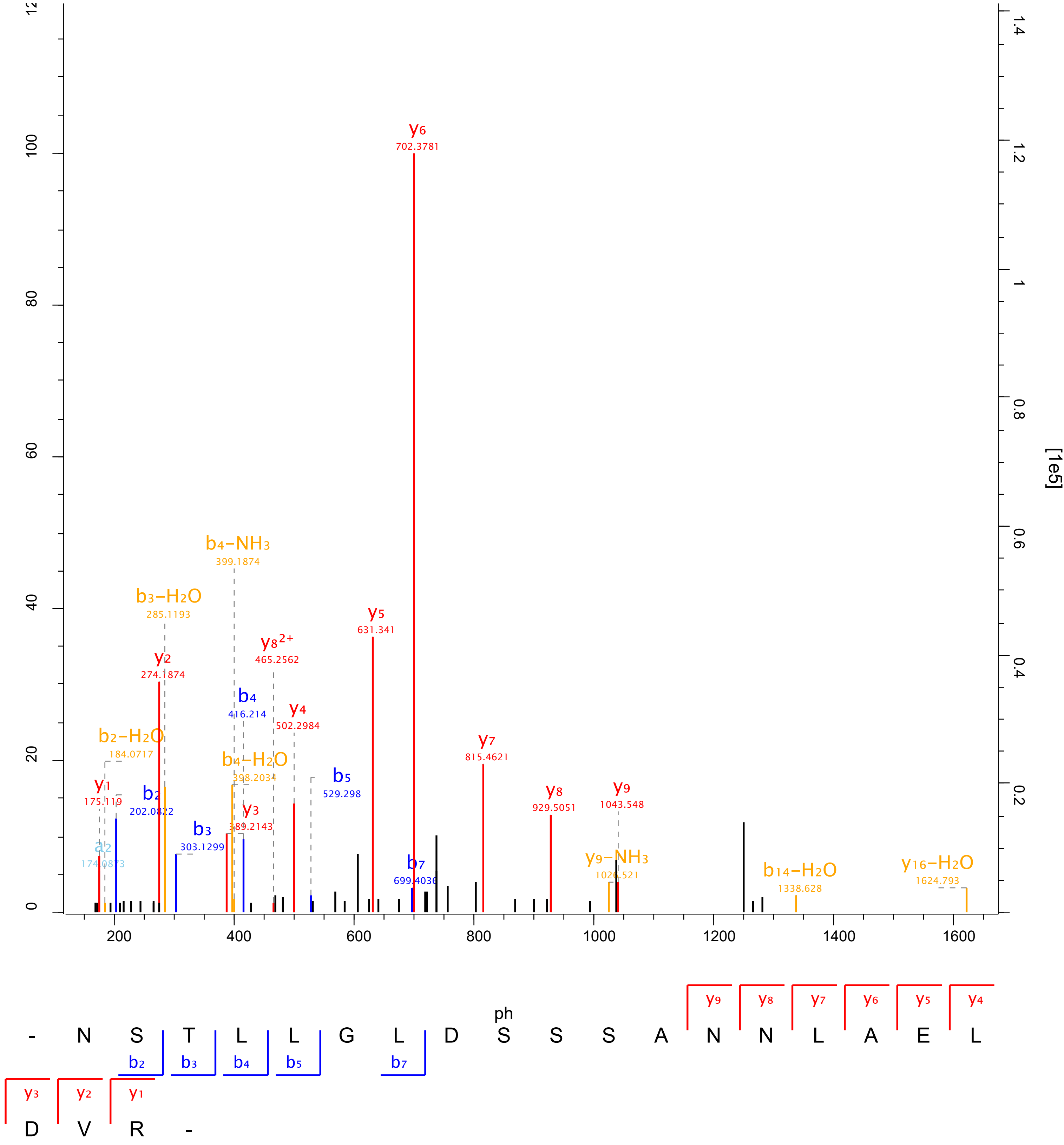

Sample output to test PDF Combine only

Raw file	Scan	Method	Score	m/z	Gene names
0523_11	24366	FTMS; HCD	167.69	754.35	LRR-RLK

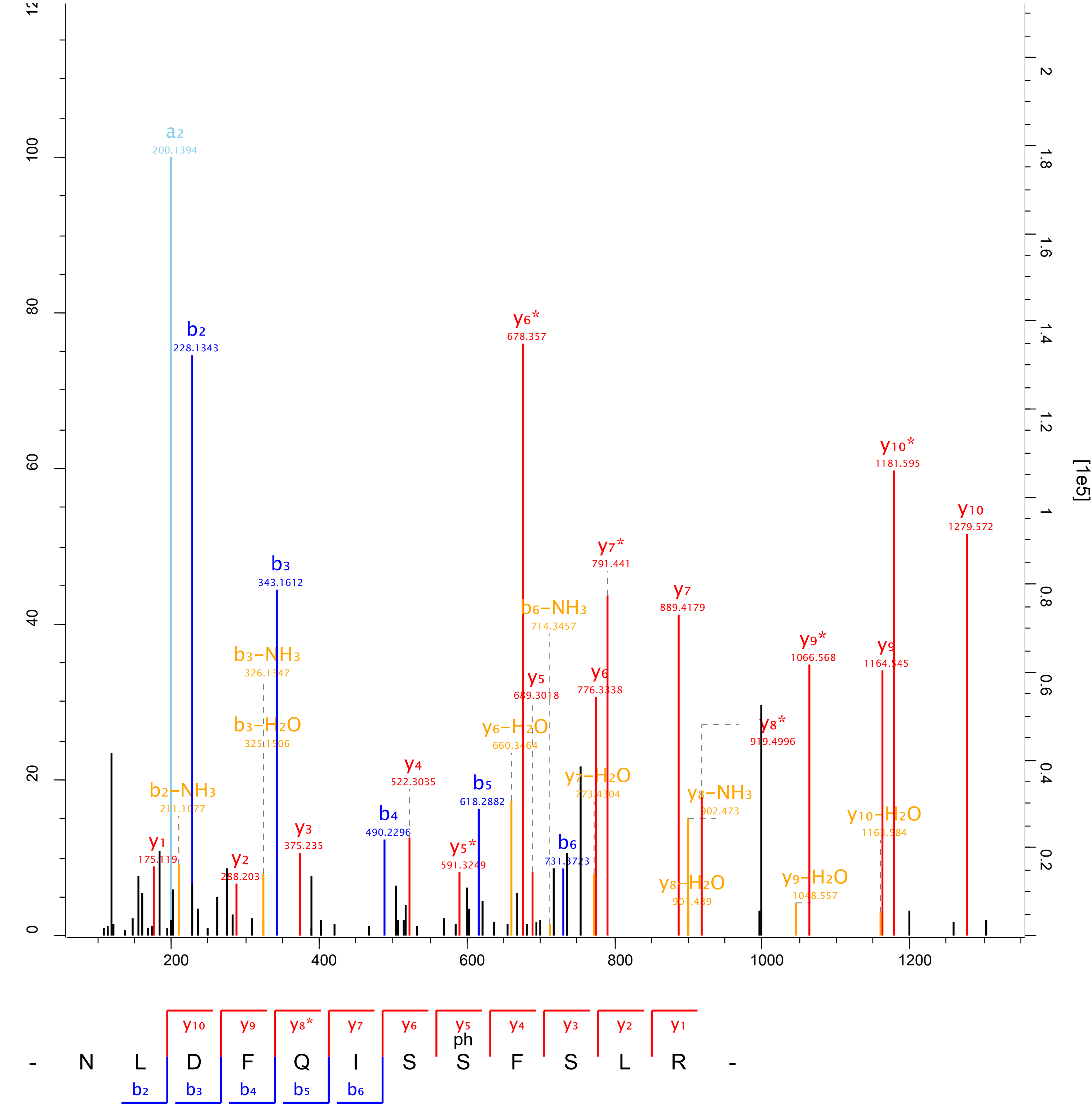

Sample output to test PDF Combine only

Raw file	Scan	Method	Score	m/z	Gene names
0523_11	24657	FTMS; HCD	230.29	1097.89	MKK2

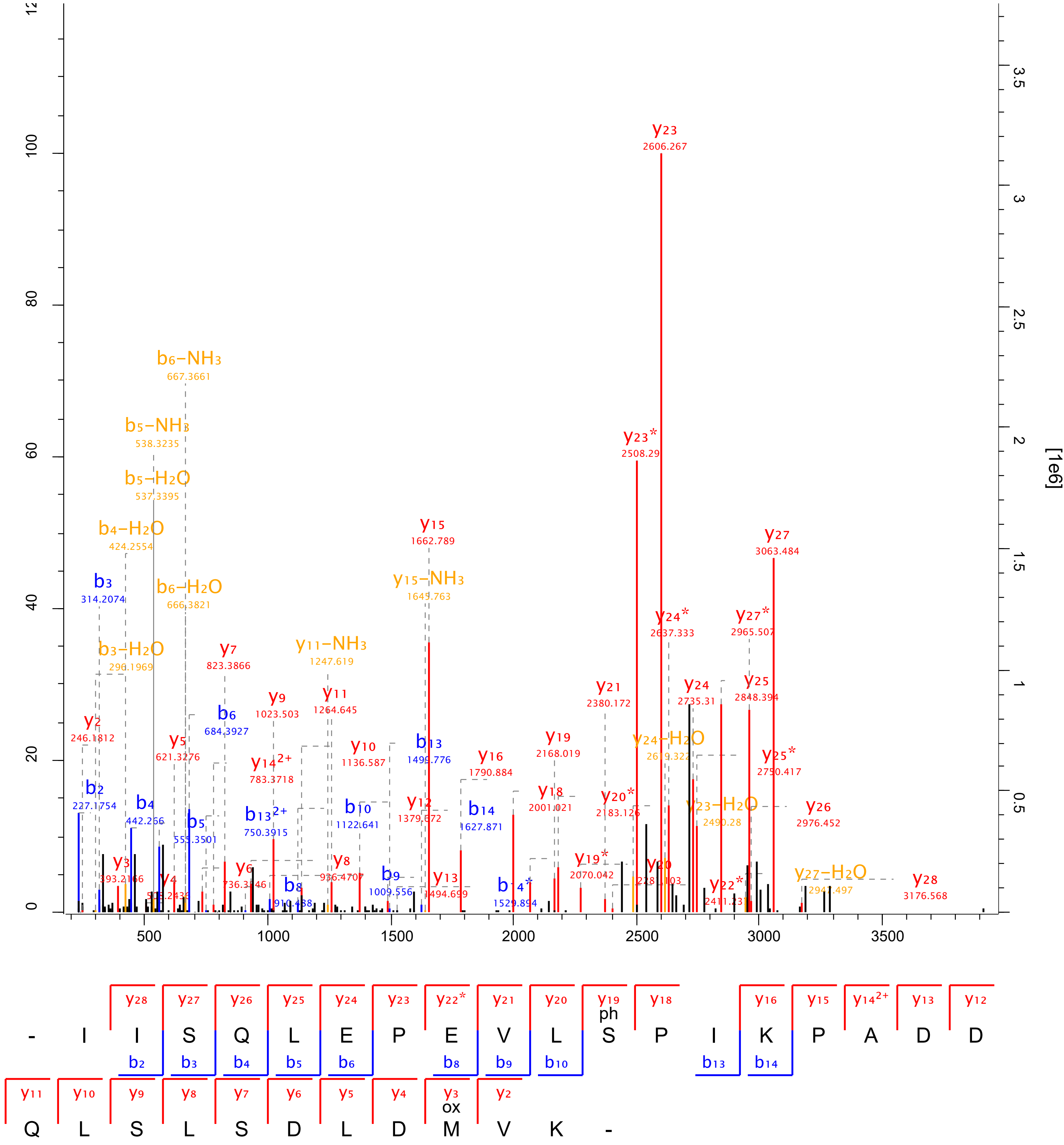

Sample output to test PDF Combine only

Raw file	Scan	Method	Score	m/z	Gene names
0523_11	24675	FTMS; HCD	72.93	753.34	APUM6

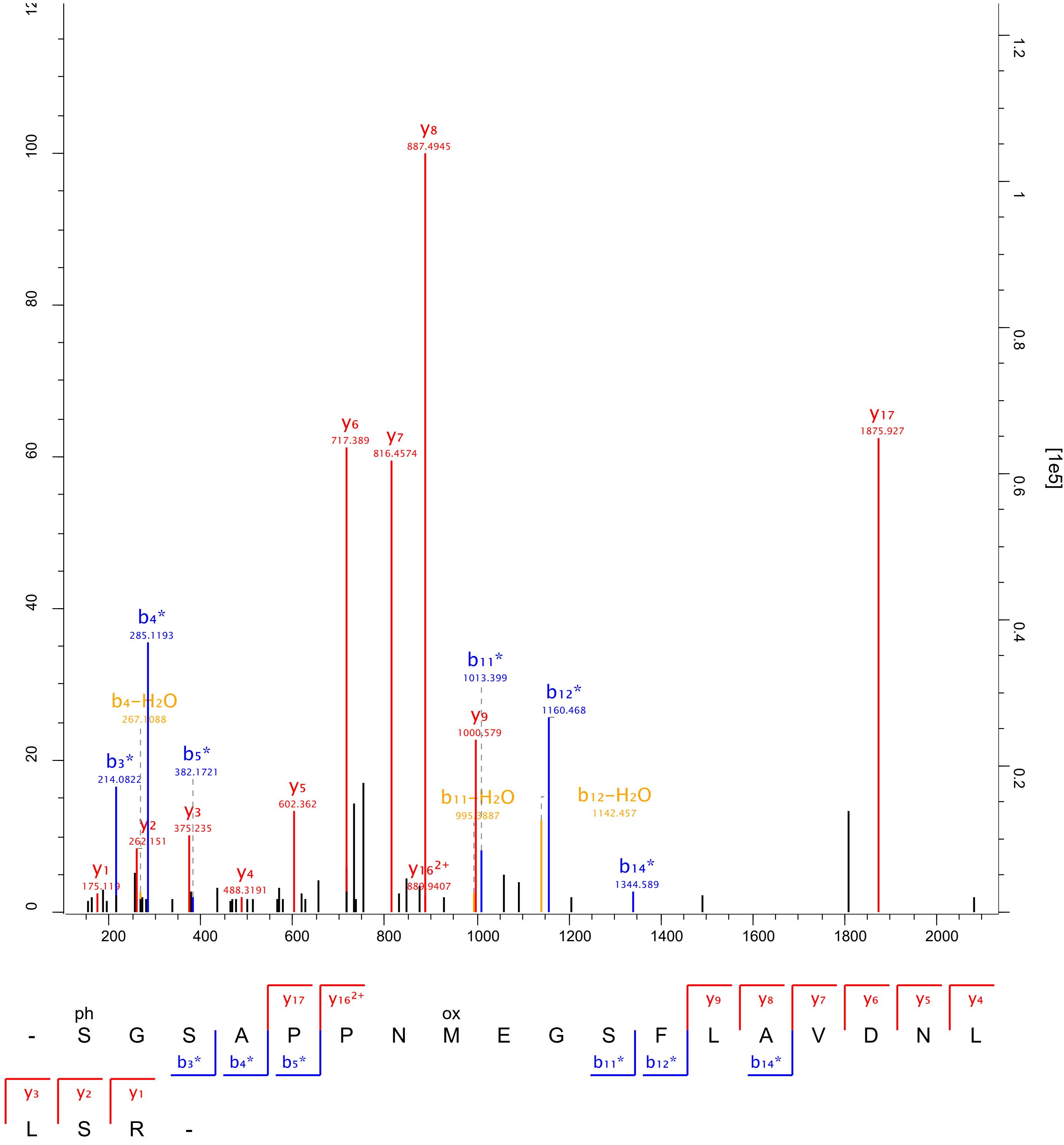

Sample output to test PDF Combine only

Raw file	Scan	Method	Score	m/z	Gene names
0523_11	25143	FTMS; HCD	350.62	1054.18	VHA-G1

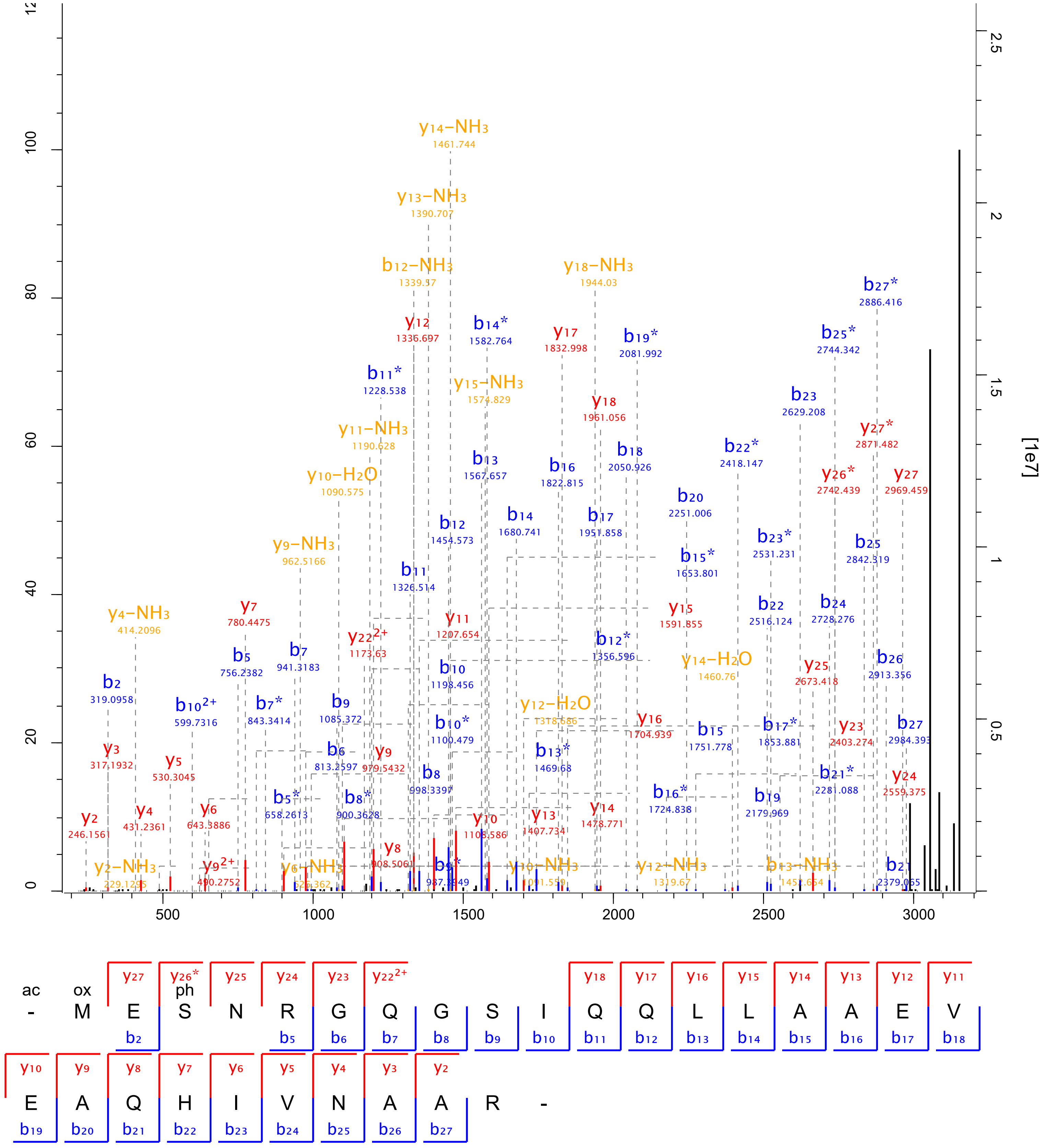

Sample output to test PDF Combine only

Raw file	Scan	Method	Score	m/z	Gene names
0523_11	25224	FTMS; HCD	41.48	766.01	PI4KA1

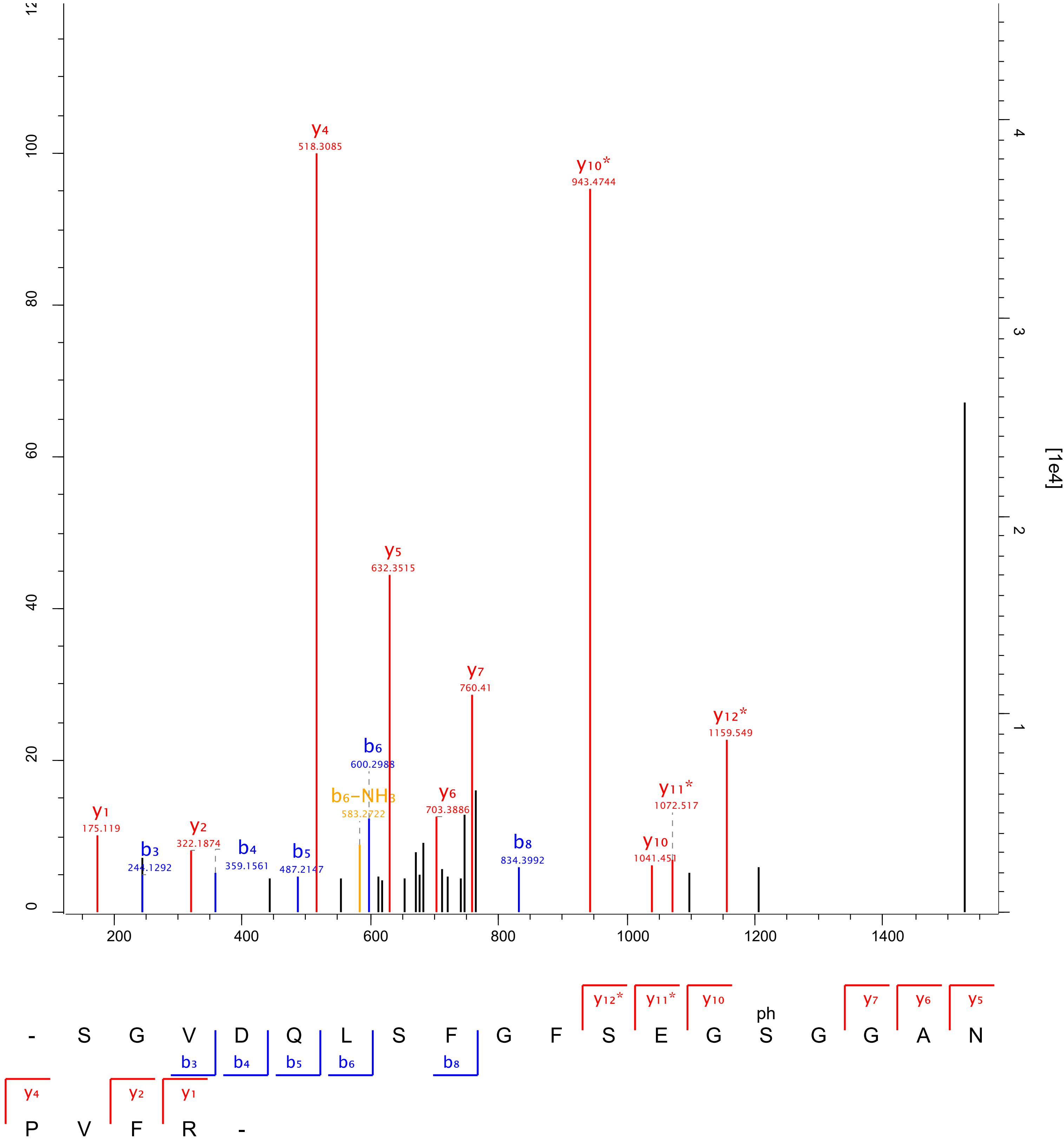

Sample output to test PDF Combine only

Raw file	Scan	Method	Score	m/z	Gene names
0523_11	25872	FTMS; HCD	272.38	1048.51	VHA-G1

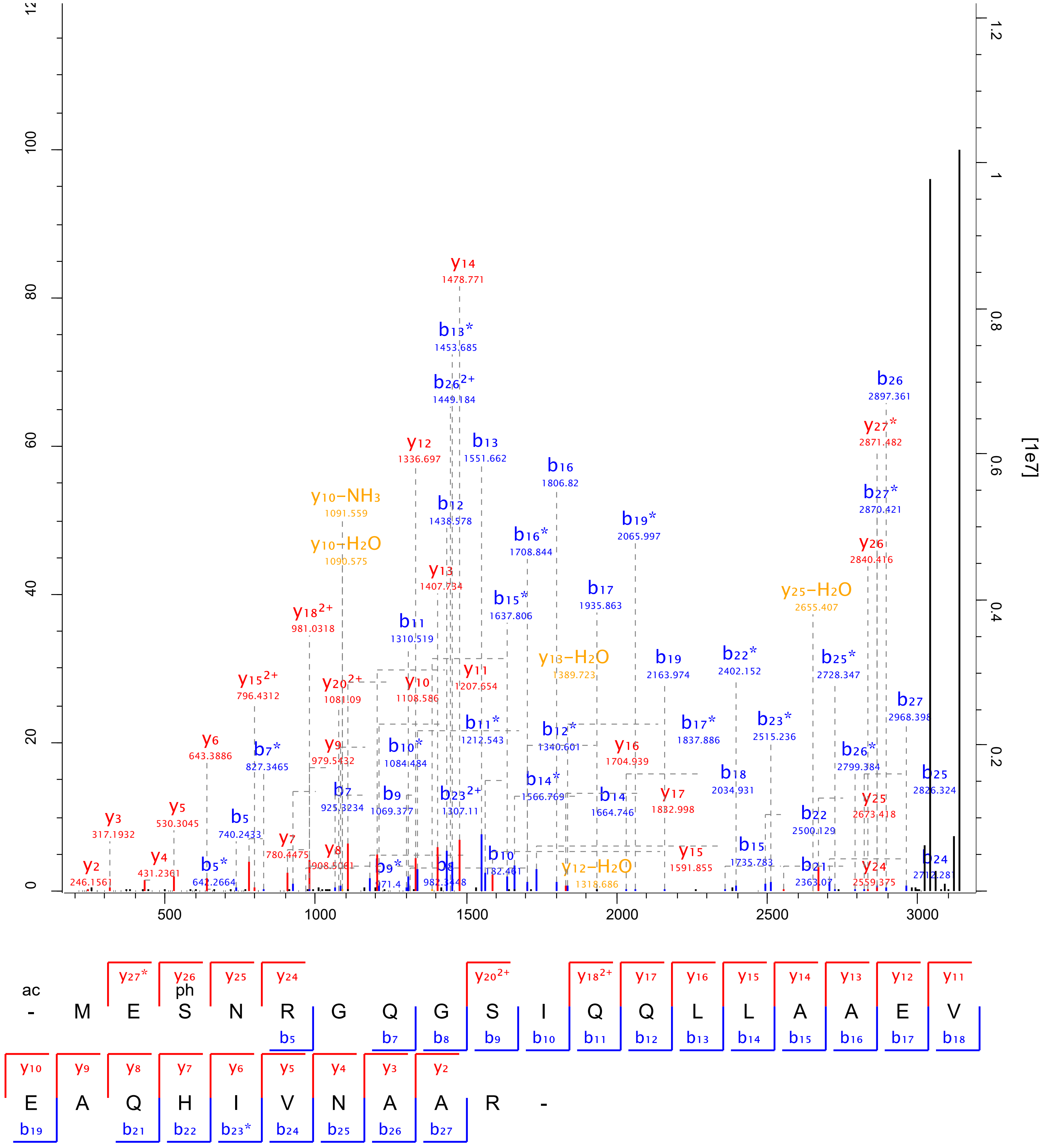

Sample output to test PDF Combine only

Raw file	Scan	Method	Score	m/z	Gene names
0523_11	25890	FTMS; HCD	184.81	1078.46	AP5M;At2g20790

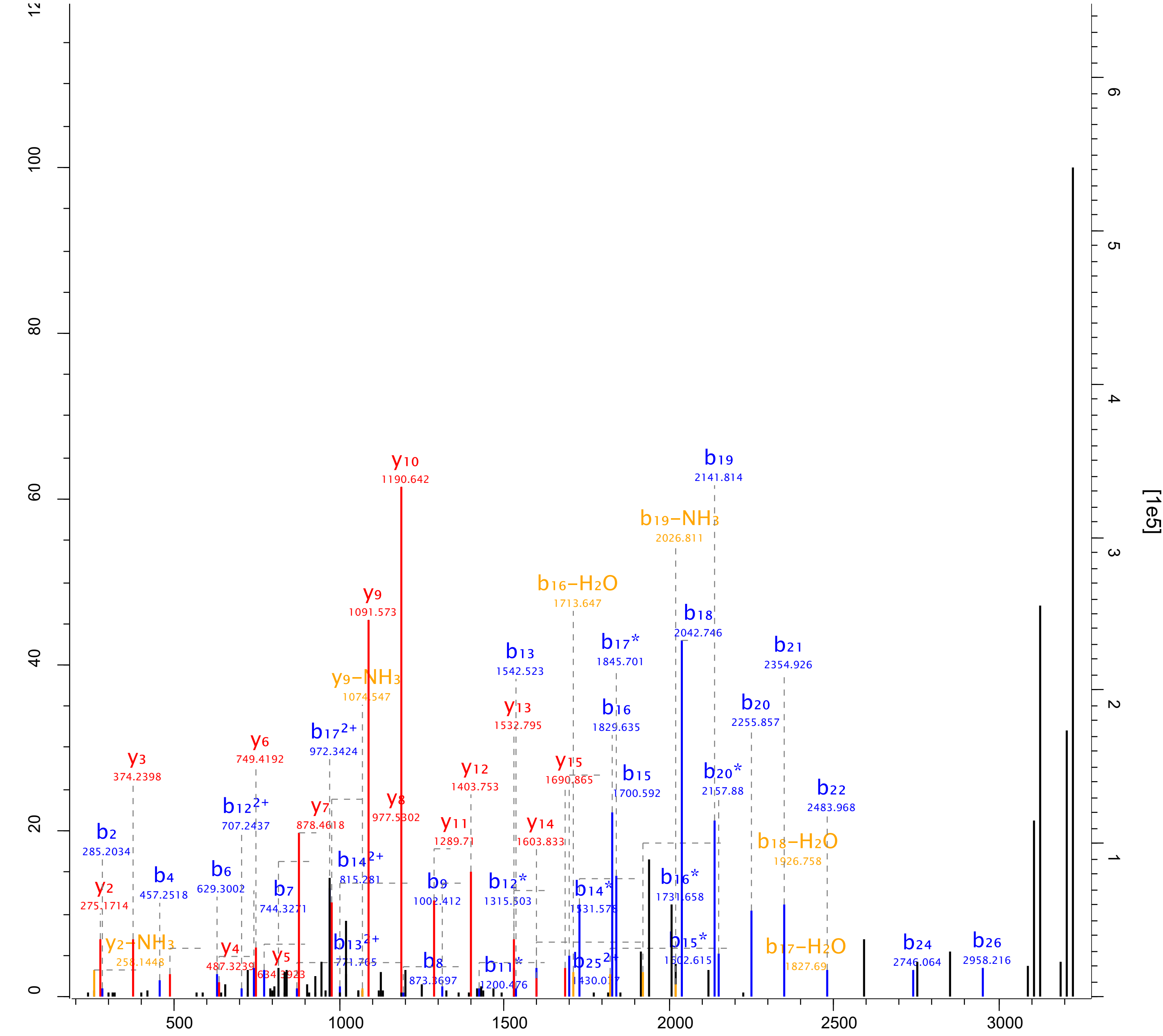

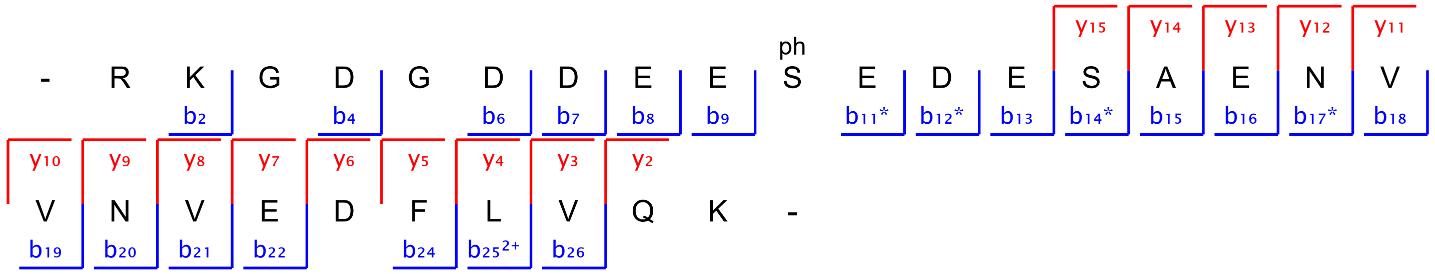

Sample output to test PDF Combine only

Raw file	Scan	Method	Score	m/z	Gene names
0523_11	26188	FTMS; HCD	116.59	1092.22	MKK2

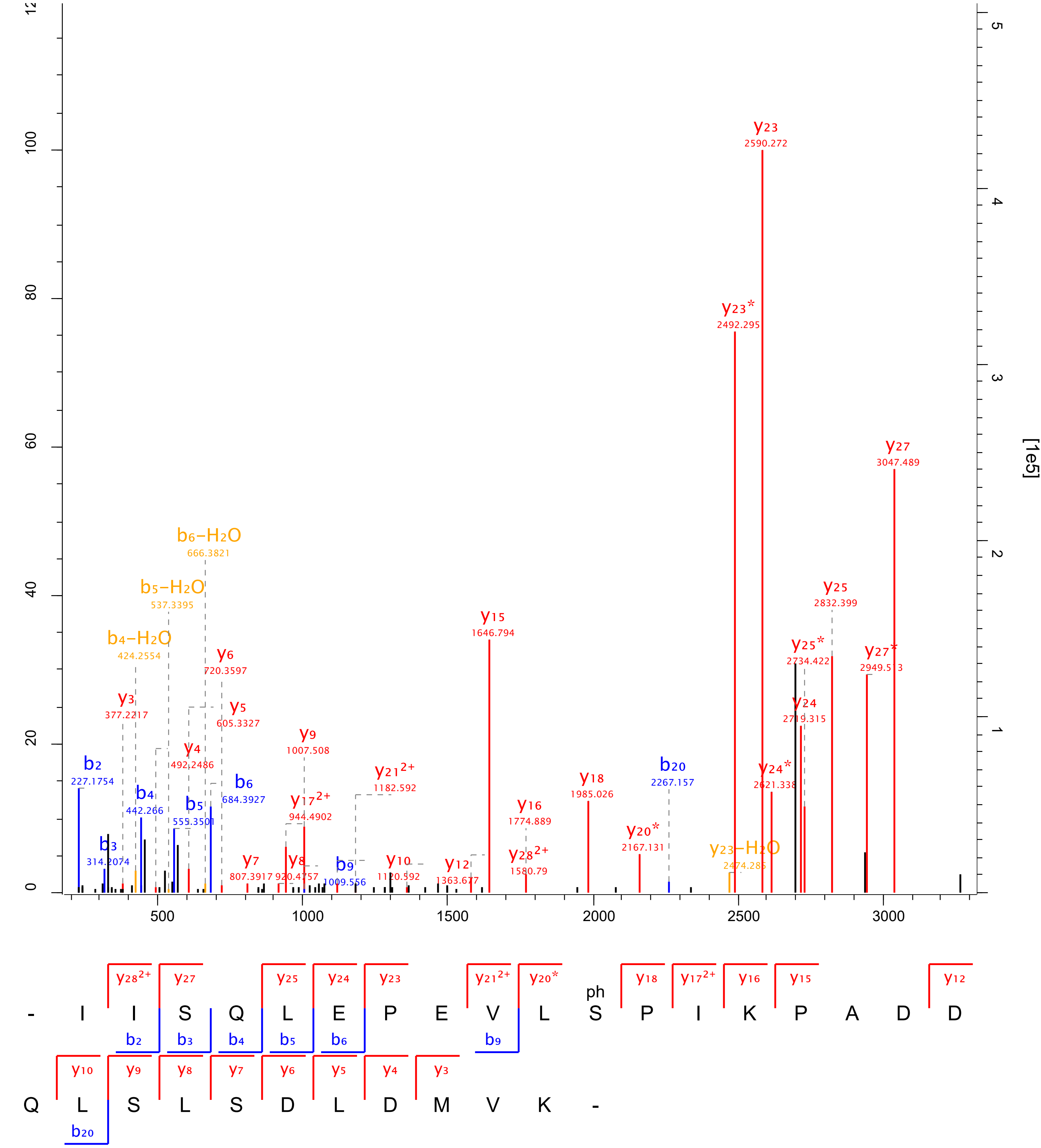

Sample output to test PDF Combine only

Raw file	Scan	Method	Score	m/z	Gene names
0523_11	26750	FTMS; HCD	108.13	1035.5	F23M19.10;At1g34220

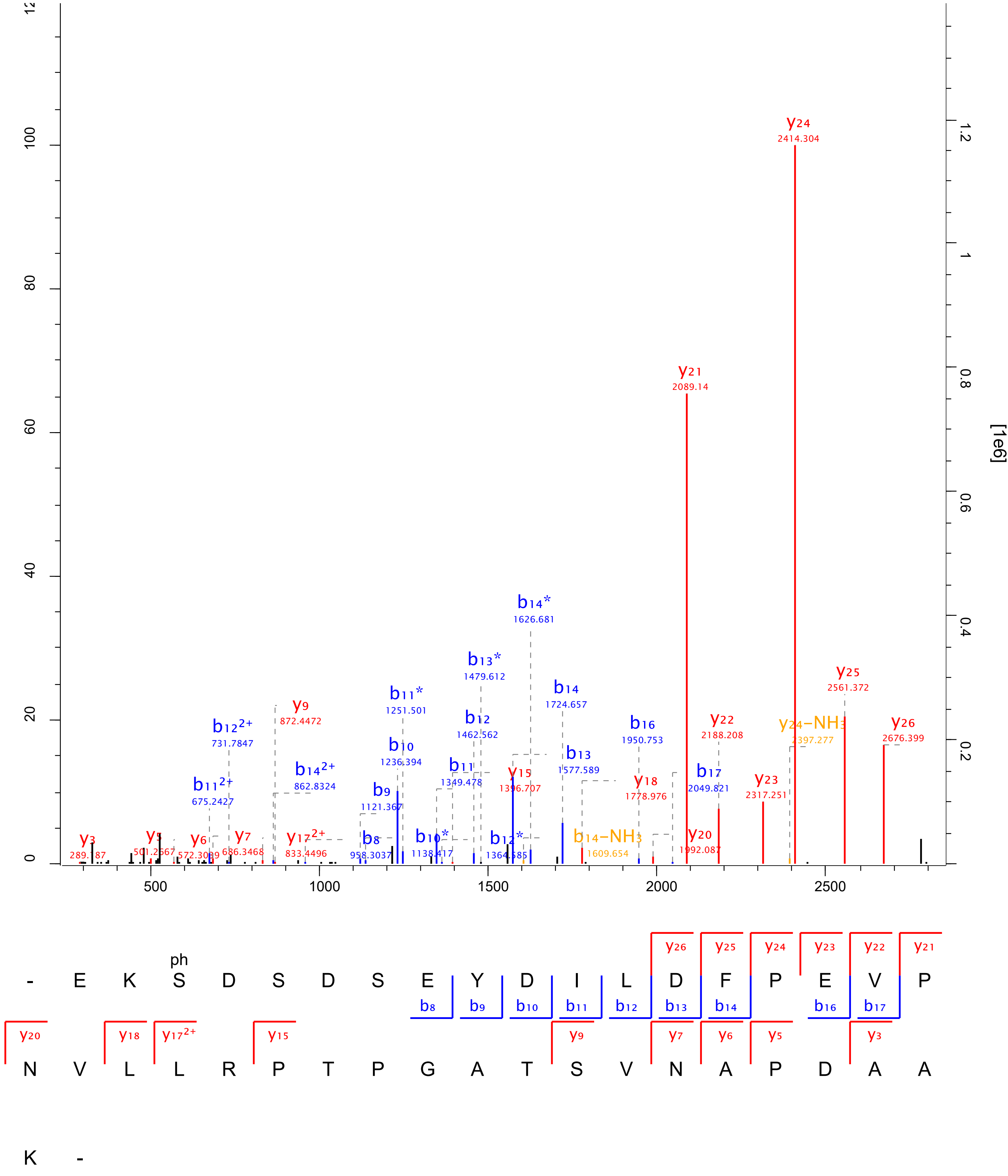

Sample output to test PDF Combine only

Raw file	Scan	Method	Score	m/z	Gene names
0523_11	31121	FTMS; HCD	134.14	842.35	RABC1

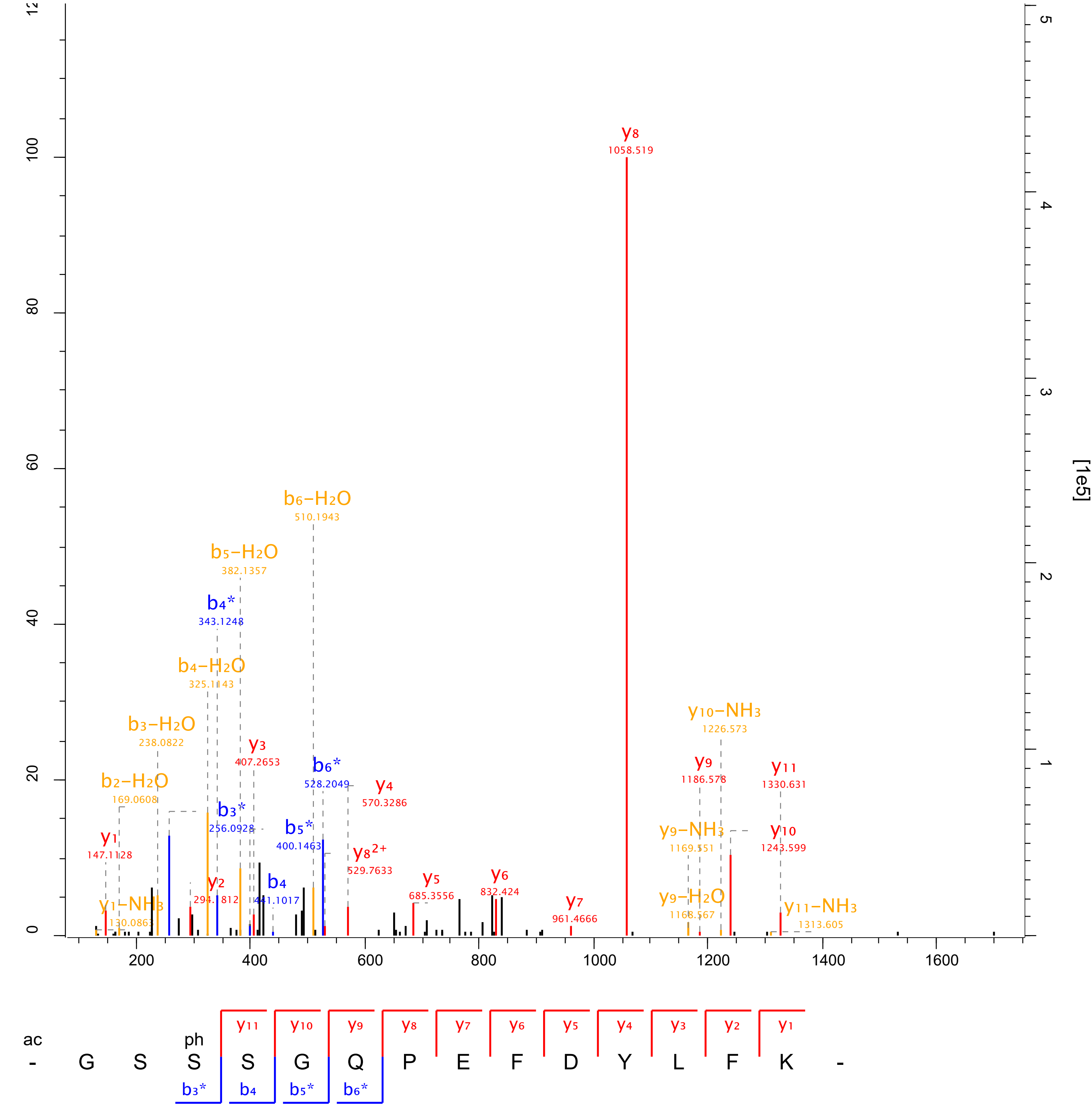

Sample output to test PDF Combine only

Raw file	Scan	Method	Score	m/z	Gene names
0523_12	1447	FTMS; HCD	173.36	625.93	APK2A

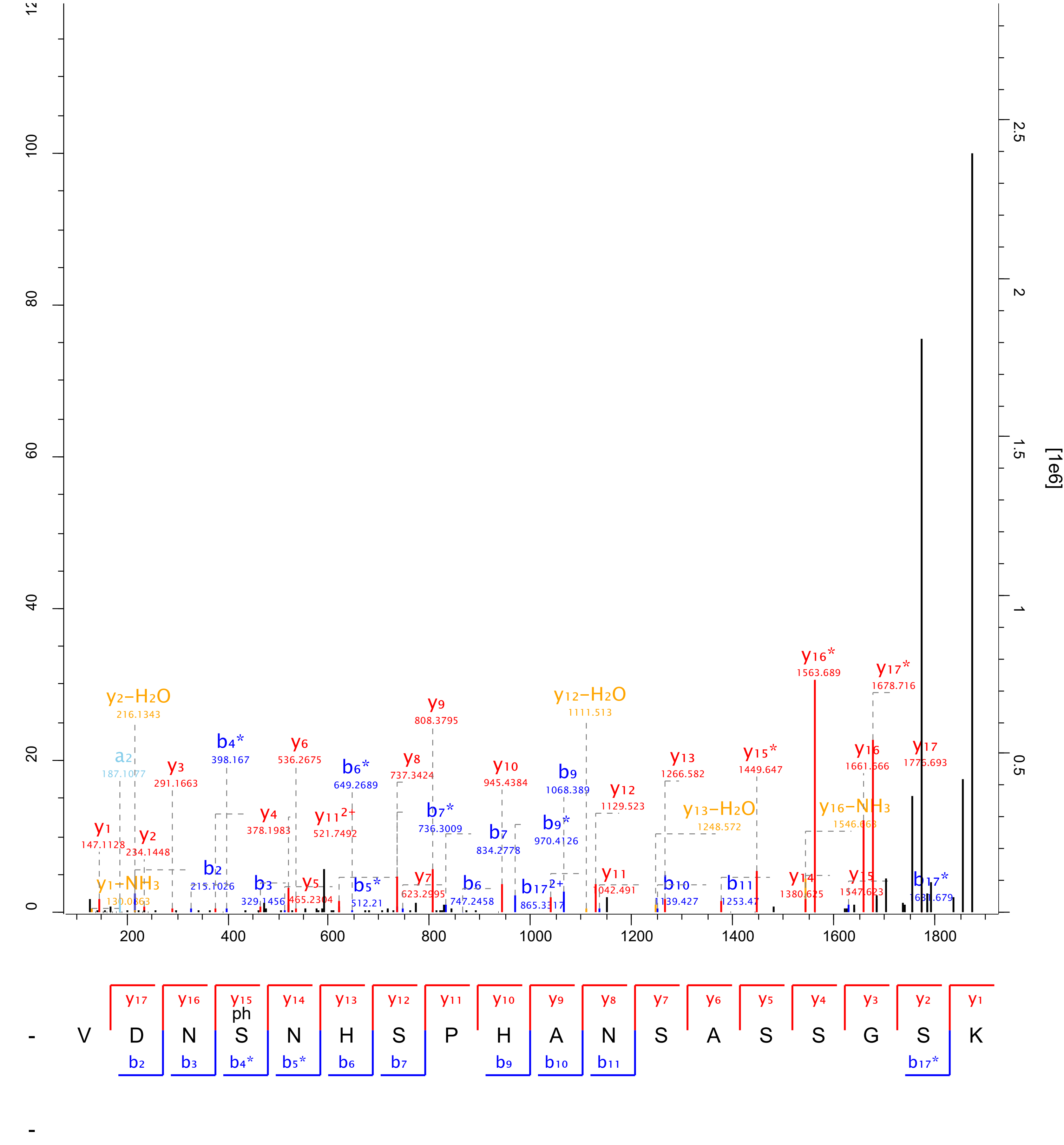

Sample output to test PDF Combine only

Raw file	Scan	Method	Score	m/z	Gene names
0523_12	1459	FTMS; HCD	64.1	367.5	At4g35785

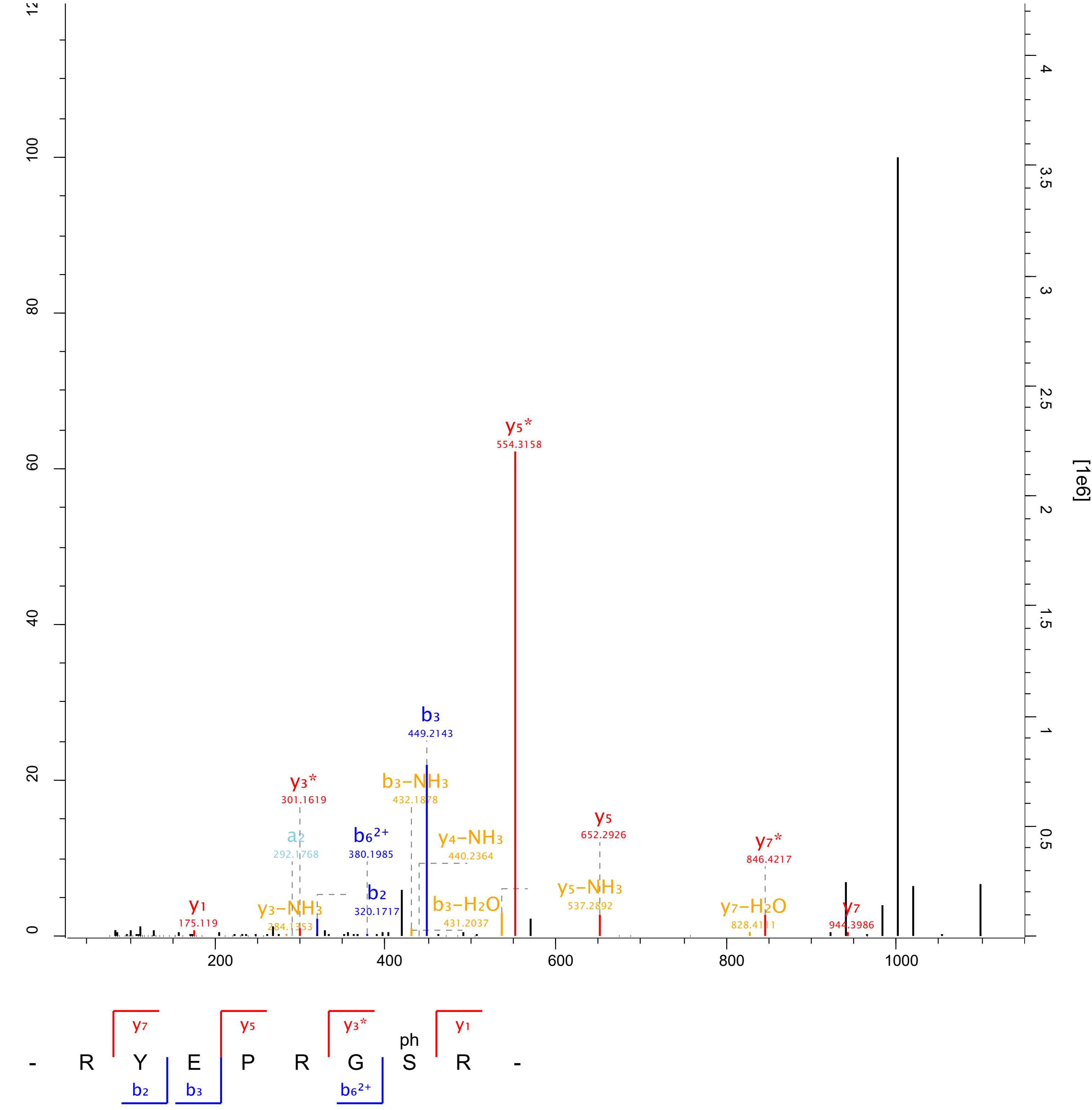

Sample output to test PDF Combine only

Raw file	Scan	Method	Score	m/z	Gene names
0523_12	1461	FTMS; HCD	89.03	362.83	RS31

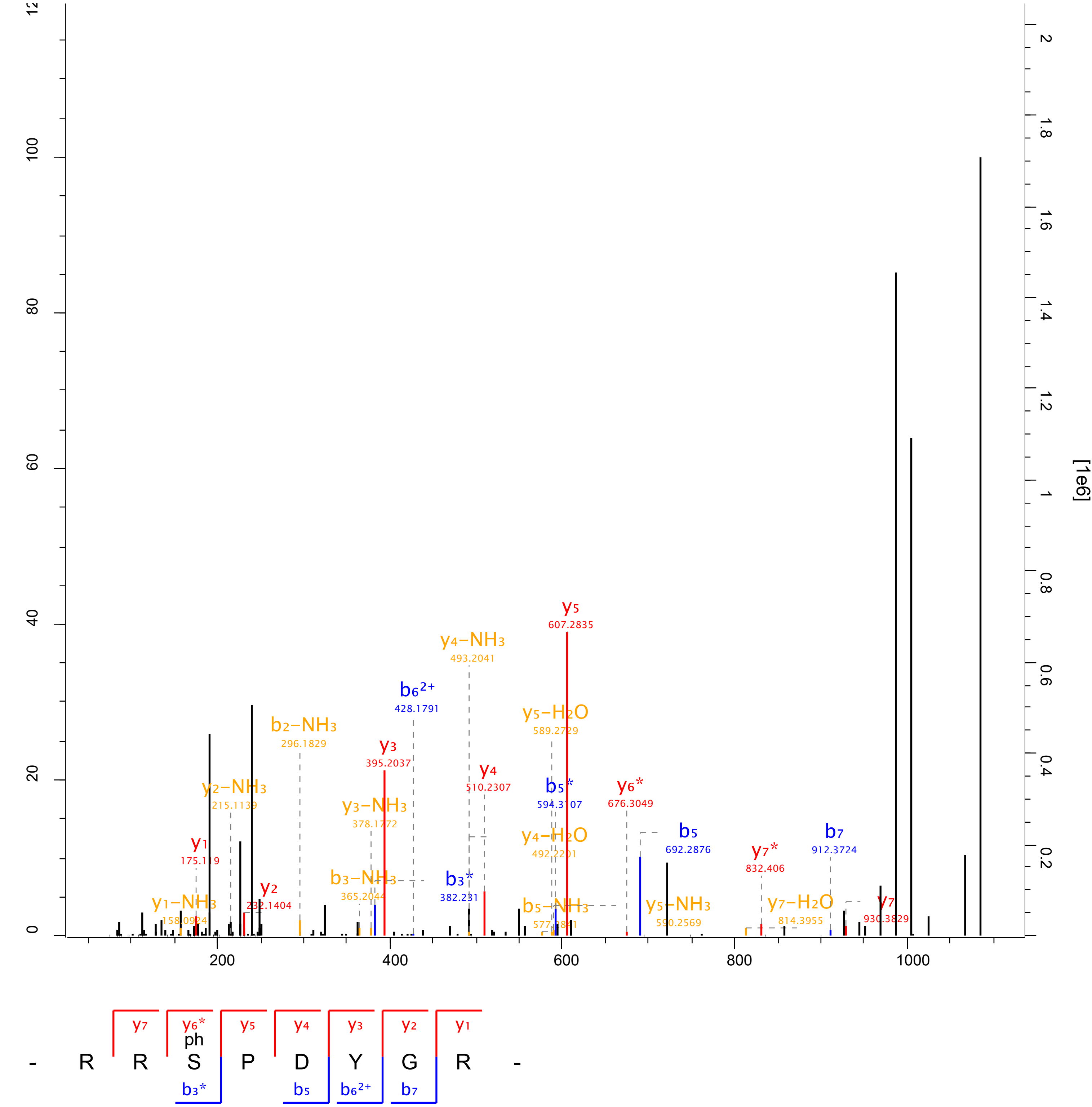

Sample output to test PDF Combine only

Raw file	Scan	Method	Score	m/z	Gene names
0523_12	1474	FTMS; HCD	133.91	479.24	RSZ22A;RSZ22

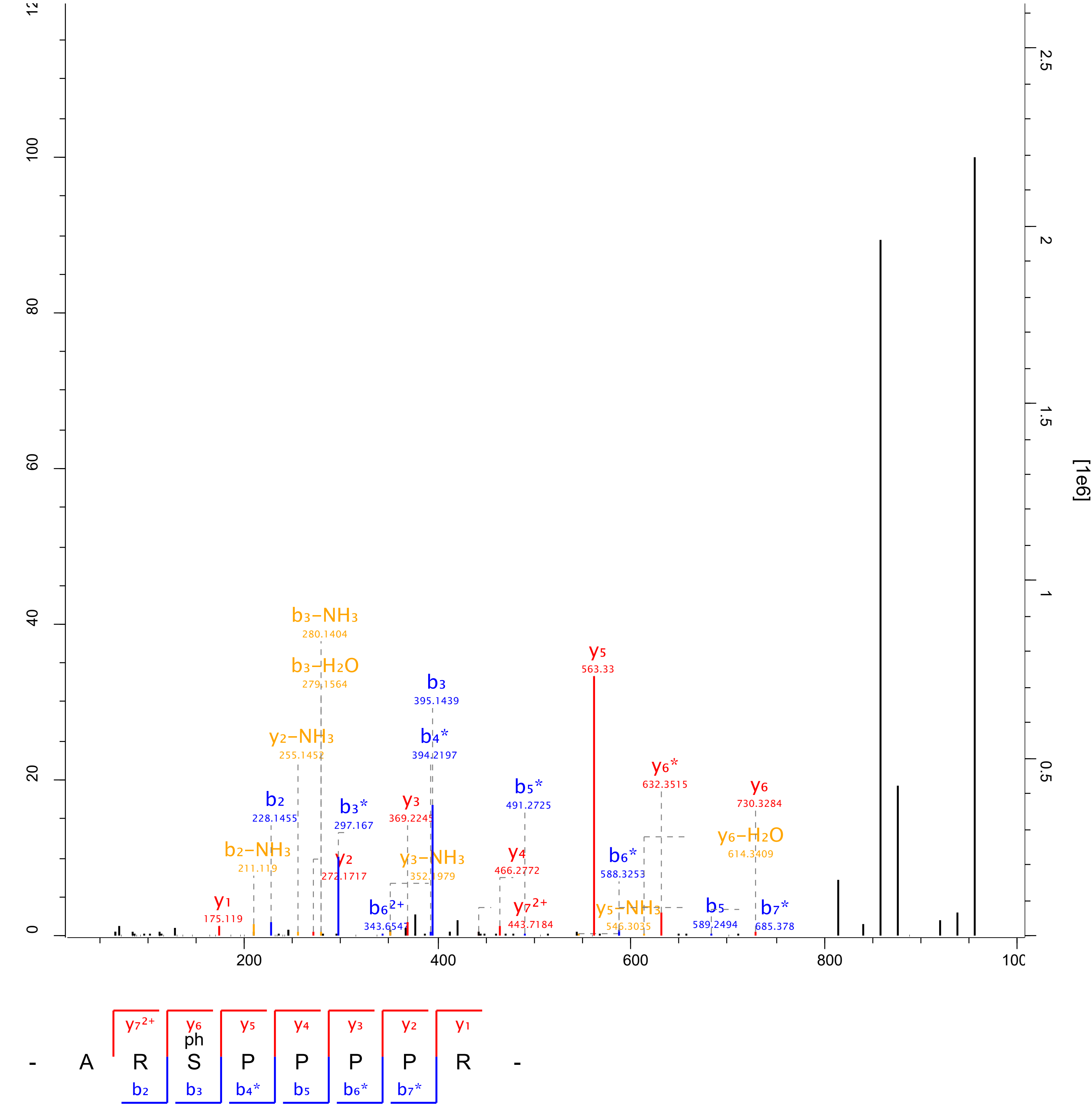

Sample output to test PDF Combine only

Raw file	Scan	Method	Score	m/z	Gene names
0523_12	1484	FTMS; HCD	123.68	527.74	F18B13.26

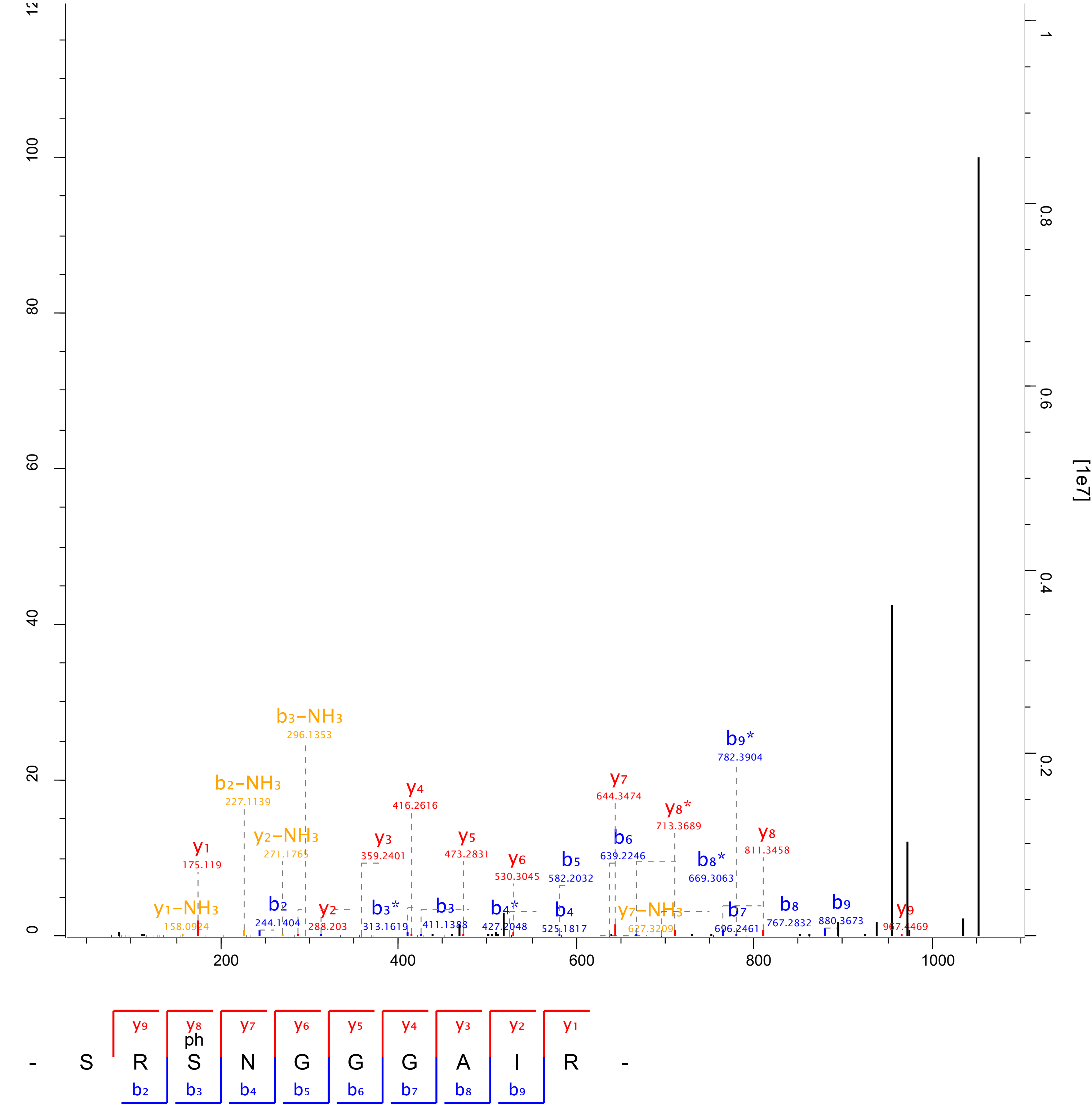

Sample output to test PDF Combine only

Raw file	Scan	Method	Score	m/z	Gene names
0523_12	1496	FTMS; HCD	75.48	381.18	RPS6B;EMB3010;RPS6A;RPS6

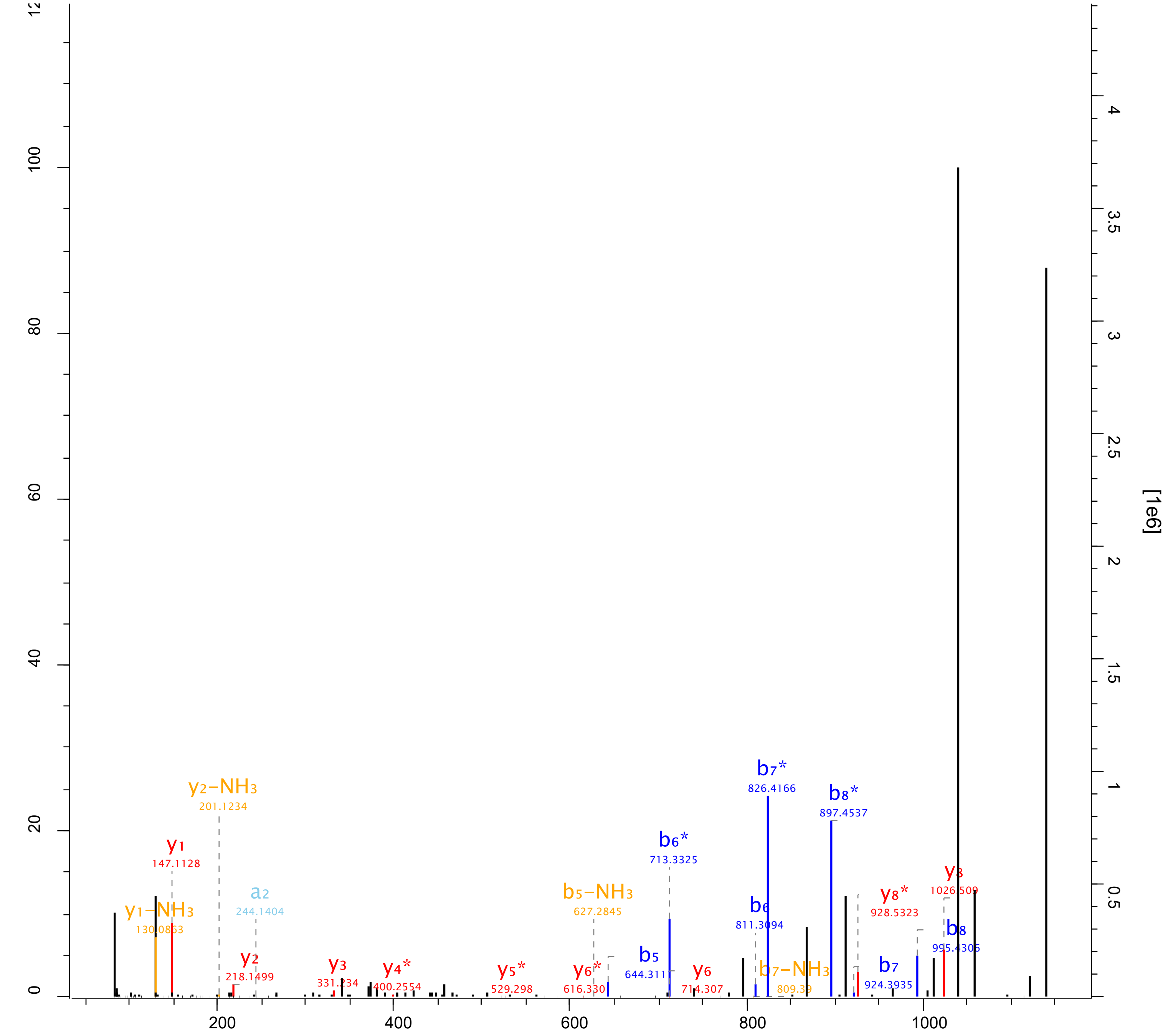

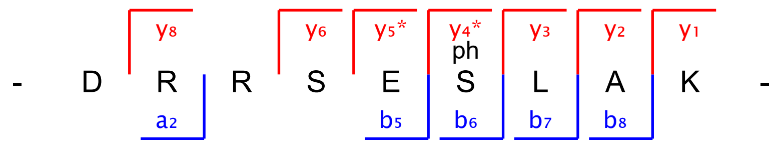

Sample output to test PDF Combine only

Raw file	Scan	Method	Score	m/z	Gene names
0523_12	1497	FTMS; HCD	79.65	451.87	SR45

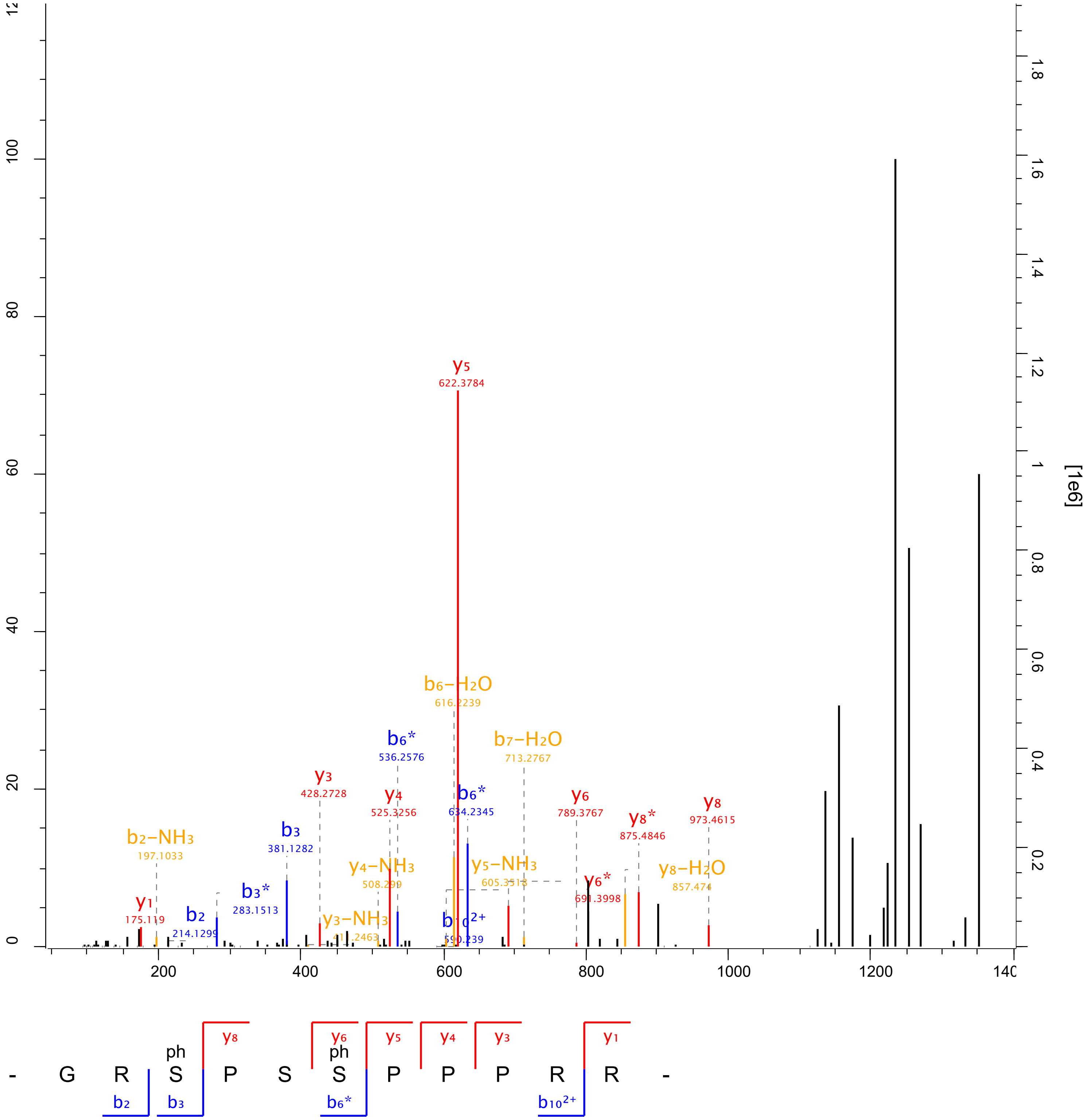

Sample output to test PDF Combine only

Raw file	Scan	Method	Score	m/z	Gene names
0523_12	1529	FTMS; HCD	78.32	621.75	At5g57370

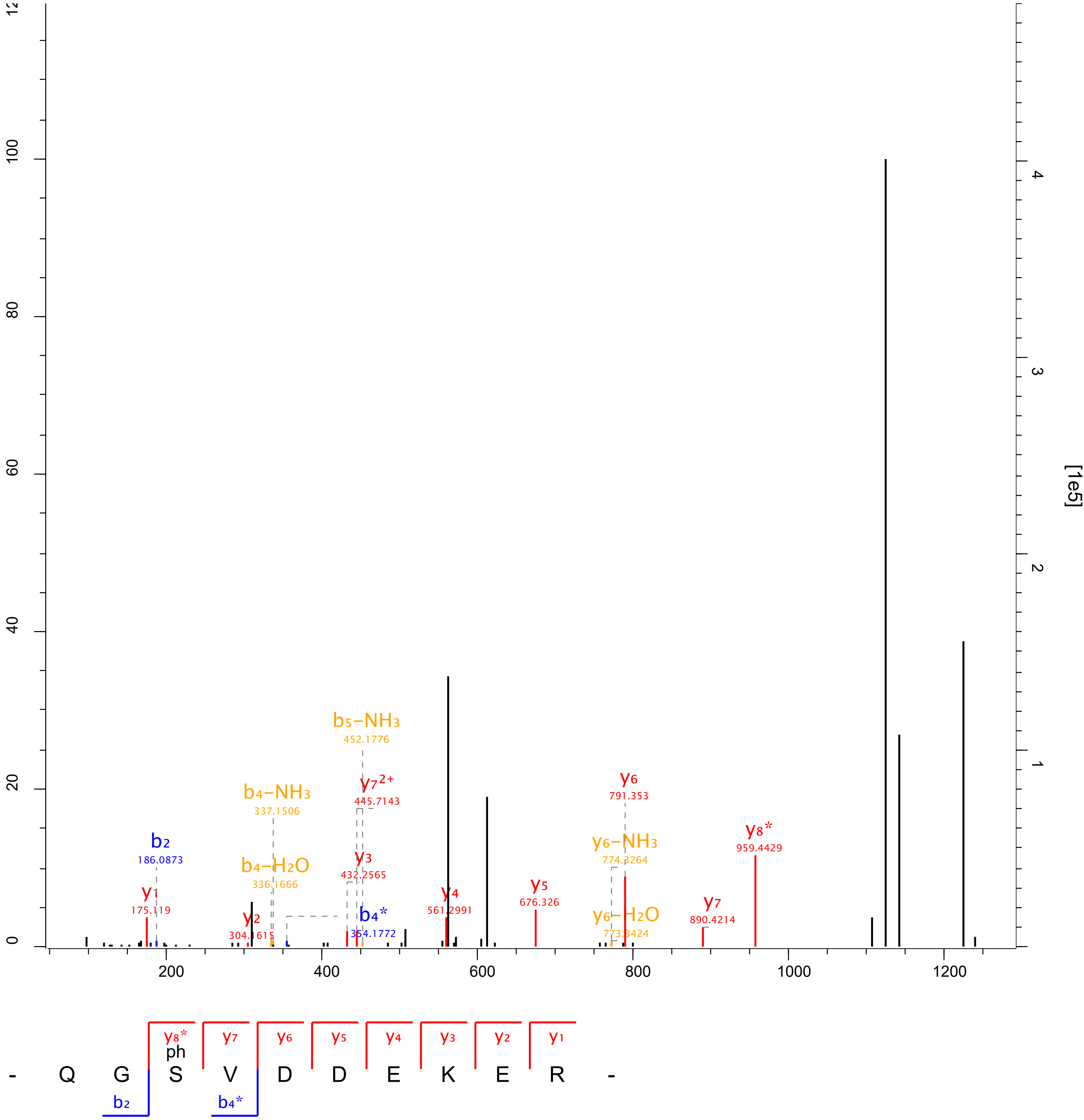

Sample output to test PDF Combine only

Raw file	Scan	Method	Score	m/z	Gene names
0523_12	1532	FTMS; HCD	117.83	535.77	RBOHG;At4g25090

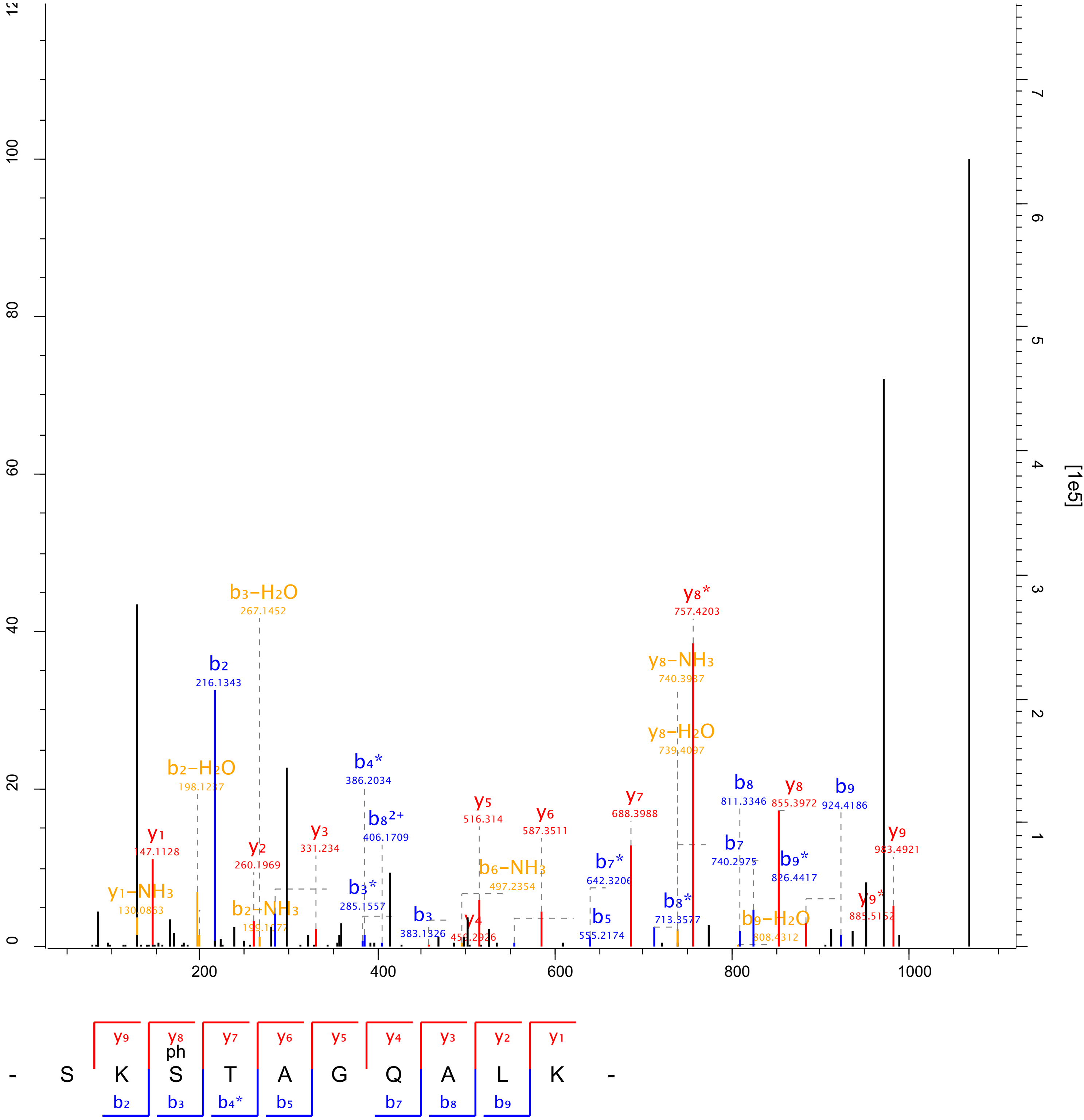

Sample output to test PDF Combine only

Raw file	Scan	Method	Score	m/z	Gene names
0523_12	1541	FTMS; HCD	52.58	585.76	PLP1

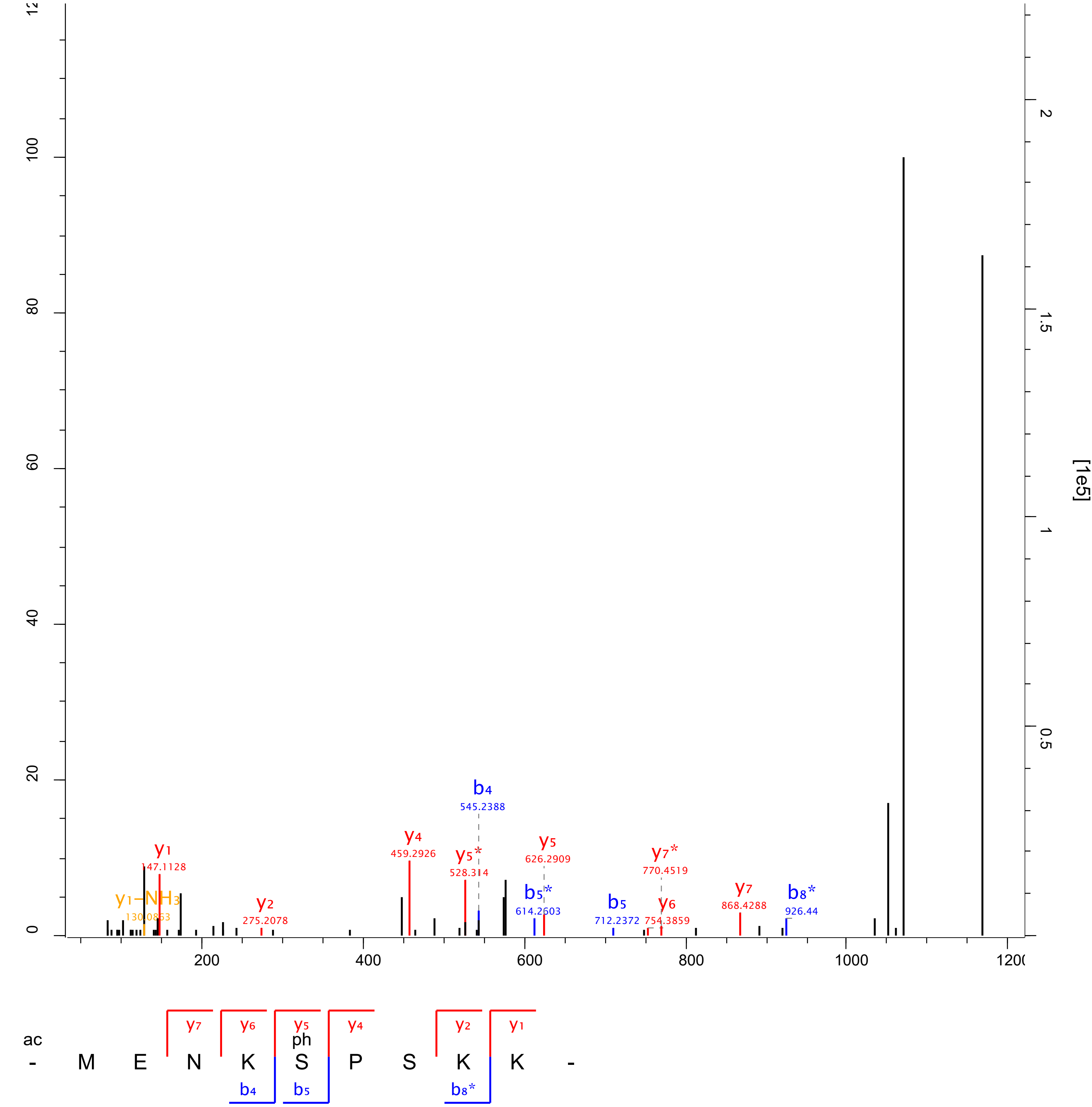

Sample output to test PDF Combine only

Raw file	Scan	Method	Score	m/z
0523_12	4274	FTMS; HCD	284.86	698.24

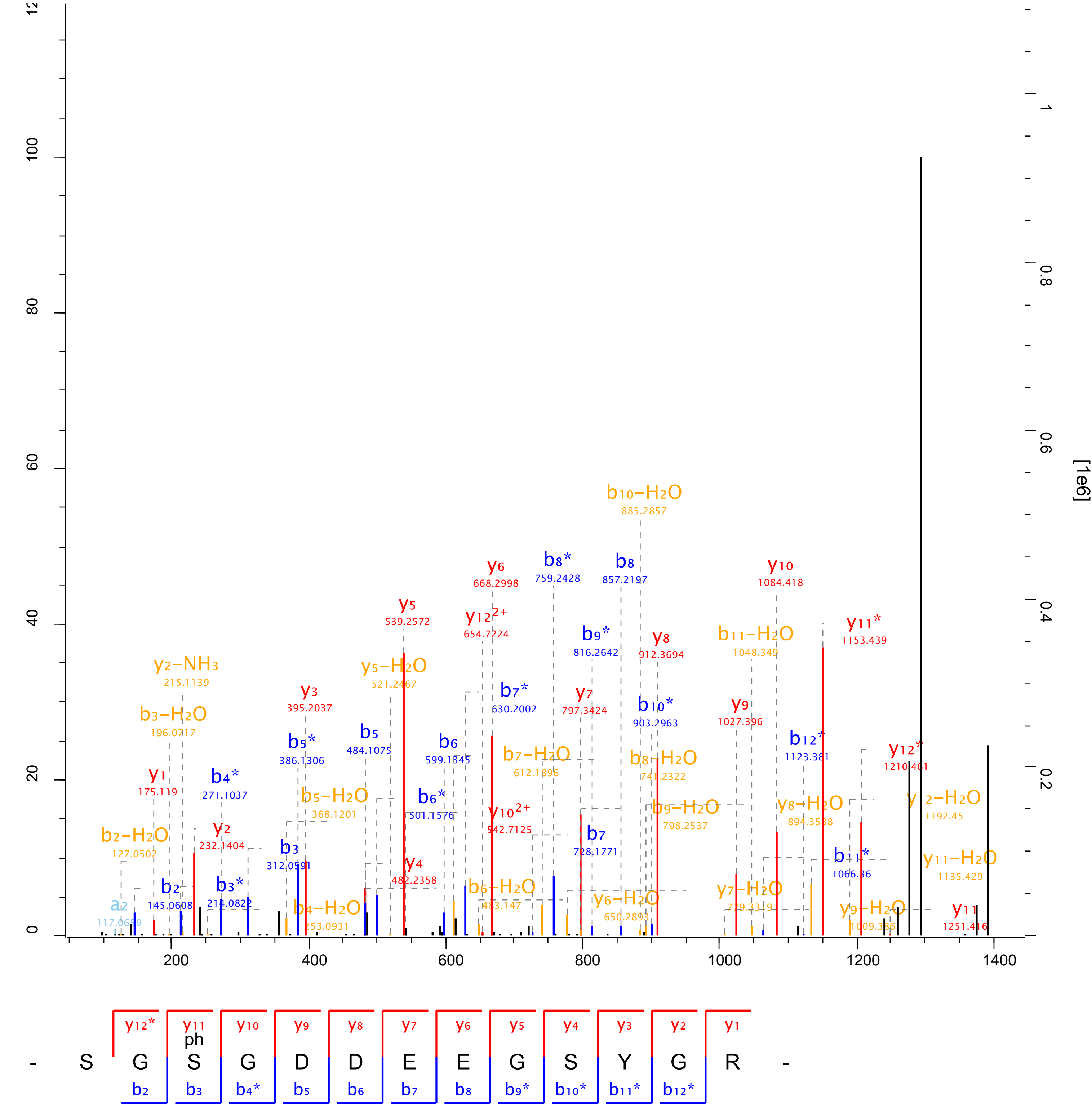

Sample output to test PDF Combine only

Raw file	Scan	Method	Score	m/z	Gene names
0523_12	7662	FTMS; HCD	66.83	575.22	F23A5.29

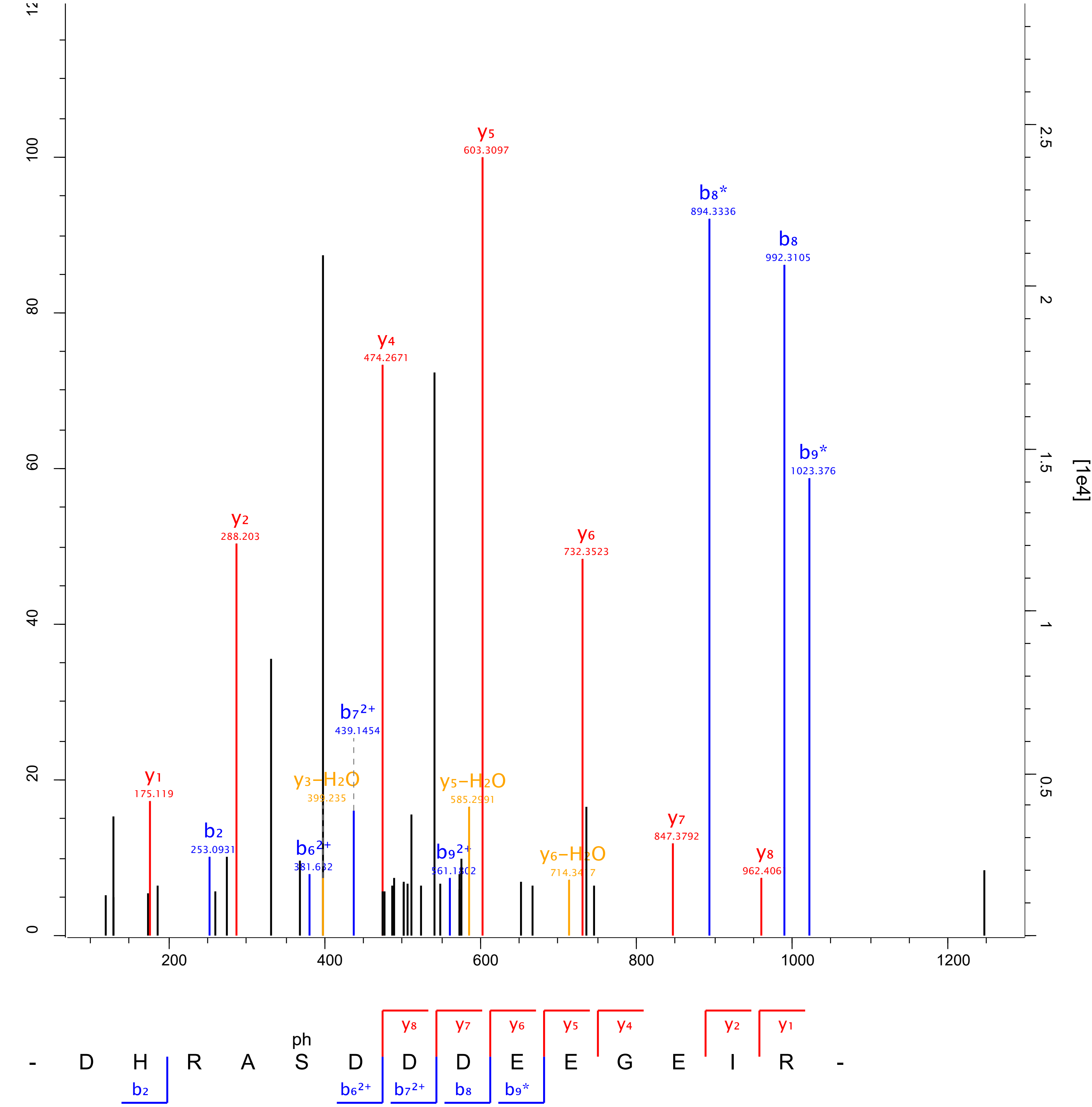

Sample output to test PDF Combine only

Raw file	Scan	Method	Score	m/z
0523_12	8122	FTMS; HCD	162.26	543.92

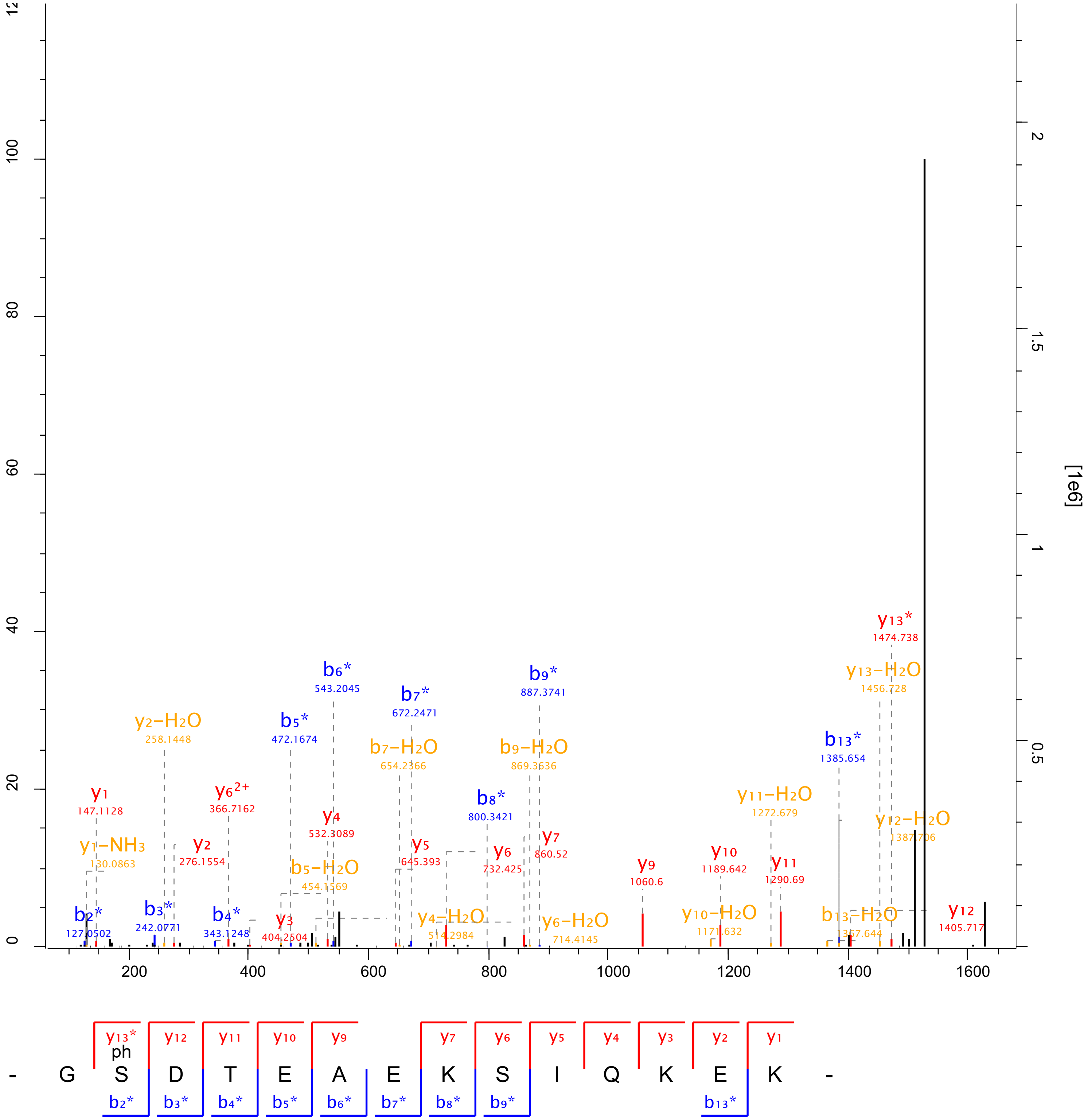

Sample output to test PDF Combine only

Raw file	Scan	Method	Score	m/z	Gene names
0523_12	8292	FTMS; HCD	153.21	540.59	PIP2-4

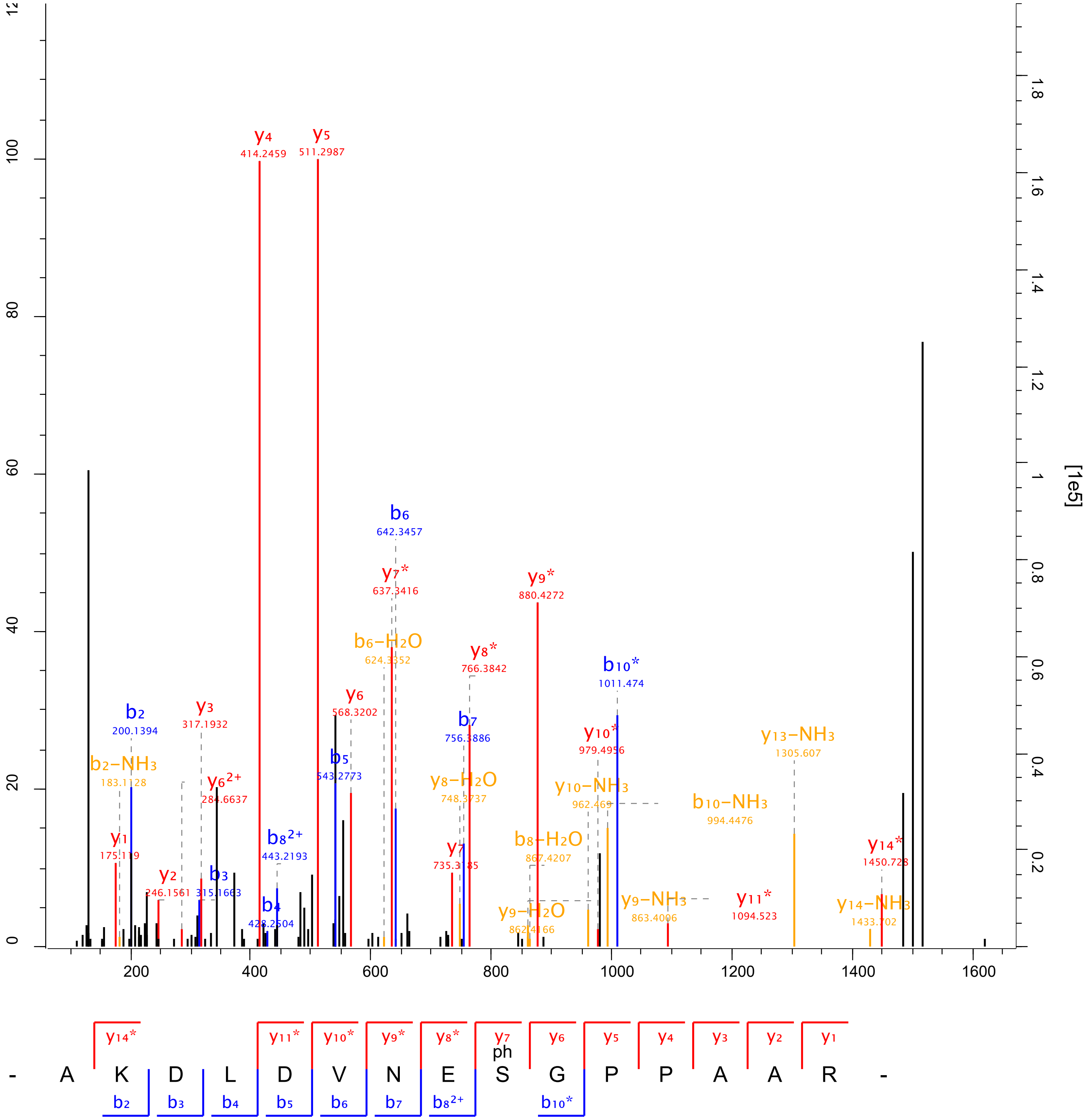

Sample output to test PDF Combine only

Raw file	Scan	Method	Score	m/z	Gene names
0523_12	8313	FTMS; HCD	139.46	704.32	AMT1-1

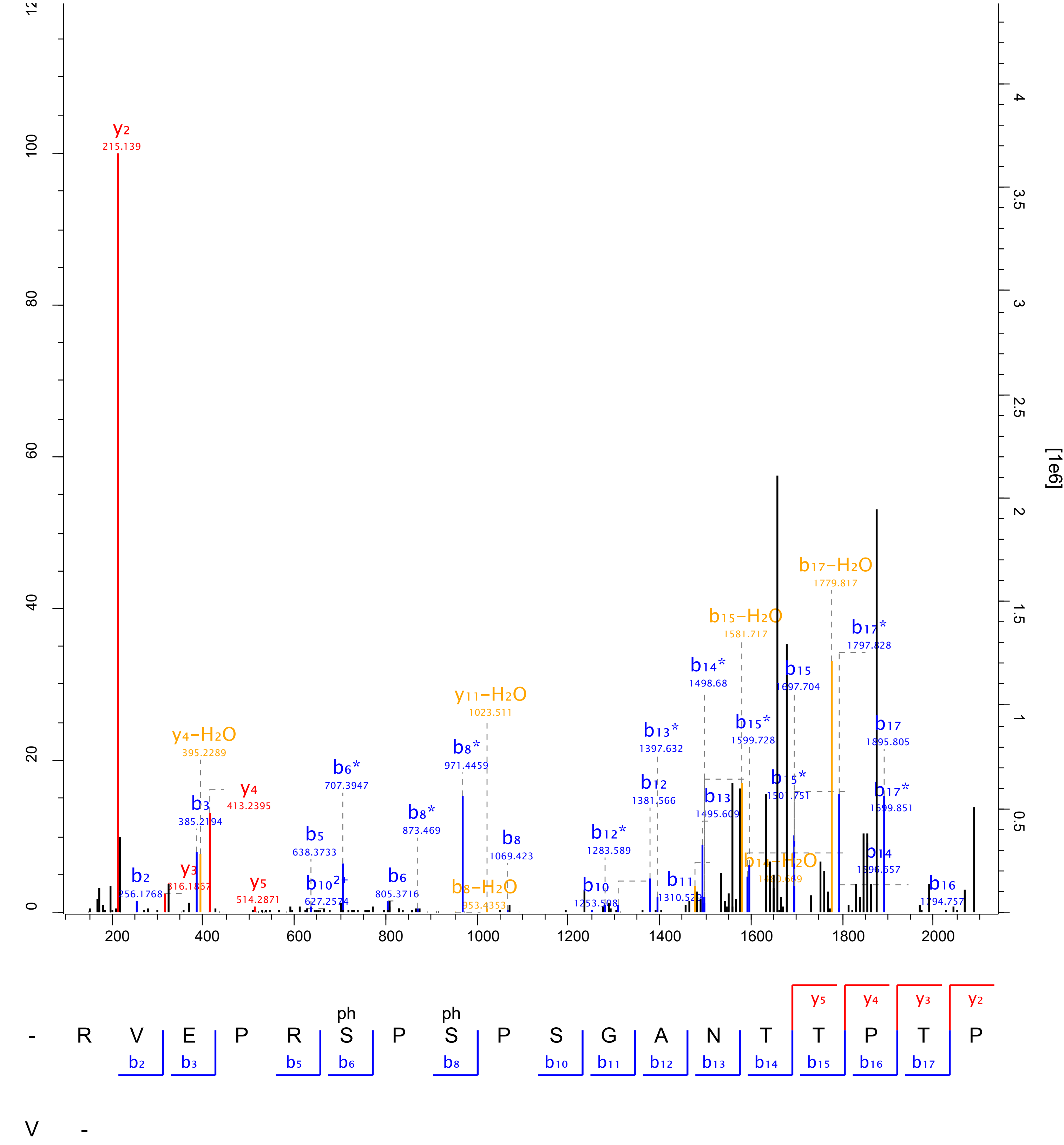

Sample output to test PDF Combine only

Raw file	Scan	Method	Score	m/z	Gene names
0523_12	8337	FTMS; HCD	43.33	521.24	CYP63

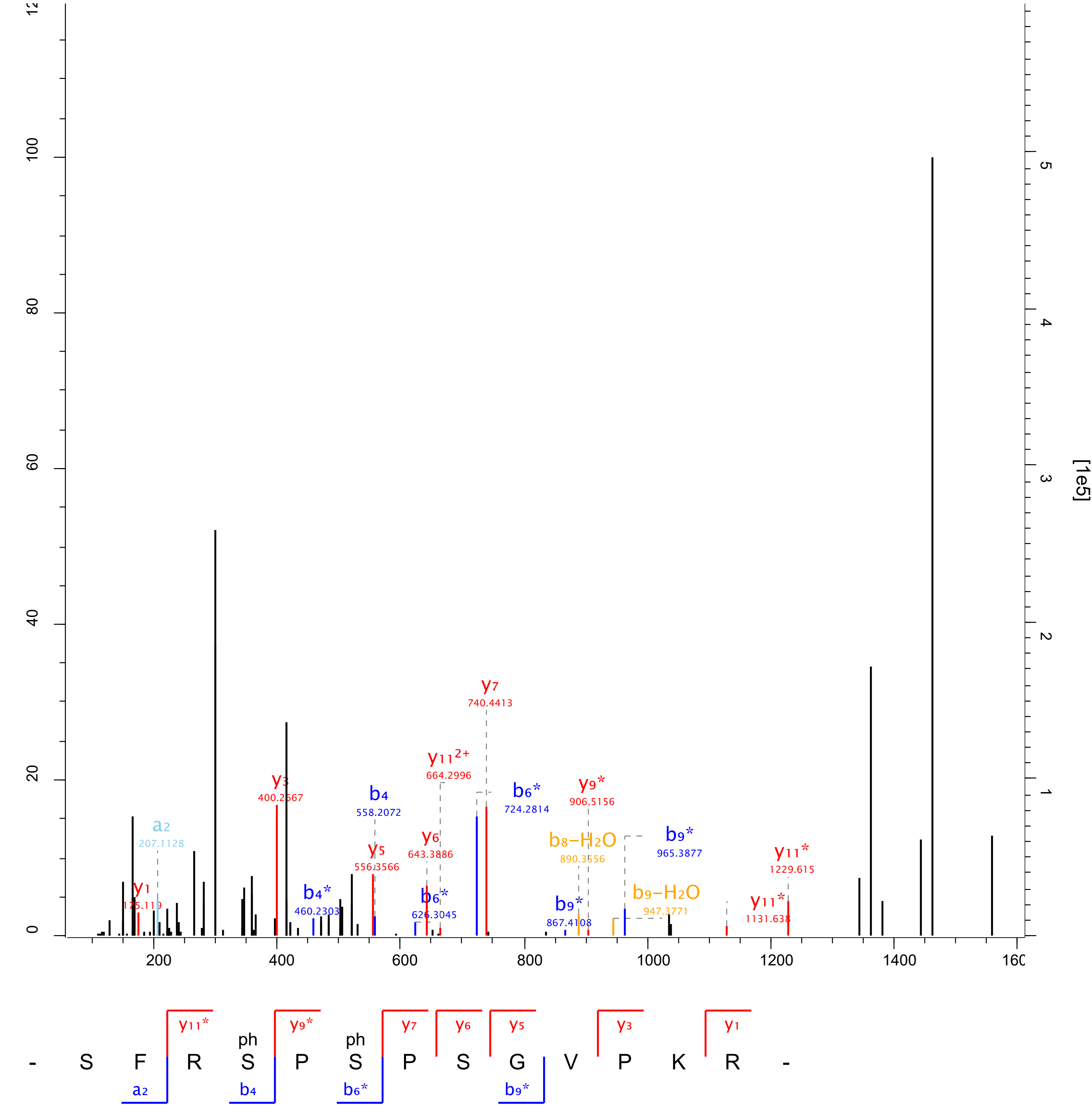

Sample output to test PDF Combine only

Raw file	Scan	Method	Score	m/z	Gene names
0523_12	8833	FTMS; HCD	158.37	693.29	SYP122

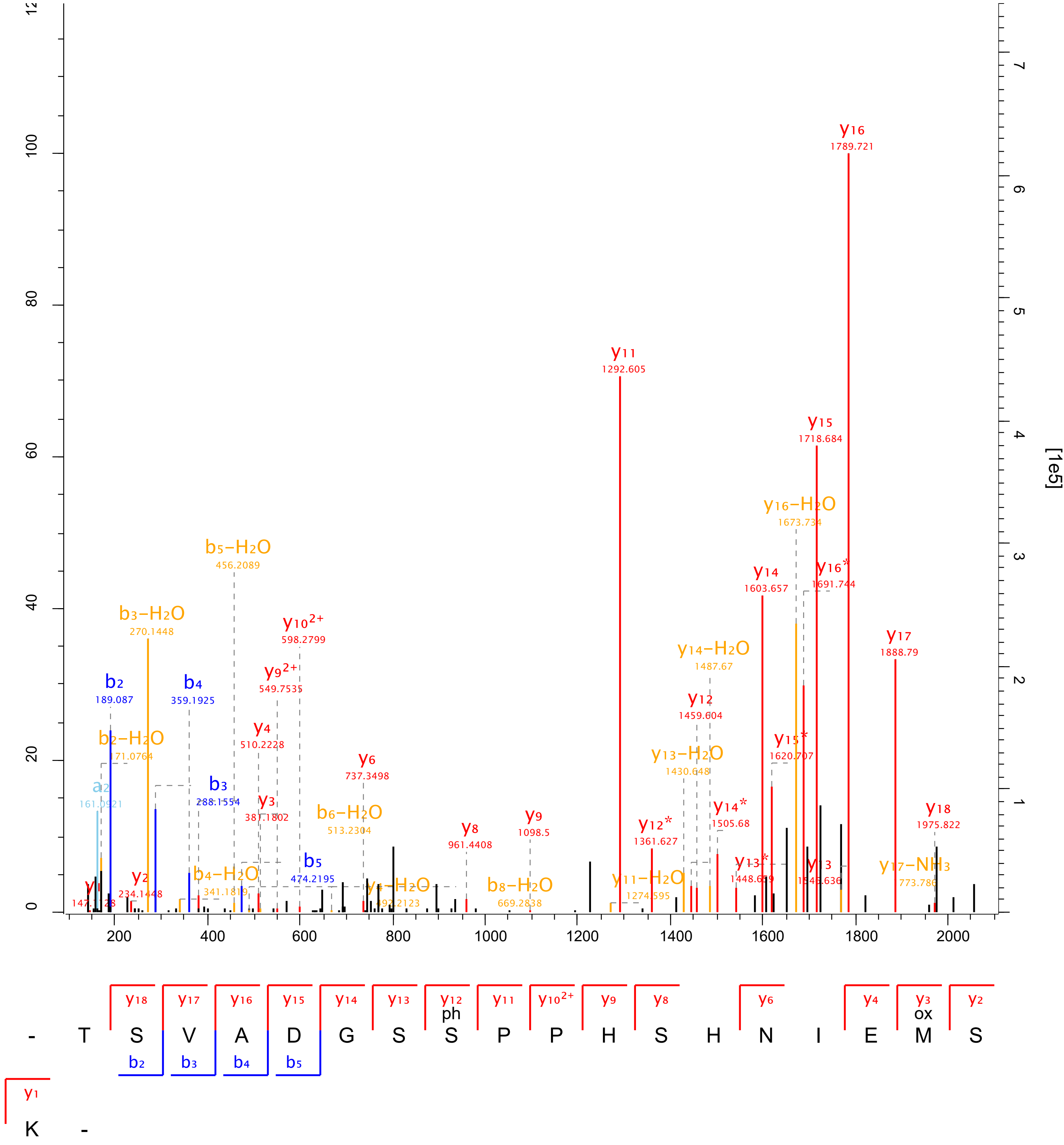

Sample output to test PDF Combine only

Raw file	Scan	Method	Score	m/z	Gene names
0523_12	9046	FTMS; HCD	121.44	589.26	KING1

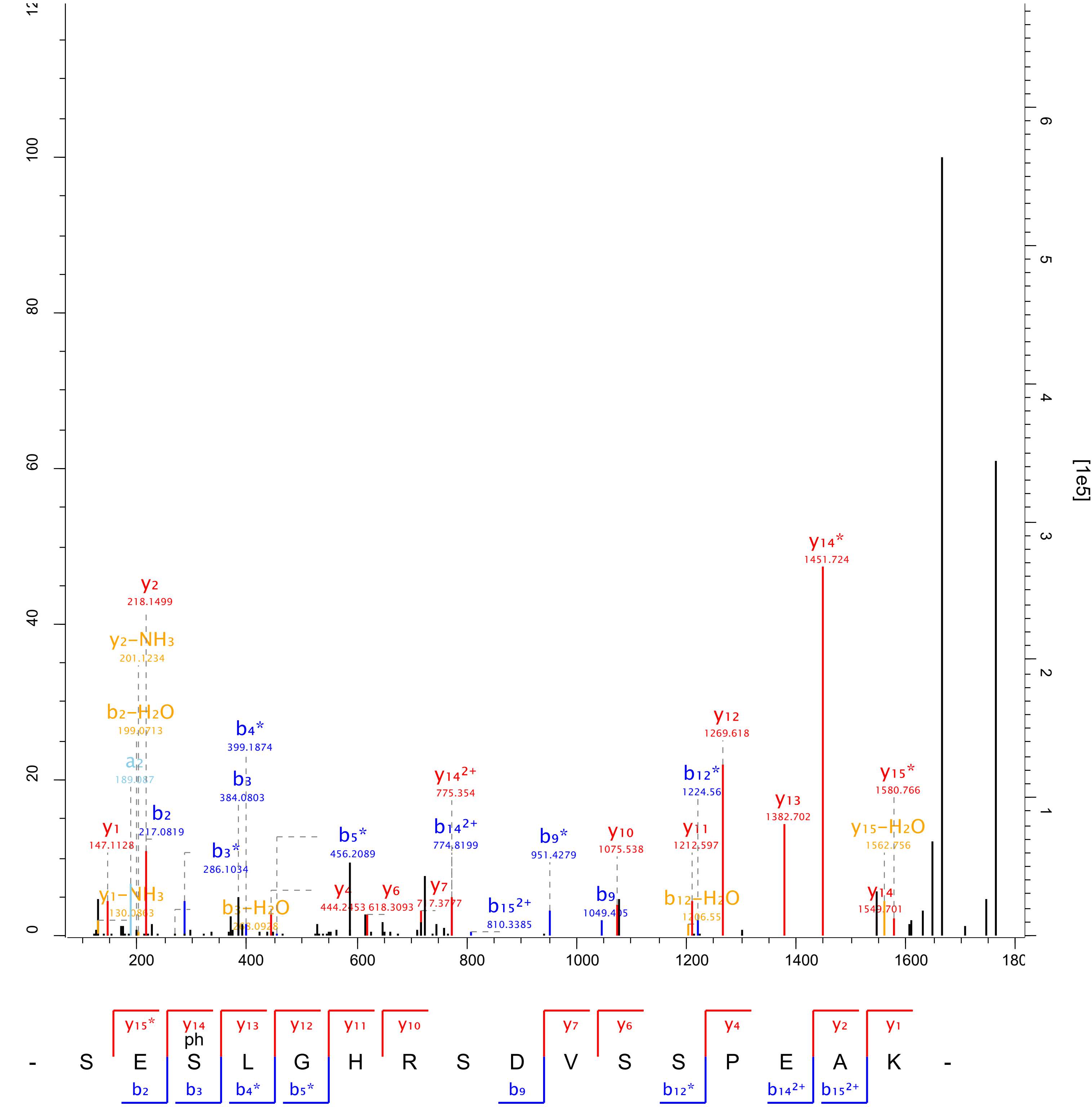

Sample output to test PDF Combine only

Raw file	Scan	Method	Score	m/z	Gene names
0523_12	9144	FTMS; HCD	108.14	625.28	ABCG36

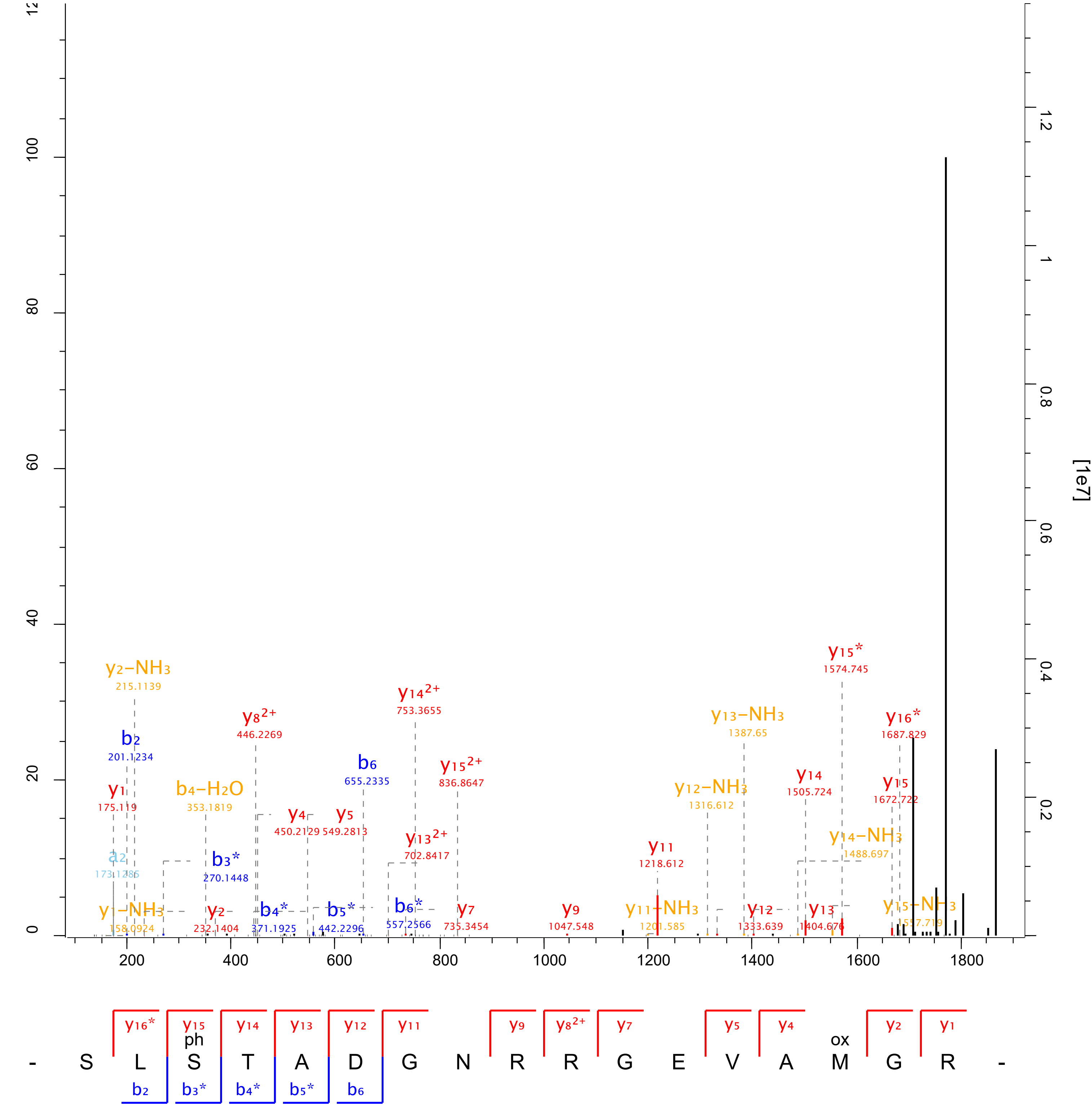

Sample output to test PDF Combine only

Raw file	Scan	Method	Score	m/z	Gene names
0523_12	9166	FTMS; HCD	48.91	534.29	SR45

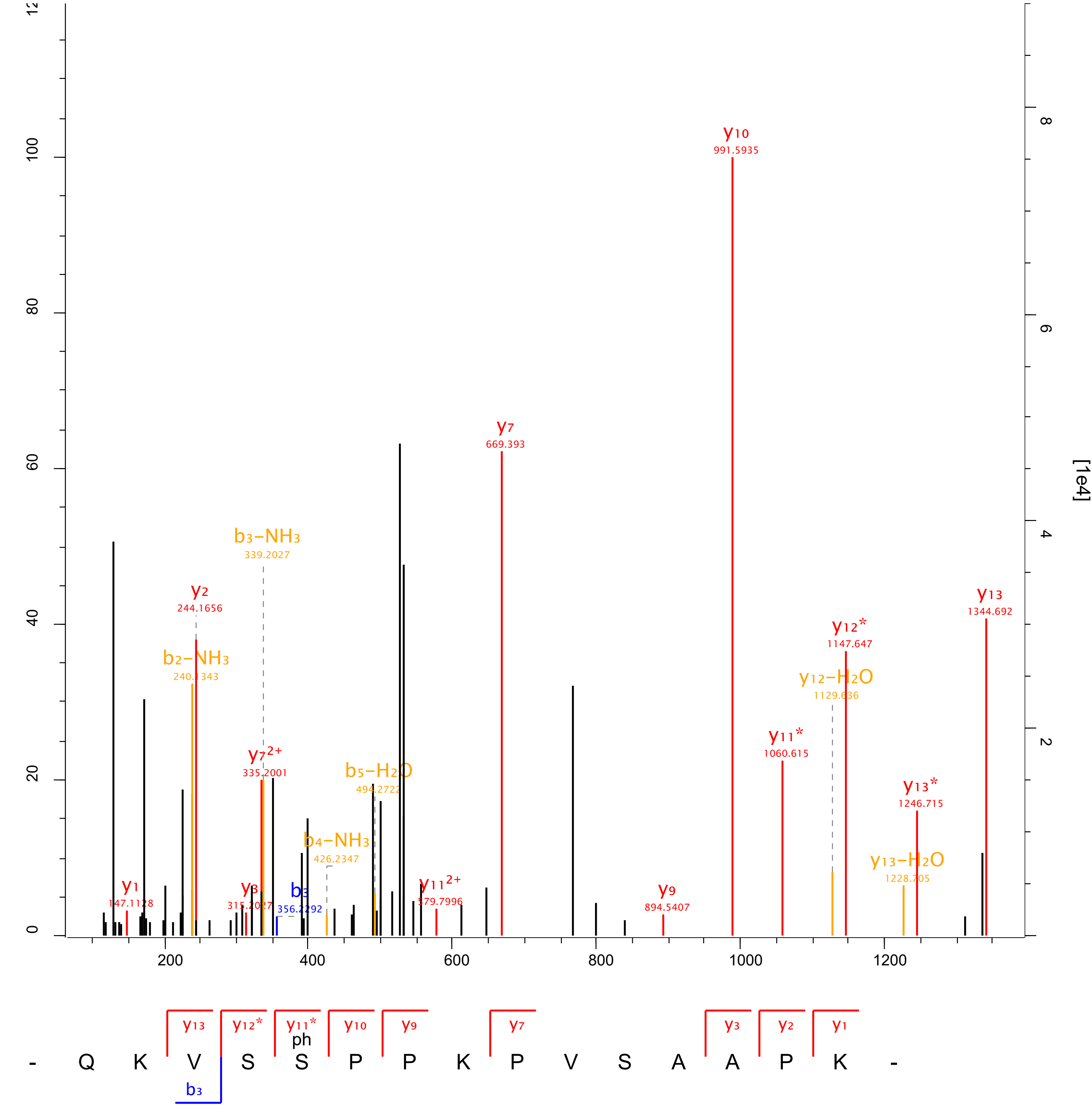

Sample output to test PDF Combine only

Raw file	Scan	Method	Score	m/z
0523_12	9256	FTMS; HCD	71.38	661.28

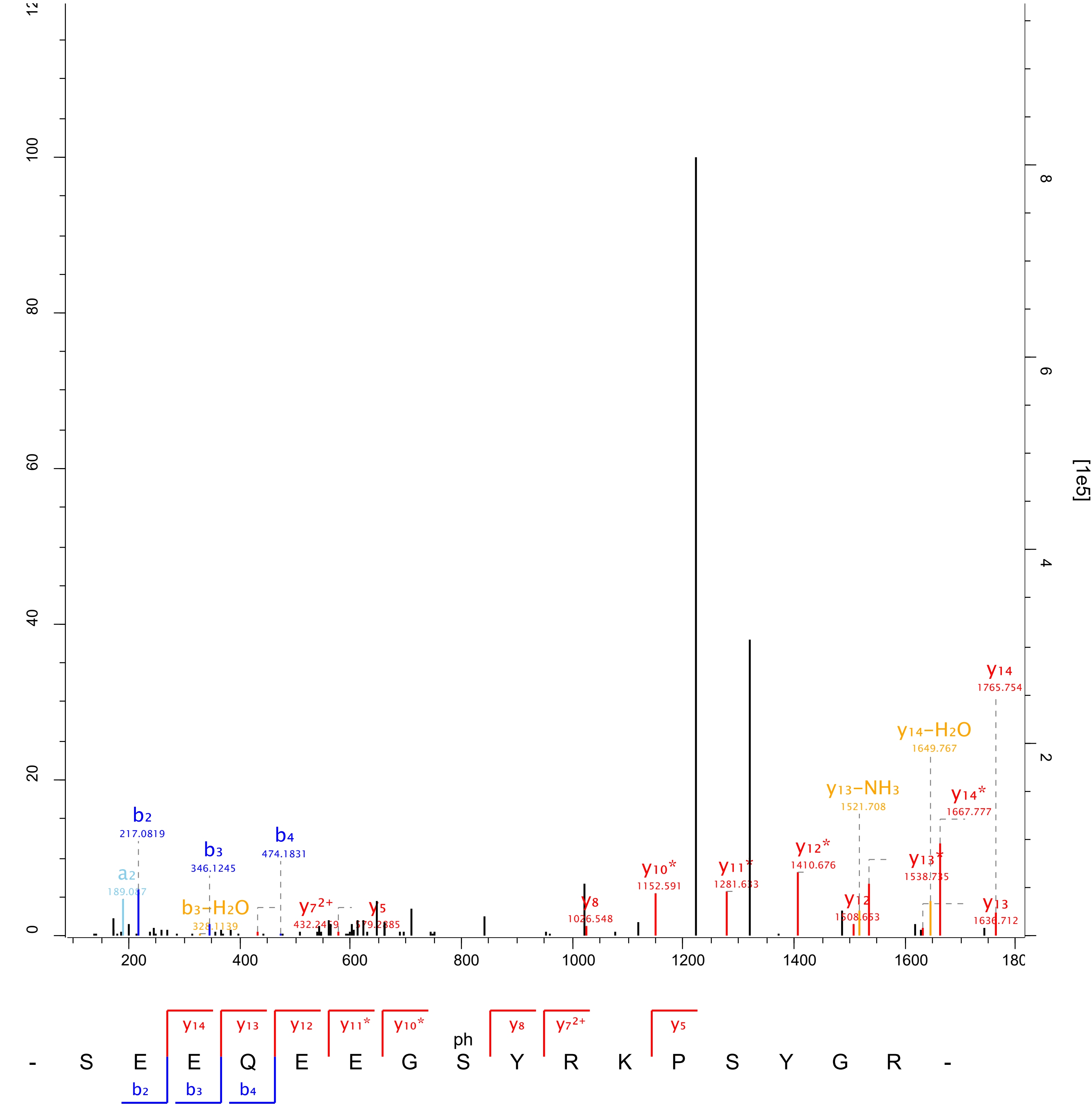

Sample output to test PDF Combine only

Raw file	Scan	Method	Score	m/z
0523_12	9604	FTMS; HCD	92.52	697.96

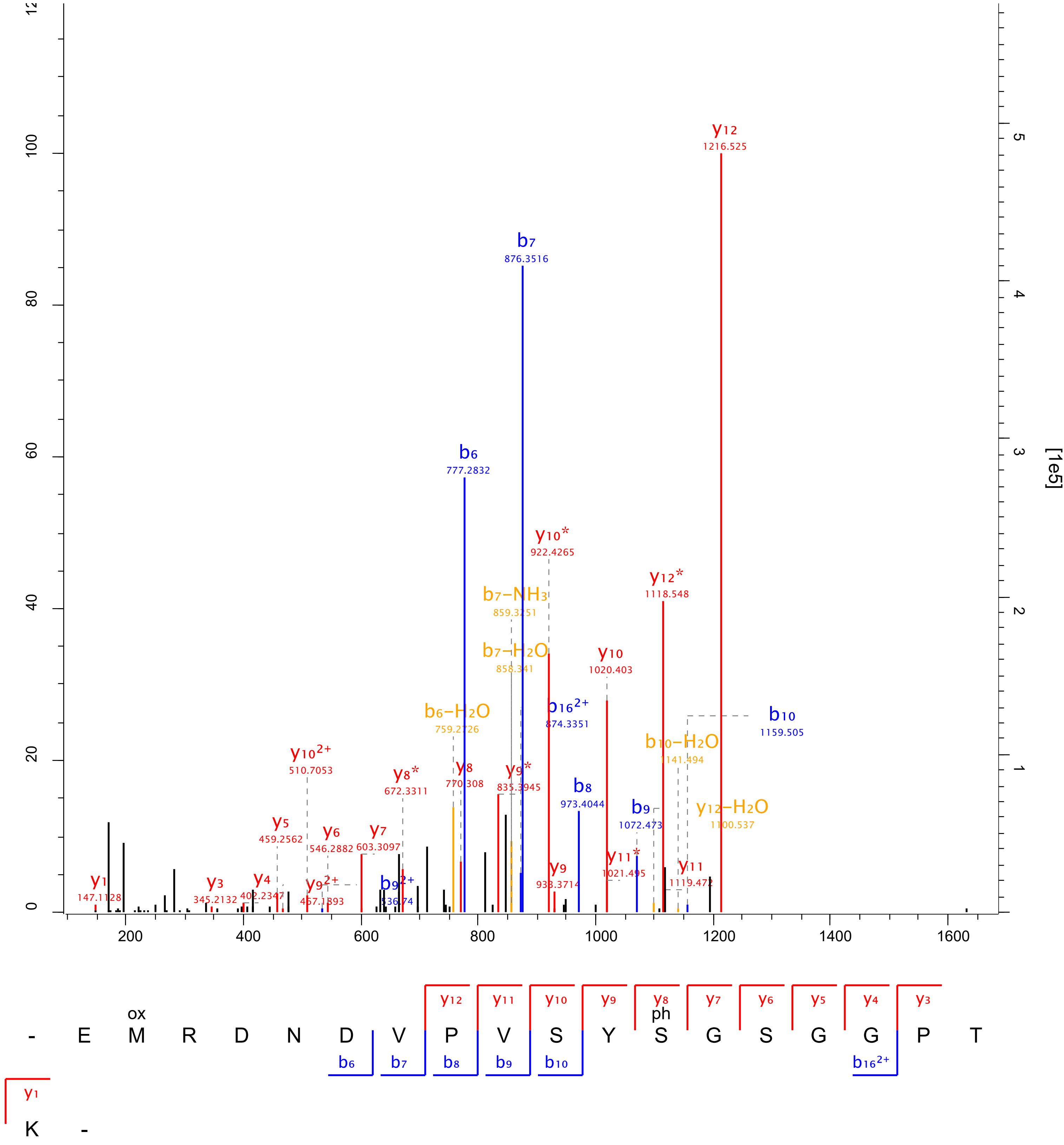

Sample output to test PDF Combine only

Raw file	Scan	Method	Score	m/z	Gene names
0523_12	9715	FTMS; HCD	92.15	914.69	T23K23.8

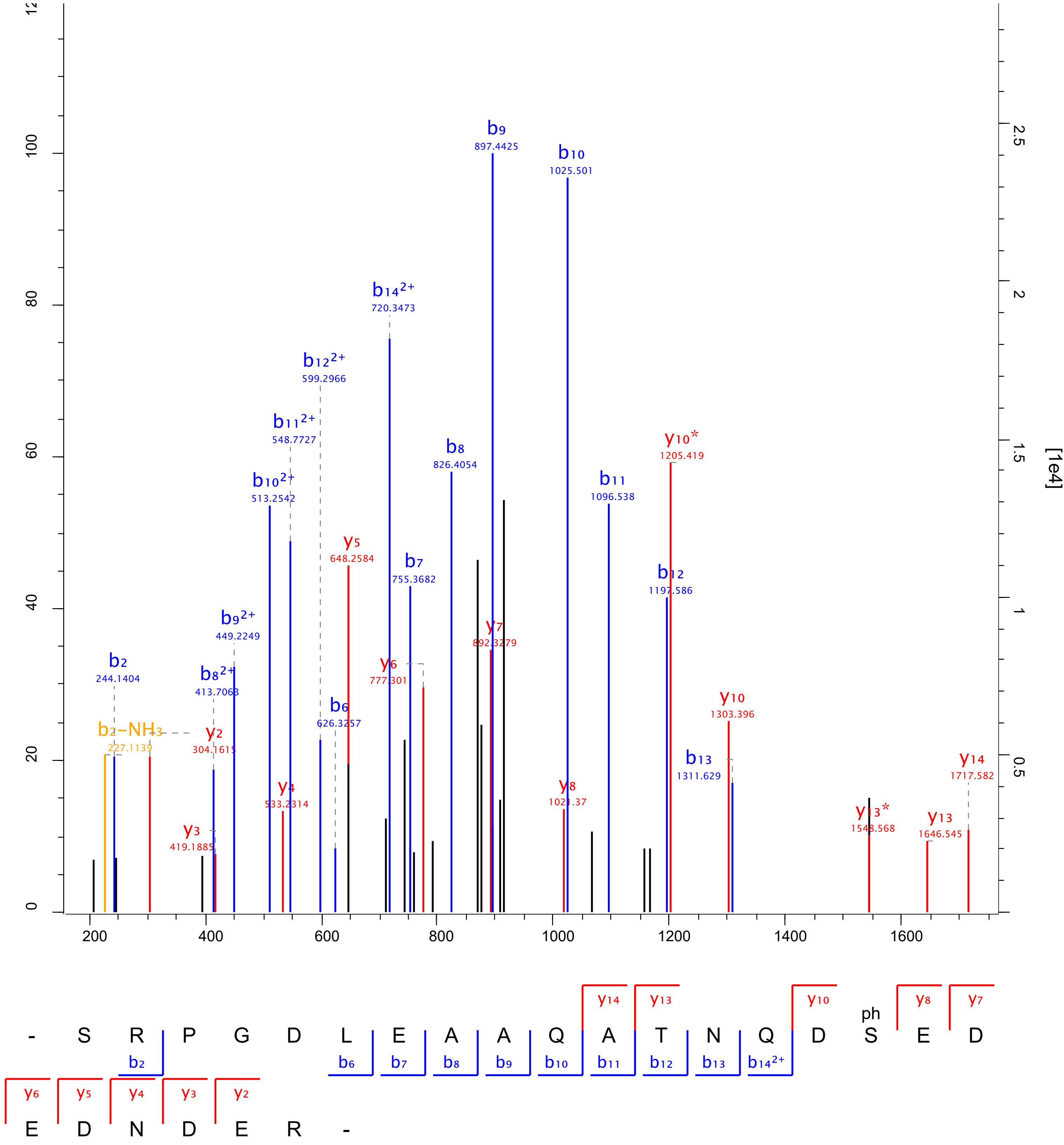

Sample output to test PDF Combine only

Raw file	Scan	Method	Score	m/z	Gene names
0523_12	9742	FTMS; HCD	42.47	541.21	NHX7

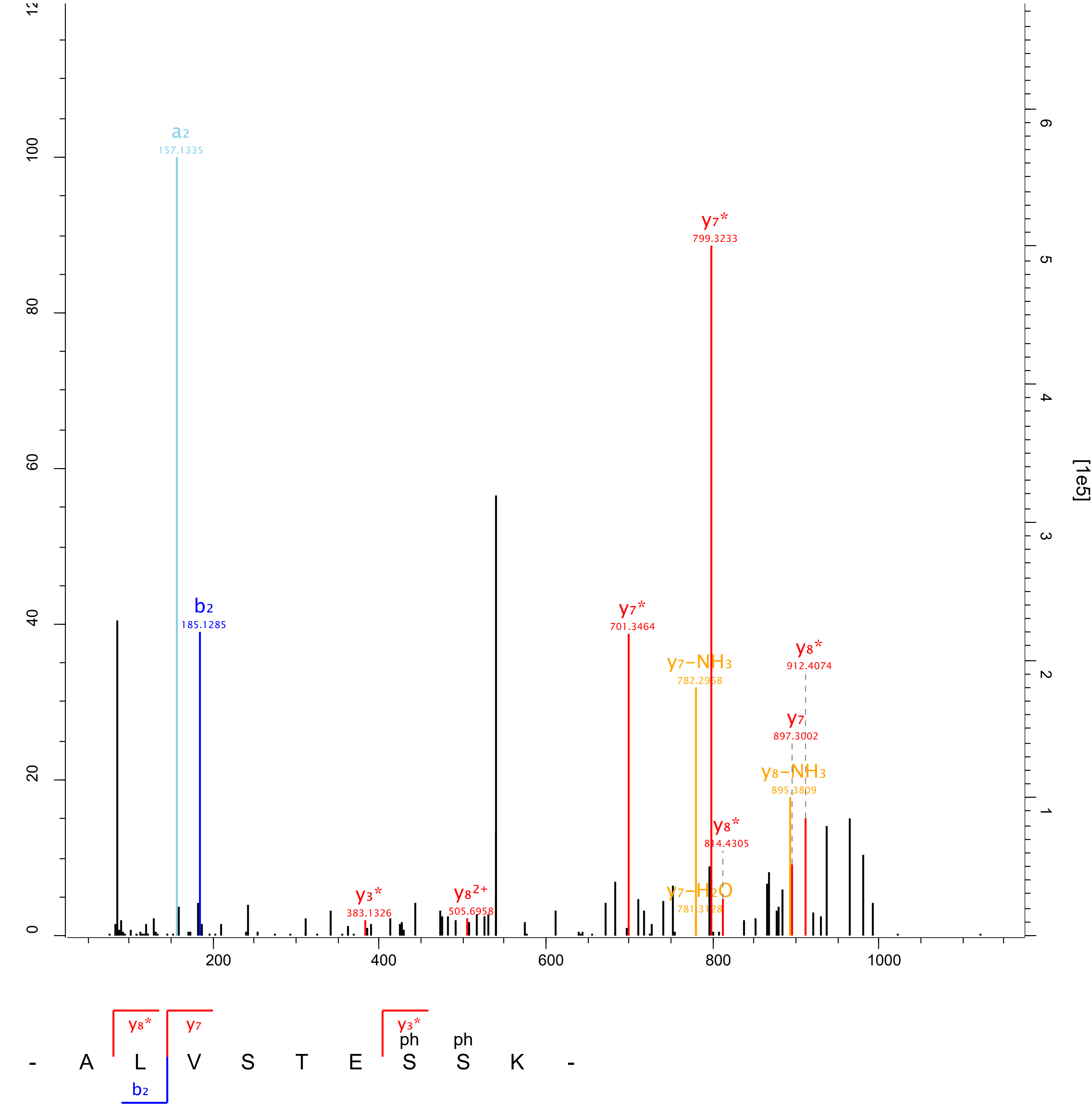

Sample output to test PDF Combine only

Raw file	Scan	Method	Score	m/z	Gene names
0523_12	10124	FTMS; HCD	133.47	726.33	PLP1

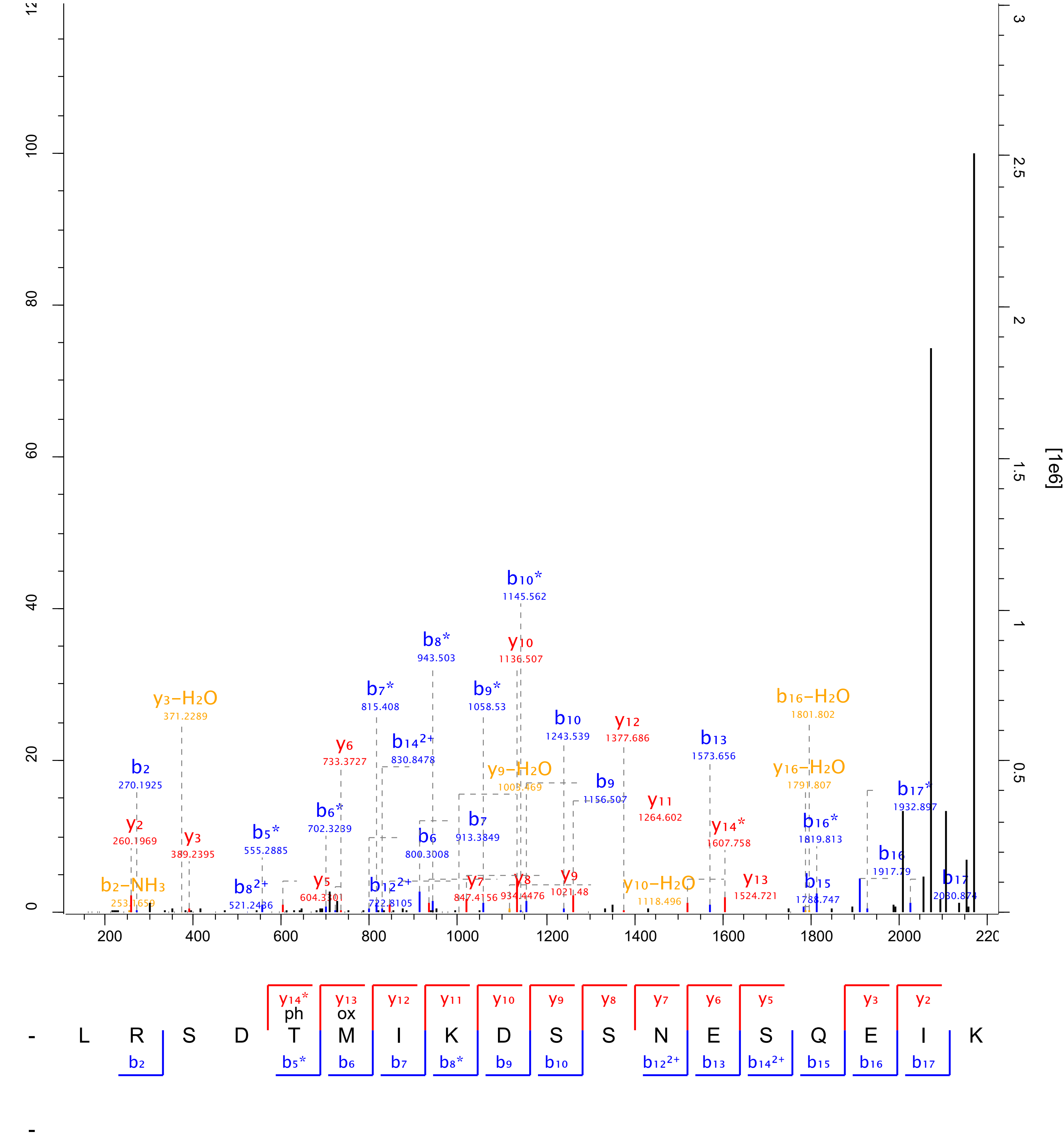

Sample output to test PDF Combine only

Raw file	Scan	Method	Score	m/z	Gene names
0523_12	10171	FTMS; HCD	42.47	631.26	DDL

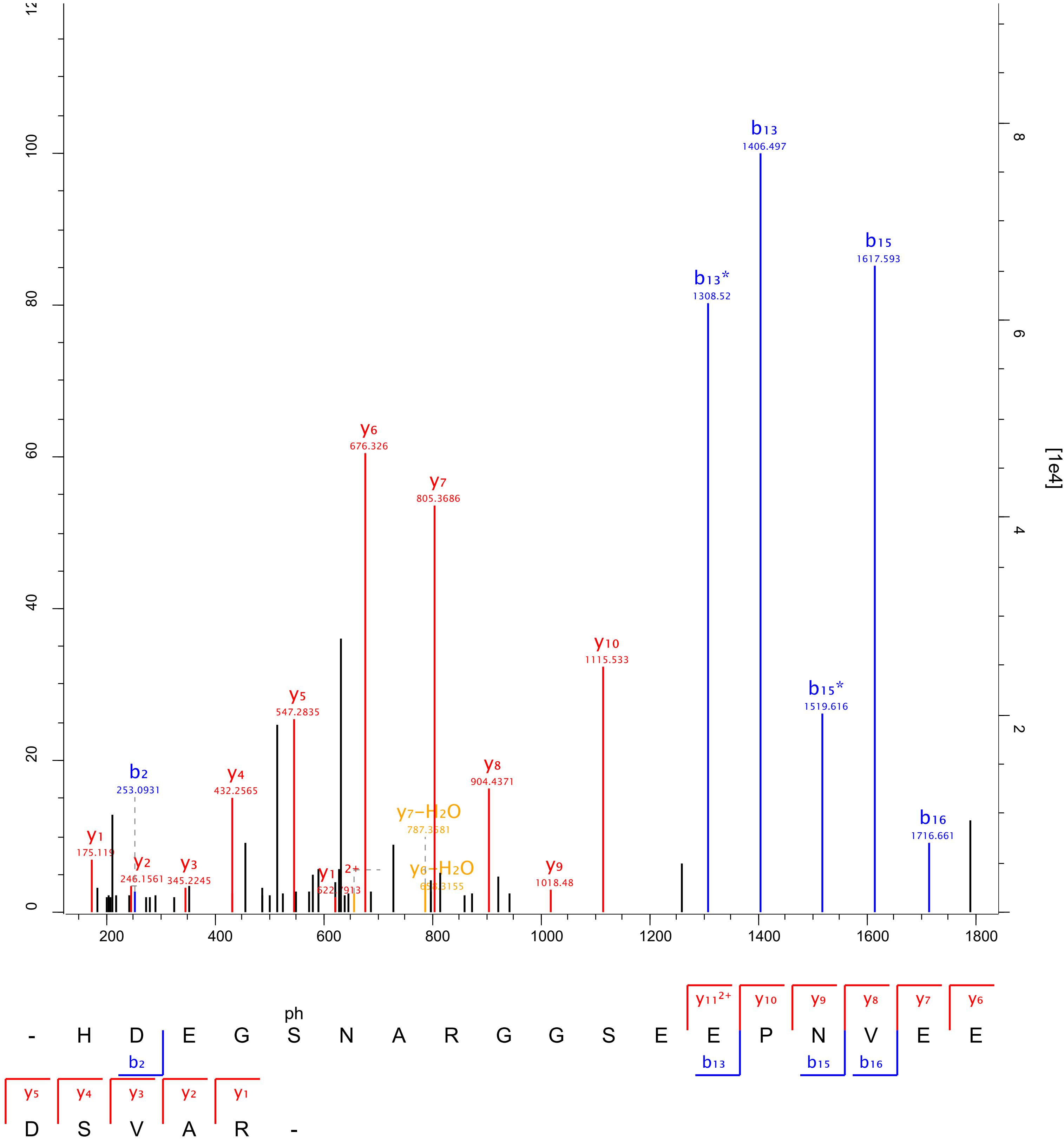

Sample output to test PDF Combine only

Raw file	Scan	Method	Score	m/z	Gene names
0523_12	10466	FTMS; HCD	125.03	546.94	PSD3

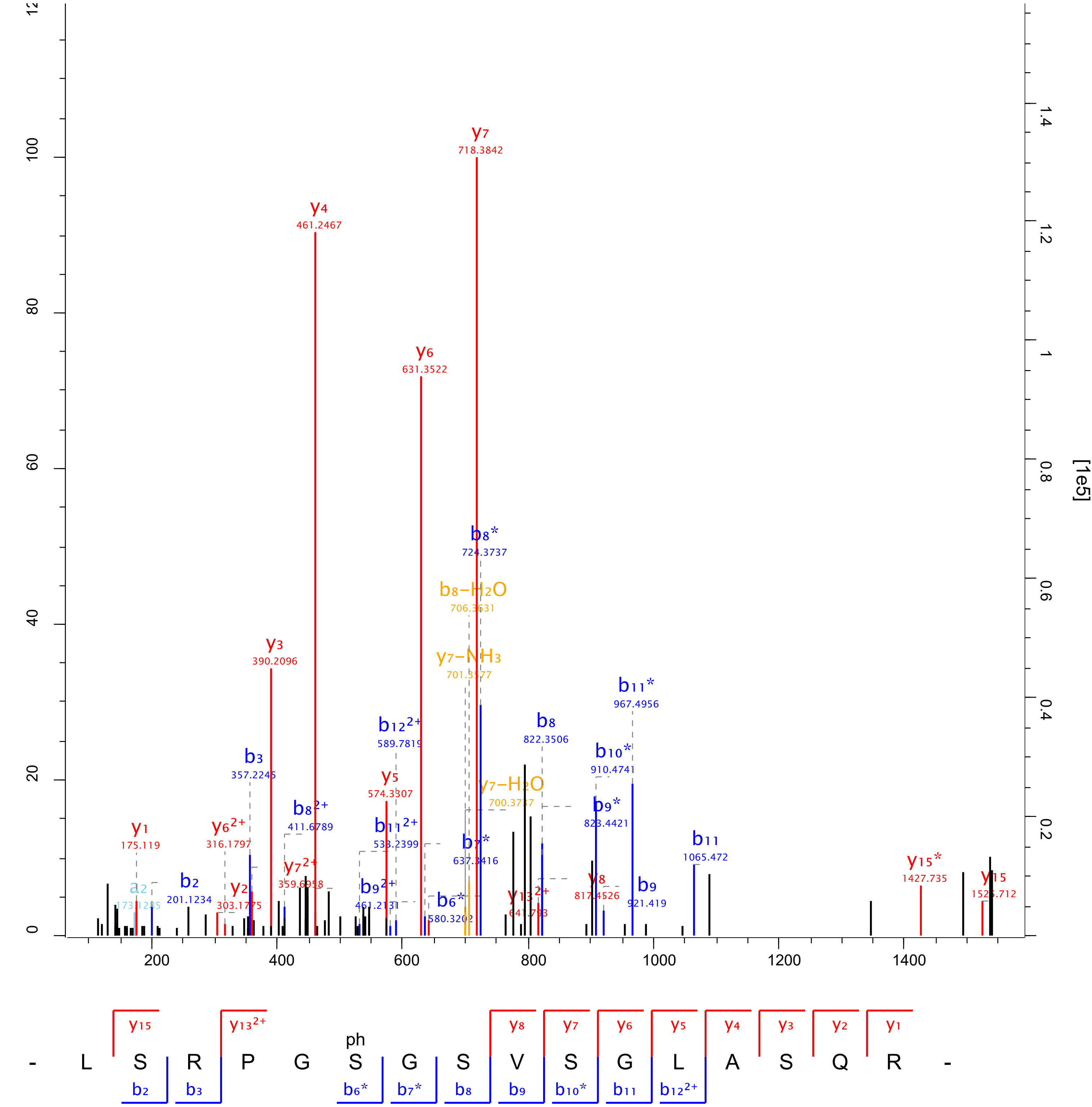

Sample output to test PDF Combine only

Raw file	Scan	Method	Score	m/z
0523_12	10488	FTMS; HCD	85.94	649.27

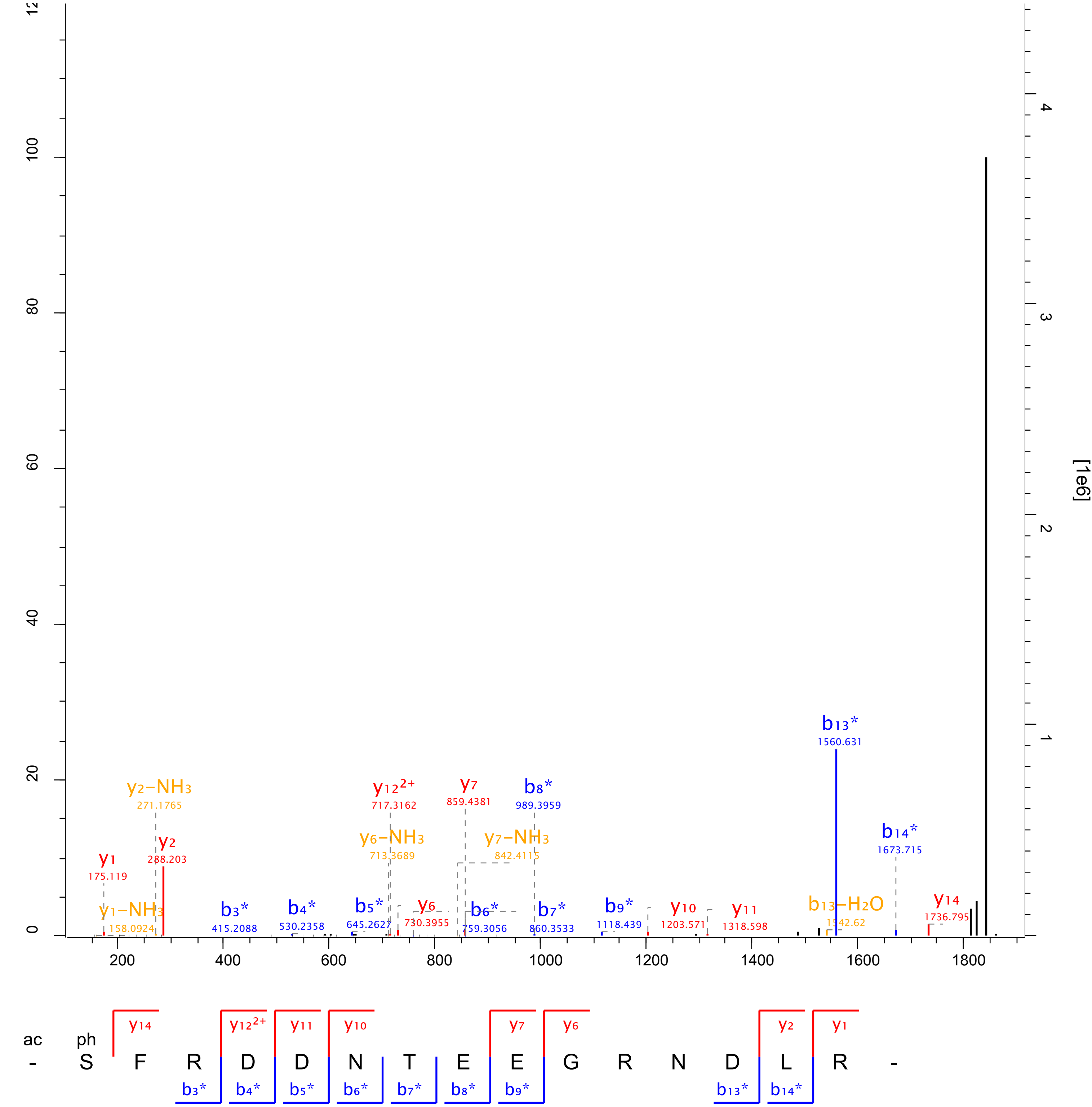

Sample output to test PDF Combine only

Raw file	Scan	Method	Score	m/z	Gene names
0523_12	10623	FTMS; HCD	43.24	811.38	At2g38410

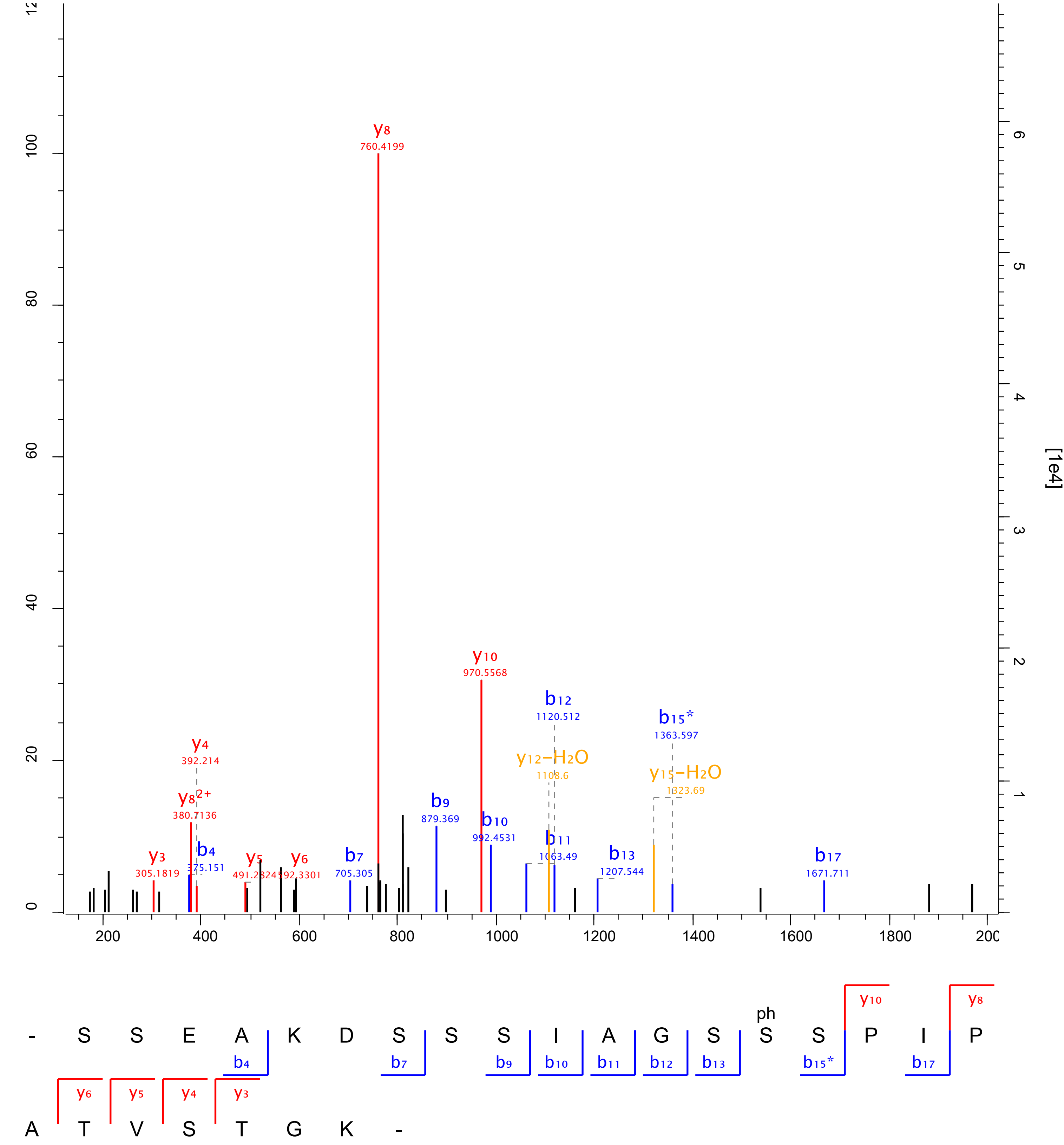

Sample output to test PDF Combine only

Raw file	Scan	Method	Score	m/z	Gene names
0523_12	10909	FTMS; HCD	47.54	515.9	DRP2B;DRP2A

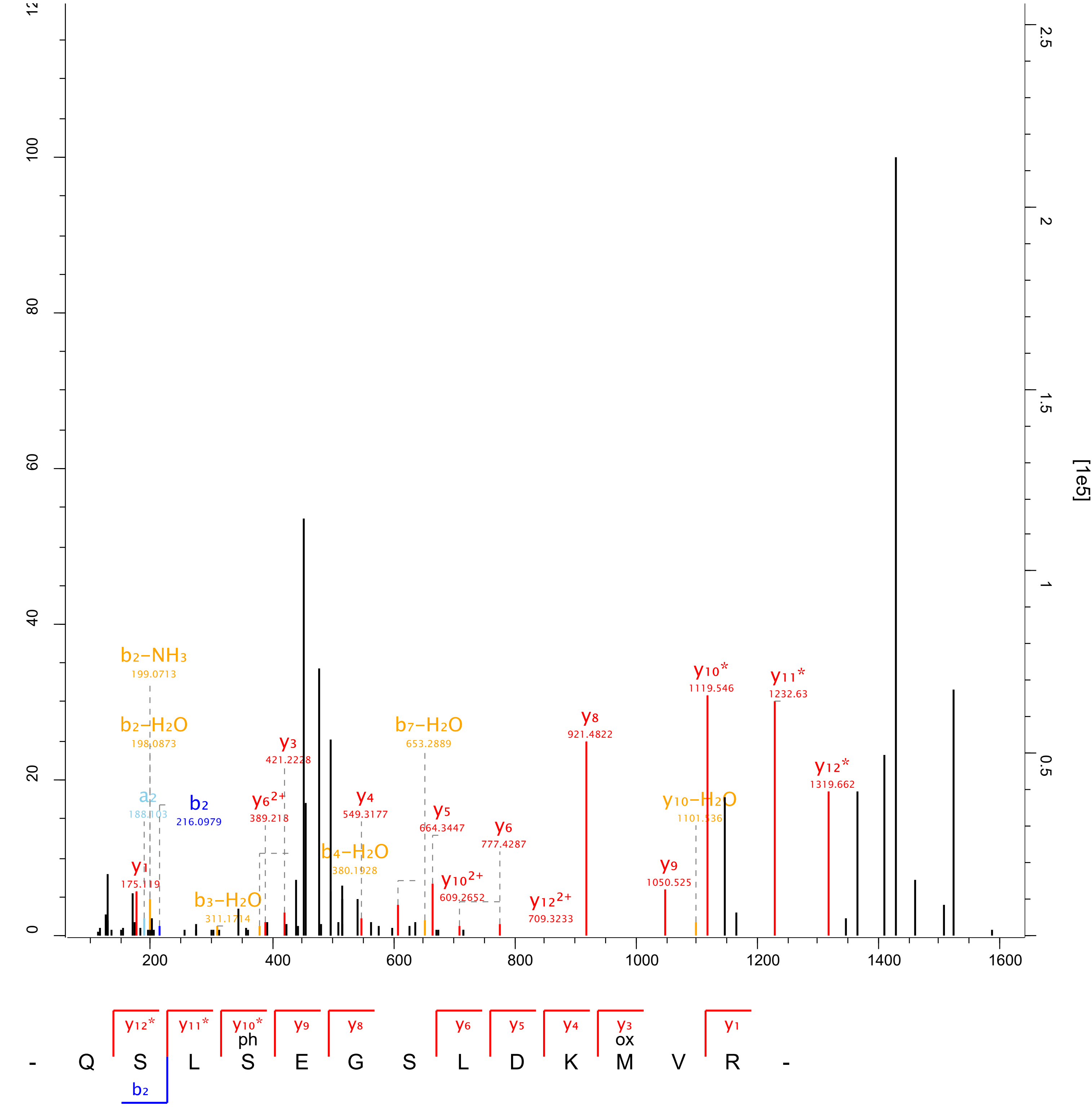

Sample output to test PDF Combine only

Raw file	Scan	Method	Score	m/z	Gene names
0523_12	10980	FTMS; HCD	114.68	652.54	RIN4

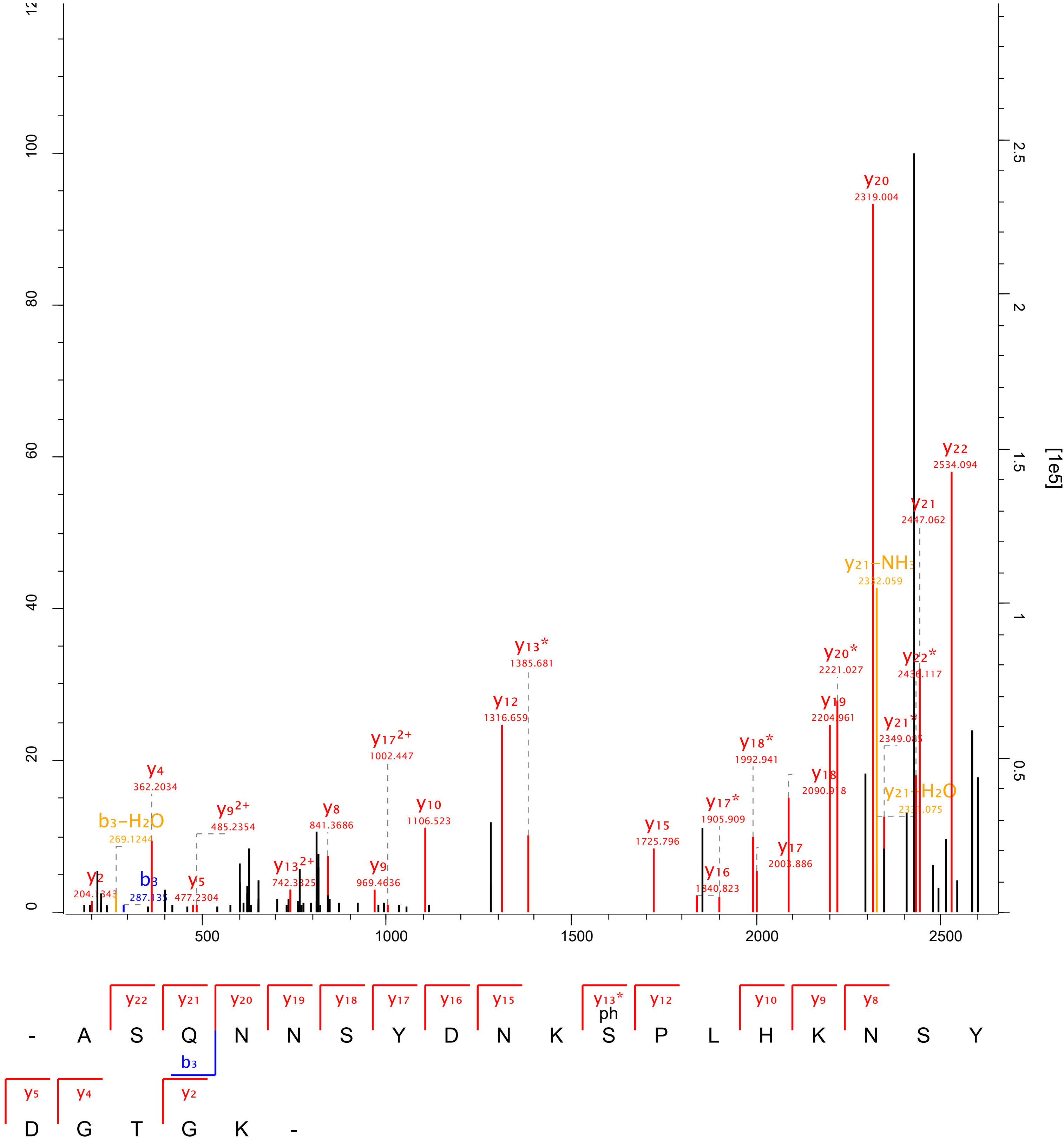

Sample output to test PDF Combine only

Raw file	Scan	Method	Score	m/z	Gene names
0523_12	11382	FTMS; HCD	50.2	711.32	At2g40980

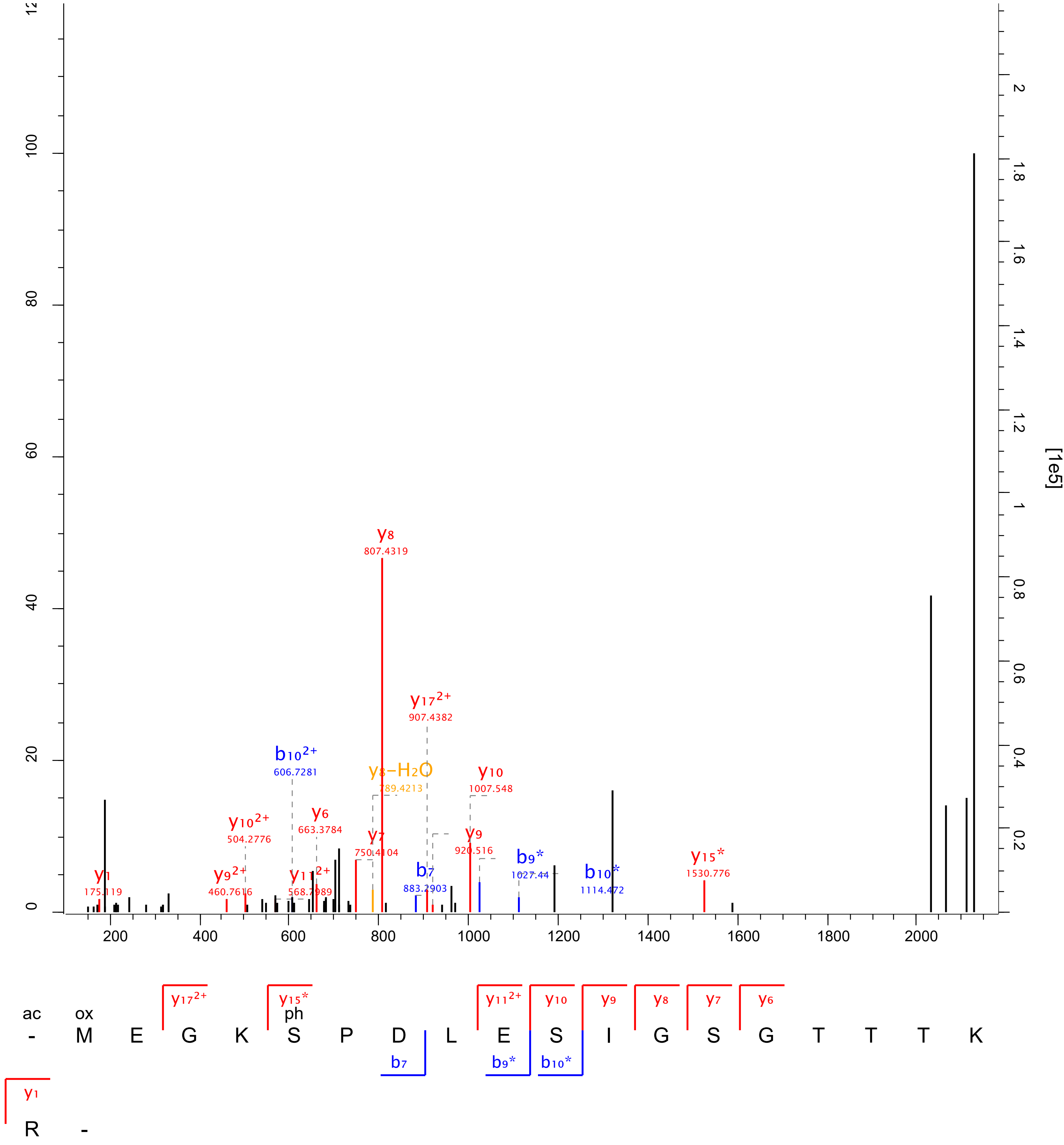

Sample output to test PDF Combine only

Raw file	Scan	Method	Score	m/z	Gene names
0523_12	11582	FTMS; HCD	219.57	882.73	PCR8

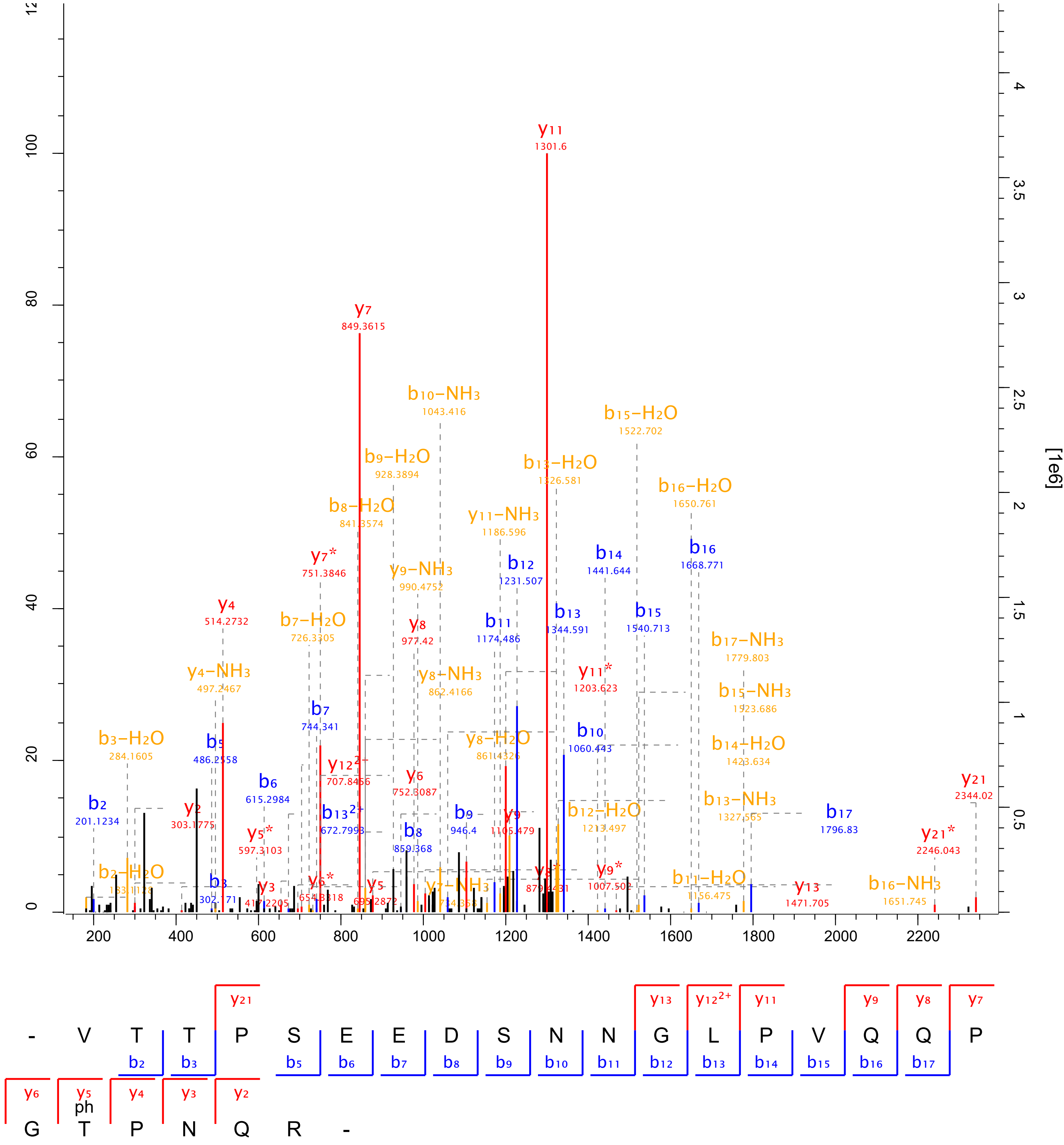

Sample output to test PDF Combine only

Raw file	Scan	Method	Score	m/z
0523_12	11885	FTMS; HCD	110.03	820.03

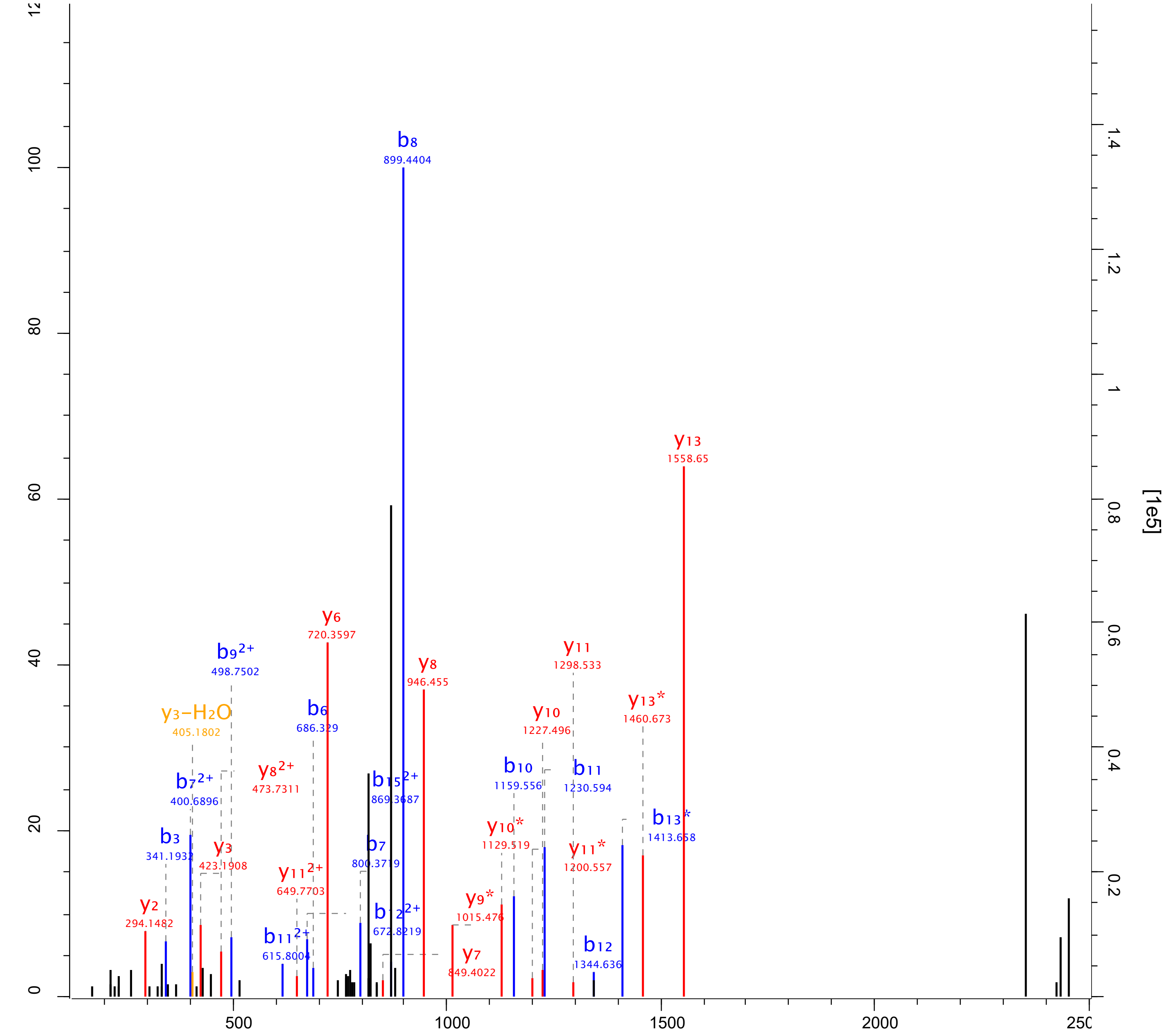

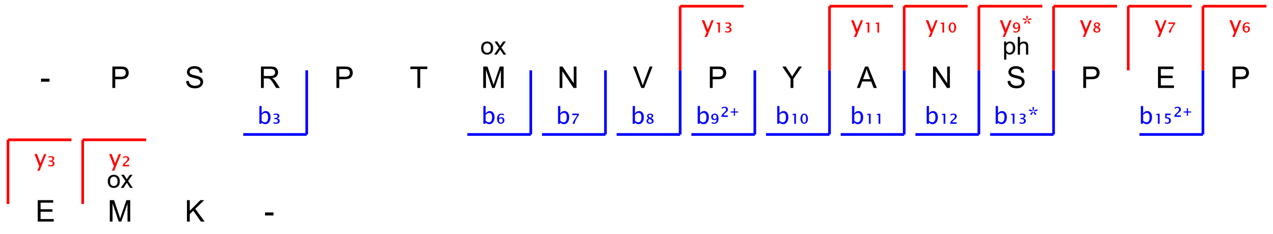

Sample output to test PDF Combine only

Raw file	Scan	Method	Score	m/z	Gene names
0523_12	12028	FTMS; HCD	239.02	750.02	PUP18

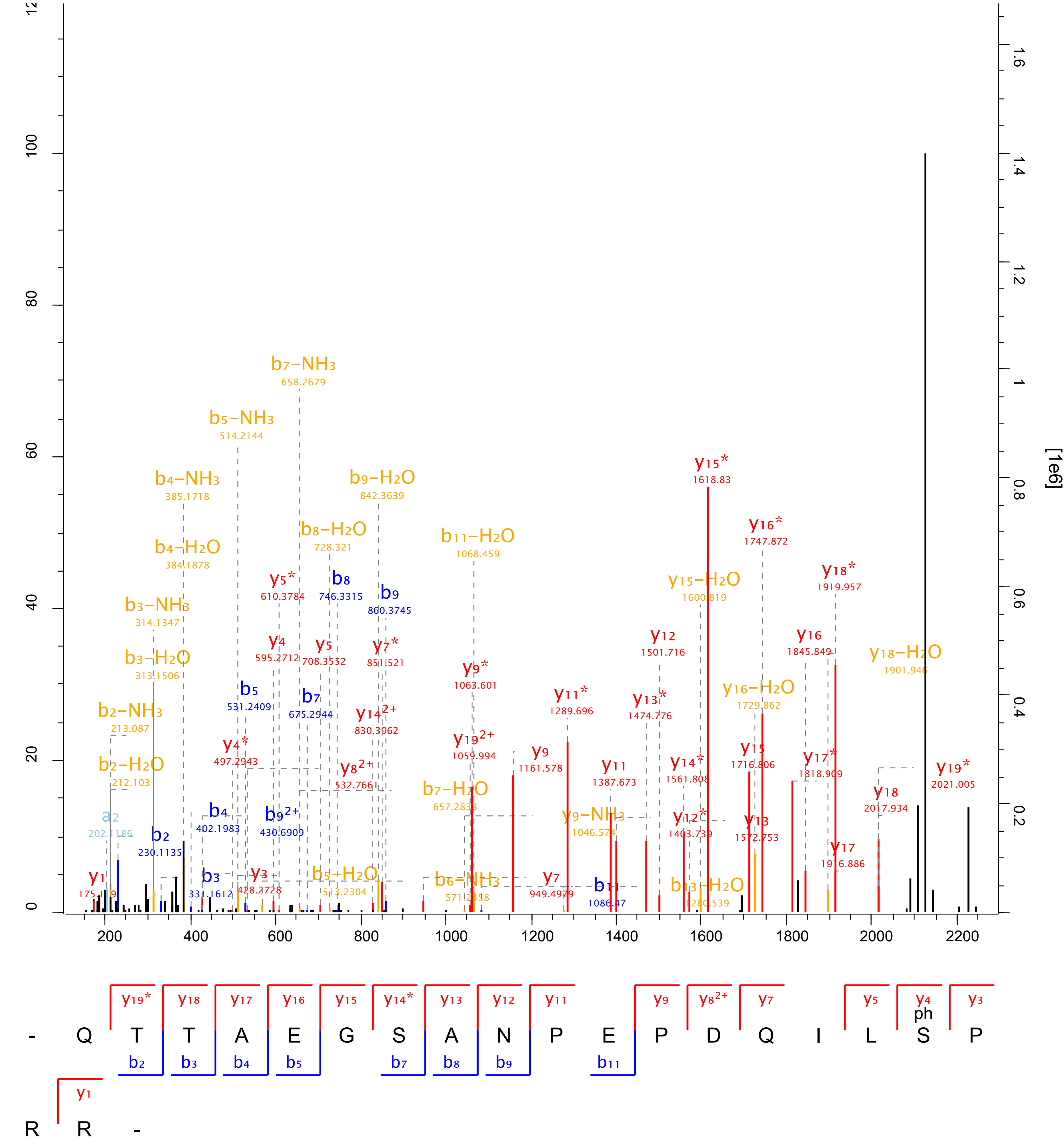

Sample output to test PDF Combine only

Raw file	Scan	Method	Score	m/z	Gene names
0523_12	12074	FTMS; HCD	73.57	718.83	PRP39

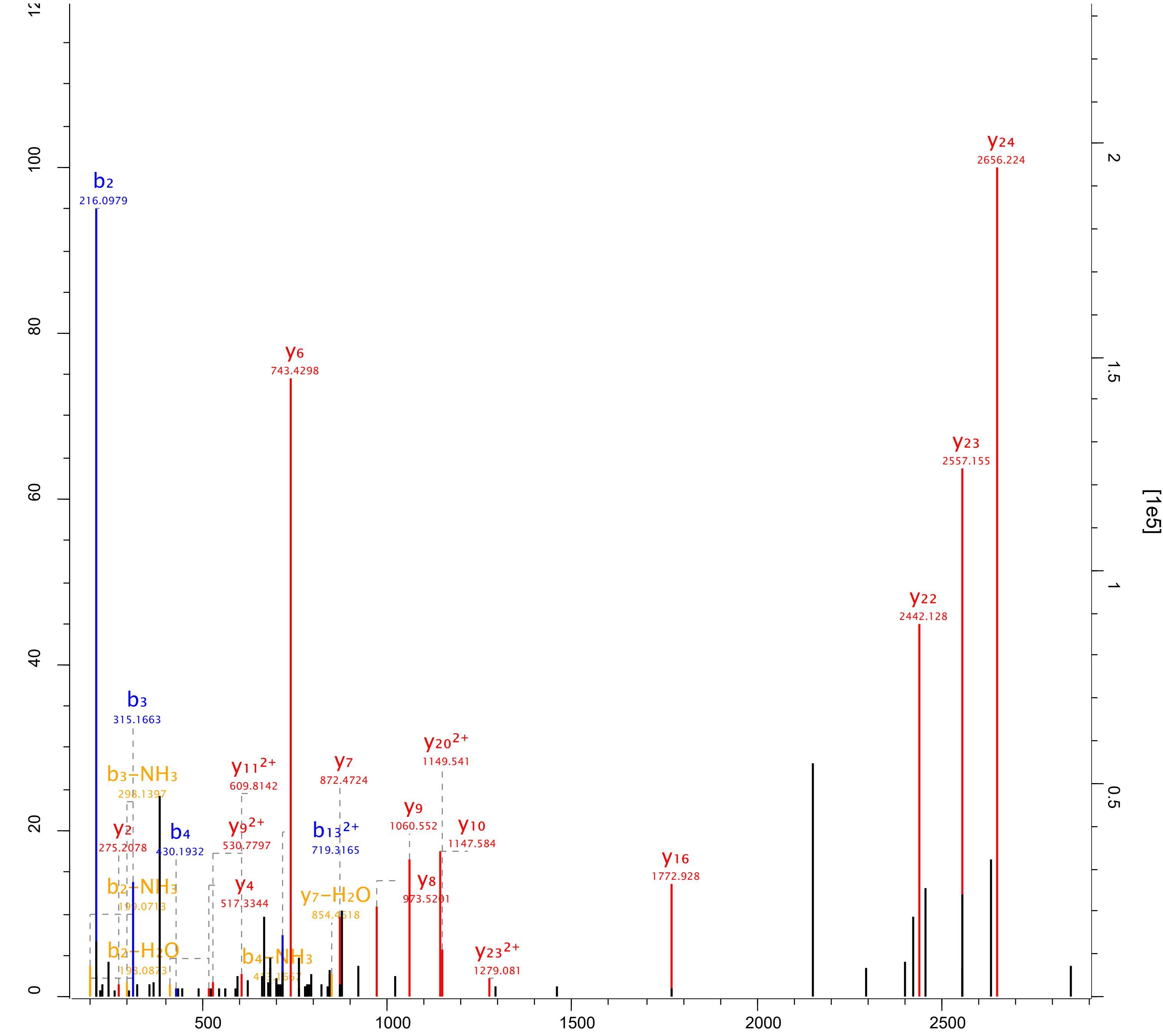

- S Q V D G S T E Q S P K L E S A S S

T E P E E L K K -

y24 y23 y22 y20 2+ (ph) y16 y11 2+ y10 y9 y8 y7 y6 y4 y2

b2 b3 b4 b13 2+

Sample output to test PDF Combine only

Raw file	Scan	Method	Score	m/z	Gene names
0523_12	12084	FTMS; HCD	54.01	628.28	F19C24.3

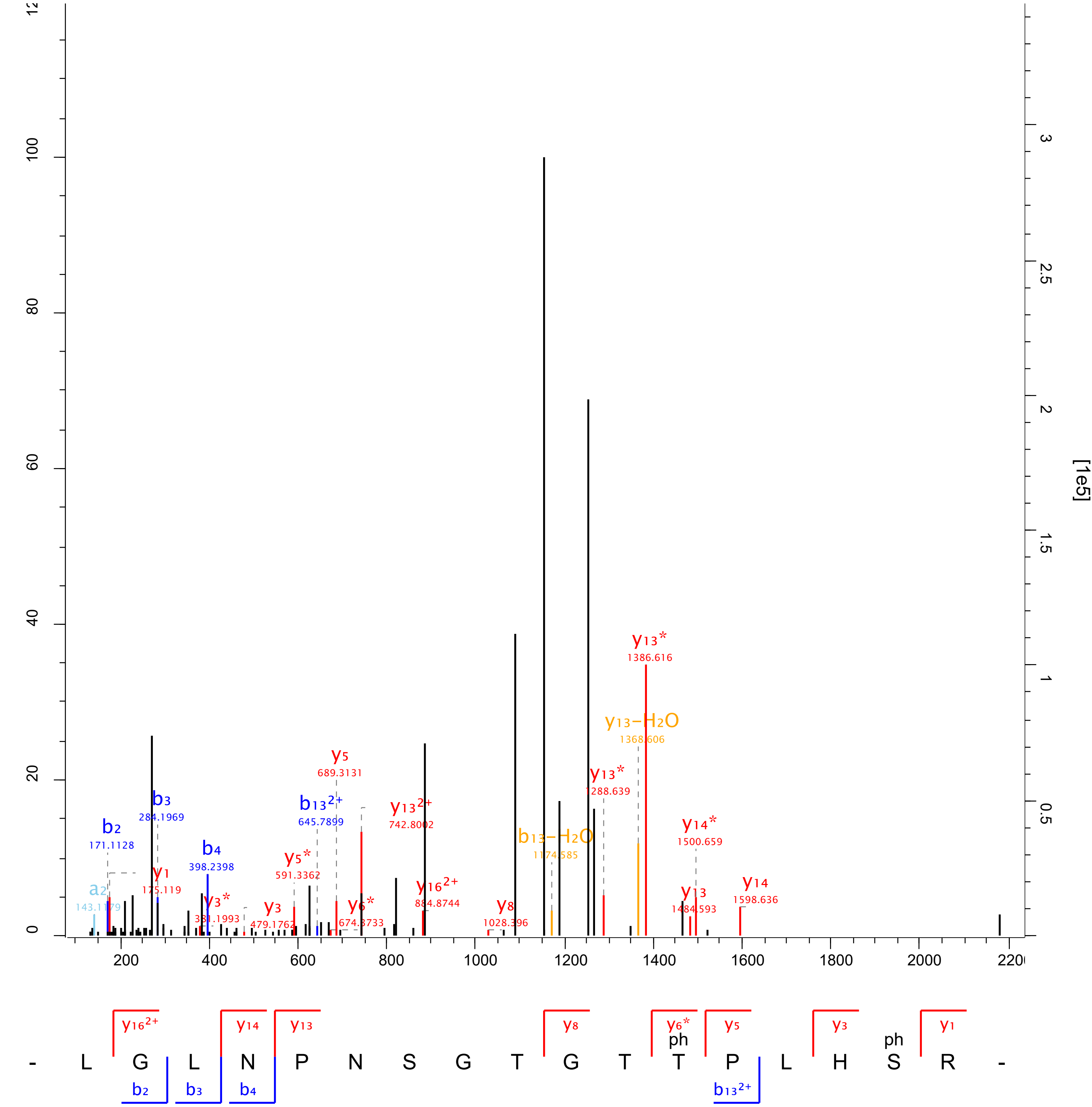

Sample output to test PDF Combine only

Raw file	Scan	Method	Score	m/z
0523_12	12094	FTMS; HCD	65.2	725.32

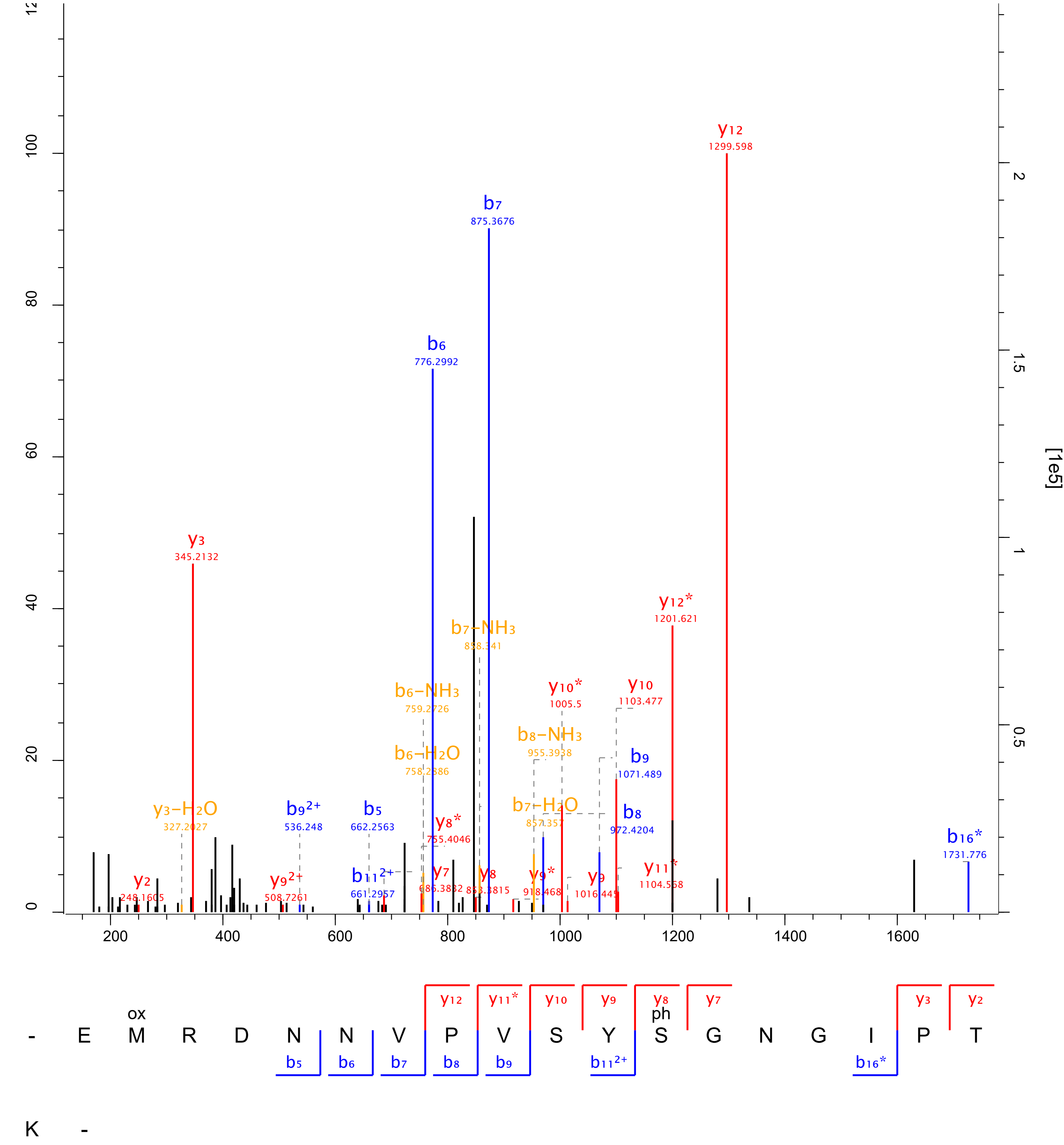

Sample output to test PDF Combine only

Raw file	Scan	Method	Score	m/z	Gene names
0523_12	12681	FTMS; HCD	60.73	708.99	CPK9

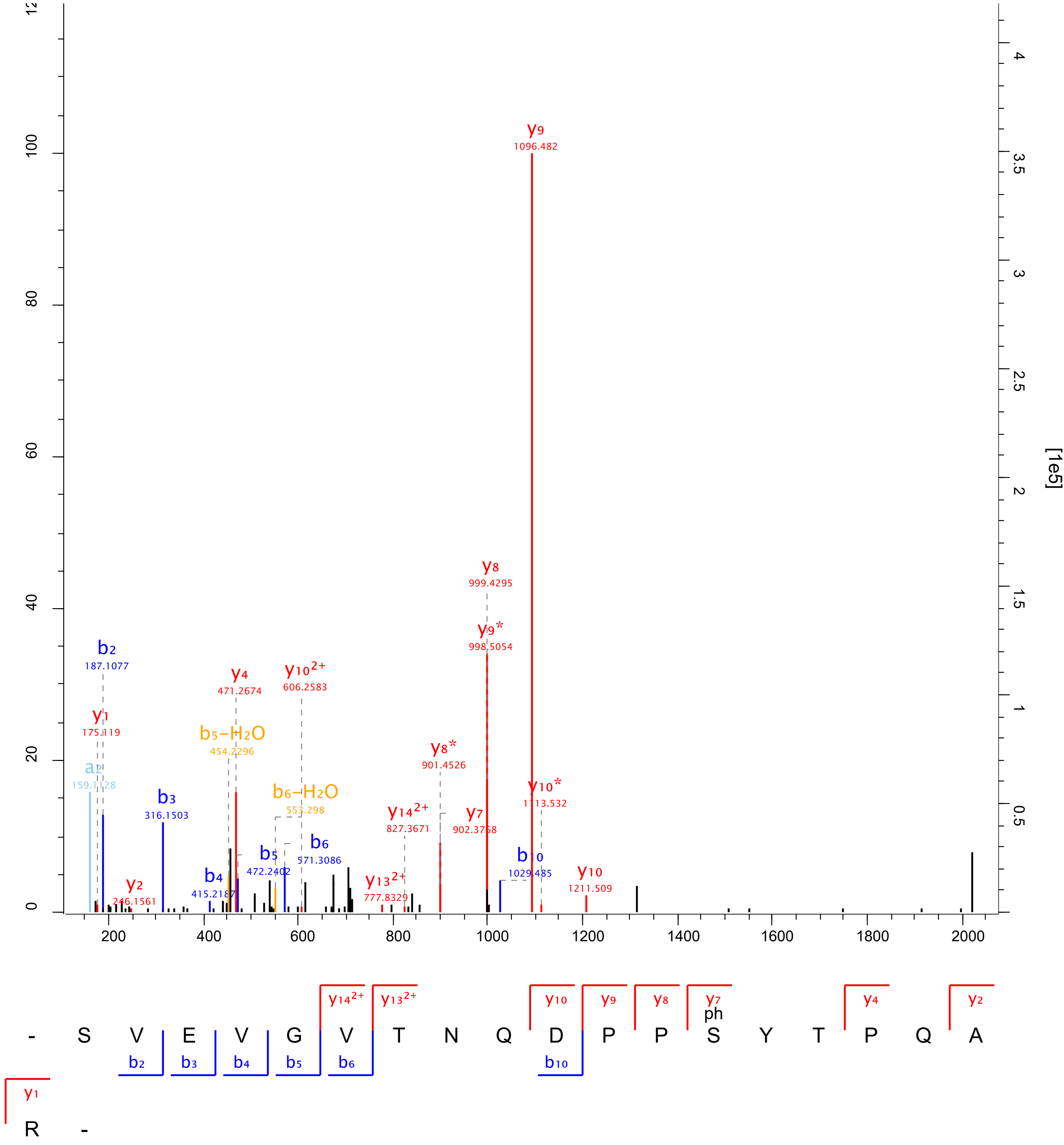

Sample output to test PDF Combine only

Raw file	Scan	Method	Score	m/z	Gene names
0523_12	12782	FTMS; HCD	58.27	852.37	STP1

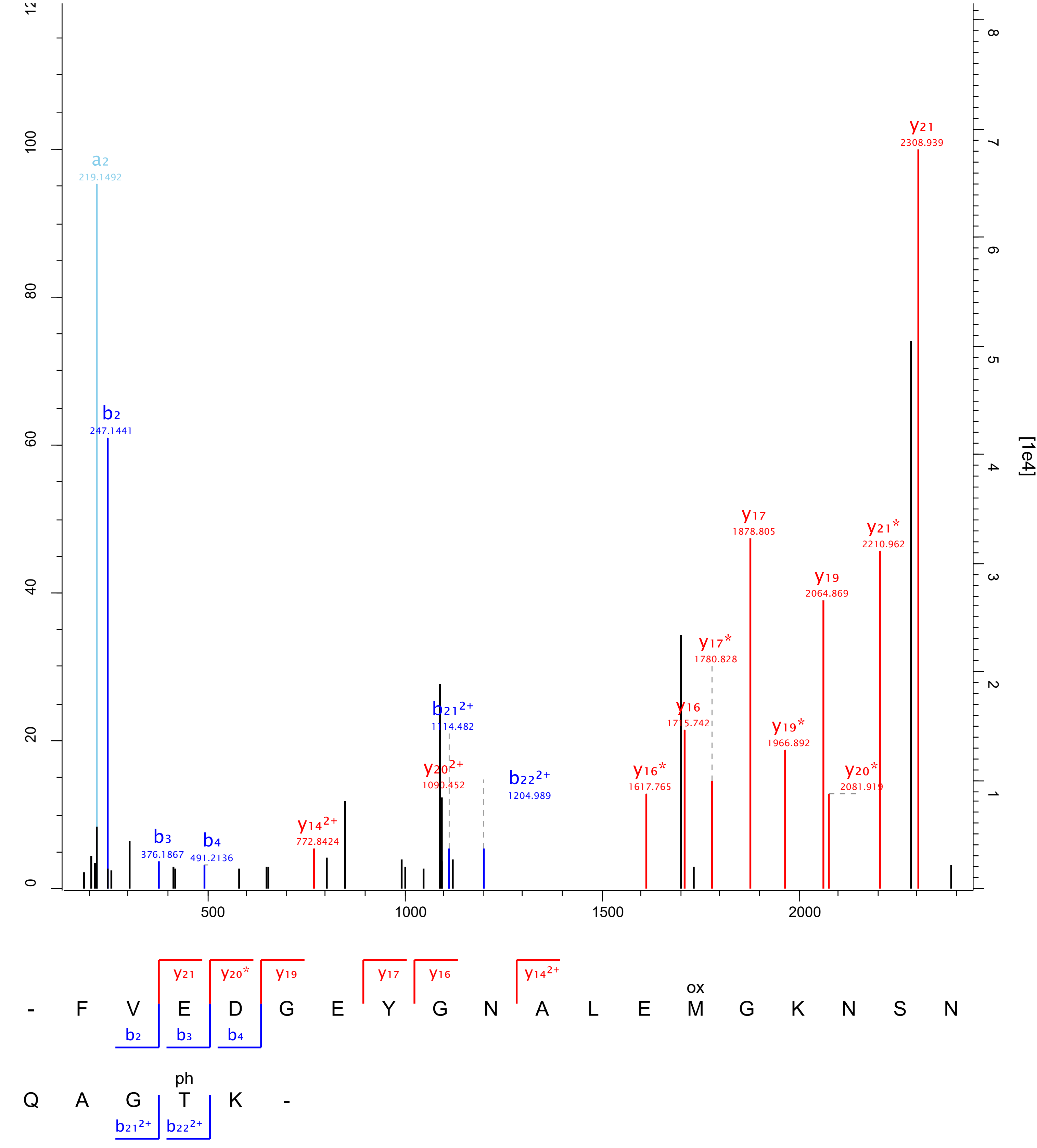

Sample output to test PDF Combine only

Raw file	Scan	Method	Score	m/z	Gene names
0523_12	12792	FTMS; HCD	81.48	769.32	EIF3B-2;TIF3B1

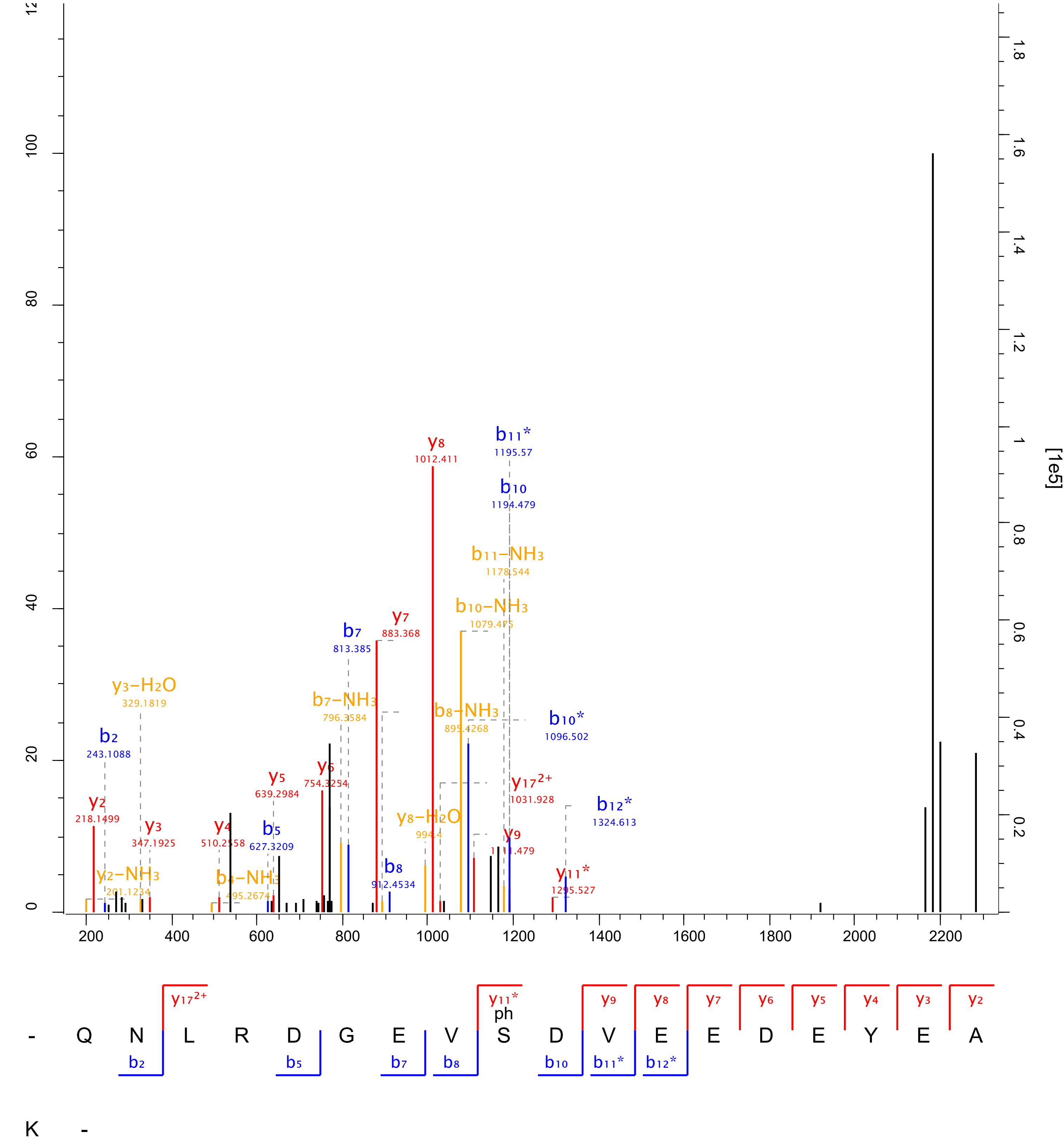

Sample output to test PDF Combine only

Raw file	Scan	Method	Score	m/z	Gene names
0523_12	12894	FTMS; HCD	256.75	596.62	SYP121

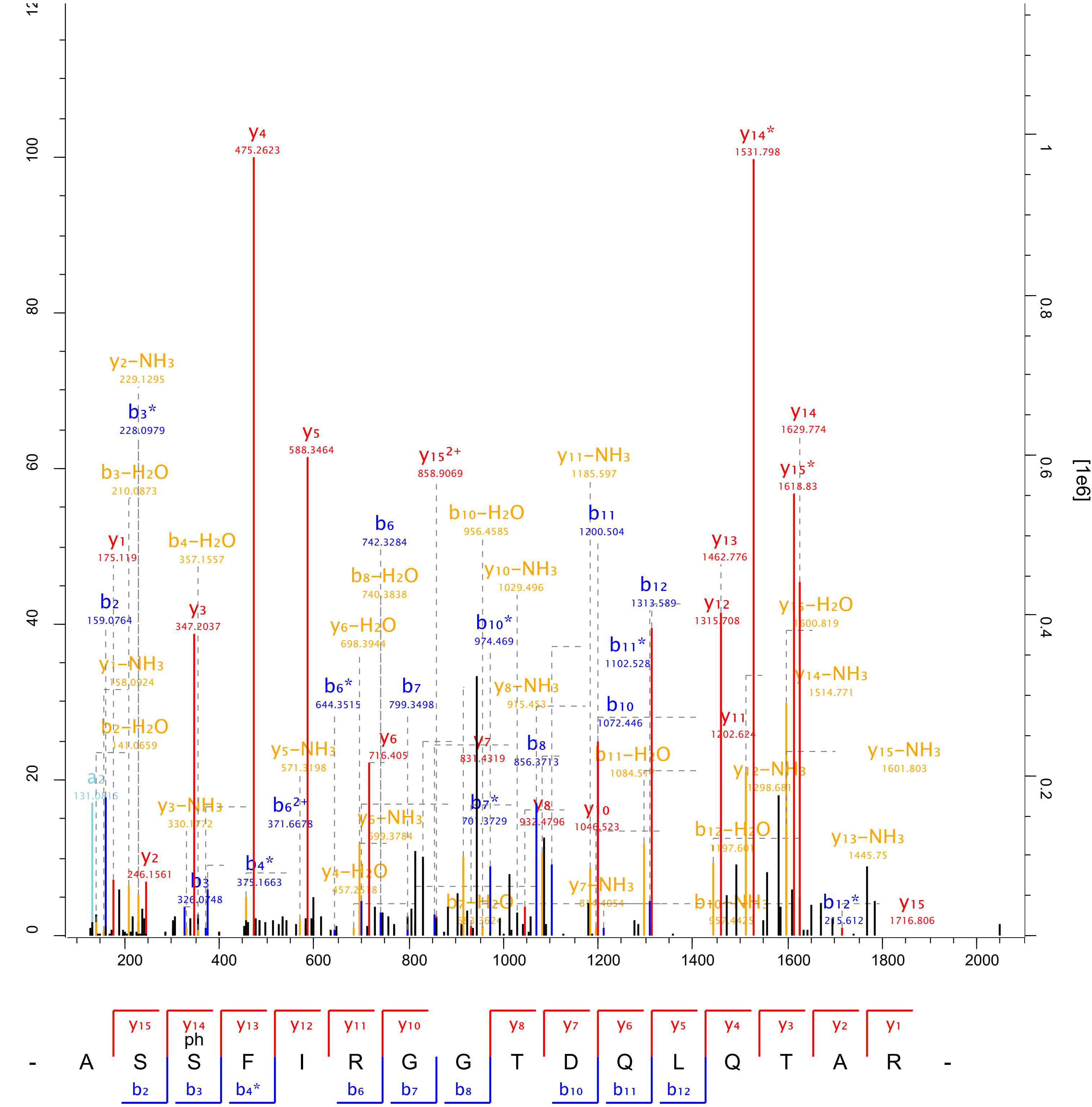

Sample output to test PDF Combine only

Raw file	Scan	Method	Score	m/z	Gene names
0523_12	13083	FTMS; HCD	93.63	872.37	PLDBETA1

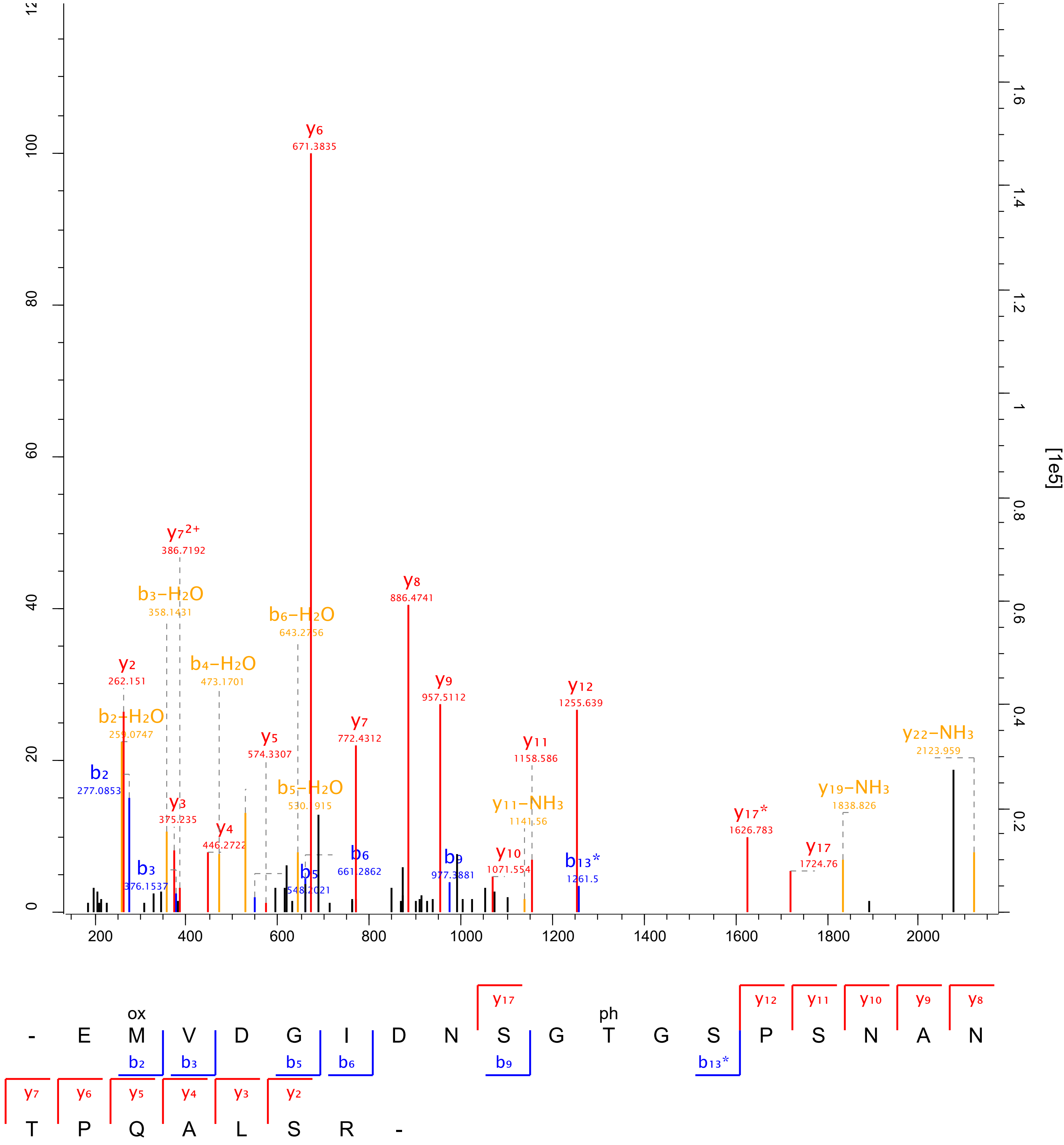

Sample output to test PDF Combine only

Raw file	Scan	Method	Score	m/z	Gene names
0523_12	13247	FTMS; HCD	77.58	675.63	PHT1-2;PHT1-1

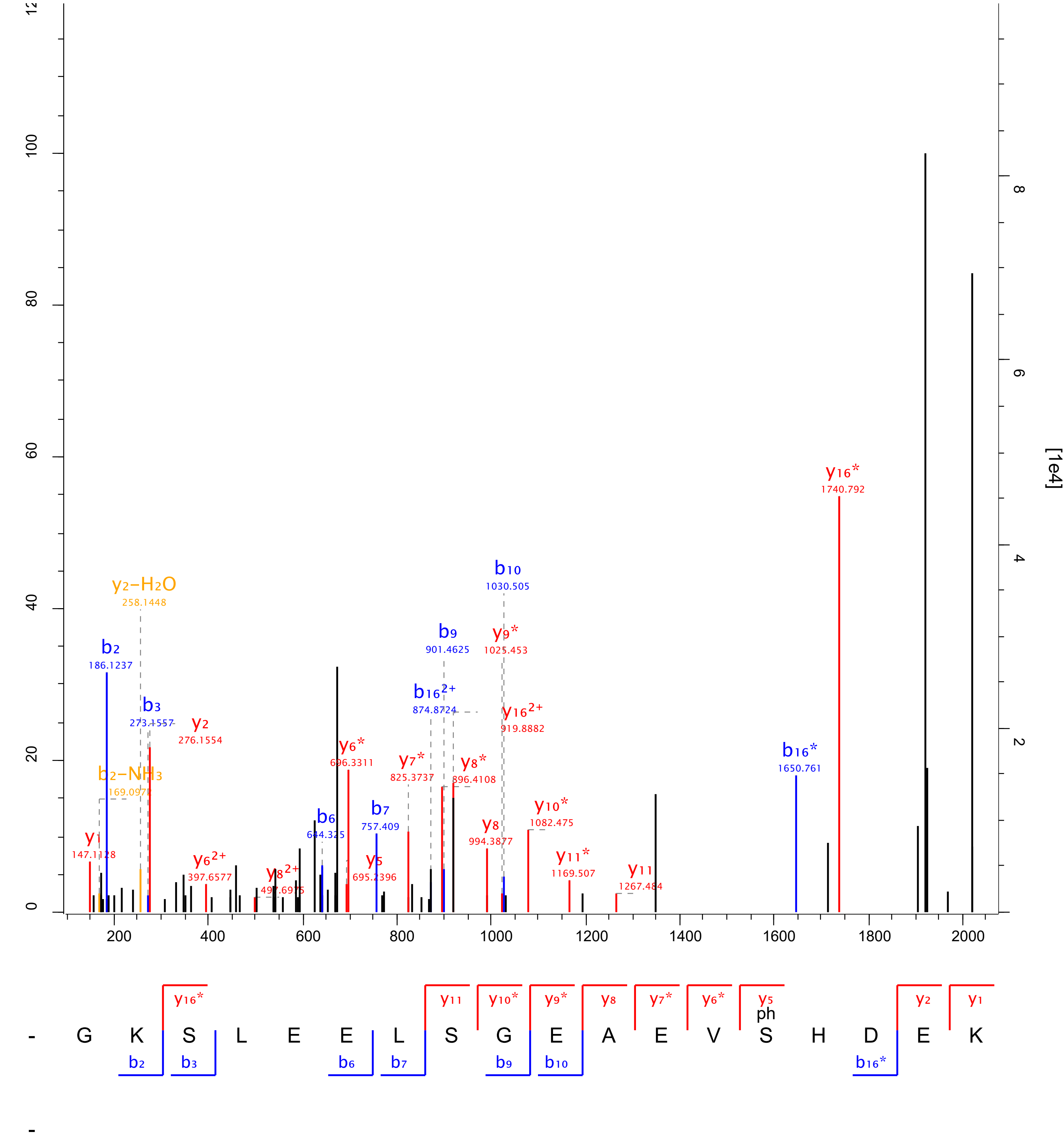

Sample output to test PDF Combine only

Raw file	Scan	Method	Score	m/z
0523_12	13355	FTMS; HCD	48.57	452.87

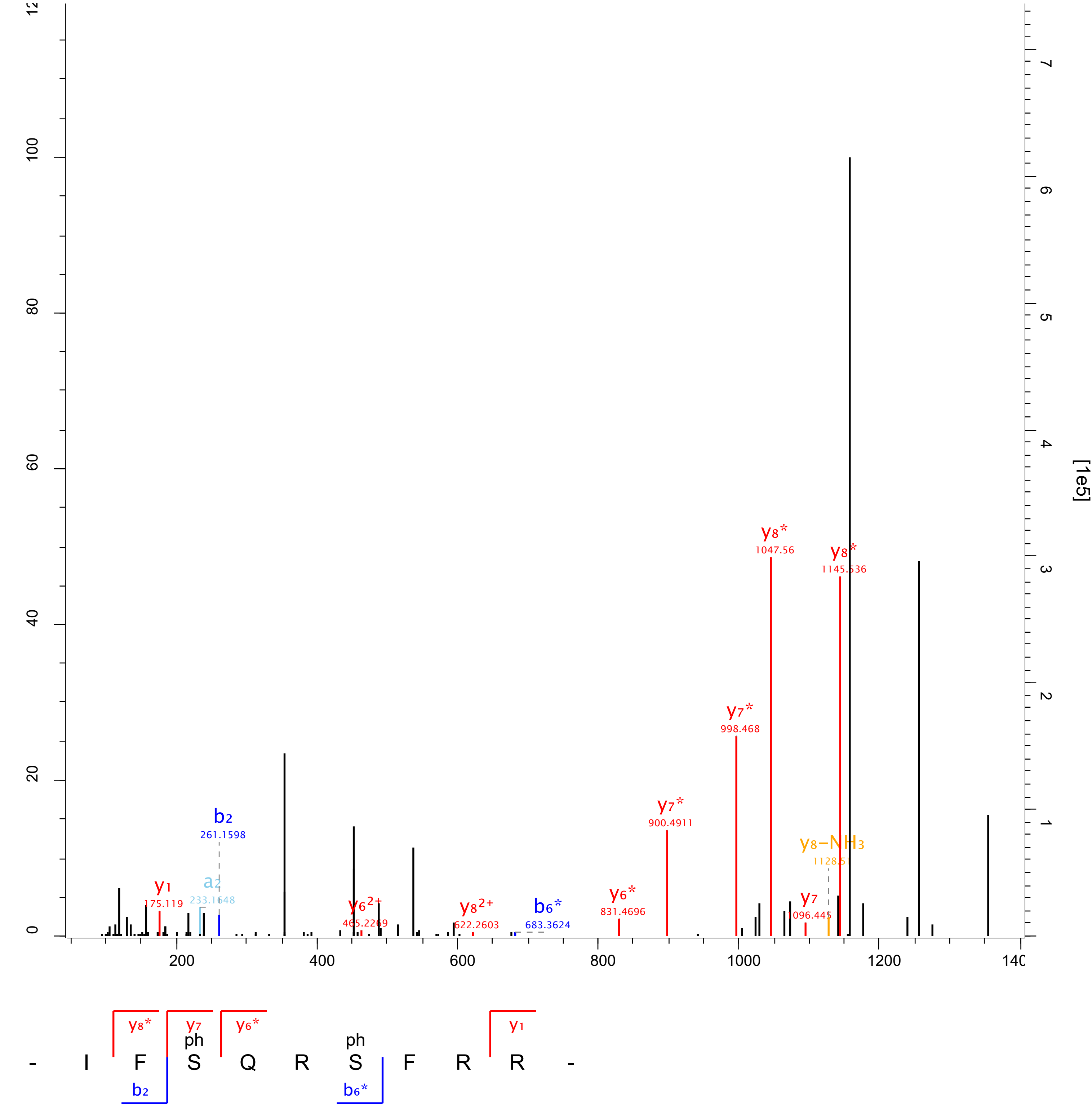

Sample output to test PDF Combine only

Raw file	Scan	Method	Score	m/z	Gene names
0523_12	13426	FTMS; HCD	117.93	600.76	CPK5

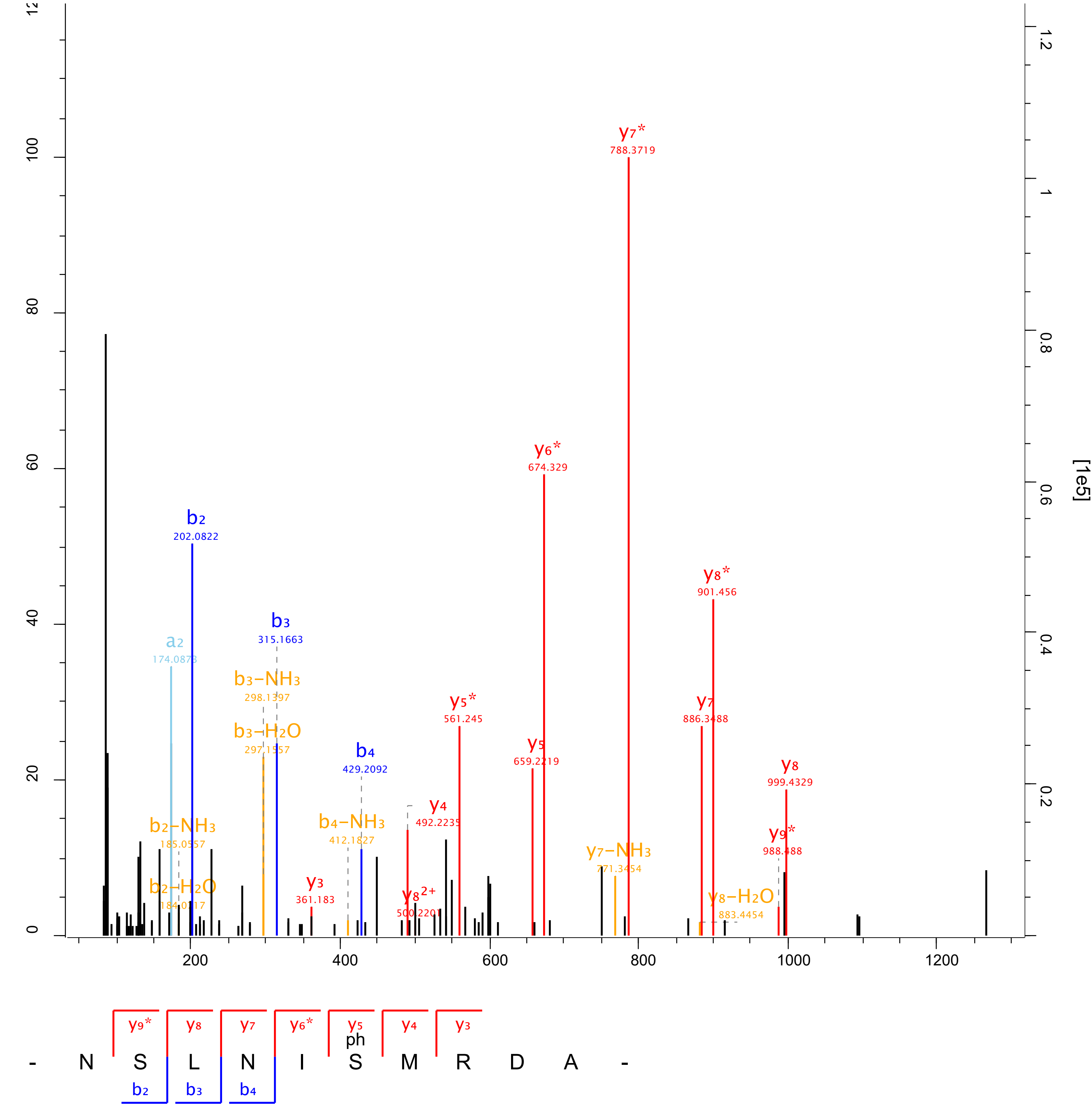

Sample output to test PDF Combine only

Raw file	Scan	Method	Score	m/z	Gene names
0523_12	13627	FTMS; HCD	58.69	671.95	CPK7

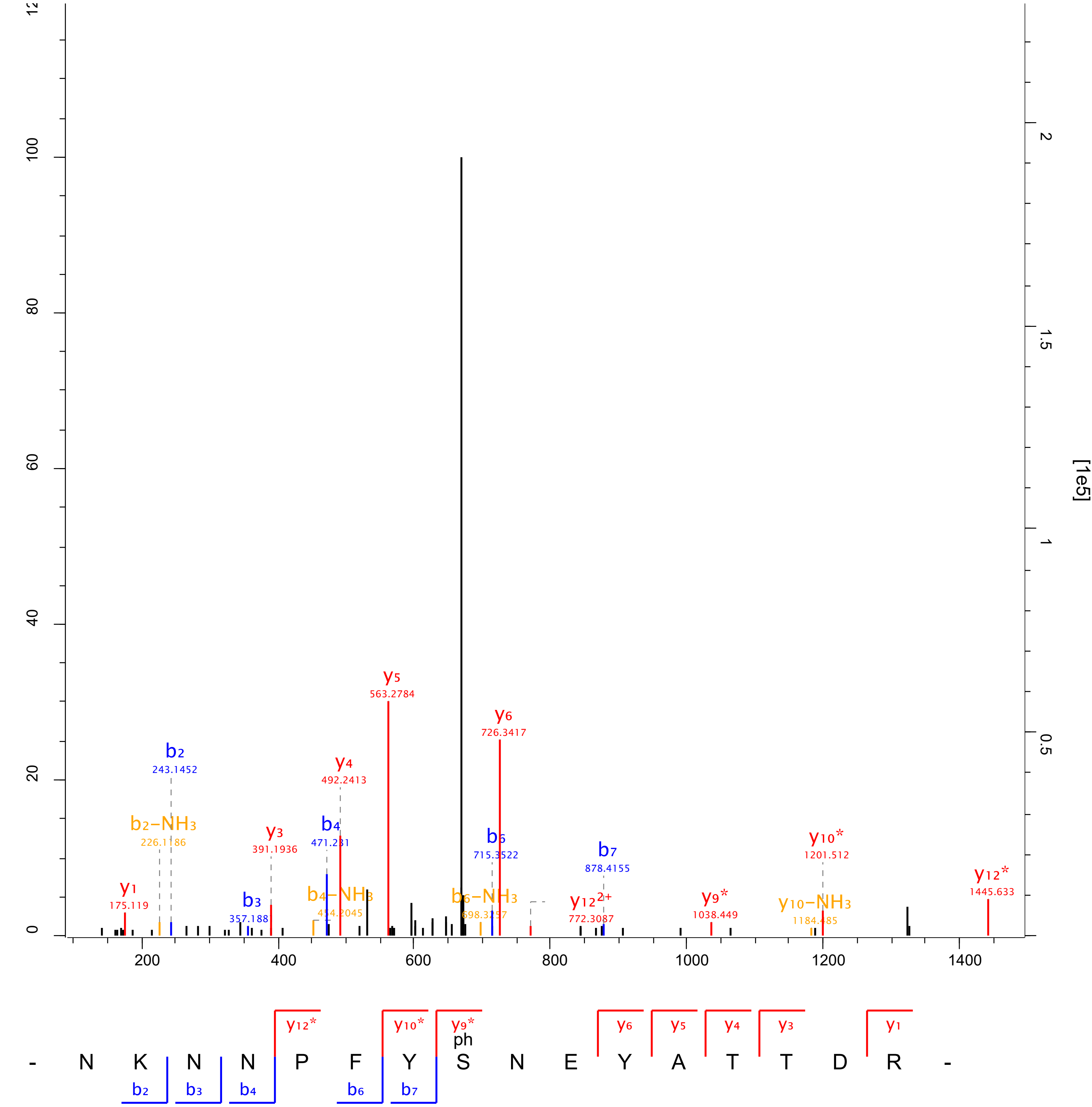

Sample output to test PDF Combine only

Raw file	Scan	Method	Score	m/z	Gene names
0523_12	14107	FTMS; HCD	83.6	669.31	PATL3

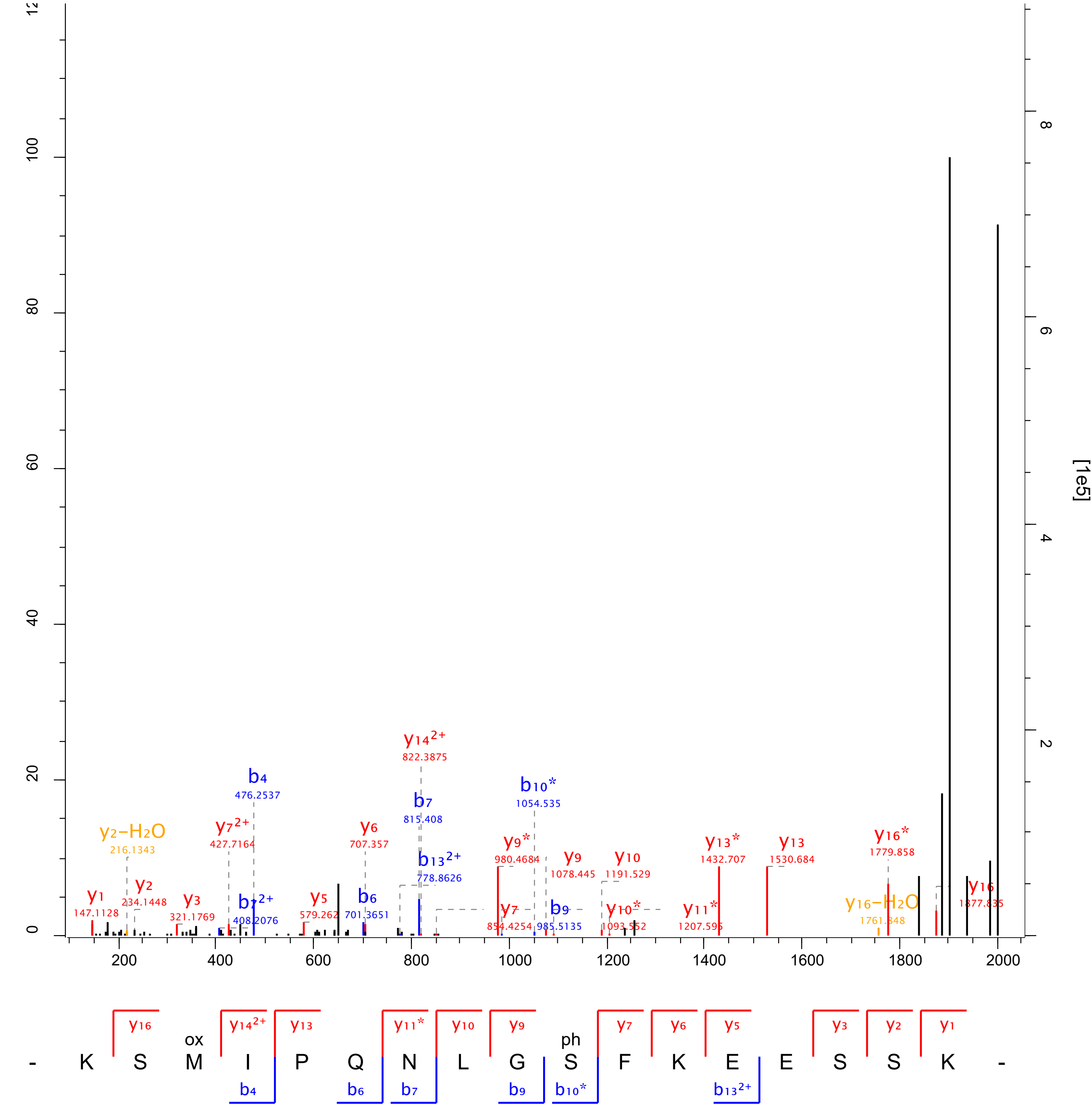

Sample output to test PDF Combine only

Raw file	Scan	Method	Score	m/z	Gene names
0523_12	14298	FTMS; HCD	89.27	826.71	F13I12.260

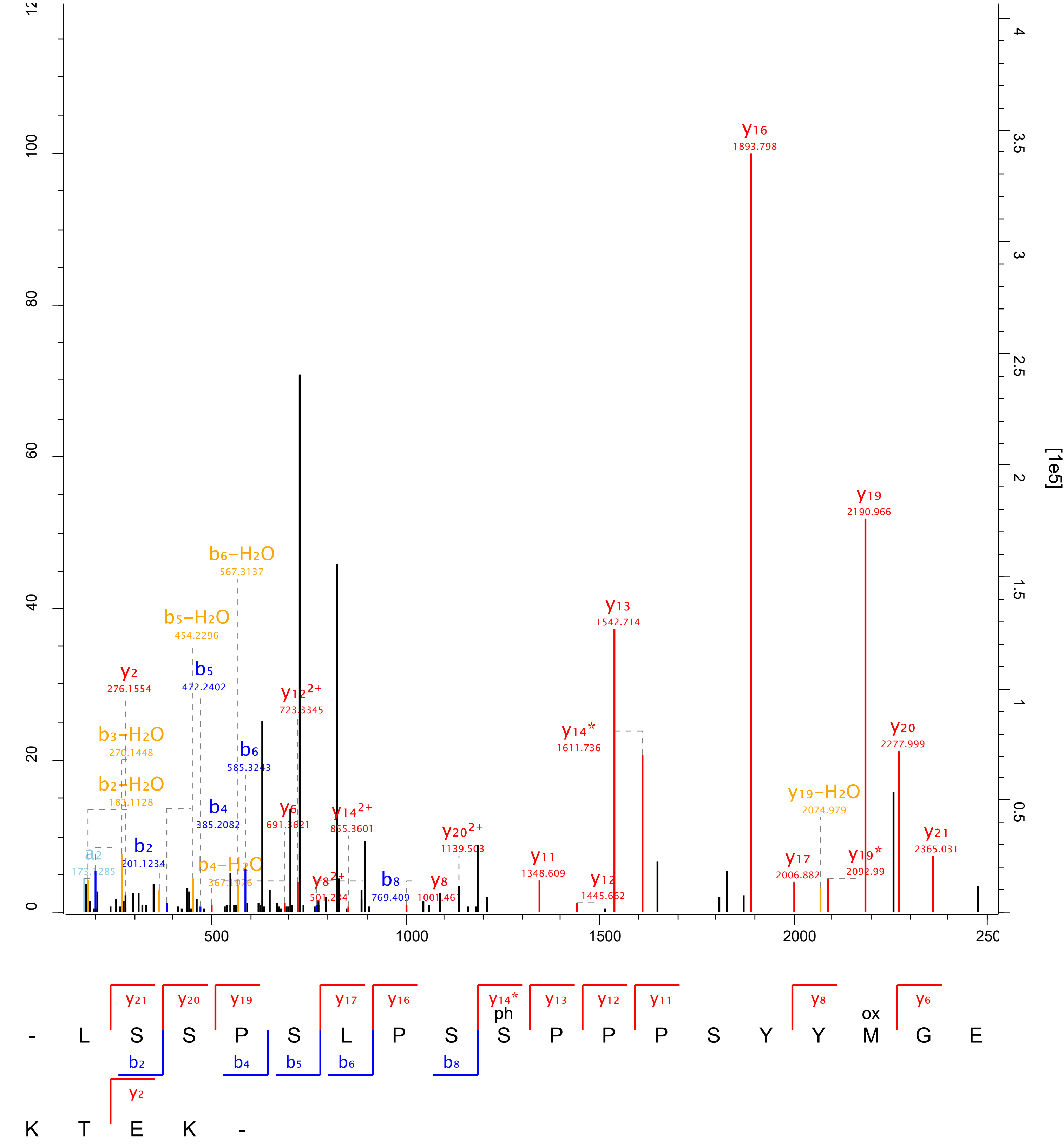

Sample output to test PDF Combine only

Raw file	Scan	Method	Score	m/z	Gene names
0523_12	14544	FTMS; HCD	139.47	1025.11	PUP18

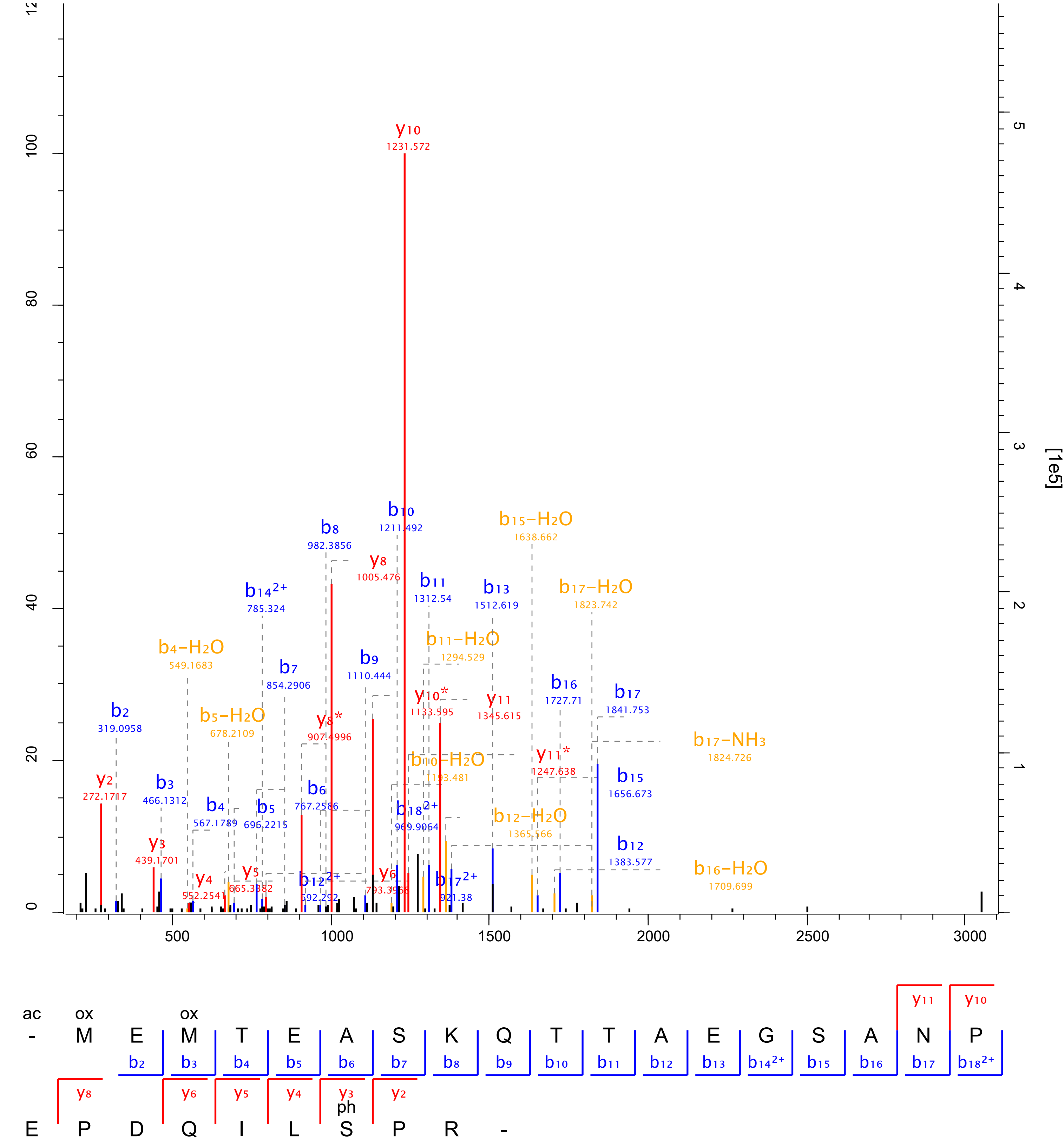

Sample output to test PDF Combine only

Raw file	Scan	Method	Score	m/z
0523_12	15014	FTMS; HCD	103.91	600.75

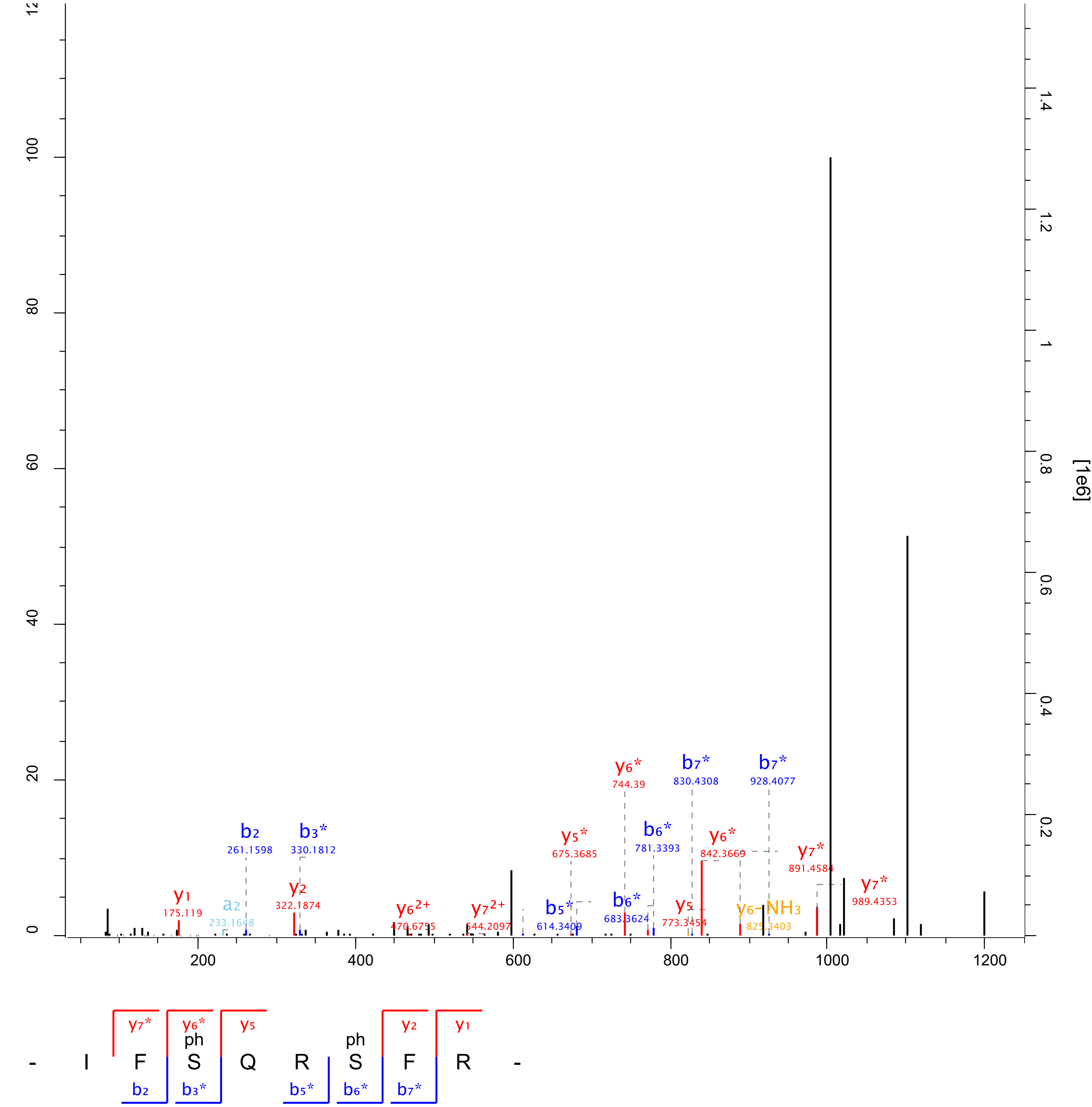

Sample output to test PDF Combine only

Raw file	Scan	Method	Score	m/z	Gene names
0523_12	15153	FTMS; HCD	110.47	1016.45	ABCB21

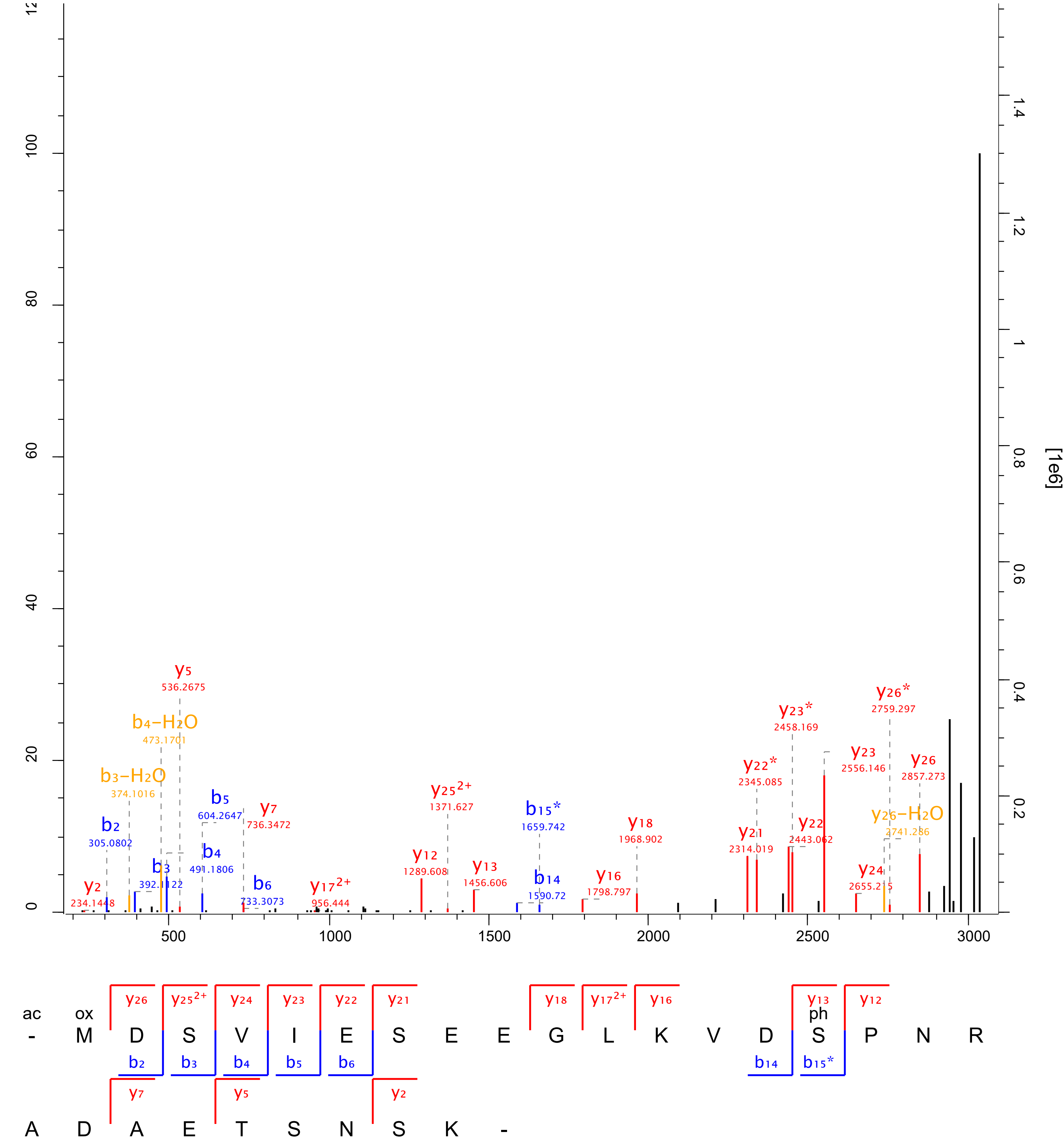

Sample output to test PDF Combine only

Raw file	Scan	Method	Score	m/z	Gene names
0523_12	15173	FTMS; HCD	58.35	802.38	At3g05900

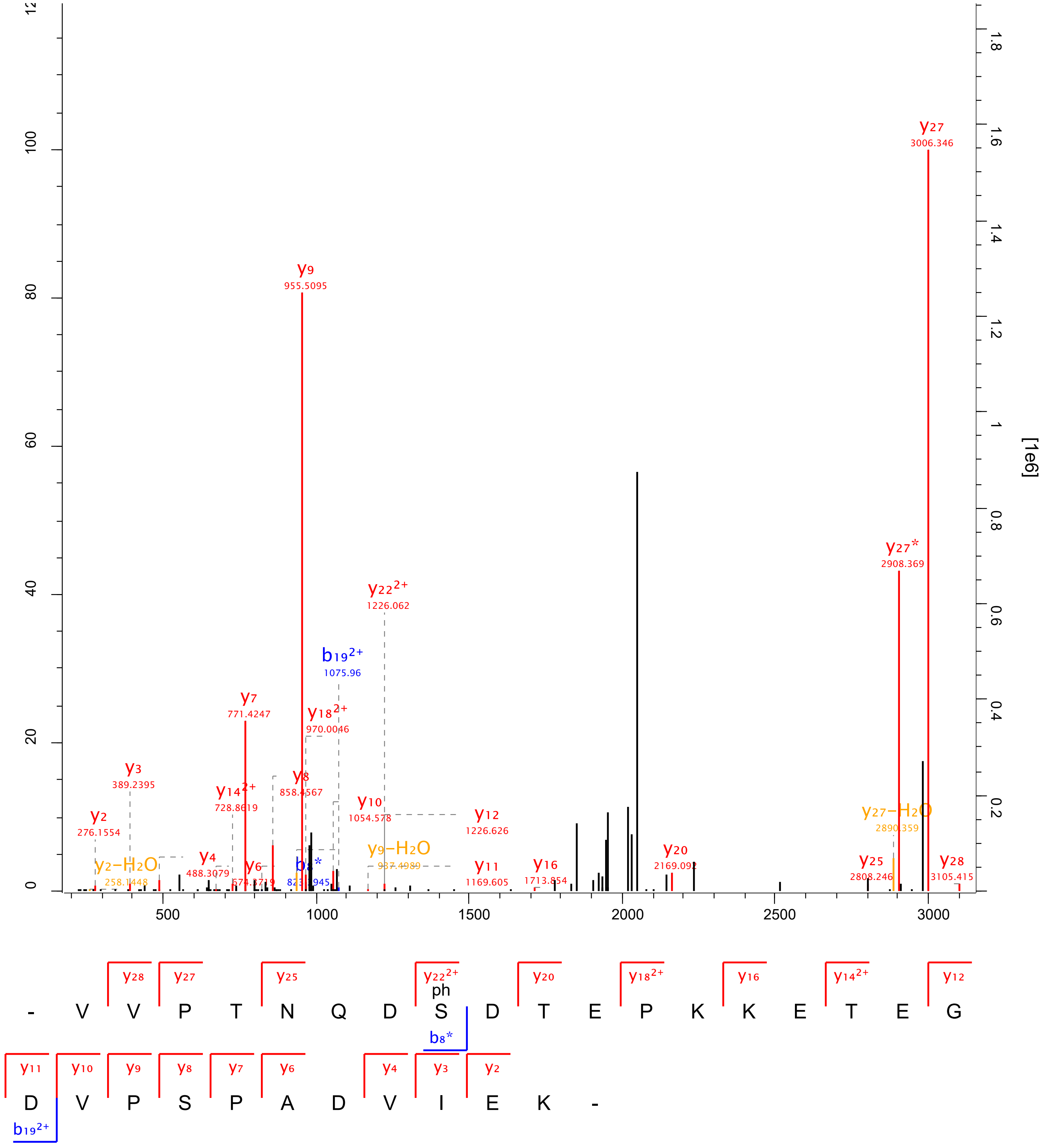

Sample output to test PDF Combine only

Raw file	Scan	Method	Score	m/z
0523_12	15282	FTMS; HCD	231.07	833.06

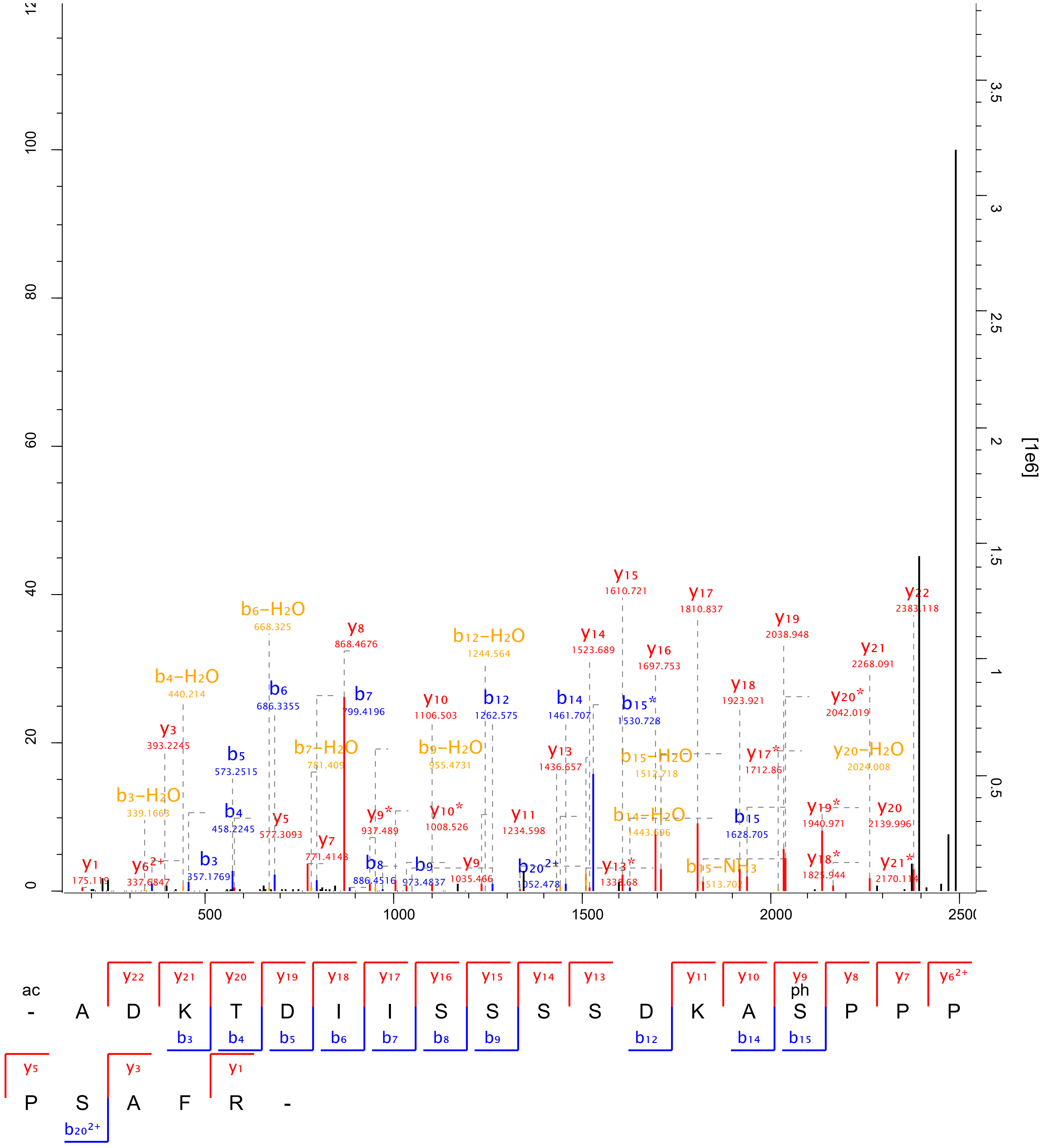

Sample output to test PDF Combine only

Raw file	Scan	Method	Score	m/z	Gene names
0523_12	15356	FTMS; HCD	55.78	741.6	PVA11

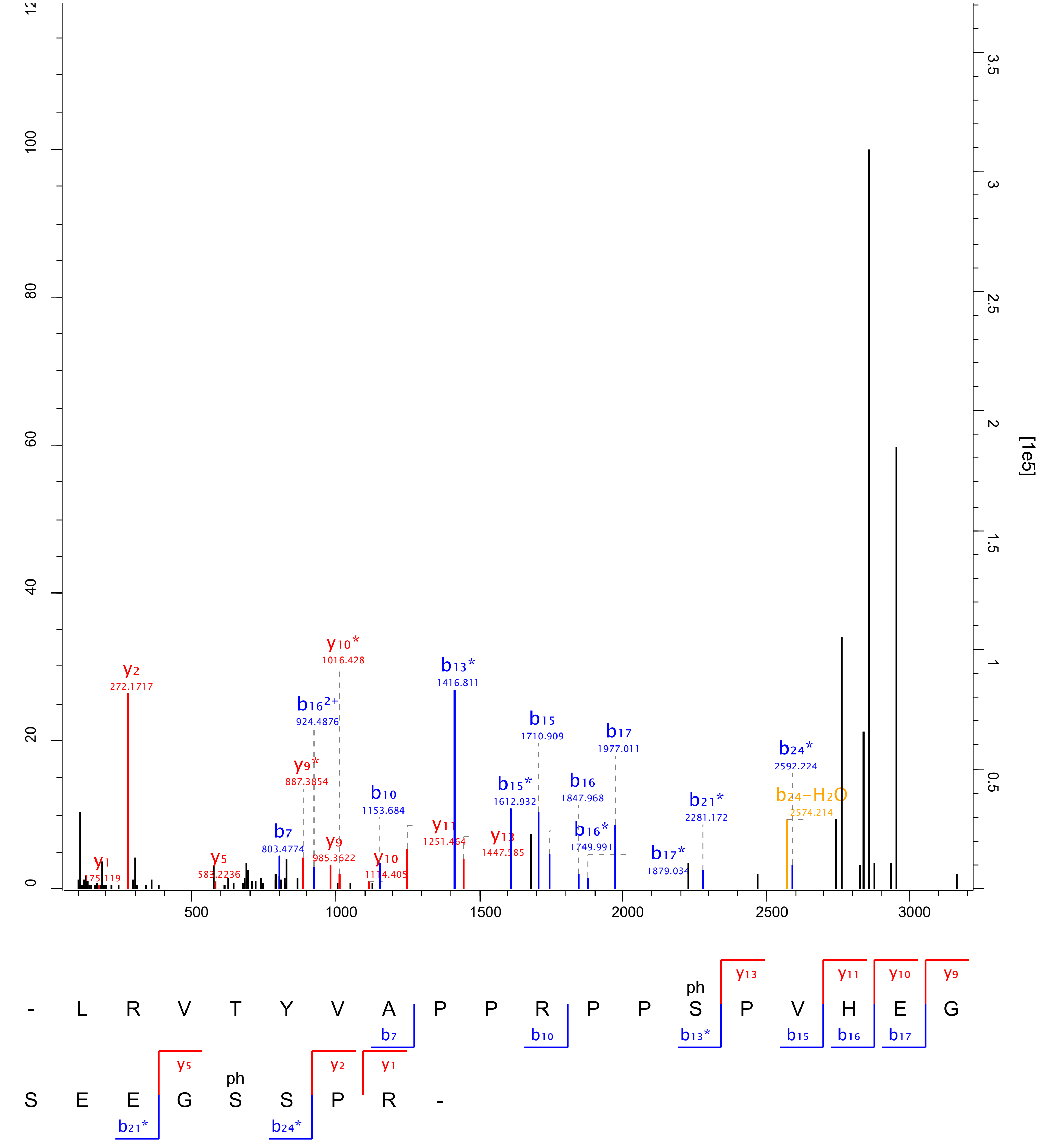

Sample output to test PDF Combine only

Raw file	Scan	Method	Score	m/z	Gene names
0523_12	15544	FTMS; HCD	41.91	603.63	At2g39130

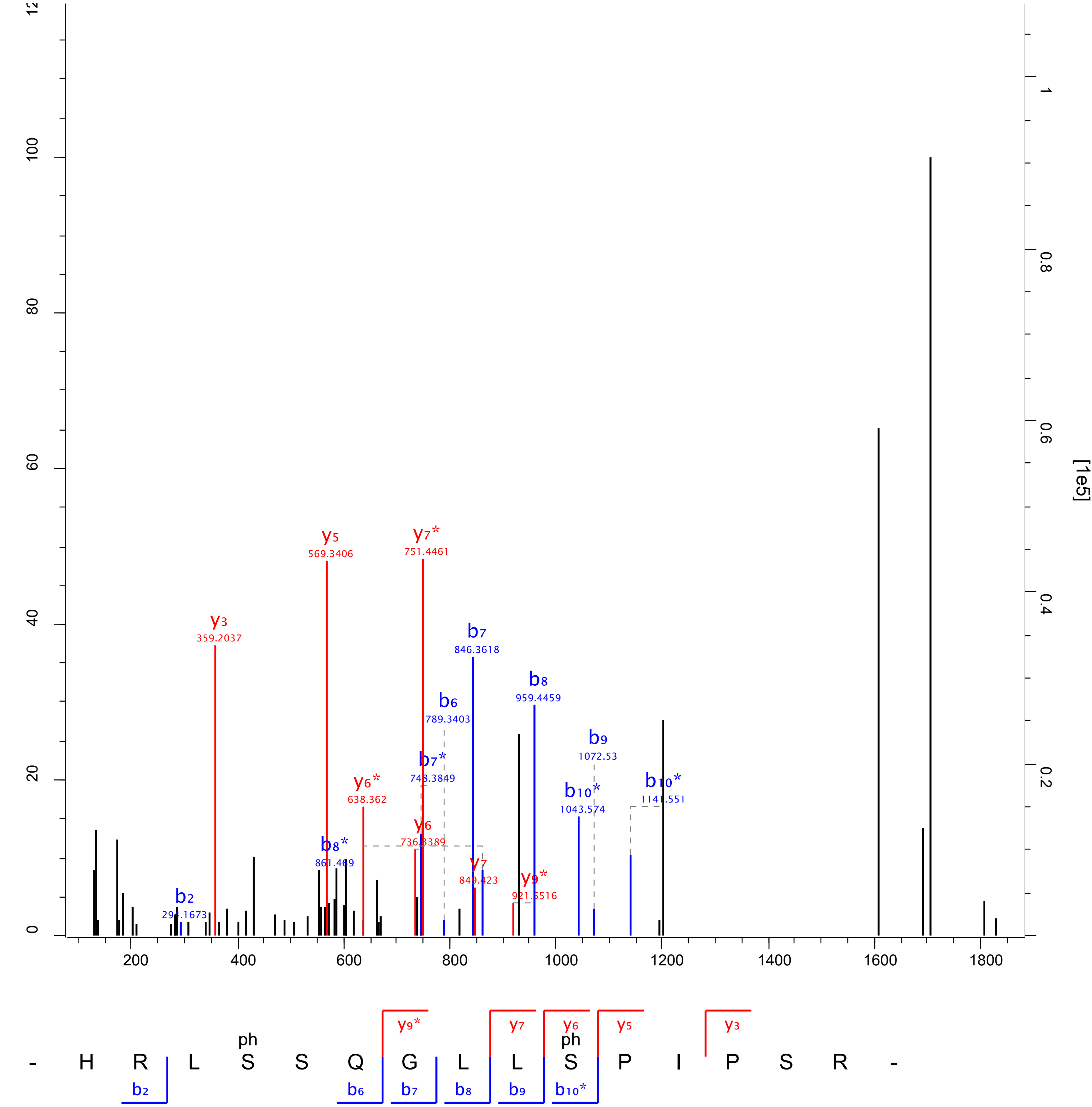

Sample output to test PDF Combine only

Raw file	Scan	Method	Score	m/z
0523_12	15596	FTMS; HCD	47.81	822.33

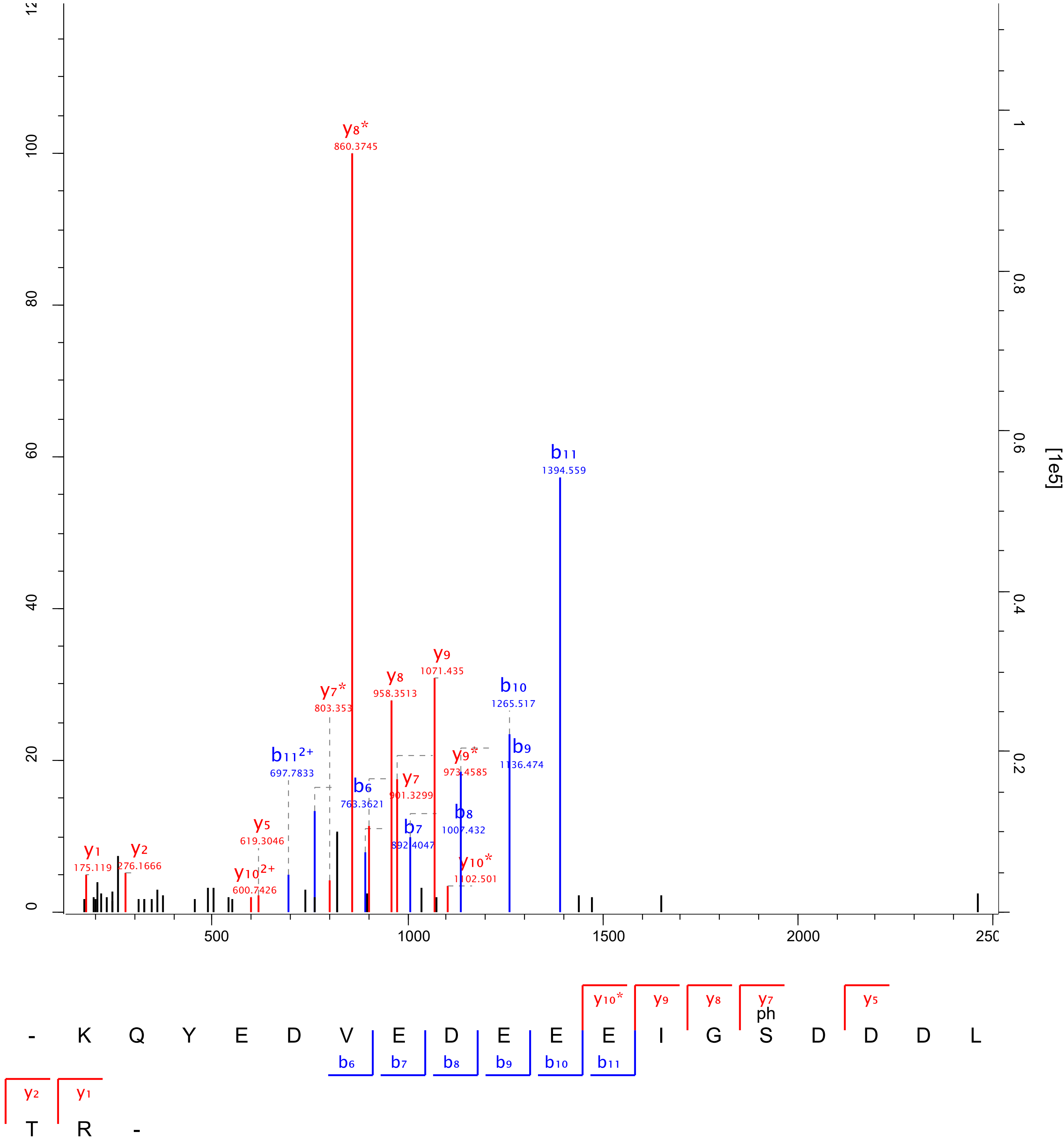

Sample output to test PDF Combine only

Raw file	Scan	Method	Score	m/z	Gene names
0523_12	15946	FTMS; HCD	99.06	843.41	At1g48210;At3g17410

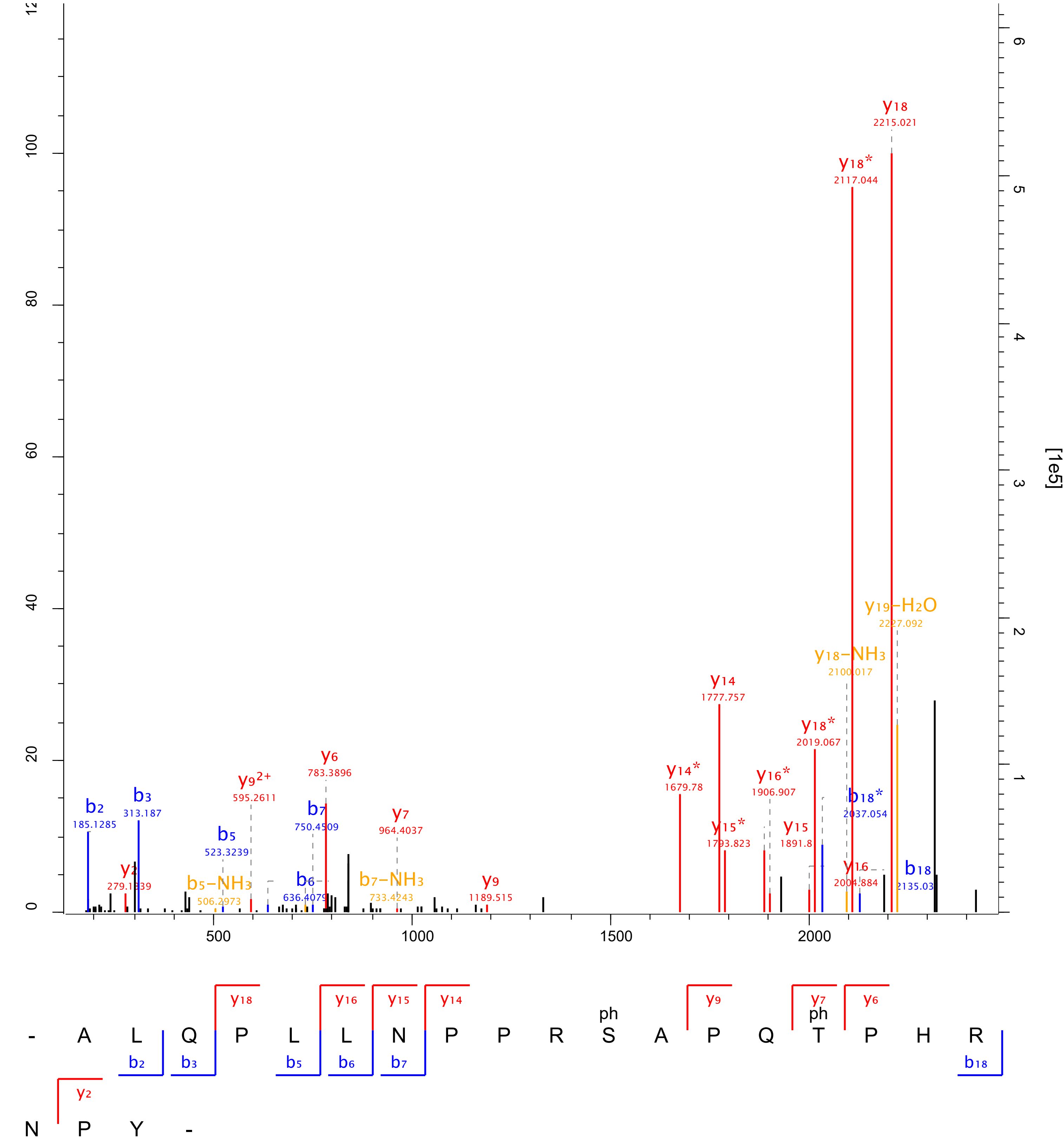

Sample output to test PDF Combine only

Raw file	Scan	Method	Score	m/z	Gene names
0523_12	15949	FTMS; HCD	56.37	673.31	IQD32

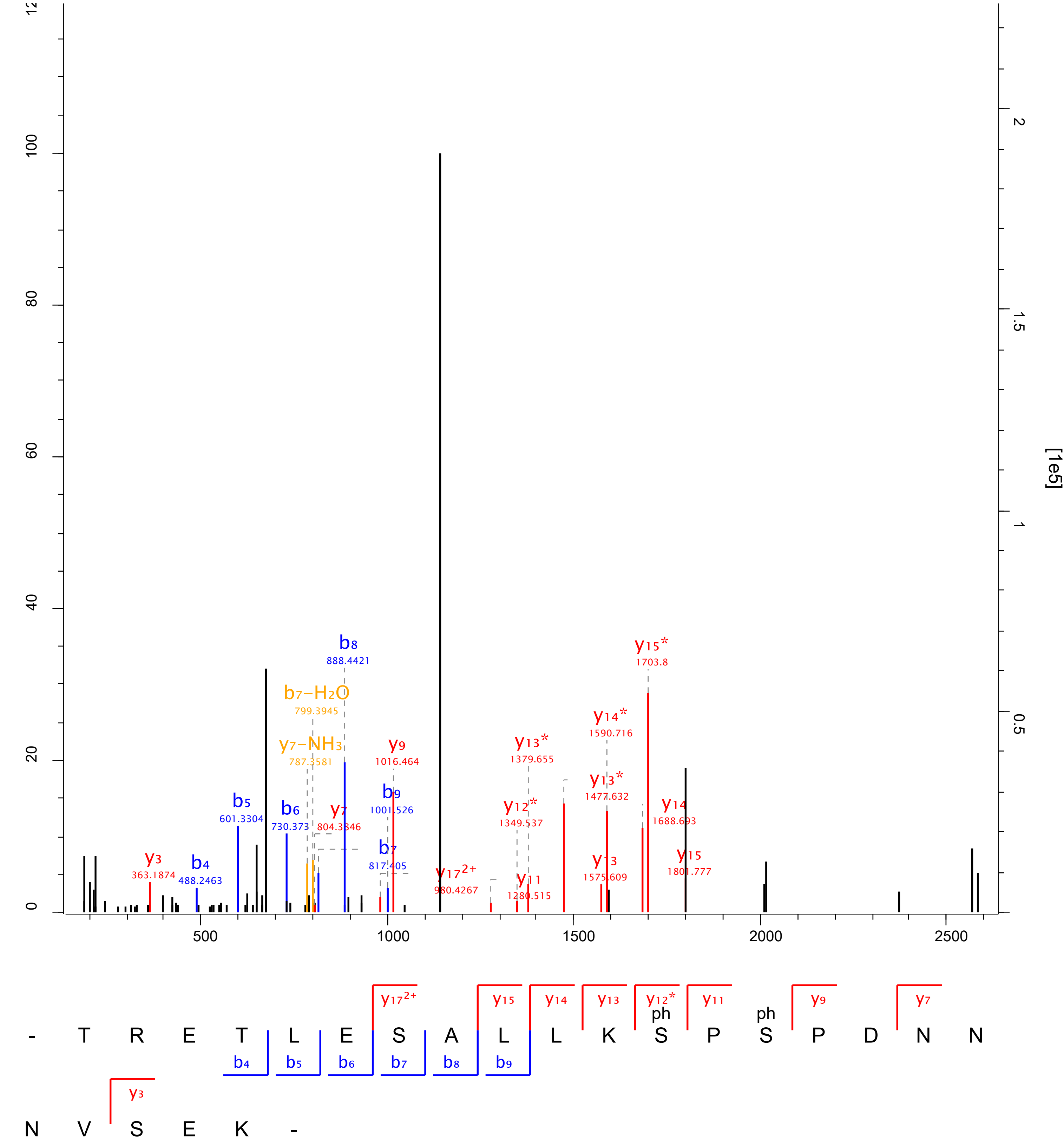

Sample output to test PDF Combine only

Raw file	Scan	Method	Score	m/z	Gene names
0523_12	16221	FTMS; HCD	82.09	813.03	APUM5

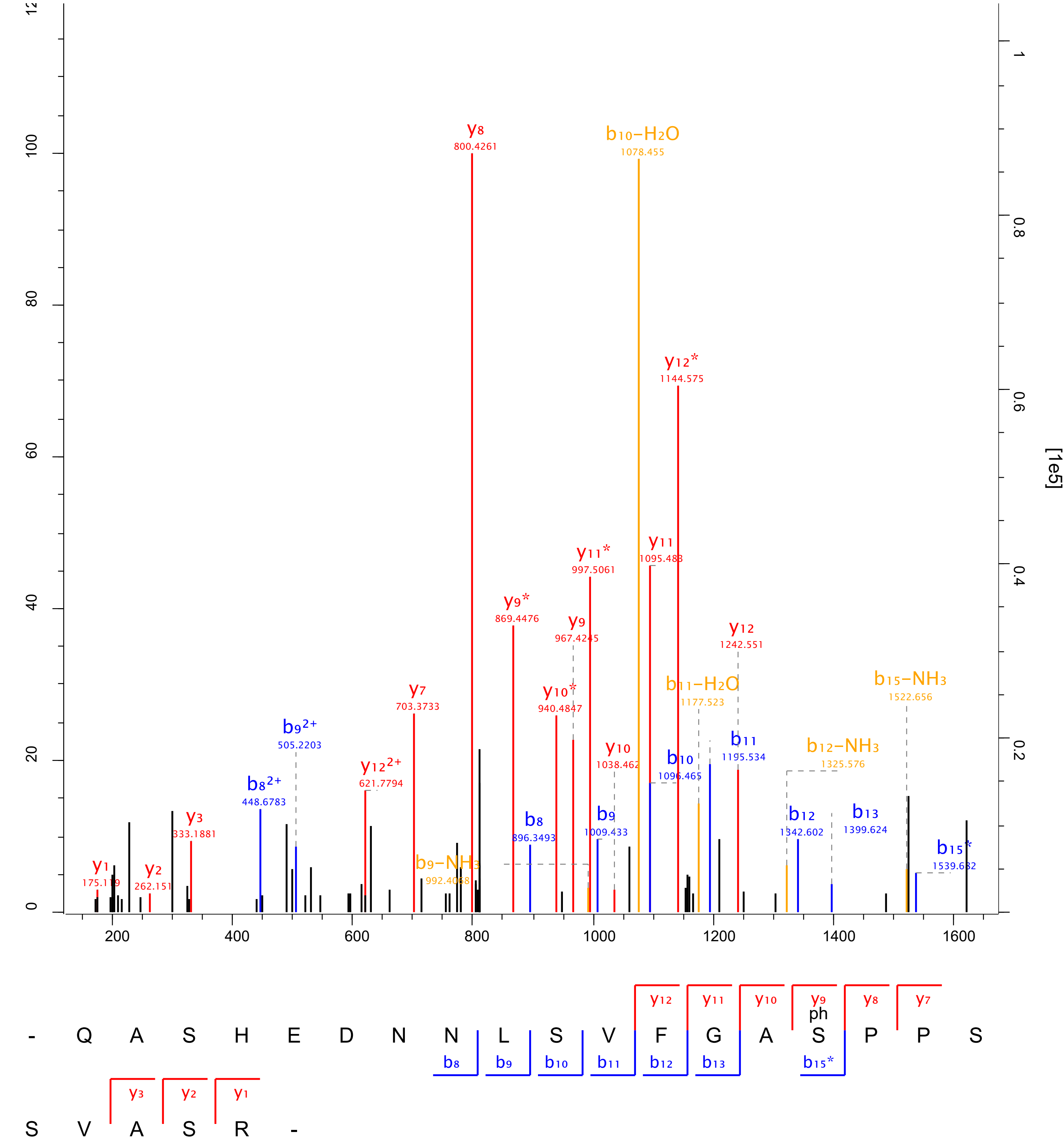

Sample output to test PDF Combine only

Raw file	Scan	Method	Score	m/z	Gene names
0523_12	16259	FTMS; HCD	60.95	868.68	BAG1

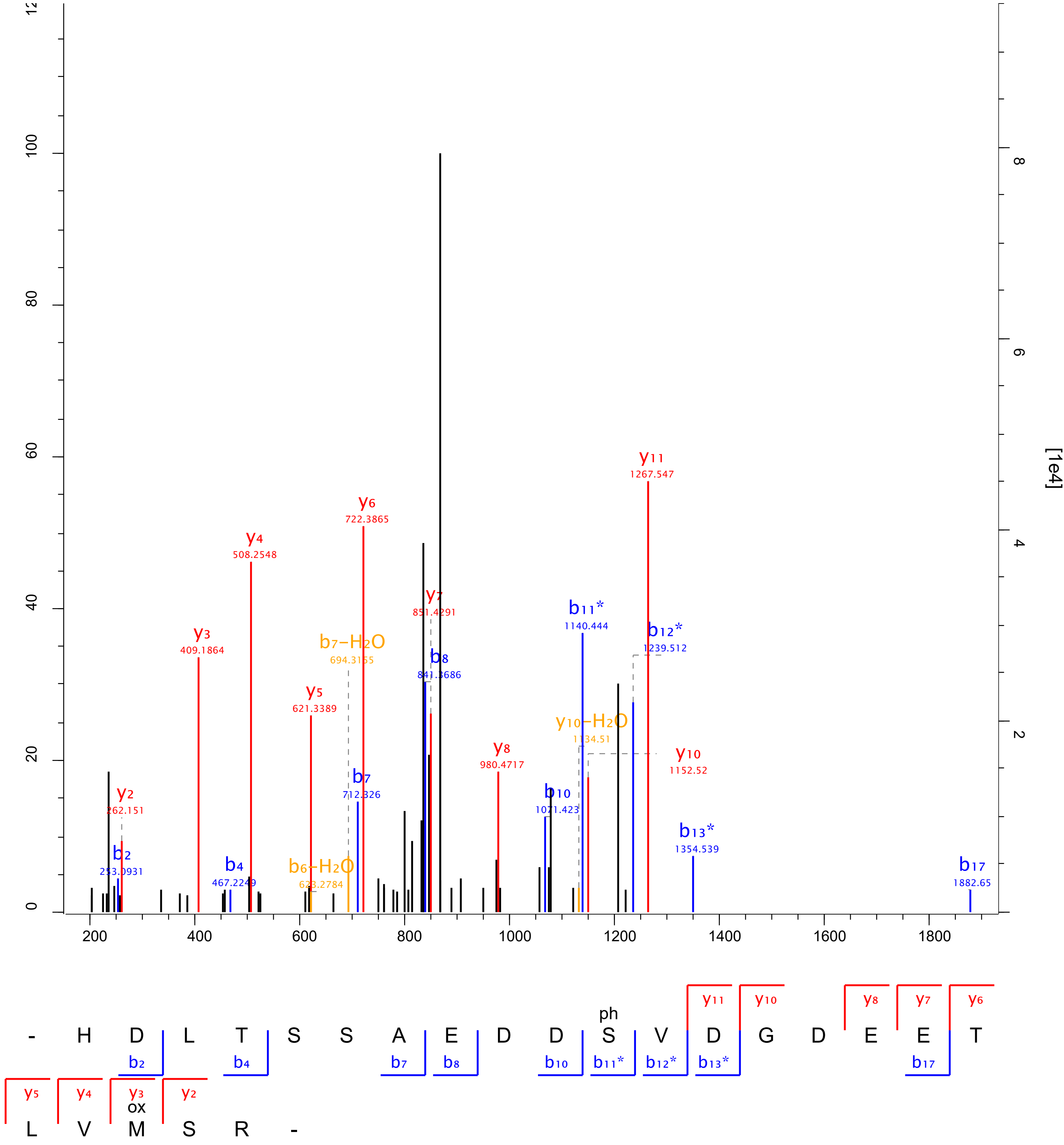

Sample output to test PDF Combine only

Raw file	Scan	Method	Score	m/z	Gene names
0523_12	16447	FTMS; HCD	197.89	856.86	ERD7

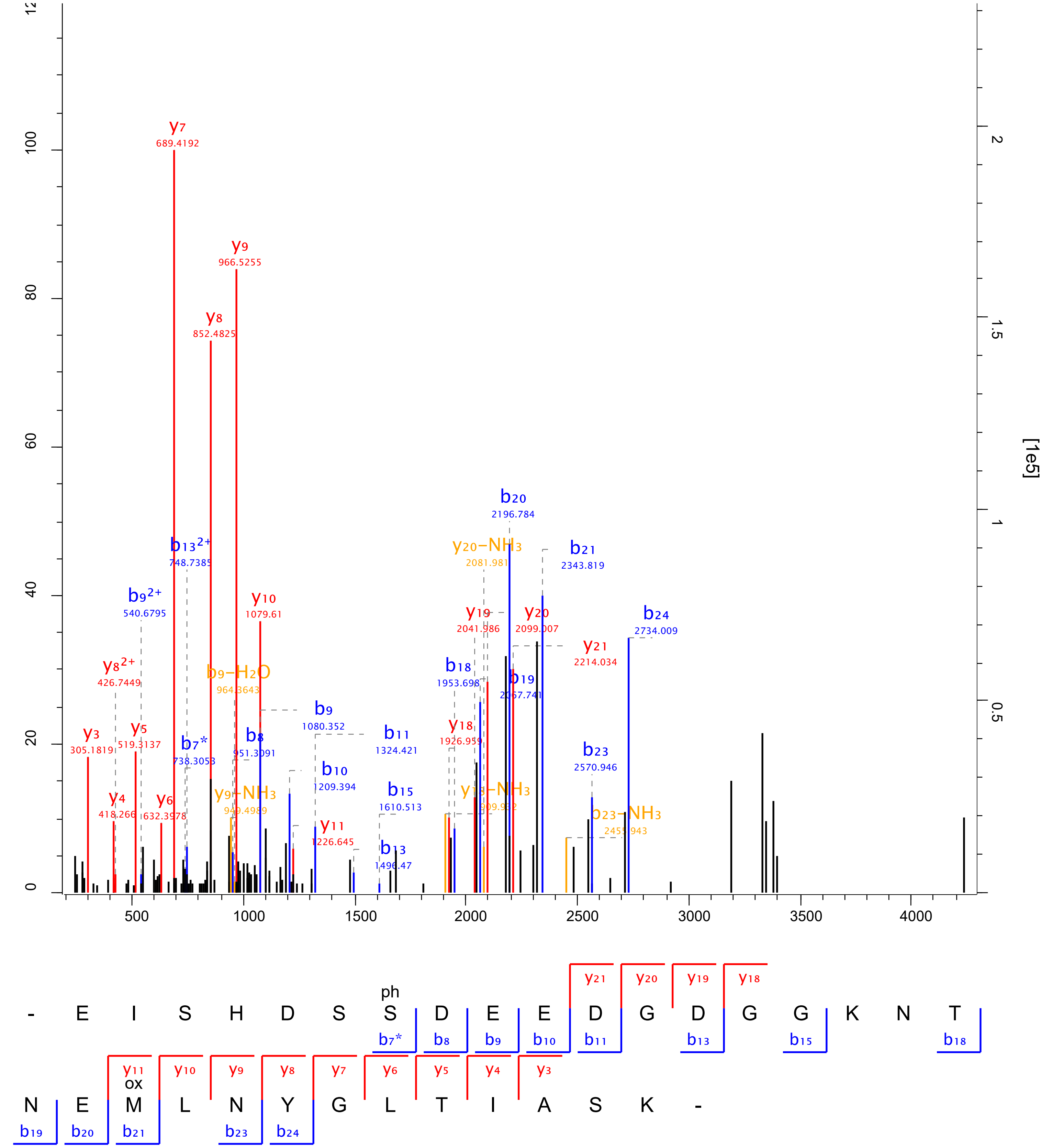

Sample output to test PDF Combine only

Raw file	Scan	Method	Score	m/z	Gene names
0523_12	16585	FTMS; HCD	59.37	712.99	EIN2

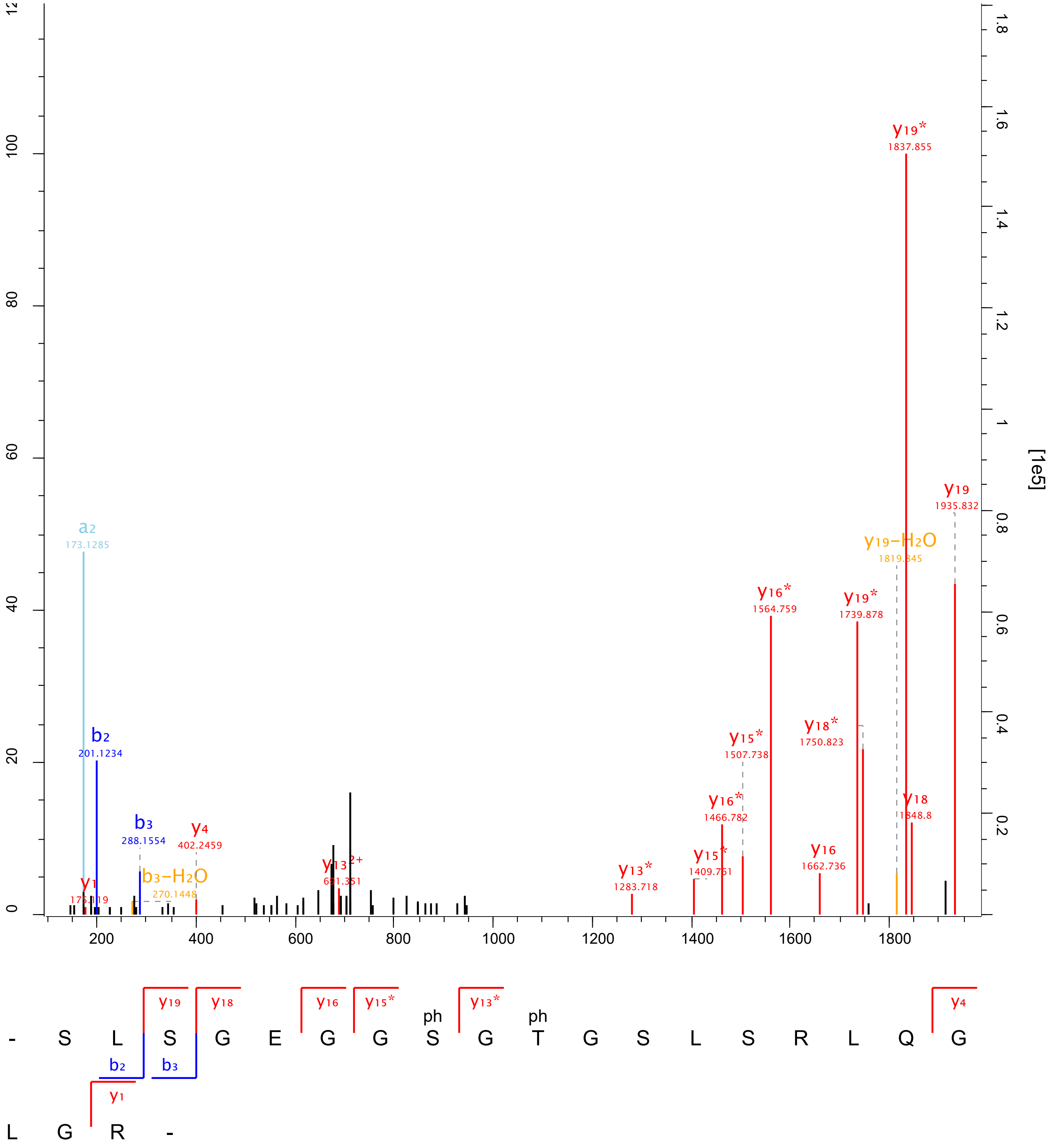

Sample output to test PDF Combine only

Raw file	Scan	Method	Score	m/z	Gene names
0523_12	16800	FTMS; HCD	119.45	772.84	BSL1

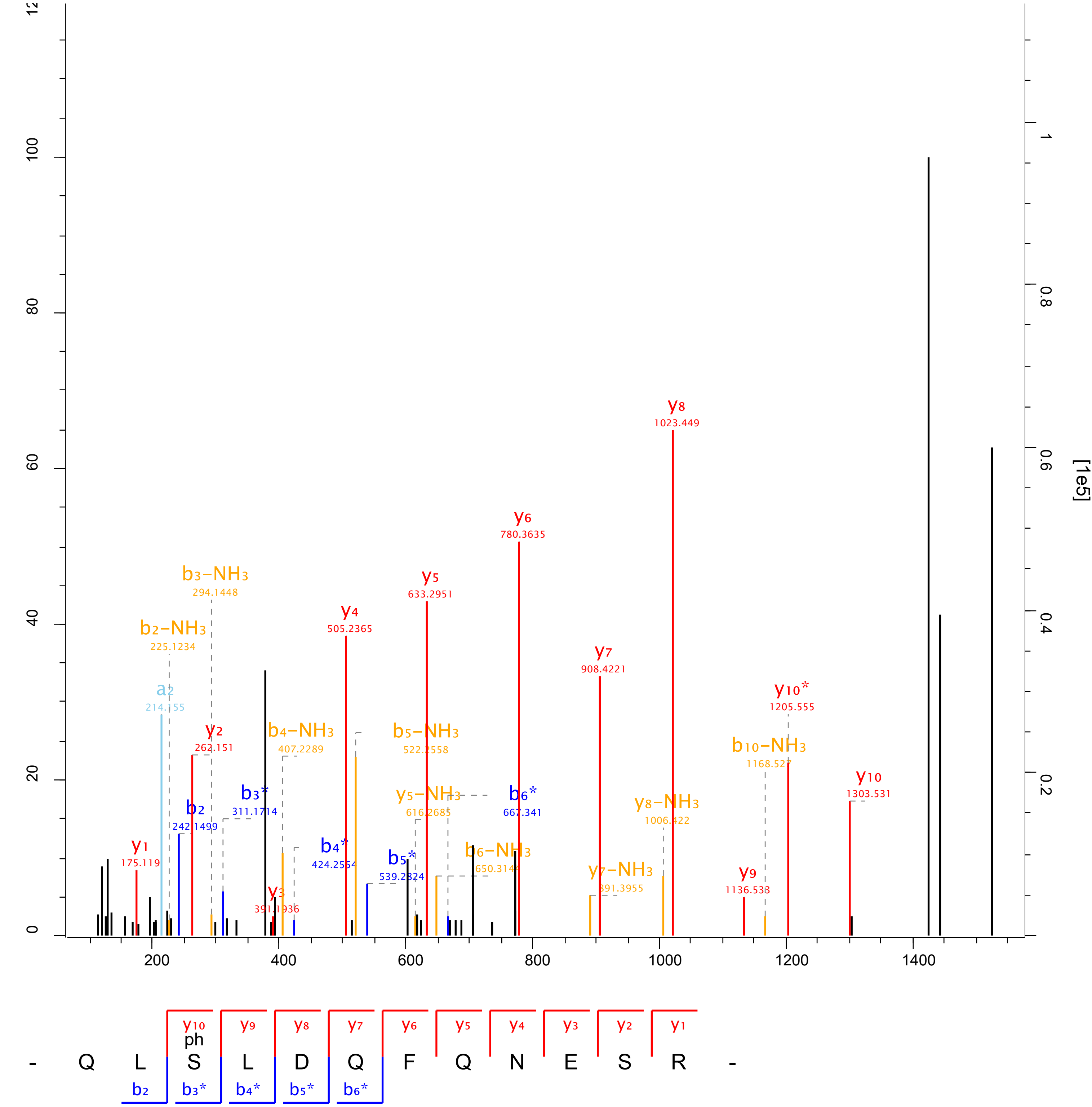

Sample output to test PDF Combine only

Raw file	Scan	Method	Score	m/z	Gene names
0523_12	16831	FTMS; HCD	193.53	908.42	At1g06890

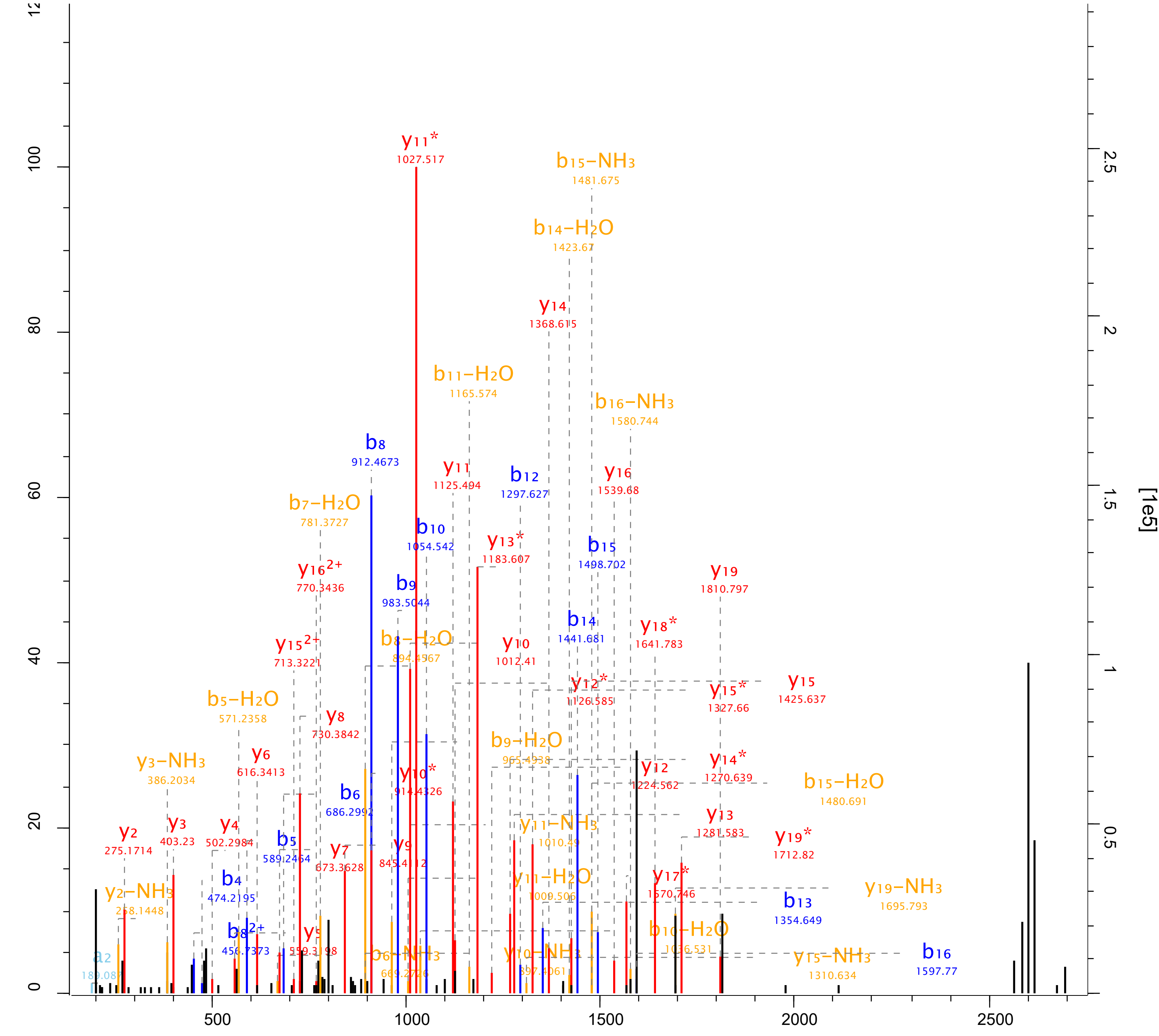

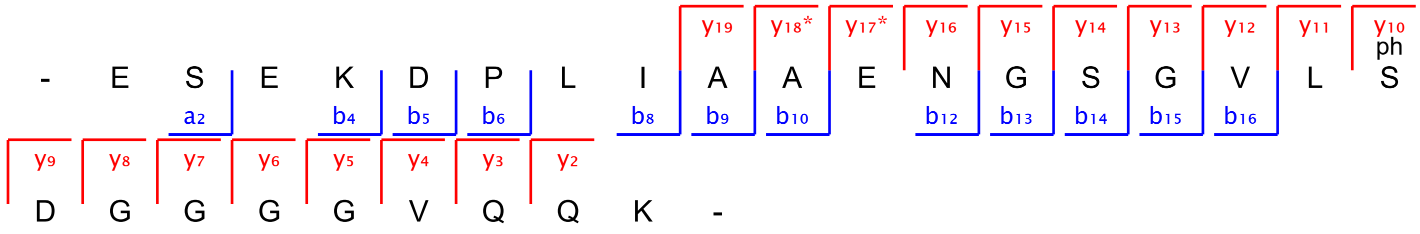

Sample output to test PDF Combine only

Raw file	Scan	Method	Score	m/z	Gene names
0523_12	16905	FTMS; HCD	252.99	827.85	SYP122

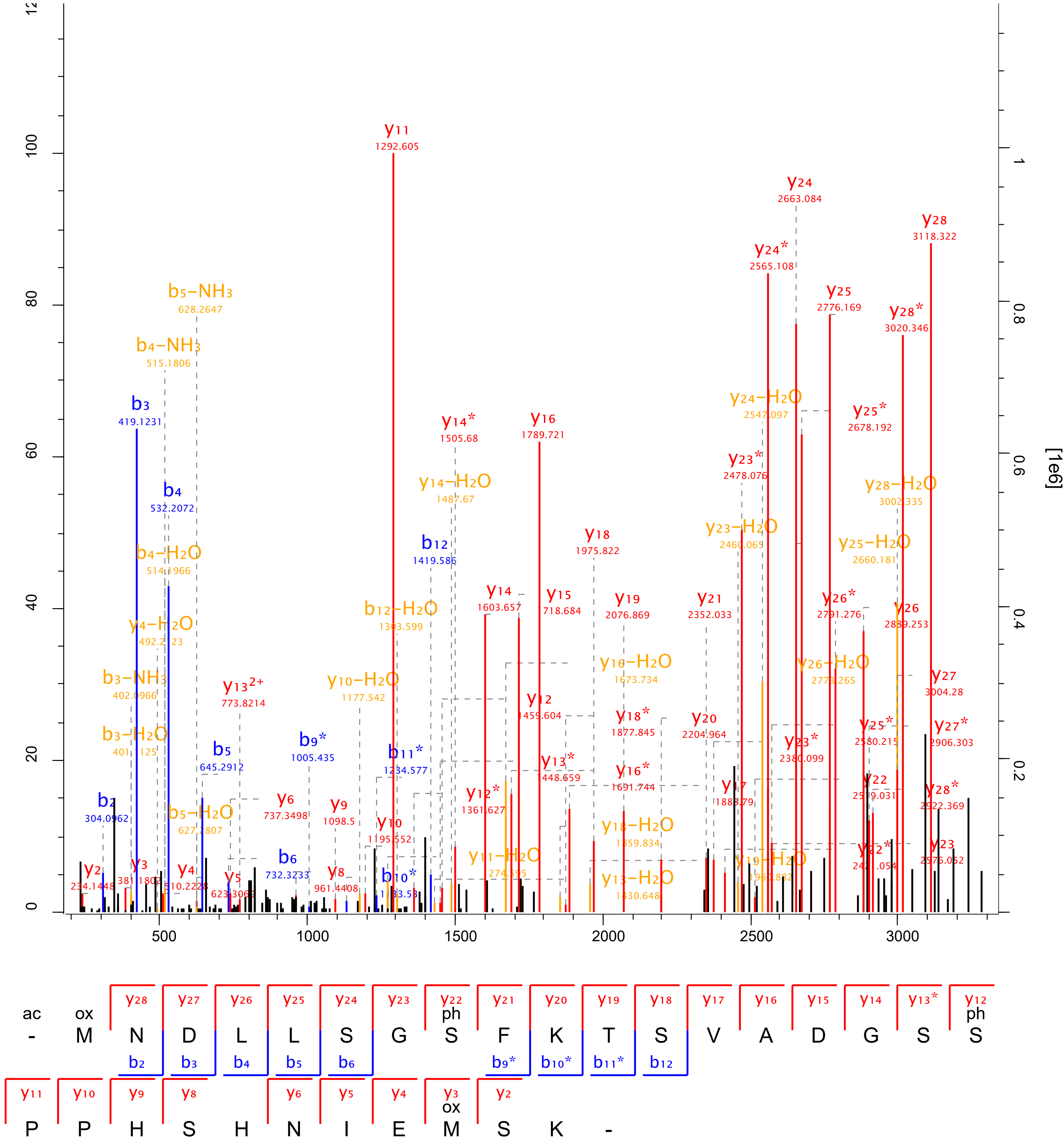

Sample output to test PDF Combine only

Raw file	Scan	Method	Score	m/z	Gene names
0523_12	17333	FTMS; HCD	186.13	802.35	RH24

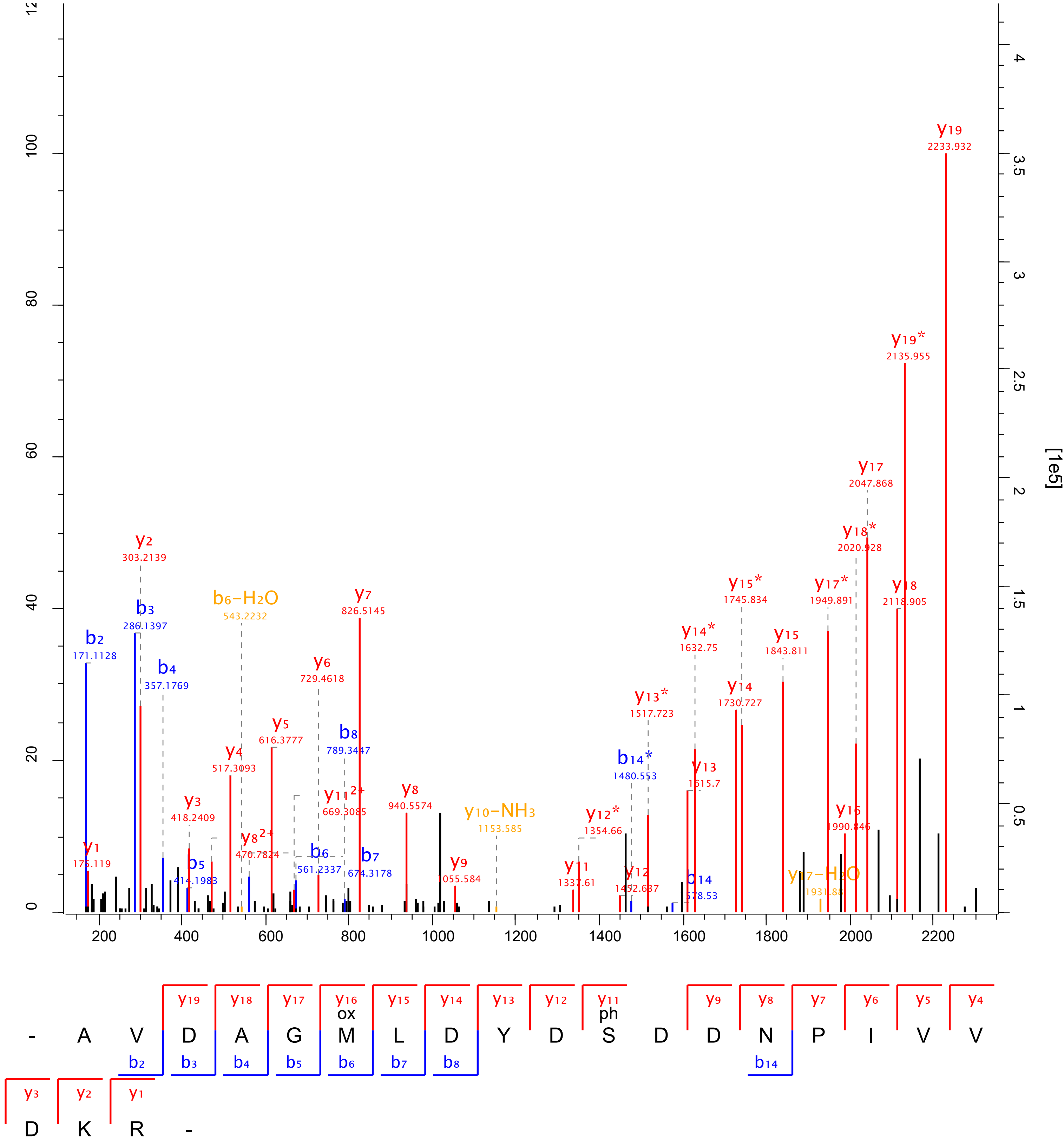

Sample output to test PDF Combine only

Raw file	Scan	Method	Score	m/z	Gene names
0523_12	17436	FTMS; HCD	112.85	764.66	CMTA4

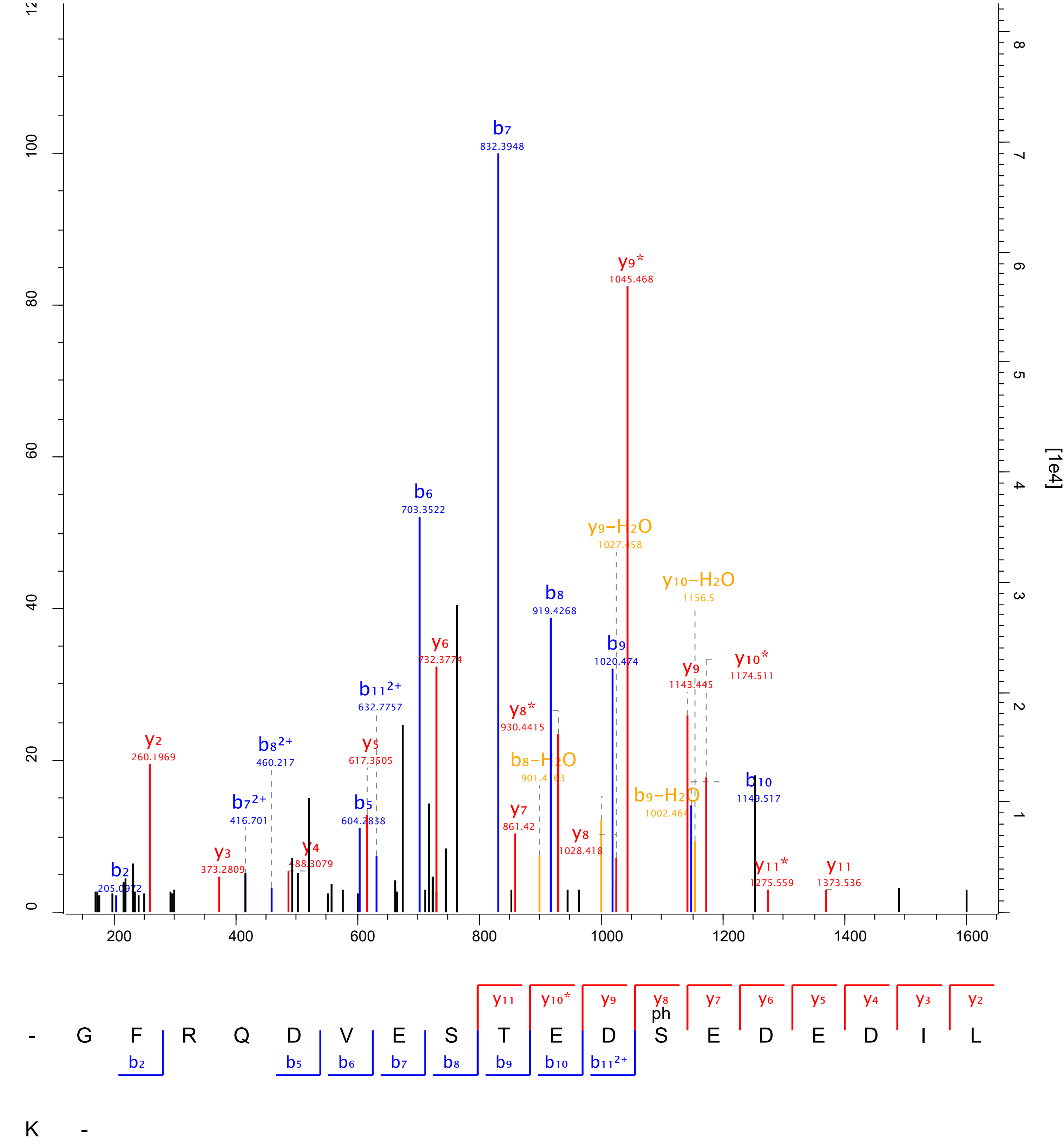

Sample output to test PDF Combine only

Raw file	Scan	Method	Score	m/z	Gene names
0523_12	17530	FTMS; HCD	77.51	717.87	At2g39130

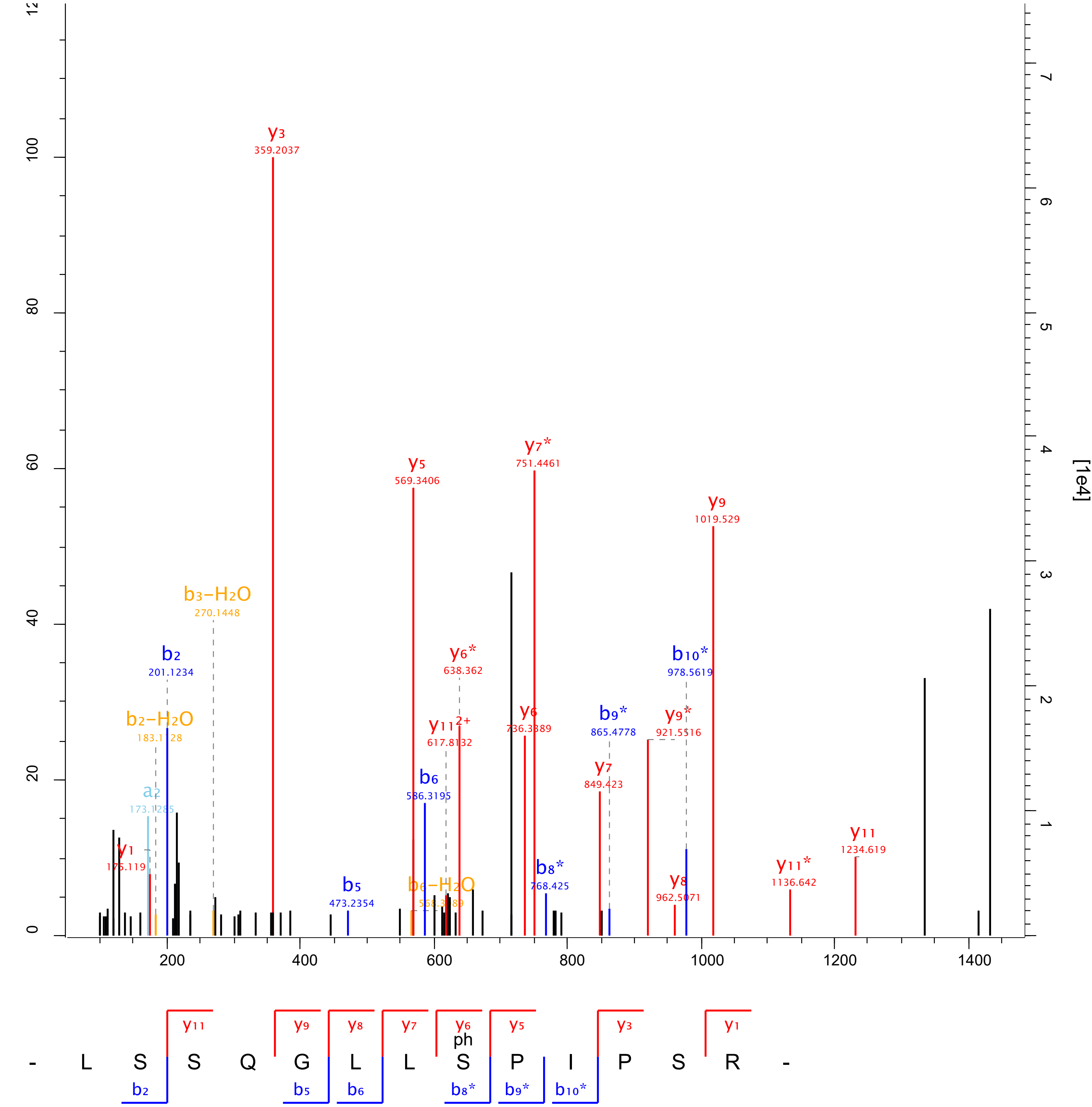

Sample output to test PDF Combine only

Raw file	Scan	Method	Score	m/z	Gene names
0523_12	17541	FTMS; HCD	99.6	955.45	At3g53180

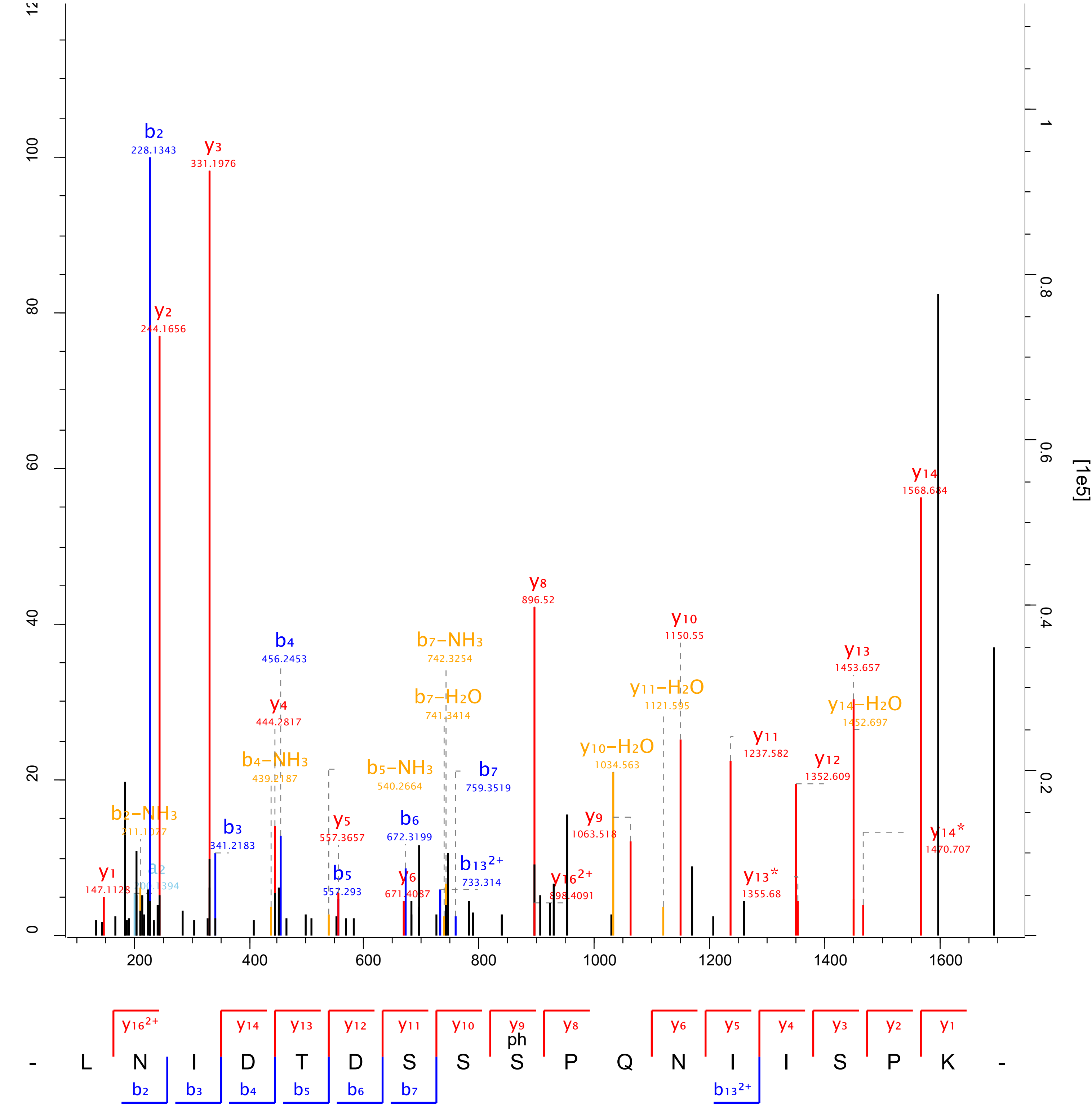

Sample output to test PDF Combine only

Raw file	Scan	Method	Score	m/z	Gene names
0523_12	18068	FTMS; HCD	83.54	692.82	At1g56230;F14G9.16

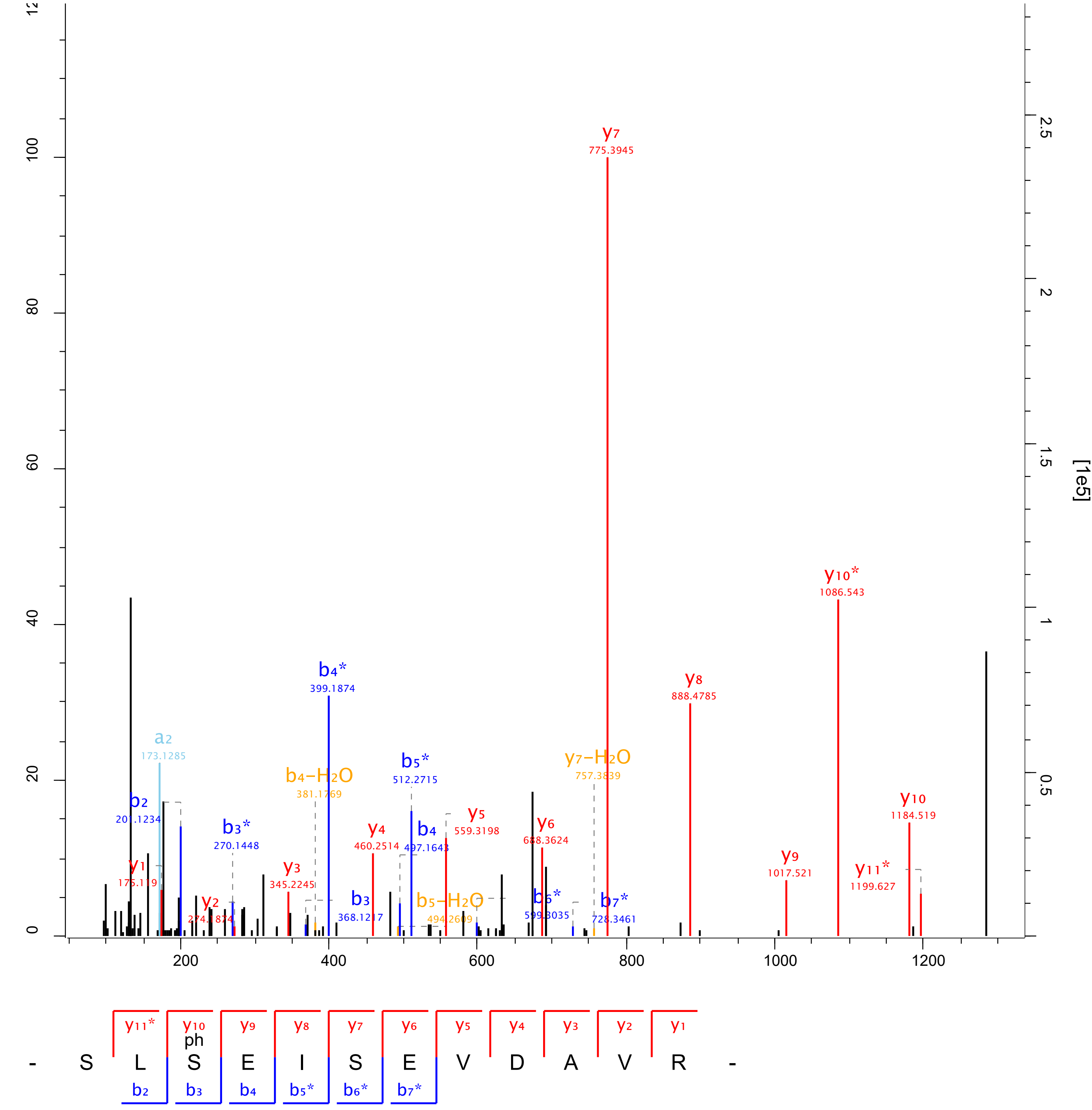

Sample output to test PDF Combine only

Raw file	Scan	Method	Score	m/z
0523_12	18084	FTMS; HCD	71.38	861.38

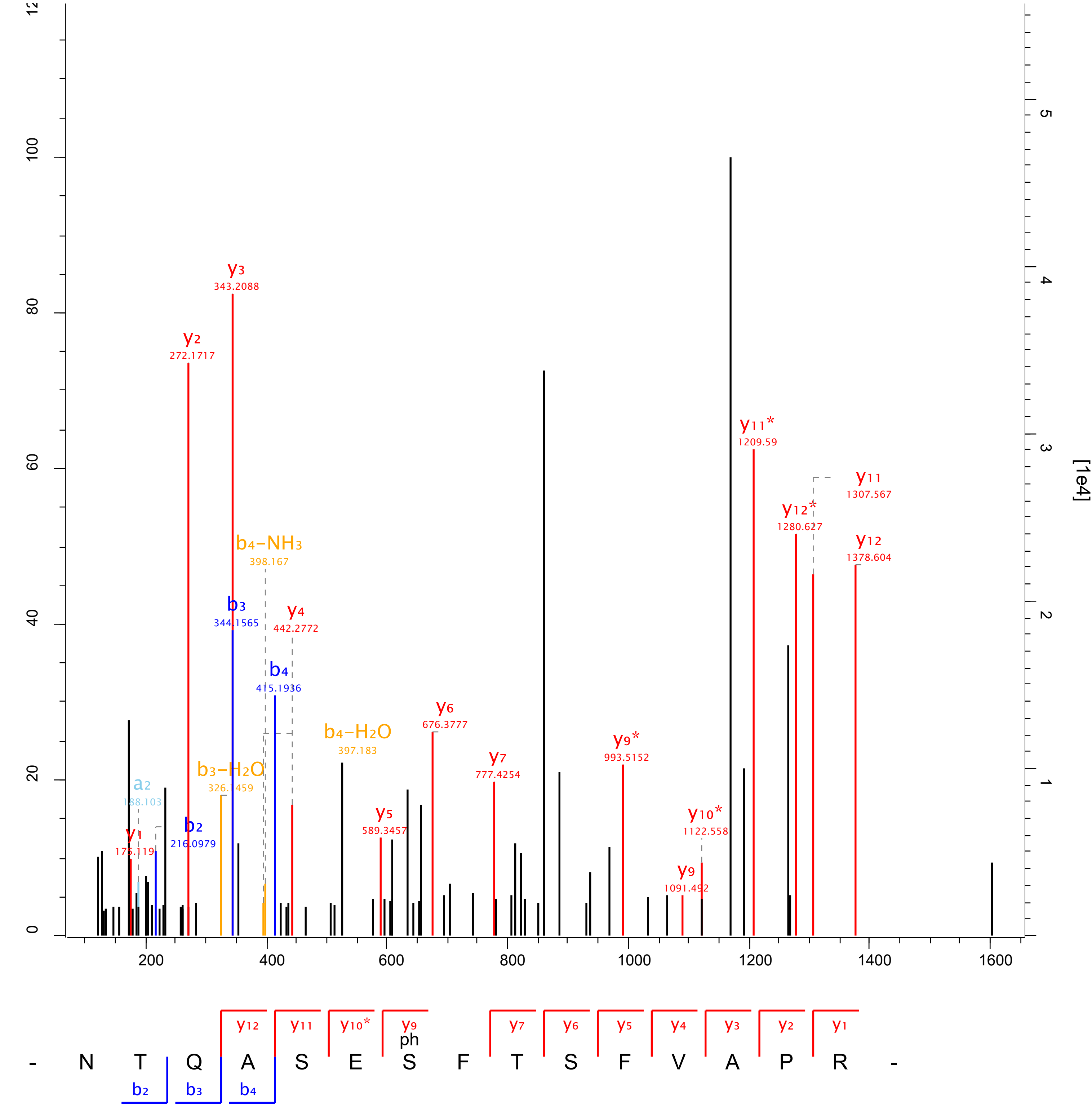

Sample output to test PDF Combine only

Raw file	Scan	Method	Score	m/z	Gene names
0523_12	18797	FTMS; HCD	214.91	811.69	IQD32

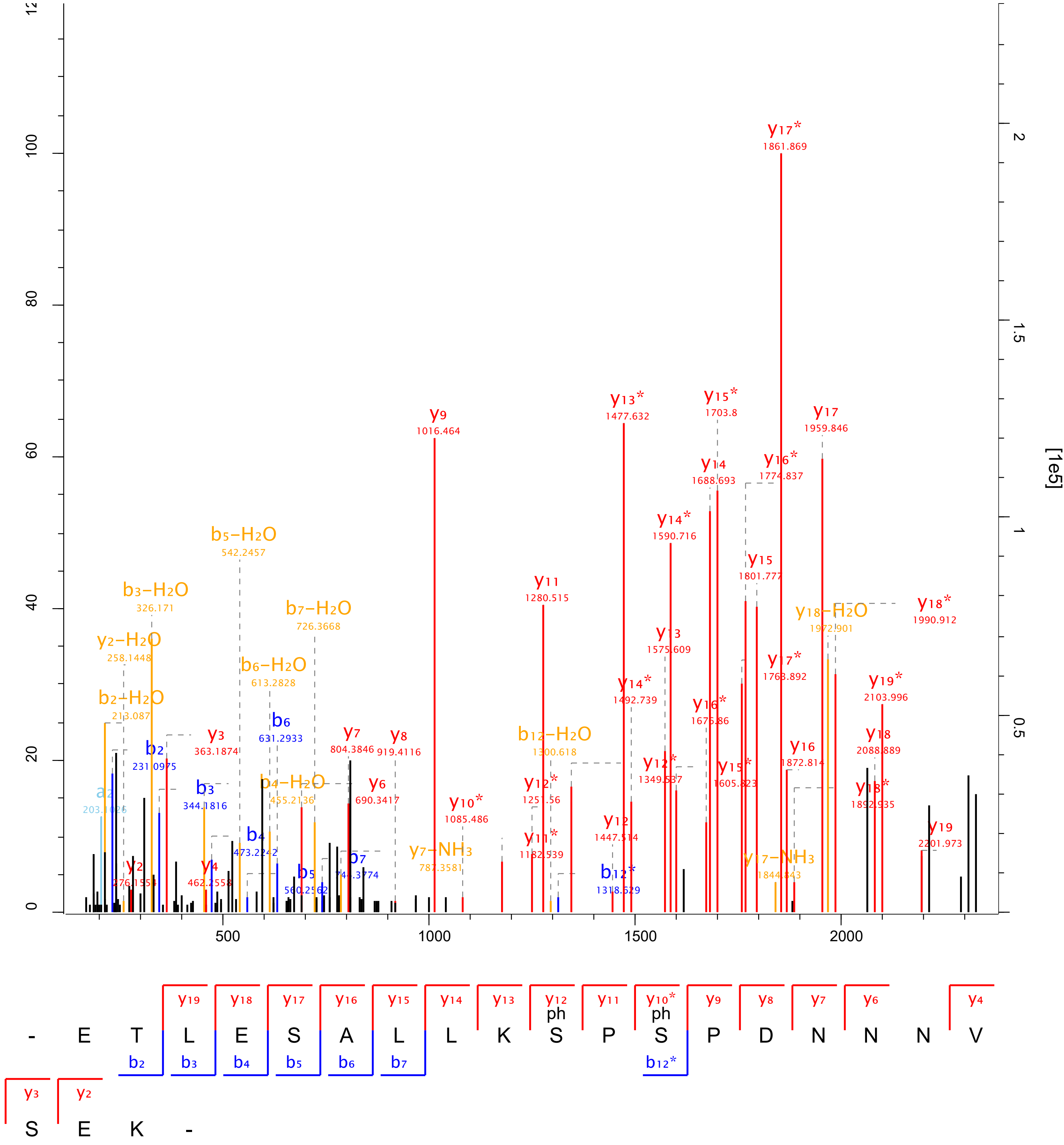

Sample output to test PDF Combine only

Raw file	Scan	Method	Score	m/z	Gene names
0523_12	19315	FTMS; HCD	97.54	752.88	At2g39130

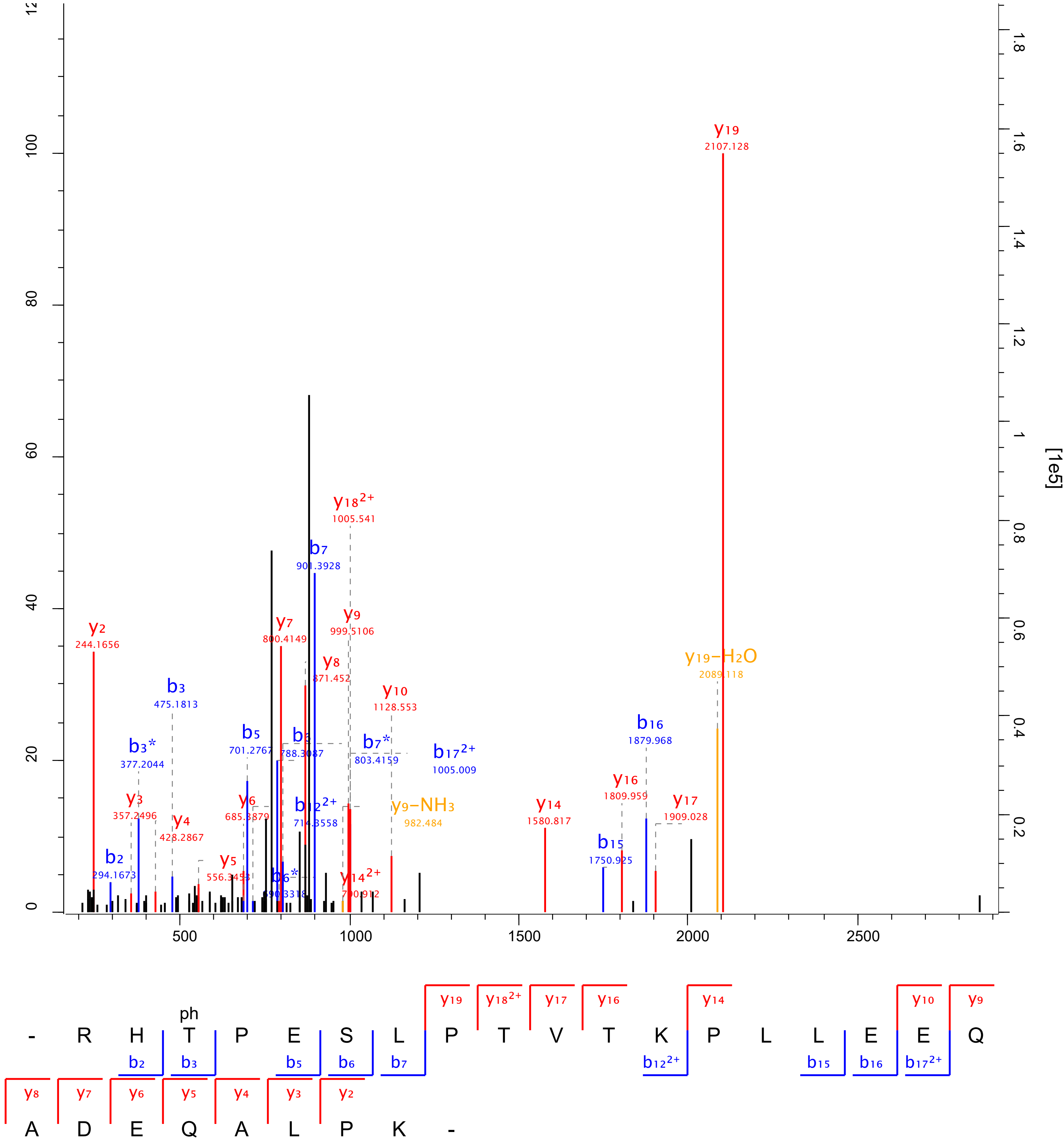

Sample output to test PDF Combine only

Raw file	Scan	Method	Score	m/z	Gene names
0523_12	20146	FTMS; HCD	52.86	785.38	At1g66880

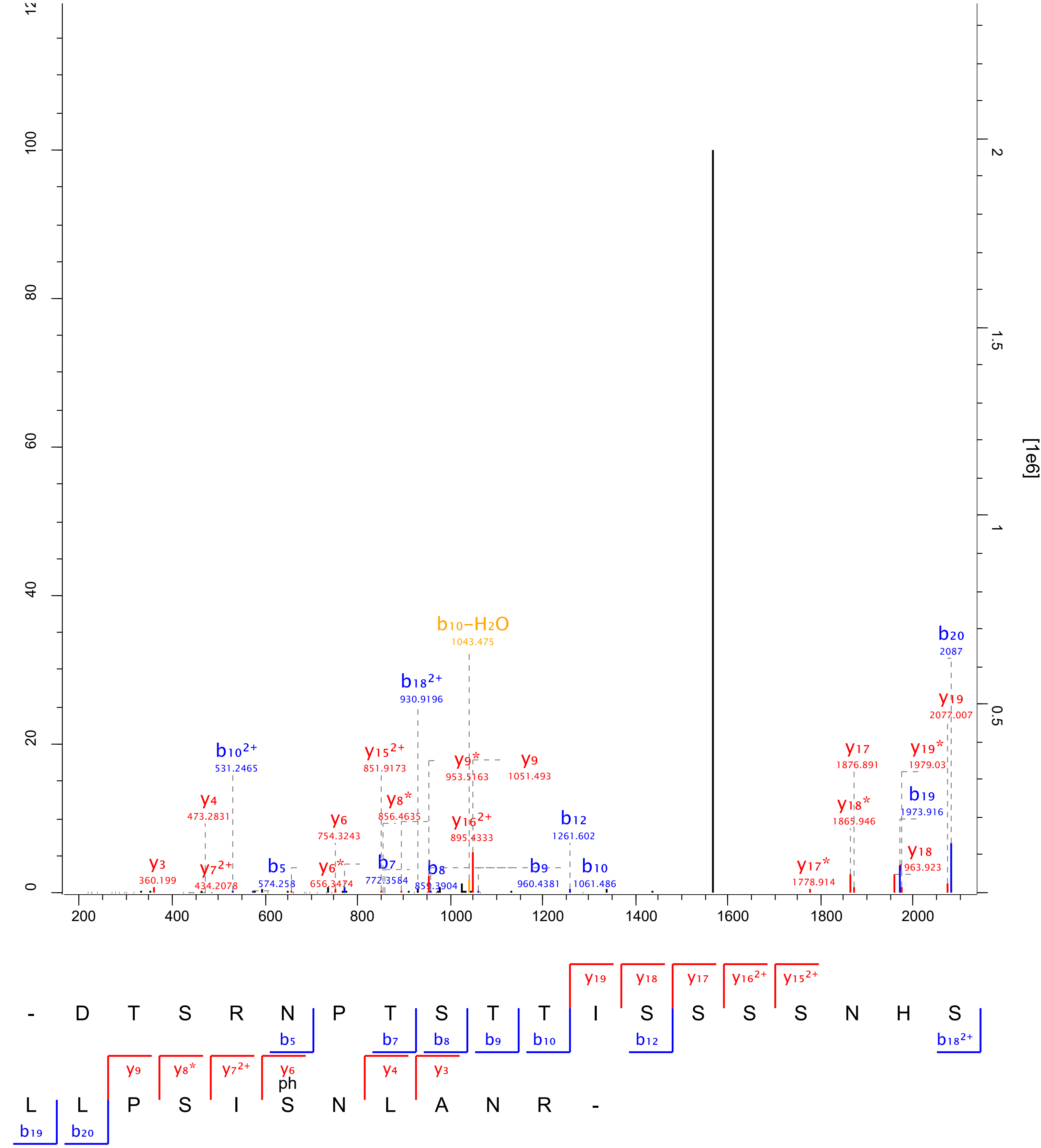

Sample output to test PDF Combine only

Raw file	Scan	Method	Score	m/z	Gene names
0523_12	20287	FTMS; HCD	144.26	720.36	HIR3

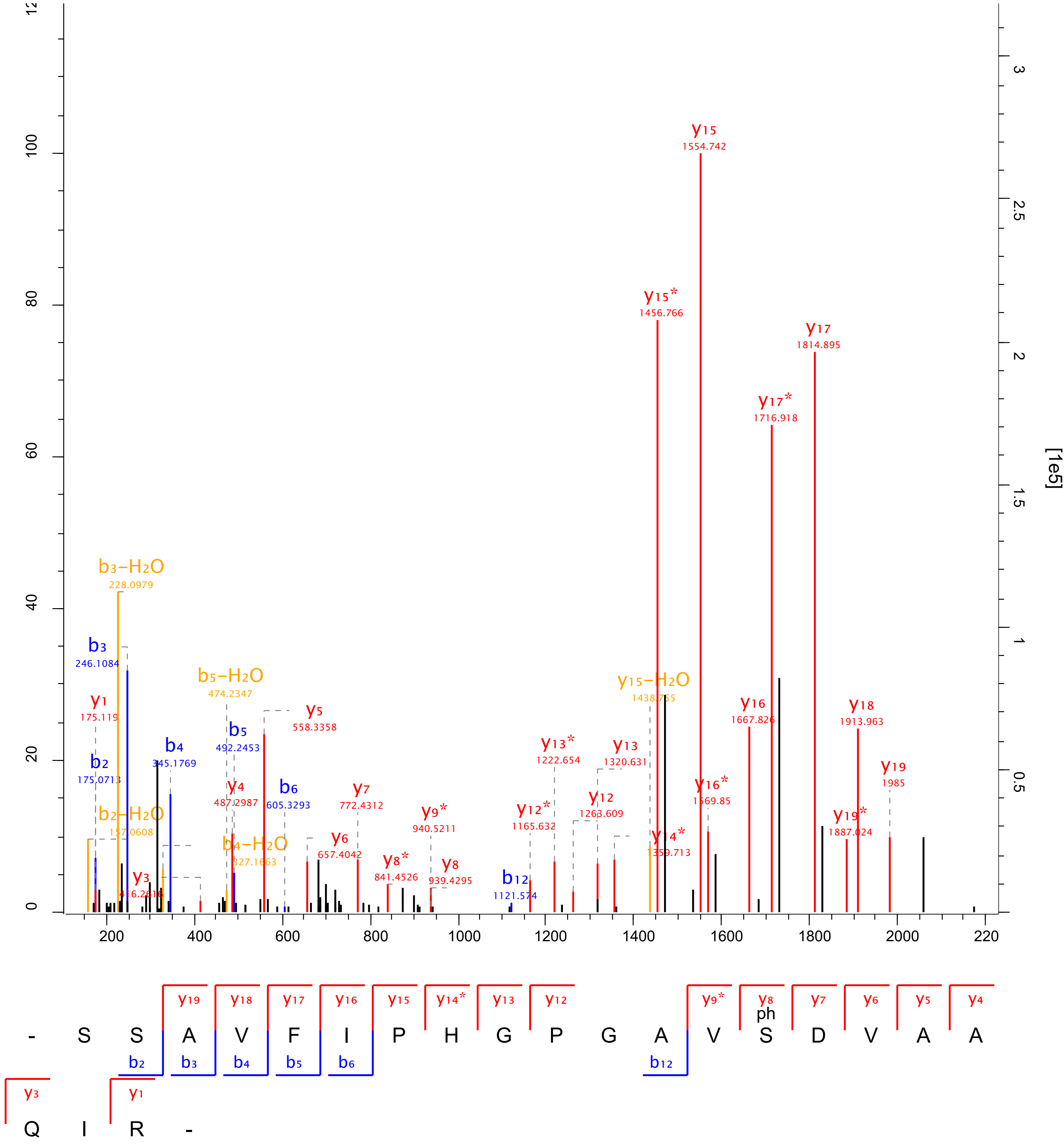

Sample output to test PDF Combine only

Raw file	Scan	Method	Score	m/z	Gene names
0523_12	20326	FTMS; HCD	51.03	872.9	IQD32

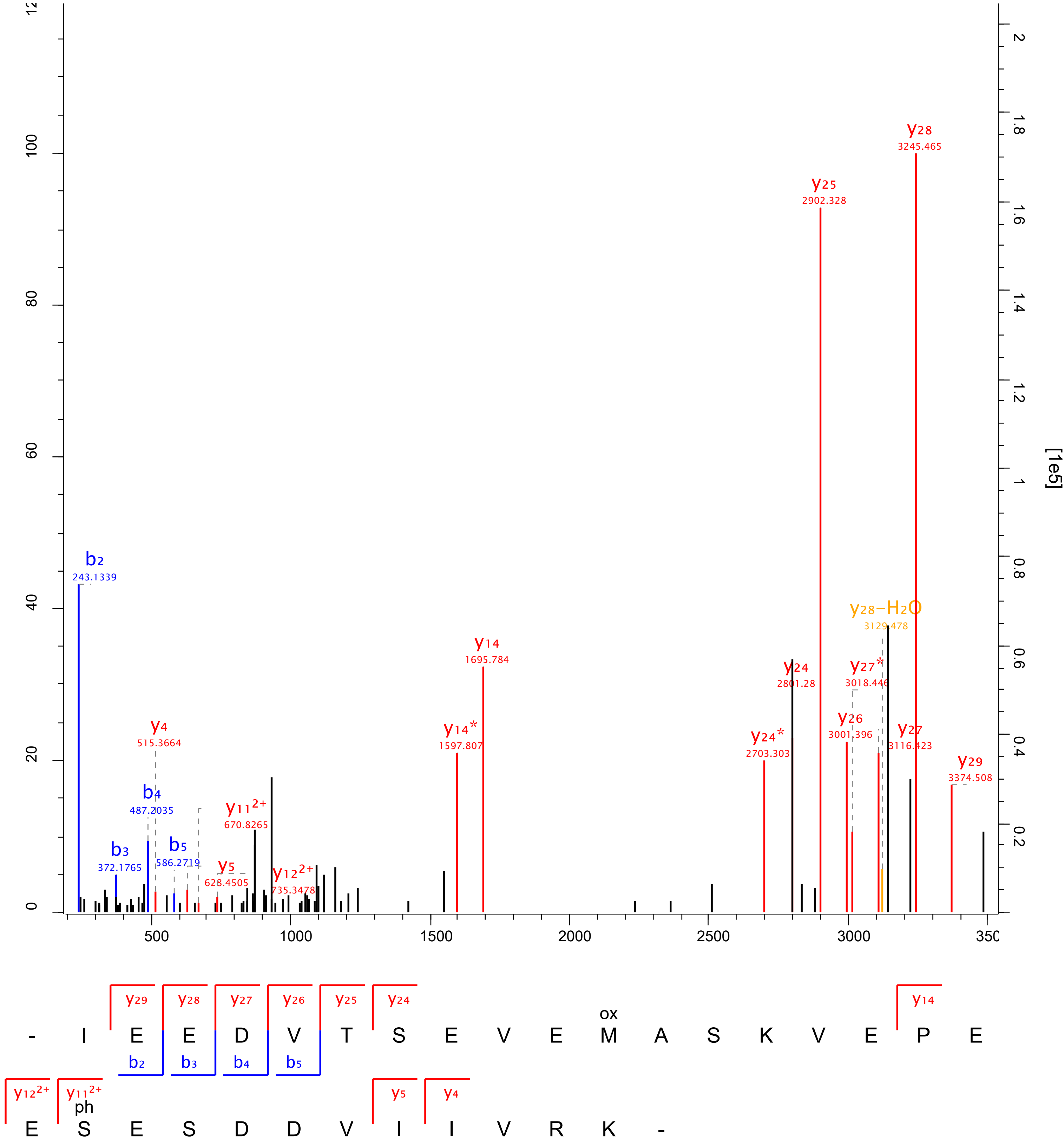

Sample output to test PDF Combine only

Raw file	Scan	Method	Score	m/z	Gene names
0523_12	20597	FTMS; HCD	55.78	973.71	At5g47690

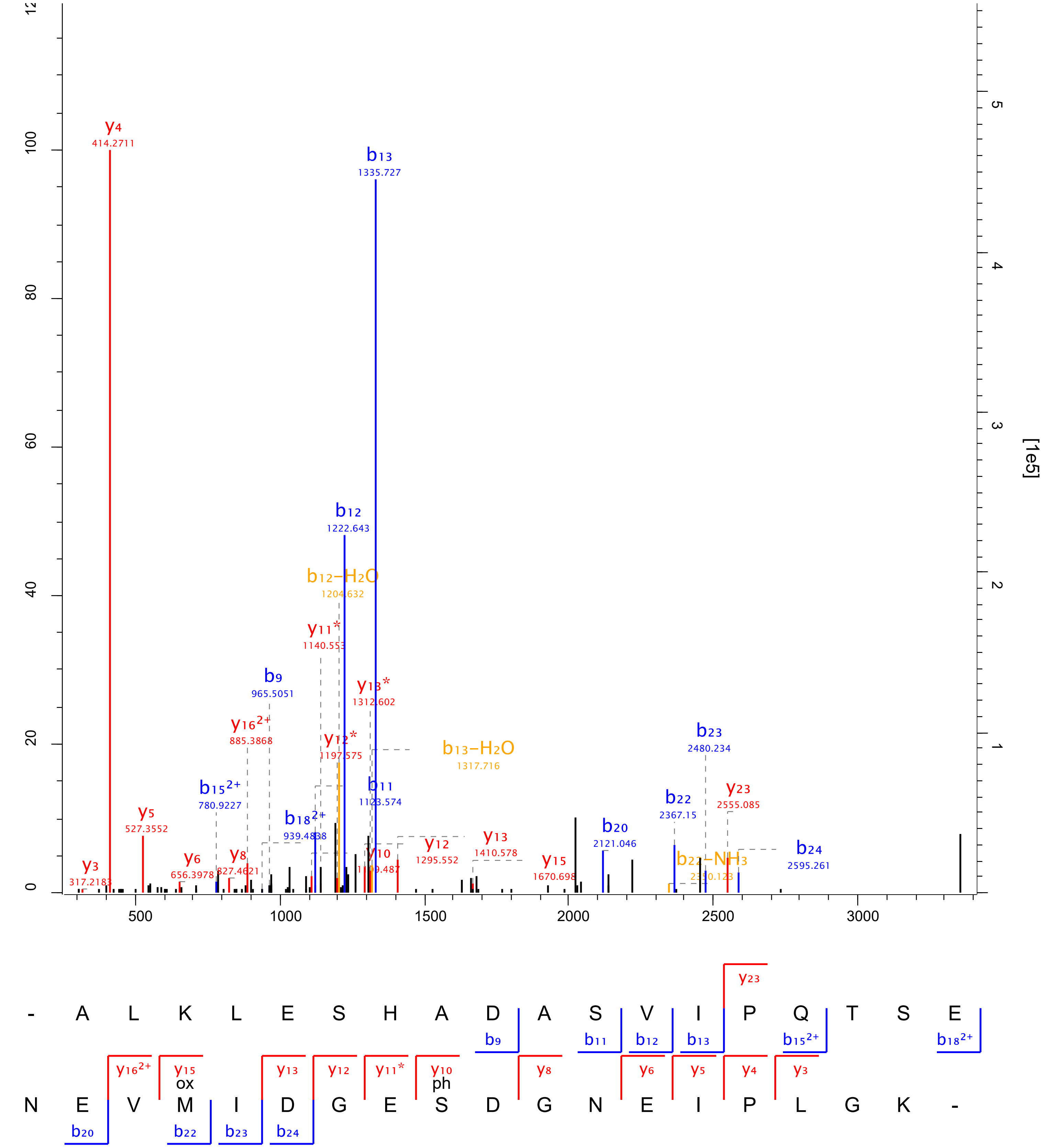

Sample output to test PDF Combine only

Raw file	Scan	Method	Score	m/z	Gene names
0523_12	20856	FTMS; HCD	200.1	1120.17	XLG3

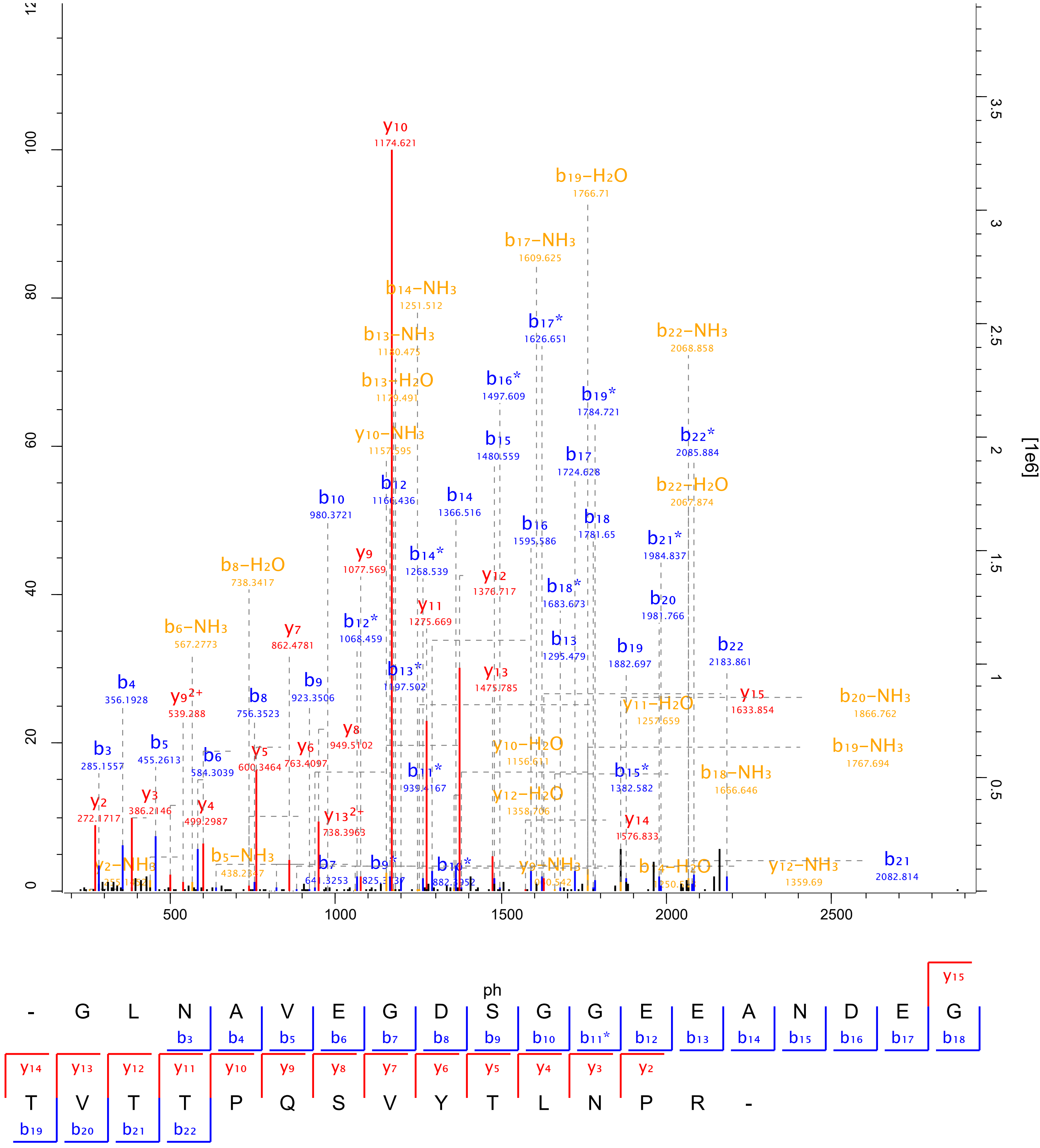

Sample output to test PDF Combine only

Raw file	Scan	Method	Score	m/z	Gene names
0523_12	20942	FTMS; HCD	118.01	973.07	PWP2

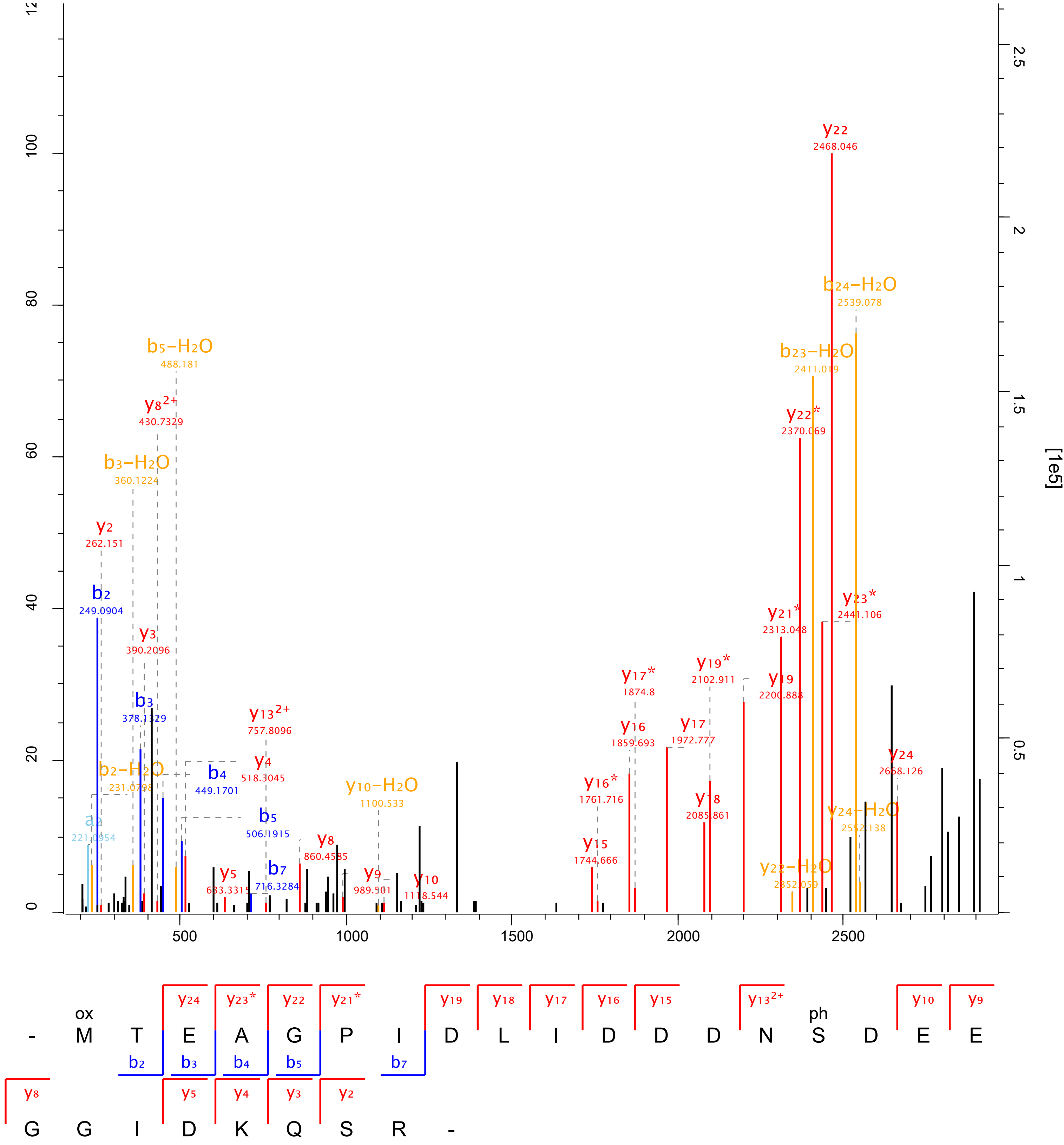

Sample output to test PDF Combine only

Raw file	Scan	Method	Score	m/z
0523_12	20984	FTMS; HCD	50.83	1129

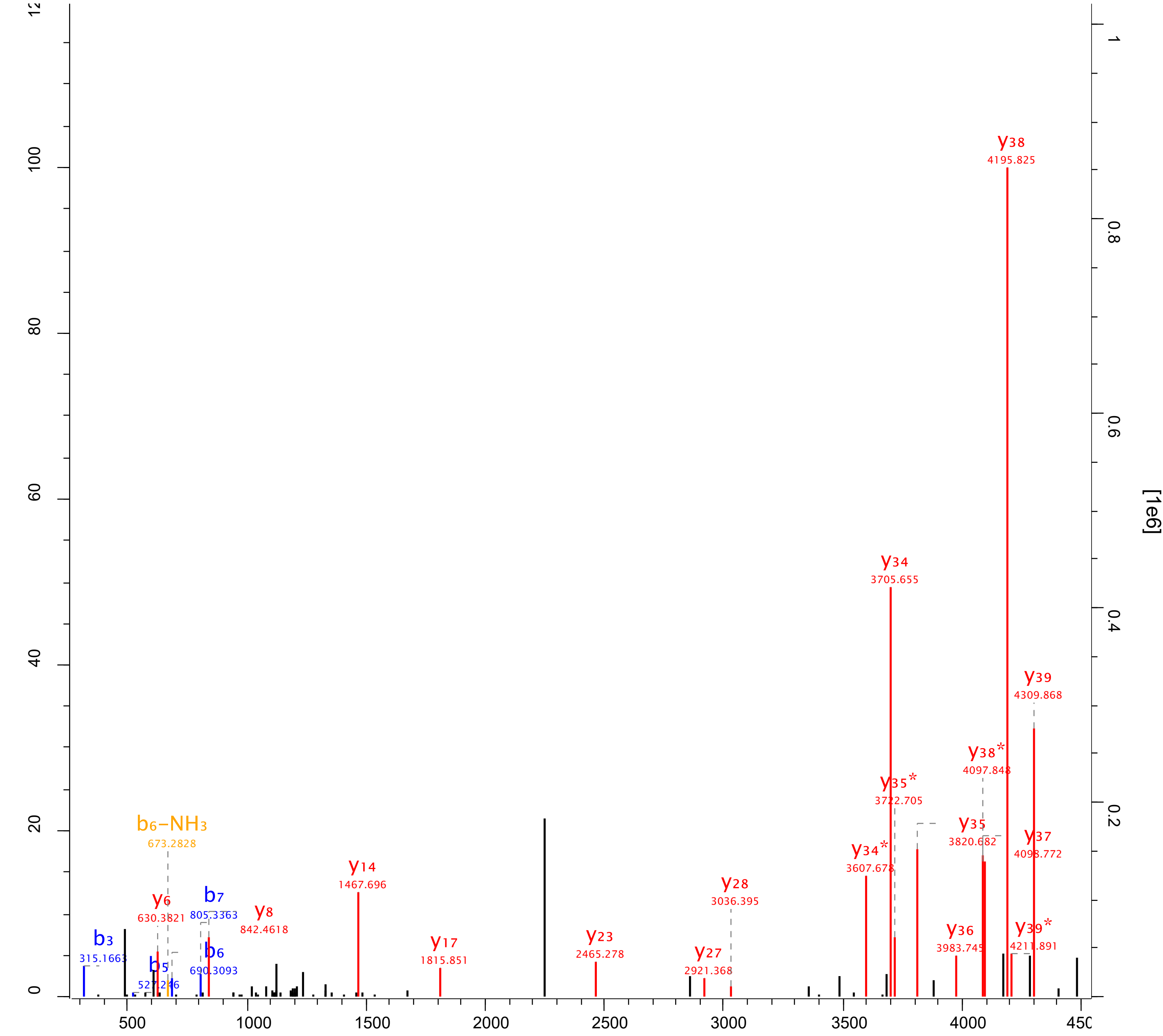

- S L N P D Y D P D D Q D V D S S S D

V R A P L I P N H P P D N S D P D P T

T A I K -

Sample output to test PDF Combine only

Raw file	Scan	Method	Score	m/z	Gene names
0523_12	21090	FTMS; HCD	199.36	795.67	T8B10_110

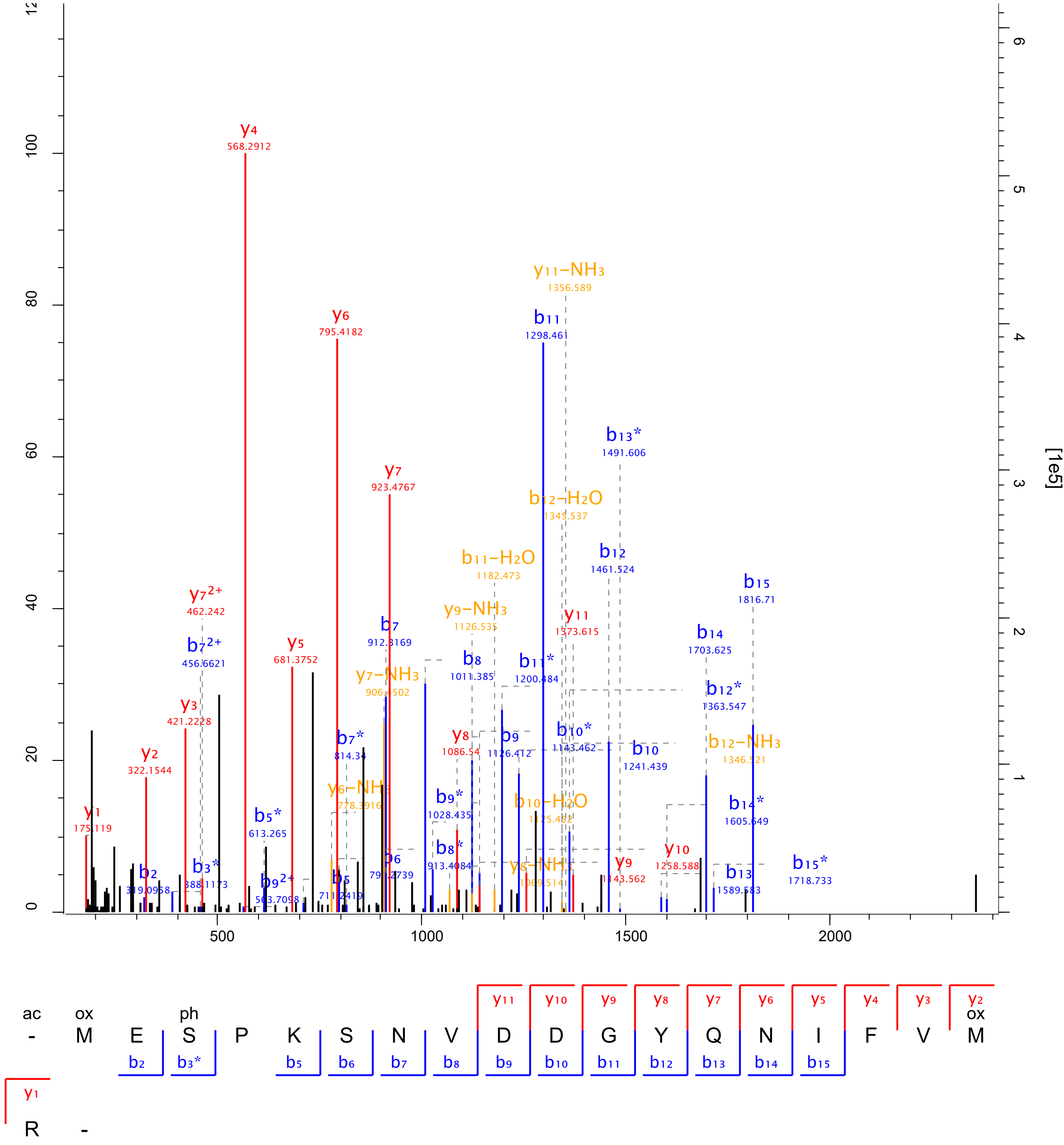

Sample output to test PDF Combine only

Raw file	Scan	Method	Score	m/z	Gene names
0523_12	21501	FTMS; HCD	141.19	765.04	NHX1

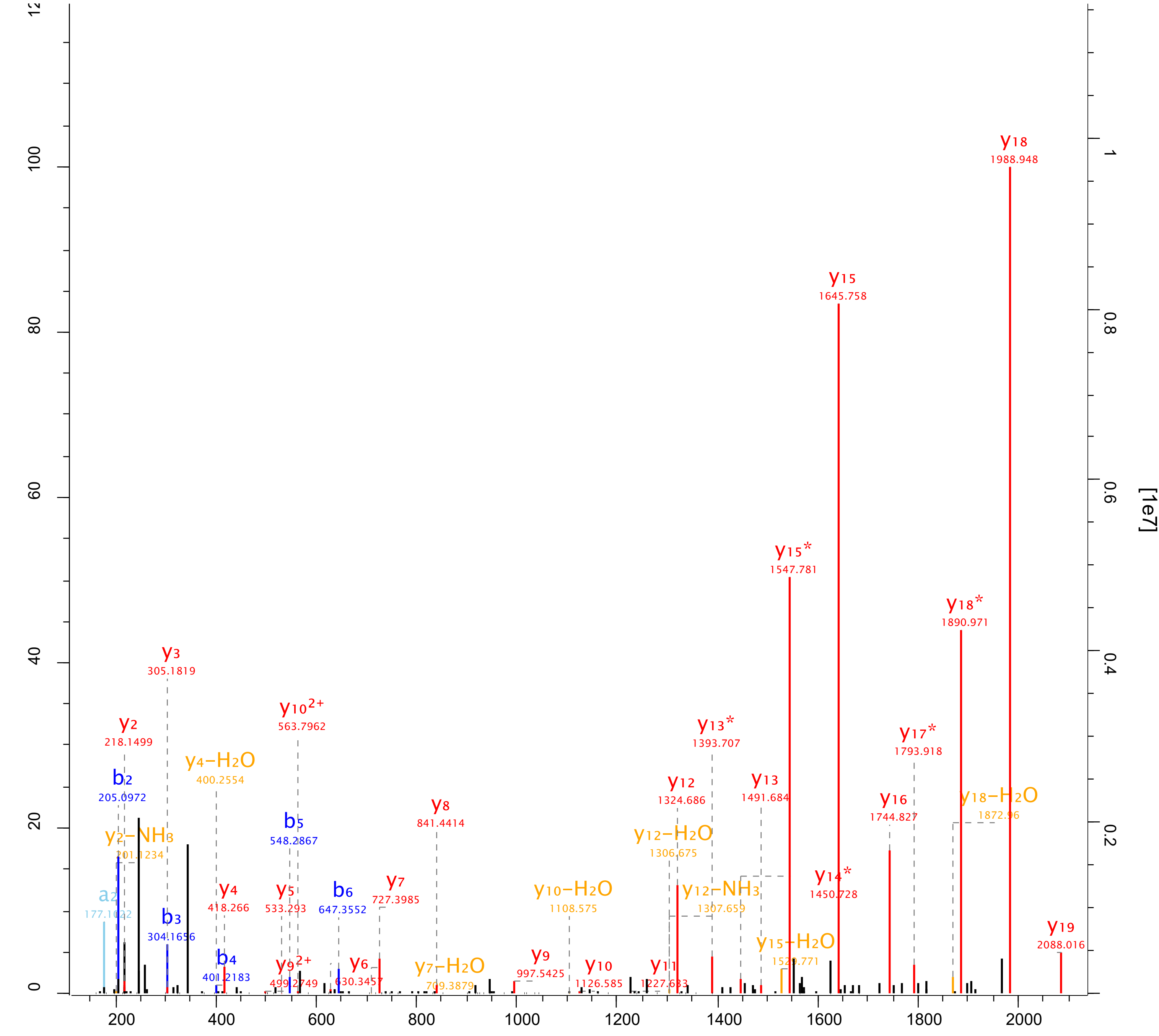

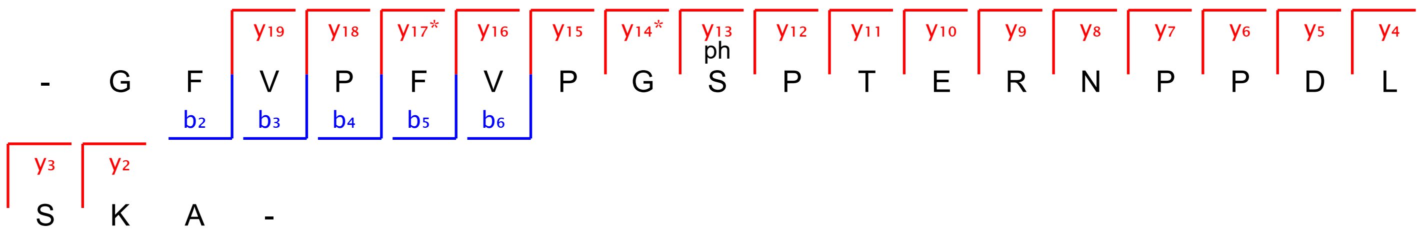

Sample output to test PDF Combine only

Raw file	Scan	Method	Score	m/z	Gene names
0523_12	21836	FTMS; HCD	47.56	714	F6N23.9;At1g01740;At5g59010;F5K20_330;At1g63500

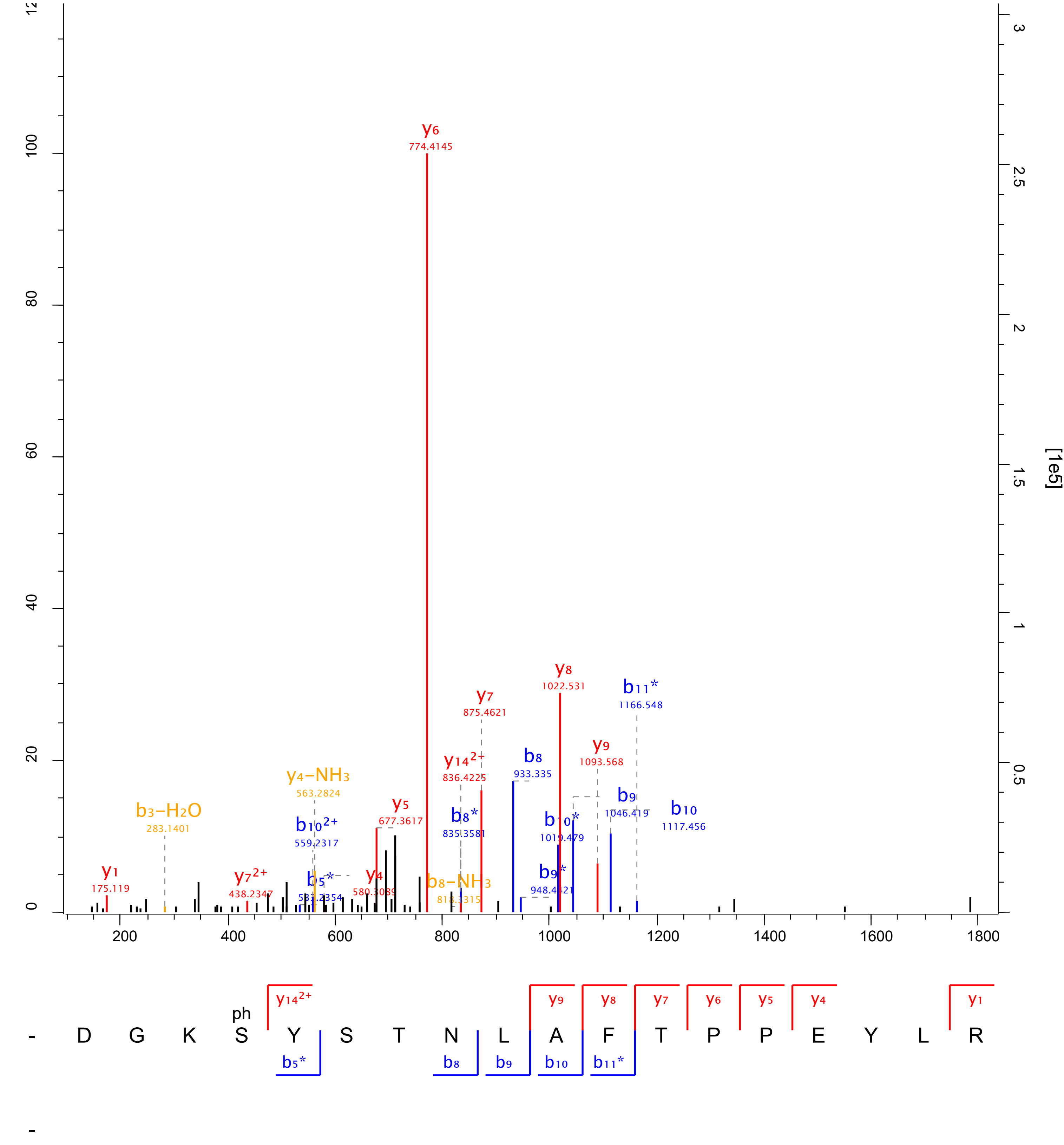

Sample output to test PDF Combine only
