# Supplementary material for: Sucrose-induced Receptor Kinase 1 is Modulated by an Interacting Kinase with Short Extracellular Domain
Source: Mol Cell Proteomics. 2019 May 30;18(8):1556–71. doi: 10.1074/mcp.RA119.001336 (PMC6683012; doi:10.1074/mcp.RA119.001336)
Supplement: Supplementry Figure 6f [file 143141_1_supp_311903_ps52ky.pdf]

Figure S6:

Spectra of all identified phosphopeptides.

Raw file	Scan	Method	Score	m/z
0523_12	22142	FTMS; HCD	71.7	764.71

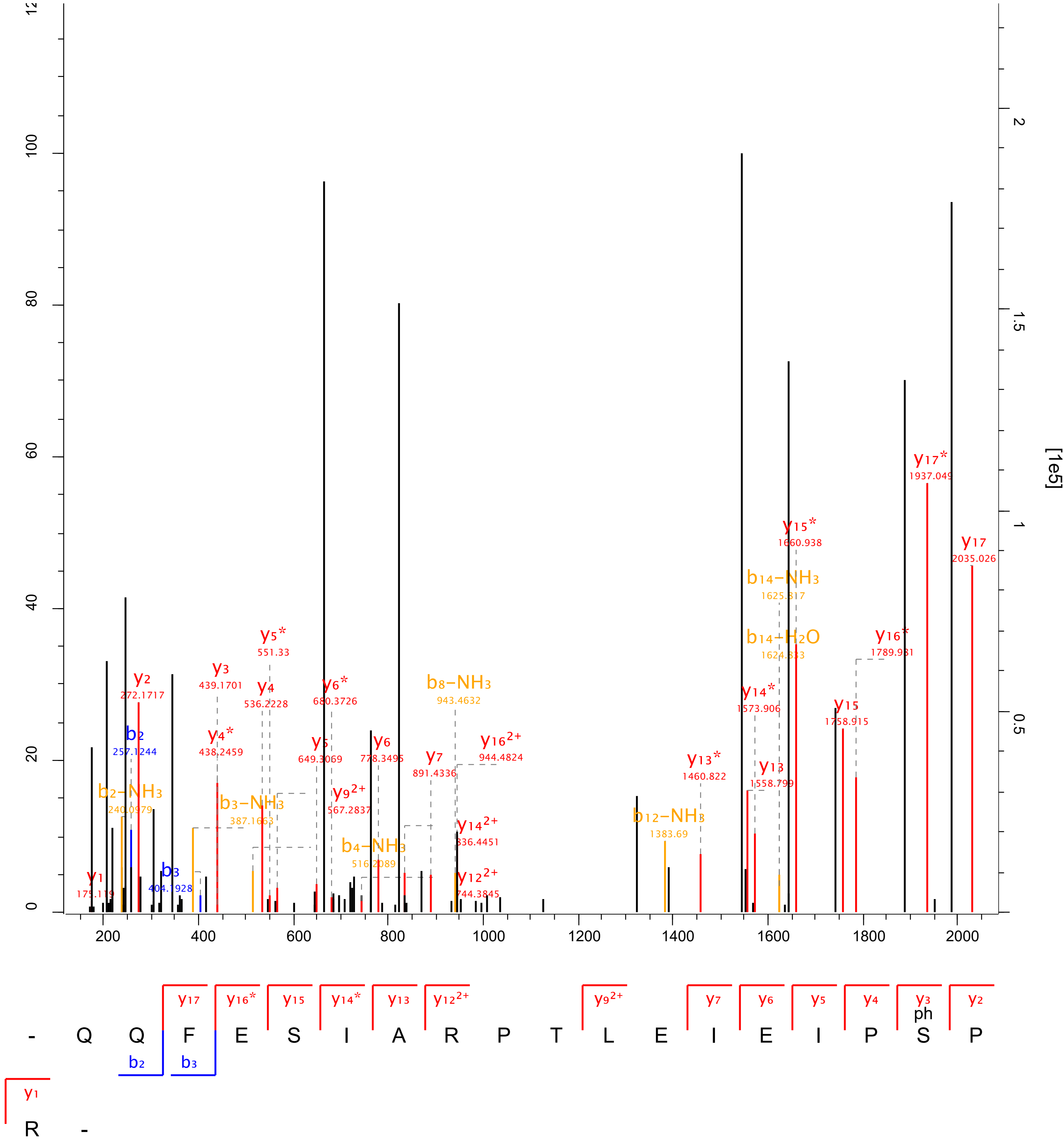

Sample output to test PDF Combine only

Raw file	Scan	Method	Score	m/z	Gene names
0523_12	22350	FTMS; HCD	280.93	1066.41	RPP2D;RPP2A;At2g27720;RPP2B;At2g27710

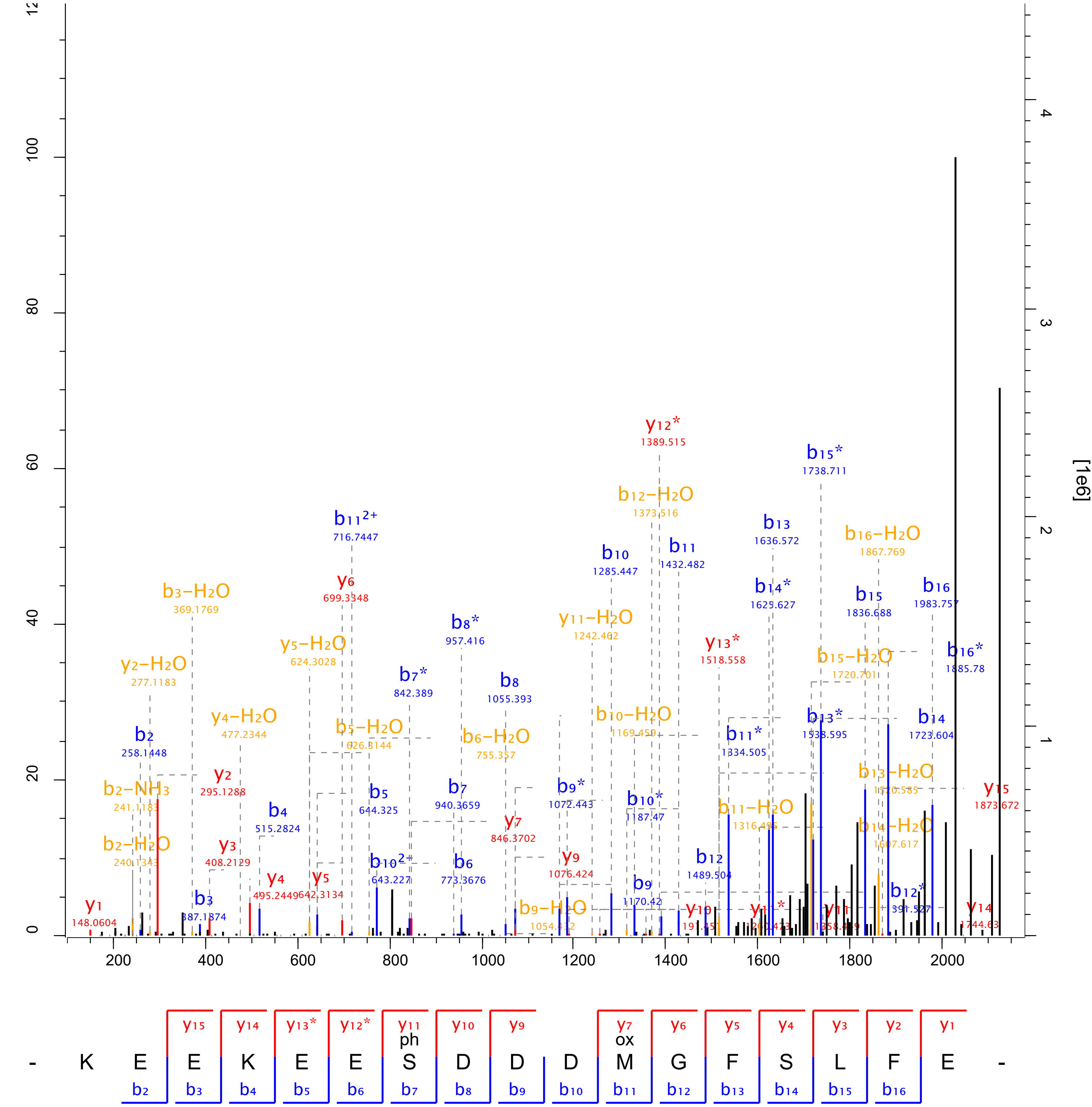

Sample output to test PDF Combine only

Raw file	Scan	Method	Score	m/z	Gene names
0523_12	23289	FTMS; HCD	147.11	898.4	BAG7

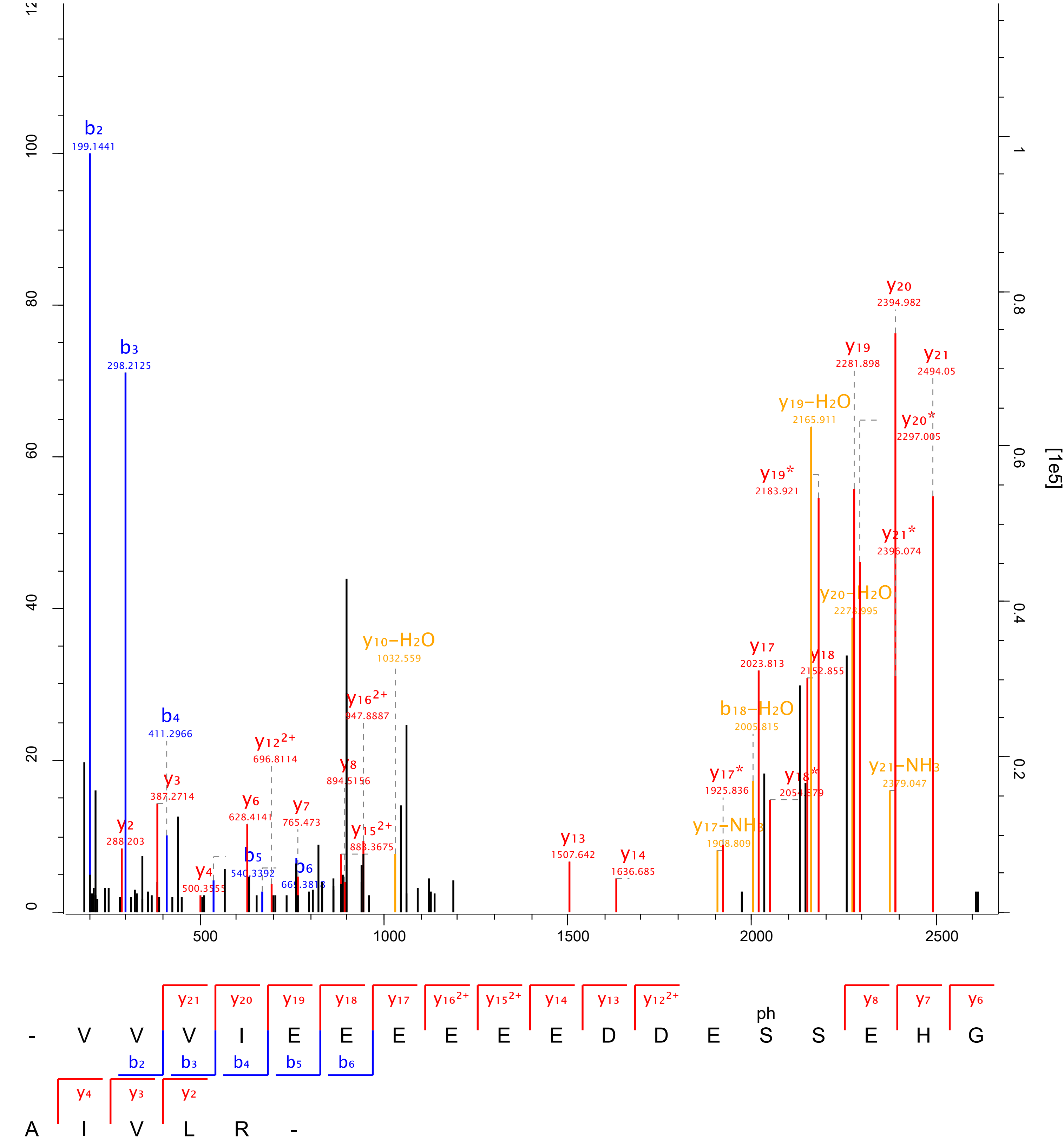

Sample output to test PDF Combine only

Raw file	Scan	Method	Score	m/z	Gene names
0523_12	23557	FTMS; HCD	162.02	1165.89	M3KE1

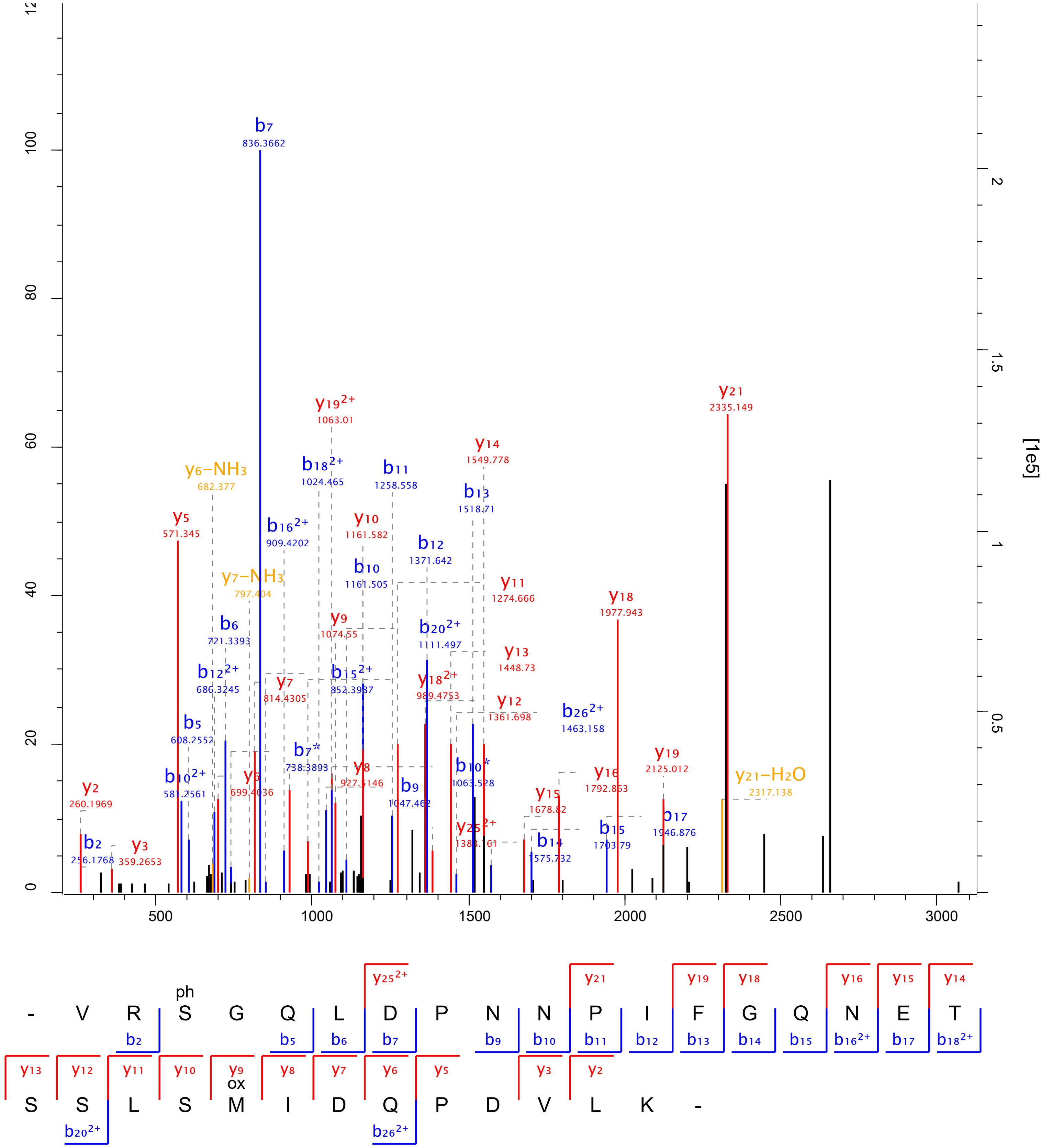

Sample output to test PDF Combine only

Raw file	Scan	Method	Score	m/z	Gene names
0523_12	23598	FTMS; HCD	239.93	973.74	At1g76970

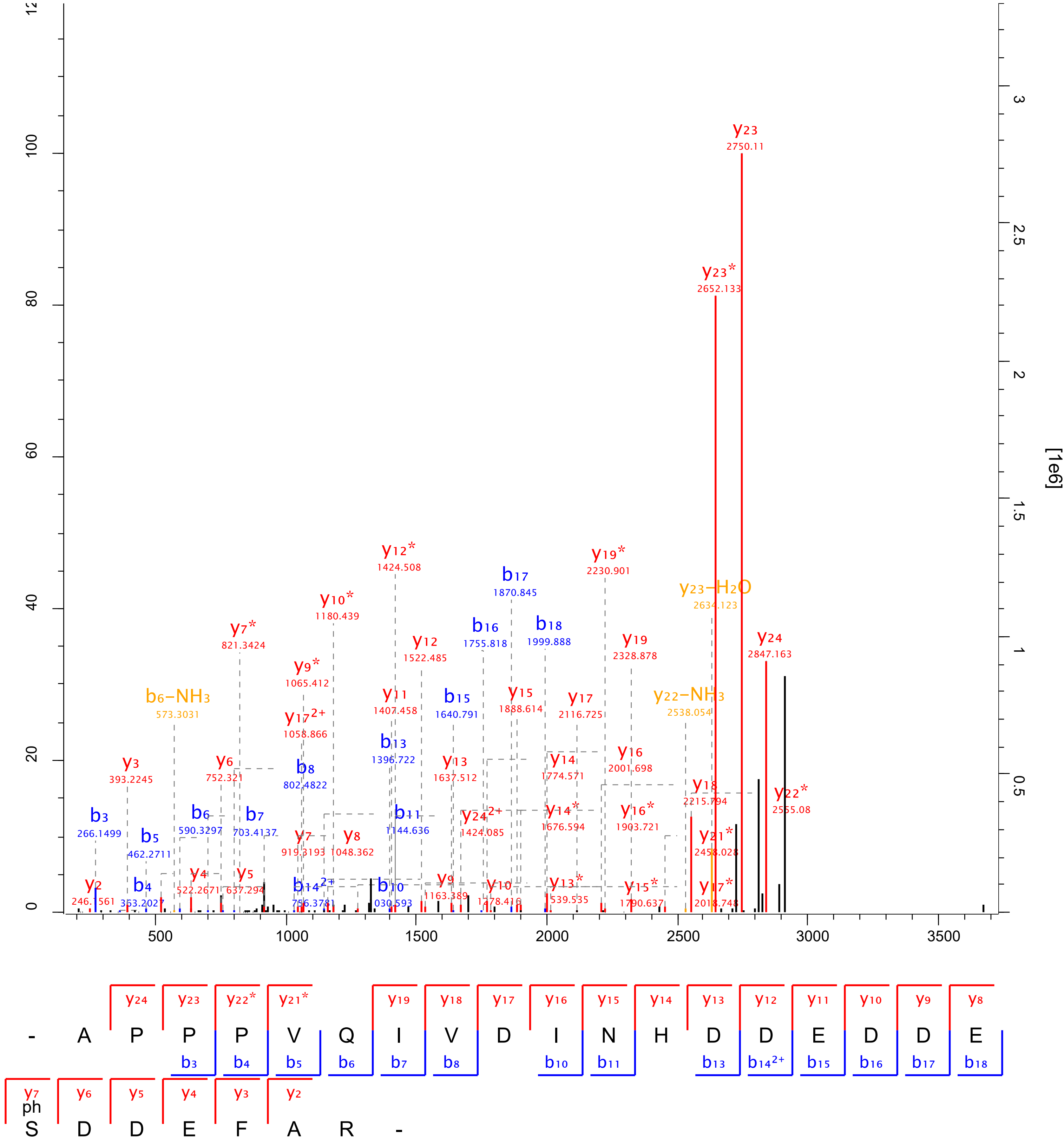

Sample output to test PDF Combine only

Raw file	Scan	Method	Score	m/z	Gene names
0523_12	24049	FTMS; HCD	77.07	675.83	At2g39130

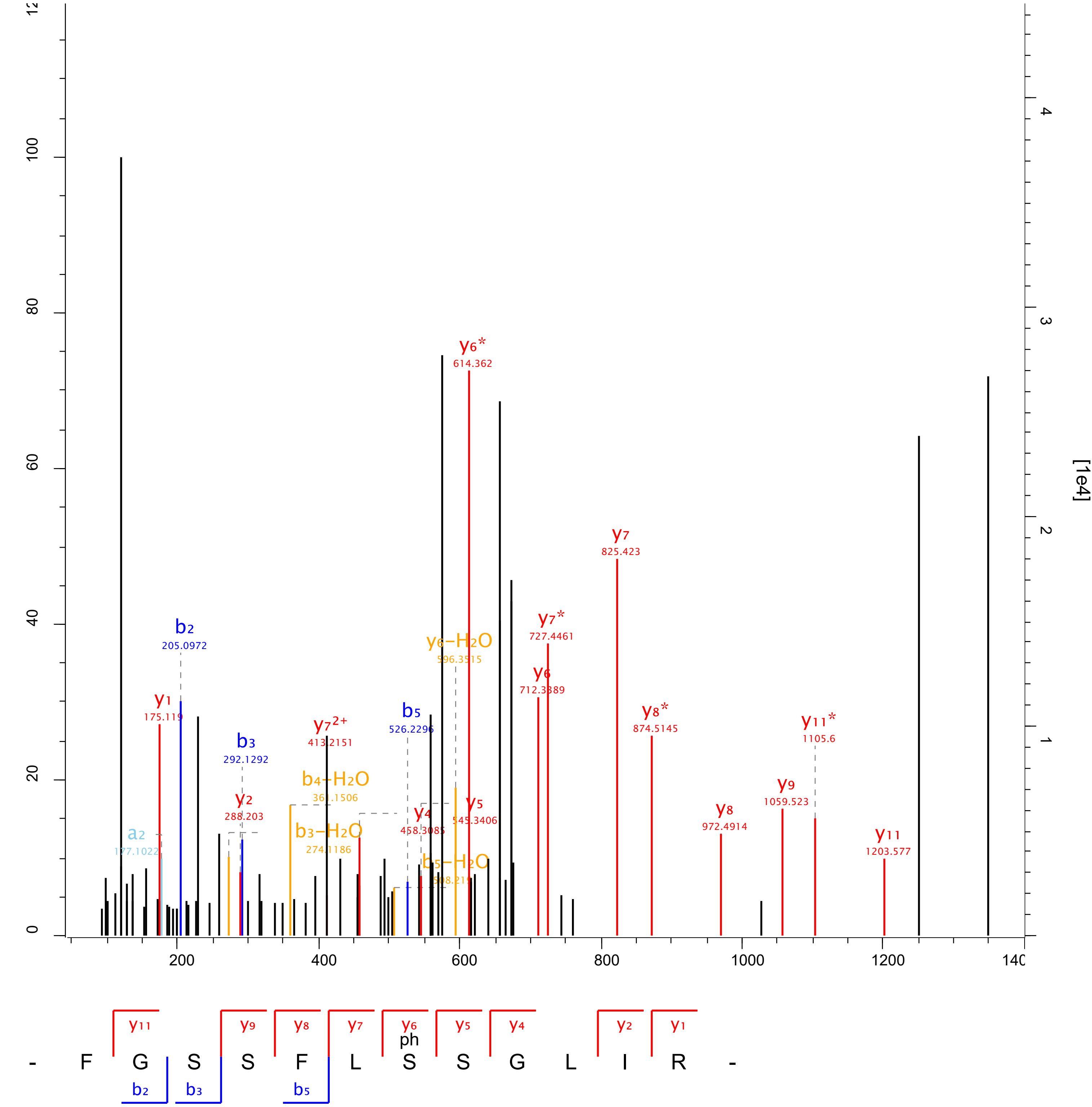

Sample output to test PDF Combine only

Raw file	Scan	Method	Score	m/z
0523_12	24060	FTMS; HCD	47.99	970.11

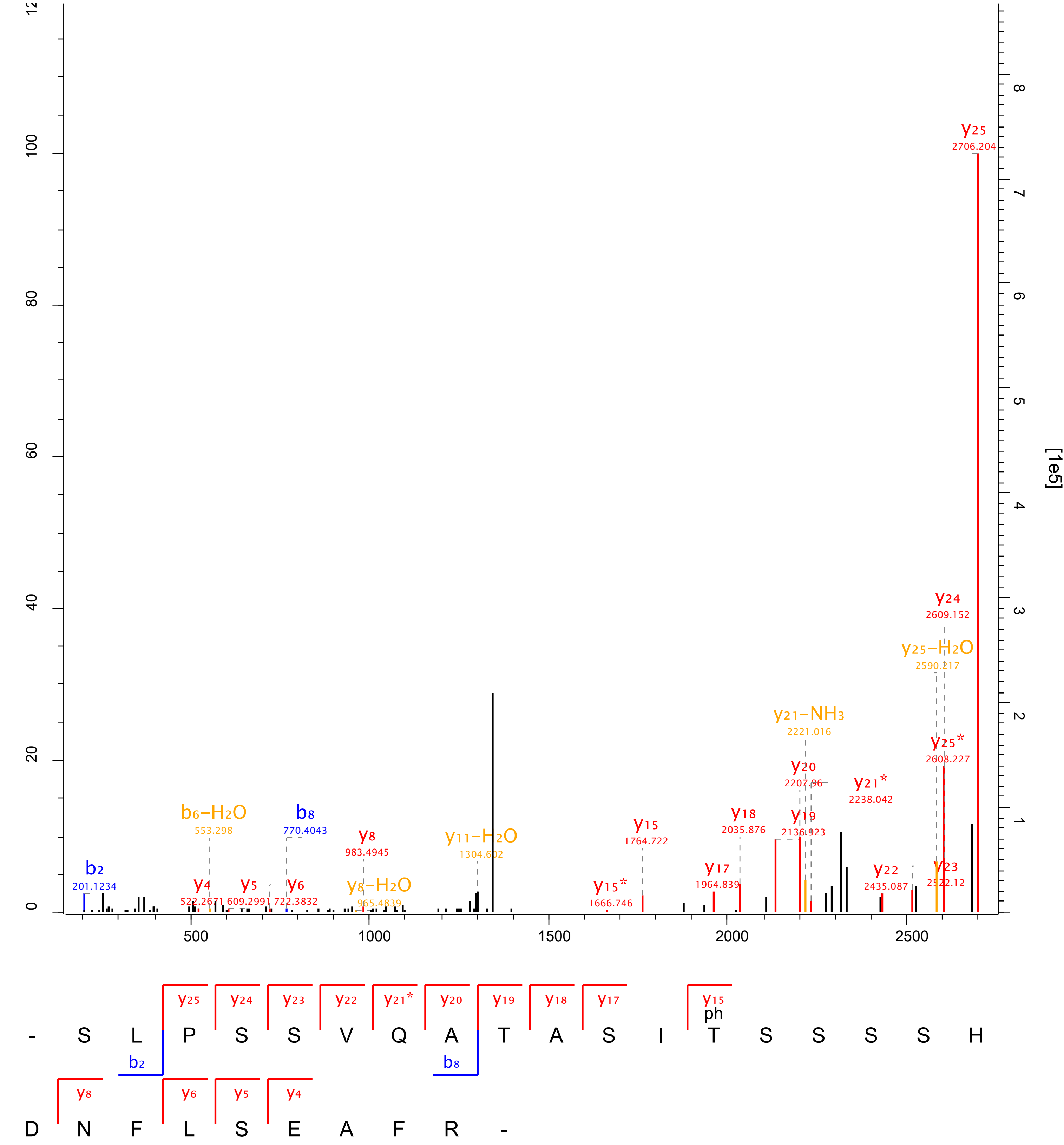

Sample output to test PDF Combine only

Raw file	Scan	Method	Score	m/z	Gene names
0523_12	24352	FTMS; HCD	61.1	732.08	At5g14120

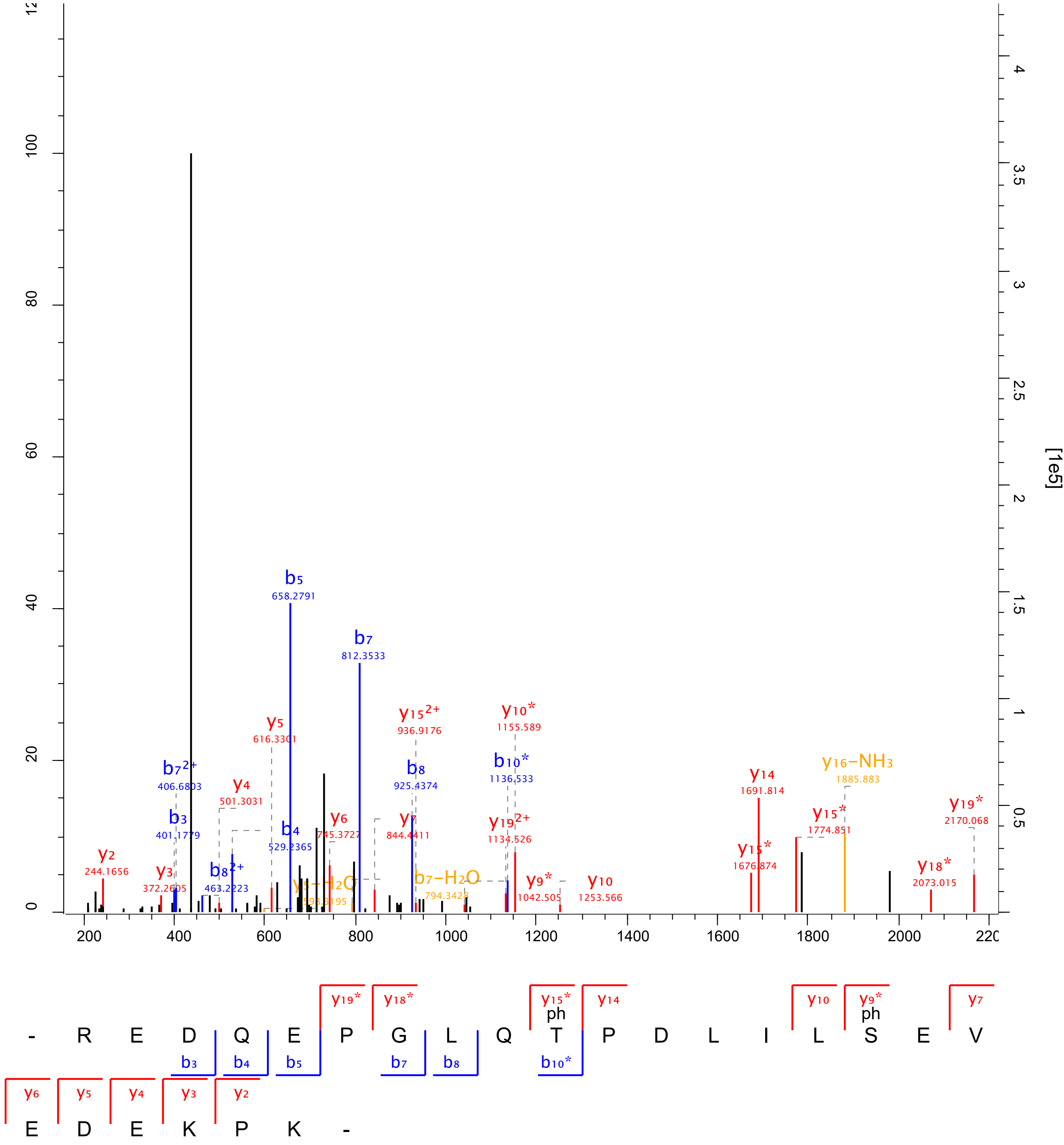

Sample output to test PDF Combine only

Raw file	Scan	Method	Score	m/z
0523_12	24523	FTMS; HCD	49.04	681.34

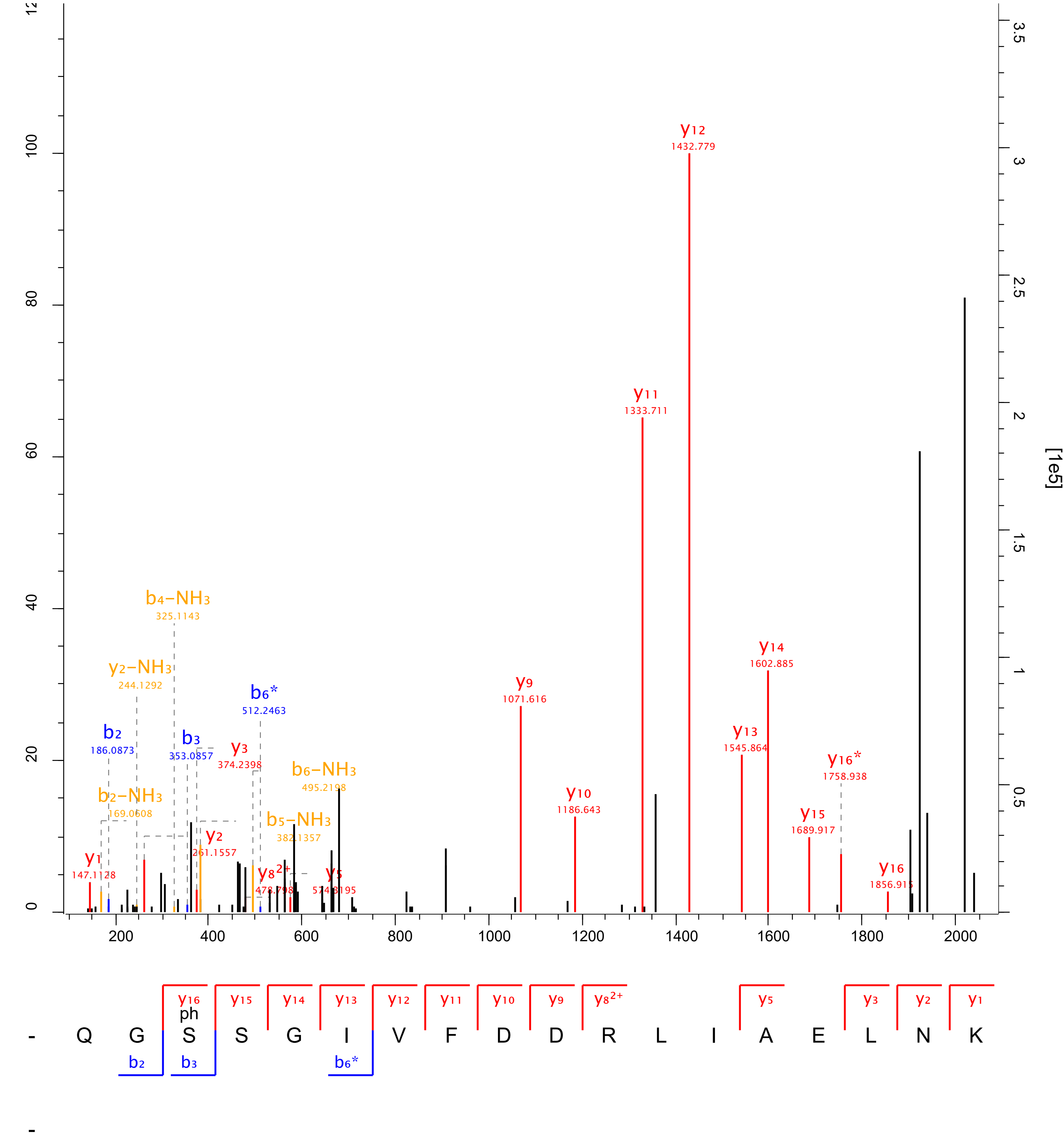

Raw file	Scan	Method	Score	m/z	Gene names
0523_12	24896	FTMS; HCD	208.47	714.78	SYP121

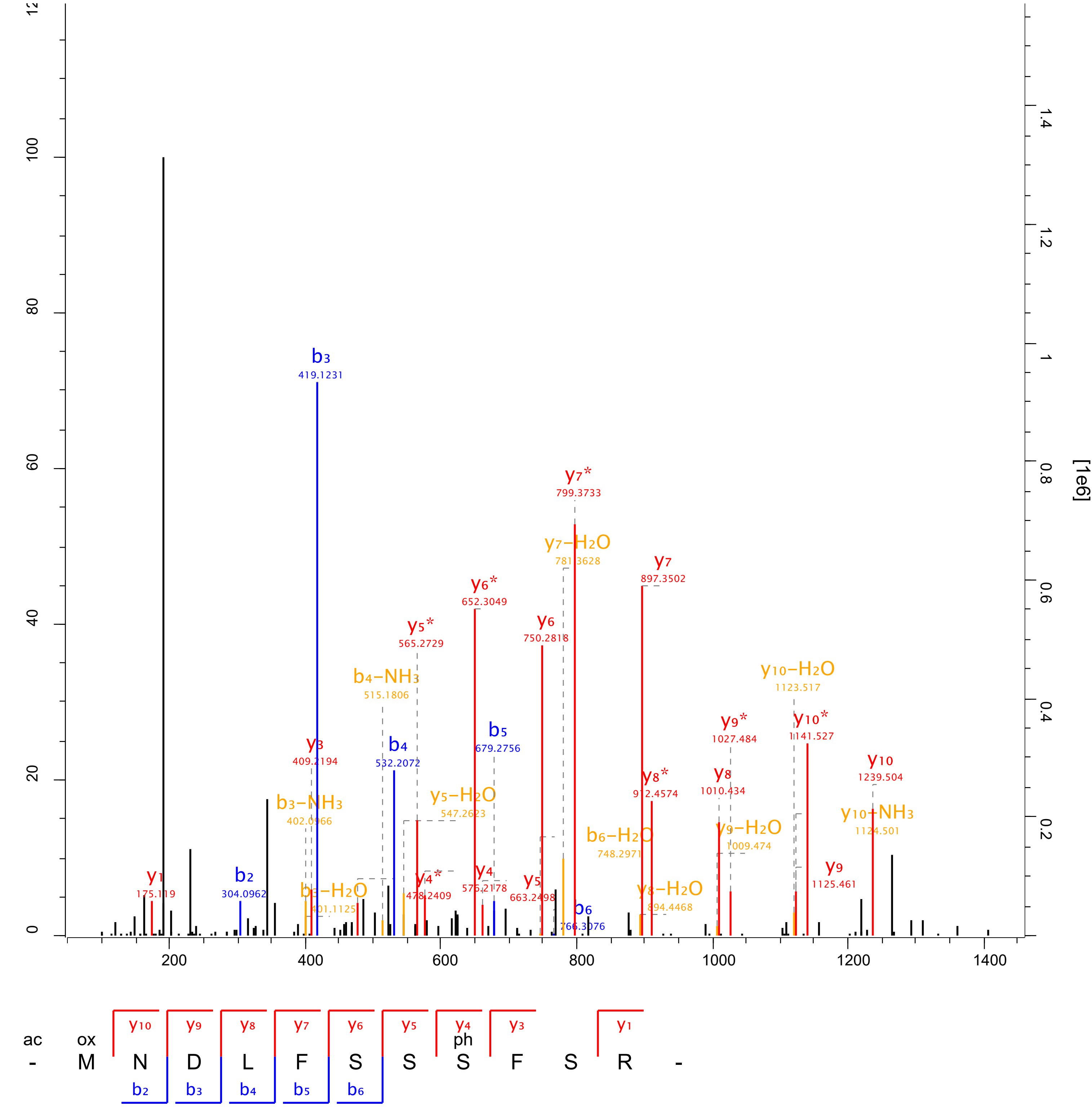

Sample output to test PDF Combine only

Raw file	Scan	Method	Score	m/z
0523_12	24901	FTMS; HCD	252.55	926.43

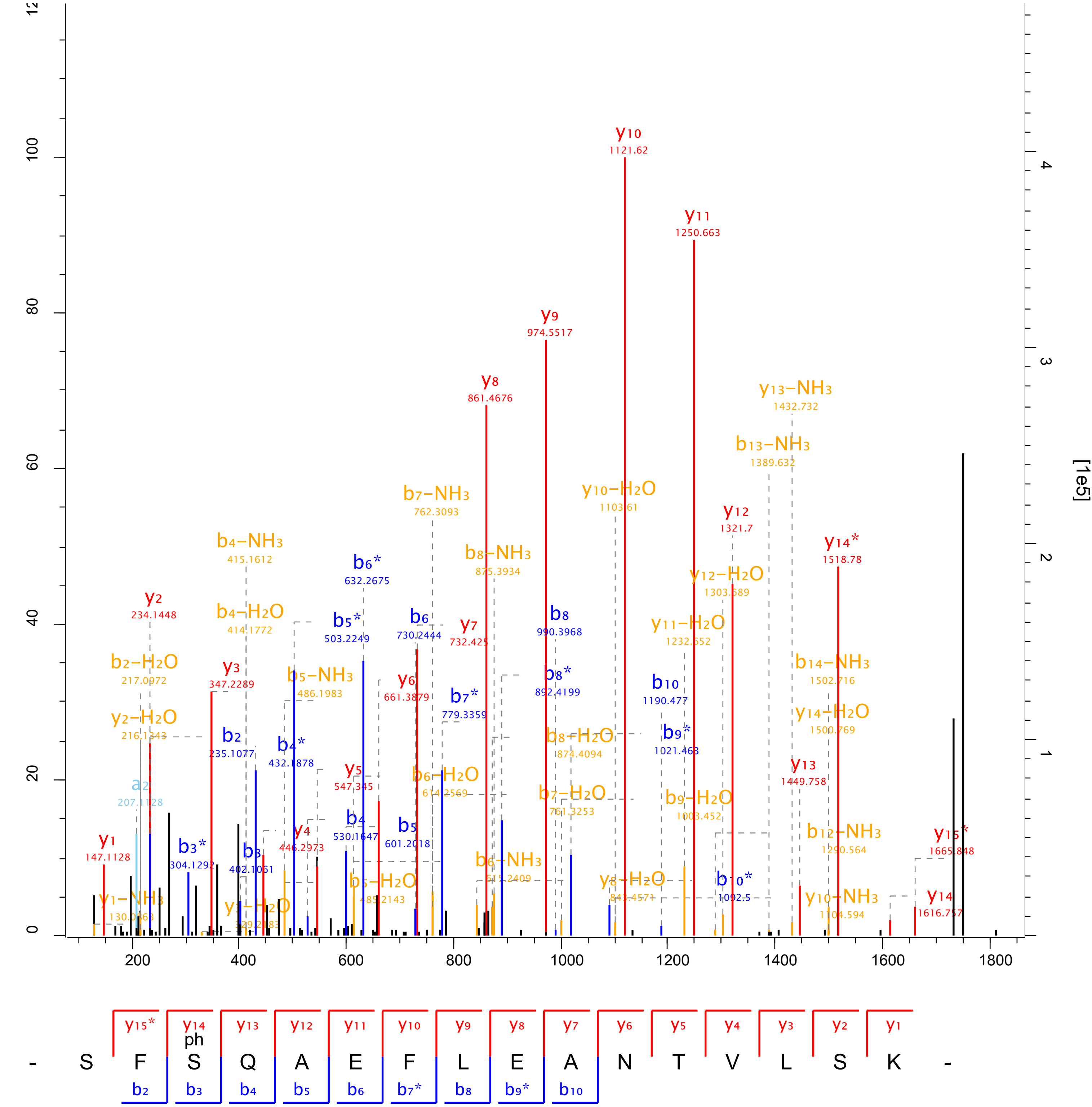

Sample output to test PDF Combine only

Raw file	Scan	Method	Score	m/z	Gene names
0523_12	26199	FTMS; HCD	50.63	1020.51	PPC3

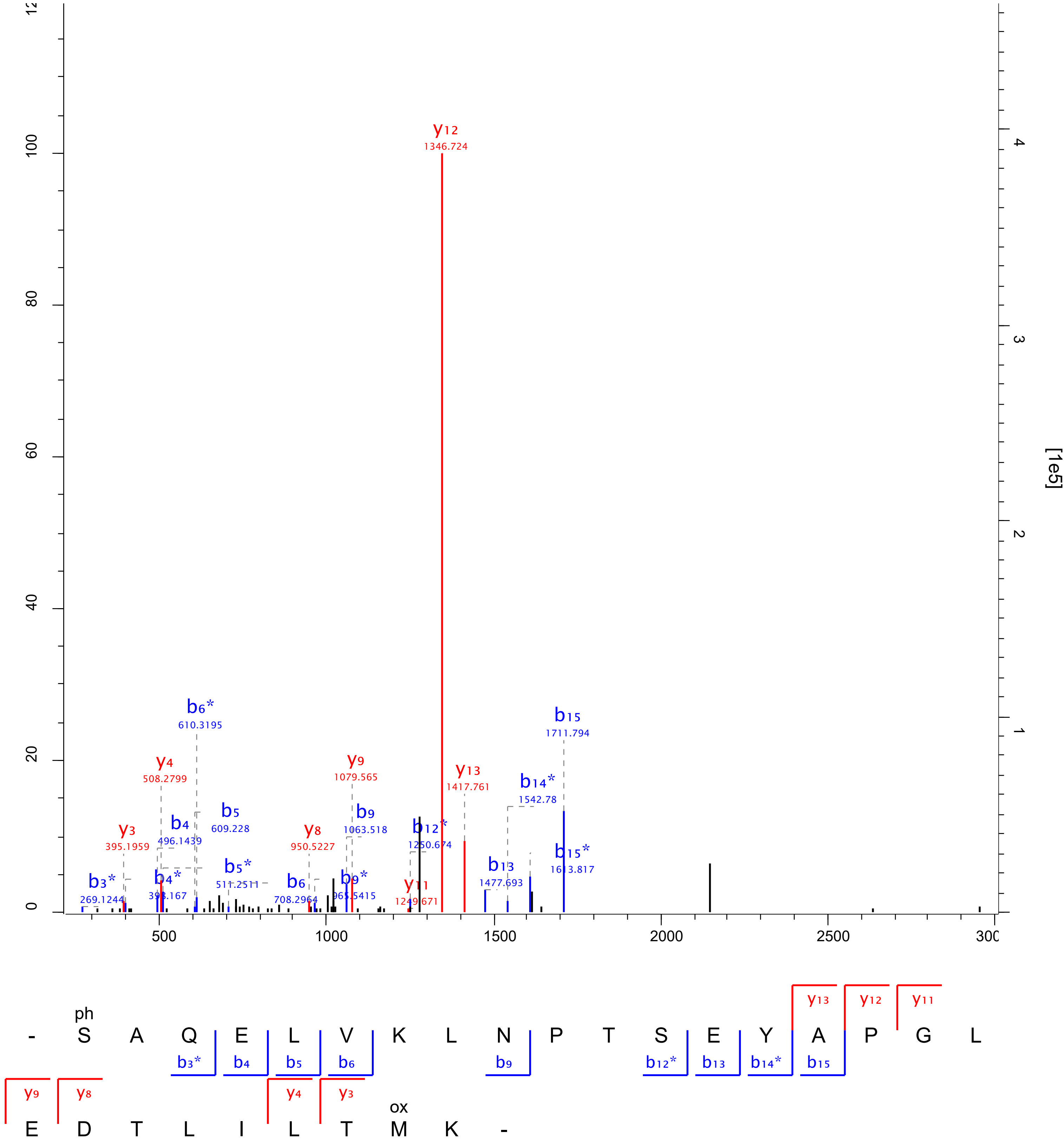

Sample output to test PDF Combine only

Raw file	Scan	Method	Score	m/z	Gene names
0523_12	26986	FTMS; HCD	234.49	1148.85	LRR-RLK

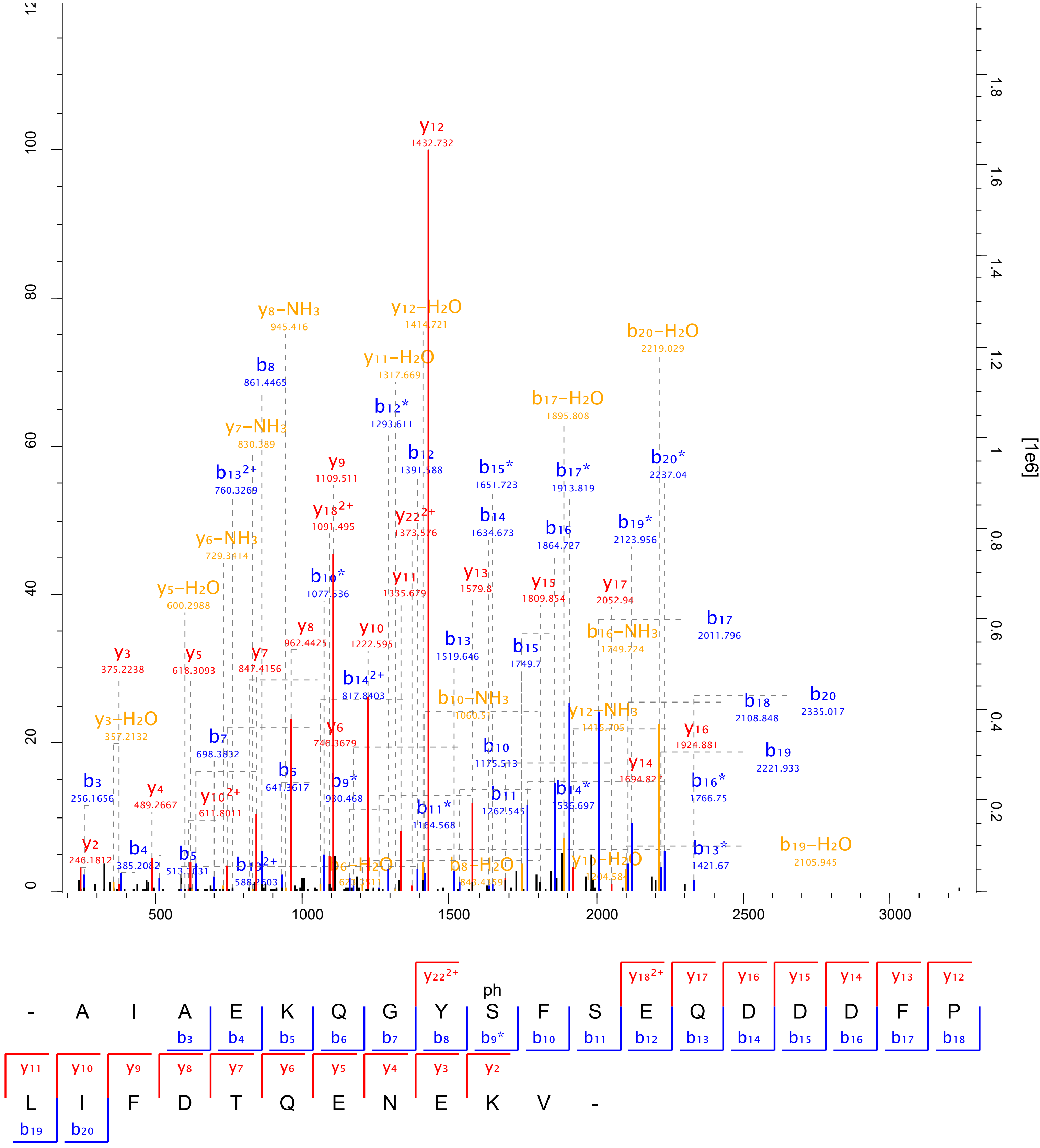

Sample output to test PDF Combine only

Raw file	Scan	Method	Score	m/z	Gene names
0523_12	27104	FTMS; HCD	240.29	945.48	ACA10

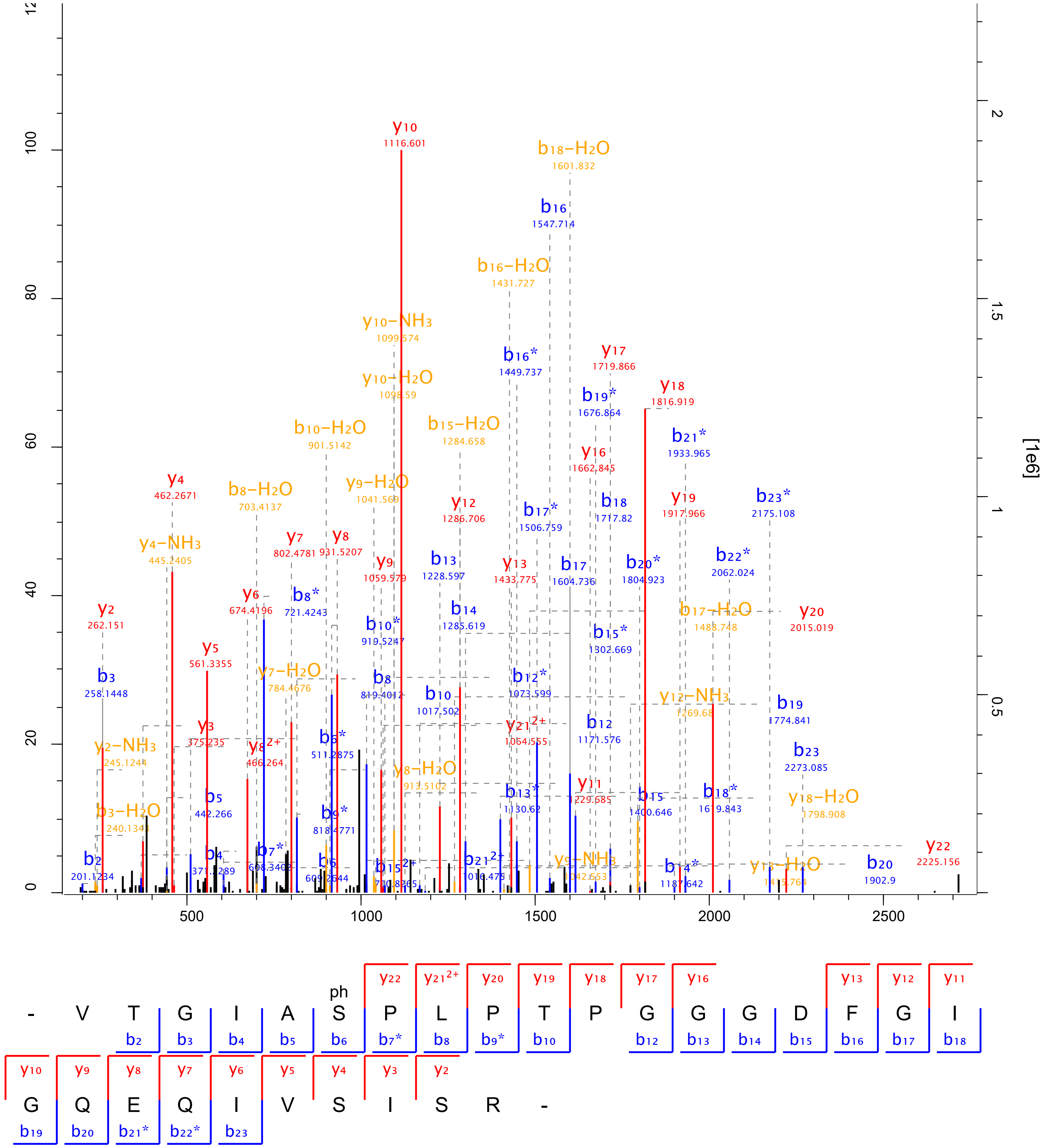

Sample output to test PDF Combine only

Raw file	Scan	Method	Score	m/z	Gene names
0523_12	27777	FTMS; HCD	347.35	930.7	RPP0C

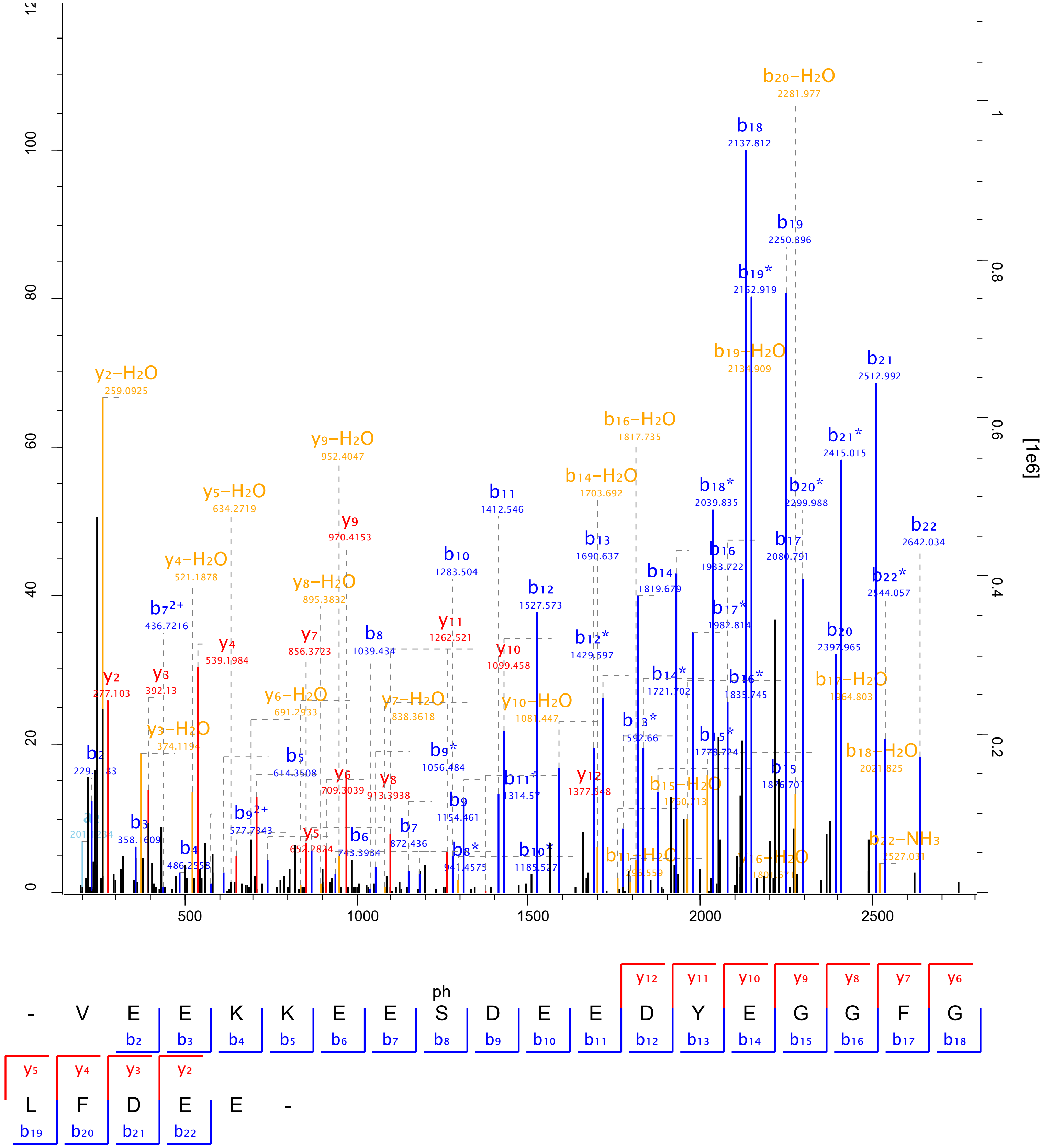

Sample output to test PDF Combine only

Raw file	Scan	Method	Score	m/z	Gene names
0523_12	28080	FTMS; HCD	58.37	758.01	BAG7

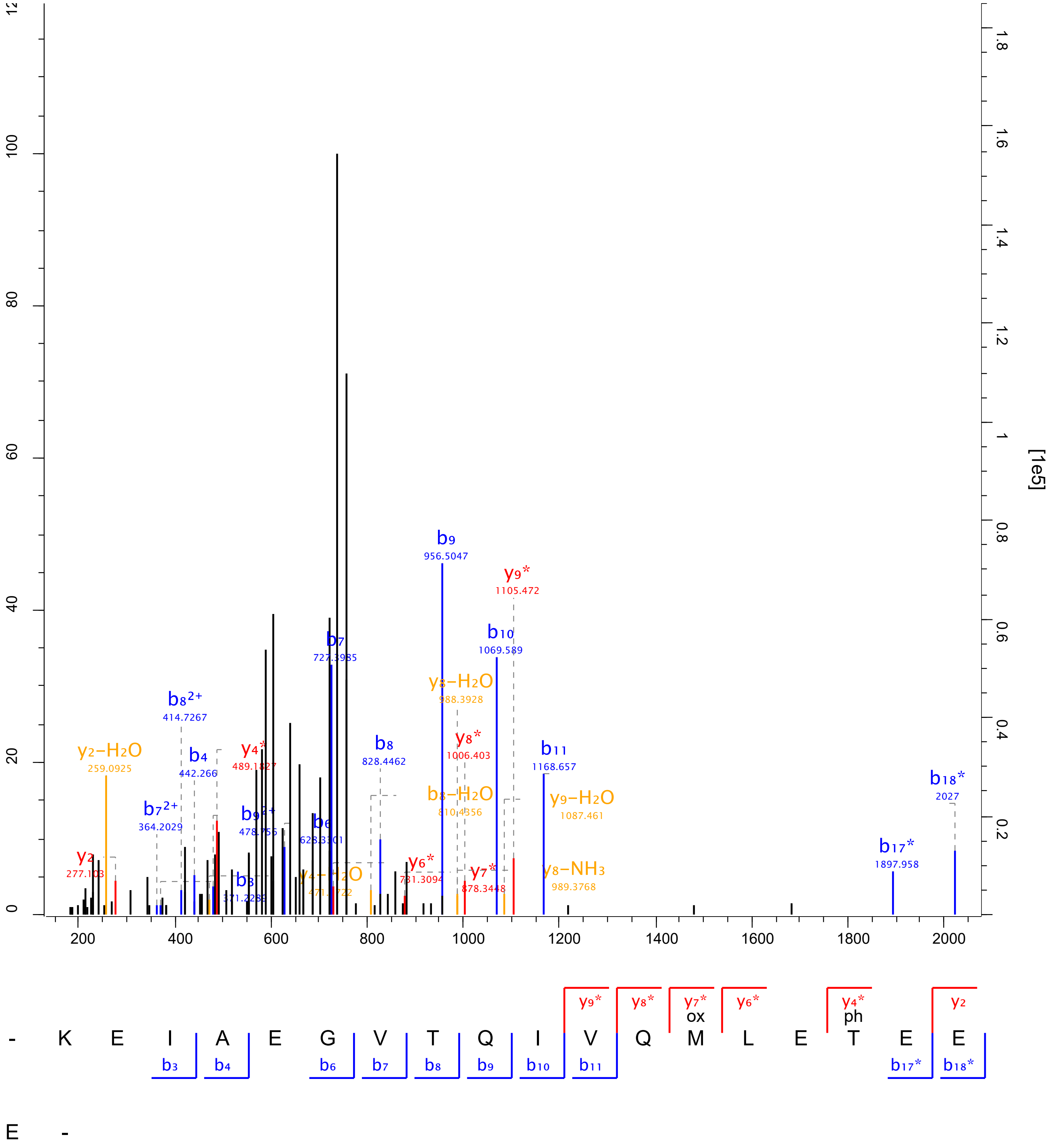

Sample output to test PDF Combine only

Raw file	Scan	Method	Score	m/z	Gene names
0523_12	28762	FTMS; HCD	76.57	1134.18	ASY2

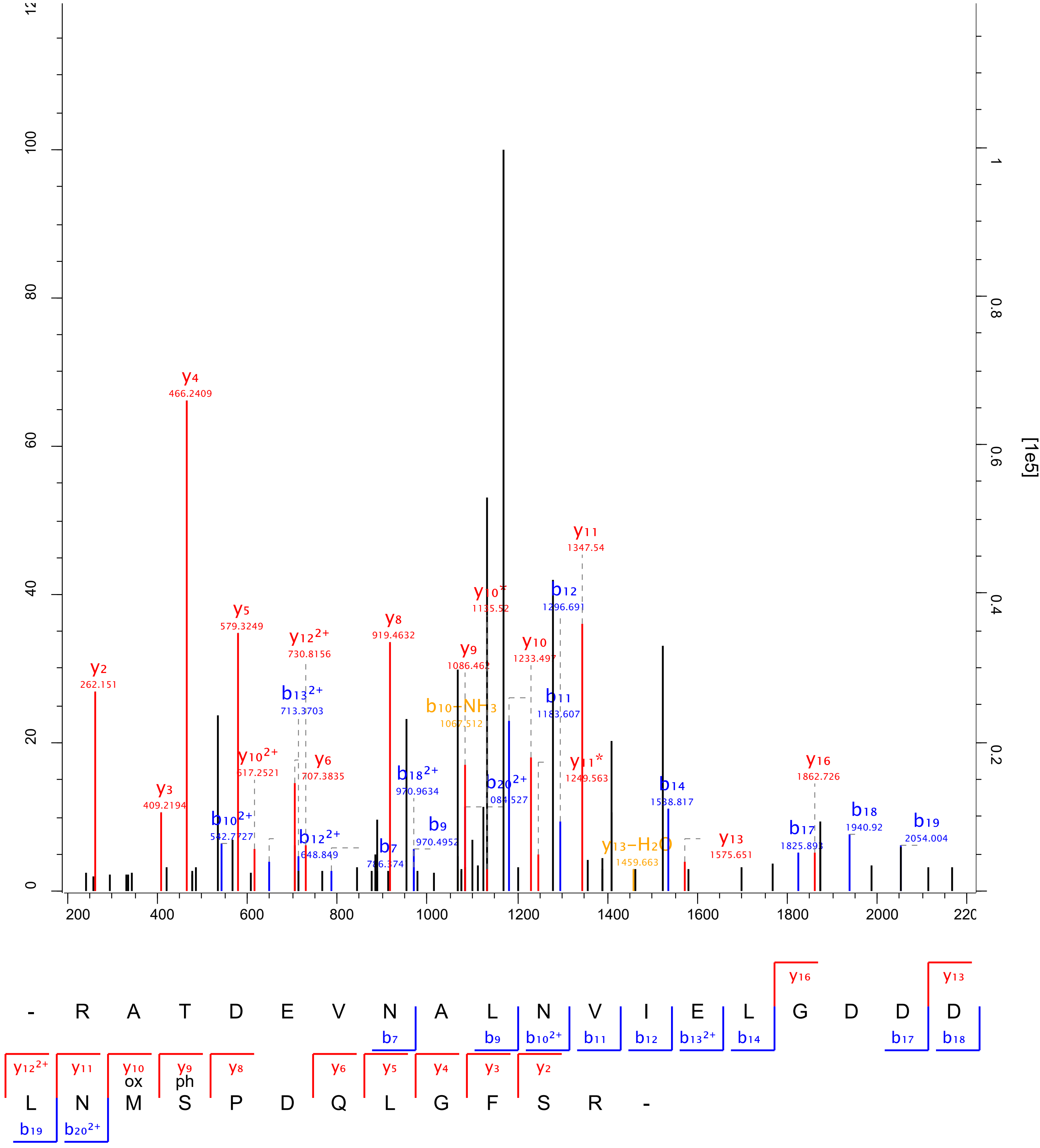

Sample output to test PDF Combine only

Raw file	Scan	Method	Score	m/z	Gene names
0523_12	28927	FTMS; HCD	43.68	647.76	PDX11

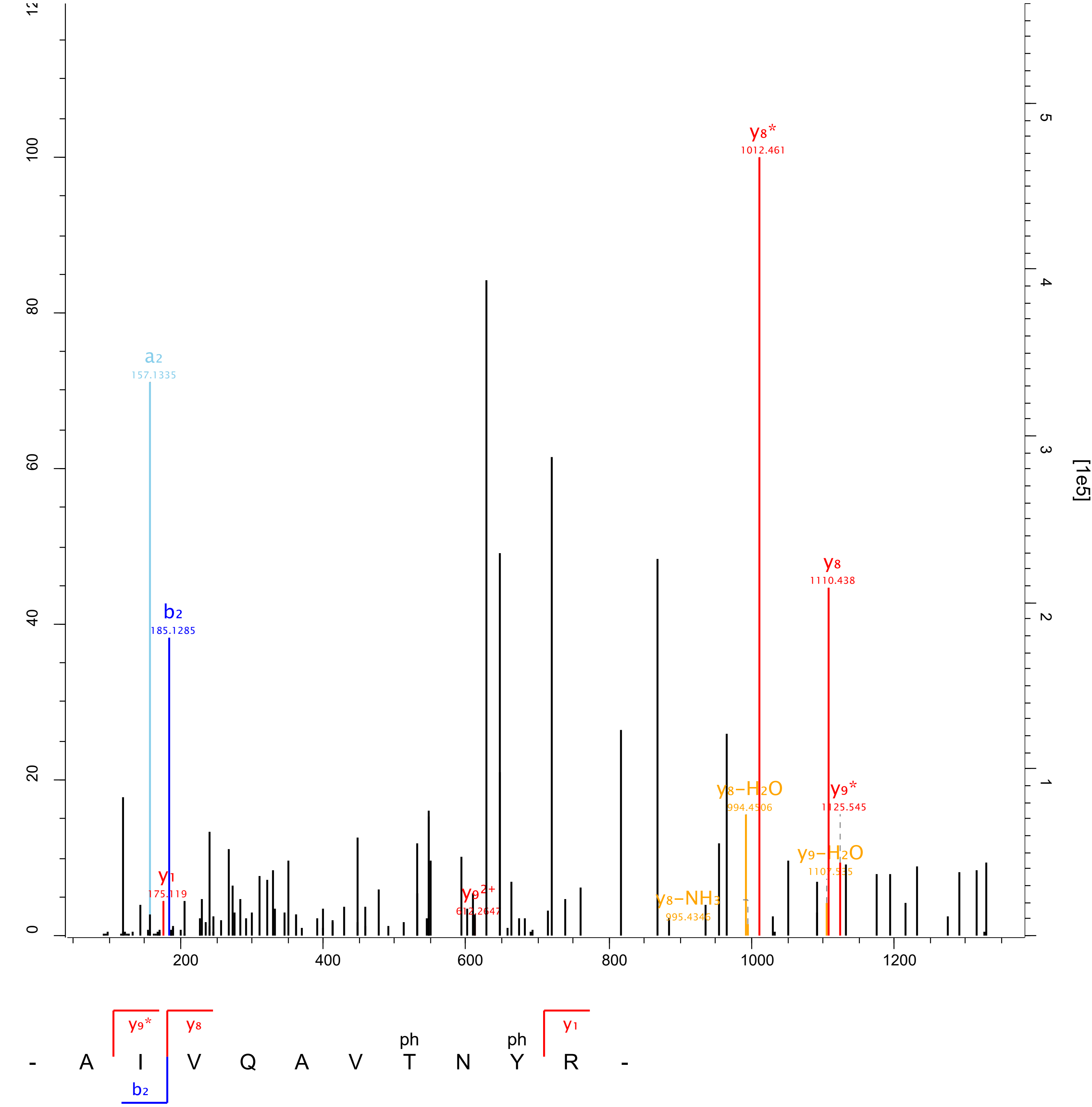

Sample output to test PDF Combine only

Raw file	Scan	Method	Score	m/z	Gene names
0523_12	29126	FTMS; HCD	73.58	1072.3	ACA10

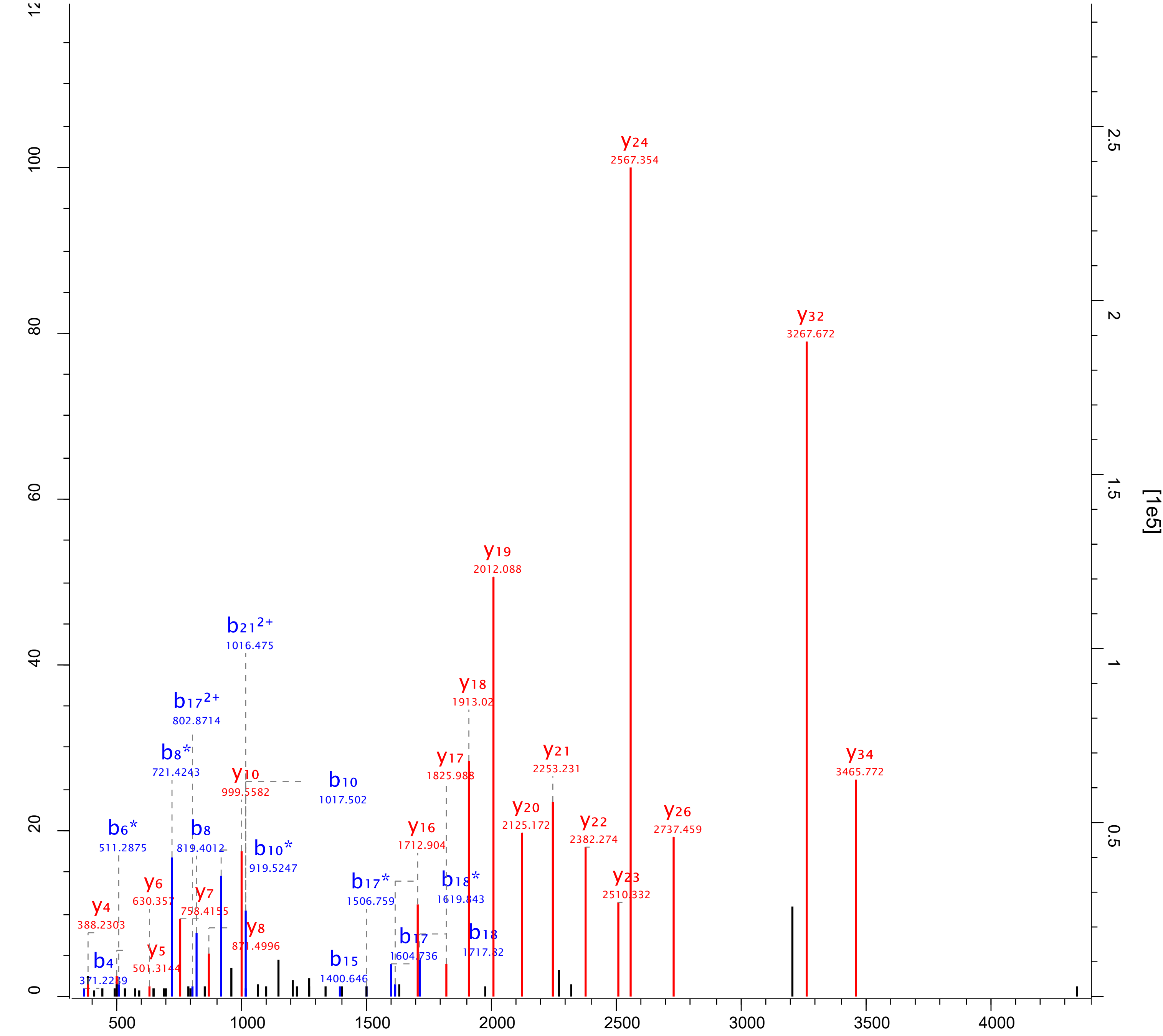

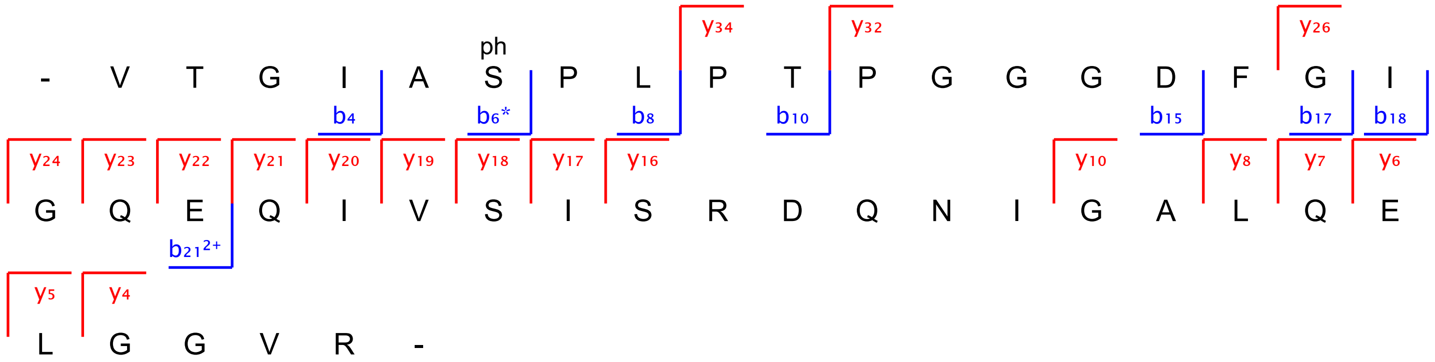

Sample output to test PDF Combine only

Raw file	Scan	Method	Score	m/z	Gene names
0523_12	29203	FTMS; HCD	171.36	1080.46	At5g59010

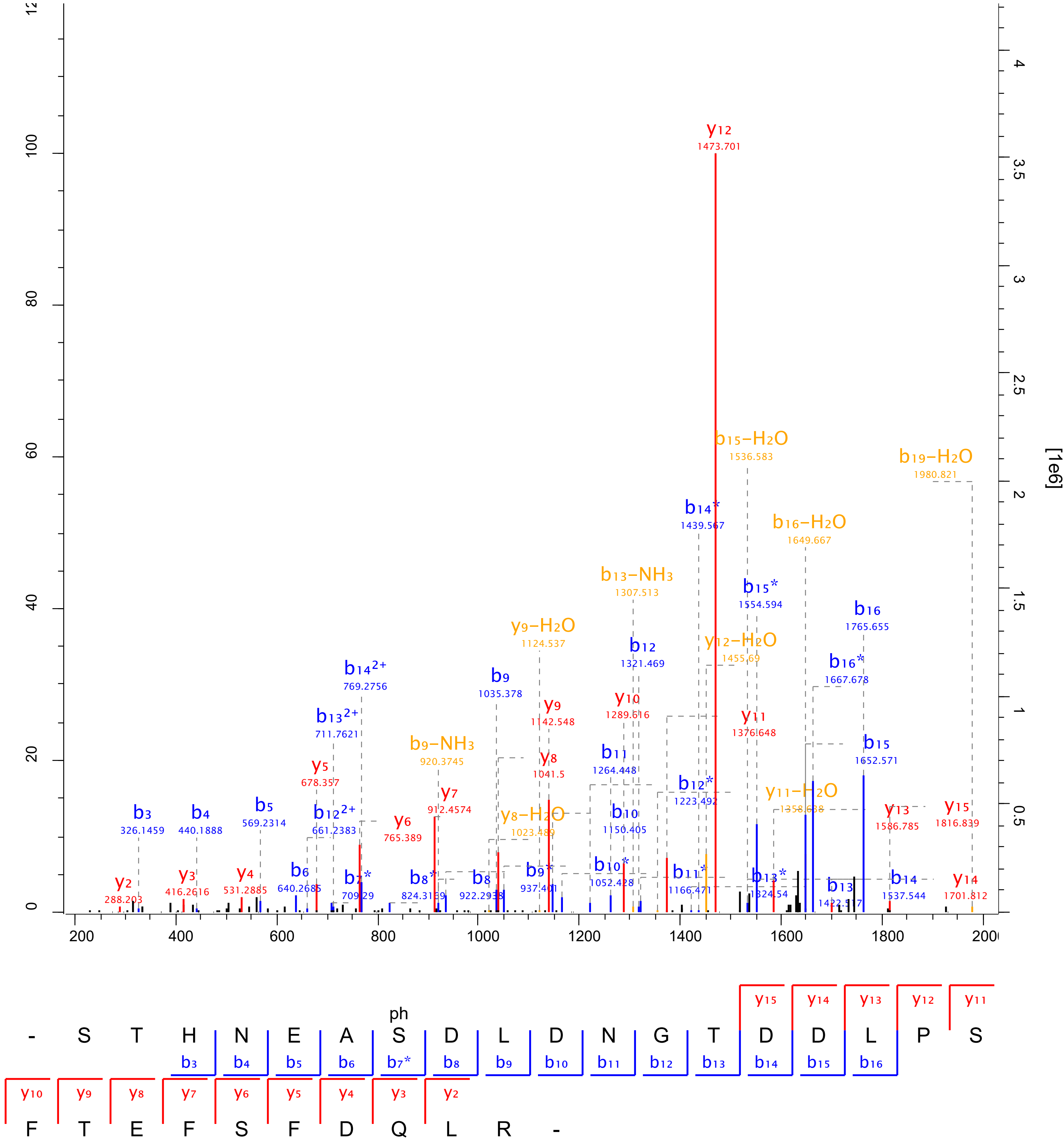

Sample output to test PDF Combine only

Raw file	Scan	Method	Score	m/z	Gene names
0523_12	29634	FTMS; HCD	77.96	967.7	At5g14120

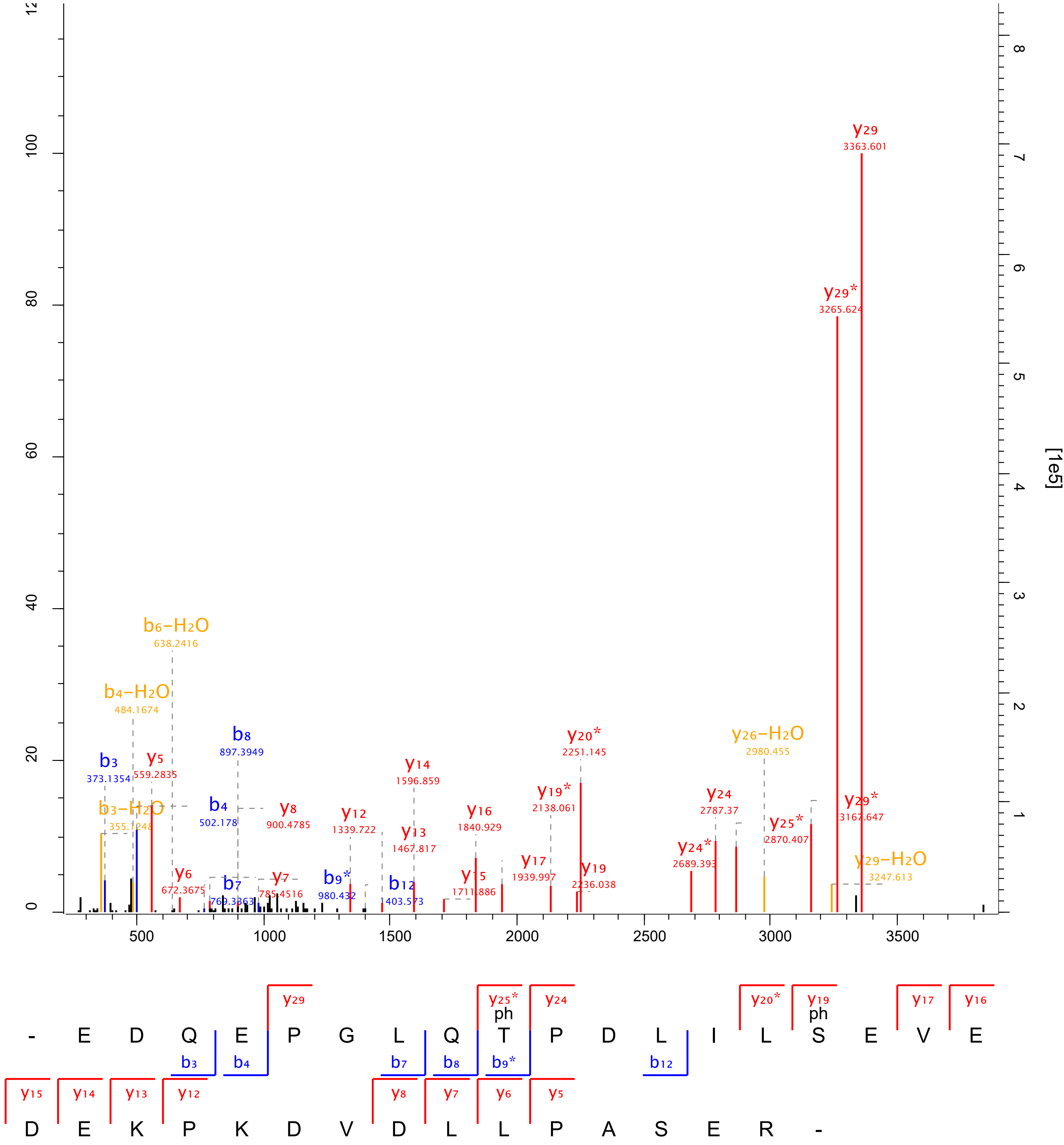

Sample output to test PDF Combine only

Raw file	Scan	Method	Score	m/z	Gene names
0523_12	29650	FTMS; HCD	198.64	1061.22	F9K20.7

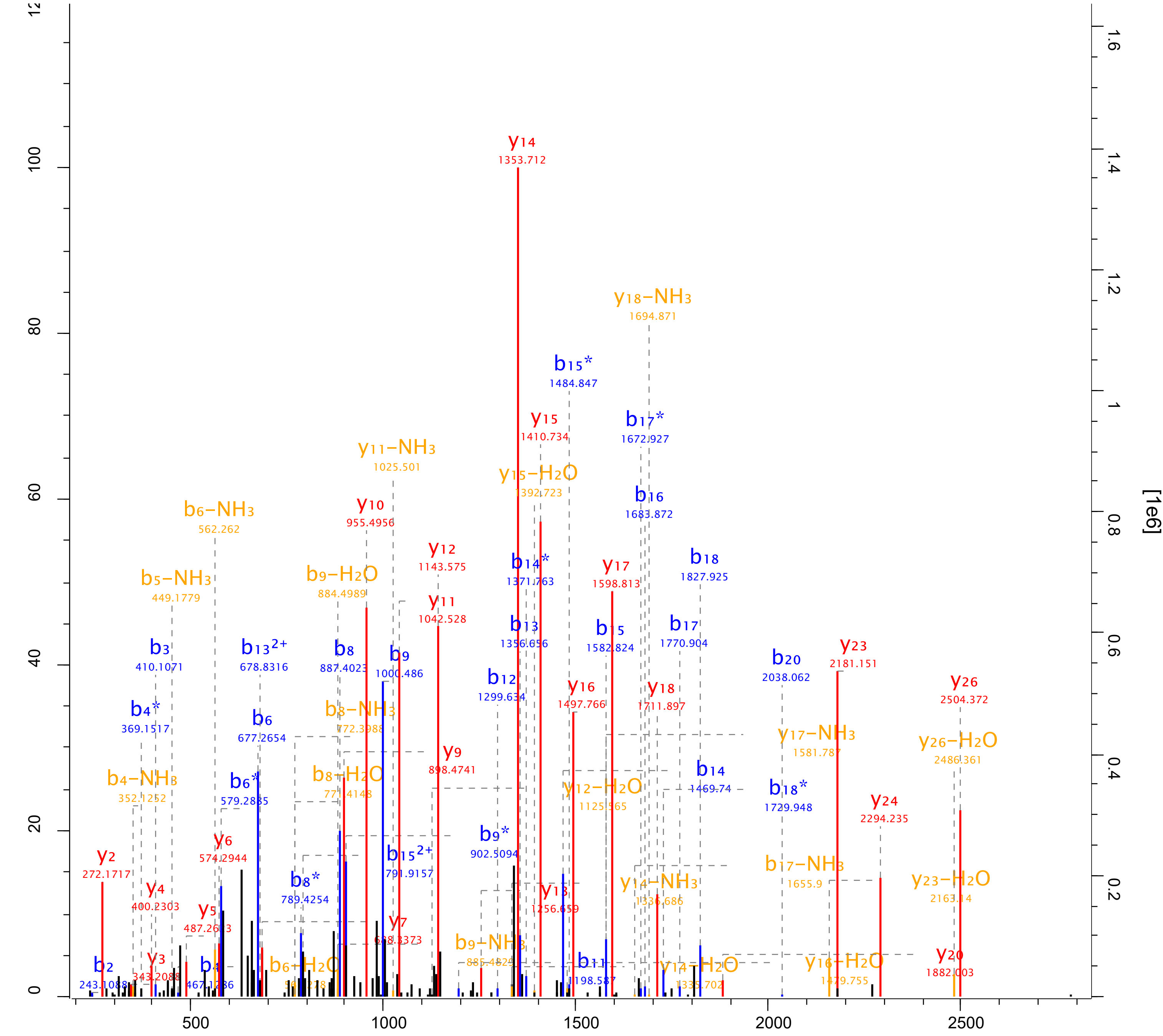

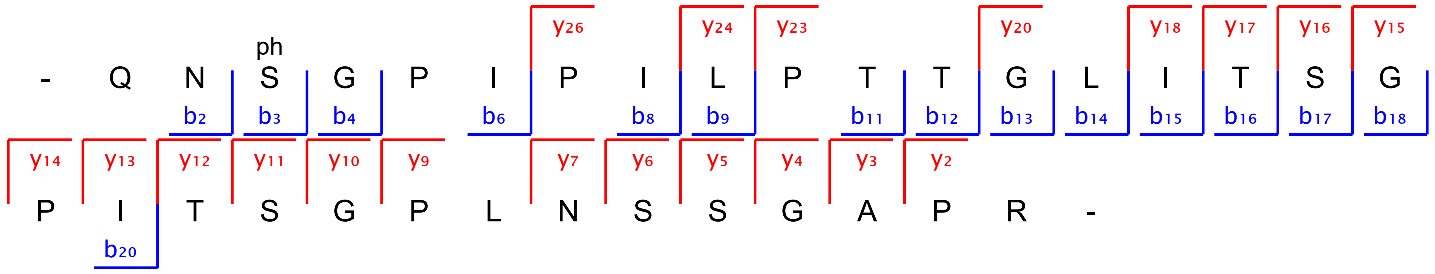

Sample output to test PDF Combine only

Raw file	Scan	Method	Score	m/z	Gene names
0523_12	30541	FTMS; HCD	82.89	906.08	LYK4

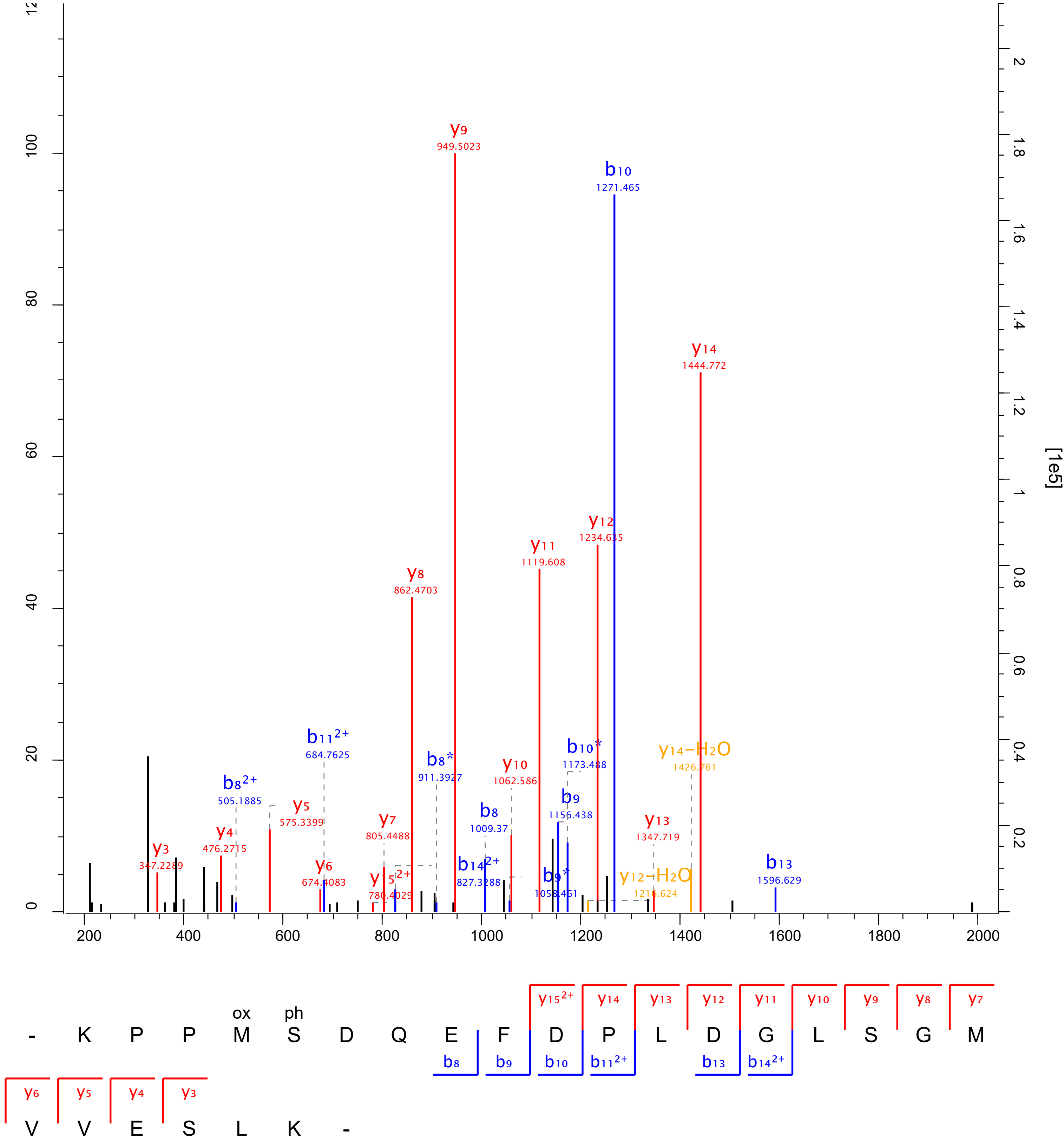

Sample output to test PDF Combine only

Raw file	Scan	Method	Score	m/z	Gene names
0523_12	30821	FTMS; HCD	65.86	866.4	TPS7

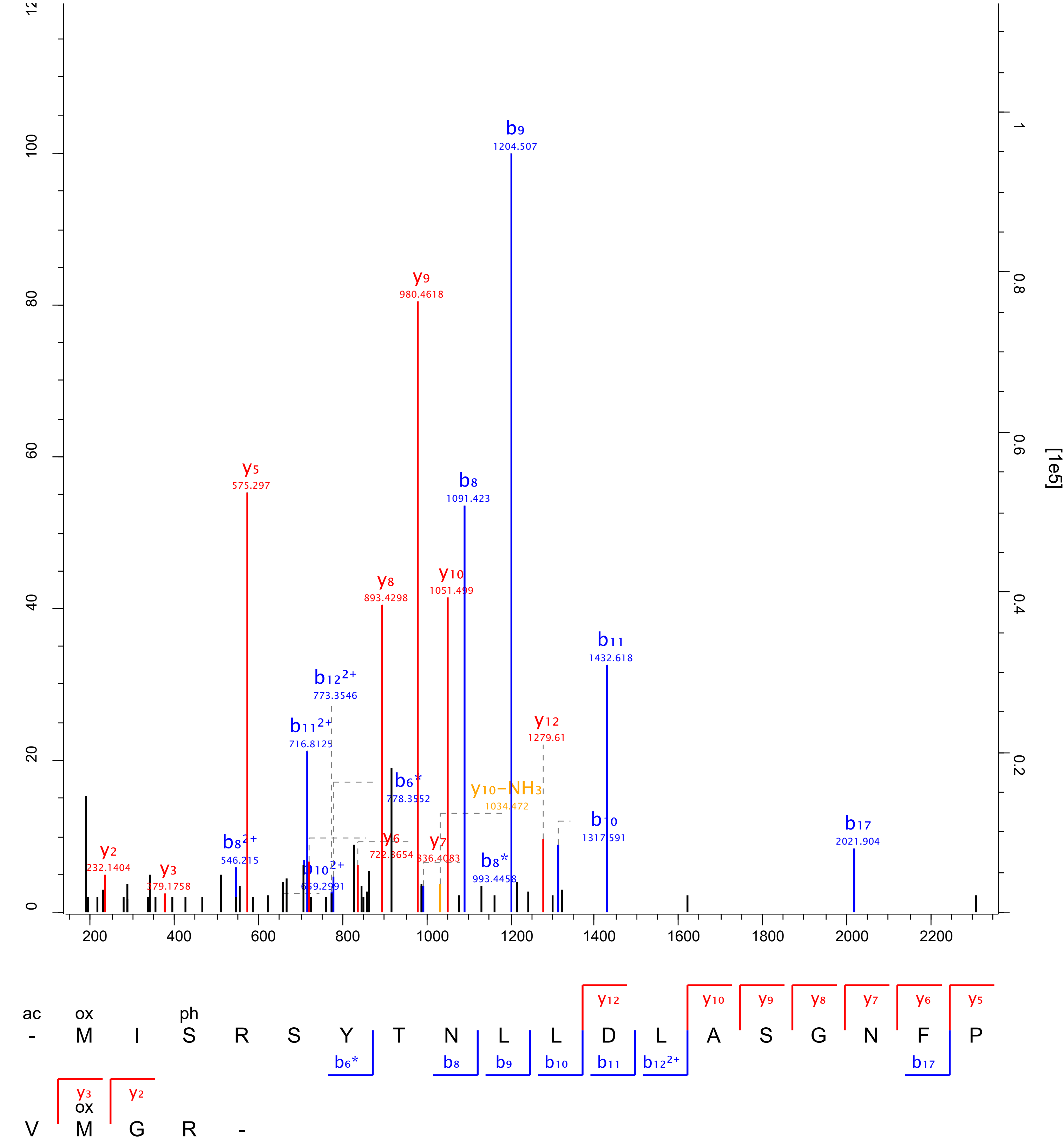

Sample output to test PDF Combine only

Raw file	Scan	Method	Score	m/z	Gene names
0523_12	31094	FTMS; HCD	137.25	1157.17	At5g63830

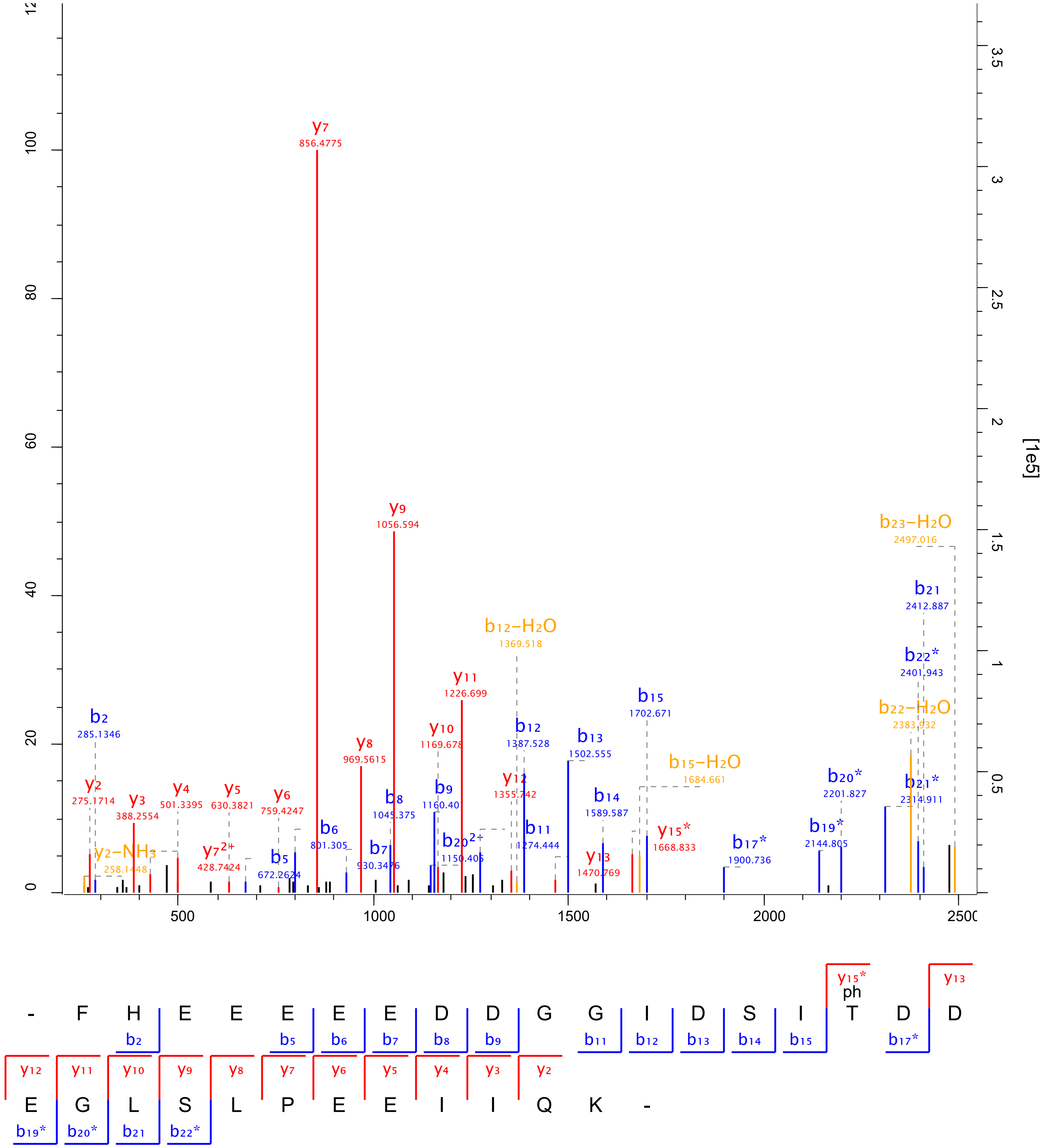

Raw file	Scan	Method	Score	m/z
0523_12	31290	FTMS; HCD	124.1	964.81

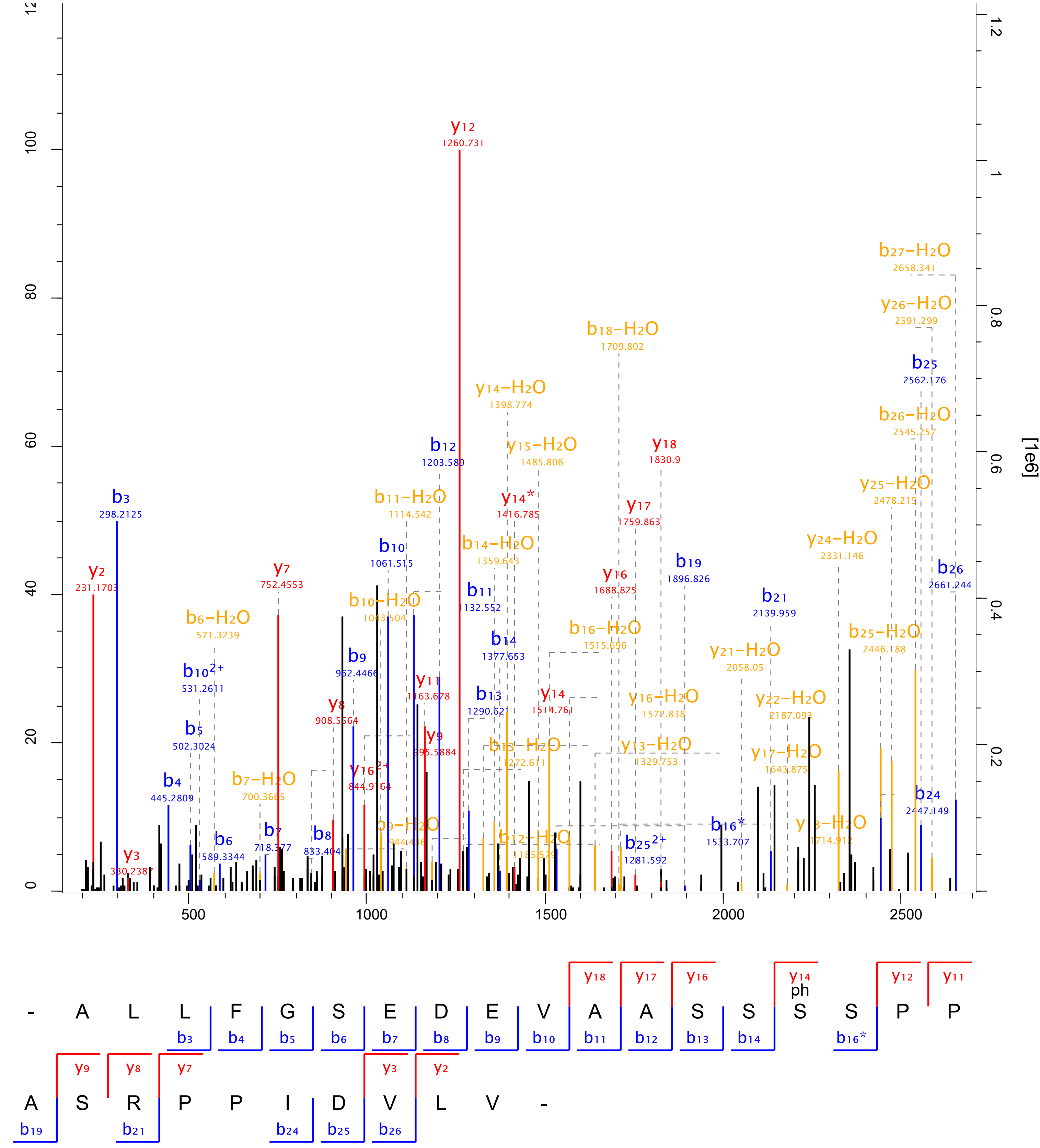

Sample output to test PDF Combine only

Raw file	Scan	Method	Score	m/z	Gene names
0523_12	32127	FTMS; HCD	56.69	928.77	At2g25800

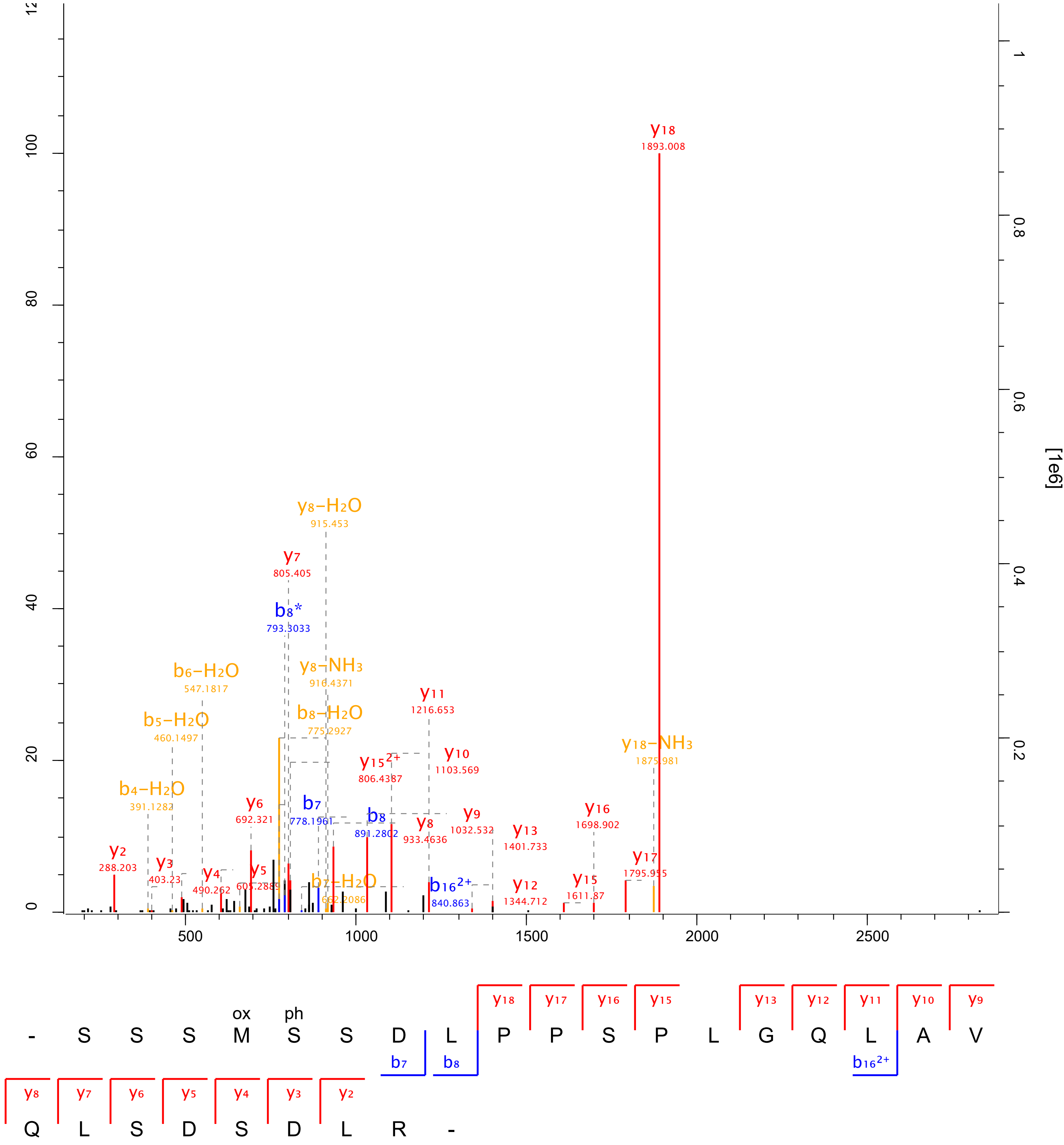

Sample output to test PDF Combine only

Raw file	Scan	Method	Score	m/z	Gene names
0523_12	32743	FTMS; HCD	49.09	1150.22	OCT5

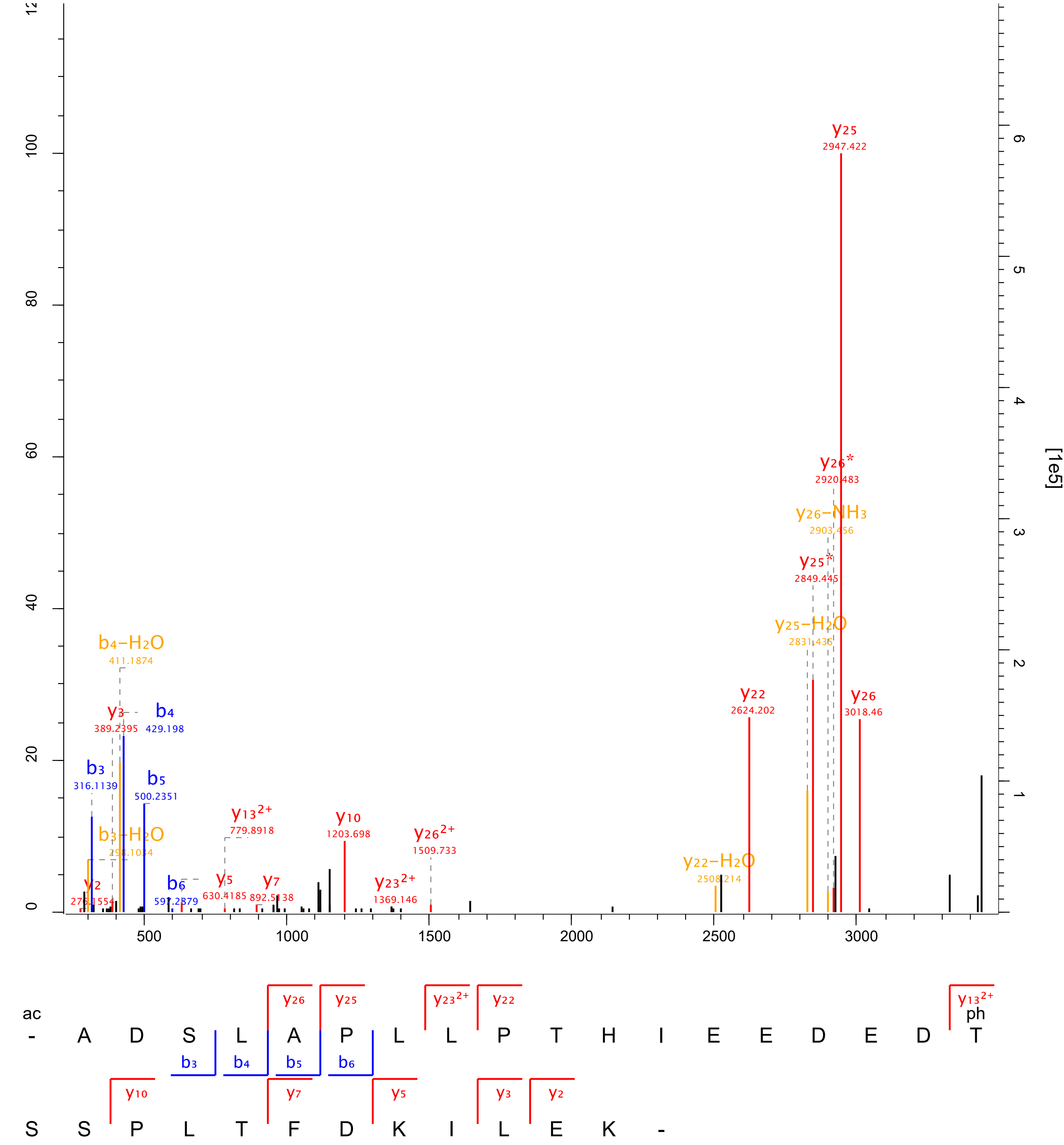

Sample output to test PDF Combine only

Raw file	Scan	Method	Score	m/z	Gene names
0523_12	32804	FTMS; HCD	121.9	946.14	LRR-RLK

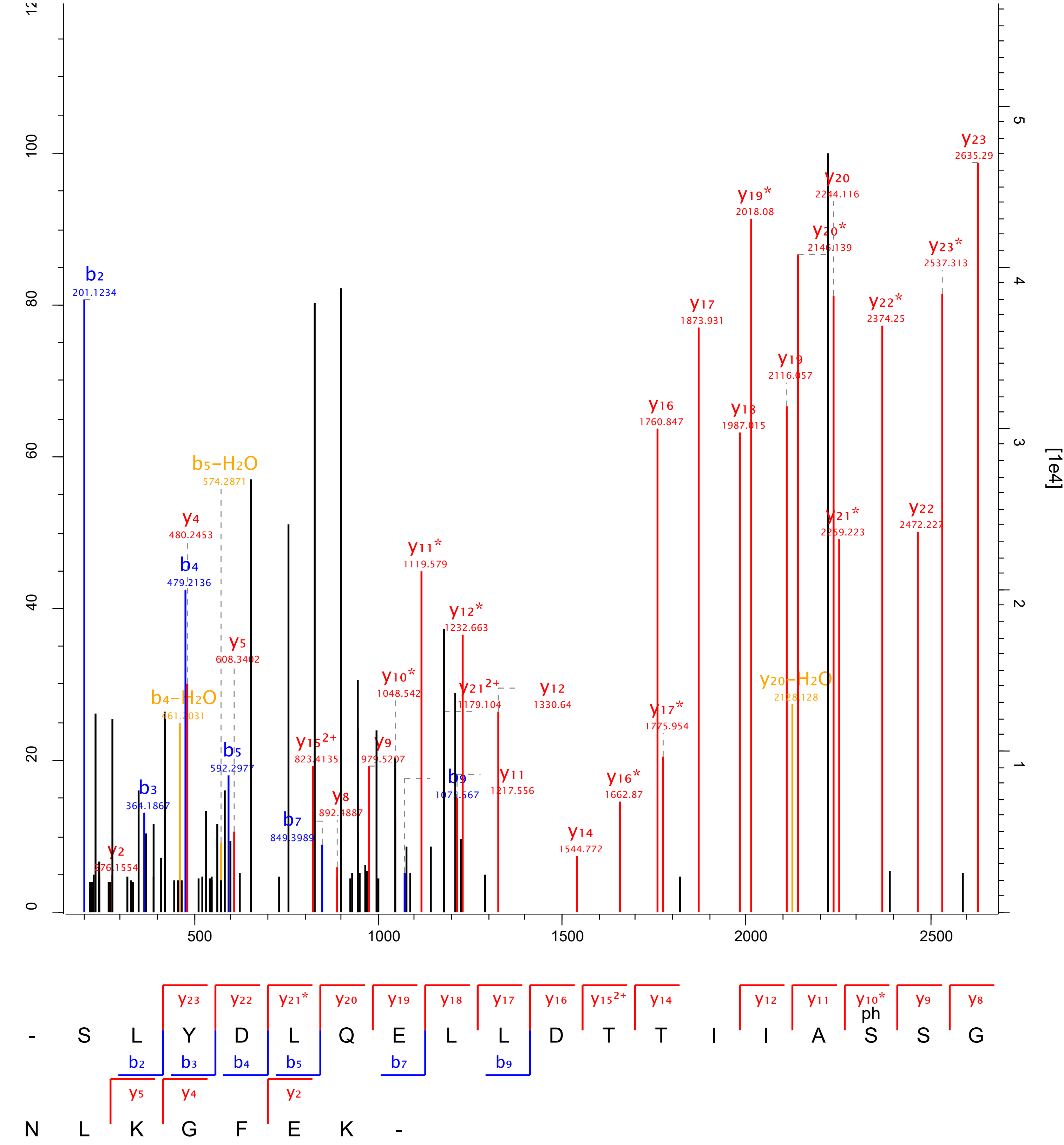

Sample output to test PDF Combine only

Raw file	Scan	Method	Score	m/z	Gene names
0523_13	1438	FTMS; HCD	177.71	704.62	COR47

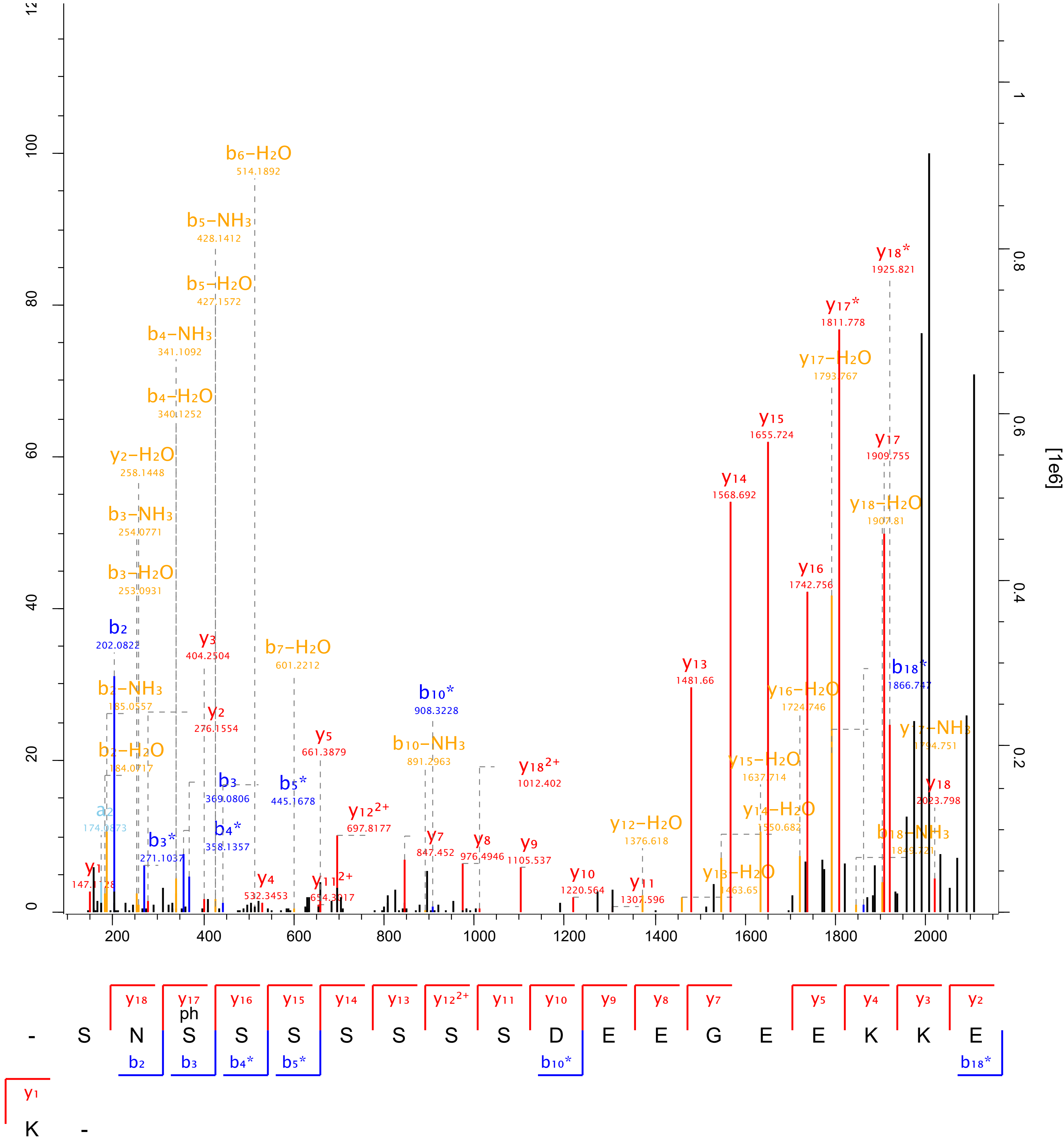

Sample output to test PDF Combine only

Raw file	Scan	Method	Score	m/z	Gene names
0523_13	1576	FTMS; HCD	61.16	433.23	SR45

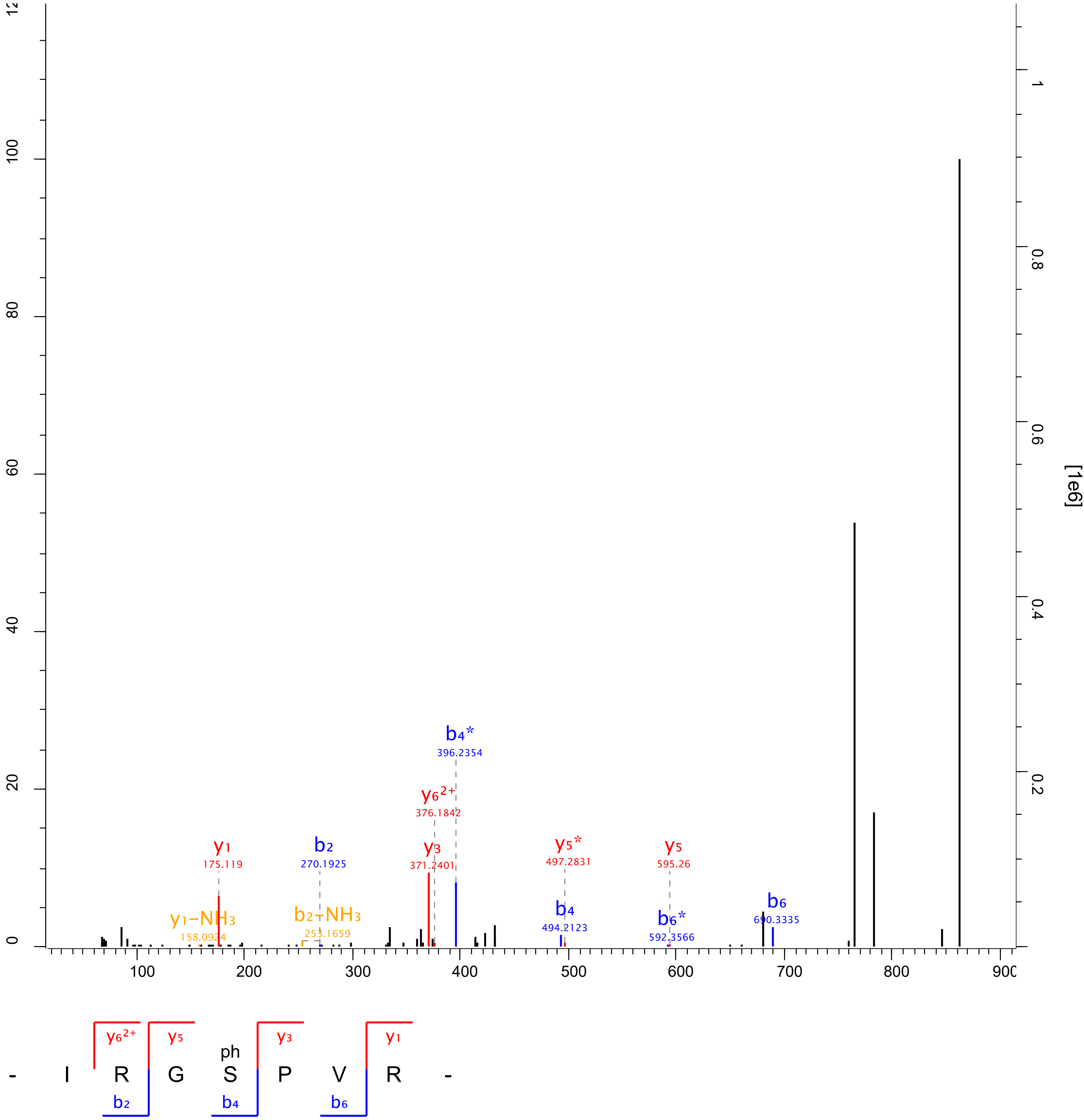

Sample output to test PDF Combine only

Raw file	Scan	Method	Score	m/z	Gene names
0523_13	1736	FTMS; HCD	98.04	453.71	AHA1

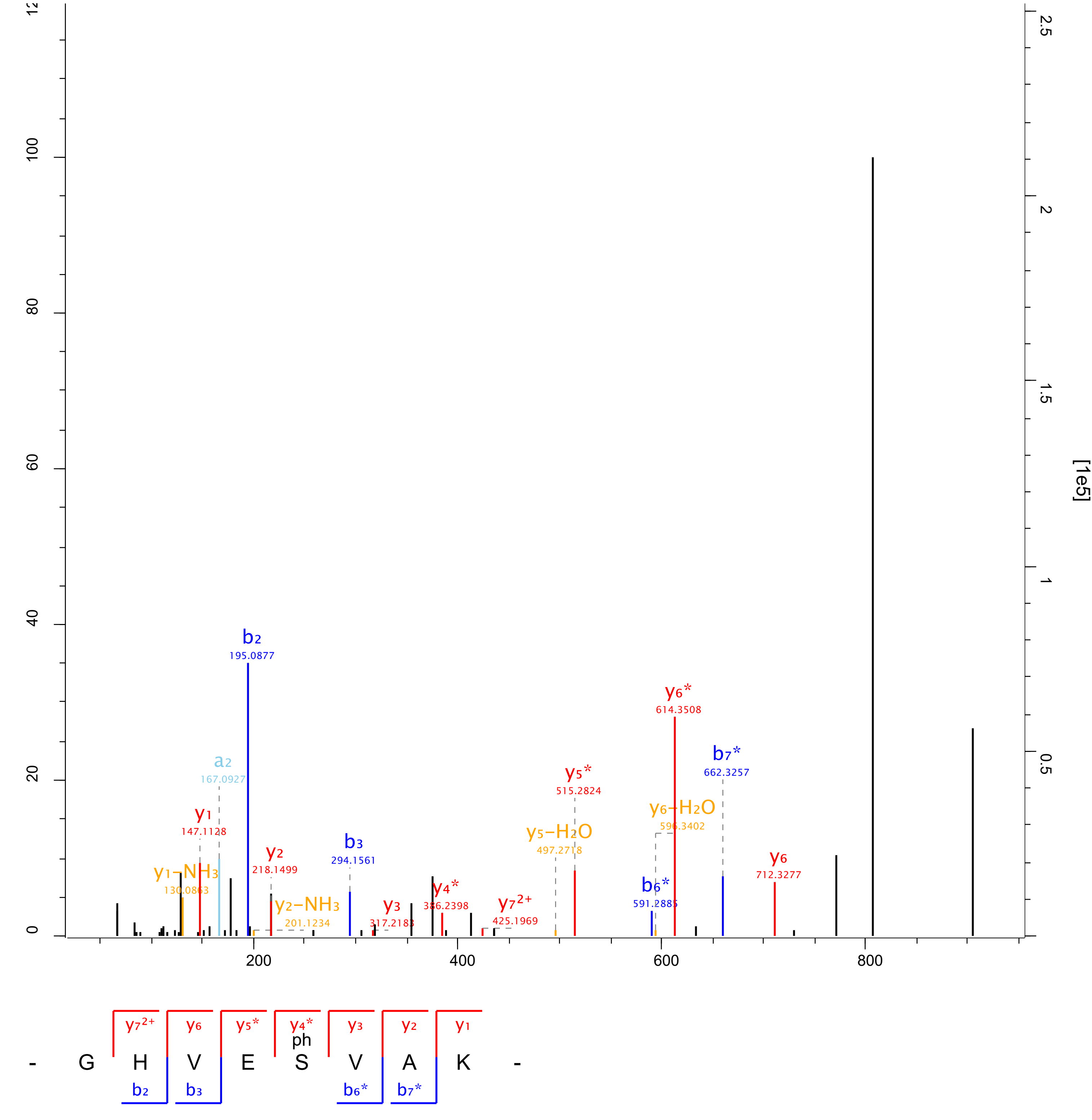

Sample output to test PDF Combine only

Raw file	Scan	Method	Score	m/z	Gene names
0523_13	2520	FTMS; HCD	106.32	561.25	HDT2

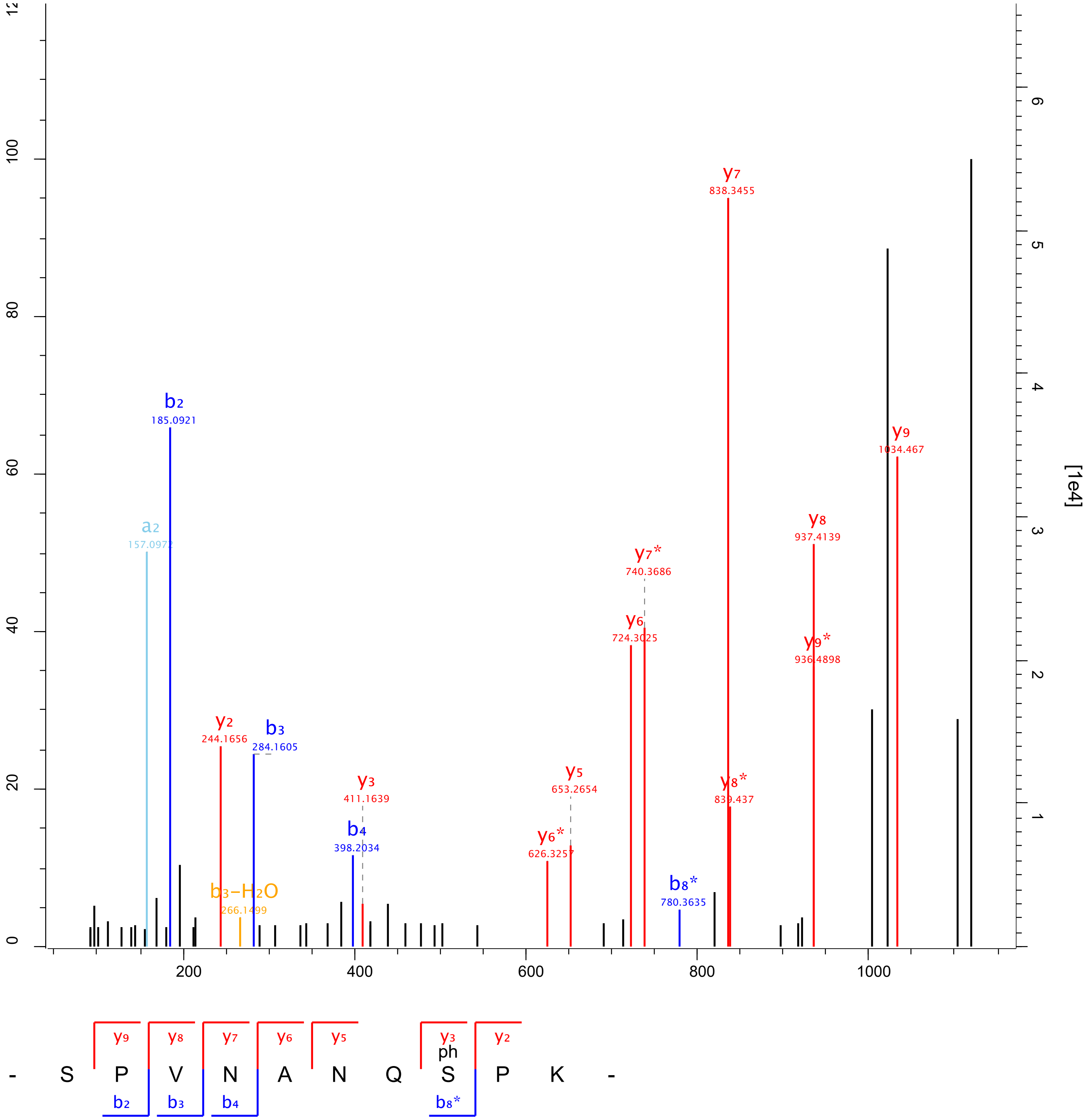

Sample output to test PDF Combine only

Raw file	Scan	Method	Score	m/z	Gene names
0523_13	2934	FTMS; HCD	94.12	557.74	PIP2-7

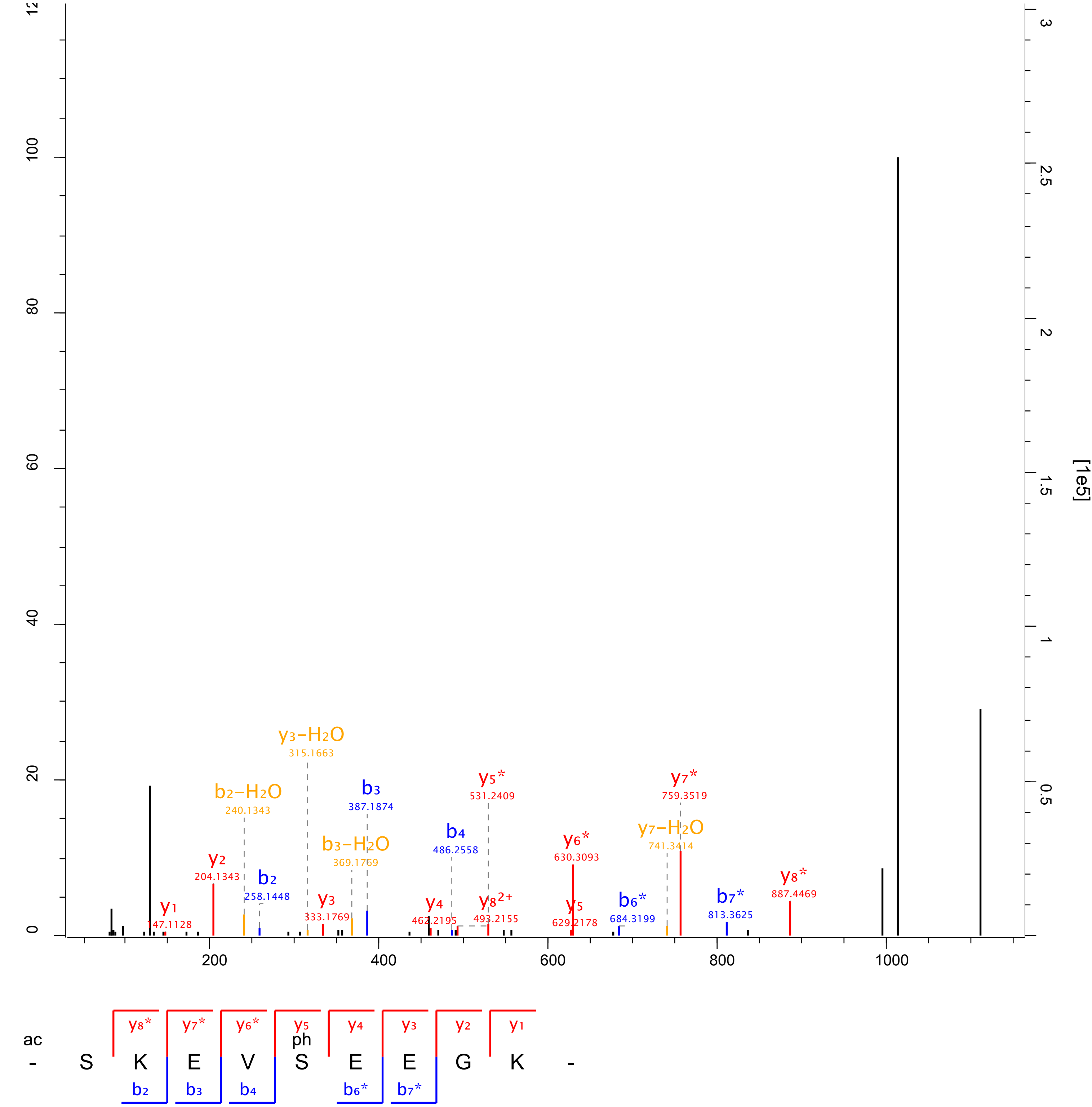

Sample output to test PDF Combine only

Raw file	Scan	Method	Score	m/z
0523_13	4306	FTMS; HCD	80.39	711.79

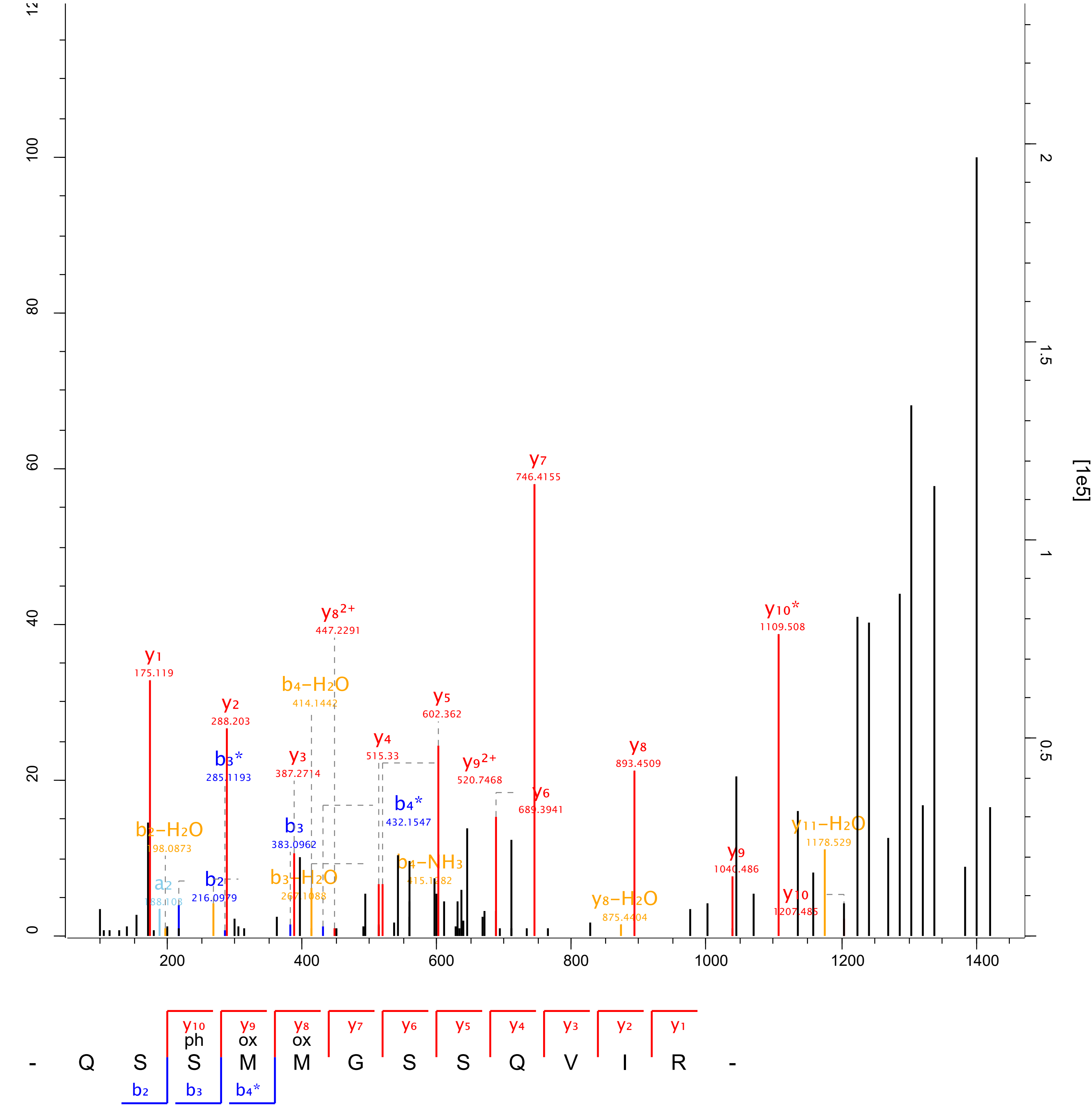

Sample output to test PDF Combine only

Raw file	Scan	Method	Score	m/z	Gene names
0523_13	4698	FTMS; HCD	135.55	427.7	PPC3

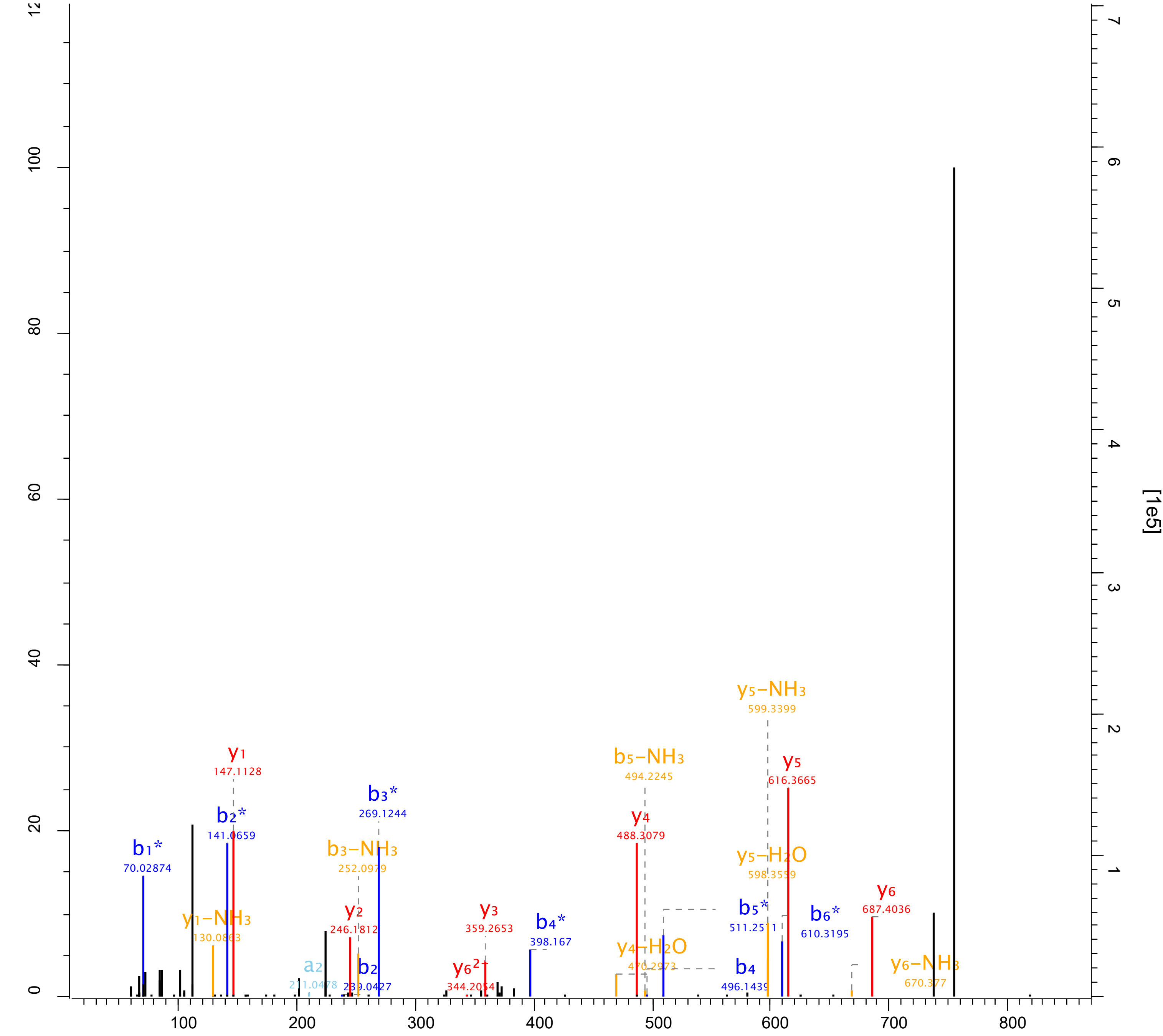

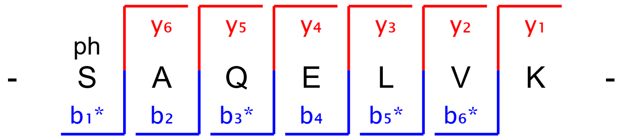

Sample output to test PDF Combine only

Raw file	Scan	Method	Score	m/z	Gene names
0523_13	4879	FTMS; HCD	83.31	421.7	DRP2B;DRP2A

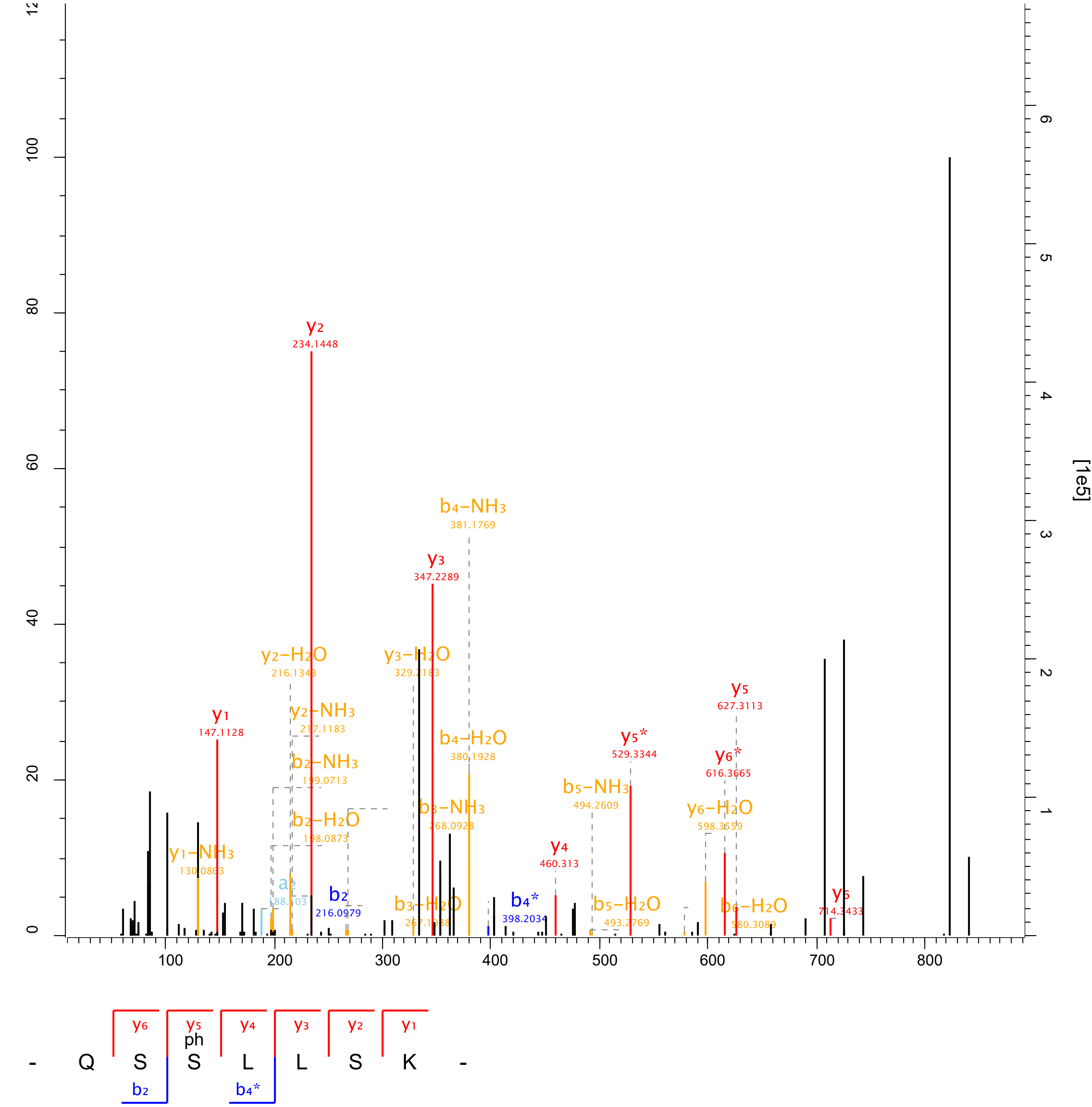

Sample output to test PDF Combine only

Raw file	Scan	Method	Score	m/z	Gene names
0523_13	5097	FTMS; HCD	78.5	699.78	At1g10140

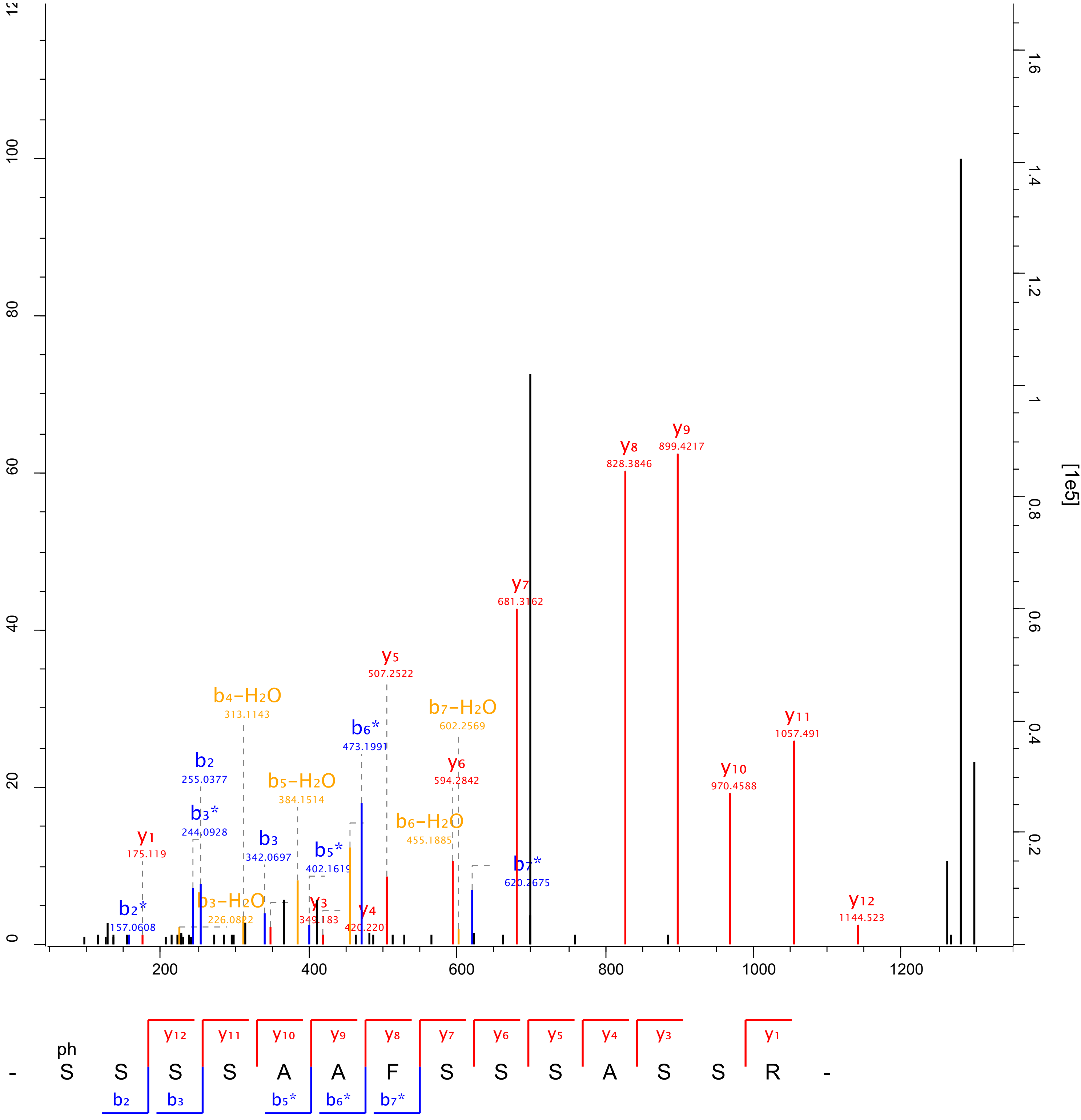

Sample output to test PDF Combine only

Raw file	Scan	Method	Score	m/z	Gene names
0523_13	5163	FTMS; HCD	71.18	564.74	ACBP4

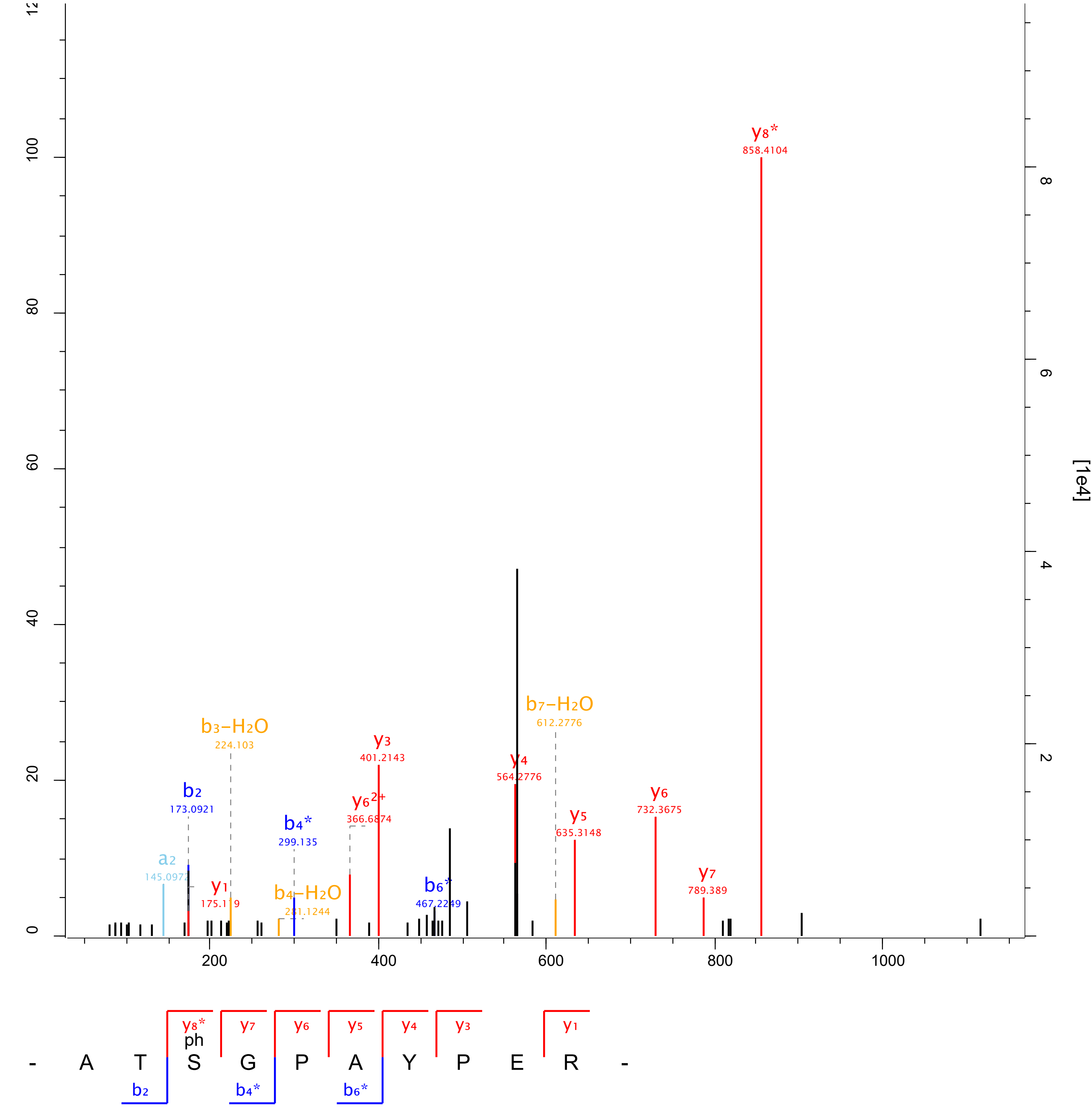

Sample output to test PDF Combine only

Raw file	Scan	Method	Score	m/z	Gene names
0523_13	5435	FTMS; HCD	102.5	609.26	F26G5_140

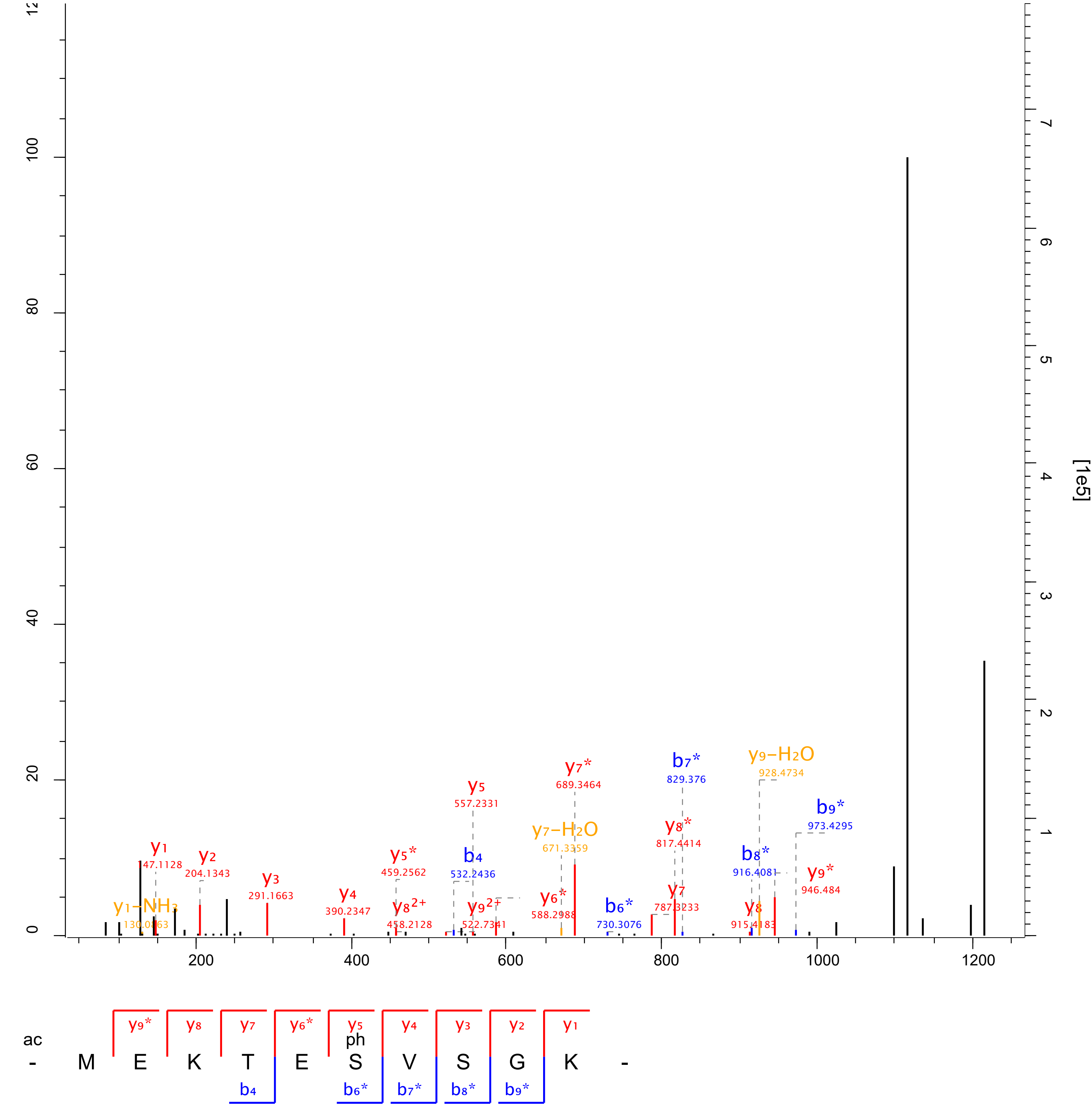

Sample output to test PDF Combine only

Raw file	Scan	Method	Score	m/z	Gene names
0523_13	5961	FTMS; HCD	61.34	489.72	At2g44010

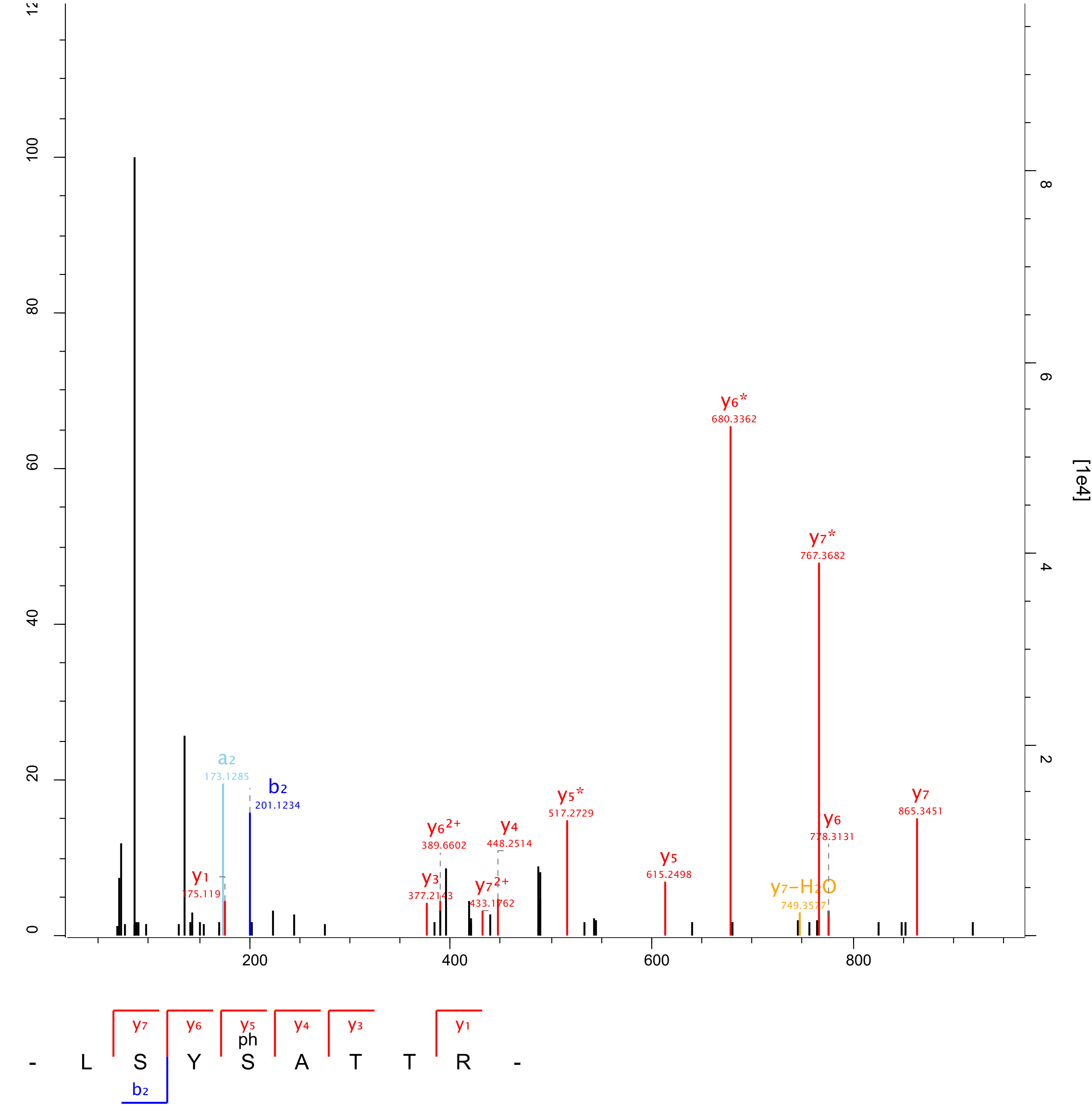

Sample output to test PDF Combine only

Raw file	Scan	Method	Score	m/z	Gene names
0523_13	6201	FTMS; HCD	184.78	843.3	AMT1-1

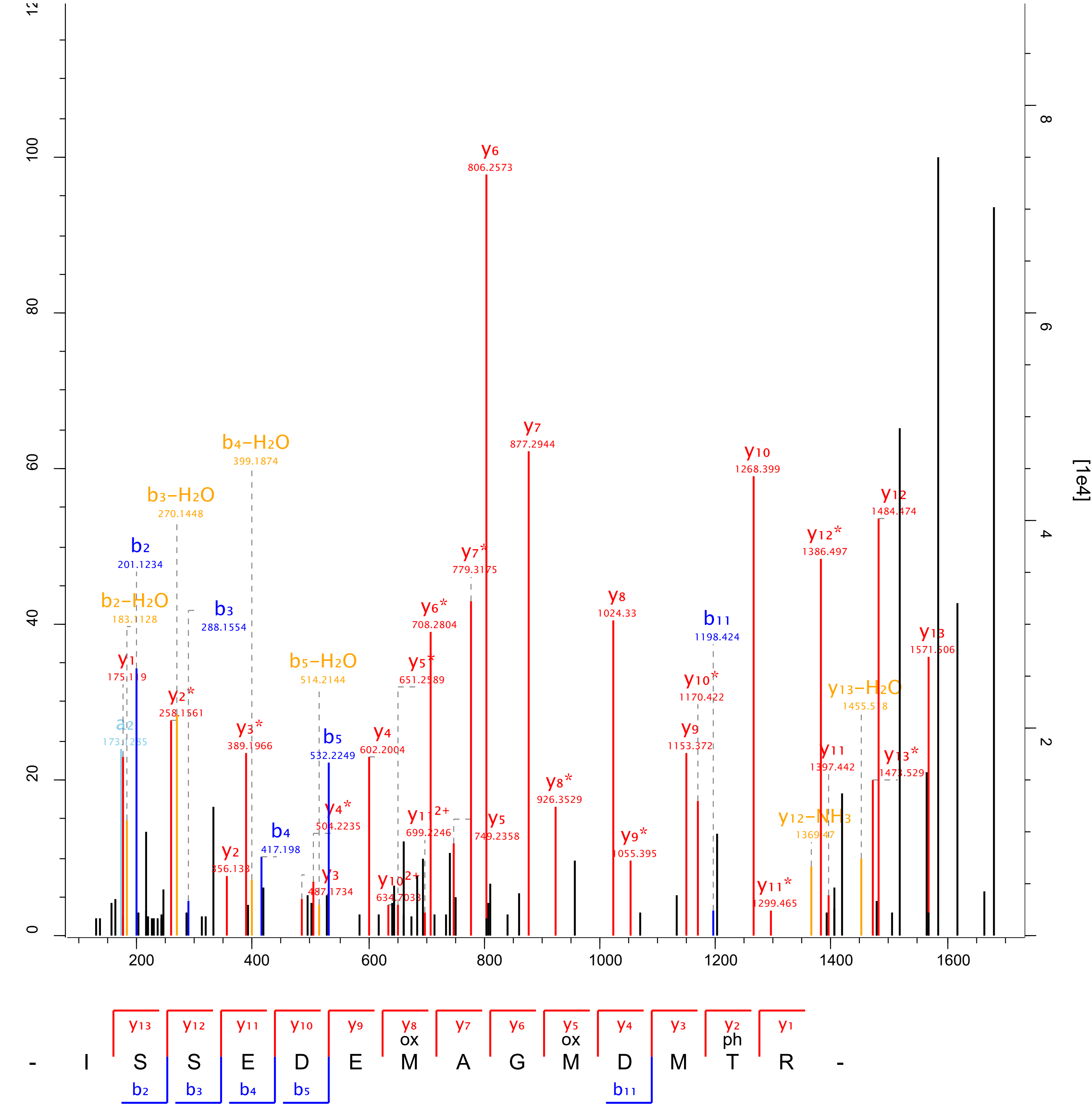

Sample output to test PDF Combine only

Raw file	Scan	Method	Score	m/z	Gene names
0523_13	6585	FTMS; HCD	179.79	700.3	MLO2

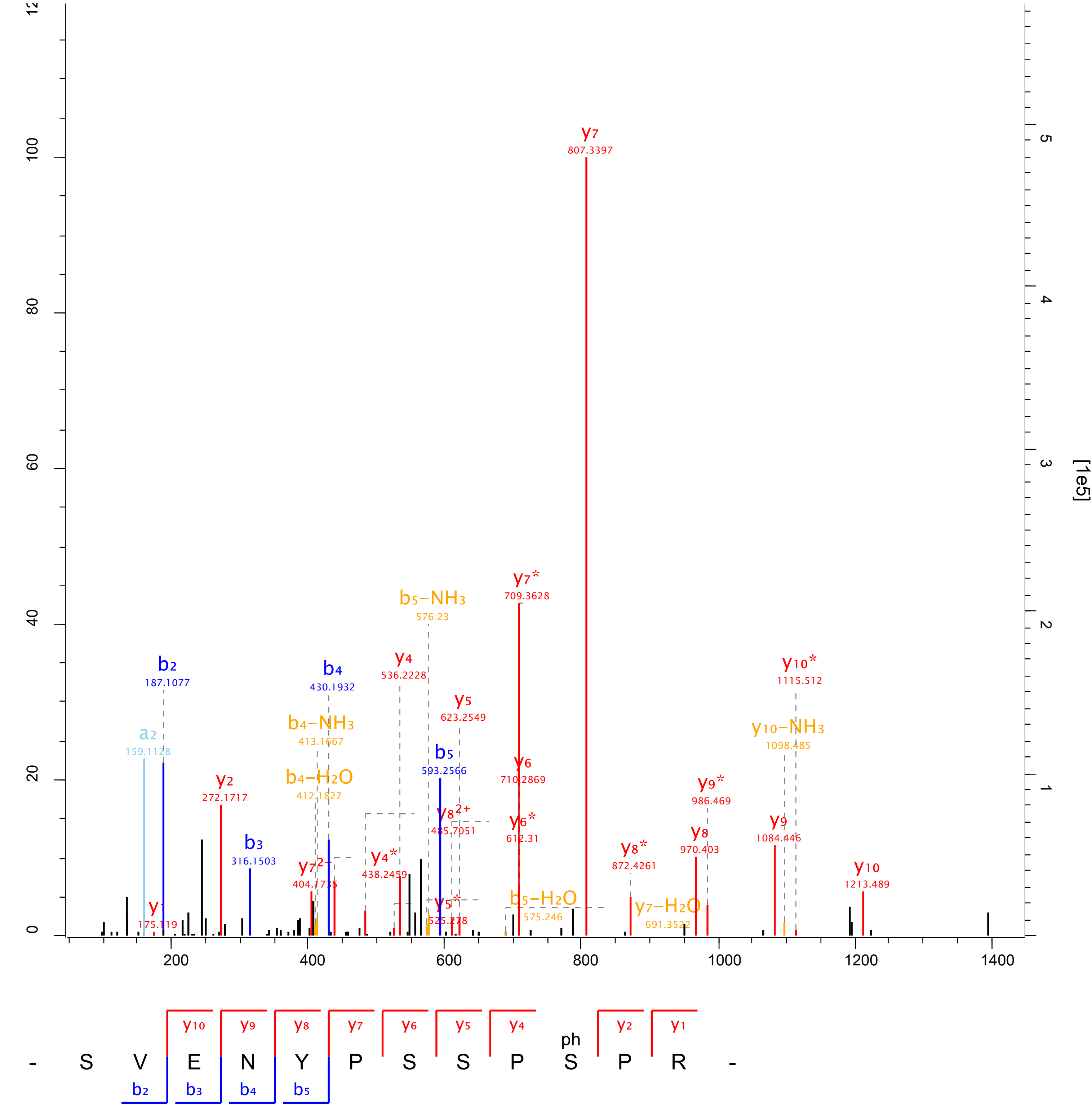

Sample output to test PDF Combine only

Raw file	Scan	Method	Score	m/z
0523_13	6789	FTMS; HCD	159.22	706.33

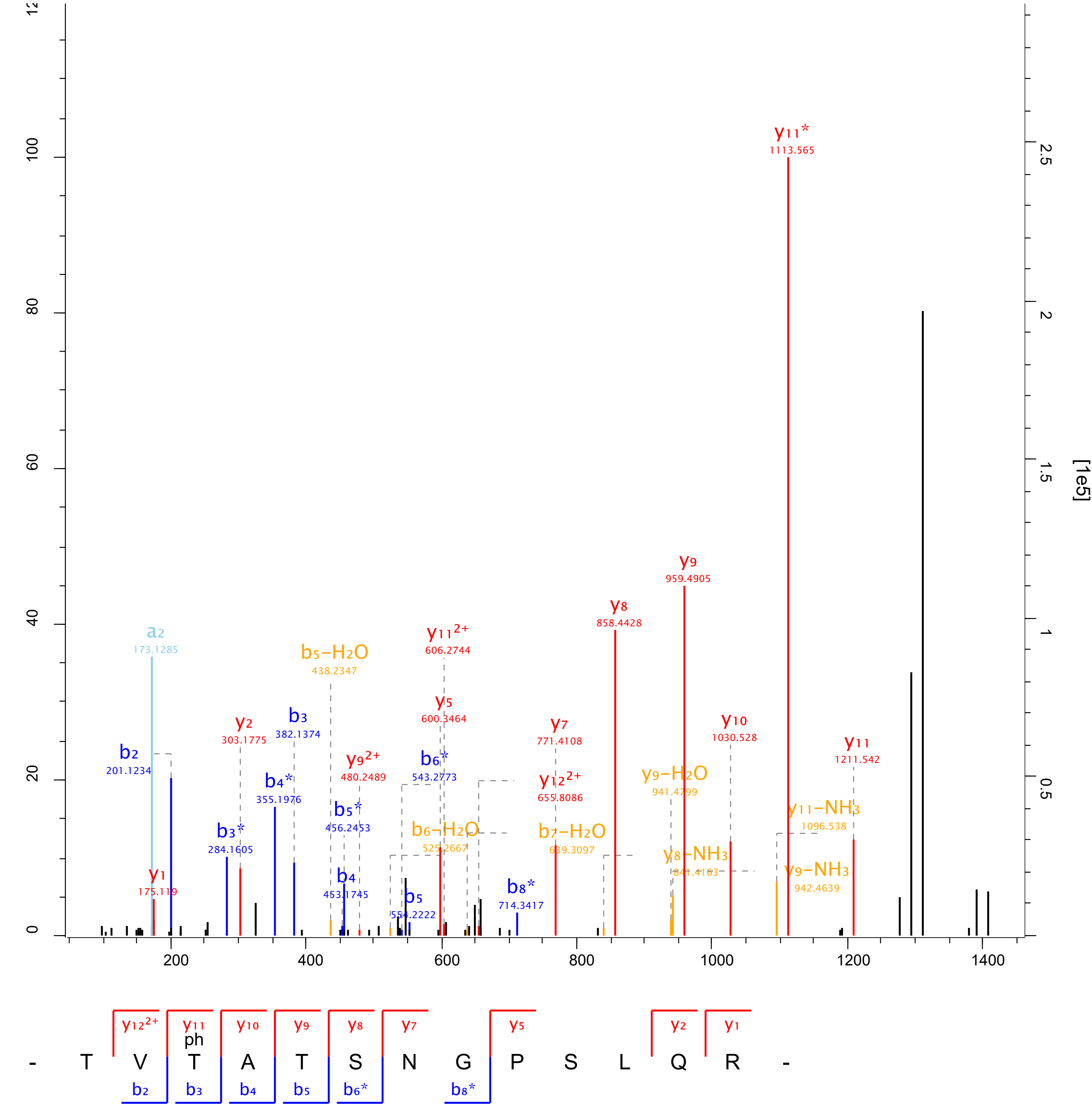

Sample output to test PDF Combine only

Raw file	Scan	Method	Score	m/z	Gene names
0523_13	7335	FTMS; HCD	211.83	572.25	DRP2B;DRP2A

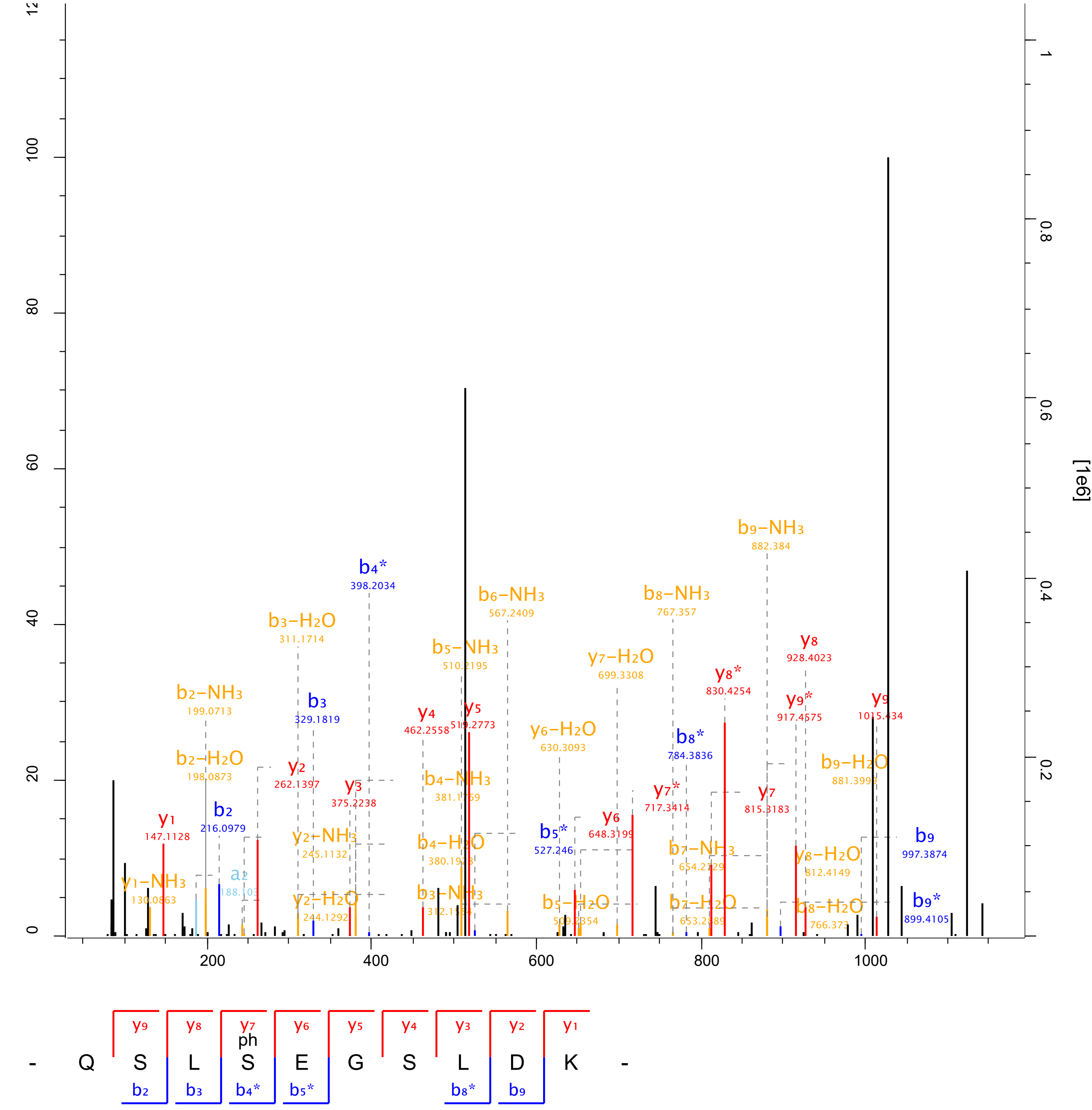

Sample output to test PDF Combine only

Raw file	Scan	Method	Score	m/z
0523_13	7726	FTMS; HCD	178.15	602.73

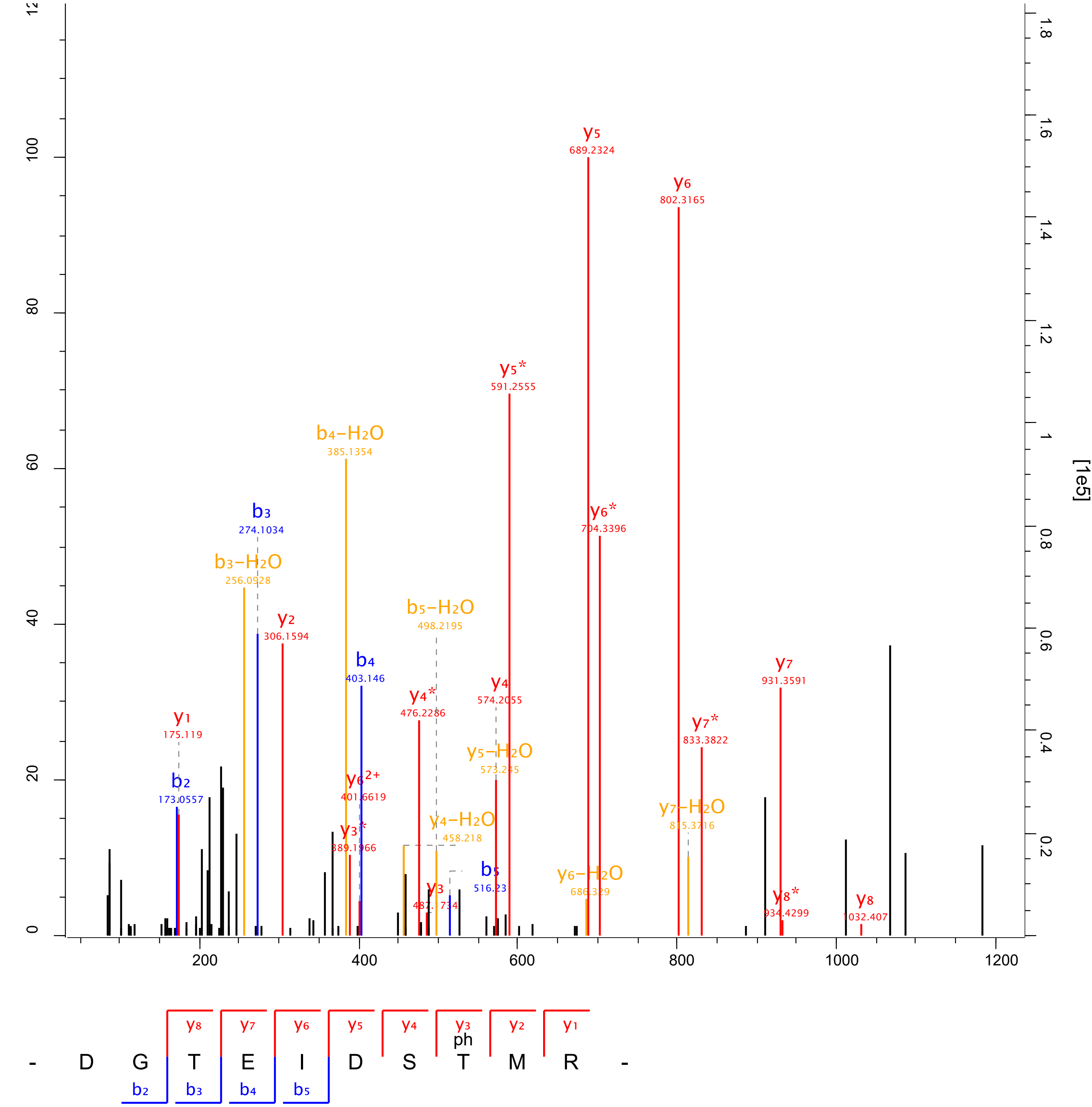

Sample output to test PDF Combine only

Raw file	Scan	Method	Score	m/z	Gene names
0523_13	7902	FTMS; HCD	119.96	672.8	At2g38410

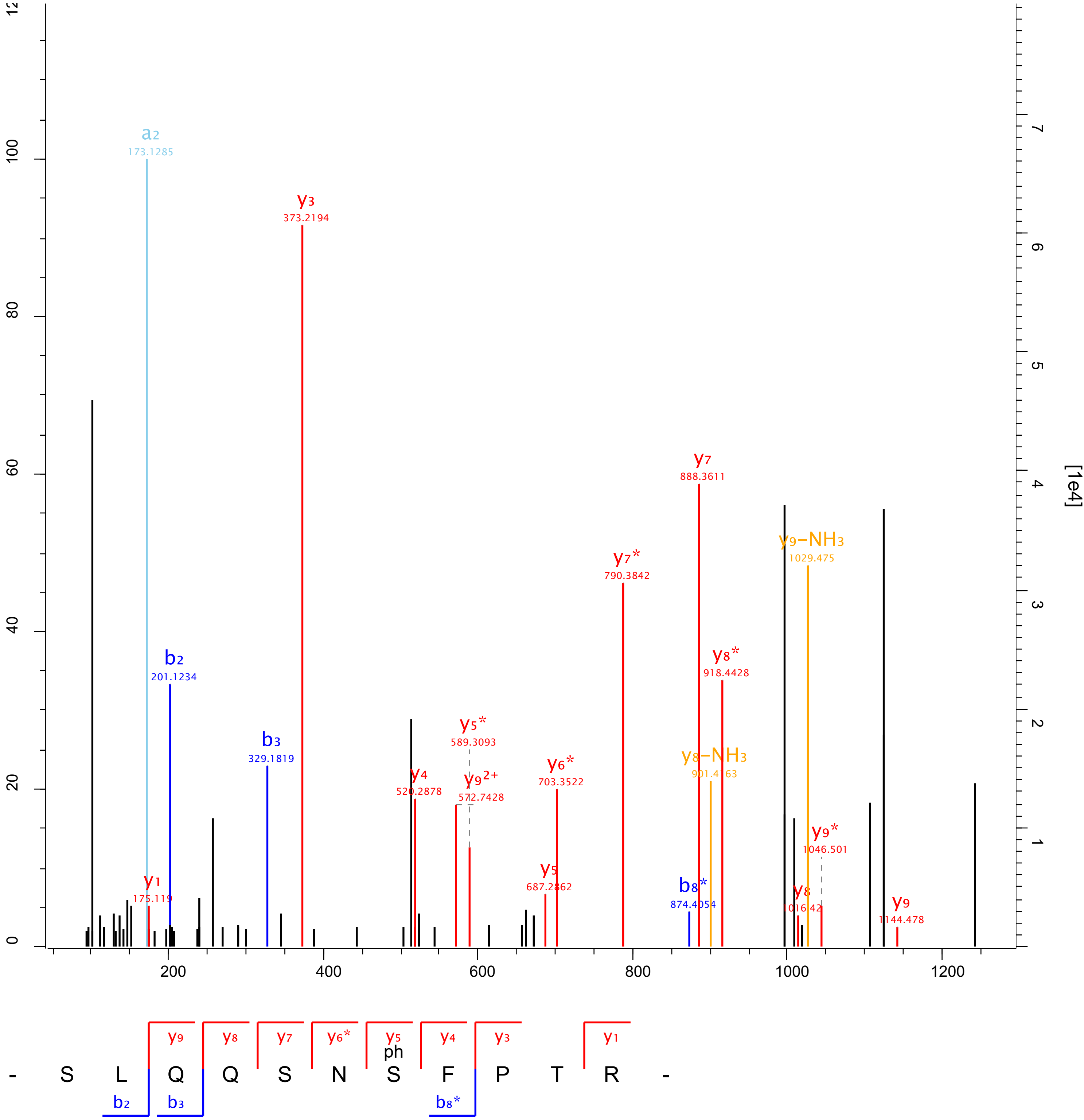

Sample output to test PDF Combine only

Raw file	Scan	Method	Score	m/z	Gene names
0523_13	8104	FTMS; HCD	177.76	636.29	POT3

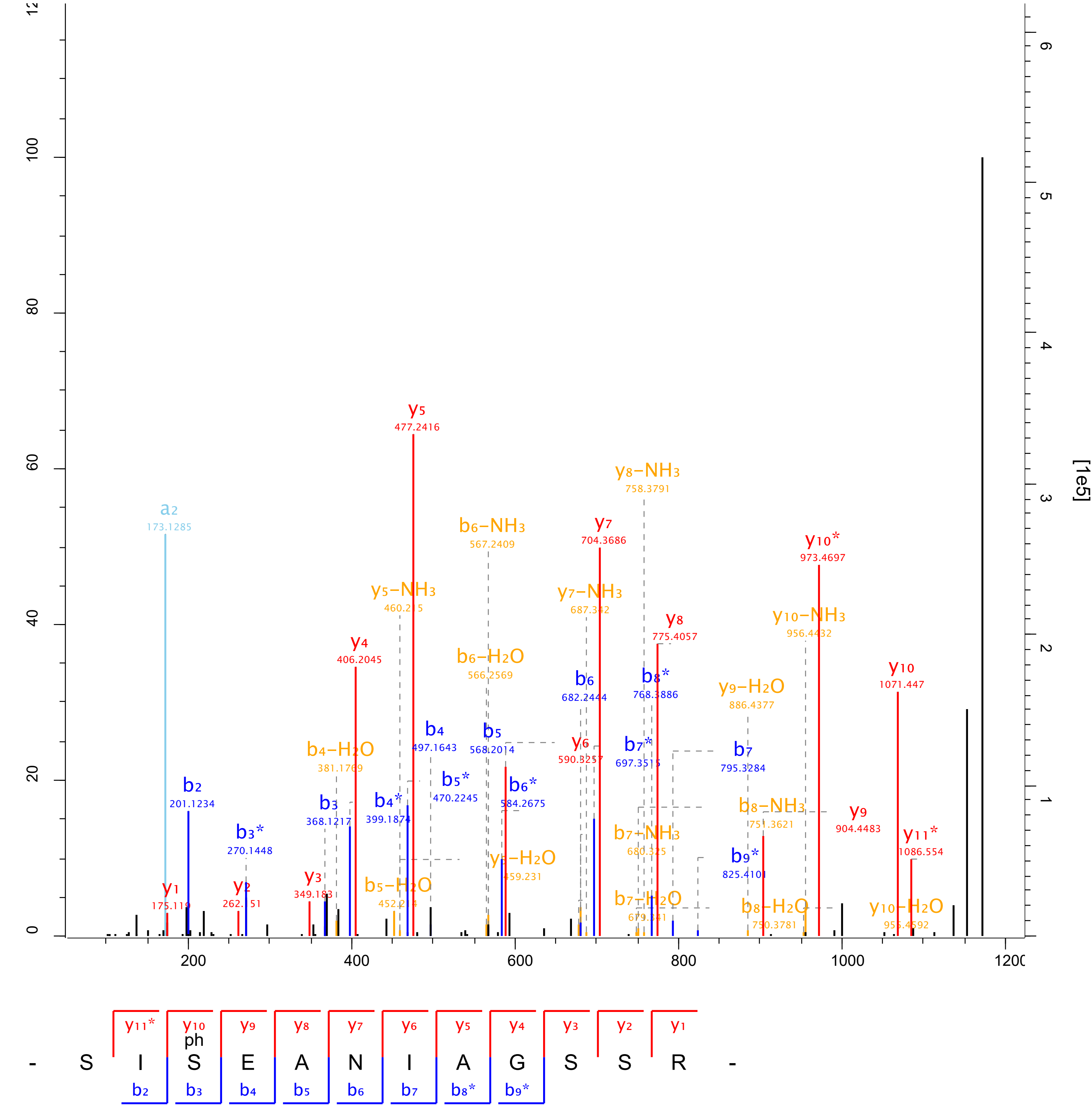

Sample output to test PDF Combine only

Raw file	Scan	Method	Score	m/z	Gene names
0523_13	8105	FTMS; HCD	195.6	740.28	MLO2

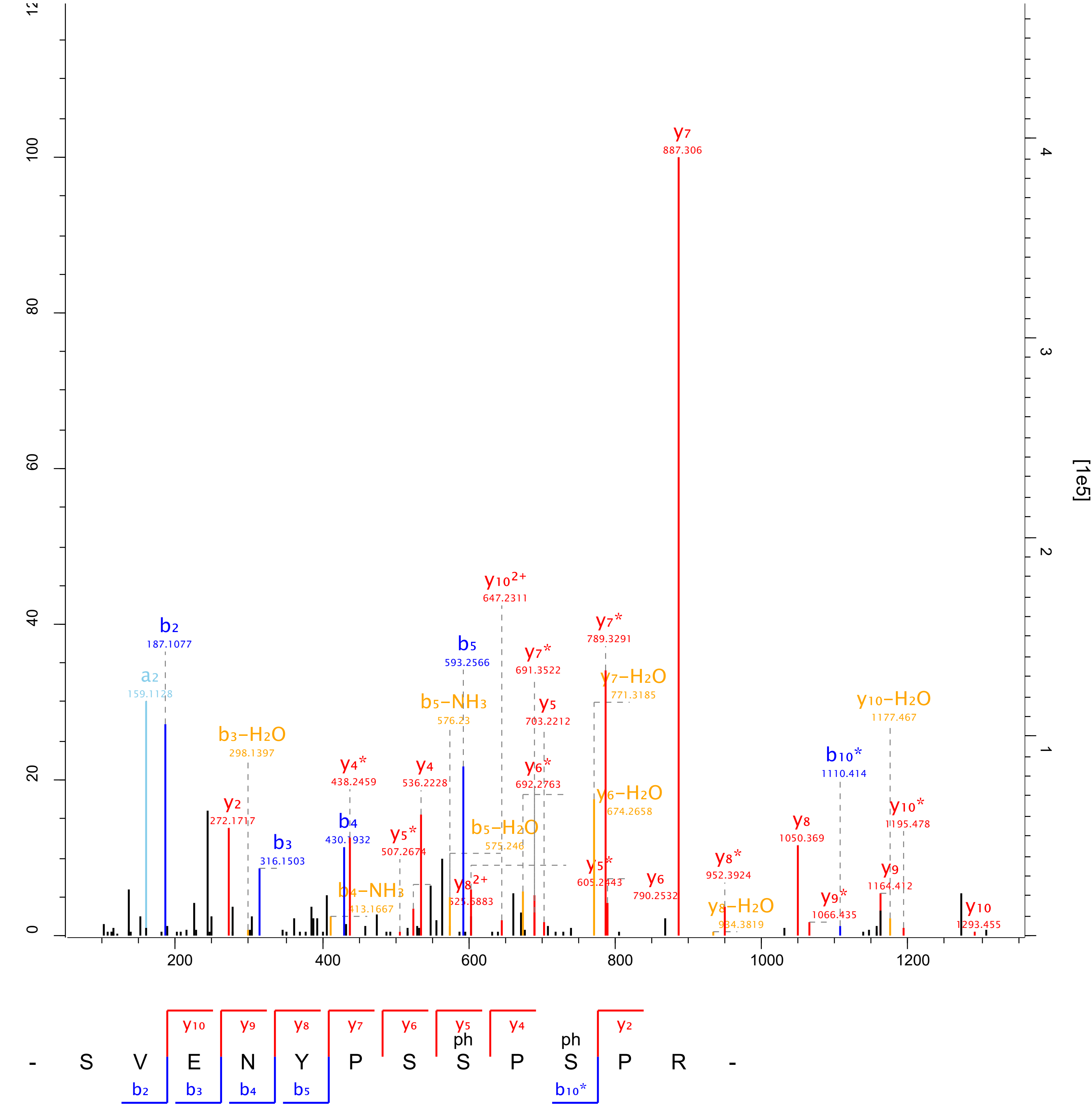

Sample output to test PDF Combine only

Raw file	Scan	Method	Score	m/z	Gene names
0523_13	8144	FTMS; HCD	176.21	786.83	At1g20760

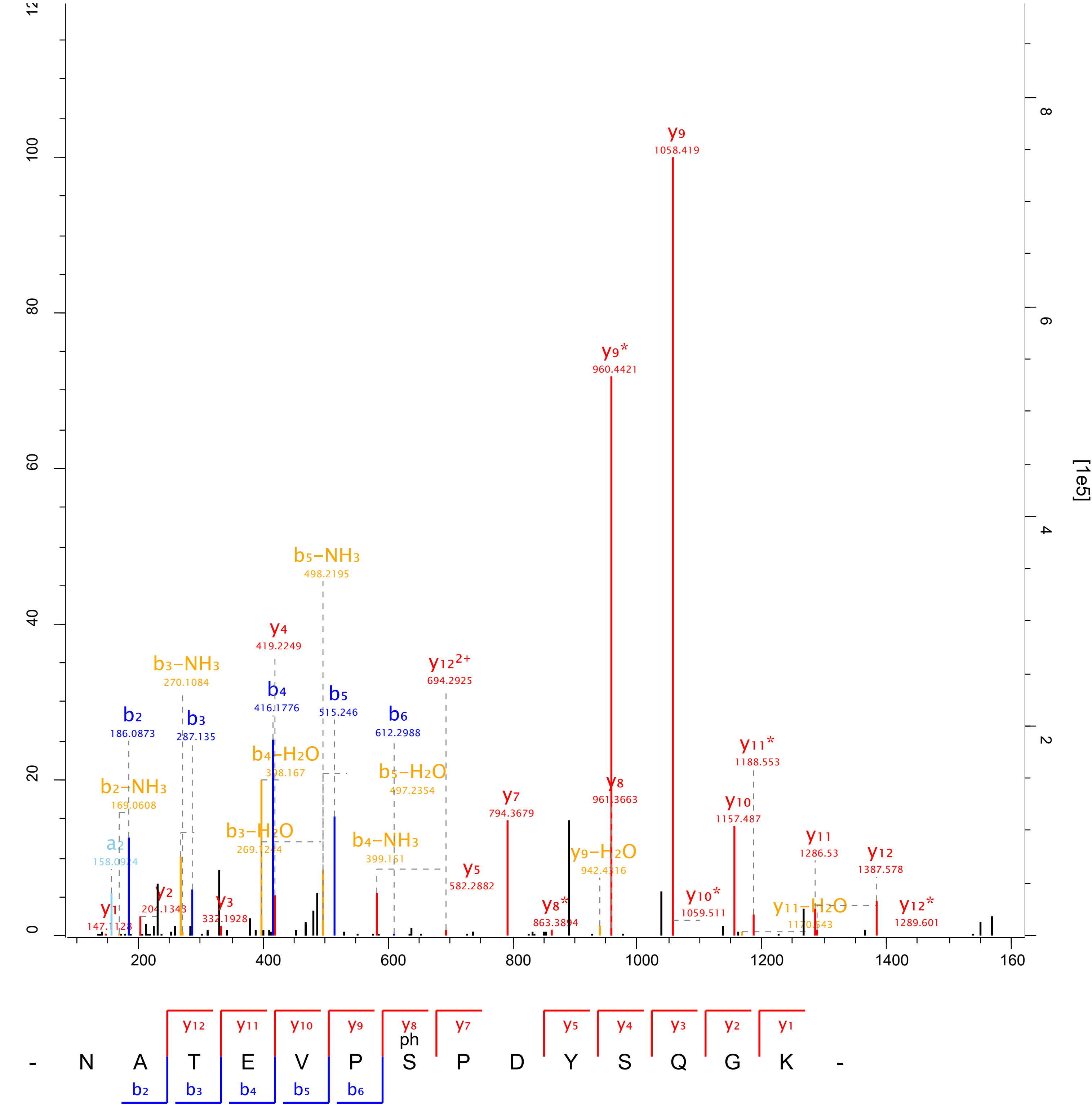

Sample output to test PDF Combine only

Raw file	Scan	Method	Score	m/z	Gene names
0523_13	8998	FTMS; HCD	85.84	629.28	PIP2-8

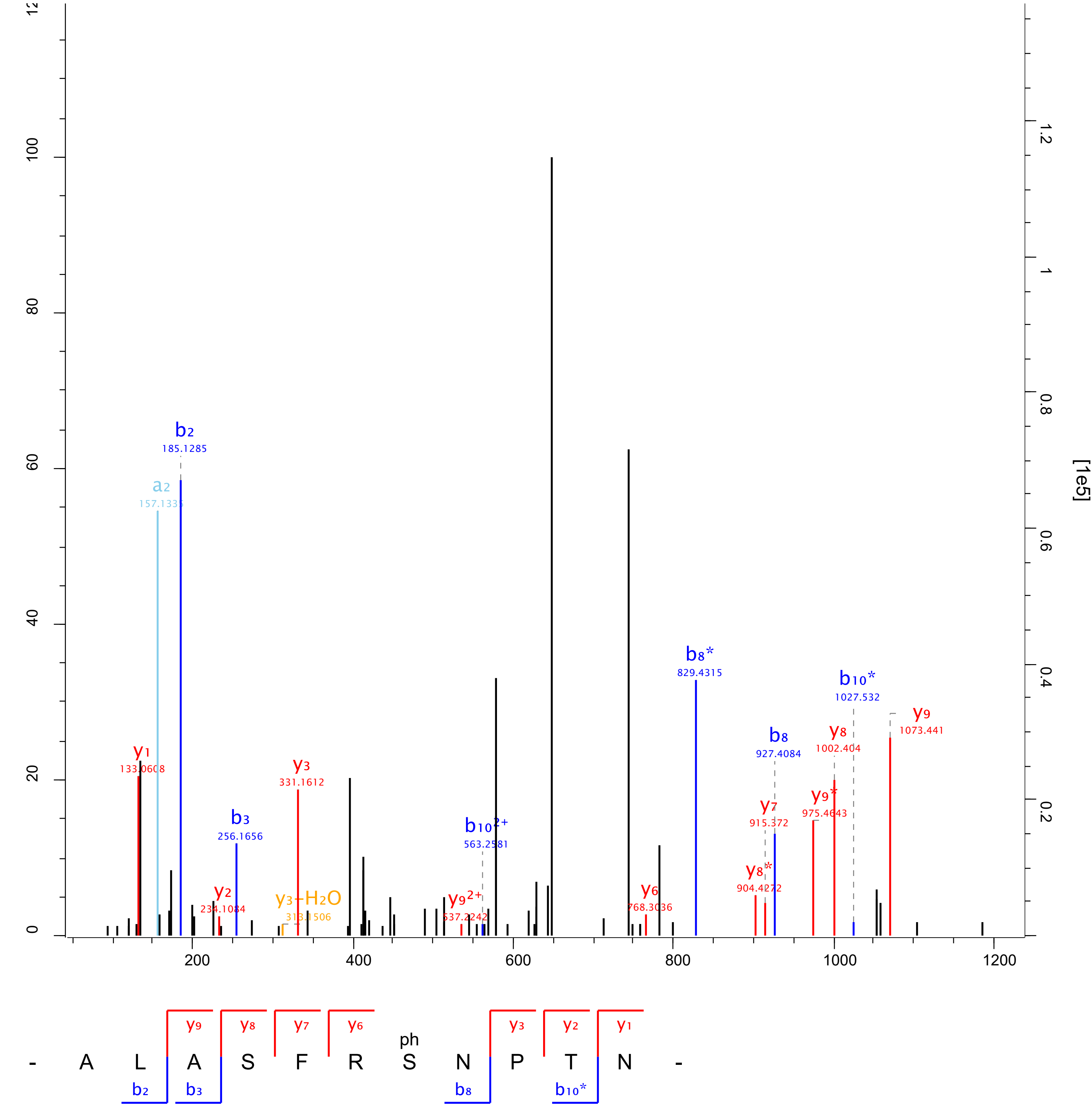

Sample output to test PDF Combine only

Raw file	Scan	Method	Score	m/z	Gene names
0523_13	9777	FTMS; HCD	99.09	834.8	AMT1-1

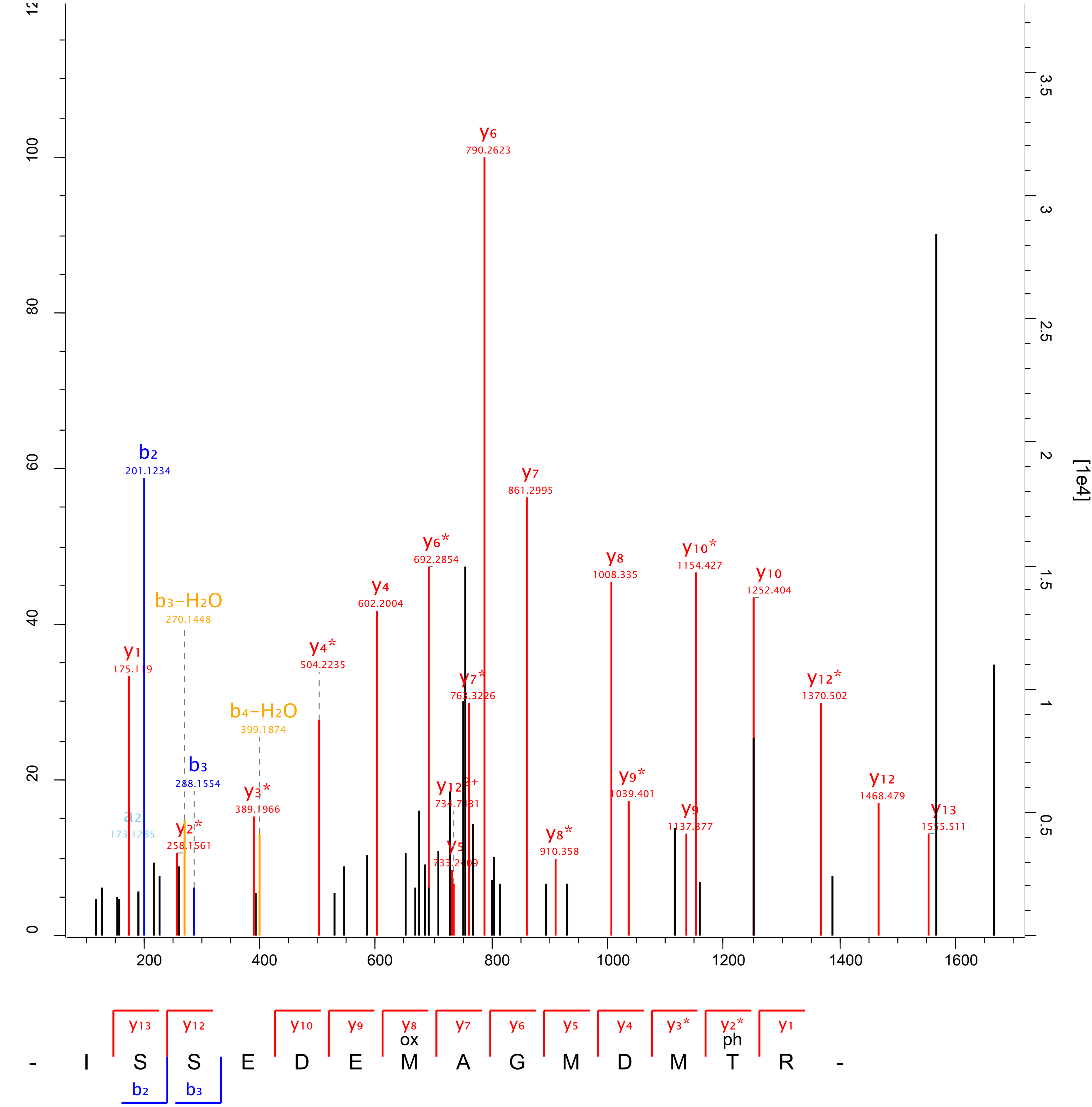

Sample output to test PDF Combine only

Raw file	Scan	Method	Score	m/z	Gene names
0523_13	10002	FTMS; HCD	65.04	561.79	ACA10;ACA9

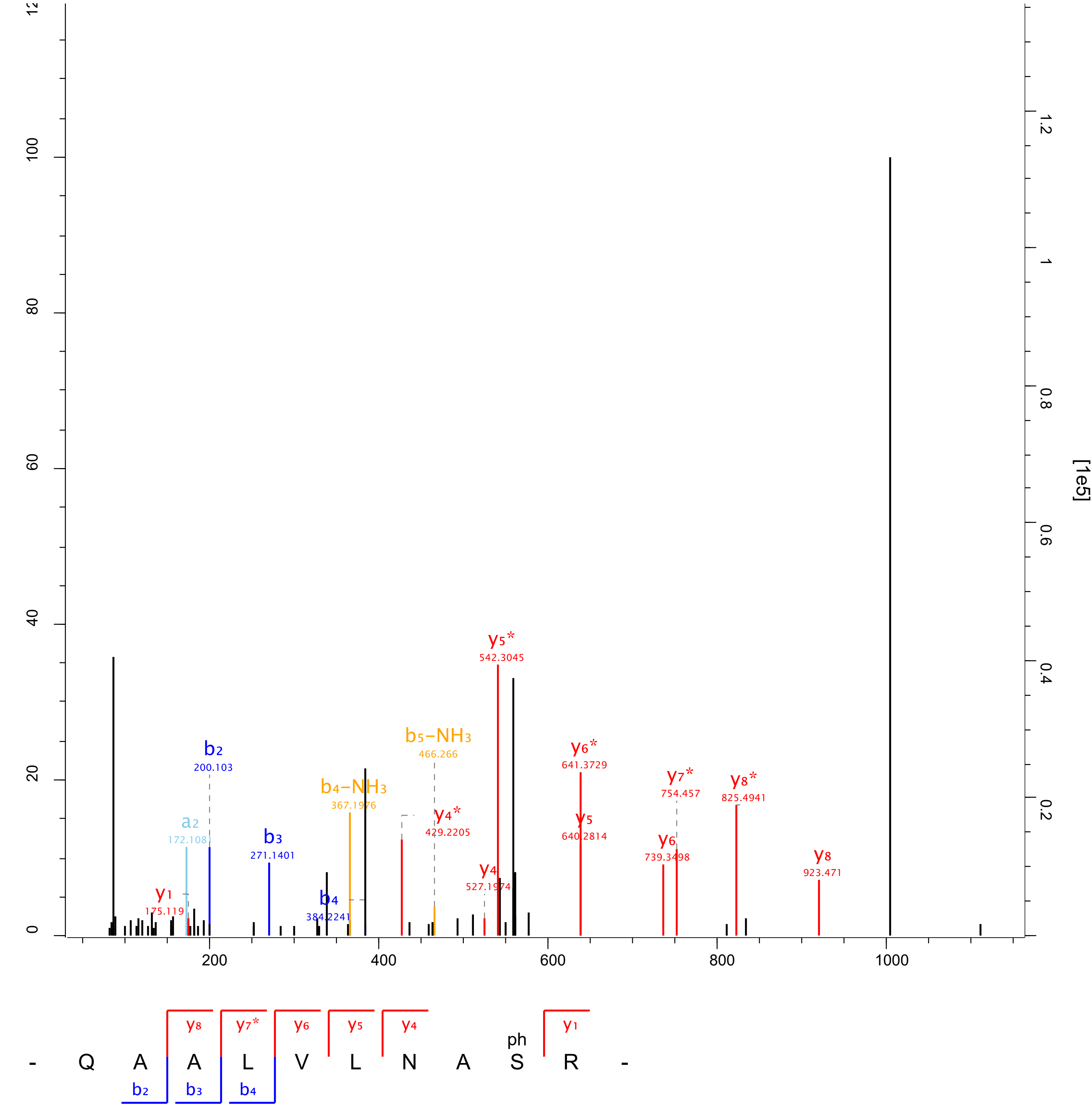

Sample output to test PDF Combine only

Raw file	Scan	Method	Score	m/z	Gene names
0523_13	10195	FTMS; HCD	222.64	710.81	PIP2-4

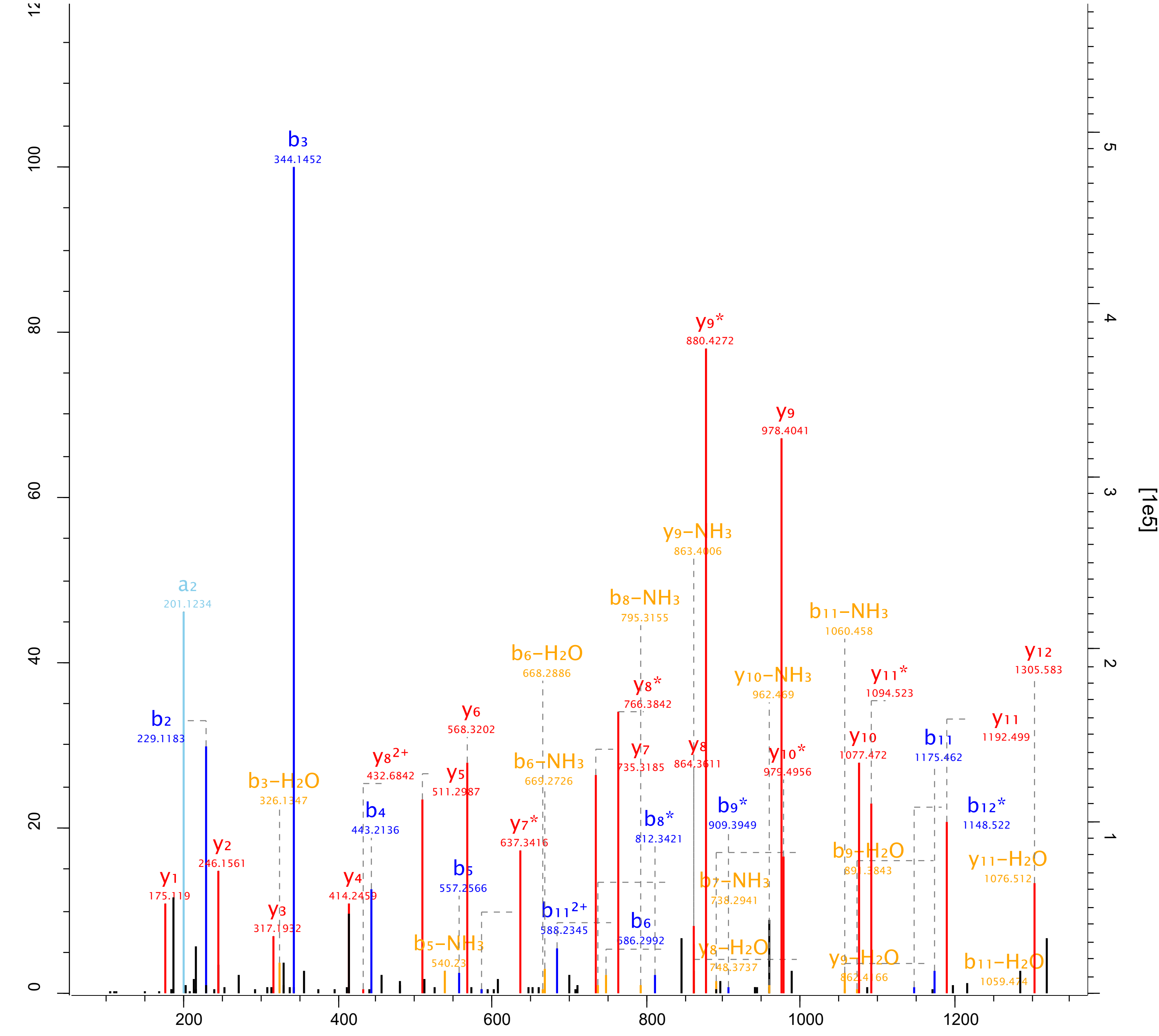

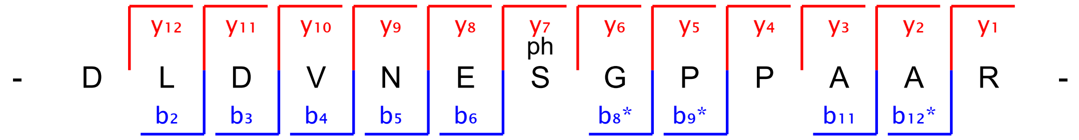

Sample output to test PDF Combine only

Raw file	Scan	Method	Score	m/z
0523_13	10294	FTMS; HCD	136.26	806.85

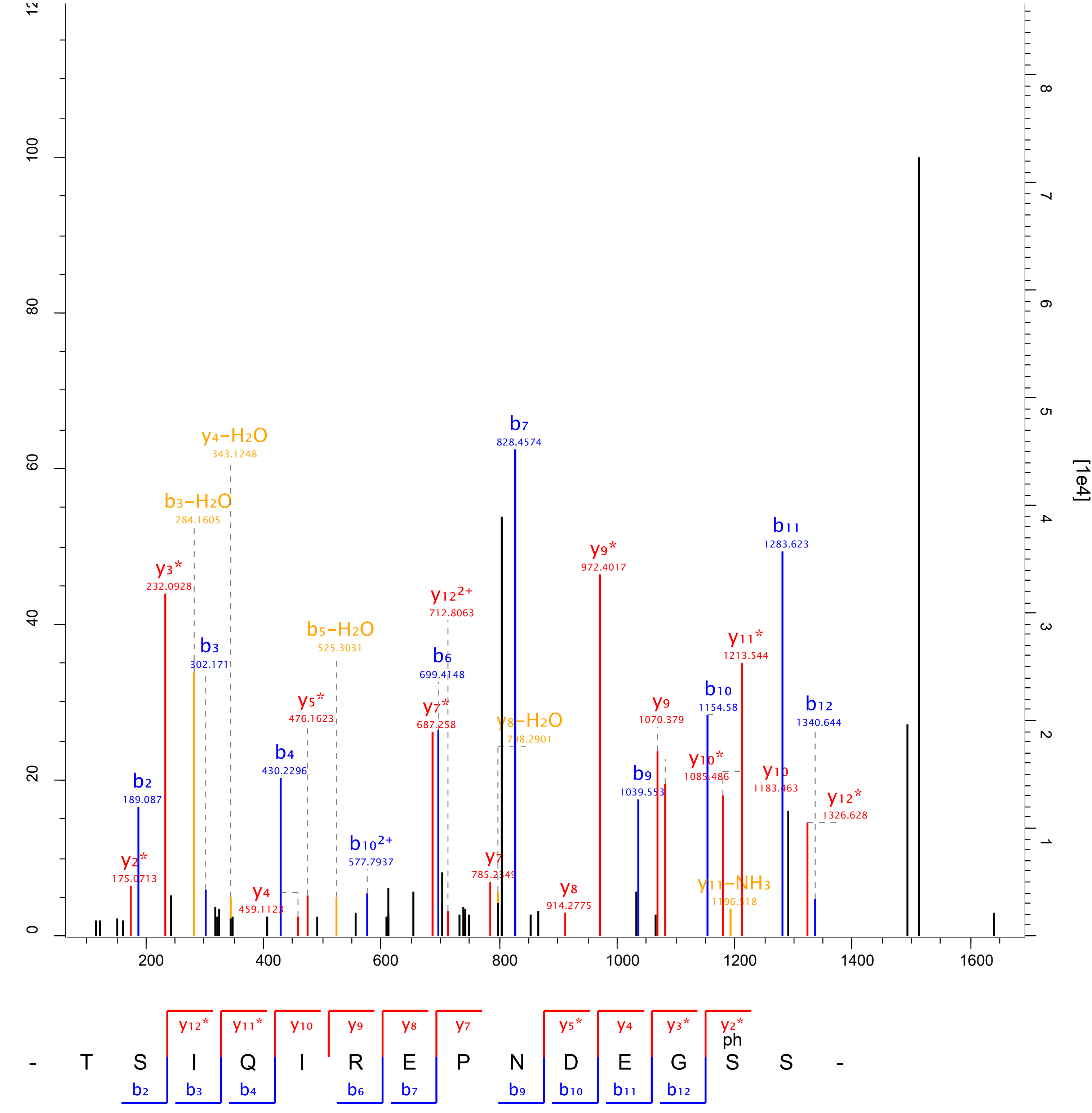

Sample output to test PDF Combine only

Raw file	Scan	Method	Score	m/z	Gene names
0523_13	10664	FTMS; HCD	240.44	941.38	LYK4

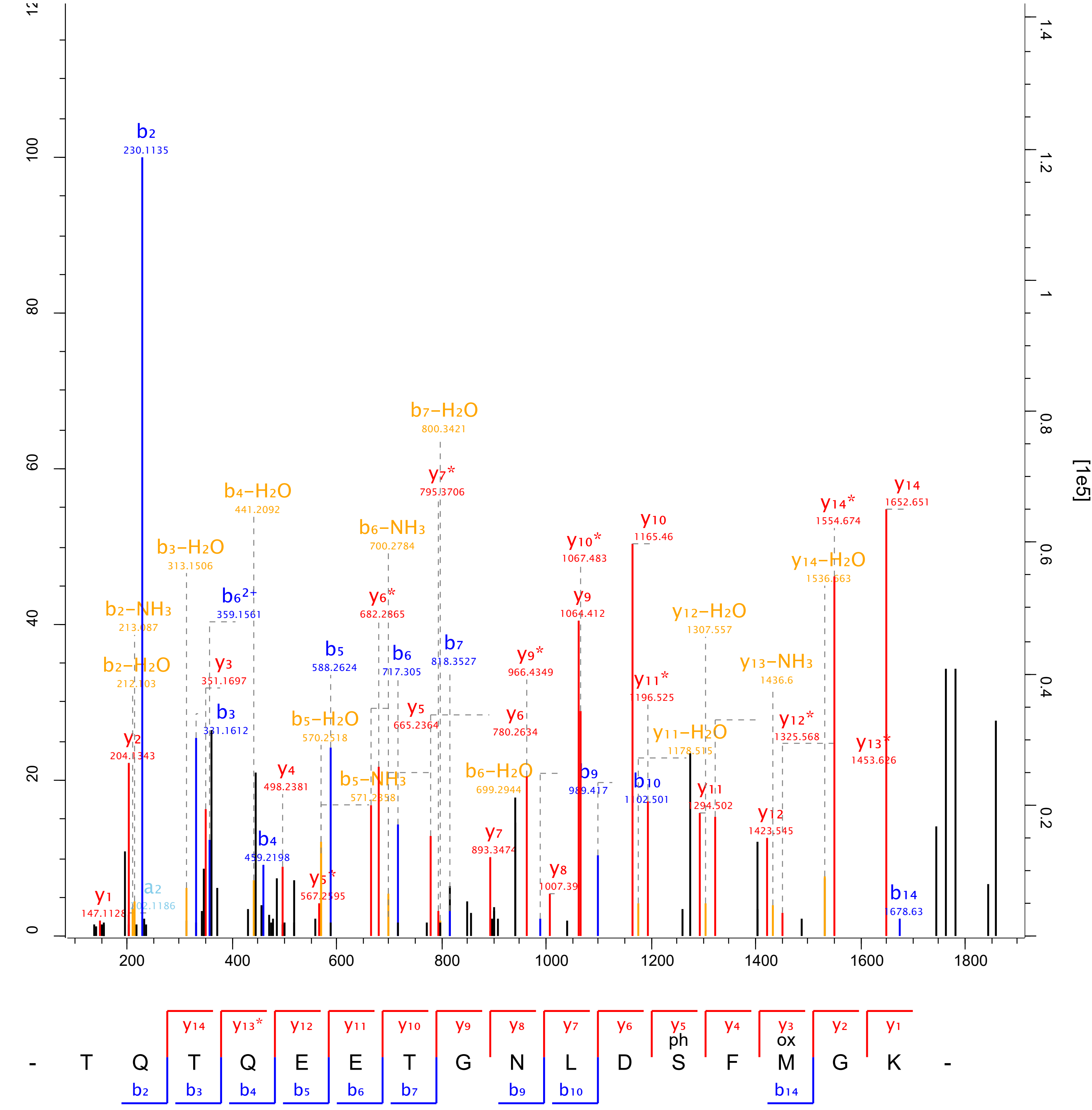

Sample output to test PDF Combine only

Raw file	Scan	Method	Score	m/z	Gene names
0523_13	10753	FTMS; HCD	246.38	735.8	PME1

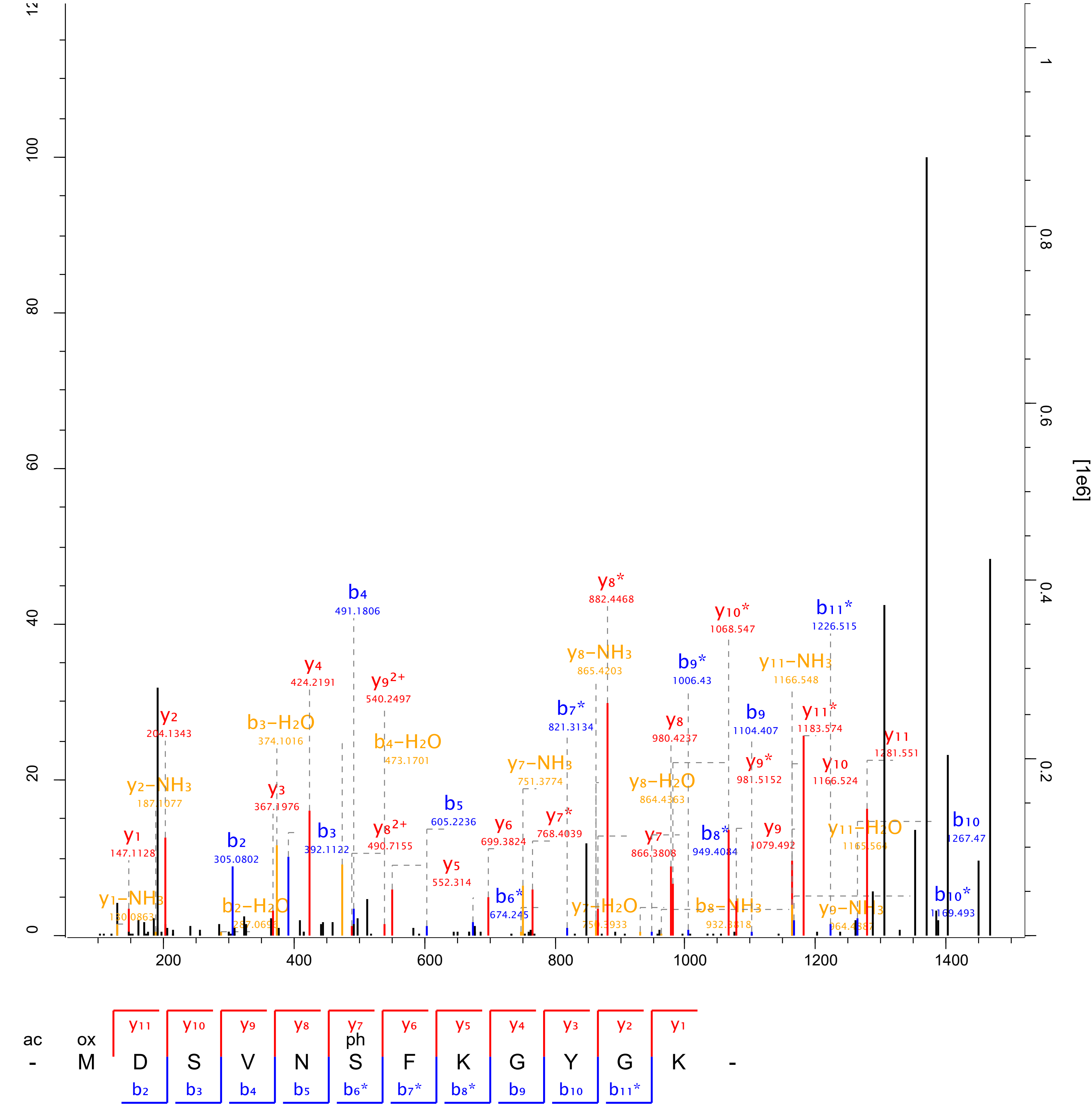

Sample output to test PDF Combine only

Raw file	Scan	Method	Score	m/z	Gene names
0523_13	10771	FTMS; HCD	198.98	831.88	PIP2-4

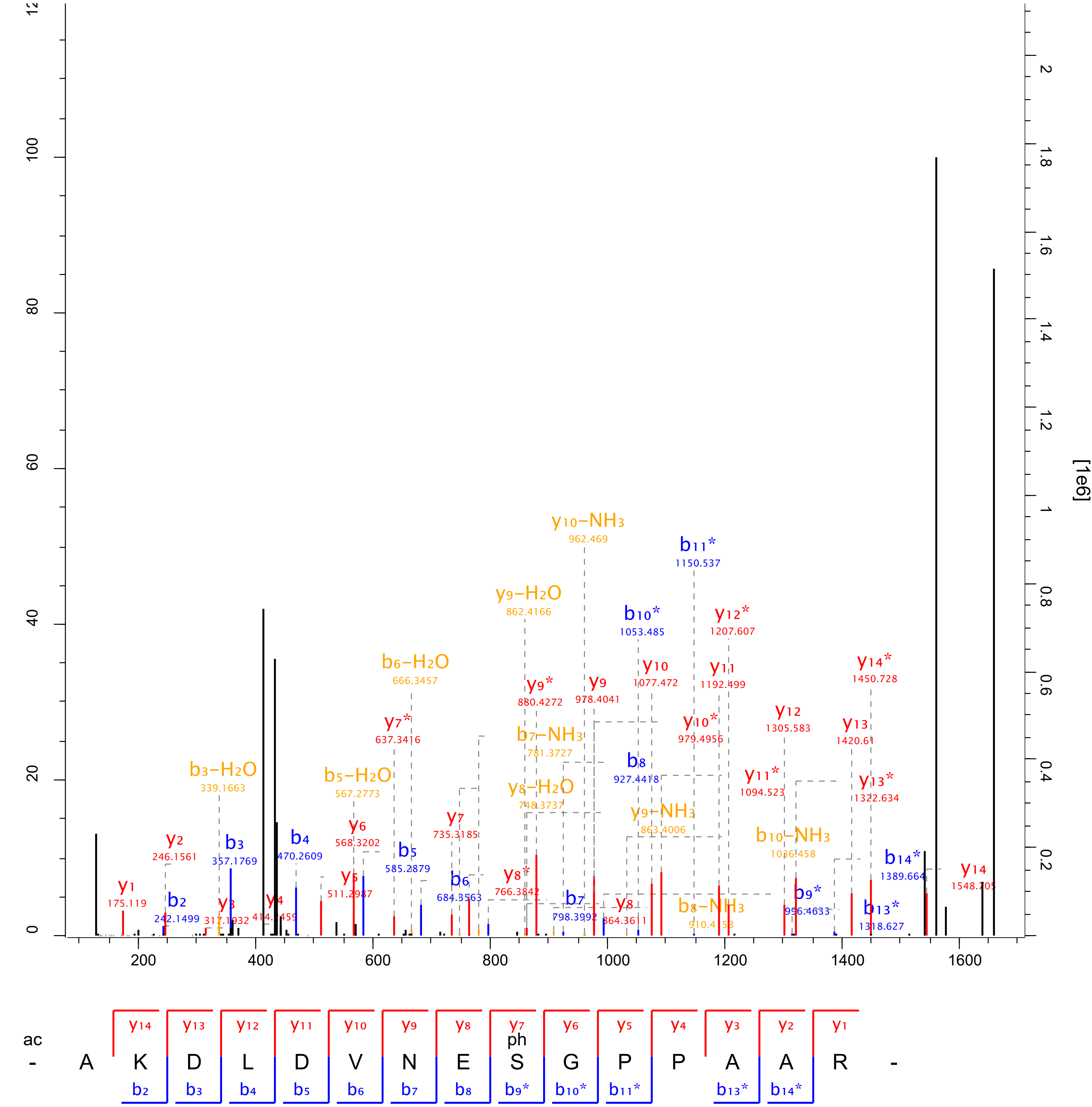

Sample output to test PDF Combine only

Raw file	Scan	Method	Score	m/z	Gene names
0523_13	10889	FTMS; HCD	199.98	649.25	PIP2-7

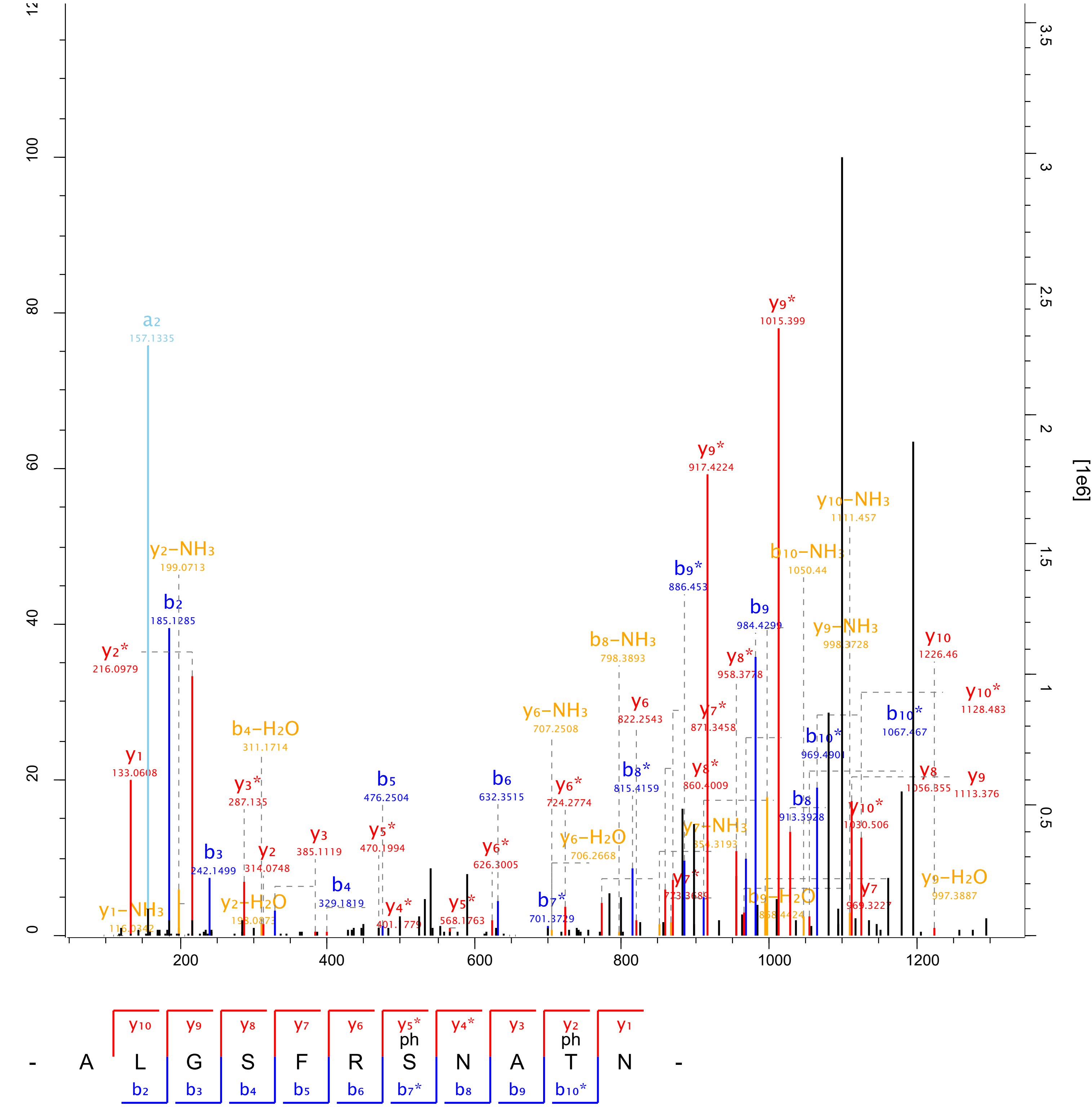

Sample output to test PDF Combine only

Raw file	Scan	Method	Score	m/z
0523_13	11348	FTMS; HCD	72.89	570.28

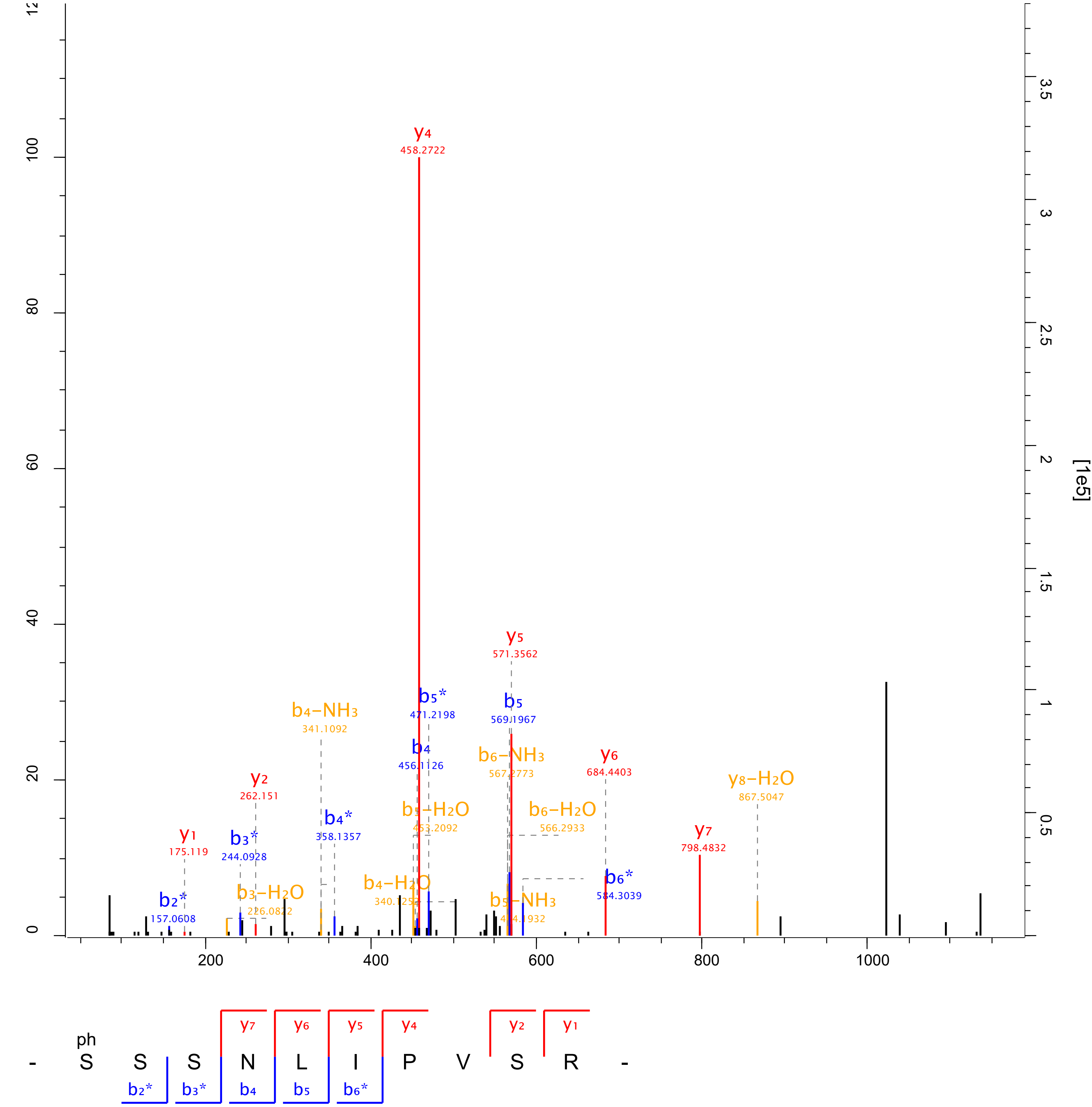

Sample output to test PDF Combine only

Raw file	Scan	Method	Score	m/z	Gene names
0523_13	12677	FTMS; HCD	428.89	1045.97	PUP18

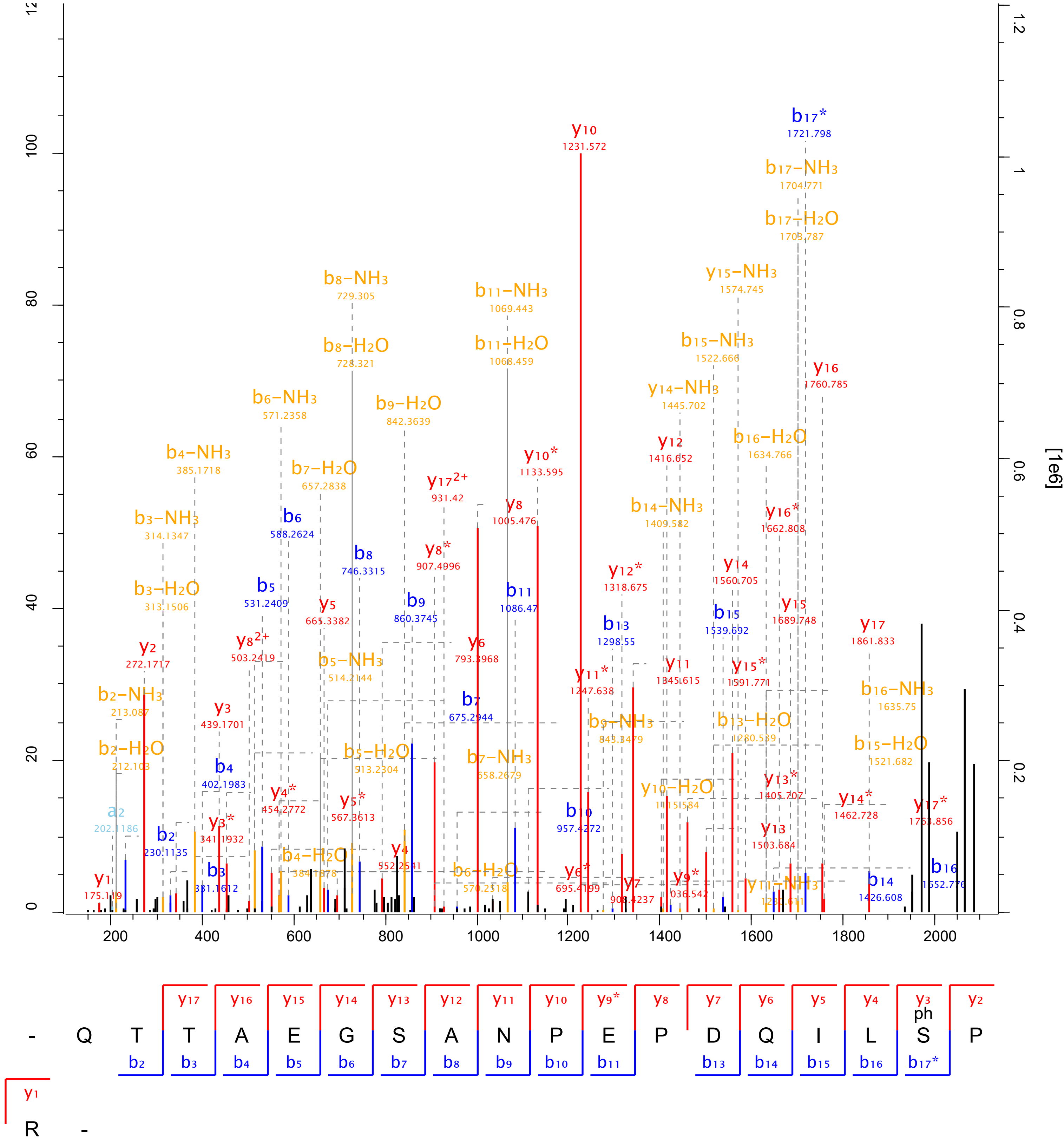

Sample output to test PDF Combine only

Raw file	Scan	Method	Score	m/z	Gene names
0523_13	12736	FTMS; HCD	167.61	611.81	COPT5

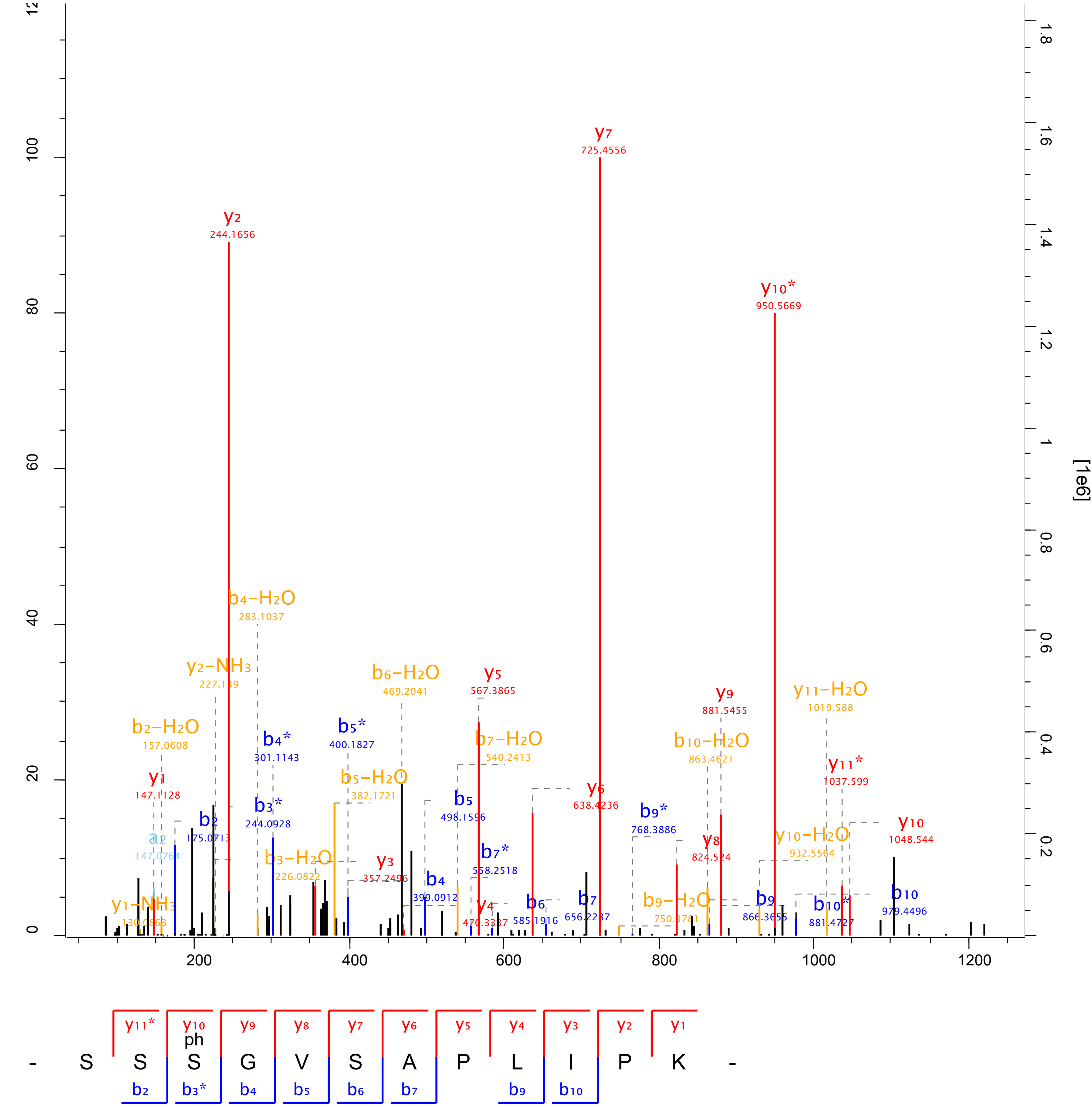

Sample output to test PDF Combine only

Raw file	Scan	Method	Score	m/z	Gene names
0523_13	13182	FTMS; HCD	150.12	693.33	At1g74910;F9E10.24

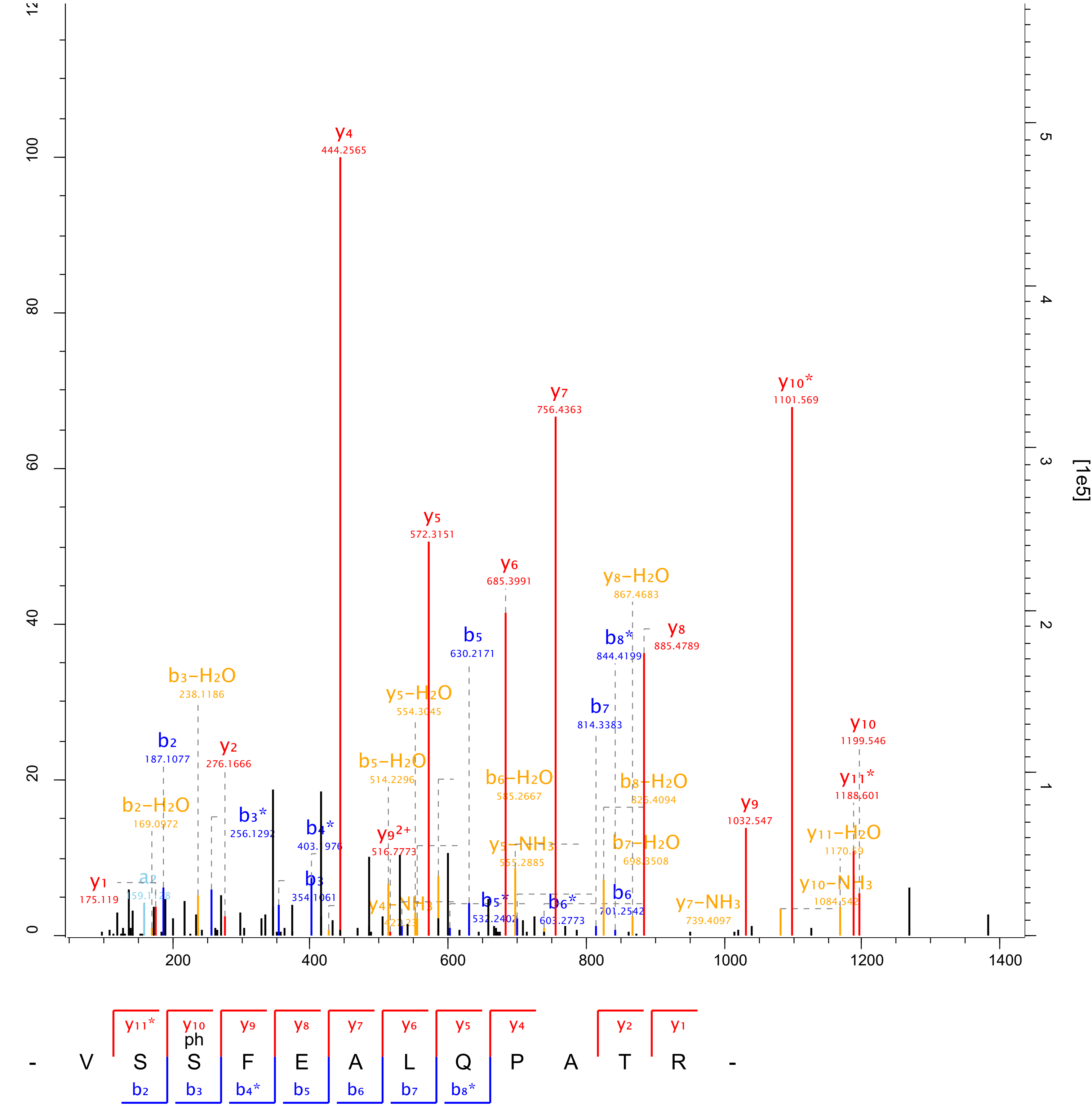

Raw file	Scan	Method	Score	m/z	Gene names
0523_13	13521	FTMS; HCD	99.04	718.32	T17J13.200

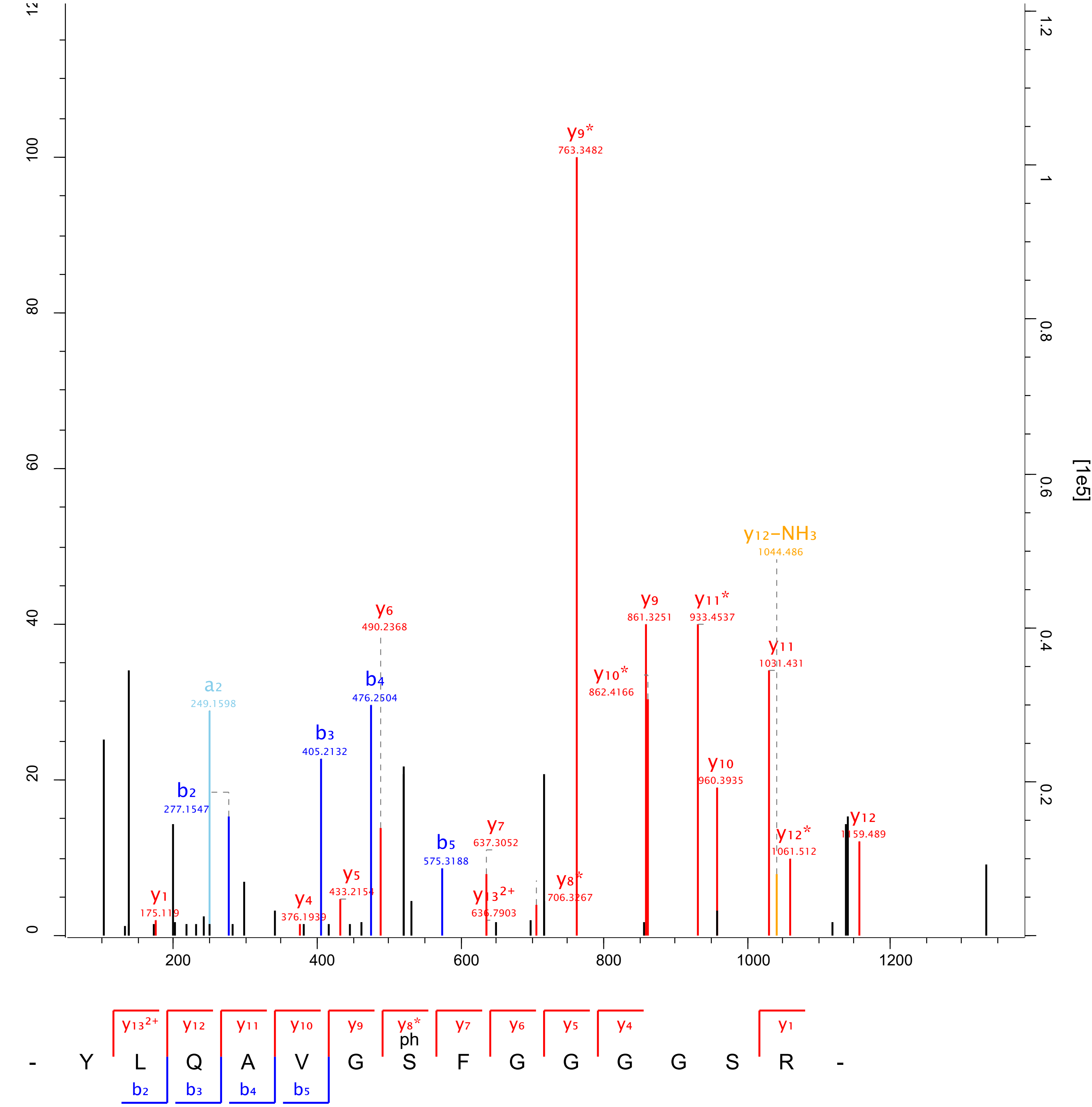

Sample output to test PDF Combine only

Raw file	Scan	Method	Score	m/z	Gene names
0523_13	13780	FTMS; HCD	119.54	718.32	SYP123

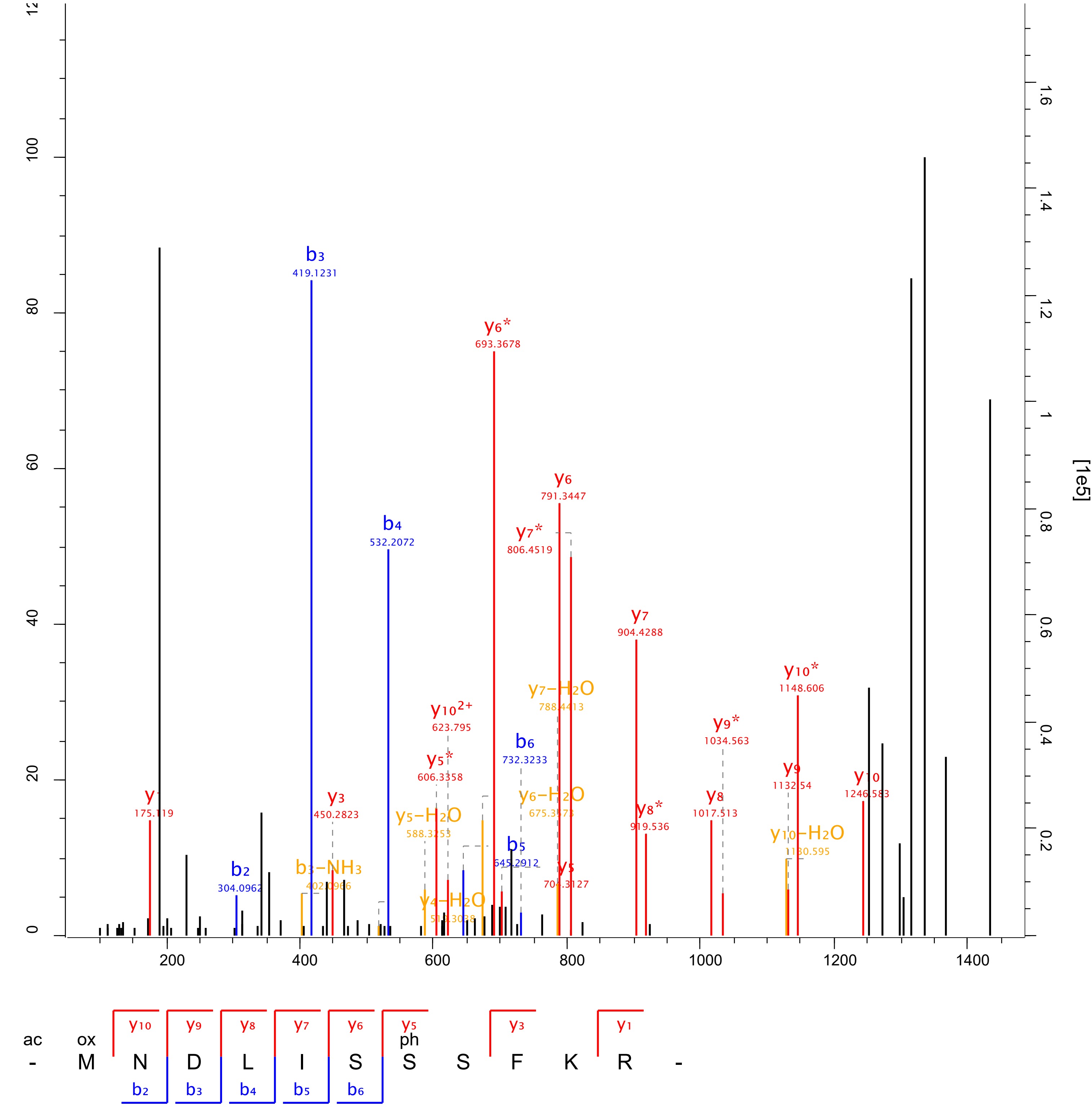

Sample output to test PDF Combine only

Raw file	Scan	Method	Score	m/z	Gene names
0523_13	13875	FTMS; HCD	112.17	750.31	VHA-B3;VHA-B1

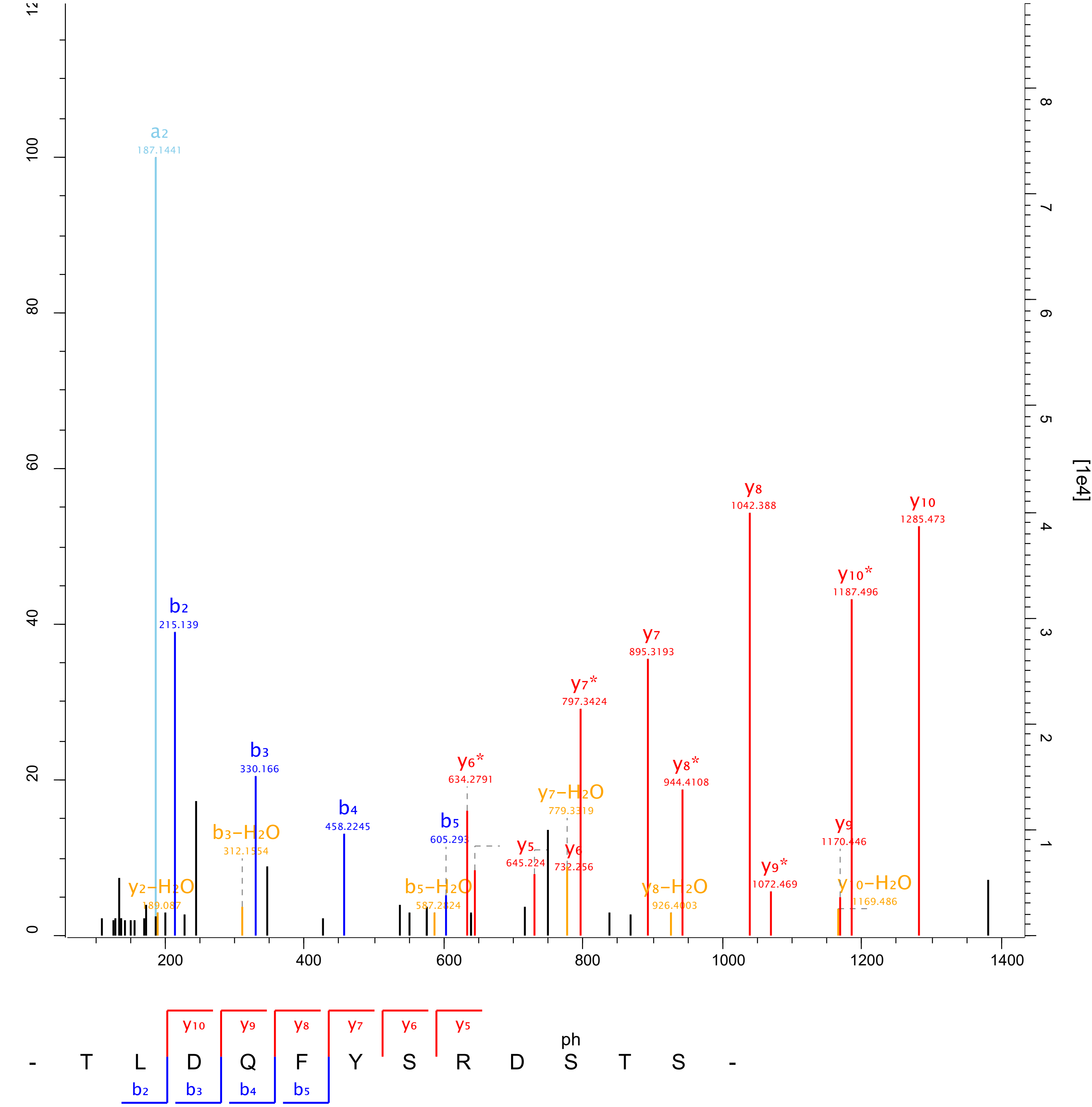

Sample output to test PDF Combine only

Raw file	Scan	Method	Score	m/z	Gene names
0523_13	14103	FTMS; HCD	101.9	724.8	RFS5

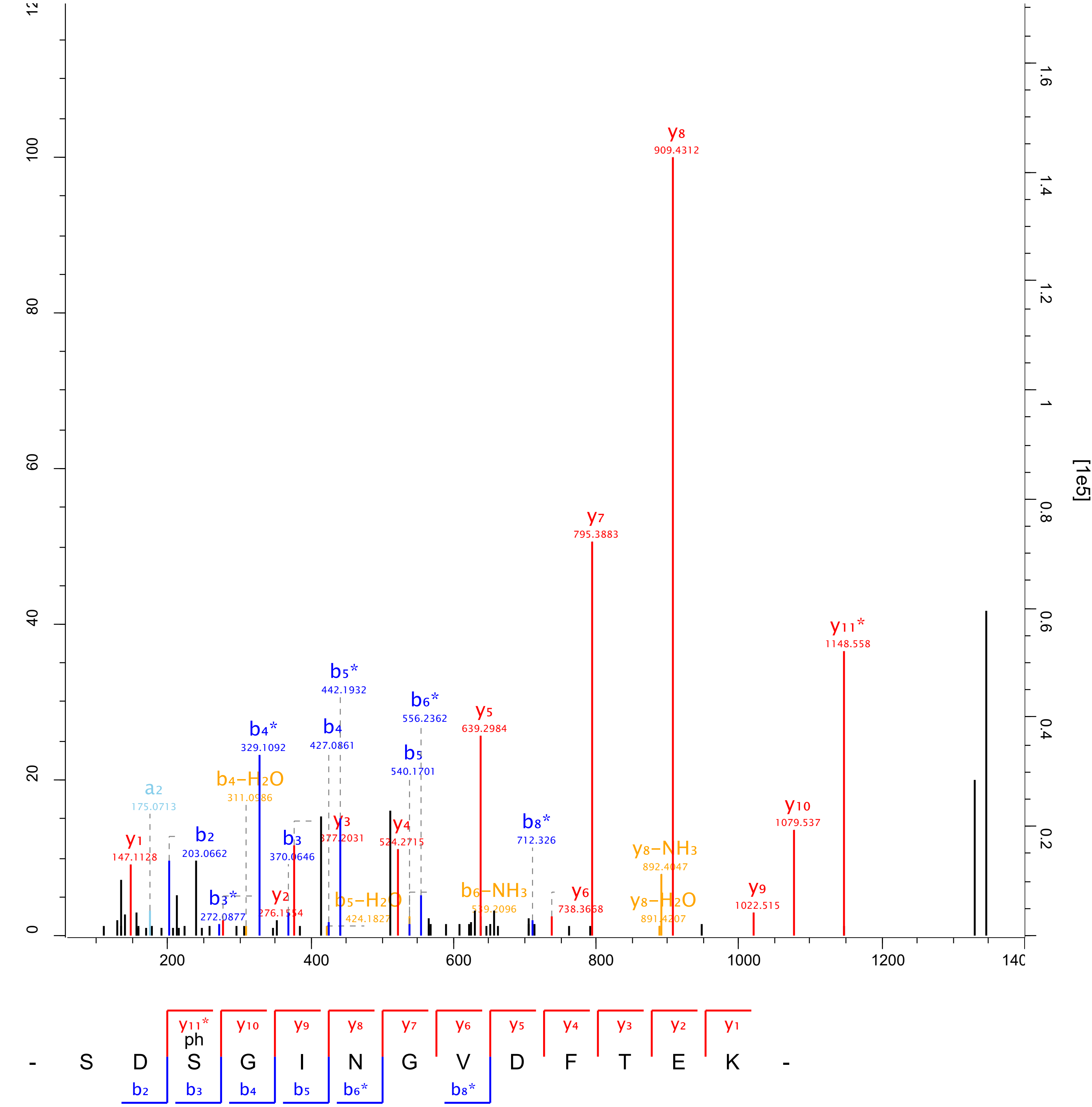

Sample output to test PDF Combine only

Raw file	Scan	Method	Score	m/z	Gene names
0523_13	15456	FTMS; HCD	176.29	727.36	LRR-RLK

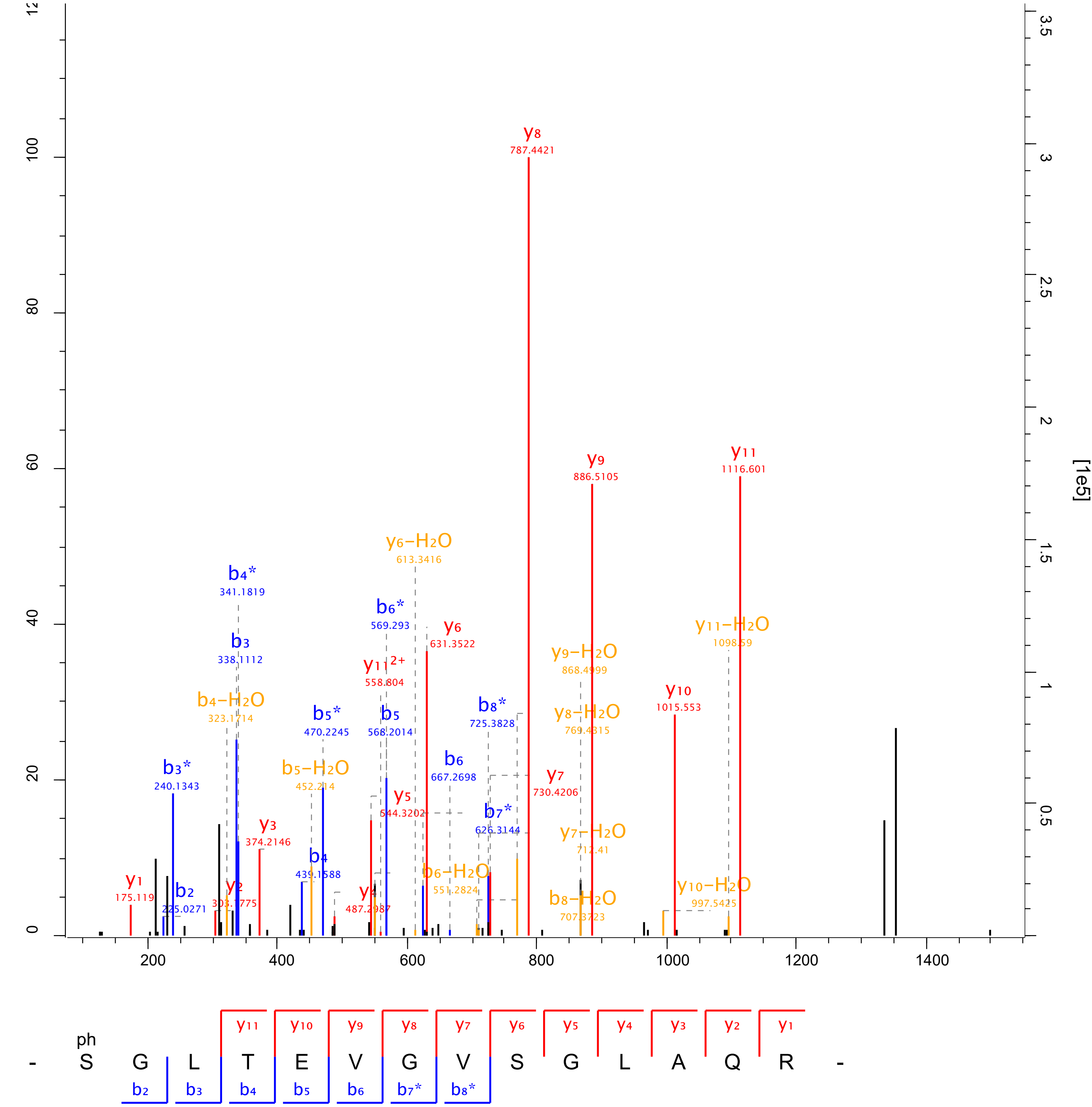

Sample output to test PDF Combine only

Raw file	Scan	Method	Score	m/z	Gene names
0523_13	15480	FTMS; HCD	104.88	806.36	At1g03740

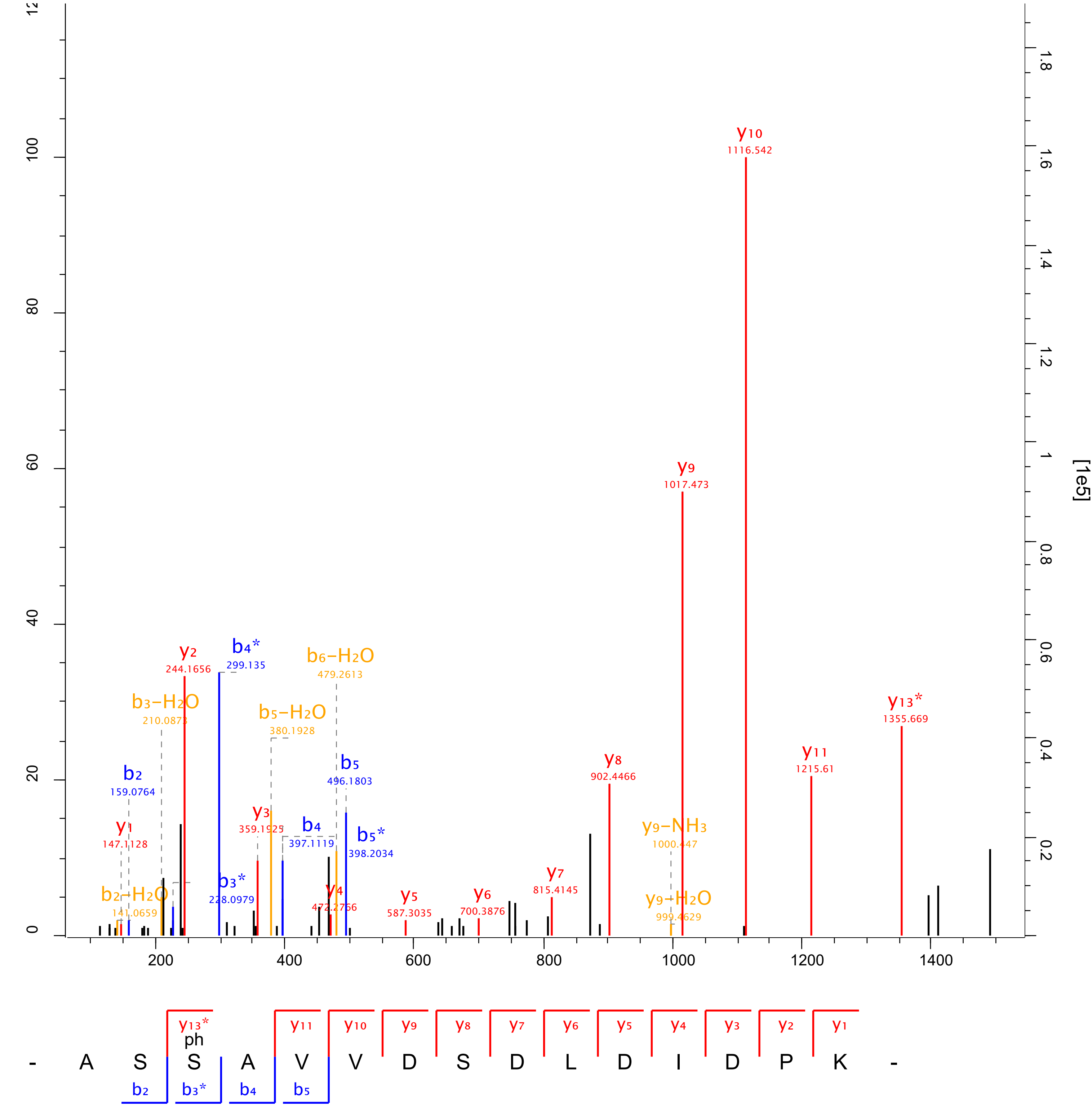

Sample output to test PDF Combine only

Raw file	Scan	Method	Score	m/z	Gene names
0523_13	17035	FTMS; HCD	68.42	1003.41	SNAP33

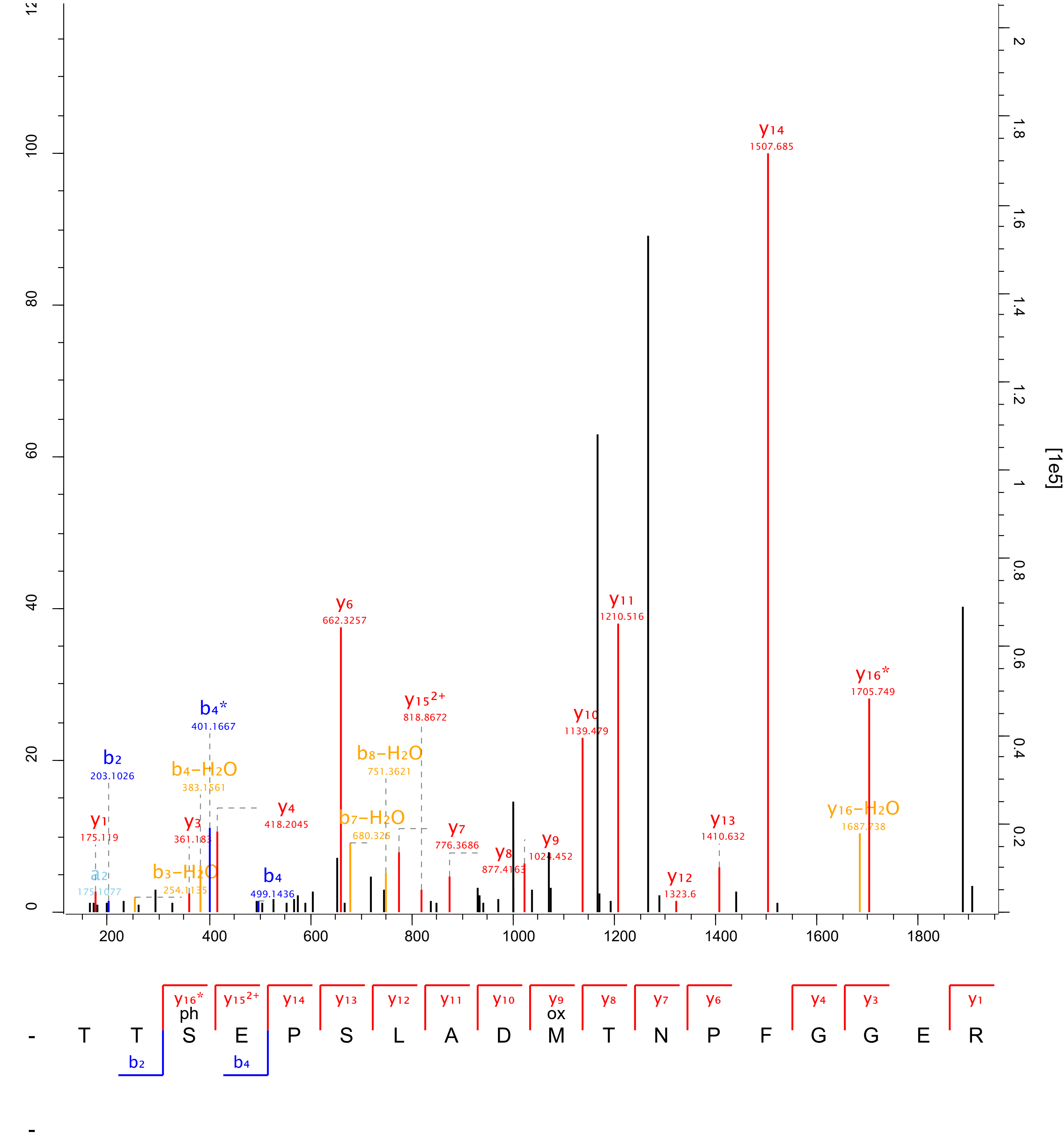

Sample output to test PDF Combine only

Raw file	Scan	Method	Score	m/z
0523_13	18488	FTMS; HCD	114.68	826.36

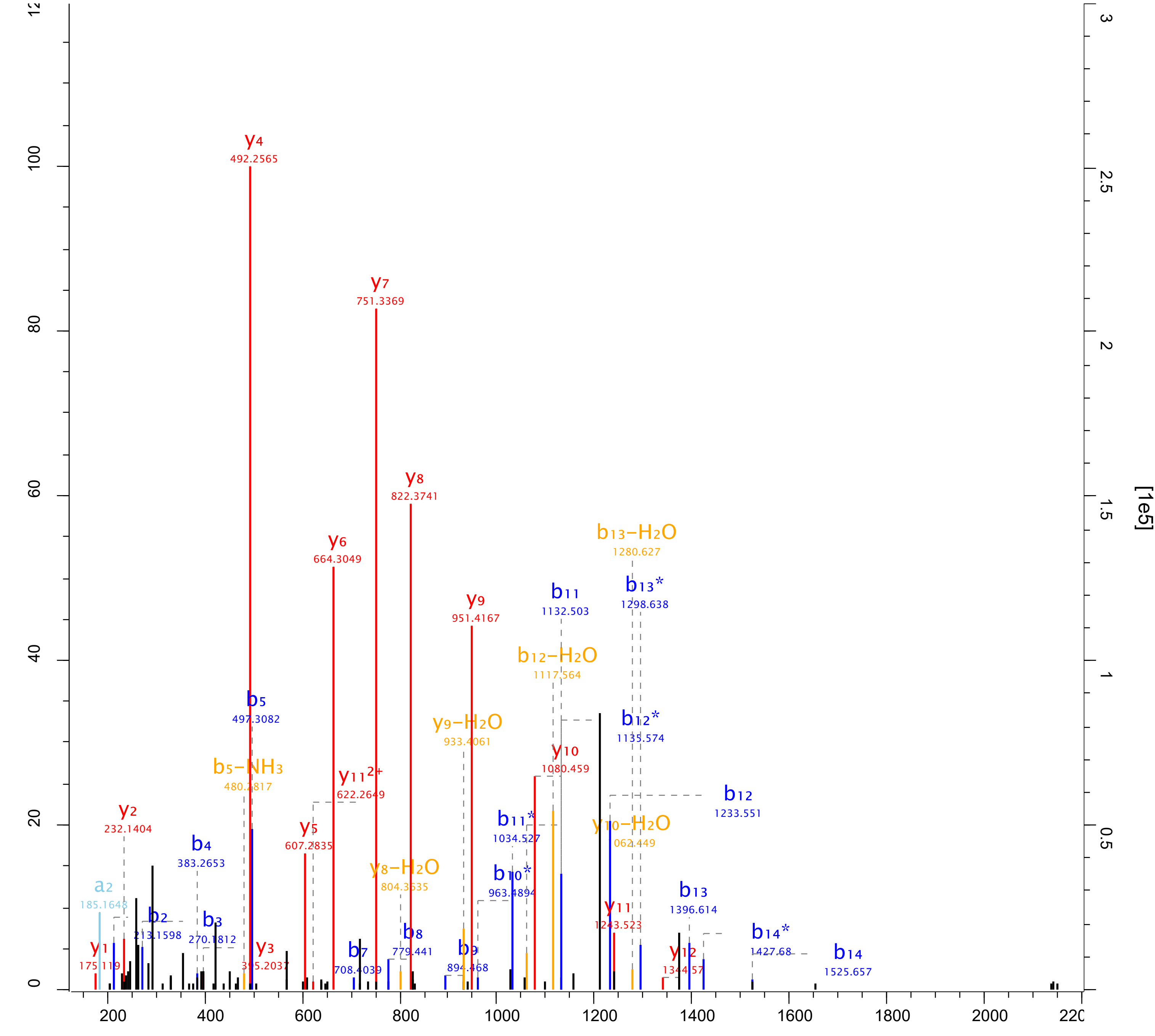

- L V G L N P N A D S(ph) A T Y E E A S G

b2 b3 b4 b5 b7 b8 b9 b10* b11 b12 b13 b14

y12 y11 y10 y9 y8 y7 y6

y5 y4 y3 y2 y1

D P Y G R -

Sample output to test PDF Combine only

Raw file	Scan	Method	Score	m/z
0523_13	19236	FTMS; HCD	138.91	757.36

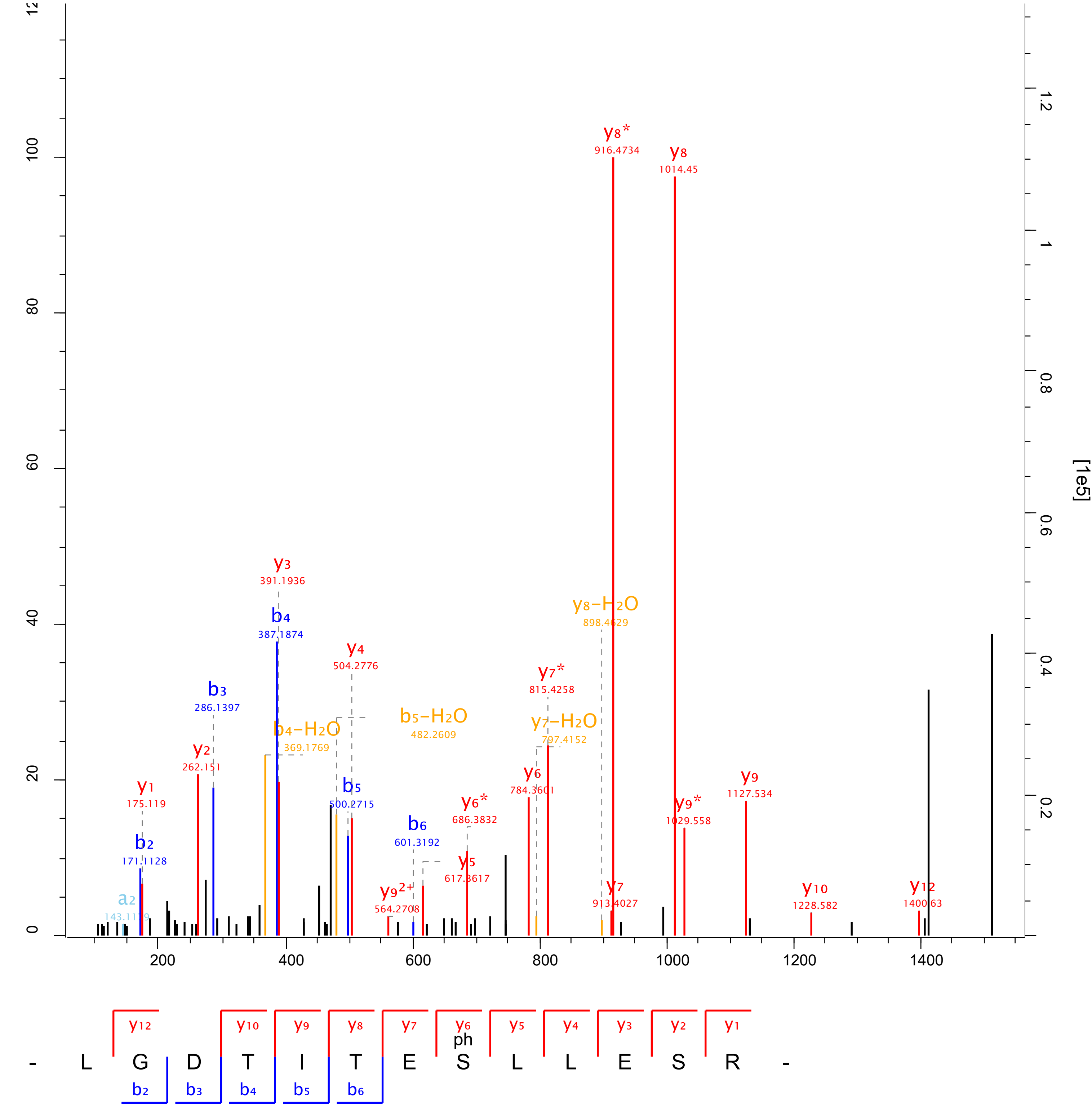

Sample output to test PDF Combine only

Raw file	Scan	Method	Score	m/z	Gene names
0523_13	20632	FTMS; HCD	68.64	757.85	At2g39130

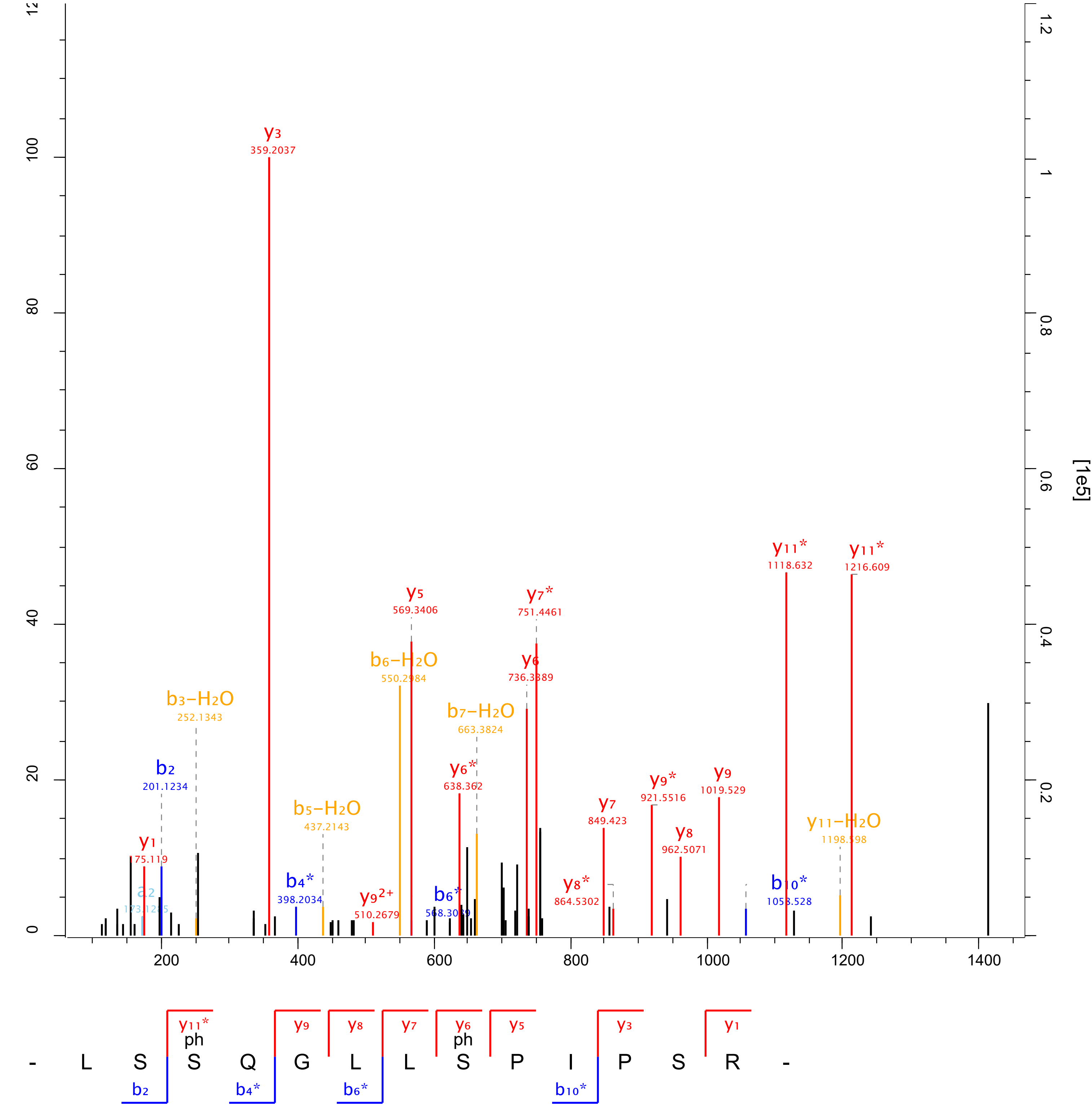

Sample output to test PDF Combine only

Raw file	Scan	Method	Score	m/z	Gene names
0523_13	20677	FTMS; HCD	111.22	797.89	At3g58640

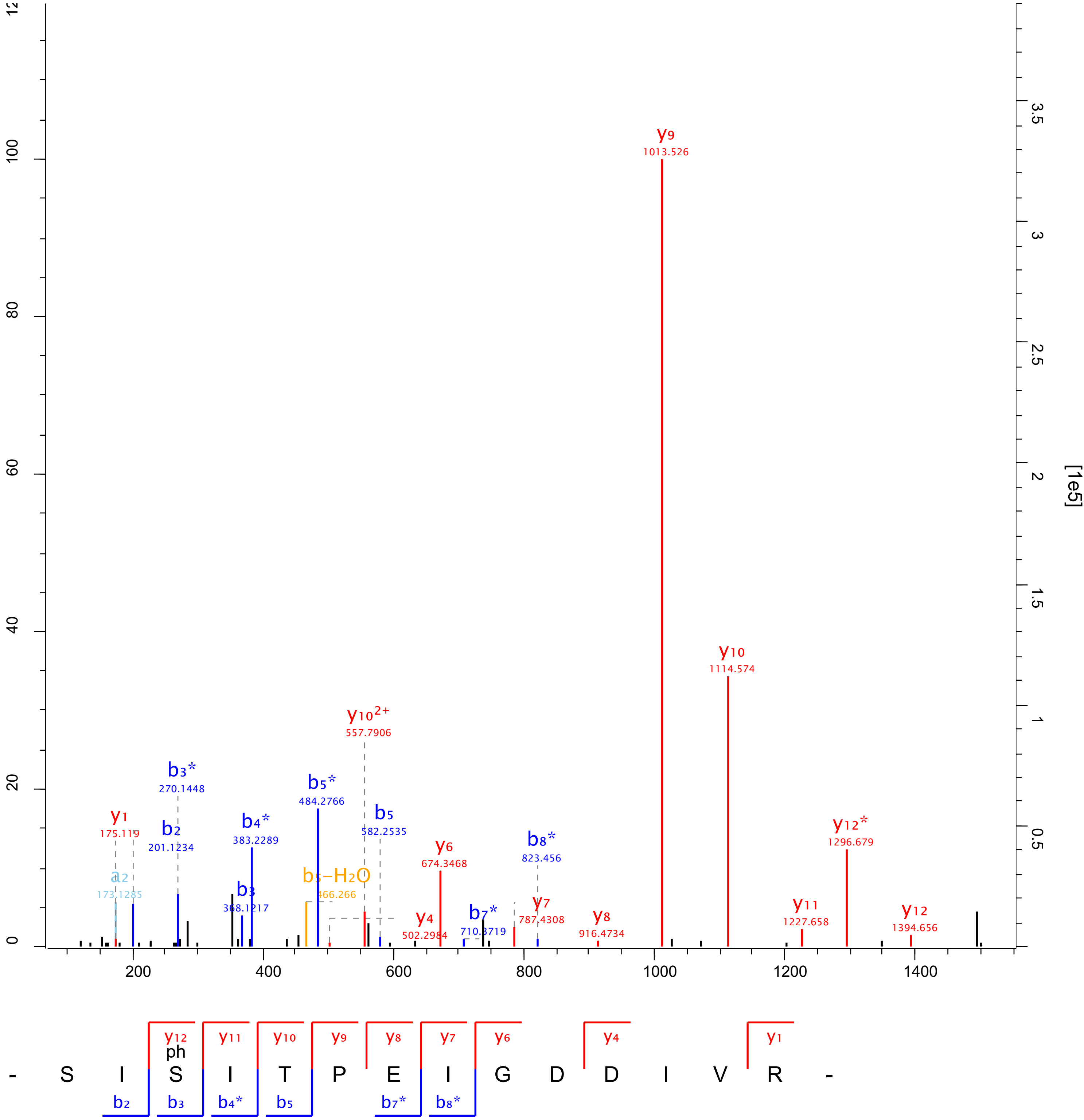

Sample output to test PDF Combine only

Raw file	Scan	Method	Score	m/z	Gene names
0523_13	20912	FTMS; HCD	148.57	669.36	CLC-A;CLC-B

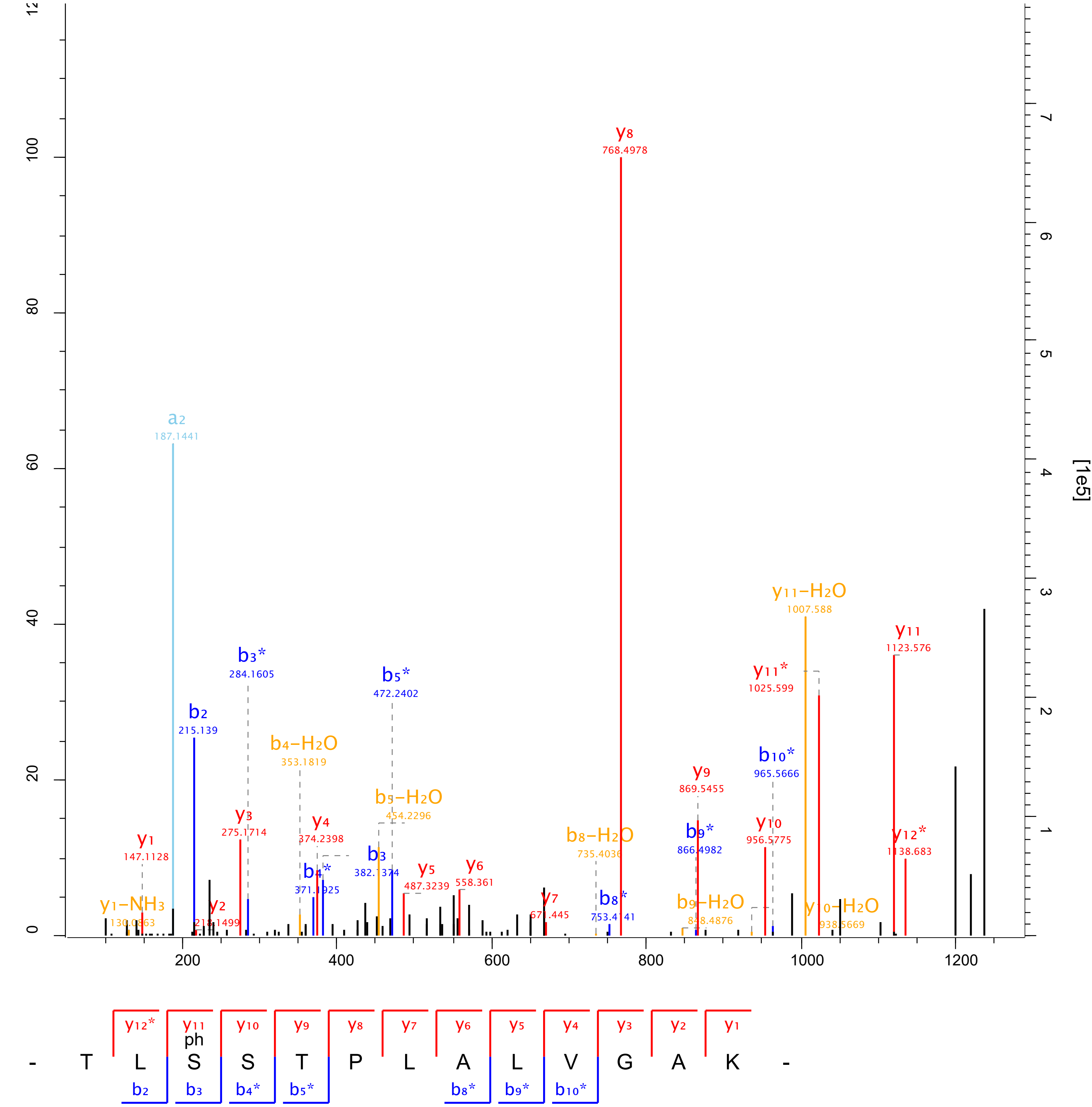

Sample output to test PDF Combine only

Raw file	Scan	Method	Score	m/z	Gene names
0523_13	21485	FTMS; HCD	113.7	1009.93	PLP1

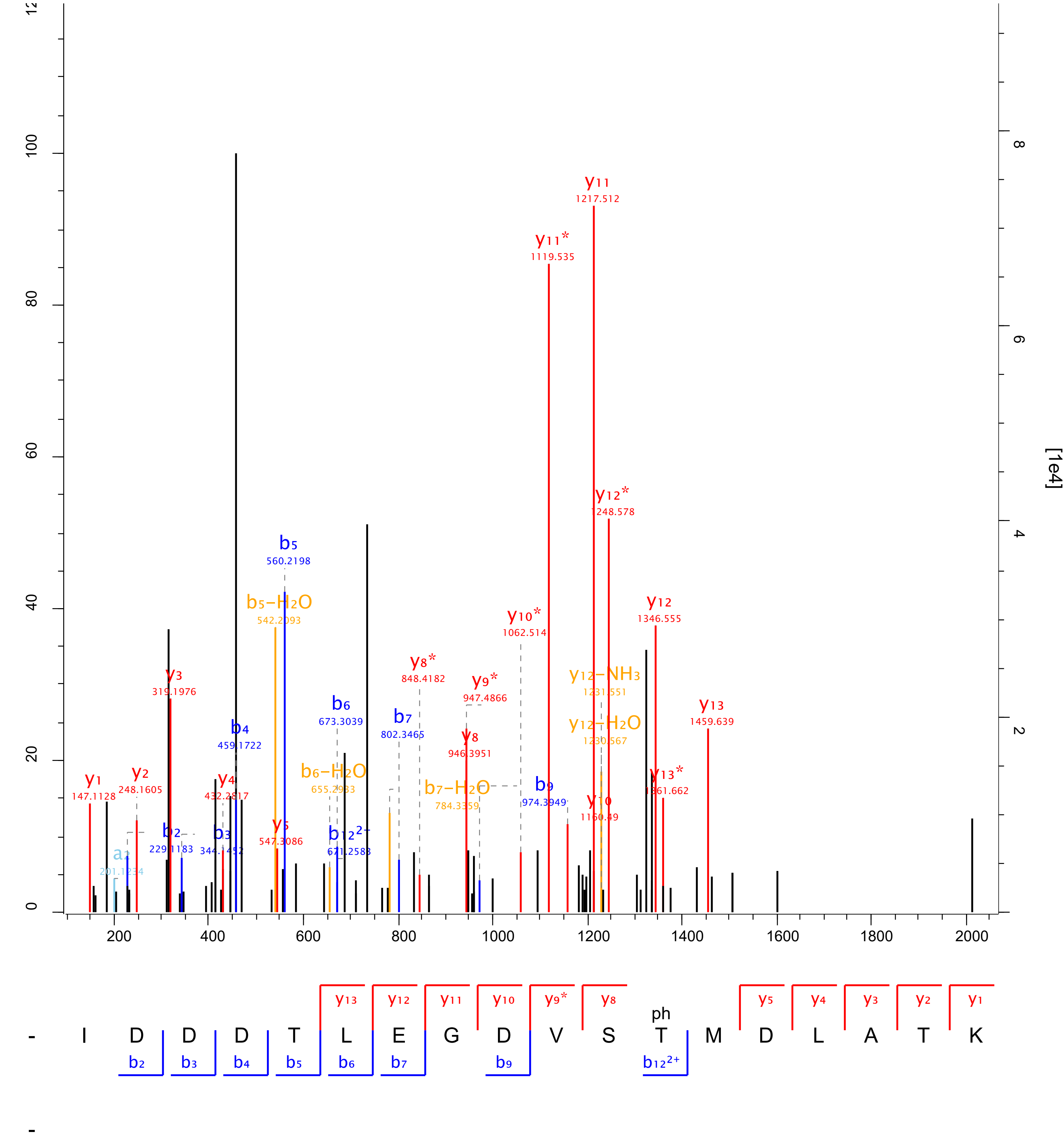

Sample output to test PDF Combine only

Raw file	Scan	Method	Score	m/z
0523_13	31682	FTMS; HCD	87.88	829.08

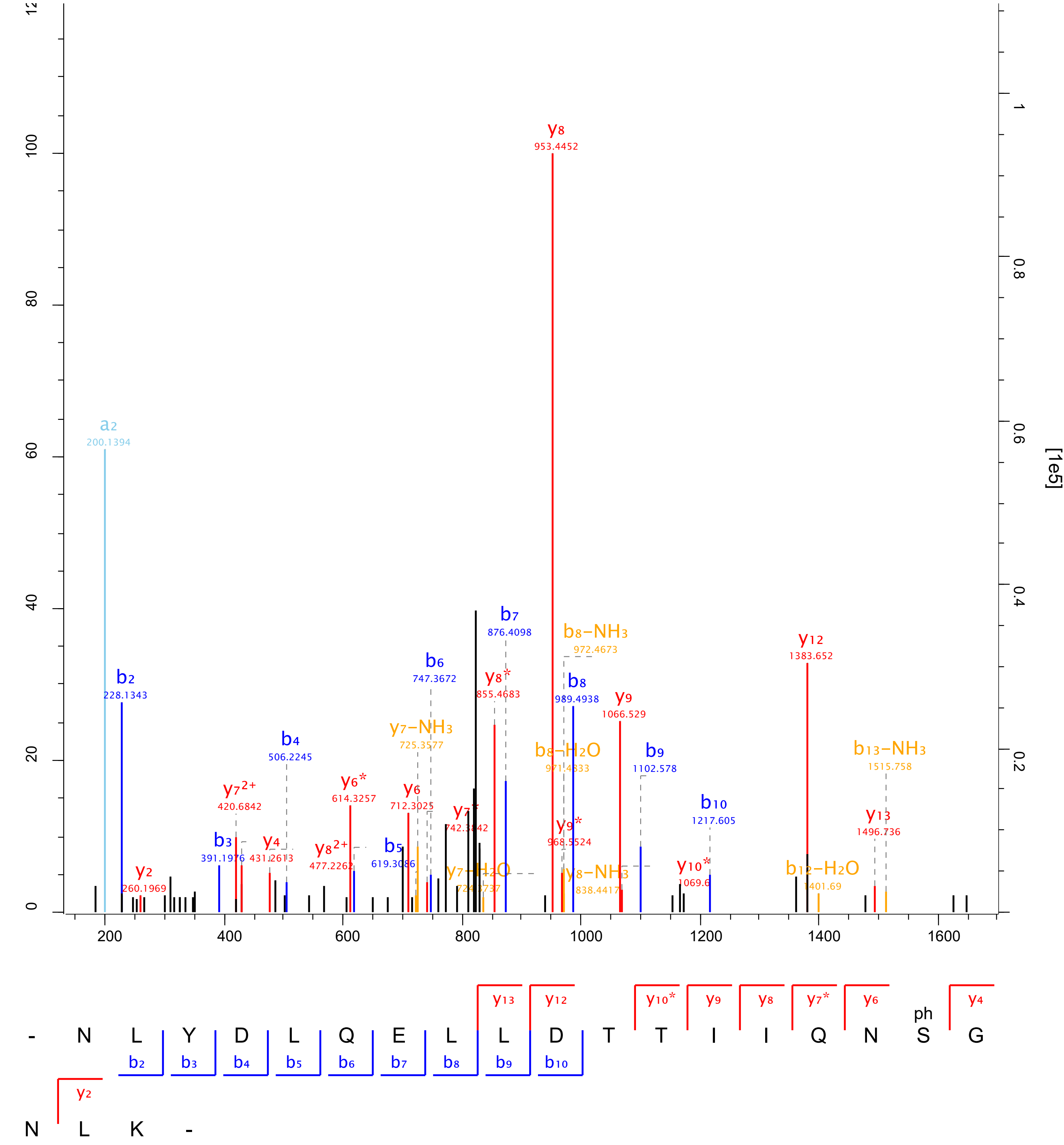

Sample output to test PDF Combine only

Raw file	Scan	Method	Score	m/z	Gene names
0523_13	31704	FTMS; HCD	226.81	889.32	PIP2-4

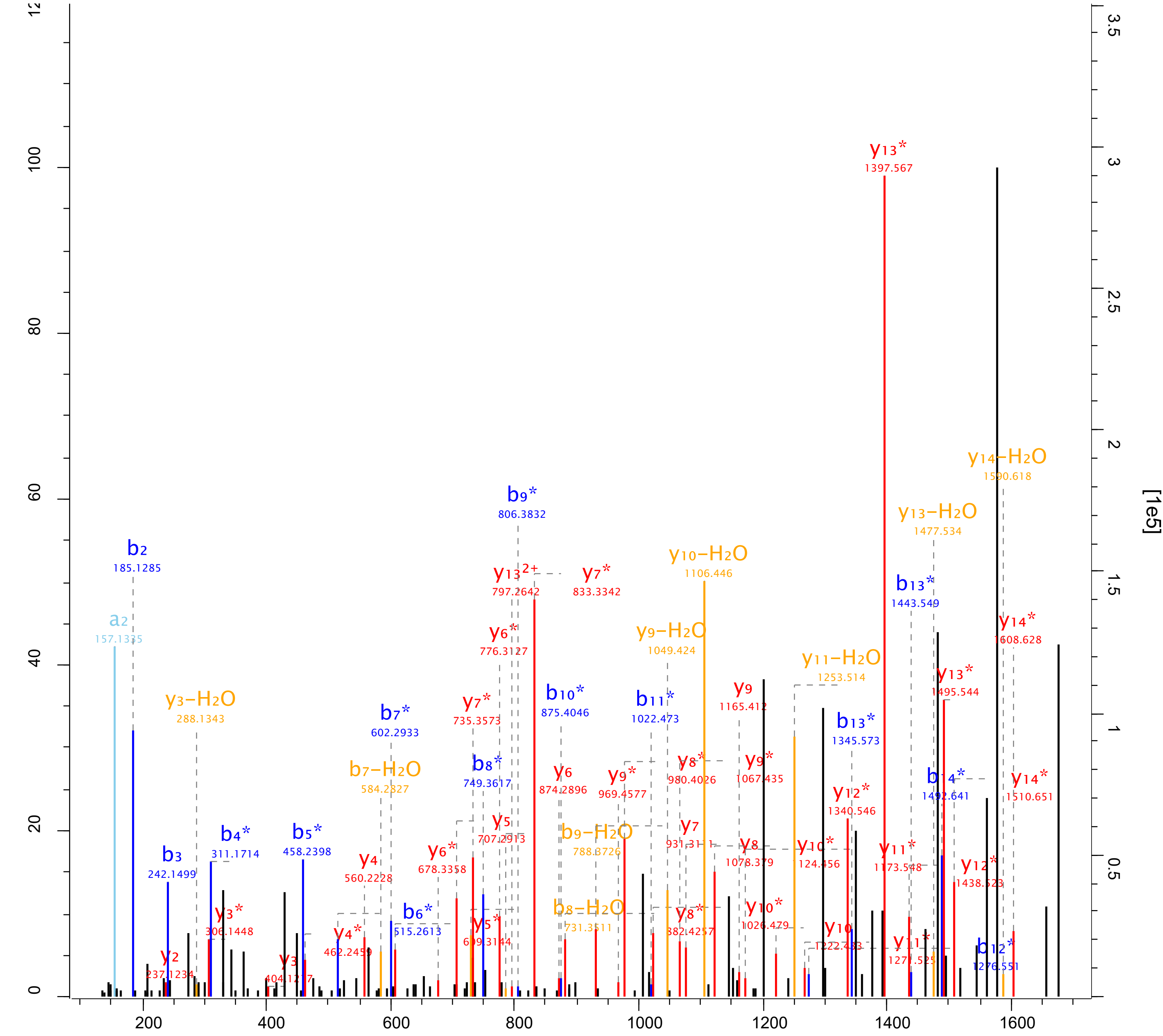

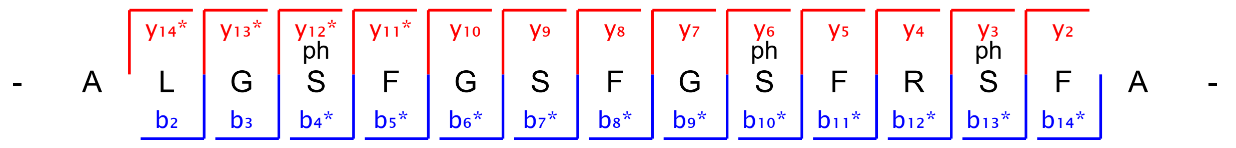

Sample output to test PDF Combine only

Raw file	Scan	Method	Score	m/z	Gene names
0523_14	1473	FTMS; HCD	70.78	521.9	RPL29A

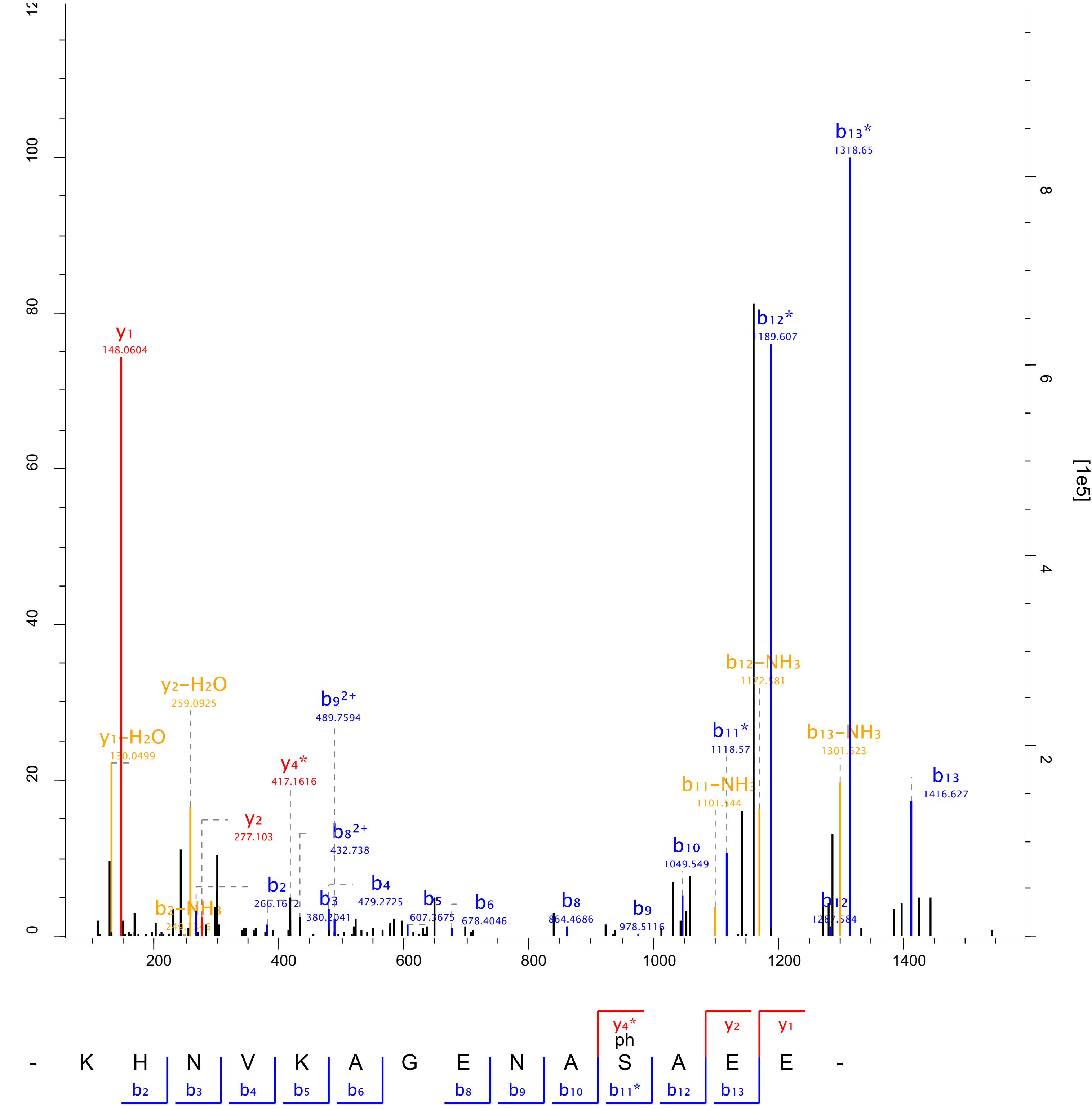

Sample output to test PDF Combine only

Raw file	Scan	Method	Score	m/z	Gene names
0523_14	1490	FTMS; HCD	43.59	523.91	At2g35880

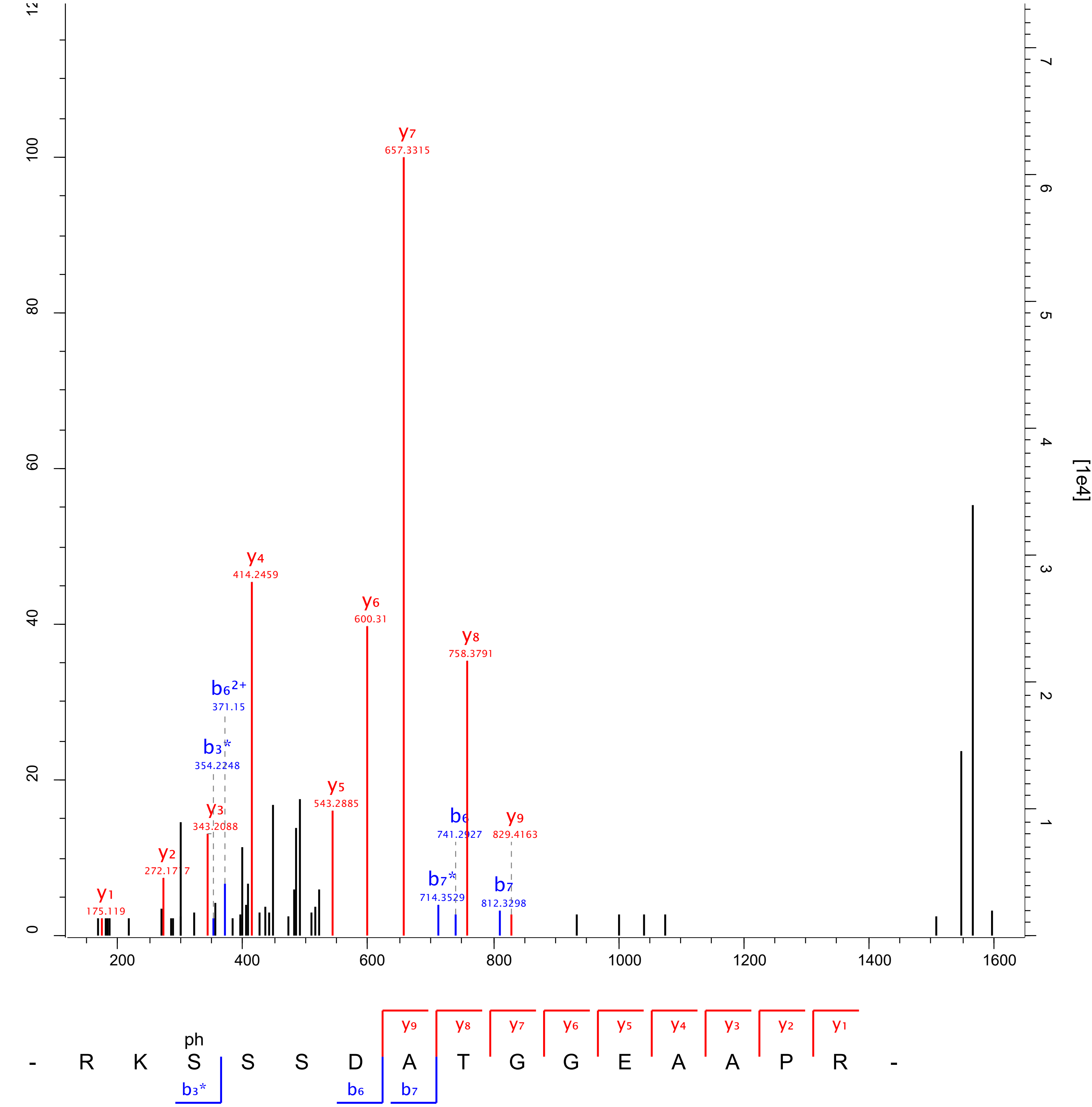

Sample output to test PDF Combine only

Raw file	Scan	Method	Score	m/z	Gene names
0523_14	1500	FTMS; HCD	96.34	339.82	INVD;CINV1;CINV2

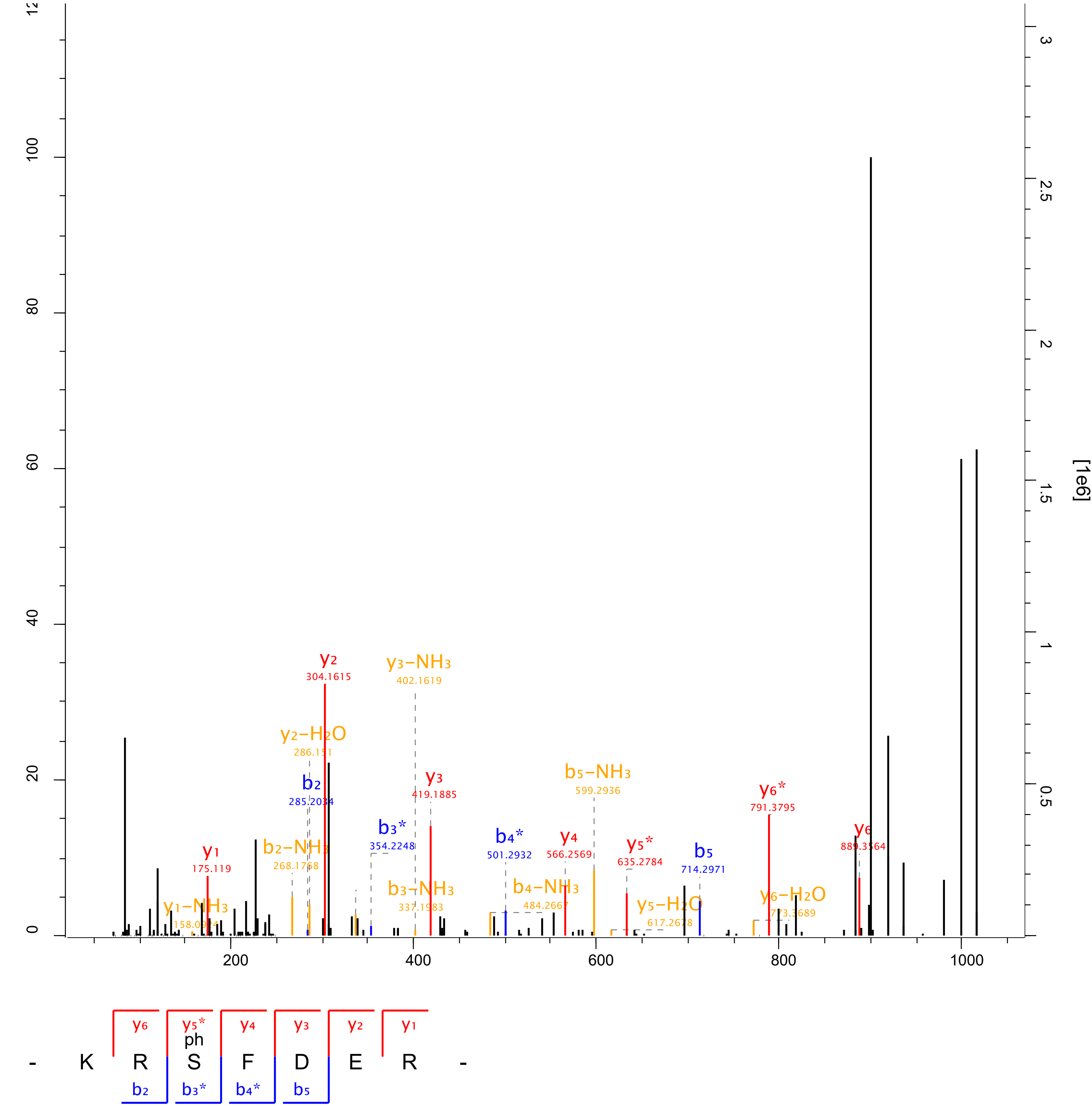

Sample output to test PDF Combine only

Raw file	Scan	Method	Score	m/z	Gene names
0523_14	9235	FTMS; HCD	49.99	615.62	At5g41950

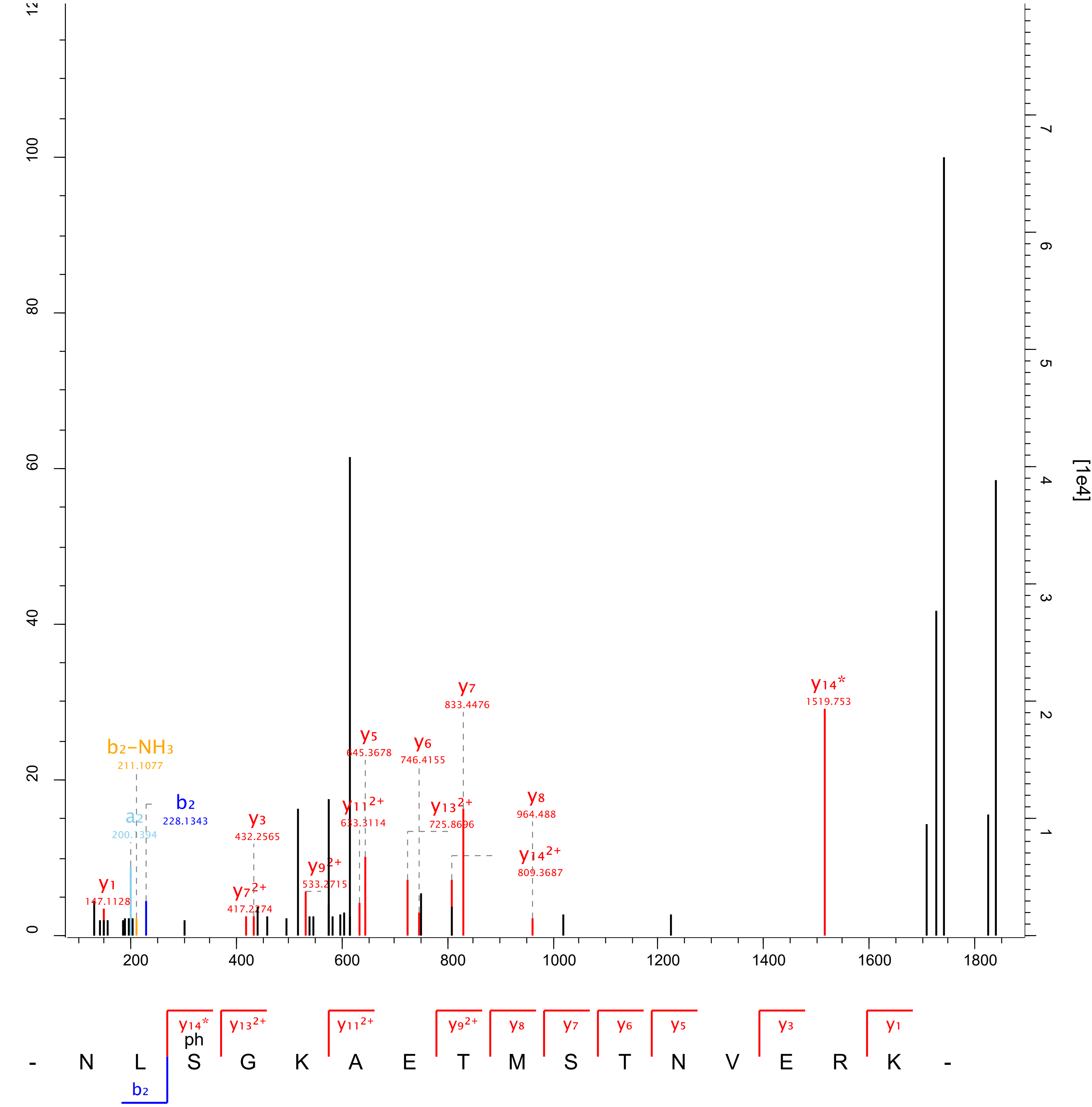

Sample output to test PDF Combine only

Raw file	Scan	Method	Score	m/z	Gene names
0523_14	12185	FTMS; HCD	131.03	939.17	PPI1

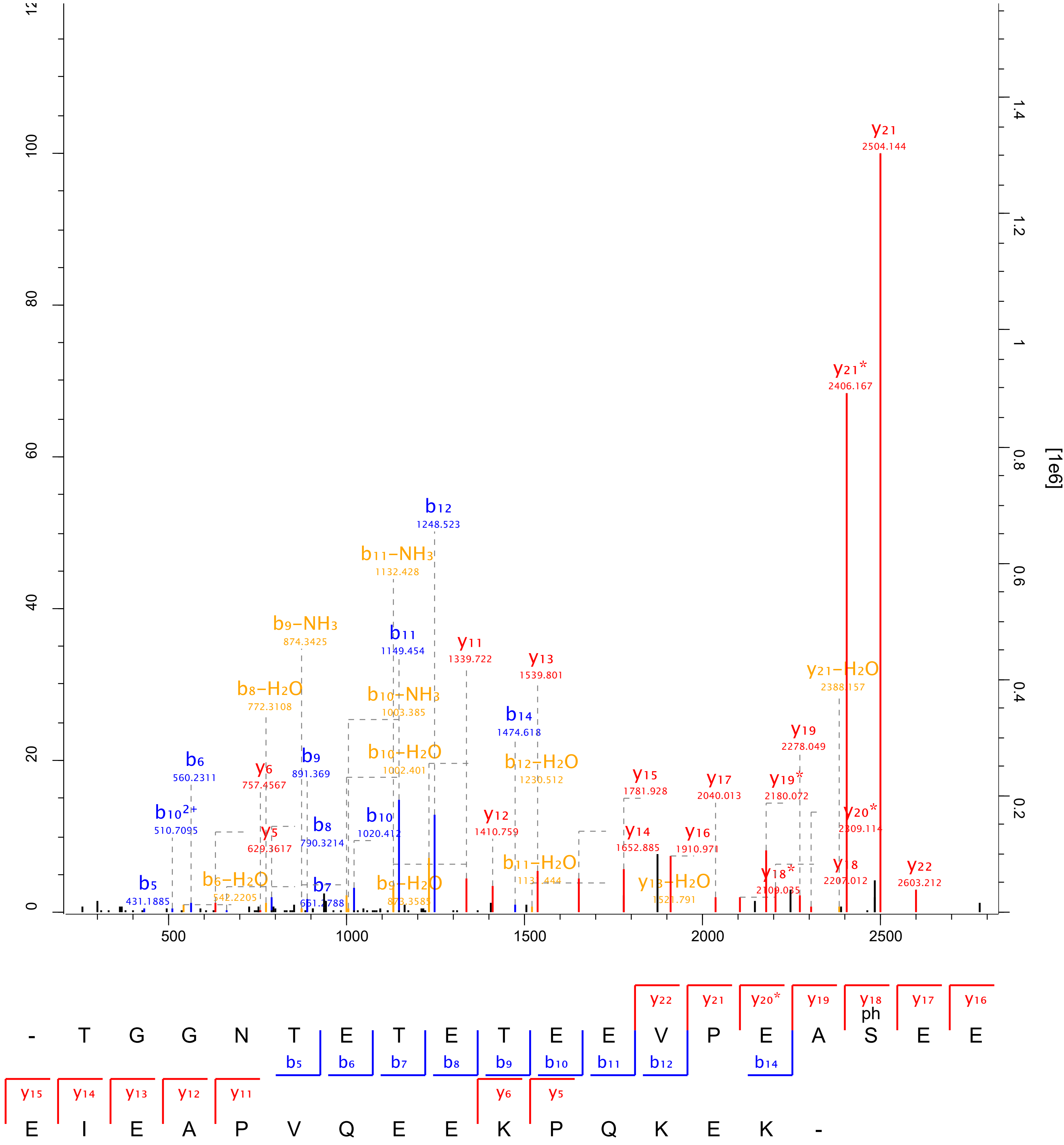

Sample output to test PDF Combine only

Raw file	Scan	Method	Score	m/z	Gene names
0523_14	12626	FTMS; HCD	60.42	734.35	SKIP

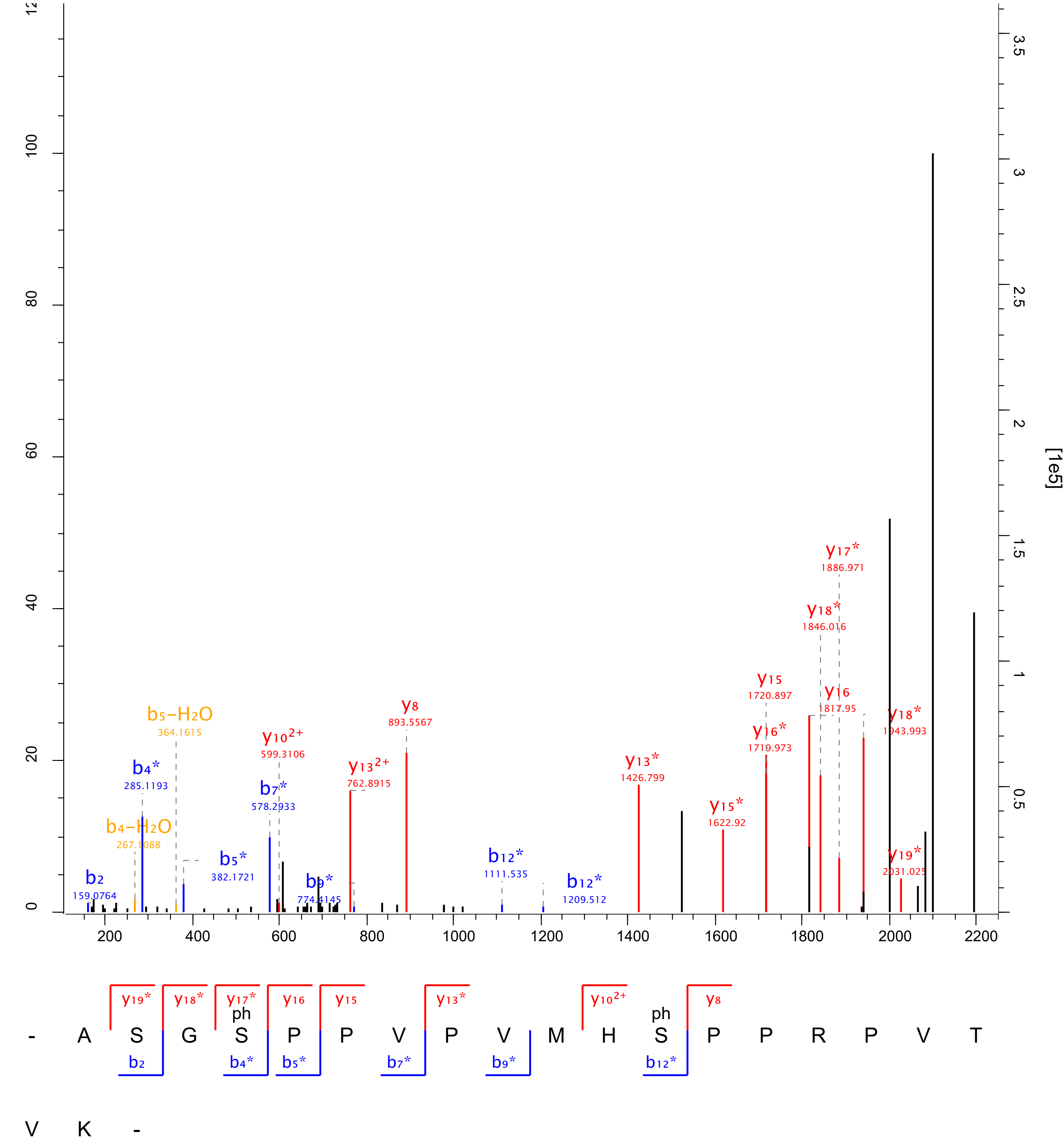

Sample output to test PDF Combine only

Raw file	Scan	Method	Score	m/z	Gene names
0523_14	13516	FTMS; HCD	55.78	839.36	At1g14170;F7A19.25

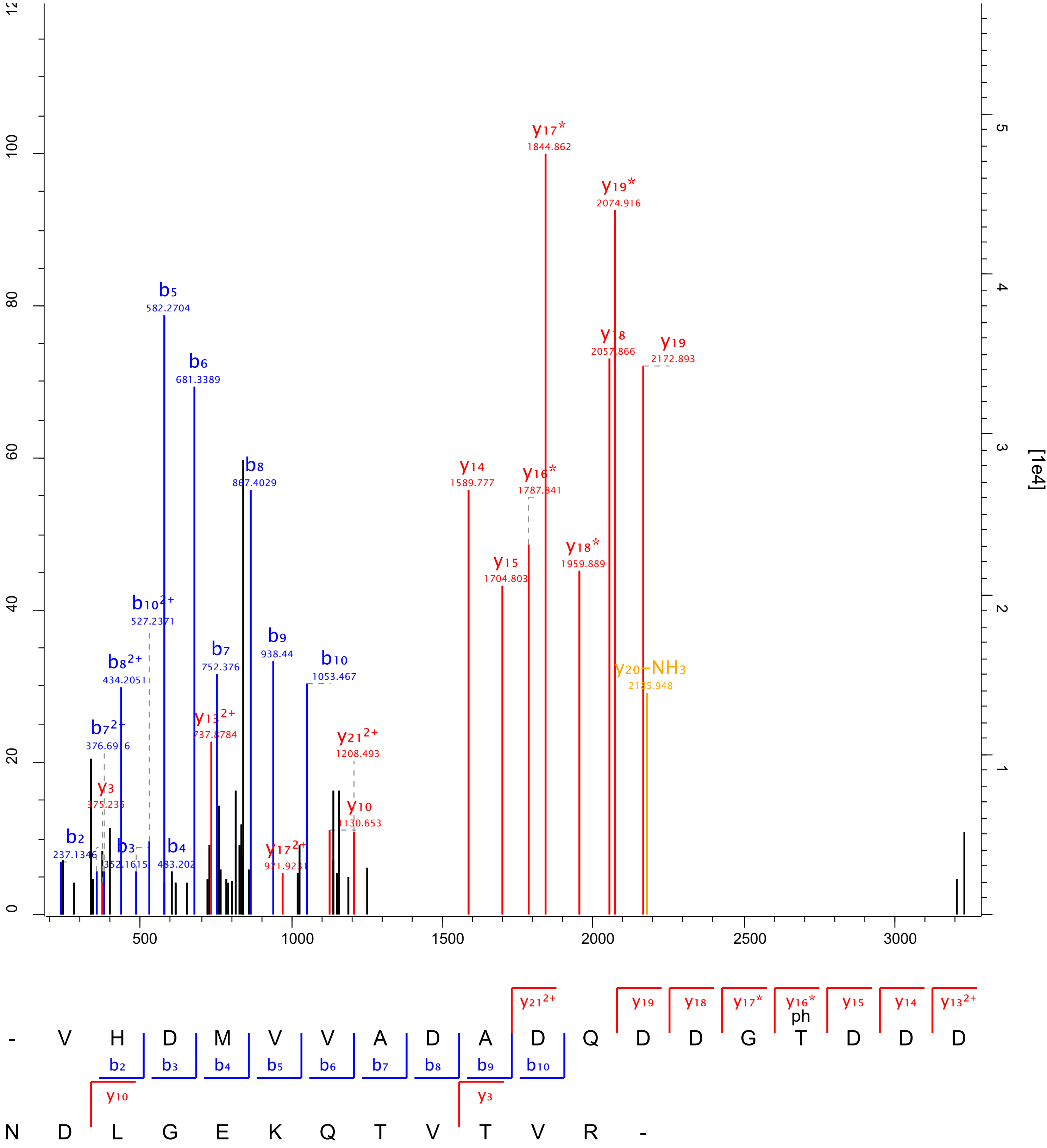

Sample output to test PDF Combine only

Raw file	Scan	Method	Score	m/z
0523_14	13924	FTMS; HCD	59.86	679.97

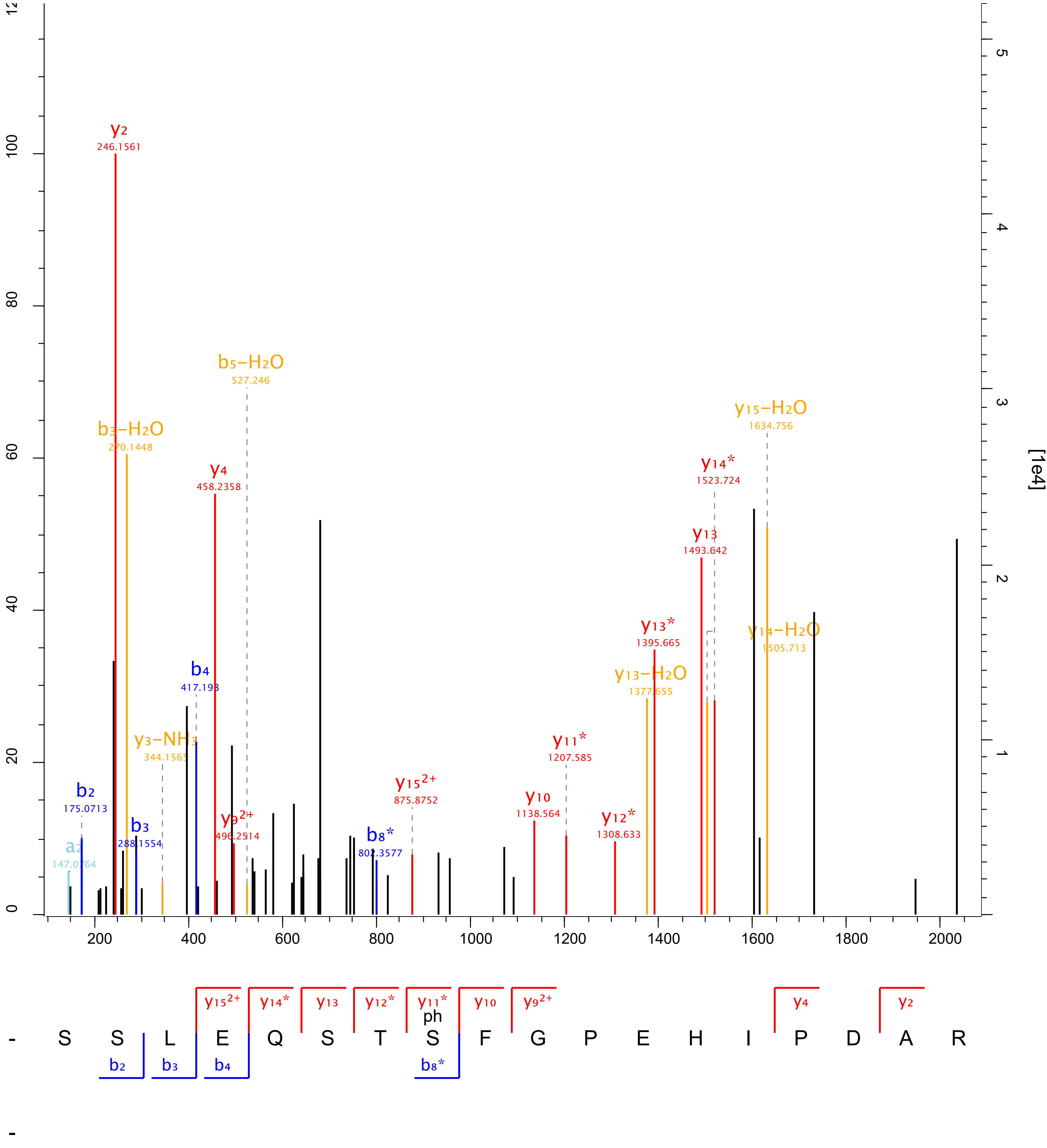

Sample output to test PDF Combine only

Raw file	Scan	Method	Score	m/z	Gene names
0523_14	14361	FTMS; HCD	61.49	798.02	EPSIN2

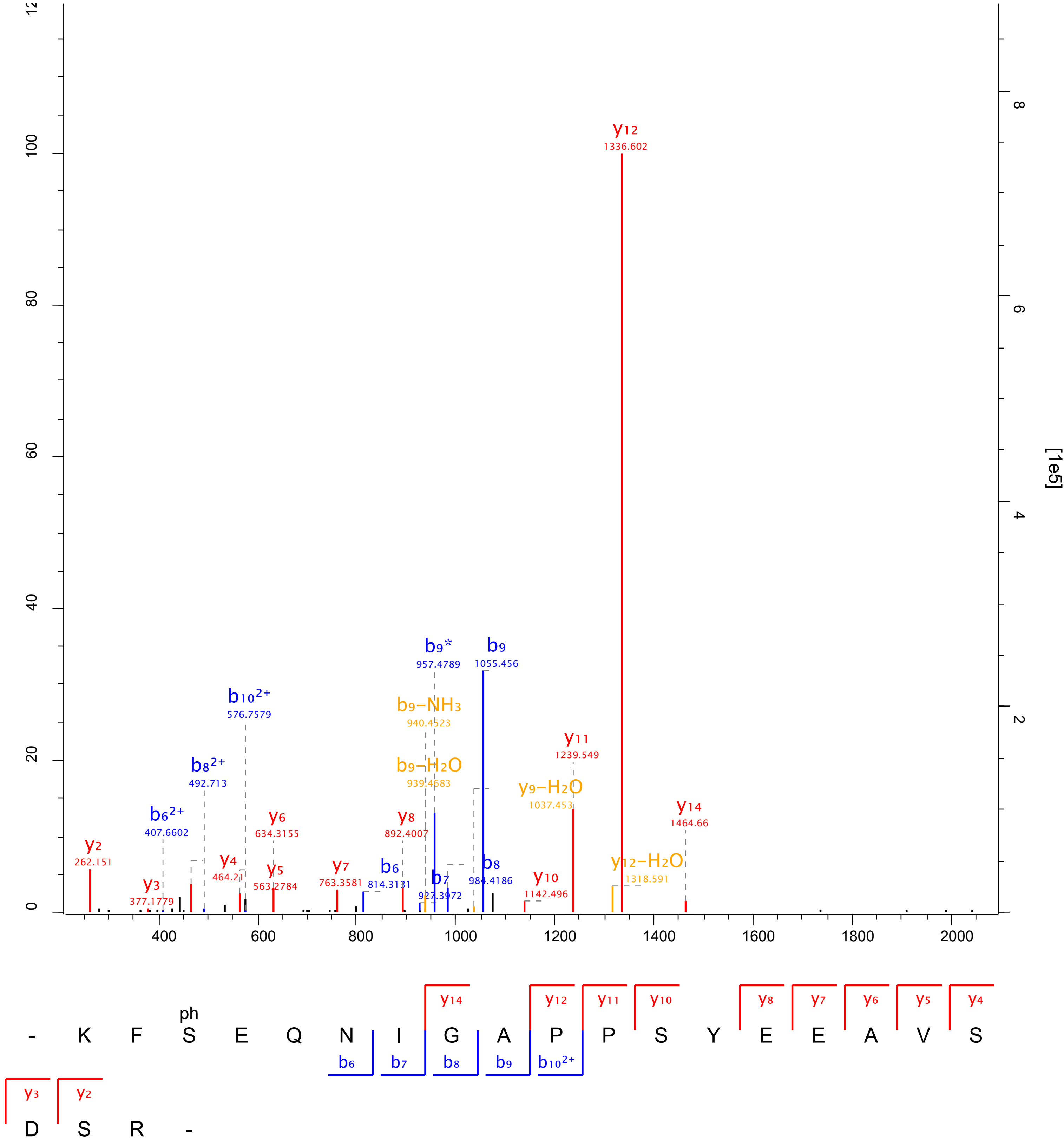

Sample output to test PDF Combine only

Raw file	Scan	Method	Score	m/z	Gene names
0523_14	14383	FTMS; HCD	92.46	775.01	TIC

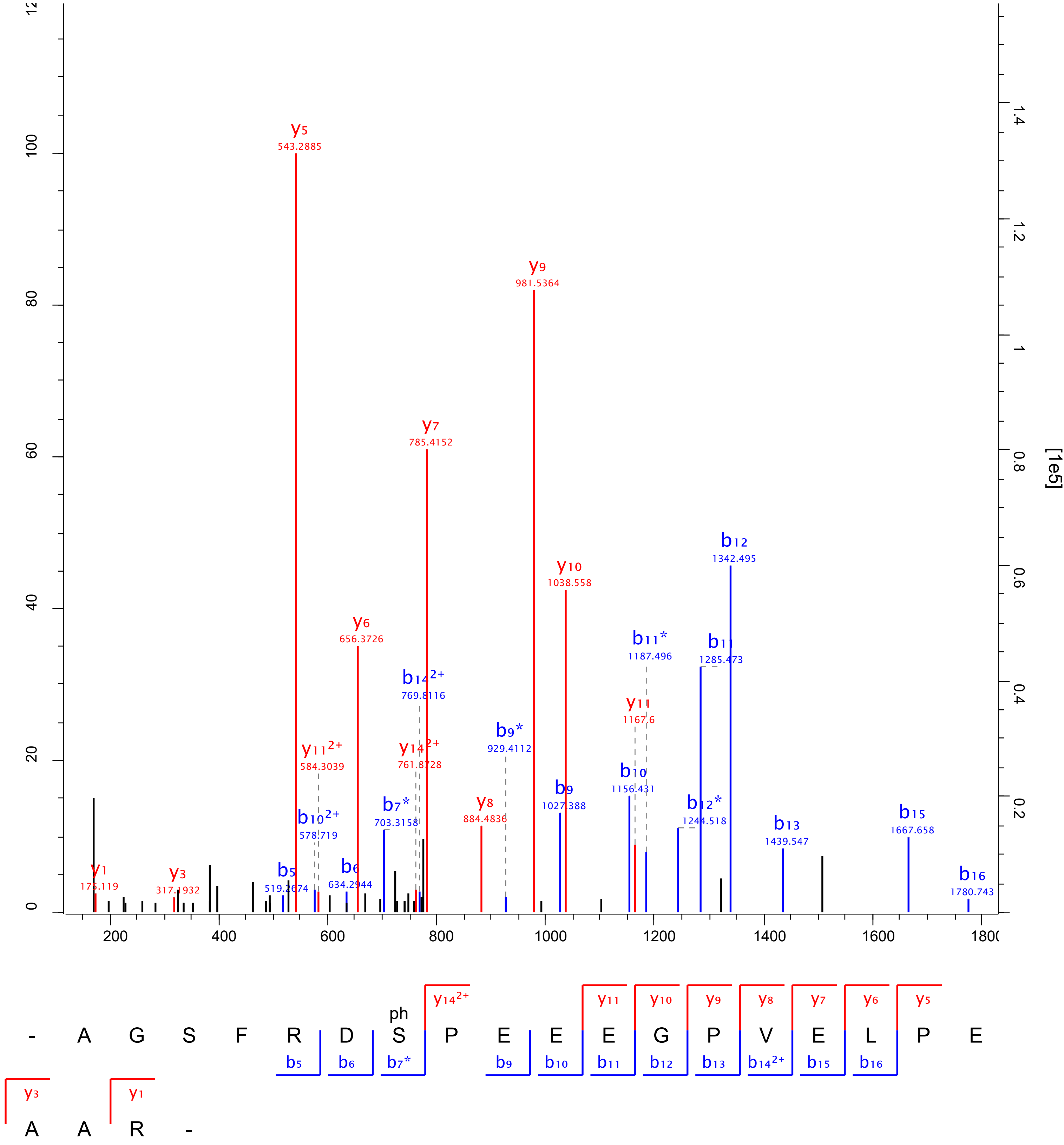

Sample output to test PDF Combine only

Raw file	Scan	Method	Score	m/z	Gene names
0523_14	14514	FTMS; HCD	55.05	698.62	At1g11360

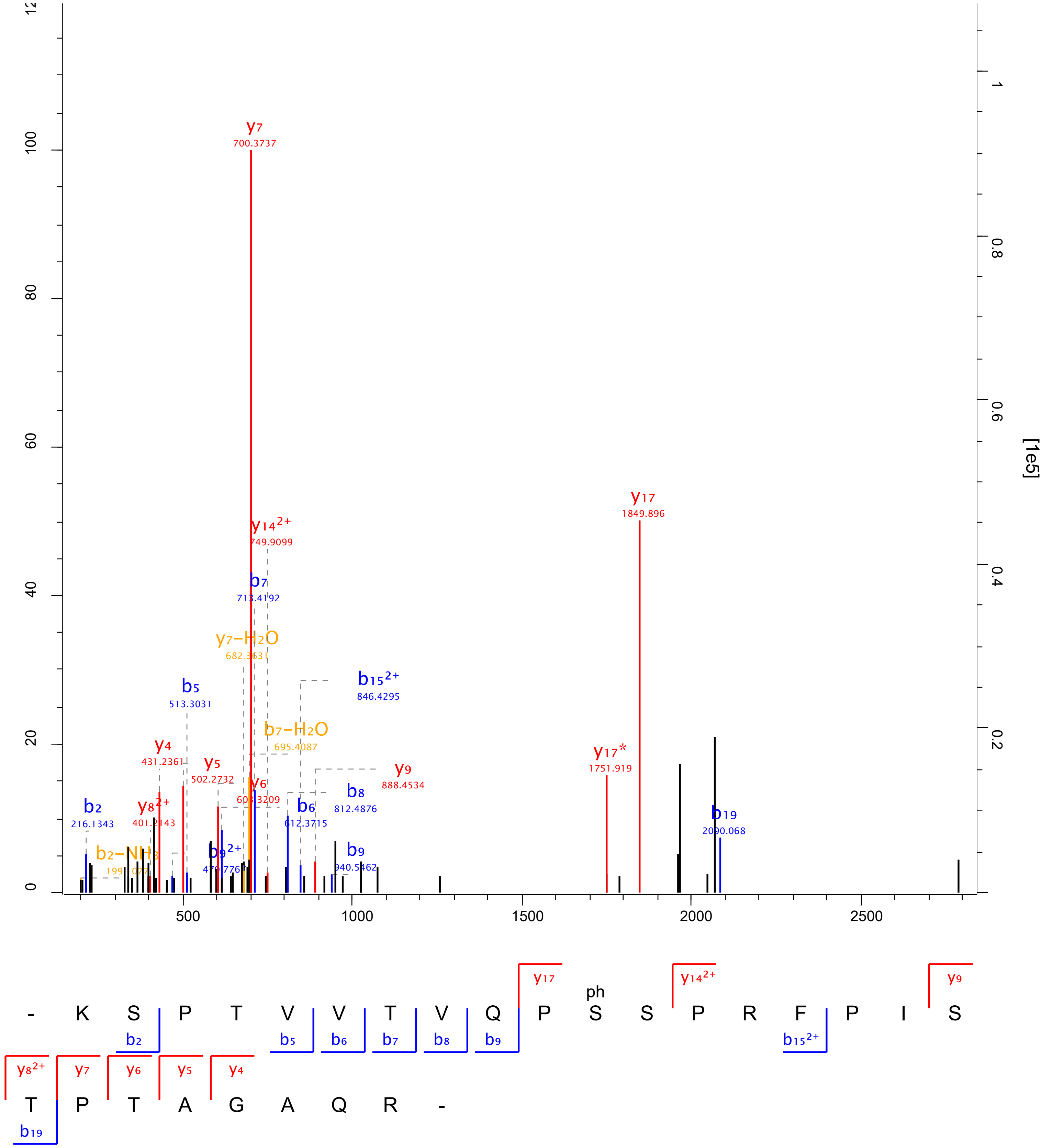

Sample output to test PDF Combine only

Raw file	Scan	Method	Score	m/z	Gene names
0523_14	14734	FTMS; HCD	71.15	627.31	At5g45190;CYCT1-5

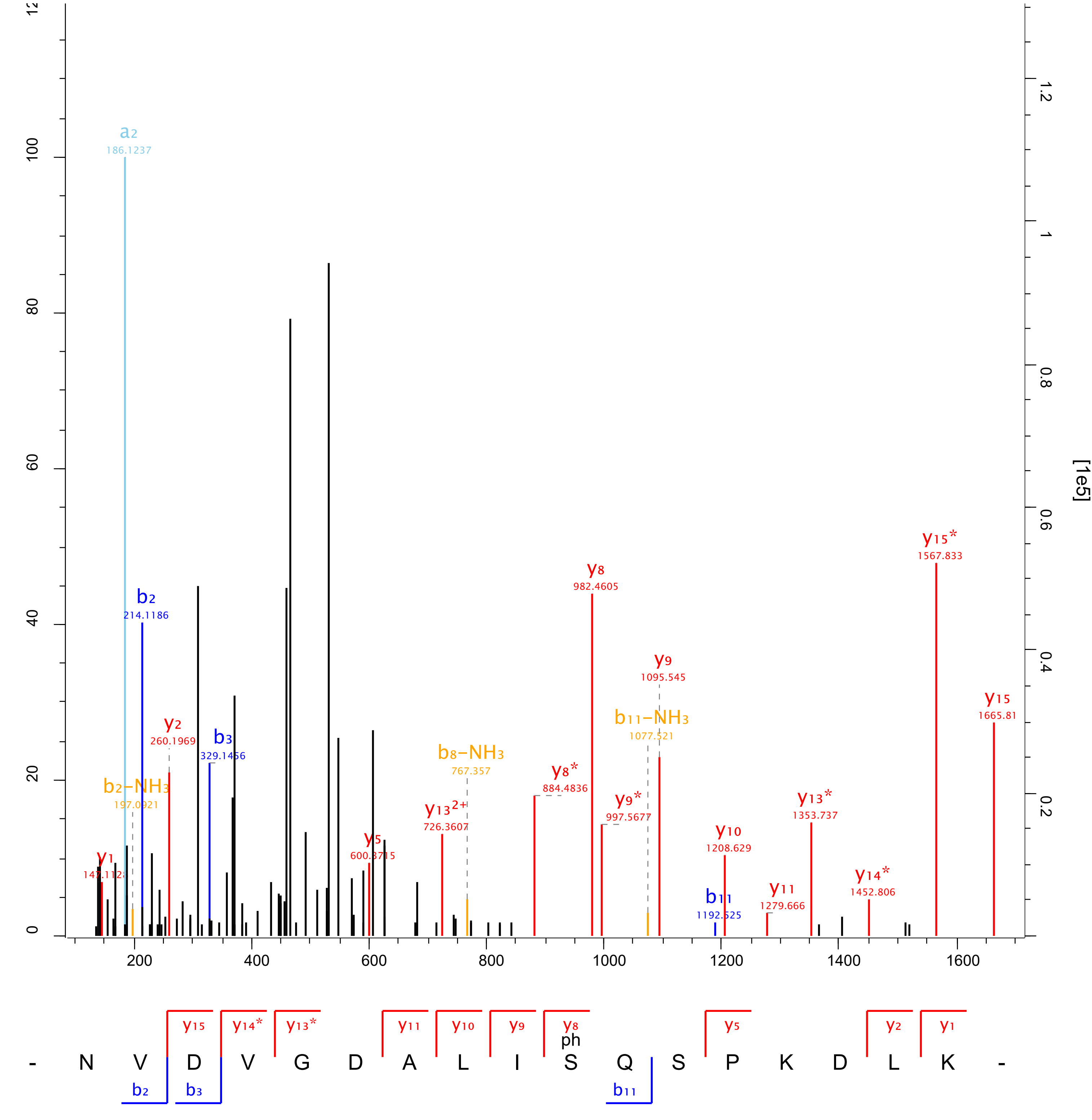

Sample output to test PDF Combine only

Raw file	Scan	Method	Score	m/z	Gene names
0523_14	15047	FTMS; HCD	72.69	1020.79	At4g38470

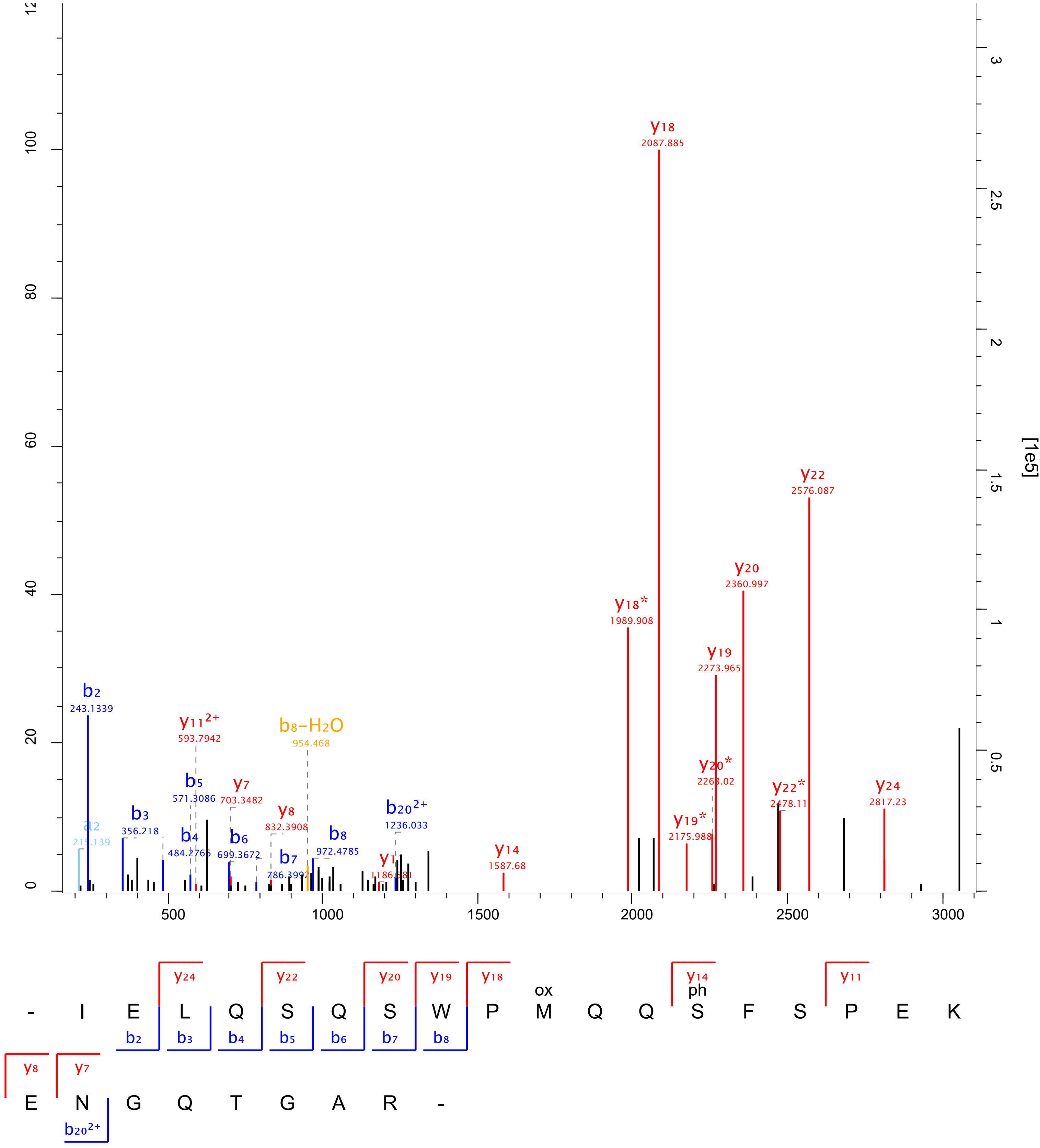

Sample output to test PDF Combine only

Raw file	Scan	Method	Score	m/z	Gene names
0523_14	15257	FTMS; HCD	185.38	832.06	UGT80A2

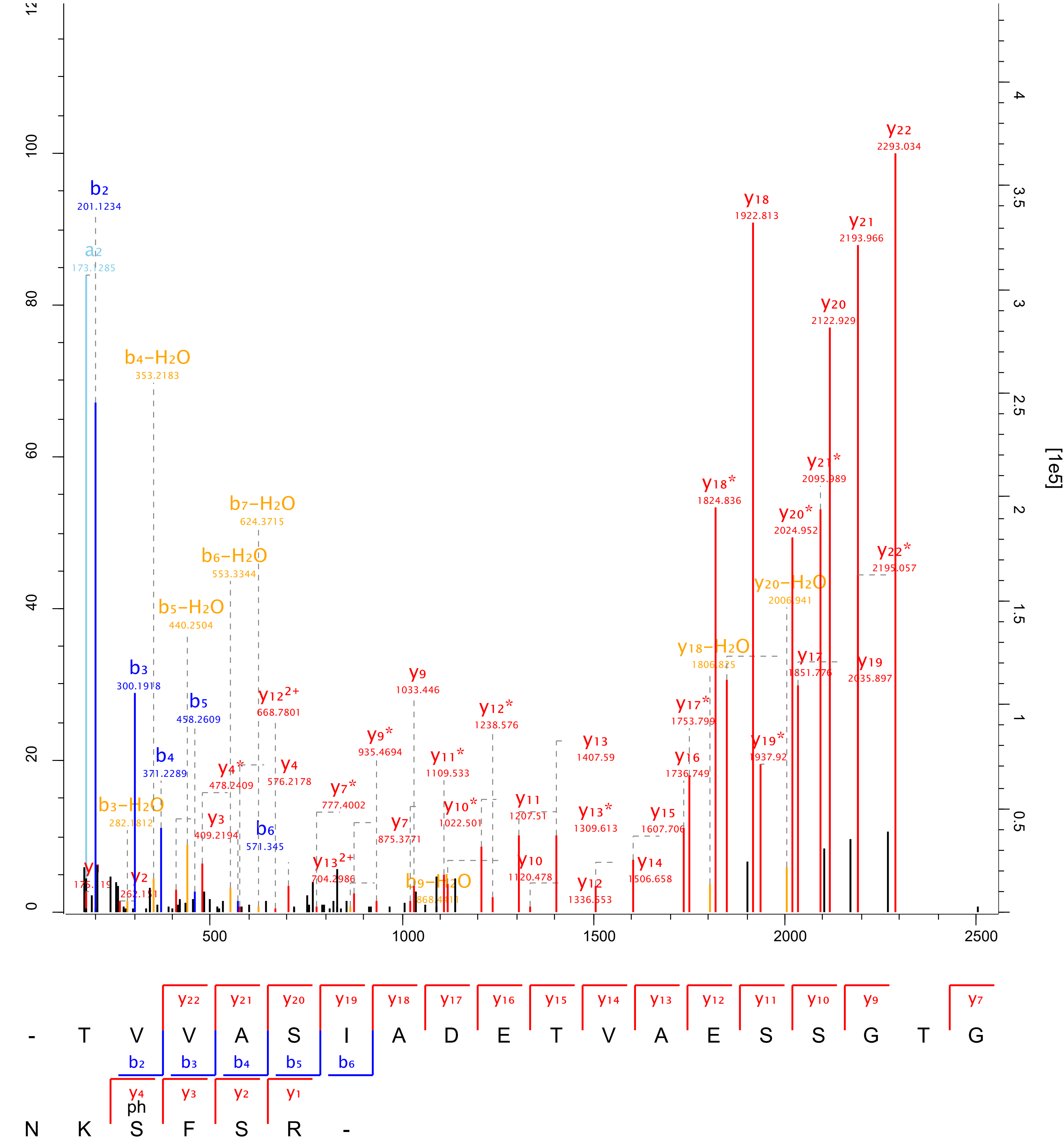

Sample output to test PDF Combine only

Raw file	Scan	Method	Score	m/z
0523_14	15341	FTMS; HCD	57.1	991.21

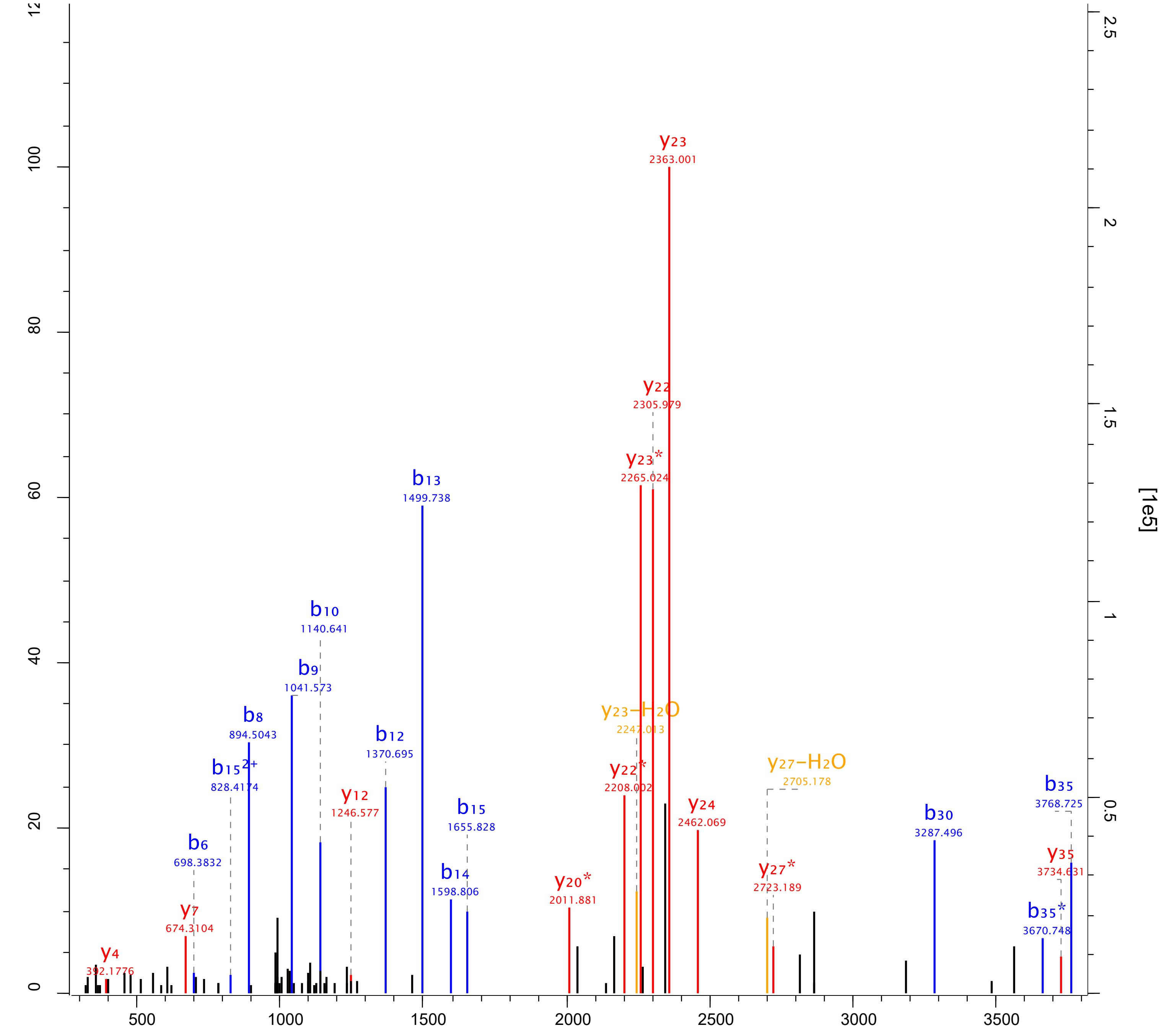

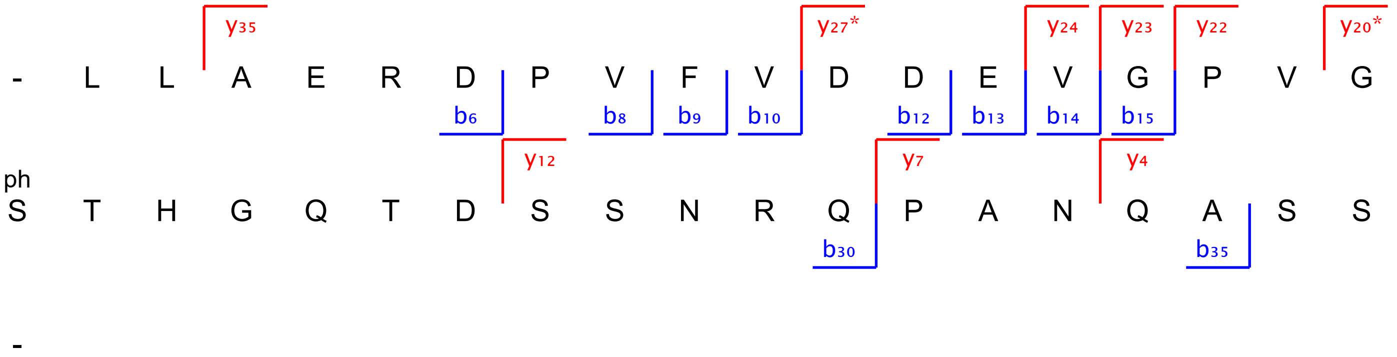

Sample output to test PDF Combine only

Raw file	Scan	Method	Score	m/z	Gene names
0523_14	15567	FTMS; HCD	53.3	487.56	ABCG36

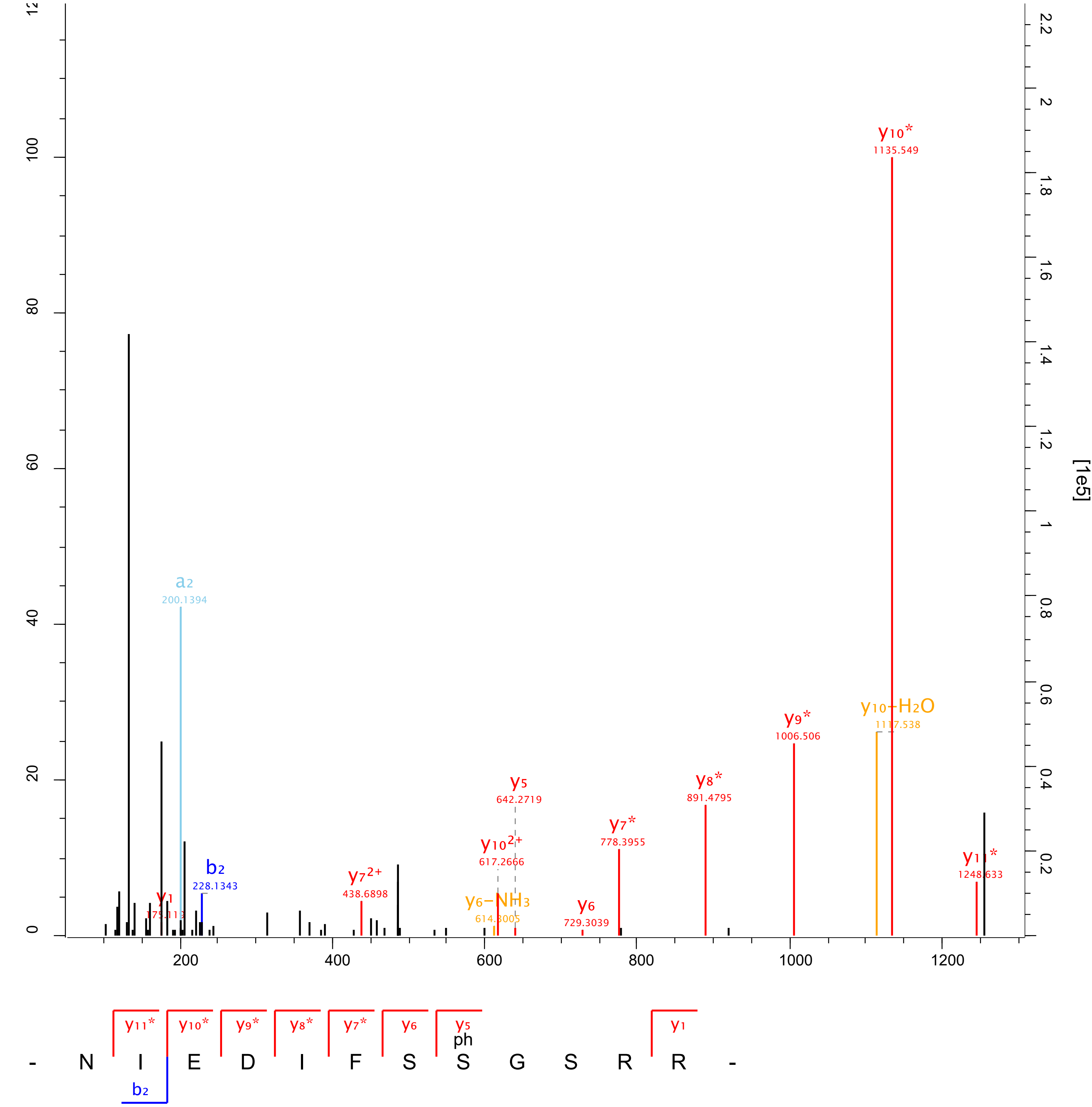

Sample output to test PDF Combine only

Raw file	Scan	Method	Score	m/z	Gene names
0523_14	16015	FTMS; HCD	80.73	888.12	At1g11360

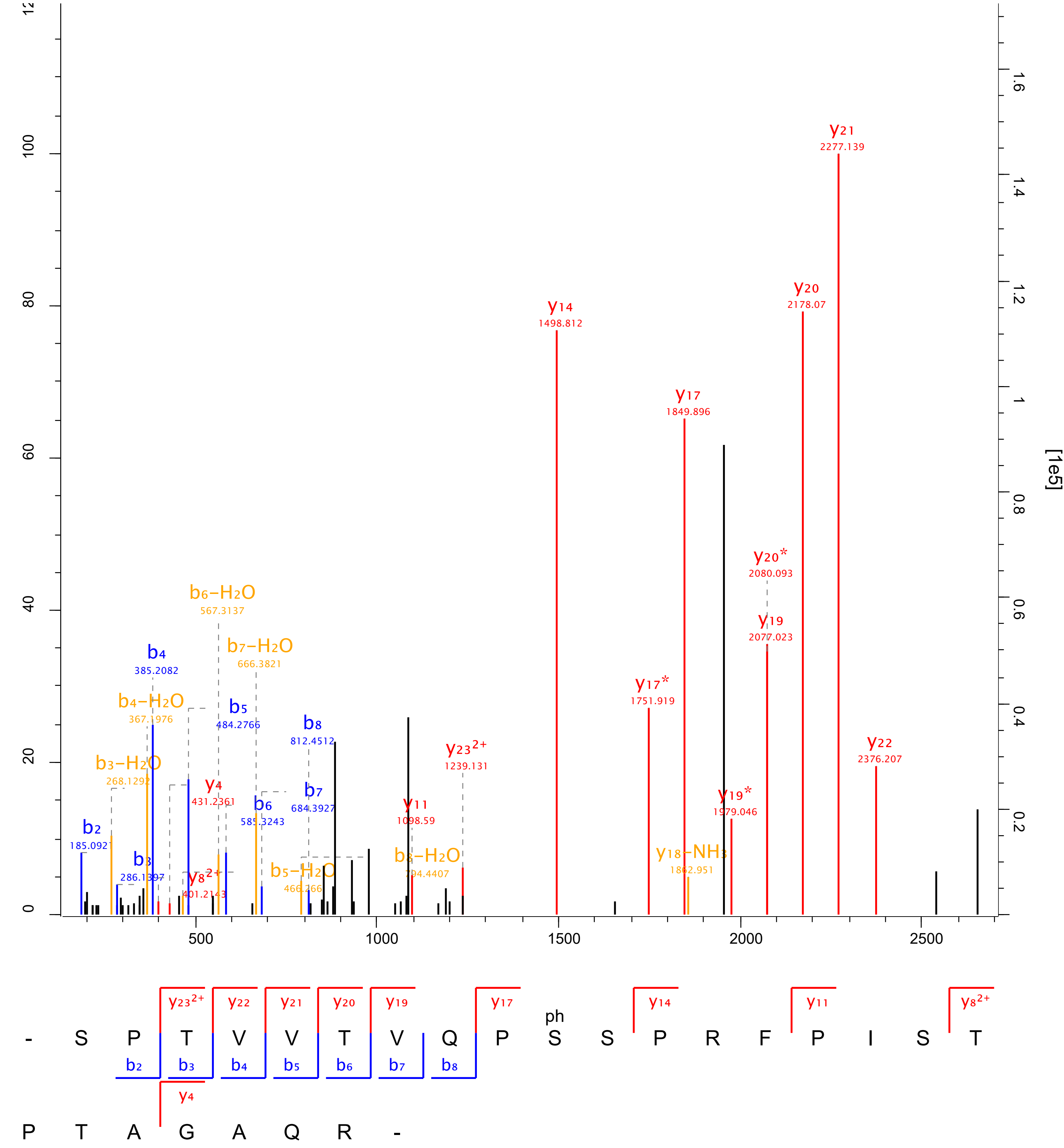

Sample output to test PDF Combine only

Raw file	Scan	Method	Score	m/z	Gene names
0523_14	16082	FTMS; HCD	157.9	944.42	EIN2

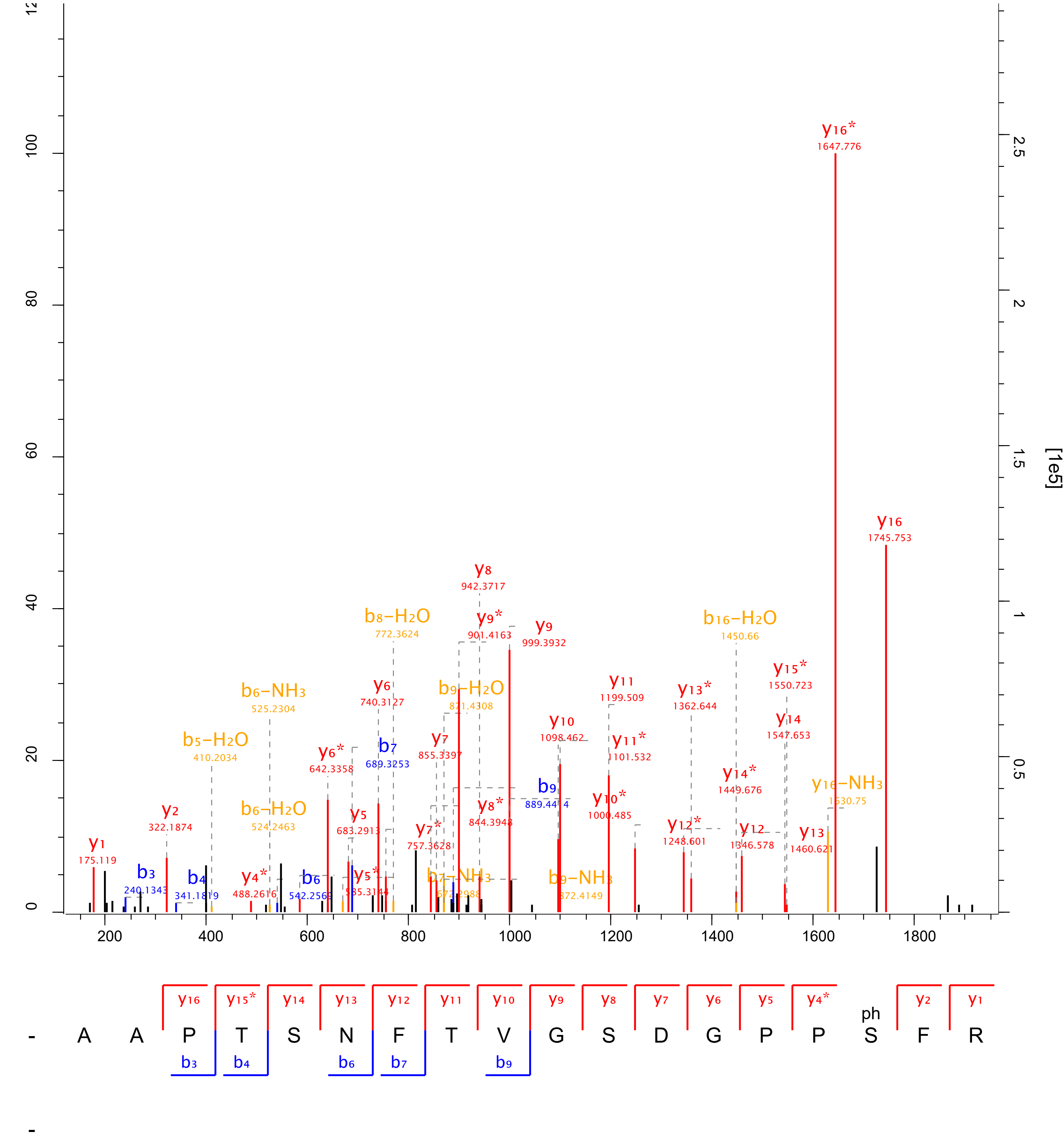

Sample output to test PDF Combine only

Raw file	Scan	Method	Score	m/z	Gene names
0523_14	16087	FTMS; HCD	80.66	848.37	F13I12.260

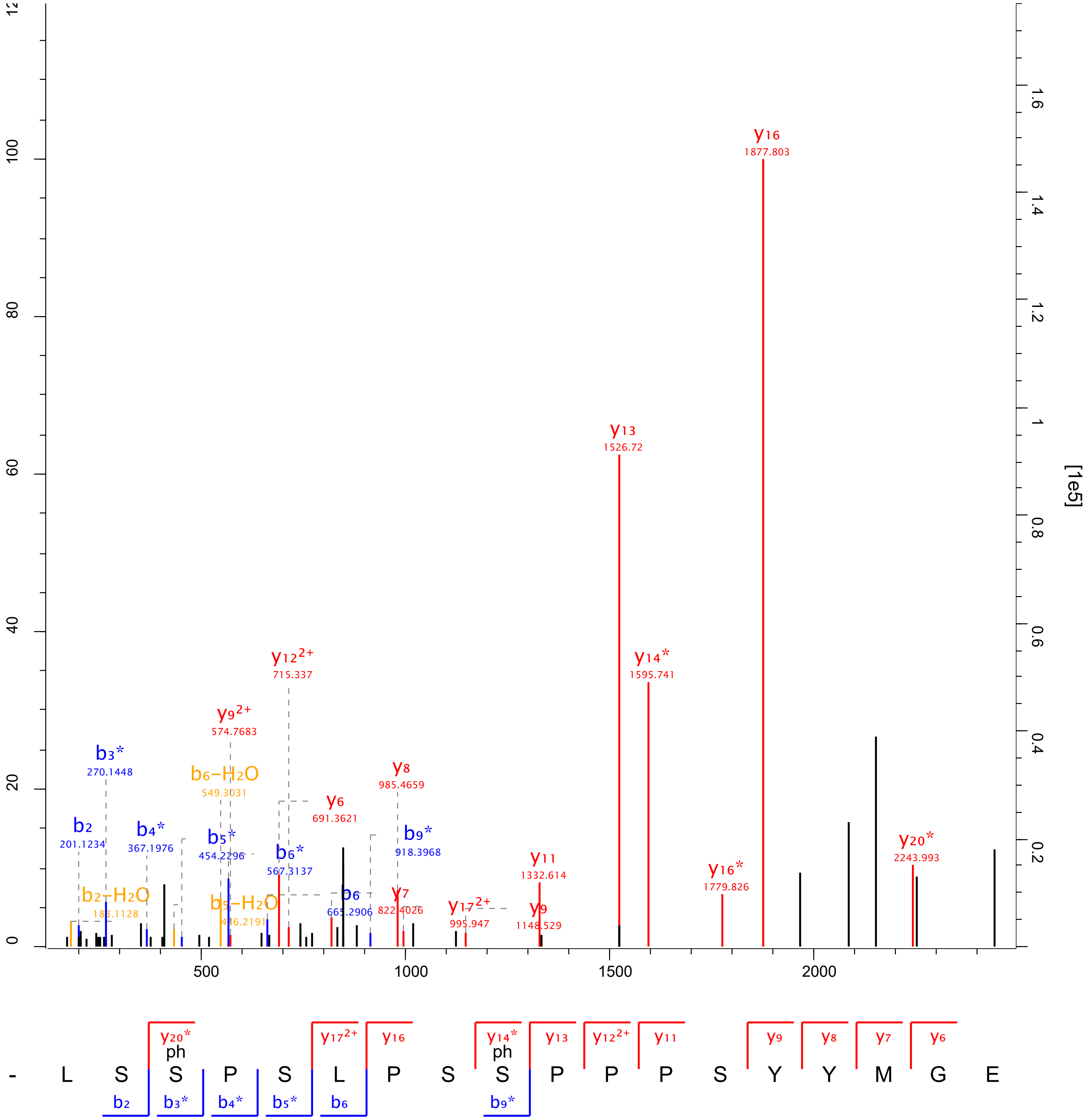

- L S S P S L P S S P P P S Y Y M G E

K T E K -

Sample output to test PDF Combine only

Raw file	Scan	Method	Score	m/z	Gene names
0523_14	16217	FTMS; HCD	103.58	777.04	At2g38000

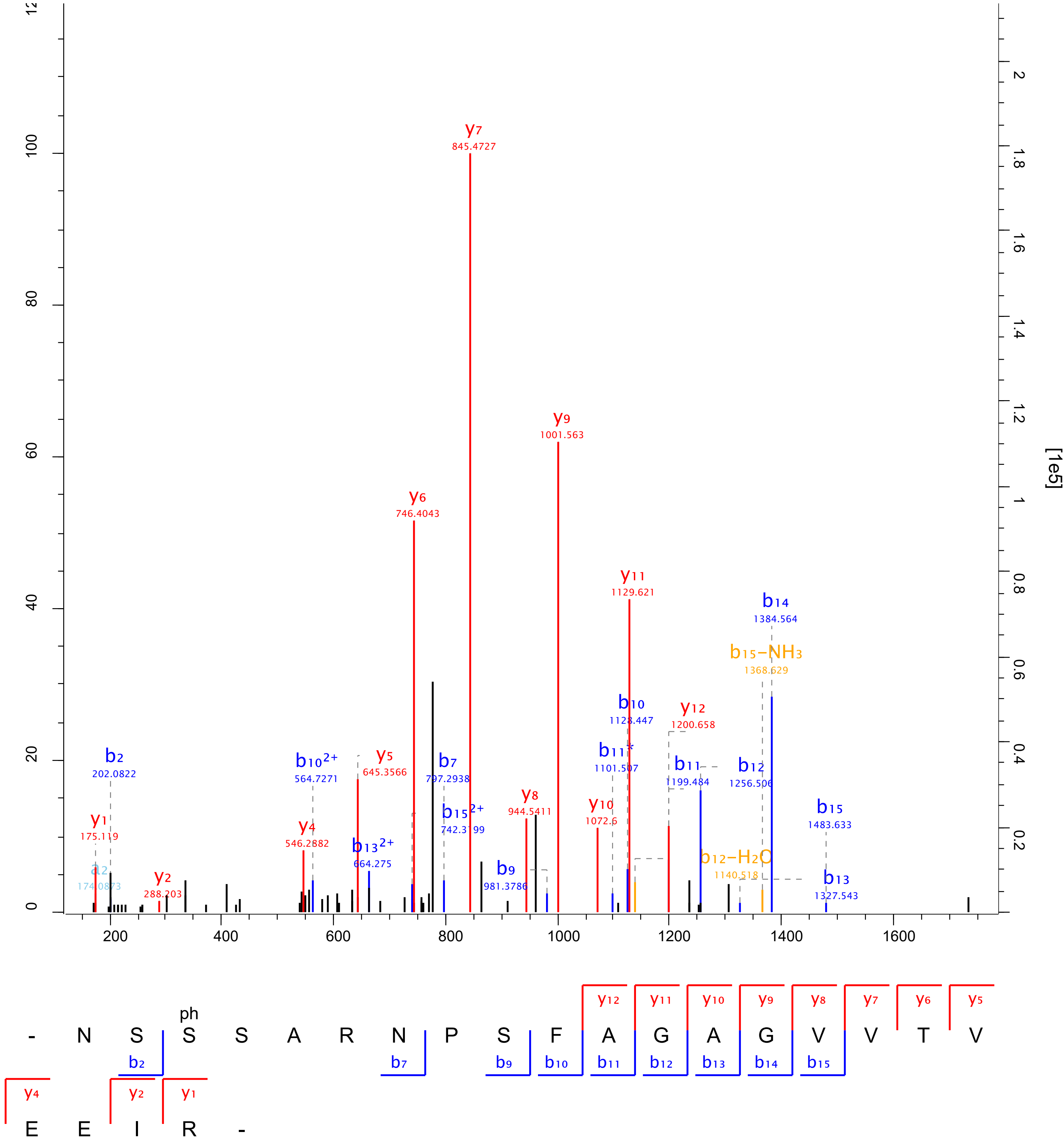

Sample output to test PDF Combine only

Raw file	Scan	Method	Score	m/z	Gene names
0523_14	16990	FTMS; HCD	53.09	721.83	Tudor1

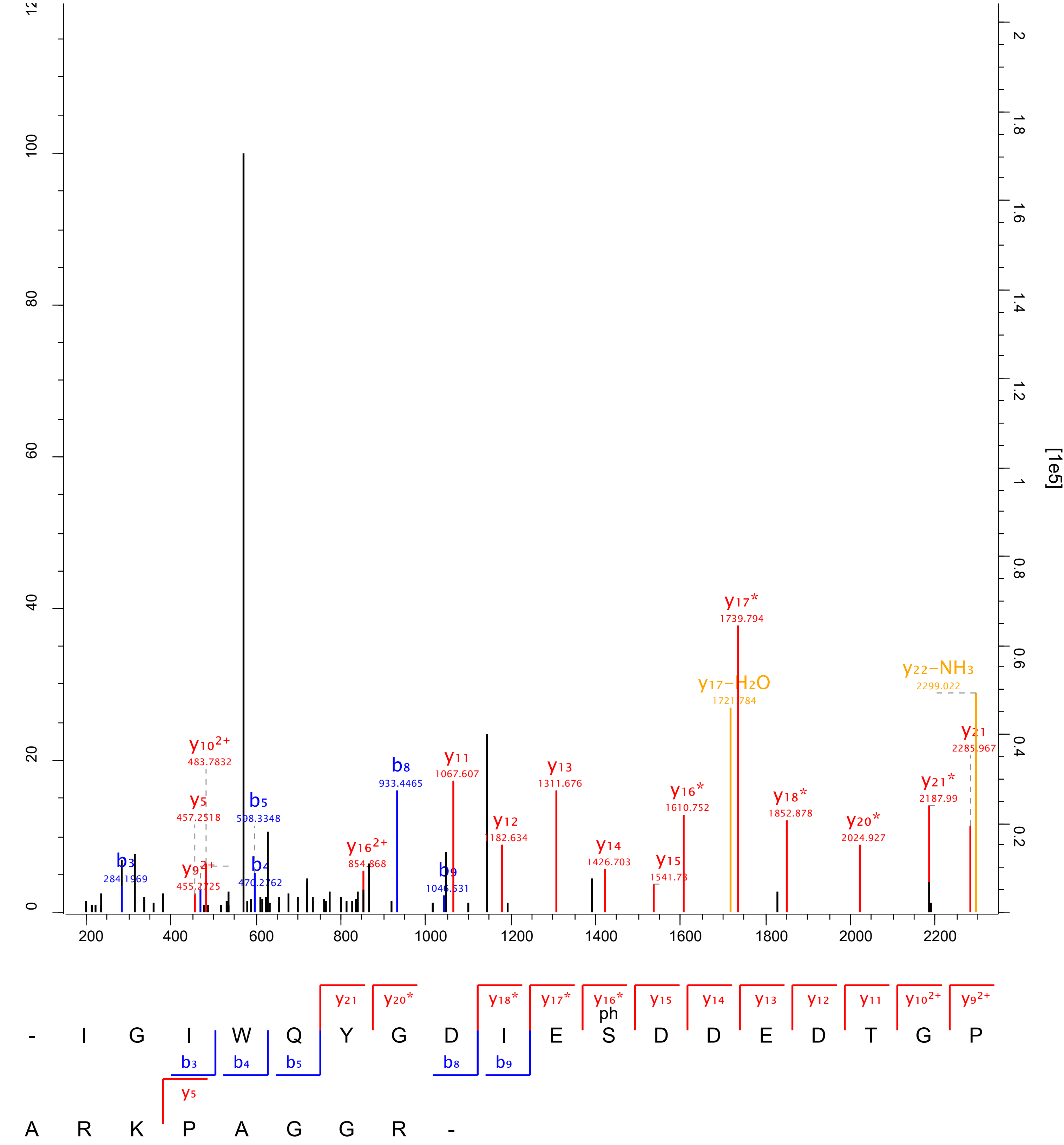

Sample output to test PDF Combine only

Raw file	Scan	Method	Score	m/z	Gene names
0523_14	17114	FTMS; HCD	51.2	1036.24	PEN2

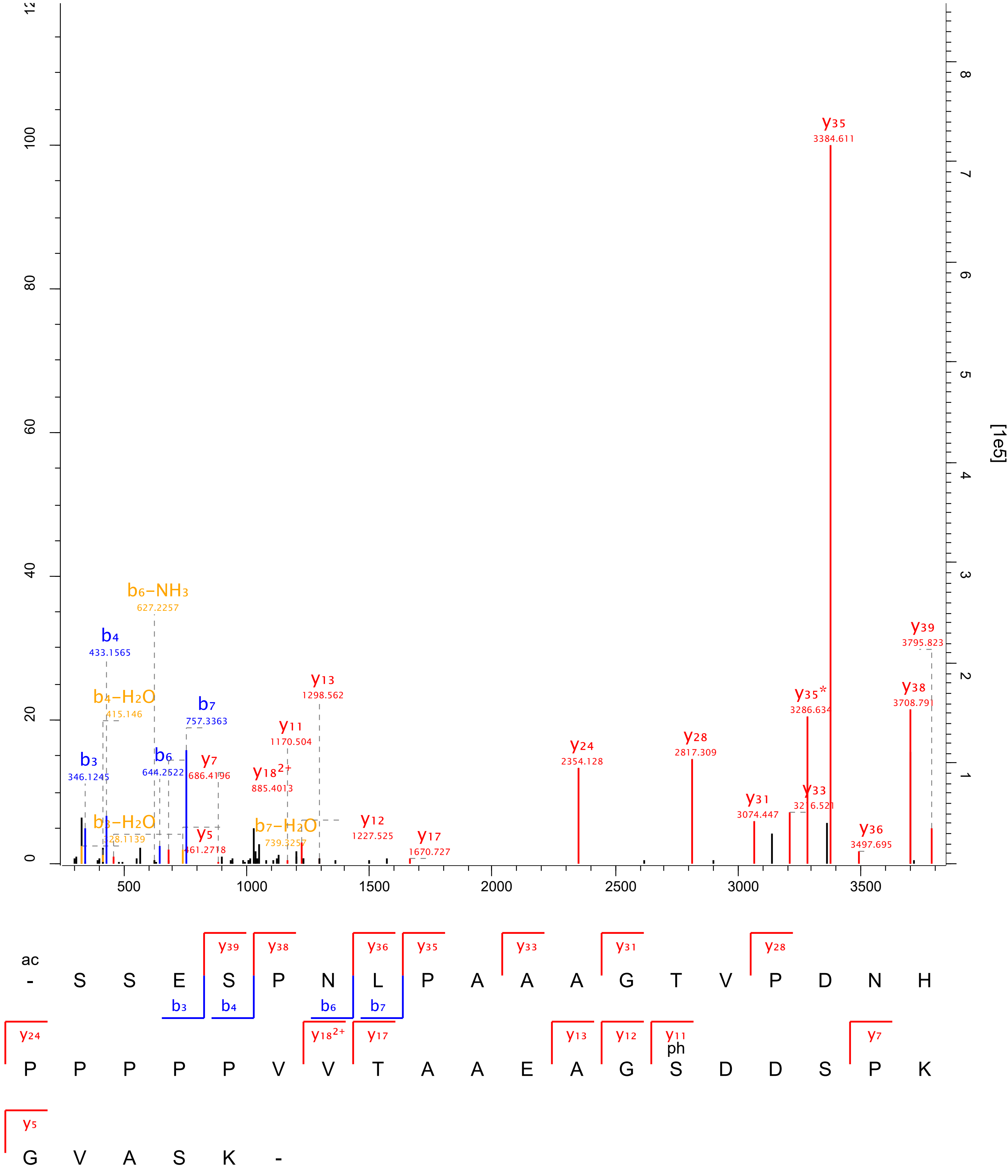

Sample output to test PDF Combine only

Raw file	Scan	Method	Score	m/z	Gene names
0523_14	17300	FTMS; HCD	185.28	1137.54	At5g09960

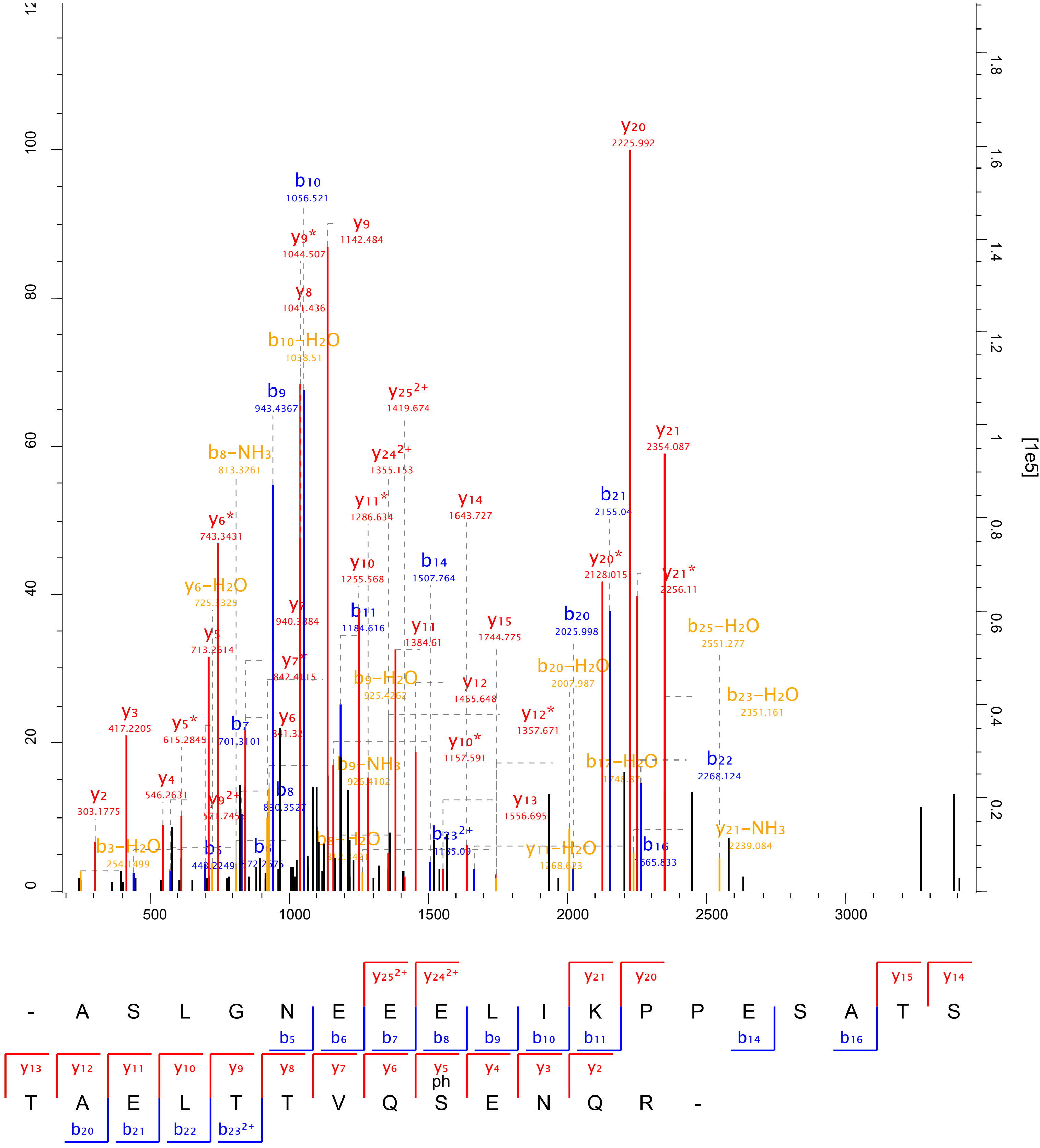

Sample output to test PDF Combine only

Raw file	Scan	Method	Score	m/z	Gene names
0523_14	17483	FTMS; HCD	169.39	967.46	At5g09960

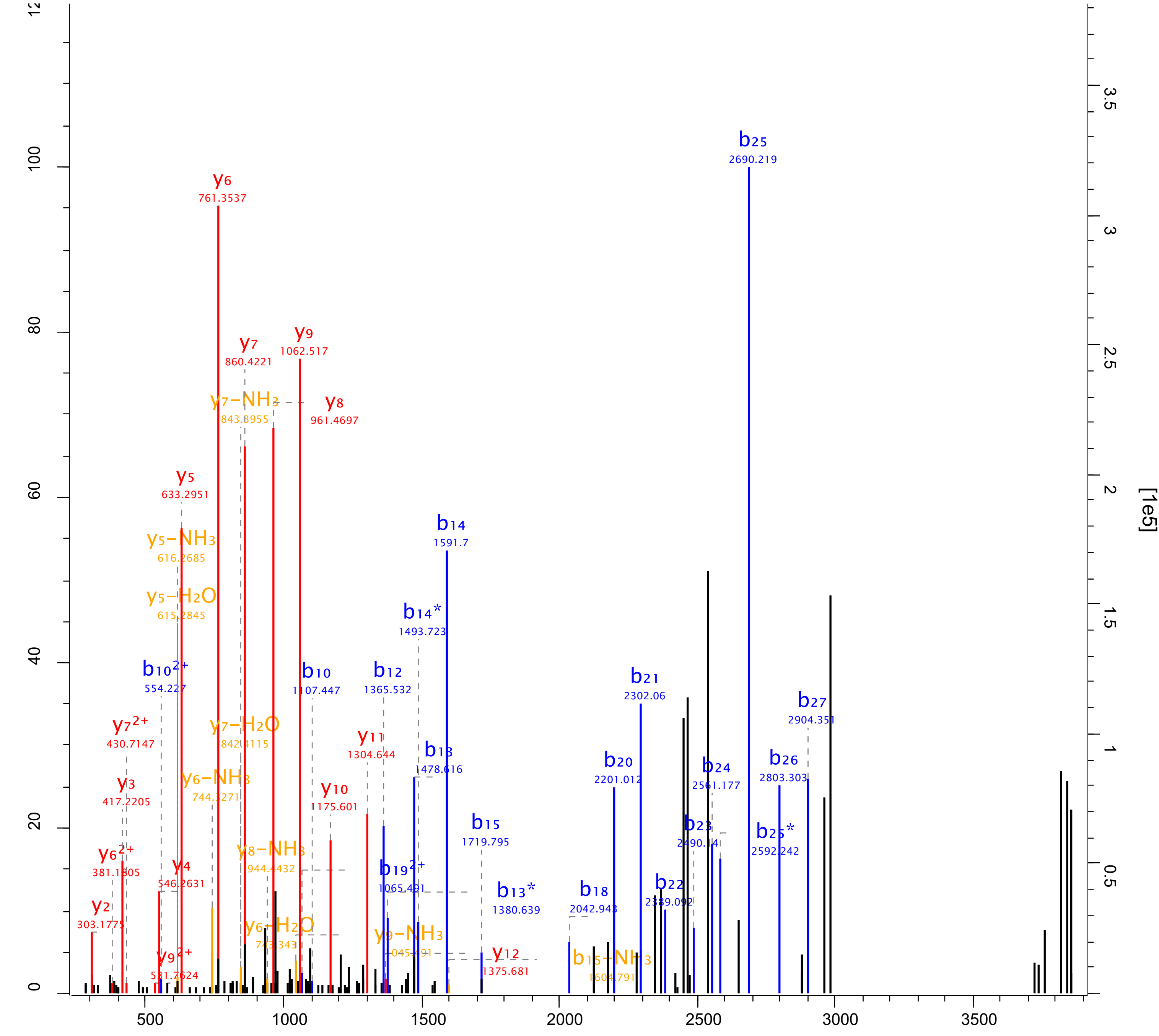

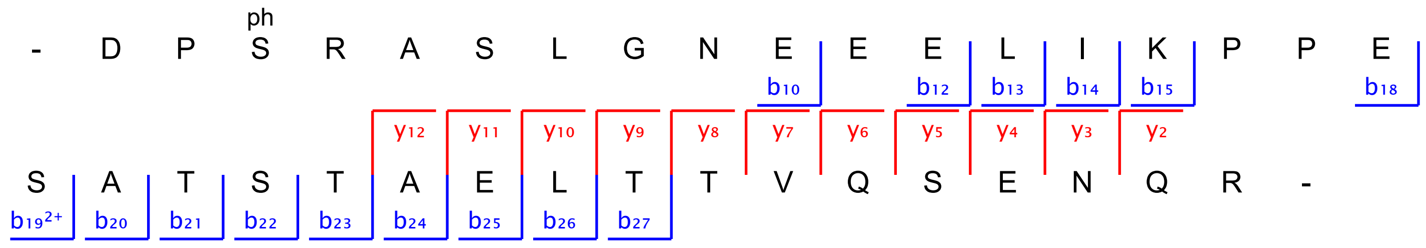

Sample output to test PDF Combine only

Raw file	Scan	Method	Score	m/z	Gene names
0523_14	17840	FTMS; HCD	108.44	853.05	EIF(ISO)4G2

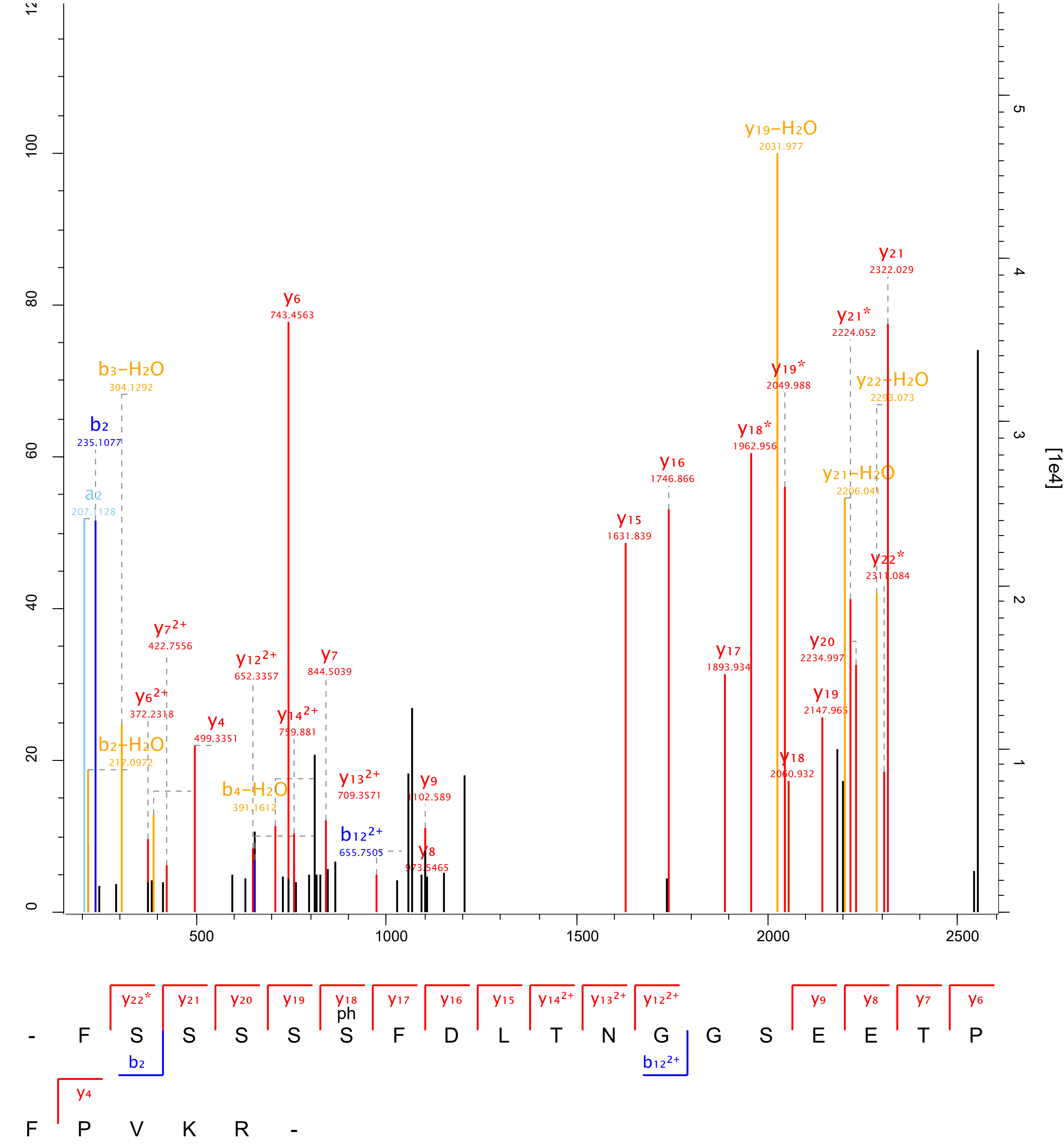

Sample output to test PDF Combine only

Raw file	Scan	Method	Score	m/z	Gene names
0523_14	18634	FTMS; HCD	111.89	875.73	PTP1

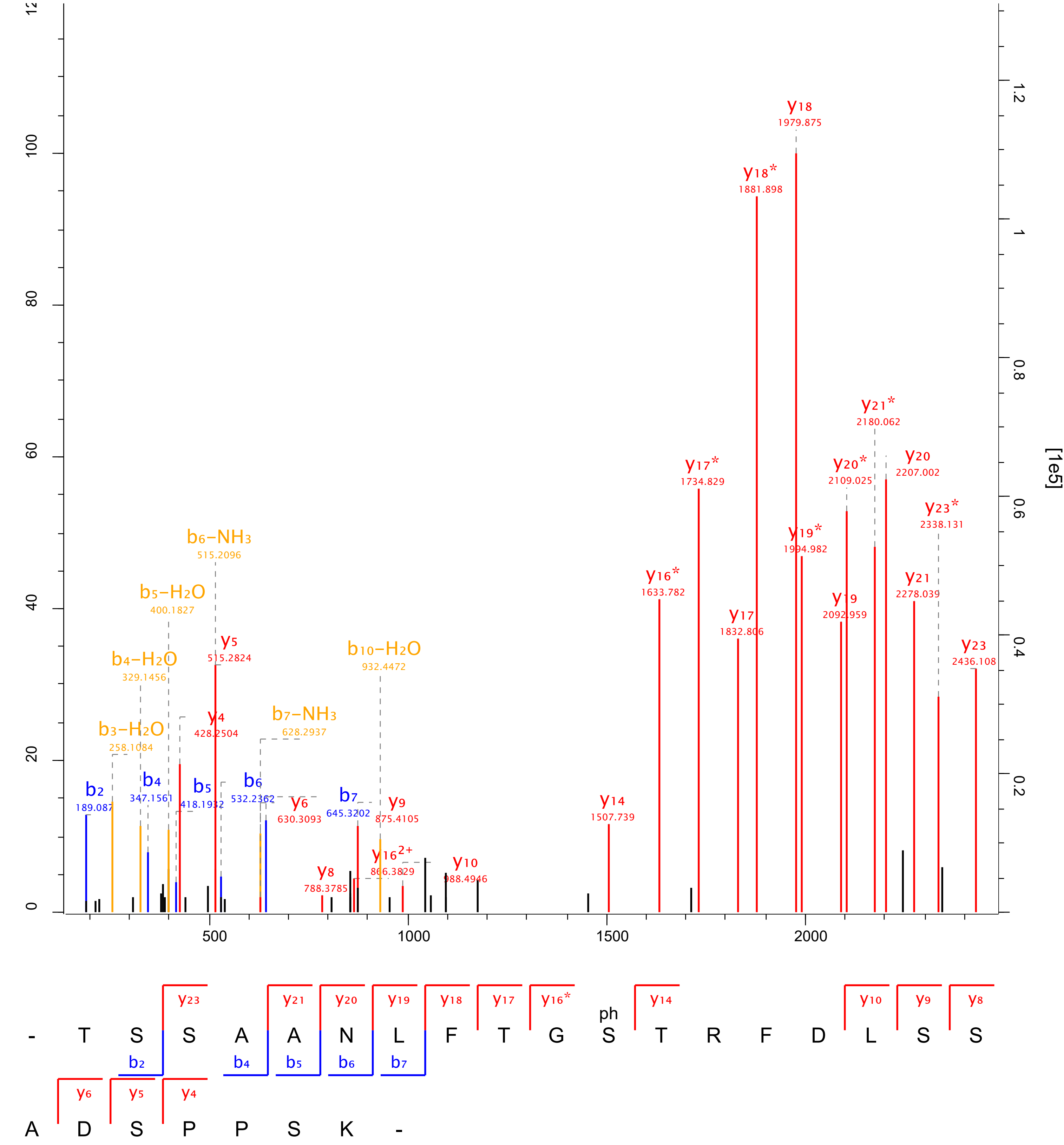

Sample output to test PDF Combine only

Raw file	Scan	Method	Score	m/z	Gene names
0523_14	18667	FTMS; HCD	165.47	797.02	RH24

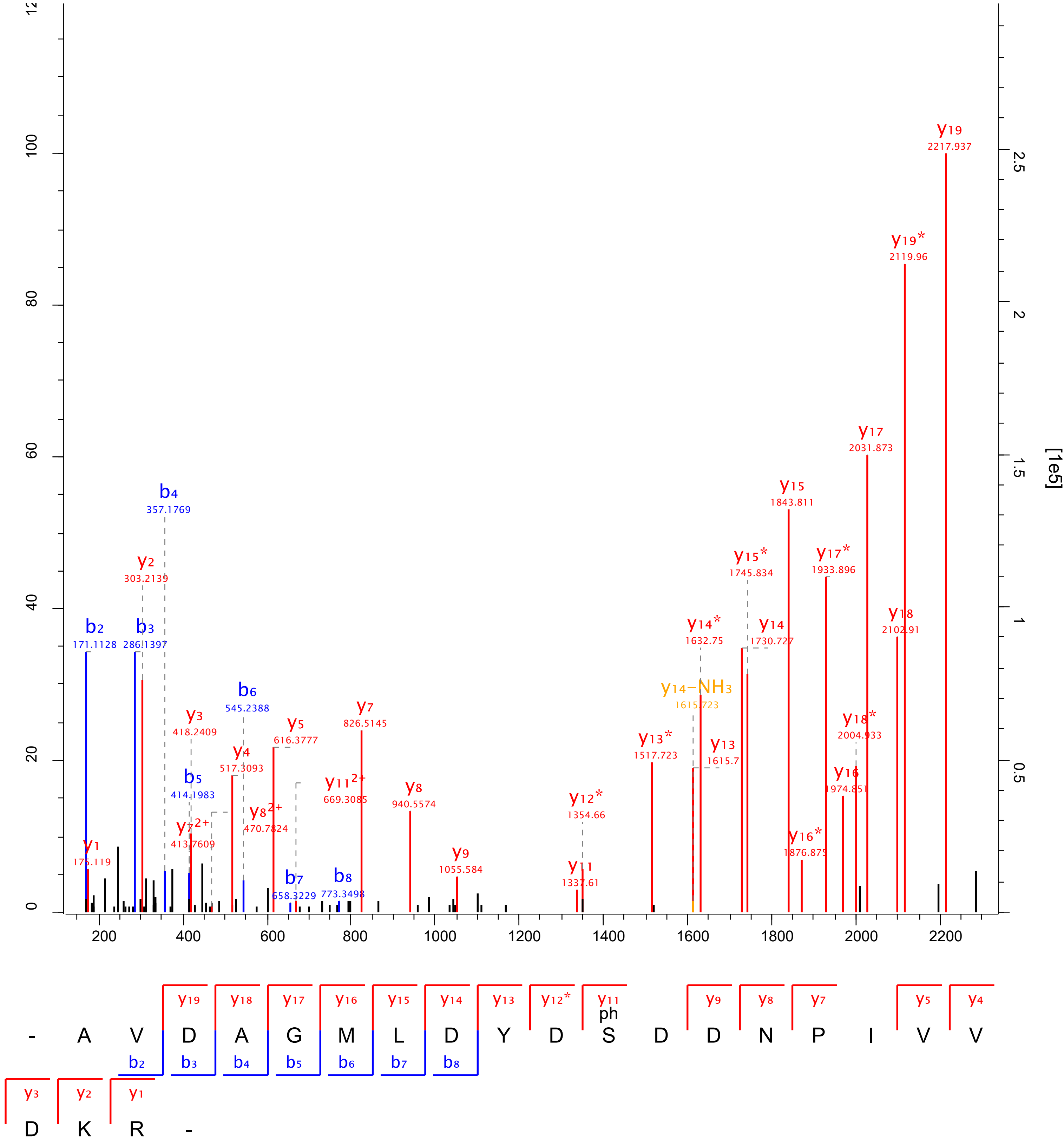

Sample output to test PDF Combine only

Raw file	Scan	Method	Score	m/z	Gene names
0523_14	19075	FTMS; HCD	98.49	797.04	NRAMP1

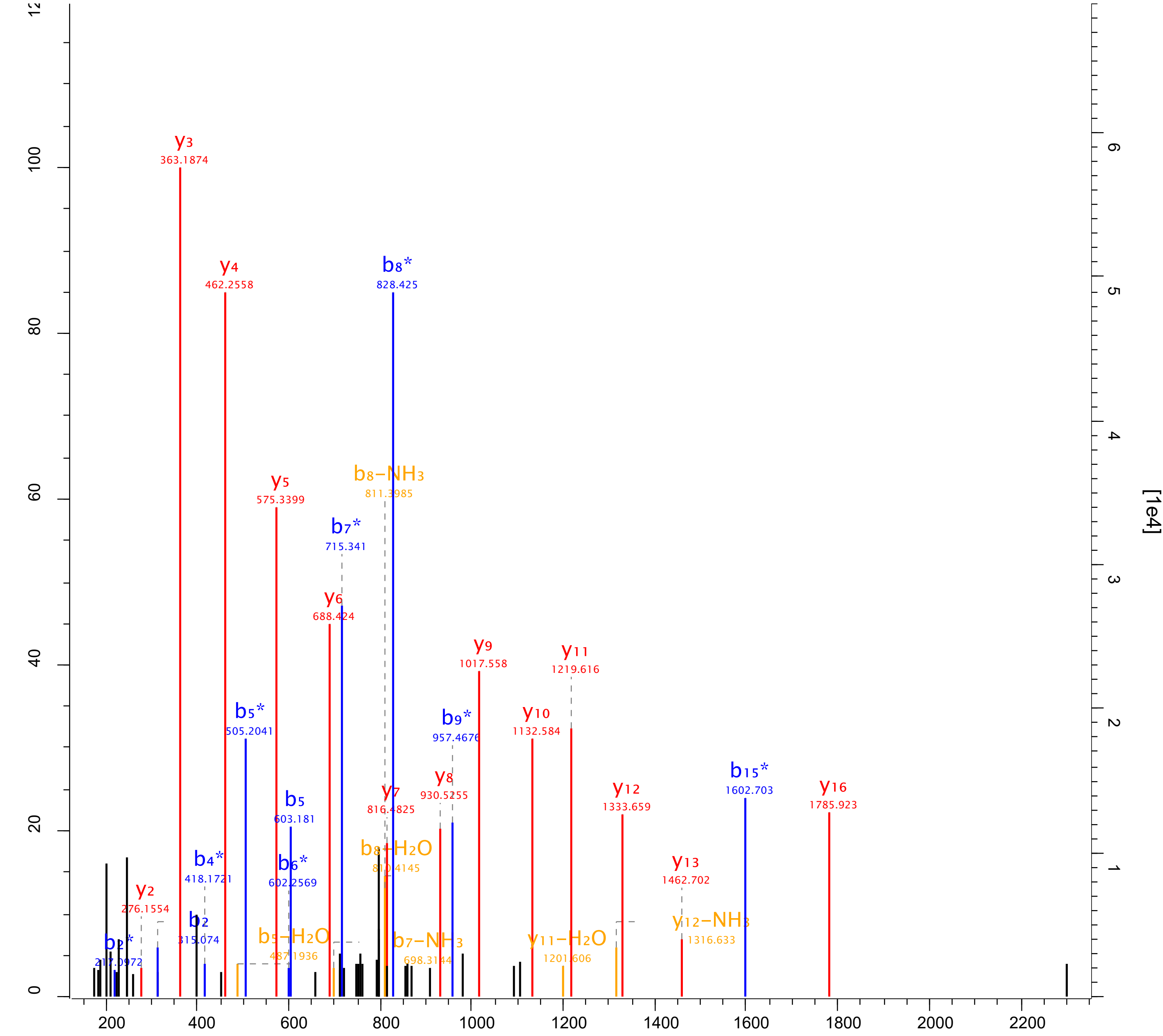

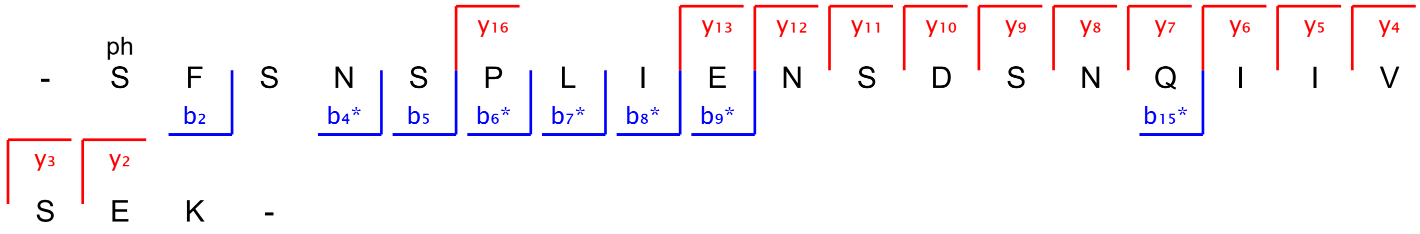

Sample output to test PDF Combine only

Raw file	Scan	Method	Score	m/z
0523_14	19427	FTMS; HCD	49.09	755.35

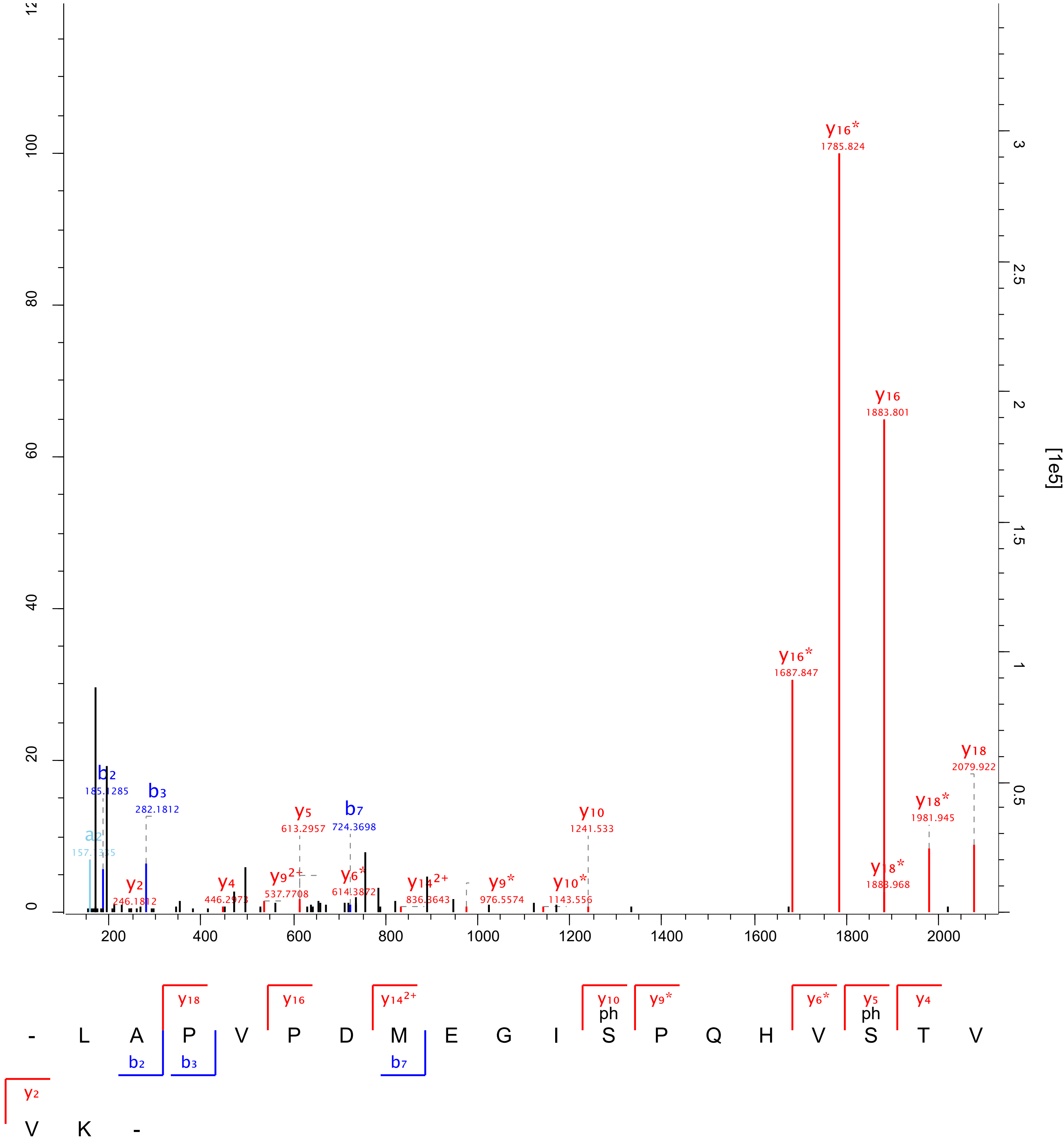

Sample output to test PDF Combine only

Raw file	Scan	Method	Score	m/z	Gene names
0523_14	19564	FTMS; HCD	49.09	897.1	AHA2;HA2

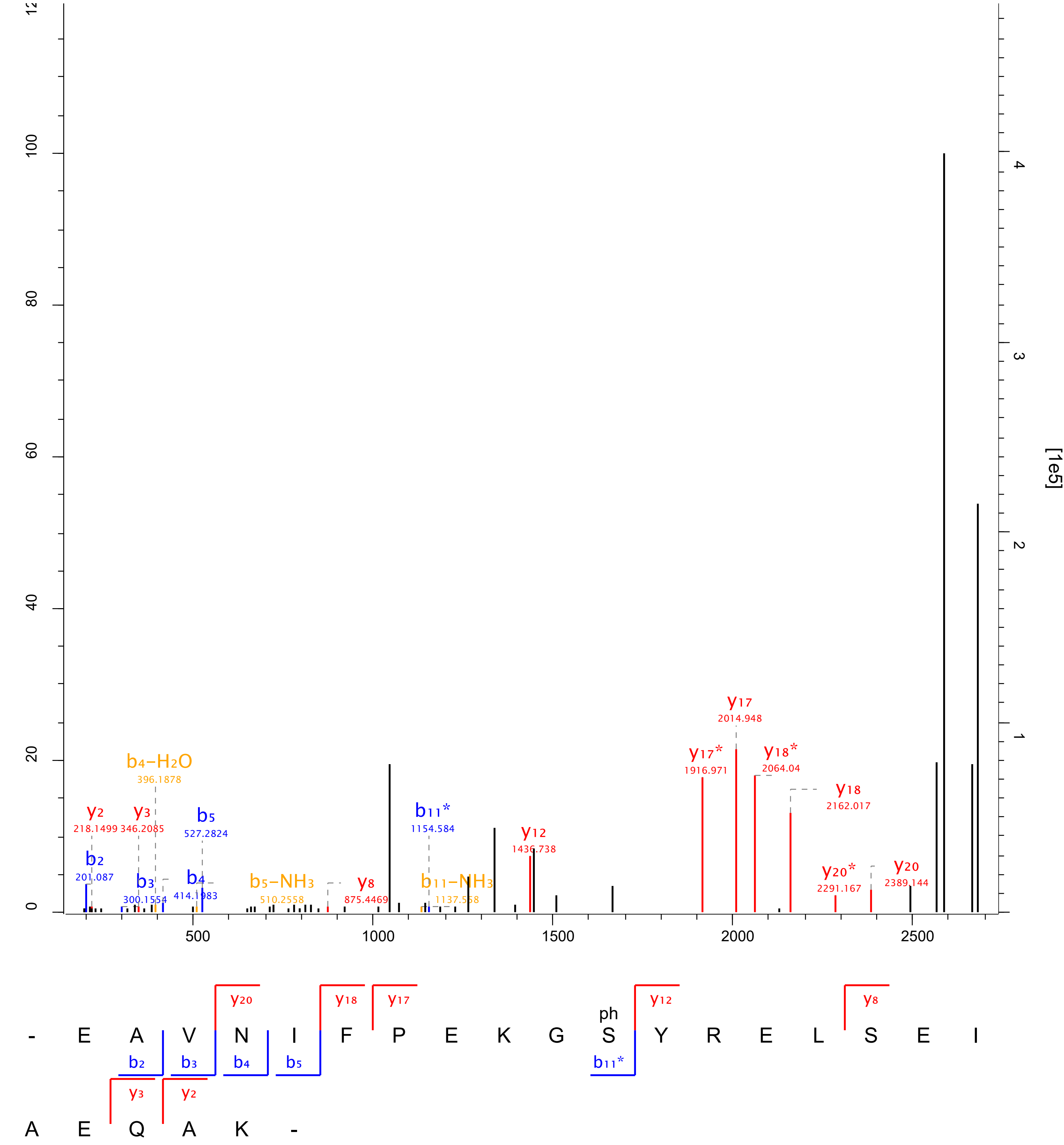

Sample output to test PDF Combine only

Raw file	Scan	Method	Score	m/z	Gene names
0523_14	20138	FTMS; HCD	160.02	819.85	SYP122

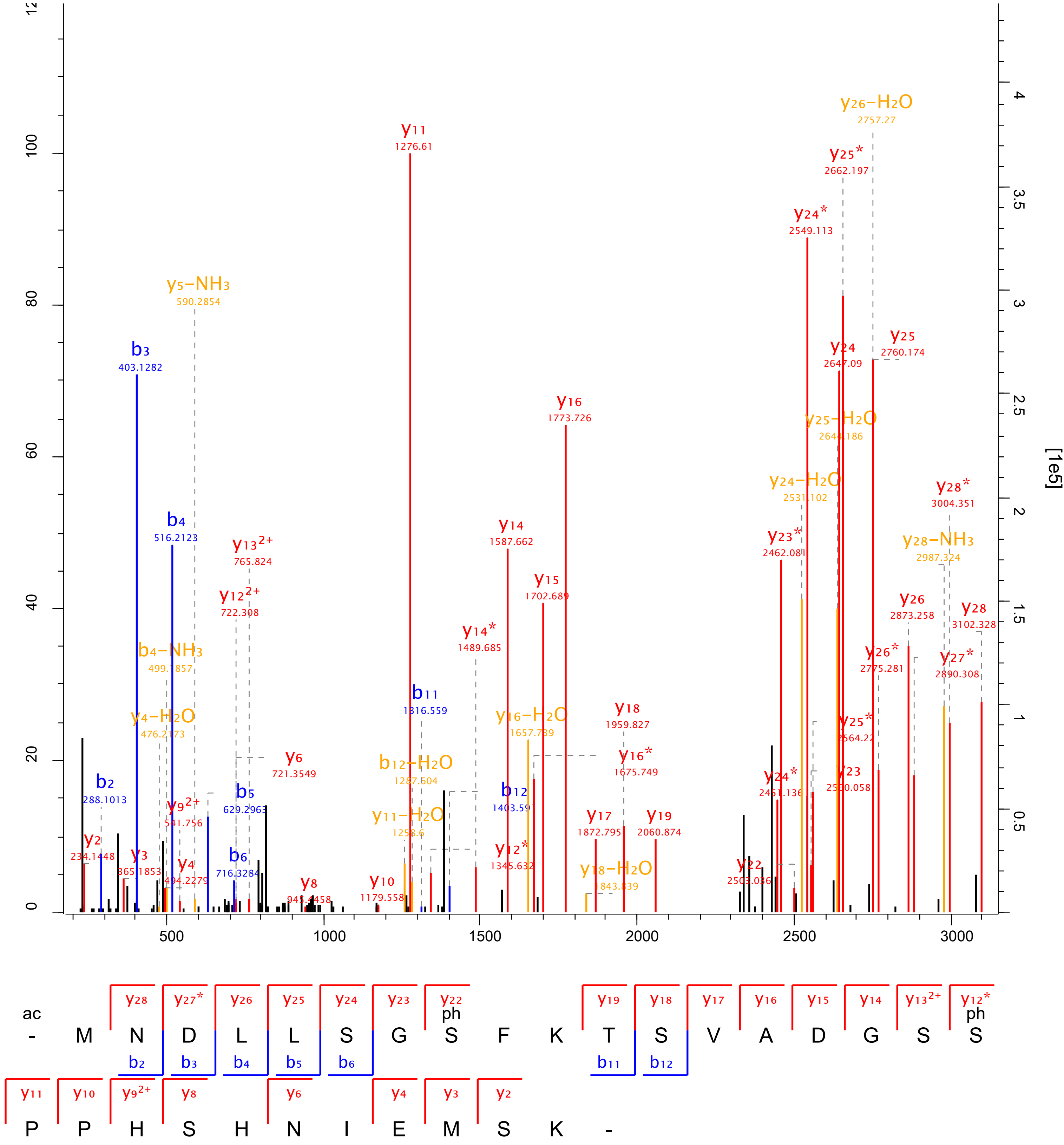

Sample output to test PDF Combine only

Raw file	Scan	Method	Score	m/z	Gene names
0523_14	20315	FTMS; HCD	51.44	883.4	AHL13

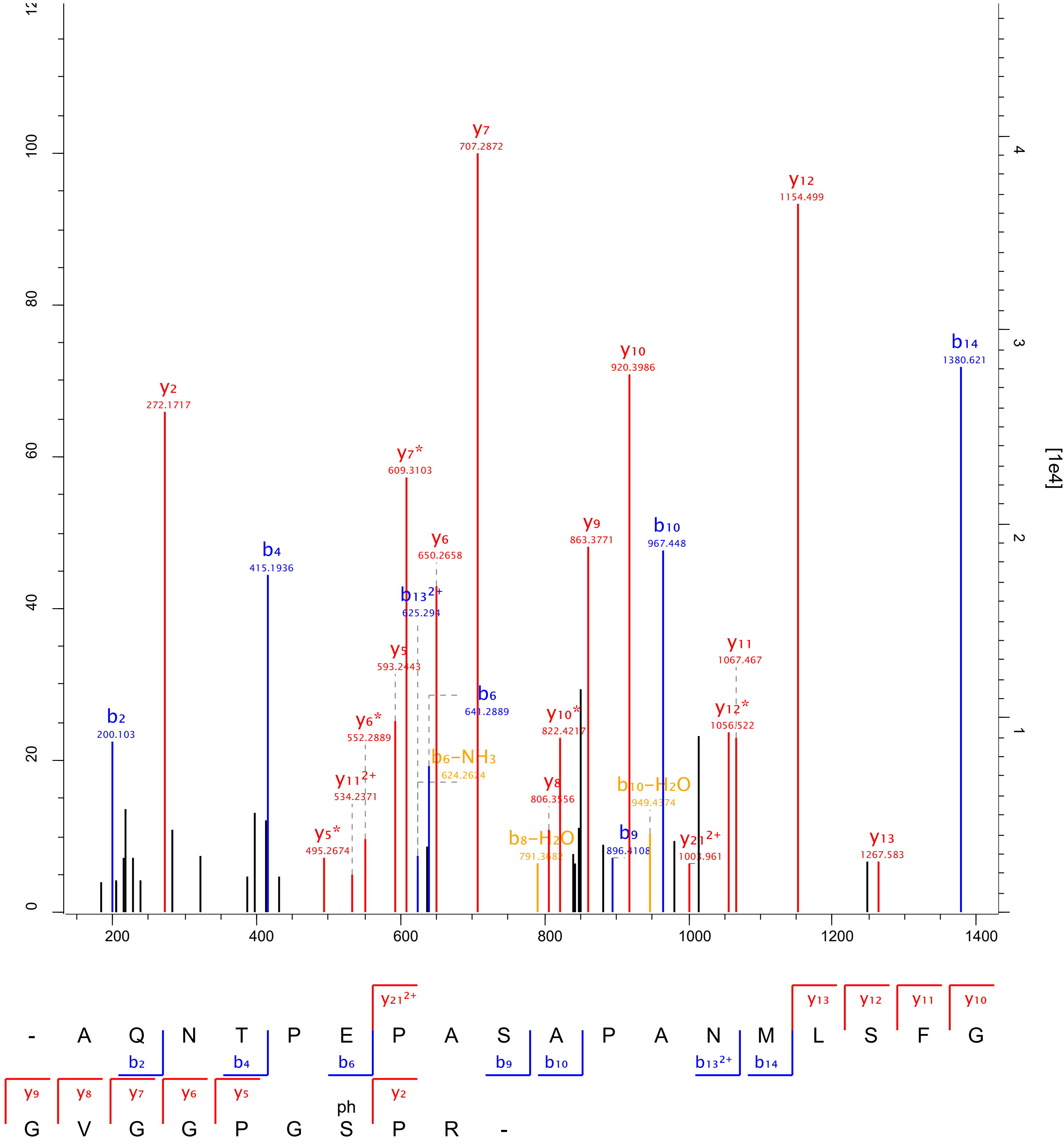

Sample output to test PDF Combine only

Raw file	Scan	Method	Score	m/z	Gene names
0523_14	20424	FTMS; HCD	126.06	1238.88	At4g31880

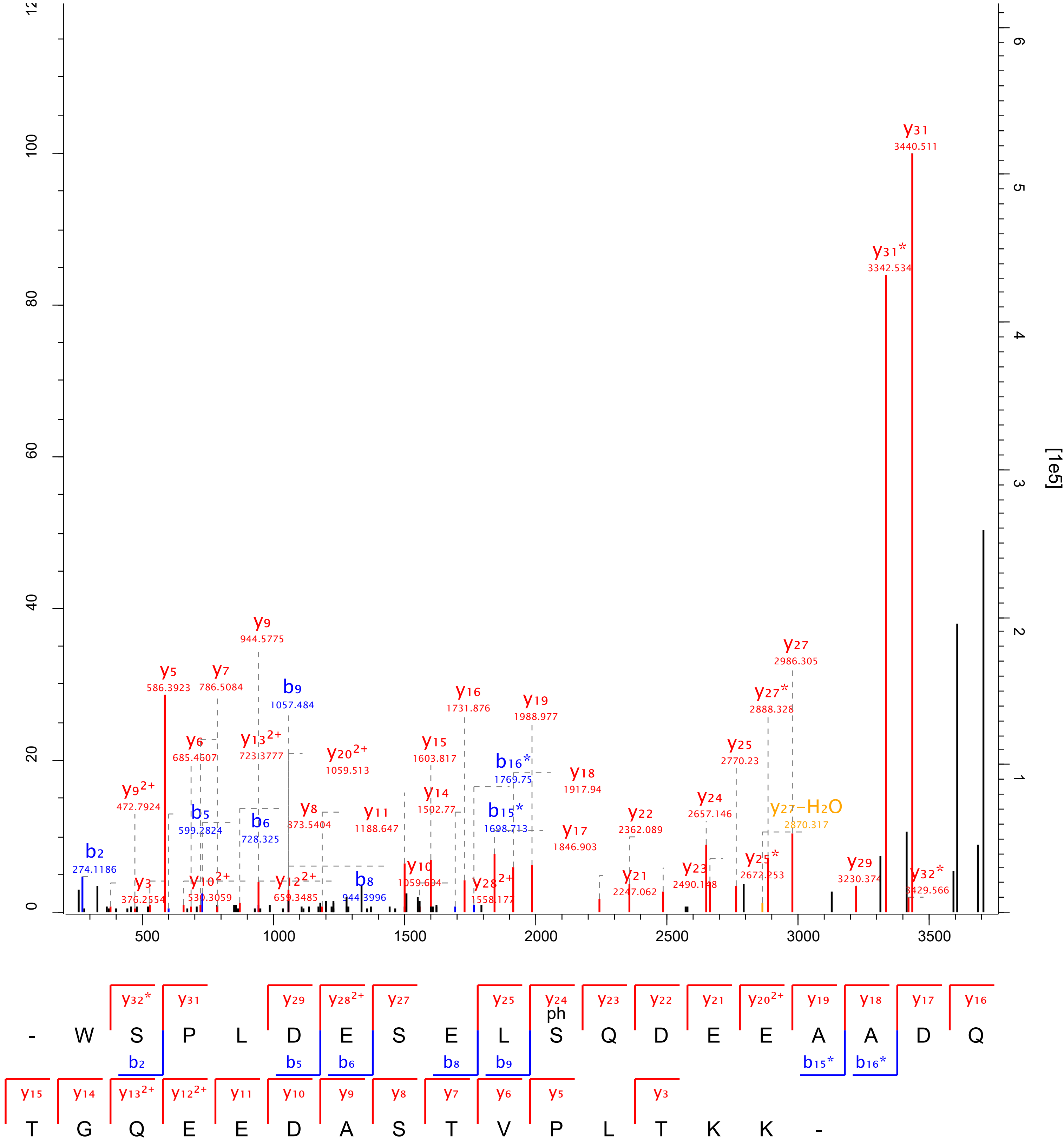

Sample output to test PDF Combine only

Raw file	Scan	Method	Score	m/z	Gene names
0523_14	21332	FTMS; HCD	85.06	790	T8B10_110

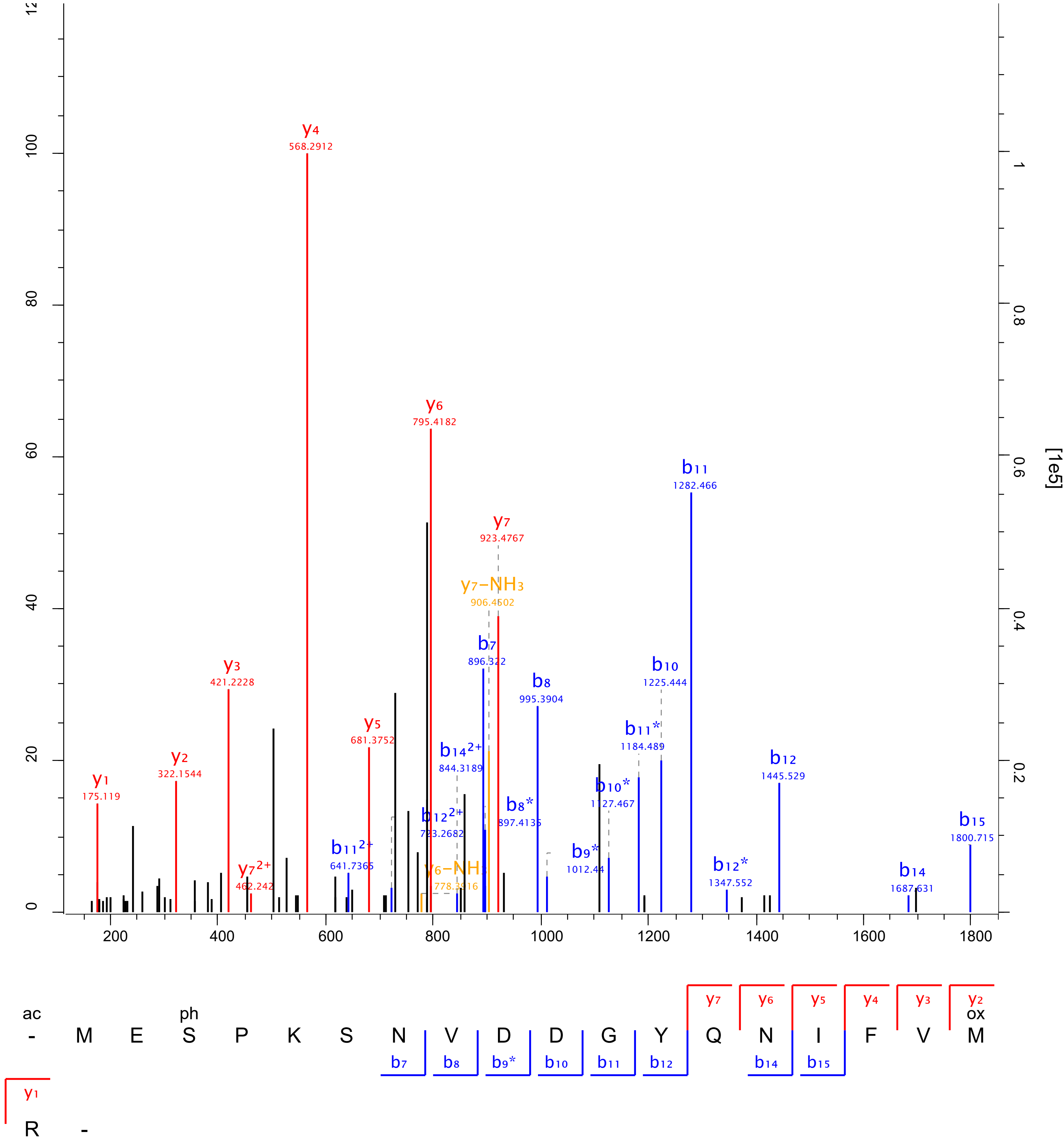

Sample output to test PDF Combine only

Raw file	Scan	Method	Score	m/z	Gene names
0523_14	21509	FTMS; HCD	132.42	952.09	IQD31

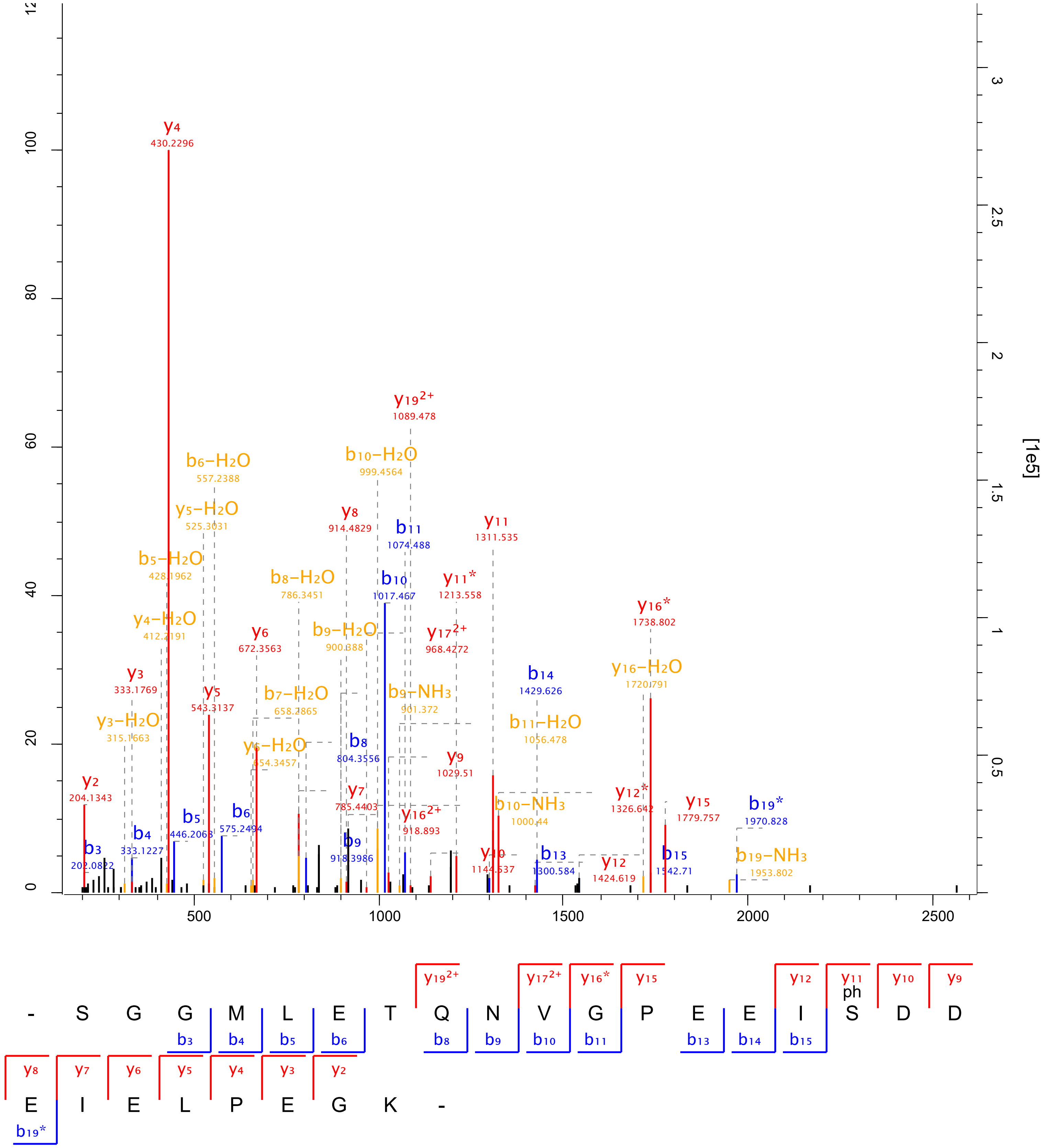

Sample output to test PDF Combine only

Raw file	Scan	Method	Score	m/z	Gene names
0523_14	22135	FTMS; HCD	63.87	774	At4g12780;At4g12770

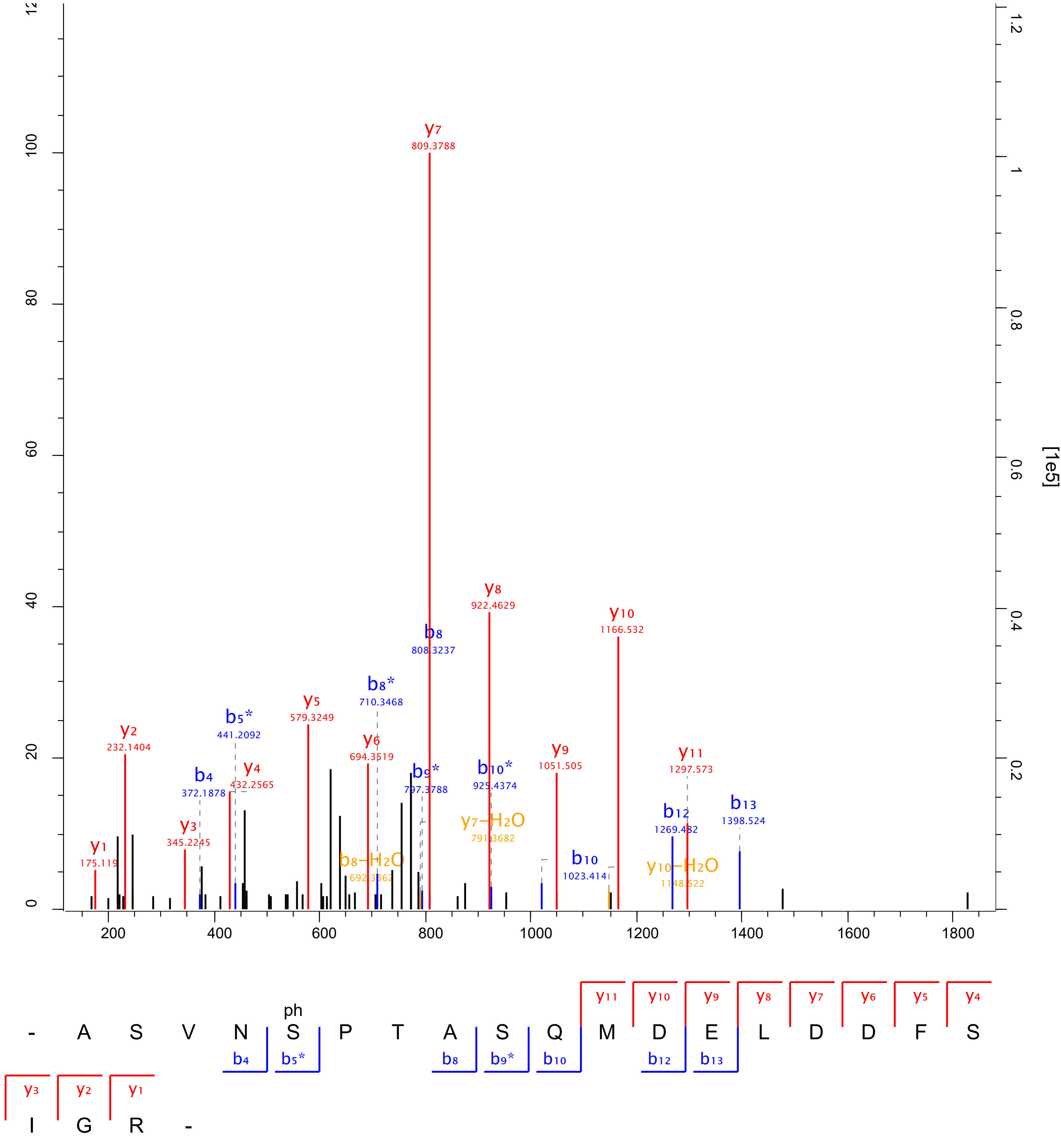

Sample output to test PDF Combine only

Raw file	Scan	Method	Score	m/z	Gene names
0523_14	22953	FTMS; HCD	74.56	1077.48	At2g34970

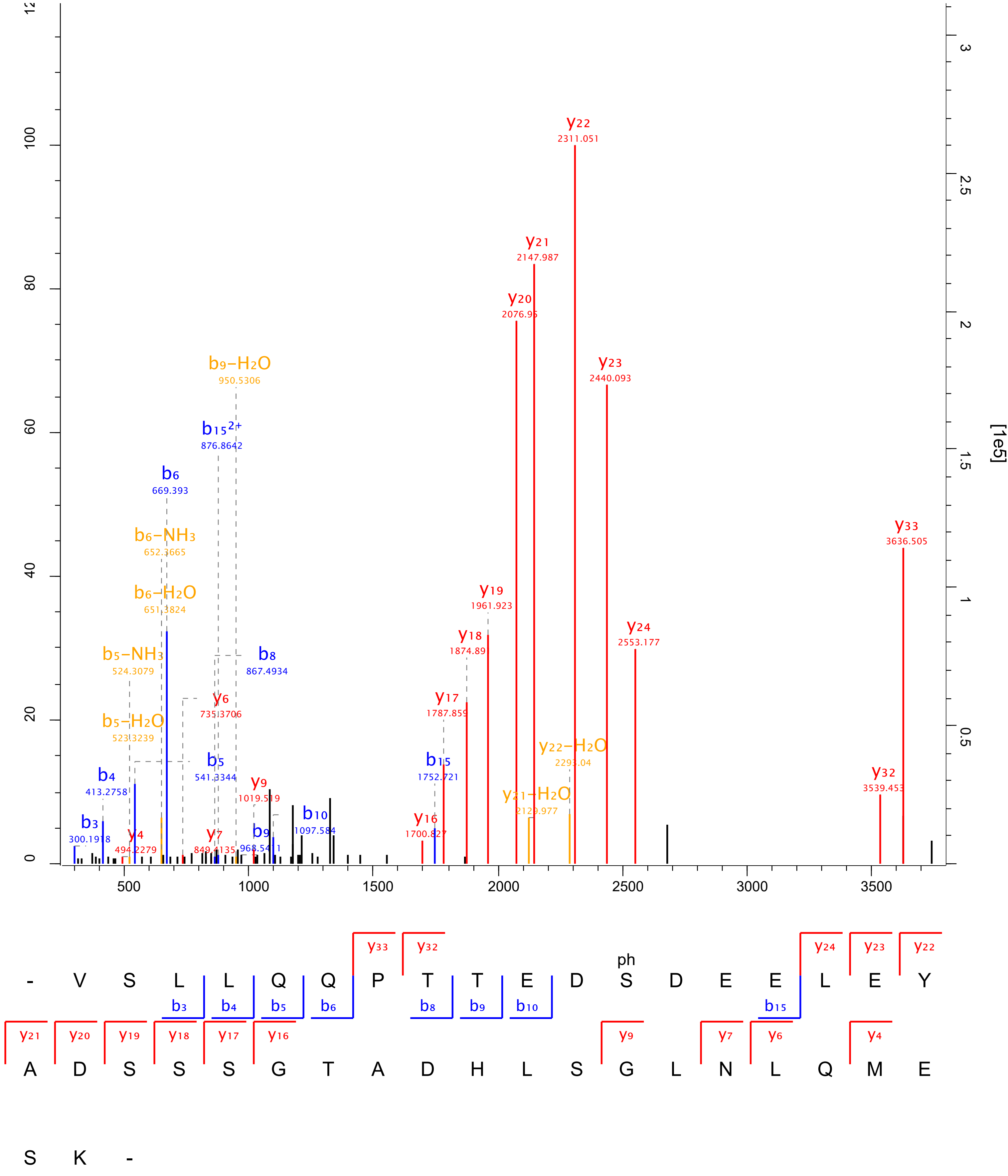

Sample output to test PDF Combine only

Raw file	Scan	Method	Score	m/z	Gene names
0523_14	26239	FTMS; HCD	248.75	696.77	PIP2-4

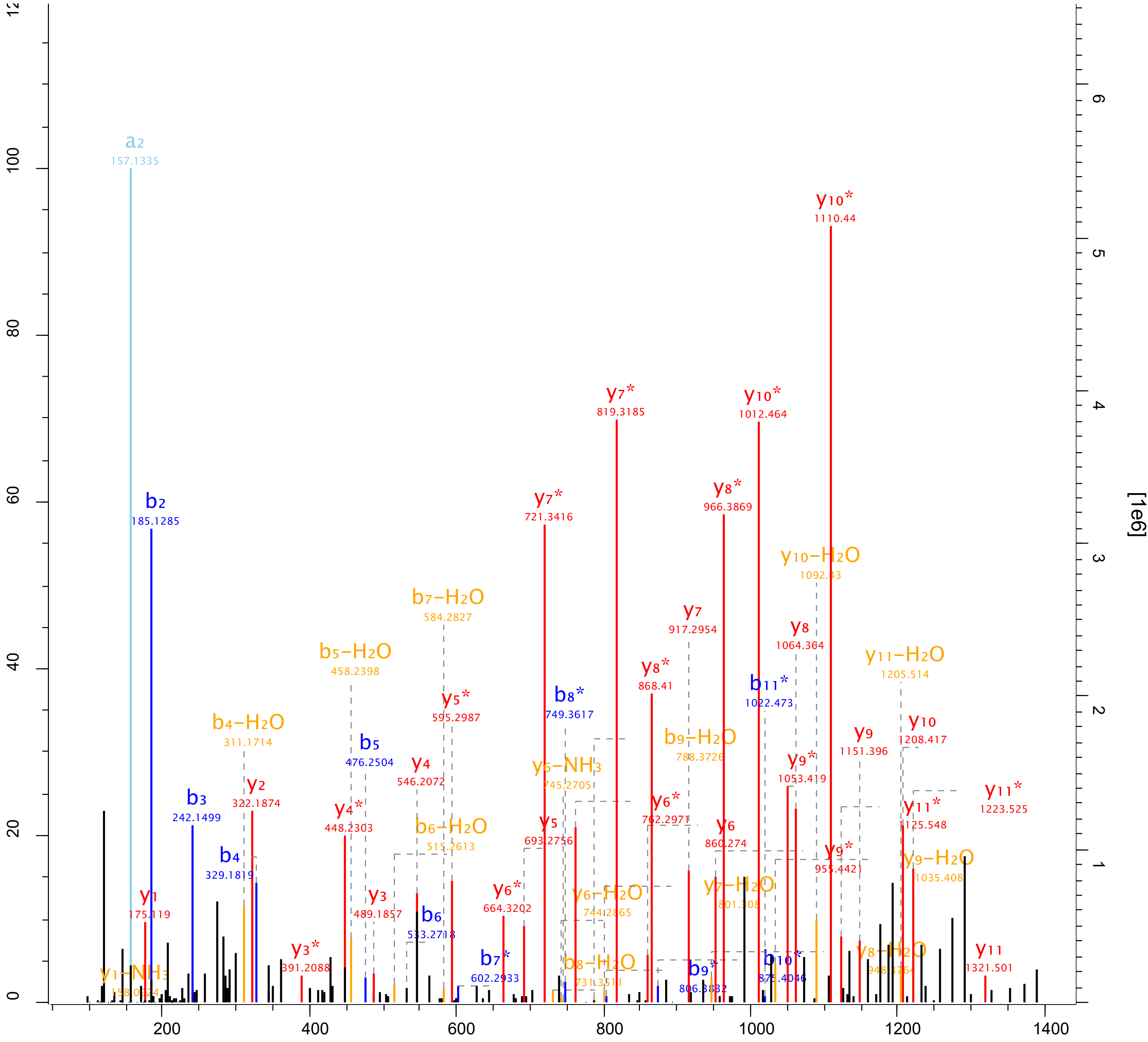

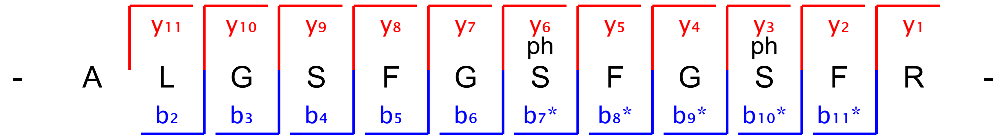

Sample output to test PDF Combine only

Raw file	Scan	Method	Score	m/z	Gene names
0523_14	28699	FTMS; HCD	130.72	706.78	SYP121

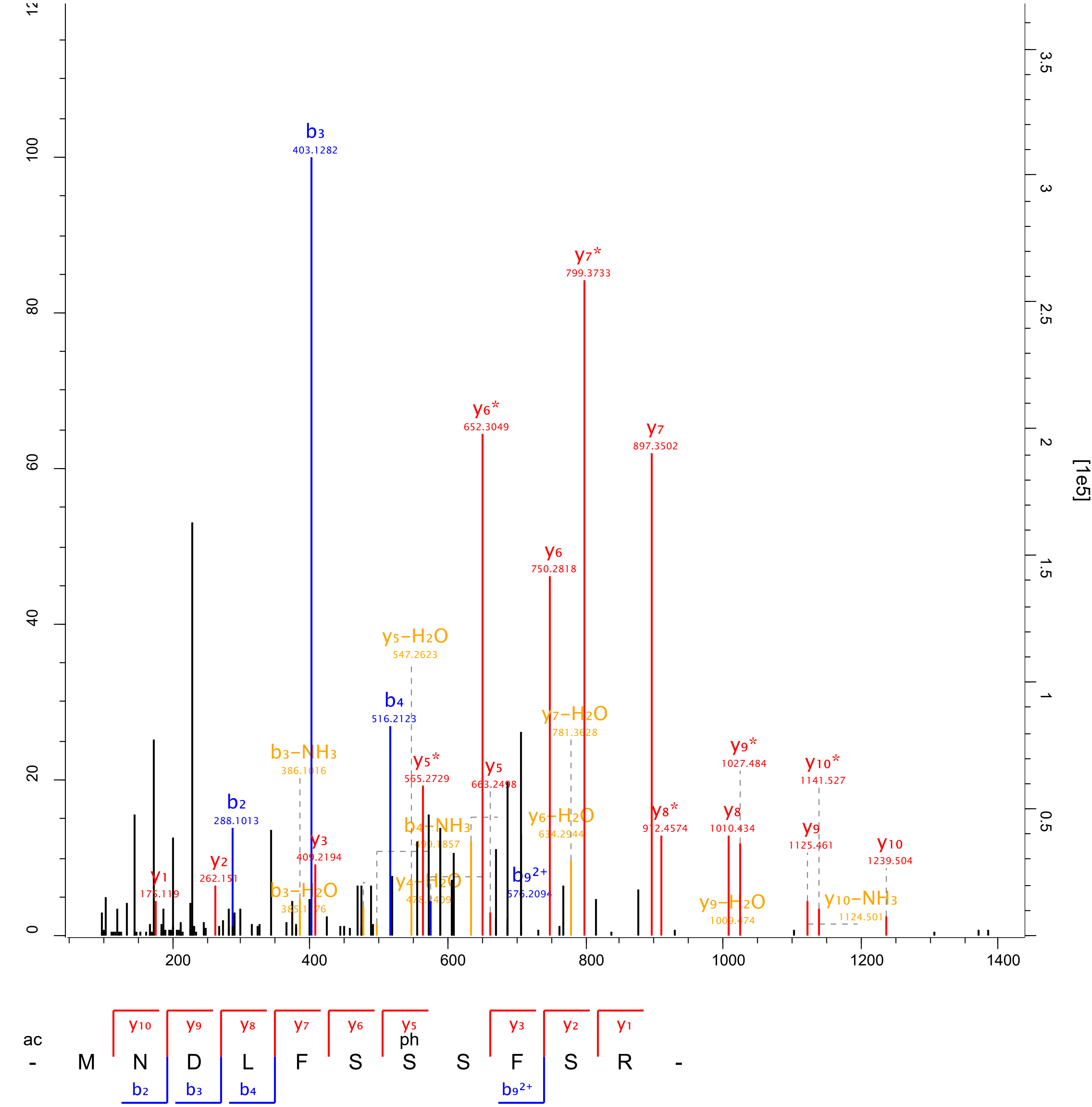

Sample output to test PDF Combine only

Raw file	Scan	Method	Score	m/z	Gene names
0523_15	1446	FTMS; HCD	86.77	426.21	IQD14

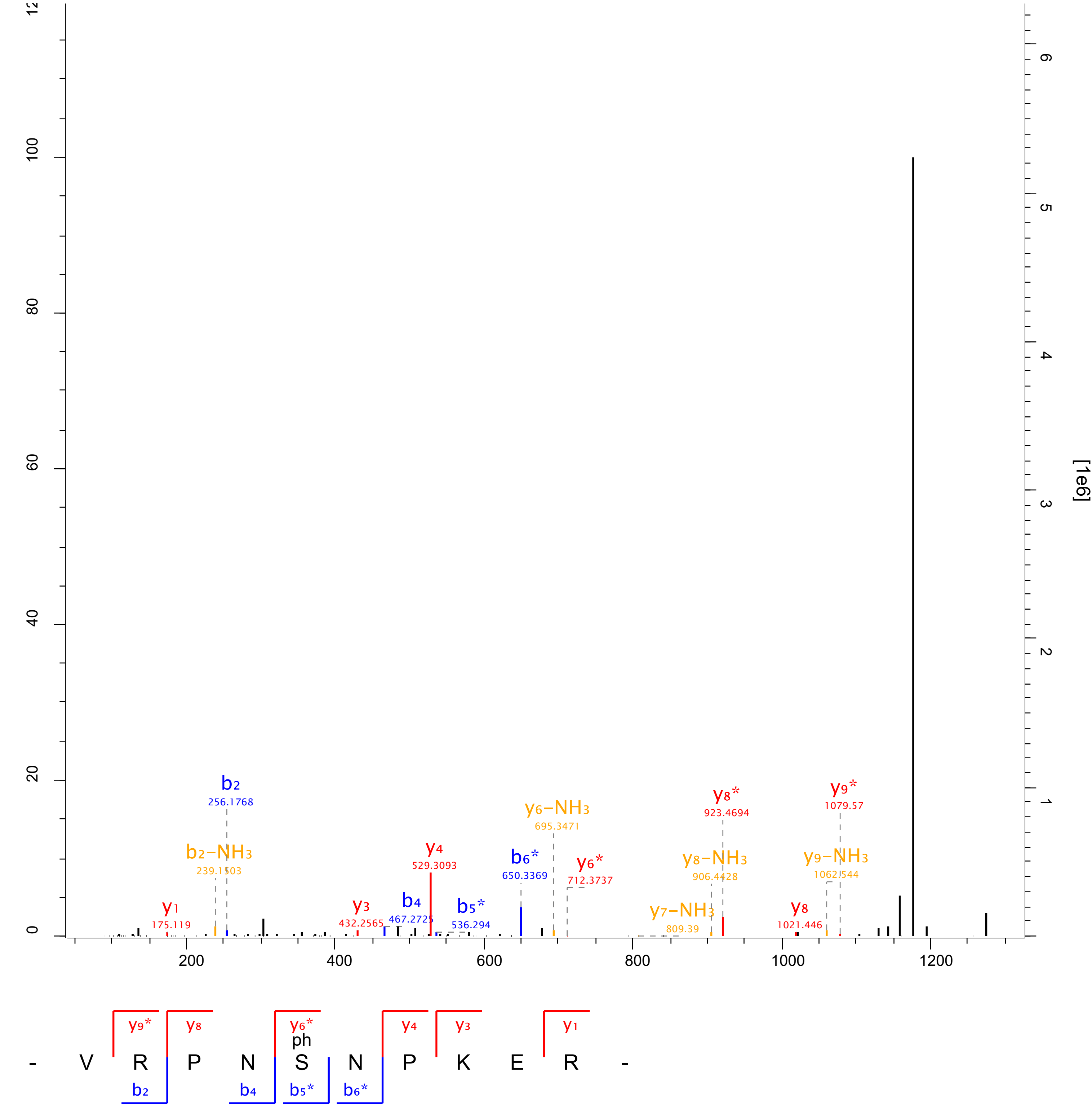

Sample output to test PDF Combine only

Raw file	Scan	Method	Score	m/z	Gene names
0523_15	1463	FTMS; HCD	105.2	365.51	F23A5.29

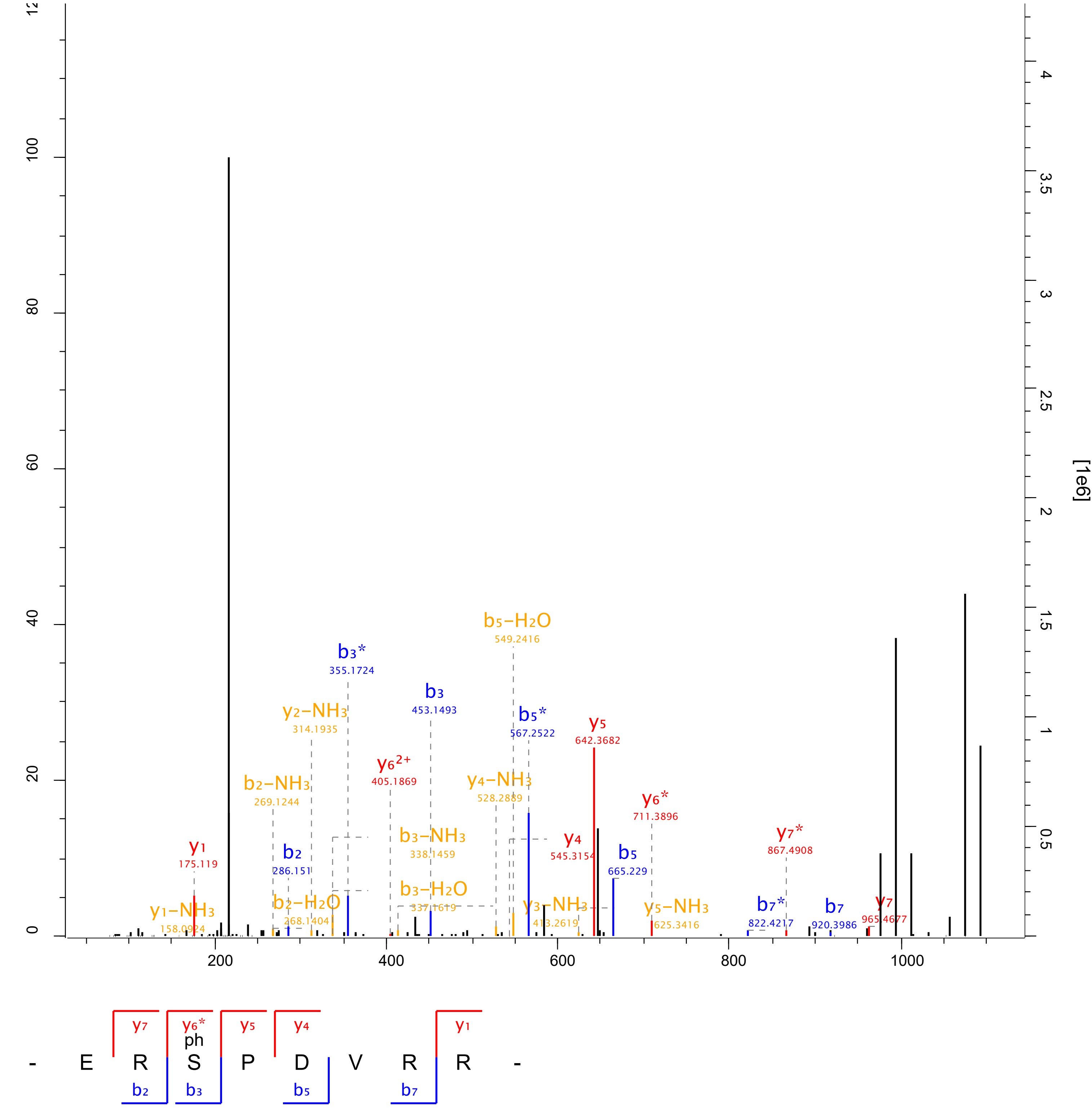

Sample output to test PDF Combine only

Raw file	Scan	Method	Score	m/z	Gene names
0523_15	1464	FTMS; HCD	43.76	397.84	PECT1

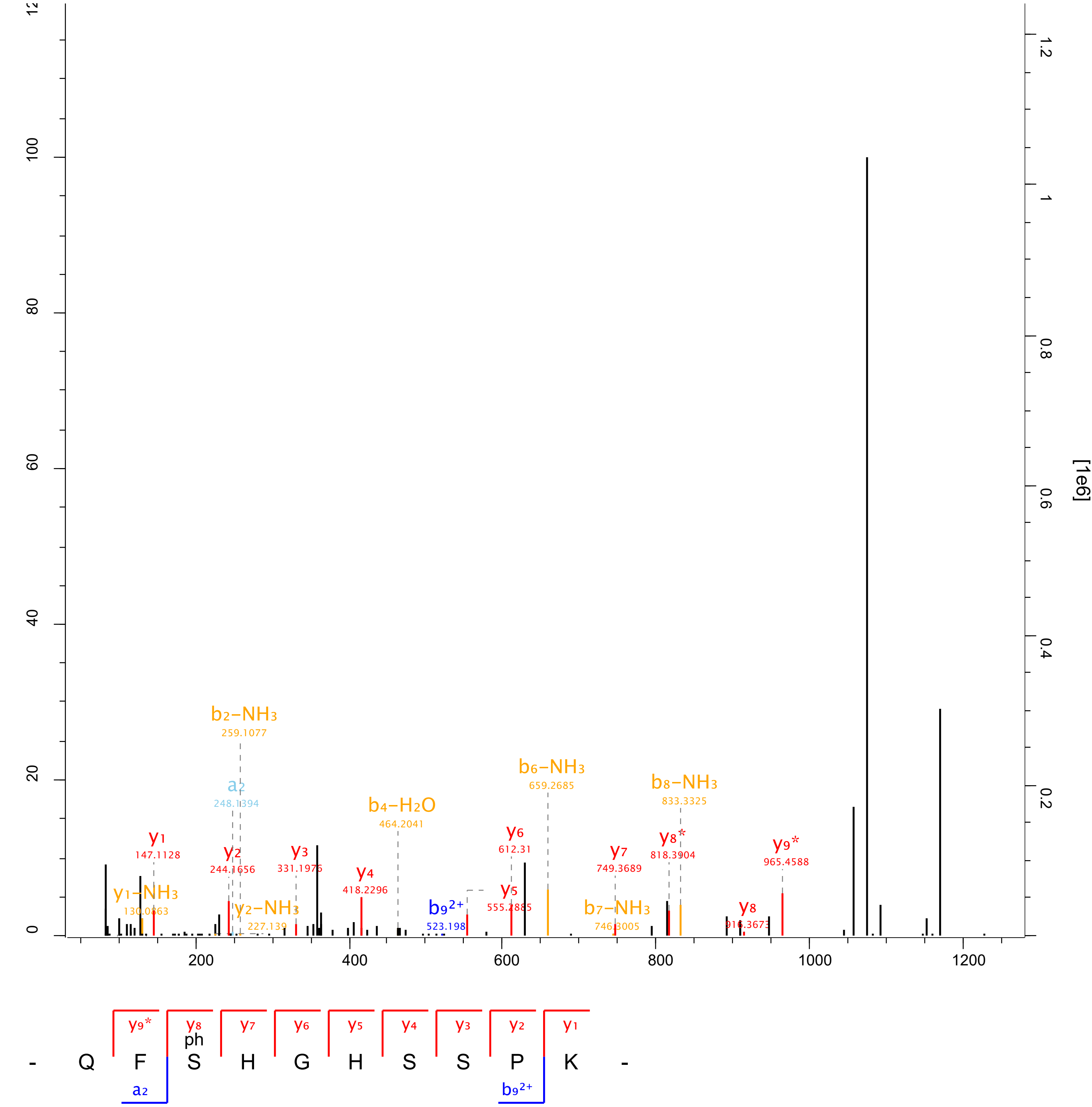

Sample output to test PDF Combine only

Raw file	Scan	Method	Score	m/z
0523_15	1469	FTMS; HCD	183.74	565.22

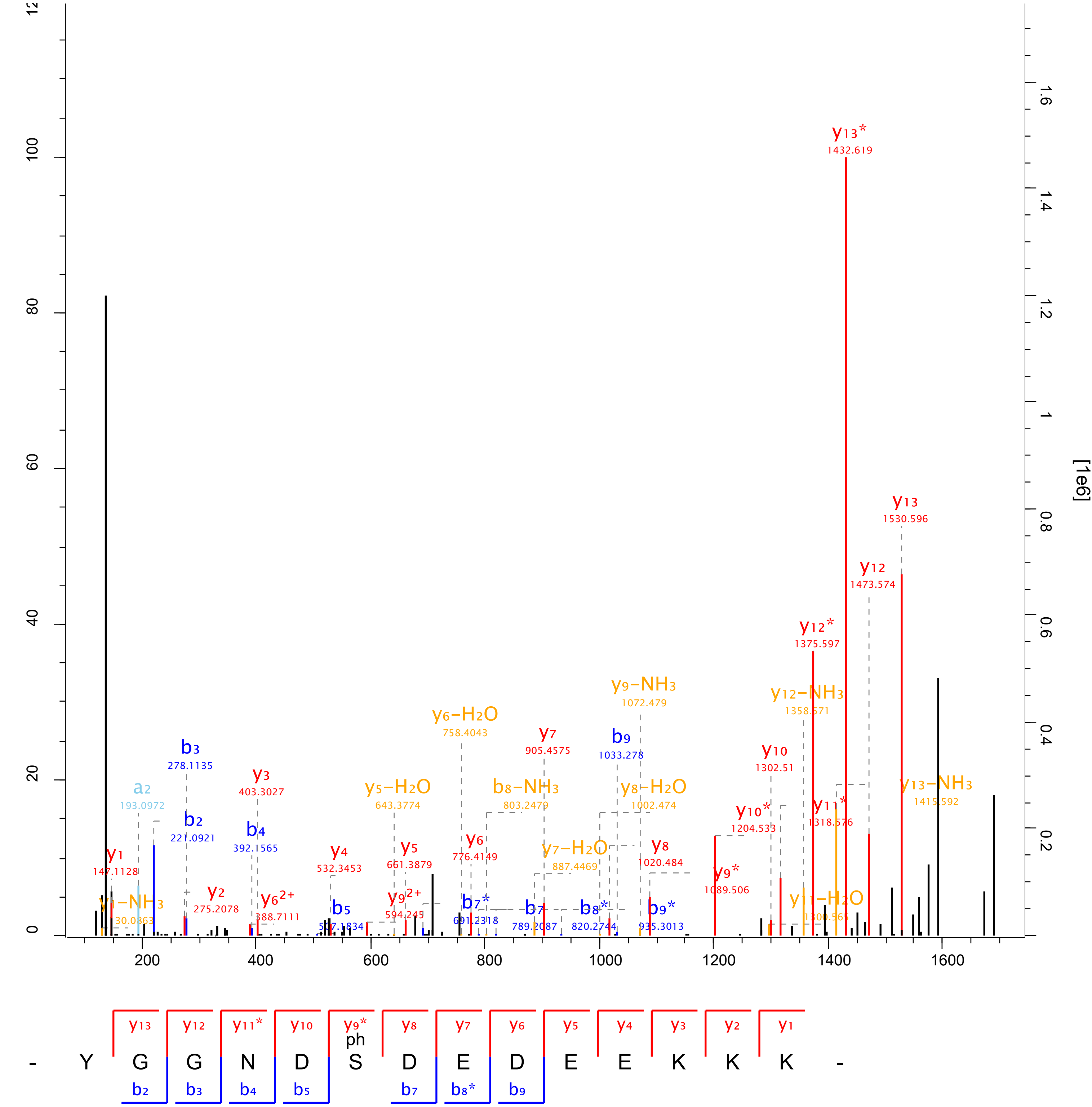

Sample output to test PDF Combine only

Raw file	Scan	Method	Score	m/z	Gene names
0523_15	1477	FTMS; HCD	117.38	593.25	CPK3

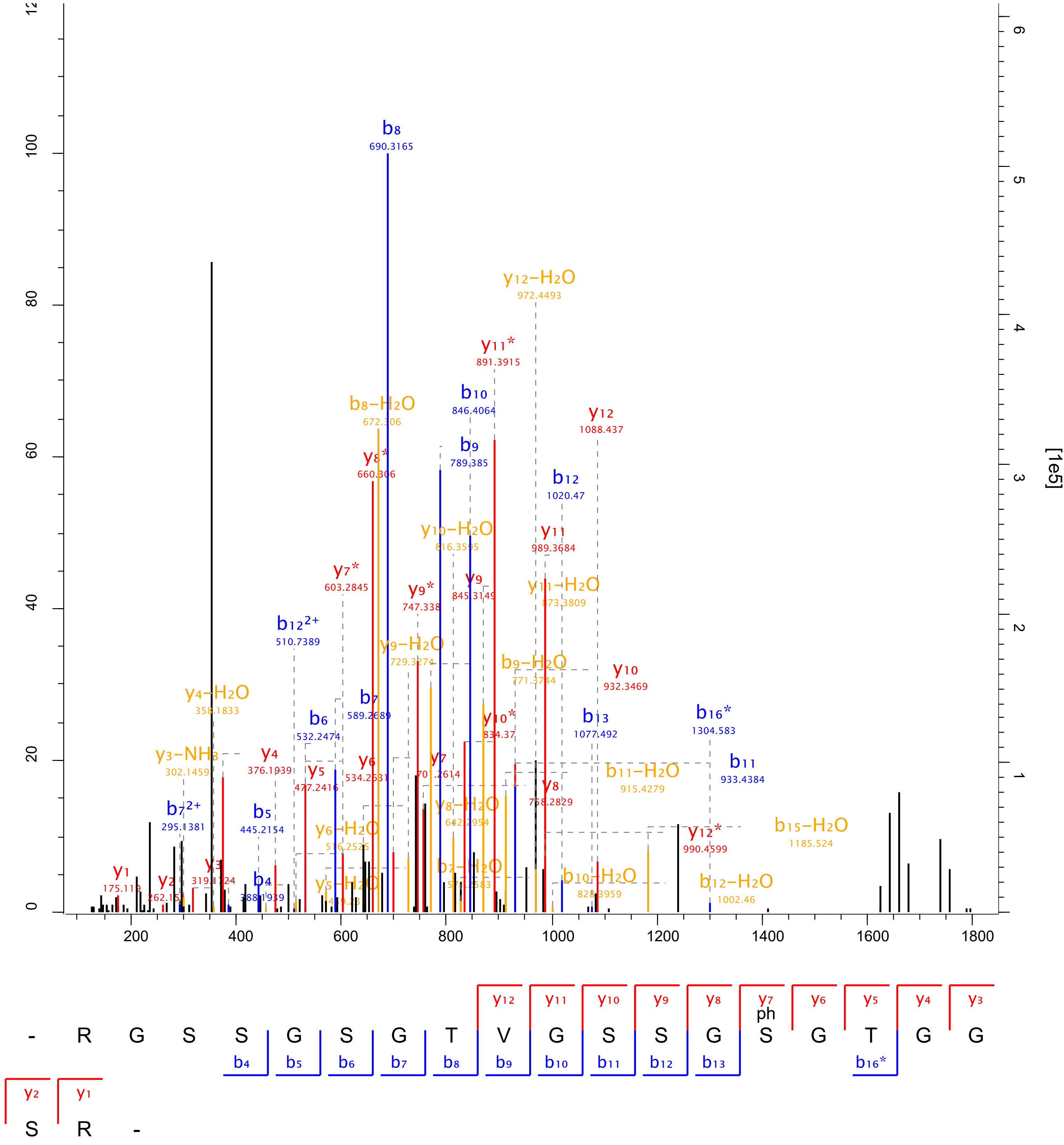

Raw file	Scan	Method	Score	m/z	Gene names
0523_15	1494	FTMS; HCD	160.46	558.24	RTNLB1

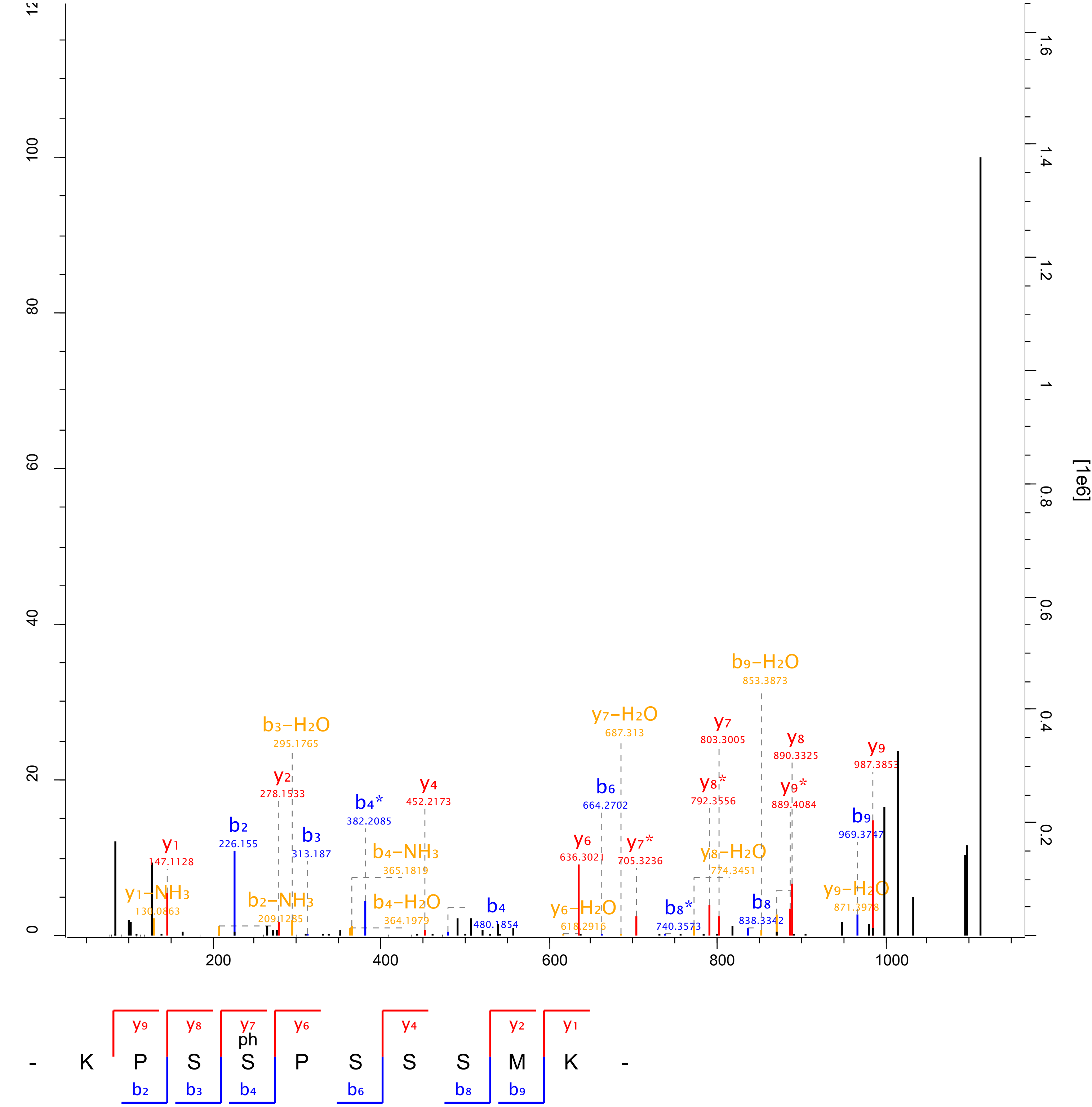

Sample output to test PDF Combine only

Raw file	Scan	Method	Score	m/z	Gene names
0523_15	1538	FTMS; HCD	72.29	396.85	At2g29210

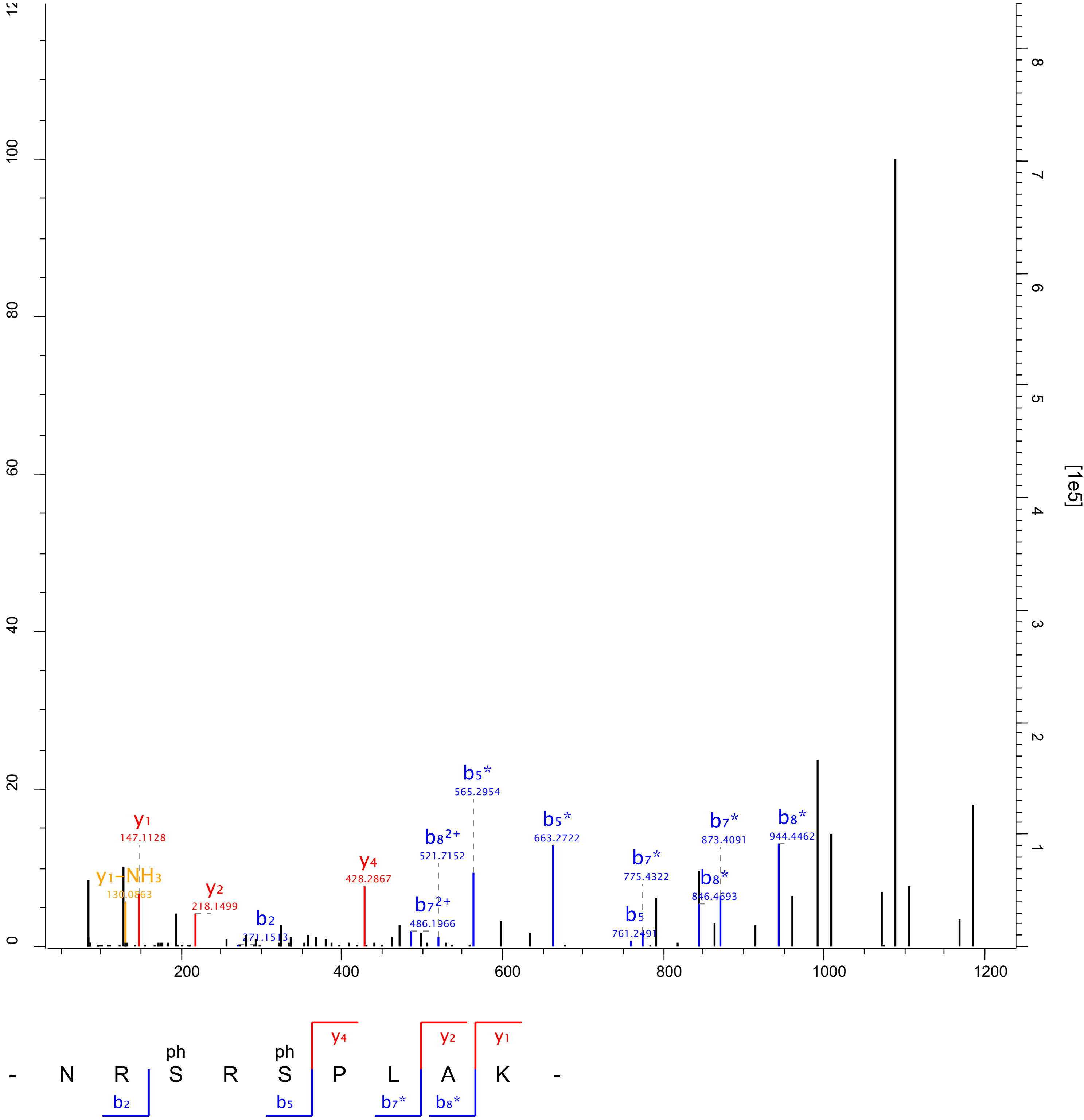

Sample output to test PDF Combine only

Raw file	Scan	Method	Score	m/z	Gene names
0523_15	1555	FTMS; HCD	59.23	506.25	F9K20.7

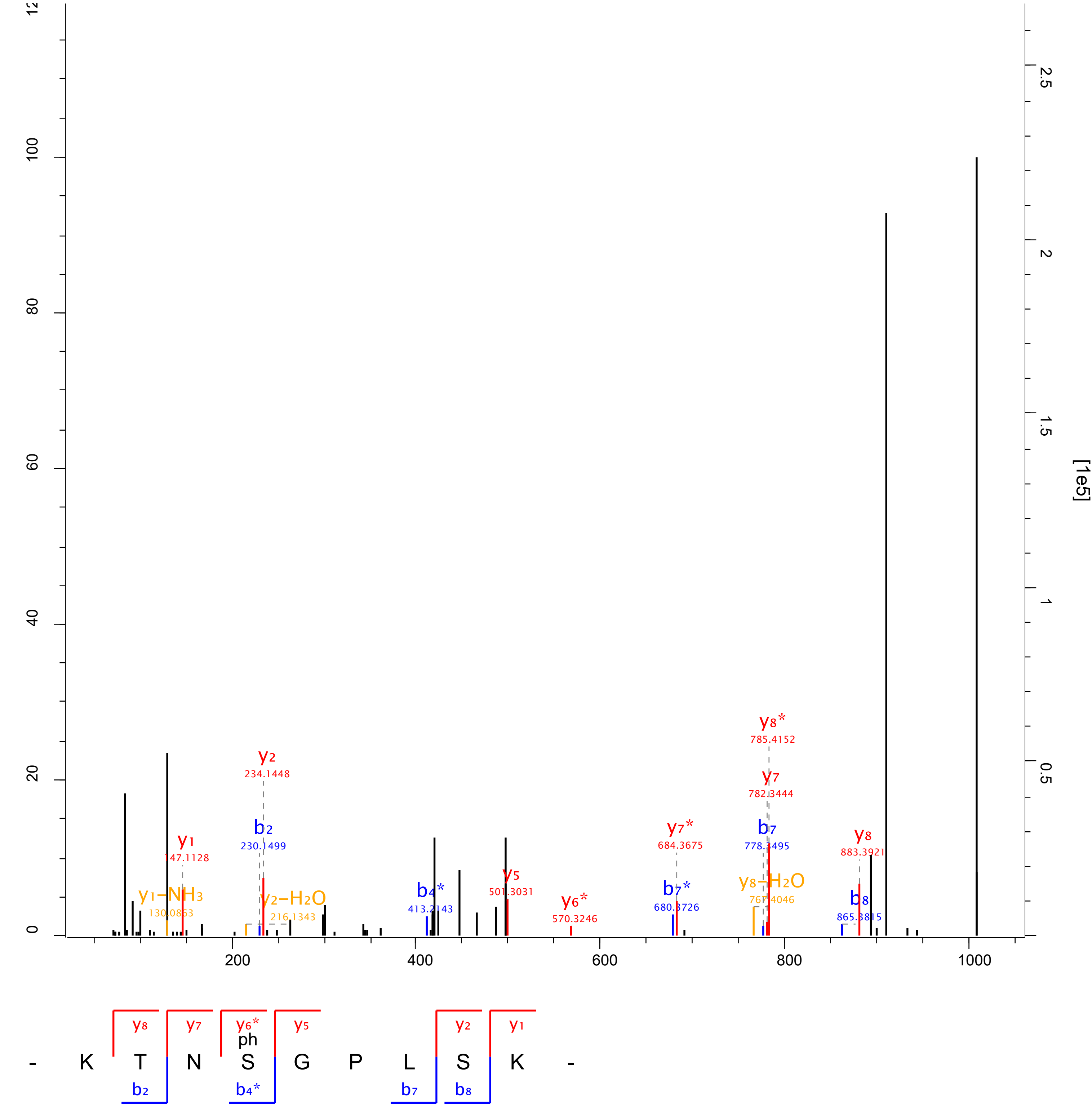

Sample output to test PDF Combine only

Raw file	Scan	Method	Score	m/z	Gene names
0523_15	1558	FTMS; HCD	76.68	433.18	KING1

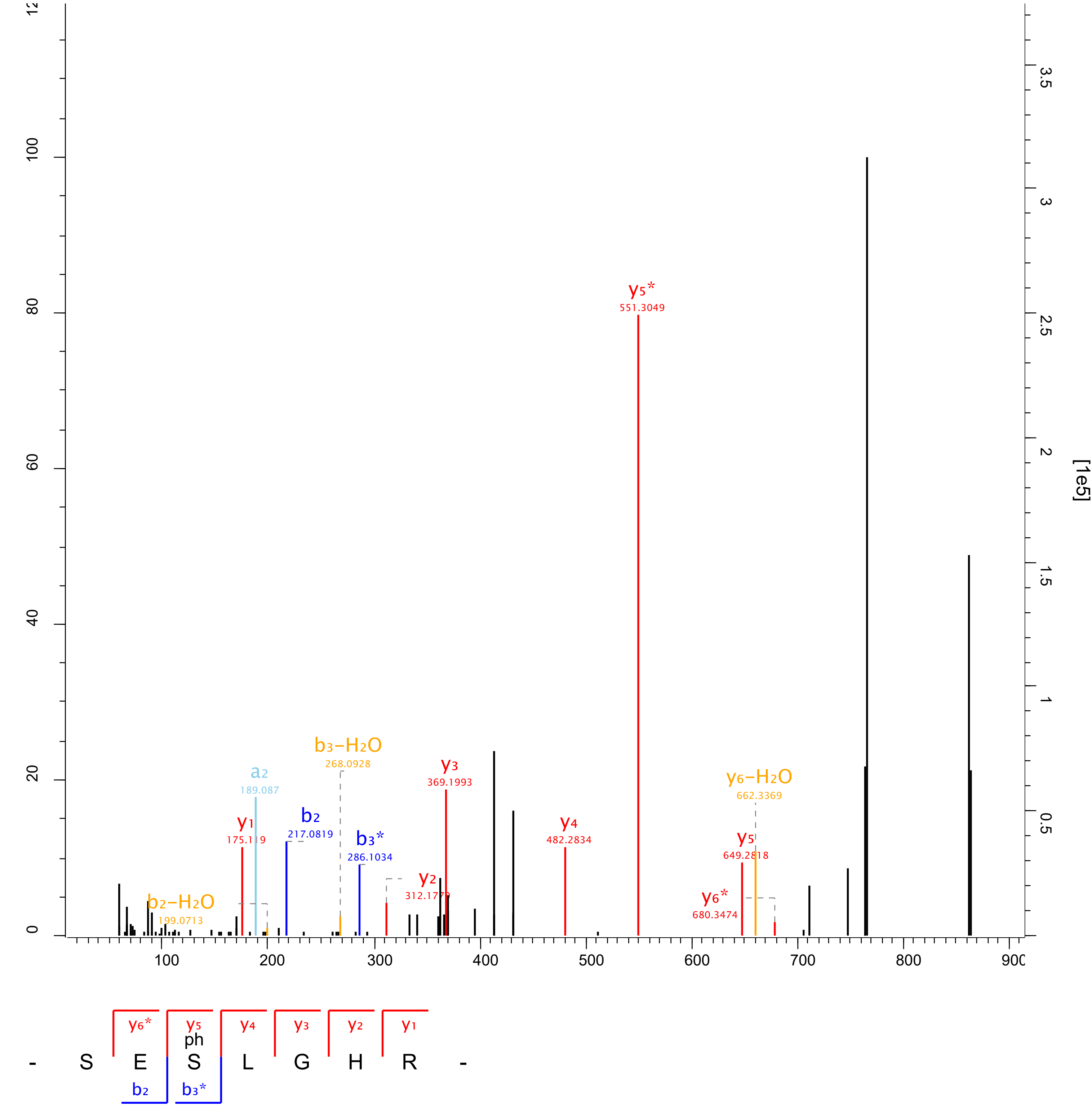

Sample output to test PDF Combine only

Raw file	Scan	Method	Score	m/z	Gene names
0523_15	9420	FTMS; HCD	46.35	609.76	STR4

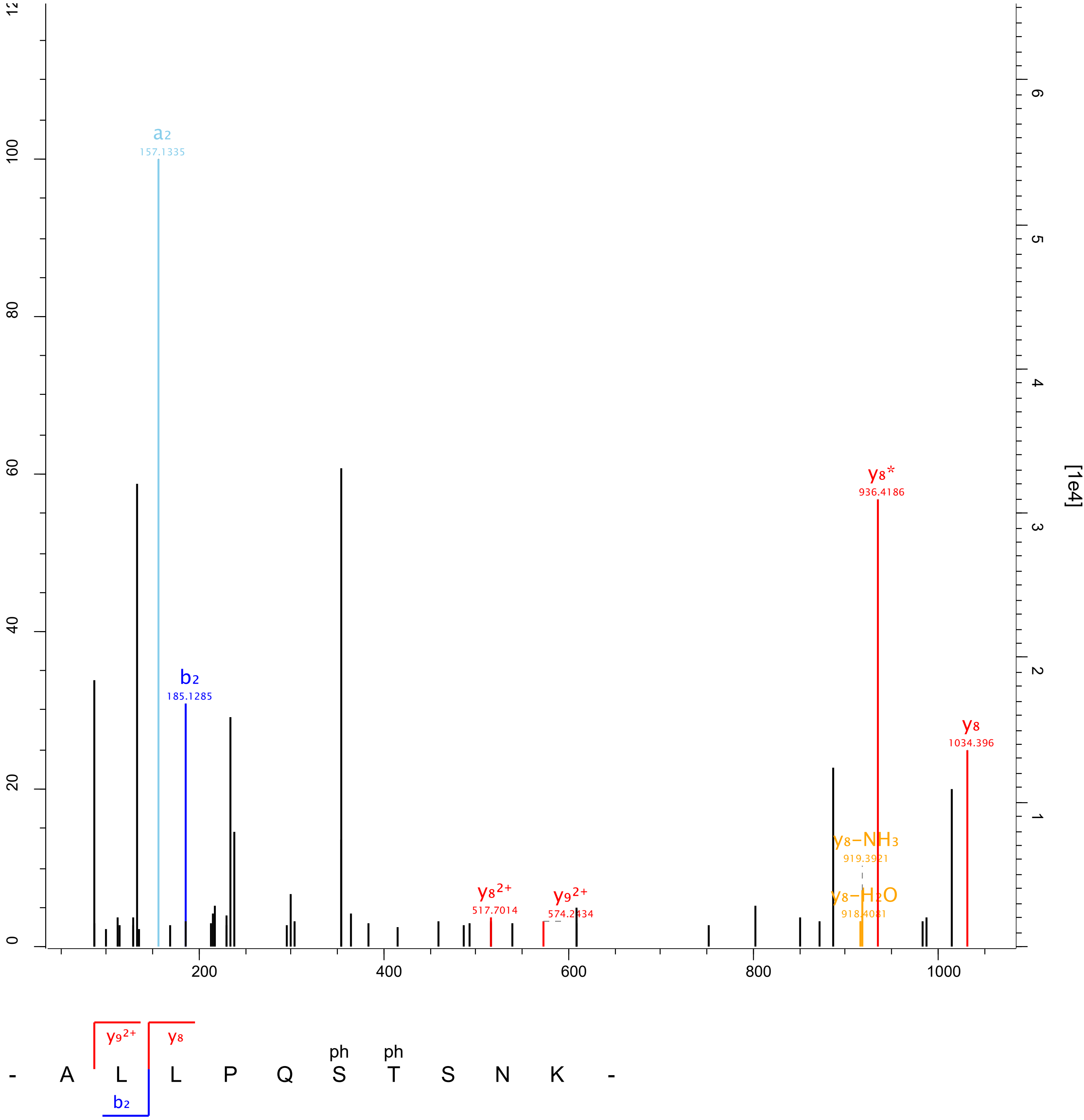

Sample output to test PDF Combine only

Raw file	Scan	Method	Score	m/z	Gene names
0523_15	14967	FTMS; HCD	78.95	606.97	ADH1

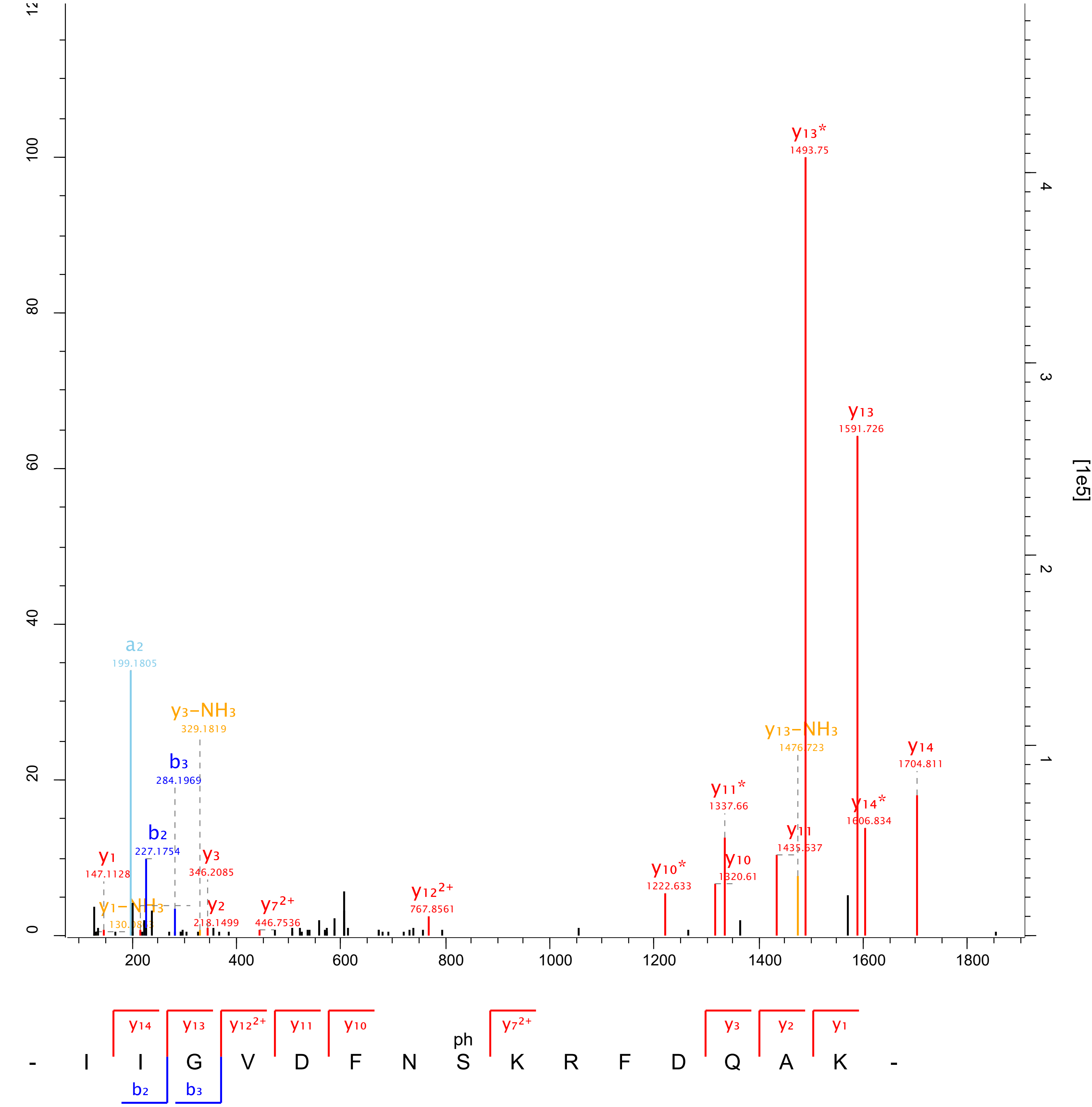

Sample output to test PDF Combine only

Raw file	Scan	Method	Score	m/z	Gene names
0523_15	15868	FTMS; HCD	84.53	706.08	BAG7

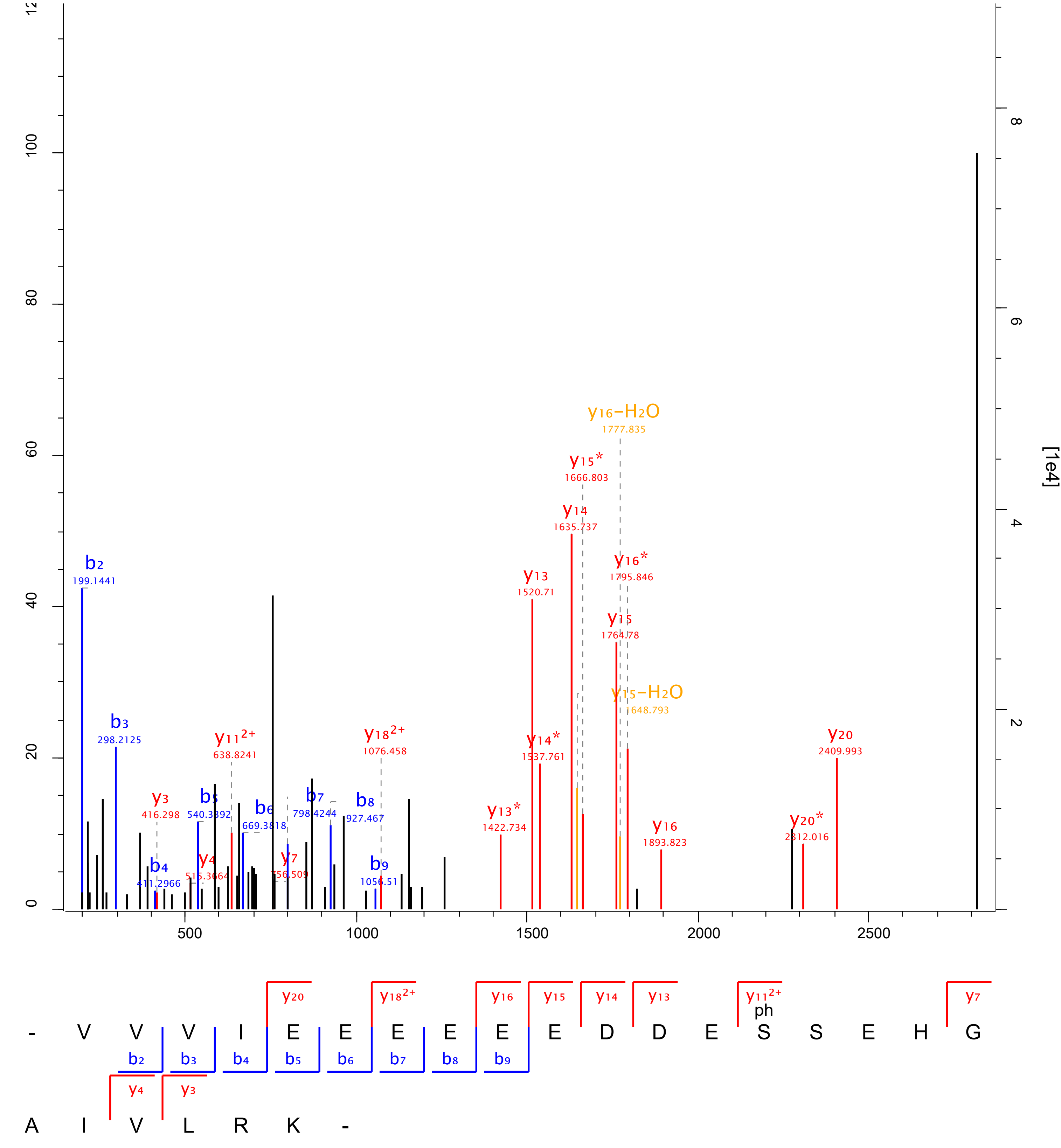

Sample output to test PDF Combine only

Raw file	Scan	Method	Score	m/z
0523_15	17520	FTMS; HCD	91.17	1011.45

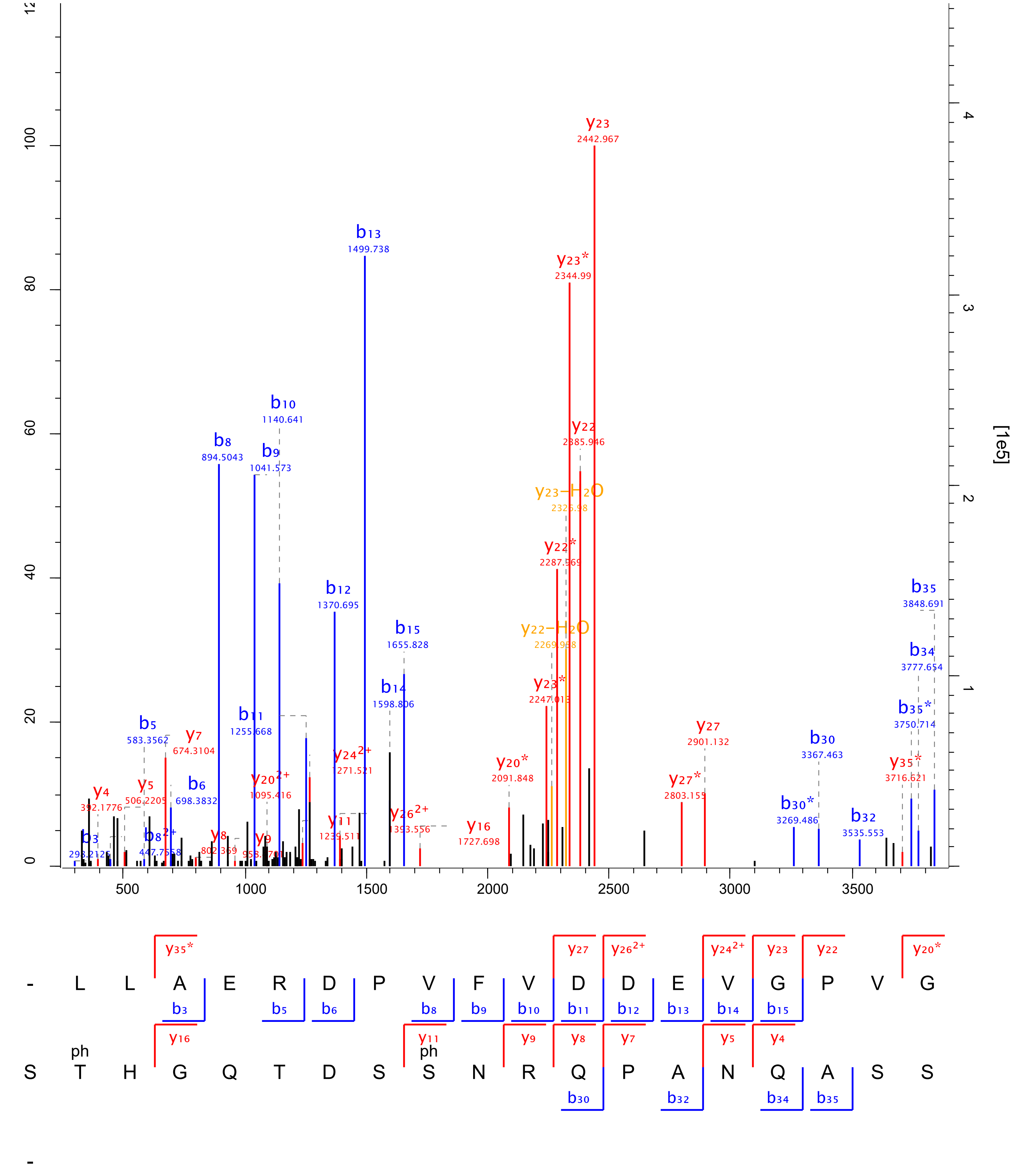

Sample output to test PDF Combine only

Raw file	Scan	Method	Score	m/z	Gene names
0523_15	19469	FTMS; HCD	205.74	824.1	SYP122

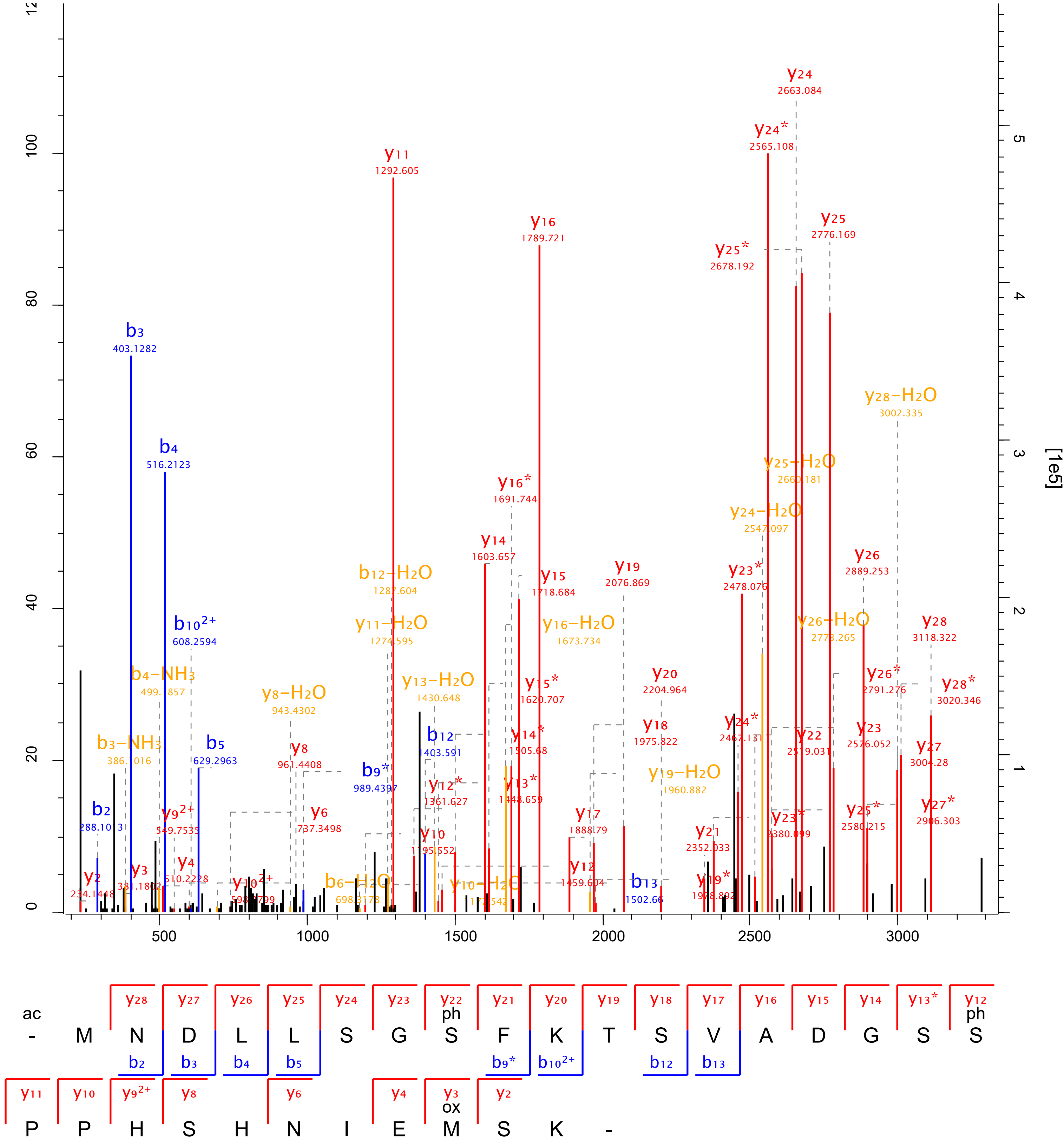

Sample output to test PDF Combine only

Raw file	Scan	Method	Score	m/z	Gene names
0523_15	19570	FTMS; HCD	158.99	1230.56	At3g57890

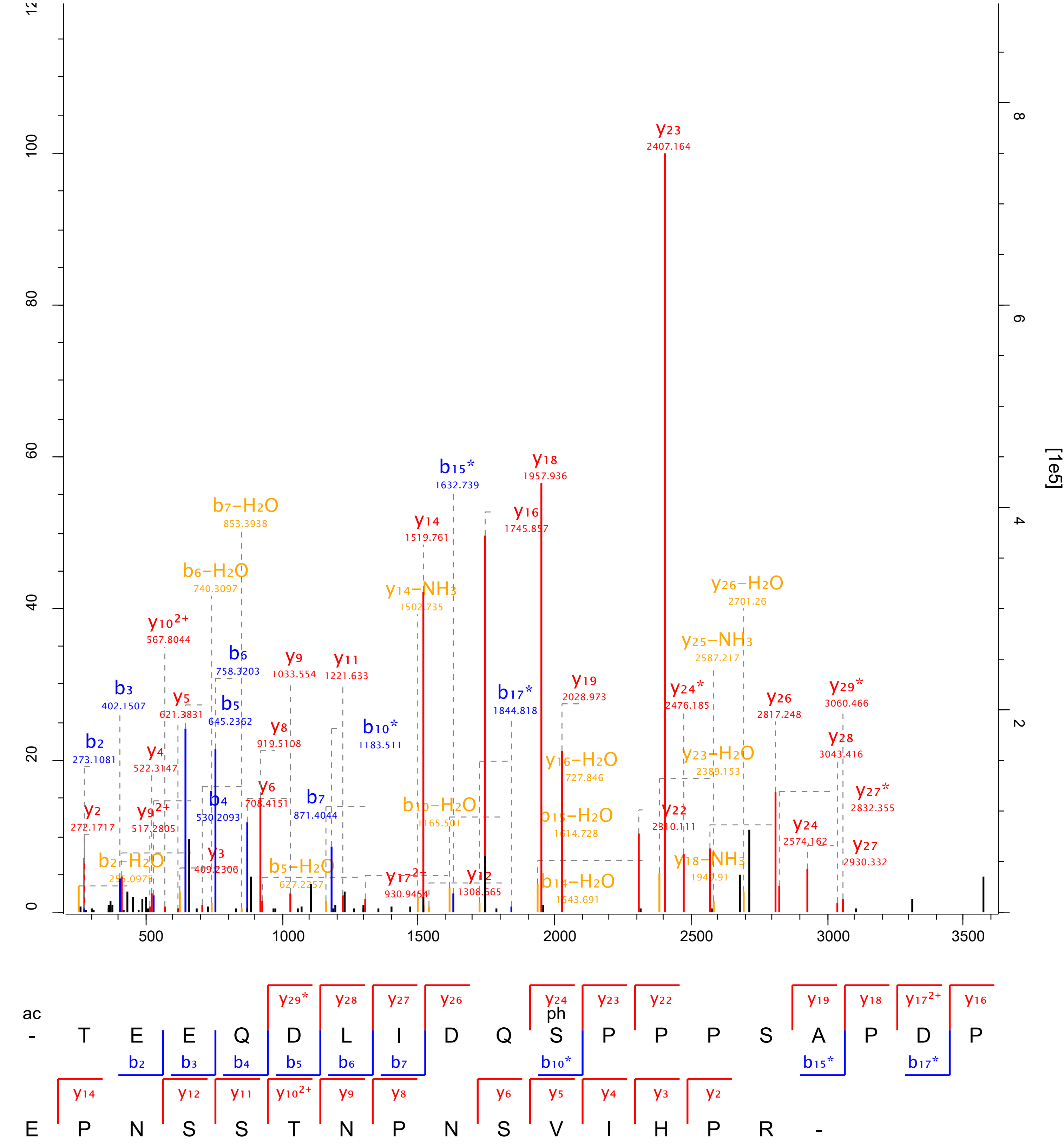

Sample output to test PDF Combine only

Raw file	Scan	Method	Score	m/z	Gene names
0523_15	19717	FTMS; HCD	153.82	794.71	At1g70730

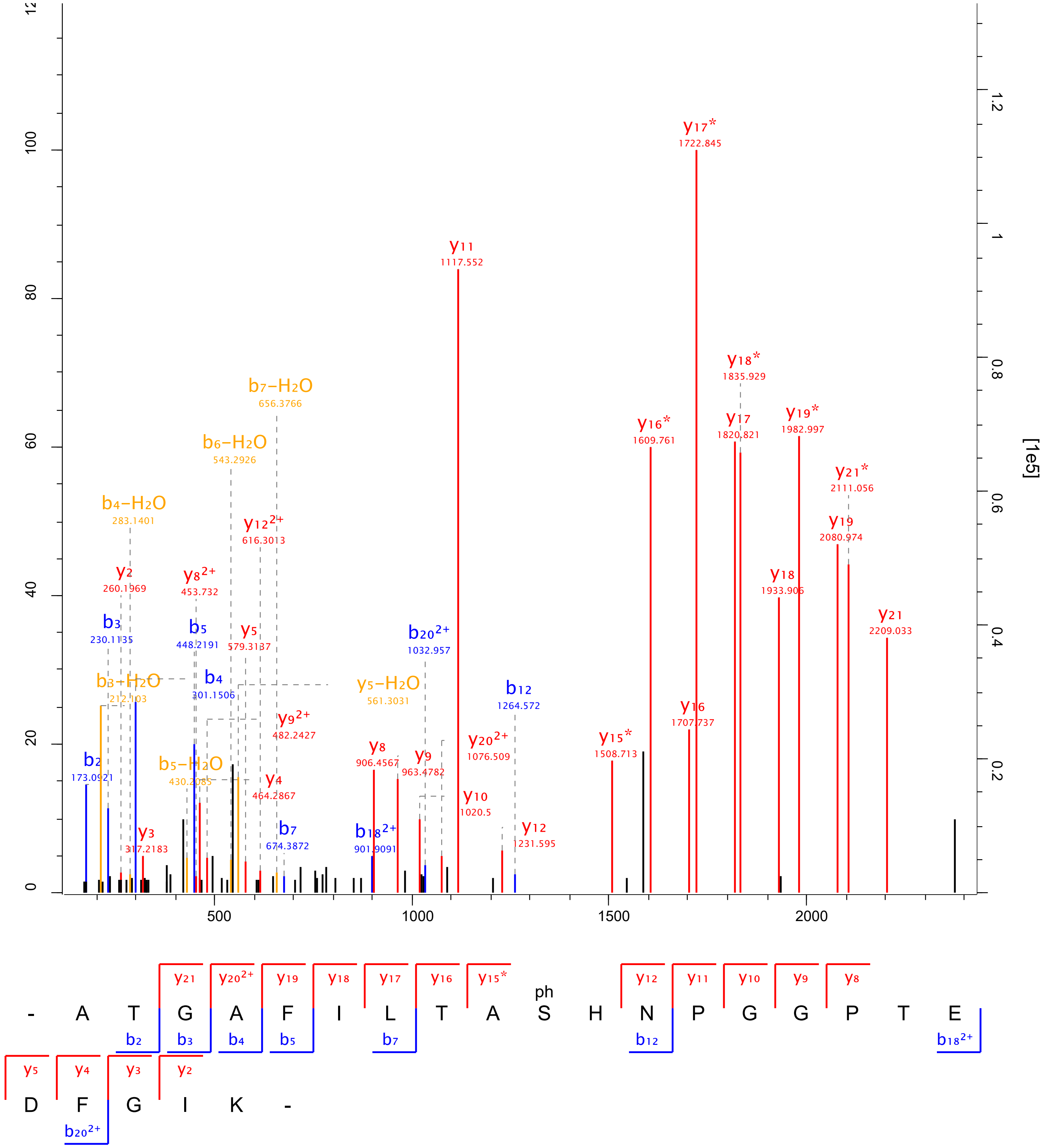

Sample output to test PDF Combine only

Raw file	Scan	Method	Score	m/z	Gene names
0523_15	19928	FTMS; HCD	155.08	880.41	At3g01310;At5g15070

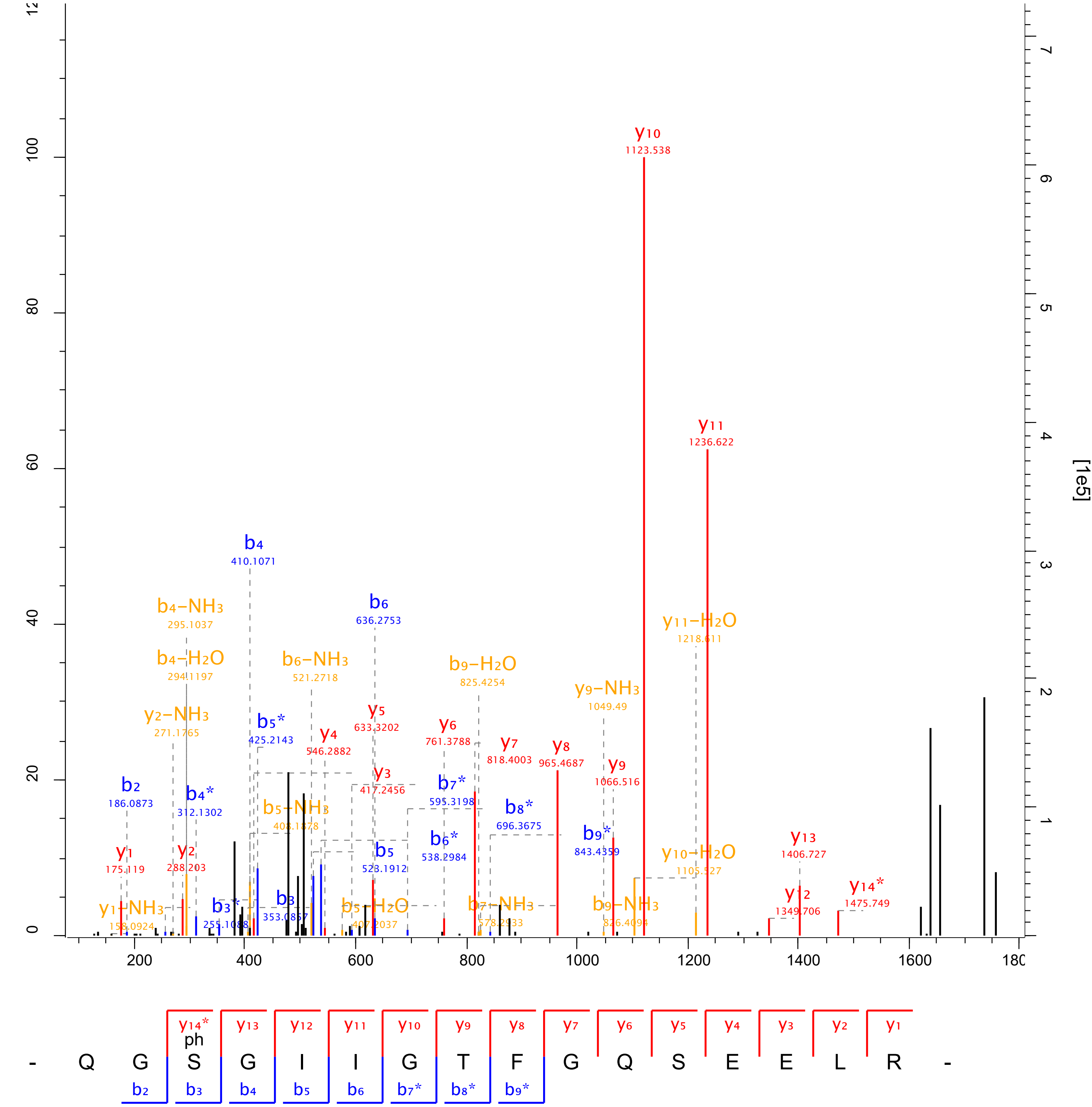

Sample output to test PDF Combine only

Raw file	Scan	Method	Score	m/z	Gene names
0523_15	20076	FTMS; HCD	92.45	951.48	RPL13B

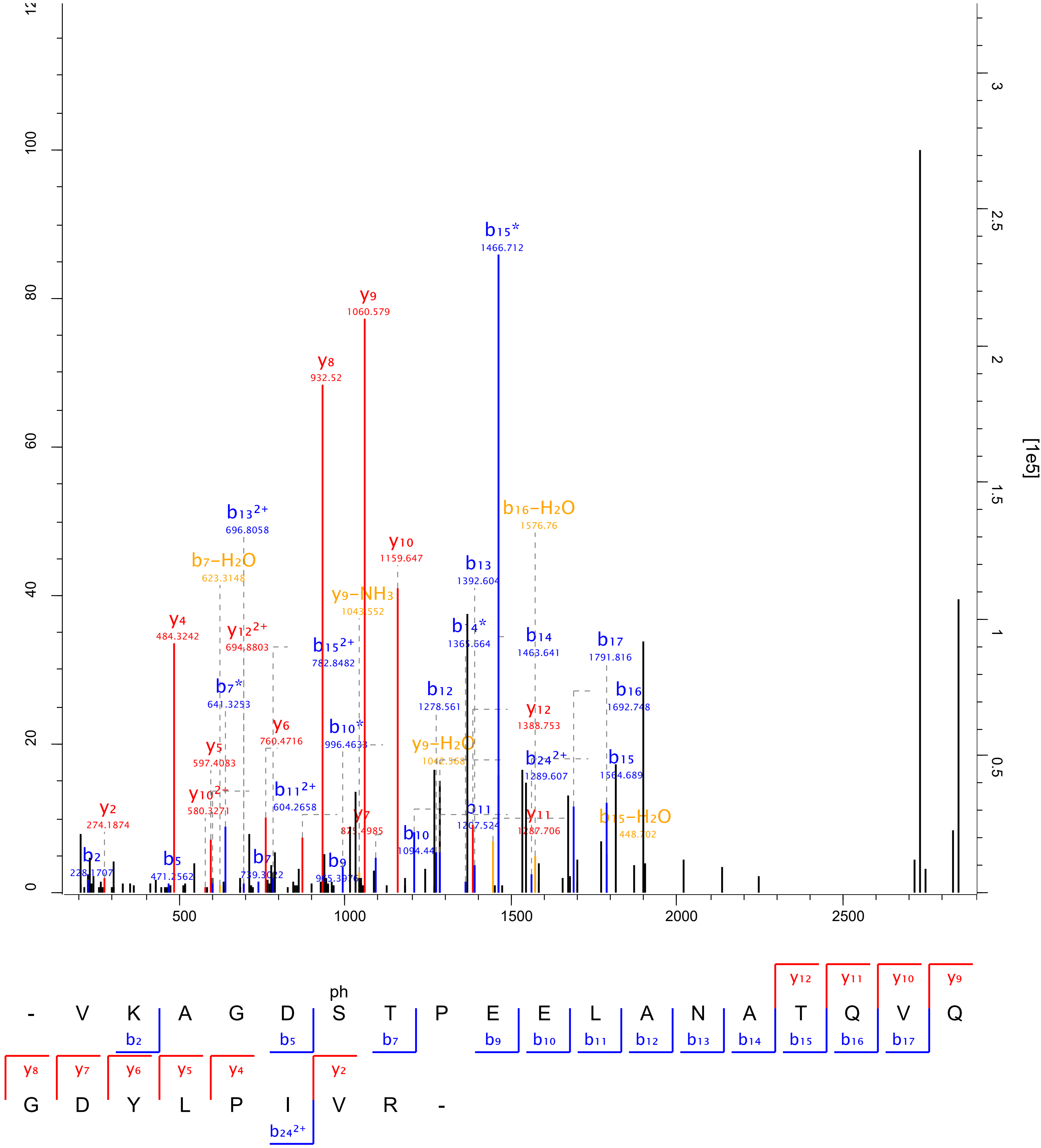

Sample output to test PDF Combine only

Raw file	Scan	Method	Score	m/z	Gene names
0523_15	20517	FTMS; HCD	132.51	710.32	LRS1

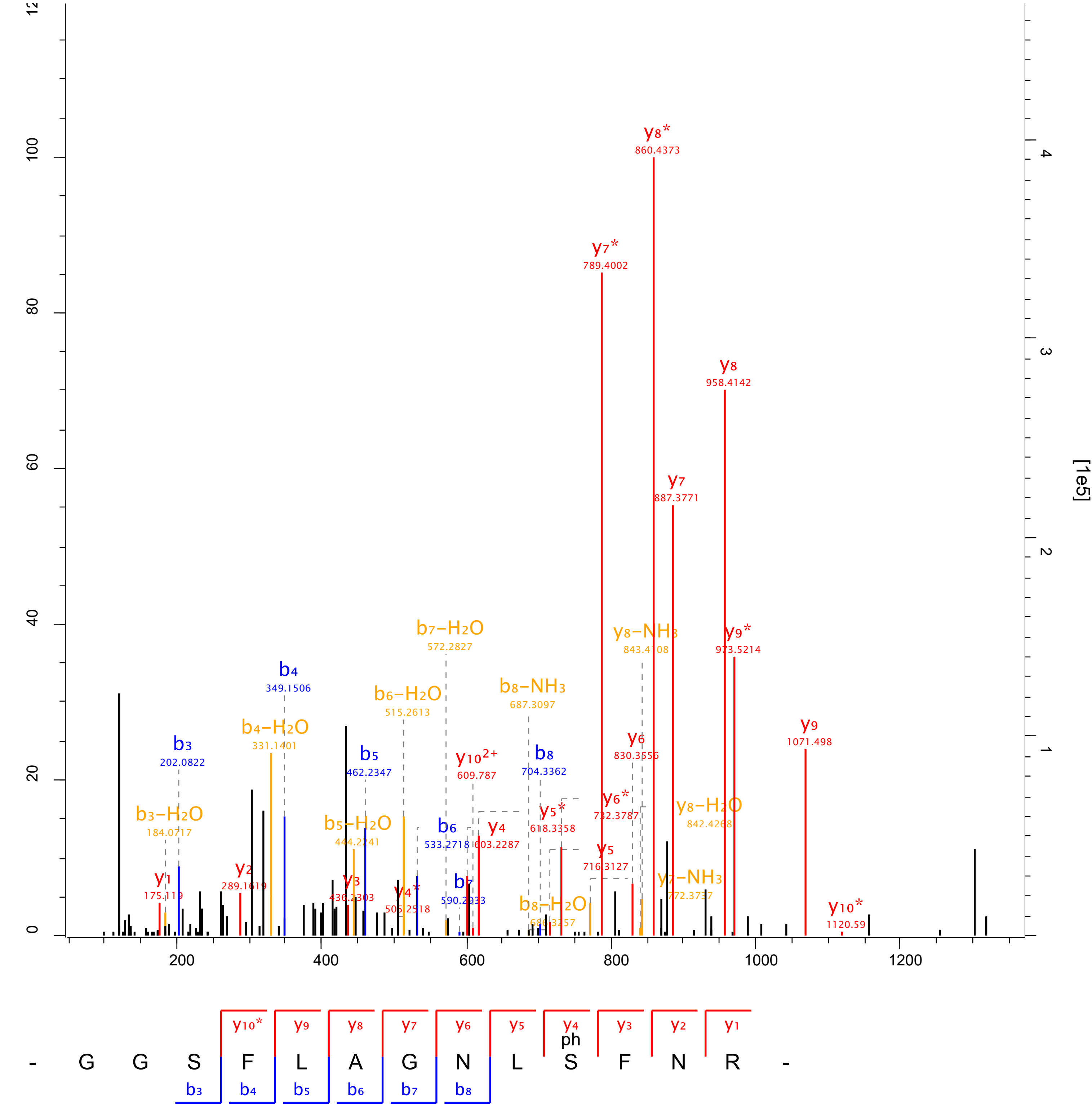

Sample output to test PDF Combine only

Raw file	Scan	Method	Score	m/z	Gene names
0523_15	21065	FTMS; HCD	102.47	778.14	FKBP53

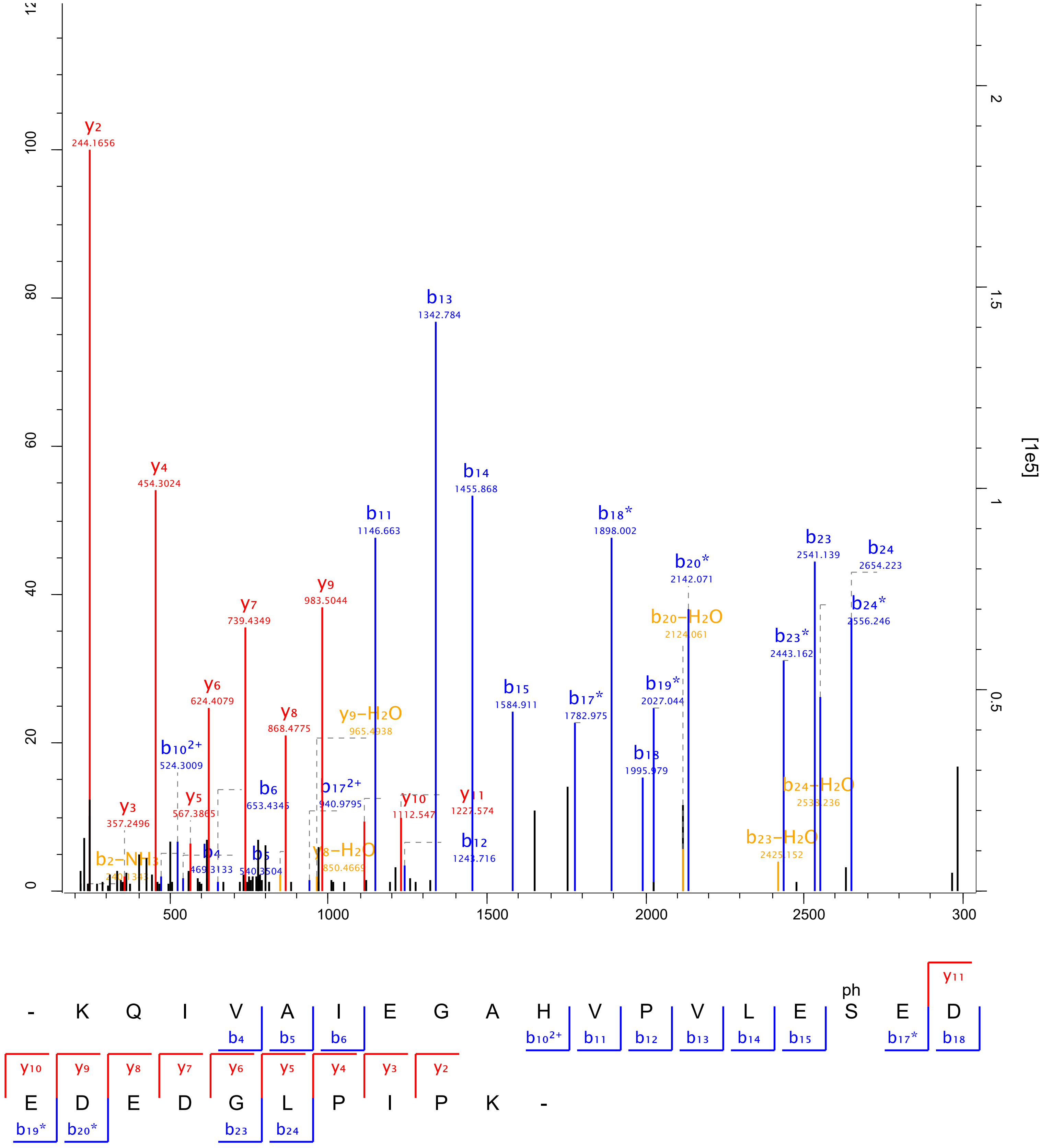

Sample output to test PDF Combine only

Raw file	Scan	Method	Score	m/z	Gene names
0523_15	21146	FTMS; HCD	55.12	897.75	JKD

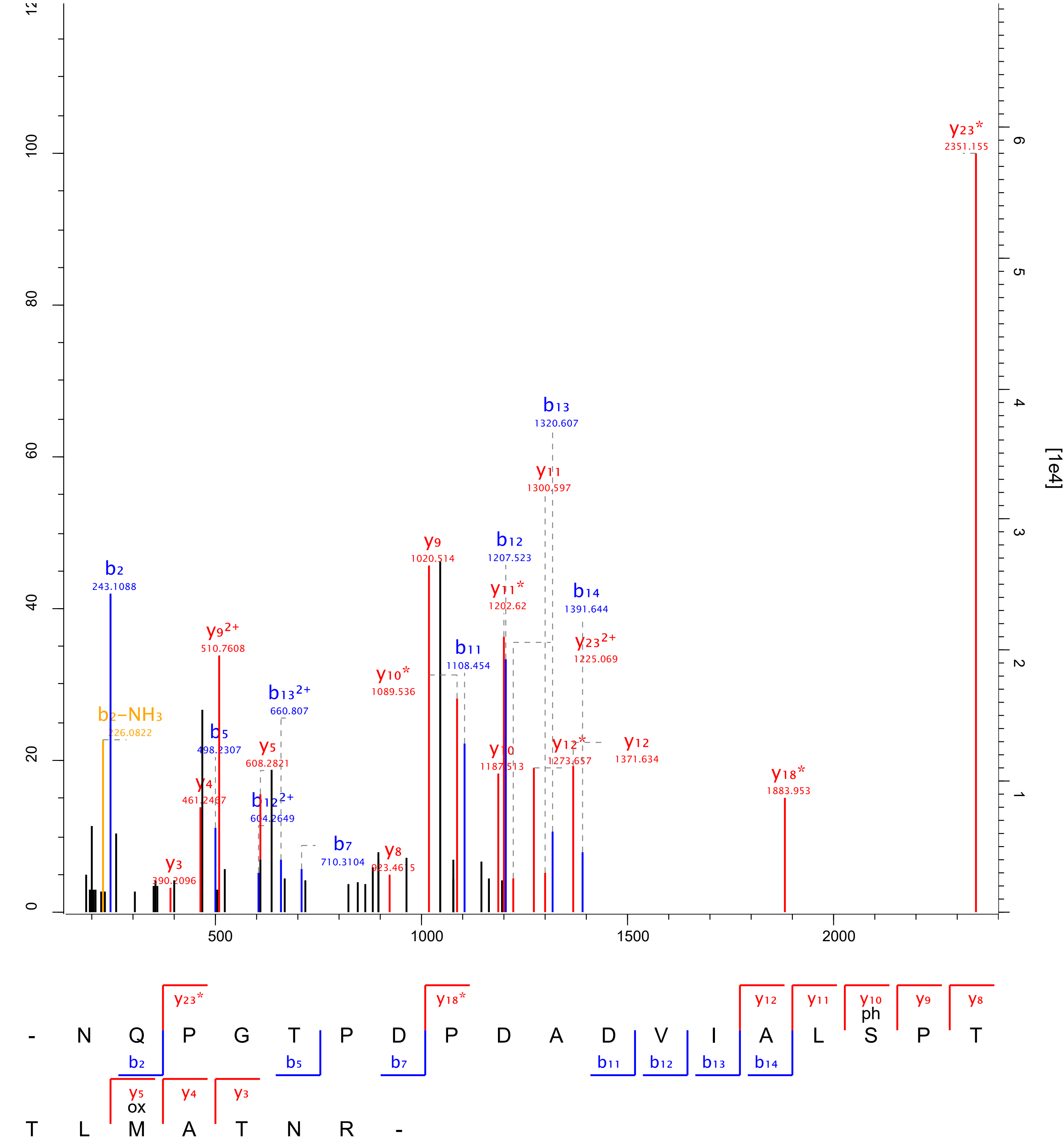

Sample output to test PDF Combine only

Raw file	Scan	Method	Score	m/z	Gene names
0523_15	21275	FTMS; HCD	64.52	796.71	SAC1

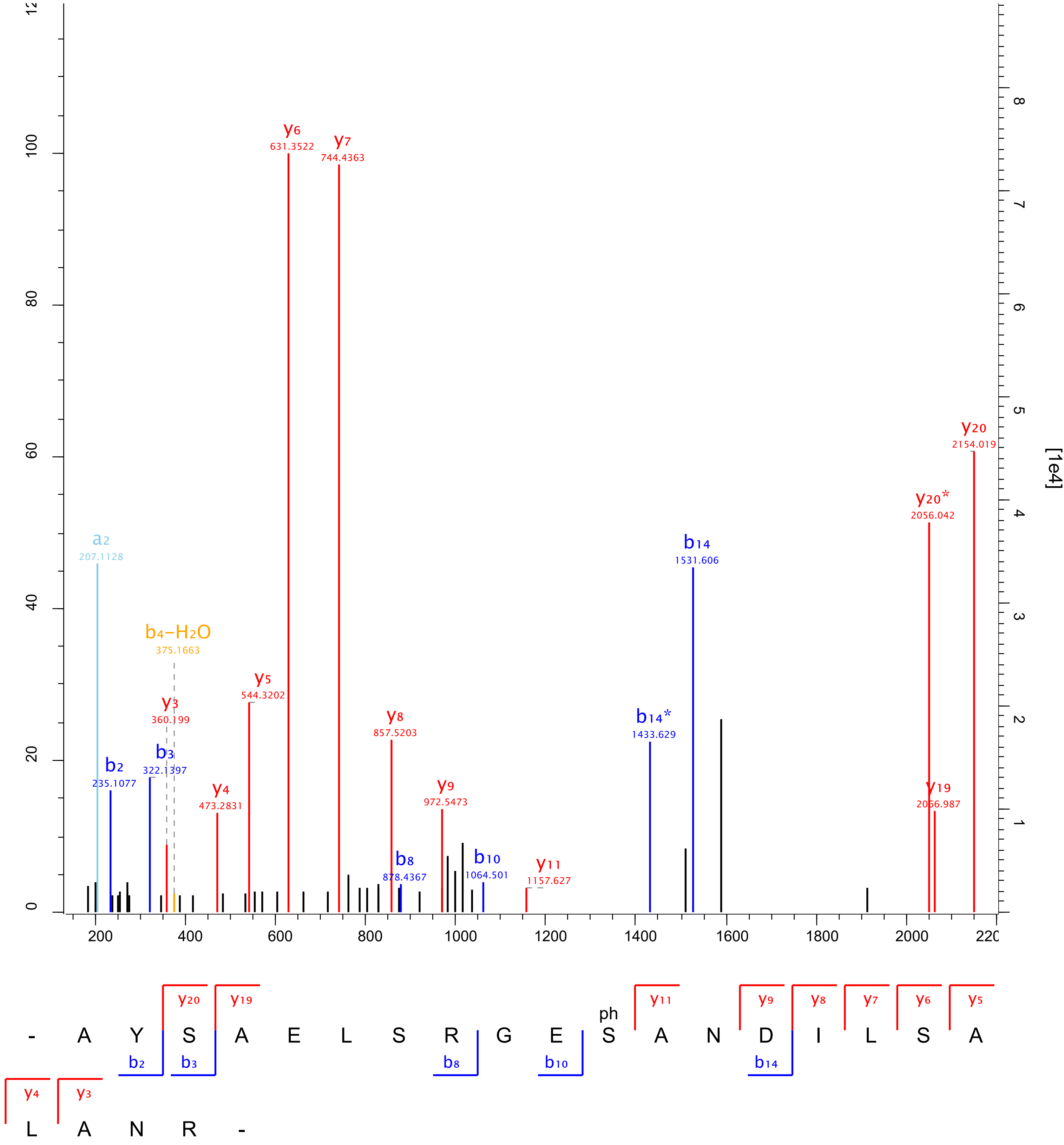

Sample output to test PDF Combine only

Raw file	Scan	Method	Score	m/z	Gene names
0523_15	21720	FTMS; HCD	179.95	1133.79	At3g58110;F9D24.20

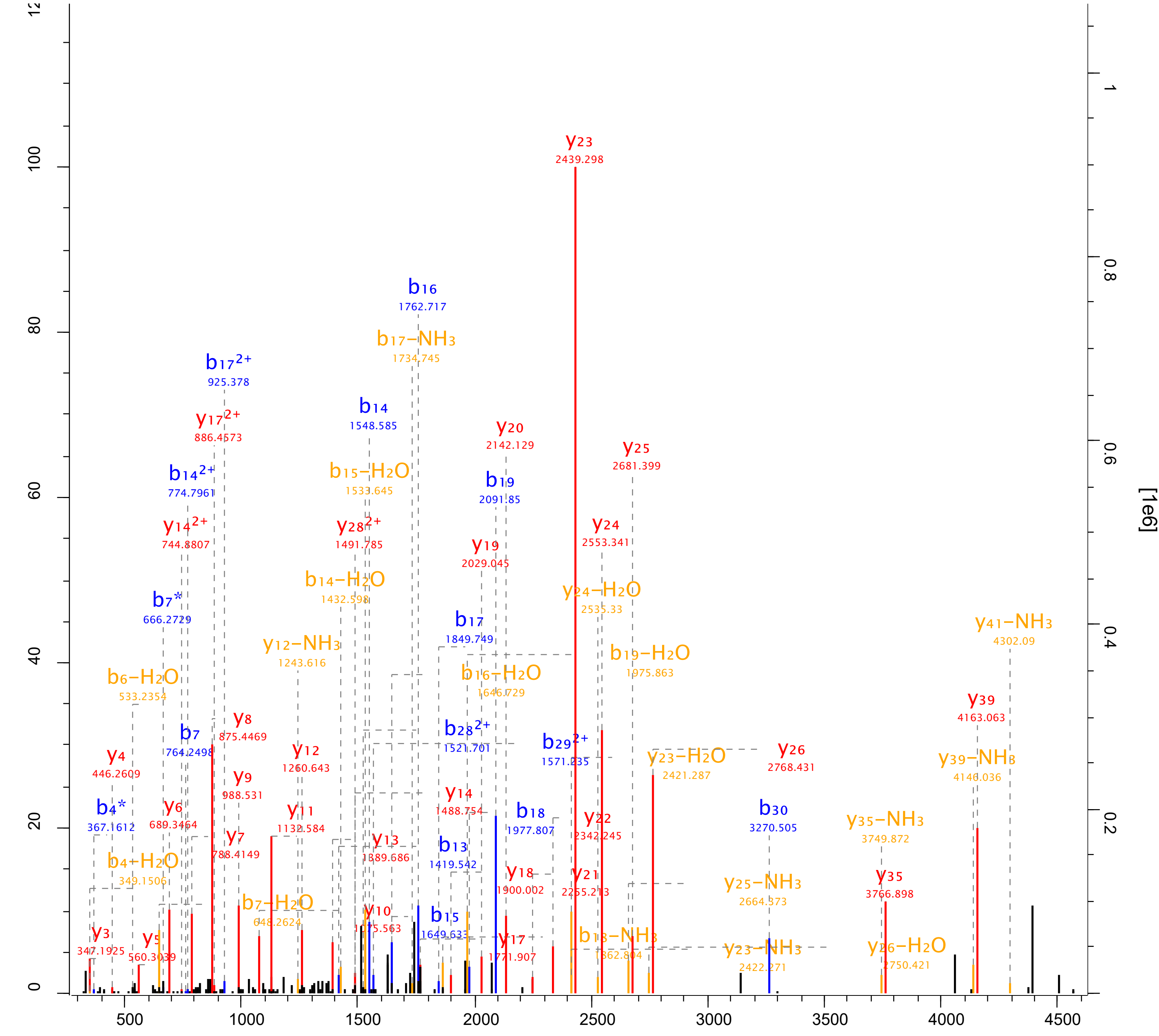

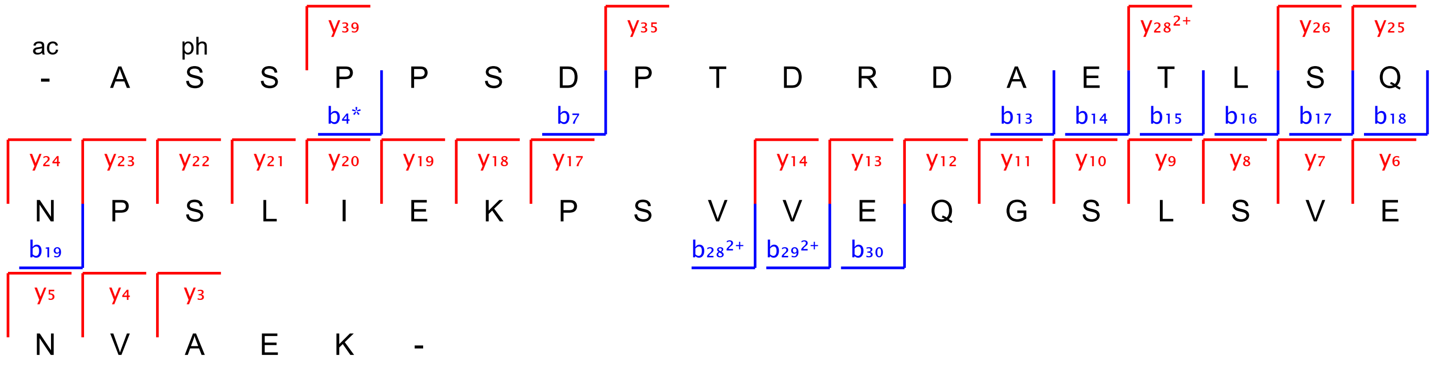

Sample output to test PDF Combine only

Raw file	Scan	Method	Score	m/z	Gene names
0523_15	21759	FTMS; HCD	88.09	1124.2	PXN

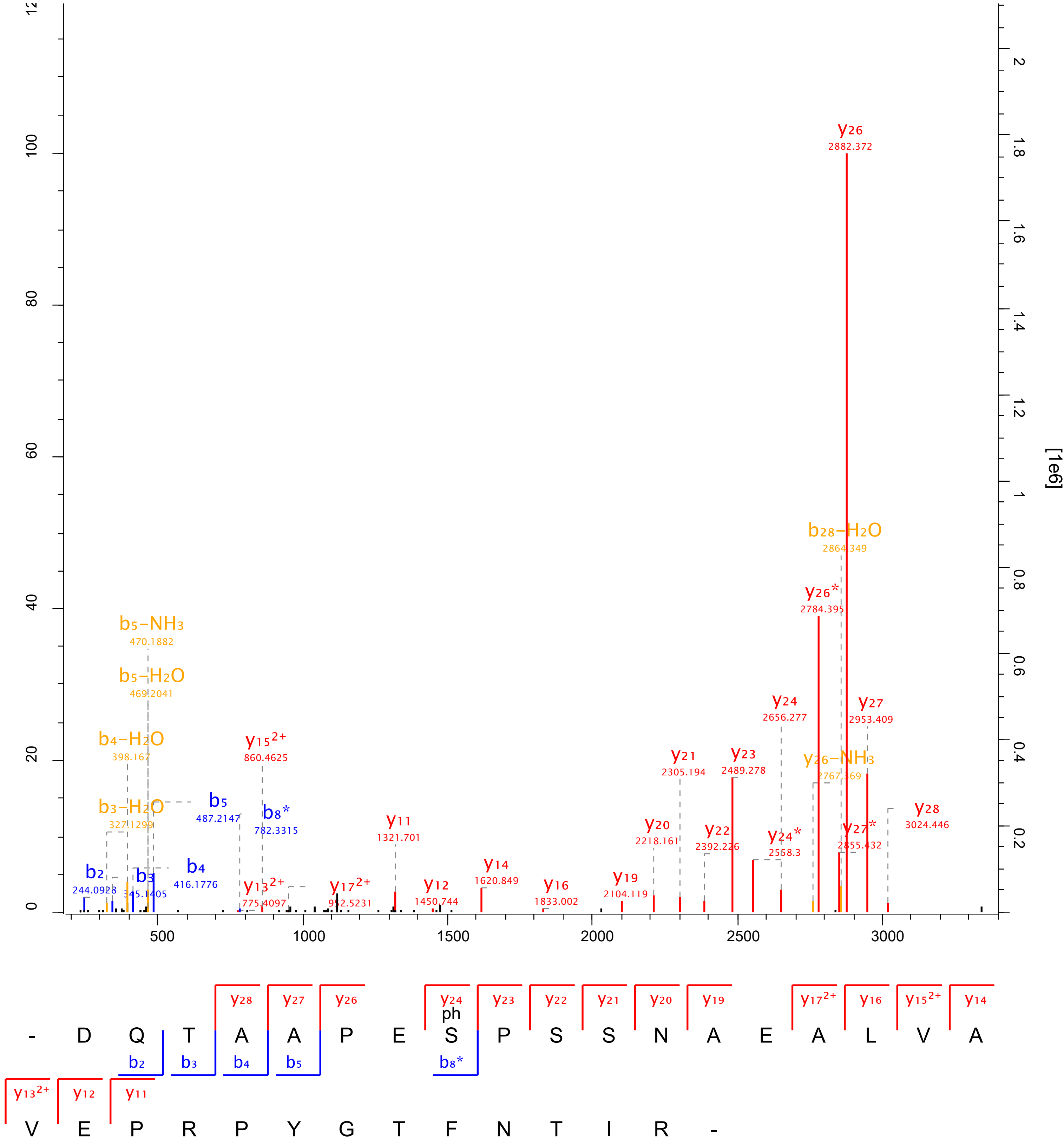

Sample output to test PDF Combine only

Raw file	Scan	Method	Score	m/z	Gene names
0523_15	21993	FTMS; HCD	66.58	957.15	PAPP2C

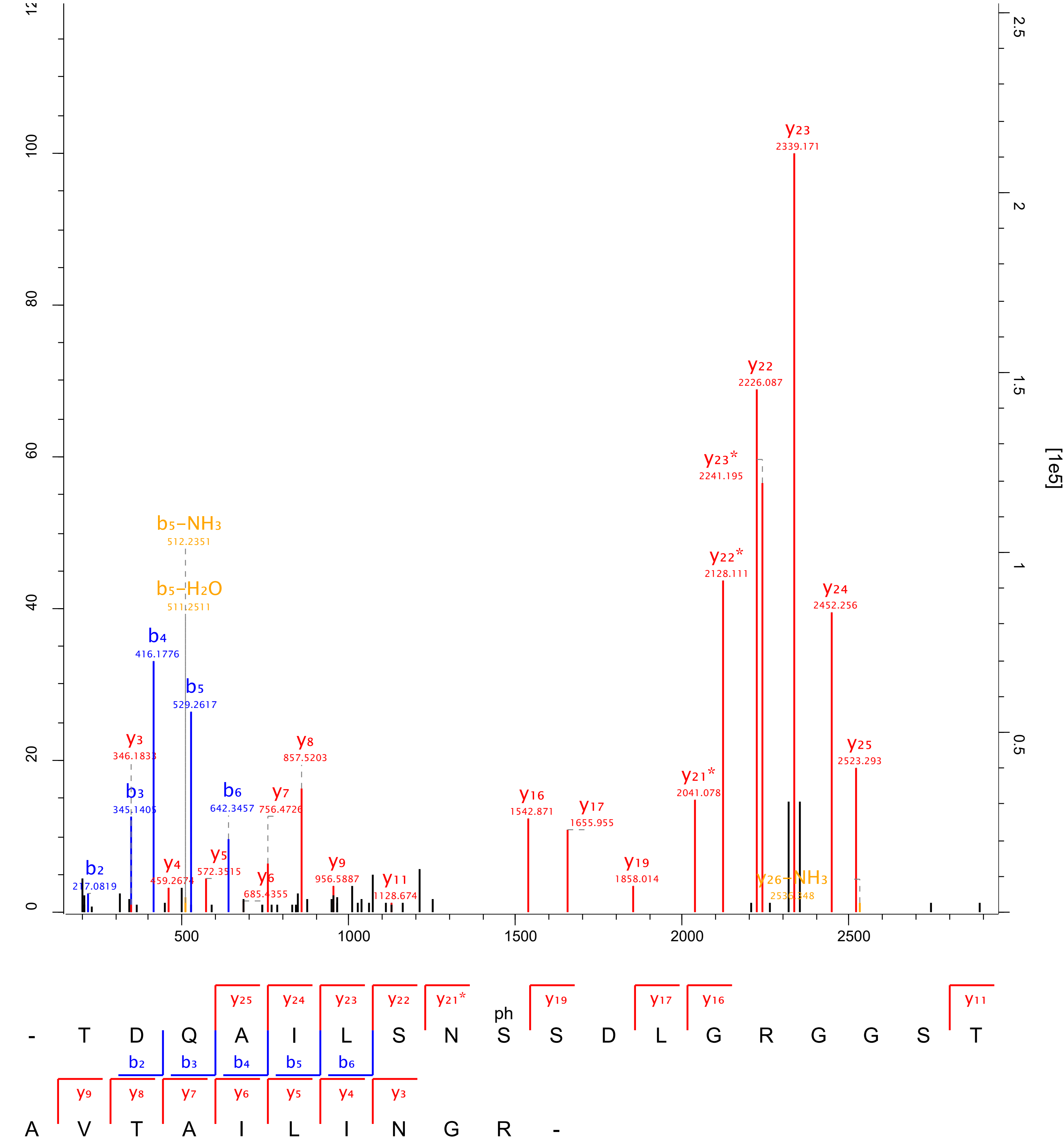

Sample output to test PDF Combine only

Raw file	Scan	Method	Score	m/z
0523_15	22054	FTMS; HCD	49.77	911.09

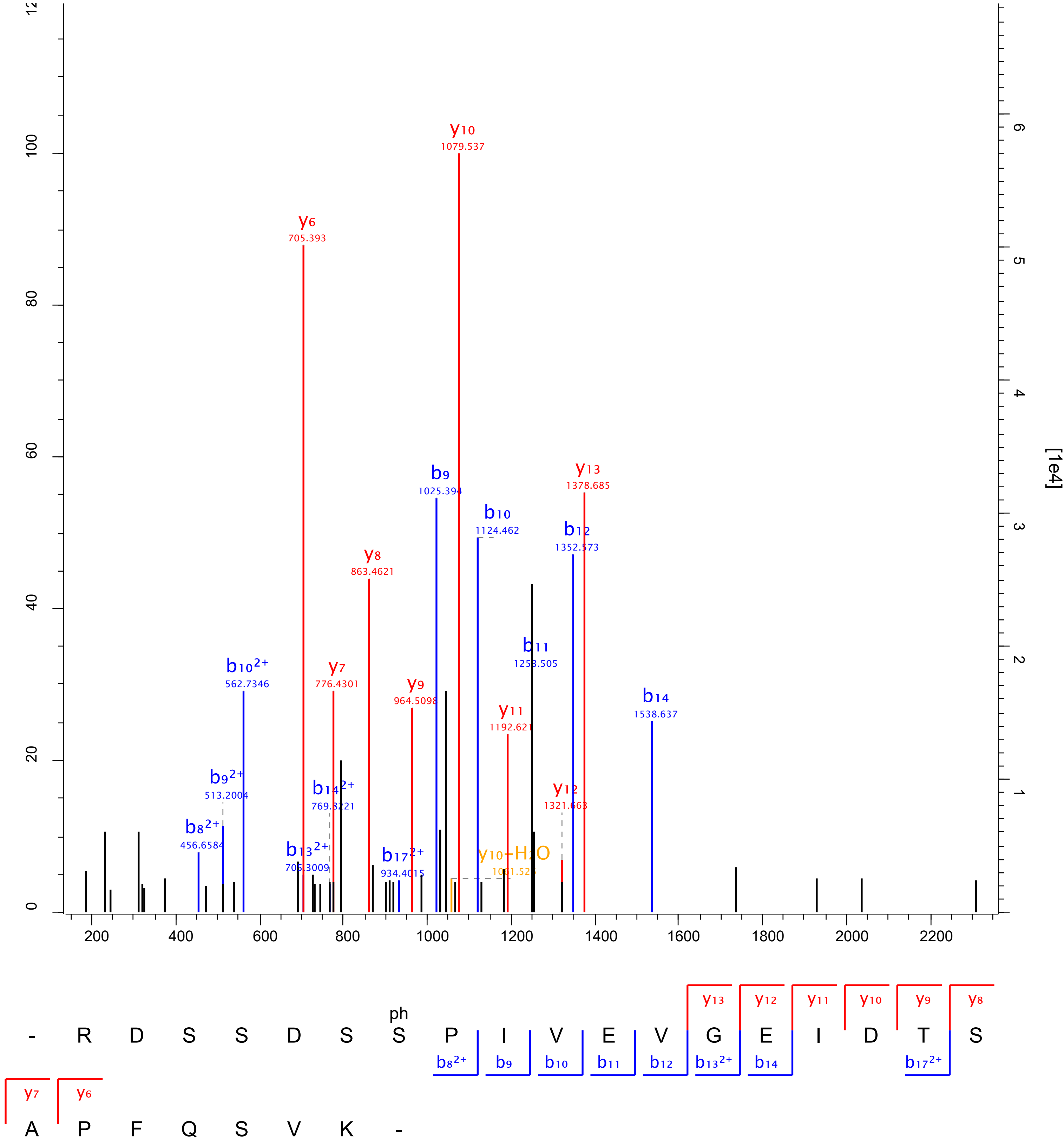

Sample output to test PDF Combine only

Raw file	Scan	Method	Score	m/z	Gene names
0523_15	22066	FTMS; HCD	121.33	913.42	PATL2

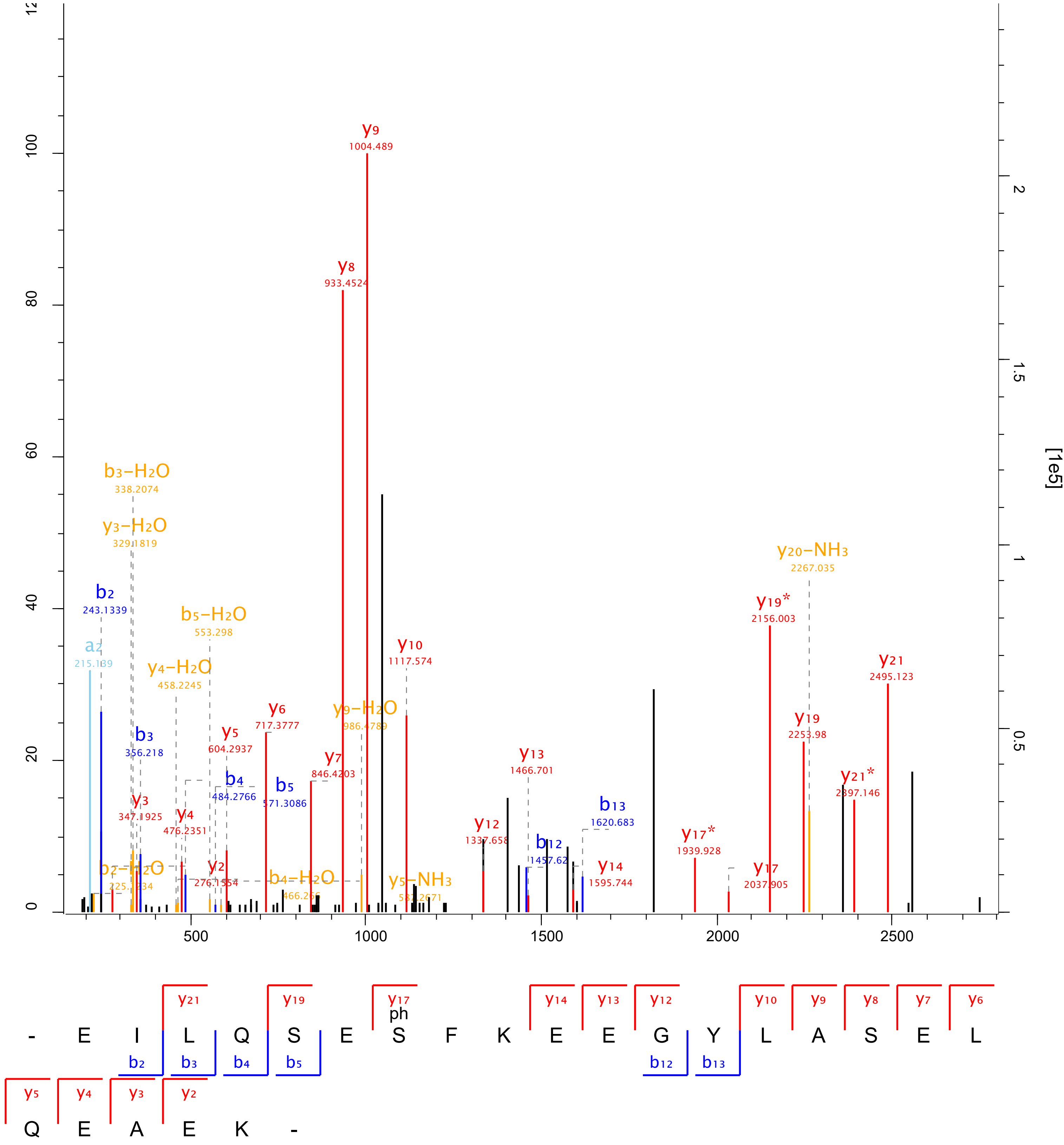

Sample output to test PDF Combine only

Raw file	Scan	Method	Score	m/z	Gene names
0523_15	22186	FTMS; HCD	101.79	1081	FRO2

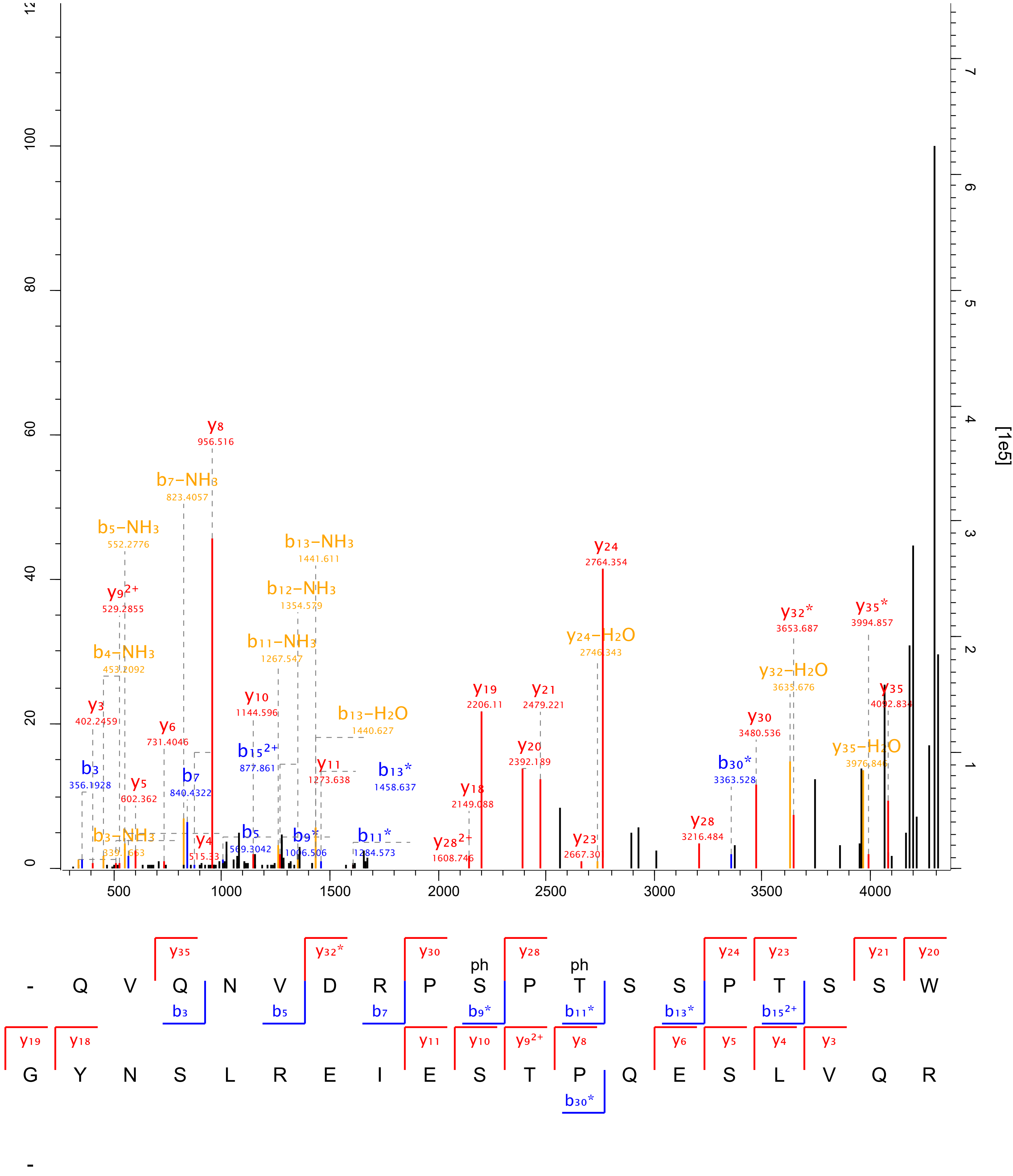

Sample output to test PDF Combine only

Raw file	Scan	Method	Score	m/z	Gene names
0523_15	22686	FTMS; HCD	129.02	886.45	AHA11;AHA4

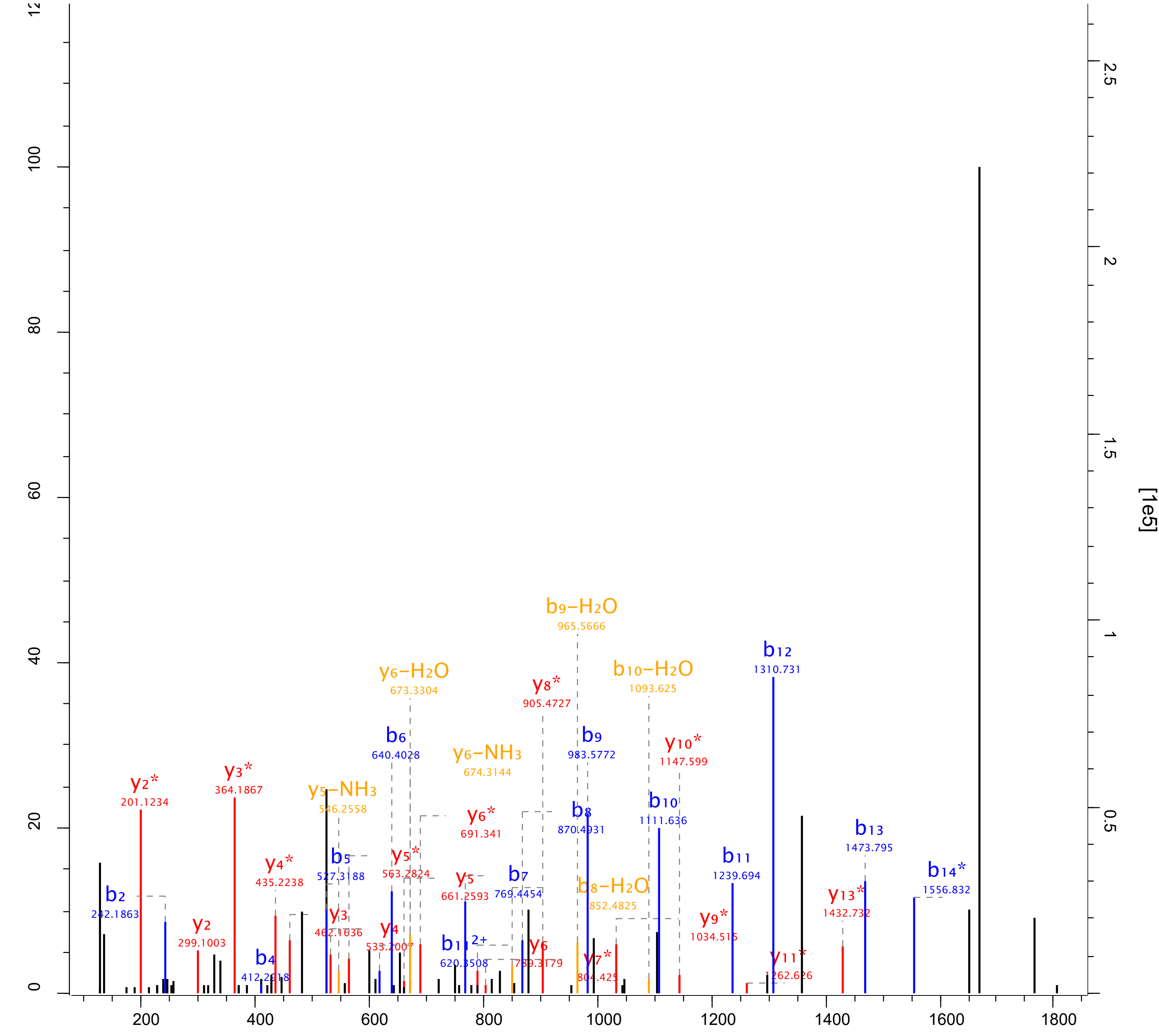

- L K G L D I E T I Q Q A Y T V -

(ph on T; y ions: y13*, y11*, y10*, y9*, y8*, y7*, y6, y5, y4, y3, y2; b ions: b2, b4, b5, b6, b7, b8, b9, b10, b11, b12, b13, b14*)

Sample output to test PDF Combine only

Raw file	Scan	Method	Score	m/z	Gene names
0523_15	23179	FTMS; HCD	140.26	902.08	At4g27450

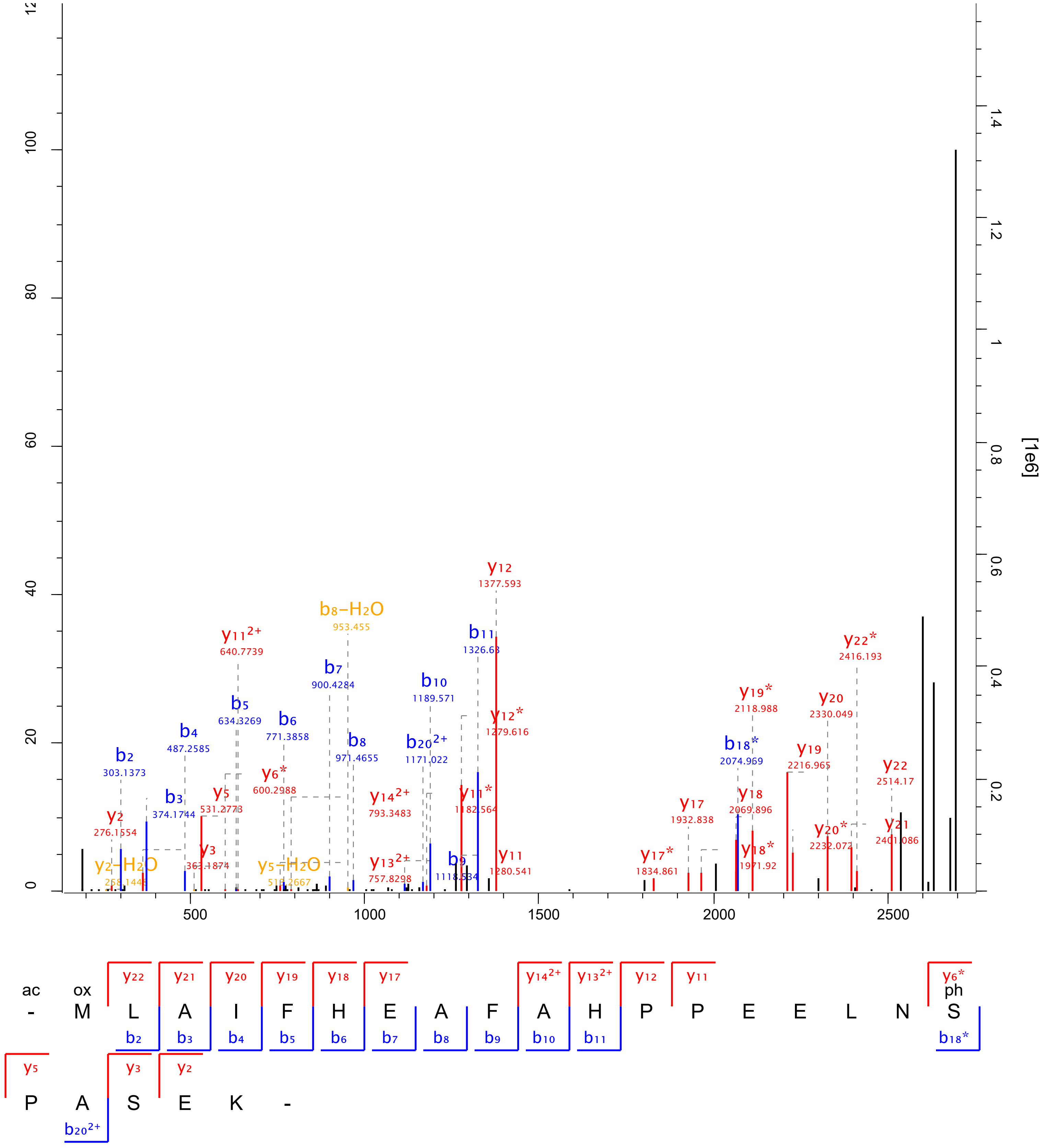

Sample output to test PDF Combine only

Raw file	Scan	Method	Score	m/z	Gene names
0523_15	23731	FTMS; HCD	60.95	856.39	F13I12.260

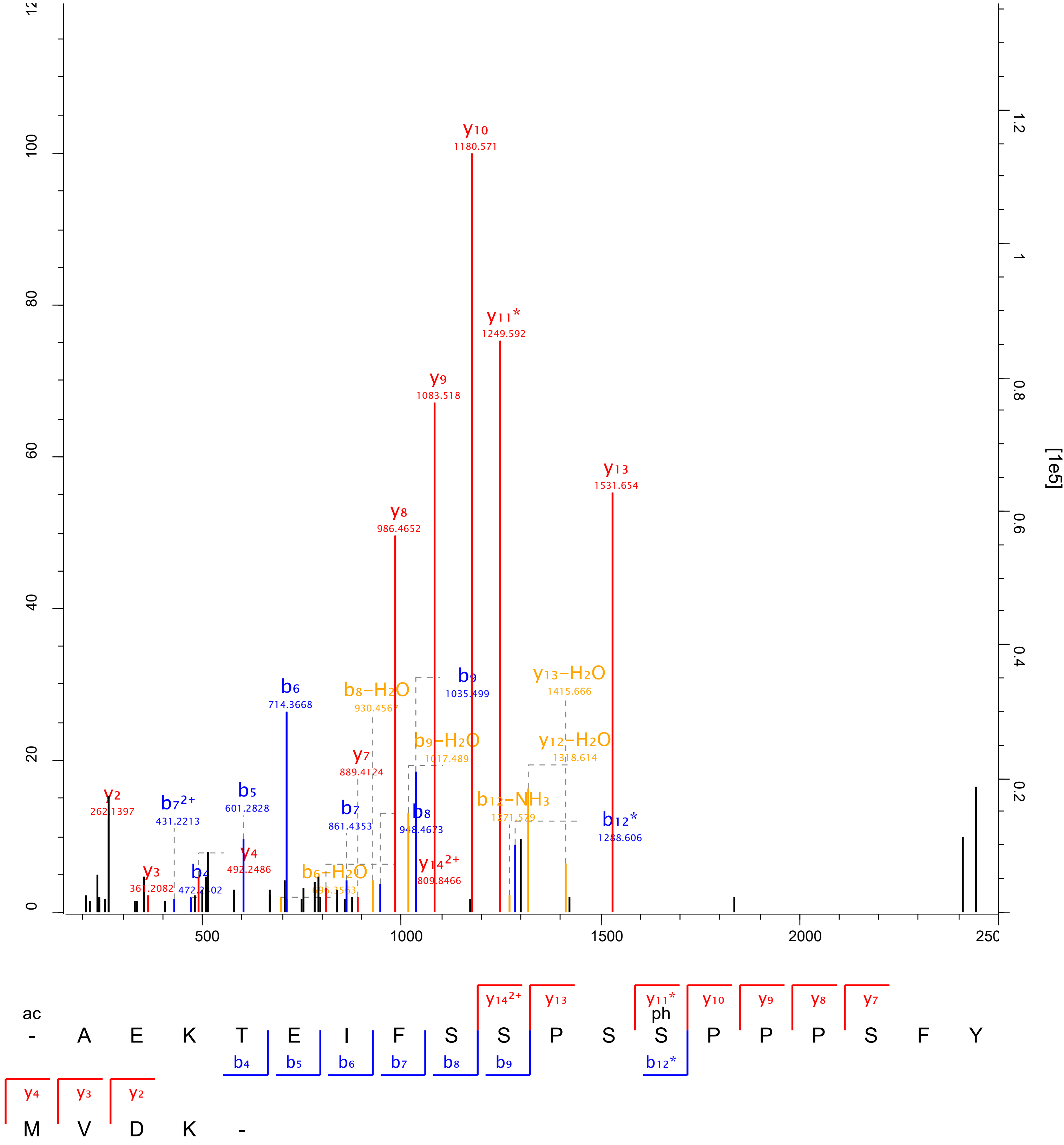

Sample output to test PDF Combine only

Raw file	Scan	Method	Score	m/z	Gene names
0523_15	23854	FTMS; HCD	129.13	934.22	PUP14

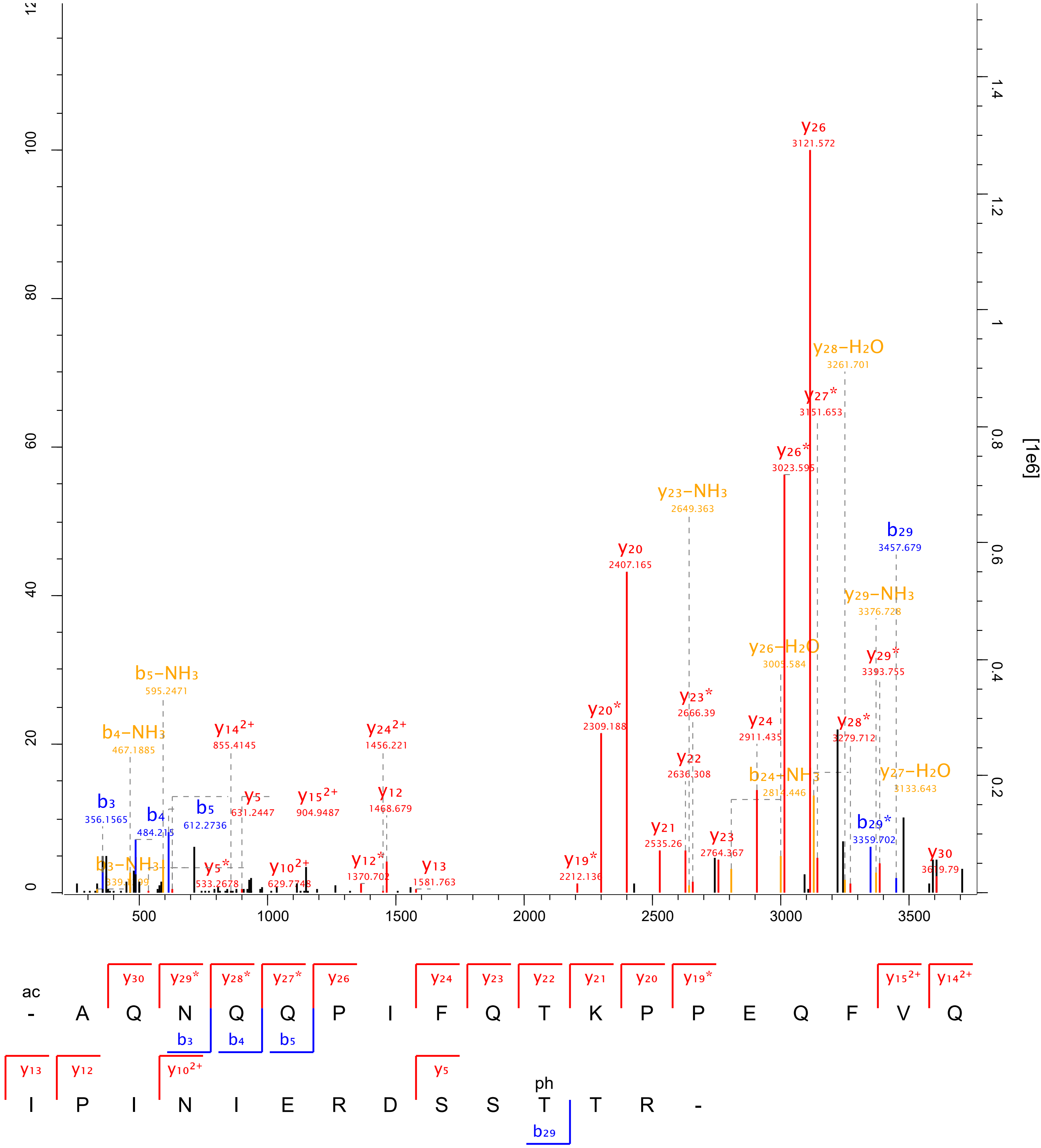

Sample output to test PDF Combine only

Raw file	Scan	Method	Score	m/z	Gene names
0523_15	24250	FTMS; HCD	316.9	994.15	FKBP53

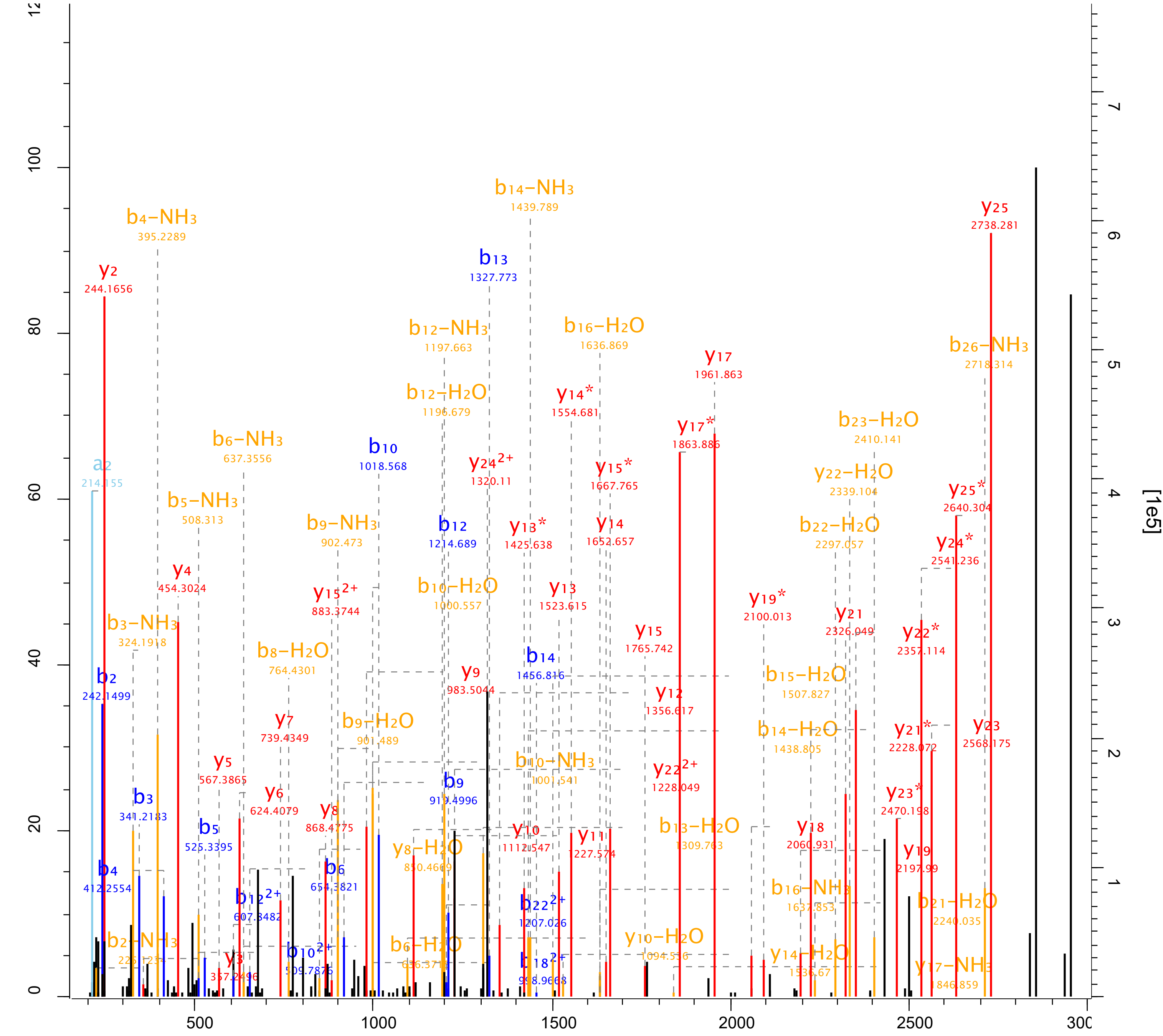

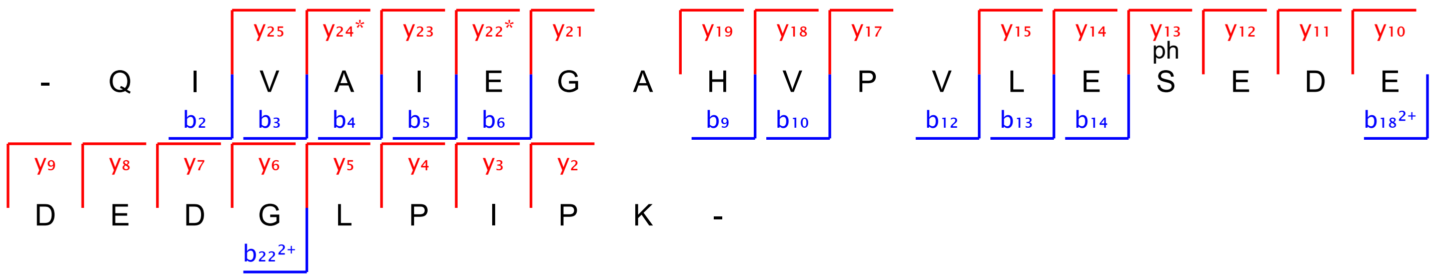

Sample output to test PDF Combine only

Raw file	Scan	Method	Score	m/z	Gene names
0523_15	24358	FTMS; HCD	78.75	955.45	RH24

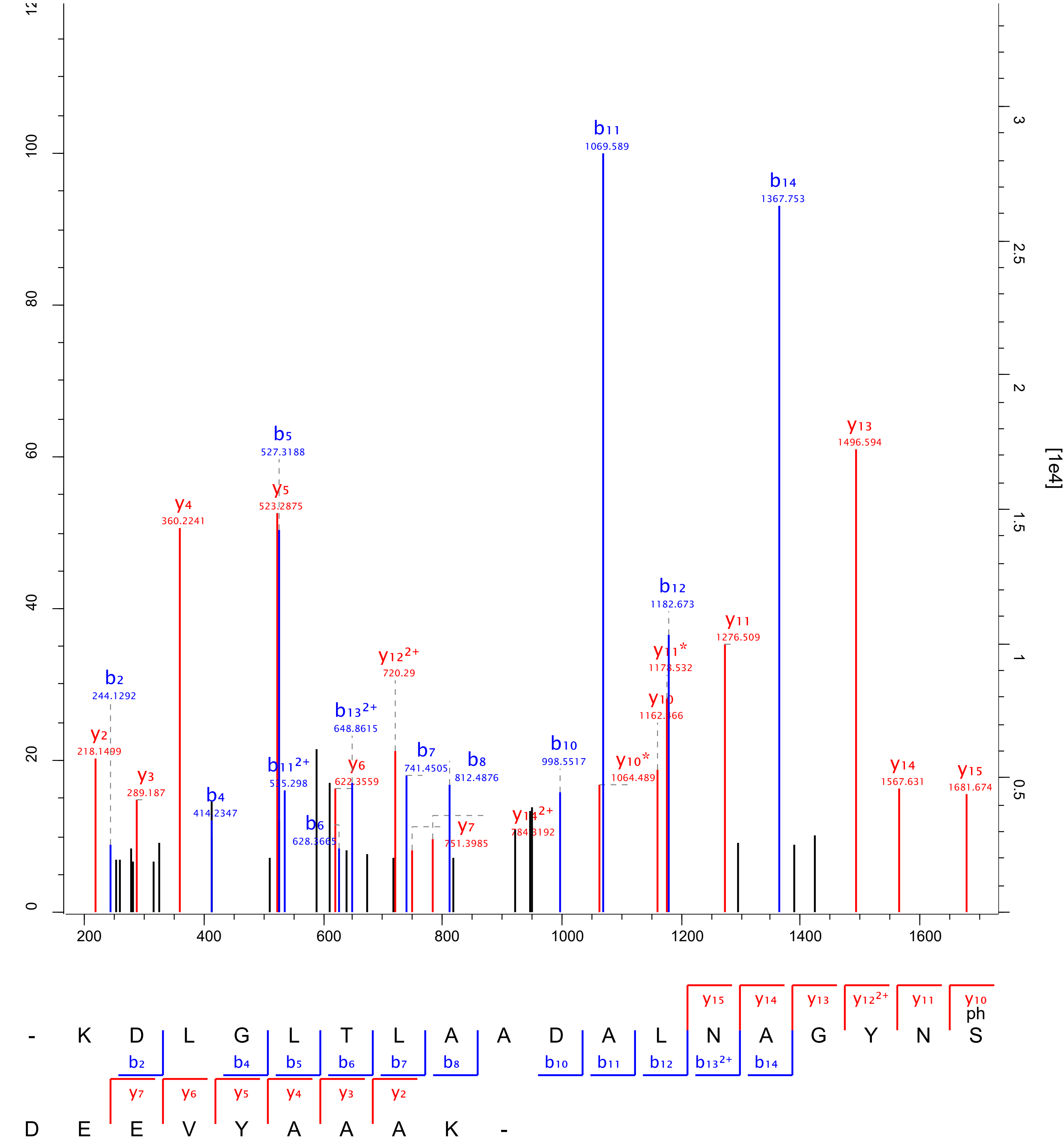

Sample output to test PDF Combine only

Raw file	Scan	Method	Score	m/z	Gene names
0523_15	24873	FTMS; HCD	134.91	911.42	LYK4

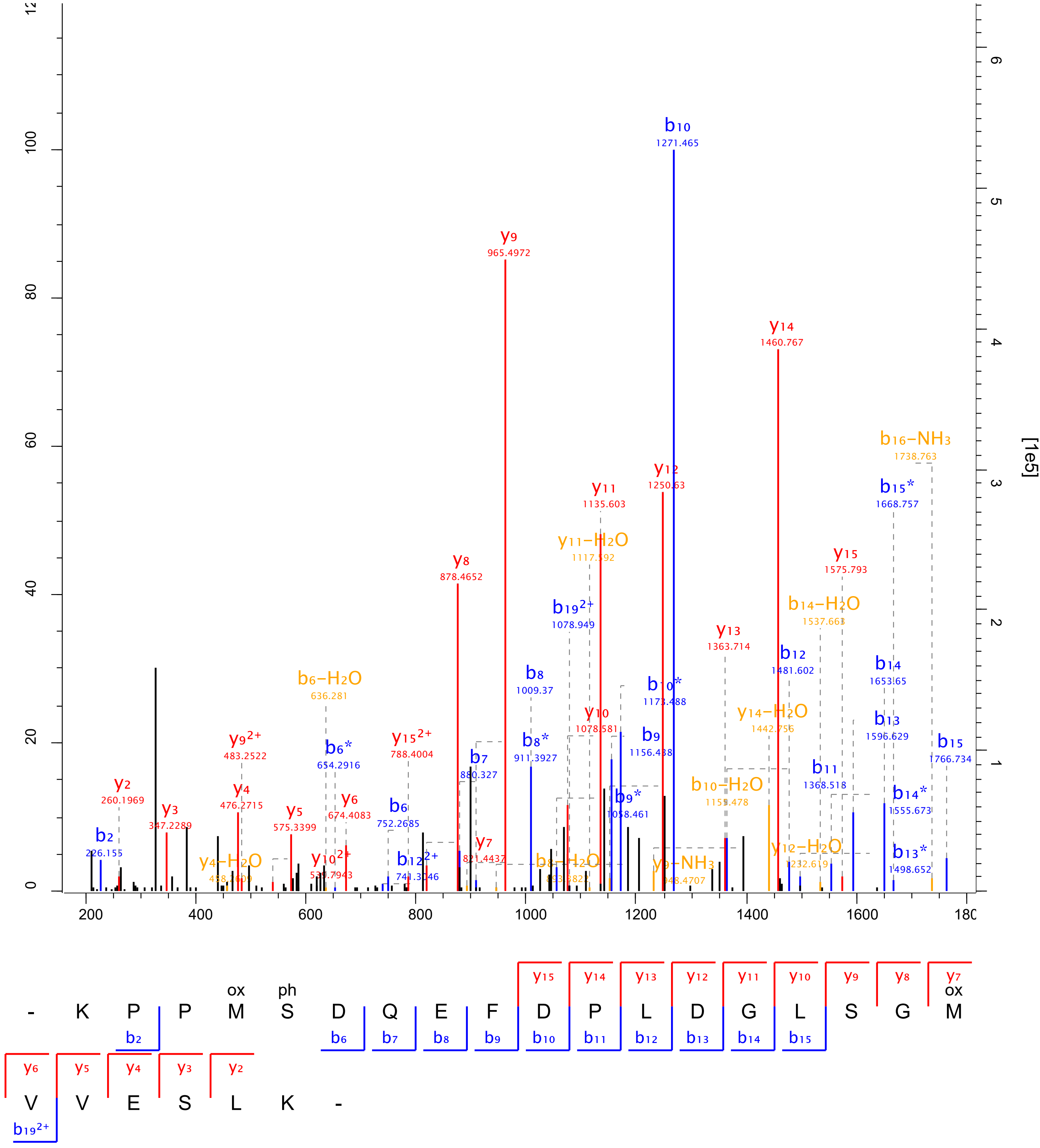

Sample output to test PDF Combine only

Raw file	Scan	Method	Score	m/z	Gene names
0523_15	25113	FTMS; HCD	46.4	815.36	At2g28360

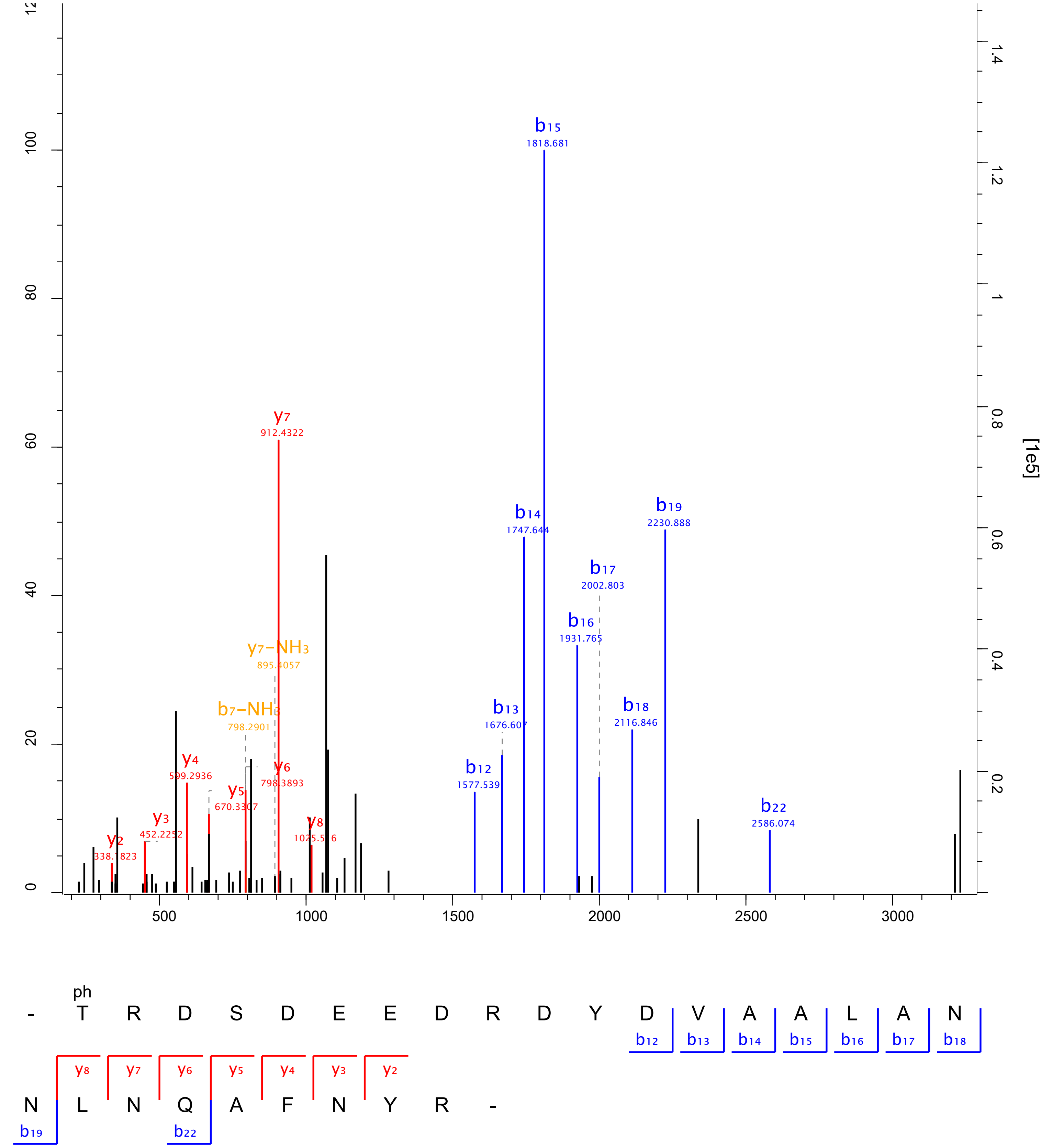

Sample output to test PDF Combine only

Raw file	Scan	Method	Score	m/z	Gene names
0523_15	25151	FTMS; HCD	112.39	911.75	PI4KA1

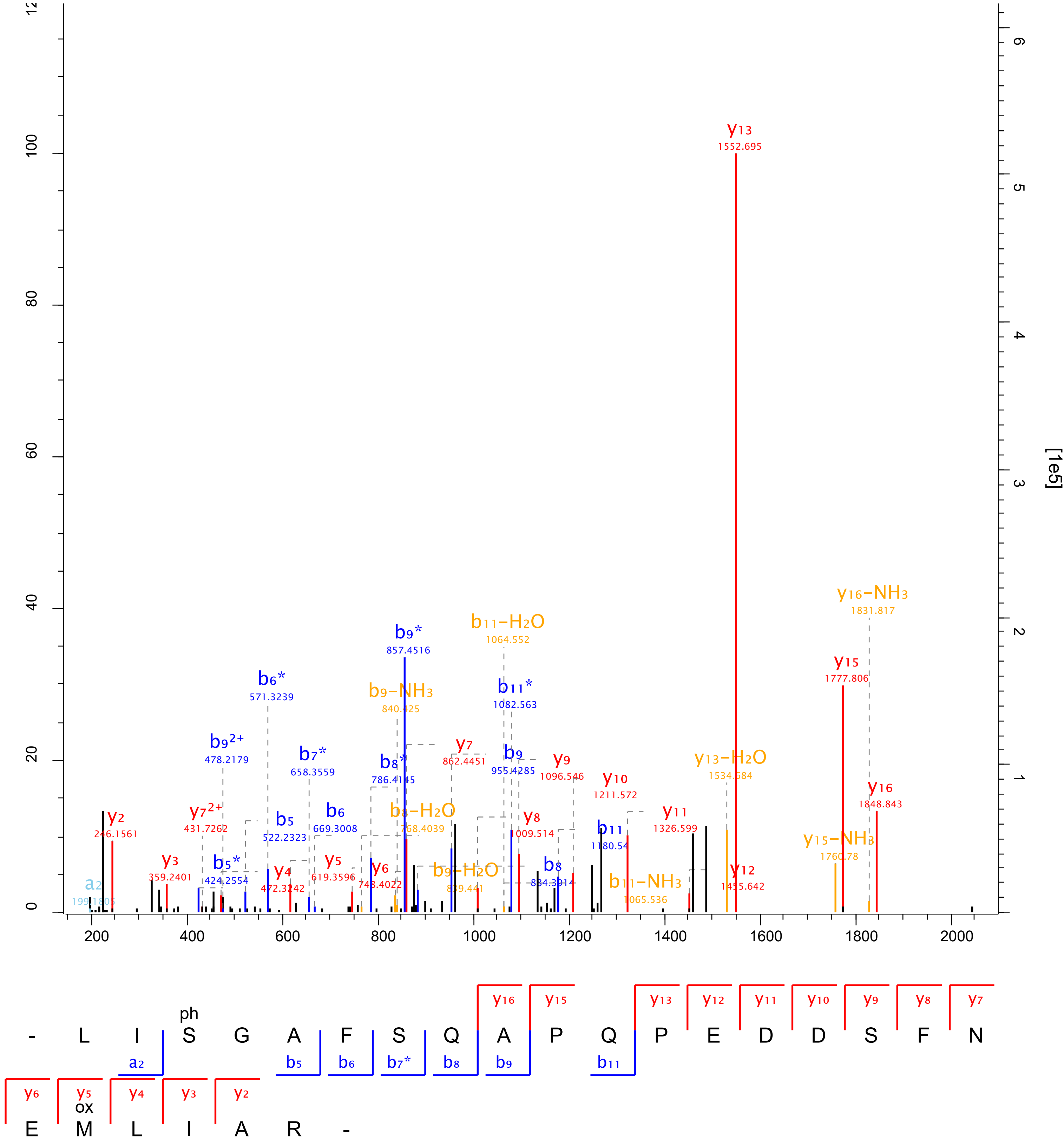

Sample output to test PDF Combine only

Raw file	Scan	Method	Score	m/z
0523_15	25220	FTMS; HCD	122.46	1348.61

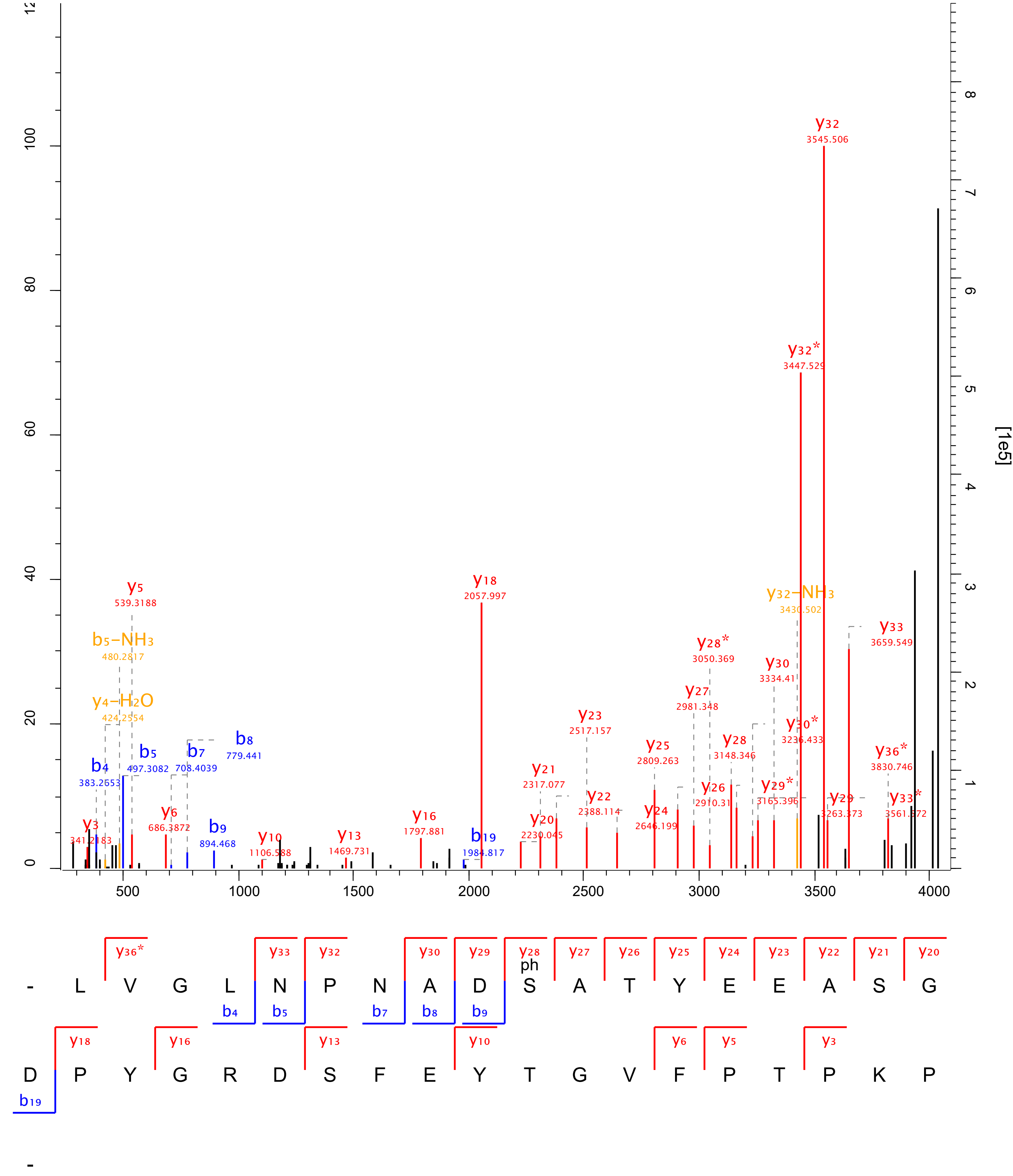

Sample output to test PDF Combine only

Raw file	Scan	Method	Score	m/z	Gene names
0523_15	25859	FTMS; HCD	144.36	1086.81	At4g26620

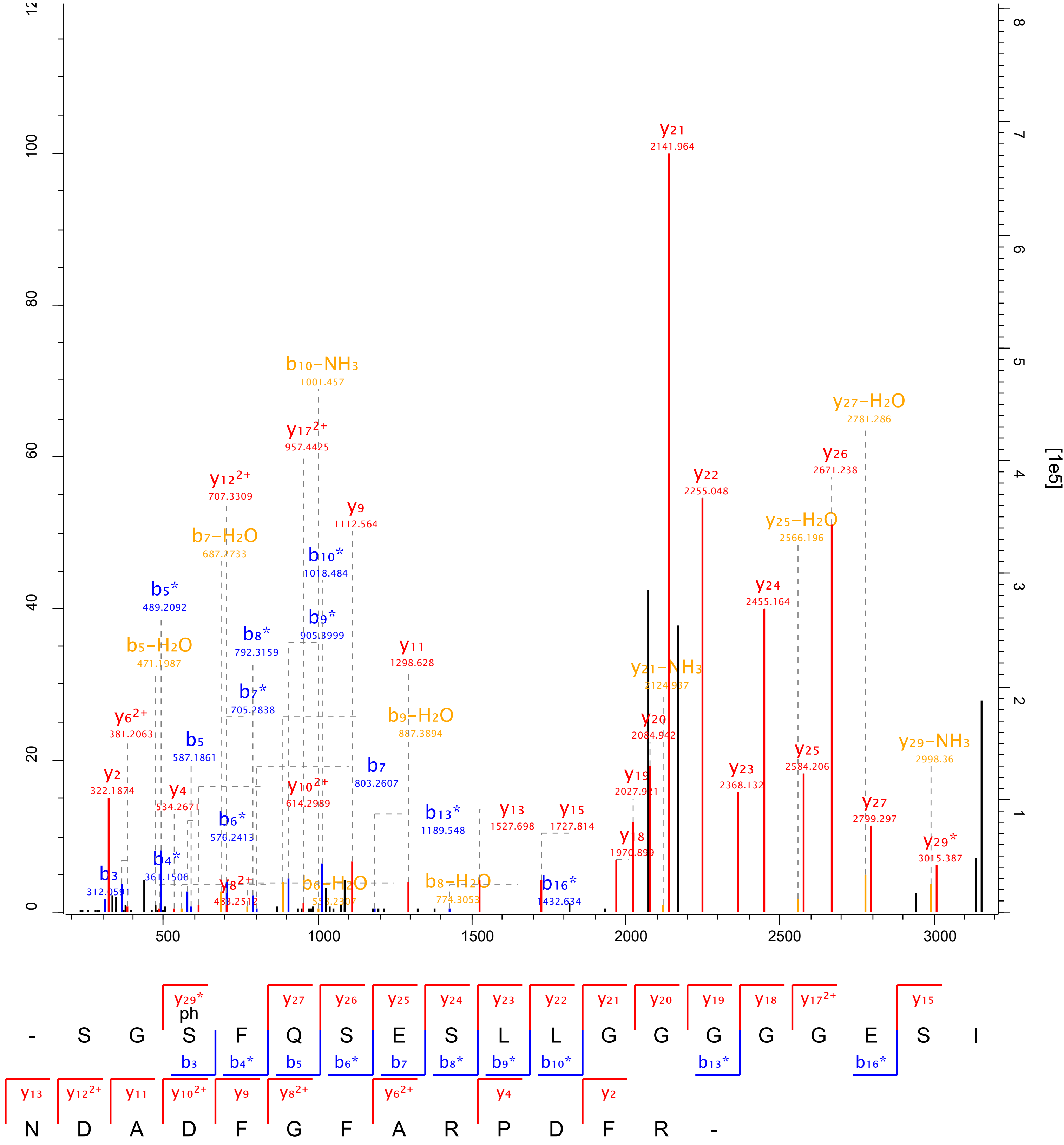

Sample output to test PDF Combine only

Raw file	Scan	Method	Score	m/z
0523_15	25968	FTMS; HCD	282.3	955.78

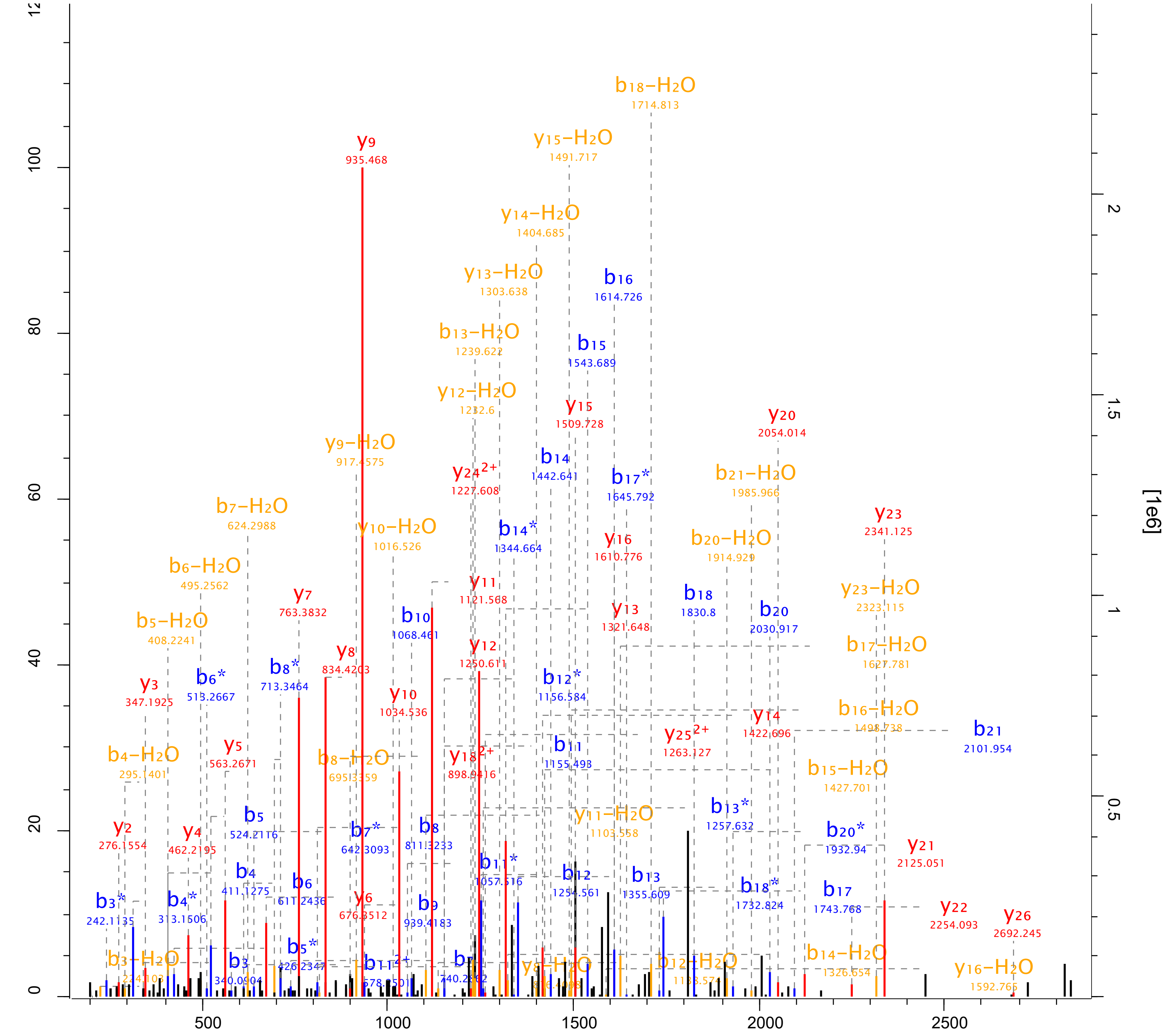

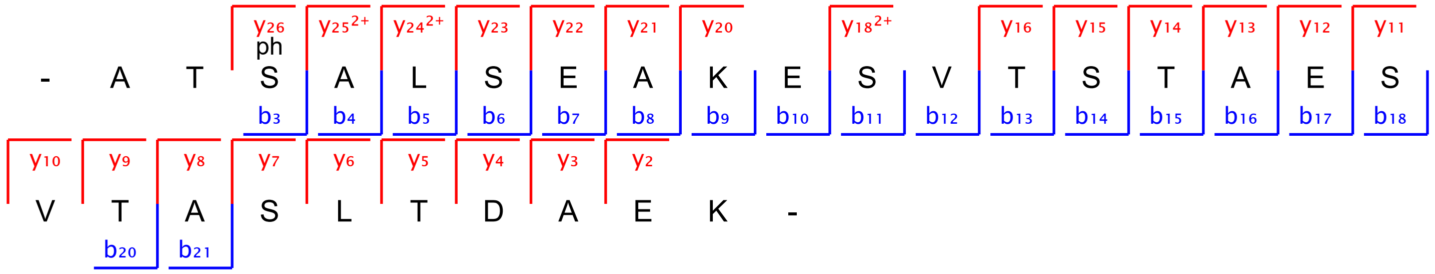

Sample output to test PDF Combine only

Raw file	Scan	Method	Score	m/z	Gene names
0523_15	26037	FTMS; HCD	81.53	739.08	At1g07990

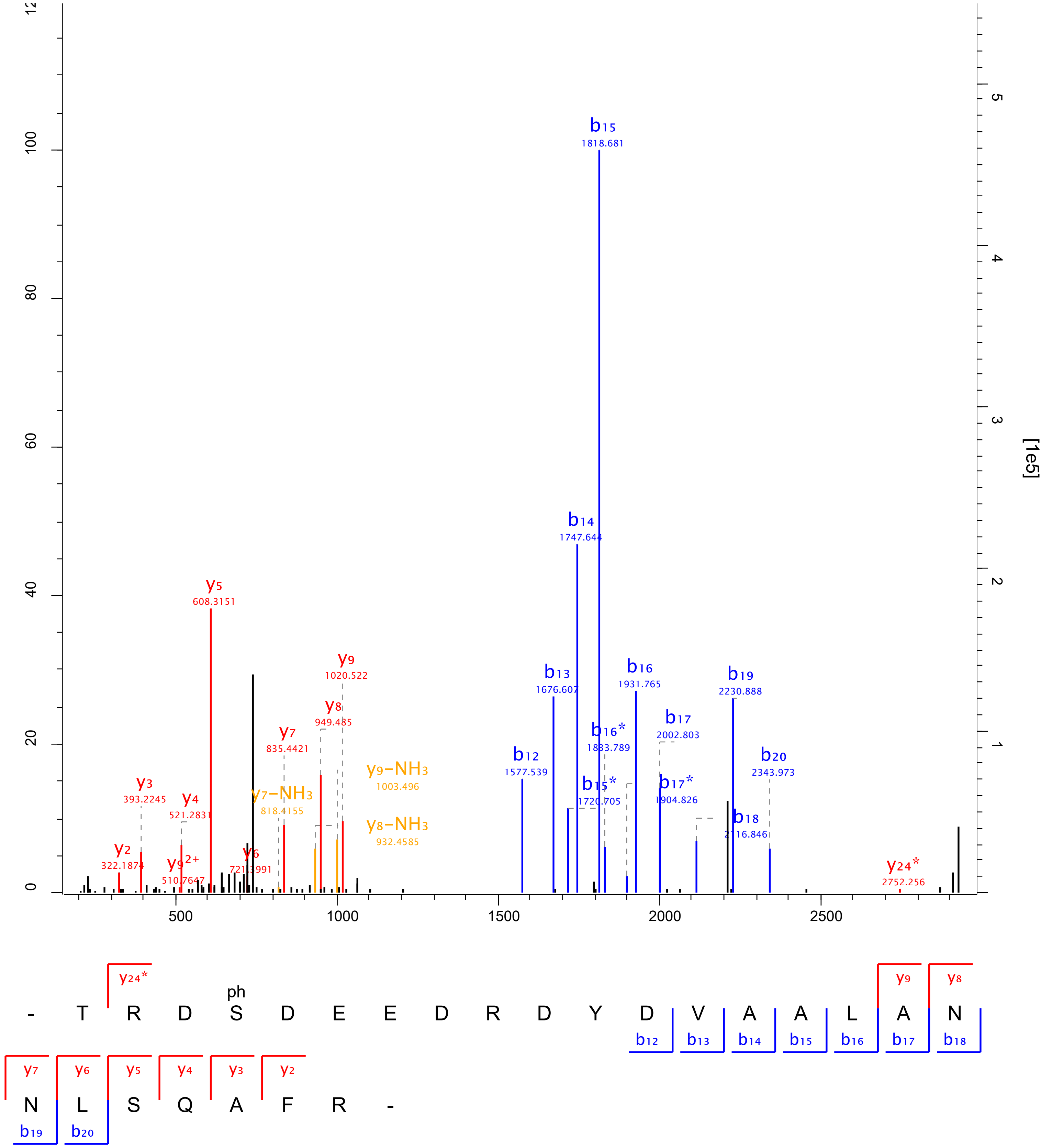

Sample output to test PDF Combine only

Raw file	Scan	Method	Score	m/z
0523_15	26377	FTMS; HCD	84.68	909.94

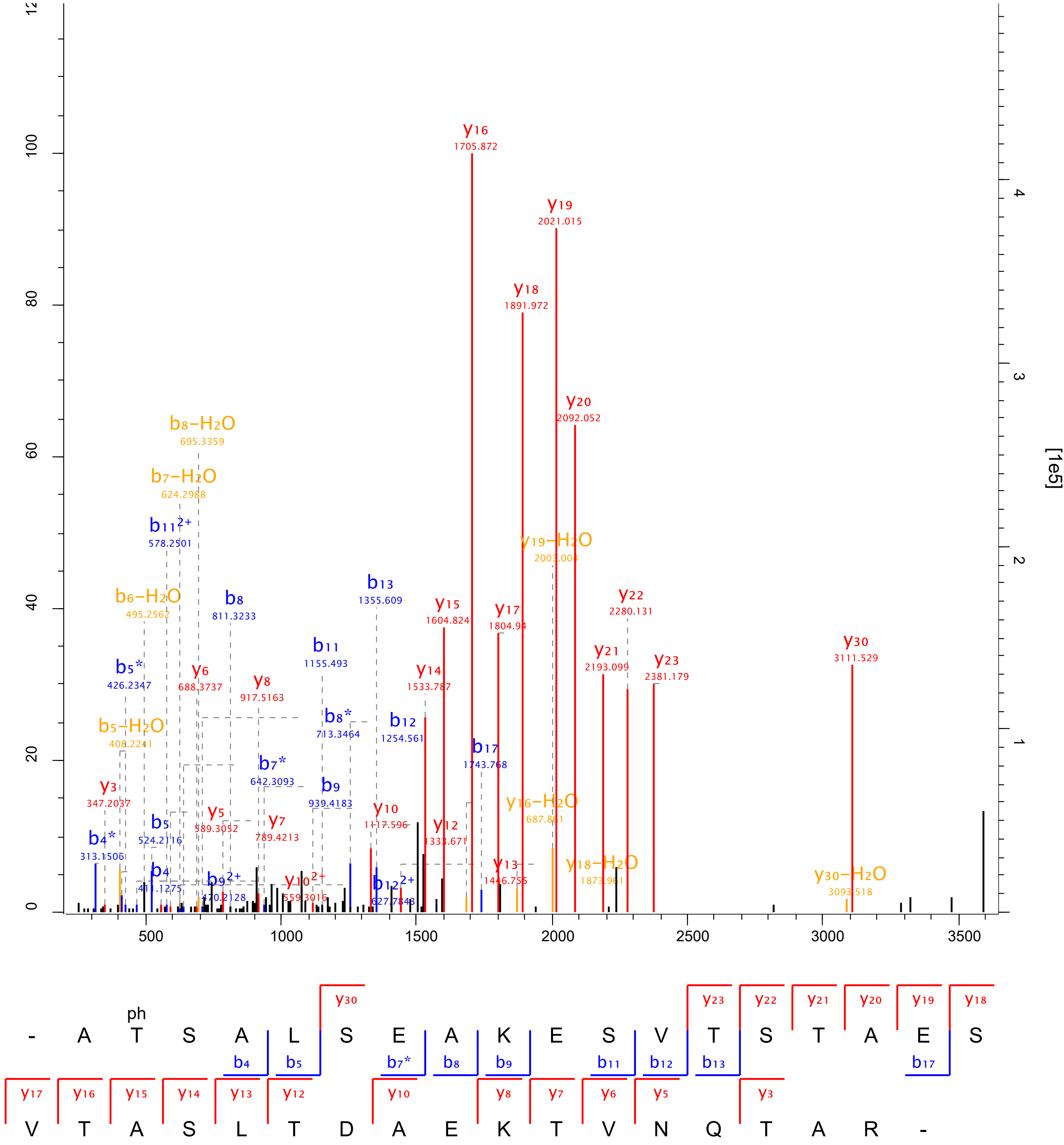

Sample output to test PDF Combine only

Raw file	Scan	Method	Score	m/z	Gene names
0523_15	27375	FTMS; HCD	50.63	1006.72	At5g42950

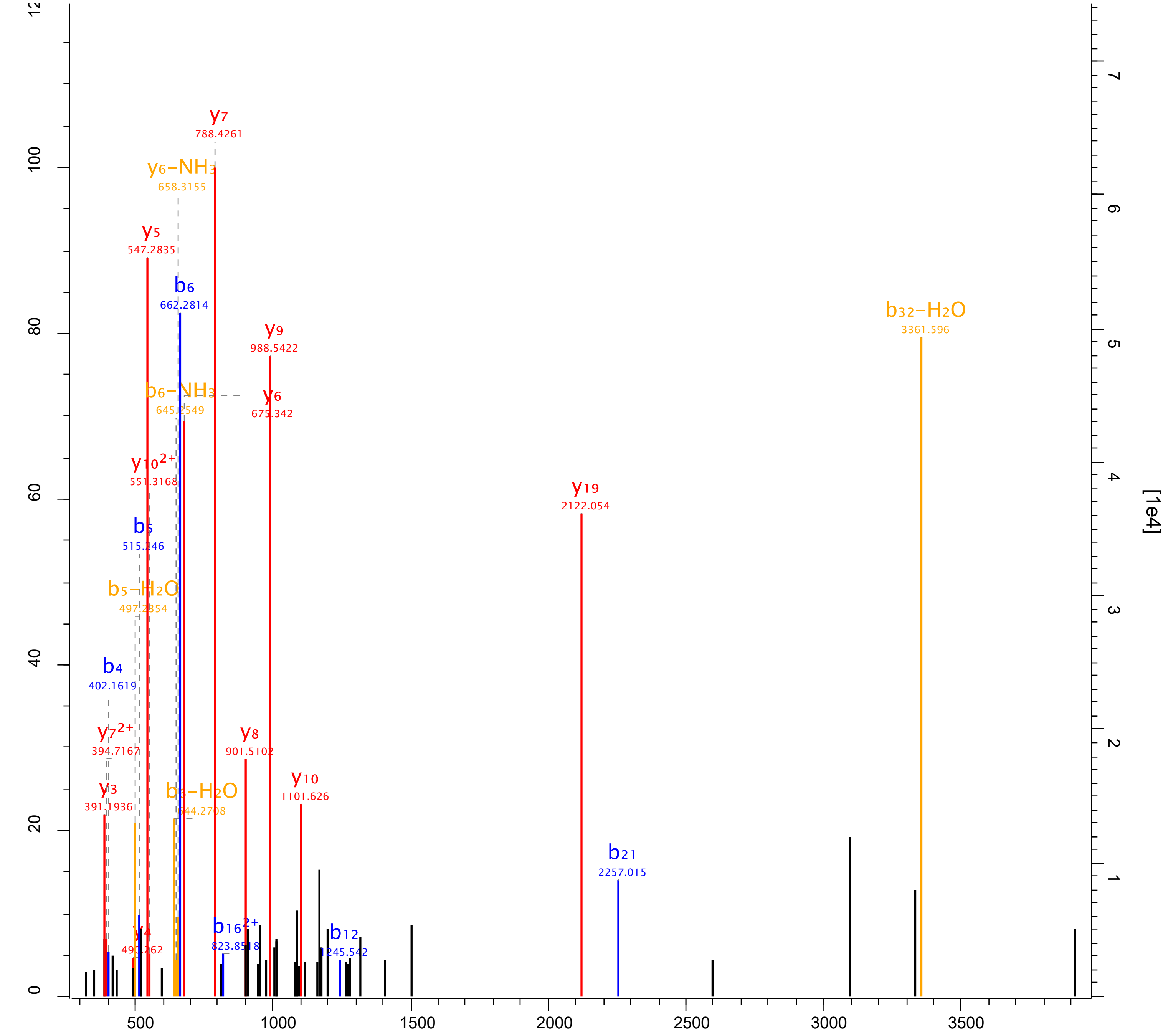

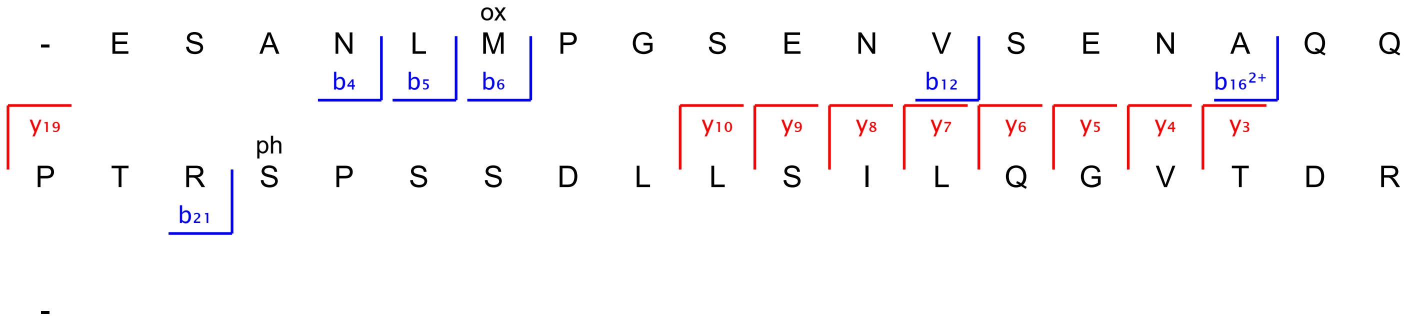

Sample output to test PDF Combine only

Raw file	Scan	Method	Score	m/z
0523_15	27735	FTMS; HCD	175.59	858.42

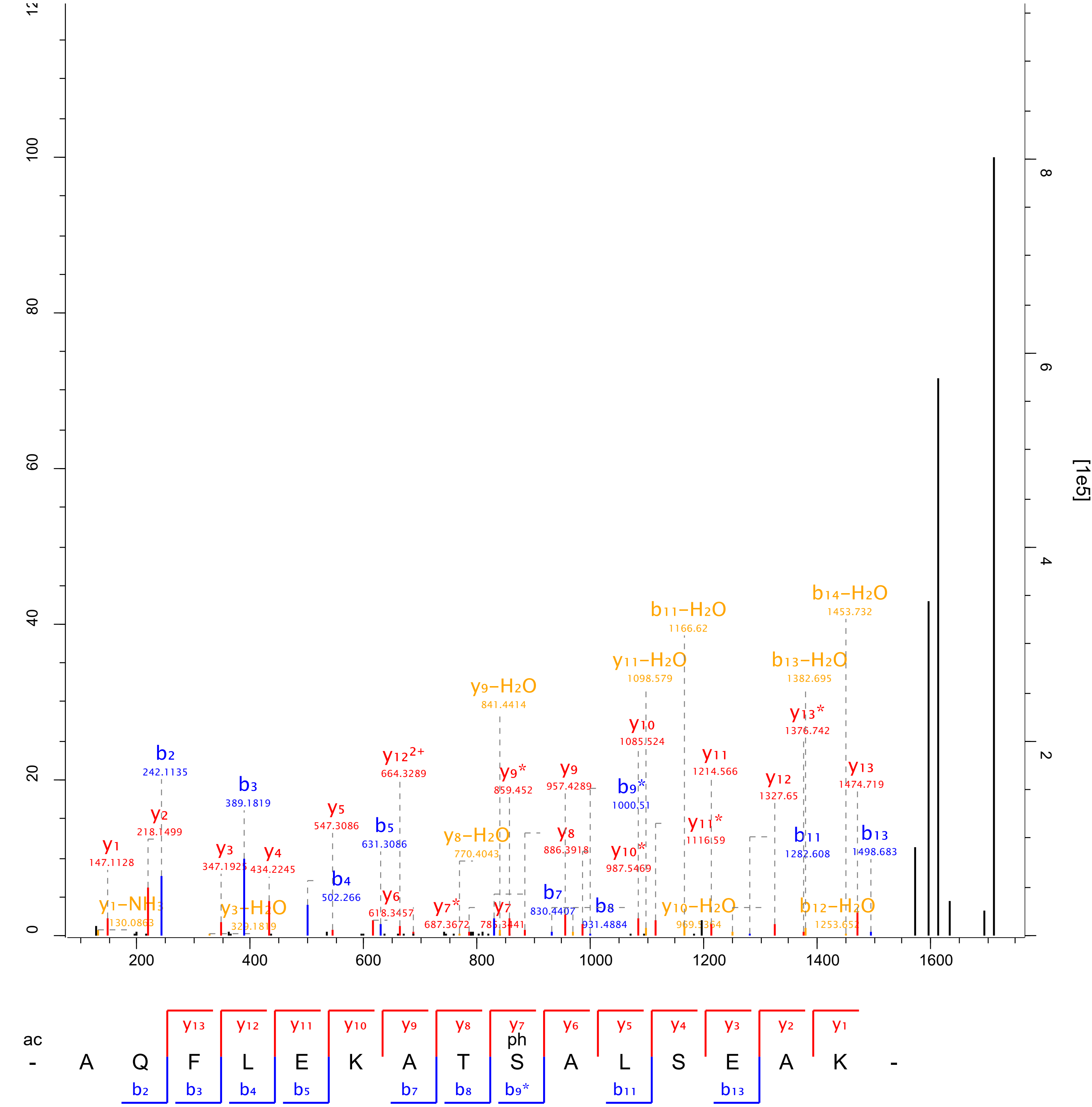

Sample output to test PDF Combine only

Raw file	Scan	Method	Score	m/z	Gene names
0523_15	28542	FTMS; HCD	115.87	870.17	PATL2

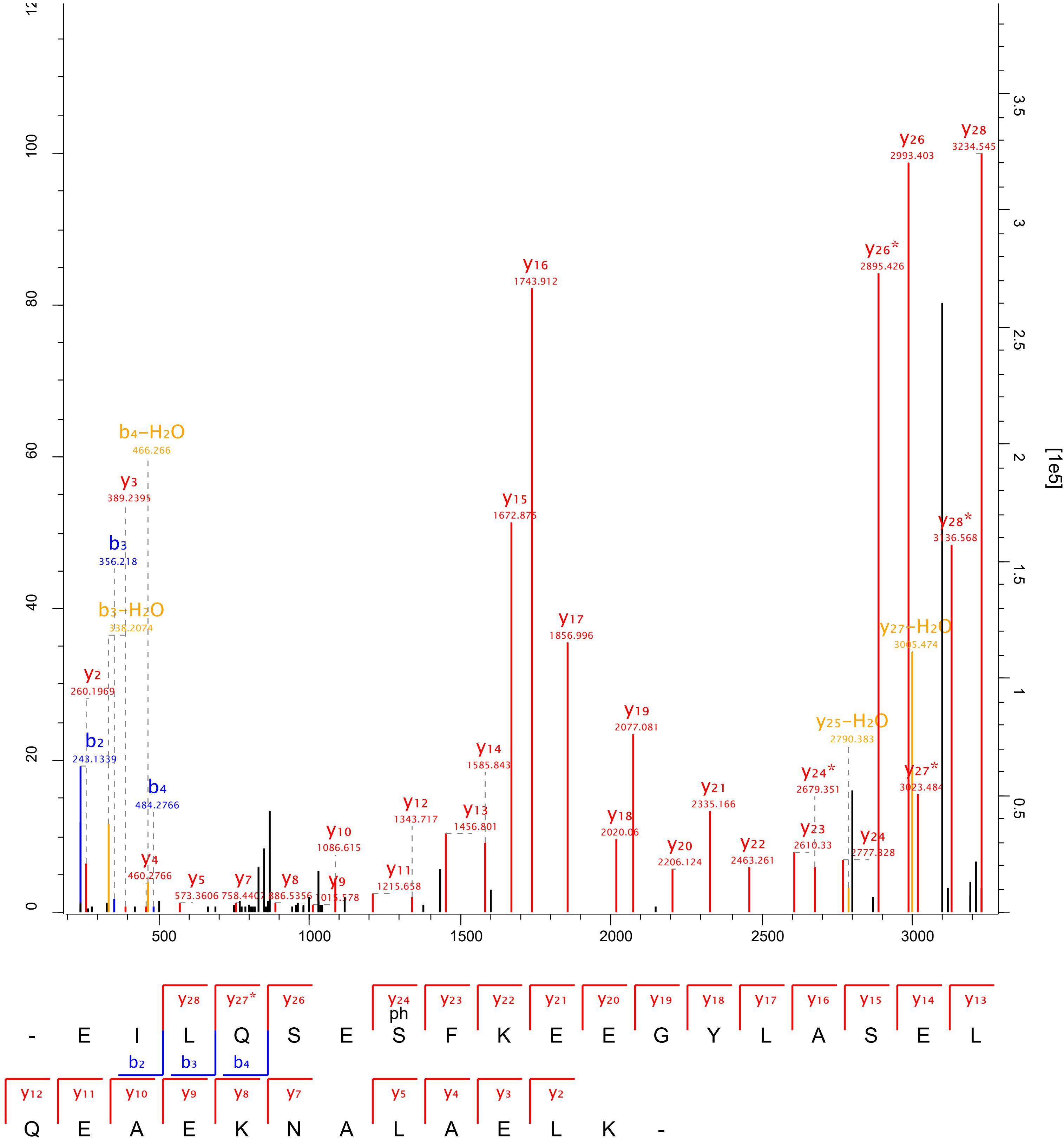

Sample output to test PDF Combine only

Raw file	Scan	Method	Score	m/z	Gene names
0523_15	28607	FTMS; HCD	129.2	1026.47	TPS7

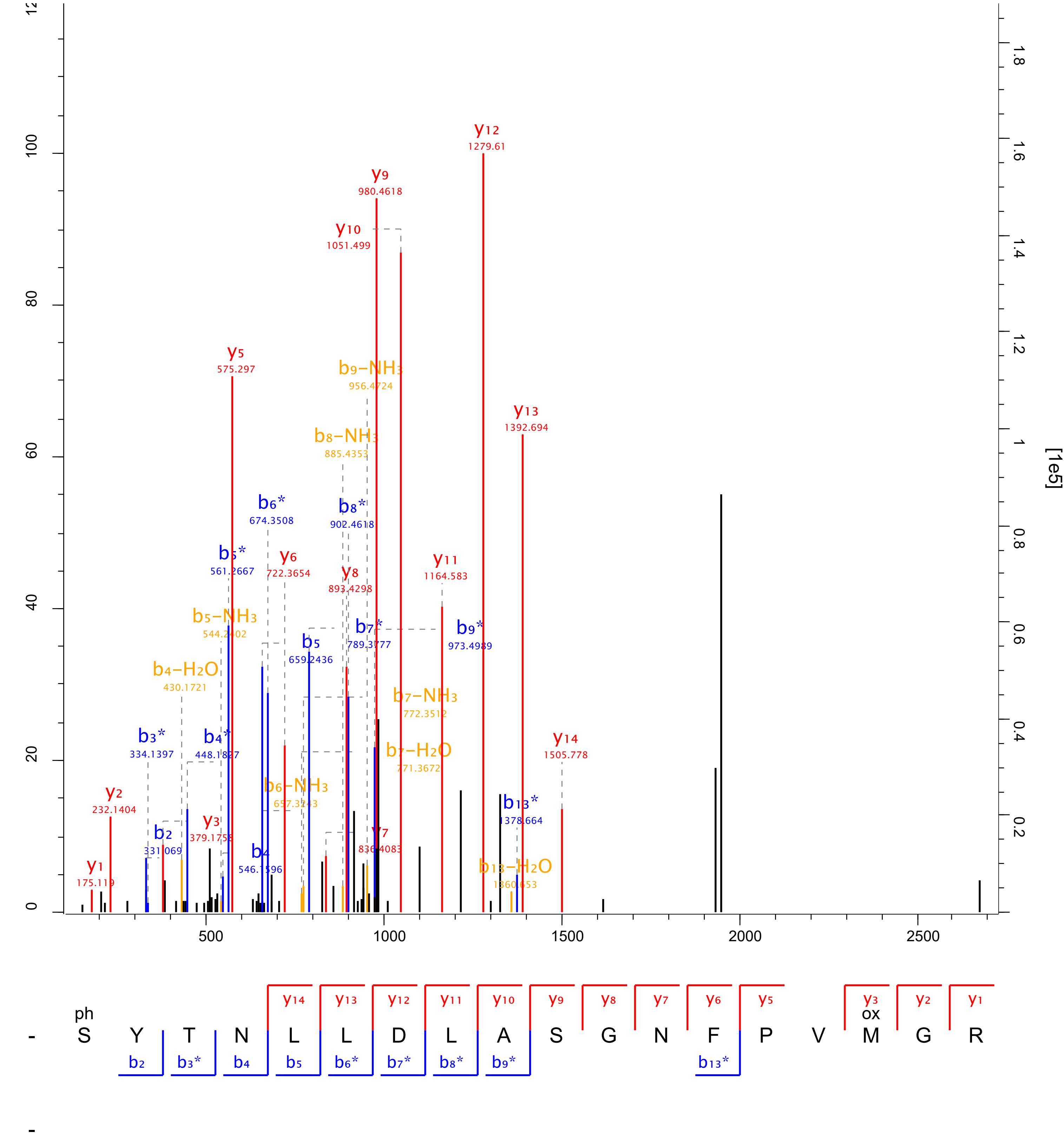

Sample output to test PDF Combine only

Raw file	Scan	Method	Score	m/z	Gene names
0523_15	28845	FTMS; HCD	69.85	896.75	At4g27450

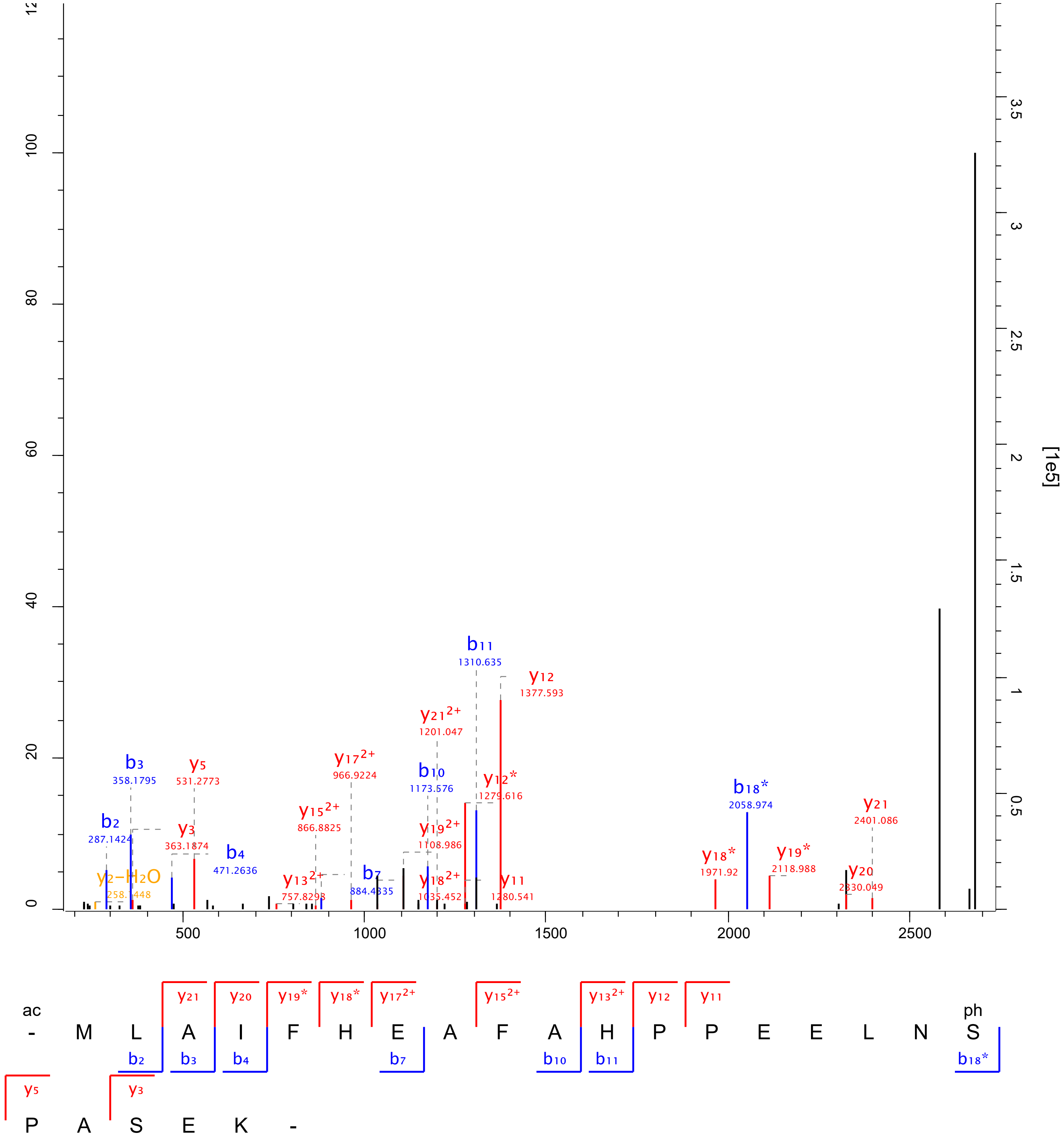

Sample output to test PDF Combine only

Raw file	Scan	Method	Score	m/z
0523_15	29296	FTMS; HCD	75.7	892.03

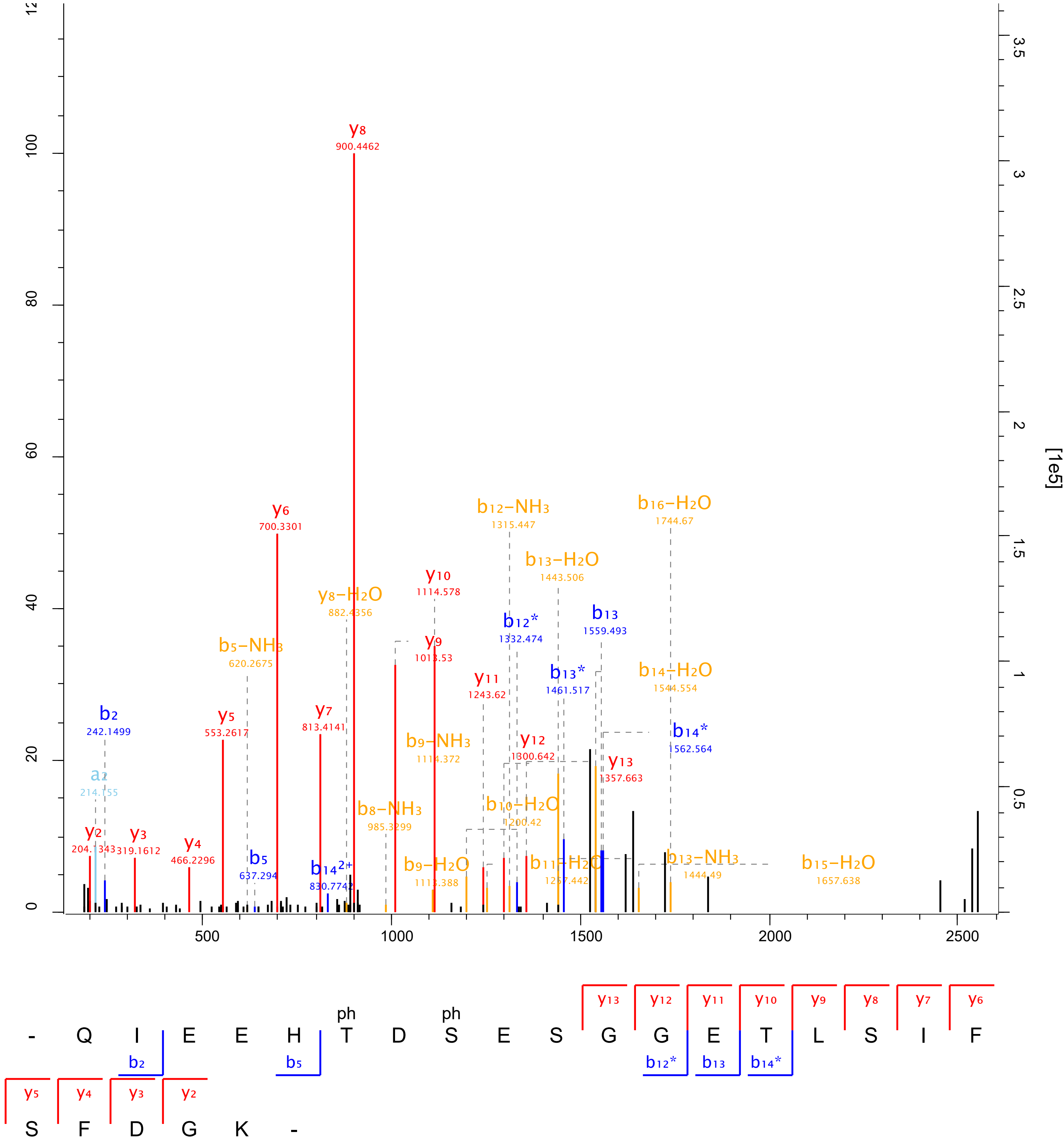

Sample output to test PDF Combine only

Raw file	Scan	Method	Score	m/z	Gene names
0523_15	29860	FTMS; HCD	80.72	984.15	F2P9.15

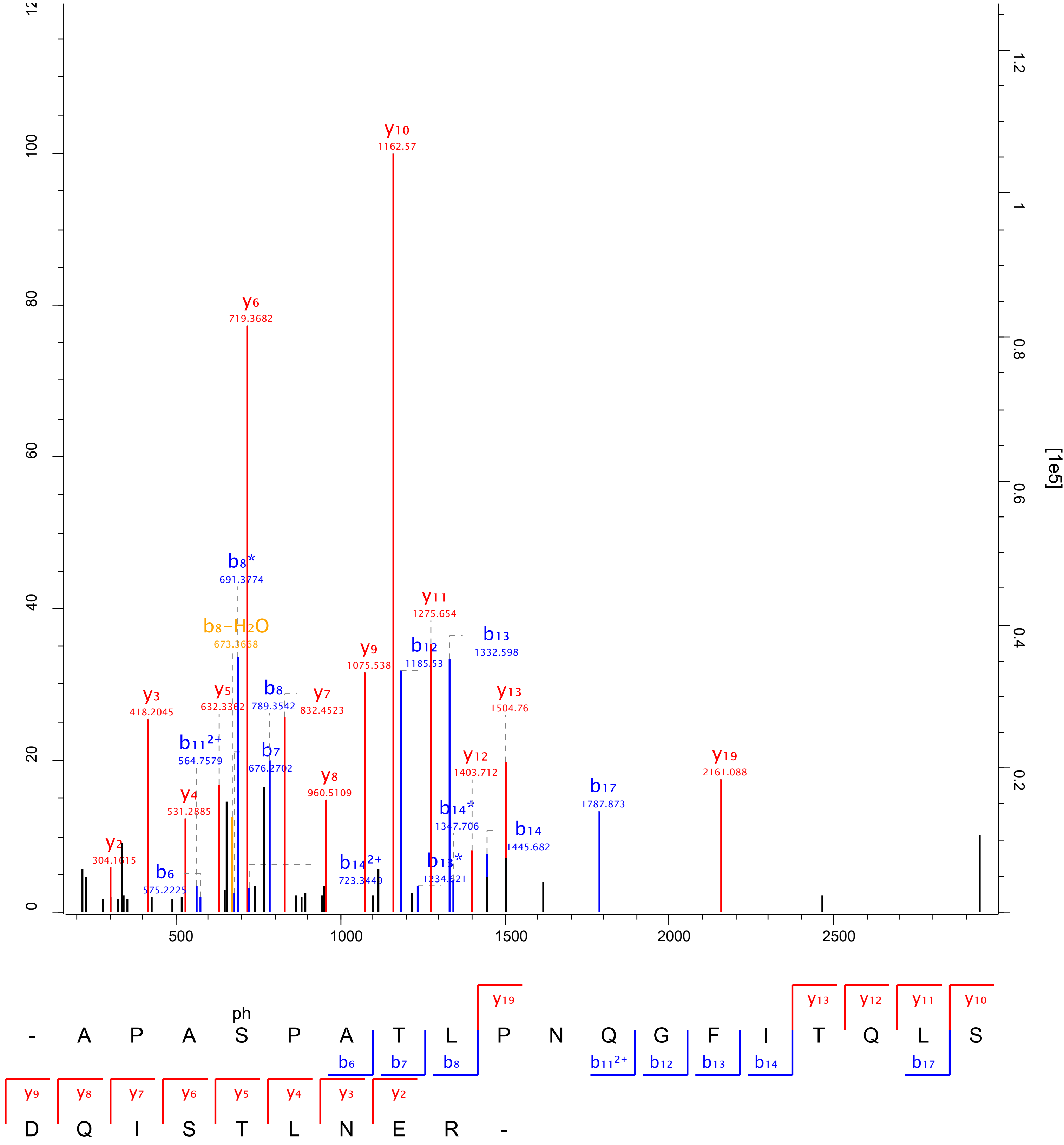

Sample output to test PDF Combine only

Raw file	Scan	Method	Score	m/z
0523_15	31275	FTMS; HCD	132.6	982.82

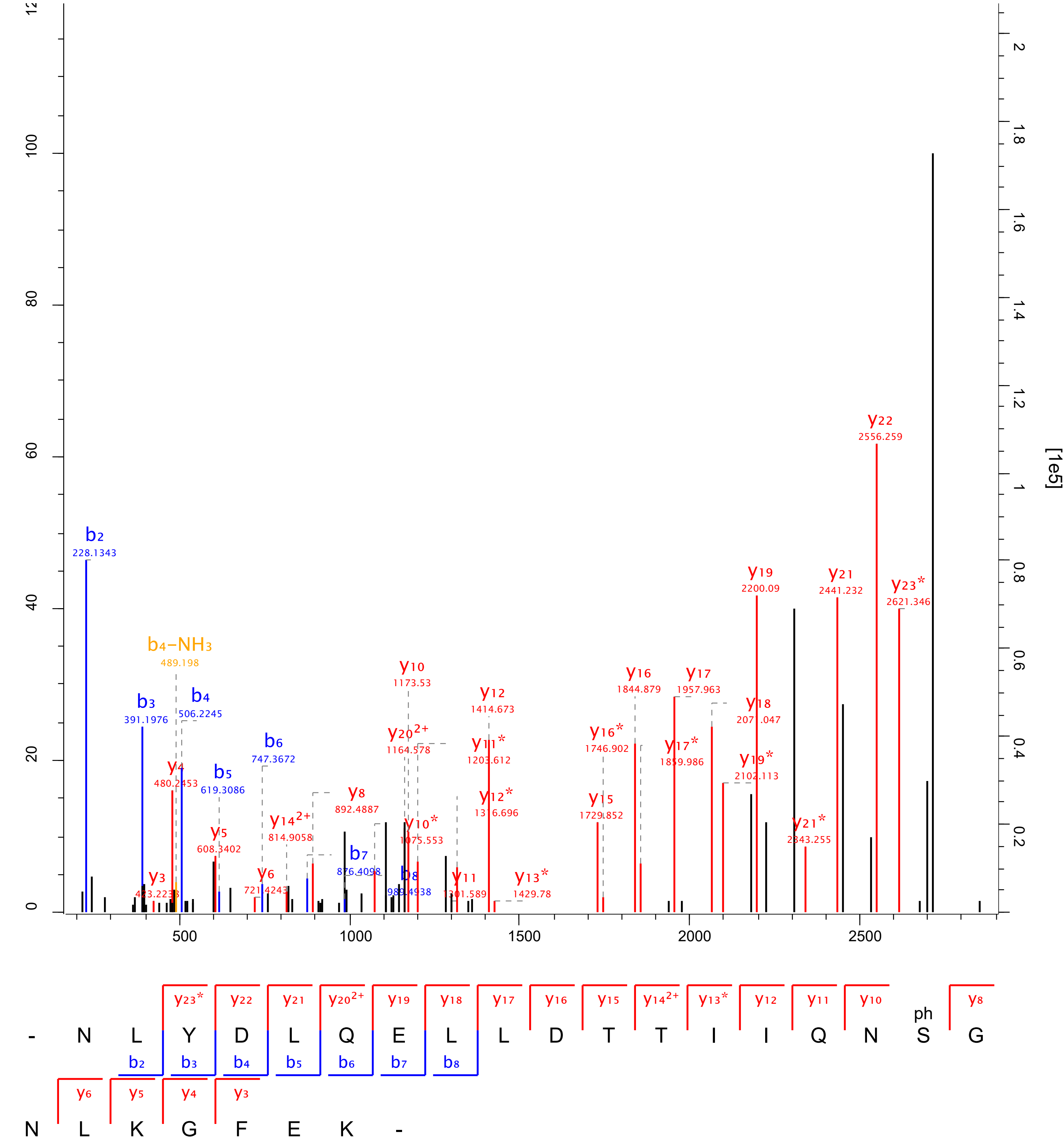

Sample output to test PDF Combine only

Raw file	Scan	Method	Score	m/z	Gene names
0523_15	31307	FTMS; HCD	118.72	910.94	CPK27

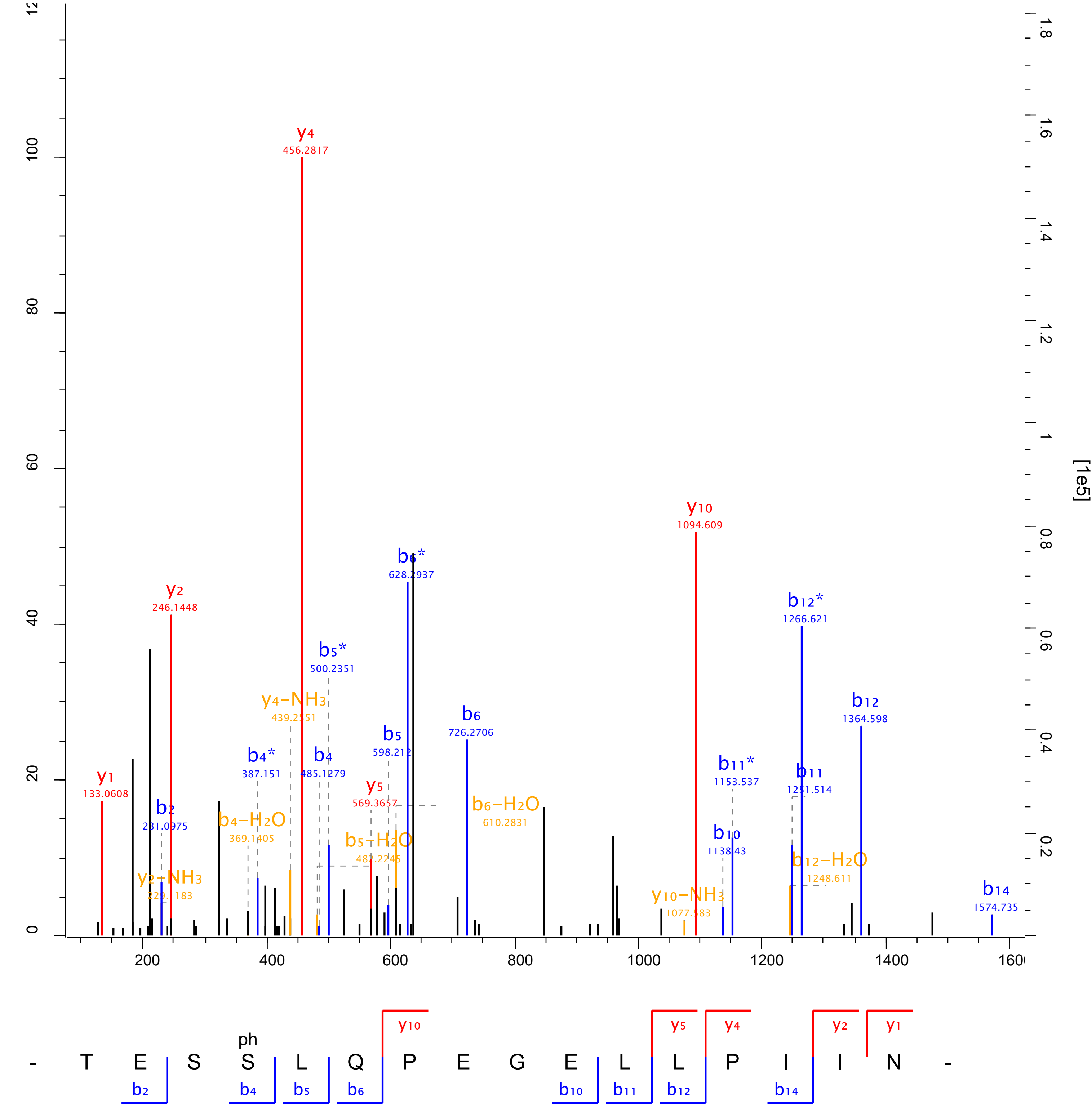

Sample output to test PDF Combine only

Raw file	Scan	Method	Score	m/z	Gene names
0523_15	32008	FTMS; HCD	88.06	1111.26	PVA13

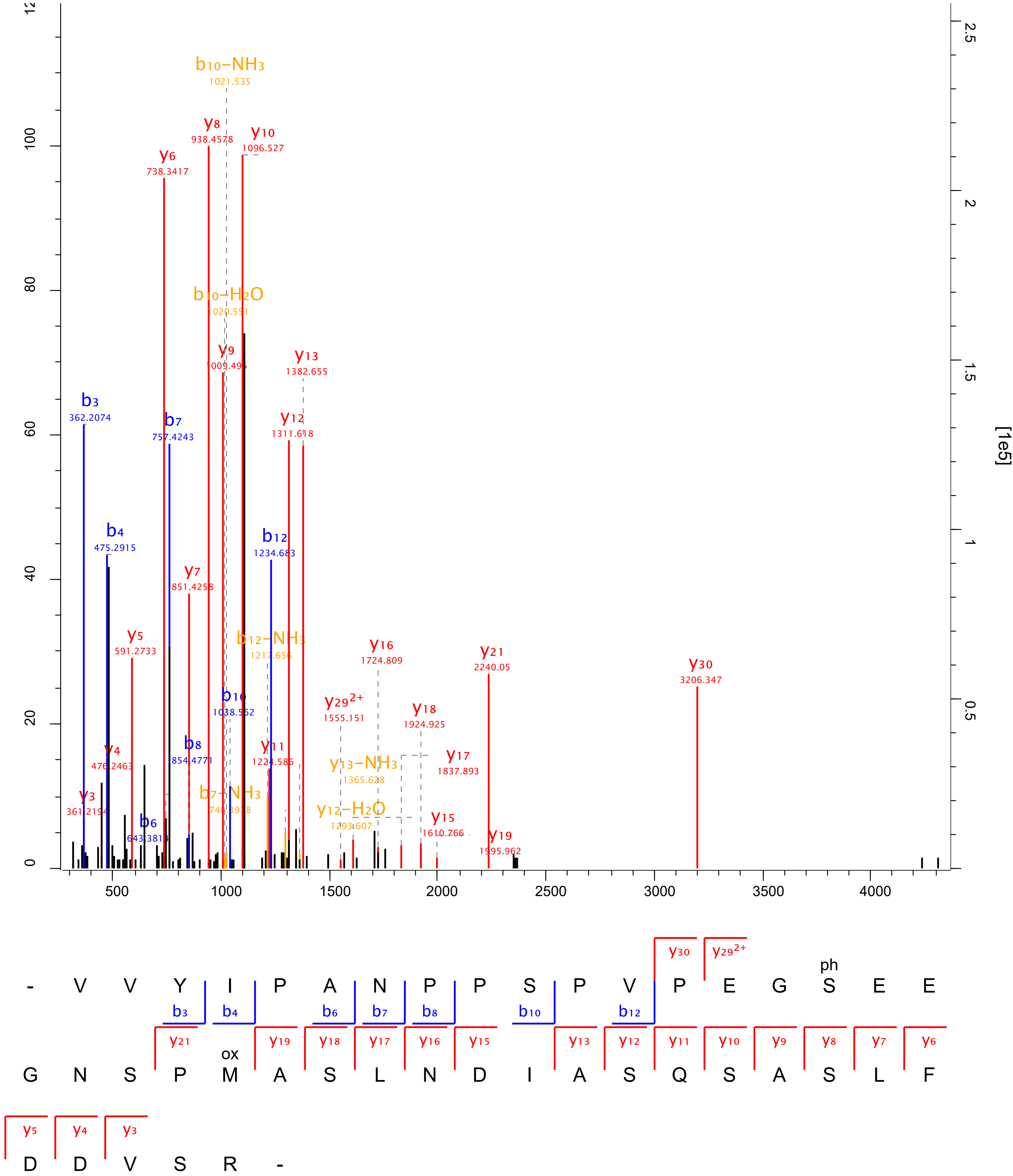

Sample output to test PDF Combine only

Raw file	Scan	Method	Score	m/z	Gene names
0523_15	32723	FTMS; HCD	79.78	1056.53	AHA2;HA2

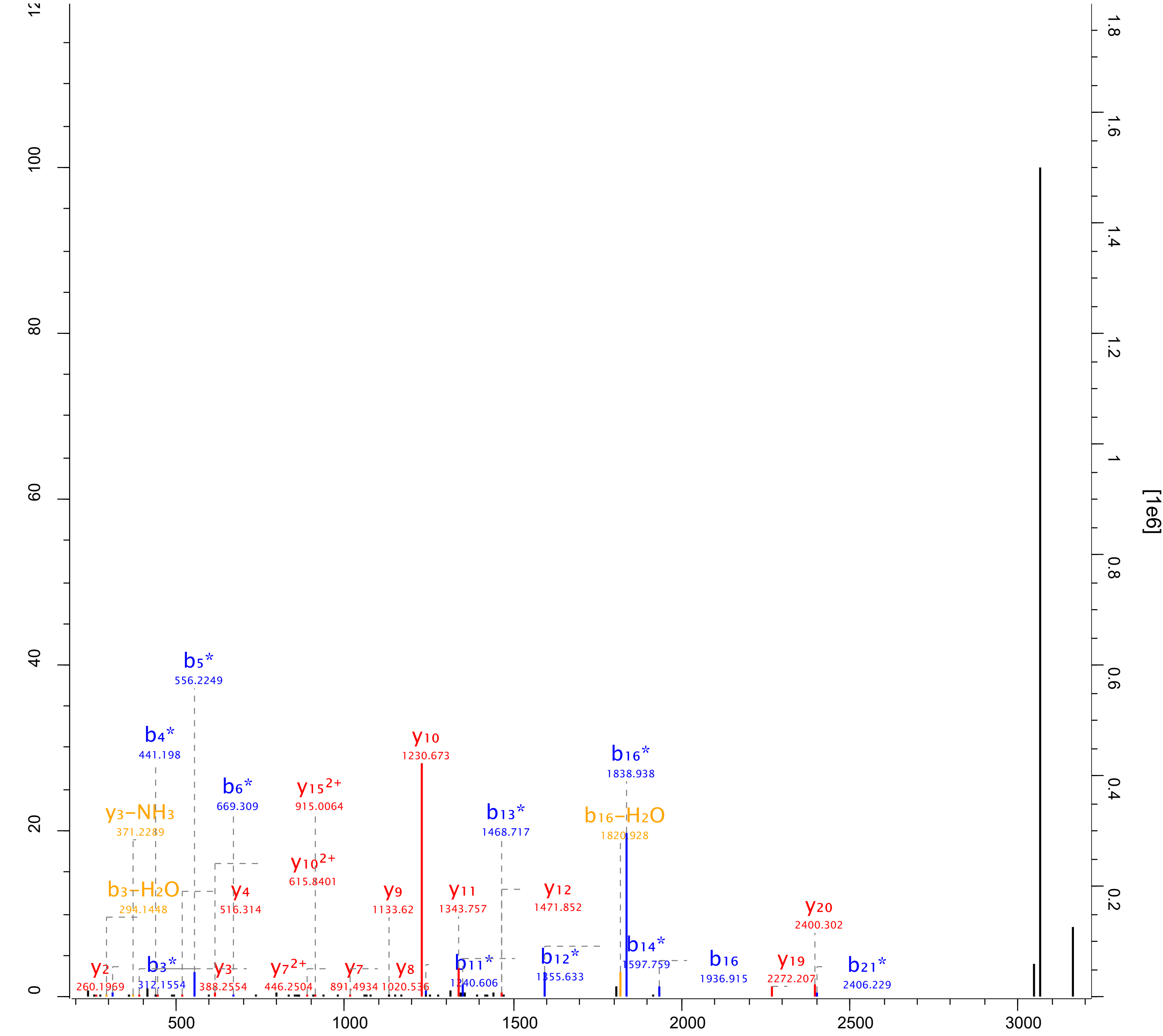

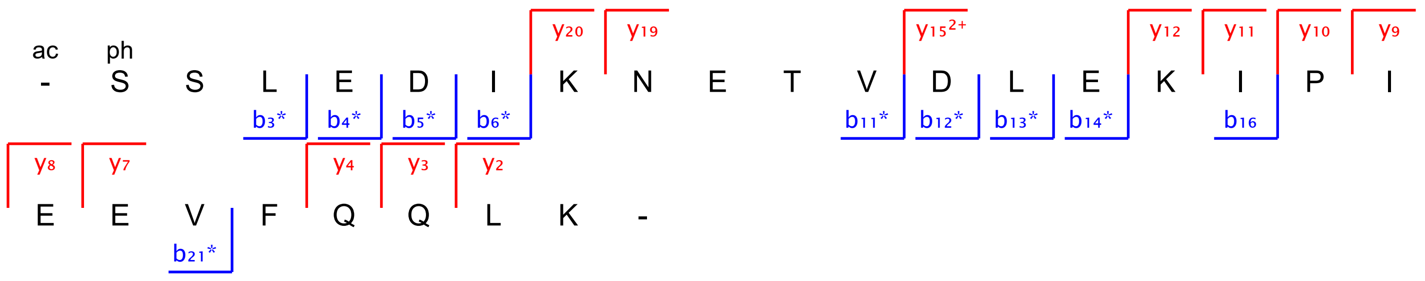

Sample output to test PDF Combine only

Raw file	Scan	Method	Score	m/z	Gene names
sirk1_sp3-mic-0-1-A	6432	FTMS; HCD	83.25	471.56	CRWN1

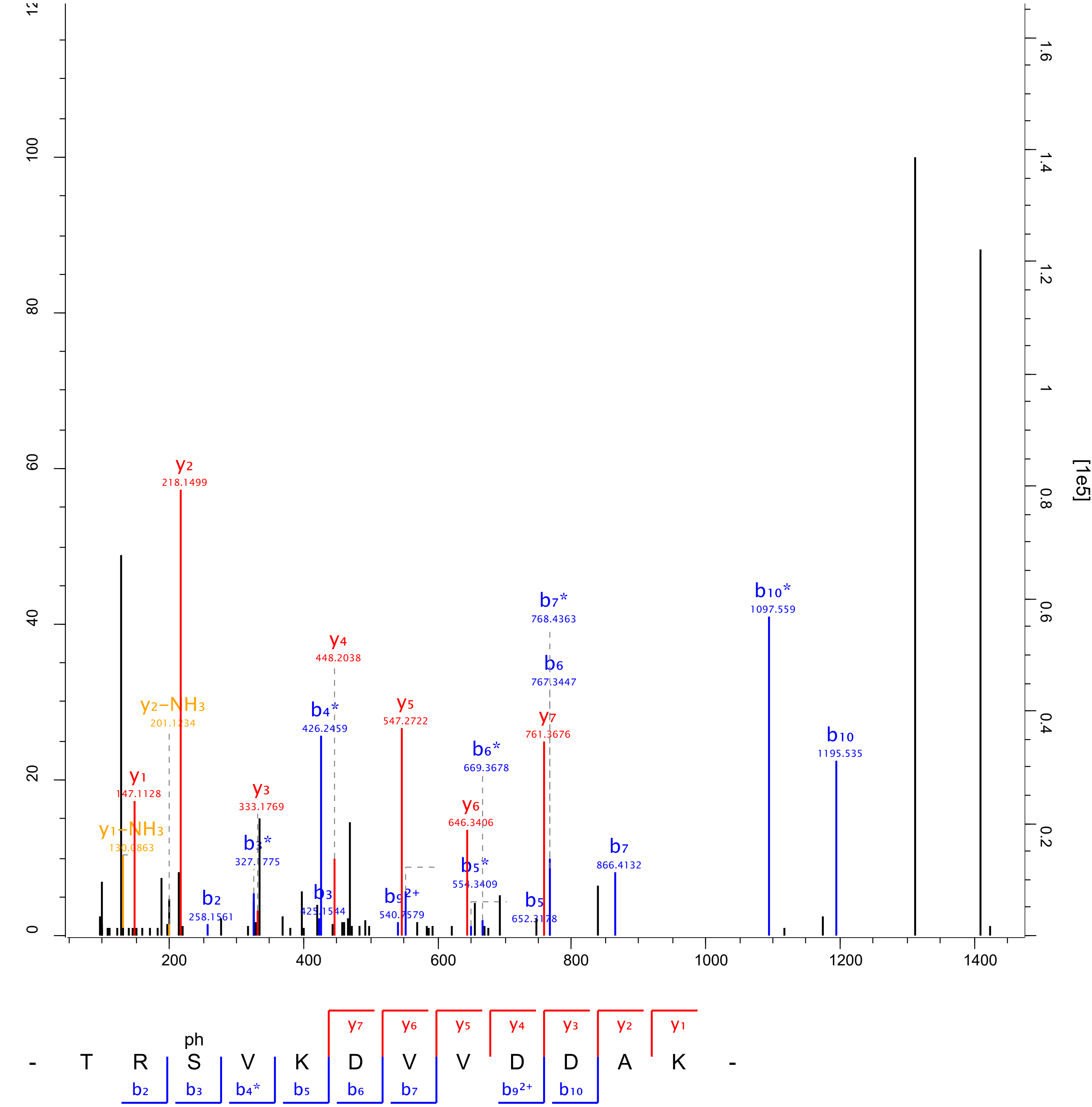

Sample output to test PDF Combine only

Raw file	Scan	Method	Score	m/z	Gene names
sirk1_sp3-mic-0-1-A	7365	FTMS; HCD	40.09	735.36	PATL1

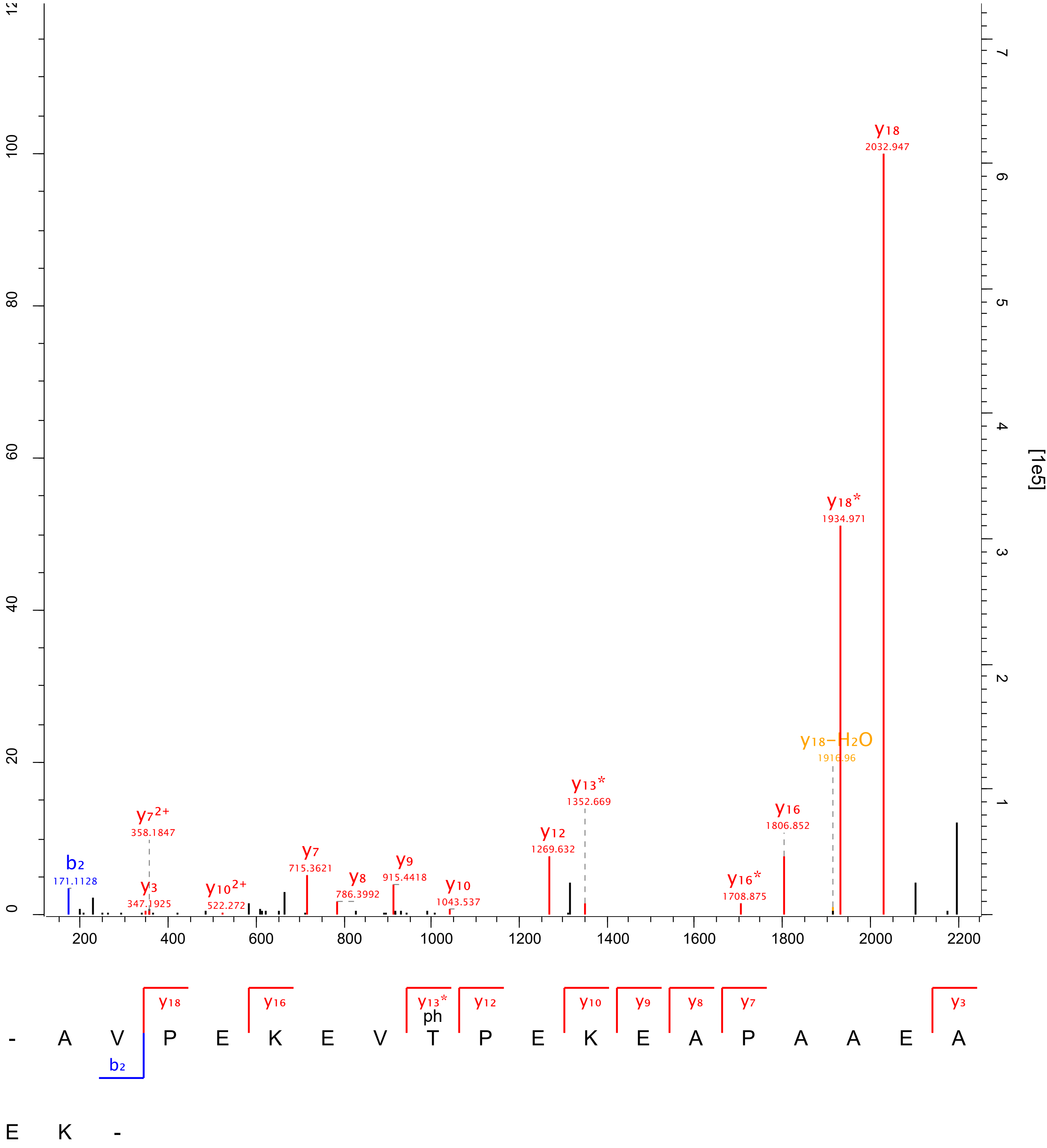

Sample output to test PDF Combine only

Raw file	Scan	Method	Score	m/z	Gene names
sirk1_sp3-mic-0-1-A	11201	FTMS; HCD	49	749.84	MYOB7

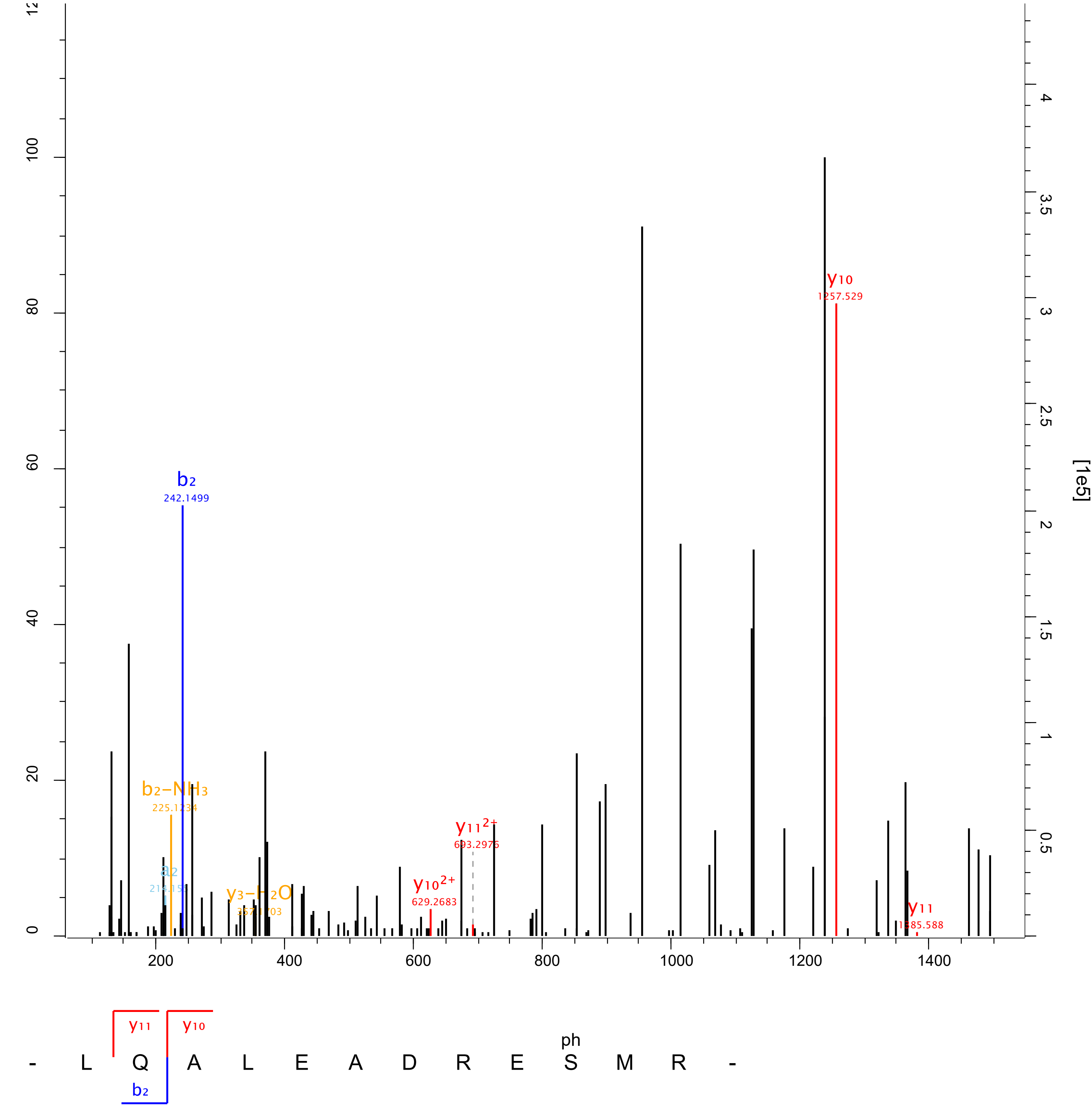

Sample output to test PDF Combine only

Raw file	Scan	Method	Score	m/z	Gene names
sirk1_sp3-mic-0-1-A	12719	FTMS; HCD	89.3	542.74	At3g22850

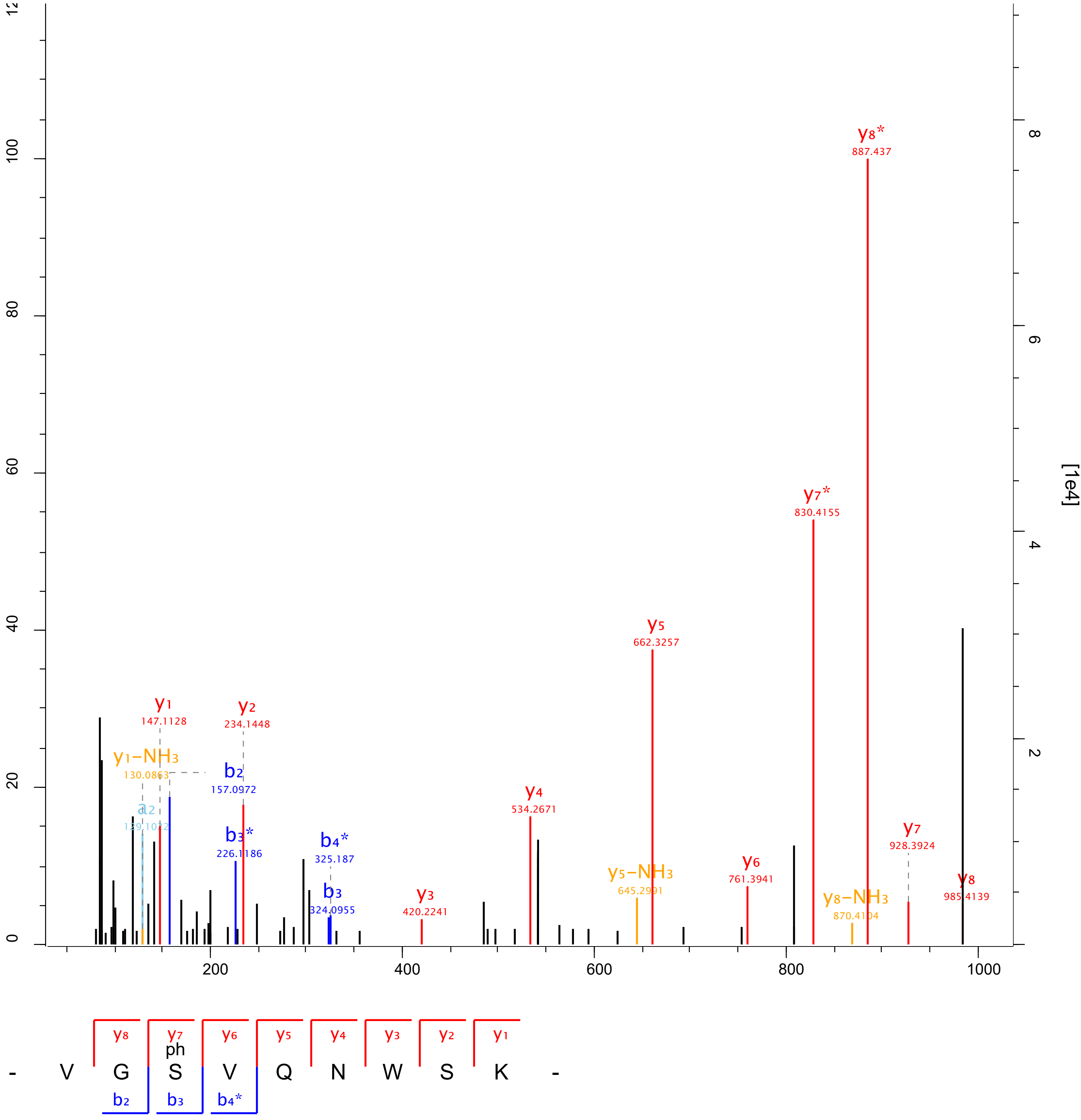

Sample output to test PDF Combine only

Raw file	Scan	Method	Score	m/z
sirk1_sp3-mic-0-1-A	18934	FTMS; HCD	80.69	553.24

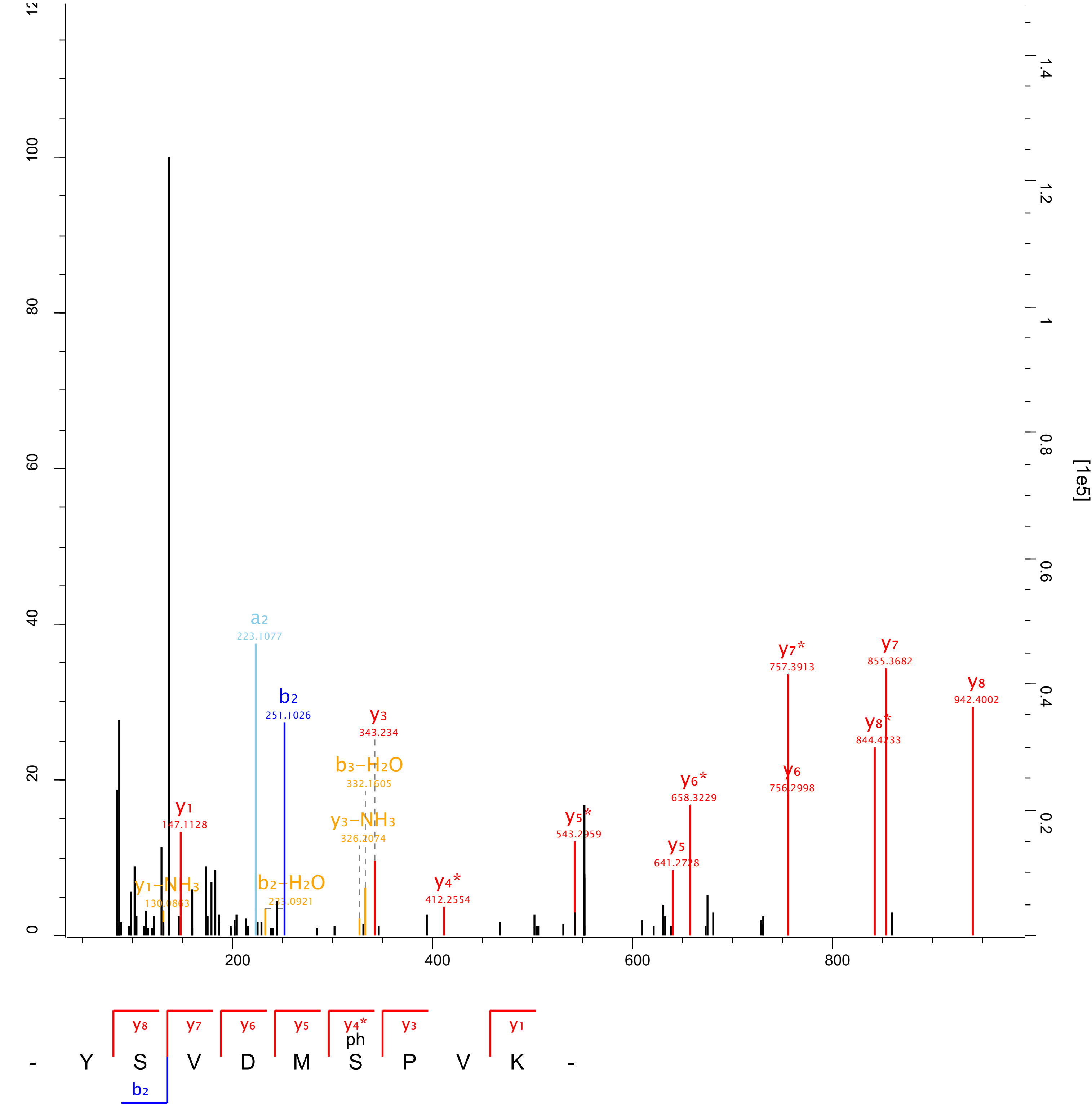

Sample output to test PDF Combine only

Raw file	Scan	Method	Score	m/z	Gene names
sirk1_sp3-mic-0-1-A	23161	FTMS; HCD	48.98	487.89	PEX2

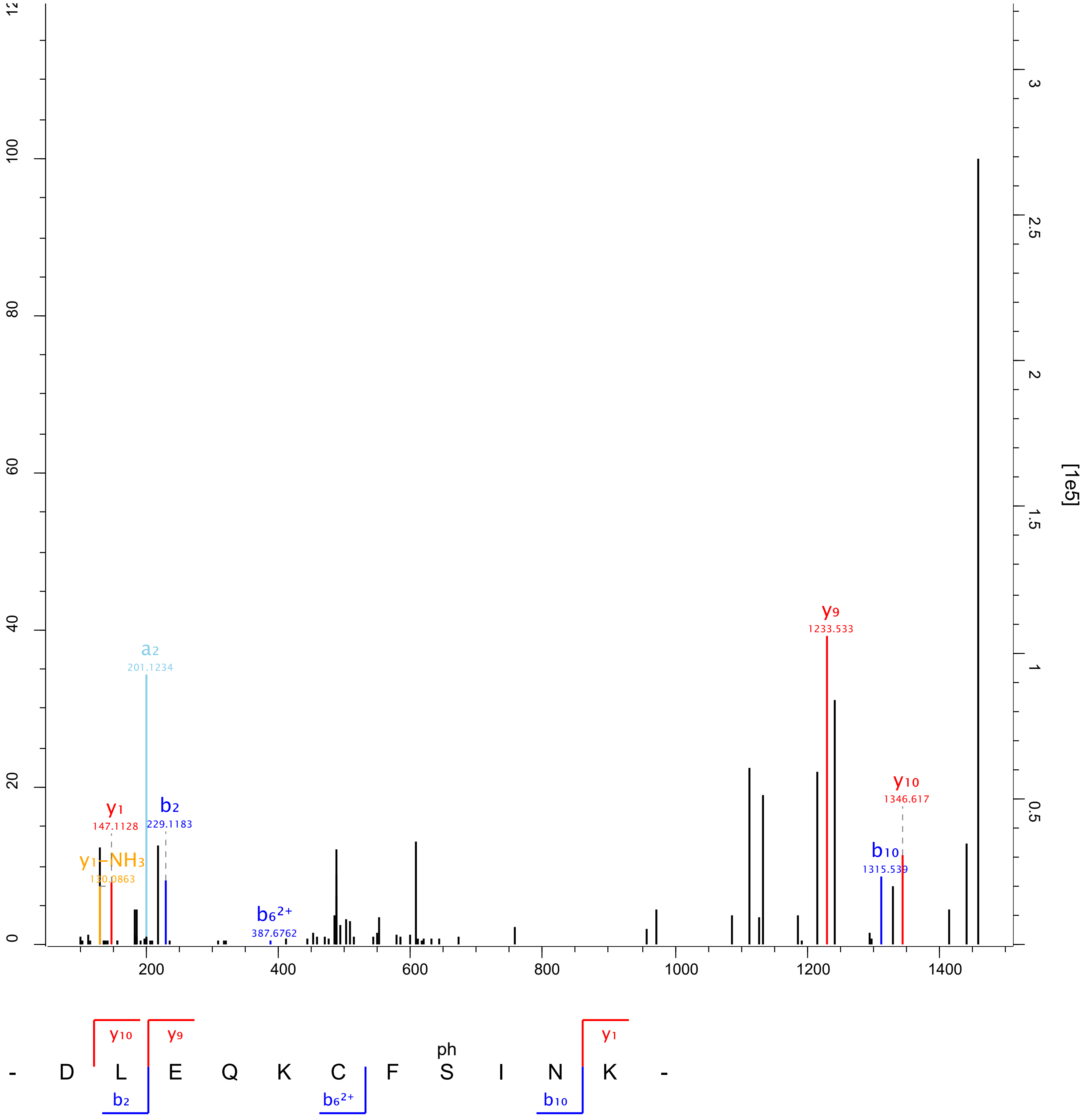

Sample output to test PDF Combine only

Raw file	Scan	Method	Score	m/z	Gene names
sirk1_sp3-mic-0-1-A	26020	FTMS; HCD	41.4	788.84	At3g28560

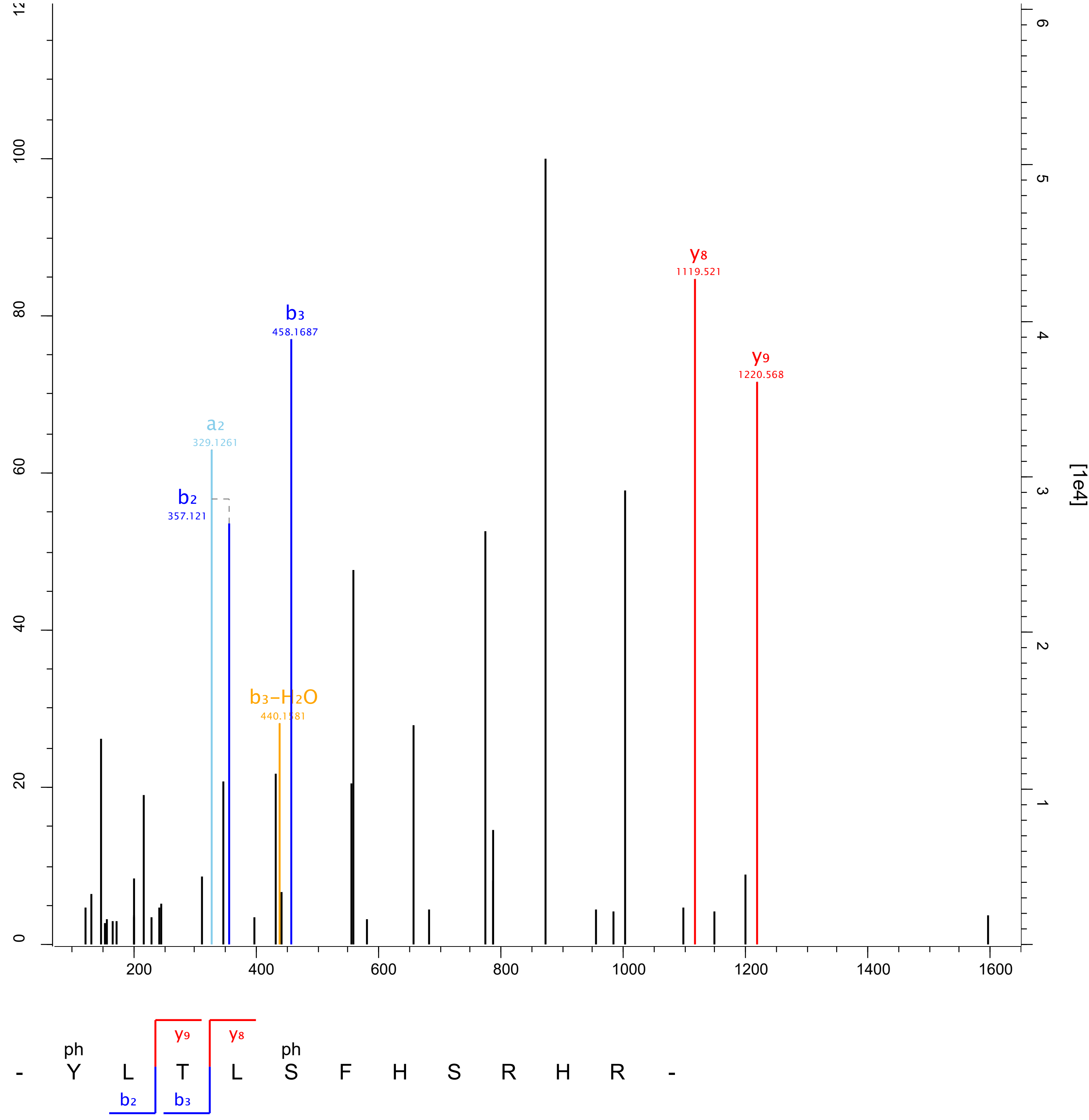

Sample output to test PDF Combine only

Raw file	Scan	Method	Score	m/z	Gene names
sirk1_sp3-mic-0-1-A	28123	FTMS; HCD	66.25	745.33	SLAH3

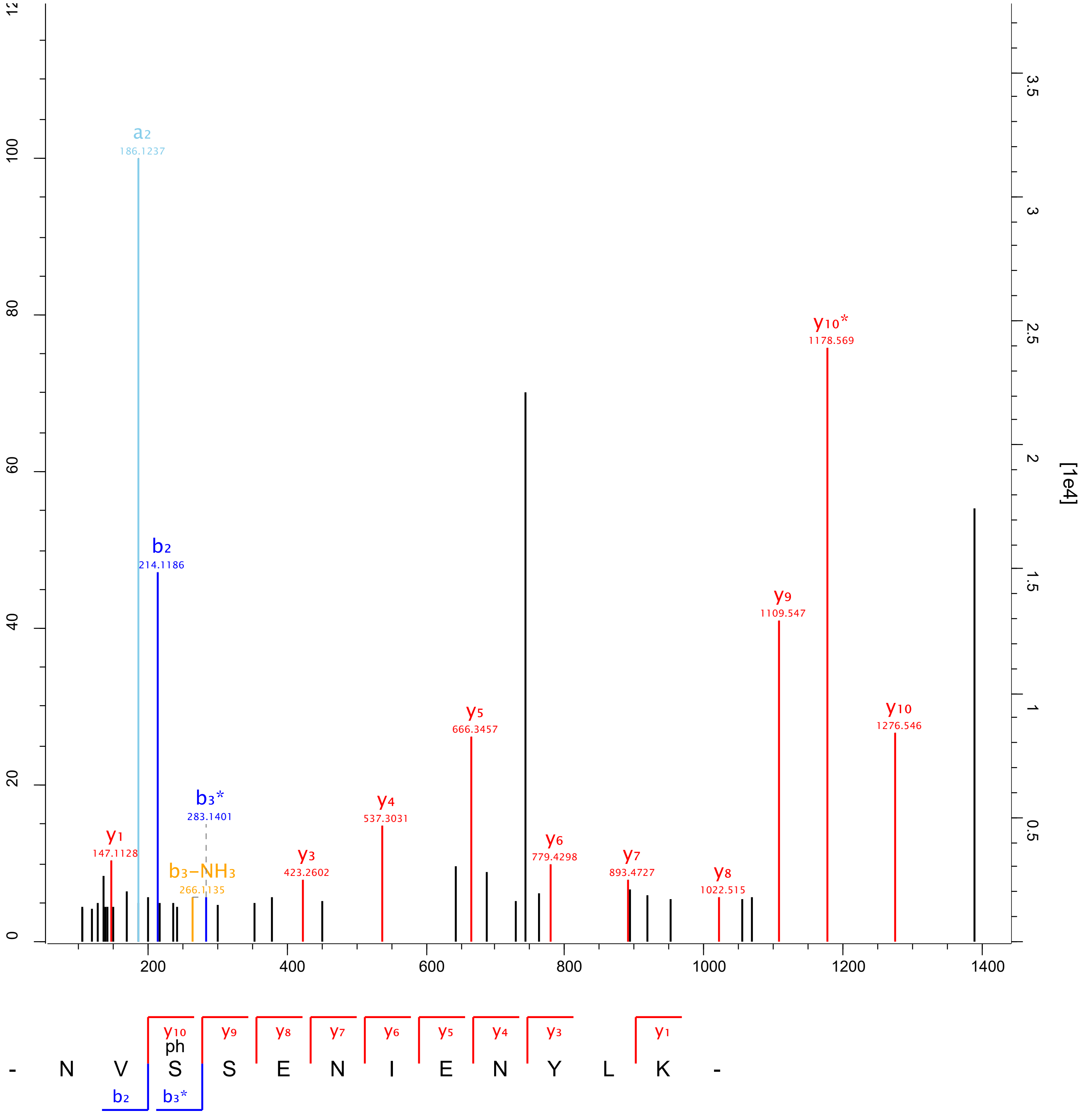

Sample output to test PDF Combine only

Raw file	Scan	Method	Score	m/z	Gene names
sirk1_sp3-mic-0-1-P	10157	FTMS; HCD	146.84	441.9	ACA8

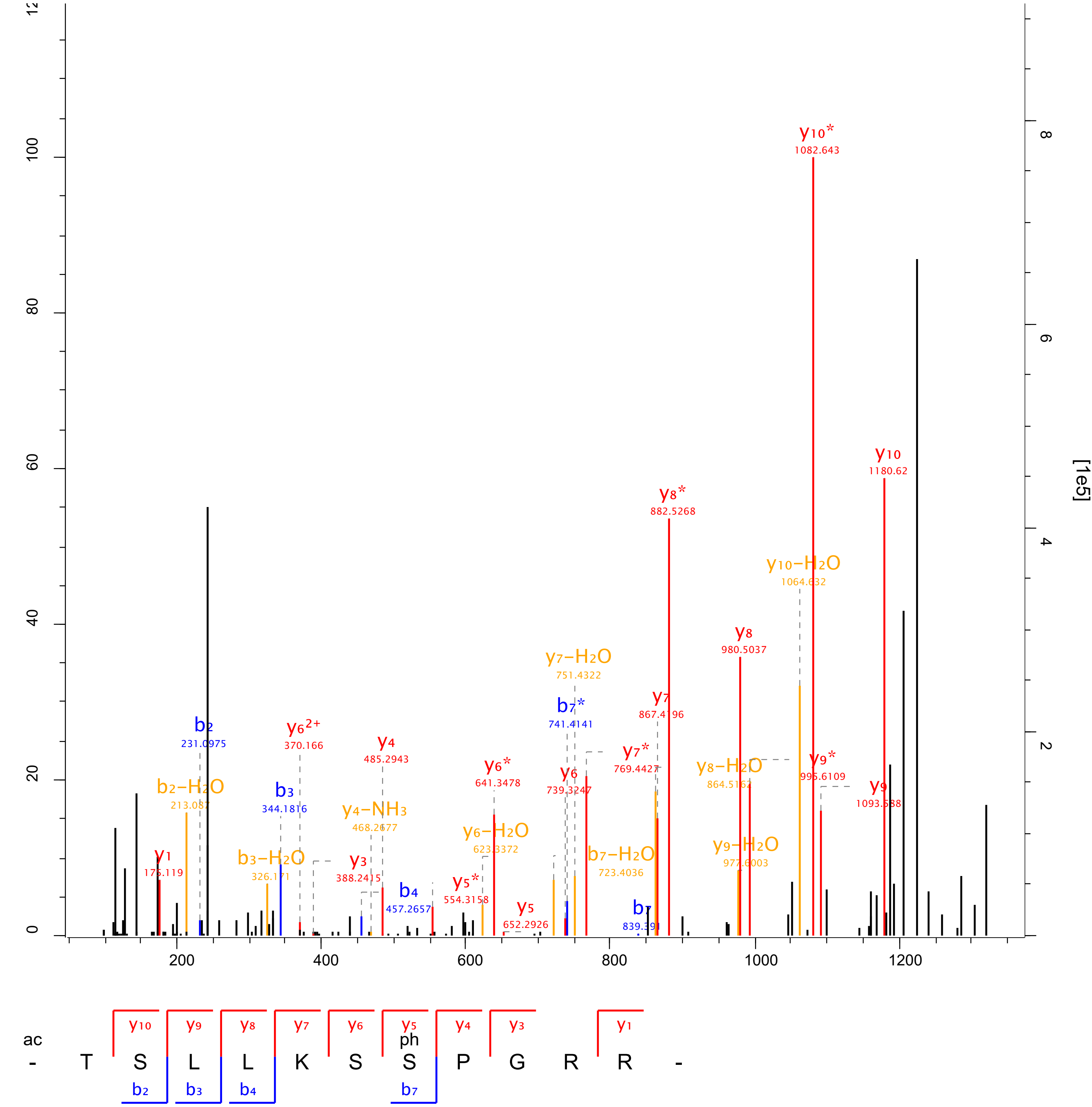

Sample output to test PDF Combine only

Raw file	Scan	Method	Score	m/z	Gene names
sirk1_sp3-mic-0-1-P	14528	FTMS; HCD	134.81	504.58	At4g27450

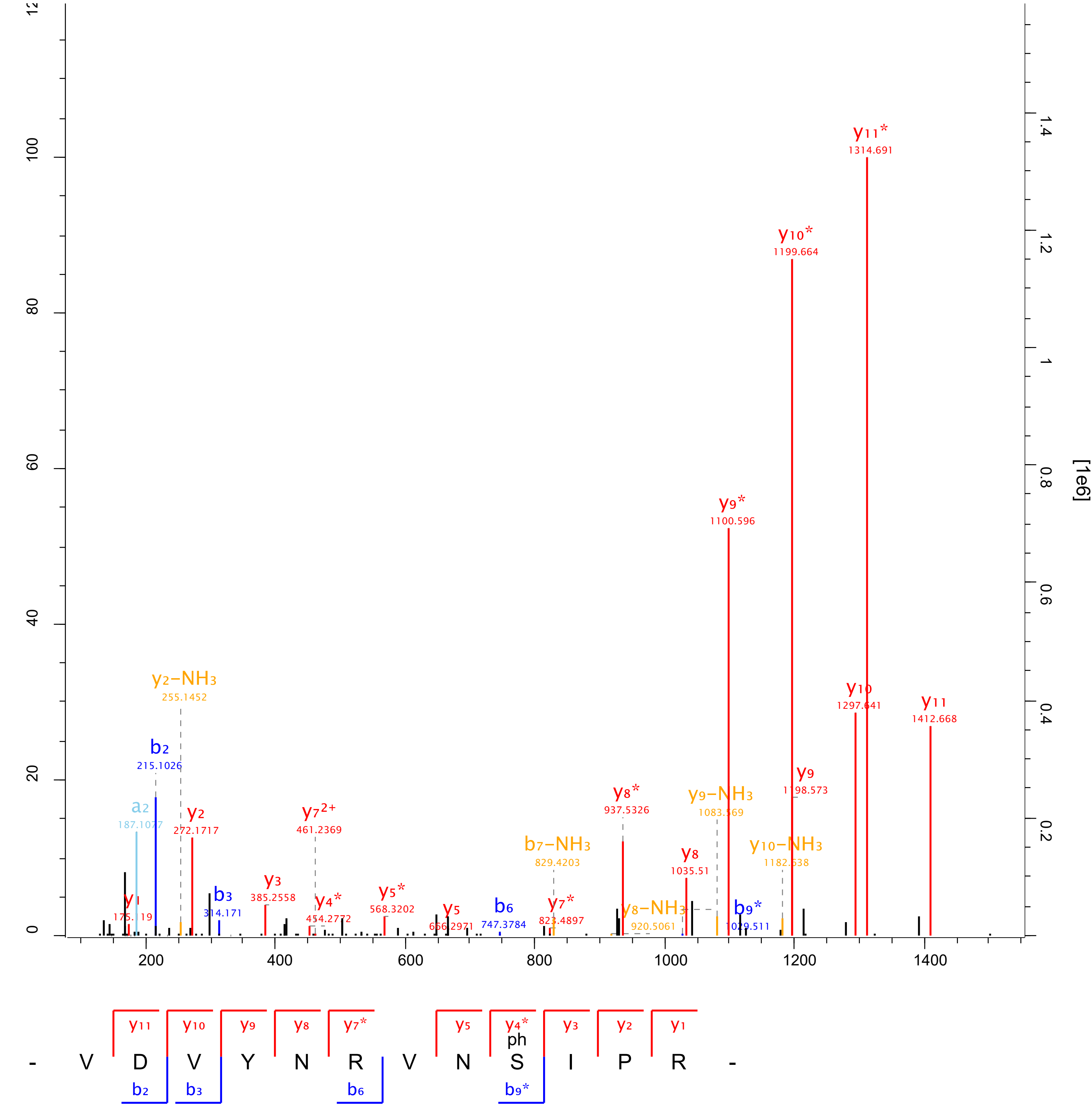

Sample output to test PDF Combine only

Raw file	Scan	Method	Score	m/z	Gene names
sirk1_sp3-mic-0-1-P	18163	FTMS; HCD	59.73	530.95	At2g39130

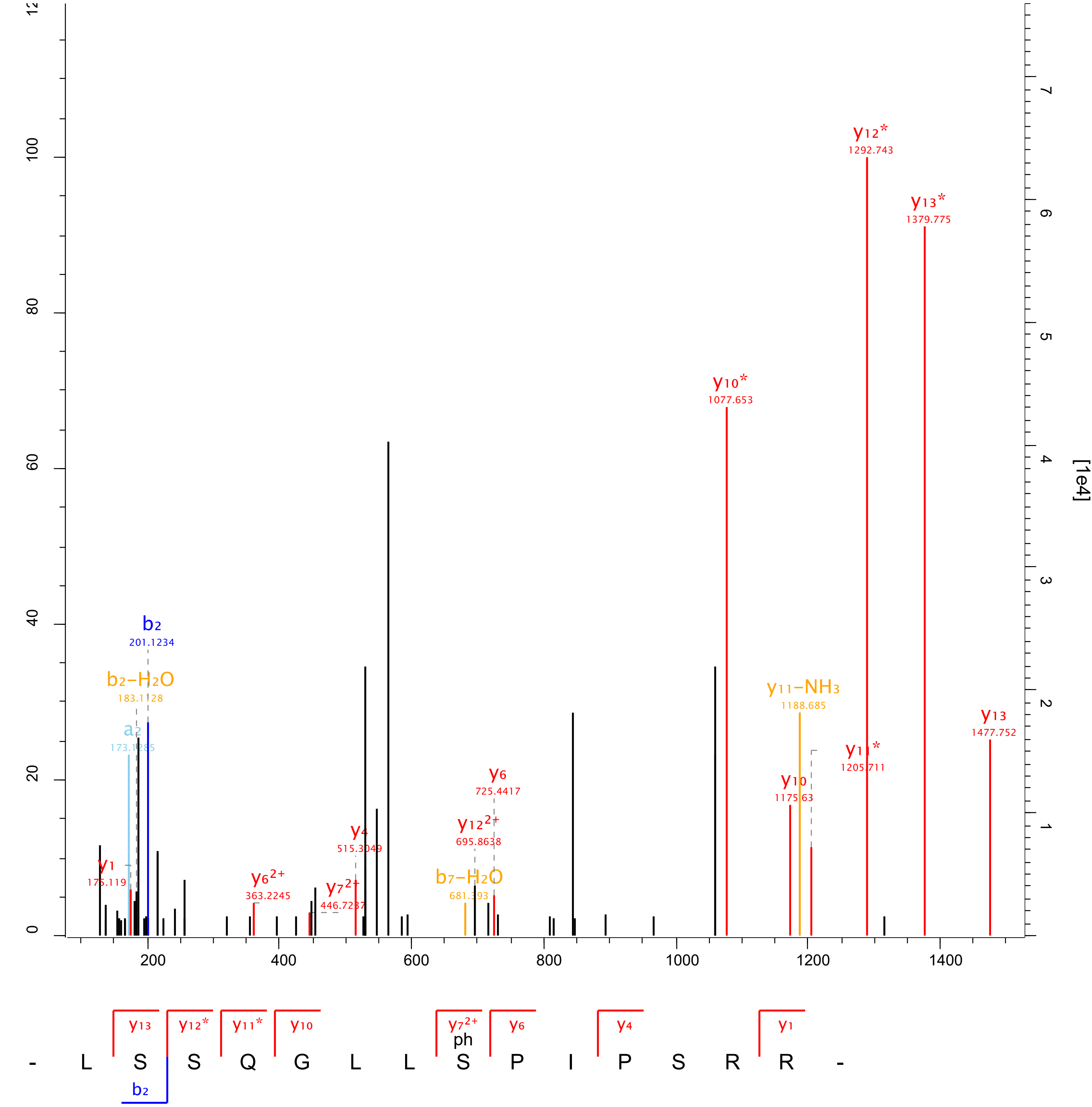

Raw file	Scan	Method	Score	m/z	Gene names
sirk1_sp3-mic-0-1-P	19092	FTMS; HCD	55.26	830.42	GC5

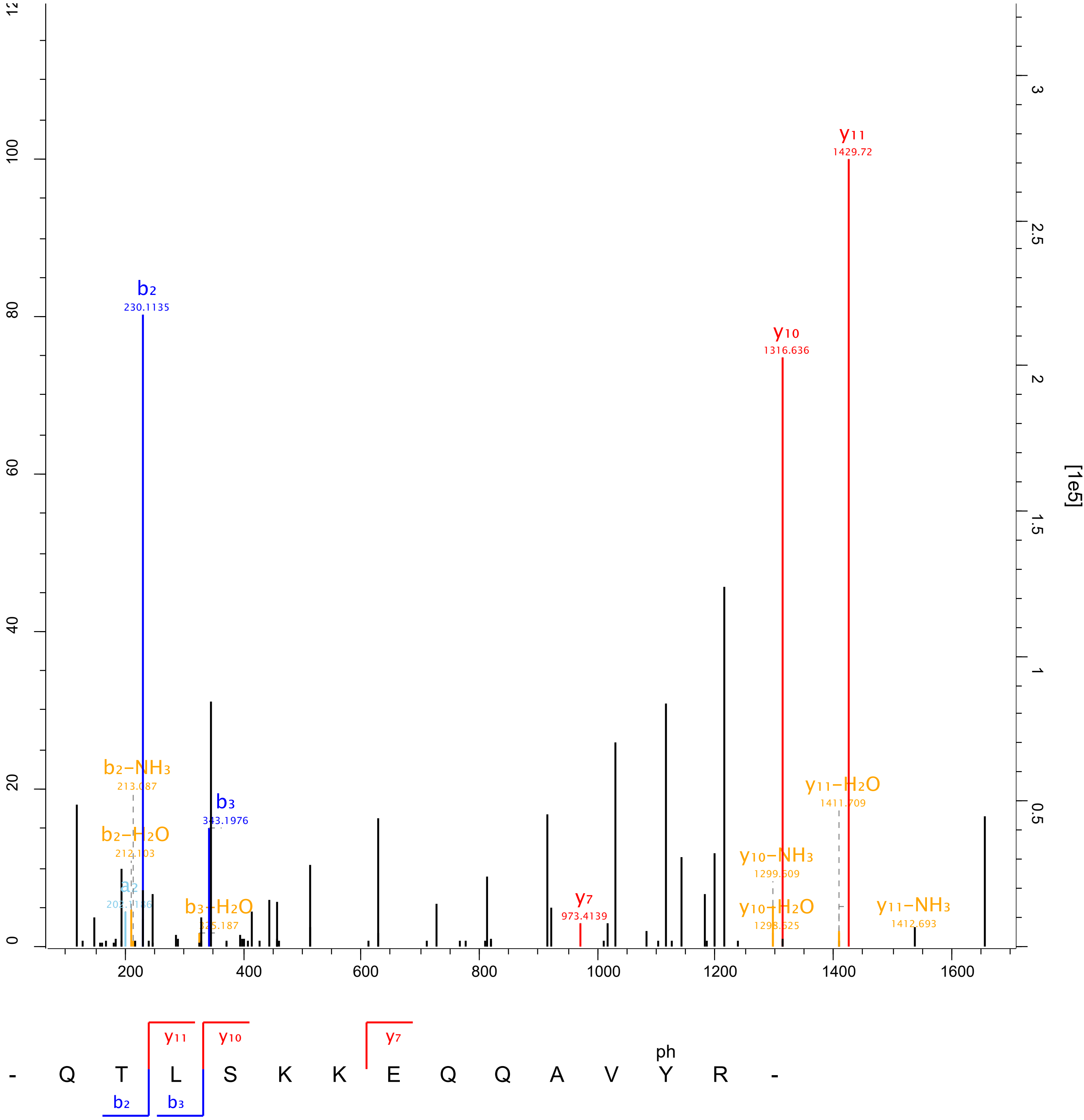

Sample output to test PDF Combine only
