# Supplementary material for: Sucrose-induced Receptor Kinase 1 is Modulated by an Interacting Kinase with Short Extracellular Domain
Source: Mol Cell Proteomics. 2019 May 30;18(8):1556–71. doi: 10.1074/mcp.RA119.001336 (PMC6683012; doi:10.1074/mcp.RA119.001336)
Supplement: Supplementary Figure S6g [file 143141_1_supp_311904_ps5fkz.pdf]

Figure S6:

Spectra of all identified phosphopeptides.

Raw file	Scan	Method	Score	m/z	Gene names
sirk1_sp3-mic-0-1-P	20419	FTMS; HCD	59.37	597.3	At3g07660

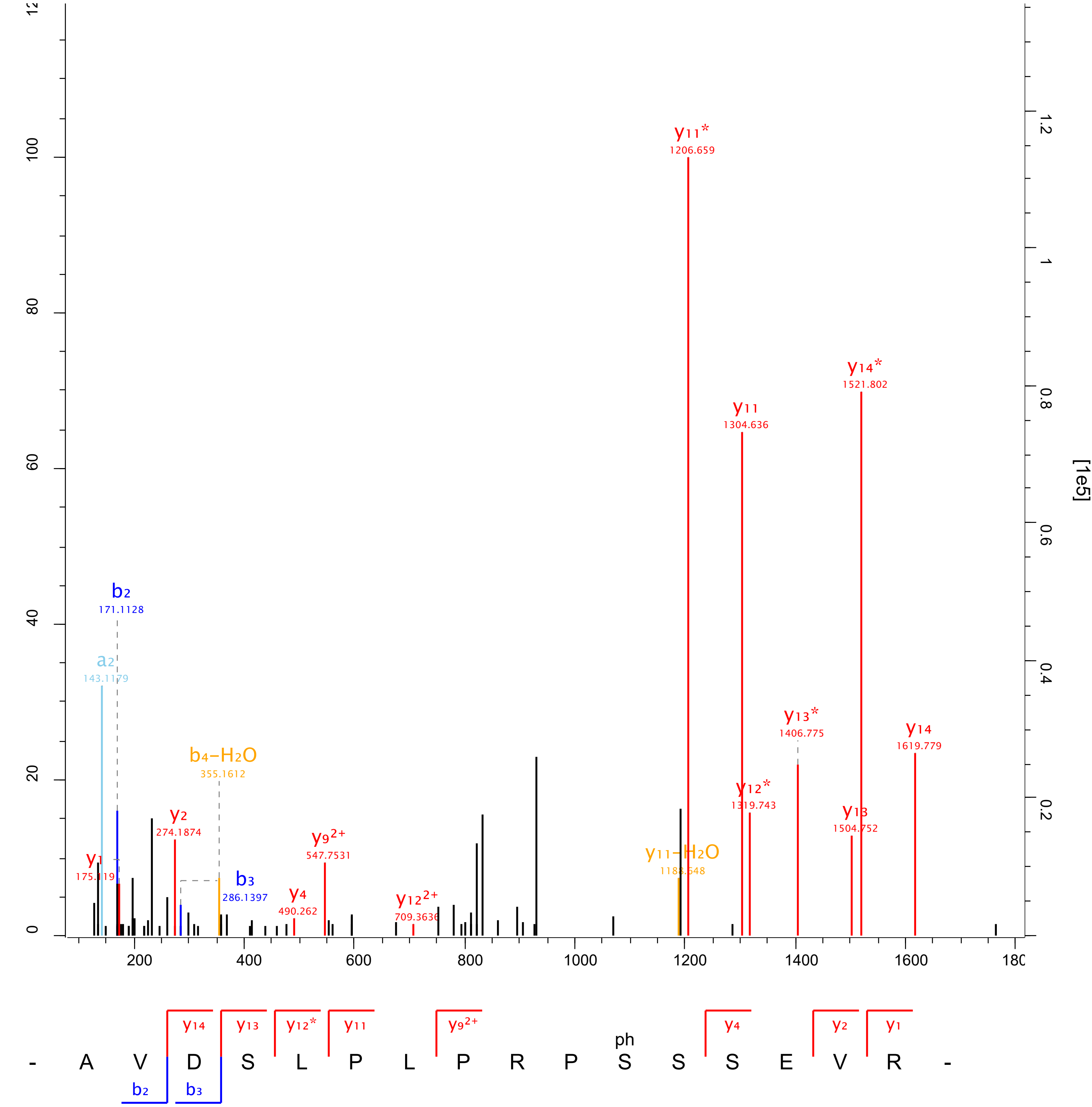

Sample output to test PDF Combine only

Raw file	Scan	Method	Score	m/z	Gene names
sirk1_sp3-mic-0-1-P	23552	FTMS; HCD	111.07	608.29	At1g27100

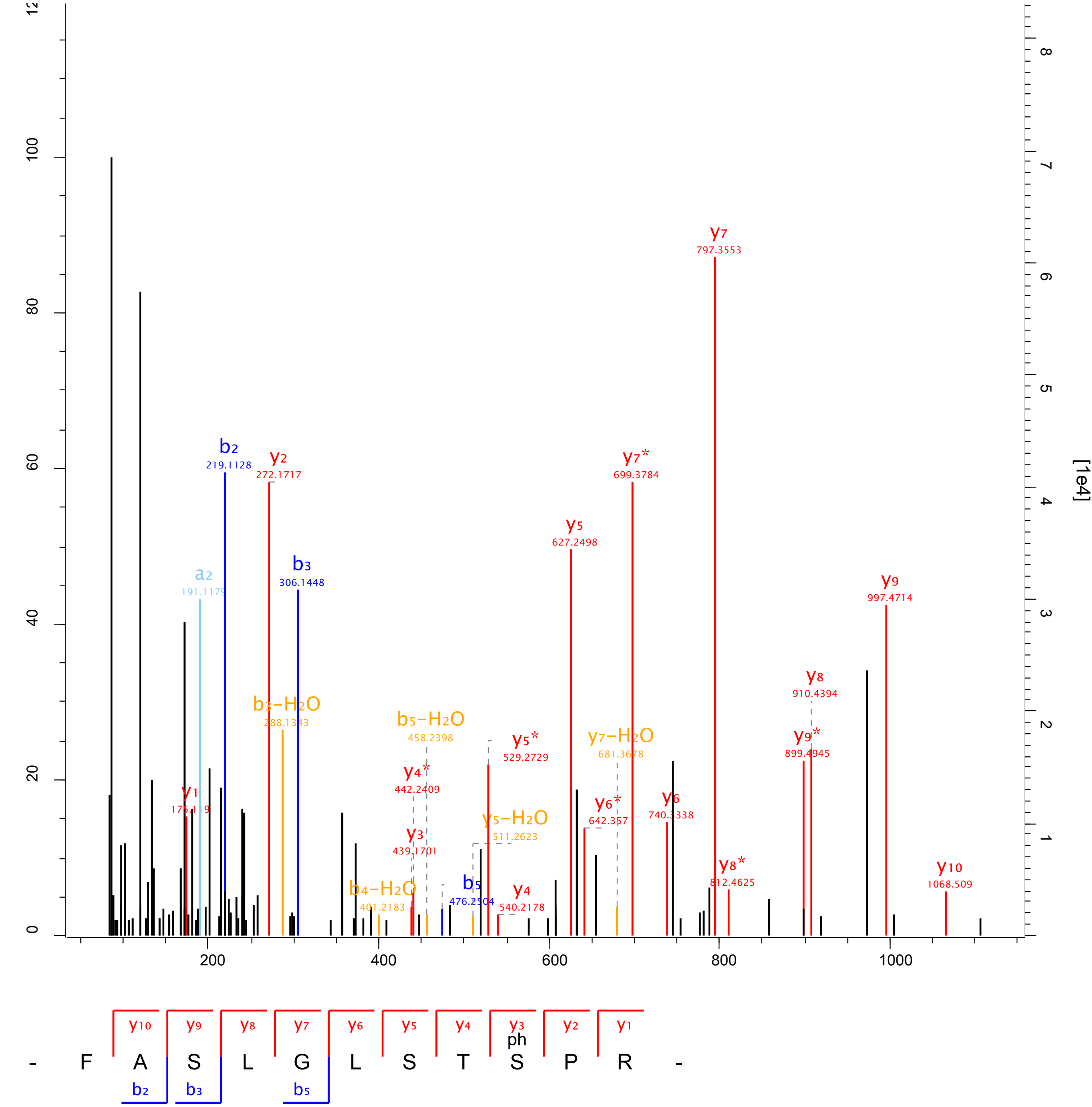

Sample output to test PDF Combine only

Raw file	Scan	Method	Score	m/z	Gene names
sirk1_sp3-mic-0-1-P	23636	FTMS; HCD	73.28	724.36	At1g10140

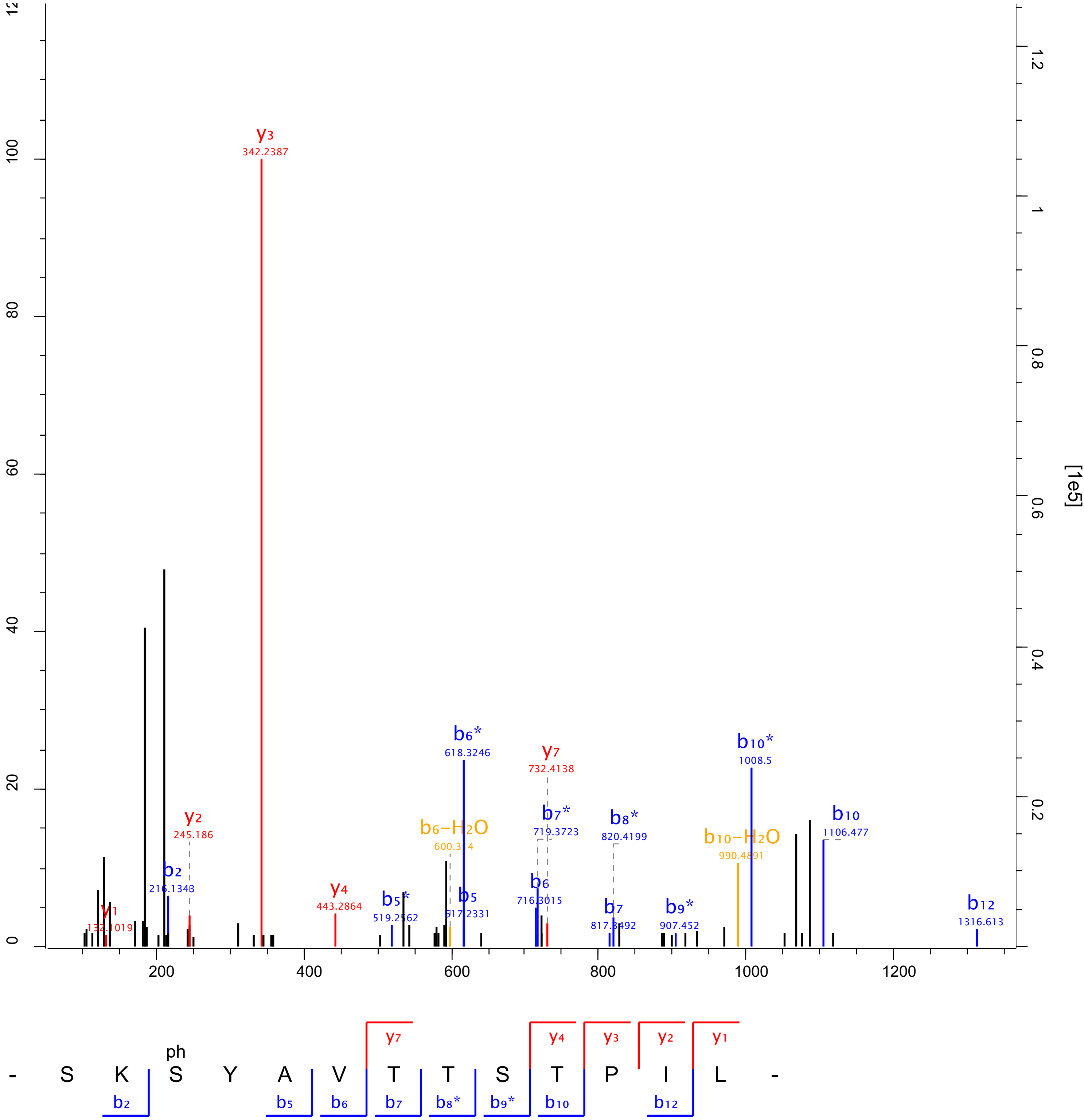

Sample output to test PDF Combine only

Raw file	Scan	Method	Score	m/z	Gene names
sirk1_sp3-mic-0-1-P	25079	FTMS; HCD	73.93	557.77	PHO1;H3

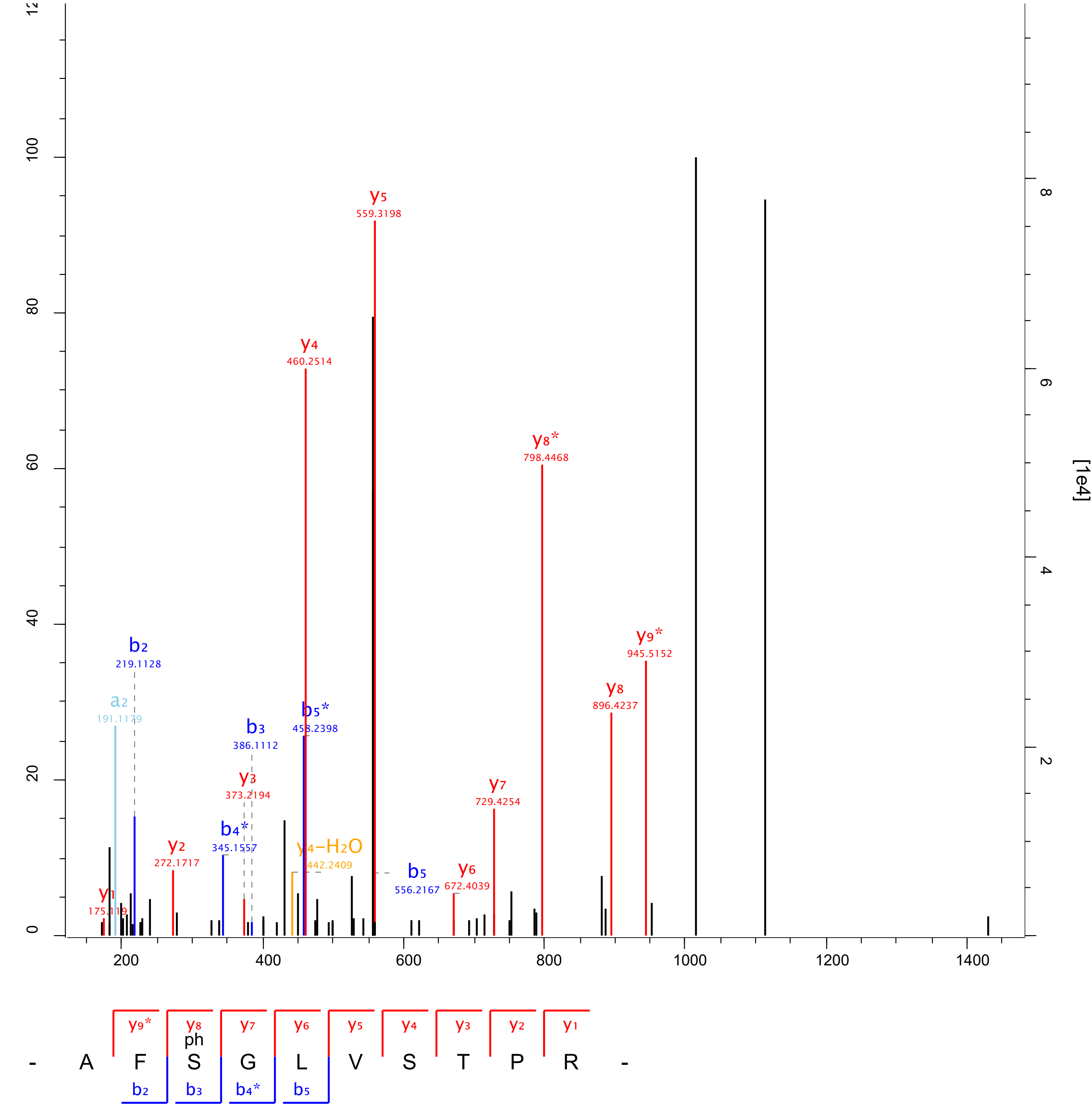

Sample output to test PDF Combine only

Raw file	Scan	Method	Score	m/z
sirk1_sp3-mic-0-1-P	27221	FTMS; HCD	68.04	498.58

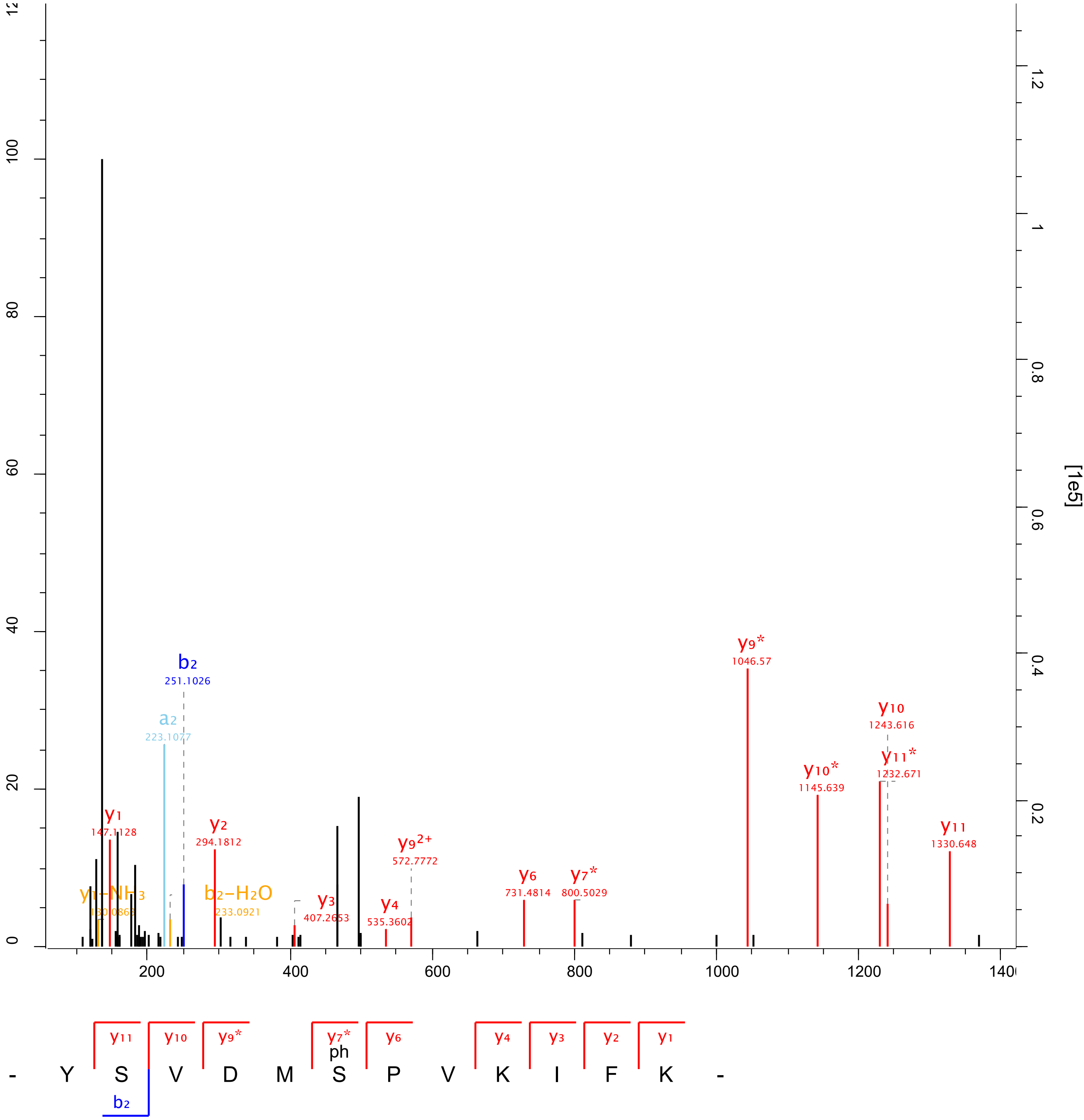

Sample output to test PDF Combine only

Raw file	Scan	Method	Score	m/z	Gene names
sirk1_sp3-mic-0-1-P	34255	FTMS; HCD	54.34	450.84	At5g52980

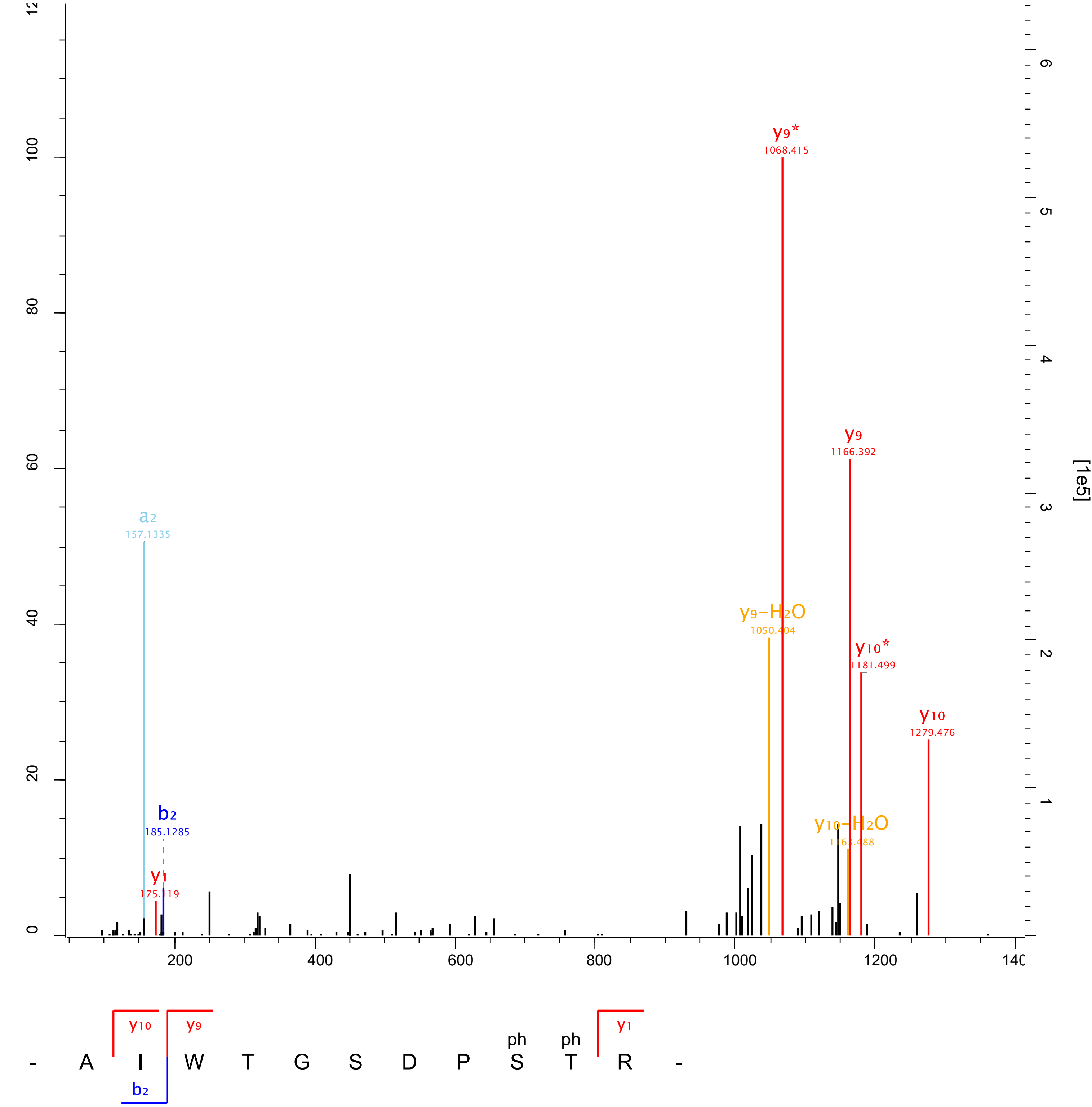

Sample output to test PDF Combine only

Raw file	Scan	Method	Score	m/z	Gene names
sirk1_sp3-mic-0-1-P	34318	FTMS; HCD	282.09	656.79	PIP2-4

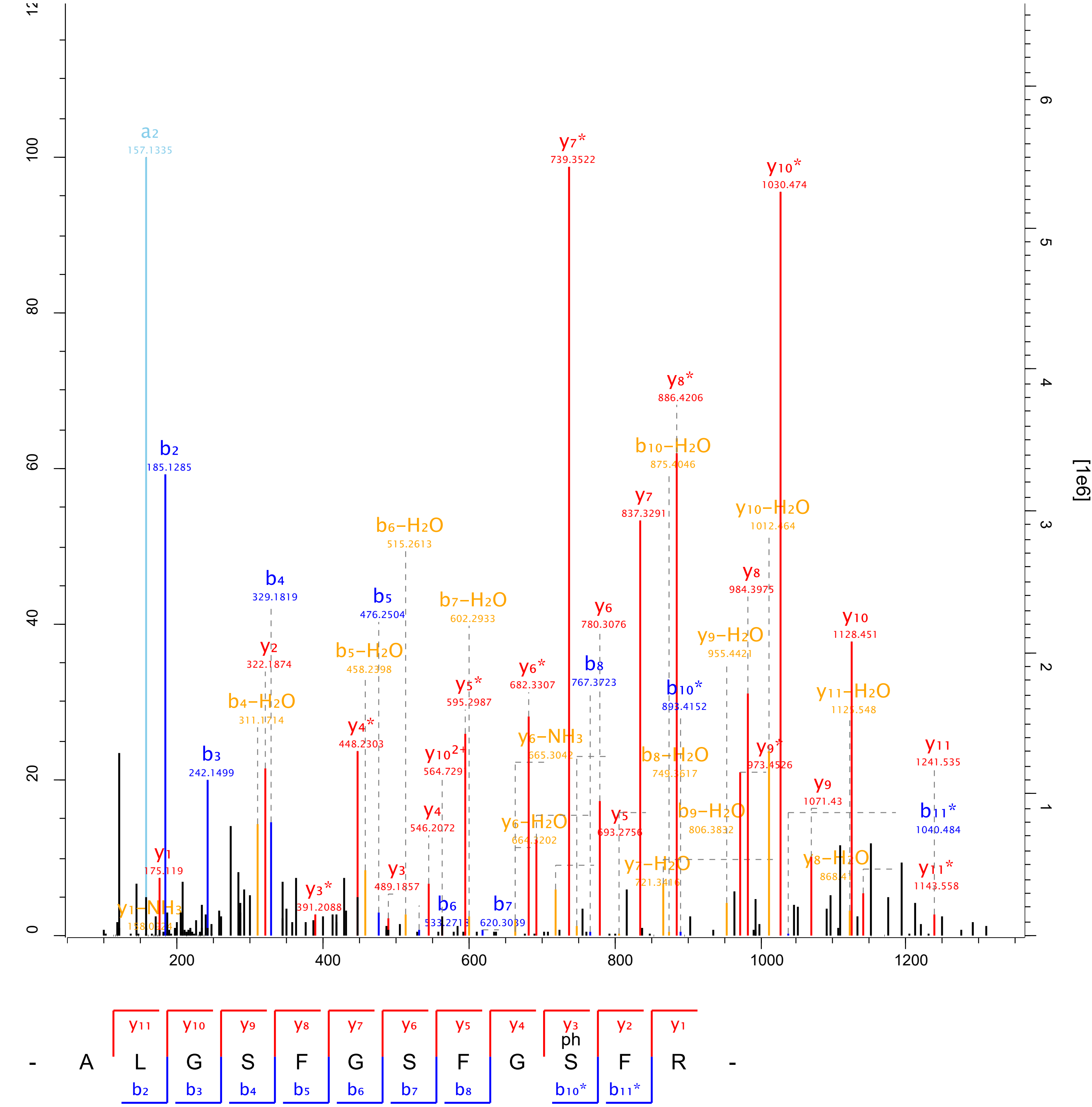

Sample output to test PDF Combine only

Raw file	Scan	Method	Score	m/z	Gene names
sirk1_sp3-mic-0-1-P	39241	FTMS; HCD	54.09	547.9	RABC1

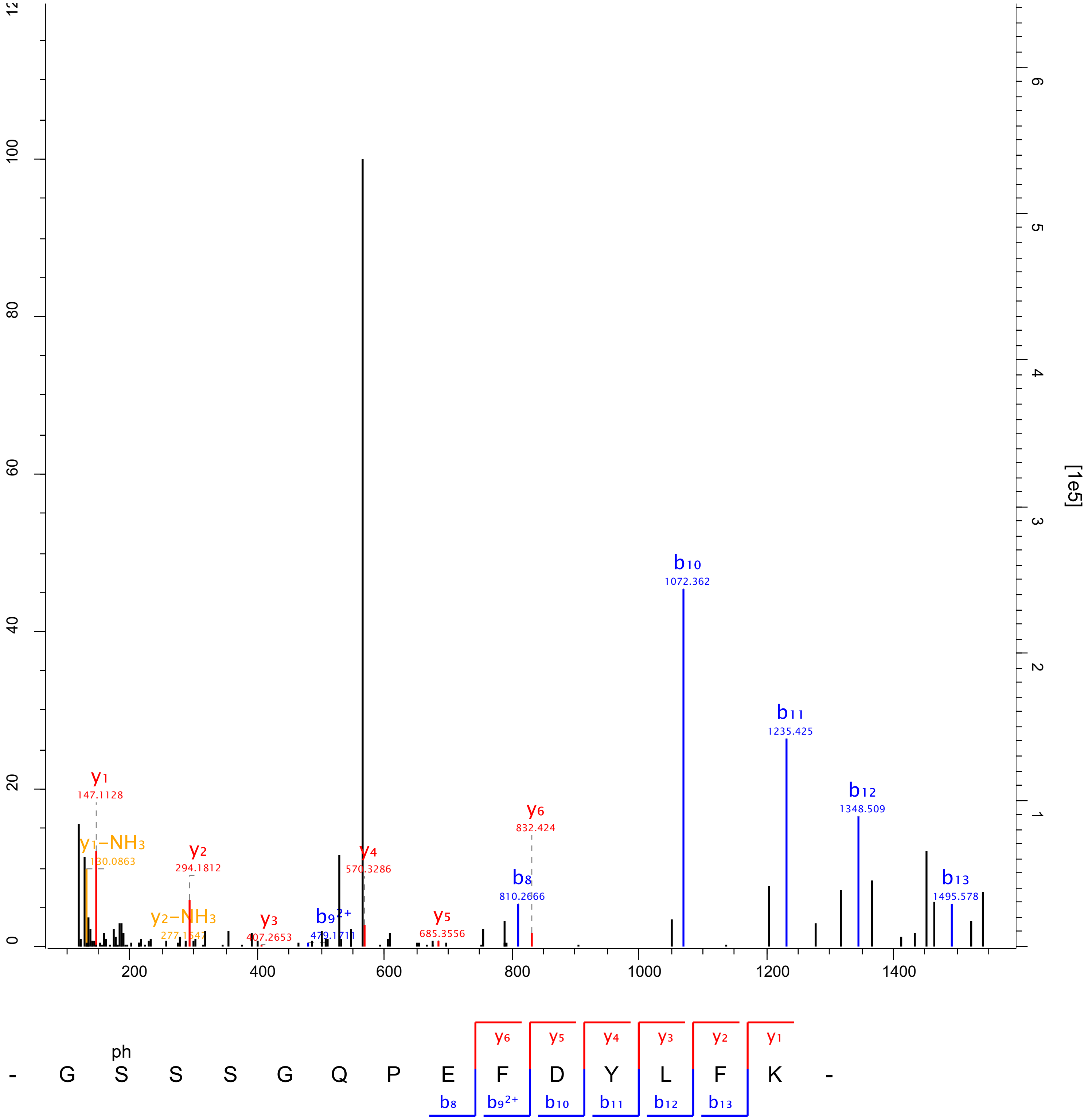

Sample output to test PDF Combine only

Raw file	Scan	Method	Score	m/z	Gene names
sirk1_sp3-mic-0-1-P	40427	FTMS; HCD	66.69	418.85	rps14

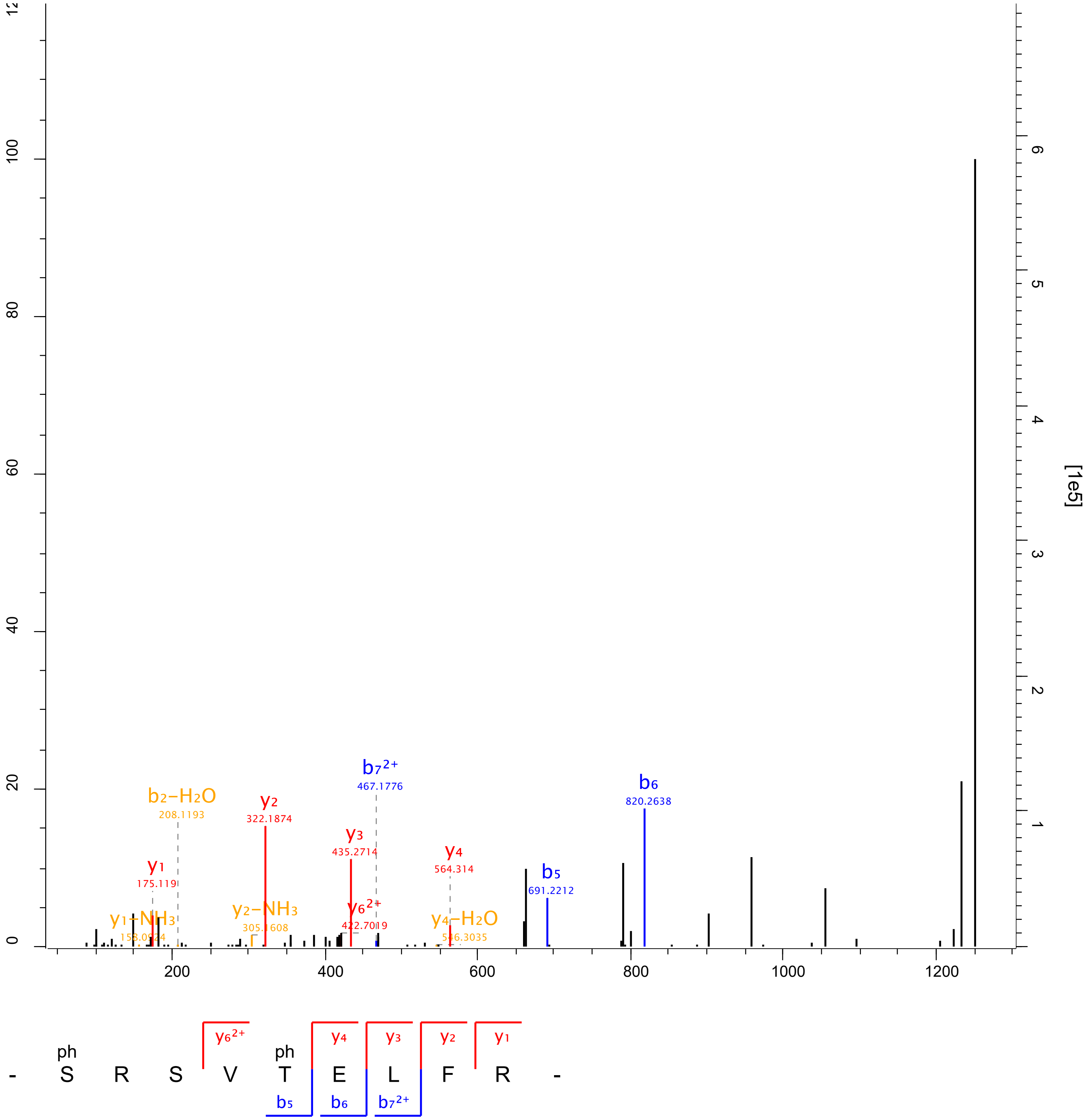

Sample output to test PDF Combine only

Raw file	Scan	Method	Score	m/z	Gene names
sirk1_sp3-mic-0-2-A	8711	FTMS; HCD	125.45	558.93	PPC3

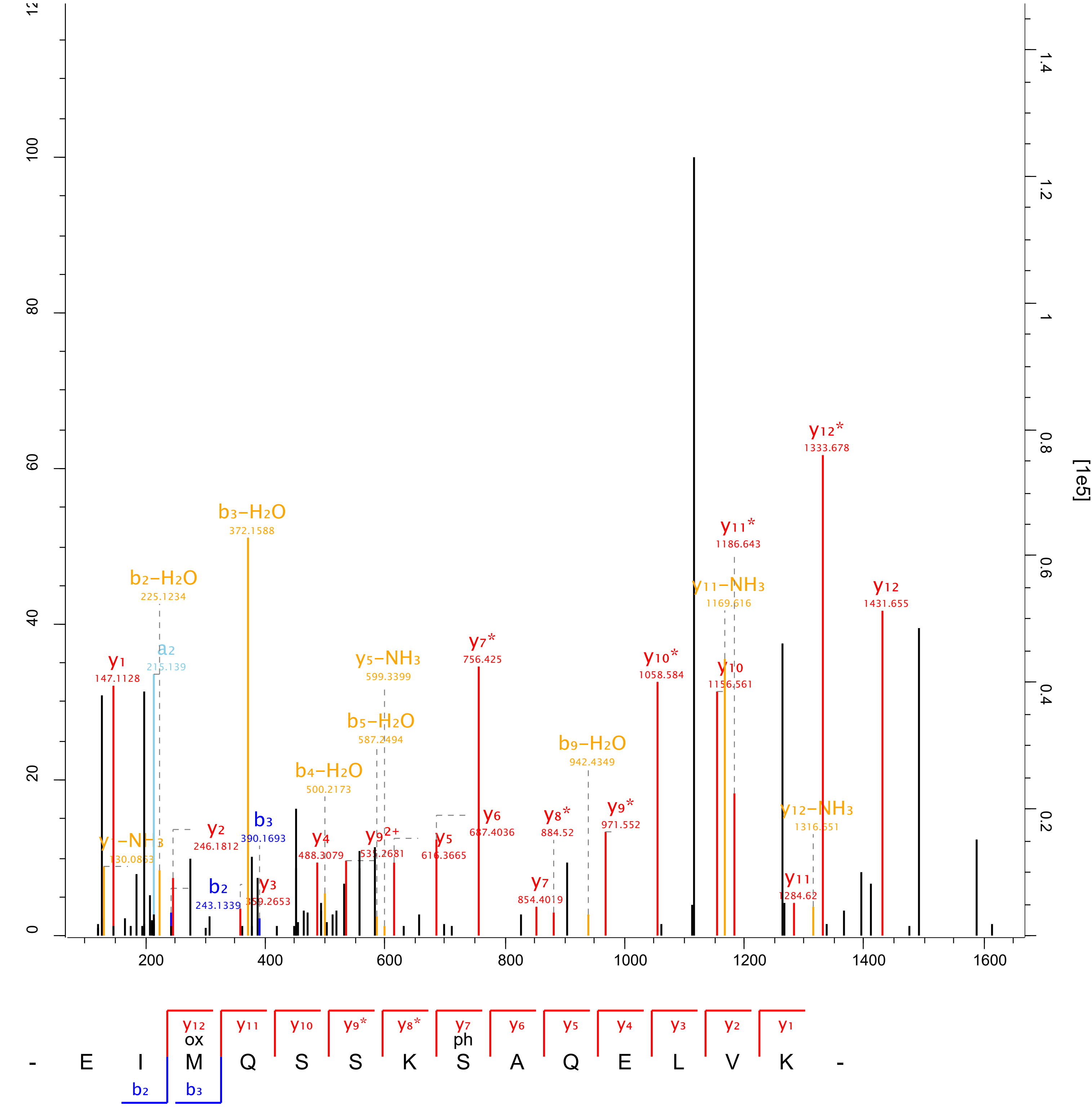

Sample output to test PDF Combine only

Raw file	Scan	Method	Score	m/z	Gene names
sirk1_sp3-mic-0-2-A	11025	FTMS; HCD	66.54	598.62	At4g38470

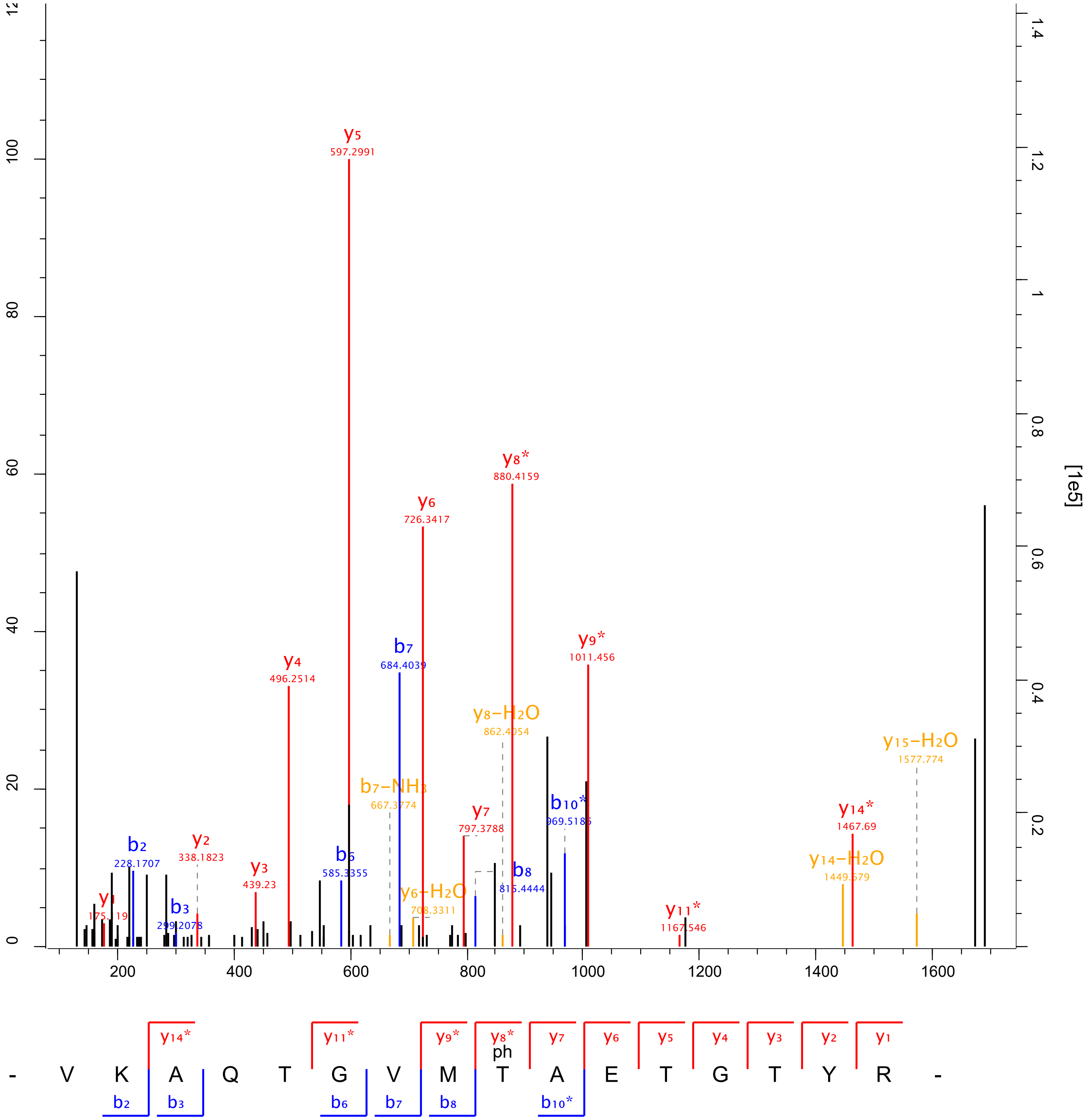

Sample output to test PDF Combine only

Raw file	Scan	Method	Score	m/z	Gene names
sirk1_sp3-mic-0-2-A	15650	FTMS; HCD	112.64	553.77	At2g29210

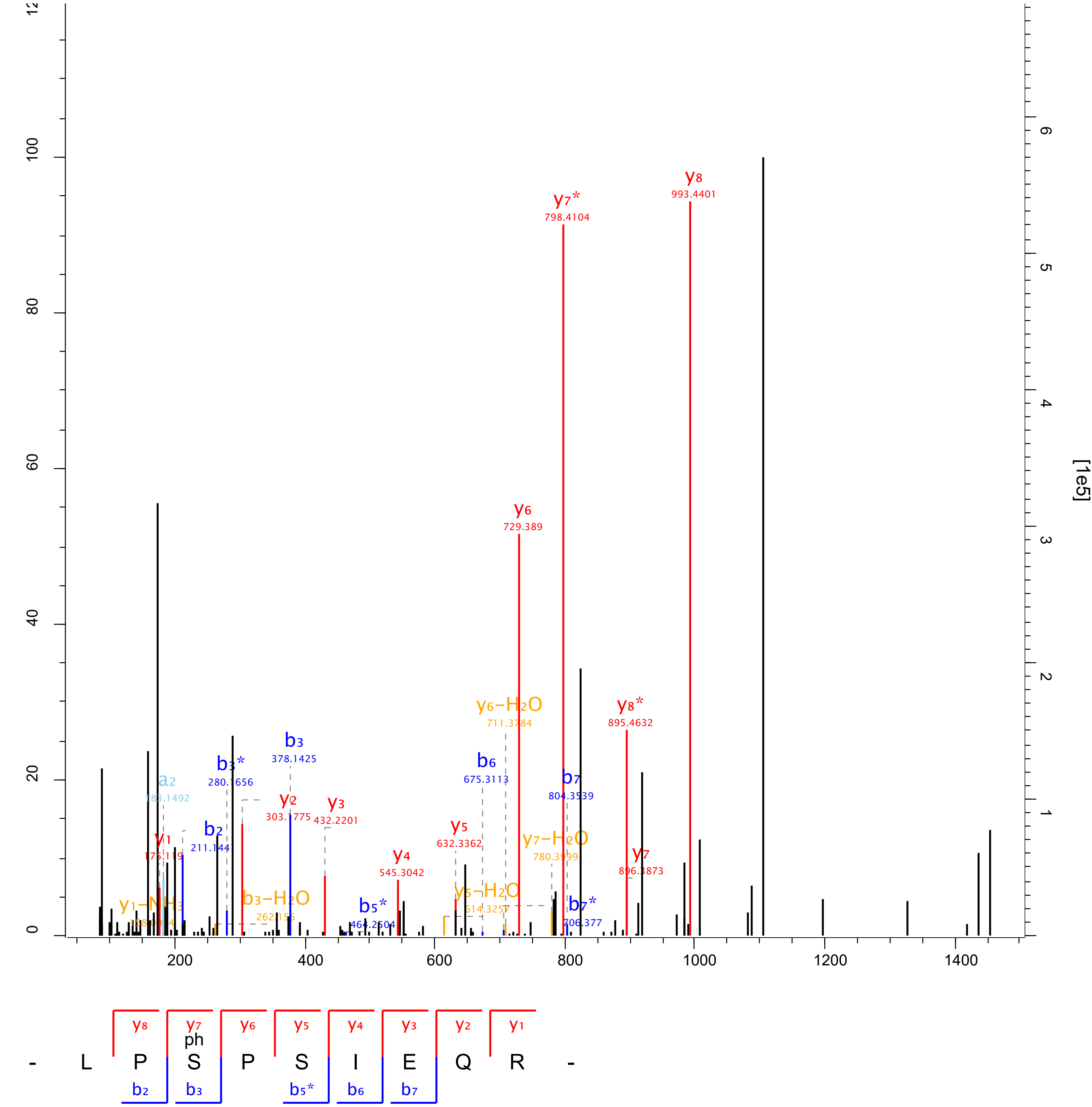

Sample output to test PDF Combine only

Raw file	Scan	Method	Score	m/z	Gene names
sirk1_sp3-mic-0-2-A	21708	FTMS; HCD	215.96	739.82	AHA1

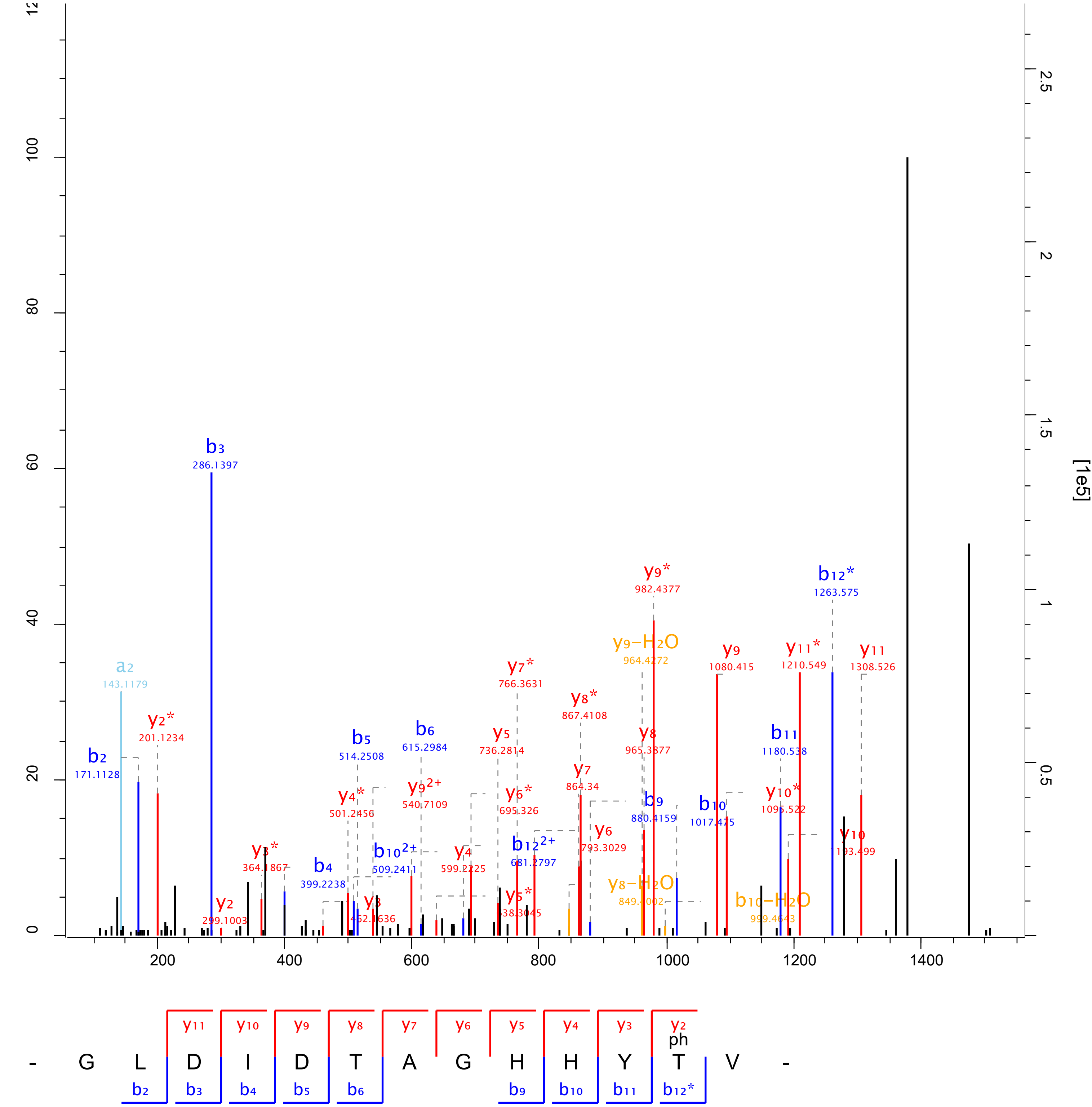

Sample output to test PDF Combine only

Raw file	Scan	Method	Score	m/z	Gene names
sirk1_sp3-mic-0-2-A	21709	FTMS; HCD	43.3	501.88	GPX2

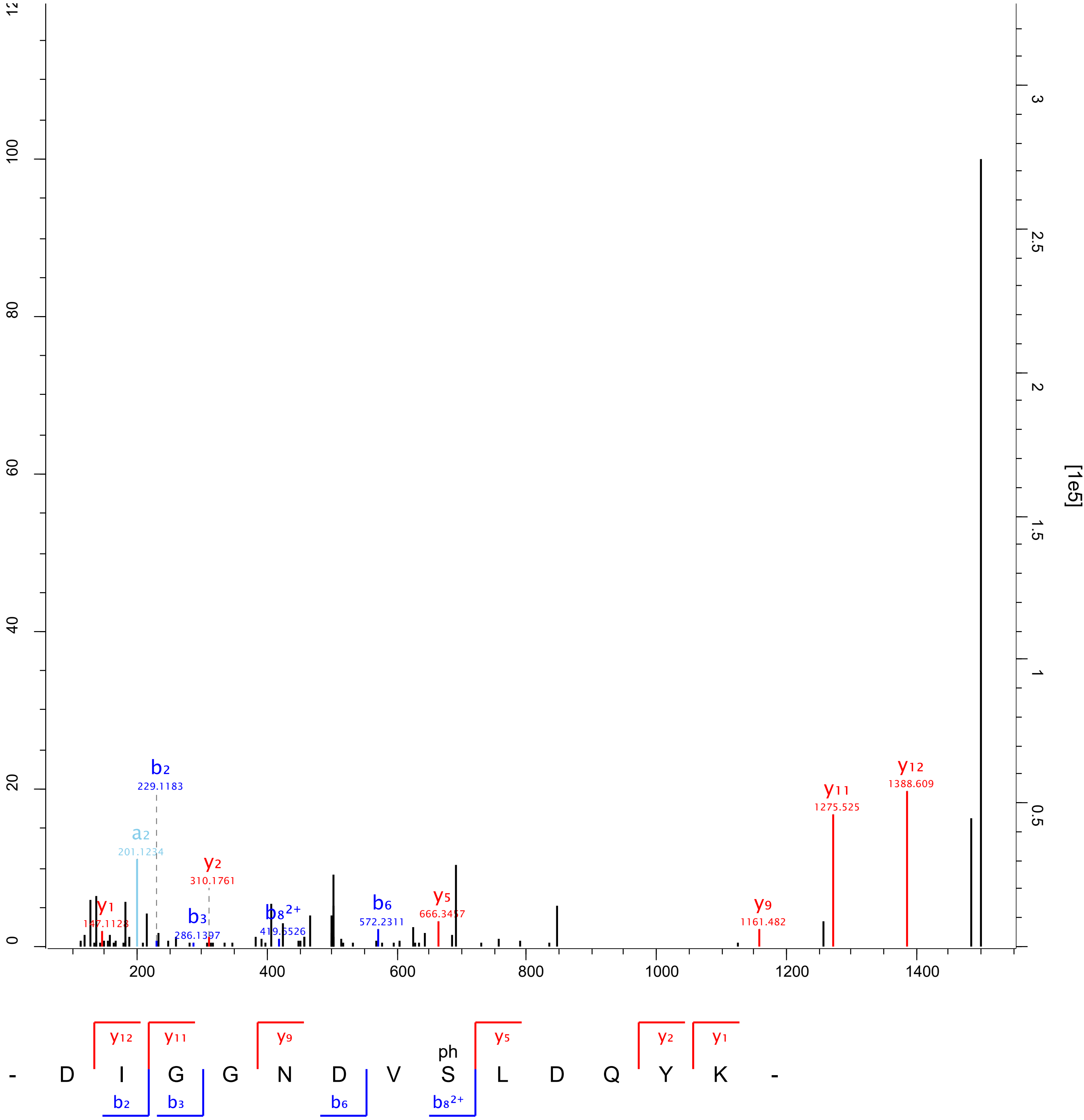

Sample output to test PDF Combine only

Raw file	Scan	Method	Score	m/z	Gene names
sirk1_sp3-mic-0-2-A	22606	FTMS; HCD	74.42	490.75	psbA

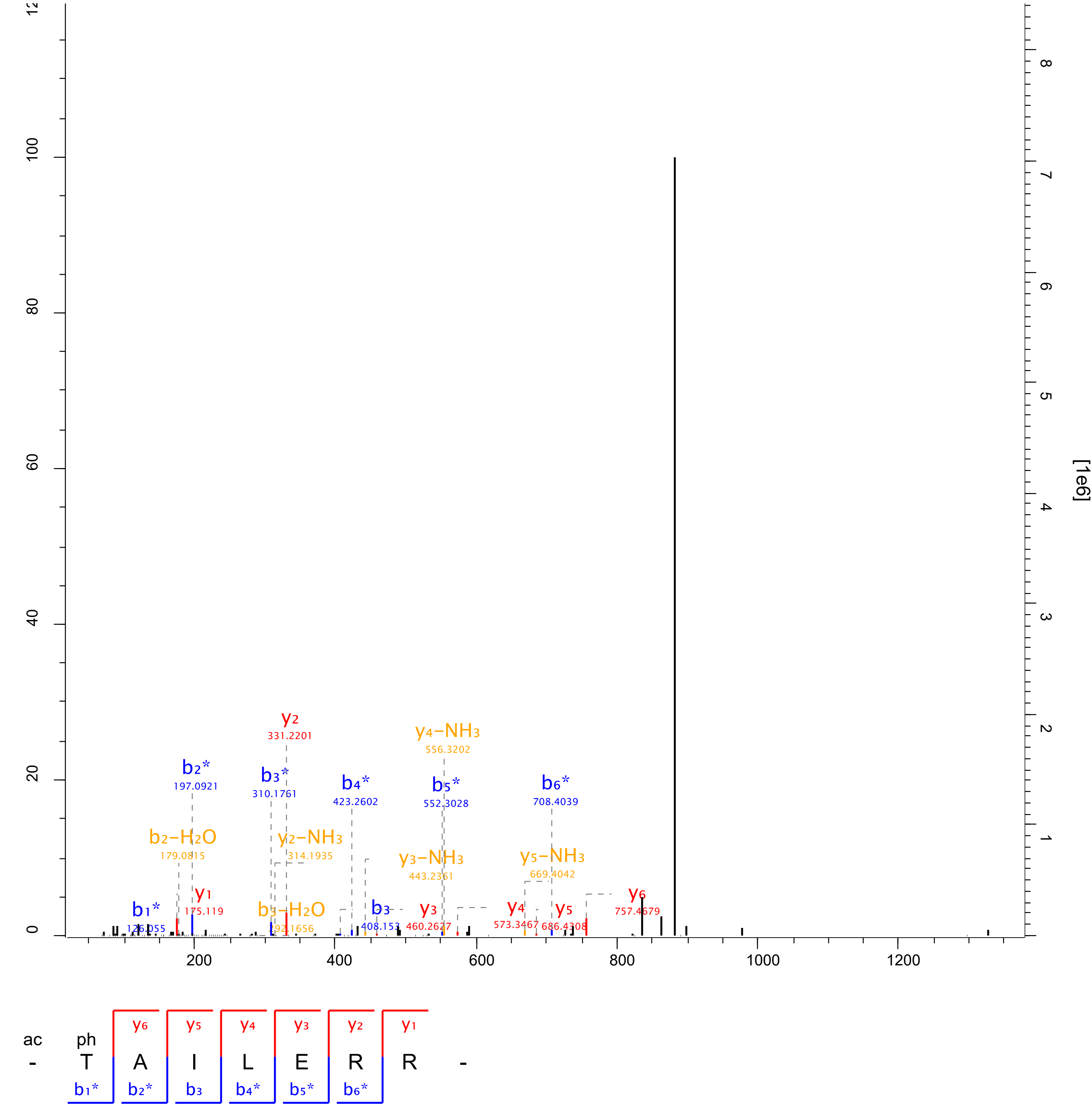

Sample output to test PDF Combine only

Raw file	Scan	Method	Score	m/z	Gene names
sirk1_sp3-mic-0-2-A	25578	FTMS; HCD	80.96	595.76	PDK1;PDPK2;PDPK1

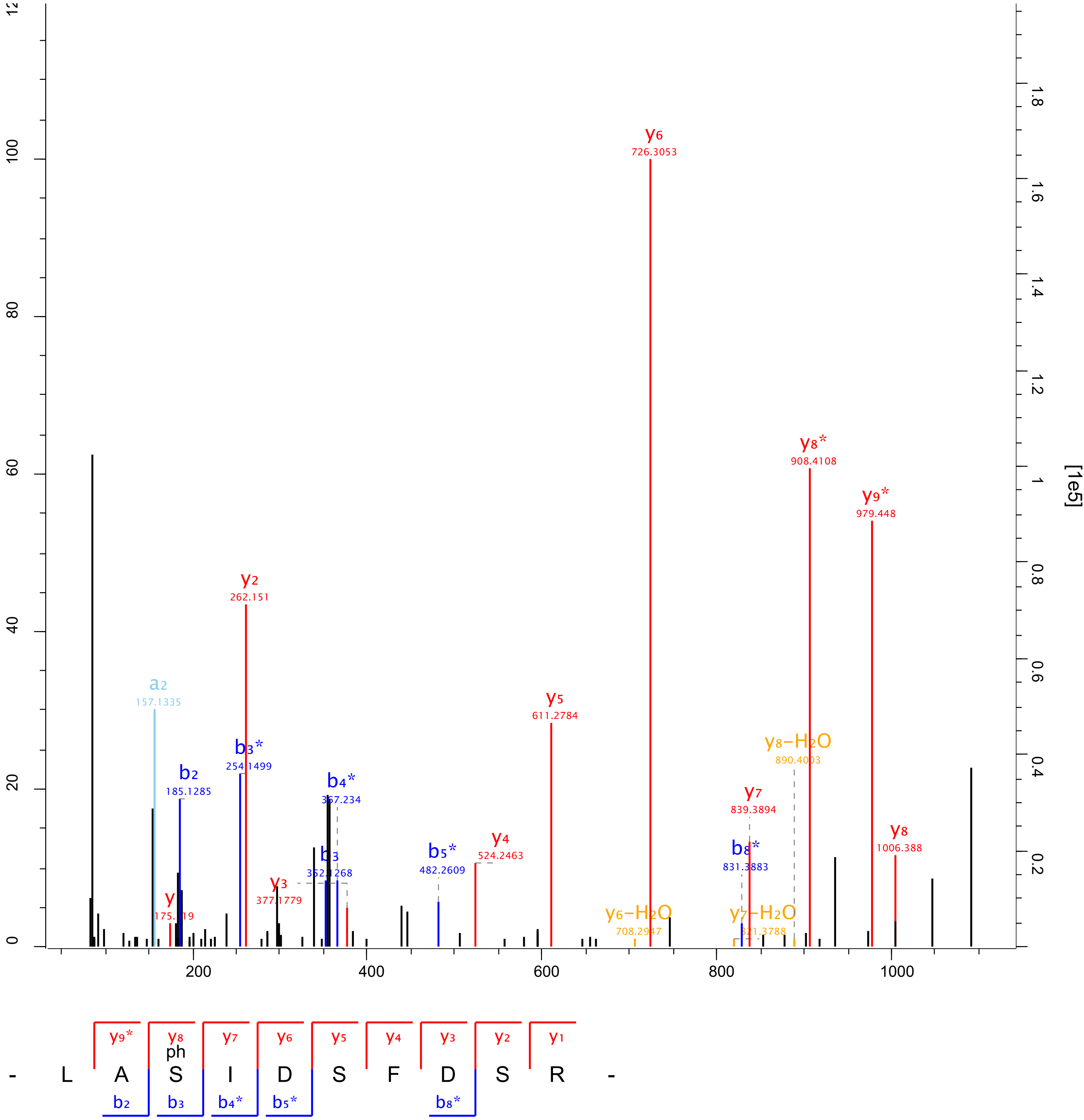

Sample output to test PDF Combine only

Raw file	Scan	Method	Score	m/z	Gene names
sirk1_sp3-mic-0-2-P	13879	FTMS; HCD	65.72	469.23	CML43;CML42

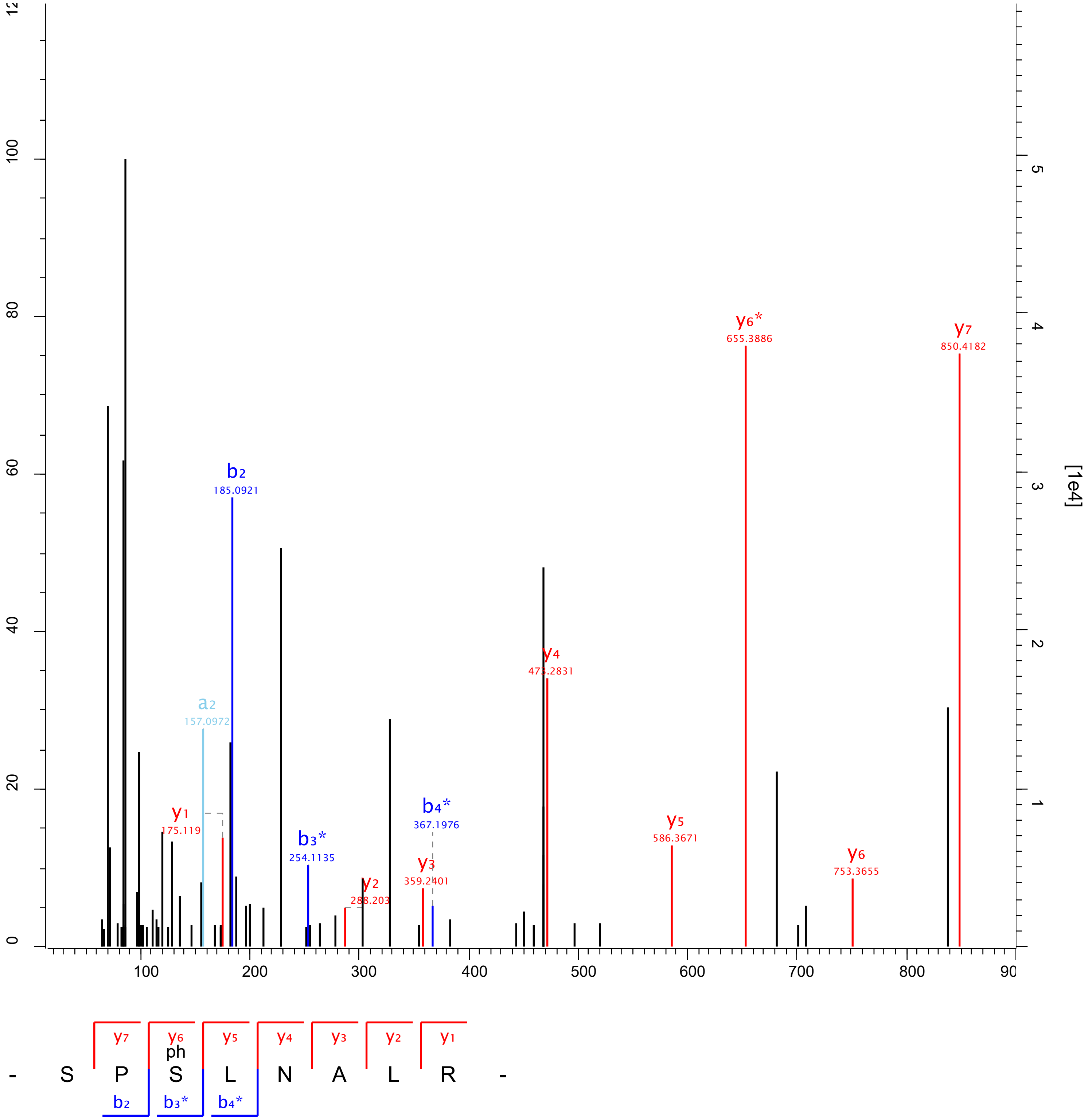

Sample output to test PDF Combine only

Raw file	Scan	Method	Score	m/z	Gene names
sirk1_sp3-mic-0-2-P	26032	FTMS; HCD	64.64	543.27	TPS8;TPS9

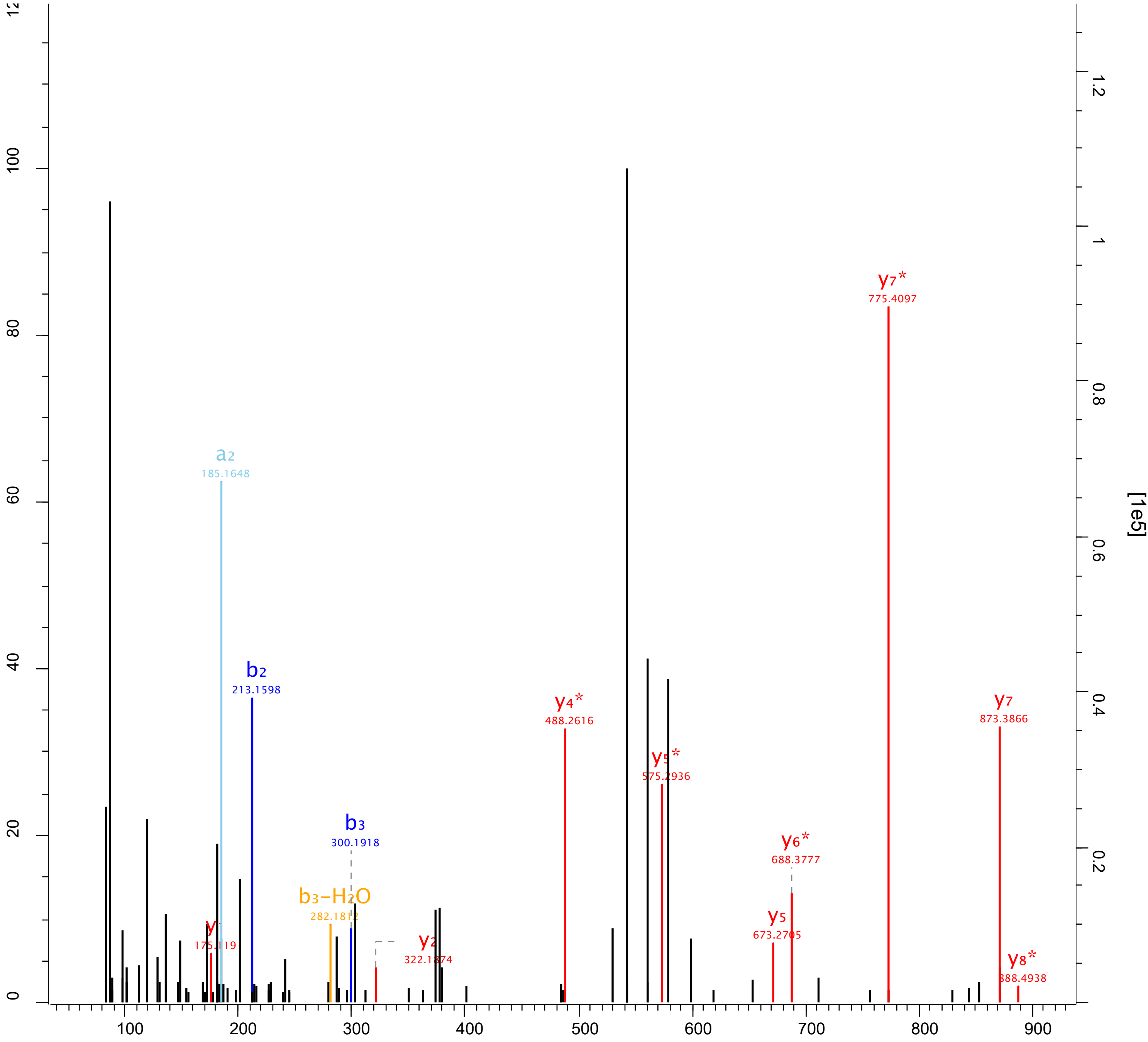

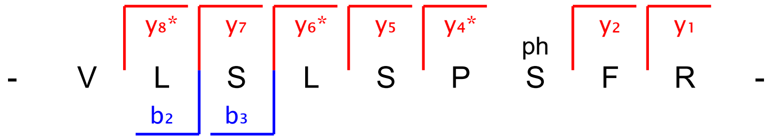

Sample output to test PDF Combine only

Raw file	Scan	Method	Score	m/z	Gene names
sirk1_sp3-mic-0-2-P	28255	FTMS; HCD	40.94	882.42	At2g34930

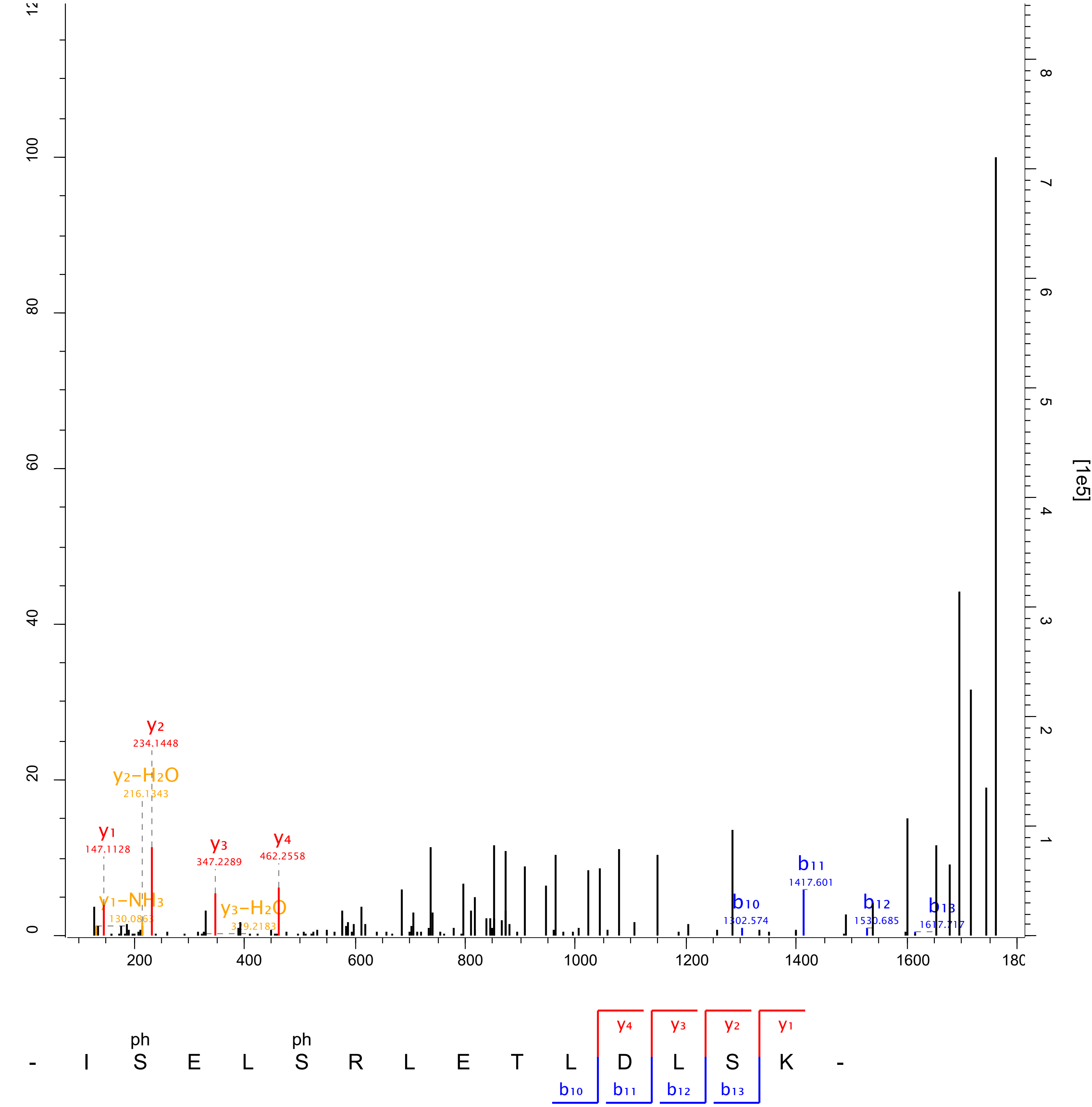

Sample output to test PDF Combine only

Raw file	Scan	Method	Score	m/z	Gene names
sirk1_sp3-mic-0-2-P	32903	FTMS; HCD	138.28	792.92	TPR1

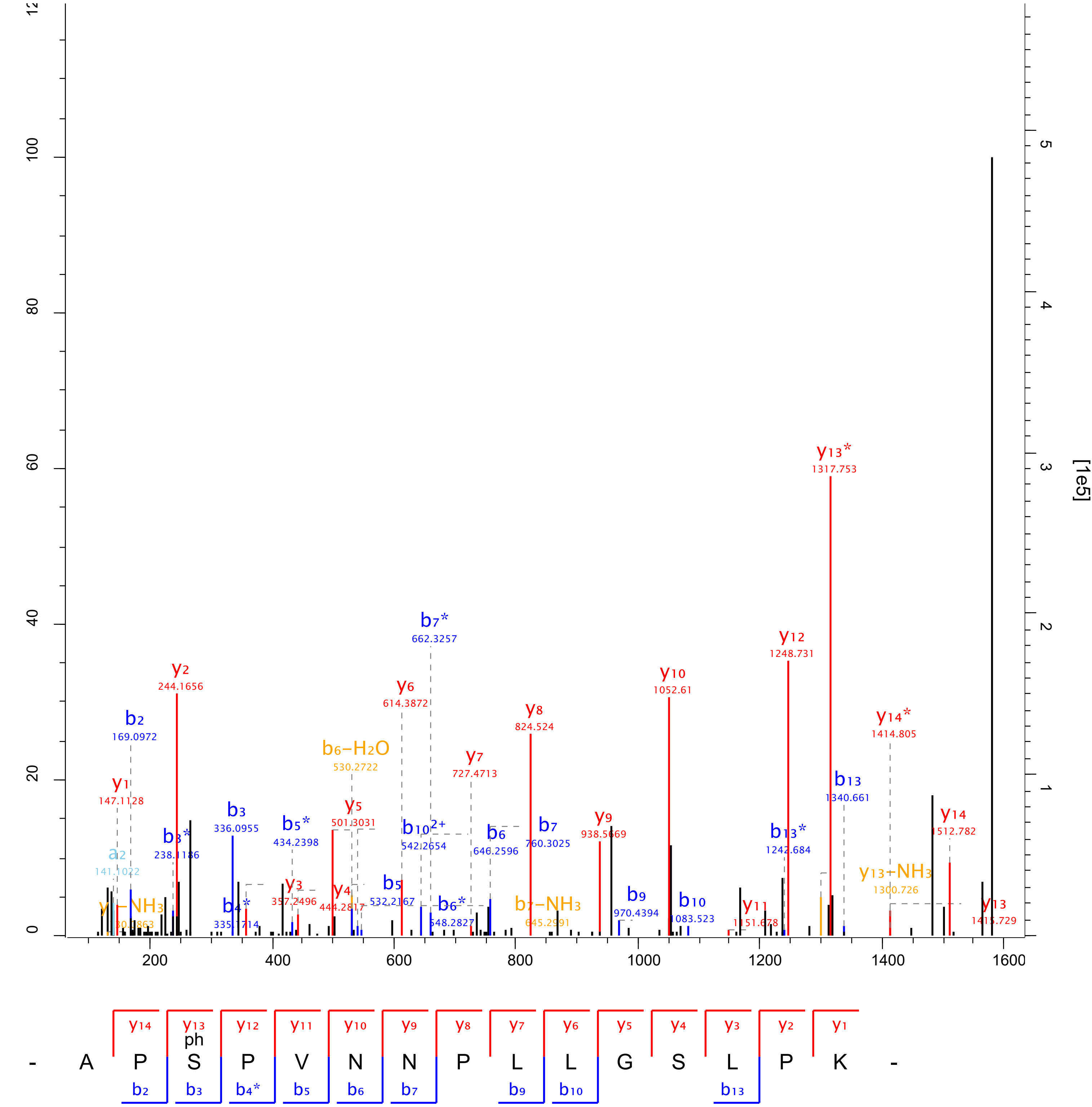

Sample output to test PDF Combine only

Raw file	Scan	Method	Score	m/z
sirk1_sp3-mic-0-2-P	33016	FTMS; HCD	63.62	758.89

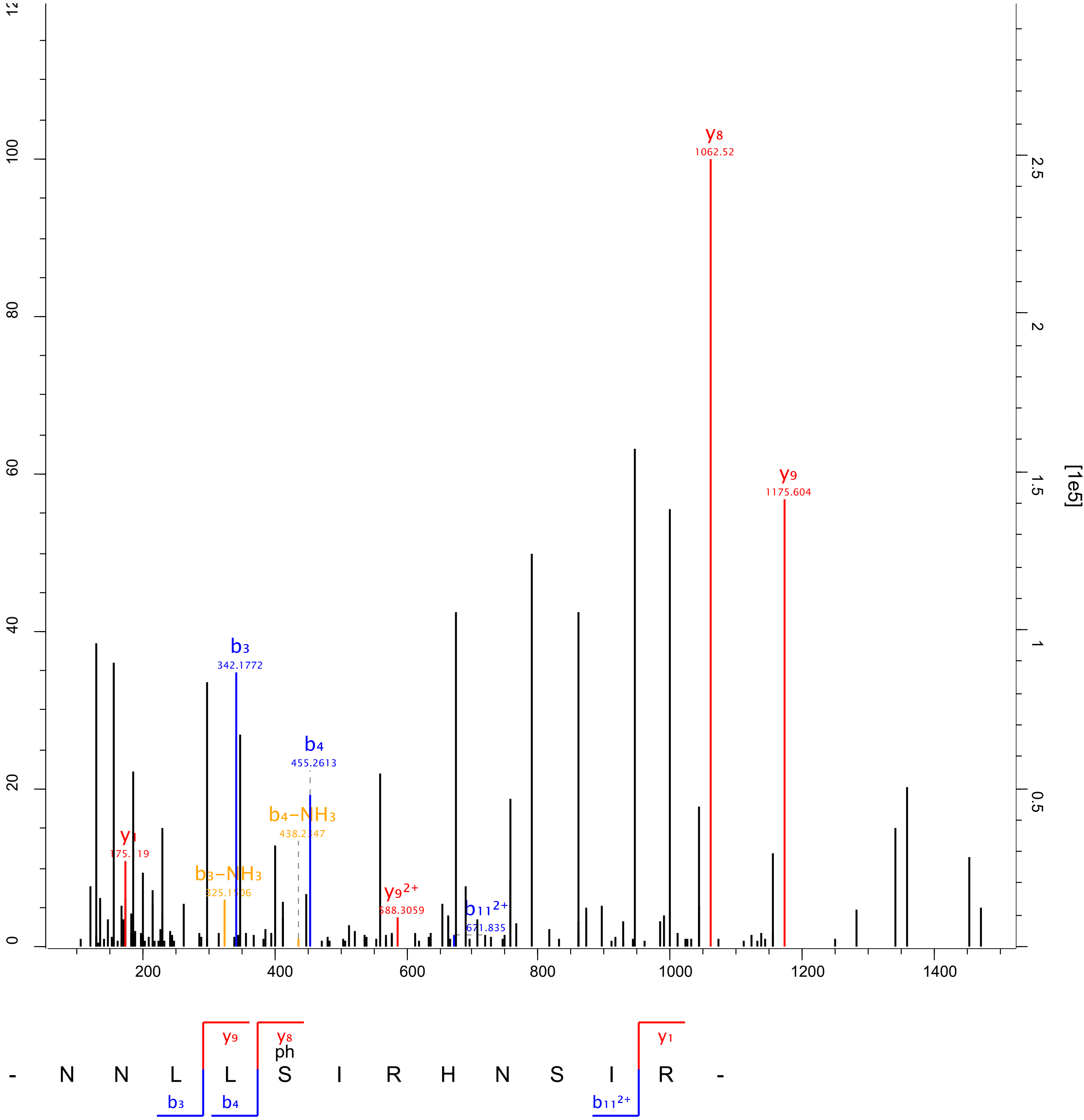

Sample output to test PDF Combine only

Raw file	Scan	Method	Score	m/z
sirk1_sp3-mic-0-2-P	38718	FTMS; HCD	50.75	925.09

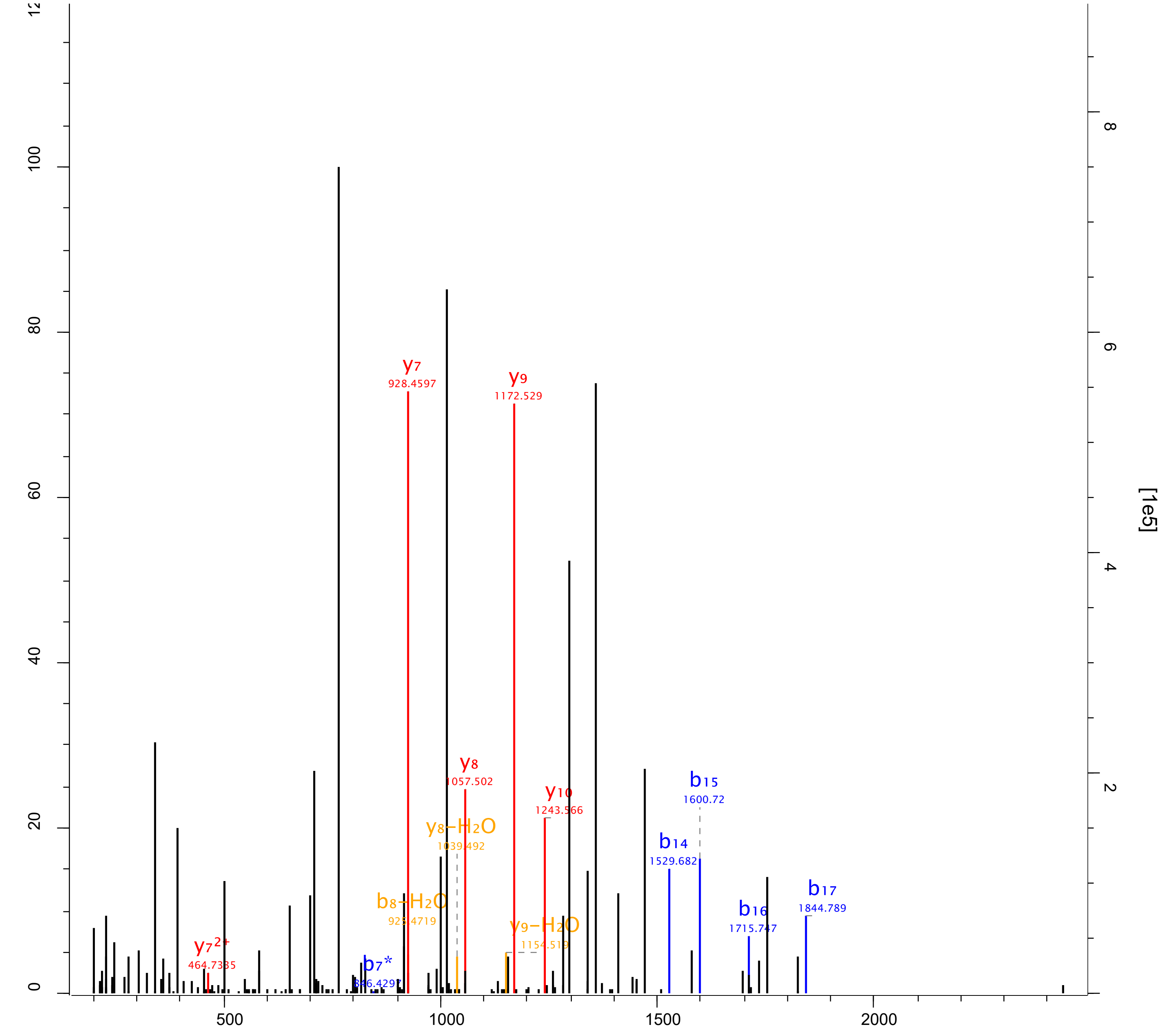

y10 y9 y8 y7

- K F P A S(ph) W F P A G T S T A A D E P

b7* b14 b15 b16 b17

F T Y C L K -

Sample output to test PDF Combine only

Raw file	Scan	Method	Score	m/z
sirk1_sp3-mic-0-3-A	8204	FTMS; HCD	40.14	747.96

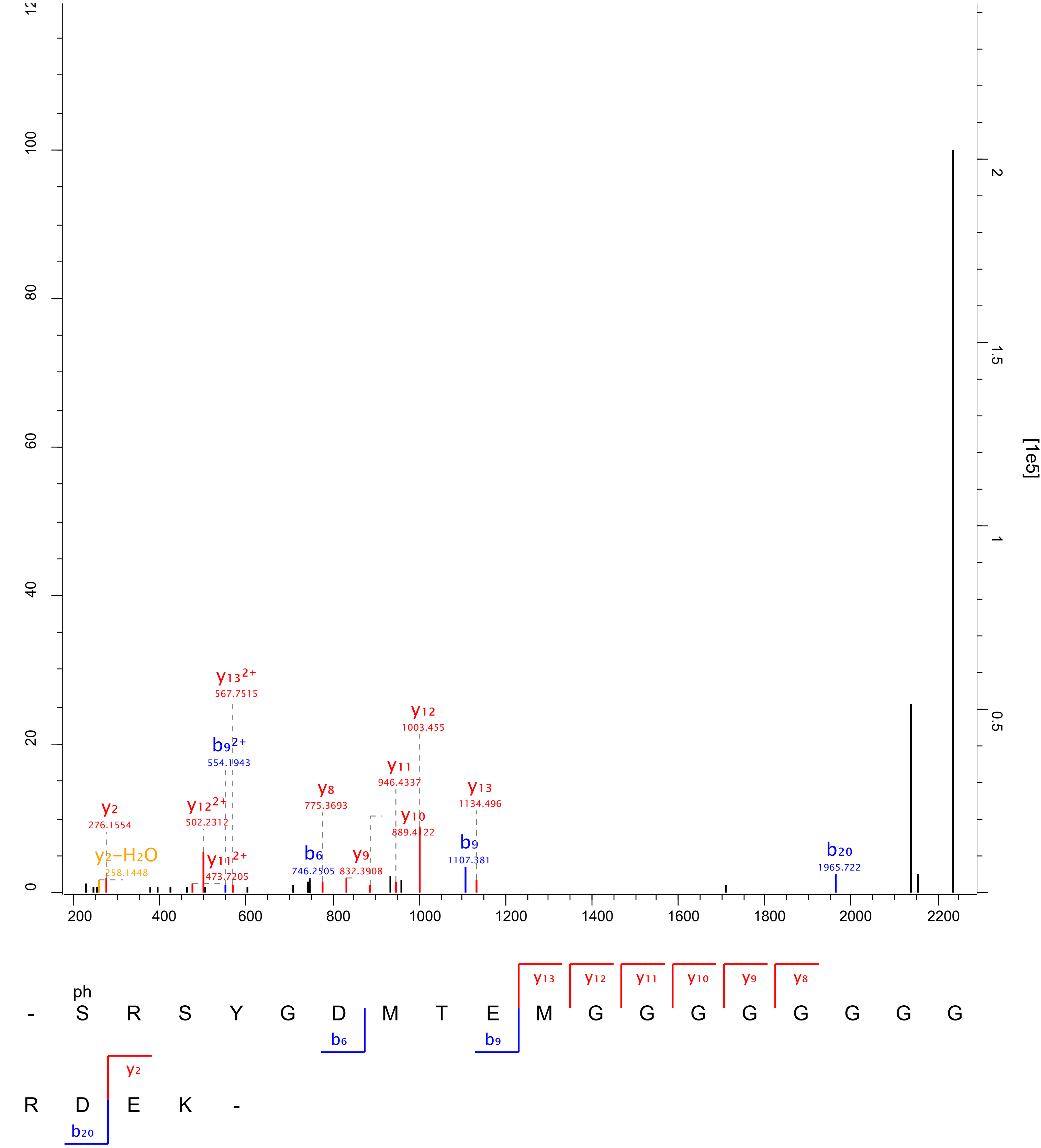

Sample output to test PDF Combine only

Raw file	Scan	Method	Score	m/z	Gene names
sirk1_sp3-mic-0-3-A	8747	FTMS; HCD	60.55	470.53	IQD32

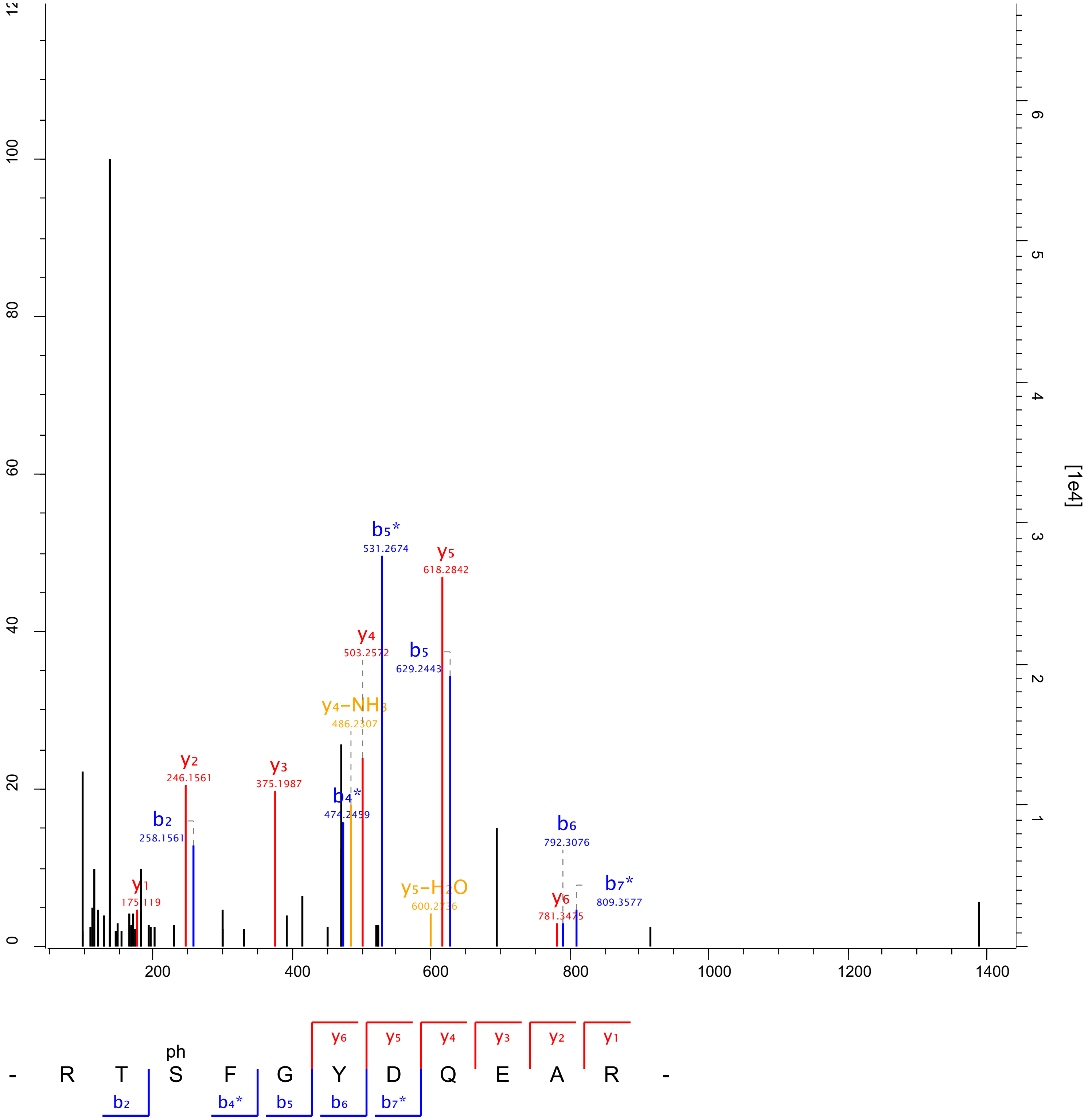

Sample output to test PDF Combine only

Raw file	Scan	Method	Score	m/z	Gene names
sirk1_sp3-mic-0-3-A	9935	FTMS; HCD	41.4	659.82	LTA3

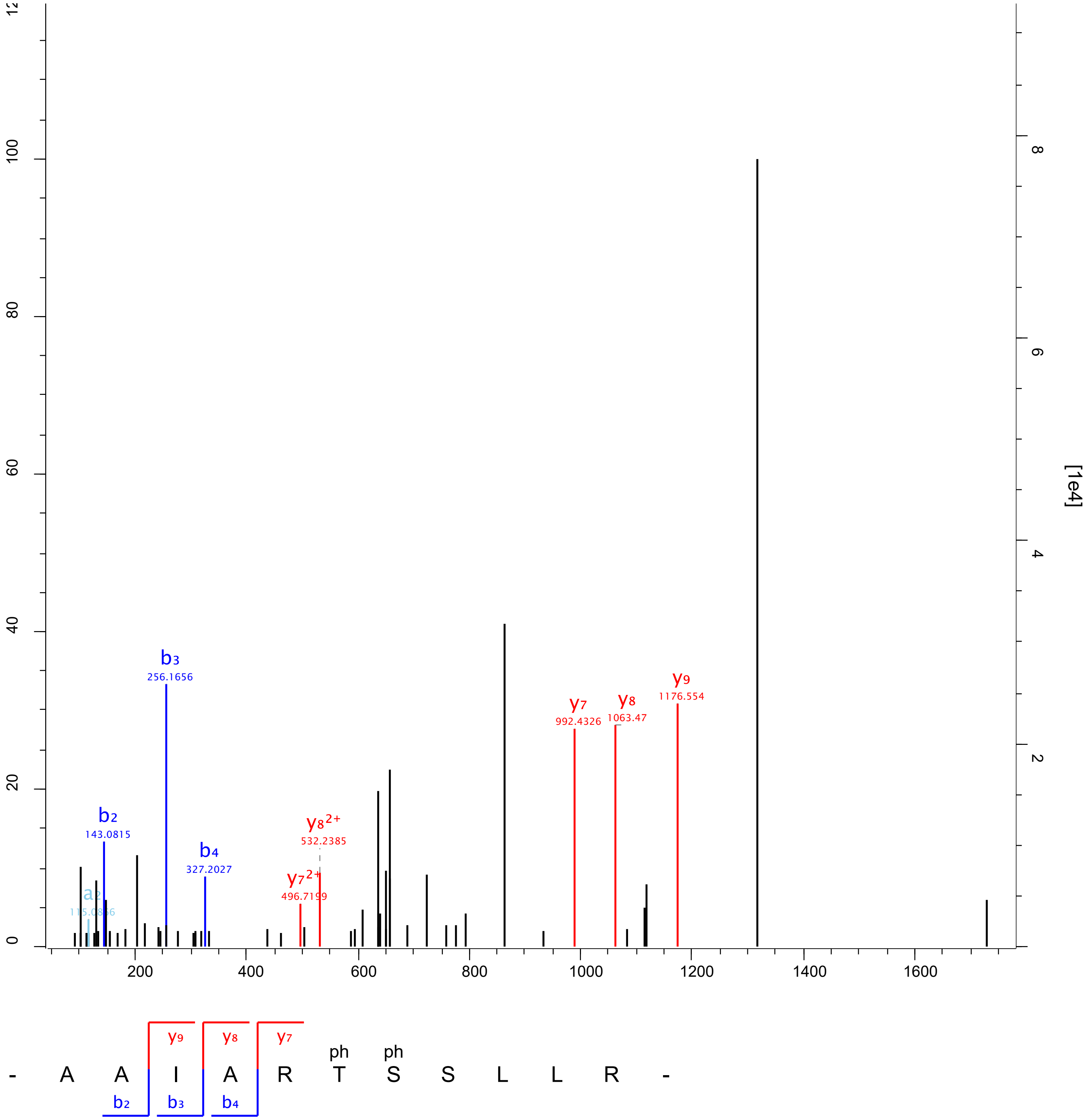

Sample output to test PDF Combine only

Raw file	Scan	Method	Score	m/z	Gene names
sirk1_sp3-mic-0-3-A	11912	FTMS; HCD	58.08	467.55	SYP132

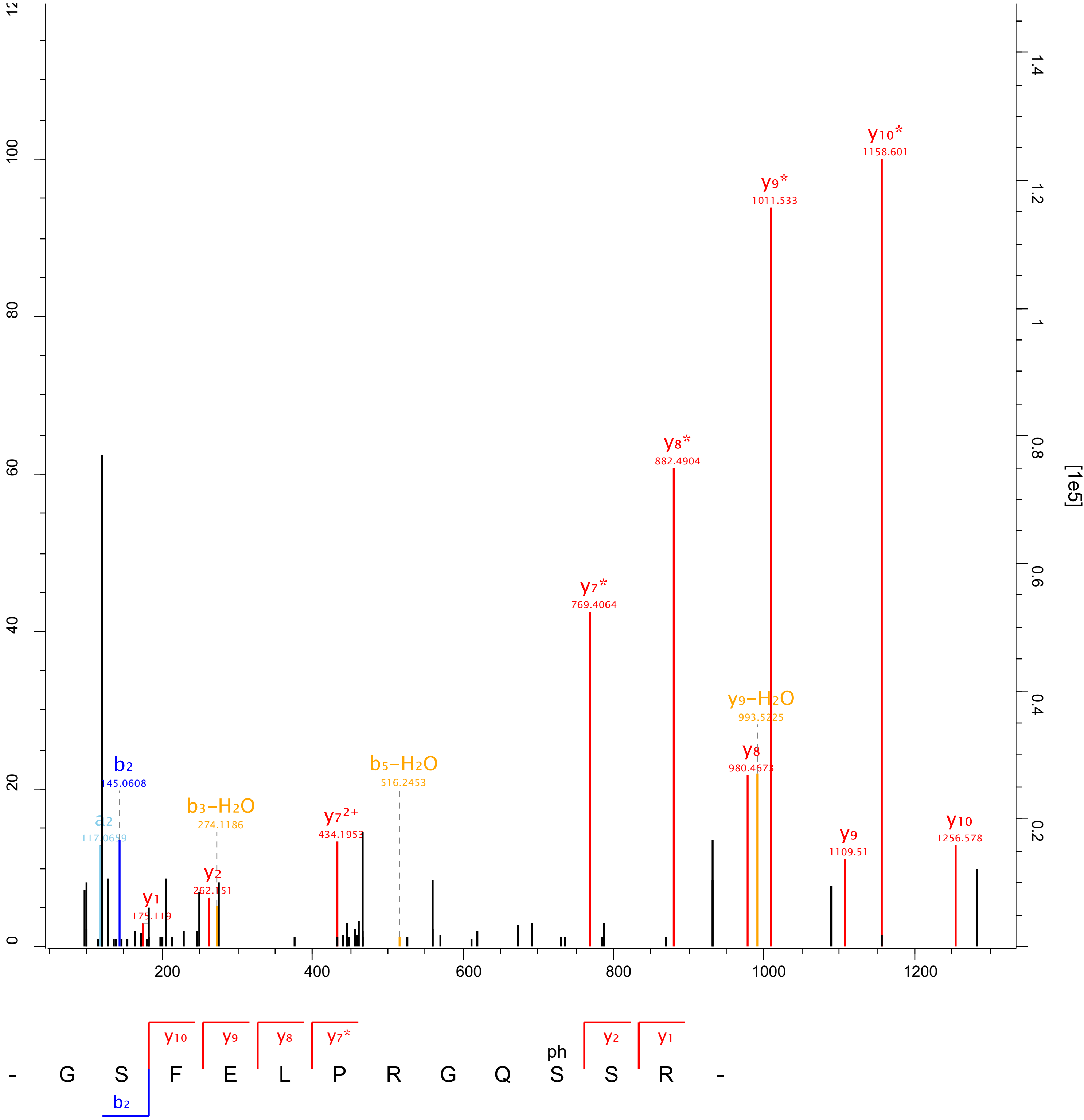

Sample output to test PDF Combine only

Raw file	Scan	Method	Score	m/z
sirk1_sp3-mic-0-3-A	14877	FTMS; HCD	78.08	811.9

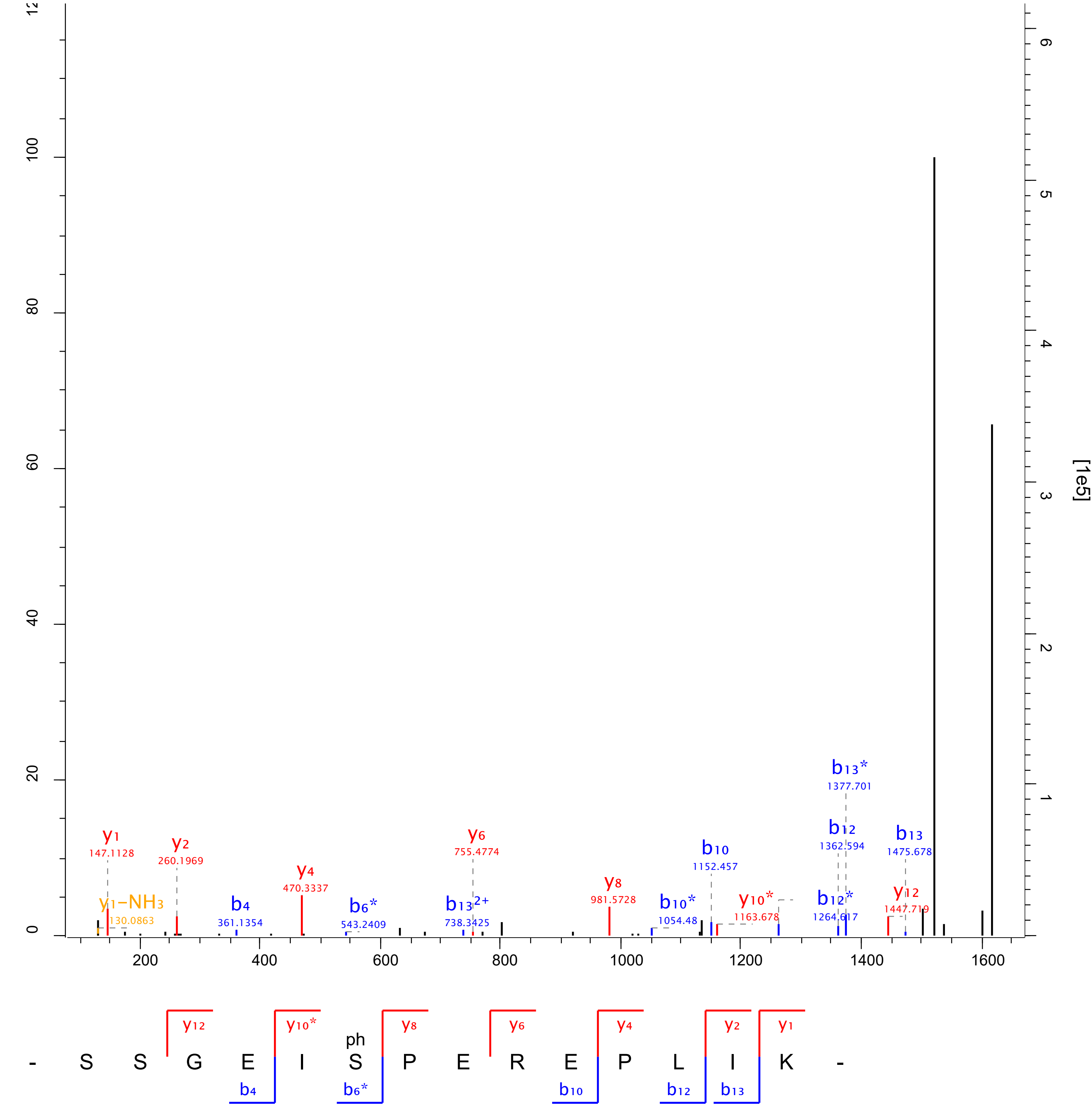

Sample output to test PDF Combine only

Raw file	Scan	Method	Score	m/z
sirk1_sp3-mic-0-3-A	15410	FTMS; HCD	54.26	684.33

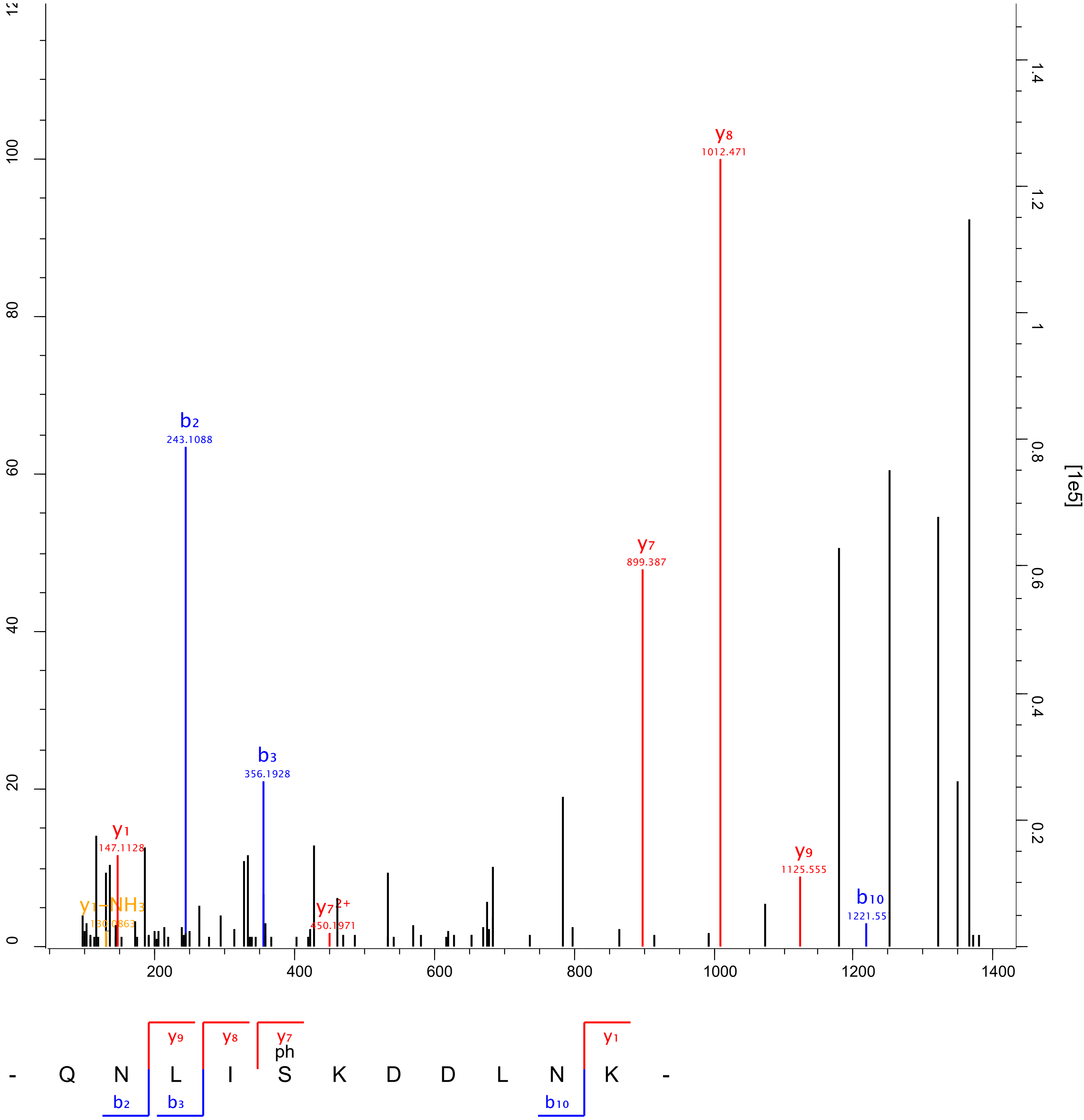

Sample output to test PDF Combine only

Raw file	Scan	Method	Score	m/z	Gene names
sirk1_sp3-mic-0-3-A	15716	FTMS; HCD	52.58	670.31	CIPK8

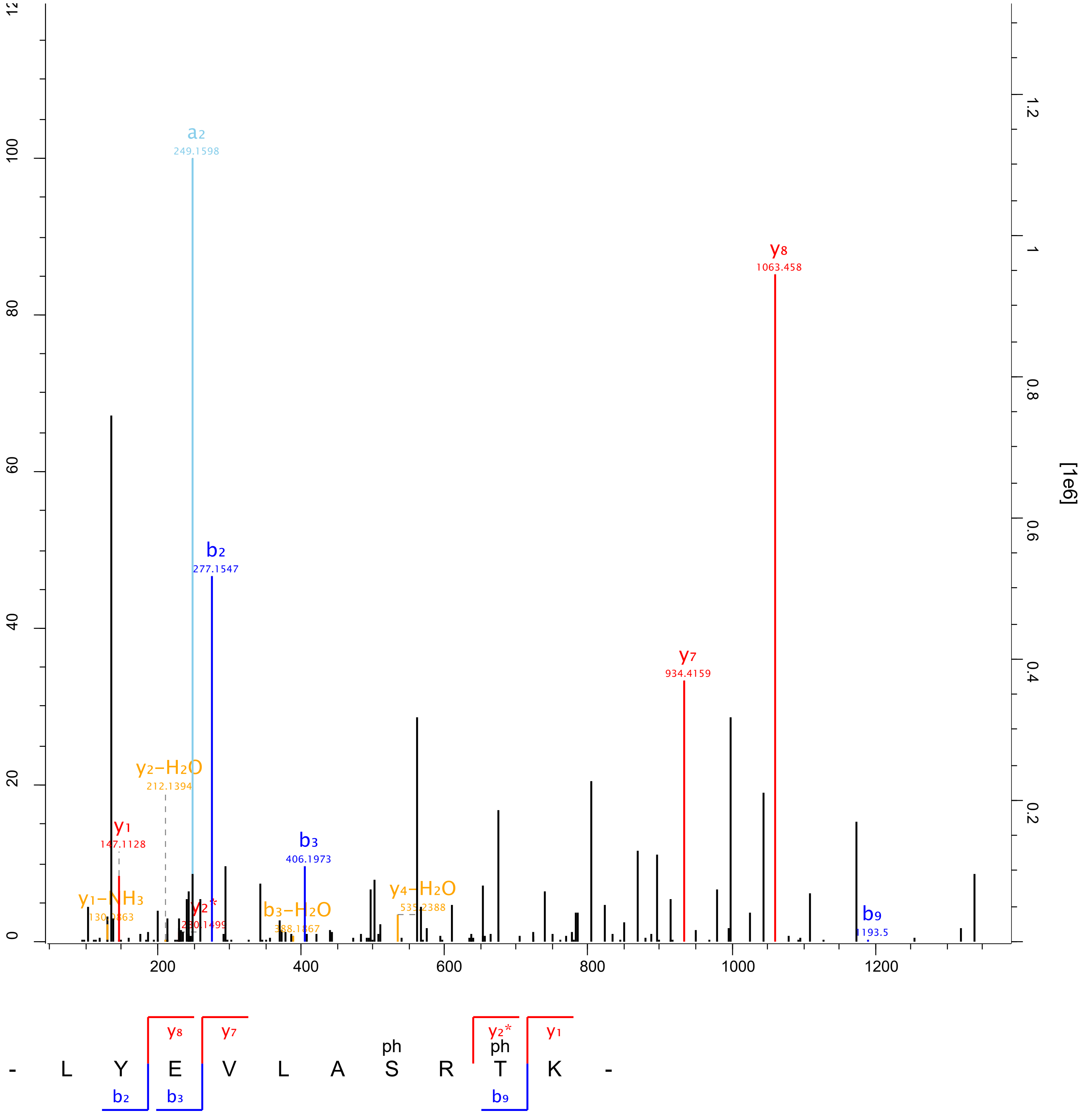

Sample output to test PDF Combine only

Raw file	Scan	Method	Score	m/z	Gene names
sirk1_sp3-mic-0-3-A	17511	FTMS; HCD	41.22	859.87	At3g08960

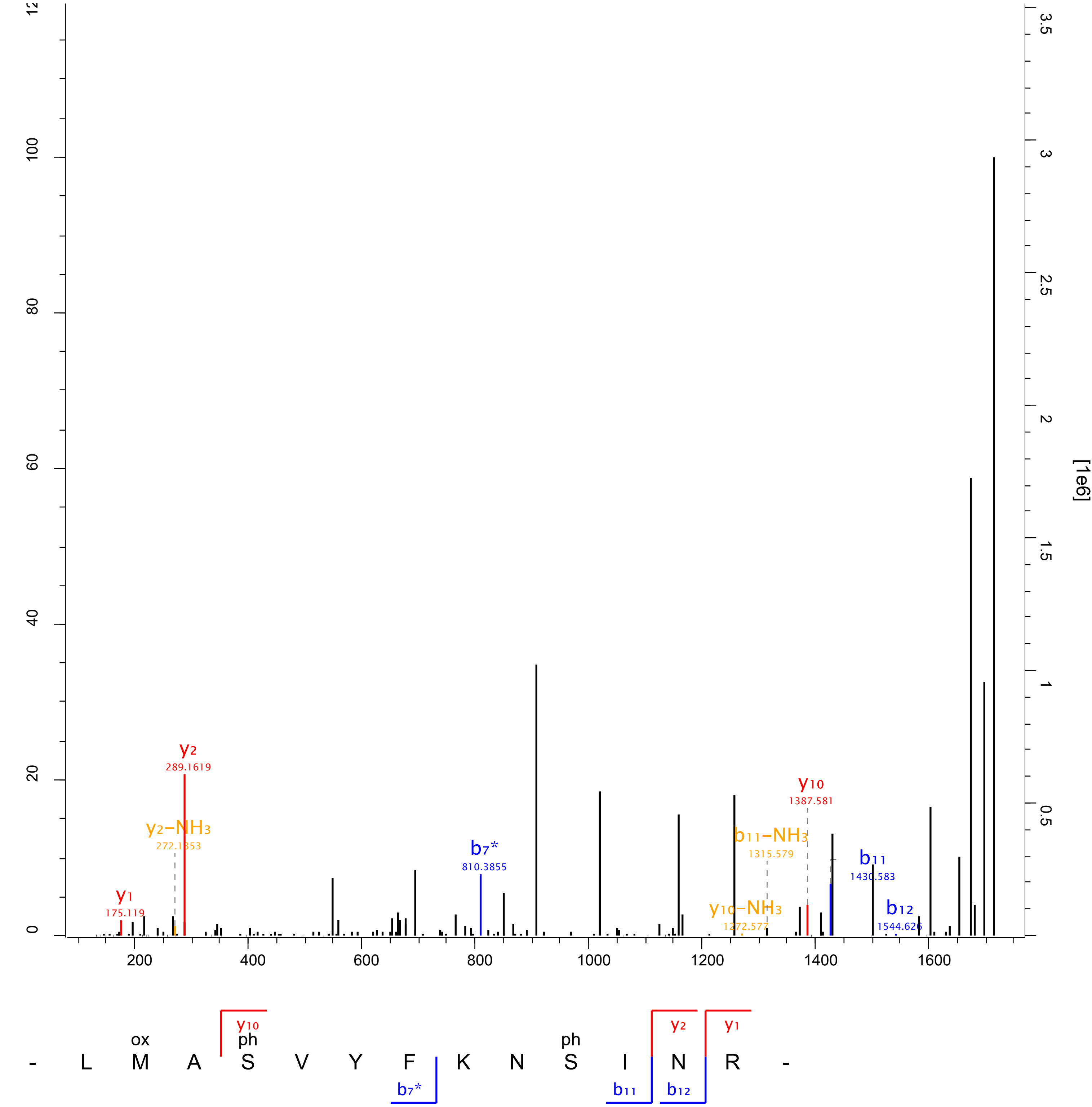

Sample output to test PDF Combine only

Raw file	Scan	Method	Score	m/z	Gene names
sirk1_sp3-mic-0-3-A	25824	FTMS; HCD	132.97	614.77	CPK6

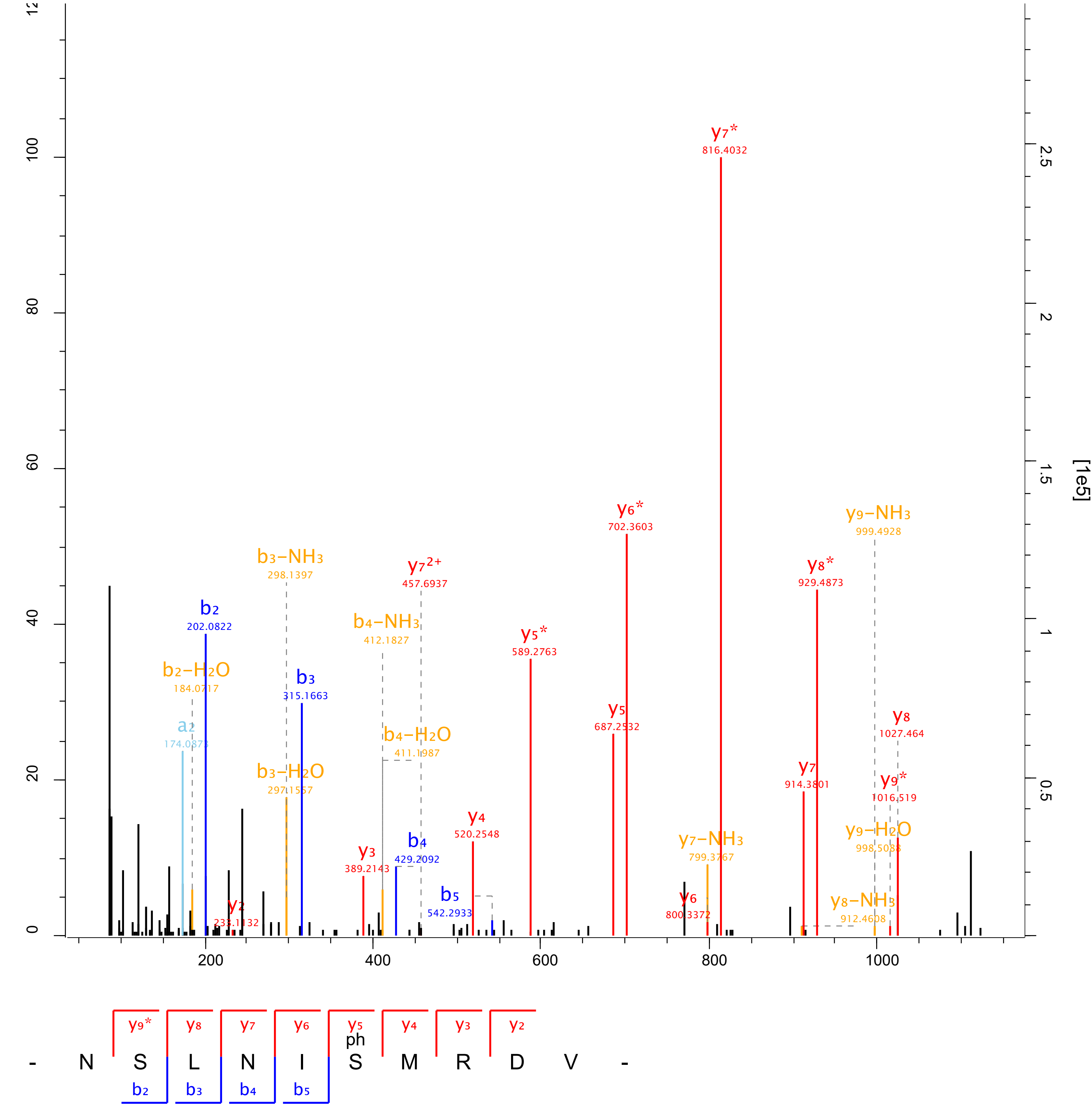

Sample output to test PDF Combine only

Raw file	Scan	Method	Score	m/z	Gene names
sirk1_sp3-mic-0-3-A	29666	FTMS; HCD	263.19	706.31	AHA2;HA2

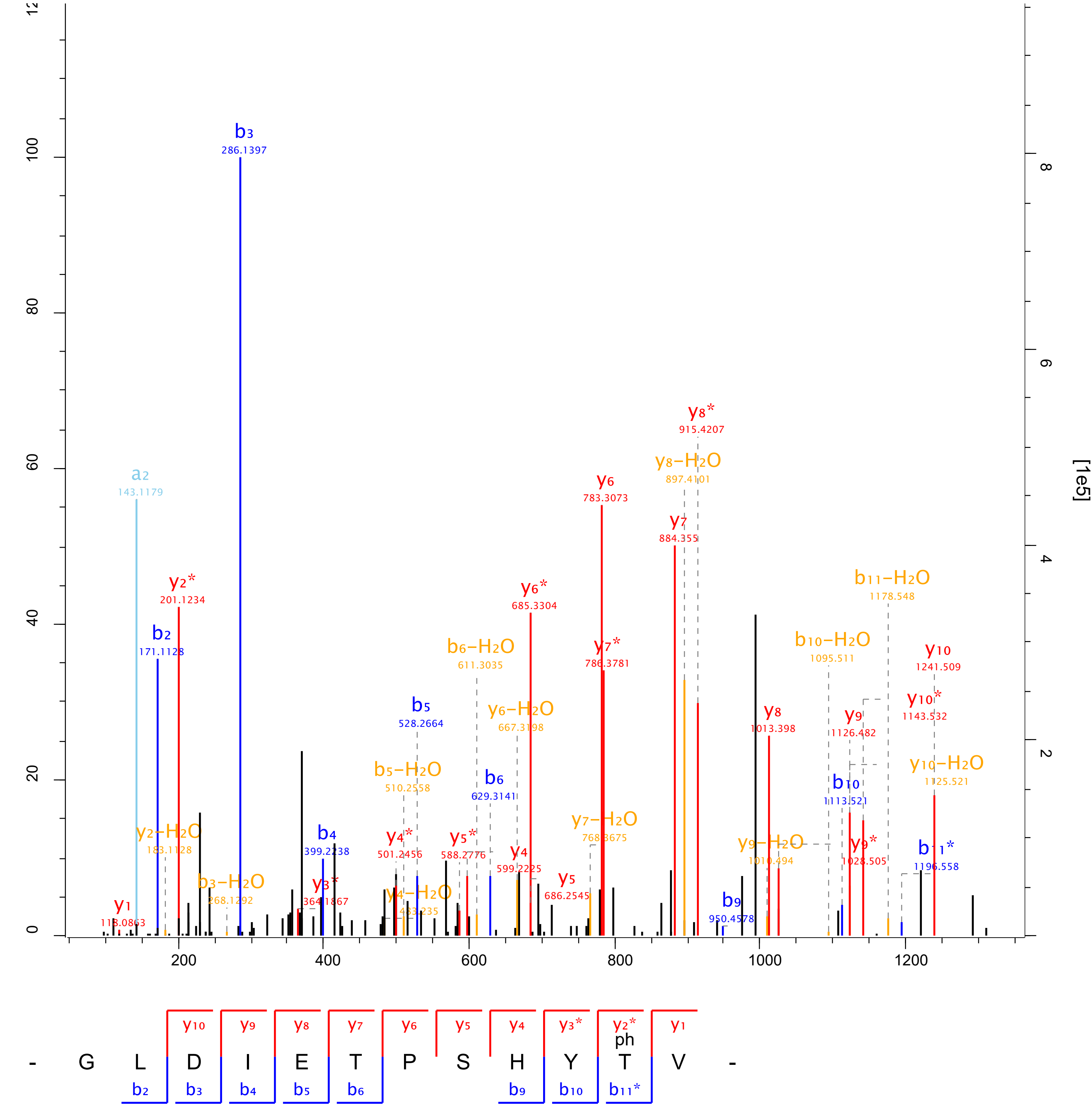

Sample output to test PDF Combine only

Raw file	Scan	Method	Score	m/z	Gene names
sirk1_sp3-mic-0-3-A	36815	FTMS; HCD	42.03	830.89	EXO84B

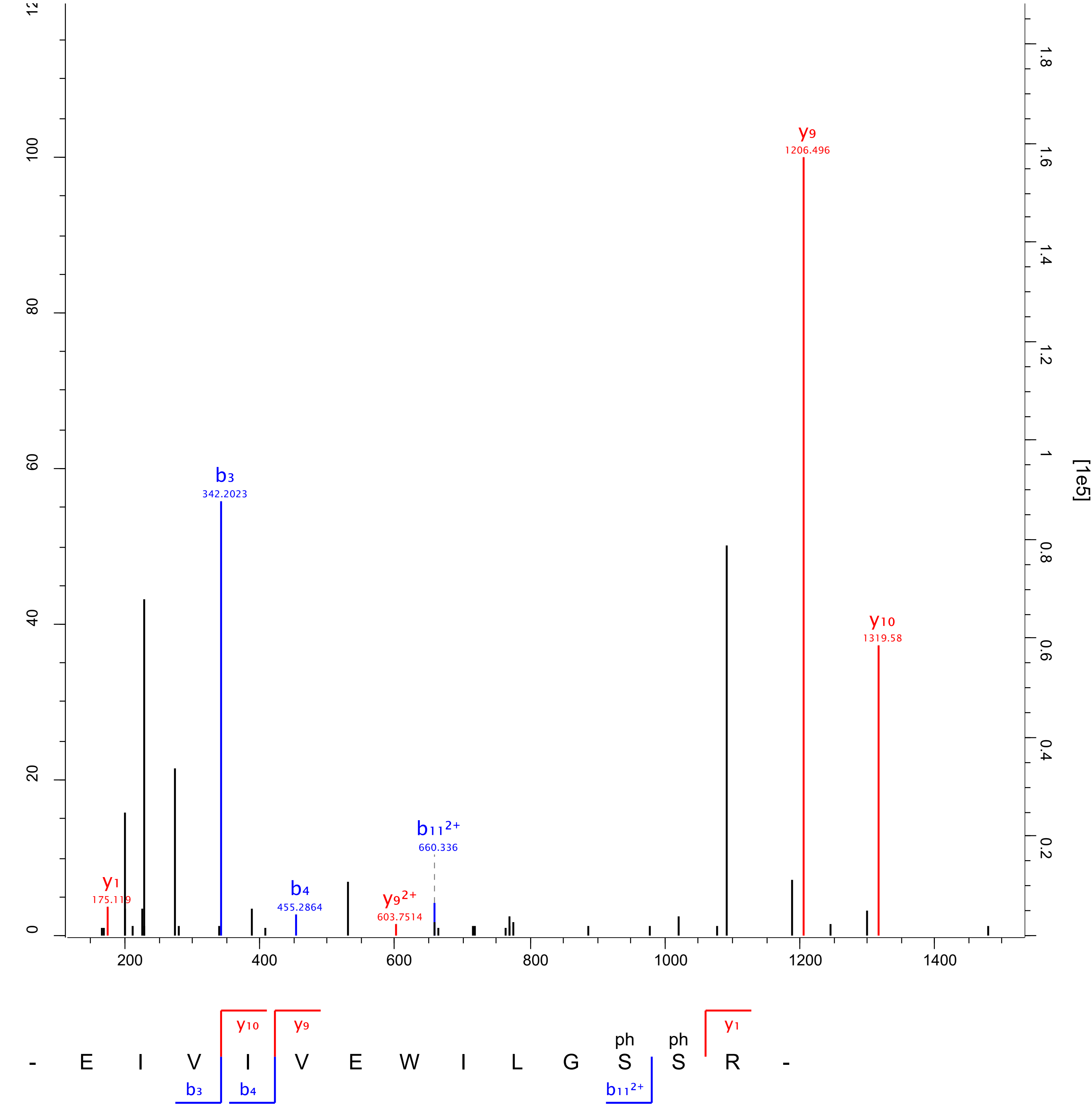

Sample output to test PDF Combine only

Raw file	Scan	Method	Score	m/z	Gene names
sirk1_sp3-mic-0-3-P	3774	FTMS; HCD	76.01	427.89	ACA8

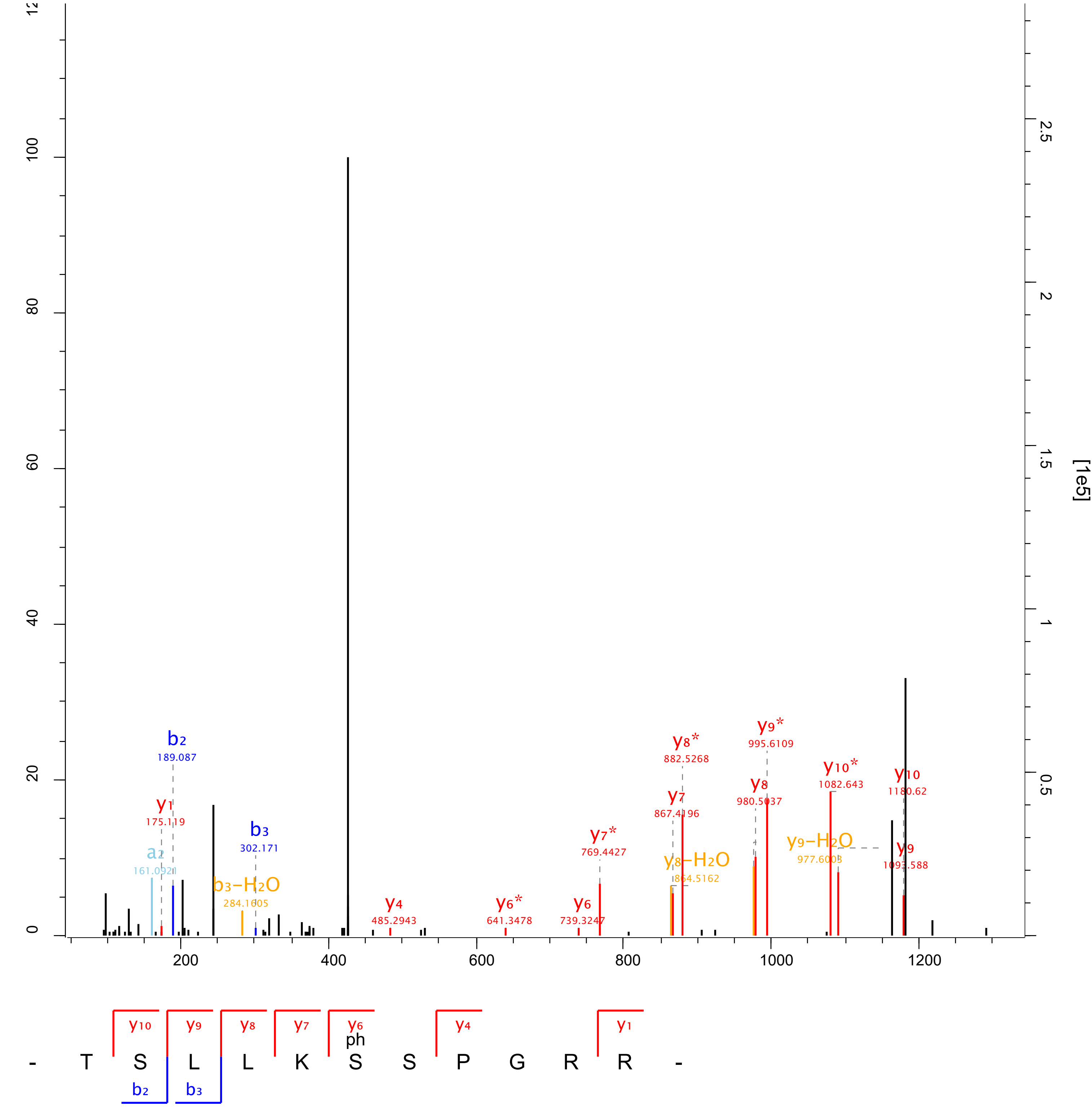

Sample output to test PDF Combine only

Raw file	Scan	Method	Score	m/z	Gene names
sirk1_sp3-mic-0-3-P	6742	FTMS; HCD	94.63	619.62	ABCG36

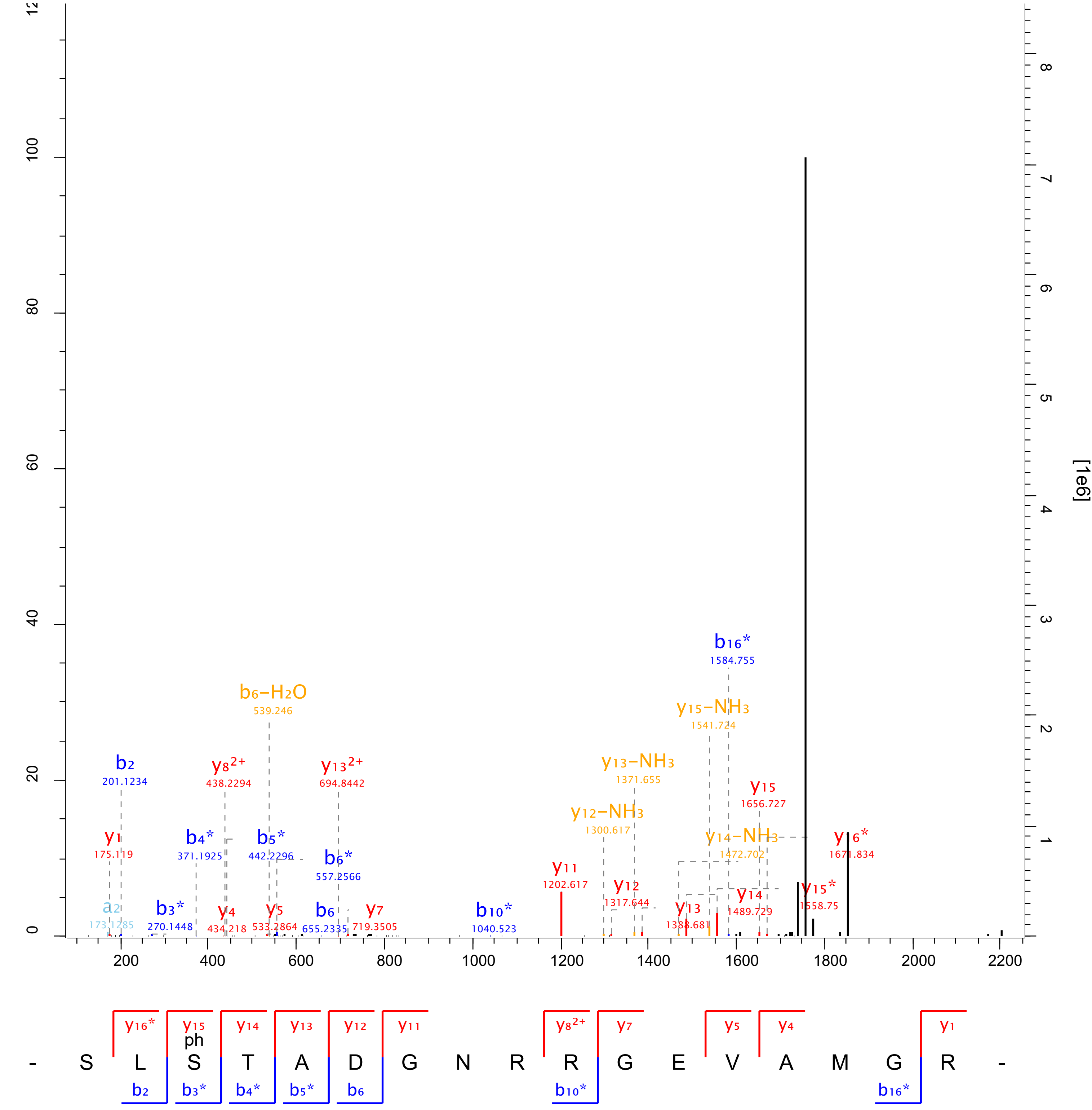

Sample output to test PDF Combine only

Raw file	Scan	Method	Score	m/z	Gene names
sirk1_sp3-mic-0-3-P	9720	FTMS; HCD	90.56	486.24	IQD32

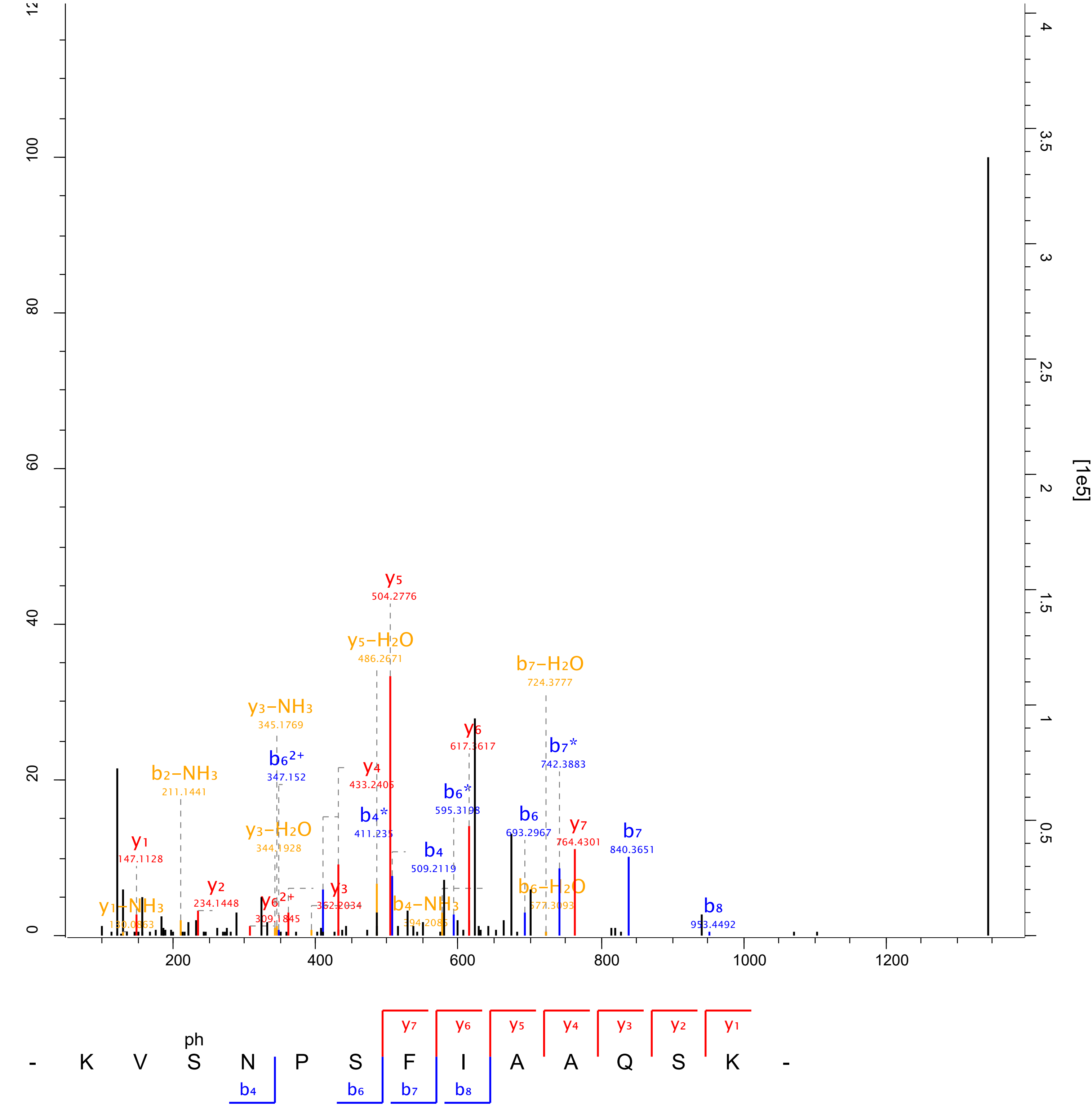

Sample output to test PDF Combine only

Raw file	Scan	Method	Score	m/z
sirk1_sp3-mic-0-3-P	10204	FTMS; HCD	52.86	545.95

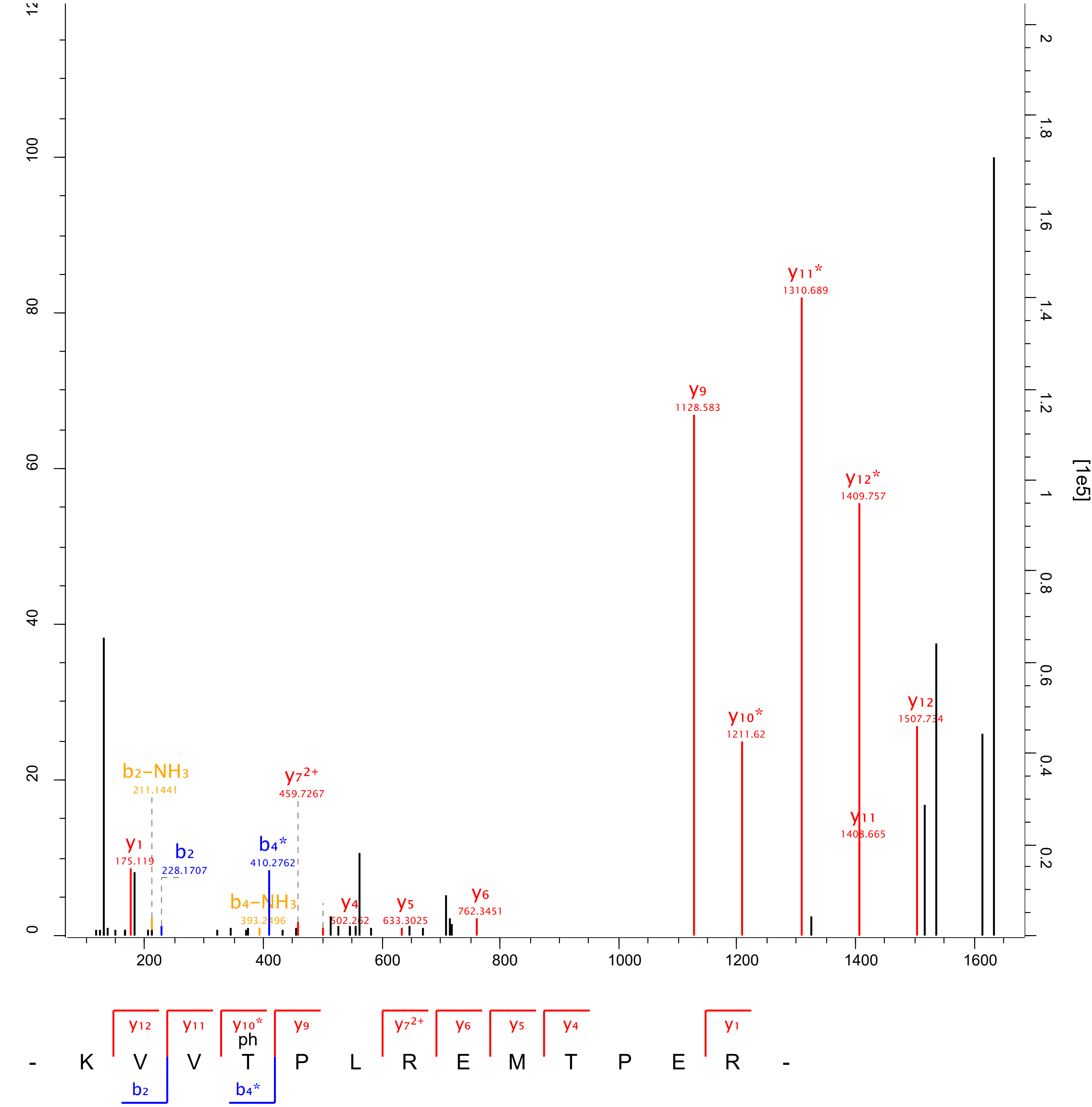

Sample output to test PDF Combine only

Raw file	Scan	Method	Score	m/z	Gene names
sirk1_sp3-mic-0-3-P	10447	FTMS; HCD	74.84	552.78	LRR-RLK

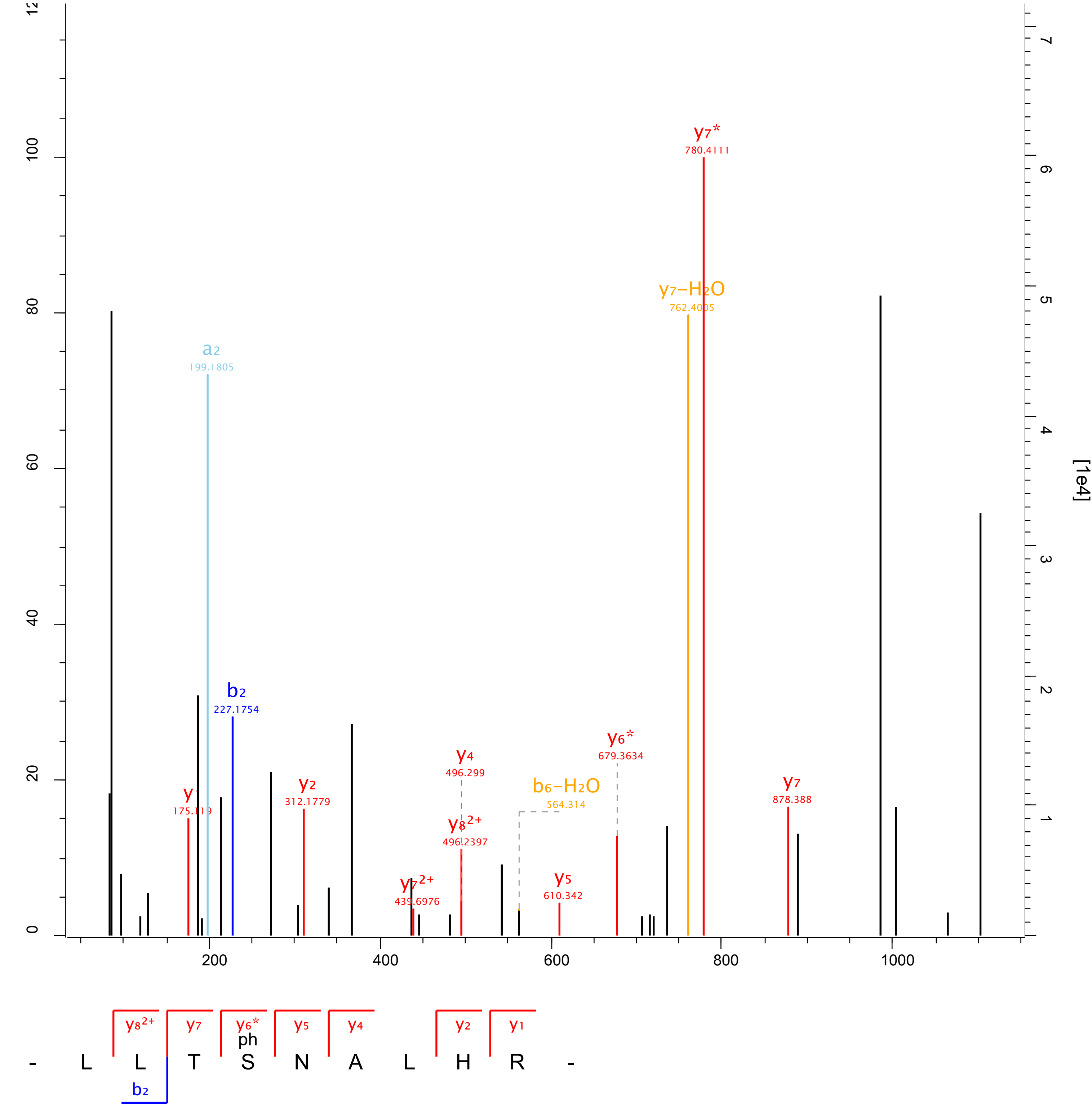

Sample output to test PDF Combine only

Raw file	Scan	Method	Score	m/z	Gene names
sirk1_sp3-mic-0-3-P	11611	FTMS; HCD	107.09	513.77	RTNLB8

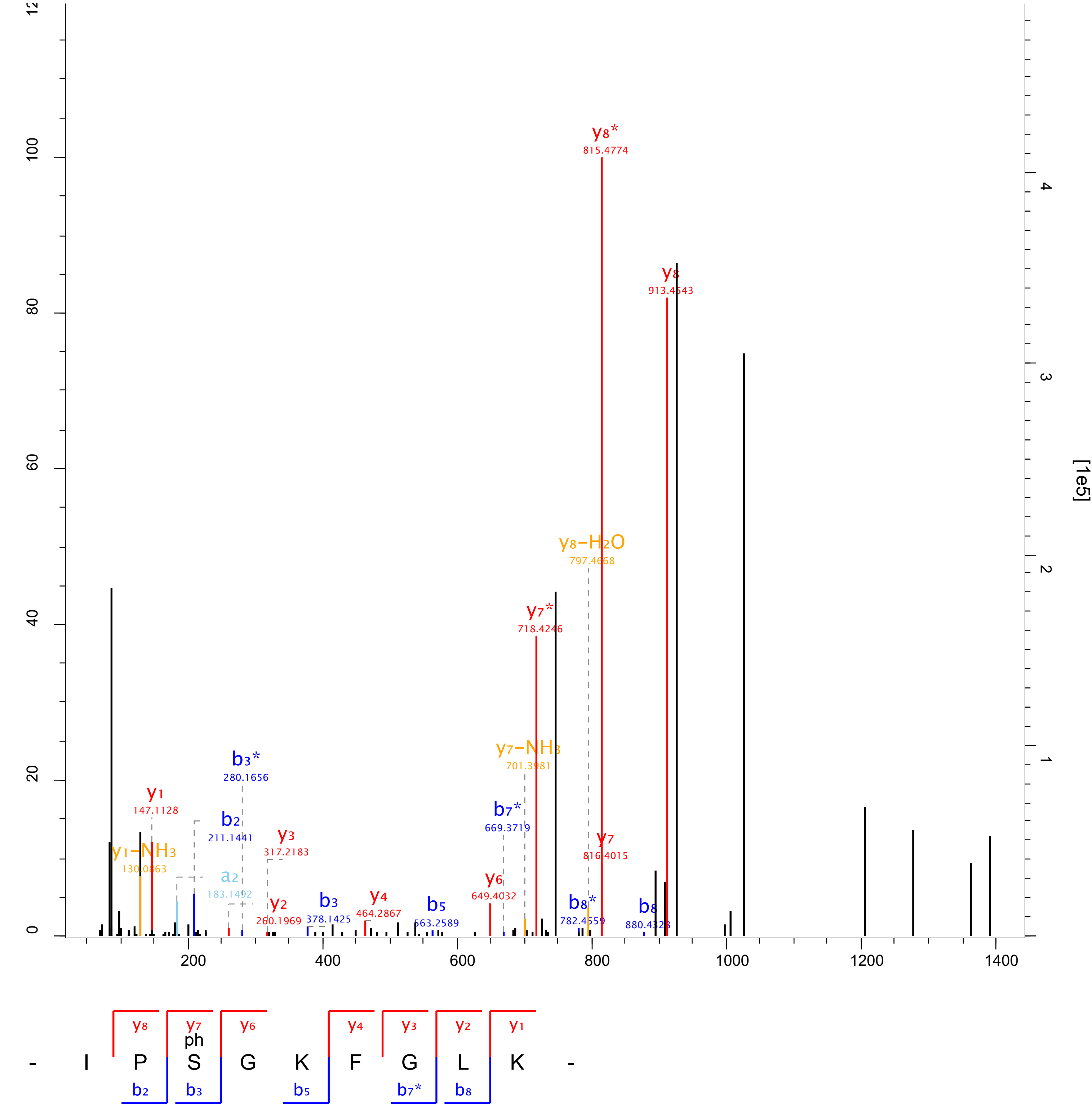

Sample output to test PDF Combine only

Raw file	Scan	Method	Score	m/z	Gene names
sirk1_sp3-mic-0-3-P	12035	FTMS; HCD	159.69	560.59	F13I12.260

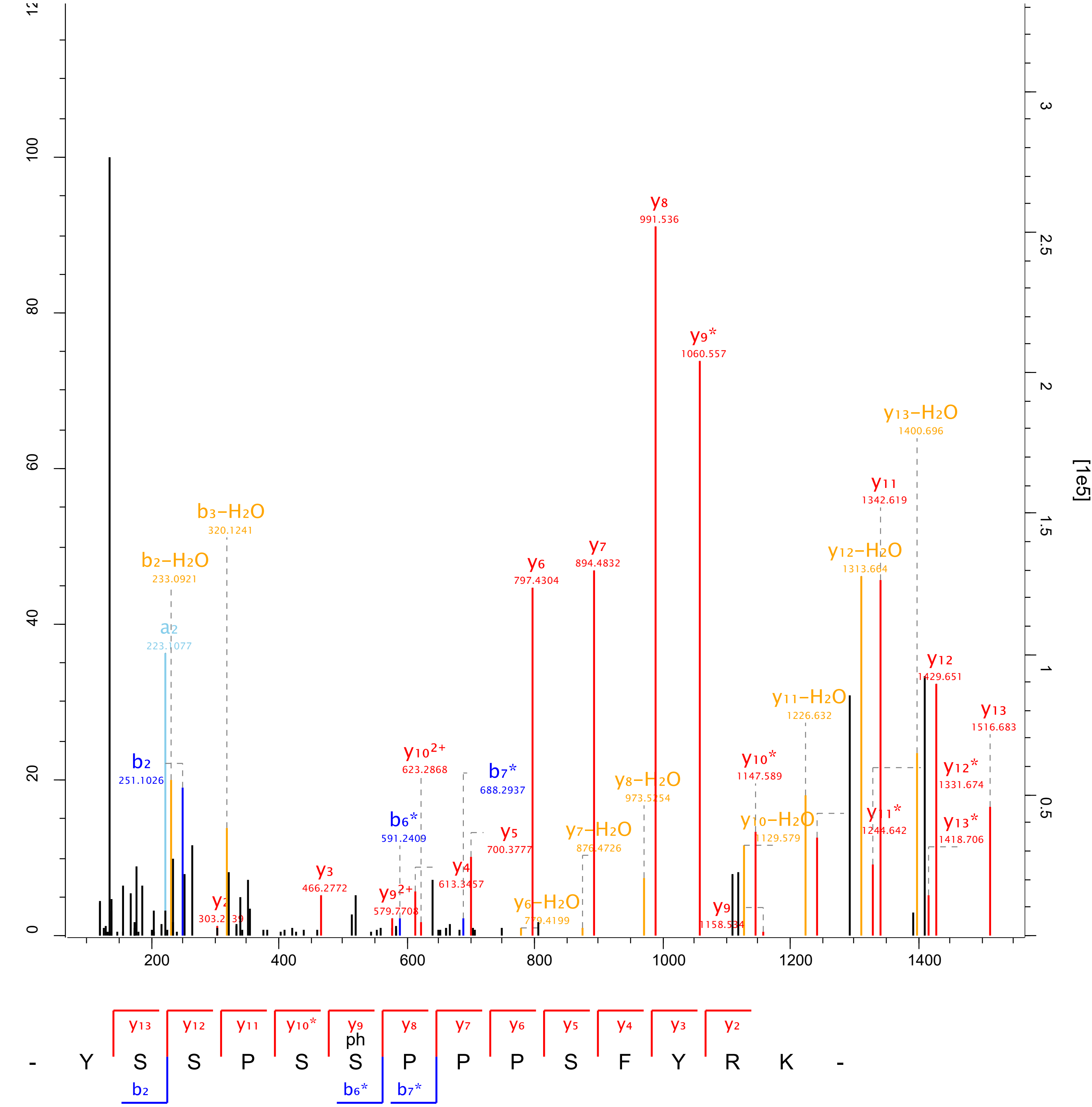

Sample output to test PDF Combine only

Raw file	Scan	Method	Score	m/z
sirk1_sp3-mic-0-3-P	12454	FTMS; HCD	82.61	631.96

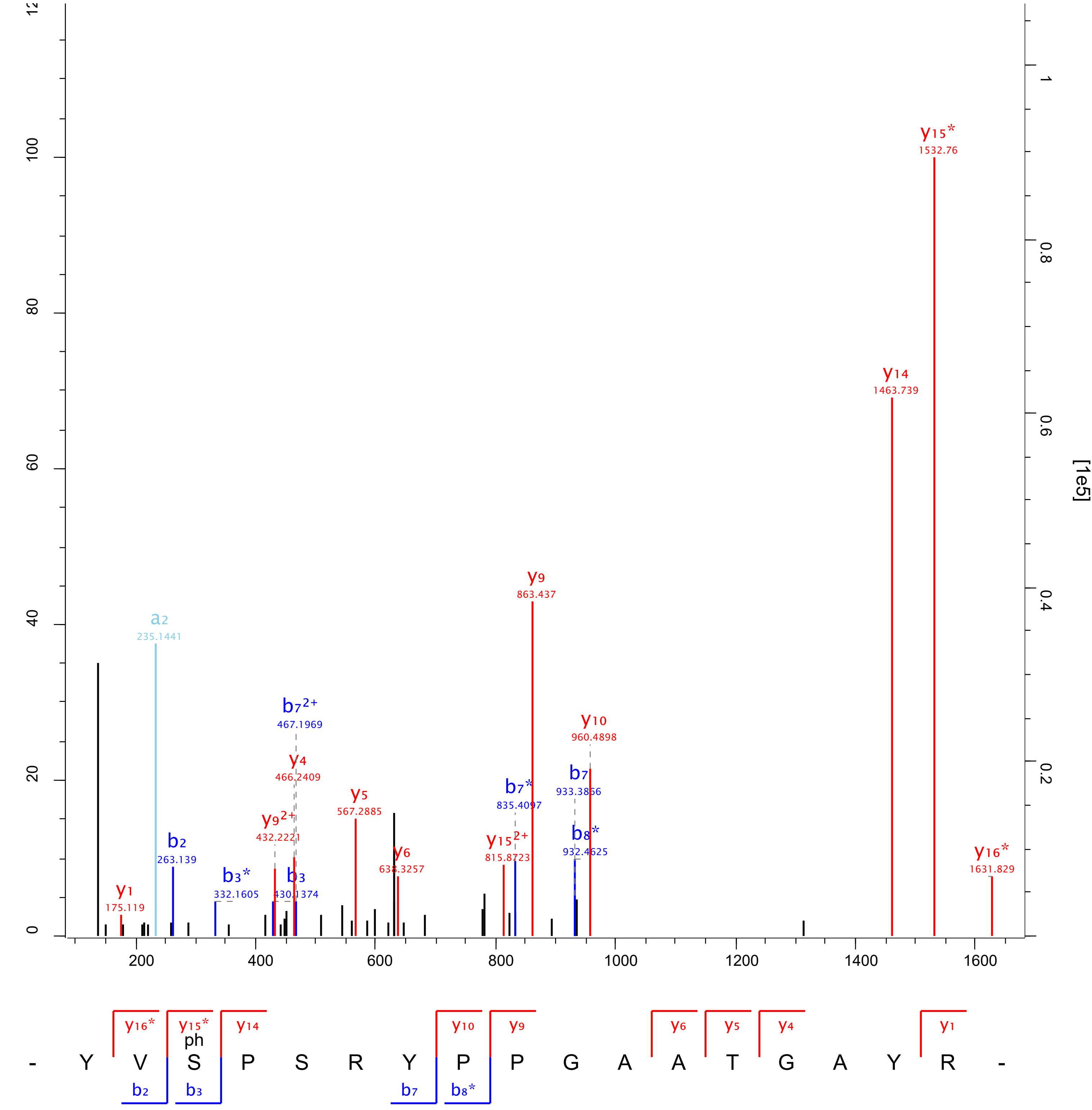

Sample output to test PDF Combine only

Raw file	Scan	Method	Score	m/z	Gene names
sirk1_sp3-mic-0-3-P	13052	FTMS; HCD	40.48	402.88	CML43;CML42

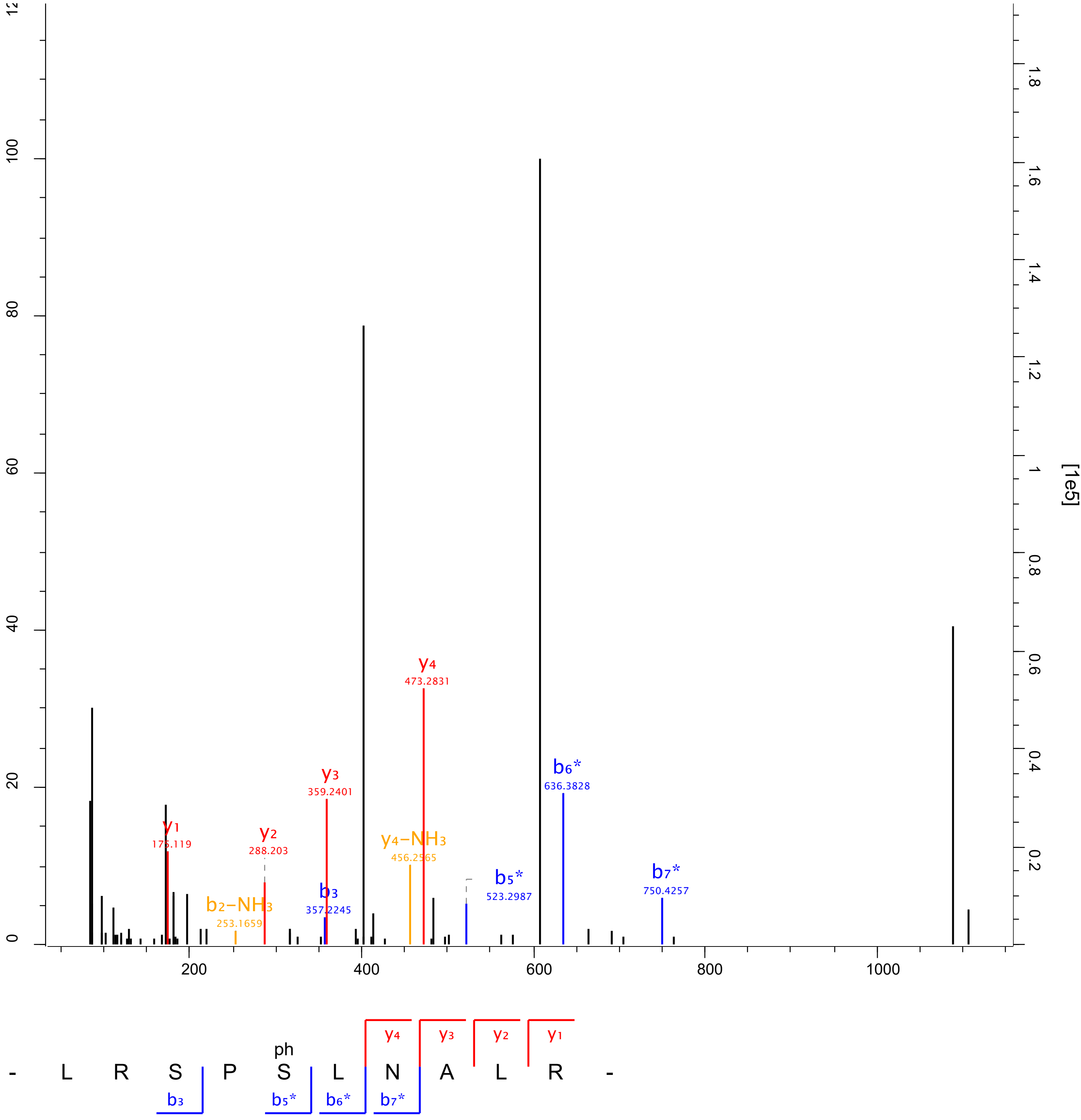

Sample output to test PDF Combine only

Raw file	Scan	Method	Score	m/z	Gene names
sirk1_sp3-mic-0-3-P	13261	FTMS; HCD	64.1	443.23	At5g15680

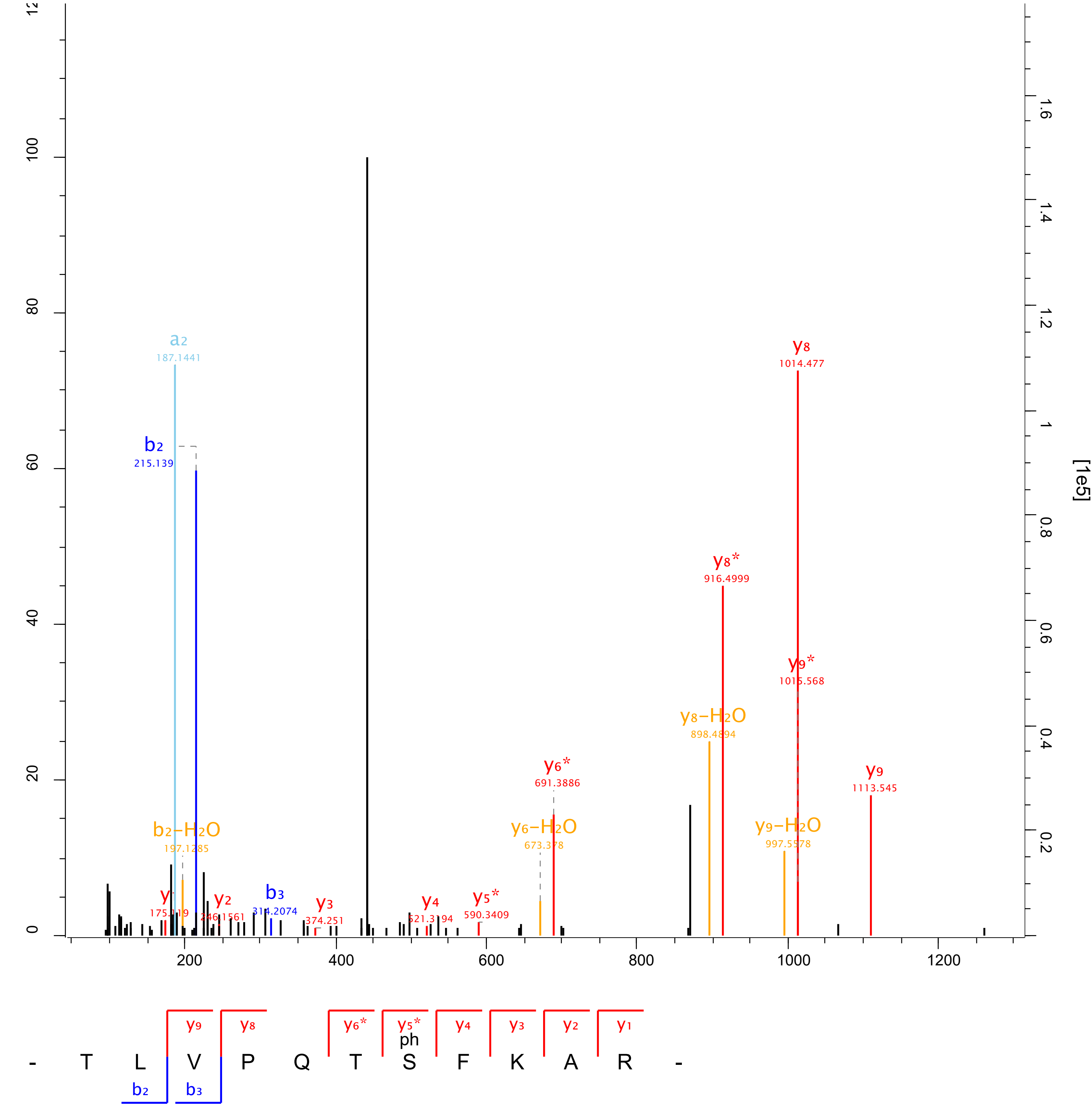

Sample output to test PDF Combine only

Raw file	Scan	Method	Score	m/z	Gene names
sirk1_sp3-mic-0-3-P	18153	FTMS; HCD	94.78	572.78	T25B24.6

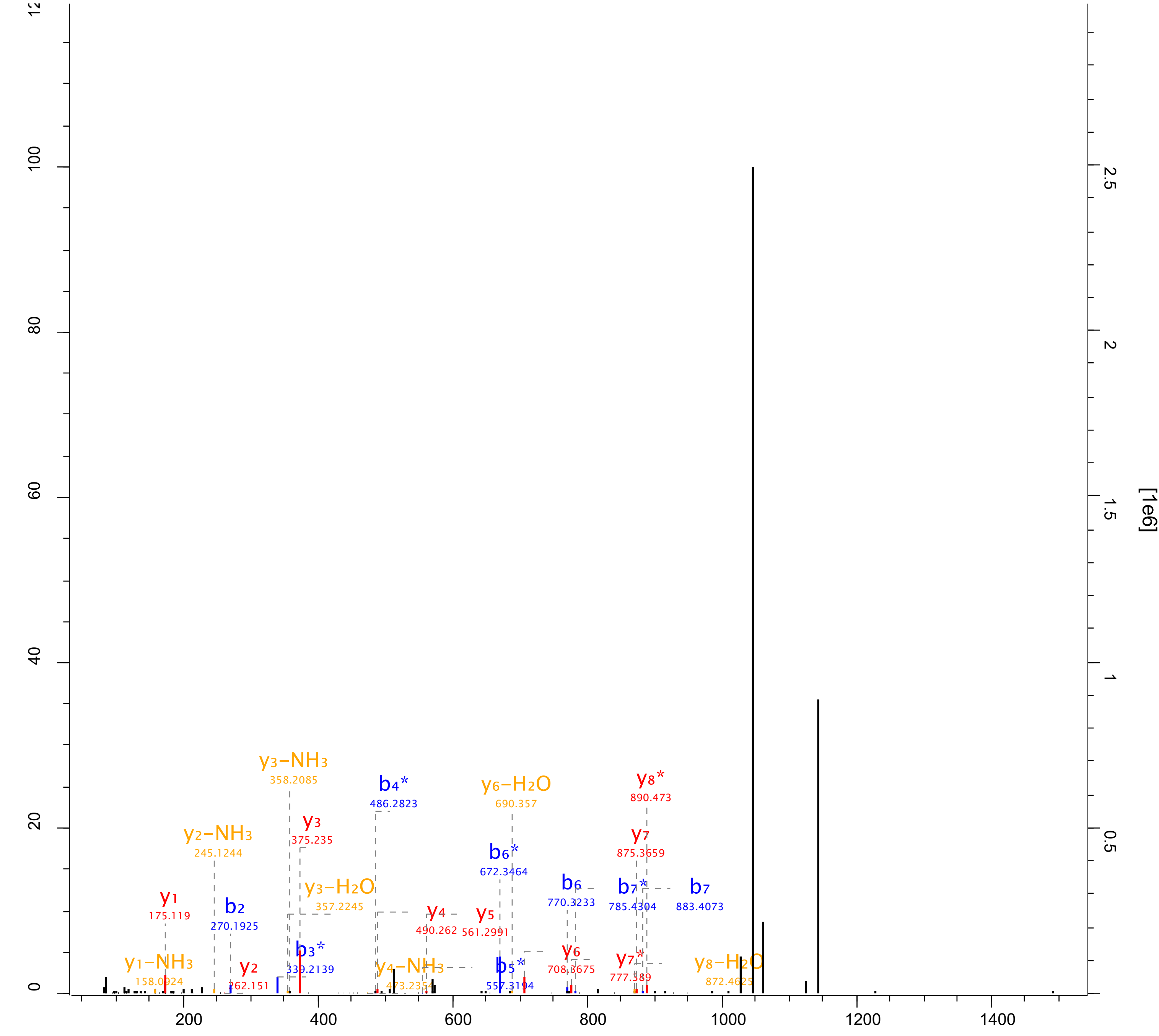

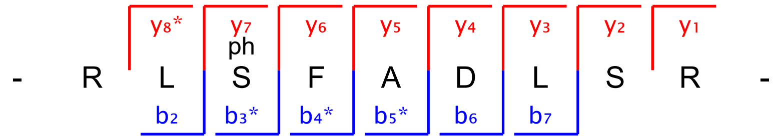

Sample output to test PDF Combine only

Raw file	Scan	Method	Score	m/z	Gene names
sirk1_sp3-mic-0-3-P	18599	FTMS; HCD	122.97	628.79	CBSX6

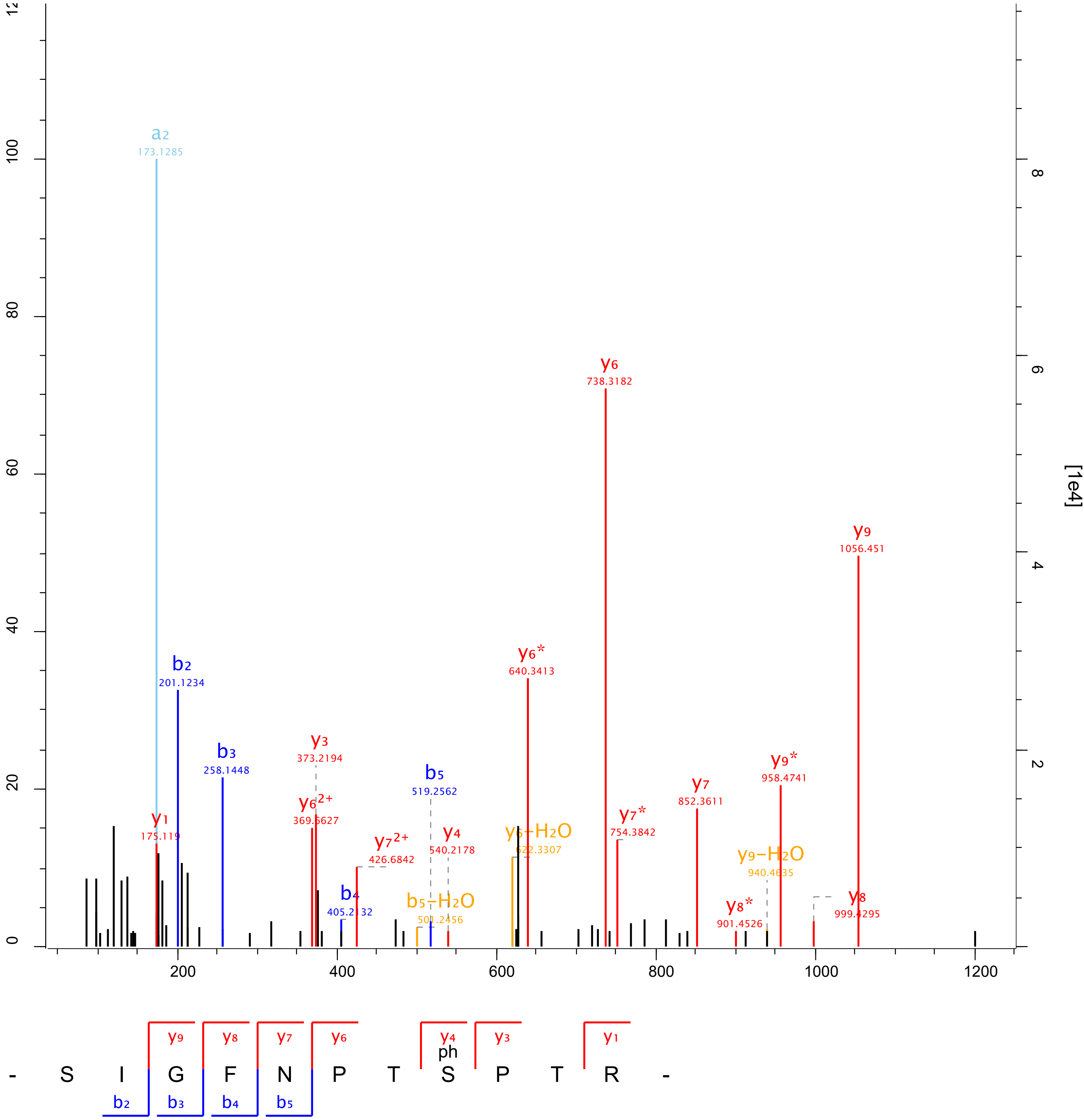

Sample output to test PDF Combine only

Raw file	Scan	Method	Score	m/z	Gene names
sirk1_sp3-mic-0-3-P	21531	FTMS; HCD	92.47	600.64	BAG7

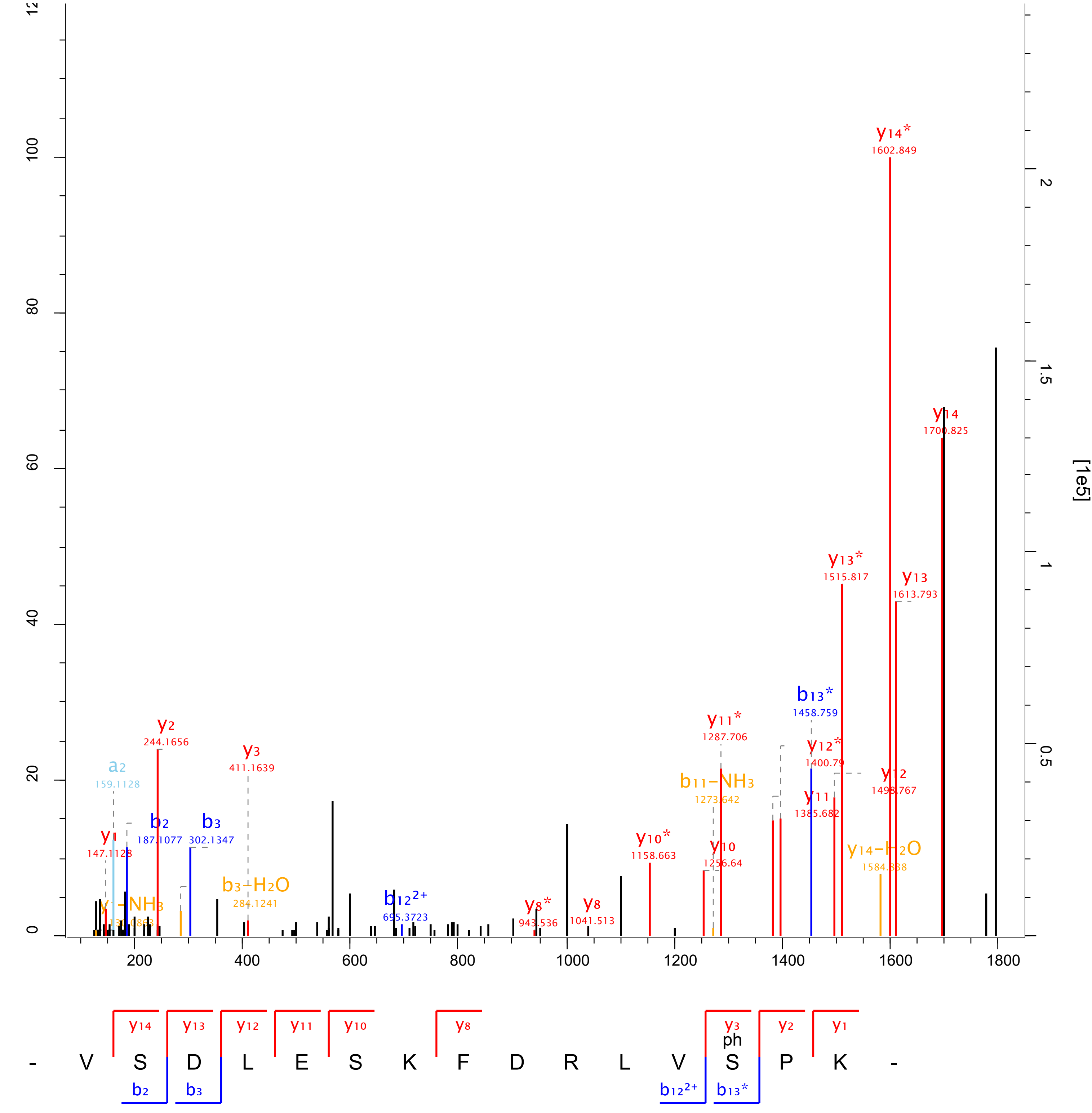

Sample output to test PDF Combine only

Raw file	Scan	Method	Score	m/z	Gene names
sirk1_sp3-mic-0-3-P	27662	FTMS; HCD	99.66	707.33	XI-K

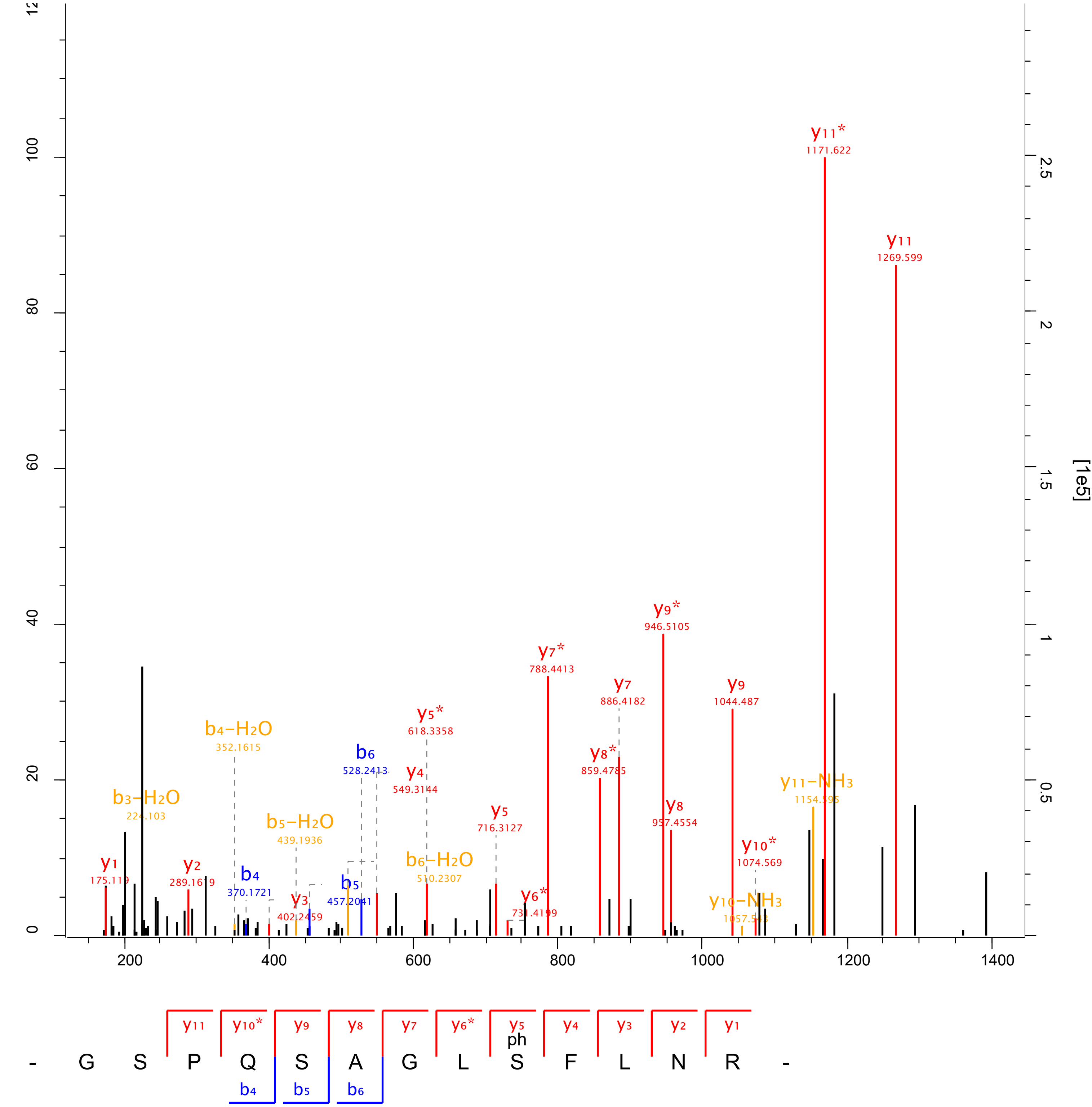

Sample output to test PDF Combine only

Raw file	Scan	Method	Score	m/z	Gene names
sirk1_sp3-mic-0-3-P	30188	FTMS; HCD	64.5	570.29	POT13

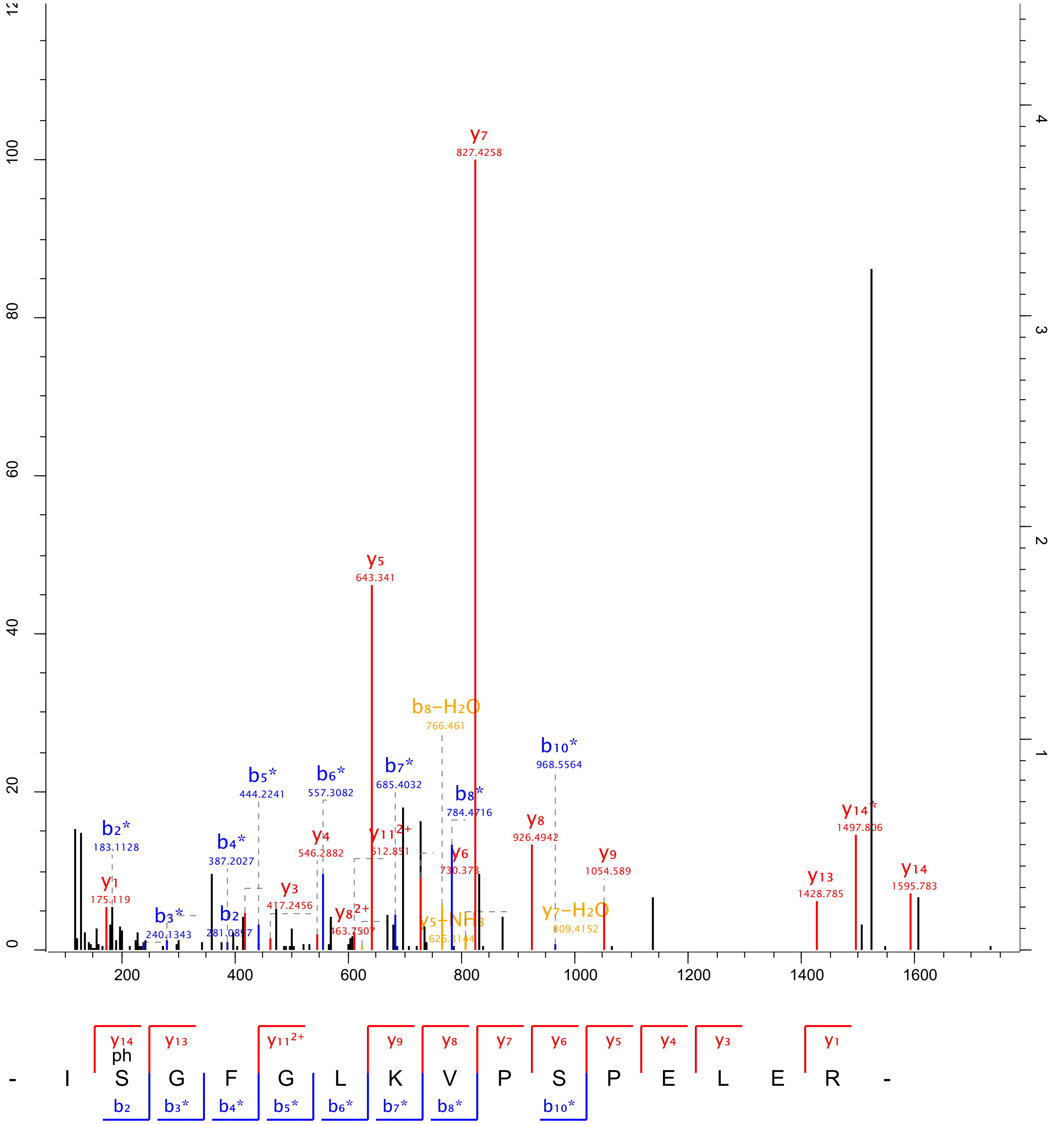

Sample output to test PDF Combine only

Raw file	Scan	Method	Score	m/z
sirk1_sp3-mic-0-3-P	30251	FTMS; HCD	60.13	557.95

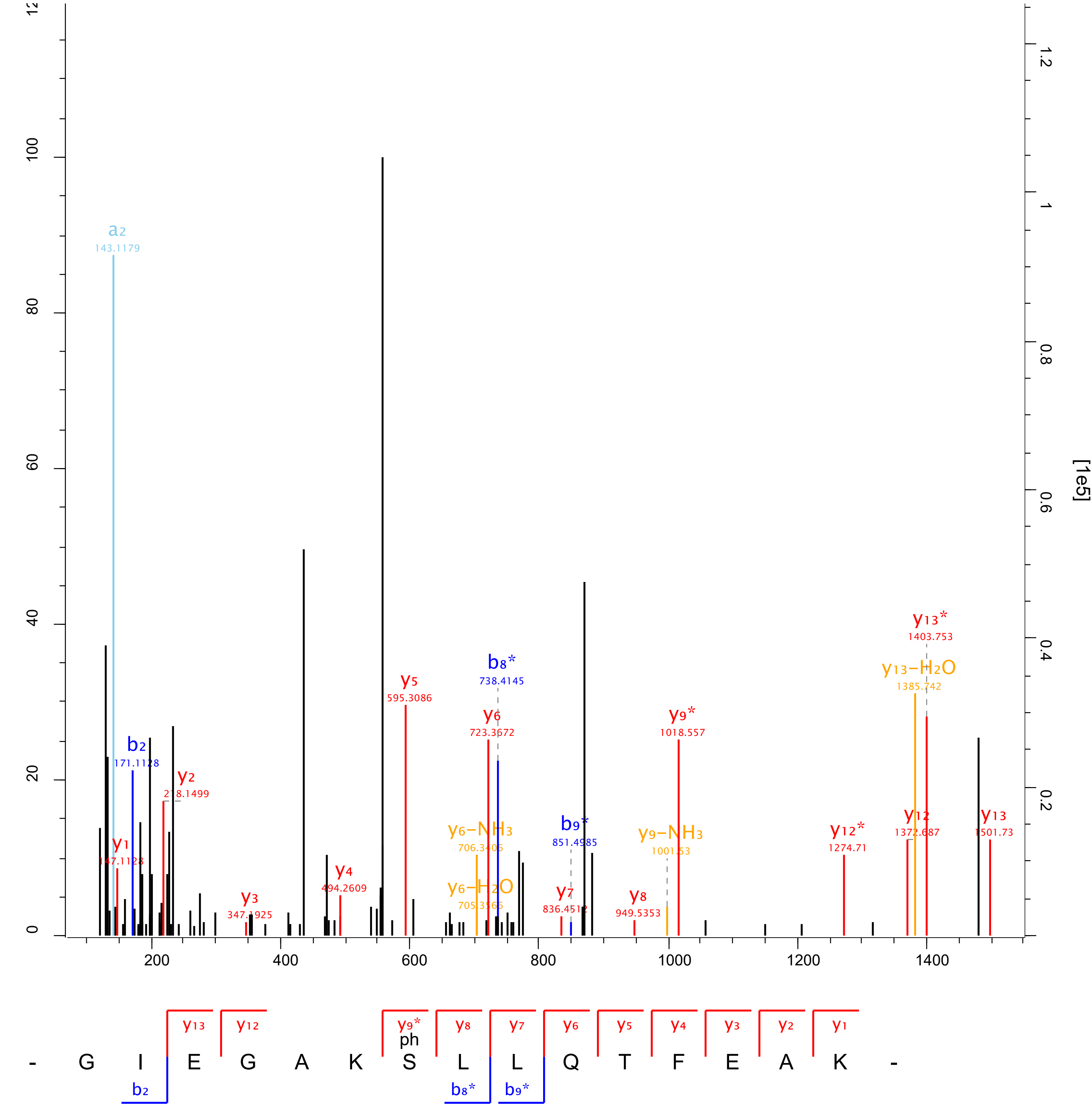

Sample output to test PDF Combine only

Raw file	Scan	Method	Score	m/z	Gene names
sirk1_sp3-mic-0-3-P	31057	FTMS; HCD	54	864.75	ABCG34

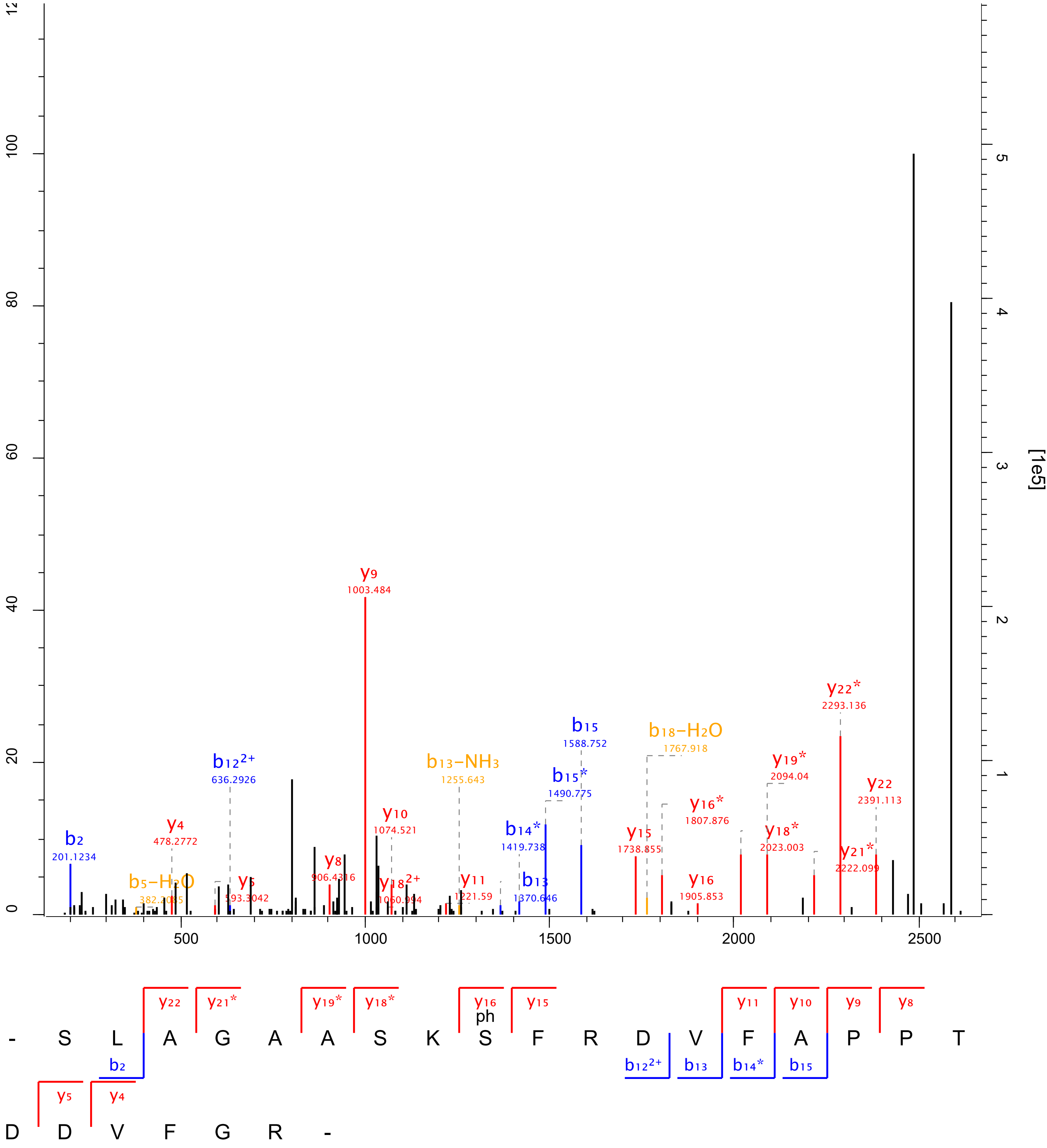

Sample output to test PDF Combine only

Raw file	Scan	Method	Score	m/z	Gene names
sirk1_sp3-mic-0-3-P	32273	FTMS; HCD	48.4	506.6	MKP1

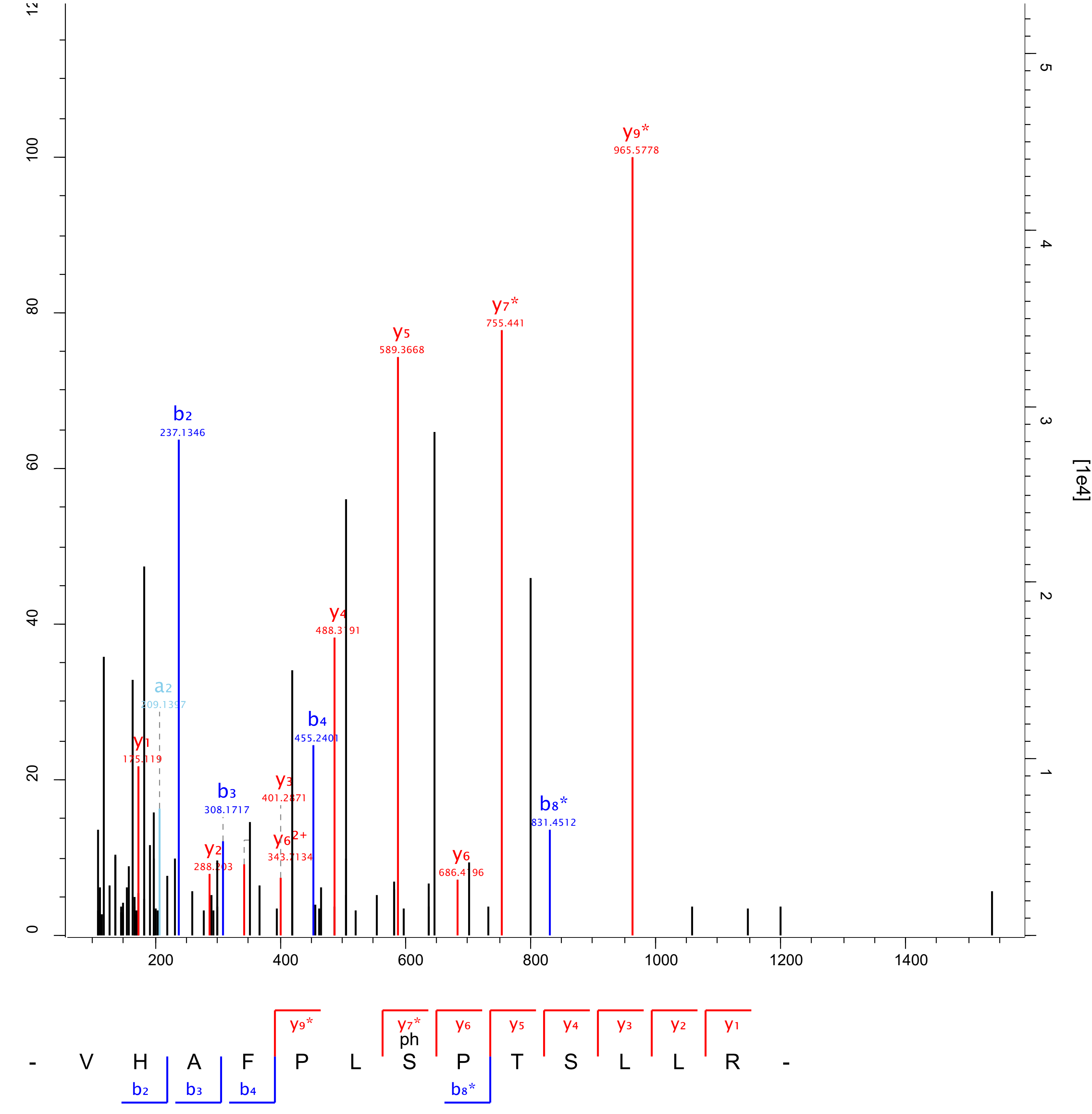

Sample output to test PDF Combine only

Raw file	Scan	Method	Score	m/z	Gene names
sirk1_sp3-mic-0-3-P	32579	FTMS; HCD	105.2	613.64	At4g17140

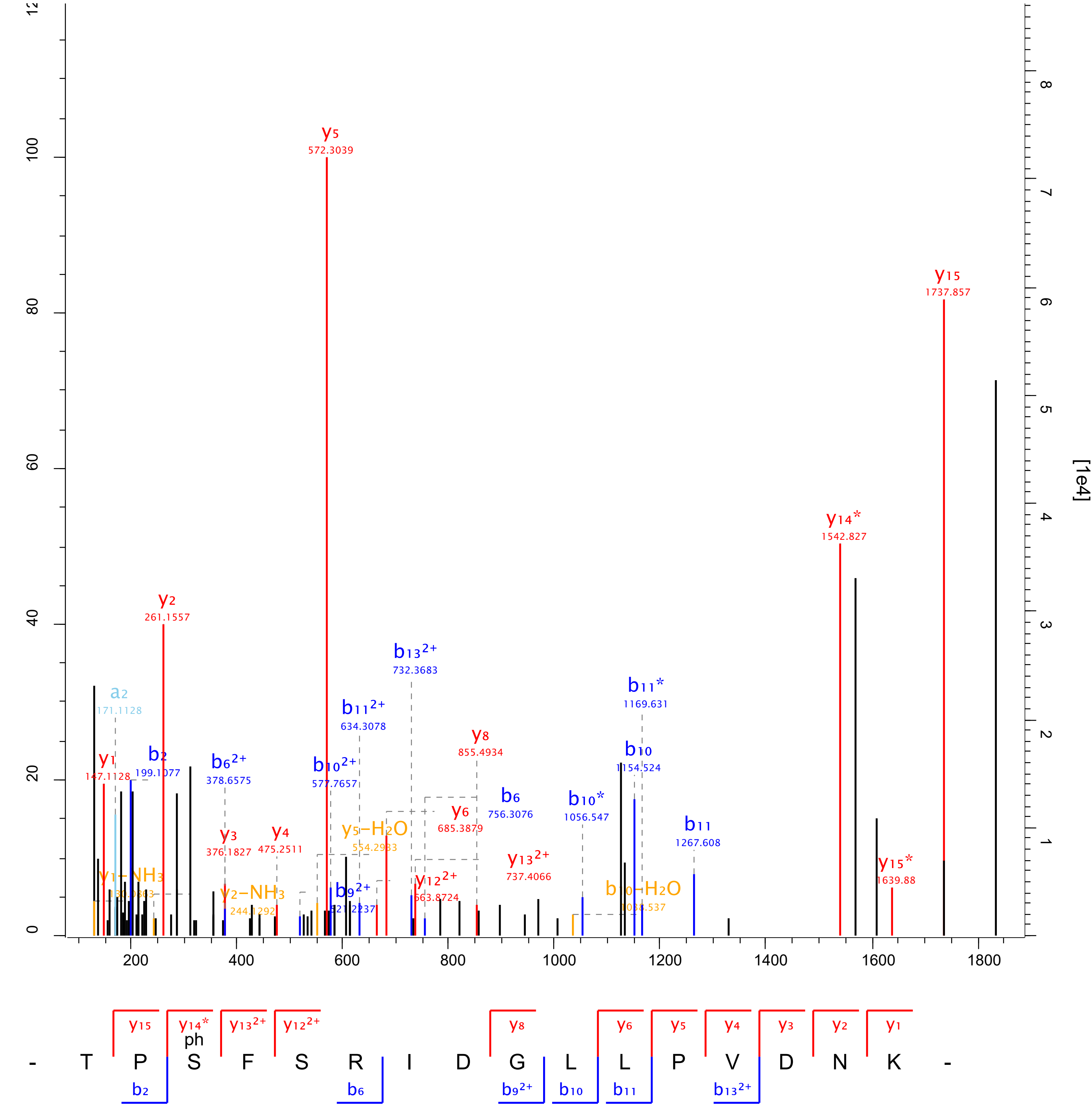

Sample output to test PDF Combine only

Raw file	Scan	Method	Score	m/z	Gene names
sirk1_sp3-mic-0-3-P	40177	FTMS; HCD	58.34	827.05	TPS5

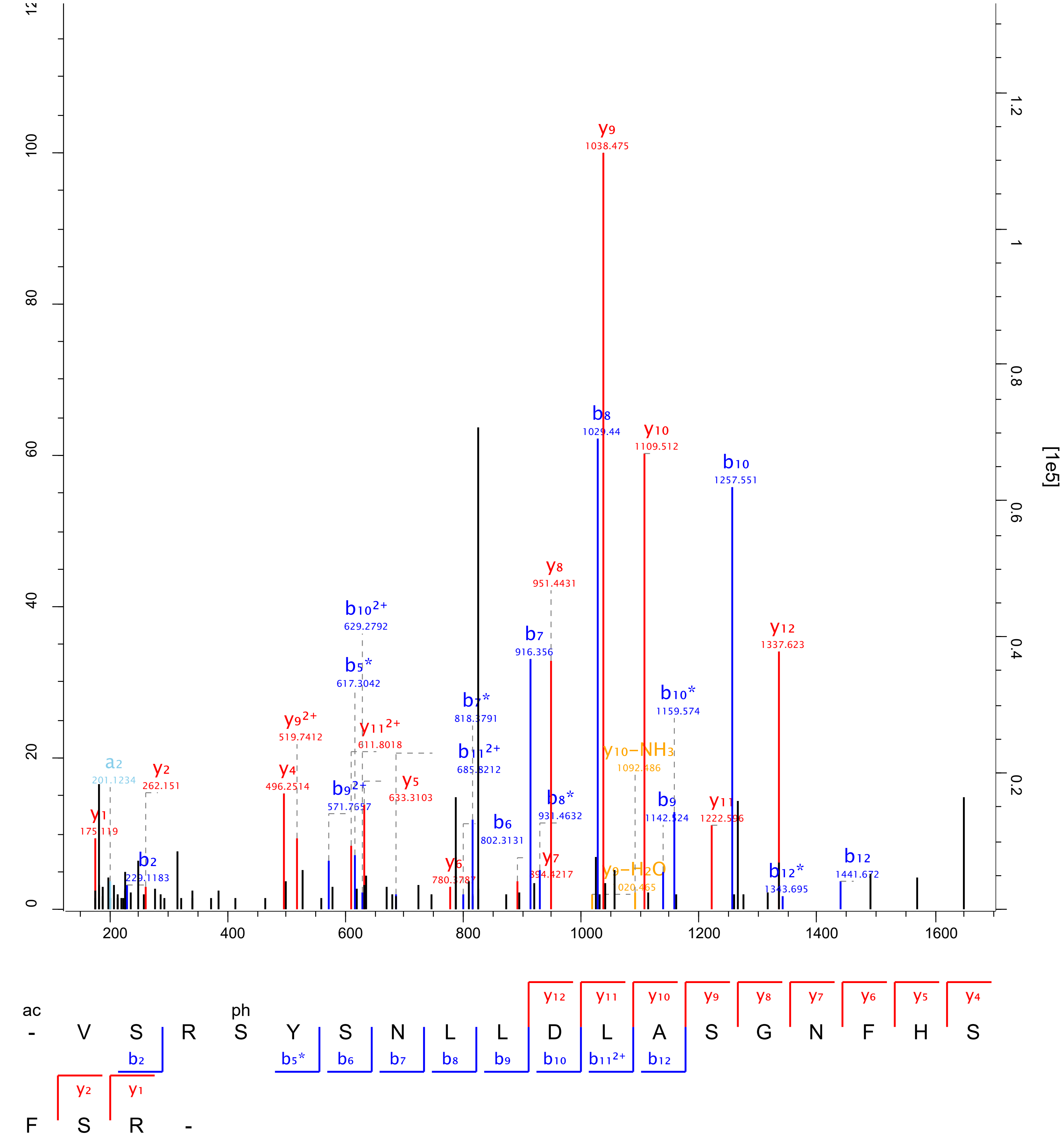

Sample output to test PDF Combine only

Raw file	Scan	Method	Score	m/z	Gene names
sirk1_sp3-mic-SUC-1-A	4760	FTMS; HCD	52.17	584.26	TIF4A-1;EIF4A1;TIF4A-2;EIF4A-2

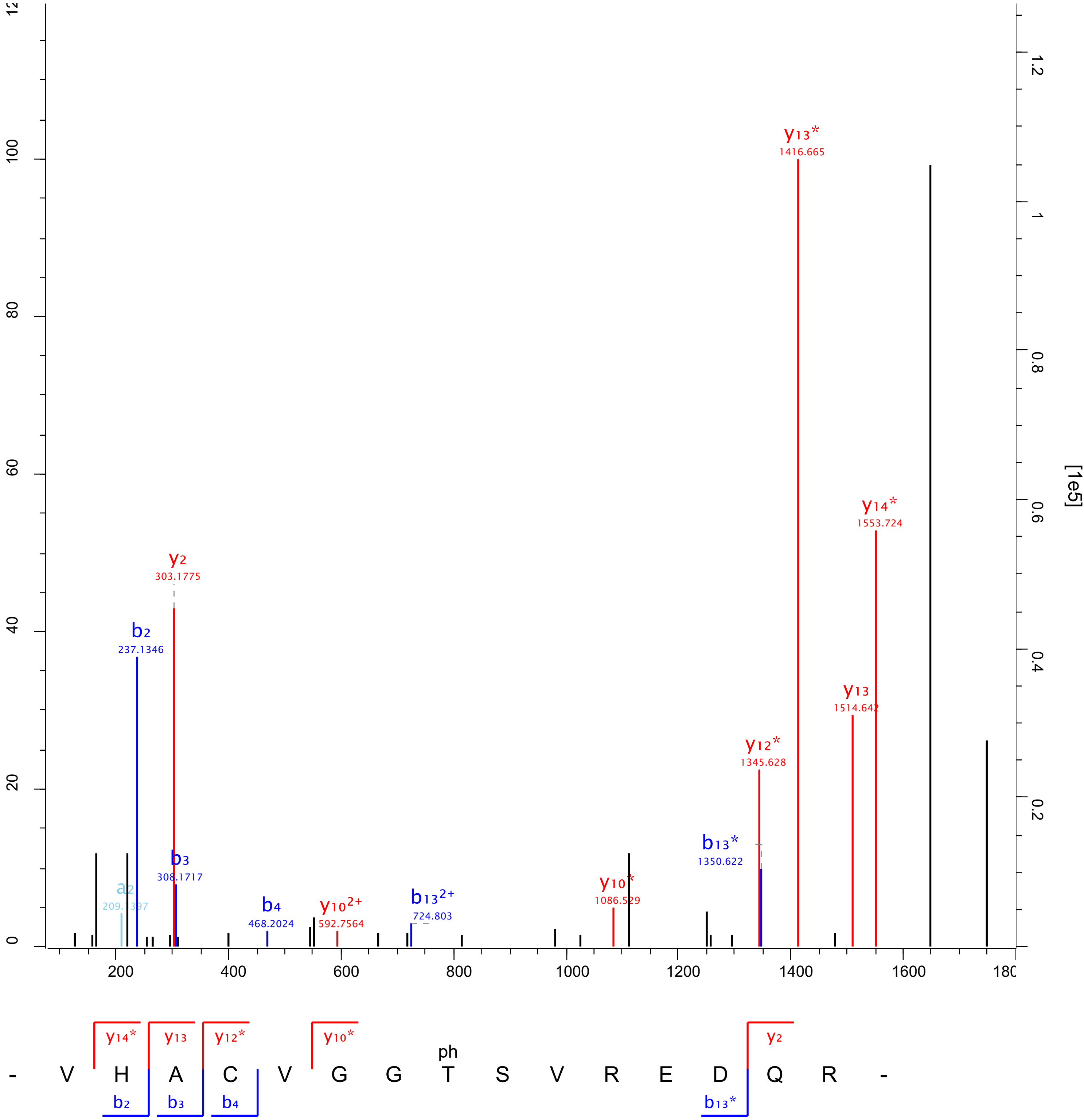

Sample output to test PDF Combine only

Raw file	Scan	Method	Score	m/z
sirk1_sp3-mic-SUC-1-A	4938	FTMS; HCD	41.62	460.55

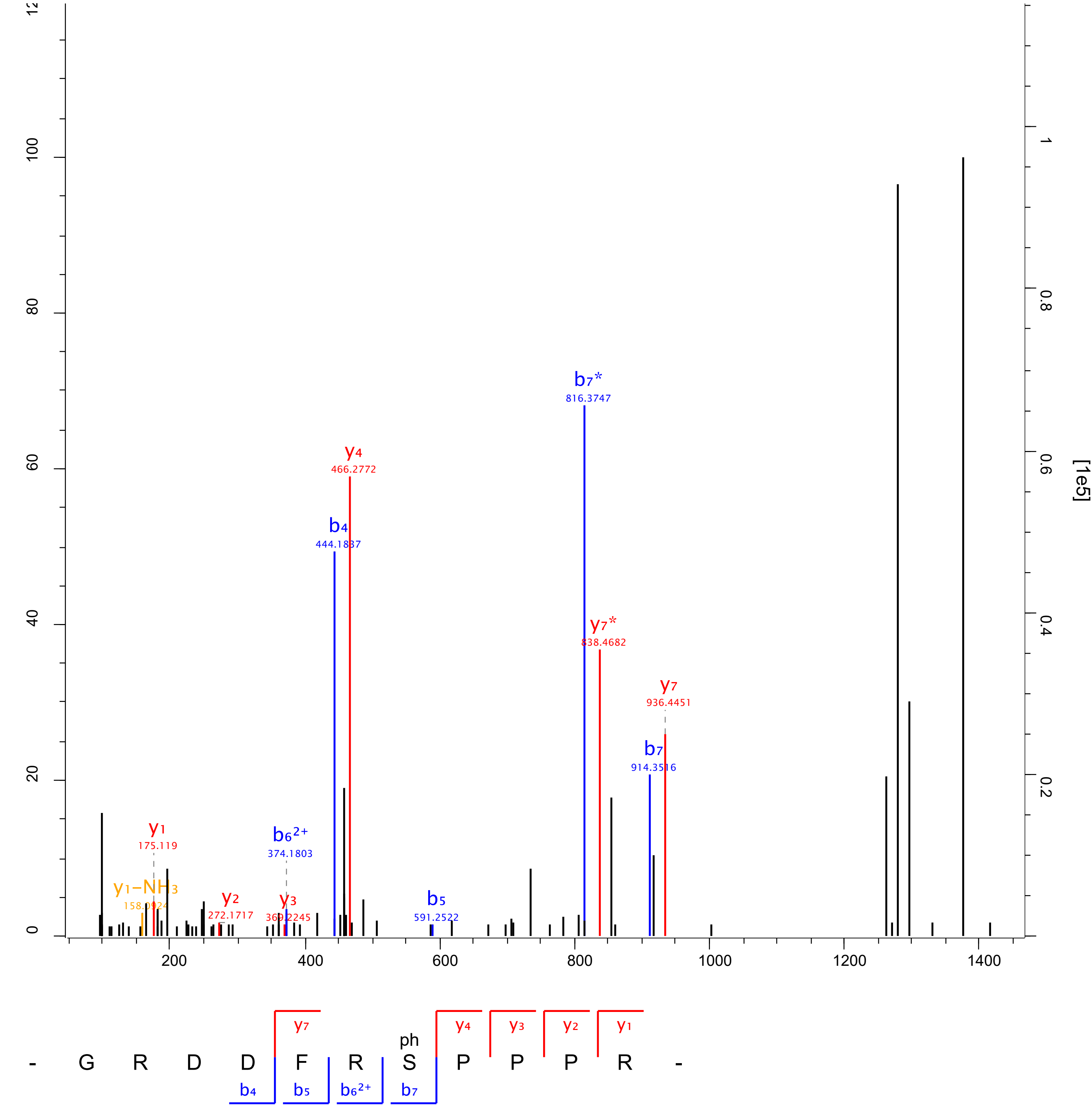

Sample output to test PDF Combine only

Raw file	Scan	Method	Score	m/z	Gene names
sirk1_sp3-mic-SUC-1-A	5747	FTMS; HCD	45	590.27	F23A5.29

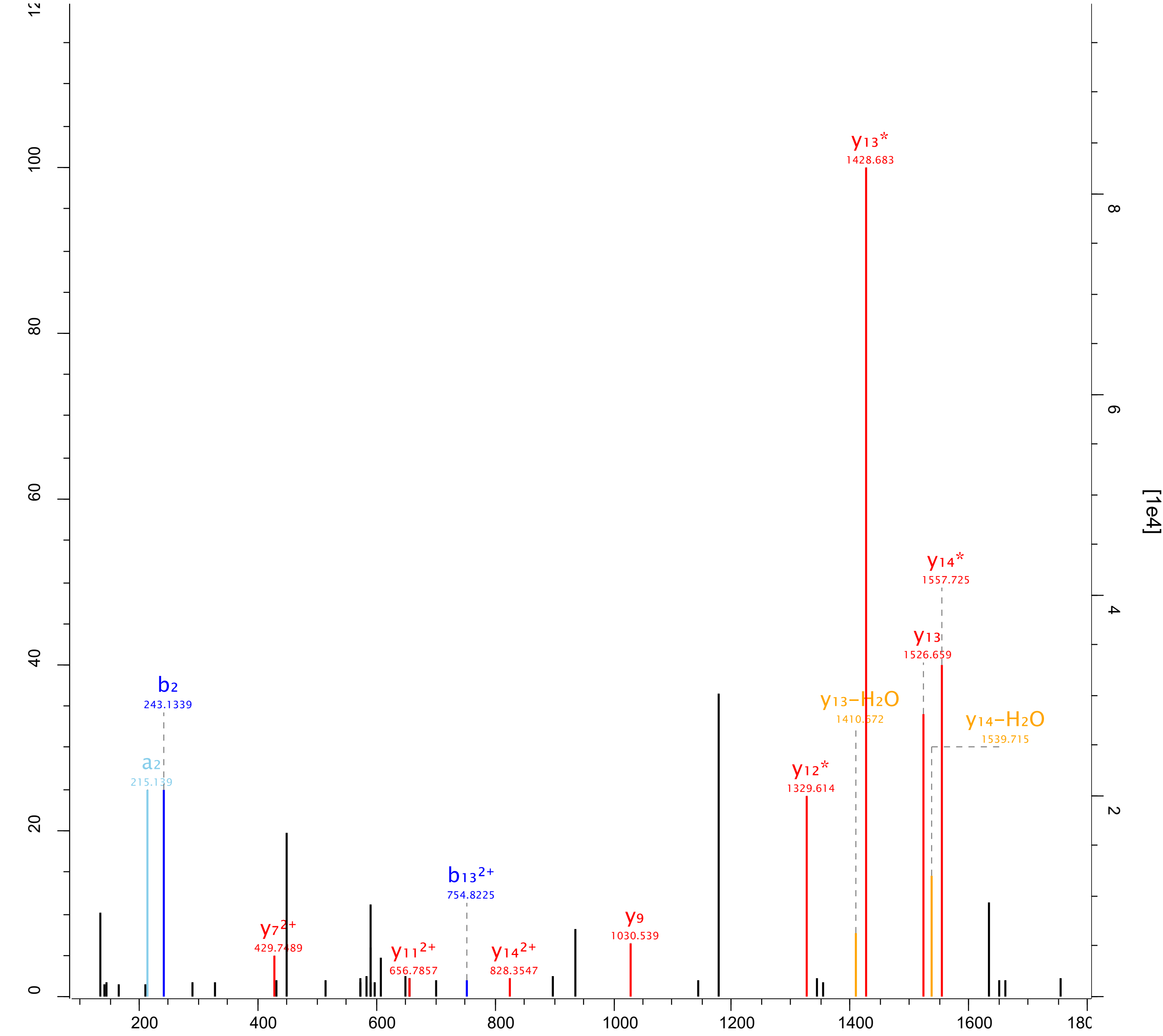

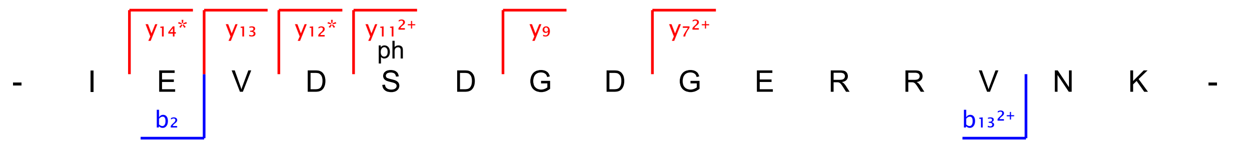

Sample output to test PDF Combine only

Raw file	Scan	Method	Score	m/z	Gene names
sirk1_sp3-mic-SUC-1-A	6404	FTMS; HCD	69.19	534.24	SPK1

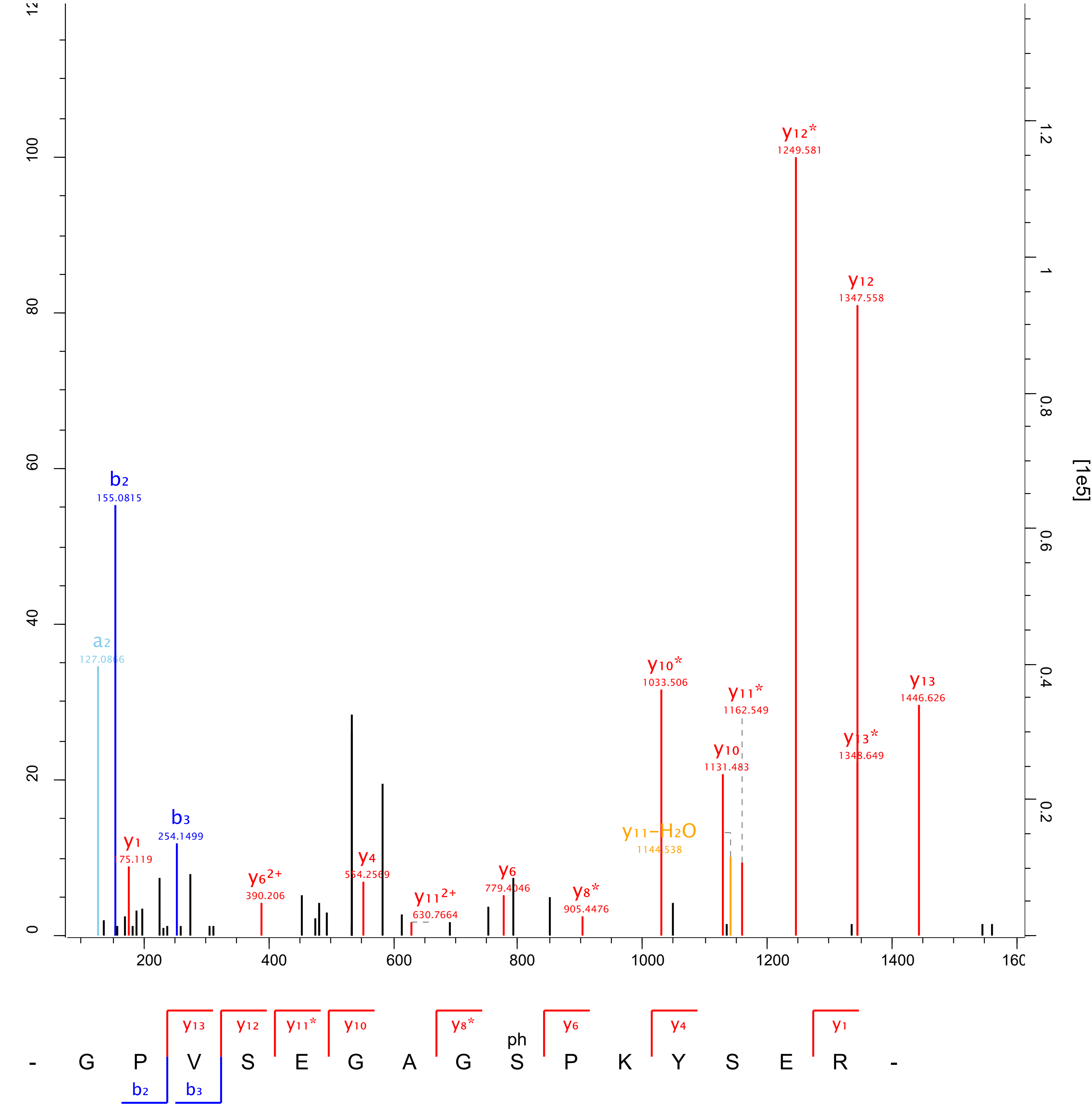

Sample output to test PDF Combine only

Raw file	Scan	Method	Score	m/z
sirk1_sp3-mic-SUC-1-A	6573	FTMS; HCD	135.47	740.32

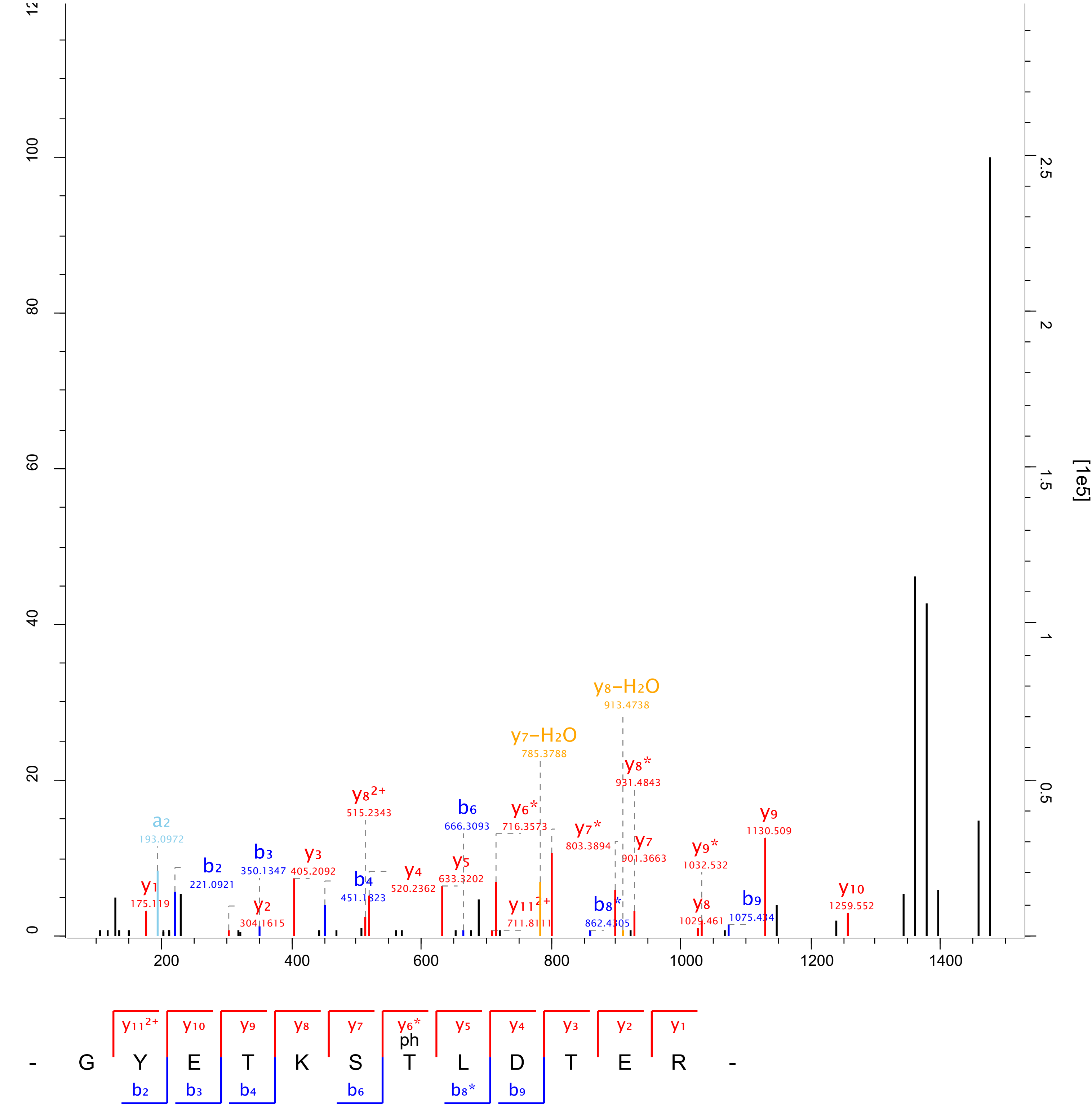

Sample output to test PDF Combine only

Raw file	Scan	Method	Score	m/z	Gene names
sirk1_sp3-mic-SUC-1-A	6856	FTMS; HCD	69.82	488.73	At4g40050

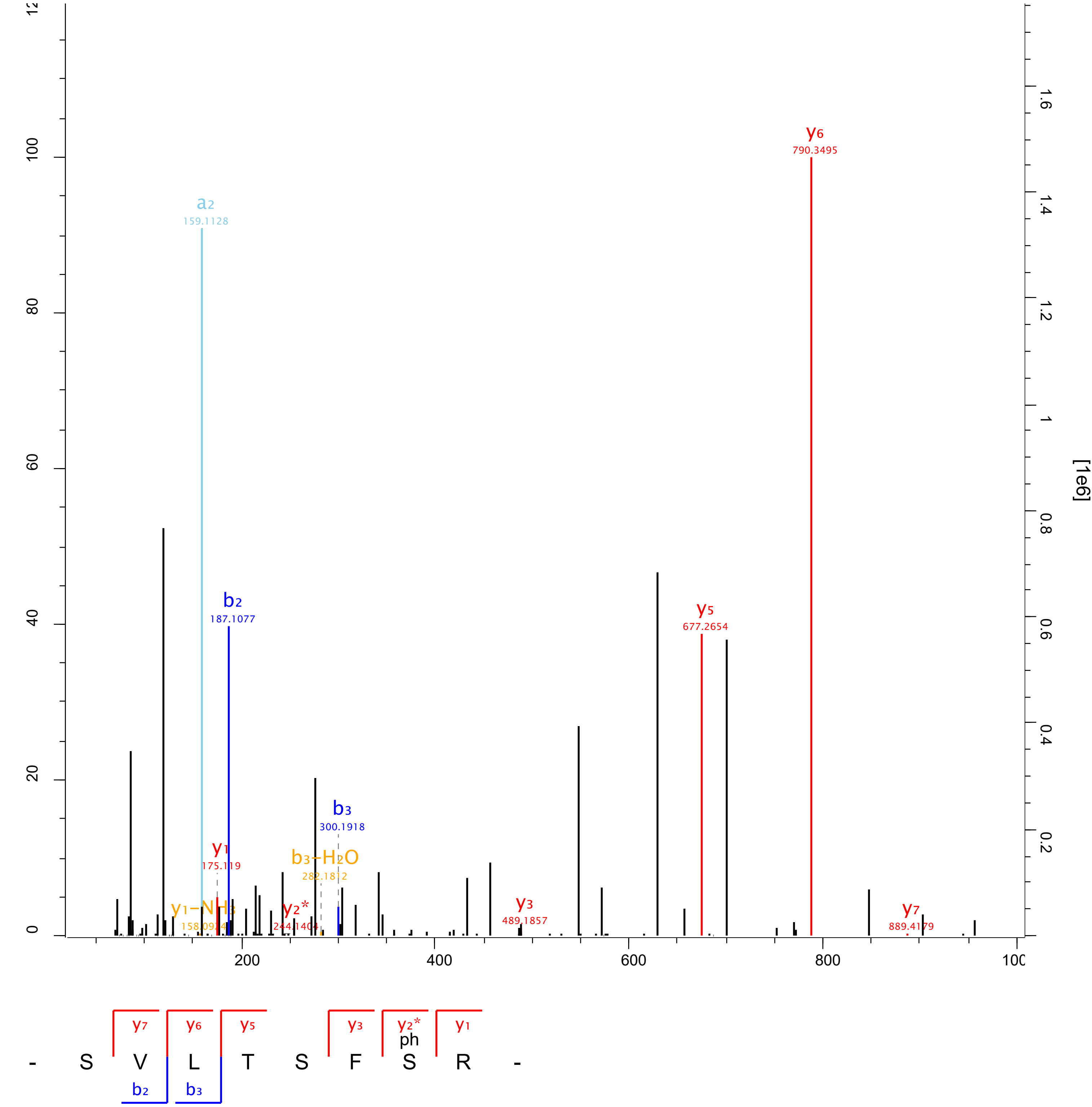

Sample output to test PDF Combine only

Raw file	Scan	Method	Score	m/z	Gene names
sirk1_sp3-mic-SUC-1-A	7568	FTMS; HCD	71.98	515.89	MEB2

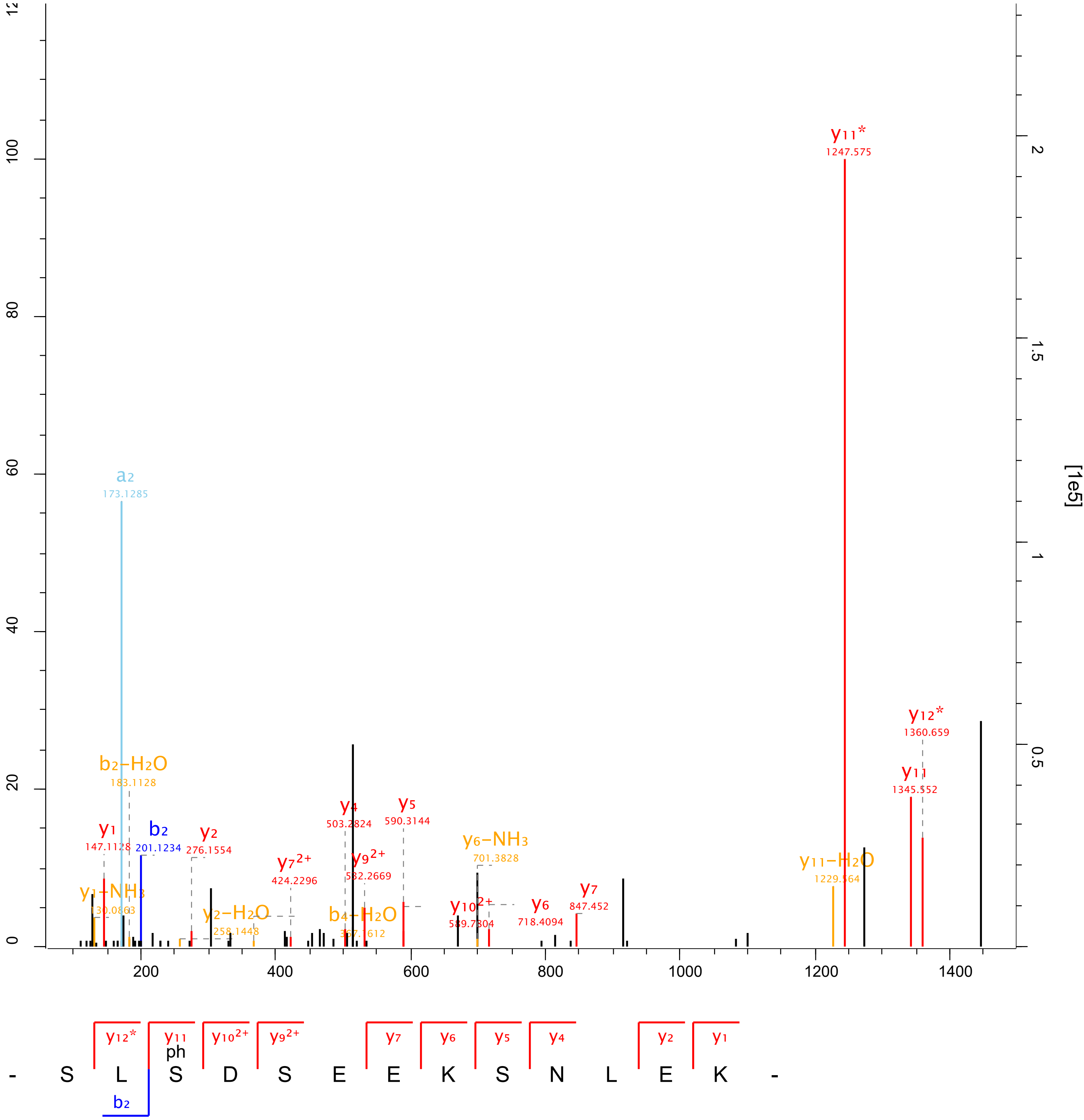

Sample output to test PDF Combine only

Raw file	Scan	Method	Score	m/z	Gene names
sirk1_sp3-mic-SUC-1-A	11773	FTMS; HCD	41.11	681.33	PATL1

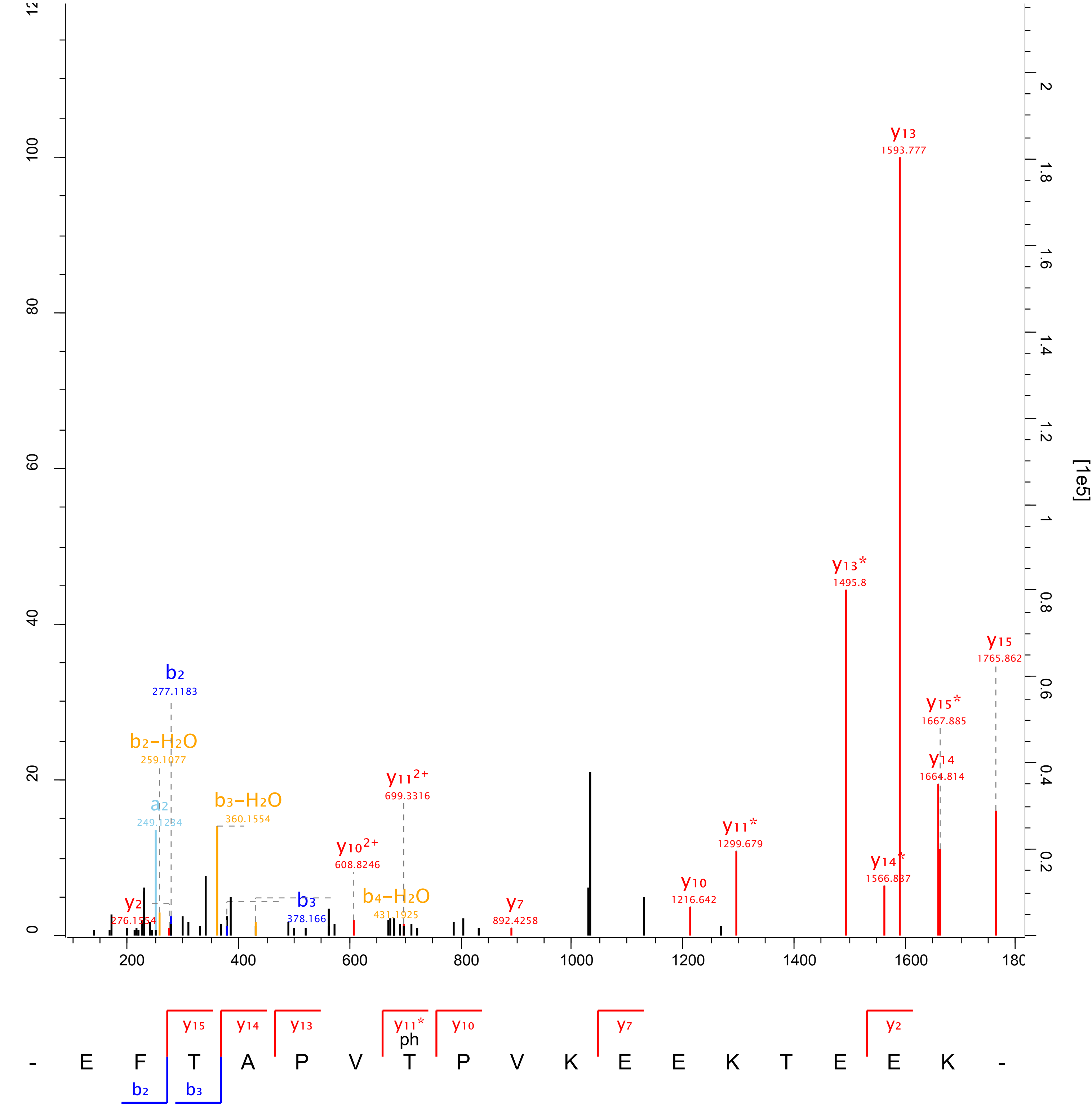

Sample output to test PDF Combine only

Raw file	Scan	Method	Score	m/z	Gene names
sirk1_sp3-mic-SUC-1-A	14558	FTMS; HCD	47.3	460.2	At3g23660;At4g14160

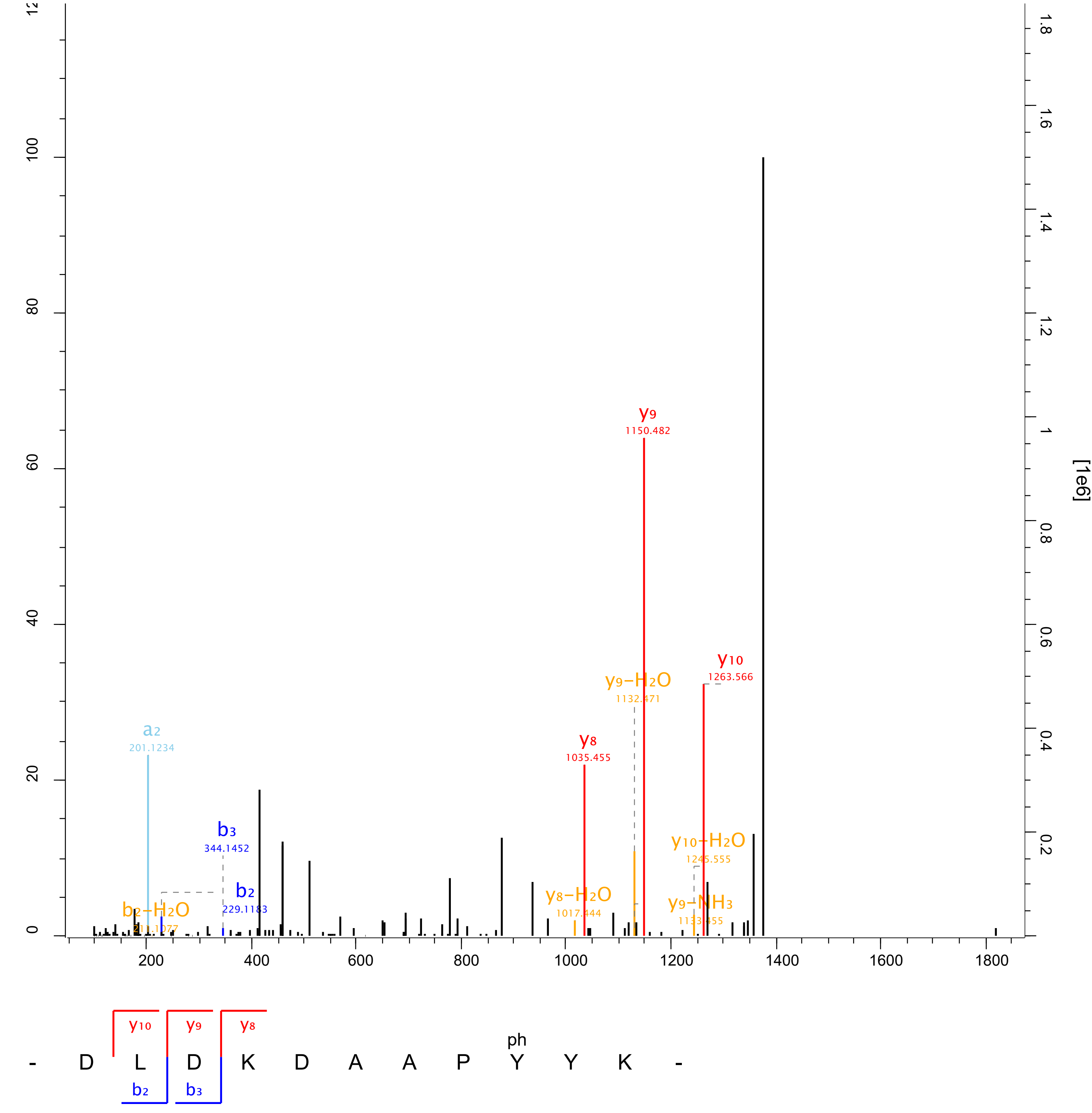

Sample output to test PDF Combine only

Raw file	Scan	Method	Score	m/z	Gene names
sirk1_sp3-mic-SUC-1-A	17137	FTMS; HCD	57.59	670.31	F28G4.18

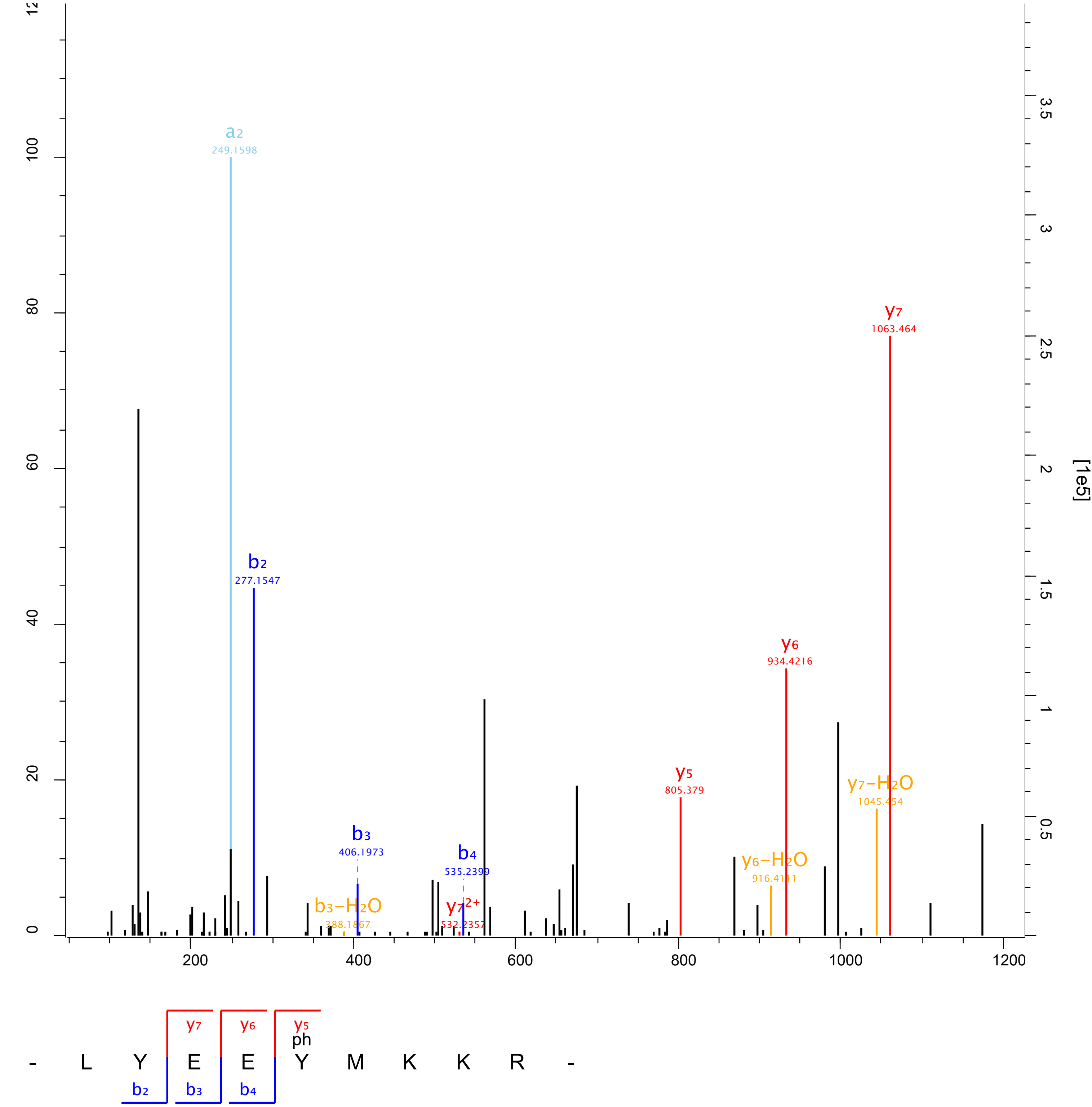

Sample output to test PDF Combine only

Raw file	Scan	Method	Score	m/z	Gene names
sirk1_sp3-mic-SUC-2-P	8673	FTMS; HCD	56.3	544.26	F18B13.26

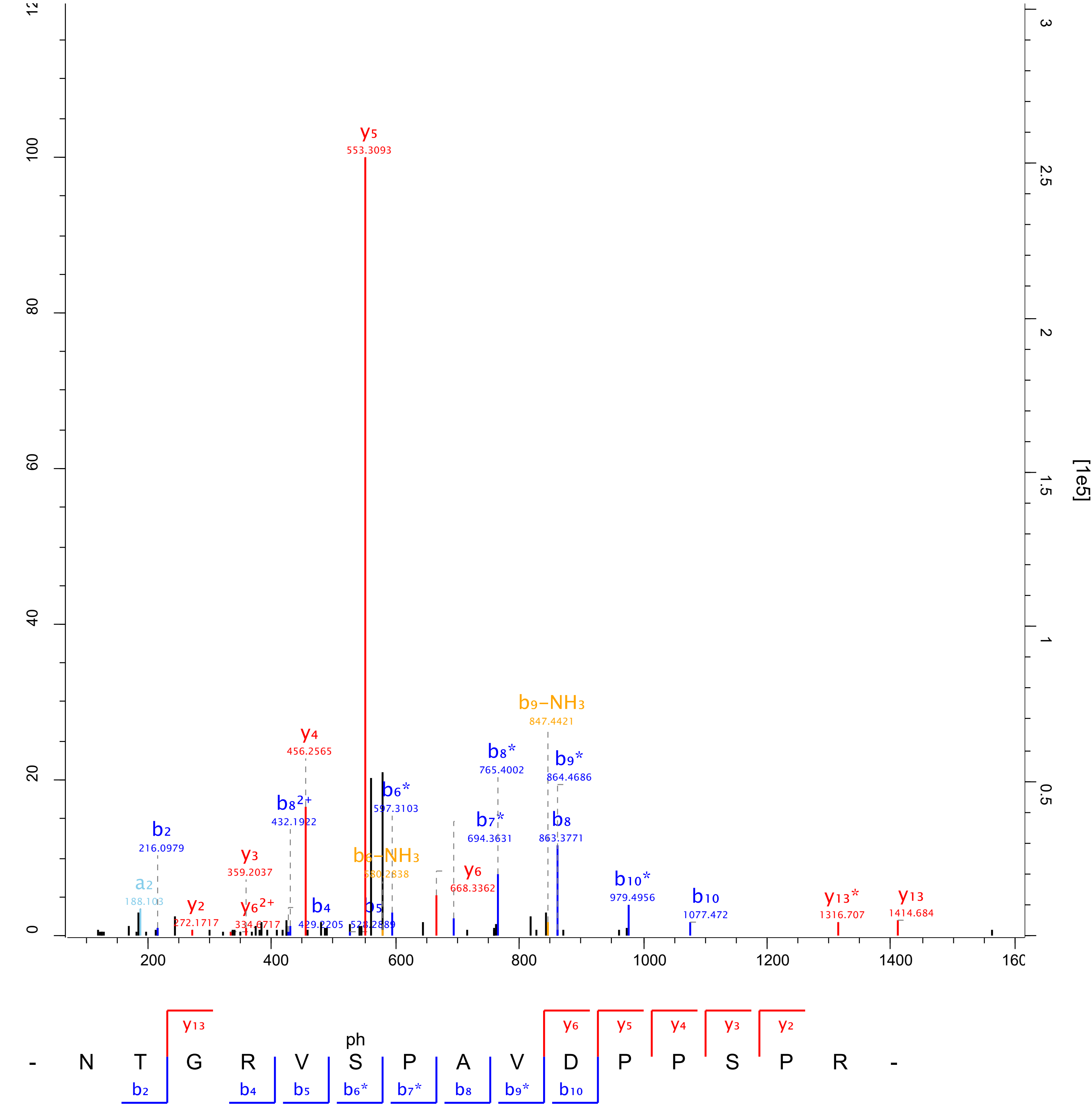

Sample output to test PDF Combine only

Raw file	Scan	Method	Score	m/z	Gene names
sirk1_sp3-mic-SUC-2-P	24569	FTMS; HCD	54.56	661.66	RZ1C

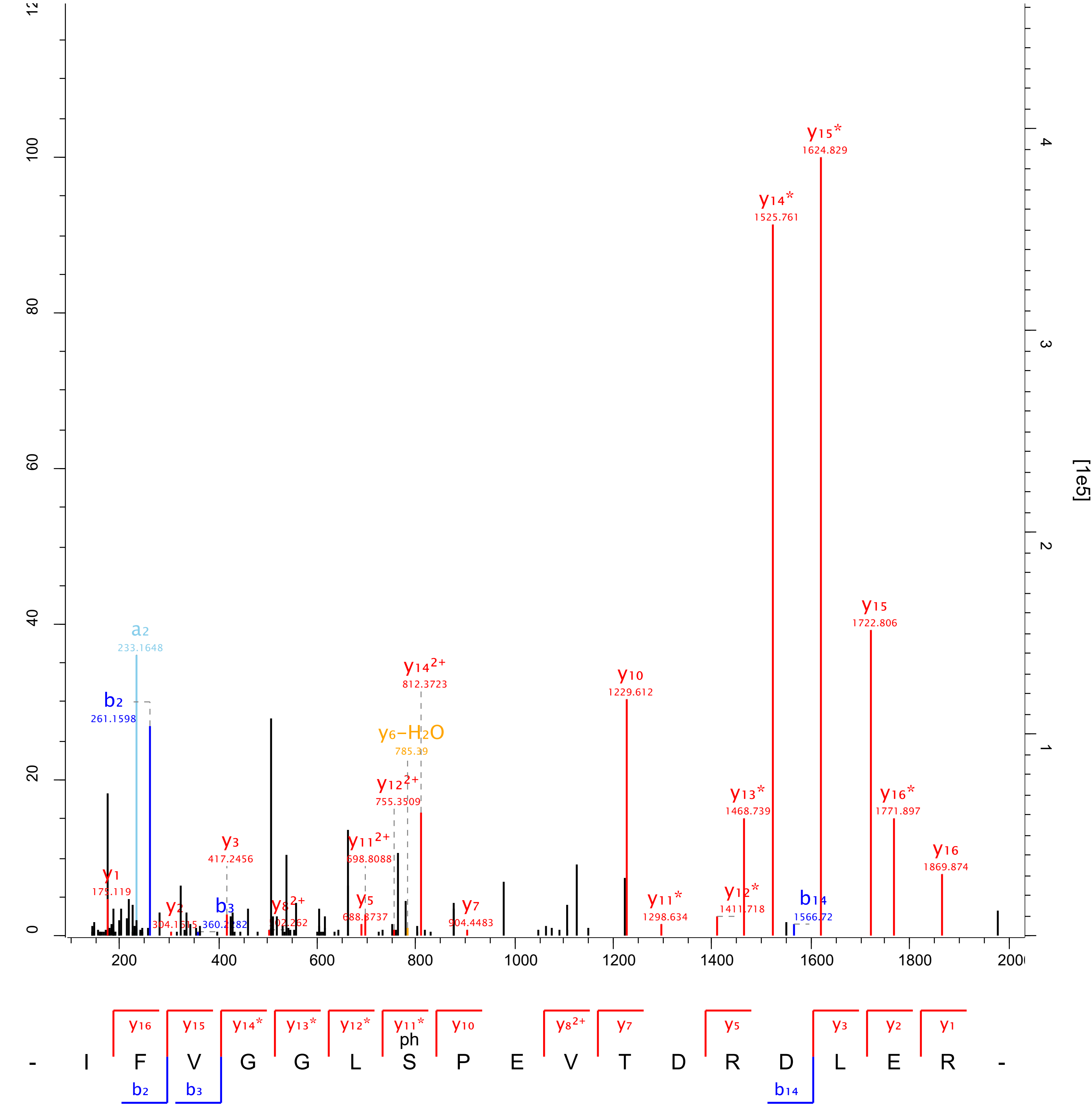

Sample output to test PDF Combine only

Raw file	Scan	Method	Score	m/z	Gene names
sirk1_sp3-mic-SUC-2-P	24812	FTMS; HCD	72.23	864.09	SC3;SCAMP3

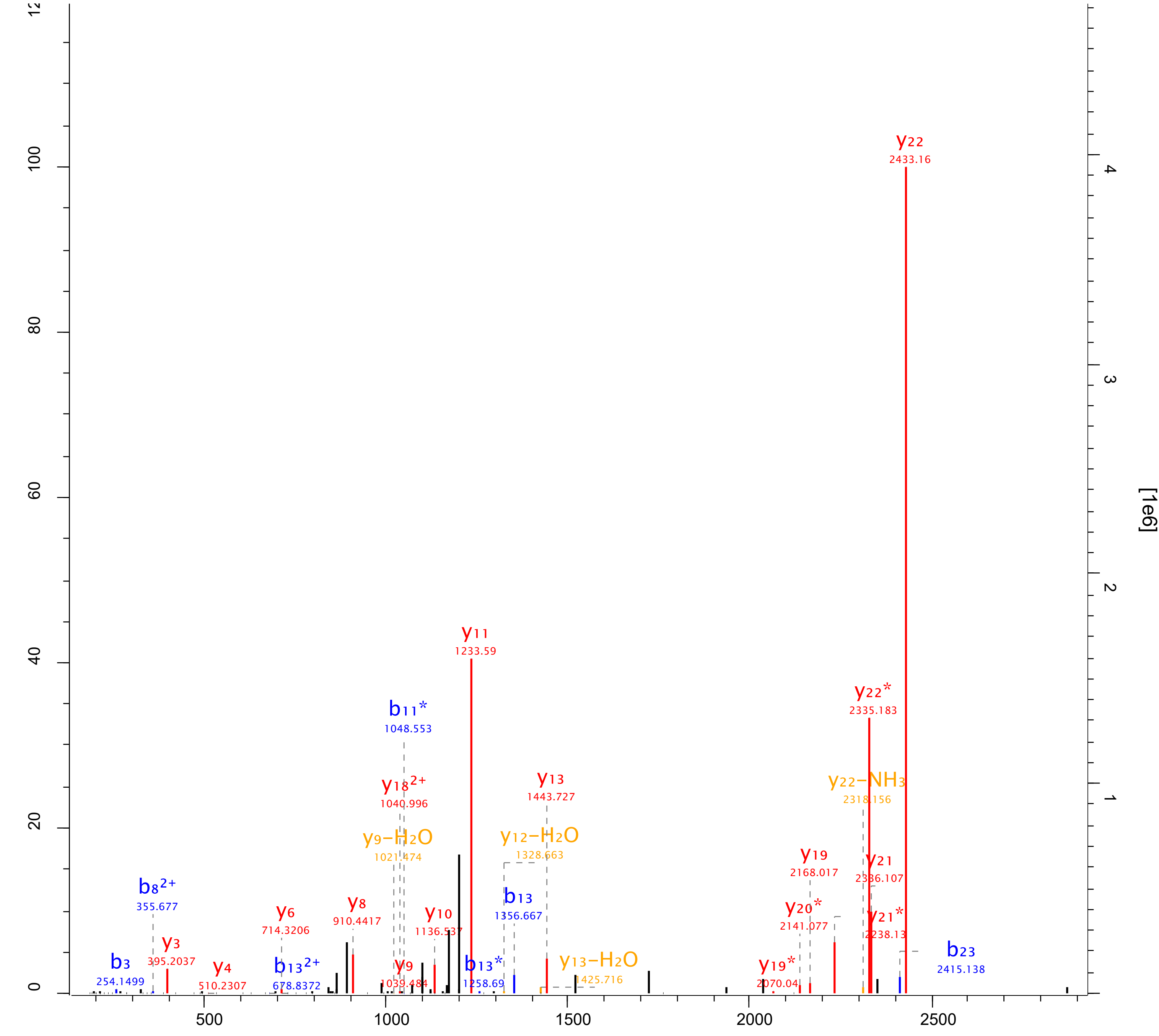

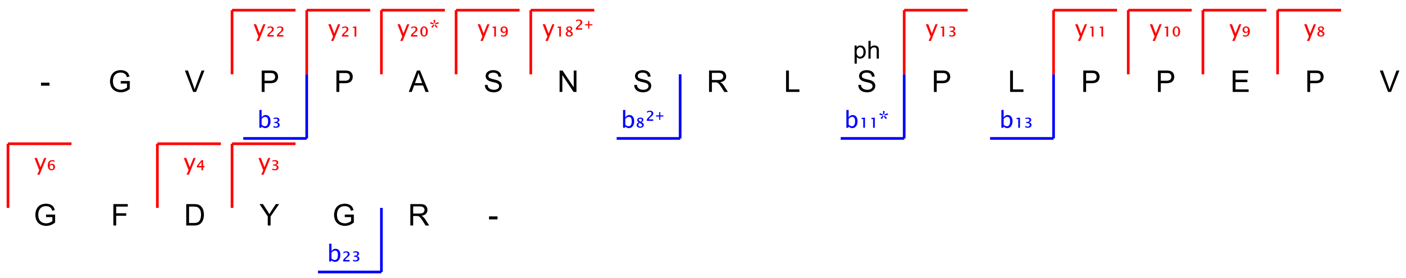

Sample output to test PDF Combine only

Raw file	Scan	Method	Score	m/z	Gene names
sirk1_sp3-mic-SUC-2-P	25185	FTMS; HCD	98.16	515.26	HIR3;HIR1

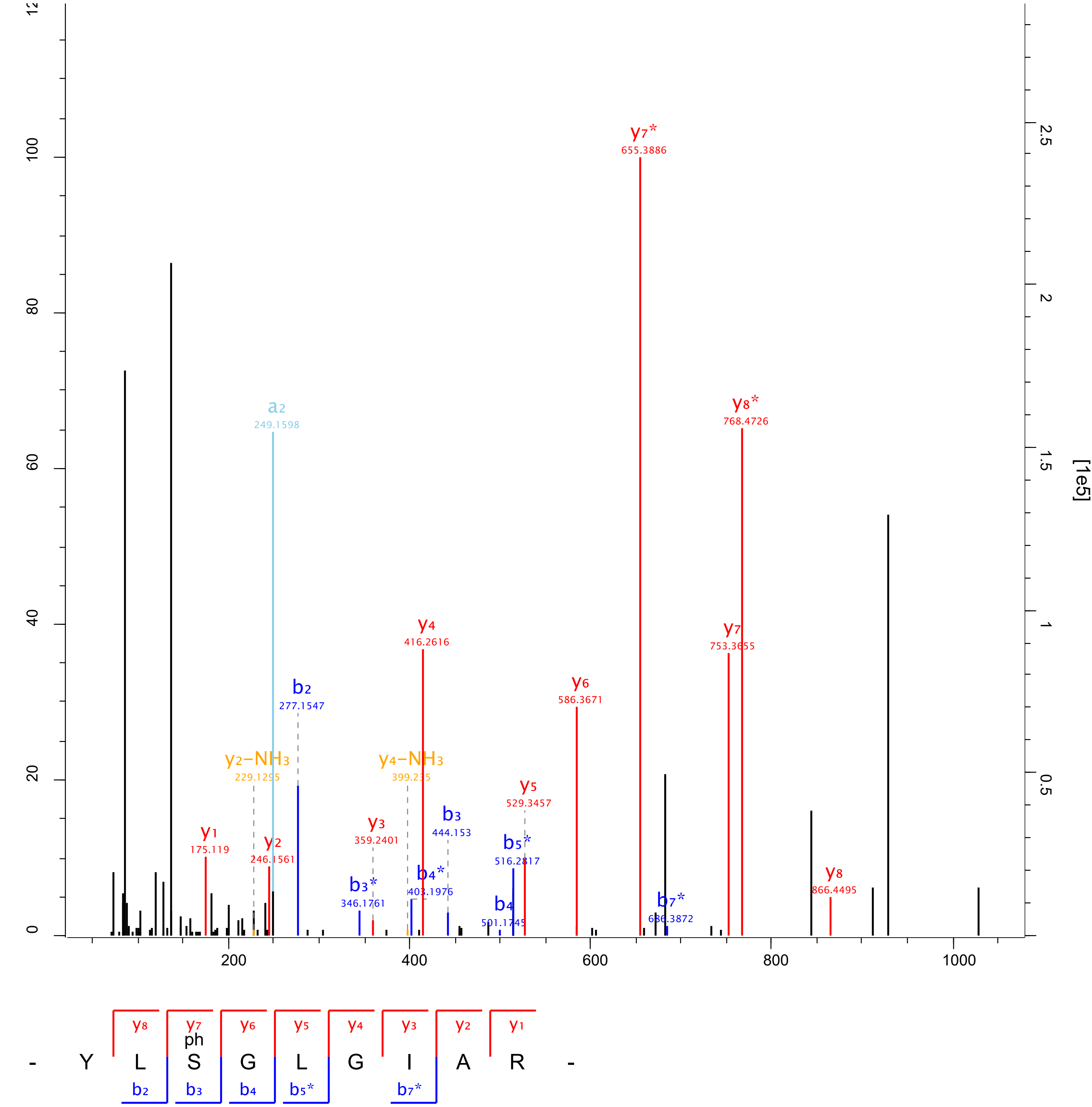

Sample output to test PDF Combine only

Raw file	Scan	Method	Score	m/z	Gene names
sirk1_sp3-mic-SUC-3-P	7147	FTMS; HCD	48.29	475.91	RPS6A;RPS6

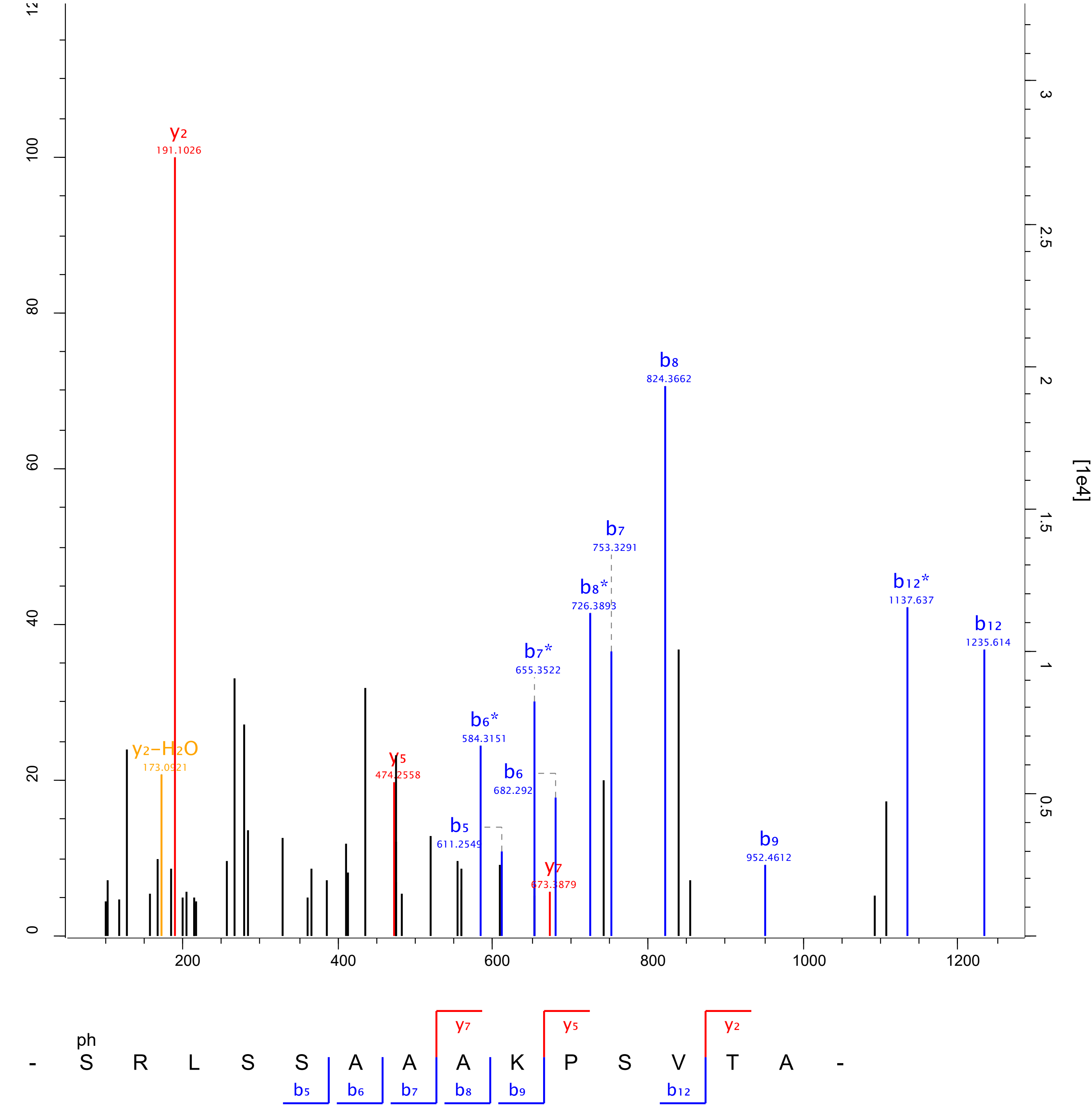

Sample output to test PDF Combine only

Raw file	Scan	Method	Score	m/z	Gene names
sirk1_sp3-mic-SUC-3-P	20635	FTMS; HCD	109.72	551.77	CLC-C

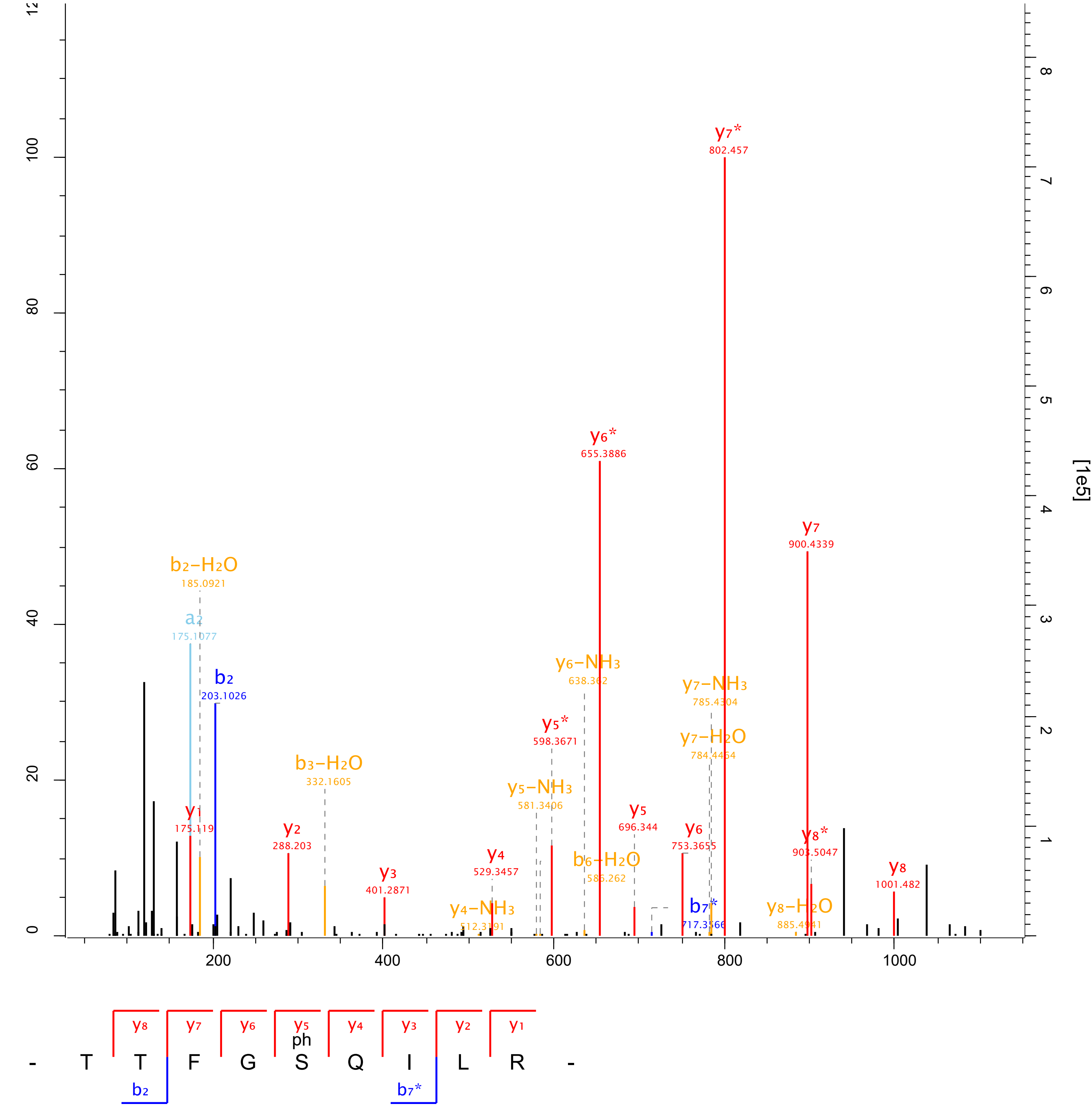

Sample output to test PDF Combine only

Raw file	Scan	Method	Score	m/z	Gene names
sirk1_sp3-mic-SUC-3-P	32194	FTMS; HCD	48.9	993.95	FH21B

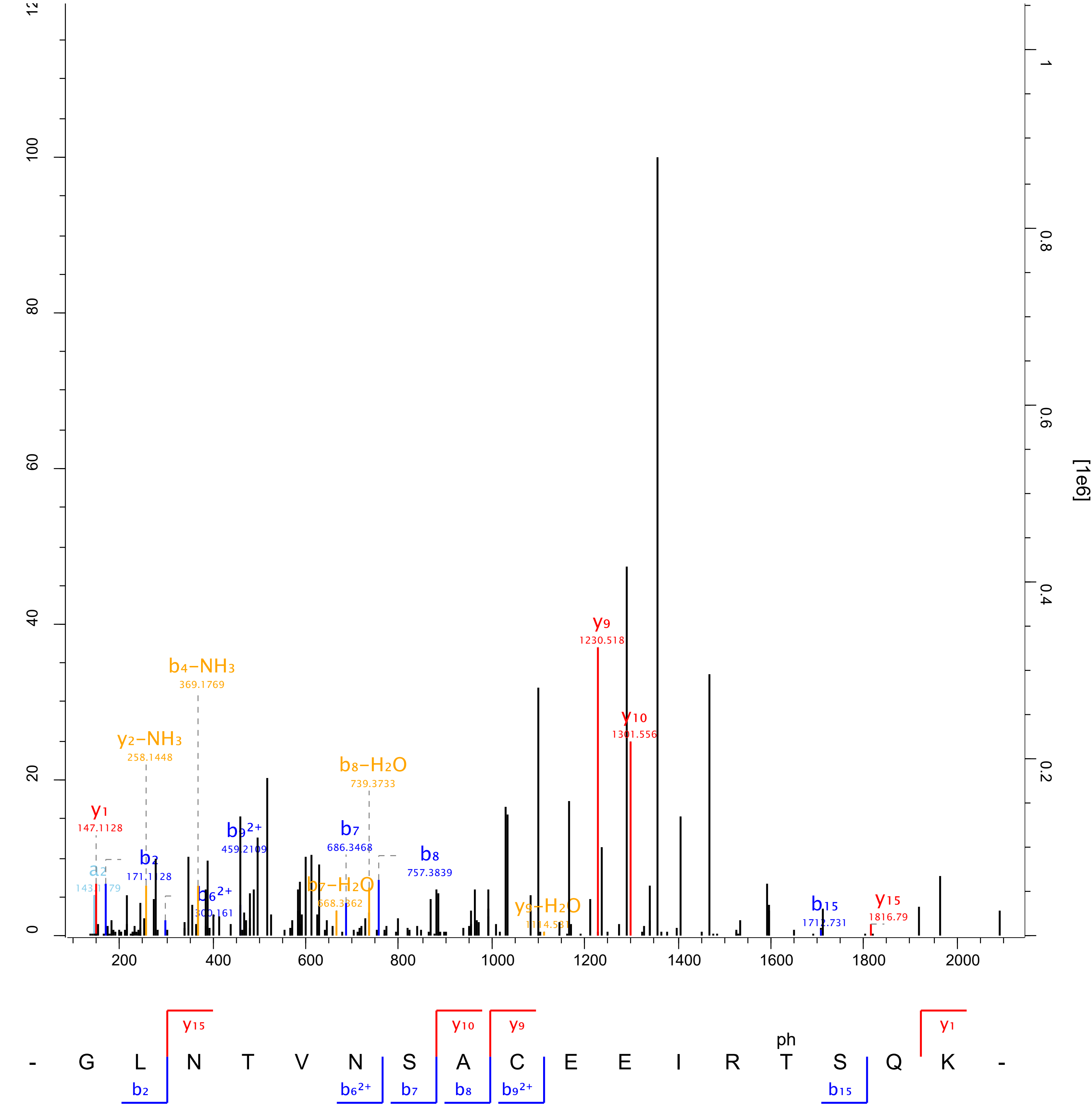

Sample output to test PDF Combine only

Raw file	Scan	Method	Score	m/z	Gene names
sirk1_sp3-solu-0-1-A	10914	FTMS; HCD	148.52	672.32	ERD14

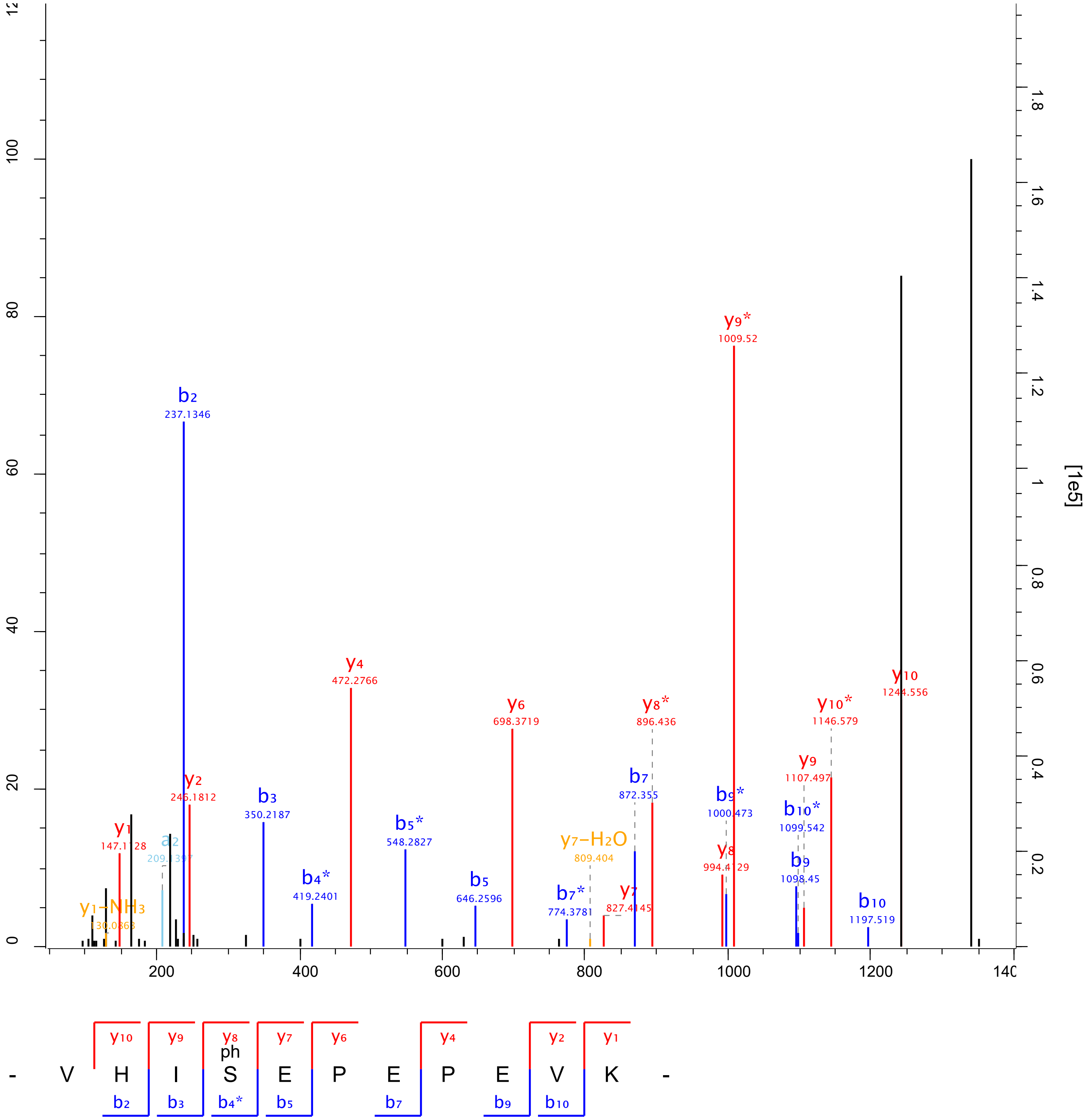

Sample output to test PDF Combine only

Raw file	Scan	Method	Score	m/z	Gene names
sirk1_sp3-solu-0-2-A	17246	FTMS; HCD	220.88	976.95	At5g63550

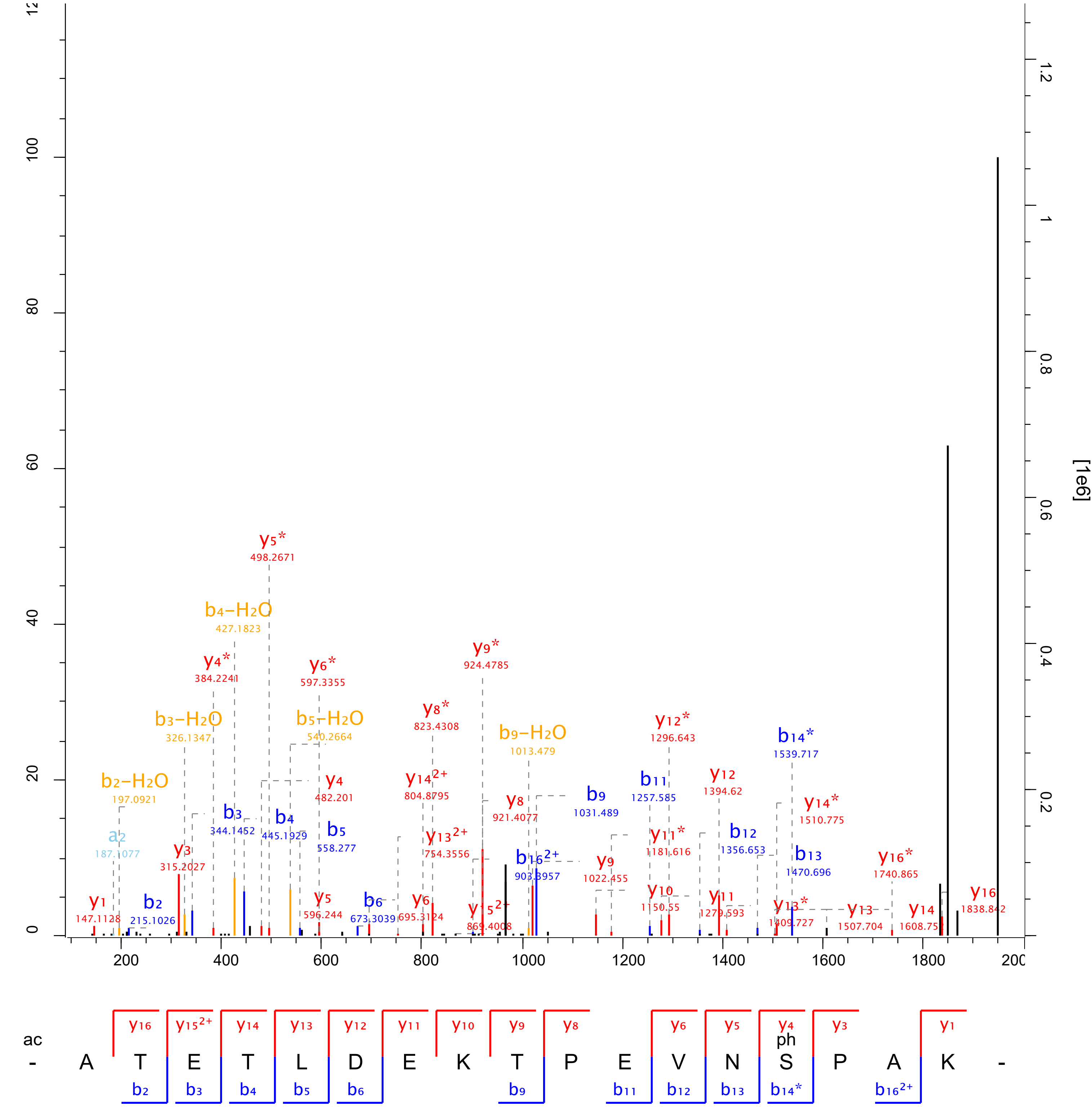

Sample output to test PDF Combine only

Raw file	Scan	Method	Score	m/z	Gene names
sirk1_sp3-solu-0-2-P	24434	FTMS; HCD	78.55	533.28	PAH2

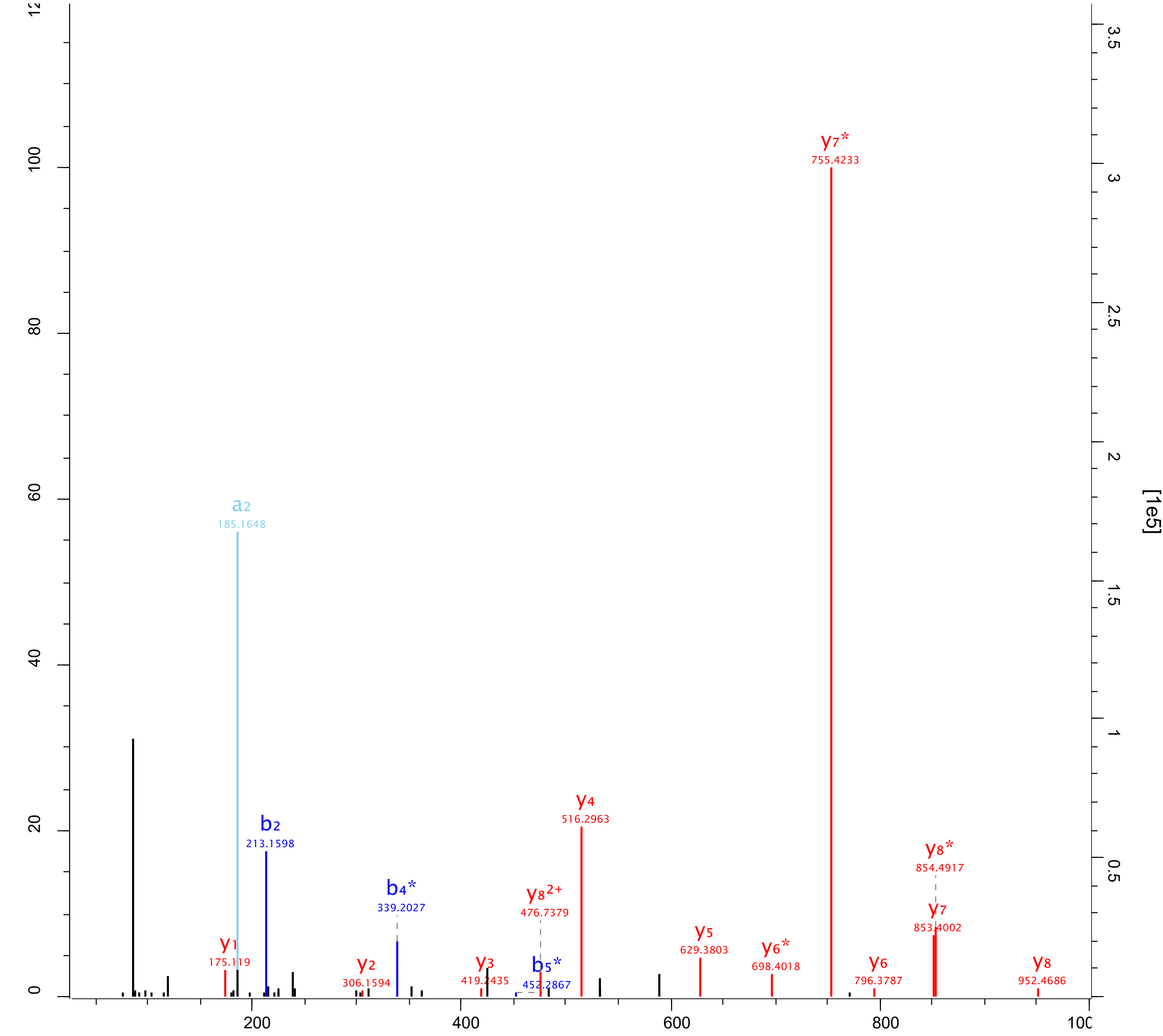

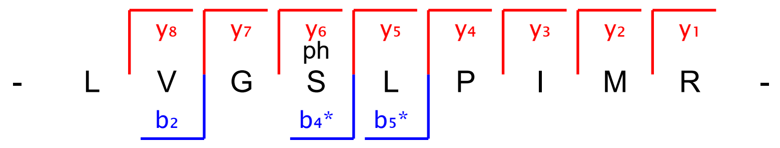

Sample output to test PDF Combine only

Raw file	Scan	Method	Score	m/z	Gene names
sirk1_sp3-solu-0-3-P	17700	FTMS; HCD	60.33	528.26	At4g24100

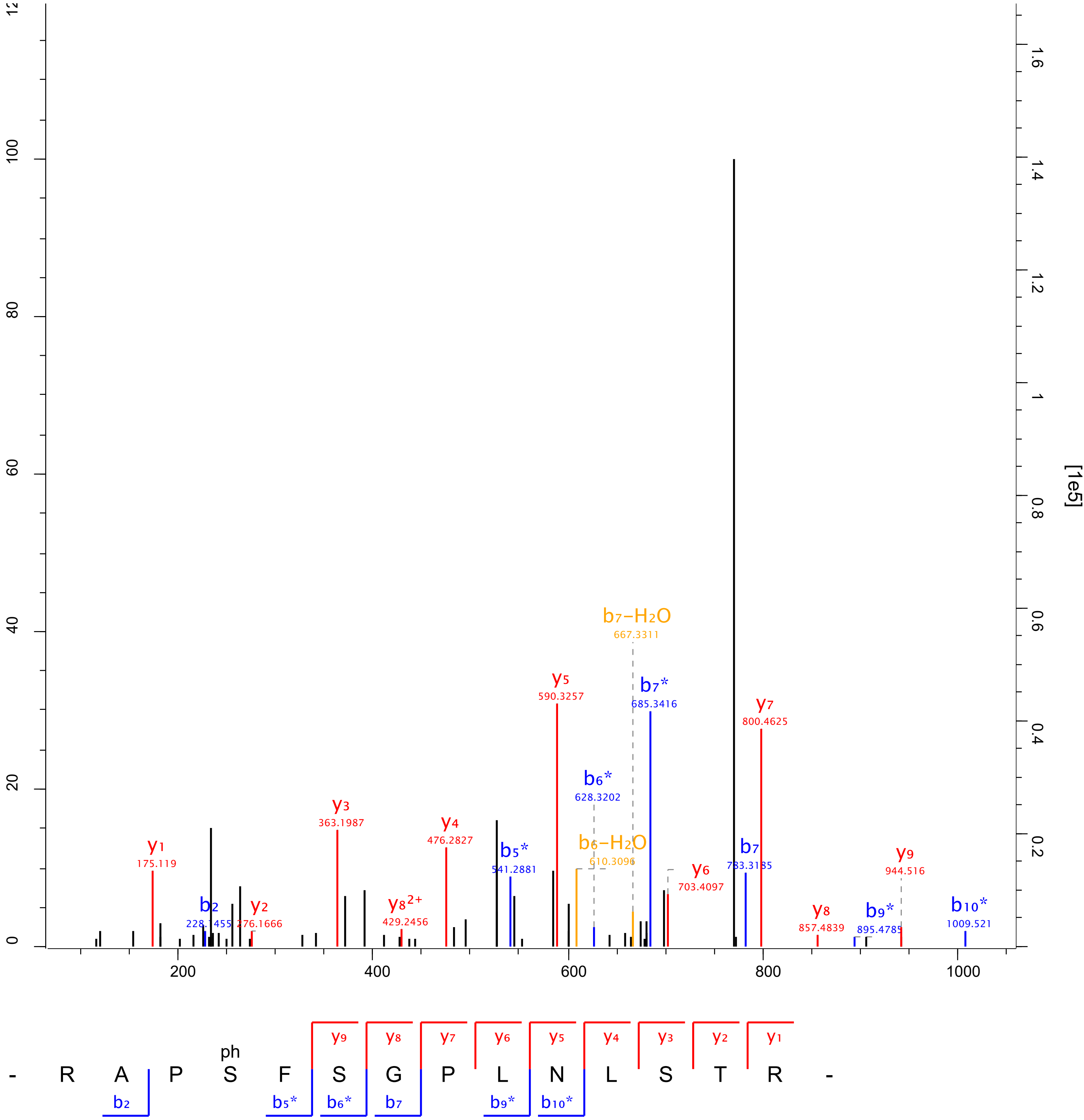

Sample output to test PDF Combine only

Raw file	Scan	Method	Score	m/z	Gene names
sirk1_sp3-solu-SUC-1-A	22396	FTMS; HCD	44.56	724.82	DUF4

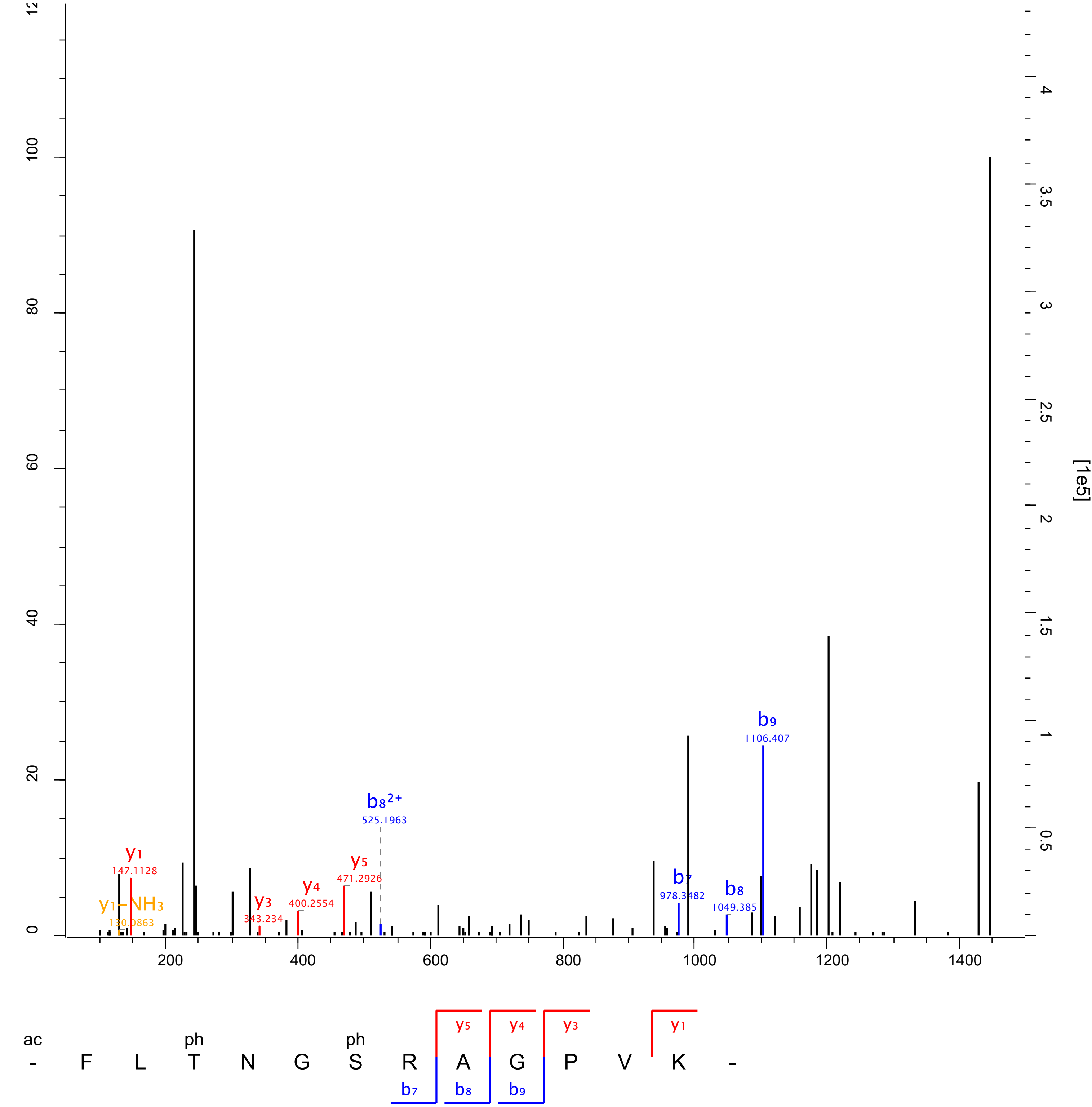

Sample output to test PDF Combine only

Raw file	Scan	Method	Score	m/z
sirk1_sp3-solu-SUC-1-P	18524	FTMS; HCD	85.35	663.31

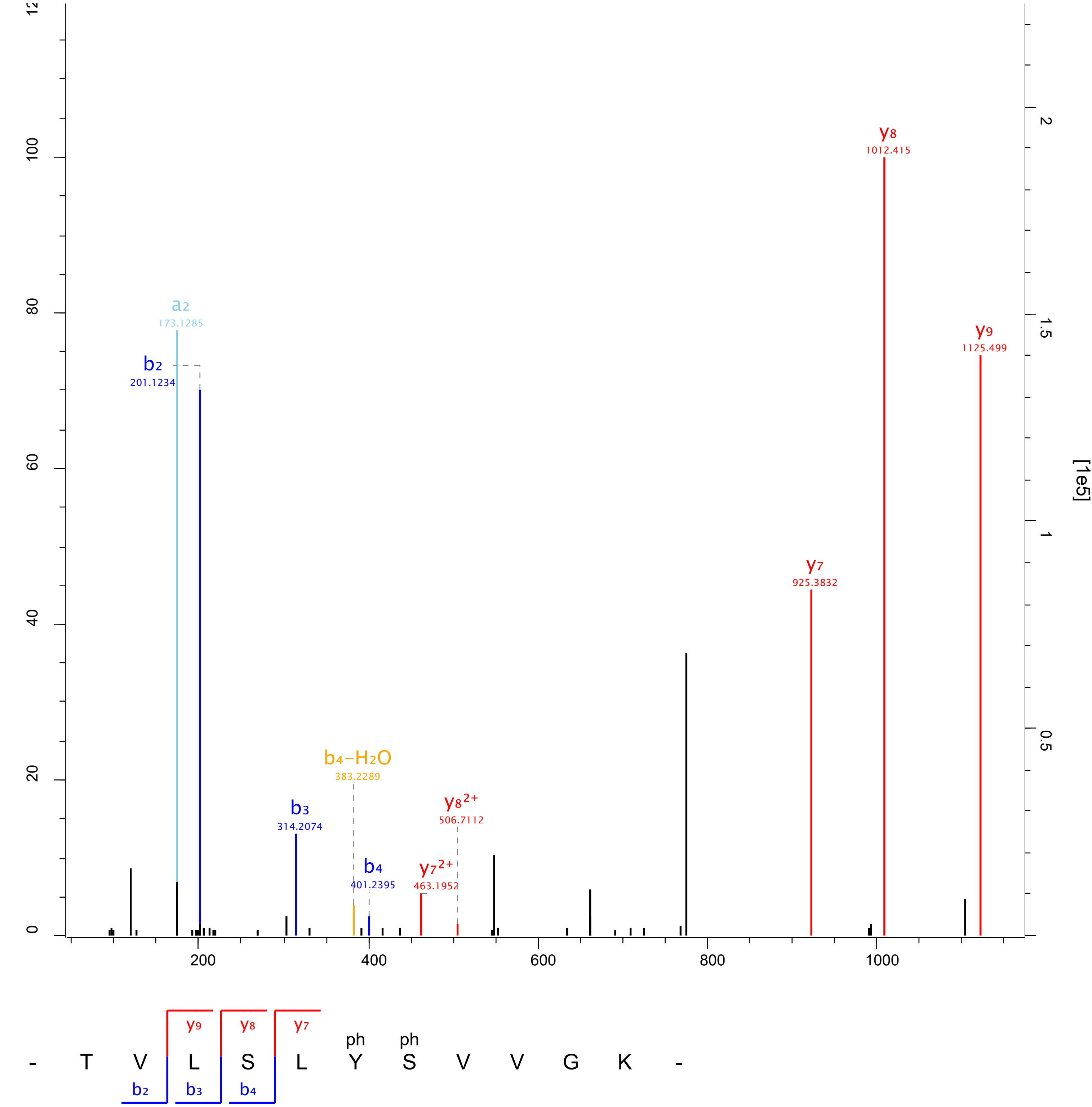

Sample output to test PDF Combine only

Raw file	Scan	Method	Score	m/z	Gene names
sirk1_sp3-solu-SUC-1-P	23266	FTMS; HCD	89.99	651.3	PATL3

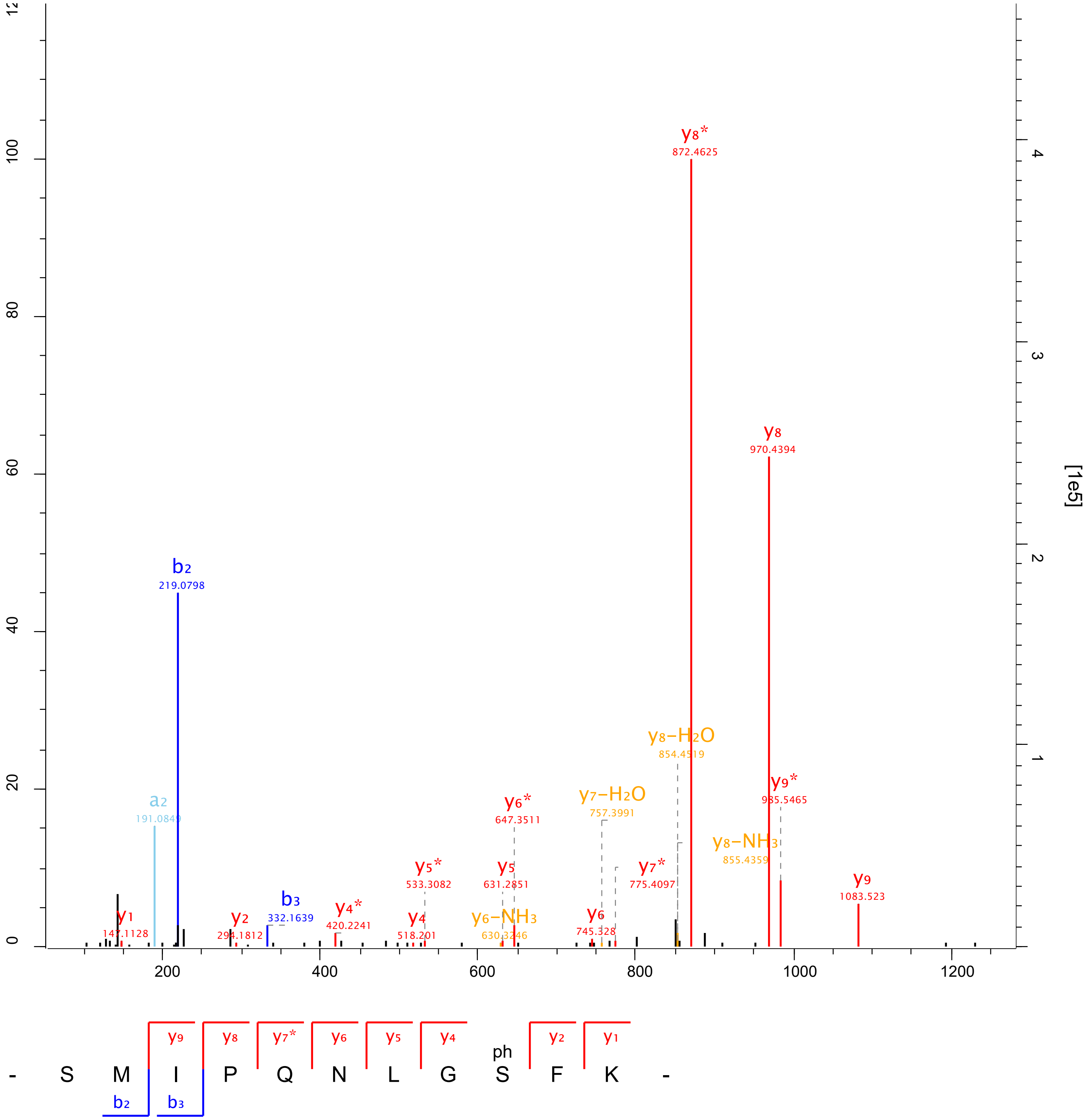

Sample output to test PDF Combine only

Raw file	Scan	Method	Score	m/z	Gene names
sirk1_sp3-solu-SUC-2-A	9465	FTMS; HCD	133.68	734.35	SUMO2

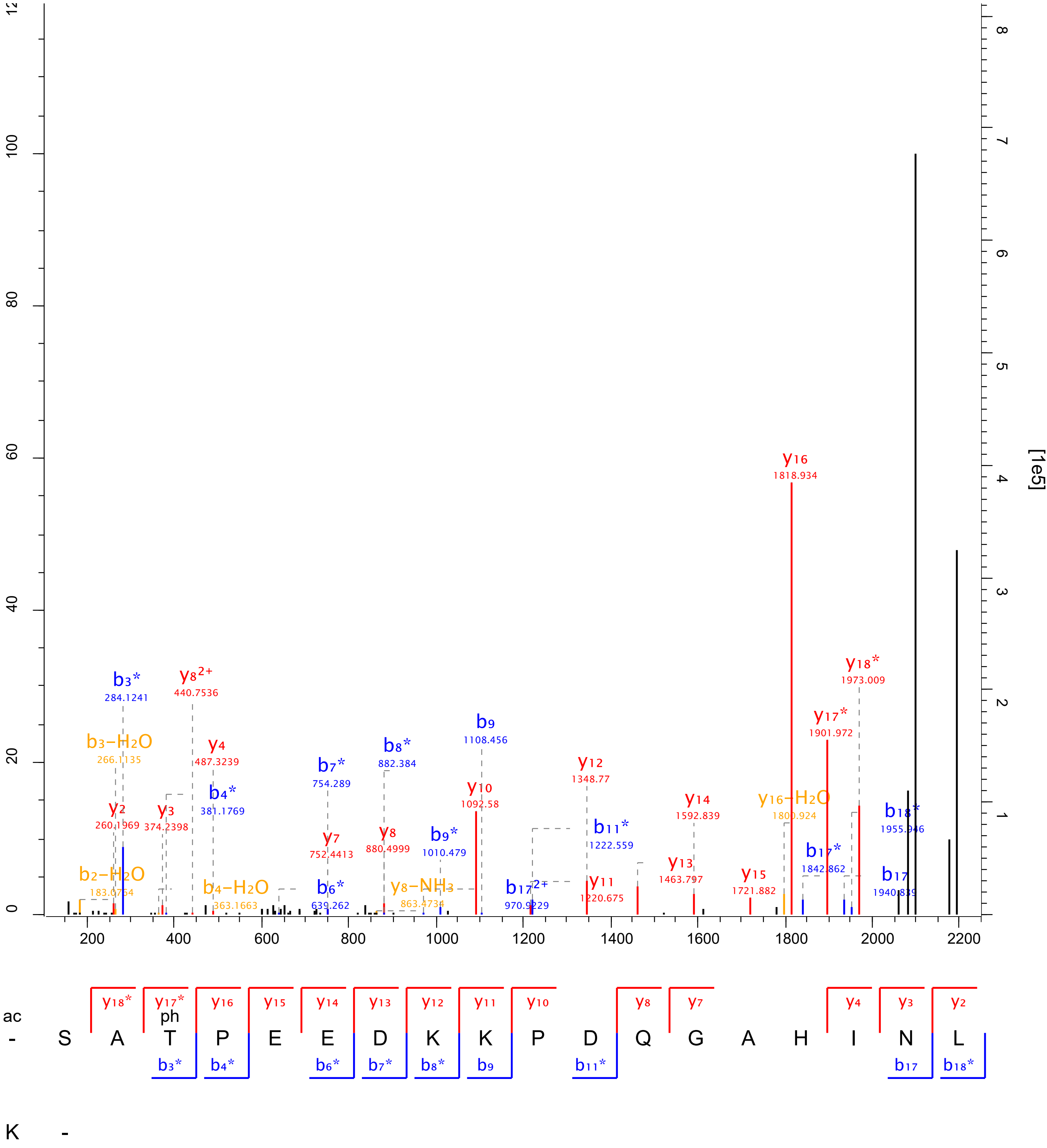

Sample output to test PDF Combine only

Raw file	Scan	Method	Score	m/z	Gene names
sirk1_sp3-solu-SUC-2-A	10406	FTMS; HCD	81.92	563.77	IQD14

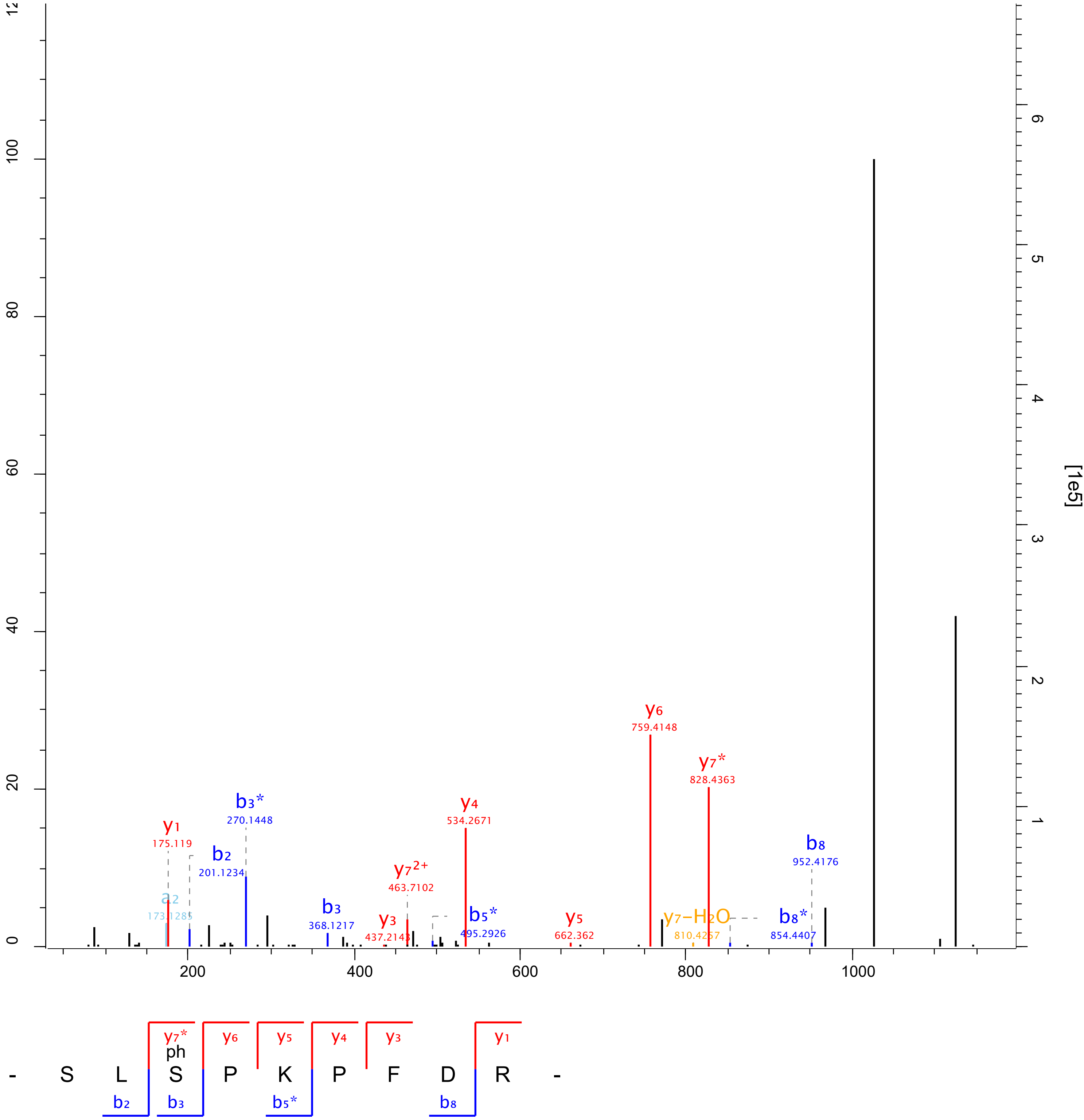

Sample output to test PDF Combine only

Raw file	Scan	Method	Score	m/z	Gene names
sirk1_sp3-solu-SUC-2-A	17470	FTMS; HCD	91.96	542.75	PPC3;PPC2;At3g42628

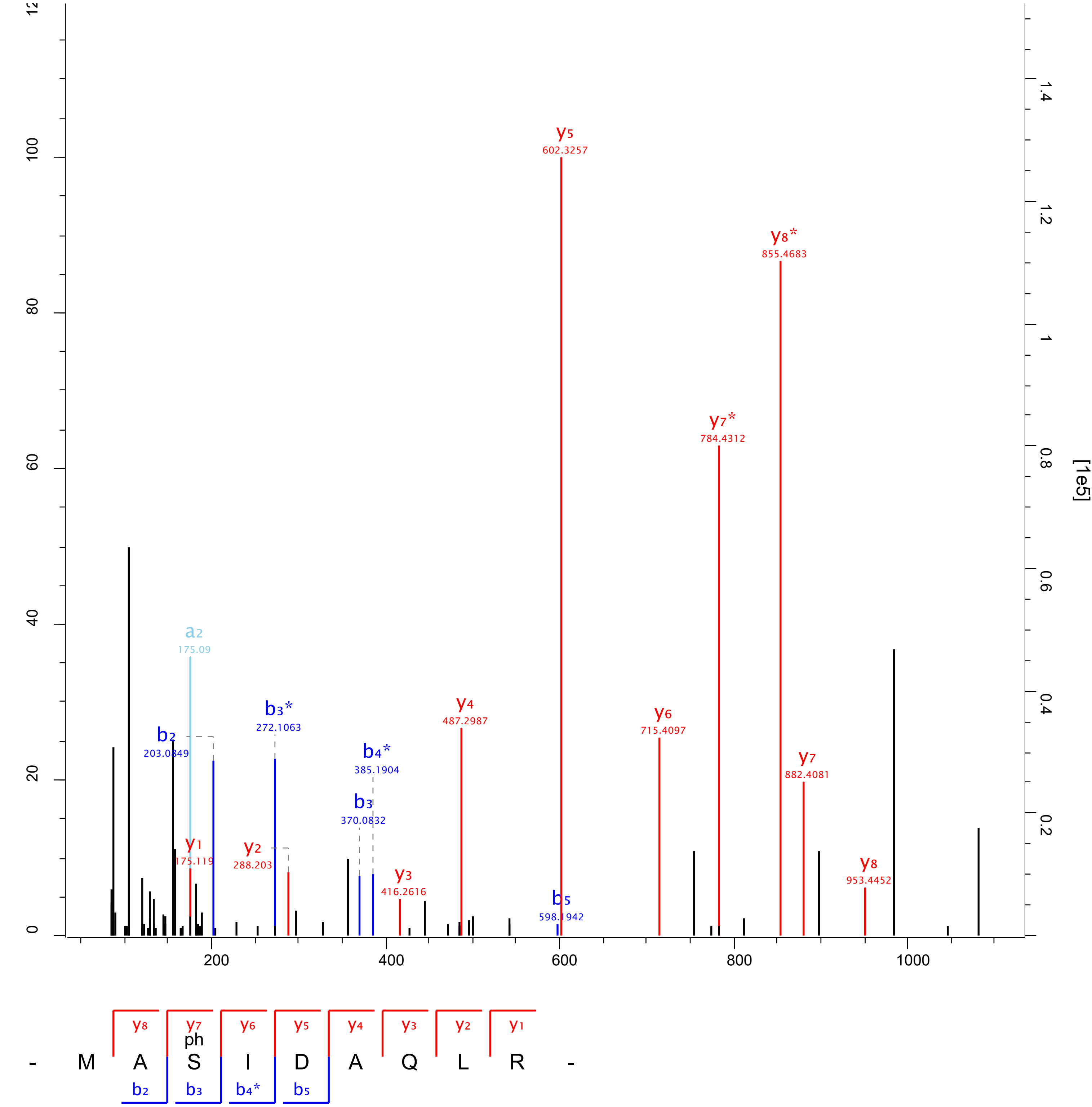

Sample output to test PDF Combine only

Raw file	Scan	Method	Score	m/z	Gene names
sirk1_sp3-solu-SUC-2-A	18256	FTMS; HCD	78.33	626.81	AHL6

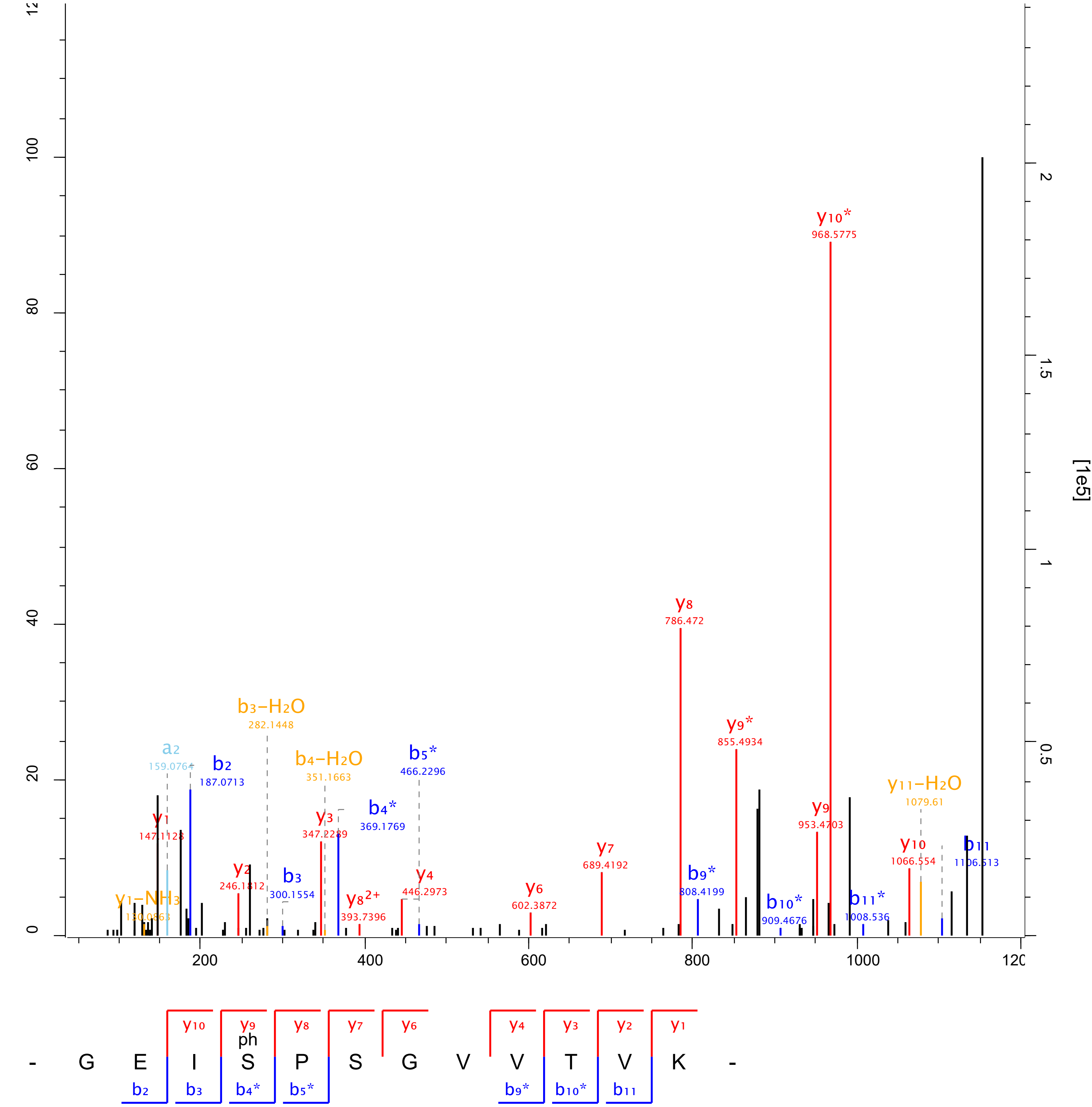

Sample output to test PDF Combine only

Raw file	Scan	Method	Score	m/z	Gene names
sirk1_sp3-solu-SUC-2-A	19486	FTMS; HCD	48.47	904.45	UGT73B2

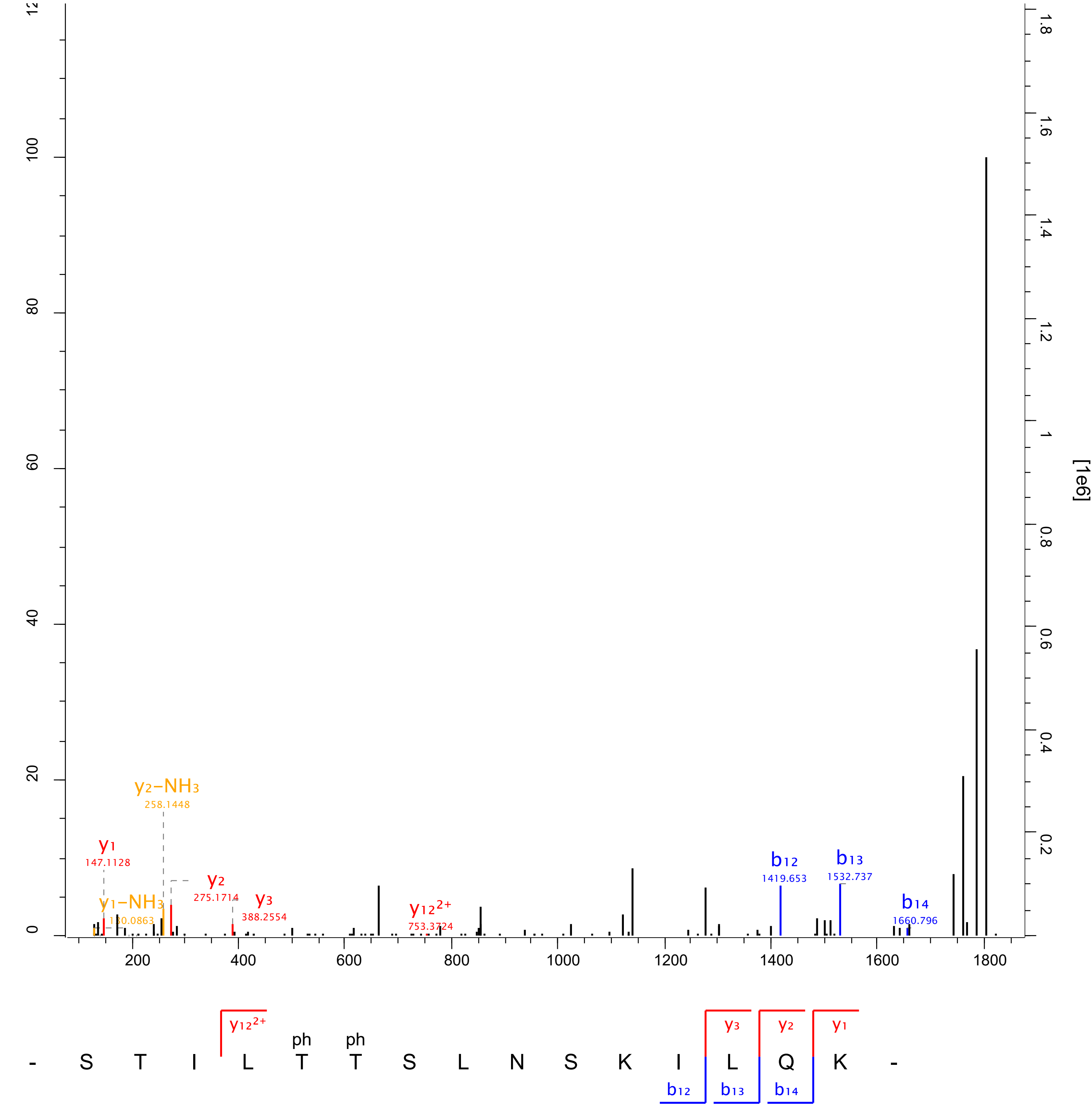

Sample output to test PDF Combine only

Raw file	Scan	Method	Score	m/z	Gene names
sirk1_sp3-solu-SUC-2-A	21042	FTMS; HCD	82.45	539.21	At5g16300

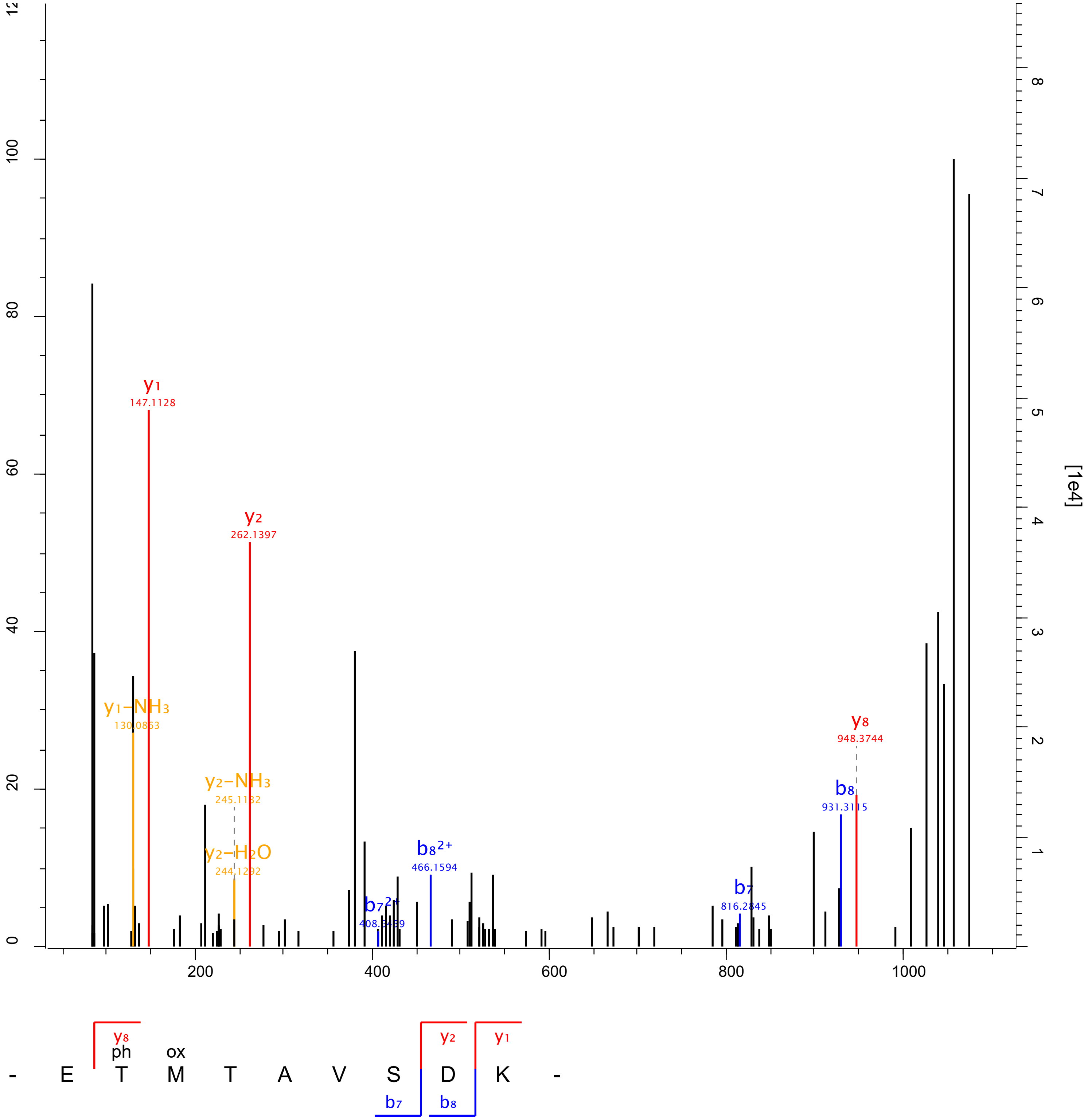

Sample output to test PDF Combine only

Raw file	Scan	Method	Score	m/z	Gene names
sirk1_sp3-solu-SUC-2-A	21161	FTMS; HCD	66.25	670.81	emb1579

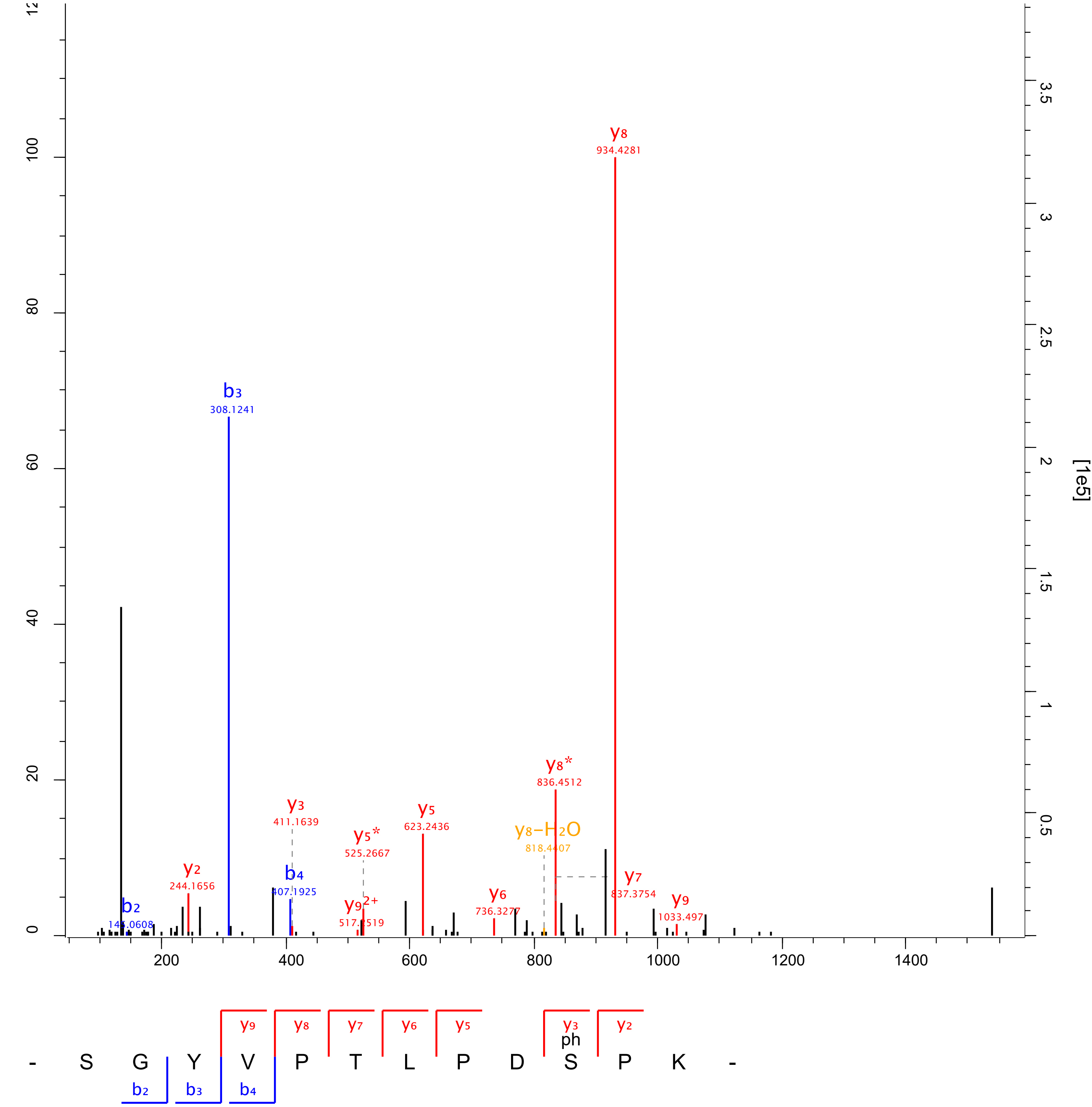

Sample output to test PDF Combine only

Raw file	Scan	Method	Score	m/z	Gene names
sirk1_sp3-solu-SUC-2-A	22186	FTMS; HCD	107.17	536.76	ADH1

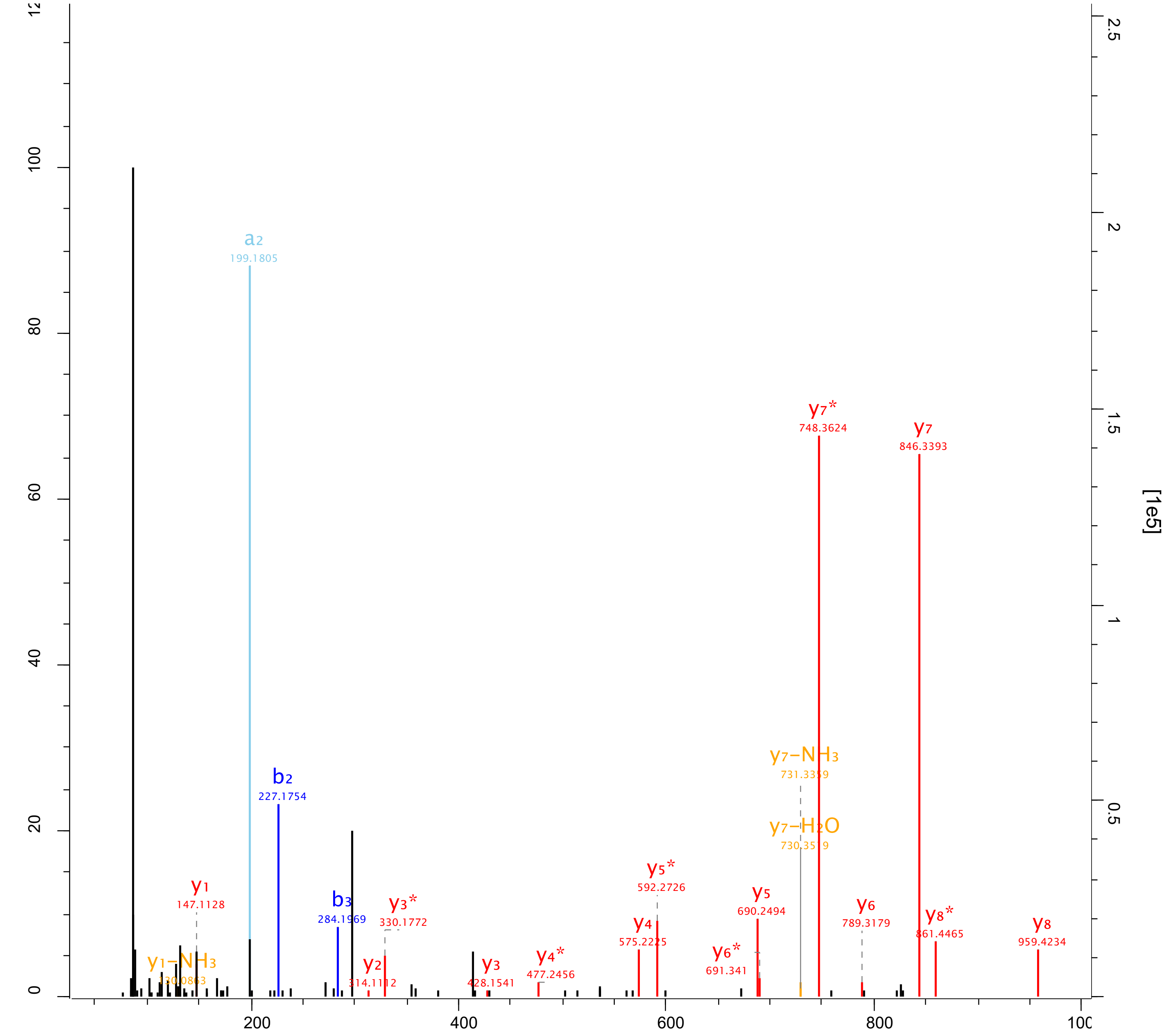

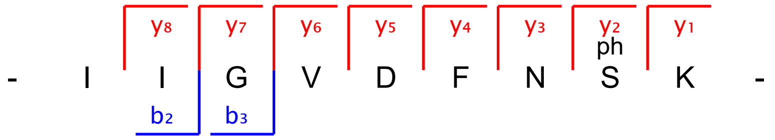

Sample output to test PDF Combine only

Raw file	Scan	Method	Score	m/z
sirk1_sp3-solu-SUC-2-A	23808	FTMS; HCD	66.83	733.86

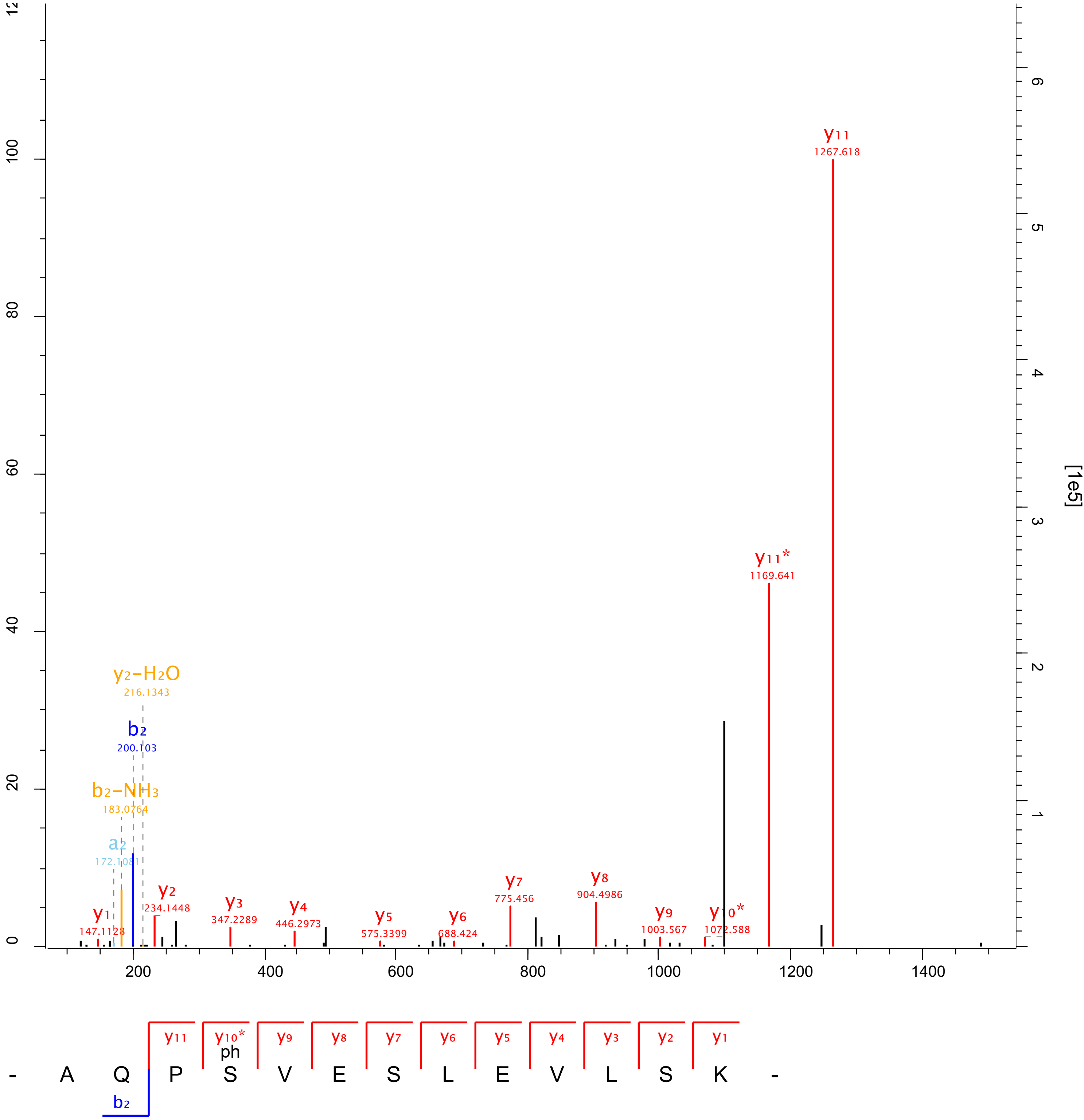

Sample output to test PDF Combine only

Raw file	Scan	Method	Score	m/z	Gene names
sirk1_sp3-solu-SUC-2-A	26261	FTMS; HCD	105.51	802.67	HAT

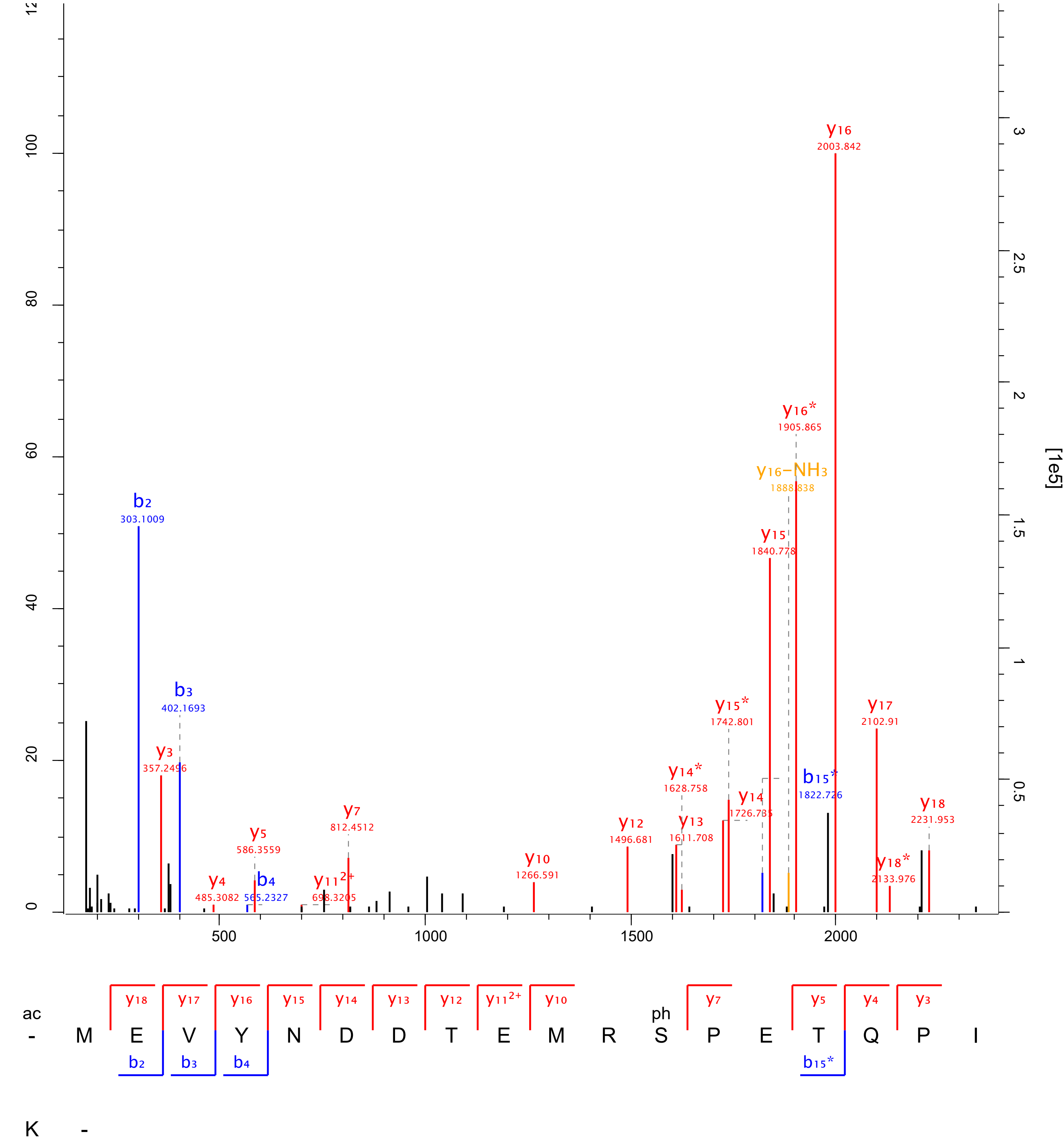

Sample output to test PDF Combine only

Raw file	Scan	Method	Score	m/z	Gene names
sirk1_sp3-solu-SUC-2-P	14647	FTMS; HCD	43.79	570.95	IQD14

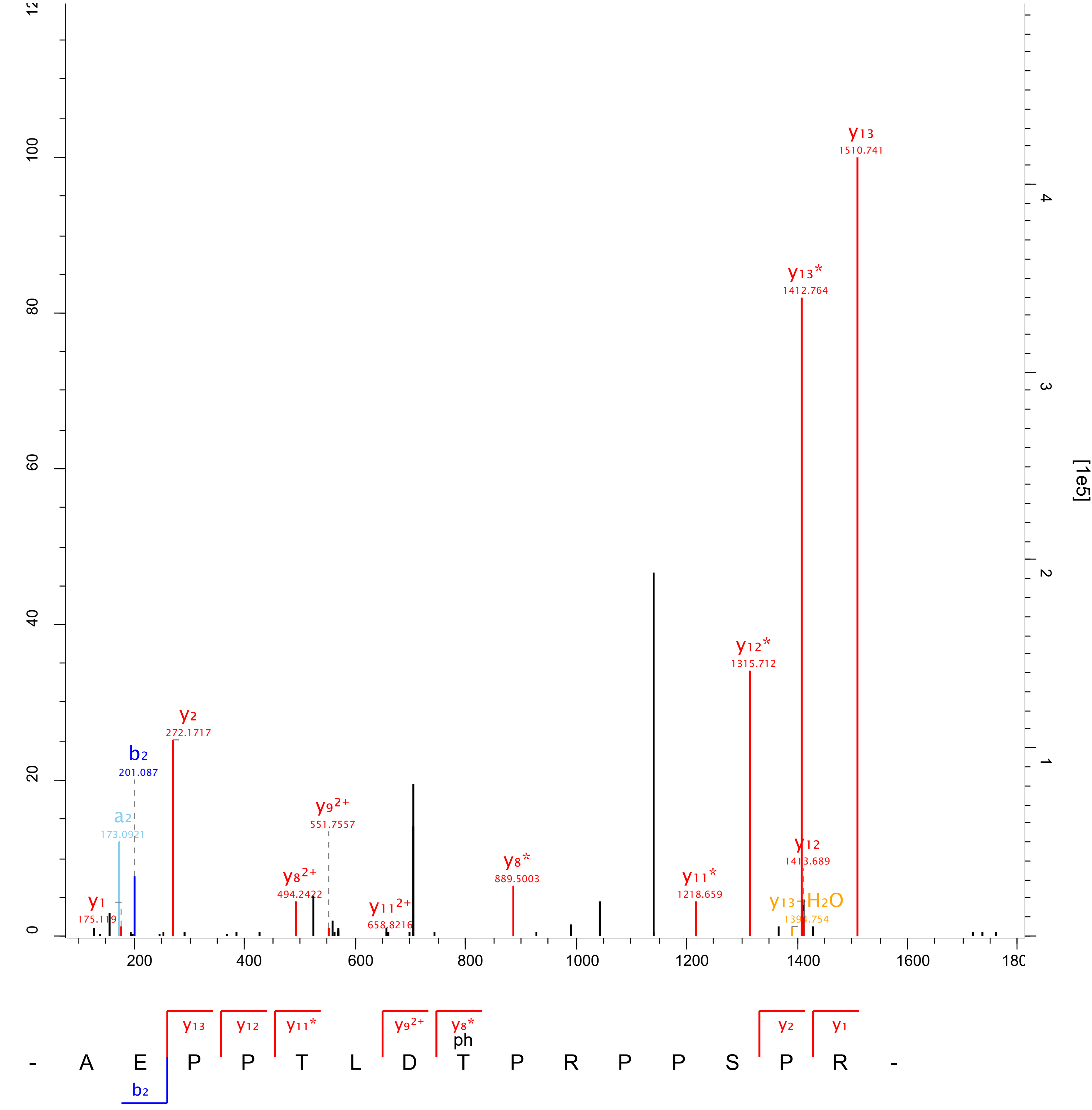

Sample output to test PDF Combine only

Raw file	Scan	Method	Score	m/z	Gene names
sirk1_sp3-solu-SUC-2-P	18006	FTMS; HCD	70.45	666	DYL1

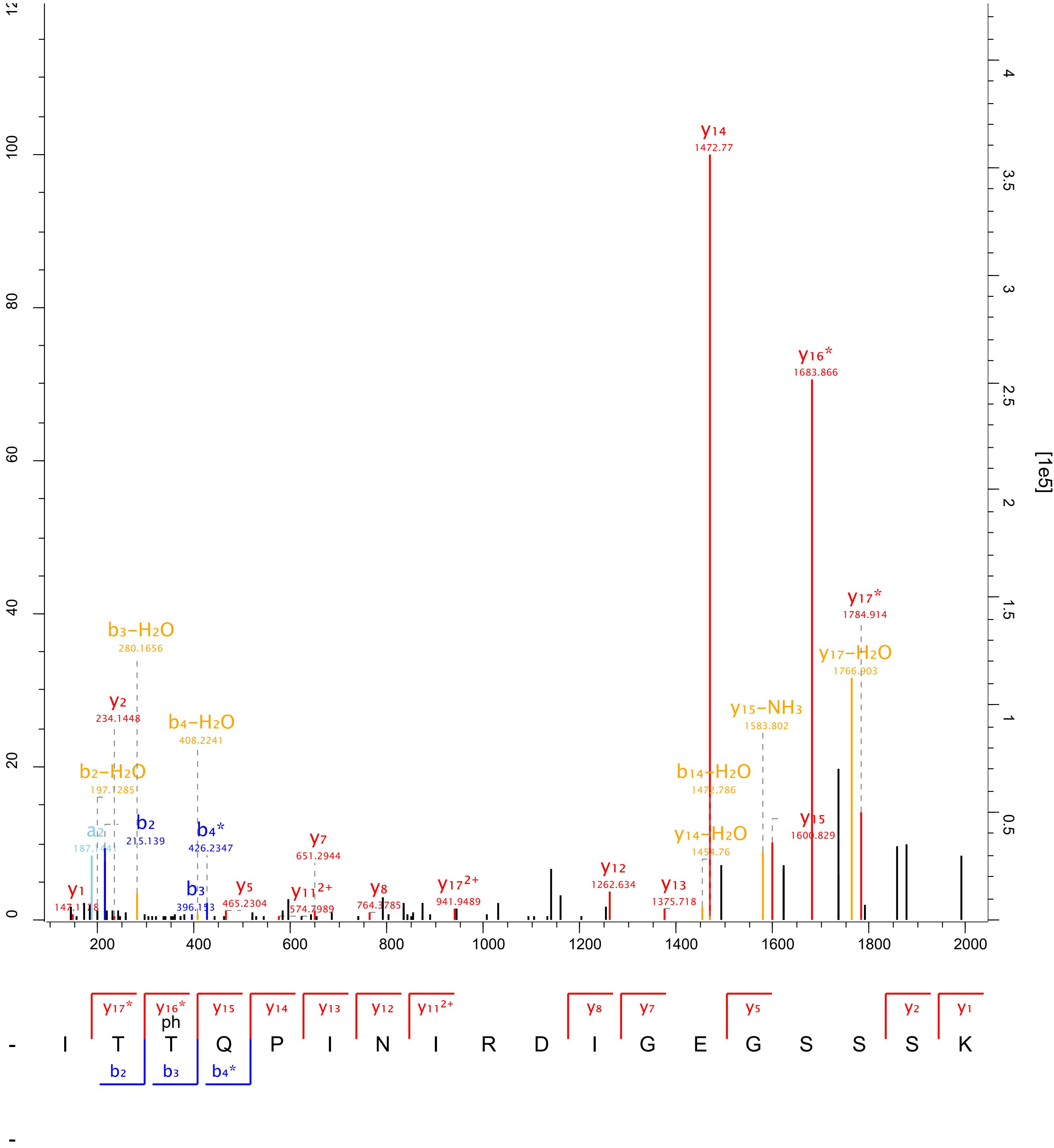

Sample output to test PDF Combine only

Raw file	Scan	Method	Score	m/z
sirk1_sp3-solu-SUC-2-P	18150	FTMS; HCD	112.74	732

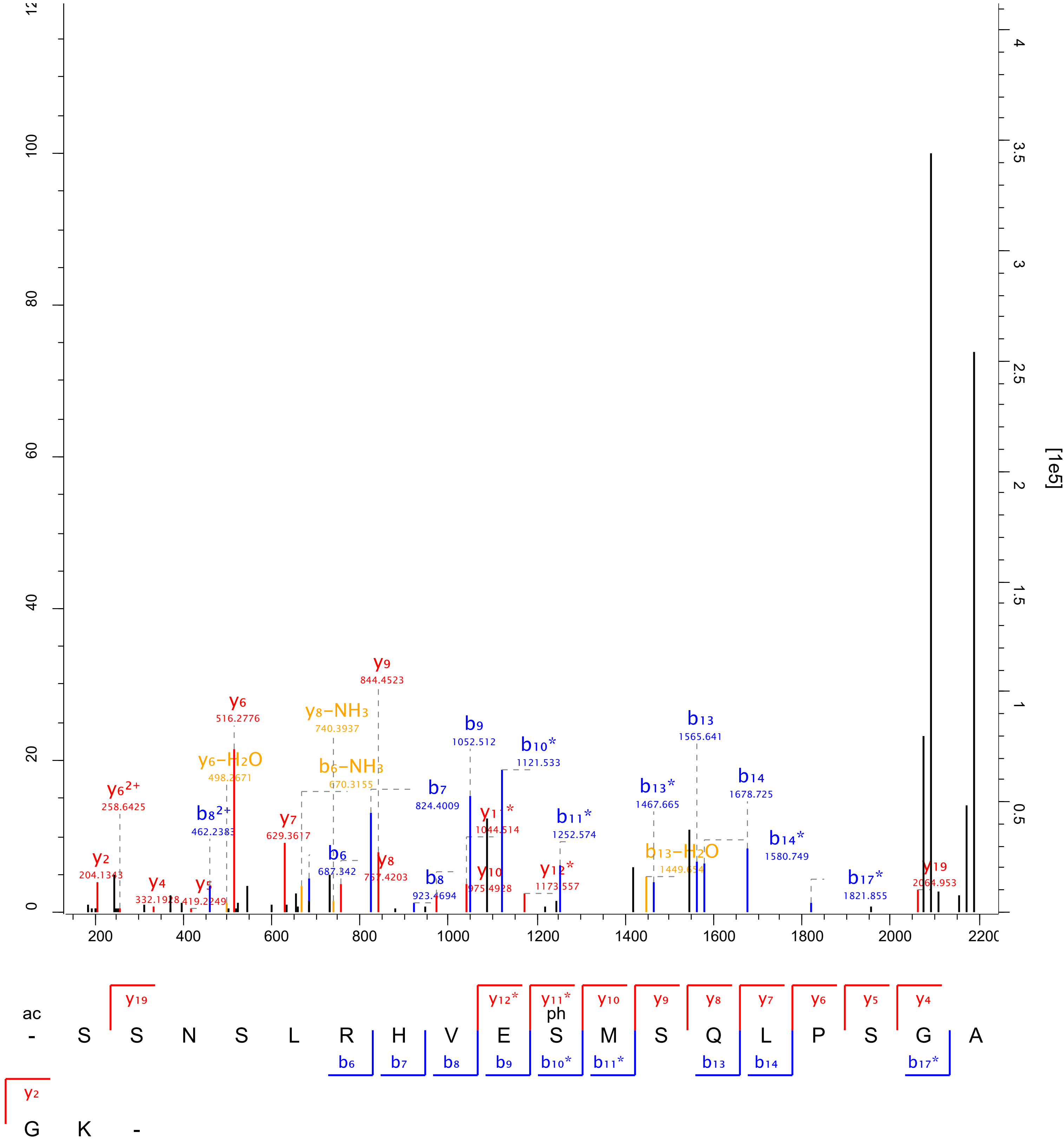

Raw file	Scan	Method	Score	m/z	Gene names
sirk1_sp3-solu-SUC-2-P	18563	FTMS; HCD	68.48	560.25	At5g43830

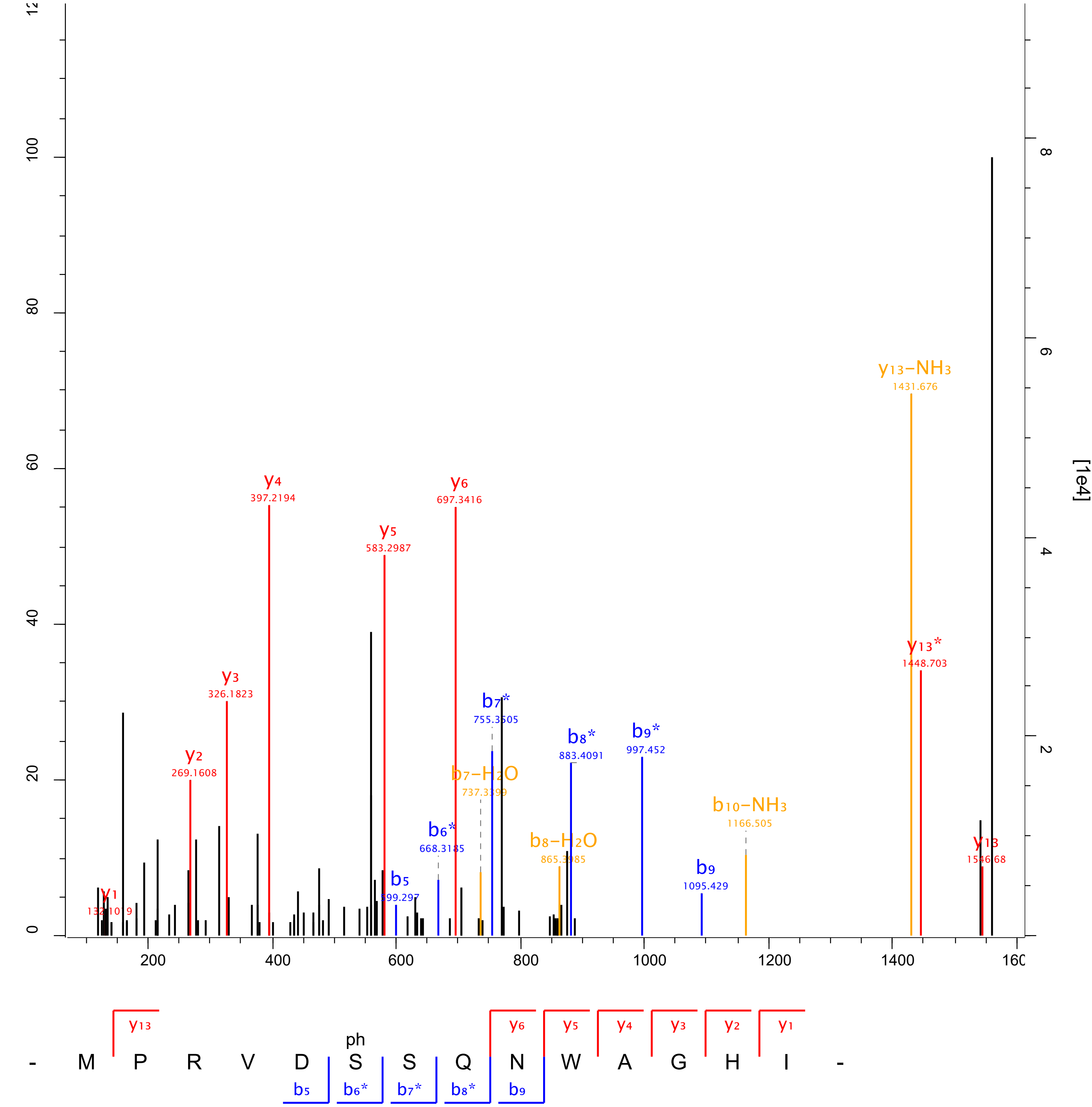

Sample output to test PDF Combine only

Raw file	Scan	Method	Score	m/z	Gene names
sirk1_sp3-solu-SUC-2-P	22198	FTMS; HCD	62.19	748.04	PCK2

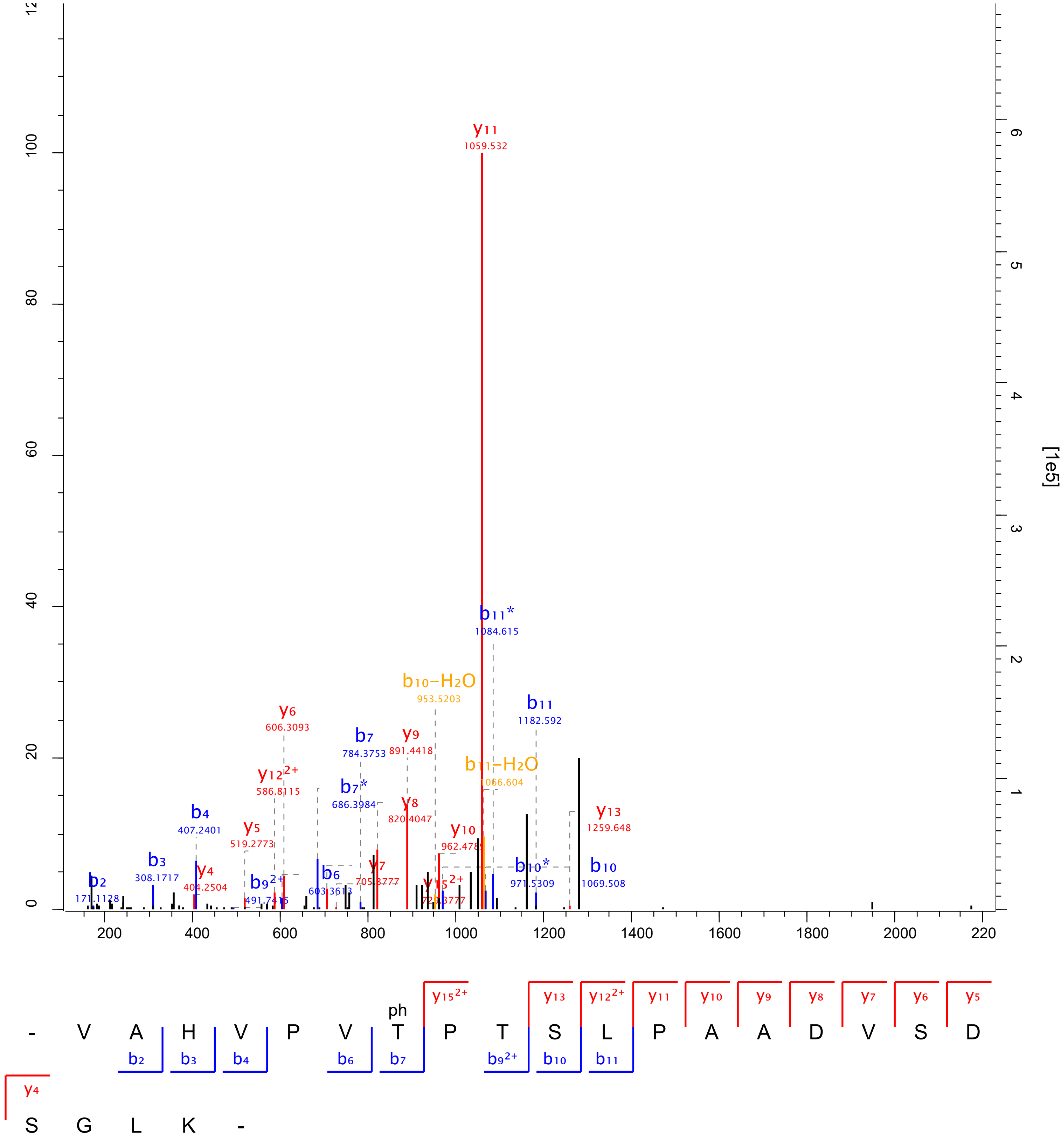

Sample output to test PDF Combine only

Raw file	Scan	Method	Score	m/z	Gene names
sirk1_sp3-solu-SUC-2-P	24617	FTMS; HCD	86.5	535.92	At1g19530

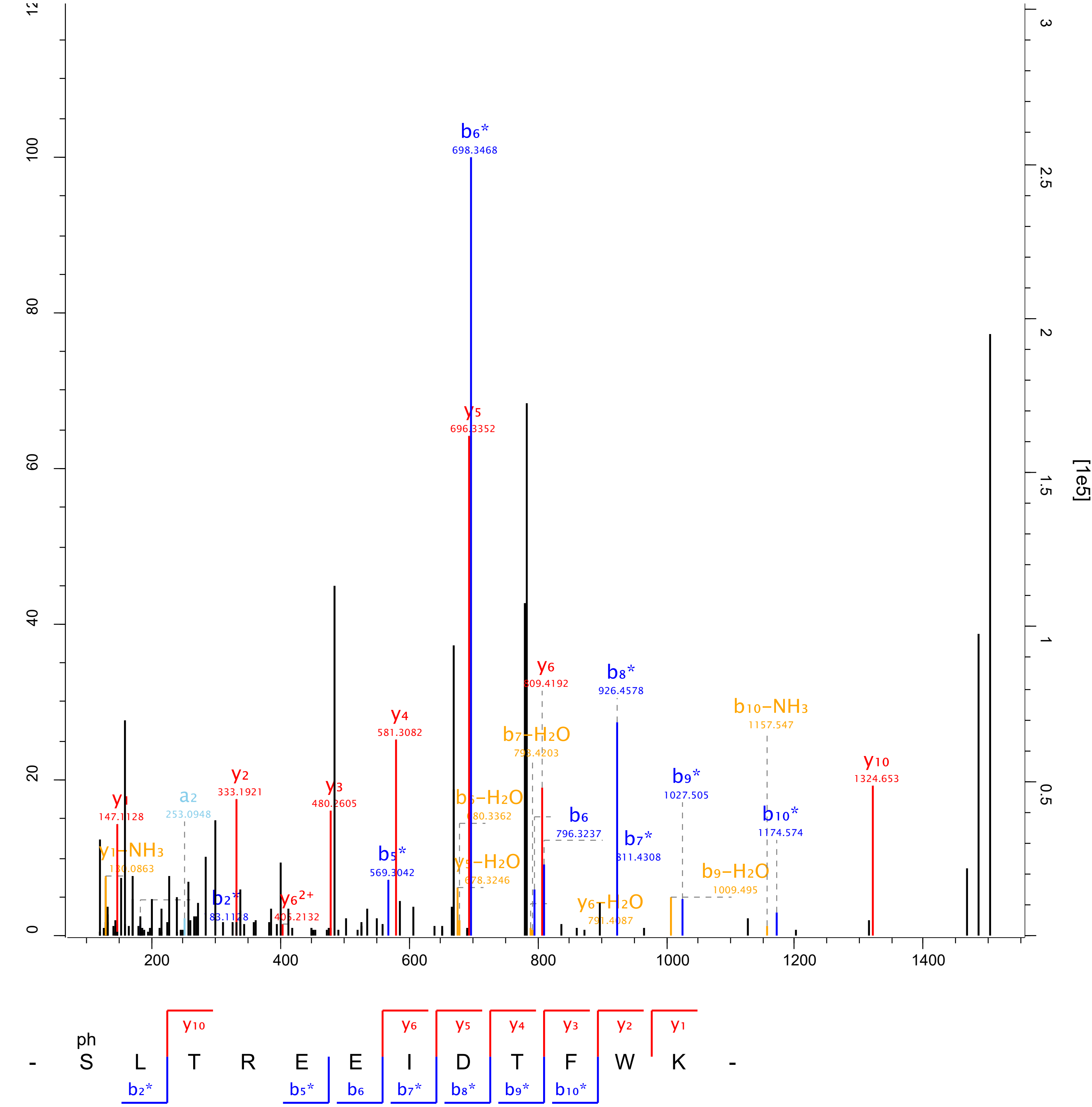

Sample output to test PDF Combine only

Raw file	Scan	Method	Score	m/z	Gene names
sirk1_sp3-solu-SUC-2-P	25354	FTMS; HCD	65.86	629.3	MYOB1

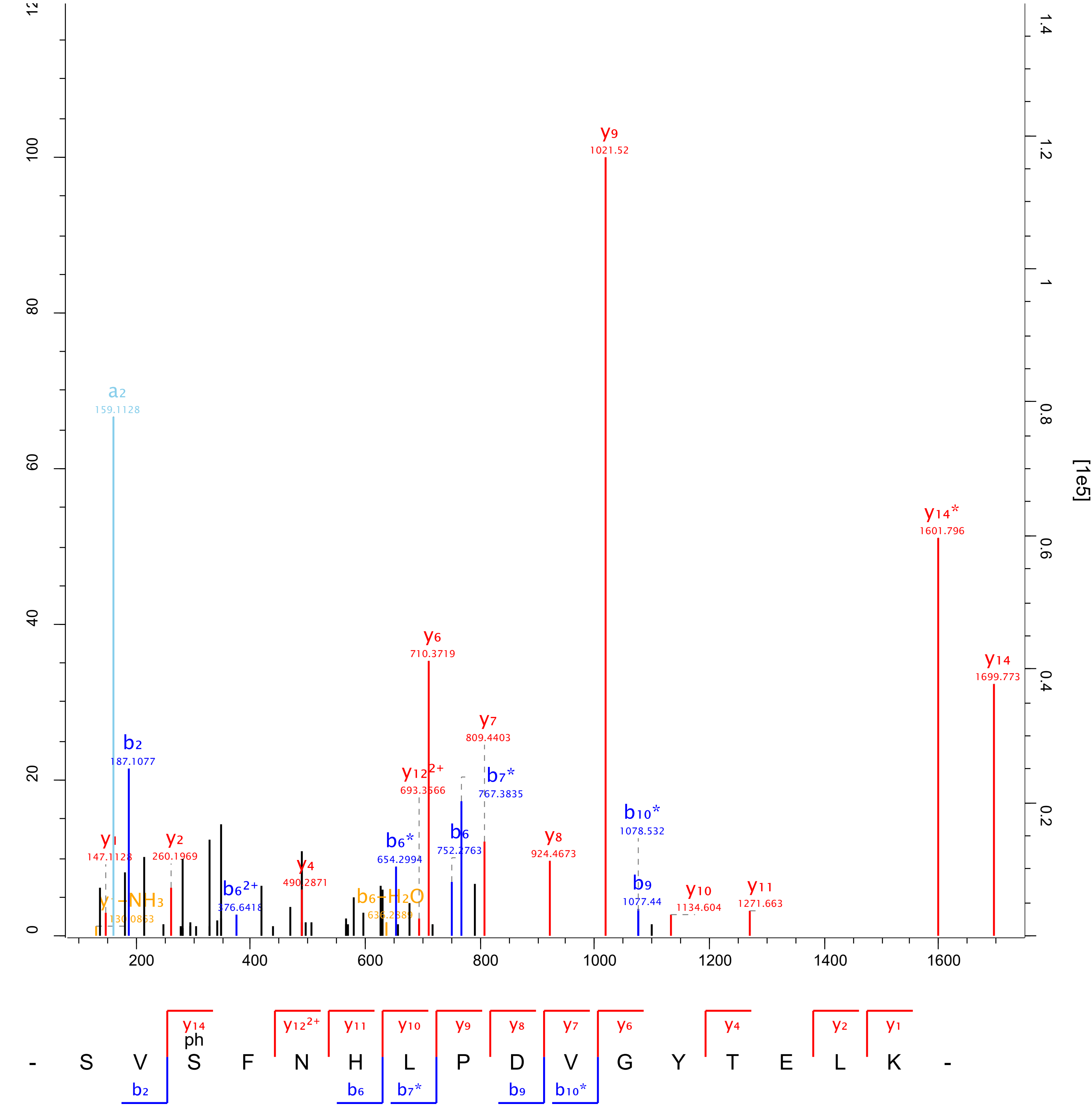

Sample output to test PDF Combine only

Raw file	Scan	Method	Score	m/z	Gene names
sirk1_sp3-solu-SUC-2-P	28250	FTMS; HCD	85.67	606.79	At1g76810

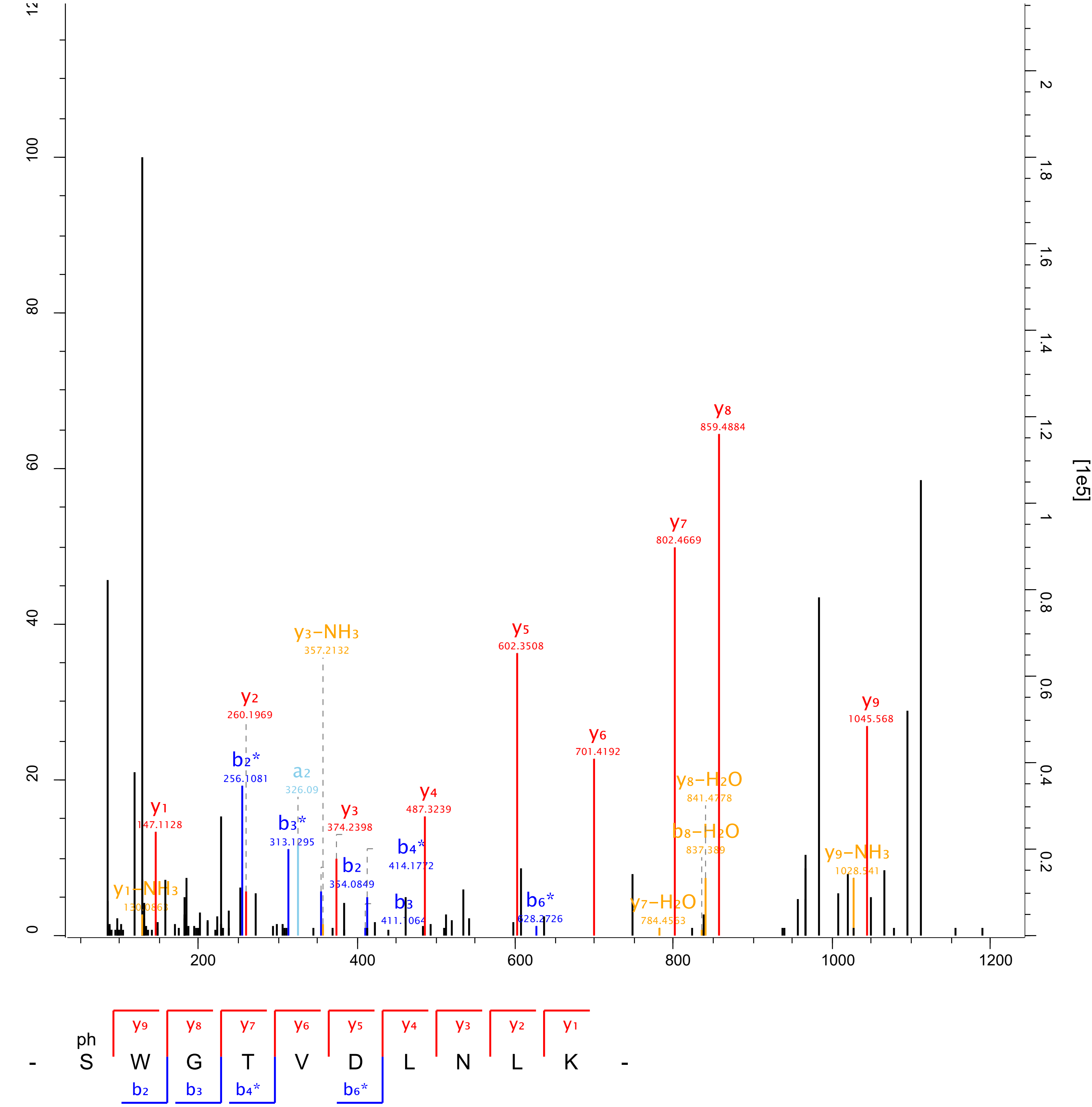

Sample output to test PDF Combine only

Raw file	Scan	Method	Score	m/z	Gene names
sirk1_sp3-solu-SUC-2-P	28609	FTMS; HCD	177.12	889.8	At4g05150

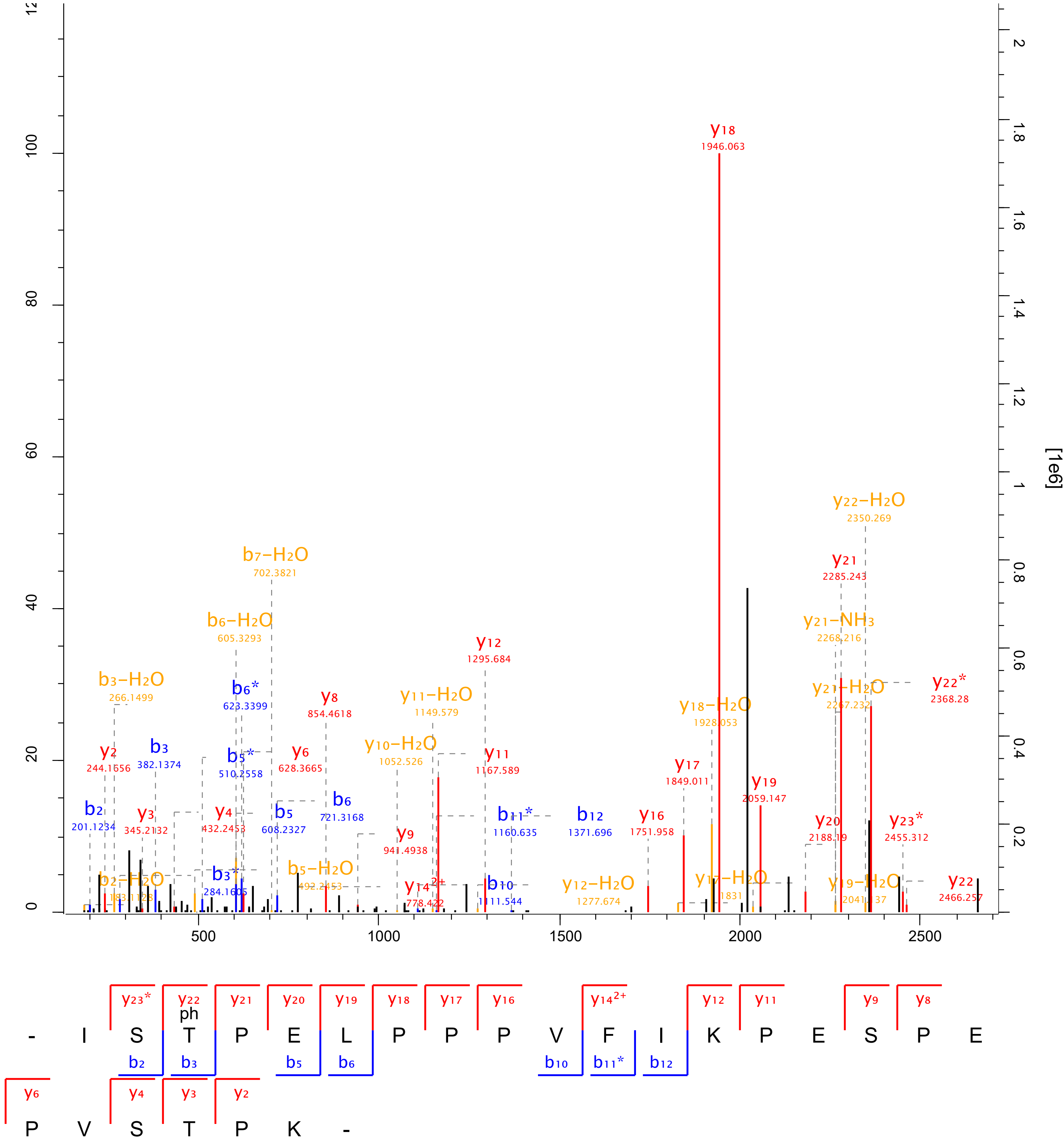

Sample output to test PDF Combine only

Raw file	Scan	Method	Score	m/z	Gene names
sirk1_sp3-solu-SUC-2-P	28662	FTMS; HCD	67.08	682.86	At2g01480

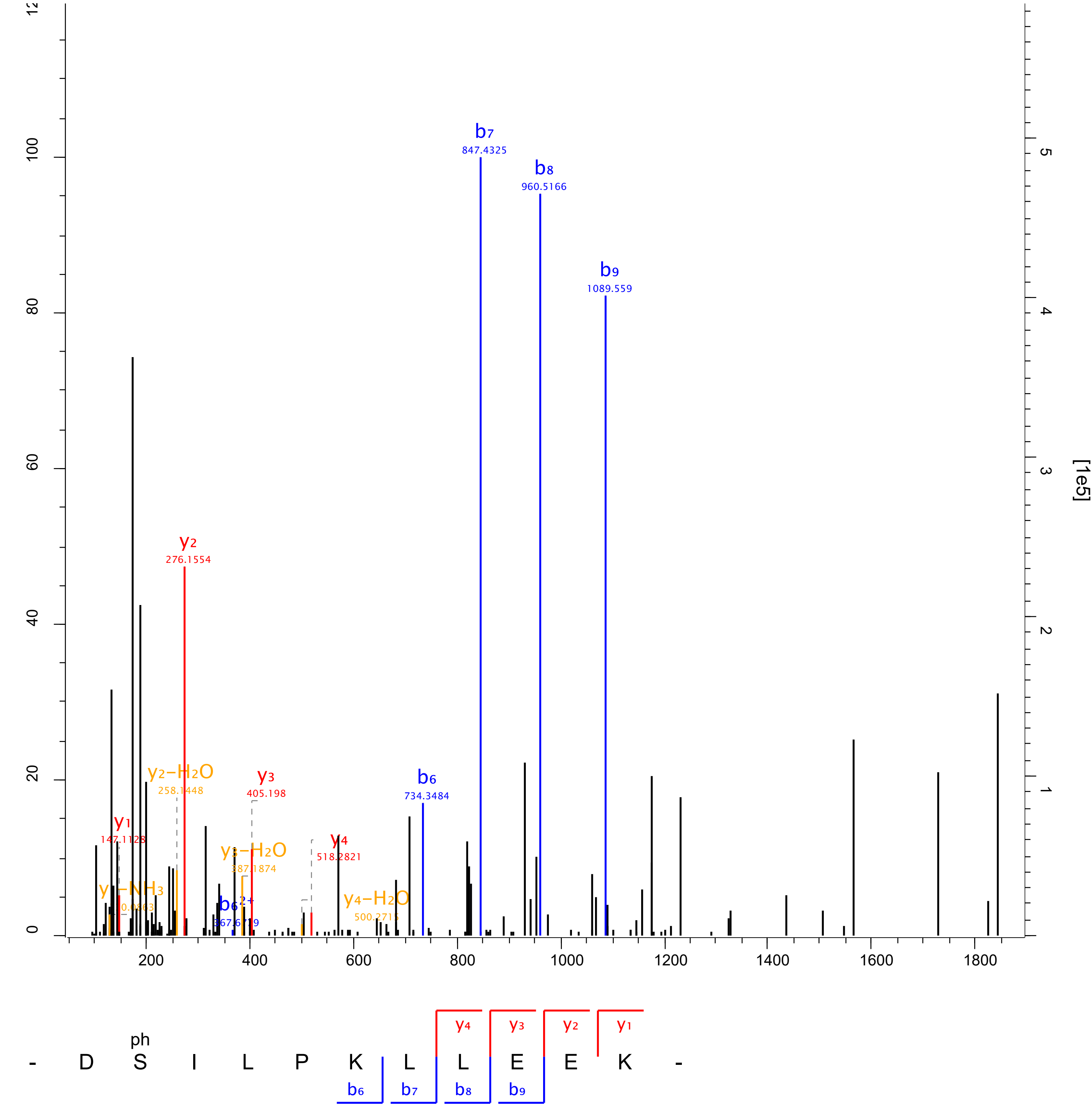

Sample output to test PDF Combine only

Raw file	Scan	Method	Score	m/z	Gene names
sirk1_sp3-solu-SUC-2-P	30898	FTMS; HCD	90.72	834.89	At5g58950

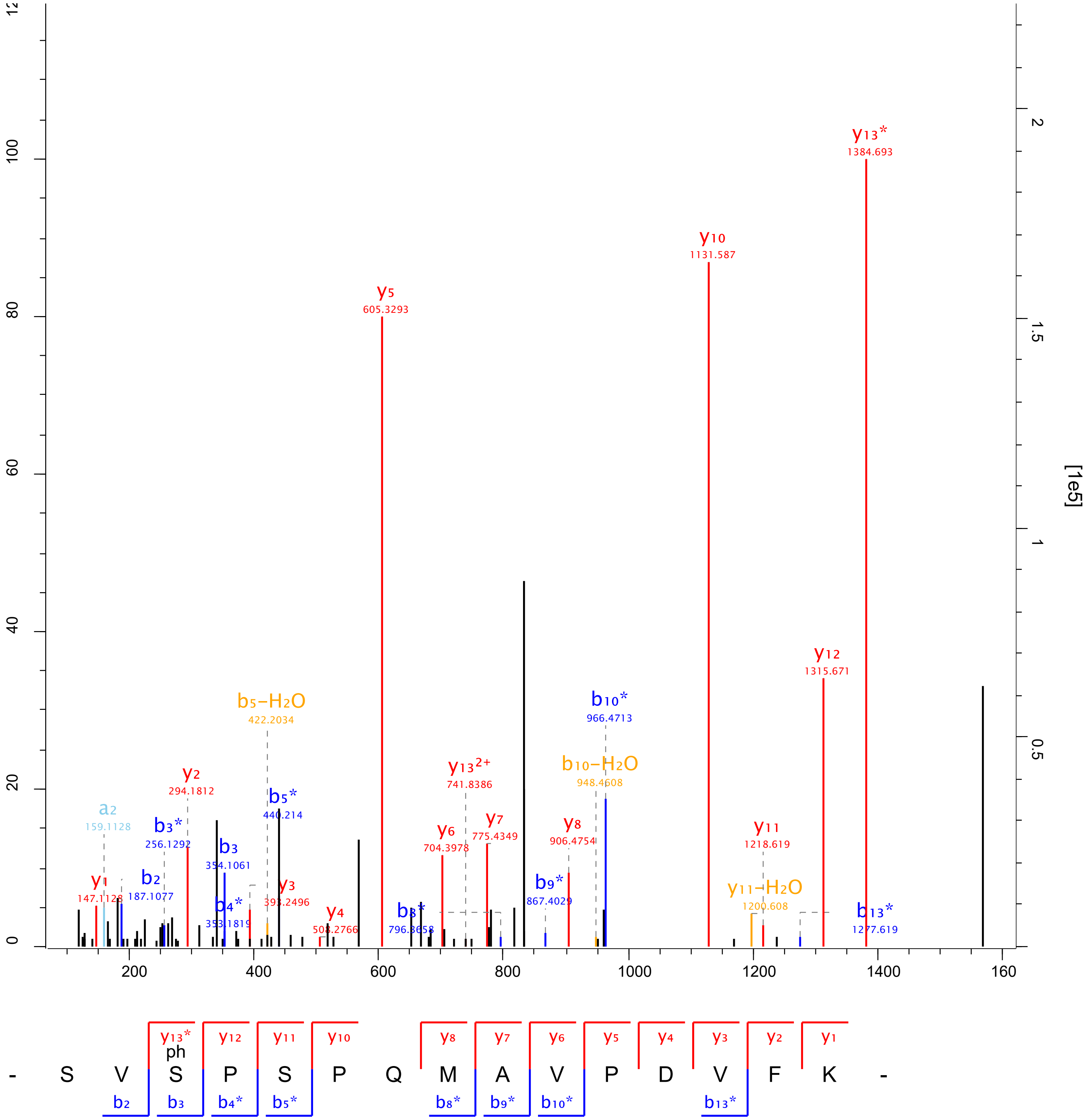

Sample output to test PDF Combine only

Raw file	Scan	Method	Score	m/z	Gene names
sirk1_sp3-solu-SUC-2-P	32740	FTMS; HCD	43.03	583.61	At3g43833

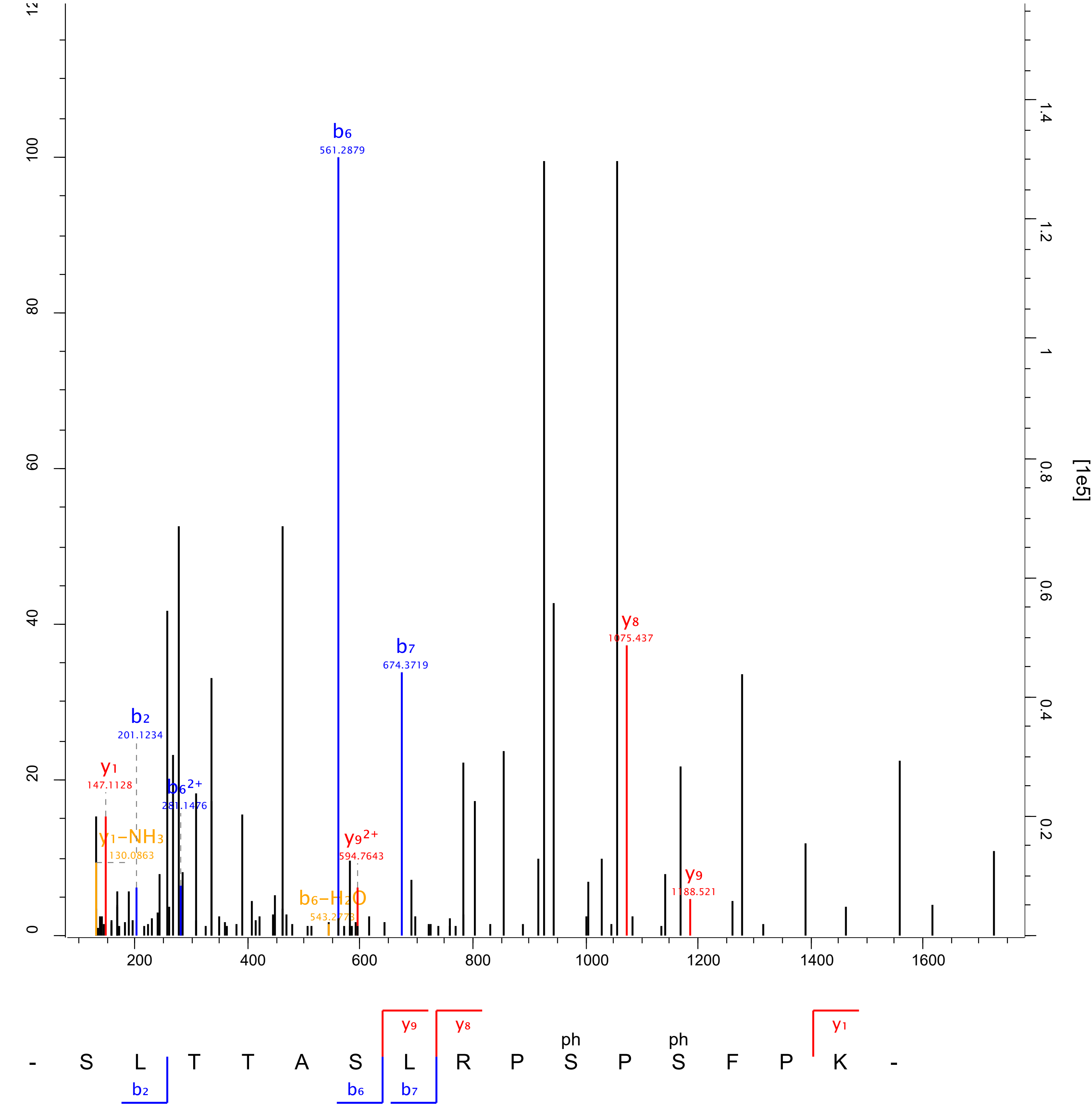

Sample output to test PDF Combine only

Raw file	Scan	Method	Score	m/z	Gene names
sirk1_sp3-solu-SUC-3-A	11772	FTMS; HCD	97.45	497.59	MBD10

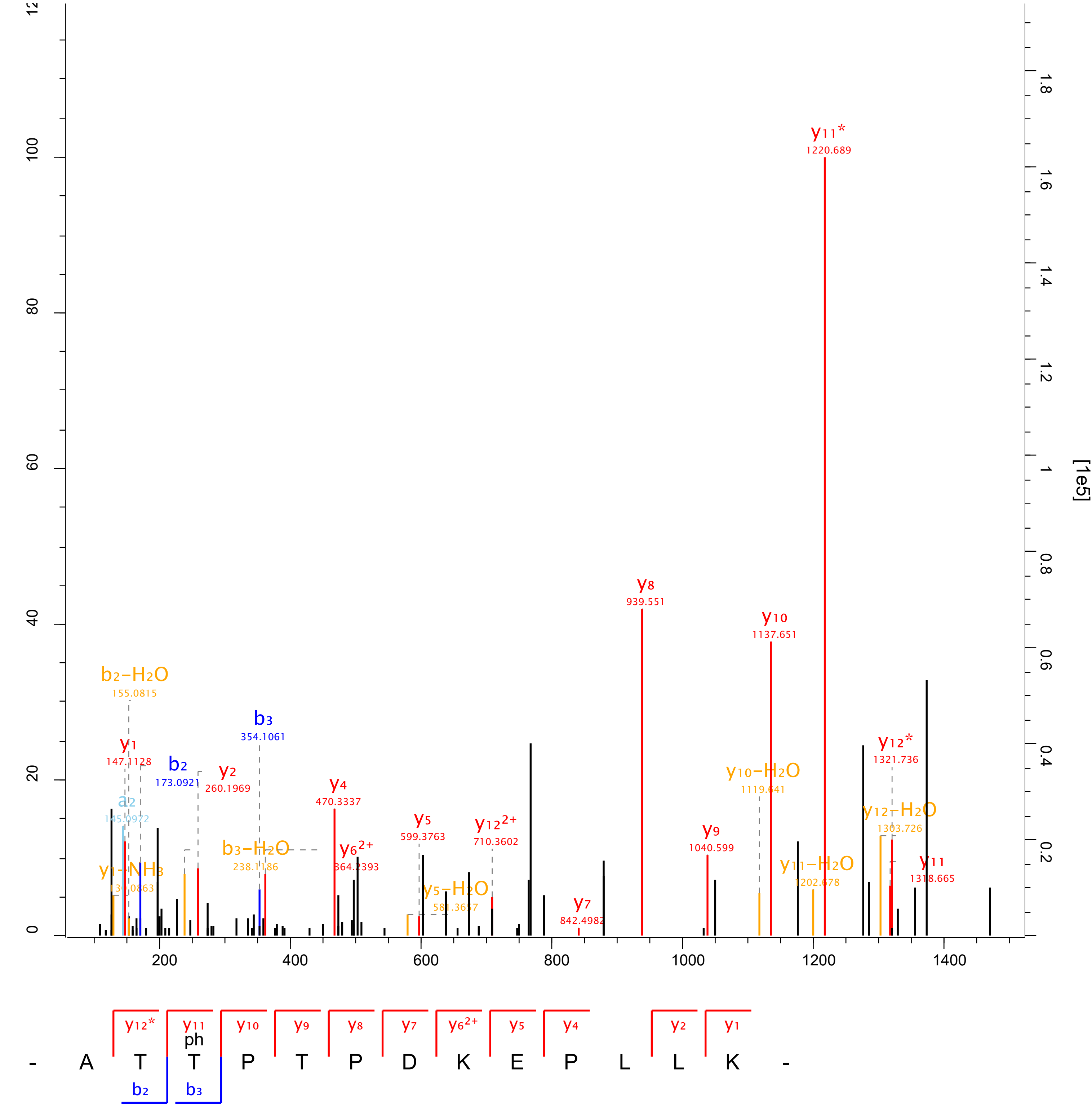

Sample output to test PDF Combine only

Raw file	Scan	Method	Score	m/z	Gene names
sirk1_sp3-solu-SUC-3-A	14857	FTMS; HCD	59.98	620.62	COR47

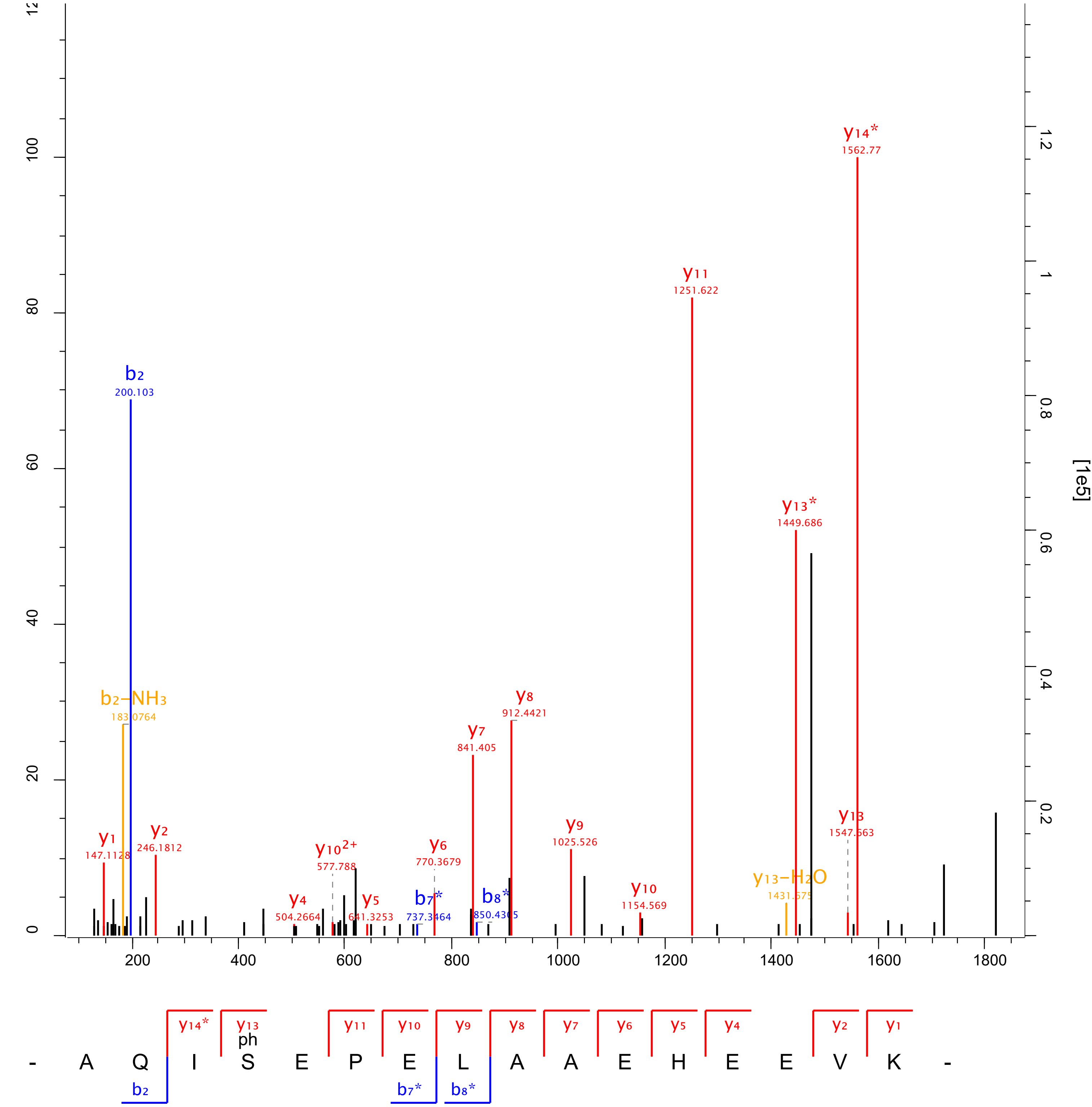

Sample output to test PDF Combine only

Raw file	Scan	Method	Score	m/z	Gene names
sirk1_sp3-solu-SUC-3-A	15938	FTMS; HCD	85.81	507.73	CPK6;CPK5

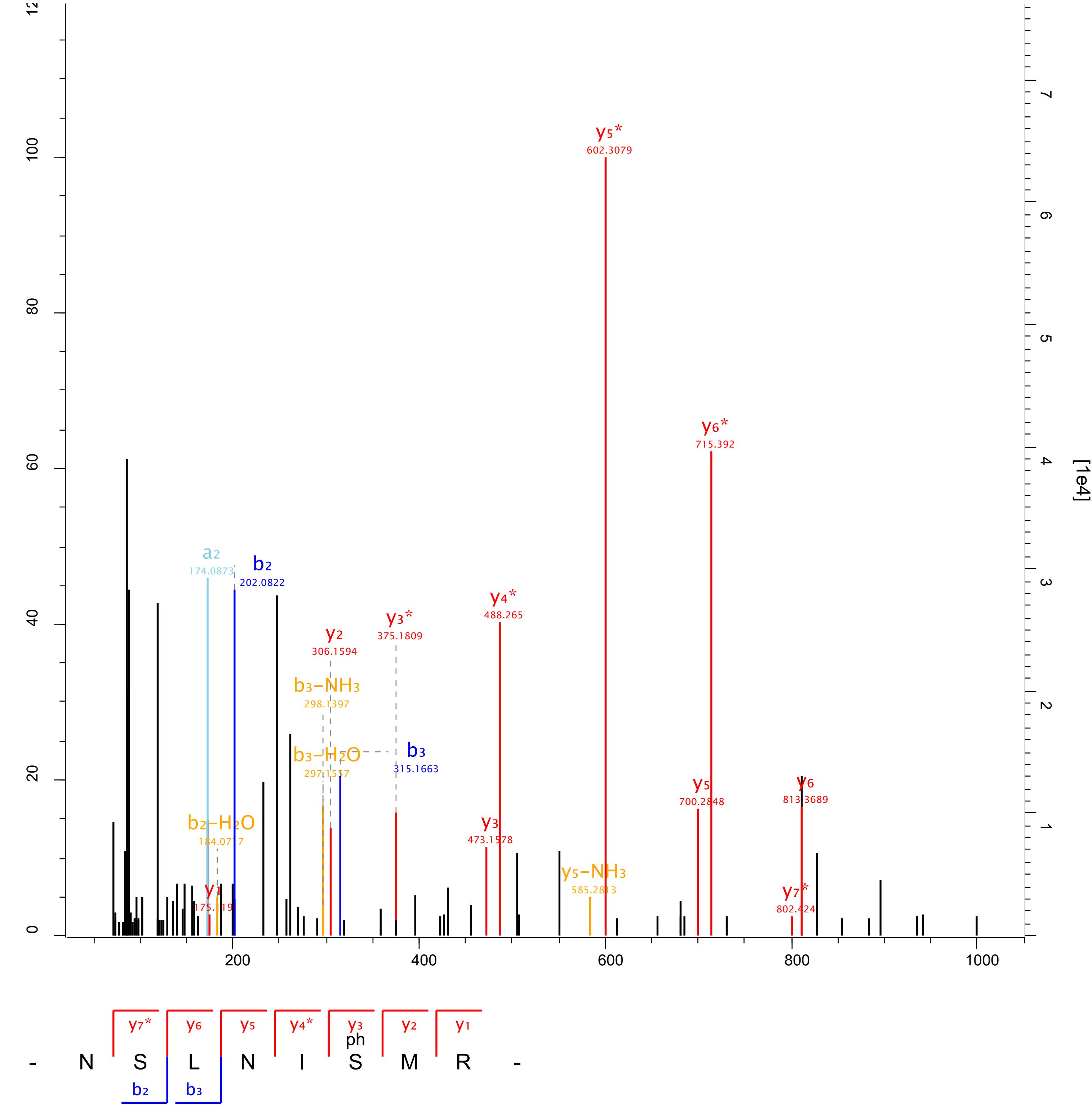

Sample output to test PDF Combine only

Raw file	Scan	Method	Score	m/z	Gene names
sirk1_sp3-solu-SUC-3-A	32069	FTMS; HCD	50.09	944.42	SKD1

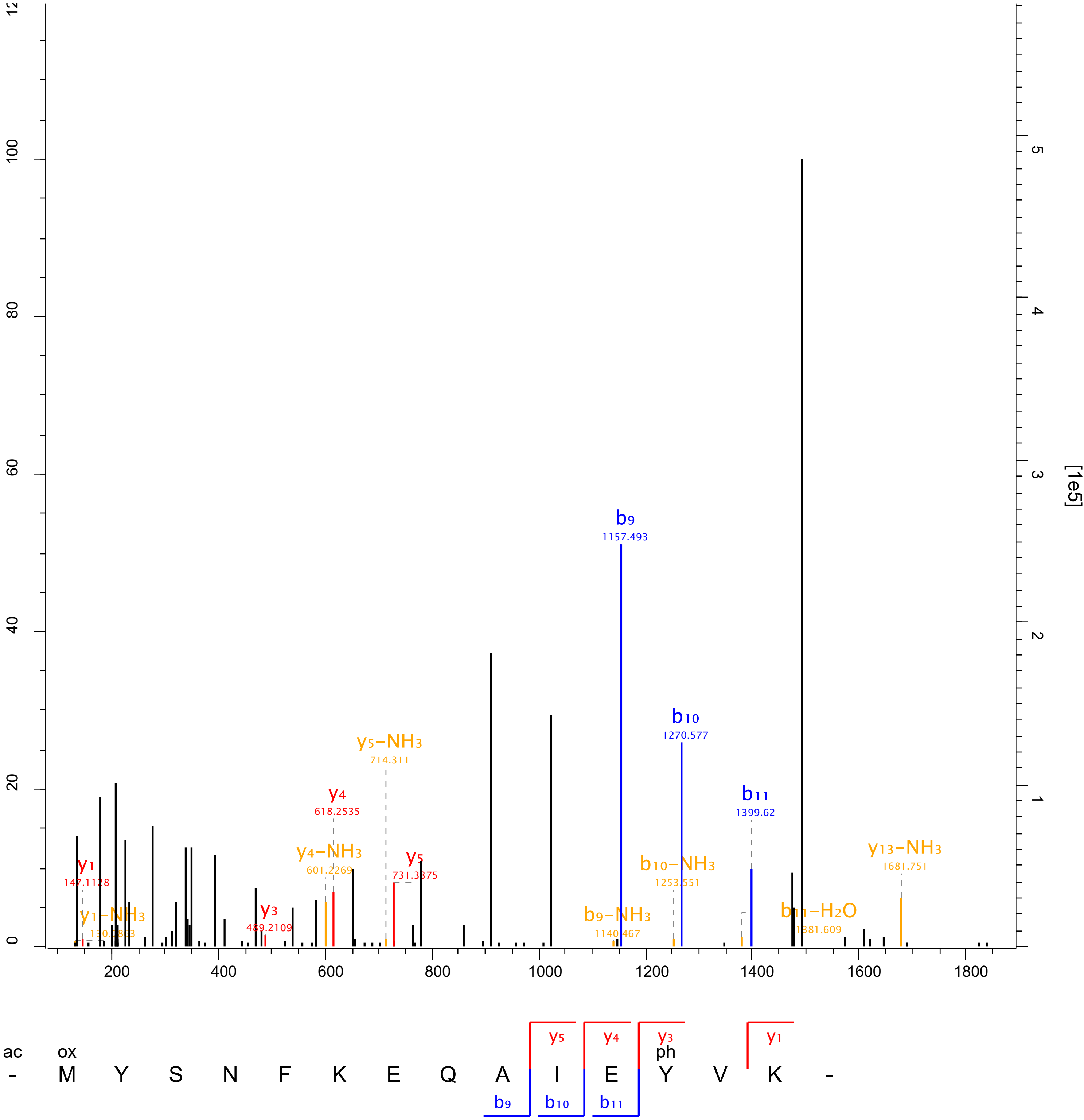

Sample output to test PDF Combine only

Raw file	Scan	Method	Score	m/z	Gene names
sirk1_sp3-solu-SUC-3-P	18443	FTMS; HCD	88.56	597.27	F2K13_30;At5g16880

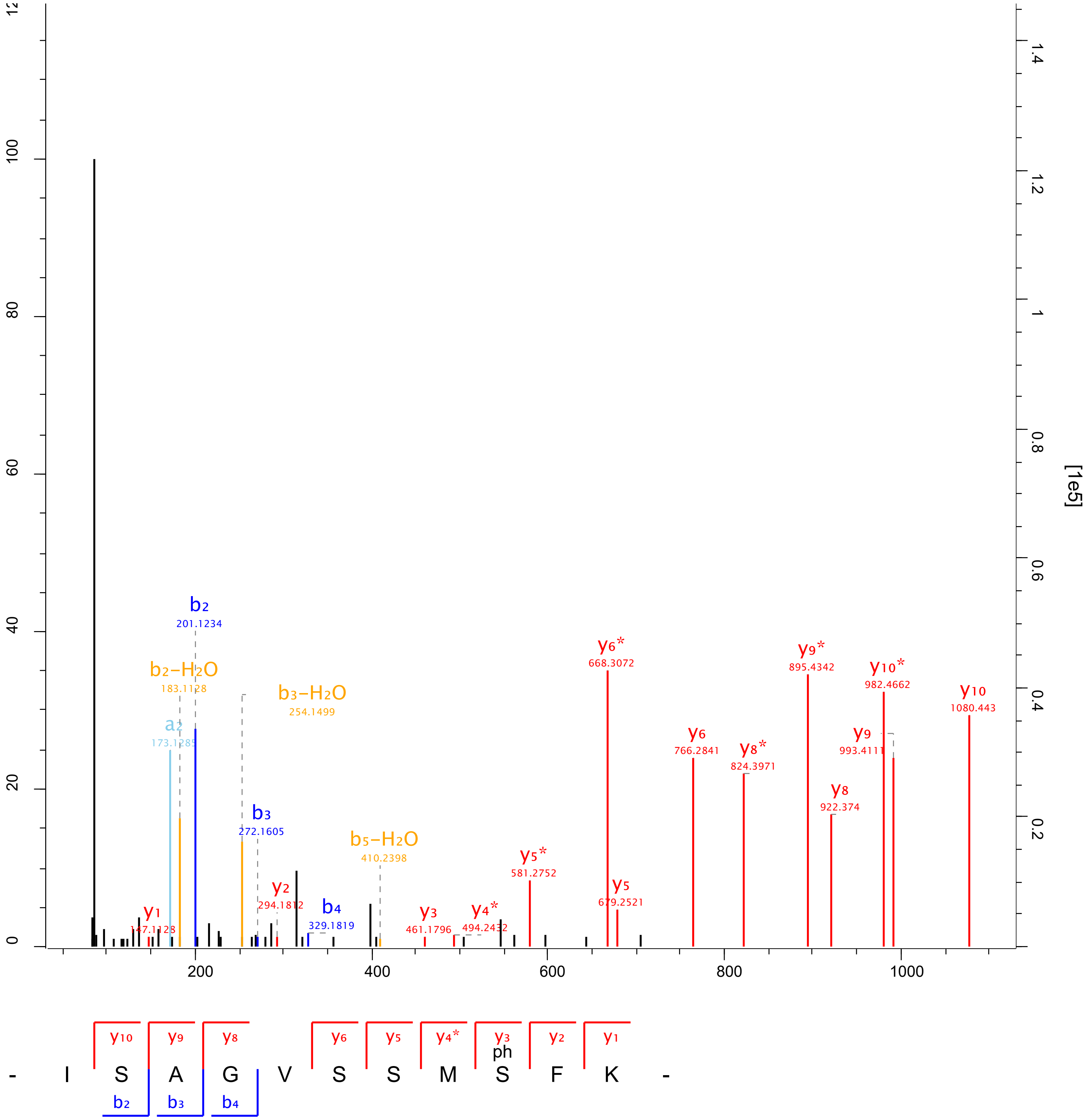

Sample output to test PDF Combine only

Raw file	Scan	Method	Score	m/z	Gene names
sirk1_sp3-solu-SUC-3-P	19147	FTMS; HCD	58.98	524.27	At5g37920

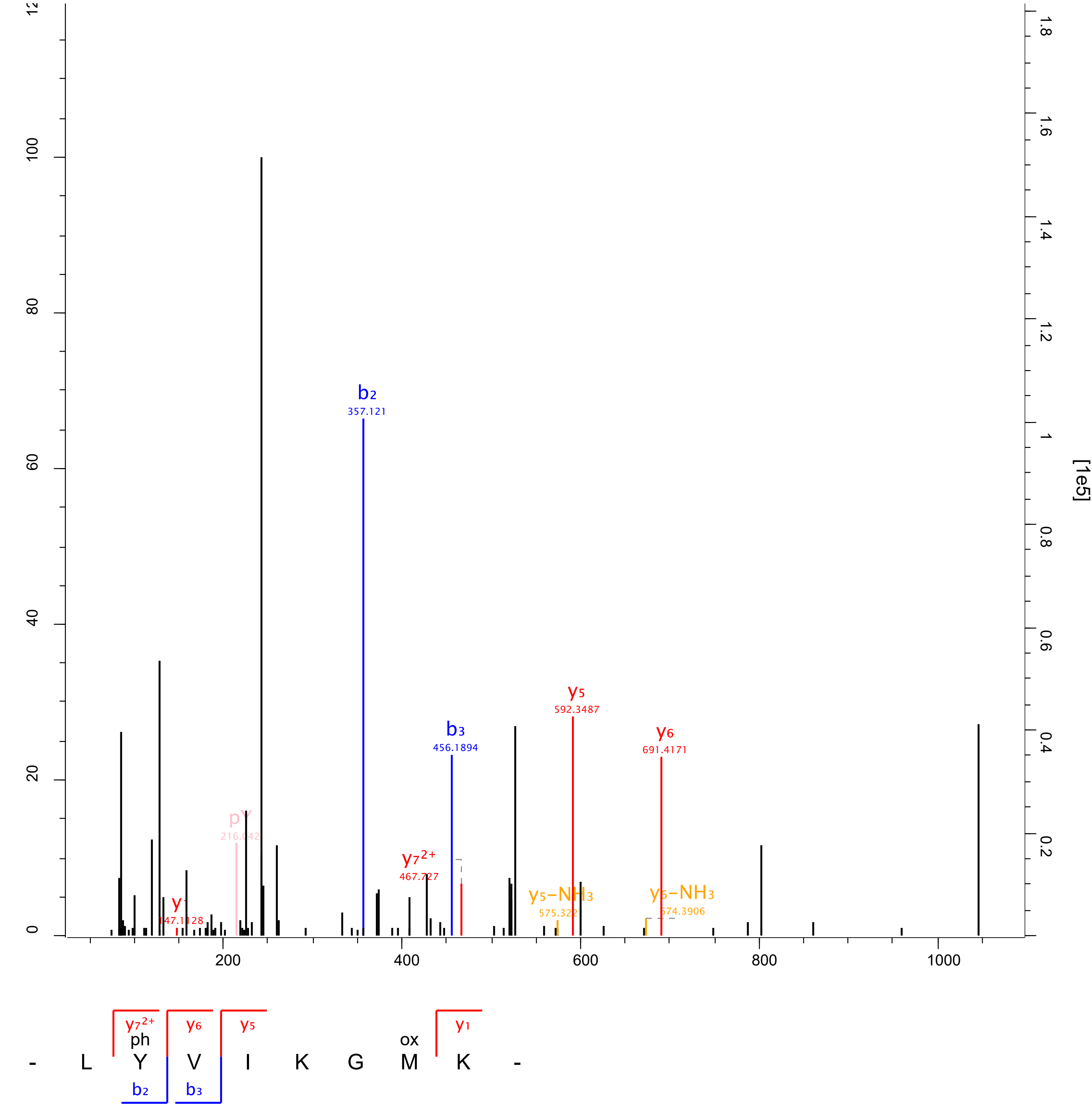

Sample output to test PDF Combine only

Raw file	Scan	Method	Score	m/z	Gene names
sirk1_sp3-solu-SUC-3-P	19606	FTMS; HCD	110.76	414.22	PPA5

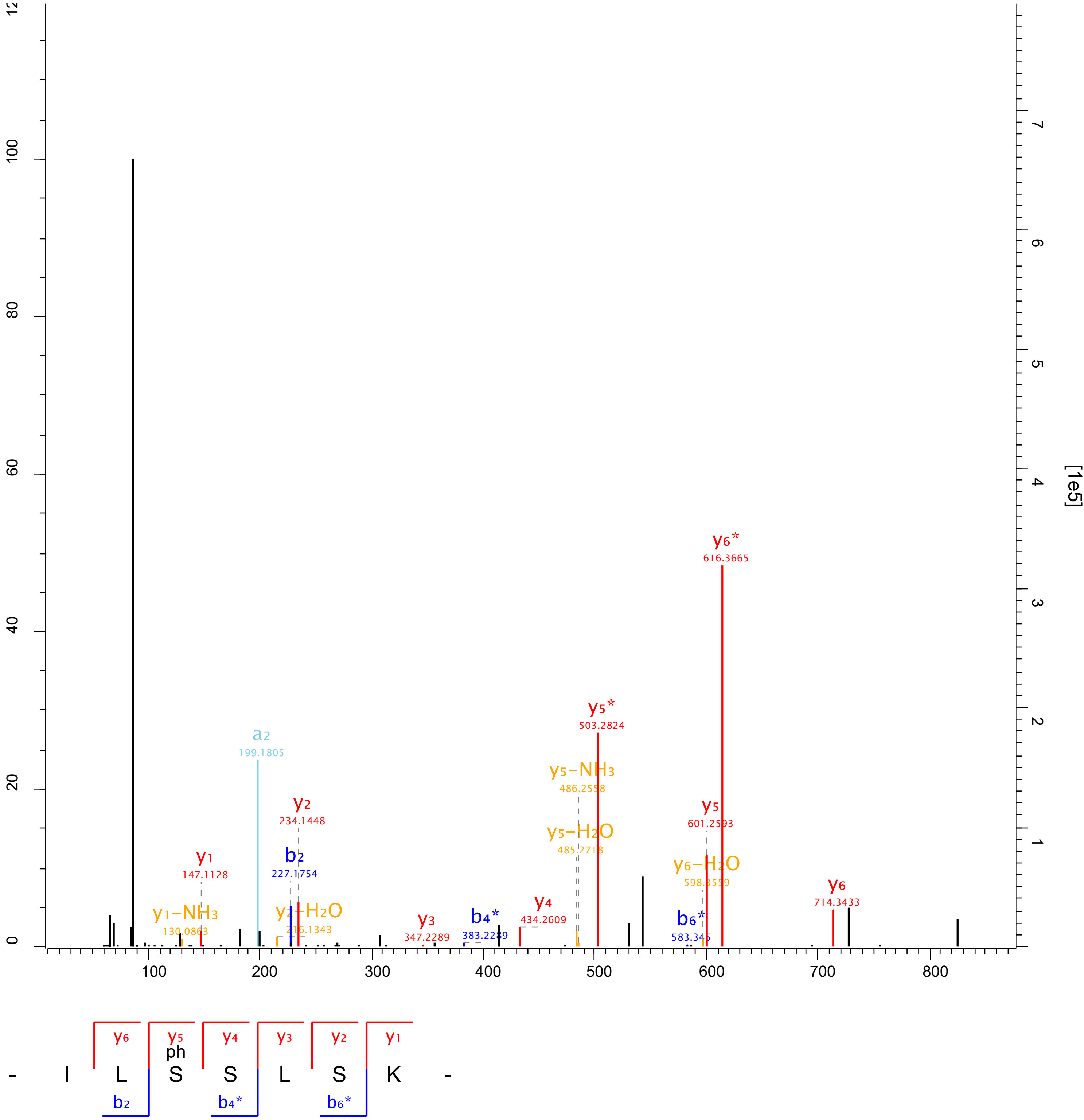

Sample output to test PDF Combine only

Raw file	Scan	Method	Score	m/z	Gene names
sirk1_sp3-solu-SUC-3-P	23915	FTMS; HCD	54.34	557.92	ASPG2

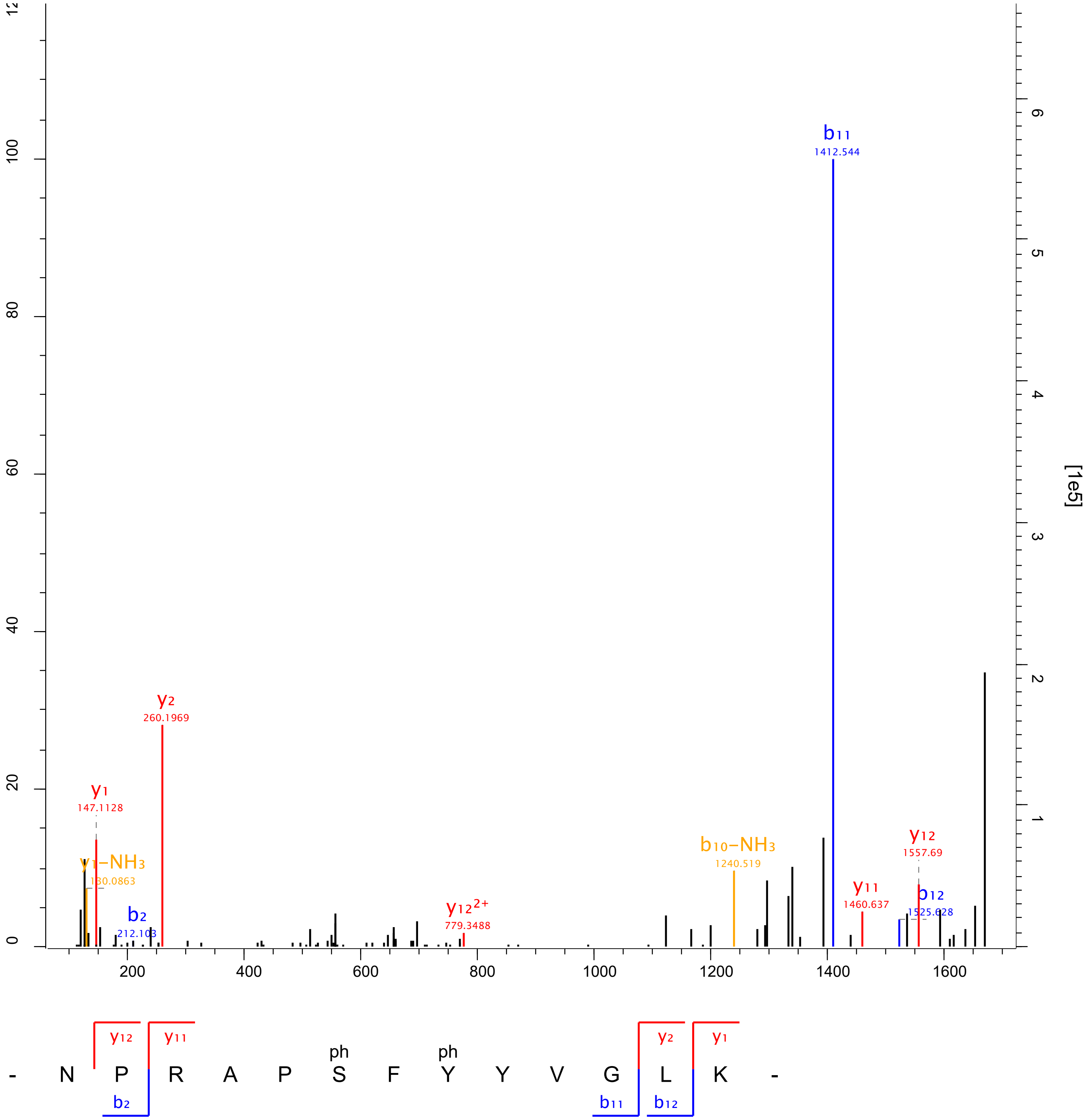

Sample output to test PDF Combine only

Raw file	Scan	Method	Score	m/z
sirk1_sp3-solu-SUC-3-P	23950	FTMS; HCD	52.79	488.69

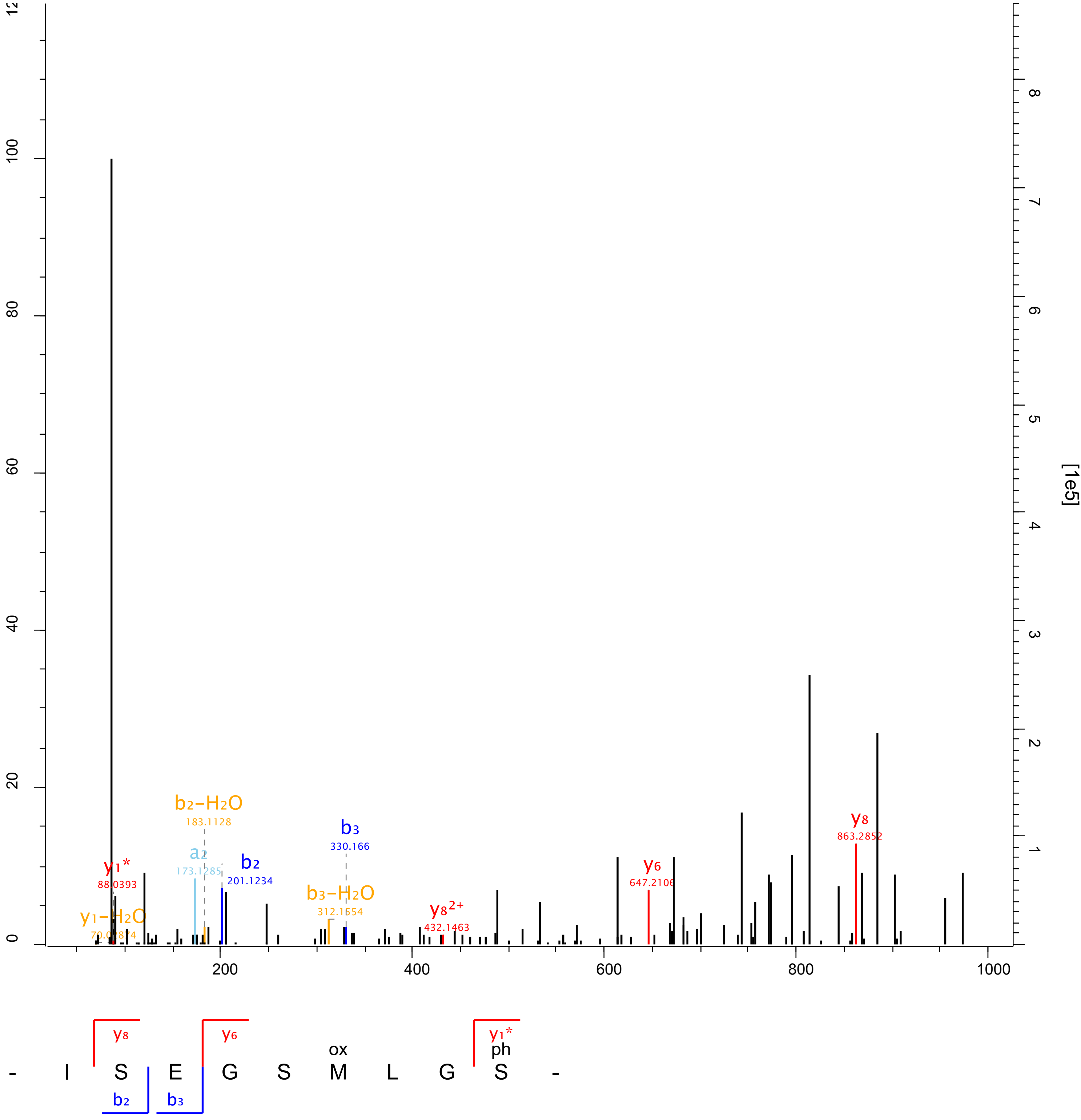

Sample output to test PDF Combine only

Raw file	Scan	Method	Score	m/z	Gene names
sirk1_sp3-solu-SUC-3-P	25893	FTMS; HCD	65.18	757.37	PLP6

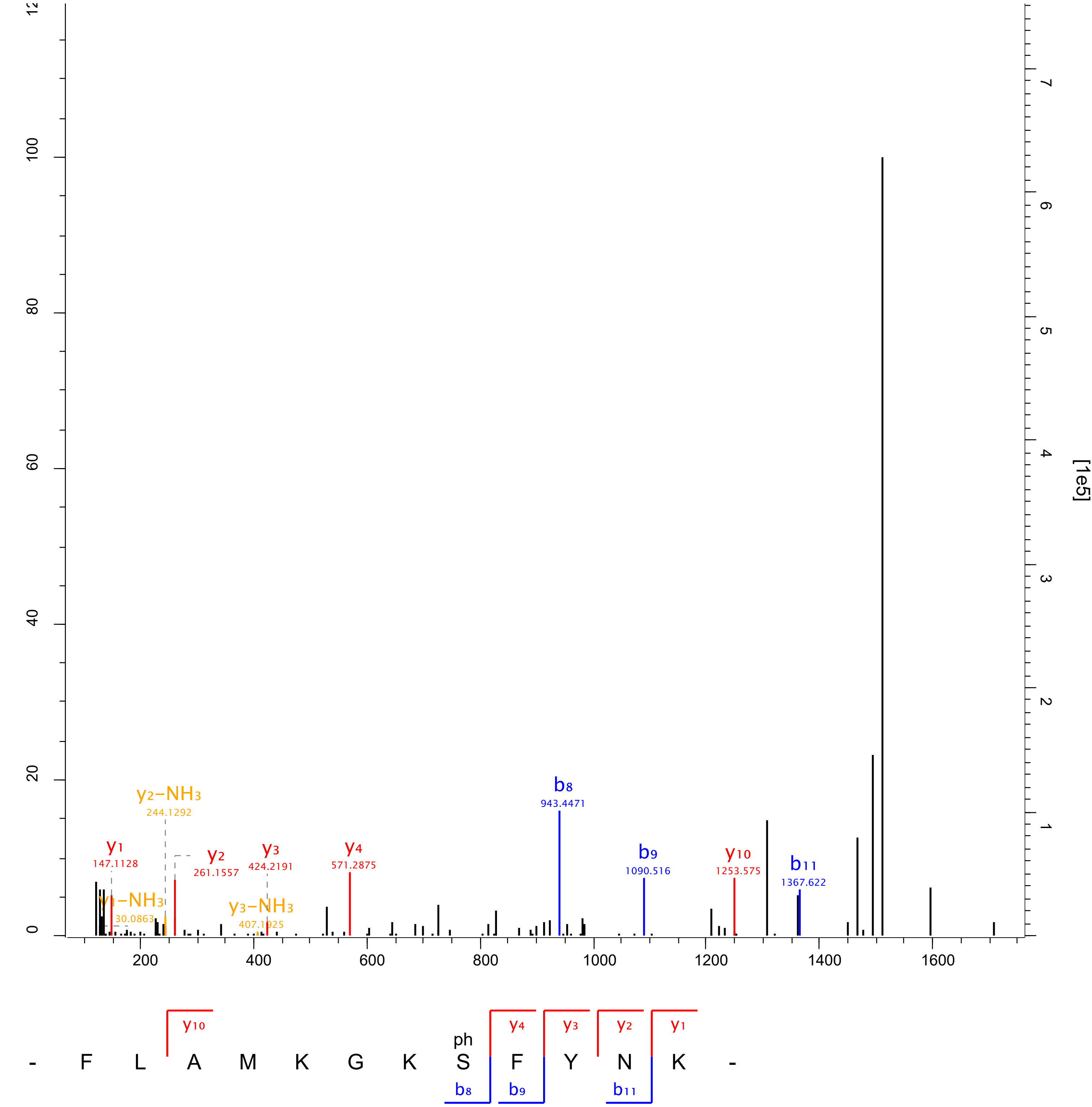

Sample output to test PDF Combine only

Raw file	Scan	Method	Score	m/z	Gene names
sirk1_sp3-solu-SUC-3-P	26344	FTMS; HCD	43.03	571.58	At5g13590

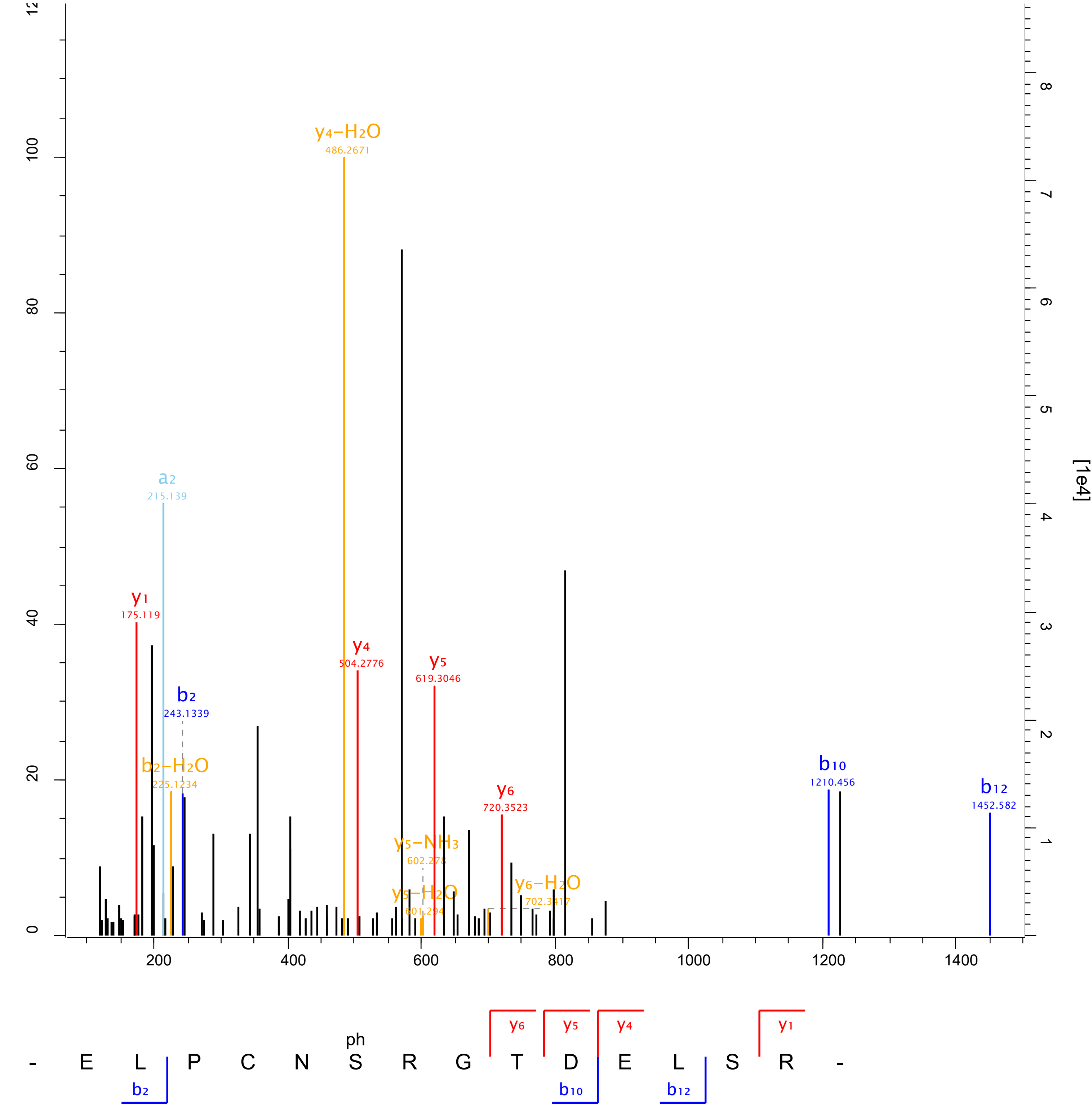

Sample output to test PDF Combine only

Raw file	Scan	Method	Score	m/z	Gene names
sirk1_sp3-solu-SUC-3-P	33097	FTMS; HCD	41.62	815.37	PCMP-H29

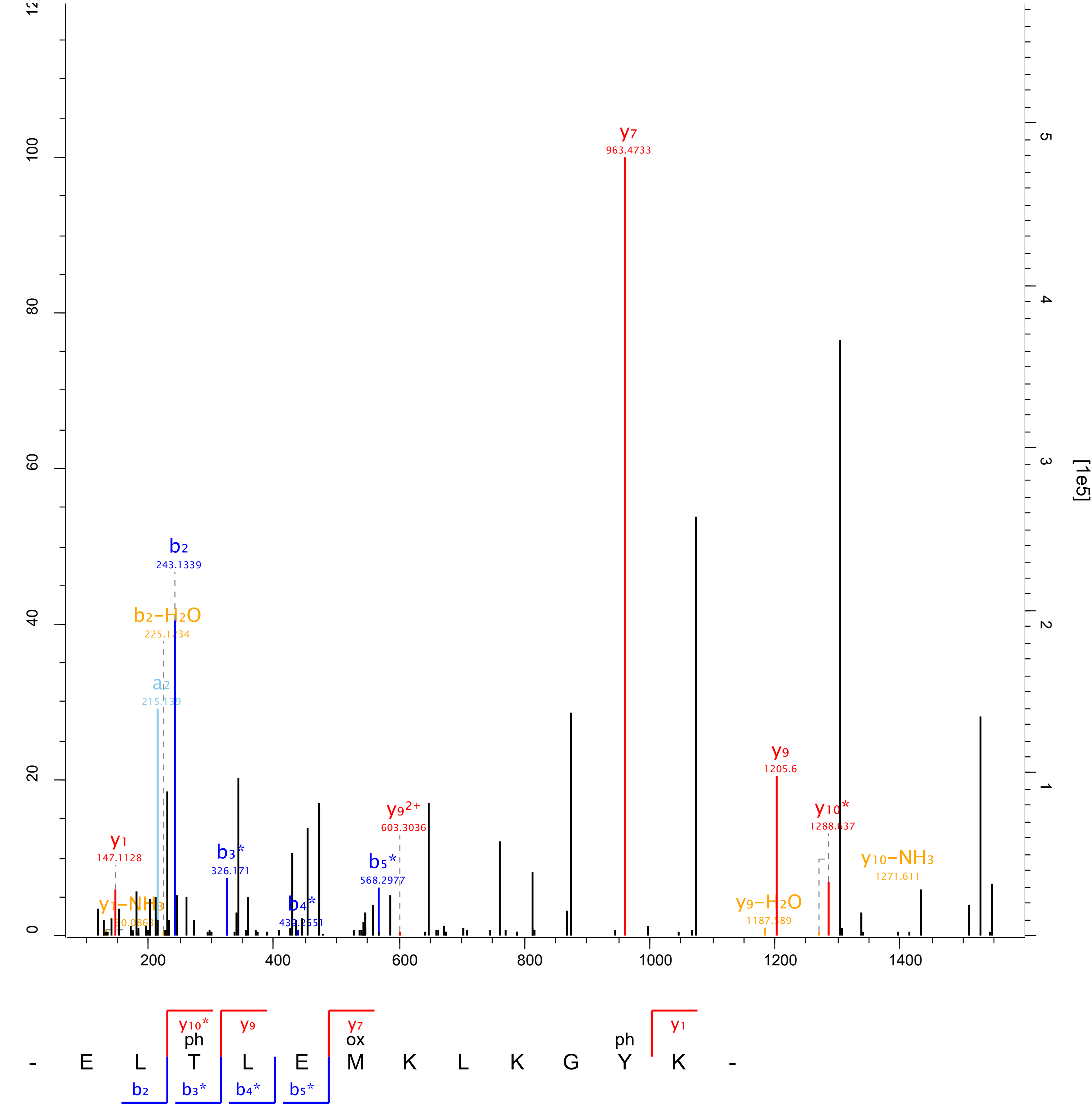

Sample output to test PDF Combine only

Raw file	Scan	Method	Score	m/z	Gene names
sirk1-mic-0-1-A	6034	FTMS; HCD	83.08	473.23	PPC3-1.2

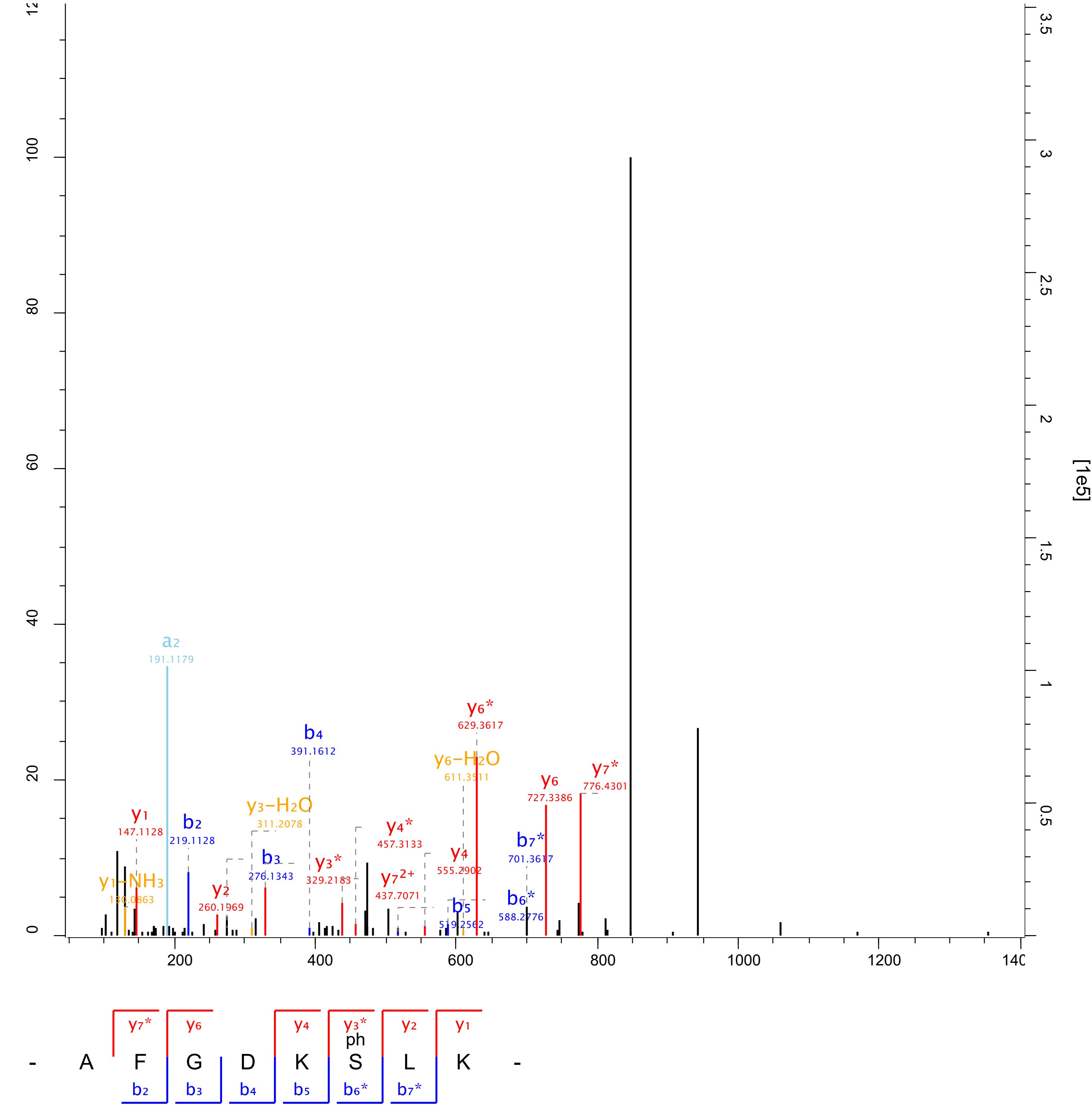

Sample output to test PDF Combine only

Raw file	Scan	Method	Score	m/z	Gene names
sirk1-mic-0-1-A	13709	FTMS; HCD	66.02	728.32	F18B13.26

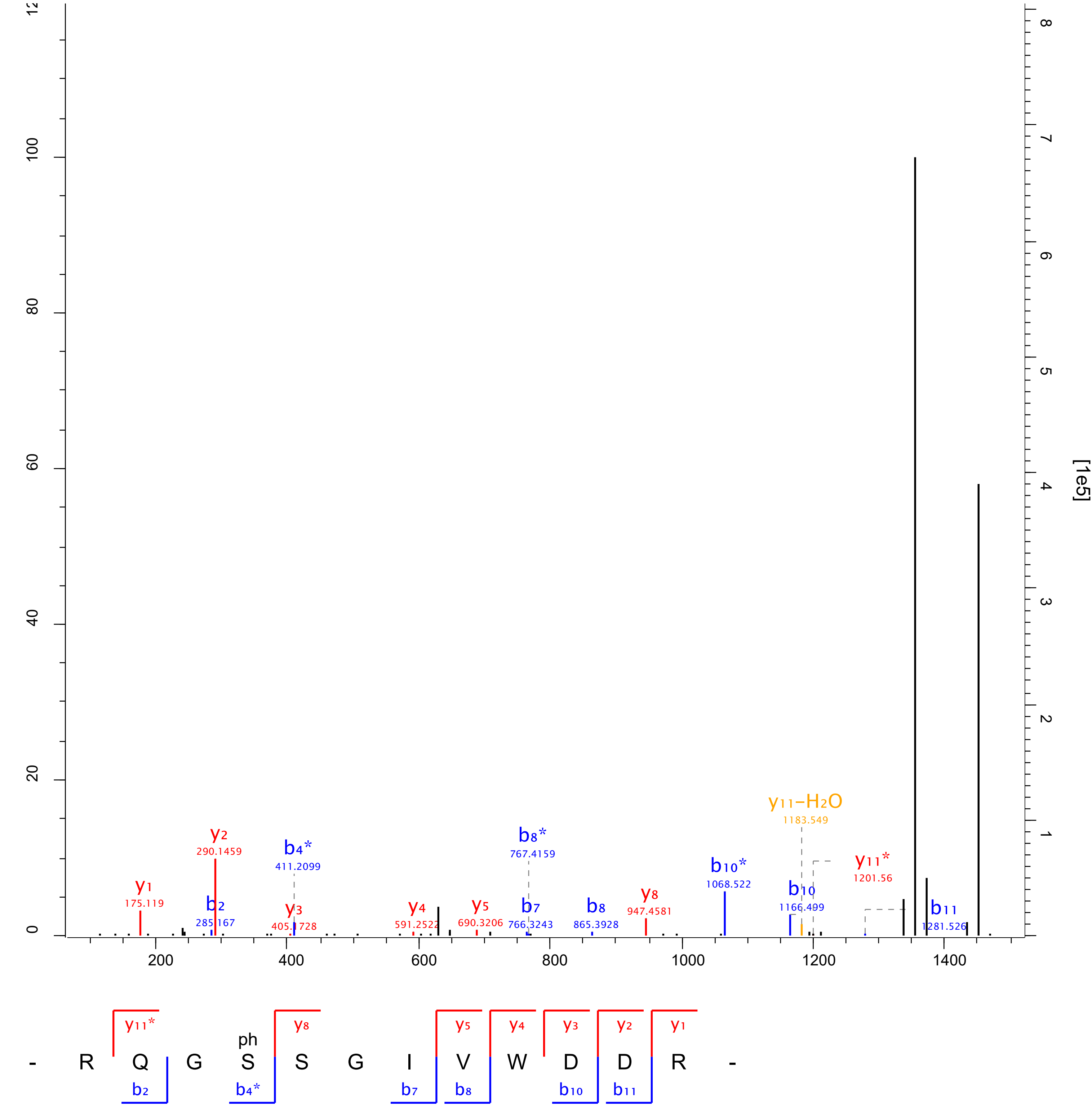

Sample output to test PDF Combine only

Raw file	Scan	Method	Score	m/z	Gene names
sirk1-mic-0-1-A	23381	FTMS; HCD	223.28	647.27	At5g43830

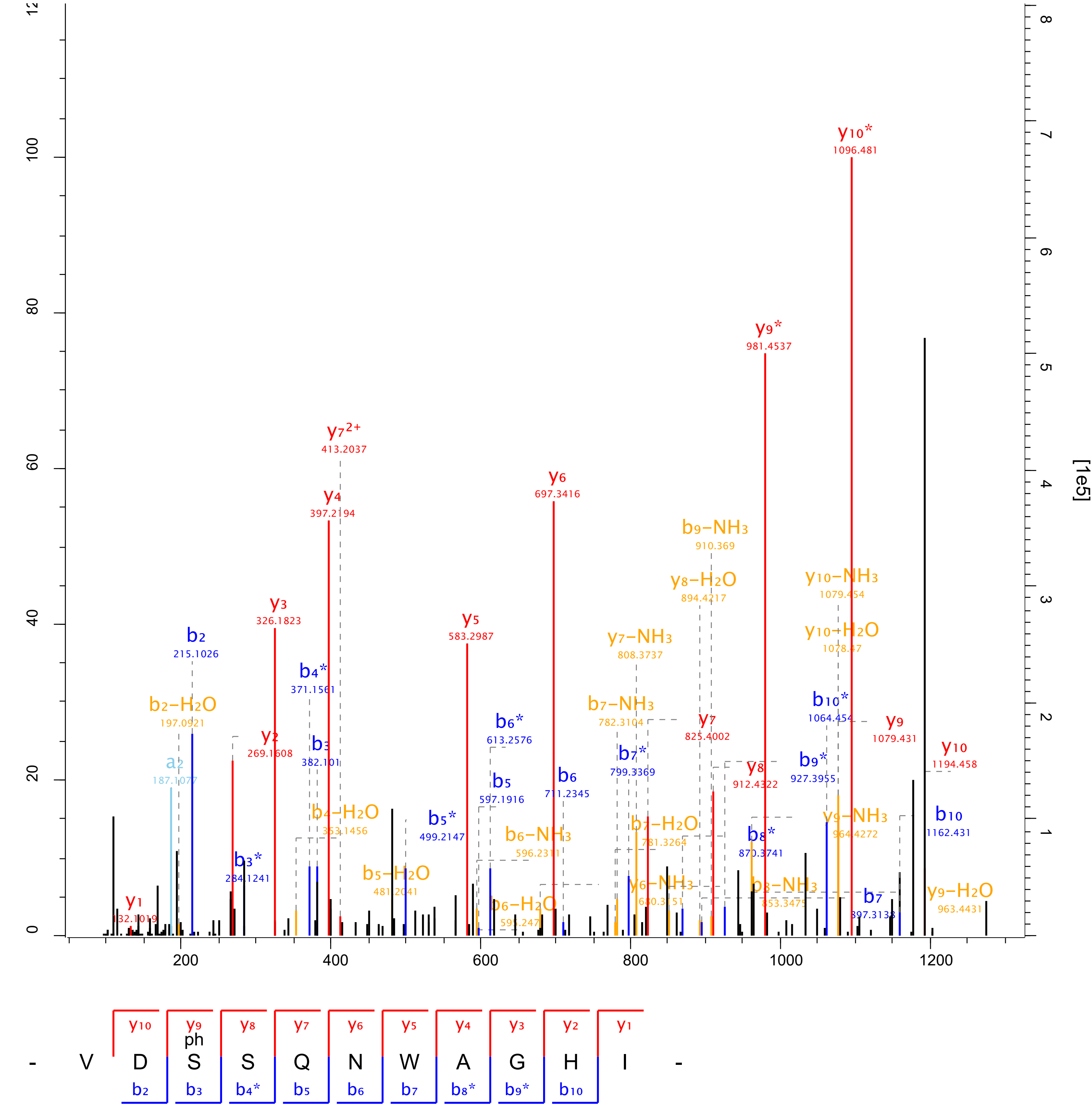

Sample output to test PDF Combine only

Raw file	Scan	Method	Score	m/z
sirk1-mic-0-1-A	27418	FTMS; HCD	91.31	558.77

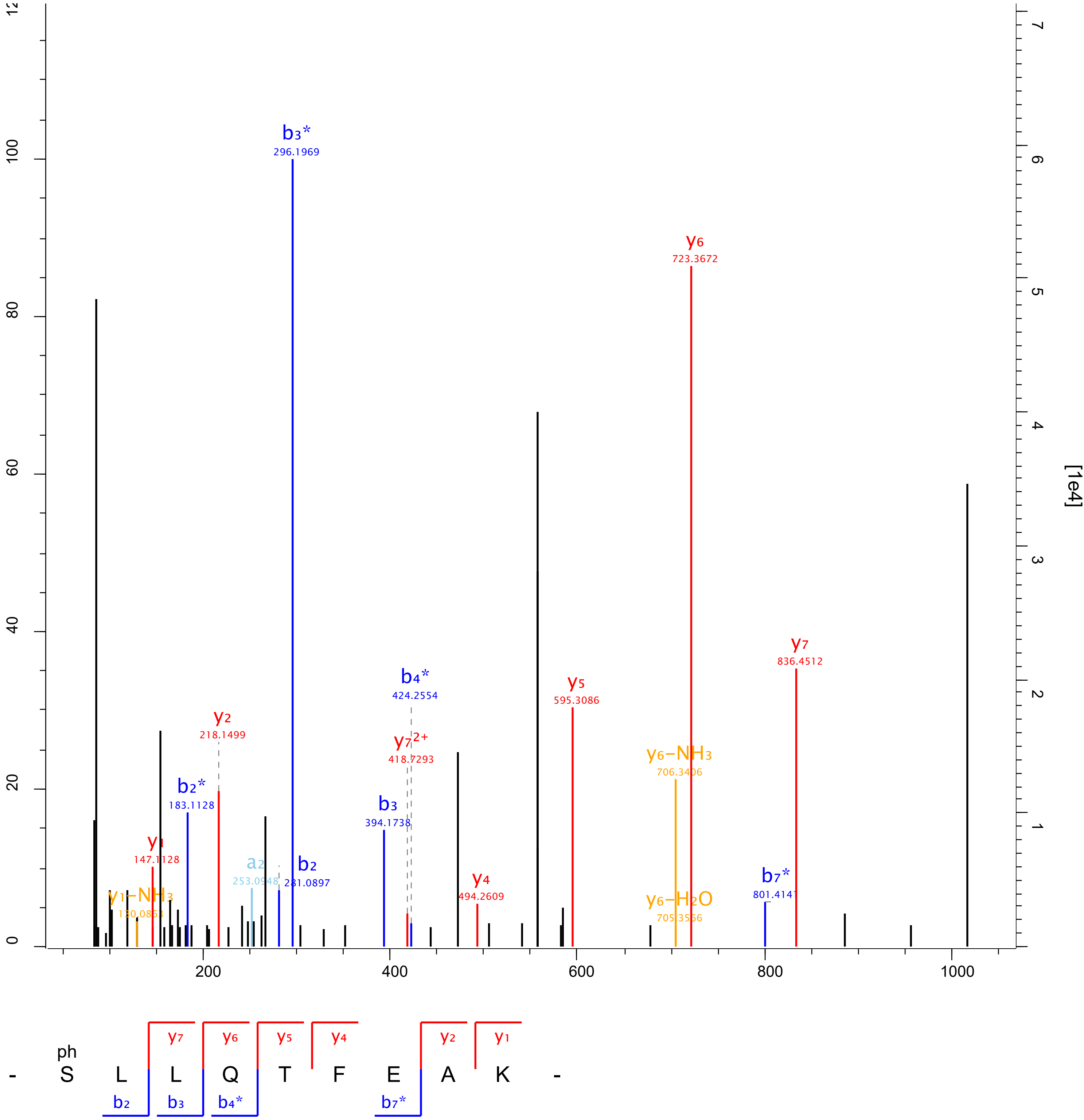

Sample output to test PDF Combine only

Raw file	Scan	Method	Score	m/z	Gene names
sirk1-mic-0-1-A	27682	FTMS; HCD	71.18	801.86	F28G4.18

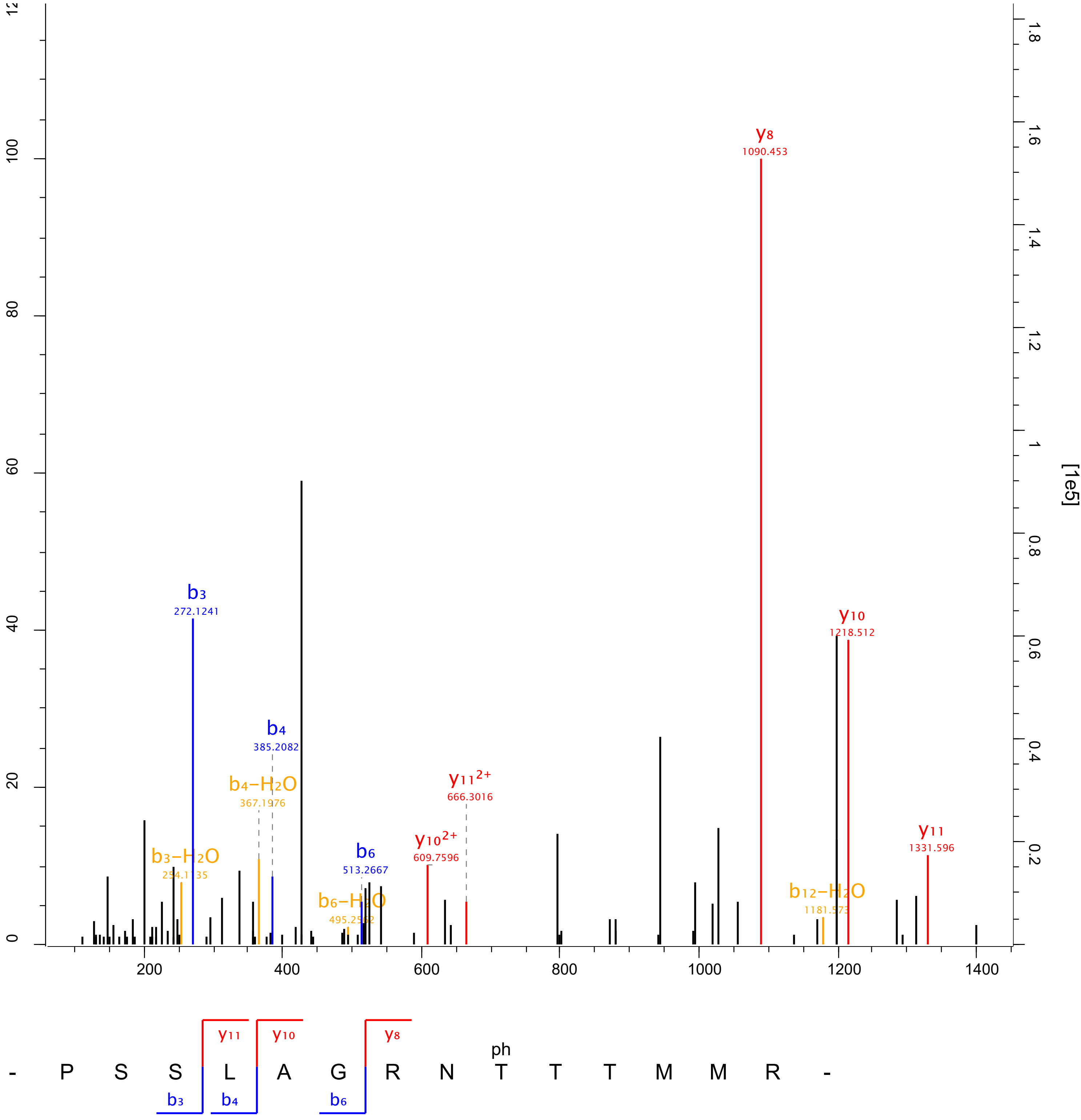

Sample output to test PDF Combine only

Raw file	Scan	Method	Score	m/z	Gene names
sirk1-mic-0-1-P	14308	FTMS; HCD	79.84	527.24	ABCB4

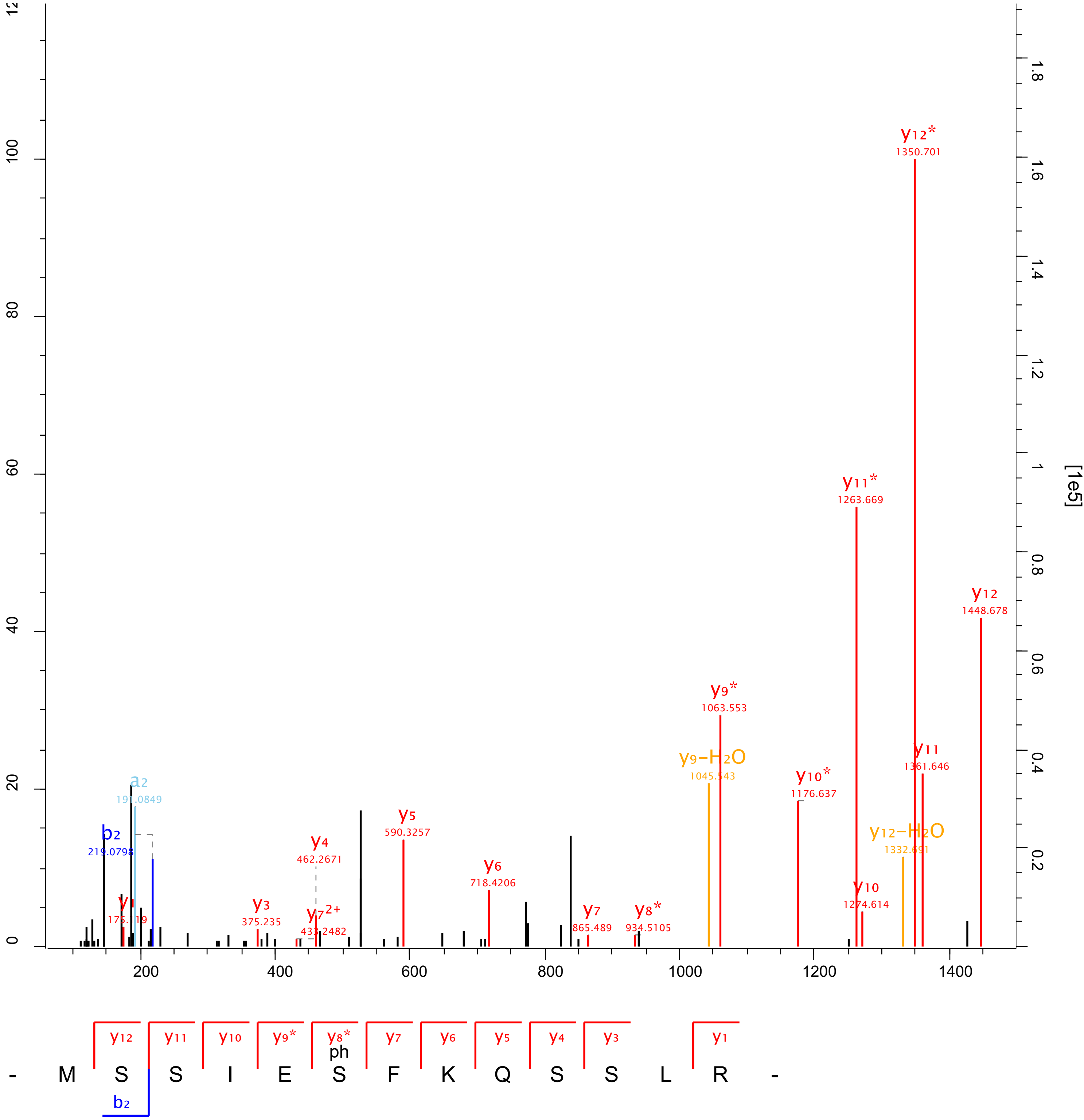

Sample output to test PDF Combine only

Raw file	Scan	Method	Score	m/z	Gene names
sirk1-mic-0-1-P	19582	FTMS; HCD	99.84	609.29	T25B24.6

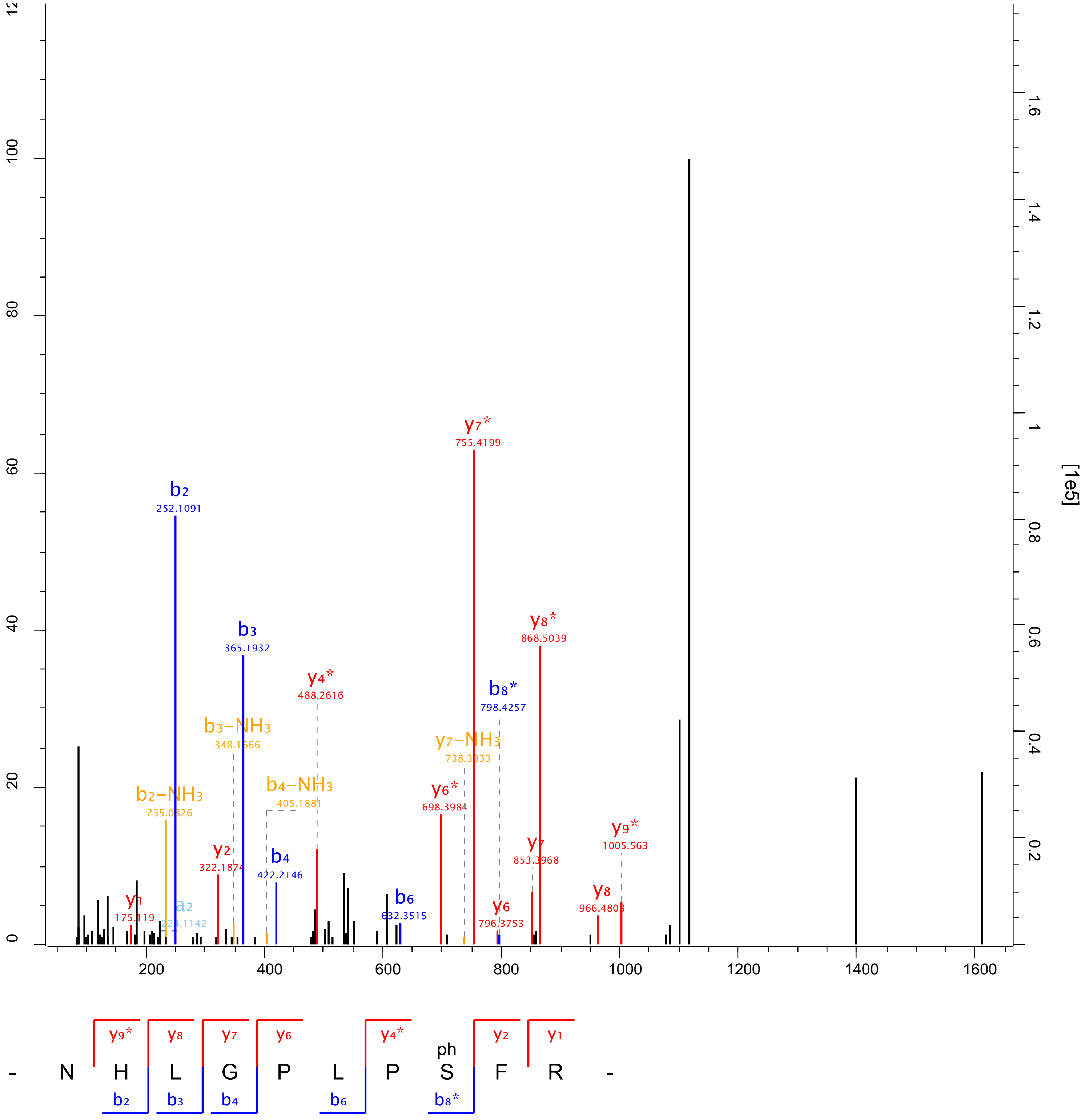

Sample output to test PDF Combine only

Raw file	Scan	Method	Score	m/z
sirk1-mic-0-1-P	31008	FTMS; HCD	92.89	737.34

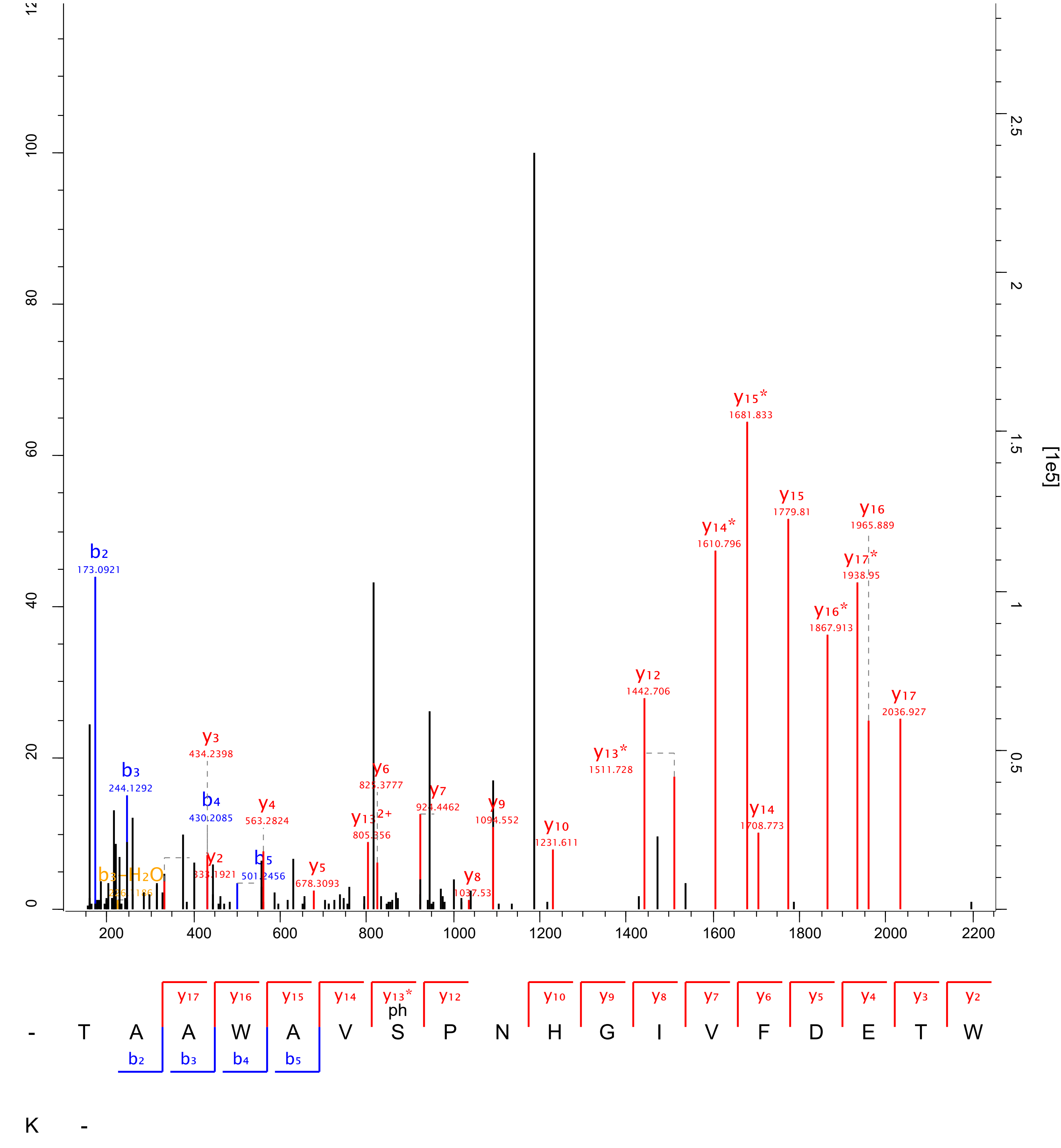

Sample output to test PDF Combine only

Raw file	Scan	Method	Score	m/z	Gene names
sirk1-mic-0-1-P	36854	FTMS; HCD	53.45	724.83	MNC6.16

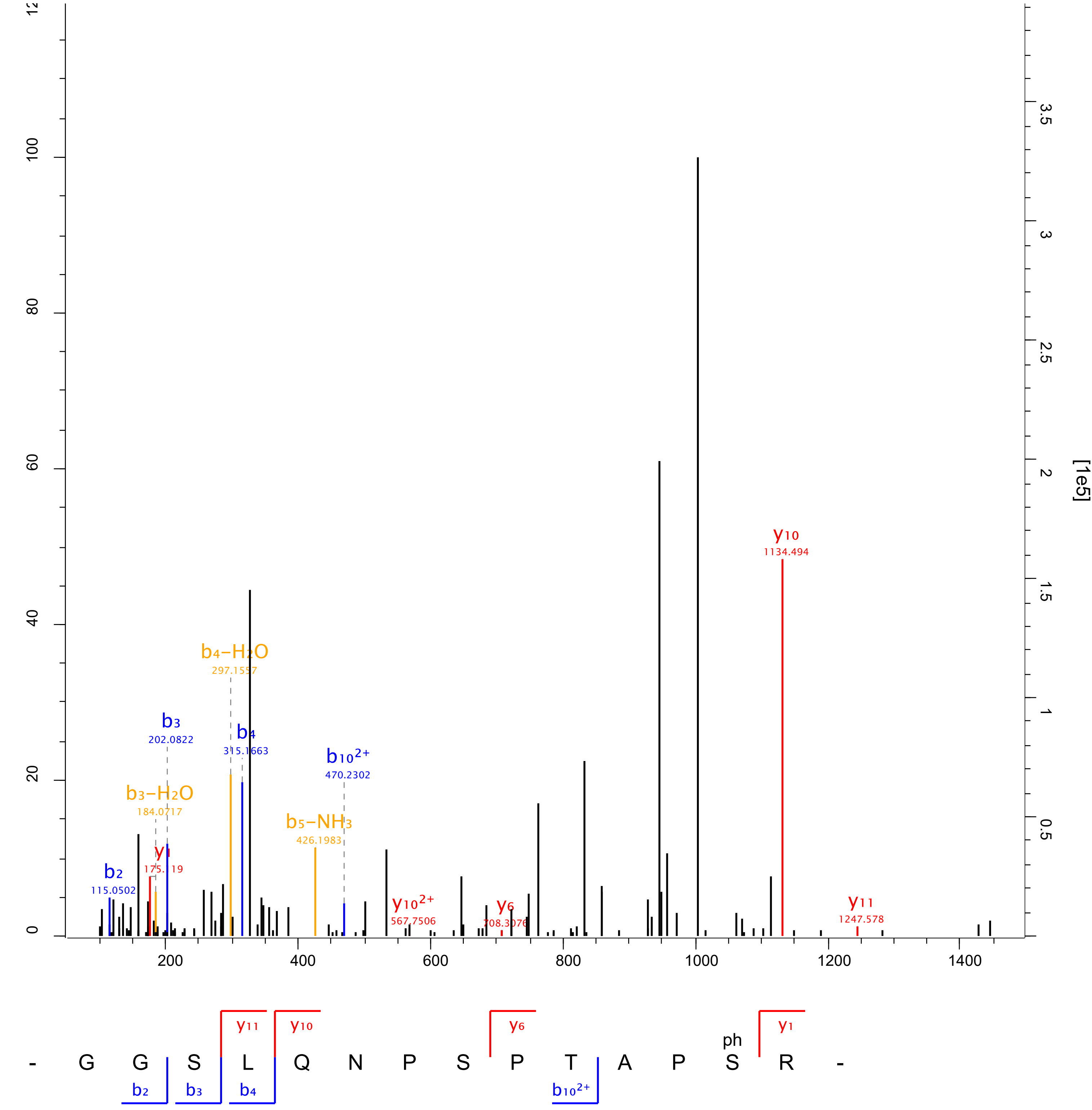

Sample output to test PDF Combine only

Raw file	Scan	Method	Score	m/z	Gene names
sirk1-mic-0-1-P	38724	FTMS; HCD	41.7	1069.98	At5g38510

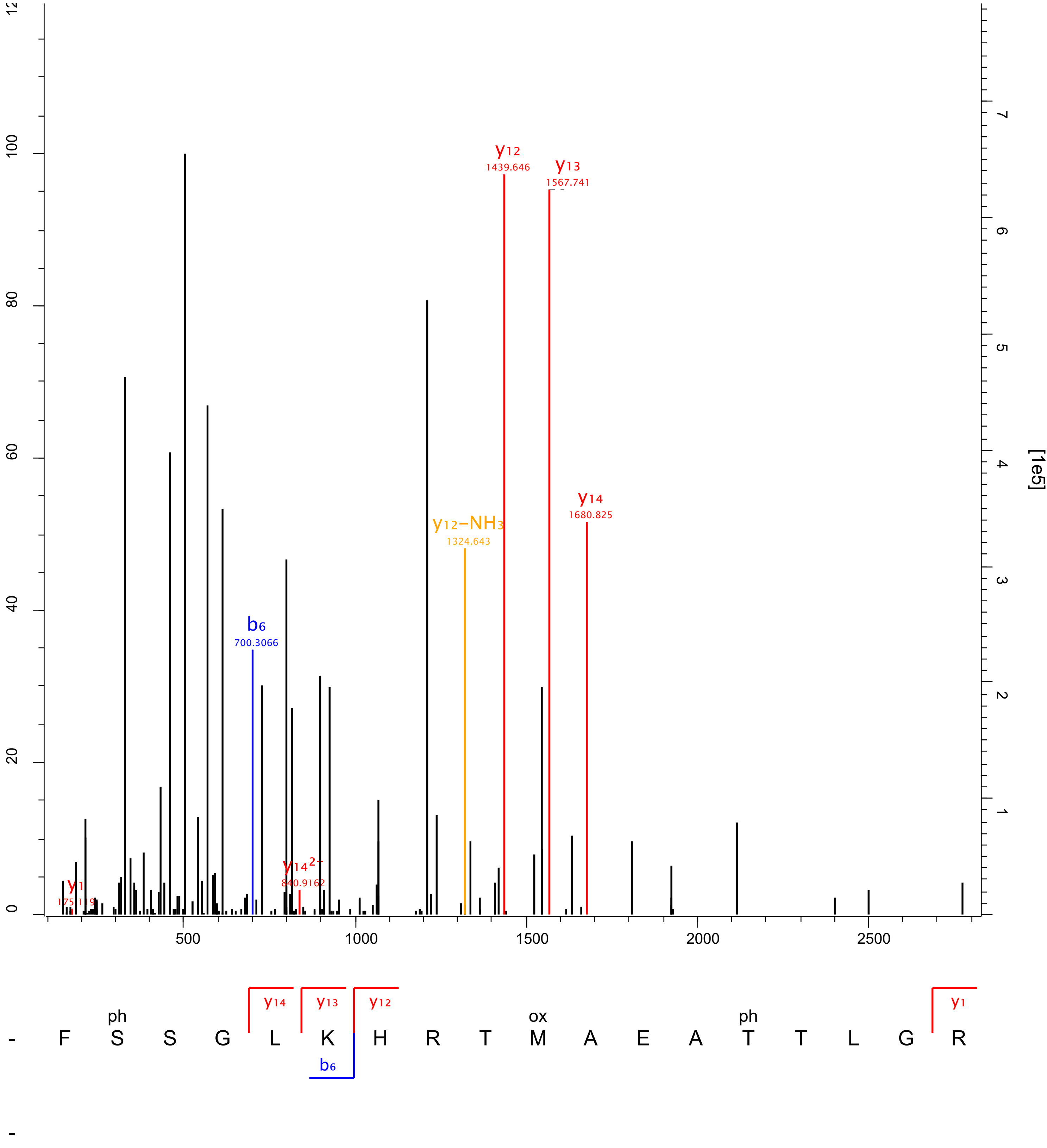

Sample output to test PDF Combine only

Raw file	Scan	Method	Score	m/z	Gene names
sirk1-mic-0-2-A	10056	FTMS; HCD	46.89	508.24	RS40

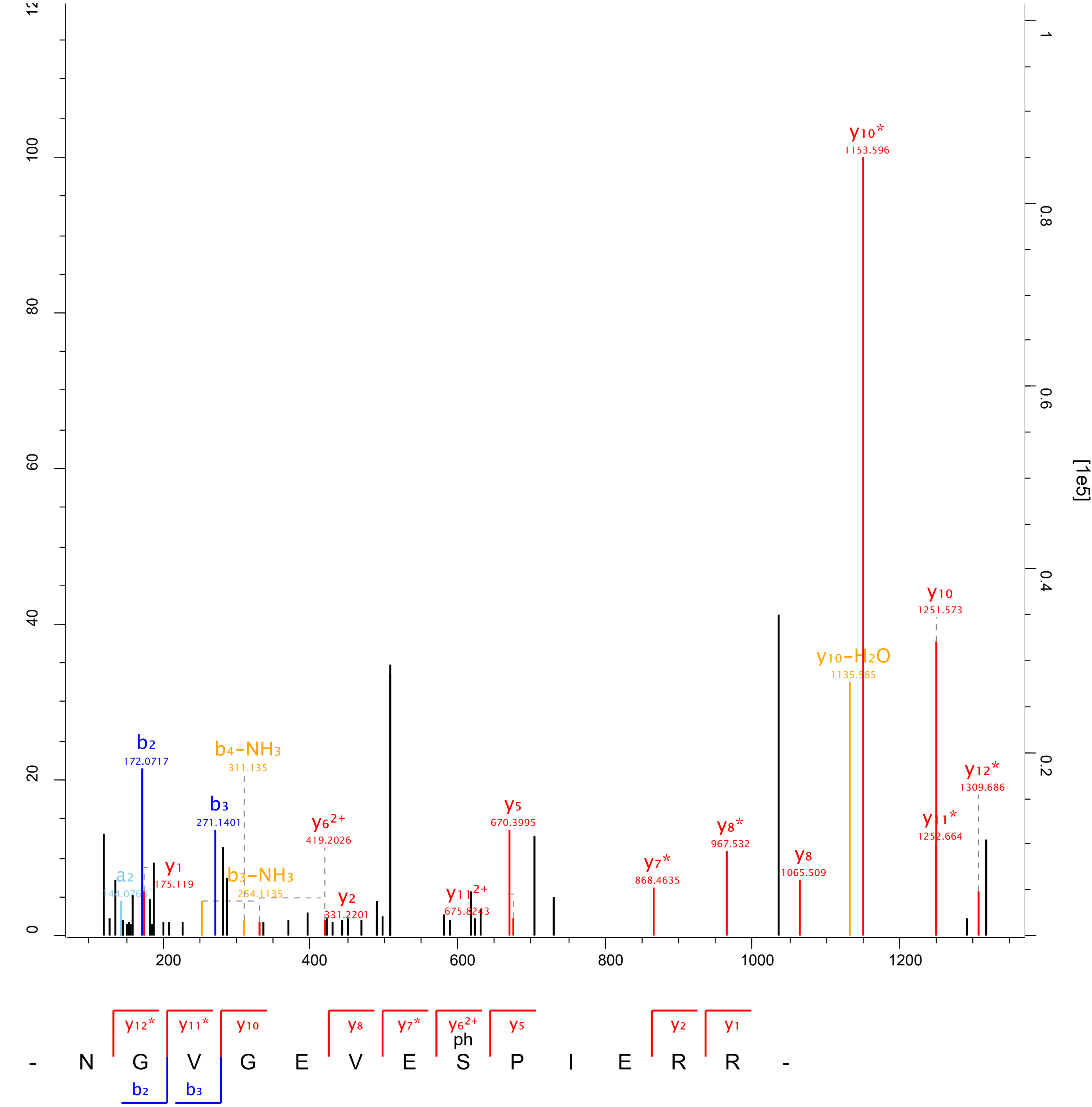

Sample output to test PDF Combine only

Raw file	Scan	Method	Score	m/z	Gene names
sirk1-mic-0-2-A	20487	FTMS; HCD	64.65	401.2	POT13

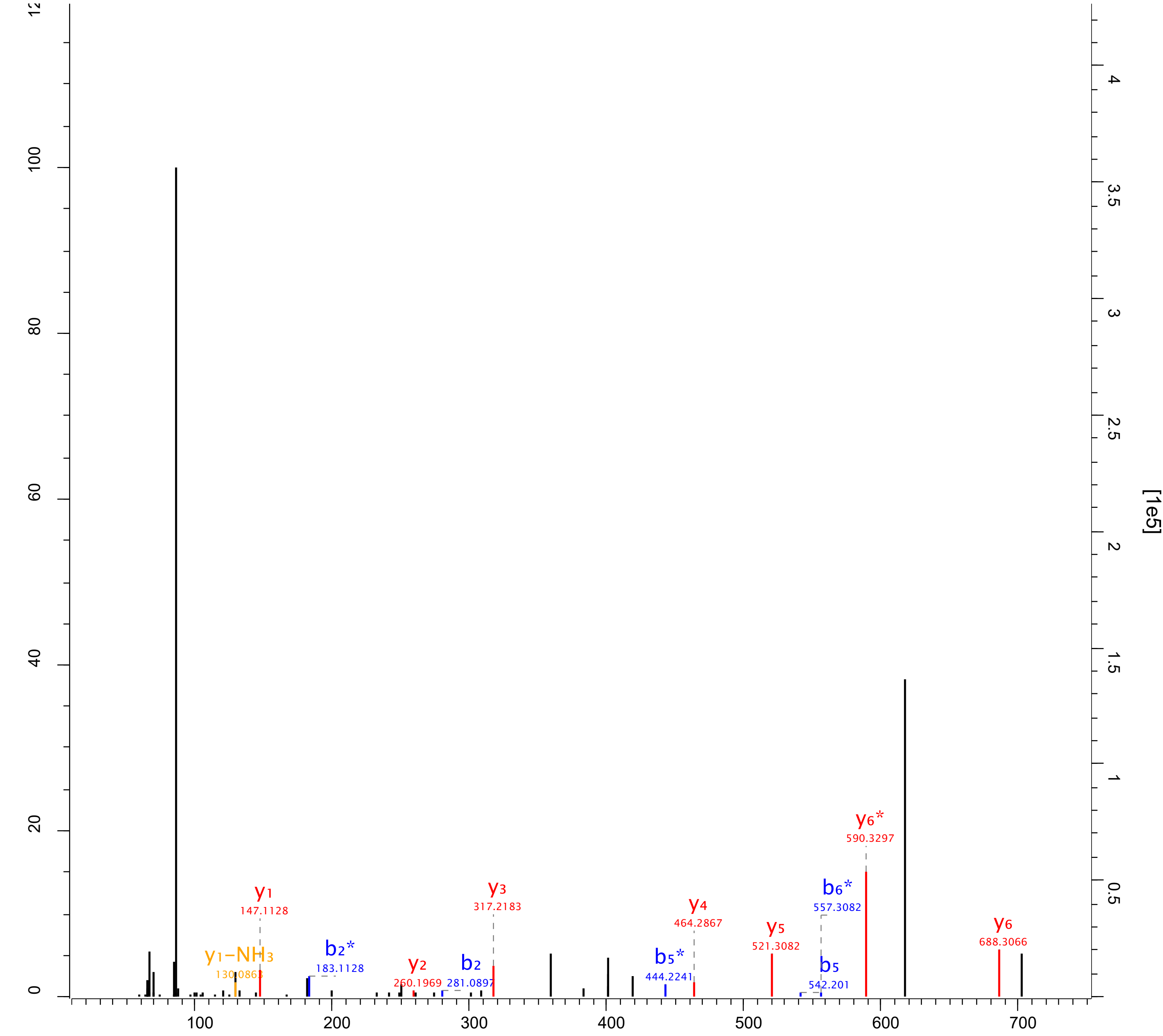

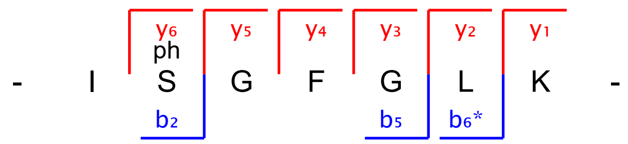

Sample output to test PDF Combine only

Raw file	Scan	Method	Score	m/z	Gene names
sirk1-mic-0-2-A	22550	FTMS; HCD	107.79	528.25	PLC2

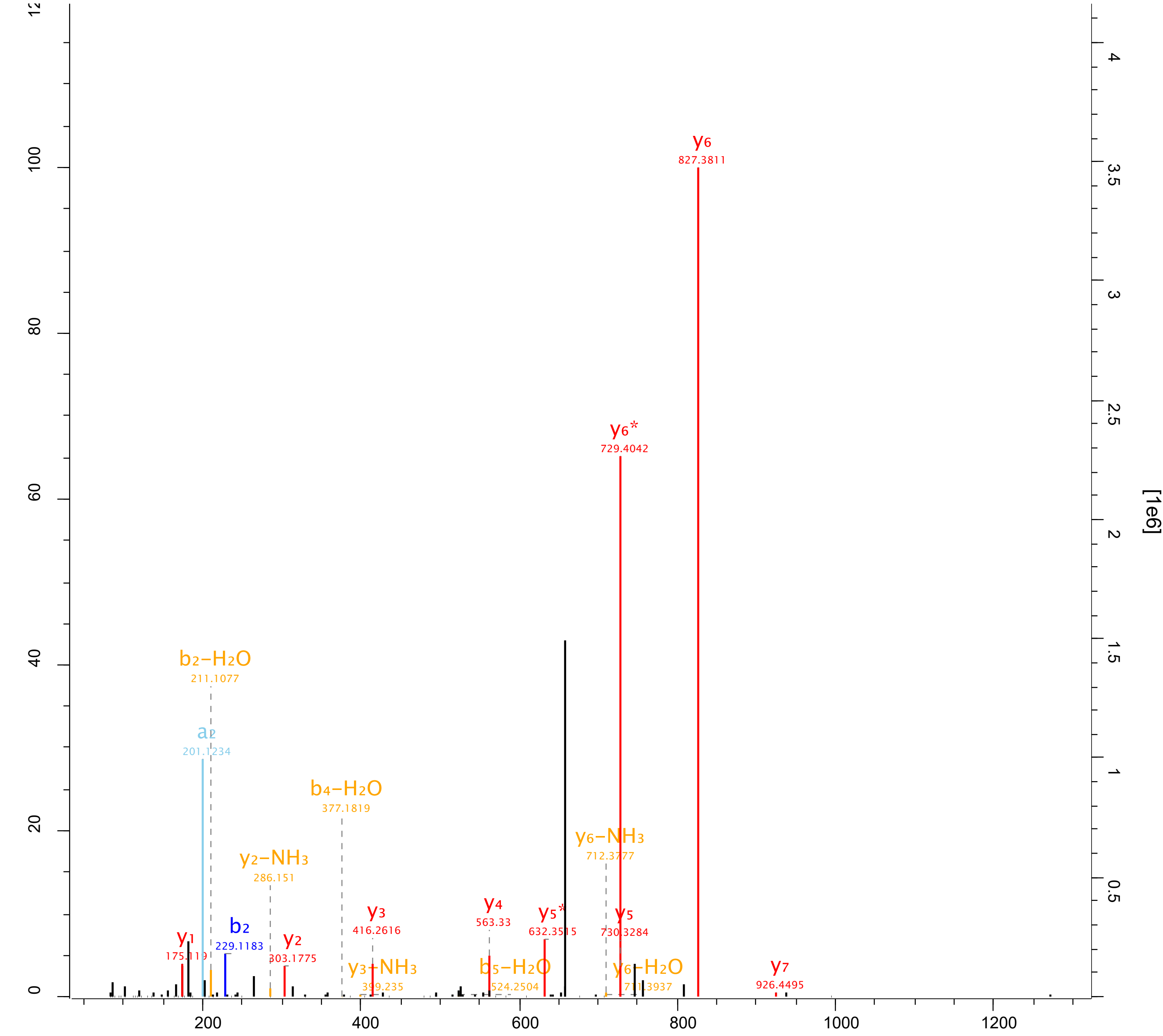

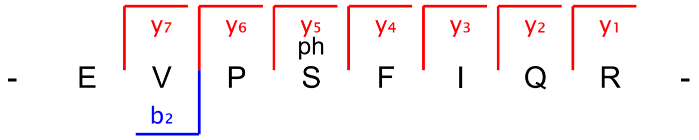

Sample output to test PDF Combine only

Raw file	Scan	Method	Score	m/z	Gene names
sirk1-mic-0-3-A	3224	FTMS; HCD	89.27	609.26	At1g21080;T22I11.9

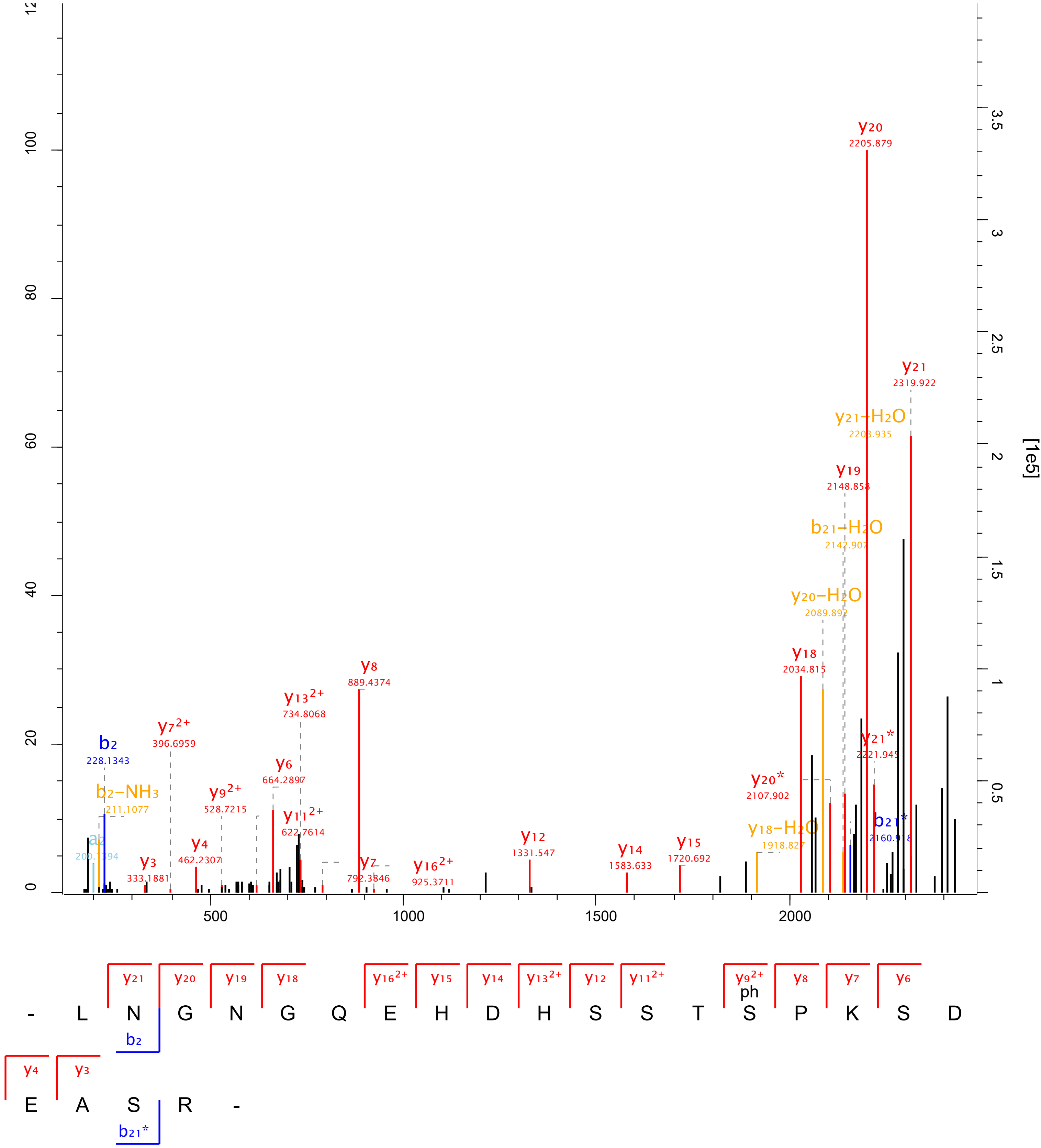

Sample output to test PDF Combine only

Raw file	Scan	Method	Score	m/z	Gene names
sirk1-mic-0-3-A	3271	FTMS; HCD	74.52	830.66	ATJ15

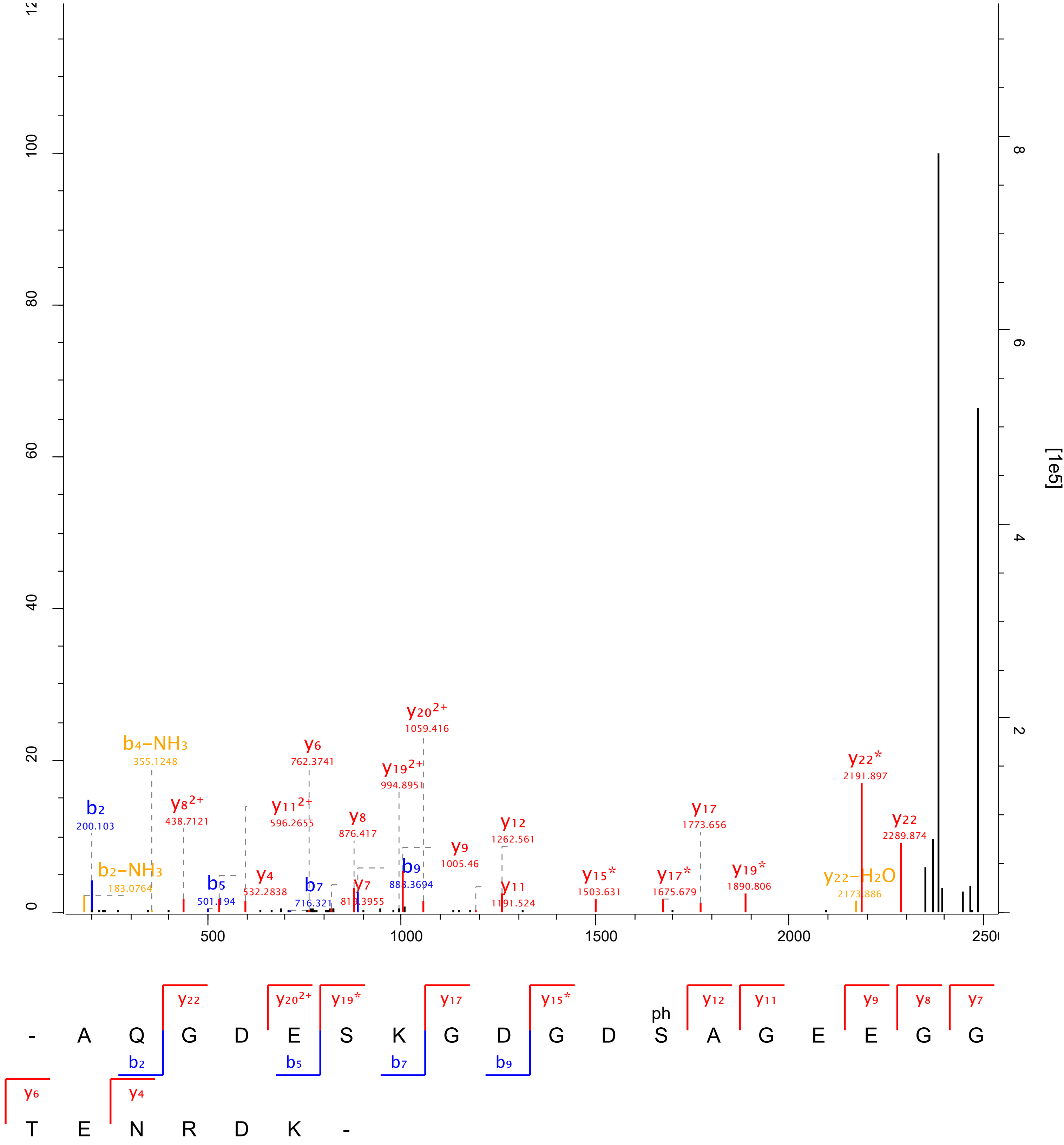

Sample output to test PDF Combine only

Raw file	Scan	Method	Score	m/z	Gene names
sirk1-mic-0-3-A	3309	FTMS; HCD	76.07	425.18	At4g35785

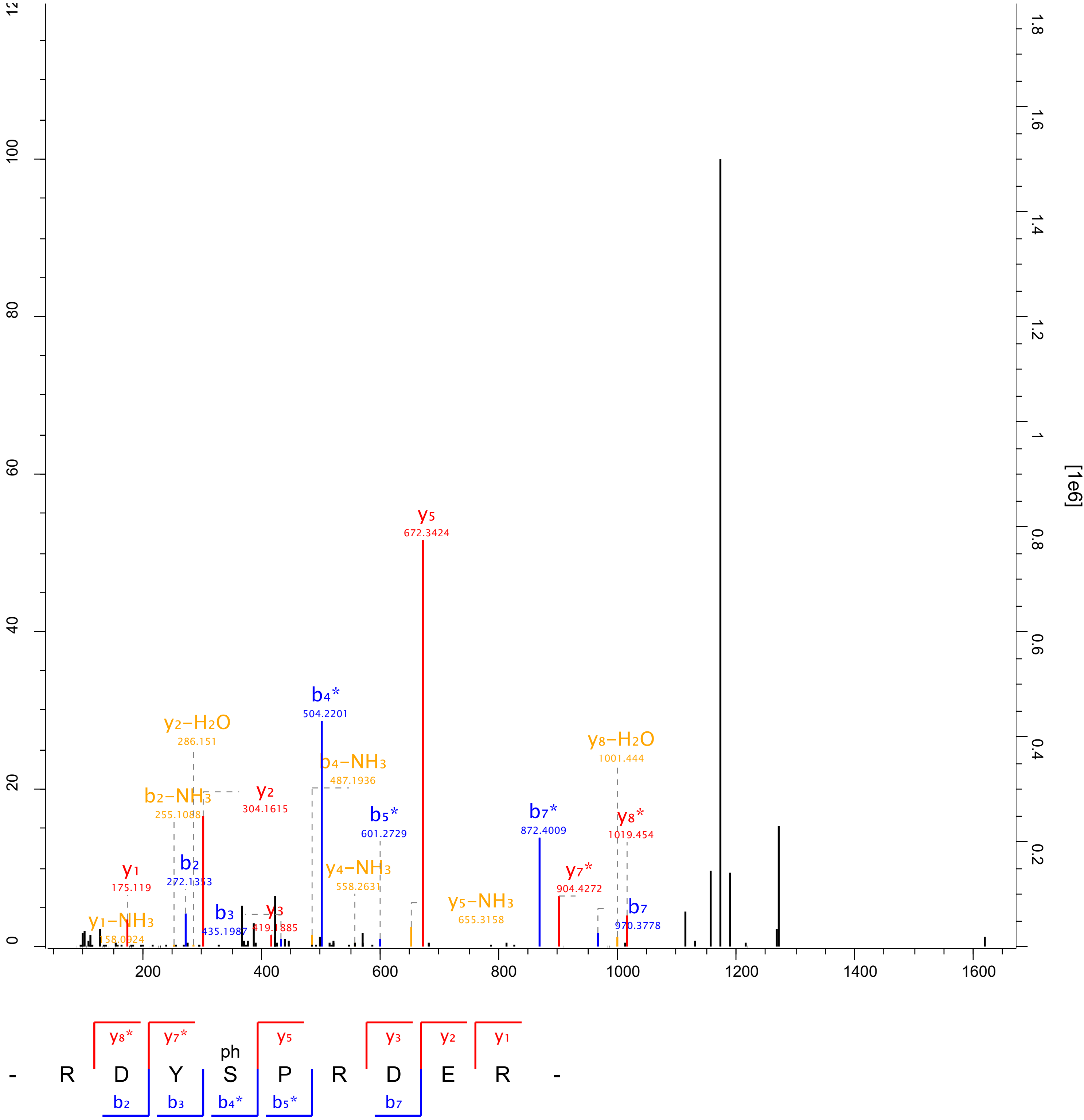

Sample output to test PDF Combine only

Raw file	Scan	Method	Score	m/z	Gene names
sirk1-mic-0-3-A	3467	FTMS; HCD	95.42	558.73	RS41;RS40

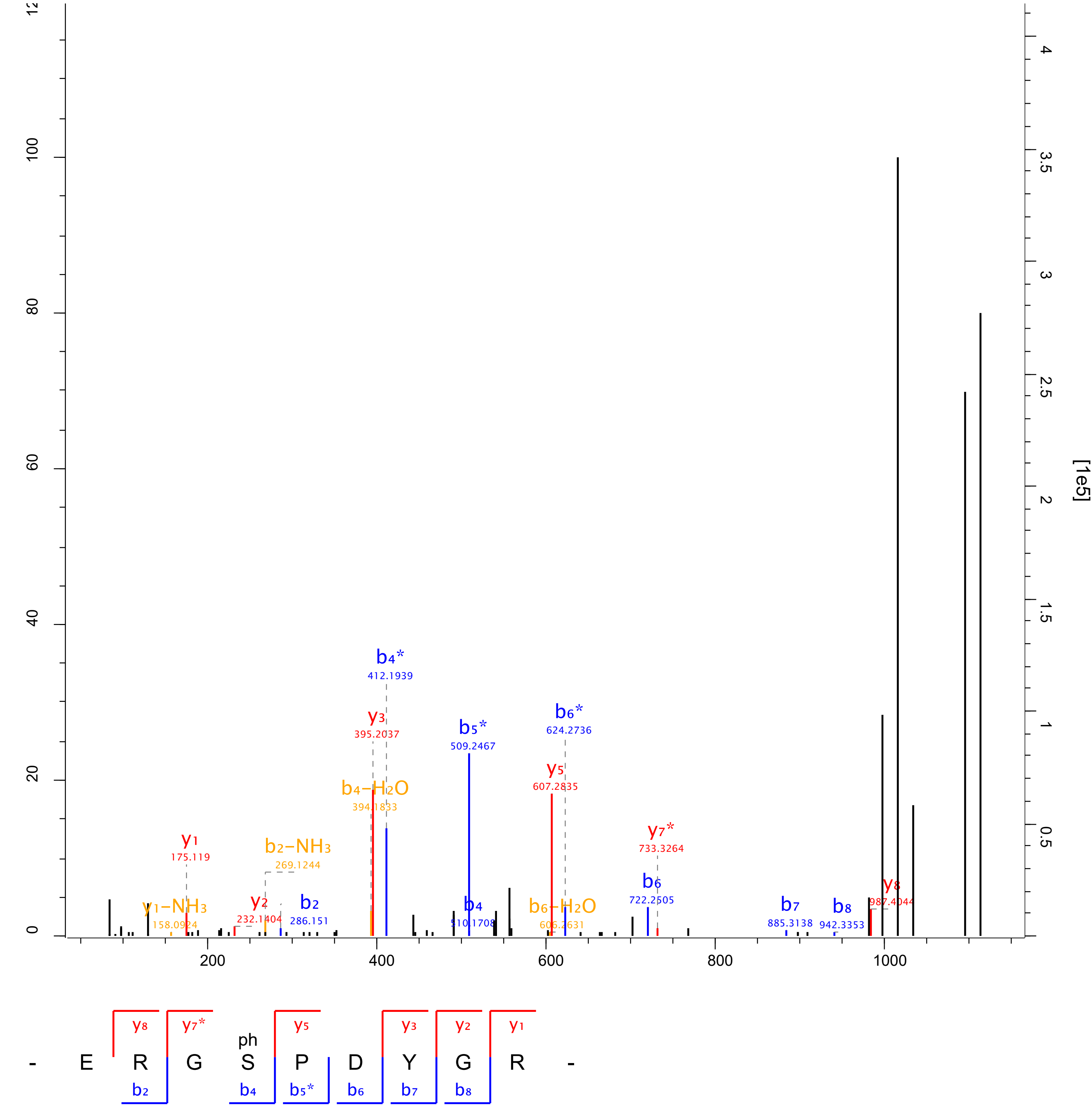

Sample output to test PDF Combine only

Raw file	Scan	Method	Score	m/z	Gene names
sirk1-mic-0-3-A	3744	FTMS; HCD	60.33	577.9	At2g29210

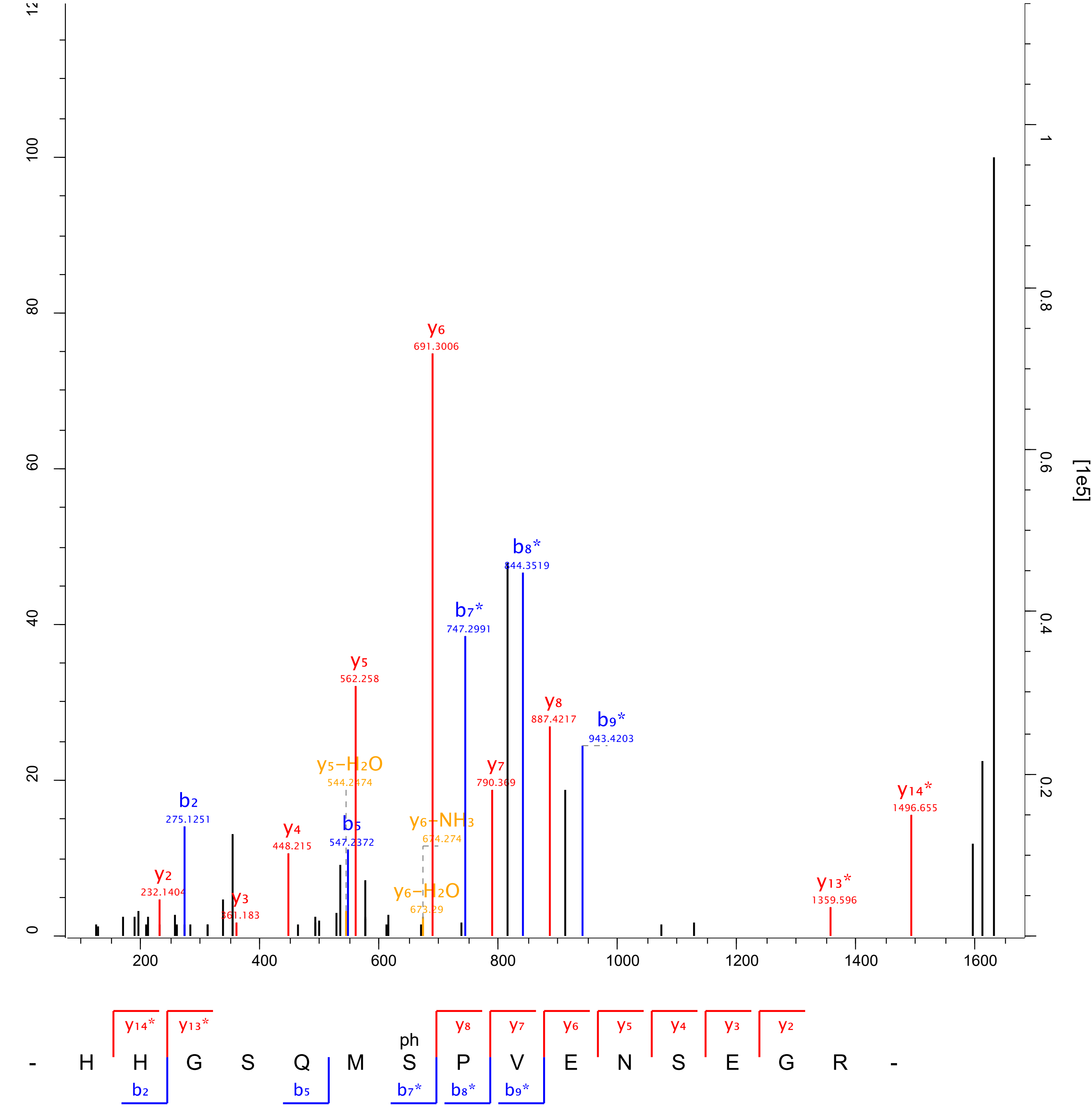

Sample output to test PDF Combine only

Raw file	Scan	Method	Score	m/z	Gene names
sirk1-mic-0-3-A	3914	FTMS; HCD	83.86	489.24	F19F18.190

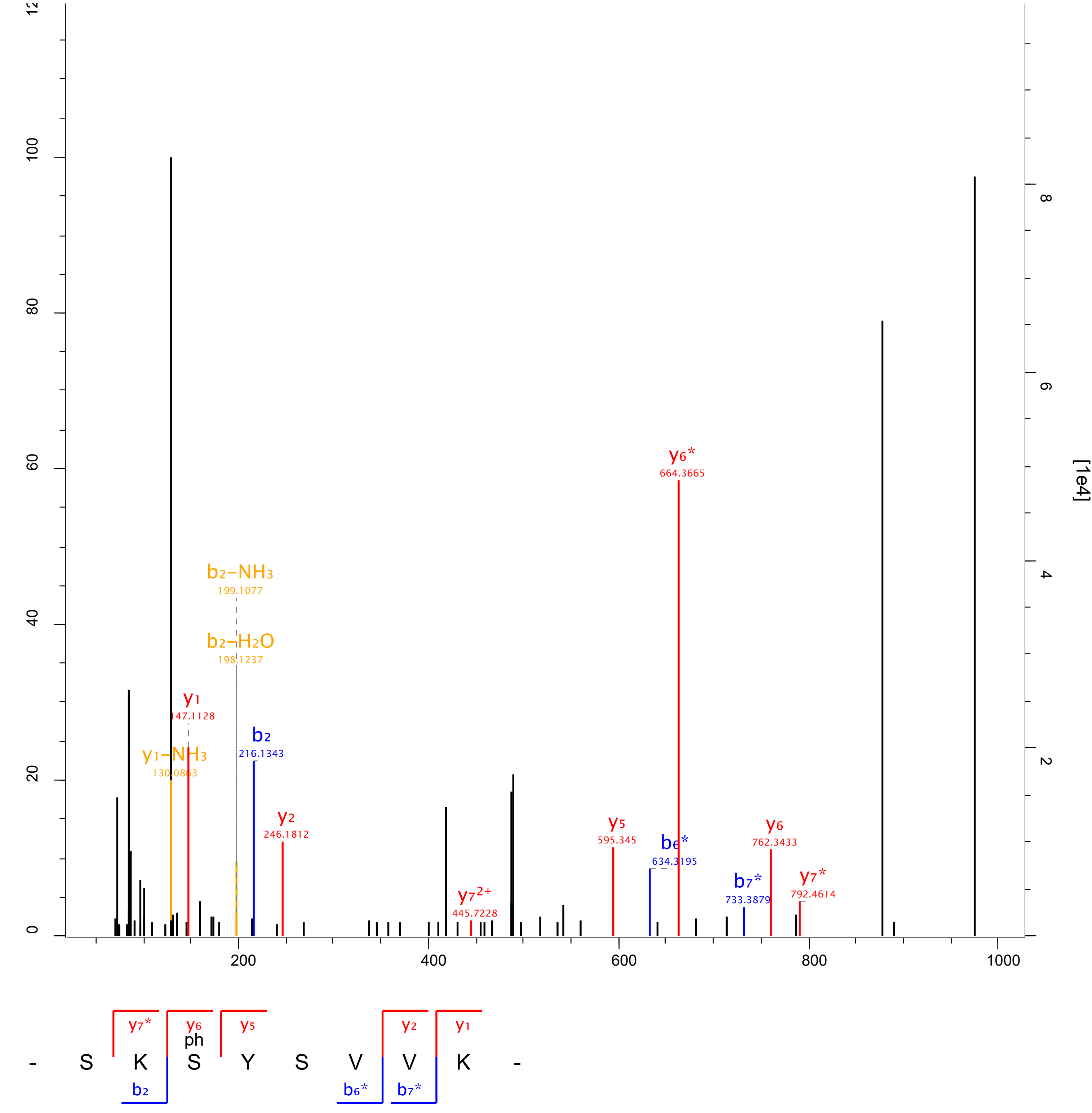

Sample output to test PDF Combine only

Raw file	Scan	Method	Score	m/z	Gene names
sirk1-mic-0-3-A	4460	FTMS; HCD	52.57	536.57	T22F8.50

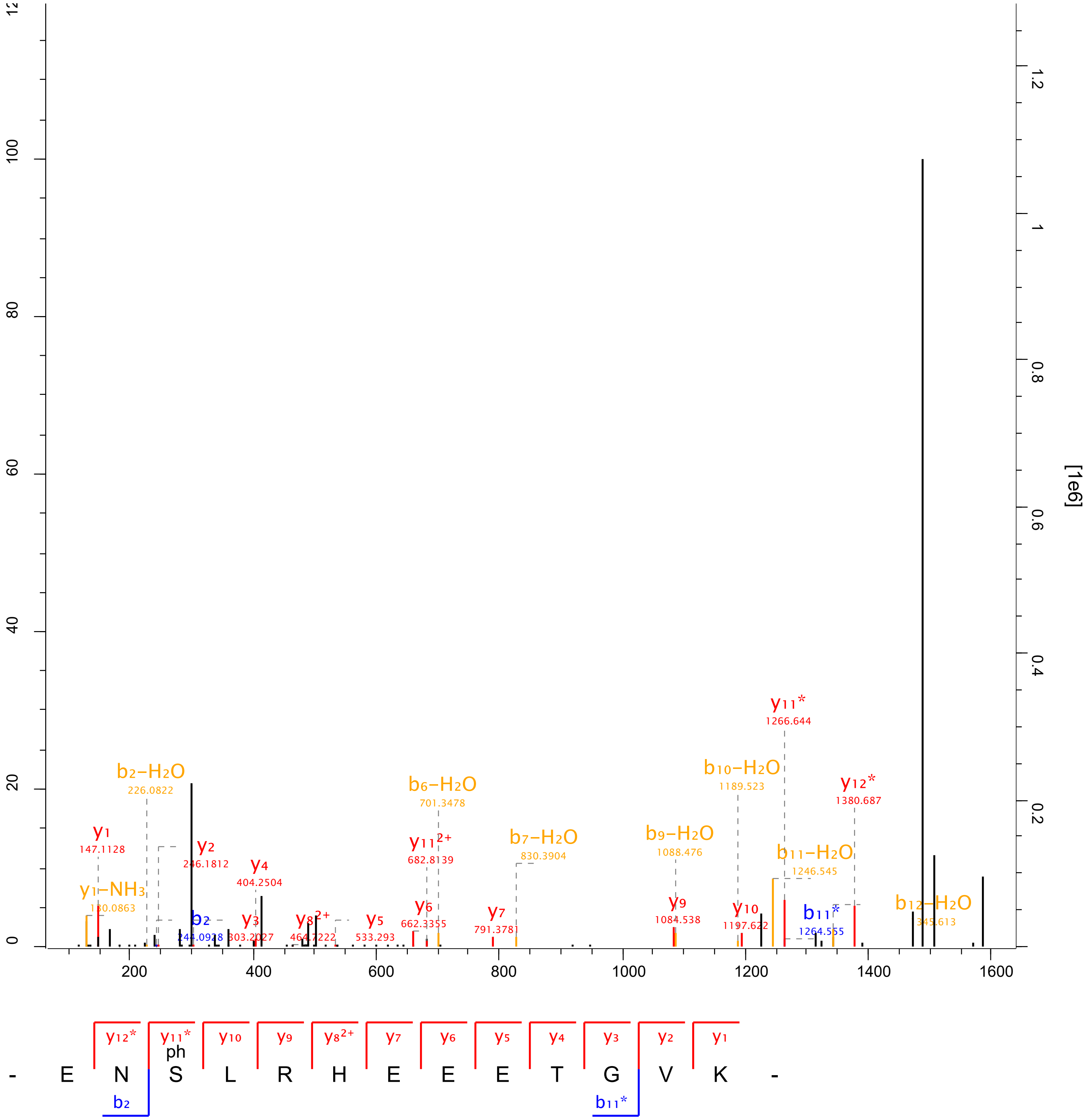

Sample output to test PDF Combine only

Raw file	Scan	Method	Score	m/z	Gene names
sirk1-mic-0-3-A	4786	FTMS; HCD	118.72	618.27	F23A5.29

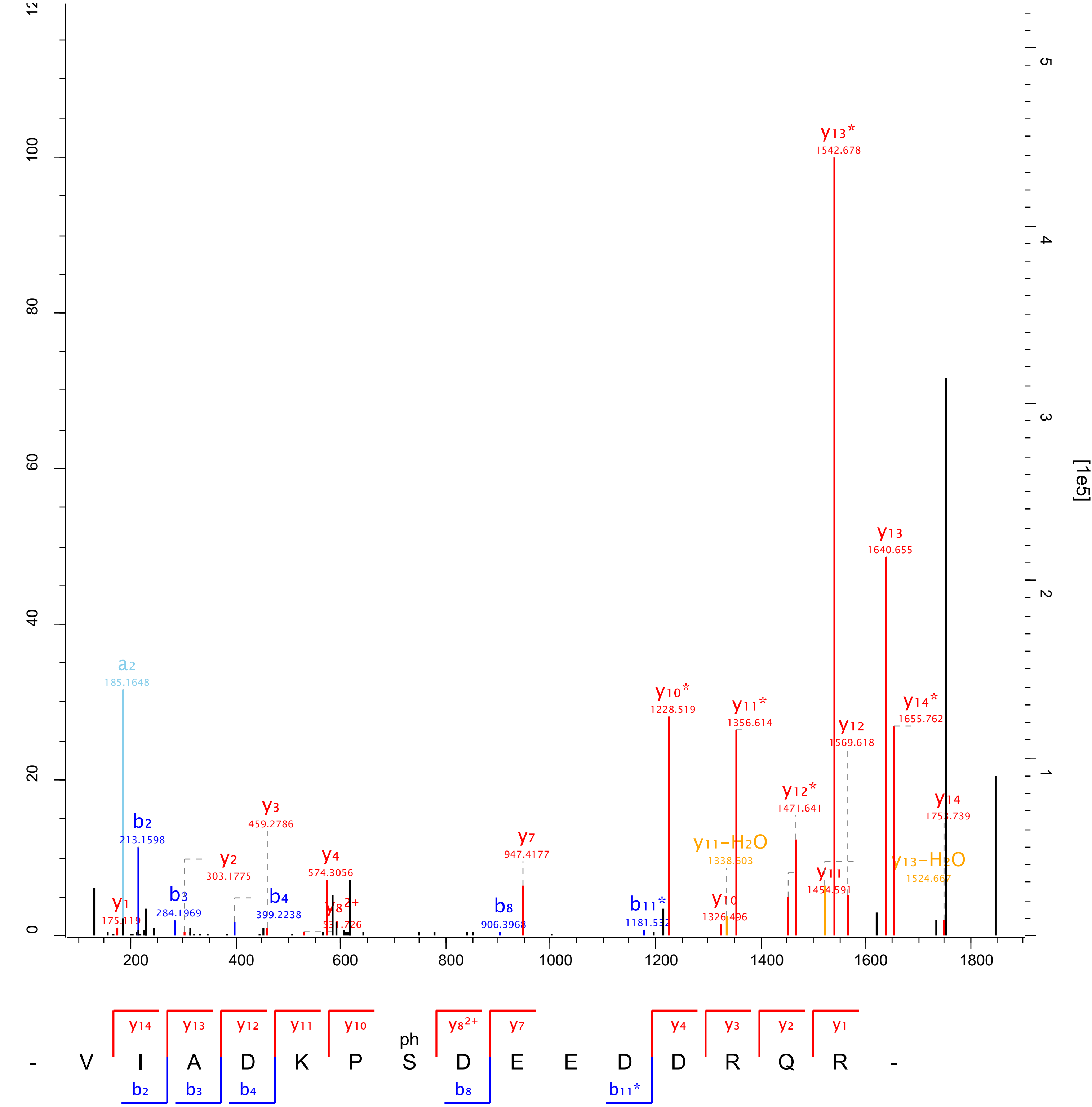

Sample output to test PDF Combine only

Raw file	Scan	Method	Score	m/z	Gene names
sirk1-mic-0-3-A	4817	FTMS; HCD	53.08	439.2	F9F8.20

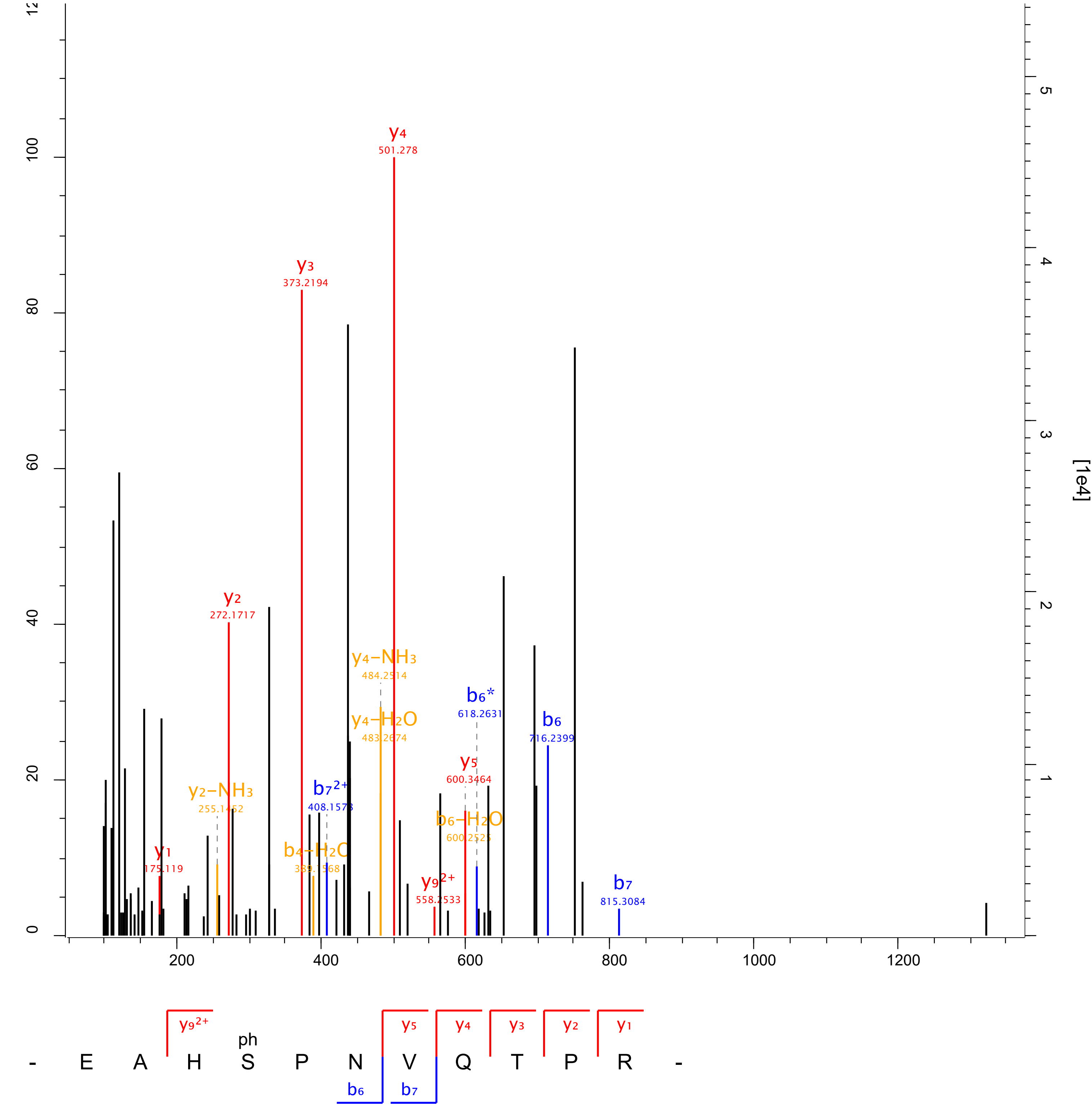

Sample output to test PDF Combine only

Raw file	Scan	Method	Score	m/z
sirk1-mic-0-3-A	6028	FTMS; HCD	71.08	506.24

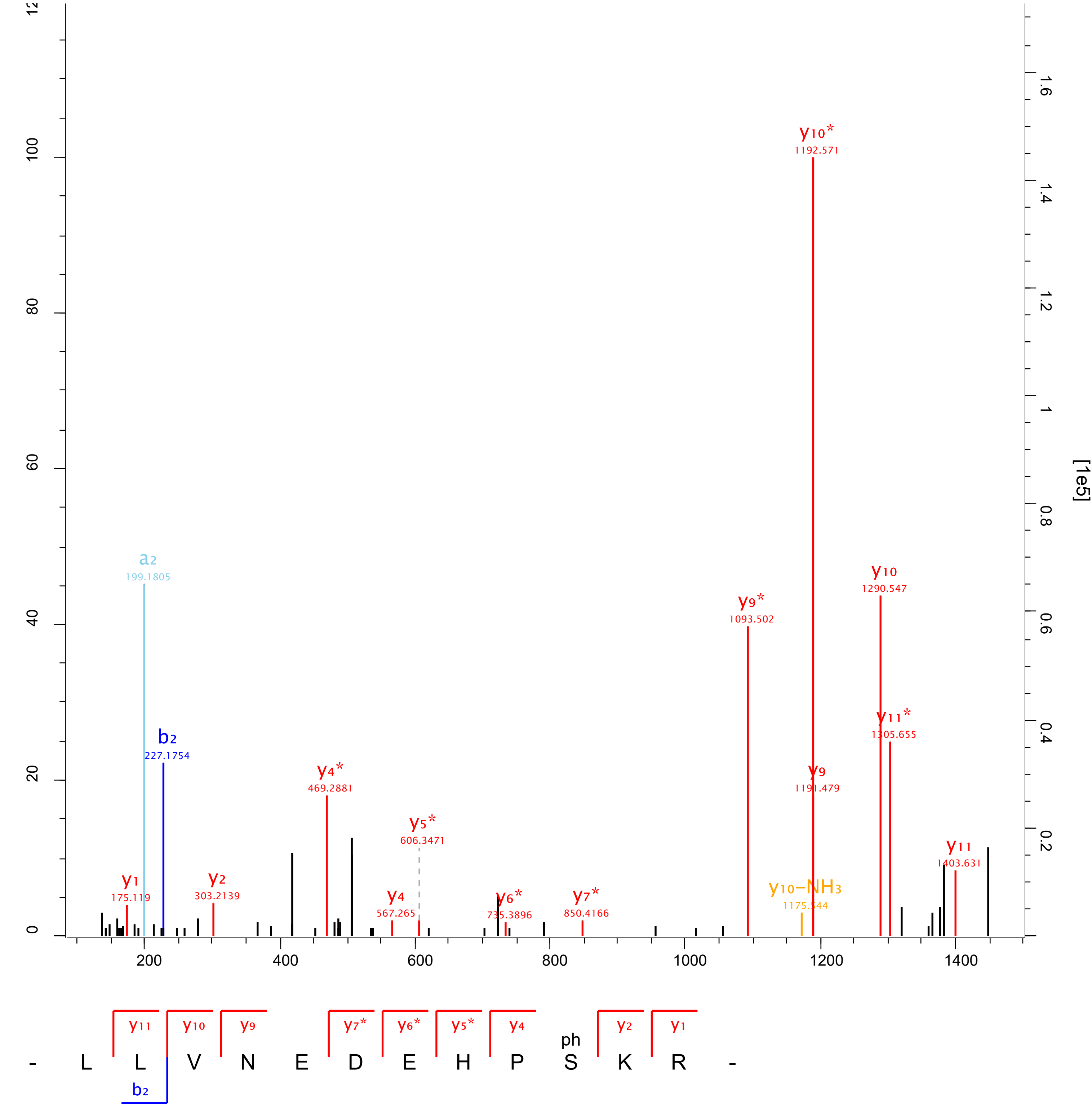

Sample output to test PDF Combine only

Raw file	Scan	Method	Score	m/z	Gene names
sirk1-mic-0-3-A	7300	FTMS; HCD	67.38	574.73	ABCB6;ABCB20

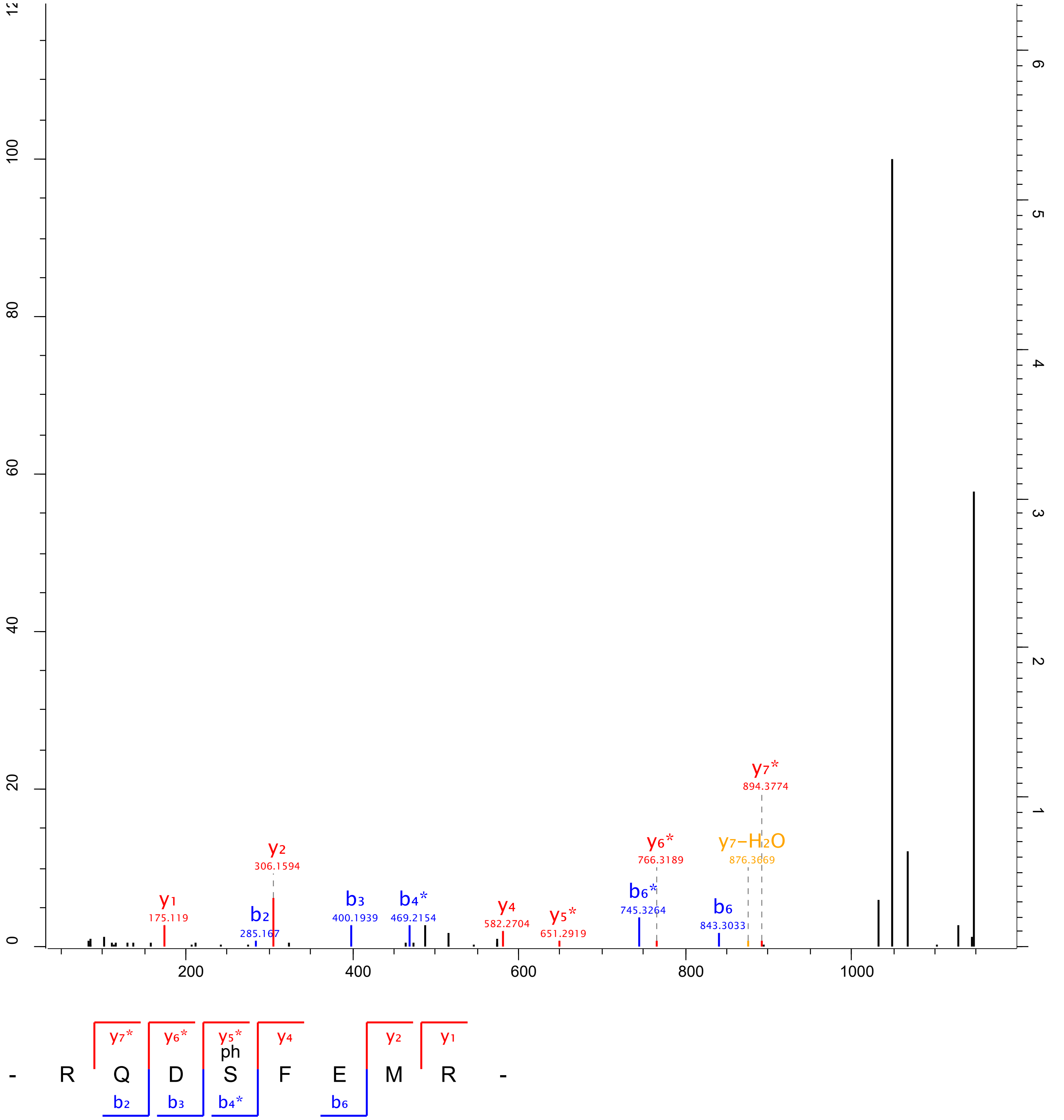

Sample output to test PDF Combine only

Raw file	Scan	Method	Score	m/z	Gene names
sirk1-mic-0-3-A	8305	FTMS; HCD	82.88	548.91	DGK3

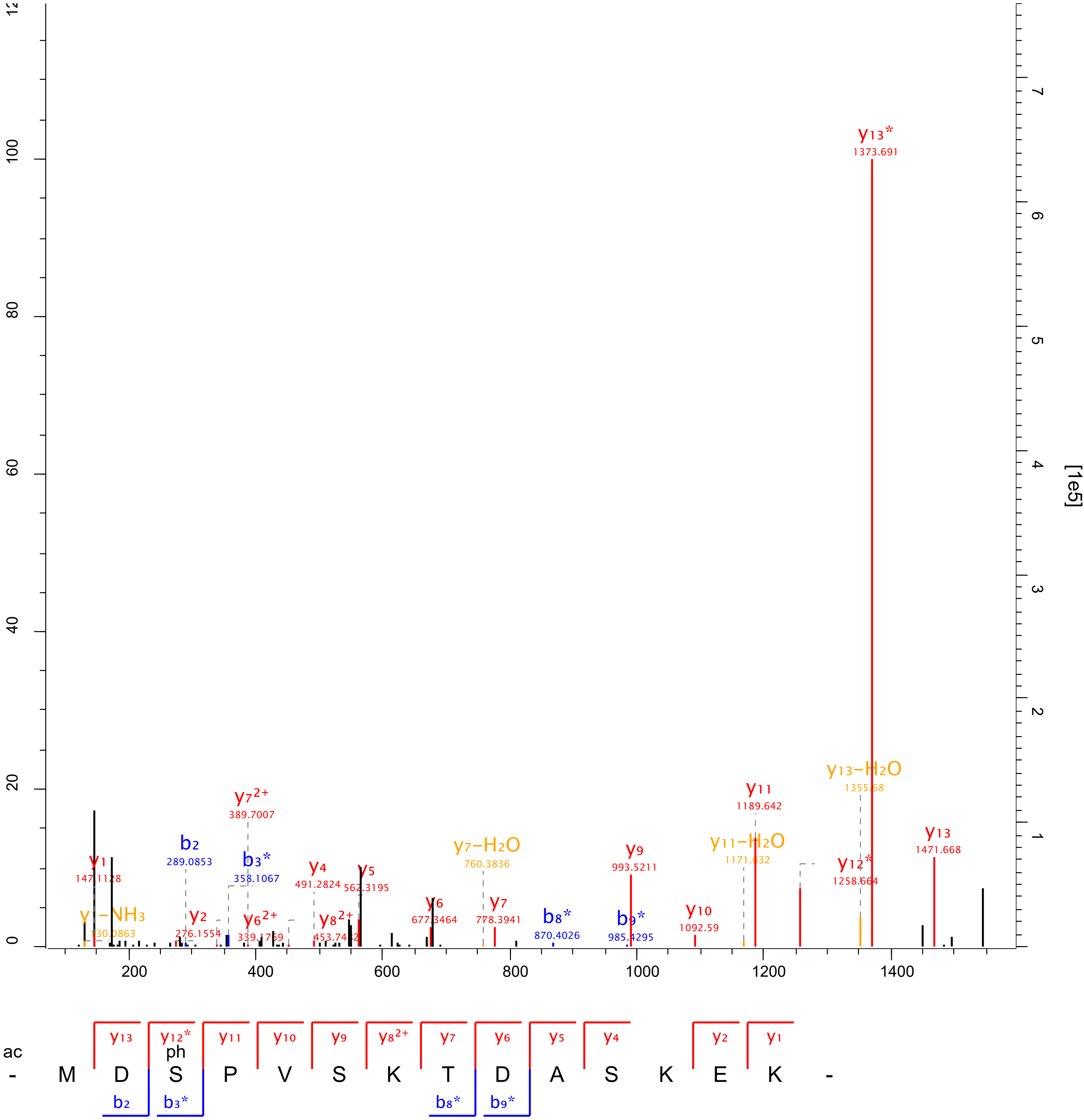

Sample output to test PDF Combine only

Raw file	Scan	Method	Score	m/z	Gene names
sirk1-mic-0-3-A	9462	FTMS; HCD	83.09	578.27	CRWN1

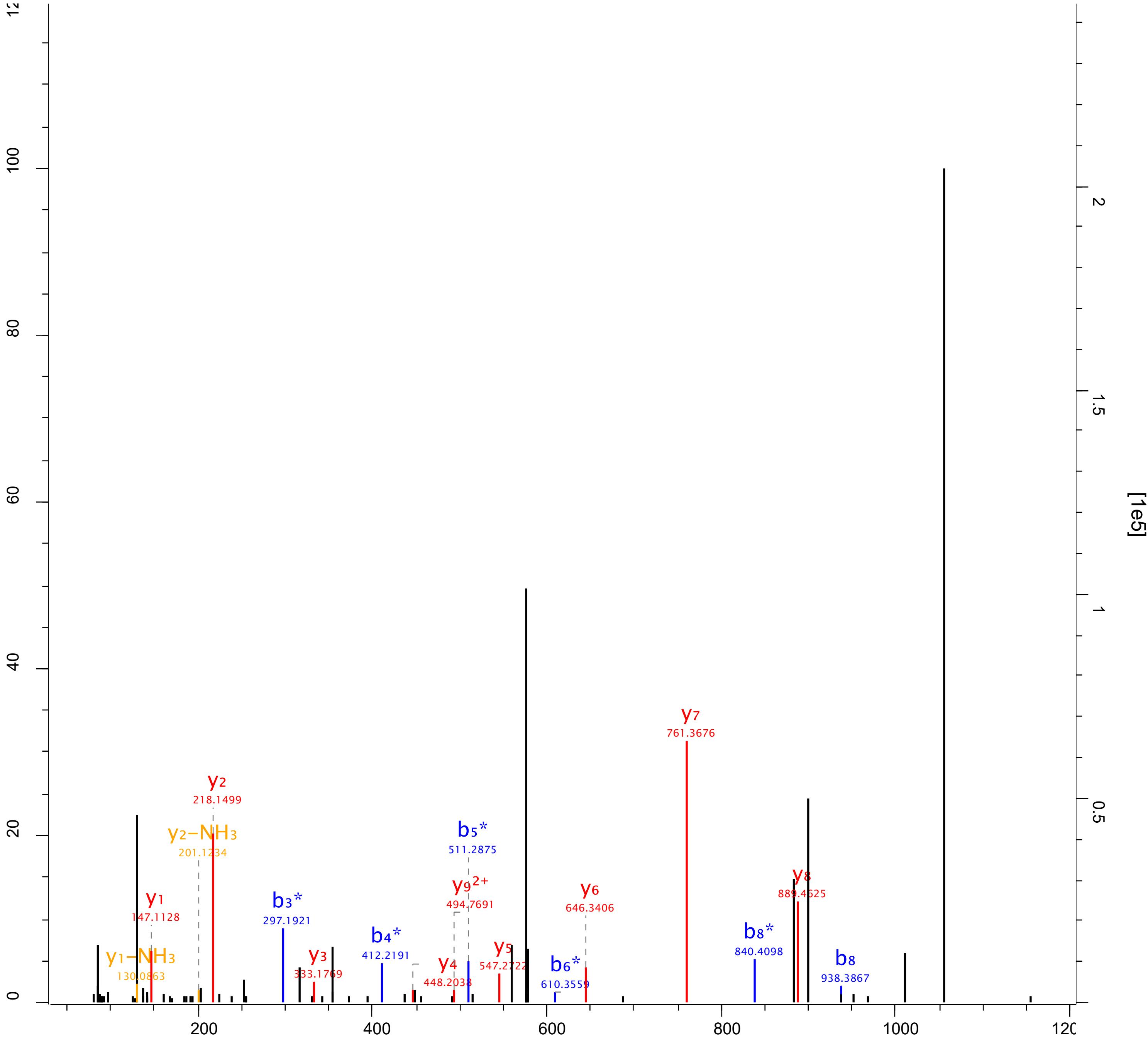

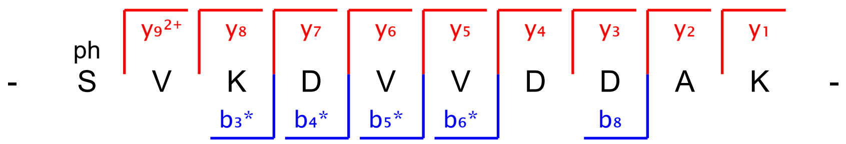

Sample output to test PDF Combine only

Raw file	Scan	Method	Score	m/z	Gene names
sirk1-mic-0-3-A	9794	FTMS; HCD	101.53	441.56	ABCC14

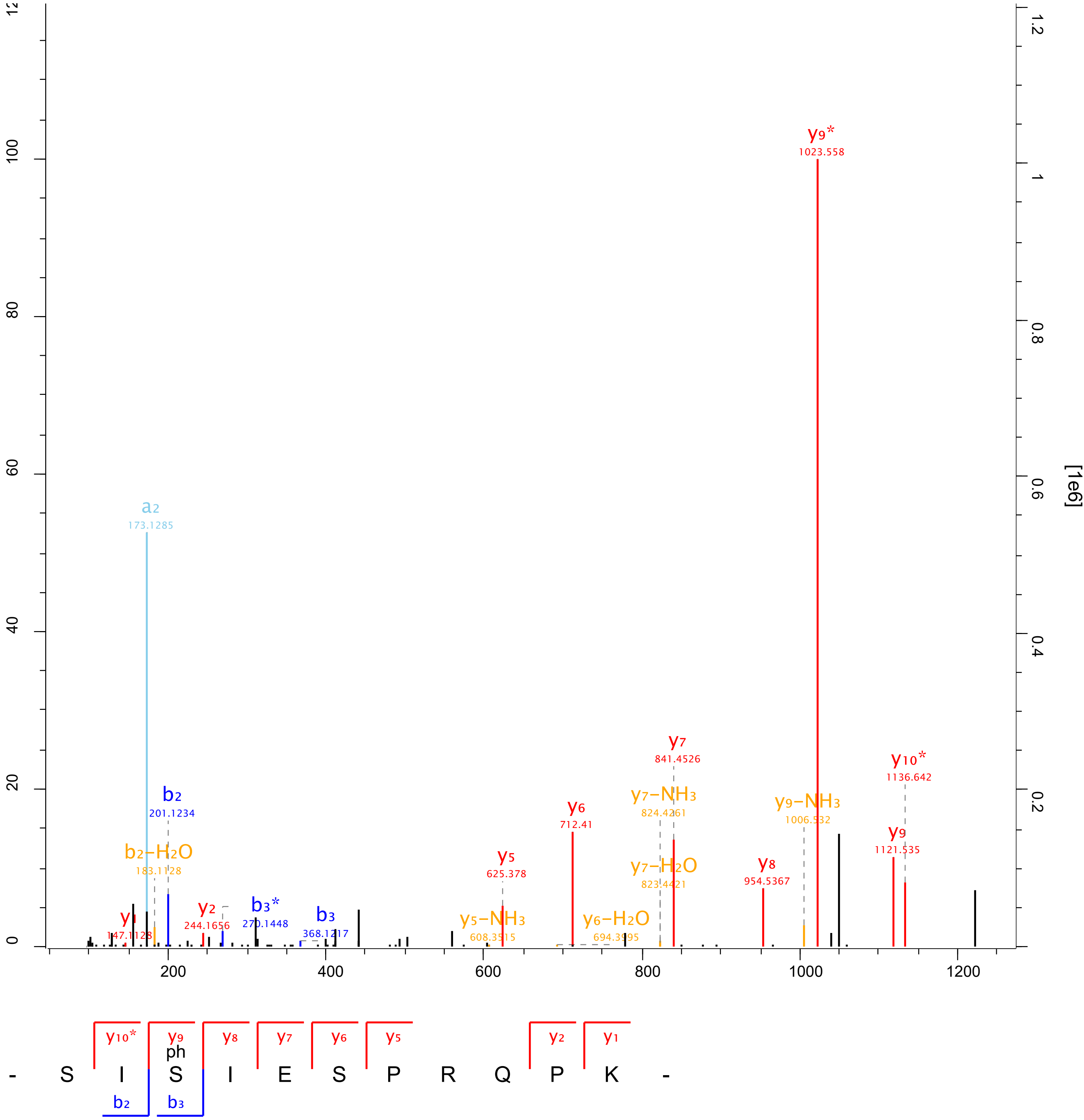

Sample output to test PDF Combine only

Raw file	Scan	Method	Score	m/z	Gene names
sirk1-mic-0-3-A	9995	FTMS; HCD	68	600.78	SOUL-1

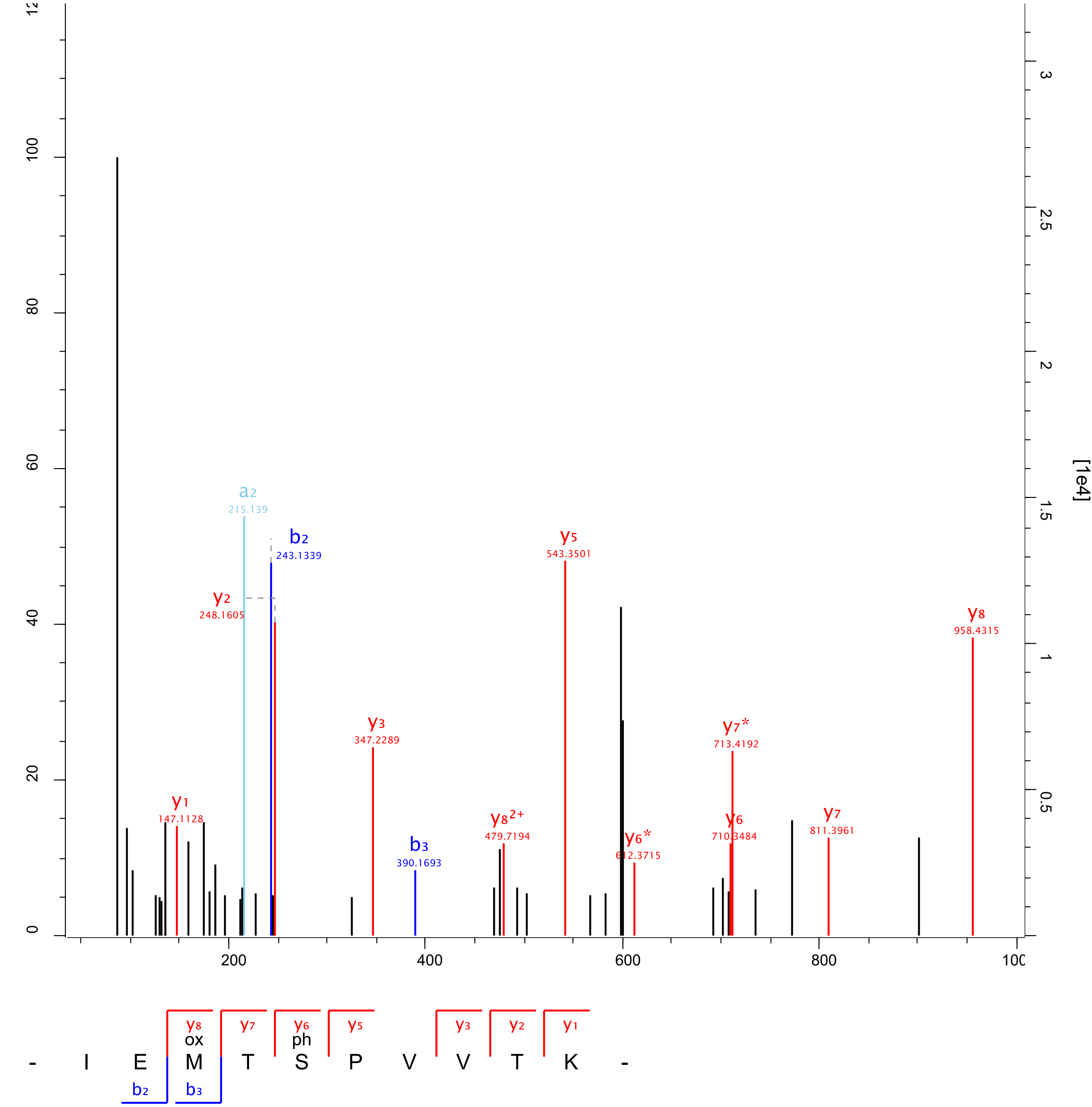

Sample output to test PDF Combine only

Raw file	Scan	Method	Score	m/z	Gene names
sirk1-mic-0-3-A	10113	FTMS; HCD	47.04	634.31	PANK2

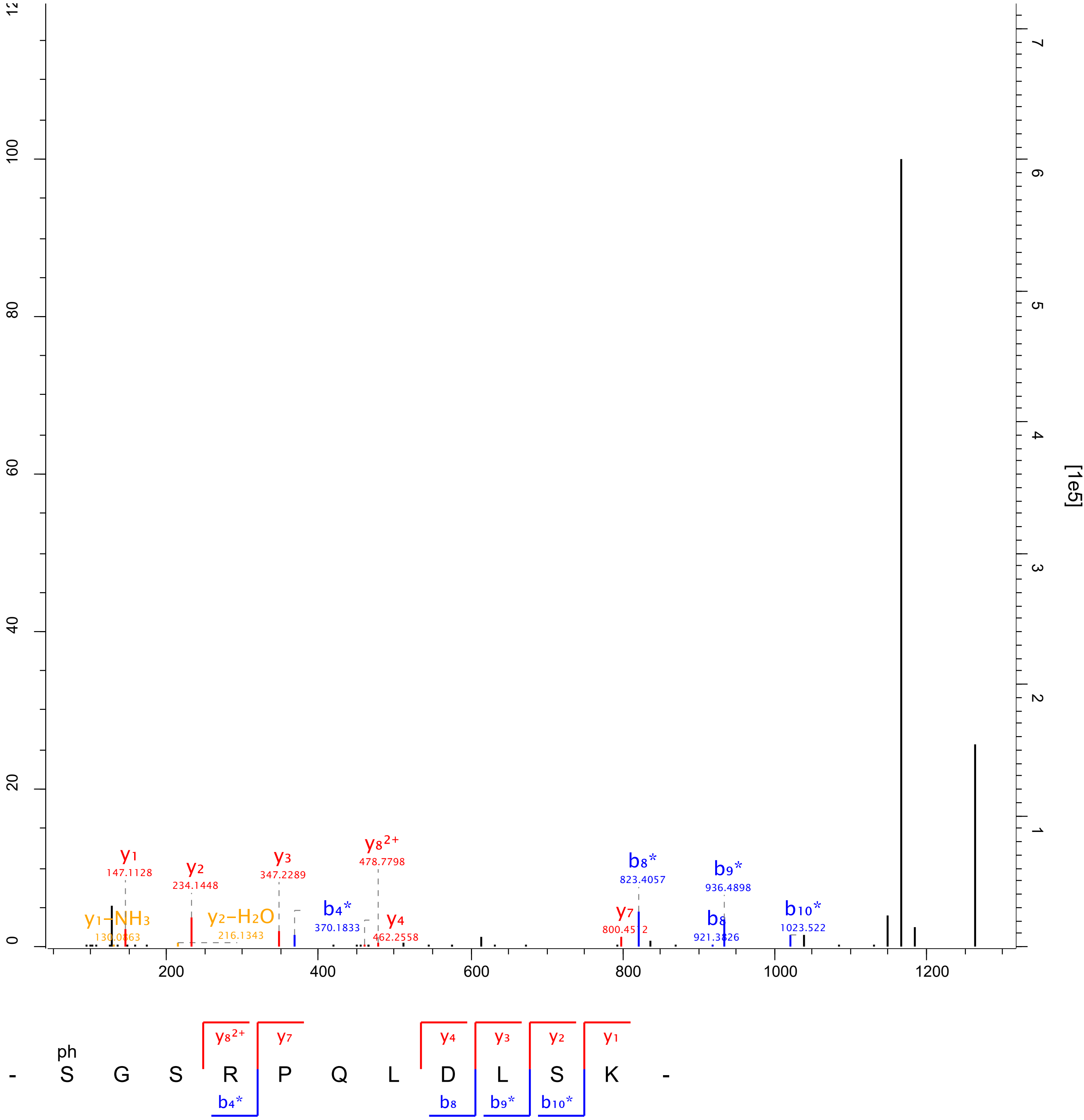

Sample output to test PDF Combine only

Raw file	Scan	Method	Score	m/z
sirk1-mic-0-3-A	11246	FTMS; HCD	44.75	385.84

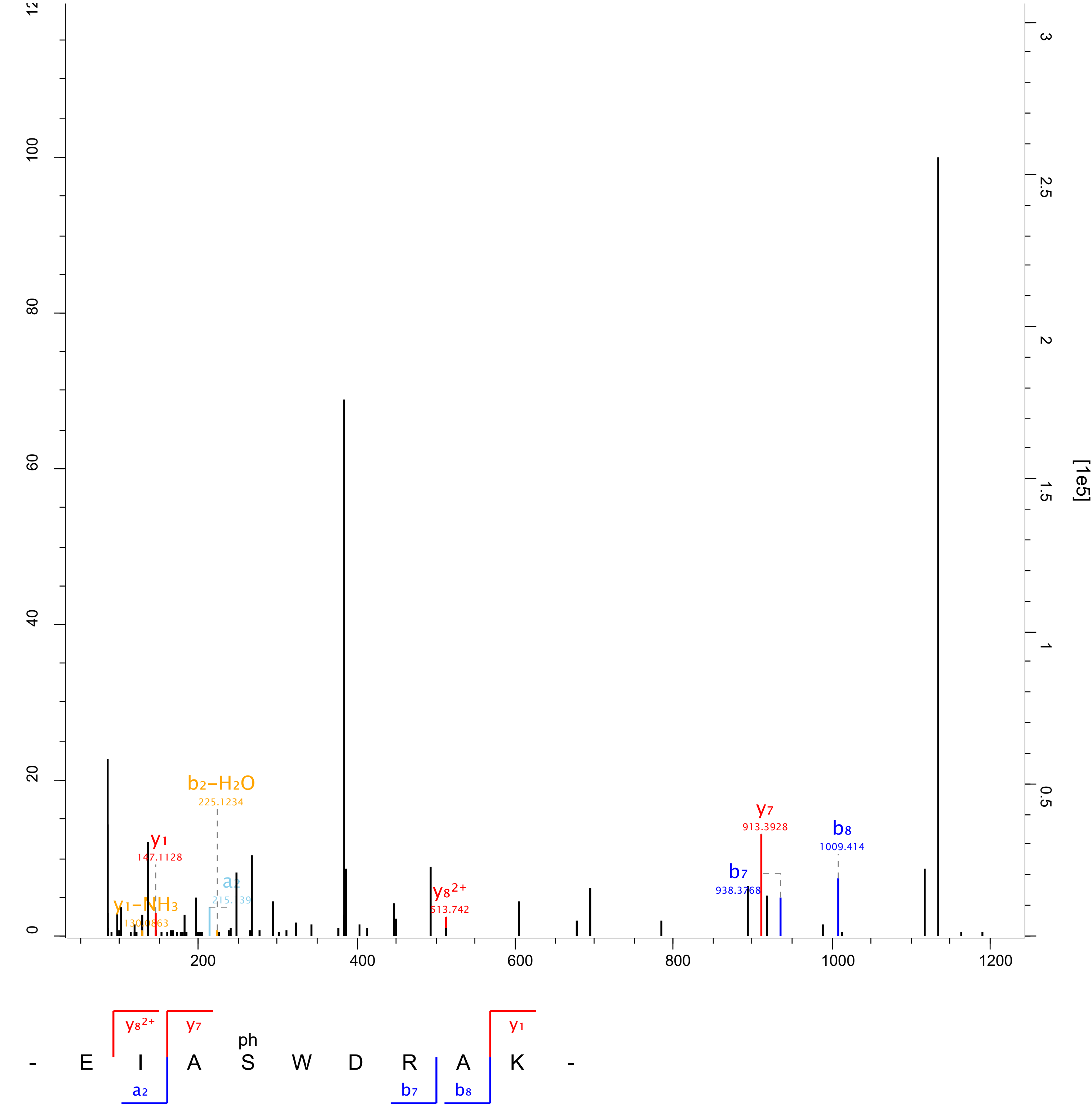

Sample output to test PDF Combine only

Raw file	Scan	Method	Score	m/z	Gene names
sirk1-mic-0-3-A	12814	FTMS; HCD	40.48	811.83	ETG1

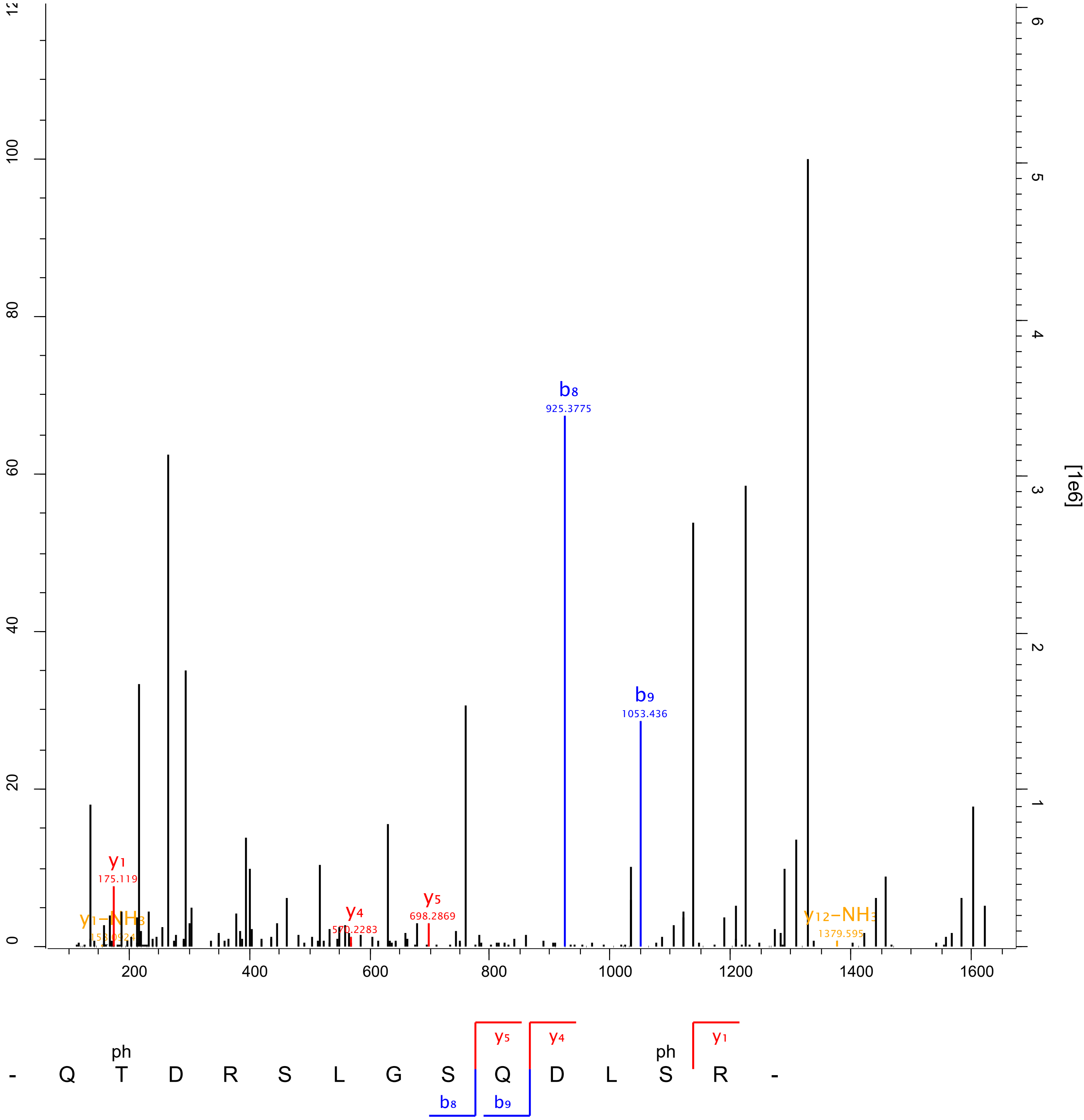

Sample output to test PDF Combine only

Raw file	Scan	Method	Score	m/z	Gene names
sirk1-mic-0-3-A	12868	FTMS; HCD	86.77	561.76	CALS12

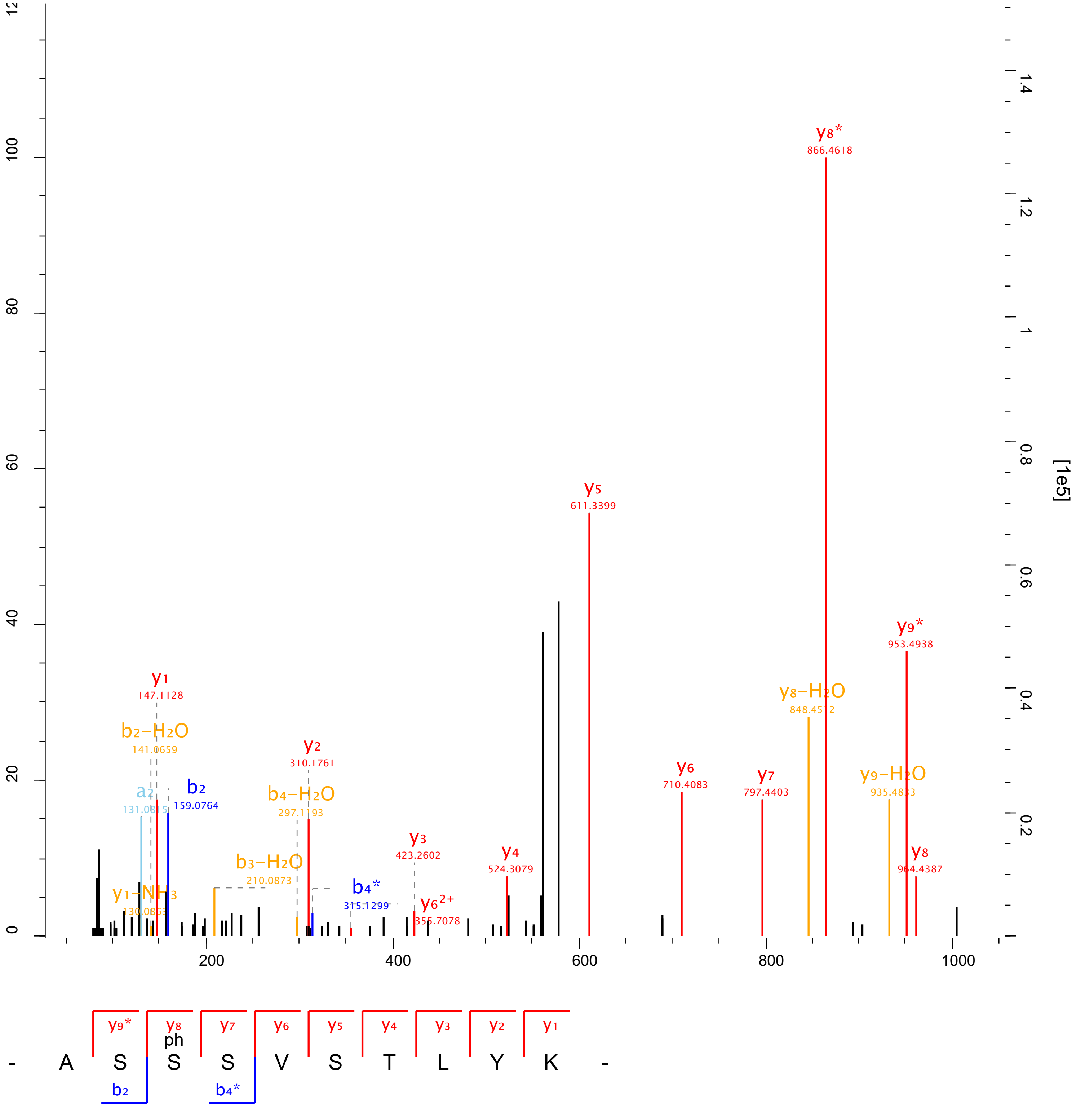

Sample output to test PDF Combine only

Raw file	Scan	Method	Score	m/z	Gene names
sirk1-mic-0-3-A	13382	FTMS; HCD	99.04	553.27	PPC3

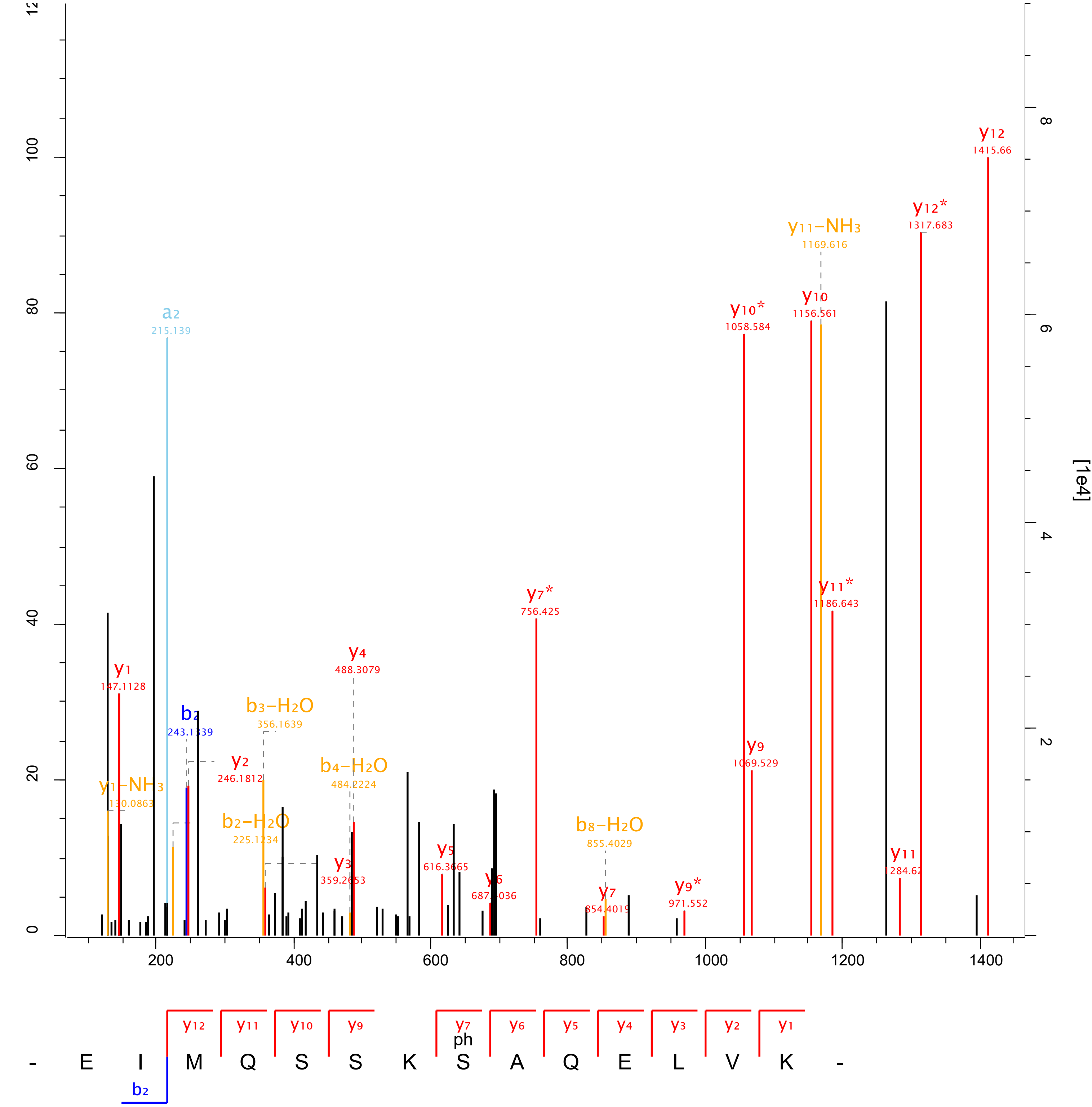

Sample output to test PDF Combine only

Raw file	Scan	Method	Score	m/z
sirk1-mic-0-3-A	14496	FTMS; HCD	82.29	634.31

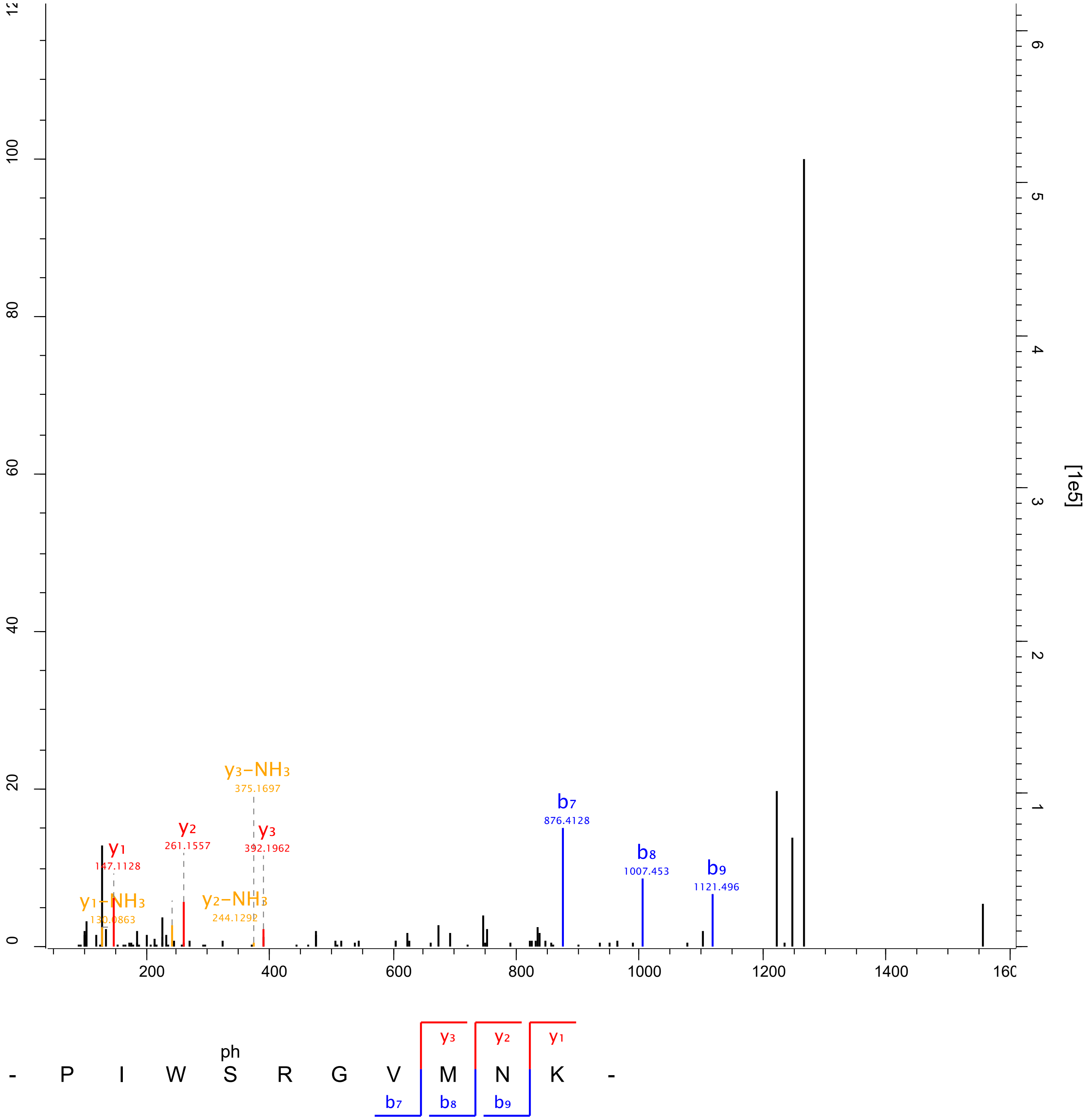

Sample output to test PDF Combine only

Raw file	Scan	Method	Score	m/z	Gene names
sirk1-mic-0-3-A	14961	FTMS; HCD	197.67	829.39	SOUL-1

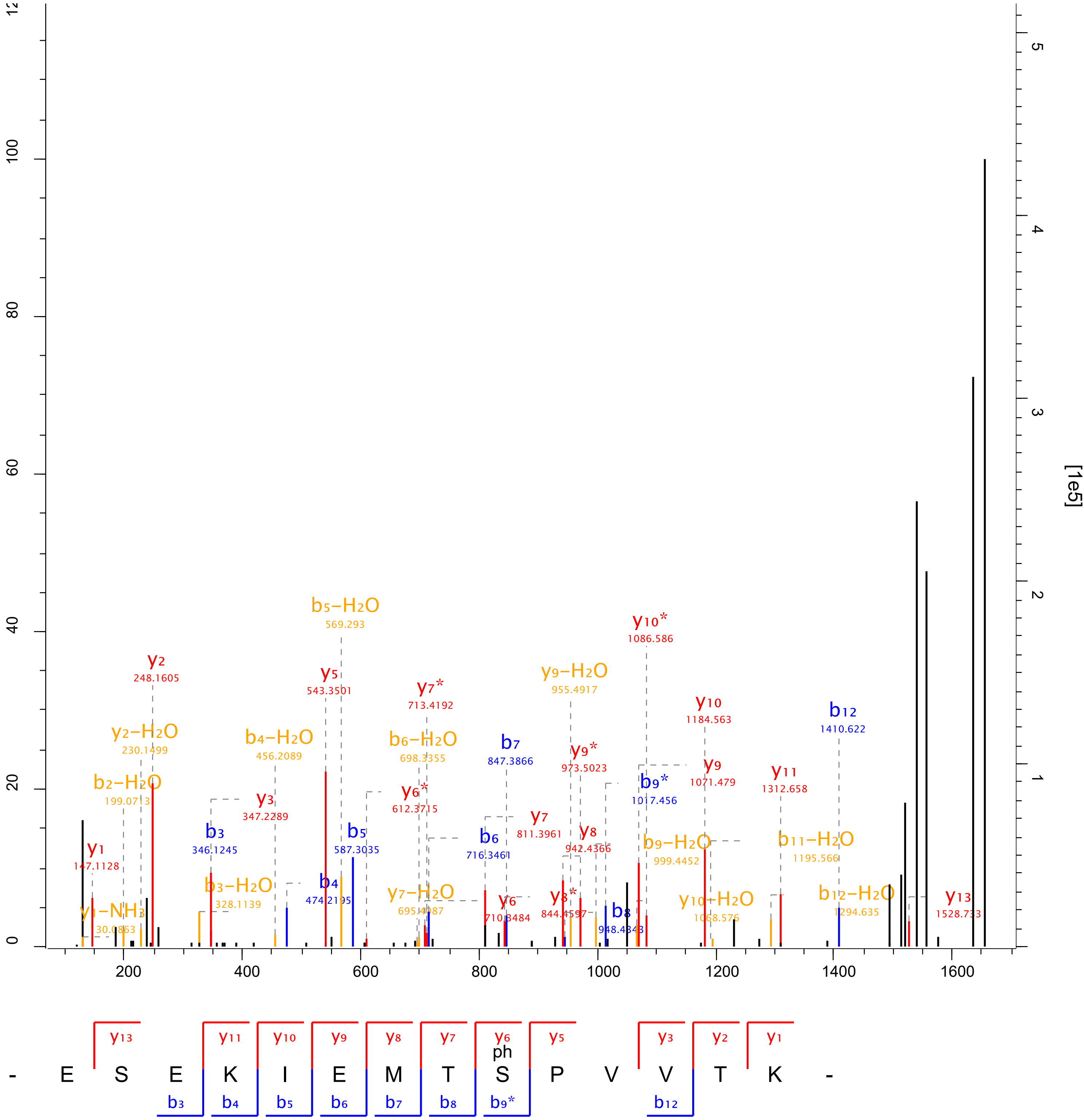

Sample output to test PDF Combine only

Raw file	Scan	Method	Score	m/z	Gene names
sirk1-mic-0-3-A	16260	FTMS; HCD	63.29	644.8	ALA1

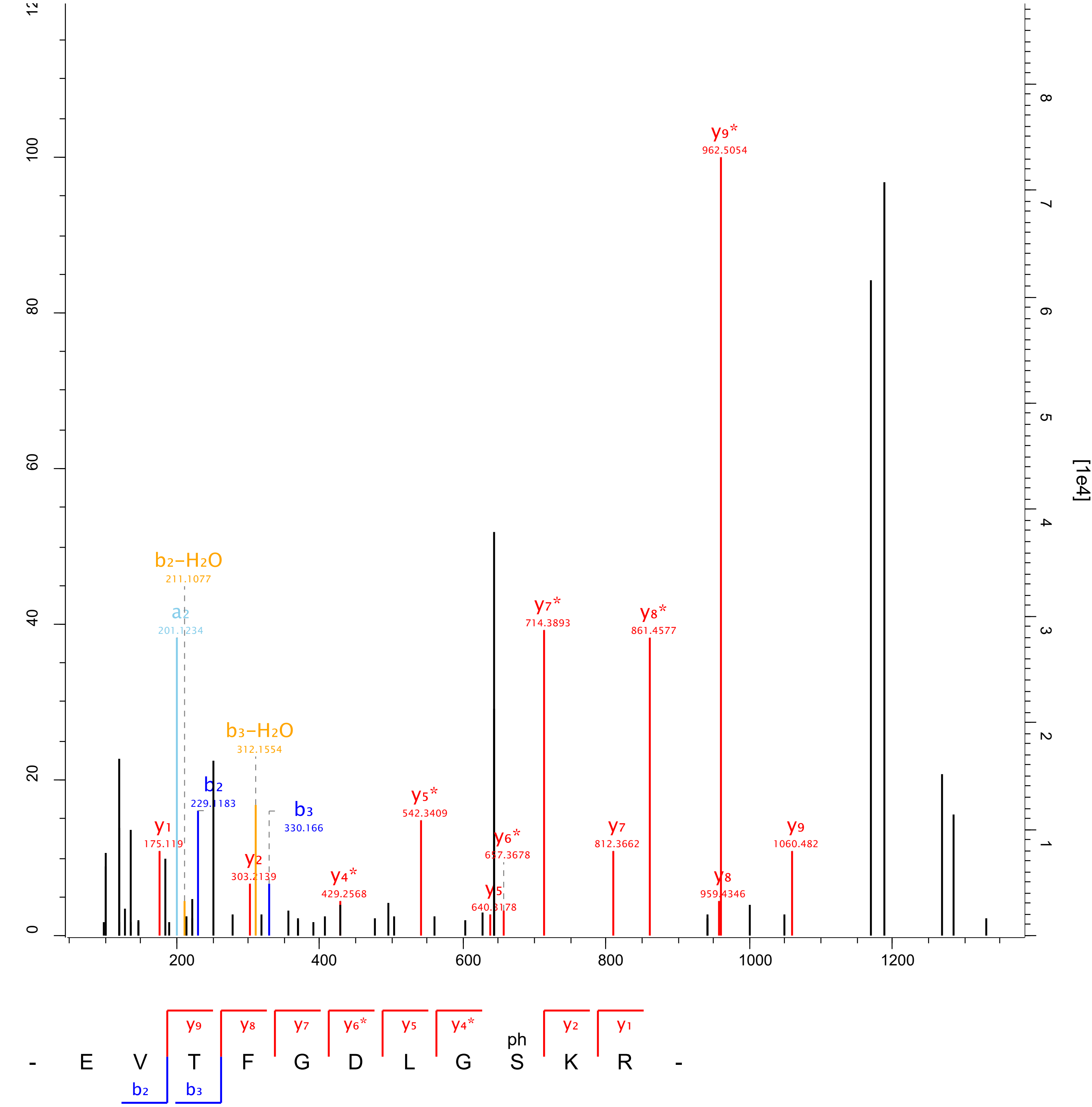

Sample output to test PDF Combine only

Raw file	Scan	Method	Score	m/z
sirk1-mic-0-3-A	16607	FTMS; HCD	42.6	546.25

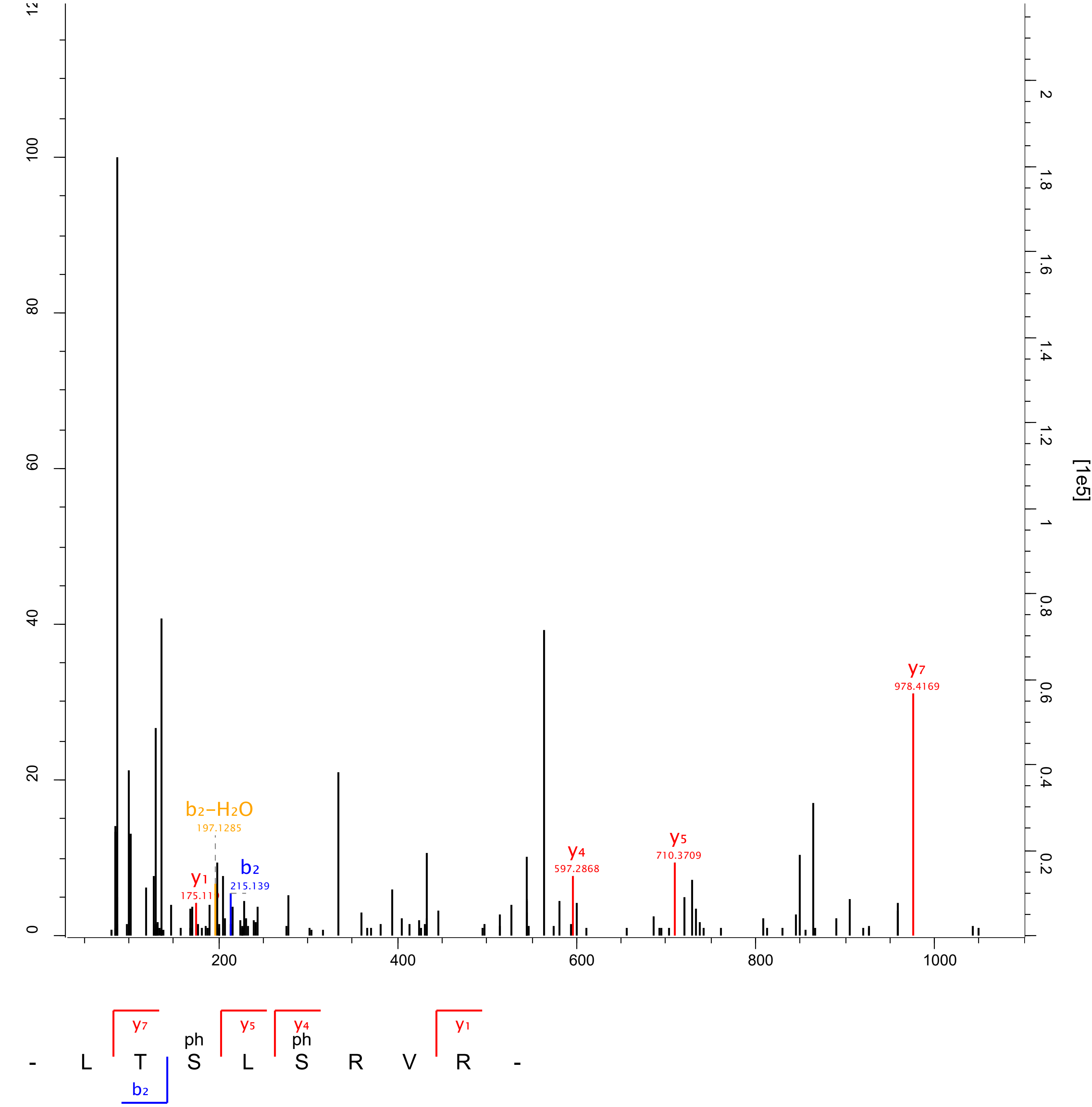

Sample output to test PDF Combine only

Raw file	Scan	Method	Score	m/z	Gene names
sirk1-mic-0-3-A	18042	FTMS; HCD	97.74	422.2	PIRL9;PIRL1

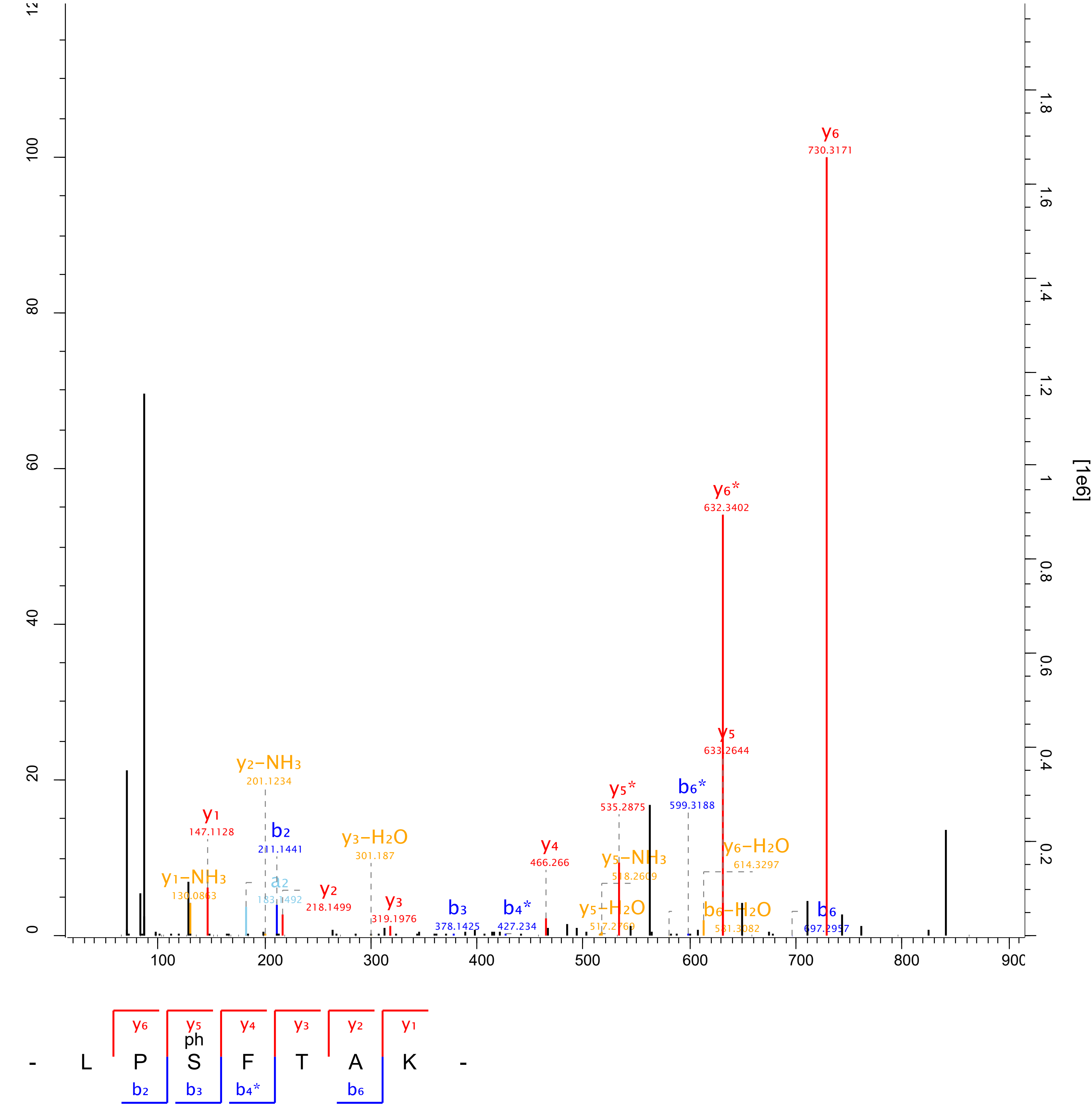

Sample output to test PDF Combine only

Raw file	Scan	Method	Score	m/z	Gene names
sirk1-mic-0-3-A	20503	FTMS; HCD	43.31	499.54	IMPDH

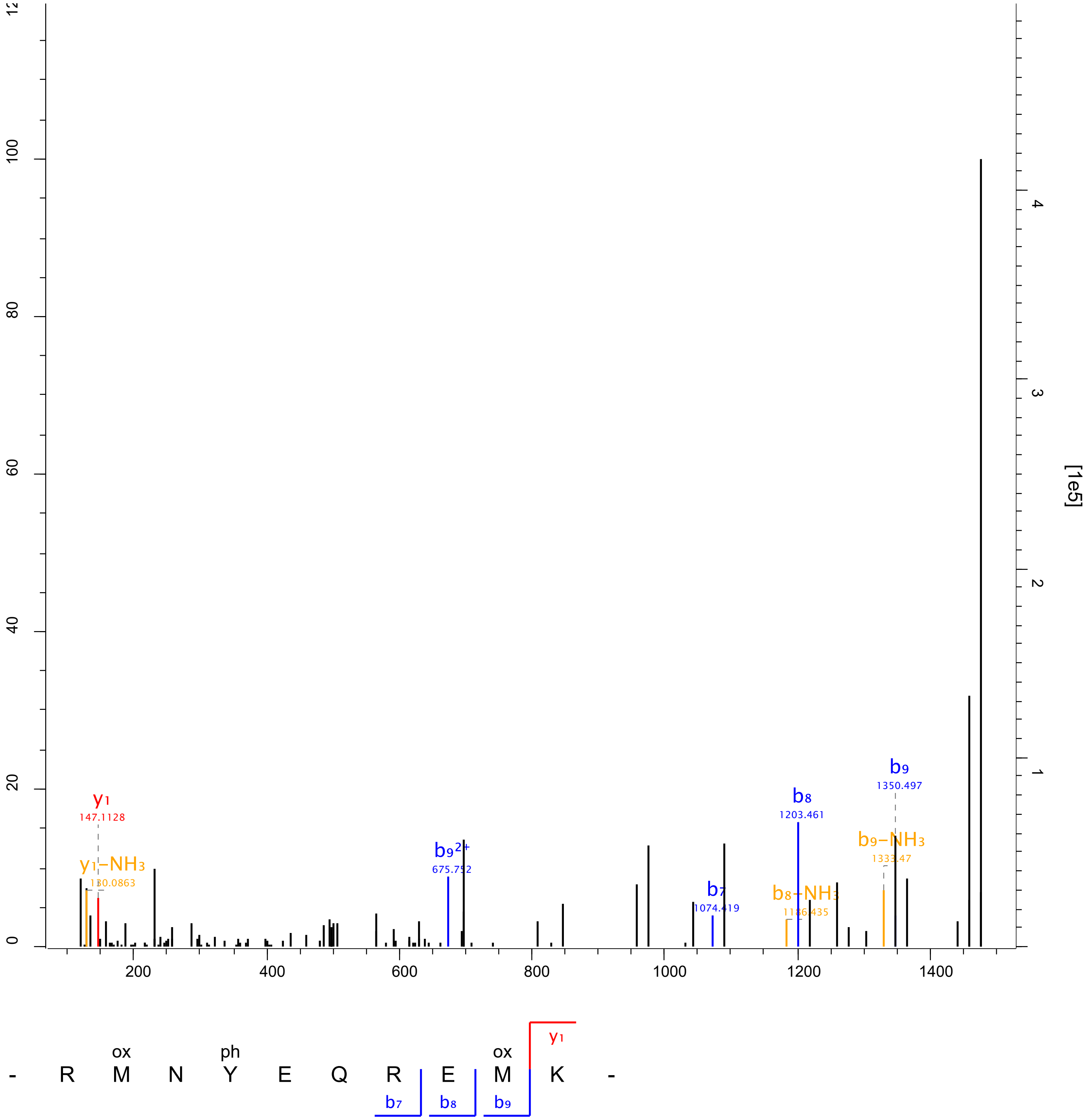

Sample output to test PDF Combine only

Raw file	Scan	Method	Score	m/z	Gene names
sirk1-mic-0-3-A	20581	FTMS; HCD	62.71	532.92	F14F18_140

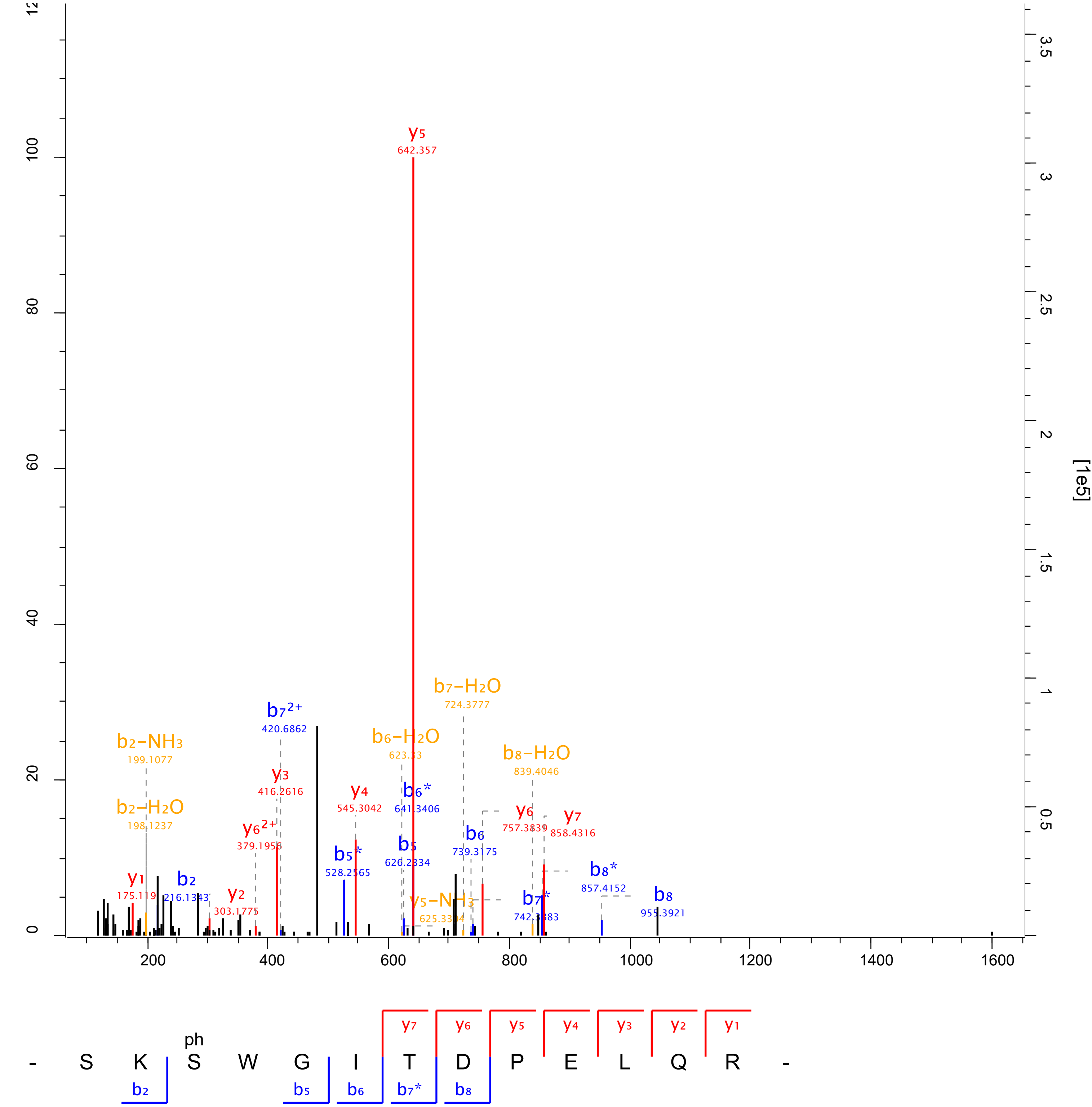

Sample output to test PDF Combine only

Raw file	Scan	Method	Score	m/z	Gene names
sirk1-mic-0-3-A	20811	FTMS; HCD	48.22	514.88	SCL30

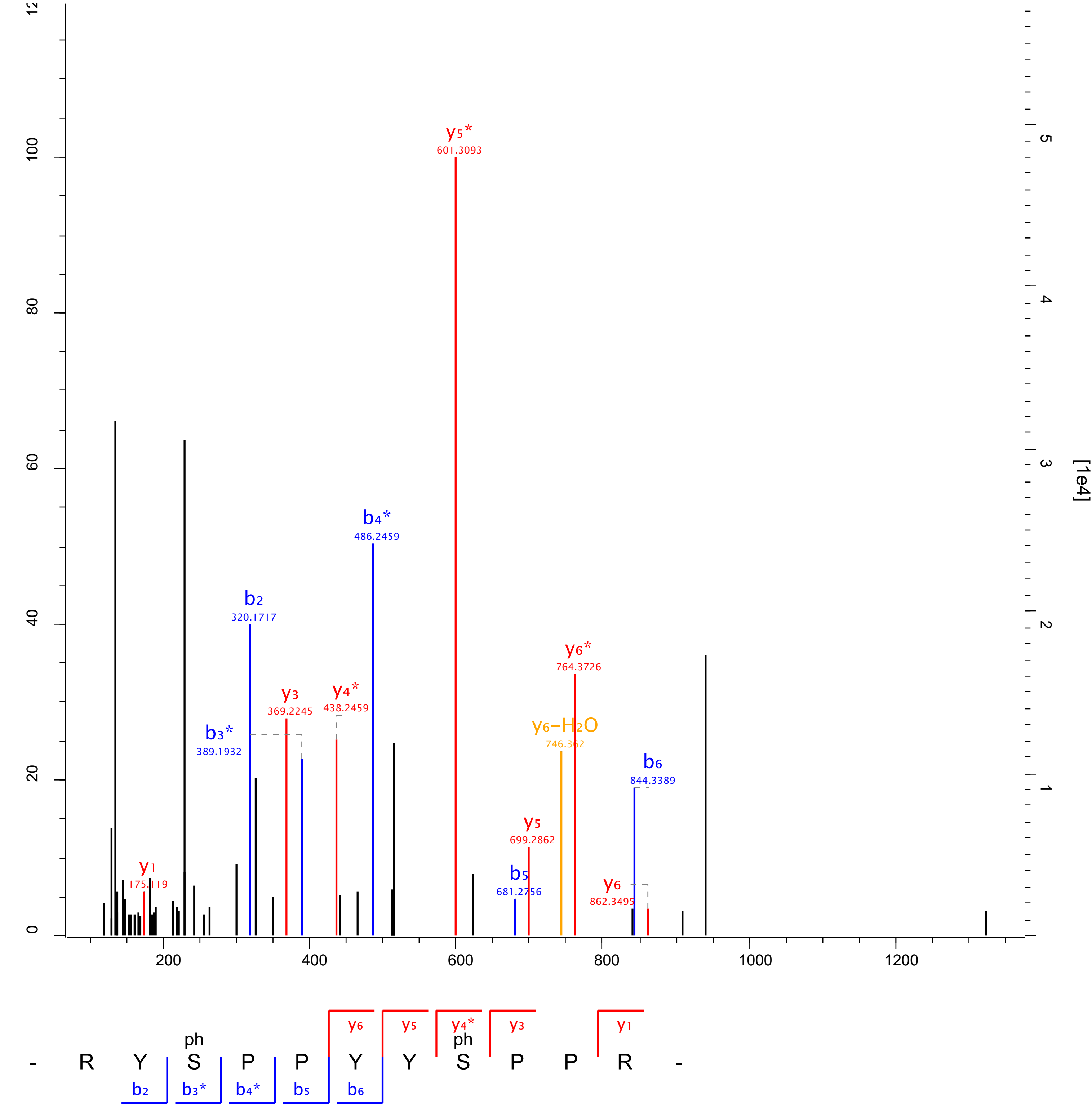

Sample output to test PDF Combine only

Raw file	Scan	Method	Score	m/z	Gene names
sirk1-mic-0-3-A	21984	FTMS; HCD	72.5	550.23	At4g27450

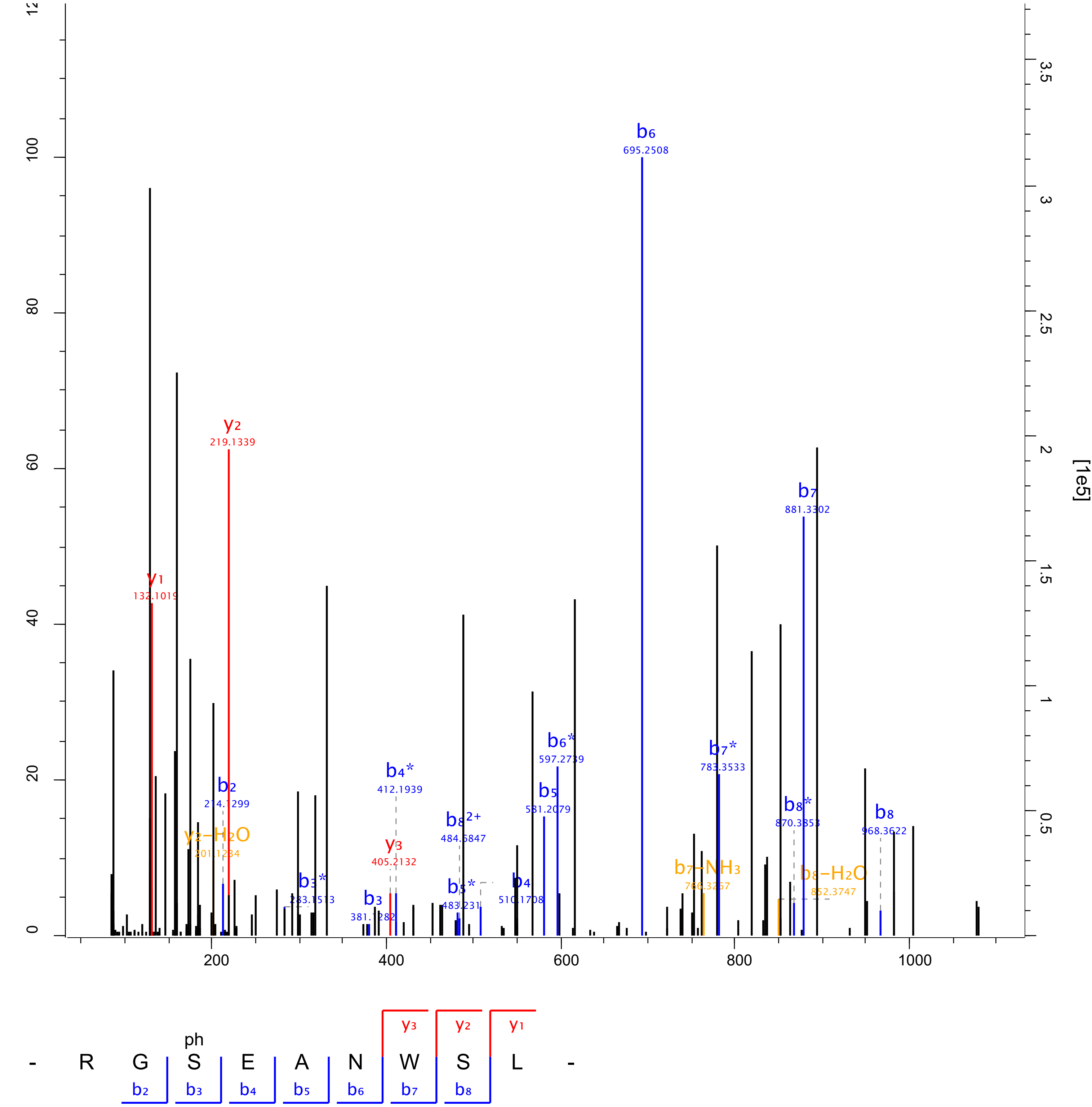

Sample output to test PDF Combine only

Raw file	Scan	Method	Score	m/z	Gene names
sirk1-mic-0-3-A	23016	FTMS; HCD	159.06	621.28	PATL3

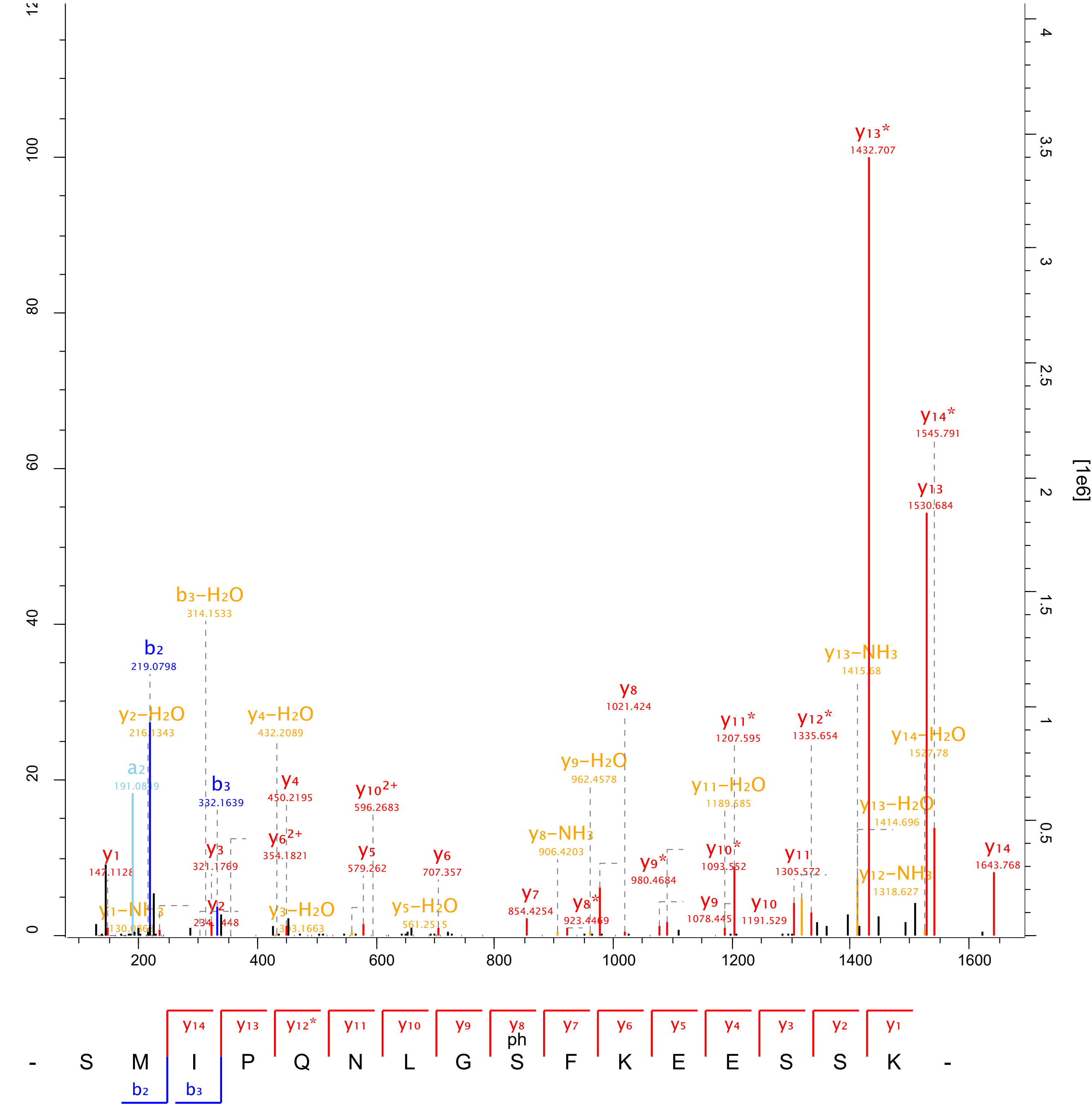

Sample output to test PDF Combine only

Raw file	Scan	Method	Score	m/z	Gene names
sirk1-mic-0-3-A	24589	FTMS; HCD	152.38	573.25	MES14

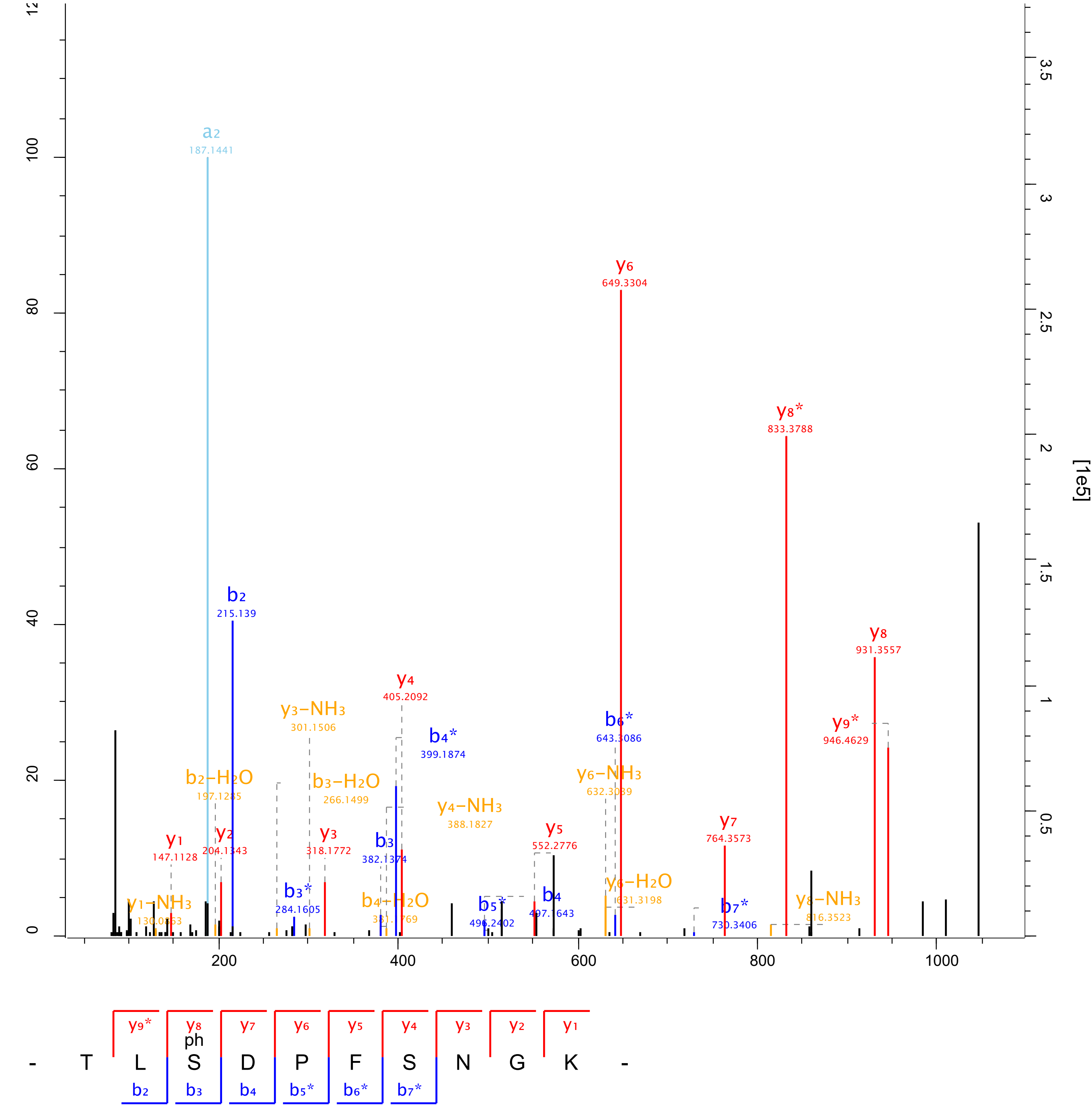

Sample output to test PDF Combine only

Raw file	Scan	Method	Score	m/z	Gene names
sirk1-mic-0-3-A	25056	FTMS; HCD	64.71	699.31	At1g72840

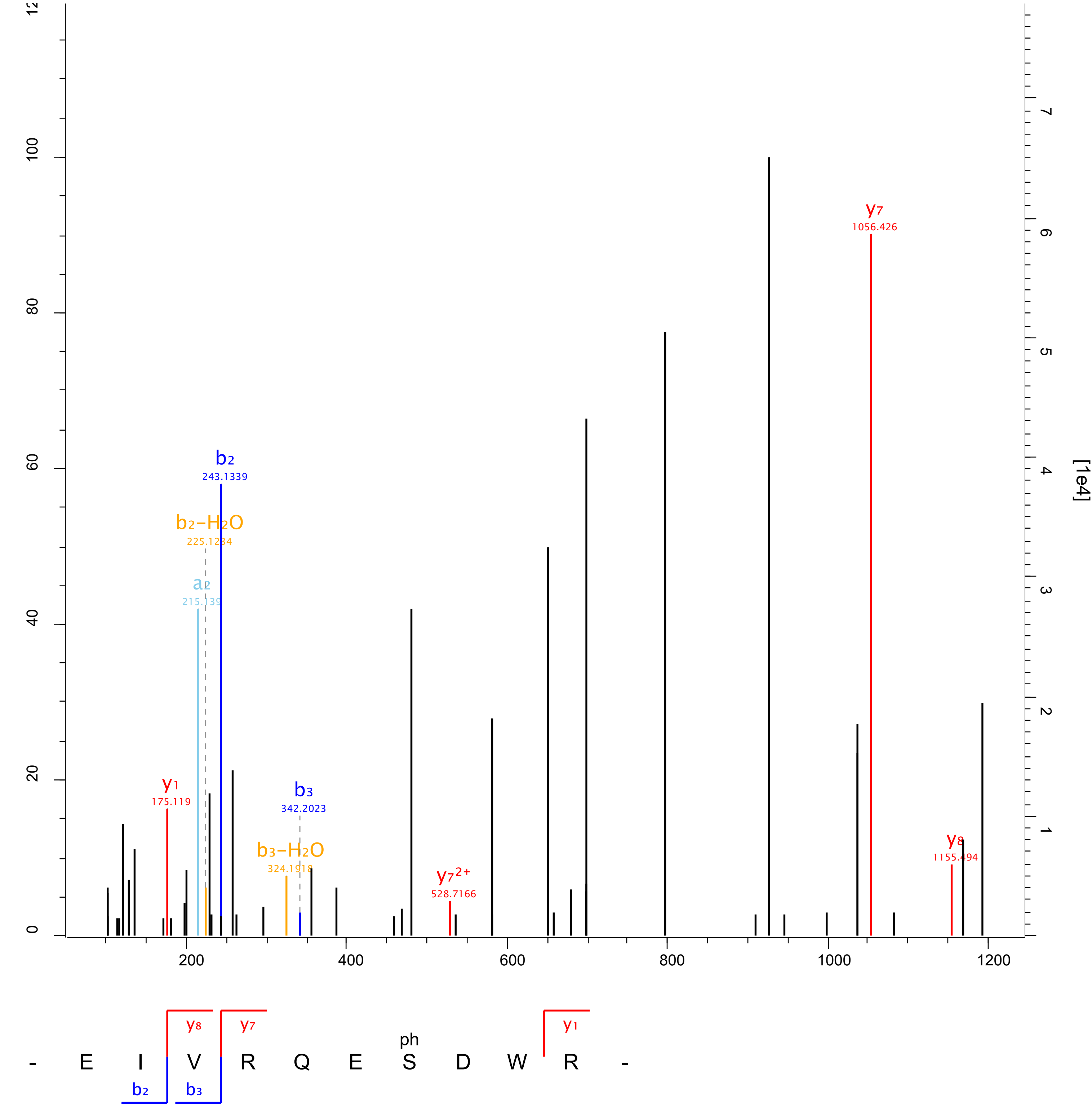

Sample output to test PDF Combine only

Raw file	Scan	Method	Score	m/z	Gene names
sirk1-mic-0-3-A	28523	FTMS; HCD	51.35	825.38	CYP71B19;CYP71B20

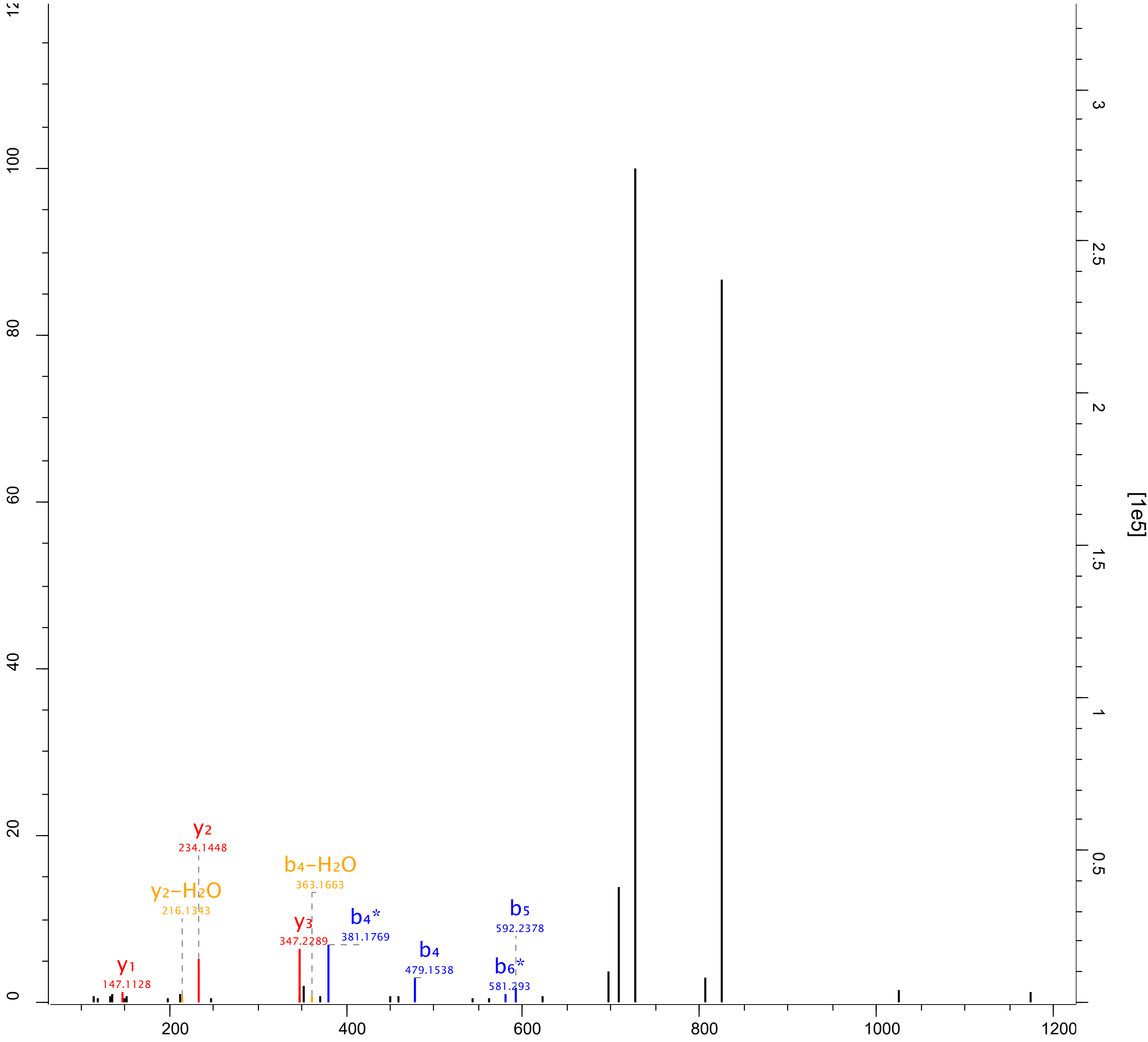

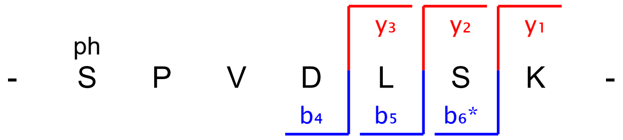

Sample output to test PDF Combine only

Raw file	Scan	Method	Score	m/z	Gene names
sirk1-mic-0-3-A	35058	FTMS; HCD	119.46	722.84	F19F18.190

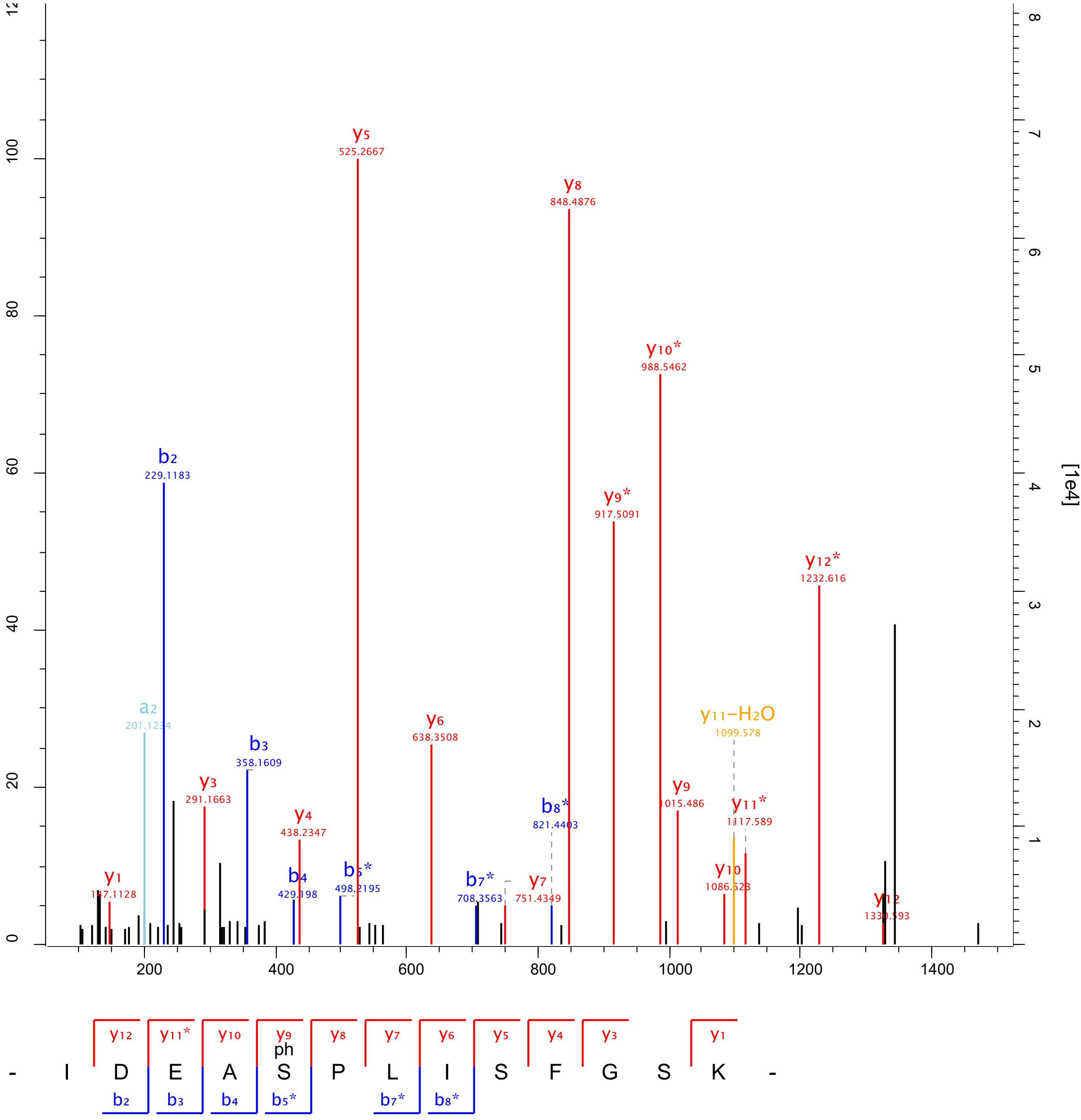

Sample output to test PDF Combine only

Raw file	Scan	Method	Score	m/z	Gene names
sirk1-mic-0-3-P	12348	FTMS; HCD	76.33	492.27	PPA5

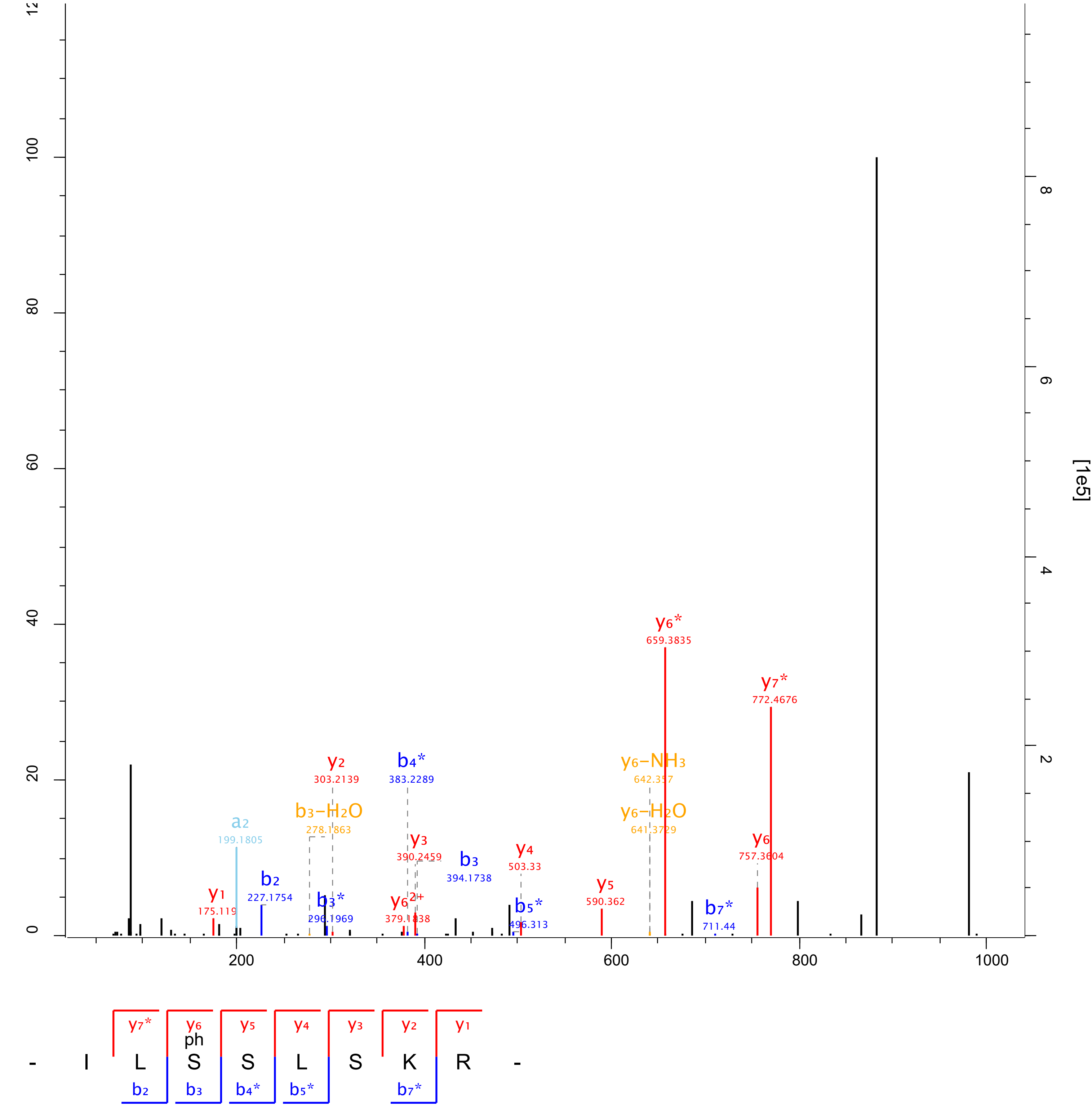

Sample output to test PDF Combine only

Raw file	Scan	Method	Score	m/z	Gene names
sirk1-mic-0-3-P	12362	FTMS; HCD	65.22	550.23	T11I11.5

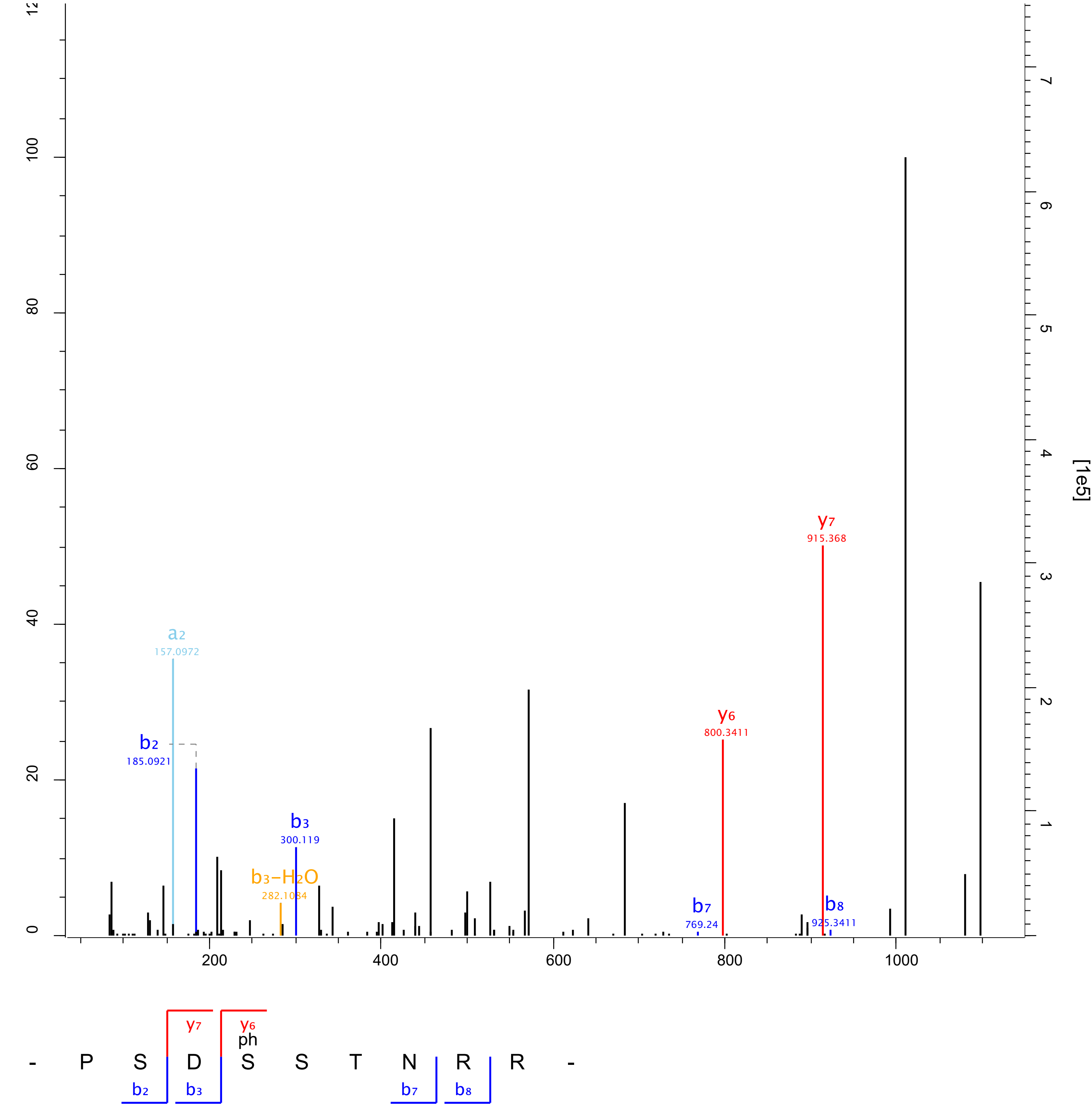

Sample output to test PDF Combine only

Raw file	Scan	Method	Score	m/z	Gene names
sirk1-mic-0-3-P	13371	FTMS; HCD	75.68	514.59	At1g74910;F9E10.24

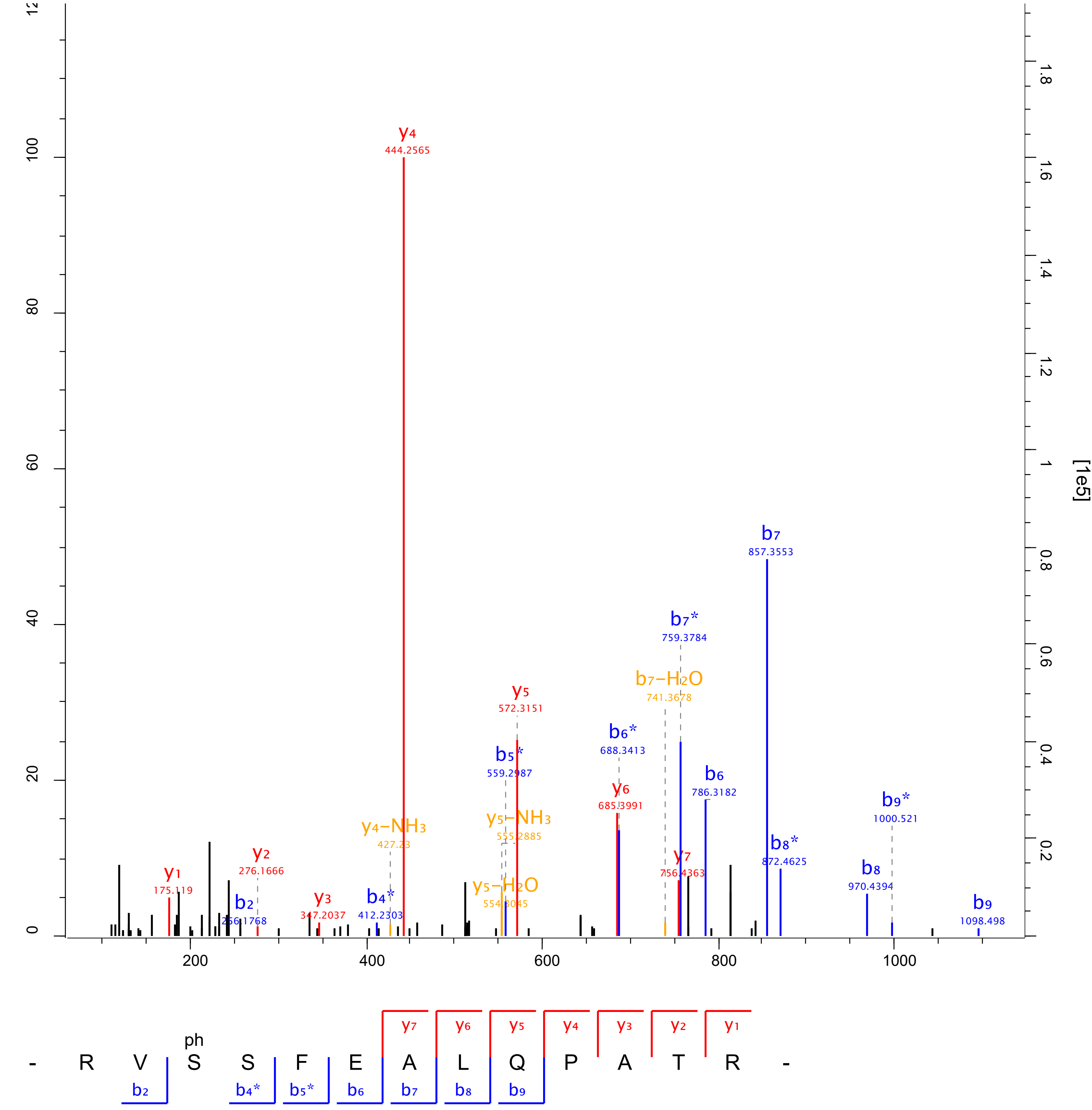

Sample output to test PDF Combine only

Raw file	Scan	Method	Score	m/z	Gene names
sirk1-mic-0-3-P	13775	FTMS; HCD	119.18	664.32	PATL3

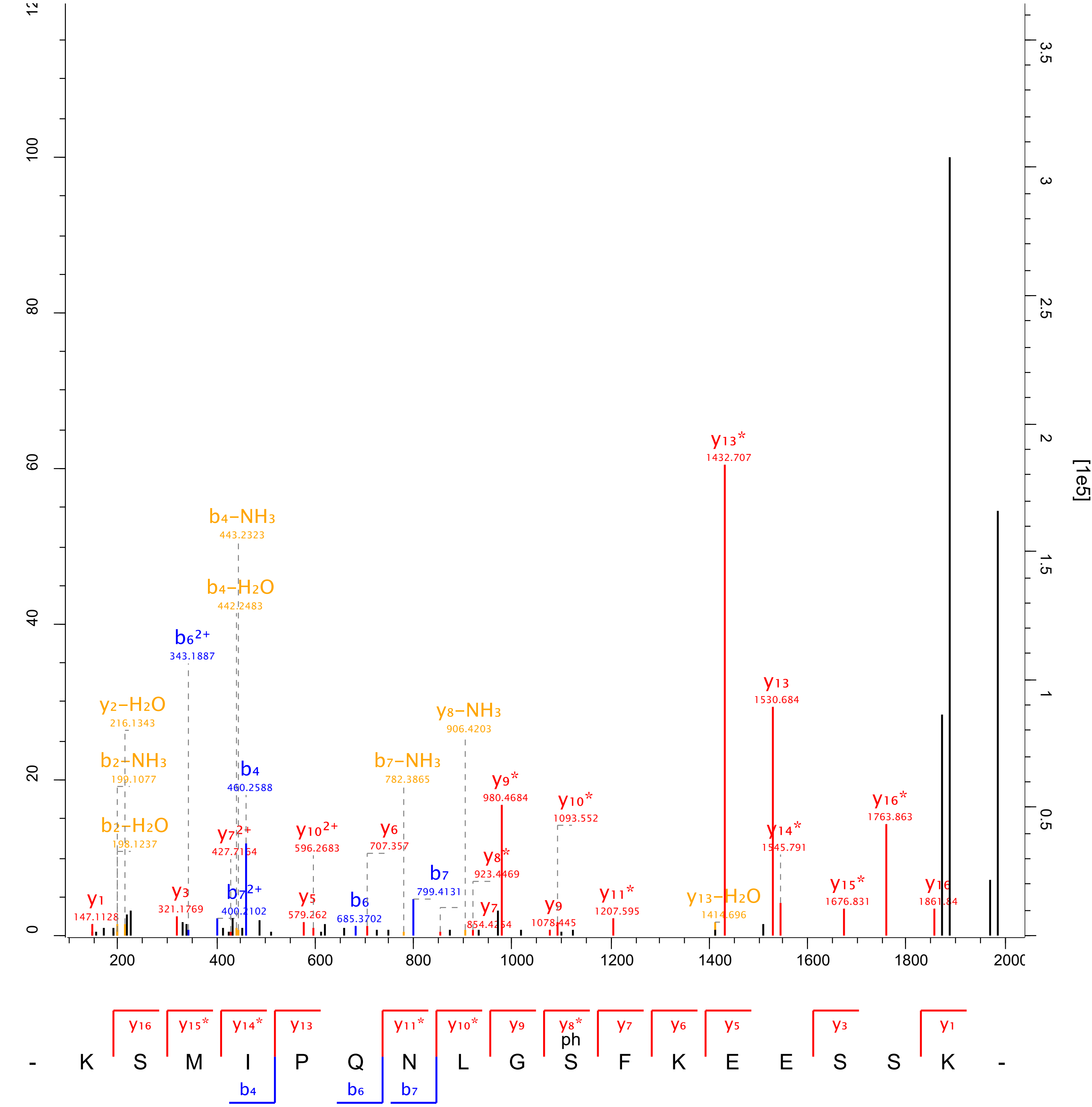

Sample output to test PDF Combine only

Raw file	Scan	Method	Score	m/z	Gene names
sirk1-mic-0-3-P	13951	FTMS; HCD	76.06	452.2	CRK4;CRK6

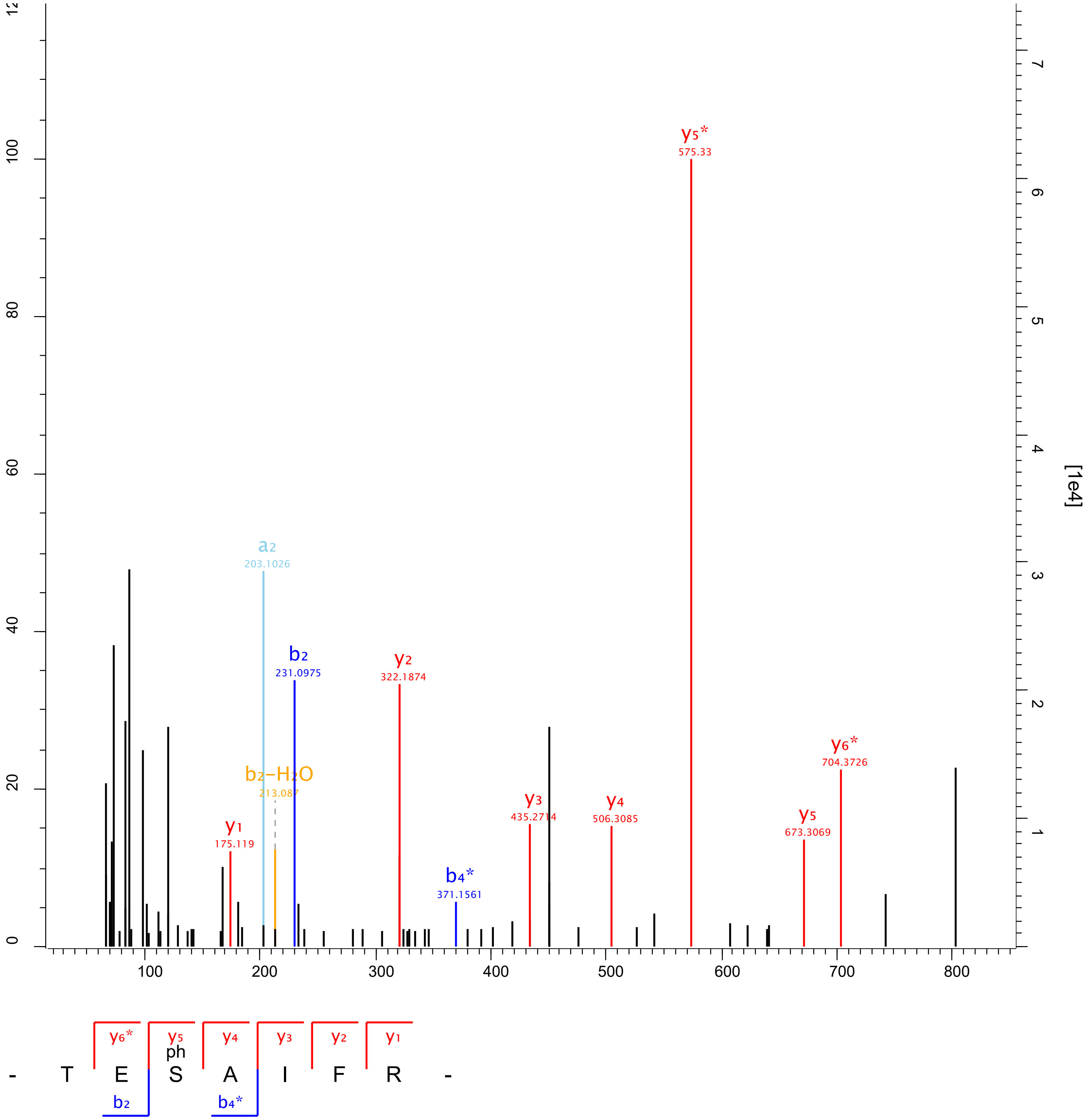

Sample output to test PDF Combine only

Raw file	Scan	Method	Score	m/z	Gene names
sirk1-mic-0-3-P	14494	FTMS; HCD	52.58	607.32	TPS8;TPS9

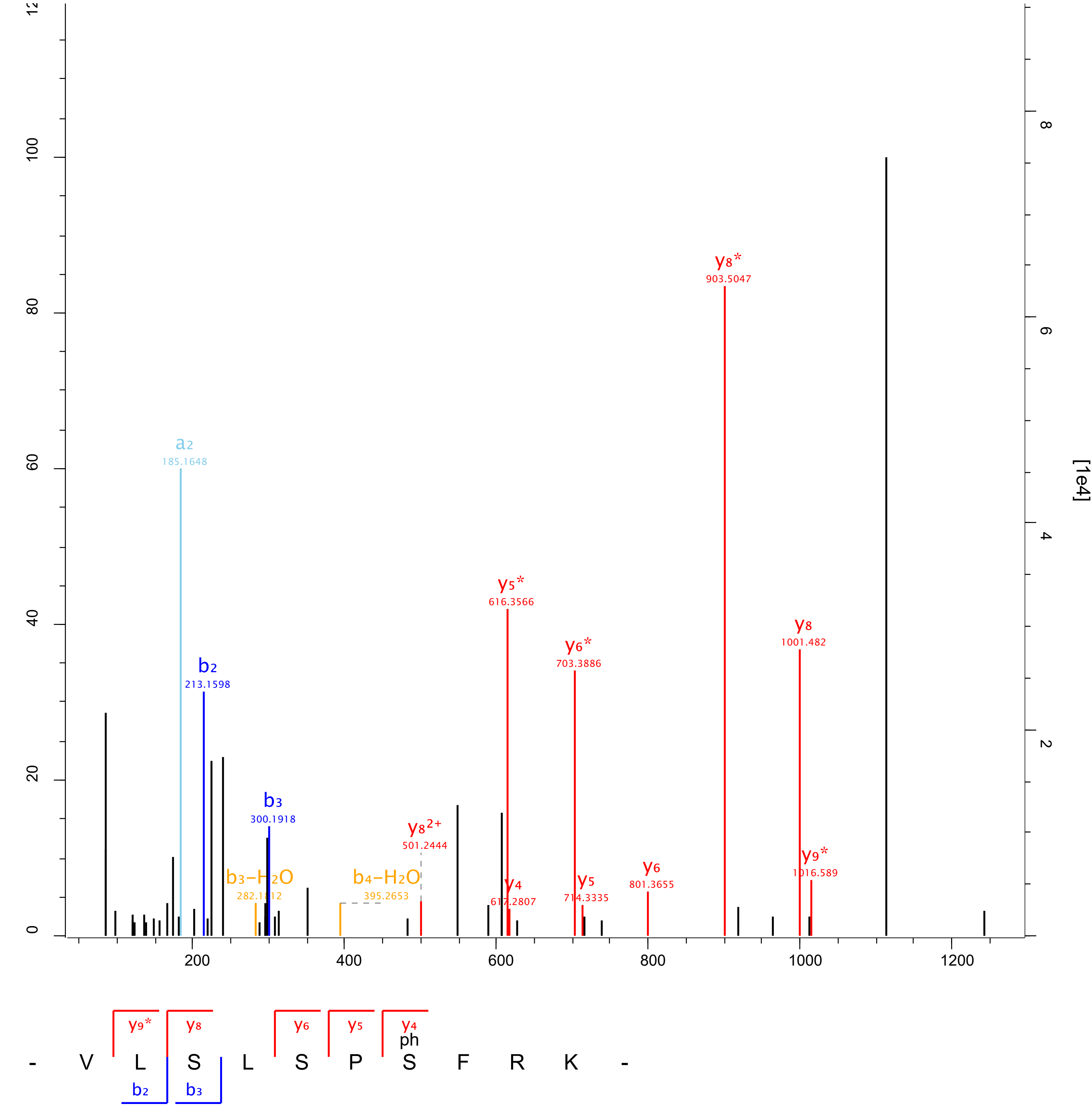

Sample output to test PDF Combine only

Raw file	Scan	Method	Score	m/z	Gene names
sirk1-mic-0-3-P	21974	FTMS; HCD	43.03	604.64	At2g20010

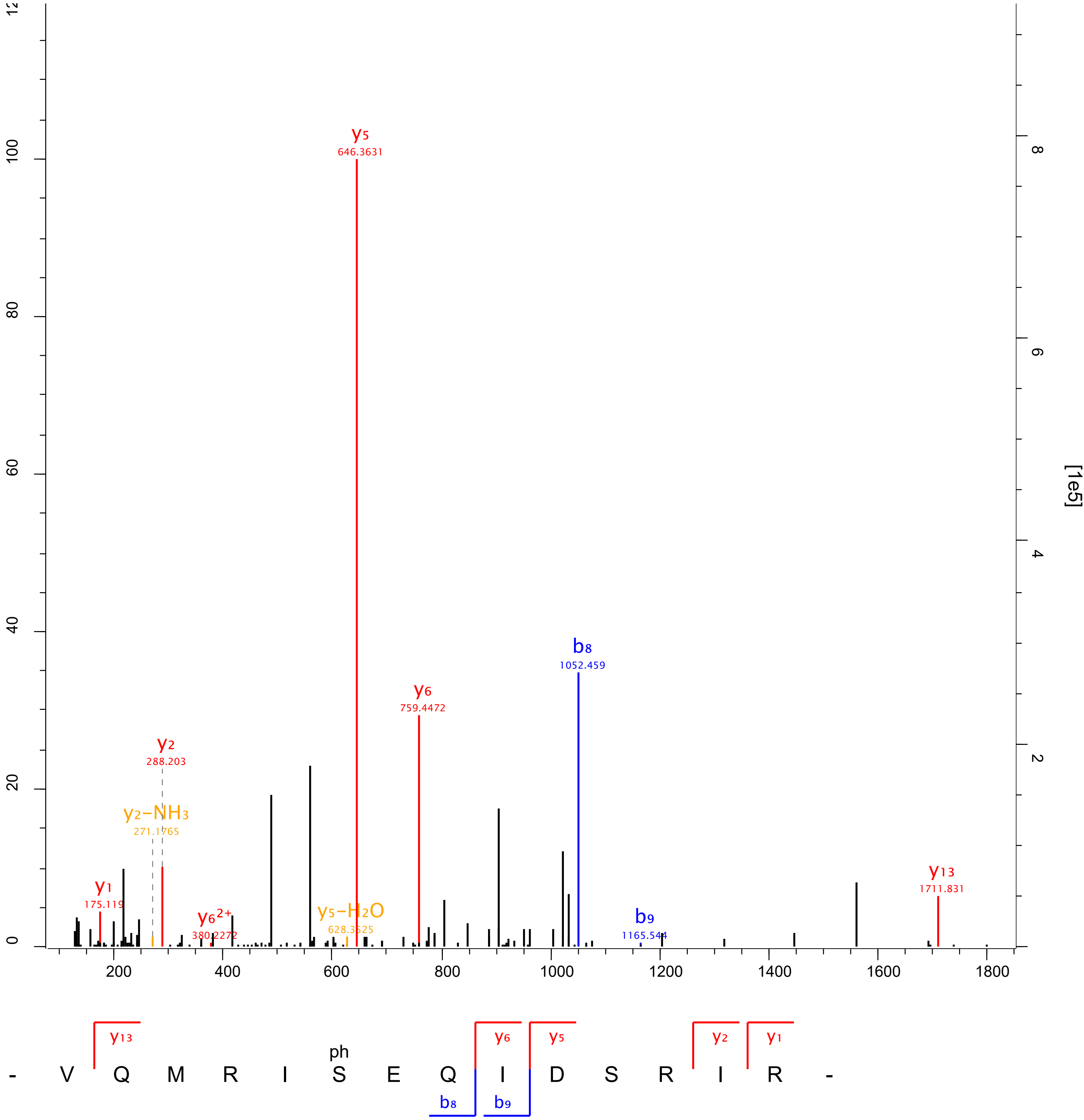

Sample output to test PDF Combine only

Raw file	Scan	Method	Score	m/z	Gene names
sirk1-mic-0-3-P	22732	FTMS; HCD	40.43	735.87	TOM2A

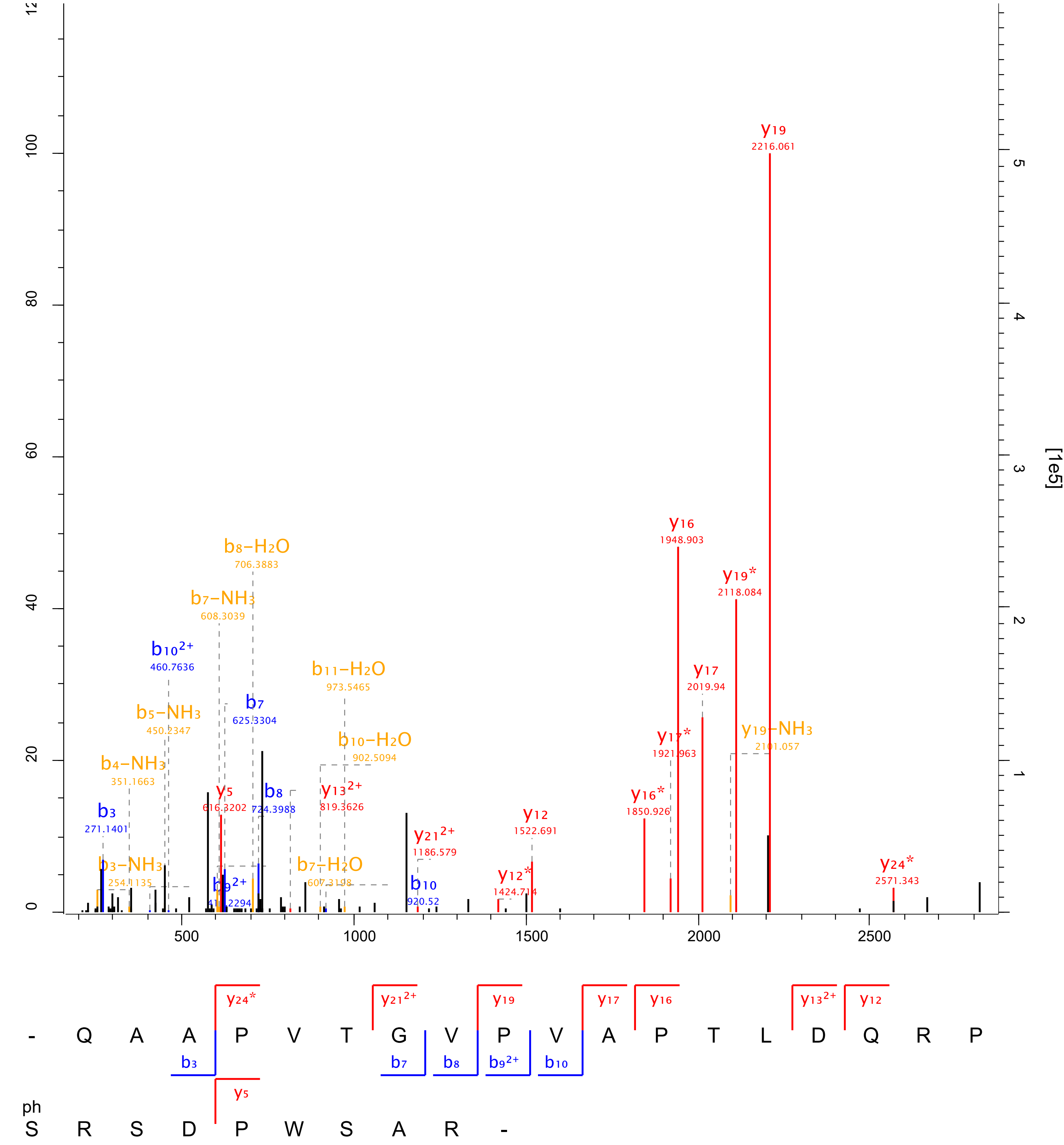

Sample output to test PDF Combine only

Raw file	Scan	Method	Score	m/z	Gene names
sirk1-mic-0-3-P	25580	FTMS; HCD	55.59	1015.8	At4g38470

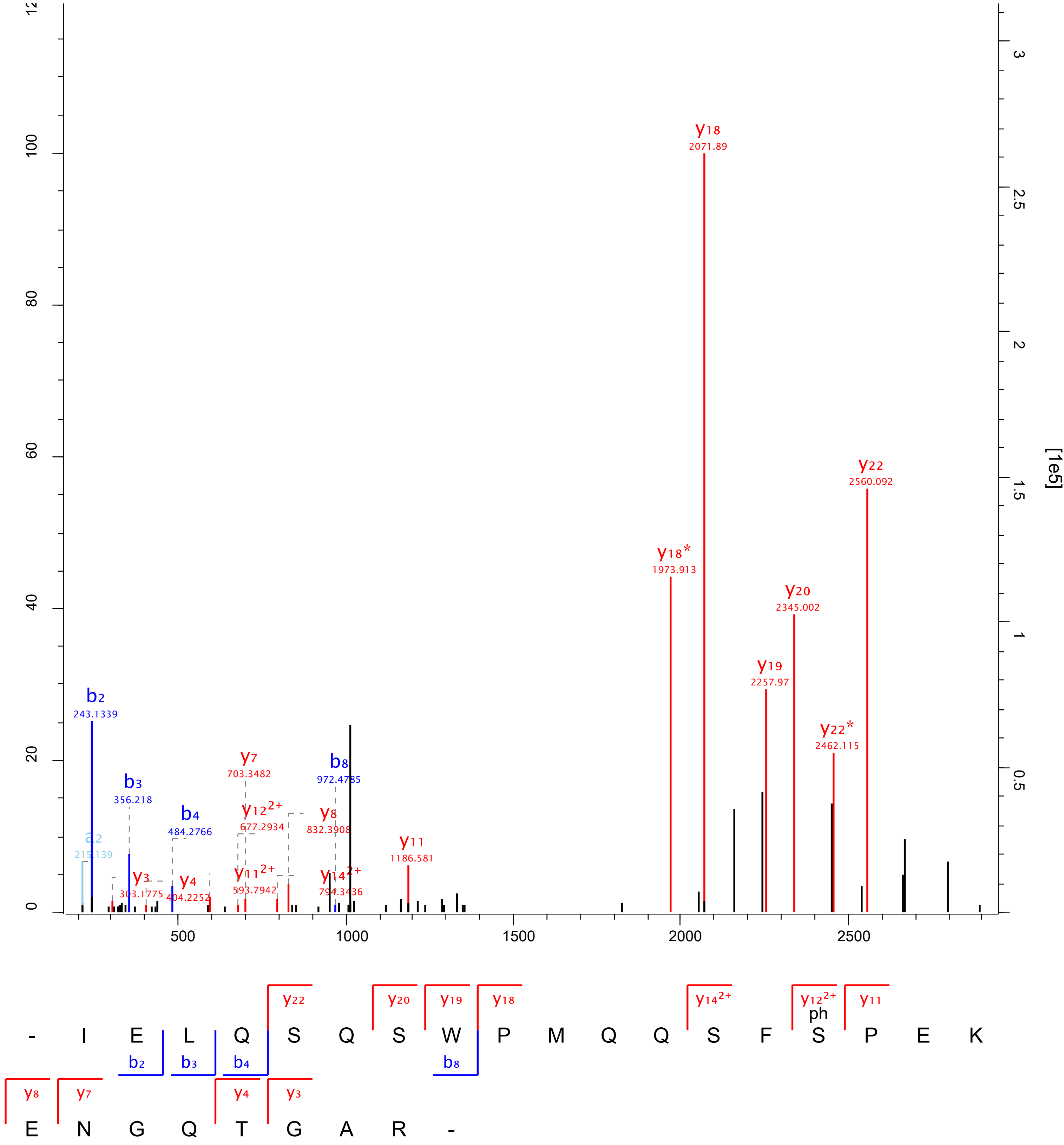

Sample output to test PDF Combine only

Raw file	Scan	Method	Score	m/z	Gene names
sirk1-mic-0-3-P	28696	FTMS; HCD	101.2	663.84	SEC5B

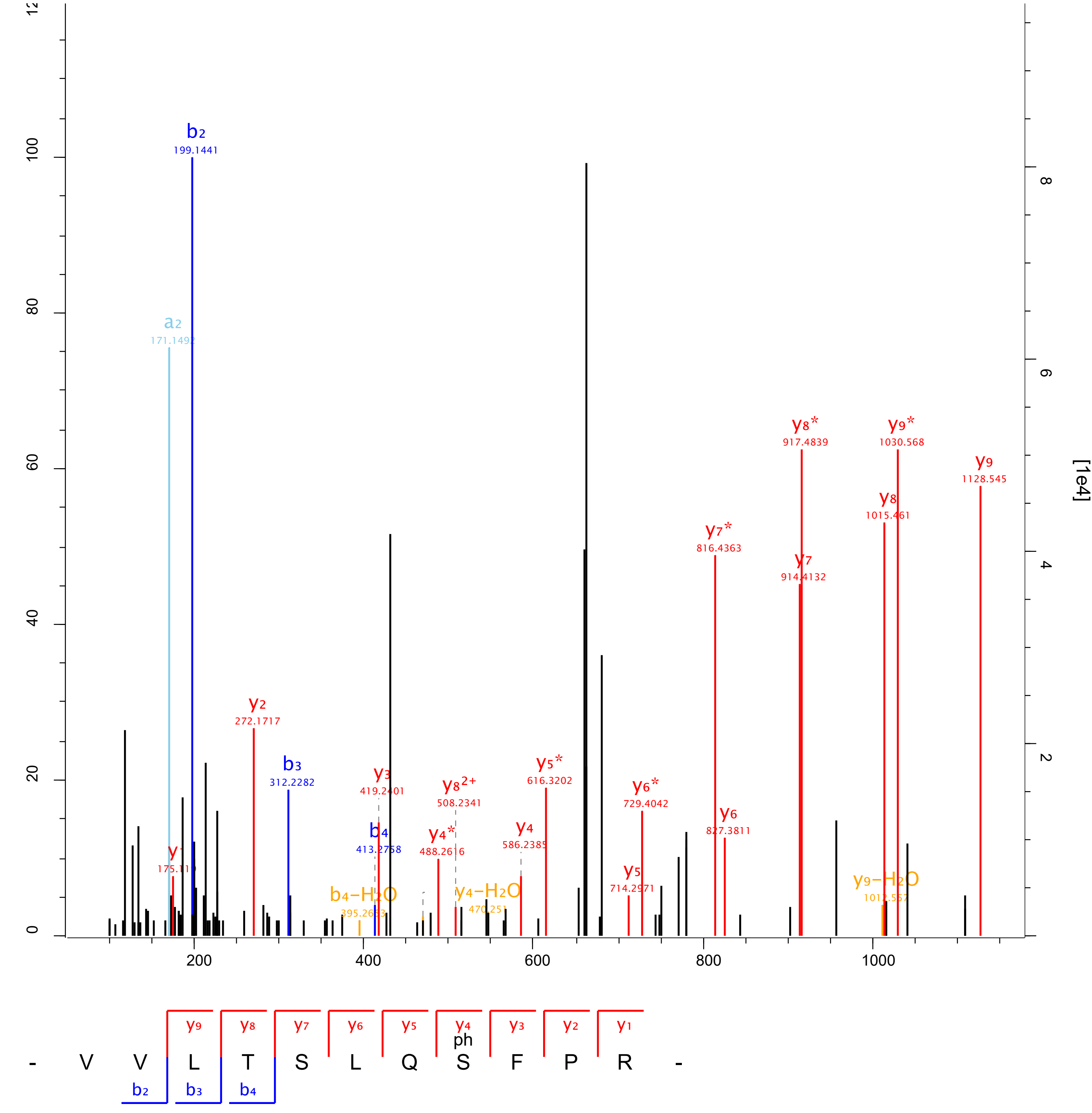

Sample output to test PDF Combine only

Raw file	Scan	Method	Score	m/z	Gene names
sirk1-mic-0-3-P	29560	FTMS; HCD	62.2	1215.09	B120

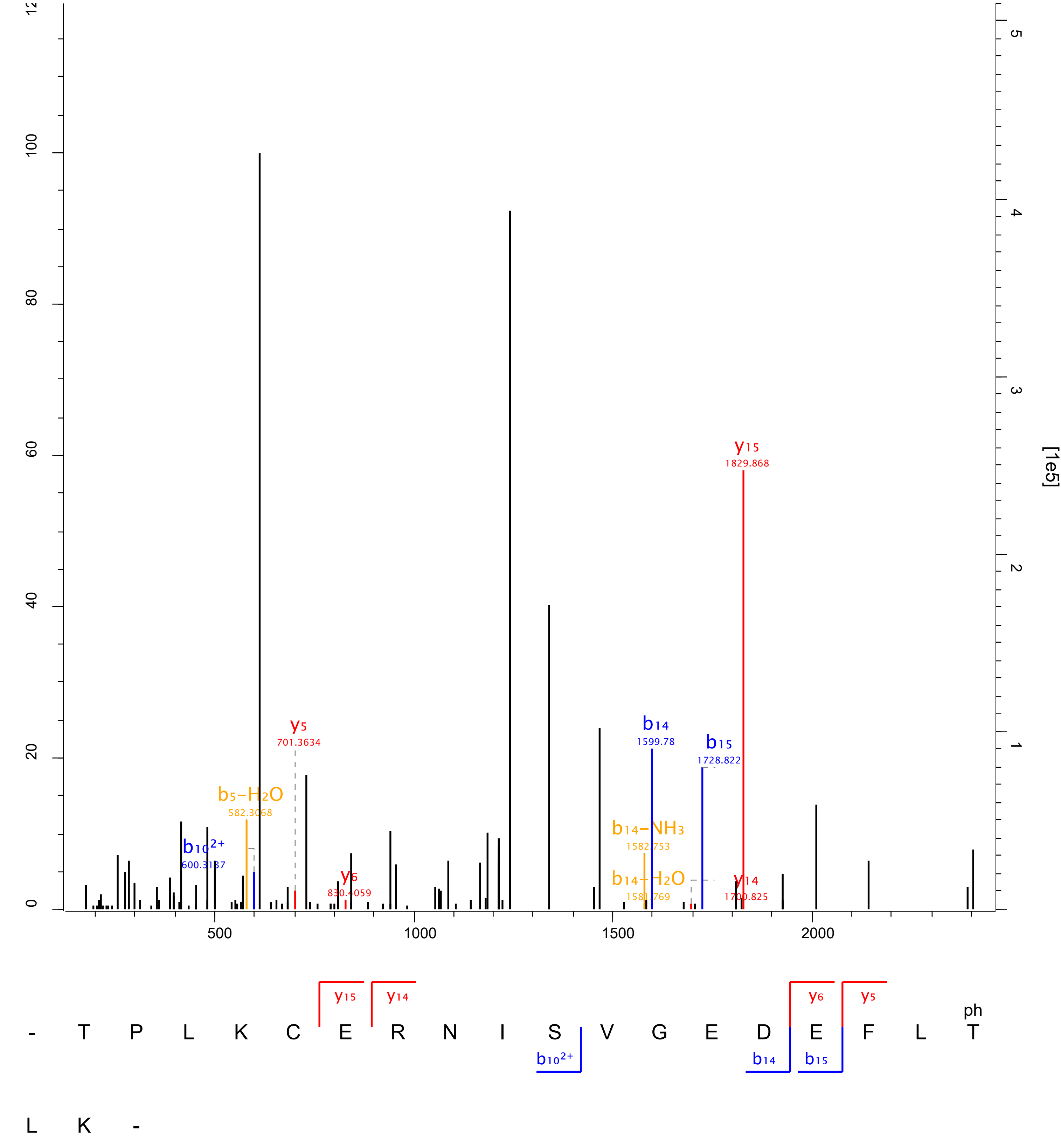

Sample output to test PDF Combine only

Raw file	Scan	Method	Score	m/z	Gene names
sirk1-mic-0-3-P	32755	FTMS; HCD	59.91	704.88	PP2A11

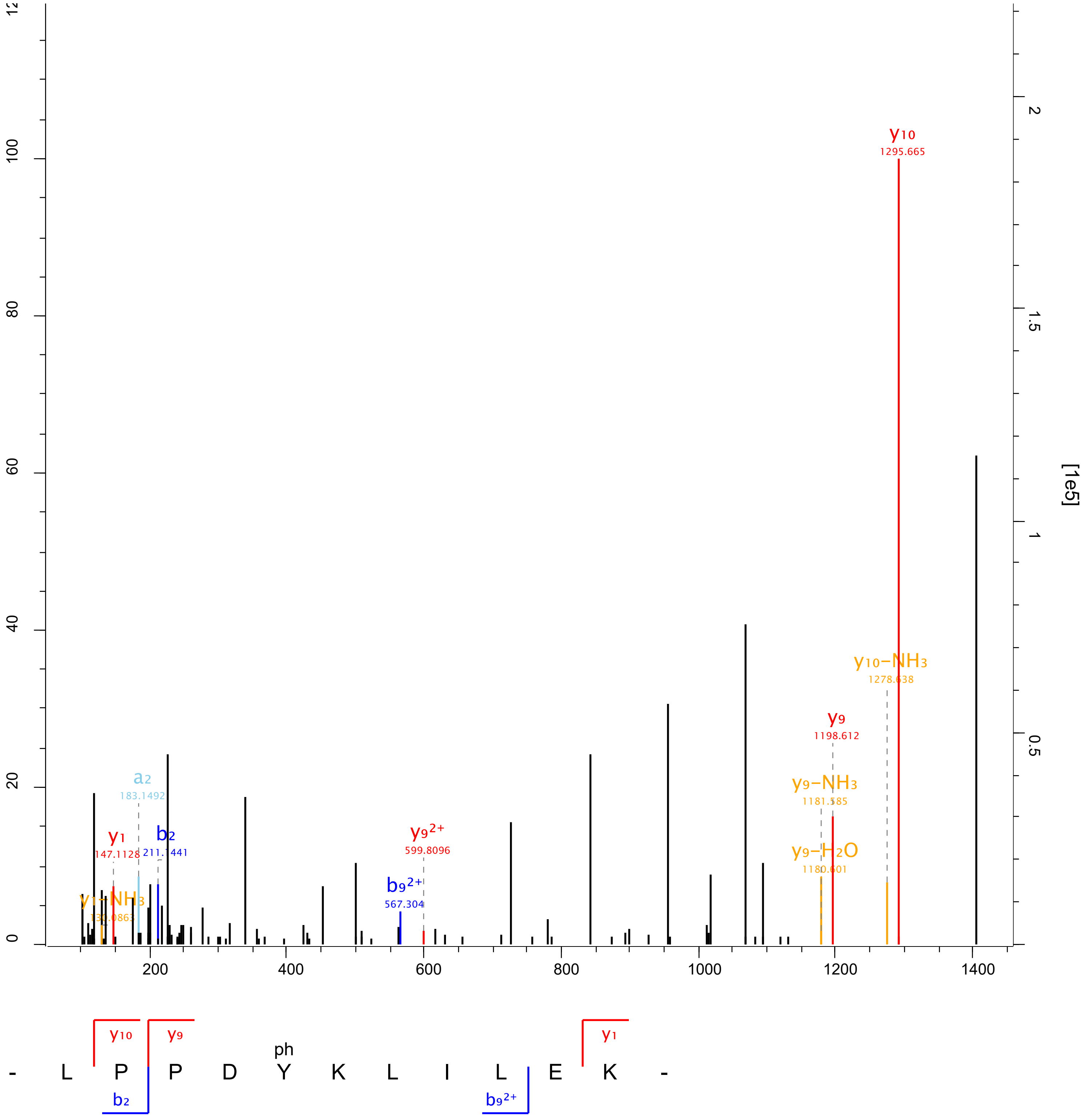

Sample output to test PDF Combine only

Raw file	Scan	Method	Score	m/z	Gene names
sirk1-mic-0-3-P	34903	FTMS; HCD	57.59	641.32	At2g17930;At4g36080

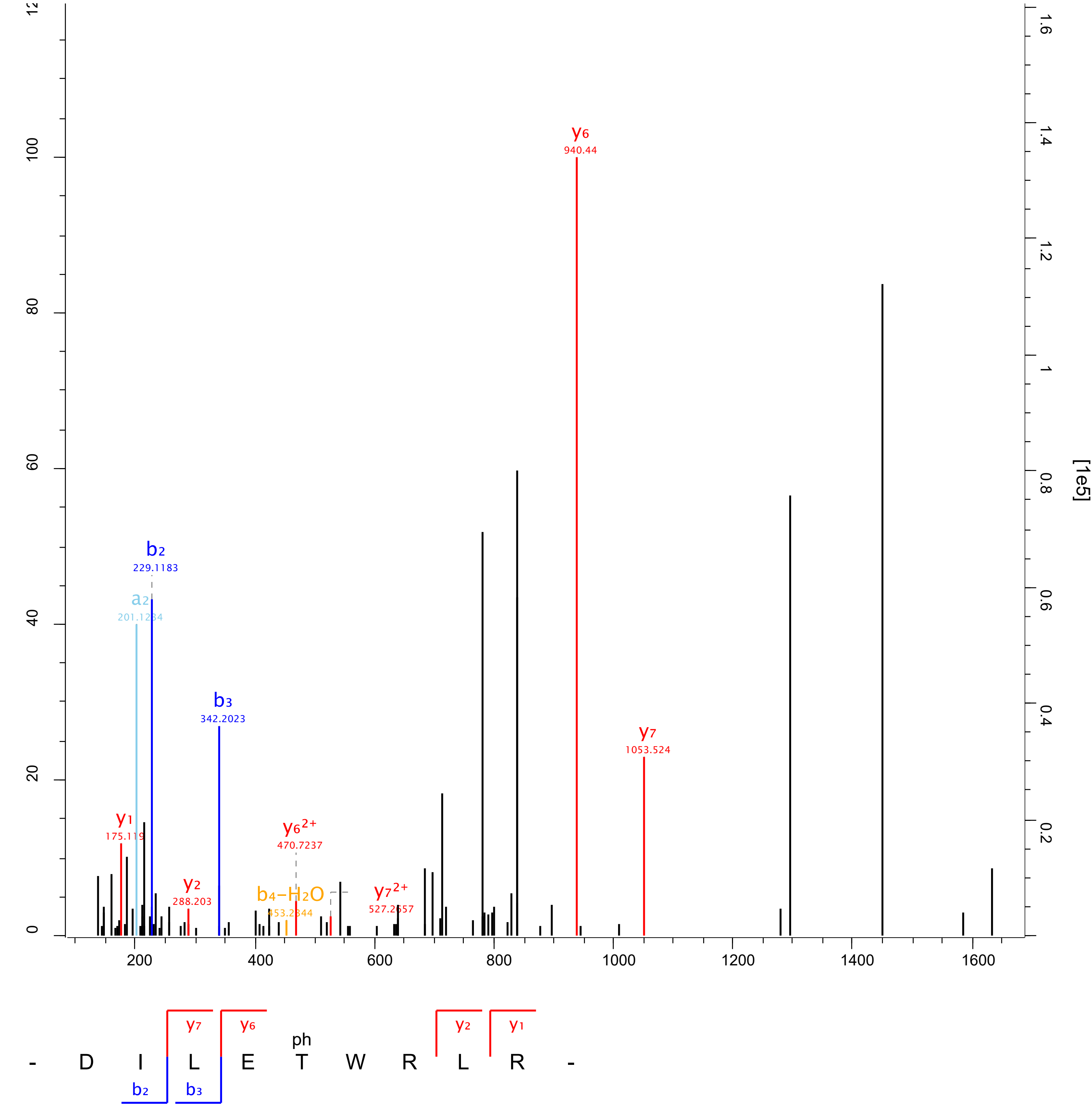

Sample output to test PDF Combine only

Raw file	Scan	Method	Score	m/z	Gene names
sirk1-mic-0-3-P	39743	FTMS; HCD	78.76	720.33	SHM4

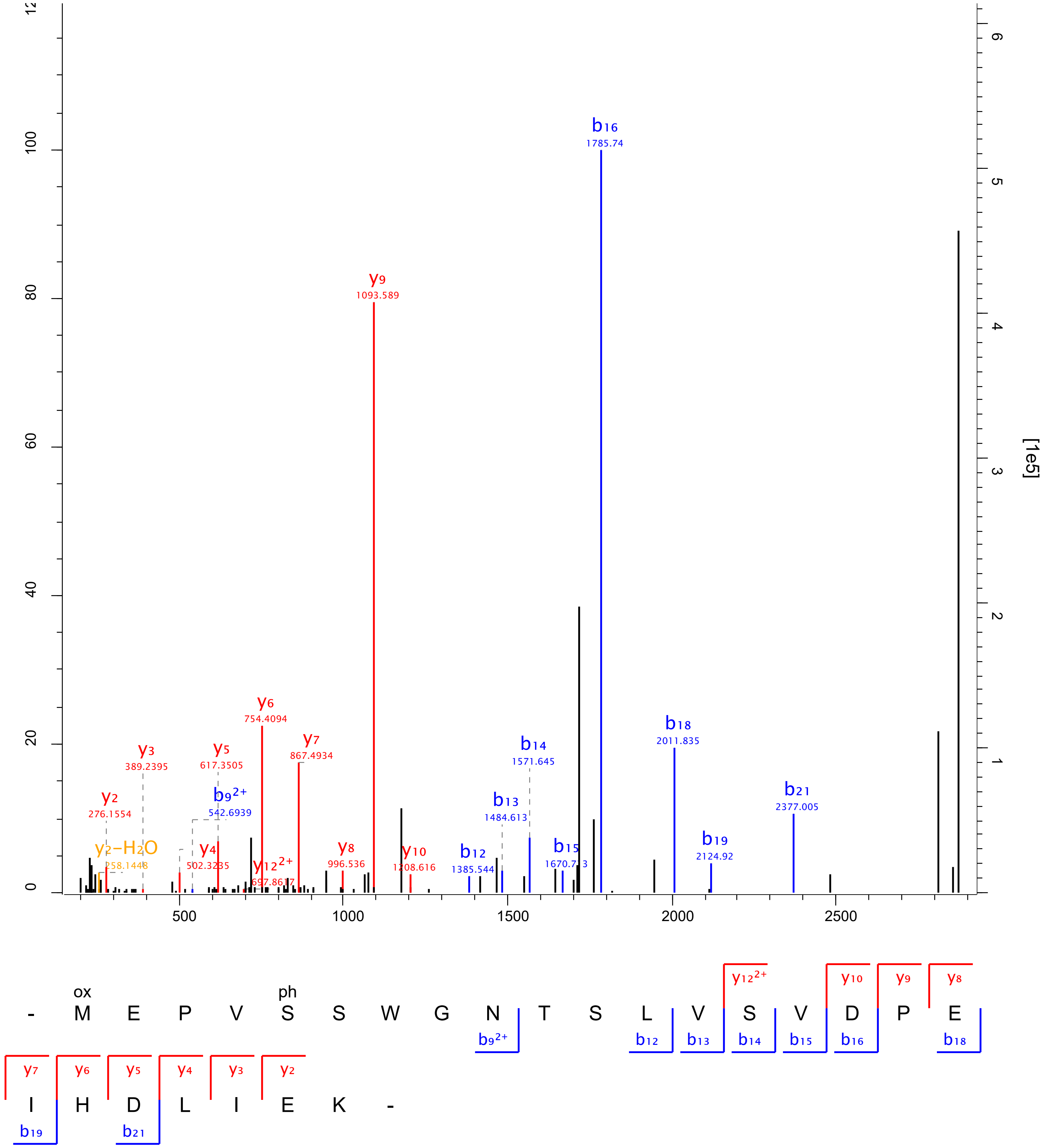

Sample output to test PDF Combine only

Raw file	Scan	Method	Score	m/z	Gene names
sirk1-mic-SUC-1-A	3268	FTMS; HCD	53.98	538.25	MRO11.3

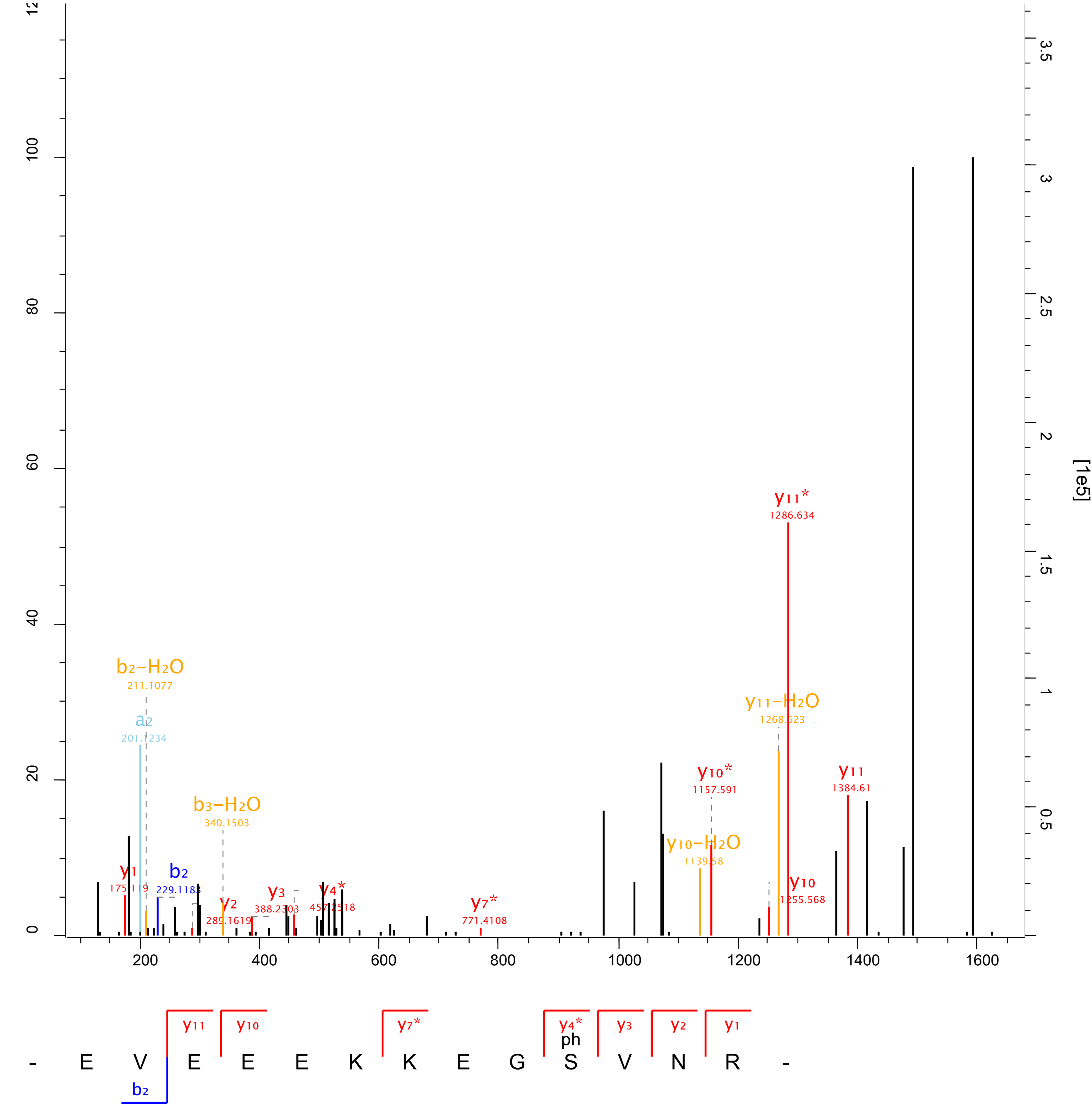

Sample output to test PDF Combine only

Raw file	Scan	Method	Score	m/z	Gene names
sirk1-mic-SUC-1-A	3576	FTMS; HCD	56.2	578.76	ABCG36

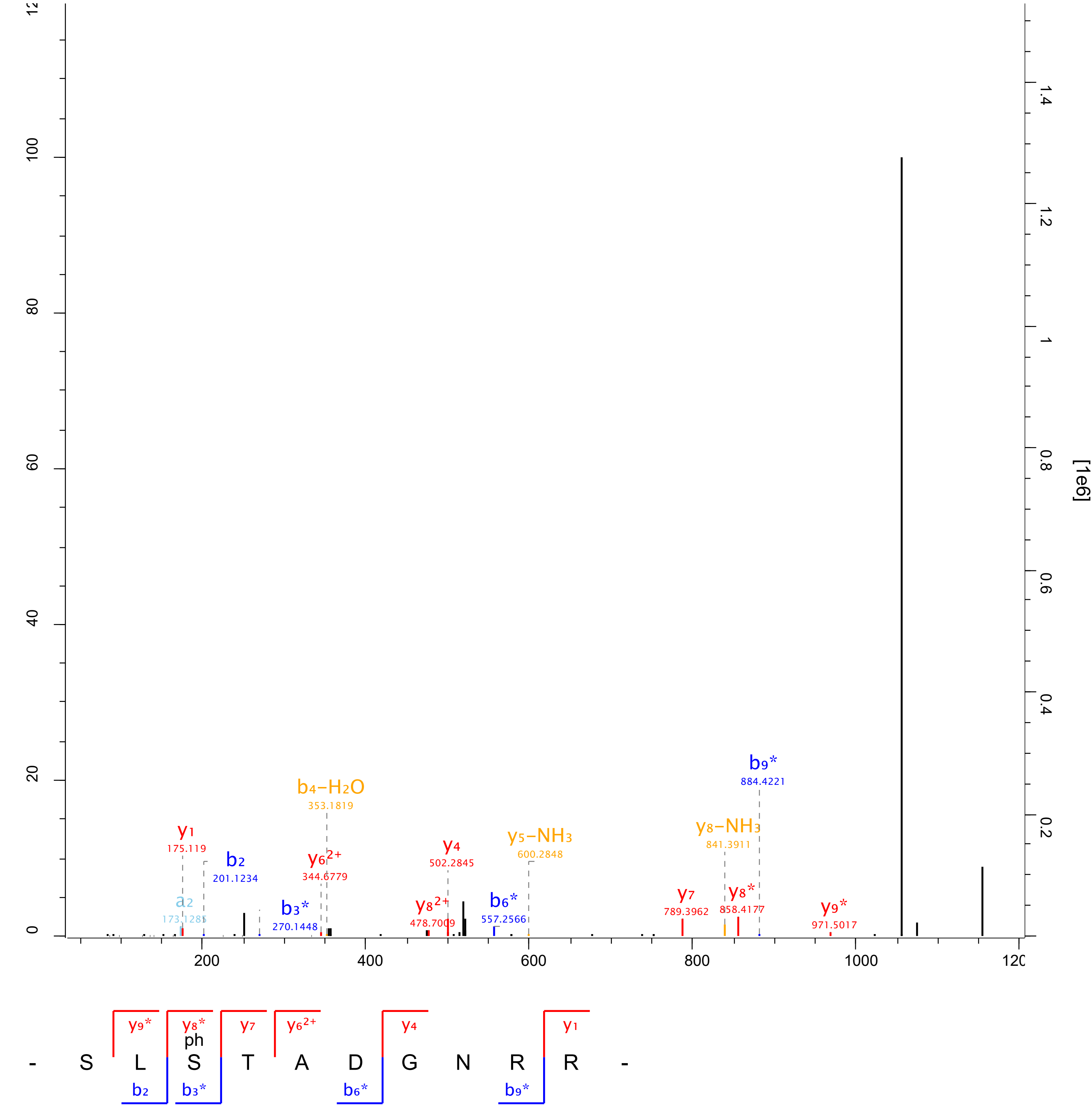

Sample output to test PDF Combine only

Raw file	Scan	Method	Score	m/z	Gene names
sirk1-mic-SUC-1-A	3995	FTMS; HCD	62.47	514.22	RS31

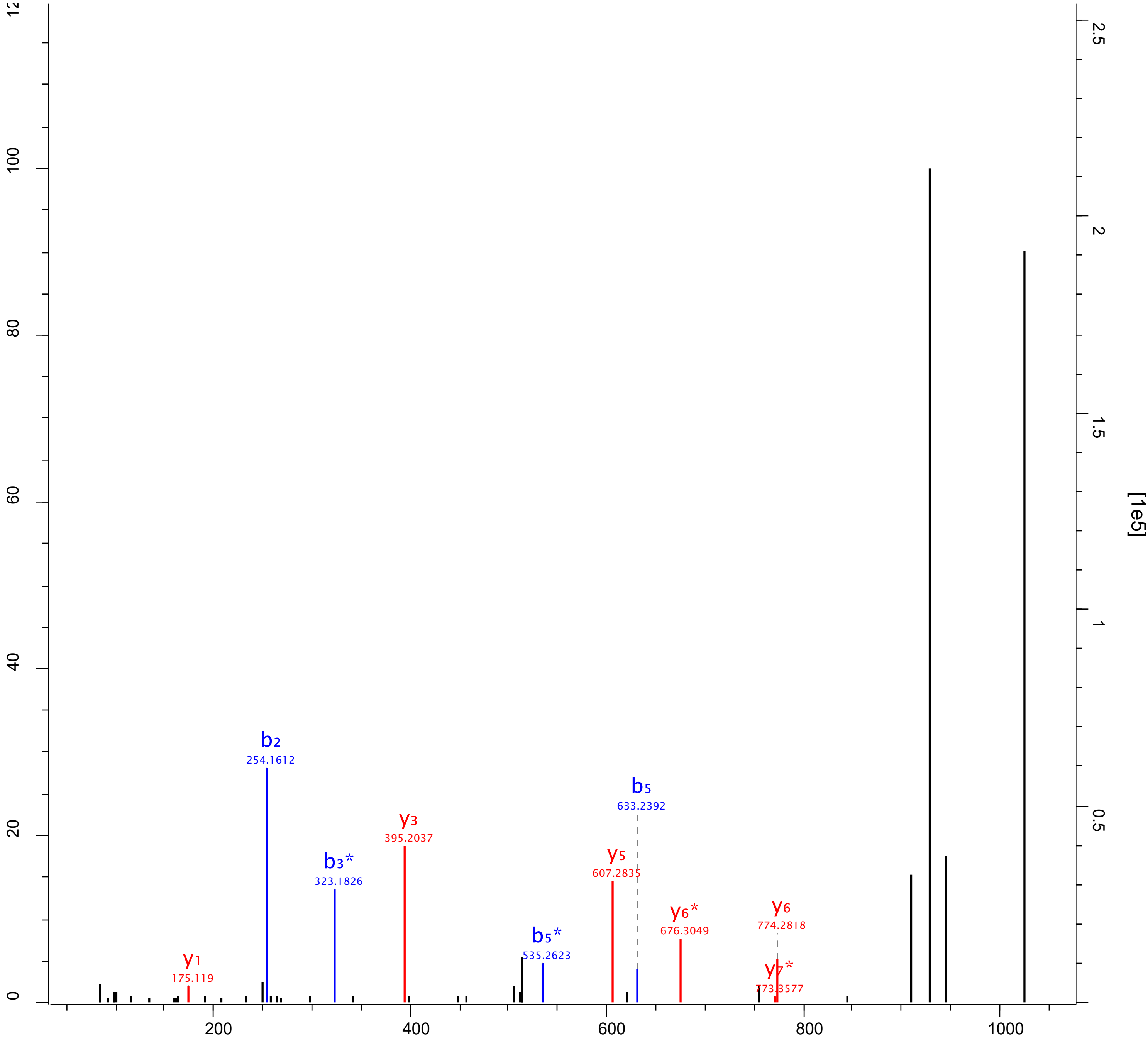

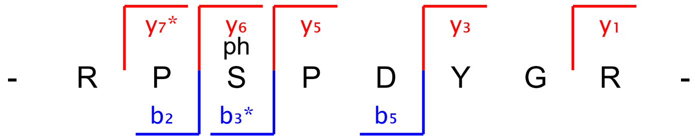

Sample output to test PDF Combine only

Raw file	Scan	Method	Score	m/z	Gene names
sirk1-mic-SUC-1-A	5201	FTMS; HCD	78.08	505.58	SEC5A

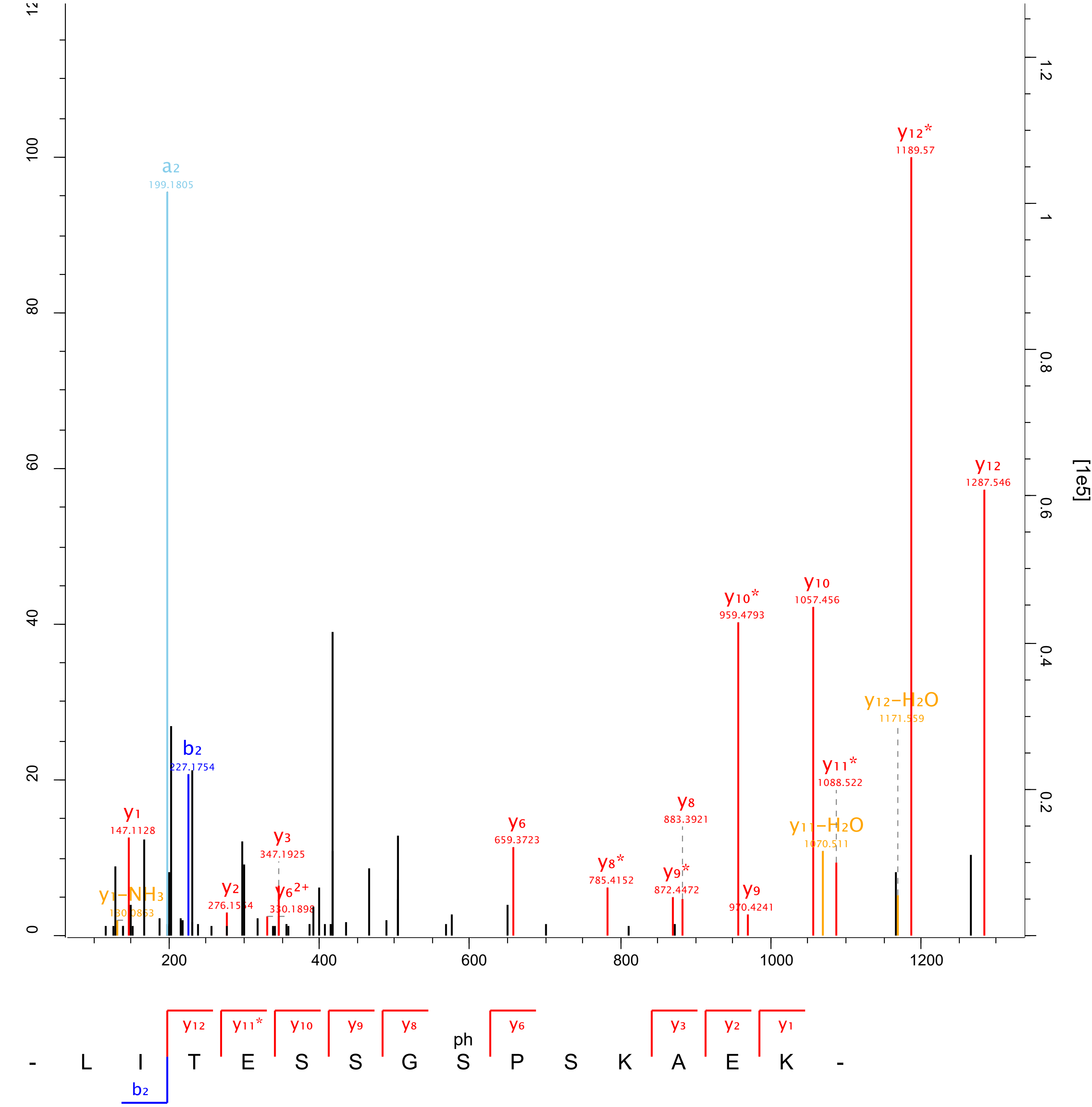

Sample output to test PDF Combine only

Raw file	Scan	Method	Score	m/z	Gene names
sirk1-mic-SUC-1-A	5786	FTMS; HCD	55.31	515.24	RS41

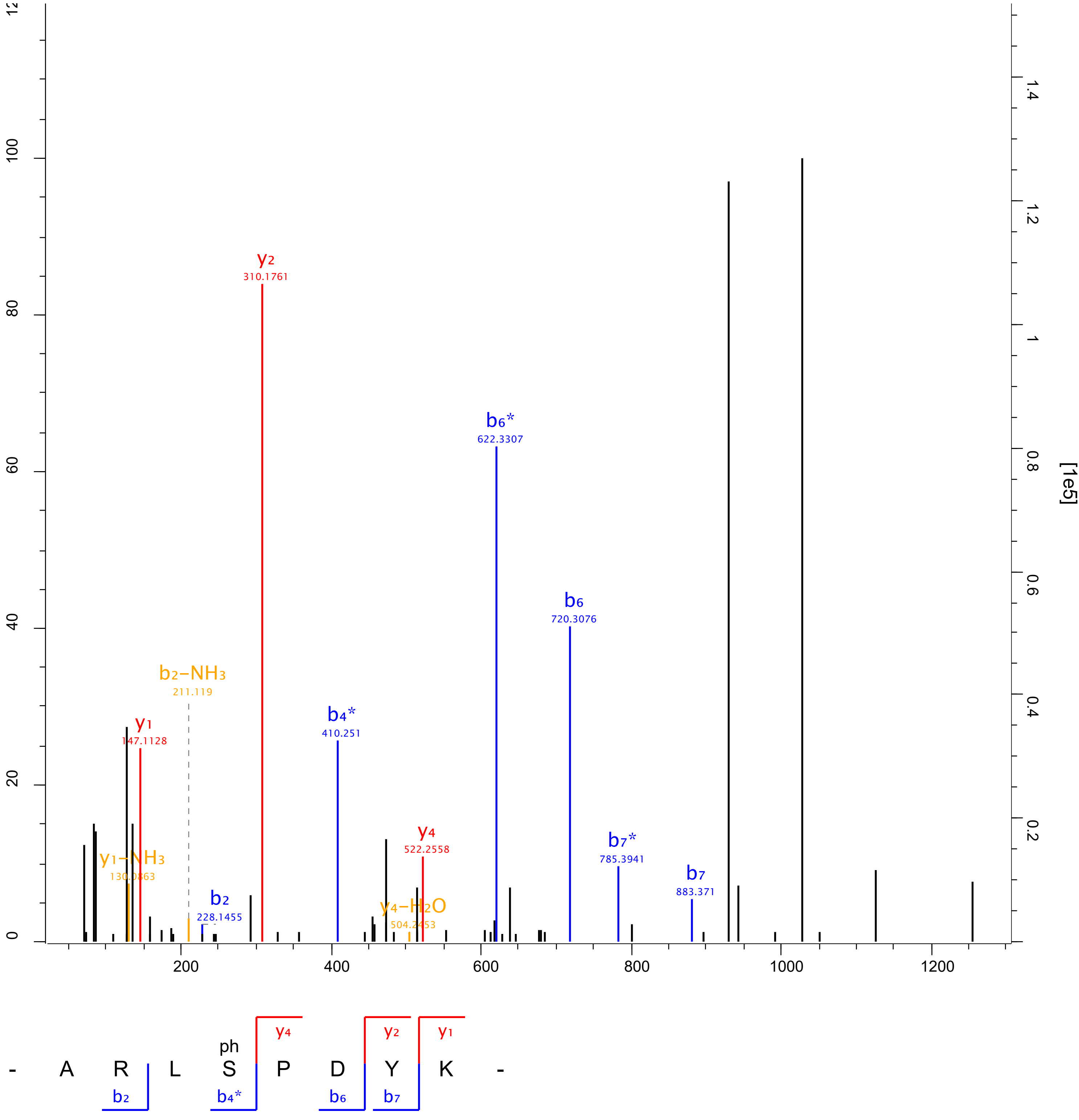

Sample output to test PDF Combine only

Raw file	Scan	Method	Score	m/z	Gene names
sirk1-mic-SUC-1-A	9771	FTMS; HCD	53.6	536.24	SYP121

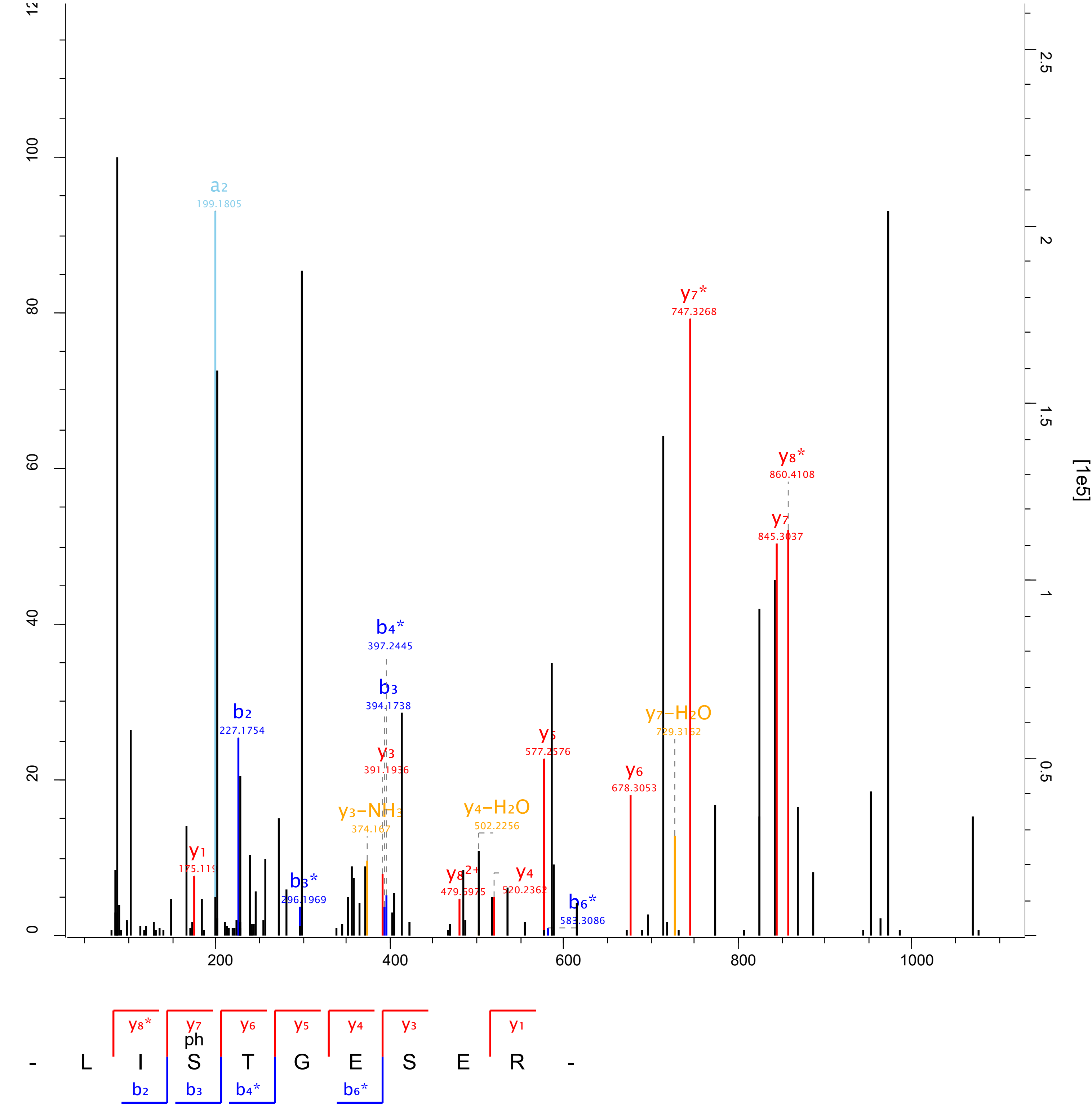

Sample output to test PDF Combine only

Raw file	Scan	Method	Score	m/z	Gene names
sirk1-mic-SUC-1-A	11683	FTMS; HCD	73.39	619.26	ABCB11

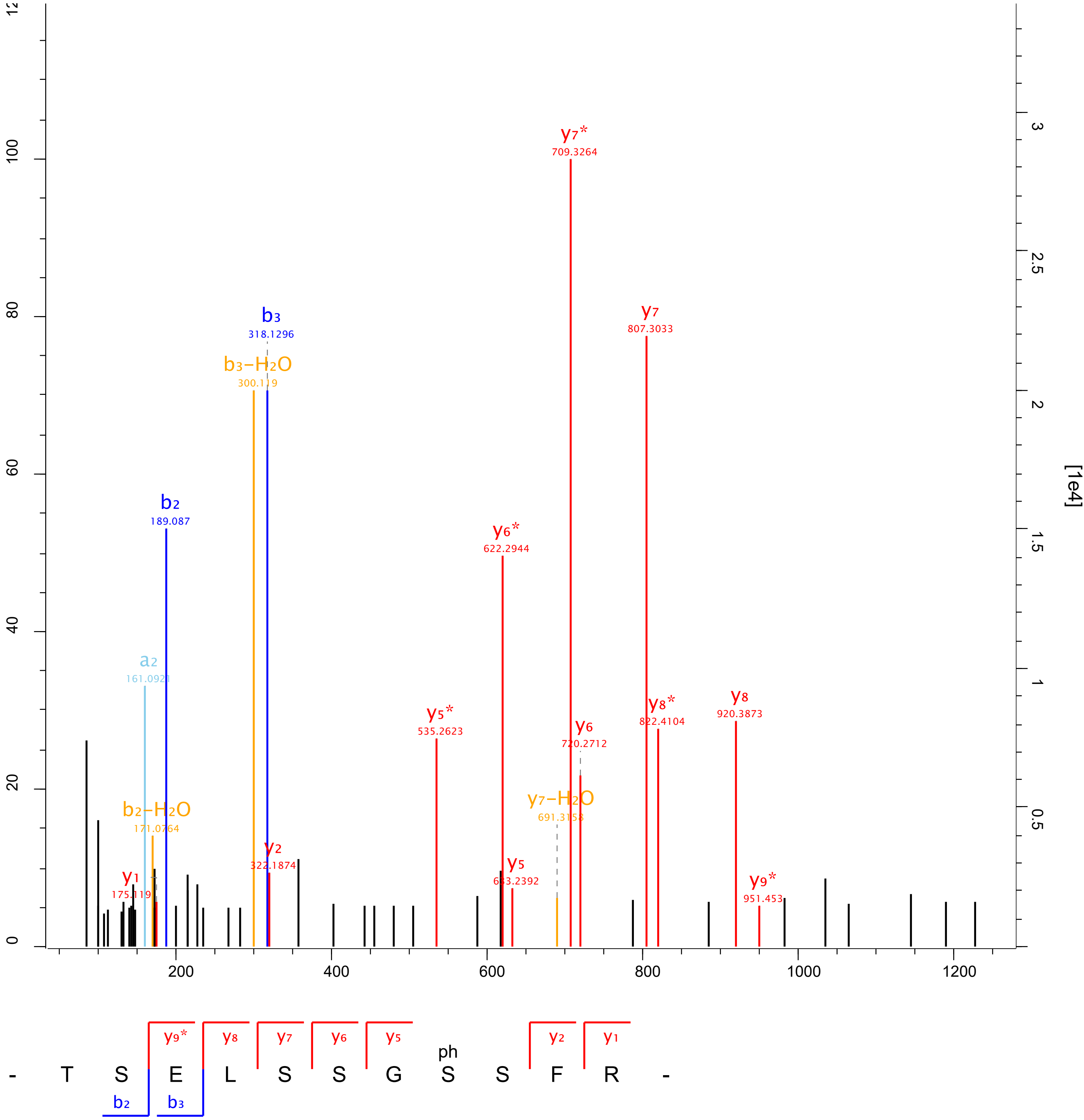

Sample output to test PDF Combine only

Raw file	Scan	Method	Score	m/z
sirk1-mic-SUC-1-A	14102	FTMS; HCD	70.06	672.31

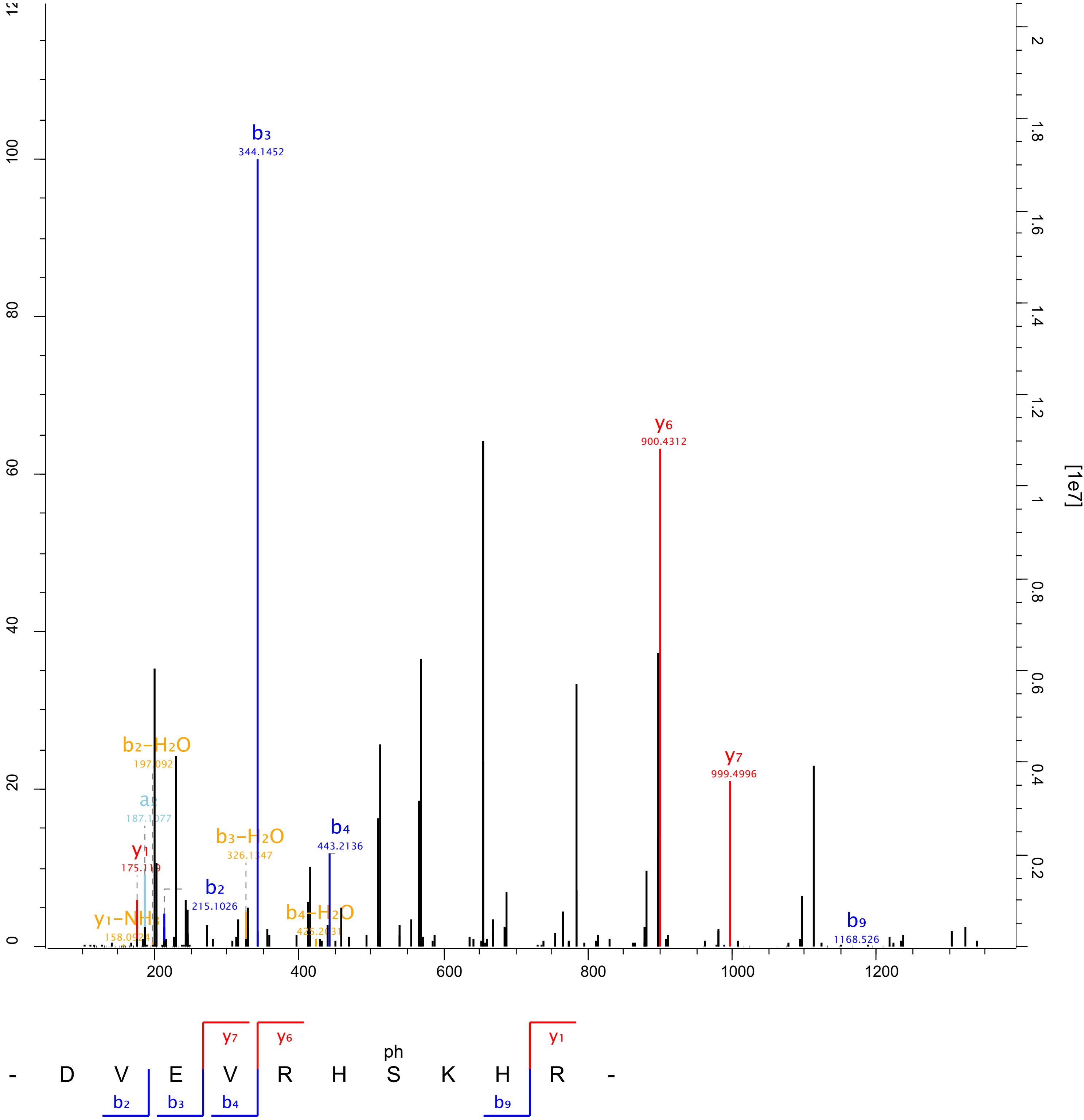

Sample output to test PDF Combine only

Raw file	Scan	Method	Score	m/z	Gene names
sirk1-mic-SUC-1-A	15314	FTMS; HCD	67.14	626.31	XI-K

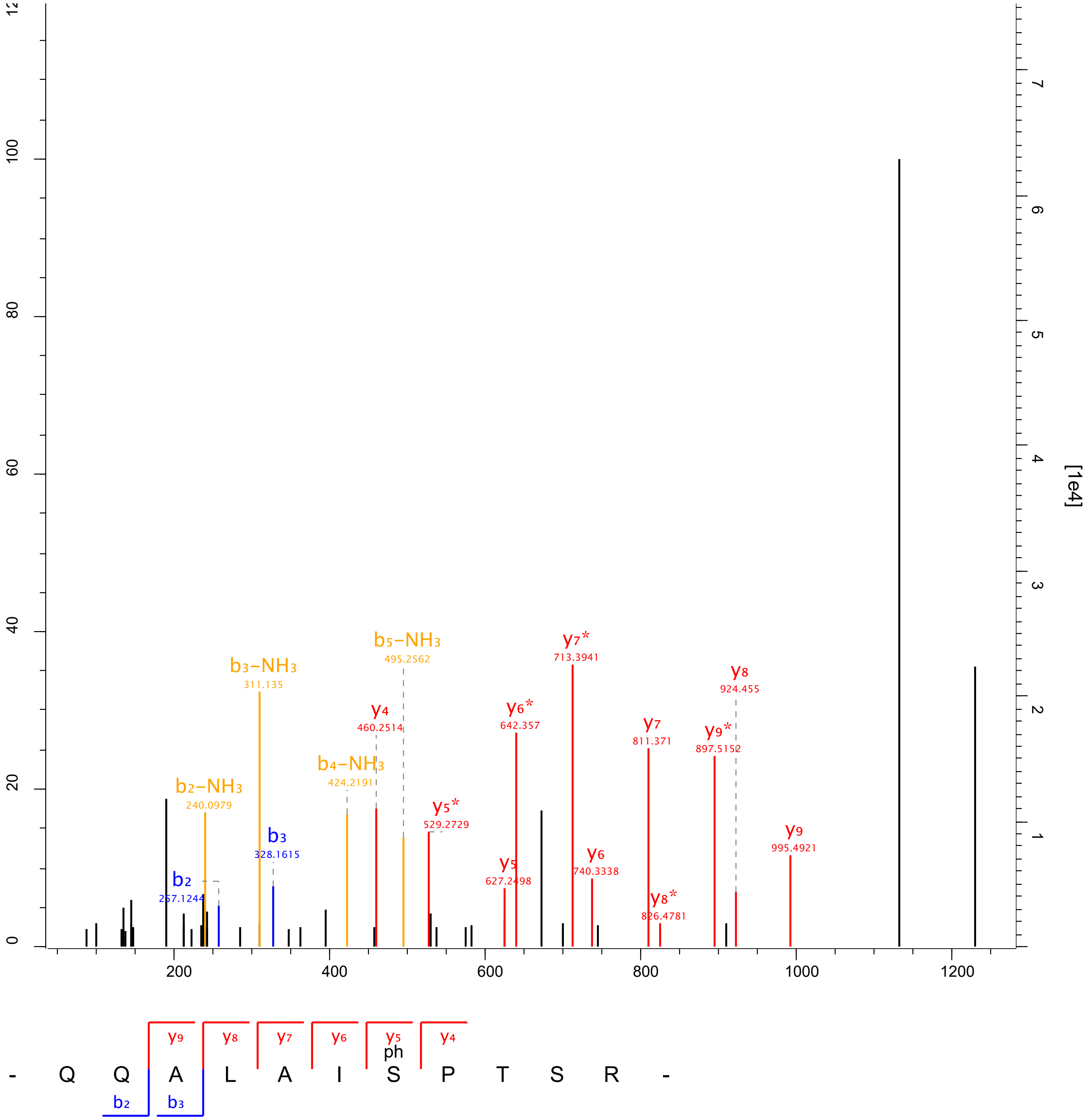

Sample output to test PDF Combine only

Raw file	Scan	Method	Score	m/z	Gene names
sirk1-mic-SUC-1-A	18014	FTMS; HCD	63.09	580.3	VLN4

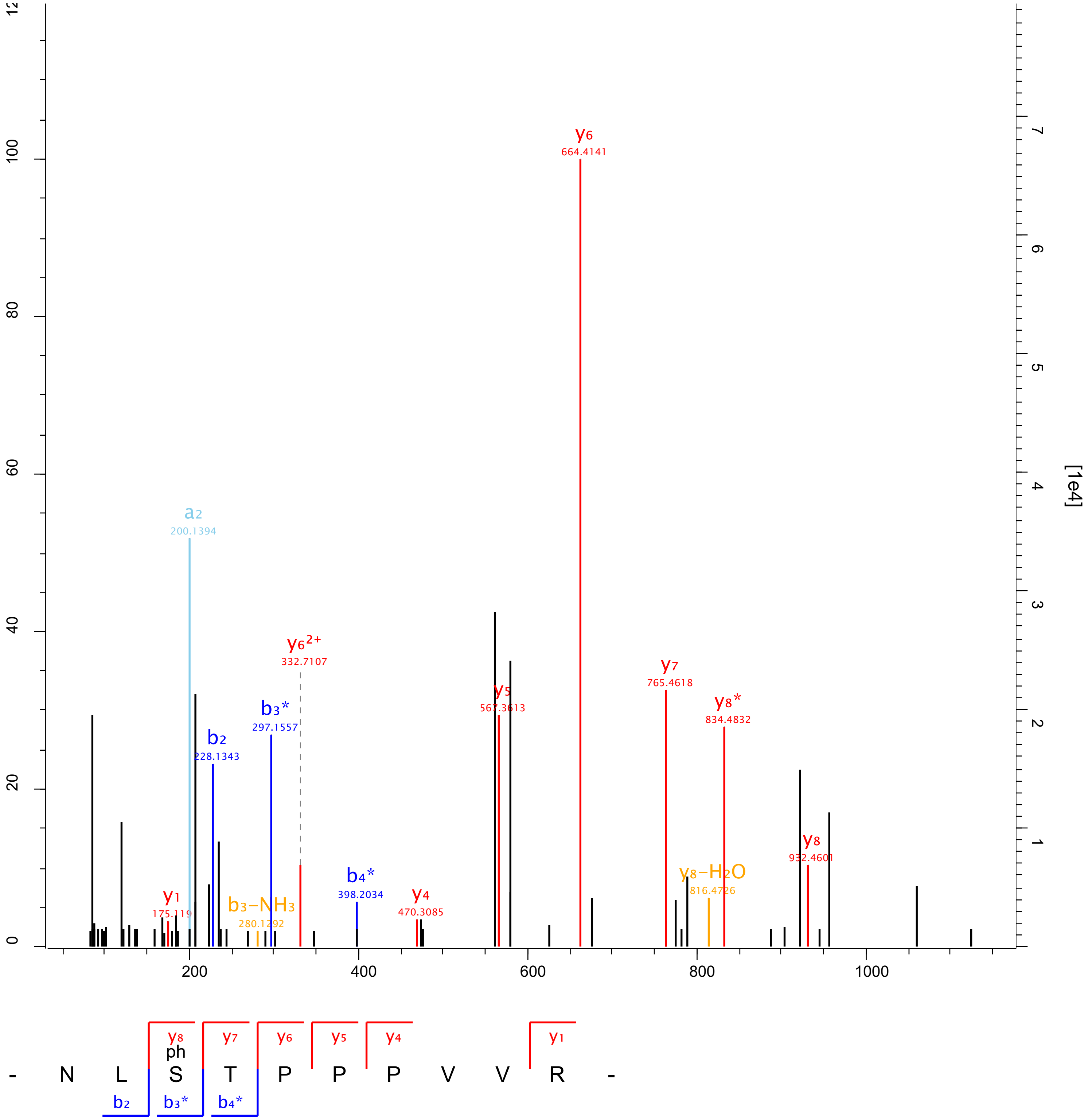

Sample output to test PDF Combine only
