# Supplementary material for: Sucrose-induced Receptor Kinase 1 is Modulated by an Interacting Kinase with Short Extracellular Domain
Source: Mol Cell Proteomics. 2019 May 30;18(8):1556–71. doi: 10.1074/mcp.RA119.001336 (PMC6683012; doi:10.1074/mcp.RA119.001336)
Supplement: Supplementay Figure S6h [file 143141_1_supp_311931_ps51kz.pdf]

Figure S6:

Spectra of all identified phosphopeptides.

Raw file	Scan	Method	Score	m/z	Gene names
sirk1-mic-SUC-1-A	20318	FTMS; HCD	42.78	628.29	POT8

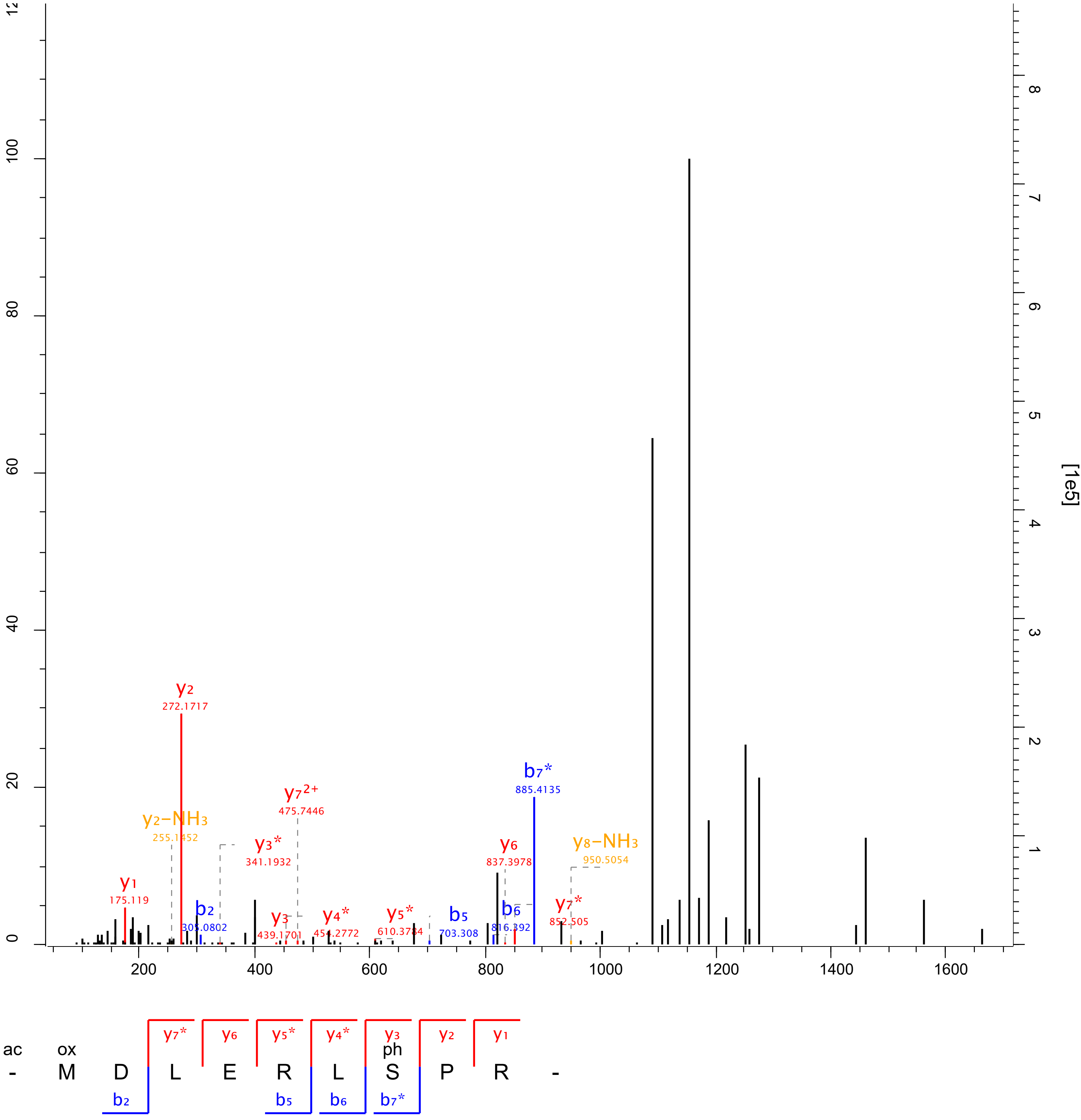

Sample output to test PDF Combine only

Raw file	Scan	Method	Score	m/z	Gene names
sirk1-mic-SUC-1-A	22318	FTMS; HCD	47.89	512.73	CINV1

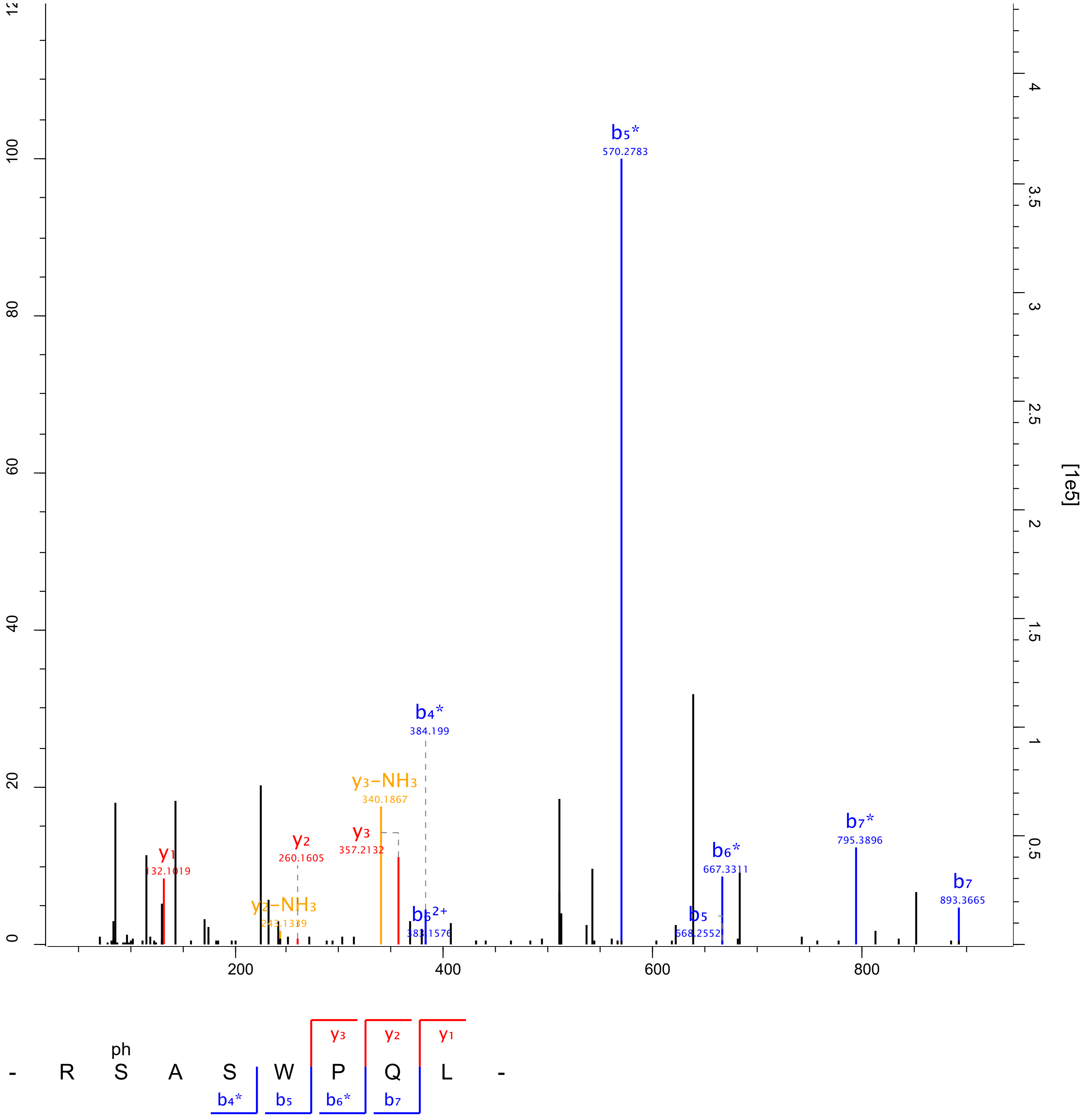

Sample output to test PDF Combine only

Raw file	Scan	Method	Score	m/z	Gene names
sirk1-mic-SUC-1-A	22930	FTMS; HCD	82.65	612.8	At4g11270

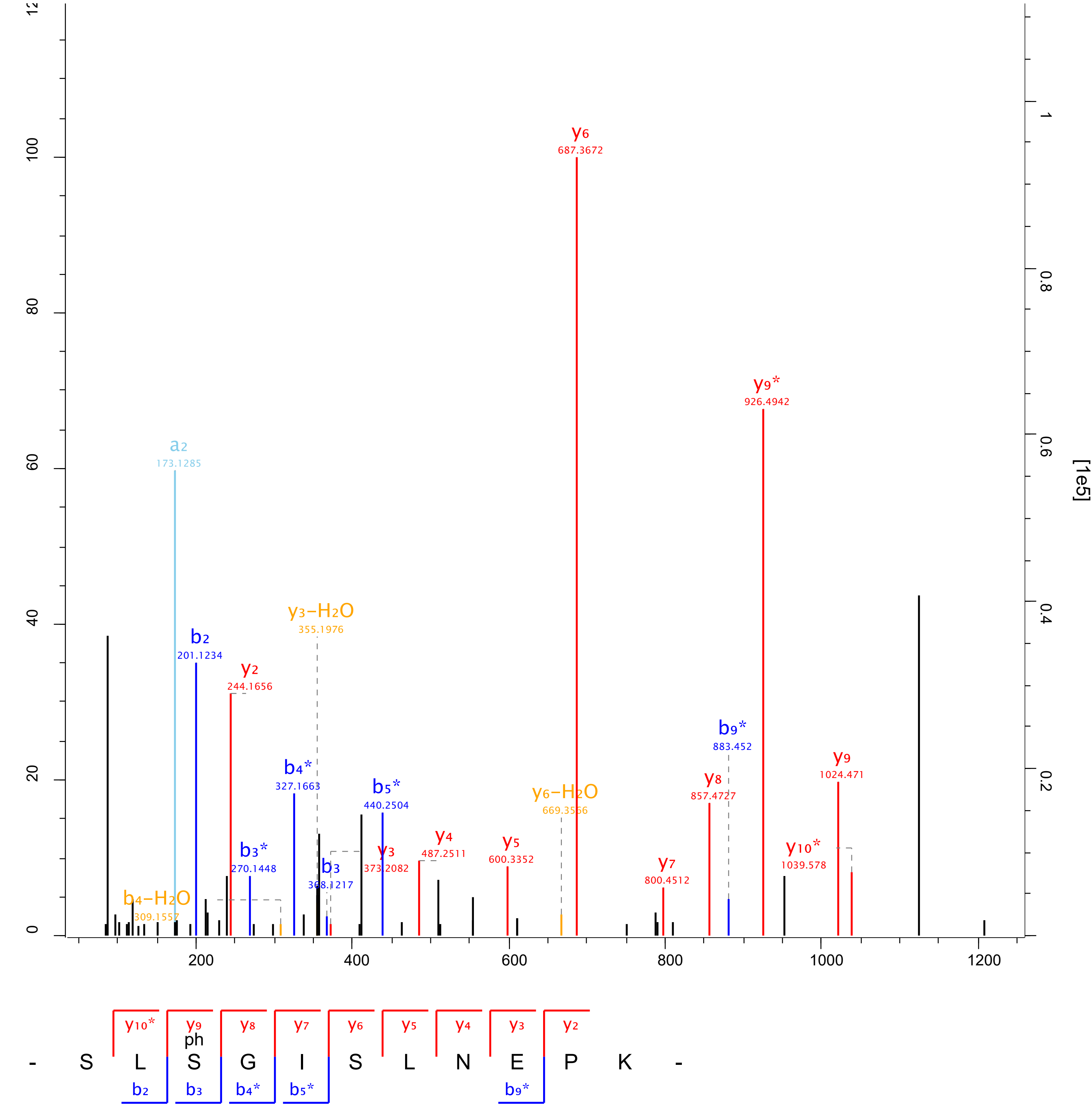

Sample output to test PDF Combine only

Raw file	Scan	Method	Score	m/z	Gene names
sirk1-mic-SUC-1-P	11409	FTMS; HCD	83.37	532.91	ABCB4

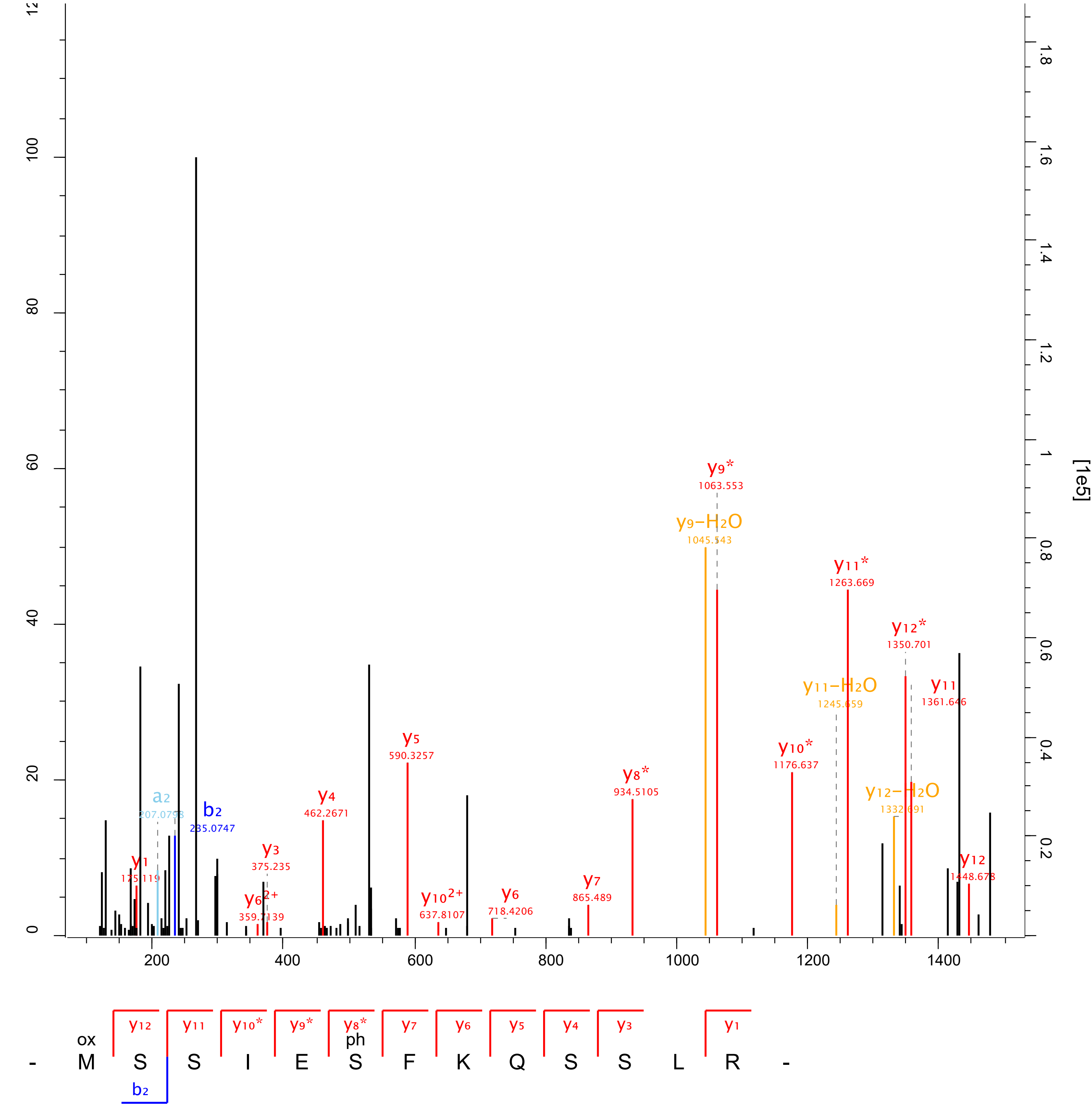

Sample output to test PDF Combine only

Raw file	Scan	Method	Score	m/z	Gene names
sirk1-mic-SUC-1-P	20021	FTMS; HCD	72.34	541.28	PAH2

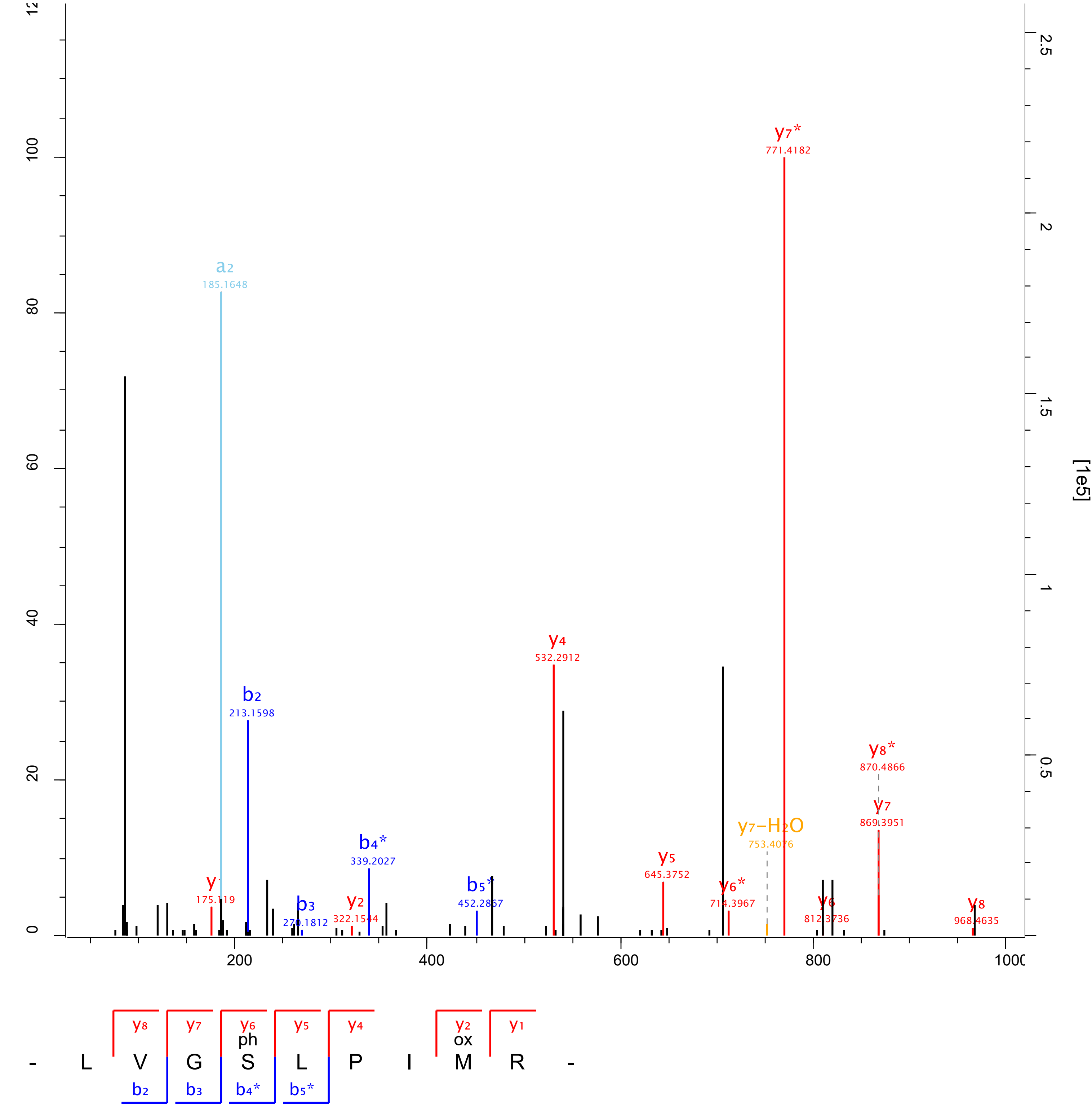

Sample output to test PDF Combine only

Raw file	Scan	Method	Score	m/z	Gene names
sirk1-mic-SUC-1-P	22254	FTMS; HCD	105.39	760.89	SWEET11

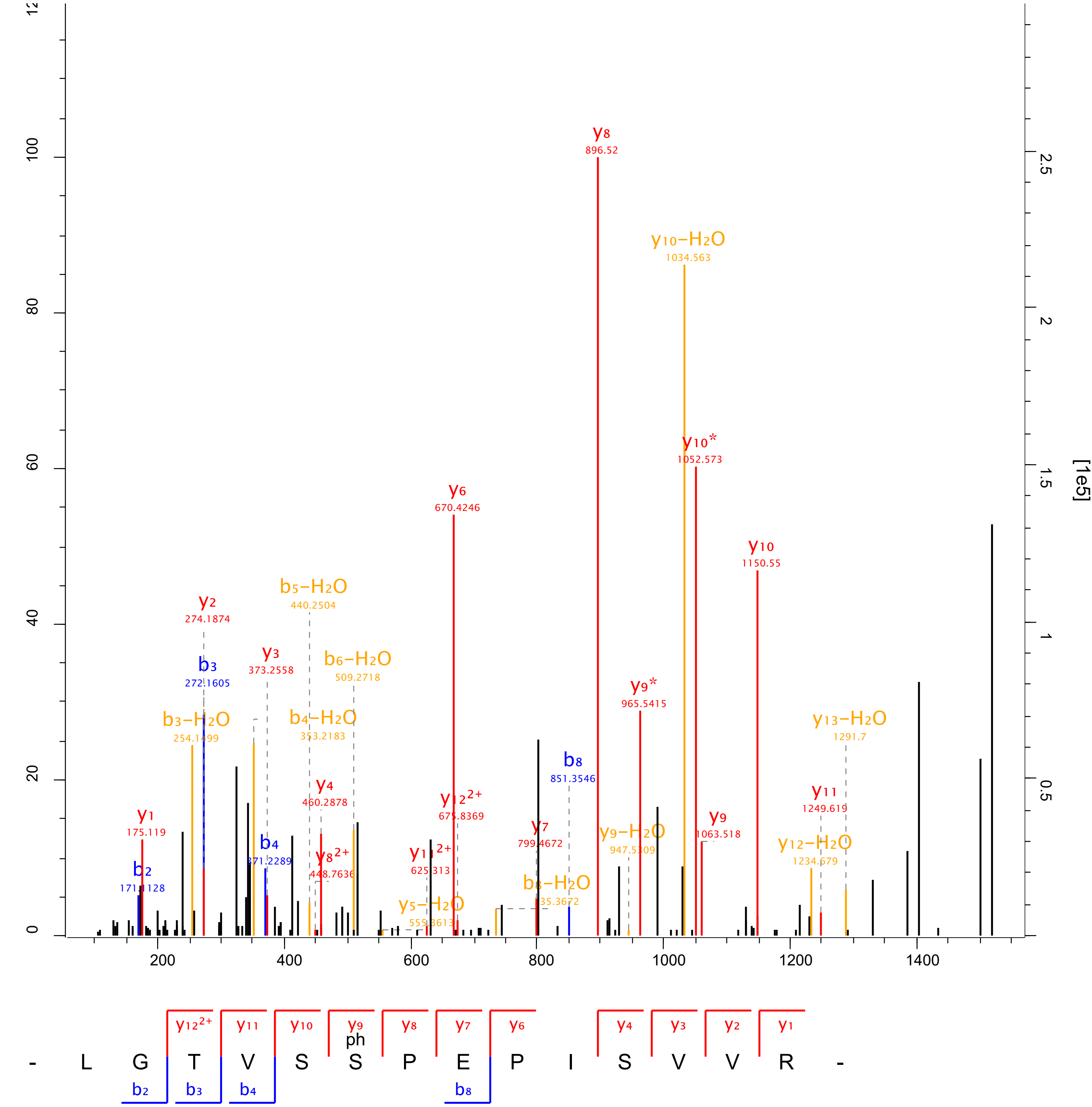

Sample output to test PDF Combine only

Raw file	Scan	Method	Score	m/z	Gene names
sirk1-mic-SUC-1-P	30152	FTMS; HCD	123.02	687.29	HIR3

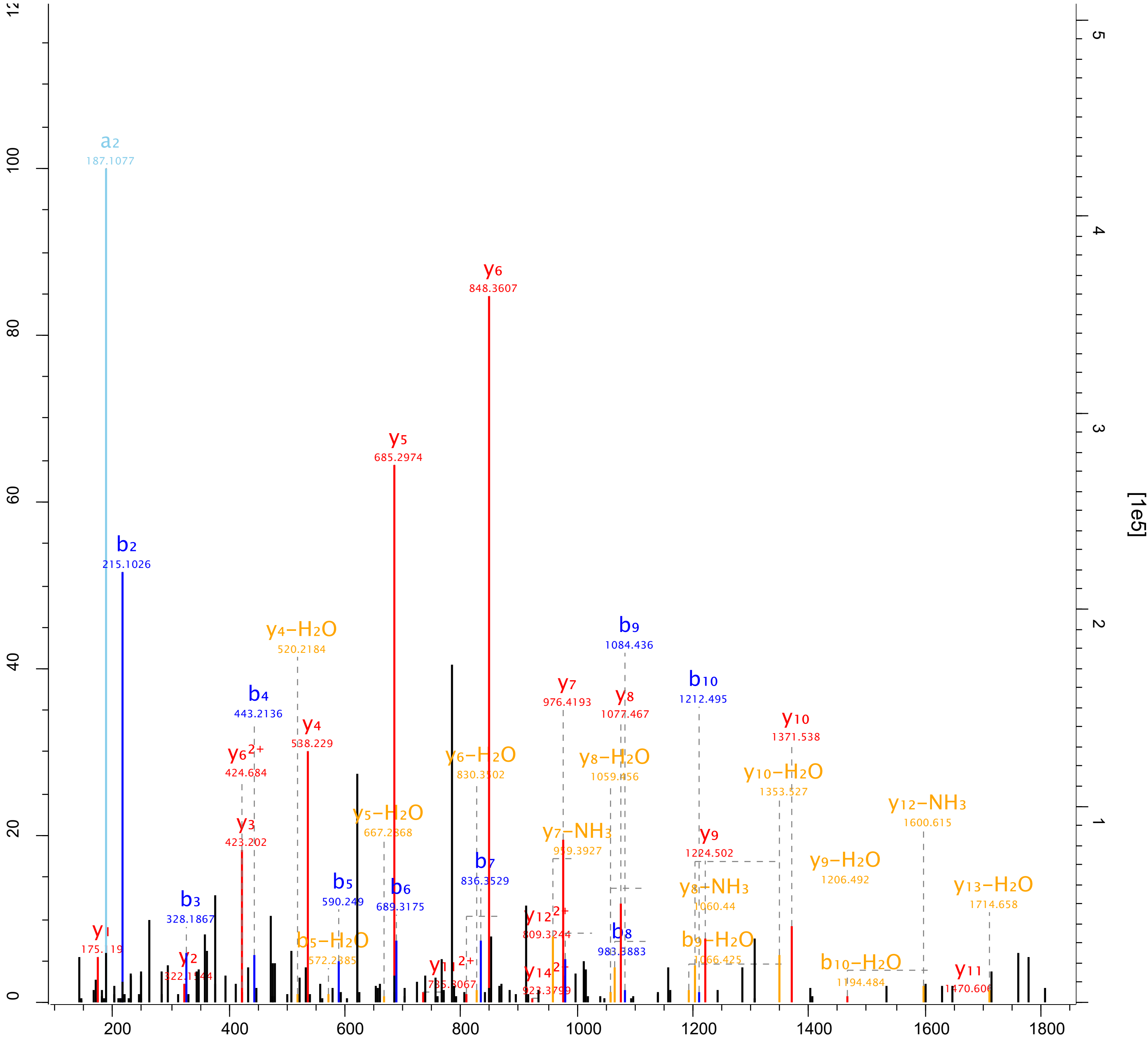

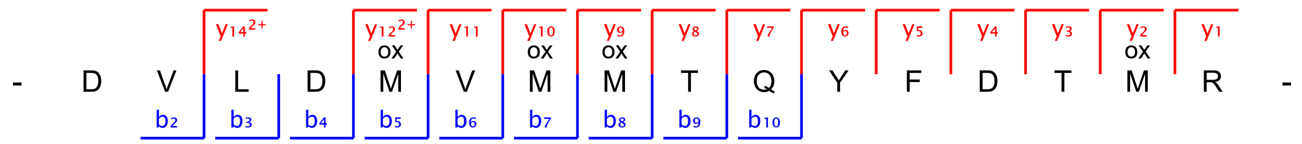

Sample output to test PDF Combine only

Raw file	Scan	Method	Score	m/z	Gene names
sirk1-mic-SUC-1-P	31716	FTMS; HCD	97.38	867.47	SWEET12

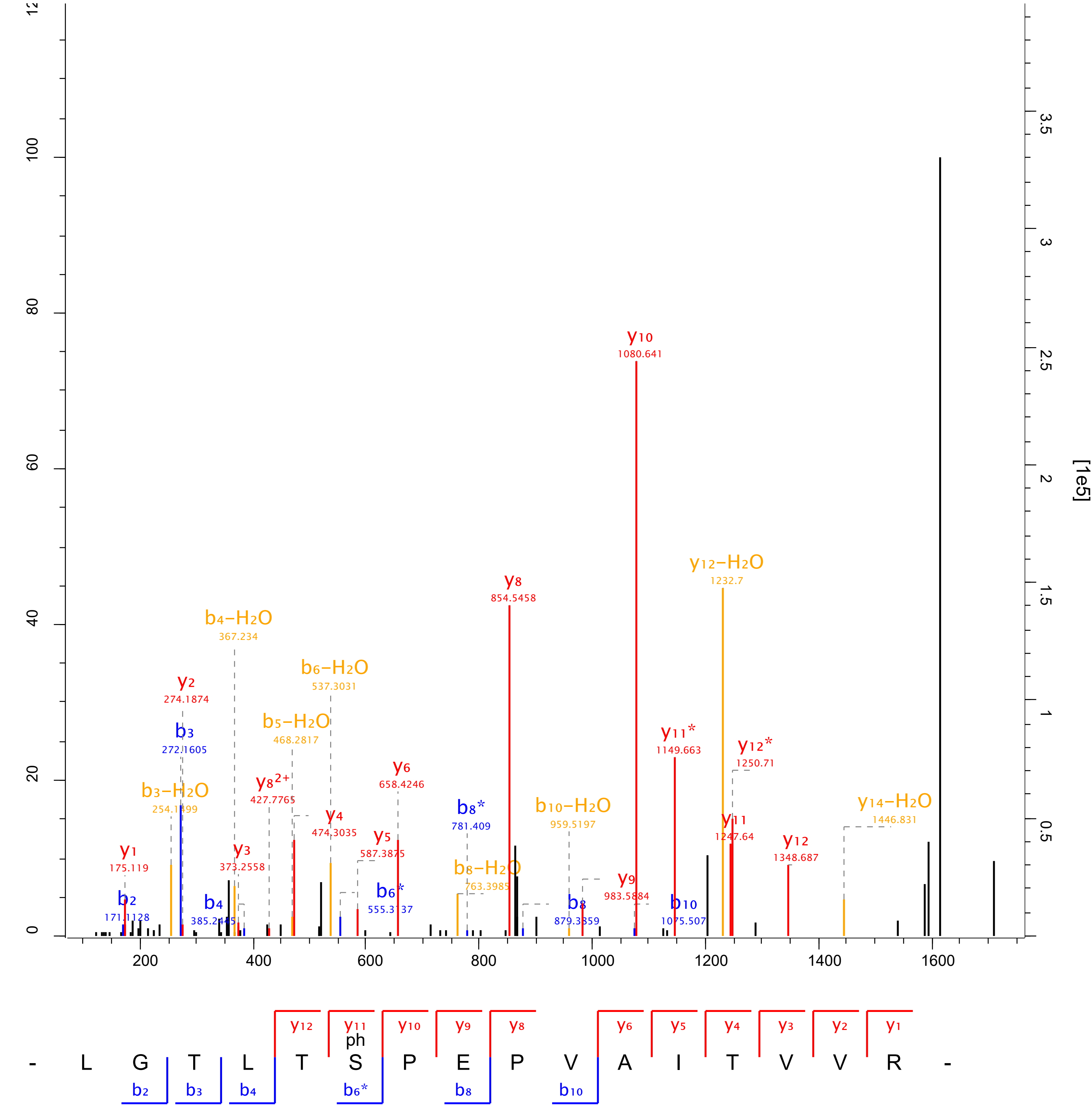

Sample output to test PDF Combine only

Raw file	Scan	Method	Score	m/z	Gene names
sirk1-mic-SUC-1-P	32254	FTMS; HCD	118.21	735.34	NHX1;NHX2

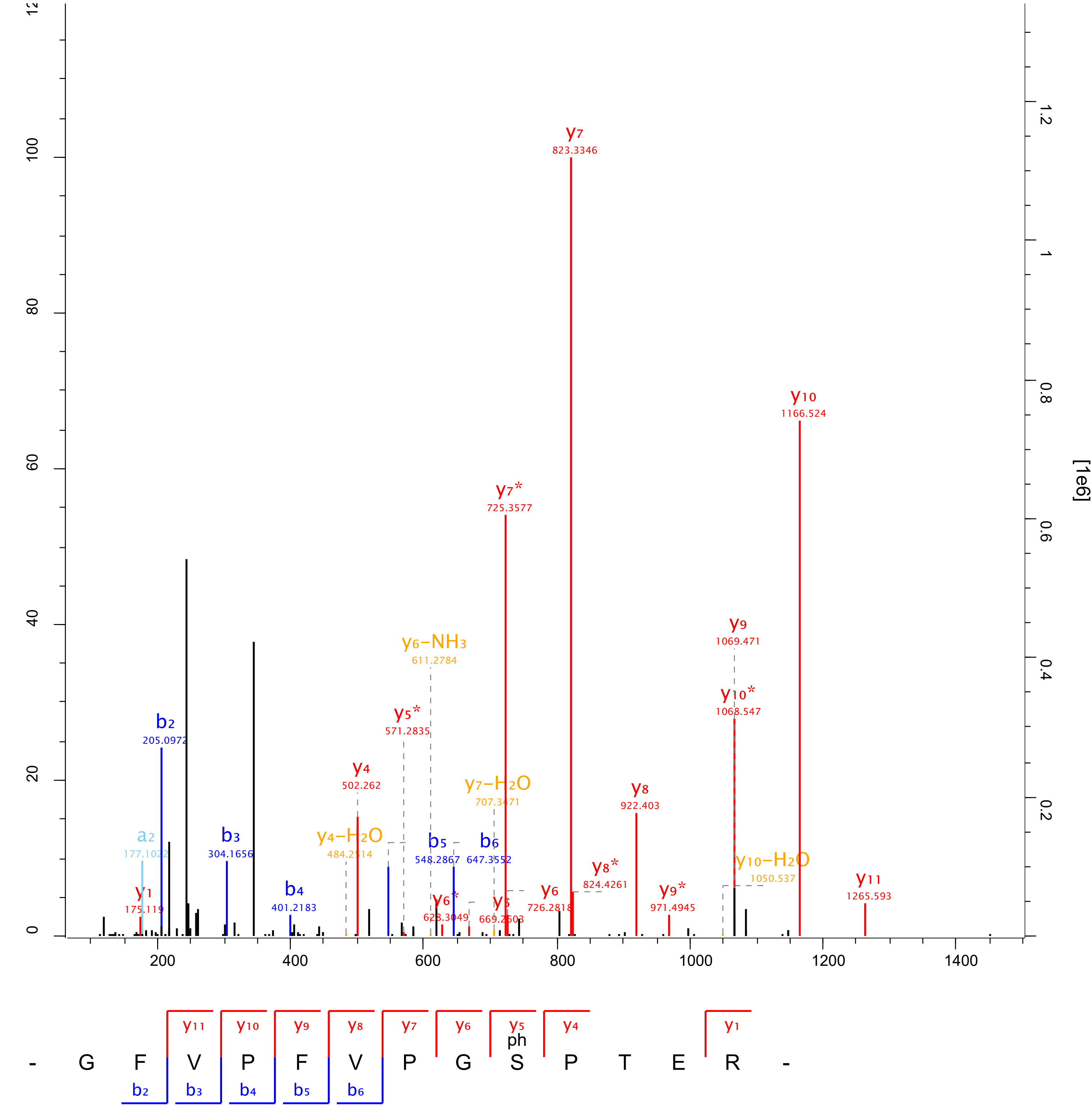

Sample output to test PDF Combine only

Raw file	Scan	Method	Score	m/z	Gene names
sirk1-mic-SUC-1-P	35040	FTMS; HCD	58.17	848.4	At5g13940

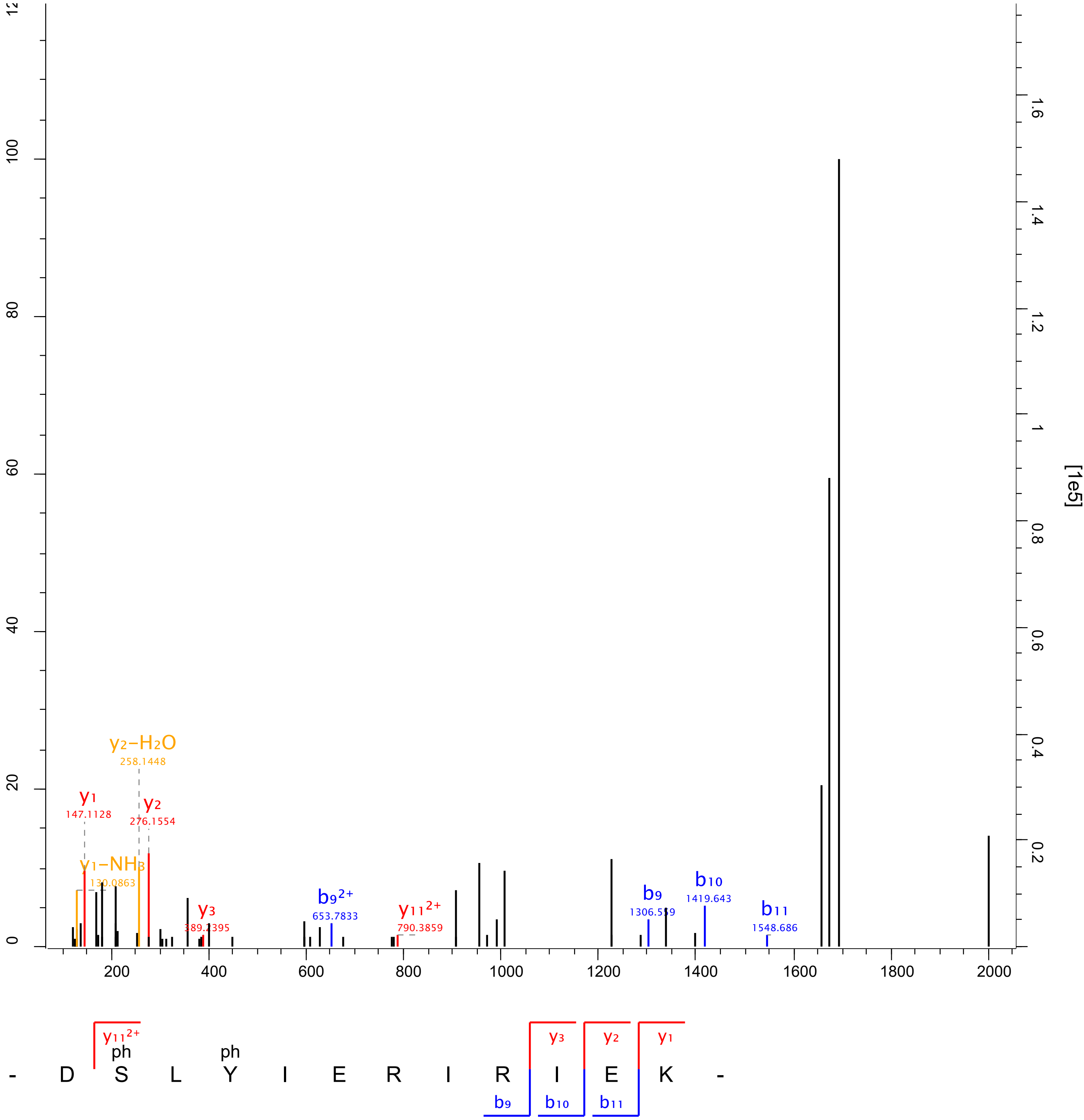

Sample output to test PDF Combine only

Raw file	Scan	Method	Score	m/z
sirk1-mic-SUC-2-A	3239	FTMS; HCD	78.27	615.28

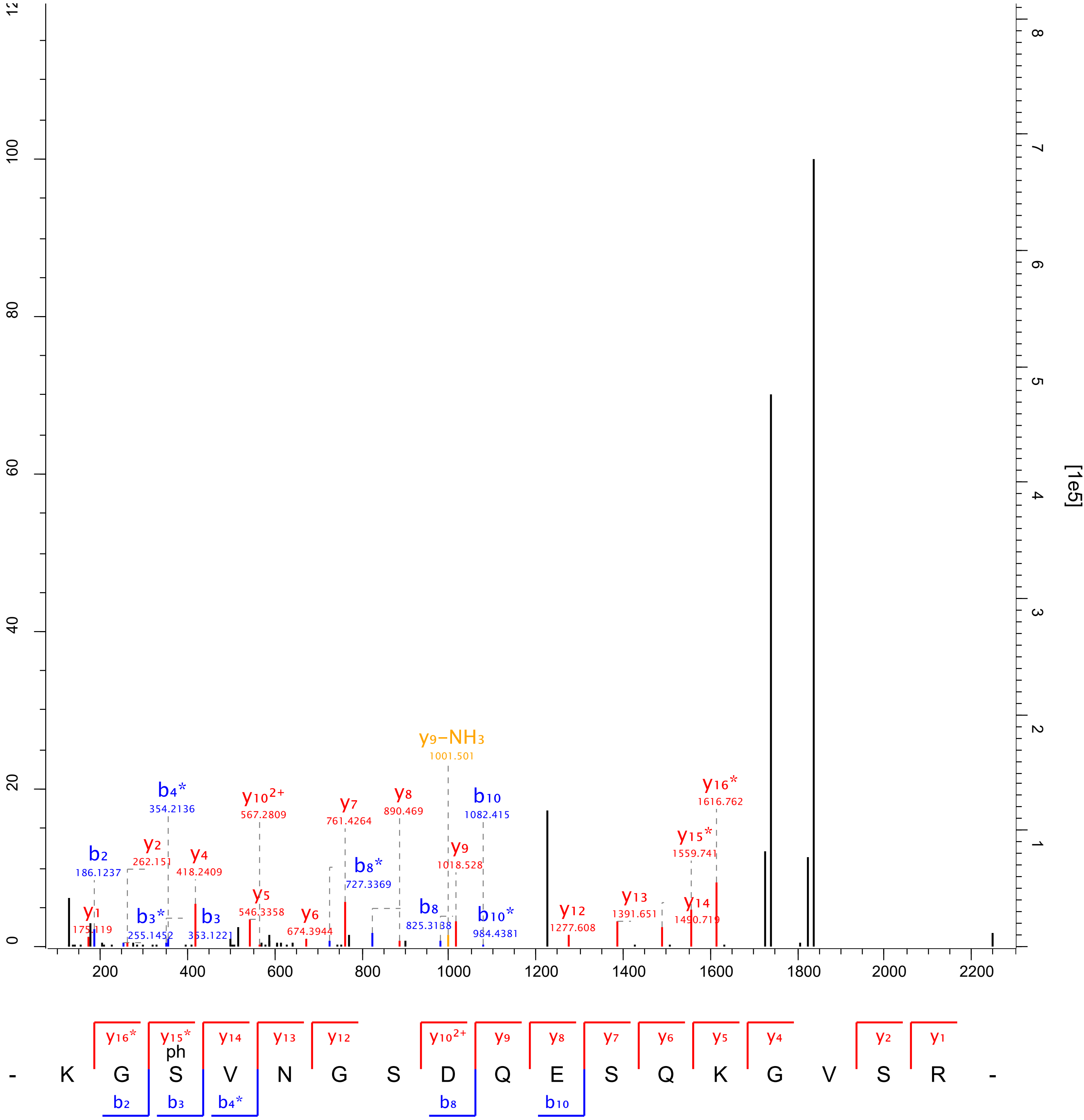

Sample output to test PDF Combine only

Raw file	Scan	Method	Score	m/z
sirk1-mic-SUC-2-A	3311	FTMS; HCD	52.32	606.56

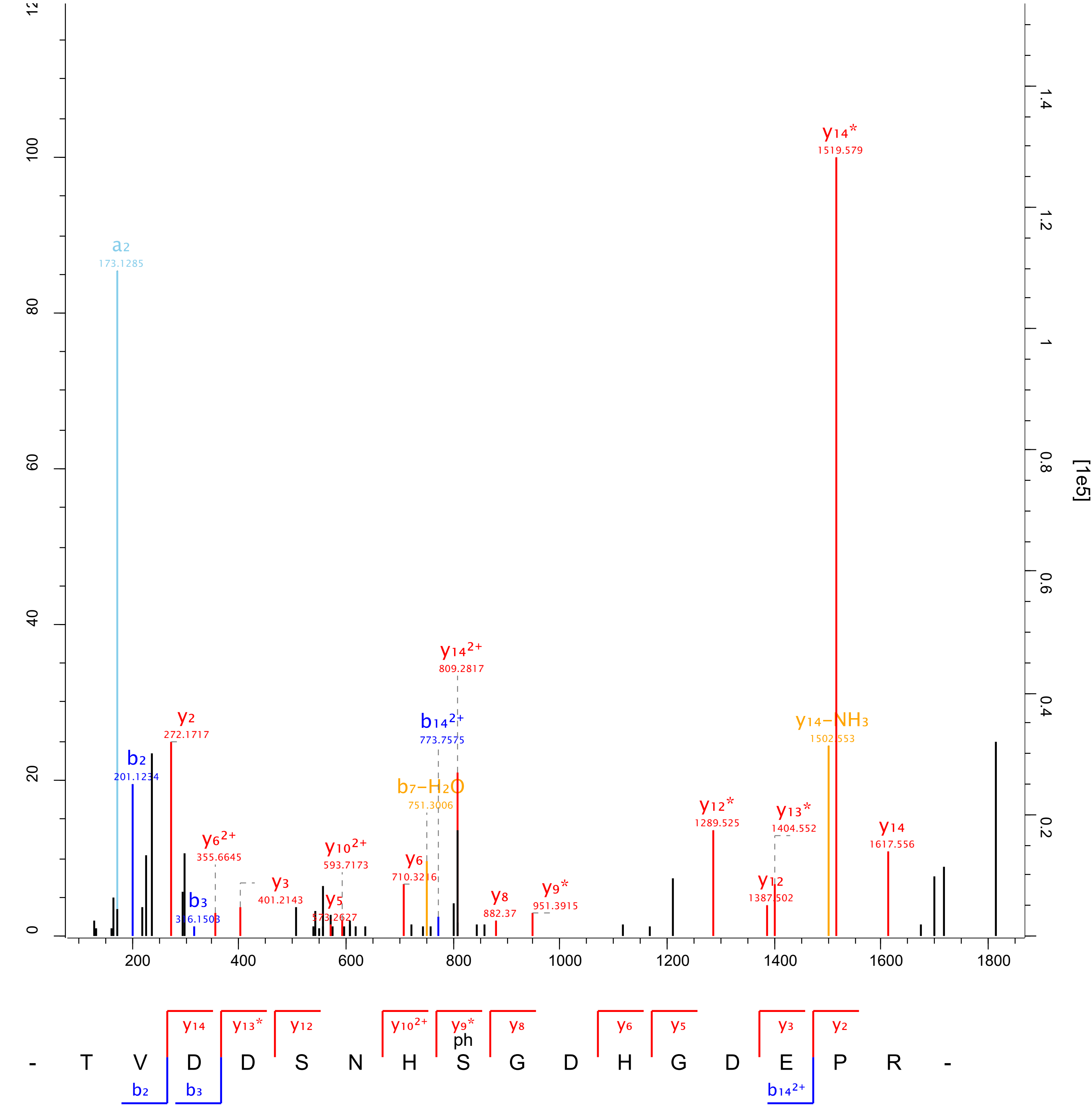

Sample output to test PDF Combine only

Raw file	Scan	Method	Score	m/z	Gene names
sirk1-mic-SUC-2-A	3317	FTMS; HCD	78.95	544.24	MSL9

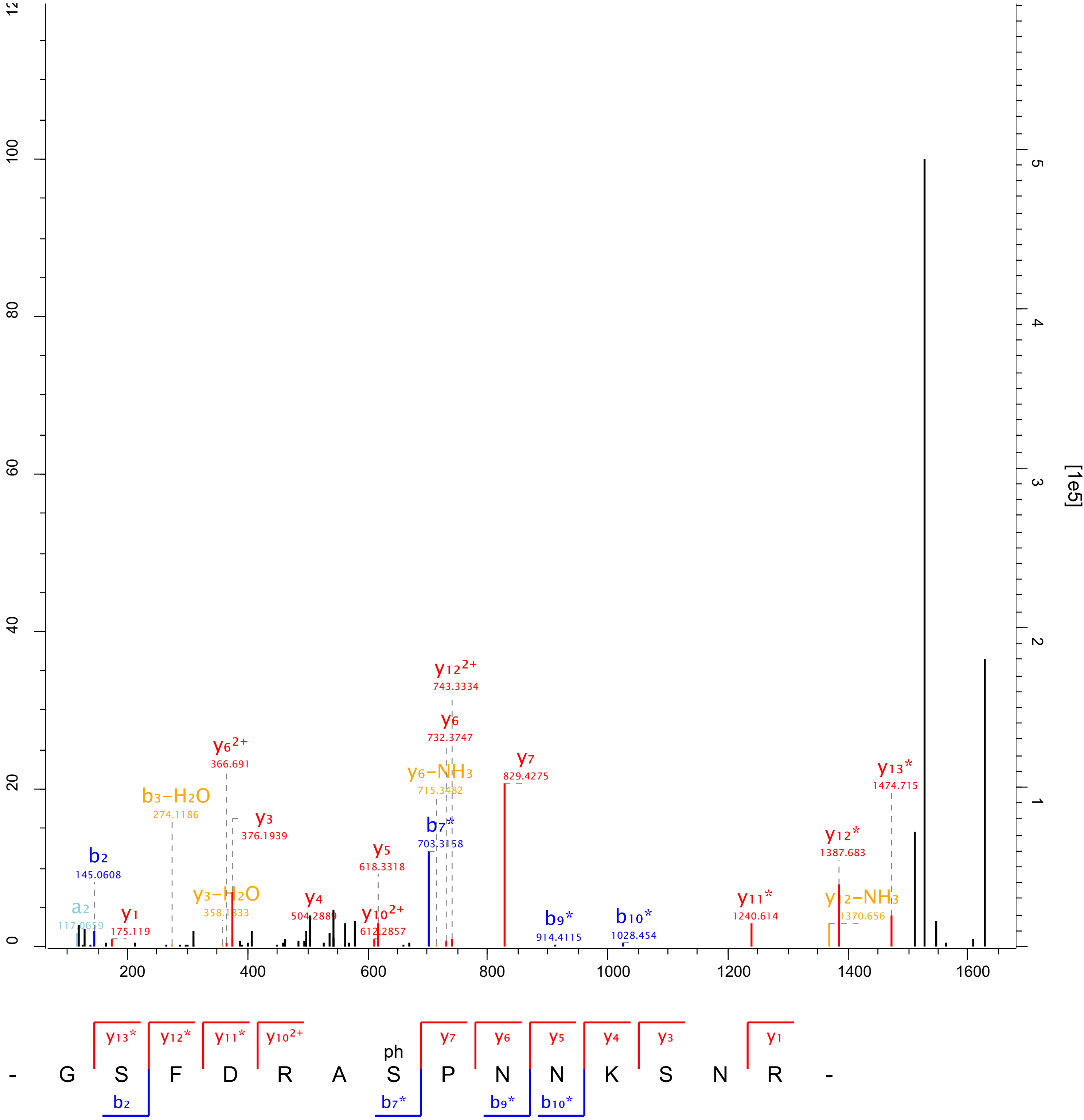

Sample output to test PDF Combine only

Raw file	Scan	Method	Score	m/z	Gene names
sirk1-mic-SUC-2-A	3347	FTMS; HCD	135.55	452.87	F16M2_110;ATMRK1

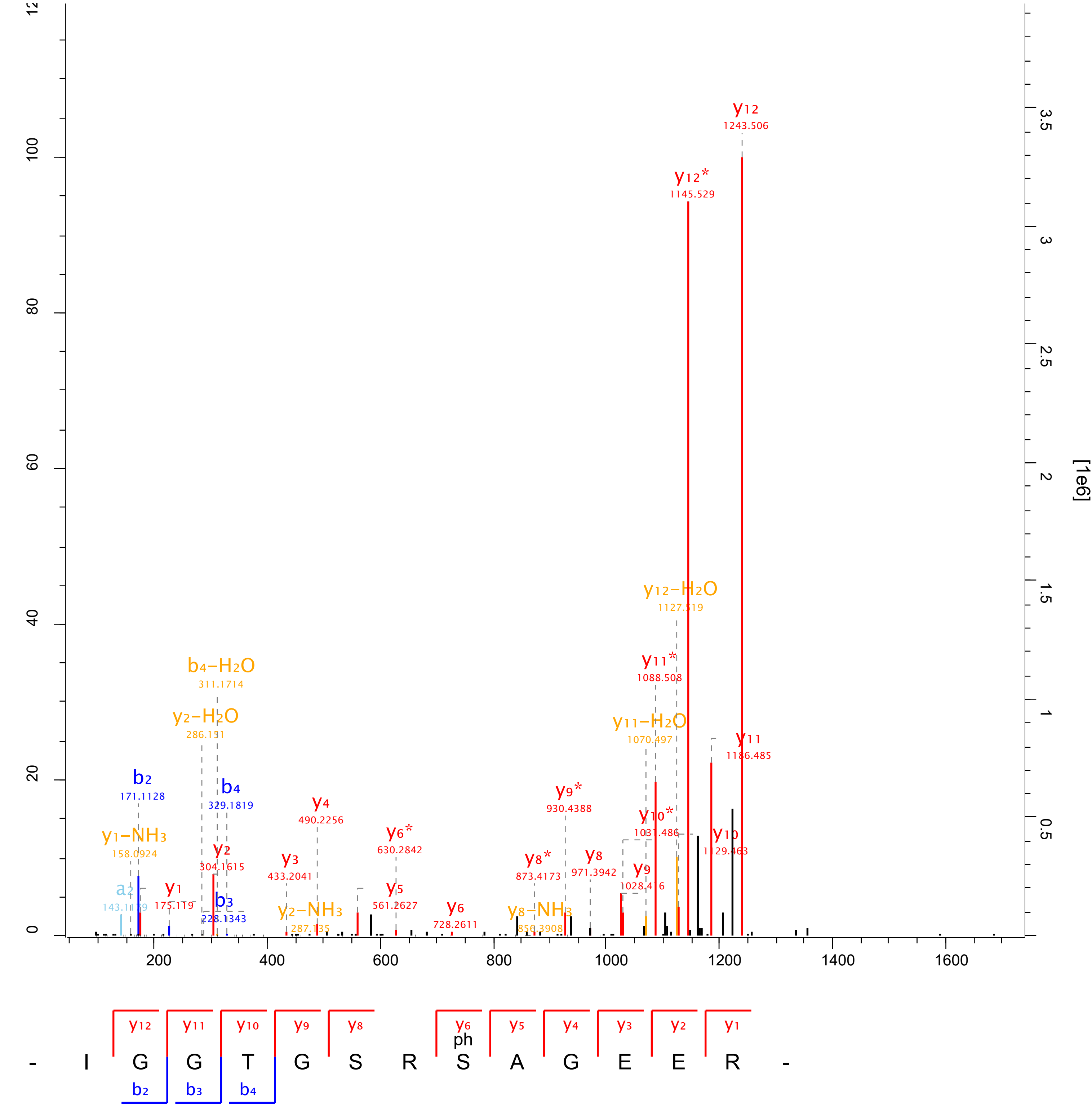

Sample output to test PDF Combine only

Raw file	Scan	Method	Score	m/z	Gene names
sirk1-mic-SUC-2-A	3399	FTMS; HCD	148.41	410.19	At5g05600;F9F8.3

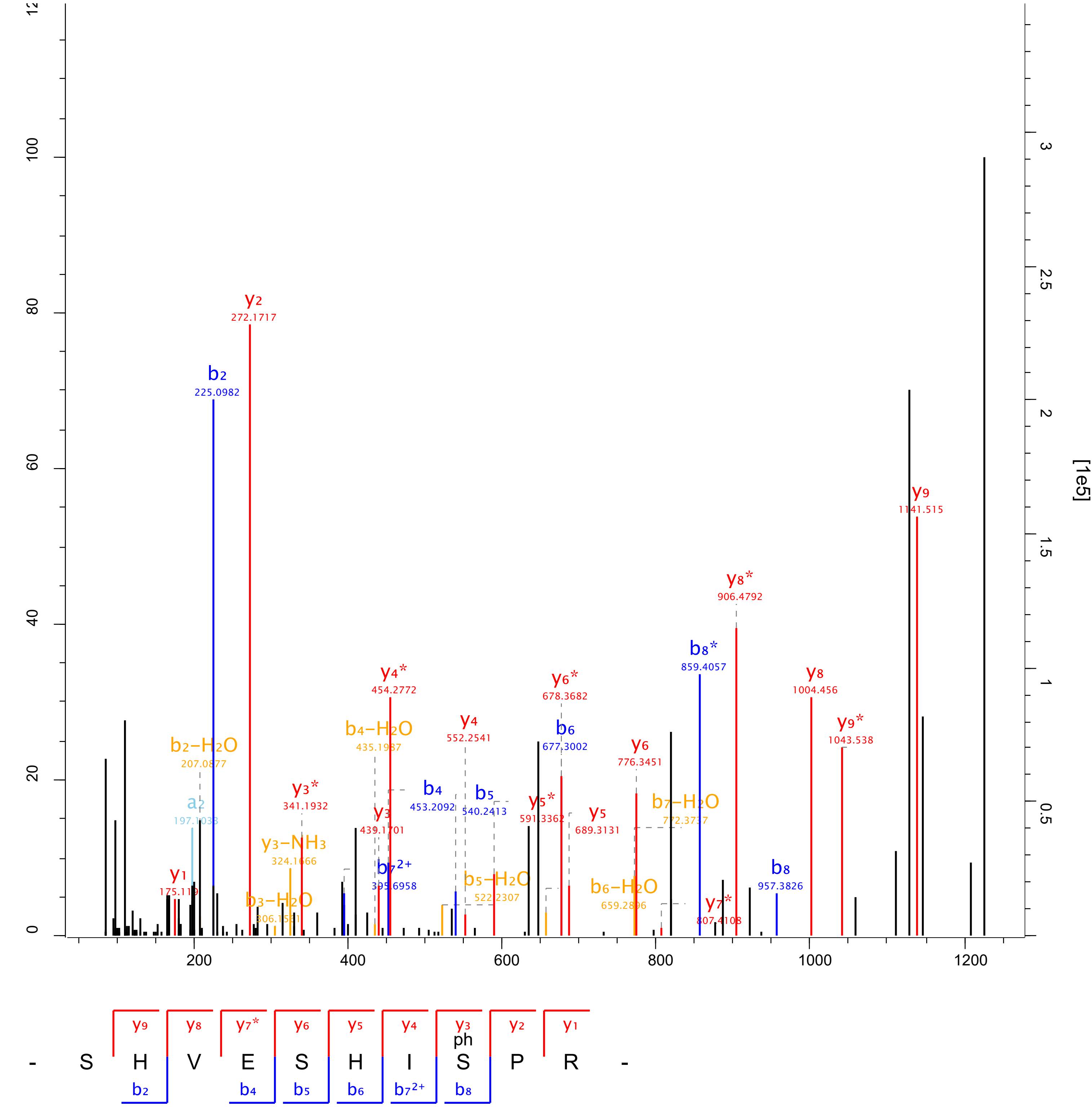

Sample output to test PDF Combine only

Raw file	Scan	Method	Score	m/z	Gene names
sirk1-mic-SUC-2-A	3444	FTMS; HCD	53.5	507.95	At5g09960

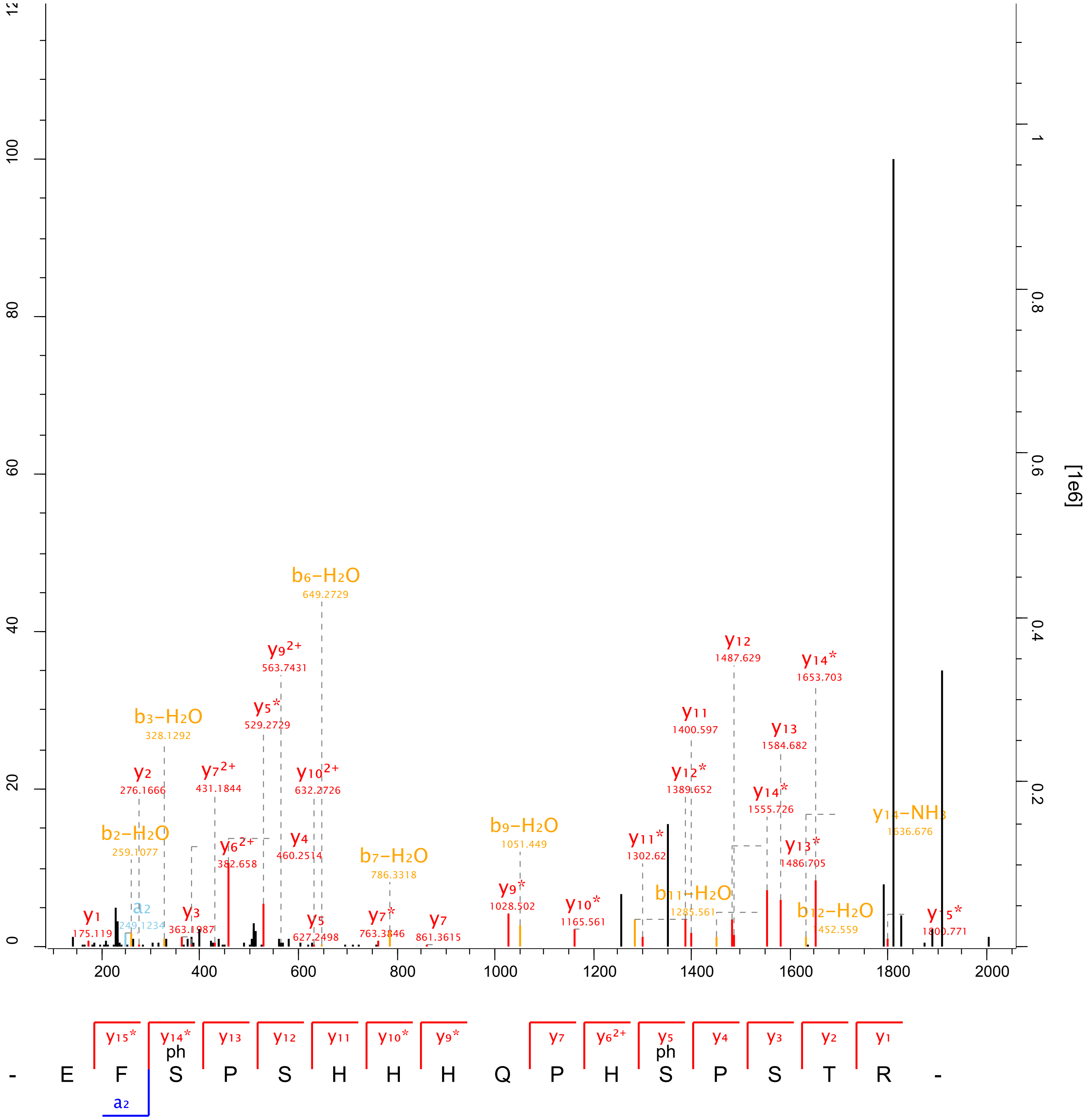

Sample output to test PDF Combine only

Raw file	Scan	Method	Score	m/z	Gene names
sirk1-mic-SUC-2-A	3576	FTMS; HCD	76.38	539.21	At4g18070

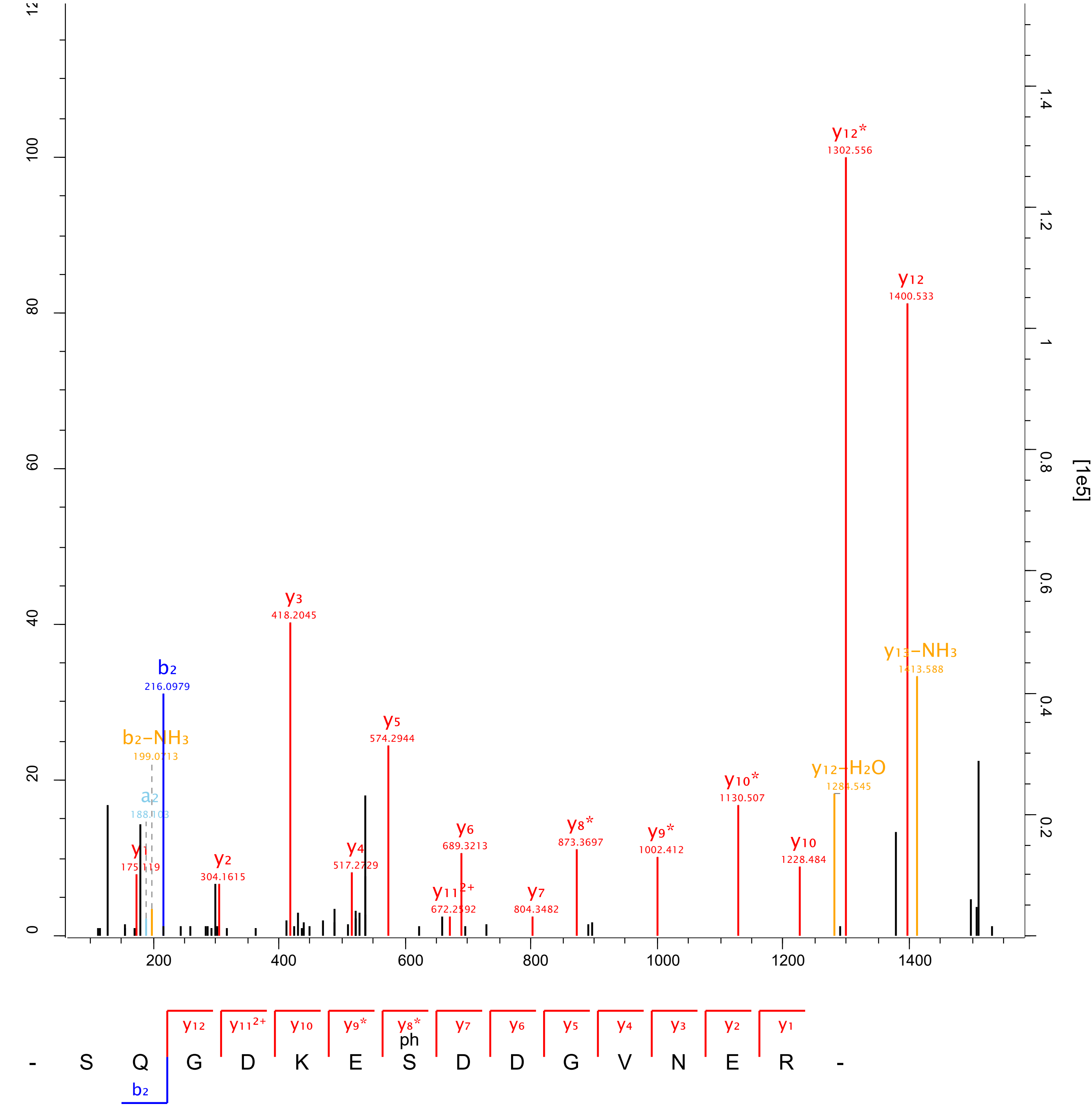

Sample output to test PDF Combine only

Raw file	Scan	Method	Score	m/z	Gene names
sirk1-mic-SUC-2-A	3684	FTMS; HCD	41.62	406.85	SEC8

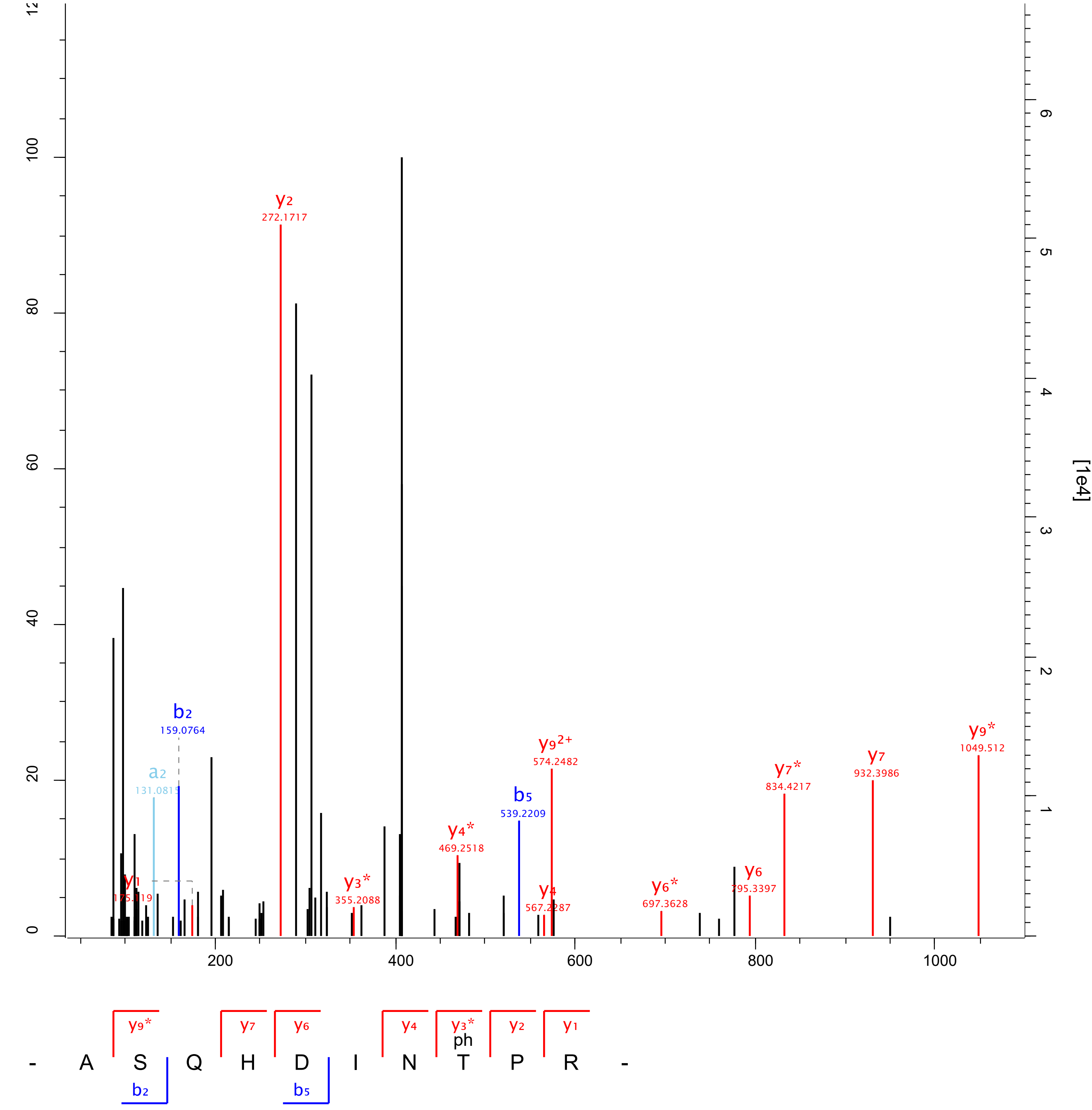

Sample output to test PDF Combine only

Raw file	Scan	Method	Score	m/z	Gene names
sirk1-mic-SUC-2-A	3695	FTMS; HCD	133.21	594.93	RIN4

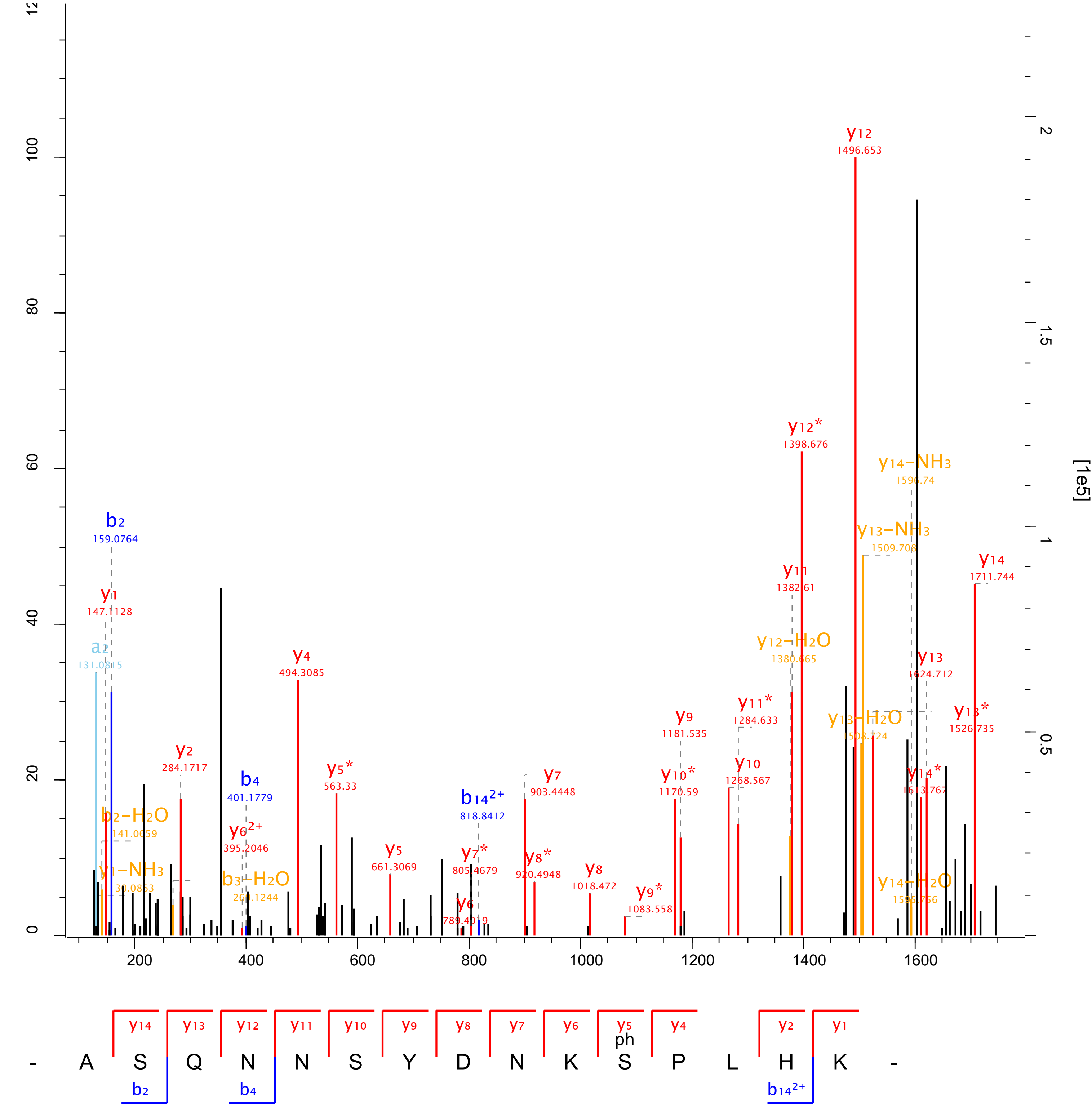

Sample output to test PDF Combine only

Raw file	Scan	Method	Score	m/z	Gene names
sirk1-mic-SUC-2-A	3738	FTMS; HCD	50.11	513.24	At5g57000

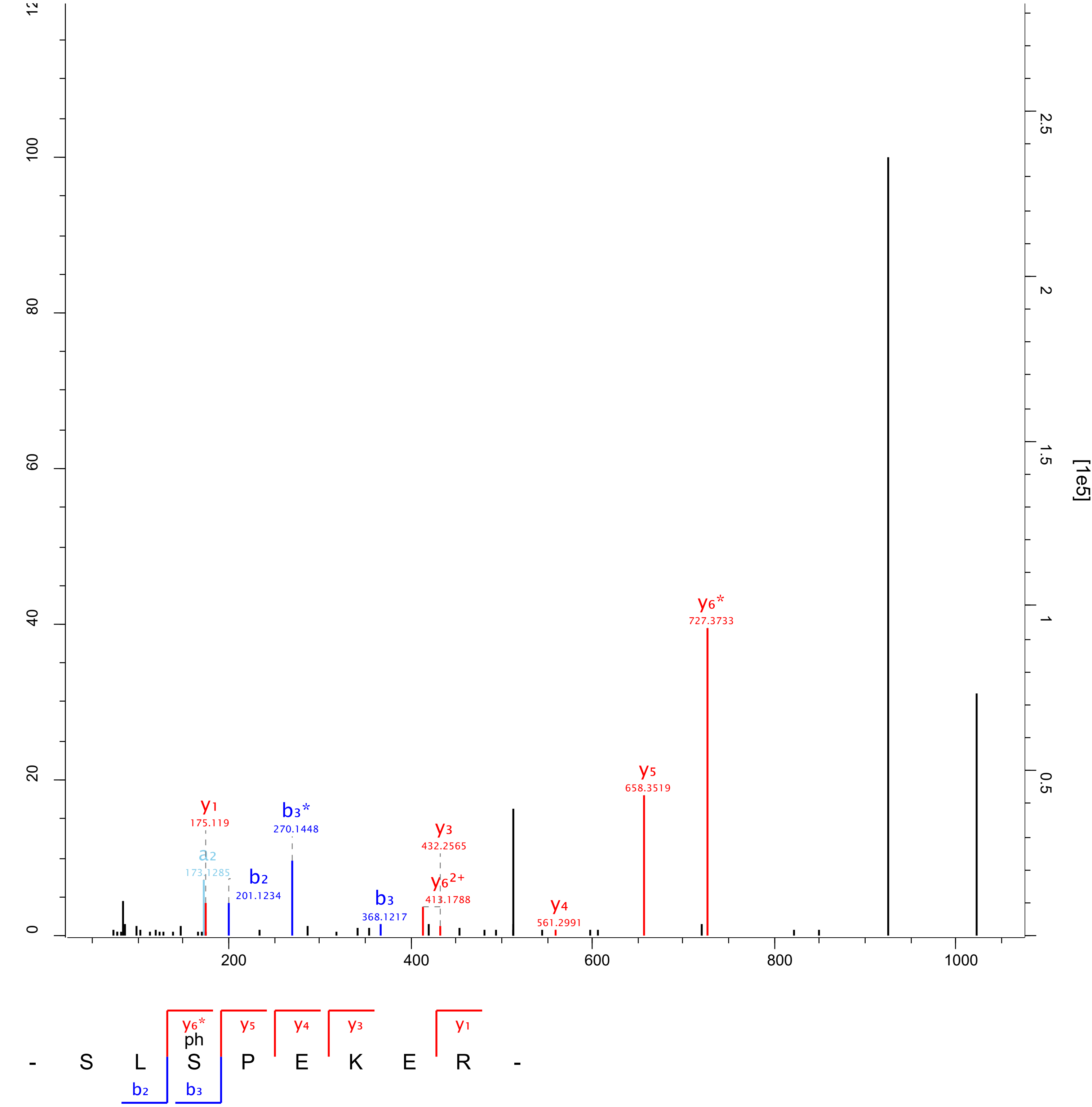

Sample output to test PDF Combine only

Raw file	Scan	Method	Score	m/z	Gene names
sirk1-mic-SUC-2-A	3888	FTMS; HCD	83.23	580.74	RS41;RS40

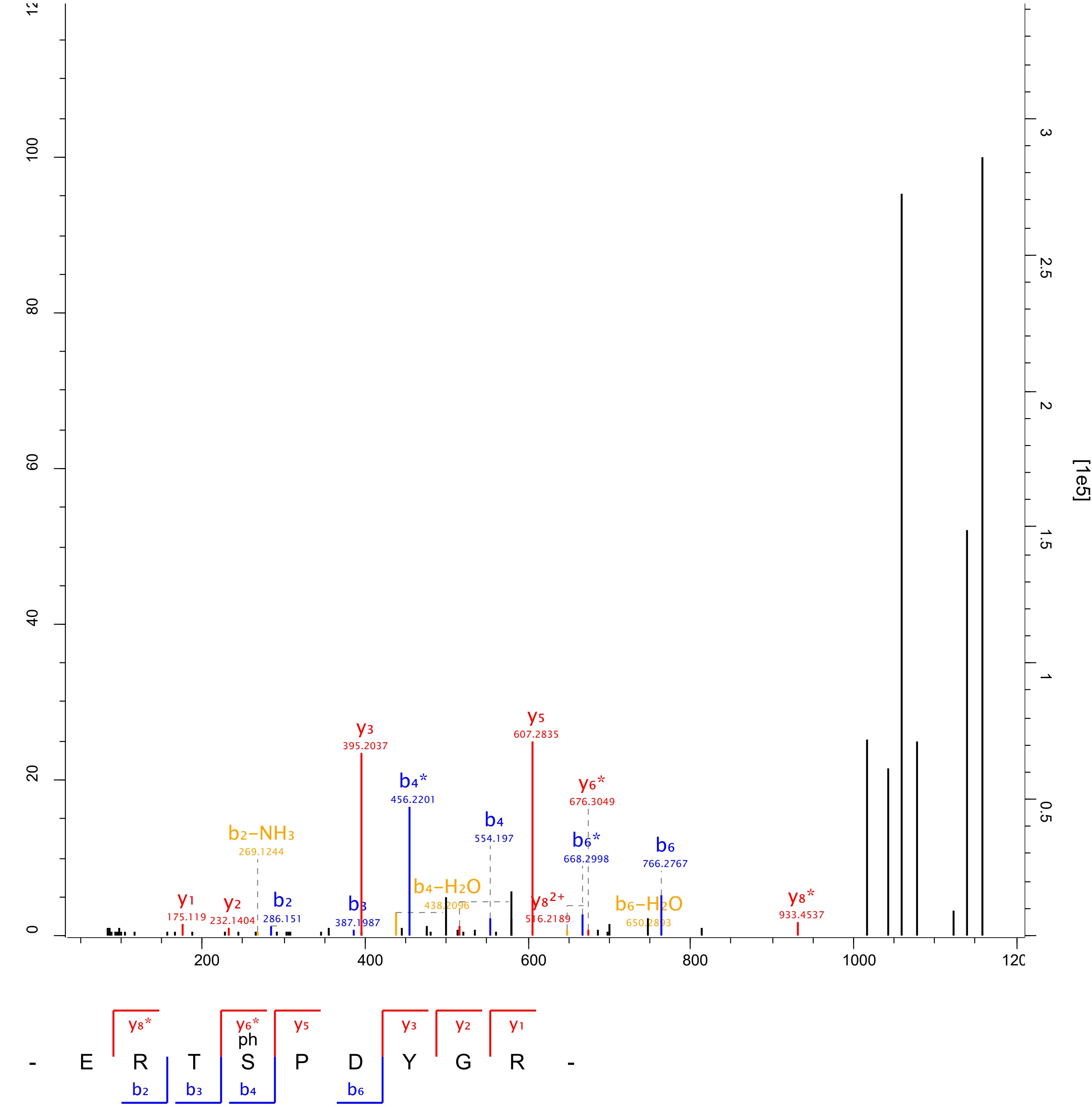

Sample output to test PDF Combine only

Raw file	Scan	Method	Score	m/z	Gene names
sirk1-mic-SUC-2-A	3902	FTMS; HCD	49.3	408.52	F15K9.5

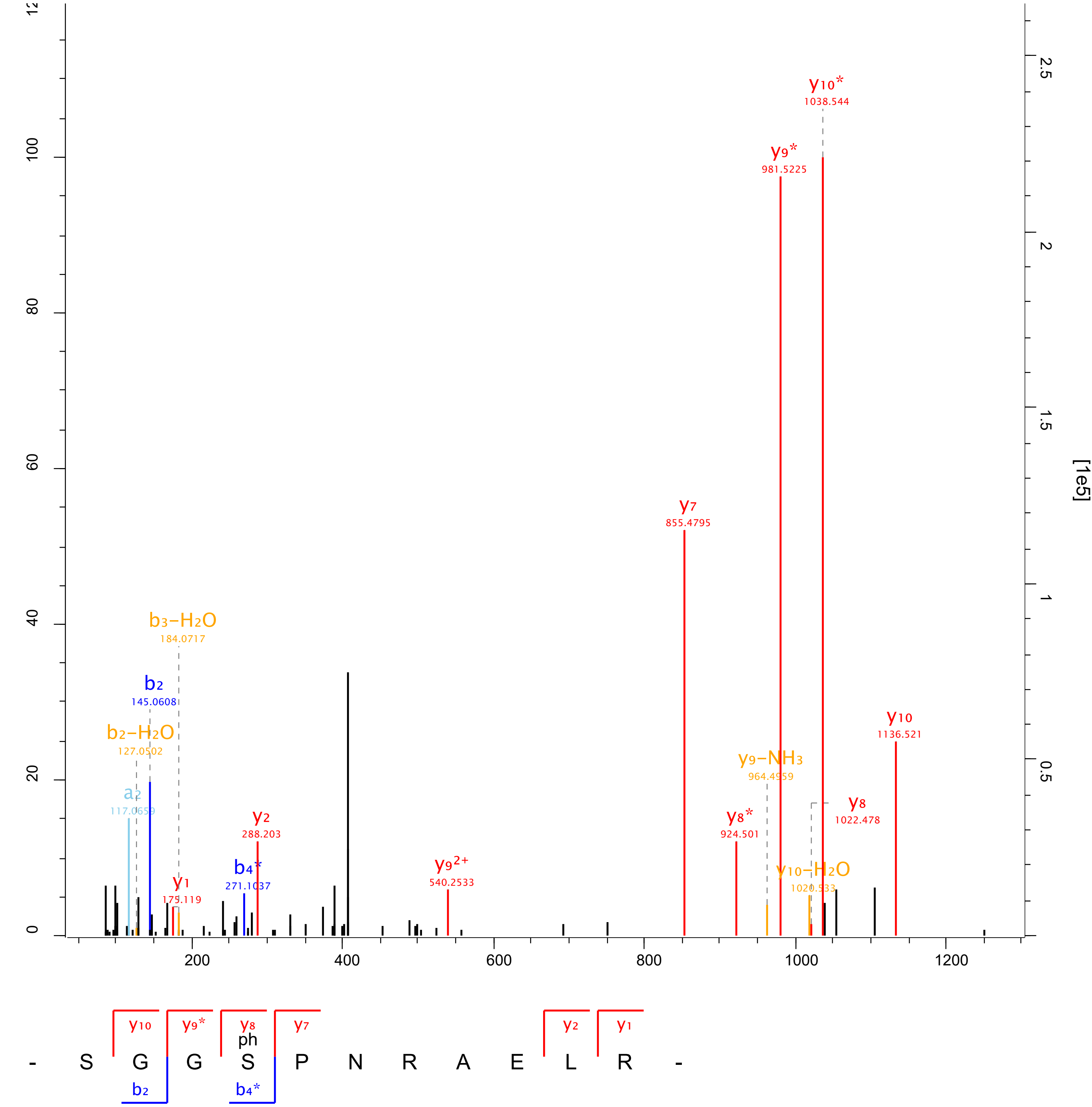

Sample output to test PDF Combine only

Raw file	Scan	Method	Score	m/z
sirk1-mic-SUC-2-A	3920	FTMS; HCD	40.94	623.29

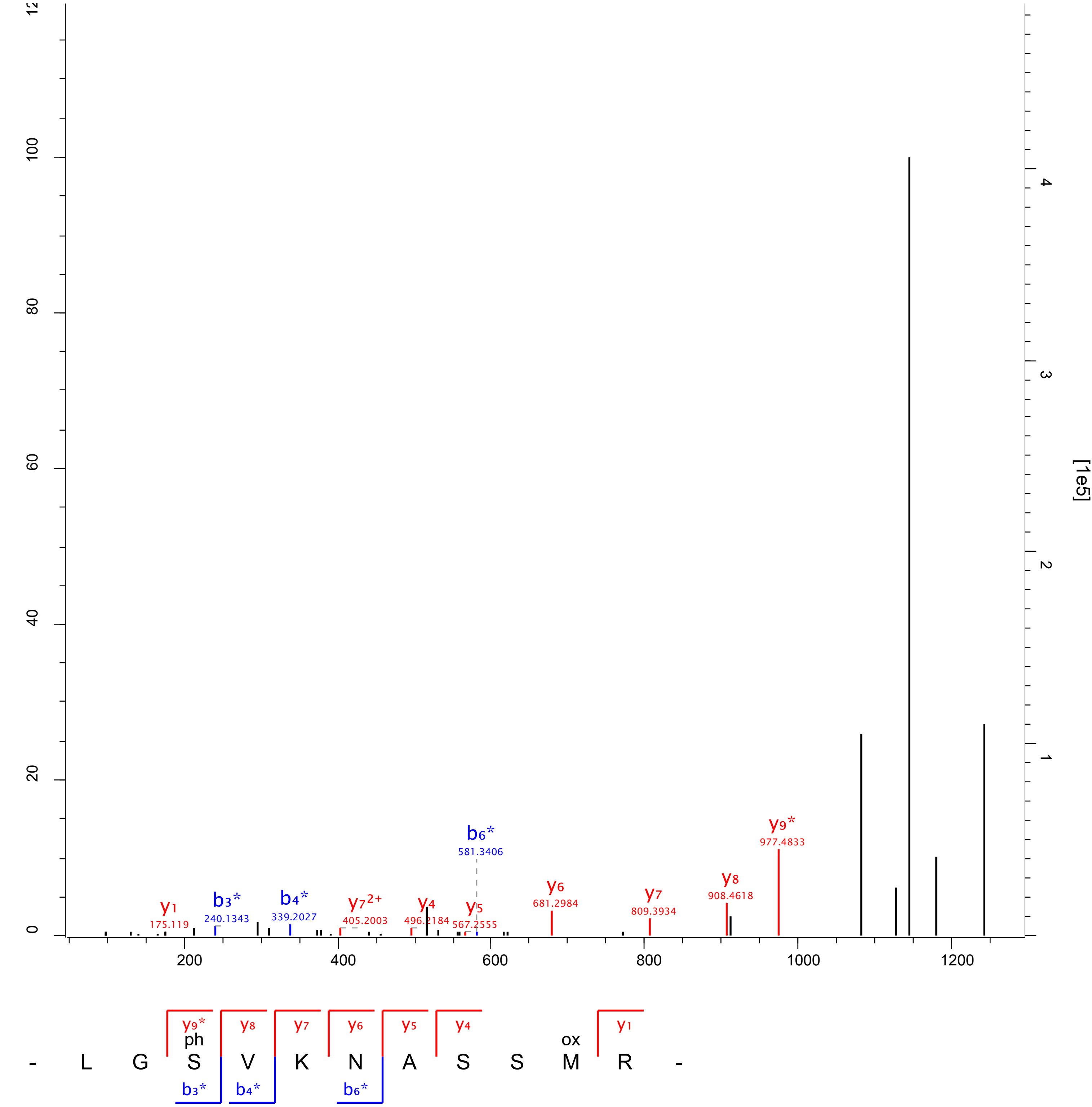

Sample output to test PDF Combine only

Raw file	Scan	Method	Score	m/z	Gene names
sirk1-mic-SUC-2-A	3929	FTMS; HCD	69.85	455.54	RS31

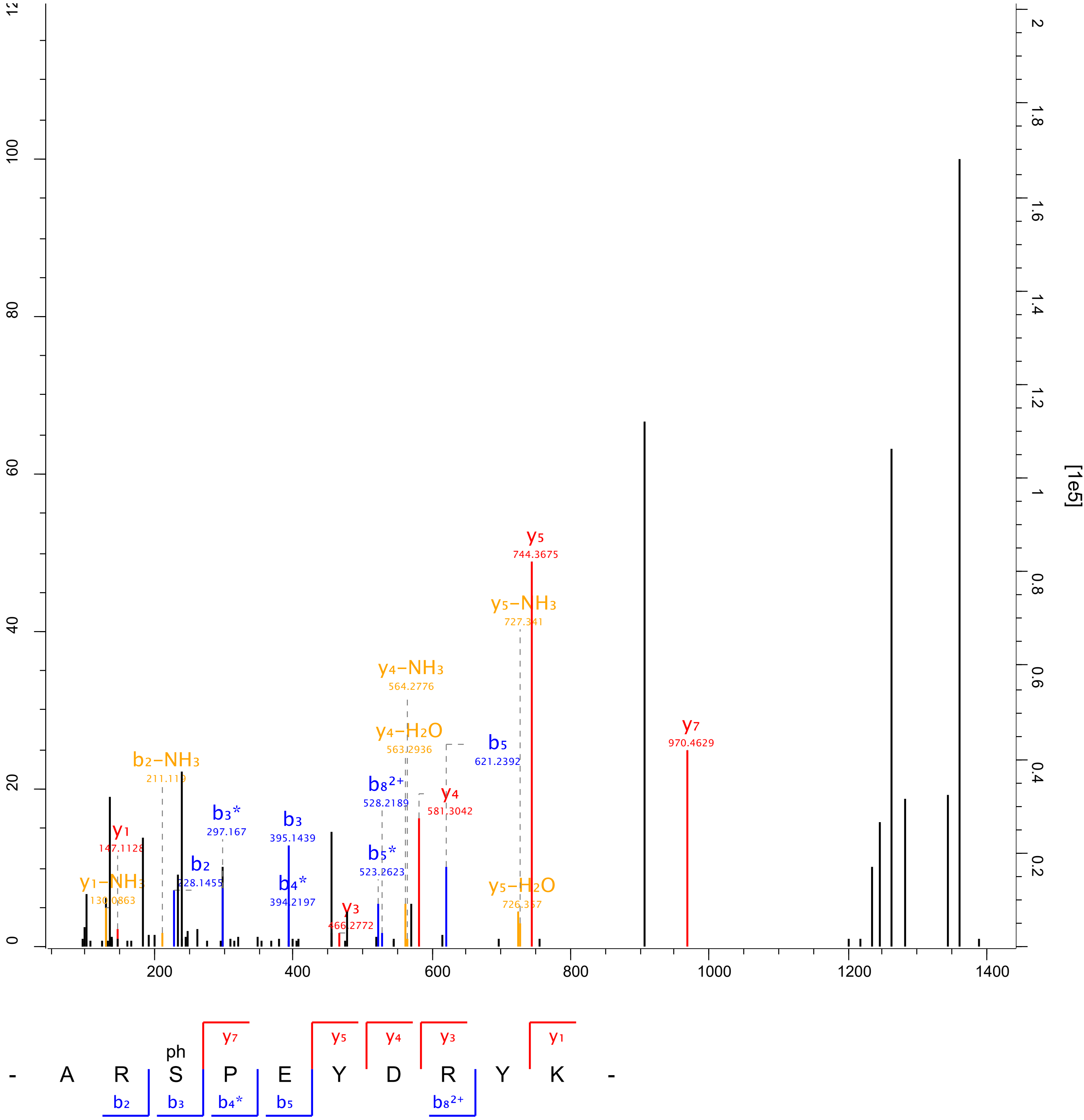

Sample output to test PDF Combine only

Raw file	Scan	Method	Score	m/z	Gene names
sirk1-mic-SUC-2-A	4225	FTMS; HCD	78.32	395.86	RS41

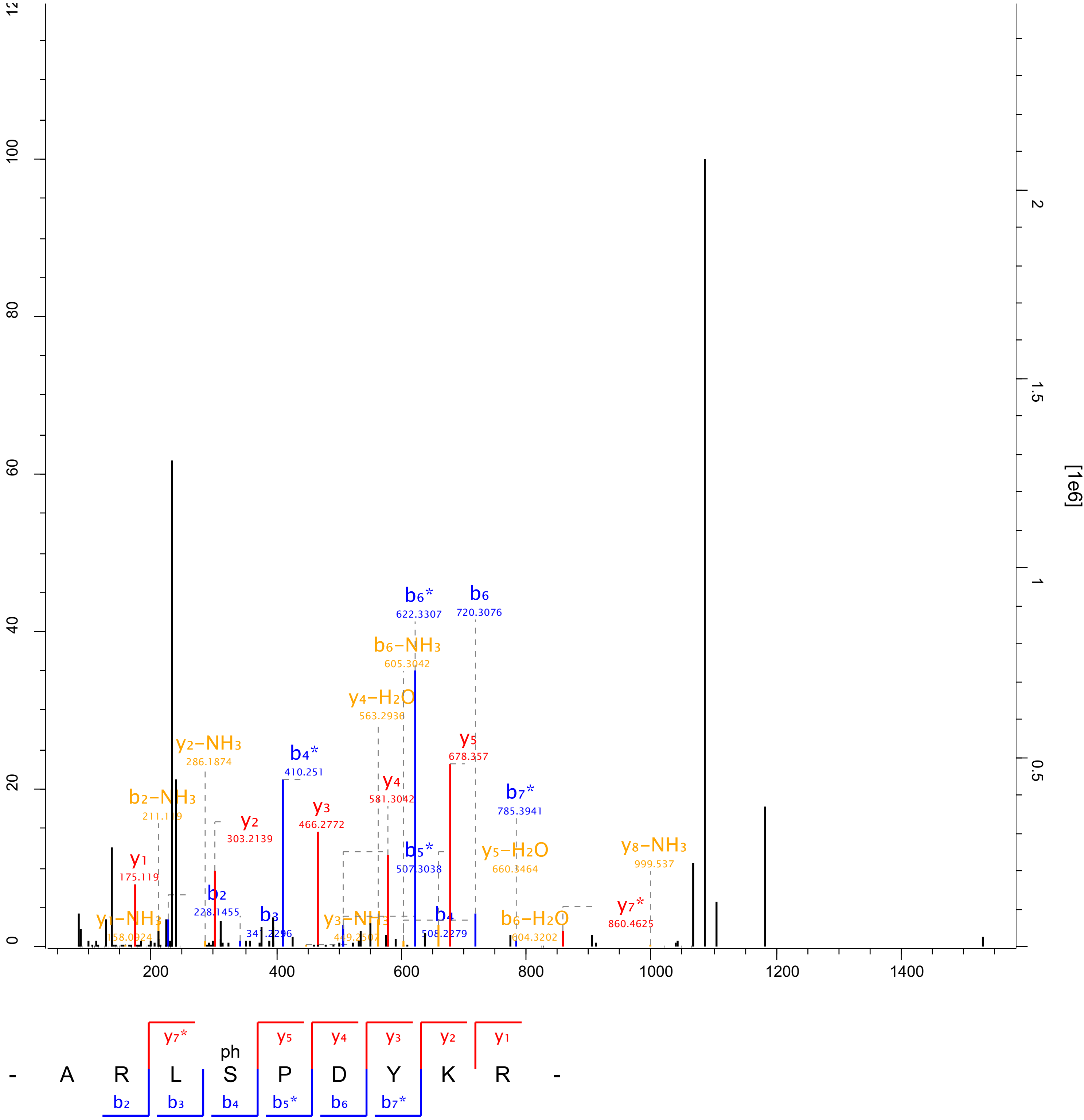

Sample output to test PDF Combine only

Raw file	Scan	Method	Score	m/z	Gene names
sirk1-mic-SUC-2-A	4242	FTMS; HCD	51.59	502.55	RS41

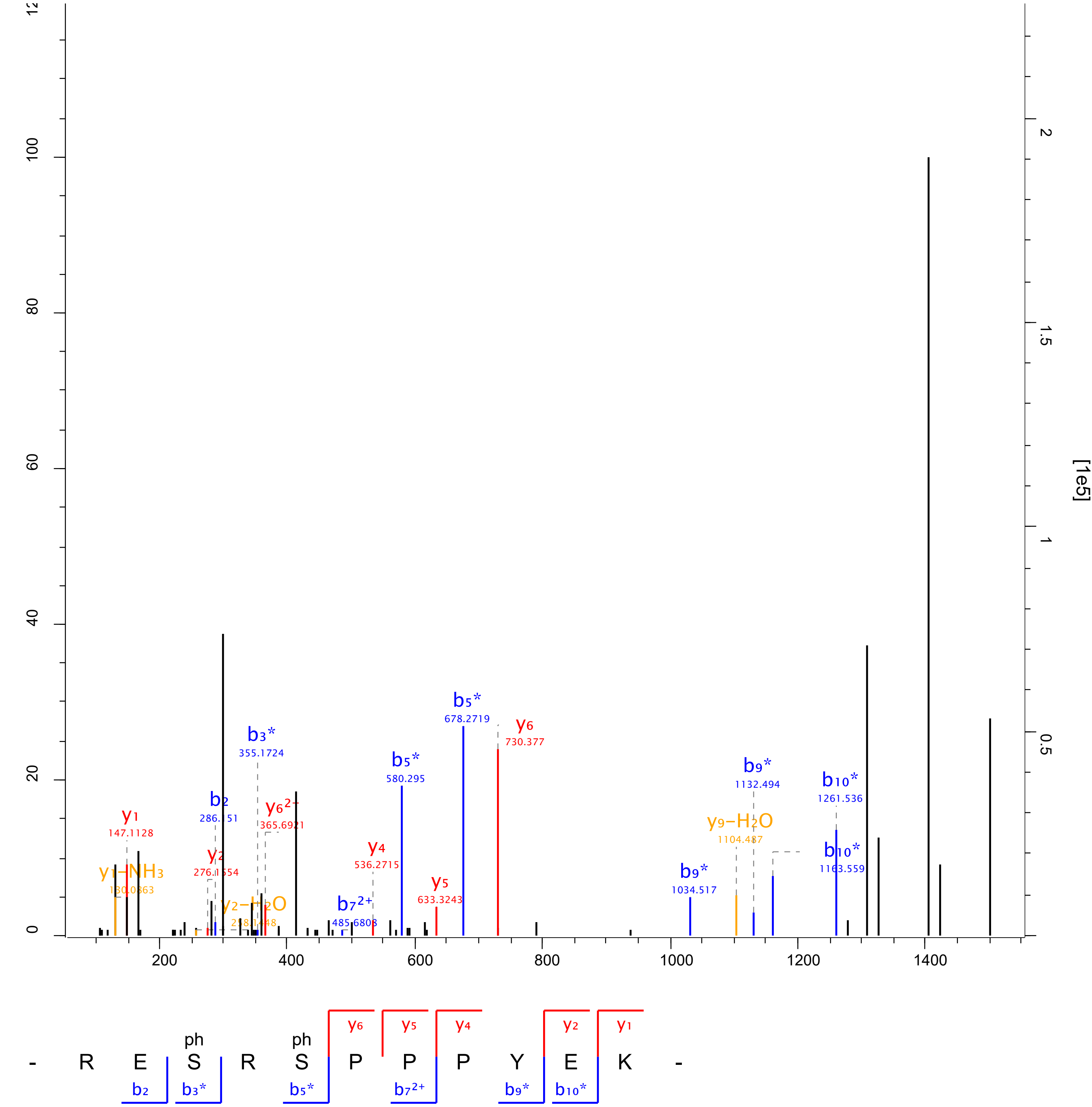

Sample output to test PDF Combine only

Raw file	Scan	Method	Score	m/z	Gene names
sirk1-mic-SUC-2-A	4333	FTMS; HCD	51.57	620.96	At5g41950

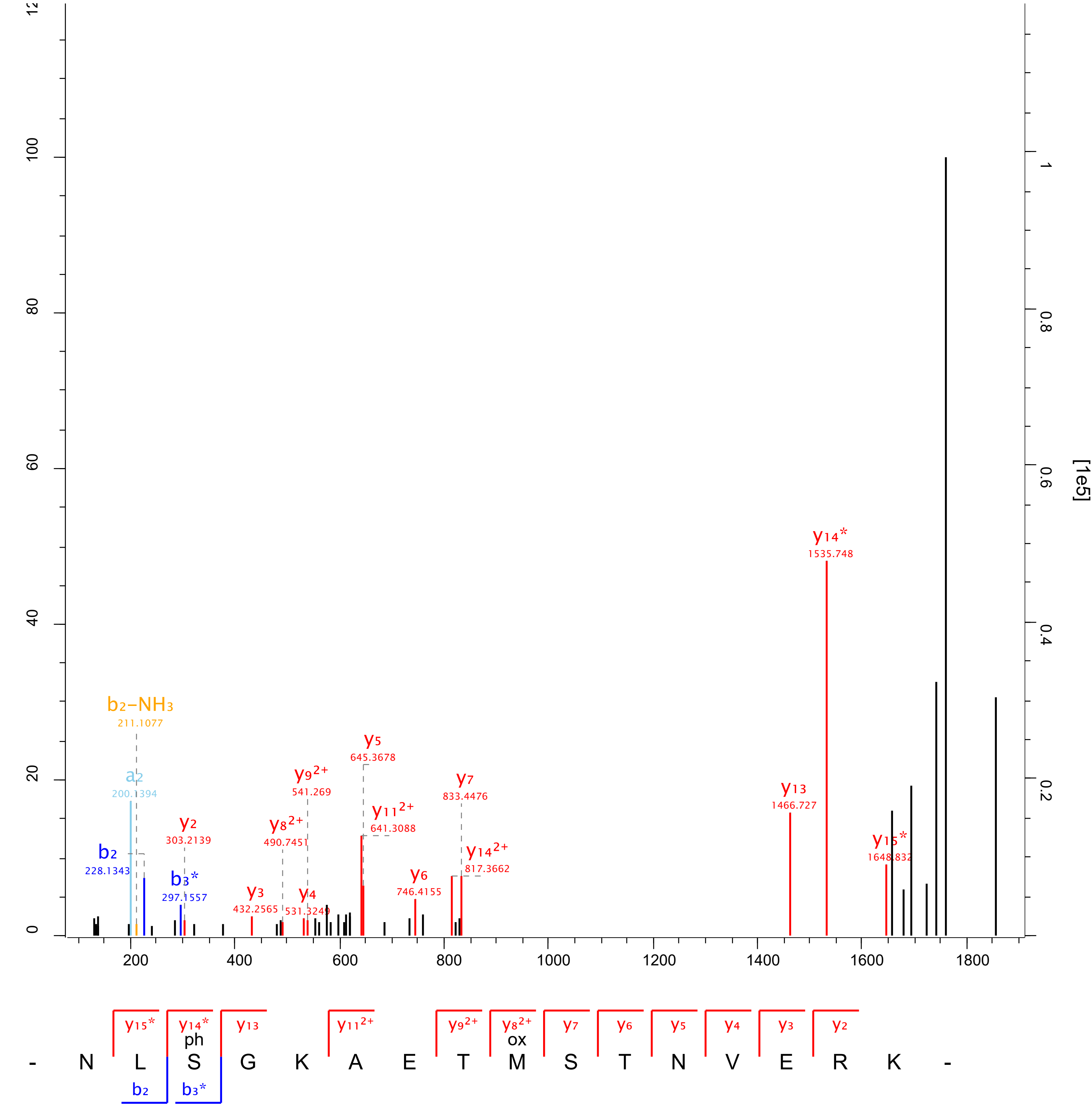

Sample output to test PDF Combine only

Raw file	Scan	Method	Score	m/z	Gene names
sirk1-mic-SUC-2-A	4460	FTMS; HCD	50.11	424.22	SE

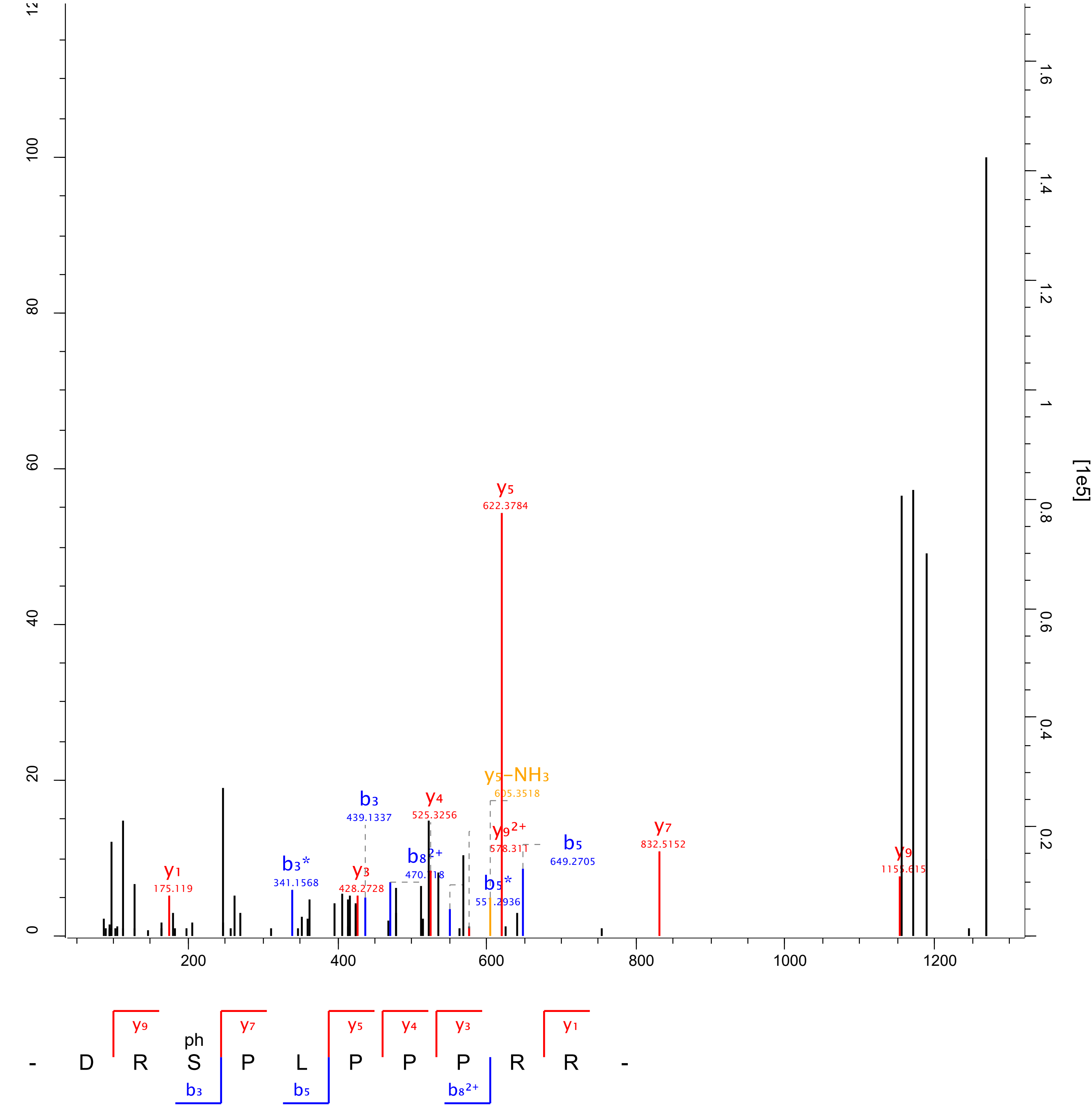

Sample output to test PDF Combine only

Raw file	Scan	Method	Score	m/z	Gene names
sirk1-mic-SUC-2-A	4705	FTMS; HCD	50.46	548.28	At3g05900

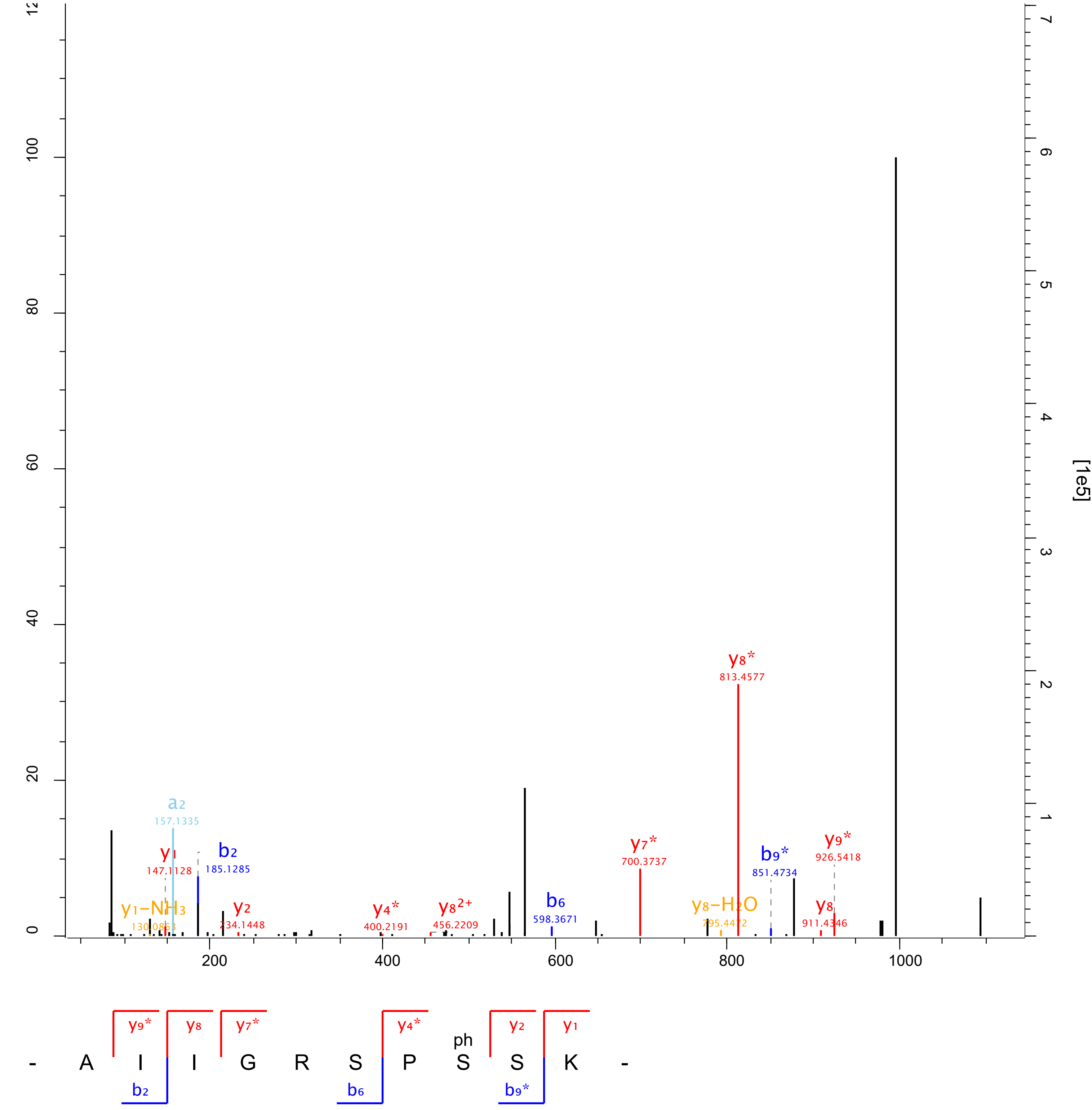

Sample output to test PDF Combine only

Raw file	Scan	Method	Score	m/z	Gene names
sirk1-mic-SUC-2-A	5013	FTMS; HCD	62.94	570.92	At5g06970

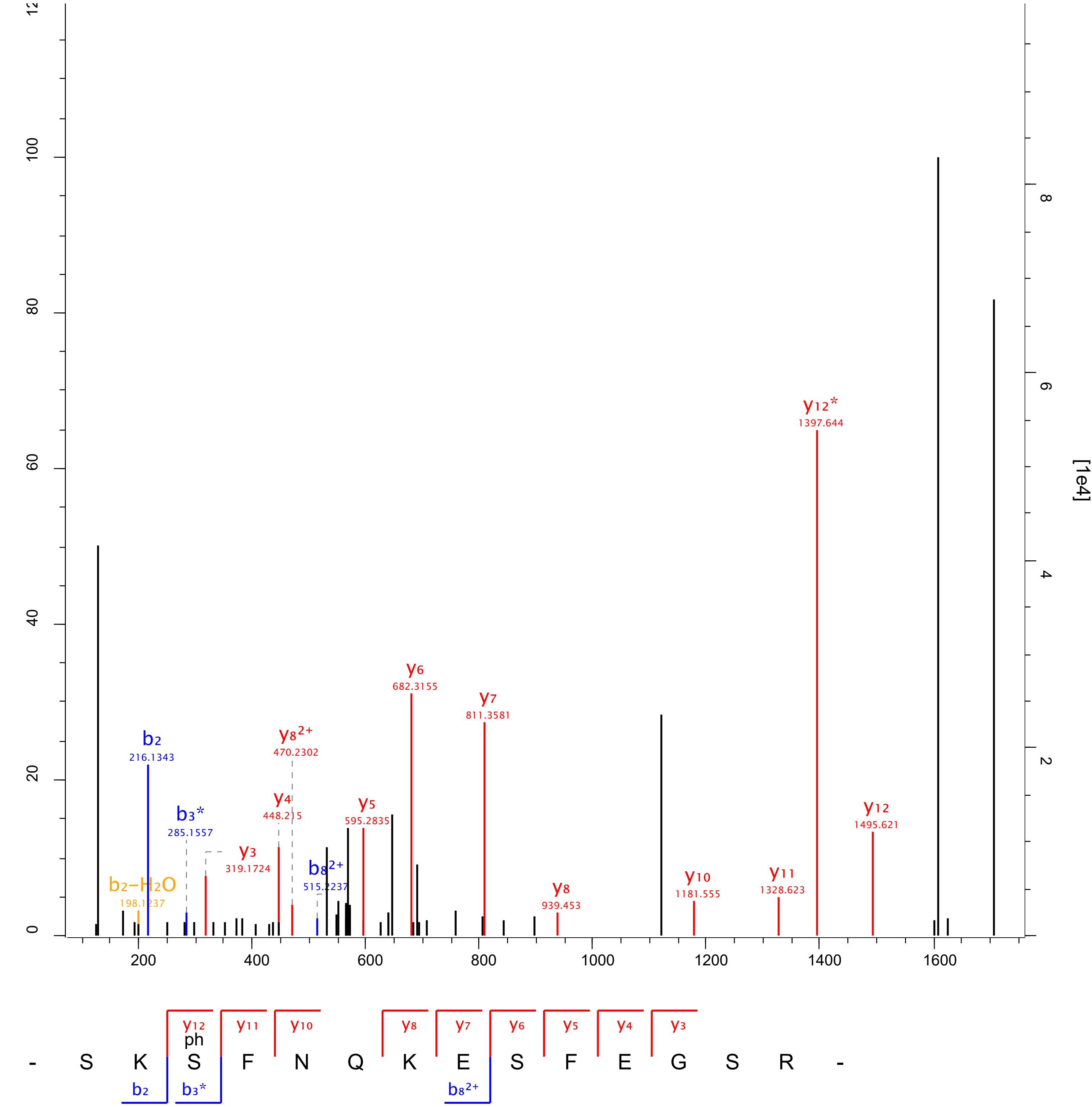

Sample output to test PDF Combine only

Raw file	Scan	Method	Score	m/z	Gene names
sirk1-mic-SUC-2-A	5268	FTMS; HCD	40.14	627.97	CBP60B

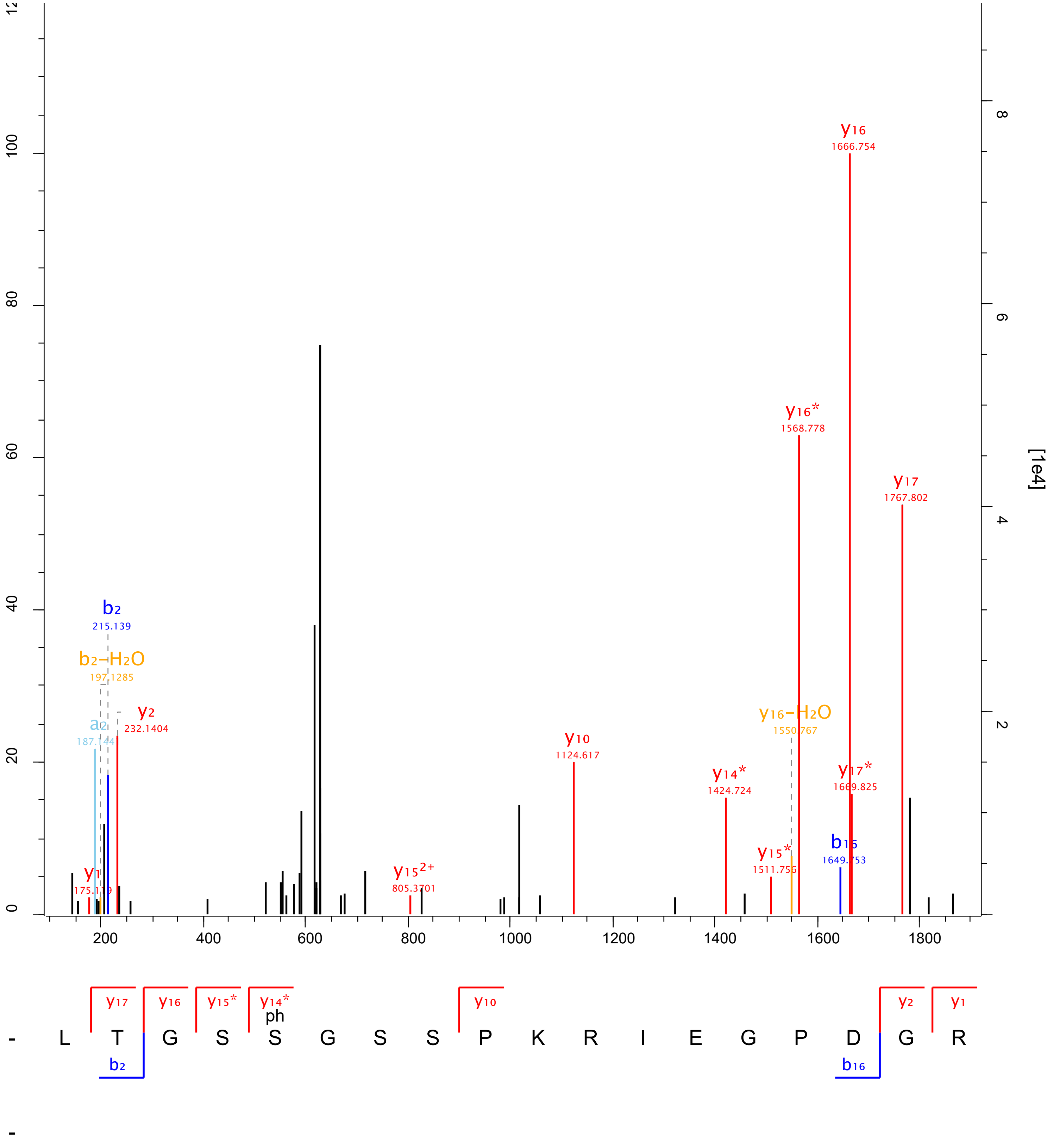

Sample output to test PDF Combine only

Raw file	Scan	Method	Score	m/z	Gene names
sirk1-mic-SUC-2-A	6448	FTMS; HCD	40.49	528.57	T12C14_30

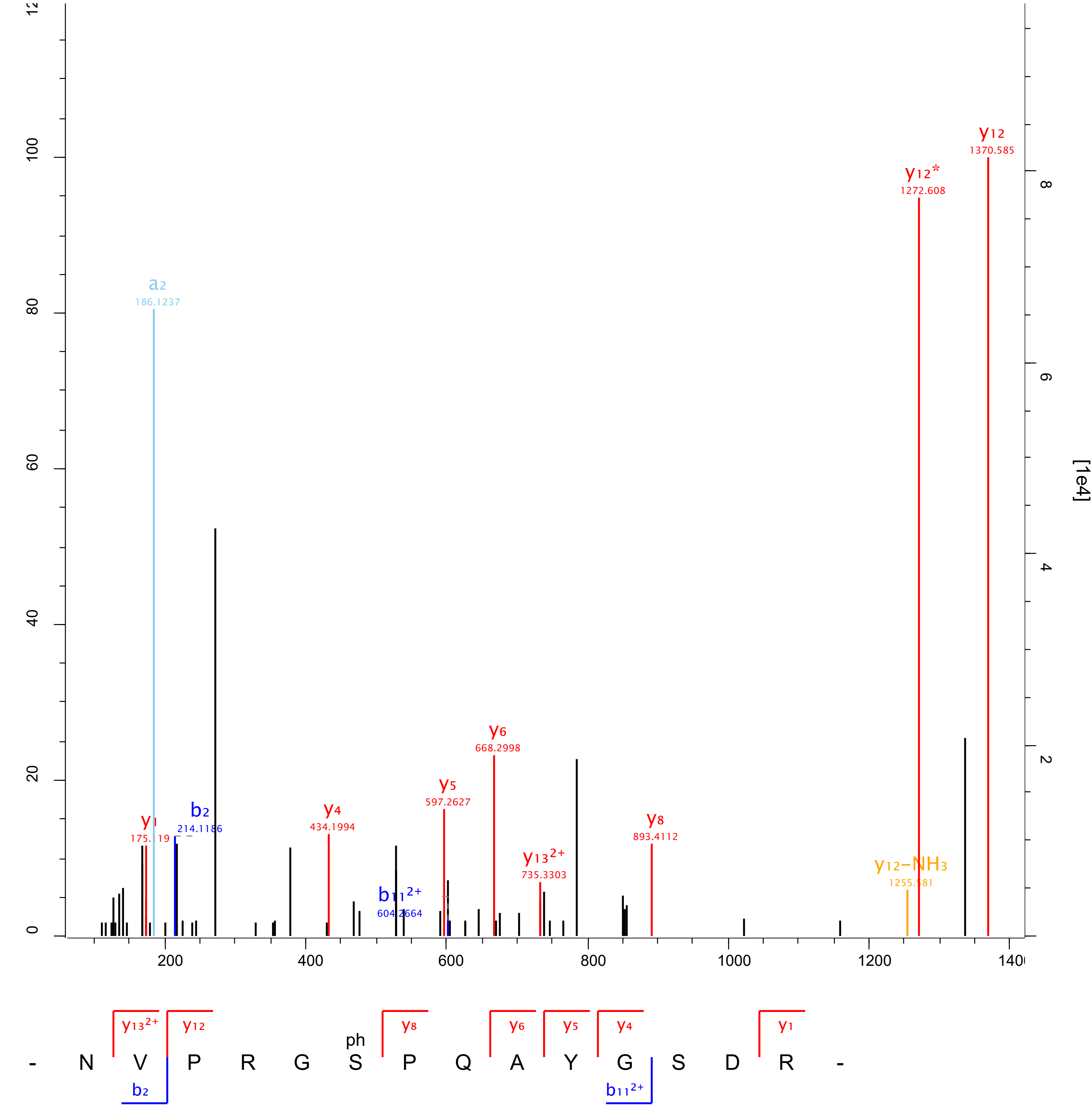

Sample output to test PDF Combine only

Raw file	Scan	Method	Score	m/z	Gene names
sirk1-mic-SUC-2-A	6749	FTMS; HCD	99.93	392.85	PIP2-1;PIP2-3;PIP2-2

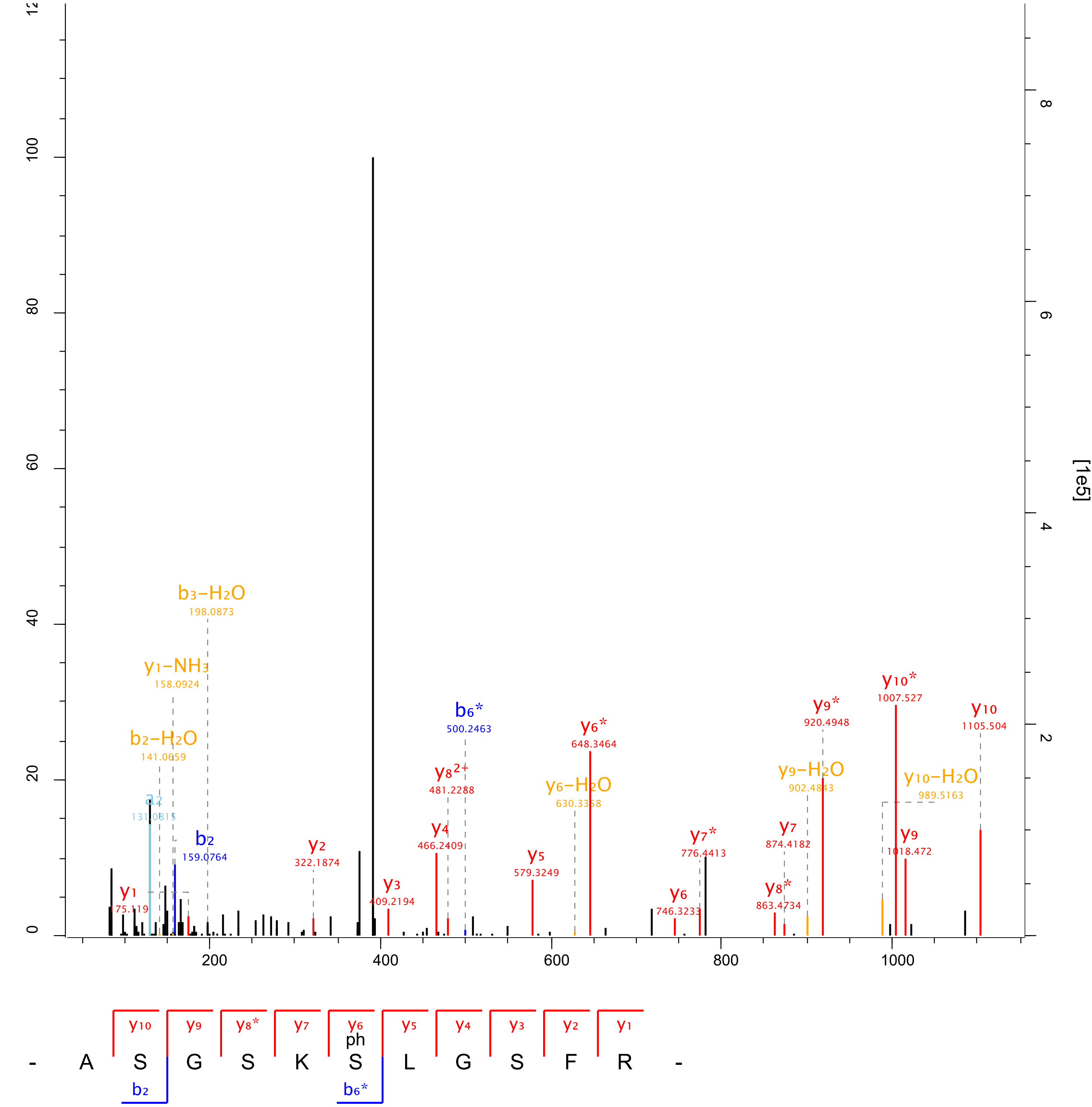

Sample output to test PDF Combine only

Raw file	Scan	Method	Score	m/z	Gene names
sirk1-mic-SUC-2-A	7353	FTMS; HCD	46.37	530.77	F15B8.100

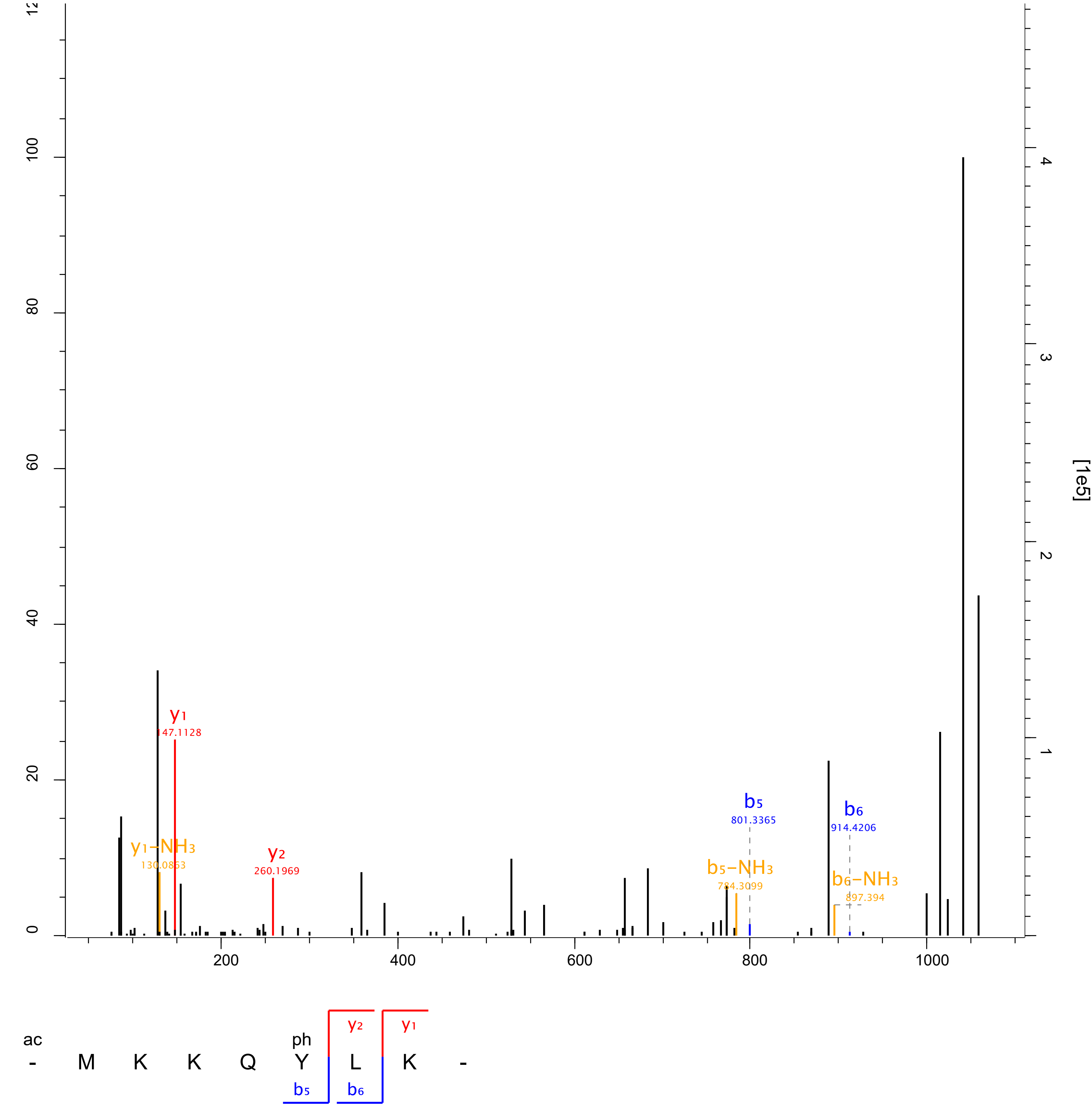

Sample output to test PDF Combine only

Raw file	Scan	Method	Score	m/z	Gene names
sirk1-mic-SUC-2-A	7649	FTMS; HCD	54.67	603.61	At4g38470

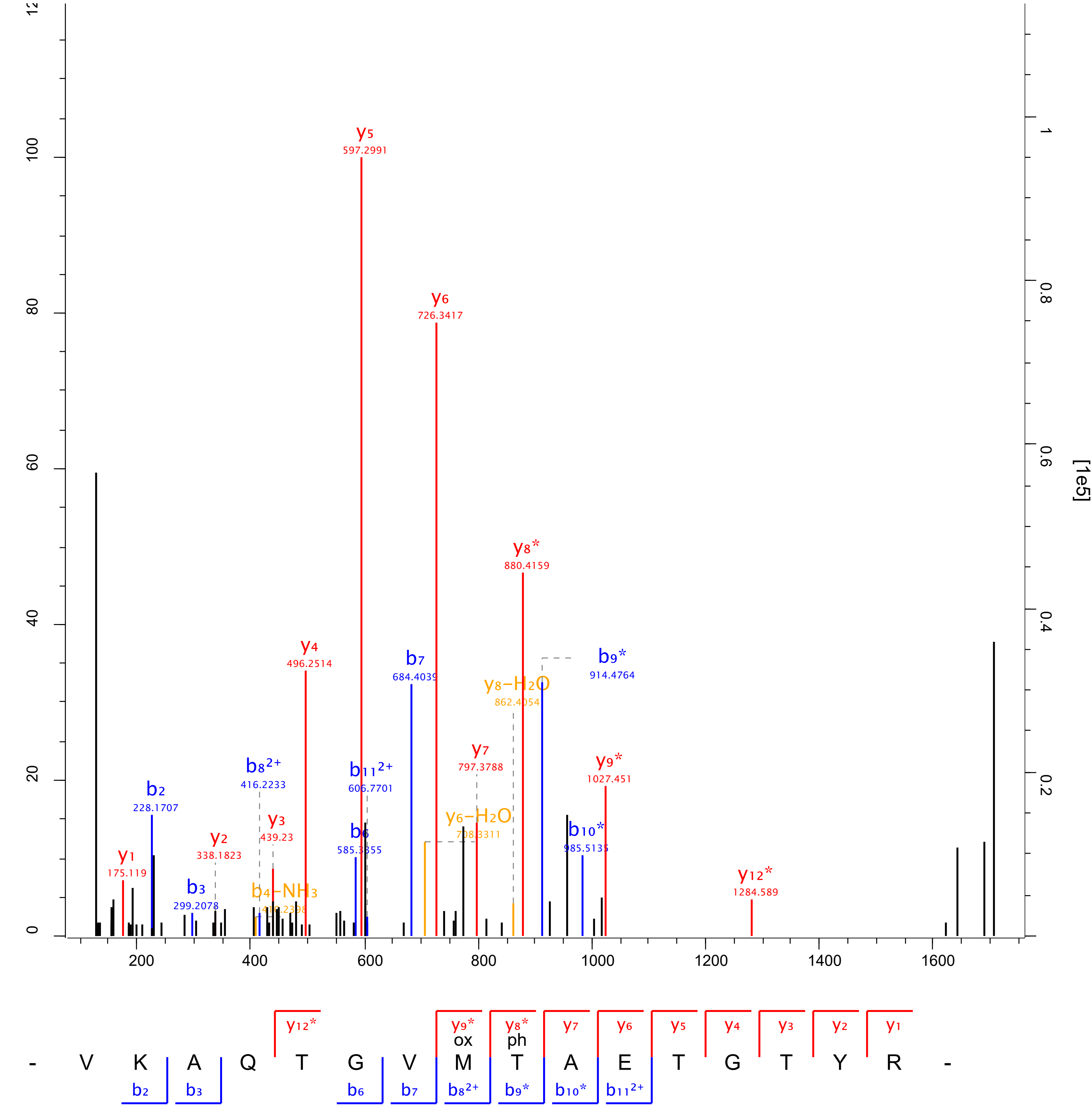

Sample output to test PDF Combine only

Raw file	Scan	Method	Score	m/z	Gene names
sirk1-mic-SUC-2-A	8466	FTMS; HCD	62.69	585.27	MLO8;MLO5;MLO7;MLO10

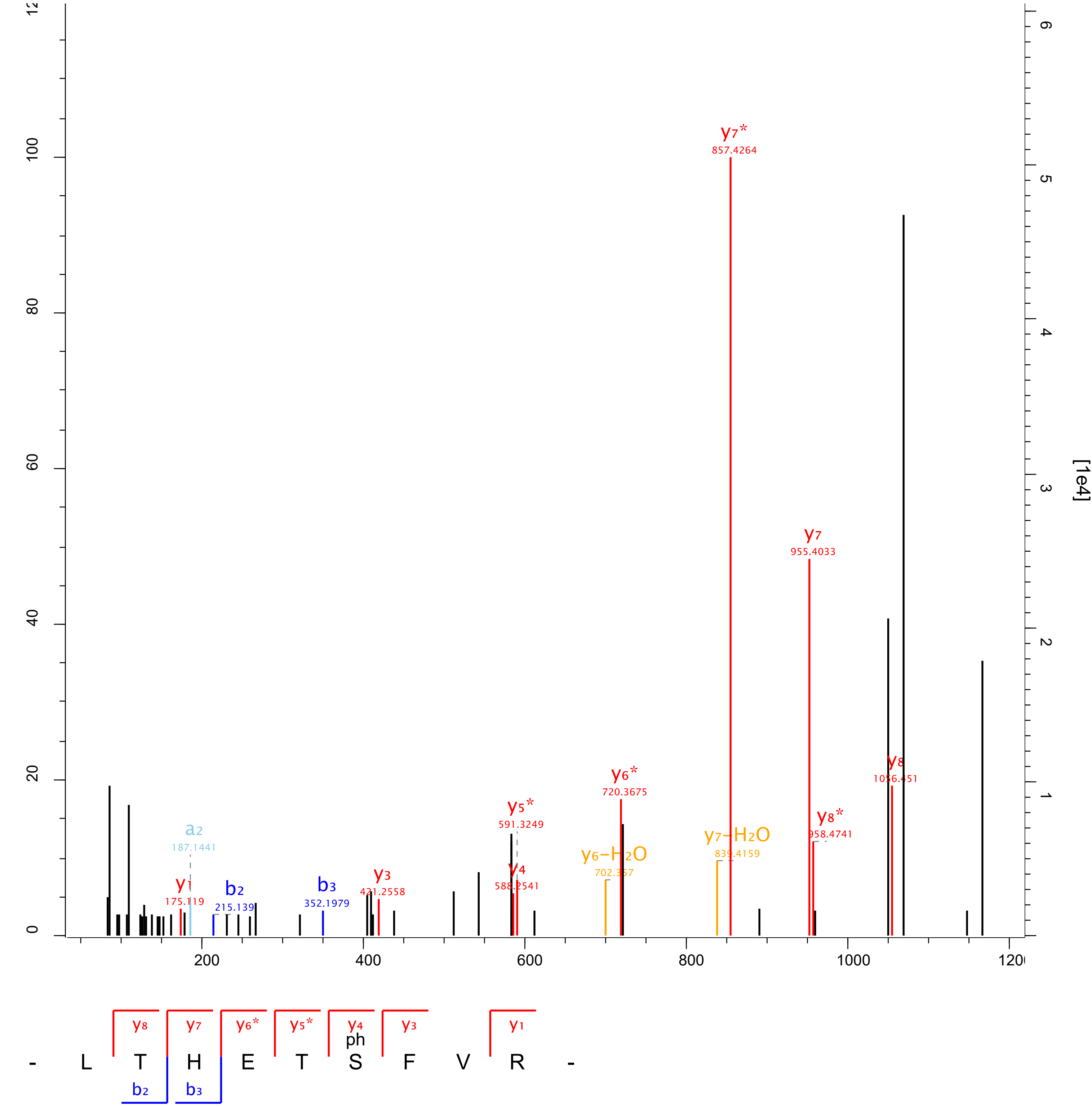

Sample output to test PDF Combine only

Raw file	Scan	Method	Score	m/z	Gene names
sirk1-mic-SUC-2-A	8805	FTMS; HCD	50.3	489.58	MRO11.3;At5g23750

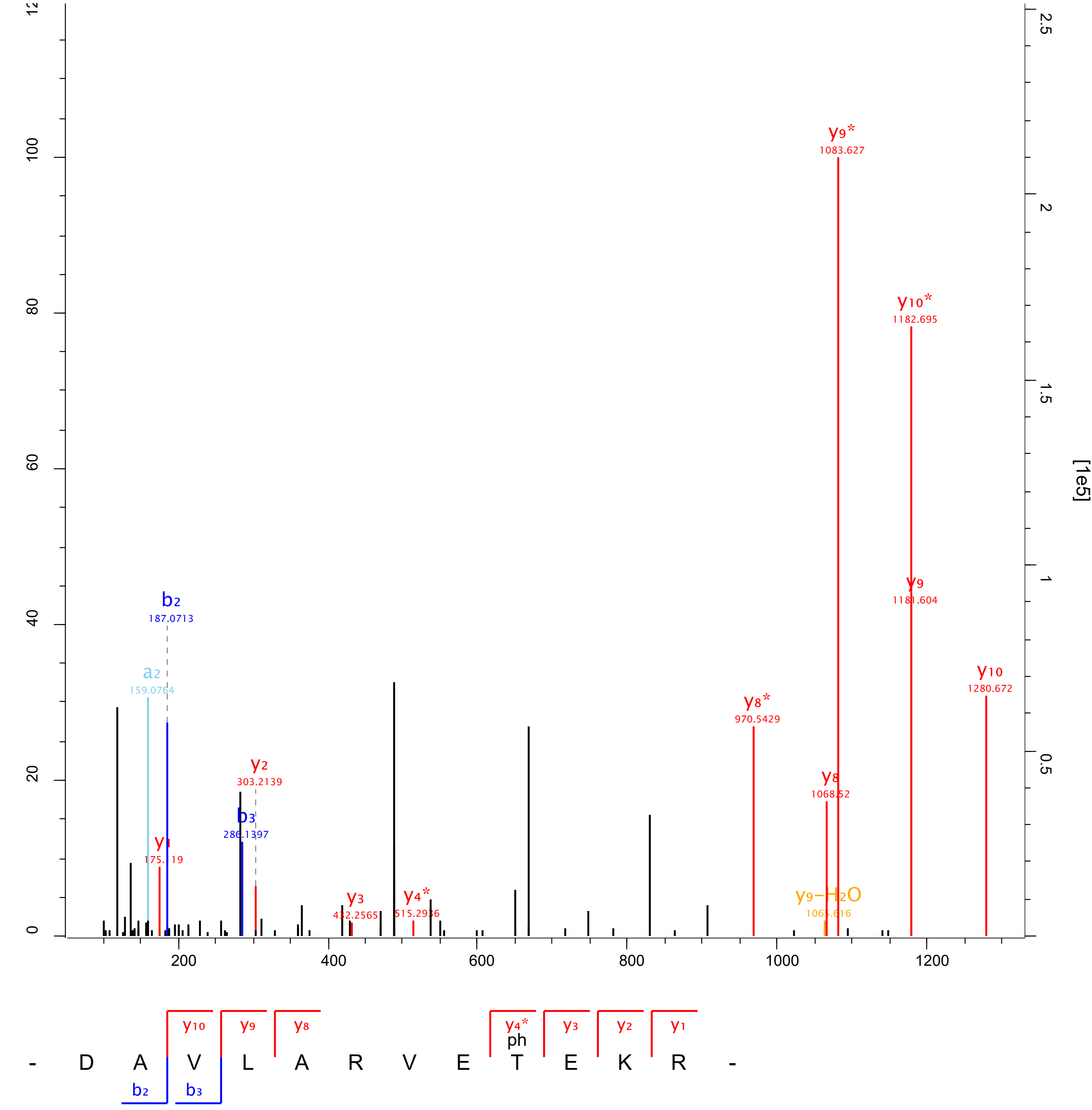

Sample output to test PDF Combine only

Raw file	Scan	Method	Score	m/z	Gene names
sirk1-mic-SUC-2-A	9010	FTMS; HCD	108.58	718.3	F19F18.190

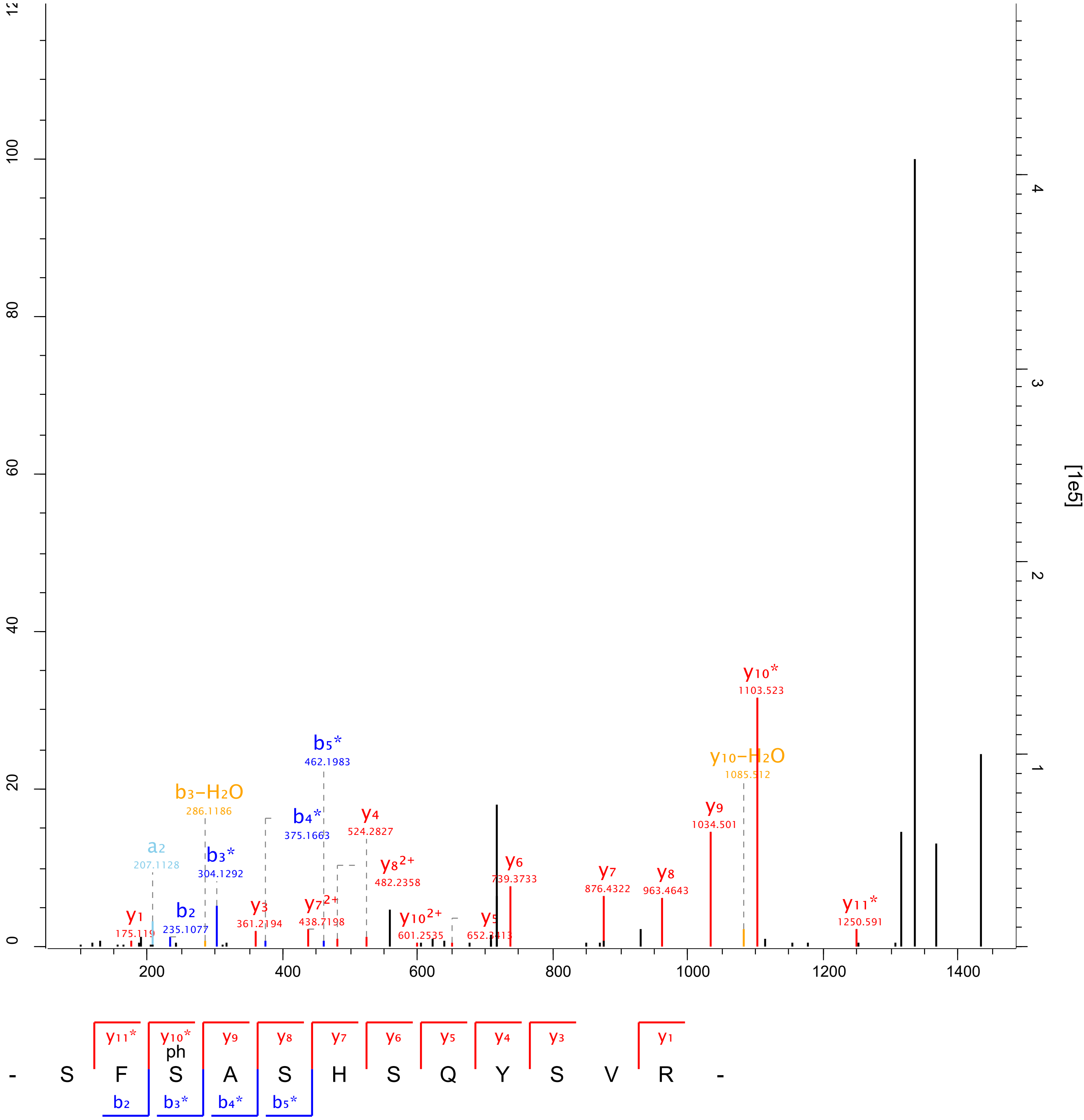

Sample output to test PDF Combine only

Raw file	Scan	Method	Score	m/z	Gene names
sirk1-mic-SUC-2-A	9864	FTMS; HCD	98.59	419.54	F28P22.12

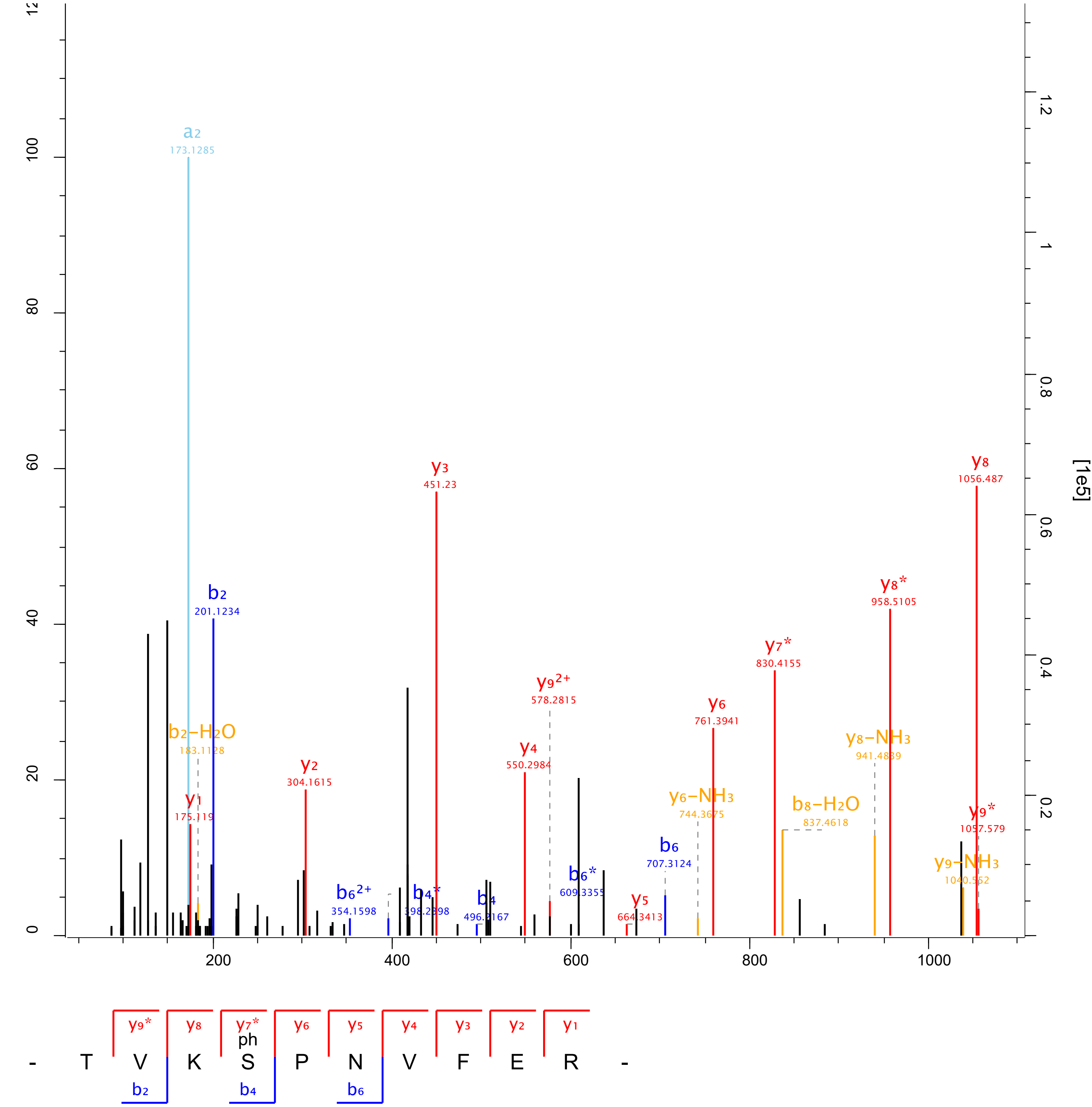

Sample output to test PDF Combine only

Raw file	Scan	Method	Score	m/z	Gene names
sirk1-mic-SUC-2-A	11922	FTMS; HCD	52.6	613.27	IQD14

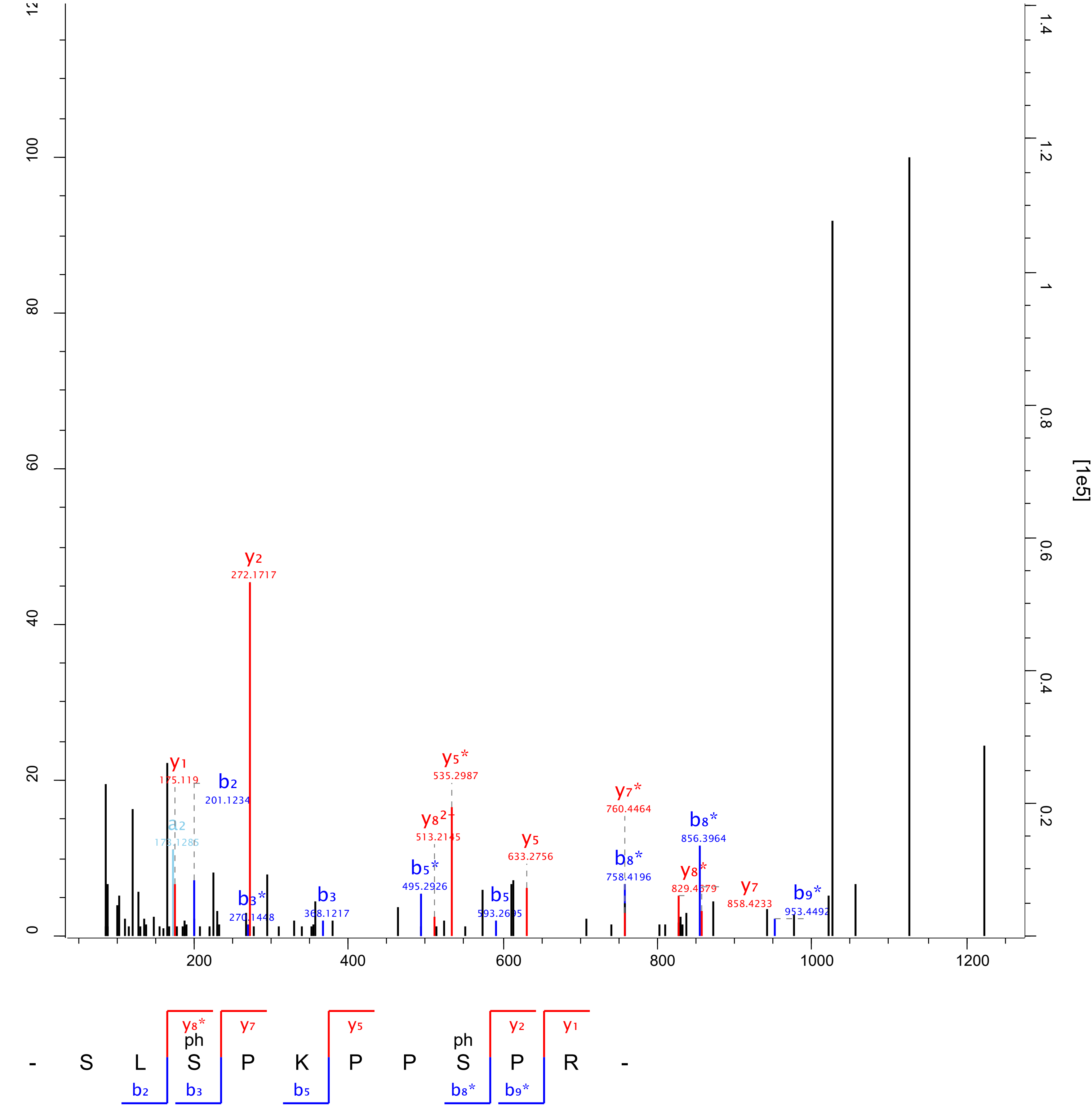

Sample output to test PDF Combine only

Raw file	Scan	Method	Score	m/z	Gene names
sirk1-mic-SUC-2-A	12449	FTMS; HCD	118.84	631.27	PLP1

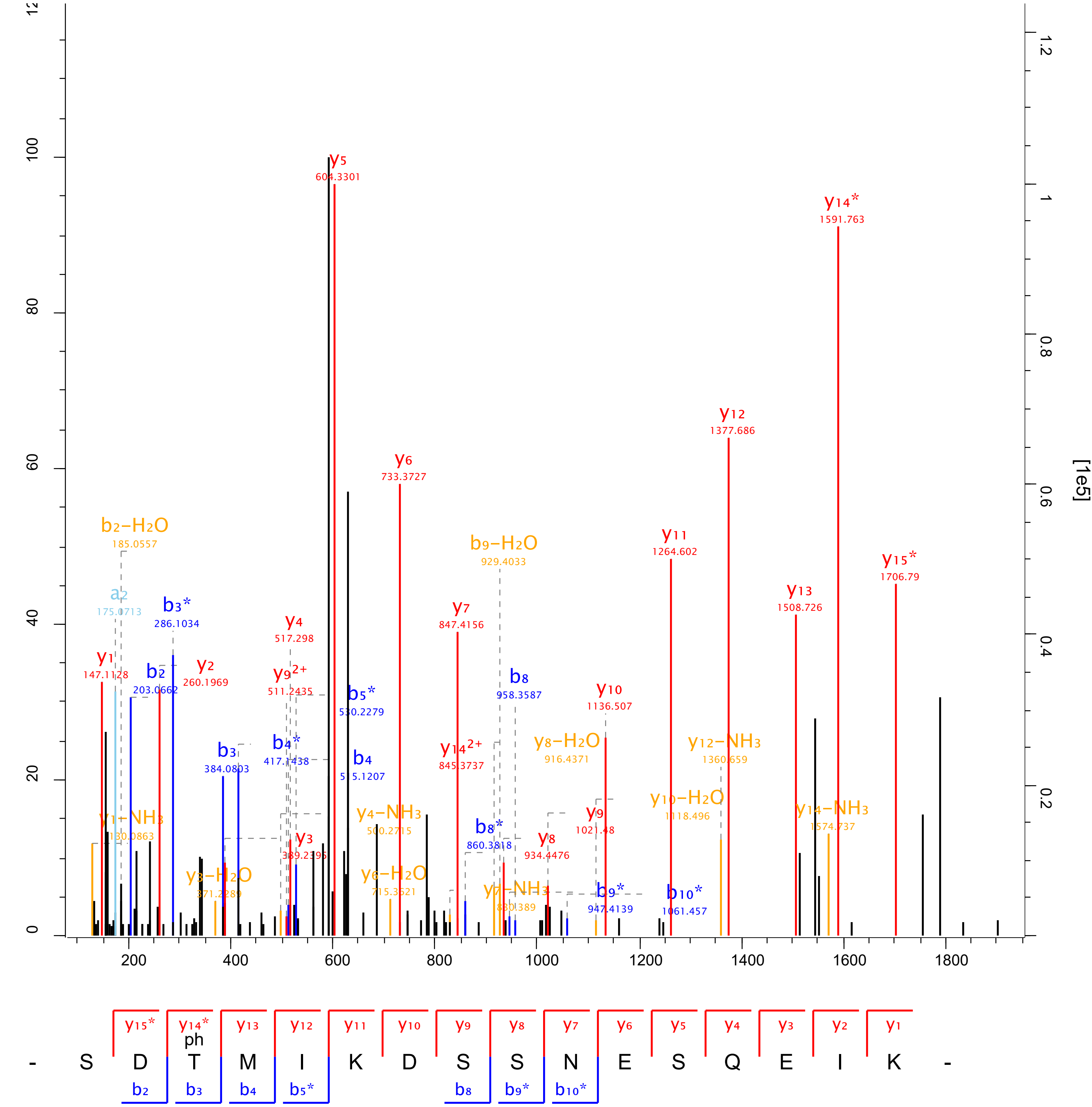

Sample output to test PDF Combine only

Raw file	Scan	Method	Score	m/z	Gene names
sirk1-mic-SUC-2-A	12466	FTMS; HCD	226.79	739.86	RIN4

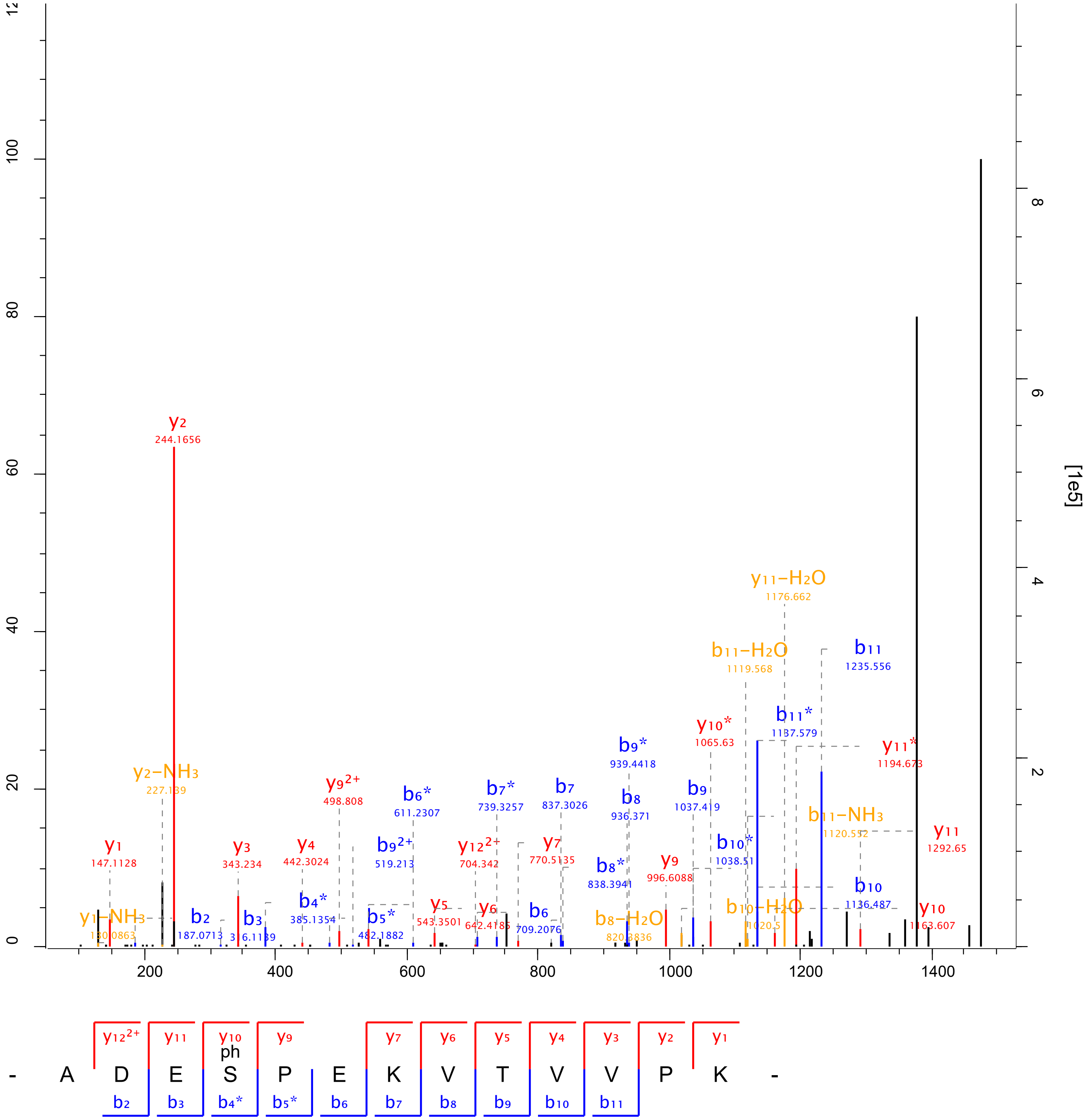

Sample output to test PDF Combine only

Raw file	Scan	Method	Score	m/z	Gene names
sirk1-mic-SUC-2-A	12549	FTMS; HCD	267.75	721.33	PLP1

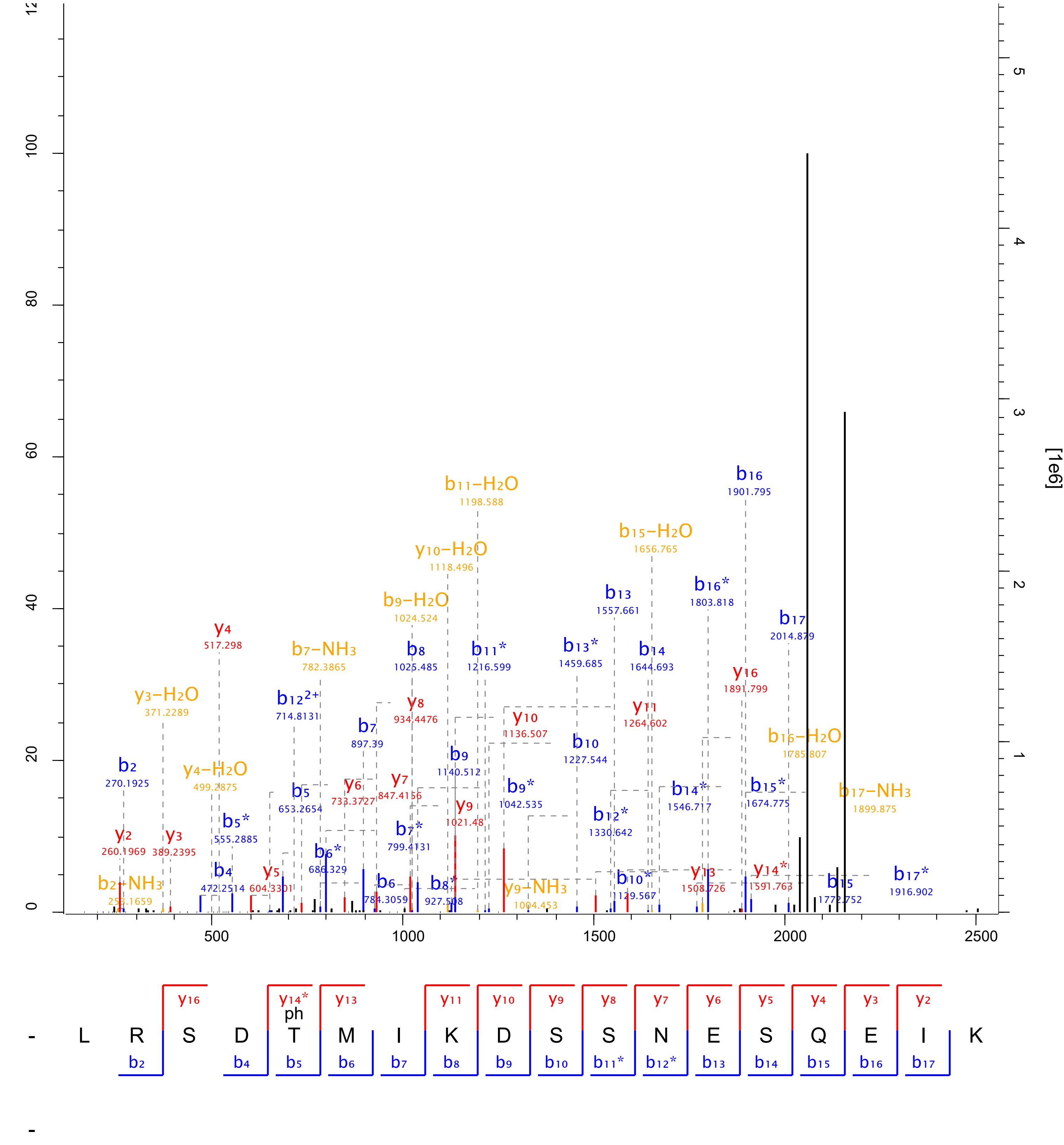

Sample output to test PDF Combine only

Raw file	Scan	Method	Score	m/z	Gene names
sirk1-mic-SUC-2-A	12564	FTMS; HCD	96.79	735.99	CPK6

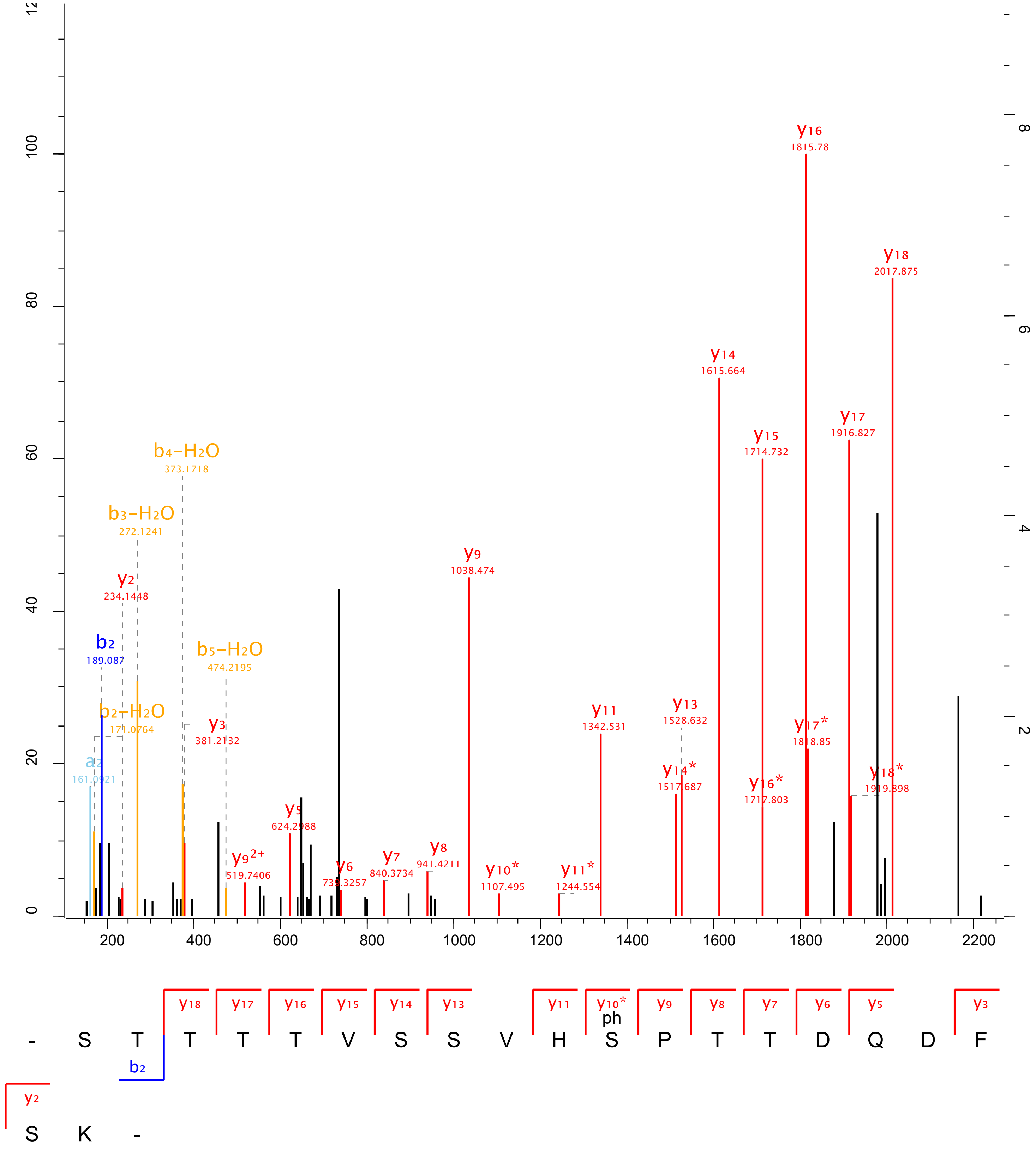

Sample output to test PDF Combine only

Raw file	Scan	Method	Score	m/z	Gene names
sirk1-mic-SUC-2-A	12576	FTMS; HCD	43.16	767.69	SOUL-1

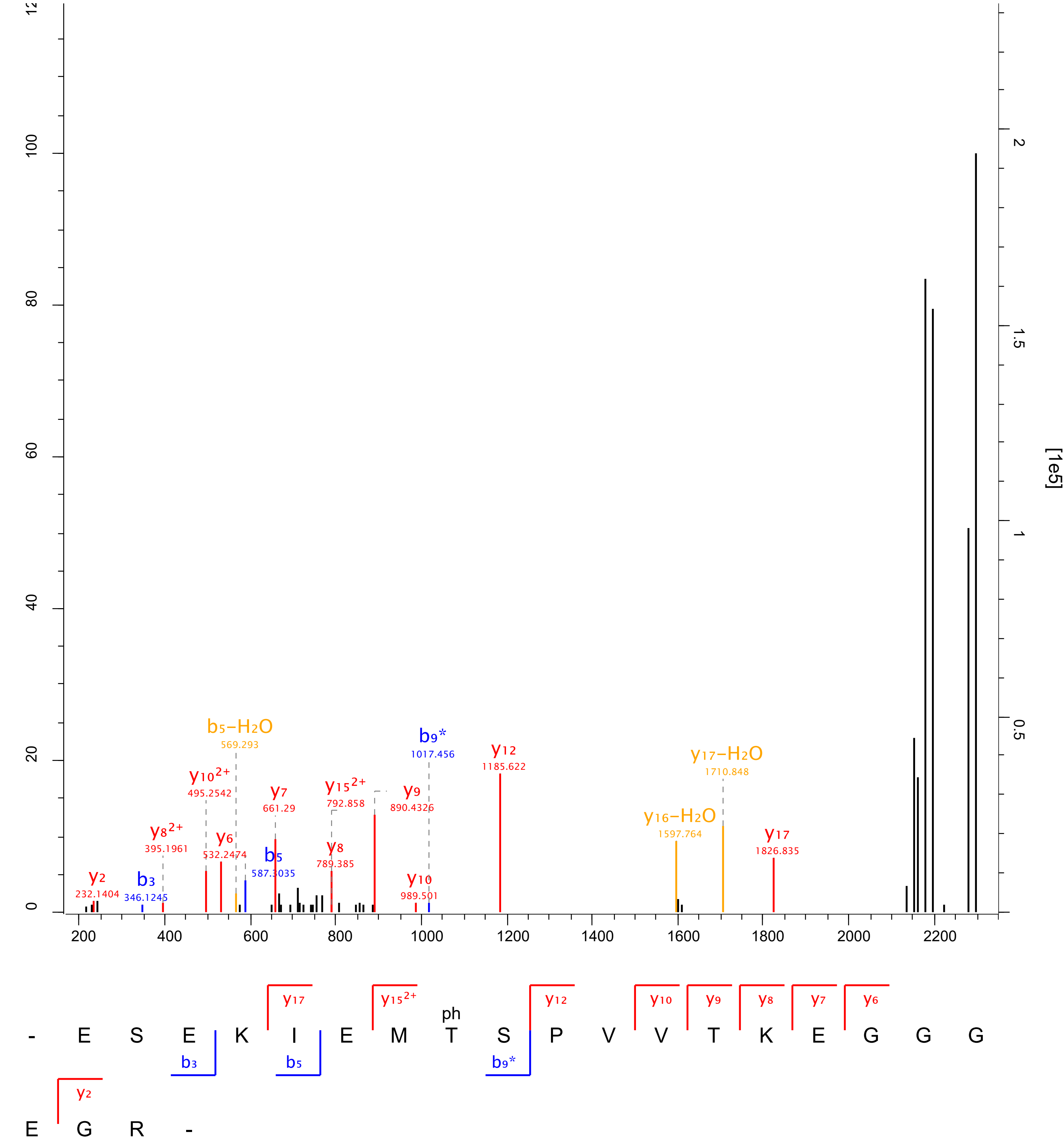

Sample output to test PDF Combine only

Raw file	Scan	Method	Score	m/z	Gene names
sirk1-mic-SUC-2-A	13496	FTMS; HCD	152.37	441.22	VHA-D

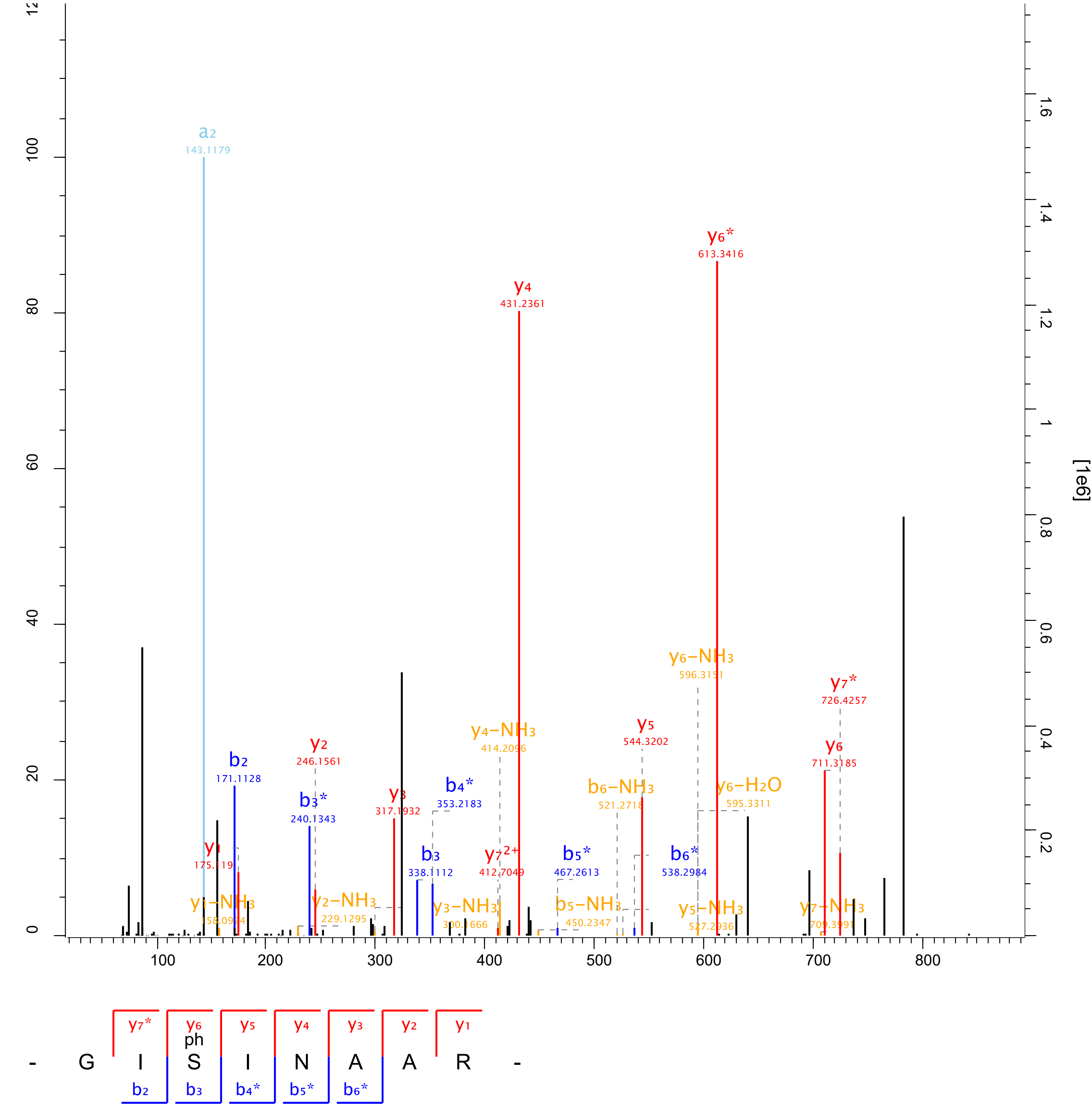

Sample output to test PDF Combine only

Raw file	Scan	Method	Score	m/z	Gene names
sirk1-mic-SUC-2-A	13539	FTMS; HCD	160.8	601.28	F18B13.26

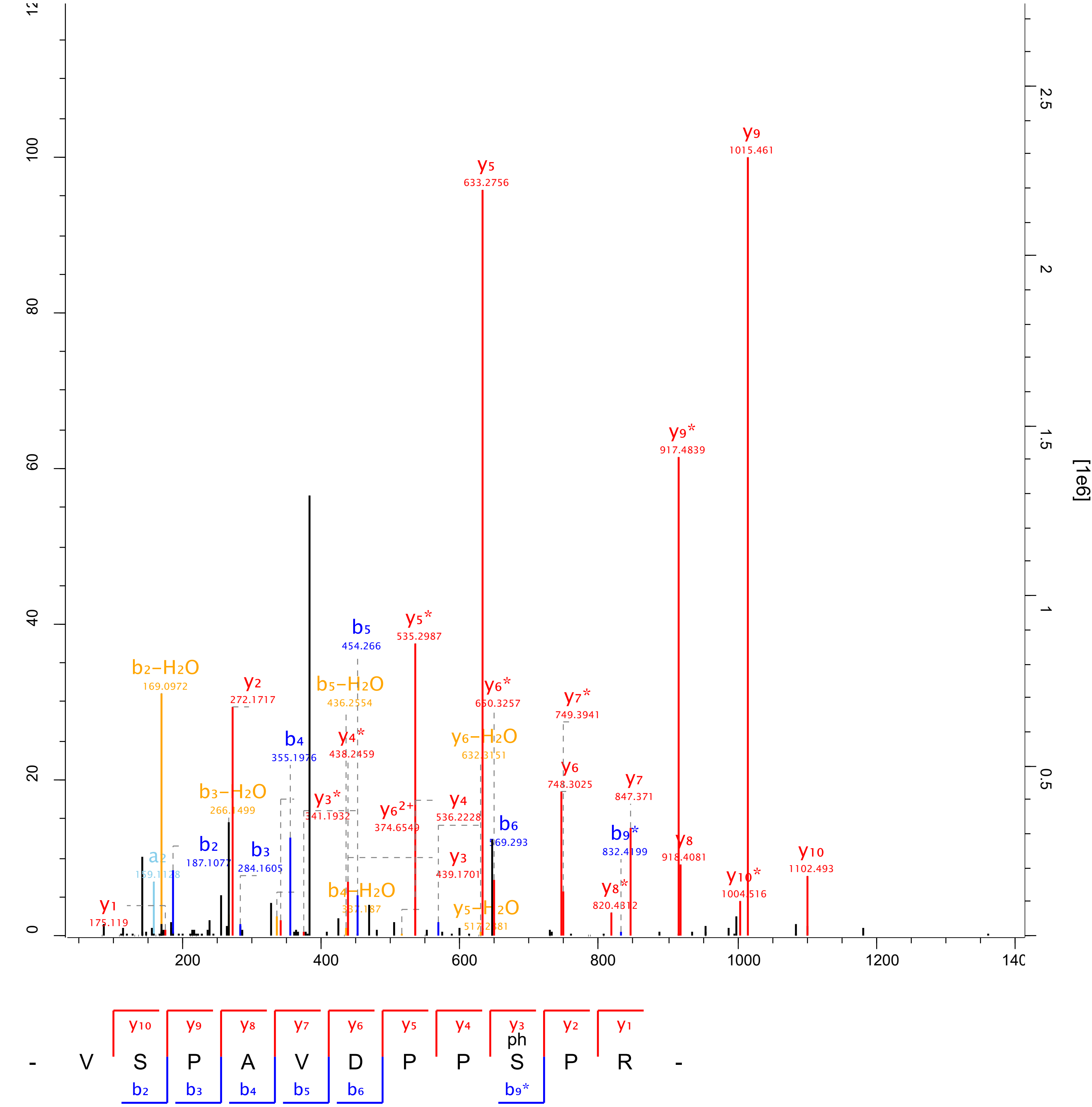

Sample output to test PDF Combine only

Raw file	Scan	Method	Score	m/z	Gene names
sirk1-mic-SUC-2-A	13552	FTMS; HCD	43.81	549.26	ALA1

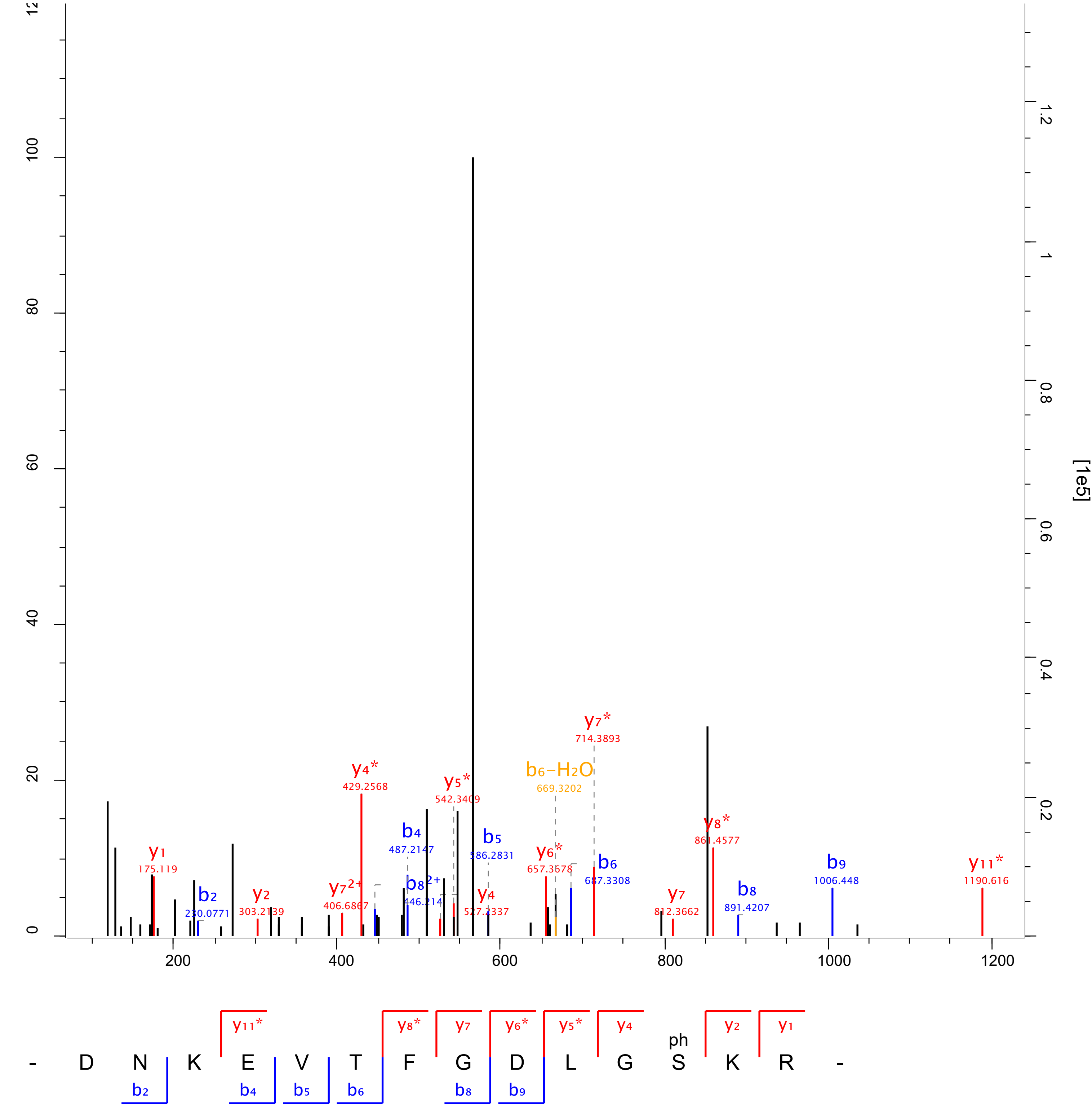

Sample output to test PDF Combine only

Raw file	Scan	Method	Score	m/z
sirk1-mic-SUC-2-A	14262	FTMS; HCD	90.23	486.89

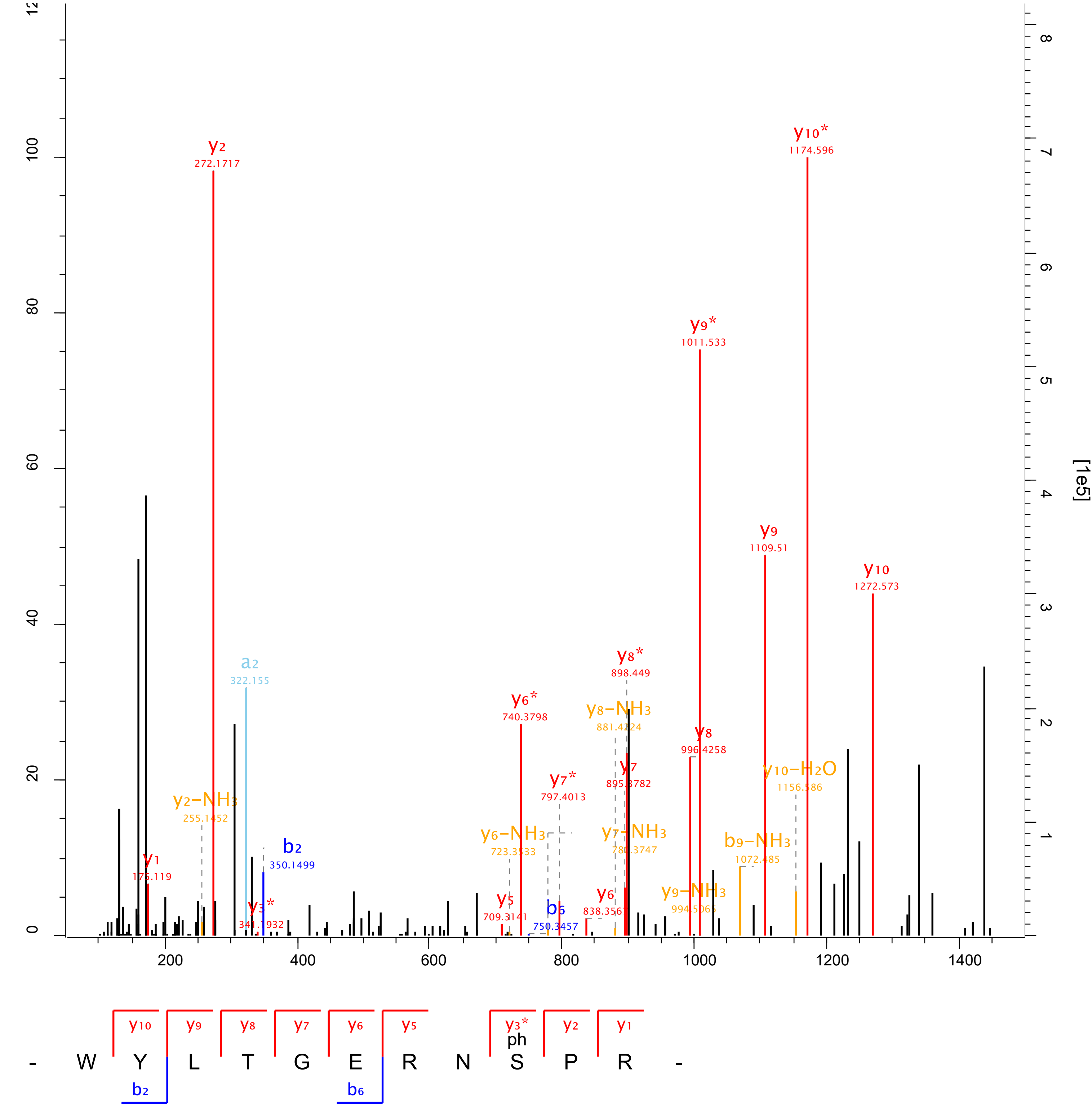

Sample output to test PDF Combine only

Raw file	Scan	Method	Score	m/z
sirk1-mic-SUC-2-A	16008	FTMS; HCD	71	692.96

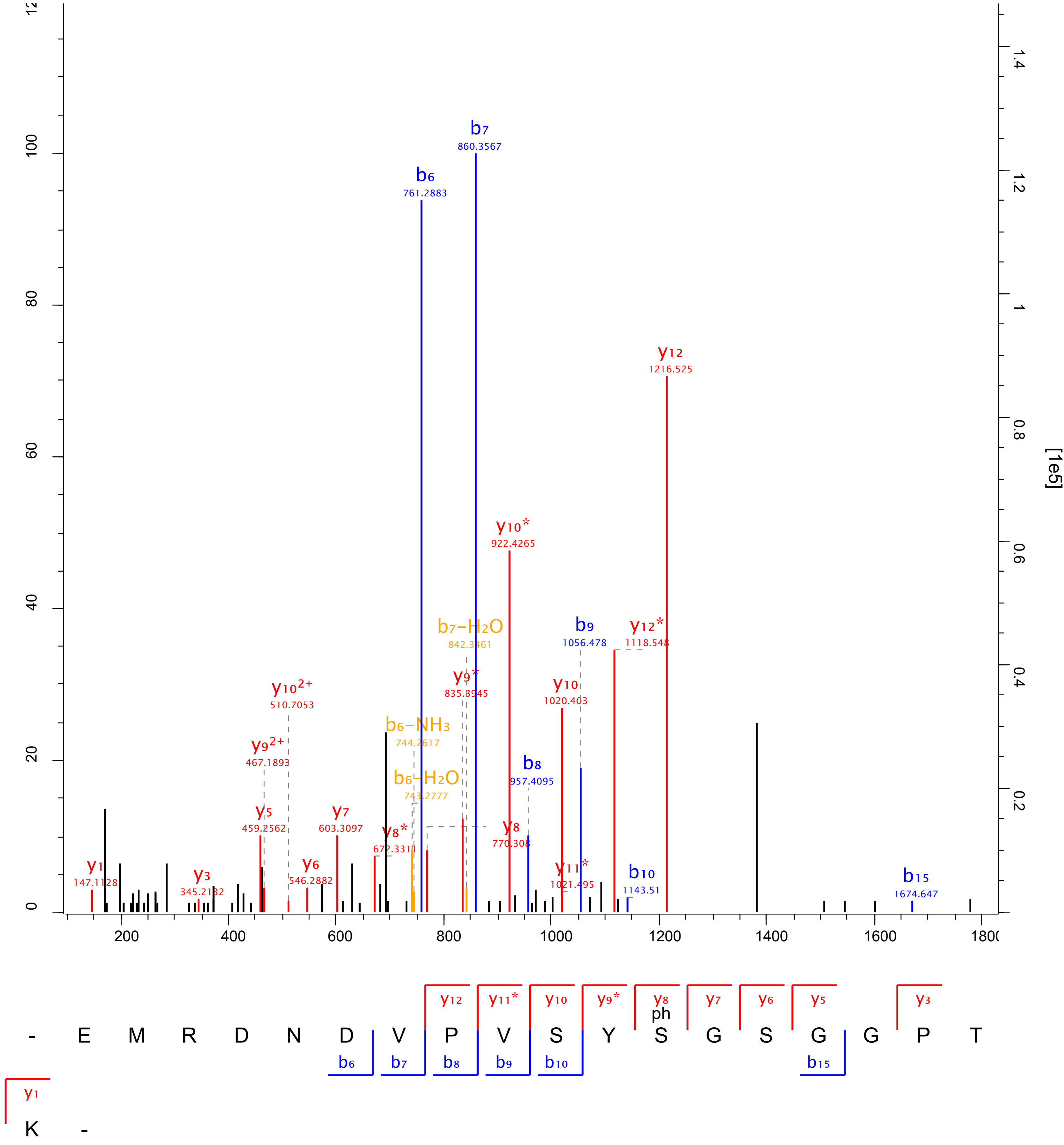

Sample output to test PDF Combine only

Raw file	Scan	Method	Score	m/z	Gene names
sirk1-mic-SUC-2-A	21073	FTMS; HCD	83.37	500.93	At3g28690

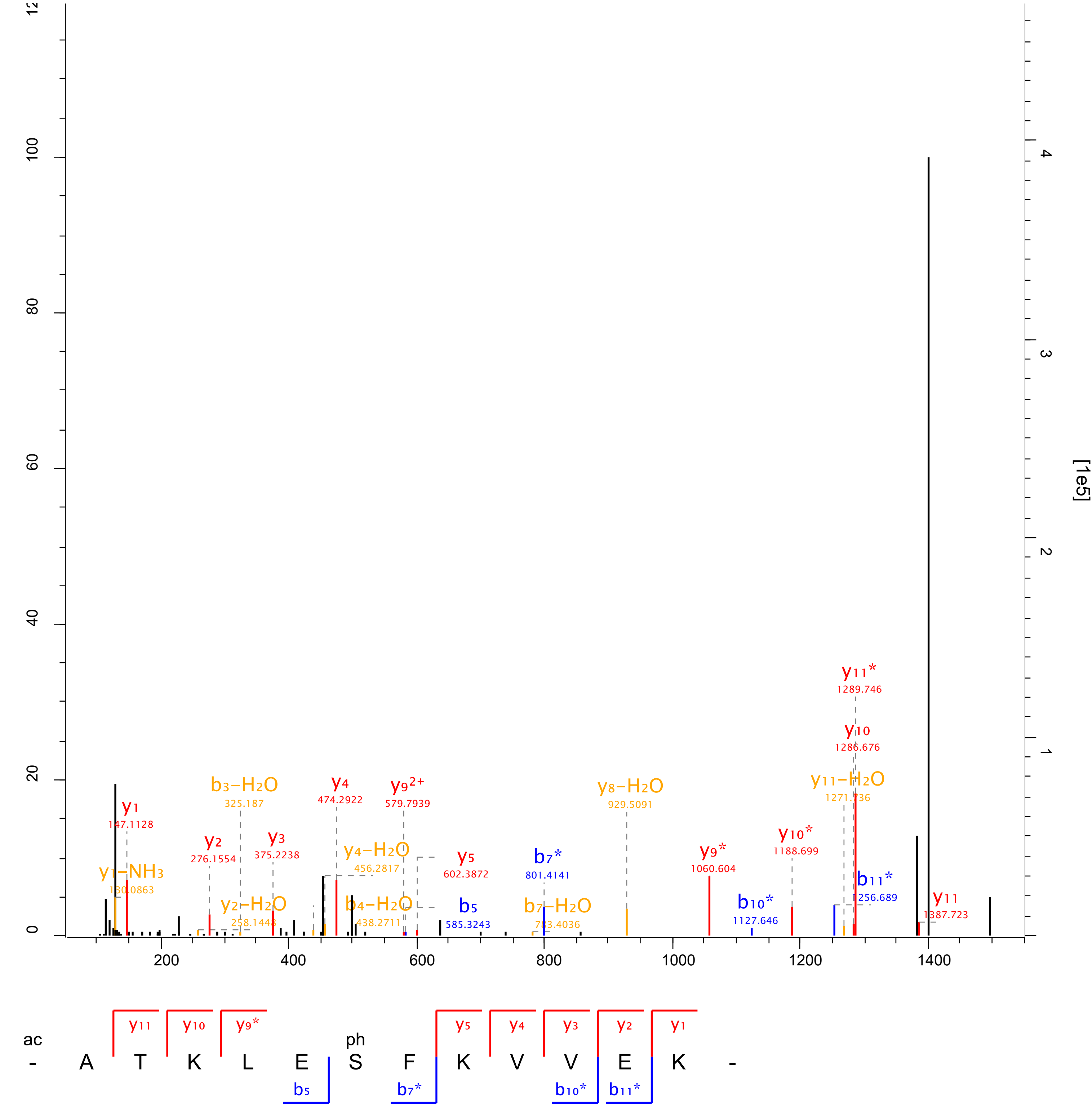

Sample output to test PDF Combine only

Raw file	Scan	Method	Score	m/z	Gene names
sirk1-mic-SUC-2-A	24370	FTMS; HCD	90.41	633.28	NHX7

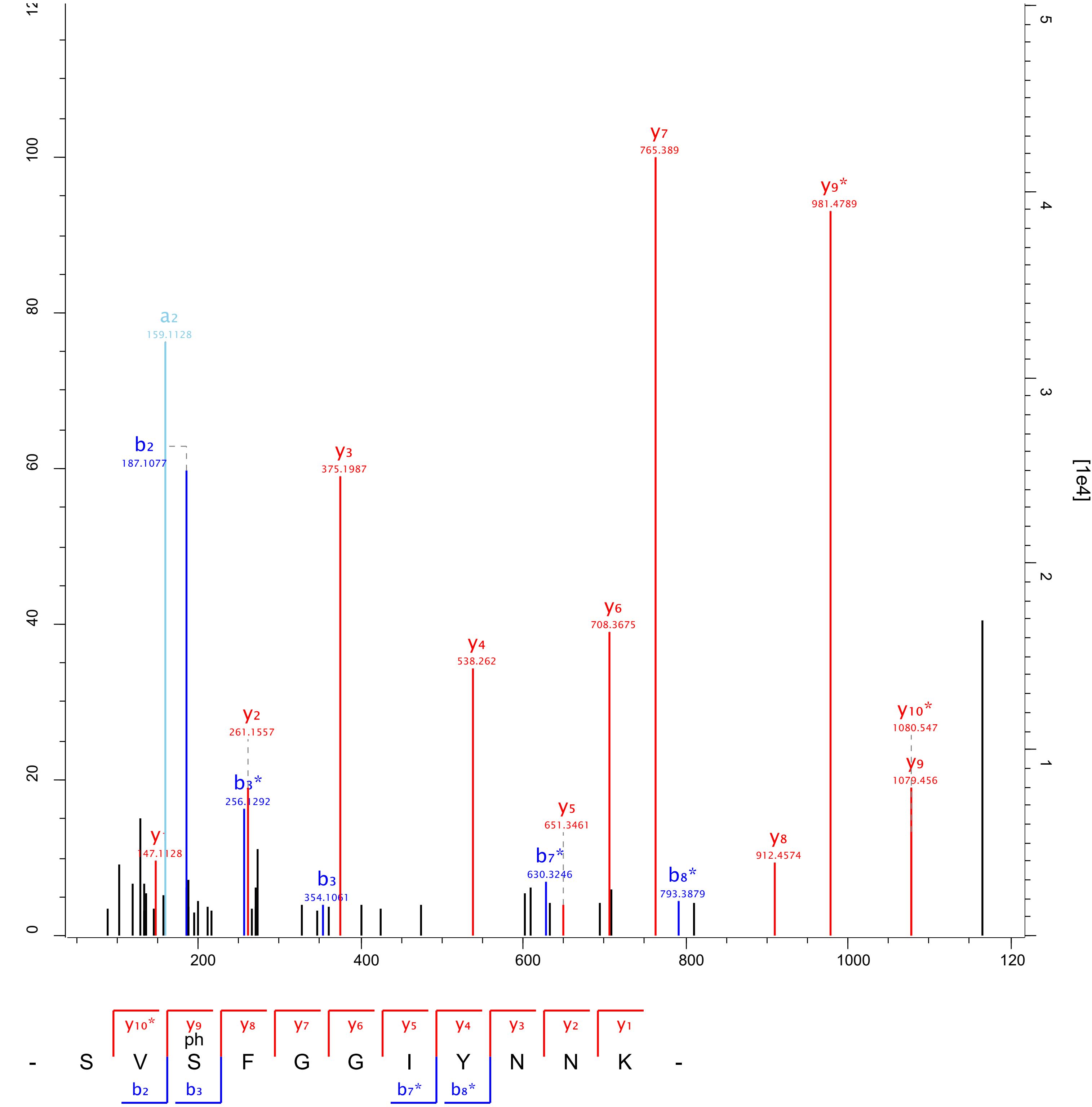

Sample output to test PDF Combine only

Raw file	Scan	Method	Score	m/z	Gene names
sirk1-mic-SUC-2-A	28623	FTMS; HCD	90.61	528.77	PLL4;PLL5

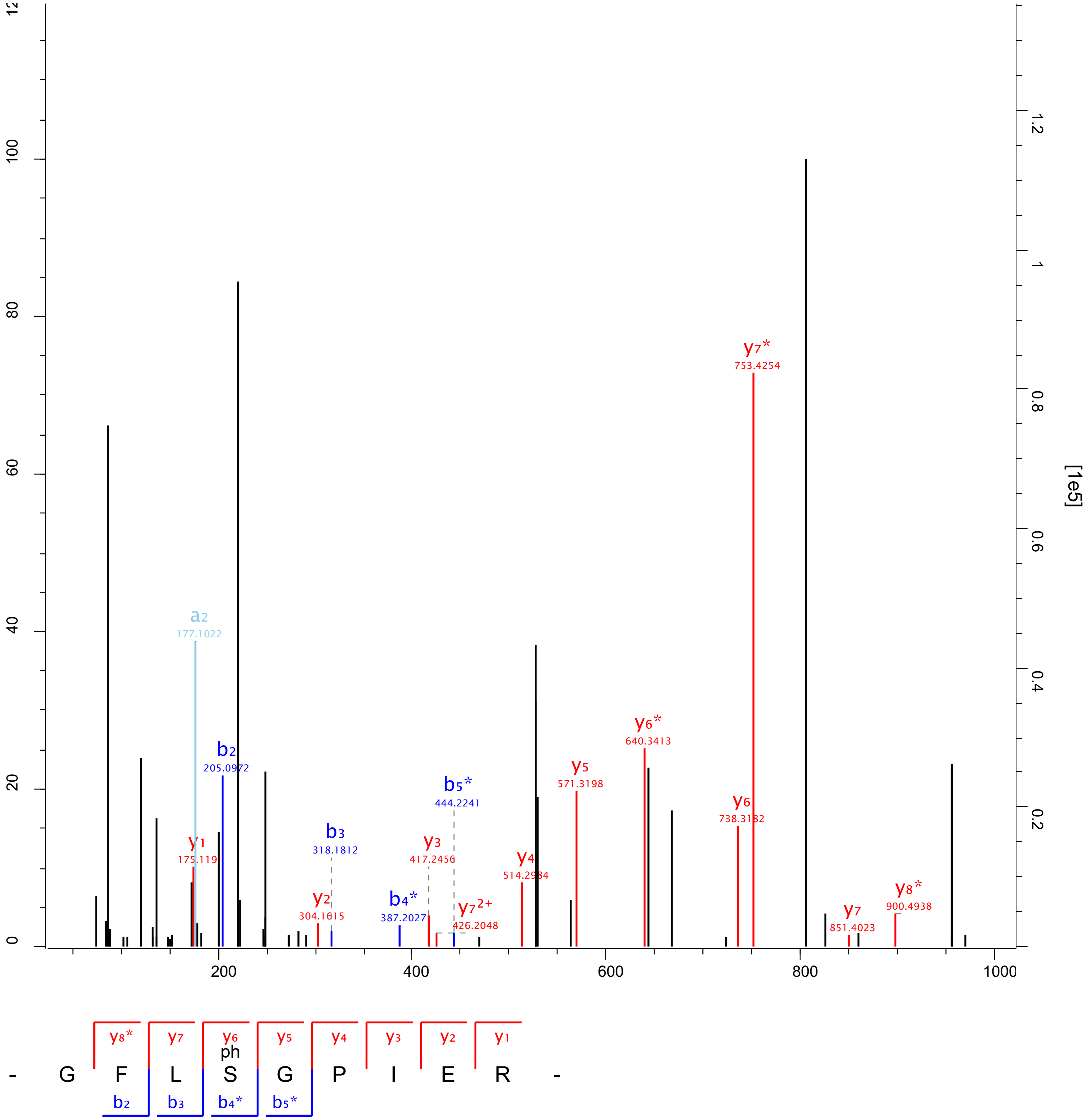

Sample output to test PDF Combine only

Raw file	Scan	Method	Score	m/z	Gene names
sirk1-mic-SUC-2-A	29235	FTMS; HCD	152.11	634.76	PIP2-1;PIP2-3;PIP2-2

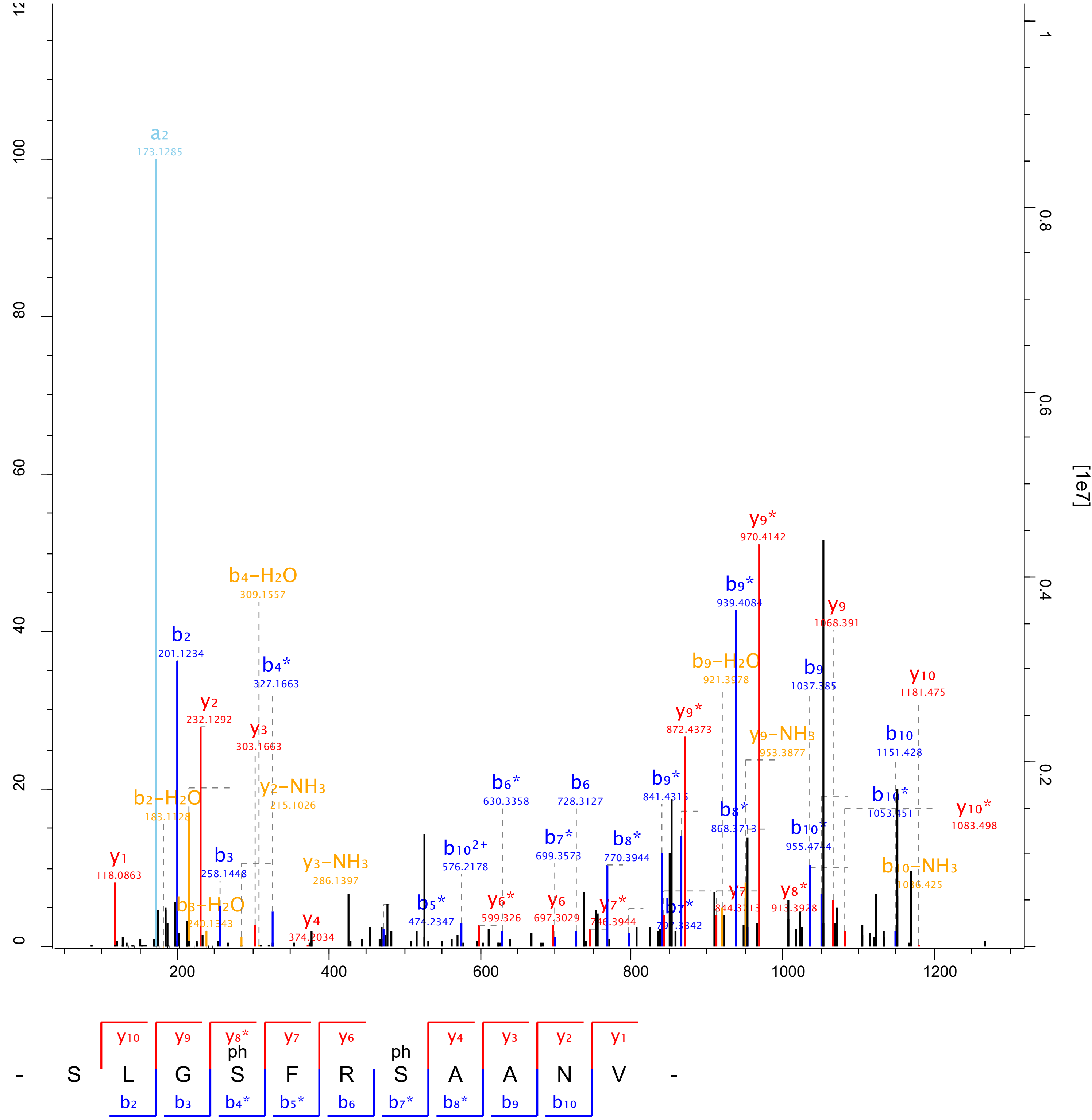

Sample output to test PDF Combine only

Raw file	Scan	Method	Score	m/z	Gene names
sirk1-mic-SUC-2-P	3512	FTMS; HCD	78.08	505.22	ABCG34

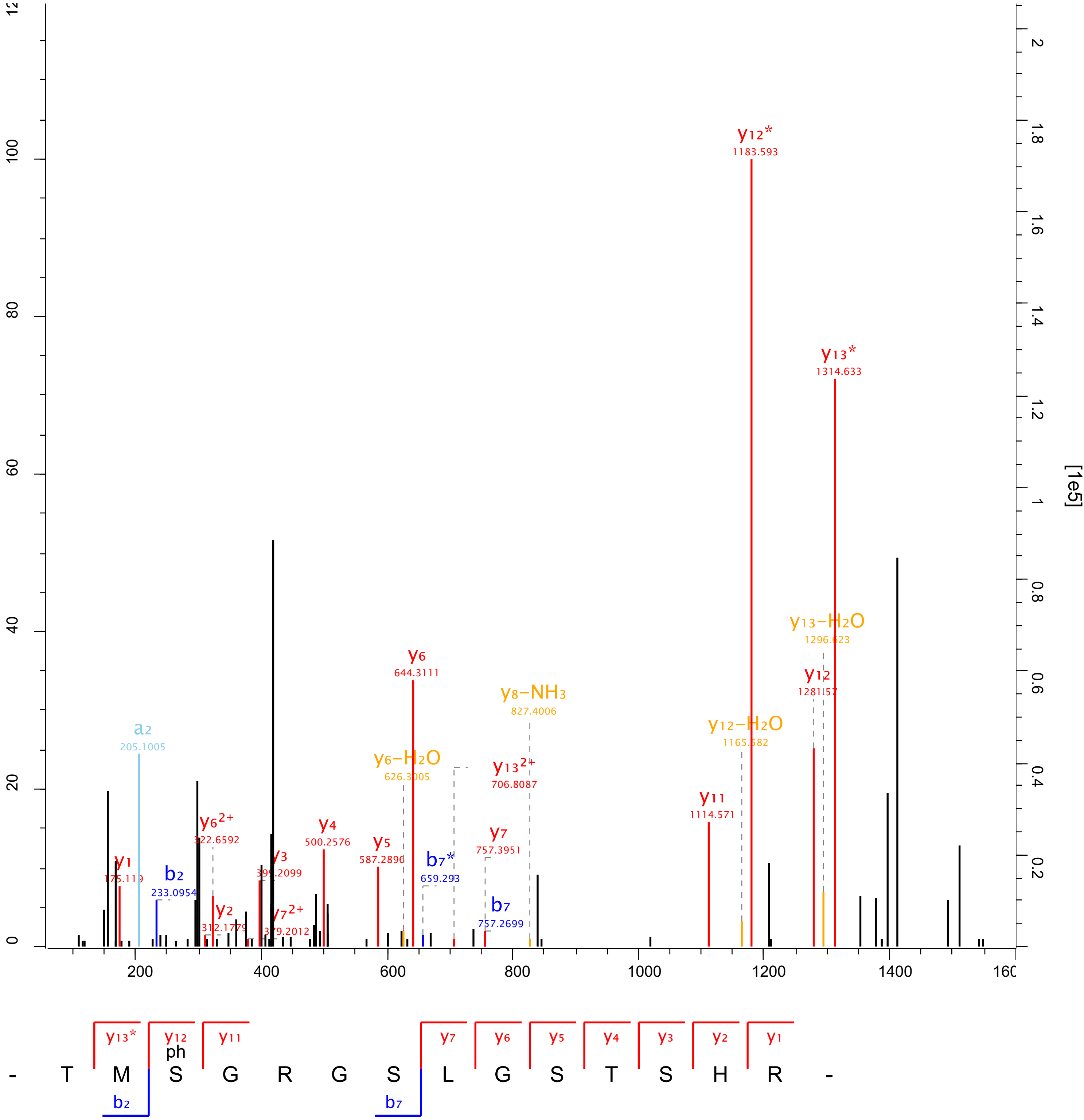

Sample output to test PDF Combine only

Raw file	Scan	Method	Score	m/z	Gene names
sirk1-mic-SUC-2-P	4361	FTMS; HCD	47.55	547.9	At1g10140

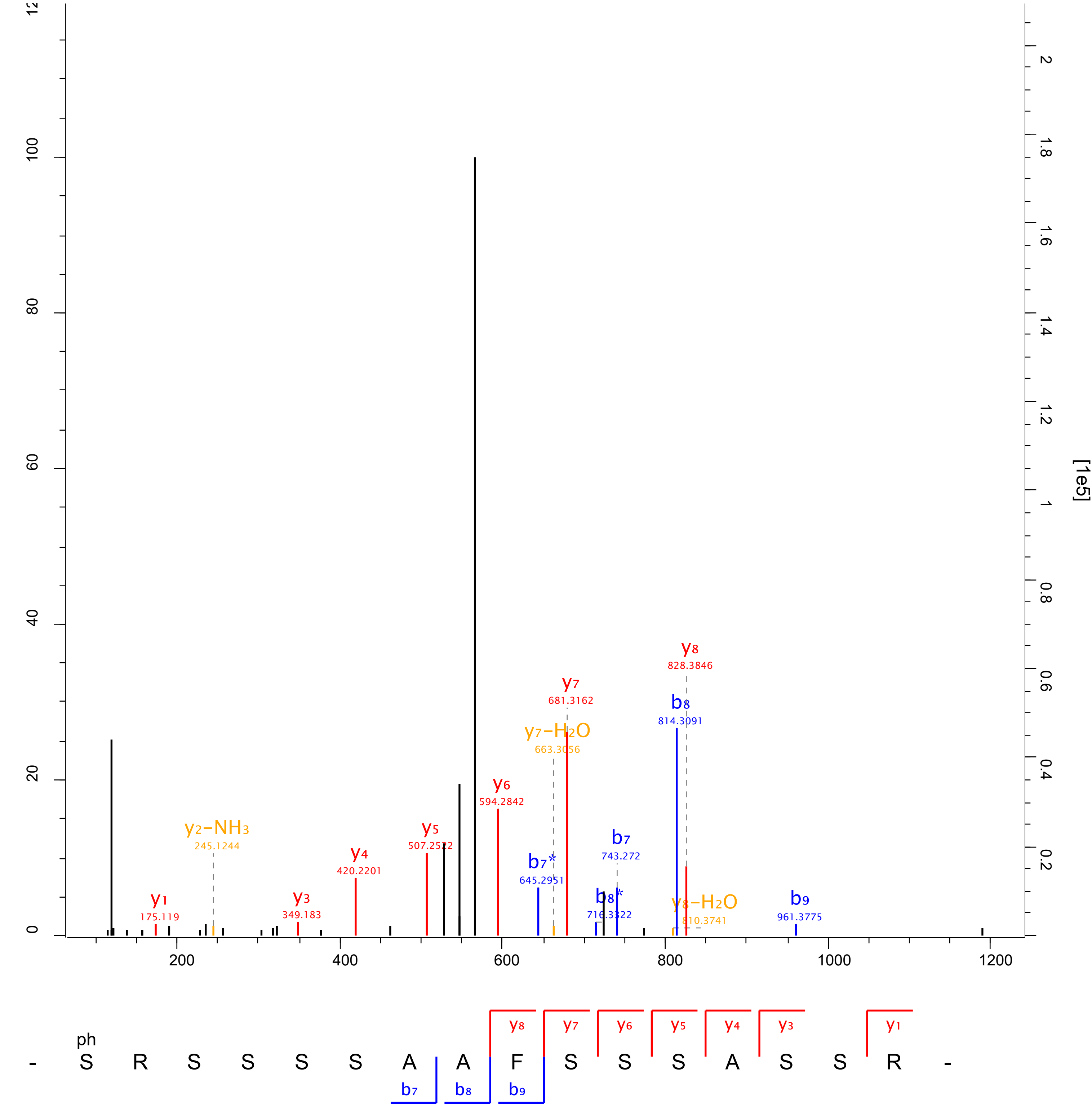

Sample output to test PDF Combine only

Raw file	Scan	Method	Score	m/z	Gene names
sirk1-mic-SUC-2-P	6866	FTMS; HCD	76.1	473.73	F18B13.26

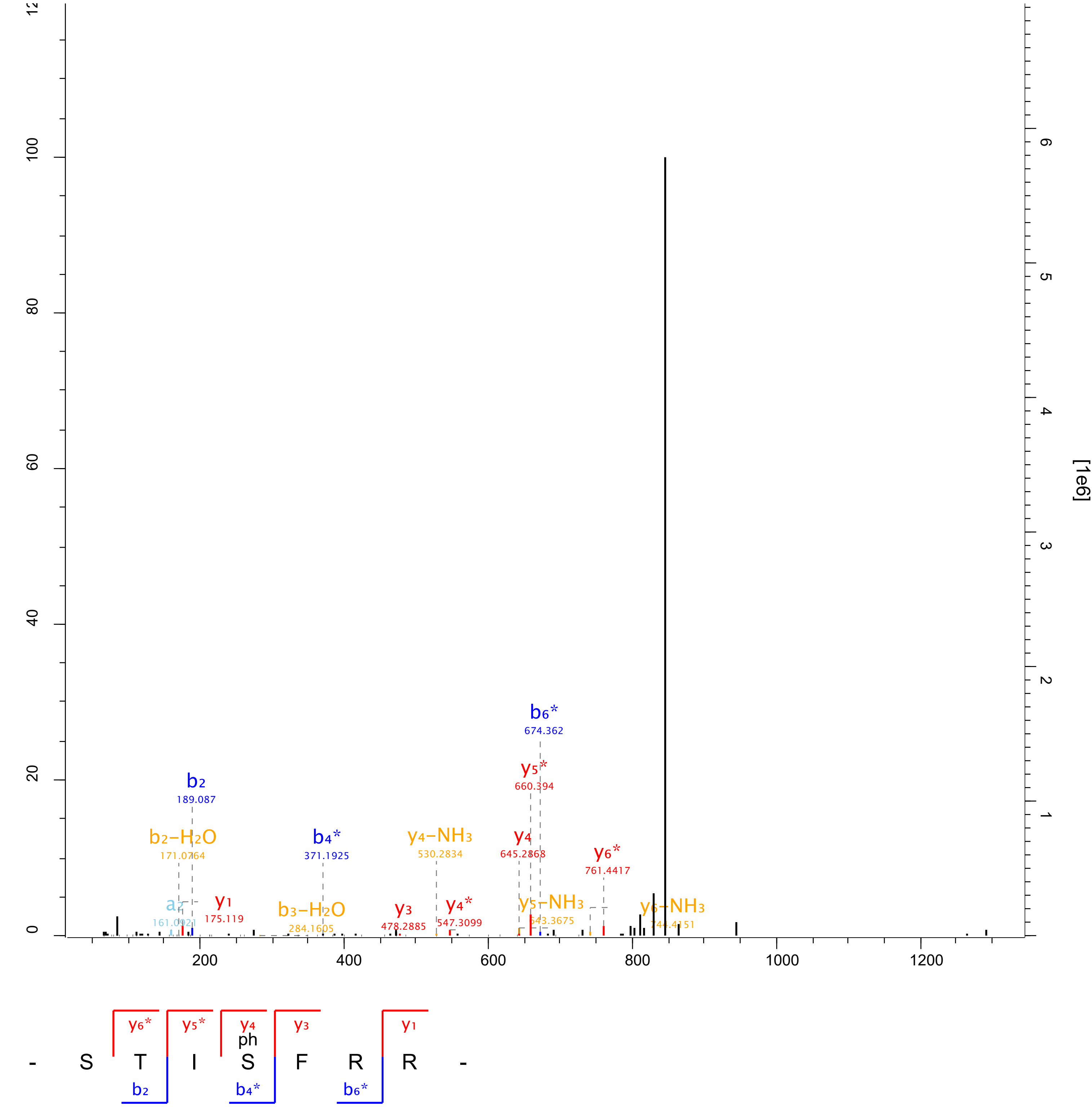

Sample output to test PDF Combine only

Raw file	Scan	Method	Score	m/z	Gene names
sirk1-mic-SUC-2-P	8390	FTMS; HCD	46.49	614.56	ELF5A-3

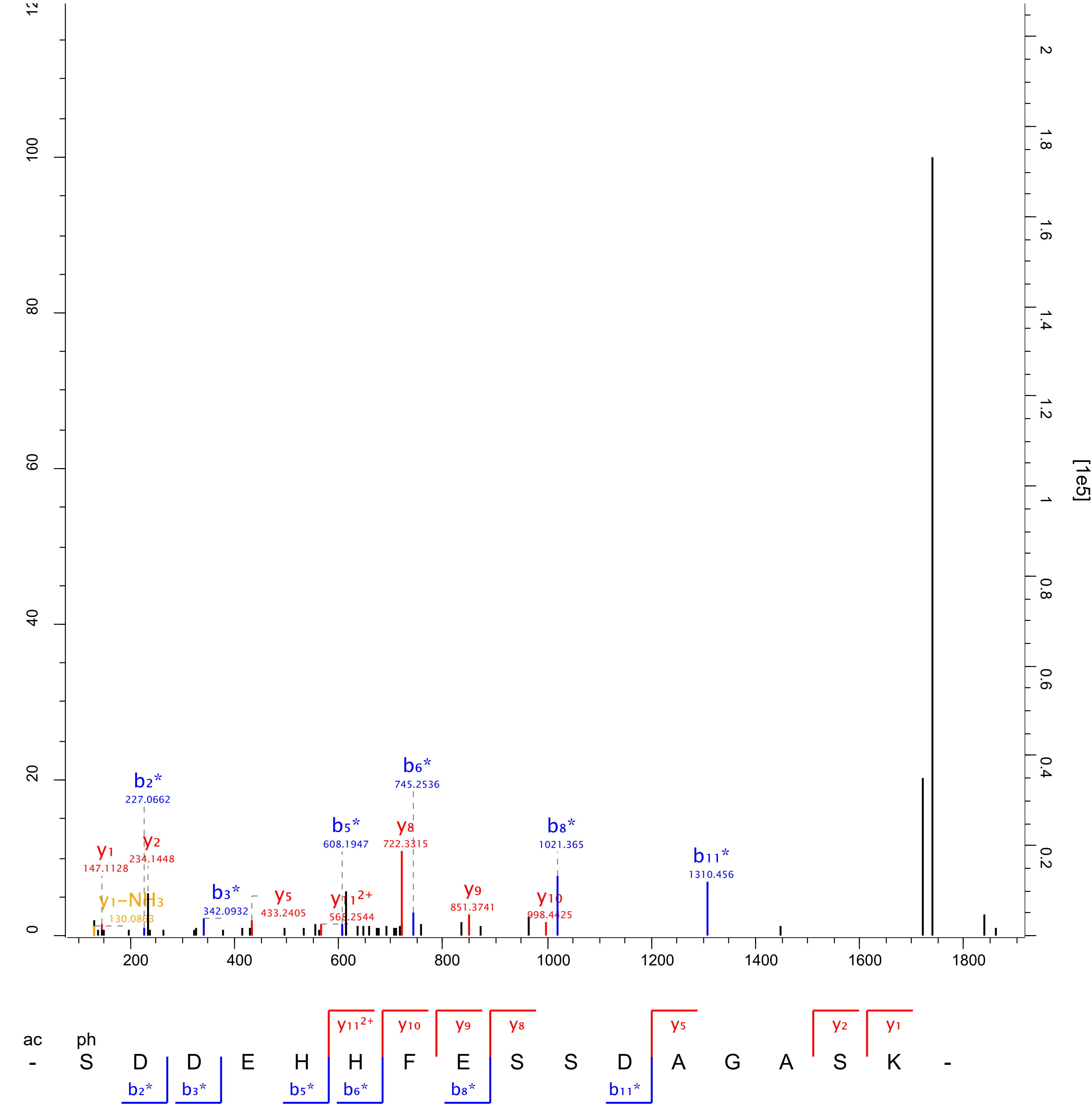

Sample output to test PDF Combine only

Raw file	Scan	Method	Score	m/z
sirk1-mic-SUC-2-P	9329	FTMS; HCD	54.1	539.6

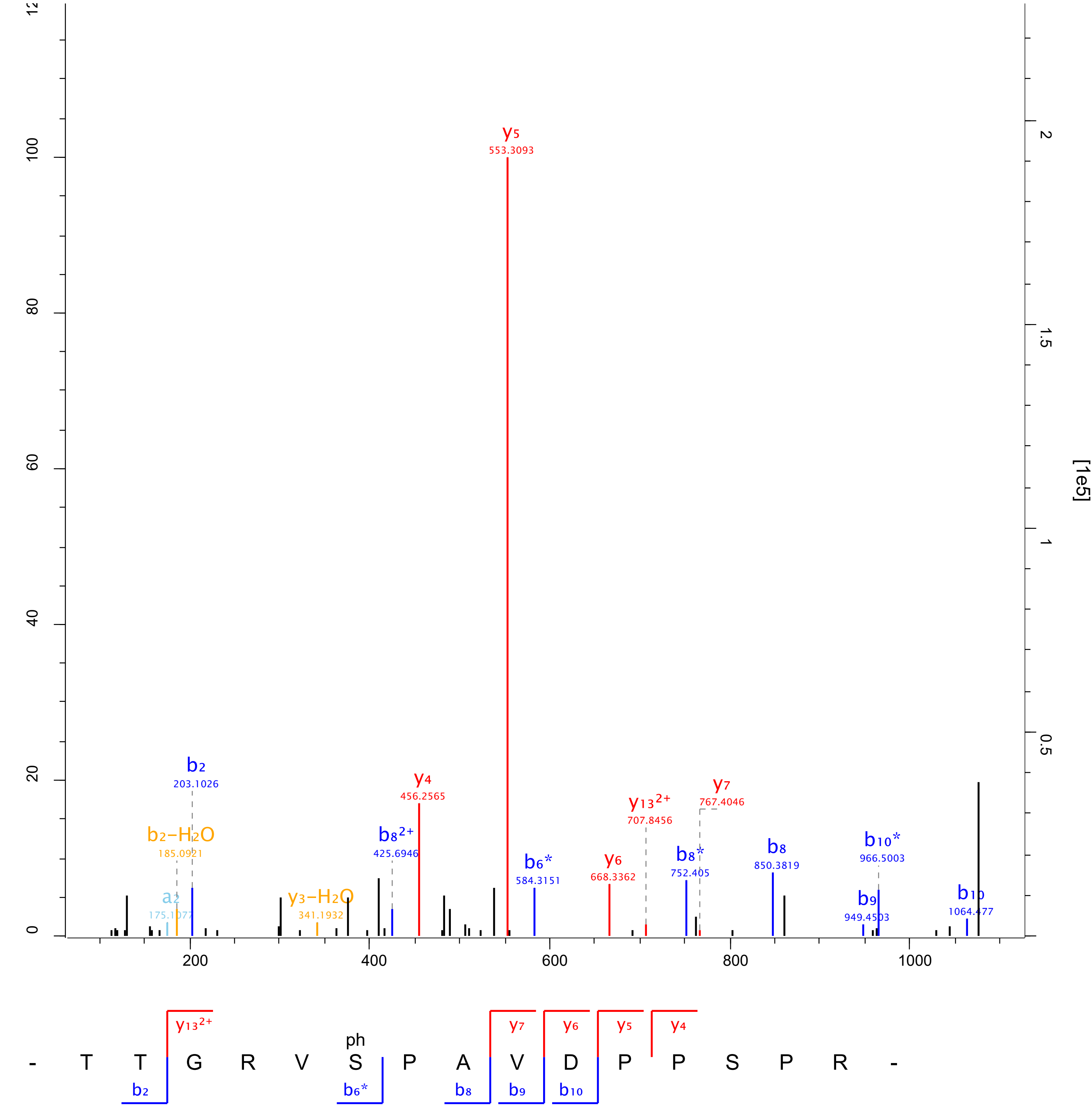

Sample output to test PDF Combine only

Raw file	Scan	Method	Score	m/z	Gene names
sirk1-mic-SUC-2-P	16985	FTMS; HCD	170.11	573.94	AHA1

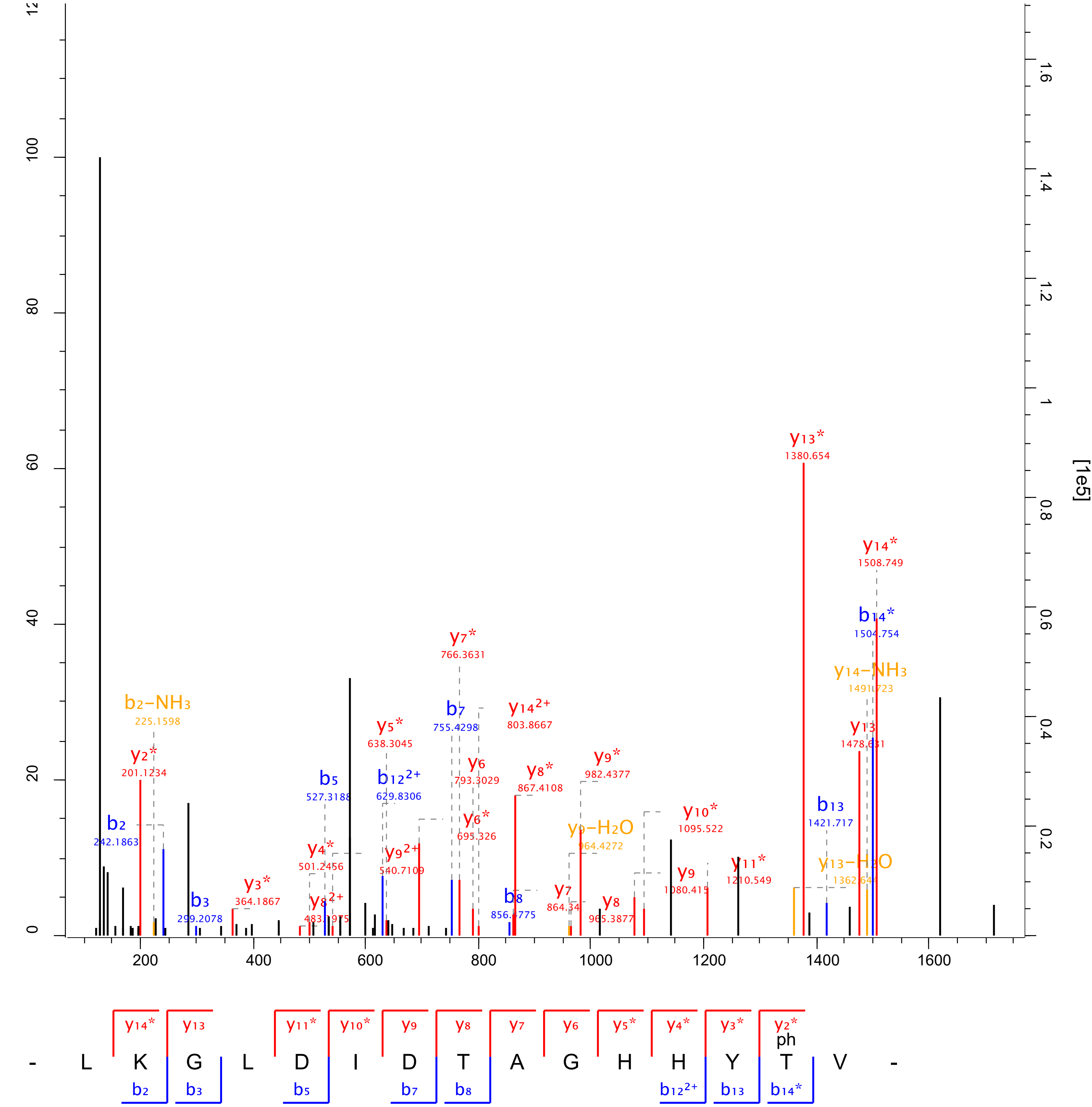

Sample output to test PDF Combine only

Raw file	Scan	Method	Score	m/z	Gene names
sirk1-mic-SUC-2-P	17186	FTMS; HCD	69.86	507.26	CAX4

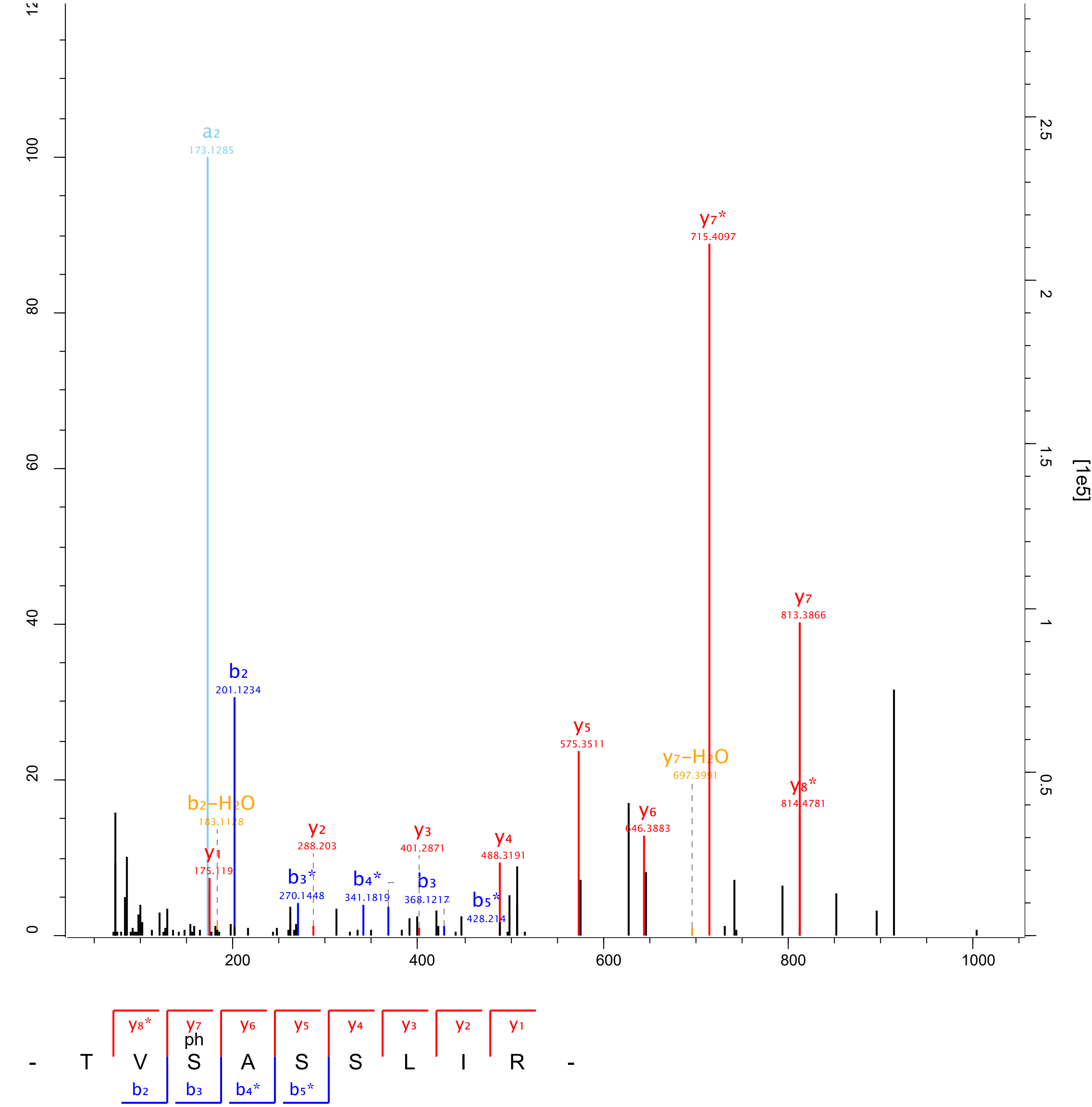

Sample output to test PDF Combine only

Raw file	Scan	Method	Score	m/z
sirk1-mic-SUC-2-P	18766	FTMS; HCD	65.04	575.31

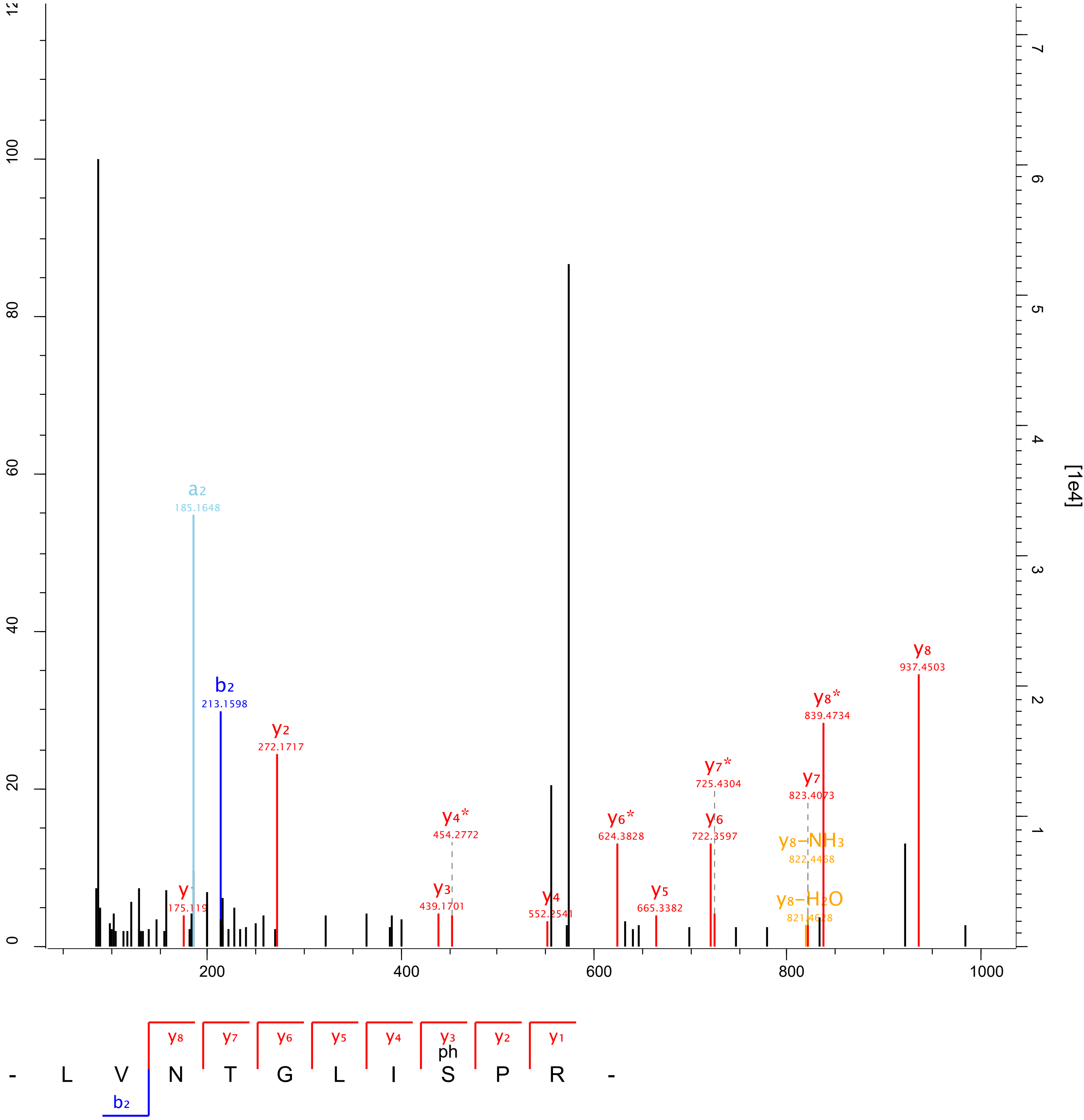

Sample output to test PDF Combine only

Raw file	Scan	Method	Score	m/z	Gene names
sirk1-mic-SUC-2-P	18959	FTMS; HCD	155.03	594.77	PIP2-1;PIP2-3;PIP2-2

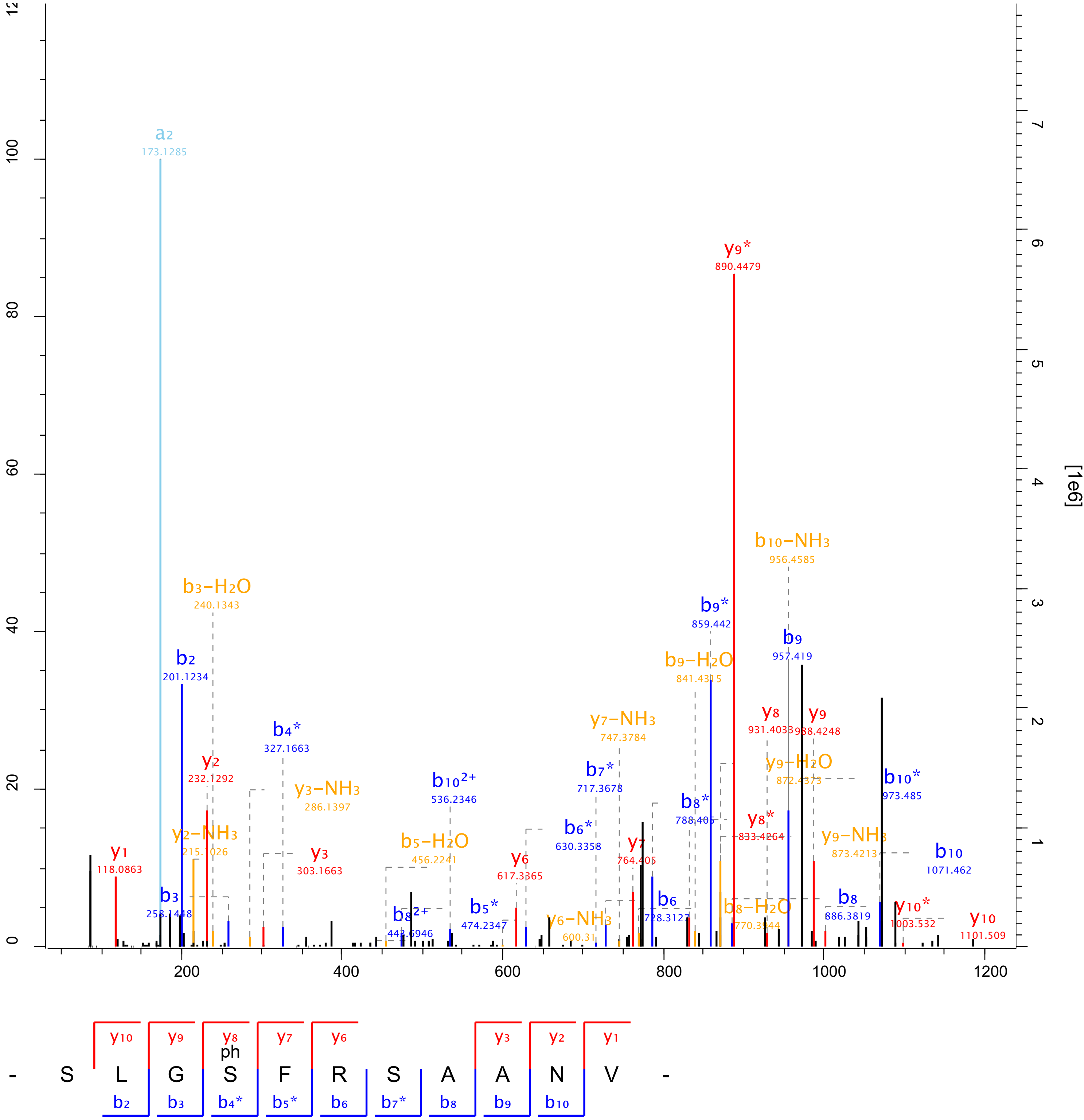

- S L G S F R S A A N V -

Sample output to test PDF Combine only

Raw file	Scan	Method	Score	m/z
sirk1-mic-SUC-2-P	26035	FTMS; HCD	59.37	802.38

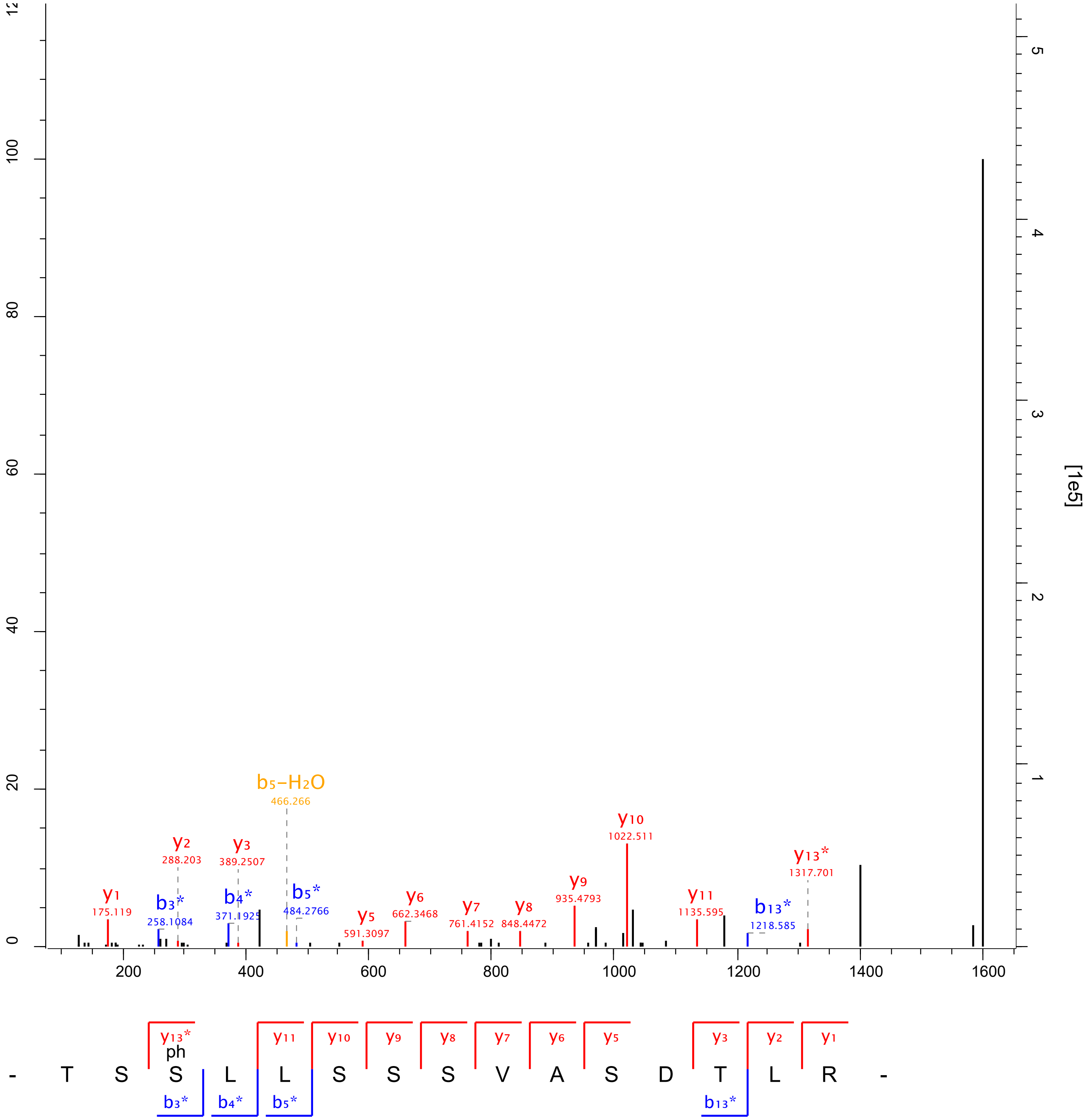

Sample output to test PDF Combine only

Raw file	Scan	Method	Score	m/z	Gene names
sirk1-mic-SUC-2-P	26113	FTMS; HCD	61.48	599.35	SAC5

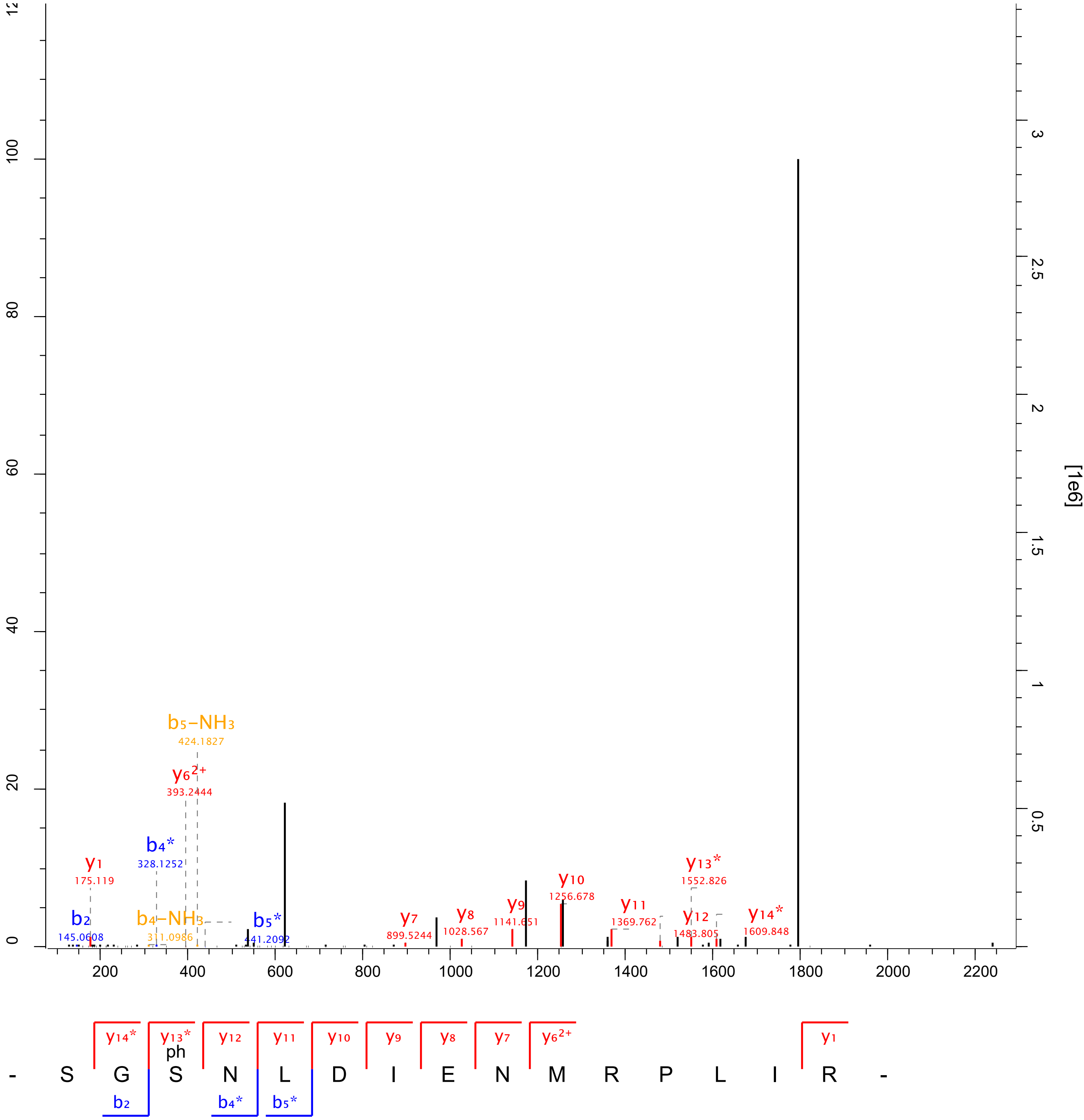

Sample output to test PDF Combine only

Raw file	Scan	Method	Score	m/z	Gene names
sirk1-mic-SUC-2-P	27677	FTMS; HCD	72.47	741.73	CLC-C

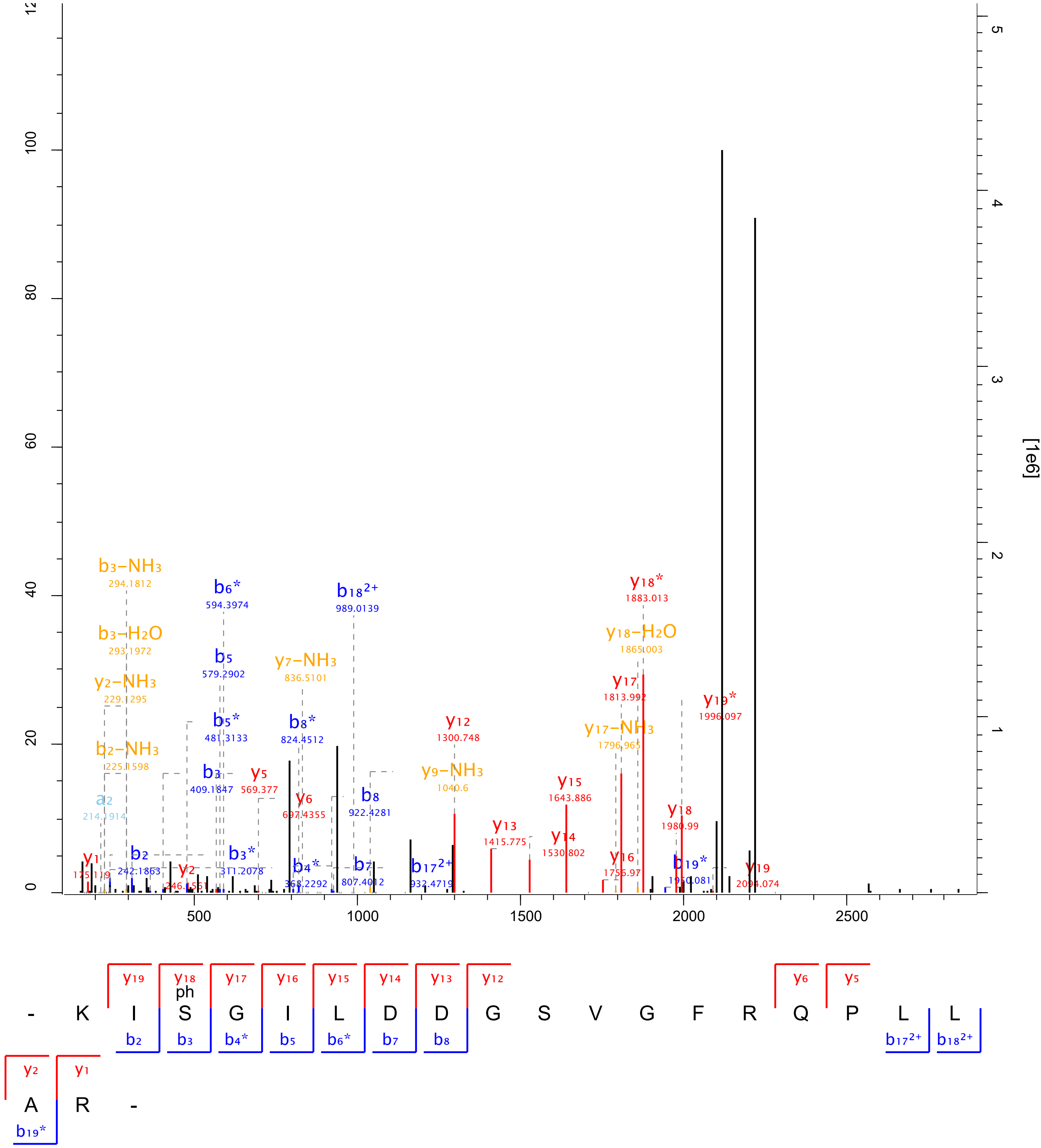

Sample output to test PDF Combine only

Raw file	Scan	Method	Score	m/z	Gene names
sirk1-mic-SUC-2-P	33224	FTMS; HCD	42.84	667.78	PAO5

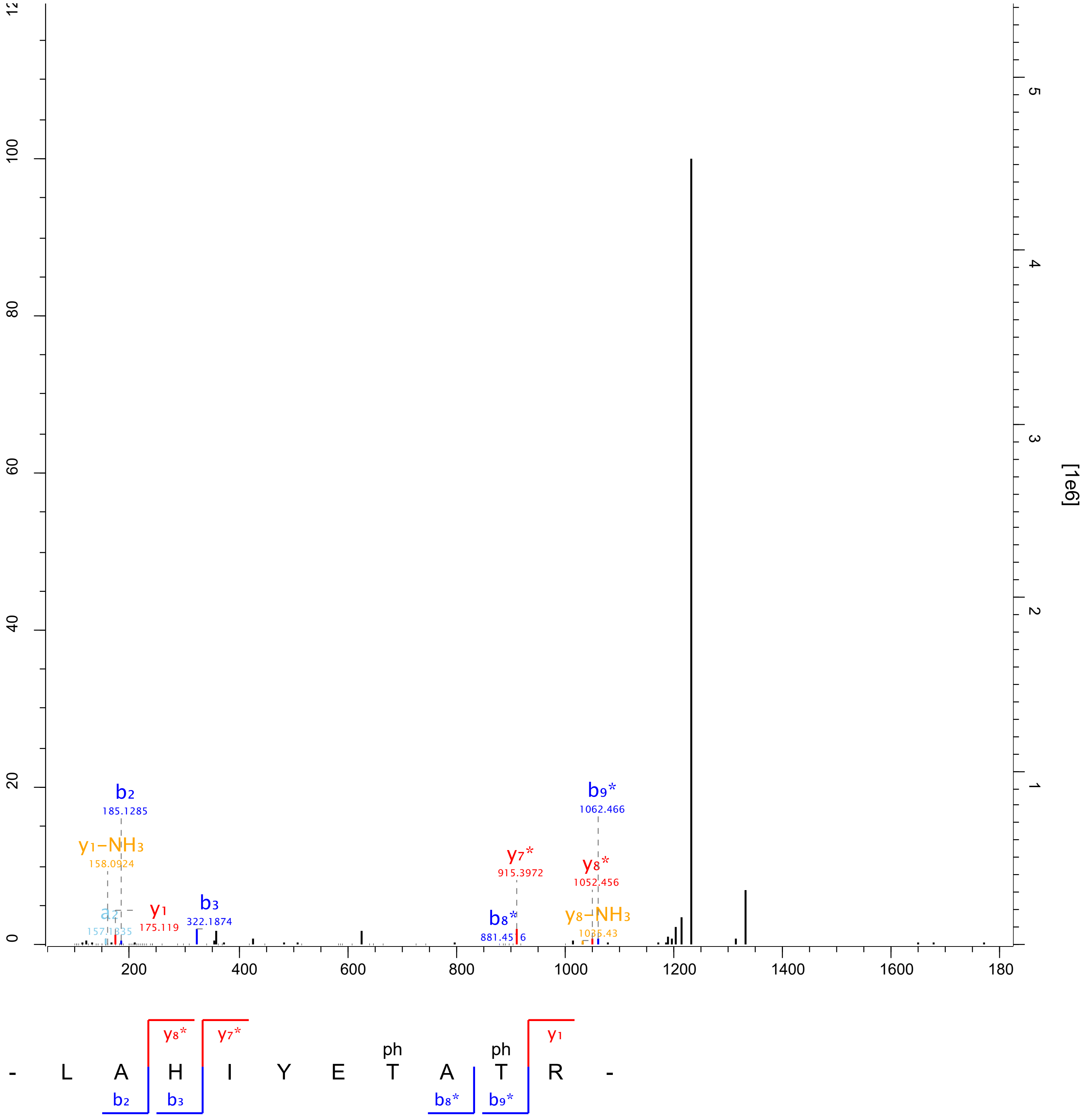

Sample output to test PDF Combine only

Raw file	Scan	Method	Score	m/z	Gene names
sirk1-mic-SUC-2-P	38992	FTMS; HCD	219.87	809.36	PIP2-4

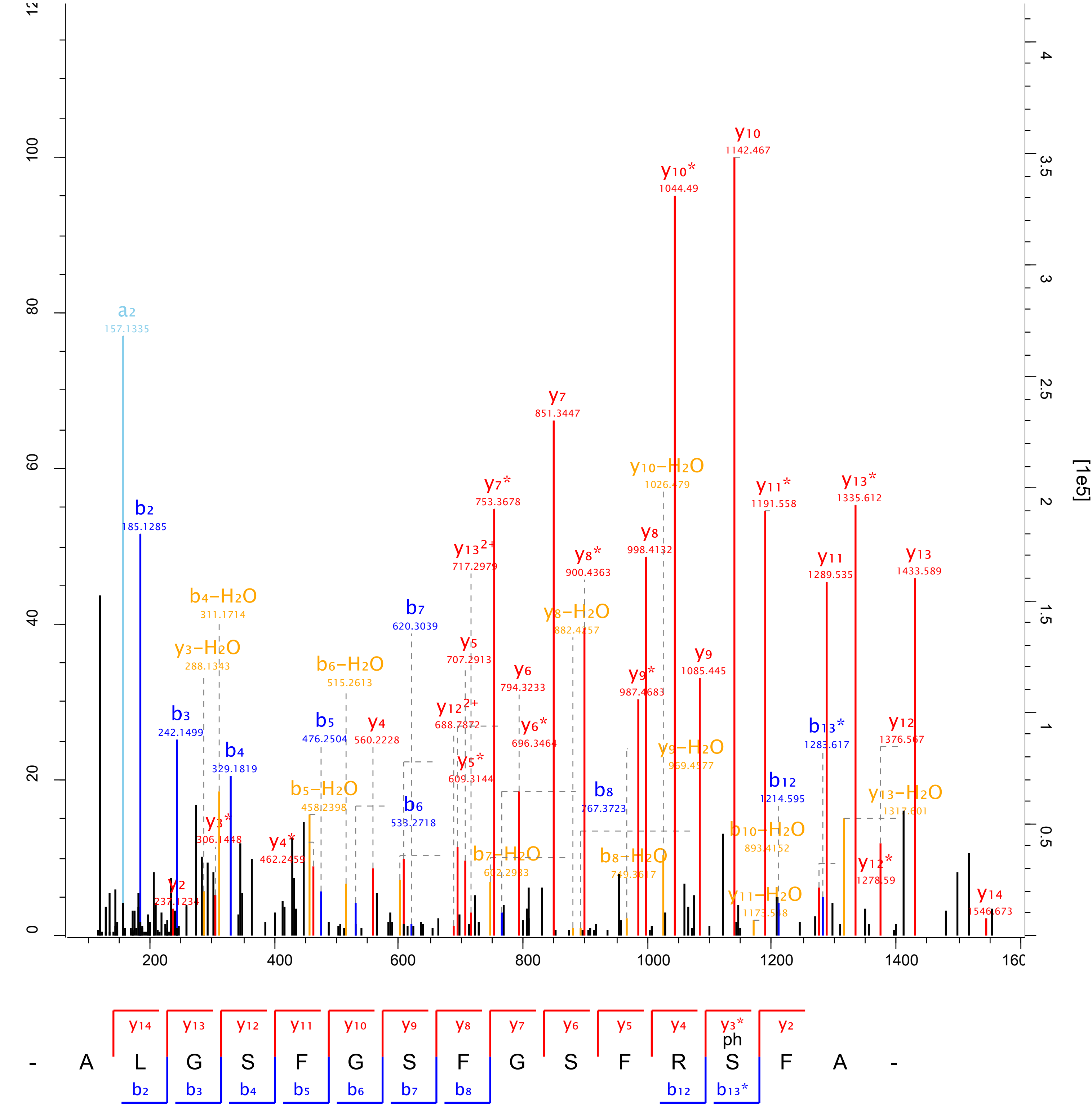

Sample output to test PDF Combine only

Raw file	Scan	Method	Score	m/z	Gene names
sirk1-mic-SUC-3-A	3380	FTMS; HCD	172.37	591.59	At2g04410

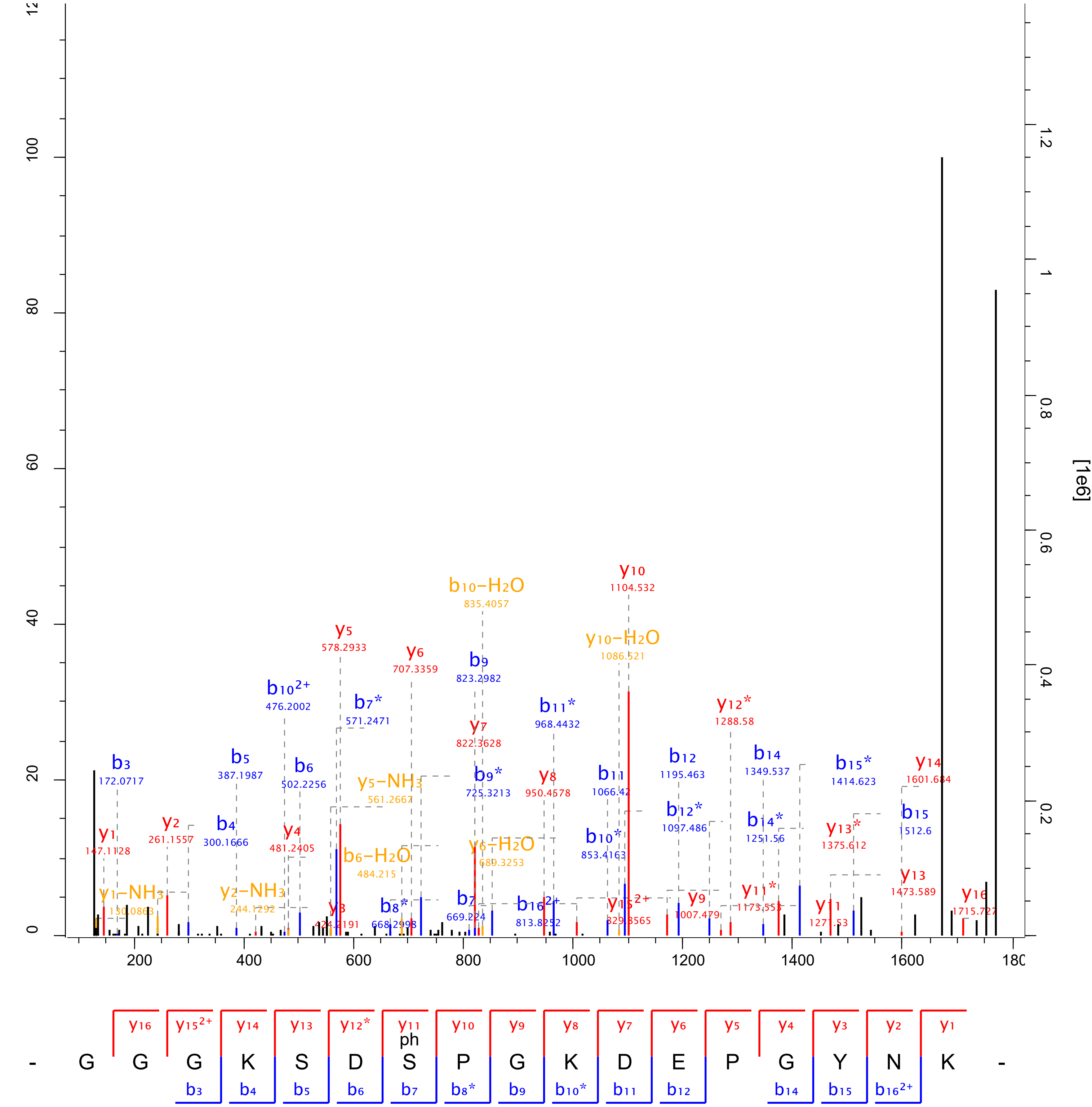

Sample output to test PDF Combine only

Raw file	Scan	Method	Score	m/z	Gene names
sirk1-mic-SUC-3-A	3540	FTMS; HCD	69.34	468.9	AGD5

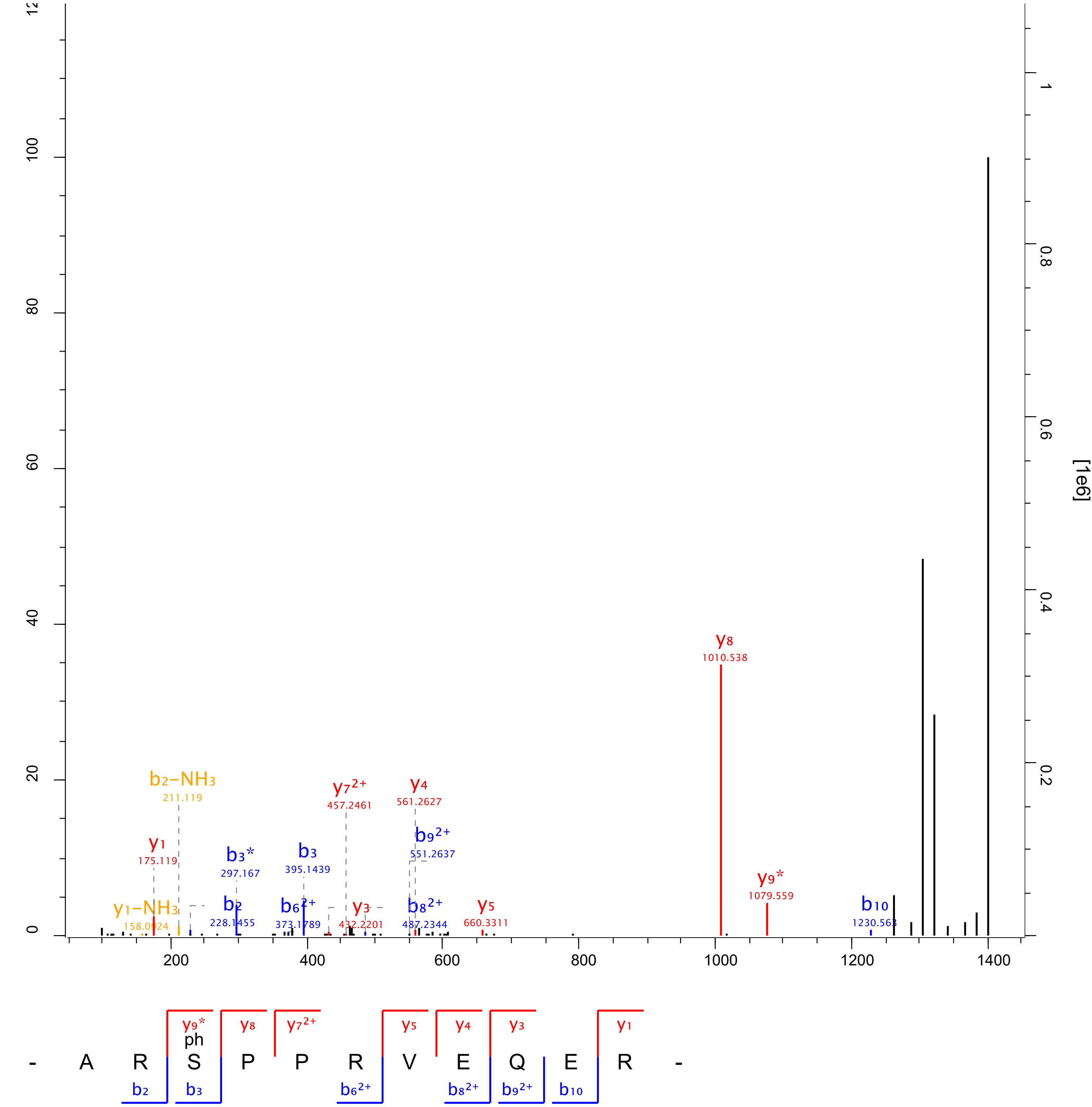

Sample output to test PDF Combine only

Raw file	Scan	Method	Score	m/z	Gene names
sirk1-mic-SUC-3-A	3570	FTMS; HCD	137.61	527.89	CDKC2;CDKC-1;CDKC-2

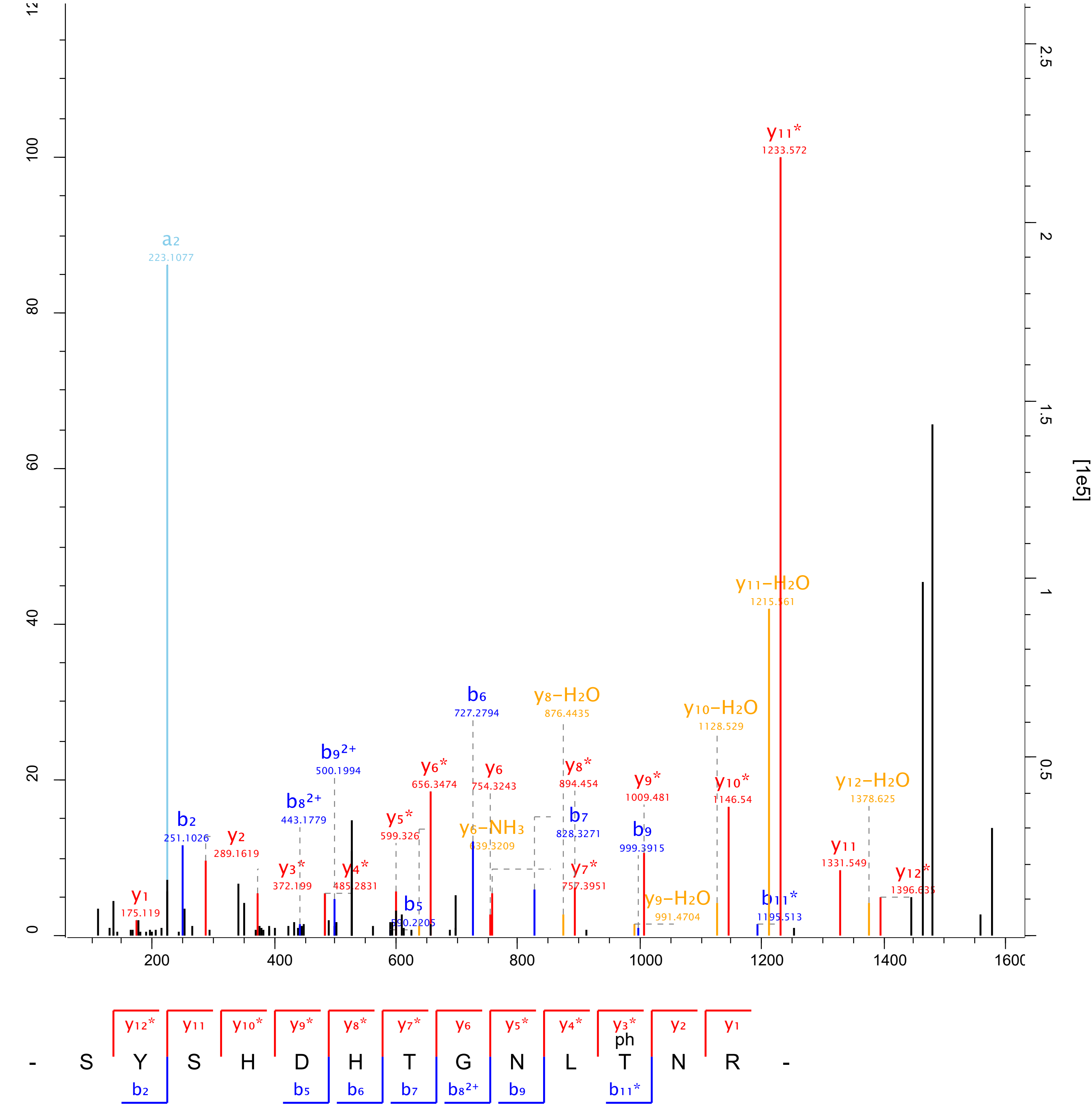

Sample output to test PDF Combine only

Raw file	Scan	Method	Score	m/z	Gene names
sirk1-mic-SUC-3-A	3733	FTMS; HCD	87.82	510.23	F23A5.29

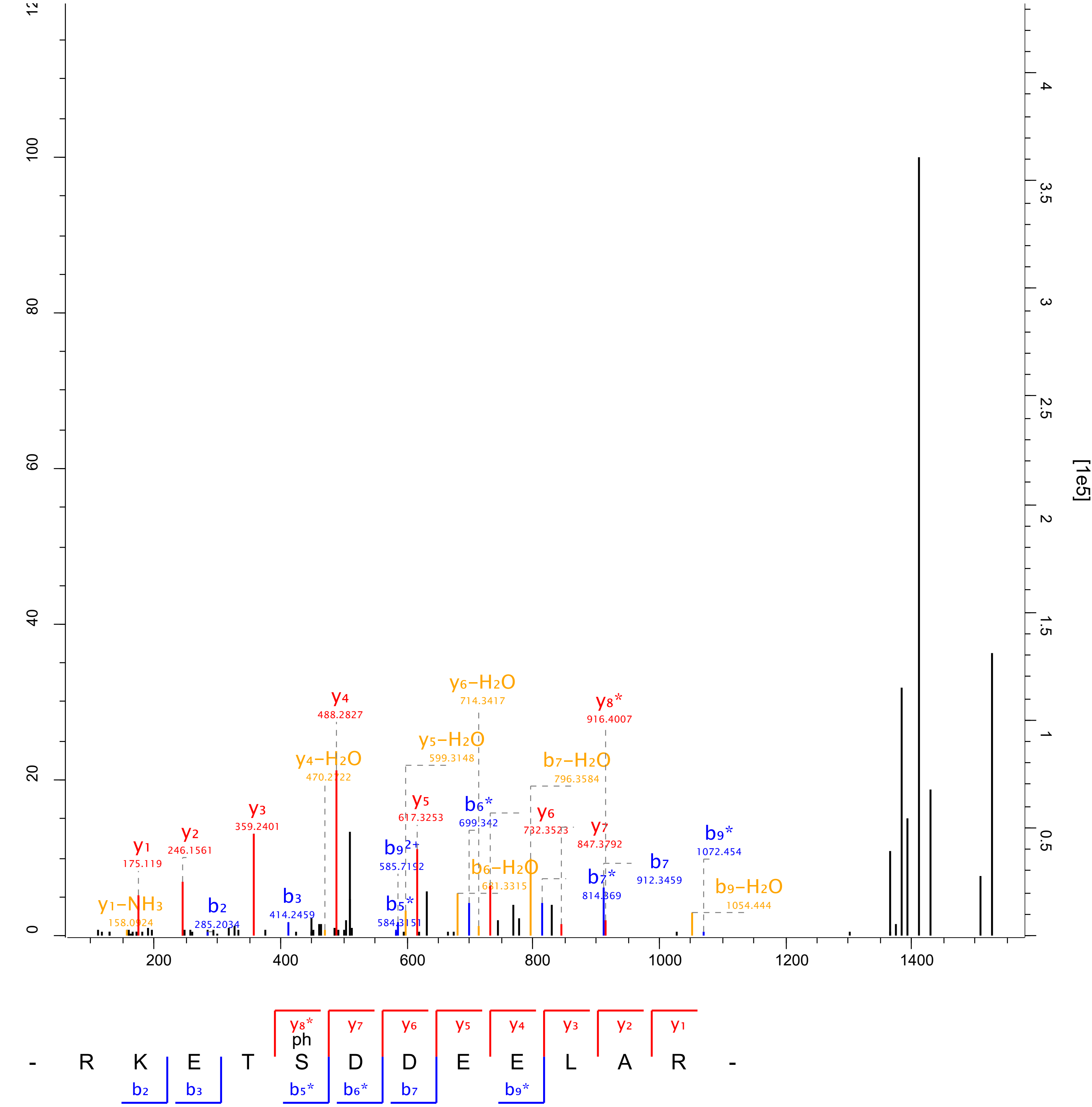

Sample output to test PDF Combine only

Raw file	Scan	Method	Score	m/z	Gene names
sirk1-mic-SUC-3-A	3918	FTMS; HCD	58.02	575.9	RS2Z33

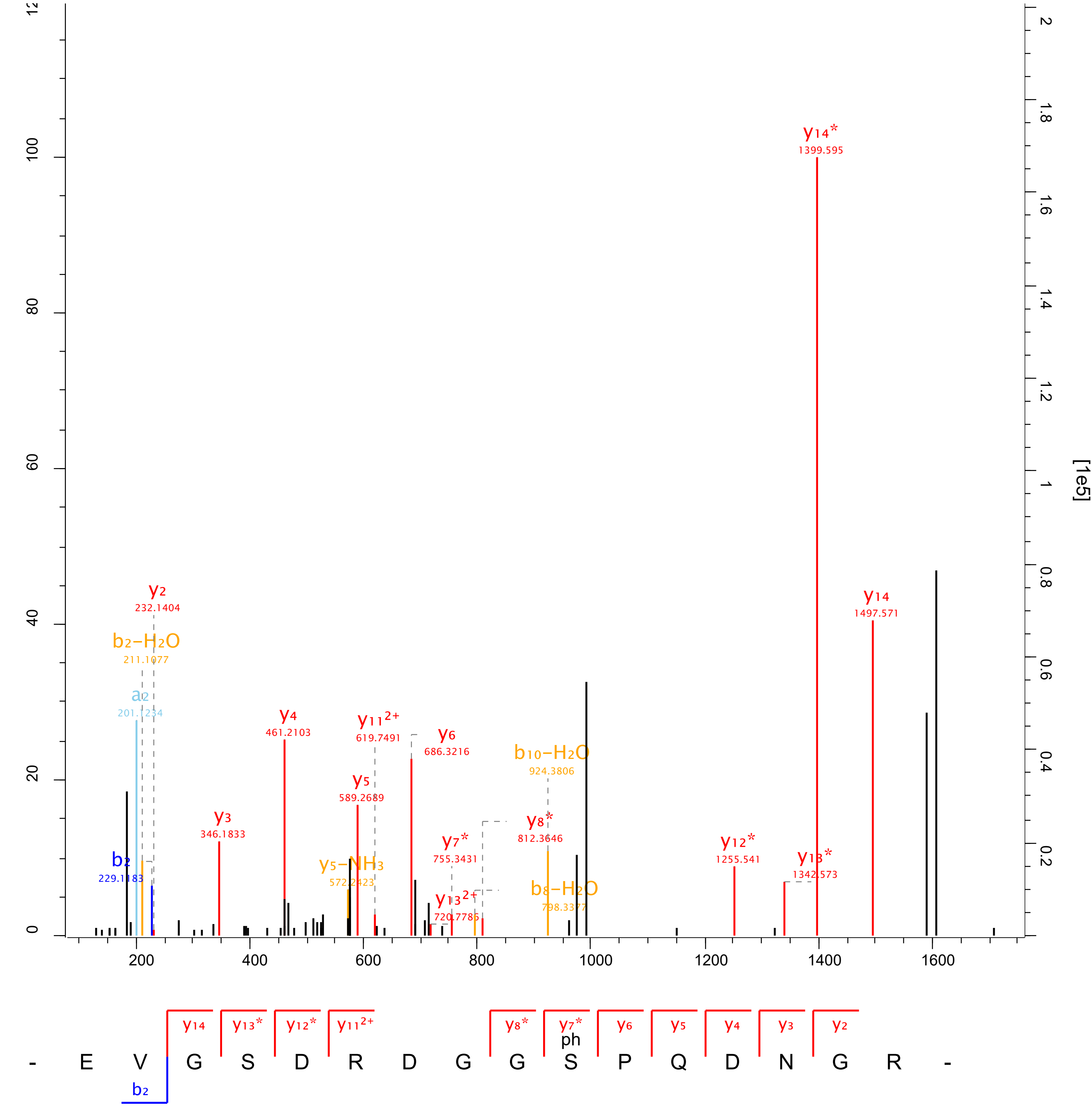

Sample output to test PDF Combine only

Raw file	Scan	Method	Score	m/z	Gene names
sirk1-mic-SUC-3-A	4141	FTMS; HCD	48.91	646.27	At2g07360

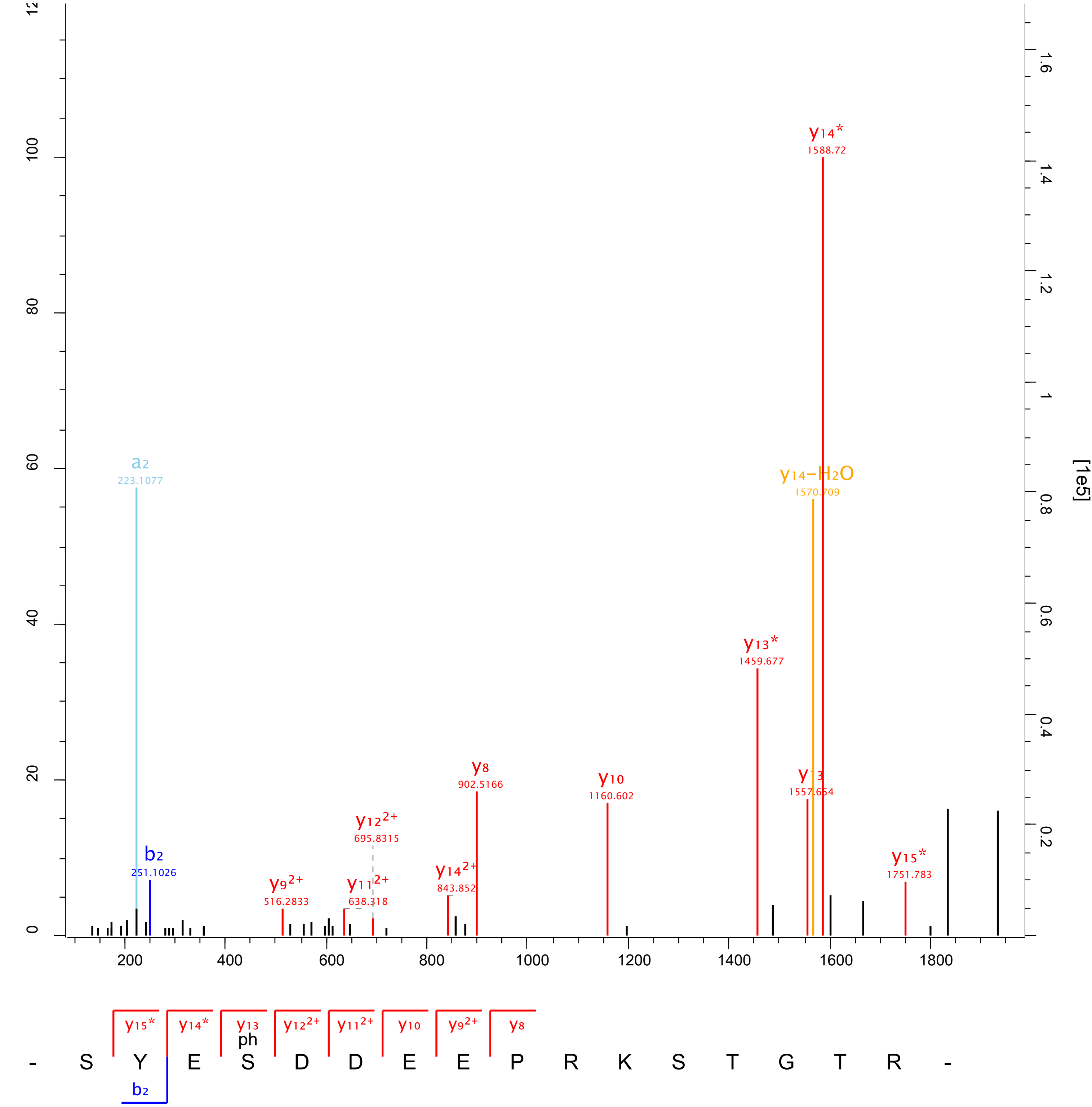

Sample output to test PDF Combine only

Raw file	Scan	Method	Score	m/z	Gene names
sirk1-mic-SUC-3-A	4979	FTMS; HCD	48.62	524.97	F23A5.29

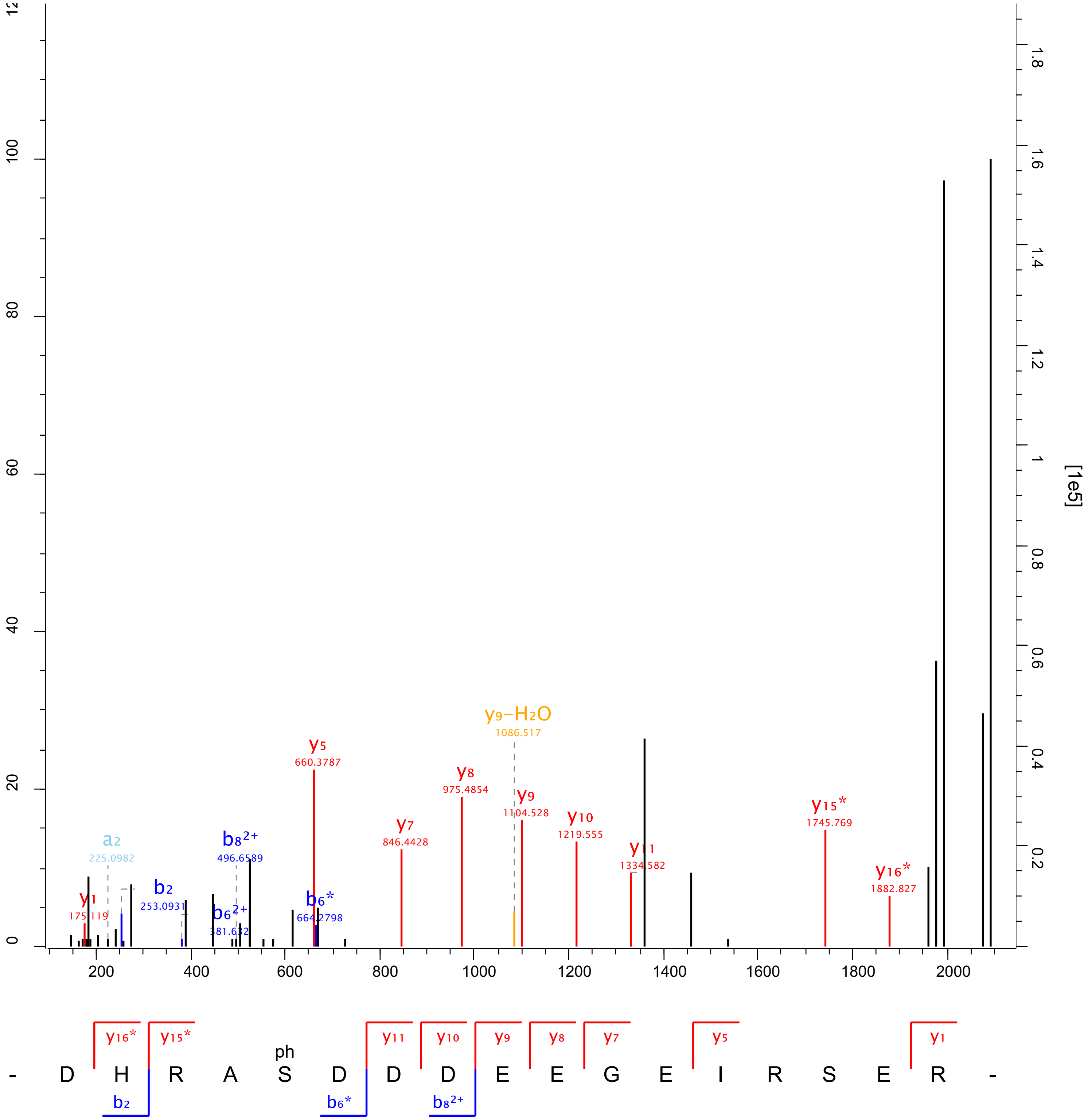

Sample output to test PDF Combine only

Raw file	Scan	Method	Score	m/z	Gene names
sirk1-mic-SUC-3-A	5166	FTMS; HCD	86.5	455.55	RS41

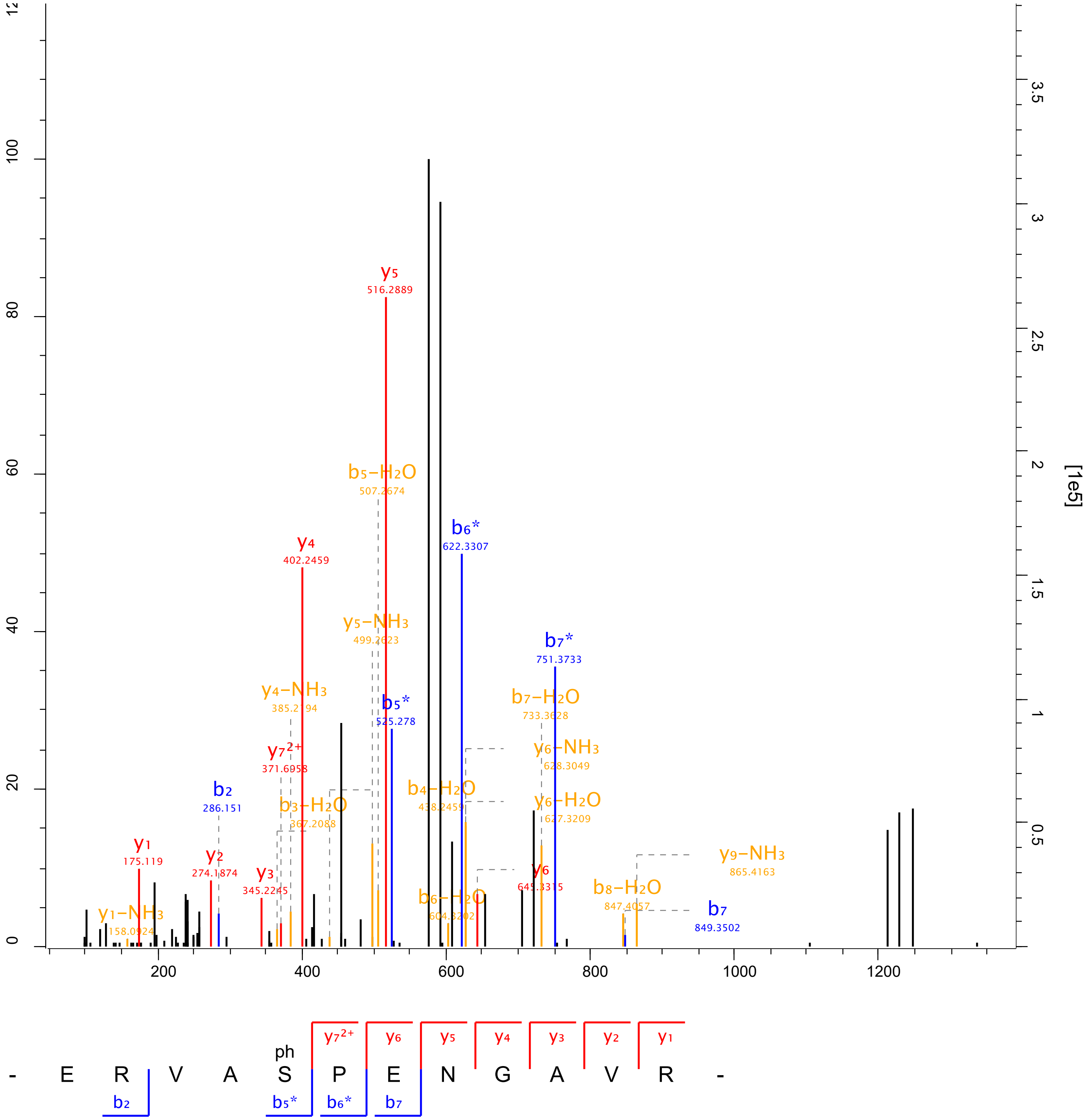

Sample output to test PDF Combine only

Raw file	Scan	Method	Score	m/z	Gene names
sirk1-mic-SUC-3-A	6134	FTMS; HCD	64.27	437.97	At5g37710

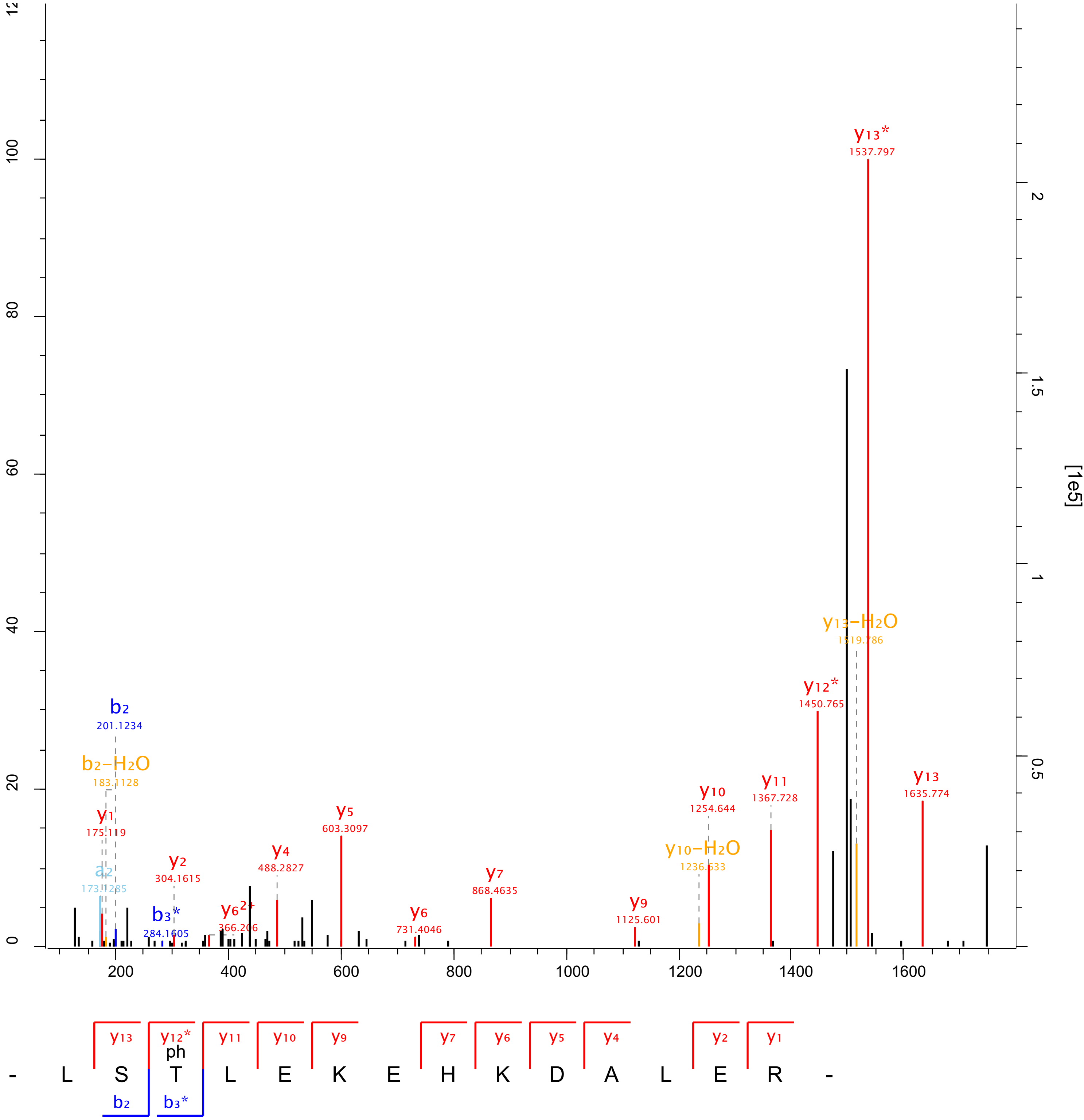

Sample output to test PDF Combine only

Raw file	Scan	Method	Score	m/z
sirk1-mic-SUC-3-A	7504	FTMS; HCD	58.09	635.29

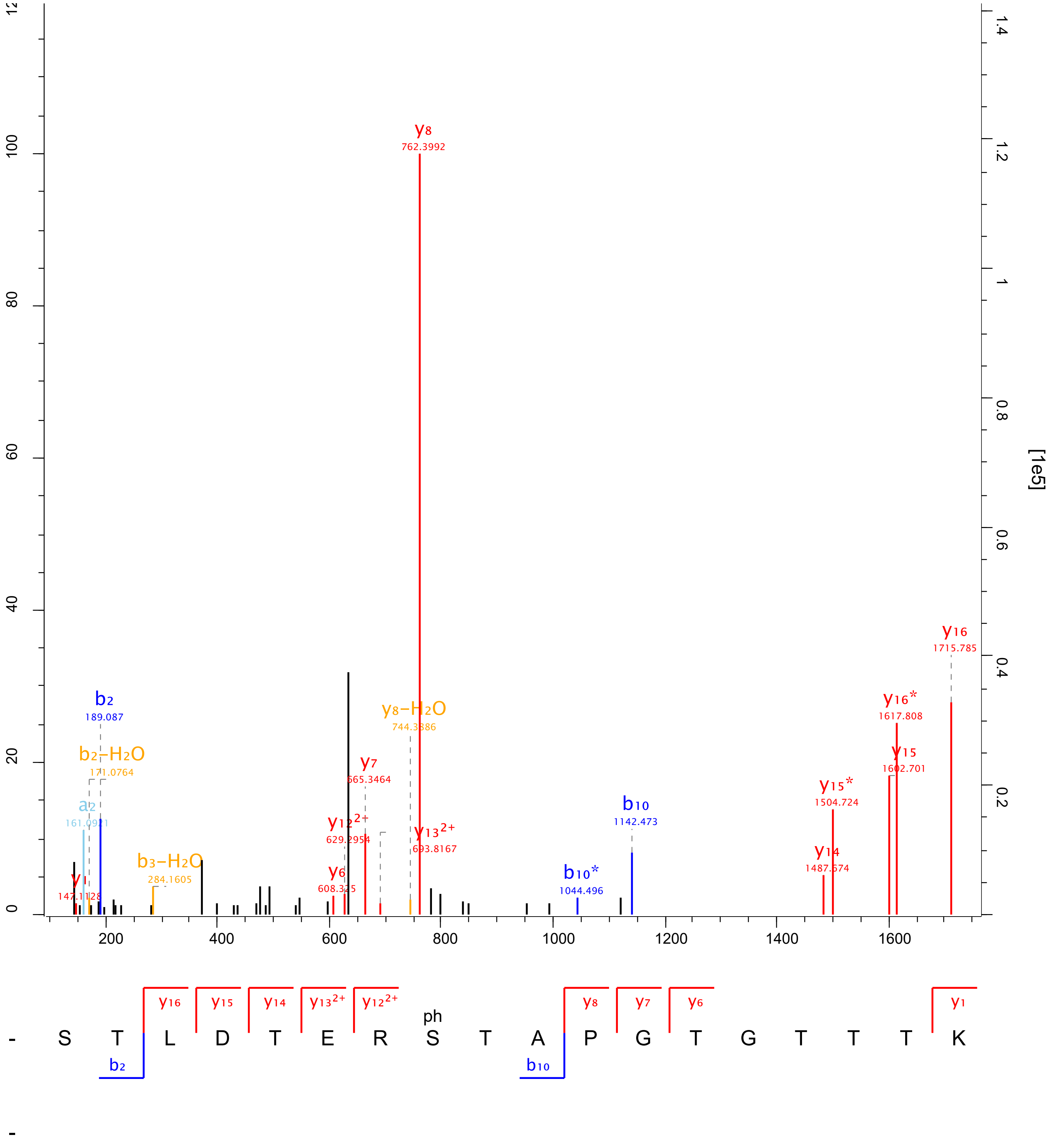

Sample output to test PDF Combine only

Raw file	Scan	Method	Score	m/z	Gene names
sirk1-mic-SUC-3-A	9907	FTMS; HCD	238.19	618.9	ELF5A-2

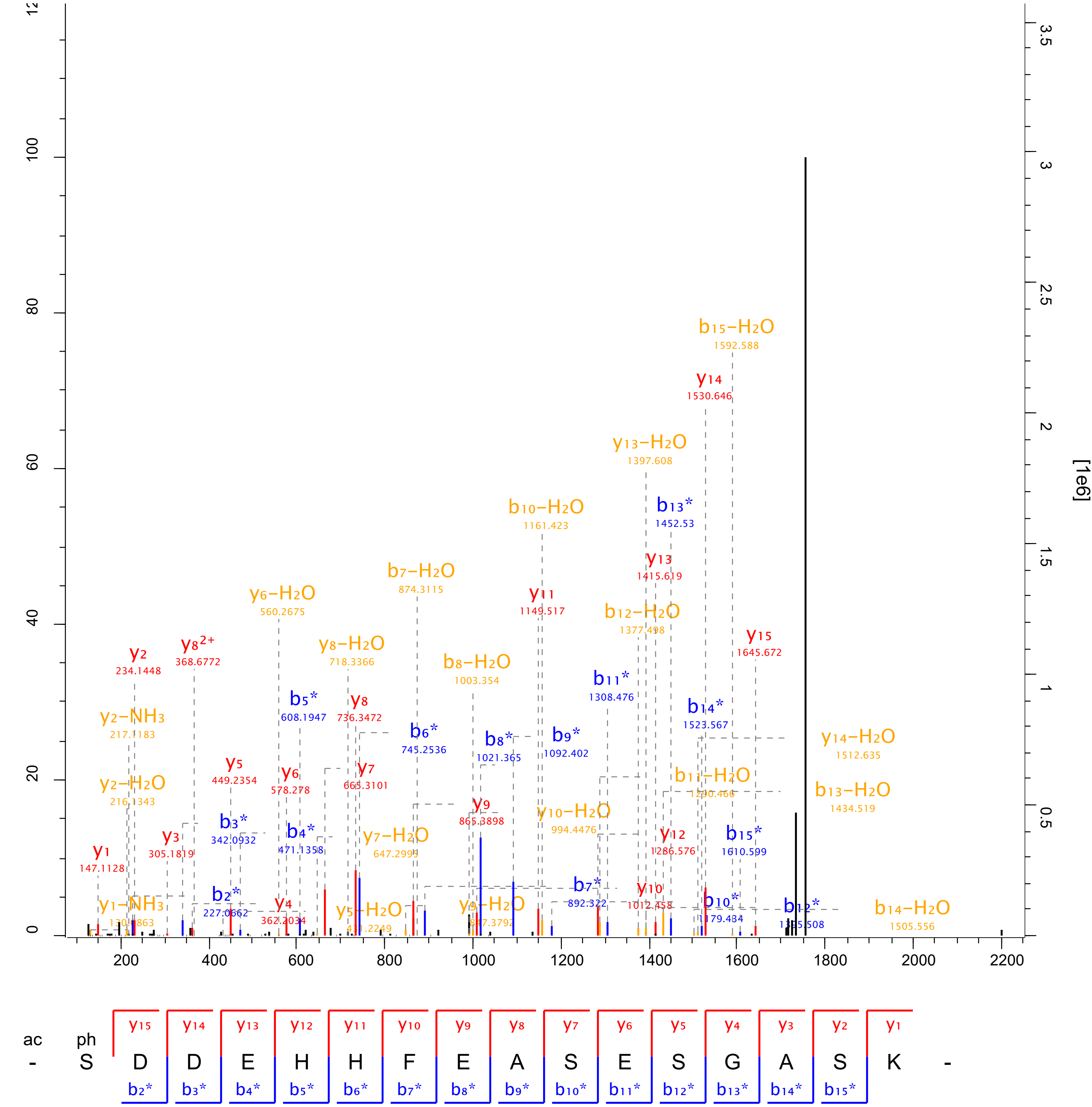

Sample output to test PDF Combine only

Raw file	Scan	Method	Score	m/z	Gene names
sirk1-mic-SUC-3-A	11551	FTMS; HCD	102.65	461.54	At2g34310

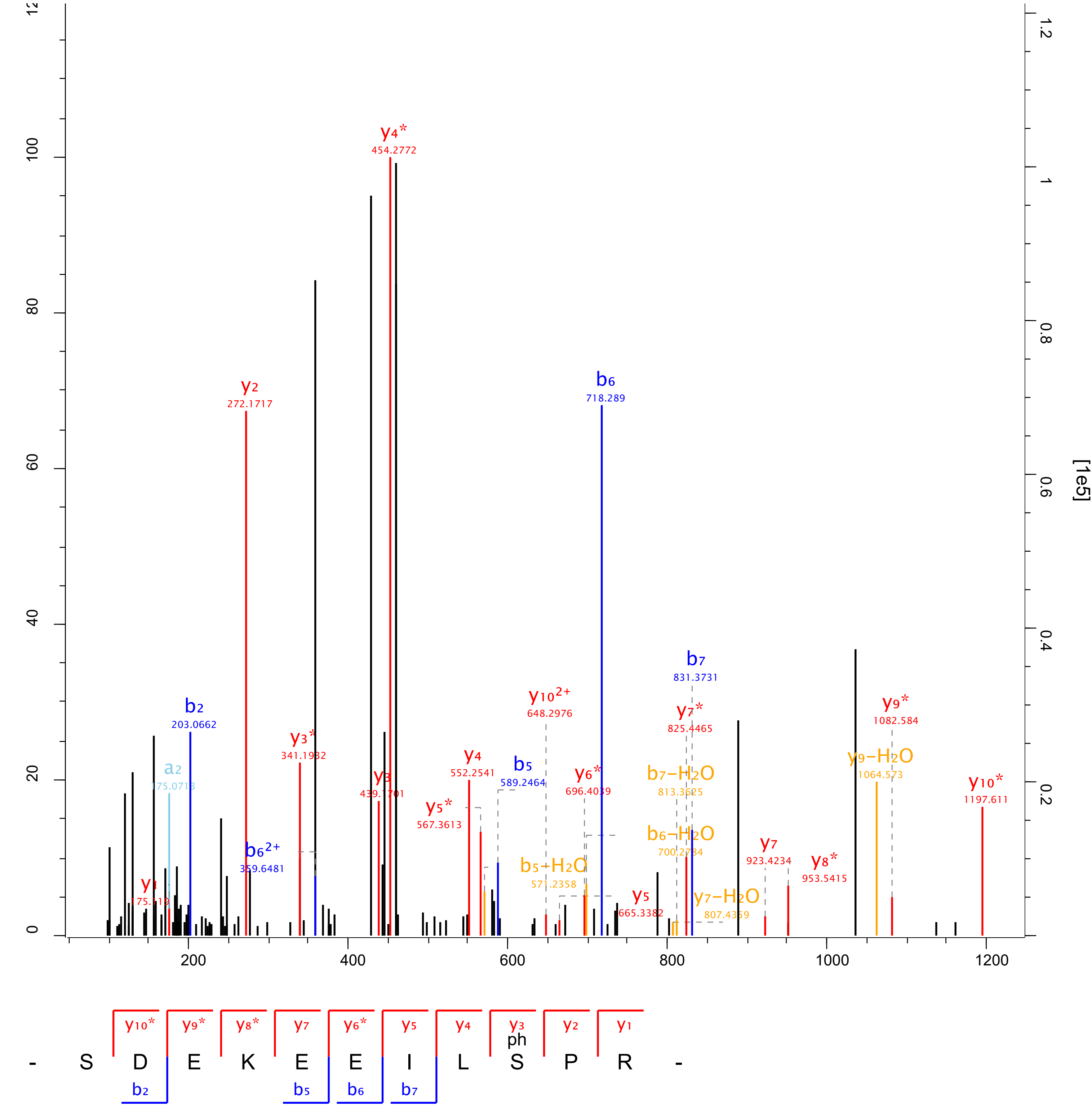

Sample output to test PDF Combine only

Raw file	Scan	Method	Score	m/z	Gene names
sirk1-solu-0-3-A	15390	FTMS; HCD	119.39	592.79	SOUL-1

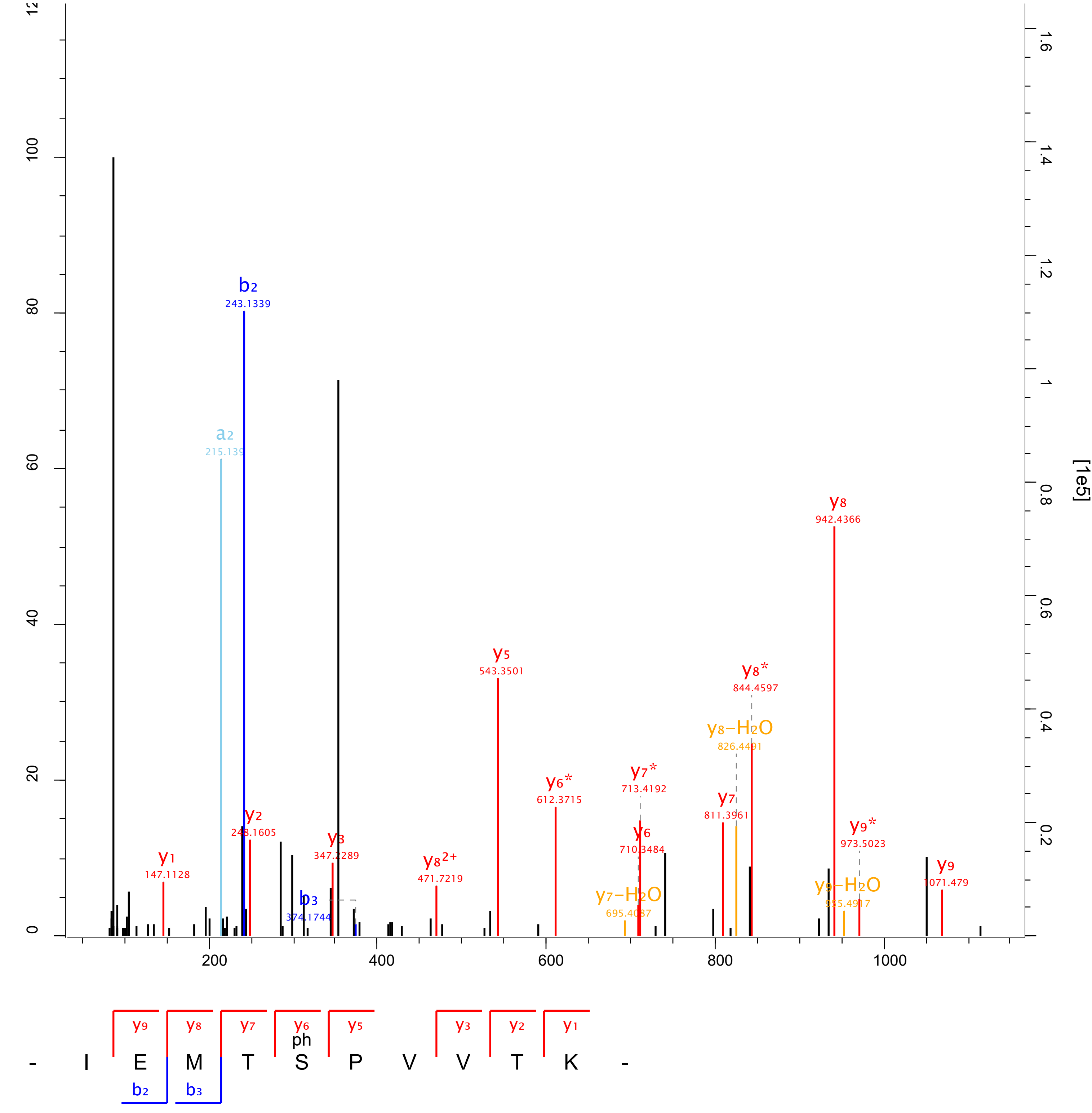

Sample output to test PDF Combine only

Raw file	Scan	Method	Score	m/z	Gene names
sirk1-solu-0-3-A	16636	FTMS; HCD	43.03	620.61	BAG1

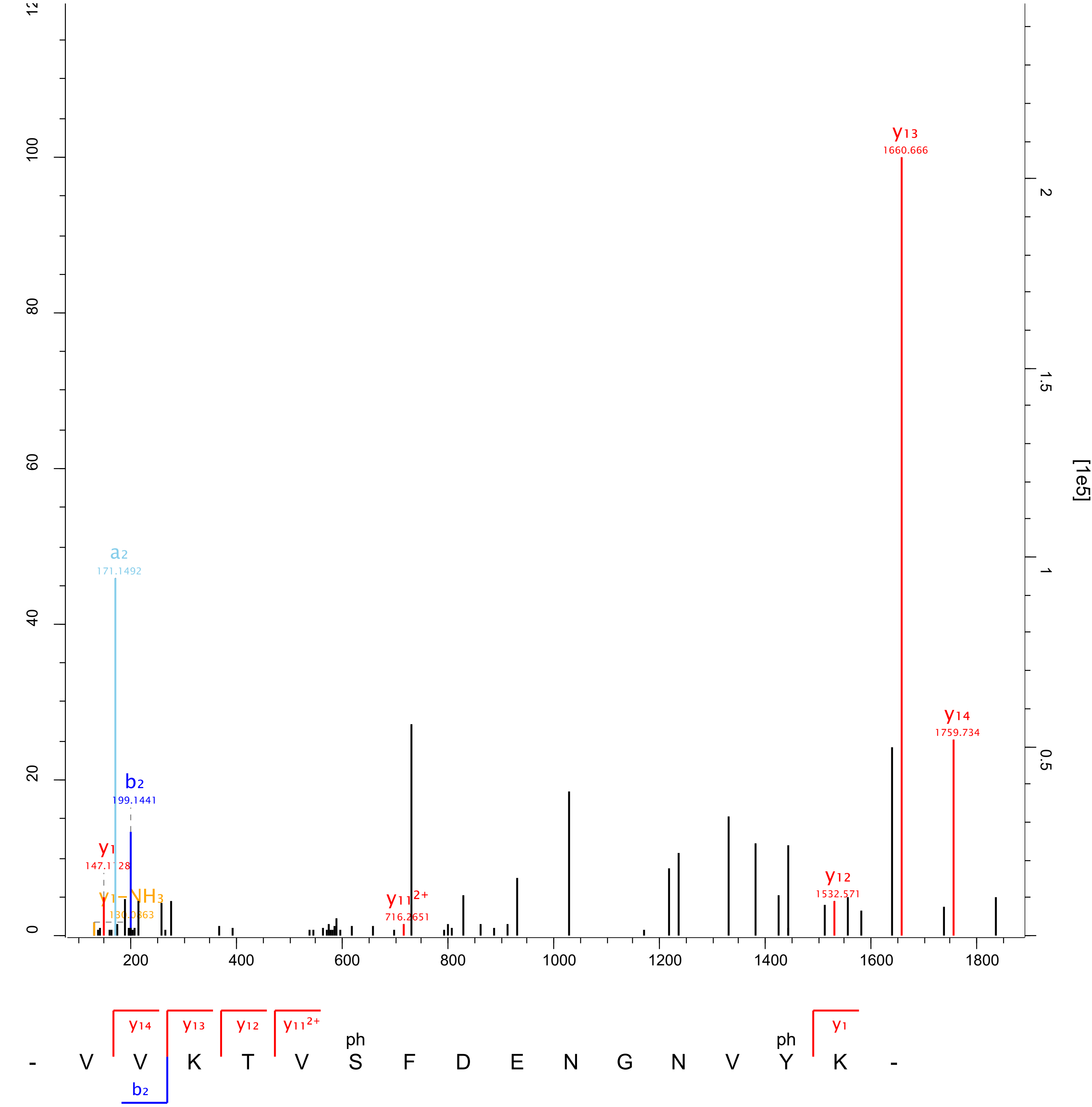

Sample output to test PDF Combine only

Raw file	Scan	Method	Score	m/z	Gene names
sirk1-solu-0-3-A	19348	FTMS; HCD	84.48	563.77	MSRB5;MSRB4;MSRB8;MSRB7

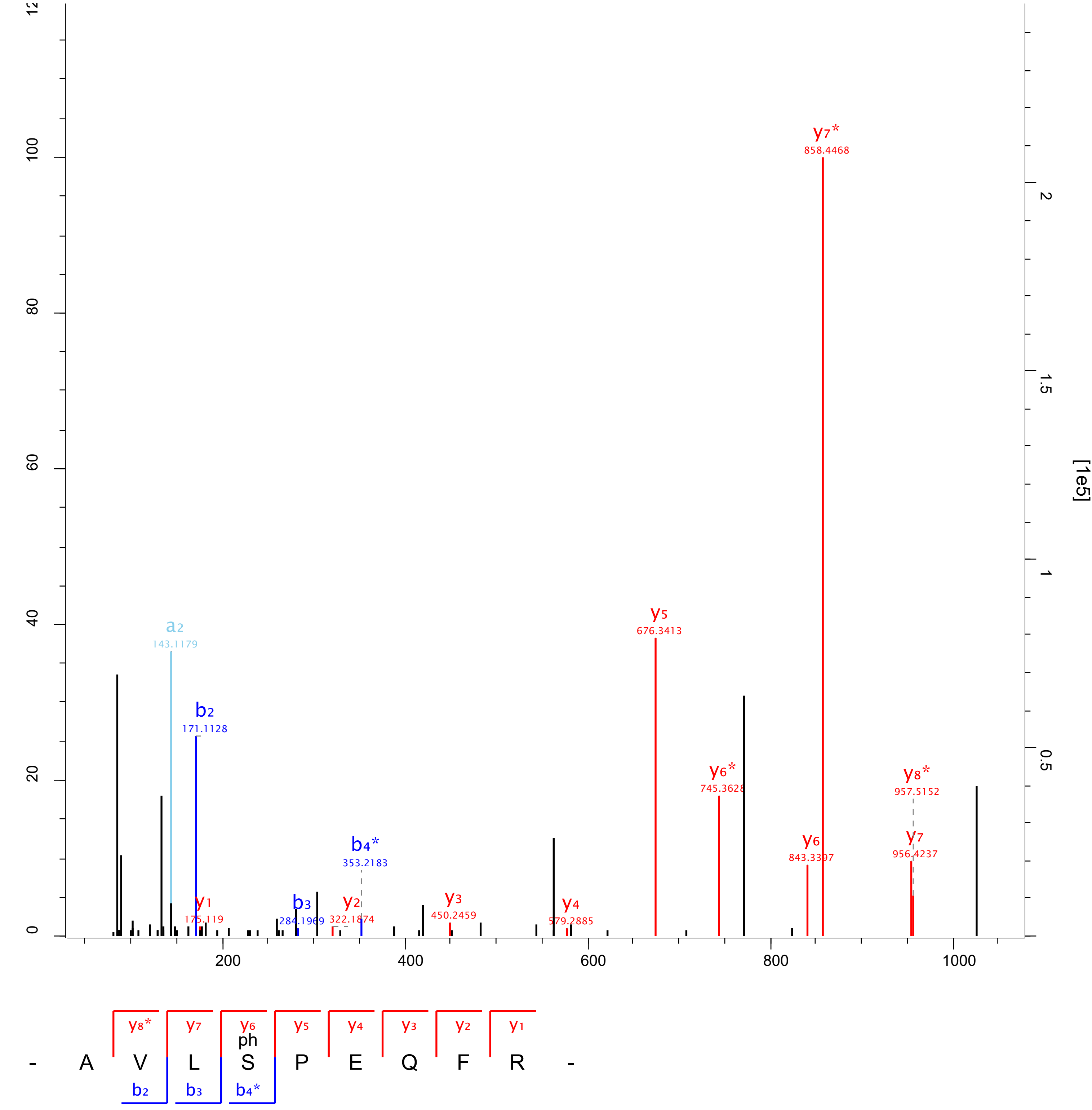

Sample output to test PDF Combine only

Raw file	Scan	Method	Score	m/z	Gene names
sirk1-solu-SUC-1-A	18477	FTMS; HCD	78.81	632.32	PATL1

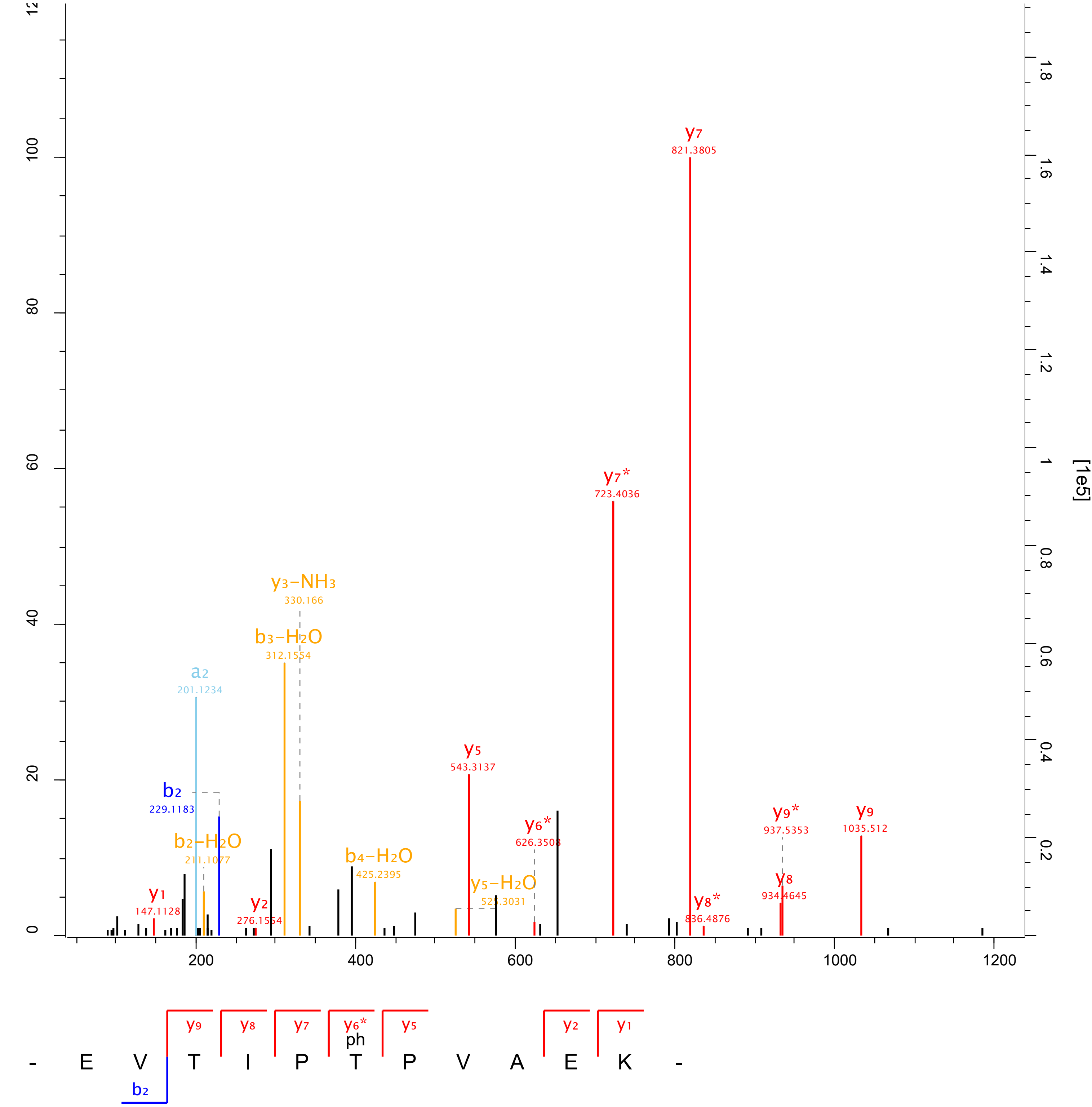

Sample output to test PDF Combine only

Raw file	Scan	Method	Score	m/z	Gene names
sirk1-solu-SUC-1-A	19251	FTMS; HCD	143.98	613.29	CINV1

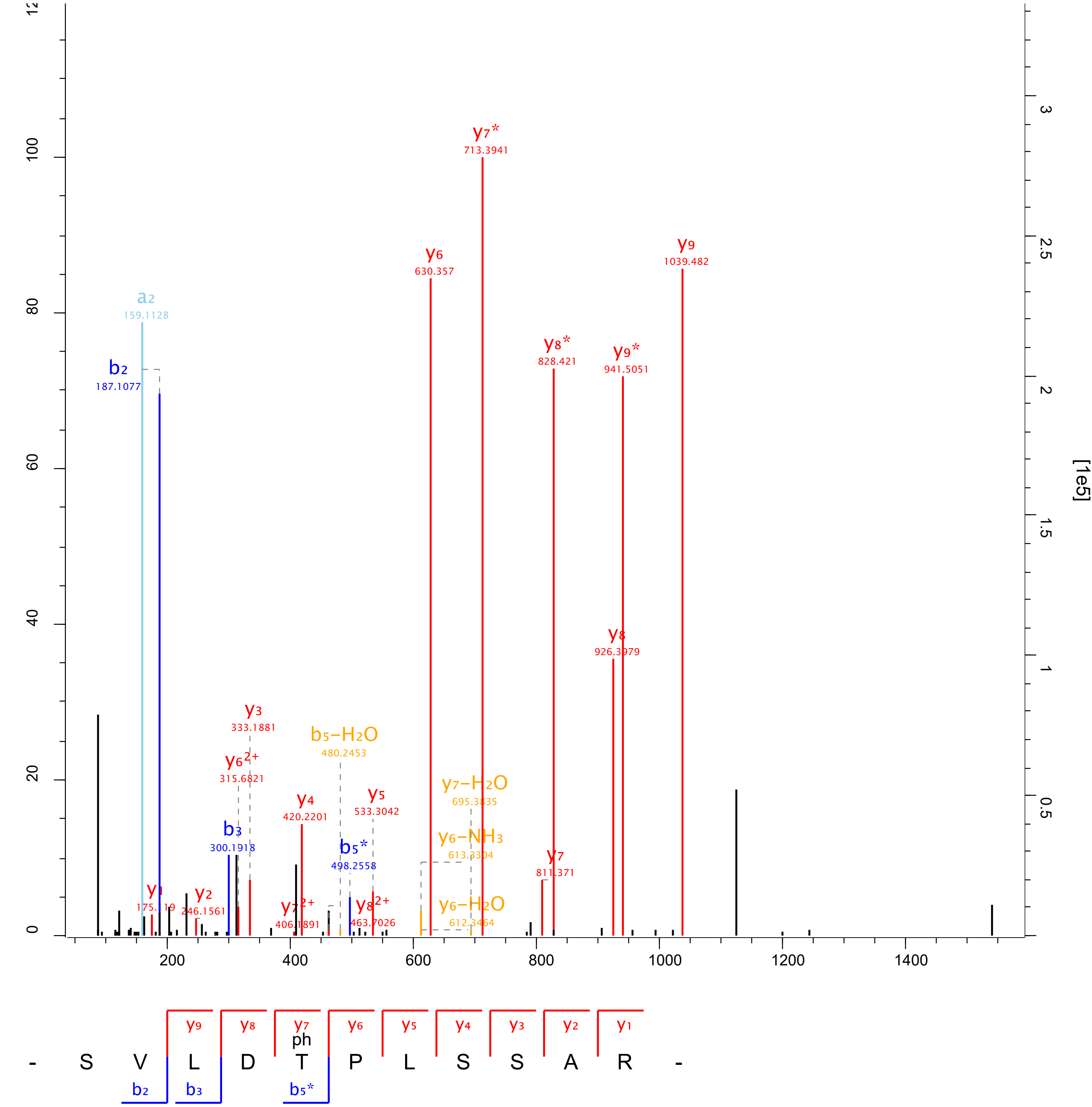

Sample output to test PDF Combine only

Raw file	Scan	Method	Score	m/z	Gene names
sirk1-solu-SUC-1-A	22614	FTMS; HCD	51.8	1049.48	PCMP-E77

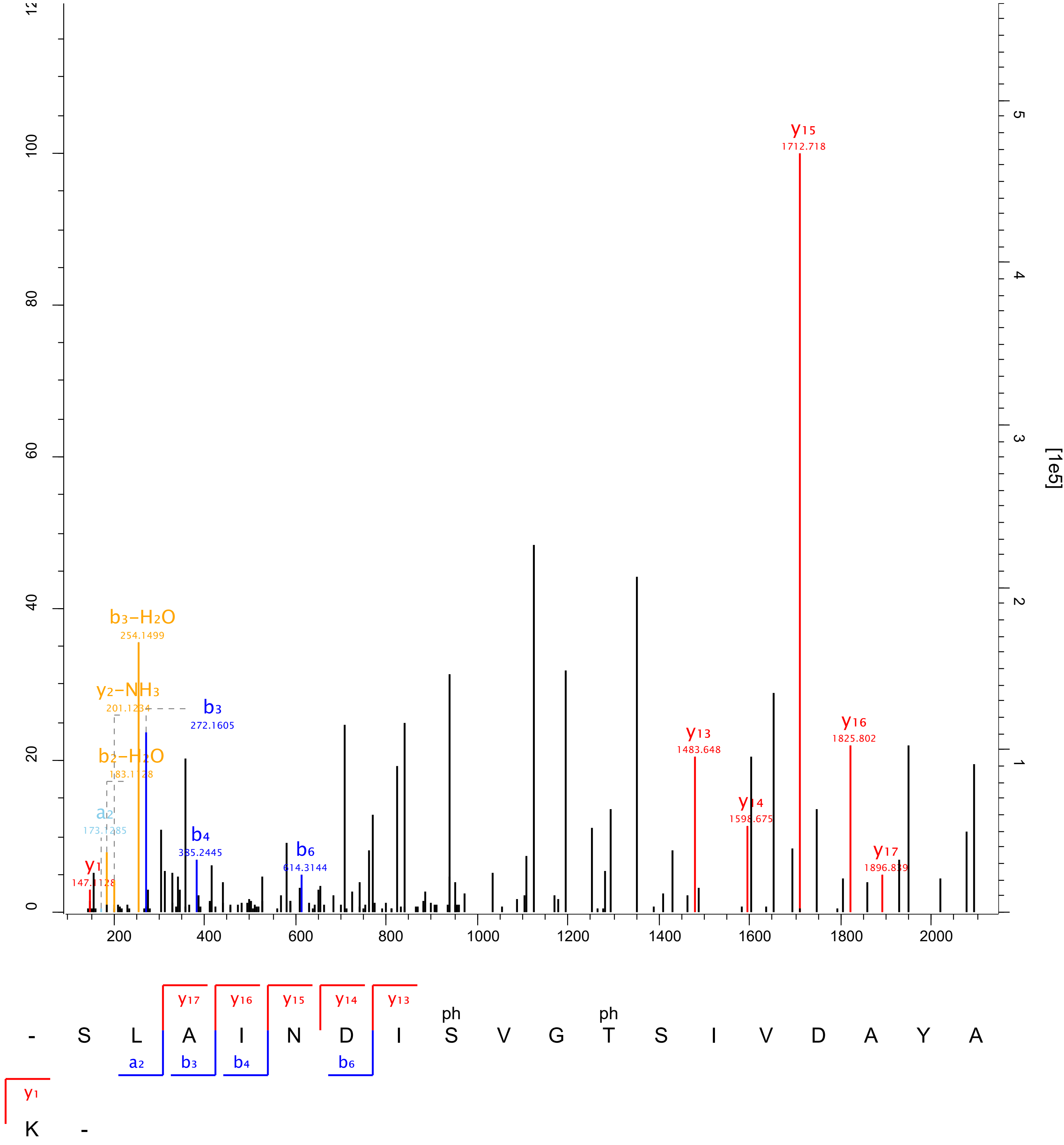

Sample output to test PDF Combine only

Raw file	Scan	Method	Score	m/z	Gene names
sirk1-solu-SUC-1-P	12371	FTMS; HCD	52.17	480.24	CINV1

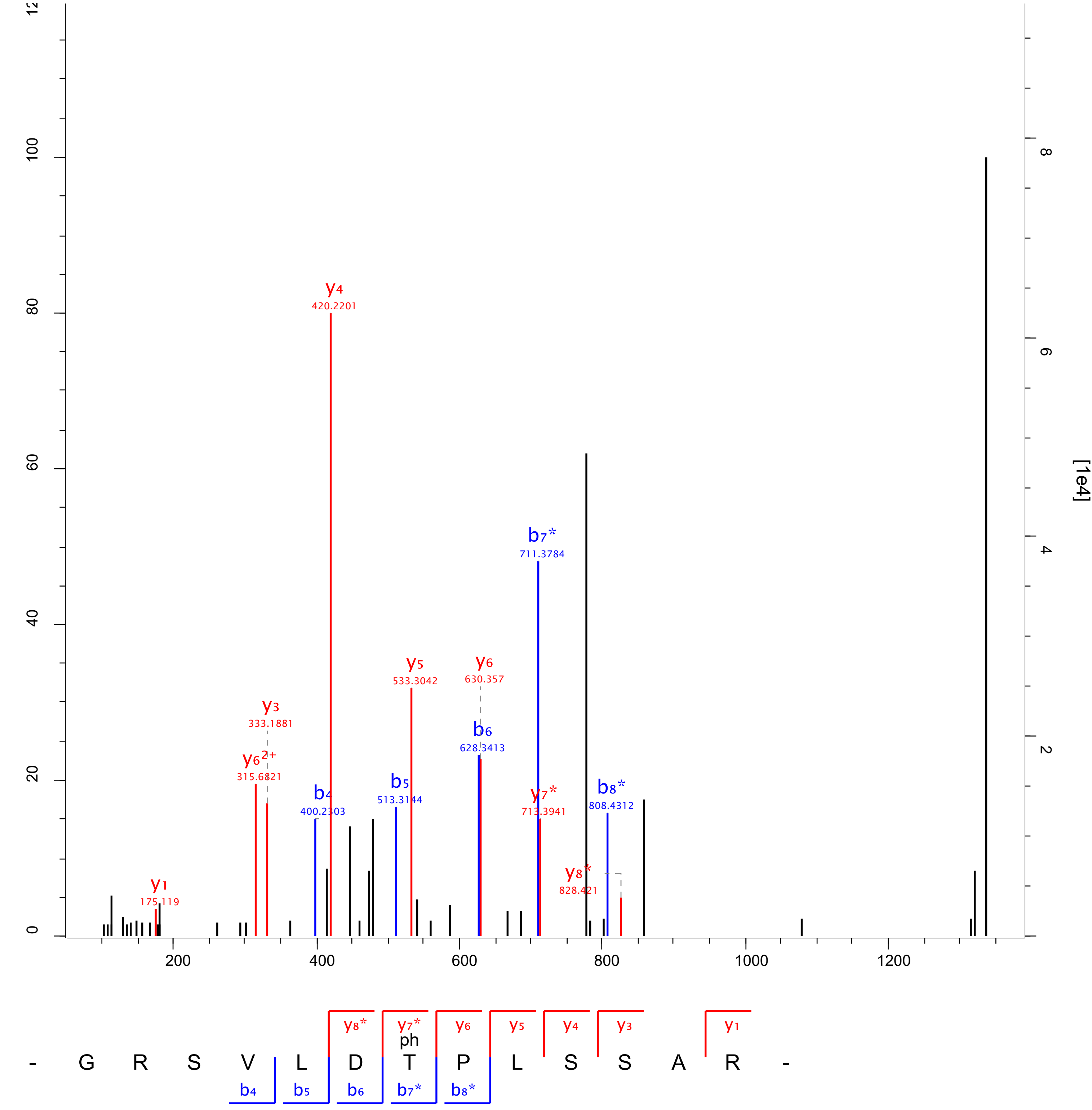

Sample output to test PDF Combine only

Raw file	Scan	Method	Score	m/z	Gene names
sirk1-solu-SUC-1-P	13842	FTMS; HCD	91.66	501.26	T9E8.90

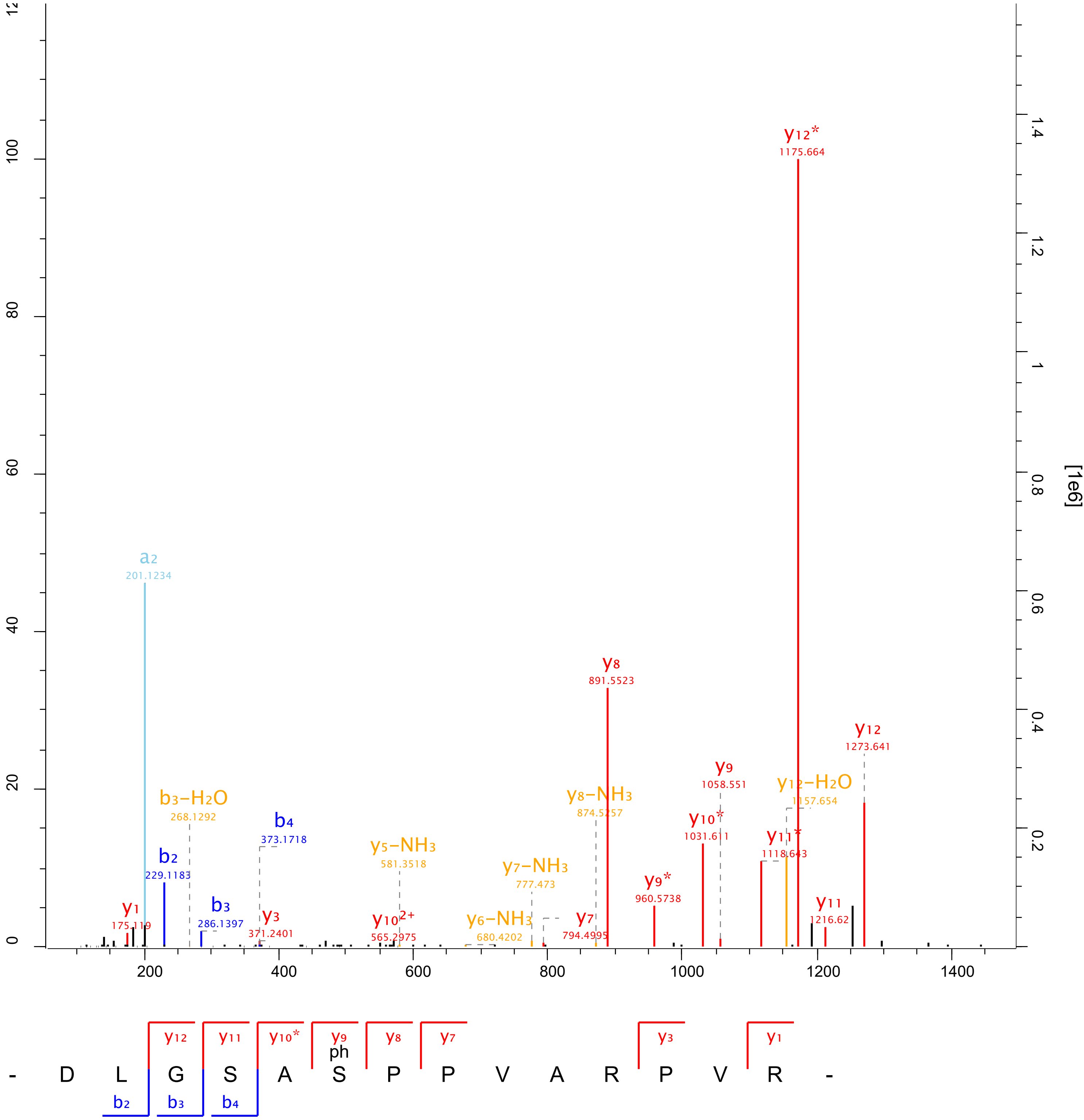

Sample output to test PDF Combine only

Raw file	Scan	Method	Score	m/z	Gene names
sirk1-solu-SUC-2-P	9142	FTMS; HCD	134.62	635.99	At2g33830

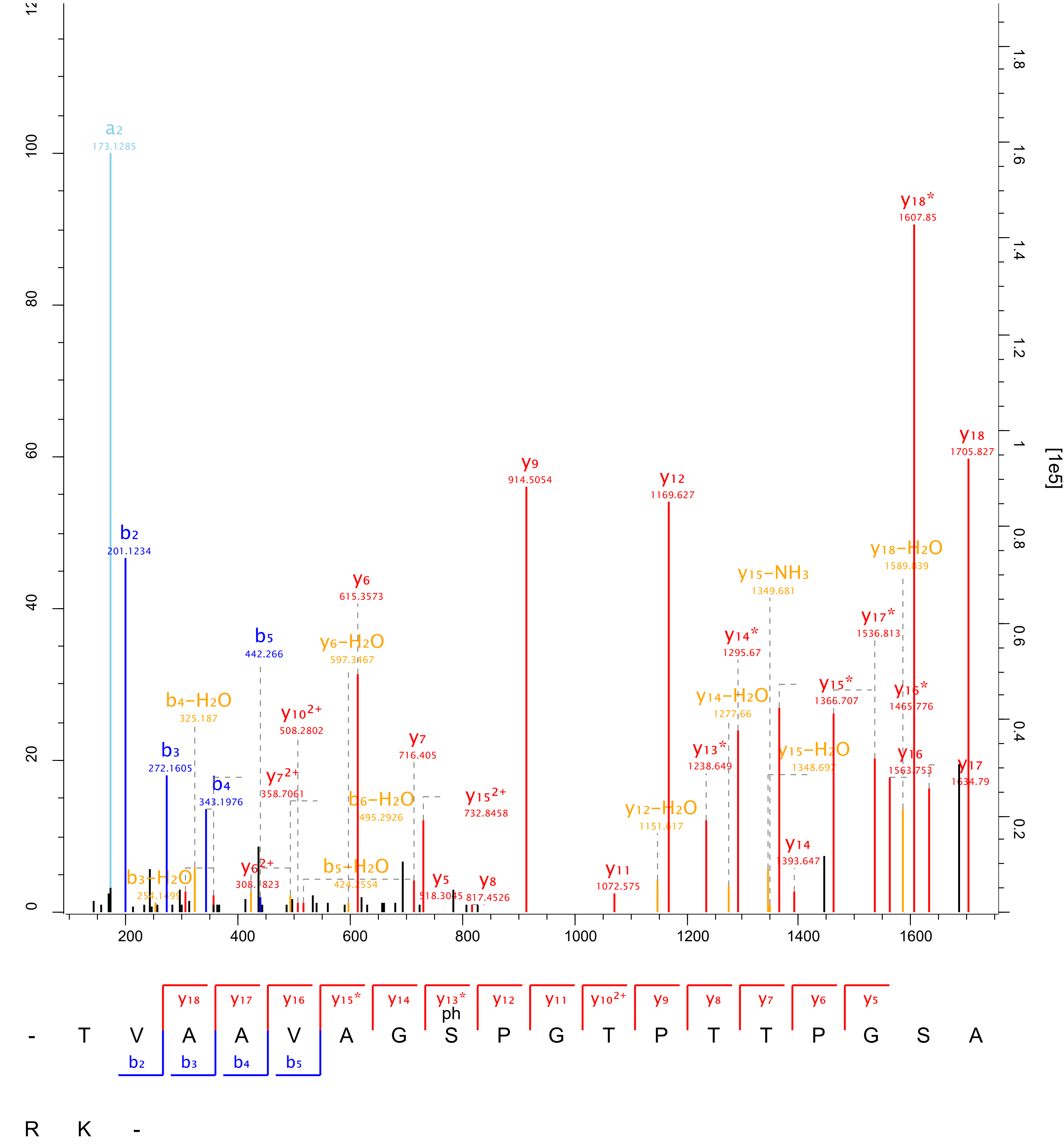

Sample output to test PDF Combine only

Raw file	Scan	Method	Score	m/z	Gene names
sirk1-solu-SUC-2-P	10933	FTMS; HCD	92.86	632.34	DYL1

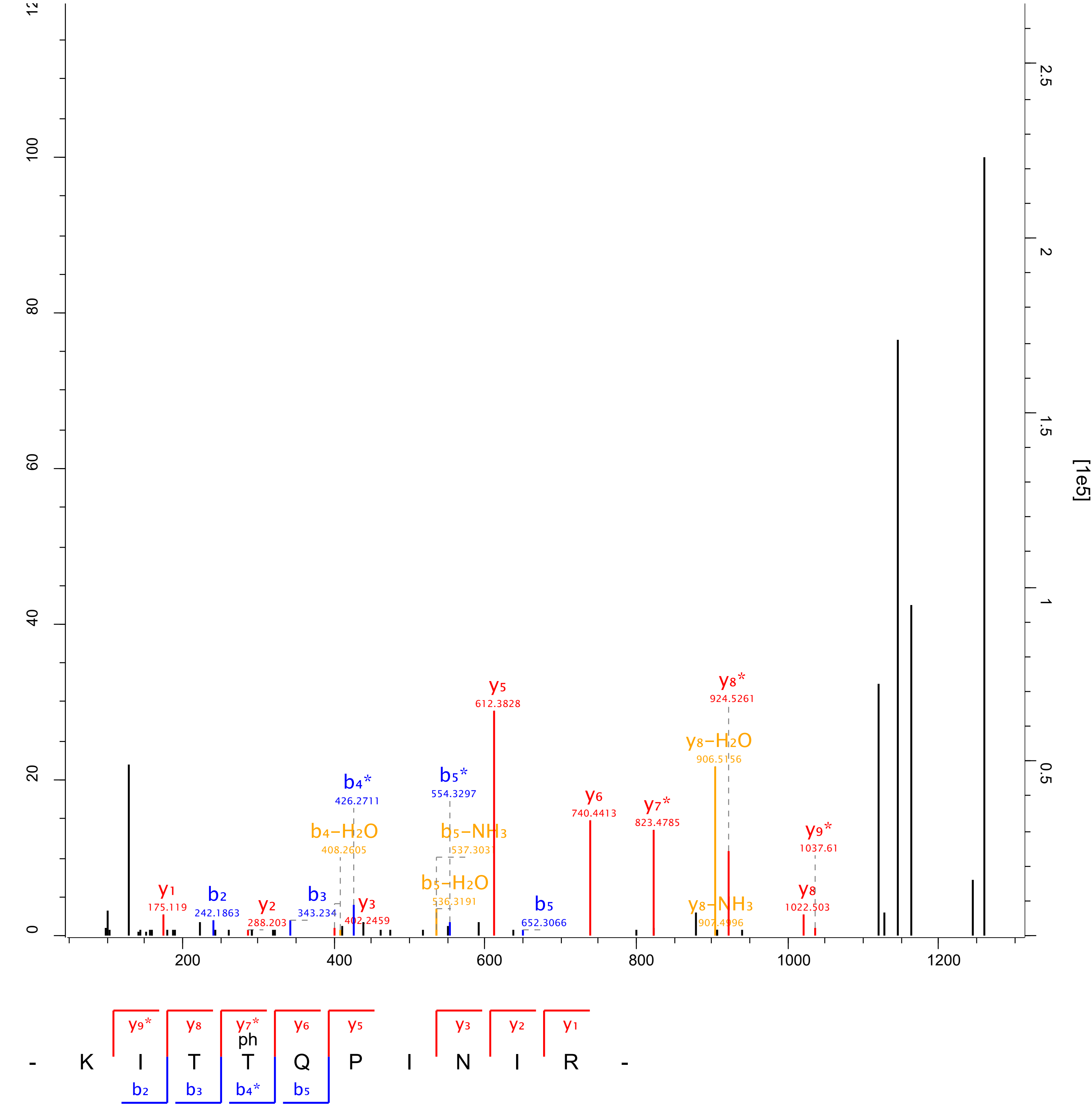

Sample output to test PDF Combine only

Raw file	Scan	Method	Score	m/z	Gene names
sirk1-solu-SUC-2-P	12363	FTMS; HCD	79.84	530.23	At1g20760

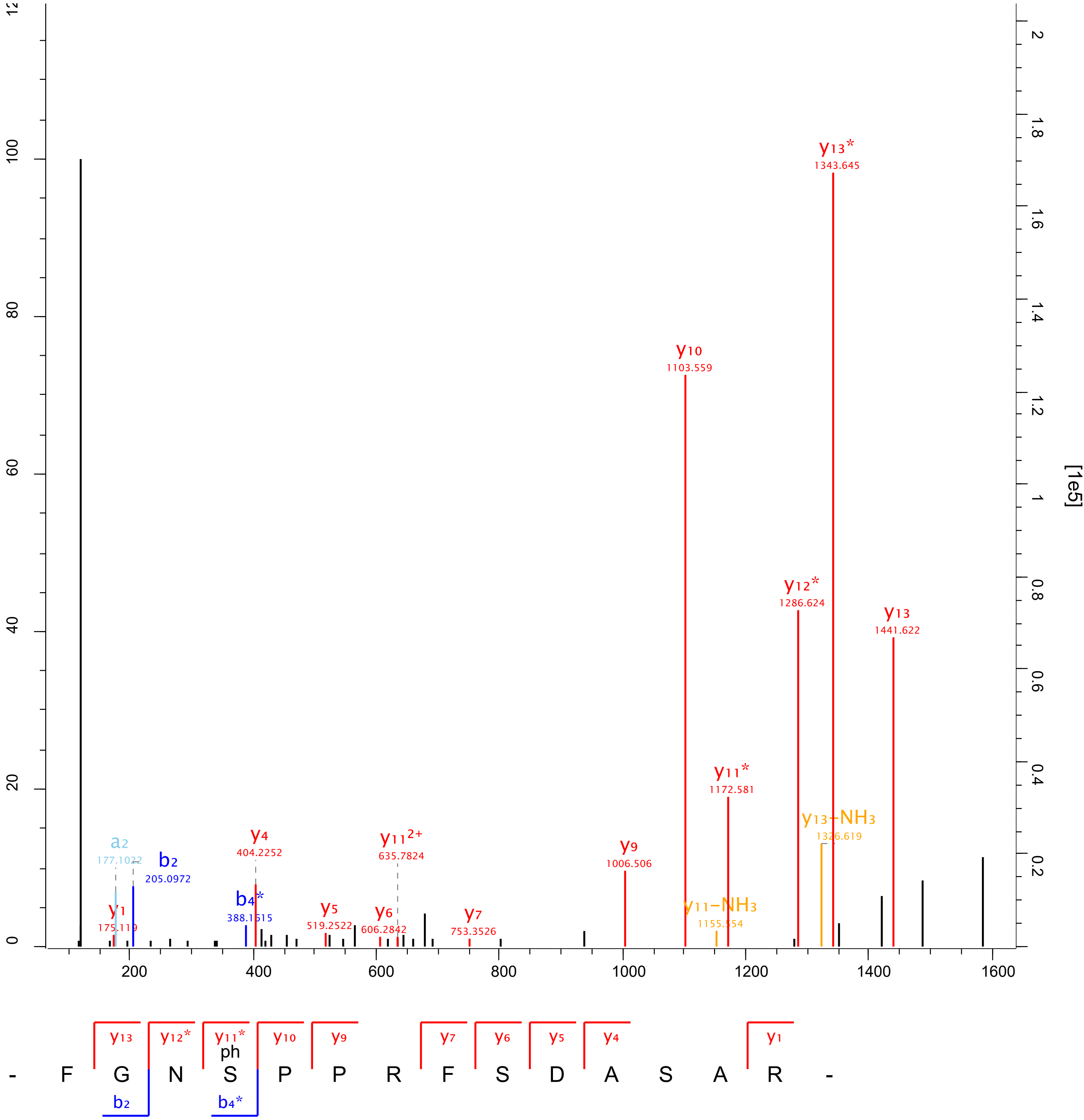

Sample output to test PDF Combine only

Raw file	Scan	Method	Score	m/z	Gene names
sirk1-solu-SUC-2-P	13326	FTMS; HCD	259.6	889.43	At2g33830

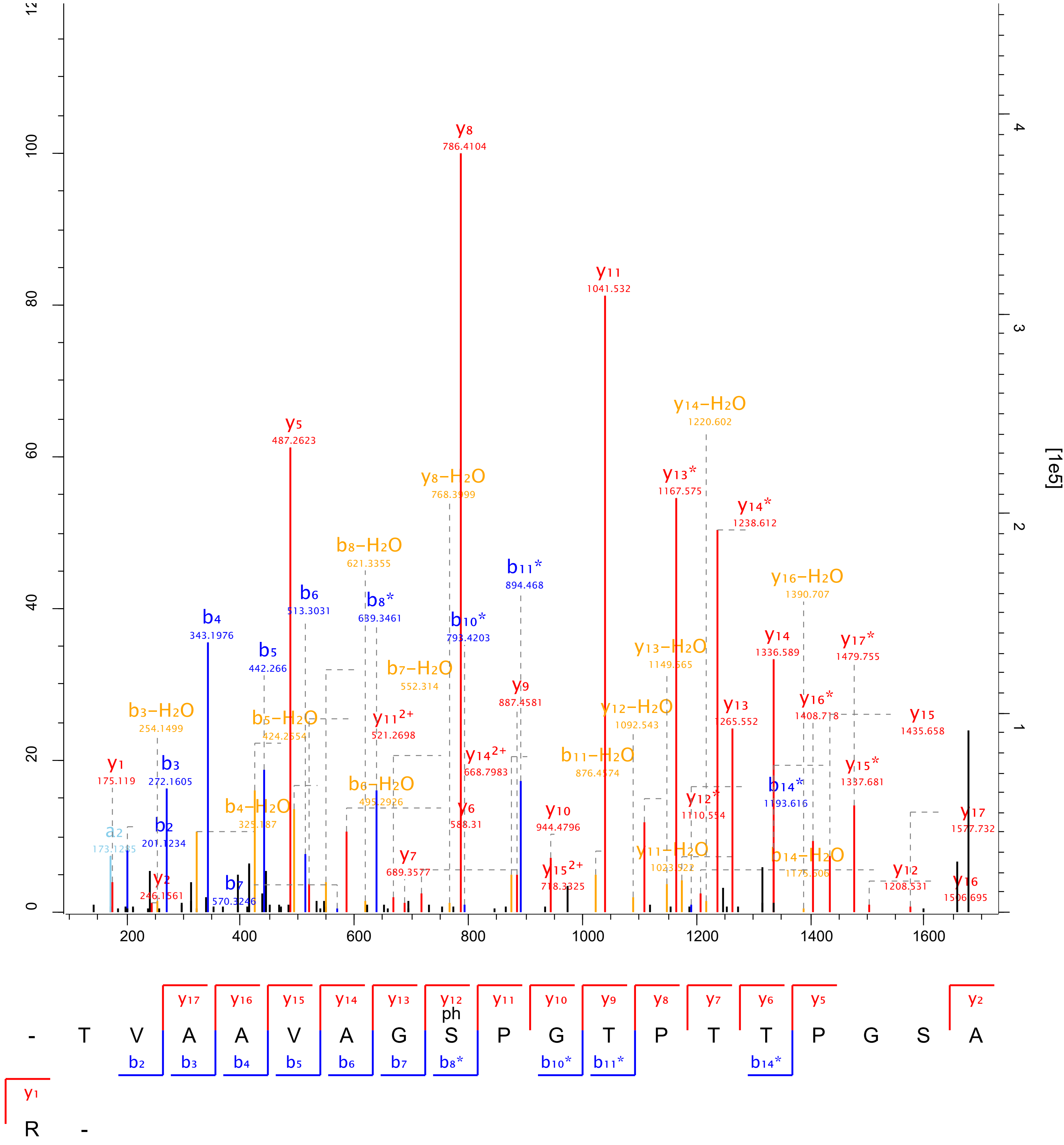

Sample output to test PDF Combine only

Raw file	Scan	Method	Score	m/z	Gene names
sirk1-solu-SUC-2-P	14078	FTMS; HCD	60.73	709.03	DYL1

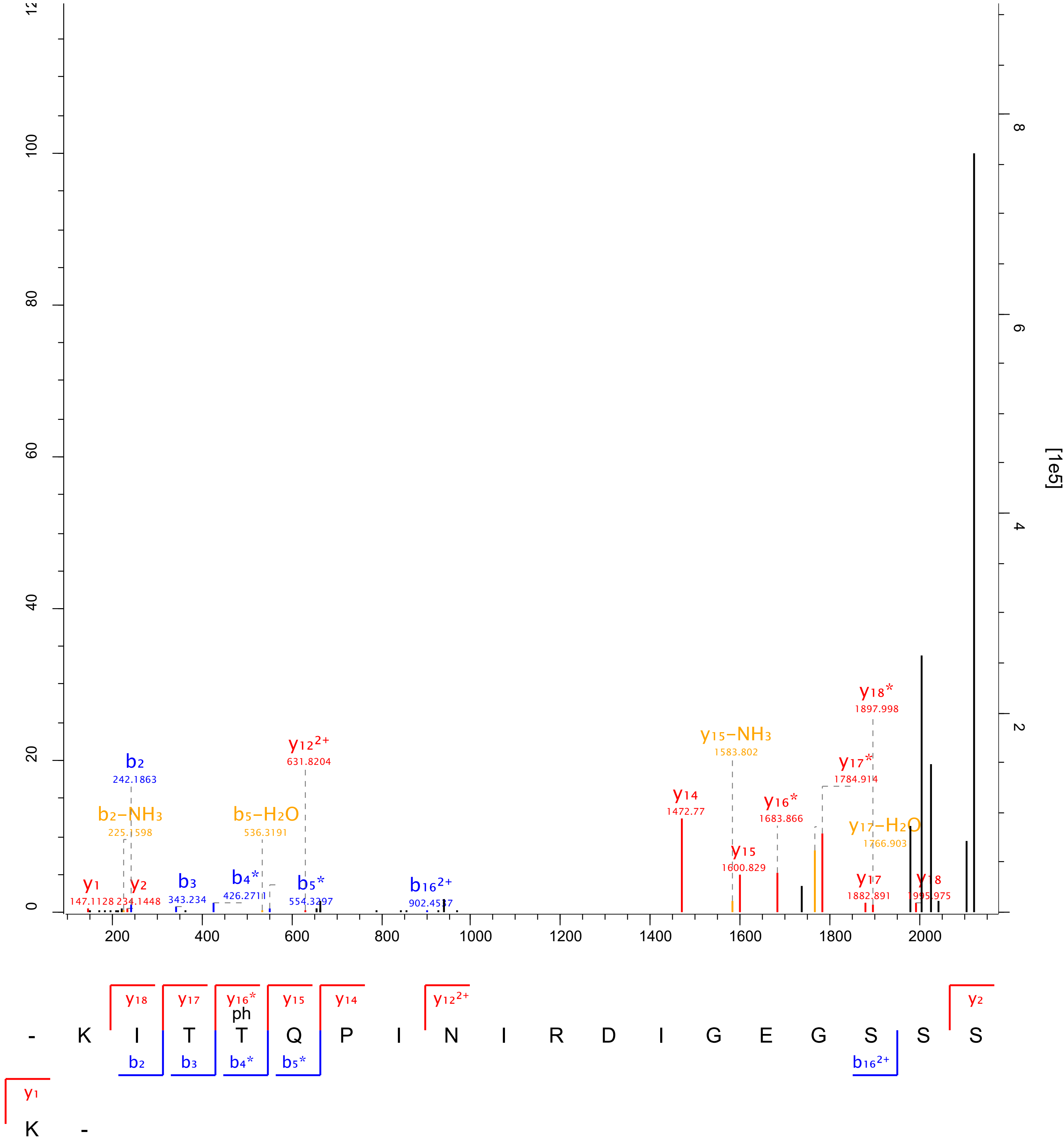

Sample output to test PDF Combine only

Raw file	Scan	Method	Score	m/z	Gene names
sirk1-solu-SUC-2-P	14342	FTMS; HCD	58.98	497.57	At1g21630

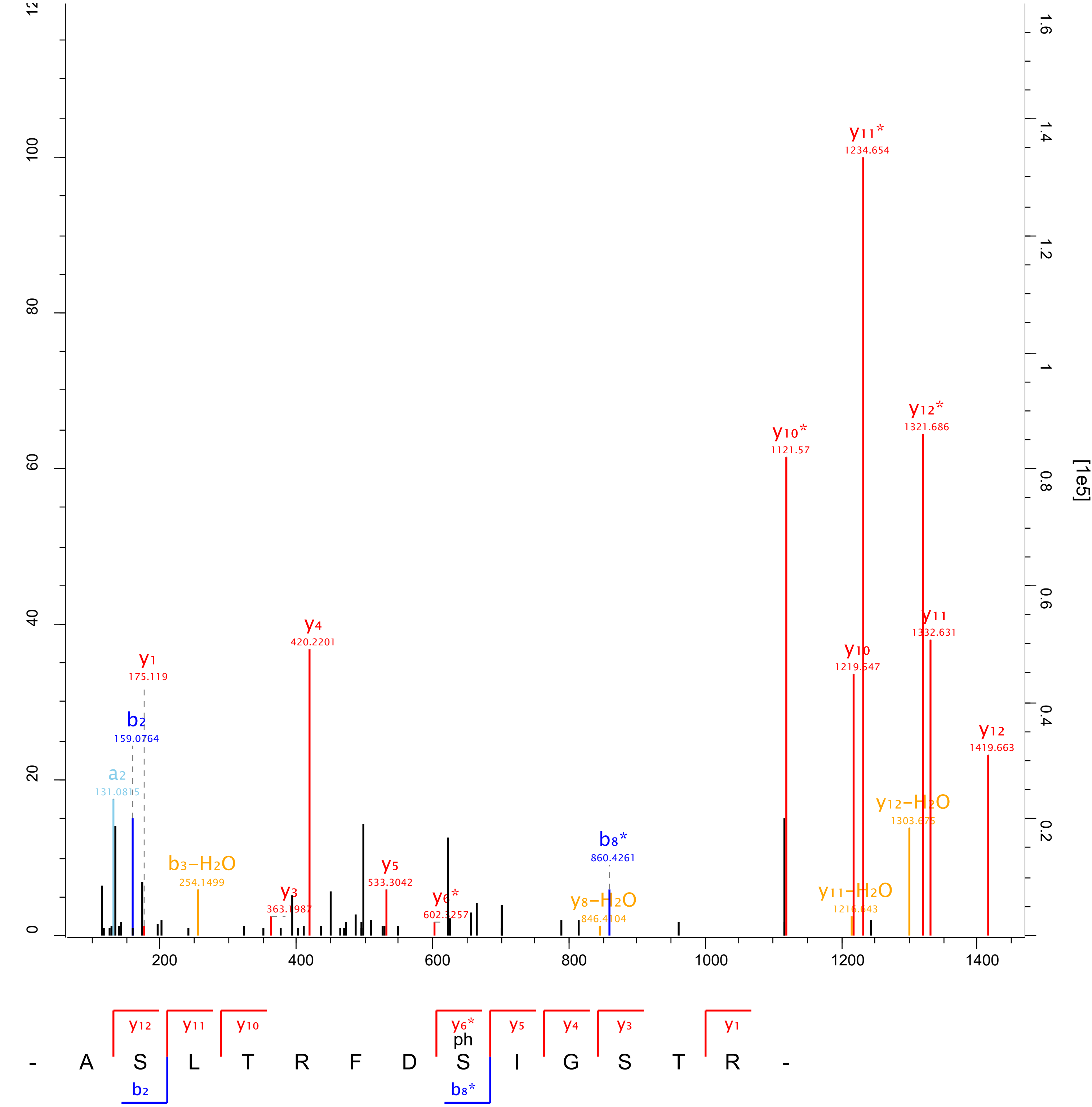

Sample output to test PDF Combine only

Raw file	Scan	Method	Score	m/z	Gene names
sirk1-solu-SUC-2-P	19782	FTMS; HCD	87.15	428.22	PPA1

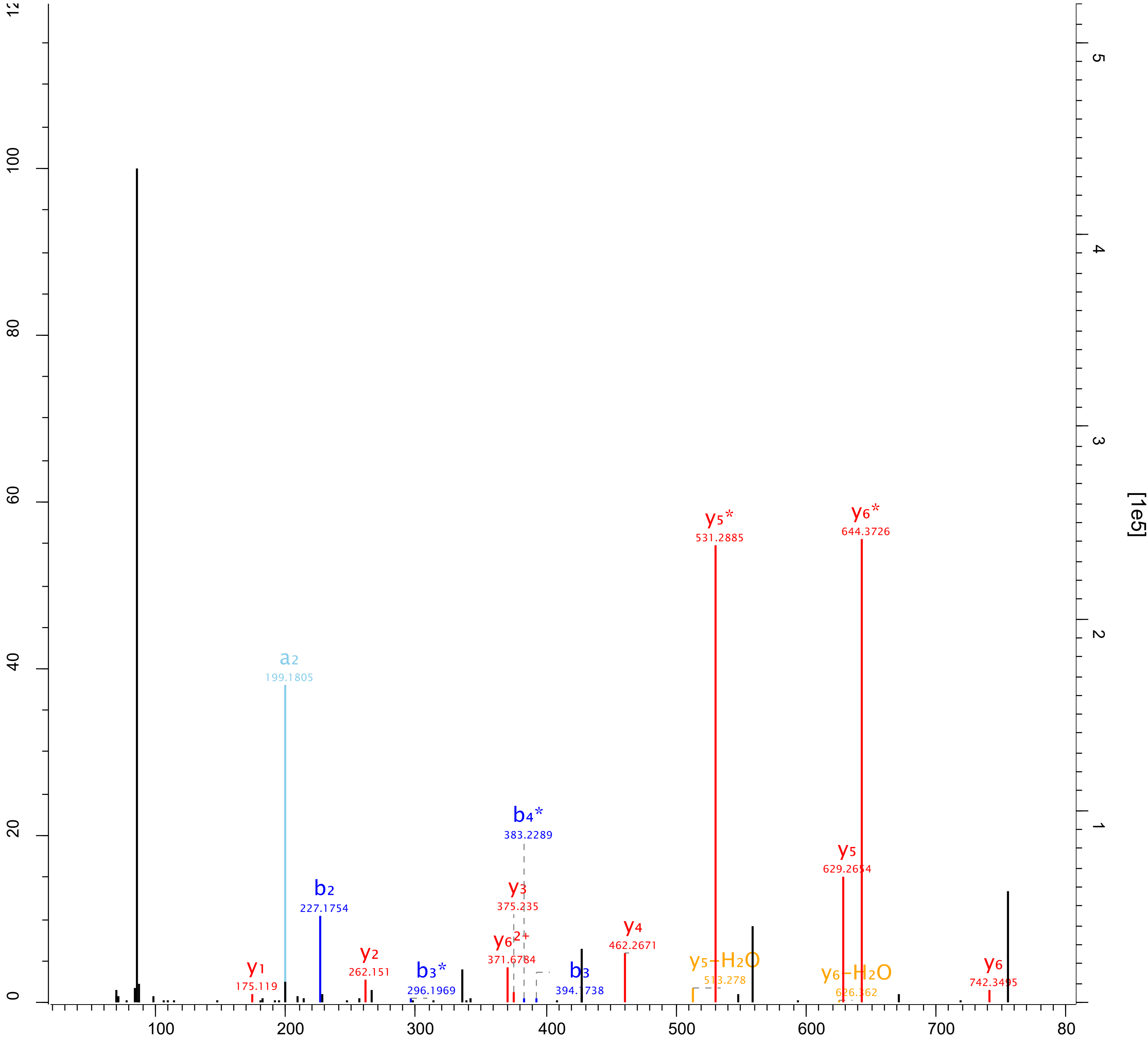

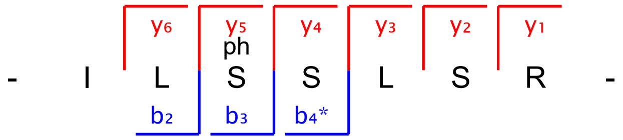

Sample output to test PDF Combine only

Raw file	Scan	Method	Score	m/z	Gene names
sirk1-solu-SUC-2-P	22478	FTMS; HCD	108.86	954.8	T9E8.90

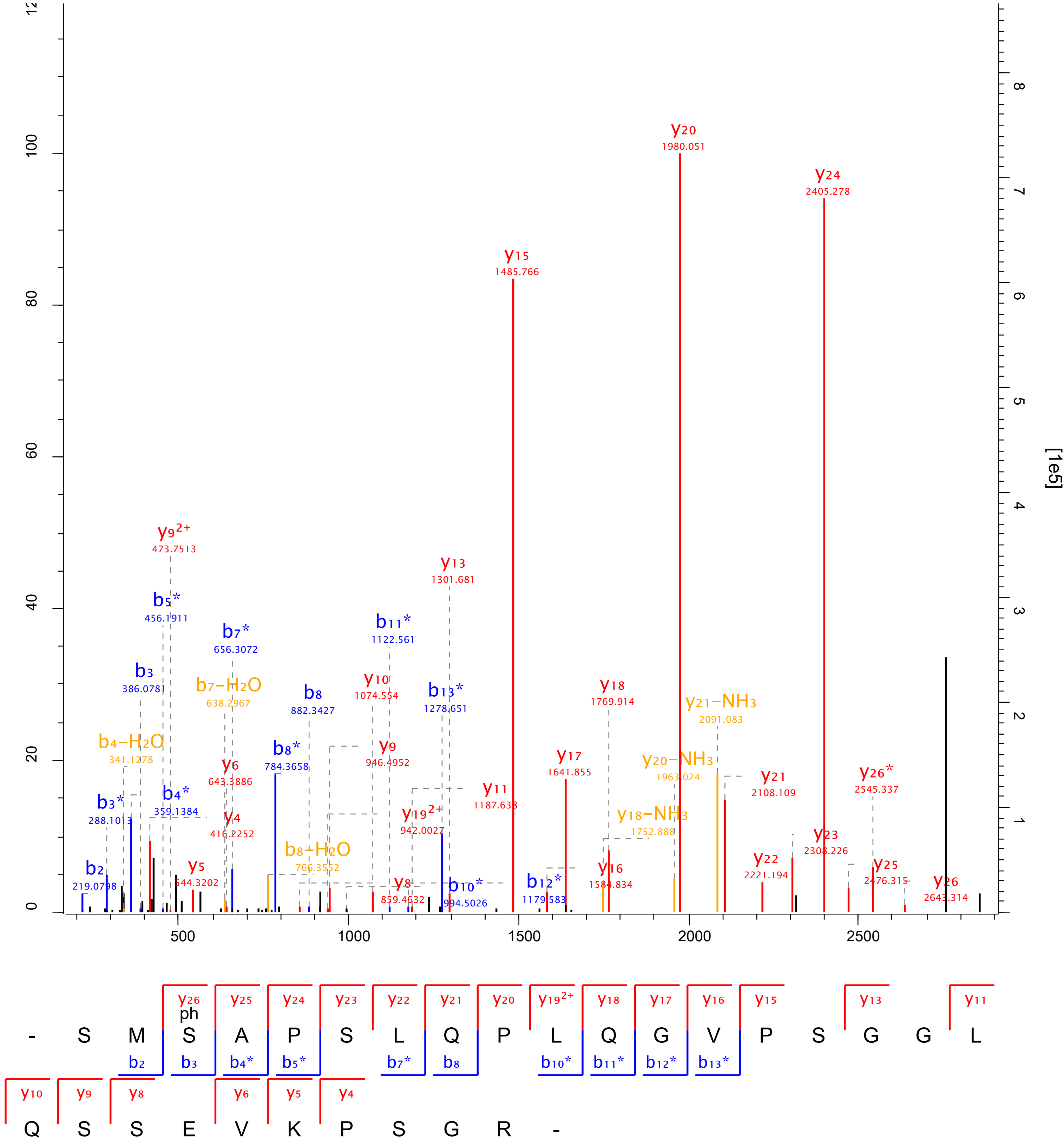

Sample output to test PDF Combine only

Raw file	Scan	Method	Score	m/z	Gene names
sirk1-solu-SUC-2-P	23921	FTMS; HCD	92.92	901.09	At4g29440

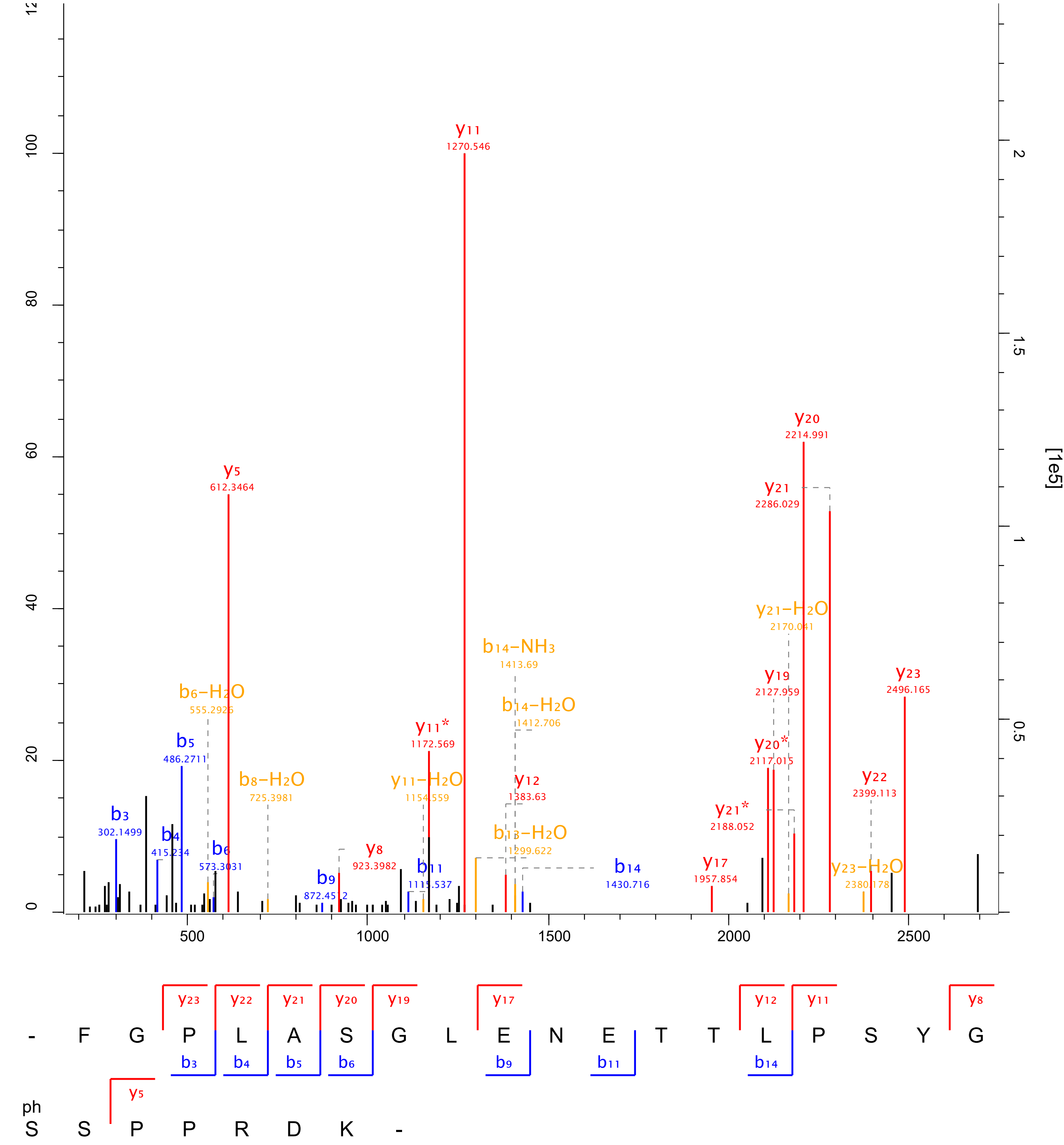

Sample output to test PDF Combine only

Raw file	Scan	Method	Score	m/z	Gene names
sirk1-solu-SUC-2-P	25573	FTMS; HCD	44.25	701.34	CYP79A2

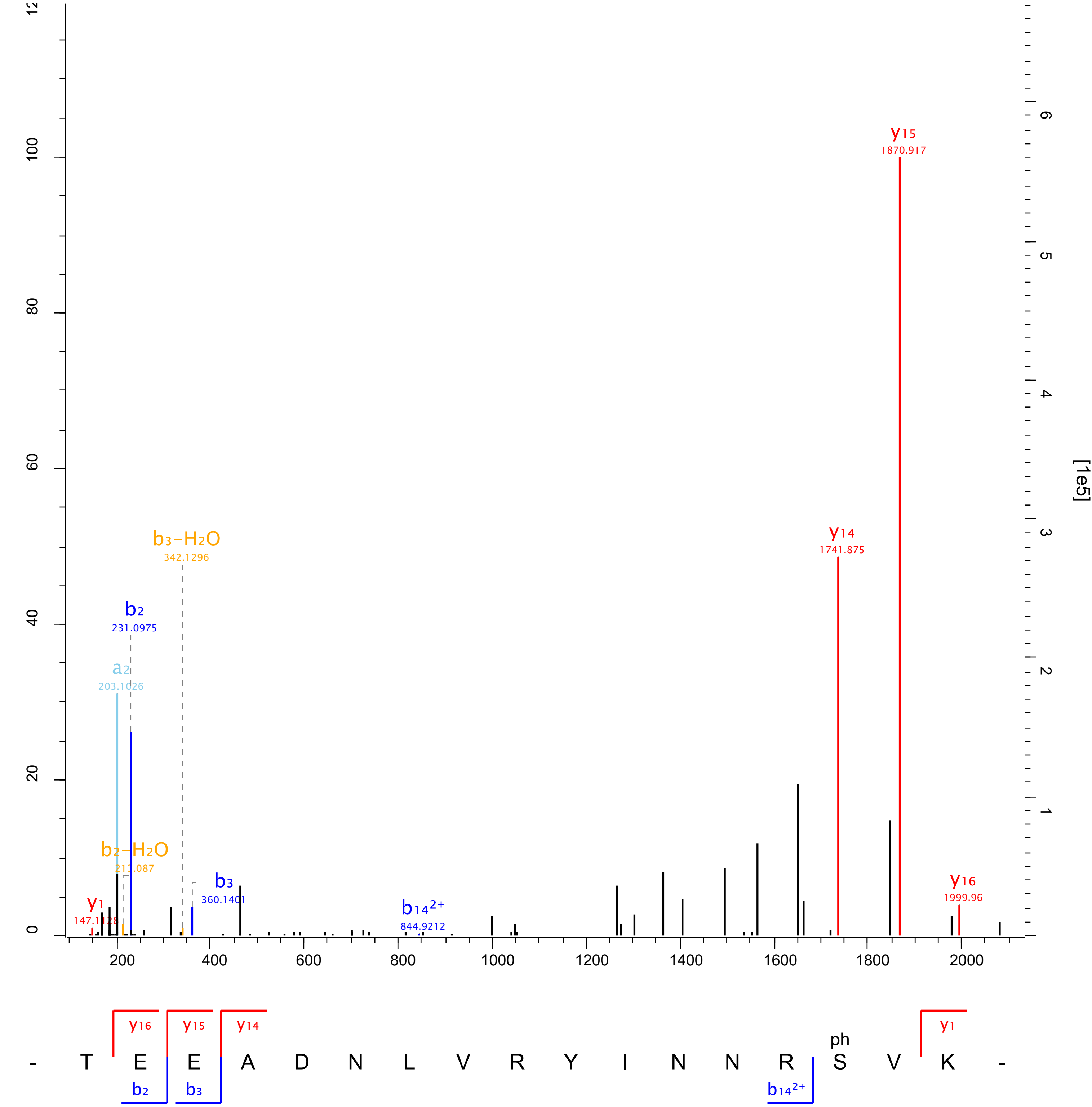

Sample output to test PDF Combine only

Raw file	Scan	Method	Score	m/z	Gene names
sirk1-solu-SUC-2-P	26580	FTMS; HCD	153.09	687.31	EDA39

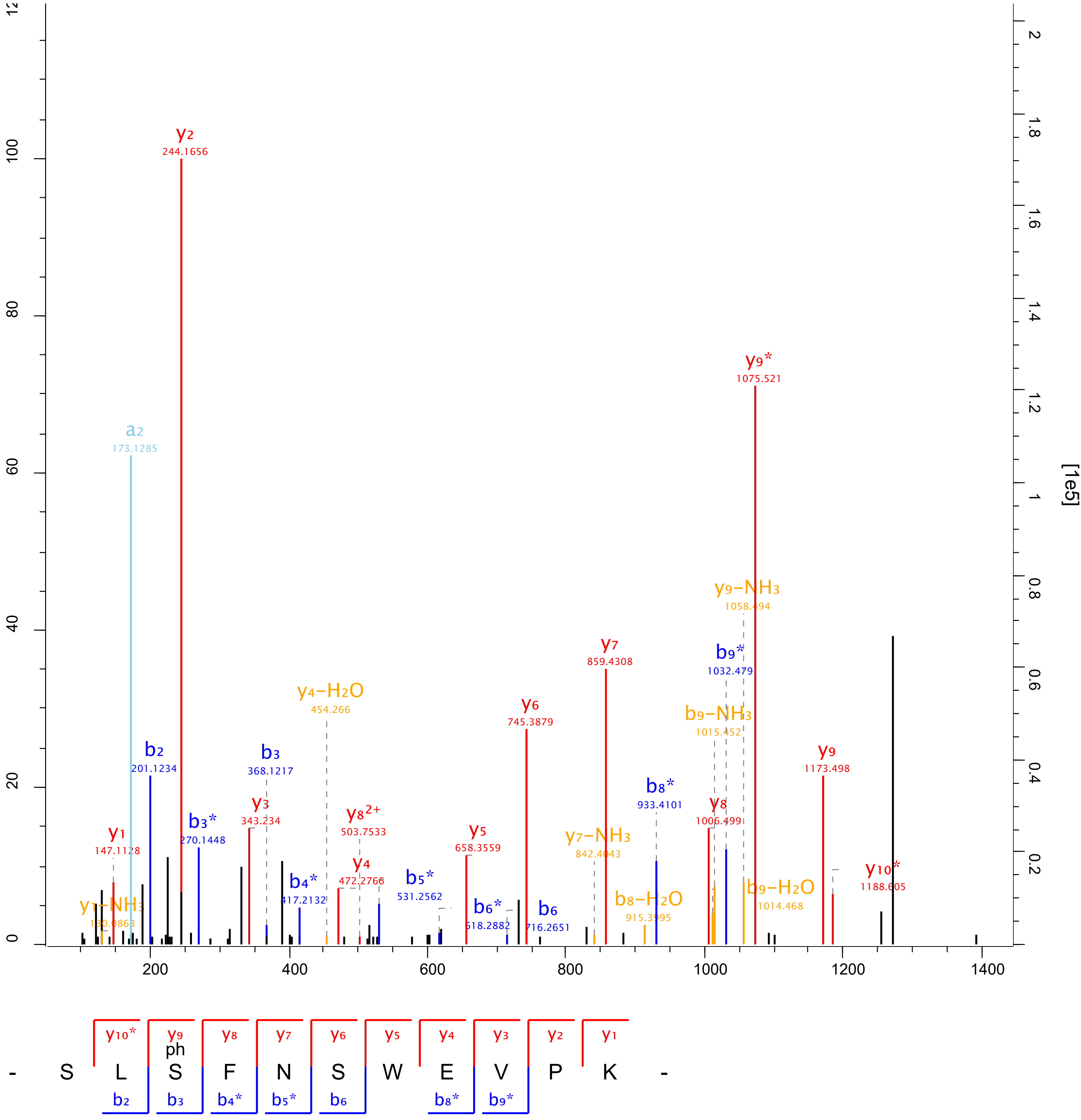

Sample output to test PDF Combine only

Raw file	Scan	Method	Score	m/z	Gene names
sirk1-solu-SUC-2-P	26793	FTMS; HCD	55.26	717.85	UPL2;UPL1

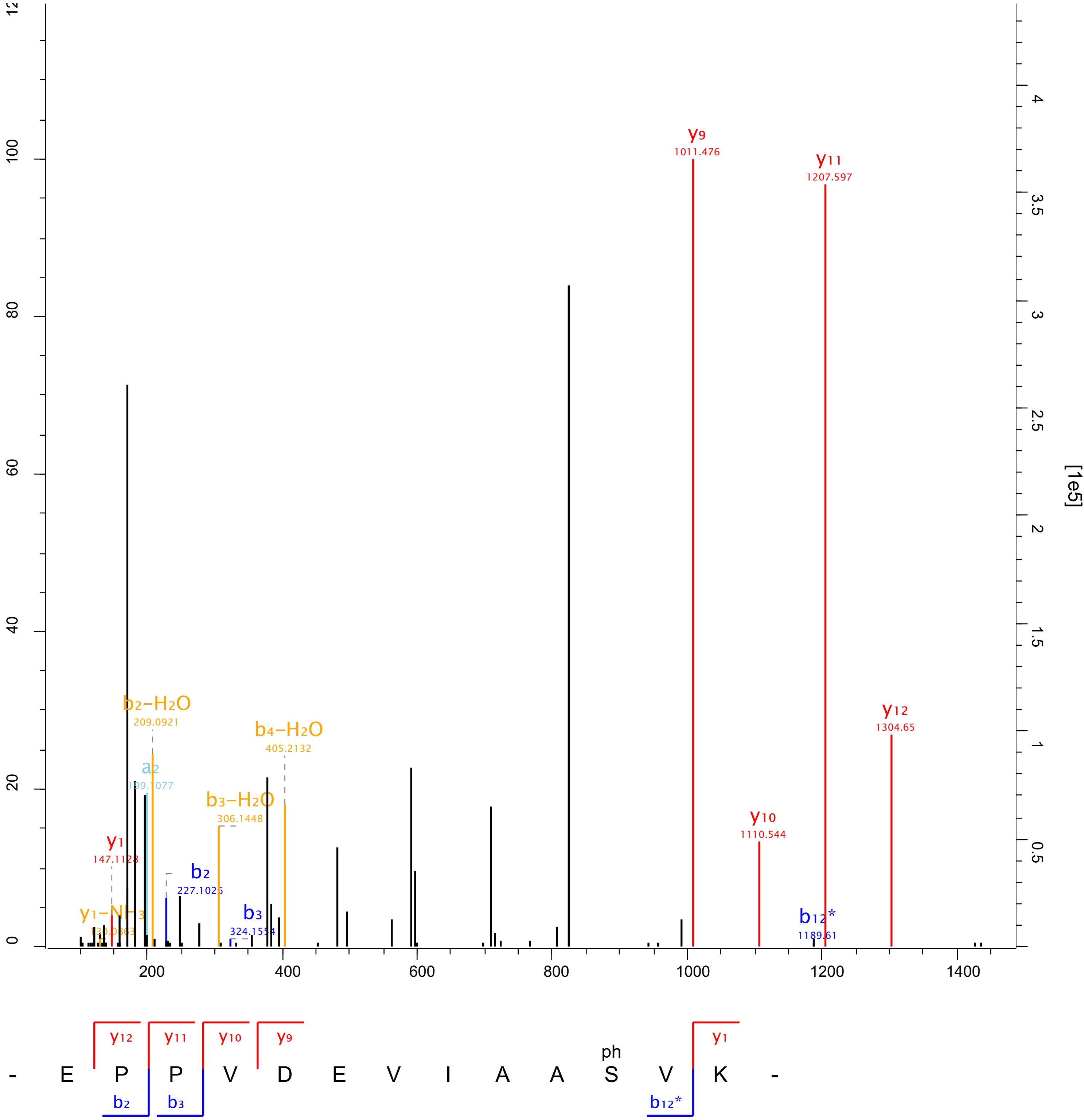

Sample output to test PDF Combine only

Raw file	Scan	Method	Score	m/z	Gene names
sirk1-solu-SUC-2-P	30391	FTMS; HCD	58.98	868.36	CINV1

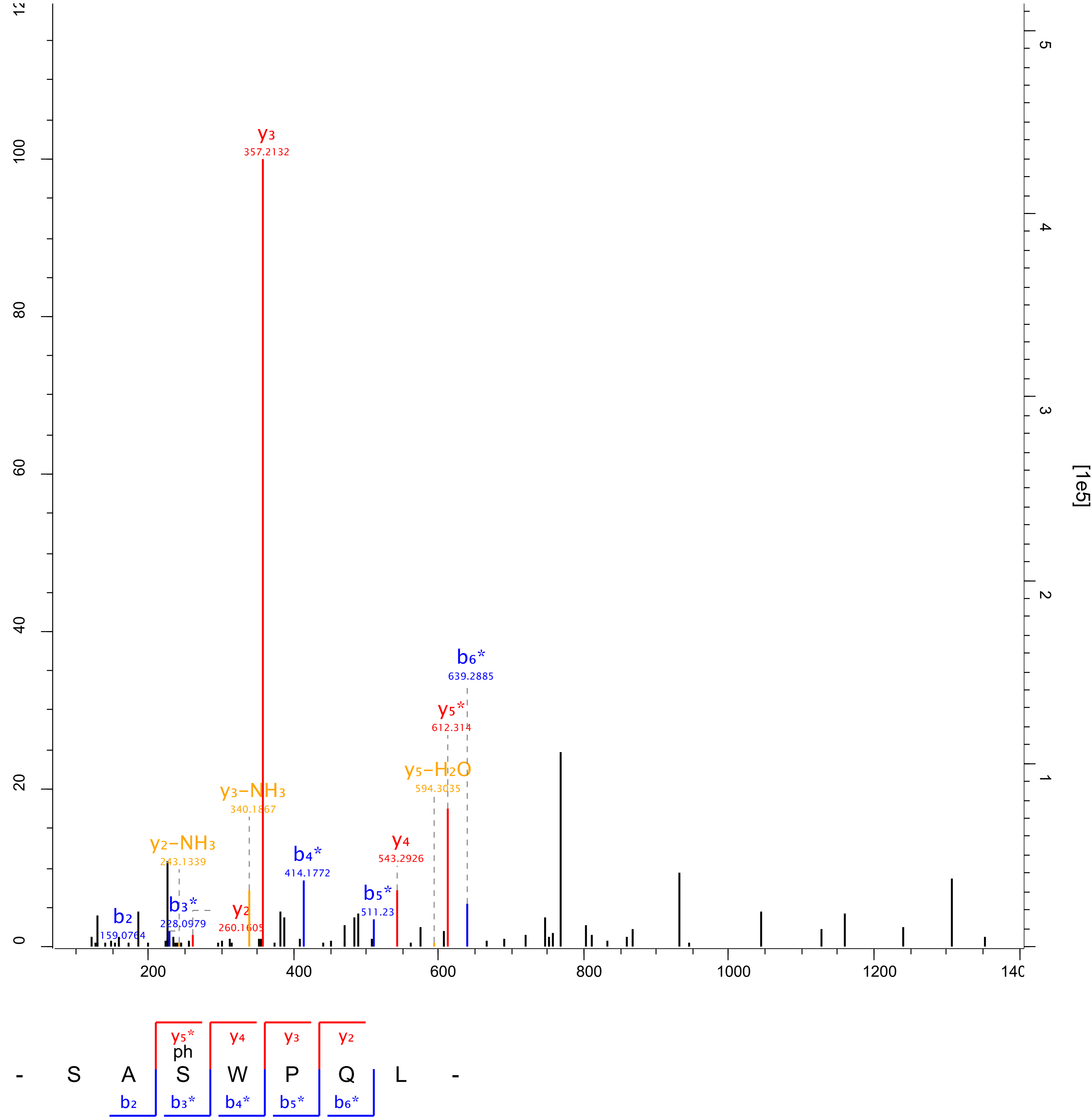

Sample output to test PDF Combine only

Raw file	Scan	Method	Score	m/z	Gene names
sirk1-solu-SUC-2-P	31151	FTMS; HCD	124.07	660.36	At1g28280;VQ4

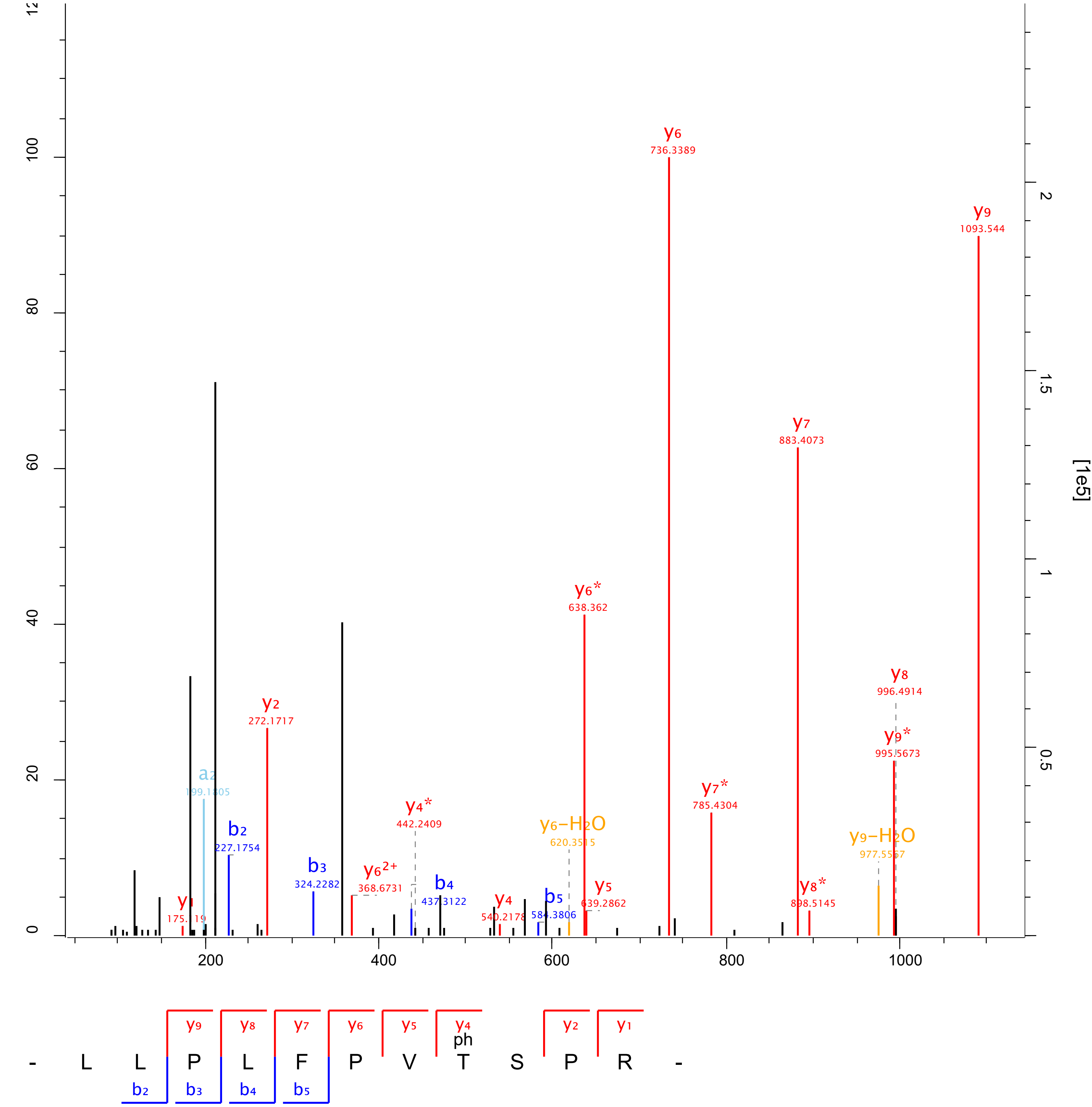

Sample output to test PDF Combine only

Raw file	Scan	Method	Score	m/z	Gene names
sp3-mic-0-1-A	7446	FTMS; HCD	72.58	499.57	PCAP1

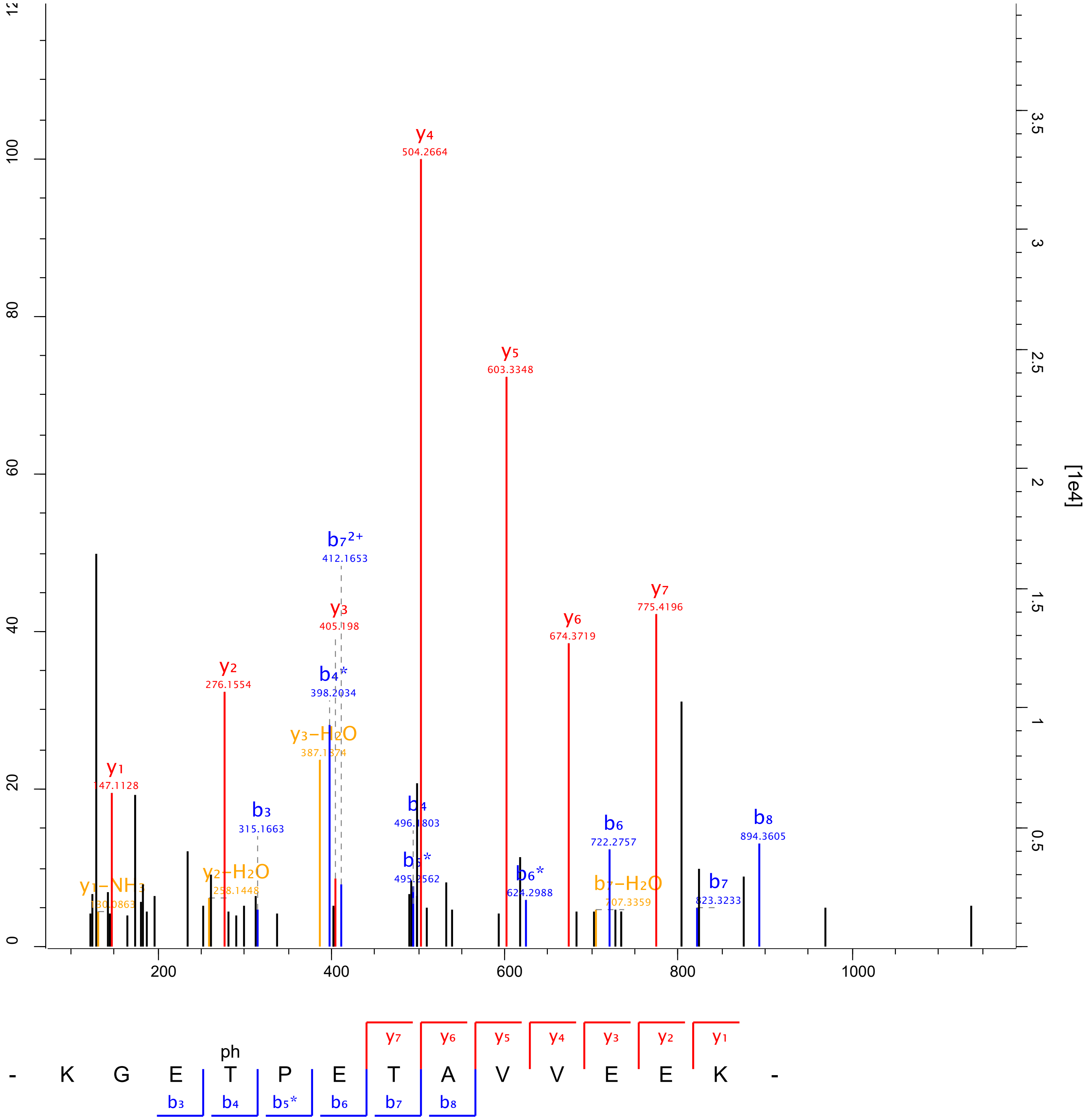

Sample output to test PDF Combine only

Raw file	Scan	Method	Score	m/z	Gene names
sp3-mic-0-1-A	9269	FTMS; HCD	41.12	596.28	At3g08840

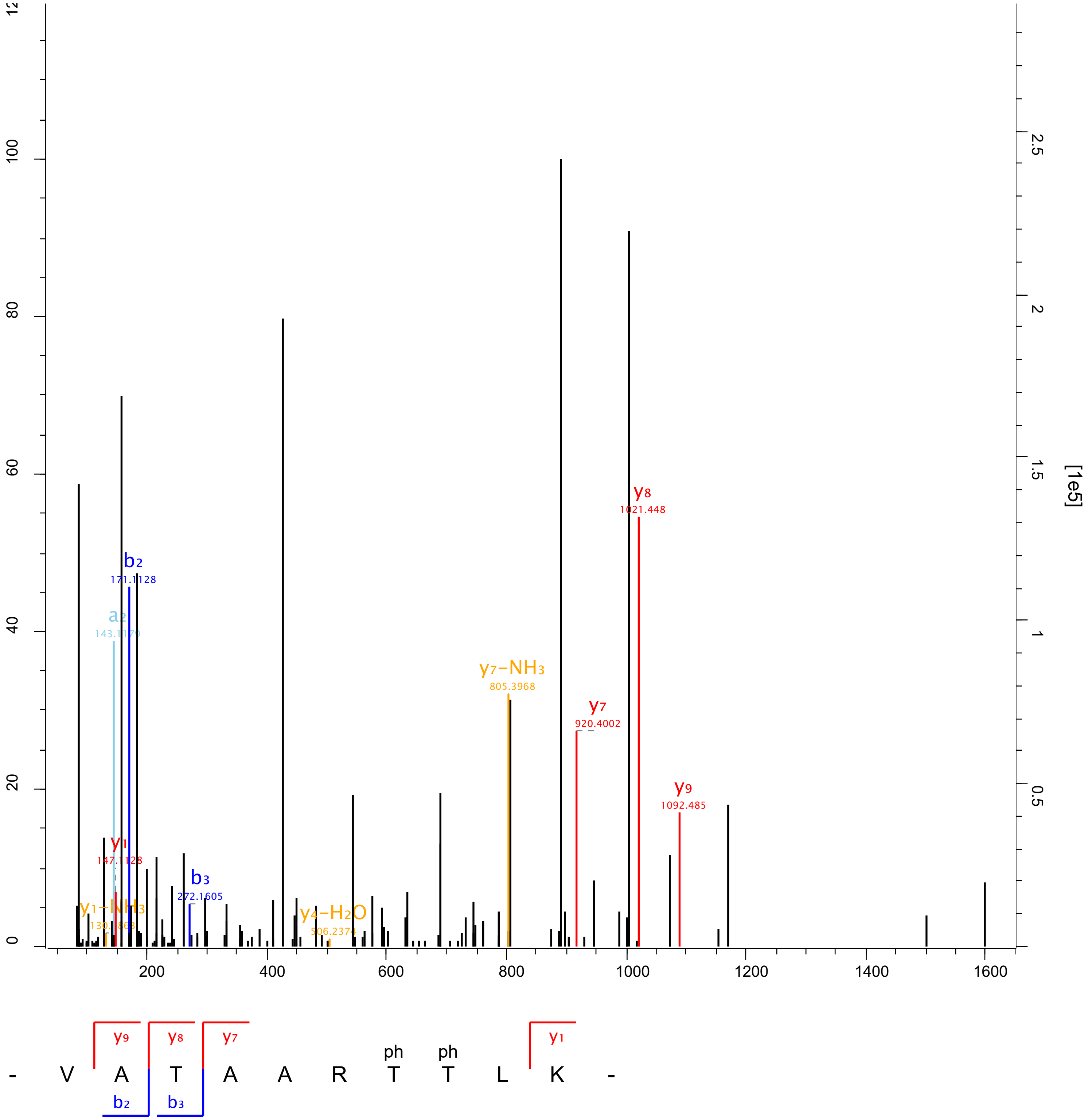

Sample output to test PDF Combine only

Raw file	Scan	Method	Score	m/z	Gene names
sp3-mic-0-1-A	14295	FTMS; HCD	104.26	777.88	PATL1

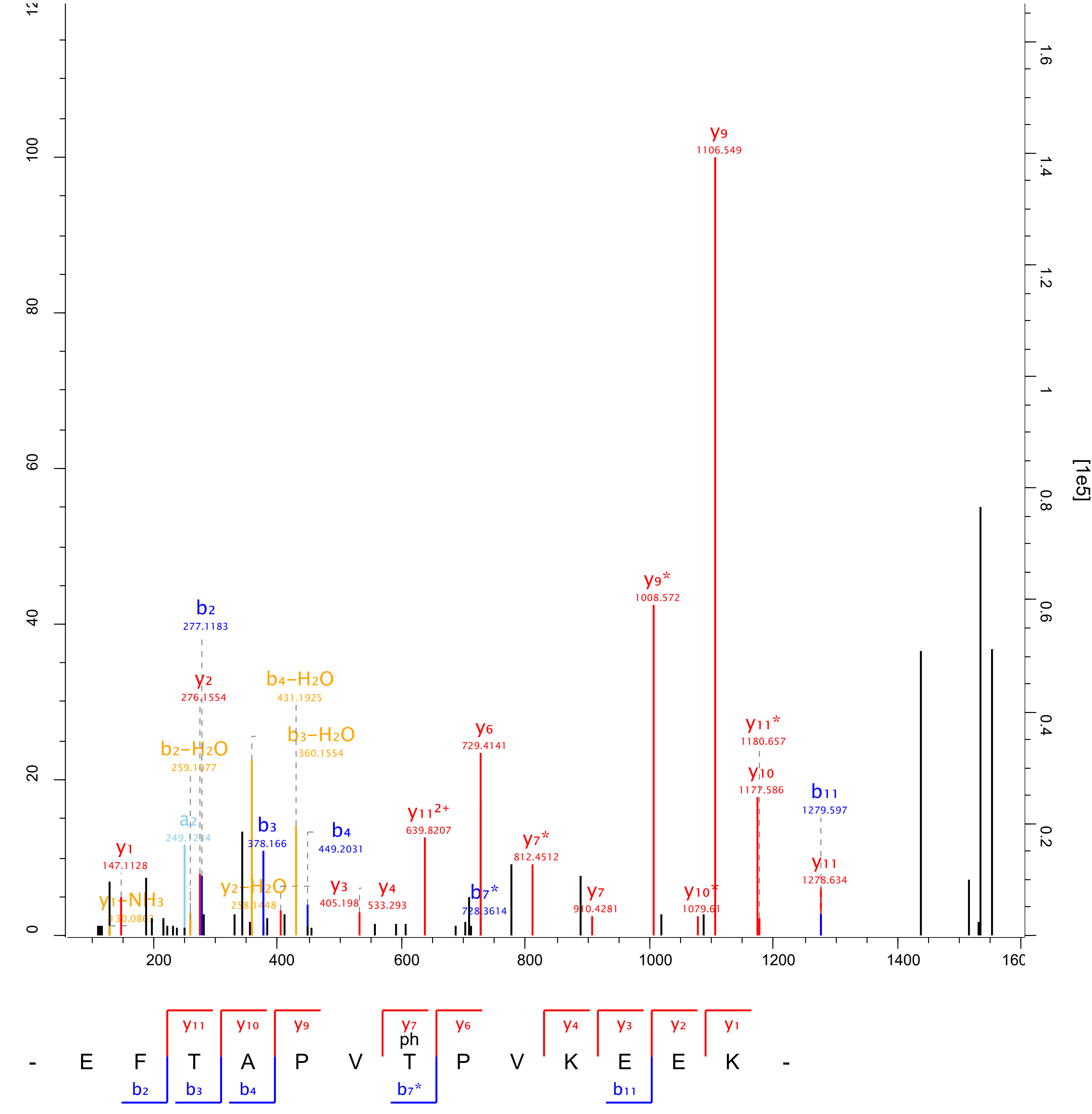

Sample output to test PDF Combine only

Raw file	Scan	Method	Score	m/z
sp3-mic-0-1-A	18782	FTMS; HCD	70.33	734.8

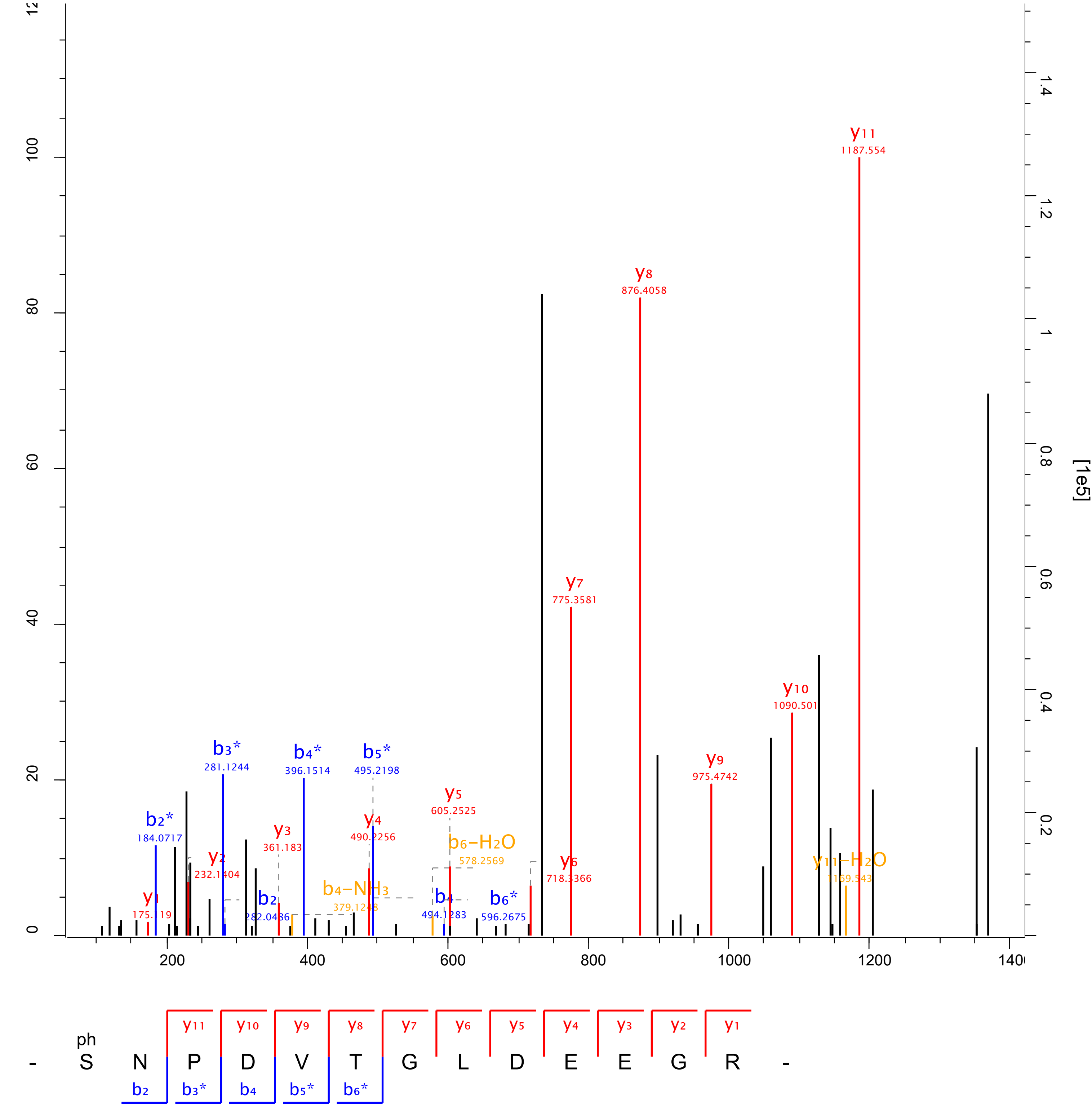

Sample output to test PDF Combine only

Raw file	Scan	Method	Score	m/z	Gene names
sp3-mic-0-1-P	31053	FTMS; HCD	45.28	797.37	At1g70550

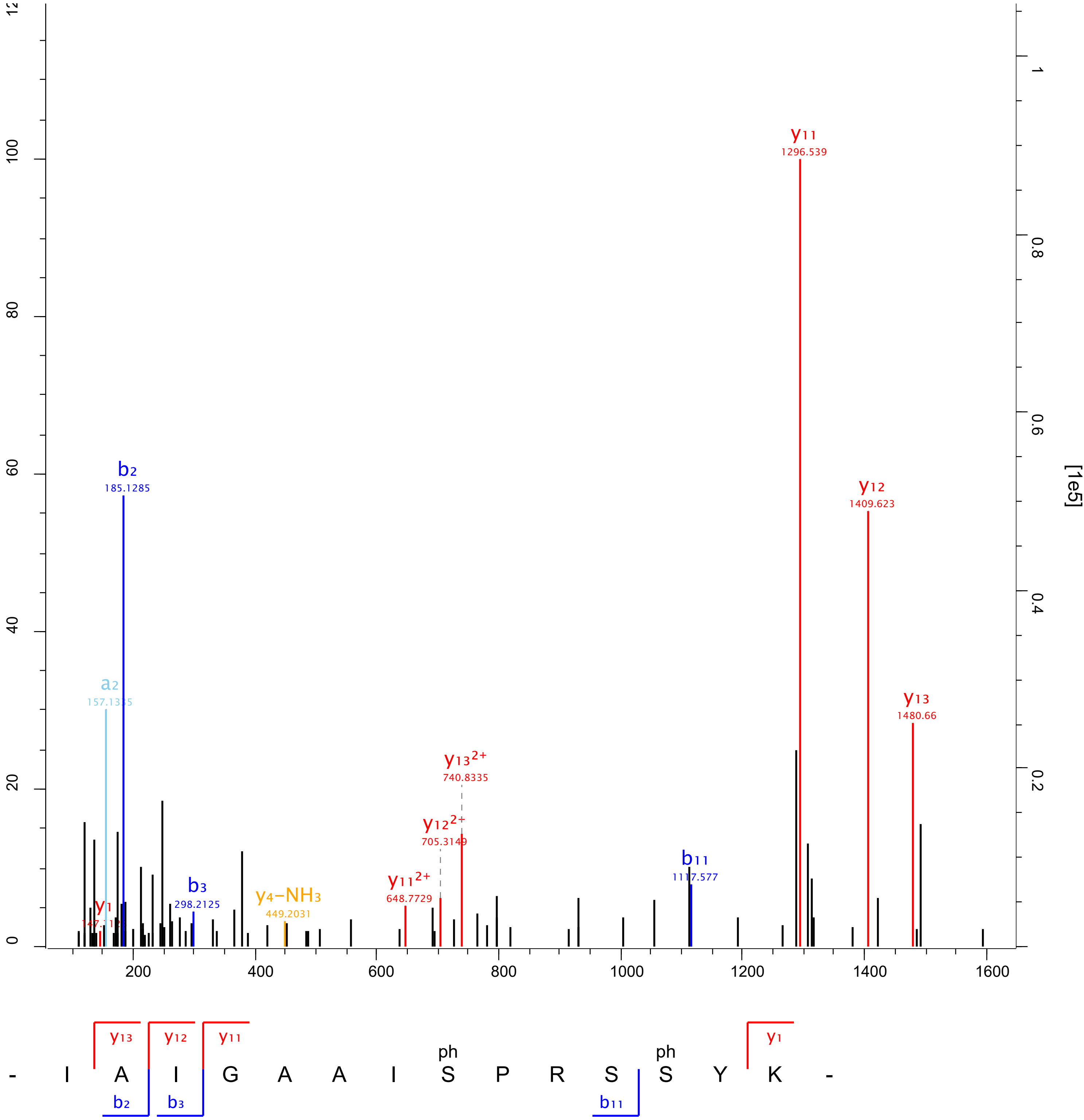

Sample output to test PDF Combine only

Raw file	Scan	Method	Score	m/z	Gene names
sp3-mic-0-1-P	40462	FTMS; HCD	42.31	1081.52	FKBP16-2;PNSL4

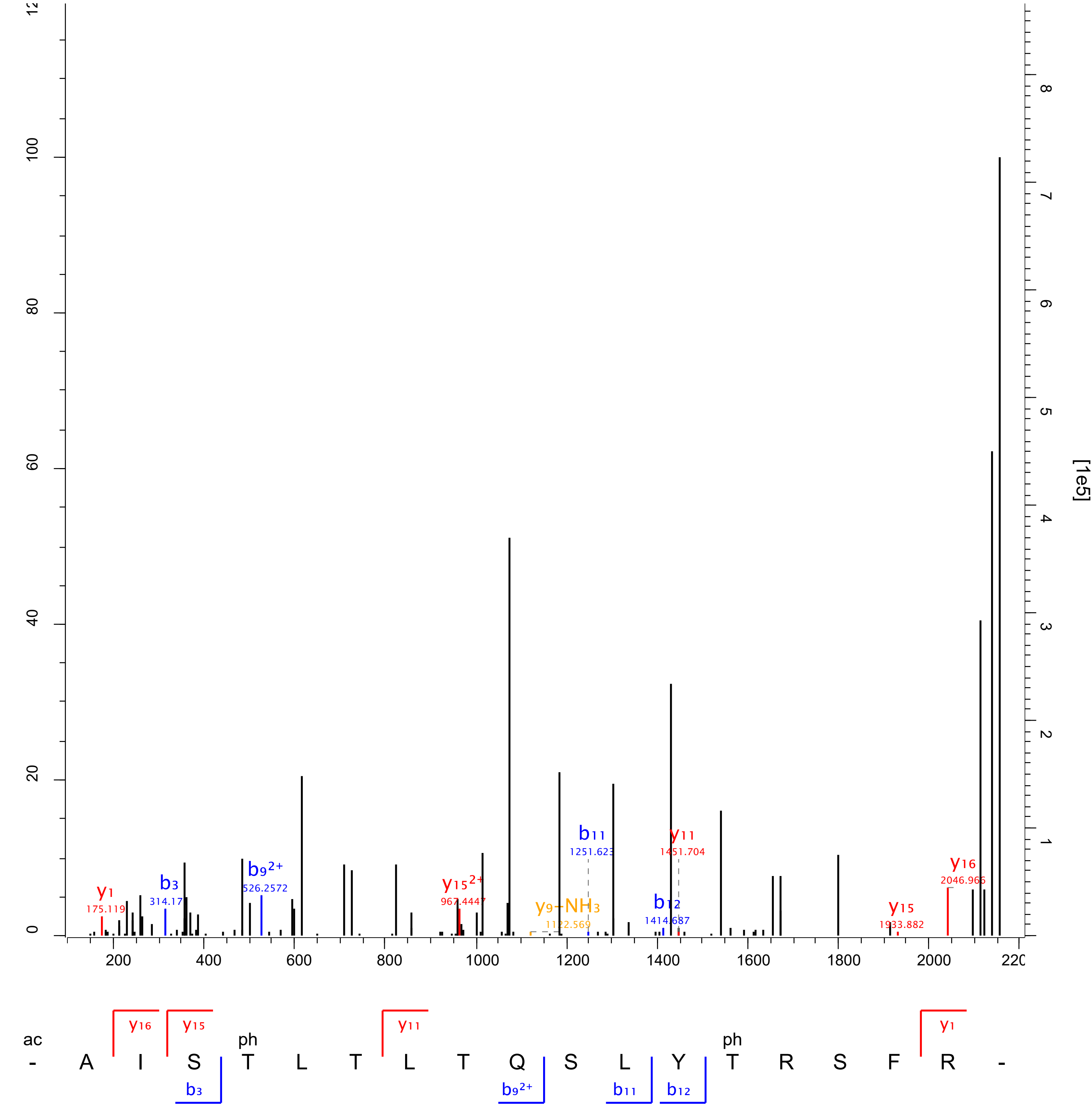

Sample output to test PDF Combine only

Raw file	Scan	Method	Score	m/z	Gene names
sp3-mic-0-2-A	5046	FTMS; HCD	96.85	733.95	At4g26630

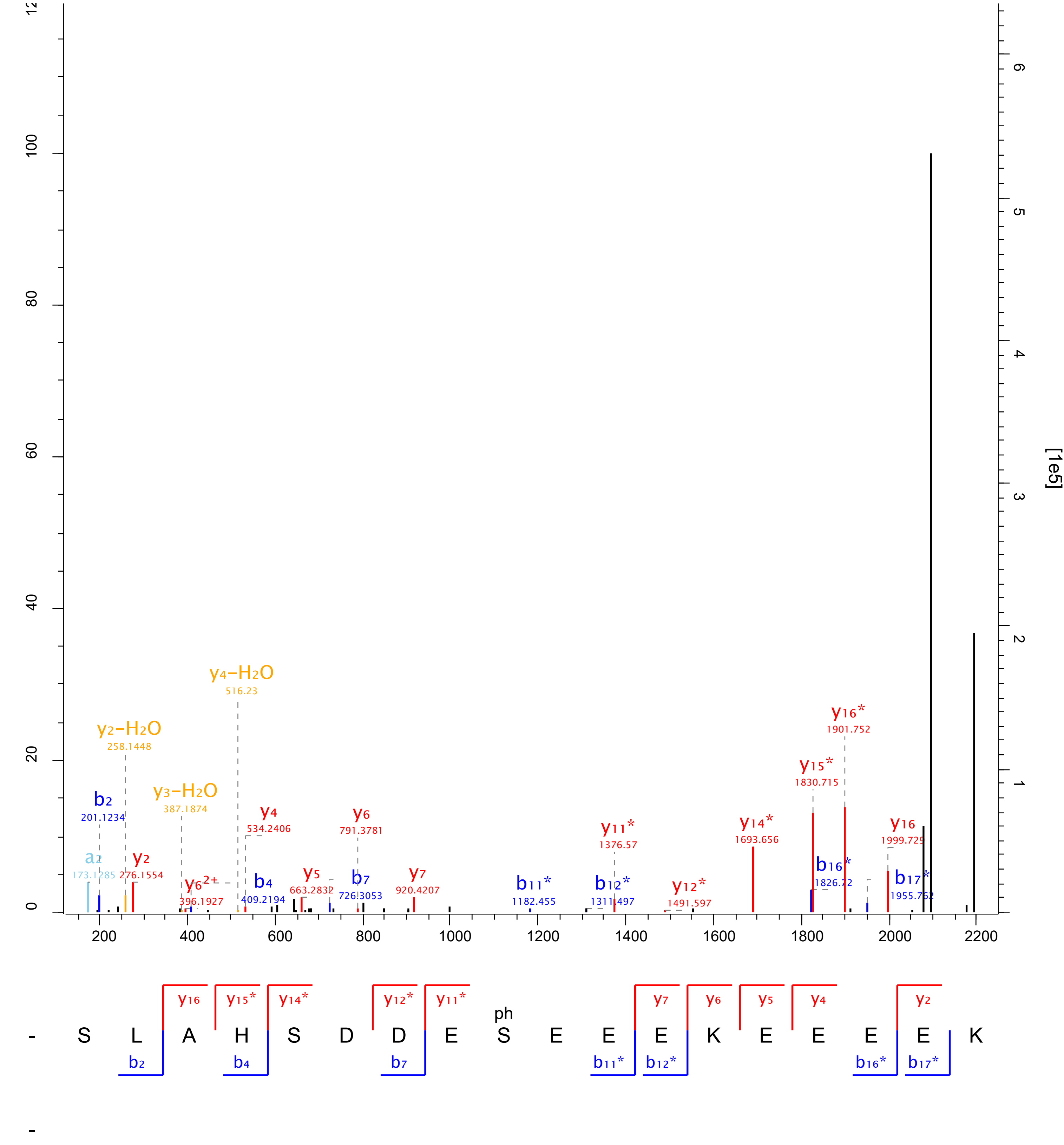

Sample output to test PDF Combine only

Raw file	Scan	Method	Score	m/z	Gene names
sp3-mic-0-2-A	7053	FTMS; HCD	92.44	783.36	At4g31880

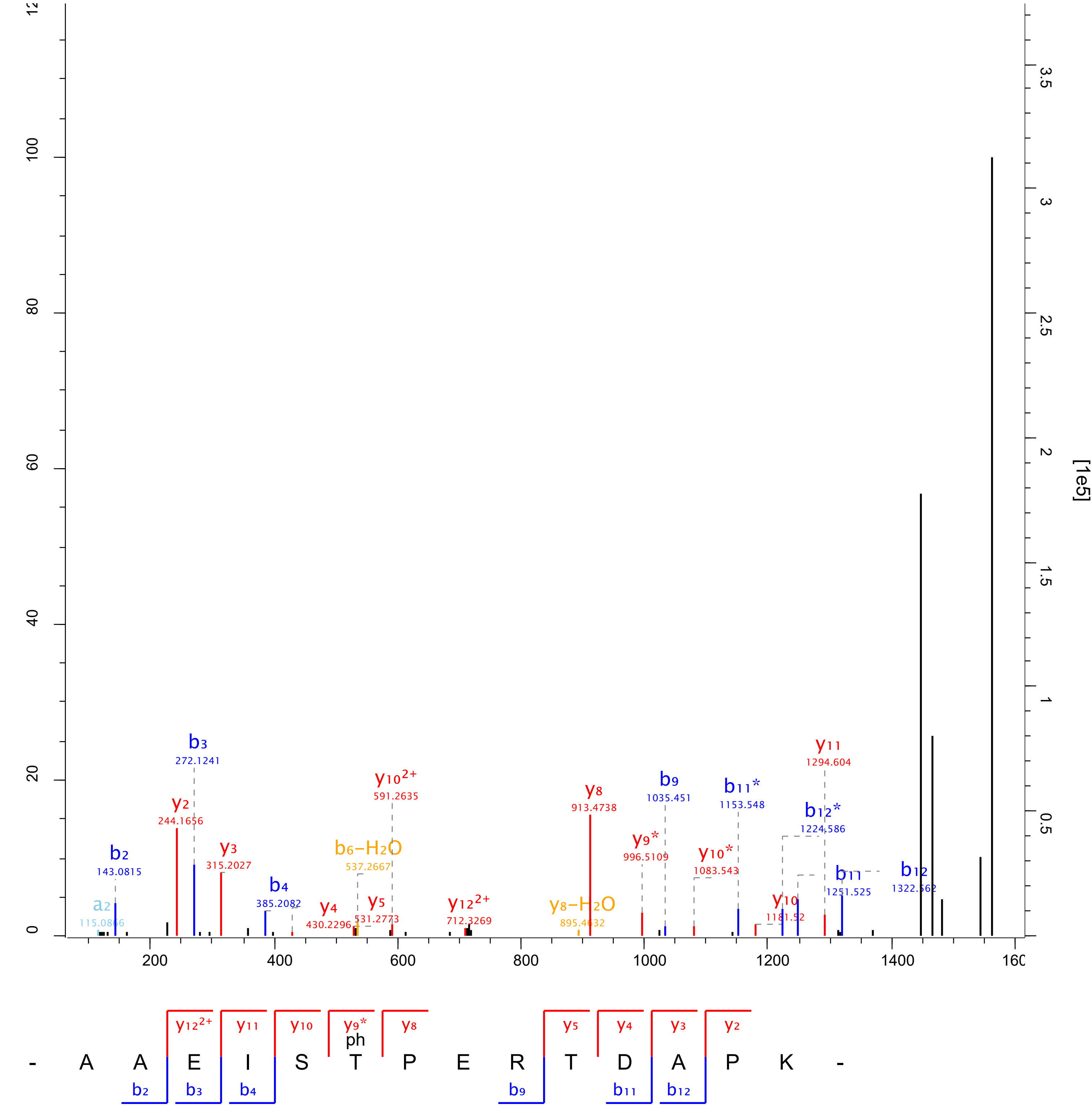

Sample output to test PDF Combine only

Raw file	Scan	Method	Score	m/z	Gene names
sp3-mic-0-2-A	7262	FTMS; HCD	118.51	687.63	SYP122

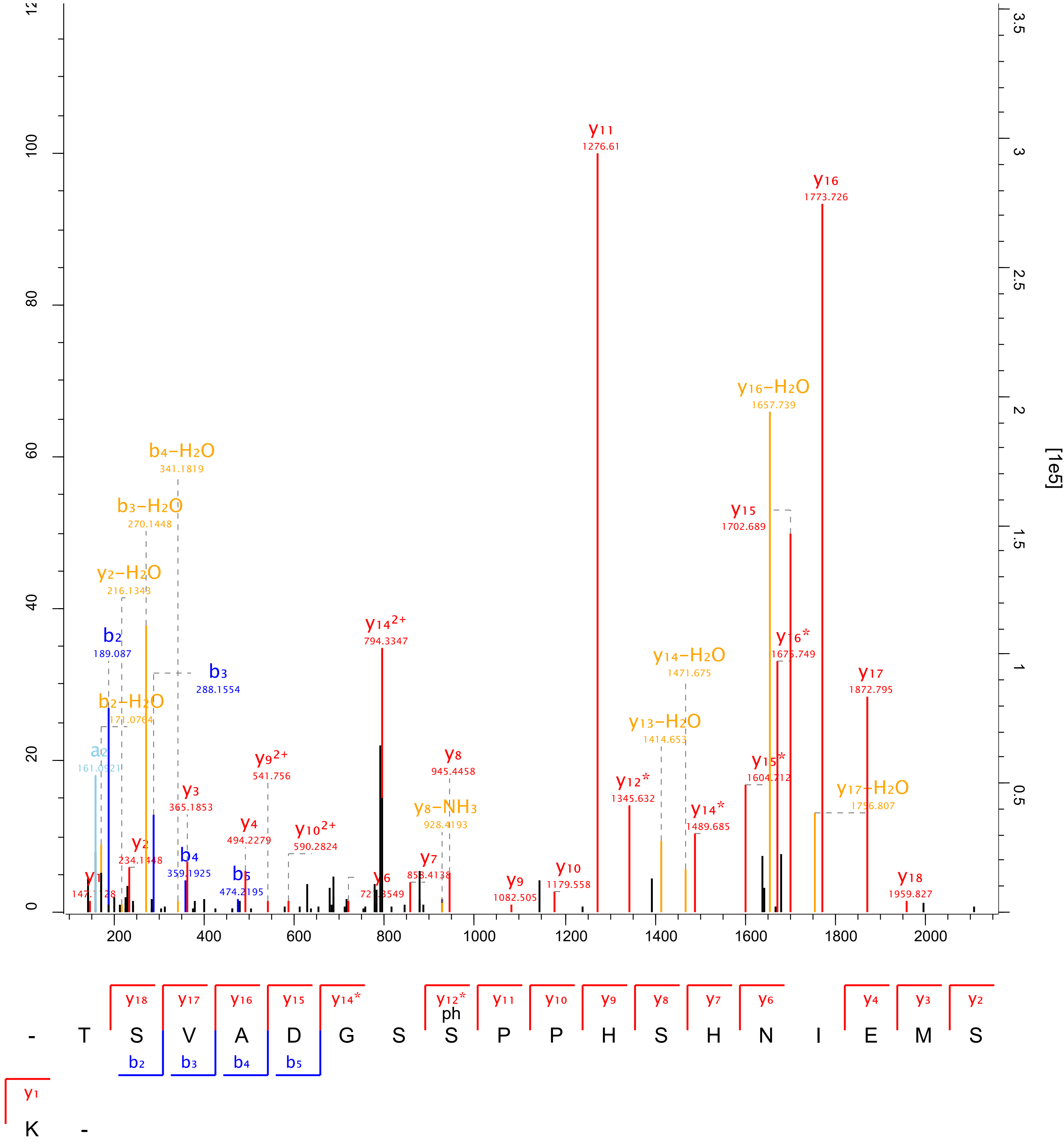

Sample output to test PDF Combine only

Raw file	Scan	Method	Score	m/z	Gene names
sp3-mic-0-2-A	7610	FTMS; HCD	81.81	634.32	At3g07030

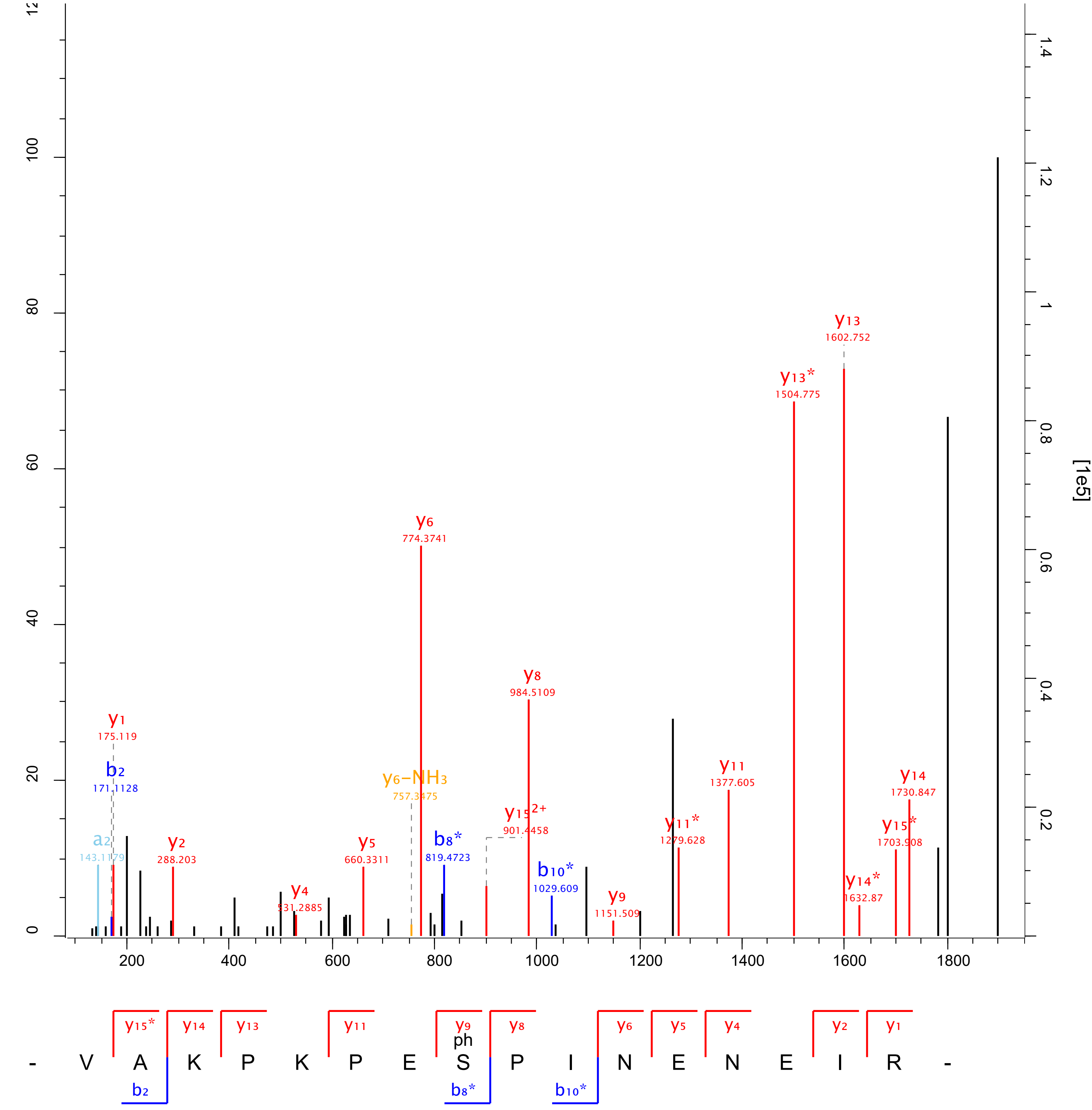

Sample output to test PDF Combine only

Raw file	Scan	Method	Score	m/z
sp3-mic-0-2-A	8945	FTMS; HCD	76.33	847.38

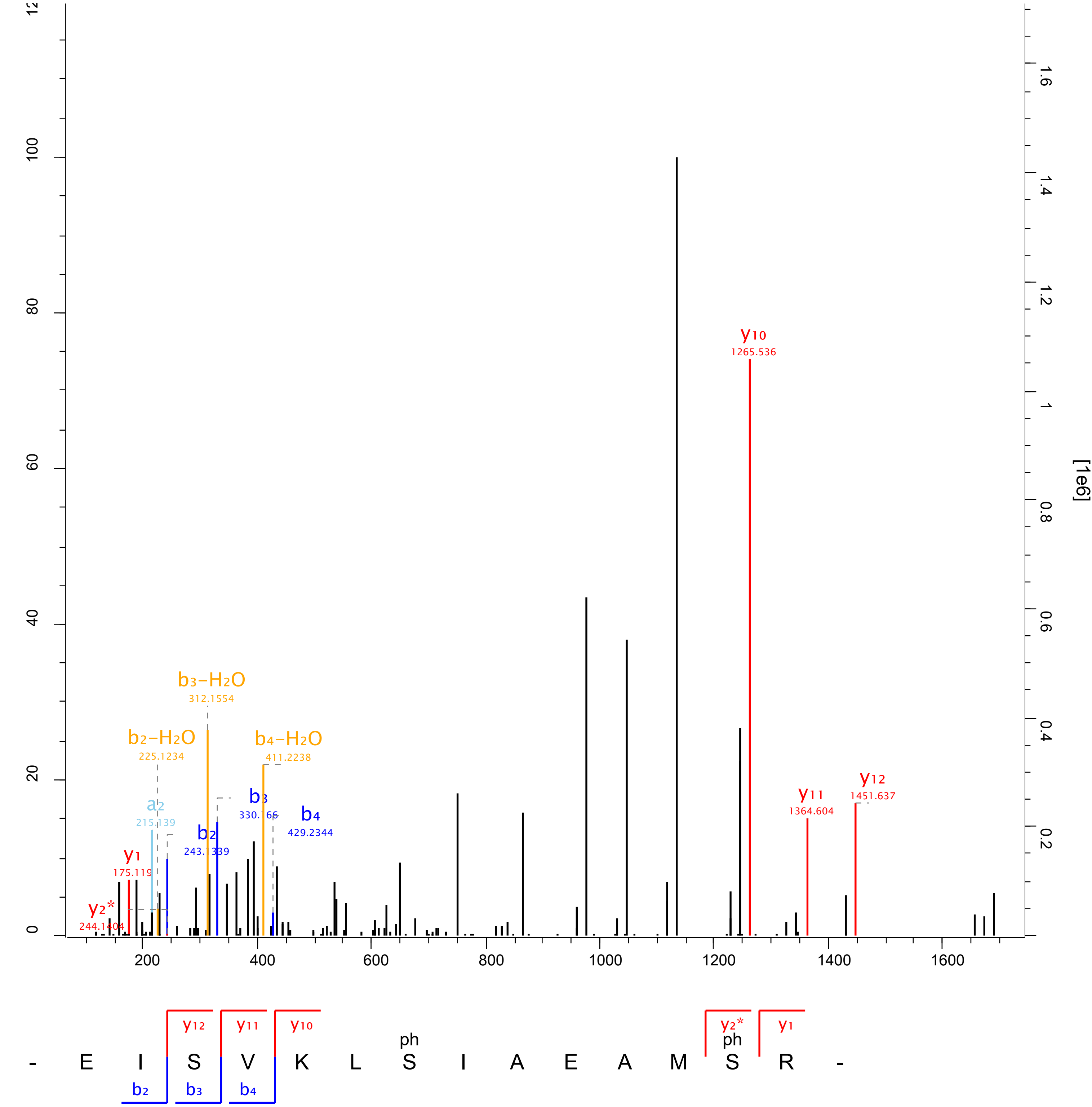

Sample output to test PDF Combine only

Raw file	Scan	Method	Score	m/z	Gene names
sp3-mic-0-2-A	9489	FTMS; HCD	46.32	456.55	MRO11.3;At5g23750

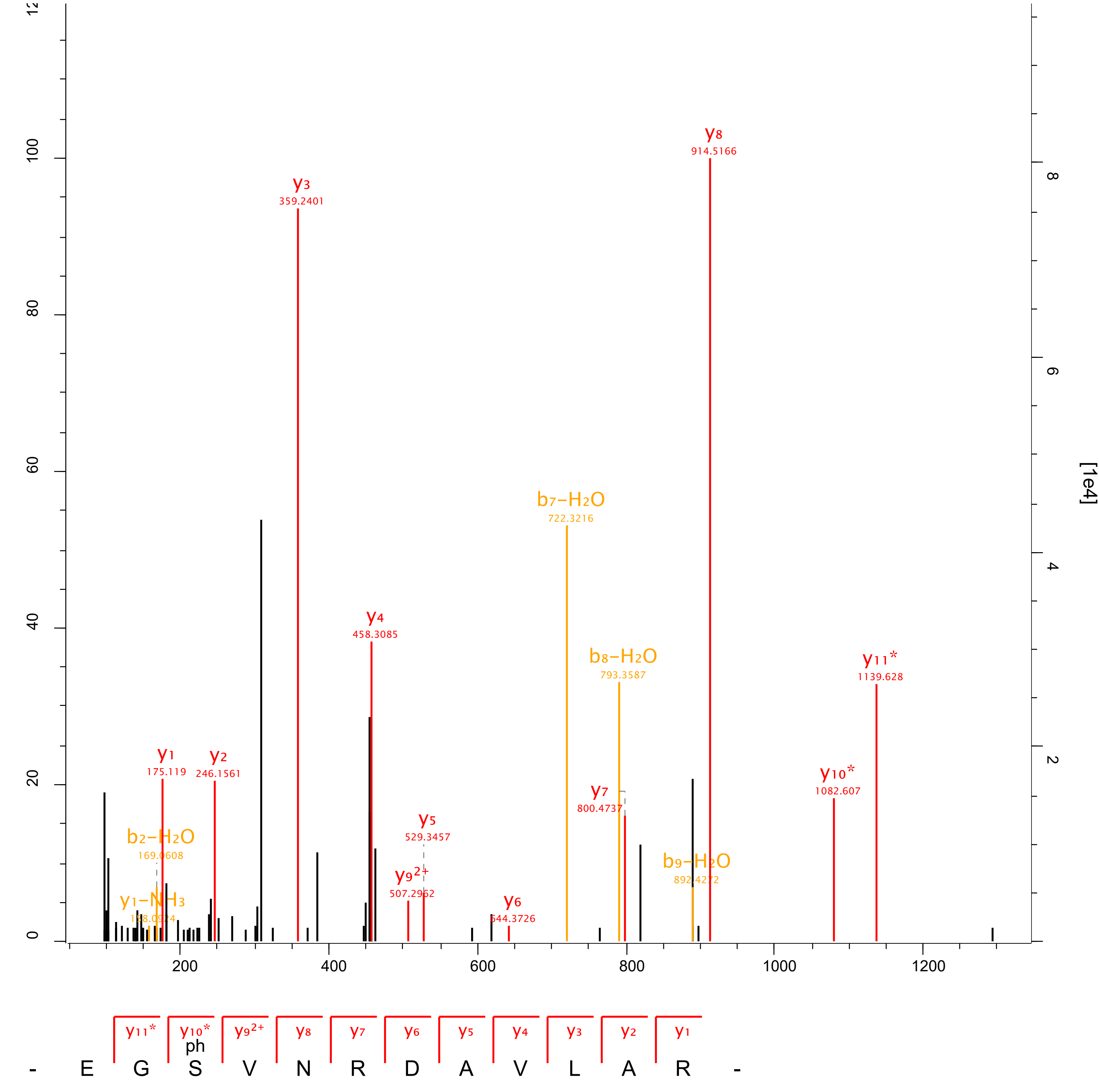

Sample output to test PDF Combine only

Raw file	Scan	Method	Score	m/z
sp3-mic-0-2-A	10127	FTMS; HCD	60.1	608.78

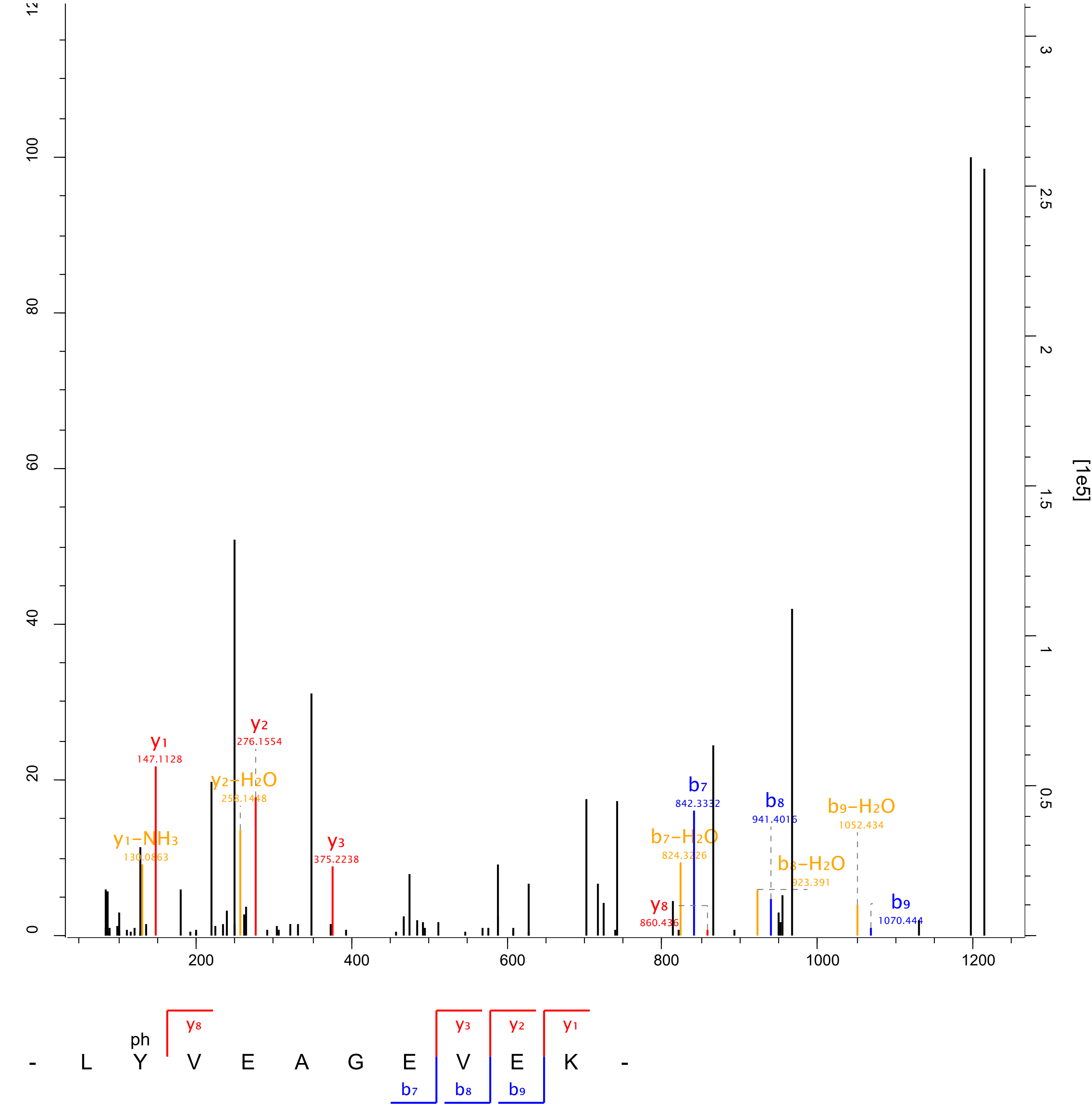

Sample output to test PDF Combine only

Raw file	Scan	Method	Score	m/z
sp3-mic-0-2-A	10595	FTMS; HCD	86.7	690.29

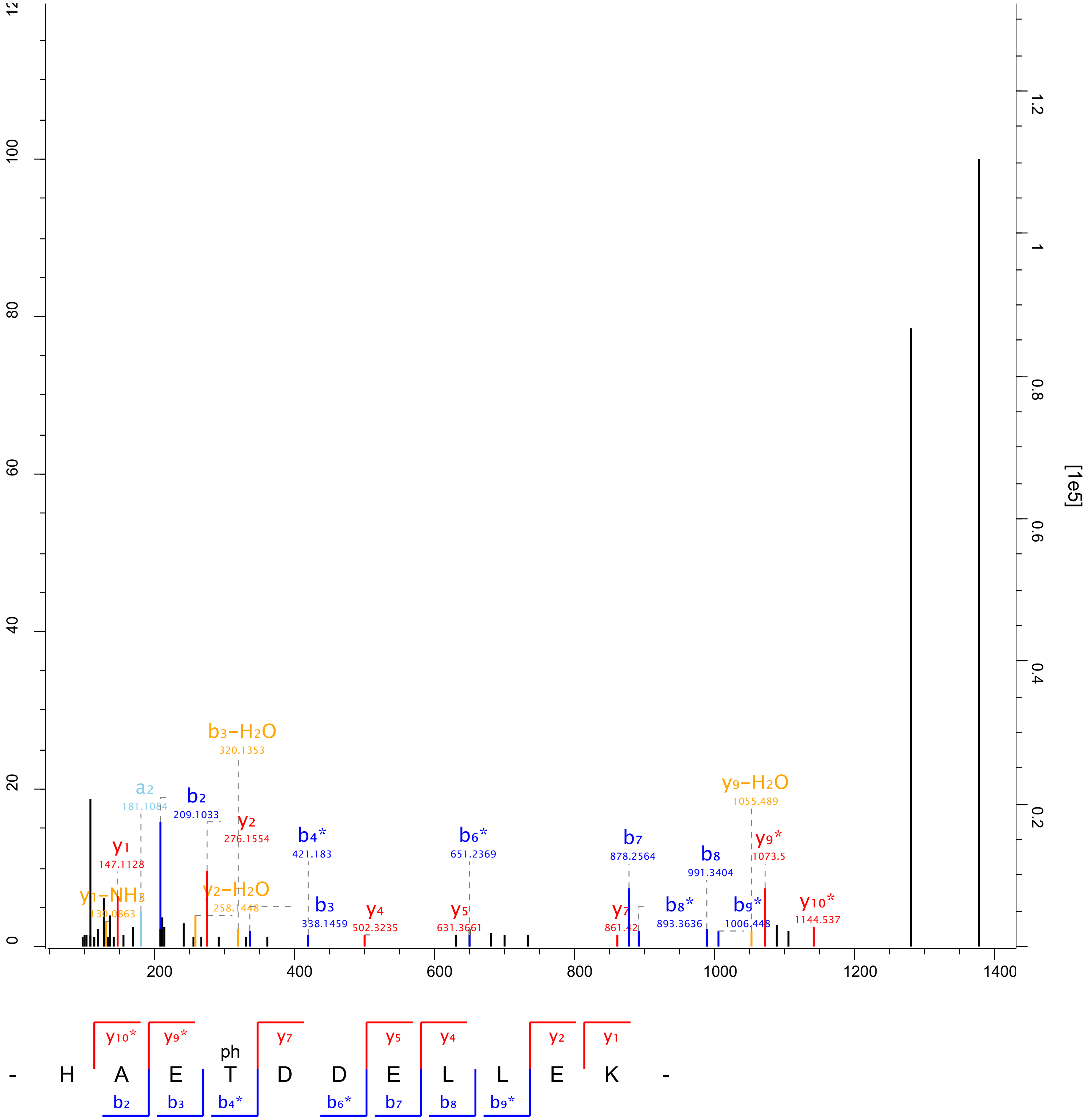

Sample output to test PDF Combine only

Raw file	Scan	Method	Score	m/z	Gene names
sp3-mic-0-2-A	10879	FTMS; HCD	138.28	580.97	DBP

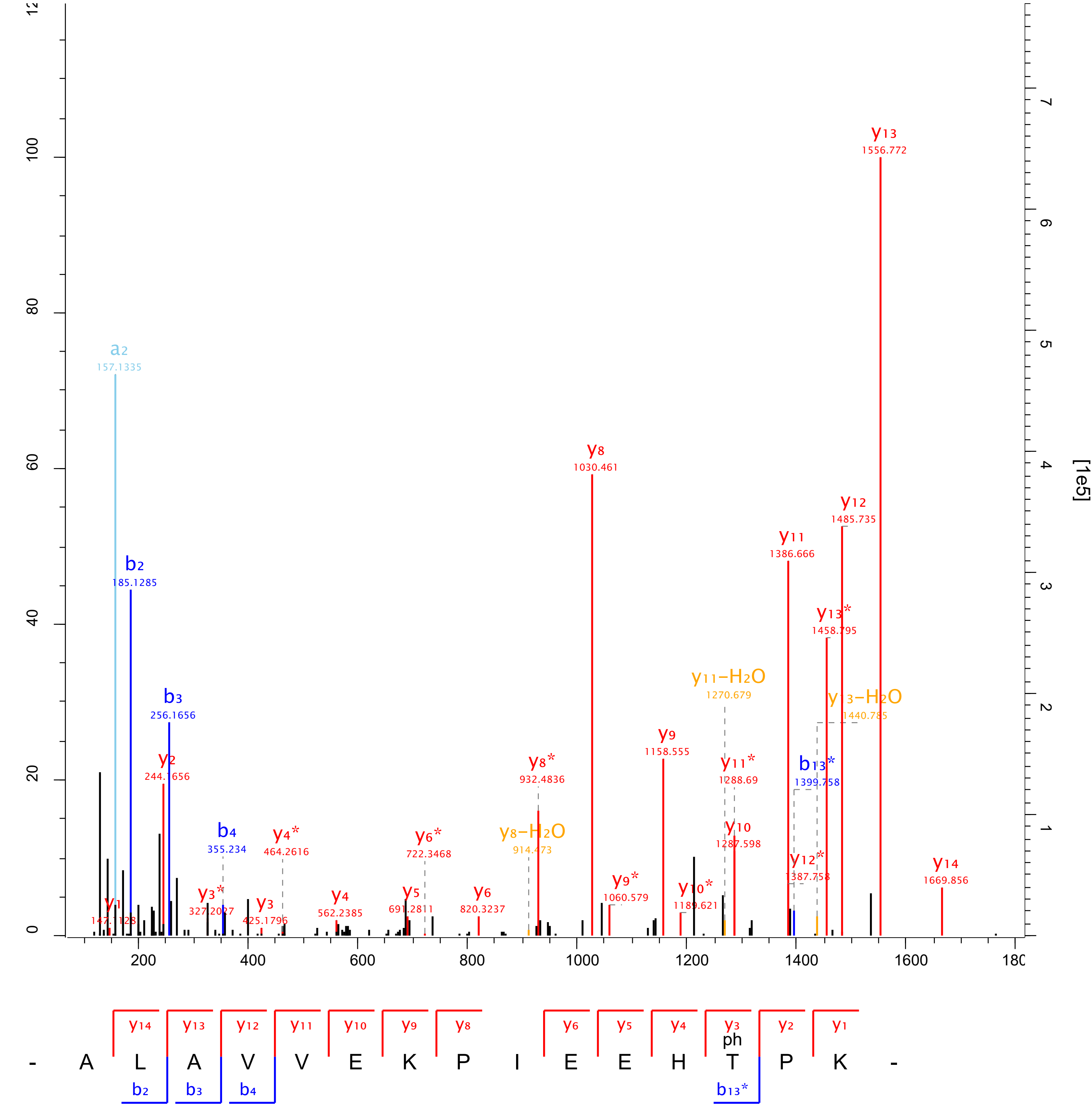

Sample output to test PDF Combine only

Raw file	Scan	Method	Score	m/z	Gene names
sp3-mic-0-2-A	12191	FTMS; HCD	62.2	930.87	DRP2B

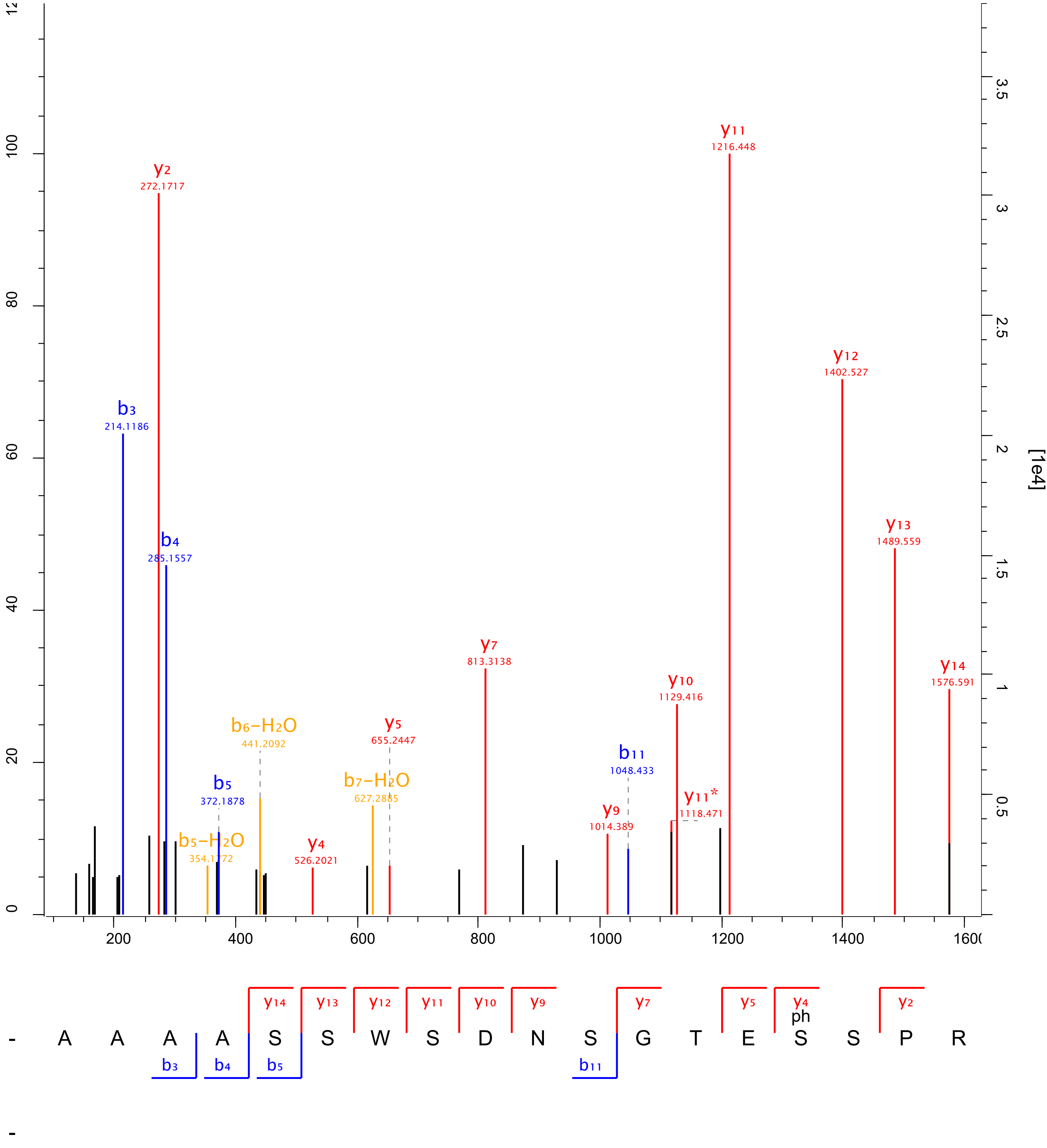

Sample output to test PDF Combine only

Raw file	Scan	Method	Score	m/z
sp3-mic-0-2-A	12465	FTMS; HCD	90.41	562.77

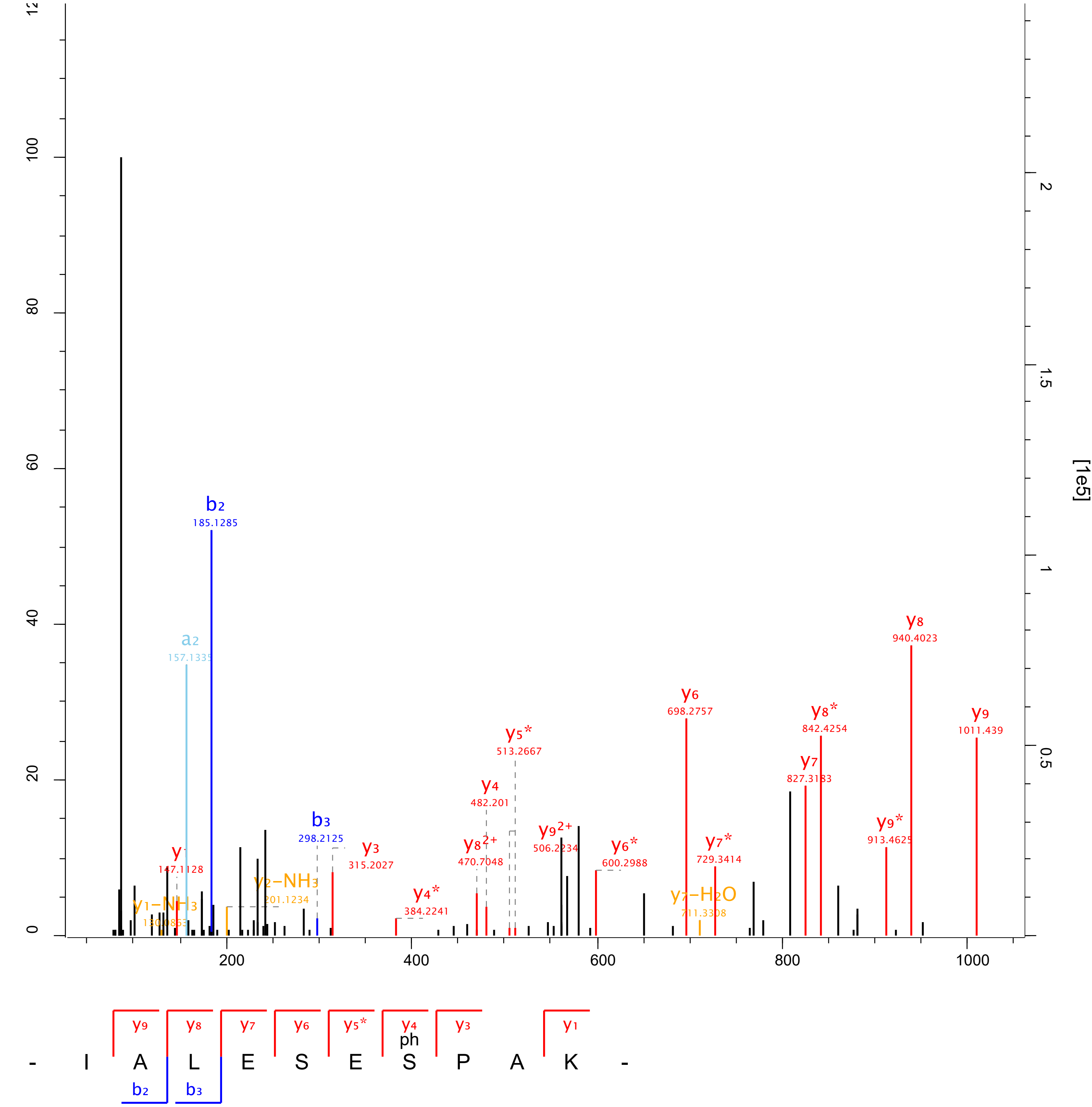

Sample output to test PDF Combine only

Raw file	Scan	Method	Score	m/z	Gene names
sp3-mic-0-2-A	13494	FTMS; HCD	56.65	513.25	ABCB4

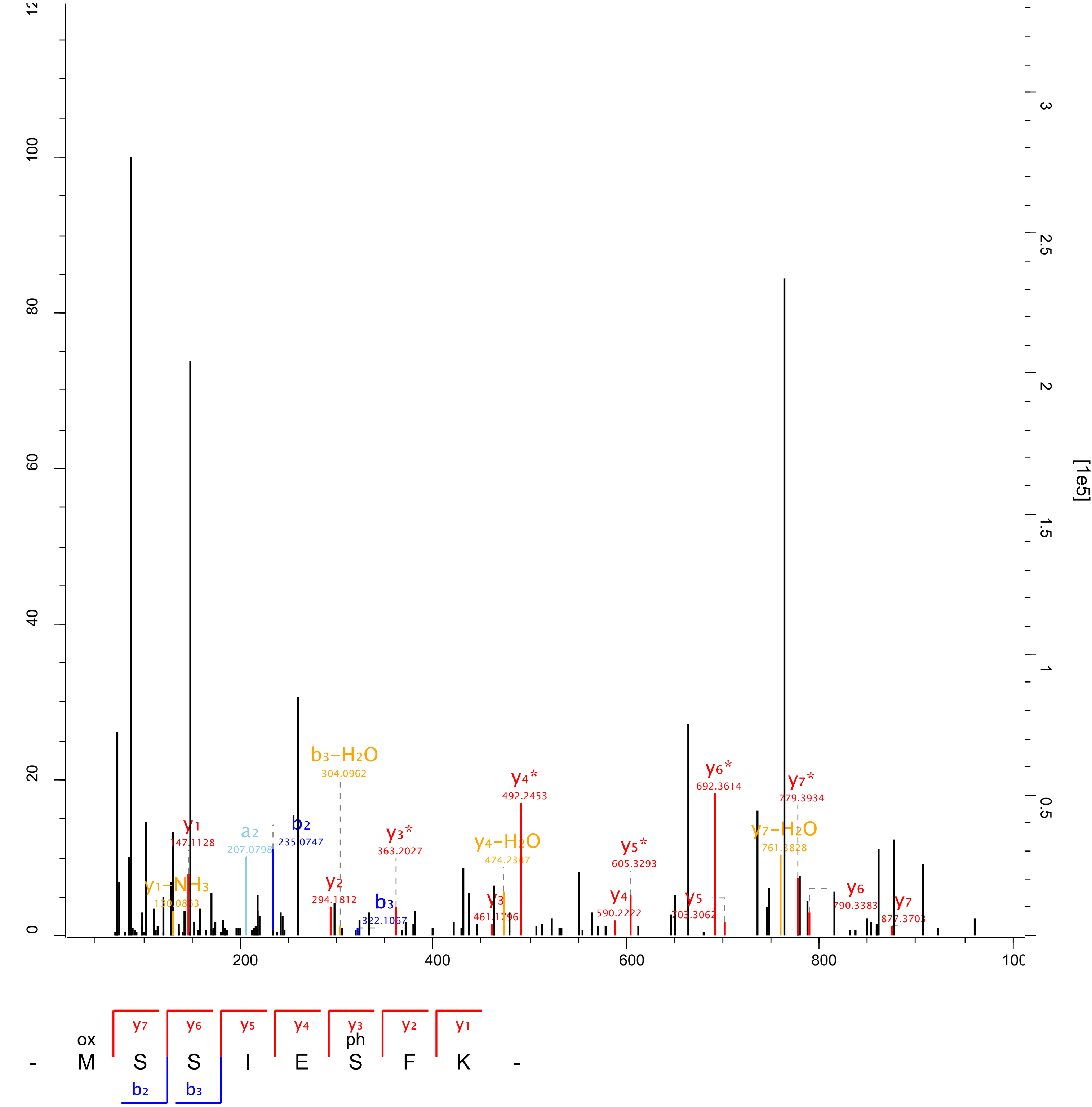

Sample output to test PDF Combine only

Raw file	Scan	Method	Score	m/z	Gene names
sp3-mic-0-2-A	17450	FTMS; HCD	58.89	712.31	RLK902

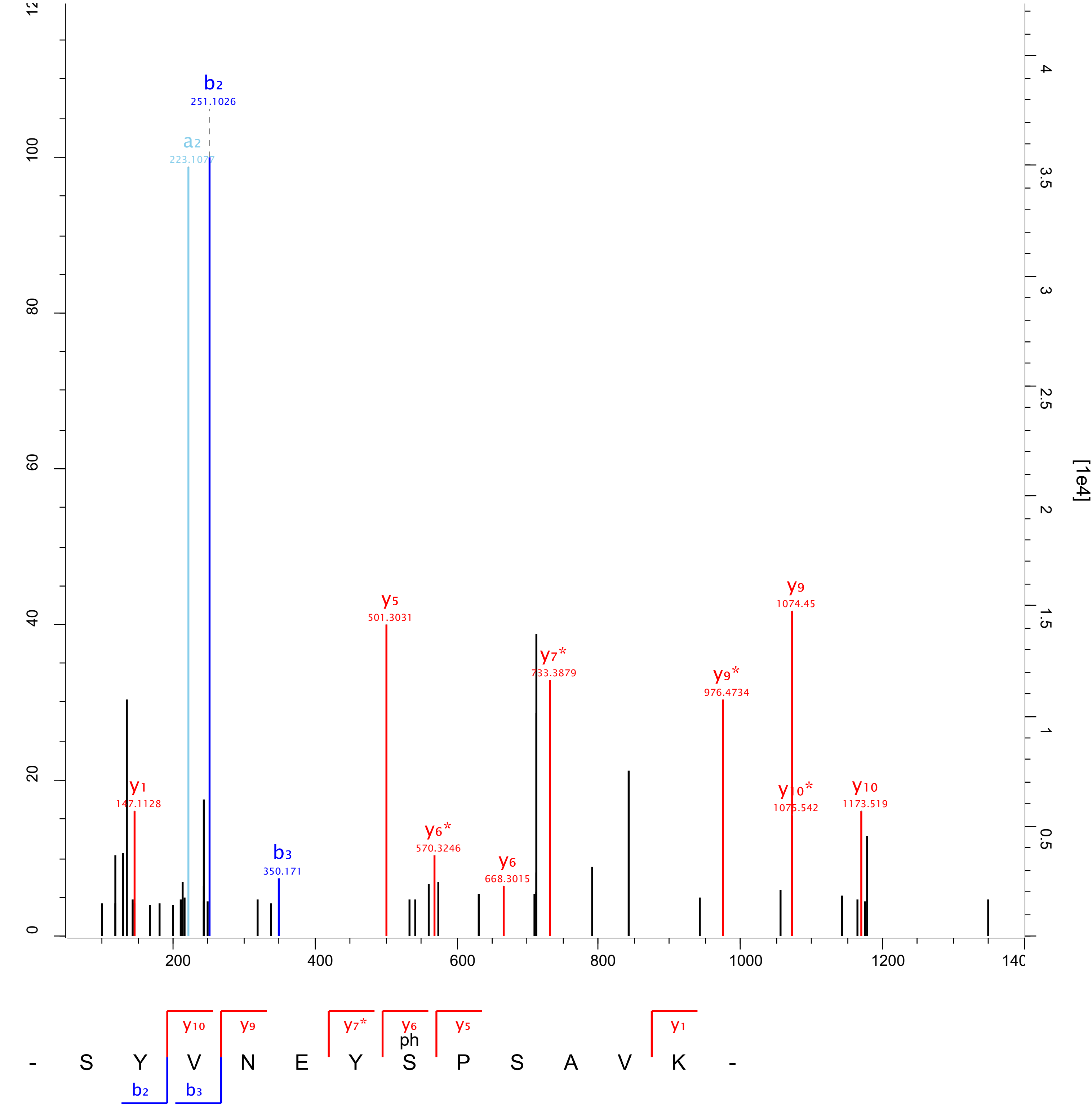

Sample output to test PDF Combine only

Raw file	Scan	Method	Score	m/z	Gene names
sp3-mic-0-2-A	18563	FTMS; HCD	73.9	504.71	ABCB4

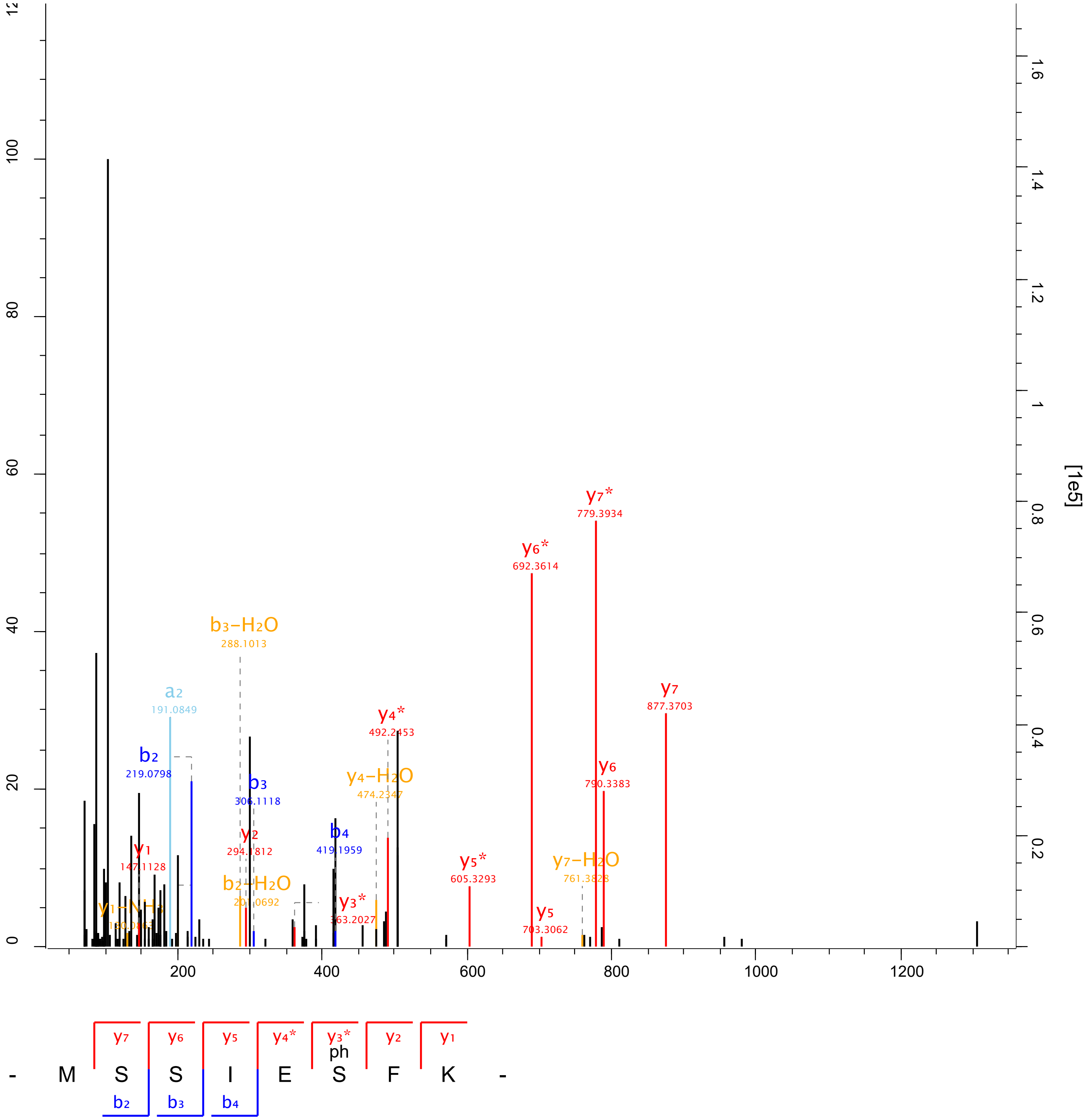

Sample output to test PDF Combine only

Raw file	Scan	Method	Score	m/z	Gene names
sp3-mic-0-2-A	19624	FTMS; HCD	136.53	770.36	RS2Z33

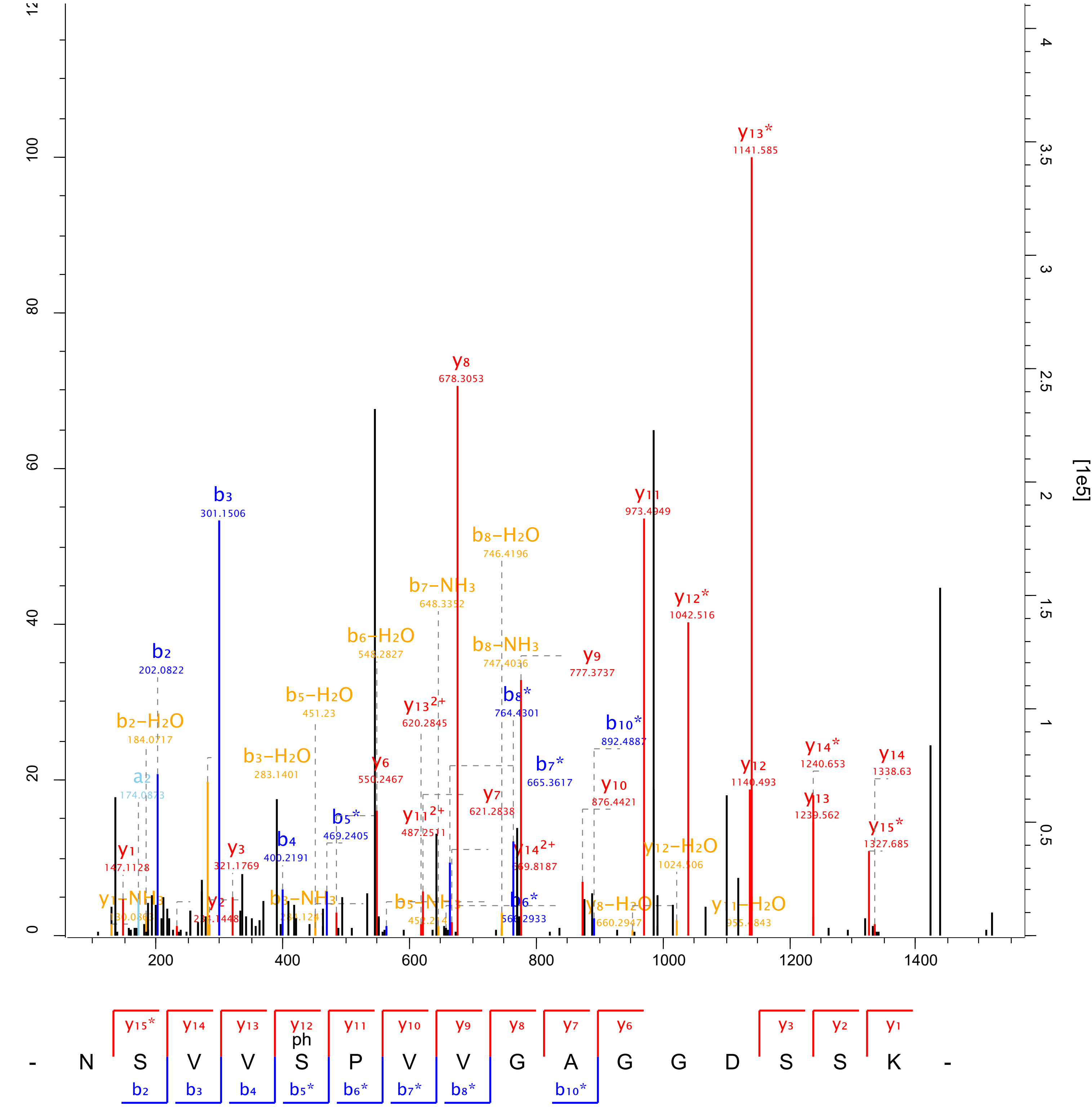

Sample output to test PDF Combine only

Raw file	Scan	Method	Score	m/z	Gene names
sp3-mic-0-2-A	20835	FTMS; HCD	197.07	580.77	PATL2

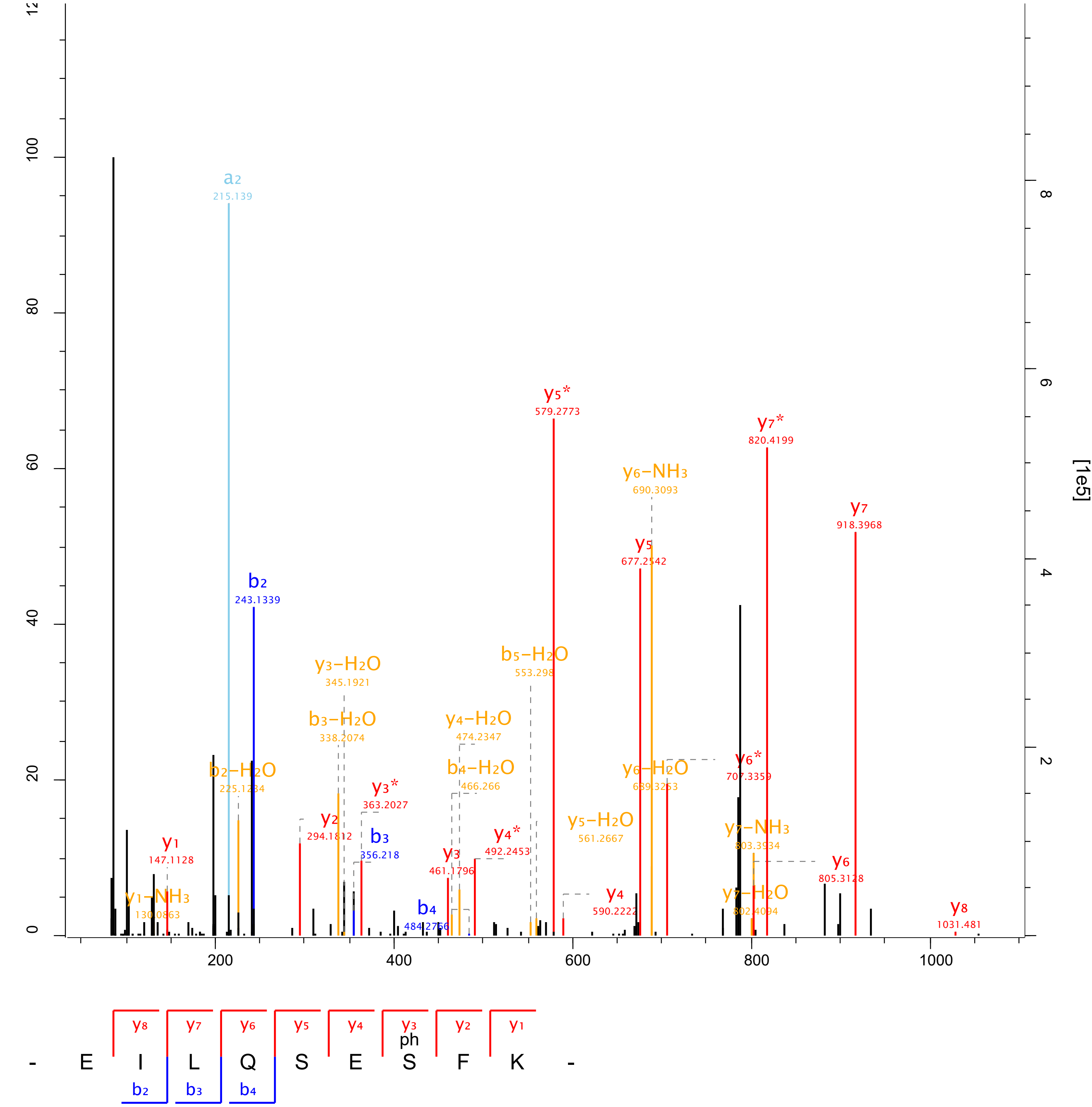

Sample output to test PDF Combine only

Raw file	Scan	Method	Score	m/z	Gene names
sp3-mic-0-2-A	21675	FTMS; HCD	46	668.81	PI4KG2

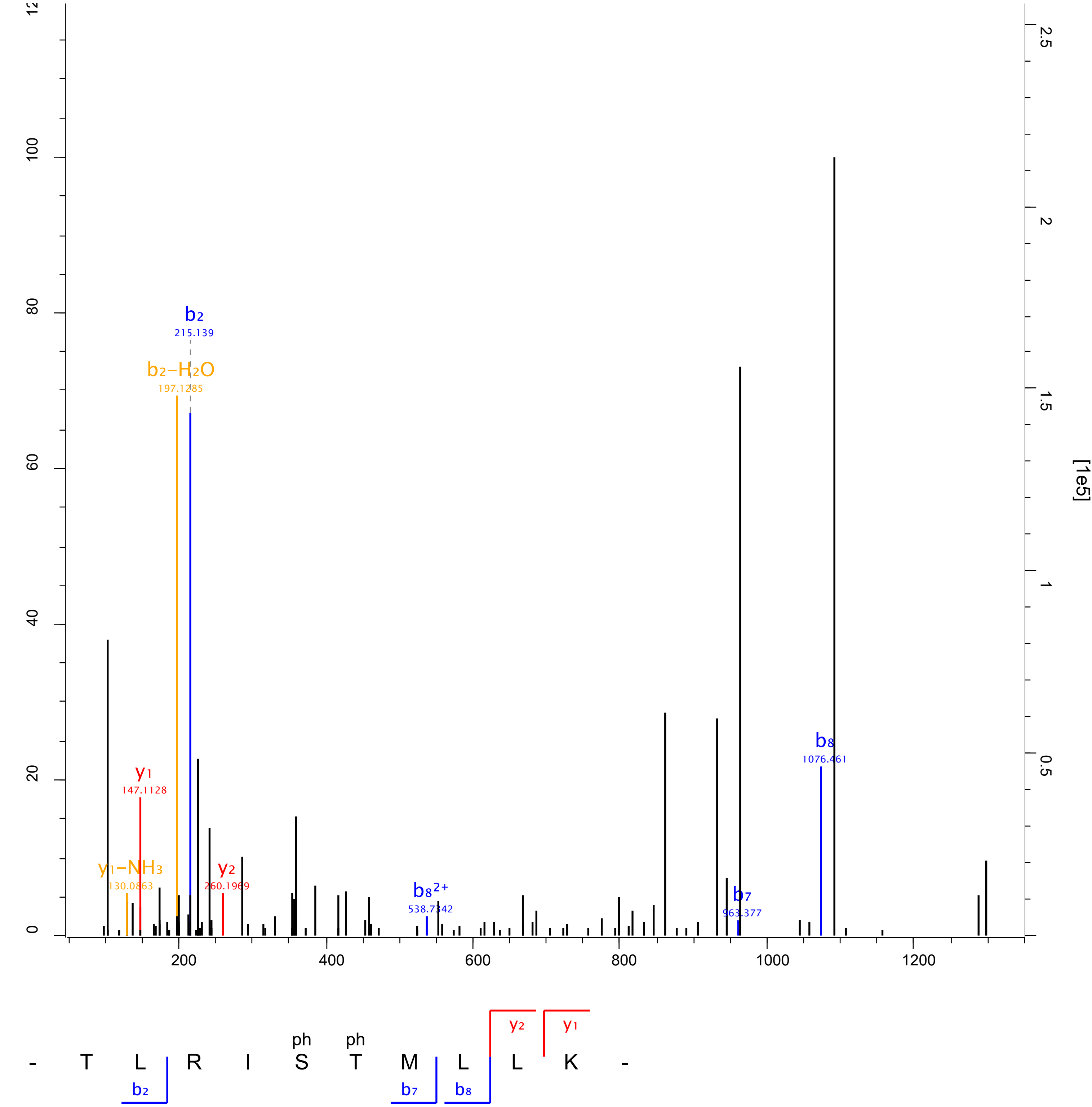

Sample output to test PDF Combine only

Raw file	Scan	Method	Score	m/z	Gene names
sp3-mic-0-2-A	22380	FTMS; HCD	114.31	655.3	CBF5

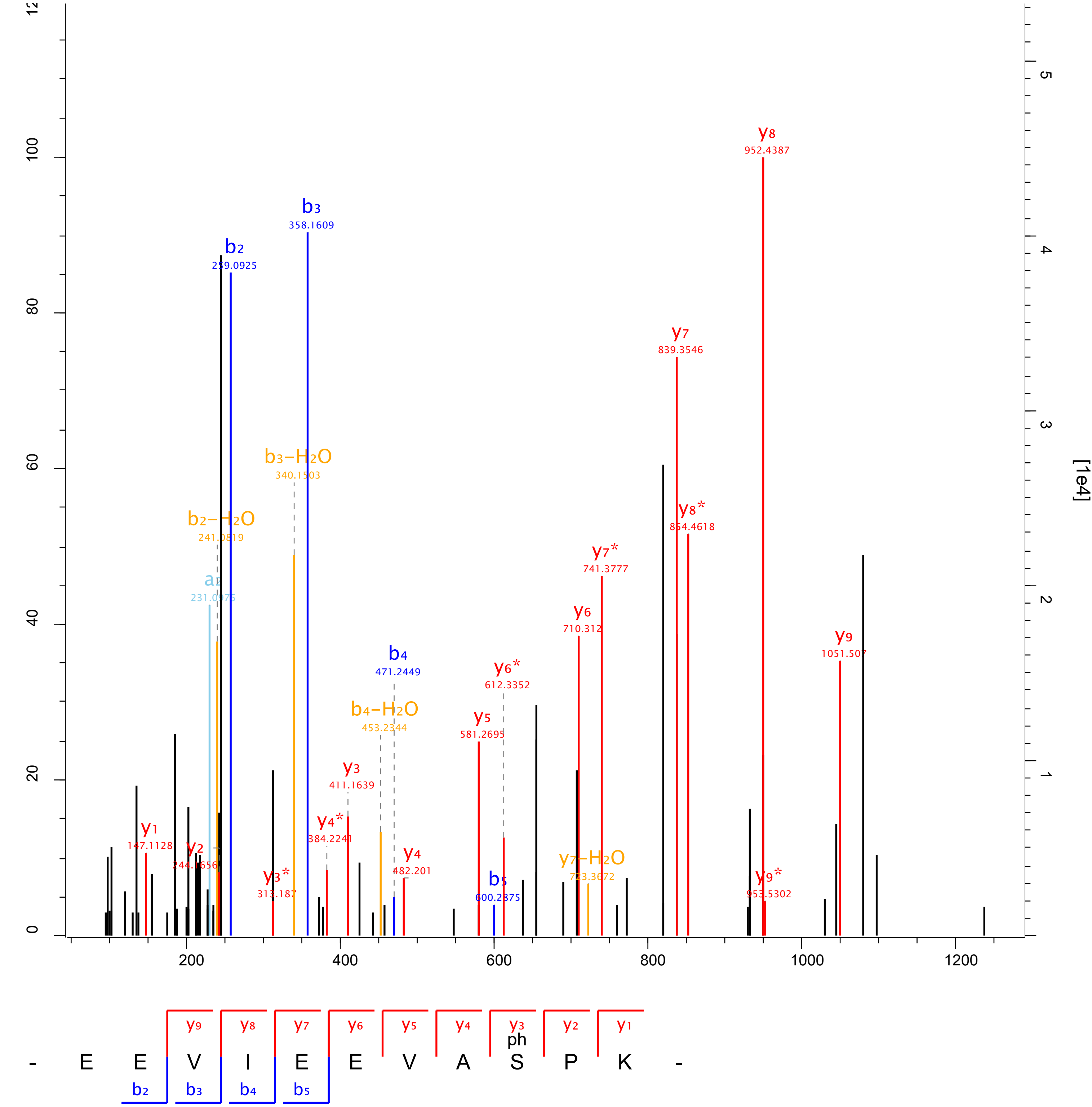

Sample output to test PDF Combine only

Raw file	Scan	Method	Score	m/z	Gene names
sp3-mic-0-2-A	22830	FTMS; HCD	46.07	855.39	POLIA

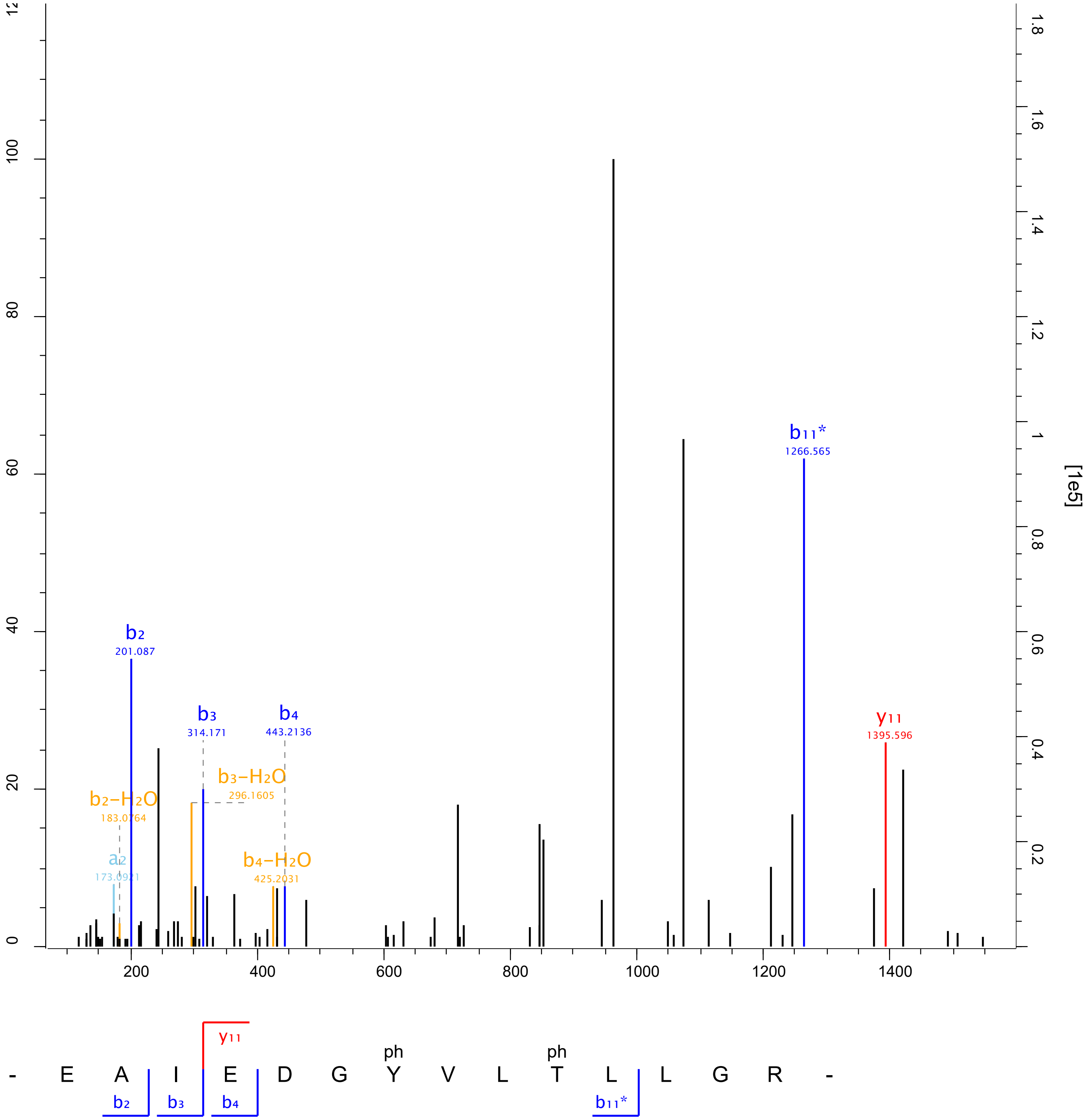

Sample output to test PDF Combine only

Raw file	Scan	Method	Score	m/z	Gene names
sp3-mic-0-2-A	24228	FTMS; HCD	50.35	757.36	EFS;ASHH2

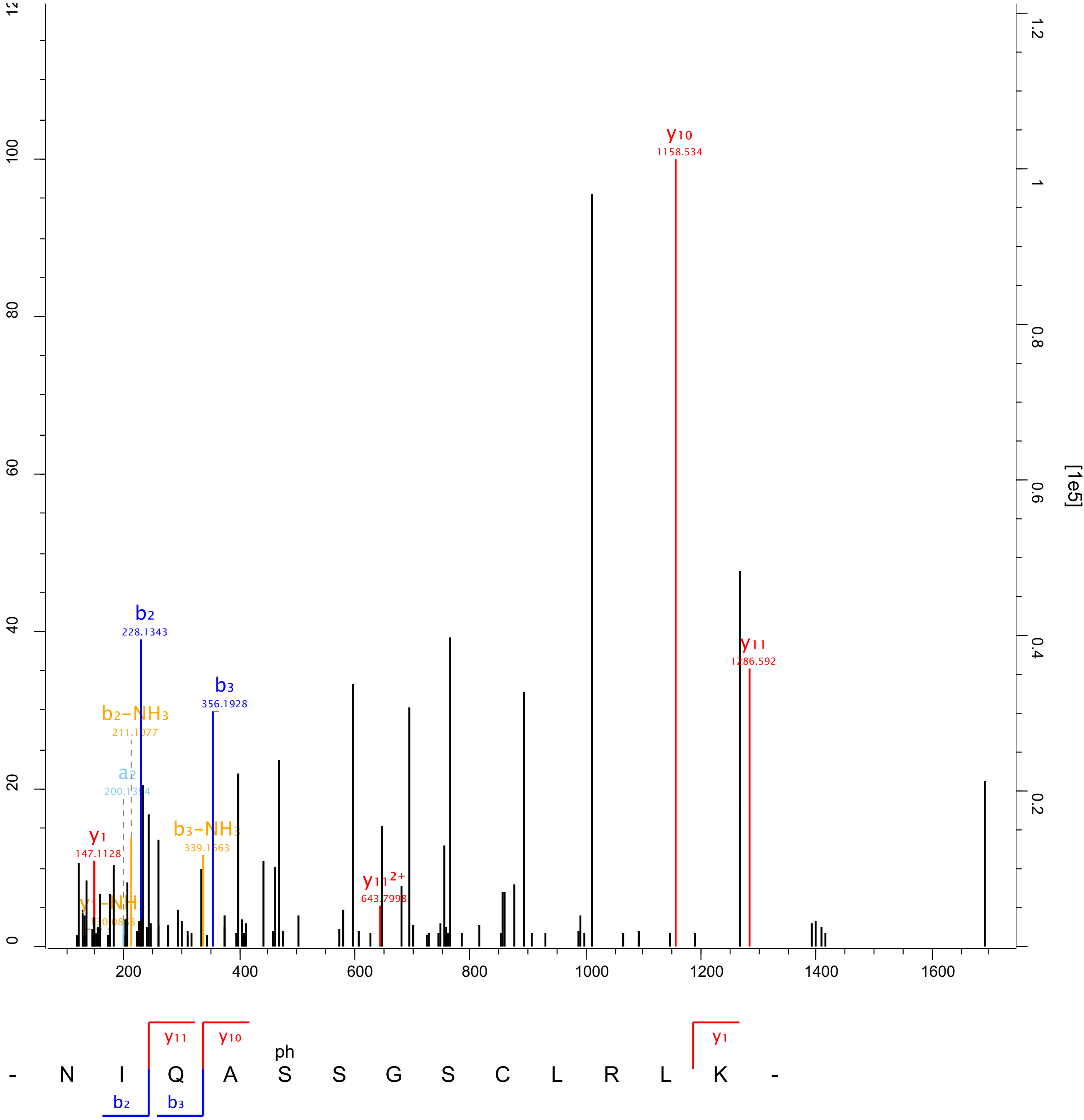

Sample output to test PDF Combine only

Raw file	Scan	Method	Score	m/z	Gene names
sp3-mic-0-2-A	30380	FTMS; HCD	63.68	675.83	At1g69790

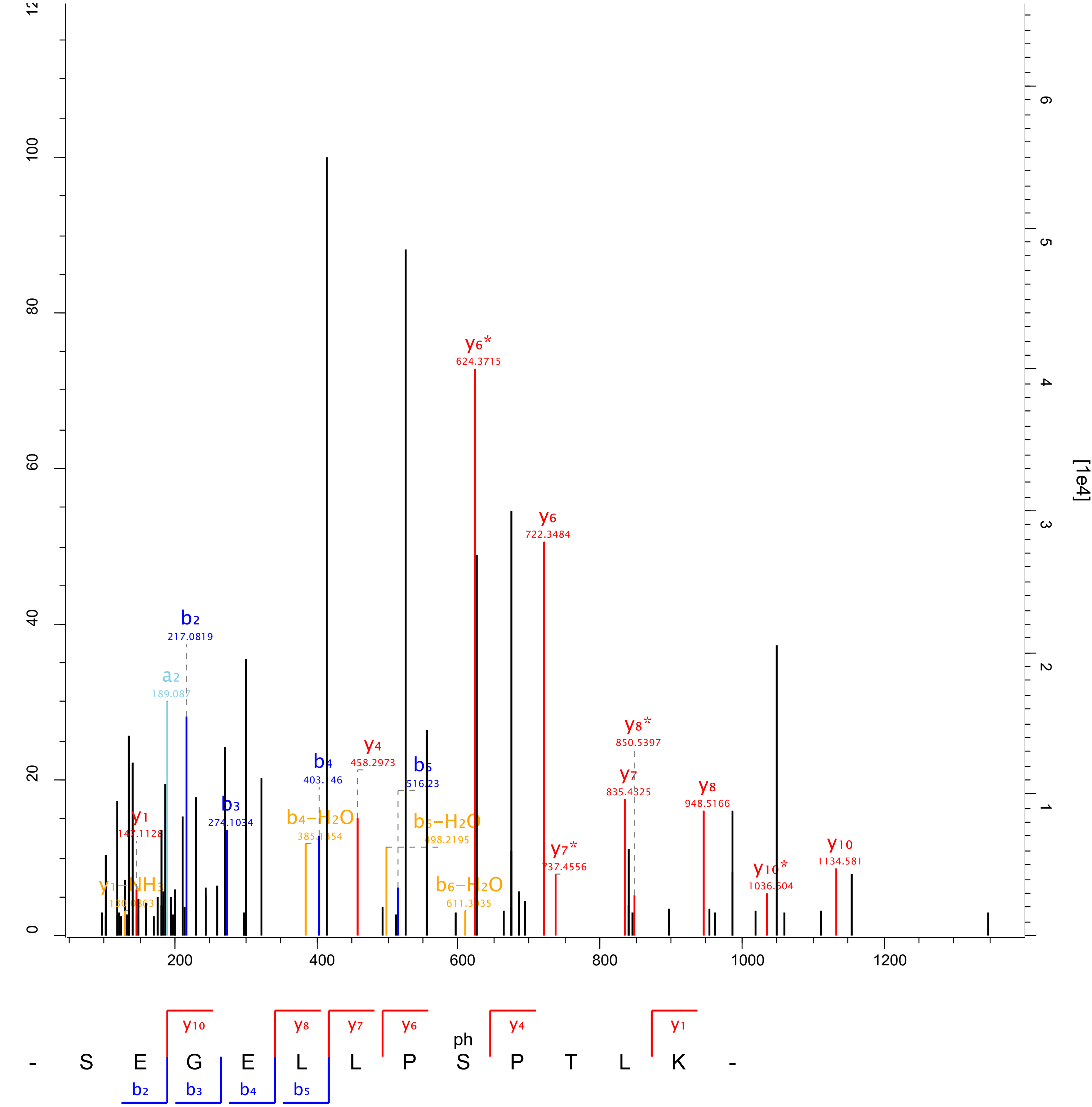

Sample output to test PDF Combine only

Raw file	Scan	Method	Score	m/z	Gene names
sp3-mic-0-2-A	35331	FTMS; HCD	68.37	712.09	At5g14120

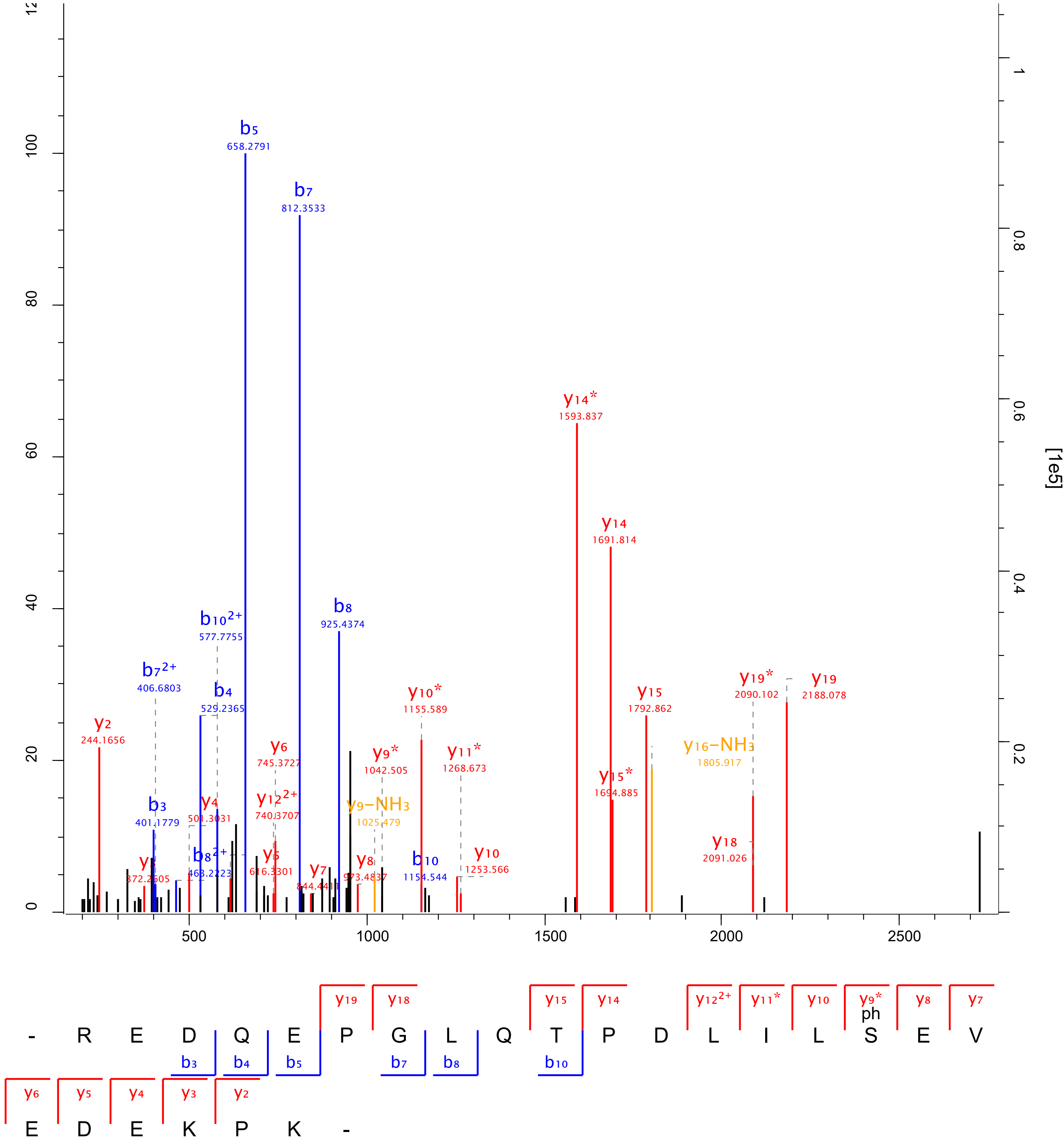

Sample output to test PDF Combine only

Raw file	Scan	Method	Score	m/z	Gene names
sp3-mic-0-2-A	39575	FTMS; HCD	86.5	619.3	F2P9.15

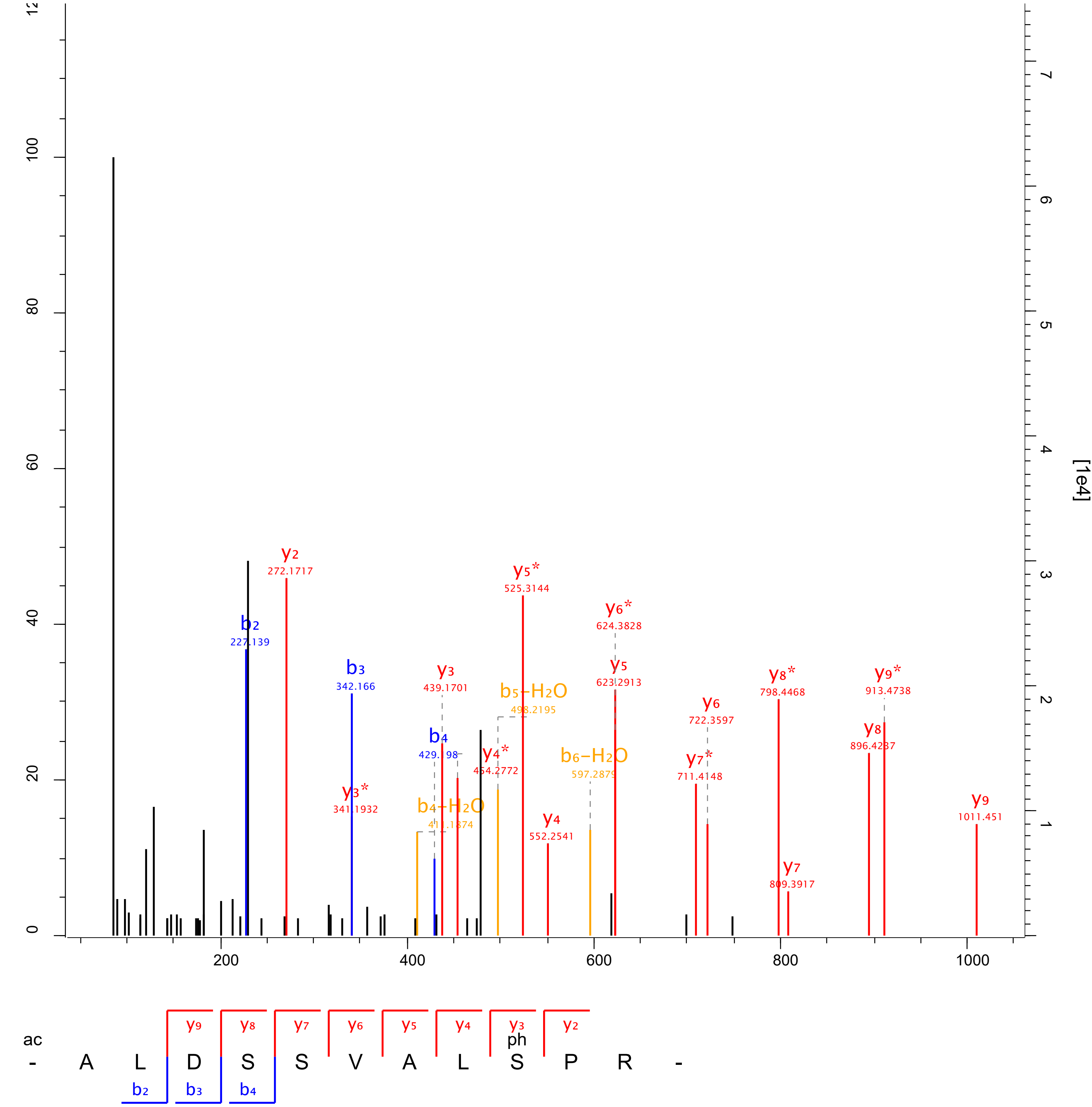

Sample output to test PDF Combine only

Raw file	Scan	Method	Score	m/z	Gene names
sp3-mic-0-2-A	40407	FTMS; HCD	128.73	625.26	SYP122

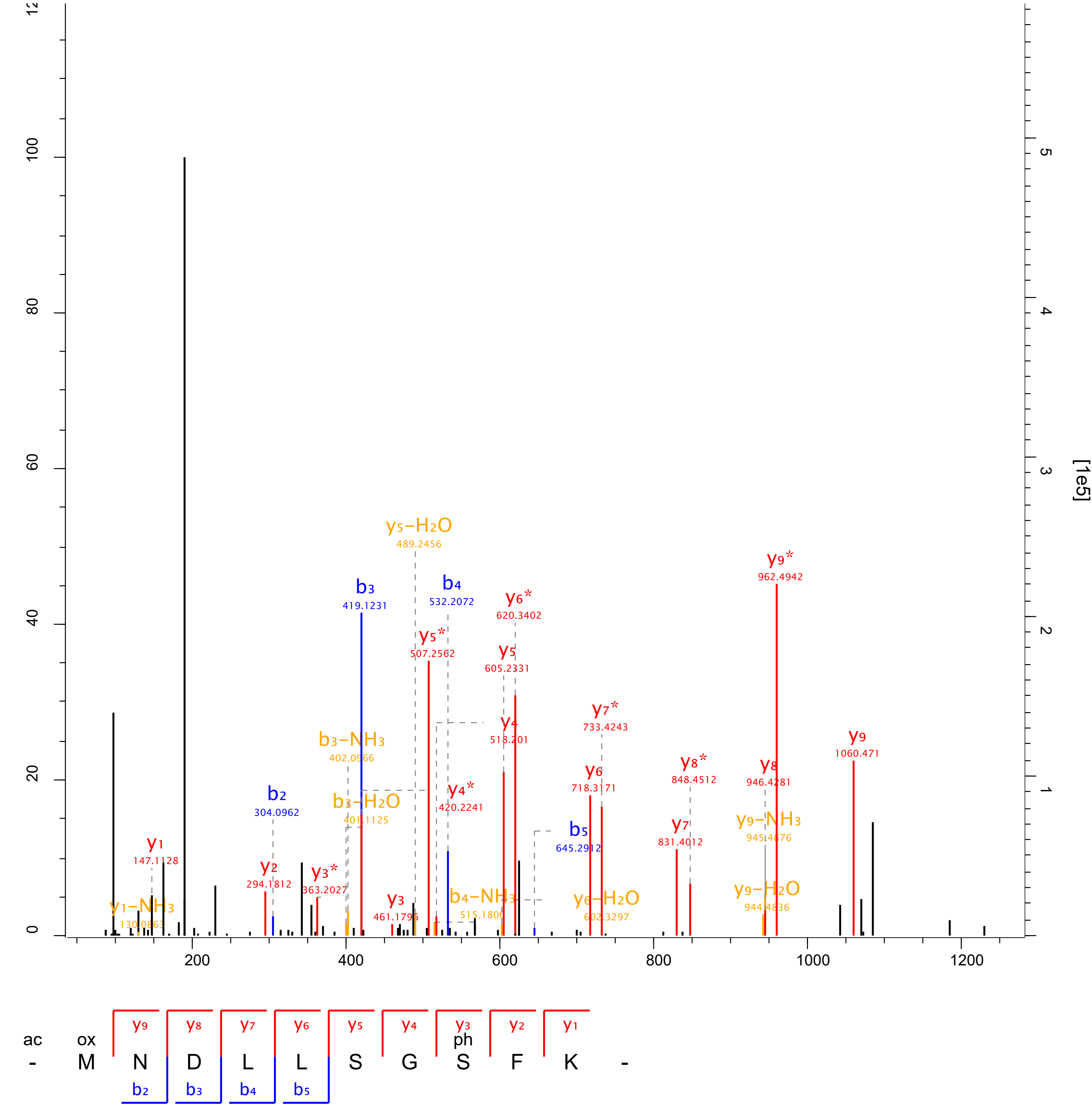

Sample output to test PDF Combine only

Raw file	Scan	Method	Score	m/z
sp3-mic-0-2-P	12028	FTMS; HCD	70.2	527.24

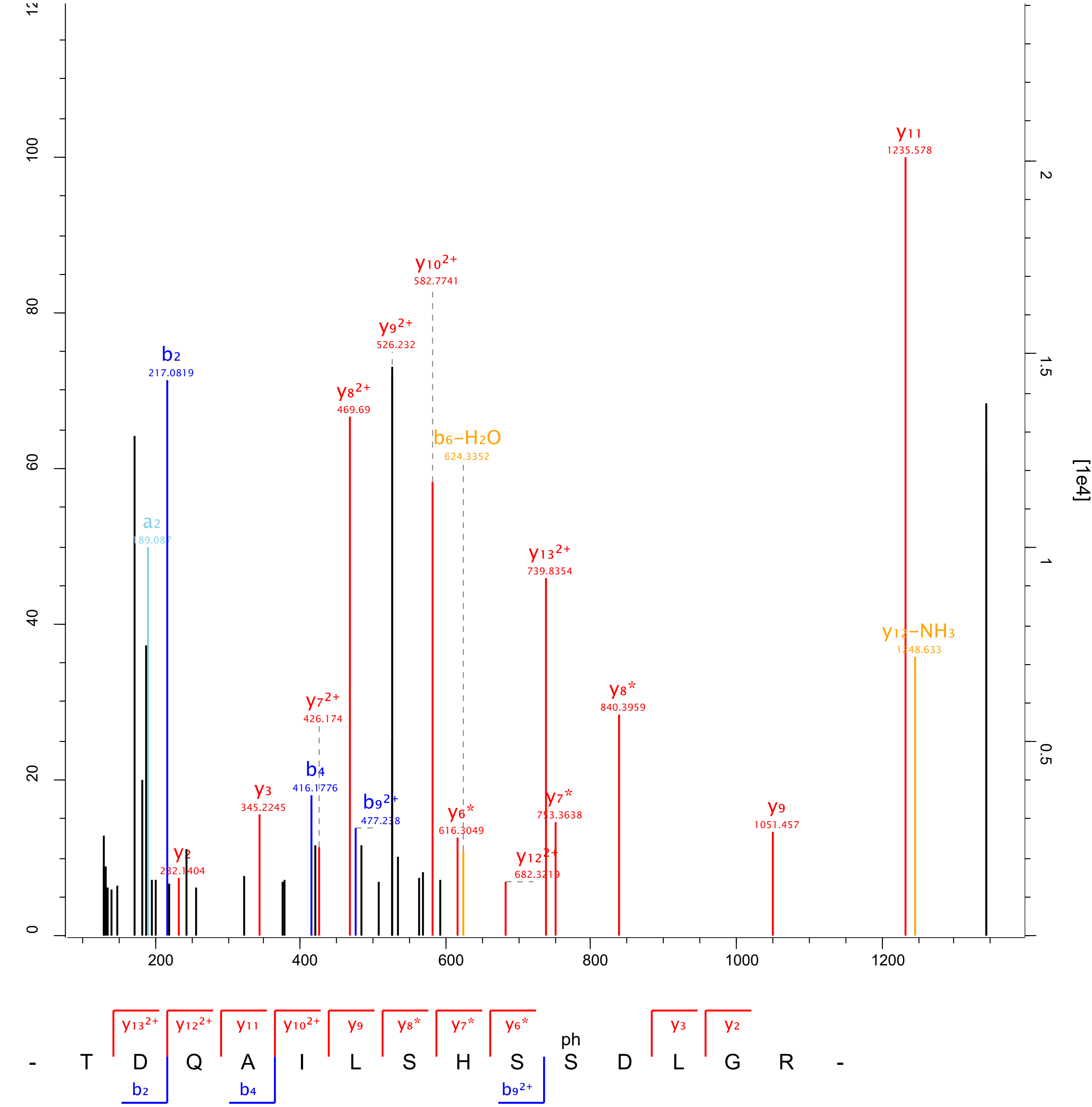

Raw file	Scan	Method	Score	m/z
sp3-mic-0-2-P	14956	FTMS; HCD	53.2	852.44

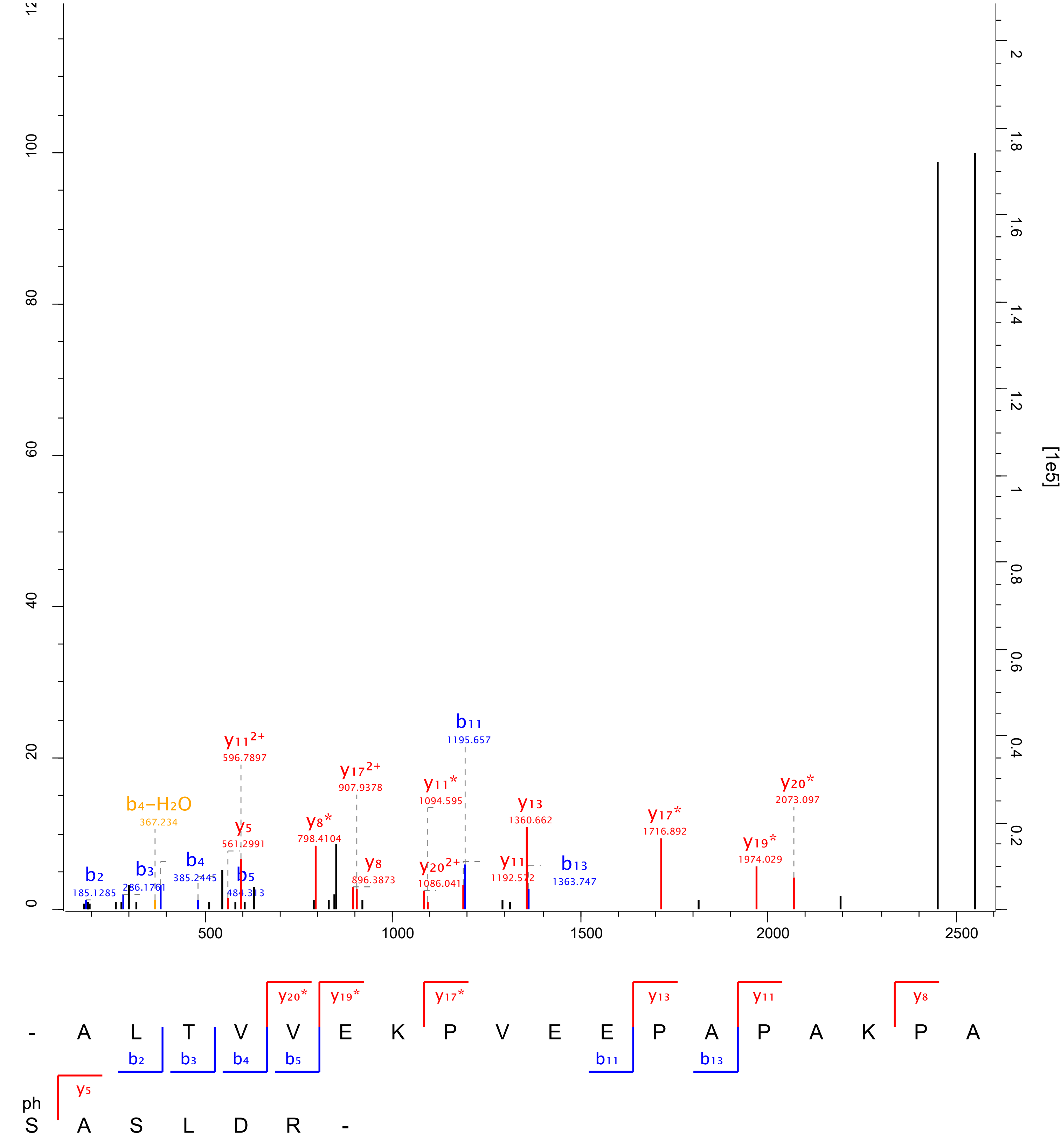

Sample output to test PDF Combine only

Raw file	Scan	Method	Score	m/z	Gene names
sp3-mic-0-2-P	16042	FTMS; HCD	110.12	538.62	PVA12

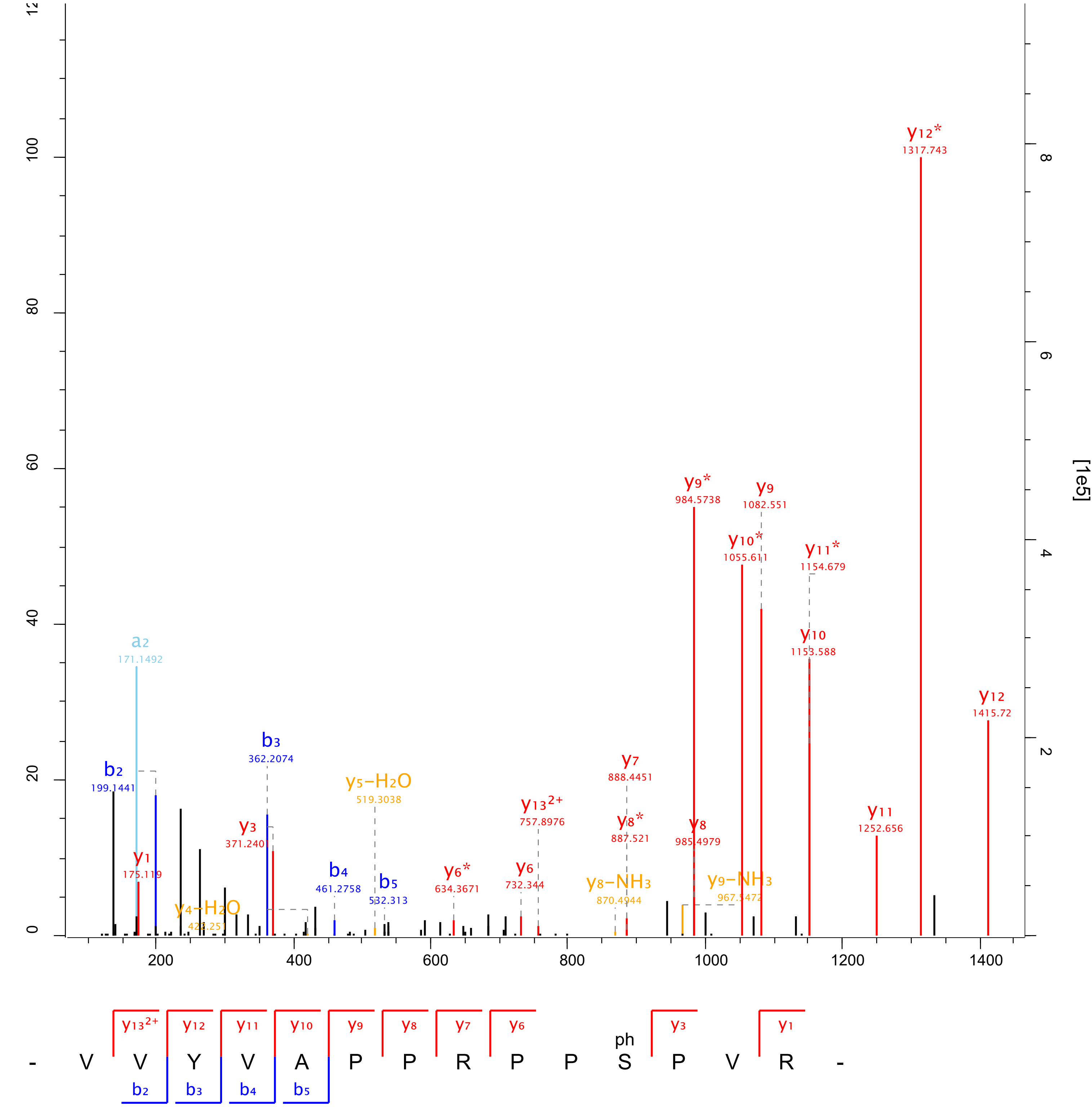

Sample output to test PDF Combine only

Raw file	Scan	Method	Score	m/z	Gene names
sp3-mic-0-2-P	20215	FTMS; HCD	62.58	714.03	TOM2A

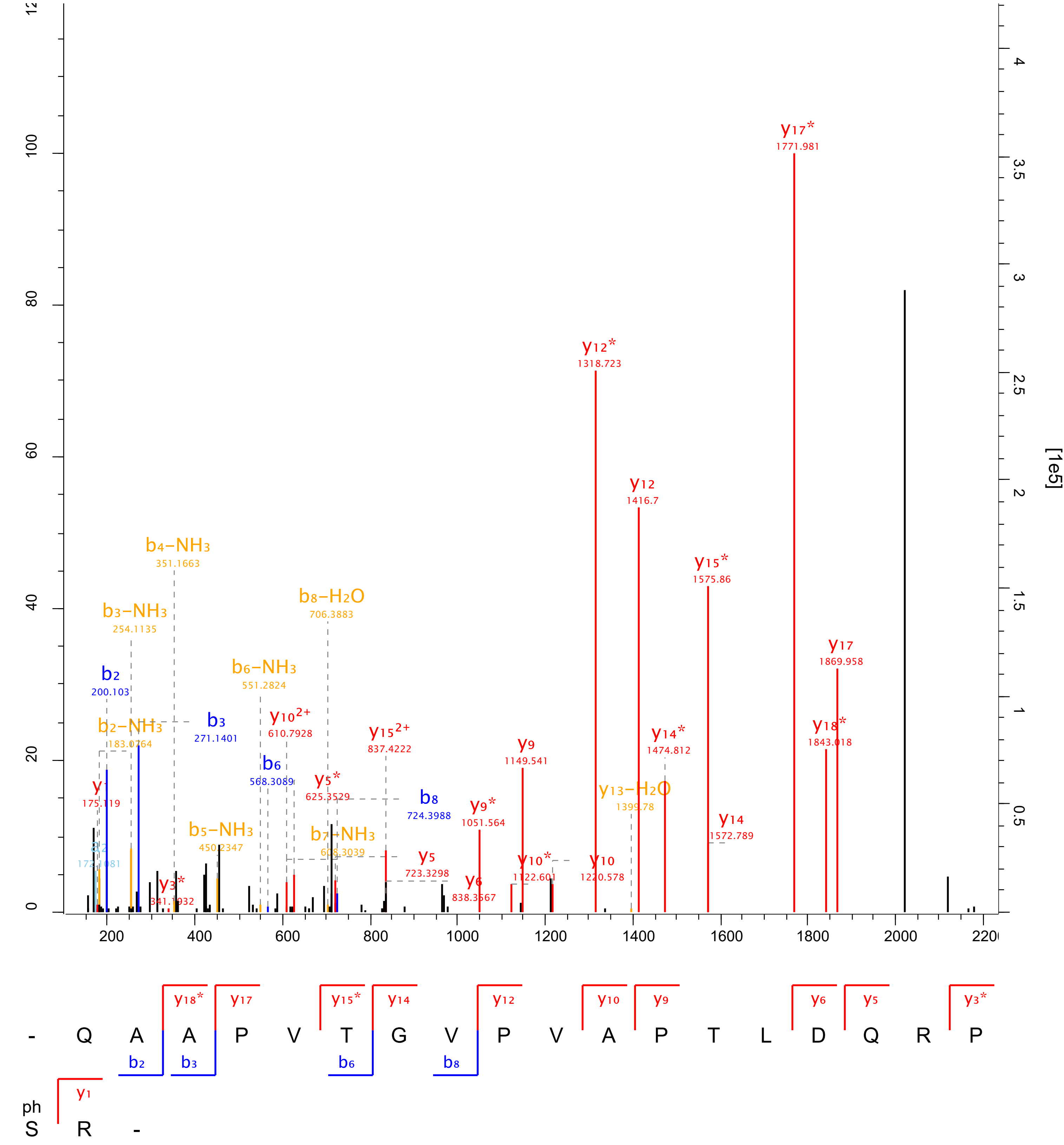

Sample output to test PDF Combine only

Raw file	Scan	Method	Score	m/z	Gene names
sp3-mic-0-2-P	20895	FTMS; HCD	42.52	659.3	PATL3

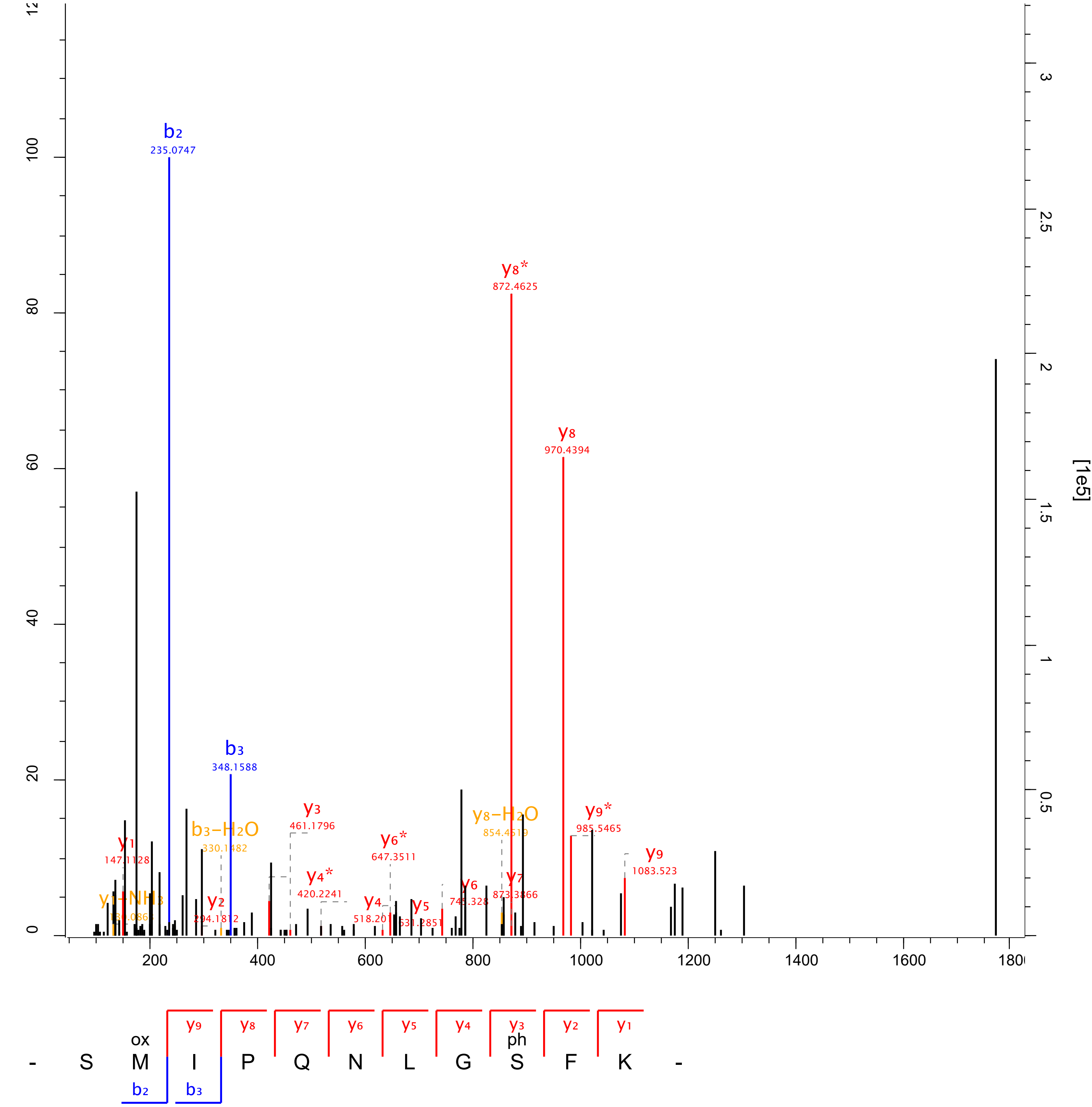

Sample output to test PDF Combine only

Raw file	Scan	Method	Score	m/z	Gene names
sp3-mic-0-2-P	25069	FTMS; HCD	56.04	721.36	At5g37920

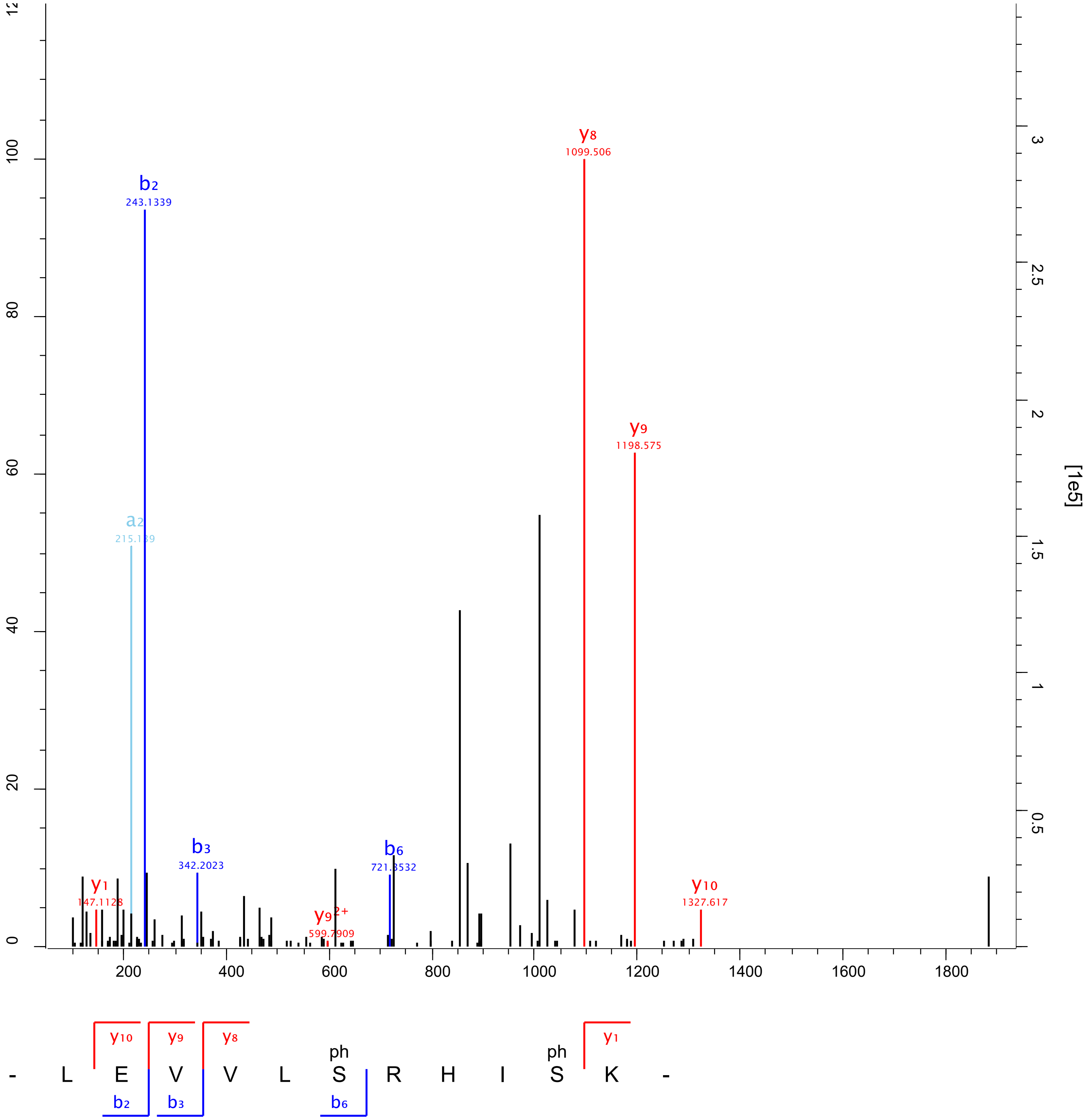

Sample output to test PDF Combine only

Raw file	Scan	Method	Score	m/z
sp3-mic-0-2-P	33553	FTMS; HCD	51.5	1207.54

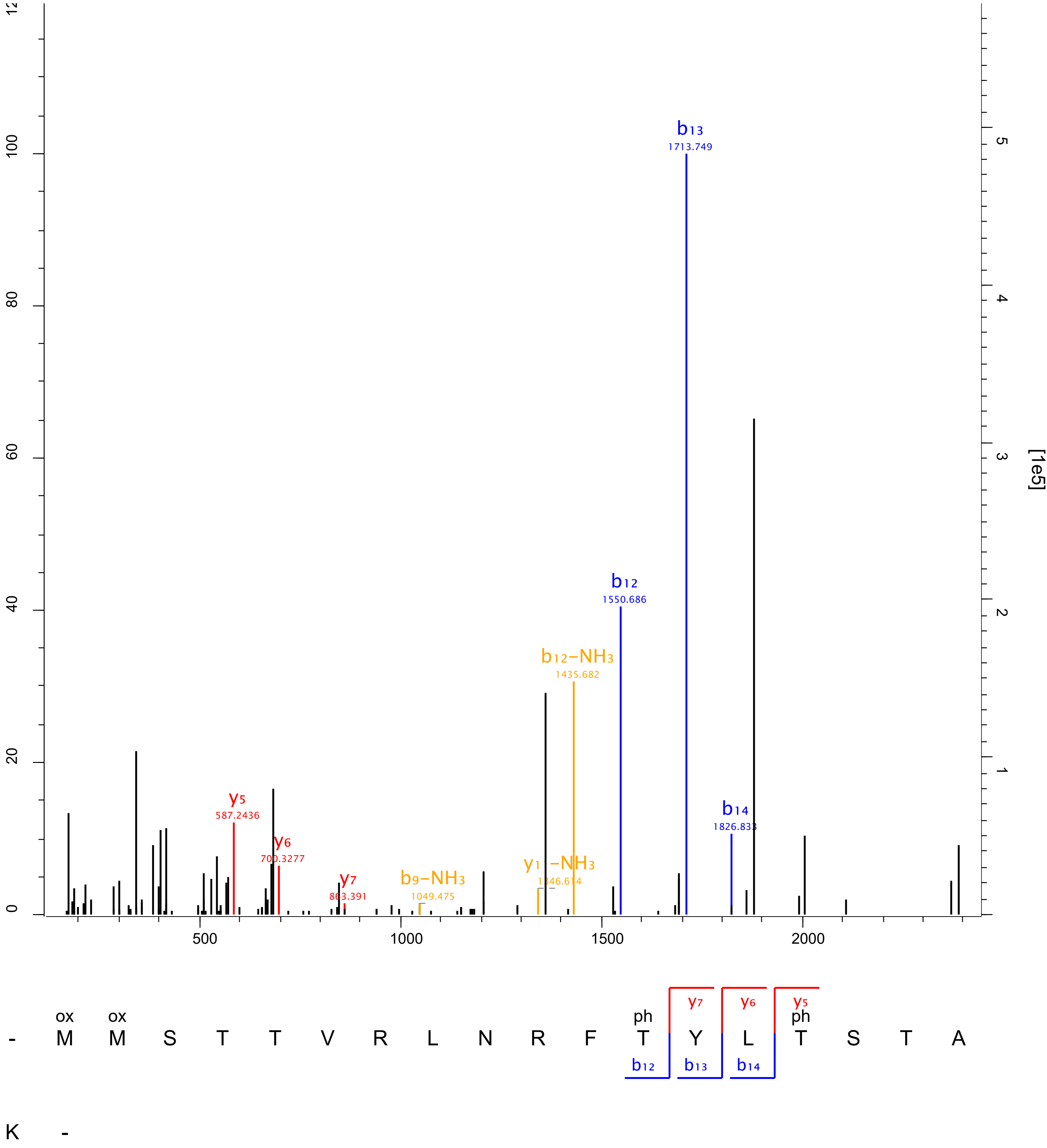

Sample output to test PDF Combine only

Raw file	Scan	Method	Score	m/z	Gene names
sp3-mic-0-2-P	40668	FTMS; HCD	56.29	551.92	TOM40-2

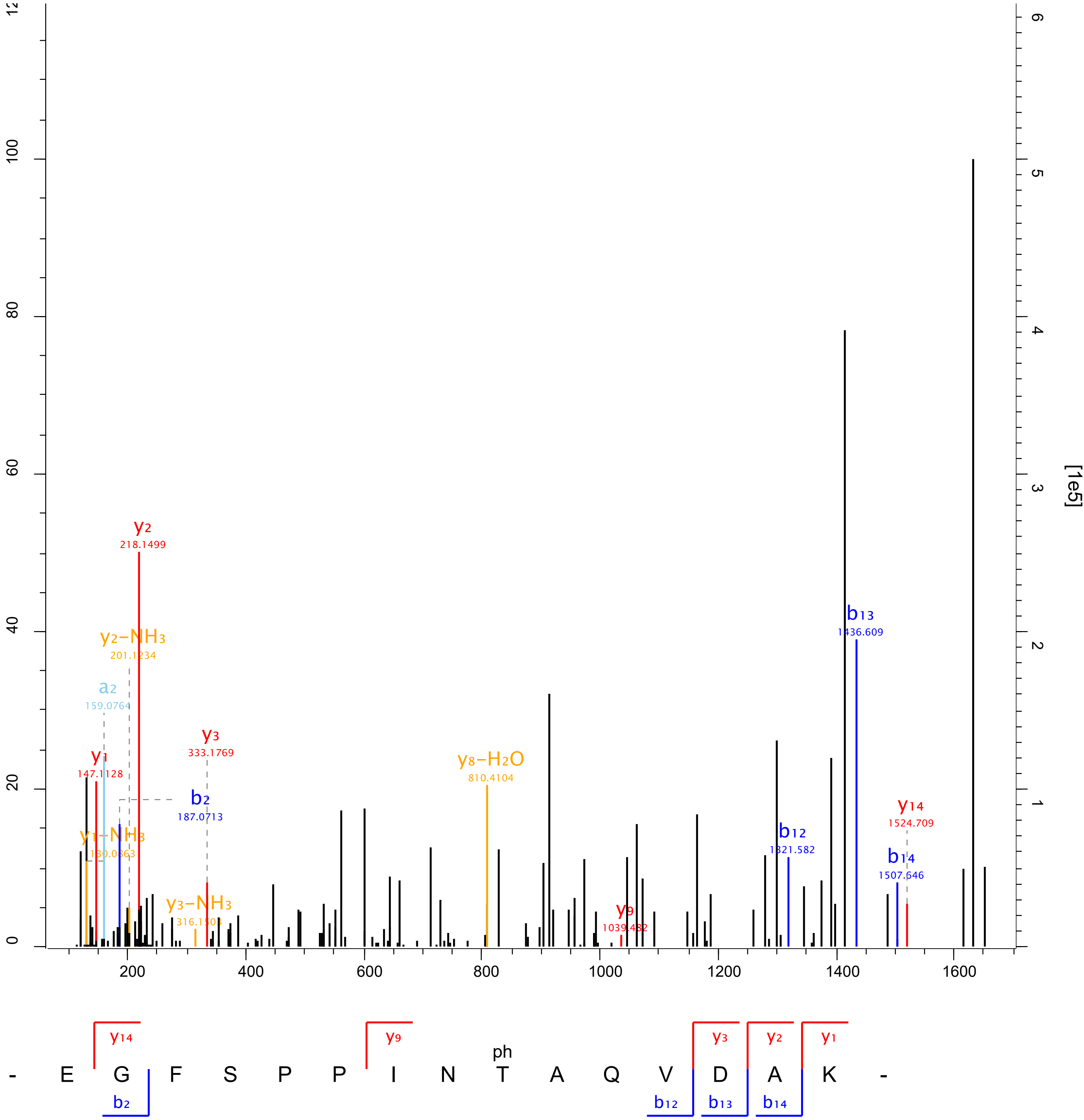

Sample output to test PDF Combine only

Raw file	Scan	Method	Score	m/z	Gene names
sp3-mic-0-3-A	5044	FTMS; HCD	50.96	568.25	BTR1

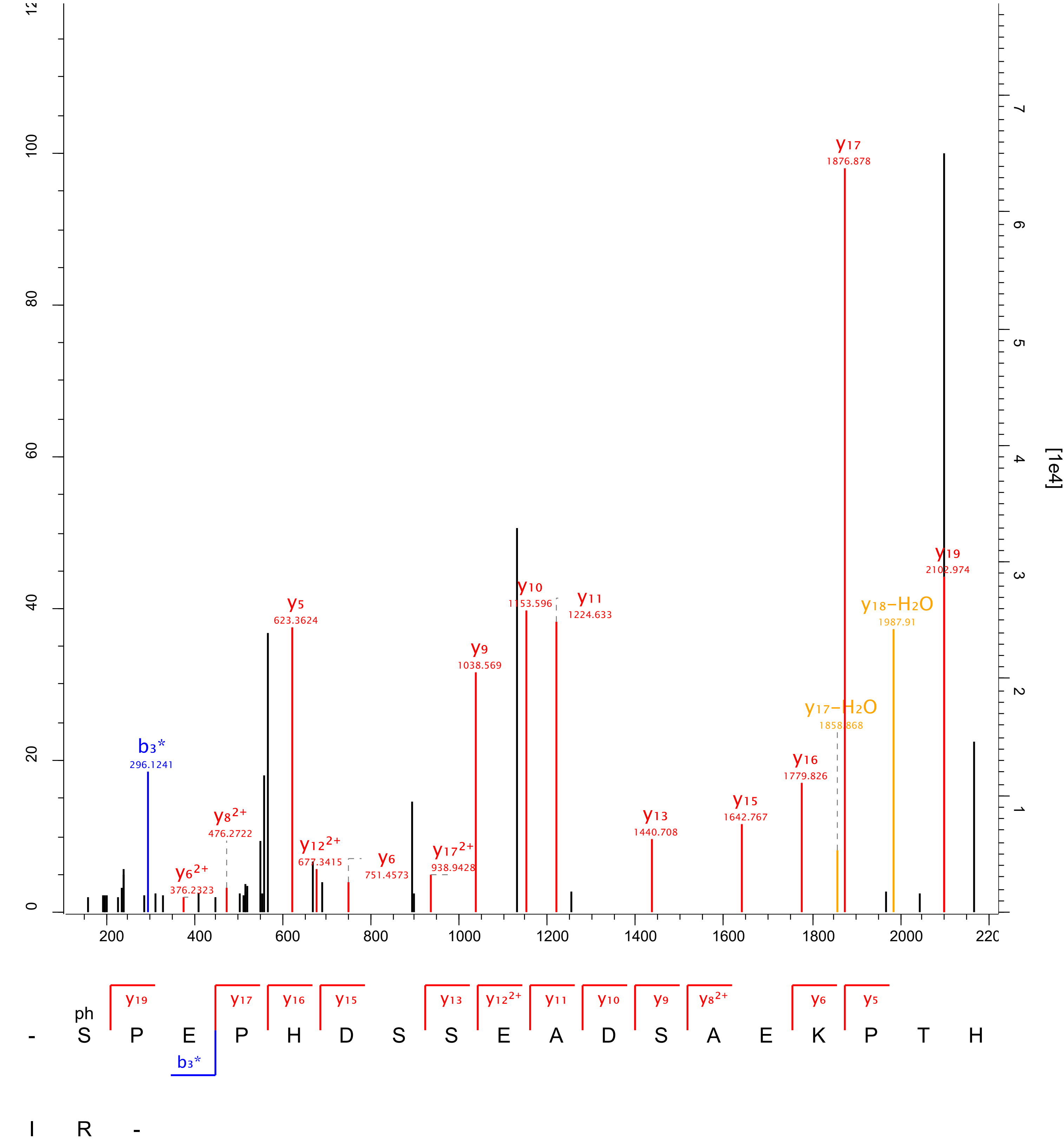

Sample output to test PDF Combine only

Raw file	Scan	Method	Score	m/z	Gene names
sp3-mic-0-3-A	5866	FTMS; HCD	183.65	706.95	HSP90-4;HSP90-2;HSP81-2;HSP90-3

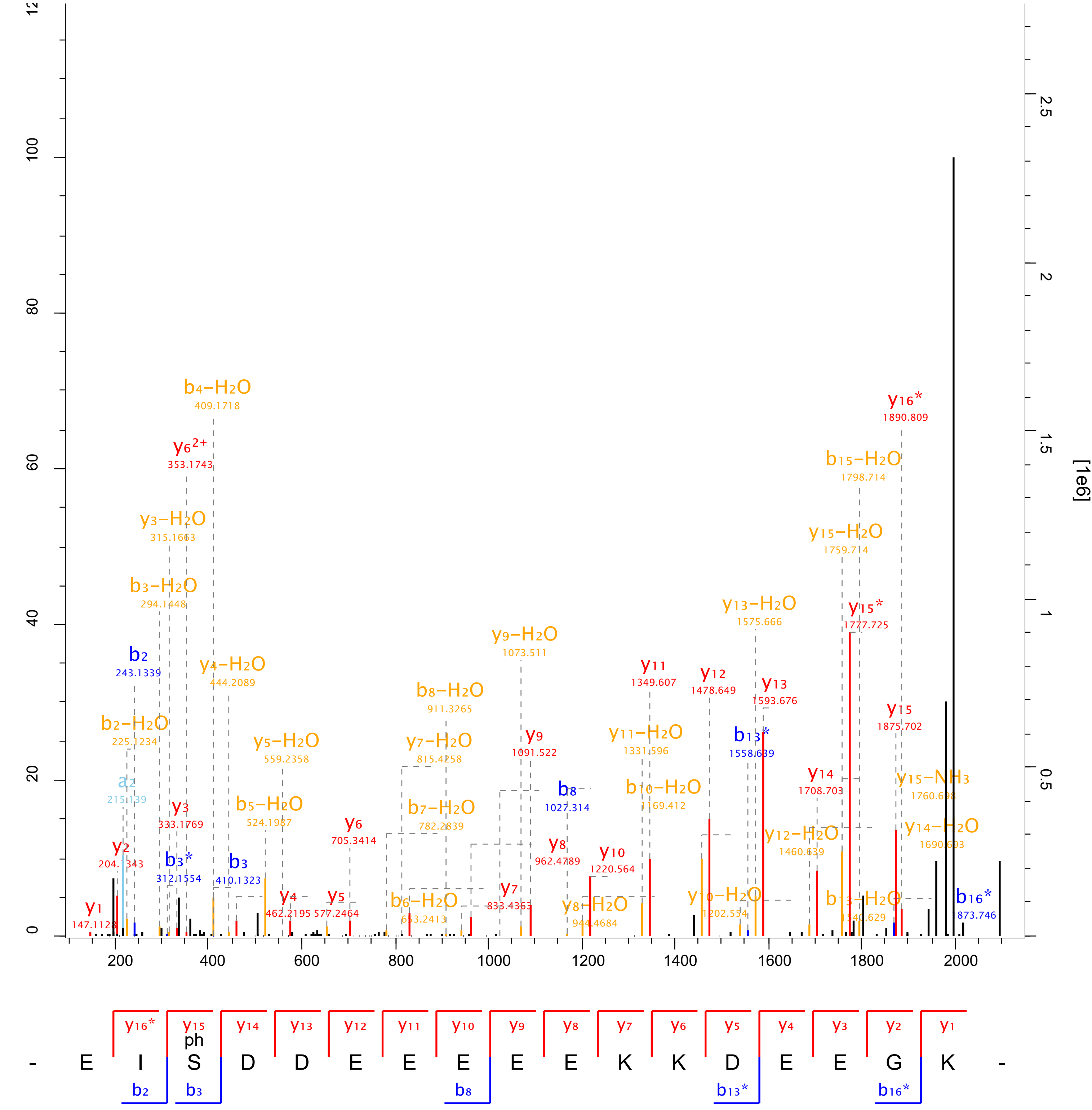

Raw file	Scan	Method	Score	m/z	Gene names
sp3-mic-0-3-A	5928	FTMS; HCD	116.45	694.65	At4g31880

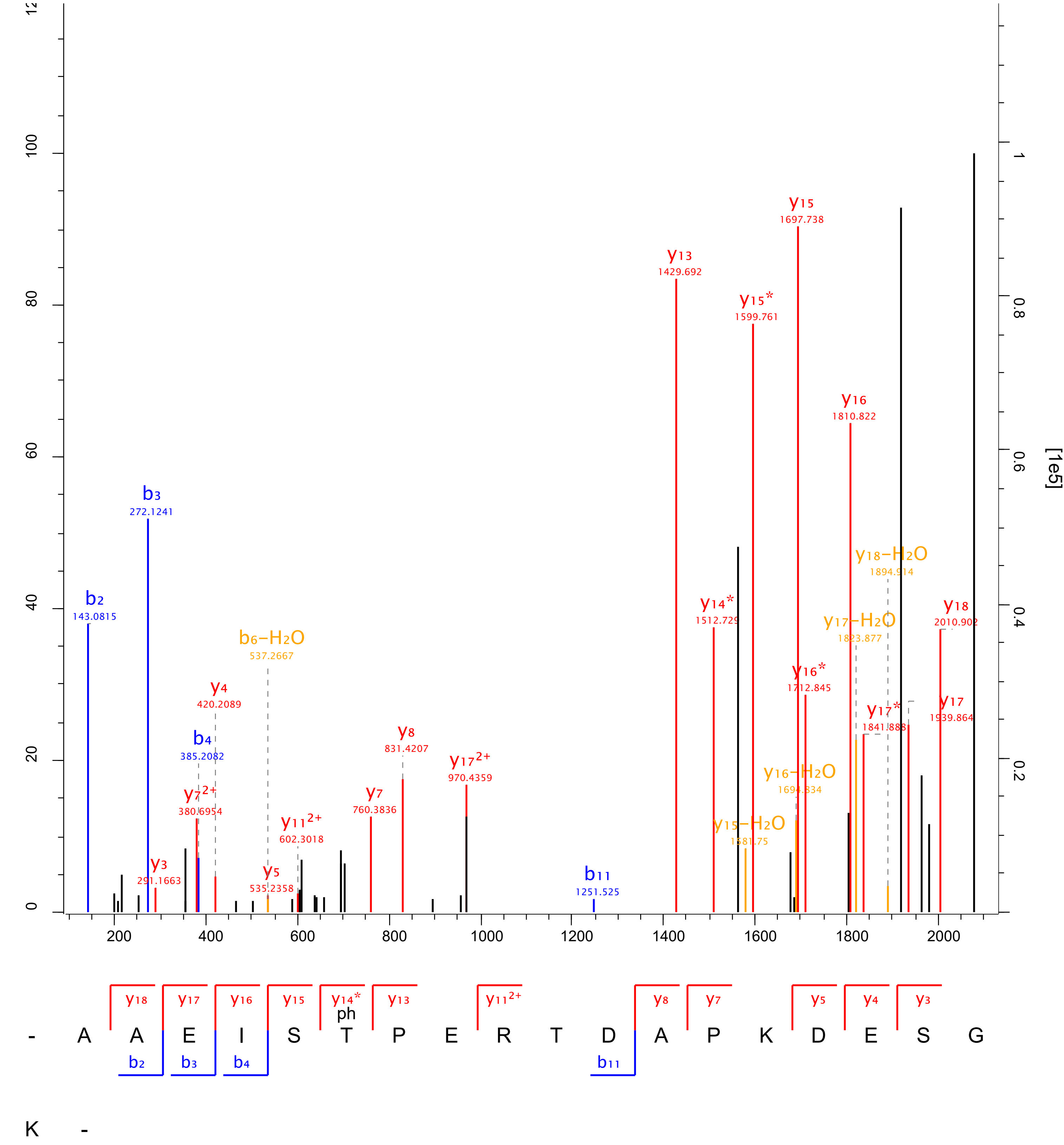

Sample output to test PDF Combine only

Raw file	Scan	Method	Score	m/z	Gene names
sp3-mic-0-3-A	7382	FTMS; HCD	63.22	576.78	TFIIS

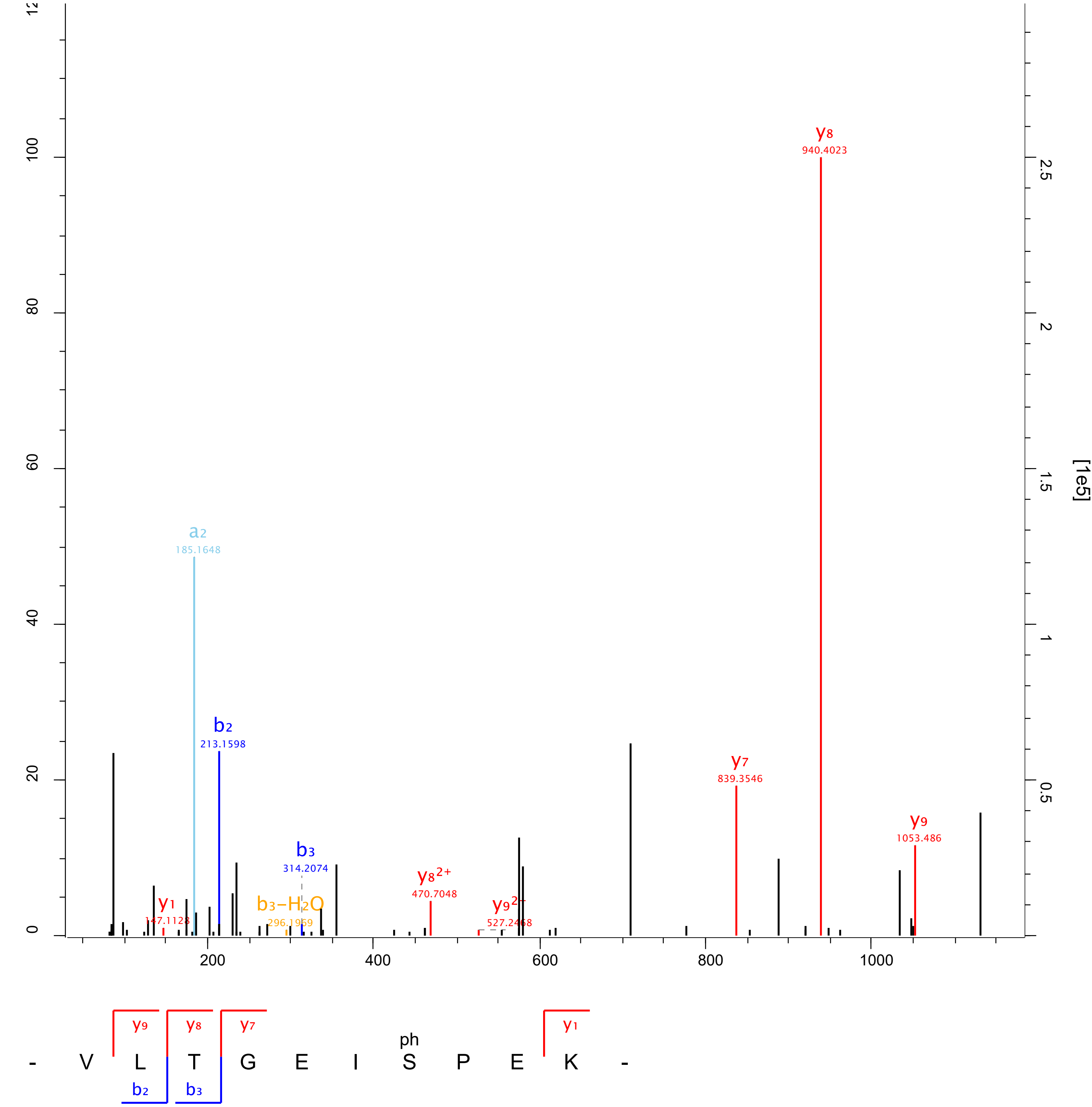

Sample output to test PDF Combine only

Raw file	Scan	Method	Score	m/z	Gene names
sp3-mic-0-3-A	11644	FTMS; HCD	42.4	555.75	HAP6;RPN2

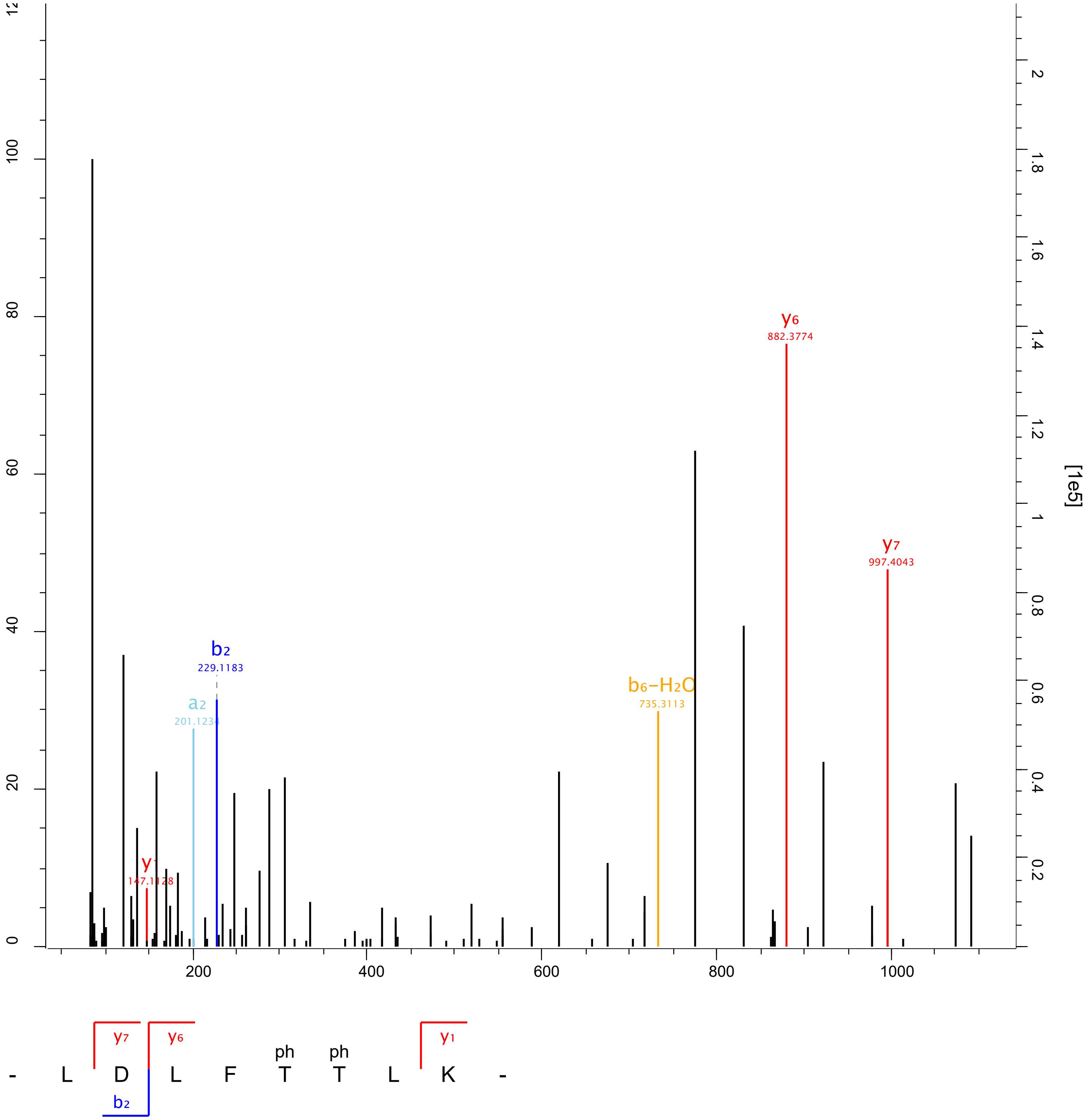

Sample output to test PDF Combine only

Raw file	Scan	Method	Score	m/z	Gene names
sp3-mic-0-3-A	12241	FTMS; HCD	45.37	694.85	At5g17980

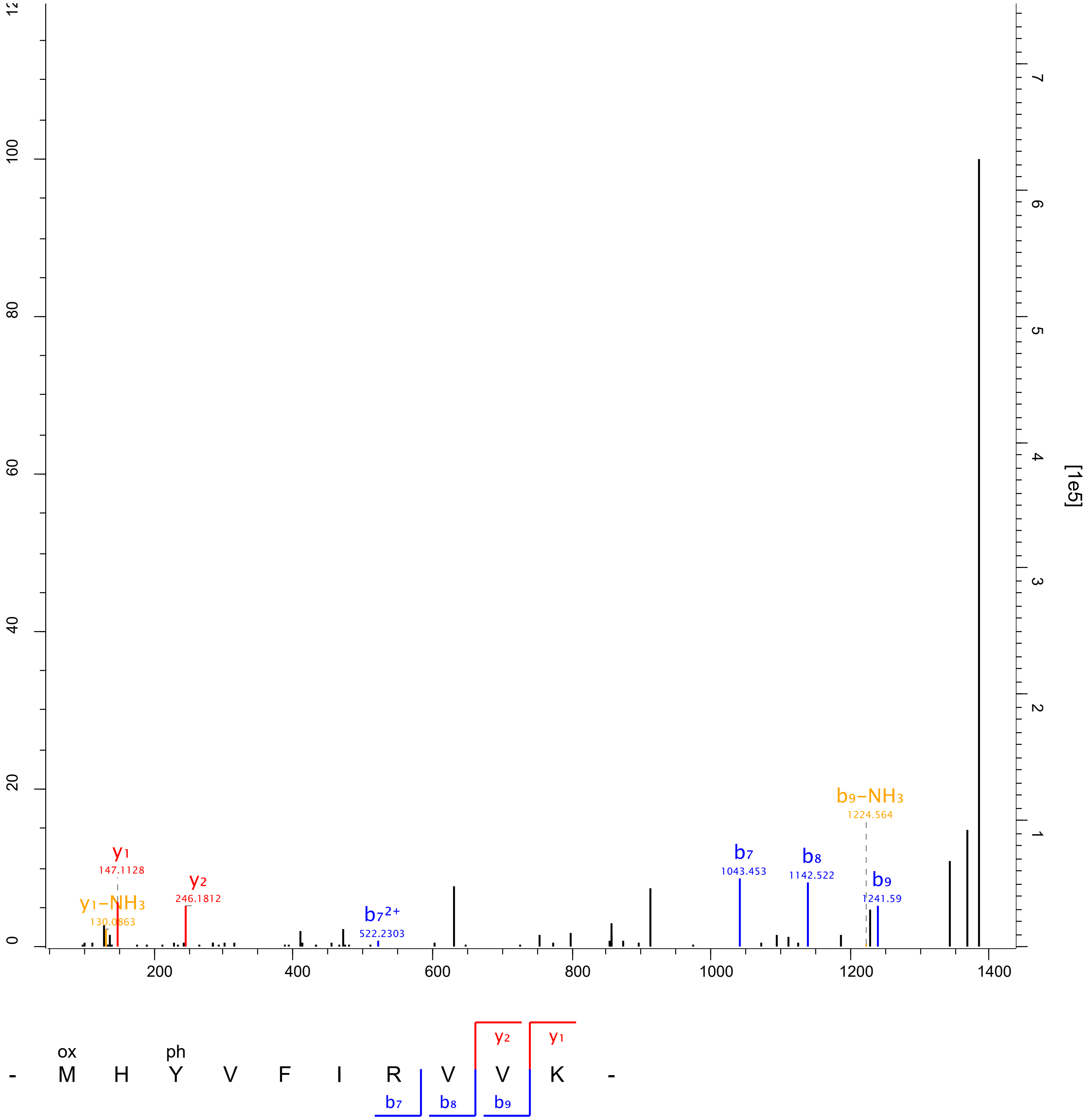

Sample output to test PDF Combine only

Raw file	Scan	Method	Score	m/z	Gene names
sp3-mic-0-3-A	23539	FTMS; HCD	49.3	573.29	MLO6

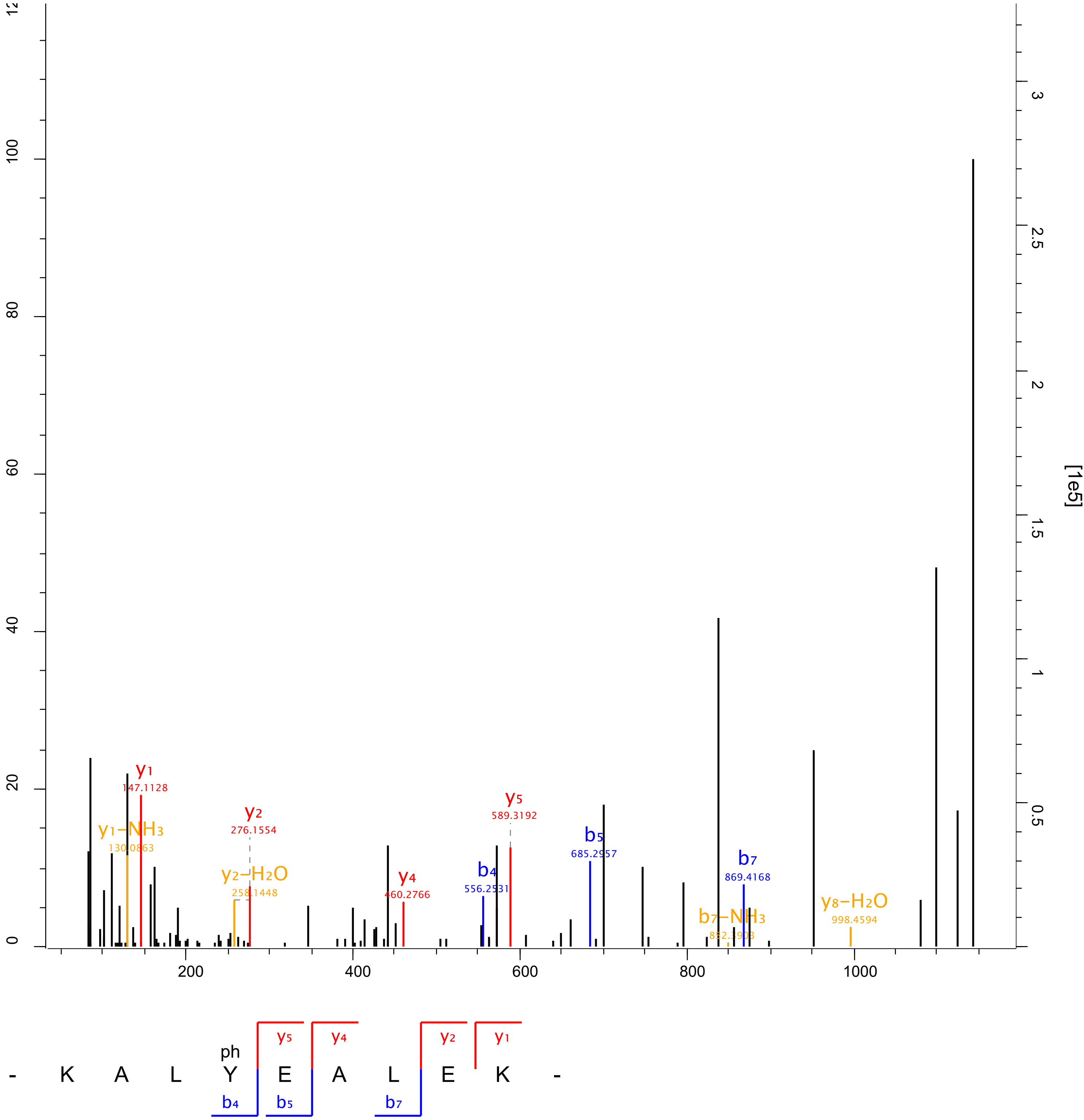

Sample output to test PDF Combine only

Raw file	Scan	Method	Score	m/z	Gene names
sp3-mic-0-3-P	15155	FTMS; HCD	42.72	693.82	RLP31

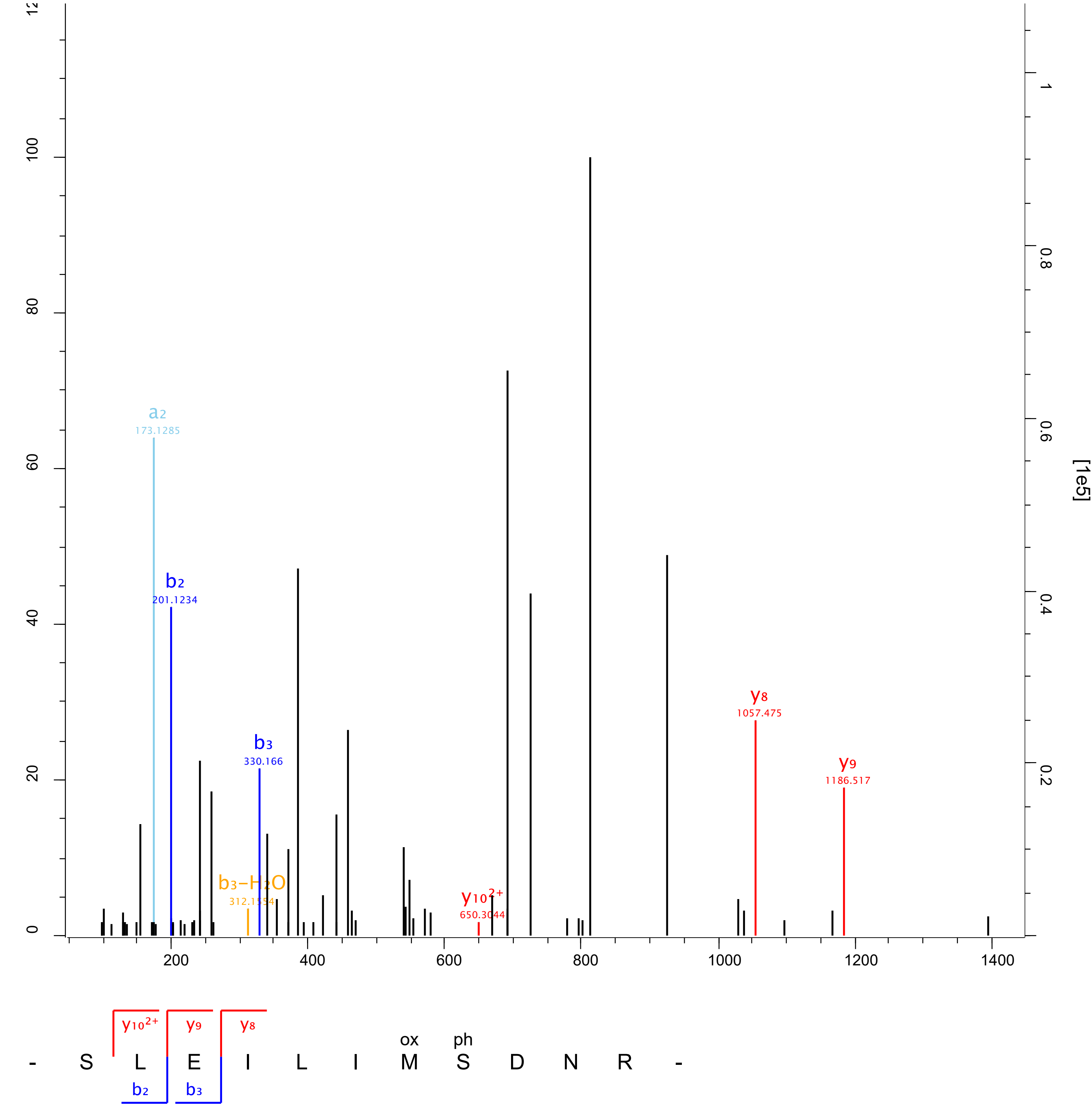

Sample output to test PDF Combine only

Raw file	Scan	Method	Score	m/z	Gene names
sp3-mic-0-3-P	38227	FTMS; HCD	41.88	571.74	PIP2-8

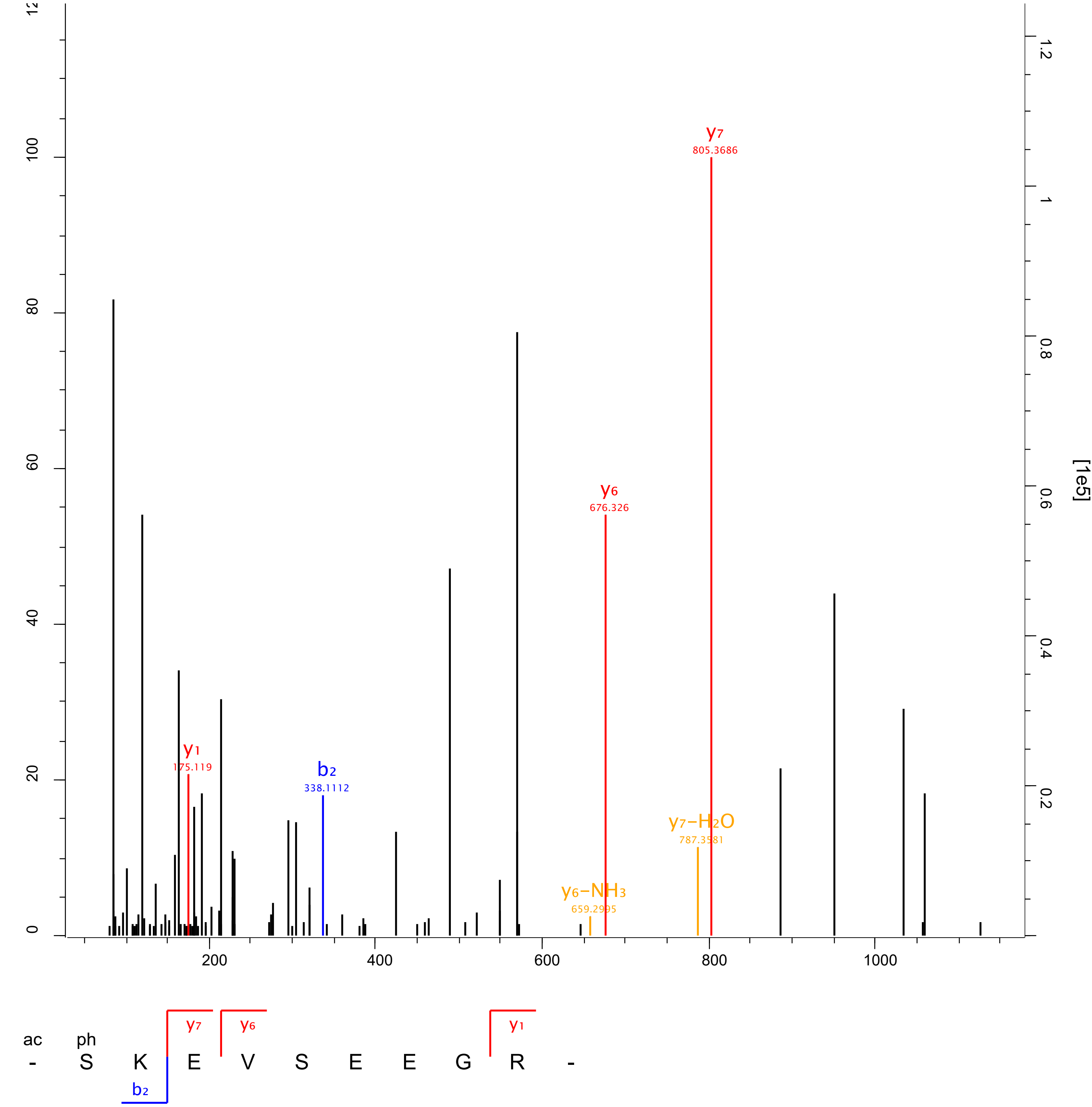

Sample output to test PDF Combine only

Raw file	Scan	Method	Score	m/z	Gene names
sp3-mic-SUC-1-A	4171	FTMS; HCD	72.9	499.22	DOT2

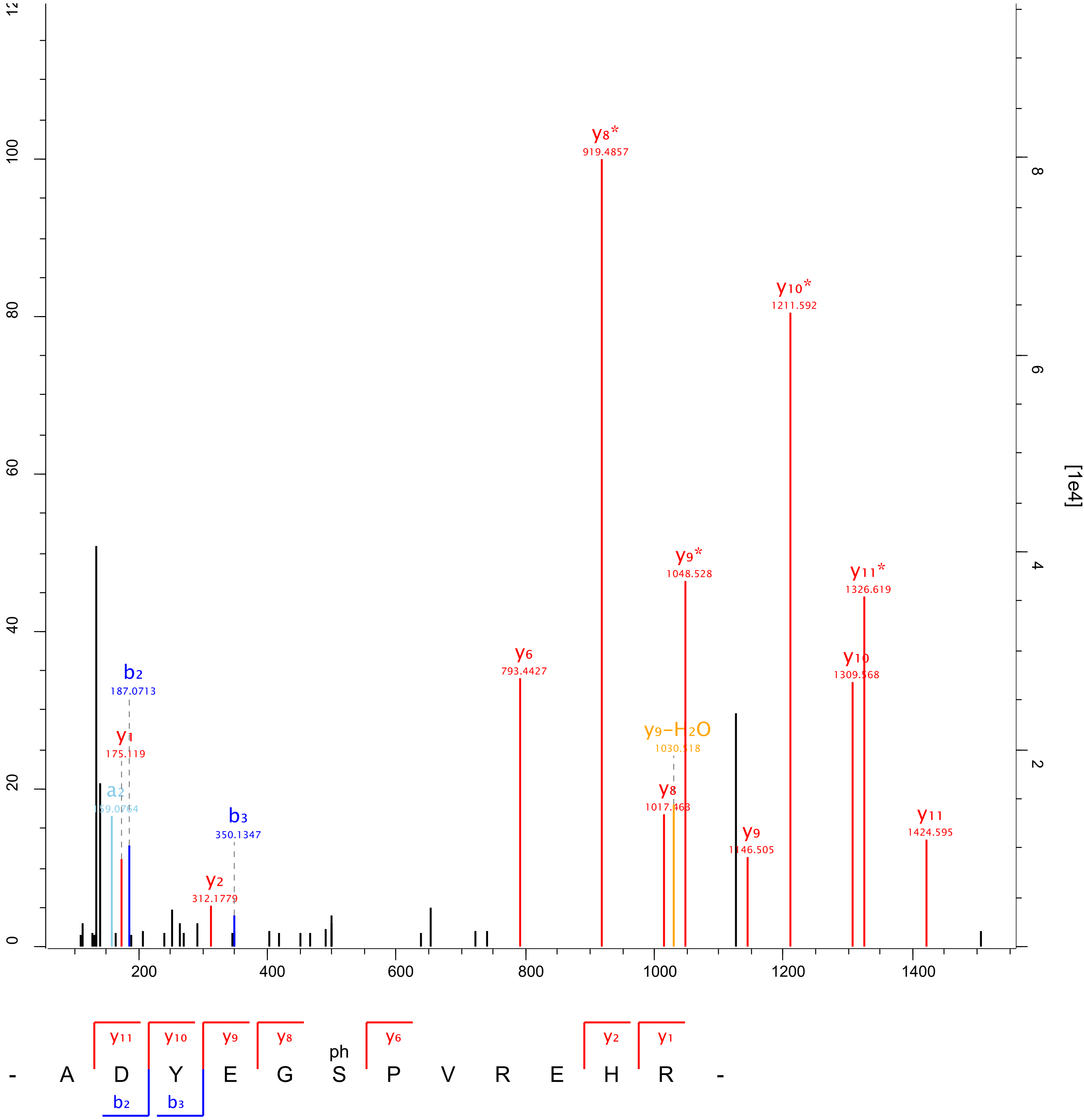

Sample output to test PDF Combine only

Raw file	Scan	Method	Score	m/z	Gene names
sp3-mic-SUC-1-A	18812	FTMS; HCD	53.3	634.31	IQD32

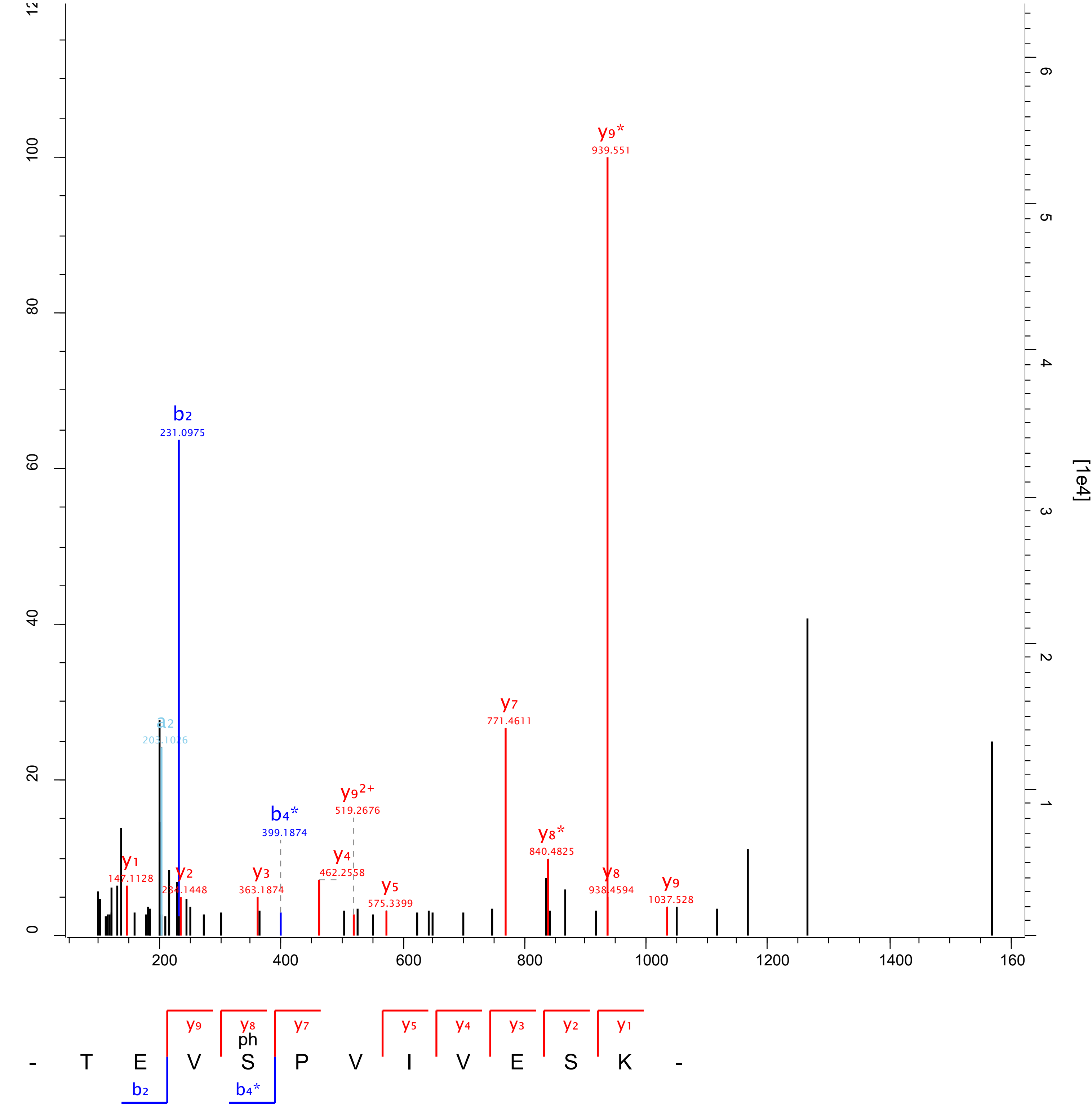

Sample output to test PDF Combine only

Raw file	Scan	Method	Score	m/z	Gene names
sp3-mic-SUC-1-A	22377	FTMS; HCD	57.86	531.77	At4g38900;BZIP29

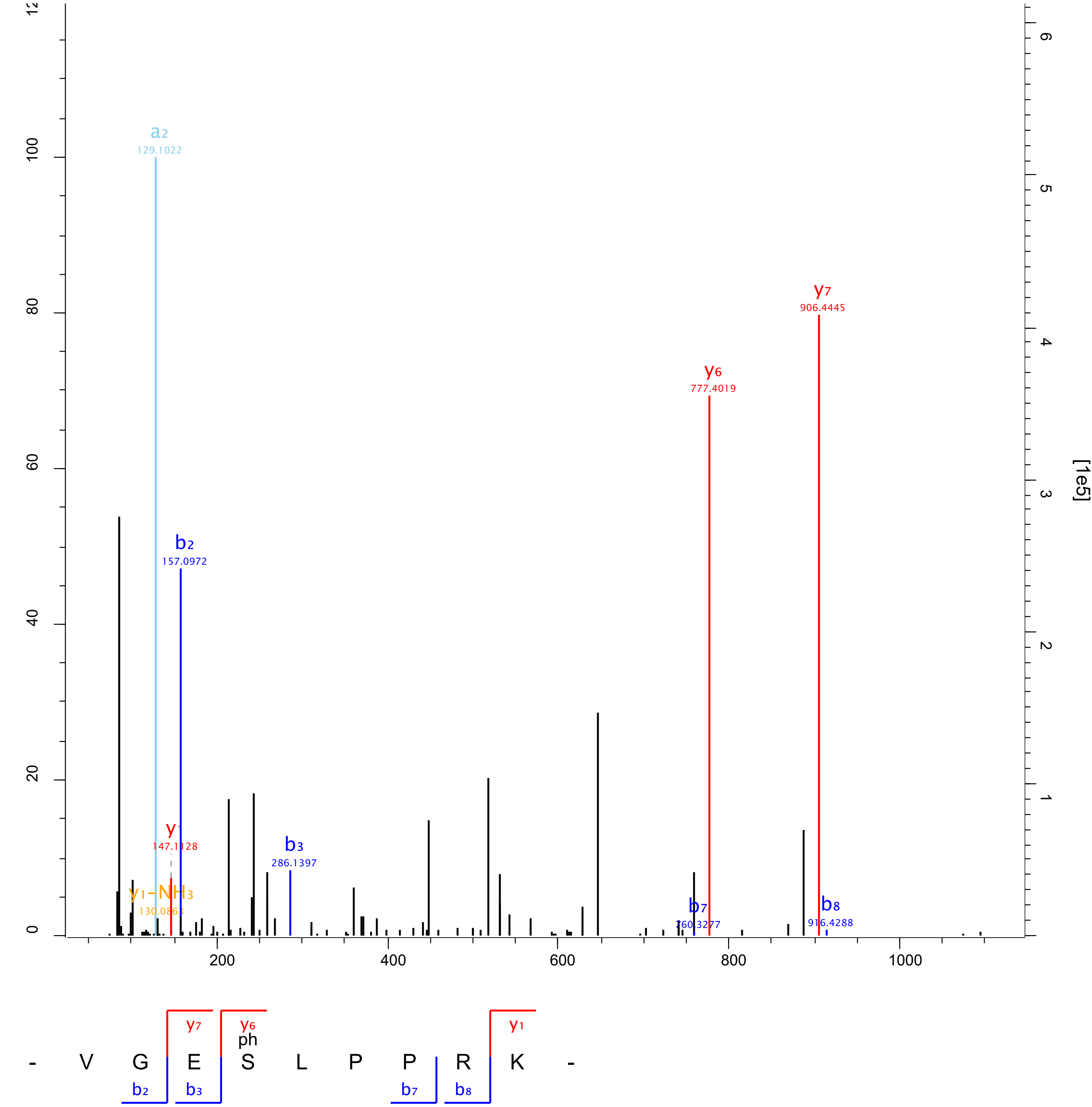

Sample output to test PDF Combine only

Raw file	Scan	Method	Score	m/z	Gene names
sp3-mic-SUC-1-A	24718	FTMS; HCD	59.34	712.66	KING1

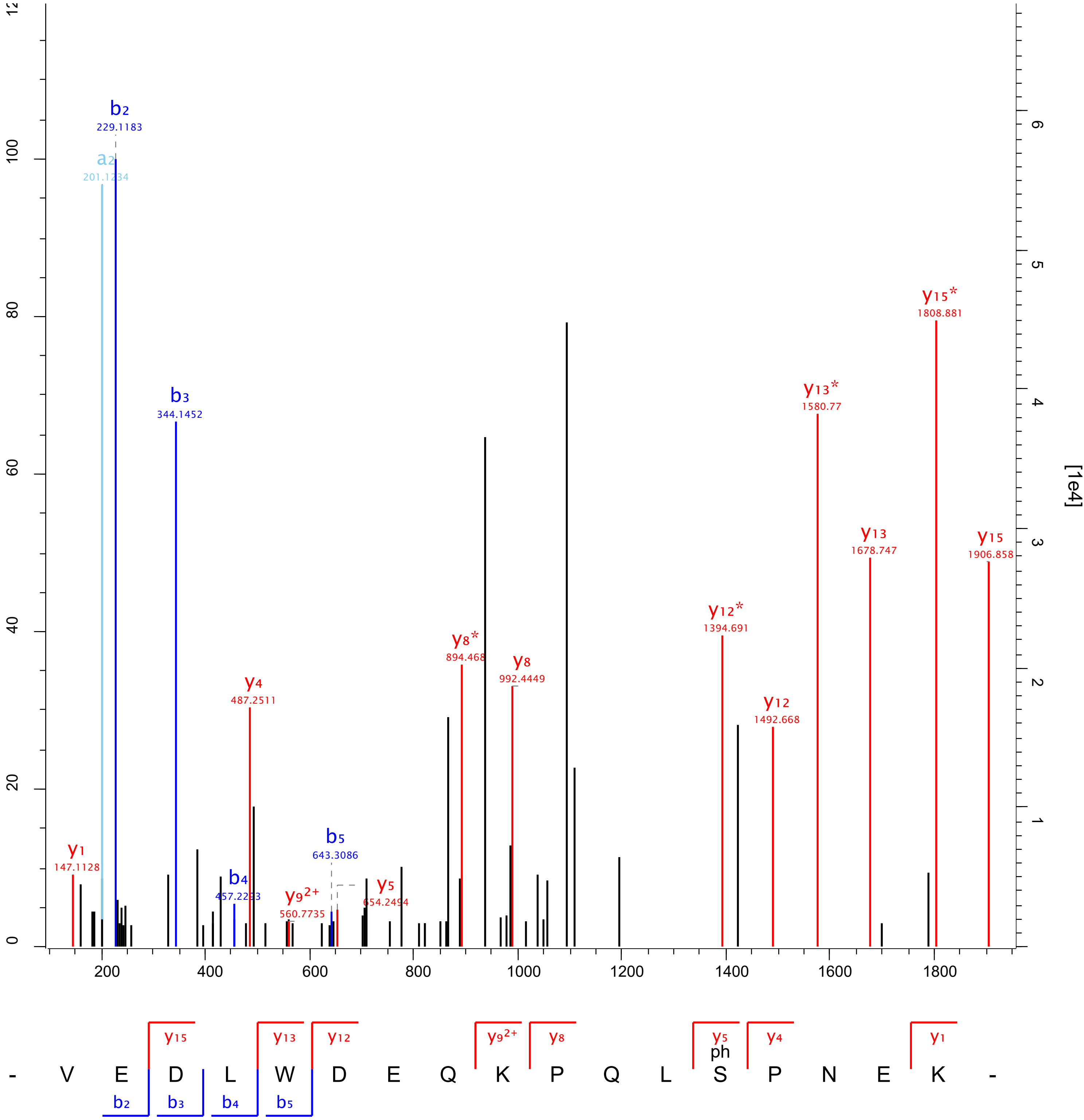

Sample output to test PDF Combine only

Raw file	Scan	Method	Score	m/z	Gene names
sp3-mic-SUC-1-A	29045	FTMS; HCD	86.5	814.86	SPK1

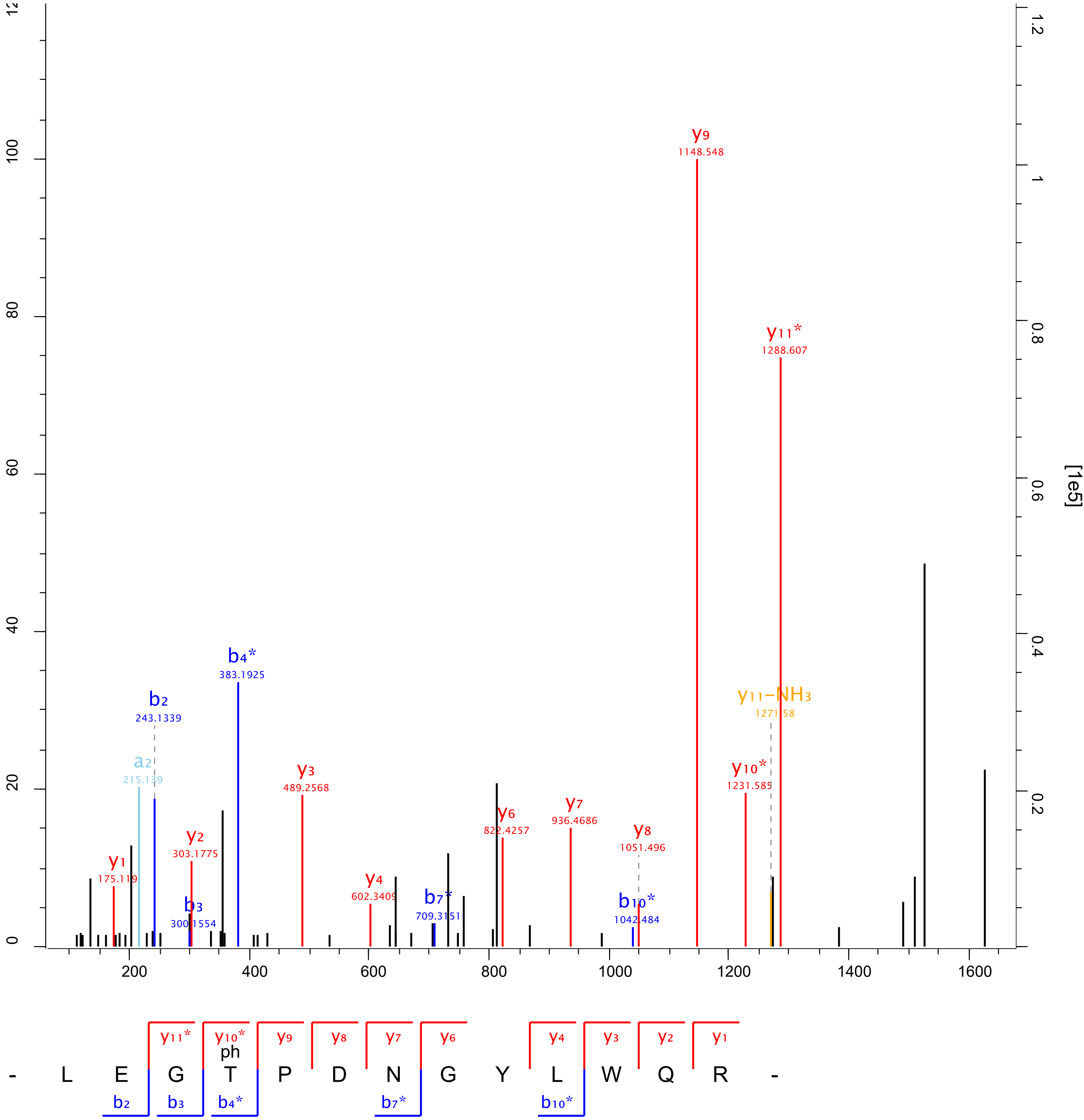

Sample output to test PDF Combine only

Raw file	Scan	Method	Score	m/z	Gene names
sp3-mic-SUC-2-A	9040	FTMS; HCD	91.96	576.78	At2g16940

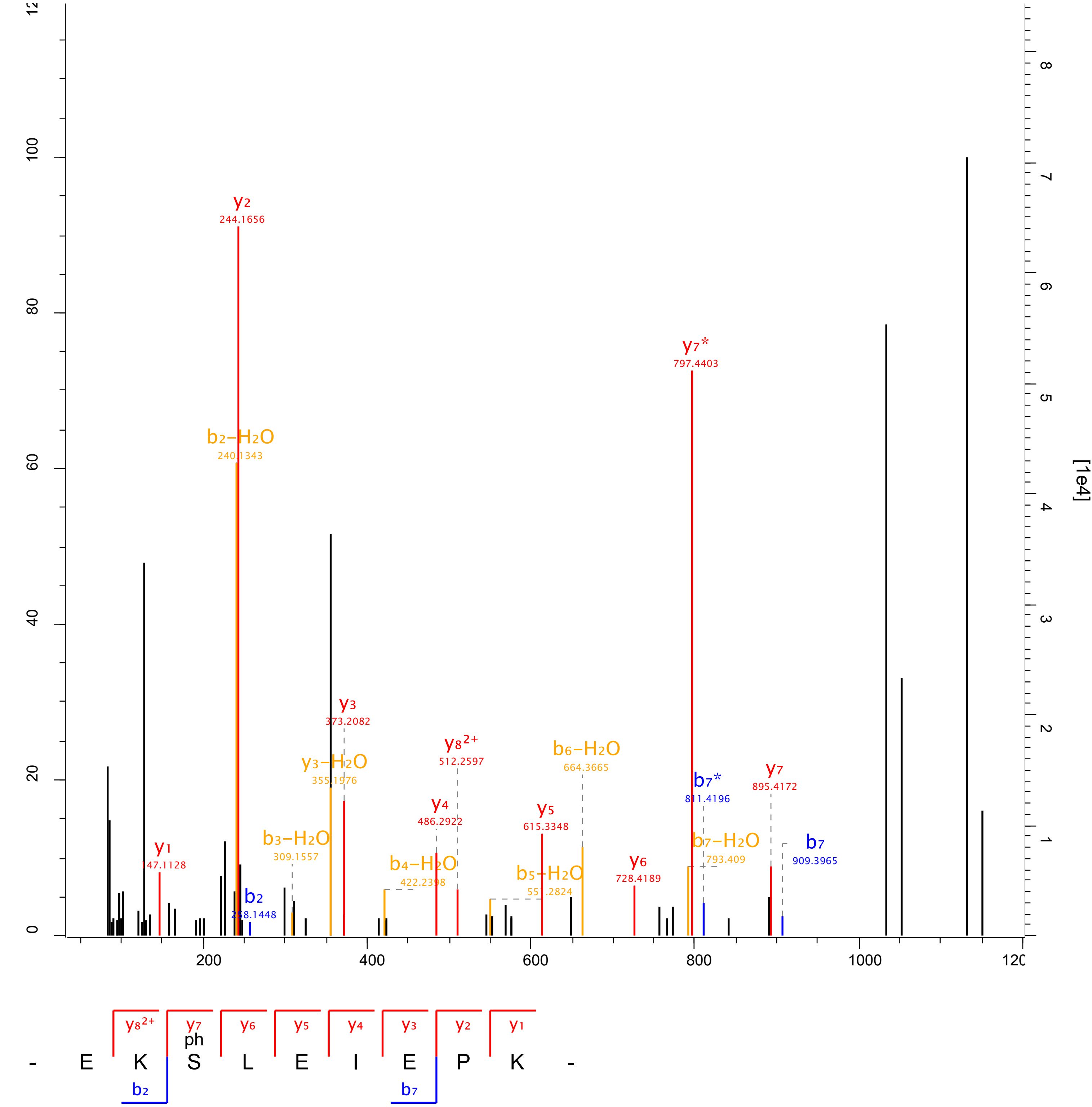

Sample output to test PDF Combine only

Raw file	Scan	Method	Score	m/z	Gene names
sp3-mic-SUC-2-A	13940	FTMS; HCD	52.25	569.26	PPC1

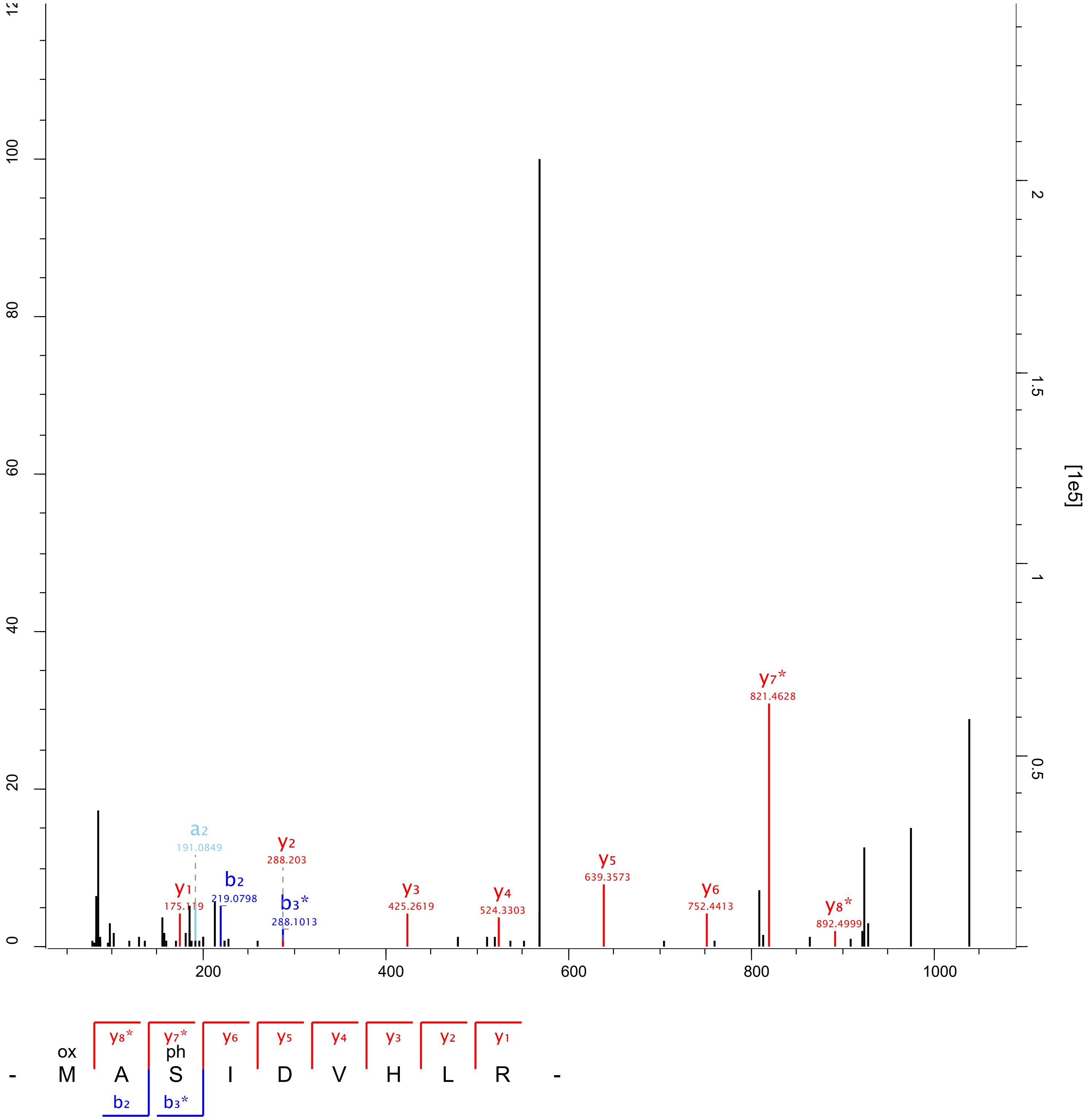

Sample output to test PDF Combine only

Raw file	Scan	Method	Score	m/z	Gene names
sp3-mic-SUC-2-A	15573	FTMS; HCD	180.31	783.34	At4g38470

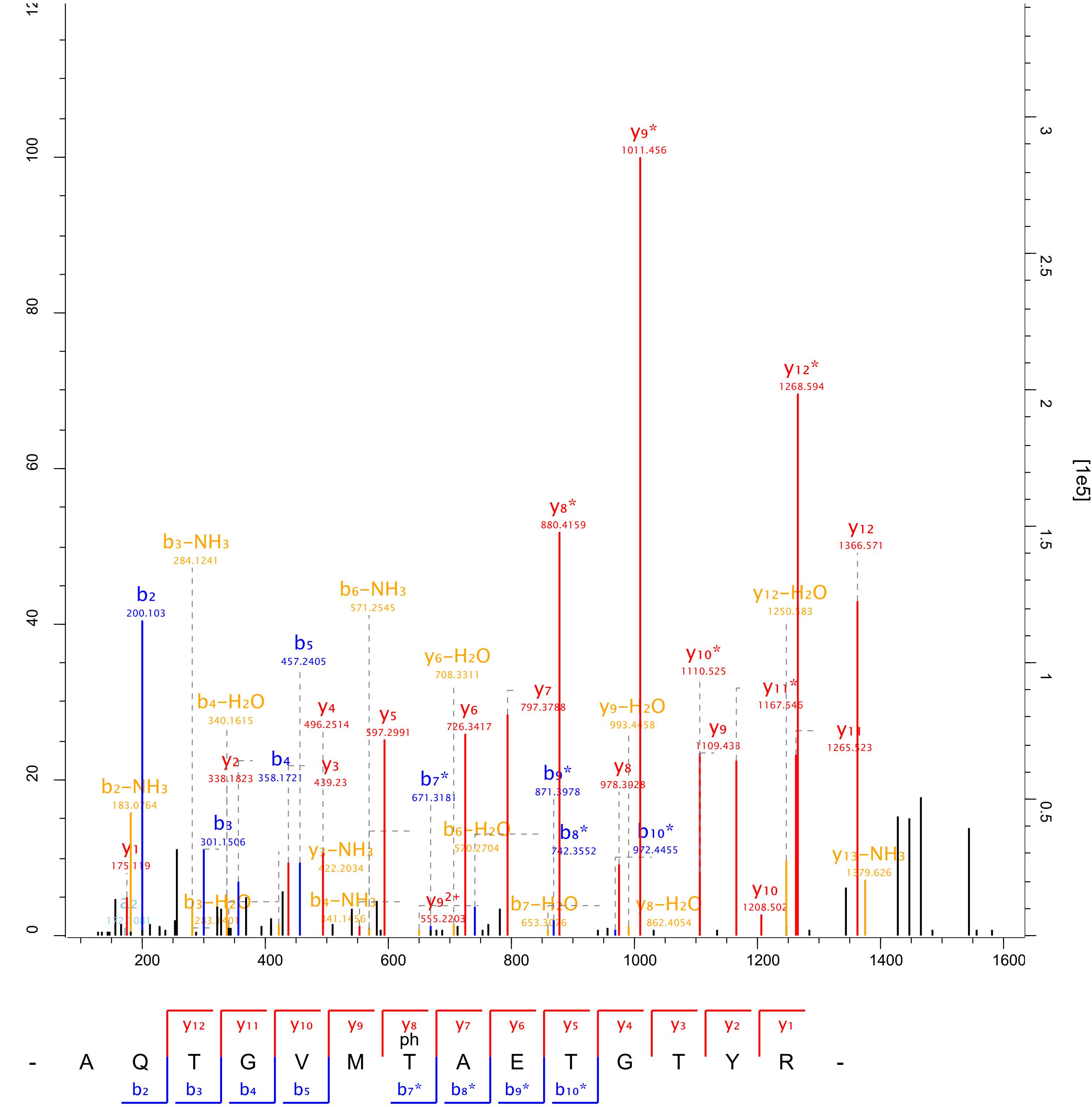

Sample output to test PDF Combine only

Raw file	Scan	Method	Score	m/z	Gene names
sp3-mic-SUC-2-P	20697	FTMS; HCD	41.24	828.89	At5g41550

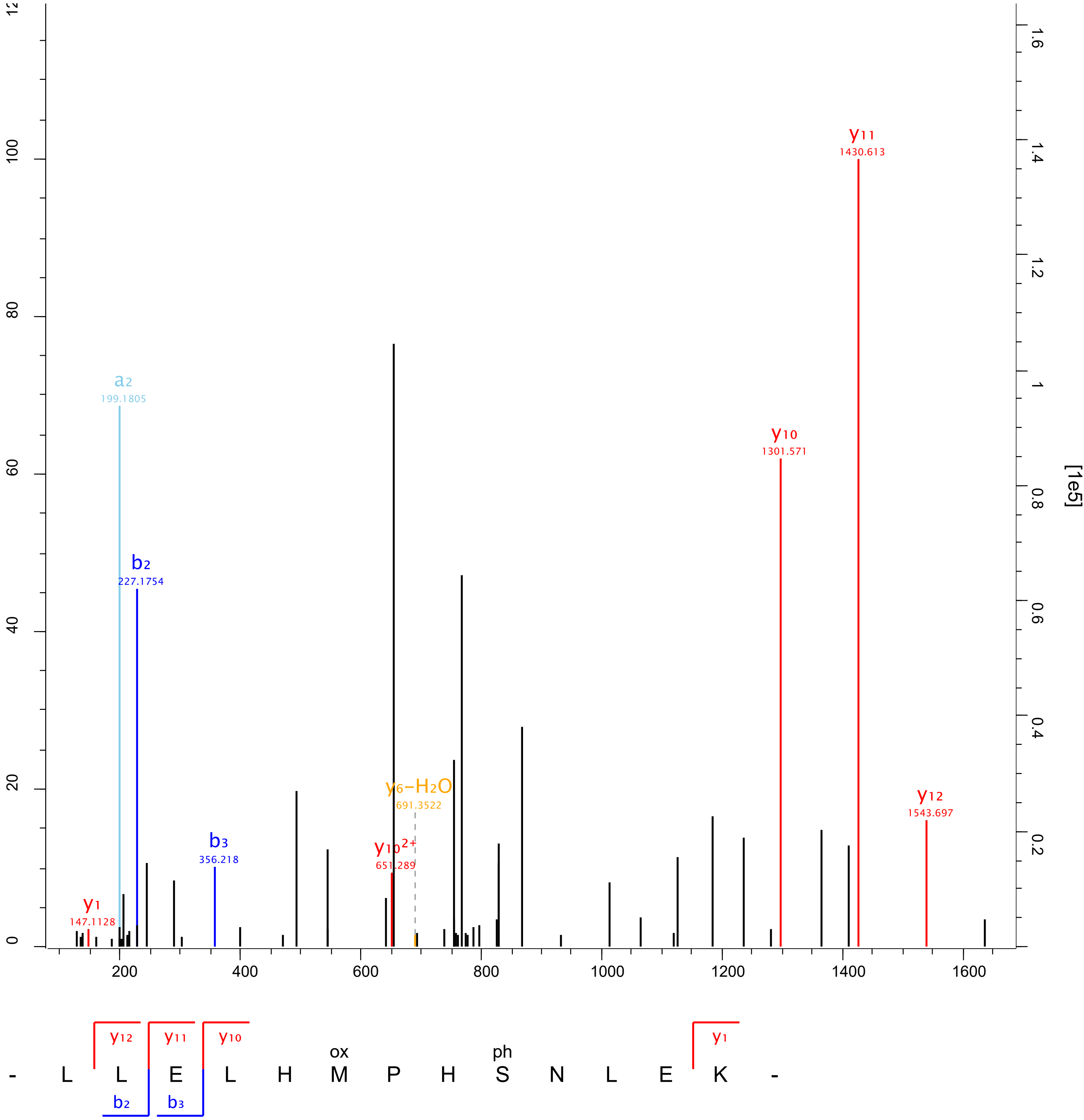

Sample output to test PDF Combine only

Raw file	Scan	Method	Score	m/z	Gene names
sp3-mic-SUC-2-P	29616	FTMS; HCD	59.54	712.02	CMTA3

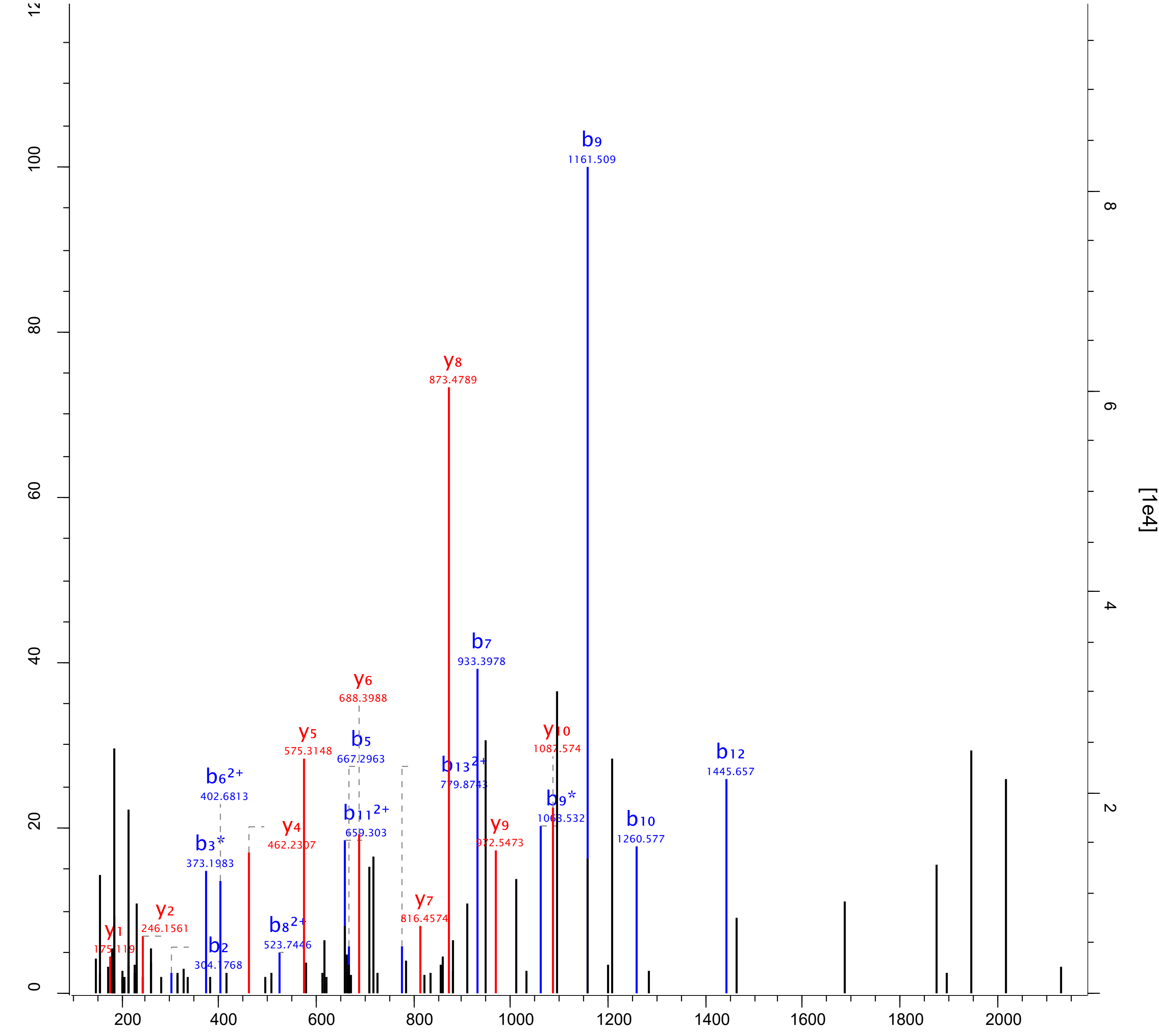

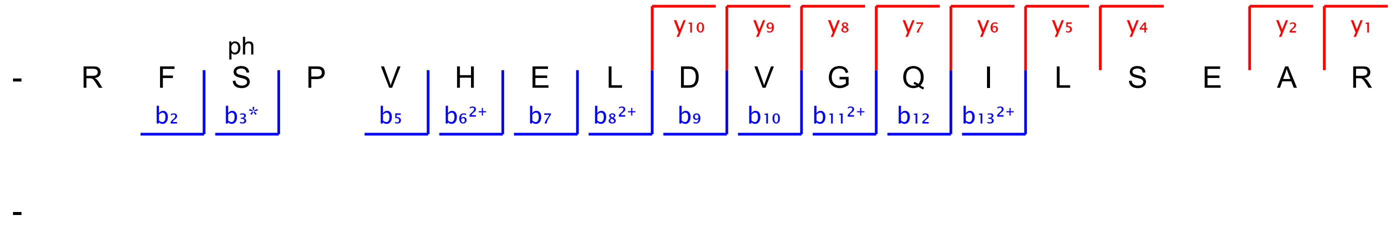

Sample output to test PDF Combine only

Raw file	Scan	Method	Score	m/z	Gene names
sp3-mic-SUC-3-A	9932	FTMS; HCD	49.45	693.3	At1g61690

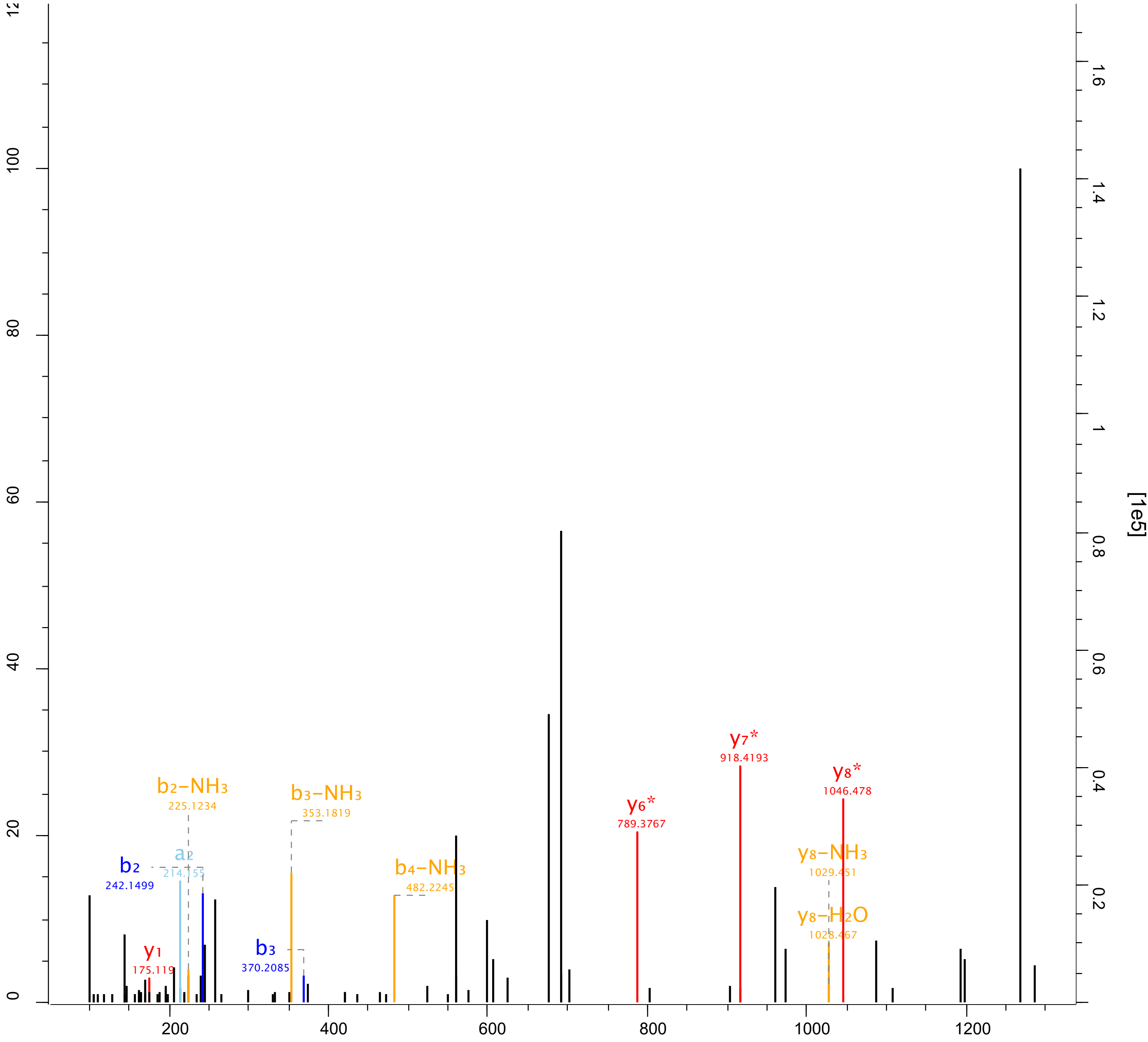

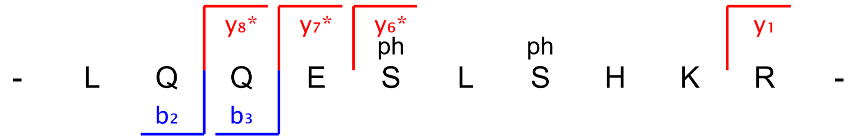

Sample output to test PDF Combine only

Raw file	Scan	Method	Score	m/z	Gene names
sp3-mic-SUC-3-A	29498	FTMS; HCD	48.57	619.78	POT8

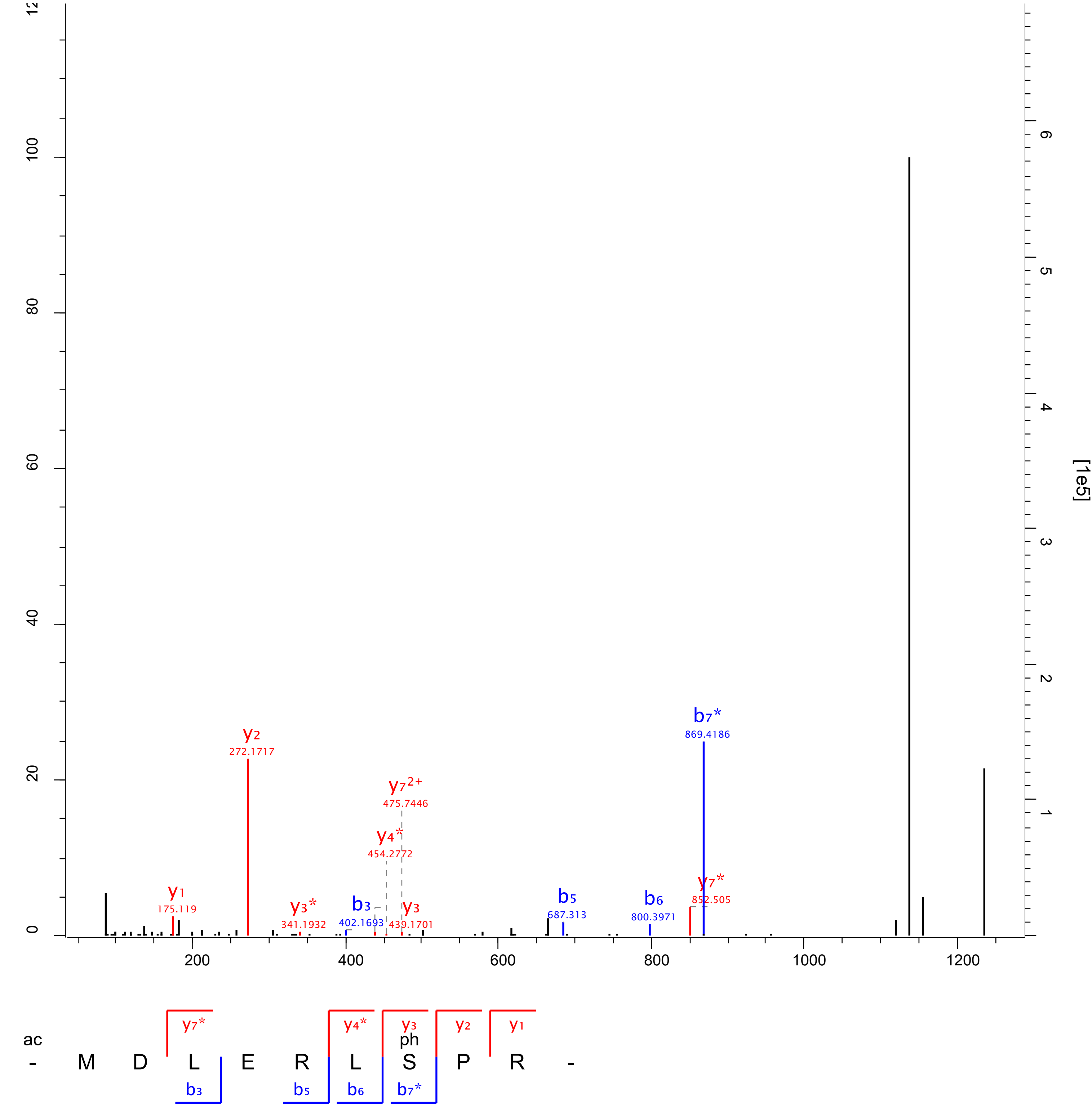

Sample output to test PDF Combine only

Raw file	Scan	Method	Score	m/z	Gene names
sp3-mic-SUC-3-P	12436	FTMS; HCD	54.9	478.58	At2g44010

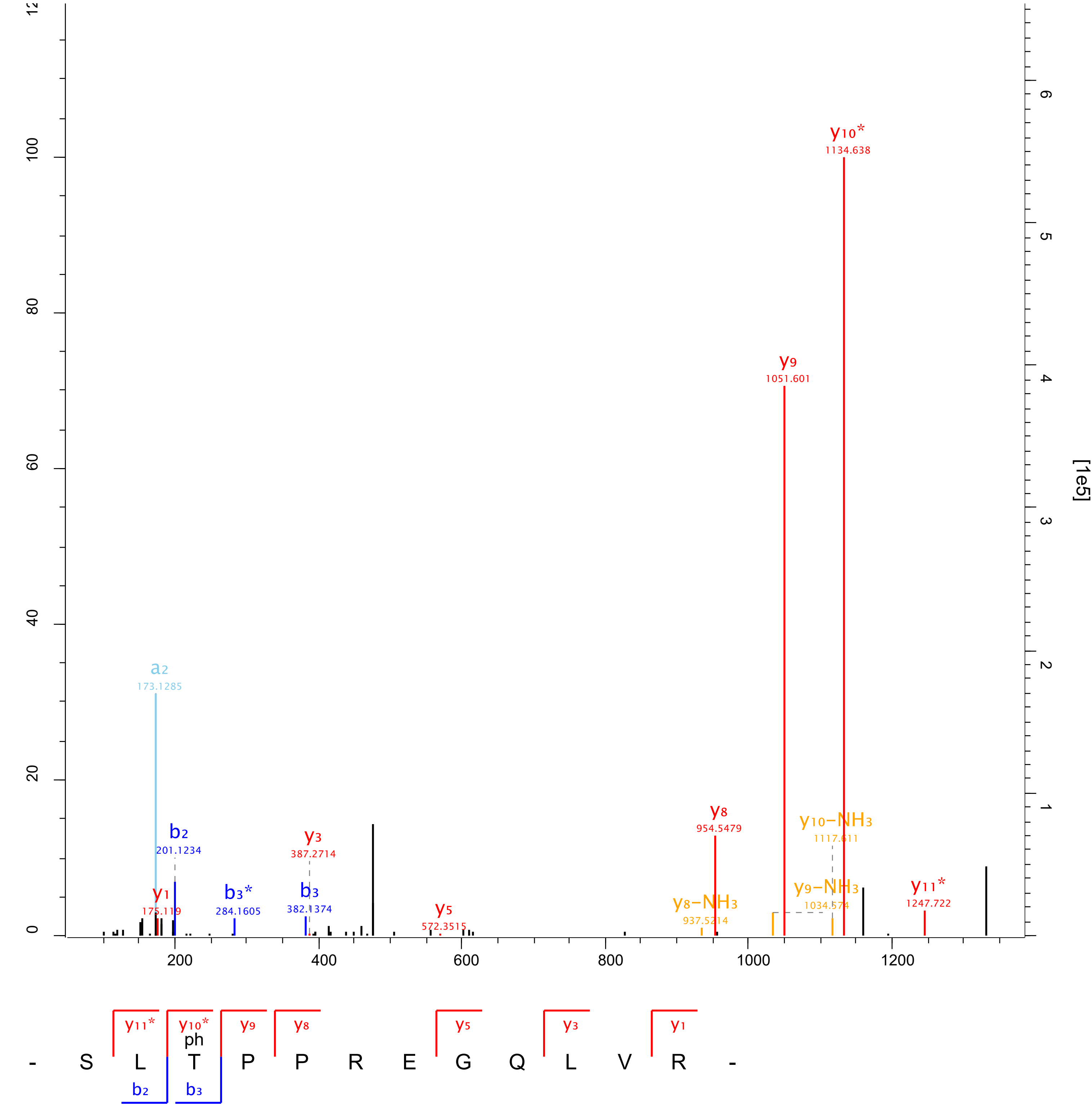

Sample output to test PDF Combine only

Raw file	Scan	Method	Score	m/z	Gene names
sp3-mic-SUC-3-P	14141	FTMS; HCD	68.28	413.21	F20B18.240

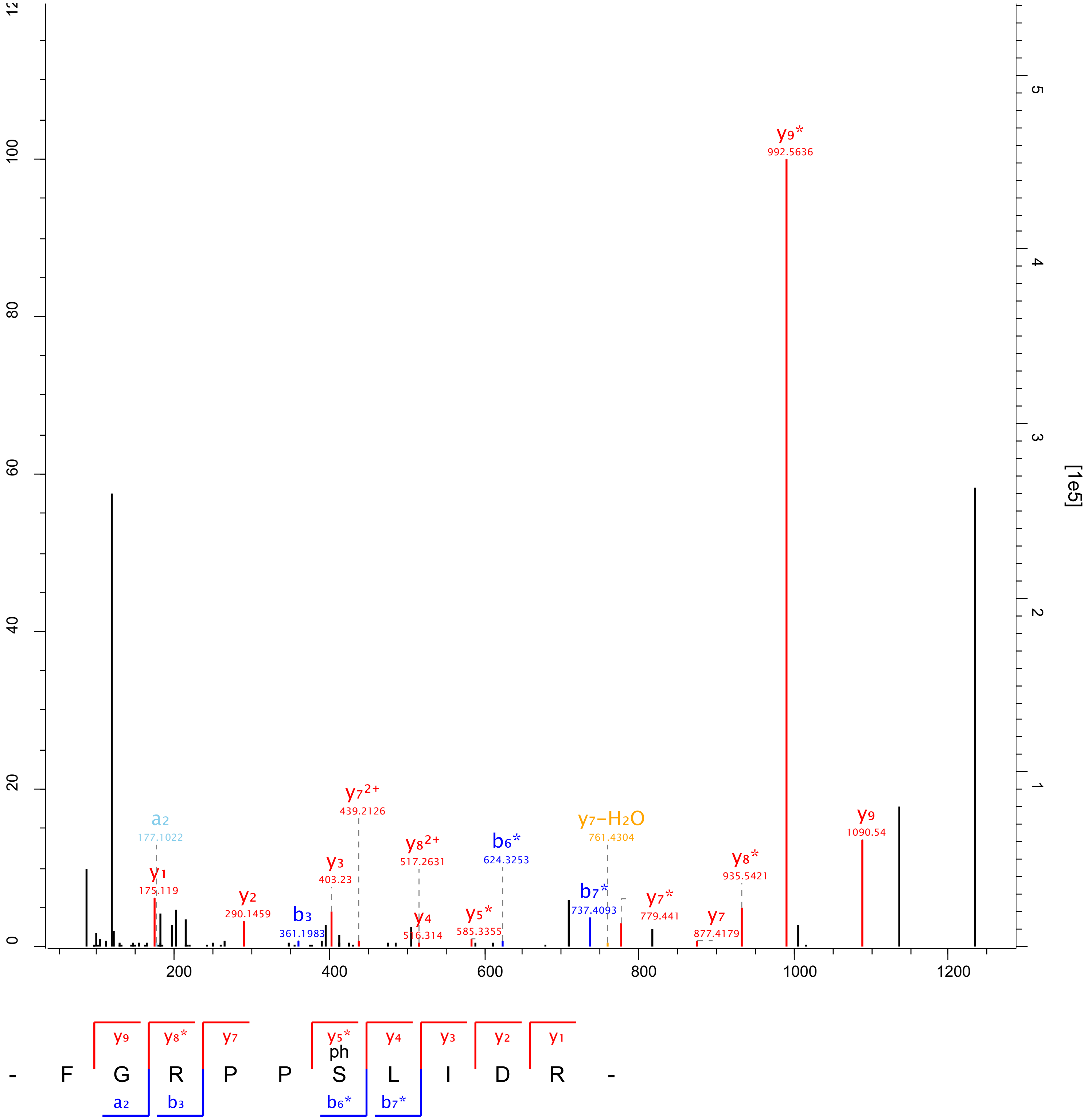

Sample output to test PDF Combine only

Raw file	Scan	Method	Score	m/z	Gene names
sp3-mic-SUC-3-P	17149	FTMS; HCD	233.19	584.29	ACA8

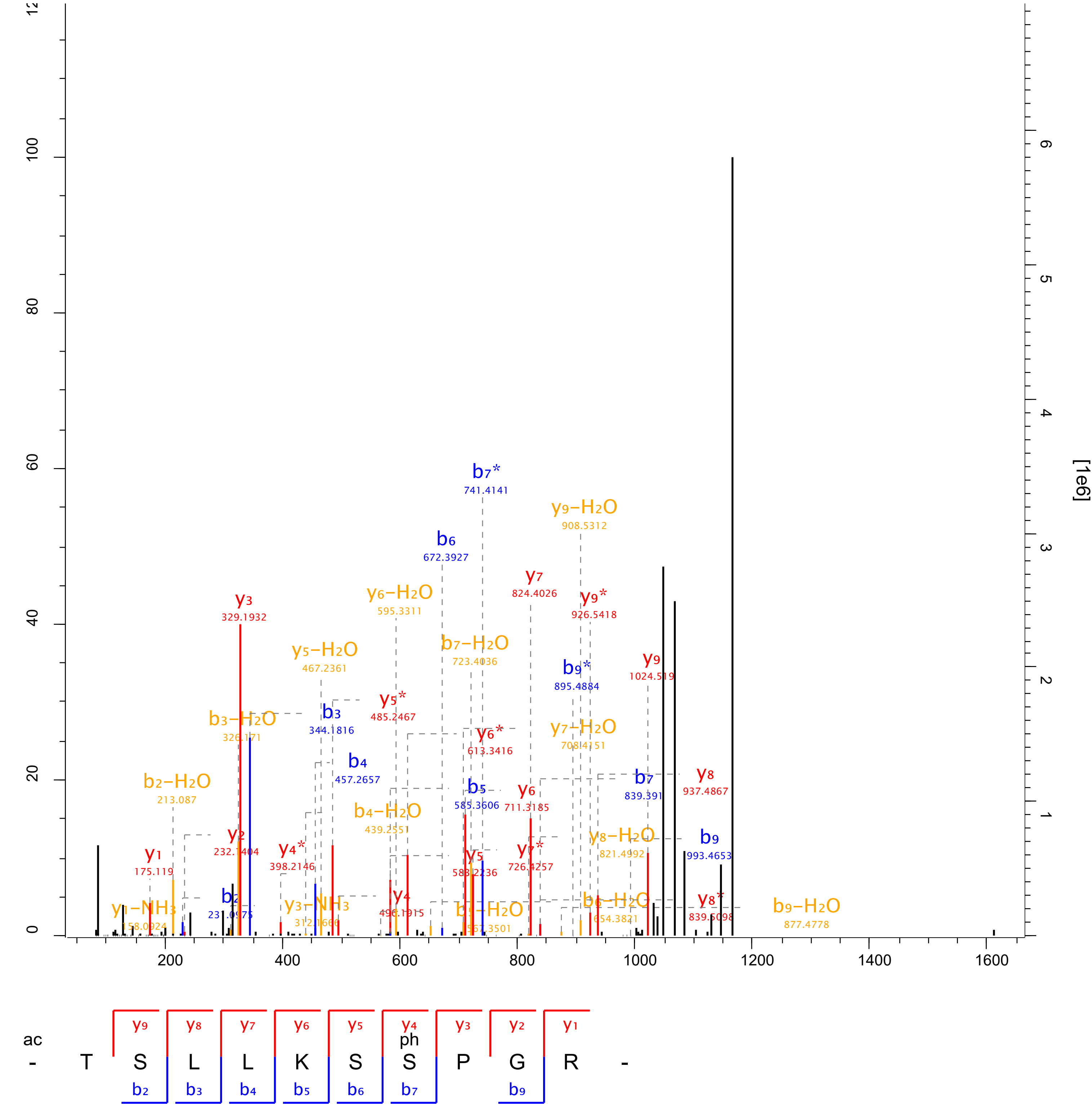

Sample output to test PDF Combine only

Raw file	Scan	Method	Score	m/z	Gene names
sp3-mic-SUC-3-P	18035	FTMS; HCD	78.11	450.73	NDA2

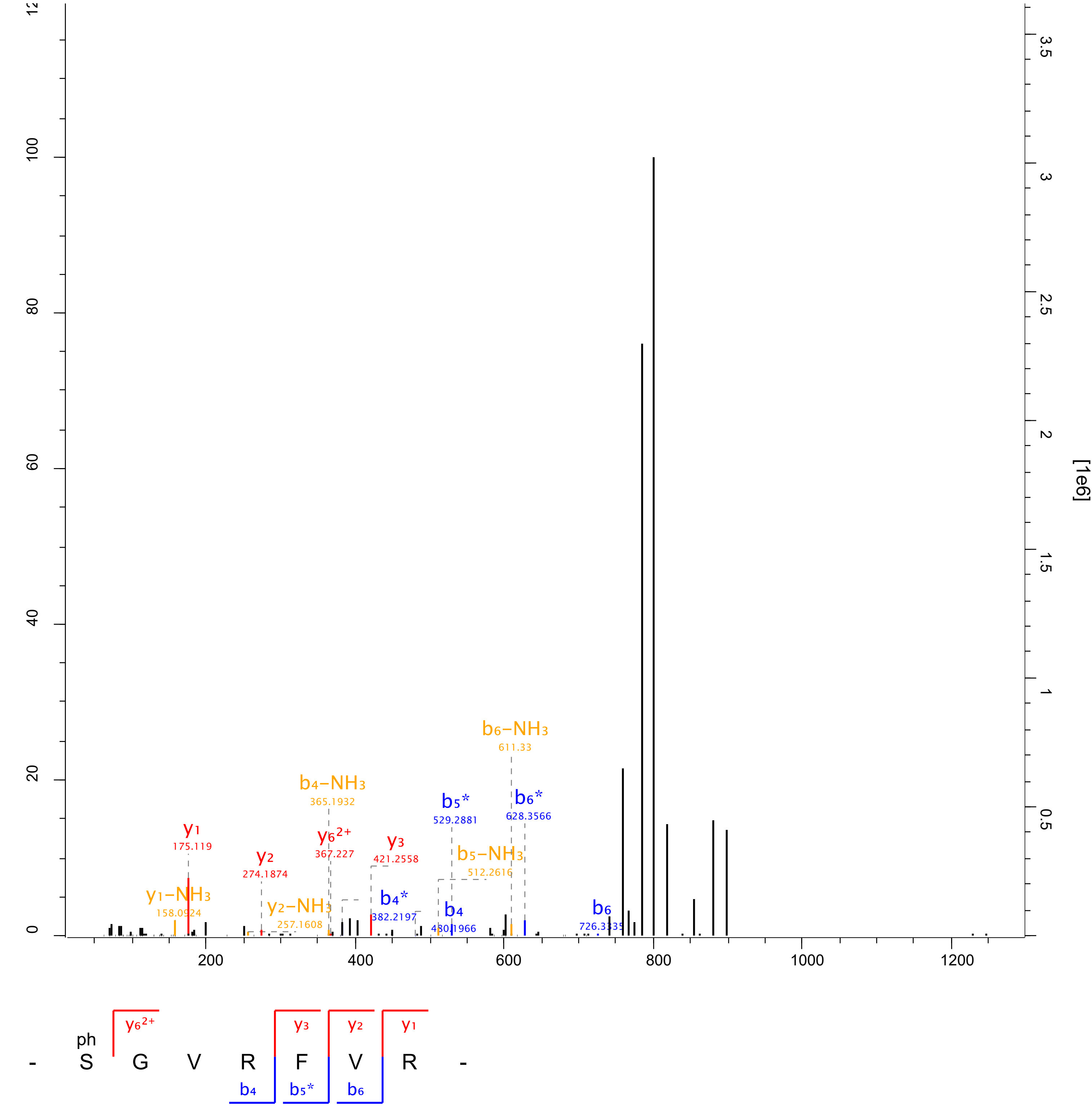

Sample output to test PDF Combine only

Raw file	Scan	Method	Score	m/z	Gene names
sp3-mic-SUC-3-P	18266	FTMS; HCD	115.38	776.33	F13I12.260

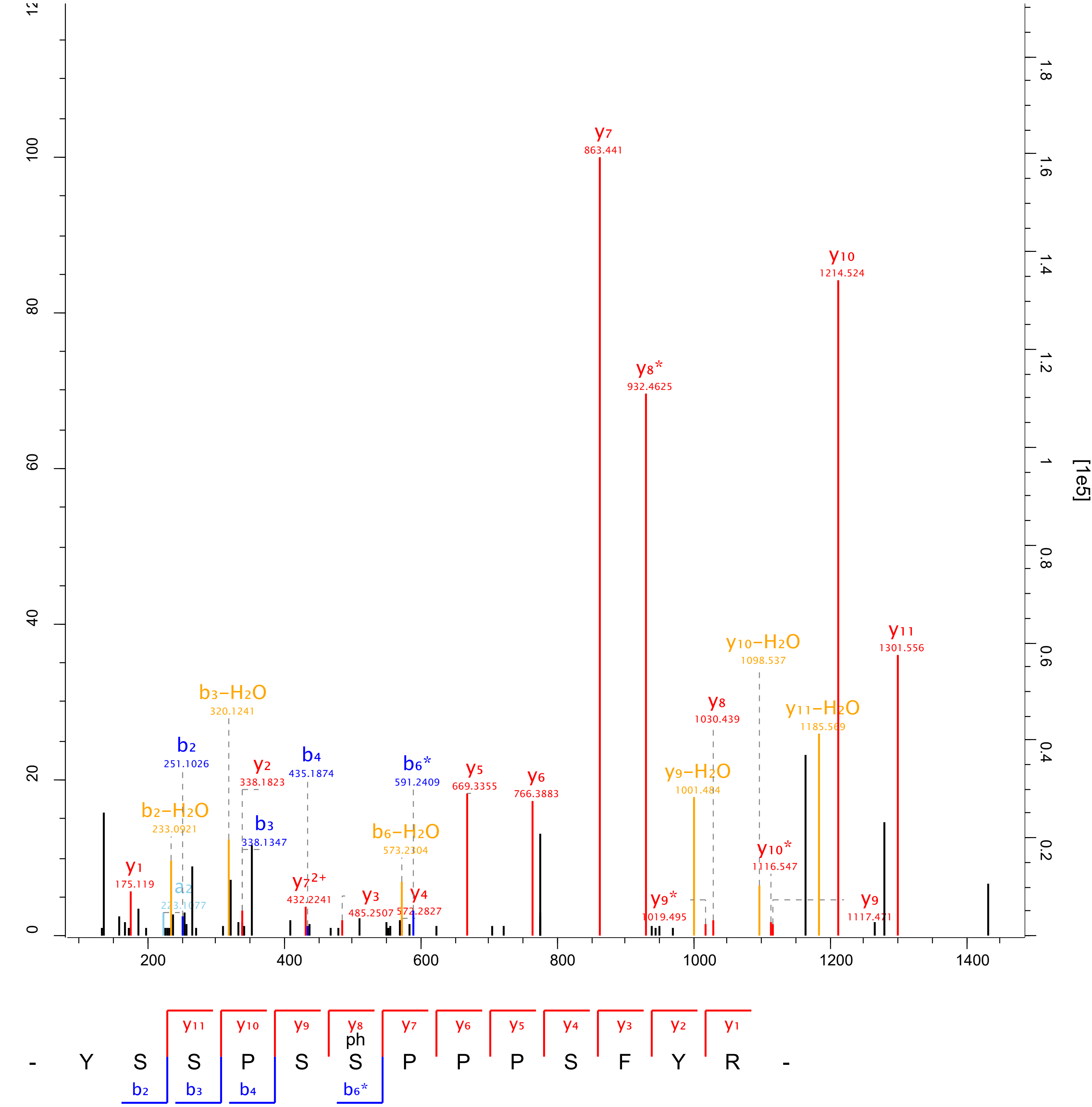

Sample output to test PDF Combine only

Raw file	Scan	Method	Score	m/z	Gene names
sp3-mic-SUC-3-P	19109	FTMS; HCD	69.36	863.91	SPK1

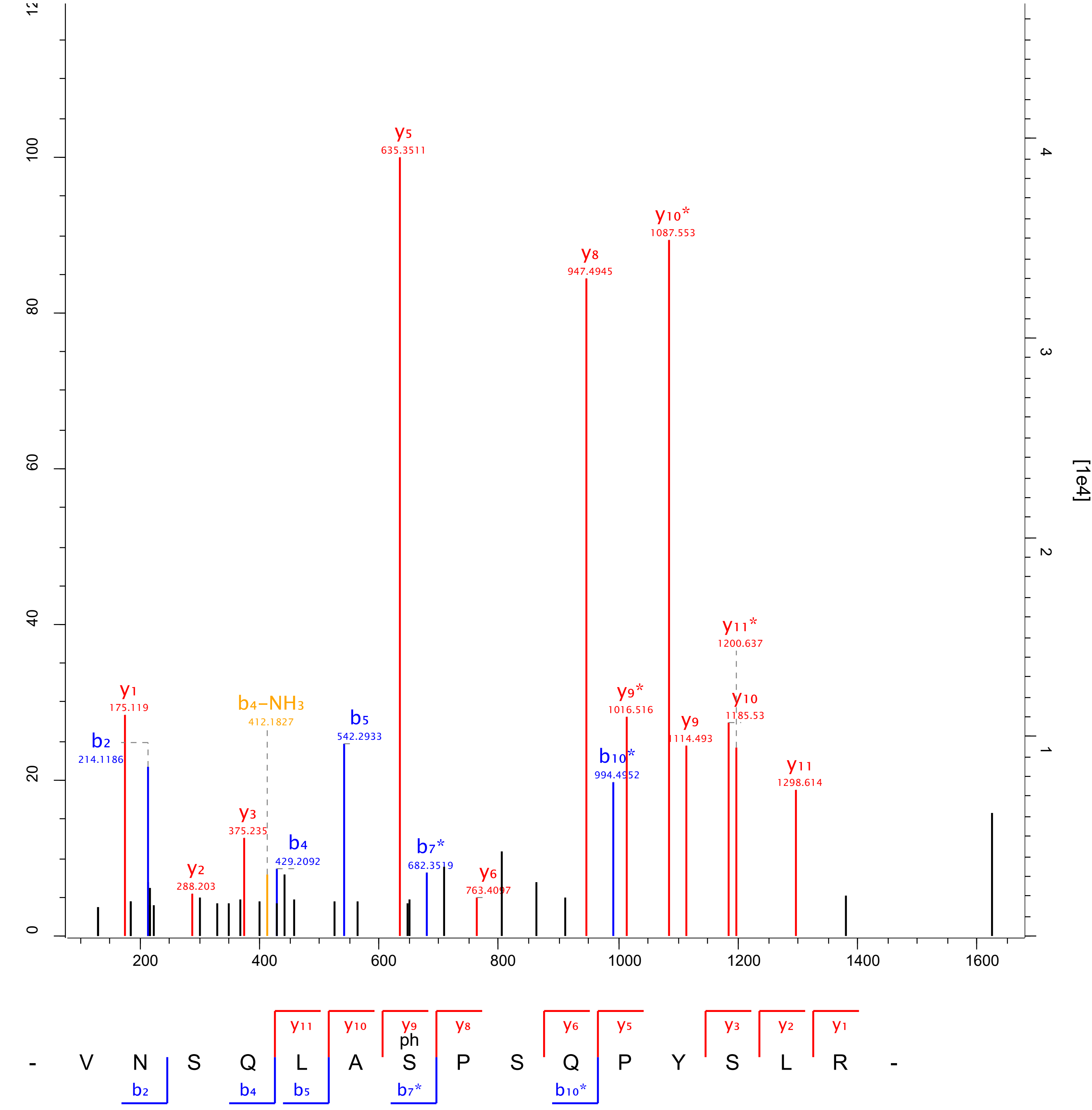

Sample output to test PDF Combine only

Raw file	Scan	Method	Score	m/z	Gene names
sp3-mic-SUC-3-P	24777	FTMS; HCD	122.44	515.25	CLC-C

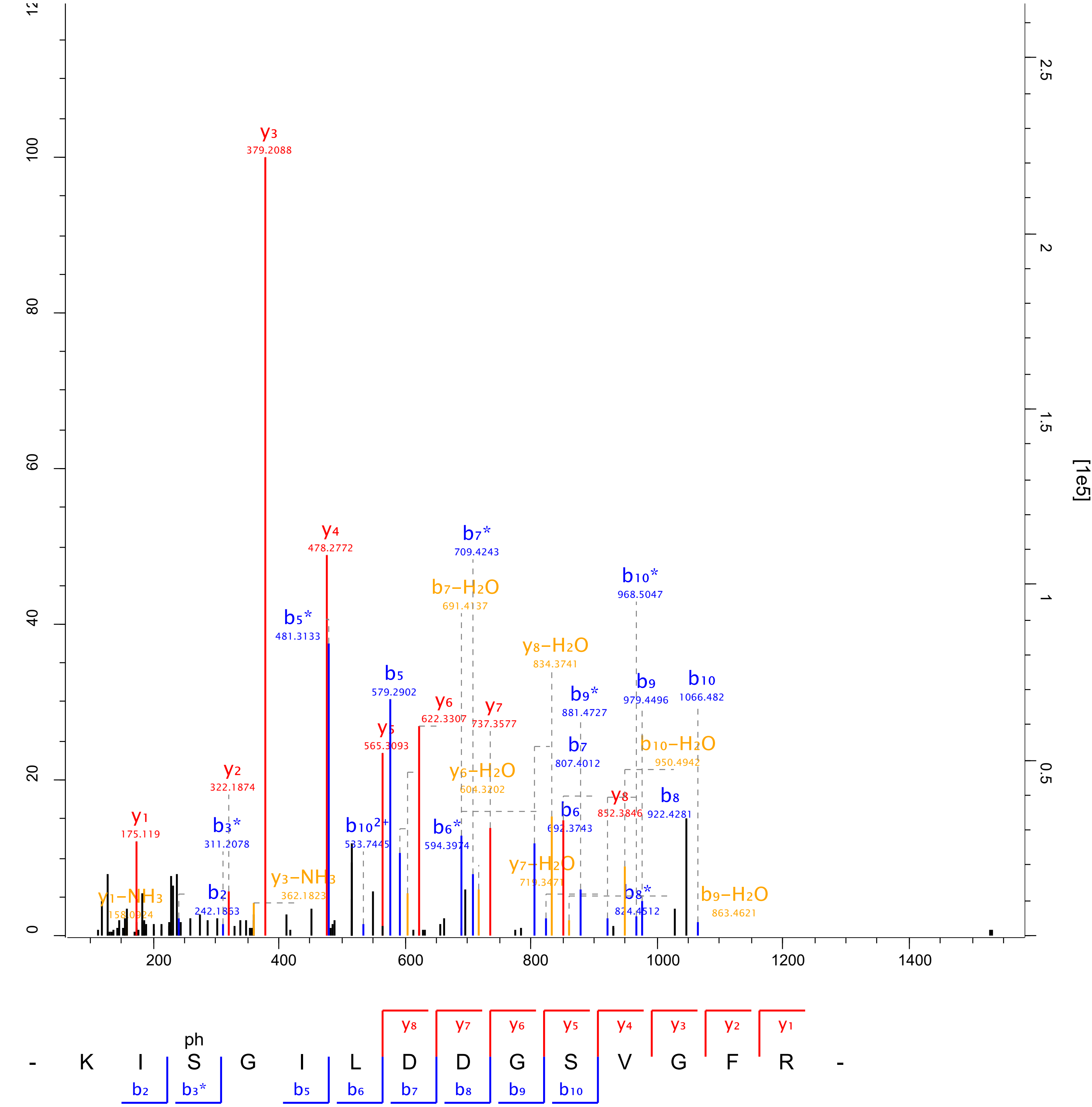

Sample output to test PDF Combine only

Raw file	Scan	Method	Score	m/z	Gene names
sp3-mic-SUC-3-P	25943	FTMS; HCD	216.54	632.84	SEC5A

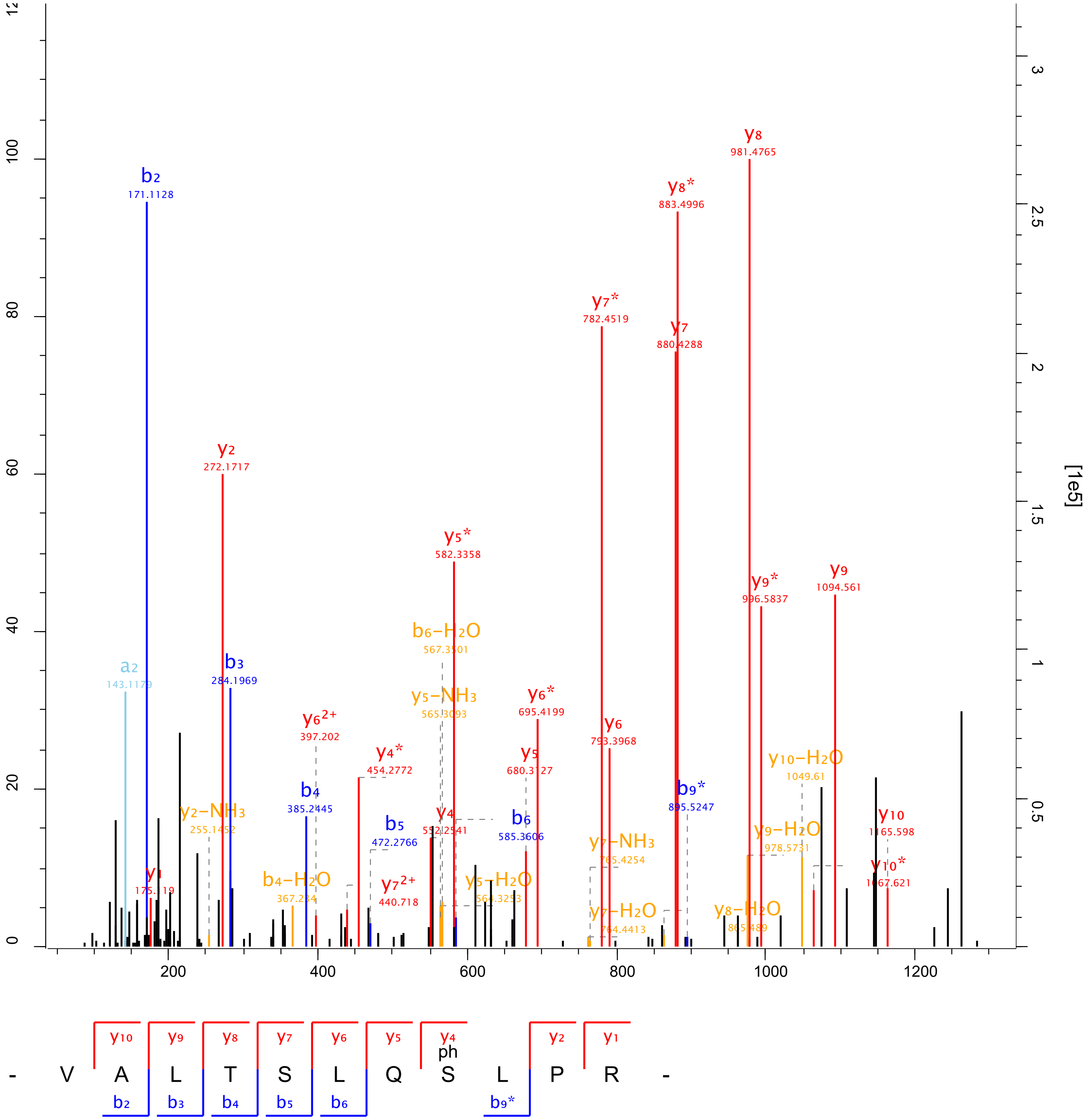

Sample output to test PDF Combine only

Raw file	Scan	Method	Score	m/z
sp3-mic-SUC-3-P	31568	FTMS; HCD	116.6	781.4

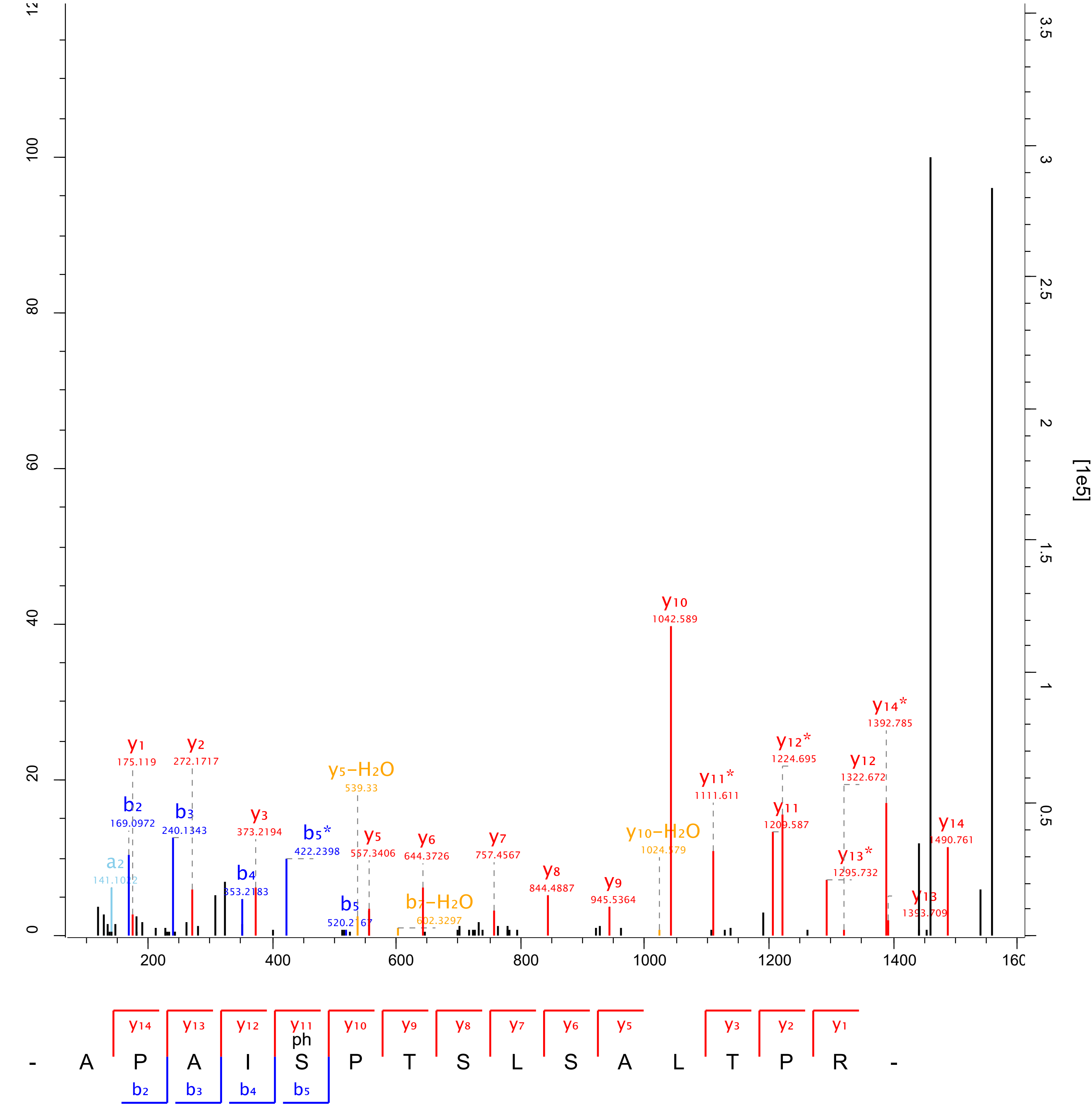

Sample output to test PDF Combine only

Raw file	Scan	Method	Score	m/z
sp3-mic-SUC-3-P	35532	FTMS; HCD	60.2	887.47

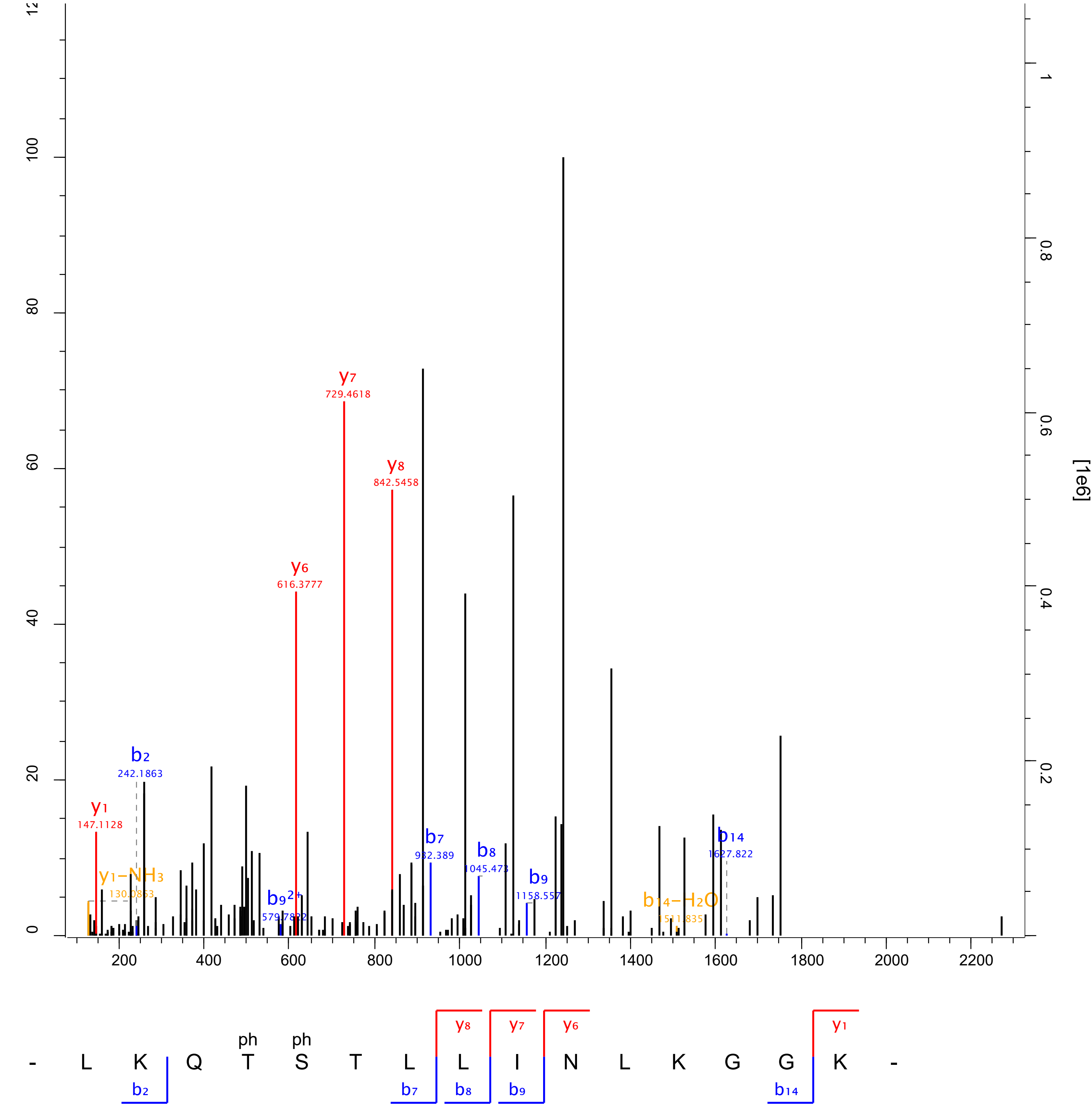

Sample output to test PDF Combine only

Raw file	Scan	Method	Score	m/z	Gene names
sp3-mic-SUC-3-P	36594	FTMS; HCD	67.23	635.96	ABCG34

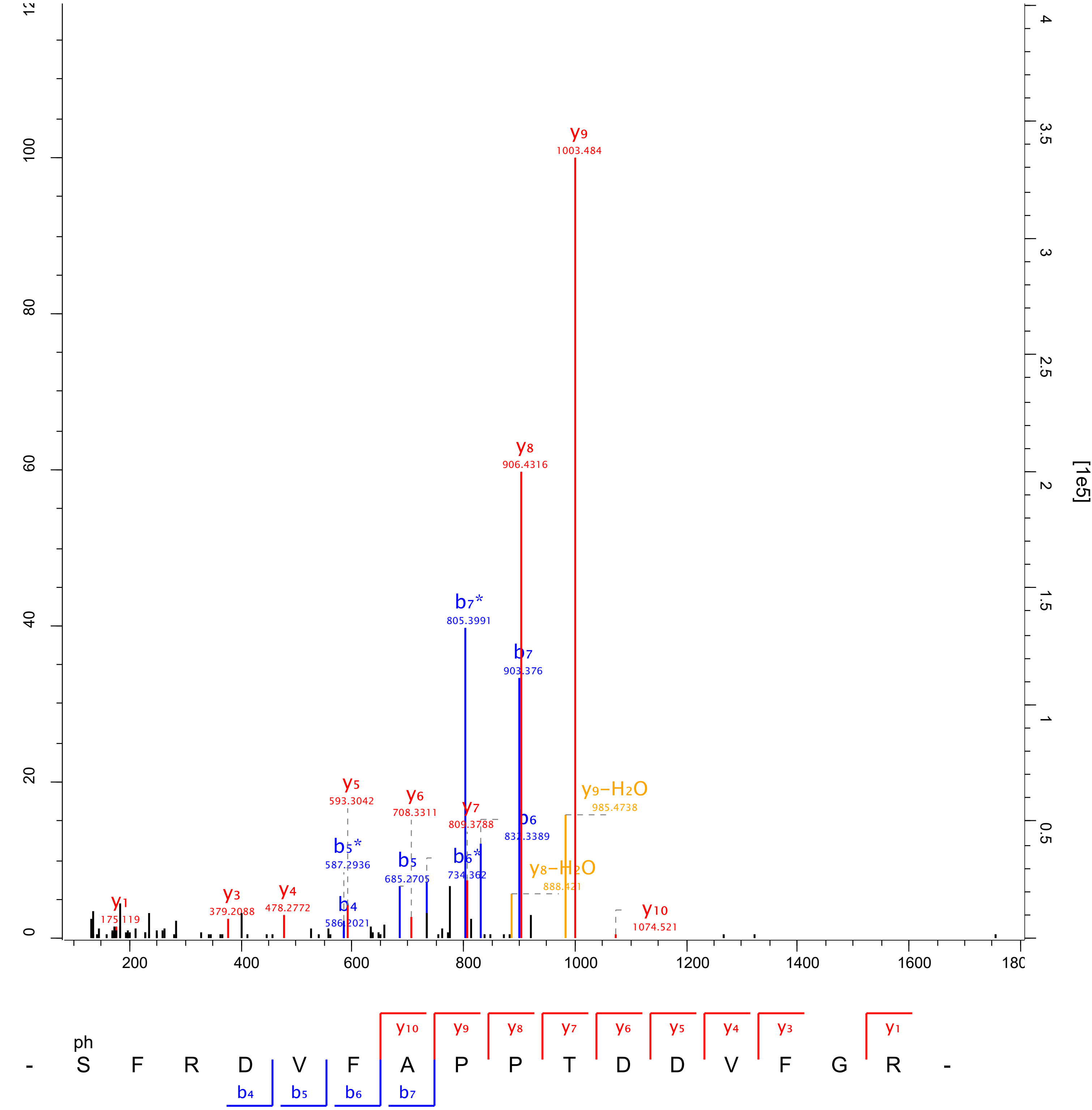

Sample output to test PDF Combine only

Raw file	Scan	Method	Score	m/z	Gene names
sp3-mic-SUC-3-P	39434	FTMS; HCD	118.32	791.11	CCT4

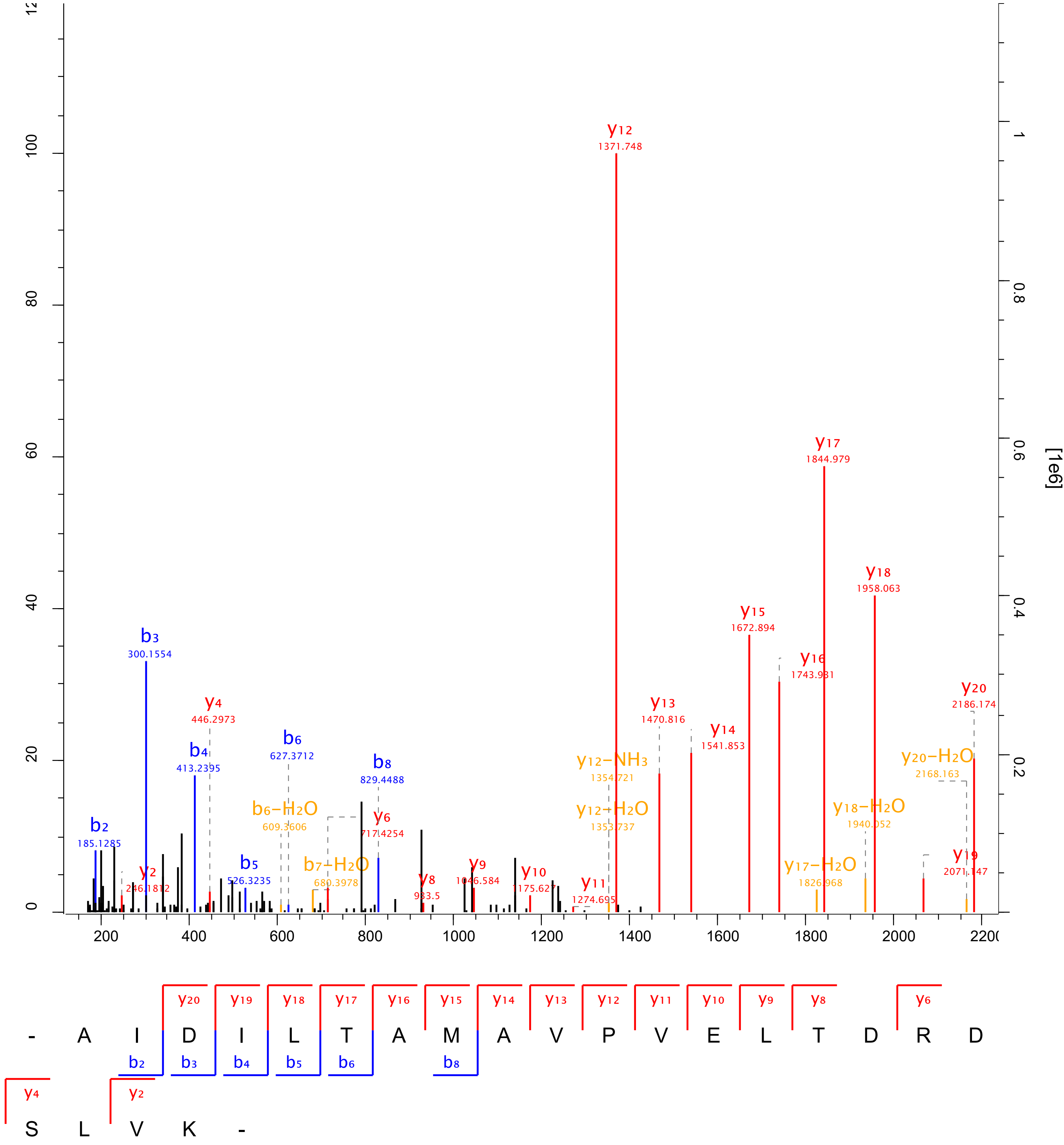

Sample output to test PDF Combine only

Raw file	Scan	Method	Score	m/z
sp3-solu-0-3-P	17129	FTMS; HCD	47.71	831.4

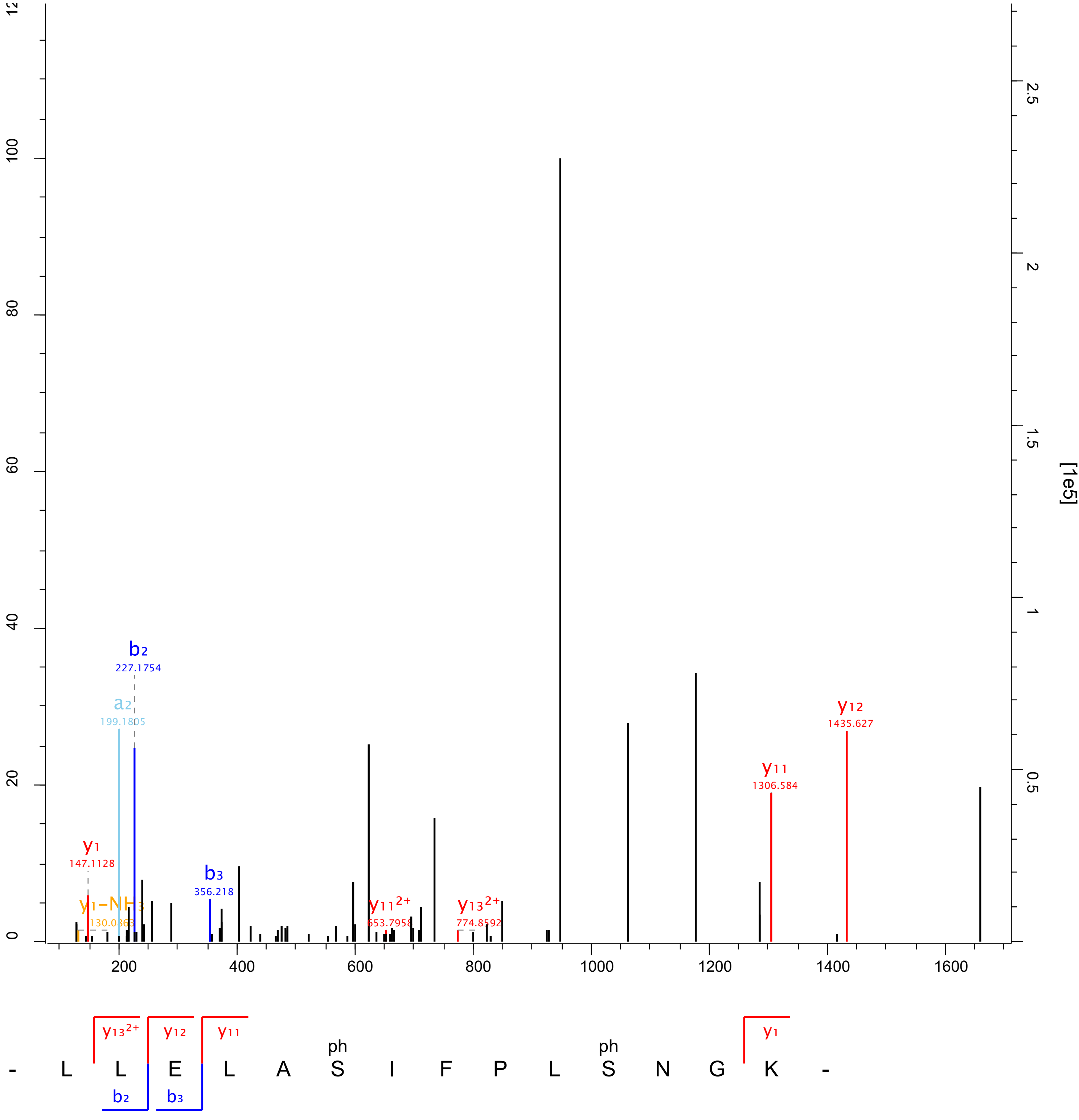

Sample output to test PDF Combine only

Raw file	Scan	Method	Score	m/z	Gene names
sp3-solu-SUC-1-A	16082	FTMS; HCD	73.88	709.81	CYS1

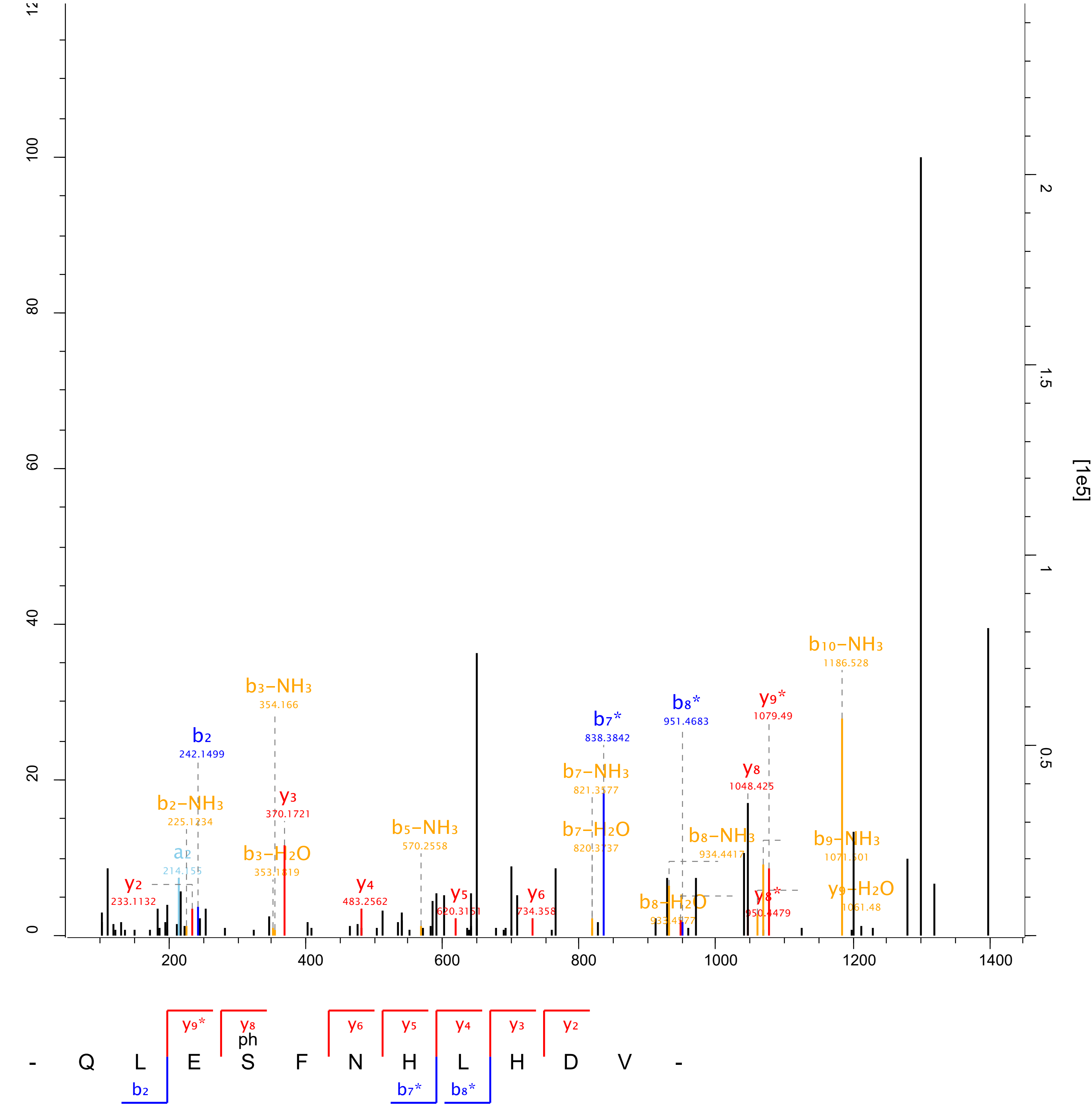

Sample output to test PDF Combine only

Raw file	Scan	Method	Score	m/z	Gene names
sp3-solu-SUC-1-A	16510	FTMS; HCD	130.05	644.65	At5g63550

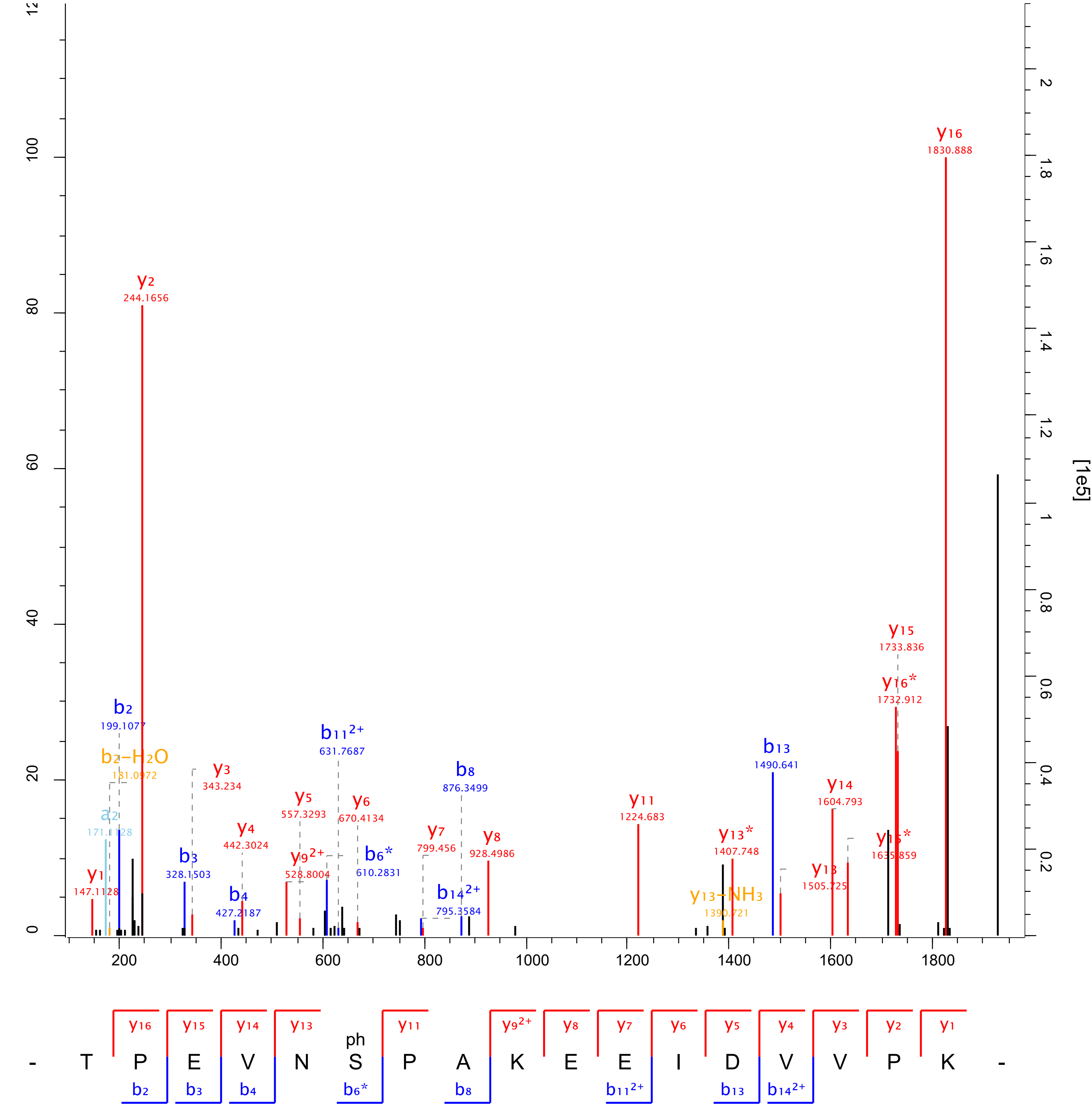

Sample output to test PDF Combine only

Raw file	Scan	Method	Score	m/z	Gene names
sp3-solu-SUC-1-A	17525	FTMS; HCD	55.88	705.85	At5g63550

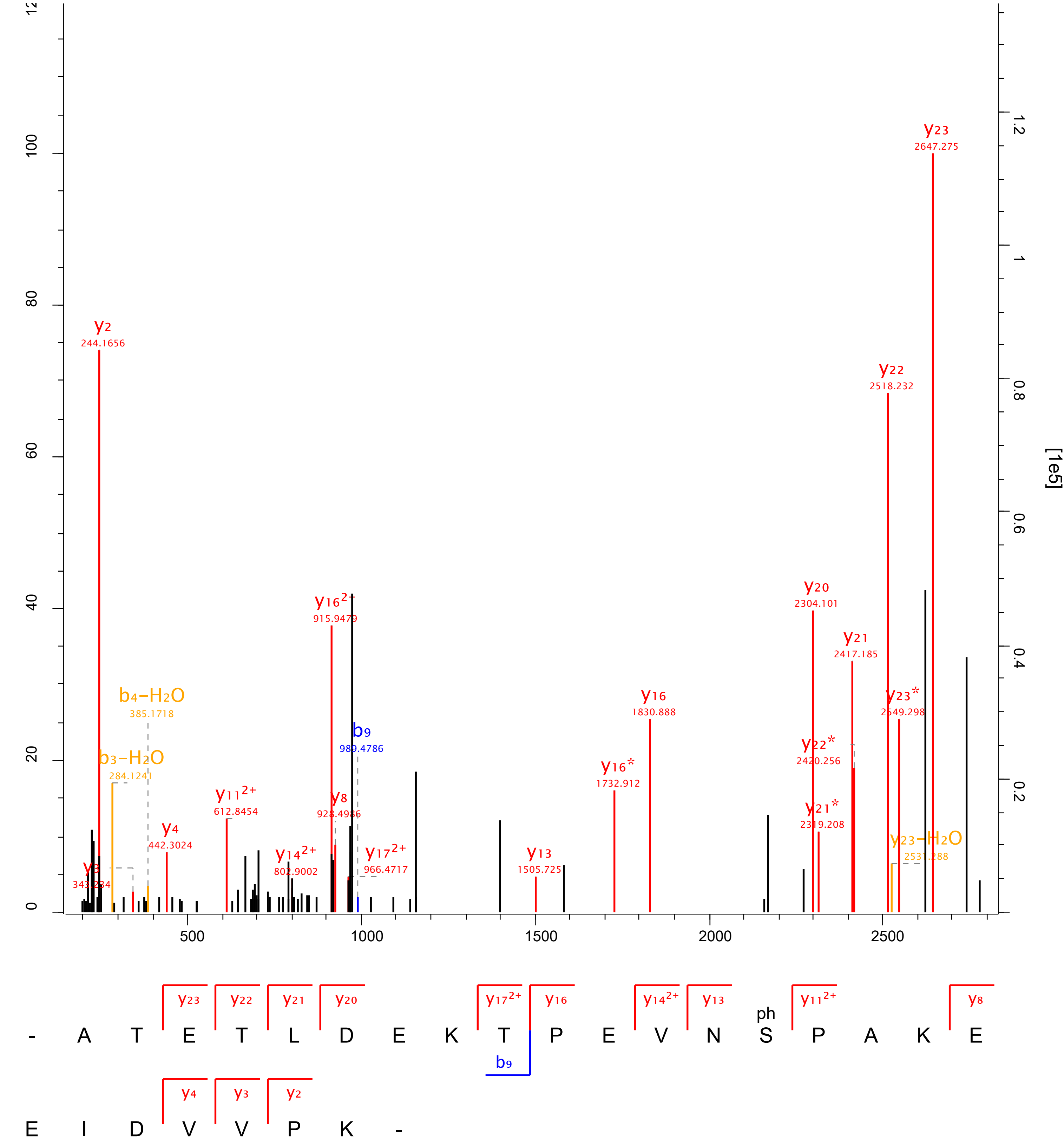

Sample output to test PDF Combine only

Raw file	Scan	Method	Score	m/z	Gene names
sp3-solu-SUC-1-A	17528	FTMS; HCD	40.99	681.32	NSN1

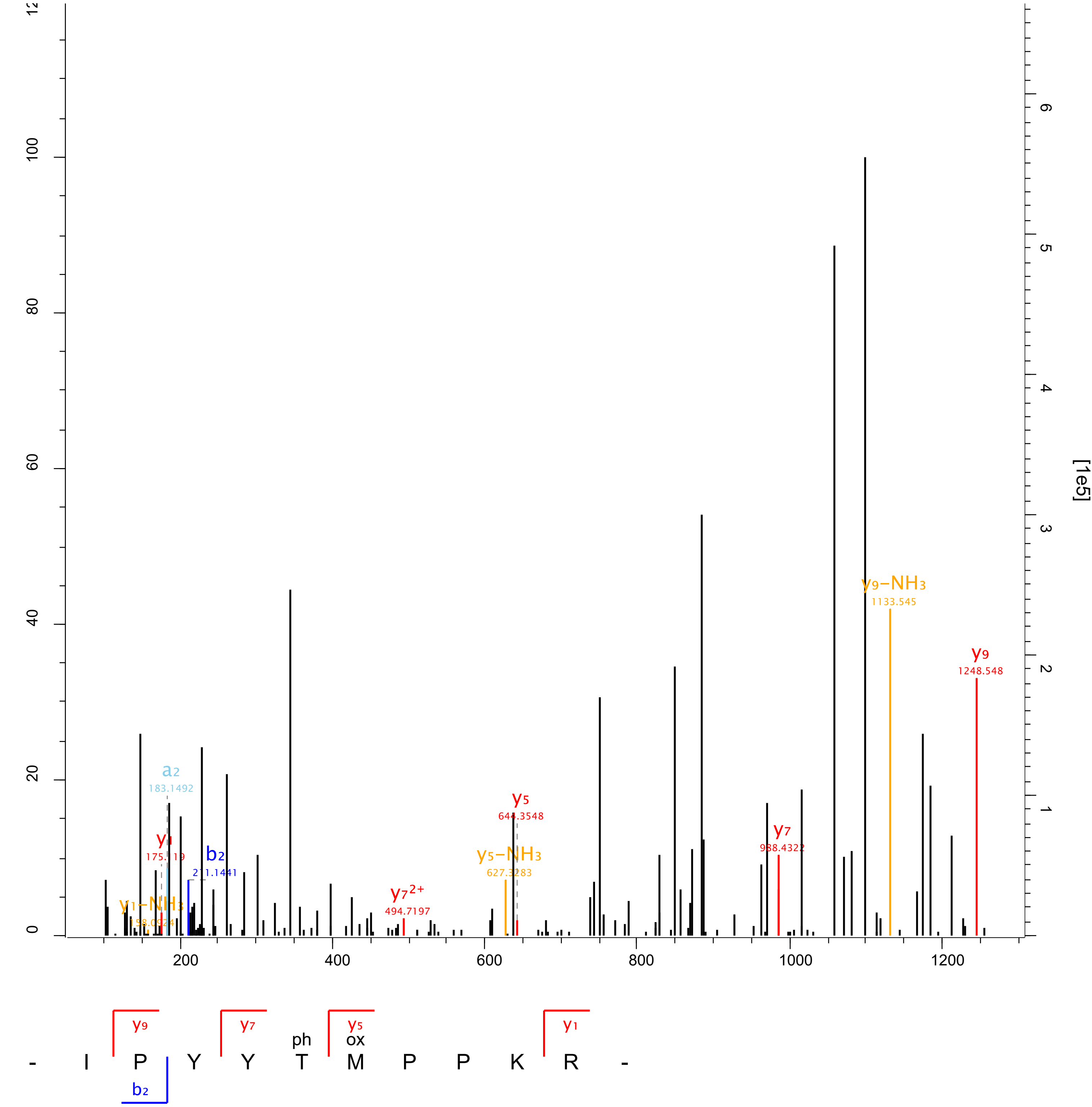

Sample output to test PDF Combine only

Raw file	Scan	Method	Score	m/z	Gene names
sp3-solu-SUC-1-A	22567	FTMS; HCD	43.03	888.91	At5g10010

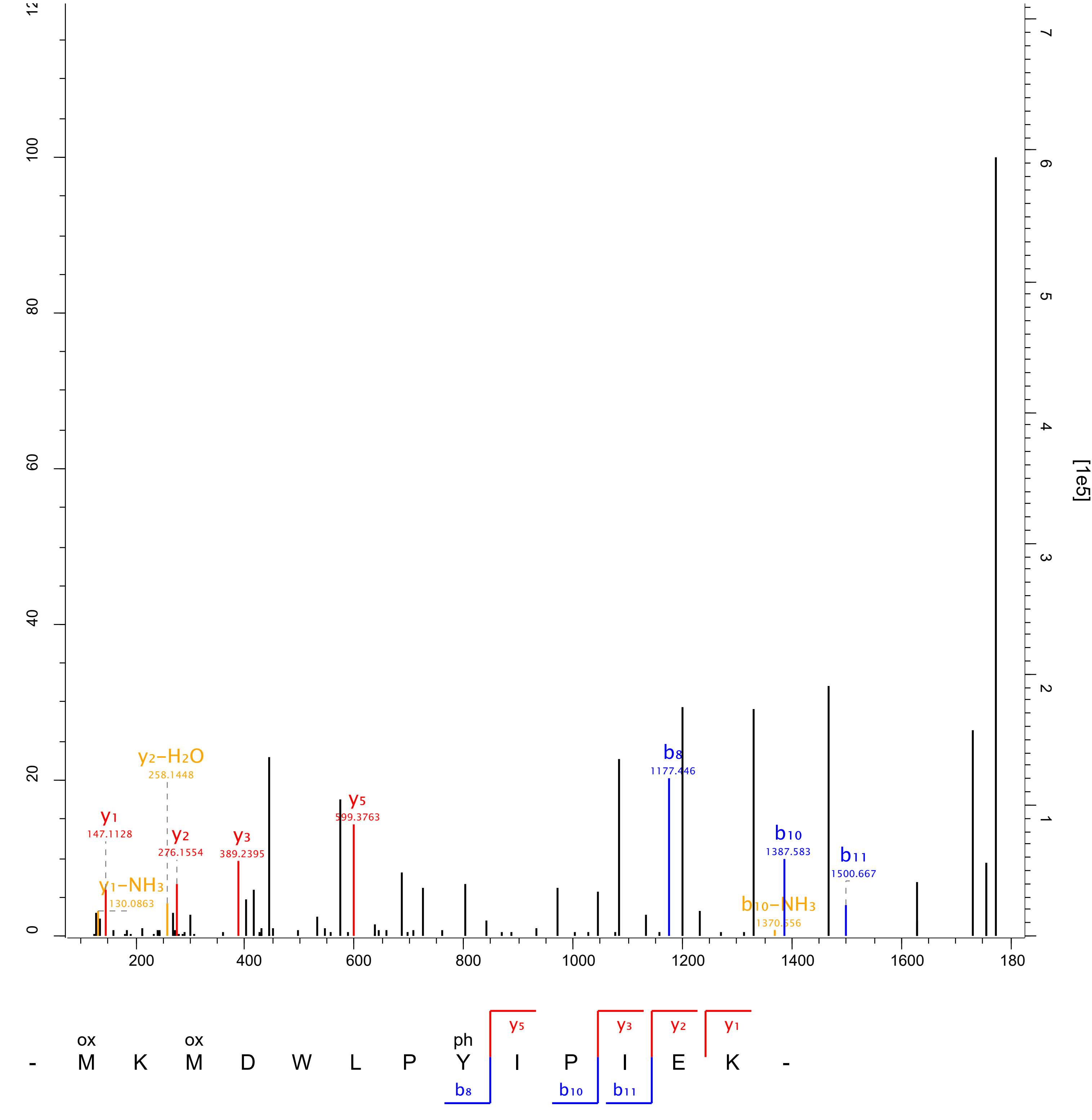

Sample output to test PDF Combine only

Raw file	Scan	Method	Score	m/z	Gene names
sp3-solu-SUC-1-A	22975	FTMS; HCD	125.31	954.8	At5g63550

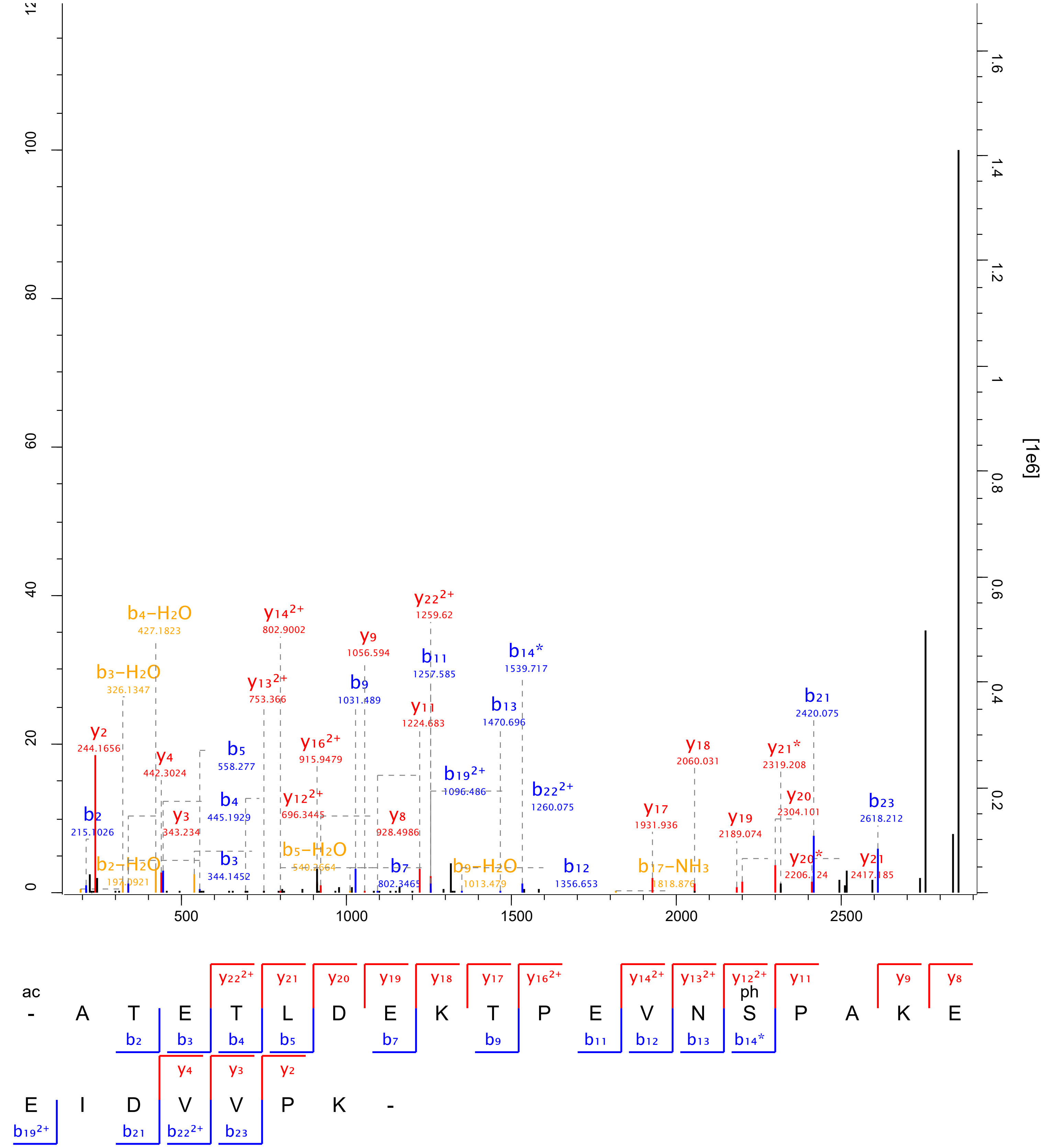

Sample output to test PDF Combine only

Raw file	Scan	Method	Score	m/z	Gene names
sp3-solu-SUC-1-P	26255	FTMS; HCD	66.47	738.7	At1g74910;F9E10.24

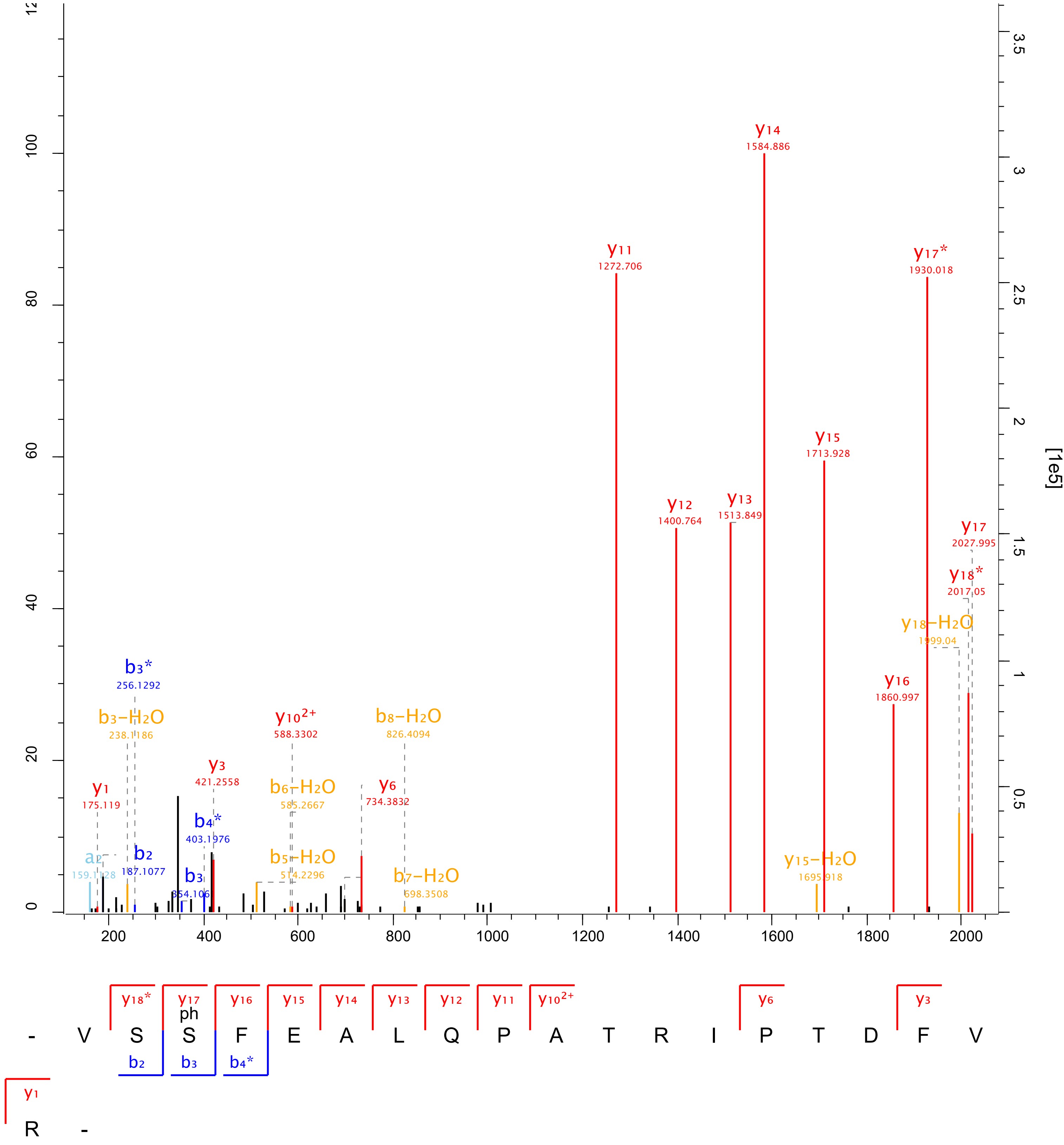

Sample output to test PDF Combine only

Raw file	Scan	Method	Score	m/z	Gene names
sp3-solu-SUC-1-P	30418	FTMS; HCD	47.04	962.01	BST1

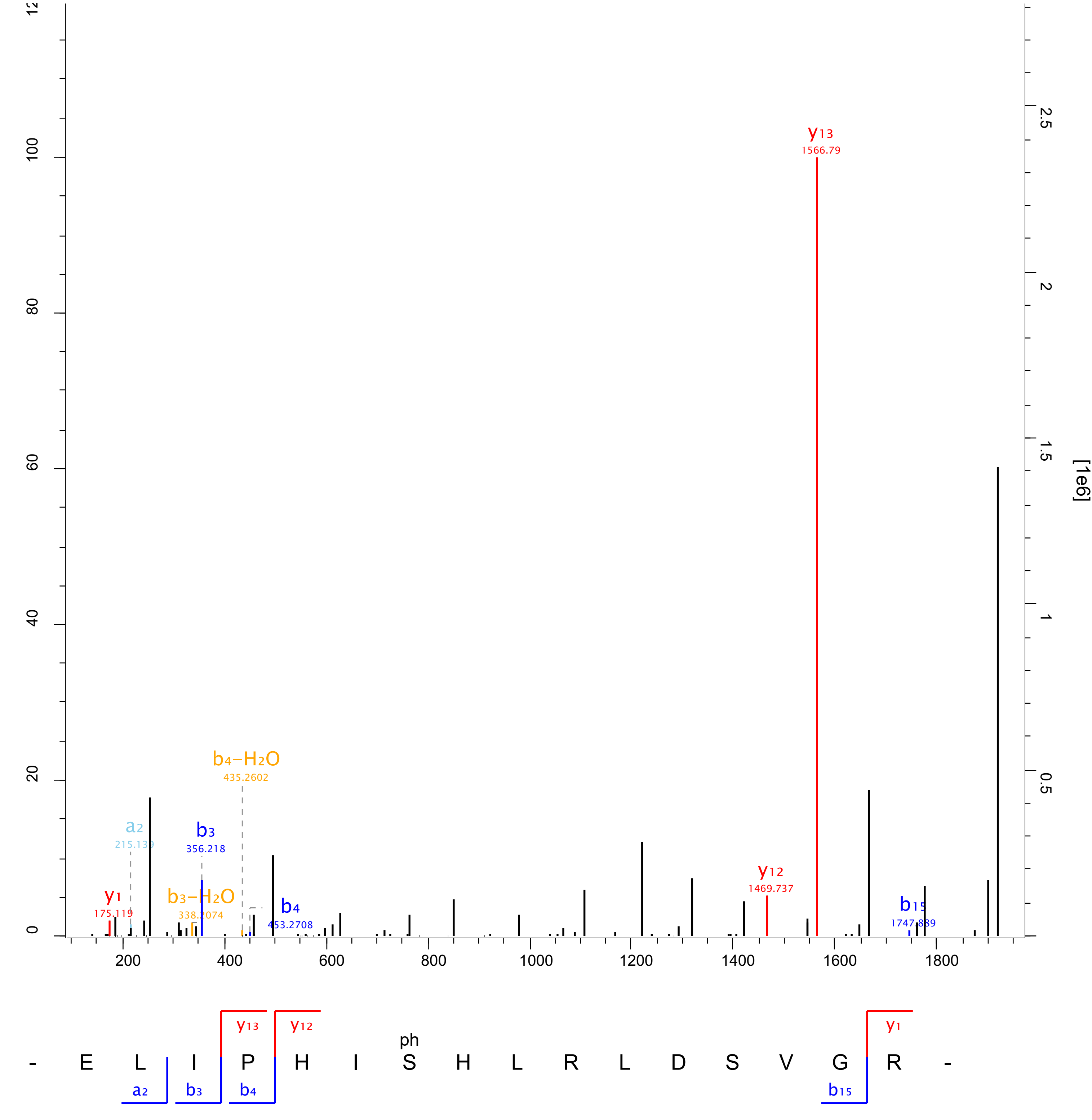

Sample output to test PDF Combine only
