# Supplementary material for: Sucrose-induced Receptor Kinase 1 is Modulated by an Interacting Kinase with Short Extracellular Domain
Source: Mol Cell Proteomics. 2019 May 30;18(8):1556–71. doi: 10.1074/mcp.RA119.001336 (PMC6683012; doi:10.1074/mcp.RA119.001336)
Supplement: Supplementary Figure S6i [file 143141_1_supp_311932_ps5kkz.pdf]

Figure S6:

Spectra of all identified phosphopeptides.

Raw file	Scan	Method	Score	m/z	Gene names
sp3-solu-SUC-1-P	30485	FTMS; HCD	40.46	899.15	T9E8.90

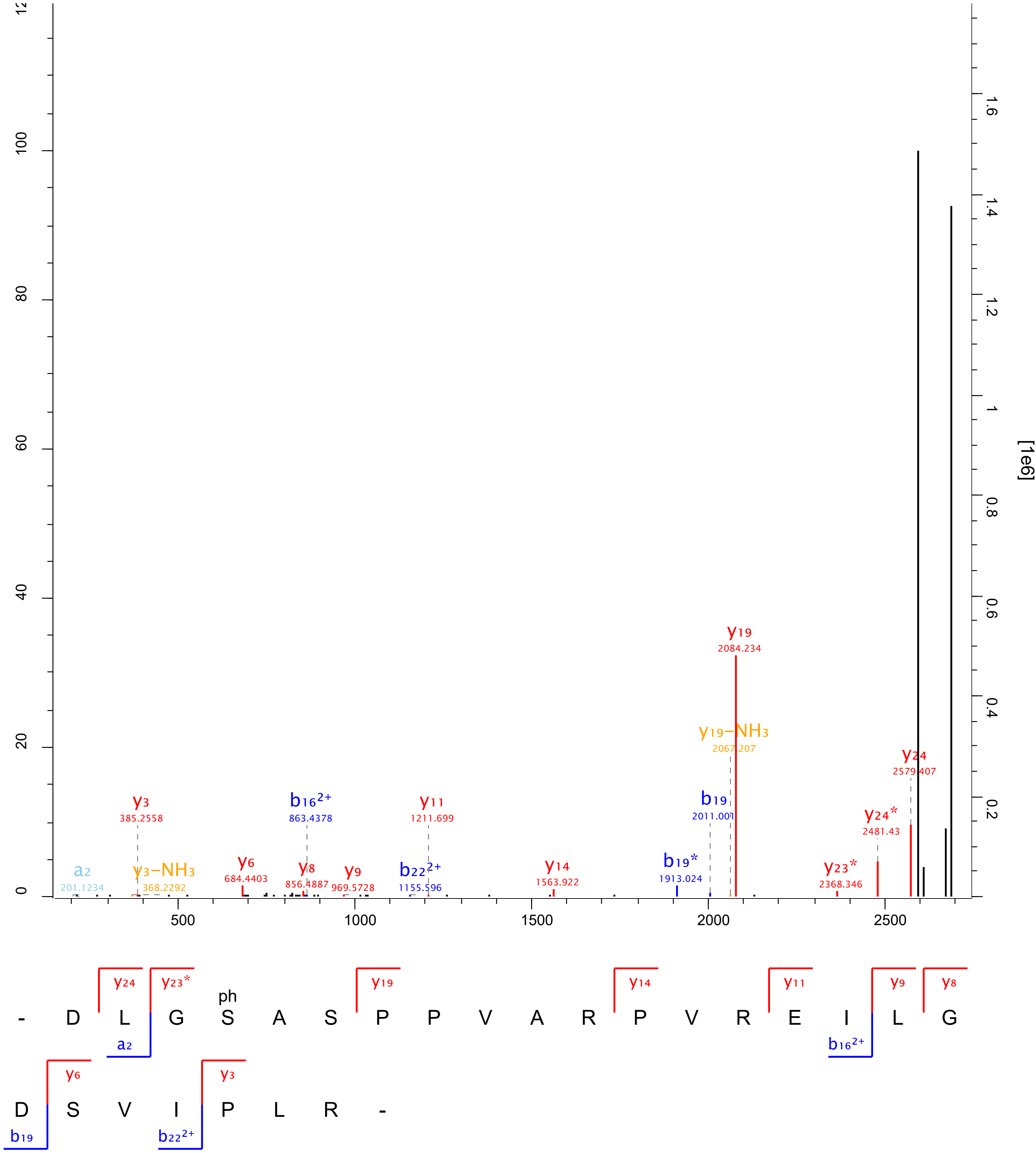

Sample output to test PDF Combine only

Raw file	Scan	Method	Score	m/z	Gene names
sp3-solu-SUC-2-P	17938	FTMS; HCD	78.81	871.43	F1C12.140

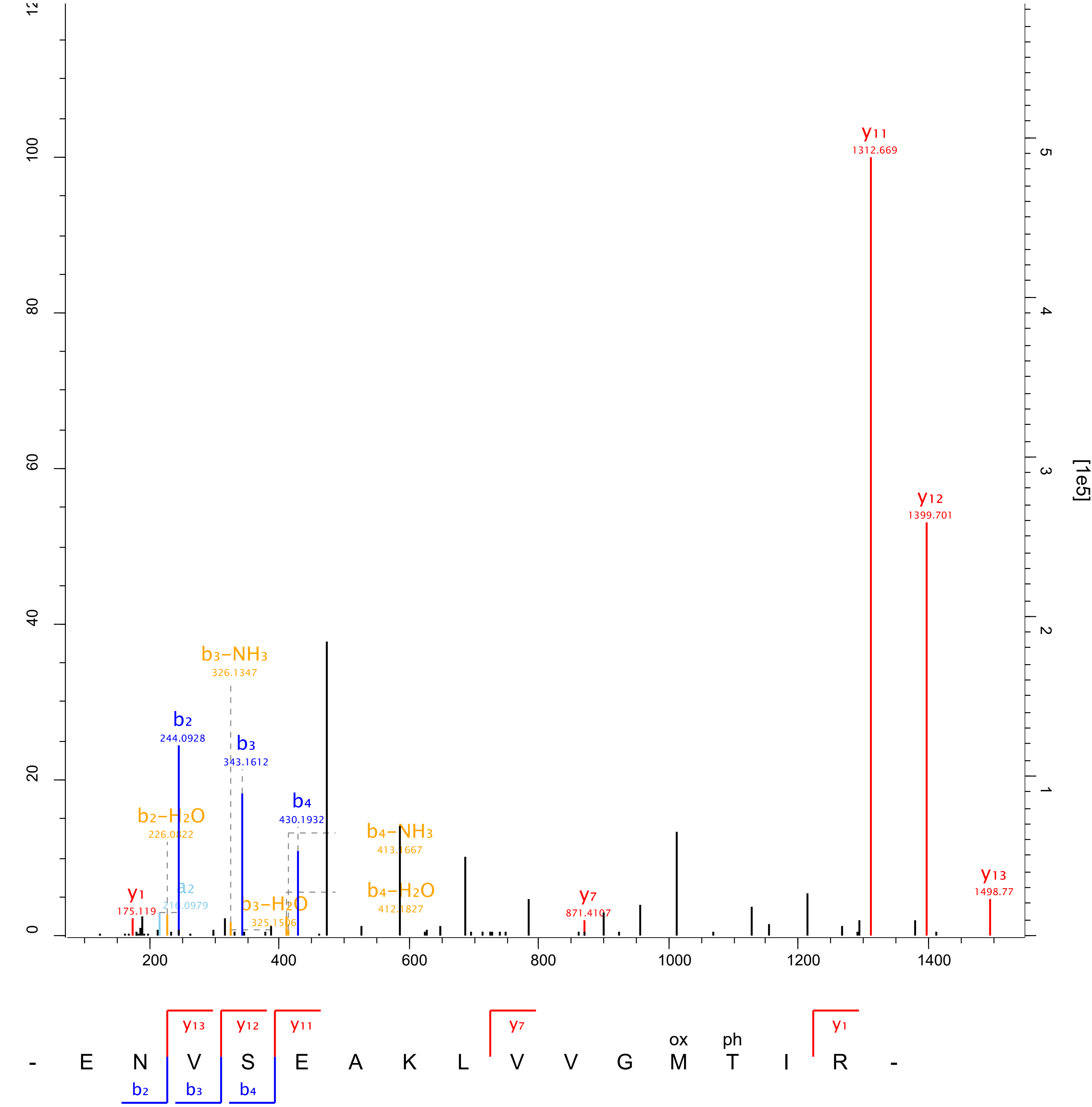

Sample output to test PDF Combine only

Raw file	Scan	Method	Score	m/z	Gene names
sp3-solu-SUC-3-P	15006	FTMS; HCD	108.9	568.3	DYL1

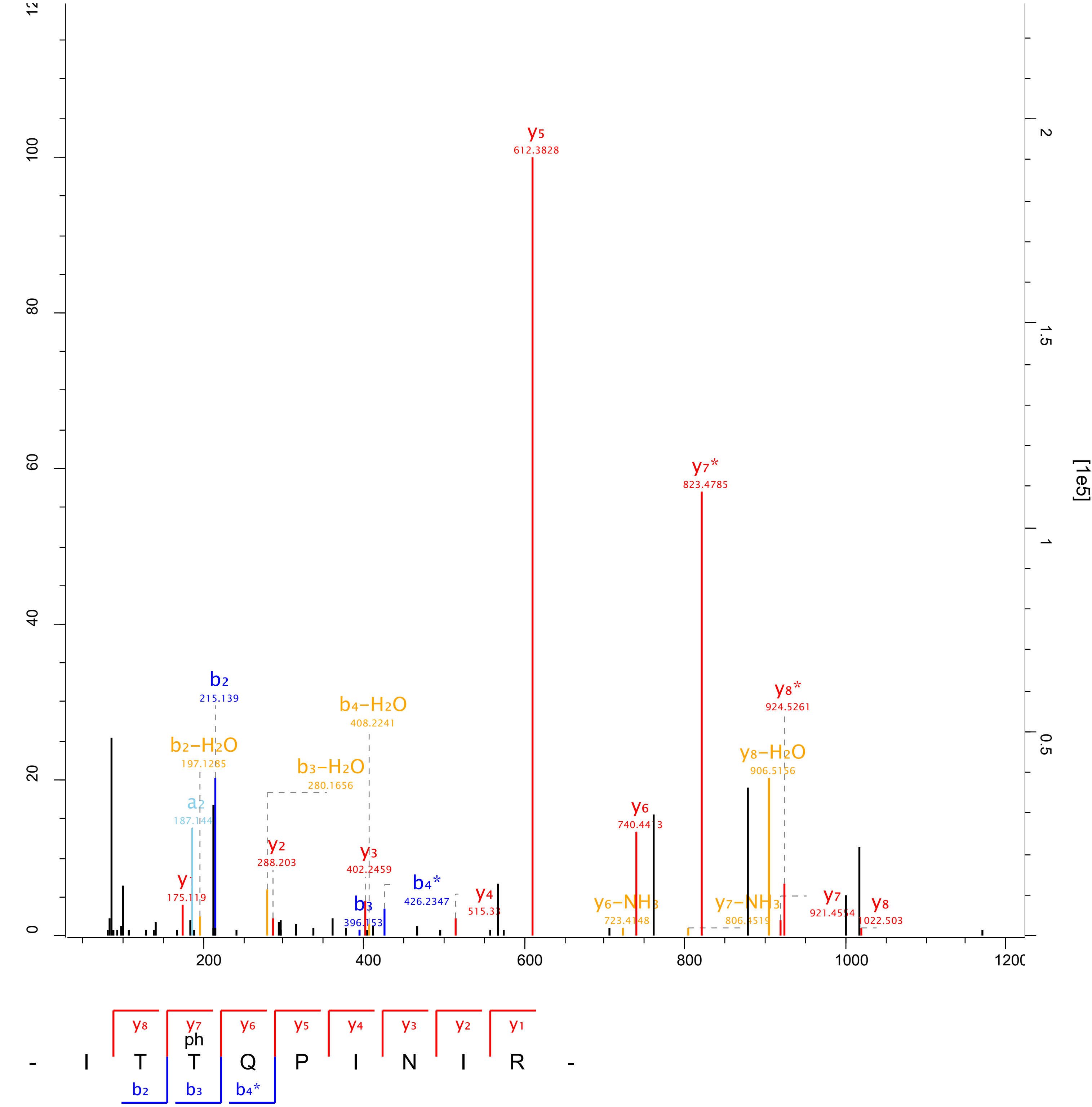

Sample output to test PDF Combine only
